# Global, regional, and national comparative risk assessment of 84 behavioural, environmental and occupational, and metabolic risks or clusters of risks, 1990–2016: a systematic analysis for the Global Burden of Disease Study 2016

**DOI:** 10.1016/S0140-6736(17)32366-8

**Published:** 2017-09-16

**Authors:** GBD 2016 Risk Factors Collaborators, Emmanuela Gakidou, Ashkan Afshin, Amanuel Alemu Abajobir, Kalkidan Hassen Abate, Cristiana Abbafati, Kaja M Abbas, Foad Abd-Allah, Abdishakur M Abdulle, Semaw Ferede Abera, Victor Aboyans, Laith J Abu-Raddad, Niveen M E Abu-Rmeileh, Gebre Yitayih Abyu, Isaac Akinkunmi Adedeji, Olatunji Adetokunboh, Mohsen Afarideh, Anurag Agrawal, Sutapa Agrawal, Hamid Ahmadieh, Muktar Beshir Ahmed, Miloud Taki Eddine Aichour, Amani Nidhal Aichour, Ibtihel Aichour, Rufus Olusola Akinyemi, Nadia Akseer, Fares Alahdab, Ziyad Al-Aly, Khurshid Alam, Noore Alam, Tahiya Alam, Deena Alasfoor, Kefyalew Addis Alene, Komal Ali, Reza Alizadeh-Navaei, Ala'a Alkerwi, François Alla, Peter Allebeck, Rajaa Al-Raddadi, Ubai Alsharif, Khalid A Altirkawi, Nelson Alvis-Guzman, Azmeraw T Amare, Erfan Amini, Walid Ammar, Yaw Ampem Amoako, Hossein Ansari, Josep M Antó, Carl Abelardo T Antonio, Palwasha Anwari, Nicholas Arian, Johan Ärnlöv, Al Artaman, Krishna Kumar Aryal, Hamid Asayesh, Solomon Weldegebreal Asgedom, Tesfay Mehari Atey, Leticia Avila-Burgos, Euripide Frinel G Arthur Avokpaho, Ashish Awasthi, Peter Azzopardi, Umar Bacha, Alaa Badawi, Kalpana Balakrishnan, Shoshana H Ballew, Aleksandra Barac, Ryan M Barber, Suzanne L Barker-Collo, Till Bärnighausen, Simon Barquera, Lars Barregard, Lope H Barrero, Carolina Batis, Katherine E Battle, Blair R Baumgarner, Bernhard T Baune, Justin Beardsley, Neeraj Bedi, Ettore Beghi, Michelle L Bell, Derrick A Bennett, James R Bennett, Isabela M Bensenor, Adugnaw Berhane, Derbew Fikadu Berhe, Eduardo Bernabé, Balem Demtsu Betsu, Mircea Beuran, Addisu Shunu Beyene, Anil Bhansali, Zulfiqar A Bhutta, Burcu Kucuk Bicer, Boris Bikbov, Charles Birungi, Stan Biryukov, Christopher D Blosser, Dube Jara Boneya, Ibrahim R Bou-Orm, Michael Brauer, Nicholas J K Breitborde, Hermann Brenner, Traolach S Brugha, Lemma Negesa Bulto Bulto, Zahid A Butt, Lucero Cahuana-Hurtado, Rosario Cárdenas, Juan Jesus Carrero, Carlos A Castañeda-Orjuela, Ferrán Catalá-López, Kelly Cercy, Hsing-Yi Chang, Fiona J Charlson, Odgerel Chimed-Ochir, Vesper Hichilombwe Chisumpa, Abdulaal A Chitheer, Hanne Christensen, Devasahayam Jesudas Christopher, Massimo Cirillo, Aaron J Cohen, Haley Comfort, Cyrus Cooper, Josef Coresh, Leslie Cornaby, Paolo Angelo Cortesi, Michael H Criqui, John A Crump, Lalit Dandona, Rakhi Dandona, José das Neves, Gail Davey, Dragos V Davitoiu, Kairat Davletov, Barbora de Courten, Barthelemy Kuate Defo, Louisa Degenhardt, Selina Deiparine, Robert P Dellavalle, Kebede Deribe, Aniruddha Deshpande, Samath D Dharmaratne, Eric L Ding, Shirin Djalalinia, Huyen Phuc Do, Klara Dokova, David Teye Doku, Aaron van Donkelaar, E Ray Dorsey, Tim R Driscoll, Manisha Dubey, Bruce Bartholow Duncan, Sarah Duncan, Hedyeh Ebrahimi, Ziad Ziad El-Khatib, Ahmadali Enayati, Aman Yesuf Endries, Sergey Petrovich Ermakov, Holly E Erskine, Babak Eshrati, Sharareh Eskandarieh, Alireza Esteghamati, Kara Estep, Emerito Jose Aquino Faraon, Carla Sofia e Sa Farinha, André Faro, Farshad Farzadfar, Kairsten Fay, Valery L Feigin, Seyed-Mohammad Fereshtehnejad, João C Fernandes, Alize J Ferrari, Tesfaye Regassa Feyissa, Irina Filip, Florian Fischer, Christina Fitzmaurice, Abraham D Flaxman, Nataliya Foigt, Kyle J Foreman, Joseph J Frostad, Nancy Fullman, Thomas Fürst, Joao M Furtado, Morsaleh Ganji, Alberto L Garcia-Basteiro, Tsegaye Tewelde Gebrehiwot, Johanna M Geleijnse, Ayele Geleto, Bikila Lencha Gemechu, Hailay Abrha Gesesew, Peter W Gething, Alireza Ghajar, Katherine B Gibney, Paramjit Singh Gill, Richard F Gillum, Ababi Zergaw Giref, Melkamu Dedefo Gishu, Giorgia Giussani, William W Godwin, Philimon N Gona, Amador Goodridge, Sameer Vali Gopalani, Yevgeniy Goryakin, Alessandra Carvalho Goulart, Nicholas Graetz, Harish Chander Gugnani, Jingwen Guo, Rajeev Gupta, Tanush Gupta, Vipin Gupta, Reyna A Gutiérrez, Vladimir Hachinski, Nima Hafezi-Nejad, Gessessew Bugssa Hailu, Randah Ribhi Hamadeh, Samer Hamidi, Mouhanad Hammami, Alexis J Handal, Graeme J Hankey, Sarah Wulf Hanson, Hilda L Harb, Habtamu Abera Hareri, Mohammad Sadegh Hassanvand, Rasmus Havmoeller, Caitlin Hawley, Simon I Hay, Mohammad T Hedayati, Delia Hendrie, Ileana Beatriz Heredia-Pi, Julio Cesar Montañez Hernandez, Hans W Hoek, Nobuyuki Horita, H Dean Hosgood, Sorin Hostiuc, Damian G Hoy, Mohamed Hsairi, Guoqing Hu, John J Huang, Hsiang Huang, Norlinah Mohamed Ibrahim, Kim Moesgaard Iburg, Chad Ikeda, Manami Inoue, Caleb Mackay Salpeter Irvine, Maria Delores Jackson, Kathryn H Jacobsen, Nader Jahanmehr, Mihajlo B Jakovljevic, Alejandra Jauregui, Mehdi Javanbakht, Panniyammakal Jeemon, Lars R K Johansson, Catherine O Johnson, Jost B Jonas, Mikk Jürisson, Zubair Kabir, Rajendra Kadel, Amaha Kahsay, Ritul Kamal, André Karch, Corine Kakizi Karema, Amir Kasaeian, Nicholas J Kassebaum, Anshul Kastor, Srinivasa Vittal Katikireddi, Norito Kawakami, Peter Njenga Keiyoro, Sefonias Getachew Kelbore, Laura Kemmer, Andre Pascal Kengne, Chandrasekharan Nair Kesavachandran, Yousef Saleh Khader, Ibrahim A Khalil, Ejaz Ahmad Khan, Young-Ho Khang, Ardeshir Khosravi, Jagdish Khubchandani, Aliasghar Ahmad Kiadaliri, Christian Kieling, Jun Y Kim, Yun Jin Kim, Daniel Kim, Ruth W Kimokoti, Yohannes Kinfu, Adnan Kisa, Katarzyna A Kissimova-Skarbek, Mika Kivimaki, Luke D Knibbs, Ann Kristin Knudsen, Jacek A Kopec, Soewarta Kosen, Parvaiz A Koul, Ai Koyanagi, Michael Kravchenko, Kristopher J Krohn, Hans Kromhout, G Anil Kumar, Michael Kutz, Hmwe H Kyu, Dharmesh Kumar Lal, Ratilal Lalloo, Tea Lallukka, Qing Lan, Van C Lansingh, Anders Larsson, Paul H Lee, Alexander Lee, James Leigh, Janni Leung, Miriam Levi, Teresa Shamah Levy, Yichong Li, Yongmei Li, Xiaofeng Liang, Misgan Legesse Liben, Shai Linn, Patrick Liu, Rakesh Lodha, Giancarlo Logroscino, Katherine J Looker, Alan D Lopez, Stefan Lorkowski, Paulo A Lotufo, Rafael Lozano, Raimundas Lunevicius, Erlyn Rachelle King Macarayan, Hassan Magdy Abd El Razek, Mohammed Magdy Abd El Razek, Marek Majdan, Reza Majdzadeh, Azeem Majeed, Reza Malekzadeh, Rajesh Malhotra, Deborah Carvalho Malta, Abdullah A Mamun, Helena Manguerra, Lorenzo G Mantovani, Chabila C Mapoma, Randall V Martin, Jose Martinez-Raga, Francisco Rogerlândio Martins-Melo, Manu Raj Mathur, Kunihiro Matsushita, Richard Matzopoulos, Mohsen Mazidi, Colm McAlinden, John J McGrath, Suresh Mehata, Man Mohan Mehndiratta, Toni Meier, Yohannes Adama Melaku, Peter Memiah, Ziad A Memish, Walter Mendoza, Melkamu Merid Mengesha, George A Mensah, Gert B M Mensink, Seid Tiku Mereta, Tuomo J Meretoja, Atte Meretoja, Haftay Berhane Mezgebe, Renata Micha, Anoushka Millear, Ted R Miller, Shawn Minnig, Mojde Mirarefin, Erkin M Mirrakhimov, Awoke Misganaw, Shiva Raj Mishra, Karzan Abdulmuhsin Mohammad, Kedir Endris Mohammed, Shafiu Mohammed, Murali B V Mohan, Ali H Mokdad, Lorenzo Monasta, Marcella Montico, Maziar Moradi-Lakeh, Paula Moraga, Lidia Morawska, Shane D Morrison, Cliff Mountjoy-Venning, Ulrich O Mueller, Erin C Mullany, Kate Muller, Gudlavalleti Venkata Satyanarayana Murthy, Kamarul Imran Musa, Mohsen Naghavi, Aliya Naheed, Vinay Nangia, Gopalakrishnan Natarajan, Ruxandra Irina Negoi, Ionut Negoi, Cuong Tat Nguyen, Quyen Le Nguyen, Trang Huyen Nguyen, Grant Nguyen, Minh Nguyen, Emma Nichols, Dina Nur Anggraini Ningrum, Marika Nomura, Vuong Minh Nong, Ole F Norheim, Bo Norrving, Jean Jacques N Noubiap, Carla Makhlouf Obermeyer, Felix Akpojene Ogbo, In-Hwan Oh, Olanrewaju Oladimeji, Andrew Toyin Olagunju, Tinuke Oluwasefunmi Olagunju, Pedro R Olivares, Helen E Olsen, Bolajoko Olubukunola Olusanya, Jacob Olusegun Olusanya, John Nelson Opio, Eyal Oren, Alberto Ortiz, Erika Ota, Mayowa O Owolabi, Mahesh PA, Rosana E Pacella, Adrian Pana, Basant Kumar Panda, Songhomitra Panda-Jonas, Jeyaraj D Pandian, Christina Papachristou, Eun-Kee Park, Charles D Parry, Scott B Patten, George C Patton, David M Pereira, Norberto Perico, Konrad Pesudovs, Max Petzold, Michael Robert Phillips, Julian David Pillay, Michael A Piradov, Farhad Pishgar, Dietrich Plass, Martin A Pletcher, Suzanne Polinder, Svetlana Popova, Richie G Poulton, Farshad Pourmalek, Narayan Prasad, Carrie Purcell, Mostafa Qorbani, Amir Radfar, Anwar Rafay, Afarin Rahimi-Movaghar, Vafa Rahimi-Movaghar, Mohammad Hifz Ur Rahman, Muhammad Aziz Rahman, Mahfuzar Rahman, Rajesh Kumar Rai, Sasa Rajsic, Usha Ram, Salman Rawaf, Colin D Rehm, Jürgen Rehm, Robert C Reiner, Marissa B Reitsma, Giuseppe Remuzzi, Andre M N Renzaho, Serge Resnikoff, Luz Myriam Reynales-Shigematsu, Satar Rezaei, Antonio L Ribeiro, Juan A Rivera, Kedir Teji Roba, David Rojas-Rueda, Yesenia Roman, Robin Room, Gholamreza Roshandel, Gregory A Roth, Dietrich Rothenbacher, Enrico Rubagotti, Lesley Rushton, Nafis Sadat, Mahdi Safdarian, Sare Safi, Saeid Safiri, Ramesh Sahathevan, Joseph Salama, Joshua A Salomon, Abdallah M Samy, Juan Ramon Sanabria, Maria Dolores Sanchez-Niño, Tania G Sánchez-Pimienta, Damian Santomauro, Itamar S Santos, Milena M Santric Milicevic, Benn Sartorius, Maheswar Satpathy, Monika Sawhney, Sonia Saxena, Maria Inês Schmidt, Ione J C Schneider, Aletta E Schutte, David C Schwebel, Falk Schwendicke, Soraya Seedat, Sadaf G Sepanlou, Berrin Serdar, Edson E Servan-Mori, Gavin Shaddick, Amira Shaheen, Saeid Shahraz, Masood Ali Shaikh, Mansour Shamsipour, Morteza Shamsizadeh, Sheikh Mohammed Shariful Islam, Jayendra Sharma, Rajesh Sharma, Jun She, Jiabin Shen, Peilin Shi, Kenji Shibuya, Chloe Shields, Mekonnen Sisay Shiferaw, Mika Shigematsu, Min-Jeong Shin, Rahman Shiri, Reza Shirkoohi, Kawkab Shishani, Haitham Shoman, Mark G Shrime, Inga Dora Sigfusdottir, Diego Augusto Santos Silva, João Pedro Silva, Dayane Gabriele Alves Silveira, Jasvinder A Singh, Virendra Singh, Dhirendra Narain Sinha, Eirini Skiadaresi, Erica Leigh Slepak, David L Smith, Mari Smith, Badr H A Sobaih, Eugene Sobngwi, Samir Soneji, Reed J D Sorensen, Luciano A Sposato, Chandrashekhar T Sreeramareddy, Vinay Srinivasan, Nicholas Steel, Dan J Stein, Caitlyn Steiner, Sabine Steinke, Mark Andrew Stokes, Bryan Strub, Michelle Subart, Muawiyyah Babale Sufiyan, Rizwan Abdulkader Suliankatchi, Patrick J Sur, Soumya Swaminathan, Bryan L Sykes, Cassandra E I Szoeke, Rafael Tabarés-Seisdedos, Santosh Kumar Tadakamadla, Ken Takahashi, Jukka S Takala, Nikhil Tandon, Marcel Tanner, Yihunie L Tarekegn, Mohammad Tavakkoli, Teketo Kassaw Tegegne, Arash Tehrani-Banihashemi, Abdullah Sulieman Terkawi, Belay Tesssema, JS Thakur, Ornwipa Thamsuwan, Kavumpurathu Raman Thankappan, Andrew M Theis, Matthew Lloyd Thomas, Alan J Thomson, Amanda G Thrift, Taavi Tillmann, Ruoyan Tobe-Gai, Myriam Tobollik, Mette C Tollanes, Marcello Tonelli, Roman Topor-Madry, Anna Torre, Miguel Tortajada, Mathilde Touvier, Bach Xuan Tran, Thomas Truelsen, Kald Beshir Tuem, Emin Murat Tuzcu, Stefanos Tyrovolas, Kingsley Nnanna Ukwaja, Chigozie Jesse Uneke, Rachel Updike, Olalekan A Uthman, Job F M van Boven, Santosh Varughese, Tommi Vasankari, Lennert J Veerman, Vidhya Venkateswaran, Narayanaswamy Venketasubramanian, Francesco S Violante, Sergey K Vladimirov, Vasiliy Victorovich Vlassov, Stein Emil Vollset, Theo Vos, Fiseha Wadilo, Tolassa Wakayo, Mitchell T Wallin, Yuan-Pang Wang, Scott Weichenthal, Elisabete Weiderpass, Robert G Weintraub, Daniel J Weiss, Andrea Werdecker, Ronny Westerman, Harvey A Whiteford, Charles Shey Wiysonge, Belete Getahun Woldeyes, Charles D A Wolfe, Rachel Woodbrook, Abdulhalik Workicho, Denis Xavier, Gelin Xu, Simon Yadgir, Bereket Yakob, Lijing L Yan, Mehdi Yaseri, Hassen Hamid Yimam, Paul Yip, Naohiro Yonemoto, Seok-Jun Yoon, Marcel Yotebieng, Mustafa Z Younis, Zoubida Zaidi, Maysaa El Sayed Zaki, Luis Zavala-Arciniega, Xueying Zhang, Stephanie Raman M Zimsen, Ben Zipkin, Sanjay Zodpey, Stephen S Lim, Christopher J L Murray

## Abstract

**Background:**

The Global Burden of Diseases, Injuries, and Risk Factors Study 2016 (GBD 2016) provides a comprehensive assessment of risk factor exposure and attributable burden of disease. By providing estimates over a long time series, this study can monitor risk exposure trends critical to health surveillance and inform policy debates on the importance of addressing risks in context.

**Methods:**

We used the comparative risk assessment framework developed for previous iterations of GBD to estimate levels and trends in exposure, attributable deaths, and attributable disability-adjusted life-years (DALYs), by age group, sex, year, and location for 84 behavioural, environmental and occupational, and metabolic risks or clusters of risks from 1990 to 2016. This study included 481 risk-outcome pairs that met the GBD study criteria for convincing or probable evidence of causation. We extracted relative risk (RR) and exposure estimates from 22 717 randomised controlled trials, cohorts, pooled cohorts, household surveys, census data, satellite data, and other sources, according to the GBD 2016 source counting methods. Using the counterfactual scenario of theoretical minimum risk exposure level (TMREL), we estimated the portion of deaths and DALYs that could be attributed to a given risk. Finally, we explored four drivers of trends in attributable burden: population growth, population ageing, trends in risk exposure, and all other factors combined.

**Findings:**

Since 1990, exposure increased significantly for 30 risks, did not change significantly for four risks, and decreased significantly for 31 risks. Among risks that are leading causes of burden of disease, child growth failure and household air pollution showed the most significant declines, while metabolic risks, such as body-mass index and high fasting plasma glucose, showed significant increases. In 2016, at Level 3 of the hierarchy, the three leading risk factors in terms of attributable DALYs at the global level for men were smoking (124·1 million DALYs [95% UI 111·2 million to 137·0 million]), high systolic blood pressure (122·2 million DALYs [110·3 million to 133·3 million], and low birthweight and short gestation (83·0 million DALYs [78·3 million to 87·7 million]), and for women, were high systolic blood pressure (89·9 million DALYs [80·9 million to 98·2 million]), high body-mass index (64·8 million DALYs [44·4 million to 87·6 million]), and high fasting plasma glucose (63·8 million DALYs [53·2 million to 76·3 million]). In 2016 in 113 countries, the leading risk factor in terms of attributable DALYs was a metabolic risk factor. Smoking remained among the leading five risk factors for DALYs for 109 countries, while low birthweight and short gestation was the leading risk factor for DALYs in 38 countries, particularly in sub-Saharan Africa and South Asia. In terms of important drivers of change in trends of burden attributable to risk factors, between 2006 and 2016 exposure to risks explains an 9·3% (6·9–11·6) decline in deaths and a 10·8% (8·3–13·1) decrease in DALYs at the global level, while population ageing accounts for 14·9% (12·7–17·5) of deaths and 6·2% (3·9–8·7) of DALYs, and population growth for 12·4% (10·1–14·9) of deaths and 12·4% (10·1–14·9) of DALYs. The largest contribution of trends in risk exposure to disease burden is seen between ages 1 year and 4 years, where a decline of 27·3% (24·9–29·7) of the change in DALYs between 2006 and 2016 can be attributed to declines in exposure to risks.

**Interpretation:**

Increasingly detailed understanding of the trends in risk exposure and the RRs for each risk-outcome pair provide insights into both the magnitude of health loss attributable to risks and how modification of risk exposure has contributed to health trends. Metabolic risks warrant particular policy attention, due to their large contribution to global disease burden, increasing trends, and variable patterns across countries at the same level of development. GBD 2016 findings show that, while it has huge potential to improve health, risk modification has played a relatively small part in the past decade.

**Funding:**

The Bill & Melinda Gates Foundation, Bloomberg Philanthropies.

**Research in context**

**Evidence before this study**

The Global Burden of Diseases, Injuries, and Risk Factors Study 2016 (GBD 2016) remains the most comprehensive effort to conduct a population-level comparative risk assessment across countries and risks. Other sources of population-level estimates of risk include WHO and UNICEF reports as well as independent scientific publications. Notable differences in methods and definitions produce variation in results, although in several cases there is general agreement in regional or global patterns. The GBD study remains the only peer-reviewed, comprehensive, and annual assessment of risk factor burden by age, sex, cause, and location for a long time series that complies with the Guidelines for Accurate and Transparent Health Estimates Reporting (GATHER).

**Added value of this study**

This study builds upon GBD 2015 and provides several important improvements as well as the quantification of five new risks. The innovations and improvements from last year can be summarised as follows. Across all risk factors, there were 7155 additional data sources, according to the GBD 2016 source counting methods. For diet, we included data for dietary recall, household budget, and food frequency questionnaires. We also incorporated sales data from 170 countries as well as national accounting of food available to populations in a given year. In GBD 2016, we are producing estimates for the following five new risks: smokeless tobacco, low birthweight and short gestation, low birthweight for gestation, short gestation for birthweight, and diet low in legumes. We also extended the high body-mass index (BMI) analysis to include childhood obesity. We have also added 93 new risk-outcome pairs. Major revisions to the estimation of the following risk factors were undertaken for GBD 2016. For second-hand smoke, we changed the estimation method to ensure consistency with the estimates for smoking prevalence. For alcohol, we estimated new relative risks (RRs) for all outcomes, we incorporated more data for exposure and new adjustments for tourism and unrecorded consumption, and we redefined the theoretical minimum risk exposure level (TMREL). For diet, we estimated the disease burden of dietary risks based on the absolute level of intake rather than the intake standardised to 2000 kcal per day. We developed an ensemble model of different parametric distributions to generate better fits to the distributions of continuous risk factors. Mediation evidence was reviewed and updated based on an analysis of ten pooled cohorts. We have expanded the analysis of geographic and temporal trends in risk exposure and burden by development, using the Socio-demographic Index (SDI), and have also explored where countries are in the risk transition. We also improved and modified our decomposition methods so that the results shown are additive and can be aggregated to explain trends in all-cause and cause-specific mortality, as well as trends across age groups. The decomposition analysis has been extended to examine how risk factors have contributed to trends in all-cause mortality by age and sex as well as by cause.

**Implications of all the available evidence**

Increasingly detailed understanding of the trends in risk exposure and the RRs for each risk-outcome pair provides insights into both the magnitude of health loss attributable to risks and how modification of risk exposure has contributed to health trends. This analysis shows a mismatch between the potential for risk modification to improve health and the relatively modest role that risk modification has played in the past generation in improving global health.

## Introduction

A core premise of public health is that prevention can be a powerful instrument for improving human health, one that is often cost-effective and minimises harm to individuals from ill health. The core objectives of prevention include the reduction or modification of exposure to risks including metabolic, behavioural, environmental, and occupational factors. Quantifying risks to health and thus the targets of many public health actions is an essential prerequisite for effective public health. The evidence on the relation between risk exposure and health is constantly evolving: new information about the relative risks (RRs) associated with different risks for different outcomes continues to emerge from cohort studies, randomised trials, and case-control studies. These studies can establish evidence for new risks or risk-outcome pairs or reduce the strength of evidence for existing risks. New data are also regularly collected on the levels of exposure in different populations and in different settings. Regularly updated monitoring of the evidence base on risk factors is crucial for public health and for individual risk modification through primary care and self-management.

Several studies explore risk-attributable burden for individual risks^1^, ^2^, ^3^ at the global, regional, or national level. Other studies provide assessments of exposure for selected risks. However, the Global Burden of Diseases, Injuries, and Risk Factors Study (GBD) comparative risk assessment (CRA) is the only comprehensive and comparable approach to risk factor quantification. The most recent of these assessments was GBD 2015.^4^, ^5^, ^6^ With each cycle of GBD, scientific discussions have emerged on various dimensions of risk quantification that have led to improvements and modifications of GBD. Many of these are focused on the strength of evidence supporting a causal connection for specific risk-outcome pairs, while others relate to measurement challenges.^7^, ^8^, ^9^ Further, new risk factors have been added for important health conditions included in GBD, such as neonatal outcomes and Alzheimer's dementia,^10^ which have previously not had associated risk factors. The recent trials on blood pressure control at lower levels of systolic blood pressure, including the Systolic Blood Pressure Intervention Trial (SPRINT)^11^ and Heart Outcomes Prevention Evaluation-3 (HOPE-3) trial,^12^ have also brought attention to the difference between a population health perspective on the quantification of risks and the clinical question of risk reversibility. The CRA framework provides an important insight into the role of different risks in contributing to levels of population health but does not necessarily provide all the information necessary to guide individual clinical decision making.

The GBD 2016 CRA includes 84 risk factors and an associated 481 risk-outcome pairs. In addition to new data and updated methods, we have included five new risks in the GBD 2016 CRA. The study was undertaken for 195 countries and territories and provides estimates of exposure and attributable deaths and disability-adjusted life-years (DALYs) for 1990 through to 2016. We explored how risks change with development, measured by the Socio-demographic Index (SDI), and also decomposed changes in deaths and DALYs into the contributions of population ageing, population growth, trends in risk exposure, and all other factors combined. As with previous iterations of GBD, the GBD 2016 CRA results presented here supersede all previously published GBD CRA estimates.

## Methods

### Overview

The CRA conceptual framework was developed by Murray and Lopez,^13^ who established a causal web of hierarchically organised risks or causes that contribute to health outcomes (method appendix; appendix 1 p 432), which allows quantification of risks or causes at any level in the framework. In GBD 2016, as in previous iterations of GBD, we evaluated a set of behavioural, environmental, and occupational, and metabolic risks, where risk-outcome pairs were included based on evidence rules (appendix 1 p 344). These risks were organised into five hierarchical levels as described in appendix 1 (p 374). At Level 0, the GBD 2016 provides estimates for all risk factors combined, at Level 1 the GBD 2016 provides estimates for three groups: environmental and occupational, metabolic, and behavioral risk factors. At Level 2, there are 17 risks, at Level 3 there are 50 risks, and at Level 4 there are 67 risks, for a total of 84 risks or clusters of risks. To date, we have not quantified the contribution of other classes of risk factors (appendix 1 p 376); however, using an analysis of the relation between risk exposures and socio-demographic development, measured with the use of SDI, we provide some insights into the potential magnitude of distal social, cultural, and economic factors.

Two types of risk assessment are possible within the CRA framework: attributable burden and avoidable burden.^13^ Attributable burden is the reduction in current disease burden that would have been possible if past population exposure had shifted to an alternative or counterfactual distribution of risk exposure. Avoidable burden is the potential reduction in future disease burden that could be achieved by changing the current distribution of exposure to a counterfactual distribution of exposure. Murray and Lopez^13^ identified four types of counterfactual exposure distributions: theoretical, plausible, feasible, and cost-effective minimum risk. In GBD studies, to date and in this study, we focus on attributable burden using the theoretical minimum risk exposure level, which is the distribution of risk comprising the levels of exposure that minimise risk for each individual in the population.

Overall, this analysis follows the CRA methods used in GBD 2015.^4^ The methods described in this study provide a high-level overview of the analytical logic, focusing on areas of notable change from the methods used in GBD 2015, with details provided in appendix 1 (p 10). This study complies with the Guidelines for Accurate and Transparent Health Estimates Reporting (GATHER) statement^14^ (appendix 1 p 377).

### Geographical units of analysis and years for estimation

In GBD 2016, locations are arranged as a set of hierarchical categories: seven super-regions, 21 regions nested within the seven super-regions, and 195 countries and territories nested in the 21 regions. Additionally, we present estimates at the subnational level for five countries with a population greater than 200 million in 2016: Brazil, China, India, Indonesia, and the USA. We produced a complete set of age-specific, sex-specific, cause-specific, and location-specific estimates of risk factor exposure and attributable burden for 1990–2016 for all included risk factors.

### Attributable burden estimation

Four key components are included in estimation of the burden attributable to a given risk factor: the metric of burden being assessed (number of deaths, years of life lost [YLLs], years lived with disability [YLDs], or DALYs [the sum of YLLs and YLDs]), the exposure levels for a risk factor, the relative risk of a given outcome due to exposure, and the counterfactual level of risk factor exposure. Estimates of attributable DALYs for a risk-outcome pair are equal to DALYs for the outcome multiplied by the population attributable fraction (PAF) for the risk-outcome pair for a given age, sex, location, and year. A similar logic applies for estimation of attributable deaths, YLLs, or YLDs. Risks are categorised on the basis of how exposure was measured: dichotomous, polytomous, or continuous. The PAF represents the proportion of outcome that would be reduced in a given year if the exposure to a risk factor in the past were reduced to the counterfactual level of the theoretical minimum risk exposure level (supplementary results, appendix 2 p 1).

### Causal evidence for risk-outcome pairs

In this study, as in GBD 2015, we have included risk-outcome pairs that we have assessed as meeting the World Cancer Research Fund grades of convincing or probable evidence (see appendix 1 p 10 for definitions of these grades).^15^ Table 1 provides a summary of the evidence supporting a causal relation between a risk and an outcome for each pair included in GBD 2016. For each risk-outcome pair, we used recent systematic reviews to identify independent prospective studies (randomised controlled trials, non-randomised interventions, and cohorts) that evaluated the putative relationship. For risk-outcome pairs with fewer than five prospective studies, we evaluated evidence from case-control studies as well (appendix 1 p 344). Table 1 summarises the evidence using multiple dimensions, which supports our assessment that each included risk-outcome pair meets the criteria of convincing or probable evidence (appendix 1 p 10 contains a justification of the criteria presented to support causality). In this summary of evidence, we have focused on randomised controlled trials and prospective observational studies, along with supporting evidence, like dose–response relationships and biologically plausible mechanisms.

**Table 1:** Descriptive cataloguing of the epidemiological evidence used to assess whether each risk-outcome paper meets the causal criteria for inclusion in the Global Burden of Disease Study 2016 by risk level

	**Risk**	**Outcome**	**RCTs (n)**	**RCTs with significant effect in the opposite direction (%)**	**RCTs with null findings (%)**	Prospective observational studies (n)*	**Prospective observational studies with significant association in the opposite direction (%)**	**Case-control studies assessing the risk- outcome pair relationship (n)**†	**Case-control studies that show significant association in the opposite direction (%)**	**Lower limit of RR >1·5**	**Dose–response relationship**	**Biological plausibility**‡	**Analogy**§
**2**	**Unsafe water, sanitation, and handwashing**												
3	Unsafe water source– chlorination or solar (point of use treatment)	Diarrhoeal diseases	24	0	42	6	0	··	··	Yes	··	Yes	No
3	Unsafe water source–piped	Diarrhoeal diseases	1	0	0	9	11	··	··	Yes	··	Yes	No
3	Unsafe water source–filter	Diarrhoeal diseases	11	0	45	2	0	··	··	Yes	··	Yes	No
3	Unsafe water source– improved water	Diarrhoeal diseases	0	··	··	5	0	··	··	Yes	··	Yes	No
3	Unsafe sanitation– piped	Diarrhoeal diseases	0	··	··	7	0	··	··	Yes	··	Yes	No
3	Unsafe sanitation– improved sanitation	Diarrhoeal diseases	0	··	··	9	0	··	··	Yes	··	Yes	No
3	No access to handwashing facility	Diarrhoeal diseases	19	0	42	0	··	··	··	No	··	Yes	No
3	No access to handwashing facility	Lower respiratory infections	8	0	50	11	0	··	··	No	··	Yes	No
**2**	**Air pollution**												
3	Ambient particulate matter pollution	Lower respiratory infections	0	··	··	19	0	··	··	No	Yes	Yes	No
3	Ambient particulate matter pollution	Tracheal, bronchus, and lung cancer	0	··	··	27	0	··	··	No	Yes	Yes	Yes
3	Ambient particulate matter pollution	Ischaemic heart disease	0	··	··	16	0	··	··	No	Yes	Yes	Yes
3	Ambient particulate matter pollution	Ischaemic stroke	0	··	··	25	0	··	··	No	Yes	Yes	Yes
3	Ambient particulate matter pollution	Haemorrhagic stroke	0	··	··	25	0	··	··	No	Yes	Yes	Yes
3	Ambient particulate matter pollution	Chronic obstructive pulmonary disease	0	··	··	12	0	··	··	No	Yes	Yes	Yes
3	Household air pollution from solid fuels	Lower respiratory infections	0	··	··	0	··	9	0	No	Yes	Yes	No
3	Household air pollution from solid fuels	Tracheal, bronchus, and lung cancer	0	··	··	0	··	20	0	No	Yes	Yes	Yes
3	Household air pollution from solid fuels	Ischaemic heart disease	0	··	··	16	0	··	··	No	Yes	Yes	Yes
3	Household air pollution from solid fuels	Ischaemic stroke	0	··	··	25	0	··	··	No	Yes	Yes	Yes
3	Household air pollution from solid fuels	Haemorrhagic stroke	0	··	··	25	0	··	··	No	Yes	Yes	Yes
3	Household air pollution from solid fuels	Chronic obstructive pulmonary disease	0	··	··	0	··	2	0	No	Yes	Yes	Yes
3	Household air pollution from solid fuels	Cataract	0	··	··	0	··	11	0	No	Yes	Yes	No
3	Ambient ozone pollution	Chronic obstructive pulmonary disease	0	··	··	4	0	0	0	No	Yes	Yes	No
**2**	**Other environmental risks**												
3	Residential radon	Tracheal, bronchus, and lung cancer	0	··	··	1	0	29	0	No	Yes	Yes	No
3	Lead exposure	Idiopathic developmental intellectual disability	0	··	··	8	0	··	··	No	Yes	Yes	No
3	Lead exposure	Systolic blood pressure	0	··	··	3	0	1	0	No	Yes	Yes	No
**2**	**Occupational risks**												
4	Occupational exposure to asbestos	Larynx cancer	0	··	··	27	0	··	··	No	··	Yes	Yes
4	Occupational exposure to asbestos	Tracheal, bronchus, and lung cancer	0	··	··	18	0	··	··	Yes	··	Yes	Yes
4	Occupational exposure to asbestos	Ovarian cancer	0	··	··	15	0	··	··	No	··	Yes	Yes
4	Occupational exposure to asbestos	Mesothelioma	0	··	··	5	0	··	··	Yes	··	Yes	Yes
4	Occupational exposure to arsenic	Tracheal, bronchus, and lung cancer	0	··	··	9	0	··	··	No	··	Yes	No
4	Occupational exposure to benzene	Leukaemia	0	··	··	12	0	··	··	Yes	··	Yes	No
4	Occupational exposure to beryllium	Tracheal, bronchus, and lung cancer	0	··	··	3	0	2	0	No	··	Yes	No
4	Occupational exposure to cadmium	Tracheal, bronchus, and lung cancer	0	··	··	7	0	··	··	No	··	Yes	No
4	Occupational exposure to chromium	Tracheal, bronchus, and lung cancer	0	··	··	26	0	··	··	No	··	Yes	No
4	Occupational exposure to diesel engine exhaust	Tracheal, bronchus, and lung cancer	0	··	··	17	0	··	··	No	··	Yes	No
4	Occupational exposure to second-hand smoke	Tracheal, bronchus, and lung cancer	0	··	··	25	0	··	··	No	··	Yes	No
4	Occupational exposure to formaldehyde	Nasopharynx cancer	0	··	··	2	0	6	0	No	··	Yes	Yes
4	Occupational exposure to formaldehyde	Leukaemia	0	··	··	13	0	··	··	No	··	Yes	Yes
4	Occupational exposure to nickel	Tracheal, bronchus, and lung cancer	0	··	··	6	0	··	··	No	··	Yes	No
4	Occupational exposure to polycyclic aromatic hydrocarbons	Tracheal, bronchus, and lung cancer	0	··	··	39	0	··	··	No	··	Yes	No
4	Occupational exposure to silica	Tracheal, bronchus, and lung cancer	0	··	··	17	0	··	··	No	··	Yes	No
4	Occupational exposure to sulfuric acid	Larynx cancer	0	··	··	14	0	··	··	Yes	··	Yes	No
4	Occupational exposure to trichloroethylene	Kidney cancer	0	··	··	20	0	··	··	No	··	Yes	No
3	Occupational asthmagens	Asthma	0	··	··	16	0	··	··	No	··	Yes	No
3	Occupational particulate matter, gases, and fumes	Chronic obstructive pulmonary disease	0	··	··	9	0	··	··	No	··	Yes	No
3	Occupational noise	Age-related and other hearing loss	0	··	··	5	0	··	··	Yes	··	Yes	No
3	Occupational ergonomic factors	Low back pain	0	··	··	10	0	··	··	No	··	Yes	No
**2**	**Child and maternal malnutrition**												
4	Non-exclusive breastfeeding	Diarrhoeal diseases	0	··	··	5	0	··	··	Yes	··	Yes	No
4	Non-exclusive breastfeeding	Lower respiratory infections	0	··	··	6	0	··	··	Yes	··	Yes	No
4	Discontinued breastfeeding	Diarrhoeal diseases	0	··	··	2	0	··	··	No	··	Yes	No
4	Child underweight	Diarrhoeal diseases	0	··	··	7	0	··	··	Yes	··	Yes	No
4	Child underweight	Lower respiratory infections	0	··	··	7	0	··	··	Yes	··	Yes	No
4	Child underweight	Measles	0	··	··	7	0	··	··	Yes	··	Yes	No
4	Child wasting	Diarrhoeal diseases	0	··	··	7	0	··	··	Yes	··	Yes	No
4	Child wasting	Lower respiratory infections	0	··	··	7	0	··	··	Yes	··	Yes	No
4	Child wasting	Measles	0	··	··	7	0	··	··	Yes	··	Yes	No
4	Child stunting	Diarrhoeal diseases	0	··	··	7	0	··	··	No	··	Yes	No
4	Child stunting	Lower respiratory infections	0	··	··	7	0	··	··	No	··	Yes	No
4	Child stunting	Measles	0	··	··	7	0	··	··	No	··	Yes	No
4	Short gestation for birthweight	Diarrhoeal diseases	0	··	··	20	0	··	··	Yes	Yes	Yes	Yes
4	Short gestation for birthweight	Lower respiratory infections	0	··	··	20	0	··	··	Yes	Yes	Yes	Yes
4	Short gestation for birthweight	Upper respiratory infections	0	··	··	20	0	··	··	Yes	Yes	Yes	Yes
4	Short gestation for birthweight	Otitis media	0	··	··	20	0	··	··	Yes	Yes	Yes	Yes
4	Short gestation for birthweight	Pneumococcal meningitis	0	··	··	20	0	··	··	Yes	Yes	Yes	Yes
4	Short gestation for birthweight	*Haemophilus influenzae* type B meningitis	0	··	··	20	0	··	··	Yes	Yes	Yes	Yes
4	Short gestation for birthweight	Meningococcal infection	0	··	··	20	0	··	··	Yes	Yes	Yes	Yes
4	Short gestation for birthweight	Other meningitis	0	··	··	20	0	··	··	Yes	Yes	Yes	Yes
4	Short gestation for birthweight	Encephalitis	0	··	··	20	0	··	··	Yes	Yes	Yes	Yes
4	Short gestation for birthweight	Neonatal preterm birth complications	0	··	··	20	0	··	··	Yes	Yes	Yes	Yes
4	Short gestation for birthweight	Neonatal encephalopathy due to birth asphyxia and trauma	0	··	··	20	0	··	··	Yes	Yes	Yes	Yes
4	Short gestation for birthweight	Neonatal sepsis and other neonatal infections	0	··	··	20	0	··	··	Yes	Yes	Yes	Yes
4	Short gestation for birthweight	Haemolytic disease and other neonatal jaundice	0	··	··	20	0	··	··	Yes	Yes	Yes	Yes
4	Short gestation for birthweight	Other neonatal disorders	0	··	··	20	0	··	··	Yes	Yes	Yes	Yes
4	Short gestation for birthweight	Sudden infant death syndrome	0	··	··	20	0	··	··	Yes	Yes	Yes	Yes
4	Low birthweight for gestation	Diarrhoeal diseases	0	··	··	20	0	··	··	Yes	Yes	Yes	Yes
4	Low birthweight for gestation	Lower respiratory infections	0	··	··	20	0	··	··	Yes	Yes	Yes	Yes
4	Low birthweight for gestation	Upper respiratory infections	0	··	··	20	0	··	··	Yes	Yes	Yes	Yes
4	Low birthweight for gestation	Otitis media	0	··	··	20	0	··	··	Yes	Yes	Yes	Yes
4	Low birthweight for gestation	Pneumococcal meningitis	0	··	··	20	0	··	··	Yes	Yes	Yes	Yes
4	Low birthweight for gestation	*Haemophilus influenzae* type B meningitis	0	··	··	20	0	··	··	Yes	Yes	Yes	Yes
4	Low birthweight for gestation	Meningococcal infection	0	··	··	20	0	··	··	Yes	Yes	Yes	Yes
4	Low birthweight for gestation	Other meningitis	0	··	··	20	0	··	··	Yes	Yes	Yes	Yes
4	Low birthweight for gestation	Encephalitis	0	··	··	20	0	··	··	Yes	Yes	Yes	Yes
4	Low birthweight for gestation	Neonatal preterm birth complications	0	··	··	20	0	··	··	Yes	Yes	Yes	Yes
4	Low birthweight for gestation	Neonatal encephalopathy due to birth asphyxia and trauma	0	··	··	20	0	··	··	Yes	Yes	Yes	Yes
4	Low birthweight for gestation	Neonatal sepsis and other neonatal infections	0	··	··	20	0	··	··	Yes	Yes	Yes	Yes
4	Low birthweight for gestation	Haemolytic disease and other neonatal jaundice	0	··	··	20	0	··	··	Yes	Yes	Yes	Yes
4	Low birthweight for gestation	Other neonatal disorders	0	··	··	20	0	··	··	Yes	Yes	Yes	Yes
4	Low birthweight for gestation	Sudden infant death syndrome	0	··	··	20	0	··	··	Yes	Yes	Yes	Yes
3	Vitamin A deficiency	Diarrhoeal diseases	19	0	63	0	··	··	··	No	··	Yes	No
3	Vitamin A deficiency	Measles	12	0	83	0	··	··	··	Yes	··	Yes	No
3	Zinc deficiency	Diarrhoeal diseases	14	0	29	0	··	··	··	No	··	Yes	No
3	Zinc deficiency	Lower respiratory infections	6	0	17	0	··	··	··	No	··	Yes	No
**2**	**Tobacco**												
3	Smoking	Tuberculosis	0	··	··	4	0	10	0	No	··	Yes	Yes
3	Smoking	Lip and oral cavity cancer	0	··	··	5	0	··	··	Yes	··	Yes	Yes
3	Smoking	Nasopharynx cancer	0	··	··	4	0	28	0	Yes	··	Yes	Yes
3	Smoking	Oesophageal cancer	0	··	··	5	0	··	··	Yes	··	Yes	Yes
3	Smoking	Colon and rectum cancer	0	··	··	19	0	··	··	No	··	Yes	Yes
3	Smoking	Liver cancer	0	··	··	54	0	··	··	Yes	··	Yes	Yes
3	Smoking	Gastric cancer	0	··	··	19	0	··	··	No	··	Yes	Yes
3	Smoking	Pancreatic cancer	0	··	··	19	0	··	··	Yes	··	Yes	Yes
3	Smoking	Larynx cancer	0	··	··	5	0	··	··	Yes	··	Yes	Yes
3	Smoking	Tracheal, bronchus, and lung cancer	0	··	··	38	0	··	··	Yes	··	Yes	Yes
3	Smoking	Breast cancer	0	··	··	19	0	··	··	No	··	Yes	Yes
3	Smoking	Cervical cancer	0	··	··	15	0	··	··	No	··	Yes	Yes
3	Smoking	Prostate cancer	0	··	··	19	0	··	··	No	··	Yes	Yes
3	Smoking	Kidney cancer	0	··	··	8	0	··	··	Yes	··	Yes	Yes
3	Smoking	Bladder cancer	0	··	··	37	0	··	··	Yes	··	Yes	Yes
3	Smoking	Leukaemia	0	··	··	22	0	··	··	No	··	Yes	Yes
3	Smoking	Ischaemic heart disease	0	··	··	86	··	··	··	No	··	Yes	Yes
3	Smoking	Ischaemic stroke	0	··	··	60	··	··	··	No	··	Yes	Yes
3	Smoking	Haemorrhagic stroke	0	··	··	60	··	··	··	No	··	Yes	Yes
3	Smoking	Atrial fibrillation and flutter	0	··	··	16	0	··	··	No	··	Yes	Yes
3	Smoking	Peripheral vascular disease	0	··	··	10	0	··	··	No	··	Yes	Yes
3	Smoking	Other cardiovascular and circulatory diseases	0	··	··	5	0	··	··	No	··	Yes	Yes
3	Smoking	Chronic obstructive pulmonary disease	0	··	··	42	0	··	··	Yes	··	Yes	Yes
3	Smoking	Asthma	0	··	··	8	12	··	··	No	··	Yes	Yes
3	Smoking	Other chronic respiratory diseases	0	··	··	5	0	··	··	Yes	··	Yes	Yes
3	Smoking	Peptic ulcer disease	0	··	··	7	0	··	··	No	··	Yes	No
3	Smoking	Gallbladder and biliary diseases	0	··	··	10	0	··	··	No	··	Yes	Yes
3	Smoking	Alzheimer's disease and other dementias	0	··	··	13	8	··	··	No	··	Yes	Yes
3	Smoking	Parkinson's disease	0	··	··	8	0	··	··	Yes	··	Yes	Yes
3	Smoking	Multiple sclerosis	0	··	··	6	0	··	··	No	··	Yes	Yes
3	Smoking	Diabetes mellitus	0	··	··	88	0	··	··	No	··	Yes	No
3	Smoking	Rheumatoid arthritis	0	··	··	5	0	··	··	No	··	Yes	No
3	Smoking	Low back pain	0	··	··	13	0	··	··	No	··	Yes	Yes
3	Smoking	Cataract	0	··	··	13	0	··	··	No	··	Yes	No
3	Smoking	Macular degeneration	0	··	··	5	0	··	··	No	··	Yes	No
3	Smoking	Low bone mass- related fractures	0	··	··	14	14	··	··	No	··	Yes	Yes
3	Smoking	Hip fracture	0	··	··	15	20	··	··	No	··	Yes	Yes
3	Smoking	Abdominal aortic aneurism	0	··	··	10	0	··	··	No	··	Yes	Yes
3	Smokeless tobacco	Oral cancer	0	··	··	4	0	21	5	Yes	··	Yes	Yes
3	Smokeless tobacco	Oesophageal cancer	0	··	··	2	0	10	0	Yes	··	Yes	Yes
3	Second-hand smoke	Lower respiratory infections	0	··	··	18	0	··	··	No	Yes	Yes	Yes
3	Second-hand smoke	Otitis media	0	··	··	1	0	4	0	No	··	Yes	Yes
3	Second-hand smoke	Tracheal, bronchus, and lung cancer	0	··	··	13	0	··	··	No	Yes	Yes	Yes
3	Second-hand smoke	Breast cancer	0	··	··	21	0	··	··	No	··	Yes	Yes
3	Second-hand smoke	Ischaemic heart disease	0	··	··	5	0	··	··	No	Yes	Yes	Yes
3	Second-hand smoke	Ischaemic stroke	0	··	··	4	0	3	··	No	Yes	Yes	Yes
3	Second-hand smoke	Haemorrhagic stroke	0	··	··	4	0	3	··	No	Yes	Yes	Yes
3	Second-hand smoke	Chronic obstructive pulmonary disease	0	··	··	2	0	1	0	No	Yes	Yes	Yes
3	Second-hand smoke	Diabetes mellitus	0	··	··	5	0	··	··	No	··	Yes	Yes
**2**	**Alcohol and drug use**												
3	Alcohol use	Tuberculosis	0	··	··	3	0	18	11	Yes	Yes	Yes	Yes
3	Alcohol use	Lower respiratory infections	0	··	··	2	0	2	0	Yes	Yes	Yes	Yes
3	Alcohol use	Lip and oral cavity cancer	0	··	··	6	0	··	··	Yes	Yes	Yes	Yes
3	Alcohol use	Nasopharynx cancer	0	··	··	6	0	··	··	Yes	Yes	Yes	Yes
3	Alcohol use	Other pharynx cancer	0	··	··	6	0	··	··	Yes	Yes	Yes	Yes
3	Alcohol use	Oesophageal cancer	0	··	··	10	0	··	··	Yes	Yes	Yes	Yes
3	Alcohol use	Colon and rectum cancer	0	··	··	15	13	··	··	Yes	Yes	Yes	Yes
3	Alcohol use	Liver cancer	0	··	··	9	0	··	··	Yes	Yes	Yes	Yes
3	Alcohol use	Larynx cancer	0	··	··	7	0	··	··	Yes	Yes	Yes	Yes
3	Alcohol use	Breast cancer	0	··	··	13	23	··	··	Yes	Yes	Yes	Yes
3	Alcohol use	Ischaemic heart disease	0	··	··	63	0	··	··	Yes	Yes	Yes	Yes
3	Alcohol use	Ischaemic stroke	0	··	··	20	0	··	··	Yes	Yes	Yes	Yes
3	Alcohol use	Haemorrhagic stroke	0	··	··	16	0	··	··	Yes	Yes	Yes	Yes
3	Alcohol use	Hypertensive heart disease	0	··	··	12	0	··	··	Yes	Yes	Yes	Yes
3	Alcohol use	Atrial fibrillation and flutter	0	··	··	10	10	··	··	Yes	Yes	Yes	Yes
3	Alcohol use	Cirrhosis	0	··	··	14	0	··	··	Yes	Yes	Yes	Yes
3	Alcohol use	Pancreatitis	0	··	··	4	50	3	0	Yes	Yes	Yes	No
3	Alcohol use	Epilepsy	0	··	··	1	0	2	0	No	Yes	Yes	No
3	Alcohol use	Diabetes mellitus	0	··	··	37	32	··	··	Yes	Yes	Yes	No
3	Alcohol use	Motor vehicle road injuries	0	··	··	3	0	··	··	Yes	Yes	Yes	Yes
3	Alcohol use	Unintentional injuries	0	··	··	4	0	4	0	Yes	Yes	Yes	Yes
3	Alcohol use	Self-harm	0	··	··	0	··	··	··	Yes	Yes	Yes	Yes
3	Alcohol use	Interpersonal violence	0	··	··	2	0	1	0	Yes	Yes	Yes	Yes
3	Drug use	Hepatitis B	0	··	··	6	0	··	··	Yes	··	Yes	Yes
3	Drug use	Hepatitis C	0	··	··	16	0	··	··	Yes	··	Yes	Yes
3	Drug use	Self-harm	0	··	··	1	0	0	0	No	··	Yes	No
**2**	**Dietary risks**												
3	Diet low in fruits	Lip and oral cavity cancer	0	··	··	2	0	15	0	No	Yes	Yes	Yes
3	Diet low in fruits	Nasopharynx cancer	0	··	··	2	0	15	0	No	Yes	Yes	Yes
3	Diet low in fruits	Other pharynx cancer	0	··	··	2	0	15	0	No	Yes	Yes	Yes
3	Diet low in fruits	Oesophageal cancer	0	··	··	5	0	··	··	No	Yes	Yes	Yes
3	Diet low in fruits	Larynx cancer	0	··	··	2	0	15	0	No	Yes	Yes	Yes
3	Diet low in fruits	Tracheal, bronchus, and lung cancer	0	··	··	22	0	··	··	No	Yes	Yes	Yes
3	Diet low in fruits	Ischaemic heart disease	0	··	··	9	0	··	··	No	Yes	Yes	Yes
3	Diet low in fruits	Ischaemic stroke	0	··	··	9	0	··	··	No	Yes	Yes	Yes
3	Diet low in fruits	Haemorrhagic stroke	0	··	··	5	0	··	··	No	Yes	Yes	Yes
3	Diet low in fruits	Diabetes mellitus	0	··	··	9	0	··	··	No	Yes	Yes	No
3	Diet low in vegetables	Oesophageal cancer	0	··	··	5	0	··	··	No	Yes	Yes	No
3	Diet low in vegetables	Ischaemic heart disease	0	··	··	9	0	··	··	No	Yes	Yes	Yes
3	Diet low in vegetables	Ischaemic stroke	0	··	··	8	0	··	··	No	Yes	Yes	Yes
3	Diet low in vegetables	Haemorrhagic stroke	0	··	··	5	0	··	··	No	Yes	Yes	Yes
3	Diet low in legumes	Ischaemic heart disease	0	··	··	5	0	··	··	No	Yes	Yes	No
3	Diet low in whole grains	Ischaemic heart disease	0	··	··	7	0	··	··	No	Yes	Yes	Yes
3	Diet low in whole grains	Ischaemic stroke	0	··	··	6	0	··	··	No	Yes	Yes	Yes
3	Diet low in whole grains	Haemorrhagic stroke	0	··	··	6	0	··	··	No	Yes	Yes	Yes
3	Diet low in whole grains	Diabetes mellitus	0	··	··	10	0	··	··	No	Yes	Yes	No
3	Diet low in nuts and seeds	Ischaemic heart disease	1	0	100	6	0	··	··	No	Yes	Yes	No
3	Diet low in nuts and seeds	Diabetes mellitus	1	0	100	5	0	··	··	No	Yes	Yes	No
3	Diet low in milk	Colon and rectum cancer	0	··	··	7	0	··	··	No	Yes	Yes	No
3	Diet high in red meat	Colon and rectum cancer	0	··	··	8	0	··	··	No	Yes	Yes	No
3	Diet high in red meat	Diabetes mellitus	0	··	··	9	11	··	··	No	Yes	Yes	No
3	Diet high in processed meat	Colon and rectum cancer	0	··	··	9	11	··	··	No	Yes	Yes	No
3	Diet high in processed meat	Ischaemic heart disease	0	··	··	5	0	··	··	No	Yes	Yes	No
3	Diet high in processed meat	Diabetes mellitus	0	··	··	8	0	··	··	No	Yes	Yes	No
3	Diet high in sugar-sweetened beverages	Body-mass index	10	0	60	22	0	··	··	Yes	Yes	Yes	No
3	Diet low in fibre	Colon and rectum cancer	0	··	··	15	0	··	··	No	Yes	Yes	No
3	Diet low in fibre	Ischaemic heart disease	0	··	··	12	0	··	··	No	Yes	Yes	No
3	Diet low in calcium	Colon and rectum cancer	0	··	··	13	0	··	··	No	Yes	Yes	No
3	Diet low in seafood omega 3 fatty acids	Ischaemic heart disease	17	0	94	16	0	··	··	No	Yes	Yes	No
3	Diet low in polyunsaturated fatty acids	Ischaemic heart disease	8	0	75	11	0	··	··	No	Yes	Yes	No
3	Diet high in trans fatty acids	Ischaemic heart disease	0	··	··	13	0	··	··	No	Yes	Yes	No
3	Diet high in sodium	Stomach cancer	0	··	··	10	0	··	··	No	Yes	Yes	No
3	Diet high in sodium	Systolic blood pressure	45	0	73	0	··	··	··	No	Yes	Yes	No
**2**	**Sexual abuse and violence**												
3	Childhood sexual abuse	Alcohol use disorders	0	··	··	2	0	3	0	No	··	Yes	Yes
3	Childhood sexual abuse	Depressive disorders	0	··	··	7	0	··	··	No	··	Yes	Yes
3	Intimate partner violence	HIV/AIDS	0	··	··	2	0	0	0	No	··	Yes	No
3	Intimate partner violence	Maternal abortion, miscarriage, and ectopic pregnancy	0	··	··	1	0	3	0	Yes	··	Yes	No
3	Intimate partner violence	Depressive disorders	0	··	··	4	0	0	0	No	··	Yes	Yes
**2**	**Low physical activity**												
2	Low physical activity	Colon and rectum cancer	0	··	··	20	15	··	··	No	Yes	Yes	Yes
2	Low physical activity	Breast cancer	0	··	··	35	0	··	··	No	Yes	Yes	Yes
2	Low physical activity	Ischaemic heart disease	0	··	··	45	9	··	··	No	Yes	Yes	Yes
2	Low physical activity	Ischaemic stroke	0	··	··	27	11	··	··	No	Yes	Yes	Yes
2	Low physical activity	Diabetes mellitus	0	··	··	57	7	··	··	No	Yes	Yes	No
2	High fasting plasma glucose	Tuberculosis	0	··	··	18	0	··	··	Yes	Yes	Yes	No
2	High fasting plasma glucose	Colon and rectum cancer	0	··	··	21	0	··	··	No	··	··	Yes
2	High fasting plasma glucose	Liver cancer	0	··	··	28	0	··	··	Yes	··	··	No
2	High fasting plasma glucose	Pancreatic cancer	0	··	··	35	0	··	··	Yes	··	··	Yes
2	High fasting plasma glucose	Lung cancer	0	··	··	16	6	··	··	No	··	··	Yes
2	High fasting plasma glucose	Breast cancer	0	··	··	39	0	··	··	No	··	··	Yes
2	High fasting plasma glucose	Ovarian cancer	0	··	··	11	0	··	··	No	··	··	Yes
2	High fasting plasma glucose	Bladder cancer	0	··	··	14	0	··	··	No	··	··	Yes
2	High fasting plasma glucose	Ischaemic heart disease	8	0	100	150	··	··	··	Yes	Yes	Yes	Yes
2	High fasting plasma glucose	Ischaemic stroke	9	0	100	150	··	··	··	Yes	Yes	Yes	Yes
2	High fasting plasma glucose	Haemorrhagic stroke	9	0	100	150	··	··	··	Yes	Yes	Yes	Yes
2	High fasting plasma glucose	Alzheimer's disease and other dementias	0	··	··	17	0	··	··	No	··	··	No
2	High fasting plasma glucose	Peripheral vascular disease	14	··	··	4	0	··	··	Yes	Yes	Yes	Yes
2	High fasting plasma glucose	Chronic kidney disease	5	··	··	32	··	··	··	Yes	Yes	Yes	No
2	High fasting plasma glucose	Glaucoma	0	··	··	5	0	··	··	No	··	··	Yes
2	High fasting plasma glucose	Cataract	0	··	··	1	0	1	0	No	··	··	Yes
2	High total cholesterol	Ischaemic heart disease	21	0	57	88	··	··	··	Yes	Yes	Yes	Yes
2	High total cholesterol	Ischaemic stroke	21	0	57	88	··	··	··	Yes	Yes	Yes	Yes
2	High systolic blood pressure	Rheumatic heart disease	0	··	··	62	··	··	··	Yes	Yes	Yes	Yes
2	High systolic blood pressure	Ischaemic heart disease	56	0	··	88	··	··	··	Yes	Yes	Yes	Yes
2	High systolic blood pressure	Ischaemic stroke	54	0	··	150	··	··	··	Yes	Yes	Yes	Yes
2	High systolic blood pressure	Haemorrhagic stroke	54	0	··	150	··	··	··	Yes	Yes	Yes	Yes
2	High systolic blood pressure	Cardiomyopathy and myocarditis	0	··	··	62	··	··	··	Yes	Yes	Yes	Yes
2	High systolic blood pressure	Other cardiomyopathy	0	··	··	62	··	··	··	Yes	Yes	Yes	Yes
2	High systolic blood pressure	Atrial fibrillation and flutter	20	5	60	88	··	··	··	Yes	Yes	Yes	Yes
2	High systolic blood pressure	Aortic aneurysm	0	··	··	62	··	··	··	Yes	Yes	Yes	Yes
2	High systolic blood pressure	Peripheral vascular disease	0	··	··	88	··	··	··	Yes	Yes	Yes	Yes
2	High systolic blood pressure	Endocarditis	0	··	··	62	··	··	··	Yes	Yes	Yes	Yes
2	High systolic blood pressure	Other cardiovascular and circulatory diseases	0	··	··	88	··	··	··	No	Yes	Yes	Yes
2	High systolic blood pressure	Chronic kidney disease	8	··	··	88	··	··	··	Yes	Yes	Yes	No
2	High body-mass index (adult)	Non-Hodgkin lymphoma	0	··	··	8	0	··	··	No	Yes	Yes	Yes
2	High body-mass index (adult)	Oesophageal cancer	0	··	··	16	0	··	··	··	Yes	Yes	Yes
2	High body-mass index (adult)	Colon and rectum cancer	0	··	··	38	0	··	··	No	Yes	Yes	Yes
2	High body-mass index (adult)	Liver cancer	0	··	··	34	0	··	··	No	Yes	Yes	Yes
2	High body-mass index (adult)	Gallbladder and biliary tract cancer	0	··	··	10	0	··	··	No	Yes	Yes	Yes
2	High body-mass index (adult)	Pancreatic cancer	0	··	··	20	0	··	··	No	Yes	Yes	Yes
2	High body-mass index (adult)	Breast cancer (post menopause)	0	··	··	44	2	··	··	No	Yes	Yes	Yes
2	High body-mass index (adult)	Breast cancer (pre-menopause)	0	··	··	25	8	··	··	No	Yes	Yes	No
2	High body-mass index (adult)	Uterine cancer	0	··	··	37	0	··	··	No	Yes	Yes	Yes
2	High body-mass index (adult)	Ovarian cancer	0	··	··	31	3	··	··	No	Yes	Yes	Yes
2	High body-mass index (adult)	Kidney cancer	0	··	··	28	0	··	··	No	Yes	Yes	Yes
2	High body-mass index (adult)	Thyroid cancer	0	··	··	16	0	··	··	No	Yes	Yes	Yes
2	High body-mass index (adult)	Multiple myeloma	0	··	··	20	··	··	··	··	Yes	Yes	Yes
2	High body-mass index (adult)	Leukaemia	0	··	··	17	0	··	··	No	Yes	Yes	Yes
2	High body-mass index (adult)	Ischaemic heart disease	0	··	··	129	··	··	··	No	Yes	Yes	Yes
2	High body-mass index (adult)	Ischaemic stroke	0	··	··	102	··	··	··	No	Yes	Yes	Yes
2	High body-mass index (adult)	Haemorrhagic stroke	0	··	··	129	··	··	··	No	Yes	Yes	Yes
2	High body-mass index (adult)	Hypertensive heart disease	0	··	··	85	··	··	··	No	Yes	Yes	Yes
2	High body-mass index (adult)	Atrial fibrillation and flutter	0	··	··	5	0	··	··	··	No	Yes	Yes
2	High body-mass index (adult)	Asthma	0	··	··	7	0	··	··	··	Yes	Yes	No
2	High body-mass index (adult)	Alzheimer's disease and other dementias	0	··	··	6	0	··	··	··	No	Yes	No
2	High body-mass index (adult)	Gallbladder disease	0	··	··	16	0	··	··	··	Yes	Yes	Yes
2	High body-mass index (adult)	Diabetes mellitus	0	··	··	85	··	··	··	Yes	Yes	Yes	No
2	High body-mass index (adult)	Chronic kidney disease	0	··	··	57	··	··	··	No	Yes	Yes	No
2	High body-mass index (adult)	Osteoarthritis	0	··	··	32	0	··	··	No	Yes	Yes	Yes
2	High body-mass index (adult)	Low back pain	0	··	··	5	0	··	··	No	Yes	Yes	Yes
2	High body-mass index (adult)	Gout	0	··	··	10	0	··	··	··	Yes	Yes	No
2	High body-mass index (adult)	Cataract	0	··	··	17	0	··	··	··	Yes	Yes	No
2	High body-mass index (child)	Asthma	0	··	··	5	0	··	··	No	Yes	Yes	No
2	Low bone mineral density	Injuries	0	··	··	12	··	··	··	No	Yes	Yes	Yes
2	Impaired kidney function	Ischaemic heart disease	0	··	··	6	0	··	··	Yes	··	Yes	Yes
2	Impaired kidney function	Ischaemic stroke	0	··	··	6	0	··	··	Yes	··	Yes	Yes
2	Impaired kidney function	Haemorrhagic stroke	0	··	··	8	0	··	··	Yes	··	Yes	Yes
2	Impaired kidney function	Peripheral vascular disease	0	··	··	5	0	··	··	Yes	··	Yes	Yes
2	Impaired kidney function	Gout	0	··	··	3	0	0	0	Yes	··	Yes	No

If multiple reports existed from the same study, we counted them as one study. We only assessed the dose–response relationship for continuous risks. To evaluate the magnitude of the effect size for continuous risks, we evaluated the relative risk comparing the 75th percentile with the 25th percentile of the exposure distribution at the global level. RCT=randomised controlled trial. RR=relative risk.

* Prospective cohort studies or non-randomised interventions.

† Case-control studies were included for those risk-outcome pairs where the sum of RCT and prospective observational studies included was less than five (where applicable).

‡ Whether or not any biological or mechanistic pathway exists that could potentially explain the relationship of the risk-outcome pair.

§ Whether or not the risk is associated with another outcome from the same category and whether or not any evidence exists that it can cause the current outcome through the same pathway.

### Estimation process

Information about the data sources, estimation methods, computational tools, and statistical analysis used in the derivation of our estimates are provided in appendix 1 (p 10). The analytical steps for estimation of burden attributable to single or clusters of risk-outcome pairs are summarised in appendix 1 (p 10). Table 2 provides definitions of exposure for each risk factor, the theoretical minimum risk exposure level (TMREL) used, and metrics of data availability. For each risk, we estimated effect size as a function of age and sex and exposure level, mean exposure, the distribution of exposure across individuals, and the TMREL. The approach taken is largely similar to GBD 2015 for each quantity for each risk. Some methodological improvements have been implemented and new data sources incorporated. Appendix 1 (p 34) provides details of each step by risk. Citation information for the data sources used for relative risks are provided in searchable form through an online source tool.

**Table 2:** GBD 2016 risk factor hierarchy and accompanying exposure definitions, theoretical minimum risk exposure level, and data representativeness index for each risk factor, pre-2006, 2006–16, and total (across all years)

	**Risk factors**	**Exposure definition**	**Theoretical minimum risk exposure level**	**Data representativeness index**		
				**<2006**	**2006–16**	**Total**
**0**	**All**	**··**	**··**	**100·0%**	**100·0%**	**100·0%**
**1**	**Environmental and occupational risks**	**··**	**··**	**100·0%**	**100·0%**	**100·0%**
**2**	**Unsafe water, sanitation, and handwashing**	**··**	**··**	**58·0%**	**75·4%**	**70·0%**
3	Unsafe water source	Proportion of households with access to different water sources (unimproved, improved except piped, piped water supply) and reported use of household water treatment methods (boiling or filtering, chlorinating or solar filtering, no treatment)	All households have access to water from a piped water supply that is also boiled or filtered before drinking	70·1%	88·4%	83·5%
3	Unsafe sanitation	Proportion of households with access to different sanitation facilities (unimproved, improved except sewer, sewer connection)	All households have access to toilets with sewer connection	69·5%	88·4%	83·5%
3	No access to handwashing facility	Proportion of households with access to handwashing facility with soap, water, and wash station	All households have access to handwashing facility with soap, water, and wash station	10·3%	33·3%	35·4%
**2**	**Air pollution**	**··**	**··**	**100·0%**	**100·0%**	**100·0%**
3	Ambient particulate matter pollution	Annual average daily exposure to outdoor air concentrations of PM_2·5_	Uniform distribution between 2·4 μg/m^3^ and 5·9 μg/m^3^	23·1%	56·9%	78·0%
3	Household air pollution from solid fuels	Individual exposure to PM_2·5_ due to use of solid cooking fuels	No households are exposed to excess indoor concentration of particles from solid fuel use (assuming PM_2·5_ in no fuel use is consistent with a TMREL of 2·4–5·9)	72·8%	59·5%	76·4%
3	Ambient ozone pollution	Seasonal (3 month) hourly maximum ozone concentrations, measured in ppb	Uniform distribution between 33·3 μg/m^3^ and 41·9 μg/m^3^, according to minimum/5th percent concentrations	100·0%	100·0%	100·0%
**2**	**Other environmental risks**	**··**	**··**	**48·7%**	**26·2%**	**51·8%**
3	Residential radon	Average daily exposure to indoor air radon levels measured in becquerels (radon disintegrations per second) per cubic metre (Bq/m^3^)	10 Bq/m^3^, corresponding to the outdoor concentration of radon	39·0%	0·0%	39·0%
3	Lead exposure	Blood lead levels in μg/dL of blood, bone lead levels in μg/g of bone	2 ug/dL, corresponding to lead levels in pre-industrial humans as natural sources of lead prevent the feasibility of zero exposure	37·4%	26·2%	43·6%
**2**	**Occupational risks**	**··**	**··**	**92·3%**	**90·8%**	**100·0%**
3	Occupational carcinogens	··	··	86·7%	85·6%	92·8%
4	Occupational exposure to asbestos	Proportion of the population with cumulative exposure to asbestos	No occupational exposure to asbestos	82·6%	74·9%	87·2%
4	Occupational exposure to arsenic	Proportion of the population ever exposed to arsenic at work or through their occupation	No occupational exposure to arsenic	82·6%	74·9%	87·2%
4	Occupational exposure to benzene	Proportion of the population ever exposed to benzene at work or through their occupation	No occupational exposure to benzene	82·6%	74·9%	87·2%
4	Occupational exposure to beryllium	Proportion of the population ever exposed to beryllium at work or through their occupation	No occupational exposure to beryllium	82·6%	74·9%	87·2%
4	Occupational exposure to cadmium	Proportion of the population ever exposed to cadmium at work or through their occupation	No occupational exposure to cadmium	82·6%	74·9%	87·2%
4	Occupational exposure to chromium	Proportion of the population ever exposed to chromium at work or through their occupation	No occupational exposure to chromium	82·6%	74·9%	87·2%
4	Occupational exposure to diesel engine exhaust	Proportion of the population ever exposed to diesel engine exhaust at work or through their occupation	No occupational exposure to diesel engine exhaust	82·6%	74·9%	87·2%
4	Occupational exposure to second-hand smoke	Proportion of the population ever exposed to second-hand smoke at work or through their occupation	No occupational exposure to second-hand smoke	82·6%	74·9%	87·2%
4	Occupational exposure to formaldehyde	Proportion of the population ever exposed to formaldehyde at work or through their occupation	No occupational exposure to formaldehyde	82·6%	74·9%	87·2%
4	Occupational exposure to nickel	Proportion of the population ever exposed to nickel at work or through their occupation	No occupational exposure to nickel	82·6%	74·9%	87·2%
4	Occupational exposure to polycyclic aromatic hydrocarbons	Proportion of the population ever exposed to polycyclic aromatic hydrocarbons at work or through their occupation	No occupational exposure to polycyclic aromatic hydrocarbons	82·6%	74·9%	87·2%
4	Occupational exposure to silica	Proportion of the population ever exposed to silica at work or through their occupation	No occupational exposure to silica	82·6%	74·9%	87·2%
4	Occupational exposure to sulfuric acid	Proportion of the population ever exposed to sulfuric acid at work or through their occupation	No occupational exposure to sulfuric acid	80·5%	73·3%	85·1%
4	Occupational exposure to trichloroethylene	Proportion of the population ever exposed to trichlorethylene at work or through their occupation	No occupational exposure to trichloroethylene	80·5%	73·3%	85·1%
3	Occupational asthmagens	Proportion of the population currently exposed to asthmagens at work or through their occupation	Background asthmagen exposures	82·6%	74·9%	87·2%
3	Occupational particulate matter, gases, and fumes	Proportion of the population ever exposed to particulates, gases, or fumes at work or through their occupation	No occupational exposure to particulates, gases, or fumes	83·6%	75·9%	88·2%
3	Occupational noise	Proportion of the population ever exposed to noise greater than 85 dB at work or through their occupation	Background noise exposure	83·6%	75·9%	88·2%
3	Occupational injuries	Proportion of the population at risk to injuries related to work or through their occupation	The rate of injury deaths per 100 000 person-years is zero	82·6%	75·4%	87·2%
3	Occupational ergonomic factors	Proportion of the population who are exposed to ergonomic risk factors for low back pain at work or through their occupation	All individuals have the ergonomic factors of clerical and related workers	82·6%	74·9%	87·2%
**1**	**Behavioural risks**	**··**	**··**	**100·0%**	**100·0%**	**100·0%**
**2**	**Child and maternal malnutrition**	**··**	**··**	**100·0%**	**100·0%**	**100·0%**
3	Suboptimal breastfeeding	··	··	67·1%	54·6%	73·9%
4	Non-exclusive breastfeeding	Proportion of children younger than 6 months who receive predominant, partial, or no breastfeeding	All children are exclusively breastfed for first 6 months of life	67·1%	54·6%	73·9%
4	Discontinued breastfeeding	Proportion of children aged 6–23 months who do not receive any breastmilk	All children continue to receive breastmilk until 2 years of age	68·1%	65·3%	79·2%
3	Child growth failure	··	··	5·6%	0·0%	5·6%
4	Child underweight	Proportion of children less than −3 SD, −3 to −2 SD, and −2 to −1 SDs of the WHO 2006 standard weight-for-age curve	All children are above −1 SD of WHO 2006 standard weight-for-age curve	77·4%	65·1%	81·0%
4	Child wasting	Proportion of children less than −3 SD, −3 to −2 SDs, and −2 to −1 SD of the WHO 2006 standard weight-for-length curve	All children are above −1 SD of WHO 2006 standard weight-for-height curve	78·0%	66·2%	82·1%
4	Child stunting	Proportion of children less than −3 SD, −3 to −2 SD, and −2 to −1 SD of the WHO 2006 standard height-for-age curve	All children are above −1 SD of WHO 2006 standard height-for-age curve	78·0%	66·2%	82·1%
3	Low birthweight and short gestation	··	**··**	3·6%	16·4%	18·0%
4	Short gestation for birthweight	Proportion of births occurring in 2 week bands starting from <24 weeks to 39–40 weeks	40–41 weeks gestation	3·6%	16·4%	18·0%
4	Low birthweight for gestation	Proportion of births occurring in 500 g categories starting from <500 g to 4000–4499 g	4500–4999 g birthweight	3·6%	16·4%	18·0%
3	Iron deficiency	Peripheral blood haemoglobin concentration in g/L	Counterfactual haemoglobin concentration in the abscence of iron deficiency in g/L	81·5%	44·1%	85·1%
3	Vitamin A deficiency	Proportion of children aged 0–5 years with serum retinol concentration <0·7 μmol/L	No childhood vitamin A deficiency	54·9%	44·1%	56·4%
3	Zinc deficiency	Proportion of the population with inadequate zinc intake versus loss	No inadequate zinc intake	94·9%	93·3%	94·9%
**2**	Tobacco	··	··	**98·0%**	**100·0%**	**100·0%**
3	Smoking	Smoking Impact Ratio method: cumulative exposure to smoked tobacco products, proxied by excess lung cancer mortality; direct smoking: 5 year lagged proportion of the population who currently smoke daily	All individuals are lifelong non-smokers	92·8%	96·9%	99·0%
3	Smokeless tobacco	Current use of any smokeless tobacco product	All individuals are lifelong non-users of smokeless tobacco products	34·4%	70·8%	73·3%
3	Second-hand smoke	Average daily exposure to air particulate matter in the home from second-hand smoke with an aerodynamic diameter smaller than 2·5 μg, measured in μg/m3, among non-smokers living with a current daily smoker	No second-hand smoke exposure	73·9%	67·7%	90·8%
**2**	**Alcohol and drug use**	**··**	**··**	**54·9%**	**62·6%**	**79·0%**
3	Alcohol use	Average daily alcohol consumption of pure alcohol (measured in g per day) in current drinkers who had consumed alcohol during the past 12 months; binge drinking: proportion of the population reporting binge consumption of at least 60 g for males and 48 g for females of pure alcohol on a single occasion	No alcohol consumption	52·3%	45·6%	69·7%
3	Drug use	Proportion of the population dependent upon opioids, cannabis, cocaine, or amphetamines; proportion of the population who have ever injected drugs	No drug use	20·5%	37·4%	43·1%
**2**	**Dietary risks**	**··**	**··**	**100·0%**	**100·0%**	**100·0%**
3	Diet low in fruits	Average daily consumption of fruits (fresh, frozen, cooked, canned, or dried fruits, excluding fruit juices and salted or pickled fruits)	Consumption of fruit between 200 g and 300 g per day	94·9%	94·9%	94·9%
3	Diet low in vegetables	Average daily consumption of vegetables (fresh, frozen, cooked, canned, or dried vegetables, excluding legumes and salted or pickled vegetables, juices, nuts, and seeds, and starchy vegetables such as potatoes or corn)	Consumption of vegetables between 290 g and 430 g per day	100·0%	100·0%	100·0%
3	Diet low in legumes	Average daily consumption of legumes (fresh, frozen, cooked, canned, or dried legumes)	Consumption of legumes between 50 g and 70 g per day	100·0%	100·0%	100·0%
3	Diet low in whole grains	Average daily consumption of whole grains (bran, germ, and endosperm in their natural proportion) from breakfast cereals, bread, rice, pasta, biscuits, muffins, tortillas, pancakes, and other sources	Consumption of whole grains between 100 g and 150 g per day	15·9%	13·9%	20·0%
3	Diet low in nuts and seeds	Average daily consumption of nut and seed foods	Consumption of nuts and seeds between 16 g and 25 g per day	100·0%	100·0%	100·0%
3	Diet low in milk	Average daily consumption of milk including non-fat, low-fat, and full-fat milk, excluding soy milk and other plant derivatives	Consumption of milk between 350 g and 520 g per day	100·0%	100·0%	100·0%
3	Diet high in red meat	Average daily consumption of red meat (beef, pork, lamb, and goat but excluding poultry, fish, eggs, and all processed meats)	Consumption of red meat between 18 g and 27 g per day	100·0%	100·0%	100·0%
3	Diet high in processed meat	Average daily consumption of meat preserved by smoking, curing, salting, or addition of chemical preservatives	Consumption of processed meat between 0 g and 4 g per day	100·0%	100·0%	100·0%
3	Diet high in sugar-sweetened beverages	Average daily consumption of beverages with ≥50 kcal per 226·8 g serving, including carbonated beverages, sodas, energy drinks, fruit drinks, but excluding 100% fruit and vegetable juices	Consumption of sugar-sweetened beverages between 0 g and 5 g per day	34·9%	30·3%	36·9%
3	Diet low in fibre	Average daily intake of fibre from all sources including fruits, vegetables, grains, legumes, and pulses	Consumption of fibre between 19 g and 28 g per day	100·0%	100·0%	100·0%
3	Diet low in calcium	Average daily intake of calcium from all sources, including milk, yogurt, and cheese	Consumption of calcium between 1·00 g and 1·50 g per day	100·0%	100·0%	100·0%
3	Diet low in seafood omega 3 fatty acids	Average daily intake of eicosapentaenoic acid and docosahexaenoic acid	Consumption of seafood omega 3 fatty acids between 200 mg and 300 mg per day	100·0%	100·0%	100·0%
3	Diet low in polyunsaturated fatty acids	Average daily intake of omega 6 fatty acids from all sources, mainly liquid vegetable oils, including soybean oil, corn oil, and safflower oil	Consumption of polyunsaturated fatty acids between 9% and 13% of total daily energy	96·9%	94·9%	96·9%
3	Diet high in transfatty acids	Average daily intake of transfat from all sources, mainly partially hydrogenated vegetable oils and ruminant products	Consumption of transfatty acids between 0% and 1% of total daily energy	37·4%	38·5%	38·5%
3	Diet high in sodium	24 h urinary sodium measured in g per day	24 h urinary sodium between 1 g and 5 g per day	15·9%	21·5%	26·2%
**2**	**Sexual abuse and violence**	**··**	**··**	**68·2%**	**78·0%**	**87·2%**
3	Childhood sexual abuse	Proportion of the population ever having had the experience of intercourse or other contact abuse (ie, fondling and other sexual touching) when aged 15 years or younger, and the perpetrator or partner was more than 5 years older than the victim	No childhood sexual abuse	31·8%	18·5%	38·0%
3	Intimate partner violence	Proportion of the population who have ever experienced one or more acts of physical or sexual violence by a present or former intimate partner since age 15 years	No intimate partner violence	67·2%	76·4%	86·2%
2	Unsafe sex	Proportion of the population with exposure to sexual encounters that convey the risk of disease	No exposure to a disease agent through sex	14·9%	51·3%	51·8%
2	Low physical activity	Average weekly physical activity at work, home, transport-related, and recreational measured by MET min per week	All adults experience 3000–4500 MET min per week	52·3%	35·9%	67·2%
**1**	**Metabolic risks**	**··**	**··**	**100·0%**	**100·0%**	**100·0%**
2	High fasting plasma glucose	Serum fasting plasma glucose measured in mmol/L	4·8–5·4 mmol/L	51·8%	53·3%	69·7%
2	High total cholesterol	Serum total cholesterol, measured in mmol/L	2·78–3·38 mmol/L	59·0%	48·2%	78·0%
2	High systolic blood pressure	Systolic blood pressure, measured in mmHg	110–115 mm Hg	64·1%	65·1%	83·6%
2	High body-mass index	Body-mass index, measured in kg/m^2^	25 kg/m^2^	91·3%	100·0%	100·0%
2	Low bone mineral density	Standardised mean bone mineral density values measured by dual x-ray absorptiometry at the femoral neck in g/cm^2^	99th percentile of NHANES 2005–14 by age and sex	33·3%	12·3%	35·9%
2	Impaired kidney function	Proportion of the population with ACR >30 mg/g and/or GFR <60 mL/min per 1·73m^2^, excluding end-stage renal disease	ACR <30 mg/g and GFR >60 mL/min per 1·73m^2^	10·3%	0·0%	10·3%

GBD=Global Burden of Disease. MET=metabolic equivalent. NHANES=National Health and Nutrition Examination Survey. PM_2.5_=particulate matter with an aerodynamic diameter smaller than 2·5 μm, measured in μg/m^3^. TMREL=theoretical minimum risk exposure level. ppb=parts per billion. ACR=albumin-to-creatine ratio. GFR=glomerular filtration rate.

All point estimates are reported with 95% uncertainty intervals (UIs). UIs include uncertainty from each relevant component, consisting of exposure, relative risks, TMREL, and burden rates. Where percentage change is reported (with 95% UIs), we computed it on the basis of the point estimates being compared.

In GBD 2015, we produced a summary measure of exposure for each risk, called the summary exposure value (SEV), which is a metric that captures risk-weighted exposure for a population, or risk-weighted prevalence of an exposure. The scale for SEV spans from 0% to 100%, such that an SEV of 0% reflects no risk exposure in a population and 100% indicates that an entire population is exposure to the maximum possible level for that risk. In GBD 2016, we show estimates of SEVs for each risk factor and provide details on how SEVs are computed for categorical and continuous risks in appendix 1 (p 10).

### Fitting a distribution to exposure data

The most informative data describing the distribution of risk factors within a population come from individual-level data; additional sources of data include reported means and variances. In cases when a risk factor also defines a disease, such as haemoglobin level and anaemia, the prevalence of disease is also frequently reported. To model the distribution of any particular risk factor, we seek a family of probability density functions (PDFs), a fitting method, and a model selection criterion. To make use of the most data describing most populations, we used the method of moments (MoM); the first two empirical moments from a population, the mean and variance, were used to determine the PDF describing the distribution of risk within any population, where exceptions to this rule are justified by context. We used the Kolmogorov-Smirnov test to measure the goodness of fit (GoF), but in some cases, the GoF was based on the prediction error for the prevalence of disease.

We used an ensemble technique in which a model selection algorithm is used to choose the best model for each risk factor.^16^ We drew the initial set of candidate models from commonly used PDF families. We fitted each PDF candidate family to each dataset using the MoM, and used the Kolmogorov-Smirnov test^17^ as the measure of GoF. Preliminary analysis showed that the GoF ranking of PDF families varied across datasets for any particular risk factor and that combining the predictions of differently fitted PDF families could dramatically improve the GoF for each dataset. Therefore, we developed a new model for prediction using the ensemble of candidate models, which is a weighted linear combination of all candidate models, {f}, where a set of weights {w} is chosen such that it is the sum of the weights equals to one and the values of the weights were determined by a second GoF criterion with its own validation process. Because of basic differences among risk factors, their distributions, and the risk attribution process, the model selection process was often slightly different for each risk factor. The details can be summarised by (1) the summary statistics for each dataset; (2) a table showing the Kolmogorov-Smirnov statistic for each candidate model and URD; (3) the criterion used for determining the overall GoF; (4) summary results of the validation process; and (5) the weights defining the final ensemble model for each dataset.

### New risks and risks with significant changes in the estimation methods compared with GBD 2015

We took several steps to improve the estimation of alcohol use as a risk factor. First, on the exposure side, we added 26 survey series, which contributed 12 195 datapoints in our models. Second, we developed and implemented a method that adjusts total consumption for tourism and unrecorded consumption for each location-year. Third, we calculated the TMREL. We chose TMREL as being the exposure that minimises an individual's risk of suffering burden from any given cause related to alcohol (appendix 1 p 22 for more detail). Fourth, we performed a systematic review of all cohort and case-control studies reporting a RR, hazard ratio, or odds ratio for any risk-outcome pairs studied in GBD 2016 and then modelled a dose-response relationship using DisMod ordinary differential equations (ODE).^18^ Fifth, we estimated injury PAFs from cohort studies and adjusted them to account for victims.

We made several improvements in the process of estimating the burden of disease attributable to dietary risks. To improve the quality and coverage of our dietary estimates, we systematically searched literature for nationally or subnationally representative studies providing information on consumption of each dietary factor. We also made a systematic effort to obtain individual-level data for consumption of dietary factors; re-extracted data from all available sources; and standardised the definition of dietary factors across different sources. To capture recent trends in consumption, we used data on sales of different fresh and packaged foods to inform our estimates. To address the concerns over within-person variation in intake, we estimated usual intake of each dietary factor and used that to estimate the attributable disease burden. To make the current and optimal levels of intake more comparable, we used absolute intake of each dietary factor (rather than intake standardised to 2000 kcal per day). For more detail, see appendix 1 (p 117).

There were two substantial changes in the estimation of second-hand smoke compared with GBD 2015. First, we estimated the proportion of a population exposed to second-hand smoke using information about household composition and smoking status from household surveys and censuses, rather than using questions that ask directly about exposure to second-hand smoke in surveys. Second, we modelled exposure using spatiotemporal Gaussian process regression (ST-GPR), borrowing strength across sexes and all ages, whereas in GBD 2015 we ran a DisMod model separately by sex and age. Further, we found significant evidence of associations between second-hand smoke exposure and two additional outcomes: breast cancer and diabetes, which were added to the list of risk-outcome pairs for second-hand smoke. More details on the estimation approach are presented in appendix 1 (p 98).

For the first time in the GBD study, we estimated exposure to and burden attributable to smokeless tobacco, defined as current use of any smokeless tobacco product. RR estimates were derived from prospective cohort studies and case-control studies and vary depending on the type of product used. Based on available evidence, for chewing tobacco RRs were significantly higher than one for oral cancer and oesophageal cancer, while for snus or snuff we did not find sufficient evidence of a RR greater than one for any health outcome. Additional details on the estimation methods and RRs are presented in appendix 1 (p 11, p 181).

Low birthweight for gestation and short gestation for birthweight are included as new risk factors for GBD 2016. The estimation has been parameterised to be polytomous by 500 g and 2 week categories. Low birthweight and gestational age are highly correlated risks and they are estimated in a completely interdependent manner. For each univariate analysis, identification of TMREL and calculation of PAFs is contingent on the other dimension. In other words, we found the lowest risk birthweight category for each 2 week gestational age band and, correspondingly, the lowest risk gestational age for each 500 g birthweight band. RRs were then estimated for each 500 g per 2 week bin. Exposure for each bin was estimated in three steps. First, we estimated by generating ensemble distribution estimates using modelled mean and categorical prevalence estimates for each of birthweight (mean, % <2500 g) and gestational age (mean, % <37 weeks, % <28 weeks) for each location, year, and sex. Second, we evaluated all microdata where both gestational age and birthweight were available and found a high degree of consistency in the correlation between them. Third, we took the pooled correlation coefficient from step 2 combined with univariate ensemble distributions from step 1 and used a copula linking function to simulate the joint distribution which was then summarised into each 500 g per 2 week category. Joint PAF calculation used a TMREL defined as the lowest overall risk of the entire matrix of birthweight and gestational age (see appendix 1 p 77 for more details).

### Mediation

In GBD 2016, we updated our approach for estimation of the joint effects of combinations of risk factors (appendix 1 p 23). Using individual-level data from prospective cohort studies, we estimated the proportion of the effect of behavioural risks on cardiometabolic outcomes mediated through metabolic risk factors. We also estimated the proportion of the effect of each metabolic risk factor on cardiometabolic outcomes mediated through other metabolic risks. For each mediation pathway, we only included the mediators for which sufficient evidence existed for their causal relationship with the disease endpoint.

### Explaining the drivers of trends in deaths and DALYs

As in GBD 2015, we undertook a decomposition analysis of changes in DALYs over the time period into four main components, namely, changes in DALYs due to changes in: (1) population growth; (2) population age structure; (3) exposure to all risks for a disease; and (4) all other factors combined, approximated as the risk-deleted death and DALY rates. Risk-deleted rates refers to death and DALY rates that would be observed if we removed all risk factors included in GBD 2016, estimated as DALY rates multiplied by one minus the PAF for the set of risks. We used methods developed by Das Gupta,^19^ but as the methods presented there do not result in the decomposition results being linear aggregates over time or risk, we adapted these methods further in GBD 2016. Our decomposition analysis was undertaken for each 5 year time period, at the all-risk level, taking into account risk mediation at the most detailed cause level. The contribution of changes in exposure for the individual risks was scaled to the all-risk effect at the most detailed outcome level. The contribution of risk exposures over longer time periods—eg, 2000–16—or at higher cause aggregates—eg, all cause—were calculated as the linear aggregate of the effect of individual risks at the most detailed cause level and time period.

### Risk transition with development

We explored how exposure to risks varies across levels of development using the SDI, a composite indicator of development status constructed for GBD 2015 whose components are strongly correlated with health outcomes. It is the geometric mean of 0 to 1 for indices of total fertility rate, mean education for those aged 15 years and older, and lag-distributed income per capita. More details on the estimation of SDI can be found in appendix 1 (p 32).

### Role of the funding source

The funders of the study had no role in the study design, data collection, data analysis, data interpretation, or writing of the report. The authors had full access to all data in the study and had final responsibility for the decision to submit for publication.

## Results

### Global exposure to risks

From 1990 to 2016, trends in SEVs varied across the set of risk factors included in GBD 2016. Of note, SEVs decreased by more than 40% for three risks: diet high in transfatty acids (51·3% [95% UI 34·1–70·1]), household air pollution from solid fuels (43·1% [40·7–45·6]), and unsafe sanitation (40·3% [35·5–44·7]; table 3, appendix 2 p 1399). During the same period, SEVs increased by more than 40% for high body-mass index (BMI; 60·2% [45·1–79·1]), diet high in sugar-sweetened beverages (44·7% [36·1–52·7]), occupational exposure to diesel engine exhaust (41·8% [41·3–42·2]), and occupational exposure to trichloroethylene (40·6% [40·2–41·1]).

**Table 3:** Global age-standardised SEVs for all risk factors, 1990, 2006, and 2016, with mean percent change for three time periods, between 1990 and 2006, 2006 and 2016, and 1990 and 2016, by risk level

	**Risk**	**Male**						**Female**						**Combined percent change 1990–2016**
		1990	2006	2016	Percent change 1990–2006	Percent change 2006–16	Percent change 1990–2016	1990	2006	2016	Percent change 1990–2006	Percent change 2006–16	Percent change 1990–2016	
**1**	**Environmental and occupational risks**													
**2**	**Unsafe water, sanitation, and handwashing**													
3	Unsafe water source	23·27 (15·57 to 26·55)	21·27 (14·30 to 24·21)	20·08 (13·47 to 22·83)	–8·61 (−10·54 to −6·62)*	–5·61 (−7·29 to −3·75)*	–13·74 (−15·78 to −11·37)*	22·94 (15·32 to 26·19)	21·12 (14·19 to 24·03)	20·04 (13·45 to 22·79)	–7·96 (−9·87 to −6·01)*	–5·08 (−6·76 to −3·25)*	–12·64 (−14·67 to −10·28)*	–13·14 (−15·18 to −10·78)*
3	Unsafe sanitation	56·46 (53·29 to 60·79)	42·29 (38·91 to 46·94)	33·26 (29·47 to 38·52)	–25·10 (−28·04 to −22·10)*	–21·36 (−25·52 to −17·53)*	–41·10 (−45·39 to −36·37)*	55·13 (51·99 to 59·44)	41·91 (38·53 to 46·70)	33·34 (29·49 to 38·67)	–23·97 (−27·05 to −20·77)*	–20·45 (−24·51 to −16·63)*	–39·51 (−44·05 to −34·59)*	–40·28 (−44·73 to −35·47)*
3	No access to handwashing facility	36·22 (35·56 to 36·95)	34·57 (34·00 to 35·14)	33·13 (32·66 to 33·62)	–4·57 (−6·31 to −2·72)*	–4·15 (−5·29 to −2·89)*	–8·53 (−10·53 to −6·29)*	35·82 (35·15 to 36·53)	34·54 (33·98 to 35·11)	33·34 (32·87 to 33·83)	–3·57 (−5·34 to −1·69)*	–3·46 (−4·64 to −2·22)*	–6·91 (−8·94 to −4·61)*	–7·67 (−9·69 to −5·40)*
**2**	**Air pollution**													
3	Ambient particulate matter pollution	44·42 (37·19 to 53·39)	45·74 (38·10 to 54·89)	49·56 (41·42 to 58·71)	2·96 (1·88 to 3·97)*	8·37 (6·79 to 9·43)*	11·57 (9·47 to 13·51)*	43·79 (36·57 to 52·71)	45·00 (37·47 to 54·11)	48·87 (40·79 to 58·02)	2·76 (1·69 to 3·76)*	8·58 (6·98 to 9·69)*	11·58 (9·44 to 13·55)*	11·60 (9·48 to 13·56)*
3	Household air pollution from solid fuels	34·05 (27·33 to 41·50)	25·65 (20·30 to 31·36)	18·95 (14·97 to 23·48)	–24·66 (−27·07 to −22·55)*	–26·12 (−28·68 to −23·75)*	–44·33 (−47·18 to −41·84)*	35·67 (30·59 to 40·81)	27·57 (23·56 to 31·88)	20·69 (17·54 to 24·11)	–22·71 (−24·92 to −20·85)*	–24·95 (−27·24 to −22·68)*	–42·00 (−44·45 to −39·54)*	–43·14 (−45·63 to −40·73)*
3	Ambient ozone pollution	38·49 (13·87 to 68·02)	43·30 (15·71 to 74·16)	48·75 (18·05 to 78·30)	12·50 (8·84 to 14·03)*	12·57 (5·92 to 15·54)*	26·63 (15·49 to 31·29)*	38·22 (13·78 to 67·36)	42·66 (15·45 to 73·25)	47·94 (17·71 to 77·40)	11·61 (8·35 to 12·94)*	12·39 (5·99 to 15·22)*	25·44 (15·05 to 29·65)*	26·03 (15·27 to 30·47)*
**2**	**Other environmental risks**													
3	Residential radon	26·12 (22·17 to 30·31)	26·08 (22·09 to 30·34)	26·17 (22·17 to 30·54)	–0·12 (−1·27 to 1·03)	0·34 (−0·24 to 0·98)	0·22 (−1·41 to 1·95)	26·27 (22·33 to 30·45)	26·23 (22·25 to 30·48)	26·34 (22·32 to 30·69)	–0·12 (−1·32 to 1·09)	0·41 (−0·22 to 1·10)	0·29 (−1·45 to 2·12)	0·25 (−1·43 to 2·04)
3	Lead exposure	20·01 (8·93 to 33·97)	18·57 (8·35 to 31·87)	15·01 (6·28 to 27·06)	–7·19 (−10·97 to −4·90)*	–19·20 (−25·88 to −14·37)*	–25·01 (−32·80 to −18·88)*	10·27 (2·82 to 21·64)	10·18 (3·19 to 21·15)	8·37 (2·47 to 18·17)	–0·80 (−4·67 to 13·60)	–17·86 (−24·00 to −13·50)*	–18·52 (−24·51 to −10·29)*	–22·68 (−30·06 to −17·08)*
**2**	**Occupational risks**													
**3**	**Occupational carcinogens**													
4	Occupational exposure to asbestos	4·11 (3·85 to 4·44)	4·00 (3·76 to 4·30)	3·90 (3·65 to 4·21)	–2·68 (−5·60 to −0·17)*	–2·41 (−3·52 to −1·46)*	–5·03 (−7·49 to −2·85)*	1·47 (1·36 to 1·68)	1·25 (1·17 to 1·40)	1·19 (1·11 to 1·32)	–14·74 (−16·66 to −13·21)*	–4·97 (−6·27 to −3·53)*	–18·98 (−21·23 to −17·53)*	–6·91 (−8·97 to −5·07)*
4	Occupational exposure to arsenic	0·91 (0·00 to 3·12)	0·96 (0·00 to 3·46)	1·02 (0·00 to 3·75)	6·31 (0·18 to 10·99)*	6·23 (3·89 to 8·29)*	12·94 (4·14 to 20·24)*	0·72 (0·00 to 2·37)	0·81 (0·00 to 2·84)	0·88 (0·00 to 3·16)	11·83 (2·14 to 19·69)*	8·82 (5·35 to 11·33)*	21·70 (8·33 to 33·09)*	16·81 (6·00 to 25·80)*
4	Occupational exposure to benzene	0·77 (0·36 to 1·59)	0·87 (0·44 to 1·74)	0·96 (0·51 to 1·88)	12·93 (9·26 to 21·67)*	10·25 (8·22 to 14·21)*	24·50 (18·21 to 38·83)*	0·65 (0·27 to 1·43)	0·80 (0·37 to 1·68)	0·94 (0·46 to 1·91)	22·92 (17·94 to 37·88)*	17·04 (13·69 to 24·74)*	43·86 (34·03 to 71·79)*	33·27 (25·56 to 52·63)*
4	Occupational exposure to beryllium	0·09 (0·09 to 0·09)	0·10 (0·10 to 0·10)	0·11 (0·11 to 0·11)	10·33 (10·18 to 10·46)*	6·40 (6·30 to 6·51)*	17·39 (17·17 to 17·62)*	0·07 (0·07 to 0·07)	0·08 (0·08 to 0·08)	0·09 (0·09 to 0·09)	23·36 (23·14 to 23·58)*	13·48 (13·35 to 13·61)*	39·99 (39·65 to 40·30)*	26·78 (26·60 to 26·96)*
4	Occupational exposure to cadmium	0·18 (0·18 to 0·18)	0·20 (0·20 to 0·20)	0·22 (0·22 to 0·22)	13·27 (12·96 to 13·59)*	9·35 (9·15 to 9·58)*	23·86 (23·39 to 24·33)*	0·13 (0·13 to 0·14)	0·16 (0·16 to 0·17)	0·19 (0·18 to 0·19)	22·86 (22·14 to 23·54)*	13·76 (13·16 to 14·47)*	39·76 (38·93 to 40·61)*	30·69 (30·23 to 31·19)*
4	Occupational exposure to chromium	0·38 (0·38 to 0·39)	0·45 (0·44 to 0·45)	0·50 (0·49 to 0·51)	17·37 (17·03 to 17·72)*	11·82 (11·59 to 12·06)*	31·24 (30·77 to 31·73)*	0·28 (0·28 to 0·29)	0·36 (0·35 to 0·37)	0·42 (0·41 to 0·43)	26·61 (25·62 to 27·46)*	16·00 (15·33 to 16·77)*	46·86 (45·96 to 47·74)*	37·94 (37·46 to 38·43)*
4	Occupational exposure to diesel engine exhaust	2·29 (2·26 to 2·32)	2·78 (2·74 to 2·81)	3·11 (3·07 to 3·14)	21·41 (20·94 to 21·81)*	11·86 (11·60 to 12·11)*	35·80 (35·28 to 36·30)*	1·22 (1·20 to 1·23)	1·61 (1·59 to 1·64)	1·86 (1·83 to 1·89)	32·59 (32·10 to 33·07)*	15·19 (14·80 to 15·65)*	52·73 (51·97 to 53·59)*	41·78 (41·29 to 42·22)*
4	Occupational exposure to second-hand smoke	12·58 (5·66 to 21·95)	12·96 (5·67 to 22·91)	13·77 (6·00 to 24·44)	3·02 (0·61 to 4·26)*	6·23 (5·39 to 6·83)*	9·44 (6·25 to 11·25)*	10·65 (4·83 to 18·85)	11·47 (5·11 to 20·49)	12·18 (5·39 to 21·86)	7·69 (5·75 to 8·77)*	6·17 (5·33 to 6·69)*	14·33 (11·51 to 15·87)*	11·63 (8·77 to 13·29)*
4	Occupational exposure to formaldehyde	0·79 (0·77 to 0·81)	0·91 (0·88 to 0·93)	1·01 (0·98 to 1·03)	14·91 (14·45 to 15·39)*	10·67 (10·41 to 10·94)*	27·17 (26·49 to 27·88)*	0·57 (0·55 to 0·58)	0·70 (0·67 to 0·72)	0·80 (0·77 to 0·82)	22·93 (21·84 to 23·99)*	14·63 (14·06 to 15·25)*	40·92 (39·83 to 42·05)*	32·87 (32·25 to 33·48)*
4	Occupational exposure to nickel	1·60 (0·00 to 7·78)	1·67 (0·00 to 8·37)	1·75 (0·00 to 8·89)	4·62 (−3·15 to 7·54)	4·52 (1·26 to 6·14)*	9·36 (−1·85 to 14·13)	1·07 (0·00 to 4·86)	1·18 (0·00 to 5·65)	1·27 (0·00 to 6·20)	10·38 (−1·64 to 15·93)	7·75 (3·07 to 10·22)*	18·93 (1·74 to 27·60)*	13·25 (−0·43 to 19·36)
4	Occupational exposure to polycyclic aromatic hydrocarbons	0·80 (0·79 to 0·81)	0·93 (0·92 to 0·94)	1·05 (1·03 to 1·06)	17·04 (16·72 to 17·34)*	11·89 (11·71 to 12·09)*	30·96 (30·53 to 31·40)*	0·58 (0·58 to 0·59)	0·74 (0·73 to 0·75)	0·86 (0·85 to 0·88)	26·50 (25·77 to 27·17)*	16·77 (16·27 to 17·29)*	47·71 (46·91 to 48·49)*	38·08 (37·65 to 38·52)*
4	Occupational exposure to silica	5·76 (2·34 to 14·58)	5·97 (2·59 to 14·73)	6·21 (2·78 to 15·06)	3·67 (0·97 to 10·72)*	4·05 (2·23 to 7·26)*	7·87 (3·25 to 18·79)*	3·11 (1·19 to 7·93)	3·16 (1·34 to 7·75)	3·29 (1·45 to 7·87)	1·62 (−2·33 to 12·08)	3·98 (1·53 to 8·05)*	5·66 (−0·80 to 21·02)	7·16 (1·90 to 19·77)*
4	Occupational exposure to sulfuric acid	0·93 (0·56 to 1·94)	0·98 (0·63 to 1·95)	1·03 (0·67 to 2·00)	5·70 (0·98 to 11·50)*	4·63 (2·40 to 7·07)*	10·58 (3·34 to 19·35)*	0·68 (0·39 to 1·49)	0·77 (0·48 to 1·57)	0·83 (0·54 to 1·64)	12·54 (5·65 to 22·38)*	7·87 (4·54 to 11·75)*	21·39 (10·34 to 36·35)*	15·18 (6·51 to 26·46)*
4	Occupational exposure to trichloroethylene	0·22 (0·22 to 0·22)	0·26 (0·26 to 0·27)	0·30 (0·29 to 0·30)	19·43 (19·07 to 19·87)*	11·90 (11·59 to 12·26)*	33·64 (33·15 to 34·20)*	0·16 (0·16 to 0·16)	0·21 (0·21 to 0·21)	0·24 (0·24 to 0·25)	29·39 (28·28 to 30·19)*	16·04 (15·52 to 16·69)*	50·15 (49·33 to 50·89)*	40·64 (40·23 to 41·07)*
3	Occupational asthmagens	23·14 (19·26 to 27·93)	23·44 (19·61 to 28·24)	23·97 (20·12 to 28·88)	1·30 (0·28 to 2·38)*	2·28 (1·75 to 2·92)*	3·61 (2·17 to 5·17)*	10·70 (8·71 to 13·08)	12·42 (10·13 to 15·13)	13·39 (10·96 to 16·30)	16·04 (14·27 to 17·83)*	7·80 (7·01 to 8·74)*	25·09 (22·75 to 27·86)*	10·50 (8·62 to 12·32)*
3	Occupational particulate matter, gases, and fumes	12·28 (9·40 to 16·46)	12·53 (9·64 to 16·72)	12·60 (9·72 to 16·79)	1·99 (1·40 to 2·55)*	0·58 (0·19 to 0·97)*	2·59 (1·76 to 3·42)*	5·59 (4·29 to 7·66)	6·15 (4·78 to 8·35)	6·49 (5·05 to 8·81)	10·01 (8·28 to 11·78)*	5·44 (4·56 to 6·36)*	16·00 (13·53 to 18·35)*	7·30 (5·61 to 8·97)*
3	Occupational noise	16·38 (13·89 to 19·41)	16·38 (14·00 to 19·31)	16·21 (13·92 to 18·94)	–0·01 (−0·92 to 0·79)	–1·06 (−2·04 to −0·40)*	–1·07 (−2·82 to 0·37)	7·11 (6·22 to 8·05)	7·94 (6·98 to 8·97)	8·45 (7·45 to 9·52)	11·69 (11·00 to 12·54)*	6·47 (6·09 to 6·87)*	18·92 (17·89 to 20·19)*	5·41 (3·83 to 6·85)*
3	Occupational ergonomic factors	24·56 (23·13 to 26·22)	24·62 (23·05 to 26·43)	23·44 (22·01 to 25·12)	0·27 (−0·72 to 1·19)	–4·79 (−5·10 to −4·46)*	–4·54 (−5·47 to −3·62)*	12·46 (11·73 to 13·36)	14·70 (13·80 to 15·80)	15·15 (14·21 to 16·25)	17·95 (16·56 to 19·42)*	3·06 (2·72 to 3·41)*	21·56 (19·97 to 23·25)*	4·31 (3·39 to 5·27)*
**1**	**Behavioural risks**													
**2**	**Child and maternal malnutrition**													
**3**	**Suboptimal breastfeeding**													
4	Non-exclusive breastfeeding	24·03 (17·85 to 32·14)	22·62 (16·92 to 29·93)	22·72 (17·08 to 29·99)	–5·85 (−7·72 to −3·89)*	0·43 (−1·59 to 2·60)	–5·45 (−7·72 to −2·66)*	23·99 (17·82 to 32·11)	22·60 (16·88 to 29·95)	22·70 (17·05 to 30·00)	–5·79 (−7·61 to −3·86)*	0·42 (−1·54 to 2·61)	–5·39 (−7·63 to −2·63)*	–5·42 (−7·68 to −2·65)*
4	Discontinued breastfeeding	12·15 (12·04 to 12·30)	11·93 (11·84 to 12·07)	12·75 (12·60 to 12·93)	–1·80 (−3·03 to −0·46)*	6·86 (5·55 to 8·28)*	4·94 (3·15 to 6·70)*	12·15 (12·04 to 12·30)	11·89 (11·80 to 12·03)	12·69 (12·53 to 12·86)	–2·12 (−3·35 to −0·80)*	6·71 (5·40 to 8·14)*	4·45 (2·68 to 6·18)*	4·70 (2·92 to 6·45)*
**3**	**Child growth failure**													
4	Child underweight	14·90 (13·04 to 16·56)	12·52 (10·85 to 14·08)	9·19 (7·59 to 10·69)	–15·99 (−19·77 to −12·69)*	–26·61 (−30·36 to −23·72)*	–38·34 (−43·20 to −34·46)*	14·04 (12·19 to 15·73)	11·14 (9·38 to 12·65)	8·41 (6·81 to 9·85)	–20·62 (−24·29 to −17·36)*	–24·50 (−28·14 to −21·50)*	–40·06 (−44·66 to −35·98)*	–39·14 (−43·61 to −35·50)*
4	Child wasting	8·46 (7·11 to 9·72)	8·39 (7·08 to 9·60)	7·11 (5·84 to 8·33)	–0·85 (−3·11 to 1·33)	–15·26 (−18·20 to −12·78)*	–15·98 (−19·31 to −13·01)*	8·24 (6·88 to 9·49)	7·57 (6·30 to 8·78)	6·54 (5·36 to 7·69)	–8·16 (−10·86 to −5·59)*	–13·56 (−15·99 to −11·39)*	–20·61 (−24·15 to −17·18)*	–18·19 (−21·38 to −15·64)*
4	Child stunting	24·71 (17·03 to 27·50)	21·31 (14·80 to 23·95)	17·07 (11·93 to 19·73)	–13·77 (−16·16 to −11·88)*	–19·86 (−23·55 to −17·01)*	–30·90 (−35·38 to −27·56)*	23·49 (16·28 to 26·33)	19·53 (13·62 to 22·16)	15·48 (10·78 to 18·07)	–16·82 (−19·62 to −14·76)*	–20·77 (−24·47 to −17·65)*	–34·10 (−38·80 to −30·53)*	–32·40 (−36·66 to −29·30)*
**3**	**Low birthweight and short gestation**													
4	Short gestation for birthweight	10·22 (9·52 to 11·09)	10·55 (9·81 to 11·50)	10·78 (10·00 to 11·81)	3·28 (2·71 to 3·92)*	2·16 (1·46 to 3·30)*	5·51 (4·37 to 7·01)*	10·26 (9·44 to 11·25)	10·67 (9·76 to 11·76)	10·92 (9·95 to 12·11)	3·95 (3·26 to 4·71)*	2·34 (1·67 to 3·41)*	6·39 (5·17 to 7·92)*	5·94 (4·95 to 7·21)*
4	Low birthweight for gestation	8·91 (7·92 to 10·06)	8·73 (7·80 to 9·80)	8·61 (7·71 to 9·64)	–2·04 (−3·00 to −1·29)*	–1·34 (−1·93 to −0·81)*	–3·36 (−4·65 to −2·25)*	9·23 (8·23 to 10·59)	8·93 (8·04 to 10·16)	8·83 (7·96 to 10·03)	–3·22 (−4·53 to −2·16)*	–1·14 (−1·68 to −0·61)*	–4·32 (−5·85 to −2·94)*	–3·83 (−5·10 to −2·79)*
3	Iron deficiency	··	··	··	··	··	··	8·36 (6·25 to 10·98)	8·49 (6·35 to 11·11)	8·52 (6·38 to 11·16)	1·46 (1·27 to 1·69)*	0·39 (0·28 to 0·50)*	1·86 (1·65 to 2·09)*	1·87 (1·67 to 2·11)*
3	Vitamin A deficiency	20·37 (16·63 to 24·22)	16·91 (13·58 to 20·19)	15·30 (12·25 to 18·41)	–16·99 (−18·83 to −15·00)*	–9·55 (−11·66 to −7·57)*	–24·92 (−27·67 to −22·08)*	19·12 (15·60 to 22·93)	16·03 (13·04 to 19·14)	14·44 (11·43 to 17·54)	–16·16 (−17·99 to −13·29)*	–9·90 (−12·40 to −7·72)*	–24·46 (−27·62 to −21·13)*	–24·69 (−27·48 to −21·70)*
3	Zinc deficiency	11·26 (3·33 to 21·64)	9·36 (2·93 to 18·11)	7·96 (2·45 to 15·87)	–16·89 (−20·14 to −10·22)*	–14·99 (−17·90 to −11·14)*	–29·35 (−33·23 to −21·63)*	11·31 (3·29 to 21·72)	9·35 (2·90 to 18·12)	7·96 (2·46 to 15·92)	–17·29 (−20·61 to −11·41)*	–14·85 (−17·96 to −10·84)*	–29·57 (−33·72 to −22·15)*	–29·46 (−32·76 to −23·49)*
**2**	**Tobacco**													
3	Smoking	35·72 (32·76 to 39·76)	30·16 (27·23 to 34·44)	25·14 (22·69 to 28·74)	–15·57 (−18·63 to −12·33)*	–16·63 (−20·29 to −12·87)*	–29·61 (−33·96 to −24·13)*	11·11 (9·22 to 14·19)	9·65 (7·88 to 12·63)	7·93 (6·49 to 10·55)	–13·15 (−16·68 to −7·93)*	–17·83 (−23·41 to −12·38)*	–28·63 (−34·48 to −20·87)*	–28·99 (−33·00 to −24·33)*
3	Smokeless tobacco	13·39 (12·68 to 14·11)	15·58 (15·10 to 16·07)	15·04 (14·34 to 15·80)	16·36 (9·65 to 23·26)*	–3·46 (−8·38 to 2·17)	12·33 (4·70 to 20·89)*	8·34 (7·65 to 9·00)	9·31 (8·82 to 9·80)	8·61 (7·88 to 9·37)	11·55 (1·46 to 23·48)*	–7·44 (−16·18 to 2·32)	3·25 (−8·03 to 16·93)	9·11 (2·16 to 16·49)*
3	Second-hand smoke	23·11 (22·52 to 23·63)	19·73 (19·48 to 19·96)	18·96 (18·60 to 19·28)	–14·62 (−16·80 to −12·40)*	–3·91 (−4·99 to −2·82)*	–17·96 (−20·68 to −15·11)*	43·29 (42·00 to 44·40)	35·87 (35·10 to 36·55)	33·32 (32·49 to 33·97)	–17·13 (−18·78 to −15·25)*	–7·11 (−8·22 to −6·01)*	–23·03 (−25·21 to −20·59)*	–21·39 (−23·64 to −18·82)*
**2**	**Alcohol and drug use**													
3	Alcohol use	13·82 (11·94 to 15·70)	14·27 (12·36 to 16·27)	14·05 (12·28 to 15·85)	3·26 (−1·07 to 8·20)	–1·54 (−5·49 to 2·86)	1·68 (−3·97 to 8·77)	5·68 (4·57 to 6·78)	4·90 (3·97 to 5·88)	4·83 (3·93 to 5·78)	–13·70 (−17·59 to −9·62)*	–1·49 (−6·97 to 4·13)	–14·98 (−20·47 to −8·98)*	–2·84 (−8·09 to 3·36)
3	Drug use	0·63 (0·32 to 1·13)	0·61 (0·31 to 1·09)	0·61 (0·31 to 1·10)	–2·98 (−3·70 to −2·26)*	0·41 (−0·76 to 1·52)	–2·58 (−4·08 to −0·98)*	0·38 (0·19 to 0·68)	0·35 (0·18 to 0·64)	0·36 (0·18 to 0·65)	–7·43 (−8·40 to −6·57)*	1·19 (−0·04 to 2·29)	–6·33 (−7·94 to −4·82)*	–3·84 (−5·28 to −2·42)*
**2**	**Dietary risks**													
3	Diet low in fruits	74·84 (54·78 to 91·07)	67·49 (47·24 to 86·59)	61·77 (41·88 to 80·91)	–9·81 (−13·94 to −4·92)*	–8·49 (−11·37 to −6·45)*	–17·47 (−23·57 to −11·01)*	72·47 (52·33 to 89·49)	63·10 (43·24 to 82·40)	56·95 (37·44 to 75·97)	–12·92 (−17·54 to −7·87)*	–9·75 (−12·98 to −7·61)*	–21·41 (−28·12 to −15·01)*	–19·41 (−25·83 to −13·02)*
3	Diet low in vegetables	54·19 (36·90 to 71·51)	45·87 (29·62 to 62·63)	41·55 (26·41 to 57·44)	–15·36 (−19·49 to −12·55)*	–9·41 (−11·75 to −7·67)*	–23·32 (−28·38 to −19·69)*	56·42 (38·74 to 74·29)	48·11 (31·55 to 64·93)	43·79 (28·06 to 60·10)	–14·74 (−18·82 to −12·04)*	–8·96 (−11·33 to −7·32)*	–22·38 (−27·62 to −18·87)*	–22·83 (−27·86 to −19·31)*
3	Diet low in legumes	41·50 (28·42 to 54·90)	45·97 (33·03 to 58·84)	45·13 (32·40 to 57·73)	10·78 (6·42 to 17·21)*	–1·83 (−3·69 to 0·03)	8·75 (4·50 to 14·83)*	47·78 (33·68 to 61·80)	52·23 (38·14 to 65·93)	51·61 (37·74 to 65·20)	9·33 (6·14 to 13·95)*	–1·18 (−2·64 to 0·16)	8·04 (4·89 to 12·67)*	8·18 (4·89 to 13·01)*
3	Diet low in whole grains	64·83 (45·85 to 83·18)	58·75 (41·16 to 76·64)	58·64 (41·07 to 76·49)	–9·37 (−10·53 to −7·87)*	–0·20 (−0·52 to 0·11)	–9·55 (−10·79 to −8·01)*	65·47 (46·12 to 83·94)	59·44 (41·30 to 77·68)	60·66 (42·41 to 78·76)	–9·21 (−10·80 to −7·50)*	2·06 (1·26 to 2·80)*	–7·34 (−8·50 to −6·19)*	–8·44 (−9·57 to −7·11)*
3	Diet low in nuts and seeds	88·83 (68·81 to 99·29)	83·75 (63·76 to 95·14)	81·39 (60·77 to 94·85)	–5·72 (−7·32 to −4·13)*	–2·82 (−4·85 to −0·31)*	–8·38 (−11·67 to −4·50)*	89·01 (68·93 to 99·33)	84·32 (64·20 to 95·78)	81·94 (61·27 to 95·19)	–5·27 (−6·94 to −3·53)*	–2·82 (−4·65 to −0·54)*	–7·94 (−11·18 to −4·16)*	–8·16 (−11·38 to −4·33)*
3	Diet low in milk	81·31 (63·75 to 93·81)	83·31 (65·78 to 95·69)	83·48 (65·88 to 95·94)	2·47 (1·94 to 3·09)*	0·20 (−0·23 to 0·59)	2·67 (2·11 to 3·25)*	81·43 (63·96 to 94·04)	83·40 (65·84 to 95·88)	83·62 (65·94 to 96·11)	2·42 (1·83 to 3·19)*	0·27 (−0·14 to 0·64)	2·70 (2·11 to 3·43)*	2·71 (2·19 to 3·22)*
3	Diet high in red meat	19·44 (16·17 to 22·95)	21·77 (18·13 to 25·66)	24·66 (21·03 to 28·64)	11·97 (6·60 to 17·58)*	13·28 (8·34 to 19·80)*	26·84 (20·66 to 34·20)*	8·50 (6·08 to 11·17)	8·96 (6·42 to 11·86)	10·84 (7·89 to 13·98)	5·35 (−2·82 to 16·00)	21·05 (10·94 to 34·59)*	27·53 (17·12 to 41·17)*	27·57 (21·74 to 34·70)*
3	Diet high in processed meat	7·84 (6·20 to 10·03)	7·62 (6·12 to 9·88)	6·19 (4·58 to 8·67)	–2·79 (−8·00 to 1·77)	–18·81 (−26·23 to −11·45)*	–21·08 (−29·56 to −12·44)*	5·38 (3·82 to 7·35)	5·07 (3·69 to 7·09)	4·32 (2·95 to 6·49)	–5·92 (−11·73 to 0·09)	–14·62 (−22·25 to −7·62)*	–19·68 (−27·67 to −10·91)*	–20·45 (−27·41 to −12·36)*
3	Diet high in sugar-sweetened beverages	12·19 (11·19 to 13·25)	15·70 (14·48 to 16·87)	17·90 (16·52 to 19·25)	28·79 (22·74 to 35·00)*	14·06 (11·40 to 16·81)*	46·89 (38·49 to 55·16)*	9·47 (8·47 to 10·53)	11·79 (10·79 to 12·82)	13·45 (12·28 to 14·59)	24·47 (17·25 to 31·99)*	14·10 (10·72 to 17·61)*	42·02 (31·42 to 52·44)*	44·73 (36·08 to 52·69)*
3	Diet low in fibre	59·23 (38·47 to 80·17)	56·12 (35·61 to 76·88)	53·27 (33·15 to 73·95)	–5·24 (−7·57 to −3·74)*	–5·08 (−7·43 to −3·57)*	–10·06 (−13·95 to −7·46)*	66·96 (45·54 to 87·29)	64·02 (42·94 to 84·48)	61·76 (40·67 to 82·64)	–4·39 (−6·33 to −3·04)*	–3·53 (−5·53 to −2·10)*	–7·77 (−11·04 to −5·33)*	–8·89 (−12·30 to −6·46)*
3	Diet low in calcium	63·99 (44·32 to 82·99)	60·79 (41·37 to 80·23)	57·11 (37·87 to 76·76)	–5·01 (−6·88 to −3·24)*	–6·05 (−8·50 to −4·33)*	–10·75 (−14·66 to −7·46)*	66·45 (46·60 to 85·13)	63·73 (43·96 to 83·09)	60·70 (41·09 to 80·49)	–4·09 (−5·70 to −2·36)*	–4·76 (−6·69 to −3·15)*	–8·66 (−11·87 to −5·44)*	–9·67 (−13·21 to −6·43)*
3	Diet low in seafood omega 3 fatty acids	80·95 (62·89 to 93·16)	78·84 (60·21 to 92·11)	76·66 (57·55 to 91·30)	–2·61 (−4·24 to −1·11)*	–2·76 (−4·41 to −0·86)*	–5·31 (−8·41 to −1·95)*	82·50 (64·41 to 94·45)	81·01 (62·43 to 93·88)	79·29 (60·18 to 93·34)	–1·81 (−3·21 to −0·60)*	–2·12 (−3·59 to −0·53)*	–3·89 (−6·61 to −1·16)*	–4·59 (−7·46 to −1·59)*
3	Diet low in polyunsaturated fatty acids	45·06 (43·63 to 46·41)	42·75 (41·51 to 43·97)	39·59 (38·34 to 40·95)	–5·12 (−8·80 to −1·13)*	–7·39 (−10·43 to −4·14)*	–12·13 (−15·52 to −8·13)*	42·88 (41·39 to 44·43)	42·13 (40·86 to 43·41)	39·01 (37·71 to 40·27)	–1·75 (−6·01 to 2·86)	–7·42 (−10·78 to −3·74)*	–9·03 (−13·10 to −4·82)*	–10·66 (−13·33 to −7·81)*
3	Diet high in transfatty acids	7·64 (3·38 to 13·77)	4·95 (1·69 to 10·43)	3·65 (0·96 to 8·75)	–35·13 (−52·63 to −22·20)*	–26·21 (−45·06 to −14·67)*	–52·13 (−72·97 to −33·77)*	10·53 (5·22 to 17·83)	7·03 (2·87 to 13·30)	5·21 (1·73 to 11·02)	–33·22 (−46·91 to −21·80)*	–25·92 (−42·55 to −15·38)*	–50·53 (−68·52 to −34·00)*	–51·32 (−70·08 to −34·10)*
3	Diet high in sodium	44·14 (19·26 to 76·14)	40·66 (12·48 to 76·89)	39·77 (11·77 to 76·42)	–7·89 (−35·22 to 1·07)	–2·18 (−9·26 to −0·40)*	–9·89 (−40·40 to 0·12)	43·80 (18·77 to 76·78)	37·98 (11·76 to 74·27)	36·22 (10·50 to 72·98)	–13·29 (−38·80 to −2·86)*	–4·64 (−12·09 to −1·57)*	–17·32 (−44·79 to −4·79)*	–13·63 (−42·22 to −2·33)*
**2**	**Sexual abuse and violence**													
3	Childhood sexual abuse	6·78 (5·66 to 8·02)	6·81 (5·72 to 7·98)	7·09 (5·94 to 8·33)	0·46 (−0·56 to 1·57)	4·10 (3·41 to 4·77)*	4·58 (3·92 to 5·29)*	7·78 (6·57 to 9·20)	7·51 (6·39 to 8·83)	7·68 (6·46 to 9·06)	–3·44 (−4·40 to −2·36)*	2·23 (1·23 to 3·13)*	–1·29 (−2·41 to 0·17)	1·46 (0·76 to 2·27)*
3	Intimate partner violence	··	··	··	··	··	··	11·80 (10·05 to 13·42)	10·90 (9·40 to 12·26)	10·32 (8·88 to 11·63)	–7·62 (−8·80 to −6·28)*	–5·33 (−5·90 to −4·79)*	–12·55 (−13·59 to −11·39)*	–12·80 (−13·82 to −11·65)*
2	Low physical activity	18·02 (9·66 to 28·42)	18·16 (9·81 to 28·87)	18·25 (9·81 to 28·80)	0·78 (−28·27 to 39·60)	0·51 (−27·42 to 41·11)	1·30 (0·94 to 1·72)*	15·32 (8·52 to 23·82)	15·12 (8·42 to 23·35)	15·05 (8·33 to 23·45)	–1·28 (−30·22 to 37·56)	–0·49 (−28·76 to 41·07)	–1·77 (−2·31 to −1·28)*	0·07 (−0·32 to 0·38)
**1**	**Metabolic risks**													
2	High fasting plasma glucose	2·87 (1·79 to 4·14)	3·68 (2·37 to 5·23)	3·70 (2·36 to 5·22)	28·39 (19·48 to 41·24)*	0·60 (−7·76 to 8·12)	29·17 (21·33 to 40·03)*	2·46 (1·48 to 3·73)	3·34 (2·14 to 4·76)	3·14 (1·97 to 4·55)	35·71 (20·73 to 59·93)*	–5·93 (−15·95 to 1·66)	27·66 (18·53 to 42·31)*	28·77 (21·32 to 39·87)*
2	High total cholesterol	17·43 (13·61 to 21·82)	16·87 (13·07 to 21·24)	16·75 (12·96 to 21·12)	–3·20 (−4·08 to −2·45)*	–0·74 (−1·28 to −0·21)*	–3·91 (−4·91 to −2·98)*	20·14 (16·16 to 24·66)	19·30 (15·40 to 23·74)	19·05 (15·10 to 23·49)	–4·17 (−5·13 to −3·34)*	–1·29 (−1·91 to −0·68)*	–5·41 (−6·65 to −4·34)*	–5·12 (−6·23 to −4·19)*
2	High systolic blood pressure	25·23 (23·60 to 27·13)	25·37 (23·68 to 27·32)	25·69 (23·99 to 27·69)	0·54 (0·18 to 0·91)*	1·29 (0·96 to 1·62)*	1·83 (1·40 to 2·31)*	26·03 (24·45 to 27·80)	25·03 (23·53 to 26·77)	24·69 (23·20 to 26·38)	–3·82 (−4·22 to −3·47)*	–1·35 (−1·70 to −0·98)*	–5·12 (−5·55 to −4·68)*	–1·95 (−2·28 to −1·61)*
2	High body-mass index	5·91 (3·95 to 8·57)	7·93 (5·41 to 11·27)	9·50 (6·52 to 13·51)	34·16 (25·41 to 45·65)*	19·82 (15·03 to 25·28)*	60·75 (45·26 to 81·32)*	6·62 (4·51 to 9·52)	8·89 (6·21 to 12·30)	10·64 (7·51 to 14·57)	34·22 (25·81 to 44·44)*	19·69 (15·07 to 24·73)*	60·65 (45·85 to 79·65)*	60·25 (45·14 to 79·11)*
2	Low bone mineral density	11·49 (10·42 to 12·67)	11·40 (10·34 to 12·58)	11·33 (10·29 to 12·51)	–0·75 (−1·08 to −0·46)*	–0·60 (−0·84 to −0·36)*	–1·34 (−1·79 to −0·91)*	12·59 (11·47 to 13·76)	12·59 (11·48 to 13·78)	12·65 (11·51 to 13·82)	0·06 (−0·16 to 0·27)	0·41 (0·16 to 0·65)*	0·46 (0·21 to 0·69)*	–0·51 (−0·75 to −0·26)*
2	Impaired kidney function	4·78 (2·94 to 9·15)	4·84 (2·98 to 9·27)	4·90 (3·01 to 9·36)	1·16 (0·41 to 1·83)*	1·23 (0·61 to 1·76)*	2·41 (1·11 to 3·34)*	5·46 (3·42 to 10·22)	5·48 (3·41 to 10·27)	5·54 (3·44 to 10·37)	0·35 (−0·80 to 1·78)	1·06 (0·47 to 1·73)*	1·41 (−0·05 to 3·33)	1·63 (0·44 to 3·05)*

Data in parentheses are 95% uncertainty intervals. SEVs=summary exposure values.

* Statistically significant increase or decrease.

**Figure 1:**
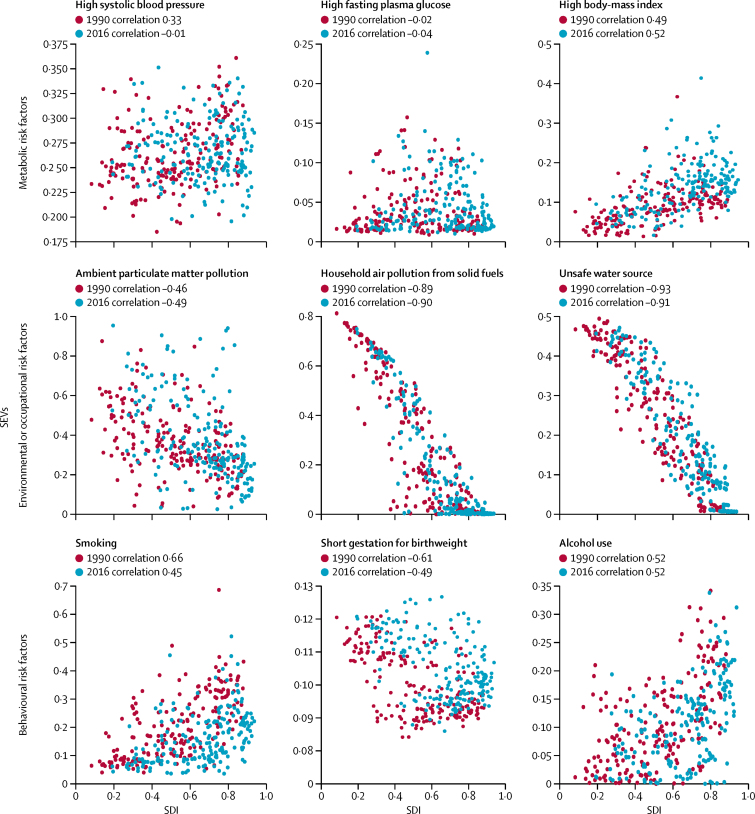
Relationship between SEVs and SDI for the three metabolic, behavioural, and environmental or occupational risk factors that are responsible for the largest number of attributable DALYs globally Each point corresponds to a country in either 1990 (red) or 2016 (blue). Pearson correlation coefficients have been estimated to summarise the relationship between SEVs and SDI in 1990 and in 2016. SEVs=summary exposure values. SDI=Socio-demographic Index. DALYs=disability-adjusted life-years.

**Figure 2:**
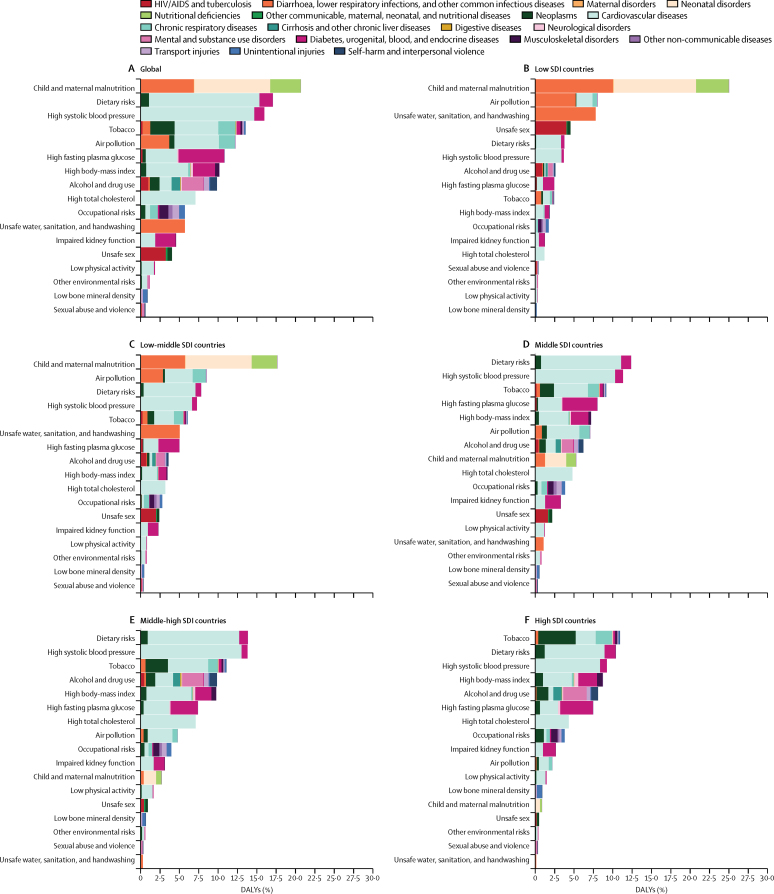
DALYs attributable to all Level 2 risk factors apportioned by Level 2 cause for each risk, both sexes combined, 2016, at the global level (A); for low SDI countries (B); for low-middle SDI countries (C); for middle SDI countries (D); for middle-high SDI countries (E); and for high SDI countries (F) DALYs from causes attributable to each risk factor are shown in different colours. Cutoffs on the SDI scale for the quintiles were selected based on examining the entire distribution of locations between 1980 and 2016. DALYs=disability-adjusted life-years. SDI=Socio-demographic Index.

Across countries there is substantial variation in risk exposure by level of SDI. Some risk factors, such as high fasting plasma glucose (FPG) and high systolic blood pressure, show similar SEVs across levels of SDI, while others, including household air pollution and unsafe water source, show marked trends with sociodemographic development. Figure 1 shows the relationship between SEVs and SDI for the leading three metabolic, behavioural, and environmental and occupational risk factors and how that changed between 1990 and 2016. Within leading metabolic risks (high BMI, high FPG, and high systolic blood pressure [SBP]), risk-weighted exposure shows an increasing trend with increasing SDI for only high BMI. Overall, the SEV for high BMI has increased during the time period. Looking at the leading three environmental risk factors (ambient air pollution, household air pollution, and unsafe water), figure 2 shows an inverse relationship with SDI for household air pollution and unsafe water, with SEVs approaching zero at high levels of SDI, while the relationship is less consistent with ambient air pollution. Finally, the relationship between SDI and the leading behavioural risk factors is more heterogeneous, with smoking and alcohol use having a positive correlation with SDI, and short gestation for birthweight having a negative correlation with SDI.

### Global attributable burden for all risk factors combined and their overlap

Globally, 59·9% (58·4–61·3) of deaths and 45·2% (43·2–47·3) of DALYs could be attributed to the risk factors assessed in GBD 2016. For deaths, non-communicable diseases (NCDs) show the largest proportion attributable to measured risk factors, at 64·4% (62·6–66·2), with communicable, maternal, neonatal, and nutritional (CMNN) causes at 57·9% (55·4–61·0), and injuries at 25·8% (23·7–27·8). The picture was different for DALYs, however, where we observed that 58·2% (56·4–60·3) of DALYs in CMNN causes are attributable to risk factors, compared with 43·5% (40·7–46·7) in NCDs and 21·0% (19·3–22·7) for injuries. Leading causes of DALYs in CMNN causes, such as diarrhoea and lower respiratory infections (LRI), also showed more than 80% of DALYs can be attributed to risk factors (appendix 2 p 1).

Within NCDs, three of the leading causes of deaths and DALYs, ischaemic heart disease (IHD; 93·3% [90·3–95·7] of deaths and 94·4% [92·6–95·8] of DALYs), haemorrhagic stroke (88·2% [84·3–91·8] of deaths and 89·5% [87·1–91·6] of DALYS), and chronic obstructive pulmonary disorder (COPD; 76·6% [69·9–82·9] of deaths and 73·8% [67·4–80·2] DALYs) all have high proportions attributable to measured risk factors. Lung cancer, a leading cause of death but not DALYs, also has a large proportion of total deaths and DALYs attributed to measured risk factors (84·1% [78·9–88·3] and 83·2% [78·0–87·6] respectively), while for Alzheimer's disease only 21·4% (11·2–34·0) of total deaths and 22·3% (11·8–35·1) of DALYs can be attributed to measured risk factors. For leading causes of DALYs that do not cause death, such as low back pain and sense organ diseases, less than a third of their total burden can be attributable to measured risk factors (23·0% [20·1–25·9] for low back and neck pain and 13·8% [12·4–15·4] for sense organ diseases). Across all cancers, 42·1% (38·9–45·3) of deaths and 39·8% (36·8–42·8) of DALYs are attributable to measured risk factors; however, there is a very large range within cancers, from cervical cancer at 100% of deaths attributable to risk factors and lung cancer at 84·08% (78·9–88·3) of deaths attributable to risk factors to several cancers at nearly zero, such as brain cancer.

Across types of risk factors, behavioural risk factors accounted for 32·7% (30·7–34·8) of attributable DALYs, followed by metabolic risk factors at 16·8% (15·7–18·0), and environmental and occupational at 13·1% (12·1–14·2). This pattern was seen in middle SDI, middle-high SDI, and high SDI locations, while in low SDI and low-middle SDI locations environmental risk factors accounted for a larger proportion of attributable DALYs than metabolic risk factors. This is a pattern that has persisted since 1990; notably, however, the importance of metabolic risk factors is growing steadily in low SDI and low-middle SDI locations, while that of environmental and occupational risks has decreased during this time period. More detail can be found in appendix 2 (p 1399).

### Levels and trends in the burden attributable to risk factors

Table 4 reports all-cause deaths and DALYs attributable to all risk factors considered here from 2006 to 2016, including detail on attributable deaths and DALYs by risk-outcome pair (appendix 2 p 1865) contains results for every location. Globally, 32·8 million (31·9 million to 33·7 million) deaths were attributable to all risk factors in 2016, a significant increase since 2006 of 2·9% (1·1–4·8); however, age-standardised attributable death rate declined from 2006 to 2016 by 18·7% (17·3–20·0). By contrast, total DALYs attributable to all risks decreased by 8·6% (6·6–10·7) since 2006, and age-standardised DALY rate attributable to all risks decreased by 21·7% (20·0–23·3). Among Level 1 risks, the largest decreases in age-standardised death rates were observed for environmental and occupational risks (24·3% [22·5–26·0]), followed by behavioural risks (21·5% [19·8–23·3]), and metabolic risks (11·9% [9·9–13·5]). Similarly, there were significant decreases in age-standardised DALY rates for all three Level 1 risk factors, although the magnitude of decrease was larger for DALY rates than death rates. In the year 2016, behavioural risk factors accounted for the largest number of deaths (21·8 million [20·5 million to 23·3 million]) and DALYs (781·1 million [737·1 million to 830·1 million]). While there were decreases in both deaths and DALYs attributable to behavioural risk factors since 2006, these decreases were significant for deaths (2·5% [0·1–4·9]) and DALYs (14·3% [11·8–16·6]). There was a significant, 17·9% (15·7–20·6), increase in number of deaths attributable to metabolic risk factors, from 14·8 million deaths (14·0 million to 15·7 million) in 2006 to 17·5 million deaths (16·4 million to 18·5 million) in 2016, with similar increases observed for DALYs. Environmental and occupational risk factors accounted for the fewest number of deaths and DALYs, and there was a significant decline in both measures since 2006.

**Table 4:** Global all-age attributable deaths and DALYs, in 2006 and 2016, and percentage change of deaths, age-standardised death rates, DALYs, and age-standardised DALY rates between 2006 and 2016, for all risk-outcome pairs, both sexes combined

	**Risk**	**2006 deaths (in thousands)**	**2016 deaths (in thousands)**	**Percentage change of deaths 2006–16**	**Percentage change of age-standardised deaths rate 2006–16**	**2006 DALYs (in thousands)**	**2016 DALYs (in thousands)**	**Percentage change of DALYs 2006–16**	**Percentage change of age-standardised DALYs rate 2006–16**
**0**	**All risk factors: all causes**	**31 848·45 (31 122·54 to 32 552·54)**	**32 756·24 (31 855·63 to 33 694·29)**	2·85 (1·12 to 4·76)*	–18·73 (−20·03 to −17·34)*	**1 182 311·16 (1 130 619·01 to 1 237 965·11)**	**1 080 115·72 (1 017 412·55 to 1 149 380·02)**	–8·64 (−10·66 to −6·56)*	–21·71 (−23·29 to −20·02)*
**1**	**Environmental and occupational risks: all causes**	**9751·57 (9103·89 to 10 482·39)**	**9293·43 (8663·33 to 9987·21)**	–4·70 (−7·01 to −2·37)*	–24·30 (−26·04 to −22·49)*	**367 198·64 (341 616·82 to 392 521·03)**	**311 970·97 (290 297·06 to 335 402·79)**	–15·04 (−18·03 to −12·10)*	–27·23 (−29·61 to −24·99)*
**2**	**Unsafe water, sanitation, and handwashing: all causes**	**2231·21 (1736·49 to 3001·11)**	**1660·77 (1253·69 to 2312·04)**	–25·57 (−32·82 to −16·38)*	–36·78 (−42·78 to −29·05)*	**118 178·24 (99 042·42 to 141 176·50)**	**75 796·04 (61 906·38 to 93 460·54)**	–35·86 (−41·64 to −29·70)*	–40·76 (−45·87 to −35·67)*
3	Unsafe water source: all causes	1570·53 (716·65 to 2364·77)	1160·16 (515·93 to 1858·37)	–26·13 (−34·62 to −15·83)*	–37·49 (−44·89 to −28·82)*	82 040·06 (38 265·29 to 110 406·22)	52 440·65 (23 552·84 to 73 900·44)	–36·08 (−42·68 to −29·41)*	–41·12 (−46·79 to −35·48)*
··	Diarrhoeal diseases	1570·53 (716·65 to 2364·77)	1160·16 (515·93 to 1858·37)	–26·13 (−34·62 to −15·83)*	–37·49 (−44·89 to −28·82)*	82 040·06 (38 265·29 to 110 406·22)	52 440·65 (23 552·84 to 73 900·44)	–36·08 (−42·68 to −29·41)*	–41·12 (−46·79 to −35·48)*
3	Unsafe sanitation: all causes	1323·65 (1010·23 to 1827·53)	898·24 (662·82 to 1307·68)	–32·14 (−39·14 to −23·02)*	–42·64 (−48·36 to −35·22)*	68 961·68 (56 942·58 to 84 299·83)	40 746·60 (32 803·83 to 52 138·77)	–40·91 (−46·85 to −34·53)*	–45·60 (−50·61 to −40·01)*
··	Diarrhoeal diseases	1323·65 (1010·23 to 1827·53)	898·24 (662·82 to 1307·68)	–32·14 (−39·14 to −23·02)*	–42·64 (−48·36 to −35·22)*	68 961·68 (56 942·58 to 84 299·83)	40 746·60 (32 803·83 to 52 138·77)	–40·91 (−46·85 to −34·53)*	–45·60 (−50·61 to −40·01)*
3	No access to handwashing facility: all causes	1015·06 (577·66 to 1507·15)	750·34 (432·56 to 1131·56)	–26·08 (−32·18 to −18·98)*	–36·78 (−41·76 to −30·81)*	55 096·20 (32 668·57 to 75 567·19)	35 254·90 (20 869·21 to 49 149·44)	–36·01 (−41·20 to −30·50)*	–40·63 (−45·29 to −35·52)*
··	Diarrhoeal diseases	792·95 (360·19 to 1257·46)	570·85 (258·99 to 952·81)	–28·01 (−35·54 to −18·65)*	–38·97 (−45·15 to −31·40)*	41 827·94 (20 281·34 to 62 434·28)	26 425·31 (12 807·57 to 39 599·17)	–36·82 (−43·37 to −30·06)*	–41·76 (−47·28 to −35·97)*
··	Lower respiratory infections	222·11 (145·63 to 295·64)	179·49 (115·61 to 242·67)	–19·19 (−24·55 to −13·84)*	–28·58 (−33·02 to −24·25)*	13 268·26 (8655·13 to 17 504·35)	8829·59 (5765·49 to 11 701·18)	–33·45 (−38·72 to −27·80)*	–37·00 (−41·97 to −31·71)*
**2**	**Air pollution: all causes**	**6219·85 (5700·42 to 6672·51)**	**6116·40 (5631·62 to 6602·60)**	**–1·66 (−4·14 to 0·71)**	–23·23 (−25·07 to −21·50)*	**186 446·12 (170 917·71 to 200 934·77)**	**162 795·90 (150 578·26 to 175 615·70)**	–12·68 (−15·73 to −9·60)*	–26·91 (−29·13 to −24·61)*
3	Ambient particulate matter pollution: all causes	3687·20 (3239·45 to 4139·59)	4092·69 (3624·44 to 4575·02)	11·00 (8·47 to 13·49)*	–13·89 (−15·70 to −12·08)*	105 732·08 (93 627·48 to 118 532·10)	105 674·02 (94 523·78 to 117 808·56)	–0·05 (−3·82 to 3·79)	–17·06 (−19·47 to −14·61)*
··	Lower respiratory infections	689·26 (521·80 to 875·27)	653·41 (493·27 to 826·93)	–5·20 (−10·38 to 0·26)	–18·07 (−22·22 to −13·78)*	37 842·21 (29 069·73 to 47 285·96)	28 517·03 (22 127·01 to 35 104·21)	–24·64 (−29·89 to −18·73)*	–29·02 (−33·86 to −23·49)*
··	Tracheal, bronchus, and lung cancer	223·57 (138·58 to 320·46)	279·72 (176·22 to 394·23)	25·11 (20·65 to 29·89)*	–3·86 (−7·22 to −0·26)*	5144·29 (3212·18 to 7331·40)	6200·23 (3930·38 to 8667·86)	20·53 (15·80 to 25·30)*	–6·26 (−9·86 to −2·55)*
··	Ischaemic heart disease	1291·11 (1080·95 to 1483·53)	1576·10 (1329·73 to 1802·54)	22·07 (18·51 to 25·96)*	–7·08 (−9·39 to −4·51)*	29 520·10 (25 239·88 to 33 875·88)	34 934·16 (29 929·72 to 40 054·61)	18·34 (14·80 to 21·99)*	–7·14 (−9·65 to −4·51)*
··	Ischaemic stroke	309·39 (245·84 to 383·15)	348·33 (280·51 to 427·60)	12·59 (8·45 to 17·60)*	–15·31 (−17·89 to −12·61)*	6437·02 (5283·80 to 7652·84)	7386·59 (6061·34 to 8749·55)	14·75 (10·75 to 19·29)*	–11·99 (−14·73 to −9·11)*
··	Haemorrhagic stroke	435·48 (366·35 to 511·88)	448·19 (377·96 to 523·91)	2·92 (0·02 to 6·19)*	–20·85 (−22·67 to −18·80)*	11 173·69 (9404·22 to 13 008·51)	11 480·35 (9697·30 to 13 306·88)	2·74 (−0·09 to 5·90)	–19·12 (−21·06 to −16·92)*
··	Chronic obstructive pulmonary disease	738·38 (436·10 to 1068·58)	786·94 (470·94 to 1144·45)	6·58 (2·97 to 11·35)*	–20·29 (−22·92 to −16·77)*	15 614·77 (9275·94 to 22 808·67)	17 155·66 (10 435·61 to 24 906·98)	9·87 (6·63 to 14·26)*	–15·81 (−18·23 to −12·39)*
3	Household air pollution from solid fuels: all causes	3260·73 (2828·54 to 3717·84)	2576·36 (2215·95 to 2968·89)	–20·99 (−23·97 to −18·17)*	–37·55 (−39·90 to −35·29)*	108 733·32 (93 447·82 to 123 249·34)	77 161·35 (66 086·37 to 88 048·87)	–29·04 (−32·28 to −25·64)*	–39·54 (−42·12 to −36·98)*
··	Lower respiratory infections	883·96 (676·61 to 1,091·63)	626·13 (474·40 to 784·88)	–29·17 (−34·70 to −24·35)*	–37·62 (−42·19 to −33·56)*	52 410·01 (39 779·96 to 64 065·04)	30 860·63 (23 269·88 to 38 522·92)	–41·12 (−46·18 to −36·08)*	–44·24 (−48·85 to −39·52)*
··	Tracheal, bronchus, and lung cancer	189·07 (129·80 to 251·56)	158·38 (104·77 to 215·35)	–16·23 (−21·84 to −10·64)*	–35·50 (−39·78 to −31·18)*	4518·13 (3103·06 to 5979·21)	3664·00 (2429·30 to 4955·47)	–18·90 (−24·24 to −13·66)*	–36·75 (−40·93 to −32·60)*
··	Ischaemic heart disease	813·36 (710·55 to 942·98)	738·11 (636·96 to 862·96)	–9·25 (−12·66 to −5·82)*	–30·16 (−32·76 to −27·49)*	20 235·31 (17 542·11 to 23 458·32)	17 906·39 (15 397·09 to 20 977·14)	–11·51 (−15·14 to −8·13)*	–30·11 (−32·73 to −27·46)*
··	Ischaemic stroke	229·91 (190·08 to 274·53)	186·00 (152·23 to 223·85)	–19·10 (−22·59 to −15·45)*	–38·70 (−41·31 to −36·03)*	5044·48 (4209·50 to 5962·85)	4157·95 (3400·32 to 4956·92)	–17·57 (−21·21 to −14·05)*	–36·61 (−39·32 to −33·88)*
··	Haemorrhagic stroke	392·09 (333·05 to 457·68)	289·08 (242·86 to 341·24)	–26·27 (−29·22 to −23·19)*	–43·24 (−45·50 to −40·97)*	10 360·06 (8799·85 to 12 047·27)	7733·95 (6482·40 to 9074·27)	–25·35 (−28·20 to −22·44)*	–41·23 (−43·47 to −38·95)*
··	Chronic obstructive pulmonary disease	752·34 (505·06 to 1102·38)	578·68 (372·08 to 886·32)	–23·08 (−28·16 to −17·61)*	–42·31 (−46·13 to −38·28)*	15 181·60 (10 127·03 to 22 826·12)	11 804·50 (7559·95 to 18 339·49)	–22·24 (−27·19 to −16·95)*	–40·36 (−44·18 to −36·26)*
··	Cataract	··	··	··	··	983·74 (689·54 to 1354·17)	1033·93 (713·92 to 1415·58)	5·10 (2·29 to 7·74)*	–19·66 (−21·81 to −17·54)*
3	Ambient ozone pollution: all causes	187·61 (71·39 to 318·15)	233·64 (90·11 to 385·30)	24·53 (20·20 to 30·74)*	–6·98 (−10·15 to −2·37)*	3159·44 (1197·45 to 5338·23)	3796·83 (1463·89 to 6257·23)	20·17 (15·72 to 26·93)*	–7·97 (−11·32 to −2·93)*
··	Chronic obstructive pulmonary disease	187·61 (71·39 to 318·15)	233·64 (90·11 to 385·30)	24·53 (20·20 to 30·74)*	–6·98 (−10·15 to −2·37)*	3159·44 (1197·45 to 5338·23)	3796·83 (1463·89 to 6257·23)	20·17 (15·72 to 26·93)*	–7·97 (−11·32 to −2·93)*
**2**	**Other environmental risks: all causes**	**518·27 (290·36 to 800·27)**	**597·74 (328·83 to 923·47)**	15·33 (11·40 to 19·50)*	–12·38 (−14·88 to −9·65)*	**14 319·52 (8496·18 to 21 426·17)**	**15 128·92 (8891·77 to 22 939·09)**	5·65 (2·11 to 8·68)*	–15·31 (−17·72 to −13·41)*
3	Residential radon: all causes	49·87 (33·90 to 66·96)	57·69 (38·12 to 77·92)	15·68 (9·62 to 21·68)*	–11·27 (−15·06 to −7·72)*	1126·44 (773·82 to 1494·91)	1255·37 (847·29 to 1677·75)	11·45 (5·90 to 16·90)*	–13·56 (−17·10 to −10·13)*
··	Tracheal, bronchus, and lung cancer	49·87 (33·90 to 66·96)	57·69 (38·12 to 77·92)	15·68 (9·62 to 21·68)*	–11·27 (−15·06 to −7·72)*	1126·44 (773·82 to 1494·91)	1255·37 (847·29 to 1677·75)	11·45 (5·90 to 16·90)*	–13·56 (−17·10 to −10·13)*
3	Lead exposure: all causes	468·39 (239·69 to 749·97)	540·04 (269·07 to 868·97)	15·30 (10·68 to 19·90)*	–12·50 (−15·42 to −9·62)*	13 193·09 (7393·18 to 20 140·03)	13 873·55 (7578·92 to 21 565·04)	5·16 (1·14 to 8·25)*	–15·47 (−18·17 to −13·45)*
··	Rheumatic heart disease	3·54 (0·91 to 8·13)	3·05 (0·68 to 7·44)	–13·92 (−31·65 to 3·67)	–31·70 (−45·03 to −20·41)*	108·53 (26·02 to 246·12)	81·79 (17·81 to 203·32)	–24·64 (−43·10 to −10·85)*	–38·40 (−52·34 to −28·96)*
··	Ischaemic heart disease	227·95 (111·53 to 383·20)	276·33 (133·04 to 465·14)	21·23 (15·40 to 26·04)*	–8·33 (−11·26 to −5·43)*	4760·44 (2328·25 to 7983·16)	5298·38 (2534·71 to 8932·87)	11·30 (6·67 to 14·95)*	–13·26 (−16·77 to −10·72)*
··	Ischaemic stroke	60·00 (28·38 to 104·80)	66·73 (30·98 to 116·68)	11·21 (5·97 to 16·91)*	–15·93 (−19·04 to −13·00)*	1285·87 (621·62 to 2199·93)	1412·53 (664·14 to 2446·24)	9·85 (4·23 to 13·89)*	–15·71 (−19·81 to −12·81)*
··	Haemorrhagic stroke	98·90 (44·03 to 168·33)	95·67 (41·01 to 165·67)	–3·26 (−9·56 to 0·71)	–25·84 (−30·31 to −23·22)*	2371·40 (1022·88 to 4002·19)	2183·12 (881·23 to 3800·89)	–7·94 (−14·84 to −3·76)*	–28·02 (−33·48 to −24·76)*
··	Hypertensive heart disease	43·68 (11·57 to 104·93)	56·00 (12·63 to 140·58)	28·21 (5·57 to 44·11)*	–4·14 (−19·03 to 6·47)	868·54 (309·66 to 1899·80)	992·22 (318·80 to 2259·29)	14·24 (−2·90 to 27·83)	–11·18 (−24·36 to −1·13)*
··	Other cardiomyopathy	1·48 (0·41 to 3·27)	1·54 (0·38 to 3·49)	3·73 (−14·57 to 16·87)	–20·52 (−32·44 to −11·60)*	35·62 (9·50 to 79·57)	33·18 (7·77 to 79·05)	–6·84 (−23·21 to 4·21)	–25·95 (−38·79 to −17·48)*
··	Atrial fibrillation and flutter	1·76 (0·65 to 3·49)	2·45 (0·88 to 4·91)	39·22 (29·86 to 46·04)*	0·69 (−2·60 to 4·79)	69·13 (28·74 to 129·13)	83·64 (32·94 to 159·75)	20·99 (12·73 to 25·56)*	–6·93 (−12·49 to −4·15)*
··	Aortic aneurysm	1·52 (0·54 to 2·93)	1·63 (0·55 to 3·25)	7·18 (−2·91 to 14·30)	–17·85 (−24·53 to −13·48)*	32·04 (11·44 to 61·16)	32·04 (10·70 to 62·83)	0·01 (−9·81 to 6·58)	–21·90 (−29·35 to −16·99)*
··	Peripheral vascular disease	0·16 (0·03 to 0·41)	0·20 (0·03 to 0·53)	19·84 (−2·64 to 35·45)	–11·54 (−24·29 to −3·03)*	5·60 (1·52 to 12·98)	6·34 (1·62 to 15·16)	13·07 (2·26 to 20·57)*	–13·53 (−21·26 to −9·06)*
··	Endocarditis	0·83 (0·29 to 1·74)	0·97 (0·32 to 2·09)	16·60 (4·99 to 26·41)*	–9·45 (−17·23 to −4·25)*	20·69 (6·81 to 43·39)	21·53 (6·70 to 47·48)	4·04 (−6·57 to 13·03)	–16·42 (−25·07 to −10·13)*
··	Other cardiovascular and circulatory diseases	5·41 (1·95 to 10·17)	5·94 (1·95 to 11·38)	9·71 (−0·49 to 16·74)	–16·12 (−23·01 to −11·62)*	152·85 (52·63 to 310·64)	153·78 (48·05 to 324·49)	0·60 (−9·13 to 6·44)	–20·84 (−28·73 to −16·05)*
··	Idiopathic developmental intellectual disability	··	··	··	··	2916·48 (1228·14 to 5089·94)	2920·47 (1234·48 to 5155·20)	0·14 (−3·18 to 2·41)	–8·99 (−12·03 to −6·90)*
··	Chronic kidney disease due to diabetes mellitus	10·10 (4·15 to 18·35)	12·65 (5·09 to 23·20)	25·28 (19·04 to 29·44)*	–4·60 (−8·89 to −1·68)*	260·59 (102·04 to 495·91)	302·16 (113·45 to 584·15)	15·96 (9·37 to 20·09)*	–10·04 (−15·36 to −6·87)*
··	Chronic kidney disease due to hypertension	6·22 (2·72 to 11·32)	8·27 (3·62 to 15·14)	33·02 (27·46 to 37·34)*	–2·02 (−5·80 to 0·78)	128·17 (54·29 to 242·87)	155·62 (64·33 to 297·32)	21·42 (15·72 to 25·19)*	–6·97 (−11·22 to −4·21)*
··	Chronic kidney disease due to glomerulonephritis	2·42 (0·93 to 4·54)	2·92 (1·08 to 5·50)	20·90 (15·70 to 26·39)*	–7·48 (−11·06 to −4·04)*	65·59 (21·46 to 133·89)	70·02 (22·73 to 143·93)	6·76 (0·61 to 11·59)*	–15·00 (−20·26 to −11·29)*
··	Chronic kidney disease due to other causes	4·42 (1·88 to 8·20)	5·69 (2·39 to 10·62)	28·68 (22·76 to 34·24)*	–2·47 (−6·62 to 1·13)	111·53 (43·93 to 213·01)	126·74 (48·88 to 248·43)	13·63 (7·40 to 18·08)*	–10·64 (−15·64 to −6·92)*
**2**	**Occupational risks: all causes**	**1409·60 (1288·25 to 1539·63)**	**1528·02 (1383·55 to 1680·97)**	8·40 (6·20 to 10·41)*	–14·80 (−16·48 to −13·37)*	**68 543·89 (60 461·38 to 77 147·09)**	**75 925·43 (66 060·97 to 86 257·10)**	10·77 (8·84 to 12·62)*	–8·98 (−10·61 to −7·49)*
3	Occupational carcinogens: all causes	628·39 (529·77 to 733·38)	746·54 (624·13 to 874·38)	18·80 (16·21 to 21·35)*	–8·62 (−10·42 to −6·83)*	17 462·68 (14 595·36 to 20 617·18)	20 682·73 (17 015·37 to 24 682·77)	18·44 (15·67 to 21·04)*	–7·56 (−9·50 to −5·67)*
4	Occupational exposure to asbestos: all causes	187·83 (142·94 to 233·46)	222·32 (168·96 to 277·92)	18·36 (15·32 to 21·47)*	–10·30 (−12·67 to −7·98)*	3197·37 (2410·48 to 4019·53)	3640·71 (2743·34 to 4594·60)	13·87 (11·05 to 16·82)*	–12·65 (−14·84 to −10·38)*
··	Larynx cancer	3·25 (1·80 to 4·82)	3·74 (2·02 to 5·53)	15·08 (11·70 to 18·64)*	–13·01 (−15·58 to −10·35)*	59·03 (32·22 to 89·00)	65·51 (35·04 to 99·12)	10·97 (7·31 to 14·61)*	–15·26 (−18·03 to −12·51)*
··	Tracheal, bronchus, and lung cancer	155·24 (111·10 to 201·47)	181·45 (128·29 to 236·62)	16·88 (13·29 to 20·48)*	–11·40 (−14·19 to −8·74)*	2539·55 (1770·09 to 3359·44)	2844·28 (1957·87 to 3803·22)	12·00 (8·53 to 15·59)*	–14·15 (−16·73 to −11·42)*
··	Ovarian cancer	5·16 (2·58 to 7·94)	6·02 (2·98 to 9·40)	16·73 (9·65 to 23·13)*	–13·33 (−18·64 to −8·71)*	82·25 (40·54 to 128·84)	93·12 (45·80 to 149·95)	13·21 (5·49 to 20·04)*	–13·97 (−19·78 to −8·87)*
··	Mesothelioma	21·29 (20·16 to 22·57)	27·61 (25·56 to 29·34)	29·68 (23·73 to 34·79)*	–1·06 (−5·59 to 2·90)	443·53 (413·23 to 481·26)	553·97 (507·29 to 597·78)	24·90 (19·28 to 29·80)*	–3·39 (−7·70 to 0·41)
··	Asbestosis	2·89 (1·92 to 3·56)	3·49 (2·43 to 4·06)	21·00 (13·33 to 30·87)*	–7·91 (−13·67 to −0·40)*	73·00 (57·24 to 86·90)	83·83 (67·86 to 97·43)	14·83 (9·18 to 21·83)*	–9·23 (−13·68 to −3·33)*
4	Occupational exposure to arsenic: all causes	6·55 (1·52 to 11·97)	8·07 (2·05 to 14·63)	23·27 (18·26 to 35·03)*	–5·27 (−9·14 to 4·00)	182·17 (43·97 to 330·51)	219·22 (57·76 to 395·48)	20·34 (15·23 to 31·54)*	–6·95 (−10·91 to 2·16)
··	Tracheal, bronchus, and lung cancer	6·55 (1·52 to 11·97)	8·07 (2·05 to 14·63)	23·27 (18·26 to 35·03)*	–5·27 (−9·14 to 4·00)	182·17 (43·97 to 330·51)	219·22 (57·76 to 395·48)	20·34 (15·23 to 31·54)*	–6·95 (−10·91 to 2·16)
4	Occupational exposure to benzene: all causes	1·63 (0·52 to 2·67)	1·90 (0·60 to 3·12)	16·21 (11·28 to 21·54)*	–1·47 (−6·03 to 3·50)	74·24 (23·12 to 121·81)	83·87 (25·51 to 138·49)	12·97 (8·01 to 18·09)*	–2·29 (−6·92 to 2·56)
··	Leukaemia	1·63 (0·52 to 2·67)	1·90 (0·60 to 3·12)	16·21 (11·28 to 21·54)*	–1·47 (−6·03 to 3·50)	74·24 (23·12 to 121·81)	83·87 (25·51 to 138·49)	12·97 (8·01 to 18·09)*	–2·29 (−6·92 to 2·56)
··	Acute lymphoid leukaemia	0·28 (0·09 to 0·46)	0·37 (0·11 to 0·62)	32·70 (19·53 to 41·21)*	14·39 (3·02 to 21·87)*	13·95 (4·29 to 22·85)	18·08 (5·39 to 29·97)	29·59 (16·43 to 37·84)*	13·73 (2·19 to 21·17)*
··	Chronic lymphoid leukaemia	0·09 (0·03 to 0·15)	0·11 (0·04 to 0·19)	24·21 (16·84 to 35·39)*	0·43 (−6·33 to 10·47)	3·30 (1·12 to 5·43)	4·07 (1·35 to 6·69)	23·32 (15·50 to 33·93)*	1·98 (−5·31 to 11·94)
··	Acute myeloid leukaemia	0·41 (0·14 to 0·67)	0·54 (0·18 to 0·90)	32·16 (24·33 to 41·56)*	11·07 (3·73 to 20·10)*	17·90 (6·04 to 29·37)	23·24 (7·47 to 38·33)	29·83 (21·43 to 39·32)*	11·51 (3·58 to 20·44)*
··	Chronic myeloid leukaemia	0·15 (0·05 to 0·24)	0·15 (0·05 to 0·25)	4·79 (−3·59 to 14·00)	–11·79 (−18·96 to −3·60)*	6·60 (2·12 to 10·80)	6·86 (2·14 to 11·39)	4·04 (−4·07 to 13·79)	–10·84 (−18·16 to −2·42)*
··	Other leukaemia	0·70 (0·21 to 1·16)	0·71 (0·21 to 1·19)	1·61 (−4·21 to 7·56)	–13·30 (−18·31 to −8·34)*	32·49 (9·76 to 53·35)	31·62 (9·61 to 52·92)	–2·70 (−8·57 to 3·32)	–15·49 (−20·60 to −10·44)*
4	Occupational exposure to beryllium: all causes	0·20 (0·17 to 0·24)	0·26 (0·21 to 0·31)	28·93 (22·38 to 34·98)*	–0·80 (−4·98 to 2·96)	5·76 (4·76 to 6·81)	7·22 (5·89 to 8·59)	25·48 (18·89 to 31·61)*	–2·63 (−6·90 to 1·11)
··	Tracheal, bronchus, and lung cancer	0·20 (0·17 to 0·24)	0·26 (0·21 to 0·31)	28·93 (22·38 to 34·98)*	–0·80 (−4·98 to 2·96)	5·76 (4·76 to 6·81)	7·22 (5·89 to 8·59)	25·48 (18·89 to 31·61)*	–2·63 (−6·90 to 1·11)
4	Occupational exposure to cadmium: all causes	0·46 (0·39 to 0·53)	0·61 (0·50 to 0·71)	31·38 (24·62 to 37·82)*	1·00 (−3·45 to 5·10)	13·15 (11·14 to 15·16)	16·83 (14·14 to 19·64)	28·00 (21·21 to 34·47)*	–0·75 (−5·34 to 3·51)
··	Tracheal, bronchus, and lung cancer	0·46 (0·39 to 0·53)	0·61 (0·50 to 0·71)	31·38 (24·62 to 37·82)*	1·00 (−3·45 to 5·10)	13·15 (11·14 to 15·16)	16·83 (14·14 to 19·64)	28·00 (21·21 to 34·47)*	–0·75 (−5·34 to 3·51)
4	Occupational exposure to chromium: all causes	0·96 (0·86 to 1·07)	1·28 (1·13 to 1·44)	33·02 (27·40 to 38·50)*	2·28 (−1·68 to 6·05)	27·33 (24·34 to 30·24)	35·45 (31·40 to 40·17)	29·71 (24·03 to 35·28)*	0·57 (−3·49 to 4·55)
··	Tracheal, bronchus, and lung cancer	0·96 (0·86 to 1·07)	1·28 (1·13 to 1·44)	33·02 (27·40 to 38·50)*	2·28 (−1·68 to 6·05)	27·33 (24·34 to 30·24)	35·45 (31·40 to 40·17)	29·71 (24·03 to 35·28)*	0·57 (−3·49 to 4·55)
4	Occupational exposure to diesel engine exhaust: all causes	13·41 (11·85 to 15·17)	17·50 (15·20 to 20·06)	30·45 (24·63 to 35·78)*	0·26 (−3·89 to 3·78)	381·69 (337·43 to 428·72)	485·69 (426·18 to 553·93)	27·25 (21·26 to 32·75)*	–1·40 (−5·65 to 2·19)
··	Tracheal, bronchus, and lung cancer	13·41 (11·85 to 15·17)	17·50 (15·20 to 20·06)	30·45 (24·63 to 35·78)*	0·26 (−3·89 to 3·78)	381·69 (337·43 to 428·72)	485·69 (426·18 to 553·93)	27·25 (21·26 to 32·75)*	–1·40 (−5·65 to 2·19)
4	Occupational exposure to second-hand smoke: all causes	364·05 (275·49 to 465·66)	433·15 (326·16 to 554·32)	18·98 (15·73 to 22·42)*	–7·67 (−9·82 to −5·44)*	12 060·36 (9008·45 to 15 202·22)	14 474·34 (10 754·05 to 18 289·00)	20·02 (16·70 to 23·11)*	–5·73 (−7·98 to −3·57)*
··	Lower respiratory infections	25·22 (11·95 to 41·26)	31·03 (14·71 to 51·31)	23·07 (18·77 to 27·30)*	–3·05 (−6·52 to 0·24)	754·30 (355·32 to 1235·87)	901·83 (424·90 to 1491·75)	19·56 (15·20 to 24·09)*	–4·55 (−7·93 to −1·02)*
··	Otitis media	0·00 (0·00 to 0·00)	0·00 (0·00 to 0·00)	–51·34 (−68·17 to −26·94)*	–53·19 (−69·51 to −29·77)*	0·00 (0·00 to 0·00)	0·00 (0·00 to 0·00)	–0·26 (−3·00 to 2·10)	–4·95 (−7·62 to −2·74)*
··	Tracheal, bronchus, and lung cancer	36·79 (17·19 to 62·63)	44·38 (20·66 to 75·46)	20·63 (16·93 to 23·85)*	–7·23 (−10·03 to −4·78)*	1009·34 (472·19 to 1717·66)	1185·42 (551·75 to 2013·66)	17·45 (13·68 to 20·74)*	–9·21 (−12·08 to −6·69)*
··	Breast cancer	3·93 (0·93 to 6·85)	4·86 (1·19 to 8·40)	23·68 (16·23 to 31·66)*	–3·23 (−9·07 to 2·90)	131·38 (30·86 to 228·23)	160·49 (39·88 to 276·83)	22·16 (14·48 to 30·64)*	–3·10 (−9·13 to 3·41)
··	Ischaemic heart disease	145·11 (108·16 to 184·75)	177·23 (131·12 to 226·23)	22·13 (16·84 to 27·55)*	–4·86 (−7·90 to −1·79)*	4427·58 (3270·63 to 5659·16)	5337·92 (3904·37 to 6856·49)	20·56 (15·58 to 25·71)*	–4·76 (−7·77 to −1·66)*
··	Ischaemic stroke	24·76 (17·40 to 32·82)	28·32 (19·67 to 38·37)	14·40 (7·34 to 21·60)*	–12·26 (−16·32 to −8·12)*	749·82 (529·06 to 995·24)	892·52 (616·96 to 1211·73)	19·03 (12·07 to 26·19)*	–8·13 (−12·07 to −4·39)*
··	Haemorrhagic stroke	52·38 (37·29 to 69·30)	56·78 (39·66 to 75·11)	8·39 (3·67 to 13·19)*	–15·19 (−17·69 to −12·61)*	1679·51 (1187·37 to 2237·60)	1799·87 (1247·86 to 2400·70)	7·17 (2·54 to 11·92)*	–14·80 (−17·22 to −12·28)*
··	Chronic obstructive pulmonary disease	48·15 (22·29 to 85·80)	51·90 (23·79 to 91·43)	7·78 (4·30 to 11·52)*	–17·01 (−19·72 to −14·14)*	1570·14 (727·09 to 2820·77)	1819·99 (831·20 to 3260·92)	15·91 (12·59 to 19·00)*	–10·63 (−13·17 to −8·26)*
··	Diabetes mellitus	27·71 (10·20 to 43·82)	38·64 (14·31 to 60·82)	39·45 (37·04 to 42·10)*	7·38 (5·52 to 9·33)*	1738·30 (616·68 to 2847·07)	2376·30 (847·55 to 3851·52)	36·70 (34·59 to 39·02)*	7·55 (5·95 to 9·37)*
4	Occupational exposure to formaldehyde: all causes	0·95 (0·78 to 1·15)	1·09 (0·90 to 1·32)	13·89 (5·40 to 22·94)*	–4·03 (−10·29 to 2·45)	42·70 (35·09 to 51·51)	46·93 (38·81 to 56·99)	9·90 (1·78 to 18·05)*	–5·67 (−12·09 to 0·77)
··	Nasopharynx cancer	0·42 (0·29 to 0·58)	0·48 (0·33 to 0·68)	13·08 (−1·13 to 29·19)	–6·37 (−16·69 to 4·83)	17·57 (11·88 to 24·10)	19·02 (12·99 to 27·09)	8·25 (−5·73 to 24·38)	–8·78 (−19·03 to 3·13)
··	Acute lymphoid leukaemia	0·09 (0·08 to 0·11)	0·13 (0·11 to 0·15)	34·88 (18·69 to 42·16)*	16·60 (3·46 to 22·11)*	4·72 (3·86 to 5·83)	6·21 (5·02 to 7·60)	31·43 (16·61 to 38·79)*	15·52 (2·59 to 21·35)*
··	Chronic lymphoid leukaemia	0·02 (0·02 to 0·03)	0·03 (0·03 to 0·04)	30·39 (20·80 to 38·61)*	7·25 (0·20 to 13·50)*	0·95 (0·79 to 1·17)	1·22 (1·02 to 1·44)	27·96 (16·27 to 37·10)*	7·86 (−0·97 to 15·12)
··	Acute myeloid leukaemia	0·11 (0·09 to 0·13)	0·15 (0·12 to 0·18)	35·38 (28·55 to 41·33)*	15·11 (9·74 to 19·69)*	5·06 (4·14 to 6·15)	6·70 (5·54 to 8·16)	32·57 (25·83 to 39·02)*	14·91 (9·32 to 20·00)*
··	Chronic myeloid leukaemia	0·04 (0·04 to 0·05)	0·05 (0·04 to 0·06)	6·23 (0·31 to 13·49)*	–9·89 (−14·91 to −4·16)*	2·05 (1·66 to 2·53)	2·15 (1·73 to 2·65)	4·77 (−1·61 to 12·57)	–9·72 (−15·13 to −3·27)*
··	Other leukaemia	0·26 (0·21 to 0·31)	0·26 (0·21 to 0·31)	–1·61 (−9·46 to 6·91)	–15·64 (−21·92 to −9·05)*	12·35 (9·66 to 14·91)	11·64 (9·27 to 14·19)	–5·80 (−13·73 to 2·60)	–17·95 (−24·52 to −11·12)*
4	Occupational exposure to nickel: all causes	6·68 (0·95 to 17·47)	8·10 (1·24 to 20·81)	21·35 (15·63 to 32·69)*	–6·73 (−11·14 to 2·03)	187·01 (27·49 to 483·87)	221·35 (34·93 to 563·34)	18·37 (12·66 to 29·20)*	–8·40 (−12·92 to 0·27)
··	Tracheal, bronchus, and lung cancer	6·68 (0·95 to 17·47)	8·10 (1·24 to 20·81)	21·35 (15·63 to 32·69)*	–6·73 (−11·14 to 2·03)	187·01 (27·49 to 483·87)	221·35 (34·93 to 563·34)	18·37 (12·66 to 29·20)*	–8·40 (−12·92 to 0·27)
4	Occupational exposure to polycyclic aromatic hydrocarbons: all causes	3·41 (2·89 to 3·92)	4·53 (3·83 to 5·29)	32·92 (26·40 to 39·18)*	2·21 (−2·06 to 6·07)	97·03 (82·37 to 111·83)	125·78 (105·37 to 145·87)	29·63 (22·78 to 35·89)*	0·51 (−4·05 to 4·55)
··	Tracheal, bronchus, and lung cancer	3·41 (2·89 to 3·92)	4·53 (3·83 to 5·29)	32·92 (26·40 to 39·18)*	2·21 (−2·06 to 6·07)	97·03 (82·37 to 111·83)	125·78 (105·37 to 145·87)	29·63 (22·78 to 35·89)*	0·51 (−4·05 to 4·55)
4	Occupational exposure to silica: all causes	50·95 (28·57 to 73·67)	58·40 (31·42 to 86·00)	14·63 (8·41 to 19·51)*	–12·09 (−16·90 to −8·34)*	1396·95 (774·36 to 2030·14)	1574·57 (860·21 to 2314·76)	12·71 (6·89 to 17·49)*	–12·64 (−17·10 to −8·94)*
··	Tracheal, bronchus, and lung cancer	40·38 (17·91 to 63·22)	48·00 (21·24 to 75·45)	18·88 (13·37 to 24·69)*	–8·64 (−12·83 to −4·21)*	1123·77 (503·32 to 1756·69)	1303·95 (576·29 to 2042·00)	16·03 (10·58 to 21·66)*	–10·26 (−14·61 to −5·78)*
··	Silicosis	10·57 (9·77 to 12·23)	10·40 (9·57 to 11·68)	–1·60 (−14·72 to 5·54)	–24·35 (−34·05 to −18·99)*	273·19 (247·13 to 310·72)	270·62 (243·58 to 301·41)	–0·94 (−14·17 to 5·65)	–22·15 (−32·16 to −17·05)*
4	Occupational exposure to sulfuric acid: all causes	2·96 (1·27 to 5·35)	3·54 (1·52 to 6·49)	19·47 (13·15 to 26·36)*	–8·14 (−12·96 to −2·92)*	89·85 (38·68 to 161·94)	105·23 (45·84 to 192·42)	17·12 (10·83 to 23·79)*	–9·04 (−13·97 to −3·84)*
··	Larynx cancer	2·96 (1·27 to 5·35)	3·54 (1·52 to 6·49)	19·47 (13·15 to 26·36)*	–8·14 (−12·96 to −2·92)*	89·85 (38·68 to 161·94)	105·23 (45·84 to 192·42)	17·12 (10·83 to 23·79)*	–9·04 (−13·97 to −3·84)*
4	Occupational exposure to trichloroethylene: all causes	0·04 (0·01 to 0·07)	0·06 (0·01 to 0·11)	48·91 (43·08 to 53·27)*	14·75 (10·28 to 18·08)*	1·17 (0·26 to 2·16)	1·72 (0·38 to 3·23)	47·21 (41·37 to 51·60)*	14·65 (10·09 to 17·96)*
··	Kidney cancer	0·04 (0·01 to 0·07)	0·06 (0·01 to 0·11)	48·91 (43·08 to 53·27)*	14·75 (10·28 to 18·08)*	1·17 (0·26 to 2·16)	1·72 (0·38 to 3·23)	47·21 (41·37 to 51·60)*	14·65 (10·09 to 17·96)*
3	Occupational asthmagens: all causes	36·83 (26·75 to 47·73)	37·57 (28·36 to 47·94)	2·02 (−6·22 to 10·50)	–19·40 (−25·79 to −12·65)*	2122·64 (1699·18 to 2619·54)	2339·48 (1860·90 to 2923·32)	10·22 (4·21 to 15·66)*	–8·91 (−14·66 to −3·71)*
··	Asthma	36·83 (26·75 to 47·73)	37·57 (28·36 to 47·94)	2·02 (−6·22 to 10·50)	–19·40 (−25·79 to −12·65)*	2122·64 (1699·18 to 2619·54)	2339·48 (1860·90 to 2923·32)	10·22 (4·21 to 15·66)*	–8·91 (−14·66 to −3·71)*
3	Occupational particulate matter, gases, and fumes: all causes	407·53 (338·66 to 479·12)	424·27 (349·98 to 507·55)	4·11 (−0·16 to 8·15)	–21·37 (−23·97 to −18·61)*	8771·11 (7497·47 to 10 068·75)	9377·10 (7972·61 to 10 789·56)	6·91 (3·78 to 10·55)*	–17·84 (−19·96 to −15·42)*
··	Chronic obstructive pulmonary disease	399·93 (331·13 to 472·15)	416·68 (342·87 to 499·76)	4·19 (−0·04 to 8·27)	–21·32 (−23·94 to −18·63)*	8557·06 (7276·89 to 9859·70)	9154·55 (7771·09 to 10 539·37)	6·98 (3·85 to 10·70)*	–17·82 (−19·97 to −15·30)*
··	Coal workers pneumoconiosis	3·03 (1·91 to 3·49)	2·68 (1·79 to 3·07)	–11·29 (−19·54 to 0·39)	–32·63 (−38·84 to −23·91)*	87·45 (65·52 to 104·62)	89·05 (70·16 to 108·86)	1·83 (−6·81 to 12·73)	–21·65 (−28·21 to −13·50)*
··	Other pneumoconiosis	4·57 (3·71 to 6·32)	4·91 (4·16 to 6·56)	7·27 (−1·26 to 15·86)	–17·49 (−24·05 to −11·05)*	126·59 (104·34 to 161·76)	133·51 (112·07 to 165·88)	5·46 (−2·23 to 13·23)	–16·68 (−22·73 to −10·63)*
3	Occupational noise: all causes	··	··	··	··	5865·39 (4107·31 to 8092·94)	7108·28 (4978·56 to 9802·69)	21·19 (19·01 to 22·96)*	–0·74 (−2·21 to 0·56)
··	Age-related and other hearing loss	··	··	··	··	5865·39 (4107·31 to 8092·94)	7108·28 (4978·56 to 9802·69)	21·19 (19·01 to 22·96)*	–0·74 (−2·21 to 0·56)
3	Occupational injuries: all causes	352·96 (344·63 to 360·98)	335·71 (328·64 to 343·27)	–4·89 (−7·71 to −1·89)*	–17·78 (−20·22 to −15·20)*	21 906·21 (20 353·14 to 23 776·95)	21 774·60 (19 810·66 to 24 090·16)	–0·60 (−4·19 to 2·98)	–12·95 (−15·95 to −9·94)*
··	Pedestrian road injuries	67·01 (62·03 to 73·85)	63·97 (59·35 to 69·72)	–4·53 (−10·63 to 0·01)	–18·09 (−23·28 to −14·24)*	3434·81 (3182·07 to 3771·04)	3278·98 (3037·91 to 3549·98)	–4·54 (−10·46 to −0·04)*	–16·52 (−21·66 to −12·63)*
··	Cyclist road injuries	10·32 (9·27 to 11·62)	9·99 (8·96 to 11·51)	–3·16 (−8·97 to 5·60)	–17·77 (−22·81 to −10·17)*	673·49 (580·35 to 787·54)	707·70 (596·26 to 850·17)	5·08 (−0·72 to 11·51)	–9·29 (−14·16 to −3·79)*
··	Motorcyclist road injuries	44·53 (40·17 to 49·15)	42·56 (38·79 to 46·82)	–4·41 (−9·93 to 0·93)	–15·65 (−20·46 to −10·89)*	2623·88 (2388·56 to 2894·57)	2549·86 (2330·54 to 2817·91)	–2·82 (−8·29 to 2·44)	–13·57 (−18·33 to −9·00)*
··	Motor vehicle road injuries	74·30 (65·78 to 85·66)	73·17 (67·36 to 83·11)	–1·51 (−6·37 to 7·20)	–13·94 (−18·25 to −6·31)*	4091·59 (3653·03 to 4667·92)	4058·76 (3712·02 to 4590·12)	–0·80 (−5·44 to 7·38)	–12·17 (−16·25 to −4·94)*
··	Other road injuries	1·98 (1·74 to 2·43)	1·88 (1·68 to 2·28)	–5·05 (−12·93 to 5·85)	–18·33 (−25·07 to −8·69)*	167·13 (137·60 to 207·71)	198·25 (158·96 to 254·29)	18·62 (9·95 to 27·66)*	2·77 (−4·57 to 10·39)
··	Other transport injuries	14·25 (12·59 to 15·77)	13·71 (12·58 to 14·97)	–3·74 (−11·07 to 5·18)	–16·70 (−22·82 to −8·99)*	970·91 (855·69 to 1109·73)	969·10 (847·30 to 1119·40)	–0·19 (−6·23 to 6·80)	–12·66 (−17·77 to −6·62)*
··	Falls	38·58 (34·48 to 40·43)	39·52 (36·06 to 41·41)	2·42 (−3·75 to 8·20)	–14·40 (−19·49 to −9·55)*	3253·95 (2718·82 to 3890·24)	3637·49 (3004·39 to 4424·49)	11·79 (6·86 to 16·48)*	–4·69 (−8·77 to −0·73)*
··	Drowning	29·91 (28·60 to 31·52)	26·74 (25·32 to 28·13)	–10·60 (−14·16 to −6·83)*	–21·41 (−24·53 to −18·10)*	1558·83 (1491·01 to 1643·06)	1365·42 (1294·39 to 1433·95)	–12·41 (−15·98 to −8·51)*	–21·39 (−24·55 to −17·92)*
··	Fire, heat, and hot substances	10·40 (9·05 to 11·29)	9·42 (8·02 to 10·46)	–9·40 (−14·52 to −4·17)*	–22·76 (−27·12 to −18·35)*	749·03 (646·32 to 879·73)	758·15 (626·89 to 922·03)	1·22 (−4·90 to 6·74)	–11·99 (−17·27 to −7·18)*
··	Poisonings	6·69 (5·21 to 7·55)	5·85 (4·37 to 6·54)	–12·57 (−23·95 to 3·86)	–24·93 (−34·60 to −11·05)*	351·08 (280·67 to 395·21)	313·70 (244·88 to 347·77)	–10·65 (−20·53 to 3·82)	–21·69 (−30·25 to −9·04)*
··	Unintentional firearm injuries	4·19 (3·23 to 4·60)	3·83 (2·80 to 4·20)	–8·61 (−17·66 to 0·01)	–19·38 (−27·38 to −11·76)*	253·51 (198·88 to 282·79)	240·12 (181·74 to 271·14)	–5·28 (−13·02 to 2·95)	–15·61 (−22·69 to −8·27)*
··	Unintentional suffocation	0·77 (0·68 to 0·90)	0·92 (0·67 to 1·04)	19·08 (−7·56 to 34·67)	4·58 (−18·86 to 18·24)	69·06 (56·28 to 86·36)	80·23 (63·30 to 101·14)	16·18 (1·23 to 25·85)*	2·43 (−10·54 to 10·82)
··	Other exposure to mechanical forces	17·58 (13·83 to 18·79)	15·29 (11·75 to 16·30)	–13·04 (−17·93 to −8·65)*	–24·91 (−29·14 to −21·17)*	1292·58 (1072·42 to 1512·50)	1290·07 (1044·64 to 1570·19)	–0·19 (−5·99 to 5·81)	–13·26 (−18·20 to −8·29)*
··	Venomous animal contact	6·92 (6·26 to 7·56)	5·66 (5·21 to 6·27)	–18·20 (−25·20 to −6·78)*	–30·45 (−36·32 to −20·64)*	446·07 (390·64 to 500·68)	389·47 (338·03 to 449·59)	–12·69 (−19·31 to −3·42)*	–23·84 (−29·63 to −15·81)*
··	Non-venomous animal contact	1·47 (1·09 to 1·80)	1·32 (0·99 to 1·71)	–9·58 (−17·89 to 0·09)	–23·14 (−30·13 to −14·72)*	122·37 (93·44 to 157·70)	116·27 (88·44 to 149·33)	–4·98 (−11·38 to 1·81)	–17·63 (−22·98 to −11·96)*
··	Pulmonary aspiration and foreign body in airway	5·70 (5·08 to 6·60)	6·15 (5·50 to 7·31)	8·00 (0·69 to 17·21)*	–8·88 (−15·06 to −1·29)*	368·39 (314·09 to 433·78)	400·88 (341·30 to 484·16)	8·82 (2·49 to 15·63)*	–5·71 (−11·16 to 0·22)
··	Foreign body in other body part	1·03 (0·70 to 1·32)	1·05 (0·76 to 1·32)	1·96 (−7·01 to 15·08)	–11·39 (−19·07 to −0·51)*	123·60 (91·48 to 162·21)	136·41 (101·25 to 180·00)	10·37 (3·84 to 16·97)*	–3·80 (−9·33 to 1·81)
··	Other unintentional injuries	17·35 (15·52 to 18·15)	14·67 (12·87 to 15·41)	–15·45 (−19·85 to −11·30)*	–26·15 (−30·00 to −22·53)*	1355·94 (1171·12 to 1583·40)	1283·74 (1074·02 to 1552·79)	–5·32 (−10·48 to −0·38)*	–17·08 (−21·56 to −12·93)*
3	Occupational ergonomic factors: all causes	··	··	··	··	13 229·58 (9255·44 to 17 770·82)	15 479·93 (10 733·37 to 20 772·45)	17·01 (14·86 to 19·35)*	–1·74 (−3·26 to −0·45)*
··	Low back pain	··	··	··	··	13 229·58 (9255·44 to 17 770·82)	15 479·93 (10 733·37 to 20 772·45)	17·01 (14·86 to 19·35)*	–1·74 (−3·26 to −0·45)*
**1**	**Behavioural risks: all causes**	**22 393·17 (21 227·31 to 23 619·19)**	**21 830·19 (20 450·24 to 23 314·12)**	–2·51 (−4·89 to −0·13)*	–21·55 (−23·25 to −19·81)*	**910 996·12 (869 496·72 to 953 010·97)**	**781 103·69 (737 052·73 to 830 058·54)**	–14·26 (−16·59 to −11·83)*	–25·18 (−27·08 to −23·22)*
**2**	**Child and maternal malnutrition: all causes**	**4301·09 (4107·68 to 4499·13)**	**2736·96 (2573·81 to 2904·34)**	–36·37 (−39·81 to −32·52)*	–36·99 (−40·42 to −33·17)*	**406 715·03 (385 244·16 to 429 424·87)**	**275 068·98 (255 117·96 to 296 600·82)**	–32·37 (−36·04 to −28·67)*	–33·64 (−37·18 to −30·01)*
3	Suboptimal breastfeeding: all causes	278·09 (223·03 to 332·55)	152·48 (124·06 to 183·65)	–45·17 (−50·75 to −38·89)*	–45·59 (−51·13 to −39·40)*	24 214·14 (19 400·12 to 28 949·80)	13 373·25 (10 878·18 to 16 087·13)	–44·77 (−50·34 to −38·57)*	–45·20 (−50·73 to −39·07)*
4	Non-exclusive breastfeeding: all causes	264·19 (210·54 to 318·37)	144·11 (116·21 to 173·92)	–45·45 (−51·08 to −39·34)*	–45·79 (−51·39 to −39·70)*	22 971·14 (18 284·60 to 27 651·42)	12 598·41 (10 160·91 to 15 194·04)	–45·16 (−50·76 to −39·06)*	–45·49 (−51·07 to −39·43)*
··	Diarrhoeal diseases	169·62 (132·18 to 206·77)	88·76 (68·74 to 111·24)	–47·67 (−54·86 to −39·28)*	–48·02 (−55·16 to −39·68)*	14 810·81 (11 518·55 to 18 077·32)	7821·54 (6057·18 to 9801·86)	–47·19 (−54·37 to −38·91)*	–47·54 (−54·67 to −39·31)*
··	Lower respiratory infections	94·57 (62·35 to 130·16)	55·35 (35·96 to 75·66)	–41·47 (−46·49 to −35·45)*	–41·79 (−46·78 to −35·80)*	8160·33 (5381·55 to 11 233·53)	4776·87 (3103·20 to 6528·56)	–41·46 (−46·48 to −35·45)*	–41·78 (−46·77 to −35·80)*
4	Discontinued breastfeeding: all causes	16·70 (5·98 to 29·32)	10·04 (3·49 to 17·76)	–39·90 (−48·41 to −29·27)*	–41·77 (−50·01 to −31·40)*	1490·66 (534·34 to 2615·99)	924·29 (322·52 to 1634·92)	–37·99 (−46·40 to −27·87)*	–39·95 (−48·10 to −30·10)*
··	Diarrhoeal diseases	16·70 (5·98 to 29·32)	10·04 (3·49 to 17·76)	–39·90 (−48·41 to −29·27)*	–41·77 (−50·01 to −31·40)*	1490·66 (534·34 to 2615·99)	924·29 (322·52 to 1634·92)	–37·99 (−46·40 to −27·87)*	–39·95 (−48·10 to −30·10)*
3	Child growth failure: all causes	1874·90 (1718·60 to 2023·15)	1010·58 (908·98 to 1119·90)	–46·10 (−51·03 to −40·34)*	–47·58 (−52·39 to −42·00)*	164 876·44 (151 738·69 to 177 603·01)	91 199·77 (82 272·24 to 100 948·47)	–44·69 (−49·42 to −39·13)*	–46·23 (−50·84 to −40·81)*
4	Child underweight: all causes	615·18 (515·40 to 776·56)	312·61 (266·20 to 389·00)	–49·18 (−55·81 to −41·66)*	–50·76 (−57·23 to −43·44)*	55 627·11 (46 807·75 to 69 301·37)	30 009·75 (25 768·76 to 36 212·38)	–46·05 (−52·86 to −37·94)*	–47·77 (−54·35 to −39·88)*
··	Diarrhoeal diseases	127·09 (100·36 to 161·58)	52·67 (40·79 to 66·71)	–58·56 (−64·86 to −51·39)*	–59·80 (−65·94 to −52·78)*	11 105·27 (8743·61 to 14 096·57)	4690·97 (3642·30 to 5935·54)	–57·76 (−64·06 to −50·68)*	–59·03 (−65·13 to −52·13)*
··	Lower respiratory infections	163·67 (110·46 to 282·27)	74·94 (50·68 to 134·75)	–54·21 (−59·84 to −48·26)*	–55·36 (−60·85 to −49·50)*	14 008·04 (9452·95 to 24 153·07)	6422·64 (4342·77 to 11 542·06)	–54·15 (−59·76 to −48·19)*	–55·29 (−60·77 to −49·43)*
··	Measles	90·51 (19·16 to 218·56)	18·86 (3·38 to 49·55)	–79·17 (−85·30 to −74·27)*	–80·02 (−85·90 to −75·33)*	7705·27 (1633·01 to 18 583·17)	1607·04 (288·75 to 4213·11)	–79·14 (−85·23 to −74·24)*	–79·99 (−85·84 to −75·30)*
··	Protein-energy malnutrition	233·90 (206·48 to 265·01)	166·14 (141·84 to 197·87)	–28·97 (−41·25 to −12·92)*	–31·28 (−43·19 to −15·72)*	22 808·52 (20 316·73 to 25 669·57)	17 289·10 (14 869·06 to 20 449·39)	–24·20 (−35·67 to −10·17)*	–26·77 (−37·78 to −13·18)*
4	Child wasting: all causes	1734·23 (1516·43 to 1927·79)	952·40 (813·72 to 1,078·99)	–45·08 (−50·29 to −39·28)*	–46·57 (−51·63 to −40·95)*	152 812·32 (134 145·84 to 169 500·58)	86 165·42 (74 409·95 to 97 423·29)	–43·61 (−48·72 to −37·94)*	–45·17 (−50·15 to −39·64)*
··	Diarrhoeal diseases	681·26 (562·68 to 775·51)	358·50 (291·19 to 415·43)	–47·38 (−54·62 to −38·82)*	–48·84 (−55·87 to −40·50)*	59 883·01 (49 482·08 to 68 365·76)	32 202·36 (26 200·00 to 37 322·59)	–46·22 (−53·39 to −38·04)*	–47·73 (−54·71 to −39·75)*
··	Lower respiratory infections	710·19 (548·14 to 830·75)	400·91 (298·47 to 479·06)	–43·55 (−49·82 to −37·17)*	–44·86 (−51·02 to −38·64)*	60 842·27 (46 968·94 to 71 152·73)	34 383·68 (25 595·42 to 41 070·89)	–43·49 (−49·75 to −37·14)*	–44·79 (−50·93 to −38·62)*
··	Measles	108·87 (22·88 to 295·48)	26·85 (4·48 to 77·75)	–75·34 (−83·92 to −69·78)*	–76·29 (−84·44 to −70·99)*	9278·52 (1953·33 to 25 167·82)	2290·28 (383·79 to 6620·03)	–75·32 (−83·90 to −69·75)*	–76·27 (−84·43 to −70·96)*
··	Protein-energy malnutrition	233·90 (206·48 to 265·01)	166·14 (141·84 to 197·87)	–28·97 (−41·25 to −12·92)*	–31·28 (−43·19 to −15·72)*	22 808·52 (20 316·73 to 25 669·57)	17 289·10 (14 869·06 to 20 449·39)	–24·20 (−35·67 to −10·17)*	–26·77 (−37·78 to −13·18)*
4	Child stunting: all causes	366·43 (184·02 to 613·94)	162·19 (74·85 to 301·18)	–55·74 (−63·28 to −48·78)*	–57·10 (−64·53 to −50·28)*	31 579·40 (15 947·91 to 52 776·94)	14 114·74 (6609·85 to 26 162·13)	–55·30 (−62·90 to −48·48)*	–56·68 (−64·19 to −50·03)*
··	Diarrhoeal diseases	133·15 (51·03 to 233·07)	60·15 (21·84 to 112·30)	–54·83 (−61·70 to −45·79)*	–56·28 (−62·91 to −47·53)*	11 661·60 (4481·93 to 20 495·06)	5381·00 (2025·40 to 10 118·70)	–53·86 (−60·50 to −44·93)*	–55·35 (−61·80 to −46·71)*
··	Lower respiratory infections	173·89 (17·78 to 415·12)	88·31 (7·20 to 226·69)	–49·22 (−56·08 to −37·47)*	–50·59 (−57·26 to −39·05)*	14 868·01 (1516·40 to 35 518·80)	7564·20 (616·04 to 19 419·68)	–49·12 (−55·96 to −37·42)*	–50·49 (−57·14 to −38·99)*
··	Measles	59·38 (5·88 to 164·35)	13·73 (1·21 to 40·90)	–76·87 (−82·40 to −71·74)*	–77·86 (−83·16 to −72·91)*	5049·80 (506·57 to 13 966·03)	1169·54 (103·24 to 3473·72)	–76·84 (−82·34 to −71·73)*	–77·82 (−83·10 to −72·90)*
3	Low birthweight and short gestation: all causes	2341·51 (2264·77 to 2427·94)	1673·60 (1589·23 to 1758·45)	–28·52 (−31·98 to −24·88)*	–28·19 (−31·66 to −24·53)*	202 783·89 (196 133·92 to 210 268·23)	144 947·75 (137 645·54 to 152 301·77)	–28·52 (−31·97 to −24·88)*	–28·19 (−31·66 to −24·53)*
4	Short gestation for birthweight: all causes	2064·01 (1949·93 to 2171·84)	1485·61 (1392·05 to 1580·00)	–28·02 (−31·59 to −24·49)*	–27·69 (−31·27 to −24·14)*	178 754·75 (168 864·65 to 188 091·13)	128 668·91 (120 565·96 to 136 862·69)	–28·02 (−31·58 to −24·49)*	–27·69 (−31·27 to −24·14)*
··	Diarrhoeal diseases	55·68 (50·20 to 61·51)	23·63 (20·92 to 26·58)	–57·57 (−62·84 to −51·19)*	–57·43 (−62·71 to −51·02)*	4820·28 (4345·76 to 5325·35)	2045·43 (1811·41 to 2301·28)	–57·57 (−62·84 to −51·19)*	–57·43 (−62·71 to −51·02)*
··	Lower respiratory infections	183·79 (162·94 to 202·96)	104·40 (89·31 to 119·24)	–43·20 (−48·74 to −37·39)*	–42·98 (−48·54 to −37·15)*	15 913·47 (14 107·71 to 17 572·78)	9039·55 (7732·87 to 10 324·80)	–43·20 (−48·74 to −37·39)*	–42·97 (−48·54 to −37·15)*
··	Upper respiratory infections	0·09 (0·06 to 0·12)	0·05 (0·04 to 0·07)	–41·66 (−60·35 to −14·21)*	–41·40 (−60·16 to −13·83)*	7·54 (5·36 to 10·34)	4·40 (3·13 to 6·32)	–41·66 (−60·35 to −14·21)*	–41·40 (−60·16 to −13·82)*
··	Otitis media	0·01 (0·01 to 0·02)	0·01 (0·00 to 0·01)	–55·23 (−72·96 to −21·17)*	–55·11 (−72·91 to −20·94)*	1·13 (0·78 to 1·72)	0·51 (0·33 to 0·83)	–55·23 (−72·96 to −21·18)*	–55·11 (−72·91 to −20·95)*
··	Pneumococcal meningitis	0·74 (0·51 to 1·02)	0·62 (0·40 to 0·93)	–15·86 (−31·66 to 6·43)	–15·56 (−31·41 to 6·80)	63·93 (43·85 to 87·89)	53·80 (35·00 to 80·84)	–15·85 (−31·66 to 6·43)	–15·56 (−31·41 to 6·80)
··	H influenzae type B meningitis	2·05 (1·48 to 2·68)	1·71 (1·22 to 2·40)	–16·55 (−32·62 to 6·20)	–16·25 (−32·37 to 6·59)	177·11 (127·74 to 231·69)	147·80 (105·37 to 207·64)	–16·55 (−32·62 to 6·20)	–16·25 (−32·37 to 6·59)
··	Meningococcal infection	7·44 (5·63 to 9·42)	4·67 (3·45 to 6·41)	–37·20 (−47·71 to −22·35)*	–36·98 (−47·52 to −22·08)*	643·78 (487·60 to 815·98)	404·31 (298·70 to 555·31)	–37·20 (−47·71 to −22·35)*	–36·98 (−47·52 to −22·08)*
··	Other meningitis	5·57 (4·16 to 7·06)	5·57 (4·05 to 8·31)	0·08 (−17·80 to 26·45)	0·43 (−17·54 to 26·88)	481·88 (360·51 to 611·08)	482·25 (350·92 to 719·69)	0·08 (−17·80 to 26·45)	0·43 (−17·53 to 26·88)
··	Encephalitis	1·34 (1·00 to 1·56)	1·00 (0·79 to 1·24)	–24·98 (−43·32 to −2·82)*	–24·73 (−43·13 to −2·52)*	115·89 (86·59 to 134·97)	86·95 (68·43 to 107·11)	–24·98 (−43·32 to −2·82)*	–24·73 (−43·13 to −2·52)*
··	Neonatal preterm birth complications	819·36 (770·29 to 909·83)	590·38 (541·05 to 643·11)	–27·95 (−33·72 to −22·15)*	–27·60 (−33·41 to −21·78)*	70 980·50 (66 730·62 to 78 805·17)	51 151·21 (46 878·45 to 55 713·15)	–27·94 (−33·70 to −22·14)*	–27·59 (−33·39 to −21·77)*
··	Neonatal encephalopathy due to birth asphyxia and trauma	477·77 (426·69 to 525·03)	370·94 (322·96 to 419·15)	–22·36 (−29·79 to −14·36)*	–21·97 (−29·43 to −13·93)*	41 371·74 (36 949·03 to 45 464·08)	32 120·93 (27 966·29 to 36 295·65)	–22·36 (−29·79 to −14·36)*	–21·97 (−29·43 to −13·93)*
··	Neonatal sepsis and other neonatal infections	170·34 (138·61 to 217·43)	151·23 (126·64 to 206·06)	–11·22 (−21·77 to 2·78)	–10·86 (−21·43 to 3·20)	14 749·24 (12 001·55 to 18 826·29)	13 094·15 (10 964·91 to 17 842·59)	–11·22 (−21·77 to 2·78)	–10·86 (−21·44 to 3·20)
··	Haemolytic disease and other neonatal jaundice	55·72 (48·90 to 64·54)	32·45 (27·90 to 38·04)	–41·77 (−49·82 to −32·96)*	–41·52 (−49·61 to −32·68)*	4824·42 (4234·14 to 5587·98)	2809·45 (2416·02 to 3293·80)	–41·77 (−49·82 to −32·96)*	–41·52 (−49·61 to −32·68)*
··	Other neonatal disorders	282·16 (250·52 to 317·48)	197·44 (173·31 to 220·92)	–30·03 (−37·01 to −21·73)*	–29·69 (−36·71 to −21·36)*	24 432·57 (21 692·72 to 27 491·34)	17 096·35 (15 006·94 to 19 130·02)	–30·03 (−37·01 to −21·73)*	–29·69 (−36·71 to −21·36)*
··	Sudden infant death syndrome	1·98 (1·47 to 2·54)	1·52 (1·16 to 1·87)	–23·02 (−39·72 to −8·37)*	–22·88 (−39·61 to −8·20)*	171·26 (127·36 to 219·51)	131·83 (100·77 to 161·72)	–23·02 (−39·72 to −8·37)*	–22·88 (−39·61 to −8·20)*
4	Low birthweight for gestation: all causes	1096·85 (1005·37 to 1207·52)	778·37 (705·63 to 864·12)	–29·04 (−33·70 to −24·31)*	–28·69 (−33·38 to −23·94)*	95 009·64 (87 086·08 to 104 596·97)	67 430·06 (61 121·27 to 74 855·14)	–29·03 (−33·69 to −24·30)*	–28·69 (−33·37 to −23·93)*
··	Diarrhoeal diseases	8·81 (6·21 to 11·88)	3·44 (2·38 to 4·75)	–60·94 (−66·12 to −55·24)*	–60·78 (−65·99 to −55·07)*	762·62 (537·68 to 1028·73)	297·89 (206·10 to 411·47)	–60·94 (−66·12 to −55·24)*	–60·78 (−65·99 to −55·07)*
··	Lower respiratory infections	35·74 (25·03 to 48·36)	19·19 (12·83 to 26·70)	–46·30 (−52·21 to −39·99)*	–46·06 (−51·99 to −39·72)*	3094·33 (2167·54 to 4187·61)	1661·65 (1111·03 to 2311·96)	–46·30 (−52·21 to −39·99)*	–46·06 (−51·99 to −39·71)*
··	Upper respiratory infections	0·02 (0·01 to 0·03)	0·01 (0·01 to 0·02)	–44·27 (−64·75 to −11·69)*	–44·00 (−64·57 to −11·28)*	1·71 (1·05 to 2·67)	0·95 (0·56 to 1·55)	–44·27 (−64·75 to −11·69)*	–44·00 (−64·57 to −11·27)*
··	Otitis media	0·00 (0·00 to 0·00)	0·00 (0·00 to 0·00)	–55·15 (−71·15 to −26·78)*	–55·00 (−71·08 to −26·52)*	0·16 (0·09 to 0·25)	0·07 (0·04 to 0·12)	–55·15 (−71·15 to −26·78)*	–55·00 (−71·08 to −26·52)*
··	Pneumococcal meningitis	0·13 (0·08 to 0·20)	0·11 (0·06 to 0·17)	–19·39 (−35·79 to 2·80)	–19·04 (−35·49 to 3·25)	11·42 (6·55 to 17·49)	9·20 (5·13 to 15·15)	–19·39 (−35·79 to 2·80)	–19·04 (−35·49 to 3·25)
··	H influenzae type B meningitis	0·36 (0·22 to 0·53)	0·29 (0·17 to 0·45)	–20·05 (−36·17 to 2·27)	–19·71 (−35·89 to 2·71)	31·35 (19·05 to 45·47)	25·07 (14·61 to 38·71)	–20·05 (−36·16 to 2·27)	–19·71 (−35·89 to 2·71)
··	Meningococcal infection	1·30 (0·81 to 1·86)	0·80 (0·46 to 1·25)	–38·84 (−50·93 to −23·22)*	–38·58 (−50·72 to −22·88)*	112·69 (69·75 to 161·06)	68·92 (39·87 to 108·45)	–38·84 (−50·93 to −23·22)*	–38·58 (−50·72 to −22·88)*
··	Other meningitis	1·00 (0·62 to 1·44)	0·94 (0·55 to 1·48)	–5·58 (−23·60 to 18·81)	–5·19 (−23·30 to 19·32)	86·49 (53·37 to 124·38)	81·66 (47·91 to 127·74)	–5·58 (−23·59 to 18·81)	–5·18 (−23·29 to 19·32)
··	Encephalitis	0·20 (0·13 to 0·28)	0·14 (0·10 to 0·21)	–29·88 (−45·21 to −11·08)*	–29·59 (−44·97 to −10·70)*	17·74 (11·22 to 24·41)	12·44 (8·28 to 17·83)	–29·88 (−45·21 to −11·08)*	–29·59 (−44·97 to −10·70)*
··	Neonatal preterm birth complications	819·36 (770·29 to 909·83)	590·38 (541·05 to 643·11)	–27·95 (−33·72 to −22·15)*	–27·60 (−33·41 to −21·78)*	70 980·50 (66 730·62 to 78 805·17)	51 151·21 (46 878·45 to 55 713·15)	–27·94 (−33·70 to −22·14)*	–27·59 (−33·39 to −21·77)*
··	Neonatal encephalopathy due to birth asphyxia and trauma	117·60 (83·00 to 156·33)	84·68 (58·86 to 116·05)	–27·99 (−35·44 to −20·42)*	–27·62 (−35·10 to −20·01)*	10 183·45 (7187·60 to 13 537·45)	7332·98 (5096·63 to 10 049·16)	–27·99 (−35·44 to −20·42)*	–27·62 (−35·10 to −20·01)*
··	Neonatal sepsis and other neonatal infections	35·50 (23·71 to 50·81)	29·56 (19·65 to 43·87)	–16·73 (−28·32 to −2·51)*	–16·34 (−27·98 to −2·04)*	3073·99 (2053·27 to 4400·09)	2559·70 (1701·26 to 3798·68)	–16·73 (−28·32 to −2·51)*	–16·34 (−27·98 to −2·04)*
··	Haemolytic disease and other neonatal jaundice	11·40 (7·99 to 15·92)	6·32 (4·34 to 9·07)	–44·53 (−53·03 to −34·47)*	–44·26 (−52·80 to −34·17)*	987·26 (692·04 to 1378·49)	547·68 (375·55 to 785·14)	–44·52 (−53·03 to −34·47)*	–44·26 (−52·80 to −34·17)*
··	Other neonatal disorders	65·24 (45·86 to 88·09)	42·37 (28·62 to 57·94)	–35·06 (−42·81 to −26·85)*	–34·73 (−42·52 to −26·48)*	5649·31 (3971·62 to 7628·08)	3668·67 (2478·27 to 5016·91)	–35·06 (−42·81 to −26·85)*	–34·73 (−42·52 to −26·48)*
··	Sudden infant death syndrome	0·19 (0·11 to 0·30)	0·14 (0·08 to 0·21)	–28·09 (−43·32 to −14·05)*	–27·96 (−43·22 to −13·89)*	16·64 (9·23 to 26·37)	11·97 (6·94 to 18·10)	–28·09 (−43·32 to −14·05)*	–27·96 (−43·22 to −13·89)*
3	Iron deficiency: all causes	27·52 (12·14 to 43·69)	20·95 (9·79 to 33·31)	–23·88 (−30·25 to −15·97)*	–31·14 (−36·96 to −24·17)*	33 835·12 (22 660·82 to 48 281·14)	35 849·87 (24 052·89 to 50 796·92)	5·95 (4·22 to 7·72)*	–3·09 (−4·68 to −1·56)*
··	Maternal haemorrhage	19·26 (7·33 to 32·35)	14·10 (5·33 to 24·29)	–26·80 (−34·82 to −17·79)*	–33·34 (−40·64 to −25·27)*	1105·24 (420·28 to 1854·49)	798·94 (301·29 to 1366·56)	–27·71 (−35·87 to −18·55)*	–33·84 (−41·20 to −25·64)*
··	Maternal sepsis and other pregnancy related infections	5·54 (2·03 to 9·37)	3·89 (1·38 to 6·67)	–29·86 (−38·95 to −20·14)*	–35·83 (−44·10 to −26·86)*	325·54 (119·05 to 544·08)	227·22 (80·46 to 386·37)	–30·20 (−38·96 to −20·38)*	–35·70 (−43·84 to −27·13)*
··	Iron-deficiency anaemia	2·72 (2·35 to 3·89)	2·96 (2·52 to 3·75)	8·94 (−9·11 to 27·26)	–11·59 (−27·78 to 4·94)	32 404·33 (21 523·57 to 46 641·55)	34 823·71 (23 073·25 to 49 667·43)	7·47 (6·17 to 8·89)*	–1·78 (−2·96 to −0·51)*
3	Vitamin A deficiency: all causes	108·40 (62·61 to 166·64)	42·18 (24·16 to 65·39)	–61·08 (−66·90 to −53·83)*	–62·78 (−68·35 to −55·79)*	9600·08 (5604·44 to 14 578·34)	3979·05 (2357·30 to 6000·15)	–58·55 (−64·51 to −51·19)*	–60·47 (−66·15 to −53·43)*
··	Diarrhoeal diseases	64·17 (32·47 to 96·79)	30·04 (14·54 to 46·71)	–53·18 (−60·01 to −44·61)*	–55·12 (−61·70 to −46·81)*	5620·97 (2833·29 to 8506·22)	2695·50 (1309·94 to 4149·27)	–52·05 (−58·86 to −43·62)*	–54·05 (−60·63 to −45·90)*
··	Measles	44·23 (13·84 to 96·01)	12·14 (3·73 to 28·13)	–72·55 (−77·30 to −67·69)*	–73·85 (−78·35 to −69·07)*	3753·95 (1185·75 to 8149·96)	1031·27 (318·97 to 2391·75)	–72·53 (−77·21 to −67·68)*	–73·83 (−78·30 to −69·05)*
··	Vitamin A deficiency	··	··	··	··	225·16 (139·62 to 348·12)	252·29 (158·71 to 388·09)	12·05 (8·70 to 15·49)*	2·64 (−0·33 to 5·60)
3	Zinc deficiency: all causes	53·32 (2·85 to 141·73)	25·09 (1·32 to 69·47)	–52·95 (−60·88 to −43·61)*	–55·43 (−62·95 to −46·59)*	4651·43 (359·57 to 12 155·34)	2245·65 (213·63 to 5993·34)	–51·72 (−59·17 to −38·63)*	–54·27 (−61·32 to −41·88)*
··	Diarrhoeal diseases	31·31 (0·00 to 87·89)	14·67 (0·00 to 42·50)	··	··	2785·75 (132·69 to 7615·90)	1359·88 (108·32 to 3778·60)	–51·18 (−58·99 to −14·97)*	–53·76 (−61·16 to −19·47)*
··	Lower respiratory infections	22·01 (0·00 to 86·72)	10·42 (0·00 to 42·20)	··	··	1865·68 (2·95 to 7331·05)	885·77 (2·26 to 3568·53)	–52·52 (−60·86 to −18·33)*	–55·03 (−62·93 to −22·65)*
**2**	**Tobacco: all causes**	**6853·45 (6227·56 to 7447·85)**	**7131·38 (6503·23 to 7780·89)**	4·06 (1·29 to 6·96)*	–20·37 (−22·48 to −18·30)*	**178 305·14 (163 133·82 to 194 298·17)**	**177 302·31 (162 327·84 to 194 250·39)**	**–0·56 (−3·34 to 2·52)**	–21·31 (−23·35 to −19·05)*
3	Smoking: all causes	6081·95 (5443·81 to 6681·35)	6321·10 (5673·66 to 6962·35)	3·93 (0·87 to 7·06)*	–20·68 (−22·98 to −18·31)*	153 365·37 (138 408·89 to 167 887·88)	155 065·75 (140 025·42 to 170 602·15)	1·11 (−1·79 to 4·20)	–20·83 (−23·12 to −18·45)*
··	Drug-susceptible tuberculosis	129·07 (66·60 to 195·37)	90·24 (44·98 to 139·18)	–30·08 (−34·42 to −26·43)*	–44·49 (−48·00 to −41·50)*	4240·53 (2168·23 to 6389·84)	2934·12 (1440·35 to 4528·89)	–30·81 (−34·75 to −27·37)*	–43·68 (−47·17 to −40·93)*
··	Multidrug-resistant tuberculosis without extensive drug resistance	14·07 (7·18 to 21·44)	8·19 (4·00 to 12·82)	–41·80 (−48·21 to −35·18)*	–53·46 (−58·72 to −48·18)*	458·79 (234·16 to 698·87)	257·57 (126·48 to 404·28)	–43·86 (−50·23 to −37·69)*	–54·10 (−59·43 to −49·05)*
··	Extensively drug-resistant tuberculosis	0·92 (0·48 to 1·40)	1·33 (0·66 to 2·11)	44·76 (19·92 to 72·63)*	16·94 (−2·69 to 38·90)	31·47 (16·42 to 48·08)	43·78 (21·82 to 68·67)	39·10 (14·10 to 68·50)*	14·83 (−5·56 to 38·75)
··	Lower respiratory infections	326·00 (257·89 to 397·51)	345·94 (270·42 to 426·72)	6·12 (0·26 to 10·80)*	–19·55 (−23·91 to −16·10)*	7002·64 (5630·01 to 8415·41)	7022·96 (5529·91 to 8607·77)	0·29 (−5·31 to 5·35)	–20·81 (−25·17 to −16·90)*
··	Lip and oral cavity cancer	51·72 (44·13 to 60·07)	64·11 (53·04 to 77·13)	23·95 (15·31 to 32·49)*	–4·81 (−11·29 to 1·58)	1393·05 (1176·02 to 1627·58)	1658·72 (1362·64 to 2015·39)	19·07 (10·04 to 28·65)*	–6·71 (−13·94 to 0·57)
··	Nasopharynx cancer	21·46 (15·13 to 28·44)	22·33 (16·02 to 30·03)	4·05 (−6·14 to 14·01)	–18·25 (−25·96 to −10·94)*	635·38 (432·94 to 851·08)	618·24 (441·07 to 838·14)	–2·70 (−13·59 to 9·00)	–22·14 (−30·47 to −13·55)*
··	Oesophageal cancer	144·04 (90·12 to 204·21)	144·40 (93·42 to 205·40)	0·25 (−5·55 to 8·89)	–23·33 (−27·74 to −17·01)*	3249·88 (2072·43 to 4607·13)	3104·99 (2025·56 to 4431·70)	–4·46 (−10·71 to 4·48)	–25·91 (−30·69 to −19·11)*
··	Stomach cancer	86·47 (50·10 to 134·64)	78·50 (45·61 to 124·13)	–9·21 (−15·82 to −2·65)*	–30·05 (−34·90 to −25·17)*	1985·62 (1151·90 to 3061·29)	1668·26 (961·69 to 2631·19)	–15·98 (−23·04 to −8·83)*	–34·01 (−39·20 to −28·67)*
··	Colon and rectum cancer	46·29 (32·90 to 59·71)	49·01 (33·90 to 64·52)	5·88 (−0·09 to 11·90)	–19·96 (−24·42 to −15·55)*	973·58 (681·64 to 1272·87)	963·80 (667·18 to 1291·65)	–1·00 (−7·15 to 5·34)	–23·19 (−27·69 to −18·37)*
··	Liver cancer due to hepatitis B	41·56 (18·21 to 77·66)	43·39 (19·66 to 81·96)	4·41 (−6·80 to 17·06)	–17·17 (−24·98 to −8·46)*	1222·27 (522·08 to 2265·72)	1164·10 (521·30 to 2235·84)	–4·76 (−17·66 to 10·74)	–22·84 (−32·30 to −11·64)*
··	Liver cancer due to hepatitis C	19·24 (10·53 to 28·42)	22·01 (12·01 to 32·79)	14·35 (7·81 to 21·02)*	–12·81 (−17·64 to −8·10)*	414·97 (225·94 to 628·23)	448·80 (238·88 to 687·22)	8·15 (0·75 to 16·28)*	–16·20 (−21·61 to −10·61)*
··	Liver cancer due to alcohol use	14·69 (8·71 to 21·74)	16·71 (9·61 to 25·36)	13·74 (5·31 to 21·66)*	–12·48 (−18·79 to −6·56)*	343·61 (201·26 to 506·60)	377·43 (217·22 to 566·00)	9·84 (0·81 to 18·67)*	–14·44 (−20·97 to −7·97)*
··	Liver cancer due to other causes	24·71 (11·36 to 45·81)	26·42 (12·15 to 49·30)	6·90 (−4·05 to 18·94)	–15·62 (−23·12 to −7·34)*	683·22 (286·75 to 1300·01)	662·38 (294·93 to 1262·06)	–3·05 (−15·91 to 13·35)	–21·80 (−31·15 to −10·21)*
··	Pancreatic cancer	61·47 (49·77 to 74·37)	70·90 (56·11 to 87·71)	15·34 (9·71 to 21·46)*	–12·20 (−16·27 to −7·87)*	1315·34 (1059·48 to 1601·29)	1431·89 (1125·82 to 1797·72)	8·86 (2·52 to 15·73)*	–15·66 (−20·48 to −10·57)*
··	Larynx cancer	60·04 (50·50 to 68·78)	64·92 (53·57 to 76·19)	8·14 (2·92 to 13·33)*	–17·06 (−21·01 to −13·10)*	1524·37 (1284·41 to 1751·12)	1596·46 (1320·70 to 1877·67)	4·73 (−0·81 to 10·00)	–18·86 (−23·02 to −14·71)*
··	Tracheal, bronchus, and lung cancer	1014·39 (875·09 to 1123·75)	1144·75 (973·82 to 1299·87)	12·85 (7·75 to 17·44)*	–13·53 (−17·47 to −10·01)*	22 094·05 (18 775·21 to 24 684·60)	23 701·45 (19 814·76 to 27 245·91)	7·28 (1·69 to 12·27)*	–16·85 (−21·09 to −13·12)*
··	Breast cancer	16·88 (5·04 to 30·02)	17·91 (5·25 to 31·90)	6·11 (−0·34 to 13·11)	–18·14 (−22·76 to −13·09)*	457·80 (129·80 to 835·33)	452·41 (126·69 to 827·14)	–1·18 (−8·24 to 6·76)	–21·43 (−26·56 to −15·69)*
··	Cervical cancer	11·03 (3·91 to 19·39)	10·85 (3·81 to 18·98)	–1·66 (−10·26 to 7·78)	–22·54 (−28·68 to −15·43)*	331·91 (114·69 to 595·93)	306·32 (105·80 to 541·94)	–7·71 (−17·35 to 2·77)	–25·19 (−32·55 to −16·79)*
··	Prostate cancer	15·29 (11·00 to 19·90)	16·68 (11·72 to 22·10)	9·09 (2·16 to 18·00)*	–19·29 (−24·05 to −12·77)*	257·95 (186·43 to 331·39)	268·27 (190·32 to 355·74)	4·00 (−3·76 to 12·92)	–21·25 (−26·88 to −14·50)*
··	Kidney cancer	20·10 (13·46 to 26·02)	22·07 (14·53 to 29·44)	9·79 (1·71 to 18·14)*	–16·07 (−21·91 to −9·92)*	464·77 (311·24 to 604·80)	480·06 (316·13 to 639·24)	3·29 (−5·41 to 12·59)	–19·84 (−26·34 to −12·84)*
··	Bladder cancer	44·33 (33·18 to 55·10)	49·84 (36·98 to 63·01)	12·42 (6·43 to 18·18)*	–15·76 (−20·05 to −11·55)*	820·50 (616·52 to 1016·68)	867·04 (639·82 to 1098·93)	5·67 (−0·75 to 11·94)	–19·01 (−23·71 to −14·36)*
··	Acute lymphoid leukaemia	2·45 (1·19 to 3·88)	2·65 (1·26 to 4·39)	8·51 (−1·79 to 18·19)	–14·18 (−21·95 to −6·75)*	74·37 (35·76 to 121·34)	77·25 (35·75 to 131·34)	3·87 (−8·05 to 15·59)	–16·04 (−25·20 to −6·72)*
··	Chronic lymphoid leukaemia	4·13 (2·06 to 6·30)	4·32 (2·09 to 6·76)	4·69 (−3·63 to 13·22)	–21·24 (−27·22 to −15·09)*	81·77 (41·39 to 125·41)	81·18 (39·93 to 126·38)	–0·73 (−9·78 to 8·35)	–23·72 (−30·52 to −17·08)*
··	Acute myeloid leukaemia	7·27 (3·54 to 11·03)	8·00 (3·82 to 12·46)	10·07 (2·74 to 16·47)*	–14·79 (−20·27 to −10·13)*	174·17 (88·65 to 265·91)	182·46 (89·93 to 285·57)	4·76 (−3·79 to 12·31)	–17·20 (−23·71 to −11·49)*
··	Chronic myeloid leukaemia	2·27 (1·10 to 3·48)	1·92 (0·89 to 3·02)	–15·62 (−22·48 to −8·88)*	–34·65 (−39·76 to −29·68)*	56·81 (27·53 to 88·46)	45·30 (21·19 to 72·34)	–20·26 (−27·97 to −13·34)*	–36·45 (−42·56 to −30·76)*
··	Other leukaemia	8·73 (4·12 to 14·42)	8·74 (4·11 to 14·48)	0·05 (−8·87 to 8·12)	–21·85 (−28·03 to −16·79)*	220·47 (100·95 to 372·96)	198·49 (92·02 to 332·58)	–9·97 (−21·62 to 1·39)	–27·29 (−35·24 to −19·78)*
··	Ischaemic heart disease	1346·04 (1125·86 to 1572·65)	1391·74 (1144·96 to 1649·56)	3·40 (−0·14 to 7·31)	–20·16 (−22·96 to −17·24)*	36 051·24 (30 135·29 to 41 836·78)	36 302·60 (29 797·02 to 42 911·24)	0·70 (−2·92 to 4·82)	–20·54 (−23·37 to −17·43)*
··	Ischaemic stroke	350·47 (292·23 to 407·20)	347·05 (290·43 to 408·73)	–0·98 (−5·13 to 3·31)	–24·72 (−27·99 to −21·50)*	8972·51 (7487·52 to 10 550·77)	9235·11 (7655·96 to 10 990·85)	2·93 (−1·37 to 6·94)	–20·73 (−24·06 to −17·62)*
··	Haemorrhagic stroke	574·87 (485·95 to 664·83)	535·26 (448·47 to 627·04)	–6·89 (−10·16 to −3·66)*	–27·91 (−30·41 to −25·36)*	16 024·57 (13 595·74 to 18 501·64)	14 873·84 (12 549·42 to 17 354·01)	–7·18 (−10·27 to −4·08)*	–26·69 (−29·11 to −24·28)*
··	Hypertensive heart disease	92·58 (69·07 to 115·52)	104·36 (75·52 to 129·77)	12·72 (−0·81 to 23·61)	–13·06 (−23·84 to −4·65)*	2418·95 (1818·67 to 3023·75)	2611·14 (1927·81 to 3211·98)	7·95 (−2·72 to 17·76)	–14·97 (−23·65 to −7·28)*
··	Atrial fibrillation and flutter	11·78 (8·34 to 15·89)	14·23 (10·02 to 19·31)	20·80 (16·34 to 24·92)*	–11·40 (−14·55 to −8·44)*	616·54 (429·29 to 846·49)	710·44 (488·75 to 984·65)	15·23 (12·91 to 17·40)*	–10·95 (−12·66 to −9·34)*
··	Aortic aneurysm	22·06 (17·20 to 26·40)	22·71 (17·69 to 27·64)	2·92 (−2·08 to 9·42)	–20·66 (−24·43 to −15·86)*	554·61 (435·81 to 658·02)	560·44 (442·97 to 678·22)	1·05 (−4·19 to 8·10)	–20·47 (−24·50 to −15·10)*
··	Peripheral vascular disease	4·59 (3·26 to 5·98)	5·12 (3·60 to 6·91)	11·65 (−0·50 to 26·59)	–15·97 (−24·80 to −5·05)*	148·14 (100·88 to 203·55)	163·17 (110·40 to 225·34)	10·14 (0·93 to 20·74)*	–16·24 (−22·89 to −8·54)*
··	Other cardiovascular and circulatory diseases	53·33 (40·77 to 70·74)	55·64 (41·83 to 73·98)	4·33 (−0·04 to 9·45)	–19·15 (−22·65 to −15·27)*	1998·90 (1531·92 to 2560·68)	2084·86 (1574·17 to 2694·49)	4·30 (0·52 to 8·39)*	–16·90 (−19·76 to −13·76)*
··	Chronic obstructive pulmonary disease	1190·52 (889·10 to 1462·49)	1253·30 (989·51 to 1520·42)	5·27 (−0·57 to 14·11)	–22·12 (−26·38 to −15·51)*	23 659·75 (18 550·88 to 28 461·63)	25 038·91 (20 395·51 to 29 918·00)	5·83 (−0·06 to 13·85)	–19·26 (−23·63 to −13·01)*
··	Asthma	65·36 (44·88 to 91·56)	56·81 (39·28 to 78·57)	–13·08 (−20·43 to −5·24)*	–32·94 (−38·71 to −26·92)*	2444·85 (1802·94 to 3242·52)	2291·51 (1694·45 to 2999·22)	–6·27 (−12·71 to −0·11)*	–25·66 (−30·95 to −20·73)*
··	Other chronic respiratory diseases	3·07 (2·06 to 4·10)	3·77 (2·53 to 5·06)	22·95 (13·58 to 33·14)*	–5·85 (−12·82 to 1·88)	103·01 (73·48 to 140·16)	126·04 (88·32 to 176·23)	22·36 (12·41 to 32·01)*	–1·70 (−9·84 to 6·67)
··	Peptic ulcer disease	43·27 (31·46 to 55·50)	36·14 (26·26 to 47·09)	–16·48 (−21·32 to −12·20)*	–35·23 (−39·05 to −31·93)*	1202·60 (882·23 to 1539·63)	1008·27 (740·13 to 1308·30)	–16·16 (−20·61 to −11·83)*	–33·35 (−36·87 to −30·04)*
··	Gallbladder and biliary diseases	2·22 (1·49 to 2·91)	2·32 (1·55 to 3·09)	4·66 (−1·51 to 10·83)	–20·29 (−25·11 to −15·43)*	54·26 (36·94 to 71·95)	55·54 (36·83 to 74·21)	2·36 (−2·84 to 7·48)	–19·57 (−23·79 to −15·50)*
··	Alzheimer's disease and other dementias	67·57 (33·10 to 106·19)	82·80 (39·50 to 132·02)	22·55 (15·91 to 27·45)*	–11·65 (−17·20 to −7·77)*	1062·73 (491·18 to 1670·22)	1256·05 (555·38 to 1982·80)	18·19 (12·39 to 22·10)*	–11·38 (−16·39 to −8·22)*
··	Parkinson's disease	–20·15 (−26·54 to −14·37)	–23·16 (−30·39 to −16·44)	14·93 (10·72 to 19·10)*	–12·99 (−16·05 to −9·98)*	–403·98 (−525·52 to −283·21)	–461·19 (−599·84 to −324·73)	14·16 (10·52 to 17·80)*	–12·22 (−14·86 to −9·59)*
··	Multiple sclerosis	1·70 (1·11 to 2·36)	1·68 (1·09 to 2·33)	–0·89 (−9·77 to 5·59)	–21·16 (−28·03 to −16·17)*	98·79 (62·61 to 138·27)	99·08 (62·16 to 140·65)	0·29 (−5·43 to 4·75)	–18·13 (−22·71 to −14·62)*
··	Diabetes mellitus	56·47 (17·07 to 99·11)	66·30 (19·12 to 117·72)	17·40 (11·86 to 21·43)*	–9·78 (−14·35 to −6·53)*	2881·41 (847·72 to 5096·99)	3192·65 (911·95 to 5662·02)	10·80 (7·07 to 13·63)*	–12·37 (−15·58 to −10·13)*
··	Rheumatoid arthritis	1·45 (0·58 to 2·38)	1·37 (0·54 to 2·27)	–6·01 (−10·76 to −0·77)*	–28·01 (−31·59 to −24·03)*	224·49 (88·79 to 401·57)	241·06 (94·19 to 434·89)	7·38 (4·34 to 9·93)*	–14·89 (−17·34 to −12·89)*
··	Low back pain	··	··	··	··	2459·39 (1047·30 to 4016·91)	2567·74 (1082·41 to 4200·46)	4·41 (1·74 to 7·02)*	–14·98 (−17·00 to −13·33)*
··	Cataract	··	··	··	··	404·92 (261·96 to 595·50)	457·74 (295·38 to 678·21)	13·05 (9·89 to 16·35)*	–12·81 (−15·31 to −10·33)*
··	Macular degeneration	··	··	··	··	35·10 (10·74 to 63·30)	43·59 (13·27 to 79·98)	24·18 (19·16 to 28·49)*	–6·46 (−10·16 to −3·27)*
··	Pedestrian road injuries	4·26 (3·12 to 5·55)	4·27 (3·07 to 5·52)	0·19 (−6·60 to 4·72)	–22·32 (−27·66 to −18·89)*	192·70 (132·72 to 266·50)	203·56 (140·13 to 285·42)	5·64 (0·50 to 9·31)*	–15·61 (−19·72 to −12·60)*
··	Cyclist road injuries	0·66 (0·46 to 0·87)	0·65 (0·46 to 0·88)	–0·71 (−7·23 to 8·03)	–22·08 (−27·08 to −15·18)*	88·95 (56·29 to 134·78)	101·22 (63·20 to 155·51)	13·79 (10·68 to 16·34)*	–8·26 (−10·82 to −6·20)*
··	Motorcyclist road injuries	1·42 (0·98 to 1·92)	1·41 (0·95 to 1·89)	–0·73 (−7·31 to 5·07)	–20·26 (−25·42 to −15·61)*	146·73 (93·93 to 217·75)	154·92 (99·00 to 232·89)	5·58 (2·29 to 8·25)*	–13·79 (−16·50 to −11·69)*
··	Motor vehicle road injuries	3·42 (2·40 to 4·58)	3·27 (2·30 to 4·35)	–4·43 (−9·10 to 3·95)	–24·68 (−28·27 to −18·07)*	220·30 (149·90 to 312·87)	222·17 (149·26 to 318·67)	0·85 (−2·50 to 5·15)	–18·53 (−21·35 to −15·12)*
··	Other road injuries	0·11 (0·08 to 0·15)	0·11 (0·08 to 0·16)	1·37 (−8·56 to 15·72)	–22·45 (−30·44 to −11·01)*	24·54 (14·89 to 38·62)	34·33 (20·60 to 54·65)	39·88 (36·47 to 42·74)*	12·99 (9·94 to 15·33)*
··	Other transport injuries	0·96 (0·71 to 1·24)	0·95 (0·69 to 1·23)	–1·50 (−7·54 to 7·47)	–22·86 (−27·55 to −15·90)*	98·02 (64·22 to 142·79)	97·82 (63·60 to 143·42)	–0·21 (−3·18 to 2·92)	–19·19 (−21·61 to −16·77)*
··	Falls	14·00 (9·94 to 18·05)	15·72 (11·19 to 20·28)	12·30 (4·47 to 19·50)*	–17·04 (−22·83 to −11·46)*	834·32 (556·29 to 1207·21)	927·35 (615·93 to 1342·67)	11·15 (8·56 to 13·31)*	–12·04 (−14·43 to −10·17)*
··	Other exposure to mechanical forces	0·72 (0·50 to 0·93)	0·68 (0·45 to 0·90)	–4·72 (−16·10 to 1·04)	–25·28 (−34·05 to −20·84)*	143·72 (85·67 to 229·57)	159·21 (93·69 to 258·36)	10·78 (8·20 to 12·76)*	–10·26 (−12·38 to −8·55)*
··	Non-venomous animal contact	0·07 (0·05 to 0·09)	0·06 (0·04 to 0·08)	–14·75 (−23·01 to −4·84)*	–33·85 (−40·10 to −26·00)*	7·11 (4·27 to 11·66)	6·44 (3·79 to 10·64)	–9·45 (−12·88 to −6·48)*	–26·94 (−29·78 to −24·44)*
··	Assault by other means	0·49 (0·30 to 0·70)	0·41 (0·27 to 0·61)	–15·12 (−27·68 to 5·98)	–32·36 (−42·05 to −16·11)*	79·58 (48·44 to 125·25)	77·53 (46·64 to 123·06)	–2·58 (−6·78 to 1·58)	–20·52 (−23·97 to −17·23)*
··	Forces of nature, conflict and terrorism, and state actor violence	0·05 (0·03 to 0·07)	0·02 (0·01 to 0·03)	–61·88 (−74·78 to −49·82)*	–68·62 (−79·42 to −58·85)*	7·32 (2·99 to 15·95)	8·92 (2·89 to 21·37)	21·82 (−10·52 to 35·55)	1·45 (−25·71 to 12·96)
3	Smokeless tobacco: all causes	39·05 (32·22 to 45·82)	48·24 (39·35 to 56·91)	23·52 (14·92 to 31·94)*	–4·58 (−11·36 to 1·82)	1063·08 (872·62 to 1258·43)	1262·17 (1016·17 to 1498·73)	18·73 (10·62 to 26·38)*	–6·49 (−12·92 to −0·38)*
··	Lip and oral cavity cancer	25·14 (19·77 to 30·36)	32·14 (24·93 to 39·24)	27·85 (17·75 to 37·18)*	–1·25 (−9·09 to 6·14)	697·47 (540·47 to 849·10)	854·15 (658·17 to 1052·56)	22·46 (12·41 to 31·49)*	–3·28 (−11·05 to 3·85)
··	Oesophageal cancer	13·91 (10·12 to 17·58)	16·10 (11·51 to 20·45)	15·71 (8·62 to 23·45)*	–10·58 (−16·11 to −4·51)*	365·61 (264·21 to 464·10)	408·02 (289·36 to 522·28)	11·60 (4·38 to 19·33)*	–12·54 (−17·96 to −6·30)*
3	Second-hand smoke: all causes	848·70 (674·54 to 1044·47)	883·93 (715·08 to 1085·10)	4·15 (0·25 to 8·62)*	–18·91 (−21·61 to −16·21)*	26 546·21 (19 817·27 to 34 362·69)	23 761·45 (18 439·15 to 29 543·70)	–10·49 (−16·84 to −2·78)*	–24·59 (−28·47 to −20·00)*
··	Lower respiratory infections	178·55 (92·47 to 275·01)	138·56 (72·78 to 213·49)	–22·40 (−26·76 to −18·06)*	–31·09 (−34·53 to −27·75)*	10 839·93 (5534·74 to 16 883·74)	6407·40 (3311·02 to 10 061·54)	–40·89 (−44·86 to −36·99)*	–43·39 (−47·17 to −39·63)*
··	Otitis media	0·09 (0·05 to 0·14)	0·04 (0·02 to 0·07)	–56·93 (−72·65 to −33·56)*	–58·53 (−73·68 to −36·05)*	219·81 (122·39 to 348·54)	205·95 (110·96 to 328·12)	–6·31 (−9·28 to −3·89)*	–10·69 (−13·55 to −8·35)*
··	Tracheal, bronchus, and lung cancer	22·02 (10·49 to 38·89)	27·35 (13·15 to 48·05)	24·20 (18·36 to 27·75)*	–4·80 (−8·93 to −2·34)*	508·53 (246·20 to 907·38)	615·93 (298·94 to 1093·44)	21·12 (14·10 to 25·46)*	–5·79 (−10·86 to −2·78)*
··	Breast cancer	9·27 (2·20 to 15·98)	10·30 (2·57 to 17·72)	11·10 (1·92 to 20·34)*	–13·39 (−20·31 to −6·37)*	287·48 (68·13 to 495·07)	313·04 (77·84 to 535·75)	8·89 (−0·35 to 19·03)	–12·87 (−20·20 to −4·98)*
··	Ischaemic heart disease	280·21 (219·44 to 344·16)	327·35 (257·71 to 402·31)	16·82 (12·22 to 21·36)*	–12·04 (−14·93 to −9·10)*	5727·36 (4517·00 to 7044·72)	6503·01 (5174·29 to 7952·09)	13·54 (9·44 to 17·40)*	–11·86 (−14·86 to −9·09)*
··	Ischaemic stroke	73·09 (53·89 to 96·52)	75·15 (54·65 to 99·65)	2·82 (−2·13 to 8·25)	–23·12 (−26·26 to −19·82)*	1420·64 (1071·30 to 1818·42)	1493·54 (1101·46 to 1909·51)	5·13 (0·25 to 10·05)*	–19·63 (−22·90 to −16·38)*
··	Haemorrhagic stroke	95·38 (73·00 to 120·43)	90·24 (68·61 to 114·07)	–5·39 (−8·89 to −1·60)*	–27·71 (−30·19 to −25·26)*	2278·60 (1754·81 to 2848·13)	2144·62 (1620·43 to 2692·11)	–5·88 (−9·72 to −2·17)*	–26·29 (−28·90 to −23·84)*
··	Chronic obstructive pulmonary disease	117·45 (54·19 to 204·35)	119·62 (57·14 to 206·04)	1·85 (−7·05 to 12·62)	–23·85 (−30·75 to −15·70)*	2373·48 (1129·66 to 4027·33)	2496·53 (1213·18 to 4231·63)	5·18 (−3·18 to 15·55)	–19·35 (−26·03 to −11·31)*
··	Diabetes mellitus	72·64 (27·70 to 111·58)	95·33 (36·10 to 146·43)	31·23 (27·87 to 34·52)*	–0·97 (−3·45 to 1·50)	2890·38 (1067·29 to 4591·29)	3581·43 (1319·94 to 5698·92)	23·91 (21·66 to 26·22)*	–3·11 (−4·92 to −1·38)*
**2**	**Alcohol and drug use: all causes**	**3001·71 (2622·51 to 3396·75)**	**3257·20 (2820·87 to 3733·04)**	8·51 (3·51 to 14·09)*	–13·22 (−17·30 to −8·58)*	**125 134·50 (113 568·68 to 136 796·97)**	**130 597·46 (117 360·41 to 144 336·48)**	4·37 (0·66 to 8·63)*	–13·06 (−16·29 to −9·26)*
3	Alcohol use: all causes	2605·72 (2228·59 to 3011·17)	2814·64 (2371·24 to 3292·68)	8·02 (2·40 to 14·06)*	–14·15 (−18·64 to −9·18)*	96 193·70 (86 180·20 to 106 743·49)	99 204·89 (88 310·44 to 111 168·34)	3·13 (−1·28 to 8·17)	–15·00 (−18·77 to −10·84)*
··	Drug-susceptible tuberculosis	304·97 (234·07 to 375·65)	253·07 (191·46 to 317·27)	–17·02 (−24·00 to −9·94)*	–33·11 (−38·92 to −27·55)*	11 260·98 (8804·92 to 13 625·09)	9208·69 (7176·84 to 11 319·15)	–18·22 (−24·75 to −11·48)*	–31·92 (−37·40 to −26·22)*
··	Multidrug-resistant tuberculosis without extensive drug resistance	33·42 (24·76 to 43·26)	23·46 (17·16 to 30·39)	–29·81 (−40·10 to −19·57)*	–42·99 (−51·14 to −34·77)*	1217·72 (916·99 to 1544·74)	820·14 (608·48 to 1048·27)	–32·65 (−41·96 to −23·08)*	–43·80 (−51·57 to −35·73)*
··	Extensively drug-resistant tuberculosis	2·44 (1·87 to 3·04)	3·98 (2·92 to 5·15)	63·02 (31·53 to 98·56)*	33·78 (8·90 to 62·02)*	91·46 (70·76 to 113·12)	140·71 (104·83 to 181·23)	53·84 (23·67 to 88·60)*	28·98 (3·78 to 57·67)*
··	Lower respiratory infections	97·10 (41·59 to 144·40)	113·58 (47·40 to 175·26)	16·97 (4·11 to 29·42)*	–9·81 (−18·30 to 1·14)	2531·19 (1331·95 to 3554·75)	2699·40 (1437·61 to 3944·68)	6·65 (−5·58 to 18·58)	–13·62 (−23·15 to −3·86)*
··	Lip and oral cavity cancer	49·44 (41·03 to 57·44)	66·24 (54·69 to 77·03)	33·98 (27·04 to 41·61)*	3·26 (−1·91 to 8·84)	1375·21 (1162·65 to 1574·04)	1769·38 (1482·47 to 2028·79)	28·66 (21·40 to 36·63)*	1·34 (−4·08 to 7·41)
··	Nasopharynx cancer	24·19 (22·28 to 26·03)	28·38 (25·63 to 31·15)	17·33 (7·75 to 26·54)*	–7·62 (−15·07 to −0·25)*	758·75 (706·19 to 809·31)	843·69 (766·09 to 922·53)	11·19 (1·77 to 20·77)*	–10·61 (−18·09 to −3·03)*
··	Other pharynx cancer	33·86 (27·61 to 39·91)	46·29 (37·28 to 55·41)	36·70 (24·71 to 47·23)*	5·77 (−3·34 to 13·91)	970·39 (799·90 to 1139·01)	1285·14 (1045·87 to 1530·24)	32·43 (20·29 to 42·99)*	3·89 (−5·37 to 12·21)
··	Oesophageal cancer	116·52 (92·63 to 140·34)	130·55 (104·87 to 157·78)	12·05 (6·25 to 19·44)*	–14·03 (−18·42 to −8·23)*	2838·08 (2305·86 to 3387·67)	3052·59 (2475·16 to 3672·35)	7·56 (1·96 to 14·53)*	–16·61 (−20·92 to −11·10)*
··	Colon and rectum cancer	97·08 (77·64 to 116·68)	116·81 (92·14 to 141·81)	20·33 (13·27 to 28·53)*	–8·92 (−14·35 to −2·95)*	2172·68 (1757·07 to 2580·89)	2544·90 (2029·33 to 3047·11)	17·13 (10·21 to 25·62)*	–9·24 (−14·62 to −2·83)*
··	Liver cancer due to alcohol use	99·05 (83·17 to 116·11)	129·18 (109·73 to 150·41)	30·41 (22·61 to 40·28)*	–0·01 (−6·03 to 7·33)	2281·36 (1911·37 to 2709·93)	2924·48 (2462·18 to 3399·46)	28·19 (20·40 to 38·54)*	–0·56 (−6·67 to 7·20)
··	Larynx cancer	26·71 (17·87 to 34·27)	29·80 (19·32 to 38·59)	11·58 (5·49 to 18·14)*	–14·18 (−18·81 to −9·19)*	709·81 (474·31 to 897·37)	764·38 (497·31 to 976·37)	7·69 (1·59 to 14·01)*	–16·32 (−20·98 to −11·38)*
··	Breast cancer	52·75 (43·43 to 63·43)	59·24 (47·43 to 72·53)	12·31 (5·75 to 20·05)*	–14·02 (−18·98 to −8·03)*	1443·76 (1178·62 to 1753·45)	1565·91 (1245·66 to 1961·00)	8·46 (1·75 to 16·23)*	–14·40 (−19·77 to −8·23)*
··	Ischaemic heart disease	–30·76 (−211·14 to 168·37)	–24·23 (−241·48 to 206·18)	–21·24 (−371·35 to 331·24)	–41·97 (−257·08 to 194·50)	767·37 (−2850·37 to 4692·06)	1084·03 (−3136·85 to 5556·97)	41·27 (−352·40 to 255·58)	35·77 (−286·02 to 364·77)
··	Ischaemic stroke	106·64 (44·65 to 173·13)	124·22 (55·72 to 200·79)	16·49 (0·38 to 42·96)*	–11·74 (−23·99 to 13·60)	2508·68 (1269·21 to 3765·46)	2930·95 (1519·90 to 4397·58)	16·83 (3·14 to 35·51)*	–10·07 (−20·90 to 5·69)
··	Haemorrhagic stroke	418·95 (317·05 to 526·94)	457·66 (345·46 to 572·96)	9·24 (3·10 to 17·61)*	–16·31 (−21·14 to −9·54)*	10 365·31 (7962·87 to 12 814·02)	10 957·49 (8335·55 to 13 516·75)	5·71 (0·00 to 13·30)	–16·87 (−21·50 to −10·55)*
··	Hypertensive heart disease	96·07 (66·61 to 126·07)	131·89 (86·83 to 176·95)	37·28 (18·72 to 50·94)*	1·92 (−11·24 to 12·00)	1987·17 (1428·04 to 2584·36)	2547·33 (1757·70 to 3394·79)	28·19 (13·24 to 39·90)*	–0·64 (−12·18 to 8·58)
··	Alcoholic cardiomyopathy	87·36 (72·97 to 97·29)	83·31 (67·17 to 102·89)	–4·64 (−21·27 to 17·01)	–24·04 (−36·79 to −7·53)*	2877·83 (2413·88 to 3220·47)	2590·34 (2055·10 to 3239·64)	–9·99 (−27·64 to 14·31)	–26·31 (−40·43 to −7·57)*
··	Atrial fibrillation and flutter	17·55 (11·75 to 24·14)	25·02 (16·86 to 35·53)	42·50 (32·14 to 53·42)*	–0·75 (−7·75 to 7·02)	542·25 (373·15 to 751·73)	722·89 (496·23 to 1010·00)	33·31 (25·87 to 42·12)*	0·33 (−5·56 to 6·94)
··	Cirrhosis and other chronic liver diseases due to alcohol use	294·43 (271·56 to 321·29)	334·68 (306·28 to 371·66)	13·67 (8·76 to 19·55)*	–10·98 (−14·62 to −6·48)*	8874·48 (8108·96 to 9683·50)	9748·69 (8868·52 to 10 855·84)	9·85 (4·80 to 15·93)*	–11·76 (−15·67 to −6·87)*
··	Pancreatitis	31·98 (25·54 to 39·43)	37·26 (28·83 to 47·01)	16·51 (5·09 to 29·89)*	–6·79 (−15·73 to 3·63)	1075·26 (892·23 to 1306·25)	1196·59 (955·03 to 1487·13)	11·28 (−1·10 to 26·14)	–8·13 (−18·00 to 3·87)
··	Epilepsy	20·88 (16·15 to 25·49)	22·02 (16·75 to 27·46)	5·48 (−0·54 to 13·84)	–11·53 (−16·52 to −4·66)*	1810·40 (1311·06 to 2355·11)	1903·17 (1362·83 to 2511·36)	5·12 (−3·66 to 14·38)	–9·55 (−17·15 to −1·60)*
··	Alcohol use disorders	171·96 (150·67 to 183·41)	173·82 (145·45 to 190·83)	1·08 (−7·15 to 10·46)	–17·62 (−24·31 to −10·21)*	15 555·03 (12 602·23 to 19 092·15)	16 237·15 (12 996·82 to 19 945·76)	4·39 (0·39 to 8·57)*	–10·98 (−14·60 to −7·48)*
··	Diabetes mellitus	6·96 (−20·42 to 34·90)	10·11 (−24·38 to 45·22)	45·21 (−231·27 to 359·63)	28·01 (−228·39 to 231·53)	529·10 (−727·58 to 1861·28)	712·23 (−881·16 to 2351·99)	34·61 (−144·45 to 218·89)	9·02 (−194·30 to 145·24)
··	Pedestrian road injuries	64·98 (37·59 to 97·10)	66·16 (38·29 to 99·80)	1·80 (−6·33 to 10·56)	–15·65 (−22·20 to −8·54)*	2844·80 (1646·88 to 4243·67)	2791·74 (1612·72 to 4240·54)	–1·87 (−9·54 to 6·31)	–16·21 (−22·63 to −9·41)*
··	Cyclist road injuries	9·71 (5·62 to 14·58)	10·09 (5·76 to 15·01)	3·95 (−4·99 to 14·63)	–14·35 (−21·62 to −5·98)*	595·27 (342·91 to 899·58)	647·09 (367·80 to 990·06)	8·71 (0·54 to 17·14)*	–8·96 (−15·42 to −1·95)*
··	Motorcyclist road injuries	32·74 (18·48 to 49·86)	32·45 (18·55 to 49·44)	–0·89 (−8·65 to 8·24)	–13·90 (−20·39 to −6·20)*	1872·33 (1066·29 to 2824·07)	1857·69 (1065·70 to 2828·52)	–0·78 (−8·22 to 7·77)	–13·07 (−19·49 to −5·77)*
··	Motor vehicle road injuries	62·76 (36·61 to 92·36)	60·04 (34·52 to 88·42)	–4·34 (−10·50 to 3·86)	–18·19 (−23·45 to −11·39)*	3304·16 (1937·56 to 4824·23)	3120·49 (1833·43 to 4562·31)	–5·56 (−11·62 to 2·27)	–17·65 (−22·82 to −11·04)*
··	Other road injuries	1·65 (0·97 to 2·49)	1·61 (0·94 to 2·45)	–2·42 (−9·91 to 8·37)	–18·54 (−24·61 to −9·67)*	131·93 (76·34 to 202·62)	160·16 (89·43 to 251·23)	21·40 (11·19 to 32·12)*	2·40 (−5·75 to 10·70)
··	Other transport injuries	11·79 (6·92 to 17·27)	11·88 (6·96 to 17·74)	0·76 (−6·67 to 10·60)	–15·17 (−21·42 to −7·08)*	696·53 (403·45 to 1034·74)	717·10 (414·64 to 1080·93)	2·95 (−3·94 to 11·46)	–11·88 (−17·72 to −5·08)*
··	Drowning	16·47 (7·29 to 27·24)	15·16 (6·74 to 24·74)	–7·94 (−14·17 to 0·87)	–21·73 (−26·65 to −14·33)*	732·89 (326·96 to 1216·93)	632·25 (274·66 to 1023·81)	–13·73 (−20·25 to −4·80)*	–24·55 (−29·67 to −17·49)*
··	Fire, heat, and hot substances	7·88 (3·53 to 12·89)	7·03 (3·24 to 11·36)	–10·85 (−19·34 to −0·76)*	–27·82 (−34·28 to −19·79)*	412·06 (184·41 to 675·60)	386·39 (172·68 to 635·92)	–6·23 (−14·00 to 2·92)	–21·30 (−27·76 to −13·73)*
··	Poisonings	3·67 (1·62 to 6·18)	3·36 (1·50 to 5·53)	–8·39 (−23·07 to 7·90)	–23·79 (−36·16 to −10·59)*	166·84 (74·30 to 278·34)	150·08 (67·35 to 244·42)	–10·05 (−22·18 to 4·15)	–23·08 (−33·23 to −11·32)*
··	Unintentional firearm injuries	1·74 (0·76 to 2·96)	1·56 (0·70 to 2·66)	–10·07 (−17·83 to −2·15)*	–23·30 (−30·13 to −16·67)*	94·71 (41·83 to 158·27)	86·98 (38·37 to 146·51)	–8·16 (−15·61 to −0·64)*	–19·85 (−26·43 to −13·45)*
··	Other unintentional injuries	7·73 (3·29 to 12·94)	6·82 (2·88 to 11·15)	–11·82 (−20·38 to −1·00)*	–24·85 (−31·85 to −16·19)*	576·20 (256·50 to 988·39)	568·51 (246·31 to 967·31)	–1·33 (−8·94 to 7·65)	–15·84 (−21·88 to −8·52)*
··	Self-harm by firearm	15·07 (8·50 to 21·95)	15·54 (8·69 to 22·61)	3·13 (−9·93 to 18·73)	–13·75 (−24·66 to −0·52)*	641·41 (369·21 to 940·23)	638·64 (368·84 to 936·40)	–0·43 (−12·27 to 14·69)	–14·04 (−24·48 to −0·99)*
··	Self-harm by other specified means	145·40 (85·30 to 202·28)	145·14 (87·17 to 202·07)	–0·18 (−9·24 to 11·19)	–16·89 (−24·27 to −7·37)*	6077·14 (3594·27 to 8488·31)	5860·44 (3530·20 to 8127·86)	–3·57 (−12·54 to 8·28)	–17·50 (−25·19 to −7·78)*
··	Assault by firearm	27·84 (14·26 to 41·44)	28·44 (14·59 to 43·20)	2·15 (−5·82 to 10·65)	–9·49 (−16·80 to −2·40)*	1493·88 (775·66 to 2210·77)	1501·73 (766·64 to 2276·30)	0·53 (−7·60 to 8·69)	–9·64 (−17·10 to −2·33)*
··	Assault by sharp object	18·56 (10·94 to 28·21)	15·99 (9·34 to 25·76)	–13·83 (−22·48 to −1·01)*	–25·17 (−32·85 to −13·74)*	975·52 (582·21 to 1474·15)	839·22 (492·92 to 1317·26)	–13·97 (−22·24 to −2·48)*	–24·22 (−31·44 to −13·76)*
··	Assault by other means	18·18 (10·48 to 27·32)	17·03 (9·98 to 26·30)	–6·33 (−19·70 to 12·35)	–20·14 (−31·48 to −4·27)*	1033·77 (611·33 to 1542·23)	996·09 (595·09 to 1505·66)	–3·64 (−15·24 to 10·31)	–16·82 (−26·73 to −4·29)*
3	Drug use: all causes	405·49 (376·08 to 438·09)	451·82 (420·40 to 486·77)	11·42 (6·47 to 17·02)*	–6·66 (−10·58 to −2·17)*	29 405·94 (25 497·29 to 33 535·95)	31 836·26 (27 445·88 to 36 580·02)	8·26 (5·09 to 11·72)*	–6·25 (−9·21 to −3·39)*
··	Drug-susceptible HIV/AIDS - Tuberculosis	17·88 (11·55 to 26·51)	9·10 (5·87 to 13·66)	–49·13 (−53·01 to −44·80)*	–56·62 (−59·99 to −52·92)*	845·64 (545·53 to 1245·37)	452·57 (301·06 to 663·93)	–46·48 (−50·70 to −41·82)*	–53·69 (−57·35 to −49·74)*
··	Multidrug-resistant HIV/AIDS - Tuberculosis without extensive drug resistance	2·54 (1·51 to 4·11)	1·20 (0·70 to 1·95)	–52·97 (−60·55 to −44·20)*	–59·62 (−66·05 to −52·09)*	118·88 (70·41 to 191·24)	56·61 (33·39 to 90·84)	–52·38 (−60·30 to −43·58)*	–58·61 (−65·43 to −50·96)*
··	Extensively drug-resistant HIV/AIDS - Tuberculosis	0·20 (0·12 to 0·33)	0·29 (0·16 to 0·47)	44·03 (18·74 to 75·38)*	25·61 (3·55 to 52·91)*	9·74 (5·65 to 16·23)	13·91 (7·92 to 22·69)	42·89 (17·38 to 74·95)*	25·59 (3·29 to 53·36)*
··	HIV/AIDS resulting in other diseases	74·37 (58·68 to 92·92)	53·21 (43·04 to 65·66)	–28·45 (−33·86 to −21·66)*	–38·29 (−42·87 to −32·58)*	3651·47 (2871·87 to 4584·05)	2670·75 (2142·39 to 3303·74)	–26·86 (−31·94 to −20·21)*	–36·29 (−40·83 to −30·65)*
··	Hepatitis B	0·32 (0·25 to 0·41)	0·31 (0·24 to 0·40)	–1·97 (−9·09 to 5·66)	–20·37 (−26·08 to −14·19)*	12·08 (9·29 to 15·29)	11·54 (8·88 to 14·71)	–4·47 (−12·19 to 3·69)	–20·80 (−27·31 to −14·08)*
··	Hepatitis C	0·42 (0·32 to 0·54)	0·48 (0·37 to 0·62)	14·60 (−1·60 to 33·24)	–6·68 (−19·61 to 8·34)	16·51 (12·58 to 21·05)	18·25 (13·84 to 23·78)	10·51 (−3·77 to 27·77)	–6·31 (−18·68 to 7·88)
··	Liver cancer due to hepatitis B	1·52 (1·16 to 1·99)	2·56 (1·97 to 3·32)	68·29 (57·54 to 79·01)*	33·74 (25·83 to 41·46)*	49·42 (37·44 to 64·43)	78·10 (59·59 to 101·47)	58·03 (46·77 to 68·88)*	28·05 (19·75 to 36·41)*
··	Liver cancer due to hepatitis C	37·35 (31·94 to 43·13)	62·46 (54·75 to 70·85)	67·23 (59·37 to 75·65)*	30·03 (23·93 to 36·56)*	998·00 (853·25 to 1148·69)	1558·54 (1357·79 to 1774·17)	56·17 (48·56 to 63·89)*	23·45 (17·44 to 29·28)*
··	Cirrhosis and other chronic liver diseases due to hepatitis B	1·54 (1·17 to 2·00)	2·44 (1·84 to 3·15)	58·98 (49·73 to 70·92)*	27·20 (20·24 to 36·57)*	53·65 (40·20 to 71·54)	82·78 (61·00 to 109·04)	54·31 (44·79 to 66·22)*	26·26 (18·84 to 35·57)*
··	Cirrhosis and other chronic liver diseases due to hepatitis C	106·76 (93·44 to 121·61)	138·75 (122·40 to 157·72)	29·96 (23·69 to 37·62)*	4·48 (−0·44 to 10·18)	3778·13 (3302·73 to 4345·35)	4702·15 (4121·52 to 5404·71)	24·46 (18·21 to 31·86)*	2·04 (−2·98 to 7·86)
··	Opioid use disorders	74·85 (60·54 to 81·38)	86·20 (72·66 to 94·65)	15·18 (2·17 to 30·71)*	–1·48 (−12·84 to 11·27)	12 811·42 (10 013·73 to 15 686·88)	14 781·97 (11 375·36 to 18 250·91)	15·38 (11·23 to 19·48)*	0·65 (−2·95 to 4·18)
··	Cocaine use disorders	8·24 (6·34 to 10·60)	8·80 (7·06 to 11·27)	6·82 (−1·13 to 16·93)	–10·56 (−17·12 to −1·95)*	1060·25 (779·98 to 1375·09)	1153·57 (846·82 to 1511·30)	8·80 (5·14 to 12·25)*	–4·91 (−8·34 to −1·70)*
··	Amphetamine use disorders	4·47 (3·56 to 5·51)	5·22 (4·30 to 6·85)	16·67 (5·26 to 32·32)*	–1·15 (−10·71 to 12·39)	833·85 (566·91 to 1189·89)	881·40 (599·29 to 1242·60)	5·70 (1·83 to 10·23)*	–3·68 (−7·47 to 0·32)
··	Cannabis use disorders	··	··	··	··	623·53 (388·95 to 904·77)	646·48 (400·64 to 944·87)	3·68 (1·23 to 5·98)*	–4·19 (−5·93 to −2·35)*
··	Other drug use disorders	37·24 (33·94 to 44·21)	43·52 (39·36 to 52·88)	16·89 (6·86 to 25·63)*	–3·22 (−11·47 to 3·81)	2671·45 (2234·42 to 3175·76)	2921·41 (2424·28 to 3502·95)	9·36 (3·77 to 14·54)*	–4·27 (−9·28 to 0·23)
··	Self-harm by firearm	4·91 (3·32 to 7·06)	5·40 (3·70 to 7·78)	10·05 (2·38 to 17·43)*	–5·02 (−11·27 to 1·34)	240·29 (162·77 to 343·62)	257·23 (178·15 to 366·05)	7·05 (−0·19 to 14·74)	–5·28 (−11·59 to 1·34)
··	Self-harm by other specified means	32·90 (22·24 to 46·44)	31·89 (21·41 to 46·12)	–3·07 (−9·44 to 4·69)	–16·34 (−21·84 to −9·54)*	1631·61 (1100·19 to 2333·27)	1549·00 (1040·55 to 2246·29)	–5·06 (−11·34 to 2·40)	–16·13 (−21·64 to −9·35)*
**2**	**Dietary risks: all causes**	**9263·92 (7965·82 to 10 628·04)**	**10 301·54 (8795·36 to 11 912·63)**	11·20 (8·54 to 13·87)*	–16·37 (−18·22 to −14·45)*	**210 958·84 (184 793·68 to 239 486·60)**	**229 065·54 (197 533·69 to 262 533·95)**	8·58 (6·07 to 11·05)*	–15·52 (−17·39 to −13·64)*
3	Diet low in fruits: all causes	2338·84 (1488·15 to 3345·70)	2361·20 (1446·10 to 3447·83)	0·96 (−4·64 to 5·30)	–22·86 (−26·87 to −19·70)*	61 173·38 (40 395·88 to 84 837·17)	60 982·39 (38 806·06 to 87 349·09)	–0·31 (−5·47 to 3·47)	–21·65 (−25·65 to −18·74)*
··	Lip and oral cavity cancer	8·96 (0·00 to 20·23)	10·98 (0·00 to 25·14)	22·53 (13·43 to 148·39)*	–5·53 (−12·25 to 73·45)	247·05 (0·01 to 557·47)	293·30 (0·01 to 670·94)	18·72 (−7·12 to 144·18)	–6·38 (−20·76 to 61·24)
··	Nasopharynx cancer	3·69 (0·00 to 8·07)	3·64 (0·00 to 8·23)	–1·26 (−12·22 to 78·86)	–22·37 (−30·72 to 41·90)	114·17 (0·00 to 249·36)	107·70 (0·00 to 242·52)	–5·67 (−16·31 to 62·03)	–24·38 (−32·90 to 46·23)
··	Other pharynx cancer	6·21 (0·00 to 13·94)	7·56 (0·00 to 16·62)	21·82 (9·01 to 33·94)*	–5·76 (−15·57 to 3·43)	173·32 (0·01 to 391·06)	205·53 (0·02 to 451·27)	18·58 (5·42 to 30·03)*	–6·90 (−17·08 to 2·37)
··	Oesophageal cancer	81·00 (18·89 to 146·29)	73·59 (16·58 to 138·27)	–9·14 (−15·59 to −4·33)*	–30·50 (−35·29 to −26·84)*	1881·66 (442·78 to 3381·15)	1670·32 (375·53 to 3114·88)	–11·23 (−17·41 to −6·63)*	–31·32 (−36·09 to −27·72)*
··	Larynx cancer	6·35 (0·00 to 13·79)	6·66 (0·00 to 14·69)	4·72 (−2·22 to 16·96)	–19·44 (−24·54 to −4·98)*	164·55 (0·01 to 358·39)	167·33 (0·01 to 367·29)	1·69 (−5·54 to 15·18)	–20·88 (−26·54 to −12·09)*
··	Tracheal, bronchus, and lung cancer	147·27 (57·31 to 247·93)	159·12 (61·41 to 273·72)	8·04 (2·20 to 12·40)*	–16·97 (−21·38 to −13·70)*	3344·09 (1317·12 to 5603·00)	3448·19 (1332·88 to 5928·34)	3·11 (−2·71 to 7·59)	–19·76 (−24·22 to −16·39)*
··	Ischaemic heart disease	892·64 (340·23 to 1554·99)	966·03 (348·99 to 1694·22)	8·22 (3·48 to 12·22)*	–18·16 (−21·67 to −15·34)*	20 722·33 (8039·33 to 35 506·28)	21 579·73 (7912·78 to 37 461·71)	4·14 (−0·42 to 7·96)	–18·07 (−21·50 to −15·19)*
··	Ischaemic stroke	425·78 (220·41 to 640·03)	409·14 (209·36 to 633·75)	–3·91 (−9·56 to 0·56)	–27·14 (−31·31 to −23·83)*	10 807·82 (5946·98 to 15 936·93)	10 769·53 (5810·94 to 16 420·82)	–0·35 (−6·08 to 3·97)	–23·01 (−27·39 to −19·74)*
··	Haemorrhagic stroke	669·49 (357·45 to 1028·72)	607·16 (318·94 to 943·73)	–9·31 (−13·97 to −5·88)*	–29·80 (−33·33 to −27·21)*	18 624·46 (10 254·35 to 28 041·51)	16 953·42 (9075·66 to 25 909·90)	–8·97 (−13·26 to −5·62)*	–27·94 (−31·29 to −25·40)*
··	Diabetes mellitus	97·44 (21·64 to 185·22)	117·31 (26·05 to 226·54)	20·39 (16·39 to 23·96)*	–7·47 (−10·40 to −4·80)*	5093·93 (1087·22 to 9951·32)	5787·36 (1191·75 to 11 487·93)	13·61 (9·08 to 16·83)*	–9·24 (−12·61 to −6·89)*
3	Diet low in vegetables: all causes	1473·57 (722·80 to 2392·73)	1519·65 (717·79 to 2507·05)	3·13 (−2·27 to 7·50)	–21·93 (−25·70 to −18·91)*	35 185·99 (17 828·73 to 55 574·27)	35 489·09 (17 454·48 to 57 174·79)	0·86 (−3·84 to 4·70)	–20·82 (−24·32 to −17·95)*
··	Ischaemic heart disease	1040·01 (407·37 to 1823·09)	1121·42 (431·72 to 2003·83)	7·83 (3·88 to 11·53)*	–18·99 (−21·88 to −16·21)*	23 371·67 (9330·74 to 40 305·40)	24 519·18 (9683·15 to 42 823·73)	4·91 (1·30 to 8·34)*	–17·79 (−20·53 to −15·19)*
··	Ischaemic stroke	166·07 (37·85 to 314·01)	158·64 (36·08 to 301·92)	–4·47 (−9·07 to 0·03)	–27·64 (−31·09 to −24·15)*	4235·33 (981·83 to 7798·25)	4145·55 (970·51 to 7782·19)	–2·12 (−6·67 to 2·32)	–24·26 (−27·67 to −20·70)*
··	Haemorrhagic stroke	267·49 (76·09 to 493·58)	239·58 (68·31 to 446·94)	–10·43 (−14·24 to −7·21)*	–30·59 (−33·61 to −28·07)*	7578·98 (2194·05 to 13 820·71)	6824·37 (1980·63 to 12 581·13)	–9·96 (−13·45 to −6·82)*	–28·54 (−31·26 to −26·01)*
3	Diet low in legumes: all causes	594·09 (262·56 to 988·59)	672·47 (288·67 to 1113·67)	13·19 (9·22 to 17·17)*	–15·35 (−18·30 to −12·39)*	13 316·19 (5884·98 to 22 031·26)	14 214·45 (6113·49 to 23 571·09)	6·75 (2·54 to 10·70)*	–16·05 (−19·24 to −12·96)*
··	Ischaemic heart disease	594·09 (262·56 to 988·59)	672·47 (288·67 to 1113·67)	13·19 (9·22 to 17·17)*	–15·35 (−18·30 to −12·39)*	13 316·19 (5884·98 to 22 031·26)	14 214·45 (6113·49 to 23 571·09)	6·75 (2·54 to 10·70)*	–16·05 (−19·24 to −12·96)*
3	Diet low in whole grains: all causes	2253·17 (1501·70 to 3156·55)	2498·69 (1662·92 to 3507·35)	10·90 (7·75 to 14·17)*	–16·06 (−18·29 to −13·58)*	57 301·21 (38 974·48 to 78 891·27)	62 596·11 (42 330·99 to 86 426·66)	9·24 (6·33 to 12·18)*	–14·31 (−16·55 to −12·03)*
··	Ischaemic heart disease	1270·67 (755·80 to 1894·10)	1457·40 (862·42 to 2171·59)	14·70 (11·16 to 18·49)*	–14·16 (−16·69 to −11·37)*	27 241·14 (16 286·00 to 40 251·87)	29 799·09 (17 817·53 to 44 415·59)	9·39 (5·80 to 13·05)*	–14·50 (−17·27 to −11·67)*
··	Ischaemic stroke	333·45 (212·24 to 475·32)	348·95 (218·28 to 502·60)	4·65 (0·84 to 8·85)*	–20·70 (−23·49 to −17·61)*	8714·95 (5616·91 to 12 308·00)	9522·32 (6076·81 to 13 506·64)	9·26 (5·36 to 13·06)*	–15·41 (−18·38 to −12·37)*
··	Haemorrhagic stroke	500·51 (320·02 to 708·04)	505·42 (325·05 to 712·14)	0·98 (−1·36 to 3·60)	–21·76 (−23·58 to −19·73)*	13 827·83 (8981·48 to 19 377·52)	13 897·80 (9065·15 to 19 424·10)	0·51 (−1·88 to 3·19)	–20·32 (−22·21 to −18·22)*
··	Diabetes mellitus	148·55 (79·68 to 232·77)	186·92 (100·27 to 288·86)	25·83 (23·33 to 28·40)*	–3·98 (−5·83 to −2·10)*	7517·29 (3992·35 to 11 904·80)	9376·90 (4975·03 to 14 870·23)	24·74 (22·77 to 26·95)*	–1·18 (−2·79 to 0·60)
3	Diet low in nuts and seeds: all causes	1879·32 (1192·82 to 2585·76)	2155·04 (1349·07 to 2965·36)	14·67 (11·69 to 17·85)*	–13·64 (−15·74 to −11·28)*	44 820·23 (29 633·64 to 60 259·65)	49 492·97 (32 430·01 to 66 636·04)	10·43 (7·52 to 13·44)*	–13·32 (−15·60 to −10·94)*
··	Ischaemic heart disease	1764·49 (1091·76 to 2446·34)	2011·41 (1232·19 to 2804·53)	13·99 (10·84 to 17·36)*	–14·23 (−16·49 to −11·77)*	38 955·49 (24 572·90 to 53 226·78)	42 449·79 (26 679·81 to 58 466·30)	8·97 (5·77 to 12·22)*	–14·60 (−17·01 to −12·09)*
··	Diabetes mellitus	114·84 (56·65 to 181·83)	143·63 (70·17 to 227·67)	25·08 (22·53 to 27·51)*	–4·23 (−6·13 to −2·42)*	5864·75 (2906·59 to 9518·13)	7043·18 (3475·81 to 11 463·51)	20·09 (17·73 to 22·23)*	–4·46 (−6·22 to −2·86)*
3	Diet low in milk: all causes	100·32 (35·93 to 172·49)	123·21 (45·00 to 213·85)	22·82 (16·96 to 27·74)*	–7·13 (−11·47 to −3·44)*	2168·24 (770·59 to 3718·76)	2581·50 (930·45 to 4435·44)	19·06 (12·71 to 24·09)*	–7·45 (−12·28 to −3·58)*
··	Colon and rectum cancer	100·32 (35·93 to 172·49)	123·21 (45·00 to 213·85)	22·82 (16·96 to 27·74)*	–7·13 (−11·47 to −3·44)*	2168·24 (770·59 to 3718·76)	2581·50 (930·45 to 4435·44)	19·06 (12·71 to 24·09)*	–7·45 (−12·28 to −3·58)*
3	Diet high in red meat: all causes	22·59 (10·56 to 36·85)	31·88 (15·08 to 51·44)	41·16 (33·97 to 50·38)*	7·35 (1·88 to 14·18)*	893·25 (363·00 to 1485·05)	1247·33 (508·19 to 2077·33)	39·64 (33·18 to 47·68)*	10·30 (4·94 to 16·49)*
··	Colon and rectum cancer	12·29 (2·60 to 22·66)	17·88 (3·78 to 32·44)	45·48 (36·21 to 56·91)*	9·72 (2·77 to 18·65)*	268·60 (57·30 to 493·26)	377·78 (80·75 to 679·93)	40·65 (32·05 to 50·68)*	9·01 (2·19 to 16·95)*
··	Diabetes mellitus	10·29 (1·45 to 18·98)	14·00 (1·98 to 25·60)	36·00 (29·34 to 43·64)*	4·40 (−0·81 to 10·51)	624·65 (87·34 to 1153·77)	869·55 (120·69 to 1598·30)	39·21 (32·22 to 47·76)*	10·89 (5·38 to 17·71)*
3	Diet high in processed meat: all causes	146·70 (29·93 to 269·63)	139·62 (29·84 to 271·40)	–4·83 (−15·30 to 5·01)	–28·85 (−36·31 to −21·48)*	3499·30 (1121·42 to 6024·02)	3196·04 (1091·35 to 5836·23)	–8·67 (−19·23 to 2·16)	–28·87 (−36·93 to −20·27)*
··	Colon and rectum cancer	9·84 (5·09 to 15·48)	10·28 (5·24 to 16·68)	4·45 (−3·10 to 11·58)	–21·45 (−26·99 to −16·30)*	196·63 (102·18 to 308·02)	194·85 (98·50 to 321·61)	–0·90 (−8·58 to 6·64)	–23·50 (−29·34 to −17·79)*
··	Ischaemic heart disease	121·78 (5·27 to 240·19)	114·54 (4·50 to 238·06)	–5·94 (−17·54 to 5·48)	–29·84 (−38·03 to −21·64)*	2421·47 (107·64 to 4745·93)	2116·23 (82·94 to 4522·45)	–12·61 (−24·84 to 0·05)	–32·11 (−41·45 to −22·48)*
··	Diabetes mellitus	15·09 (7·05 to 24·10)	14·80 (6·45 to 25·75)	–1·89 (−13·21 to 10·00)	–25·27 (−33·73 to −16·41)*	881·20 (420·66 to 1466·58)	884·96 (395·86 to 1583·28)	0·43 (−9·91 to 10·71)	–20·75 (−28·79 to −12·62)*
3	Diet high in sugar-sweetened beverages: all causes	17·80 (11·49 to 29·39)	22·56 (15·33 to 33·36)	26·77 (−20·93 to 56·21)	–4·36 (−40·34 to 19·43)	605·81 (401·43 to 932·96)	779·51 (523·90 to 1145·18)	28·67 (−13·65 to 50·53)	1·96 (−32·16 to 19·85)
··	Oesophageal cancer	0·29 (0·09 to 0·55)	0·37 (0·11 to 0·70)	28·96 (−25·95 to 56·82)	–1·48 (−44·00 to 19·43)	7·08 (2·10 to 13·48)	8·90 (2·55 to 17·04)	25·73 (−26·29 to 51·70)	–2·25 (−41·60 to 17·56)
··	Colon and rectum cancer	0·36 (0·21 to 0·69)	0·43 (0·27 to 0·65)	20·23 (−39·42 to 72·88)	–9·20 (−53·49 to 29·91)	8·10 (4·86 to 15·13)	9·67 (6·13 to 14·54)	19·38 (−40·62 to 73·22)	–7·22 (−53·29 to 33·26)
··	Liver cancer due to hepatitis B	0·11 (0·04 to 0·31)	0·16 (0·07 to 0·29)	46·97 (−48·94 to 107·46)	15·78 (−61·11 to 65·31)	3·43 (1·25 to 9·32)	4·91 (2·09 to 9·01)	43·11 (−47·86 to 105·26)	15·74 (−58·01 to 66·01)
··	Liver cancer due to hepatitis C	0·09 (0·04 to 0·17)	0·13 (0·06 to 0·22)	35·33 (−26·26 to 72·90)	2·46 (−42·04 to 30·86)	2·08 (0·95 to 3·96)	2·77 (1·23 to 4·80)	32·68 (−26·61 to 69·83)	2·19 (−45·21 to 30·56)
··	Liver cancer due to alcohol use	0·07 (0·03 to 0·14)	0·09 (0·04 to 0·15)	38·16 (−35·40 to 78·76)	5·72 (−51·04 to 33·84)	1·56 (0·67 to 3·18)	2·15 (0·98 to 3·68)	37·92 (−31·88 to 72·80)	7·05 (−47·30 to 34·90)
··	Liver cancer due to other causes	0·07 (0·03 to 0·22)	0·11 (0·05 to 0·19)	47·23 (−49·13 to 98·67)	14·78 (−59·69 to 53·95)	2·05 (0·74 to 5·72)	2·93 (1·22 to 5·21)	42·72 (−40·63 to 97·68)	14·45 (−56·86 to 57·63)
··	Gallbladder and biliary tract cancer	0·10 (0·06 to 0·17)	0·12 (0·07 to 0·20)	21·19 (−26·26 to 53·17)	–8·78 (−44·49 to 16·53)	2·17 (1·19 to 3·59)	2·56 (1·49 to 4·06)	18·44 (−25·82 to 54·57)	–8·47 (−43·46 to 17·51)
··	Pancreatic cancer	0·12 (0·04 to 0·25)	0·15 (0·05 to 0·27)	20·30 (−51·55 to 98·90)	–9·06 (−63·71 to 47·86)	2·64 (0·91 to 6·08)	3·12 (1·12 to 5·98)	18·12 (−53·72 to 96·56)	–8·66 (−65·03 to 47·86)
··	Breast cancer	0·15 (0·06 to 0·38)	0·17 (0·08 to 0·32)	14·33 (−56·82 to 109·36)	–14·46 (−67·38 to 58·17)	3·61 (1·40 to 9·75)	4·14 (1·92 to 7·86)	14·87 (−62·30 to 129·42)	–12·26 (−69·56 to 74·33)
··	Uterine cancer	0·10 (0·07 to 0·16)	0·13 (0·09 to 0·20)	31·24 (−14·19 to 56·34)	–1·19 (−31·40 to 16·65)	2·50 (1·60 to 3·91)	3·32 (2·20 to 4·84)	32·82 (−12·54 to 58·57)	2·48 (−32·80 to 21·96)
··	Ovarian cancer	0·03 (0·00 to 0·11)	0·03 (0·00 to 0·07)	–5·42 (−97·18 to 231·08)	–27·30 (−97·63 to 169·46)	0·81 (0·06 to 2·86)	0·75 (0·00 to 1·90)	–7·10 (−97·19 to 231·03)	–26·75 (−97·31 to 156·14)
··	Kidney cancer	0·13 (0·08 to 0·20)	0·17 (0·11 to 0·26)	34·51 (−0·86 to 60·98)	2·17 (−24·86 to 21·78)	3·08 (1·96 to 4·80)	4·06 (2·61 to 6·04)	31·80 (−2·65 to 56·85)	2·54 (−25·05 to 22·42)
··	Thyroid cancer	0·02 (0·01 to 0·03)	0·02 (0·01 to 0·04)	23·74 (−45·68 to 71·02)	–5·05 (−56·07 to 32·19)	0·48 (0·23 to 0·95)	0·58 (0·31 to 0·95)	22·78 (−42·37 to 72·84)	–2·72 (−53·99 to 35·78)
··	Non-Hodgkin lymphoma	0·07 (0·03 to 0·14)	0·08 (0·04 to 0·14)	14·80 (−48·12 to 89·49)	–11·36 (−59·15 to 44·86)	1·93 (0·88 to 3·84)	2·17 (1·02 to 3·70)	12·11 (−53·36 to 89·82)	–9·92 (−61·71 to 50·34)
··	Multiple myeloma	0·04 (0·02 to 0·07)	0·04 (0·02 to 0·08)	20·90 (−36·98 to 86·20)	–8·58 (−52·81 to 40·23)	0·80 (0·33 to 1·52)	0·96 (0·43 to 1·68)	19·88 (−41·55 to 79·48)	–7·25 (−53·09 to 39·99)
··	Acute lymphoid leukaemia	0·01 (0·01 to 0·03)	0·02 (0·01 to 0·03)	21·67 (−42·96 to 84·50)	–0·50 (−52·49 to 49·86)	0·60 (0·33 to 1·16)	0·72 (0·44 to 1·15)	20·31 (−42·71 to 77·73)	2·35 (−50·81 to 53·90)
··	Chronic lymphoid leukaemia	0·02 (0·01 to 0·03)	0·02 (0·01 to 0·03)	11·87 (−40·76 to 59·76)	–17·04 (−56·19 to 17·59)	0·31 (0·17 to 0·60)	0·34 (0·20 to 0·57)	9·98 (−47·66 to 65·06)	–15·46 (−59·33 to 28·08)
··	Acute myeloid leukaemia	0·04 (0·02 to 0·06)	0·04 (0·03 to 0·07)	20·64 (−33·37 to 72·72)	–5·40 (−47·60 to 32·17)	1·08 (0·62 to 1·88)	1·27 (0·79 to 2·04)	17·65 (−34·43 to 64·82)	–3·94 (−45·68 to 33·05)
··	Chronic myeloid leukaemia	0·01 (0·01 to 0·02)	0·01 (0·01 to 0·02)	–11·77 (−55·23 to 31·96)	–31·15 (−64·52 to 1·81)	0·31 (0·17 to 0·64)	0·26 (0·16 to 0·42)	–15·95 (−62·20 to 24·58)	–31·30 (−68·51 to 2·27)
··	Other leukaemia	0·04 (0·02 to 0·10)	0·04 (0·02 to 0·07)	7·12 (−62·16 to 97·24)	–16·02 (−65·44 to 48·79)	1·08 (0·54 to 3·48)	1·09 (0·64 to 1·86)	1·35 (−70·91 to 82·39)	–16·40 (−73·68 to 53·84)
··	Ischaemic heart disease	5·98 (3·78 to 9·98)	7·03 (4·58 to 10·61)	17·56 (−29·12 to 52·55)	–11·34 (−46·57 to 15·33)	150·69 (97·37 to 229·60)	176·63 (116·37 to 263·07)	17·21 (−23·17 to 43·80)	–7·75 (−40·83 to 13·00)
··	Ischaemic stroke	1·09 (0·61 to 2·32)	1·17 (0·73 to 1·80)	7·45 (−54·16 to 58·69)	–19·02 (−66·03 to 20·18)	31·26 (19·20 to 57·90)	36·59 (24·19 to 54·00)	17·06 (−40·45 to 50·57)	–9·27 (−55·99 to 17·68)
··	Haemorrhagic stroke	2·14 (1·36 to 3·88)	2·47 (1·65 to 3·69)	15·27 (−38·70 to 39·47)	–9·12 (−52·40 to 11·93)	72·43 (47·51 to 116·88)	84·66 (57·32 to 122·05)	16·90 (−28·03 to 35·60)	–4·88 (−42·13 to 11·16)
··	Hypertensive heart disease	0·94 (0·53 to 1·57)	1·33 (0·73 to 2·21)	41·34 (1·13 to 61·93)*	5·33 (−25·08 to 21·50)	22·35 (14·24 to 34·30)	30·09 (18·45 to 46·26)	34·67 (1·67 to 50·99)*	5·72 (−20·06 to 18·87)
··	Atrial fibrillation and flutter	0·18 (0·10 to 0·30)	0·25 (0·15 to 0·39)	44·03 (−1·62 to 70·54)	–0·79 (−32·88 to 17·51)	5·37 (3·13 to 8·88)	7·33 (4·22 to 11·54)	36·35 (0·50 to 59·28)*	2·11 (−24·17 to 18·00)
··	Asthma	0·15 (0·08 to 0·36)	0·14 (0·09 to 0·23)	–3·48 (−64·53 to 42·11)	–25·38 (−72·46 to 10·94)	15·00 (8·45 to 25·64)	17·32 (10·00 to 28·61)	15·47 (−28·42 to 40·68)	–3·65 (−40·85 to 18·12)
··	Gallbladder and biliary diseases	0·13 (0·08 to 0·21)	0·19 (0·12 to 0·28)	45·16 (5·28 to 65·76)*	7·18 (−20·39 to 22·76)	3·01 (1·95 to 4·61)	4·24 (2·75 to 6·19)	40·81 (3·82 to 57·89)*	10·74 (−18·83 to 24·22)
··	Alzheimer's disease and other dementias	1·09 (0·44 to 2·12)	1·57 (0·66 to 2·80)	43·79 (−31·20 to 81·61)	–1·32 (−48·85 to 25·19)	13·62 (5·90 to 27·29)	18·90 (7·82 to 34·07)	38·78 (−28·85 to 85·48)	0·35 (−48·69 to 32·06)
··	Diabetes mellitus	2·68 (1·80 to 3·83)	3·72 (2·50 to 5·30)	38·65 (13·79 to 51·31)*	6·23 (−14·28 to 15·85)	160·49 (104·01 to 235·77)	228·01 (146·36 to 338·55)	42·07 (24·20 to 52·10)*	14·03 (−0·94 to 22·21)
··	Chronic kidney disease due to diabetes mellitus	0·69 (0·34 to 1·18)	1·05 (0·53 to 1·79)	51·91 (15·47 to 70·56)*	14·19 (−12·15 to 27·71)	21·91 (9·80 to 37·27)	32·96 (15·37 to 55·88)	50·42 (16·36 to 69·24)*	15·99 (−9·49 to 29·45)
··	Chronic kidney disease due to hypertension	0·28 (0·12 to 0·51)	0·43 (0·18 to 0·78)	52·70 (−0·59 to 74·23)	10·13 (−27·47 to 28·35)	6·42 (3·12 to 10·98)	9·82 (4·86 to 16·38)	52·91 (2·60 to 73·37)*	15·09 (−21·34 to 31·55)
··	Chronic kidney disease due to glomerulonephritis	0·29 (0·14 to 0·50)	0·43 (0·20 to 0·73)	46·29 (22·10 to 61·05)*	11·21 (−7·71 to 21·11)	9·59 (3·88 to 17·35)	13·85 (5·67 to 24·73)	44·51 (18·51 to 60·36)*	13·38 (−7·28 to 24·40)
··	Chronic kidney disease due to other causes	0·30 (0·14 to 0·52)	0·45 (0·21 to 0·78)	51·23 (14·95 to 70·98)*	13·60 (−11·31 to 26·17)	9·12 (3·96 to 16·64)	13·55 (5·90 to 24·85)	48·59 (10·10 to 67·56)*	15·55 (−13·97 to 28·71)
··	Osteoarthritis	··	··	··	··	12·25 (6·32 to 21·91)	17·50 (9·15 to 29·79)	42·81 (−10·75 to 77·51)	12·04 (−30·98 to 38·72)
··	Low back pain	··	··	··	··	23·67 (12·74 to 49·51)	27·62 (16·04 to 44·45)	16·69 (−41·75 to 83·77)	–2·98 (−51·67 to 51·38)
··	Gout	··	··	··	··	1·82 (0·94 to 3·25)	2·48 (1·29 to 4·40)	36·03 (8·43 to 49·14)*	9·54 (−13·22 to 20·02)
··	Cataract	··	··	··	··	1·12 (0·43 to 3·45)	1·27 (0·64 to 2·43)	13·37 (−69·99 to 140·46)	–13·50 (−77·20 to 81·95)
3	Diet low in fibre: all causes	769·74 (446·50 to 1159·77)	877·85 (502·37 to 1337·53)	14·05 (10·69 to 17·13)*	–13·93 (−16·34 to −11·62)*	18 522·14 (10 865·99 to 27 596·25)	20 119·47 (11 653·46 to 30 430·15)	8·62 (5·17 to 11·61)*	–14·06 (−16·63 to −11·74)*
··	Colon and rectum cancer	77·72 (39·53 to 121·45)	92·53 (46·61 to 146·52)	19·05 (13·44 to 23·86)*	–10·11 (−14·25 to −6·60)*	1658·67 (852·86 to 2584·40)	1905·91 (965·68 to 3008·24)	14·91 (8·93 to 19·90)*	–10·61 (−15·08 to −6·81)*
··	Ischaemic heart disease	692·02 (395·74 to 1063·40)	785·32 (440·85 to 1225·40)	13·48 (9·90 to 16·76)*	–14·36 (−16·96 to −11·96)*	16 863·47 (9820·85 to 25 673·28)	18 213·56 (10 409·47 to 28 118·37)	8·01 (4·25 to 11·19)*	–14·42 (−17·20 to −11·94)*
3	Diet low in calcium: all causes	135·49 (86·00 to 194·76)	159·88 (101·07 to 232·62)	18·00 (11·94 to 22·51)*	–10·61 (−15·09 to −7·34)*	2935·65 (1882·89 to 4176·04)	3353·07 (2127·08 to 4832·47)	14·22 (7·84 to 18·84)*	–11·07 (−15·93 to −7·61)*
··	Colon and rectum cancer	135·49 (86·00 to 194·76)	159·88 (101·07 to 232·62)	18·00 (11·94 to 22·51)*	–10·61 (−15·09 to −7·34)*	2935·65 (1882·89 to 4176·04)	3353·07 (2127·08 to 4832·47)	14·22 (7·84 to 18·84)*	–11·07 (−15·93 to −7·61)*
3	Diet low in seafood omega 3 fatty acids: all causes	1347·53 (575·06 to 2186·21)	1538·76 (641·93 to 2518·12)	14·19 (11·23 to 17·24)*	–13·43 (−15·57 to −11·15)*	30 245·39 (13 187·67 to 48 313·74)	33 347·84 (14 222·64 to 53 678·05)	10·26 (7·23 to 13·38)*	–13·56 (−15·86 to −11·20)*
··	Ischaemic heart disease	1347·53 (575·06 to 2186·21)	1538·76 (641·93 to 2518·12)	14·19 (11·23 to 17·24)*	–13·43 (−15·57 to −11·15)*	30 245·39 (13 187·67 to 48 313·74)	33 347·84 (14 222·64 to 53 678·05)	10·26 (7·23 to 13·38)*	–13·56 (−15·86 to −11·20)*
3	Diet low in polyunsaturated fatty acids: all causes	373·71 (152·88 to 579·39)	404·13 (167·80 to 628·84)	8·14 (1·10 to 15·92)*	–18·99 (−24·21 to −13·07)*	8077·08 (3337·50 to 12 512·69)	8351·81 (3443·29 to 12 916·37)	3·40 (−2·82 to 10·23)	–18·99 (−23·72 to −13·61)*
··	Ischaemic heart disease	373·71 (152·88 to 579·39)	404·13 (167·80 to 628·84)	8·14 (1·10 to 15·92)*	–18·99 (−24·21 to −13·07)*	8077·08 (3337·50 to 12 512·69)	8351·81 (3443·29 to 12 916·37)	3·40 (−2·82 to 10·23)	–18·99 (−23·72 to −13·61)*
3	Diet high in trans fatty acids: all causes	236·27 (80·11 to 490·84)	223·64 (62·82 to 513·16)	–5·34 (−25·31 to 5·65)	–29·61 (−44·81 to −21·00)*	5426·02 (1751·02 to 11 428·66)	5111·02 (1348·61 to 11 683·02)	–5·81 (−24·90 to 4·01)	–26·47 (−41·85 to −18·49)*
··	Ischaemic heart disease	236·27 (80·11 to 490·84)	223·64 (62·82 to 513·16)	–5·34 (−25·31 to 5·65)	–29·61 (−44·81 to −21·00)*	5426·02 (1751·02 to 11 428·66)	5111·02 (1348·61 to 11 683·02)	–5·81 (−24·90 to 4·01)	–26·47 (−41·85 to −18·49)*
3	Diet high in sodium: all causes	2093·86 (641·82 to 4027·16)	2310·47 (654·70 to 4498·83)	10·35 (1·14 to 14·18)*	–17·24 (−23·87 to −14·54)*	44 080·70 (14 013·37 to 84 853·20)	47 567·08 (14 436·69 to 92 411·61)	7·91 (0·83 to 11·33)*	–16·81 (−22·41 to −14·20)*
··	Stomach cancer	87·78 (29·91 to 169·46)	82·00 (25·89 to 164·38)	–6·58 (−19·18 to 0·49)	–28·70 (−37·72 to −24·32)*	1858·76 (665·42 to 3570·13)	1677·96 (551·88 to 3313·52)	–9·73 (−21·11 to −2·91)*	–30·26 (−38·59 to −25·91)*
··	Rheumatic heart disease	18·85 (5·42 to 40·97)	16·56 (4·21 to 36·93)	–12·11 (−24·41 to −3·58)*	–32·10 (−41·39 to −26·32)*	508·22 (141·81 to 1117·27)	433·72 (110·18 to 999·89)	–14·66 (−26·19 to −7·04)*	–31·92 (−41·31 to −26·21)*
··	Ischaemic heart disease	933·02 (228·66 to 1899·86)	1097·91 (271·71 to 2220·51)	17·67 (12·33 to 22·92)*	–12·43 (−16·13 to −8·54)*	18 024·31 (4595·42 to 37 082·96)	20 494·46 (5230·42 to 41 448·18)	13·70 (9·53 to 19·27)*	–12·58 (−15·65 to −8·42)*
··	Ischaemic stroke	298·64 (87·80 to 607·24)	312·00 (88·62 to 643·83)	4·48 (−3·00 to 9·19)	–21·85 (−27·22 to −18·72)*	6331·08 (2048·13 to 12 479·99)	6939·12 (2243·57 to 13 630·21)	9·60 (4·34 to 14·91)*	–16·53 (−20·56 to −12·41)*
··	Haemorrhagic stroke	462·49 (172·54 to 842·63)	432·53 (147·88 to 808·61)	–6·48 (−16·08 to −1·80)*	–29·05 (−36·48 to −25·70)*	10 650·45 (3995·11 to 19 583·09)	9962·38 (3520·25 to 18 774·49)	–6·46 (−14·68 to −2·50)*	–27·53 (−34·08 to −24·41)*
··	Hypertensive heart disease	142·05 (27·30 to 351·99)	181·96 (33·21 to 464·61)	28·10 (2·03 to 45·37)*	–5·27 (−24·95 to 6·83)	2731·43 (670·95 to 6388·79)	3298·57 (730·16 to 7748·68)	20·76 (1·85 to 35·50)*	–7·33 (−22·43 to 3·79)
··	Other cardiomyopathy	10·66 (2·22 to 23·96)	12·52 (2·32 to 28·86)	17·39 (−3·70 to 28·91)	–11·88 (−28·39 to −2·67)*	242·84 (51·24 to 538·45)	270·46 (53·89 to 606·34)	11·38 (−5·12 to 20·11)	–12·68 (−26·01 to −5·61)*
··	Atrial fibrillation and flutter	11·26 (2·54 to 25·22)	15·56 (3·33 to 35·44)	38·19 (25·76 to 43·79)*	–1·98 (−9·57 to 1·66)	382·36 (95·58 to 800·54)	501·90 (124·45 to 1052·73)	31·26 (25·19 to 34·93)*	–0·58 (−4·89 to 2·57)
··	Aortic aneurysm	9·57 (2·18 to 20·52)	11·02 (2·31 to 24·08)	15·18 (2·55 to 21·25)*	–13·17 (−22·38 to −9·02)*	184·54 (43·83 to 391·55)	204·79 (44·97 to 436·91)	10·97 (−0·89 to 17·09)	–14·32 (−23·42 to −9·77)*
··	Peripheral vascular disease	1·88 (0·25 to 4·56)	2·51 (0·34 to 6·25)	33·19 (16·18 to 51·41)*	–3·46 (−14·44 to 10·20)	50·71 (9·14 to 121·19)	62·48 (10·88 to 148·77)	23·20 (11·72 to 32·25)*	–7·07 (−15·48 to −0·73)*
··	Endocarditis	4·12 (0·77 to 9·81)	5·16 (0·90 to 12·34)	25·41 (14·57 to 30·73)*	–5·45 (−13·78 to −1·43)*	92·56 (16·37 to 222·32)	111·10 (18·80 to 270·10)	20·04 (11·39 to 25·25)*	–5·37 (−13·40 to −1·59)*
··	Other cardiovascular and circulatory diseases	29·97 (6·67 to 64·98)	34·19 (7·06 to 77·30)	14·10 (−1·52 to 21·18)	–14·37 (−25·64 to −9·26)*	866·90 (215·87 to 1874·08)	970·65 (226·05 to 2134·15)	11·97 (0·73 to 17·31)*	–12·89 (−21·61 to −8·68)*
··	Chronic kidney disease due to diabetes mellitus	38·25 (9·51 to 82·81)	47·89 (10·61 to 105·42)	25·21 (10·95 to 29·75)*	–5·22 (−15·38 to −2·14)*	1047·19 (279·10 to 2297·95)	1269·79 (302·82 to 2829·29)	21·26 (8·49 to 25·52)*	–5·96 (−15·38 to −2·85)*
··	Chronic kidney disease due to hypertension	23·28 (5·93 to 50·23)	30·37 (7·11 to 66·16)	30·44 (17·26 to 35·06)*	–4·36 (−12·78 to −1·54)*	497·97 (135·51 to 1054·49)	626·30 (162·35 to 1355·19)	25·77 (15·42 to 29·65)*	–4·23 (−11·72 to −1·41)*
··	Chronic kidney disease due to glomerulonephritis	8·03 (1·35 to 19·34)	9·84 (1·48 to 24·04)	22·57 (8·58 to 26·59)*	–6·75 (−16·15 to −4·18)*	241·64 (42·80 to 581·91)	282·61 (47·47 to 682·09)	16·95 (5·24 to 20·87)*	–7·52 (−15·54 to −5·01)*
··	Chronic kidney disease due to other causes	14·03 (2·72 to 33·20)	18·43 (3·17 to 44·06)	31·38 (15·39 to 36·18)*	–1·84 (−13·73 to 1·01)	369·73 (73·40 to 902·50)	460·78 (84·25 to 1145·85)	24·62 (11·02 to 28·56)*	–2·97 (−13·68 to −0·33)*
**2**	**Sexual abuse and violence: all causes**	**149·42 (94·83 to 204·16)**	**73·83 (53·79 to 94·09)**	–50·59 (−54·93 to −41·98)*	–57·82 (−61·57 to −50·49)*	**11 095·59 (8127·52 to 13 985·73)**	**8201·58 (6354·86 to 10 332·83)**	–26·08 (−34·79 to −15·42)*	–36·15 (−43·53 to −27·22)*
3	Childhood sexual abuse: all causes	8·98 (6·58 to 11·81)	8·74 (6·40 to 11·74)	–2·66 (−13·60 to 10·23)	–20·18 (−28·78 to −10·11)*	2495·64 (1766·89 to 3377·91)	2748·30 (1920·53 to 3735·79)	10·12 (7·71 to 12·41)*	–6·09 (−8·31 to −4·17)*
··	Alcohol use disorders	8·98 (6·58 to 11·81)	8·74 (6·40 to 11·74)	–2·66 (−13·60 to 10·23)	–20·18 (−28·78 to −10·11)*	814·13 (574·56 to 1131·70)	854·71 (596·52 to 1200·17)	4·98 (−1·17 to 10·82)	–10·40 (−15·87 to −5·29)*
··	Major depressive disorder	··	··	··	··	1681·51 (1101·62 to 2354·77)	1893·59 (1235·31 to 2667·57)	12·61 (11·02 to 14·30)*	–4·04 (−5·26 to −2·80)*
3	Intimate partner violence: all causes	140·45 (86·78 to 194·82)	65·09 (44·85 to 85·84)	–53·65 (−57·43 to −45·90)*	–60·39 (−63·74 to −53·55)*	8702·76 (6067·70 to 11 437·85)	5575·29 (4224·54 to 7079·58)	–35·94 (−43·21 to −25·04)*	–44·53 (−50·72 to −35·17)*
··	Drug-susceptible HIV/AIDS–tuberculosis	26·36 (12·66 to 43·52)	9·23 (4·43 to 14·96)	–64·97 (−67·40 to −62·15)*	–70·87 (−72·90 to −68·73)*	1146·54 (540·62 to 1916·16)	423·59 (203·21 to 687·99)	–63·05 (−65·77 to −59·57)*	–68·63 (−70·89 to −65·77)*
··	Multidrug-resistant HIV/AIDS–tuberculosis without extensive drug resistance	2·07 (0·93 to 3·52)	0·71 (0·32 to 1·22)	–65·82 (−72·96 to −56·64)*	–71·63 (−77·56 to −64·16)*	88·64 (39·75 to 152·39)	31·67 (14·10 to 54·75)	–64·27 (−71·71 to −54·47)*	–69·71 (−75·98 to −61·51)*
··	Extensively drug-resistant HIV/AIDS –tuberculosis	0·02 (0·01 to 0·04)	0·02 (0·01 to 0·04)	13·96 (−2·84 to 32·78)	–4·23 (−18·41 to 11·39)	0·96 (0·42 to 1·70)	1·12 (0·48 to 1·97)	16·38 (−0·88 to 36·31)	–0·47 (−15·26 to 16·38)
··	HIV/AIDS resulting in other diseases	84·54 (43·40 to 129·36)	31·10 (16·08 to 47·65)	–63·22 (−66·05 to −59·87)*	–68·71 (−71·09 to −65·93)*	4027·09 (2046·13 to 6160·95)	1584·24 (814·13 to 2425·13)	–60·66 (−63·52 to −57·44)*	–66·07 (−68·50 to −63·31)*
··	Maternal abortion, miscarriage, and ectopic pregnancy	4·11 (2·45 to 6·19)	3·00 (1·75 to 4·79)	–27·02 (−36·19 to −17·03)*	–34·53 (−42·83 to −25·55)*	233·81 (137·40 to 351·12)	170·68 (97·12 to 270·75)	–27·00 (−35·84 to −17·85)*	–34·18 (−42·23 to −25·85)*
··	Major depressive disorder	··	··	··	··	1582·91 (966·13 to 2381·34)	1870·82 (1146·94 to 2801·23)	18·19 (15·62 to 21·12)*	–1·74 (−3·69 to 0·18)
··	Assault by firearm	4·73 (3·09 to 5·60)	4·77 (3·13 to 6·06)	0·75 (−8·07 to 24·07)	–10·75 (−18·39 to 9·61)	250·39 (163·69 to 295·57)	246·14 (163·72 to 308·71)	–1·70 (−10·63 to 22·32)	–10·98 (−19·00 to 10·46)
··	Assault by sharp object	6·88 (4·69 to 8·17)	6·00 (4·41 to 7·87)	–12·69 (−23·31 to 21·78)	–23·39 (−32·53 to 6·30)	367·54 (255·41 to 435·61)	314·12 (235·32 to 404·00)	–14·54 (−24·84 to 18·52)	–23·58 (−32·59 to 5·48)
··	Sexual violence	··	··	··	··	291·29 (191·88 to 418·18)	298·83 (195·75 to 428·19)	2·59 (0·69 to 4·22)*	–6·63 (−7·90 to −5·72)*
··	Assault by other means	11·73 (8·71 to 14·49)	10·26 (8·02 to 13·17)	–12·56 (−24·54 to 5·66)	–23·23 (−33·65 to −7·28)*	713·59 (558·79 to 866·06)	634·08 (509·27 to 793·77)	–11·14 (−22·12 to 4·98)	–20·79 (−30·38 to −7·10)*
2	Unsafe sex: all causes	1799·64 (1709·98 to 1892·29)	1100·90 (1048·42 to 1148·40)	–38·83 (−40·96 to −36·41)*	–47·76 (−49·54 to −45·78)*	86 860·81 (81 591·84 to 92 234·51)	54 603·03 (51 340·06 to 58 075·62)	–37·14 (−39·35 to −34·64)*	–45·34 (−47·21 to −43·21)*
··	Drug-susceptible HIV/AIDS–tuberculosis	363·54 (244·52 to 481·15)	177·41 (121·51 to 236·50)	–51·20 (−54·00 to −48·03)*	–58·56 (−60·94 to −56·00)*	17 186·87 (11 569·46 to 22 809·50)	8948·63 (6232·62 to 11 865·25)	–47·93 (−50·95 to −44·18)*	–54·73 (−57·35 to −51·55)*
··	Multidrug-resistant HIV/AIDS–tuberculosis without extensive drug resistance	30·65 (18·73 to 45·88)	14·52 (8·81 to 21·68)	–52·62 (−61·10 to −42·60)*	–59·80 (−67·03 to −51·32)*	1423·49 (866·21 to 2131·53)	714·22 (433·23 to 1064·03)	–49·83 (−58·78 to −39·15)*	–56·42 (−64·22 to −47·18)*
··	Extensively drug-resistant HIV/AIDS– tuberculosis	0·52 (0·33 to 0·79)	0·77 (0·47 to 1·20)	48·49 (29·74 to 70·40)*	27·46 (11·27 to 46·30)*	24·64 (15·40 to 37·14)	36·82 (22·60 to 56·99)	49·44 (30·40 to 72·32)*	30·37 (13·77 to 50·30)*
··	HIV/AIDS resulting in other diseases	1165·31 (1020·87 to 1330·03)	652·04 (578·82 to 729·70)	–44·05 (−46·83 to −40·95)*	–51·77 (−54·17 to −49·13)*	58 595·06 (51 311·35 to 66 970·36)	34 615·61 (30 661·75 to 38 960·02)	–40·92 (−43·71 to −37·90)*	–48·30 (−50·71 to −45·70)*
··	Syphilis	3·31 (2·85 to 3·91)	3·02 (2·55 to 3·44)	–8·89 (−18·77 to 9·37)	–24·89 (−33·13 to −9·95)*	277·24 (229·80 to 328·03)	305·18 (247·07 to 367·04)	10·08 (1·88 to 18·96)*	–8·03 (−14·11 to −0·79)*
··	Chlamydial infection	1·24 (0·99 to 1·37)	1·19 (0·98 to 1·33)	–4·51 (−11·73 to 12·18)	–20·70 (−26·52 to −7·57)*	519·31 (341·24 to 781·67)	562·13 (370·06 to 850·69)	8·25 (5·89 to 10·42)*	–3·33 (−5·56 to −1·40)*
··	Gonococcal infection	3·51 (2·81 to 3·85)	3·37 (2·76 to 3·80)	–4·05 (−11·10 to 12·51)	–20·87 (−26·48 to −7·90)*	581·90 (412·15 to 823·83)	674·77 (467·35 to 974·12)	15·96 (10·35 to 21·76)*	2·68 (−2·71 to 7·83)
··	Trichomoniasis	··	··	··	··	170·83 (65·10 to 361·77)	198·07 (75·83 to 420·49)	15·95 (14·84 to 17·09)*	1·82 (0·94 to 2·72)*
··	Genital herpes	··	··	··	··	187·73 (60·91 to 427·68)	221·21 (71·15 to 506·61)	17·84 (15·47 to 19·69)*	–0·16 (−1·64 to 1·54)
··	Other sexually transmitted diseases	1·73 (1·40 to 1·90)	1·63 (1·35 to 1·83)	–5·92 (−12·96 to 11·16)	–21·03 (−26·82 to −7·18)*	858·99 (589·04 to 1221·09)	942·39 (643·29 to 1348·57)	9·71 (7·38 to 12·17)*	–2·62 (−4·83 to −0·42)*
··	Cervical cancer	229·83 (195·46 to 245·84)	246·95 (203·95 to 263·27)	7·45 (1·21 to 15·47)*	–15·99 (−20·69 to −9·78)*	7034·76 (5873·55 to 7509·99)	7384·00 (6014·77 to 7862·78)	4·96 (−1·30 to 13·23)	–15·71 (−20·76 to −9·21)*
2	Low physical activity: all causes	1159·60 (607·84 to 1790·07)	1373·34 (717·65 to 2084·16)	18·43 (−7·89 to 55·46)	–12·88 (−31·98 to 13·86)	21 078·75 (11 156·78 to 32 368·81)	24 315·86 (12 811·32 to 36 604·69)	15·36 (−12·15 to 55·44)	–11·79 (−32·55 to 18·47)
··	Colon and rectum cancer	20·87 (1·07 to 50·40)	25·51 (1·28 to 61·93)	22·21 (−46·00 to 212·87)	–8·42 (−59·11 to 136·31)	411·01 (23·10 to 991·31)	488·61 (26·82 to 1183·40)	18·88 (−48·29 to 205·64)	–8·33 (−59·81 to 136·94)
··	Breast cancer	6·71 (0·04 to 14·74)	7·85 (0·03 to 17·15)	16·97 (−19·98 to 88·93)	–10·88 (−38·58 to 44·86)	175·07 (1·05 to 388·06)	200·14 (0·80 to 440·98)	14·33 (−24·10 to 90·78)	–10·01 (−39·82 to 49·21)
··	Ischaemic heart disease	835·44 (347·97 to 1353·66)	1005·58 (425·31 to 1640·08)	20·37 (−6·38 to 58·30)	–11·49 (−30·93 to 15·93)	14 658·37 (6114·10 to 23 953·87)	16 943·23 (7260·81 to 27 786·10)	15·59 (−11·49 to 54·73)	–11·42 (−31·91 to 18·08)
··	Ischaemic stroke	267·12 (53·52 to 511·96)	295·28 (49·52 to 562·19)	10·54 (−19·06 to 49·40)	–18·82 (−40·33 to 9·53)	4725·12 (1026·32 to 9141·81)	5268·62 (938·08 to 10 006·35)	11·50 (−18·88 to 54·06)	–15·63 (−38·34 to 16·18)
··	Diabetes mellitus	29·46 (7·36 to 52·61)	39·12 (8·65 to 71·86)	32·80 (−11·95 to 104·74)	–0·40 (−33·63 to 52·78)	1109·18 (248·01 to 2082·98)	1415·26 (312·14 to 2604·08)	27·59 (−17·68 to 104·26)	–0·58 (−35·47 to 58·06)
**1**	**Metabolic risks: all causes**	**14 834·47 (13 966·55 to 15 690·95)**	**17 493·53 (16 427·65 to 18 524·26)**	17·92 (15·73 to 20·58)*	–11·86 (−13·47 to −9·94)*	**348 438·17 (324 520·78 to 374 936·88)**	**401 813·92 (372 407·65 to 434 394·06)**	15·32 (13·16 to 17·52)*	–10·29 (−11·98 to −8·60)*
2	High fasting plasma glucose: all causes	4700·40 (3722·99 to 5906·04)	5612·45 (4457·29 to 6987·54)	19·40 (15·54 to 23·19)*	–10·32 (−13·29 to −7·61)*	123 096·04 (102 887·96 to 146 660·95)	144 088·58 (119 872·60 to 171 585·77)	17·05 (13·94 to 19·89)*	–8·83 (−11·35 to −6·55)*
··	Drug-susceptible tuberculosis	125·08 (78·55 to 175·35)	99·60 (62·39 to 140·59)	–20·36 (−24·38 to −16·57)*	–37·14 (−40·10 to −34·46)*	4013·77 (2581·12 to 5546·25)	3126·64 (2002·75 to 4288·46)	–22·10 (−25·60 to −18·55)*	–37·09 (−39·78 to −34·65)*
··	Multidrug-resistant tuberculosis without extensive drug resistance	12·50 (7·74 to 18·06)	8·80 (5·32 to 12·83)	–29·61 (−36·56 to −22·33)*	–44·21 (−49·66 to −38·40)*	394·88 (253·40 to 557·02)	266·97 (168·75 to 375·02)	–32·39 (−38·90 to −25·48)*	–45·28 (−50·42 to −39·77)*
··	Extensively drug-resistant tuberculosis	0·54 (0·33 to 0·79)	0·94 (0·58 to 1·39)	73·66 (50·32 to 101·15)*	37·94 (20·01 to 59·36)*	17·18 (10·63 to 24·33)	28·42 (17·68 to 40·36)	65·41 (42·36 to 90·02)*	34·13 (15·91 to 54·00)*
··	Colon and rectum cancer	46·54 (11·36 to 101·41)	56·18 (13·51 to 122·80)	20·70 (15·48 to 25·34)*	–9·50 (−13·53 to −5·91)*	884·79 (208·91 to 1926·46)	1047·94 (242·88 to 2300·07)	18·44 (13·03 to 23·15)*	–9·42 (−13·57 to −5·78)*
··	Liver cancer due to other causes	10·01 (2·06 to 23·07)	11·82 (2·44 to 26·96)	18·12 (13·22 to 22·67)*	–8·69 (−12·24 to −5·26)*	247·67 (51·70 to 571·25)	279·35 (58·20 to 649·12)	12·79 (7·38 to 17·65)*	–11·62 (−15·55 to −7·90)*
··	Pancreatic cancer	21·37 (4·71 to 46·72)	27·75 (6·10 to 60·79)	29·86 (26·27 to 33·12)*	–2·11 (−4·96 to 0·48)	406·22 (90·54 to 891·18)	518·70 (115·69 to 1142·16)	27·69 (24·05 to 30·83)*	–2·46 (−5·20 to 0·04)
··	Tracheal, bronchus, and lung cancer	100·11 (22·74 to 219·56)	117·06 (26·22 to 256·14)	16·93 (13·75 to 19·76)*	–10·83 (−13·19 to −8·64)*	2028·69 (457·34 to 4483·59)	2304·26 (517·29 to 5093·70)	13·58 (10·25 to 16·54)*	–12·74 (−15·22 to −10·53)*
··	Breast cancer	26·09 (4·93 to 59·11)	31·03 (5·96 to 69·25)	18·94 (11·73 to 26·02)*	–10·02 (−15·31 to −4·93)*	637·43 (120·29 to 1460·33)	749·77 (144·08 to 1694·69)	17·62 (9·76 to 25·72)*	–8·95 (−14·91 to −2·97)*
··	Ovarian cancer	8·11 (1·53 to 19·32)	10·01 (1·88 to 23·94)	23·40 (18·12 to 28·40)*	–6·84 (−10·75 to −3·13)*	182·64 (34·17 to 438·11)	226·27 (41·63 to 545·49)	23·89 (18·46 to 29·09)*	–5·00 (−9·13 to −1·10)*
··	Bladder cancer	10·79 (2·22 to 23·92)	13·47 (2·80 to 29·79)	24·87 (20·66 to 28·68)*	–6·86 (−10·10 to −3·96)*	185·65 (37·01 to 413·28)	225·37 (45·96 to 501·26)	21·39 (16·66 to 25·27)*	–7·62 (−11·16 to −4·73)*
··	Ischaemic heart disease	1576·70 (935·55 to 2479·20)	1883·33 (1104·82 to 2942·53)	19·45 (13·37 to 25·39)*	–11·29 (−15·21 to −7·64)*	29 401·46 (18 681·34 to 45 481·26)	33 937·53 (21 184·55 to 51 236·60)	15·43 (10·21 to 20·56)*	–11·40 (−15·50 to −7·55)*
··	Ischaemic stroke	449·06 (229·48 to 849·74)	472·53 (246·22 to 879·04)	5·23 (−1·90 to 12·75)	–21·44 (−26·20 to −17·05)*	8810·40 (4539·51 to 14 964·85)	9467·73 (5021·42 to 15 876·46)	7·46 (0·60 to 13·96)*	–18·27 (−23·34 to −13·54)*
··	Haemorrhagic stroke	484·34 (304·87 to 745·37)	473·30 (301·08 to 720·04)	–2·28 (−8·77 to 3·28)	–25·71 (−30·75 to −21·17)*	10 790·83 (6746·12 to 15 844·21)	10 638·08 (6692·27 to 15 613·04)	–1·42 (−7·28 to 3·80)	–23·88 (−28·89 to −19·71)*
··	Peripheral vascular disease	8·67 (6·23 to 12·26)	11·73 (8·71 to 17·62)	35·33 (24·67 to 48·64)*	–2·51 (−9·98 to 6·88)	213·89 (150·72 to 302·55)	271·68 (195·01 to 383·83)	27·02 (21·52 to 34·82)*	–4·49 (−8·52 to 1·11)
··	Alzheimer's disease and other dementias	123·02 (26·35 to 274·89)	174·35 (37·30 to 388·04)	41·73 (38·26 to 45·05)*	–1·20 (−3·44 to 1·60)	1584·88 (330·04 to 3563·61)	2138·24 (445·48 to 4857·92)	34·92 (32·10 to 37·60)*	–1·28 (−3·30 to 1·10)
··	Diabetes mellitus	1095·53 (1065·39 to 1121·32)	1436·26 (1401·25 to 1469·57)	31·10 (28·92 to 33·39)*	–0·87 (−2·52 to 0·84)	45 947·41 (38 659·07 to 54 662·94)	57 175·71 (47 919·49 to 68 211·91)	24·44 (22·70 to 26·24)*	–1·66 (−3·03 to −0·22)*
··	Chronic kidney disease due to diabetes mellitus	384·78 (349·87 to 418·93)	500·41 (452·11 to 543·57)	30·05 (26·18 to 32·84)*	–0·63 (−3·43 to 1·30)	11 723·50 (10 608·16 to 12 883·32)	14 649·82 (13 196·95 to 16 191·89)	24·96 (21·91 to 27·57)*	–1·37 (−3·51 to 0·51)
··	Chronic kidney disease due to hypertension	103·23 (70·71 to 134·57)	135·31 (92·80 to 176·80)	31·08 (24·95 to 37·33)*	–3·63 (−7·93 to 0·57)	2165·18 (1452·61 to 2857·87)	2724·97 (1827·82 to 3615·75)	25·85 (20·11 to 31·49)*	–4·20 (−8·66 to 0·03)
··	Chronic kidney disease due to glomerulonephritis	42·94 (28·57 to 57·82)	54·38 (36·92 to 73·20)	26·63 (21·53 to 31·75)*	–3·73 (−7·53 to 0·00)	1251·76 (815·12 to 1731·15)	1519·82 (1004·85 to 2100·93)	21·42 (17·17 to 25·92)*	–4·39 (−7·34 to −1·01)*
··	Chronic kidney disease due to other causes	71·01 (48·20 to 94·44)	94·20 (63·89 to 125·10)	32·66 (26·70 to 38·48)*	–0·15 (−4·43 to 3·81)	1866·69 (1234·98 to 2533·02)	2346·56 (1566·99 to 3183·01)	25·71 (20·33 to 30·88)*	–2·03 (−6·05 to 1·77)
··	Glaucoma	··	··	··	··	25·15 (5·71 to 58·51)	33·85 (7·75 to 78·65)	34·62 (31·92 to 37·57)*	1·52 (−0·42 to 3·66)
··	Cataract	··	··	··	··	315·98 (65·93 to 735·41)	410·89 (85·82 to 961·77)	30·04 (27·58 to 32·78)*	–1·16 (−3·06 to 1·06)
2	High total cholesterol: all causes	3802·10 (2971·09 to 4832·93)	4392·51 (3374·22 to 5619·87)	15·53 (11·51 to 19·77)*	–14·14 (−16·62 to −11·41)*	83 976·46 (70 004·69 to 98 804·76)	93 844·03 (78 027·31 to 111 266·48)	11·75 (8·59 to 15·13)*	–13·29 (−15·68 to −10·73)*
··	Ischaemic heart disease	3343·63 (2597·25 to 4187·22)	3896·10 (2982·29 to 4940·40)	16·52 (12·29 to 20·89)*	–13·22 (−15·68 to −10·56)*	73 403·57 (61 220·12 to 86 047·11)	82 187·03 (68 385·19 to 96 854·44)	11·97 (8·76 to 15·47)*	–12·88 (−15·33 to −10·24)*
··	Ischaemic stroke	458·46 (185·09 to 924·24)	496·40 (196·69 to 990·11)	8·28 (1·92 to 14·44)*	–20·63 (−23·82 to −17·08)*	10 572·88 (6206·10 to 17 681·57)	11 657·00 (6791·38 to 19 428·74)	10·25 (6·20 to 14·39)*	–16·02 (−19·10 to −12·79)*
2	High systolic blood pressure: all causes	9083·07 (8209·73 to 9963·14)	10 455·86 (9381·88 to 11 507·49)	15·11 (12·53 to 18·15)*	–14·05 (−15·90 to −11·81)*	188 635·23 (171 004·50 to 205 178·38)	212 105·09 (191 466·22 to 230 661·27)	12·44 (10·03 to 15·07)*	–13·27 (−15·09 to −11·25)*
··	Rheumatic heart disease	85·51 (58·24 to 126·10)	80·86 (55·41 to 124·17)	–5·43 (−12·76 to 3·81)	–26·92 (−32·20 to −20·72)*	2412·32 (1643·79 to 3500·44)	2234·54 (1547·75 to 3250·51)	–7·37 (−13·42 to 0·51)	–25·82 (−30·59 to −19·76)*
··	Ischaemic heart disease	4476·47 (3732·81 to 5193·76)	5261·72 (4374·30 to 6188·35)	17·54 (14·19 to 21·12)*	–12·69 (−14·94 to −10·11)*	85 975·47 (74 665·22 to 97 205·97)	97 886·68 (84 378·88 to 110 500·79)	13·85 (10·72 to 17·14)*	–12·44 (−14·77 to −9·97)*
··	Ischaemic stroke	1283·00 (989·81 to 1551·76)	1372·51 (1053·44 to 1670·88)	6·98 (3·05 to 11·54)*	–20·42 (−23·09 to −17·59)*	25 564·17 (20 155·09 to 29 832·63)	28 119·95 (21 993·71 to 32 960·56)	10·00 (6·27 to 13·81)*	–16·42 (−19·14 to −13·56)*
··	Haemorrhagic stroke	1636·18 (1343·27 to 1897·92)	1672·64 (1375·89 to 1947·04)	2·23 (−0·33 to 4·87)	–22·45 (−24·32 to −20·60)*	37 920·74 (31 833·70 to 43 655·27)	38 611·64 (32 491·61 to 44 204·86)	1·82 (−0·41 to 4·22)	–20·91 (−22·79 to −19·01)*
··	Hypertensive heart disease	694·18 (579·81 to 760·86)	893·14 (698·18 to 982·33)	28·66 (14·46 to 42·90)*	–4·39 (−14·79 to 5·66)	13 562·97 (11 596·54 to 15 040·61)	16 323·95 (13 447·14 to 17 832·20)	20·36 (10·19 to 32·88)*	–6·60 (−14·56 to 2·76)
··	Other cardiomyopathy	60·64 (44·14 to 77·33)	74·93 (54·83 to 95·99)	23·56 (16·20 to 31·99)*	–7·84 (−13·17 to −1·67)*	1352·53 (1021·85 to 1642·20)	1599·77 (1242·11 to 1935·40)	18·28 (11·07 to 26·29)*	–7·37 (−12·73 to −1·09)*
··	Atrial fibrillation and flutter	61·68 (45·24 to 81·70)	85·31 (62·06 to 113·83)	38·31 (33·98 to 42·56)*	–2·78 (−5·10 to −0·60)*	1865·37 (1396·49 to 2444·60)	2439·54 (1822·85 to 3211·02)	30·78 (28·89 to 32·53)*	–1·52 (−2·73 to −0·44)*
··	Aortic aneurysm	51·01 (40·98 to 60·67)	60·10 (48·02 to 72·42)	17·81 (13·84 to 22·53)*	–11·62 (−14·32 to −8·15)*	961·55 (799·00 to 1113·52)	1100·81 (930·12 to 1274·73)	14·48 (10·15 to 19·96)*	–11·71 (−14·92 to −7·65)*
··	Peripheral vascular disease	12·49 (8·26 to 18·74)	16·55 (10·89 to 25·60)	32·49 (20·21 to 47·35)*	–4·83 (−12·82 to 5·05)	290·81 (199·85 to 436·13)	360·60 (246·52 to 530·75)	24·00 (17·02 to 32·57)*	–6·80 (−11·75 to −0·54)*
··	Endocarditis	25·33 (19·02 to 32·47)	33·12 (24·85 to 42·80)	30·76 (25·24 to 36·10)*	–1·38 (−5·39 to 2·95)	589·46 (447·95 to 744·37)	745·71 (570·49 to 942·16)	26·51 (21·03 to 31·79)*	0·16 (−4·21 to 4·17)
··	Other cardiovascular and circulatory diseases	174·04 (148·57 to 214·23)	208·84 (177·87 to 255·72)	20·00 (15·44 to 25·66)*	–10·49 (−13·75 to −6·47)*	4740·48 (3978·63 to 5703·94)	5577·83 (4690·60 to 6705·53)	17·66 (14·21 to 22·09)*	–8·60 (−11·32 to −5·34)*
··	Chronic kidney disease due to diabetes mellitus	176·23 (128·84 to 227·68)	233·70 (170·75 to 301·63)	32·61 (29·00 to 35·48)*	–0·06 (−2·77 to 1·94)	4755·11 (3375·01 to 6163·36)	6154·78 (4359·36 to 7973·15)	29·44 (26·62 to 32·07)*	–0·01 (−2·10 to 1·78)
··	Chronic kidney disease due to hypertension	222·32 (199·99 to 248·86)	299·48 (268·03 to 335·26)	34·71 (30·47 to 38·02)*	–0·96 (−3·95 to 1·00)	5166·00 (4517·97 to 5842·00)	6602·34 (5756·41 to 7488·87)	27·80 (24·67 to 30·62)*	–1·02 (−3·31 to 0·87)
··	Chronic kidney disease due to glomerulonephritis	47·82 (34·20 to 62·17)	60·17 (42·74 to 78·14)	25·83 (22·33 to 29·04)*	–4·69 (−6·90 to −2·70)*	1443·70 (1010·81 to 1931·01)	1739·55 (1207·79 to 2320·30)	20·49 (17·59 to 23·37)*	–4·97 (−6·98 to −2·95)*
··	Chronic kidney disease due to other causes	76·17 (51·67 to 99·57)	102·79 (69·50 to 135·39)	34·95 (30·99 to 38·74)*	0·84 (−1·83 to 3·10)	2034·56 (1366·91 to 2688·13)	2607·39 (1738·64 to 3467·30)	28·16 (25·16 to 31·01)*	–0·11 (−2·27 to 1·77)
2	High body-mass index: all causes	3519·12 (2136·48 to 5165·34)	4525·10 (2867·22 to 6434·24)	28·59 (23·43 to 35·93)*	–2·71 (−6·52 to 2·81)	105 257·57 (65 833·95 to 150 547·40)	135 381·33 (88 608·73 to 187 363·70)	28·62 (23·09 to 36·63)*	0·88 (−3·40 to 7·02)
··	Oesophageal cancer	57·66 (18·86 to 112·99)	70·33 (22·52 to 133·63)	21·97 (11·96 to 36·08)*	–6·97 (−14·64 to 3·67)	1357·91 (431·31 to 2647·91)	1622·45 (516·51 to 3060·94)	19·48 (9·83 to 33·83)*	–7·80 (−15·24 to 3·26)
··	Colon and rectum cancer	49·63 (26·72 to 79·41)	65·11 (35·86 to 102·02)	31·19 (25·23 to 38·71)*	–0·94 (−5·50 to 4·74)	1075·85 (579·66 to 1714·70)	1394·02 (775·82 to 2160·04)	29·57 (22·96 to 37·66)*	–0·04 (−4·94 to 6·05)
··	Liver cancer due to hepatitis B	26·37 (8·91 to 55·20)	37·72 (13·44 to 74·28)	43·05 (31·18 to 64·94)*	11·96 (2·72 to 28·75)*	782·13 (261·88 to 1631·20)	1078·26 (379·06 to 2124·71)	37·86 (25·99 to 60·04)*	10·01 (0·61 to 27·39)*
··	Liver cancer due to hepatitis C	15·00 (6·11 to 28·01)	21·21 (8·95 to 38·36)	41·40 (33·58 to 52·25)*	6·97 (1·14 to 15·12)*	328·28 (134·34 to 602·47)	459·82 (197·62 to 813·07)	40·07 (31·65 to 52·24)*	7·44 (1·26 to 16·40)*
··	Liver cancer due to alcohol use	11·43 (4·52 to 21·77)	16·59 (6·67 to 31·05)	45·18 (35·92 to 58·24)*	10·99 (3·90 to 20·62)*	265·54 (104·88 to 498·00)	383·17 (157·32 to 707·88)	44·30 (34·95 to 57·95)*	11·45 (4·17 to 21·82)*
··	Liver cancer due to other causes	14·98 (4·98 to 31·65)	21·93 (7·78 to 43·51)	46·36 (35·51 to 64·87)*	13·85 (5·45 to 27·54)*	415·44 (136·60 to 884·65)	586·63 (208·44 to 1183·29)	41·21 (29·77 to 61·17)*	12·10 (3·36 to 27·26)*
··	Gallbladder and biliary tract cancer	19·19 (10·00 to 31·40)	24·23 (12·96 to 38·93)	26·31 (20·46 to 33·91)*	–5·19 (−9·47 to 0·48)	398·83 (206·71 to 659·38)	501·99 (271·35 to 804·62)	25·87 (19·54 to 33·96)*	–3·50 (−8·43 to 2·52)
··	Pancreatic cancer	17·09 (6·73 to 32·72)	23·80 (9·45 to 45·36)	39·31 (33·12 to 46·65)*	5·04 (0·00 to 10·77)	355·53 (132·75 to 689·18)	488·30 (185·48 to 937·58)	37·34 (31·04 to 44·82)*	5·41 (0·50 to 11·10)*
··	Breast cancer	24·50 (9·01 to 45·25)	34·14 (14·17 to 61·44)	39·33 (26·71 to 66·63)*	1·49 (−7·17 to 17·88)	478·48 (134·90 to 931·31)	696·82 (241·06 to 1278·23)	45·63 (28·58 to 100·78)*	4·33 (−6·57 to 30·51)
··	Uterine cancer	25·33 (16·84 to 34·86)	31·98 (22·02 to 42·77)	26·29 (17·00 to 39·60)*	–4·35 (−11·15 to 5·40)	616·37 (406·52 to 852·11)	777·06 (534·09 to 1037·37)	26·07 (16·24 to 39·81)*	–2·75 (−10·14 to 7·44)
··	Ovarian cancer	3·96 (−0·06 to 8·73)	5·16 (−0·08 to 11·22)	30·36 (21·79 to 40·84)*	–0·87 (−7·38 to 6·92)	100·08 (−1·45 to 221·53)	130·91 (−2·01 to 284·65)	30·81 (22·13 to 41·76)*	1·63 (−5·03 to 10·00)
··	Kidney cancer	18·46 (10·70 to 28·15)	24·80 (14·55 to 37·29)	34·35 (28·83 to 41·33)*	1·72 (−2·45 to 6·95)	414·68 (240·63 to 629·90)	545·45 (324·36 to 818·33)	31·53 (25·96 to 38·50)*	1·48 (−2·72 to 6·69)
··	Thyroid cancer	2·91 (1·43 to 5·04)	3·99 (2·04 to 6·86)	37·08 (28·94 to 47·41)*	4·67 (−1·57 to 12·41)	75·91 (37·37 to 132·41)	104·28 (53·14 to 180·08)	37·39 (29·21 to 48·00)*	7·53 (1·34 to 15·42)*
··	Non-Hodgkin lymphoma	8·76 (3·45 to 15·95)	12·11 (4·73 to 21·66)	38·22 (32·55 to 44·75)*	5·46 (1·16 to 10·68)*	214·81 (83·04 to 391·23)	295·53 (117·37 to 532·38)	37·58 (31·28 to 43·90)*	8·27 (3·38 to 13·37)*
··	Multiple myeloma	4·86 (2·10 to 8·71)	6·66 (2·89 to 11·78)	37·06 (31·43 to 44·71)*	3·30 (−1·17 to 9·24)	103·69 (45·00 to 186·13)	142·21 (62·40 to 249·59)	37·15 (31·41 to 45·43)*	5·30 (0·94 to 11·53)*
··	Acute lymphoid leukaemia	1·59 (0·75 to 2·73)	2·22 (1·11 to 3·78)	39·14 (29·44 to 48·47)*	10·63 (3·24 to 17·88)*	53·85 (25·63 to 93·15)	73·29 (36·49 to 125·61)	36·10 (26·03 to 46·07)*	12·10 (3·94 to 19·97)*
··	Chronic lymphoid leukaemia	2·36 (1·18 to 3·92)	2·92 (1·50 to 4·80)	23·58 (17·80 to 31·41)*	–8·23 (−13·00 to −2·59)*	45·52 (22·50 to 75·54)	55·53 (28·44 to 89·89)	21·98 (15·87 to 30·42)*	–6·69 (−11·35 to −0·57)*
··	Acute myeloid leukaemia	4·65 (2·33 to 7·66)	6·22 (3·15 to 10·05)	33·79 (28·67 to 40·45)*	3·44 (−0·54 to 8·61)	119·03 (59·43 to 197·90)	156·14 (79·64 to 253·37)	31·17 (26·04 to 38·42)*	4·62 (0·50 to 10·33)*
··	Chronic myeloid leukaemia	1·50 (0·74 to 2·54)	1·56 (0·79 to 2·62)	3·86 (−0·97 to 9·84)	–20·41 (−24·02 to −15·73)*	38·75 (18·95 to 66·00)	39·64 (20·22 to 66·66)	2·32 (−2·78 to 8·83)	–18·45 (−22·36 to −13·42)*
··	Other leukaemia	5·65 (2·63 to 9·91)	6·91 (3·43 to 11·92)	22·36 (13·74 to 33·25)*	–5·39 (−11·58 to 2·67)	150·74 (68·70 to 270·44)	175·88 (86·80 to 306·17)	16·67 (6·35 to 30·31)*	–6·03 (−13·63 to 3·83)
··	Ischaemic heart disease	1288·03 (750·54 to 1915·37)	1592·33 (949·29 to 2325·60)	23·62 (18·28 to 31·01)*	–6·63 (−10·33 to −1·34)*	30 281·04 (18 069·07 to 44 440·56)	36 991·70 (22 899·69 to 52 749·96)	22·16 (17·05 to 29·82)*	–4·63 (−8·58 to 1·24)
··	Ischaemic stroke	283·31 (157·87 to 446·42)	318·39 (179·56 to 494·72)	12·38 (6·06 to 21·03)*	–14·52 (−19·22 to −8·07)*	7636·97 (4439·93 to 11 465·75)	9139·16 (5520·94 to 13 559·93)	19·67 (13·90 to 27·73)*	–7·74 (−12·19 to −1·69)*
··	Haemorrhagic stroke	517·26 (299·18 to 797·22)	592·91 (364·88 to 872·03)	14·63 (7·65 to 24·31)*	–10·40 (−15·68 to −2·82)*	15 913·88 (9447·13 to 23 465·66)	18 284·39 (11 769·74 to 25 665·62)	14·90 (7·94 to 24·58)*	–8·36 (−13·89 to −0·19)*
··	Hypertensive heart disease	215·62 (115·71 to 340·33)	300·81 (162·11 to 482·35)	39·51 (23·49 to 54·97)*	4·14 (−6·93 to 14·90)	4745·65 (2865·31 to 6909·68)	6328·03 (3954·75 to 8998·62)	33·34 (20·77 to 46·93)*	3·67 (−6·03 to 13·90)
··	Atrial fibrillation and flutter	30·66 (15·59 to 50·28)	46·15 (23·86 to 74·25)	50·50 (44·68 to 57·96)*	4·01 (0·15 to 9·07)*	847·22 (424·79 to 1434·95)	1206·94 (614·35 to 2008·58)	42·46 (38·94 to 47·40)*	6·27 (3·63 to 10·00)*
··	Asthma	50·50 (26·16 to 85·76)	60·27 (33·27 to 99·29)	19·33 (9·60 to 32·81)*	–7·72 (−15·43 to 2·78)	3096·98 (1685·64 to 5065·91)	3888·00 (2214·88 to 6203·97)	25·54 (19·00 to 34·22)*	4·23 (−2·03 to 11·64)
··	Gallbladder and biliary diseases	22·65 (14·01 to 33·32)	31·11 (20·15 to 44·30)	37·32 (30·32 to 47·23)*	1·35 (−3·54 to 8·56)	478·24 (295·15 to 708·79)	634·28 (413·77 to 904·27)	32·63 (25·47 to 42·16)*	3·09 (−2·47 to 10·47)
··	Alzheimer's disease and other dementias	185·54 (67·16 to 358·86)	286·44 (106·50 to 545·02)	54·38 (48·77 to 62·72)*	6·35 (2·04 to 13·00)*	2357·11 (900·89 to 4607·68)	3493·12 (1387·03 to 6739·85)	48·20 (43·46 to 55·36)*	7·68 (4·04 to 13·61)*
··	Diabetes mellitus	390·47 (263·53 to 530·40)	553·44 (386·74 to 727·93)	41·74 (35·95 to 49·03)*	8·24 (3·84 to 14·15)*	20 585·42 (13 617·44 to 29 152·97)	28 645·74 (19 660·88 to 39 287·38)	39·16 (33·19 to 47·36)*	10·31 (5·57 to 16·73)*
··	Chronic kidney disease due to diabetes mellitus	98·46 (43·76 to 164·31)	146·40 (65·81 to 237·24)	48·69 (39·03 to 60·84)*	12·65 (7·32 to 19·88)*	3124·61 (1338·04 to 5247·93)	4566·00 (2050·51 to 7410·10)	46·13 (38·14 to 58·53)*	13·04 (7·74 to 20·08)*
··	Chronic kidney disease due to hypertension	47·69 (18·11 to 89·54)	73·45 (26·32 to 135·91)	54·03 (38·50 to 66·89)*	12·85 (7·10 to 23·44)*	1176·40 (503·88 to 2012·81)	1785·47 (805·84 to 2934·09)	51·77 (41·13 to 65·00)*	15·47 (9·33 to 24·63)*
··	Chronic kidney disease due to glomerulonephritis	30·93 (13·32 to 52·67)	41·71 (18·41 to 69·06)	34·87 (25·53 to 45·01)*	2·81 (−1·47 to 7·98)	1032·52 (417·03 to 1809·69)	1358·82 (567·29 to 2326·03)	31·60 (25·38 to 40·49)*	3·51 (−0·63 to 8·87)
··	Chronic kidney disease due to other causes	42·14 (18·81 to 71·07)	62·11 (26·60 to 104·77)	47·39 (32·26 to 59·95)*	11·26 (5·17 to 18·01)*	1301·30 (581·61 to 2232·05)	1867·87 (883·35 to 3113·60)	43·54 (35·86 to 54·17)*	11·97 (7·12 to 18·59)*
··	Osteoarthritis	··	··	··	··	2173·98 (1045·13 to 3867·70)	3225·98 (1624·93 to 5586·80)	48·39 (42·88 to 57·06)*	15·18 (11·04 to 21·86)*
··	Low back pain	··	··	··	··	2684·27 (1366·73 to 4685·98)	3630·51 (1919·27 to 6254·44)	35·25 (30·07 to 42·46)*	9·39 (5·70 to 14·83)*
··	Gout	··	··	··	··	229·32 (105·94 to 410·88)	321·88 (154·53 to 568·22)	40·37 (35·33 to 47·42)*	10·69 (6·98 to 16·07)*
··	Cataract	··	··	··	··	201·26 (82·62 to 397·74)	306·06 (132·21 to 586·28)	52·08 (45·72 to 61·98)*	15·83 (10·93 to 23·62)*
2	Low bone mineral density: all causes	341·07 (288·70 to 360·77)	441·23 (374·93 to 466·70)	29·37 (24·07 to 34·07)*	–5·78 (−9·63 to −2·34)*	9412·32 (8030·50 to 11 131·37)	11 955·49 (10 090·79 to 14 196·27)	27·02 (23·65 to 29·65)*	–3·07 (−5·68 to −1·03)*
··	Pedestrian road injuries	40·74 (38·39 to 43·41)	46·93 (44·13 to 49·88)	15·20 (9·02 to 18·80)*	–13·11 (−17·62 to −10·45)*	1094·99 (979·92 to 1226·14)	1293·53 (1143·70 to 1463·75)	18·13 (12·36 to 21·58)*	–8·69 (−12·96 to −6·11)*
··	Cyclist road injuries	5·17 (4·64 to 5·66)	6·03 (5·44 to 6·73)	16·64 (11·21 to 23·63)*	–10·21 (−14·32 to −5·00)*	343·04 (267·55 to 438·99)	447·83 (343·84 to 583·35)	30·55 (26·30 to 34·15)*	1·26 (−1·80 to 3·89)
··	Motorcyclist road injuries	8·60 (7·58 to 9·40)	10·61 (9·08 to 11·55)	23·37 (16·31 to 29·85)*	–3·30 (−8·74 to 1·71)	516·37 (422·92 to 630·17)	665·90 (537·39 to 824·92)	28·96 (24·64 to 32·55)*	1·63 (−1·65 to 4·24)
··	Motor vehicle road injuries	29·15 (26·52 to 32·31)	33·42 (30·60 to 37·18)	14·65 (10·86 to 20·30)*	–12·30 (−15·19 to −8·05)*	1053·30 (916·84 to 1212·20)	1227·50 (1055·45 to 1420·62)	16·54 (13·17 to 21·00)*	–9·15 (−11·70 to −5·87)*
··	Other road injuries	1·11 (0·97 to 1·42)	1·34 (1·19 to 1·71)	20·66 (10·56 to 35·98)*	–10·20 (−17·75 to 1·23)	85·10 (62·48 to 115·48)	129·35 (92·12 to 178·79)	51·99 (46·52 to 56·32)*	16·84 (12·28 to 20·37)*
··	Other transport injuries	7·41 (6·74 to 7·98)	8·91 (8·22 to 10·00)	20·29 (13·83 to 28·79)*	–8·50 (−13·44 to −2·28)*	330·22 (273·06 to 402·51)	394·33 (321·20 to 482·74)	19·41 (15·51 to 24·18)*	–7·29 (−10·26 to −3·74)*
··	Falls	237·28 (186·55 to 254·10)	321·08 (254·50 to 344·23)	35·32 (28·08 to 41·49)*	–3·51 (−8·60 to 0·81)	5306·53 (4397·57 to 6284·03)	6968·79 (5750·97 to 8276·40)	31·32 (26·75 to 34·67)*	–1·34 (−4·93 to 1·31)
··	Other exposure to mechanical forces	6·42 (5·21 to 6·94)	7·62 (5·85 to 8·28)	18·75 (11·69 to 23·63)*	–11·15 (−16·26 to −7·33)*	401·92 (305·39 to 523·87)	513·05 (380·93 to 681·19)	27·65 (23·66 to 30·49)*	–1·40 (−4·33 to 0·74)
··	Non-venomous animal contact	0·68 (0·53 to 0·88)	0·76 (0·59 to 1·02)	12·06 (4·71 to 23·04)*	–15·81 (−21·13 to −7·87)*	21·55 (16·66 to 27·50)	23·45 (18·15 to 30·34)	8·80 (3·65 to 14·68)*	–15·77 (−19·71 to −11·18)*
··	Assault by other means	3·91 (3·13 to 4·64)	4·14 (3·51 to 5·31)	5·95 (−3·67 to 21·09)	–18·19 (−25·57 to −7·12)*	236·94 (182·44 to 304·08)	268·81 (204·14 to 351·16)	13·45 (7·54 to 19·57)*	–11·45 (−15·88 to −6·82)*
··	Forces of nature, conflict and terrorism, and state actor violence	0·60 (0·40 to 0·81)	0·37 (0·21 to 0·57)	–38·05 (−56·98 to −20·83)*	–51·96 (−66·57 to −38·70)*	22·35 (13·90 to 36·70)	22·95 (10·58 to 47·82)	2·68 (−28·79 to 30·32)	–19·58 (−43·89 to 1·64)
2	Impaired kidney function: all causes	2108·45 (1943·12 to 2277·00)	2554·21 (2346·59 to 2766·51)	21·14 (18·37 to 23·96)*	–9·08 (−10·89 to −7·16)*	52 009·54 (48 088·99 to 55 861·74)	60 482·18 (55 678·63 to 65 319·35)	16·29 (13·87 to 18·61)*	–8·10 (−9·94 to −6·30)*
··	Ischaemic heart disease	753·35 (627·96 to 868·81)	906·02 (749·80 to 1056·12)	20·27 (15·84 to 24·87)*	–11·91 (−14·40 to −9·02)*	13 095·90 (11 202·99 to 14 872·74)	15 068·46 (12 896·50 to 17 267·64)	15·06 (11·42 to 18·84)*	–11·75 (−14·21 to −9·06)*
··	Ischaemic stroke	201·59 (153·59 to 247·89)	219·00 (164·95 to 274·84)	8·63 (2·78 to 14·64)*	–19·04 (−22·23 to −15·40)*	4041·29 (3235·99 to 4810·89)	4478·73 (3577·63 to 5417·18)	10·82 (6·20 to 15·37)*	–15·45 (−18·71 to −12·11)*
··	Haemorrhagic stroke	227·29 (185·54 to 269·78)	236·16 (191·40 to 283·30)	3·91 (0·62 to 7·40)*	–20·50 (−22·52 to −18·46)*	5431·74 (4452·60 to 6455·91)	5578·78 (4537·14 to 6686·22)	2·71 (−0·10 to 5·74)	–19·77 (−21·70 to −17·83)*
··	Peripheral vascular disease	5·64 (3·81 to 8·18)	7·32 (4·82 to 11·42)	29·76 (16·19 to 45·98)*	–4·33 (−12·92 to 6·28)	170·24 (121·08 to 237·00)	210·83 (147·41 to 296·25)	23·84 (16·07 to 32·93)*	–5·65 (−11·04 to 0·58)
··	Chronic kidney disease due to diabetes mellitus	384·78 (349·87 to 418·93)	500·41 (452·11 to 543·57)	30·05 (26·18 to 32·84)*	–0·63 (−3·43 to 1·30)	11 723·50 (10 608·16 to 12 883·32)	14 649·82 (13 196·95 to 16 191·89)	24·96 (21·91 to 27·57)*	–1·37 (−3·51 to 0·51)
··	Chronic kidney disease due to hypertension	222·32 (199·99 to 248·86)	299·48 (268·03 to 335·26)	34·71 (30·47 to 38·02)*	–0·96 (−3·95 to 1·00)	5166·00 (4517·97 to 5842·00)	6602·34 (5756·41 to 7488·87)	27·80 (24·67 to 30·62)*	–1·02 (−3·31 to 0·87)
··	Chronic kidney disease due to glomerulonephritis	127·88 (114·79 to 143·00)	149·99 (133·07 to 168·74)	17·29 (13·77 to 20·69)*	–6·33 (−8·54 to −4·22)*	5463·57 (4839·64 to 6152·24)	5927·94 (5222·09 to 6740·39)	8·50 (5·53 to 11·81)*	–7·67 (−9·80 to −5·37)*
··	Chronic kidney disease due to other causes	185·61 (164·22 to 208·15)	235·84 (206·86 to 266·27)	27·06 (23·30 to 30·93)*	–0·92 (−3·46 to 1·41)	6815·19 (6057·76 to 7656·73)	7827·49 (6911·39 to 8843·22)	14·85 (11·59 to 18·40)*	–3·98 (−6·26 to −1·43)*
··	Gout	··	··	··	··	102·11 (70·04 to 141·30)	137·78 (94·34 to 190·02)	34·93 (32·98 to 36·86)*	2·87 (1·60 to 4·23)*

Data in parentheses are 95% uncertainty intervals. DALYs=disability-adjusted life-years.

* Statistically significant increase or decrease.

### Global patterns of burden attributable to risk factors across quintiles of SDI

**Figure 3:**
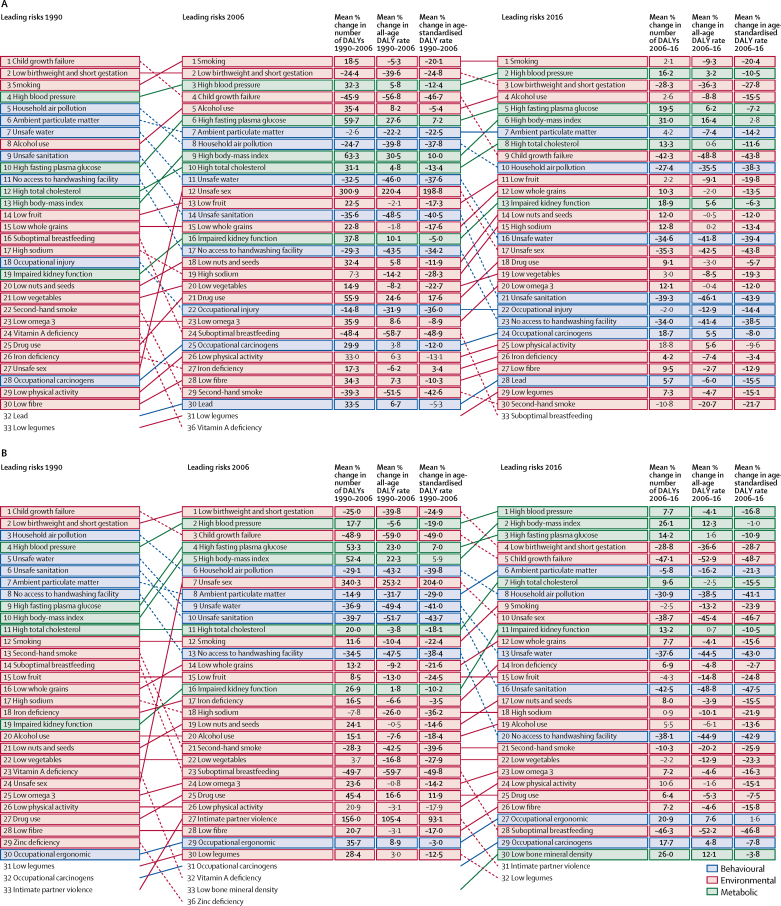
Leading 30 Level 3 risk factors by attributable DALYs at the global level, 1990, 2006, and 2016, for males (A) and females (B) Risks are connected by lines between time periods. Behavioural risk factors are shown in red, environmental risks in blue, and metabolic risks in green. For the time period of 1990 to 2006 and for 2006–16, three measures of change are shown: percent change in the number of DALYs, percent change in the all-age DALY rate, and percent change in the age-standardised DALY rate. Statistically significant increases or decreases are shown in bold (p<0·05). DALYs=disability-adjusted life-years.

Figure 2 shows that in 2016, the leading Level 2 risk factors in terms of attributable DALYs at the global level for both sexes combined were malnutrition (11·5% [10·8–12·3] of DALYs), diet (9·6% [8·2–11·1] of DALYs), high blood pressure (8·9% [7·9–9·9] of DALYs), tobacco (7·4% [6·7–8·3] of DALYs), and air pollution (6·8% [6·1–7·6] of DALYs). The list at this level of aggregation is similar for both sexes combined, with a notable difference being that alcohol and drug use is the fifth-leading risk factor for men, with 7·9% (7·2–8·6) of DALYs, but is at eleventh place for women (2·6% [2·3–2·9] of DALYs). More detail can be found in appendix 2 (p 1399). The patterns of risks vary by development, as seen across the panels of figure 3. At the lowest level of SDI, the leading risk is malnutrition with 25·0% (23·2–26·6) of DALYs, followed by air pollution (8·0% [7·1–9·0] of DALYs), unsafe water, sanitation, and handwashing (7·8% [6·6–9·4] of DALYs), and unsafe sex (4·7% [4·3–5·2] of DALYs). While malnutrition remains the leading risk factor at the low-middle level of SDI, diet (7·8% [6·8–9·0] of DALYs), high systolic blood pressure (7·2% [6·8–8·1] of DALYs), and tobacco use (5·9% [5·3–6·6] of DALYs) get included among the leading five causes as well. At the middle SDI level, diet is among the leading five risks with 12·5% (10·6–14·6) of DALYs while high systolic blood pressure and tobacco follow in importance. At the top three levels of SDI, high BMI increases in importance and makes it to the leading five risks, with 7·2% (4·7–10·0) of DALYs in middle SDI locations, with 9·8% (6·5–13·2) of DALYs in high-middle SDI locations, and 8·7% (5·9–11·7) of DALYs in high SDI locations. The panels in figure 3 clearly show the risk transition across levels of development.

### Changes in leading risk factors in 1990, 2006, and 2016

Figure 3 shows the leading 30 risk factors at Level 3 of the hierarchy and the median change in DALYs between 1990, 2006, and 2016. In terms of rates, among the top ten leading risks in 1990, child growth failure, unsafe sanitation, and unsafe water have experienced the largest declines over the period of 1990–2016. While these three risks have remained in the top 30 in 2016 for men, their ranks have fallen by several places to 9th (child growth failure), 21st (unsafe sanitation), and 16th (unsafe water). For women, their ranks have fallen to 5th (child growth failure), 16th (unsafe sanitation), and 13th (unsafe water). Between 1990 and 2006, median age-standardised DALY rates decreased by 46·7% (42·1–51·1) for men and 49·0% (45·0–53·0) for women, and in the most recent period child growth failure demonstrated further declines by 43·8% (36·9–49·8) for men and 48·7% (42·3–54·6) for women.

The risk factor of low birthweight for gestation and short gestation for birthweight remains among the leading risks (second position in 1990 for both sexes; third position in 2016 for men and fourth position for women), despite declines in both the number of DALYs and the age-standardised DALY rates since 1990. Smoking is another risk where there has been a consistent decline since 1990 in both SEVs and age-standardised DALY rates, yet it has consistently been ranked among the leading three risk factors for men in DALYs since 1990.

The trend in unsafe sex coincides with the HIV/AIDS epidemic. Figure 3 shows that unsafe sex experienced large increases between 1990 and 2006, by 198·8% (170·45–228·2) for men and 204·0% (170·0–236·4) for women, resulting in a higher rank in 2006, followed by declines of 43·8% (41·7–45·7) for men and 46·7% (44·1–49·0) for women since 2006 resulting in a lower rank in 2016. On the other hand, drug use follows a different trend, and increased for men by 17·6% (13·0–25·5) between 1990 and 2006 and resulted in a higher rank in 2006, and decreased 5·7% (2·2–9·0) since 2006. Despite declines, drug use rose from the 25th leading risk to the 18th leading risk for men between 1990 and 2016.

Air pollution, both household air pollution and ambient particulate matter, were among the leading ten risk factors for men and women in 1990 and have remained important in 2016. The median percent change in age-standardised DALY rates showed important declines in both time periods for men and women. Specifically, in the most recent time period household air pollution declined by 38·3% (35·3–41·4) for men and 41·1% (37·8–44·2) for women, and ambient air pollution decreased by 14·2% (11·5–17·1) for men and 21·3% (17·8–24·5) for women, in terms of median age-standardised DALY rates.

The metabolic risk factors have increased in both rank and in the absolute number of DALYs between 1990 and 2016 for both men and women. High blood pressure was the fourth-leading risk factor for both men and women in 1990 and had risen to be the second leading risk factor for men and the leading risk factor for women by 2016. In terms of the number of DALYs, men showed an increase of 16·2% (13·1–19·4) since 2006, while for women the increase was less steep at 7·7% (4·5–11·7). In terms of the median change in age-standardised DALY rates since 2006, both sexes showed a decline, 10·5% (8·2–12·7) for men and 16·8% (13·7–19·3) for women. Other leading metabolic risk factors, including high BMI, high FPG, and high total cholesterol, exhibited similar trends to high blood pressure over this time period. All four of these metabolic risk factors are within the leading ten risk factors globally for men and women in 2016.

**Figure 4:**
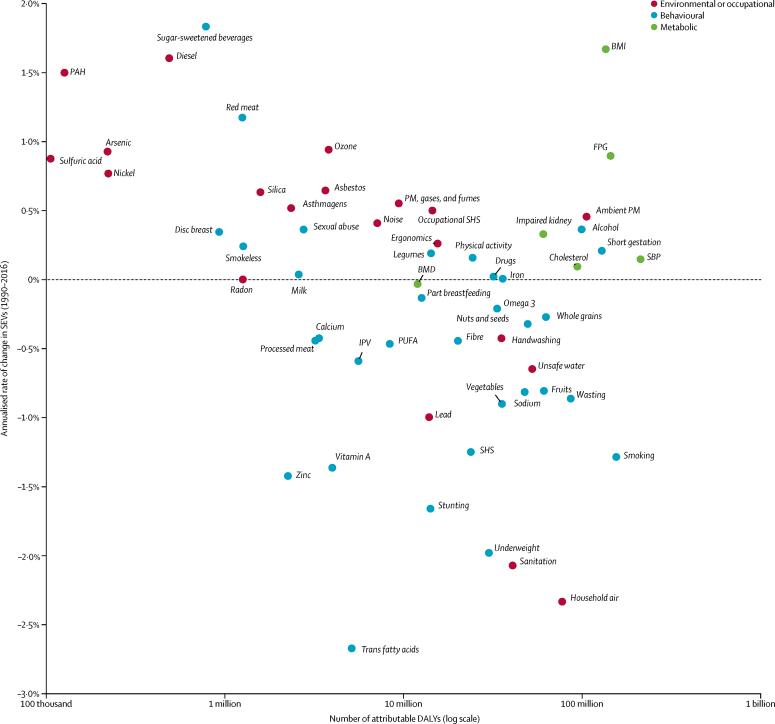
Relationship between attributable DALYs in 2016 for Level 3 risk factors and annualised rate of change in SEV, at the global level, both sexes combined, 1990–2016 DALYs are represented on a logarithmic scale. Risks shown exhibited a statistically significant change in SEV between 1990 and 2016. The following six risks, each of which is responsible for fewer than 100 thousand DALYs, are not shown: occupational exposure to benzene, beryllium, cadmium, chromium, formaldehyde, and trichloroethylene. DALYs=disability-adjusted life-years. SEV=summary exposure value. Ambient PM=ambient particulate matter pollution. Alcohol=alcohol use. Arsenic=occupational exposure to arsenic. Asbestos=occupational exposure to asbestos. Asthmagens=occupational asthmagens. BMD=low bone mineral density. BMI=high body-mass index. Calcium=diet low in calcium. Cholesterol=high total cholesterol. Diesel=occupational exposure to diesel engine exhaust. Disc breast=discontinued breastfeeding. Drugs=drug use. Ergonomics=occupational ergonomic factors. Fibre=diet low in fibre. FPG=high fasting plasma glucose. Fruits=diet low in fruits. Handwashing=no access to handwashing facility. Household air=household air pollution from solid fuels. Impaired kidney=impaired kidney function. IPV=intimate partner violence. Iron=iron deficiency. Lead=lead exposure. Legumes=diet low in legumes. Milk=diet low in milk. Nickel=occupational exposure to nickel. Noise=occupational noise. Nuts and seeds=diet low in nuts and seeds. Occupational SHS=occupational exposure to second-hand smoke. Omega 3=diet low in seafood omega 3 fatty acids. Ozone=ambient ozone pollution. PAH=occupational exposure to polycyclic aromatic hydrocarbons. Part breastfeeding=non-exclusive breastfeeding. Physical activity=low physical activity. PM, gases, and fumes=occupational particulate matter, gases, and fumes. Processed meat=diet high in processed meat. PUFA=diet low in polyunsaturated fatty acids. Radon=residential radon. Red meat=diet high in red meat. Sanitation=unsafe sanitation. SBP=high systolic blood pressure. Sexual abuse=childhood sexual abuse. SHS=second-hand smoke. Silica=occupational exposure to silica. Smokeless=smokeless tobacco. Sodium=diet high in sodium. Stunting=child stunting. Sugar-sweetened beverages=diet high in sugar-sweetened beverages. Sulfuric acid=occupational exposure to sulfuric acid. Transfatty acids=diet high in transfatty acids. Underweight=child underweight. Vegetables=diet low in vegetables. Vitamin A=vitamin A deficiency. Wasting=child wasting. Water=unsafe water source. Whole grains=diet low in whole grains. Zinc=zinc deficiency.

Among the leading risk factors in terms of DALYs, high BMI and high FPG have the fastest increases in SEVs with annualised rates of change of 1·7% (1·5–1·9) and 0·9% (0·6–1·3), respectively, since 1990 (figure 4). On the other hand, other leading risk factors in 2016 such as smoking and household air pollution exhibited significant and fast declines in SEVs, with a −1·3% (–1·6 to −1·1) annualised rate of change for smoking and −2·3% (–2·5 to −2·2) for household air pollution between 1990 and 2016 (figure 4).

### Drivers of changes in risk-attributable deaths and DALYs

**Figure 5:**
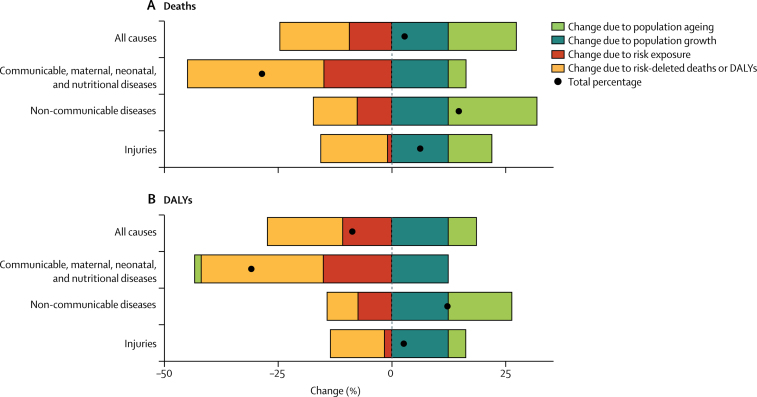
Percent change in deaths (A) and DALYs (B) at the global level, 2006–16, due to population growth, population ageing, trends in exposure to all risks included in GBD 2016, and and all other (risk-deleted or residual) factors Results are shown for all causes combined; communicable, maternal, neonatal, and nutritional diseases; non-communicable diseases; and injuries. DALYs=disability-adjusted life-years.

Figure 5 shows the relative contributions to changes in deaths and DALYs of important drivers grouped into four mutually exclusive categories: population growth, population ageing, trends in exposure to all risk factors measured in GBD 2016, and all other factors combined. Globally, trends in exposure to all risk factors combined would have led to a decrease of deaths by 9·3% (6·9–11·6) and DALYs by 10·8% (8·3–13·1). Risk factors play a larger part in CMNN causes, where trends in exposure to risks would have resulted in a decrease of deaths by 14·9% (12·4–17·1) and DALYs by 15·0% (12·7–17·6). Overall, population ageing and population growth are both driving deaths and DALYs to increase significantly. At the global level, across all causes, population growth alone would have resulted in 12·4% (10·1–14·9) more deaths and 12·4% (10·1–14·9) more DALYs, while population ageing would have contributed 14·9% (12·7–17·5) more deaths and 12·4% (10·1–14·9) more DALYs. The contribution of population ageing in NCDs is noteworthy as it is the largest driver of trends in NCDs, and accounts for 19·5% (17·3–22·0) more deaths and 14·0% (11·6–16·3) more DALYs between 2006 and 2016. The residual category, which includes improvements in treatment along with other factors, accounts for a decrease of 15·3% (12·9–17·7) for deaths and 16·5% (14·1–18·8) for DALYs across all causes and is particularly large for CMNN causes accounting for a 30·0% (27·5–32·4) decline in deaths and a 26·8% (24·4–29·2) decline in DALYs since 2006.

**Figure 6:**
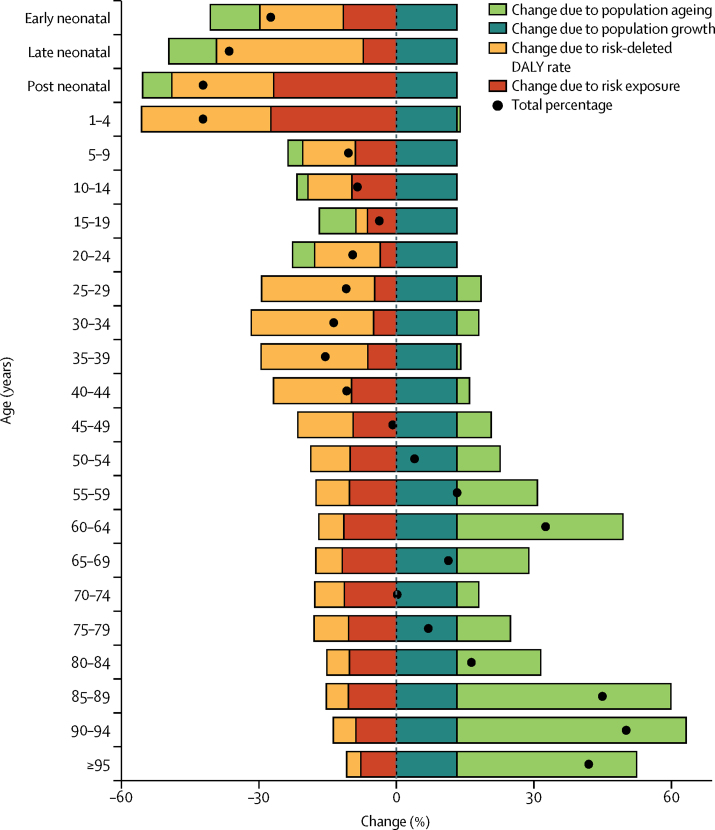
Percent change in all-cause DALYs, by age, at the global level, 2006-2016, due to the following drivers: population growth, population ageing, trends in exposure to all risks included in GBD 2016, and all other factors DALYs=disability-adjusted life-years.

Figure 6 shows the contribution of these drivers across age groups for DALYs. Across age groups, the contributions of the four drivers differ greatly. Changes in risk exposure have played a major part in the declines in DALYs younger than 5 years, accounting for 26·7% (24·3–29·7) of the trend in DALYs in the post-neonatal period and 27·3% (24·9–29·7) among ages 1–4 years. Trends in risks account for a decline of 8·7% (6·3–11·1) of DALYs in older children (ages 5–9 years) and 9·0% (6·5–11·4) of DALYs in young adolescents (ages 10–14 years). As expected, population ageing is a more significant driver among older age groups, accounting for up to 51·4% (49·1–53·9) of the change in DALYs since 2006 among the age group 90–94 years. Finally, the proportion of the change in DALYs that is due to all other factors—ie, not explained by these three major drivers—also shows large variation across age groups, ranging from a decrease of 3·5% (1·1–6·0) in the age group 15–19 years to a decrease of 28·2% (25·8–30·5) in the age group 1–4 years.

### Key results for new risks, leading risks, and risks with significant changes in GBD 2016

In 2016, for Level 3 risks factors, more DALYs were attributable to increased SBP than any other risk factor. Increased SBP was the second leading risk factor for men and leading risk factor for women globally, accounting for 89·9 million (80·9 million to 98·2 million) DALYs among women and 124·1 million (111·2 million to 138·0 million) DALYs among men. IHD was the largest source of DALYs attributable to increased SBP, followed by haemorrhagic stroke and ischaemic stroke. Since 1990, the SEV for increased SBP rose for men (22·9 [21·5–24·6] in 1990 to 24·6 [23·0–26·6] in 2016, a 7·5% increase [7·0–8·0]), and increased for women (24·2 [22·7–25·8] in 1990 to 24·2 [22·7–25·8] in 2016, a 0·7% increase [0·2–1·2]).

In 2016, 7·1 million (6·5 million to 7·8 million) deaths and 177·3 million (162·3 million to 194·3 million) DALYs were attributable to tobacco, most of which is attributable to smoking tobacco. Smoking-attributable deaths have increased by 20·1% (15·3–25·2) since 1990, with most deaths occurring in China, India, the USA, and Russia. Smoking is the second-leading risk factor for men for deaths and leading for DALYs, accounting for 16·3% (14·6–17·9) of deaths and 9·5% (8·5–10·7) of DALYs, and the sixth for women for deaths and ninth for DALYs, with 5·8% (5·0–6·7) of deaths and 2·9% (2·5–2·94) of DALYs. In 2016, there were 177·3 million (162·3 million to 194·3 million) smoking-attributable DALYs globally. Overall, in 2016 chronic respiratory diseases (30·3% [25·2–36·0]), neoplasms (19·2% [16·0–22·8]), and cardiovascular diseases (18·0% [16·0–20·0] were the three leading causes of smoking-attributable age-standardised DALYs across both sexes. For women, the leading cause of DALYs was COPD, whereas the leading cause for men was IHD.

Second-hand smoke exposure is highest in eastern Asia and Oceania and higher among women and children compared with men. The distribution of DALYs attributable to second-hand smoke exposure is bimodal, with peaks in the post-neonatal period and again in older age groups. Globally 0·9 million (0·7 million to 1·1 million) deaths were attributable to second-hand smoke exposure, of which 56 340 (28 951–89 043) occurred among children younger than age 10 years.

In estimating the burden attributable to smokeless tobacco, we found that the risk varies by the toxicity of the type used; there is sufficient evidence that chewing tobacco and other products of similar toxicity cause excess risk of oral and oesophageal cancer while, at this time, existing evidence does not support attributing burden to snus or similar smokeless tobacco products. Globally, smoking tobacco causes far more burden than smokeless tobacco; nonetheless, smokeless tobacco is an important risk factor for oral and oesophageal cancer in India, where more than half of the 32 141 (24 930–39 243) global deaths attributable to smokeless tobacco occur.

Low birthweight and short gestation, new risk factors in GBD 2016, were the third-ranked Level 3 risk factor globally for all-ages DALYs in 2016, which reflects a 61·6% (59·3–64·0) decrease in all-ages DALY rates from 5112·8 (4934·2–5389·6) DALYs per 100 000 in 1990 to 1960·8 (1862·0–2060·3) DALYs in 2016. In 1990, this risk factor was the second-ranked Level 3 risk factor globally for all-age DALYs; most of the decrease from 1990 to 2016 is due to a lower mortality burden in the causes attributable to low birthweight and short gestation rather than changes in exposure itself. Increasing SDI was associated with decreasing exposure, but the exposure gradient between SDI quintiles was not as large as the differential between high and low SDI in attributable burden. Exposure was highest in South Asia, eastern sub-Saharan Africa, and parts of the western Sahel zone, while attributable burden was highest in South Asia and parts of the western Sahel zone. The trend in exposure to low birthweight for gestation decreased at the global level from 1990 to 2016, reflective of the overall decrease in DALYs burden during the same time period. The biggest improvements were seen in Colombia, Brunei, and Zimbabwe, with broad improvements also seen across much of eastern sub-Saharan Africa.

In 2016, high FPG was the third-leading risk factor for deaths and the fourth-leading risk factor for DALYs globally among Level 3 risk factors, accounting for more than 5·6 million deaths (4·5 million to 7·0 million) and 144·1 million DALYs (119·9 million to 171·6 million). Since 1990, the age-standardised percent of deaths and DALYs attributable to high FPG has increased globally from 7·8% (6·0–10·1) to 10·5% (8·3–13·1) and 4·4% (3·7–5·3) to 6·2% (5·3–7·3), respectively. Diabetes was the largest source of DALYs attributable to increased FPG, followed by ischaemic heart disease and chronic kidney disease. We re-evaluated epidemiological evidence supporting the causal relationship between high FPG and disease endpoints and found sufficient evidence to include ten new outcomes for high FPG. These new outcomes included glaucoma, cataracts, dementia, liver cancer, lung cancer, ovarian cancer, breast cancer, bladder cancer, colorectal cancer, and pancreatic cancer. The new outcomes together contributed to 174 352 (37 297–388 039) additional deaths and 2·6 million (0·6 million to 5·7 million) additional DALYs beyond the causes that were included in GBD 2015.

In 2016, BMI was the fifth-ranked Level 3 risk factor for death globally, accounting for more than 4·5 million (2·9 million to 6·4 million) deaths and 135·4 million (88·6 million to 187·4 million) DALYs. Among Level 3 risk factors with more than 10 million attributable DALYs, high BMI had the fastest annualised rate of increase in SEV since 1990 (appendix 2 p 1399). Despite this significant increase in risk exposure, increases in attributable burden were attenuated by significant decreases in risk-deleted DALY rates, mainly due to reductions in cardiovascular disease mortality rates. We find that the burden attributable to high BMI increases with increasing development, with the lowest rates of disease attributable to high BMI found in sub-Saharan Africa, yet development is not the only predictor. We conducted a systematic search of health outcomes caused by excess bodyweight and added eight new causes for GBD 2016, which together contributed to 442 750 (191 407–796 350) additional deaths beyond the causes that were included in GBD 2015. Additionally, we included childhood overweight and childhood obesity as new risk factors, allowing us to better capture the health effects of excess bodyweight across the life course. Within the CRA framework, the only childhood overweight and obesity outcome eligible for inclusion was asthma. We found that 10·4% (3·1–21·2) of asthma can be attributed to childhood excess bodyweight globally, a total of 1128 (311–2354) deaths and 642 532·1 (180 916·3 to 1 456 342·7) DALYs. While childhood burden is much smaller compared with adult burden, estimating exposure for children is crucially important in view of the well described effects of childhood overweight and obesity on adult health outcomes.

Air pollution was ranked sixth in terms of attributable DALYs in 2016. We found that 7·5% (6·6–8·4) of deaths globally were attributable to ambient air pollution in 2016 (4·1 million [3·6 million to 4·6 million] deaths, 1·3 million [1·1 million to 1·5 million] in South Asia). Countries with notably high levels of attributable deaths include China (11·1% [9·7–12·7] of all deaths attributable to ambient particulate matter) and India (10·6% [9·2–11·9] of all deaths). The diseases with the largest proportion of burden attributable to air pollution are LRI and COPD; ambient particulate matter is responsible for 27·5% (21·4–34·4) of all LRI and 26·8% (16·1–38·6) of COPD deaths and 33·3% (26·3–40·5) of LRI deaths in children younger than 5 years. In terms of overall ranking, ambient particulate matter has increased from seventh in 1990 with 115·2 million (99·1 million to 132·9 million) DALYs to sixth in 2016 with 105·7 million (94·2 million to 117·8 million) DALYs. For deaths, it is among the top ten ranked risk factors in 195 countries and territories, including India and China, where it was in third and fourth place, respectively. Also of note is that updated satellite data indicate increased ambient air pollution in 2015–16 in West Africa that is driven by wind-blown dust from the Sahara. This effect has profound effect on disease burden in this region, as intense particulate matter with an aerodynamic diameter smaller than 2·5 μm (PM_2·5_) events affect Africa's densest region.

Globally, alcohol is estimated to be the seventh-leading risk factor in 2016 in terms of DALYs. In the same year, alcohol use was estimated to have caused 99·2 million DALYs (88·3 million to 111·2 million), accounting for 4·2% (3·7–4·6) of total DALYs. This is a larger share of total burden than previously reported, driven primarily by changes made to both the exposure and RR models. This burden is distributed unequally among the sexes and regions. When decomposed by sex, alcohol use accounts for 6·2% (5·6–6·9) of total DALYs among men and 1·7% (1·4–2·0) of total DALYs among women. When decomposed by region in 2016, alcohol use accounts for 13·9% (11·5–16·8) of age-standardised DALYs in eastern Europe, 4·0% (3·4–4·6) of age-standardised DALYs in Southeast Asia, but only 0·8% (0·6–1·0) of age-standardised DALYs in the Middle East. Alcohol use attributable DALYs have also increased by more than 25% over the years 1990–2016, driven primarily by increased consumption in South Asia, Southeast Asia, and Central Asia, among both men and women. Globally, alcohol use exposure has increased by 15·2% (8·7–22·6) over that time frame among men and decreased by 3·2% (–9·1 to 3·1) among women. However, the largest increases in exposure have been in countries in the low-middle quintile of SDI. Globally, alcohol use is the leading risk factor in DALYS between the ages of 15 years and 49 years in 2016. However, unlike tobacco or drugs, governments have been discouraged from efforts to limit alcohol's availability by trade agreements and disputes. Given alcohol's health burden within these age groups, an increased focus on alcohol control policies is needed to effectively address this risk factor.

It is worth noting some key results for dietary risks as well. In 2016, suboptimal diet was the second-leading risk factor for deaths and DALYs globally, accounting for 18·8% (16·0–21·7) of all deaths and 9·6% (8·2–11·1) of all DALYs. Comparing men and women, suboptimal diet accounts for the greatest percentage of total deaths in men (19·0% [16·3–21·8]) and the second largest in women (18·6% [15·7–21·7]). Meanwhile, suboptimal diet accounts for the second-largest percent of total DALYs in both men (10·6% [9·1–12·2]) and women (8·4% [7·0–9·9]). More than 50% of deaths (51·5% [44·2–59·2]) and DALYs (54·1% [47·1–61·5]) attributable to suboptimal diet were due to cardiovascular diseases. Among the individual dietary risks, a diet low in whole grains accounted for the largest number of deaths (4·6% [3·0–6·4]), followed by a diet low in fruits (4·3% [2·7–6·3]) and a diet high in sodium (4·2% [1·2–8·3]). Leading dietary risks for DALYs were low intakes of whole grains (2·6% [1·8–3·6]), fruits (2·6% [1·6–3·7]), and nuts and seeds (2·1% [1·4–2·8]). The greatest increase in attributable deaths and DALYs between 1990 and 2016 occurred for a diet high in red meat, followed by a diet high in sugar-sweetened beverages and a diet low in milk, respectively.

## Discussion

### General findings

Based on the analysis of 22717 sources, we estimated disease burden attributable to 84 metabolic, environmental, occupational, and behavioural risk factors or clusters of risks from 1990 to 2016 in 195 countries and territories. In 2016, all risks combined contributed to 59·9% (58·4–61·3) of deaths and 45·2% (43·2–47·3) of DALYs worldwide, compared with 60·3% (59·0–61·6) of deaths and 49·6% (47·6–51·7) of DALYs in 1990. The role of changes in risk factors in explaining changes in deaths and DALYs varies considerably across causes and ages, with the largest effects noted in children due to infectious diseases. Since 1990, exposure increased significantly for 30 risks, did not change significantly for four risks, and decreased significantly for 31 risks. The risks with the highest increases in SEVs include high body-mass index, occupational exposure to diesel engine exhaust, and occupational exposure to trichloroethylene, while the risks with the largest decreases in exposure are diet high in transfatty acids, household air pollution from solid fuels, and unsafe sanitation.

We found substantial heterogeneity across countries in the leading risk factors. Some notable patterns are the role of unsafe sexual practices as a driver of the HIV epidemic in Eastern and Southern Africa and the role of alcohol consumption in Eastern Europe and Central Asia. There are also marked spatial patterns for other risks such as high BMI in Central America, North Africa and the Middle East, and Oceania. Interpreting spatial patterns needs to take into account the fact that some risks have a strong relationship with socioeconomic development. Several environmental and behavioural risks, including water, sanitation, handwashing, household air pollution, and childhood growth failure decline profoundly with development. Another cluster of risks tends to increase with socioeconomic development, including high BMI, high SBP, red meat consumption, sugar-sweetened beverages, alcohol, and high FPG.

### Cross-cutting themes

Many factors should determine government priorities for action including the size of the problem, inequalities related to the problem, likely future trends, the availability of effective policy options, and the opportunity cost of tackling a particular problem. In this analysis, we provided information about the size of the problem, trends in exposure in the last 27 years, and the range of exposure at given levels of socioeconomic development. Problems that are large, increasing, and variable across countries at the same level of development likely warrant particular policy attention. Our analysis showed that components of diet, obesity, FPG, and SBP are the most prominent global risks fulfilling these criteria. Because of the strong interrelationships between these risks, the true driver of this cluster is likely diet, the risk in BMI, or both, with knock-on consequences for FPG and SBP. The rise of obesity and the associated increases in FPG and SBP warrant considerable global policy attention. Other major risks that should continue to receive attention—even intensified attention in some locations—such as smoking, are nevertheless declining at the global level. The unique combination of large current effect and increasing exposure puts obesity in a special category of risks. Obesity is likely to not only influence future population health in many locations, but will have considerable financial implications for health systems, given what we know about treatment costs for the associated diseases. Since important drivers of obesity such as physical activity and diet patterns are adopted in childhood and adolescence, more work is needed to proactively address the adoption of these risks in these younger age groups.

For the first time, we assess the contribution of changes of risk exposures to the overall global trend for deaths and DALYs; for example, in the past 10 years, changes in all risk exposures contributed to an 10·8% (8·3–13·1) decline in DALYs, while other factors contributed to a 16·5% (14·1–18·8) decrease in DALYs. More detailed assessments show large declines in CMNN causes and increases in injuries and non-communicable DALYs. In each case, the contribution of other factors was substantially larger than the contribution of risk reduction. Our findings of the relatively small contribution of risk reduction to the declines in NCDs are not at odds with published studies for the UK and the USA,^20^, ^21^, ^22^ because we are reporting at the global level; our results at the national level suggest a larger role for risk reduction in some high-SDI locations. These observations lead to two directions for further analysis. First, what is the explanation for the declines driven by other factors? Some of this effect might be social policy working through various causal channels, and some is likely due to improvements in access to high-quality health care. This is particularly true for conditions such as selected cancers, ischaemic heart disease, cerebrovascular disease, chronic kidney diseases, HIV/AIDS, tuberculosis, and maternal mortality, for which health care is known to have large effects. Second, in view of the enormous potential of risk reduction to change health outcomes as documented in this and many other studies, why has progress on many risks been comparatively slow? For example, even though global tobacco consumption is declining in terms of rates, the pace of decline has been remarkably slow on average, despite more than 50 years of good evidence on the harms of tobacco. The relatively poor track record for global risk reduction might in part reflect the low rate of investment in risk reduction compared with curative health care. It might also reflect the continuing challenge of changing many risky behaviours. Relatively little funding for research on changing behaviours compared with new diagnostics and therapeutics might also be part of the explanation of the prevention paradox.^23^, ^24^ Changing behavioural risks could also require more than government action; harnessing the private sector to facilitate behavioural change might also be crucial.

### Important changes in GBD 2016 compared with in GBD 2015 (risks ordered by global rank)

#### Systolic blood pressure

Increased SBP remains the leading global risk at Level 3 in the GBD risk hierarchy. Highly effective interventions exist to manage blood pressure at the primary care level, as do a range of public health interventions, so it is quite remarkable that global exposure to increased SBP is increasing. Part of this increase might be tied to the global rise in high BMI, but the increase in SBP represents significant missed opportunity for the world's health systems. In 54 countries high SBP is actually declining, while its increase in China is now well documented in a series of population-based surveys.^25^, ^26^, ^27^ Tackling rising SBP is a global concern, but this is particularly important in those locations where rates are increasing. In view of the effect of the risk and the large array of available, effective interventions, health systems and the global health community need to mobilise increased resources and policy attention to tackle this problem. It might be necessary to design a variety of public policies including food reformulation to reduce sodium content and efforts to incentivise primary care providers to give priority to the management of SBP.^28^, ^29^, ^30^

#### Tobacco

In moving toward developing a comprehensive picture of tobacco use globally, in GBD 2016, we have for the first time included smokeless tobacco use as a risk factor. While the burden of smokeless tobacco is minimal in the majority of countries, it is of huge importance in south Asia, where the highest risk-weighted exposure is observed in Bangladesh (risk-weighted exposure of 0·75 [0·61–0·87]), Bhutan (0·53 [0·44–0·62]), Myanmar (0·50 [0·42–0·59]), Nepal (0·50 [0·42–0·58]), and India (0·45 [0·43–0·47]). In these countries more women use smokeless tobacco products than smoked tobacco products, and we find that use of any tobacco products, smoked or smokeless, continuously increases with age, a regional age pattern that differs from the global and male regional age pattern. The combination of high exposure and large population results in a majority of global deaths attributable to smokeless tobacco in 2016 occurring in India, where it is also the leading risk factor for oral cancer.

In GBD 2016, we also improved the estimation of burden attributable to second-hand smoke. At the global level, while the burden of second-hand smoke remains substantial, exposure to second-hand smoke has been declining significantly at an annualised rate of change of 1·9% (1·5–2·4). These reductions are likely attributable to a wide range of public health measures to control tobacco, which have accelerated in a large number of countries since the implementation of the Framework Convention on Tobacco Control (FCTC).^31^

Progress combatting the tobacco epidemic has resulted in global declines in prevalence of tobacco use and second-hand smoke exposure, yet the number of deaths and DALYs attributable to tobacco has increased since 1990. Increases in burden were driven by a combination of population growth and population ageing, along with persistently high smoking prevalence in some of the most populous countries of the world. Taken together, we can expect the burden of tobacco to remain high in years to come, unless the rate of progress is significantly accelerated. Many countries with persistently high levels of daily smoking recorded marginal progress in the past decade, and smoking remains a leading risk factor in most countries. The fact that tobacco use patterns diverge by location, level of development, and sex highlights the need for more tailored approaches to change smoking behaviours in the future. Particularly worrisome are the trends among young men and women. For example, in Indonesia, a country that has not yet ratified the FCTC,^31^ more than half of men aged 20–24 years are daily smokers. Understanding what works—and what does not—for tobacco control across contexts and within subpopulations (ie, men and women, younger and older individuals, various socioeconomic groups) is of growing priority. To significantly and permanently change the toll of tobacco, a renewed and sustained focus is needed on comprehensive tobacco control policies around the world.

#### Fasting plasma glucose

The global increase in FPG is likely tied to the increase in BMI. While exposure is increasing, age-standardised attributable mortaliy rate is not; a related pattern is that the prevalence of diabetes is increasing, but deaths from diabetes have been declining, likely because clinical management of the macrovascular complications of diabetes has improved in many (but not all) locations. Prevention trials show that with intensive resources devoted to weight loss and physical activity, reductions in FPG can be achieved; however, these interventions have not been implemented at a national scale and adherence in the long run is challenging. Systematic efforts to screen for high FPG implemented in some countries may increase awareness and action in more patients but can be resource-intensive. Clinical interventions to reduce FPG can be effective, although there are more recent debates on the appropriate targets for treatment in some cases. With FPG increasing in many settings, it is difficult to determine the population effect of treatment of blood sugar on population FPG. FPG remains one of the risk factors that is most likely influenced at the primary health-care level, emphasising the role of universal coverage for primary care in a multipronged response to this increasing problem.

#### Body-mass index

One of the most alarming risks in the analysis is increased BMI, because its burden is large and increasing, and it is prevalent across all levels of SDI.^32^, ^33^ The potential drivers of this global epidemic include changes in food industries and systems, which increase availability, accessibility, and affordability of energy-dense foods, along with intense marketing of such foods, as well as reduced opportunities for physical activity.^34^ A range of interventions have been proposed to reduce obesity, including restricting the advertisement of unhealthy foods to children, improving school meals, taxation of sugar-sweetened beverages, and taxation to reduce consumption of other unhealthy foods and subsidies to increase intake of healthy foods, and using supply-chain incentives to increase production of healthy foods.^35^ However, the evidence base that many of these interventions can affect trends in obesity at scale is currently weak.^36^ What we know without a doubt is that obesity rates continue to increase in almost all locations. Low-SDI and middle-SDI countries generally have little financial resources for nutrition programs and mostly rely on external donors whose programmes often preferentially target undernutrition.^37^ The increase in exposure to high BMI is greater than the increase in attributable burden largely because cardiovascular disease death rates continue to decline because of other changes, particularly improvements in treatment and declines in smoking and high cholesterol. Proposed policies, even if fully implemented, are unlikely to rapidly reduce the prevalence of obesity. While not a solution to the rise of overweight and obesity, clinical interventions that control high SBP, cholesterol, and FPG (the major risk factors for cardiovascular disease) can be used to mitigate some of the cardiovascular ill-effects.^20^ Expanded use of such interventions among obese people could effectively reduce the disease burden of high BMI. Sustained progress, however, will require policies that effectively control weight in childhood and in young and middle-aged adults.

#### Diet

In GBD 2016, poor dietary habits were the second leading risk factor at Level 2 of the hierarchy for mortality globally, accounting for nearly one in every five deaths. The overall burden of dietary risks at the global level was 14·8% (11·7–18·5) lower than in GBD 2015. Additionally, important differences were observed in the attributable burden and the ranking of individual dietary risks. Multiple factors have contributed to these differences, including using more data sources, as well as improving the method of estimation of the mean and distribution of intake for each dietary factor. In GBD 2016, for the first time, we used sales data to inform our estimates of consumption for most dietary factors. Using sales data, in addition to improving our overall data coverage, allowed us to capture recent trends in consumption. This was particularly important for specific dietary factors, such as sugar-sweetened beverages, which have been the target of dietary policies in several countries.^38^, ^39^, ^40^, ^41^, ^42^, ^43^ Additionally, to improve the consistency of definitions of dietary risk factors across surveys, we made a systematic effort to obtain and re-extract individual-level data from nutrition surveys. To make the current level of intake and optimal level of intake more comparable, we used the absolute level of intake (rather than the intake standardised to 2000 kcal per day) as the primary exposure in GBD 2016. We also corrected our estimated daily intake of each individual dietary factor for within-person variation and characterised the usual intake at the population level. Finally, given the differences in the health effects and patterns of intake for legumes and vegetables, we estimated the burden of disease attributable to low intake of legumes and low intake of vegetables separately.

The decade of 2016–25 has been declared as the Decade of Action on Nutrition by the United Nations General Assembly.^44^ GBD 2016 provides a comprehensive picture of various forms of malnutrition (ie, undernutrition, overweight or obesity, and poor dietary habits) across all countries at the start of the Decade of Action on Nutrition and can inform priorities for evidence-based interventions in each country. GBD also provides an independent avenue to annually monitor the progress of countries toward achieving their nutrition-related goals in a comparable and consistent manner. Our results show that among all forms of malnutrition, poor dietary habits, particularly low intake of healthy foods, is the leading risk factor for mortality. This finding has important implications for national governments and international organisations aiming at ending malnutrition over the next decade, highlighting the need for comprehensive food system interventions to promote the production, distribution, and consumption of healthy foods across nations.

#### Low birthweight and short gestation

Low birthweight and short gestation have been added for GBD 2016; they are the third-leading global risk at Level 3 in the GBD risk hierarchy. Improvements in burden attributable to low birthweight and short gestation have been largely driven by other factors influencing neonatal death rates, given that exposure to low birthweight and short gestation have not improved much over the past 27 years. Little progress in exposure suggests suboptimal coverage of interventions and programmes that can prevent low birthweight and short gestation. These include women-centred services for optimising nutrition (including minimising obesity), infection control, smoking cessation, and preventive care for pregnant women or those contemplating pregnancy.^45^, ^46^, ^47^ Efforts should also focus on maximising the quality of antenatal care services to identify and appropriately manage at-risk and high-risk pregnancies,^48^ including avoidance of provider-initiated preterm delivery. If evidence-based interventions are employed, it should be possible even in resource-limited settings to shift the risk curve for those babies who will be born early, small, or both, despite best efforts. Before birth, this includes potentially antenatal steroid administration to promote lung development;^49^ at birth, this requires presence of adequately trained and equipped neonatal resuscitation services;^50^, ^51^ post-delivery, it should include physicians with neonatal specialisation and availability of supportive equipment such as continuous positive airway pressure.^52^ Facility-based infection control measures are crucial to prevent nosocomial transmission, as such events are highly lethal in low birthweight or short gestation neonates.^53^ The inclusion of this risk for a major cause of DALYs—namely, neonatal mortality—also expands the share of overall burden that can be attributed to risks in general. More work remains, however, to understand the relationship between low birthweight and short gestation and childhood growth failure after 1 month. Our analysis to date may actually underestimate the importance of this risk if the share of childhood growth failure that can be traced to low birthweight and gestational age is fully established.

#### Alcohol

Globally, alcohol is estimated to be the seventh-leading risk factor in 2016 in both DALYs (4·2% [3·7–4·6]) and deaths (5·2% [4·4–6·0]). Previous studies have noted the possibility that the preventive effects of alcohol might have been overstated due to selection bias and choice of the reference population.^2^, ^54^, ^55^, ^56^ Our findings lend further credence to these hypotheses; with the exception of IHD, our results show either a minor or non-significant preventive effect for causes previously estimated to have large preventive effects. Further, our analysis noted a much larger risk of neoplasms due to alcohol use than previously reported. Combined with our new data for alcohol use exposure, alcohol use is ranked as one of the leading risk factors, surpassing cholesterol as a share of total DALYs, comparied with previous iterations of GBD.^4^, ^5^, ^6^

#### Ensemble distributions

In GBD 2016 we have introduced a more accurate method for developing the distributions of exposure for many risk factors. Our work on distributions and the shift to ensemble distributions shows that the assessment of attributable burden is sensitive to distributional assumptions. Given that a number of risks, such as BMI, SBP, cholesterol, and FPG, rise exponentially as a function of exposure, the estimation of the tail of the distribution has an important effect on the results. The ensemble modelling approach can provide more accurate estimation of the full distribution, including the tails of the distribution. In general, we believe that the assessment of the distribution of the risks deserves more careful attention in future research.

### Comparison of GBD 2016 to other estimates

The GBD study is the most comprehensive effort to conduct a population-level CRA across countries and risks. Differences between GBD 2016 estimates and other global estimates are generally related to approaches to data processing, access to data sources, and analysis decisions. For several risks, including smoking,^57^ ambient ozone pollution, household air pollution from solid fuels, lead exposure,^58^ intimate partner violence,^59^ unsafe water source,^60^ and breastfeeding, GBD estimates were lower than published WHO estimates.^57^, ^58^, ^59^, ^60^ These discrepancies can be attributed to different definitions, methodological decisions, granularity, and input data. For some findings, annual estimates might disagree, but regional patterns were consistent between WHO and GBD. UNICEF^61^ produces estimates for child stunting that are lower than GBD estimates with some disagreement where progress has been made globally. There is more consistency in estimates between UNICEF and GBD for child wasting and child underweight.^61^ GBD estimates for the prevalence of low birthweight and short gestation are slightly lower when compared with WHO estimates, but show similar geographical patterns.^62^ Scientific literature reveals similar results to GBD for impaired kidney function^63^ and low birthweight and short gestation;^64^, ^65^ research analysing ambient air pollution^66^ differed from GBD estimates due to older methods and less granularity. Research published on iron-deficiency anaemia^67^ differs from GBD in methods and definitions, resulting in generally higher GBD estimates. GBD estimates were much lower than published research on occupational estimates,^3^, ^68^, ^69^ largely due to different cause-outcome pairs and GBD's application of the CRA approach (see appendix 1 p 10).

### Future directions

Interpretation of our results and prioritisation at the national level might also need to take into account the variable strength of evidence supporting the causal connection for each risk-outcome pair. In GBD 2016, we have continued to use the World Cancer Research Fund criteria of convincing or probable evidence to select risk-outcome pairs for inclusion. Some aspects of these definitions are subjective. Not all researchers would agree on the interpretation of the available evidence as fulfilling these criteria. For example, there are six studies on non-exclusive breastfeeding and LRI; there are two studies on discontinued breastfeeding and diarrhoeal diseases. We have sought to quantify the number of studies of different kinds that are available to support these judgements in table 1, but not all studies support causality to the same extent. Randomised trials, if well conducted, provide the strongest evidence of causality, because they are likely not affected by confounding. But even randomised trials can have biases when there are missing observations, as is often the case. Randomised trials are also not feasible in many cases, or if feasible, not representative for many risks, including environmental risks. Cohort studies can provide compelling evidence, but many cohorts do not adequately control for socioeconomic confounders and can suffer from many other issues related to the quality of exposure measurement or outcome ascertainment. To go beyond, the quantification of the number of studies of each type we have provided here will necessitate a deeper analysis of the potential limitations of all 2579 studies used across the risk-outcome pairs. In future work, we plan to evaluate the quality of each of these studies with a standardised approach and work toward an overall evidence summary. There is also a more fundamental philosophical question about the presentation of risk information. Should decision makers only pay attention to risk factor quantification for those risks supported by the strongest causal evidence such as randomised trials? Or do notions such as the precautionary principle suggest that we should pay attention to risk quantification even for risk-outcome pairs where the evidence is less definitive.^70^, ^71^, ^72^ Because the social response to risks, particularly risks that might be emerging, can take considerable time, ignoring risks for which the evidence is less definitive might actually lead to worse outcomes for society. Conversely, in a world of scarce political and financial resources, devoting attention to risks that might turn out not to be causal might lead to less action on more well documented risks.

As part of future iterations of GBD, we plan to quantify the burden attributable to some distal social risks. We have embarked on this work, but it proves to have challenges that are qualitatively different than many of the risks included here. For nearly all risk-outcome pairs, we assume in the absence of other evidence that the RRs by age and sex are generalisable across populations (the exception is for BMI in Asian and non-Asian populations for breast cancer). In principle, if there is evidence of statistically significant RRs for different population groups, we would incorporate these into the CRA. For distal social risks, the pathways to outcomes can be modified in many ways by other risks or by health-system interventions. We expect that the RR due to low education for 40-year-old men would be different in Norway than in Kenya. Given the greater potential for variation in RRs for distal risks, inclusion in GBD will require more local quantification of RRs and then a further modelling step to estimate RRs for these determinants for all locations. Our first planned target for this quantification is educational attainment.

Given the global policy focus on the potential health effects of climate change driven by rising levels of greenhouse gases, and consequently temperature, we will add temperature and precipitation as risk factors that are quantified on an annual basis in future iterations of GBD. Even though most of the potential harm that might come from rising temperatures or extreme weather events will occur in the future, in some locations, we might already find significant attributable burden.^73^ This analysis will need to examine the relationship between disease and mortality risk and temperature for each relevant outcome. For some outcomes, these relationships are likely to be U shaped, with an optimal temperature for minimum risk. These U-shaped relationships could mean that for some outcomes in some locations, rising temperature might reduce harm, even if in most locations it will increase burden. Likewise, a major issue in understanding the temperature and health outcome relationships is that we would expect these to be attenuated in high-SDI settings, where many individuals can protect themselves from some of the consequences. In other words, generalising from studies in high-SDI locations to other locations might underestimate the risk relationships.

In the GBD CRA approach, the TMREL is the level of risk exposure that leads to minimum risk for individuals. In principle, the TMREL could vary by location, age, and sex. To date, the TMREL in the GBD work has been selected to be universal. For more detail on TMREL, see appendix 1 (p 22). The analysis of alcohol, where for IHD there is a protective effect at mild to moderate consumption but a harmful effect for neoplasms and injuries, is a good example of where it would be desirable to vary TMREL by age. In younger ages, injuries will be more important than cardiovascular diseases, pushing the TMREL toward zero consumption of alcohol, whereas at older ages, the TMREL might be higher. Letting the TMREL vary by age and sex and even location will add an extra analytical step to GBD; like all other estimation steps, this can have estimation error. To date, we have thought the estimation error associated with a TMREL that varies may not make the effort worthwhile. As evidence accumulates on some risks like alcohol, we will carefully evaluate this position.

### Limitations

A study of this scope has many limitations. Here we discuss the limitations that apply to the overall risk factor analytical framework and limitations in the estimation approach for new risks and risks that have undertaken significant revisions from GBD 2015. More details and limitations of the analytical approach for each risk factor are presented in appendix 1 (p 43). First, we continue to include risk-outcome pairs that meet the World Cancer Research Fund criteria of convincing or probable evidence for causality. While these criteria have proven a useful bar for inclusion, there is an important subjective element to their interpretation. Some risk-outcome pairs included in this study might not meet these criteria or alternative criteria that are developed as new randomised trials, cohort studies, or case-control studies are published. Second, we used published cohort studies to evaluate the degree to which different risks are mediated through other risks. Estimates of pathways of mediation are used to compute the burden attributable to aggregates of risk factors such as all behavioural risks or all risks combined. While we have conducted pooled cohort analyses to strengthen the assessment of mediation, this work was not yet ready for inclusion in this assessment. Pooled cohort studies have the advantage of providing a more standardised framework for assessing mediation across multiple risks. A related issue is the validation of the aggregation of risks in GBD. Pooled cohort studies will allow (in some circumstances) the opportunity to estimate if the aggregation of GBD RRs is as predictive of outcomes as suggested by the risk-by-risk analysis with mediation. Third, we have used the Das Gupta formula applied for each 5-year interval and for GBD Level 3 causes. Aggregations at higher levels of causes and for longer periods of time are based on these more granular analyses to guarantee consistency. Given the non-linear nature of the Das Gupta decomposition formula, however, alternative results are possible using different time periods and causes in the formula. Fourth, we have introduced the use of ensemble distributions to improve the empirical fitting of distributions of risk exposure in settings where only mean and standard deviation are known or where we use models to predict the mean and standard deviation of exposure. Ensemble models provide more accurate fits as assessed out of sample for settings with microdata. The underlying assumption is that the same ensemble weights are applicable across all settings. It is possible that the shape of distributions of risk exposure might vary across locations, for example because of the effects of access to treatment.

Limitations that apply to new risks in GBD 2016 or risks with significant estimation updates are presented here. For low birthweight and short gestation, we have included the effect of low birthweight and short gestation only on neonatal outcomes; we have not found the evidence to meet our inclusion criteria for the link between low birthweight and short gestation and NCDs in adult age groups. Our analysis of RRs has used a very large US-linked birth cohort dataset and much more limited data from middle-SDI and low-SDI populations. Given the large number of observations from the USA, our results are heavily influenced by the pattern of RRs across birthweight and gestational age in that population. The microdata used to develop the ensemble distributions for birthweight and gestational age are largely from middle-SDI and high-SDI locations. The estimation of alcohol use relies heavily on sales data, which are limited and whose quality we cannot easily assess. Also, the estimation of unrecorded consumption of alcohol is based on limited data and has significant uncertainty; nevertheless, we feel it is important to include it and plan to continue to look for additional sources of information to improve the estimation of unrecorded consumption in future iterations of GBD. Lastly, methods for calculating TMREL rely on observed DALYs for a given time rather than on the expected share of DALYs estimated from alcohol use alone. Future iterations of GBD will likely need to test this assumption further and determine if separate TMREL by age and sex should be calculated.

### Conclusion

Understanding the levels and trends of major risks for human health is essential to prioritise public health action and evaluate the success of different programmes and policies. This study provides a comprehensive and comparable assessment of 84 metabolic, environmental, occupational, and behavioural risks across locations and time. Our findings show that risk modification has been an important contributor to reductions in communicable, maternal, neonatal, and nutritional causes, but has played a relatively small part in trends in NCDs. Conflicting trends in risks for NCDs at the global level, such as the decline in smoking prevalence coupled with the rise in obesity, FPG, and SBP, account for this finding. By contrast with trends in diseases and injuries at the global level and even at the national level, there is much greater heterogeneity of global trends across risks and considerable geographical variation in leading risks as well. Public health action in each country and region needs to focus on the major risks in that community. Our findings reinforce the crucial need for robust monitoring of the exposure to risks to health and assessment of the evidence supporting causal effects for each risk-outcome pair; GBD provides the main global mechanism for this monitoring function.

Correspondence to: Prof Emmanuela Gakidou, Institute for Health Metrics and Evaluation, Seattle, WA 98121, USA gakidou@uw.edu

For **SHARE Project** see www.share-project.org

For **HBSC** see http://www.hbsc.org

**This online publication has been corrected. The corrected version first appeared at thelancet.com on September 18, 2017**
