# Department of Error

**DOI:** 10.1016/S0140-6736(17)32485-6

**Published:** 2017-10-14

*GBD 2016 Risk Factors Collaborators. Global, regional, and national comparative risk assessment of 84 behavioural, environmental and occupational, and metabolic risks or clusters of risks, 1990–2016: a systematic analysis for the Global Burden of Disease Study 2016.* Lancet *2017; **390**: 1343–420*—Data in Figure 5 have been amended. These corrections have been made to the online version as of Sept 18.

