# Supplementary material for: Global, regional, and national comparative risk assessment of 84 behavioural, environmental and occupational, and metabolic risks or clusters of risks, 1990–2016: a systematic analysis for the Global Burden of Disease Study 2016
Source: Lancet. 2017 Sep 16;390(10100):1345–422. doi: 10.1016/S0140-6736(17)32366-8 (PMC5614451; doi:10.1016/S0140-6736(17)32366-8)
Supplement: Supplementary appendix 2 [file mmc2.pdf]

THE LANCET

Supplementary appendix 2

This appendix formed part of the original submission and has been peer reviewed. We post it as supplied by the authors.

Supplement to: GBD 2016 Risk Factors Collaborators. Global, regional, and national comparative risk assessment of 84 behavioural, environmental and occupational, and metabolic risks or clusters of risks, 1990–2016: a systematic analysis for the Global Burden of Disease Study 2016. *Lancet* 2017; **390:** 1345–422.

Supplemental Results Appendix to Global, regional, and national comparative risk assessment of 84 behavioural, environmental and occupational, and metabolic risks or clusters of risks, 1990-2016: a systematic analysis for the Global Burden of Disease Study 2016

Contents

Supplemental Results Appendix to Global, regional, and national comparative risk assessment of 84 behavioural, environmental and occupational, and metabolic risks or clusters of risks, 1990-2016: a systematic analysis for the Global Burden of Disease Study 2016 1

Supplementary Results Table 1. Age-standardised PAFs attributable to the joint distribution of all risk factors for all ages, both sexes combined for each GBD level 1 and level 2 cause and all causes in 2016 for Global. Results are shown for deaths, YLLs, YLDs, and DALYs =. Data in parenthesis are 95% uncertainty intervals. PAFs=population attributable fractions. GBD=Global Burden of Disease. YLLs=years of life lost. YLDs=years live with disability. DALYs=disability-adjusted life-years 2

Supplementary Results Table 2. Age-standardised Summary Exposure Values (SEVs) for all risk factors and percentage change, by sex, 1990, 2005, and 2016. 1400

Supplementary Results Table 3. All-age deaths, death PAFs, DALYs, DALY PAFs, and percentage change for each risk factor and outcome for both sexes combined in 2006 and 2016 for Central Sub-Saharan Africa. Data in parenthesis are 95% uncertainty intervals. PAFs=population attributate fractions. DALYs=disability-adjusted life-years. 1866

Supplemental Results Figure 1: Leading 30 level 3 Risk factors for DALYs by SDI level and sex, 1990, 2006 and 2016. Risks are connected by lines between time periods. Behavioural risk factors are shown in red, environmental risks in blue and metabolic risks in green. For the time period 1990 to 2006 and for 2006 to 2016 3497

Supplemental Results Figure 2: Leading 10 level 3 risk factors for DALYs and deaths in 2016 by location. Each risk is coloured by the percetage change in all-age DALYs from 2006 to 2016. DALYs=disability-adjusted life-years............ 3507

Supplementary Results Table 1. Age-standardised PAFs attributable to the joint distribution of all risk factors for all ages, both sexes combined for each GBD level 1 and level 2 cause and all causes in 2016 for Global. Results are shown for deaths, YLLs, YLDs, and DALYs
Data in parenthesis are 95% uncertainty intervals. PAFs=population attributable fractions. GBD=Global Burden of Disease. YLLs=years of life lost. YLDs=years live with disability. DALYs=disability-adjusted life-years

	Deaths 2016	YLLs 2016	YLDs 2016	DALYs 2016
**All causes**	60.2% (58.7% to 61.7%)	55.2% (54.2% to 56.3%)	26.5% (25.5% to 27.6%)	45.8% (43.7% to 47.9%)
**HIV/AIDS and tuberculosis**	56.1% (52.7% to 59.3%)	57.9% (55.1% to 60.5%)	64.5% (60.5% to 69.0%)	58.3% (55.7% to 60.9%)
Tuberculosis	34.9% (29.2% to 40.5%)	33.4% (28.2% to 38.7%)	35.2% (29.8% to 40.3%)	33.6% (28.2% to 38.8%)
HIV/AIDS	82.9% (81.5% to 84.1%)	77.0% (75.5% to 78.5%)	85.9% (84.4% to 87.3%)	77.6% (76.1% to 79.1%)
**Diarrhoea, lower respiratory infections, and other common infectious diseases**	67.1% (62.4% to 71.8%)	69.7% (65.6% to 73.7%)	34.0% (27.9% to 39.6%)	66.6% (63.0% to 70.5%)
Diarrhoeal diseases	91.2% (81.6% to 96.3%)	95.4% (89.7% to 98.4%)	84.8% (71.7% to 93.0%)	94.3% (87.7% to 97.8%)
Lower respiratory infections	68.3% (63.9% to 73.2%)	83.1% (79.9% to 86.3%)	67.3% (62.1% to 72.4%)	83.1% (79.8% to 86.2%)
Upper respiratory infections	2.4% (1.7% to 3.4%)	3.8% (2.6% to 5.7%)	0.0% (0.0% to 0.0%)	0.1% (0.0% to 0.2%)
Otitis media	3.9% (2.6% to 5.4%)	7.2% (5.0% to 9.7%)	6.6% (4.4% to 8.6%)	6.6% (4.4% to 8.6%)
Meningitis	4.3% (3.1% to 5.3%)	5.8% (4.5% to 7.0%)	0.0% (0.0% to 0.0%)	5.4% (4.2% to 6.6%)
Encephalitis	1.0% (0.8% to 1.3%)	1.9% (1.4% to 2.3%)	0.0% (0.0% to 0.0%)	1.4% (1.1% to 1.8%)
Measles	69.3% (57.3% to 83.9%)	70.3% (58.0% to 85.0%)	67.2% (55.6% to 81.4%)	70.3% (58.0% to 85.0%)
**Maternal disorders**	9.1% (4.1% to 14.4%)	9.1% (4.1% to 14.4%)	2.7% (1.4% to 4.3%)	8.7% (3.9% to 13.8%)
Maternal haemorrhage	19.4% (7.1% to 31.4%)	19.6% (7.2% to 31.6%)	16.2% (5.9% to 26.1%)	19.6% (7.2% to 31.5%)
Maternal sepsis and other pregnancy related infections	19.9% (7.2% to 32.1%)	20.1% (7.3% to 32.4%)	16.3% (5.9% to 26.6%)	20.0% (7.3% to 32.2%)
Maternal abortion, miscarriage, and ectopic pregnancy	15.1% (9.6% to 20.8%)	14.9% (9.4% to 20.5%)	12.7% (7.0% to 18.7%)	14.8% (9.3% to 20.4%)
**Neonatal disorders**	80.1% (78.4% to 82.0%)	80.2% (78.5% to 82.0%)	0.2% (0.2% to 0.3%)	73.9% (71.2% to 76.4%)
Neonatal preterm birth complications	95.2% (94.5% to 96.1%)	95.2% (94.5% to 96.1%)	0.4% (0.3% to 0.4%)	83.4% (79.0% to 86.9%)
Neonatal encephalopathy due to birth asphyxia and trauma	75.0% (72.2% to 78.0%)	75.0% (72.2% to 78.0%)	0.0% (0.0% to 0.0%)	72.7% (69.0% to 76.3%)
Neonatal sepsis and other neonatal infections	65.8% (63.3% to 68.9%)	65.9% (63.3% to 68.9%)	0.0% (0.0% to 0.0%)	59.1% (50.0% to 64.7%)
Hemolytic disease and other neonatal jaundice	69.4% (66.6% to 72.1%)	69.5% (66.7% to 72.2%)	0.0% (0.0% to 0.0%)	60.8% (57.8% to 64.1%)

Supplementary Results Table 1. Age-standardised PAFs attributable to the joint distribution of all risk factors for all ages, both sexes combined for each GBD level 1 and level 2 cause and all causes in 2016 for Global. Results are shown for deaths, YLLs, YLDs, and DALYs
Data in parenthesis are 95% uncertainty intervals. PAFs=population attributable fractions. GBD=Global Burden of Disease. YLLs=years of life lost. YLDs=years live with disability. DALYs=disability-adjusted life-years

	**Deaths 2016**	**YLLs 2016**	**YLDs 2016**	**DALYs 2016**
Other neonatal disorders	71.2% (68.4% to 73.7%)	71.2% (68.5% to 73.8%)	0.0% (0.0% to 0.0%)	69.8% (67.2% to 72.5%)
**Nutritional deficiencies**	43.8% (40.1% to 47.5%)	73.4% (70.3% to 76.1%)	92.0% (90.8% to 93.1%)	85.8% (83.6% to 87.7%)
Protein-energy malnutrition	51.3% (47.5% to 55.3%)	80.9% (78.2% to 83.4%)	97.6% (97.5% to 97.7%)	83.5% (81.2% to 85.5%)
Vitamin A deficiency	-	-	99.9% (99.9% to 99.9%)	99.9% (99.9% to 99.9%)
Iron-deficiency anaemia	99.9% (99.9% to 100.0%)	100.0% (99.9% to 100.0%)	99.9% (99.9% to 99.9%)	99.9% (99.9% to 99.9%)
**Other communicable, maternal, neonatal, and nutritional diseases**	2.9% (2.4% to 3.5%)	1.9% (1.5% to 2.5%)	59.9% (55.6% to 64.4%)	11.8% (8.1% to 17.1%)
Sexually transmitted diseases excluding HIV	8.2% (5.0% to 13.4%)	3.8% (2.2% to 6.3%)	99.9% (99.9% to 99.9%)	23.3% (13.9% to 36.1%)
Hepatitis	0.6% (0.5% to 0.7%)	0.5% (0.4% to 0.6%)	0.4% (0.3% to 0.6%)	0.5% (0.4% to 0.6%)
**Neoplasms**	42.1% (39.0% to 45.4%)	40.1% (37.1% to 43.2%)	35.5% (32.5% to 38.8%)	40.0% (37.0% to 43.1%)
Oesophageal cancer	69.6% (59.7% to 79.0%)	70.0% (60.5% to 79.0%)	68.9% (59.0% to 78.5%)	70.0% (60.5% to 79.0%)
Stomach cancer	18.5% (10.5% to 27.9%)	17.6% (10.4% to 26.4%)	18.8% (10.7% to 28.4%)	17.6% (10.4% to 26.4%)
Liver cancer	41.7% (34.0% to 50.6%)	39.9% (32.0% to 49.1%)	41.2% (33.3% to 50.5%)	39.9% (32.0% to 49.1%)
Larynx cancer	71.6% (63.8% to 78.4%)	71.7% (63.8% to 78.4%)	75.4% (67.9% to 81.8%)	71.8% (63.9% to 78.5%)
Tracheal, bronchus, and lung cancer	84.2% (79.1% to 88.3%)	83.3% (78.2% to 87.7%)	83.4% (78.1% to 87.7%)	83.3% (78.2% to 87.7%)
Breast cancer	27.2% (20.7% to 34.5%)	24.6% (18.7% to 31.3%)	29.0% (22.6% to 36.1%)	24.9% (18.9% to 31.6%)
Cervical cancer	99.9% (99.9% to 99.9%)	99.9% (99.9% to 99.9%)	99.9% (99.9% to 99.9%)	99.9% (99.9% to 99.9%)
Uterine cancer	36.5% (25.2% to 48.4%)	36.3% (25.1% to 48.1%)	42.2% (29.9% to 54.7%)	36.7% (25.5% to 48.6%)
Prostate cancer	4.4% (3.2% to 5.7%)	4.3% (3.2% to 5.6%)	5.4% (4.0% to 6.9%)	4.4% (3.2% to 5.7%)
Colon and rectum cancer	53.2% (44.8% to 61.6%)	53.1% (45.1% to 60.9%)	53.9% (45.6% to 62.1%)	53.1% (45.1% to 60.9%)
Lip and oral cavity cancer	69.5% (64.3% to 74.4%)	69.6% (64.6% to 74.3%)	68.6% (63.5% to 73.3%)	69.5% (64.6% to 74.3%)
Nasopharynx cancer	64.0% (58.2% to 69.7%)	62.4% (57.2% to 68.0%)	62.1% (56.6% to 67.9%)	62.4% (57.2% to 68.0%)

Supplementary Results Table 1. Age-standardised PAFs attributable to the joint distribution of all risk factors for all ages, both sexes combined for each GBD level 1 and level 2 cause and all causes in 2016 for Global. Results are shown for deaths, YLLs, YLDs, and DALYs
Data in parenthesis are 95% uncertainty intervals. PAFs=population attributable fractions. GBD=Global Burden of Disease. YLLs=years of life lost. YLDs=years live with disability. DALYs=disability-adjusted life-years

	Deaths 2016	YLLs 2016	YLDs 2016	DALYs 2016
Other pharynx cancer	42.6% (34.2% to 50.4%)	43.7% (35.6% to 51.3%)	47.7% (39.3% to 55.3%)	43.7% (35.7% to 51.3%)
Gallbladder and biliary tract cancer	15.0% (7.9% to 24.4%)	15.1% (8.1% to 24.4%)	15.3% (8.2% to 24.9%)	15.1% (8.1% to 24.4%)
Pancreatic cancer	27.5% (20.7% to 35.5%)	27.1% (20.1% to 35.0%)	27.3% (20.6% to 35.3%)	27.1% (20.1% to 35.0%)
Ovarian cancer	12.7% (6.1% to 21.1%)	10.5% (4.5% to 18.1%)	10.8% (4.8% to 18.3%)	10.5% (4.5% to 18.2%)
Kidney cancer	32.2% (23.6% to 41.6%)	30.7% (22.6% to 39.5%)	32.2% (23.9% to 41.4%)	30.8% (22.6% to 39.5%)
Bladder cancer	32.1% (23.3% to 41.1%)	31.2% (22.9% to 39.9%)	33.5% (24.8% to 42.5%)	31.3% (23.0% to 40.1%)
Thyroid cancer	9.3% (4.8% to 16.0%)	9.2% (4.6% to 15.7%)	11.8% (6.4% to 19.2%)	9.3% (4.8% to 16.0%)
Mesothelioma	92.3% (90.4% to 94.0%)	85.0% (81.8% to 87.8%)	90.3% (88.0% to 92.2%)	85.1% (81.9% to 87.9%)
Non-Hodgkin's lymphoma	5.1% (2.1% to 9.2%)	4.4% (1.8% to 7.8%)	6.0% (2.4% to 10.4%)	4.4% (1.8% to 7.9%)
Multiple myeloma	6.8% (3.0% to 12.1%)	6.8% (3.0% to 12.0%)	7.3% (3.2% to 13.0%)	6.8% (3.0% to 12.0%)
Leukaemia	15.1% (9.5% to 21.0%)	11.3% (7.4% to 15.6%)	14.8% (9.4% to 20.5%)	11.4% (7.5% to 15.7%)
**Cardiovascular diseases**	86.0% (83.9% to 87.9%)	87.1% (85.6% to 88.4%)	75.5% (72.6% to 78.2%)	86.0% (84.4% to 87.4%)
Rheumatic heart disease	25.7% (17.4% to 40.3%)	23.4% (16.5% to 34.8%)	19.7% (12.4% to 28.4%)	22.9% (15.9% to 33.8%)
Ischaemic heart disease	93.0% (89.7% to 95.5%)	94.3% (92.4% to 95.8%)	90.7% (88.2% to 93.2%)	94.1% (92.2% to 95.7%)
Cerebrovascular disease	85.5% (81.7% to 89.1%)	88.5% (86.1% to 90.8%)	86.3% (82.7% to 89.6%)	88.3% (85.8% to 90.6%)
Hypertensive heart disease	99.9% (99.9% to 99.9%)	99.9% (99.9% to 99.9%)	99.9% (99.9% to 99.9%)	99.9% (99.9% to 99.9%)
Cardiomyopathy and myocarditis	47.6% (41.2% to 55.1%)	50.1% (44.7% to 55.7%)	39.8% (34.6% to 45.8%)	49.4% (44.1% to 55.0%)
Atrial fibrillation and flutter	50.5% (43.5% to 57.3%)	54.4% (48.2% to 60.0%)	59.9% (55.2% to 64.3%)	57.6% (52.3% to 62.5%)
Aortic aneurysm	43.2% (36.4% to 50.4%)	48.9% (43.1% to 54.3%)	-	48.9% (43.1% to 54.3%)
Peripheral vascular disease	50.9% (44.4% to 58.3%)	58.0% (52.6% to 63.2%)	59.3% (54.3% to 64.1%)	58.5% (53.5% to 63.3%)
Endocarditis	38.1% (29.4% to 48.3%)	34.7% (27.7% to 42.4%)	37.5% (29.0% to 46.8%)	34.8% (27.7% to 42.5%)

Supplementary Results Table 1. Age-standardised PAFs attributable to the joint distribution of all risk factors for all ages, both sexes combined for each GBD level 1 and level 2 cause and all causes in 2016 for Global. Results are shown for deaths, YLLs, YLDs, and DALYs
Data in parenthesis are 95% uncertainty intervals. PAFs=population attributable fractions. GBD=Global Burden of Disease. YLLs=years of life lost. YLDs=years live with disability. DALYs=disability-adjusted life-years

	Deaths 2016	YLLs 2016	YLDs 2016	DALYs 2016
Other cardiovascular and circulatory diseases	44.5% (39.4% to 49.6%)	45.3% (41.3% to 49.1%)	44.6% (40.0% to 49.2%)	45.1% (41.1% to 49.0%)
**Chronic respiratory diseases**	68.2% (62.2% to 73.7%)	65.2% (59.6% to 70.5%)	49.2% (43.6% to 55.0%)	60.0% (55.0% to 65.3%)
Chronic obstructive pulmonary disease	76.6% (69.9% to 82.9%)	76.0% (69.6% to 82.3%)	67.6% (60.6% to 74.6%)	74.0% (67.6% to 80.4%)
Pneumoconiosis	100.0% (100.0% to 100.0%)	100.0% (100.0% to 100.0%)	100.0% (100.0% to 100.0%)	100.0% (100.0% to 100.0%)
Asthma	31.6% (25.4% to 38.9%)	33.1% (27.2% to 39.9%)	29.1% (22.7% to 36.6%)	31.0% (25.1% to 37.7%)
Other chronic respiratory diseases	9.9% (7.6% to 12.4%)	7.3% (5.3% to 9.4%)	4.6% (3.2% to 6.2%)	6.1% (4.6% to 8.0%)
**Cirrhosis and other chronic liver diseases**	37.5% (35.5% to 39.4%)	37.5% (35.3% to 39.6%)	30.3% (28.8% to 31.8%)	37.2% (35.1% to 39.3%)
Cirrhosis and other chronic liver diseases due to hepatitis B	0.6% (0.5% to 0.8%)	0.7% (0.5% to 0.9%)	0.7% (0.5% to 0.9%)	0.7% (0.5% to 0.9%)
Cirrhosis and other chronic liver diseases due to hepatitis C	41.0% (37.7% to 44.2%)	47.2% (43.5% to 50.7%)	50.1% (46.7% to 53.4%)	47.3% (43.6% to 50.8%)
Cirrhosis and other chronic liver diseases due to alcohol use	99.9% (99.9% to 99.9%)	99.9% (99.9% to 99.9%)	99.9% (99.9% to 99.9%)	99.9% (99.9% to 99.9%)
**Digestive diseases**	9.5% (7.9% to 11.2%)	9.6% (8.2% to 11.1%)	3.2% (2.6% to 4.0%)	8.3% (7.0% to 9.7%)
Peptic ulcer disease	14.3% (10.4% to 18.7%)	15.2% (11.1% to 19.7%)	9.6% (6.9% to 12.6%)	14.1% (10.2% to 18.4%)
Gallbladder and biliary diseases	32.3% (21.4% to 45.0%)	31.7% (21.2% to 43.7%)	36.8% (25.6% to 49.9%)	32.2% (21.7% to 44.1%)
Pancreatitis	32.4% (25.7% to 39.3%)	35.4% (29.4% to 42.3%)	29.5% (22.7% to 36.6%)	35.3% (29.2% to 42.1%)
**Neurological disorders**	18.1% (9.3% to 29.0%)	16.5% (9.2% to 25.2%)	3.9% (2.5% to 5.5%)	8.5% (5.0% to 12.9%)
Alzheimer's disease and other dementias	21.3% (11.1% to 34.0%)	22.2% (11.7% to 34.9%)	22.2% (11.7% to 34.8%)	22.2% (11.7% to 34.9%)
Parkinson's disease	-10.7% (-12.6% to -8.4%)	-13.5% (-16.1% to -10.5%)	-14.8% (-17.7% to -11.5%)	-13.8% (-16.4% to -10.7%)
Epilepsy	17.4% (13.2% to 21.3%)	14.5% (11.1% to 17.7%)	13.5% (10.2% to 16.9%)	14.0% (10.6% to 17.3%)
Multiple sclerosis	8.7% (5.6% to 11.9%)	9.3% (6.0% to 12.8%)	7.8% (5.1% to 10.6%)	8.5% (5.6% to 11.6%)
**Mental and substance use disorders**	99.8% (99.7% to 99.8%)	99.7% (99.7% to 99.7%)	20.5% (18.4% to 22.6%)	26.4% (23.8% to 29.1%)
Alcohol use disorders	100.0% (99.9% to 100.0%)	100.0% (100.0% to 100.0%)	99.9% (99.9% to 99.9%)	100.0% (99.9% to 100.0%)

Supplementary Results Table 1. Age-standardised PAFs attributable to the joint distribution of all risk factors for all ages, both sexes combined for each GBD level 1 and level 2 cause and all causes in 2016 for Global. Results are shown for deaths, YLLs, YLDs, and DALYs
Data in parenthesis are 95% uncertainty intervals. PAFs=population attributable fractions. GBD=Global Burden of Disease. YLLs=years of life lost. YLDs=years live with disability. DALYs=disability-adjusted life-years

	Deaths 2016	YLLs 2016	YLDs 2016	DALYs 2016
Drug use disorders	100.0% (100.0% to 100.0%)	100.0% (100.0% to 100.0%)	99.9% (99.9% to 99.9%)	100.0% (100.0% to 100.0%)
Depressive disorders	-	-	8.1% (6.9% to 9.6%)	8.1% (6.9% to 9.6%)
Idiopathic developmental intellectual disability	-	-	63.7% (33.4% to 82.5%)	63.7% (33.4% to 82.5%)
**Diabetes, urogenital, blood, and endocrine diseases**	82.2% (81.6% to 82.8%)	77.1% (75.8% to 78.0%)	60.7% (56.9% to 64.4%)	69.6% (67.1% to 71.8%)
Diabetes mellitus	99.9% (99.9% to 99.9%)	99.9% (99.9% to 99.9%)	99.9% (99.9% to 99.9%)	99.9% (99.9% to 99.9%)
Chronic kidney disease	99.9% (99.9% to 99.9%)	99.9% (99.9% to 99.9%)	99.9% (99.9% to 99.9%)	99.9% (99.9% to 99.9%)
**Musculoskeletal disorders**	1.5% (0.6% to 2.6%)	1.3% (0.6% to 2.2%)	16.9% (14.5% to 19.8%)	16.7% (14.3% to 19.5%)
Rheumatoid arthritis	4.3% (1.8% to 7.2%)	4.8% (2.0% to 8.0%)	4.2% (1.7% to 7.0%)	4.3% (1.8% to 7.1%)
Osteoarthritis	-	-	19.7% (11.0% to 29.4%)	19.7% (11.0% to 29.4%)
Low back and neck pain	-	-	22.4% (19.6% to 25.4%)	22.4% (19.6% to 25.4%)
Gout	-	-	39.7% (27.7% to 53.6%)	39.7% (27.7% to 53.6%)
**Other non-communicable diseases**	0.2% (0.2% to 0.3%)	0.3% (0.2% to 0.4%)	6.1% (5.3% to 7.0%)	4.7% (4.1% to 5.4%)
Sense organ diseases	-	-	13.4% (12.0% to 14.9%)	13.4% (12.0% to 14.9%)
Sudden infant death syndrome	5.5% (4.5% to 6.4%)	5.5% (4.6% to 6.4%)	-	5.5% (4.6% to 6.4%)
**Transport injuries**	30.8% (26.8% to 35.4%)	27.1% (23.4% to 31.4%)	36.4% (32.5% to 41.0%)	28.6% (24.9% to 33.0%)
Road injuries	30.7% (26.6% to 35.3%)	27.1% (23.3% to 31.4%)	38.6% (34.4% to 43.4%)	28.7% (25.0% to 33.1%)
Other transport injuries	32.7% (28.8% to 37.1%)	27.7% (24.0% to 31.9%)	27.4% (24.2% to 30.9%)	27.6% (24.1% to 31.5%)
**Unintentional injuries**	28.1% (25.2% to 30.1%)	15.6% (14.5% to 16.9%)	20.5% (19.6% to 21.5%)	17.3% (16.3% to 18.5%)
Falls	54.4% (47.2% to 56.6%)	32.3% (30.0% to 33.4%)	26.8% (26.0% to 27.6%)	29.4% (28.2% to 30.4%)
Drowning	12.5% (10.0% to 15.2%)	10.6% (8.9% to 12.5%)	15.8% (13.0% to 19.4%)	10.7% (8.9% to 12.6%)
Fire, heat, and hot substances	11.2% (8.6% to 14.8%)	10.5% (8.5% to 13.3%)	17.7% (14.6% to 21.4%)	12.7% (10.2% to 15.7%)

Supplementary Results Table 1. Age-standardised PAFs attributable to the joint distribution of all risk factors for all ages, both sexes combined for each GBD level 1 and level 2 cause and all causes in 2016 for Global. Results are shown for deaths, YLLs, YLDs, and DALYs
Data in parenthesis are 95% uncertainty intervals. PAFs=population attributable fractions. GBD=Global Burden of Disease. YLLs=years of life lost. YLDs=years live with disability. DALYs=disability-adjusted life-years

	**Deaths 2016**	**YLLs 2016**	**YLDs 2016**	**DALYs 2016**
Poisonings	14.4% (11.5% to 18.0%)	12.7% (10.3% to 15.7%)	17.8% (14.6% to 21.8%)	13.2% (10.7% to 16.1%)
Exposure to mechanical forces	18.0% (17.2% to 18.8%)	14.7% (13.7% to 15.6%)	21.7% (21.0% to 22.4%)	17.3% (16.3% to 18.2%)
Animal contact	8.0% (7.1% to 10.1%)	7.5% (6.6% to 9.5%)	11.0% (10.5% to 11.6%)	8.4% (7.6% to 10.1%)
Foreign body	5.8% (5.2% to 6.8%)	6.2% (5.2% to 7.0%)	9.7% (9.1% to 10.2%)	7.2% (6.3% to 7.9%)
Other unintentional injuries	19.7% (16.3% to 23.2%)	19.5% (16.5% to 22.5%)	16.5% (13.6% to 20.2%)	18.0% (15.1% to 21.4%)
**Self-harm and interpersonal violence**	22.4% (16.8% to 27.4%)	21.4% (16.5% to 25.9%)	25.9% (23.0% to 28.9%)	21.7% (17.0% to 26.1%)
Self-harm	22.9% (15.3% to 29.9%)	22.3% (15.2% to 28.9%)	20.7% (12.8% to 28.0%)	22.3% (15.2% to 28.9%)
Interpersonal violence	21.4% (16.0% to 27.3%)	19.8% (14.7% to 25.5%)	26.7% (23.8% to 29.9%)	20.8% (16.1% to 26.1%)
**Forces of nature, conflict and terrorism, and executions and police conflict**	0.3% (0.1% to 0.5%)	0.1% (0.0% to 0.2%)	1.0% (0.7% to 1.6%)	0.2% (0.1% to 0.4%)
Forces of nature, conflict and terrorism, and state actor violence	5.9% (5.2% to 6.5%)	2.4% (2.0% to 2.7%)	5.9% (4.3% to 8.2%)	3.8% (2.8% to 5.4%)

Supplementary Results Table 1. Age-standardised PAFs attributable to the joint distribution of all risk factors for all ages, both sexes combined for each GBD level 1 and level 2 cause and all causes in 2016 for High SDI. Results are shown for deaths, YLLs, YLDs, and DALYs
Data in parenthesis are 95% uncertainty intervals. PAFs=population attributable fractions. GBD=Global Burden of Disease. YLLs=years of life lost. YLDs=years live with disability. DALYs=disability-adjusted life-years

	Deaths 2016	YLLs 2016	YLDs 2016	DALYs 2016
**All causes**	56.0% (53.9% to 58.0%)	53.8% (52.3% to 55.3%)	22.8% (21.4% to 24.2%)	37.6% (35.1% to 40.2%)
**HIV/AIDS and tuberculosis**	70.5% (65.9% to 74.7%)	78.0% (75.7% to 80.3%)	87.0% (83.6% to 89.2%)	80.8% (78.1% to 83.0%)
Tuberculosis	46.7% (36.8% to 55.9%)	51.7% (43.1% to 59.4%)	47.7% (39.3% to 55.5%)	51.1% (42.5% to 58.8%)
HIV/AIDS	89.9% (89.0% to 90.5%)	87.4% (86.5% to 88.1%)	92.0% (91.0% to 92.7%)	88.9% (87.9% to 89.9%)
**Diarrhoea, lower respiratory infections, and other common infectious diseases**	35.6% (28.6% to 43.3%)	41.2% (35.2% to 48.2%)	9.2% (6.6% to 12.2%)	29.3% (24.2% to 35.0%)
Diarrhoeal diseases	20.8% (13.9% to 31.3%)	37.2% (30.6% to 45.8%)	40.2% (29.7% to 51.5%)	38.8% (30.5% to 48.9%)
Lower respiratory infections	38.0% (30.3% to 46.7%)	45.8% (38.6% to 53.9%)	43.2% (35.6% to 52.0%)	45.7% (38.6% to 53.9%)
Upper respiratory infections	0.6% (0.4% to 0.8%)	1.7% (1.2% to 2.4%)	0.0% (0.0% to 0.0%)	0.0% (0.0% to 0.0%)
Otitis media	3.5% (2.5% to 4.7%)	7.4% (5.3% to 9.5%)	7.0% (4.7% to 9.1%)	7.0% (4.8% to 9.1%)
Meningitis	1.9% (1.6% to 2.2%)	3.6% (3.1% to 3.9%)	0.0% (0.0% to 0.0%)	3.0% (2.5% to 3.3%)
Encephalitis	0.5% (0.5% to 0.6%)	1.1% (1.0% to 1.3%)	0.0% (0.0% to 0.0%)	0.7% (0.6% to 0.9%)
Measles	15.9% (5.4% to 43.7%)	17.0% (5.8% to 46.9%)	21.3% (7.4% to 58.0%)	17.2% (6.0% to 47.8%)
**Maternal disorders**	1.8% (0.9% to 2.8%)	1.8% (0.8% to 2.8%)	2.8% (1.3% to 4.6%)	2.1% (1.0% to 3.3%)
Maternal haemorrhage	10.4% (3.8% to 17.0%)	10.4% (3.8% to 16.9%)	10.1% (3.7% to 16.4%)	10.3% (3.7% to 16.6%)
Maternal sepsis and other pregnancy related infections	10.0% (3.6% to 16.3%)	10.0% (3.6% to 16.4%)	9.9% (3.6% to 16.1%)	10.0% (3.6% to 16.2%)
Maternal abortion, miscarriage, and ectopic pregnancy	2.7% (1.6% to 4.0%)	2.8% (1.7% to 4.2%)	4.4% (2.3% to 7.0%)	3.3% (1.9% to 5.0%)
**Neonatal disorders**	78.4% (77.1% to 79.4%)	78.5% (77.2% to 79.5%)	0.1% (0.1% to 0.1%)	55.8% (50.1% to 60.7%)
Neonatal preterm birth complications	90.6% (89.8% to 91.1%)	90.6% (89.8% to 91.2%)	0.2% (0.1% to 0.2%)	63.1% (55.2% to 69.8%)
Neonatal encephalopathy due to birth asphyxia and trauma	64.0% (62.2% to 66.0%)	64.1% (62.2% to 66.1%)	0.0% (0.0% to 0.0%)	44.7% (37.7% to 51.2%)
Neonatal sepsis and other neonatal infections	57.8% (55.6% to 60.4%)	57.8% (55.6% to 60.4%)	0.0% (0.0% to 0.0%)	29.2% (16.7% to 41.4%)
Hemolytic disease and other neonatal jaundice	55.4% (52.4% to 58.2%)	55.5% (52.6% to 58.4%)	0.0% (0.0% to 0.0%)	16.6% (12.8% to 21.0%)

Supplementary Results Table 1. Age-standardised PAFs attributable to the joint distribution of all risk factors for all ages, both sexes combined for each GBD level 1 and level 2 cause and all causes in 2016 for High SDI. Results are shown for deaths, YLLs, YLDs, and DALYs
Data in parenthesis are 95% uncertainty intervals. PAFs=population attributable fractions. GBD=Global Burden of Disease. YLLs=years of life lost. YLDs=years live with disability. DALYs=disability-adjusted life-years

	Deaths 2016	YLLs 2016	YLDs 2016	DALYs 2016
Other neonatal disorders	64.2% (62.3% to 66.1%)	64.2% (62.4% to 66.2%)	0.0% (0.0% to 0.0%)	60.9% (58.7% to 63.0%)
**Nutritional deficiencies**	2.6% (1.5% to 3.1%)	7.9% (6.6% to 8.8%)	79.2% (75.2% to 82.7%)	72.7% (67.5% to 77.4%)
Protein-energy malnutrition	0.6% (0.6% to 0.8%)	4.6% (4.1% to 5.7%)	98.2% (98.1% to 98.3%)	57.2% (47.4% to 66.2%)
Vitamin A deficiency	-	-	0.0% (0.0% to 0.0%)	0.0% (0.0% to 0.0%)
Iron-deficiency anaemia	100.0% (100.0% to 100.0%)	100.0% (100.0% to 100.0%)	100.0% (100.0% to 100.0%)	100.0% (100.0% to 100.0%)
**Other communicable, maternal, neonatal, and nutritional diseases**	5.8% (5.2% to 6.6%)	5.3% (4.7% to 6.1%)	72.7% (67.4% to 77.9%)	32.7% (24.6% to 43.6%)
Sexually transmitted diseases excluding HIV	88.3% (79.9% to 93.9%)	70.1% (54.8% to 82.5%)	100.0% (100.0% to 100.0%)	97.2% (94.3% to 98.9%)
Hepatitis	7.2% (5.7% to 8.9%)	8.0% (6.4% to 10.1%)	3.6% (2.8% to 4.7%)	7.7% (6.1% to 9.7%)
**Neoplasms**	46.7% (44.2% to 49.5%)	46.2% (43.9% to 48.8%)	34.2% (31.4% to 37.3%)	45.7% (43.4% to 48.3%)
Oesophageal cancer	82.2% (75.0% to 88.2%)	83.1% (76.0% to 88.9%)	80.0% (72.4% to 86.4%)	83.1% (75.9% to 88.9%)
Stomach cancer	21.7% (13.1% to 31.5%)	20.8% (12.7% to 29.7%)	20.7% (12.2% to 30.2%)	20.8% (12.7% to 29.8%)
Liver cancer	58.2% (51.5% to 64.4%)	59.9% (53.2% to 66.0%)	56.7% (50.0% to 63.2%)	59.8% (53.2% to 65.9%)
Larynx cancer	89.5% (84.2% to 93.2%)	89.3% (84.1% to 92.9%)	87.6% (81.8% to 91.6%)	89.2% (84.0% to 92.8%)
Tracheal, bronchus, and lung cancer	88.3% (86.4% to 90.1%)	87.8% (85.9% to 89.5%)	87.1% (85.1% to 89.0%)	87.8% (85.9% to 89.5%)
Breast cancer	35.9% (29.3% to 43.3%)	34.7% (28.5% to 41.5%)	34.9% (28.6% to 42.0%)	34.8% (28.5% to 41.5%)
Cervical cancer	100.0% (100.0% to 100.0%)	100.0% (100.0% to 100.0%)	100.0% (100.0% to 100.0%)	100.0% (100.0% to 100.0%)
Uterine cancer	43.7% (31.1% to 56.5%)	44.7% (32.1% to 57.5%)	46.5% (33.6% to 59.2%)	45.0% (32.3% to 57.7%)
Prostate cancer	6.5% (4.7% to 8.5%)	6.7% (4.9% to 8.4%)	6.7% (5.0% to 8.5%)	6.7% (4.9% to 8.4%)
Colon and rectum cancer	55.8% (47.5% to 64.0%)	56.7% (48.6% to 64.1%)	55.8% (47.5% to 63.6%)	56.6% (48.6% to 64.1%)
Lip and oral cavity cancer	81.2% (76.4% to 85.3%)	82.5% (78.0% to 86.2%)	80.1% (75.3% to 84.3%)	82.4% (77.9% to 86.2%)
Nasopharynx cancer	80.9% (77.4% to 84.1%)	79.1% (75.8% to 82.1%)	77.8% (74.3% to 80.9%)	79.1% (75.8% to 82.1%)

Supplementary Results Table 1. Age-standardised PAFs attributable to the joint distribution of all risk factors for all ages, both sexes combined for each GBD level 1 and level 2 cause and all causes in 2016 for High SDI. Results are shown for deaths, YLLs, YLDs, and DALYs
Data in parenthesis are 95% uncertainty intervals. PAFs=population attributable fractions. GBD=Global Burden of Disease. YLLs=years of life lost. YLDs=years live with disability. DALYs=disability-adjusted life-years

	Deaths 2016	YLLs 2016	YLDs 2016	DALYs 2016
Other pharynx cancer	63.2% (53.9% to 71.4%)	65.5% (56.0% to 73.3%)	63.1% (53.3% to 71.5%)	65.4% (55.9% to 73.3%)
Gallbladder and biliary tract cancer	15.6% (8.1% to 25.9%)	16.4% (8.7% to 26.8%)	16.3% (8.6% to 26.6%)	16.4% (8.7% to 26.8%)
Pancreatic cancer	33.9% (26.8% to 41.7%)	34.2% (27.3% to 41.7%)	33.0% (26.0% to 40.7%)	34.2% (27.3% to 41.7%)
Ovarian cancer	15.0% (7.8% to 23.5%)	12.3% (5.7% to 19.8%)	12.0% (5.8% to 19.2%)	12.3% (5.7% to 19.8%)
Kidney cancer	38.0% (28.6% to 48.3%)	38.2% (28.9% to 48.1%)	38.4% (29.1% to 48.3%)	38.2% (28.9% to 48.1%)
Bladder cancer	40.6% (30.7% to 50.0%)	41.0% (31.6% to 50.1%)	39.9% (30.5% to 49.0%)	40.9% (31.6% to 50.0%)
Thyroid cancer	13.1% (6.5% to 21.7%)	14.1% (7.0% to 23.2%)	14.4% (7.6% to 23.1%)	14.1% (7.1% to 23.2%)
Mesothelioma	96.5% (95.6% to 97.3%)	93.0% (91.4% to 94.4%)	95.5% (94.4% to 96.4%)	93.0% (91.5% to 94.4%)
Non-Hodgkin's lymphoma	7.0% (2.9% to 12.2%)	7.2% (3.0% to 12.4%)	7.4% (3.1% to 12.5%)	7.2% (3.0% to 12.4%)
Multiple myeloma	8.1% (3.6% to 14.3%)	8.6% (3.8% to 14.9%)	8.6% (3.8% to 14.8%)	8.6% (3.8% to 14.9%)
Leukaemia	21.1% (13.4% to 28.5%)	18.1% (11.7% to 24.3%)	18.7% (11.9% to 25.3%)	18.1% (11.7% to 24.3%)
**Cardiovascular diseases**	81.4% (78.6% to 83.7%)	83.4% (81.6% to 85.0%)	73.4% (70.5% to 76.0%)	81.6% (79.7% to 83.4%)
Rheumatic heart disease	25.2% (15.2% to 45.2%)	24.2% (16.6% to 37.9%)	22.9% (15.2% to 35.2%)	24.2% (16.5% to 37.8%)
Ischaemic heart disease	92.1% (88.3% to 95.2%)	94.0% (91.7% to 96.0%)	90.6% (87.9% to 93.2%)	93.8% (91.4% to 95.8%)
Cerebrovascular disease	81.1% (75.3% to 86.4%)	85.7% (82.3% to 88.9%)	83.9% (79.8% to 87.8%)	85.2% (81.7% to 88.5%)
Hypertensive heart disease	100.0% (100.0% to 100.0%)	100.0% (100.0% to 100.0%)	100.0% (100.0% to 100.0%)	100.0% (100.0% to 100.0%)
Cardiomyopathy and myocarditis	44.0% (37.2% to 52.5%)	45.6% (39.3% to 51.6%)	42.6% (37.1% to 48.9%)	45.3% (39.1% to 51.2%)
Atrial fibrillation and flutter	53.0% (45.9% to 60.0%)	57.1% (51.0% to 63.2%)	61.6% (56.8% to 66.5%)	59.9% (54.5% to 65.1%)
Aortic aneurysm	41.5% (34.5% to 48.8%)	47.1% (41.1% to 52.7%)	-	47.1% (41.1% to 52.7%)
Peripheral vascular disease	51.5% (45.5% to 58.3%)	58.0% (53.2% to 62.8%)	58.0% (53.1% to 62.8%)	58.0% (53.2% to 62.8%)
Endocarditis	37.4% (28.1% to 49.0%)	36.7% (29.0% to 45.6%)	38.8% (29.9% to 48.7%)	36.8% (29.0% to 45.6%)

Supplementary Results Table 1. Age-standardised PAFs attributable to the joint distribution of all risk factors for all ages, both sexes combined for each GBD level 1 and level 2 cause and all causes in 2016 for High SDI. Results are shown for deaths, YLLs, YLDs, and DALYs
Data in parenthesis are 95% uncertainty intervals. PAFs=population attributable fractions. GBD=Global Burden of Disease. YLLs=years of life lost. YLDs=years live with disability. DALYs=disability-adjusted life-years

	Deaths 2016	YLLs 2016	YLDs 2016	DALYs 2016
Other cardiovascular and circulatory diseases	42.9% (37.5% to 48.4%)	47.1% (42.6% to 51.5%)	49.2% (44.5% to 54.1%)	48.0% (43.3% to 52.6%)
**Chronic respiratory diseases**	70.6% (67.4% to 75.0%)	68.4% (65.5% to 72.5%)	49.1% (43.0% to 55.4%)	58.5% (53.9% to 63.1%)
Chronic obstructive pulmonary disease	81.9% (78.7% to 85.0%)	82.0% (78.9% to 84.9%)	74.7% (70.7% to 78.6%)	79.6% (76.3% to 82.7%)
Pneumoconiosis	100.0% (100.0% to 100.0%)	100.0% (100.0% to 100.0%)	100.0% (100.0% to 100.0%)	100.0% (100.0% to 100.0%)
Asthma	38.6% (30.1% to 48.5%)	43.4% (35.4% to 52.5%)	34.2% (26.4% to 43.3%)	35.1% (27.3% to 44.3%)
Other chronic respiratory diseases	20.1% (16.5% to 23.8%)	14.5% (11.3% to 17.6%)	9.8% (7.8% to 12.4%)	12.4% (9.8% to 15.3%)
**Cirrhosis and other chronic liver diseases**	61.3% (58.8% to 63.7%)	63.9% (61.6% to 66.5%)	53.9% (51.9% to 56.0%)	63.4% (61.1% to 65.9%)
Cirrhosis and other chronic liver diseases due to hepatitis B	2.8% (2.0% to 3.7%)	3.4% (2.4% to 4.6%)	2.8% (2.0% to 3.8%)	3.4% (2.4% to 4.5%)
Cirrhosis and other chronic liver diseases due to hepatitis C	73.4% (71.0% to 75.5%)	81.2% (79.7% to 82.5%)	78.2% (76.4% to 79.8%)	81.1% (79.6% to 82.4%)
Cirrhosis and other chronic liver diseases due to alcohol use	100.0% (100.0% to 100.0%)	100.0% (100.0% to 100.0%)	100.0% (100.0% to 100.0%)	100.0% (100.0% to 100.0%)
**Digestive diseases**	9.9% (8.0% to 12.1%)	12.2% (10.1% to 14.5%)	3.6% (2.8% to 4.4%)	9.2% (7.5% to 11.1%)
Peptic ulcer disease	11.5% (8.2% to 15.4%)	15.5% (11.1% to 20.4%)	12.6% (8.9% to 16.9%)	14.7% (10.5% to 19.4%)
Gallbladder and biliary diseases	36.7% (24.8% to 50.5%)	39.2% (26.9% to 52.5%)	44.6% (32.0% to 58.2%)	40.2% (27.9% to 53.6%)
Pancreatitis	38.1% (27.3% to 49.2%)	42.4% (32.3% to 53.5%)	32.9% (21.1% to 44.9%)	41.7% (31.5% to 52.9%)
**Neurological disorders**	19.2% (9.6% to 30.7%)	17.9% (10.1% to 27.1%)	4.6% (3.0% to 6.6%)	9.2% (5.5% to 13.9%)
Alzheimer's disease and other dementias	22.7% (11.3% to 36.2%)	24.0% (12.6% to 37.5%)	24.0% (12.5% to 37.5%)	24.0% (12.7% to 37.5%)
Parkinson's disease	-7.0% (-8.1% to -5.8%)	-9.0% (-10.4% to -7.4%)	-10.2% (-11.7% to -8.4%)	-9.2% (-10.7% to -7.6%)
Epilepsy	34.6% (26.3% to 41.7%)	32.0% (24.5% to 38.5%)	25.2% (18.4% to 31.4%)	27.3% (20.3% to 33.5%)
Multiple sclerosis	9.1% (6.0% to 12.5%)	9.9% (6.5% to 13.6%)	8.5% (5.5% to 11.7%)	9.1% (5.9% to 12.5%)
**Mental and substance use disorders**	99.6% (99.6% to 99.7%)	99.5% (99.5% to 99.6%)	24.1% (21.8% to 26.5%)	32.1% (29.1% to 35.4%)
Alcohol use disorders	100.0% (100.0% to 100.0%)	100.0% (100.0% to 100.0%)	100.0% (100.0% to 100.0%)	100.0% (100.0% to 100.0%)

Supplementary Results Table 1. Age-standardised PAFs attributable to the joint distribution of all risk factors for all ages, both sexes combined for each GBD level 1 and level 2 cause and all causes in 2016 for High SDI. Results are shown for deaths, YLLs, YLDs, and DALYs
Data in parenthesis are 95% uncertainty intervals. PAFs=population attributable fractions. GBD=Global Burden of Disease. YLLs=years of life lost. YLDs=years live with disability. DALYs=disability-adjusted life-years

	Deaths 2016	YLLs 2016	YLDs 2016	DALYs 2016
Drug use disorders	100.0% (100.0% to 100.0%)	100.0% (100.0% to 100.0%)	100.0% (100.0% to 100.0%)	100.0% (100.0% to 100.0%)
Depressive disorders	-	-	8.6% (7.3% to 10.3%)	8.6% (7.3% to 10.3%)
Idiopathic developmental intellectual disability	-	-	53.6% (27.4% to 71.4%)	53.6% (27.4% to 71.4%)
**Diabetes, urogenital, blood, and endocrine diseases**	76.0% (75.2% to 78.4%)	71.1% (69.7% to 74.0%)	65.2% (61.2% to 68.9%)	67.6% (65.1% to 70.0%)
Diabetes mellitus	100.0% (100.0% to 100.0%)	100.0% (100.0% to 100.0%)	100.0% (100.0% to 100.0%)	100.0% (100.0% to 100.0%)
Chronic kidney disease	100.0% (100.0% to 100.0%)	100.0% (100.0% to 100.0%)	100.0% (100.0% to 100.0%)	100.0% (100.0% to 100.0%)
**Musculoskeletal disorders**	1.1% (0.4% to 1.8%)	1.1% (0.4% to 1.8%)	16.8% (14.2% to 19.6%)	16.5% (14.0% to 19.4%)
Rheumatoid arthritis	3.8% (1.5% to 6.4%)	4.7% (1.9% to 7.9%)	5.3% (2.1% to 8.8%)	5.2% (2.1% to 8.8%)
Osteoarthritis	-	-	26.3% (14.9% to 38.1%)	26.3% (14.9% to 38.1%)
Low back and neck pain	-	-	20.3% (17.7% to 23.1%)	20.3% (17.7% to 23.1%)
Gout	-	-	47.6% (31.6% to 64.1%)	47.6% (31.6% to 64.1%)
**Other non-communicable diseases**	0.4% (0.3% to 0.5%)	0.6% (0.5% to 0.6%)	2.4% (2.1% to 2.7%)	2.2% (1.9% to 2.4%)
Sense organ diseases	-	-	7.6% (6.9% to 8.5%)	7.6% (6.9% to 8.5%)
Sudden infant death syndrome	3.8% (3.5% to 4.1%)	3.8% (3.5% to 4.1%)	-	3.8% (3.5% to 4.1%)
**Transport injuries**	36.2% (28.9% to 44.2%)	33.3% (25.8% to 41.4%)	40.8% (34.0% to 47.8%)	35.4% (28.2% to 43.4%)
Road injuries	35.9% (28.7% to 43.9%)	33.0% (25.6% to 41.1%)	41.3% (34.5% to 48.3%)	35.3% (28.0% to 43.3%)
Other transport injuries	39.5% (32.2% to 47.3%)	36.2% (28.8% to 44.2%)	37.1% (30.8% to 43.9%)	36.5% (29.6% to 43.9%)
**Unintentional injuries**	35.1% (31.2% to 37.3%)	21.5% (19.5% to 23.5%)	22.4% (21.1% to 24.0%)	22.1% (20.6% to 23.7%)
Falls	61.3% (57.6% to 63.2%)	40.6% (37.3% to 42.1%)	24.9% (23.9% to 26.1%)	28.5% (26.9% to 30.2%)
Drowning	17.9% (13.1% to 23.8%)	17.7% (13.5% to 22.5%)	17.3% (12.4% to 24.0%)	17.6% (13.5% to 22.5%)
Fire, heat, and hot substances	15.4% (10.2% to 21.7%)	16.7% (12.4% to 21.8%)	22.5% (17.2% to 29.1%)	19.8% (14.9% to 25.7%)

Supplementary Results Table 1. Age-standardised PAFs attributable to the joint distribution of all risk factors for all ages, both sexes combined for each GBD level 1 and level 2 cause and all causes in 2016 for High SDI. Results are shown for deaths, YLLs, YLDs, and DALYs
Data in parenthesis are 95% uncertainty intervals. PAFs=population attributable fractions. GBD=Global Burden of Disease. YLLs=years of life lost. YLDs=years live with disability. DALYs=disability-adjusted life-years

	Deaths 2016	YLLs 2016	YLDs 2016	DALYs 2016
Poisonings	21.6% (16.2% to 27.9%)	21.9% (16.8% to 27.7%)	20.1% (15.3% to 26.0%)	21.2% (16.2% to 26.9%)
Exposure to mechanical forces	17.3% (15.9% to 19.3%)	13.7% (12.2% to 16.4%)	21.7% (20.4% to 23.0%)	18.7% (17.3% to 20.4%)
Animal contact	13.9% (12.9% to 15.0%)	11.8% (10.8% to 13.0%)	12.0% (11.3% to 12.9%)	12.0% (11.3% to 12.8%)
Foreign body	5.7% (5.0% to 7.0%)	8.3% (7.5% to 9.5%)	8.8% (7.8% to 9.8%)	8.5% (7.7% to 9.5%)
Other unintentional injuries	25.8% (19.6% to 32.8%)	26.6% (20.9% to 33.2%)	21.2% (15.8% to 28.2%)	22.0% (16.5% to 28.8%)
**Self-harm and interpersonal violence**	32.7% (22.5% to 42.2%)	33.5% (23.9% to 42.7%)	31.2% (26.5% to 36.1%)	33.3% (24.2% to 42.2%)
Self-harm	33.2% (20.7% to 44.7%)	34.9% (22.6% to 46.1%)	29.0% (16.5% to 40.4%)	34.8% (22.5% to 46.0%)
Interpersonal violence	30.0% (22.9% to 37.3%)	28.2% (21.5% to 35.4%)	32.0% (27.1% to 36.9%)	29.0% (22.6% to 35.5%)
**Forces of nature, conflict and terrorism, and executions and police conflict**	2.9% (1.7% to 3.9%)	1.1% (0.6% to 1.5%)	3.5% (2.1% to 5.3%)	2.1% (1.2% to 3.2%)
Forces of nature, conflict and terrorism, and state actor violence	7.0% (6.1% to 7.9%)	2.9% (2.6% to 3.2%)	7.9% (5.2% to 11.7%)	5.1% (3.3% to 7.9%)

Supplementary Results Table 1. Age-standardised PAFs attributable to the joint distribution of all risk factors for all ages, both sexes combined for each GBD level 1 and level 2 cause and all causes in 2016 for High-middle SDI. Results are shown for deaths, YLLs, YLDs, and DALYs
Data in parenthesis are 95% uncertainty intervals. PAFs=population attributable fractions. GBD=Global Burden of Disease. YLLs=years of life lost. YLDs=years live with disability. DALYs=disability-adjusted life-years

	Deaths 2016	YLLs 2016	YLDs 2016	DALYs 2016
**All causes**	66.6% (64.6% to 68.5%)	60.6% (59.0% to 62.1%)	24.6% (23.3% to 25.8%)	45.4% (42.8% to 48.4%)
**HIV/AIDS and tuberculosis**	67.6% (64.2% to 70.7%)	68.0% (65.3% to 70.5%)	61.2% (55.8% to 66.3%)	67.3% (64.6% to 69.7%)
Tuberculosis	56.6% (49.7% to 62.7%)	57.0% (50.1% to 63.1%)	42.2% (36.1% to 47.9%)	55.1% (48.6% to 61.3%)
HIV/AIDS	78.4% (77.4% to 79.3%)	75.5% (74.4% to 76.5%)	80.1% (77.8% to 82.2%)	75.9% (74.8% to 76.9%)
**Diarrhoea, lower respiratory infections, and other common infectious diseases**	52.6% (47.1% to 58.6%)	57.5% (52.7% to 62.3%)	25.1% (18.4% to 31.9%)	48.5% (43.4% to 53.5%)
Diarrhoeal diseases	66.4% (47.4% to 81.5%)	81.4% (68.3% to 91.3%)	69.8% (51.0% to 85.0%)	74.2% (57.4% to 87.4%)
Lower respiratory infections	56.5% (50.3% to 63.3%)	67.2% (61.6% to 73.1%)	58.1% (51.7% to 65.0%)	67.1% (61.5% to 73.0%)
Upper respiratory infections	1.1% (0.6% to 1.9%)	2.7% (1.5% to 4.7%)	0.0% (0.0% to 0.0%)	0.0% (0.0% to 0.0%)
Otitis media	4.5% (2.0% to 11.0%)	8.2% (4.2% to 15.6%)	8.6% (5.9% to 11.1%)	8.6% (5.9% to 11.2%)
Meningitis	2.0% (1.6% to 2.5%)	3.3% (2.7% to 3.9%)	0.0% (0.0% to 0.0%)	2.6% (2.2% to 3.2%)
Encephalitis	1.1% (0.8% to 1.3%)	1.7% (1.3% to 2.1%)	0.0% (0.0% to 0.0%)	1.0% (0.8% to 1.3%)
Measles	53.7% (36.5% to 78.8%)	54.5% (37.1% to 80.0%)	42.5% (25.2% to 71.7%)	54.2% (36.9% to 79.8%)
**Maternal disorders**	5.7% (2.7% to 8.7%)	5.6% (2.6% to 8.7%)	3.9% (1.9% to 6.2%)	5.1% (2.4% to 7.9%)
Maternal haemorrhage	14.7% (5.3% to 23.8%)	14.7% (5.3% to 23.8%)	12.7% (4.6% to 20.6%)	14.4% (5.2% to 23.4%)
Maternal sepsis and other pregnancy related infections	14.2% (5.1% to 23.2%)	14.3% (5.1% to 23.2%)	12.6% (4.5% to 20.7%)	13.8% (5.0% to 22.7%)
Maternal abortion, miscarriage, and ectopic pregnancy	8.8% (5.4% to 12.8%)	8.6% (5.1% to 12.5%)	7.7% (4.1% to 11.5%)	8.4% (5.0% to 12.1%)
**Neonatal disorders**	78.5% (76.9% to 80.2%)	78.6% (76.9% to 80.3%)	0.1% (0.1% to 0.1%)	65.6% (60.9% to 69.8%)
Neonatal preterm birth complications	93.1% (91.7% to 94.6%)	93.1% (91.8% to 94.6%)	0.2% (0.2% to 0.2%)	77.4% (71.2% to 82.6%)
Neonatal encephalopathy due to birth asphyxia and trauma	66.8% (64.0% to 69.8%)	66.9% (64.1% to 69.9%)	0.0% (0.0% to 0.0%)	56.8% (50.3% to 62.3%)
Neonatal sepsis and other neonatal infections	57.1% (54.2% to 60.1%)	57.2% (54.2% to 60.1%)	0.0% (0.0% to 0.0%)	40.7% (27.0% to 49.6%)
Hemolytic disease and other neonatal jaundice	63.2% (59.6% to 66.5%)	63.2% (59.7% to 66.5%)	0.0% (0.0% to 0.0%)	34.9% (29.3% to 40.2%)

Supplementary Results Table 1. Age-standardised PAFs attributable to the joint distribution of all risk factors for all ages, both sexes combined for each GBD level 1 and level 2 cause and all causes in 2016 for High-middle SDI. Results are shown for deaths, YLLs, YLDs, and DALYs
Data in parenthesis are 95% uncertainty intervals. PAFs=population attributable fractions. GBD=Global Burden of Disease. YLLs=years of life lost. YLDs=years live with disability. DALYs=disability-adjusted life-years

	**Deaths 2016**	**YLLs 2016**	**YLDs 2016**	**DALYs 2016**
Other neonatal disorders	63.3% (60.6% to 66.2%)	63.4% (60.7% to 66.2%)	0.0% (0.0% to 0.0%)	61.3% (58.6% to 64.1%)
**Nutritional deficiencies**	8.8% (7.8% to 10.0%)	33.5% (30.2% to 37.0%)	86.7% (84.3% to 88.7%)	82.1% (78.9% to 85.0%)
Protein-energy malnutrition	8.5% (7.2% to 10.0%)	38.3% (34.3% to 43.1%)	95.2% (94.7% to 95.6%)	66.5% (60.3% to 72.1%)
Vitamin A deficiency	-	-	102.3% (101.2% to 103.3%)	102.3% (101.2% to 103.3%)
Iron-deficiency anaemia	101.7% (101.2% to 102.4%)	101.6% (100.9% to 102.2%)	99.4% (99.2% to 99.6%)	99.4% (99.2% to 99.6%)
**Other communicable, maternal, neonatal, and nutritional diseases**	5.7% (5.0% to 6.5%)	4.5% (3.8% to 5.3%)	78.3% (74.7% to 81.4%)	41.9% (33.8% to 51.6%)
Sexually transmitted diseases excluding HIV	47.3% (27.3% to 70.0%)	26.8% (12.7% to 47.7%)	99.4% (98.9% to 99.8%)	87.4% (76.5% to 95.1%)
Hepatitis	2.0% (1.6% to 2.5%)	2.0% (1.6% to 2.5%)	1.2% (0.9% to 1.8%)	1.9% (1.5% to 2.4%)
**Neoplasms**	45.6% (41.6% to 49.7%)	44.2% (40.5% to 48.1%)	35.8% (32.4% to 39.4%)	44.0% (40.3% to 47.9%)
Oesophageal cancer	75.3% (64.3% to 86.4%)	77.2% (66.6% to 87.9%)	69.6% (59.1% to 80.7%)	77.1% (66.5% to 87.8%)
Stomach cancer	20.8% (11.7% to 31.2%)	21.0% (12.2% to 30.7%)	19.3% (11.0% to 29.3%)	21.0% (12.2% to 30.7%)
Liver cancer	47.6% (38.4% to 58.8%)	44.7% (35.4% to 56.3%)	42.0% (32.9% to 53.4%)	44.7% (35.4% to 56.2%)
Larynx cancer	87.0% (80.3% to 92.7%)	87.5% (81.4% to 92.8%)	82.6% (75.9% to 88.2%)	87.3% (81.2% to 92.6%)
Tracheal, bronchus, and lung cancer	89.0% (81.8% to 94.7%)	88.6% (81.8% to 94.4%)	83.2% (75.3% to 89.7%)	88.5% (81.7% to 94.3%)
Breast cancer	29.7% (22.6% to 37.5%)	28.4% (21.8% to 35.4%)	27.5% (20.9% to 34.5%)	28.4% (21.8% to 35.4%)
Cervical cancer	103.5% (102.5% to 104.4%)	103.8% (102.6% to 104.9%)	99.2% (98.1% to 99.9%)	103.7% (102.5% to 104.7%)
Uterine cancer	48.1% (34.5% to 61.5%)	48.5% (34.7% to 61.9%)	46.4% (33.5% to 59.2%)	48.3% (34.6% to 61.6%)
Prostate cancer	5.3% (3.8% to 7.0%)	6.0% (4.3% to 8.0%)	5.5% (4.0% to 7.3%)	6.0% (4.3% to 7.9%)
Colon and rectum cancer	56.7% (47.8% to 65.2%)	57.6% (48.9% to 65.8%)	55.2% (46.9% to 63.0%)	57.5% (48.8% to 65.7%)
Lip and oral cavity cancer	79.7% (74.4% to 84.4%)	82.1% (77.4% to 86.3%)	72.9% (67.6% to 77.7%)	81.8% (77.2% to 86.0%)
Nasopharynx cancer	70.9% (64.1% to 78.7%)	69.2% (62.8% to 76.7%)	64.2% (57.9% to 71.3%)	69.1% (62.7% to 76.6%)

Supplementary Results Table 1. Age-standardised PAFs attributable to the joint distribution of all risk factors for all ages, both sexes combined for each GBD level 1 and level 2 cause and all causes in 2016 for High-middle SDI. Results are shown for deaths, YLLs, YLDs, and DALYs
Data in parenthesis are 95% uncertainty intervals. PAFs=population attributable fractions. GBD=Global Burden of Disease. YLLs=years of life lost. YLDs=years live with disability. DALYs=disability-adjusted life-years

	Deaths 2016	YLLs 2016	YLDs 2016	DALYs 2016
Other pharynx cancer	66.4% (58.3% to 73.2%)	68.6% (60.8% to 75.2%)	64.5% (56.8% to 70.7%)	68.5% (60.7% to 75.1%)
Gallbladder and biliary tract cancer	21.5% (12.0% to 33.4%)	21.8% (12.1% to 33.8%)	20.3% (11.2% to 31.3%)	21.7% (12.1% to 33.7%)
Pancreatic cancer	29.0% (21.2% to 37.9%)	30.3% (22.5% to 39.0%)	27.5% (20.2% to 35.8%)	30.3% (22.5% to 39.0%)
Ovarian cancer	12.5% (5.4% to 20.8%)	11.0% (4.5% to 18.5%)	10.3% (4.3% to 17.5%)	10.9% (4.5% to 18.5%)
Kidney cancer	36.9% (27.0% to 47.2%)	36.5% (27.0% to 46.3%)	34.0% (25.1% to 43.3%)	36.4% (26.9% to 46.2%)
Bladder cancer	34.8% (25.0% to 45.0%)	37.0% (27.2% to 47.1%)	33.5% (24.6% to 42.7%)	36.8% (27.0% to 46.9%)
Thyroid cancer	14.3% (7.6% to 23.2%)	14.7% (7.7% to 24.1%)	13.0% (7.0% to 20.6%)	14.5% (7.6% to 23.7%)
Mesothelioma	91.8% (88.7% to 94.3%)	84.1% (79.6% to 88.0%)	84.9% (81.6% to 87.7%)	84.1% (79.6% to 88.0%)
Non-Hodgkin's lymphoma	6.6% (2.7% to 11.6%)	6.1% (2.5% to 10.6%)	6.1% (2.4% to 10.8%)	6.1% (2.5% to 10.6%)
Multiple myeloma	8.3% (3.7% to 14.6%)	8.4% (3.7% to 14.6%)	7.7% (3.4% to 13.7%)	8.4% (3.7% to 14.6%)
Leukaemia	17.1% (11.1% to 23.7%)	13.2% (8.7% to 18.3%)	13.0% (8.3% to 18.5%)	13.2% (8.7% to 18.3%)
**Cardiovascular diseases**	91.0% (88.6% to 93.3%)	92.8% (91.2% to 94.3%)	81.1% (78.5% to 83.6%)	91.5% (89.8% to 93.1%)
Rheumatic heart disease	28.9% (19.2% to 46.5%)	27.6% (19.6% to 41.4%)	20.1% (13.2% to 28.7%)	27.0% (19.3% to 40.1%)
Ischaemic heart disease	96.9% (93.6% to 99.7%)	98.5% (96.4% to 100.2%)	90.8% (88.4% to 93.0%)	98.1% (96.0% to 99.9%)
Cerebrovascular disease	90.4% (85.4% to 95.2%)	95.4% (92.1% to 98.5%)	89.3% (85.5% to 92.7%)	94.3% (91.0% to 97.3%)
Hypertensive heart disease	102.4% (101.4% to 105.3%)	102.4% (101.4% to 105.2%)	99.7% (99.3% to 100.0%)	102.1% (101.2% to 104.5%)
Cardiomyopathy and myocarditis	63.0% (55.8% to 70.9%)	69.2% (63.5% to 75.5%)	47.0% (42.3% to 52.0%)	68.3% (62.6% to 74.5%)
Atrial fibrillation and flutter	54.7% (47.3% to 62.1%)	59.6% (52.7% to 65.8%)	64.1% (59.4% to 68.5%)	62.2% (56.7% to 67.3%)
Aortic aneurysm	49.6% (42.9% to 56.5%)	55.9% (50.1% to 61.1%)	-	55.9% (50.1% to 61.1%)
Peripheral vascular disease	53.3% (46.2% to 60.5%)	61.2% (55.2% to 66.8%)	59.1% (53.9% to 64.2%)	60.6% (55.1% to 66.0%)
Endocarditis	40.9% (32.0% to 50.7%)	38.7% (31.1% to 46.6%)	38.7% (30.4% to 47.2%)	38.7% (31.1% to 46.6%)

Supplementary Results Table 1. Age-standardised PAFs attributable to the joint distribution of all risk factors for all ages, both sexes combined for each GBD level 1 and level 2 cause and all causes in 2016 for High-middle SDI. Results are shown for deaths, YLLs, YLDs, and DALYs
Data in parenthesis are 95% uncertainty intervals. PAFs=population attributable fractions. GBD=Global Burden of Disease. YLLs=years of life lost. YLDs=years live with disability. DALYs=disability-adjusted life-years

	Deaths 2016	YLLs 2016	YLDs 2016	DALYs 2016
Other cardiovascular and circulatory diseases	48.0% (43.1% to 53.1%)	50.2% (45.8% to 54.4%)	46.8% (42.3% to 51.5%)	49.1% (44.9% to 53.2%)
**Chronic respiratory diseases**	71.9% (63.9% to 78.4%)	70.2% (64.0% to 75.7%)	45.6% (39.3% to 52.3%)	60.0% (54.0% to 66.1%)
Chronic obstructive pulmonary disease	77.3% (68.3% to 84.2%)	77.6% (70.3% to 84.0%)	67.6% (59.2% to 75.3%)	75.2% (67.6% to 81.8%)
Pneumoconiosis	117.4% (107.8% to 122.5%)	118.7% (109.1% to 123.9%)	124.5% (121.3% to 127.9%)	121.0% (115.3% to 124.4%)
Asthma	40.8% (31.9% to 51.0%)	44.0% (36.0% to 53.3%)	33.0% (25.3% to 42.0%)	34.8% (27.1% to 43.7%)
Other chronic respiratory diseases	14.5% (10.8% to 18.5%)	11.8% (8.6% to 15.4%)	4.8% (3.2% to 6.8%)	9.1% (6.7% to 12.1%)
**Cirrhosis and other chronic liver diseases**	49.3% (46.8% to 51.5%)	50.2% (47.7% to 52.4%)	34.7% (32.9% to 36.4%)	49.4% (47.1% to 51.7%)
Cirrhosis and other chronic liver diseases due to hepatitis B	1.5% (1.1% to 2.1%)	1.9% (1.3% to 2.6%)	1.2% (0.9% to 1.6%)	1.9% (1.3% to 2.6%)
Cirrhosis and other chronic liver diseases due to hepatitis C	69.9% (66.6% to 72.7%)	76.1% (73.3% to 78.5%)	71.8% (69.1% to 74.3%)	76.0% (73.1% to 78.4%)
Cirrhosis and other chronic liver diseases due to alcohol use	101.3% (101.1% to 101.8%)	101.3% (101.0% to 101.7%)	97.8% (97.2% to 98.3%)	101.2% (100.9% to 101.6%)
**Digestive diseases**	16.8% (14.2% to 19.7%)	21.2% (18.0% to 24.6%)	4.1% (3.2% to 4.9%)	15.5% (12.9% to 18.7%)
Peptic ulcer disease	18.0% (13.1% to 23.1%)	21.9% (16.2% to 28.0%)	11.5% (8.3% to 14.9%)	18.4% (13.5% to 23.8%)
Gallbladder and biliary diseases	41.3% (28.7% to 55.3%)	42.4% (29.9% to 56.1%)	42.7% (30.1% to 56.4%)	42.5% (29.9% to 56.2%)
Pancreatitis	50.7% (40.8% to 59.9%)	57.2% (47.7% to 66.0%)	36.7% (29.3% to 43.8%)	56.8% (47.3% to 65.5%)
**Neurological disorders**	20.7% (10.3% to 33.3%)	19.4% (10.5% to 29.9%)	4.1% (2.6% to 5.9%)	9.3% (5.2% to 14.3%)
Alzheimer's disease and other dementias	24.3% (12.4% to 38.7%)	25.7% (13.7% to 40.4%)	24.6% (13.1% to 38.8%)	25.5% (13.5% to 40.0%)
Parkinson's disease	-11.5% (-14.0% to -8.9%)	-15.1% (-18.5% to -11.5%)	-16.4% (-20.0% to -12.5%)	-15.4% (-19.0% to -11.7%)
Epilepsy	24.0% (18.9% to 28.9%)	20.4% (16.1% to 24.7%)	15.0% (11.0% to 19.1%)	16.8% (12.7% to 20.7%)
Multiple sclerosis	11.5% (7.4% to 15.8%)	12.2% (8.0% to 16.7%)	8.9% (5.9% to 12.0%)	10.4% (6.9% to 14.1%)
**Mental and substance use disorders**	101.4% (101.0% to 102.4%)	101.2% (100.8% to 102.1%)	21.2% (19.2% to 23.4%)	29.9% (26.7% to 33.9%)
Alcohol use disorders	100.6% (100.4% to 101.4%)	100.6% (100.4% to 101.3%)	98.1% (97.9% to 98.3%)	99.3% (99.0% to 99.6%)

Supplementary Results Table 1. Age-standardised PAFs attributable to the joint distribution of all risk factors for all ages, both sexes combined for each GBD level 1 and level 2 cause and all causes in 2016 for High-middle SDI. Results are shown for deaths, YLLs, YLDs, and DALYs
Data in parenthesis are 95% uncertainty intervals. PAFs=population attributable fractions. GBD=Global Burden of Disease. YLLs=years of life lost. YLDs=years live with disability. DALYs=disability-adjusted life-years

	Deaths 2016	YLLs 2016	YLDs 2016	DALYs 2016
Drug use disorders	102.7% (102.0% to 104.1%)	102.1% (101.5% to 103.4%)	99.0% (98.9% to 99.2%)	100.1% (99.8% to 100.7%)
Depressive disorders	-	-	6.9% (5.7% to 8.3%)	6.9% (5.7% to 8.3%)
Idiopathic developmental intellectual disability	-	-	63.0% (32.9% to 82.7%)	63.0% (32.9% to 82.7%)
**Diabetes, urogenital, blood, and endocrine diseases**	83.3% (82.6% to 84.3%)	79.5% (78.3% to 81.2%)	60.3% (56.6% to 63.9%)	68.4% (65.4% to 71.1%)
Diabetes mellitus	104.4% (103.8% to 105.1%)	104.7% (104.0% to 105.4%)	100.2% (99.8% to 100.7%)	102.0% (101.5% to 102.5%)
Chronic kidney disease	103.2% (102.8% to 104.0%)	103.6% (103.0% to 104.4%)	99.0% (98.8% to 99.2%)	101.9% (101.5% to 102.6%)
**Musculoskeletal disorders**	1.6% (0.6% to 2.6%)	1.3% (0.5% to 2.1%)	17.5% (14.5% to 20.8%)	17.2% (14.3% to 20.5%)
Rheumatoid arthritis	4.9% (2.0% to 8.1%)	5.6% (2.3% to 9.3%)	4.9% (2.1% to 8.2%)	5.0% (2.1% to 8.2%)
Osteoarthritis	-	-	25.2% (14.4% to 36.4%)	25.2% (14.4% to 36.4%)
Low back and neck pain	-	-	20.5% (17.6% to 23.4%)	20.5% (17.6% to 23.4%)
Gout	-	-	43.3% (29.4% to 58.6%)	43.3% (29.4% to 58.6%)
**Other non-communicable diseases**	0.2% (0.1% to 0.2%)	0.2% (0.2% to 0.3%)	4.8% (4.2% to 5.4%)	3.8% (3.4% to 4.3%)
Sense organ diseases	-	-	11.1% (10.0% to 12.3%)	11.1% (10.0% to 12.3%)
Sudden infant death syndrome	5.8% (4.9% to 6.7%)	5.8% (5.0% to 6.7%)	-	5.8% (5.0% to 6.7%)
**Transport injuries**	34.8% (29.6% to 40.5%)	31.0% (26.1% to 36.4%)	38.7% (34.0% to 43.9%)	32.6% (27.9% to 37.9%)
Road injuries	34.5% (29.3% to 40.2%)	30.8% (26.0% to 36.1%)	39.8% (35.1% to 45.2%)	32.5% (27.8% to 37.8%)
Other transport injuries	39.5% (33.4% to 46.3%)	35.1% (29.1% to 41.8%)	31.0% (27.2% to 35.1%)	33.6% (28.4% to 39.2%)
**Unintentional injuries**	26.8% (22.5% to 30.1%)	17.5% (15.2% to 19.7%)	20.8% (19.5% to 22.2%)	19.0% (17.3% to 20.7%)
Falls	51.7% (41.6% to 55.9%)	31.0% (27.9% to 33.4%)	25.0% (23.9% to 26.2%)	27.0% (25.6% to 28.5%)
Drowning	17.8% (13.2% to 22.8%)	16.0% (12.2% to 20.0%)	19.1% (15.2% to 23.8%)	16.0% (12.3% to 20.1%)
Fire, heat, and hot substances	16.5% (11.4% to 21.9%)	16.9% (12.3% to 21.7%)	19.6% (15.5% to 24.5%)	17.9% (13.5% to 22.6%)

Supplementary Results Table 1. Age-standardised PAFs attributable to the joint distribution of all risk factors for all ages, both sexes combined for each GBD level 1 and level 2 cause and all causes in 2016 for High-middle SDI. Results are shown for deaths, YLLs, YLDs, and DALYs
Data in parenthesis are 95% uncertainty intervals. PAFs=population attributable fractions. GBD=Global Burden of Disease. YLLs=years of life lost. YLDs=years live with disability. DALYs=disability-adjusted life-years

	**Deaths 2016**	**YLLs 2016**	**YLDs 2016**	**DALYs 2016**
Poisonings	18.0% (13.2% to 23.1%)	16.3% (12.3% to 20.6%)	18.0% (14.4% to 22.3%)	16.5% (12.7% to 20.7%)
Exposure to mechanical forces	20.1% (18.0% to 22.1%)	17.4% (14.8% to 19.6%)	22.0% (20.7% to 23.4%)	19.8% (18.1% to 21.4%)
Animal contact	11.4% (10.4% to 12.8%)	9.6% (8.5% to 11.0%)	12.4% (11.5% to 13.5%)	11.4% (10.4% to 12.4%)
Foreign body	6.8% (5.7% to 8.5%)	7.0% (5.9% to 8.3%)	9.4% (8.6% to 10.4%)	7.6% (6.6% to 8.8%)
Other unintentional injuries	24.8% (19.5% to 30.4%)	24.6% (19.8% to 29.8%)	17.6% (14.0% to 21.7%)	20.5% (16.3% to 24.9%)
**Self-harm and interpersonal violence**	34.0% (26.7% to 40.7%)	33.4% (26.4% to 39.8%)	30.3% (26.1% to 34.5%)	33.1% (26.6% to 39.4%)
Self-harm	36.7% (26.1% to 46.2%)	37.3% (27.0% to 46.6%)	27.2% (19.0% to 35.2%)	37.2% (27.0% to 46.5%)
Interpersonal violence	28.3% (21.0% to 36.0%)	26.5% (19.5% to 34.2%)	30.7% (26.3% to 35.3%)	27.2% (20.4% to 34.3%)
**Forces of nature, conflict and terrorism, and executions and police conflict**	0.3% (0.1% to 0.6%)	0.1% (0.0% to 0.2%)	0.6% (0.3% to 0.9%)	0.3% (0.1% to 0.5%)
Forces of nature, conflict and terrorism, and state actor violence	7.1% (6.0% to 7.7%)	3.1% (2.5% to 3.4%)	5.4% (3.7% to 7.6%)	4.5% (3.4% to 6.4%)

Supplementary Results Table 1. Age-standardised PAFs attributable to the joint distribution of all risk factors for all ages, both sexes combined for each GBD level 1 and level 2 cause and all causes in 2016 for Middle SDI. Results are shown for deaths, YLLs, YLDs, and DALYs
Data in parenthesis are 95% uncertainty intervals. PAFs=population attributable fractions. GBD=Global Burden of Disease. YLLs=years of life lost. YLDs=years live with disability. DALYs=disability-adjusted life-years

	Deaths 2016	YLLs 2016	YLDs 2016	DALYs 2016
**All causes**	60.1% (58.2% to 62.0%)	54.6% (53.2% to 56.0%)	25.8% (24.7% to 27.0%)	44.8% (42.7% to 47.0%)
**HIV/AIDS and tuberculosis**	57.8% (54.1% to 61.6%)	59.1% (55.6% to 62.8%)	62.7% (58.2% to 67.4%)	59.5% (56.1% to 62.9%)
Tuberculosis	36.4% (30.2% to 42.3%)	36.5% (30.8% to 41.8%)	37.8% (32.4% to 43.0%)	36.6% (31.1% to 41.8%)
HIV/AIDS	79.3% (76.0% to 82.0%)	71.6% (67.4% to 75.4%)	88.8% (87.2% to 90.3%)	72.9% (68.8% to 76.5%)
**Diarrhoea, lower respiratory infections, and other common infectious diseases**	66.1% (59.2% to 73.7%)	62.9% (57.9% to 68.1%)	32.0% (24.6% to 38.5%)	58.2% (53.1% to 63.6%)
Diarrhoeal diseases	110.9% (80.4% to 138.8%)	99.9% (82.3% to 114.6%)	81.6% (63.2% to 94.0%)	95.5% (78.7% to 108.9%)
Lower respiratory infections	63.9% (58.3% to 69.6%)	71.4% (66.4% to 75.9%)	64.1% (58.4% to 69.7%)	71.3% (66.3% to 75.8%)
Upper respiratory infections	1.3% (0.9% to 1.9%)	3.0% (2.0% to 4.4%)	0.0% (0.0% to 0.0%)	0.0% (0.0% to 0.1%)
Otitis media	2.3% (1.4% to 3.3%)	4.8% (3.1% to 6.8%)	7.5% (5.1% to 9.8%)	7.5% (5.1% to 9.7%)
Meningitis	1.6% (1.2% to 2.2%)	2.6% (2.1% to 3.2%)	0.0% (0.0% to 0.0%)	2.3% (1.8% to 2.9%)
Encephalitis	0.8% (0.6% to 1.1%)	1.5% (1.2% to 1.9%)	0.0% (0.0% to 0.0%)	1.1% (0.8% to 1.4%)
Measles	60.1% (46.3% to 78.1%)	61.0% (47.0% to 79.3%)	63.0% (48.7% to 82.1%)	61.0% (47.0% to 79.4%)
**Maternal disorders**	7.1% (3.1% to 11.3%)	7.1% (3.1% to 11.4%)	3.1% (1.6% to 4.8%)	6.6% (2.9% to 10.7%)
Maternal haemorrhage	16.4% (6.0% to 26.7%)	16.5% (6.0% to 26.9%)	14.9% (5.4% to 24.2%)	16.4% (6.0% to 26.8%)
Maternal sepsis and other pregnancy related infections	16.2% (5.8% to 26.3%)	16.3% (5.9% to 26.6%)	15.0% (5.4% to 24.3%)	16.2% (5.8% to 26.4%)
Maternal abortion, miscarriage, and ectopic pregnancy	9.4% (5.6% to 13.6%)	9.2% (5.5% to 13.3%)	10.1% (5.5% to 15.3%)	9.3% (5.6% to 13.4%)
**Neonatal disorders**	72.8% (70.5% to 75.3%)	72.9% (70.6% to 75.3%)	0.2% (0.1% to 0.2%)	64.9% (61.5% to 68.1%)
Neonatal preterm birth complications	87.3% (84.3% to 90.6%)	87.3% (84.3% to 90.6%)	0.2% (0.2% to 0.3%)	74.0% (68.6% to 78.6%)
Neonatal encephalopathy due to birth asphyxia and trauma	64.6% (61.2% to 68.4%)	64.7% (61.2% to 68.4%)	0.0% (0.0% to 0.0%)	62.2% (58.1% to 66.6%)
Neonatal sepsis and other neonatal infections	58.7% (55.9% to 61.5%)	58.8% (55.9% to 61.5%)	0.0% (0.0% to 0.0%)	50.8% (41.4% to 56.4%)
Hemolytic disease and other neonatal jaundice	52.6% (47.5% to 57.8%)	52.6% (47.6% to 57.8%)	0.0% (0.0% to 0.0%)	39.0% (34.6% to 43.6%)

Supplementary Results Table 1. Age-standardised PAFs attributable to the joint distribution of all risk factors for all ages, both sexes combined for each GBD level 1 and level 2 cause and all causes in 2016 for Middle SDI. Results are shown for deaths, YLLs, YLDs, and DALYs
Data in parenthesis are 95% uncertainty intervals. PAFs=population attributable fractions. GBD=Global Burden of Disease. YLLs=years of life lost. YLDs=years live with disability. DALYs=disability-adjusted life-years

	Deaths 2016	YLLs 2016	YLDs 2016	DALYs 2016
Other neonatal disorders	58.1% (53.7% to 62.4%)	58.2% (53.7% to 62.5%)	0.0% (0.0% to 0.0%)	56.6% (52.2% to 60.8%)
**Nutritional deficiencies**	11.3% (10.3% to 12.3%)	41.4% (38.8% to 44.2%)	96.1% (94.6% to 97.6%)	85.8% (81.7% to 89.2%)
Protein-energy malnutrition	12.4% (11.3% to 13.6%)	47.9% (45.0% to 51.2%)	99.2% (96.3% to 101.4%)	65.7% (60.7% to 70.6%)
Vitamin A deficiency	-	-	102.4% (101.3% to 103.5%)	102.5% (101.4% to 103.5%)
Iron-deficiency anaemia	86.4% (77.5% to 107.2%)	91.7% (86.5% to 103.0%)	107.5% (106.7% to 108.4%)	107.5% (106.6% to 108.3%)
**Other communicable, maternal, neonatal, and nutritional diseases**	3.7% (3.3% to 4.2%)	3.1% (2.6% to 3.6%)	71.4% (67.2% to 74.7%)	24.7% (18.6% to 32.7%)
Sexually transmitted diseases excluding HIV	31.3% (19.3% to 46.0%)	15.6% (8.7% to 25.7%)	101.1% (100.4% to 101.9%)	69.6% (53.5% to 83.6%)
Hepatitis	0.6% (0.5% to 0.8%)	0.6% (0.5% to 0.8%)	0.5% (0.3% to 0.8%)	0.6% (0.5% to 0.8%)
**Neoplasms**	38.4% (33.7% to 43.4%)	36.3% (31.9% to 41.0%)	35.3% (31.2% to 39.9%)	36.3% (31.9% to 41.0%)
Oesophageal cancer	64.2% (51.9% to 76.2%)	64.7% (53.1% to 75.8%)	66.1% (53.2% to 78.9%)	64.7% (53.2% to 75.8%)
Stomach cancer	18.5% (9.5% to 29.1%)	17.4% (9.4% to 27.3%)	19.4% (10.2% to 30.7%)	17.4% (9.4% to 27.4%)
Liver cancer	35.8% (27.5% to 46.0%)	34.6% (26.2% to 45.3%)	36.8% (28.1% to 48.5%)	34.6% (26.2% to 45.3%)
Larynx cancer	62.2% (51.5% to 70.8%)	62.2% (52.1% to 70.5%)	66.2% (54.7% to 75.4%)	62.3% (52.2% to 70.6%)
Tracheal, bronchus, and lung cancer	71.1% (62.2% to 78.0%)	70.7% (61.8% to 78.1%)	75.6% (65.7% to 83.8%)	70.8% (61.8% to 78.1%)
Breast cancer	23.1% (15.6% to 31.6%)	21.5% (14.5% to 29.2%)	23.8% (16.0% to 32.7%)	21.6% (14.6% to 29.4%)
Cervical cancer	96.7% (95.1% to 98.9%)	96.8% (95.2% to 99.0%)	101.7% (100.5% to 103.6%)	96.9% (95.4% to 99.1%)
Uterine cancer	29.3% (19.0% to 40.7%)	29.9% (19.6% to 41.4%)	32.1% (20.8% to 44.8%)	30.0% (19.6% to 41.6%)
Prostate cancer	2.7% (1.7% to 4.1%)	2.6% (1.8% to 4.0%)	2.7% (1.8% to 4.2%)	2.6% (1.8% to 4.0%)
Colon and rectum cancer	45.9% (38.2% to 53.6%)	46.7% (39.1% to 54.3%)	49.7% (41.5% to 57.9%)	46.8% (39.2% to 54.4%)
Lip and oral cavity cancer	57.6% (51.9% to 63.0%)	59.2% (53.6% to 64.5%)	59.7% (54.0% to 65.2%)	59.2% (53.6% to 64.5%)
Nasopharynx cancer	58.6% (51.3% to 66.2%)	57.3% (50.8% to 64.2%)	58.9% (52.0% to 66.8%)	57.4% (50.8% to 64.3%)

Supplementary Results Table 1. Age-standardised PAFs attributable to the joint distribution of all risk factors for all ages, both sexes combined for each GBD level 1 and level 2 cause and all causes in 2016 for Middle SDI. Results are shown for deaths, YLLs, YLDs, and DALYs
Data in parenthesis are 95% uncertainty intervals. PAFs=population attributable fractions. GBD=Global Burden of Disease. YLLs=years of life lost. YLDs=years live with disability. DALYs=disability-adjusted life-years

	Deaths 2016	YLLs 2016	YLDs 2016	DALYs 2016
Other pharynx cancer	33.0% (25.9% to 40.3%)	33.8% (26.9% to 40.9%)	37.5% (29.9% to 44.5%)	33.9% (26.9% to 41.0%)
Gallbladder and biliary tract cancer	13.2% (6.9% to 21.7%)	13.7% (7.3% to 22.1%)	14.0% (7.2% to 22.9%)	13.7% (7.3% to 22.1%)
Pancreatic cancer	19.3% (12.1% to 28.2%)	18.8% (11.8% to 27.5%)	20.1% (12.7% to 29.4%)	18.8% (11.8% to 27.5%)
Ovarian cancer	10.1% (3.9% to 18.4%)	8.8% (3.3% to 16.2%)	9.1% (3.5% to 16.7%)	8.8% (3.3% to 16.2%)
Kidney cancer	20.8% (13.7% to 29.8%)	19.4% (12.8% to 27.6%)	20.7% (13.6% to 29.4%)	19.4% (12.9% to 27.6%)
Bladder cancer	21.8% (12.9% to 32.1%)	20.9% (12.4% to 30.6%)	21.9% (12.7% to 32.8%)	21.0% (12.4% to 30.7%)
Thyroid cancer	8.0% (3.9% to 14.0%)	8.2% (4.1% to 14.4%)	8.6% (4.3% to 14.5%)	8.3% (4.1% to 14.4%)
Mesothelioma	80.8% (76.9% to 84.2%)	71.3% (66.3% to 75.9%)	82.1% (77.7% to 85.9%)	71.5% (66.5% to 76.0%)
Non-Hodgkin's lymphoma	3.8% (1.4% to 7.1%)	3.6% (1.4% to 6.6%)	4.0% (1.5% to 7.4%)	3.6% (1.4% to 6.6%)
Multiple myeloma	5.3% (2.3% to 9.8%)	5.5% (2.3% to 9.8%)	5.4% (2.3% to 9.9%)	5.5% (2.3% to 9.8%)
Leukaemia	9.9% (6.0% to 14.8%)	7.5% (4.7% to 11.2%)	9.1% (5.4% to 14.0%)	7.6% (4.7% to 11.2%)
**Cardiovascular diseases**	82.2% (79.9% to 84.2%)	83.8% (82.1% to 85.4%)	77.4% (75.0% to 79.7%)	83.2% (81.5% to 84.8%)
Rheumatic heart disease	25.6% (16.7% to 41.1%)	23.6% (16.6% to 35.7%)	22.4% (14.5% to 32.1%)	23.4% (16.5% to 35.4%)
Ischaemic heart disease	89.0% (85.7% to 91.8%)	91.6% (89.4% to 93.6%)	91.2% (88.7% to 93.7%)	91.6% (89.4% to 93.5%)
Cerebrovascular disease	78.1% (74.6% to 81.5%)	81.1% (78.9% to 83.4%)	84.5% (81.3% to 87.4%)	81.5% (79.2% to 83.8%)
Hypertensive heart disease	96.5% (94.5% to 98.1%)	96.7% (95.0% to 98.3%)	100.0% (99.6% to 100.4%)	97.0% (95.4% to 98.4%)
Cardiomyopathy and myocarditis	32.1% (26.5% to 38.4%)	30.0% (26.0% to 34.6%)	26.8% (22.1% to 31.7%)	29.8% (25.9% to 34.4%)
Atrial fibrillation and flutter	44.3% (37.9% to 50.4%)	49.0% (43.2% to 54.3%)	58.9% (54.3% to 63.4%)	54.7% (49.6% to 59.4%)
Aortic aneurysm	42.5% (36.2% to 48.8%)	47.3% (42.1% to 52.1%)	-	47.3% (42.1% to 52.1%)
Peripheral vascular disease	52.9% (47.0% to 59.6%)	60.4% (55.6% to 65.1%)	59.9% (54.8% to 64.7%)	60.0% (55.1% to 64.8%)
Endocarditis	35.4% (27.7% to 44.5%)	30.6% (24.2% to 37.2%)	36.1% (28.3% to 44.4%)	30.7% (24.3% to 37.3%)

Supplementary Results Table 1. Age-standardised PAFs attributable to the joint distribution of all risk factors for all ages, both sexes combined for each GBD level 1 and level 2 cause and all causes in 2016 for Middle SDI. Results are shown for deaths, YLLs, YLDs, and DALYs
Data in parenthesis are 95% uncertainty intervals. PAFs=population attributable fractions. GBD=Global Burden of Disease. YLLs=years of life lost. YLDs=years live with disability. DALYs=disability-adjusted life-years

	Deaths 2016	YLLs 2016	YLDs 2016	DALYs 2016
Other cardiovascular and circulatory diseases	42.6% (38.1% to 47.5%)	42.7% (39.0% to 46.4%)	43.7% (39.2% to 48.3%)	43.0% (39.2% to 46.7%)
**Chronic respiratory diseases**	69.5% (60.0% to 76.6%)	66.8% (58.8% to 73.4%)	47.2% (40.9% to 53.8%)	60.8% (53.7% to 67.2%)
Chronic obstructive pulmonary disease	75.0% (64.4% to 82.9%)	74.4% (65.0% to 82.5%)	63.9% (55.0% to 72.1%)	72.1% (62.7% to 79.8%)
Pneumoconiosis	96.3% (94.5% to 99.5%)	96.8% (95.3% to 99.4%)	93.4% (92.5% to 94.3%)	95.7% (94.6% to 97.6%)
Asthma	32.2% (25.7% to 39.9%)	34.5% (28.5% to 41.5%)	27.8% (20.9% to 35.8%)	30.5% (24.3% to 37.8%)
Other chronic respiratory diseases	6.3% (3.8% to 9.8%)	4.4% (2.9% to 6.7%)	3.6% (2.1% to 5.6%)	4.1% (2.6% to 6.2%)
**Cirrhosis and other chronic liver diseases**	29.9% (28.2% to 31.7%)	32.4% (30.4% to 34.4%)	26.6% (25.1% to 28.0%)	32.2% (30.2% to 34.2%)
Cirrhosis and other chronic liver diseases due to hepatitis B	0.4% (0.3% to 0.5%)	0.5% (0.4% to 0.6%)	0.5% (0.4% to 0.6%)	0.5% (0.4% to 0.6%)
Cirrhosis and other chronic liver diseases due to hepatitis C	31.4% (27.2% to 36.0%)	42.2% (37.3% to 47.3%)	47.3% (42.5% to 51.9%)	42.3% (37.5% to 47.4%)
Cirrhosis and other chronic liver diseases due to alcohol use	96.4% (94.8% to 98.0%)	96.6% (94.5% to 98.6%)	100.5% (99.3% to 101.5%)	96.7% (94.6% to 98.6%)
**Digestive diseases**	10.2% (8.4% to 12.1%)	10.7% (9.1% to 12.5%)	3.3% (2.5% to 4.0%)	8.8% (7.3% to 10.3%)
Peptic ulcer disease	16.0% (11.6% to 20.8%)	18.0% (13.2% to 23.1%)	9.5% (6.8% to 12.4%)	15.9% (11.5% to 20.4%)
Gallbladder and biliary diseases	30.0% (19.9% to 41.9%)	32.5% (21.9% to 44.0%)	34.3% (22.6% to 48.3%)	32.7% (22.1% to 44.5%)
Pancreatitis	27.8% (21.3% to 34.9%)	30.3% (24.1% to 37.2%)	25.1% (19.5% to 30.5%)	30.2% (24.0% to 36.9%)
**Neurological disorders**	16.1% (8.2% to 25.9%)	15.5% (8.7% to 23.9%)	4.1% (2.7% to 5.9%)	8.6% (5.1% to 13.1%)
Alzheimer's disease and other dementias	18.6% (9.7% to 29.8%)	19.8% (10.5% to 31.0%)	21.0% (11.2% to 33.0%)	20.1% (10.7% to 31.5%)
Parkinson's disease	-14.9% (-17.8% to -11.6%)	-17.8% (-21.4% to -13.6%)	-19.5% (-23.5% to -14.8%)	-18.2% (-21.8% to -13.9%)
Epilepsy	17.6% (13.1% to 21.8%)	15.4% (11.5% to 19.2%)	13.0% (9.6% to 16.6%)	14.0% (10.5% to 17.4%)
Multiple sclerosis	8.2% (5.0% to 11.6%)	8.1% (5.0% to 11.4%)	6.2% (3.9% to 8.6%)	7.3% (4.5% to 10.2%)
**Mental and substance use disorders**	99.2% (95.6% to 101.2%)	98.5% (95.3% to 100.6%)	18.8% (16.8% to 20.8%)	23.5% (21.1% to 26.0%)
Alcohol use disorders	103.8% (97.7% to 107.1%)	103.2% (97.0% to 106.6%)	100.9% (100.4% to 101.5%)	101.7% (99.5% to 103.1%)

Supplementary Results Table 1. Age-standardised PAFs attributable to the joint distribution of all risk factors for all ages, both sexes combined for each GBD level 1 and level 2 cause and all causes in 2016 for Middle SDI. Results are shown for deaths, YLLs, YLDs, and DALYs
Data in parenthesis are 95% uncertainty intervals. PAFs=population attributable fractions. GBD=Global Burden of Disease. YLLs=years of life lost. YLDs=years live with disability. DALYs=disability-adjusted life-years

	**Deaths 2016**	**YLLs 2016**	**YLDs 2016**	**DALYs 2016**
Drug use disorders	94.9% (93.3% to 96.0%)	94.0% (92.5% to 95.4%)	101.1% (100.6% to 101.5%)	99.3% (98.5% to 99.9%)
Depressive disorders	-	-	6.8% (5.7% to 8.2%)	6.8% (5.7% to 8.2%)
Idiopathic developmental intellectual disability	-	-	67.7% (35.9% to 87.3%)	67.7% (35.9% to 87.3%)
**Diabetes, urogenital, blood, and endocrine diseases**	83.6% (82.6% to 84.8%)	81.6% (80.5% to 82.7%)	63.1% (59.2% to 66.5%)	73.7% (71.2% to 76.0%)
Diabetes mellitus	95.7% (94.5% to 97.2%)	96.4% (95.3% to 97.7%)	99.1% (98.7% to 99.5%)	97.6% (96.9% to 98.4%)
Chronic kidney disease	95.8% (94.7% to 98.7%)	97.1% (95.8% to 98.7%)	102.9% (102.5% to 103.2%)	98.2% (97.2% to 99.6%)
**Musculoskeletal disorders**	2.2% (0.9% to 3.6%)	1.6% (0.7% to 2.6%)	15.7% (13.2% to 18.6%)	15.4% (12.9% to 18.2%)
Rheumatoid arthritis	5.1% (2.2% to 8.5%)	5.4% (2.3% to 9.0%)	3.9% (1.6% to 6.4%)	4.1% (1.7% to 6.7%)
Osteoarthritis	-	-	18.2% (10.0% to 27.8%)	18.2% (10.0% to 27.8%)
Low back and neck pain	-	-	21.1% (18.4% to 24.1%)	21.1% (18.4% to 24.1%)
Gout	-	-	33.4% (22.6% to 46.8%)	33.4% (22.6% to 46.8%)
**Other non-communicable diseases**	0.2% (0.1% to 0.2%)	0.2% (0.2% to 0.3%)	6.6% (5.8% to 7.6%)	5.1% (4.4% to 5.8%)
Sense organ diseases	-	-	13.8% (12.4% to 15.3%)	13.8% (12.4% to 15.3%)
Sudden infant death syndrome	6.4% (5.5% to 7.2%)	6.4% (5.5% to 7.3%)	-	6.4% (5.5% to 7.3%)
**Transport injuries**	31.0% (26.7% to 36.0%)	27.5% (23.4% to 32.2%)	36.3% (32.6% to 40.8%)	28.7% (24.8% to 33.4%)
Road injuries	31.0% (26.6% to 36.0%)	27.5% (23.4% to 32.3%)	38.8% (34.9% to 43.5%)	28.8% (24.8% to 33.6%)
Other transport injuries	31.9% (28.0% to 36.2%)	27.0% (23.2% to 31.3%)	27.0% (23.9% to 30.6%)	27.0% (23.5% to 31.1%)
**Unintentional injuries**	29.7% (25.0% to 32.0%)	16.8% (15.5% to 18.2%)	20.1% (19.1% to 21.1%)	17.8% (16.6% to 19.0%)
Falls	53.5% (42.5% to 56.8%)	33.1% (29.6% to 34.7%)	28.4% (27.5% to 29.3%)	31.2% (28.9% to 32.4%)
Drowning	11.3% (8.9% to 14.1%)	9.8% (8.0% to 11.8%)	15.0% (12.4% to 18.3%)	9.9% (8.1% to 11.9%)
Fire, heat, and hot substances	10.5% (8.0% to 13.8%)	10.9% (8.7% to 13.9%)	16.7% (14.1% to 19.6%)	12.7% (10.3% to 15.8%)

Supplementary Results Table 1. Age-standardised PAFs attributable to the joint distribution of all risk factors for all ages, both sexes combined for each GBD level 1 and level 2 cause and all causes in 2016 for Middle SDI. Results are shown for deaths, YLLs, YLDs, and DALYs
Data in parenthesis are 95% uncertainty intervals. PAFs=population attributable fractions. GBD=Global Burden of Disease. YLLs=years of life lost. YLDs=years live with disability. DALYs=disability-adjusted life-years

	**Deaths 2016**	**YLLs 2016**	**YLDs 2016**	**DALYs 2016**
Poisonings	14.2% (11.1% to 17.7%)	14.1% (11.6% to 16.9%)	16.6% (14.1% to 19.6%)	14.3% (11.8% to 17.0%)
Exposure to mechanical forces	17.8% (17.0% to 19.1%)	14.7% (13.8% to 16.2%)	21.9% (21.0% to 22.7%)	16.7% (15.7% to 18.2%)
Animal contact	11.7% (10.8% to 13.3%)	10.8% (9.8% to 12.2%)	11.4% (10.8% to 12.1%)	11.0% (10.3% to 12.0%)
Foreign body	5.7% (5.1% to 6.6%)	5.1% (4.5% to 5.9%)	10.1% (9.4% to 10.8%)	6.4% (5.7% to 7.2%)
Other unintentional injuries	21.9% (17.7% to 26.7%)	22.2% (18.3% to 26.5%)	17.0% (14.0% to 20.4%)	19.9% (16.5% to 23.9%)
**Self-harm and interpersonal violence**	20.9% (16.2% to 25.5%)	20.2% (15.7% to 24.5%)	25.1% (22.2% to 28.3%)	20.5% (16.0% to 24.7%)
Self-harm	20.3% (13.6% to 26.9%)	19.9% (13.3% to 26.3%)	17.9% (11.8% to 24.0%)	19.9% (13.3% to 26.2%)
Interpersonal violence	21.9% (16.0% to 28.4%)	20.6% (14.9% to 27.2%)	25.9% (22.8% to 29.4%)	21.2% (15.8% to 27.3%)
**Forces of nature, conflict and terrorism, and executions and police conflict**	1.9% (0.8% to 2.9%)	0.6% (0.3% to 1.0%)	2.0% (1.5% to 2.8%)	1.1% (0.7% to 1.5%)
Forces of nature, conflict and terrorism, and state actor violence	6.3% (5.8% to 6.7%)	2.4% (2.1% to 2.6%)	5.9% (4.4% to 8.0%)	3.7% (2.8% to 5.0%)

Supplementary Results Table 1. Age-standardised PAFs attributable to the joint distribution of all risk factors for all ages, both sexes combined for each GBD level 1 and level 2 cause and all causes in 2016 for Low-middle SDI. Results are shown for deaths, YLLs, YLDs, and DALYs
Data in parenthesis are 95% uncertainty intervals. PAFs=population attributable fractions. GBD=Global Burden of Disease. YLLs=years of life lost. YLDs=years live with disability. DALYs=disability-adjusted life-years

	Deaths 2016	YLLs 2016	YLDs 2016	DALYs 2016
**All causes**	64.2% (62.5% to 66.0%)	57.6% (56.2% to 59.0%)	28.6% (27.6% to 29.7%)	49.1% (47.2% to 51.1%)
**HIV/AIDS and tuberculosis**	47.6% (43.0% to 52.3%)	52.5% (48.8% to 56.1%)	55.9% (51.4% to 60.4%)	52.7% (49.1% to 56.3%)
Tuberculosis	33.2% (26.5% to 39.6%)	32.9% (27.1% to 38.5%)	33.4% (27.3% to 38.9%)	32.9% (27.0% to 38.5%)
HIV/AIDS	80.0% (77.5% to 82.3%)	76.0% (73.3% to 78.5%)	79.1% (77.4% to 81.0%)	76.2% (73.6% to 78.5%)
**Diarrhoea, lower respiratory infections, and other common infectious diseases**	73.0% (67.0% to 77.7%)	71.0% (66.6% to 75.5%)	42.5% (36.3% to 47.8%)	69.1% (64.8% to 73.5%)
Diarrhoeal diseases	86.5% (75.8% to 93.1%)	91.8% (84.4% to 96.8%)	88.9% (77.2% to 95.0%)	91.6% (83.9% to 96.5%)
Lower respiratory infections	76.3% (71.5% to 80.8%)	85.2% (81.6% to 88.3%)	76.4% (71.4% to 81.4%)	85.1% (81.6% to 88.3%)
Upper respiratory infections	2.1% (1.2% to 3.5%)	4.1% (2.3% to 6.9%)	0.0% (0.0% to 0.0%)	0.1% (0.1% to 0.3%)
Otitis media	2.5% (1.5% to 4.2%)	6.4% (4.0% to 10.1%)	6.3% (4.3% to 8.3%)	6.3% (4.3% to 8.3%)
Meningitis	3.4% (2.2% to 4.8%)	5.3% (3.8% to 7.2%)	0.0% (0.0% to 0.0%)	4.9% (3.5% to 6.7%)
Encephalitis	0.6% (0.5% to 0.7%)	1.5% (1.2% to 1.7%)	0.0% (0.0% to 0.0%)	1.2% (1.0% to 1.4%)
Measles	70.2% (58.1% to 84.1%)	71.3% (58.9% to 85.4%)	67.8% (57.2% to 79.9%)	71.2% (58.9% to 85.3%)
**Maternal disorders**	10.4% (4.6% to 16.5%)	10.5% (4.6% to 16.8%)	2.2% (1.1% to 3.6%)	10.0% (4.4% to 16.0%)
Maternal haemorrhage	20.2% (7.5% to 32.3%)	20.4% (7.6% to 32.6%)	20.0% (7.3% to 32.3%)	20.4% (7.6% to 32.6%)
Maternal sepsis and other pregnancy related infections	22.5% (8.2% to 36.1%)	22.8% (8.4% to 36.4%)	19.4% (7.0% to 31.2%)	22.7% (8.3% to 36.2%)
Maternal abortion, miscarriage, and ectopic pregnancy	13.2% (8.4% to 18.6%)	13.1% (8.1% to 18.5%)	14.9% (8.3% to 21.9%)	13.2% (8.1% to 18.5%)
**Neonatal disorders**	83.2% (81.0% to 85.5%)	83.3% (81.0% to 85.6%)	0.3% (0.2% to 0.3%)	77.5% (74.3% to 80.3%)
Neonatal preterm birth complications	100.4% (97.3% to 103.7%)	100.4% (97.4% to 103.8%)	0.4% (0.3% to 0.5%)	87.8% (82.7% to 92.6%)
Neonatal encephalopathy due to birth asphyxia and trauma	76.5% (73.2% to 79.9%)	76.5% (73.3% to 79.9%)	0.0% (0.0% to 0.0%)	75.2% (71.5% to 78.8%)
Neonatal sepsis and other neonatal infections	67.3% (63.8% to 70.7%)	67.3% (63.9% to 70.7%)	0.0% (0.0% to 0.0%)	60.5% (50.2% to 66.6%)
Hemolytic disease and other neonatal jaundice	71.7% (68.1% to 75.0%)	71.7% (68.1% to 75.0%)	0.0% (0.0% to 0.0%)	64.5% (61.1% to 67.9%)

Supplementary Results Table 1. Age-standardised PAFs attributable to the joint distribution of all risk factors for all ages, both sexes combined for each GBD level 1 and level 2 cause and all causes in 2016 for Low-middle SDI. Results are shown for deaths, YLLs, YLDs, and DALYs
Data in parenthesis are 95% uncertainty intervals. PAFs=population attributable fractions. GBD=Global Burden of Disease. YLLs=years of life lost. YLDs=years live with disability. DALYs=disability-adjusted life-years

	**Deaths 2016**	**YLLs 2016**	**YLDs 2016**	**DALYs 2016**
Other neonatal disorders	76.3% (73.1% to 79.6%)	76.3% (73.1% to 79.6%)	0.0% (0.0% to 0.0%)	75.0% (71.8% to 78.3%)
**Nutritional deficiencies**	31.2% (26.7% to 37.1%)	64.3% (59.0% to 69.9%)	91.1% (90.2% to 92.0%)	84.6% (82.2% to 86.8%)
Protein-energy malnutrition	41.5% (36.2% to 48.4%)	76.9% (72.8% to 81.6%)	95.9% (95.1% to 97.1%)	81.1% (77.9% to 84.5%)
Vitamin A deficiency	-	-	100.0% (98.8% to 101.2%)	100.0% (98.8% to 101.2%)
Iron-deficiency anaemia	110.3% (97.0% to 118.7%)	105.1% (97.7% to 108.3%)	97.0% (96.8% to 97.2%)	97.0% (96.8% to 97.2%)
**Other communicable, maternal, neonatal, and nutritional diseases**	3.2% (2.7% to 3.8%)	2.5% (2.0% to 3.0%)	47.7% (43.4% to 52.6%)	10.4% (7.6% to 14.4%)
Sexually transmitted diseases excluding HIV	17.2% (10.4% to 27.3%)	7.4% (4.2% to 12.5%)	98.8% (98.1% to 99.6%)	29.4% (17.7% to 44.2%)
Hepatitis	0.2% (0.2% to 0.3%)	0.2% (0.2% to 0.3%)	0.2% (0.1% to 0.3%)	0.2% (0.2% to 0.3%)
**Neoplasms**	41.3% (38.5% to 44.1%)	38.6% (36.0% to 41.3%)	37.1% (34.6% to 40.0%)	38.5% (35.9% to 41.2%)
Oesophageal cancer	68.7% (58.6% to 78.0%)	68.8% (58.8% to 77.8%)	65.9% (56.1% to 74.9%)	68.8% (58.8% to 77.8%)
Stomach cancer	13.9% (7.5% to 22.1%)	13.9% (7.6% to 21.6%)	13.3% (7.1% to 21.2%)	13.9% (7.6% to 21.6%)
Liver cancer	41.0% (33.1% to 48.7%)	41.5% (33.4% to 49.7%)	39.6% (32.0% to 47.3%)	41.5% (33.4% to 49.7%)
Larynx cancer	62.1% (52.6% to 71.3%)	64.8% (55.2% to 74.0%)	61.5% (52.1% to 70.4%)	64.8% (55.2% to 74.0%)
Tracheal, bronchus, and lung cancer	113.1% (105.7% to 118.3%)	107.1% (101.6% to 111.9%)	104.2% (98.1% to 108.9%)	107.1% (101.6% to 111.8%)
Breast cancer	18.8% (12.2% to 27.1%)	17.1% (11.0% to 24.7%)	17.3% (11.2% to 24.7%)	17.2% (11.0% to 24.7%)
Cervical cancer	101.4% (98.8% to 103.6%)	101.0% (98.5% to 103.1%)	98.0% (95.3% to 100.1%)	101.0% (98.5% to 103.1%)
Uterine cancer	25.9% (16.2% to 37.3%)	27.6% (17.6% to 39.2%)	26.6% (16.8% to 38.0%)	27.6% (17.6% to 39.2%)
Prostate cancer	2.1% (1.5% to 2.8%)	2.1% (1.5% to 2.7%)	2.2% (1.6% to 2.9%)	2.1% (1.5% to 2.7%)
Colon and rectum cancer	58.9% (48.9% to 69.0%)	54.7% (45.4% to 64.1%)	55.7% (46.1% to 65.8%)	54.7% (45.5% to 64.1%)
Lip and oral cavity cancer	71.8% (65.0% to 78.4%)	70.1% (63.5% to 76.3%)	66.9% (60.4% to 72.9%)	70.0% (63.4% to 76.3%)
Nasopharynx cancer	59.6% (52.1% to 67.1%)	60.4% (53.0% to 67.9%)	58.2% (50.8% to 65.2%)	60.4% (52.9% to 67.8%)

**Supplementary Results Table 1. Age-standardised PAFs attributable to the joint distribution of all risk factors for all ages, both sexes combined for each GBD level 1 and level 2 cause and all causes in 2016 for Low-middle SDI. Results are shown for deaths, YLLs, YLDs, and DALYs** Data in parenthesis are 95% uncertainty intervals. PAFs=population attributable fractions. GBD=Global Burden of Disease. YLLs=years of life lost. YLDs=years live with disability. DALYs=disability-adjusted life-years	**Deaths 2016**	**YLLs 2016**	**YLDs 2016**	**DALYs 2016**
Other pharynx cancer	34.7% (25.3% to 44.3%)	36.7% (27.3% to 46.2%)	34.0% (25.0% to 43.0%)	36.7% (27.3% to 46.2%)
Gallbladder and biliary tract cancer	11.1% (5.4% to 19.3%)	11.8% (5.8% to 20.3%)	10.6% (5.2% to 18.5%)	11.7% (5.8% to 20.3%)
Pancreatic cancer	21.1% (14.5% to 29.6%)	21.1% (14.6% to 29.1%)	20.2% (13.8% to 28.3%)	21.1% (14.6% to 29.1%)
Ovarian cancer	10.7% (4.2% to 20.3%)	9.4% (3.4% to 18.2%)	9.0% (3.3% to 17.3%)	9.4% (3.4% to 18.2%)
Kidney cancer	19.2% (13.3% to 26.2%)	18.6% (12.8% to 25.1%)	18.3% (12.7% to 24.8%)	18.6% (12.8% to 25.1%)
Bladder cancer	24.5% (16.6% to 34.0%)	25.2% (17.4% to 34.5%)	24.8% (17.1% to 33.9%)	25.2% (17.4% to 34.5%)
Thyroid cancer	6.6% (3.1% to 11.8%)	6.9% (3.3% to 12.2%)	7.0% (3.4% to 12.2%)	6.9% (3.3% to 12.2%)
Mesothelioma	88.1% (83.0% to 92.8%)	78.9% (72.4% to 85.0%)	81.5% (76.1% to 86.7%)	78.9% (72.4% to 85.0%)
Non-Hodgkin's lymphoma	3.3% (1.2% to 6.3%)	3.1% (1.2% to 5.8%)	3.2% (1.2% to 6.0%)	3.1% (1.2% to 5.8%)
Multiple myeloma	3.8% (1.5% to 7.2%)	4.0% (1.6% to 7.5%)	3.7% (1.5% to 7.0%)	4.0% (1.6% to 7.5%)
Leukaemia	9.6% (6.1% to 13.6%)	8.2% (5.4% to 11.6%)	9.0% (5.7% to 12.7%)	8.2% (5.4% to 11.6%)
**Cardiovascular diseases**	92.4% (90.0% to 94.8%)	91.8% (90.0% to 93.6%)	75.2% (71.6% to 78.2%)	90.7% (89.0% to 92.6%)
Rheumatic heart disease	25.7% (17.7% to 40.7%)	24.1% (17.0% to 35.9%)	20.5% (13.2% to 29.4%)	23.6% (16.5% to 35.0%)
Ischaemic heart disease	95.9% (92.8% to 98.7%)	95.8% (93.6% to 98.0%)	89.5% (86.8% to 92.0%)	95.6% (93.4% to 97.7%)
Cerebrovascular disease	96.4% (92.3% to 100.4%)	98.0% (95.2% to 100.8%)	91.5% (88.2% to 94.6%)	97.6% (94.7% to 100.3%)
Hypertensive heart disease	105.5% (102.3% to 110.6%)	104.1% (101.5% to 108.5%)	101.7% (100.8% to 102.4%)	103.9% (101.5% to 107.9%)
Cardiomyopathy and myocarditis	43.0% (35.9% to 50.9%)	35.6% (30.4% to 41.3%)	31.0% (25.3% to 36.5%)	35.3% (30.1% to 40.9%)
Atrial fibrillation and flutter	50.4% (42.3% to 57.6%)	53.3% (46.6% to 59.1%)	55.8% (51.3% to 60.3%)	54.8% (49.4% to 59.6%)
Aortic aneurysm	47.2% (40.2% to 54.6%)	51.0% (44.9% to 56.9%)	-	51.0% (44.9% to 56.9%)
Peripheral vascular disease	52.7% (46.2% to 59.5%)	58.5% (53.1% to 63.6%)	60.0% (55.1% to 64.9%)	59.7% (54.8% to 64.5%)
Endocarditis	42.3% (33.1% to 52.3%)	38.5% (30.6% to 46.5%)	37.2% (28.9% to 46.3%)	38.5% (30.6% to 46.5%)

Supplementary Results Table 1. Age-standardised PAFs attributable to the joint distribution of all risk factors for all ages, both sexes combined for each GBD level 1 and level 2 cause and all causes in 2016 for Low-middle SDI. Results are shown for deaths, YLLs, YLDs, and DALYs
Data in parenthesis are 95% uncertainty intervals. PAFs=population attributable fractions. GBD=Global Burden of Disease. YLLs=years of life lost. YLDs=years live with disability. DALYs=disability-adjusted life-years

	Deaths 2016	YLLs 2016	YLDs 2016	DALYs 2016
Other cardiovascular and circulatory diseases	50.9% (45.5% to 56.5%)	48.6% (44.1% to 53.0%)	43.5% (38.6% to 48.3%)	47.4% (43.0% to 51.8%)
**Chronic respiratory diseases**	66.2% (59.5% to 72.6%)	64.3% (58.3% to 70.2%)	54.2% (47.8% to 60.6%)	61.8% (56.0% to 67.3%)
Chronic obstructive pulmonary disease	75.5% (68.3% to 82.9%)	75.2% (68.2% to 82.1%)	67.6% (59.4% to 75.4%)	73.5% (66.3% to 80.3%)
Pneumoconiosis	96.7% (94.6% to 98.5%)	97.2% (95.2% to 98.8%)	106.5% (104.9% to 108.4%)	98.6% (96.8% to 100.1%)
Asthma	29.4% (22.8% to 36.6%)	32.4% (26.0% to 39.3%)	26.5% (21.4% to 32.6%)	30.5% (24.9% to 36.9%)
Other chronic respiratory diseases	7.4% (5.3% to 10.0%)	6.2% (4.3% to 8.3%)	4.5% (3.2% to 6.2%)	5.5% (3.9% to 7.4%)
**Cirrhosis and other chronic liver diseases**	30.3% (28.2% to 32.7%)	29.9% (27.6% to 32.3%)	22.4% (20.7% to 24.2%)	29.6% (27.4% to 31.9%)
Cirrhosis and other chronic liver diseases due to hepatitis B	0.2% (0.1% to 0.3%)	0.2% (0.2% to 0.3%)	0.2% (0.1% to 0.3%)	0.2% (0.2% to 0.3%)
Cirrhosis and other chronic liver diseases due to hepatitis C	21.2% (17.6% to 25.0%)	26.9% (22.2% to 31.9%)	22.2% (17.8% to 27.0%)	26.8% (22.1% to 31.7%)
Cirrhosis and other chronic liver diseases due to alcohol use	102.2% (100.0% to 104.2%)	101.4% (99.1% to 103.7%)	98.6% (97.4% to 99.8%)	101.3% (99.1% to 103.6%)
**Digestive diseases**	7.3% (5.9% to 8.9%)	7.1% (5.8% to 8.6%)	2.5% (1.9% to 3.2%)	6.6% (5.3% to 7.9%)
Peptic ulcer disease	14.8% (10.6% to 19.3%)	14.8% (10.8% to 19.3%)	8.2% (5.9% to 10.7%)	14.1% (10.3% to 18.4%)
Gallbladder and biliary diseases	23.8% (14.4% to 35.8%)	23.7% (14.7% to 34.8%)	24.7% (15.9% to 35.7%)	23.8% (14.8% to 35.0%)
Pancreatitis	19.8% (13.6% to 26.9%)	22.1% (16.4% to 29.0%)	20.5% (15.4% to 26.6%)	22.1% (16.4% to 29.0%)
**Neurological disorders**	16.1% (8.2% to 26.2%)	14.0% (8.0% to 21.6%)	2.4% (1.5% to 3.5%)	6.1% (3.5% to 9.7%)
Alzheimer's disease and other dementias	19.5% (9.8% to 31.8%)	20.0% (10.4% to 32.2%)	18.9% (9.7% to 30.2%)	19.8% (10.2% to 31.8%)
Parkinson's disease	-14.3% (-17.1% to -11.1%)	-16.1% (-19.3% to -12.4%)	-16.2% (-19.5% to -12.4%)	-16.1% (-19.4% to -12.3%)
Epilepsy	11.7% (8.4% to 15.2%)	10.3% (7.5% to 13.3%)	8.8% (6.2% to 12.0%)	9.6% (7.0% to 12.5%)
Multiple sclerosis	7.6% (4.3% to 11.6%)	7.4% (4.1% to 11.3%)	4.9% (2.9% to 6.9%)	6.3% (3.7% to 9.4%)
**Mental and substance use disorders**	97.0% (94.5% to 101.6%)	98.1% (95.5% to 102.3%)	19.8% (17.7% to 22.0%)	24.0% (21.5% to 26.5%)
Alcohol use disorders	93.5% (90.3% to 99.5%)	94.4% (91.1% to 100.6%)	98.4% (97.8% to 99.0%)	96.9% (95.4% to 99.2%)

Supplementary Results Table 1. Age-standardised PAFs attributable to the joint distribution of all risk factors for all ages, both sexes combined for each GBD level 1 and level 2 cause and all causes in 2016 for Low-middle SDI. Results are shown for deaths, YLLs, YLDs, and DALYs
Data in parenthesis are 95% uncertainty intervals. PAFs=population attributable fractions. GBD=Global Burden of Disease. YLLs=years of life lost. YLDs=years live with disability. DALYs=disability-adjusted life-years

	Deaths 2016	YLLs 2016	YLDs 2016	DALYs 2016
Drug use disorders	104.4% (102.2% to 107.6%)	105.4% (103.1% to 107.9%)	99.3% (98.8% to 99.9%)	100.6% (99.8% to 101.3%)
Depressive disorders	-	-	9.5% (8.1% to 11.1%)	9.5% (8.1% to 11.1%)
Idiopathic developmental intellectual disability	-	-	62.5% (32.4% to 80.5%)	62.5% (32.4% to 80.5%)
**Diabetes, urogenital, blood, and endocrine diseases**	87.1% (85.3% to 88.8%)	80.5% (78.6% to 82.1%)	58.9% (54.9% to 62.5%)	72.1% (69.2% to 74.6%)
Diabetes mellitus	103.8% (101.8% to 105.8%)	102.9% (101.1% to 104.5%)	102.7% (102.1% to 103.2%)	102.8% (101.7% to 103.8%)
Chronic kidney disease	106.3% (99.9% to 108.7%)	103.0% (99.7% to 105.1%)	96.6% (96.2% to 97.1%)	101.6% (99.1% to 103.3%)
**Musculoskeletal disorders**	1.9% (0.8% to 3.3%)	1.5% (0.6% to 2.6%)	16.7% (14.4% to 19.3%)	16.5% (14.3% to 19.1%)
Rheumatoid arthritis	4.0% (1.7% to 6.7%)	4.2% (1.7% to 7.1%)	3.3% (1.4% to 5.4%)	3.4% (1.4% to 5.6%)
Osteoarthritis	-	-	12.0% (5.9% to 19.7%)	12.0% (5.9% to 19.7%)
Low back and neck pain	-	-	26.3% (23.6% to 29.3%)	26.3% (23.6% to 29.3%)
Gout	-	-	32.3% (23.7% to 43.6%)	32.3% (23.7% to 43.6%)
**Other non-communicable diseases**	0.3% (0.2% to 0.4%)	0.3% (0.3% to 0.4%)	8.8% (7.5% to 10.3%)	6.8% (5.8% to 7.8%)
Sense organ diseases	-	-	16.5% (14.6% to 18.6%)	16.5% (14.6% to 18.6%)
Sudden infant death syndrome	6.1% (4.7% to 7.6%)	6.1% (4.7% to 7.6%)	-	6.1% (4.7% to 7.6%)
**Transport injuries**	28.9% (25.6% to 32.7%)	24.6% (21.6% to 28.2%)	31.1% (28.5% to 34.3%)	25.6% (22.6% to 29.2%)
Road injuries	28.5% (25.3% to 32.4%)	24.5% (21.5% to 28.2%)	33.8% (31.2% to 37.2%)	25.7% (22.7% to 29.5%)
Other transport injuries	32.7% (29.6% to 36.3%)	25.4% (22.4% to 29.0%)	23.2% (21.0% to 25.8%)	24.6% (22.0% to 27.7%)
**Unintentional injuries**	32.2% (26.0% to 35.7%)	16.5% (15.1% to 18.2%)	17.8% (17.0% to 18.8%)	16.9% (15.8% to 18.3%)
Falls	60.6% (47.5% to 64.7%)	37.3% (33.0% to 39.3%)	26.9% (26.0% to 27.8%)	32.9% (30.3% to 34.7%)
Drowning	10.1% (8.3% to 12.5%)	9.0% (7.5% to 10.8%)	12.8% (10.9% to 15.2%)	9.0% (7.5% to 10.9%)
Fire, heat, and hot substances	7.5% (6.0% to 9.7%)	7.7% (6.4% to 9.7%)	12.0% (10.4% to 14.0%)	8.8% (7.4% to 10.9%)

Supplementary Results Table 1. Age-standardised PAFs attributable to the joint distribution of all risk factors for all ages, both sexes combined for each GBD level 1 and level 2 cause and all causes in 2016 for Low-middle SDI. Results are shown for deaths, YLLs, YLDs, and DALYs
Data in parenthesis are 95% uncertainty intervals. PAFs=population attributable fractions. GBD=Global Burden of Disease. YLLs=years of life lost. YLDs=years live with disability. DALYs=disability-adjusted life-years

	**Deaths 2016**	**YLLs 2016**	**YLDs 2016**	**DALYs 2016**
Poisonings	12.0% (9.3% to 14.9%)	10.6% (8.2% to 13.1%)	13.1% (11.3% to 15.2%)	10.8% (8.5% to 13.2%)
Exposure to mechanical forces	17.5% (16.4% to 18.5%)	13.2% (11.7% to 14.5%)	19.2% (18.3% to 20.1%)	15.1% (14.0% to 16.2%)
Animal contact	5.8% (5.0% to 7.7%)	6.1% (5.2% to 8.2%)	8.8% (8.1% to 9.6%)	6.8% (6.0% to 8.5%)
Foreign body	5.6% (4.6% to 6.4%)	5.5% (4.0% to 6.5%)	8.0% (7.4% to 8.7%)	6.5% (5.3% to 7.3%)
Other unintentional injuries	14.5% (12.0% to 17.4%)	15.8% (13.4% to 18.4%)	12.3% (10.6% to 14.5%)	14.1% (12.2% to 16.5%)
**Self-harm and interpersonal violence**	13.5% (10.1% to 17.2%)	12.9% (9.7% to 16.3%)	21.4% (19.6% to 23.3%)	13.4% (10.3% to 16.7%)
Self-harm	12.7% (8.3% to 17.3%)	12.3% (8.1% to 16.5%)	11.3% (7.1% to 15.6%)	12.3% (8.1% to 16.4%)
Interpersonal violence	15.7% (12.1% to 19.7%)	14.3% (10.8% to 18.2%)	23.3% (21.3% to 25.3%)	15.8% (12.6% to 19.4%)
**Forces of nature, conflict and terrorism, and executions and police conflict**	0.2% (0.1% to 0.4%)	0.1% (0.0% to 0.1%)	1.3% (0.8% to 2.1%)	0.2% (0.1% to 0.4%)
Forces of nature, conflict and terrorism, and state actor violence	7.9% (7.0% to 8.8%)	3.3% (2.8% to 3.8%)	6.4% (4.5% to 9.0%)	4.9% (3.8% to 6.7%)

Supplementary Results Table 1. Age-standardised PAFs attributable to the joint distribution of all risk factors for all ages, both sexes combined for each GBD level 1 and level 2 cause and all causes in 2016 for Low SDI. Results are shown for deaths, YLLs, YLDs, and DALYs

Data in parenthesis are 95% uncertainty intervals. PAFs=population attributable fractions. GBD=Global Burden of Disease. YLLs=years of life lost. YLDs=years live with disability. DALYs=disability-adjusted life-years

	**Deaths 2016**	**YLLs 2016**	**YLDs 2016**	**DALYs 2016**
**All causes**	58.4% (56.6% to 60.3%)	54.3% (52.8% to 56.0%)	28.8% (27.8% to 29.9%)	48.4% (46.6% to 50.3%)
**HIV/AIDS and tuberculosis**	56.7% (51.7% to 61.7%)	64.2% (60.1% to 68.3%)	83.6% (79.3% to 88.3%)	65.0% (61.1% to 69.0%)
Tuberculosis	32.9% (25.3% to 40.0%)	34.4% (27.4% to 41.6%)	32.3% (25.7% to 39.2%)	34.3% (27.3% to 41.5%)
HIV/AIDS	98.4% (95.6% to 101.3%)	93.4% (90.5% to 96.5%)	98.7% (96.5% to 101.2%)	93.8% (90.9% to 96.7%)
**Diarrhoea, lower respiratory infections, and other common infectious diseases**	79.5% (74.4% to 84.5%)	75.6% (71.2% to 80.1%)	46.5% (41.2% to 51.3%)	74.1% (70.0% to 78.6%)
Diarrhoeal diseases	96.3% (88.3% to 100.0%)	97.2% (91.5% to 100.2%)	95.6% (88.6% to 98.9%)	97.1% (91.3% to 100.1%)
Lower respiratory infections	82.9% (77.6% to 87.7%)	89.0% (84.9% to 92.3%)	79.6% (74.6% to 84.3%)	88.9% (84.9% to 92.3%)
Upper respiratory infections	1.4% (0.8% to 2.3%)	2.6% (1.5% to 4.2%)	0.0% (0.0% to 0.0%)	0.2% (0.1% to 0.4%)
Otitis media	2.0% (1.2% to 3.1%)	5.1% (3.3% to 7.0%)	4.0% (2.6% to 5.2%)	4.0% (2.7% to 5.3%)
Meningitis	2.5% (1.7% to 3.4%)	4.6% (3.3% to 5.7%)	0.0% (0.0% to 0.0%)	4.4% (3.2% to 5.5%)
Encephalitis	1.5% (0.7% to 2.6%)	2.6% (1.5% to 4.0%)	0.0% (0.0% to 0.0%)	1.8% (1.1% to 2.8%)
Measles	68.2% (56.2% to 82.8%)	69.4% (57.0% to 84.2%)	68.1% (56.4% to 82.2%)	69.4% (57.0% to 84.2%)
**Maternal disorders**	8.5% (4.1% to 13.1%)	8.5% (4.1% to 13.2%)	2.4% (1.3% to 3.7%)	8.3% (4.0% to 12.7%)
Maternal haemorrhage	19.1% (6.9% to 30.9%)	19.3% (7.0% to 31.2%)	18.7% (6.8% to 30.4%)	19.3% (7.0% to 31.2%)
Maternal sepsis and other pregnancy related infections	17.7% (6.4% to 28.9%)	17.8% (6.4% to 29.1%)	18.1% (6.6% to 29.5%)	17.9% (6.4% to 29.2%)
Maternal abortion, miscarriage, and ectopic pregnancy	20.1% (13.2% to 27.1%)	19.8% (12.8% to 26.6%)	20.5% (12.3% to 28.6%)	19.8% (12.8% to 26.6%)
**Neonatal disorders**	79.2% (76.3% to 82.2%)	79.2% (76.4% to 82.2%)	0.4% (0.2% to 0.6%)	75.3% (72.0% to 78.5%)
Neonatal preterm birth complications	92.9% (87.7% to 97.5%)	93.0% (87.7% to 97.6%)	0.9% (0.7% to 1.1%)	87.1% (82.1% to 92.0%)
Neonatal encephalopathy due to birth asphyxia and trauma	77.8% (74.3% to 81.1%)	77.8% (74.4% to 81.1%)	0.0% (0.0% to 0.0%)	75.4% (70.5% to 79.4%)
Neonatal sepsis and other neonatal infections	67.5% (63.9% to 71.3%)	67.5% (63.9% to 71.3%)	0.0% (0.0% to 0.0%)	62.7% (55.1% to 68.3%)
Hemolytic disease and other neonatal jaundice	70.8% (67.5% to 74.0%)	70.8% (67.5% to 74.1%)	0.0% (0.0% to 0.0%)	63.7% (60.0% to 67.7%)

Supplementary Results Table 1. Age-standardised PAFs attributable to the joint distribution of all risk factors for all ages, both sexes combined for each GBD level 1 and level 2 cause and all causes in 2016 for Low SDI. Results are shown for deaths, YLLs, YLDs, and DALYs
Data in parenthesis are 95% uncertainty intervals. PAFs=population attributable fractions. GBD=Global Burden of Disease. YLLs=years of life lost. YLDs=years live with disability. DALYs=disability-adjusted life-years

	Deaths 2016	YLLs 2016	YLDs 2016	DALYs 2016
Other neonatal disorders	69.4% (66.1% to 72.6%)	69.5% (66.1% to 72.7%)	0.0% (0.0% to 0.0%)	68.2% (64.9% to 71.3%)
**Nutritional deficiencies**	26.2% (20.7% to 30.7%)	60.4% (53.3% to 65.8%)	85.6% (84.0% to 87.0%)	72.3% (66.7% to 75.9%)
Protein-energy malnutrition	31.5% (25.8% to 36.3%)	67.3% (60.4% to 72.3%)	96.6% (96.0% to 97.3%)	68.7% (61.9% to 73.4%)
Vitamin A deficiency	-	-	95.4% (94.0% to 96.8%)	95.4% (94.0% to 96.8%)
Iron-deficiency anaemia	99.7% (98.4% to 101.6%)	99.8% (98.6% to 101.2%)	94.3% (93.9% to 94.7%)	94.4% (94.0% to 94.8%)
**Other communicable, maternal, neonatal, and nutritional diseases**	3.9% (3.0% to 5.0%)	2.5% (1.8% to 3.4%)	62.4% (57.9% to 67.3%)	9.1% (6.1% to 13.2%)
Sexually transmitted diseases excluding HIV	9.7% (6.0% to 15.4%)	4.3% (2.6% to 7.0%)	99.5% (98.2% to 100.6%)	15.7% (9.3% to 25.2%)
Hepatitis	0.3% (0.2% to 0.4%)	0.3% (0.2% to 0.5%)	0.3% (0.2% to 0.5%)	0.3% (0.2% to 0.5%)
**Neoplasms**	32.1% (29.4% to 35.6%)	32.2% (29.6% to 35.2%)	33.2% (30.4% to 37.1%)	32.2% (29.6% to 35.2%)
Oesophageal cancer	59.3% (47.8% to 69.4%)	61.6% (50.6% to 71.6%)	60.0% (48.2% to 70.0%)	61.6% (50.6% to 71.6%)
Stomach cancer	7.5% (3.0% to 15.7%)	8.1% (3.5% to 15.6%)	7.5% (3.0% to 15.3%)	8.1% (3.5% to 15.6%)
Liver cancer	30.1% (23.6% to 37.5%)	30.4% (23.9% to 37.8%)	29.8% (23.4% to 37.1%)	30.4% (23.9% to 37.8%)
Larynx cancer	54.0% (43.5% to 64.2%)	57.3% (46.9% to 67.0%)	54.0% (43.4% to 64.2%)	57.2% (46.8% to 66.9%)
Tracheal, bronchus, and lung cancer	82.8% (78.4% to 86.8%)	82.9% (78.7% to 86.7%)	82.2% (77.8% to 86.1%)	82.9% (78.7% to 86.7%)
Breast cancer	18.5% (12.9% to 25.6%)	17.4% (12.1% to 23.8%)	17.6% (12.3% to 24.0%)	17.4% (12.1% to 23.8%)
Cervical cancer	101.7% (100.5% to 102.8%)	101.6% (100.5% to 102.8%)	102.1% (101.0% to 102.9%)	101.6% (100.5% to 102.8%)
Uterine cancer	24.9% (15.3% to 35.6%)	25.8% (16.3% to 36.8%)	25.1% (15.7% to 35.8%)	25.8% (16.3% to 36.8%)
Prostate cancer	1.1% (0.7% to 1.5%)	1.2% (0.8% to 1.7%)	1.1% (0.7% to 1.6%)	1.2% (0.8% to 1.7%)
Colon and rectum cancer	46.8% (37.9% to 55.8%)	46.7% (38.3% to 55.3%)	46.7% (38.2% to 55.6%)	46.7% (38.3% to 55.3%)
Lip and oral cavity cancer	57.6% (49.4% to 65.1%)	59.6% (51.9% to 66.3%)	57.7% (49.7% to 65.1%)	59.6% (51.8% to 66.3%)
Nasopharynx cancer	48.3% (41.0% to 55.8%)	49.6% (42.8% to 56.7%)	49.0% (42.0% to 55.6%)	49.6% (42.8% to 56.7%)

Supplementary Results Table 1. Age-standardised PAFs attributable to the joint distribution of all risk factors for all ages, both sexes combined for each GBD level 1 and level 2 cause and all causes in 2016 for Low SDI. Results are shown for deaths, YLLs, YLDs, and DALYs
Data in parenthesis are 95% uncertainty intervals. PAFs=population attributable fractions. GBD=Global Burden of Disease. YLLs=years of life lost. YLDs=years live with disability. DALYs=disability-adjusted life-years

	Deaths 2016	YLLs 2016	YLDs 2016	DALYs 2016
Other pharynx cancer	37.1% (26.0% to 47.6%)	39.0% (28.2% to 49.5%)	37.4% (26.3% to 48.0%)	39.0% (28.2% to 49.5%)
Gallbladder and biliary tract cancer	10.5% (5.1% to 18.4%)	10.8% (5.3% to 18.9%)	10.1% (4.9% to 17.7%)	10.8% (5.3% to 18.9%)
Pancreatic cancer	14.2% (8.6% to 21.9%)	14.5% (9.1% to 21.5%)	14.0% (8.5% to 21.4%)	14.5% (9.1% to 21.5%)
Ovarian cancer	8.3% (3.5% to 15.3%)	7.3% (3.0% to 13.5%)	7.4% (3.1% to 13.6%)	7.3% (3.0% to 13.5%)
Kidney cancer	14.6% (9.5% to 21.3%)	13.7% (8.9% to 19.7%)	13.7% (9.0% to 19.8%)	13.7% (8.9% to 19.7%)
Bladder cancer	13.2% (7.5% to 20.3%)	13.8% (8.1% to 20.6%)	13.2% (7.6% to 20.2%)	13.8% (8.1% to 20.6%)
Thyroid cancer	5.6% (2.6% to 10.0%)	5.7% (2.7% to 10.2%)	5.6% (2.6% to 9.9%)	5.7% (2.7% to 10.2%)
Mesothelioma	85.8% (80.3% to 89.7%)	77.2% (70.2% to 82.6%)	82.6% (76.4% to 87.0%)	77.3% (70.3% to 82.7%)
Non-Hodgkin's lymphoma	2.5% (0.9% to 5.0%)	2.2% (0.8% to 4.2%)	2.4% (0.9% to 4.7%)	2.2% (0.8% to 4.2%)
Multiple myeloma	3.7% (1.4% to 7.0%)	3.8% (1.5% to 7.2%)	3.7% (1.4% to 7.0%)	3.8% (1.5% to 7.2%)
Leukaemia	7.7% (4.6% to 11.2%)	7.0% (4.3% to 9.9%)	7.4% (4.5% to 10.8%)	7.0% (4.3% to 9.9%)
**Cardiovascular diseases**	82.7% (80.3% to 84.7%)	83.2% (81.1% to 84.9%)	63.1% (59.2% to 66.8%)	82.0% (80.0% to 83.8%)
Rheumatic heart disease	25.7% (17.4% to 39.7%)	24.7% (17.1% to 36.9%)	22.2% (14.3% to 31.4%)	24.2% (16.9% to 35.2%)
Ischaemic heart disease	89.8% (87.0% to 92.2%)	90.5% (88.6% to 92.2%)	86.6% (83.9% to 89.2%)	90.4% (88.5% to 92.1%)
Cerebrovascular disease	86.5% (83.2% to 89.5%)	88.6% (86.4% to 90.6%)	85.4% (82.3% to 88.3%)	88.4% (86.2% to 90.5%)
Hypertensive heart disease	100.1% (99.0% to 101.3%)	100.0% (98.7% to 101.1%)	99.8% (99.4% to 100.2%)	100.0% (98.8% to 101.1%)
Cardiomyopathy and myocarditis	39.3% (32.1% to 47.4%)	37.1% (30.5% to 43.9%)	40.7% (34.3% to 47.3%)	37.3% (30.9% to 44.0%)
Atrial fibrillation and flutter	42.5% (35.6% to 49.3%)	47.0% (40.6% to 52.7%)	53.2% (48.5% to 57.8%)	50.1% (44.5% to 55.2%)
Aortic aneurysm	41.6% (35.1% to 48.0%)	46.2% (40.5% to 51.7%)	-	46.2% (40.5% to 51.7%)
Peripheral vascular disease	51.9% (45.3% to 59.7%)	59.2% (53.8% to 65.2%)	59.1% (54.0% to 64.3%)	59.1% (54.1% to 64.0%)
Endocarditis	38.1% (29.8% to 47.8%)	36.4% (29.0% to 44.3%)	36.2% (27.4% to 46.4%)	36.4% (29.0% to 44.3%)

Supplementary Results Table 1. Age-standardised PAFs attributable to the joint distribution of all risk factors for all ages, both sexes combined for each GBD level 1 and level 2 cause and all causes in 2016 for Low SDI. Results are shown for deaths, YLLs, YLDs, and DALYs
Data in parenthesis are 95% uncertainty intervals. PAFs=population attributable fractions. GBD=Global Burden of Disease. YLLs=years of life lost. YLDs=years live with disability. DALYs=disability-adjusted life-years

	**Deaths 2016**	**YLLs 2016**	**YLDs 2016**	**DALYs 2016**
Other cardiovascular and circulatory diseases	44.5% (39.6% to 49.6%)	46.7% (42.5% to 51.0%)	39.9% (34.7% to 45.3%)	45.7% (41.4% to 49.7%)
**Chronic respiratory diseases**	64.8% (57.5% to 71.5%)	62.0% (55.5% to 67.9%)	46.7% (40.3% to 53.8%)	58.1% (52.2% to 63.9%)
Chronic obstructive pulmonary disease	79.1% (71.5% to 86.9%)	79.2% (72.3% to 86.5%)	71.0% (62.0% to 80.3%)	77.5% (70.5% to 85.0%)
Pneumoconiosis	98.1% (97.2% to 99.0%)	98.2% (97.3% to 99.1%)	94.5% (94.0% to 95.1%)	97.9% (96.9% to 98.6%)
Asthma	25.2% (19.4% to 32.3%)	28.2% (22.8% to 35.0%)	22.5% (17.5% to 28.5%)	26.2% (20.9% to 32.7%)
Other chronic respiratory diseases	7.3% (4.8% to 10.4%)	7.2% (4.6% to 10.3%)	2.8% (1.6% to 4.3%)	6.2% (3.9% to 8.9%)
**Cirrhosis and other chronic liver diseases**	28.9% (26.4% to 31.4%)	28.3% (25.9% to 30.7%)	22.3% (20.5% to 24.3%)	28.1% (25.8% to 30.5%)
Cirrhosis and other chronic liver diseases due to hepatitis B	0.1% (0.1% to 0.2%)	0.1% (0.1% to 0.2%)	0.1% (0.1% to 0.2%)	0.1% (0.1% to 0.2%)
Cirrhosis and other chronic liver diseases due to hepatitis C	9.9% (7.6% to 12.9%)	13.4% (10.3% to 17.1%)	13.7% (10.6% to 17.3%)	13.5% (10.3% to 17.1%)
Cirrhosis and other chronic liver diseases due to alcohol use	104.3% (102.6% to 106.2%)	104.6% (102.8% to 106.8%)	103.6% (103.1% to 104.1%)	104.6% (102.8% to 106.7%)
**Digestive diseases**	4.3% (3.3% to 5.6%)	4.7% (3.7% to 5.9%)	1.6% (1.2% to 2.1%)	4.5% (3.5% to 5.6%)
Peptic ulcer disease	6.7% (4.5% to 9.0%)	7.6% (5.2% to 10.3%)	6.2% (4.3% to 8.4%)	7.6% (5.2% to 10.2%)
Gallbladder and biliary diseases	21.2% (12.4% to 32.4%)	22.1% (13.2% to 33.1%)	23.1% (14.3% to 34.3%)	22.1% (13.2% to 33.1%)
Pancreatitis	24.6% (16.2% to 33.9%)	28.4% (20.1% to 37.6%)	22.8% (15.6% to 30.8%)	28.4% (20.0% to 37.6%)
**Neurological disorders**	12.8% (6.2% to 21.2%)	13.1% (7.7% to 19.6%)	3.0% (2.0% to 4.3%)	6.9% (4.2% to 10.4%)
Alzheimer's disease and other dementias	13.9% (6.4% to 23.9%)	14.9% (7.0% to 25.0%)	14.6% (6.9% to 24.5%)	14.8% (7.0% to 25.0%)
Parkinson's disease	-5.4% (-6.7% to -4.2%)	-6.4% (-7.8% to -5.0%)	-6.9% (-8.4% to -5.5%)	-6.5% (-7.9% to -5.1%)
Epilepsy	14.9% (10.5% to 19.4%)	14.6% (10.3% to 18.8%)	12.6% (8.7% to 17.1%)	13.7% (9.7% to 17.7%)
Multiple sclerosis	5.0% (2.7% to 8.0%)	5.0% (2.6% to 8.3%)	3.8% (2.3% to 5.4%)	4.6% (2.6% to 7.3%)
**Mental and substance use disorders**	103.1% (101.5% to 105.3%)	102.8% (100.8% to 105.3%)	20.1% (17.7% to 22.4%)	23.6% (21.1% to 26.3%)
Alcohol use disorders	103.3% (101.6% to 105.8%)	103.2% (101.4% to 105.8%)	102.0% (101.8% to 102.2%)	102.4% (101.8% to 103.3%)

Supplementary Results Table 1. Age-standardised PAFs attributable to the joint distribution of all risk factors for all ages, both sexes combined for each GBD level 1 and level 2 cause and all causes in 2016 for Low SDI. Results are shown for deaths, YLLs, YLDs, and DALYs
Data in parenthesis are 95% uncertainty intervals. PAFs=population attributable fractions. GBD=Global Burden of Disease. YLLs=years of life lost. YLDs=years live with disability. DALYs=disability-adjusted life-years

	Deaths 2016	YLLs 2016	YLDs 2016	DALYs 2016
Drug use disorders	103.0% (99.3% to 106.7%)	102.7% (98.7% to 107.0%)	96.7% (96.1% to 97.3%)	97.8% (96.9% to 98.8%)
Depressive disorders	-	-	10.5% (8.9% to 12.3%)	10.5% (8.9% to 12.3%)
Idiopathic developmental intellectual disability	-	-	62.7% (31.6% to 82.1%)	62.7% (31.6% to 82.1%)
**Diabetes, urogenital, blood, and endocrine diseases**	82.5% (81.0% to 83.7%)	75.2% (73.1% to 76.7%)	58.2% (54.2% to 61.8%)	69.1% (66.5% to 71.4%)
Diabetes mellitus	100.7% (99.8% to 101.7%)	100.5% (99.6% to 101.5%)	98.3% (97.8% to 98.7%)	99.8% (99.2% to 100.5%)
Chronic kidney disease	97.7% (95.1% to 100.0%)	98.3% (95.9% to 100.6%)	99.2% (98.9% to 99.6%)	98.6% (96.9% to 100.2%)
**Musculoskeletal disorders**	0.9% (0.3% to 1.6%)	0.7% (0.3% to 1.2%)	22.3% (19.8% to 25.2%)	21.9% (19.5% to 24.8%)
Rheumatoid arthritis	2.2% (0.8% to 4.0%)	2.2% (0.9% to 4.1%)	2.4% (1.0% to 4.2%)	2.4% (0.9% to 4.2%)
Osteoarthritis	-	-	12.8% (6.1% to 21.1%)	12.8% (6.1% to 21.1%)
Low back and neck pain	-	-	30.2% (27.6% to 33.0%)	30.2% (27.6% to 33.0%)
Gout	-	-	38.0% (30.5% to 48.1%)	38.0% (30.5% to 48.1%)
**Other non-communicable diseases**	0.2% (0.1% to 0.3%)	0.3% (0.1% to 0.5%)	9.0% (7.6% to 10.4%)	6.7% (5.6% to 7.8%)
Sense organ diseases	-	-	17.0% (15.1% to 19.0%)	17.0% (15.1% to 19.0%)
Sudden infant death syndrome	4.7% (3.0% to 6.3%)	4.8% (3.1% to 6.3%)	-	4.8% (3.1% to 6.3%)
**Transport injuries**	31.7% (28.5% to 35.4%)	28.0% (25.0% to 31.4%)	37.0% (33.6% to 40.9%)	29.0% (26.0% to 32.5%)
Road injuries	31.5% (28.3% to 35.2%)	27.8% (24.9% to 31.3%)	39.4% (35.8% to 43.4%)	29.0% (26.0% to 32.5%)
Other transport injuries	35.8% (32.2% to 39.7%)	31.0% (27.3% to 35.0%)	28.4% (25.4% to 31.7%)	30.1% (26.5% to 33.8%)
**Unintentional injuries**	30.8% (26.8% to 33.8%)	18.7% (17.2% to 20.5%)	24.2% (22.9% to 25.6%)	20.1% (18.8% to 21.7%)
Falls	61.9% (51.7% to 66.3%)	40.7% (36.9% to 43.3%)	33.2% (31.7% to 34.6%)	37.6% (35.3% to 39.5%)
Drowning	14.2% (12.1% to 16.8%)	13.4% (11.5% to 15.5%)	18.5% (15.8% to 21.9%)	13.5% (11.6% to 15.5%)
Fire, heat, and hot substances	11.1% (8.6% to 14.4%)	12.3% (10.1% to 15.4%)	21.3% (18.7% to 24.5%)	14.0% (11.6% to 17.0%)

Supplementary Results Table 1. Age-standardised PAFs attributable to the joint distribution of all risk factors for all ages, both sexes combined for each GBD level 1 and level 2 cause and all causes in 2016 for Low SDI. Results are shown for deaths, YLLs, YLDs, and DALYs
Data in parenthesis are 95% uncertainty intervals. PAFs=population attributable fractions. GBD=Global Burden of Disease. YLLs=years of life lost. YLDs=years live with disability. DALYs=disability-adjusted life-years

	**Deaths 2016**	**YLLs 2016**	**YLDs 2016**	**DALYs 2016**
Poisonings	14.9% (12.1% to 18.6%)	14.2% (11.3% to 17.8%)	20.6% (18.1% to 23.5%)	14.5% (11.6% to 18.1%)
Exposure to mechanical forces	23.7% (22.1% to 25.4%)	20.0% (18.2% to 22.0%)	26.5% (24.7% to 28.3%)	21.6% (19.9% to 23.5%)
Animal contact	10.7% (9.4% to 12.8%)	12.0% (10.5% to 14.6%)	15.7% (14.2% to 17.4%)	12.7% (11.3% to 15.1%)
Foreign body	8.9% (6.8% to 11.1%)	9.0% (5.8% to 12.0%)	14.2% (12.9% to 15.6%)	10.6% (7.9% to 12.8%)
Other unintentional injuries	18.6% (16.0% to 21.8%)	19.8% (17.4% to 22.6%)	18.0% (15.6% to 20.9%)	19.0% (16.8% to 21.8%)
**Self-harm and interpersonal violence**	16.2% (11.2% to 21.2%)	15.9% (11.7% to 20.2%)	22.2% (20.0% to 24.5%)	16.4% (12.4% to 20.4%)
Self-harm	15.9% (8.6% to 23.5%)	16.7% (9.4% to 23.8%)	15.0% (8.1% to 22.5%)	16.7% (9.3% to 23.8%)
Interpersonal violence	16.6% (12.1% to 21.1%)	14.9% (10.5% to 19.4%)	22.9% (20.7% to 25.4%)	16.1% (12.2% to 20.2%)
**Forces of nature, conflict and terrorism, and executions and police conflict**	0.3% (0.2% to 0.5%)	0.1% (0.1% to 0.1%)	0.5% (0.2% to 0.8%)	0.2% (0.1% to 0.3%)
Forces of nature, conflict and terrorism, and state actor violence	8.1% (7.6% to 8.6%)	3.4% (3.0% to 3.6%)	8.1% (5.9% to 10.3%)	4.8% (3.7% to 6.4%)

Supplementary Results Table 1. Age-standardised PAFs attributable to the joint distribution of all risk factors for all ages, both sexes combined for each GBD level 1 and level 2 cause and all causes in 2016 for Central Europe, Eastern Europe, and Central Asia. Results are shown for deaths,
Data in parenthesis are 95% uncertainty intervals. PAFs=population attributable fractions. GBD=Global Burden of Disease. YLLs=years of life lost. YLDs=years live with disability. DALYs=disability-adjusted life-years

	Deaths 2016	YLLs 2016	YLDs 2016	DALYs 2016
**All causes**	70.3% (68.6% to 71.7%)	64.5% (63.1% to 65.8%)	26.5% (25.0% to 27.9%)	51.3% (48.5% to 54.3%)
**HIV/AIDS and tuberculosis**	69.8% (66.5% to 73.0%)	68.8% (65.8% to 71.6%)	67.9% (63.8% to 71.5%)	68.8% (65.8% to 71.5%)
Tuberculosis	66.4% (59.1% to 73.2%)	65.9% (58.7% to 72.5%)	55.3% (48.6% to 61.6%)	65.5% (58.3% to 72.1%)
HIV/AIDS	72.8% (71.5% to 74.1%)	70.7% (69.3% to 72.1%)	74.2% (72.8% to 75.5%)	70.9% (69.5% to 72.3%)
**Diarrhoea, lower respiratory infections, and other common infectious diseases**	61.7% (56.4% to 67.0%)	67.6% (62.5% to 72.0%)	27.0% (20.7% to 33.6%)	60.2% (55.2% to 64.9%)
Diarrhoeal diseases	85.5% (75.9% to 93.3%)	92.8% (87.1% to 97.0%)	74.2% (58.8% to 87.5%)	82.5% (71.1% to 91.9%)
Lower respiratory infections	66.6% (60.9% to 72.3%)	74.6% (69.1% to 79.7%)	56.6% (49.8% to 63.9%)	74.5% (68.9% to 79.6%)
Upper respiratory infections	1.1% (0.5% to 2.1%)	2.5% (1.2% to 4.8%)	0.0% (0.0% to 0.0%)	0.0% (0.0% to 0.0%)
Otitis media	1.8% (1.1% to 2.6%)	4.3% (2.9% to 6.2%)	9.0% (6.1% to 11.5%)	9.0% (6.1% to 11.5%)
Meningitis	2.9% (2.4% to 3.7%)	4.4% (3.7% to 5.3%)	0.0% (0.0% to 0.0%)	3.5% (2.9% to 4.3%)
Encephalitis	1.6% (1.0% to 2.5%)	2.4% (1.4% to 3.6%)	0.0% (0.0% to 0.0%)	2.0% (1.2% to 3.1%)
Measles	31.5% (11.6% to 70.0%)	32.2% (11.8% to 71.7%)	33.0% (13.2% to 71.8%)	32.3% (11.8% to 71.7%)
**Maternal disorders**	6.3% (3.2% to 9.6%)	6.2% (3.0% to 9.5%)	5.4% (2.4% to 8.8%)	5.9% (2.9% to 9.1%)
Maternal haemorrhage	16.0% (5.8% to 25.9%)	16.0% (5.8% to 25.9%)	12.8% (4.7% to 20.9%)	15.0% (5.5% to 24.3%)
Maternal sepsis and other pregnancy related infections	15.6% (5.6% to 25.5%)	15.6% (5.7% to 25.5%)	14.0% (5.0% to 22.8%)	14.8% (5.4% to 24.1%)
Maternal abortion, miscarriage, and ectopic pregnancy	8.3% (5.0% to 12.2%)	8.1% (4.8% to 12.0%)	7.2% (3.6% to 11.4%)	7.9% (4.6% to 11.8%)
**Neonatal disorders**	75.8% (73.9% to 77.8%)	75.8% (74.0% to 77.8%)	0.1% (0.1% to 0.1%)	62.2% (57.6% to 66.5%)
Neonatal preterm birth complications	93.1% (91.7% to 94.3%)	93.1% (91.7% to 94.3%)	0.2% (0.2% to 0.3%)	77.6% (71.8% to 82.7%)
Neonatal encephalopathy due to birth asphyxia and trauma	67.9% (64.9% to 71.4%)	67.9% (64.9% to 71.5%)	0.0% (0.0% to 0.0%)	55.8% (48.2% to 62.2%)
Neonatal sepsis and other neonatal infections	56.0% (52.5% to 59.6%)	56.0% (52.5% to 59.7%)	0.0% (0.0% to 0.0%)	39.3% (27.3% to 48.3%)
Hemolytic disease and other neonatal jaundice	66.2% (62.9% to 69.7%)	66.2% (63.0% to 69.7%)	0.0% (0.0% to 0.0%)	24.5% (19.2% to 30.9%)

Supplementary Results Table 1. Age-standardised PAFs attributable to the joint distribution of all risk factors for all ages, both sexes combined for each GBD level 1 and level 2 cause and all causes in 2016 for Central Europe, Eastern Europe, and Central Asia. Results are shown for deaths,
Data in parenthesis are 95% uncertainty intervals. PAFs=population attributable fractions. GBD=Global Burden of Disease. YLLs=years of life lost. YLDs=years live with disability. DALYs=disability-adjusted life-years

	Deaths 2016	YLLs 2016	YLDs 2016	DALYs 2016
Other neonatal disorders	63.0% (60.1% to 66.0%)	63.1% (60.2% to 66.1%)	0.0% (0.0% to 0.0%)	60.2% (57.3% to 63.4%)
**Nutritional deficiencies**	16.6% (13.2% to 21.1%)	33.5% (27.4% to 41.0%)	91.2% (89.3% to 93.0%)	88.6% (86.0% to 90.7%)
Protein-energy malnutrition	24.3% (17.3% to 33.7%)	49.8% (39.6% to 61.1%)	96.9% (96.4% to 97.5%)	83.3% (77.7% to 88.5%)
Vitamin A deficiency	-	-	100.0% (100.0% to 100.0%)	100.0% (100.0% to 100.0%)
Iron-deficiency anaemia	100.0% (100.0% to 100.0%)	100.0% (100.0% to 100.0%)	100.0% (100.0% to 100.0%)	100.0% (100.0% to 100.0%)
**Other communicable, maternal, neonatal, and nutritional diseases**	7.1% (5.5% to 9.4%)	4.5% (3.3% to 6.3%)	72.3% (68.1% to 76.2%)	34.7% (26.6% to 44.4%)
Sexually transmitted diseases excluding HIV	60.5% (43.6% to 75.6%)	40.3% (24.7% to 57.4%)	100.0% (100.0% to 100.0%)	91.2% (84.0% to 95.9%)
Hepatitis	3.2% (2.5% to 4.0%)	2.4% (1.9% to 3.1%)	1.3% (0.9% to 1.9%)	2.3% (1.8% to 3.0%)
**Neoplasms**	46.6% (43.5% to 49.7%)	46.6% (43.7% to 49.5%)	39.5% (36.2% to 42.8%)	46.4% (43.5% to 49.4%)
Oesophageal cancer	85.0% (78.4% to 90.7%)	86.9% (81.0% to 91.9%)	85.4% (79.0% to 91.0%)	86.9% (81.0% to 91.9%)
Stomach cancer	22.6% (13.3% to 32.9%)	24.1% (14.6% to 34.1%)	21.9% (13.4% to 31.2%)	24.0% (14.6% to 34.0%)
Liver cancer	68.5% (61.5% to 74.7%)	67.9% (60.7% to 74.2%)	68.2% (61.3% to 74.4%)	67.9% (60.7% to 74.2%)
Larynx cancer	90.9% (86.6% to 94.1%)	91.9% (87.8% to 94.8%)	91.2% (87.1% to 94.3%)	91.9% (87.8% to 94.8%)
Tracheal, bronchus, and lung cancer	88.2% (85.8% to 90.2%)	89.3% (87.1% to 91.2%)	87.5% (85.1% to 89.8%)	89.2% (87.1% to 91.2%)
Breast cancer	31.1% (24.9% to 38.1%)	30.5% (24.4% to 37.1%)	30.7% (24.7% to 37.3%)	30.5% (24.4% to 37.1%)
Cervical cancer	100.0% (100.0% to 100.0%)	100.0% (100.0% to 100.0%)	100.0% (100.0% to 100.0%)	100.0% (100.0% to 100.0%)
Uterine cancer	51.9% (37.6% to 65.4%)	52.4% (37.8% to 65.9%)	53.2% (38.5% to 66.5%)	52.5% (37.9% to 65.9%)
Prostate cancer	7.1% (5.2% to 9.4%)	8.5% (6.2% to 11.2%)	8.6% (6.3% to 11.1%)	8.5% (6.2% to 11.1%)
Colon and rectum cancer	55.4% (47.0% to 63.0%)	57.1% (48.9% to 64.1%)	56.4% (48.1% to 63.8%)	57.0% (48.8% to 64.1%)
Lip and oral cavity cancer	84.9% (80.7% to 88.5%)	87.6% (84.1% to 90.6%)	82.1% (77.5% to 86.1%)	87.5% (83.9% to 90.5%)
Nasopharynx cancer	82.6% (79.6% to 85.4%)	80.0% (77.2% to 82.7%)	80.5% (77.6% to 82.9%)	80.0% (77.3% to 82.7%)

Supplementary Results Table 1. Age-standardised PAFs attributable to the joint distribution of all risk factors for all ages, both sexes combined for each GBD level 1 and level 2 cause and all causes in 2016 for Central Europe, Eastern Europe, and Central Asia. Results are shown for deaths,
Data in parenthesis are 95% uncertainty intervals. PAFs=population attributable fractions. GBD=Global Burden of Disease. YLLs=years of life lost. YLDs=years live with disability. DALYs=disability-adjusted life-years

	Deaths 2016	YLLs 2016	YLDs 2016	DALYs 2016
Other pharynx cancer	73.5% (65.6% to 79.8%)	74.9% (67.3% to 80.9%)	73.7% (65.9% to 80.0%)	74.9% (67.2% to 80.9%)
Gallbladder and biliary tract cancer	25.8% (15.0% to 38.6%)	25.8% (15.0% to 38.4%)	26.2% (15.2% to 39.1%)	25.8% (15.0% to 38.4%)
Pancreatic cancer	33.7% (26.4% to 41.5%)	36.5% (28.8% to 44.4%)	33.9% (26.7% to 41.4%)	36.4% (28.8% to 44.4%)
Ovarian cancer	11.7% (5.0% to 19.2%)	10.4% (4.2% to 17.4%)	10.2% (4.2% to 17.2%)	10.3% (4.2% to 17.4%)
Kidney cancer	42.1% (31.6% to 52.4%)	42.7% (32.2% to 52.8%)	41.4% (31.2% to 51.4%)	42.7% (32.1% to 52.7%)
Bladder cancer	39.1% (29.6% to 48.2%)	43.6% (33.7% to 53.1%)	41.0% (31.8% to 49.5%)	43.5% (33.5% to 52.9%)
Thyroid cancer	16.1% (8.6% to 25.4%)	16.3% (8.7% to 26.1%)	15.6% (8.7% to 24.1%)	16.2% (8.7% to 25.8%)
Mesothelioma	88.9% (85.6% to 91.5%)	81.7% (77.1% to 85.5%)	86.6% (83.0% to 89.6%)	81.8% (77.2% to 85.6%)
Non-Hodgkin's lymphoma	7.4% (3.0% to 12.6%)	6.6% (2.7% to 11.4%)	7.4% (2.9% to 12.6%)	6.7% (2.7% to 11.4%)
Multiple myeloma	9.5% (4.0% to 16.4%)	9.6% (4.2% to 16.4%)	9.8% (4.1% to 17.0%)	9.6% (4.2% to 16.4%)
Leukaemia	21.8% (14.0% to 29.5%)	17.6% (11.5% to 23.7%)	20.1% (13.0% to 27.3%)	17.6% (11.6% to 23.7%)
**Cardiovascular diseases**	89.4% (87.2% to 91.3%)	91.0% (89.7% to 92.3%)	84.1% (81.7% to 86.3%)	90.5% (89.1% to 91.8%)
Rheumatic heart disease	31.8% (22.5% to 46.9%)	30.0% (21.0% to 42.2%)	21.9% (14.4% to 30.9%)	29.2% (20.5% to 41.3%)
Ischaemic heart disease	94.2% (91.1% to 96.6%)	95.7% (93.8% to 97.1%)	93.2% (91.0% to 95.1%)	95.6% (93.7% to 97.0%)
Cerebrovascular disease	86.3% (81.5% to 90.8%)	90.5% (87.7% to 93.1%)	88.7% (85.1% to 92.0%)	90.3% (87.4% to 93.0%)
Hypertensive heart disease	100.0% (100.0% to 100.0%)	100.0% (100.0% to 100.0%)	100.0% (100.0% to 100.0%)	100.0% (100.0% to 100.0%)
Cardiomyopathy and myocarditis	68.8% (61.8% to 76.7%)	77.1% (72.4% to 82.5%)	59.4% (54.7% to 64.5%)	76.6% (71.9% to 81.9%)
Atrial fibrillation and flutter	56.9% (49.2% to 64.3%)	61.9% (55.2% to 68.0%)	69.4% (64.6% to 73.9%)	66.3% (60.5% to 71.5%)
Aortic aneurysm	55.2% (48.6% to 61.2%)	60.9% (55.2% to 65.9%)	-	60.9% (55.2% to 65.9%)
Peripheral vascular disease	54.8% (47.3% to 61.8%)	63.0% (56.9% to 68.4%)	60.3% (54.5% to 65.9%)	62.5% (56.5% to 68.0%)
Endocarditis	46.6% (38.1% to 55.5%)	44.4% (35.1% to 53.3%)	44.3% (35.9% to 52.6%)	44.4% (35.1% to 53.3%)

Supplementary Results Table 1. Age-standardised PAFs attributable to the joint distribution of all risk factors for all ages, both sexes combined for each GBD level 1 and level 2 cause and all causes in 2016 for Central Europe, Eastern Europe, and Central Asia. Results are shown for deaths,
Data in parenthesis are 95% uncertainty intervals. PAFs=population attributable fractions. GBD=Global Burden of Disease. YLLs=years of life lost. YLDs=years live with disability. DALYs=disability-adjusted life-years

	Deaths 2016	YLLs 2016	YLDs 2016	DALYs 2016
Other cardiovascular and circulatory diseases	55.6% (51.0% to 60.1%)	60.6% (56.4% to 64.4%)	58.0% (53.5% to 62.4%)	59.9% (55.7% to 63.9%)
**Chronic respiratory diseases**	67.8% (62.6% to 72.4%)	69.3% (64.9% to 73.3%)	43.1% (36.8% to 49.6%)	58.4% (53.3% to 63.3%)
Chronic obstructive pulmonary disease	72.9% (67.2% to 77.9%)	76.4% (71.4% to 80.7%)	70.6% (64.6% to 76.0%)	75.4% (70.5% to 79.8%)
Pneumoconiosis	100.0% (100.0% to 100.0%)	100.0% (100.0% to 100.0%)	100.0% (100.0% to 100.0%)	100.0% (100.0% to 100.0%)
Asthma	45.0% (35.0% to 55.7%)	47.7% (38.5% to 57.6%)	35.5% (27.8% to 44.4%)	37.3% (29.5% to 46.2%)
Other chronic respiratory diseases	18.1% (12.8% to 24.1%)	17.8% (12.3% to 23.7%)	7.2% (5.1% to 9.7%)	13.8% (9.7% to 19.0%)
**Cirrhosis and other chronic liver diseases**	54.9% (52.3% to 57.3%)	54.0% (51.4% to 56.4%)	40.8% (38.4% to 43.2%)	53.5% (51.0% to 56.0%)
Cirrhosis and other chronic liver diseases due to hepatitis B	2.2% (1.5% to 3.1%)	2.5% (1.6% to 3.5%)	2.1% (1.4% to 2.9%)	2.5% (1.6% to 3.5%)
Cirrhosis and other chronic liver diseases due to hepatitis C	86.5% (85.2% to 87.6%)	88.1% (87.3% to 88.8%)	86.3% (85.2% to 87.2%)	88.1% (87.3% to 88.7%)
Cirrhosis and other chronic liver diseases due to alcohol use	100.0% (100.0% to 100.0%)	100.0% (100.0% to 100.0%)	100.0% (100.0% to 100.0%)	100.0% (100.0% to 100.0%)
**Digestive diseases**	22.2% (18.9% to 25.8%)	28.5% (24.2% to 32.8%)	5.3% (4.2% to 6.5%)	22.3% (18.4% to 26.5%)
Peptic ulcer disease	19.2% (13.9% to 25.0%)	23.0% (17.0% to 29.6%)	16.2% (11.7% to 21.4%)	21.5% (15.7% to 27.8%)
Gallbladder and biliary diseases	46.9% (32.9% to 61.0%)	47.2% (33.6% to 60.9%)	50.0% (36.6% to 63.8%)	47.8% (34.4% to 61.4%)
Pancreatitis	58.3% (47.8% to 68.0%)	63.7% (53.1% to 72.7%)	46.1% (36.0% to 55.4%)	63.5% (52.9% to 72.4%)
**Neurological disorders**	20.4% (10.1% to 32.8%)	19.5% (11.9% to 28.5%)	3.8% (2.5% to 5.4%)	8.7% (5.4% to 12.7%)
Alzheimer's disease and other dementias	24.9% (11.8% to 39.9%)	26.3% (13.3% to 41.3%)	26.3% (13.2% to 41.4%)	26.3% (13.3% to 41.3%)
Parkinson's disease	-9.5% (-11.7% to -7.3%)	-12.9% (-16.1% to -9.9%)	-15.0% (-18.3% to -11.5%)	-13.4% (-16.5% to -10.3%)
Epilepsy	32.7% (26.2% to 38.8%)	28.8% (22.9% to 34.2%)	25.6% (19.6% to 30.9%)	27.2% (21.1% to 32.5%)
Multiple sclerosis	13.4% (8.7% to 18.2%)	14.1% (9.2% to 19.2%)	11.0% (7.3% to 14.8%)	12.7% (8.4% to 17.2%)
**Mental and substance use disorders**	100.0% (100.0% to 100.0%)	100.0% (100.0% to 100.0%)	28.7% (26.1% to 31.7%)	45.9% (41.0% to 51.9%)
Alcohol use disorders	100.0% (100.0% to 100.0%)	100.0% (100.0% to 100.0%)	100.0% (100.0% to 100.0%)	100.0% (100.0% to 100.0%)

Supplementary Results Table 1. Age-standardised PAFs attributable to the joint distribution of all risk factors for all ages, both sexes combined for each GBD level 1 and level 2 cause and all causes in 2016 for Central Europe, Eastern Europe, and Central Asia. Results are shown for deaths,
Data in parenthesis are 95% uncertainty intervals. PAFs=population attributable fractions. GBD=Global Burden of Disease. YLLs=years of life lost. YLDs=years live with disability. DALYs=disability-adjusted life-years

	Deaths 2016	YLLs 2016	YLDs 2016	DALYs 2016
Drug use disorders	100.0% (100.0% to 100.0%)	100.0% (100.0% to 100.0%)	100.0% (100.0% to 100.0%)	100.0% (100.0% to 100.0%)
Depressive disorders	-	-	8.5% (7.3% to 9.9%)	8.5% (7.3% to 9.9%)
Idiopathic developmental intellectual disability	-	-	56.5% (27.8% to 76.6%)	56.5% (27.8% to 76.6%)
**Diabetes, urogenital, blood, and endocrine diseases**	78.3% (77.4% to 79.3%)	73.9% (72.9% to 75.4%)	52.6% (48.6% to 56.5%)	61.6% (58.1% to 64.5%)
Diabetes mellitus	100.0% (100.0% to 100.0%)	100.0% (100.0% to 100.0%)	100.0% (100.0% to 100.0%)	100.0% (100.0% to 100.0%)
Chronic kidney disease	100.0% (100.0% to 100.0%)	100.0% (100.0% to 100.0%)	100.0% (100.0% to 100.0%)	100.0% (100.0% to 100.0%)
**Musculoskeletal disorders**	2.0% (0.8% to 3.3%)	2.0% (0.8% to 3.4%)	23.8% (19.8% to 27.8%)	23.6% (19.6% to 27.5%)
Rheumatoid arthritis	4.8% (1.9% to 8.0%)	5.8% (2.3% to 9.5%)	6.2% (2.5% to 10.1%)	6.1% (2.5% to 10.0%)
Osteoarthritis	-	-	30.2% (16.5% to 43.4%)	30.2% (16.5% to 43.4%)
Low back and neck pain	-	-	24.6% (21.2% to 28.1%)	24.6% (21.2% to 28.1%)
Gout	-	-	49.1% (33.8% to 64.7%)	49.1% (33.8% to 64.7%)
**Other non-communicable diseases**	0.3% (0.2% to 0.4%)	0.3% (0.2% to 0.4%)	4.0% (3.5% to 4.5%)	3.3% (2.9% to 3.7%)
Sense organ diseases	-	-	9.0% (8.1% to 10.1%)	9.0% (8.1% to 10.1%)
Sudden infant death syndrome	5.5% (4.5% to 6.7%)	5.5% (4.5% to 6.7%)	-	5.5% (4.5% to 6.7%)
**Transport injuries**	37.3% (29.2% to 46.3%)	34.5% (26.3% to 43.1%)	39.6% (32.5% to 47.2%)	35.6% (27.8% to 44.1%)
Road injuries	36.9% (28.9% to 45.9%)	34.2% (26.1% to 42.9%)	40.0% (33.0% to 47.7%)	35.5% (27.7% to 44.0%)
Other transport injuries	40.4% (31.9% to 49.3%)	37.2% (28.7% to 46.0%)	32.6% (25.6% to 40.1%)	36.5% (28.2% to 45.3%)
**Unintentional injuries**	19.9% (16.3% to 23.4%)	14.6% (11.4% to 17.9%)	18.7% (17.2% to 20.4%)	16.6% (14.2% to 18.9%)
Falls	39.0% (34.6% to 42.7%)	21.9% (19.3% to 24.7%)	21.9% (20.5% to 23.4%)	21.9% (20.2% to 23.7%)
Drowning	20.0% (13.3% to 27.4%)	17.9% (12.2% to 24.3%)	18.8% (12.6% to 26.1%)	17.9% (12.2% to 24.3%)
Fire, heat, and hot substances	19.2% (12.6% to 26.6%)	18.8% (12.7% to 25.9%)	18.5% (12.6% to 25.2%)	18.7% (12.6% to 25.3%)

Supplementary Results Table 1. Age-standardised PAFs attributable to the joint distribution of all risk factors for all ages, both sexes combined for each GBD level 1 and level 2 cause and all causes in 2016 for Central Europe, Eastern Europe, and Central Asia. Results are shown for deaths,
Data in parenthesis are 95% uncertainty intervals. PAFs=population attributable fractions. GBD=Global Burden of Disease. YLLs=years of life lost. YLDs=years live with disability. DALYs=disability-adjusted life-years

	**Deaths 2016**	**YLLs 2016**	**YLDs 2016**	**DALYs 2016**
Poisonings	19.6% (12.8% to 26.8%)	17.5% (11.7% to 23.4%)	17.9% (12.5% to 24.0%)	17.5% (11.7% to 23.4%)
Exposure to mechanical forces	15.3% (12.6% to 18.2%)	13.0% (10.6% to 15.7%)	16.1% (14.5% to 18.0%)	14.8% (12.8% to 17.0%)
Animal contact	10.7% (9.2% to 12.5%)	9.3% (7.9% to 11.0%)	7.7% (6.4% to 9.3%)	8.3% (7.0% to 9.8%)
Foreign body	7.5% (5.9% to 9.5%)	7.2% (5.7% to 9.1%)	6.3% (5.2% to 7.8%)	7.0% (5.7% to 8.7%)
Other unintentional injuries	23.1% (15.9% to 31.0%)	22.4% (15.7% to 29.8%)	17.3% (11.8% to 23.9%)	19.7% (13.7% to 26.3%)
**Self-harm and interpersonal violence**	40.1% (31.2% to 48.0%)	40.0% (31.4% to 47.8%)	31.9% (26.2% to 37.4%)	39.6% (31.2% to 47.2%)
Self-harm	42.8% (30.8% to 53.4%)	43.4% (31.3% to 53.7%)	35.0% (24.0% to 45.5%)	43.3% (31.3% to 53.5%)
Interpersonal violence	33.0% (23.9% to 42.7%)	31.7% (22.7% to 41.4%)	31.3% (25.4% to 37.6%)	31.6% (23.0% to 40.8%)
**Forces of nature, conflict and terrorism, and executions and police conflict**	0.0% (0.0% to 0.0%)	0.0% (0.0% to 0.0%)	1.0% (0.7% to 1.5%)	0.8% (0.5% to 1.2%)
Forces of nature, conflict and terrorism, and state actor violence	0.0% (0.0% to 0.0%)	0.0% (0.0% to 0.0%)	4.9% (3.4% to 7.0%)	4.9% (3.4% to 7.0%)

Supplementary Results Table 1. Age-standardised PAFs attributable to the joint distribution of all risk factors for all ages, both sexes combined for each GBD level 1 and level 2 cause and all causes in 2016 for High-income. Results are shown for deaths, YLLs, YLDs, and DALYs
Data in parenthesis are 95% uncertainty intervals. PAFs=population attributable fractions. GBD=Global Burden of Disease. YLLs=years of life lost. YLDs=years live with disability. DALYs=disability-adjusted life-years

	Deaths 2016	YLLs 2016	YLDs 2016	DALYs 2016
**All causes**	55.1% (53.0% to 57.1%)	53.0% (51.5% to 54.5%)	22.4% (21.0% to 23.7%)	36.9% (34.5% to 39.5%)
**HIV/AIDS and tuberculosis**	72.9% (68.7% to 76.6%)	79.3% (77.1% to 81.4%)	88.2% (85.7% to 89.9%)	82.1% (79.7% to 84.2%)
Tuberculosis	46.0% (35.8% to 55.5%)	50.0% (40.7% to 57.8%)	48.4% (39.5% to 56.5%)	49.8% (40.7% to 57.6%)
HIV/AIDS	90.3% (89.4% to 91.0%)	87.9% (86.9% to 88.6%)	91.6% (90.7% to 92.5%)	89.1% (88.1% to 90.1%)
**Diarrhoea, lower respiratory infections, and other common infectious diseases**	35.2% (28.2% to 42.8%)	41.0% (35.0% to 47.8%)	7.3% (5.3% to 9.6%)	29.1% (24.0% to 35.0%)
Diarrhoeal diseases	21.4% (14.4% to 32.2%)	38.9% (32.2% to 47.8%)	34.8% (26.1% to 45.0%)	36.9% (29.5% to 46.4%)
Lower respiratory infections	37.6% (29.9% to 46.2%)	45.5% (38.3% to 53.6%)	42.8% (35.2% to 51.8%)	45.5% (38.2% to 53.6%)
Upper respiratory infections	0.7% (0.5% to 1.1%)	2.0% (1.3% to 3.0%)	0.0% (0.0% to 0.0%)	0.0% (0.0% to 0.0%)
Otitis media	3.6% (2.6% to 4.8%)	7.4% (5.3% to 9.5%)	7.0% (4.8% to 9.2%)	7.0% (4.8% to 9.2%)
Meningitis	1.9% (1.7% to 2.2%)	3.5% (3.1% to 3.9%)	0.0% (0.0% to 0.0%)	3.1% (2.7% to 3.4%)
Encephalitis	0.5% (0.4% to 0.6%)	1.1% (0.9% to 1.3%)	0.0% (0.0% to 0.0%)	0.8% (0.6% to 0.9%)
Measles	11.9% (2.9% to 38.4%)	12.9% (3.1% to 41.8%)	12.1% (2.7% to 43.6%)	12.9% (3.1% to 41.8%)
**Maternal disorders**	2.4% (1.1% to 3.7%)	2.4% (1.1% to 3.7%)	2.7% (1.3% to 4.3%)	2.5% (1.2% to 3.8%)
Maternal haemorrhage	10.5% (3.8% to 17.1%)	10.5% (3.8% to 17.1%)	9.7% (3.5% to 15.7%)	10.2% (3.7% to 16.6%)
Maternal sepsis and other pregnancy related infections	10.9% (3.9% to 17.7%)	10.8% (3.9% to 17.7%)	9.8% (3.5% to 15.8%)	10.5% (3.8% to 17.1%)
Maternal abortion, miscarriage, and ectopic pregnancy	3.4% (2.0% to 5.0%)	3.5% (2.1% to 5.3%)	4.8% (2.5% to 7.5%)	3.9% (2.2% to 5.8%)
**Neonatal disorders**	78.4% (77.0% to 79.5%)	78.5% (77.1% to 79.5%)	0.1% (0.1% to 0.1%)	57.4% (52.0% to 62.1%)
Neonatal preterm birth complications	90.9% (90.2% to 91.5%)	91.0% (90.2% to 91.6%)	0.2% (0.1% to 0.2%)	65.1% (57.4% to 71.4%)
Neonatal encephalopathy due to birth asphyxia and trauma	63.8% (61.9% to 65.8%)	63.9% (61.9% to 65.9%)	0.0% (0.0% to 0.0%)	46.1% (39.0% to 52.3%)
Neonatal sepsis and other neonatal infections	57.6% (55.4% to 60.3%)	57.6% (55.4% to 60.3%)	0.0% (0.0% to 0.0%)	33.2% (20.5% to 44.5%)
Hemolytic disease and other neonatal jaundice	57.0% (54.0% to 59.6%)	57.1% (54.1% to 59.7%)	0.0% (0.0% to 0.0%)	15.6% (12.2% to 19.7%)

Supplementary Results Table 1. Age-standardised PAFs attributable to the joint distribution of all risk factors for all ages, both sexes combined for each GBD level 1 and level 2 cause and all causes in 2016 for High-income. Results are shown for deaths, YLLs, YLDs, and DALYs
Data in parenthesis are 95% uncertainty intervals. PAFs=population attributable fractions. GBD=Global Burden of Disease. YLLs=years of life lost. YLDs=years live with disability. DALYs=disability-adjusted life-years

	**Deaths 2016**	**YLLs 2016**	**YLDs 2016**	**DALYs 2016**
Other neonatal disorders	63.6% (61.7% to 65.7%)	63.7% (61.8% to 65.7%)	0.0% (0.0% to 0.0%)	60.6% (58.5% to 62.7%)
**Nutritional deficiencies**	3.9% (2.6% to 4.7%)	15.1% (13.1% to 17.4%)	78.0% (73.8% to 81.6%)	70.8% (65.5% to 75.8%)
Protein-energy malnutrition	2.4% (1.9% to 3.1%)	14.7% (12.1% to 18.6%)	98.3% (98.2% to 98.4%)	55.0% (46.2% to 63.7%)
Vitamin A deficiency	-	-	0.0% (0.0% to 0.0%)	0.0% (0.0% to 0.0%)
Iron-deficiency anaemia	100.0% (100.0% to 100.0%)	100.0% (100.0% to 100.0%)	100.0% (100.0% to 100.0%)	100.0% (100.0% to 100.0%)
**Other communicable, maternal, neonatal, and nutritional diseases**	5.8% (5.1% to 6.6%)	5.1% (4.2% to 6.0%)	70.1% (64.2% to 76.1%)	28.1% (20.2% to 39.4%)
Sexually transmitted diseases excluding HIV	53.9% (26.8% to 88.5%)	30.6% (10.5% to 71.2%)	99.9% (99.9% to 100.0%)	79.6% (56.5% to 97.0%)
Hepatitis	7.3% (5.8% to 9.1%)	8.2% (6.4% to 10.3%)	3.4% (2.6% to 4.4%)	7.7% (6.1% to 9.7%)
**Neoplasms**	46.0% (43.5% to 48.8%)	45.5% (43.2% to 48.0%)	34.1% (31.2% to 37.1%)	45.0% (42.7% to 47.6%)
Oesophageal cancer	81.9% (74.6% to 88.0%)	82.8% (75.6% to 88.8%)	79.7% (72.0% to 86.1%)	82.8% (75.6% to 88.8%)
Stomach cancer	20.8% (12.2% to 30.5%)	20.0% (11.9% to 28.8%)	20.2% (11.8% to 29.7%)	20.0% (11.9% to 28.9%)
Liver cancer	59.1% (52.7% to 65.1%)	61.2% (54.8% to 67.1%)	57.5% (51.0% to 63.9%)	61.1% (54.8% to 67.1%)
Larynx cancer	88.9% (83.4% to 92.8%)	88.7% (83.0% to 92.5%)	87.4% (81.5% to 91.5%)	88.6% (82.9% to 92.4%)
Tracheal, bronchus, and lung cancer	87.9% (86.0% to 89.7%)	87.4% (85.5% to 89.1%)	86.8% (84.8% to 88.7%)	87.3% (85.5% to 89.1%)
Breast cancer	35.6% (29.0% to 42.9%)	34.4% (28.2% to 41.0%)	34.7% (28.4% to 41.6%)	34.5% (28.2% to 41.0%)
Cervical cancer	100.0% (100.0% to 100.0%)	100.0% (100.0% to 100.0%)	100.0% (100.0% to 100.0%)	100.0% (100.0% to 100.0%)
Uterine cancer	43.7% (31.3% to 56.5%)	44.8% (32.1% to 57.5%)	46.6% (33.8% to 59.2%)	45.0% (32.3% to 57.8%)
Prostate cancer	6.2% (4.5% to 8.2%)	6.4% (4.7% to 8.0%)	6.6% (4.9% to 8.3%)	6.4% (4.7% to 8.1%)
Colon and rectum cancer	56.0% (47.6% to 64.1%)	56.8% (48.7% to 64.3%)	55.9% (47.7% to 63.7%)	56.7% (48.7% to 64.3%)
Lip and oral cavity cancer	80.2% (75.1% to 84.5%)	81.5% (76.7% to 85.5%)	79.7% (74.8% to 84.0%)	81.4% (76.6% to 85.5%)
Nasopharynx cancer	81.2% (77.7% to 84.2%)	79.3% (76.0% to 82.1%)	77.9% (74.7% to 80.9%)	79.2% (76.0% to 82.0%)

**Supplementary Results Table 1. Age-standardised PAFs attributable to the joint distribution of all risk factors for all ages, both sexes combined for each GBD level 1 and level 2 cause and all causes in 2016 for High-income. Results are shown for deaths, YLLs, YLDs, and DALYs** Data in parenthesis are 95% uncertainty intervals. PAFs=population attributable fractions. GBD=Global Burden of Disease. YLLs=years of life lost. YLDs=years live with disability. DALYs=disability-adjusted life-years	**Deaths 2016**	**YLLs 2016**	**YLDs 2016**	**DALYs 2016**
Other pharynx cancer	62.5% (53.1% to 70.9%)	64.8% (55.1% to 72.9%)	62.7% (52.9% to 71.1%)	64.7% (55.1% to 72.9%)
Gallbladder and biliary tract cancer	16.1% (8.4% to 26.4%)	17.1% (9.2% to 27.5%)	16.7% (8.8% to 27.2%)	17.1% (9.2% to 27.5%)
Pancreatic cancer	33.3% (26.3% to 41.3%)	33.6% (26.7% to 41.2%)	32.5% (25.5% to 40.2%)	33.6% (26.6% to 41.2%)
Ovarian cancer	15.1% (7.9% to 23.7%)	12.3% (5.8% to 20.0%)	12.1% (5.8% to 19.4%)	12.3% (5.8% to 20.0%)
Kidney cancer	37.5% (28.2% to 47.8%)	37.6% (28.4% to 47.5%)	38.1% (28.8% to 48.0%)	37.6% (28.4% to 47.5%)
Bladder cancer	39.7% (29.7% to 49.1%)	40.0% (30.7% to 49.1%)	39.3% (30.1% to 48.5%)	39.9% (30.6% to 49.1%)
Thyroid cancer	13.3% (6.7% to 22.0%)	14.3% (7.1% to 23.5%)	14.5% (7.7% to 23.3%)	14.3% (7.2% to 23.5%)
Mesothelioma	96.5% (95.6% to 97.3%)	92.9% (91.4% to 94.4%)	95.4% (94.3% to 96.4%)	93.0% (91.4% to 94.4%)
Non-Hodgkin's lymphoma	7.1% (3.0% to 12.4%)	7.2% (3.0% to 12.4%)	7.4% (3.2% to 12.7%)	7.2% (3.0% to 12.4%)
Multiple myeloma	8.2% (3.6% to 14.4%)	8.6% (3.9% to 14.9%)	8.6% (3.8% to 14.9%)	8.6% (3.9% to 14.9%)
Leukaemia	20.7% (13.2% to 28.1%)	17.6% (11.4% to 23.7%)	18.6% (11.9% to 25.2%)	17.7% (11.4% to 23.7%)
**Cardiovascular diseases**	80.6% (77.9% to 82.9%)	82.5% (80.6% to 84.1%)	72.7% (69.7% to 75.4%)	80.8% (78.8% to 82.5%)
Rheumatic heart disease	25.3% (15.1% to 45.7%)	24.1% (16.3% to 38.0%)	22.6% (15.1% to 35.1%)	24.0% (16.3% to 37.9%)
Ischaemic heart disease	91.9% (88.1% to 95.1%)	93.8% (91.5% to 95.8%)	90.3% (87.5% to 93.0%)	93.6% (91.2% to 95.6%)
Cerebrovascular disease	80.8% (75.0% to 86.2%)	85.1% (81.6% to 88.3%)	83.6% (79.4% to 87.5%)	84.7% (81.1% to 88.0%)
Hypertensive heart disease	100.0% (100.0% to 100.0%)	100.0% (100.0% to 100.0%)	100.0% (100.0% to 100.0%)	100.0% (100.0% to 100.0%)
Cardiomyopathy and myocarditis	42.2% (35.3% to 50.8%)	42.7% (36.6% to 48.4%)	42.0% (36.4% to 48.2%)	42.6% (36.8% to 48.4%)
Atrial fibrillation and flutter	53.3% (46.2% to 60.4%)	57.3% (51.2% to 63.4%)	61.8% (57.0% to 66.7%)	60.1% (54.7% to 65.4%)
Aortic aneurysm	41.3% (34.1% to 48.6%)	46.6% (40.7% to 52.4%)	-	46.6% (40.7% to 52.4%)
Peripheral vascular disease	51.0% (45.0% to 57.8%)	57.3% (52.4% to 62.1%)	57.8% (52.9% to 62.7%)	57.5% (52.7% to 62.2%)
Endocarditis	37.5% (28.3% to 49.1%)	36.6% (28.8% to 45.4%)	38.8% (29.8% to 48.8%)	36.6% (28.9% to 45.5%)

Supplementary Results Table 1. Age-standardised PAFs attributable to the joint distribution of all risk factors for all ages, both sexes combined for each GBD level 1 and level 2 cause and all causes in 2016 for High-income. Results are shown for deaths, YLLs, YLDs, and DALYs
Data in parenthesis are 95% uncertainty intervals. PAFs=population attributable fractions. GBD=Global Burden of Disease. YLLs=years of life lost. YLDs=years live with disability. DALYs=disability-adjusted life-years

	Deaths 2016	YLLs 2016	YLDs 2016	DALYs 2016
Other cardiovascular and circulatory diseases	42.4% (37.0% to 48.1%)	46.2% (41.6% to 50.7%)	48.6% (43.9% to 53.5%)	47.2% (42.4% to 51.8%)
**Chronic respiratory diseases**	69.1% (65.8% to 73.5%)	67.1% (64.0% to 71.2%)	48.3% (42.1% to 54.8%)	57.6% (53.0% to 62.2%)
Chronic obstructive pulmonary disease	80.4% (76.9% to 83.7%)	80.8% (77.5% to 83.8%)	74.3% (70.2% to 78.2%)	78.7% (75.3% to 81.9%)
Pneumoconiosis	100.0% (100.0% to 100.0%)	100.0% (100.0% to 100.0%)	100.0% (100.0% to 100.0%)	100.0% (100.0% to 100.0%)
Asthma	38.9% (30.3% to 48.9%)	43.6% (35.5% to 52.8%)	34.4% (26.5% to 43.6%)	35.2% (27.3% to 44.5%)
Other chronic respiratory diseases	19.3% (15.5% to 23.0%)	14.2% (11.1% to 17.5%)	9.8% (7.7% to 12.5%)	12.5% (9.9% to 15.5%)
**Cirrhosis and other chronic liver diseases**	63.2% (60.8% to 65.5%)	66.1% (63.9% to 68.6%)	55.7% (53.7% to 57.6%)	65.6% (63.3% to 68.0%)
Cirrhosis and other chronic liver diseases due to hepatitis B	3.0% (2.1% to 4.0%)	3.7% (2.6% to 5.0%)	2.9% (2.1% to 4.0%)	3.7% (2.6% to 5.0%)
Cirrhosis and other chronic liver diseases due to hepatitis C	71.8% (69.3% to 73.9%)	80.0% (78.4% to 81.5%)	77.0% (75.1% to 78.7%)	79.9% (78.2% to 81.3%)
Cirrhosis and other chronic liver diseases due to alcohol use	100.0% (100.0% to 100.0%)	100.0% (100.0% to 100.0%)	99.9% (99.9% to 99.9%)	100.0% (100.0% to 100.0%)
**Digestive diseases**	9.6% (7.6% to 11.8%)	11.3% (9.2% to 13.7%)	3.4% (2.7% to 4.2%)	8.7% (7.0% to 10.7%)
Peptic ulcer disease	10.7% (7.5% to 14.4%)	14.3% (10.2% to 19.0%)	11.8% (8.4% to 15.9%)	13.7% (9.7% to 18.2%)
Gallbladder and biliary diseases	37.7% (25.4% to 51.4%)	40.1% (27.3% to 53.6%)	45.1% (32.4% to 58.6%)	40.9% (28.4% to 54.3%)
Pancreatitis	36.8% (26.2% to 48.0%)	40.2% (29.8% to 51.6%)	32.6% (20.6% to 44.7%)	39.7% (29.1% to 51.1%)
**Neurological disorders**	19.3% (9.6% to 30.9%)	17.9% (10.0% to 27.2%)	4.6% (3.0% to 6.6%)	9.2% (5.4% to 13.9%)
Alzheimer's disease and other dementias	22.8% (11.4% to 36.4%)	24.1% (12.7% to 37.6%)	24.1% (12.5% to 37.6%)	24.1% (12.7% to 37.6%)
Parkinson's disease	-6.8% (-7.8% to -5.6%)	-8.7% (-10.0% to -7.2%)	-9.9% (-11.4% to -8.2%)	-8.9% (-10.3% to -7.4%)
Epilepsy	34.1% (25.8% to 41.4%)	31.4% (23.9% to 37.9%)	25.5% (18.7% to 31.5%)	27.3% (20.3% to 33.6%)
Multiple sclerosis	8.9% (5.8% to 12.2%)	9.7% (6.3% to 13.3%)	8.6% (5.5% to 11.8%)	9.0% (5.9% to 12.4%)
**Mental and substance use disorders**	99.6% (99.6% to 99.6%)	99.5% (99.5% to 99.6%)	23.6% (21.4% to 26.0%)	31.0% (28.1% to 34.1%)
Alcohol use disorders	100.0% (100.0% to 100.0%)	100.0% (100.0% to 100.0%)	100.0% (99.9% to 100.0%)	100.0% (100.0% to 100.0%)

Supplementary Results Table 1. Age-standardised PAFs attributable to the joint distribution of all risk factors for all ages, both sexes combined for each GBD level 1 and level 2 cause and all causes in 2016 for High-income. Results are shown for deaths, YLLs, YLDs, and DALYs
Data in parenthesis are 95% uncertainty intervals. PAFs=population attributable fractions. GBD=Global Burden of Disease. YLLs=years of life lost. YLDs=years live with disability. DALYs=disability-adjusted life-years

	Deaths 2016	YLLs 2016	YLDs 2016	DALYs 2016
Drug use disorders	100.0% (100.0% to 100.0%)	100.0% (100.0% to 100.0%)	100.0% (100.0% to 100.0%)	100.0% (100.0% to 100.0%)
Depressive disorders	-	-	8.6% (7.3% to 10.3%)	8.6% (7.3% to 10.3%)
Idiopathic developmental intellectual disability	-	-	54.7% (28.1% to 72.8%)	54.7% (28.1% to 72.8%)
**Diabetes, urogenital, blood, and endocrine diseases**	75.5% (74.5% to 77.9%)	70.4% (68.9% to 73.2%)	65.3% (61.3% to 69.0%)	67.4% (64.8% to 69.8%)
Diabetes mellitus	100.0% (100.0% to 100.0%)	100.0% (100.0% to 100.0%)	100.0% (100.0% to 100.0%)	100.0% (100.0% to 100.0%)
Chronic kidney disease	100.0% (100.0% to 100.0%)	100.0% (100.0% to 100.0%)	100.0% (100.0% to 100.0%)	100.0% (100.0% to 100.0%)
**Musculoskeletal disorders**	1.0% (0.4% to 1.8%)	1.1% (0.4% to 1.8%)	16.5% (13.9% to 19.3%)	16.2% (13.8% to 19.0%)
Rheumatoid arthritis	3.7% (1.5% to 6.3%)	4.7% (1.9% to 7.8%)	5.4% (2.2% to 9.0%)	5.3% (2.1% to 8.9%)
Osteoarthritis	-	-	26.4% (14.8% to 38.3%)	26.4% (14.8% to 38.3%)
Low back and neck pain	-	-	20.2% (17.6% to 23.0%)	20.2% (17.6% to 23.0%)
Gout	-	-	48.0% (31.8% to 64.6%)	48.0% (31.8% to 64.6%)
**Other non-communicable diseases**	0.4% (0.3% to 0.5%)	0.5% (0.5% to 0.6%)	2.3% (2.1% to 2.7%)	2.1% (1.8% to 2.3%)
Sense organ diseases	-	-	7.5% (6.8% to 8.4%)	7.5% (6.8% to 8.4%)
Sudden infant death syndrome	3.9% (3.6% to 4.3%)	3.9% (3.6% to 4.3%)	-	3.9% (3.6% to 4.3%)
**Transport injuries**	37.2% (29.8% to 45.1%)	34.3% (26.9% to 42.5%)	41.5% (34.8% to 48.3%)	36.2% (28.9% to 44.1%)
Road injuries	36.9% (29.6% to 44.9%)	34.1% (26.6% to 42.2%)	42.0% (35.2% to 48.9%)	36.1% (28.8% to 44.0%)
Other transport injuries	40.5% (33.3% to 48.3%)	37.3% (29.9% to 45.4%)	37.8% (31.4% to 44.7%)	37.5% (30.6% to 44.9%)
**Unintentional injuries**	34.9% (31.2% to 37.0%)	21.7% (19.7% to 23.8%)	22.9% (21.5% to 24.5%)	22.5% (21.0% to 24.1%)
Falls	62.3% (59.0% to 64.3%)	41.9% (39.0% to 43.5%)	25.4% (24.3% to 26.5%)	29.2% (27.5% to 30.9%)
Drowning	18.3% (13.8% to 24.0%)	18.0% (14.2% to 22.5%)	17.9% (13.0% to 24.6%)	18.0% (14.1% to 22.6%)
Fire, heat, and hot substances	15.7% (10.9% to 21.9%)	17.1% (13.0% to 22.1%)	22.9% (17.7% to 29.5%)	20.2% (15.4% to 26.2%)

Supplementary Results Table 1. Age-standardised PAFs attributable to the joint distribution of all risk factors for all ages, both sexes combined for each GBD level 1 and level 2 cause and all causes in 2016 for High-income. Results are shown for deaths, YLLs, YLDs, and DALYs
Data in parenthesis are 95% uncertainty intervals. PAFs=population attributable fractions. GBD=Global Burden of Disease. YLLs=years of life lost. YLDs=years live with disability. DALYs=disability-adjusted life-years

	**Deaths 2016**	**YLLs 2016**	**YLDs 2016**	**DALYs 2016**
Poisonings	21.9% (16.5% to 28.3%)	21.9% (16.9% to 27.9%)	20.5% (15.7% to 26.3%)	21.4% (16.5% to 26.9%)
Exposure to mechanical forces	18.7% (17.1% to 21.0%)	15.3% (13.5% to 18.3%)	22.9% (21.6% to 24.3%)	20.0% (18.4% to 21.8%)
Animal contact	14.7% (13.6% to 15.9%)	12.4% (11.1% to 13.9%)	12.6% (11.8% to 13.5%)	12.6% (11.7% to 13.5%)
Foreign body	6.5% (5.6% to 8.0%)	9.3% (8.2% to 10.6%)	9.4% (8.3% to 10.5%)	9.3% (8.3% to 10.4%)
Other unintentional injuries	28.2% (22.5% to 35.1%)	28.2% (22.9% to 34.8%)	21.9% (16.5% to 28.6%)	22.8% (17.5% to 29.3%)
**Self-harm and interpersonal violence**	32.5% (22.2% to 42.0%)	33.4% (23.7% to 42.3%)	31.4% (26.7% to 36.3%)	33.3% (24.2% to 41.7%)
Self-harm	33.2% (20.6% to 44.8%)	35.0% (22.6% to 46.4%)	29.1% (16.5% to 40.6%)	34.9% (22.4% to 46.2%)
Interpersonal violence	29.7% (22.4% to 37.2%)	27.9% (20.9% to 35.3%)	32.2% (27.1% to 37.0%)	28.8% (22.1% to 35.5%)
**Forces of nature, conflict and terrorism, and executions and police conflict**	2.2% (0.8% to 3.3%)	0.7% (0.3% to 1.1%)	3.0% (1.8% to 4.6%)	1.8% (1.0% to 3.1%)
Forces of nature, conflict and terrorism, and state actor violence	8.2% (7.7% to 8.6%)	3.2% (2.9% to 3.4%)	8.1% (5.3% to 11.9%)	6.1% (3.9% to 9.3%)

Supplementary Results Table 1. Age-standardised PAFs attributable to the joint distribution of all risk factors for all ages, both sexes combined for each GBD level 1 and level 2 cause and all causes in 2016 for Latin America and Caribbean. Results are shown for deaths, YLLs, YLDs, and
Data in parenthesis are 95% uncertainty intervals. PAFs=population attributable fractions. GBD=Global Burden of Disease. YLLs=years of life lost. YLDs=years live with disability. DALYs=disability-adjusted life-years

	Deaths 2016	YLLs 2016	YLDs 2016	DALYs 2016
**All causes**	56.3% (54.7% to 57.9%)	51.9% (50.7% to 53.0%)	24.3% (23.1% to 25.6%)	41.4% (39.3% to 43.5%)
**HIV/AIDS and tuberculosis**	75.1% (72.4% to 77.9%)	76.0% (73.7% to 78.4%)	83.3% (79.2% to 87.0%)	76.4% (74.0% to 78.8%)
Tuberculosis	47.0% (39.9% to 54.4%)	47.6% (41.1% to 54.5%)	44.5% (38.0% to 50.9%)	47.5% (41.0% to 54.3%)
HIV/AIDS	88.2% (85.9% to 89.8%)	84.7% (82.2% to 86.5%)	89.8% (87.4% to 91.7%)	85.0% (82.5% to 86.9%)
**Diarrhoea, lower respiratory infections, and other common infectious diseases**	51.3% (46.0% to 57.7%)	60.5% (56.0% to 65.3%)	39.9% (31.1% to 48.4%)	56.6% (52.2% to 61.3%)
Diarrhoeal diseases	78.3% (65.1% to 89.1%)	87.4% (79.3% to 93.7%)	76.2% (61.9% to 88.6%)	82.9% (72.4% to 91.7%)
Lower respiratory infections	50.8% (44.7% to 58.1%)	63.5% (57.5% to 69.4%)	53.6% (47.5% to 60.9%)	63.4% (57.4% to 69.3%)
Upper respiratory infections	1.4% (0.9% to 2.2%)	2.8% (1.8% to 4.5%)	0.0% (0.0% to 0.0%)	0.0% (0.0% to 0.0%)
Otitis media	3.3% (2.4% to 4.2%)	6.2% (4.7% to 7.8%)	5.1% (3.4% to 6.7%)	5.1% (3.4% to 6.7%)
Meningitis	2.4% (1.8% to 3.3%)	3.5% (2.7% to 4.7%)	0.0% (0.0% to 0.0%)	3.2% (2.5% to 4.4%)
Encephalitis	1.3% (1.1% to 1.5%)	1.9% (1.7% to 2.2%)	0.0% (0.0% to 0.0%)	1.6% (1.4% to 1.9%)
Measles	23.5% (8.8% to 56.8%)	24.7% (9.2% to 59.8%)	20.2% (5.5% to 58.0%)	24.6% (9.1% to 59.7%)
**Maternal disorders**	5.8% (2.8% to 8.8%)	5.6% (2.8% to 8.7%)	3.6% (2.0% to 5.6%)	5.4% (2.7% to 8.2%)
Maternal haemorrhage	15.2% (5.5% to 24.8%)	15.1% (5.4% to 24.6%)	14.1% (5.0% to 22.9%)	15.0% (5.4% to 24.5%)
Maternal sepsis and other pregnancy related infections	15.0% (5.4% to 24.4%)	14.9% (5.3% to 24.1%)	13.9% (5.0% to 22.7%)	14.7% (5.3% to 23.9%)
Maternal abortion, miscarriage, and ectopic pregnancy	11.4% (7.2% to 15.9%)	11.2% (7.1% to 15.9%)	10.5% (5.9% to 15.4%)	11.1% (7.0% to 15.7%)
**Neonatal disorders**	73.7% (72.2% to 75.2%)	73.7% (72.2% to 75.3%)	0.1% (0.1% to 0.2%)	65.7% (62.7% to 68.4%)
Neonatal preterm birth complications	92.3% (91.5% to 93.0%)	92.3% (91.6% to 93.1%)	0.2% (0.2% to 0.3%)	80.4% (76.0% to 84.0%)
Neonatal encephalopathy due to birth asphyxia and trauma	63.3% (61.0% to 65.9%)	63.4% (61.1% to 65.9%)	0.0% (0.0% to 0.0%)	59.3% (54.6% to 63.2%)
Neonatal sepsis and other neonatal infections	56.4% (54.3% to 59.0%)	56.5% (54.4% to 59.0%)	0.0% (0.0% to 0.0%)	49.0% (40.7% to 54.2%)
Hemolytic disease and other neonatal jaundice	59.8% (55.2% to 63.0%)	59.9% (55.3% to 63.1%)	0.0% (0.0% to 0.0%)	28.6% (23.9% to 34.4%)

Supplementary Results Table 1. Age-standardised PAFs attributable to the joint distribution of all risk factors for all ages, both sexes combined for each GBD level 1 and level 2 cause and all causes in 2016 for Latin America and Caribbean. Results are shown for deaths, YLLs, YLDs, and
Data in parenthesis are 95% uncertainty intervals. PAFs=population attributable fractions. GBD=Global Burden of Disease. YLLs=years of life lost. YLDs=years live with disability. DALYs=disability-adjusted life-years

	Deaths 2016	YLLs 2016	YLDs 2016	DALYs 2016
Other neonatal disorders	58.8% (56.7% to 61.1%)	58.9% (56.7% to 61.1%)	0.0% (0.0% to 0.0%)	57.2% (55.0% to 59.6%)
**Nutritional deficiencies**	11.2% (10.2% to 12.5%)	42.0% (39.6% to 44.8%)	91.9% (90.6% to 93.0%)	74.1% (68.9% to 78.7%)
Protein-energy malnutrition	11.9% (10.8% to 13.6%)	45.6% (42.7% to 49.2%)	96.2% (95.9% to 96.6%)	51.1% (48.0% to 54.7%)
Vitamin A deficiency	-	-	99.7% (99.7% to 99.7%)	99.7% (99.7% to 99.7%)
Iron-deficiency anaemia	99.8% (99.6% to 99.9%)	99.7% (99.5% to 99.8%)	99.5% (99.4% to 99.5%)	99.5% (99.4% to 99.5%)
**Other communicable, maternal, neonatal, and nutritional diseases**	7.6% (6.5% to 8.6%)	5.7% (4.4% to 6.9%)	64.9% (59.3% to 71.0%)	24.2% (17.1% to 33.5%)
Sexually transmitted diseases excluding HIV	28.5% (17.8% to 42.0%)	13.6% (7.8% to 21.9%)	99.6% (99.5% to 99.7%)	50.3% (33.2% to 68.9%)
Hepatitis	2.0% (1.5% to 2.7%)	2.2% (1.7% to 2.9%)	1.6% (1.0% to 2.4%)	2.2% (1.6% to 2.8%)
**Neoplasms**	34.1% (31.4% to 36.9%)	33.4% (31.0% to 36.0%)	29.2% (26.6% to 32.0%)	33.3% (30.9% to 36.0%)
Oesophageal cancer	71.6% (62.3% to 80.2%)	73.6% (64.7% to 82.0%)	72.0% (62.7% to 80.8%)	73.6% (64.7% to 82.0%)
Stomach cancer	11.0% (5.0% to 19.1%)	10.5% (4.8% to 18.0%)	10.9% (5.0% to 18.7%)	10.5% (4.8% to 18.0%)
Liver cancer	49.3% (41.7% to 57.8%)	50.0% (42.4% to 58.8%)	49.5% (41.9% to 58.1%)	50.0% (42.4% to 58.7%)
Larynx cancer	75.3% (66.3% to 82.1%)	76.2% (67.7% to 82.9%)	76.2% (67.5% to 82.8%)	76.2% (67.7% to 82.9%)
Tracheal, bronchus, and lung cancer	77.0% (73.4% to 80.4%)	76.3% (72.8% to 79.7%)	76.4% (72.7% to 79.8%)	76.3% (72.8% to 79.8%)
Breast cancer	24.2% (17.3% to 32.4%)	22.2% (15.4% to 30.0%)	23.2% (16.4% to 31.2%)	22.3% (15.4% to 30.1%)
Cervical cancer	99.5% (99.4% to 99.5%)	99.5% (99.4% to 99.6%)	99.6% (99.5% to 99.6%)	99.5% (99.4% to 99.6%)
Uterine cancer	44.7% (31.7% to 58.3%)	46.5% (33.1% to 60.3%)	48.0% (34.4% to 61.7%)	46.6% (33.2% to 60.4%)
Prostate cancer	2.6% (1.8% to 3.5%)	2.6% (1.9% to 3.4%)	2.7% (1.9% to 3.5%)	2.6% (1.9% to 3.4%)
Colon and rectum cancer	51.5% (42.5% to 60.0%)	52.6% (44.1% to 60.7%)	51.8% (43.0% to 60.3%)	52.6% (44.1% to 60.7%)
Lip and oral cavity cancer	67.5% (60.2% to 73.7%)	70.5% (63.8% to 76.2%)	66.3% (59.1% to 72.5%)	70.4% (63.6% to 76.0%)
Nasopharynx cancer	66.8% (62.2% to 71.1%)	65.1% (61.2% to 68.6%)	64.6% (60.7% to 68.2%)	65.1% (61.2% to 68.6%)

Supplementary Results Table 1. Age-standardised PAFs attributable to the joint distribution of all risk factors for all ages, both sexes combined for each GBD level 1 and level 2 cause and all causes in 2016 for Latin America and Caribbean. Results are shown for deaths, YLLs, YLDs, and
Data in parenthesis are 95% uncertainty intervals. PAFs=population attributable fractions. GBD=Global Burden of Disease. YLLs=years of life lost. YLDs=years live with disability. DALYs=disability-adjusted life-years

	**Deaths 2016**	**YLLs 2016**	**YLDs 2016**	**DALYs 2016**
Other pharynx cancer	53.0% (41.9% to 62.9%)	57.1% (46.0% to 66.6%)	55.2% (44.5% to 64.9%)	57.0% (46.0% to 66.6%)
Gallbladder and biliary tract cancer	23.5% (13.6% to 35.7%)	24.6% (14.4% to 37.1%)	23.8% (13.8% to 36.0%)	24.6% (14.4% to 37.1%)
Pancreatic cancer	23.5% (16.4% to 32.4%)	23.3% (16.3% to 32.2%)	23.3% (16.2% to 32.3%)	23.3% (16.3% to 32.2%)
Ovarian cancer	12.4% (5.0% to 21.3%)	11.0% (4.1% to 19.3%)	10.8% (4.2% to 18.8%)	11.0% (4.1% to 19.3%)
Kidney cancer	28.1% (19.7% to 38.0%)	26.6% (18.5% to 35.9%)	27.5% (19.2% to 37.1%)	26.6% (18.6% to 36.0%)
Bladder cancer	24.4% (16.7% to 32.9%)	23.7% (16.3% to 32.0%)	23.9% (16.5% to 32.2%)	23.7% (16.4% to 32.0%)
Thyroid cancer	14.1% (7.6% to 22.8%)	14.9% (7.9% to 23.9%)	15.1% (8.4% to 23.4%)	14.9% (8.0% to 23.9%)
Mesothelioma	88.1% (85.2% to 90.6%)	80.3% (76.1% to 84.0%)	85.6% (82.3% to 88.4%)	80.4% (76.2% to 84.0%)
Non-Hodgkin's lymphoma	6.7% (2.7% to 11.6%)	6.3% (2.6% to 10.8%)	6.8% (2.8% to 11.7%)	6.3% (2.6% to 10.8%)
Multiple myeloma	8.1% (3.6% to 14.2%)	8.5% (3.8% to 14.7%)	8.4% (3.7% to 14.7%)	8.5% (3.8% to 14.7%)
Leukaemia	12.5% (8.1% to 17.4%)	9.4% (6.2% to 13.0%)	11.7% (7.7% to 16.4%)	9.5% (6.3% to 13.0%)
**Cardiovascular diseases**	83.4% (80.9% to 85.5%)	83.2% (81.4% to 84.7%)	72.8% (70.2% to 75.2%)	82.2% (80.4% to 83.7%)
Rheumatic heart disease	24.6% (16.6% to 38.7%)	21.2% (14.7% to 31.7%)	16.6% (10.5% to 24.3%)	20.2% (14.0% to 30.0%)
Ischaemic heart disease	91.5% (87.9% to 94.4%)	92.9% (90.7% to 94.8%)	90.2% (87.7% to 92.7%)	92.7% (90.5% to 94.7%)
Cerebrovascular disease	81.1% (76.4% to 85.5%)	82.7% (79.5% to 85.4%)	82.7% (78.2% to 86.7%)	82.7% (79.3% to 85.6%)
Hypertensive heart disease	99.4% (99.3% to 99.5%)	99.3% (99.2% to 99.5%)	99.5% (99.5% to 99.5%)	99.4% (99.3% to 99.5%)
Cardiomyopathy and myocarditis	42.1% (34.4% to 50.5%)	40.1% (34.3% to 46.7%)	37.0% (31.0% to 43.3%)	40.0% (34.2% to 46.5%)
Atrial fibrillation and flutter	50.3% (43.2% to 57.1%)	54.9% (48.4% to 60.6%)	61.5% (56.6% to 66.4%)	58.9% (53.7% to 64.1%)
Aortic aneurysm	43.4% (36.7% to 50.0%)	47.3% (41.4% to 52.8%)	-	47.3% (41.4% to 52.8%)
Peripheral vascular disease	50.8% (44.9% to 57.4%)	56.8% (51.8% to 61.5%)	59.4% (54.4% to 64.2%)	57.8% (52.8% to 62.4%)
Endocarditis	35.9% (28.0% to 45.3%)	28.9% (22.1% to 36.0%)	34.8% (27.3% to 42.6%)	29.0% (22.1% to 36.1%)

Supplementary Results Table 1. Age-standardised PAFs attributable to the joint distribution of all risk factors for all ages, both sexes combined for each GBD level 1 and level 2 cause and all causes in 2016 for Latin America and Caribbean. Results are shown for deaths, YLLs, YLDs, and Data in parenthesis are 95% uncertainty intervals. PAFs=population attributable fractions. GBD=Global Burden of Disease. YLLs=years of life lost. YLDs=years live with disability. DALYs=disability-adjusted life-years	Deaths 2016	YLLs 2016	YLDs 2016	DALYs 2016
Other cardiovascular and circulatory diseases	43.9% (39.2% to 48.6%)	42.8% (38.6% to 46.7%)	43.0% (38.2% to 47.7%)	42.8% (38.7% to 46.8%)
**Chronic respiratory diseases**	59.6% (54.4% to 64.9%)	56.6% (52.0% to 61.4%)	36.3% (29.3% to 43.9%)	49.8% (45.0% to 54.7%)
Chronic obstructive pulmonary disease	67.0% (60.9% to 72.8%)	66.6% (60.8% to 72.1%)	59.7% (53.3% to 66.0%)	65.6% (59.9% to 71.2%)
Pneumoconiosis	99.9% (99.8% to 99.9%)	99.9% (99.8% to 99.9%)	99.8% (99.8% to 99.8%)	99.9% (99.8% to 99.9%)
Asthma	35.4% (26.7% to 45.5%)	34.7% (26.7% to 43.8%)	28.7% (20.2% to 39.0%)	29.8% (21.4% to 39.8%)
Other chronic respiratory diseases	7.6% (5.5% to 9.7%)	4.4% (3.1% to 5.9%)	2.8% (1.8% to 4.2%)	4.0% (2.9% to 5.5%)
**Cirrhosis and other chronic liver diseases**	52.8% (50.6% to 55.1%)	55.3% (53.0% to 57.6%)	46.2% (44.2% to 48.1%)	55.0% (52.7% to 57.3%)
Cirrhosis and other chronic liver diseases due to hepatitis B	0.8% (0.6% to 1.2%)	1.0% (0.6% to 1.4%)	0.9% (0.6% to 1.3%)	1.0% (0.6% to 1.4%)
Cirrhosis and other chronic liver diseases due to hepatitis C	40.3% (35.8% to 44.9%)	49.2% (43.9% to 54.0%)	44.5% (39.9% to 49.0%)	49.1% (43.9% to 53.9%)
Cirrhosis and other chronic liver diseases due to alcohol use	99.7% (99.7% to 99.7%)	99.7% (99.7% to 99.7%)	99.6% (99.6% to 99.7%)	99.7% (99.7% to 99.7%)
**Digestive diseases**	12.2% (9.8% to 14.7%)	13.3% (11.0% to 15.8%)	3.7% (2.8% to 4.6%)	11.0% (9.0% to 13.1%)
Peptic ulcer disease	8.9% (6.1% to 11.9%)	10.0% (6.9% to 13.4%)	7.7% (5.3% to 10.4%)	9.3% (6.4% to 12.5%)
Gallbladder and biliary diseases	42.7% (29.6% to 56.8%)	44.0% (30.8% to 58.0%)	48.5% (35.1% to 63.1%)	44.4% (31.2% to 58.3%)
Pancreatitis	39.9% (29.9% to 50.8%)	46.0% (36.8% to 55.7%)	35.4% (26.8% to 44.2%)	45.8% (36.6% to 55.5%)
**Neurological disorders**	22.0% (11.1% to 34.5%)	21.0% (12.0% to 31.4%)	5.2% (3.5% to 7.2%)	10.7% (6.4% to 15.9%)
Alzheimer's disease and other dementias	24.3% (12.1% to 38.4%)	25.2% (13.1% to 39.0%)	25.2% (13.0% to 39.3%)	25.2% (13.1% to 39.1%)
Parkinson's disease	-6.6% (-7.6% to -5.4%)	-7.8% (-9.0% to -6.4%)	-8.4% (-9.6% to -6.9%)	-7.9% (-9.2% to -6.5%)
Epilepsy	25.4% (19.1% to 31.2%)	23.3% (17.6% to 28.5%)	19.8% (14.8% to 24.7%)	21.0% (15.8% to 25.9%)
Multiple sclerosis	6.1% (3.9% to 8.6%)	6.2% (4.0% to 8.7%)	5.4% (3.5% to 7.5%)	6.0% (3.9% to 8.3%)
**Mental and substance use disorders**	99.4% (99.3% to 99.4%)	99.3% (99.2% to 99.3%)	20.6% (18.7% to 22.8%)	26.1% (23.5% to 28.7%)
Alcohol use disorders	99.7% (99.6% to 99.7%)	99.7% (99.7% to 99.8%)	99.8% (99.8% to 99.8%)	99.8% (99.7% to 99.8%)

Supplementary Results Table 1. Age-standardised PAFs attributable to the joint distribution of all risk factors for all ages, both sexes combined for each GBD level 1 and level 2 cause and all causes in 2016 for Latin America and Caribbean. Results are shown for deaths, YLLs, YLDs, and
Data in parenthesis are 95% uncertainty intervals. PAFs=population attributable fractions. GBD=Global Burden of Disease. YLLs=years of life lost. YLDs=years live with disability. DALYs=disability-adjusted life-years

	Deaths 2016	YLLs 2016	YLDs 2016	DALYs 2016
Drug use disorders	99.6% (99.6% to 99.7%)	99.7% (99.6% to 99.7%)	99.7% (99.7% to 99.8%)	99.7% (99.7% to 99.8%)
Depressive disorders	-	-	7.0% (5.8% to 8.3%)	7.0% (5.8% to 8.3%)
Idiopathic developmental intellectual disability	-	-	68.7% (37.9% to 87.5%)	68.7% (37.9% to 87.5%)
**Diabetes, urogenital, blood, and endocrine diseases**	85.4% (84.8% to 86.0%)	83.9% (83.2% to 84.9%)	74.8% (70.9% to 78.3%)	80.8% (79.0% to 82.2%)
Diabetes mellitus	99.5% (99.4% to 99.5%)	99.5% (99.5% to 99.5%)	99.6% (99.5% to 99.6%)	99.5% (99.5% to 99.6%)
Chronic kidney disease	99.7% (99.7% to 99.7%)	99.7% (99.7% to 99.7%)	99.7% (99.7% to 99.7%)	99.7% (99.7% to 99.7%)
**Musculoskeletal disorders**	1.0% (0.4% to 1.7%)	0.7% (0.3% to 1.2%)	17.2% (14.3% to 20.4%)	16.7% (13.9% to 19.9%)
Rheumatoid arthritis	3.0% (1.2% to 5.1%)	3.3% (1.3% to 5.6%)	3.6% (1.4% to 6.0%)	3.5% (1.4% to 5.9%)
Osteoarthritis	-	-	27.4% (15.2% to 40.0%)	27.4% (15.2% to 40.0%)
Low back and neck pain	-	-	21.8% (19.1% to 24.8%)	21.8% (19.1% to 24.8%)
Gout	-	-	49.3% (35.1% to 64.1%)	49.3% (35.1% to 64.1%)
**Other non-communicable diseases**	0.1% (0.1% to 0.2%)	0.2% (0.1% to 0.2%)	5.4% (4.7% to 6.3%)	4.1% (3.6% to 4.8%)
Sense organ diseases	-	-	12.1% (10.7% to 13.7%)	12.1% (10.7% to 13.7%)
Sudden infant death syndrome	6.6% (5.6% to 7.4%)	6.6% (5.6% to 7.4%)	-	6.6% (5.6% to 7.4%)
**Transport injuries**	36.9% (31.1% to 43.8%)	33.8% (27.9% to 40.4%)	42.2% (36.7% to 49.0%)	34.6% (28.8% to 41.3%)
Road injuries	36.7% (30.9% to 43.6%)	33.7% (27.8% to 40.3%)	42.5% (36.9% to 49.2%)	34.6% (28.7% to 41.3%)
Other transport injuries	40.3% (34.6% to 47.0%)	35.9% (30.2% to 42.6%)	35.7% (30.3% to 42.1%)	35.9% (30.1% to 42.6%)
**Unintentional injuries**	29.6% (25.8% to 31.6%)	18.2% (16.1% to 20.1%)	23.2% (22.0% to 24.4%)	20.1% (18.3% to 21.7%)
Falls	57.1% (53.4% to 59.1%)	36.7% (33.4% to 38.3%)	28.2% (27.1% to 29.2%)	31.2% (29.5% to 32.6%)
Drowning	18.7% (14.9% to 23.3%)	16.8% (13.6% to 20.7%)	18.6% (14.2% to 23.8%)	16.8% (13.6% to 20.7%)
Fire, heat, and hot substances	12.8% (9.6% to 16.7%)	13.7% (10.9% to 17.1%)	20.6% (16.7% to 25.1%)	16.1% (12.9% to 19.9%)

Supplementary Results Table 1. Age-standardised PAFs attributable to the joint distribution of all risk factors for all ages, both sexes combined for each GBD level 1 and level 2 cause and all causes in 2016 for Latin America and Caribbean. Results are shown for deaths, YLLs, YLDs, and
Data in parenthesis are 95% uncertainty intervals. PAFs=population attributable fractions. GBD=Global Burden of Disease. YLLs=years of life lost. YLDs=years live with disability. DALYs=disability-adjusted life-years

	**Deaths 2016**	**YLLs 2016**	**YLDs 2016**	**DALYs 2016**
Poisonings	17.4% (14.0% to 21.6%)	16.4% (13.3% to 20.0%)	20.0% (16.6% to 24.2%)	16.7% (13.7% to 20.4%)
Exposure to mechanical forces	22.8% (19.9% to 25.3%)	20.9% (17.2% to 23.8%)	24.3% (22.5% to 26.0%)	22.0% (19.3% to 24.4%)
Animal contact	12.2% (10.9% to 13.2%)	11.2% (9.6% to 12.3%)	14.7% (13.4% to 15.9%)	12.4% (11.0% to 13.6%)
Foreign body	6.9% (6.2% to 8.1%)	5.9% (5.3% to 7.2%)	9.1% (7.9% to 10.2%)	6.5% (5.8% to 7.5%)
Other unintentional injuries	23.9% (19.5% to 29.0%)	24.7% (20.3% to 29.7%)	19.0% (14.8% to 23.8%)	22.2% (18.1% to 27.1%)
**Self-harm and interpersonal violence**	23.7% (17.7% to 29.9%)	22.9% (17.0% to 29.2%)	23.1% (18.8% to 27.8%)	22.9% (17.1% to 29.1%)
Self-harm	30.2% (20.3% to 39.8%)	30.7% (20.8% to 40.0%)	26.8% (17.5% to 35.8%)	30.6% (20.8% to 39.9%)
Interpersonal violence	21.8% (14.9% to 29.5%)	20.9% (14.3% to 28.3%)	22.9% (18.4% to 27.8%)	21.0% (14.5% to 28.3%)
**Forces of nature, conflict and terrorism, and executions and police conflict**	3.9% (2.9% to 5.0%)	1.4% (1.0% to 1.9%)	3.5% (2.4% to 4.7%)	1.9% (1.4% to 2.4%)
Forces of nature, conflict and terrorism, and state actor violence	5.2% (4.5% to 5.7%)	2.0% (1.7% to 2.2%)	4.8% (3.4% to 6.5%)	2.6% (2.1% to 3.3%)

Supplementary Results Table 1. Age-standardised PAFs attributable to the joint distribution of all risk factors for all ages, both sexes combined for each GBD level 1 and level 2 cause and all causes in 2016 for North Africa and Middle East. Results are shown for deaths, YLLs, YLDs, and
Data in parenthesis are 95% uncertainty intervals. PAFs=population attributable fractions. GBD=Global Burden of Disease. YLLs=years of life lost. YLDs=years live with disability. DALYs=disability-adjusted life-years

	**Deaths 2016**	**YLLs 2016**	**YLDs 2016**	**DALYs 2016**
**All causes**	61.1% (59.2% to 62.9%)	52.7% (51.1% to 54.5%)	26.2% (24.8% to 27.7%)	42.8% (40.6% to 45.0%)
**HIV/AIDS and tuberculosis**	27.4% (21.5% to 34.1%)	29.7% (24.4% to 36.2%)	37.5% (30.2% to 46.9%)	30.0% (24.7% to 36.4%)
Tuberculosis	22.8% (16.5% to 29.4%)	22.2% (16.8% to 27.6%)	21.2% (16.3% to 26.0%)	22.1% (16.8% to 27.5%)
HIV/AIDS	80.5% (77.0% to 84.3%)	78.1% (74.2% to 82.4%)	81.6% (78.5% to 85.1%)	78.3% (74.5% to 82.5%)
**Diarrhoea, lower respiratory infections, and other common infectious diseases**	61.5% (57.0% to 66.1%)	68.0% (63.4% to 72.4%)	35.5% (28.2% to 42.0%)	62.9% (58.5% to 67.3%)
Diarrhoeal diseases	90.0% (80.7% to 96.2%)	95.6% (91.3% to 98.3%)	80.8% (65.8% to 91.9%)	92.0% (85.0% to 96.8%)
Lower respiratory infections	64.9% (59.3% to 70.6%)	78.0% (73.0% to 82.6%)	62.0% (56.2% to 67.8%)	77.9% (72.9% to 82.4%)
Upper respiratory infections	2.2% (0.9% to 4.3%)	4.3% (1.7% to 8.7%)	0.0% (0.0% to 0.0%)	0.1% (0.0% to 0.1%)
Otitis media	3.4% (1.2% to 6.9%)	7.3% (3.9% to 11.0%)	7.3% (4.9% to 9.6%)	7.3% (5.0% to 9.6%)
Meningitis	4.5% (2.8% to 6.8%)	6.3% (4.2% to 9.1%)	0.0% (0.0% to 0.0%)	5.6% (3.7% to 8.2%)
Encephalitis	1.9% (0.9% to 3.5%)	3.2% (1.6% to 5.6%)	0.0% (0.0% to 0.0%)	1.7% (0.8% to 3.3%)
Measles	62.4% (48.9% to 79.2%)	63.6% (50.0% to 80.5%)	43.4% (25.4% to 73.5%)	63.5% (49.8% to 80.4%)
**Maternal disorders**	9.4% (4.1% to 14.9%)	9.2% (3.9% to 14.8%)	3.1% (1.4% to 5.2%)	8.7% (3.7% to 13.9%)
Maternal haemorrhage	18.5% (6.7% to 30.2%)	18.5% (6.7% to 30.1%)	15.9% (5.7% to 26.0%)	18.4% (6.7% to 30.0%)
Maternal sepsis and other pregnancy related infections	18.8% (6.8% to 30.5%)	18.8% (6.8% to 30.4%)	16.1% (5.8% to 26.2%)	18.6% (6.8% to 30.2%)
Maternal abortion, miscarriage, and ectopic pregnancy	18.2% (11.7% to 25.7%)	17.7% (11.5% to 25.1%)	13.9% (8.0% to 20.1%)	17.4% (11.3% to 24.7%)
**Neonatal disorders**	77.8% (75.4% to 80.0%)	77.9% (75.4% to 80.1%)	0.1% (0.1% to 0.2%)	70.2% (64.2% to 74.0%)
Neonatal preterm birth complications	89.7% (87.3% to 91.8%)	89.8% (87.3% to 91.8%)	0.3% (0.2% to 0.3%)	83.0% (79.0% to 86.5%)
Neonatal encephalopathy due to birth asphyxia and trauma	63.8% (59.3% to 67.9%)	63.9% (59.5% to 68.0%)	0.0% (0.0% to 0.0%)	59.9% (53.2% to 65.4%)
Neonatal sepsis and other neonatal infections	57.6% (52.4% to 62.3%)	57.6% (52.5% to 62.4%)	0.0% (0.0% to 0.0%)	45.4% (28.3% to 54.7%)
Hemolytic disease and other neonatal jaundice	67.8% (63.4% to 71.8%)	67.8% (63.5% to 71.8%)	0.0% (0.0% to 0.0%)	39.0% (31.0% to 48.0%)

Supplementary Results Table 1. Age-standardised PAFs attributable to the joint distribution of all risk factors for all ages, both sexes combined for each GBD level 1 and level 2 cause and all causes in 2016 for North Africa and Middle East. Results are shown for deaths, YLLs, YLDs, and
Data in parenthesis are 95% uncertainty intervals. PAFs=population attributable fractions. GBD=Global Burden of Disease. YLLs=years of life lost. YLDs=years live with disability. DALYs=disability-adjusted life-years

	**Deaths 2016**	**YLLs 2016**	**YLDs 2016**	**DALYs 2016**
Other neonatal disorders	61.6% (57.4% to 65.5%)	61.7% (57.5% to 65.5%)	0.0% (0.0% to 0.0%)	59.9% (55.4% to 63.8%)
**Nutritional deficiencies**	16.2% (12.8% to 20.6%)	47.2% (40.6% to 54.2%)	87.9% (85.5% to 90.0%)	85.2% (82.5% to 87.6%)
Protein-energy malnutrition	17.0% (12.8% to 22.3%)	52.2% (44.6% to 59.9%)	97.3% (96.9% to 97.5%)	74.6% (68.6% to 79.2%)
Vitamin A deficiency	-	-	99.9% (99.9% to 99.9%)	99.9% (99.9% to 99.9%)
Iron-deficiency anaemia	99.9% (99.9% to 99.9%)	99.9% (99.9% to 99.9%)	99.9% (99.9% to 99.9%)	99.9% (99.9% to 99.9%)
**Other communicable, maternal, neonatal, and nutritional diseases**	1.6% (1.1% to 2.2%)	1.0% (0.6% to 1.6%)	78.3% (73.1% to 83.0%)	26.5% (16.6% to 37.8%)
Sexually transmitted diseases excluding HIV	3.2% (1.6% to 6.0%)	1.3% (0.6% to 2.5%)	99.9% (99.9% to 99.9%)	38.6% (22.9% to 57.6%)
Hepatitis	1.3% (0.9% to 1.7%)	1.3% (1.0% to 1.8%)	1.0% (0.6% to 1.5%)	1.3% (1.0% to 1.8%)
**Neoplasms**	35.4% (31.8% to 39.3%)	33.9% (30.6% to 37.4%)	29.4% (25.9% to 33.4%)	33.8% (30.5% to 37.4%)
Oesophageal cancer	60.2% (46.7% to 72.9%)	61.7% (48.4% to 74.4%)	60.1% (46.4% to 72.9%)	61.7% (48.4% to 74.4%)
Stomach cancer	14.4% (7.2% to 22.5%)	15.2% (8.1% to 23.1%)	14.4% (7.3% to 22.2%)	15.2% (8.1% to 23.1%)
Liver cancer	44.7% (34.3% to 55.7%)	47.6% (37.1% to 58.3%)	45.4% (35.0% to 56.6%)	47.6% (37.1% to 58.3%)
Larynx cancer	68.6% (59.3% to 76.6%)	69.9% (61.0% to 77.4%)	69.0% (60.4% to 76.6%)	69.9% (61.0% to 77.4%)
Tracheal, bronchus, and lung cancer	84.0% (80.4% to 87.2%)	84.5% (81.2% to 87.5%)	83.4% (80.0% to 86.6%)	84.5% (81.2% to 87.5%)
Breast cancer	19.1% (10.6% to 29.0%)	15.5% (7.6% to 24.4%)	16.7% (8.5% to 26.0%)	15.5% (7.6% to 24.4%)
Cervical cancer	99.9% (99.9% to 99.9%)	99.9% (99.9% to 99.9%)	99.9% (99.9% to 99.9%)	99.9% (99.9% to 99.9%)
Uterine cancer	50.0% (37.1% to 63.0%)	50.6% (37.7% to 63.3%)	53.1% (39.5% to 66.1%)	50.7% (37.8% to 63.4%)
Prostate cancer	3.6% (2.4% to 4.9%)	3.9% (2.7% to 5.3%)	4.1% (3.0% to 5.5%)	3.9% (2.7% to 5.3%)
Colon and rectum cancer	46.7% (36.7% to 57.0%)	46.8% (37.2% to 56.4%)	46.5% (36.6% to 56.6%)	46.8% (37.2% to 56.4%)
Lip and oral cavity cancer	47.0% (40.4% to 53.5%)	49.1% (43.0% to 55.4%)	44.2% (38.4% to 50.7%)	49.0% (42.9% to 55.2%)
Nasopharynx cancer	51.0% (44.0% to 57.4%)	50.7% (44.1% to 56.4%)	49.2% (43.1% to 55.2%)	50.6% (44.1% to 56.3%)

Supplementary Results Table 1. Age-standardised PAFs attributable to the joint distribution of all risk factors for all ages, both sexes combined for each GBD level 1 and level 2 cause and all causes in 2016 for North Africa and Middle East. Results are shown for deaths, YLLs, YLDs, and
Data in parenthesis are 95% uncertainty intervals. PAFs=population attributable fractions. GBD=Global Burden of Disease. YLLs=years of life lost. YLDs=years live with disability. DALYs=disability-adjusted life-years

	Deaths 2016	YLLs 2016	YLDs 2016	DALYs 2016
Other pharynx cancer	16.2% (10.0% to 23.4%)	17.6% (11.2% to 24.4%)	16.6% (10.5% to 23.6%)	17.6% (11.2% to 24.3%)
Gallbladder and biliary tract cancer	24.9% (14.9% to 36.6%)	25.7% (15.6% to 37.8%)	25.4% (15.3% to 37.2%)	25.7% (15.6% to 37.8%)
Pancreatic cancer	28.5% (19.3% to 38.9%)	29.8% (21.1% to 39.8%)	28.6% (19.6% to 38.9%)	29.8% (21.0% to 39.8%)
Ovarian cancer	15.7% (6.5% to 26.9%)	14.1% (5.6% to 24.6%)	14.0% (5.6% to 24.1%)	14.1% (5.6% to 24.6%)
Kidney cancer	33.6% (24.4% to 43.8%)	32.6% (23.8% to 42.2%)	33.4% (24.8% to 43.3%)	32.6% (23.9% to 42.3%)
Bladder cancer	28.0% (18.5% to 38.7%)	28.6% (19.4% to 39.2%)	28.7% (19.5% to 39.0%)	28.6% (19.4% to 39.1%)
Thyroid cancer	15.4% (8.7% to 24.3%)	15.7% (8.8% to 24.8%)	16.6% (9.6% to 25.1%)	15.7% (8.8% to 24.9%)
Mesothelioma	91.2% (88.7% to 93.1%)	84.9% (81.1% to 88.1%)	89.5% (86.8% to 91.8%)	85.0% (81.2% to 88.1%)
Non-Hodgkin's lymphoma	7.6% (3.3% to 13.0%)	7.1% (3.0% to 12.1%)	7.8% (3.3% to 13.2%)	7.1% (3.0% to 12.2%)
Multiple myeloma	9.2% (4.2% to 15.6%)	9.5% (4.3% to 16.0%)	9.6% (4.4% to 16.3%)	9.5% (4.3% to 16.0%)
Leukaemia	16.1% (10.5% to 22.2%)	13.4% (8.9% to 18.4%)	15.7% (10.2% to 21.4%)	13.4% (8.9% to 18.5%)
**Cardiovascular diseases**	87.7% (85.3% to 89.9%)	88.4% (86.8% to 89.9%)	74.5% (71.7% to 77.3%)	87.3% (85.6% to 88.8%)
Rheumatic heart disease	26.3% (18.4% to 39.8%)	23.4% (16.2% to 33.9%)	21.4% (13.7% to 30.7%)	22.9% (15.5% to 32.9%)
Ischaemic heart disease	93.8% (90.9% to 96.1%)	95.0% (93.3% to 96.5%)	92.0% (89.7% to 94.2%)	94.9% (93.2% to 96.4%)
Cerebrovascular disease	84.1% (79.7% to 89.0%)	86.2% (83.5% to 89.0%)	86.3% (82.4% to 90.3%)	86.2% (83.4% to 89.1%)
Hypertensive heart disease	99.9% (99.9% to 99.9%)	99.9% (99.9% to 99.9%)	99.9% (99.9% to 99.9%)	99.9% (99.9% to 99.9%)
Cardiomyopathy and myocarditis	38.2% (31.6% to 45.7%)	31.8% (26.3% to 37.1%)	31.7% (26.0% to 37.9%)	31.8% (26.4% to 37.1%)
Atrial fibrillation and flutter	47.0% (39.7% to 54.3%)	51.6% (44.8% to 57.8%)	58.4% (53.5% to 63.6%)	55.5% (49.6% to 60.9%)
Aortic aneurysm	44.7% (38.0% to 51.4%)	50.8% (45.4% to 56.1%)	-	50.8% (45.4% to 56.1%)
Peripheral vascular disease	55.7% (47.6% to 62.6%)	63.9% (55.8% to 69.4%)	65.9% (61.0% to 70.7%)	65.1% (59.6% to 69.9%)
Endocarditis	38.8% (29.8% to 50.2%)	36.2% (28.8% to 43.9%)	39.5% (30.7% to 48.9%)	36.2% (28.8% to 44.0%)

Supplementary Results Table 1. Age-standardised PAFs attributable to the joint distribution of all risk factors for all ages, both sexes combined for each GBD level 1 and level 2 cause and all causes in 2016 for North Africa and Middle East. Results are shown for deaths, YLLs, YLDs, and
Data in parenthesis are 95% uncertainty intervals. PAFs=population attributable fractions. GBD=Global Burden of Disease. YLLs=years of life lost. YLDs=years live with disability. DALYs=disability-adjusted life-years

	Deaths 2016	YLLs 2016	YLDs 2016	DALYs 2016
Other cardiovascular and circulatory diseases	43.4% (38.5% to 48.2%)	44.0% (40.0% to 47.9%)	45.8% (41.1% to 50.4%)	44.5% (40.5% to 48.5%)
**Chronic respiratory diseases**	59.3% (53.3% to 65.2%)	57.8% (52.7% to 63.2%)	45.4% (38.9% to 52.0%)	53.1% (48.1% to 58.8%)
Chronic obstructive pulmonary disease	70.1% (63.0% to 77.3%)	71.1% (64.7% to 77.7%)	65.7% (59.1% to 72.8%)	69.8% (63.4% to 76.5%)
Pneumoconiosis	99.9% (99.9% to 99.9%)	99.9% (99.9% to 99.9%)	99.9% (99.9% to 99.9%)	99.9% (99.9% to 99.9%)
Asthma	41.4% (32.1% to 51.9%)	42.2% (33.5% to 51.8%)	36.0% (27.2% to 45.7%)	38.3% (29.9% to 47.9%)
Other chronic respiratory diseases	10.4% (7.4% to 13.7%)	9.4% (6.5% to 12.2%)	6.1% (4.2% to 8.3%)	8.7% (6.1% to 11.4%)
**Cirrhosis and other chronic liver diseases**	17.6% (15.0% to 20.5%)	22.9% (19.5% to 26.6%)	20.9% (18.3% to 23.6%)	22.8% (19.5% to 26.5%)
Cirrhosis and other chronic liver diseases due to hepatitis B	0.3% (0.2% to 0.4%)	0.3% (0.2% to 0.5%)	0.3% (0.2% to 0.5%)	0.3% (0.2% to 0.5%)
Cirrhosis and other chronic liver diseases due to hepatitis C	20.7% (15.7% to 26.9%)	33.3% (26.2% to 41.1%)	48.0% (41.6% to 54.0%)	33.7% (26.6% to 41.4%)
Cirrhosis and other chronic liver diseases due to alcohol use	99.9% (99.9% to 99.9%)	99.9% (99.9% to 99.9%)	99.9% (99.9% to 99.9%)	99.9% (99.9% to 99.9%)
**Digestive diseases**	11.2% (7.9% to 14.3%)	11.0% (7.8% to 14.0%)	2.9% (2.2% to 3.6%)	8.3% (6.0% to 10.4%)
Peptic ulcer disease	11.7% (8.1% to 15.5%)	13.5% (9.5% to 17.7%)	7.6% (5.4% to 10.1%)	11.6% (8.1% to 15.3%)
Gallbladder and biliary diseases	46.2% (33.2% to 59.3%)	46.9% (33.7% to 59.8%)	49.0% (35.8% to 62.1%)	47.1% (34.0% to 60.0%)
Pancreatitis	9.1% (4.6% to 15.4%)	11.2% (6.4% to 18.1%)	10.2% (5.9% to 15.9%)	11.2% (6.4% to 18.0%)
**Neurological disorders**	25.2% (12.8% to 39.2%)	22.7% (12.1% to 34.9%)	3.7% (2.0% to 5.8%)	10.3% (5.3% to 16.3%)
Alzheimer's disease and other dementias	28.9% (15.0% to 44.7%)	29.9% (16.1% to 45.5%)	30.1% (15.7% to 46.0%)	30.0% (16.1% to 45.7%)
Parkinson's disease	-9.4% (-11.4% to -7.4%)	-11.5% (-13.8% to -9.0%)	-12.1% (-14.4% to -9.4%)	-11.6% (-13.9% to -9.1%)
Epilepsy	4.8% (3.0% to 6.7%)	4.0% (2.6% to 5.6%)	3.5% (2.1% to 5.4%)	3.7% (2.3% to 5.4%)
Multiple sclerosis	8.1% (4.8% to 11.7%)	8.3% (4.9% to 11.9%)	6.4% (4.0% to 9.0%)	7.1% (4.3% to 10.2%)
**Mental and substance use disorders**	99.7% (99.6% to 99.7%)	99.6% (99.5% to 99.7%)	20.3% (17.7% to 23.1%)	22.5% (19.7% to 25.5%)
Alcohol use disorders	99.9% (99.9% to 99.9%)	99.9% (99.9% to 99.9%)	99.9% (99.9% to 99.9%)	99.9% (99.9% to 99.9%)

Supplementary Results Table 1. Age-standardised PAFs attributable to the joint distribution of all risk factors for all ages, both sexes combined for each GBD level 1 and level 2 cause and all causes in 2016 for North Africa and Middle East. Results are shown for deaths, YLLs, YLDs, and
Data in parenthesis are 95% uncertainty intervals. PAFs=population attributable fractions. GBD=Global Burden of Disease. YLLs=years of life lost. YLDs=years live with disability. DALYs=disability-adjusted life-years

	Deaths 2016	YLLs 2016	YLDs 2016	DALYs 2016
Drug use disorders	99.9% (99.9% to 99.9%)	99.9% (99.9% to 99.9%)	99.9% (99.9% to 99.9%)	99.9% (99.9% to 99.9%)
Depressive disorders	-	-	8.2% (6.6% to 9.9%)	8.2% (6.6% to 9.9%)
Idiopathic developmental intellectual disability	-	-	66.8% (35.5% to 84.9%)	66.8% (35.5% to 84.9%)
**Diabetes, urogenital, blood, and endocrine diseases**	89.4% (88.4% to 90.4%)	85.1% (83.3% to 86.5%)	70.5% (66.2% to 74.3%)	78.1% (75.5% to 80.4%)
Diabetes mellitus	99.9% (99.9% to 99.9%)	99.9% (99.9% to 99.9%)	99.9% (99.9% to 99.9%)	99.9% (99.9% to 99.9%)
Chronic kidney disease	99.9% (99.9% to 99.9%)	99.9% (99.9% to 99.9%)	99.9% (99.9% to 99.9%)	99.9% (99.9% to 99.9%)
**Musculoskeletal disorders**	0.7% (0.3% to 1.2%)	0.5% (0.2% to 0.9%)	16.8% (13.7% to 20.2%)	16.6% (13.6% to 20.0%)
Rheumatoid arthritis	3.3% (1.3% to 5.6%)	3.4% (1.4% to 5.7%)	3.4% (1.4% to 5.6%)	3.4% (1.4% to 5.6%)
Osteoarthritis	-	-	31.9% (18.3% to 45.2%)	31.9% (18.3% to 45.2%)
Low back and neck pain	-	-	19.7% (16.8% to 22.9%)	19.7% (16.8% to 22.9%)
Gout	-	-	55.6% (41.8% to 69.9%)	55.6% (41.8% to 69.9%)
**Other non-communicable diseases**	0.1% (0.1% to 0.2%)	0.2% (0.1% to 0.2%)	5.5% (4.6% to 6.7%)	3.8% (3.1% to 4.6%)
Sense organ diseases	-	-	11.5% (9.8% to 13.6%)	11.5% (9.8% to 13.6%)
Sudden infant death syndrome	4.9% (3.5% to 6.5%)	4.9% (3.5% to 6.5%)	-	4.9% (3.5% to 6.5%)
**Transport injuries**	22.4% (21.0% to 24.0%)	19.0% (17.6% to 20.6%)	27.6% (25.9% to 29.4%)	19.9% (18.4% to 21.5%)
Road injuries	22.3% (20.9% to 23.9%)	19.0% (17.6% to 20.6%)	29.9% (28.2% to 31.8%)	19.8% (18.4% to 21.5%)
Other transport injuries	27.6% (25.9% to 29.5%)	20.9% (19.1% to 22.9%)	20.1% (18.5% to 21.8%)	20.5% (19.0% to 22.1%)
**Unintentional injuries**	22.3% (19.3% to 24.2%)	13.3% (12.2% to 14.4%)	17.3% (16.3% to 18.5%)	14.8% (13.8% to 15.9%)
Falls	45.6% (37.3% to 49.2%)	24.3% (21.2% to 27.0%)	25.2% (24.0% to 26.4%)	24.7% (22.9% to 26.4%)
Drowning	12.0% (10.6% to 13.4%)	10.2% (9.0% to 11.6%)	12.5% (11.2% to 13.9%)	10.3% (9.0% to 11.6%)
Fire, heat, and hot substances	8.3% (7.0% to 9.5%)	8.9% (7.8% to 10.1%)	11.5% (10.3% to 12.9%)	9.6% (8.6% to 10.9%)

Supplementary Results Table 1. Age-standardised PAFs attributable to the joint distribution of all risk factors for all ages, both sexes combined for each GBD level 1 and level 2 cause and all causes in 2016 for North Africa and Middle East. Results are shown for deaths, YLLs, YLDs, and
Data in parenthesis are 95% uncertainty intervals. PAFs=population attributable fractions. GBD=Global Burden of Disease. YLLs=years of life lost. YLDs=years live with disability. DALYs=disability-adjusted life-years

	Deaths 2016	YLLs 2016	YLDs 2016	DALYs 2016
Poisonings	9.4% (7.8% to 10.9%)	8.8% (7.1% to 10.3%)	11.8% (10.7% to 13.2%)	9.1% (7.6% to 10.6%)
Exposure to mechanical forces	19.0% (16.3% to 21.1%)	16.3% (13.1% to 18.5%)	22.2% (20.8% to 23.8%)	18.4% (16.2% to 20.3%)
Animal contact	11.3% (9.6% to 13.0%)	7.7% (6.3% to 9.6%)	11.2% (10.2% to 12.3%)	9.8% (8.7% to 10.9%)
Foreign body	6.2% (5.2% to 7.1%)	5.6% (4.6% to 6.5%)	9.1% (8.1% to 10.2%)	7.0% (6.1% to 7.9%)
Other unintentional injuries	16.0% (13.9% to 18.3%)	15.5% (13.4% to 17.9%)	11.6% (10.5% to 13.0%)	13.2% (11.8% to 14.8%)
**Self-harm and interpersonal violence**	10.4% (8.1% to 13.0%)	10.3% (8.0% to 13.0%)	22.1% (20.5% to 23.8%)	11.3% (9.1% to 13.8%)
Self-harm	10.9% (7.6% to 14.9%)	11.7% (8.1% to 15.7%)	9.7% (6.6% to 13.3%)	11.6% (8.0% to 15.7%)
Interpersonal violence	9.6% (7.0% to 12.8%)	8.5% (6.0% to 11.6%)	23.7% (22.2% to 25.5%)	11.1% (8.8% to 14.7%)
**Forces of nature, conflict and terrorism, and executions and police conflict**	0.0% (0.0% to 0.0%)	0.0% (0.0% to 0.0%)	0.2% (0.1% to 0.3%)	0.0% (0.0% to 0.0%)
Forces of nature, conflict and terrorism, and state actor violence	4.8% (4.4% to 5.1%)	1.8% (1.6% to 2.0%)	6.6% (4.6% to 9.5%)	5.3% (3.3% to 7.6%)

Supplementary Results Table 1. Age-standardised PAFs attributable to the joint distribution of all risk factors for all ages, both sexes combined for each GBD level 1 and level 2 cause and all causes in 2016 for South Asia. Results are shown for deaths, YLLs, YLDs, and DALYs
Data in parenthesis are 95% uncertainty intervals. PAFs=population attributable fractions. GBD=Global Burden of Disease. YLLs=years of life lost. YLDs=years live with disability. DALYs=disability-adjusted life-years

	Deaths 2016	YLLs 2016	YLDs 2016	DALYs 2016
**All causes**	63.7% (61.8% to 65.6%)	58.6% (57.2% to 60.1%)	30.0% (28.7% to 31.2%)	49.8% (47.8% to 51.9%)
**HIV/AIDS and tuberculosis**	35.9% (30.3% to 41.3%)	39.3% (34.5% to 43.8%)	41.2% (35.8% to 46.1%)	39.4% (34.7% to 43.9%)
Tuberculosis	30.3% (23.9% to 36.2%)	30.5% (24.7% to 36.0%)	32.2% (26.3% to 38.2%)	30.6% (24.8% to 36.1%)
HIV/AIDS	78.1% (75.4% to 80.5%)	74.7% (71.8% to 77.2%)	78.6% (75.6% to 81.2%)	74.9% (72.1% to 77.5%)
**Diarrhoea, lower respiratory infections, and other common infectious diseases**	76.2% (68.7% to 82.4%)	70.9% (64.9% to 76.6%)	43.2% (36.4% to 48.7%)	68.9% (63.0% to 74.5%)
Diarrhoeal diseases	91.2% (78.8% to 97.4%)	93.3% (83.9% to 98.1%)	90.4% (77.7% to 97.1%)	93.0% (83.4% to 98.0%)
Lower respiratory infections	77.3% (72.9% to 81.9%)	86.6% (83.7% to 89.5%)	74.2% (69.3% to 79.1%)	86.5% (83.6% to 89.4%)
Upper respiratory infections	2.4% (1.3% to 3.9%)	4.9% (2.8% to 8.0%)	0.0% (0.0% to 0.0%)	0.1% (0.1% to 0.3%)
Otitis media	2.4% (1.3% to 4.0%)	5.8% (3.5% to 8.4%)	7.2% (4.8% to 9.3%)	7.2% (4.8% to 9.3%)
Meningitis	2.8% (1.5% to 4.2%)	4.7% (2.9% to 6.8%)	0.0% (0.0% to 0.0%)	4.3% (2.7% to 6.2%)
Encephalitis	0.6% (0.5% to 0.7%)	1.4% (1.2% to 1.6%)	0.0% (0.0% to 0.0%)	1.2% (1.0% to 1.4%)
Measles	75.4% (66.6% to 85.2%)	76.6% (67.7% to 86.5%)	72.2% (63.1% to 82.4%)	76.5% (67.7% to 86.4%)
**Maternal disorders**	13.7% (5.9% to 21.6%)	13.7% (5.8% to 21.7%)	2.6% (1.3% to 4.1%)	13.0% (5.6% to 20.7%)
Maternal haemorrhage	22.5% (8.5% to 35.8%)	22.6% (8.6% to 36.0%)	24.0% (8.9% to 38.0%)	22.7% (8.6% to 36.0%)
Maternal sepsis and other pregnancy related infections	24.3% (9.0% to 38.6%)	24.4% (9.1% to 38.7%)	22.7% (8.5% to 36.0%)	24.4% (9.1% to 38.7%)
Maternal abortion, miscarriage, and ectopic pregnancy	16.1% (10.5% to 22.0%)	16.1% (10.2% to 22.1%)	16.6% (8.9% to 24.8%)	16.2% (10.2% to 22.0%)
**Neonatal disorders**	82.6% (80.5% to 84.9%)	82.6% (80.5% to 84.9%)	0.3% (0.2% to 0.3%)	75.6% (72.5% to 78.5%)
Neonatal preterm birth complications	97.1% (95.9% to 98.1%)	97.1% (95.9% to 98.2%)	0.4% (0.3% to 0.4%)	80.7% (75.3% to 85.2%)
Neonatal encephalopathy due to birth asphyxia and trauma	76.7% (73.6% to 80.1%)	76.7% (73.7% to 80.1%)	0.0% (0.0% to 0.0%)	75.4% (71.5% to 79.1%)
Neonatal sepsis and other neonatal infections	71.4% (68.3% to 74.3%)	71.4% (68.3% to 74.3%)	0.0% (0.0% to 0.0%)	60.4% (47.6% to 68.8%)
Hemolytic disease and other neonatal jaundice	70.4% (67.0% to 73.4%)	70.4% (67.0% to 73.4%)	0.0% (0.0% to 0.0%)	65.0% (61.7% to 68.1%)

Supplementary Results Table 1. Age-standardised PAFs attributable to the joint distribution of all risk factors for all ages, both sexes combined for each GBD level 1 and level 2 cause and all causes in 2016 for South Asia. Results are shown for deaths, YLLs, YLDs, and DALYs
Data in parenthesis are 95% uncertainty intervals. PAFs=population attributable fractions. GBD=Global Burden of Disease. YLLs=years of life lost. YLDs=years live with disability. DALYs=disability-adjusted life-years

	Deaths 2016	YLLs 2016	YLDs 2016	DALYs 2016
Other neonatal disorders	76.1% (73.0% to 78.9%)	76.1% (73.0% to 78.9%)	0.0% (0.0% to 0.0%)	75.0% (71.9% to 78.0%)
**Nutritional deficiencies**	25.9% (23.1% to 29.5%)	55.6% (52.2% to 59.5%)	94.1% (93.1% to 94.9%)	88.4% (86.0% to 90.3%)
Protein-energy malnutrition	46.6% (42.3% to 51.0%)	81.0% (78.3% to 83.5%)	97.7% (97.6% to 97.8%)	88.3% (86.1% to 90.3%)
Vitamin A deficiency	-	-	100.0% (100.0% to 100.0%)	100.0% (100.0% to 100.0%)
Iron-deficiency anaemia	100.0% (100.0% to 100.0%)	100.0% (100.0% to 100.0%)	100.0% (100.0% to 100.0%)	100.0% (100.0% to 100.0%)
**Other communicable, maternal, neonatal, and nutritional diseases**	2.7% (2.2% to 3.2%)	2.3% (1.8% to 2.8%)	38.6% (33.4% to 44.3%)	8.5% (6.2% to 11.3%)
Sexually transmitted diseases excluding HIV	22.7% (12.8% to 37.0%)	10.3% (5.4% to 18.6%)	100.0% (100.0% to 100.0%)	34.3% (20.0% to 51.6%)
Hepatitis	0.1% (0.1% to 0.2%)	0.1% (0.1% to 0.2%)	0.1% (0.1% to 0.2%)	0.1% (0.1% to 0.2%)
**Neoplasms**	32.8% (30.2% to 35.6%)	31.9% (29.3% to 34.4%)	33.1% (30.4% to 35.8%)	31.9% (29.4% to 34.4%)
Oesophageal cancer	60.1% (49.4% to 70.0%)	61.8% (51.3% to 71.2%)	61.0% (50.2% to 70.6%)	61.7% (51.3% to 71.2%)
Stomach cancer	8.8% (3.6% to 16.7%)	9.0% (3.9% to 15.9%)	9.3% (4.0% to 16.9%)	9.0% (3.9% to 15.9%)
Liver cancer	26.6% (20.4% to 33.3%)	27.3% (21.1% to 34.1%)	27.1% (20.7% to 34.0%)	27.3% (21.1% to 34.1%)
Larynx cancer	53.6% (43.8% to 63.1%)	57.2% (47.5% to 66.4%)	55.3% (45.7% to 64.3%)	57.1% (47.5% to 66.4%)
Tracheal, bronchus, and lung cancer	82.5% (78.5% to 86.4%)	82.7% (78.8% to 86.4%)	83.0% (79.2% to 86.7%)	82.7% (78.8% to 86.4%)
Breast cancer	13.8% (7.4% to 22.2%)	12.3% (6.4% to 20.0%)	12.8% (6.7% to 20.9%)	12.3% (6.4% to 20.0%)
Cervical cancer	100.0% (100.0% to 100.0%)	100.0% (100.0% to 100.0%)	100.0% (100.0% to 100.0%)	100.0% (100.0% to 100.0%)
Uterine cancer	22.0% (13.1% to 32.2%)	23.6% (14.4% to 34.2%)	23.5% (14.4% to 33.6%)	23.6% (14.4% to 34.2%)
Prostate cancer	1.4% (1.0% to 1.9%)	1.6% (1.1% to 2.1%)	1.6% (1.1% to 2.2%)	1.6% (1.1% to 2.1%)
Colon and rectum cancer	45.4% (36.2% to 54.9%)	44.8% (36.0% to 54.0%)	45.5% (36.4% to 54.9%)	44.8% (36.0% to 54.0%)
Lip and oral cavity cancer	63.4% (56.7% to 69.5%)	64.4% (57.7% to 70.4%)	62.1% (55.8% to 68.2%)	64.3% (57.7% to 70.3%)
Nasopharynx cancer	44.6% (37.2% to 52.0%)	45.3% (38.0% to 52.1%)	45.4% (38.2% to 51.9%)	45.3% (38.0% to 52.1%)

Supplementary Results Table 1. Age-standardised PAFs attributable to the joint distribution of all risk factors for all ages, both sexes combined for each GBD level 1 and level 2 cause and all causes in 2016 for South Asia. Results are shown for deaths, YLLs, YLDs, and DALYs
Data in parenthesis are 95% uncertainty intervals. PAFs=population attributable fractions. GBD=Global Burden of Disease. YLLs=years of life lost. YLDs=years live with disability. DALYs=disability-adjusted life-years

	**Deaths 2016**	**YLLs 2016**	**YLDs 2016**	**DALYs 2016**
Other pharynx cancer	29.9% (21.1% to 38.8%)	31.3% (22.6% to 39.9%)	30.1% (21.5% to 38.7%)	31.3% (22.6% to 39.9%)
Gallbladder and biliary tract cancer	9.3% (4.4% to 16.4%)	10.0% (4.8% to 17.4%)	9.4% (4.4% to 16.4%)	10.0% (4.8% to 17.4%)
Pancreatic cancer	15.1% (8.6% to 23.8%)	15.7% (9.6% to 23.9%)	15.7% (9.2% to 24.4%)	15.7% (9.6% to 23.9%)
Ovarian cancer	9.3% (3.2% to 18.8%)	8.6% (2.9% to 17.2%)	8.4% (2.9% to 16.9%)	8.5% (2.9% to 17.2%)
Kidney cancer	14.0% (9.1% to 20.1%)	14.6% (9.7% to 20.8%)	14.9% (9.8% to 21.0%)	14.6% (9.7% to 20.8%)
Bladder cancer	18.1% (11.0% to 27.5%)	19.5% (12.4% to 28.5%)	19.4% (12.2% to 28.7%)	19.5% (12.4% to 28.5%)
Thyroid cancer	5.4% (2.5% to 9.7%)	5.6% (2.6% to 10.1%)	5.8% (2.8% to 10.1%)	5.6% (2.6% to 10.1%)
Mesothelioma	85.3% (80.0% to 89.2%)	76.1% (68.9% to 81.7%)	82.4% (76.3% to 86.9%)	76.2% (69.0% to 81.7%)
Non-Hodgkin's lymphoma	2.6% (1.0% to 4.9%)	2.5% (0.9% to 4.8%)	2.7% (1.0% to 5.0%)	2.5% (0.9% to 4.8%)
Multiple myeloma	3.1% (1.2% to 5.9%)	3.4% (1.4% to 6.3%)	3.2% (1.3% to 6.1%)	3.4% (1.4% to 6.3%)
Leukaemia	7.1% (4.4% to 10.4%)	6.4% (4.0% to 9.4%)	7.0% (4.4% to 10.2%)	6.4% (4.0% to 9.4%)
**Cardiovascular diseases**	87.7% (85.5% to 89.6%)	88.7% (87.2% to 90.1%)	74.8% (71.6% to 77.6%)	88.0% (86.4% to 89.5%)
Rheumatic heart disease	25.2% (17.2% to 39.4%)	23.6% (16.5% to 34.9%)	20.8% (13.4% to 30.2%)	23.3% (16.2% to 34.3%)
Ischaemic heart disease	92.9% (89.9% to 95.2%)	94.2% (92.4% to 95.7%)	89.4% (86.5% to 92.2%)	94.1% (92.2% to 95.6%)
Cerebrovascular disease	86.3% (82.9% to 89.6%)	89.7% (87.3% to 92.0%)	85.9% (82.5% to 89.1%)	89.5% (87.1% to 91.8%)
Hypertensive heart disease	100.0% (100.0% to 100.0%)	100.0% (100.0% to 100.0%)	100.0% (100.0% to 100.0%)	100.0% (100.0% to 100.0%)
Cardiomyopathy and myocarditis	33.2% (26.5% to 41.0%)	26.9% (20.5% to 35.1%)	17.8% (13.3% to 23.0%)	26.2% (20.0% to 34.0%)
Atrial fibrillation and flutter	41.1% (34.3% to 47.7%)	45.6% (39.5% to 51.1%)	52.4% (47.8% to 57.1%)	49.5% (44.4% to 54.1%)
Aortic aneurysm	41.5% (35.1% to 47.9%)	46.1% (40.6% to 51.3%)	-	46.1% (40.6% to 51.3%)
Peripheral vascular disease	52.3% (46.2% to 58.9%)	59.2% (54.1% to 64.5%)	59.4% (54.6% to 64.2%)	59.4% (54.6% to 64.1%)
Endocarditis	38.6% (29.9% to 48.3%)	37.1% (29.0% to 45.2%)	35.7% (27.4% to 45.0%)	37.1% (29.0% to 45.2%)

Supplementary Results Table 1. Age-standardised PAFs attributable to the joint distribution of all risk factors for all ages, both sexes combined for each GBD level 1 and level 2 cause and all causes in 2016 for South Asia. Results are shown for deaths, YLLs, YLDs, and DALYs
Data in parenthesis are 95% uncertainty intervals. PAFs=population attributable fractions. GBD=Global Burden of Disease. YLLs=years of life lost. YLDs=years live with disability. DALYs=disability-adjusted life-years

	Deaths 2016	YLLs 2016	YLDs 2016	DALYs 2016
Other cardiovascular and circulatory diseases	42.8% (37.7% to 47.9%)	47.7% (43.2% to 52.0%)	43.5% (39.1% to 48.1%)	46.9% (42.4% to 51.2%)
**Chronic respiratory diseases**	64.5% (57.4% to 71.6%)	63.8% (57.1% to 70.3%)	55.4% (47.8% to 62.5%)	61.6% (55.3% to 68.1%)
Chronic obstructive pulmonary disease	73.8% (65.8% to 82.0%)	74.8% (67.1% to 82.5%)	64.8% (56.0% to 73.5%)	72.3% (64.3% to 79.9%)
Pneumoconiosis	100.0% (100.0% to 100.0%)	100.0% (100.0% to 100.0%)	100.0% (100.0% to 100.0%)	100.0% (100.0% to 100.0%)
Asthma	27.2% (21.2% to 34.5%)	30.5% (24.5% to 37.5%)	27.4% (22.5% to 33.1%)	29.7% (24.1% to 36.4%)
Other chronic respiratory diseases	5.3% (3.6% to 7.3%)	5.5% (3.7% to 8.0%)	3.1% (1.9% to 4.8%)	4.3% (2.8% to 6.3%)
**Cirrhosis and other chronic liver diseases**	29.1% (26.9% to 31.3%)	28.1% (25.9% to 30.5%)	22.5% (20.6% to 24.5%)	27.9% (25.6% to 30.2%)
Cirrhosis and other chronic liver diseases due to hepatitis B	0.1% (0.1% to 0.1%)	0.1% (0.1% to 0.2%)	0.1% (0.1% to 0.2%)	0.1% (0.1% to 0.2%)
Cirrhosis and other chronic liver diseases due to hepatitis C	10.3% (7.0% to 14.3%)	14.7% (9.9% to 20.5%)	14.9% (10.0% to 20.7%)	14.7% (10.0% to 20.5%)
Cirrhosis and other chronic liver diseases due to alcohol use	100.0% (100.0% to 100.0%)	100.0% (100.0% to 100.0%)	100.0% (100.0% to 100.0%)	100.0% (100.0% to 100.0%)
**Digestive diseases**	5.7% (4.5% to 7.1%)	5.7% (4.5% to 7.1%)	2.2% (1.6% to 2.8%)	5.3% (4.2% to 6.7%)
Peptic ulcer disease	12.6% (9.0% to 16.7%)	12.8% (9.1% to 17.0%)	7.6% (5.4% to 10.1%)	12.3% (8.8% to 16.2%)
Gallbladder and biliary diseases	19.7% (11.6% to 30.1%)	20.2% (12.2% to 30.4%)	21.3% (12.7% to 31.6%)	20.3% (12.2% to 30.5%)
Pancreatitis	14.8% (9.5% to 21.0%)	16.9% (11.7% to 23.4%)	15.0% (10.7% to 20.6%)	16.9% (11.7% to 23.4%)
**Neurological disorders**	12.6% (6.1% to 20.8%)	10.6% (5.8% to 16.8%)	1.5% (0.9% to 2.4%)	4.2% (2.4% to 7.0%)
Alzheimer's disease and other dementias	15.8% (7.7% to 26.2%)	16.7% (8.4% to 27.3%)	16.8% (8.5% to 27.3%)	16.8% (8.4% to 27.4%)
Parkinson's disease	-10.0% (-12.1% to -7.8%)	-11.3% (-13.5% to -8.8%)	-11.8% (-14.1% to -9.1%)	-11.4% (-13.6% to -8.8%)
Epilepsy	7.8% (5.5% to 10.4%)	6.6% (4.7% to 8.6%)	5.9% (4.1% to 8.1%)	6.3% (4.4% to 8.2%)
Multiple sclerosis	6.6% (3.6% to 10.2%)	6.4% (3.4% to 10.0%)	4.7% (2.8% to 6.7%)	5.6% (3.2% to 8.4%)
**Mental and substance use disorders**	99.9% (99.8% to 99.9%)	99.8% (99.7% to 99.9%)	20.7% (18.4% to 23.0%)	24.8% (22.2% to 27.5%)
Alcohol use disorders	100.0% (100.0% to 100.0%)	100.0% (100.0% to 100.0%)	100.0% (100.0% to 100.0%)	100.0% (100.0% to 100.0%)

Supplementary Results Table 1. Age-standardised PAFs attributable to the joint distribution of all risk factors for all ages, both sexes combined for each GBD level 1 and level 2 cause and all causes in 2016 for South Asia. Results are shown for deaths, YLLs, YLDs, and DALYs
Data in parenthesis are 95% uncertainty intervals. PAFs=population attributable fractions. GBD=Global Burden of Disease. YLLs=years of life lost. YLDs=years live with disability. DALYs=disability-adjusted life-years

	Deaths 2016	YLLs 2016	YLDs 2016	DALYs 2016
Drug use disorders	100.0% (100.0% to 100.0%)	100.0% (100.0% to 100.0%)	100.0% (100.0% to 100.0%)	100.0% (100.0% to 100.0%)
Depressive disorders	-	-	11.3% (9.7% to 13.1%)	11.3% (9.7% to 13.1%)
Idiopathic developmental intellectual disability	-	-	65.3% (33.8% to 84.2%)	65.3% (33.8% to 84.2%)
**Diabetes, urogenital, blood, and endocrine diseases**	84.5% (83.6% to 85.2%)	82.1% (80.8% to 83.0%)	59.0% (55.2% to 62.6%)	73.2% (70.6% to 75.8%)
Diabetes mellitus	100.0% (100.0% to 100.0%)	100.0% (100.0% to 100.0%)	100.0% (100.0% to 100.0%)	100.0% (100.0% to 100.0%)
Chronic kidney disease	100.0% (100.0% to 100.0%)	100.0% (100.0% to 100.0%)	100.0% (100.0% to 100.0%)	100.0% (100.0% to 100.0%)
**Musculoskeletal disorders**	1.7% (0.6% to 2.9%)	1.5% (0.6% to 2.6%)	15.2% (13.0% to 17.6%)	15.0% (12.9% to 17.5%)
Rheumatoid arthritis	3.3% (1.3% to 5.6%)	3.4% (1.3% to 5.8%)	3.2% (1.3% to 5.3%)	3.2% (1.3% to 5.4%)
Osteoarthritis	-	-	11.0% (5.1% to 18.5%)	11.0% (5.1% to 18.5%)
Low back and neck pain	-	-	26.6% (23.9% to 29.7%)	26.6% (23.9% to 29.7%)
Gout	-	-	31.7% (23.5% to 42.5%)	31.7% (23.5% to 42.5%)
**Other non-communicable diseases**	0.4% (0.2% to 0.5%)	0.5% (0.3% to 0.7%)	9.0% (7.7% to 10.6%)	7.2% (6.1% to 8.3%)
Sense organ diseases	-	-	16.4% (14.4% to 18.5%)	16.4% (14.4% to 18.5%)
Sudden infant death syndrome	6.5% (4.9% to 8.3%)	6.6% (4.9% to 8.4%)	-	6.6% (4.9% to 8.4%)
**Transport injuries**	25.0% (22.5% to 28.0%)	21.3% (18.8% to 24.2%)	28.1% (25.7% to 30.9%)	22.4% (20.0% to 25.3%)
Road injuries	24.6% (22.0% to 27.5%)	21.1% (18.6% to 24.1%)	30.7% (28.2% to 33.6%)	22.5% (20.0% to 25.6%)
Other transport injuries	28.7% (26.2% to 31.6%)	22.4% (19.8% to 25.5%)	20.9% (19.0% to 23.3%)	21.9% (19.6% to 24.7%)
**Unintentional injuries**	34.2% (29.5% to 37.6%)	17.1% (15.7% to 19.2%)	17.2% (16.3% to 18.2%)	17.1% (16.0% to 18.7%)
Falls	60.5% (52.1% to 62.8%)	38.4% (35.5% to 40.2%)	27.4% (26.4% to 28.4%)	34.1% (31.8% to 35.8%)
Drowning	7.7% (6.4% to 9.3%)	6.9% (5.8% to 8.1%)	9.8% (8.3% to 11.7%)	6.9% (5.8% to 8.2%)
Fire, heat, and hot substances	5.3% (4.3% to 7.0%)	5.7% (4.7% to 7.4%)	8.5% (7.3% to 9.9%)	6.4% (5.3% to 8.2%)

Supplementary Results Table 1. Age-standardised PAFs attributable to the joint distribution of all risk factors for all ages, both sexes combined for each GBD level 1 and level 2 cause and all causes in 2016 for South Asia. Results are shown for deaths, YLLs, YLDs, and DALYs
Data in parenthesis are 95% uncertainty intervals. PAFs=population attributable fractions. GBD=Global Burden of Disease. YLLs=years of life lost. YLDs=years live with disability. DALYs=disability-adjusted life-years

	Deaths 2016	YLLs 2016	YLDs 2016	DALYs 2016
Poisonings	7.7% (6.3% to 9.5%)	6.9% (5.5% to 8.5%)	10.3% (8.9% to 12.0%)	7.3% (5.9% to 8.9%)
Exposure to mechanical forces	15.9% (15.2% to 16.6%)	11.1% (10.4% to 12.1%)	16.9% (16.0% to 17.8%)	13.2% (12.3% to 14.2%)
Animal contact	5.7% (4.8% to 7.9%)	6.0% (5.0% to 8.4%)	8.4% (7.7% to 9.2%)	6.7% (5.7% to 8.5%)
Foreign body	2.9% (2.4% to 3.7%)	3.3% (2.8% to 4.2%)	6.0% (5.5% to 6.6%)	4.8% (4.2% to 5.5%)
Other unintentional injuries	12.4% (10.4% to 15.0%)	14.1% (12.1% to 16.8%)	10.6% (9.0% to 12.6%)	12.3% (10.6% to 14.7%)
**Self-harm and interpersonal violence**	11.5% (8.3% to 15.0%)	11.0% (7.8% to 14.2%)	19.4% (17.9% to 20.8%)	11.4% (8.4% to 14.6%)
Self-harm	10.7% (6.6% to 14.9%)	10.4% (6.6% to 14.3%)	10.2% (6.3% to 14.3%)	10.4% (6.6% to 14.3%)
Interpersonal violence	14.7% (12.0% to 18.1%)	13.0% (10.4% to 16.1%)	21.5% (20.1% to 23.0%)	14.7% (12.4% to 17.5%)
**Forces of nature, conflict and terrorism, and executions and police conflict**	4.0% (3.2% to 4.7%)	1.7% (1.6% to 1.9%)	3.9% (2.7% to 5.6%)	2.7% (2.1% to 3.9%)
Forces of nature, conflict and terrorism, and state actor violence	6.8% (6.4% to 7.1%)	2.7% (2.5% to 2.9%)	5.7% (4.0% to 8.1%)	4.1% (3.2% to 5.9%)

Supplementary Results Table 1. Age-standardised PAFs attributable to the joint distribution of all risk factors for all ages, both sexes combined for each GBD level 1 and level 2 cause and all causes in 2016 for Southeast Asia, East Asia, and Oceania. Results are shown for deaths, YLLs,
Data in parenthesis are 95% uncertainty intervals. PAFs=population attributable fractions. GBD=Global Burden of Disease. YLLs=years of life lost. YLDs=years live with disability. DALYs=disability-adjusted life-years

	Deaths 2016	YLLs 2016	YLDs 2016	DALYs 2016
**All causes**	63.1% (60.7% to 65.4%)	57.0% (55.3% to 58.7%)	24.5% (23.3% to 25.7%)	45.3% (42.9% to 47.9%)
**HIV/AIDS and tuberculosis**	47.4% (42.1% to 52.3%)	49.3% (45.2% to 53.3%)	47.9% (42.6% to 53.5%)	49.1% (45.0% to 53.0%)
Tuberculosis	38.2% (31.5% to 44.8%)	35.9% (30.1% to 41.6%)	37.9% (32.7% to 42.8%)	36.3% (30.5% to 41.8%)
HIV/AIDS	78.9% (77.4% to 80.4%)	75.7% (73.9% to 77.4%)	81.7% (80.2% to 83.1%)	76.4% (74.6% to 78.1%)
**Diarrhoea, lower respiratory infections, and other common infectious diseases**	64.2% (58.8% to 69.3%)	64.4% (59.4% to 69.3%)	26.8% (19.9% to 32.5%)	58.1% (53.3% to 62.7%)
Diarrhoeal diseases	83.0% (60.9% to 95.0%)	89.5% (75.4% to 96.9%)	80.1% (58.8% to 92.8%)	86.9% (71.0% to 95.8%)
Lower respiratory infections	70.7% (65.2% to 76.3%)	80.7% (76.1% to 84.7%)	70.7% (64.9% to 76.1%)	80.6% (76.0% to 84.6%)
Upper respiratory infections	1.4% (0.9% to 2.2%)	3.2% (2.0% to 4.8%)	0.0% (0.0% to 0.0%)	0.0% (0.0% to 0.1%)
Otitis media	4.1% (1.4% to 10.2%)	8.8% (3.8% to 16.9%)	8.9% (6.1% to 11.5%)	8.9% (6.1% to 11.5%)
Meningitis	1.8% (1.4% to 2.2%)	2.5% (2.1% to 3.1%)	0.0% (0.0% to 0.0%)	2.2% (1.8% to 2.7%)
Encephalitis	0.8% (0.6% to 0.9%)	1.1% (0.9% to 1.3%)	0.0% (0.0% to 0.0%)	0.6% (0.5% to 0.8%)
Measles	65.7% (51.0% to 83.6%)	66.6% (51.8% to 84.7%)	60.6% (44.7% to 81.1%)	66.6% (51.7% to 84.7%)
**Maternal disorders**	6.9% (3.1% to 10.9%)	6.8% (3.1% to 10.8%)	2.9% (1.4% to 4.7%)	6.4% (2.9% to 10.2%)
Maternal haemorrhage	15.3% (5.5% to 24.8%)	15.2% (5.5% to 24.6%)	14.5% (5.2% to 23.6%)	15.2% (5.5% to 24.6%)
Maternal sepsis and other pregnancy related infections	15.1% (5.5% to 24.6%)	15.1% (5.5% to 24.5%)	14.2% (5.1% to 23.3%)	15.0% (5.5% to 24.5%)
Maternal abortion, miscarriage, and ectopic pregnancy	9.6% (5.5% to 14.1%)	9.3% (5.2% to 13.7%)	8.5% (4.4% to 13.0%)	9.3% (5.2% to 13.6%)
**Neonatal disorders**	77.7% (76.1% to 79.5%)	77.7% (76.2% to 79.5%)	0.2% (0.1% to 0.2%)	69.6% (66.6% to 72.3%)
Neonatal preterm birth complications	93.8% (93.0% to 95.0%)	93.9% (93.1% to 95.0%)	0.2% (0.2% to 0.3%)	79.7% (74.4% to 83.9%)
Neonatal encephalopathy due to birth asphyxia and trauma	67.7% (65.0% to 70.7%)	67.7% (65.1% to 70.7%)	0.0% (0.0% to 0.0%)	65.7% (62.5% to 69.0%)
Neonatal sepsis and other neonatal infections	59.7% (56.5% to 62.9%)	59.8% (56.5% to 62.9%)	0.0% (0.0% to 0.0%)	53.2% (45.3% to 58.3%)
Hemolytic disease and other neonatal jaundice	53.2% (50.2% to 56.4%)	53.3% (50.2% to 56.5%)	0.0% (0.0% to 0.0%)	33.4% (29.6% to 37.2%)

Supplementary Results Table 1. Age-standardised PAFs attributable to the joint distribution of all risk factors for all ages, both sexes combined for each GBD level 1 and level 2 cause and all causes in 2016 for Southeast Asia, East Asia, and Oceania. Results are shown for deaths, YLLs,
Data in parenthesis are 95% uncertainty intervals. PAFs=population attributable fractions. GBD=Global Burden of Disease. YLLs=years of life lost. YLDs=years live with disability. DALYs=disability-adjusted life-years

	Deaths 2016	YLLs 2016	YLDs 2016	DALYs 2016
Other neonatal disorders	55.7% (52.8% to 58.4%)	55.8% (52.9% to 58.5%)	0.0% (0.0% to 0.0%)	53.8% (50.9% to 56.6%)
**Nutritional deficiencies**	8.9% (7.7% to 10.2%)	37.0% (33.4% to 40.9%)	87.8% (86.0% to 89.3%)	78.3% (74.2% to 81.9%)
Protein-energy malnutrition	8.2% (7.0% to 9.7%)	39.5% (35.3% to 43.9%)	96.7% (96.3% to 97.1%)	59.4% (53.7% to 64.9%)
Vitamin A deficiency	-	-	99.8% (99.8% to 99.8%)	99.9% (99.9% to 99.9%)
Iron-deficiency anaemia	99.9% (99.8% to 99.9%)	99.9% (99.9% to 99.9%)	99.8% (99.7% to 99.8%)	99.8% (99.8% to 99.8%)
**Other communicable, maternal, neonatal, and nutritional diseases**	4.0% (3.3% to 4.6%)	2.9% (2.3% to 3.6%)	78.2% (74.8% to 81.2%)	26.0% (18.8% to 34.9%)
Sexually transmitted diseases excluding HIV	19.1% (10.6% to 30.6%)	8.4% (4.3% to 14.4%)	99.7% (99.7% to 99.8%)	55.6% (38.3% to 72.5%)
Hepatitis	0.9% (0.7% to 1.1%)	1.0% (0.8% to 1.2%)	0.7% (0.5% to 1.0%)	0.9% (0.8% to 1.2%)
**Neoplasms**	45.0% (38.9% to 51.6%)	41.7% (36.3% to 47.8%)	38.1% (33.1% to 43.6%)	41.7% (36.2% to 47.7%)
Oesophageal cancer	67.1% (53.8% to 80.4%)	67.5% (55.1% to 80.4%)	65.4% (52.3% to 79.3%)	67.5% (55.1% to 80.4%)
Stomach cancer	21.3% (11.3% to 32.8%)	20.0% (11.2% to 30.7%)	20.6% (10.9% to 32.1%)	20.1% (11.2% to 30.7%)
Liver cancer	37.4% (28.4% to 49.2%)	35.9% (26.7% to 47.8%)	36.4% (27.5% to 48.4%)	35.9% (26.7% to 47.8%)
Larynx cancer	73.6% (59.0% to 84.9%)	72.5% (58.8% to 84.0%)	72.8% (57.7% to 84.9%)	72.5% (58.8% to 84.0%)
Tracheal, bronchus, and lung cancer	81.5% (70.5% to 90.1%)	79.8% (69.1% to 88.8%)	80.3% (68.9% to 89.5%)	79.9% (69.1% to 88.8%)
Breast cancer	27.9% (17.9% to 39.4%)	26.9% (17.5% to 37.6%)	26.9% (17.3% to 38.0%)	26.9% (17.5% to 37.7%)
Cervical cancer	99.9% (99.9% to 99.9%)	99.9% (99.9% to 99.9%)	99.9% (99.8% to 99.9%)	99.9% (99.9% to 99.9%)
Uterine cancer	23.2% (12.9% to 35.6%)	24.6% (14.1% to 37.3%)	25.6% (14.3% to 38.8%)	24.7% (14.1% to 37.4%)
Prostate cancer	3.8% (2.1% to 6.6%)	3.4% (2.0% to 6.0%)	3.3% (1.7% to 6.0%)	3.4% (2.0% to 6.0%)
Colon and rectum cancer	53.3% (45.0% to 61.6%)	52.9% (44.6% to 60.8%)	53.1% (44.9% to 61.3%)	52.9% (44.6% to 60.8%)
Lip and oral cavity cancer	72.3% (65.2% to 79.1%)	71.0% (64.6% to 77.5%)	69.4% (62.3% to 76.6%)	71.0% (64.5% to 77.5%)
Nasopharynx cancer	65.3% (57.9% to 73.7%)	64.5% (57.9% to 72.1%)	62.4% (55.9% to 70.5%)	64.4% (57.9% to 72.0%)

Supplementary Results Table 1. Age-standardised PAFs attributable to the joint distribution of all risk factors for all ages, both sexes combined for each GBD level 1 and level 2 cause and all causes in 2016 for Southeast Asia, East Asia, and Oceania. Results are shown for deaths, YLLs, Data in parenthesis are 95% uncertainty intervals. PAFs=population attributable fractions. GBD=Global Burden of Disease. YLLs=years of life lost. YLDs=years live with disability. DALYs=disability-adjusted life-years				
	**Deaths 2016**	**YLLs 2016**	**YLDs 2016**	**DALYs 2016**
Other pharynx cancer	52.0% (44.0% to 60.0%)	54.0% (46.2% to 61.8%)	54.9% (47.0% to 62.7%)	54.1% (46.3% to 61.8%)
Gallbladder and biliary tract cancer	10.0% (4.3% to 18.1%)	10.7% (4.8% to 19.2%)	10.4% (4.5% to 18.7%)	10.7% (4.8% to 19.2%)
Pancreatic cancer	21.5% (12.9% to 32.7%)	20.2% (12.2% to 30.7%)	20.9% (12.3% to 32.0%)	20.2% (12.2% to 30.7%)
Ovarian cancer	9.9% (3.9% to 18.6%)	8.5% (3.1% to 16.2%)	8.3% (3.0% to 15.7%)	8.5% (3.1% to 16.2%)
Kidney cancer	19.5% (11.4% to 29.8%)	17.4% (10.2% to 26.6%)	17.7% (10.2% to 26.9%)	17.4% (10.2% to 26.6%)
Bladder cancer	27.2% (15.2% to 41.1%)	25.0% (13.9% to 38.1%)	24.7% (13.2% to 38.0%)	25.0% (13.9% to 38.1%)
Thyroid cancer	6.3% (2.5% to 12.3%)	6.7% (2.8% to 12.8%)	7.2% (3.1% to 13.3%)	6.7% (2.8% to 12.9%)
Mesothelioma	81.3% (76.6% to 85.2%)	70.4% (64.3% to 75.9%)	77.4% (72.3% to 81.9%)	70.5% (64.4% to 75.9%)
Non-Hodgkin's lymphoma	3.0% (1.0% to 6.2%)	2.8% (1.0% to 5.7%)	3.2% (1.1% to 6.5%)	2.8% (1.0% to 5.8%)
Multiple myeloma	3.6% (1.3% to 7.3%)	3.8% (1.4% to 7.4%)	3.8% (1.3% to 7.5%)	3.8% (1.4% to 7.4%)
Leukaemia	9.7% (5.6% to 15.8%)	7.1% (4.3% to 11.2%)	8.1% (4.6% to 13.4%)	7.2% (4.3% to 11.3%)
**Cardiovascular diseases**	86.6% (84.3% to 88.7%)	87.6% (86.1% to 89.1%)	79.2% (76.5% to 81.7%)	86.7% (85.1% to 88.2%)
Rheumatic heart disease	25.6% (16.6% to 42.0%)	23.5% (16.3% to 35.9%)	19.7% (13.1% to 28.6%)	23.2% (16.3% to 35.5%)
Ischaemic heart disease	92.4% (89.0% to 95.2%)	93.9% (91.7% to 95.6%)	90.3% (87.8% to 92.7%)	93.7% (91.6% to 95.4%)
Cerebrovascular disease	85.6% (81.7% to 89.2%)	88.8% (86.2% to 91.1%)	86.5% (83.2% to 89.5%)	88.4% (86.0% to 90.8%)
Hypertensive heart disease	99.9% (99.9% to 100.0%)	99.9% (99.9% to 100.0%)	99.9% (99.9% to 99.9%)	99.9% (99.9% to 100.0%)
Cardiomyopathy and myocarditis	31.0% (25.7% to 36.9%)	28.6% (24.4% to 32.8%)	23.8% (19.5% to 28.1%)	28.3% (24.2% to 32.4%)
Atrial fibrillation and flutter	48.0% (40.8% to 54.2%)	52.5% (46.4% to 57.8%)	59.0% (54.5% to 63.4%)	56.2% (51.1% to 60.9%)
Aortic aneurysm	47.1% (40.5% to 53.8%)	52.4% (46.8% to 57.6%)	-	52.4% (46.8% to 57.6%)
Peripheral vascular disease	52.3% (46.1% to 58.8%)	59.5% (54.5% to 64.2%)	58.4% (53.2% to 63.3%)	58.7% (53.6% to 63.4%)
Endocarditis	38.2% (29.8% to 47.3%)	33.4% (26.5% to 40.7%)	37.1% (29.3% to 45.6%)	33.5% (26.6% to 40.8%)

Supplementary Results Table 1. Age-standardised PAFs attributable to the joint distribution of all risk factors for all ages, both sexes combined for each GBD level 1 and level 2 cause and all causes in 2016 for Southeast Asia, East Asia, and Oceania. Results are shown for deaths, YLLs,
Data in parenthesis are 95% uncertainty intervals. PAFs=population attributable fractions. GBD=Global Burden of Disease. YLLs=years of life lost. YLDs=years live with disability. DALYs=disability-adjusted life-years

	Deaths 2016	YLLs 2016	YLDs 2016	DALYs 2016
Other cardiovascular and circulatory diseases	46.6% (41.7% to 51.5%)	45.3% (41.5% to 49.4%)	43.6% (39.0% to 48.1%)	44.8% (40.8% to 48.8%)
**Chronic respiratory diseases**	75.1% (63.8% to 83.3%)	71.5% (61.7% to 79.0%)	49.0% (42.4% to 56.3%)	64.8% (56.3% to 71.8%)
Chronic obstructive pulmonary disease	79.6% (67.0% to 88.5%)	77.9% (66.6% to 86.6%)	69.5% (59.7% to 77.7%)	76.1% (64.9% to 84.7%)
Pneumoconiosis	100.0% (100.0% to 100.0%)	100.0% (100.0% to 100.0%)	100.0% (100.0% to 100.0%)	100.0% (100.0% to 100.0%)
Asthma	32.8% (26.5% to 40.1%)	34.9% (29.1% to 41.4%)	24.4% (18.9% to 31.1%)	28.7% (23.3% to 35.1%)
Other chronic respiratory diseases	10.8% (6.5% to 16.5%)	7.4% (4.9% to 10.9%)	5.2% (3.5% to 8.0%)	6.3% (4.3% to 9.4%)
**Cirrhosis and other chronic liver diseases**	25.9% (24.2% to 27.8%)	27.6% (25.8% to 29.7%)	22.0% (20.6% to 23.5%)	27.4% (25.6% to 29.4%)
Cirrhosis and other chronic liver diseases due to hepatitis B	0.5% (0.4% to 0.7%)	0.6% (0.5% to 0.8%)	0.6% (0.5% to 0.8%)	0.6% (0.5% to 0.8%)
Cirrhosis and other chronic liver diseases due to hepatitis C	49.2% (44.9% to 54.0%)	59.1% (54.7% to 63.6%)	63.9% (59.8% to 68.1%)	59.3% (54.9% to 63.7%)
Cirrhosis and other chronic liver diseases due to alcohol use	99.9% (99.9% to 99.9%)	99.9% (99.9% to 99.9%)	99.9% (99.9% to 99.9%)	99.9% (99.9% to 99.9%)
**Digestive diseases**	11.0% (8.9% to 13.1%)	11.1% (9.2% to 13.1%)	3.2% (2.5% to 4.0%)	8.7% (7.1% to 10.4%)
Peptic ulcer disease	19.4% (14.3% to 24.8%)	21.2% (15.8% to 26.7%)	10.1% (7.3% to 13.0%)	18.5% (13.6% to 23.6%)
Gallbladder and biliary diseases	21.2% (12.1% to 33.5%)	23.0% (13.9% to 35.0%)	26.1% (15.2% to 39.8%)	23.4% (14.1% to 35.5%)
Pancreatitis	29.4% (22.4% to 36.8%)	30.0% (23.6% to 36.6%)	24.4% (19.4% to 29.8%)	29.8% (23.5% to 36.4%)
**Neurological disorders**	15.3% (7.5% to 25.3%)	14.9% (8.0% to 23.8%)	4.3% (2.7% to 6.5%)	8.8% (5.0% to 14.0%)
Alzheimer's disease and other dementias	18.0% (9.2% to 28.9%)	19.3% (10.1% to 30.5%)	19.2% (9.9% to 30.6%)	19.2% (10.0% to 30.5%)
Parkinson's disease	-19.8% (-23.6% to -15.3%)	-23.3% (-28.1% to -17.8%)	-24.2% (-29.4% to -18.3%)	-23.5% (-28.4% to -17.9%)
Epilepsy	18.6% (13.8% to 22.9%)	15.8% (11.8% to 19.4%)	12.0% (8.9% to 15.2%)	13.3% (9.9% to 16.6%)
Multiple sclerosis	11.3% (6.9% to 15.8%)	11.0% (6.6% to 15.4%)	7.9% (4.9% to 10.9%)	9.8% (6.0% to 13.7%)
**Mental and substance use disorders**	99.8% (99.7% to 99.8%)	99.7% (99.6% to 99.7%)	17.1% (15.2% to 19.0%)	21.7% (19.4% to 24.0%)
Alcohol use disorders	100.0% (100.0% to 100.0%)	100.0% (100.0% to 100.0%)	99.9% (99.9% to 99.9%)	100.0% (99.9% to 100.0%)

**Supplementary Results Table 1. Age-standardised PAFs attributable to the joint distribution of all risk factors for all ages, both sexes combined for each GBD level 1 and level 2 cause and all causes in 2016 for Southeast Asia, East Asia, and Oceania. Results are shown for deaths, YLLs,** Data in parenthesis are 95% uncertainty intervals. PAFs=population attributable fractions. GBD=Global Burden of Disease. YLLs=years of life lost. YLDs=years live with disability. DALYs=disability-adjusted life-years	**Deaths 2016**	**YLLs 2016**	**YLDs 2016**	**DALYs 2016**
Drug use disorders	100.0% (100.0% to 100.0%)	100.0% (100.0% to 100.0%)	99.9% (99.9% to 100.0%)	100.0% (100.0% to 100.0%)
Depressive disorders	-	-	4.7% (3.8% to 5.7%)	4.7% (3.8% to 5.7%)
Idiopathic developmental intellectual disability	-	-	65.0% (33.4% to 84.0%)	65.0% (33.4% to 84.0%)
**Diabetes, urogenital, blood, and endocrine diseases**	86.5% (85.8% to 87.1%)	83.9% (83.0% to 84.7%)	57.5% (54.0% to 61.0%)	70.8% (67.5% to 73.7%)
Diabetes mellitus	99.9% (99.9% to 99.9%)	99.9% (99.9% to 99.9%)	99.9% (99.9% to 99.9%)	99.9% (99.9% to 99.9%)
Chronic kidney disease	99.9% (99.9% to 99.9%)	99.9% (99.9% to 99.9%)	99.9% (99.9% to 99.9%)	99.9% (99.9% to 99.9%)
**Musculoskeletal disorders**	2.7% (1.1% to 4.6%)	1.9% (0.8% to 3.2%)	15.1% (12.6% to 18.1%)	14.9% (12.4% to 17.8%)
Rheumatoid arthritis	6.1% (2.6% to 10.1%)	6.4% (2.8% to 10.6%)	4.3% (1.8% to 7.0%)	4.7% (1.9% to 7.6%)
Osteoarthritis	-	-	14.0% (6.0% to 24.5%)	14.0% (6.0% to 24.5%)
Low back and neck pain	-	-	20.5% (17.8% to 23.6%)	20.5% (17.8% to 23.6%)
Gout	-	-	28.1% (17.7% to 41.7%)	28.1% (17.7% to 41.7%)
**Other non-communicable diseases**	0.2% (0.1% to 0.2%)	0.2% (0.2% to 0.2%)	6.9% (6.1% to 7.9%)	5.4% (4.8% to 6.1%)
Sense organ diseases	-	-	14.6% (13.2% to 16.0%)	14.6% (13.2% to 16.0%)
Sudden infant death syndrome	6.9% (5.3% to 8.6%)	6.9% (5.3% to 8.6%)	-	6.9% (5.3% to 8.6%)
**Transport injuries**	34.8% (30.0% to 40.3%)	30.9% (26.5% to 36.0%)	38.9% (35.1% to 43.4%)	32.2% (27.9% to 37.2%)
Road injuries	34.6% (29.8% to 40.1%)	30.9% (26.4% to 36.0%)	41.4% (37.5% to 46.1%)	32.3% (27.9% to 37.4%)
Other transport injuries	37.7% (33.2% to 42.8%)	31.8% (27.6% to 36.7%)	30.1% (26.5% to 34.0%)	31.0% (27.2% to 35.3%)
**Unintentional injuries**	30.6% (22.0% to 33.8%)	17.5% (15.7% to 19.0%)	22.4% (21.3% to 23.6%)	18.8% (17.4% to 20.2%)
Falls	54.5% (34.3% to 60.5%)	33.1% (27.3% to 35.4%)	31.2% (30.2% to 32.3%)	32.4% (28.8% to 34.0%)
Drowning	11.5% (9.1% to 14.2%)	9.7% (8.0% to 11.6%)	17.0% (14.3% to 20.0%)	9.8% (8.1% to 11.7%)
Fire, heat, and hot substances	13.0% (10.0% to 16.5%)	14.3% (12.0% to 17.2%)	22.0% (19.1% to 25.4%)	17.4% (14.8% to 20.4%)

Supplementary Results Table 1. Age-standardised PAFs attributable to the joint distribution of all risk factors for all ages, both sexes combined for each GBD level 1 and level 2 cause and all causes in 2016 for Southeast Asia, East Asia, and Oceania. Results are shown for deaths, YLLs,
Data in parenthesis are 95% uncertainty intervals. PAFs=population attributable fractions. GBD=Global Burden of Disease. YLLs=years of life lost. YLDs=years live with disability. DALYs=disability-adjusted life-years

	**Deaths 2016**	**YLLs 2016**	**YLDs 2016**	**DALYs 2016**
Poisonings	15.5% (12.3% to 19.3%)	15.6% (12.9% to 18.8%)	21.1% (18.2% to 24.5%)	15.9% (13.1% to 19.1%)
Exposure to mechanical forces	18.5% (17.6% to 20.3%)	14.9% (13.9% to 17.1%)	25.0% (24.0% to 26.3%)	17.9% (16.6% to 19.9%)
Animal contact	15.5% (14.4% to 17.1%)	14.4% (13.3% to 15.9%)	18.0% (16.9% to 19.2%)	15.6% (14.6% to 16.8%)
Foreign body	6.4% (5.7% to 7.4%)	5.8% (5.2% to 6.6%)	13.4% (12.6% to 14.2%)	7.9% (7.0% to 8.9%)
Other unintentional injuries	24.9% (20.1% to 30.1%)	25.1% (20.7% to 29.7%)	20.4% (17.2% to 24.3%)	23.2% (19.3% to 27.5%)
**Self-harm and interpersonal violence**	21.8% (16.0% to 27.5%)	20.9% (15.8% to 25.9%)	28.0% (24.6% to 31.5%)	21.7% (16.9% to 26.2%)
Self-harm	21.8% (14.6% to 29.0%)	21.6% (14.6% to 28.4%)	18.2% (12.1% to 24.4%)	21.6% (14.6% to 28.3%)
Interpersonal violence	21.6% (16.4% to 26.9%)	19.2% (14.2% to 24.2%)	28.8% (25.3% to 32.6%)	21.9% (17.7% to 26.5%)
**Forces of nature, conflict and terrorism, and executions and police conflict**	2.8% (1.0% to 4.2%)	1.0% (0.4% to 1.6%)	2.0% (1.4% to 2.7%)	1.4% (1.0% to 1.9%)
Forces of nature, conflict and terrorism, and state actor violence	7.3% (6.5% to 7.8%)	2.9% (2.5% to 3.2%)	5.7% (4.2% to 7.7%)	4.3% (3.4% to 5.7%)

Supplementary Results Table 1. Age-standardised PAFs attributable to the joint distribution of all risk factors for all ages, both sexes combined for each GBD level 1 and level 2 cause and all causes in 2016 for Sub-Saharan Africa. Results are shown for deaths, YLLs, YLDs, and DALYs
Data in parenthesis are 95% uncertainty intervals. PAFs=population attributable fractions. GBD=Global Burden of Disease. YLLs=years of life lost. YLDs=years live with disability. DALYs=disability-adjusted life-years

	Deaths 2016	YLLs 2016	YLDs 2016	DALYs 2016
**All causes**	57.6% (55.9% to 59.5%)	54.4% (52.9% to 56.1%)	29.4% (28.2% to 30.6%)	48.9% (47.1% to 50.7%)
**HIV/AIDS and tuberculosis**	65.8% (61.9% to 69.7%)	70.6% (67.9% to 73.3%)	85.1% (82.5% to 87.6%)	71.5% (68.9% to 74.0%)
Tuberculosis	36.6% (29.0% to 44.2%)	38.1% (30.8% to 45.5%)	36.3% (29.6% to 43.0%)	38.1% (30.7% to 45.4%)
HIV/AIDS	90.8% (89.7% to 91.7%)	86.8% (85.5% to 87.9%)	92.2% (91.0% to 93.4%)	87.2% (86.0% to 88.3%)
**Diarrhoea, lower respiratory infections, and other common infectious diseases**	75.7% (71.2% to 80.7%)	73.9% (69.9% to 78.3%)	45.7% (40.3% to 50.5%)	72.6% (68.7% to 76.9%)
Diarrhoeal diseases	95.1% (87.6% to 98.7%)	96.6% (91.2% to 99.1%)	94.5% (86.6% to 98.4%)	96.5% (90.9% to 99.1%)
Lower respiratory infections	78.1% (72.8% to 82.8%)	84.6% (80.4% to 88.2%)	77.6% (72.2% to 82.5%)	84.6% (80.4% to 88.1%)
Upper respiratory infections	1.5% (0.9% to 2.5%)	2.6% (1.5% to 4.4%)	0.0% (0.0% to 0.0%)	0.2% (0.1% to 0.3%)
Otitis media	2.1% (1.3% to 3.1%)	5.3% (3.6% to 7.2%)	3.3% (2.2% to 4.4%)	3.3% (2.2% to 4.5%)
Meningitis	2.5% (1.7% to 3.2%)	4.5% (3.4% to 5.6%)	0.0% (0.0% to 0.0%)	4.3% (3.2% to 5.3%)
Encephalitis	1.9% (1.4% to 2.7%)	2.9% (2.2% to 4.0%)	0.0% (0.0% to 0.0%)	1.9% (1.3% to 2.7%)
Measles	65.7% (52.2% to 82.0%)	66.7% (52.9% to 83.2%)	64.3% (50.9% to 81.0%)	66.7% (52.9% to 83.2%)
**Maternal disorders**	7.3% (3.5% to 11.4%)	7.3% (3.5% to 11.4%)	2.0% (1.0% to 3.1%)	7.0% (3.4% to 10.9%)
Maternal haemorrhage	17.7% (6.4% to 28.9%)	17.8% (6.4% to 29.1%)	17.8% (6.4% to 28.9%)	17.8% (6.4% to 29.1%)
Maternal sepsis and other pregnancy related infections	17.5% (6.3% to 28.7%)	17.7% (6.4% to 28.9%)	17.4% (6.2% to 28.4%)	17.7% (6.4% to 28.9%)
Maternal abortion, miscarriage, and ectopic pregnancy	17.1% (11.0% to 23.2%)	16.8% (10.7% to 22.9%)	18.8% (11.3% to 26.6%)	16.8% (10.7% to 23.0%)
**Neonatal disorders**	80.1% (78.1% to 82.1%)	80.1% (78.1% to 82.1%)	0.4% (0.2% to 0.7%)	77.0% (74.2% to 79.6%)
Neonatal preterm birth complications	96.7% (95.7% to 97.8%)	96.8% (95.8% to 97.8%)	1.3% (0.9% to 1.6%)	92.7% (90.6% to 94.4%)
Neonatal encephalopathy due to birth asphyxia and trauma	77.4% (74.4% to 80.6%)	77.4% (74.4% to 80.7%)	0.0% (0.0% to 0.0%)	75.5% (71.5% to 79.2%)
Neonatal sepsis and other neonatal infections	67.3% (64.5% to 70.4%)	67.3% (64.5% to 70.4%)	0.0% (0.0% to 0.0%)	63.1% (56.0% to 67.9%)
Hemolytic disease and other neonatal jaundice	69.9% (66.7% to 73.3%)	69.9% (66.7% to 73.3%)	0.0% (0.0% to 0.0%)	64.6% (61.1% to 68.5%)

Supplementary Results Table 1. Age-standardised PAFs attributable to the joint distribution of all risk factors for all ages, both sexes combined for each GBD level 1 and level 2 cause and all causes in 2016 for Sub-Saharan Africa. Results are shown for deaths, YLLs, YLDs, and DALYs
Data in parenthesis are 95% uncertainty intervals. PAFs=population attributable fractions. GBD=Global Burden of Disease. YLLs=years of life lost. YLDs=years live with disability. DALYs=disability-adjusted life-years

	Deaths 2016	YLLs 2016	YLDs 2016	DALYs 2016
Other neonatal disorders	69.8% (66.9% to 73.0%)	69.8% (67.0% to 73.0%)	0.0% (0.0% to 0.0%)	68.4% (65.6% to 71.7%)
**Nutritional deficiencies**	28.1% (22.5% to 32.6%)	62.3% (55.5% to 67.1%)	89.3% (87.7% to 90.9%)	73.4% (68.0% to 77.2%)
Protein-energy malnutrition	32.8% (27.5% to 37.5%)	68.1% (62.1% to 72.7%)	97.8% (97.6% to 98.0%)	69.1% (63.3% to 73.5%)
Vitamin A deficiency	-	-	100.0% (100.0% to 100.0%)	100.0% (100.0% to 100.0%)
Iron-deficiency anaemia	100.0% (100.0% to 100.0%)	100.0% (100.0% to 100.0%)	100.0% (100.0% to 100.0%)	100.0% (100.0% to 100.0%)
**Other communicable, maternal, neonatal, and nutritional diseases**	4.4% (3.4% to 5.8%)	2.7% (2.0% to 3.8%)	68.2% (64.2% to 72.8%)	11.0% (7.4% to 16.1%)
Sexually transmitted diseases excluding HIV	11.0% (6.8% to 17.5%)	4.8% (2.9% to 7.8%)	100.0% (100.0% to 100.0%)	18.9% (11.3% to 29.9%)
Hepatitis	0.4% (0.3% to 0.5%)	0.4% (0.3% to 0.5%)	0.3% (0.2% to 0.5%)	0.4% (0.3% to 0.5%)
**Neoplasms**	32.1% (29.5% to 35.5%)	32.3% (29.8% to 35.4%)	32.4% (29.7% to 36.2%)	32.3% (29.8% to 35.4%)
Oesophageal cancer	62.2% (51.3% to 71.8%)	64.5% (53.9% to 73.9%)	63.5% (52.9% to 73.0%)	64.5% (53.9% to 73.9%)
Stomach cancer	6.5% (2.5% to 14.8%)	6.7% (2.7% to 14.5%)	6.6% (2.6% to 14.7%)	6.7% (2.7% to 14.5%)
Liver cancer	30.9% (24.6% to 38.1%)	30.6% (24.4% to 37.8%)	31.1% (25.0% to 38.4%)	30.6% (24.4% to 37.8%)
Larynx cancer	56.7% (46.8% to 65.6%)	59.0% (49.3% to 68.0%)	59.0% (49.6% to 67.6%)	59.0% (49.3% to 68.0%)
Tracheal, bronchus, and lung cancer	82.2% (78.1% to 86.1%)	82.4% (78.5% to 86.2%)	81.9% (77.8% to 85.6%)	82.4% (78.5% to 86.1%)
Breast cancer	20.4% (14.8% to 27.3%)	18.7% (13.4% to 25.0%)	20.0% (14.2% to 26.6%)	18.8% (13.4% to 25.0%)
Cervical cancer	100.0% (100.0% to 100.0%)	100.0% (100.0% to 100.0%)	100.0% (100.0% to 100.0%)	100.0% (100.0% to 100.0%)
Uterine cancer	29.0% (19.0% to 39.8%)	29.8% (19.8% to 40.8%)	33.3% (23.2% to 44.4%)	29.8% (19.8% to 40.8%)
Prostate cancer	1.2% (0.8% to 1.6%)	1.2% (0.9% to 1.7%)	1.5% (1.1% to 2.2%)	1.3% (0.9% to 1.7%)
Colon and rectum cancer	47.3% (38.9% to 55.9%)	47.6% (39.6% to 55.8%)	48.3% (40.1% to 56.7%)	47.6% (39.6% to 55.8%)
Lip and oral cavity cancer	58.5% (50.5% to 65.6%)	60.8% (53.3% to 67.1%)	59.5% (52.2% to 66.2%)	60.8% (53.3% to 67.1%)
Nasopharynx cancer	52.5% (45.6% to 59.3%)	53.5% (47.1% to 59.9%)	53.6% (47.3% to 59.5%)	53.5% (47.1% to 59.9%)

Supplementary Results Table 1. Age-standardised PAFs attributable to the joint distribution of all risk factors for all ages, both sexes combined for each GBD level 1 and level 2 cause and all causes in 2016 for Sub-Saharan Africa. Results are shown for deaths, YLLs, YLDs, and DALYs
Data in parenthesis are 95% uncertainty intervals. PAFs=population attributable fractions. GBD=Global Burden of Disease. YLLs=years of life lost. YLDs=years live with disability. DALYs=disability-adjusted life-years

	Deaths 2016	YLLs 2016	YLDs 2016	DALYs 2016
Other pharynx cancer	42.8% (31.4% to 52.8%)	44.9% (34.1% to 54.7%)	44.3% (33.0% to 54.0%)	44.9% (34.1% to 54.7%)
Gallbladder and biliary tract cancer	14.6% (7.8% to 23.7%)	15.2% (8.2% to 24.4%)	15.1% (8.1% to 24.3%)	15.2% (8.2% to 24.4%)
Pancreatic cancer	15.5% (9.7% to 23.1%)	15.5% (10.0% to 22.6%)	15.6% (9.8% to 23.1%)	15.5% (10.0% to 22.6%)
Ovarian cancer	9.0% (3.8% to 16.2%)	7.8% (3.1% to 14.0%)	8.1% (3.3% to 14.5%)	7.8% (3.1% to 14.0%)
Kidney cancer	17.3% (11.7% to 24.2%)	15.7% (10.7% to 22.0%)	17.2% (12.0% to 23.9%)	15.7% (10.7% to 22.0%)
Bladder cancer	14.1% (8.6% to 21.5%)	14.1% (8.8% to 20.9%)	15.9% (10.4% to 23.3%)	14.1% (8.9% to 20.9%)
Thyroid cancer	6.7% (3.3% to 11.5%)	6.8% (3.3% to 11.7%)	8.1% (4.3% to 13.3%)	6.8% (3.3% to 11.7%)
Mesothelioma	88.5% (85.3% to 91.1%)	80.7% (76.2% to 84.5%)	85.8% (82.0% to 88.7%)	80.8% (76.3% to 84.5%)
Non-Hodgkin's lymphoma	3.1% (1.1% to 5.8%)	2.5% (1.0% to 4.8%)	3.1% (1.2% to 5.7%)	2.5% (1.0% to 4.8%)
Multiple myeloma	4.6% (1.9% to 8.4%)	4.7% (1.9% to 8.5%)	4.9% (2.0% to 8.9%)	4.7% (1.9% to 8.5%)
Leukaemia	8.9% (5.6% to 12.9%)	7.8% (5.0% to 11.2%)	9.0% (5.7% to 12.9%)	7.8% (5.0% to 11.2%)
**Cardiovascular diseases**	84.1% (81.8% to 86.0%)	84.5% (82.7% to 86.0%)	63.4% (59.5% to 66.9%)	82.8% (81.0% to 84.4%)
Rheumatic heart disease	28.7% (19.5% to 44.3%)	27.0% (18.8% to 39.5%)	23.9% (15.6% to 33.8%)	25.9% (18.1% to 37.7%)
Ischaemic heart disease	93.3% (90.4% to 95.6%)	94.5% (92.7% to 96.0%)	90.2% (87.6% to 92.6%)	94.3% (92.5% to 95.9%)
Cerebrovascular disease	87.5% (84.1% to 90.5%)	89.5% (87.3% to 91.4%)	87.7% (84.5% to 90.7%)	89.4% (87.2% to 91.3%)
Hypertensive heart disease	99.9% (99.9% to 100.0%)	100.0% (100.0% to 100.0%)	100.0% (100.0% to 100.0%)	100.0% (100.0% to 100.0%)
Cardiomyopathy and myocarditis	42.2% (34.7% to 50.9%)	40.3% (33.7% to 46.9%)	44.8% (38.5% to 51.4%)	40.6% (34.1% to 47.2%)
Atrial fibrillation and flutter	46.9% (39.5% to 54.0%)	51.1% (44.6% to 57.3%)	58.1% (53.3% to 62.5%)	54.8% (49.1% to 59.8%)
Aortic aneurysm	43.6% (36.4% to 50.5%)	48.1% (42.1% to 53.8%)	-	48.1% (42.1% to 53.8%)
Peripheral vascular disease	57.6% (51.5% to 64.2%)	65.8% (61.2% to 70.5%)	60.7% (55.5% to 66.0%)	62.8% (58.1% to 67.6%)
Endocarditis	39.2% (30.8% to 48.6%)	36.6% (29.2% to 44.1%)	36.4% (27.9% to 46.6%)	36.6% (29.3% to 44.1%)

Supplementary Results Table 1. Age-standardised PAFs attributable to the joint distribution of all risk factors for all ages, both sexes combined for each GBD level 1 and level 2 cause and all causes in 2016 for Sub-Saharan Africa. Results are shown for deaths, YLLs, YLDs, and DALYs
Data in parenthesis are 95% uncertainty intervals. PAFs=population attributable fractions. GBD=Global Burden of Disease. YLLs=years of life lost. YLDs=years live with disability. DALYs=disability-adjusted life-years

	Deaths 2016	YLLs 2016	YLDs 2016	DALYs 2016
Other cardiovascular and circulatory diseases	45.1% (40.2% to 50.1%)	47.0% (42.9% to 51.1%)	40.0% (34.9% to 45.1%)	45.7% (41.5% to 49.7%)
**Chronic respiratory diseases**	60.7% (54.0% to 66.9%)	57.5% (51.4% to 63.2%)	41.3% (35.5% to 47.9%)	52.4% (46.8% to 58.2%)
Chronic obstructive pulmonary disease	75.0% (67.4% to 82.5%)	75.0% (68.0% to 82.4%)	66.3% (57.5% to 75.2%)	73.1% (66.0% to 80.5%)
Pneumoconiosis	100.0% (100.0% to 100.0%)	100.0% (100.0% to 100.0%)	100.0% (100.0% to 100.0%)	100.0% (100.0% to 100.0%)
Asthma	28.5% (21.8% to 36.7%)	30.9% (24.9% to 38.4%)	24.1% (18.6% to 30.7%)	27.8% (22.2% to 34.6%)
Other chronic respiratory diseases	4.3% (2.8% to 6.0%)	3.9% (2.4% to 6.0%)	3.0% (1.8% to 4.6%)	3.8% (2.3% to 5.7%)
**Cirrhosis and other chronic liver diseases**	29.0% (26.7% to 31.5%)	28.1% (25.8% to 30.5%)	22.4% (20.5% to 24.4%)	28.0% (25.7% to 30.4%)
Cirrhosis and other chronic liver diseases due to hepatitis B	0.1% (0.1% to 0.2%)	0.1% (0.1% to 0.2%)	0.1% (0.1% to 0.2%)	0.1% (0.1% to 0.2%)
Cirrhosis and other chronic liver diseases due to hepatitis C	7.7% (6.0% to 9.8%)	10.3% (7.8% to 13.2%)	12.1% (9.4% to 15.3%)	10.3% (7.8% to 13.2%)
Cirrhosis and other chronic liver diseases due to alcohol use	100.0% (100.0% to 100.0%)	100.0% (100.0% to 100.0%)	100.0% (100.0% to 100.0%)	100.0% (100.0% to 100.0%)
**Digestive diseases**	4.7% (3.6% to 6.0%)	5.2% (4.1% to 6.4%)	1.9% (1.4% to 2.5%)	4.9% (3.9% to 6.1%)
Peptic ulcer disease	6.3% (4.3% to 8.5%)	7.1% (4.8% to 9.6%)	5.9% (4.1% to 8.0%)	7.0% (4.8% to 9.5%)
Gallbladder and biliary diseases	24.0% (15.0% to 35.4%)	24.7% (15.8% to 35.6%)	28.4% (18.7% to 40.0%)	24.8% (15.9% to 35.8%)
Pancreatitis	30.8% (21.8% to 40.6%)	35.5% (26.2% to 44.8%)	32.3% (24.2% to 41.6%)	35.4% (26.2% to 44.8%)
**Neurological disorders**	15.3% (7.9% to 24.7%)	16.0% (10.1% to 22.8%)	4.2% (2.9% to 5.6%)	8.8% (5.8% to 12.6%)
Alzheimer's disease and other dementias	16.2% (7.8% to 27.2%)	16.9% (8.2% to 28.0%)	17.3% (8.5% to 28.8%)	17.0% (8.3% to 28.2%)
Parkinson's disease	-5.2% (-6.0% to -4.1%)	-6.0% (-7.0% to -4.8%)	-6.4% (-7.5% to -5.1%)	-6.1% (-7.1% to -4.8%)
Epilepsy	21.3% (15.4% to 27.8%)	20.5% (15.0% to 26.4%)	16.0% (11.5% to 20.8%)	18.4% (13.4% to 23.4%)
Multiple sclerosis	4.4% (2.4% to 6.7%)	4.4% (2.4% to 6.9%)	3.6% (2.2% to 5.1%)	4.1% (2.4% to 6.2%)
**Mental and substance use disorders**	99.8% (99.7% to 99.9%)	99.7% (99.6% to 99.8%)	19.8% (17.6% to 21.9%)	23.4% (20.9% to 26.1%)
Alcohol use disorders	100.0% (100.0% to 100.0%)	100.0% (100.0% to 100.0%)	100.0% (100.0% to 100.0%)	100.0% (100.0% to 100.0%)

**Supplementary Results Table 1. Age-standardised PAFs attributable to the joint distribution of all risk factors for all ages, both sexes combined for each GBD level 1 and level 2 cause and all causes in 2016 for Sub-Saharan Africa. Results are shown for deaths, YLLs, YLDs, and DALYs** Data in parenthesis are 95% uncertainty intervals. PAFs=population attributable fractions. GBD=Global Burden of Disease. YLLs=years of life lost. YLDs=years live with disability. DALYs=disability-adjusted life-years	**Deaths 2016**	**YLLs 2016**	**YLDs 2016**	**DALYs 2016**
Drug use disorders	100.0% (100.0% to 100.0%)	100.0% (100.0% to 100.0%)	100.0% (100.0% to 100.0%)	100.0% (100.0% to 100.0%)
Depressive disorders	-	-	10.8% (9.2% to 12.7%)	10.8% (9.2% to 12.7%)
Idiopathic developmental intellectual disability	-	-	61.3% (30.8% to 80.7%)	61.3% (30.8% to 80.7%)
**Diabetes, urogenital, blood, and endocrine diseases**	79.0% (77.8% to 80.1%)	68.8% (67.0% to 70.7%)	59.4% (55.2% to 63.1%)	65.3% (63.1% to 67.3%)
Diabetes mellitus	100.0% (100.0% to 100.0%)	100.0% (100.0% to 100.0%)	100.0% (100.0% to 100.0%)	100.0% (100.0% to 100.0%)
Chronic kidney disease	100.0% (100.0% to 100.0%)	100.0% (100.0% to 100.0%)	100.0% (100.0% to 100.0%)	100.0% (100.0% to 100.0%)
**Musculoskeletal disorders**	0.8% (0.3% to 1.4%)	0.7% (0.3% to 1.1%)	23.8% (21.1% to 26.8%)	23.4% (20.7% to 26.4%)
Rheumatoid arthritis	2.3% (0.9% to 4.1%)	2.5% (1.0% to 4.5%)	2.4% (1.0% to 4.0%)	2.4% (1.0% to 4.1%)
Osteoarthritis	-	-	16.7% (8.7% to 26.2%)	16.7% (8.7% to 26.2%)
Low back and neck pain	-	-	29.6% (27.0% to 32.6%)	29.6% (27.0% to 32.6%)
Gout	-	-	43.2% (34.8% to 53.8%)	43.2% (34.8% to 53.8%)
**Other non-communicable diseases**	0.2% (0.1% to 0.3%)	0.3% (0.1% to 0.5%)	8.9% (7.5% to 10.2%)	6.6% (5.5% to 7.7%)
Sense organ diseases	-	-	17.2% (15.3% to 19.1%)	17.2% (15.3% to 19.1%)
Sudden infant death syndrome	4.8% (2.9% to 6.6%)	4.8% (2.9% to 6.7%)	-	4.8% (2.9% to 6.7%)
**Transport injuries**	34.1% (30.0% to 38.9%)	29.7% (25.8% to 34.1%)	40.0% (35.6% to 45.0%)	31.0% (26.9% to 35.4%)
Road injuries	34.0% (29.8% to 38.8%)	29.6% (25.7% to 34.1%)	41.7% (37.2% to 46.9%)	31.0% (27.0% to 35.4%)
Other transport injuries	36.9% (32.5% to 41.8%)	31.3% (26.8% to 36.3%)	31.9% (27.8% to 36.3%)	31.5% (27.4% to 36.0%)
**Unintentional injuries**	29.3% (25.0% to 32.5%)	17.6% (16.0% to 19.5%)	24.3% (22.8% to 25.6%)	19.4% (18.0% to 21.0%)
Falls	60.2% (49.1% to 66.5%)	40.7% (36.0% to 44.1%)	32.7% (31.3% to 34.0%)	37.0% (34.5% to 39.4%)
Drowning	13.9% (11.6% to 16.8%)	13.1% (11.1% to 15.4%)	18.9% (15.6% to 22.6%)	13.2% (11.2% to 15.5%)
Fire, heat, and hot substances	11.3% (8.6% to 15.1%)	12.7% (10.2% to 15.9%)	21.9% (18.8% to 25.6%)	14.5% (11.9% to 17.7%)

Supplementary Results Table 1. Age-standardised PAFs attributable to the joint distribution of all risk factors for all ages, both sexes combined for each GBD level 1 and level 2 cause and all causes in 2016 for Sub-Saharan Africa. Results are shown for deaths, YLLs, YLDs, and DALYs
Data in parenthesis are 95% uncertainty intervals. PAFs=population attributable fractions. GBD=Global Burden of Disease. YLLs=years of life lost. YLDs=years live with disability. DALYs=disability-adjusted life-years

	**Deaths 2016**	**YLLs 2016**	**YLDs 2016**	**DALYs 2016**
Poisonings	14.8% (11.9% to 18.9%)	14.2% (11.1% to 18.2%)	21.1% (17.9% to 24.5%)	14.5% (11.4% to 18.5%)
Exposure to mechanical forces	22.2% (20.6% to 24.1%)	17.9% (15.9% to 20.5%)	25.1% (23.5% to 26.8%)	19.8% (18.0% to 21.9%)
Animal contact	10.3% (9.2% to 11.7%)	11.5% (10.4% to 13.2%)	16.6% (15.3% to 18.1%)	12.5% (11.3% to 14.0%)
Foreign body	8.0% (5.7% to 10.0%)	8.0% (4.8% to 10.6%)	13.8% (12.5% to 15.2%)	9.6% (6.9% to 11.7%)
Other unintentional injuries	16.6% (13.8% to 19.8%)	17.7% (15.1% to 20.5%)	19.1% (16.0% to 22.8%)	18.3% (15.6% to 21.3%)
**Self-harm and interpersonal violence**	19.9% (14.6% to 25.5%)	20.3% (15.6% to 25.3%)	23.4% (20.9% to 26.3%)	20.6% (16.0% to 25.3%)
Self-harm	18.9% (11.0% to 27.4%)	21.0% (13.0% to 29.3%)	20.0% (11.8% to 28.7%)	20.9% (13.0% to 29.3%)
Interpersonal violence	21.4% (16.3% to 26.7%)	19.7% (14.6% to 25.1%)	23.8% (21.1% to 26.7%)	20.3% (15.4% to 25.3%)
**Forces of nature, conflict and terrorism, and executions and police conflict**	0.2% (0.1% to 0.2%)	0.1% (0.0% to 0.1%)	0.1% (0.0% to 0.1%)	0.1% (0.0% to 0.1%)
Forces of nature, conflict and terrorism, and state actor violence	6.7% (6.2% to 7.1%)	2.7% (2.4% to 2.9%)	7.0% (4.7% to 10.2%)	3.6% (2.8% to 5.1%)

Supplementary Results Table 1. Age-standardised PAFs attributable to the joint distribution of all risk factors for all ages, both sexes combined for each GBD level 1 and level 2 cause and all causes in 2016 for Andean Latin America. Results are shown for deaths, YLLs, YLDs, and DALYs
Data in parenthesis are 95% uncertainty intervals. PAFs=population attributable fractions. GBD=Global Burden of Disease. YLLs=years of life lost. YLDs=years live with disability. DALYs=disability-adjusted life-years

	Deaths 2016	YLLs 2016	YLDs 2016	DALYs 2016
**All causes**	51.3% (49.5% to 53.1%)	47.9% (46.4% to 49.3%)	23.0% (21.8% to 24.3%)	37.6% (35.4% to 39.7%)
**HIV/AIDS and tuberculosis**	59.2% (51.0% to 68.7%)	64.1% (57.0% to 73.2%)	73.9% (66.9% to 80.9%)	64.7% (57.8% to 73.4%)
Tuberculosis	42.8% (33.7% to 52.1%)	43.6% (35.6% to 52.1%)	40.3% (31.8% to 48.9%)	43.5% (35.5% to 52.0%)
HIV/AIDS	94.5% (93.3% to 95.4%)	92.0% (89.9% to 93.5%)	95.5% (94.5% to 96.2%)	92.3% (90.4% to 93.6%)
**Diarrhoea, lower respiratory infections, and other common infectious diseases**	50.2% (43.9% to 57.3%)	58.8% (53.1% to 64.3%)	39.3% (29.9% to 48.5%)	55.9% (50.5% to 61.5%)
Diarrhoeal diseases	75.6% (59.1% to 89.4%)	83.7% (71.6% to 93.2%)	75.2% (58.7% to 89.1%)	79.3% (65.5% to 91.0%)
Lower respiratory infections	50.7% (43.5% to 58.7%)	62.4% (55.3% to 69.0%)	53.9% (46.7% to 61.2%)	62.4% (55.3% to 69.0%)
Upper respiratory infections	1.5% (0.4% to 3.7%)	3.1% (0.8% to 7.5%)	0.0% (0.0% to 0.0%)	0.0% (0.0% to 0.1%)
Otitis media	2.3% (0.8% to 5.4%)	4.6% (2.2% to 8.2%)	3.3% (2.2% to 4.5%)	3.3% (2.2% to 4.5%)
Meningitis	2.3% (1.7% to 3.1%)	3.5% (2.5% to 4.5%)	0.0% (0.0% to 0.0%)	3.1% (2.3% to 4.1%)
Encephalitis	1.3% (1.1% to 1.7%)	2.0% (1.6% to 2.6%)	0.0% (0.0% to 0.0%)	1.8% (1.5% to 2.3%)
Measles	26.4% (10.7% to 58.2%)	27.6% (11.1% to 60.7%)	34.8% (14.5% to 71.8%)	27.6% (11.1% to 60.7%)
**Maternal disorders**	8.7% (4.2% to 13.4%)	8.6% (4.1% to 13.3%)	4.6% (2.3% to 7.3%)	8.1% (3.9% to 12.5%)
Maternal haemorrhage	15.2% (5.5% to 25.0%)	15.1% (5.4% to 24.8%)	13.9% (5.0% to 22.6%)	15.1% (5.4% to 24.7%)
Maternal sepsis and other pregnancy related infections	14.8% (5.3% to 24.2%)	14.7% (5.3% to 24.0%)	13.8% (4.9% to 22.6%)	14.6% (5.3% to 23.9%)
Maternal abortion, miscarriage, and ectopic pregnancy	12.3% (7.8% to 17.4%)	12.3% (7.7% to 17.3%)	13.2% (7.4% to 18.9%)	12.3% (7.7% to 17.3%)
**Neonatal disorders**	77.0% (74.5% to 79.4%)	77.1% (74.6% to 79.5%)	0.1% (0.1% to 0.2%)	66.7% (61.6% to 70.8%)
Neonatal preterm birth complications	92.7% (90.1% to 94.6%)	92.7% (90.2% to 94.6%)	0.3% (0.3% to 0.4%)	82.6% (77.8% to 86.6%)
Neonatal encephalopathy due to birth asphyxia and trauma	66.6% (63.2% to 70.2%)	66.7% (63.2% to 70.2%)	0.0% (0.0% to 0.0%)	62.2% (56.0% to 67.3%)
Neonatal sepsis and other neonatal infections	61.0% (57.2% to 64.8%)	61.1% (57.2% to 64.8%)	0.0% (0.0% to 0.0%)	47.0% (34.3% to 56.1%)
Hemolytic disease and other neonatal jaundice	66.5% (62.9% to 70.1%)	66.5% (62.9% to 70.1%)	0.0% (0.0% to 0.0%)	20.5% (15.0% to 26.8%)

Supplementary Results Table 1. Age-standardised PAFs attributable to the joint distribution of all risk factors for all ages, both sexes combined for each GBD level 1 and level 2 cause and all causes in 2016 for Andean Latin America. Results are shown for deaths, YLLs, YLDs, and DALYs
Data in parenthesis are 95% uncertainty intervals. PAFs=population attributable fractions. GBD=Global Burden of Disease. YLLs=years of life lost. YLDs=years live with disability. DALYs=disability-adjusted life-years

	**Deaths 2016**	**YLLs 2016**	**YLDs 2016**	**DALYs 2016**
Other neonatal disorders	59.0% (54.2% to 63.4%)	59.1% (54.2% to 63.4%)	0.0% (0.0% to 0.0%)	54.3% (49.3% to 59.1%)
**Nutritional deficiencies**	11.6% (8.8% to 15.0%)	44.2% (37.4% to 51.6%)	99.0% (98.8% to 99.2%)	79.8% (74.1% to 84.7%)
Protein-energy malnutrition	12.9% (9.8% to 16.7%)	49.1% (41.9% to 56.9%)	97.1% (95.8% to 98.0%)	51.1% (44.4% to 58.4%)
Vitamin A deficiency	-	-	100.0% (100.0% to 100.0%)	100.0% (100.0% to 100.0%)
Iron-deficiency anaemia	100.0% (100.0% to 100.0%)	100.0% (100.0% to 100.0%)	100.0% (100.0% to 100.0%)	100.0% (100.0% to 100.0%)
**Other communicable, maternal, neonatal, and nutritional diseases**	6.4% (5.0% to 8.3%)	4.2% (2.8% to 5.8%)	60.1% (54.0% to 66.3%)	21.5% (14.4% to 31.0%)
Sexually transmitted diseases excluding HIV	18.9% (10.2% to 30.7%)	7.8% (3.9% to 13.8%)	100.0% (100.0% to 100.0%)	39.7% (23.2% to 58.1%)
Hepatitis	2.0% (1.5% to 2.7%)	2.0% (1.5% to 2.6%)	1.5% (0.9% to 2.5%)	2.0% (1.5% to 2.6%)
**Neoplasms**	28.6% (25.2% to 32.0%)	28.0% (24.8% to 31.2%)	28.8% (25.7% to 31.8%)	28.0% (24.8% to 31.1%)
Oesophageal cancer	64.9% (53.0% to 76.2%)	66.4% (54.7% to 77.3%)	65.1% (53.2% to 76.1%)	66.4% (54.7% to 77.3%)
Stomach cancer	9.0% (3.2% to 17.9%)	8.0% (2.6% to 16.0%)	8.5% (2.9% to 17.0%)	8.0% (2.6% to 16.0%)
Liver cancer	40.2% (31.3% to 50.5%)	40.3% (31.0% to 50.8%)	40.2% (31.4% to 50.6%)	40.3% (31.0% to 50.8%)
Larynx cancer	65.4% (53.5% to 75.0%)	64.4% (52.7% to 74.1%)	64.8% (53.1% to 74.2%)	64.5% (52.7% to 74.1%)
Tracheal, bronchus, and lung cancer	70.0% (63.7% to 75.7%)	67.3% (60.5% to 73.8%)	68.5% (62.2% to 74.4%)	67.3% (60.5% to 73.8%)
Breast cancer	24.9% (18.3% to 31.8%)	23.5% (16.8% to 30.1%)	23.7% (17.2% to 30.4%)	23.5% (16.8% to 30.1%)
Cervical cancer	100.0% (100.0% to 100.0%)	100.0% (100.0% to 100.0%)	100.0% (100.0% to 100.0%)	100.0% (100.0% to 100.0%)
Uterine cancer	46.2% (31.3% to 60.6%)	48.8% (33.4% to 62.8%)	49.3% (34.0% to 63.3%)	48.8% (33.5% to 62.9%)
Prostate cancer	1.4% (0.9% to 2.1%)	1.2% (0.7% to 1.8%)	1.2% (0.7% to 1.8%)	1.2% (0.7% to 1.8%)
Colon and rectum cancer	51.6% (42.7% to 59.9%)	52.5% (44.0% to 60.5%)	51.0% (42.2% to 59.4%)	52.5% (43.8% to 60.5%)
Lip and oral cavity cancer	57.6% (47.5% to 67.0%)	59.6% (49.6% to 68.8%)	56.8% (46.8% to 66.1%)	59.5% (49.5% to 68.7%)
Nasopharynx cancer	59.6% (52.6% to 66.1%)	57.3% (51.3% to 63.3%)	56.6% (51.0% to 62.0%)	57.3% (51.3% to 63.3%)

**Supplementary Results Table 1. Age-standardised PAFs attributable to the joint distribution of all risk factors for all ages, both sexes combined for each GBD level 1 and level 2 cause and all causes in 2016 for Andean Latin America. Results are shown for deaths, YLLs, YLDs, and DALYs** Data in parenthesis are 95% uncertainty intervals. PAFs=population attributable fractions. GBD=Global Burden of Disease. YLLs=years of life lost. YLDs=years live with disability. DALYs=disability-adjusted life-years				
	**Deaths 2016**	**YLLs 2016**	**YLDs 2016**	**DALYs 2016**
Other pharynx cancer	39.9% (28.6% to 50.8%)	44.4% (33.2% to 55.3%)	42.2% (30.8% to 53.0%)	44.3% (33.1% to 55.2%)
Gallbladder and biliary tract cancer	23.7% (12.9% to 35.9%)	25.3% (14.1% to 37.9%)	24.0% (13.2% to 36.5%)	25.3% (14.1% to 37.9%)
Pancreatic cancer	18.5% (11.6% to 27.2%)	17.8% (10.9% to 26.4%)	18.1% (11.3% to 26.7%)	17.8% (10.9% to 26.5%)
Ovarian cancer	11.3% (4.9% to 18.8%)	9.8% (3.7% to 17.0%)	9.5% (3.6% to 16.5%)	9.8% (3.7% to 17.0%)
Kidney cancer	24.9% (15.8% to 35.6%)	23.9% (15.3% to 34.0%)	24.9% (16.0% to 35.1%)	24.0% (15.3% to 34.1%)
Bladder cancer	16.3% (10.0% to 23.7%)	14.4% (8.6% to 21.4%)	14.5% (8.7% to 21.6%)	14.4% (8.6% to 21.4%)
Thyroid cancer	14.1% (7.3% to 22.6%)	15.0% (7.9% to 23.9%)	14.9% (8.0% to 23.0%)	15.0% (7.9% to 23.9%)
Mesothelioma	86.3% (82.7% to 89.3%)	75.0% (69.9% to 79.8%)	82.5% (78.3% to 86.1%)	75.1% (70.0% to 79.9%)
Non-Hodgkin's lymphoma	6.9% (2.7% to 12.0%)	6.5% (2.6% to 11.4%)	6.9% (2.7% to 12.0%)	6.6% (2.6% to 11.4%)
Multiple myeloma	8.4% (3.5% to 14.8%)	8.9% (3.7% to 15.4%)	8.6% (3.6% to 15.3%)	8.9% (3.7% to 15.4%)
Leukaemia	10.6% (6.6% to 15.4%)	8.0% (4.9% to 11.5%)	10.1% (6.2% to 14.6%)	8.0% (4.9% to 11.6%)
**Cardiovascular diseases**	81.6% (78.6% to 84.2%)	79.6% (77.4% to 81.5%)	65.4% (62.0% to 68.4%)	77.8% (75.5% to 79.8%)
Rheumatic heart disease	20.9% (12.8% to 36.2%)	16.7% (11.2% to 26.9%)	13.8% (8.6% to 20.5%)	15.8% (10.7% to 25.1%)
Ischaemic heart disease	90.2% (86.1% to 93.8%)	91.1% (88.2% to 93.4%)	88.5% (85.2% to 91.6%)	90.9% (88.0% to 93.2%)
Cerebrovascular disease	77.8% (72.8% to 82.6%)	77.9% (74.5% to 81.2%)	79.7% (74.9% to 84.2%)	78.1% (74.7% to 81.4%)
Hypertensive heart disease	100.0% (100.0% to 100.0%)	100.0% (100.0% to 100.0%)	100.0% (100.0% to 100.0%)	100.0% (100.0% to 100.0%)
Cardiomyopathy and myocarditis	28.7% (22.7% to 36.3%)	21.6% (16.9% to 26.8%)	23.3% (18.4% to 28.9%)	21.7% (17.1% to 27.0%)
Atrial fibrillation and flutter	46.5% (39.0% to 53.8%)	49.7% (42.9% to 56.1%)	54.5% (48.7% to 60.3%)	52.6% (46.2% to 58.6%)
Aortic aneurysm	33.7% (27.3% to 40.6%)	35.7% (30.2% to 40.9%)	-	35.7% (30.2% to 40.9%)
Peripheral vascular disease	43.2% (36.5% to 51.1%)	47.7% (42.5% to 53.3%)	50.2% (45.2% to 55.2%)	49.8% (44.9% to 54.7%)
Endocarditis	29.4% (21.5% to 39.5%)	19.9% (14.4% to 26.3%)	30.3% (22.7% to 38.6%)	20.0% (14.5% to 26.6%)

Supplementary Results Table 1. Age-standardised PAFs attributable to the joint distribution of all risk factors for all ages, both sexes combined for each GBD level 1 and level 2 cause and all causes in 2016 for Andean Latin America. Results are shown for deaths, YLLs, YLDs, and DALYs
Data in parenthesis are 95% uncertainty intervals. PAFs=population attributable fractions. GBD=Global Burden of Disease. YLLs=years of life lost. YLDs=years live with disability. DALYs=disability-adjusted life-years

	**Deaths 2016**	**YLLs 2016**	**YLDs 2016**	**DALYs 2016**
Other cardiovascular and circulatory diseases	34.4% (29.6% to 38.9%)	28.4% (24.6% to 32.2%)	32.2% (27.7% to 36.9%)	29.7% (25.9% to 33.6%)
**Chronic respiratory diseases**	44.5% (39.1% to 50.6%)	39.2% (34.3% to 44.0%)	31.4% (24.0% to 40.1%)	36.1% (30.8% to 41.1%)
Chronic obstructive pulmonary disease	67.2% (60.0% to 73.6%)	62.3% (54.7% to 68.9%)	54.2% (45.5% to 61.7%)	60.9% (53.1% to 67.6%)
Pneumoconiosis	100.0% (100.0% to 100.0%)	100.0% (100.0% to 100.0%)	100.0% (100.0% to 100.0%)	100.0% (100.0% to 100.0%)
Asthma	35.6% (25.6% to 46.8%)	35.2% (26.1% to 45.5%)	27.7% (18.7% to 39.2%)	28.4% (19.5% to 39.5%)
Other chronic respiratory diseases	5.2% (3.4% to 7.5%)	2.8% (1.6% to 4.3%)	1.5% (0.6% to 2.8%)	2.5% (1.4% to 3.9%)
**Cirrhosis and other chronic liver diseases**	50.2% (47.2% to 53.1%)	51.2% (48.2% to 54.1%)	41.8% (39.0% to 44.8%)	50.9% (47.9% to 53.8%)
Cirrhosis and other chronic liver diseases due to hepatitis B	0.5% (0.3% to 0.8%)	0.6% (0.3% to 1.0%)	0.5% (0.3% to 0.8%)	0.6% (0.3% to 1.0%)
Cirrhosis and other chronic liver diseases due to hepatitis C	50.3% (41.1% to 59.1%)	62.8% (54.0% to 70.5%)	53.8% (45.4% to 62.1%)	62.6% (53.7% to 70.2%)
Cirrhosis and other chronic liver diseases due to alcohol use	100.0% (100.0% to 100.0%)	100.0% (100.0% to 100.0%)	100.0% (100.0% to 100.0%)	100.0% (100.0% to 100.0%)
**Digestive diseases**	11.3% (8.5% to 14.2%)	11.8% (9.2% to 14.5%)	3.1% (2.3% to 4.0%)	9.5% (7.4% to 11.9%)
Peptic ulcer disease	5.8% (3.9% to 8.1%)	7.0% (4.7% to 9.7%)	4.3% (2.9% to 5.9%)	6.1% (4.1% to 8.4%)
Gallbladder and biliary diseases	41.6% (27.1% to 57.1%)	43.6% (28.9% to 58.2%)	47.2% (32.2% to 61.9%)	44.0% (29.3% to 58.8%)
Pancreatitis	34.8% (22.8% to 46.6%)	38.9% (27.0% to 50.7%)	33.1% (22.0% to 44.8%)	38.8% (26.9% to 50.6%)
**Neurological disorders**	18.9% (9.2% to 30.9%)	18.6% (10.3% to 28.4%)	4.2% (2.5% to 6.3%)	8.2% (4.9% to 12.2%)
Alzheimer's disease and other dementias	21.4% (10.2% to 35.2%)	22.6% (11.1% to 36.5%)	22.6% (11.1% to 36.7%)	22.6% (11.1% to 36.5%)
Parkinson's disease	-3.7% (-4.5% to -2.9%)	-4.5% (-5.4% to -3.6%)	-4.8% (-5.6% to -3.8%)	-4.6% (-5.5% to -3.7%)
Epilepsy	23.0% (16.0% to 30.0%)	21.0% (14.7% to 27.3%)	20.3% (13.5% to 27.3%)	20.5% (14.1% to 27.2%)
Multiple sclerosis	3.9% (2.3% to 5.8%)	4.1% (2.5% to 6.1%)	3.2% (2.0% to 4.5%)	3.8% (2.3% to 5.5%)
**Mental and substance use disorders**	99.8% (99.7% to 99.8%)	99.6% (99.5% to 99.7%)	19.8% (18.0% to 22.0%)	24.0% (21.6% to 26.8%)
Alcohol use disorders	100.0% (100.0% to 100.0%)	100.0% (100.0% to 100.0%)	100.0% (100.0% to 100.0%)	100.0% (100.0% to 100.0%)

Supplementary Results Table 1. Age-standardised PAFs attributable to the joint distribution of all risk factors for all ages, both sexes combined for each GBD level 1 and level 2 cause and all causes in 2016 for Andean Latin America. Results are shown for deaths, YLLs, YLDs, and DALYs
Data in parenthesis are 95% uncertainty intervals. PAFs=population attributable fractions. GBD=Global Burden of Disease. YLLs=years of life lost. YLDs=years live with disability. DALYs=disability-adjusted life-years

	**Deaths 2016**	**YLLs 2016**	**YLDs 2016**	**DALYs 2016**
Drug use disorders	100.0% (100.0% to 100.0%)	100.0% (100.0% to 100.0%)	100.0% (100.0% to 100.0%)	100.0% (100.0% to 100.0%)
Depressive disorders	-	-	10.5% (9.0% to 12.2%)	10.5% (9.0% to 12.2%)
Idiopathic developmental intellectual disability	-	-	69.4% (38.5% to 88.4%)	69.4% (38.5% to 88.4%)
**Diabetes, urogenital, blood, and endocrine diseases**	88.9% (87.0% to 89.8%)	87.4% (84.7% to 88.5%)	65.9% (61.1% to 70.1%)	79.4% (76.5% to 81.7%)
Diabetes mellitus	100.0% (100.0% to 100.0%)	100.0% (100.0% to 100.0%)	100.0% (100.0% to 100.0%)	100.0% (100.0% to 100.0%)
Chronic kidney disease	100.0% (100.0% to 100.0%)	100.0% (100.0% to 100.0%)	100.0% (100.0% to 100.0%)	100.0% (100.0% to 100.0%)
**Musculoskeletal disorders**	0.6% (0.2% to 1.1%)	0.5% (0.2% to 0.8%)	22.3% (18.7% to 25.9%)	22.0% (18.4% to 25.5%)
Rheumatoid arthritis	1.5% (0.6% to 2.6%)	1.8% (0.7% to 3.0%)	2.0% (0.8% to 3.4%)	2.0% (0.8% to 3.4%)
Osteoarthritis	-	-	28.3% (15.0% to 41.5%)	28.3% (15.0% to 41.5%)
Low back and neck pain	-	-	27.2% (24.1% to 30.3%)	27.2% (24.1% to 30.3%)
Gout	-	-	48.5% (32.5% to 64.7%)	48.5% (32.5% to 64.7%)
**Other non-communicable diseases**	0.1% (0.1% to 0.1%)	0.1% (0.1% to 0.1%)	6.0% (5.0% to 7.1%)	4.8% (4.1% to 5.6%)
Sense organ diseases	-	-	13.0% (11.4% to 14.7%)	13.0% (11.4% to 14.7%)
Sudden infant death syndrome	6.1% (4.2% to 8.6%)	6.1% (4.2% to 8.6%)	-	6.1% (4.2% to 8.6%)
**Transport injuries**	34.4% (28.4% to 41.3%)	29.9% (24.1% to 36.6%)	40.1% (34.0% to 47.2%)	31.0% (25.0% to 37.8%)
Road injuries	34.2% (28.2% to 41.1%)	29.8% (23.9% to 36.3%)	40.4% (34.3% to 47.5%)	30.9% (25.0% to 37.7%)
Other transport injuries	37.4% (30.8% to 44.4%)	32.2% (25.6% to 39.5%)	33.7% (27.6% to 40.7%)	32.3% (25.8% to 39.7%)
**Unintentional injuries**	20.7% (17.9% to 23.1%)	12.4% (10.4% to 14.6%)	21.2% (19.0% to 24.0%)	15.3% (13.1% to 17.6%)
Falls	50.9% (47.7% to 53.9%)	31.7% (29.0% to 35.1%)	28.5% (26.3% to 31.2%)	29.7% (27.4% to 32.3%)
Drowning	15.9% (11.9% to 20.5%)	13.7% (10.4% to 17.5%)	17.7% (13.1% to 23.1%)	13.8% (10.5% to 17.6%)
Fire, heat, and hot substances	12.0% (8.6% to 16.3%)	11.9% (8.9% to 15.3%)	19.7% (15.0% to 25.3%)	14.7% (11.0% to 18.7%)

Supplementary Results Table 1. Age-standardised PAFs attributable to the joint distribution of all risk factors for all ages, both sexes combined for each GBD level 1 and level 2 cause and all causes in 2016 for Andean Latin America. Results are shown for deaths, YLLs, YLDs, and DALYs
Data in parenthesis are 95% uncertainty intervals. PAFs=population attributable fractions. GBD=Global Burden of Disease. YLLs=years of life lost. YLDs=years live with disability. DALYs=disability-adjusted life-years

	**Deaths 2016**	**YLLs 2016**	**YLDs 2016**	**DALYs 2016**
Poisonings	16.3% (12.2% to 21.8%)	14.4% (10.6% to 19.1%)	18.6% (14.1% to 23.8%)	14.8% (11.0% to 19.5%)
Exposure to mechanical forces	18.6% (15.2% to 22.3%)	15.9% (12.4% to 19.8%)	20.9% (18.1% to 24.8%)	17.6% (14.4% to 21.3%)
Animal contact	11.1% (9.2% to 13.4%)	9.8% (7.9% to 12.2%)	12.7% (10.2% to 16.1%)	11.1% (9.1% to 13.8%)
Foreign body	6.2% (4.8% to 7.8%)	4.8% (3.7% to 6.2%)	9.5% (7.5% to 12.3%)	5.5% (4.3% to 7.1%)
Other unintentional injuries	22.1% (16.8% to 28.0%)	22.7% (17.3% to 28.6%)	18.0% (13.5% to 23.3%)	19.9% (15.3% to 25.1%)
**Self-harm and interpersonal violence**	25.4% (18.9% to 31.6%)	24.6% (18.6% to 30.8%)	33.2% (29.7% to 36.9%)	25.1% (19.2% to 31.1%)
Self-harm	26.4% (16.7% to 35.5%)	26.4% (17.1% to 35.4%)	25.5% (15.5% to 35.3%)	26.3% (17.1% to 35.4%)
Interpersonal violence	24.6% (16.1% to 33.3%)	23.4% (15.4% to 31.9%)	33.8% (30.1% to 37.7%)	24.4% (16.8% to 32.4%)
**Forces of nature, conflict and terrorism, and executions and police conflict**	6.6% (6.1% to 7.0%)	2.7% (2.4% to 2.9%)	2.9% (2.0% to 4.2%)	2.7% (2.4% to 3.0%)
Forces of nature, conflict and terrorism, and state actor violence	6.6% (6.2% to 7.0%)	2.7% (2.5% to 3.0%)	4.7% (3.0% to 7.1%)	2.8% (2.5% to 3.2%)

Supplementary Results Table 1. Age-standardised PAFs attributable to the joint distribution of all risk factors for all ages, both sexes combined for each GBD level 1 and level 2 cause and all causes in 2016 for Australasia. Results are shown for deaths, YLLs, YLDs, and DALYs
Data in parenthesis are 95% uncertainty intervals. PAFs=population attributable fractions. GBD=Global Burden of Disease. YLLs=years of life lost. YLDs=years live with disability. DALYs=disability-adjusted life-years

	Deaths 2016	YLLs 2016	YLDs 2016	DALYs 2016
**All causes**	54.1% (51.9% to 56.2%)	51.1% (49.3% to 52.8%)	20.2% (18.9% to 21.7%)	33.4% (31.0% to 36.2%)
**HIV/AIDS and tuberculosis**	76.1% (71.2% to 80.5%)	82.3% (79.7% to 84.6%)	91.9% (88.3% to 94.1%)	86.6% (83.4% to 89.4%)
Tuberculosis	53.0% (41.9% to 62.4%)	54.5% (45.2% to 62.8%)	53.3% (43.1% to 62.5%)	54.3% (44.7% to 62.7%)
HIV/AIDS	94.5% (94.0% to 94.9%)	91.8% (91.0% to 92.4%)	96.5% (96.0% to 96.9%)	94.0% (93.1% to 94.9%)
**Diarrhoea, lower respiratory infections, and other common infectious diseases**	26.5% (19.4% to 34.7%)	35.9% (29.9% to 43.0%)	5.2% (3.6% to 7.6%)	20.9% (16.2% to 26.6%)
Diarrhoeal diseases	29.7% (16.0% to 50.6%)	45.3% (33.2% to 62.0%)	33.4% (19.8% to 52.9%)	39.3% (26.8% to 57.7%)
Lower respiratory infections	27.4% (19.2% to 36.3%)	39.9% (32.5% to 48.3%)	40.7% (33.2% to 49.4%)	39.9% (32.6% to 48.3%)
Upper respiratory infections	0.7% (0.1% to 2.0%)	2.0% (0.3% to 5.7%)	0.0% (0.0% to 0.0%)	0.0% (0.0% to 0.0%)
Otitis media	0.6% (0.3% to 0.9%)	3.0% (1.8% to 4.4%)	6.7% (4.4% to 8.8%)	6.7% (4.4% to 8.8%)
Meningitis	3.6% (2.8% to 4.4%)	6.0% (4.8% to 7.1%)	0.0% (0.0% to 0.0%)	5.0% (3.8% to 6.2%)
Encephalitis	0.4% (0.3% to 0.6%)	1.0% (0.8% to 1.3%)	0.0% (0.0% to 0.0%)	0.6% (0.4% to 0.8%)
Measles	5.0% (0.5% to 20.3%)	5.8% (0.6% to 23.6%)	12.7% (1.2% to 50.9%)	5.9% (0.6% to 23.8%)
**Maternal disorders**	2.2% (0.9% to 3.5%)	2.1% (0.9% to 3.5%)	3.3% (1.4% to 5.6%)	2.8% (1.2% to 4.5%)
Maternal haemorrhage	9.7% (3.5% to 15.9%)	9.7% (3.5% to 15.7%)	9.8% (3.5% to 15.9%)	9.8% (3.5% to 15.9%)
Maternal sepsis and other pregnancy related infections	9.8% (3.6% to 16.1%)	9.8% (3.6% to 16.0%)	9.7% (3.5% to 15.7%)	9.7% (3.5% to 15.8%)
Maternal abortion, miscarriage, and ectopic pregnancy	3.4% (1.9% to 5.2%)	3.5% (1.9% to 5.4%)	3.8% (1.9% to 6.1%)	3.7% (2.0% to 5.8%)
**Neonatal disorders**	75.7% (73.4% to 78.1%)	75.8% (73.5% to 78.2%)	0.1% (0.1% to 0.1%)	53.1% (46.8% to 59.0%)
Neonatal preterm birth complications	87.7% (85.0% to 90.0%)	87.7% (85.1% to 90.1%)	0.2% (0.1% to 0.2%)	54.6% (45.6% to 62.7%)
Neonatal encephalopathy due to birth asphyxia and trauma	66.3% (63.0% to 69.8%)	66.4% (63.1% to 69.8%)	0.0% (0.0% to 0.0%)	56.4% (50.3% to 62.0%)
Neonatal sepsis and other neonatal infections	62.4% (58.4% to 66.7%)	62.5% (58.5% to 66.8%)	0.0% (0.0% to 0.0%)	22.2% (10.6% to 39.3%)
Hemolytic disease and other neonatal jaundice	61.1% (55.3% to 66.4%)	61.2% (55.5% to 66.5%)	0.0% (0.0% to 0.0%)	19.9% (14.0% to 27.7%)

Supplementary Results Table 1. Age-standardised PAFs attributable to the joint distribution of all risk factors for all ages, both sexes combined for each GBD level 1 and level 2 cause and all causes in 2016 for Australasia. Results are shown for deaths, YLLs, YLDs, and DALYs
Data in parenthesis are 95% uncertainty intervals. PAFs=population attributable fractions. GBD=Global Burden of Disease. YLLs=years of life lost. YLDs=years live with disability. DALYs=disability-adjusted life-years

	Deaths 2016	YLLs 2016	YLDs 2016	DALYs 2016
Other neonatal disorders	66.4% (62.7% to 70.0%)	66.5% (62.7% to 70.0%)	0.0% (0.0% to 0.0%)	63.1% (59.0% to 66.7%)
**Nutritional deficiencies**	2.9% (1.7% to 3.6%)	10.8% (8.3% to 12.6%)	74.4% (69.4% to 79.0%)	69.3% (63.7% to 74.8%)
Protein-energy malnutrition	0.8% (0.5% to 1.1%)	5.7% (3.6% to 7.9%)	98.0% (97.4% to 98.5%)	72.5% (63.3% to 80.5%)
Vitamin A deficiency	-	-	0.0% (0.0% to 0.0%)	0.0% (0.0% to 0.0%)
Iron-deficiency anaemia	100.0% (100.0% to 100.0%)	100.0% (100.0% to 100.0%)	100.0% (100.0% to 100.0%)	100.0% (100.0% to 100.0%)
**Other communicable, maternal, neonatal, and nutritional diseases**	4.5% (3.7% to 5.9%)	4.0% (3.2% to 5.3%)	75.6% (69.5% to 81.5%)	30.5% (21.4% to 42.9%)
Sexually transmitted diseases excluding HIV	89.8% (82.2% to 95.6%)	70.0% (54.6% to 84.9%)	100.0% (100.0% to 100.0%)	98.3% (96.4% to 99.5%)
Hepatitis	6.0% (4.2% to 8.2%)	6.2% (4.3% to 8.7%)	3.8% (2.4% to 5.5%)	6.1% (4.2% to 8.5%)
**Neoplasms**	43.4% (40.9% to 46.0%)	42.5% (40.1% to 44.9%)	27.8% (25.2% to 30.5%)	41.7% (39.3% to 44.2%)
Oesophageal cancer	85.7% (78.9% to 91.4%)	86.6% (80.3% to 92.1%)	85.5% (78.8% to 91.3%)	86.6% (80.3% to 92.1%)
Stomach cancer	16.9% (10.1% to 25.1%)	15.8% (9.5% to 23.1%)	16.5% (9.8% to 24.6%)	15.8% (9.5% to 23.2%)
Liver cancer	76.6% (70.3% to 82.1%)	76.3% (70.1% to 81.5%)	76.9% (70.4% to 82.5%)	76.3% (70.1% to 81.5%)
Larynx cancer	90.4% (84.9% to 94.2%)	89.7% (83.9% to 93.5%)	90.0% (84.2% to 93.9%)	89.7% (84.0% to 93.5%)
Tracheal, bronchus, and lung cancer	88.1% (85.9% to 90.1%)	86.6% (84.3% to 88.7%)	87.5% (85.1% to 89.5%)	86.6% (84.3% to 88.7%)
Breast cancer	36.0% (28.9% to 43.2%)	34.8% (28.1% to 41.5%)	35.3% (28.5% to 42.1%)	34.8% (28.2% to 41.5%)
Cervical cancer	100.0% (100.0% to 100.0%)	100.0% (100.0% to 100.0%)	100.0% (100.0% to 100.0%)	100.0% (100.0% to 100.0%)
Uterine cancer	50.6% (36.4% to 63.4%)	52.1% (37.9% to 64.6%)	52.6% (38.5% to 64.9%)	52.1% (38.0% to 64.6%)
Prostate cancer	5.1% (3.5% to 6.7%)	4.8% (3.4% to 6.3%)	4.7% (3.4% to 6.2%)	4.8% (3.4% to 6.3%)
Colon and rectum cancer	59.6% (51.3% to 67.0%)	61.0% (53.0% to 68.1%)	60.3% (52.4% to 67.8%)	60.9% (52.9% to 68.1%)
Lip and oral cavity cancer	83.1% (76.9% to 87.5%)	84.2% (78.6% to 88.4%)	84.4% (78.7% to 88.6%)	84.2% (78.7% to 88.4%)
Nasopharynx cancer	86.4% (82.2% to 89.8%)	85.2% (81.3% to 88.4%)	84.4% (80.2% to 87.9%)	85.2% (81.3% to 88.3%)

Supplementary Results Table 1. Age-standardised PAFs attributable to the joint distribution of all risk factors for all ages, both sexes combined for each GBD level 1 and level 2 cause and all causes in 2016 for Australasia. Results are shown for deaths, YLLs, YLDs, and DALYs
Data in parenthesis are 95% uncertainty intervals. PAFs=population attributable fractions. GBD=Global Burden of Disease. YLLs=years of life lost. YLDs=years live with disability. DALYs=disability-adjusted life-years

	Deaths 2016	YLLs 2016	YLDs 2016	DALYs 2016
Other pharynx cancer	65.9% (54.9% to 74.5%)	69.8% (59.9% to 77.8%)	68.9% (58.8% to 77.0%)	69.7% (59.9% to 77.8%)
Gallbladder and biliary tract cancer	23.8% (13.6% to 36.0%)	24.5% (14.2% to 36.7%)	25.4% (14.8% to 37.8%)	24.6% (14.2% to 36.7%)
Pancreatic cancer	31.9% (24.9% to 39.5%)	31.1% (24.2% to 38.8%)	31.7% (24.7% to 39.3%)	31.1% (24.2% to 38.8%)
Ovarian cancer	18.8% (11.1% to 27.1%)	14.8% (8.0% to 22.4%)	15.2% (8.5% to 22.5%)	14.8% (8.1% to 22.4%)
Kidney cancer	37.0% (27.3% to 47.5%)	36.4% (26.8% to 46.6%)	36.6% (26.7% to 46.7%)	36.4% (26.8% to 46.6%)
Bladder cancer	35.0% (25.7% to 44.4%)	33.8% (24.9% to 42.8%)	33.7% (24.9% to 42.7%)	33.8% (24.9% to 42.8%)
Thyroid cancer	16.5% (8.7% to 26.4%)	17.3% (9.1% to 27.6%)	16.9% (9.6% to 25.9%)	17.2% (9.2% to 27.2%)
Mesothelioma	98.3% (97.9% to 98.7%)	96.3% (95.4% to 97.1%)	97.7% (97.1% to 98.3%)	96.3% (95.5% to 97.1%)
Non-Hodgkin's lymphoma	7.9% (3.3% to 13.1%)	8.1% (3.4% to 13.4%)	8.1% (3.5% to 13.6%)	8.1% (3.4% to 13.4%)
Multiple myeloma	8.9% (4.0% to 15.4%)	9.4% (4.3% to 16.1%)	9.3% (4.2% to 15.9%)	9.4% (4.3% to 16.1%)
Leukaemia	20.3% (12.8% to 27.8%)	17.5% (11.3% to 23.8%)	18.4% (11.7% to 25.2%)	17.5% (11.3% to 23.9%)
**Cardiovascular diseases**	79.0% (75.4% to 81.9%)	81.6% (79.3% to 83.6%)	73.0% (70.0% to 75.7%)	80.1% (77.8% to 82.2%)
Rheumatic heart disease	25.0% (15.6% to 43.7%)	24.4% (16.8% to 37.8%)	21.4% (14.6% to 31.9%)	24.4% (16.8% to 37.7%)
Ischaemic heart disease	90.5% (85.9% to 94.3%)	93.1% (90.1% to 95.5%)	90.6% (87.7% to 93.1%)	92.9% (89.9% to 95.3%)
Cerebrovascular disease	76.8% (69.3% to 83.5%)	82.9% (77.9% to 87.3%)	82.8% (78.1% to 87.1%)	82.8% (78.2% to 87.1%)
Hypertensive heart disease	100.0% (100.0% to 100.0%)	100.0% (100.0% to 100.0%)	100.0% (100.0% to 100.0%)	100.0% (100.0% to 100.0%)
Cardiomyopathy and myocarditis	52.6% (45.8% to 59.9%)	53.5% (46.9% to 59.1%)	49.8% (45.0% to 54.8%)	53.1% (46.9% to 58.5%)
Atrial fibrillation and flutter	52.1% (44.4% to 59.6%)	57.0% (50.0% to 63.3%)	64.7% (59.6% to 69.8%)	61.7% (55.9% to 67.3%)
Aortic aneurysm	37.6% (30.2% to 45.2%)	43.1% (36.8% to 49.1%)	-	43.1% (36.8% to 49.1%)
Peripheral vascular disease	43.3% (36.1% to 51.3%)	48.5% (42.5% to 54.7%)	53.9% (48.6% to 59.2%)	49.9% (44.1% to 55.5%)
Endocarditis	35.9% (27.4% to 46.6%)	36.4% (28.9% to 44.5%)	37.3% (29.0% to 46.5%)	36.4% (28.9% to 44.5%)

Supplementary Results Table 1. Age-standardised PAFs attributable to the joint distribution of all risk factors for all ages, both sexes combined for each GBD level 1 and level 2 cause and all causes in 2016 for Australasia. Results are shown for deaths, YLLs, YLDs, and DALYs
Data in parenthesis are 95% uncertainty intervals. PAFs=population attributable fractions. GBD=Global Burden of Disease. YLLs=years of life lost. YLDs=years live with disability. DALYs=disability-adjusted life-years

	Deaths 2016	YLLs 2016	YLDs 2016	DALYs 2016
Other cardiovascular and circulatory diseases	39.8% (34.4% to 45.5%)	45.6% (40.9% to 50.1%)	48.1% (43.2% to 53.0%)	46.7% (41.8% to 51.3%)
**Chronic respiratory diseases**	66.8% (62.7% to 73.1%)	64.5% (60.7% to 70.6%)	42.4% (35.1% to 50.6%)	51.5% (45.8% to 57.8%)
Chronic obstructive pulmonary disease	76.7% (72.8% to 80.5%)	76.2% (72.3% to 79.8%)	71.3% (67.2% to 75.5%)	75.1% (71.3% to 78.8%)
Pneumoconiosis	100.0% (100.0% to 100.0%)	100.0% (100.0% to 100.0%)	100.0% (100.0% to 100.0%)	100.0% (100.0% to 100.0%)
Asthma	41.9% (31.4% to 53.1%)	44.0% (35.3% to 53.9%)	37.4% (28.8% to 47.4%)	37.8% (29.3% to 47.7%)
Other chronic respiratory diseases	15.6% (12.5% to 18.9%)	10.8% (8.4% to 13.6%)	7.2% (4.9% to 10.4%)	9.3% (7.0% to 12.2%)
**Cirrhosis and other chronic liver diseases**	51.1% (48.4% to 53.9%)	53.6% (50.8% to 56.4%)	42.8% (38.8% to 47.1%)	53.0% (50.2% to 55.9%)
Cirrhosis and other chronic liver diseases due to hepatitis B	2.2% (1.0% to 3.7%)	2.7% (1.2% to 4.5%)	2.5% (1.1% to 4.2%)	2.7% (1.2% to 4.5%)
Cirrhosis and other chronic liver diseases due to hepatitis C	81.0% (76.0% to 84.8%)	87.1% (84.8% to 88.7%)	85.3% (82.4% to 87.5%)	87.1% (84.7% to 88.7%)
Cirrhosis and other chronic liver diseases due to alcohol use	100.0% (100.0% to 100.0%)	100.0% (100.0% to 100.0%)	100.0% (100.0% to 100.0%)	100.0% (100.0% to 100.0%)
**Digestive diseases**	9.2% (7.0% to 11.5%)	11.6% (9.2% to 14.2%)	5.4% (4.1% to 6.6%)	9.6% (7.8% to 11.8%)
Peptic ulcer disease	6.5% (4.4% to 9.0%)	9.3% (6.4% to 12.8%)	10.3% (7.0% to 14.3%)	9.7% (6.7% to 13.3%)
Gallbladder and biliary diseases	45.3% (31.0% to 59.5%)	46.3% (32.3% to 60.3%)	52.9% (39.4% to 66.1%)	47.6% (33.7% to 61.5%)
Pancreatitis	40.1% (25.0% to 54.3%)	49.1% (34.3% to 62.2%)	39.3% (24.4% to 53.4%)	48.4% (33.8% to 61.5%)
**Neurological disorders**	19.6% (9.8% to 31.0%)	17.6% (10.4% to 26.3%)	3.6% (2.0% to 5.8%)	8.2% (4.7% to 12.7%)
Alzheimer's disease and other dementias	24.4% (11.9% to 38.8%)	25.3% (12.9% to 39.6%)	25.4% (12.9% to 39.8%)	25.3% (12.9% to 39.6%)
Parkinson's disease	-4.6% (-5.4% to -3.8%)	-5.8% (-6.9% to -4.8%)	-7.1% (-8.3% to -5.9%)	-6.1% (-7.2% to -5.1%)
Epilepsy	37.5% (28.6% to 45.5%)	35.5% (27.0% to 43.1%)	28.8% (20.7% to 36.5%)	32.1% (23.9% to 39.7%)
Multiple sclerosis	6.9% (4.4% to 9.7%)	7.7% (4.9% to 10.9%)	7.4% (4.7% to 10.4%)	7.6% (4.8% to 10.5%)
**Mental and substance use disorders**	99.6% (99.5% to 99.7%)	99.5% (99.4% to 99.6%)	21.0% (18.7% to 23.5%)	26.5% (23.8% to 29.5%)
Alcohol use disorders	100.0% (100.0% to 100.0%)	100.0% (100.0% to 100.0%)	100.0% (100.0% to 100.0%)	100.0% (100.0% to 100.0%)

Supplementary Results Table 1. Age-standardised PAFs attributable to the joint distribution of all risk factors for all ages, both sexes combined for each GBD level 1 and level 2 cause and all causes in 2016 for Australasia. Results are shown for deaths, YLLs, YLDs, and DALYs
Data in parenthesis are 95% uncertainty intervals. PAFs=population attributable fractions. GBD=Global Burden of Disease. YLLs=years of life lost. YLDs=years live with disability. DALYs=disability-adjusted life-years

	**Deaths 2016**	**YLLs 2016**	**YLDs 2016**	**DALYs 2016**
Drug use disorders	100.0% (100.0% to 100.0%)	100.0% (100.0% to 100.0%)	100.0% (100.0% to 100.0%)	100.0% (100.0% to 100.0%)
Depressive disorders	-	-	8.5% (7.2% to 10.0%)	8.5% (7.2% to 10.0%)
Idiopathic developmental intellectual disability	-	-	64.2% (34.4% to 83.1%)	64.2% (34.4% to 83.1%)
**Diabetes, urogenital, blood, and endocrine diseases**	74.0% (72.6% to 77.3%)	66.2% (63.8% to 69.9%)	62.2% (58.4% to 65.9%)	64.0% (61.6% to 66.3%)
Diabetes mellitus	100.0% (100.0% to 100.0%)	100.0% (100.0% to 100.0%)	100.0% (100.0% to 100.0%)	100.0% (100.0% to 100.0%)
Chronic kidney disease	100.0% (100.0% to 100.0%)	100.0% (100.0% to 100.0%)	100.0% (100.0% to 100.0%)	100.0% (100.0% to 100.0%)
**Musculoskeletal disorders**	0.7% (0.3% to 1.3%)	0.9% (0.3% to 1.6%)	17.3% (14.5% to 20.2%)	17.0% (14.3% to 19.9%)
Rheumatoid arthritis	2.8% (1.1% to 4.8%)	3.5% (1.4% to 6.1%)	4.5% (1.8% to 7.9%)	4.5% (1.8% to 7.7%)
Osteoarthritis	-	-	30.9% (17.6% to 44.1%)	30.9% (17.6% to 44.1%)
Low back and neck pain	-	-	22.7% (19.7% to 25.6%)	22.7% (19.7% to 25.6%)
Gout	-	-	52.5% (34.3% to 70.4%)	52.5% (34.3% to 70.4%)
**Other non-communicable diseases**	0.4% (0.3% to 0.5%)	0.6% (0.4% to 0.8%)	2.1% (1.8% to 2.3%)	1.9% (1.6% to 2.1%)
Sense organ diseases	-	-	6.8% (6.1% to 7.6%)	6.8% (6.1% to 7.6%)
Sudden infant death syndrome	3.6% (2.7% to 4.8%)	3.6% (2.7% to 4.8%)	-	3.6% (2.7% to 4.8%)
**Transport injuries**	37.8% (29.0% to 47.5%)	33.4% (24.7% to 43.2%)	42.6% (34.4% to 51.6%)	36.4% (28.0% to 46.1%)
Road injuries	37.2% (28.5% to 47.0%)	32.8% (24.3% to 42.7%)	43.2% (34.9% to 52.1%)	36.1% (27.8% to 45.9%)
Other transport injuries	42.6% (33.3% to 52.7%)	38.4% (29.1% to 48.3%)	39.7% (31.4% to 48.7%)	39.0% (30.4% to 48.1%)
**Unintentional injuries**	46.8% (41.7% to 49.9%)	24.3% (20.7% to 27.3%)	20.1% (17.6% to 22.9%)	21.4% (18.6% to 24.3%)
Falls	71.1% (67.6% to 74.3%)	48.4% (44.5% to 51.5%)	22.5% (20.4% to 24.9%)	28.4% (25.6% to 31.6%)
Drowning	19.4% (12.8% to 26.4%)	16.4% (11.0% to 22.0%)	19.1% (12.3% to 27.1%)	16.5% (11.0% to 22.2%)
Fire, heat, and hot substances	15.2% (9.2% to 22.8%)	16.3% (11.0% to 22.7%)	21.6% (14.4% to 29.7%)	19.9% (13.3% to 27.4%)

Supplementary Results Table 1. Age-standardised PAFs attributable to the joint distribution of all risk factors for all ages, both sexes combined for each GBD level 1 and level 2 cause and all causes in 2016 for Australasia. Results are shown for deaths, YLLs, YLDs, and DALYs
Data in parenthesis are 95% uncertainty intervals. PAFs=population attributable fractions. GBD=Global Burden of Disease. YLLs=years of life lost. YLDs=years live with disability. DALYs=disability-adjusted life-years

	**Deaths 2016**	**YLLs 2016**	**YLDs 2016**	**DALYs 2016**
Poisonings	20.6% (13.6% to 28.7%)	20.1% (13.6% to 27.4%)	18.3% (12.1% to 25.2%)	19.1% (12.8% to 26.0%)
Exposure to mechanical forces	15.4% (12.5% to 19.5%)	10.9% (8.1% to 15.1%)	19.6% (16.7% to 23.2%)	16.7% (13.8% to 20.6%)
Animal contact	12.7% (10.1% to 16.1%)	9.8% (6.9% to 13.4%)	9.7% (7.3% to 12.7%)	9.7% (7.3% to 12.8%)
Foreign body	4.6% (3.2% to 6.8%)	6.5% (4.7% to 9.1%)	6.1% (4.4% to 8.4%)	6.4% (4.6% to 8.7%)
Other unintentional injuries	24.4% (16.5% to 33.5%)	24.3% (16.7% to 33.1%)	20.5% (13.4% to 28.0%)	20.8% (13.7% to 28.5%)
**Self-harm and interpersonal violence**	42.4% (30.3% to 53.3%)	42.2% (30.3% to 53.3%)	36.4% (31.0% to 42.1%)	41.8% (30.4% to 52.4%)
Self-harm	43.1% (29.4% to 55.1%)	43.4% (29.6% to 55.6%)	34.1% (20.0% to 46.3%)	43.2% (29.4% to 55.3%)
Interpersonal violence	36.6% (29.1% to 43.7%)	33.4% (26.1% to 39.9%)	37.2% (32.5% to 42.0%)	34.6% (28.3% to 40.3%)
**Forces of nature, conflict and terrorism, and executions and police conflict**	0.0% (0.0% to 0.0%)	0.0% (0.0% to 0.0%)	3.5% (2.2% to 5.3%)	1.7% (0.6% to 3.3%)
Forces of nature, conflict and terrorism, and state actor violence	0.0% (0.0% to 0.0%)	0.0% (0.0% to 0.0%)	7.5% (4.9% to 11.2%)	7.5% (4.9% to 11.2%)

Supplementary Results Table 1. Age-standardised PAFs attributable to the joint distribution of all risk factors for all ages, both sexes combined for each GBD level 1 and level 2 cause and all causes in 2016 for Caribbean. Results are shown for deaths, YLLs, YLDs, and DALYs
Data in parenthesis are 95% uncertainty intervals. PAFs=population attributable fractions. GBD=Global Burden of Disease. YLLs=years of life lost. YLDs=years live with disability. DALYs=disability-adjusted life-years

	Deaths 2016	YLLs 2016	YLDs 2016	DALYs 2016
**All causes**	60.4% (58.9% to 61.8%)	56.0% (54.6% to 57.2%)	24.0% (22.8% to 25.2%)	45.0% (42.8% to 47.4%)
**HIV/AIDS and tuberculosis**	72.3% (65.6% to 77.2%)	68.8% (61.5% to 74.2%)	73.6% (66.2% to 79.3%)	69.1% (61.8% to 74.4%)
Tuberculosis	43.7% (35.9% to 51.8%)	42.8% (35.6% to 50.8%)	40.4% (32.9% to 47.4%)	42.7% (35.5% to 50.6%)
HIV/AIDS	77.4% (69.1% to 82.8%)	72.3% (64.0% to 78.2%)	75.4% (67.7% to 81.2%)	72.5% (64.3% to 78.5%)
**Diarrhoea, lower respiratory infections, and other common infectious diseases**	59.7% (54.0% to 65.7%)	68.2% (60.5% to 74.2%)	41.4% (33.1% to 48.9%)	65.2% (58.4% to 71.1%)
Diarrhoeal diseases	94.6% (87.3% to 98.5%)	97.3% (93.2% to 99.3%)	84.1% (70.1% to 92.2%)	95.1% (89.4% to 98.3%)
Lower respiratory infections	58.1% (51.4% to 65.0%)	74.4% (67.9% to 80.4%)	56.8% (51.0% to 63.0%)	74.2% (67.8% to 80.3%)
Upper respiratory infections	2.0% (0.4% to 5.8%)	3.6% (0.6% to 10.8%)	0.0% (0.0% to 0.0%)	0.1% (0.0% to 0.2%)
Otitis media	2.1% (0.7% to 4.9%)	4.2% (1.5% to 8.7%)	3.7% (2.5% to 4.9%)	3.7% (2.5% to 4.9%)
Meningitis	5.6% (3.7% to 8.6%)	6.9% (4.6% to 10.2%)	0.0% (0.0% to 0.0%)	6.6% (4.4% to 9.8%)
Encephalitis	1.2% (0.9% to 1.6%)	1.8% (1.3% to 2.3%)	0.0% (0.0% to 0.0%)	1.5% (1.1% to 1.9%)
Measles	18.2% (6.2% to 47.2%)	19.8% (6.6% to 51.0%)	19.8% (5.2% to 57.4%)	19.8% (6.4% to 52.3%)
**Maternal disorders**	5.5% (2.6% to 8.9%)	5.5% (2.6% to 8.8%)	3.2% (1.7% to 5.0%)	5.3% (2.5% to 8.4%)
Maternal haemorrhage	20.0% (7.3% to 32.3%)	19.9% (7.3% to 32.2%)	16.9% (6.1% to 27.3%)	19.9% (7.3% to 32.1%)
Maternal sepsis and other pregnancy related infections	19.0% (6.9% to 30.8%)	19.0% (6.9% to 30.8%)	16.5% (6.0% to 26.7%)	18.7% (6.8% to 30.4%)
Maternal abortion, miscarriage, and ectopic pregnancy	11.1% (6.7% to 16.3%)	11.2% (6.7% to 16.5%)	9.0% (4.8% to 13.6%)	10.9% (6.6% to 16.1%)
**Neonatal disorders**	72.7% (69.1% to 76.2%)	72.8% (69.1% to 76.2%)	0.1% (0.1% to 0.2%)	64.3% (58.9% to 68.6%)
Neonatal preterm birth complications	95.4% (92.6% to 97.2%)	95.4% (92.6% to 97.2%)	0.3% (0.2% to 0.3%)	80.1% (72.8% to 85.7%)
Neonatal encephalopathy due to birth asphyxia and trauma	64.6% (59.3% to 69.5%)	64.7% (59.3% to 69.5%)	0.0% (0.0% to 0.0%)	63.7% (58.3% to 68.8%)
Neonatal sepsis and other neonatal infections	59.9% (55.0% to 64.4%)	59.9% (55.0% to 64.4%)	0.0% (0.0% to 0.0%)	46.8% (33.4% to 55.9%)
Hemolytic disease and other neonatal jaundice	57.4% (45.4% to 65.4%)	57.5% (45.5% to 65.4%)	0.0% (0.0% to 0.0%)	43.9% (34.4% to 52.8%)

Supplementary Results Table 1. Age-standardised PAFs attributable to the joint distribution of all risk factors for all ages, both sexes combined for each GBD level 1 and level 2 cause and all causes in 2016 for Caribbean. Results are shown for deaths, YLLs, YLDs, and DALYs
Data in parenthesis are 95% uncertainty intervals. PAFs=population attributable fractions. GBD=Global Burden of Disease. YLLs=years of life lost. YLDs=years live with disability. DALYs=disability-adjusted life-years

	Deaths 2016	YLLs 2016	YLDs 2016	DALYs 2016
Other neonatal disorders	60.6% (54.8% to 65.5%)	60.7% (54.8% to 65.6%)	0.0% (0.0% to 0.0%)	59.1% (53.1% to 64.1%)
**Nutritional deficiencies**	36.8% (24.0% to 50.1%)	67.5% (52.2% to 78.3%)	94.7% (94.3% to 95.1%)	87.5% (80.5% to 90.5%)
Protein-energy malnutrition	41.0% (27.5% to 54.8%)	73.1% (58.5% to 83.2%)	93.6% (92.8% to 94.3%)	74.8% (60.9% to 83.9%)
Vitamin A deficiency	-	-	96.6% (96.6% to 96.7%)	96.6% (96.6% to 96.7%)
Iron-deficiency anaemia	100.0% (100.0% to 100.0%)	100.0% (100.0% to 100.0%)	96.4% (96.4% to 96.5%)	96.4% (96.4% to 96.5%)
**Other communicable, maternal, neonatal, and nutritional diseases**	7.2% (4.5% to 10.6%)	4.7% (2.6% to 7.6%)	61.2% (53.9% to 69.2%)	17.1% (9.7% to 26.3%)
Sexually transmitted diseases excluding HIV	14.1% (6.3% to 26.9%)	6.7% (2.9% to 13.6%)	96.5% (96.4% to 96.7%)	27.1% (12.9% to 47.4%)
Hepatitis	7.0% (5.1% to 9.4%)	6.8% (4.8% to 9.4%)	4.9% (3.3% to 7.0%)	6.7% (4.8% to 9.2%)
**Neoplasms**	38.8% (36.2% to 41.9%)	38.7% (36.2% to 41.4%)	30.4% (27.7% to 33.5%)	38.5% (36.0% to 41.2%)
Oesophageal cancer	74.4% (66.2% to 81.9%)	76.4% (68.4% to 83.6%)	71.2% (63.3% to 78.5%)	76.4% (68.4% to 83.5%)
Stomach cancer	14.1% (8.0% to 21.4%)	14.3% (8.3% to 21.3%)	13.9% (8.0% to 20.9%)	14.3% (8.3% to 21.3%)
Liver cancer	56.1% (49.0% to 62.9%)	56.3% (49.5% to 63.1%)	53.6% (47.3% to 60.1%)	56.3% (49.5% to 63.1%)
Larynx cancer	83.3% (75.9% to 89.0%)	83.9% (76.9% to 89.2%)	80.0% (73.2% to 85.0%)	83.8% (76.8% to 89.1%)
Tracheal, bronchus, and lung cancer	84.4% (81.8% to 87.1%)	84.3% (81.8% to 86.8%)	79.9% (77.3% to 82.4%)	84.2% (81.7% to 86.7%)
Breast cancer	24.0% (16.9% to 32.5%)	22.3% (15.4% to 30.1%)	22.4% (15.5% to 30.0%)	22.3% (15.4% to 30.1%)
Cervical cancer	100.0% (100.0% to 100.0%)	100.0% (100.0% to 100.0%)	95.5% (95.4% to 95.5%)	99.9% (99.9% to 99.9%)
Uterine cancer	38.9% (26.7% to 51.9%)	41.4% (28.6% to 54.4%)	43.5% (30.2% to 56.7%)	41.5% (28.8% to 54.5%)
Prostate cancer	4.2% (3.0% to 5.5%)	4.2% (3.0% to 5.5%)	4.3% (3.1% to 5.6%)	4.2% (3.0% to 5.5%)
Colon and rectum cancer	50.4% (40.9% to 59.7%)	51.4% (42.4% to 60.3%)	48.1% (39.5% to 56.9%)	51.3% (42.3% to 60.1%)
Lip and oral cavity cancer	72.0% (65.2% to 77.9%)	74.4% (67.9% to 79.8%)	67.5% (61.5% to 73.2%)	74.1% (67.7% to 79.6%)
Nasopharynx cancer	69.4% (63.7% to 74.2%)	67.4% (62.5% to 71.6%)	64.4% (60.1% to 68.2%)	67.3% (62.4% to 71.5%)

Supplementary Results Table 1. Age-standardised PAFs attributable to the joint distribution of all risk factors for all ages, both sexes combined for each GBD level 1 and level 2 cause and all causes in 2016 for Caribbean. Results are shown for deaths, YLLs, YLDs, and DALYs
Data in parenthesis are 95% uncertainty intervals. PAFs=population attributable fractions. GBD=Global Burden of Disease. YLLs=years of life lost. YLDs=years live with disability. DALYs=disability-adjusted life-years

	Deaths 2016	YLLs 2016	YLDs 2016	DALYs 2016
Other pharynx cancer	47.8% (36.0% to 58.5%)	51.8% (40.3% to 61.6%)	47.6% (36.5% to 57.2%)	51.7% (40.2% to 61.5%)
Gallbladder and biliary tract cancer	17.4% (9.2% to 27.7%)	18.5% (10.1% to 29.1%)	16.9% (9.2% to 26.6%)	18.5% (10.1% to 29.0%)
Pancreatic cancer	29.6% (21.8% to 38.4%)	30.1% (22.6% to 38.5%)	28.2% (21.0% to 36.4%)	30.1% (22.6% to 38.5%)
Ovarian cancer	12.2% (4.4% to 22.3%)	11.2% (3.9% to 20.4%)	10.3% (3.5% to 18.7%)	11.2% (3.9% to 20.4%)
Kidney cancer	30.0% (22.1% to 39.1%)	28.1% (20.6% to 36.7%)	28.2% (20.7% to 36.7%)	28.2% (20.6% to 36.7%)
Bladder cancer	35.3% (25.9% to 45.4%)	35.4% (26.2% to 45.2%)	33.9% (25.2% to 43.4%)	35.4% (26.1% to 45.1%)
Thyroid cancer	10.8% (5.6% to 18.3%)	11.5% (6.0% to 19.3%)	12.8% (6.9% to 20.5%)	11.6% (6.1% to 19.4%)
Mesothelioma	80.1% (75.0% to 84.5%)	69.7% (62.9% to 75.8%)	72.8% (67.3% to 77.7%)	69.8% (63.0% to 75.9%)
Non-Hodgkin's lymphoma	5.4% (2.1% to 9.6%)	5.1% (2.1% to 9.1%)	5.6% (2.2% to 9.9%)	5.1% (2.1% to 9.1%)
Multiple myeloma	6.5% (2.7% to 11.8%)	6.9% (3.0% to 12.4%)	6.7% (2.9% to 12.1%)	6.9% (3.0% to 12.4%)
Leukaemia	14.1% (9.0% to 19.6%)	10.9% (7.0% to 15.2%)	13.0% (8.3% to 17.9%)	11.0% (7.1% to 15.3%)
**Cardiovascular diseases**	83.4% (80.8% to 85.7%)	82.5% (80.4% to 84.3%)	67.2% (64.4% to 69.8%)	81.3% (79.2% to 83.2%)
Rheumatic heart disease	22.3% (15.2% to 35.7%)	17.6% (11.8% to 26.2%)	16.5% (10.1% to 24.4%)	17.4% (11.3% to 26.0%)
Ischaemic heart disease	91.5% (87.7% to 94.5%)	93.2% (91.0% to 95.2%)	85.2% (82.4% to 87.9%)	92.8% (90.5% to 94.8%)
Cerebrovascular disease	81.6% (77.1% to 85.9%)	81.6% (78.1% to 84.8%)	78.3% (74.0% to 82.3%)	81.4% (77.9% to 84.6%)
Hypertensive heart disease	100.0% (100.0% to 100.0%)	100.0% (100.0% to 100.0%)	95.1% (95.1% to 95.2%)	99.6% (99.5% to 99.8%)
Cardiomyopathy and myocarditis	41.4% (35.5% to 47.2%)	39.7% (33.0% to 45.5%)	35.8% (31.0% to 40.8%)	39.5% (33.0% to 45.3%)
Atrial fibrillation and flutter	46.4% (39.3% to 53.5%)	51.2% (44.7% to 57.2%)	55.2% (50.3% to 59.8%)	53.6% (48.2% to 58.8%)
Aortic aneurysm	41.8% (35.3% to 48.5%)	46.3% (40.1% to 52.0%)	-	46.3% (40.1% to 52.0%)
Peripheral vascular disease	49.4% (41.5% to 56.4%)	56.2% (49.2% to 62.0%)	56.1% (51.5% to 60.7%)	56.2% (50.0% to 61.5%)
Endocarditis	35.0% (27.3% to 44.1%)	30.1% (23.4% to 37.3%)	32.6% (25.5% to 40.3%)	30.1% (23.5% to 37.3%)

Supplementary Results Table 1. Age-standardised PAFs attributable to the joint distribution of all risk factors for all ages, both sexes combined for each GBD level 1 and level 2 cause and all causes in 2016 for Caribbean. Results are shown for deaths, YLLs, YLDs, and DALYs
Data in parenthesis are 95% uncertainty intervals. PAFs=population attributable fractions. GBD=Global Burden of Disease. YLLs=years of life lost. YLDs=years live with disability. DALYs=disability-adjusted life-years

	Deaths 2016	YLLs 2016	YLDs 2016	DALYs 2016
Other cardiovascular and circulatory diseases	42.3% (37.8% to 46.8%)	39.5% (34.8% to 44.1%)	40.8% (36.3% to 45.3%)	39.7% (35.3% to 44.3%)
**Chronic respiratory diseases**	63.9% (59.8% to 68.1%)	56.0% (51.5% to 60.6%)	29.8% (23.5% to 37.0%)	44.8% (39.5% to 50.3%)
Chronic obstructive pulmonary disease	76.8% (72.0% to 81.5%)	75.6% (71.0% to 79.9%)	65.9% (61.2% to 70.6%)	74.1% (69.6% to 78.4%)
Pneumoconiosis	100.0% (100.0% to 100.0%)	100.0% (100.0% to 100.0%)	95.4% (95.3% to 95.4%)	98.2% (97.7% to 98.6%)
Asthma	29.7% (22.7% to 38.0%)	27.2% (20.4% to 35.3%)	23.8% (17.1% to 31.9%)	24.8% (18.3% to 32.5%)
Other chronic respiratory diseases	13.3% (9.3% to 17.2%)	10.1% (6.7% to 13.6%)	4.8% (3.2% to 6.6%)	9.5% (6.3% to 12.7%)
**Cirrhosis and other chronic liver diseases**	64.5% (62.0% to 66.9%)	64.6% (62.2% to 66.9%)	50.9% (48.5% to 53.2%)	64.0% (61.7% to 66.3%)
Cirrhosis and other chronic liver diseases due to hepatitis B	5.7% (3.6% to 8.5%)	7.3% (4.6% to 10.9%)	5.5% (3.4% to 8.3%)	7.2% (4.5% to 10.8%)
Cirrhosis and other chronic liver diseases due to hepatitis C	76.5% (72.8% to 79.8%)	82.3% (79.6% to 84.6%)	75.0% (72.1% to 77.7%)	82.1% (79.4% to 84.4%)
Cirrhosis and other chronic liver diseases due to alcohol use	100.0% (100.0% to 100.0%)	100.0% (100.0% to 100.0%)	95.4% (95.3% to 95.5%)	99.8% (99.8% to 99.9%)
**Digestive diseases**	7.8% (6.2% to 9.5%)	8.0% (6.1% to 10.1%)	2.9% (2.1% to 3.8%)	6.8% (5.3% to 8.4%)
Peptic ulcer disease	9.7% (6.7% to 13.1%)	9.6% (6.7% to 12.9%)	8.6% (5.9% to 11.8%)	9.4% (6.6% to 12.6%)
Gallbladder and biliary diseases	33.4% (22.2% to 46.5%)	34.1% (23.1% to 47.1%)	40.5% (28.2% to 54.2%)	35.0% (23.7% to 48.0%)
Pancreatitis	33.6% (22.7% to 45.1%)	39.9% (29.1% to 51.1%)	27.6% (19.5% to 37.0%)	39.7% (29.0% to 50.8%)
**Neurological disorders**	18.3% (9.5% to 29.0%)	17.9% (10.8% to 26.3%)	3.5% (2.2% to 5.1%)	8.1% (5.0% to 12.0%)
Alzheimer's disease and other dementias	21.1% (10.7% to 33.8%)	22.5% (11.7% to 35.1%)	21.4% (11.2% to 33.5%)	22.3% (11.6% to 34.7%)
Parkinson's disease	-8.3% (-10.0% to -6.5%)	-9.8% (-11.8% to -7.6%)	-9.9% (-11.8% to -7.9%)	-9.8% (-11.8% to -7.7%)
Epilepsy	24.7% (18.6% to 30.9%)	23.7% (17.7% to 30.1%)	19.5% (14.3% to 24.4%)	21.4% (16.3% to 26.9%)
Multiple sclerosis	6.7% (4.4% to 9.6%)	6.4% (4.1% to 9.1%)	5.3% (3.4% to 7.4%)	6.1% (3.9% to 8.6%)
**Mental and substance use disorders**	99.9% (99.8% to 99.9%)	99.8% (99.7% to 99.8%)	17.4% (15.5% to 19.5%)	22.0% (19.6% to 24.6%)
Alcohol use disorders	100.0% (100.0% to 100.0%)	100.0% (100.0% to 100.0%)	96.5% (96.4% to 96.6%)	97.7% (97.4% to 98.0%)

Supplementary Results Table 1. Age-standardised PAFs attributable to the joint distribution of all risk factors for all ages, both sexes combined for each GBD level 1 and level 2 cause and all causes in 2016 for Caribbean. Results are shown for deaths, YLLs, YLDs, and DALYs
Data in parenthesis are 95% uncertainty intervals. PAFs=population attributable fractions. GBD=Global Burden of Disease. YLLs=years of life lost. YLDs=years live with disability. DALYs=disability-adjusted life-years

	**Deaths 2016**	**YLLs 2016**	**YLDs 2016**	**DALYs 2016**
Drug use disorders	100.0% (100.0% to 100.0%)	100.0% (100.0% to 100.0%)	96.5% (96.4% to 96.6%)	97.2% (97.0% to 97.4%)
Depressive disorders	-	-	6.1% (5.0% to 7.3%)	6.1% (5.0% to 7.3%)
Idiopathic developmental intellectual disability	-	-	65.8% (36.0% to 84.2%)	65.8% (36.0% to 84.2%)
**Diabetes, urogenital, blood, and endocrine diseases**	87.2% (85.7% to 87.9%)	82.0% (79.6% to 83.7%)	67.8% (64.0% to 71.2%)	76.7% (74.3% to 78.7%)
Diabetes mellitus	100.0% (100.0% to 100.0%)	100.0% (100.0% to 100.0%)	95.4% (95.4% to 95.5%)	98.1% (97.7% to 98.5%)
Chronic kidney disease	100.0% (100.0% to 100.0%)	100.0% (100.0% to 100.0%)	95.5% (95.4% to 95.6%)	99.2% (99.0% to 99.4%)
**Musculoskeletal disorders**	0.7% (0.3% to 1.2%)	0.4% (0.2% to 0.7%)	12.9% (10.5% to 15.7%)	12.5% (10.1% to 15.2%)
Rheumatoid arthritis	3.1% (1.2% to 5.3%)	3.2% (1.3% to 5.4%)	3.7% (1.5% to 6.3%)	3.7% (1.5% to 6.2%)
Osteoarthritis	-	-	21.8% (11.6% to 33.3%)	21.8% (11.6% to 33.3%)
Low back and neck pain	-	-	17.4% (14.9% to 20.2%)	17.4% (14.9% to 20.2%)
Gout	-	-	40.3% (27.9% to 54.2%)	40.3% (27.9% to 54.2%)
**Other non-communicable diseases**	0.2% (0.1% to 0.3%)	0.2% (0.1% to 0.3%)	4.9% (4.1% to 5.8%)	3.6% (3.0% to 4.3%)
Sense organ diseases	-	-	11.4% (10.0% to 13.1%)	11.4% (10.0% to 13.1%)
Sudden infant death syndrome	4.8% (2.4% to 7.9%)	4.9% (2.4% to 7.9%)	-	4.9% (2.4% to 7.9%)
**Transport injuries**	32.2% (25.2% to 39.5%)	27.6% (20.8% to 35.2%)	40.2% (33.0% to 47.6%)	28.9% (22.0% to 36.5%)
Road injuries	31.8% (24.8% to 38.9%)	27.3% (20.3% to 34.6%)	40.6% (33.3% to 47.9%)	28.7% (21.9% to 36.2%)
Other transport injuries	38.8% (27.9% to 52.9%)	32.5% (20.7% to 48.5%)	33.6% (27.0% to 40.3%)	32.7% (21.4% to 47.4%)
**Unintentional injuries**	26.5% (18.2% to 30.8%)	14.4% (10.3% to 18.4%)	24.9% (20.3% to 29.5%)	18.1% (13.8% to 22.1%)
Falls	62.7% (53.1% to 67.1%)	40.5% (29.4% to 47.4%)	31.3% (26.7% to 35.7%)	34.3% (29.0% to 39.4%)
Drowning	14.5% (9.1% to 19.8%)	12.4% (7.5% to 17.3%)	19.3% (13.2% to 25.3%)	12.4% (7.6% to 17.3%)
Fire, heat, and hot substances	9.2% (6.4% to 12.6%)	8.3% (5.4% to 12.2%)	24.2% (17.3% to 30.8%)	13.1% (8.9% to 17.7%)

Supplementary Results Table 1. Age-standardised PAFs attributable to the joint distribution of all risk factors for all ages, both sexes combined for each GBD level 1 and level 2 cause and all causes in 2016 for Caribbean. Results are shown for deaths, YLLs, YLDs, and DALYs
Data in parenthesis are 95% uncertainty intervals. PAFs=population attributable fractions. GBD=Global Burden of Disease. YLLs=years of life lost. YLDs=years live with disability. DALYs=disability-adjusted life-years

	Deaths 2016	YLLs 2016	YLDs 2016	DALYs 2016
Poisonings	11.6% (7.0% to 16.8%)	10.0% (5.1% to 15.1%)	24.4% (17.4% to 30.7%)	11.4% (6.2% to 16.5%)
Exposure to mechanical forces	17.4% (13.1% to 22.0%)	14.6% (10.1% to 19.5%)	24.4% (18.1% to 30.9%)	17.7% (12.8% to 22.8%)
Animal contact	14.0% (11.0% to 18.6%)	13.7% (9.9% to 17.7%)	17.3% (12.1% to 22.5%)	15.2% (11.0% to 19.5%)
Foreign body	6.7% (4.4% to 10.0%)	6.3% (3.7% to 9.7%)	12.0% (8.3% to 15.6%)	7.6% (4.8% to 10.9%)
Other unintentional injuries	21.4% (12.7% to 29.4%)	19.3% (11.3% to 26.9%)	22.9% (16.4% to 29.1%)	20.9% (13.6% to 27.5%)
**Self-harm and interpersonal violence**	26.1% (20.6% to 31.8%)	26.5% (21.2% to 32.3%)	27.7% (24.9% to 30.9%)	26.5% (21.5% to 32.2%)
Self-harm	26.7% (17.2% to 36.3%)	28.8% (19.0% to 38.7%)	22.5% (13.9% to 31.6%)	28.7% (18.9% to 38.7%)
Interpersonal violence	25.5% (19.6% to 32.7%)	24.9% (18.8% to 32.1%)	28.2% (25.3% to 31.6%)	25.1% (19.4% to 32.0%)
**Forces of nature, conflict and terrorism, and executions and police conflict**	3.5% (3.2% to 3.8%)	1.4% (1.2% to 1.5%)	4.1% (2.9% to 5.5%)	2.1% (1.5% to 2.9%)
Forces of nature, conflict and terrorism, and state actor violence	3.6% (3.3% to 3.9%)	1.4% (1.3% to 1.6%)	4.1% (3.0% to 5.6%)	2.1% (1.6% to 2.9%)

Supplementary Results Table 1. Age-standardised PAFs attributable to the joint distribution of all risk factors for all ages, both sexes combined for each GBD level 1 and level 2 cause and all causes in 2016 for Central Asia. Results are shown for deaths, YLLs, YLDs, and DALYs
Data in parenthesis are 95% uncertainty intervals. PAFs=population attributable fractions. GBD=Global Burden of Disease. YLLs=years of life lost. YLDs=years live with disability. DALYs=disability-adjusted life-years

	Deaths 2016	YLLs 2016	YLDs 2016	DALYs 2016
**All causes**	72.1% (70.5% to 73.6%)	65.0% (63.8% to 66.1%)	27.9% (26.7% to 29.1%)	53.3% (50.8% to 55.7%)
**HIV/AIDS and tuberculosis**	56.6% (49.8% to 62.9%)	56.3% (50.1% to 62.2%)	51.6% (44.9% to 58.5%)	56.1% (49.9% to 61.9%)
Tuberculosis	53.4% (45.5% to 61.0%)	52.8% (45.3% to 60.1%)	43.8% (36.2% to 51.0%)	52.4% (44.9% to 59.7%)
HIV/AIDS	72.3% (69.8% to 74.1%)	70.0% (67.4% to 71.9%)	71.7% (69.4% to 73.6%)	70.1% (67.5% to 72.0%)
**Diarrhoea, lower respiratory infections, and other common infectious diseases**	65.3% (60.1% to 70.2%)	70.6% (65.3% to 75.3%)	33.1% (27.2% to 39.1%)	67.2% (62.0% to 72.2%)
Diarrhoeal diseases	93.0% (86.2% to 97.4%)	95.2% (90.3% to 98.3%)	81.8% (68.6% to 92.3%)	91.0% (83.3% to 96.5%)
Lower respiratory infections	71.3% (65.5% to 77.0%)	77.8% (71.7% to 83.0%)	59.5% (53.0% to 66.7%)	77.7% (71.6% to 82.9%)
Upper respiratory infections	1.5% (0.6% to 3.4%)	3.2% (1.2% to 7.0%)	0.0% (0.0% to 0.0%)	0.1% (0.0% to 0.2%)
Otitis media	2.8% (1.8% to 4.2%)	6.2% (4.0% to 8.5%)	6.9% (4.6% to 8.9%)	6.9% (4.6% to 8.9%)
Meningitis	3.0% (2.3% to 4.1%)	4.3% (3.3% to 5.7%)	0.0% (0.0% to 0.0%)	3.4% (2.6% to 4.6%)
Encephalitis	1.9% (1.1% to 3.0%)	2.6% (1.4% to 4.0%)	0.0% (0.0% to 0.0%)	2.4% (1.3% to 3.8%)
Measles	32.7% (12.1% to 71.8%)	33.3% (12.3% to 73.0%)	32.9% (13.1% to 71.5%)	33.3% (12.3% to 73.0%)
**Maternal disorders**	8.3% (4.0% to 12.9%)	8.1% (3.8% to 12.8%)	4.9% (2.2% to 8.4%)	7.3% (3.5% to 11.6%)
Maternal haemorrhage	18.3% (6.6% to 29.8%)	18.3% (6.6% to 29.7%)	18.2% (6.6% to 29.4%)	18.3% (6.6% to 29.7%)
Maternal sepsis and other pregnancy related infections	18.9% (6.9% to 30.3%)	18.9% (6.9% to 30.3%)	18.0% (6.5% to 29.1%)	18.5% (6.8% to 29.8%)
Maternal abortion, miscarriage, and ectopic pregnancy	10.3% (6.3% to 15.0%)	10.0% (6.0% to 14.5%)	9.0% (4.3% to 14.8%)	9.8% (5.8% to 14.4%)
**Neonatal disorders**	75.3% (73.0% to 77.8%)	75.3% (73.1% to 77.8%)	0.1% (0.1% to 0.2%)	67.9% (64.3% to 71.2%)
Neonatal preterm birth complications	93.8% (91.7% to 95.5%)	93.8% (91.8% to 95.5%)	0.3% (0.2% to 0.4%)	83.6% (79.2% to 87.7%)
Neonatal encephalopathy due to birth asphyxia and trauma	67.9% (64.7% to 71.6%)	67.9% (64.7% to 71.7%)	0.0% (0.0% to 0.0%)	63.5% (57.5% to 68.2%)
Neonatal sepsis and other neonatal infections	50.4% (46.0% to 54.5%)	50.5% (46.1% to 54.6%)	0.0% (0.0% to 0.0%)	40.6% (31.2% to 47.8%)
Hemolytic disease and other neonatal jaundice	64.0% (60.0% to 67.9%)	64.0% (60.1% to 67.9%)	0.0% (0.0% to 0.0%)	23.2% (17.9% to 29.2%)

Supplementary Results Table 1. Age-standardised PAFs attributable to the joint distribution of all risk factors for all ages, both sexes combined for each GBD level 1 and level 2 cause and all causes in 2016 for Central Asia. Results are shown for deaths, YLLs, YLDs, and DALYs
Data in parenthesis are 95% uncertainty intervals. PAFs=population attributable fractions. GBD=Global Burden of Disease. YLLs=years of life lost. YLDs=years live with disability. DALYs=disability-adjusted life-years

	Deaths 2016	YLLs 2016	YLDs 2016	DALYs 2016
Other neonatal disorders	61.1% (57.1% to 65.2%)	61.1% (57.1% to 65.2%)	0.0% (0.0% to 0.0%)	59.3% (55.5% to 63.3%)
**Nutritional deficiencies**	26.3% (20.3% to 34.1%)	44.2% (35.7% to 54.2%)	93.7% (92.4% to 95.0%)	92.2% (90.4% to 93.6%)
Protein-energy malnutrition	39.5% (28.8% to 51.2%)	63.8% (52.9% to 74.1%)	95.6% (94.2% to 96.6%)	84.3% (79.6% to 88.3%)
Vitamin A deficiency	-	-	100.0% (100.0% to 100.0%)	100.0% (100.0% to 100.0%)
Iron-deficiency anaemia	100.0% (100.0% to 100.0%)	100.0% (100.0% to 100.0%)	100.0% (100.0% to 100.0%)	100.0% (100.0% to 100.0%)
**Other communicable, maternal, neonatal, and nutritional diseases**	5.1% (3.8% to 8.1%)	3.2% (2.1% to 5.2%)	63.6% (58.3% to 68.4%)	26.5% (18.7% to 34.8%)
Sexually transmitted diseases excluding HIV	56.3% (38.7% to 74.1%)	34.8% (20.5% to 53.5%)	100.0% (100.0% to 100.0%)	89.9% (81.8% to 95.7%)
Hepatitis	2.4% (1.9% to 3.1%)	1.9% (1.4% to 2.5%)	1.1% (0.7% to 1.6%)	1.8% (1.4% to 2.4%)
**Neoplasms**	42.5% (39.7% to 45.3%)	40.9% (38.5% to 43.5%)	37.8% (35.0% to 40.8%)	40.9% (38.4% to 43.5%)
Oesophageal cancer	73.3% (62.7% to 82.5%)	75.4% (65.5% to 84.0%)	73.9% (63.2% to 82.9%)	75.4% (65.5% to 84.0%)
Stomach cancer	19.7% (11.6% to 28.7%)	20.3% (12.3% to 29.0%)	19.7% (11.6% to 28.7%)	20.3% (12.3% to 29.0%)
Liver cancer	62.2% (55.2% to 68.8%)	62.4% (55.7% to 68.8%)	62.3% (55.4% to 68.9%)	62.4% (55.7% to 68.8%)
Larynx cancer	77.7% (70.1% to 84.0%)	80.7% (73.6% to 86.4%)	79.3% (71.8% to 85.3%)	80.7% (73.6% to 86.4%)
Tracheal, bronchus, and lung cancer	84.3% (81.3% to 87.1%)	85.3% (82.7% to 87.8%)	84.0% (81.0% to 86.8%)	85.3% (82.6% to 87.8%)
Breast cancer	27.0% (21.0% to 33.9%)	25.5% (19.7% to 32.0%)	26.1% (20.3% to 32.7%)	25.5% (19.8% to 32.0%)
Cervical cancer	100.0% (100.0% to 100.0%)	100.0% (100.0% to 100.0%)	100.0% (100.0% to 100.0%)	100.0% (100.0% to 100.0%)
Uterine cancer	46.8% (33.6% to 60.5%)	46.8% (33.0% to 60.5%)	47.8% (34.0% to 61.6%)	46.8% (33.0% to 60.5%)
Prostate cancer	4.1% (2.9% to 5.3%)	5.2% (3.8% to 6.7%)	5.3% (3.8% to 6.8%)	5.2% (3.8% to 6.7%)
Colon and rectum cancer	49.3% (41.0% to 57.4%)	50.2% (42.1% to 57.9%)	50.1% (41.9% to 57.9%)	50.2% (42.1% to 57.9%)
Lip and oral cavity cancer	70.0% (62.2% to 76.8%)	72.3% (65.6% to 78.4%)	68.4% (60.7% to 75.1%)	72.2% (65.4% to 78.3%)
Nasopharynx cancer	67.5% (61.9% to 72.6%)	63.5% (58.7% to 68.2%)	64.2% (59.4% to 68.9%)	63.5% (58.8% to 68.2%)

Supplementary Results Table 1. Age-standardised PAFs attributable to the joint distribution of all risk factors for all ages, both sexes combined for each GBD level 1 and level 2 cause and all causes in 2016 for Central Asia. Results are shown for deaths, YLLs, YLDs, and DALYs
Data in parenthesis are 95% uncertainty intervals. PAFs=population attributable fractions. GBD=Global Burden of Disease. YLLs=years of life lost. YLDs=years live with disability. DALYs=disability-adjusted life-years

	Deaths 2016	YLLs 2016	YLDs 2016	DALYs 2016
Other pharynx cancer	58.8% (46.6% to 68.6%)	60.4% (48.8% to 69.8%)	59.7% (47.8% to 69.4%)	60.4% (48.8% to 69.8%)
Gallbladder and biliary tract cancer	22.8% (13.0% to 35.1%)	23.0% (13.1% to 35.0%)	23.1% (13.2% to 35.0%)	23.0% (13.1% to 35.0%)
Pancreatic cancer	24.9% (18.3% to 32.1%)	27.2% (20.2% to 34.2%)	25.3% (18.8% to 32.5%)	27.1% (20.2% to 34.2%)
Ovarian cancer	9.7% (3.9% to 16.6%)	8.6% (3.1% to 15.1%)	8.5% (3.1% to 14.8%)	8.6% (3.1% to 15.0%)
Kidney cancer	32.4% (23.1% to 42.3%)	31.5% (22.6% to 41.0%)	31.8% (22.8% to 41.2%)	31.5% (22.7% to 41.0%)
Bladder cancer	29.3% (21.3% to 38.0%)	33.1% (24.8% to 41.8%)	31.7% (23.7% to 39.7%)	33.1% (24.7% to 41.7%)
Thyroid cancer	13.4% (7.1% to 22.1%)	13.5% (7.1% to 22.2%)	13.6% (7.3% to 21.3%)	13.5% (7.1% to 22.2%)
Mesothelioma	81.2% (76.8% to 85.0%)	69.8% (63.9% to 75.1%)	77.9% (73.2% to 82.1%)	69.9% (64.1% to 75.2%)
Non-Hodgkin's lymphoma	5.9% (2.3% to 10.4%)	5.1% (2.1% to 9.1%)	5.8% (2.3% to 10.2%)	5.2% (2.1% to 9.1%)
Multiple myeloma	7.7% (3.2% to 13.9%)	7.7% (3.3% to 13.7%)	7.8% (3.3% to 13.9%)	7.7% (3.3% to 13.7%)
Leukaemia	14.8% (9.5% to 20.3%)	11.1% (7.2% to 15.2%)	14.0% (9.1% to 19.2%)	11.1% (7.3% to 15.2%)
**Cardiovascular diseases**	90.3% (88.0% to 92.3%)	91.0% (89.4% to 92.4%)	80.7% (78.0% to 83.0%)	90.4% (88.9% to 91.8%)
Rheumatic heart disease	29.0% (20.8% to 43.2%)	26.7% (18.6% to 38.6%)	23.1% (15.0% to 32.9%)	26.2% (17.9% to 37.5%)
Ischaemic heart disease	93.1% (89.9% to 95.7%)	94.5% (92.4% to 96.1%)	91.6% (89.1% to 94.1%)	94.4% (92.3% to 96.1%)
Cerebrovascular disease	86.6% (82.7% to 90.1%)	90.3% (87.7% to 92.4%)	87.5% (83.8% to 90.6%)	90.0% (87.4% to 92.2%)
Hypertensive heart disease	100.0% (100.0% to 100.0%)	100.0% (100.0% to 100.0%)	100.0% (100.0% to 100.0%)	100.0% (100.0% to 100.0%)
Cardiomyopathy and myocarditis	58.6% (53.0% to 63.8%)	55.0% (49.8% to 59.8%)	45.4% (40.4% to 50.4%)	54.6% (49.5% to 59.3%)
Atrial fibrillation and flutter	53.0% (45.3% to 60.5%)	57.1% (50.2% to 63.3%)	63.3% (58.1% to 68.4%)	60.8% (54.8% to 66.2%)
Aortic aneurysm	51.1% (44.3% to 57.6%)	56.2% (50.4% to 61.3%)	-	56.2% (50.4% to 61.3%)
Peripheral vascular disease	52.0% (45.0% to 59.4%)	60.0% (54.5% to 65.4%)	59.2% (53.6% to 65.0%)	59.4% (53.9% to 64.9%)
Endocarditis	41.1% (33.0% to 49.5%)	34.9% (27.3% to 42.6%)	37.4% (29.7% to 45.7%)	34.9% (27.3% to 42.6%)

Supplementary Results Table 1. Age-standardised PAFs attributable to the joint distribution of all risk factors for all ages, both sexes combined for each GBD level 1 and level 2 cause and all causes in 2016 for Central Asia. Results are shown for deaths, YLLs, YLDs, and DALYs
Data in parenthesis are 95% uncertainty intervals. PAFs=population attributable fractions. GBD=Global Burden of Disease. YLLs=years of life lost. YLDs=years live with disability. DALYs=disability-adjusted life-years

	Deaths 2016	YLLs 2016	YLDs 2016	DALYs 2016
Other cardiovascular and circulatory diseases	52.0% (47.6% to 56.3%)	54.4% (50.2% to 58.4%)	48.3% (43.4% to 53.2%)	53.1% (48.9% to 57.3%)
**Chronic respiratory diseases**	59.1% (53.2% to 65.2%)	60.7% (55.7% to 65.8%)	39.8% (33.6% to 46.5%)	54.1% (49.0% to 59.5%)
Chronic obstructive pulmonary disease	66.6% (59.4% to 73.9%)	69.9% (63.8% to 76.2%)	66.1% (59.4% to 72.5%)	69.4% (63.2% to 75.6%)
Pneumoconiosis	100.0% (100.0% to 100.0%)	100.0% (100.0% to 100.0%)	100.0% (100.0% to 100.0%)	100.0% (100.0% to 100.0%)
Asthma	39.5% (30.3% to 50.2%)	42.6% (33.8% to 52.7%)	33.0% (25.0% to 42.3%)	35.7% (27.5% to 45.1%)
Other chronic respiratory diseases	13.0% (9.3% to 17.4%)	14.3% (10.2% to 19.1%)	4.9% (3.1% to 6.9%)	12.0% (8.5% to 16.4%)
**Cirrhosis and other chronic liver diseases**	42.6% (40.4% to 44.9%)	42.7% (40.5% to 44.8%)	30.3% (28.4% to 32.3%)	42.3% (40.2% to 44.5%)
Cirrhosis and other chronic liver diseases due to hepatitis B	1.4% (0.9% to 2.0%)	1.7% (1.1% to 2.5%)	1.3% (0.9% to 1.9%)	1.7% (1.1% to 2.4%)
Cirrhosis and other chronic liver diseases due to hepatitis C	75.7% (72.1% to 79.1%)	81.3% (78.6% to 83.7%)	77.8% (74.7% to 80.9%)	81.2% (78.5% to 83.7%)
Cirrhosis and other chronic liver diseases due to alcohol use	100.0% (100.0% to 100.0%)	100.0% (100.0% to 100.0%)	100.0% (100.0% to 100.0%)	100.0% (100.0% to 100.0%)
**Digestive diseases**	16.1% (12.9% to 19.7%)	16.8% (13.4% to 20.5%)	3.0% (2.3% to 3.7%)	13.0% (10.3% to 16.1%)
Peptic ulcer disease	15.0% (10.7% to 19.5%)	16.0% (11.4% to 20.7%)	9.6% (6.9% to 12.5%)	15.1% (10.9% to 19.7%)
Gallbladder and biliary diseases	41.8% (29.1% to 55.8%)	41.2% (28.6% to 54.9%)	45.1% (32.1% to 59.8%)	41.8% (29.1% to 55.7%)
Pancreatitis	44.2% (28.7% to 58.8%)	47.7% (33.2% to 61.3%)	36.1% (22.7% to 48.7%)	47.6% (33.1% to 61.2%)
**Neurological disorders**	18.6% (9.8% to 29.0%)	18.5% (12.4% to 25.2%)	4.2% (2.7% to 6.1%)	9.0% (6.1% to 12.4%)
Alzheimer's disease and other dementias	21.9% (10.2% to 35.1%)	22.7% (11.2% to 36.1%)	22.8% (11.1% to 36.2%)	22.7% (11.2% to 36.1%)
Parkinson's disease	-8.3% (-10.4% to -6.3%)	-10.5% (-13.1% to -8.0%)	-11.3% (-14.2% to -8.5%)	-10.7% (-13.3% to -8.1%)
Epilepsy	25.8% (18.8% to 32.4%)	22.9% (16.5% to 29.1%)	21.2% (14.5% to 27.0%)	22.2% (15.6% to 28.0%)
Multiple sclerosis	7.7% (4.3% to 11.3%)	8.9% (5.2% to 12.7%)	5.9% (3.5% to 8.2%)	7.2% (4.3% to 10.1%)
**Mental and substance use disorders**	100.0% (100.0% to 100.0%)	100.0% (100.0% to 100.0%)	22.2% (19.9% to 24.8%)	32.1% (28.8% to 35.9%)
Alcohol use disorders	100.0% (100.0% to 100.0%)	100.0% (100.0% to 100.0%)	100.0% (100.0% to 100.0%)	100.0% (100.0% to 100.0%)

Supplementary Results Table 1. Age-standardised PAFs attributable to the joint distribution of all risk factors for all ages, both sexes combined for each GBD level 1 and level 2 cause and all causes in 2016 for Central Asia. Results are shown for deaths, YLLs, YLDs, and DALYs
Data in parenthesis are 95% uncertainty intervals. PAFs=population attributable fractions. GBD=Global Burden of Disease. YLLs=years of life lost. YLDs=years live with disability. DALYs=disability-adjusted life-years

	Deaths 2016	YLLs 2016	YLDs 2016	DALYs 2016
Drug use disorders	100.0% (100.0% to 100.0%)	100.0% (100.0% to 100.0%)	100.0% (100.0% to 100.0%)	100.0% (100.0% to 100.0%)
Depressive disorders	-	-	3.6% (2.8% to 4.5%)	3.6% (2.8% to 4.5%)
Idiopathic developmental intellectual disability	-	-	56.9% (27.7% to 77.0%)	56.9% (27.7% to 77.0%)
**Diabetes, urogenital, blood, and endocrine diseases**	83.3% (82.7% to 83.9%)	79.6% (78.7% to 80.7%)	56.7% (52.4% to 60.8%)	69.9% (66.7% to 72.6%)
Diabetes mellitus	100.0% (100.0% to 100.0%)	100.0% (100.0% to 100.0%)	100.0% (100.0% to 100.0%)	100.0% (100.0% to 100.0%)
Chronic kidney disease	100.0% (100.0% to 100.0%)	100.0% (100.0% to 100.0%)	100.0% (100.0% to 100.0%)	100.0% (100.0% to 100.0%)
**Musculoskeletal disorders**	1.4% (0.5% to 2.4%)	1.2% (0.5% to 2.0%)	24.8% (21.2% to 28.7%)	24.5% (20.9% to 28.5%)
Rheumatoid arthritis	2.7% (1.0% to 4.5%)	2.9% (1.2% to 5.0%)	3.2% (1.3% to 5.2%)	3.2% (1.3% to 5.1%)
Osteoarthritis	-	-	26.3% (14.4% to 38.8%)	26.3% (14.4% to 38.8%)
Low back and neck pain	-	-	27.1% (23.8% to 30.5%)	27.1% (23.8% to 30.5%)
Gout	-	-	48.0% (34.8% to 62.3%)	48.0% (34.8% to 62.3%)
**Other non-communicable diseases**	0.2% (0.2% to 0.3%)	0.3% (0.2% to 0.3%)	5.7% (5.0% to 6.5%)	4.6% (4.0% to 5.2%)
Sense organ diseases	-	-	12.0% (10.7% to 13.3%)	12.0% (10.7% to 13.3%)
Sudden infant death syndrome	6.1% (4.6% to 7.7%)	6.1% (4.7% to 7.7%)	-	6.1% (4.7% to 7.7%)
**Transport injuries**	32.6% (25.6% to 40.2%)	29.2% (22.3% to 36.4%)	37.9% (31.1% to 45.5%)	30.7% (24.0% to 38.0%)
Road injuries	32.6% (25.5% to 40.2%)	29.2% (22.3% to 36.4%)	38.7% (31.9% to 46.4%)	30.8% (24.0% to 38.1%)
Other transport injuries	34.2% (27.4% to 41.5%)	29.6% (22.8% to 36.4%)	28.6% (22.3% to 35.4%)	29.3% (22.9% to 36.2%)
**Unintentional injuries**	15.6% (13.1% to 18.7%)	11.7% (9.5% to 14.4%)	18.6% (17.2% to 20.2%)	14.7% (12.9% to 16.8%)
Falls	30.9% (27.8% to 32.9%)	17.3% (15.5% to 18.9%)	22.6% (21.4% to 23.9%)	21.3% (20.1% to 22.6%)
Drowning	14.9% (10.9% to 19.8%)	12.2% (9.0% to 16.3%)	17.0% (12.1% to 23.3%)	12.3% (9.0% to 16.4%)
Fire, heat, and hot substances	15.8% (11.2% to 21.4%)	14.1% (10.3% to 18.8%)	16.6% (11.9% to 22.5%)	15.0% (10.8% to 19.9%)

Supplementary Results Table 1. Age-standardised PAFs attributable to the joint distribution of all risk factors for all ages, both sexes combined for each GBD level 1 and level 2 cause and all causes in 2016 for Central Asia. Results are shown for deaths, YLLs, YLDs, and DALYs
Data in parenthesis are 95% uncertainty intervals. PAFs=population attributable fractions. GBD=Global Burden of Disease. YLLs=years of life lost. YLDs=years live with disability. DALYs=disability-adjusted life-years

	**Deaths 2016**	**YLLs 2016**	**YLDs 2016**	**DALYs 2016**
Poisonings	18.4% (13.4% to 25.0%)	16.6% (12.2% to 22.1%)	16.0% (11.5% to 21.9%)	16.6% (12.2% to 22.1%)
Exposure to mechanical forces	14.5% (12.4% to 17.4%)	11.8% (9.9% to 14.5%)	16.9% (15.7% to 18.3%)	14.7% (13.2% to 16.6%)
Animal contact	11.4% (10.4% to 12.6%)	8.1% (7.2% to 9.3%)	8.5% (7.7% to 9.6%)	8.4% (7.6% to 9.3%)
Foreign body	6.1% (5.2% to 7.1%)	4.9% (4.1% to 5.8%)	7.1% (6.3% to 8.0%)	5.5% (4.7% to 6.4%)
Other unintentional injuries	20.2% (15.3% to 26.6%)	18.5% (14.1% to 24.4%)	16.3% (11.5% to 22.5%)	17.5% (12.8% to 23.4%)
**Self-harm and interpersonal violence**	29.5% (21.2% to 37.7%)	28.6% (20.5% to 36.6%)	26.2% (21.7% to 30.9%)	28.4% (20.7% to 36.2%)
Self-harm	30.9% (19.2% to 42.6%)	30.3% (18.9% to 41.3%)	27.6% (16.1% to 39.1%)	30.3% (18.8% to 41.3%)
Interpersonal violence	26.0% (18.9% to 34.1%)	24.6% (17.5% to 32.4%)	26.0% (21.2% to 31.3%)	24.8% (18.1% to 32.2%)
**Forces of nature, conflict and terrorism, and executions and police conflict**	0.0% (0.0% to 0.0%)	0.0% (0.0% to 0.0%)	2.9% (2.0% to 4.0%)	2.6% (1.8% to 3.6%)
Forces of nature, conflict and terrorism, and state actor violence	0.0% (0.0% to 0.0%)	0.0% (0.0% to 0.0%)	7.1% (5.0% to 9.8%)	7.1% (5.0% to 9.8%)

Supplementary Results Table 1. Age-standardised PAFs attributable to the joint distribution of all risk factors for all ages, both sexes combined for each GBD level 1 and level 2 cause and all causes in 2016 for Central Europe. Results are shown for deaths, YLLs, YLDs, and DALYs
Data in parenthesis are 95% uncertainty intervals. PAFs=population attributable fractions. GBD=Global Burden of Disease. YLLs=years of life lost. YLDs=years live with disability. DALYs=disability-adjusted life-years

	Deaths 2016	YLLs 2016	YLDs 2016	DALYs 2016
**All causes**	67.8% (65.9% to 69.7%)	63.3% (61.6% to 65.0%)	25.2% (23.9% to 26.6%)	46.7% (43.5% to 49.7%)
**HIV/AIDS and tuberculosis**	69.2% (60.7% to 76.0%)	69.9% (61.6% to 76.3%)	65.3% (58.1% to 71.2%)	69.5% (61.5% to 75.7%)
Tuberculosis	67.0% (56.4% to 75.4%)	68.3% (57.1% to 77.1%)	56.1% (46.3% to 63.9%)	67.3% (56.2% to 76.0%)
HIV/AIDS	78.5% (77.3% to 79.7%)	73.9% (72.6% to 75.3%)	83.2% (81.7% to 84.5%)	74.9% (73.4% to 76.3%)
**Diarrhoea, lower respiratory infections, and other common infectious diseases**	55.7% (49.5% to 62.2%)	62.1% (57.0% to 67.1%)	34.8% (26.2% to 44.0%)	53.6% (48.6% to 58.6%)
Diarrhoeal diseases	58.6% (38.8% to 78.0%)	75.4% (63.1% to 87.2%)	67.0% (49.9% to 82.7%)	68.3% (51.8% to 83.1%)
Lower respiratory infections	58.7% (51.8% to 65.6%)	68.5% (62.4% to 74.3%)	60.9% (54.2% to 68.2%)	68.4% (62.3% to 74.2%)
Upper respiratory infections	0.5% (0.3% to 1.0%)	1.6% (0.8% to 2.9%)	0.0% (0.0% to 0.0%)	0.0% (0.0% to 0.0%)
Otitis media	4.7% (2.9% to 6.8%)	10.4% (7.1% to 13.9%)	9.6% (6.6% to 12.4%)	9.6% (6.6% to 12.4%)
Meningitis	3.0% (2.5% to 3.6%)	5.0% (4.3% to 5.8%)	0.0% (0.0% to 0.0%)	3.6% (3.0% to 4.3%)
Encephalitis	0.8% (0.6% to 1.1%)	1.4% (1.0% to 1.9%)	0.0% (0.0% to 0.0%)	1.0% (0.8% to 1.4%)
Measles	33.6% (16.3% to 66.2%)	35.1% (17.1% to 68.9%)	32.8% (14.6% to 70.6%)	34.9% (16.9% to 68.9%)
**Maternal disorders**	5.4% (2.9% to 8.1%)	5.3% (2.9% to 8.0%)	6.0% (2.5% to 9.7%)	5.6% (2.7% to 8.7%)
Maternal haemorrhage	13.1% (4.7% to 21.6%)	13.2% (4.8% to 21.7%)	13.2% (4.7% to 22.0%)	13.2% (4.7% to 22.0%)
Maternal sepsis and other pregnancy related infections	12.8% (4.6% to 21.0%)	12.9% (4.7% to 21.2%)	13.1% (4.7% to 21.6%)	13.0% (4.7% to 21.4%)
Maternal abortion, miscarriage, and ectopic pregnancy	6.5% (4.0% to 9.5%)	6.4% (3.8% to 9.5%)	5.6% (2.8% to 9.1%)	6.2% (3.5% to 9.3%)
**Neonatal disorders**	78.5% (77.0% to 79.8%)	78.5% (77.0% to 79.9%)	0.1% (0.0% to 0.1%)	55.4% (48.9% to 61.7%)
Neonatal preterm birth complications	90.7% (89.3% to 91.8%)	90.7% (89.4% to 91.8%)	0.2% (0.1% to 0.2%)	71.1% (64.0% to 76.8%)
Neonatal encephalopathy due to birth asphyxia and trauma	61.7% (59.2% to 64.4%)	61.8% (59.3% to 64.5%)	0.0% (0.0% to 0.0%)	30.5% (21.0% to 41.5%)
Neonatal sepsis and other neonatal infections	54.7% (51.3% to 57.9%)	54.7% (51.4% to 58.0%)	0.0% (0.0% to 0.0%)	24.1% (12.3% to 36.7%)
Hemolytic disease and other neonatal jaundice	64.8% (62.0% to 67.6%)	64.8% (62.1% to 67.6%)	0.0% (0.0% to 0.0%)	20.3% (15.2% to 26.3%)

Supplementary Results Table 1. Age-standardised PAFs attributable to the joint distribution of all risk factors for all ages, both sexes combined for each GBD level 1 and level 2 cause and all causes in 2016 for Central Europe. Results are shown for deaths, YLLs, YLDs, and DALYs
Data in parenthesis are 95% uncertainty intervals. PAFs=population attributable fractions. GBD=Global Burden of Disease. YLLs=years of life lost. YLDs=years live with disability. DALYs=disability-adjusted life-years

	Deaths 2016	YLLs 2016	YLDs 2016	DALYs 2016
Other neonatal disorders	61.7% (59.3% to 64.2%)	61.7% (59.4% to 64.2%)	0.0% (0.0% to 0.0%)	58.3% (55.7% to 61.0%)
**Nutritional deficiencies**	8.6% (7.4% to 10.6%)	21.1% (18.3% to 25.5%)	92.1% (89.8% to 93.9%)	89.9% (87.2% to 92.2%)
Protein-energy malnutrition	7.3% (5.7% to 10.7%)	25.1% (20.4% to 34.3%)	96.9% (96.0% to 97.5%)	78.2% (71.1% to 85.2%)
Vitamin A deficiency	-	-	0.0% (0.0% to 0.0%)	0.0% (0.0% to 0.0%)
Iron-deficiency anaemia	100.0% (100.0% to 100.0%)	100.0% (100.0% to 100.0%)	100.0% (100.0% to 100.0%)	100.0% (100.0% to 100.0%)
**Other communicable, maternal, neonatal, and nutritional diseases**	7.4% (6.6% to 8.3%)	6.0% (5.3% to 6.8%)	71.7% (66.5% to 76.7%)	49.8% (42.0% to 59.1%)
Sexually transmitted diseases excluding HIV	74.5% (58.2% to 87.6%)	53.3% (34.1% to 72.9%)	100.0% (100.0% to 100.0%)	97.2% (94.2% to 99.1%)
Hepatitis	5.0% (3.7% to 6.7%)	4.5% (3.3% to 6.0%)	2.1% (1.4% to 3.1%)	4.3% (3.2% to 5.8%)
**Neoplasms**	50.6% (47.9% to 53.4%)	51.5% (49.1% to 54.0%)	41.0% (37.9% to 44.2%)	51.3% (48.8% to 53.7%)
Oesophageal cancer	90.1% (85.3% to 93.9%)	91.5% (87.0% to 94.8%)	90.4% (85.6% to 94.1%)	91.5% (87.0% to 94.8%)
Stomach cancer	28.5% (18.9% to 38.3%)	30.7% (20.7% to 40.1%)	28.5% (18.9% to 38.0%)	30.6% (20.7% to 40.1%)
Liver cancer	72.1% (64.7% to 78.3%)	72.8% (65.1% to 79.1%)	72.2% (64.6% to 78.5%)	72.8% (65.1% to 79.1%)
Larynx cancer	93.5% (90.2% to 96.0%)	94.2% (91.2% to 96.4%)	93.8% (90.8% to 96.2%)	94.1% (91.2% to 96.4%)
Tracheal, bronchus, and lung cancer	91.2% (89.7% to 92.6%)	92.0% (90.7% to 93.3%)	91.0% (89.6% to 92.5%)	92.0% (90.7% to 93.2%)
Breast cancer	33.3% (26.9% to 40.4%)	33.0% (26.6% to 39.6%)	33.2% (26.9% to 39.7%)	33.0% (26.6% to 39.6%)
Cervical cancer	100.0% (100.0% to 100.0%)	100.0% (100.0% to 100.0%)	100.0% (100.0% to 100.0%)	100.0% (100.0% to 100.0%)
Uterine cancer	48.1% (34.0% to 61.7%)	48.6% (34.6% to 62.0%)	48.7% (34.4% to 62.2%)	48.6% (34.5% to 62.0%)
Prostate cancer	7.5% (5.5% to 9.5%)	8.9% (6.7% to 11.1%)	9.1% (6.8% to 11.3%)	9.0% (6.7% to 11.2%)
Colon and rectum cancer	58.8% (51.1% to 66.3%)	60.5% (53.0% to 67.7%)	60.1% (52.6% to 67.3%)	60.5% (53.0% to 67.7%)
Lip and oral cavity cancer	90.0% (87.4% to 92.2%)	91.9% (89.8% to 93.7%)	89.0% (86.1% to 91.3%)	91.8% (89.7% to 93.6%)
Nasopharynx cancer	87.5% (84.7% to 89.8%)	85.9% (83.1% to 88.2%)	85.9% (82.8% to 88.5%)	85.9% (83.1% to 88.2%)

Supplementary Results Table 1. Age-standardised PAFs attributable to the joint distribution of all risk factors for all ages, both sexes combined for each GBD level 1 and level 2 cause and all causes in 2016 for Central Europe. Results are shown for deaths, YLLs, YLDs, and DALYs
Data in parenthesis are 95% uncertainty intervals. PAFs=population attributable fractions. GBD=Global Burden of Disease. YLLs=years of life lost. YLDs=years live with disability. DALYs=disability-adjusted life-years

	Deaths 2016	YLLs 2016	YLDs 2016	DALYs 2016
Other pharynx cancer	76.2% (70.3% to 81.6%)	76.9% (71.1% to 82.3%)	75.8% (69.7% to 81.3%)	76.9% (71.1% to 82.2%)
Gallbladder and biliary tract cancer	25.1% (14.3% to 37.9%)	25.3% (14.5% to 38.1%)	25.2% (14.5% to 37.8%)	25.3% (14.5% to 38.1%)
Pancreatic cancer	37.6% (30.5% to 44.8%)	40.9% (33.9% to 47.9%)	38.2% (31.1% to 45.4%)	40.9% (33.9% to 47.9%)
Ovarian cancer	10.6% (4.4% to 17.9%)	9.4% (3.5% to 16.1%)	9.3% (3.5% to 15.9%)	9.4% (3.5% to 16.1%)
Kidney cancer	43.5% (33.6% to 54.0%)	44.8% (34.9% to 54.9%)	43.0% (33.2% to 53.0%)	44.7% (34.8% to 54.8%)
Bladder cancer	42.6% (33.4% to 51.1%)	47.4% (37.9% to 55.9%)	45.2% (36.3% to 53.4%)	47.3% (37.8% to 55.8%)
Thyroid cancer	15.4% (8.2% to 24.6%)	15.8% (8.4% to 25.4%)	14.2% (7.8% to 22.1%)	15.6% (8.3% to 25.0%)
Mesothelioma	89.6% (86.7% to 91.8%)	83.5% (79.7% to 86.8%)	87.4% (84.3% to 89.9%)	83.6% (79.7% to 86.8%)
Non-Hodgkin's lymphoma	7.4% (3.0% to 12.8%)	6.9% (2.8% to 11.9%)	7.2% (2.9% to 12.3%)	6.9% (2.8% to 11.9%)
Multiple myeloma	9.0% (3.9% to 15.7%)	9.2% (4.0% to 15.9%)	9.2% (4.0% to 16.0%)	9.2% (4.0% to 15.9%)
Leukaemia	24.3% (15.6% to 32.5%)	21.3% (14.0% to 28.1%)	22.1% (14.1% to 29.6%)	21.3% (14.0% to 28.2%)
**Cardiovascular diseases**	86.5% (84.0% to 88.7%)	88.5% (86.9% to 90.0%)	81.6% (79.2% to 83.8%)	87.6% (86.0% to 89.2%)
Rheumatic heart disease	33.4% (22.7% to 51.2%)	33.3% (23.3% to 48.5%)	29.0% (20.5% to 40.4%)	33.0% (23.2% to 48.5%)
Ischaemic heart disease	94.0% (90.8% to 96.7%)	95.6% (93.6% to 97.2%)	93.2% (91.0% to 95.1%)	95.5% (93.5% to 97.0%)
Cerebrovascular disease	86.6% (81.9% to 91.1%)	90.2% (87.3% to 92.8%)	88.3% (84.8% to 91.6%)	89.9% (87.0% to 92.6%)
Hypertensive heart disease	100.0% (100.0% to 100.0%)	100.0% (100.0% to 100.0%)	100.0% (100.0% to 100.0%)	100.0% (100.0% to 100.0%)
Cardiomyopathy and myocarditis	47.5% (39.0% to 58.2%)	54.4% (47.9% to 61.2%)	49.8% (43.7% to 56.5%)	54.1% (47.7% to 60.9%)
Atrial fibrillation and flutter	59.3% (51.7% to 66.7%)	64.3% (57.9% to 70.3%)	71.9% (67.2% to 76.4%)	68.7% (63.3% to 73.7%)
Aortic aneurysm	54.7% (47.9% to 61.1%)	60.6% (54.9% to 65.7%)	-	60.6% (54.9% to 65.7%)
Peripheral vascular disease	53.5% (46.1% to 60.8%)	61.8% (56.1% to 67.0%)	60.1% (53.9% to 65.8%)	61.4% (55.7% to 66.5%)
Endocarditis	48.6% (39.9% to 57.7%)	46.8% (38.9% to 54.7%)	46.2% (37.5% to 54.8%)	46.8% (38.8% to 54.6%)

Supplementary Results Table 1. Age-standardised PAFs attributable to the joint distribution of all risk factors for all ages, both sexes combined for each GBD level 1 and level 2 cause and all causes in 2016 for Central Europe. Results are shown for deaths, YLLs, YLDs, and DALYs
Data in parenthesis are 95% uncertainty intervals. PAFs=population attributable fractions. GBD=Global Burden of Disease. YLLs=years of life lost. YLDs=years live with disability. DALYs=disability-adjusted life-years

	Deaths 2016	YLLs 2016	YLDs 2016	DALYs 2016
Other cardiovascular and circulatory diseases	55.2% (50.3% to 59.9%)	61.1% (57.0% to 65.0%)	60.0% (55.5% to 64.5%)	60.7% (56.6% to 64.6%)
**Chronic respiratory diseases**	74.5% (70.3% to 78.3%)	76.4% (73.1% to 79.5%)	48.2% (41.8% to 54.2%)	64.0% (59.5% to 68.4%)
Chronic obstructive pulmonary disease	79.7% (75.3% to 83.8%)	83.3% (79.9% to 86.6%)	80.0% (76.7% to 83.4%)	82.6% (79.2% to 85.8%)
Pneumoconiosis	100.0% (100.0% to 100.0%)	100.0% (100.0% to 100.0%)	100.0% (100.0% to 100.0%)	100.0% (100.0% to 100.0%)
Asthma	43.7% (33.4% to 54.7%)	47.2% (37.8% to 57.0%)	35.2% (27.5% to 43.8%)	36.3% (28.7% to 45.1%)
Other chronic respiratory diseases	18.9% (14.9% to 23.6%)	17.5% (13.3% to 22.3%)	10.7% (8.2% to 13.5%)	13.5% (10.4% to 17.1%)
**Cirrhosis and other chronic liver diseases**	59.7% (55.3% to 63.7%)	59.9% (55.5% to 64.1%)	46.7% (43.0% to 50.5%)	59.4% (55.1% to 63.6%)
Cirrhosis and other chronic liver diseases due to hepatitis B	2.3% (1.3% to 3.5%)	2.6% (1.5% to 4.0%)	2.2% (1.3% to 3.3%)	2.6% (1.5% to 4.0%)
Cirrhosis and other chronic liver diseases due to hepatitis C	85.0% (82.2% to 87.1%)	87.6% (85.9% to 88.8%)	85.3% (82.8% to 87.1%)	87.5% (85.8% to 88.7%)
Cirrhosis and other chronic liver diseases due to alcohol use	100.0% (100.0% to 100.0%)	100.0% (100.0% to 100.0%)	100.0% (100.0% to 100.0%)	100.0% (100.0% to 100.0%)
**Digestive diseases**	18.7% (16.3% to 21.7%)	23.7% (20.8% to 27.0%)	5.5% (4.3% to 6.9%)	17.0% (14.4% to 19.9%)
Peptic ulcer disease	16.1% (11.7% to 21.0%)	21.3% (15.6% to 27.6%)	19.2% (13.8% to 25.1%)	20.6% (15.0% to 26.7%)
Gallbladder and biliary diseases	44.0% (29.7% to 58.9%)	44.8% (30.9% to 59.2%)	47.5% (34.1% to 61.3%)	45.5% (31.6% to 59.7%)
Pancreatitis	56.8% (47.1% to 67.1%)	60.0% (50.7% to 69.4%)	48.7% (39.5% to 57.3%)	59.7% (50.5% to 69.1%)
**Neurological disorders**	20.0% (10.2% to 31.6%)	19.4% (11.7% to 28.1%)	4.0% (2.6% to 5.7%)	8.7% (5.5% to 12.8%)
Alzheimer's disease and other dementias	24.4% (12.1% to 39.1%)	26.0% (13.9% to 40.5%)	26.0% (13.8% to 40.6%)	26.0% (13.9% to 40.6%)
Parkinson's disease	-9.1% (-10.8% to -7.4%)	-12.4% (-14.7% to -10.0%)	-15.1% (-17.9% to -12.0%)	-13.0% (-15.4% to -10.3%)
Epilepsy	39.1% (32.0% to 45.7%)	35.2% (28.1% to 41.5%)	26.9% (20.3% to 33.1%)	30.4% (23.4% to 36.4%)
Multiple sclerosis	13.8% (9.3% to 18.7%)	14.6% (9.8% to 19.7%)	12.0% (8.0% to 16.2%)	13.5% (9.1% to 18.3%)
**Mental and substance use disorders**	99.9% (99.8% to 99.9%)	99.8% (99.8% to 99.8%)	19.5% (17.6% to 21.7%)	28.2% (25.4% to 31.5%)
Alcohol use disorders	100.0% (100.0% to 100.0%)	100.0% (100.0% to 100.0%)	100.0% (100.0% to 100.0%)	100.0% (100.0% to 100.0%)

Supplementary Results Table 1. Age-standardised PAFs attributable to the joint distribution of all risk factors for all ages, both sexes combined for each GBD level 1 and level 2 cause and all causes in 2016 for Central Europe. Results are shown for deaths, YLLs, YLDs, and DALYs
Data in parenthesis are 95% uncertainty intervals. PAFs=population attributable fractions. GBD=Global Burden of Disease. YLLs=years of life lost. YLDs=years live with disability. DALYs=disability-adjusted life-years

	Deaths 2016	YLLs 2016	YLDs 2016	DALYs 2016
Drug use disorders	100.0% (100.0% to 100.0%)	100.0% (100.0% to 100.0%)	100.0% (100.0% to 100.0%)	100.0% (100.0% to 100.0%)
Depressive disorders	-	-	6.4% (5.4% to 7.6%)	6.4% (5.4% to 7.6%)
Idiopathic developmental intellectual disability	-	-	55.7% (27.9% to 75.2%)	55.7% (27.9% to 75.2%)
**Diabetes, urogenital, blood, and endocrine diseases**	85.7% (85.1% to 86.4%)	82.1% (81.0% to 83.2%)	54.3% (50.2% to 58.4%)	65.6% (61.9% to 68.9%)
Diabetes mellitus	100.0% (100.0% to 100.0%)	100.0% (100.0% to 100.0%)	100.0% (100.0% to 100.0%)	100.0% (100.0% to 100.0%)
Chronic kidney disease	100.0% (100.0% to 100.0%)	100.0% (100.0% to 100.0%)	100.0% (100.0% to 100.0%)	100.0% (100.0% to 100.0%)
**Musculoskeletal disorders**	2.2% (0.9% to 3.8%)	2.3% (0.9% to 3.9%)	24.3% (20.4% to 28.4%)	24.1% (20.2% to 28.1%)
Rheumatoid arthritis	5.9% (2.4% to 9.9%)	7.4% (3.1% to 12.2%)	7.8% (3.2% to 12.8%)	7.7% (3.2% to 12.8%)
Osteoarthritis	-	-	28.9% (15.7% to 42.1%)	28.9% (15.7% to 42.1%)
Low back and neck pain	-	-	25.3% (21.7% to 28.9%)	25.3% (21.7% to 28.9%)
Gout	-	-	47.2% (30.8% to 63.6%)	47.2% (30.8% to 63.6%)
**Other non-communicable diseases**	0.3% (0.2% to 0.3%)	0.3% (0.2% to 0.4%)	3.6% (3.1% to 4.0%)	3.1% (2.7% to 3.5%)
Sense organ diseases	-	-	8.3% (7.4% to 9.2%)	8.3% (7.4% to 9.2%)
Sudden infant death syndrome	5.7% (4.7% to 6.9%)	5.7% (4.7% to 6.9%)	-	5.7% (4.7% to 6.9%)
**Transport injuries**	37.5% (30.2% to 44.7%)	33.6% (26.4% to 40.7%)	39.6% (33.0% to 46.3%)	35.6% (28.5% to 42.4%)
Road injuries	37.1% (29.8% to 44.2%)	33.2% (26.2% to 40.3%)	39.8% (33.2% to 46.5%)	35.5% (28.5% to 42.4%)
Other transport injuries	40.9% (33.2% to 48.0%)	36.8% (29.1% to 43.9%)	34.2% (27.3% to 40.9%)	36.4% (28.8% to 43.5%)
**Unintentional injuries**	32.2% (29.7% to 35.1%)	19.5% (17.2% to 22.2%)	20.6% (19.2% to 22.0%)	20.2% (18.8% to 22.0%)
Falls	53.0% (49.0% to 55.9%)	31.8% (29.6% to 34.1%)	22.7% (21.3% to 24.1%)	24.3% (22.7% to 25.8%)
Drowning	20.5% (14.1% to 27.3%)	18.7% (13.3% to 24.5%)	18.6% (12.7% to 25.1%)	18.7% (13.3% to 24.5%)
Fire, heat, and hot substances	18.4% (11.9% to 25.3%)	19.1% (13.2% to 25.4%)	19.4% (13.8% to 25.7%)	19.3% (13.6% to 25.2%)

Supplementary Results Table 1. Age-standardised PAFs attributable to the joint distribution of all risk factors for all ages, both sexes combined for each GBD level 1 and level 2 cause and all causes in 2016 for Central Europe. Results are shown for deaths, YLLs, YLDs, and DALYs
Data in parenthesis are 95% uncertainty intervals. PAFs=population attributable fractions. GBD=Global Burden of Disease. YLLs=years of life lost. YLDs=years live with disability. DALYs=disability-adjusted life-years

	Deaths 2016	YLLs 2016	YLDs 2016	DALYs 2016
Poisonings	18.7% (13.1% to 25.4%)	18.1% (13.2% to 24.4%)	18.5% (13.5% to 24.1%)	18.3% (13.4% to 24.2%)
Exposure to mechanical forces	17.5% (15.8% to 19.4%)	14.8% (13.1% to 16.9%)	17.4% (15.9% to 19.2%)	16.7% (15.2% to 18.4%)
Animal contact	12.6% (11.3% to 13.8%)	11.4% (10.0% to 12.8%)	8.7% (7.6% to 9.9%)	9.5% (8.4% to 10.8%)
Foreign body	7.6% (6.4% to 9.0%)	8.0% (6.7% to 9.4%)	6.4% (5.6% to 7.5%)	7.6% (6.4% to 8.9%)
Other unintentional injuries	23.7% (17.1% to 30.6%)	23.4% (17.2% to 30.0%)	18.0% (12.7% to 23.9%)	19.8% (14.4% to 26.0%)
**Self-harm and interpersonal violence**	38.3% (28.9% to 47.0%)	36.7% (27.3% to 45.4%)	30.5% (25.3% to 35.6%)	36.2% (27.3% to 44.5%)
Self-harm	39.2% (28.8% to 48.7%)	37.9% (27.5% to 47.8%)	31.5% (21.8% to 40.9%)	37.8% (27.4% to 47.8%)
Interpersonal violence	32.2% (25.1% to 38.9%)	29.2% (22.7% to 35.6%)	30.3% (24.9% to 35.6%)	29.6% (23.4% to 35.5%)
**Forces of nature, conflict and terrorism, and executions and police conflict**	0.0% (0.0% to 0.0%)	0.0% (0.0% to 0.0%)	0.3% (0.2% to 0.4%)	0.2% (0.2% to 0.4%)
Forces of nature, conflict and terrorism, and state actor violence	0.0% (0.0% to 0.0%)	0.0% (0.0% to 0.0%)	4.5% (3.0% to 6.6%)	4.5% (3.0% to 6.6%)

Supplementary Results Table 1. Age-standardised PAFs attributable to the joint distribution of all risk factors for all ages, both sexes combined for each GBD level 1 and level 2 cause and all causes in 2016 for Central Latin America. Results are shown for deaths, YLLs, YLDs, and DALYs
Data in parenthesis are 95% uncertainty intervals. PAFs=population attributable fractions. GBD=Global Burden of Disease. YLLs=years of life lost. YLDs=years live with disability. DALYs=disability-adjusted life-years

	Deaths 2016	YLLs 2016	YLDs 2016	DALYs 2016
**All causes**	59.0% (57.3% to 60.7%)	53.7% (52.5% to 54.9%)	25.3% (23.9% to 26.8%)	42.9% (40.7% to 45.1%)
**HIV/AIDS and tuberculosis**	78.8% (75.9% to 81.8%)	81.3% (79.5% to 83.3%)	88.7% (84.9% to 92.1%)	81.8% (80.0% to 83.7%)
Tuberculosis	49.3% (41.8% to 57.3%)	48.8% (42.3% to 55.8%)	42.8% (36.1% to 48.9%)	48.6% (42.0% to 55.5%)
HIV/AIDS	95.5% (94.9% to 95.9%)	92.7% (92.0% to 93.2%)	97.1% (96.3% to 97.7%)	93.0% (92.2% to 93.6%)
**Diarrhoea, lower respiratory infections, and other common infectious diseases**	56.7% (51.3% to 62.6%)	64.3% (59.8% to 68.9%)	30.9% (22.6% to 39.4%)	57.7% (52.7% to 62.5%)
Diarrhoeal diseases	73.4% (57.4% to 86.6%)	83.8% (73.6% to 92.1%)	70.7% (53.5% to 85.5%)	79.5% (67.1% to 90.0%)
Lower respiratory infections	57.9% (51.4% to 64.7%)	68.6% (62.5% to 74.4%)	57.7% (50.9% to 64.8%)	68.5% (62.4% to 74.3%)
Upper respiratory infections	1.3% (0.7% to 2.1%)	2.7% (1.5% to 4.5%)	0.0% (0.0% to 0.0%)	0.0% (0.0% to 0.0%)
Otitis media	2.3% (1.6% to 2.9%)	4.8% (3.4% to 6.1%)	5.6% (3.7% to 7.3%)	5.6% (3.7% to 7.3%)
Meningitis	1.8% (1.5% to 2.1%)	2.9% (2.5% to 3.5%)	0.0% (0.0% to 0.0%)	2.6% (2.2% to 3.1%)
Encephalitis	1.2% (1.0% to 1.6%)	1.9% (1.7% to 2.3%)	0.0% (0.0% to 0.0%)	1.7% (1.4% to 2.1%)
Measles	26.1% (10.9% to 54.1%)	28.0% (11.8% to 57.7%)	31.8% (11.0% to 70.1%)	28.0% (11.8% to 57.7%)
**Maternal disorders**	5.0% (2.4% to 7.5%)	4.8% (2.4% to 7.4%)	3.3% (1.8% to 5.0%)	4.6% (2.3% to 6.9%)
Maternal haemorrhage	13.4% (4.8% to 22.0%)	13.4% (4.7% to 21.8%)	13.3% (4.7% to 21.7%)	13.4% (4.7% to 21.8%)
Maternal sepsis and other pregnancy related infections	13.2% (4.7% to 21.7%)	13.1% (4.7% to 21.5%)	13.0% (4.7% to 21.3%)	13.1% (4.7% to 21.5%)
Maternal abortion, miscarriage, and ectopic pregnancy	9.6% (5.9% to 13.8%)	9.5% (5.9% to 13.6%)	8.9% (4.9% to 13.2%)	9.4% (5.7% to 13.4%)
**Neonatal disorders**	75.2% (73.7% to 76.8%)	75.2% (73.8% to 76.8%)	0.1% (0.1% to 0.1%)	67.8% (65.0% to 70.3%)
Neonatal preterm birth complications	92.9% (92.1% to 93.9%)	92.9% (92.1% to 93.9%)	0.2% (0.2% to 0.2%)	83.2% (79.5% to 86.2%)
Neonatal encephalopathy due to birth asphyxia and trauma	63.5% (61.3% to 66.0%)	63.6% (61.4% to 66.0%)	0.0% (0.0% to 0.0%)	58.3% (52.7% to 62.7%)
Neonatal sepsis and other neonatal infections	55.7% (53.4% to 58.1%)	55.8% (53.5% to 58.1%)	0.0% (0.0% to 0.0%)	50.9% (44.8% to 54.6%)
Hemolytic disease and other neonatal jaundice	60.2% (57.7% to 62.9%)	60.2% (57.8% to 62.9%)	0.0% (0.0% to 0.0%)	20.6% (16.4% to 25.8%)

Supplementary Results Table 1. Age-standardised PAFs attributable to the joint distribution of all risk factors for all ages, both sexes combined for each GBD level 1 and level 2 cause and all causes in 2016 for Central Latin America. Results are shown for deaths, YLLs, YLDs, and DALYs
Data in parenthesis are 95% uncertainty intervals. PAFs=population attributable fractions. GBD=Global Burden of Disease. YLLs=years of life lost. YLDs=years live with disability. DALYs=disability-adjusted life-years

	Deaths 2016	YLLs 2016	YLDs 2016	DALYs 2016
Other neonatal disorders	57.8% (55.6% to 60.2%)	57.9% (55.6% to 60.3%)	0.0% (0.0% to 0.0%)	56.1% (53.8% to 58.5%)
**Nutritional deficiencies**	9.6% (8.6% to 10.5%)	38.0% (35.4% to 40.6%)	75.6% (71.8% to 79.2%)	55.0% (50.3% to 59.6%)
Protein-energy malnutrition	10.1% (9.0% to 11.5%)	41.1% (38.0% to 44.3%)	96.8% (96.5% to 97.1%)	45.1% (42.0% to 48.6%)
Vitamin A deficiency	-	-	100.0% (100.0% to 100.0%)	100.0% (100.0% to 100.0%)
Iron-deficiency anaemia	100.0% (100.0% to 100.0%)	100.0% (100.0% to 100.0%)	100.0% (100.0% to 100.0%)	100.0% (100.0% to 100.0%)
**Other communicable, maternal, neonatal, and nutritional diseases**	9.5% (8.3% to 10.5%)	7.7% (6.3% to 8.8%)	71.3% (65.6% to 77.1%)	30.2% (22.2% to 40.9%)
Sexually transmitted diseases excluding HIV	46.8% (31.9% to 62.1%)	26.0% (15.6% to 38.9%)	100.0% (100.0% to 100.0%)	69.3% (52.1% to 83.6%)
Hepatitis	3.1% (2.3% to 4.0%)	3.2% (2.4% to 4.1%)	2.0% (1.2% to 3.2%)	3.1% (2.3% to 4.0%)
**Neoplasms**	32.0% (28.9% to 35.4%)	30.7% (27.9% to 33.9%)	26.6% (23.8% to 29.7%)	30.6% (27.8% to 33.8%)
Oesophageal cancer	68.2% (57.5% to 78.2%)	69.9% (59.4% to 79.7%)	68.5% (57.8% to 78.5%)	69.9% (59.4% to 79.6%)
Stomach cancer	8.7% (3.5% to 16.7%)	8.1% (3.3% to 15.3%)	8.8% (3.6% to 16.4%)	8.2% (3.3% to 15.3%)
Liver cancer	52.5% (45.0% to 61.0%)	53.7% (46.3% to 62.4%)	52.8% (45.4% to 61.3%)	53.7% (46.3% to 62.4%)
Larynx cancer	69.4% (59.9% to 77.4%)	70.5% (61.2% to 78.1%)	70.6% (61.2% to 78.3%)	70.5% (61.2% to 78.1%)
Tracheal, bronchus, and lung cancer	72.7% (67.6% to 77.5%)	71.8% (66.7% to 76.5%)	72.1% (67.0% to 76.8%)	71.8% (66.7% to 76.5%)
Breast cancer	25.5% (17.7% to 34.9%)	22.6% (14.9% to 31.4%)	23.9% (16.5% to 33.1%)	22.6% (15.0% to 31.5%)
Cervical cancer	100.0% (100.0% to 100.0%)	100.0% (100.0% to 100.0%)	100.0% (100.0% to 100.0%)	100.0% (100.0% to 100.0%)
Uterine cancer	48.1% (33.9% to 62.2%)	49.8% (35.5% to 63.9%)	51.1% (36.7% to 65.1%)	49.9% (35.6% to 63.9%)
Prostate cancer	1.8% (1.2% to 2.4%)	1.8% (1.2% to 2.4%)	1.9% (1.3% to 2.5%)	1.8% (1.2% to 2.4%)
Colon and rectum cancer	52.8% (43.6% to 62.1%)	53.5% (44.9% to 62.0%)	52.9% (43.9% to 62.1%)	53.5% (44.9% to 62.0%)
Lip and oral cavity cancer	61.4% (53.7% to 68.4%)	62.5% (55.4% to 68.7%)	58.4% (51.0% to 65.4%)	62.3% (55.2% to 68.6%)
Nasopharynx cancer	65.4% (59.9% to 70.5%)	62.9% (59.0% to 67.1%)	62.8% (58.4% to 67.1%)	62.9% (59.0% to 67.1%)

**Supplementary Results Table 1. Age-standardised PAFs attributable to the joint distribution of all risk factors for all ages, both sexes combined for each GBD level 1 and level 2 cause and all causes in 2016 for Central Latin America. Results are shown for deaths, YLLs, YLDs, and DALYs** Data in parenthesis are 95% uncertainty intervals. PAFs=population attributable fractions. GBD=Global Burden of Disease. YLLs=years of life lost. YLDs=years live with disability. DALYs=disability-adjusted life-years	**Deaths 2016**	**YLLs 2016**	**YLDs 2016**	**DALYs 2016**
Other pharynx cancer	50.2% (40.8% to 59.1%)	51.7% (42.3% to 60.3%)	51.5% (41.9% to 60.3%)	51.7% (42.2% to 60.3%)
Gallbladder and biliary tract cancer	25.1% (14.6% to 37.7%)	26.5% (15.2% to 39.4%)	25.4% (14.7% to 38.2%)	26.5% (15.2% to 39.4%)
Pancreatic cancer	22.7% (14.3% to 33.7%)	22.4% (14.1% to 33.3%)	22.6% (14.0% to 33.7%)	22.4% (14.1% to 33.3%)
Ovarian cancer	13.6% (5.2% to 24.0%)	12.5% (4.5% to 22.3%)	12.1% (4.3% to 21.5%)	12.5% (4.5% to 22.3%)
Kidney cancer	27.5% (18.5% to 37.9%)	26.4% (17.8% to 36.2%)	27.5% (18.6% to 37.5%)	26.5% (17.8% to 36.3%)
Bladder cancer	20.5% (12.5% to 30.9%)	19.8% (12.1% to 29.9%)	20.1% (12.2% to 30.3%)	19.9% (12.1% to 29.9%)
Thyroid cancer	15.1% (8.2% to 24.0%)	15.9% (8.7% to 25.2%)	16.2% (9.0% to 24.6%)	16.0% (8.7% to 25.2%)
Mesothelioma	87.3% (84.3% to 90.0%)	79.5% (75.2% to 83.3%)	85.1% (81.5% to 88.0%)	79.6% (75.3% to 83.4%)
Non-Hodgkin's lymphoma	7.3% (3.0% to 12.5%)	6.9% (2.9% to 11.6%)	7.4% (3.1% to 12.6%)	6.9% (2.9% to 11.6%)
Multiple myeloma	9.0% (3.9% to 15.6%)	9.5% (4.2% to 16.0%)	9.3% (4.0% to 16.0%)	9.4% (4.2% to 16.0%)
Leukaemia	11.7% (7.4% to 16.4%)	9.0% (5.8% to 12.5%)	11.0% (7.0% to 15.4%)	9.0% (5.8% to 12.5%)
**Cardiovascular diseases**	87.2% (84.7% to 89.4%)	86.4% (84.7% to 87.9%)	74.1% (71.6% to 76.4%)	85.1% (83.4% to 86.6%)
Rheumatic heart disease	26.2% (17.8% to 40.5%)	23.7% (16.4% to 35.9%)	16.5% (10.4% to 24.0%)	22.5% (15.3% to 32.9%)
Ischaemic heart disease	93.1% (89.6% to 95.8%)	94.2% (92.2% to 96.0%)	91.8% (89.4% to 94.0%)	94.1% (92.0% to 95.9%)
Cerebrovascular disease	83.3% (78.7% to 87.4%)	82.8% (80.0% to 85.6%)	84.2% (80.2% to 87.9%)	83.0% (80.1% to 85.7%)
Hypertensive heart disease	100.0% (100.0% to 100.0%)	100.0% (100.0% to 100.0%)	100.0% (100.0% to 100.0%)	100.0% (100.0% to 100.0%)
Cardiomyopathy and myocarditis	39.0% (32.1% to 46.9%)	32.0% (26.7% to 37.5%)	31.0% (25.4% to 36.6%)	32.0% (26.8% to 37.4%)
Atrial fibrillation and flutter	52.1% (44.7% to 58.8%)	56.5% (49.9% to 62.4%)	62.8% (57.7% to 67.8%)	60.3% (54.7% to 65.5%)
Aortic aneurysm	43.4% (36.2% to 50.5%)	47.3% (40.9% to 52.8%)	-	47.3% (40.9% to 52.8%)
Peripheral vascular disease	56.7% (50.5% to 63.5%)	63.3% (58.4% to 68.1%)	65.1% (60.4% to 69.7%)	64.3% (59.7% to 68.8%)
Endocarditis	36.1% (28.1% to 44.8%)	28.2% (21.5% to 35.2%)	35.6% (28.0% to 43.5%)	28.3% (21.6% to 35.3%)

Supplementary Results Table 1. Age-standardised PAFs attributable to the joint distribution of all risk factors for all ages, both sexes combined for each GBD level 1 and level 2 cause and all causes in 2016 for Central Latin America. Results are shown for deaths, YLLs, YLDs, and DALYs
Data in parenthesis are 95% uncertainty intervals. PAFs=population attributable fractions. GBD=Global Burden of Disease. YLLs=years of life lost. YLDs=years live with disability. DALYs=disability-adjusted life-years

	**Deaths 2016**	**YLLs 2016**	**YLDs 2016**	**DALYs 2016**
Other cardiovascular and circulatory diseases	44.7% (39.9% to 49.2%)	43.4% (39.3% to 47.2%)	44.2% (39.4% to 49.0%)	43.7% (39.5% to 47.7%)
**Chronic respiratory diseases**	55.3% (48.9% to 61.9%)	51.9% (46.3% to 57.8%)	37.4% (30.5% to 44.7%)	46.6% (41.4% to 52.0%)
Chronic obstructive pulmonary disease	61.5% (54.0% to 68.5%)	61.2% (54.1% to 68.3%)	54.3% (46.7% to 61.9%)	60.0% (52.9% to 67.2%)
Pneumoconiosis	100.0% (100.0% to 100.0%)	100.0% (100.0% to 100.0%)	100.0% (100.0% to 100.0%)	100.0% (100.0% to 100.0%)
Asthma	38.2% (28.5% to 49.4%)	38.3% (29.3% to 48.6%)	31.3% (22.4% to 41.9%)	32.5% (23.8% to 42.7%)
Other chronic respiratory diseases	4.0% (2.4% to 5.6%)	2.1% (1.1% to 3.3%)	1.9% (1.0% to 3.1%)	2.0% (1.2% to 3.3%)
**Cirrhosis and other chronic liver diseases**	60.2% (57.3% to 63.0%)	64.4% (61.6% to 67.0%)	53.3% (50.9% to 55.6%)	64.0% (61.3% to 66.5%)
Cirrhosis and other chronic liver diseases due to hepatitis B	1.6% (1.0% to 2.3%)	1.9% (1.2% to 2.8%)	1.2% (0.8% to 1.8%)	1.9% (1.2% to 2.8%)
Cirrhosis and other chronic liver diseases due to hepatitis C	52.6% (47.2% to 58.2%)	64.4% (59.1% to 69.4%)	54.3% (49.0% to 59.4%)	64.1% (59.0% to 69.2%)
Cirrhosis and other chronic liver diseases due to alcohol use	100.0% (100.0% to 100.0%)	100.0% (100.0% to 100.0%)	100.0% (100.0% to 100.0%)	100.0% (100.0% to 100.0%)
**Digestive diseases**	11.9% (9.5% to 14.3%)	13.0% (10.6% to 15.3%)	3.9% (3.0% to 4.9%)	10.8% (8.7% to 12.8%)
Peptic ulcer disease	9.1% (6.4% to 12.2%)	10.2% (7.1% to 13.6%)	6.8% (4.7% to 9.3%)	9.1% (6.3% to 12.2%)
Gallbladder and biliary diseases	44.4% (30.7% to 58.8%)	46.3% (32.6% to 60.6%)	51.3% (36.5% to 66.3%)	46.8% (33.0% to 61.1%)
Pancreatitis	42.6% (33.2% to 53.1%)	45.9% (37.2% to 55.5%)	34.8% (27.2% to 43.2%)	45.7% (37.0% to 55.3%)
**Neurological disorders**	24.0% (12.8% to 37.4%)	22.4% (13.0% to 33.5%)	5.4% (3.7% to 7.3%)	11.0% (6.7% to 16.2%)
Alzheimer's disease and other dementias	26.8% (14.0% to 42.0%)	27.9% (15.1% to 43.2%)	27.9% (14.7% to 43.2%)	27.9% (15.0% to 43.2%)
Parkinson's disease	-7.7% (-9.1% to -6.2%)	-9.0% (-10.6% to -7.2%)	-9.3% (-10.8% to -7.5%)	-9.1% (-10.6% to -7.3%)
Epilepsy	24.5% (18.2% to 30.8%)	21.6% (16.2% to 27.0%)	18.0% (13.2% to 23.0%)	19.2% (14.2% to 24.2%)
Multiple sclerosis	6.4% (4.1% to 9.0%)	6.3% (4.1% to 8.9%)	5.0% (3.2% to 6.9%)	6.0% (3.9% to 8.4%)
**Mental and substance use disorders**	99.7% (99.6% to 99.7%)	99.5% (99.4% to 99.5%)	20.6% (18.7% to 22.9%)	26.6% (24.0% to 29.3%)
Alcohol use disorders	100.0% (100.0% to 100.0%)	100.0% (100.0% to 100.0%)	100.0% (100.0% to 100.0%)	100.0% (100.0% to 100.0%)

Supplementary Results Table 1. Age-standardised PAFs attributable to the joint distribution of all risk factors for all ages, both sexes combined for each GBD level 1 and level 2 cause and all causes in 2016 for Central Latin America. Results are shown for deaths, YLLs, YLDs, and DALYs
Data in parenthesis are 95% uncertainty intervals. PAFs=population attributable fractions. GBD=Global Burden of Disease. YLLs=years of life lost. YLDs=years live with disability. DALYs=disability-adjusted life-years

	Deaths 2016	YLLs 2016	YLDs 2016	DALYs 2016
Drug use disorders	100.0% (100.0% to 100.0%)	100.0% (100.0% to 100.0%)	100.0% (100.0% to 100.0%)	100.0% (100.0% to 100.0%)
Depressive disorders	-	-	8.7% (7.2% to 10.5%)	8.7% (7.2% to 10.5%)
Idiopathic developmental intellectual disability	-	-	69.8% (38.7% to 88.6%)	69.8% (38.7% to 88.6%)
**Diabetes, urogenital, blood, and endocrine diseases**	90.6% (90.1% to 91.1%)	89.4% (88.8% to 90.1%)	81.9% (78.6% to 84.7%)	86.8% (85.5% to 87.9%)
Diabetes mellitus	100.0% (100.0% to 100.0%)	100.0% (100.0% to 100.0%)	100.0% (100.0% to 100.0%)	100.0% (100.0% to 100.0%)
Chronic kidney disease	100.0% (100.0% to 100.0%)	100.0% (100.0% to 100.0%)	100.0% (100.0% to 100.0%)	100.0% (100.0% to 100.0%)
**Musculoskeletal disorders**	1.3% (0.5% to 2.2%)	0.9% (0.4% to 1.6%)	15.5% (12.3% to 18.9%)	14.9% (11.9% to 18.2%)
Rheumatoid arthritis	3.1% (1.2% to 5.2%)	3.3% (1.3% to 5.6%)	3.4% (1.3% to 5.8%)	3.4% (1.4% to 5.7%)
Osteoarthritis	-	-	29.3% (16.2% to 42.1%)	29.3% (16.2% to 42.1%)
Low back and neck pain	-	-	18.5% (15.8% to 21.5%)	18.5% (15.8% to 21.5%)
Gout	-	-	54.9% (41.2% to 68.7%)	54.9% (41.2% to 68.7%)
**Other non-communicable diseases**	0.2% (0.2% to 0.3%)	0.3% (0.2% to 0.4%)	6.0% (5.1% to 7.2%)	4.6% (4.0% to 5.4%)
Sense organ diseases	-	-	12.9% (11.2% to 14.9%)	12.9% (11.2% to 14.9%)
Sudden infant death syndrome	7.7% (6.6% to 9.0%)	7.7% (6.6% to 9.1%)	-	7.7% (6.6% to 9.1%)
**Transport injuries**	39.9% (34.0% to 46.9%)	36.6% (31.0% to 43.2%)	44.9% (38.9% to 52.0%)	37.6% (32.0% to 44.2%)
Road injuries	39.7% (33.7% to 46.7%)	36.5% (30.8% to 43.0%)	45.2% (39.1% to 52.3%)	37.5% (31.9% to 44.2%)
Other transport injuries	43.8% (37.5% to 50.6%)	39.6% (33.5% to 46.2%)	38.0% (32.2% to 44.6%)	39.5% (33.5% to 46.0%)
**Unintentional injuries**	29.6% (27.0% to 31.8%)	20.5% (18.3% to 22.5%)	26.2% (24.6% to 27.6%)	22.4% (20.5% to 24.2%)
Falls	54.9% (52.3% to 56.8%)	36.6% (34.1% to 38.5%)	29.7% (28.3% to 30.9%)	32.2% (30.7% to 33.7%)
Drowning	21.1% (16.8% to 26.1%)	19.3% (15.6% to 23.3%)	18.4% (14.0% to 23.9%)	19.3% (15.6% to 23.3%)
Fire, heat, and hot substances	15.0% (11.3% to 19.6%)	17.3% (13.9% to 21.2%)	21.9% (18.2% to 26.0%)	18.9% (15.5% to 23.0%)

Supplementary Results Table 1. Age-standardised PAFs attributable to the joint distribution of all risk factors for all ages, both sexes combined for each GBD level 1 and level 2 cause and all causes in 2016 for Central Latin America. Results are shown for deaths, YLLs, YLDs, and DALYs
Data in parenthesis are 95% uncertainty intervals. PAFs=population attributable fractions. GBD=Global Burden of Disease. YLLs=years of life lost. YLDs=years live with disability. DALYs=disability-adjusted life-years

	**Deaths 2016**	**YLLs 2016**	**YLDs 2016**	**DALYs 2016**
Poisonings	21.4% (17.5% to 26.1%)	20.6% (17.0% to 24.6%)	21.2% (17.7% to 25.3%)	20.7% (17.1% to 24.5%)
Exposure to mechanical forces	27.3% (23.0% to 30.8%)	26.3% (20.8% to 30.3%)	27.9% (25.0% to 30.4%)	26.8% (22.6% to 30.3%)
Animal contact	13.1% (11.0% to 14.6%)	11.8% (9.0% to 13.7%)	15.9% (14.1% to 17.6%)	13.4% (11.0% to 15.1%)
Foreign body	9.5% (8.6% to 10.9%)	8.0% (7.0% to 10.0%)	10.7% (9.2% to 12.0%)	8.4% (7.6% to 10.1%)
Other unintentional injuries	30.0% (24.8% to 35.4%)	30.4% (25.3% to 35.4%)	23.5% (19.0% to 28.7%)	28.1% (23.3% to 32.9%)
**Self-harm and interpersonal violence**	22.9% (16.5% to 29.4%)	21.7% (15.6% to 27.9%)	21.9% (16.6% to 27.5%)	21.7% (15.6% to 28.0%)
Self-harm	30.6% (20.5% to 40.2%)	29.5% (19.6% to 39.0%)	26.7% (17.6% to 35.6%)	29.4% (19.6% to 38.9%)
Interpersonal violence	20.9% (14.0% to 28.9%)	19.8% (13.3% to 27.4%)	21.6% (16.2% to 27.6%)	19.8% (13.4% to 27.4%)
**Forces of nature, conflict and terrorism, and executions and police conflict**	1.5% (0.8% to 4.0%)	0.5% (0.3% to 1.5%)	2.3% (1.6% to 3.3%)	1.3% (0.7% to 2.2%)
Forces of nature, conflict and terrorism, and state actor violence	7.0% (6.6% to 7.4%)	2.8% (2.5% to 3.0%)	5.0% (3.4% to 7.1%)	4.3% (3.2% to 6.0%)

Supplementary Results Table 1. Age-standardised PAFs attributable to the joint distribution of all risk factors for all ages, both sexes combined for each GBD level 1 and level 2 cause and all causes in 2016 for Central Sub-Saharan Africa. Results are shown for deaths, YLLs, YLDs, and
Data in parenthesis are 95% uncertainty intervals. PAFs=population attributable fractions. GBD=Global Burden of Disease. YLLs=years of life lost. YLDs=years live with disability. DALYs=disability-adjusted life-years

	Deaths 2016	YLLs 2016	YLDs 2016	DALYs 2016
**All causes**	53.9% (49.9% to 57.0%)	50.6% (47.8% to 53.0%)	25.4% (24.2% to 26.7%)	45.0% (42.5% to 47.3%)
**HIV/AIDS and tuberculosis**	49.0% (39.2% to 58.7%)	56.2% (47.4% to 64.4%)	72.9% (65.7% to 79.3%)	56.7% (48.0% to 64.7%)
Tuberculosis	35.5% (24.1% to 47.5%)	38.2% (26.9% to 49.8%)	36.4% (25.7% to 48.1%)	38.2% (26.9% to 49.7%)
HIV/AIDS	93.7% (92.5% to 94.6%)	90.3% (88.4% to 91.9%)	93.2% (91.4% to 94.9%)	90.5% (88.6% to 92.0%)
**Diarrhoea, lower respiratory infections, and other common infectious diseases**	78.2% (73.0% to 83.3%)	75.5% (69.4% to 80.7%)	45.8% (40.4% to 50.9%)	73.9% (67.9% to 79.0%)
Diarrhoeal diseases	95.6% (88.1% to 98.9%)	96.7% (91.0% to 99.2%)	96.0% (89.4% to 99.0%)	96.7% (90.8% to 99.2%)
Lower respiratory infections	78.2% (72.7% to 83.5%)	84.8% (80.2% to 89.0%)	78.5% (72.9% to 83.5%)	84.8% (80.2% to 89.0%)
Upper respiratory infections	1.3% (0.2% to 4.1%)	2.4% (0.4% to 8.3%)	0.0% (0.0% to 0.0%)	0.2% (0.0% to 0.6%)
Otitis media	1.5% (0.6% to 3.2%)	4.2% (2.2% to 6.8%)	3.9% (2.6% to 5.2%)	3.9% (2.6% to 5.2%)
Meningitis	2.3% (1.2% to 3.9%)	3.9% (2.2% to 6.2%)	0.0% (0.0% to 0.0%)	3.6% (2.1% to 6.0%)
Encephalitis	0.8% (0.5% to 1.1%)	1.3% (0.8% to 1.8%)	0.0% (0.0% to 0.0%)	0.7% (0.5% to 1.1%)
Measles	64.9% (50.8% to 81.9%)	66.1% (51.7% to 83.3%)	61.2% (46.6% to 79.8%)	66.1% (51.7% to 83.3%)
**Maternal disorders**	6.0% (2.8% to 9.6%)	6.1% (2.8% to 9.8%)	3.0% (1.5% to 4.8%)	6.1% (2.7% to 9.7%)
Maternal haemorrhage	16.0% (5.9% to 26.3%)	16.5% (6.0% to 27.0%)	17.5% (6.3% to 28.5%)	16.5% (6.0% to 27.0%)
Maternal sepsis and other pregnancy related infections	14.9% (5.3% to 24.9%)	15.6% (5.6% to 26.0%)	17.0% (6.1% to 27.8%)	15.6% (5.6% to 26.1%)
Maternal abortion, miscarriage, and ectopic pregnancy	30.7% (21.4% to 40.4%)	30.5% (21.5% to 39.8%)	29.9% (19.4% to 39.7%)	30.5% (21.6% to 39.8%)
**Neonatal disorders**	76.7% (73.0% to 80.1%)	76.7% (73.0% to 80.1%)	0.6% (0.3% to 1.1%)	73.8% (70.1% to 77.8%)
Neonatal preterm birth complications	95.7% (92.2% to 98.1%)	95.8% (92.3% to 98.1%)	1.8% (1.2% to 2.4%)	92.0% (87.8% to 95.0%)
Neonatal encephalopathy due to birth asphyxia and trauma	70.6% (66.6% to 74.3%)	70.6% (66.6% to 74.4%)	0.0% (0.0% to 0.0%)	68.7% (63.6% to 72.9%)
Neonatal sepsis and other neonatal infections	66.0% (61.5% to 69.7%)	66.0% (61.5% to 69.7%)	0.0% (0.0% to 0.0%)	62.4% (54.6% to 68.0%)
Hemolytic disease and other neonatal jaundice	68.4% (62.2% to 73.0%)	68.4% (62.3% to 73.0%)	0.0% (0.0% to 0.0%)	60.7% (52.6% to 67.0%)

Supplementary Results Table 1. Age-standardised PAFs attributable to the joint distribution of all risk factors for all ages, both sexes combined for each GBD level 1 and level 2 cause and all causes in 2016 for Central Sub-Saharan Africa. Results are shown for deaths, YLLs, YLDs, and
Data in parenthesis are 95% uncertainty intervals. PAFs=population attributable fractions. GBD=Global Burden of Disease. YLLs=years of life lost. YLDs=years live with disability. DALYs=disability-adjusted life-years

	Deaths 2016	YLLs 2016	YLDs 2016	DALYs 2016
Other neonatal disorders	67.4% (62.9% to 71.4%)	67.4% (63.0% to 71.4%)	0.0% (0.0% to 0.0%)	66.3% (61.9% to 70.6%)
**Nutritional deficiencies**	20.3% (9.0% to 31.0%)	52.2% (30.7% to 66.7%)	80.3% (77.6% to 83.1%)	61.5% (41.8% to 71.1%)
Protein-energy malnutrition	25.0% (11.4% to 37.1%)	59.3% (36.5% to 73.2%)	97.8% (97.2% to 98.3%)	60.3% (37.5% to 73.7%)
Vitamin A deficiency	-	-	100.0% (100.0% to 100.0%)	100.0% (100.0% to 100.0%)
Iron-deficiency anaemia	100.0% (100.0% to 100.0%)	100.0% (100.0% to 100.0%)	100.0% (100.0% to 100.0%)	100.0% (100.0% to 100.0%)
**Other communicable, maternal, neonatal, and nutritional diseases**	3.0% (1.7% to 5.8%)	1.8% (0.9% to 3.3%)	65.9% (61.2% to 70.6%)	7.5% (4.3% to 12.1%)
Sexually transmitted diseases excluding HIV	8.3% (4.2% to 14.8%)	3.3% (1.6% to 6.2%)	100.0% (100.0% to 100.0%)	13.7% (7.1% to 23.8%)
Hepatitis	0.0% (0.0% to 0.0%)	0.0% (0.0% to 0.0%)	0.0% (0.0% to 0.1%)	0.0% (0.0% to 0.0%)
**Neoplasms**	34.0% (30.7% to 37.9%)	35.7% (32.2% to 39.3%)	35.7% (31.2% to 40.3%)	35.7% (32.2% to 39.3%)
Oesophageal cancer	63.4% (51.8% to 74.1%)	66.4% (55.0% to 76.7%)	64.2% (52.6% to 74.8%)	66.4% (54.9% to 76.7%)
Stomach cancer	8.3% (2.4% to 18.6%)	8.7% (2.9% to 18.6%)	8.4% (2.6% to 18.5%)	8.7% (2.9% to 18.6%)
Liver cancer	26.9% (20.3% to 34.6%)	27.7% (20.8% to 35.6%)	27.0% (20.3% to 34.6%)	27.7% (20.8% to 35.6%)
Larynx cancer	58.9% (47.4% to 70.3%)	63.2% (51.9% to 73.8%)	59.7% (48.1% to 70.8%)	63.1% (51.8% to 73.8%)
Tracheal, bronchus, and lung cancer	82.1% (76.8% to 86.3%)	83.1% (78.1% to 87.2%)	82.3% (77.1% to 86.6%)	83.1% (78.1% to 87.2%)
Breast cancer	20.4% (14.5% to 27.4%)	19.6% (14.1% to 26.3%)	19.8% (14.1% to 26.5%)	19.7% (14.1% to 26.3%)
Cervical cancer	100.0% (100.0% to 100.0%)	100.0% (100.0% to 100.0%)	100.0% (100.0% to 100.0%)	100.0% (100.0% to 100.0%)
Uterine cancer	24.8% (14.4% to 36.9%)	25.6% (15.4% to 37.8%)	25.7% (15.3% to 37.8%)	25.6% (15.4% to 37.8%)
Prostate cancer	0.8% (0.5% to 1.3%)	1.1% (0.7% to 1.7%)	1.0% (0.6% to 1.4%)	1.1% (0.7% to 1.7%)
Colon and rectum cancer	50.6% (41.7% to 59.6%)	50.9% (42.3% to 59.5%)	50.8% (42.0% to 59.5%)	50.9% (42.3% to 59.5%)
Lip and oral cavity cancer	61.1% (49.8% to 71.7%)	65.1% (54.0% to 75.2%)	61.9% (50.6% to 72.1%)	65.0% (54.0% to 75.1%)
Nasopharynx cancer	55.9% (45.5% to 65.6%)	57.2% (47.0% to 66.1%)	55.5% (45.8% to 64.7%)	57.2% (47.0% to 66.1%)

Supplementary Results Table 1. Age-standardised PAFs attributable to the joint distribution of all risk factors for all ages, both sexes combined for each GBD level 1 and level 2 cause and all causes in 2016 for Central Sub-Saharan Africa. Results are shown for deaths, YLLs, YLDs, and
Data in parenthesis are 95% uncertainty intervals. PAFs=population attributable fractions. GBD=Global Burden of Disease. YLLs=years of life lost. YLDs=years live with disability. DALYs=disability-adjusted life-years

	Deaths 2016	YLLs 2016	YLDs 2016	DALYs 2016
Other pharynx cancer	42.4% (27.6% to 56.8%)	45.1% (30.1% to 59.4%)	43.5% (28.1% to 57.5%)	45.1% (30.1% to 59.4%)
Gallbladder and biliary tract cancer	12.2% (6.1% to 21.0%)	12.7% (6.5% to 21.7%)	12.0% (5.9% to 20.8%)	12.7% (6.5% to 21.7%)
Pancreatic cancer	15.3% (9.2% to 23.6%)	16.0% (10.1% to 24.0%)	15.3% (9.2% to 23.5%)	16.0% (10.1% to 24.0%)
Ovarian cancer	9.4% (3.8% to 17.4%)	8.4% (3.4% to 15.7%)	8.3% (3.3% to 15.5%)	8.4% (3.4% to 15.7%)
Kidney cancer	16.1% (10.3% to 23.4%)	15.8% (10.2% to 22.8%)	15.6% (10.1% to 22.6%)	15.8% (10.2% to 22.7%)
Bladder cancer	13.5% (7.4% to 22.2%)	15.2% (8.9% to 23.1%)	14.3% (8.0% to 22.4%)	15.2% (8.9% to 23.1%)
Thyroid cancer	6.8% (3.2% to 11.7%)	7.0% (3.3% to 11.8%)	7.0% (3.3% to 11.9%)	7.0% (3.3% to 11.8%)
Mesothelioma	86.1% (80.2% to 89.9%)	77.9% (70.4% to 83.3%)	82.5% (75.8% to 87.1%)	77.9% (70.5% to 83.4%)
Non-Hodgkin's lymphoma	3.4% (1.3% to 6.3%)	3.1% (1.2% to 5.9%)	3.2% (1.2% to 6.1%)	3.1% (1.2% to 5.9%)
Multiple myeloma	4.3% (1.7% to 8.0%)	4.4% (1.8% to 8.2%)	4.3% (1.7% to 8.0%)	4.4% (1.8% to 8.2%)
Leukaemia	6.8% (3.9% to 10.3%)	5.7% (3.4% to 8.6%)	6.2% (3.6% to 9.4%)	5.7% (3.4% to 8.7%)
**Cardiovascular diseases**	84.5% (82.2% to 86.6%)	85.5% (83.8% to 87.1%)	60.1% (55.7% to 64.3%)	83.8% (81.8% to 85.7%)
Rheumatic heart disease	27.6% (18.7% to 42.2%)	27.5% (19.2% to 40.7%)	24.3% (15.5% to 34.3%)	26.4% (18.2% to 37.7%)
Ischaemic heart disease	93.7% (90.8% to 96.0%)	95.0% (93.1% to 96.6%)	90.2% (87.3% to 92.9%)	94.9% (92.9% to 96.5%)
Cerebrovascular disease	87.0% (83.4% to 90.5%)	89.8% (87.3% to 92.0%)	87.2% (83.7% to 90.6%)	89.7% (87.1% to 92.0%)
Hypertensive heart disease	100.0% (100.0% to 100.0%)	100.0% (100.0% to 100.0%)	100.0% (100.0% to 100.0%)	100.0% (100.0% to 100.0%)
Cardiomyopathy and myocarditis	40.3% (32.6% to 49.4%)	39.3% (32.2% to 47.0%)	42.8% (35.9% to 50.0%)	39.5% (32.5% to 47.2%)
Atrial fibrillation and flutter	41.5% (34.1% to 48.7%)	46.4% (39.8% to 52.6%)	53.3% (47.9% to 58.6%)	49.8% (43.9% to 55.4%)
Aortic aneurysm	40.9% (34.2% to 47.0%)	46.3% (40.2% to 51.8%)	-	46.3% (40.2% to 51.8%)
Peripheral vascular disease	52.1% (46.1% to 58.4%)	59.2% (54.3% to 64.1%)	58.7% (53.7% to 63.8%)	58.9% (54.2% to 63.7%)
Endocarditis	39.1% (30.2% to 49.5%)	39.9% (31.7% to 48.8%)	35.0% (26.3% to 45.6%)	39.8% (31.6% to 48.7%)

Supplementary Results Table 1. Age-standardised PAFs attributable to the joint distribution of all risk factors for all ages, both sexes combined for each GBD level 1 and level 2 cause and all causes in 2016 for Central Sub-Saharan Africa. Results are shown for deaths, YLLs, YLDs, and
Data in parenthesis are 95% uncertainty intervals. PAFs=population attributable fractions. GBD=Global Burden of Disease. YLLs=years of life lost. YLDs=years live with disability. DALYs=disability-adjusted life-years

	Deaths 2016	YLLs 2016	YLDs 2016	DALYs 2016
Other cardiovascular and circulatory diseases	42.6% (37.8% to 47.5%)	46.4% (42.2% to 50.6%)	39.2% (34.1% to 44.5%)	45.1% (40.9% to 49.4%)
**Chronic respiratory diseases**	61.3% (51.5% to 68.7%)	58.6% (50.3% to 65.1%)	36.9% (30.5% to 43.9%)	52.0% (45.2% to 58.4%)
Chronic obstructive pulmonary disease	76.6% (68.1% to 85.2%)	77.6% (70.2% to 85.5%)	67.6% (58.3% to 77.2%)	75.6% (68.1% to 83.5%)
Pneumoconiosis	100.0% (100.0% to 100.0%)	100.0% (100.0% to 100.0%)	100.0% (100.0% to 100.0%)	100.0% (100.0% to 100.0%)
Asthma	21.1% (13.7% to 30.3%)	23.1% (16.4% to 31.1%)	18.9% (13.5% to 25.5%)	21.1% (15.3% to 28.2%)
Other chronic respiratory diseases	4.3% (2.5% to 6.8%)	4.4% (2.4% to 7.4%)	2.6% (1.3% to 4.4%)	3.8% (2.1% to 6.4%)
**Cirrhosis and other chronic liver diseases**	24.1% (22.4% to 25.7%)	23.0% (21.3% to 24.7%)	18.4% (16.4% to 20.5%)	22.9% (21.2% to 24.6%)
Cirrhosis and other chronic liver diseases due to hepatitis B	0.0% (0.0% to 0.0%)	0.0% (0.0% to 0.0%)	0.0% (0.0% to 0.0%)	0.0% (0.0% to 0.0%)
Cirrhosis and other chronic liver diseases due to hepatitis C	0.9% (0.5% to 1.5%)	1.3% (0.6% to 2.1%)	1.8% (0.8% to 3.2%)	1.3% (0.6% to 2.2%)
Cirrhosis and other chronic liver diseases due to alcohol use	100.0% (100.0% to 100.0%)	100.0% (100.0% to 100.0%)	100.0% (100.0% to 100.0%)	100.0% (100.0% to 100.0%)
**Digestive diseases**	4.6% (3.3% to 6.3%)	5.2% (3.8% to 6.8%)	2.0% (1.4% to 2.6%)	5.0% (3.7% to 6.5%)
Peptic ulcer disease	6.1% (3.8% to 8.7%)	7.7% (4.8% to 10.9%)	7.1% (4.9% to 9.7%)	7.7% (4.9% to 10.8%)
Gallbladder and biliary diseases	23.0% (13.3% to 35.3%)	23.5% (13.9% to 35.4%)	24.9% (15.2% to 36.8%)	23.5% (13.9% to 35.4%)
Pancreatitis	25.9% (11.9% to 45.9%)	30.0% (14.9% to 51.1%)	27.1% (13.5% to 45.7%)	30.0% (14.9% to 51.1%)
**Neurological disorders**	14.4% (6.8% to 24.5%)	14.7% (8.1% to 23.2%)	3.7% (2.1% to 5.5%)	8.5% (4.8% to 13.3%)
Alzheimer's disease and other dementias	15.1% (6.7% to 26.5%)	15.9% (7.1% to 27.2%)	15.8% (7.0% to 27.2%)	15.9% (7.1% to 27.2%)
Parkinson's disease	-4.0% (-5.3% to -2.8%)	-5.0% (-6.6% to -3.8%)	-5.7% (-7.3% to -4.1%)	-5.2% (-6.7% to -3.8%)
Epilepsy	17.8% (10.5% to 25.4%)	16.5% (9.9% to 23.4%)	15.0% (8.8% to 21.7%)	15.8% (9.7% to 22.5%)
Multiple sclerosis	4.2% (1.8% to 7.5%)	4.5% (1.9% to 8.1%)	3.4% (1.9% to 5.1%)	4.2% (2.0% to 7.1%)
**Mental and substance use disorders**	99.8% (99.6% to 99.9%)	99.7% (99.5% to 99.9%)	19.2% (16.9% to 21.5%)	22.7% (20.0% to 26.1%)
Alcohol use disorders	100.0% (100.0% to 100.0%)	100.0% (100.0% to 100.0%)	100.0% (100.0% to 100.0%)	100.0% (100.0% to 100.0%)

Supplementary Results Table 1. Age-standardised PAFs attributable to the joint distribution of all risk factors for all ages, both sexes combined for each GBD level 1 and level 2 cause and all causes in 2016 for Central Sub-Saharan Africa. Results are shown for deaths, YLLs, YLDs, and
Data in parenthesis are 95% uncertainty intervals. PAFs=population attributable fractions. GBD=Global Burden of Disease. YLLs=years of life lost. YLDs=years live with disability. DALYs=disability-adjusted life-years

	Deaths 2016	YLLs 2016	YLDs 2016	DALYs 2016
Drug use disorders	100.0% (100.0% to 100.0%)	100.0% (100.0% to 100.0%)	100.0% (100.0% to 100.0%)	100.0% (100.0% to 100.0%)
Depressive disorders	-	-	10.0% (8.4% to 11.9%)	10.0% (8.4% to 11.9%)
Idiopathic developmental intellectual disability	-	-	62.8% (31.4% to 82.2%)	62.8% (31.4% to 82.2%)
**Diabetes, urogenital, blood, and endocrine diseases**	81.6% (78.7% to 84.1%)	74.0% (68.6% to 77.6%)	59.7% (55.4% to 63.5%)	67.8% (63.8% to 71.0%)
Diabetes mellitus	100.0% (100.0% to 100.0%)	100.0% (100.0% to 100.0%)	100.0% (100.0% to 100.0%)	100.0% (100.0% to 100.0%)
Chronic kidney disease	100.0% (100.0% to 100.0%)	100.0% (100.0% to 100.0%)	100.0% (100.0% to 100.0%)	100.0% (100.0% to 100.0%)
**Musculoskeletal disorders**	0.6% (0.2% to 1.1%)	0.5% (0.2% to 1.0%)	17.9% (15.4% to 20.6%)	17.6% (15.1% to 20.3%)
Rheumatoid arthritis	1.5% (0.5% to 2.8%)	1.7% (0.6% to 3.3%)	2.1% (0.9% to 3.6%)	2.1% (0.8% to 3.5%)
Osteoarthritis	-	-	14.5% (7.1% to 23.7%)	14.5% (7.1% to 23.7%)
Low back and neck pain	-	-	21.7% (19.1% to 24.5%)	21.7% (19.1% to 24.5%)
Gout	-	-	40.2% (31.5% to 51.1%)	40.2% (31.5% to 51.1%)
**Other non-communicable diseases**	0.2% (0.1% to 0.3%)	0.3% (0.1% to 0.5%)	6.8% (5.6% to 8.0%)	5.1% (4.2% to 6.1%)
Sense organ diseases	-	-	12.6% (10.5% to 14.5%)	12.6% (10.5% to 14.5%)
Sudden infant death syndrome	5.3% (2.5% to 9.1%)	5.3% (2.6% to 9.2%)	-	5.3% (2.6% to 9.2%)
**Transport injuries**	39.9% (33.3% to 45.2%)	34.6% (27.8% to 40.1%)	47.2% (40.9% to 52.7%)	36.0% (29.4% to 41.5%)
Road injuries	39.6% (33.0% to 45.0%)	34.4% (27.6% to 39.8%)	50.2% (43.6% to 55.8%)	35.8% (29.2% to 41.2%)
Other transport injuries	46.6% (36.5% to 54.2%)	40.6% (30.3% to 48.6%)	38.5% (32.9% to 43.7%)	39.7% (31.5% to 46.1%)
**Unintentional injuries**	34.0% (28.7% to 38.2%)	22.6% (18.3% to 26.9%)	33.3% (28.4% to 36.9%)	25.2% (21.1% to 29.3%)
Falls	68.8% (53.6% to 73.3%)	48.9% (42.0% to 54.3%)	41.4% (36.7% to 45.0%)	45.3% (40.7% to 49.5%)
Drowning	19.8% (15.2% to 25.5%)	15.4% (11.1% to 21.5%)	28.6% (23.8% to 33.4%)	15.6% (11.2% to 21.5%)
Fire, heat, and hot substances	16.8% (12.3% to 22.0%)	18.4% (13.3% to 23.7%)	31.3% (24.8% to 36.3%)	20.8% (15.9% to 25.9%)

Supplementary Results Table 1. Age-standardised PAFs attributable to the joint distribution of all risk factors for all ages, both sexes combined for each GBD level 1 and level 2 cause and all causes in 2016 for Central Sub-Saharan Africa. Results are shown for deaths, YLLs, YLDs, and
Data in parenthesis are 95% uncertainty intervals. PAFs=population attributable fractions. GBD=Global Burden of Disease. YLLs=years of life lost. YLDs=years live with disability. DALYs=disability-adjusted life-years

	Deaths 2016	YLLs 2016	YLDs 2016	DALYs 2016
Poisonings	22.8% (16.2% to 29.6%)	22.0% (14.0% to 30.1%)	28.6% (23.1% to 33.3%)	22.3% (14.5% to 30.2%)
Exposure to mechanical forces	33.1% (26.2% to 39.2%)	27.3% (19.2% to 35.2%)	38.7% (31.1% to 44.1%)	30.3% (22.3% to 36.8%)
Animal contact	21.2% (16.7% to 26.4%)	22.5% (17.0% to 28.7%)	26.9% (21.4% to 31.2%)	23.5% (18.1% to 29.0%)
Foreign body	15.9% (10.3% to 21.6%)	14.6% (7.4% to 22.0%)	22.5% (17.4% to 26.3%)	16.9% (10.6% to 22.6%)
Other unintentional injuries	26.2% (18.8% to 33.2%)	27.4% (19.4% to 34.5%)	28.2% (22.7% to 32.7%)	27.8% (21.7% to 33.2%)
**Self-harm and interpersonal violence**	16.7% (9.9% to 25.1%)	17.1% (11.2% to 24.4%)	21.1% (18.3% to 24.1%)	17.5% (12.0% to 24.4%)
Self-harm	16.5% (6.9% to 27.5%)	18.0% (8.2% to 28.7%)	17.2% (7.5% to 28.2%)	17.9% (8.2% to 28.7%)
Interpersonal violence	17.2% (11.9% to 22.8%)	15.8% (10.6% to 21.1%)	21.6% (18.7% to 24.5%)	16.9% (12.3% to 21.8%)
**Forces of nature, conflict and terrorism, and executions and police conflict**	0.3% (0.3% to 0.3%)	0.1% (0.1% to 0.1%)	0.0% (0.0% to 0.0%)	0.1% (0.0% to 0.1%)
Forces of nature, conflict and terrorism, and state actor violence	6.2% (5.8% to 6.6%)	2.6% (2.3% to 2.8%)	5.6% (3.5% to 8.5%)	2.8% (2.4% to 3.5%)

Supplementary Results Table 1. Age-standardised PAFs attributable to the joint distribution of all risk factors for all ages, both sexes combined for each GBD level 1 and level 2 cause and all causes in 2016 for East Asia. Results are shown for deaths, YLLs, YLDs, and DALYs
Data in parenthesis are 95% uncertainty intervals. PAFs=population attributable fractions. GBD=Global Burden of Disease. YLLs=years of life lost. YLDs=years live with disability. DALYs=disability-adjusted life-years

	Deaths 2016	YLLs 2016	YLDs 2016	DALYs 2016
**All causes**	63.8% (60.7% to 66.8%)	56.7% (54.4% to 59.1%)	23.3% (22.0% to 24.7%)	44.0% (41.3% to 47.0%)
**HIV/AIDS and tuberculosis**	60.5% (56.5% to 64.7%)	62.1% (58.9% to 65.2%)	43.7% (38.1% to 49.4%)	57.3% (53.9% to 61.0%)
Tuberculosis	48.2% (41.5% to 54.8%)	46.8% (41.0% to 52.6%)	38.4% (33.3% to 43.3%)	43.5% (37.9% to 49.1%)
HIV/AIDS	79.0% (77.0% to 81.0%)	75.2% (72.4% to 77.7%)	81.2% (79.5% to 83.0%)	75.6% (73.0% to 78.1%)
**Diarrhoea, lower respiratory infections, and other common infectious diseases**	66.3% (61.4% to 71.2%)	68.4% (64.1% to 72.5%)	18.6% (13.8% to 23.0%)	54.6% (49.6% to 59.7%)
Diarrhoeal diseases	83.9% (68.3% to 94.1%)	91.0% (81.3% to 96.8%)	75.2% (53.7% to 90.0%)	82.5% (66.8% to 93.0%)
Lower respiratory infections	71.5% (66.2% to 77.1%)	80.6% (75.7% to 84.7%)	71.7% (65.8% to 77.5%)	80.5% (75.6% to 84.6%)
Upper respiratory infections	1.0% (0.6% to 1.7%)	2.5% (1.4% to 4.1%)	0.0% (0.0% to 0.0%)	0.0% (0.0% to 0.0%)
Otitis media	2.2% (1.4% to 3.0%)	5.9% (4.2% to 7.7%)	9.1% (6.2% to 11.7%)	9.1% (6.2% to 11.7%)
Meningitis	1.2% (0.8% to 1.6%)	2.0% (1.5% to 2.4%)	0.0% (0.0% to 0.0%)	1.7% (1.3% to 2.1%)
Encephalitis	0.9% (0.8% to 1.1%)	1.2% (1.0% to 1.4%)	0.0% (0.0% to 0.0%)	0.5% (0.4% to 0.7%)
Measles	39.1% (19.6% to 72.5%)	39.7% (19.9% to 73.6%)	33.7% (14.3% to 69.4%)	39.2% (19.6% to 73.1%)
**Maternal disorders**	6.6% (3.0% to 10.0%)	6.4% (2.9% to 9.9%)	3.3% (1.7% to 5.0%)	5.6% (2.7% to 8.6%)
Maternal haemorrhage	15.0% (5.3% to 24.3%)	14.9% (5.3% to 24.2%)	14.1% (5.0% to 22.9%)	14.8% (5.3% to 24.1%)
Maternal sepsis and other pregnancy related infections	14.0% (5.0% to 22.8%)	14.2% (5.0% to 23.0%)	13.9% (4.9% to 22.8%)	14.1% (5.0% to 23.0%)
Maternal abortion, miscarriage, and ectopic pregnancy	9.7% (5.7% to 14.2%)	9.4% (5.4% to 13.7%)	8.3% (4.0% to 13.1%)	9.1% (5.1% to 13.5%)
**Neonatal disorders**	75.7% (74.0% to 77.4%)	75.7% (74.1% to 77.5%)	0.1% (0.1% to 0.2%)	65.9% (62.5% to 68.8%)
Neonatal preterm birth complications	91.6% (90.6% to 93.3%)	91.7% (90.7% to 93.3%)	0.2% (0.1% to 0.2%)	76.0% (70.5% to 80.3%)
Neonatal encephalopathy due to birth asphyxia and trauma	64.4% (61.8% to 67.8%)	64.5% (61.9% to 67.9%)	0.0% (0.0% to 0.0%)	61.5% (58.0% to 65.1%)
Neonatal sepsis and other neonatal infections	49.4% (46.3% to 53.6%)	49.5% (46.4% to 53.7%)	0.0% (0.0% to 0.0%)	35.0% (24.6% to 43.3%)
Hemolytic disease and other neonatal jaundice	46.4% (43.3% to 49.6%)	46.5% (43.3% to 49.7%)	0.0% (0.0% to 0.0%)	31.2% (27.8% to 34.8%)

Supplementary Results Table 1. Age-standardised PAFs attributable to the joint distribution of all risk factors for all ages, both sexes combined for each GBD level 1 and level 2 cause and all causes in 2016 for East Asia. Results are shown for deaths, YLLs, YLDs, and DALYs
Data in parenthesis are 95% uncertainty intervals. PAFs=population attributable fractions. GBD=Global Burden of Disease. YLLs=years of life lost. YLDs=years live with disability. DALYs=disability-adjusted life-years

	Deaths 2016	YLLs 2016	YLDs 2016	DALYs 2016
Other neonatal disorders	48.4% (45.7% to 51.2%)	48.5% (45.8% to 51.3%)	0.0% (0.0% to 0.0%)	46.8% (44.1% to 49.4%)
**Nutritional deficiencies**	5.9% (5.1% to 7.0%)	29.0% (25.6% to 34.5%)	77.6% (74.6% to 80.3%)	70.6% (66.4% to 74.6%)
Protein-energy malnutrition	5.7% (4.8% to 7.1%)	35.6% (31.3% to 40.8%)	97.7% (97.5% to 97.9%)	55.7% (49.8% to 61.3%)
Vitamin A deficiency	-	-	99.8% (99.8% to 99.9%)	100.0% (100.0% to 100.0%)
Iron-deficiency anaemia	100.0% (100.0% to 100.0%)	100.0% (100.0% to 100.0%)	99.9% (99.9% to 99.9%)	100.0% (100.0% to 100.0%)
**Other communicable, maternal, neonatal, and nutritional diseases**	4.4% (3.5% to 4.8%)	3.9% (3.3% to 4.3%)	85.3% (82.7% to 87.6%)	47.7% (38.6% to 57.5%)
Sexually transmitted diseases excluding HIV	68.6% (50.7% to 82.1%)	43.8% (26.2% to 61.2%)	99.7% (99.7% to 99.8%)	95.8% (91.7% to 98.3%)
Hepatitis	1.1% (0.9% to 1.4%)	1.2% (0.9% to 1.5%)	0.9% (0.7% to 1.2%)	1.2% (0.9% to 1.5%)
**Neoplasms**	44.9% (37.7% to 52.6%)	41.4% (35.0% to 48.8%)	37.4% (31.7% to 43.4%)	41.3% (34.9% to 48.6%)
Oesophageal cancer	66.6% (53.1% to 80.3%)	67.1% (54.3% to 80.4%)	64.9% (51.3% to 79.2%)	67.0% (54.3% to 80.4%)
Stomach cancer	21.8% (11.1% to 33.8%)	20.5% (11.0% to 31.9%)	20.9% (10.8% to 32.6%)	20.5% (11.0% to 31.9%)
Liver cancer	35.5% (26.2% to 48.5%)	33.8% (24.3% to 46.5%)	35.0% (25.7% to 47.7%)	33.8% (24.3% to 46.5%)
Larynx cancer	73.7% (54.5% to 88.5%)	72.3% (54.2% to 87.1%)	73.1% (54.9% to 87.7%)	72.3% (54.2% to 87.1%)
Tracheal, bronchus, and lung cancer	80.3% (67.5% to 90.3%)	78.7% (66.1% to 89.2%)	79.4% (66.8% to 89.8%)	78.7% (66.1% to 89.2%)
Breast cancer	26.9% (16.8% to 39.1%)	25.7% (16.4% to 36.8%)	26.1% (16.4% to 37.2%)	25.7% (16.5% to 36.8%)
Cervical cancer	100.0% (100.0% to 100.0%)	100.0% (100.0% to 100.0%)	100.0% (100.0% to 100.0%)	100.0% (100.0% to 100.0%)
Uterine cancer	22.8% (11.6% to 36.3%)	23.6% (12.2% to 37.3%)	24.9% (13.3% to 38.7%)	23.7% (12.3% to 37.3%)
Prostate cancer	3.4% (1.2% to 7.5%)	2.9% (1.0% to 6.4%)	2.9% (1.0% to 6.4%)	2.9% (1.0% to 6.4%)
Colon and rectum cancer	52.5% (44.0% to 60.9%)	52.4% (44.3% to 60.4%)	52.7% (44.5% to 61.0%)	52.4% (44.3% to 60.4%)
Lip and oral cavity cancer	70.6% (60.2% to 80.8%)	70.8% (61.7% to 80.0%)	68.6% (58.8% to 78.8%)	70.8% (61.6% to 79.9%)
Nasopharynx cancer	66.3% (57.7% to 76.8%)	65.4% (57.6% to 74.8%)	62.8% (55.2% to 72.4%)	65.4% (57.6% to 74.7%)

Supplementary Results Table 1. Age-standardised PAFs attributable to the joint distribution of all risk factors for all ages, both sexes combined for each GBD level 1 and level 2 cause and all causes in 2016 for East Asia. Results are shown for deaths, YLLs, YLDs, and DALYs
Data in parenthesis are 95% uncertainty intervals. PAFs=population attributable fractions. GBD=Global Burden of Disease. YLLs=years of life lost. YLDs=years live with disability. DALYs=disability-adjusted life-years

	Deaths 2016	YLLs 2016	YLDs 2016	DALYs 2016
Other pharynx cancer	57.5% (48.5% to 65.2%)	60.0% (51.4% to 67.4%)	61.4% (52.8% to 69.0%)	60.0% (51.5% to 67.4%)
Gallbladder and biliary tract cancer	9.8% (4.0% to 18.0%)	10.4% (4.4% to 18.9%)	10.2% (4.2% to 18.6%)	10.4% (4.4% to 18.9%)
Pancreatic cancer	20.1% (10.1% to 32.8%)	18.6% (9.5% to 30.8%)	19.6% (10.0% to 32.0%)	18.6% (9.5% to 30.8%)
Ovarian cancer	9.3% (3.7% to 17.5%)	8.0% (2.9% to 15.2%)	8.0% (3.0% to 15.0%)	8.0% (2.9% to 15.2%)
Kidney cancer	18.6% (9.3% to 30.8%)	16.0% (8.1% to 26.5%)	16.5% (8.5% to 27.0%)	16.0% (8.1% to 26.5%)
Bladder cancer	25.9% (11.8% to 42.7%)	23.1% (10.1% to 38.5%)	23.4% (10.4% to 39.0%)	23.2% (10.1% to 38.6%)
Thyroid cancer	6.7% (2.3% to 13.7%)	7.0% (2.5% to 14.3%)	7.1% (2.9% to 13.7%)	7.0% (2.5% to 14.2%)
Mesothelioma	80.4% (75.4% to 84.5%)	69.2% (63.0% to 74.6%)	76.7% (71.6% to 81.1%)	69.3% (63.2% to 74.7%)
Non-Hodgkin's lymphoma	3.1% (1.0% to 6.5%)	2.9% (0.9% to 6.0%)	3.3% (1.0% to 6.7%)	2.9% (0.9% to 6.0%)
Multiple myeloma	3.6% (1.2% to 7.4%)	3.7% (1.2% to 7.5%)	3.8% (1.3% to 7.6%)	3.7% (1.2% to 7.5%)
Leukaemia	8.3% (4.3% to 14.9%)	5.9% (3.2% to 10.2%)	6.8% (3.5% to 11.9%)	5.9% (3.2% to 10.2%)
**Cardiovascular diseases**	86.2% (83.7% to 88.5%)	87.4% (85.8% to 89.0%)	79.6% (76.9% to 82.2%)	86.5% (84.8% to 88.2%)
Rheumatic heart disease	25.4% (16.1% to 42.9%)	24.4% (16.4% to 37.7%)	20.2% (13.4% to 29.6%)	24.1% (16.3% to 37.7%)
Ischaemic heart disease	91.8% (88.0% to 94.9%)	93.3% (90.9% to 95.2%)	89.6% (86.8% to 92.2%)	93.2% (90.8% to 95.1%)
Cerebrovascular disease	85.0% (81.1% to 88.7%)	88.4% (85.7% to 90.8%)	86.1% (82.7% to 89.2%)	88.0% (85.5% to 90.5%)
Hypertensive heart disease	100.0% (100.0% to 100.0%)	100.0% (100.0% to 100.0%)	100.0% (100.0% to 100.0%)	100.0% (100.0% to 100.0%)
Cardiomyopathy and myocarditis	23.8% (19.0% to 30.6%)	21.2% (17.8% to 25.9%)	18.8% (15.1% to 22.7%)	21.0% (17.7% to 25.4%)
Atrial fibrillation and flutter	46.7% (39.6% to 53.1%)	51.3% (45.3% to 56.8%)	58.2% (53.7% to 62.6%)	55.4% (50.2% to 60.1%)
Aortic aneurysm	46.0% (39.7% to 52.5%)	52.0% (46.4% to 57.2%)	-	52.0% (46.4% to 57.2%)
Peripheral vascular disease	51.4% (45.4% to 57.9%)	58.4% (53.6% to 63.3%)	56.7% (51.5% to 61.7%)	57.1% (52.0% to 61.9%)
Endocarditis	38.7% (29.1% to 50.5%)	32.7% (24.5% to 40.6%)	39.2% (30.6% to 48.1%)	32.8% (24.6% to 40.7%)

Supplementary Results Table 1. Age-standardised PAFs attributable to the joint distribution of all risk factors for all ages, both sexes combined for each GBD level 1 and level 2 cause and all causes in 2016 for East Asia. Results are shown for deaths, YLLs, YLDs, and DALYs
Data in parenthesis are 95% uncertainty intervals. PAFs=population attributable fractions. GBD=Global Burden of Disease. YLLs=years of life lost. YLDs=years live with disability. DALYs=disability-adjusted life-years

	Deaths 2016	YLLs 2016	YLDs 2016	DALYs 2016
Other cardiovascular and circulatory diseases	44.1% (39.0% to 49.2%)	41.1% (37.2% to 45.3%)	42.7% (38.2% to 47.3%)	41.7% (37.8% to 45.8%)
**Chronic respiratory diseases**	78.3% (64.5% to 87.9%)	76.3% (63.7% to 85.8%)	48.9% (40.3% to 58.2%)	69.1% (57.9% to 78.2%)
Chronic obstructive pulmonary disease	80.0% (65.7% to 90.1%)	78.5% (65.1% to 88.7%)	69.6% (55.4% to 81.6%)	77.0% (63.5% to 87.3%)
Pneumoconiosis	100.0% (100.0% to 100.0%)	100.0% (100.0% to 100.0%)	100.0% (100.0% to 100.0%)	100.0% (100.0% to 100.0%)
Asthma	29.3% (22.4% to 37.7%)	33.4% (26.5% to 41.4%)	23.6% (17.8% to 30.8%)	25.3% (19.5% to 32.5%)
Other chronic respiratory diseases	9.1% (2.6% to 19.8%)	5.3% (1.7% to 11.7%)	3.6% (1.1% to 7.9%)	4.3% (1.4% to 9.4%)
**Cirrhosis and other chronic liver diseases**	22.8% (21.5% to 24.2%)	24.9% (23.4% to 26.5%)	21.6% (20.3% to 23.0%)	24.7% (23.2% to 26.3%)
Cirrhosis and other chronic liver diseases due to hepatitis B	0.6% (0.5% to 0.7%)	0.7% (0.5% to 0.9%)	0.6% (0.5% to 0.8%)	0.7% (0.5% to 0.9%)
Cirrhosis and other chronic liver diseases due to hepatitis C	59.7% (54.9% to 64.5%)	71.3% (67.3% to 75.4%)	71.3% (67.2% to 75.2%)	71.3% (67.3% to 75.4%)
Cirrhosis and other chronic liver diseases due to alcohol use	100.0% (100.0% to 100.0%)	100.0% (100.0% to 100.0%)	100.0% (100.0% to 100.0%)	100.0% (100.0% to 100.0%)
**Digestive diseases**	11.6% (9.3% to 14.0%)	12.3% (10.2% to 14.7%)	2.9% (2.2% to 3.6%)	7.9% (6.2% to 9.6%)
Peptic ulcer disease	19.8% (14.5% to 25.3%)	22.4% (16.7% to 28.2%)	9.4% (6.7% to 12.1%)	17.2% (12.6% to 22.1%)
Gallbladder and biliary diseases	20.6% (10.9% to 33.6%)	21.5% (11.9% to 34.4%)	25.5% (14.5% to 39.6%)	22.3% (12.4% to 35.3%)
Pancreatitis	35.6% (26.9% to 44.3%)	39.0% (31.0% to 47.5%)	24.0% (19.0% to 29.6%)	37.8% (30.1% to 45.9%)
**Neurological disorders**	15.3% (7.5% to 25.4%)	14.8% (7.8% to 23.9%)	4.6% (2.8% to 6.9%)	9.1% (5.1% to 14.5%)
Alzheimer's disease and other dementias	18.1% (9.2% to 29.3%)	19.3% (10.0% to 30.3%)	19.2% (9.9% to 30.5%)	19.2% (10.0% to 30.4%)
Parkinson's disease	-19.6% (-23.5% to -15.1%)	-23.3% (-28.3% to -17.7%)	-24.3% (-29.5% to -18.3%)	-23.5% (-28.6% to -17.8%)
Epilepsy	19.7% (15.0% to 24.2%)	16.3% (12.2% to 20.0%)	13.9% (10.4% to 17.5%)	14.8% (11.2% to 18.4%)
Multiple sclerosis	11.7% (7.0% to 16.4%)	11.3% (6.7% to 15.9%)	8.0% (5.0% to 11.1%)	9.9% (6.0% to 13.8%)
**Mental and substance use disorders**	99.9% (99.9% to 99.9%)	99.8% (99.8% to 99.9%)	17.7% (15.8% to 19.7%)	22.7% (20.3% to 25.1%)
Alcohol use disorders	100.0% (100.0% to 100.0%)	100.0% (100.0% to 100.0%)	100.0% (100.0% to 100.0%)	100.0% (100.0% to 100.0%)

Supplementary Results Table 1. Age-standardised PAFs attributable to the joint distribution of all risk factors for all ages, both sexes combined for each GBD level 1 and level 2 cause and all causes in 2016 for East Asia. Results are shown for deaths, YLLs, YLDs, and DALYs
Data in parenthesis are 95% uncertainty intervals. PAFs=population attributable fractions. GBD=Global Burden of Disease. YLLs=years of life lost. YLDs=years live with disability. DALYs=disability-adjusted life-years

	Deaths 2016	YLLs 2016	YLDs 2016	DALYs 2016
Drug use disorders	100.0% (100.0% to 100.0%)	100.0% (100.0% to 100.0%)	100.0% (100.0% to 100.0%)	100.0% (100.0% to 100.0%)
Depressive disorders	-	-	4.6% (3.7% to 5.6%)	4.6% (3.7% to 5.6%)
Idiopathic developmental intellectual disability	-	-	64.9% (33.2% to 83.9%)	64.9% (33.2% to 83.9%)
**Diabetes, urogenital, blood, and endocrine diseases**	85.9% (85.2% to 86.8%)	81.7% (80.5% to 83.0%)	53.0% (49.5% to 56.3%)	64.3% (60.7% to 67.6%)
Diabetes mellitus	100.0% (100.0% to 100.0%)	100.0% (100.0% to 100.0%)	100.0% (100.0% to 100.0%)	100.0% (100.0% to 100.0%)
Chronic kidney disease	100.0% (100.0% to 100.0%)	100.0% (100.0% to 100.0%)	100.0% (100.0% to 100.0%)	100.0% (100.0% to 100.0%)
**Musculoskeletal disorders**	3.1% (1.3% to 5.2%)	2.3% (1.0% to 3.8%)	13.2% (10.7% to 16.3%)	13.0% (10.5% to 16.0%)
Rheumatoid arthritis	6.1% (2.6% to 10.0%)	6.4% (2.8% to 10.6%)	4.2% (1.8% to 6.8%)	4.6% (1.9% to 7.5%)
Osteoarthritis	-	-	14.1% (5.7% to 25.1%)	14.1% (5.7% to 25.1%)
Low back and neck pain	-	-	17.2% (14.7% to 20.1%)	17.2% (14.7% to 20.1%)
Gout	-	-	27.7% (17.0% to 41.8%)	27.7% (17.0% to 41.8%)
**Other non-communicable diseases**	0.1% (0.1% to 0.1%)	0.1% (0.1% to 0.1%)	6.7% (5.9% to 7.5%)	5.2% (4.6% to 5.9%)
Sense organ diseases	-	-	14.1% (12.9% to 15.5%)	14.1% (12.9% to 15.5%)
Sudden infant death syndrome	7.0% (5.9% to 8.0%)	7.0% (5.9% to 8.1%)	-	7.0% (5.9% to 8.1%)
**Transport injuries**	34.1% (28.7% to 40.1%)	30.0% (25.3% to 35.4%)	38.3% (34.0% to 43.2%)	31.3% (26.7% to 36.6%)
Road injuries	33.9% (28.6% to 39.9%)	29.9% (25.2% to 35.3%)	40.6% (36.2% to 45.7%)	31.4% (26.7% to 36.8%)
Other transport injuries	37.0% (31.9% to 42.9%)	31.5% (26.6% to 37.1%)	29.6% (25.6% to 33.8%)	30.5% (26.1% to 35.4%)
**Unintentional injuries**	28.2% (20.7% to 31.3%)	15.5% (13.9% to 16.9%)	21.4% (20.2% to 22.8%)	17.0% (15.7% to 18.4%)
Falls	51.9% (33.6% to 58.4%)	30.2% (25.0% to 32.7%)	30.0% (28.9% to 31.3%)	30.1% (26.8% to 31.9%)
Drowning	9.6% (7.5% to 12.2%)	7.6% (6.3% to 9.3%)	14.9% (12.2% to 18.1%)	7.7% (6.4% to 9.4%)
Fire, heat, and hot substances	12.1% (8.8% to 16.3%)	13.5% (10.7% to 16.8%)	21.2% (18.0% to 24.9%)	16.9% (13.9% to 20.5%)

Supplementary Results Table 1. Age-standardised PAFs attributable to the joint distribution of all risk factors for all ages, both sexes combined for each GBD level 1 and level 2 cause and all causes in 2016 for East Asia. Results are shown for deaths, YLLs, YLDs, and DALYs
Data in parenthesis are 95% uncertainty intervals. PAFs=population attributable fractions. GBD=Global Burden of Disease. YLLs=years of life lost. YLDs=years live with disability. DALYs=disability-adjusted life-years

	**Deaths 2016**	**YLLs 2016**	**YLDs 2016**	**DALYs 2016**
Poisonings	14.0% (10.9% to 17.9%)	13.4% (11.0% to 16.9%)	19.9% (16.8% to 23.6%)	13.7% (11.2% to 17.2%)
Exposure to mechanical forces	16.5% (15.4% to 18.8%)	12.7% (11.5% to 15.5%)	23.9% (22.7% to 25.6%)	15.6% (14.0% to 18.6%)
Animal contact	12.5% (11.5% to 14.7%)	11.3% (10.3% to 13.3%)	16.2% (14.9% to 17.8%)	13.4% (12.4% to 15.0%)
Foreign body	3.5% (3.2% to 4.3%)	3.0% (2.6% to 3.6%)	11.1% (10.3% to 12.2%)	5.5% (4.5% to 6.6%)
Other unintentional injuries	23.8% (19.0% to 29.3%)	23.6% (19.3% to 28.5%)	18.9% (15.2% to 23.3%)	21.8% (17.8% to 26.4%)
**Self-harm and interpersonal violence**	22.0% (15.5% to 28.5%)	21.0% (15.0% to 26.5%)	29.6% (26.1% to 33.1%)	21.9% (16.5% to 27.0%)
Self-harm	21.7% (14.4% to 29.0%)	21.1% (14.0% to 28.0%)	18.9% (12.5% to 25.3%)	21.1% (13.9% to 28.0%)
Interpersonal violence	24.0% (19.2% to 28.7%)	20.1% (16.0% to 24.3%)	30.5% (26.9% to 34.5%)	24.0% (20.0% to 28.3%)
**Forces of nature, conflict and terrorism, and executions and police conflict**	4.5% (1.9% to 5.7%)	1.7% (0.7% to 2.2%)	1.1% (0.7% to 1.6%)	1.5% (0.9% to 2.0%)
Forces of nature, conflict and terrorism, and state actor violence	6.9% (6.5% to 7.3%)	2.7% (2.4% to 2.9%)	5.4% (3.6% to 7.8%)	3.3% (2.7% to 4.5%)

Supplementary Results Table 1. Age-standardised PAFs attributable to the joint distribution of all risk factors for all ages, both sexes combined for each GBD level 1 and level 2 cause and all causes in 2016 for Eastern Europe. Results are shown for deaths, YLLs, YLDs, and DALYs
Data in parenthesis are 95% uncertainty intervals. PAFs=population attributable fractions. GBD=Global Burden of Disease. YLLs=years of life lost. YLDs=years live with disability. DALYs=disability-adjusted life-years

	Deaths 2016	YLLs 2016	YLDs 2016	DALYs 2016
**All causes**	71.5% (69.7% to 73.1%)	65.5% (63.8% to 67.1%)	26.3% (24.4% to 28.0%)	52.6% (49.4% to 55.9%)
**HIV/AIDS and tuberculosis**	72.1% (69.0% to 75.1%)	70.9% (68.1% to 73.5%)	71.4% (68.5% to 73.7%)	70.9% (68.2% to 73.5%)
Tuberculosis	71.5% (63.2% to 79.0%)	71.9% (63.8% to 79.7%)	62.9% (55.8% to 70.1%)	71.6% (63.5% to 79.4%)
HIV/AIDS	72.5% (71.2% to 73.8%)	70.4% (68.9% to 71.7%)	74.0% (72.5% to 75.3%)	70.5% (69.0% to 71.9%)
**Diarrhoea, lower respiratory infections, and other common infectious diseases**	58.8% (52.2% to 65.1%)	60.8% (54.8% to 66.4%)	22.8% (17.2% to 28.9%)	49.6% (43.8% to 55.7%)
Diarrhoeal diseases	78.4% (63.0% to 90.1%)	84.9% (73.9% to 93.4%)	75.7% (59.4% to 88.6%)	77.2% (61.6% to 89.5%)
Lower respiratory infections	64.5% (57.6% to 71.5%)	68.9% (62.1% to 75.1%)	53.6% (46.3% to 61.7%)	68.6% (61.9% to 74.9%)
Upper respiratory infections	0.5% (0.1% to 1.3%)	1.3% (0.3% to 3.4%)	0.0% (0.0% to 0.0%)	0.0% (0.0% to 0.0%)
Otitis media	1.0% (0.5% to 1.6%)	2.4% (1.3% to 3.9%)	10.1% (7.0% to 12.9%)	10.1% (7.0% to 12.9%)
Meningitis	2.3% (1.7% to 3.0%)	3.8% (3.0% to 4.7%)	0.0% (0.0% to 0.0%)	3.0% (2.4% to 3.8%)
Encephalitis	1.0% (0.7% to 1.4%)	1.8% (1.2% to 2.5%)	0.0% (0.0% to 0.0%)	1.3% (0.9% to 1.8%)
Measles	15.2% (3.4% to 46.0%)	16.7% (3.8% to 49.5%)	21.8% (5.7% to 61.7%)	16.7% (3.8% to 49.6%)
**Maternal disorders**	4.3% (2.4% to 6.4%)	4.2% (2.3% to 6.2%)	5.3% (2.3% to 8.7%)	4.6% (2.4% to 7.1%)
Maternal haemorrhage	11.8% (4.2% to 19.4%)	11.8% (4.2% to 19.2%)	11.7% (4.2% to 18.8%)	11.7% (4.2% to 19.1%)
Maternal sepsis and other pregnancy related infections	11.8% (4.2% to 19.1%)	11.7% (4.2% to 19.0%)	11.5% (4.1% to 18.6%)	11.6% (4.2% to 18.8%)
Maternal abortion, miscarriage, and ectopic pregnancy	7.5% (4.4% to 11.3%)	7.3% (4.2% to 11.0%)	7.0% (3.5% to 11.1%)	7.2% (4.1% to 11.0%)
**Neonatal disorders**	76.0% (73.8% to 78.3%)	76.0% (73.8% to 78.3%)	0.1% (0.0% to 0.1%)	53.9% (47.5% to 60.2%)
Neonatal preterm birth complications	92.7% (90.7% to 94.4%)	92.7% (90.8% to 94.4%)	0.2% (0.2% to 0.3%)	68.3% (59.6% to 75.6%)
Neonatal encephalopathy due to birth asphyxia and trauma	69.0% (65.7% to 72.4%)	69.0% (65.8% to 72.4%)	0.0% (0.0% to 0.0%)	44.5% (34.9% to 54.5%)
Neonatal sepsis and other neonatal infections	63.0% (58.7% to 67.0%)	63.1% (58.7% to 67.1%)	0.0% (0.0% to 0.0%)	40.3% (25.8% to 52.1%)
Hemolytic disease and other neonatal jaundice	69.2% (64.6% to 73.6%)	69.2% (64.6% to 73.6%)	0.0% (0.0% to 0.0%)	23.6% (16.8% to 32.7%)

Supplementary Results Table 1. Age-standardised PAFs attributable to the joint distribution of all risk factors for all ages, both sexes combined for each GBD level 1 and level 2 cause and all causes in 2016 for Eastern Europe. Results are shown for deaths, YLLs, YLDs, and DALYs
Data in parenthesis are 95% uncertainty intervals. PAFs=population attributable fractions. GBD=Global Burden of Disease. YLLs=years of life lost. YLDs=years live with disability. DALYs=disability-adjusted life-years

	Deaths 2016	YLLs 2016	YLDs 2016	DALYs 2016
Other neonatal disorders	66.3% (62.7% to 69.8%)	66.3% (62.8% to 69.9%)	0.0% (0.0% to 0.0%)	62.1% (58.4% to 66.1%)
**Nutritional deficiencies**	10.3% (7.6% to 15.4%)	20.1% (15.0% to 27.4%)	82.3% (77.3% to 86.8%)	76.8% (70.9% to 81.6%)
Protein-energy malnutrition	14.8% (8.3% to 24.0%)	30.4% (19.9% to 44.5%)	98.3% (97.8% to 98.8%)	83.2% (74.6% to 89.9%)
Vitamin A deficiency	-	-	0.0% (0.0% to 0.0%)	0.0% (0.0% to 0.0%)
Iron-deficiency anaemia	100.0% (100.0% to 100.0%)	100.0% (100.0% to 100.0%)	100.0% (100.0% to 100.0%)	100.0% (100.0% to 100.0%)
**Other communicable, maternal, neonatal, and nutritional diseases**	10.0% (7.0% to 13.7%)	6.5% (4.2% to 9.4%)	81.2% (77.5% to 84.3%)	40.9% (32.1% to 51.6%)
Sexually transmitted diseases excluding HIV	65.3% (43.4% to 83.0%)	46.3% (24.9% to 68.5%)	100.0% (100.0% to 100.0%)	91.2% (82.2% to 96.9%)
Hepatitis	4.2% (3.2% to 5.5%)	3.6% (2.7% to 4.8%)	1.9% (1.3% to 2.7%)	3.5% (2.6% to 4.7%)
**Neoplasms**	44.7% (40.5% to 48.7%)	44.9% (40.8% to 49.0%)	38.6% (35.0% to 42.3%)	44.7% (40.6% to 48.8%)
Oesophageal cancer	86.7% (79.7% to 92.4%)	89.3% (83.2% to 94.0%)	87.3% (80.7% to 92.6%)	89.3% (83.2% to 93.9%)
Stomach cancer	20.9% (11.0% to 32.0%)	22.8% (12.7% to 34.1%)	20.1% (11.2% to 29.8%)	22.8% (12.6% to 34.1%)
Liver cancer	68.7% (60.9% to 75.5%)	67.9% (59.7% to 75.1%)	68.1% (60.5% to 74.7%)	67.9% (59.7% to 75.1%)
Larynx cancer	91.1% (86.2% to 94.7%)	92.4% (87.9% to 95.6%)	91.2% (86.2% to 94.8%)	92.4% (87.9% to 95.5%)
Tracheal, bronchus, and lung cancer	86.0% (82.0% to 89.3%)	87.5% (83.9% to 90.6%)	84.9% (81.1% to 88.3%)	87.5% (83.9% to 90.5%)
Breast cancer	30.5% (23.5% to 38.0%)	30.3% (23.2% to 37.6%)	30.0% (23.2% to 37.2%)	30.3% (23.3% to 37.5%)
Cervical cancer	100.0% (100.0% to 100.0%)	100.0% (100.0% to 100.0%)	100.0% (100.0% to 100.0%)	100.0% (100.0% to 100.0%)
Uterine cancer	54.3% (39.1% to 67.7%)	54.9% (39.8% to 68.1%)	55.4% (40.3% to 68.5%)	54.9% (39.9% to 68.1%)
Prostate cancer	7.1% (4.7% to 10.0%)	8.5% (5.7% to 11.9%)	8.5% (5.7% to 11.7%)	8.5% (5.7% to 11.9%)
Colon and rectum cancer	53.6% (45.0% to 61.3%)	55.6% (47.2% to 63.3%)	54.4% (46.0% to 62.3%)	55.6% (47.2% to 63.3%)
Lip and oral cavity cancer	84.1% (78.5% to 88.8%)	87.8% (83.2% to 91.7%)	80.8% (74.7% to 85.9%)	87.6% (83.0% to 91.5%)
Nasopharynx cancer	84.7% (80.4% to 88.3%)	84.4% (80.6% to 87.6%)	84.2% (80.8% to 87.0%)	84.4% (80.6% to 87.6%)

Supplementary Results Table 1. Age-standardised PAFs attributable to the joint distribution of all risk factors for all ages, both sexes combined for each GBD level 1 and level 2 cause and all causes in 2016 for Eastern Europe. Results are shown for deaths, YLLs, YLDs, and DALYs
Data in parenthesis are 95% uncertainty intervals. PAFs=population attributable fractions. GBD=Global Burden of Disease. YLLs=years of life lost. YLDs=years live with disability. DALYs=disability-adjusted life-years

	Deaths 2016	YLLs 2016	YLDs 2016	DALYs 2016
Other pharynx cancer	73.4% (63.5% to 81.0%)	75.4% (66.2% to 82.5%)	73.6% (63.5% to 81.2%)	75.3% (66.1% to 82.5%)
Gallbladder and biliary tract cancer	26.9% (15.9% to 39.7%)	26.6% (15.6% to 39.4%)	27.6% (16.1% to 40.9%)	26.7% (15.6% to 39.4%)
Pancreatic cancer	32.0% (23.4% to 41.1%)	34.9% (26.0% to 44.3%)	32.1% (24.4% to 40.3%)	34.9% (26.0% to 44.2%)
Ovarian cancer	12.4% (5.5% to 20.2%)	11.0% (4.5% to 18.4%)	10.9% (4.5% to 18.0%)	11.0% (4.5% to 18.4%)
Kidney cancer	42.4% (31.1% to 53.1%)	43.2% (31.6% to 53.7%)	41.8% (31.2% to 52.2%)	43.1% (31.6% to 53.6%)
Bladder cancer	37.3% (26.6% to 48.3%)	42.3% (30.9% to 53.6%)	38.8% (28.8% to 48.2%)	42.1% (30.8% to 53.3%)
Thyroid cancer	16.8% (9.1% to 26.4%)	17.0% (9.1% to 26.9%)	16.3% (9.1% to 25.1%)	16.9% (9.1% to 26.8%)
Mesothelioma	89.5% (85.6% to 92.5%)	82.5% (76.9% to 87.0%)	87.1% (83.1% to 90.5%)	82.6% (77.0% to 87.1%)
Non-Hodgkin's lymphoma	7.7% (3.2% to 13.1%)	6.9% (2.8% to 11.9%)	7.6% (3.1% to 13.1%)	6.9% (2.8% to 11.9%)
Multiple myeloma	10.1% (4.3% to 17.7%)	10.1% (4.4% to 17.5%)	10.3% (4.4% to 17.8%)	10.1% (4.4% to 17.5%)
Leukaemia	22.1% (14.0% to 30.8%)	18.4% (11.8% to 25.6%)	18.7% (11.8% to 25.8%)	18.4% (11.8% to 25.6%)
**Cardiovascular diseases**	90.3% (88.1% to 92.4%)	92.1% (90.7% to 93.4%)	86.7% (84.3% to 89.0%)	91.8% (90.3% to 93.1%)
Rheumatic heart disease	34.0% (23.5% to 49.0%)	33.7% (23.0% to 47.1%)	32.6% (21.8% to 47.2%)	33.7% (23.0% to 47.1%)
Ischaemic heart disease	94.4% (91.4% to 96.8%)	96.0% (94.2% to 97.4%)	93.3% (91.1% to 95.2%)	95.9% (94.1% to 97.4%)
Cerebrovascular disease	86.0% (80.9% to 90.8%)	90.7% (87.5% to 93.5%)	89.0% (85.3% to 92.2%)	90.4% (87.3% to 93.3%)
Hypertensive heart disease	100.0% (100.0% to 100.0%)	100.0% (100.0% to 100.0%)	100.0% (100.0% to 100.0%)	100.0% (100.0% to 100.0%)
Cardiomyopathy and myocarditis	82.9% (78.1% to 89.4%)	85.3% (81.9% to 89.7%)	71.9% (67.5% to 75.9%)	85.0% (81.6% to 89.4%)
Atrial fibrillation and flutter	55.8% (47.9% to 63.6%)	61.0% (53.9% to 67.6%)	69.0% (64.0% to 73.8%)	65.7% (59.7% to 71.1%)
Aortic aneurysm	56.3% (49.7% to 62.0%)	62.0% (56.2% to 67.0%)	-	62.0% (56.2% to 67.0%)
Peripheral vascular disease	55.5% (47.7% to 62.7%)	63.7% (57.1% to 69.4%)	60.6% (54.8% to 66.1%)	63.2% (57.1% to 68.7%)
Endocarditis	46.9% (38.0% to 56.1%)	45.9% (35.3% to 56.3%)	44.5% (36.2% to 53.0%)	45.9% (35.3% to 56.2%)

Supplementary Results Table 1. Age-standardised PAFs attributable to the joint distribution of all risk factors for all ages, both sexes combined for each GBD level 1 and level 2 cause and all causes in 2016 for Eastern Europe. Results are shown for deaths, YLLs, YLDs, and DALYs
Data in parenthesis are 95% uncertainty intervals. PAFs=population attributable fractions. GBD=Global Burden of Disease. YLLs=years of life lost. YLDs=years live with disability. DALYs=disability-adjusted life-years

	**Deaths 2016**	**YLLs 2016**	**YLDs 2016**	**DALYs 2016**
Other cardiovascular and circulatory diseases	57.9% (53.2% to 62.5%)	62.6% (57.8% to 67.0%)	56.3% (51.6% to 60.8%)	61.8% (57.1% to 66.1%)
**Chronic respiratory diseases**	65.8% (58.8% to 71.5%)	69.1% (63.1% to 74.0%)	40.8% (34.1% to 47.6%)	56.6% (50.6% to 62.6%)
Chronic obstructive pulmonary disease	69.5% (61.8% to 75.9%)	74.8% (68.3% to 80.1%)	63.8% (54.8% to 72.0%)	73.0% (66.4% to 78.7%)
Pneumoconiosis	100.0% (100.0% to 100.0%)	100.0% (100.0% to 100.0%)	100.0% (100.0% to 100.0%)	100.0% (100.0% to 100.0%)
Asthma	48.0% (37.7% to 58.9%)	50.6% (41.4% to 60.4%)	36.2% (28.5% to 44.9%)	38.1% (30.4% to 46.9%)
Other chronic respiratory diseases	20.5% (13.4% to 28.4%)	20.8% (13.5% to 28.7%)	6.1% (3.8% to 9.2%)	15.5% (9.7% to 22.6%)
**Cirrhosis and other chronic liver diseases**	58.2% (55.6% to 60.7%)	57.3% (54.6% to 60.0%)	44.5% (41.6% to 47.4%)	56.9% (54.3% to 59.6%)
Cirrhosis and other chronic liver diseases due to hepatitis B	2.6% (1.5% to 4.0%)	2.9% (1.7% to 4.5%)	2.6% (1.5% to 3.9%)	2.9% (1.7% to 4.5%)
Cirrhosis and other chronic liver diseases due to hepatitis C	89.9% (89.8% to 90.0%)	89.9% (89.9% to 90.0%)	89.0% (88.7% to 89.2%)	89.9% (89.9% to 90.0%)
Cirrhosis and other chronic liver diseases due to alcohol use	100.0% (100.0% to 100.0%)	100.0% (100.0% to 100.0%)	100.0% (100.0% to 100.0%)	100.0% (100.0% to 100.0%)
**Digestive diseases**	25.6% (21.1% to 30.4%)	33.8% (28.0% to 39.6%)	5.8% (4.6% to 7.0%)	27.2% (21.8% to 32.7%)
Peptic ulcer disease	22.4% (16.1% to 29.2%)	26.5% (19.5% to 33.9%)	15.6% (11.1% to 20.7%)	24.1% (17.7% to 31.0%)
Gallbladder and biliary diseases	49.8% (35.5% to 63.8%)	50.2% (36.1% to 63.9%)	52.0% (38.3% to 65.6%)	50.6% (36.8% to 64.1%)
Pancreatitis	60.5% (47.9% to 71.8%)	66.4% (53.6% to 77.2%)	45.7% (34.2% to 56.6%)	66.1% (53.4% to 76.9%)
**Neurological disorders**	21.0% (9.9% to 33.9%)	20.1% (11.5% to 30.3%)	3.6% (2.0% to 5.6%)	8.4% (4.7% to 12.8%)
Alzheimer's disease and other dementias	25.7% (12.1% to 41.7%)	27.0% (13.6% to 42.6%)	27.0% (13.2% to 42.9%)	27.0% (13.5% to 42.6%)
Parkinson's disease	-10.1% (-13.2% to -7.3%)	-13.8% (-18.3% to -10.0%)	-15.7% (-19.7% to -11.7%)	-14.2% (-18.5% to -10.5%)
Epilepsy	44.4% (34.3% to 53.7%)	42.7% (33.0% to 51.8%)	30.9% (22.8% to 38.7%)	35.8% (27.2% to 44.5%)
Multiple sclerosis	13.7% (8.8% to 18.9%)	14.1% (9.1% to 19.4%)	11.1% (7.4% to 15.0%)	12.9% (8.5% to 17.5%)
**Mental and substance use disorders**	100.0% (100.0% to 100.0%)	100.0% (100.0% to 100.0%)	34.4% (31.5% to 37.7%)	54.8% (49.2% to 61.5%)
Alcohol use disorders	100.0% (100.0% to 100.0%)	100.0% (100.0% to 100.0%)	100.0% (100.0% to 100.0%)	100.0% (100.0% to 100.0%)

Supplementary Results Table 1. Age-standardised PAFs attributable to the joint distribution of all risk factors for all ages, both sexes combined for each GBD level 1 and level 2 cause and all causes in 2016 for Eastern Europe. Results are shown for deaths, YLLs, YLDs, and DALYs
Data in parenthesis are 95% uncertainty intervals. PAFs=population attributable fractions. GBD=Global Burden of Disease. YLLs=years of life lost. YLDs=years live with disability. DALYs=disability-adjusted life-years

	Deaths 2016	YLLs 2016	YLDs 2016	DALYs 2016
Drug use disorders	100.0% (100.0% to 100.0%)	100.0% (100.0% to 100.0%)	100.0% (100.0% to 100.0%)	100.0% (100.0% to 100.0%)
Depressive disorders	-	-	10.7% (9.2% to 12.4%)	10.7% (9.2% to 12.4%)
Idiopathic developmental intellectual disability	-	-	57.2% (27.8% to 77.9%)	57.2% (27.8% to 77.9%)
**Diabetes, urogenital, blood, and endocrine diseases**	68.2% (67.0% to 69.5%)	64.9% (63.5% to 67.1%)	50.7% (46.6% to 54.8%)	55.5% (52.2% to 58.4%)
Diabetes mellitus	100.0% (100.0% to 100.0%)	100.0% (100.0% to 100.0%)	100.0% (100.0% to 100.0%)	100.0% (100.0% to 100.0%)
Chronic kidney disease	100.0% (100.0% to 100.0%)	100.0% (100.0% to 100.0%)	100.0% (100.0% to 100.0%)	100.0% (100.0% to 100.0%)
**Musculoskeletal disorders**	1.9% (0.7% to 3.4%)	2.0% (0.8% to 3.4%)	23.2% (19.1% to 27.5%)	23.0% (18.8% to 27.3%)
Rheumatoid arthritis	4.7% (1.8% to 7.9%)	5.9% (2.3% to 9.8%)	6.1% (2.5% to 10.0%)	6.1% (2.5% to 10.0%)
Osteoarthritis	-	-	31.7% (17.5% to 45.2%)	31.7% (17.5% to 45.2%)
Low back and neck pain	-	-	23.4% (20.0% to 27.1%)	23.4% (20.0% to 27.1%)
Gout	-	-	50.7% (35.2% to 66.2%)	50.7% (35.2% to 66.2%)
**Other non-communicable diseases**	0.3% (0.2% to 0.5%)	0.4% (0.3% to 0.5%)	3.6% (3.2% to 4.2%)	3.0% (2.7% to 3.5%)
Sense organ diseases	-	-	8.5% (7.6% to 9.5%)	8.5% (7.6% to 9.5%)
Sudden infant death syndrome	5.1% (3.7% to 6.9%)	5.2% (3.7% to 6.9%)	-	5.2% (3.7% to 6.9%)
**Transport injuries**	38.9% (29.2% to 49.8%)	36.8% (26.9% to 47.5%)	39.9% (31.7% to 49.1%)	37.4% (27.9% to 47.9%)
Road injuries	38.6% (29.0% to 49.6%)	36.6% (26.8% to 47.3%)	40.3% (32.1% to 49.5%)	37.3% (27.9% to 47.7%)
Other transport injuries	40.8% (30.8% to 51.8%)	38.4% (28.2% to 49.3%)	33.7% (25.8% to 42.8%)	37.7% (28.1% to 48.4%)
**Unintentional injuries**	17.1% (12.8% to 21.5%)	14.8% (10.6% to 19.3%)	17.5% (15.2% to 20.2%)	15.9% (12.7% to 19.4%)
Falls	29.6% (25.3% to 34.1%)	18.9% (15.6% to 23.1%)	20.8% (18.8% to 23.5%)	20.2% (17.8% to 23.3%)
Drowning	22.5% (14.0% to 32.3%)	21.1% (13.2% to 30.0%)	20.1% (11.9% to 29.4%)	21.1% (13.2% to 29.9%)
Fire, heat, and hot substances	20.6% (12.7% to 29.5%)	21.2% (13.3% to 30.0%)	19.2% (11.9% to 27.5%)	20.6% (13.0% to 29.2%)

Supplementary Results Table 1. Age-standardised PAFs attributable to the joint distribution of all risk factors for all ages, both sexes combined for each GBD level 1 and level 2 cause and all causes in 2016 for Eastern Europe. Results are shown for deaths, YLLs, YLDs, and DALYs

Data in parenthesis are 95% uncertainty intervals. PAFs=population attributable fractions. GBD=Global Burden of Disease. YLLs=years of life lost. YLDs=years live with disability. DALYs=disability-adjusted life-years

	**Deaths 2016**	**YLLs 2016**	**YLDs 2016**	**DALYs 2016**
Poisonings	20.1% (12.5% to 28.7%)	17.9% (11.3% to 25.3%)	18.1% (11.2% to 25.7%)	17.9% (11.3% to 25.3%)
Exposure to mechanical forces	15.0% (11.4% to 19.5%)	13.2% (9.6% to 17.6%)	15.1% (12.5% to 18.7%)	14.1% (11.0% to 17.8%)
Animal contact	10.6% (8.4% to 13.4%)	10.0% (7.6% to 12.9%)	7.1% (5.3% to 9.8%)	8.2% (6.2% to 10.7%)
Foreign body	8.1% (5.8% to 11.0%)	8.1% (5.9% to 10.9%)	6.0% (4.2% to 8.6%)	7.7% (5.6% to 10.3%)
Other unintentional injuries	24.2% (15.4% to 34.3%)	24.0% (15.5% to 33.7%)	17.3% (10.6% to 24.9%)	20.6% (13.1% to 28.8%)
**Self-harm and interpersonal violence**	42.4% (32.6% to 51.0%)	42.9% (33.2% to 51.4%)	33.7% (26.6% to 40.4%)	42.5% (33.0% to 50.7%)
Self-harm	46.0% (32.5% to 57.7%)	47.5% (34.2% to 58.9%)	37.9% (25.5% to 48.7%)	47.4% (34.2% to 58.8%)
Interpersonal violence	34.3% (24.2% to 45.4%)	33.2% (23.1% to 44.4%)	32.9% (25.5% to 41.0%)	33.2% (23.4% to 44.1%)
**Forces of nature, conflict and terrorism, and executions and police conflict**	0.0% (0.0% to 0.0%)	0.0% (0.0% to 0.0%)	0.8% (0.5% to 1.2%)	0.5% (0.2% to 0.9%)
Forces of nature, conflict and terrorism, and state actor violence	0.0% (0.0% to 0.0%)	0.0% (0.0% to 0.0%)	3.8% (2.5% to 5.7%)	3.8% (2.5% to 5.7%)

Supplementary Results Table 1. Age-standardised PAFs attributable to the joint distribution of all risk factors for all ages, both sexes combined for each GBD level 1 and level 2 cause and all causes in 2016 for Eastern Sub-Saharan Africa. Results are shown for deaths, YLLs, YLDs, and
Data in parenthesis are 95% uncertainty intervals. PAFs=population attributable fractions. GBD=Global Burden of Disease. YLLs=years of life lost. YLDs=years live with disability. DALYs=disability-adjusted life-years

	Deaths 2016	YLLs 2016	YLDs 2016	DALYs 2016
**All causes**	57.5% (55.6% to 59.6%)	55.9% (54.3% to 57.9%)	29.6% (28.4% to 30.9%)	50.0% (48.2% to 52.2%)
**HIV/AIDS and tuberculosis**	61.0% (56.3% to 65.4%)	66.1% (62.6% to 69.5%)	83.5% (80.4% to 86.3%)	67.1% (63.8% to 70.4%)
Tuberculosis	34.7% (27.2% to 41.9%)	36.1% (28.9% to 43.0%)	32.5% (25.6% to 39.7%)	36.0% (28.8% to 42.9%)
HIV/AIDS	89.4% (87.7% to 91.0%)	84.9% (82.9% to 86.8%)	91.7% (89.9% to 93.2%)	85.4% (83.5% to 87.3%)
**Diarrhoea, lower respiratory infections, and other common infectious diseases**	76.4% (70.7% to 81.6%)	73.1% (68.2% to 78.1%)	47.0% (41.7% to 51.7%)	71.8% (67.2% to 76.7%)
Diarrhoeal diseases	95.1% (87.4% to 98.8%)	96.0% (89.3% to 99.0%)	95.4% (88.3% to 98.8%)	96.0% (89.2% to 99.0%)
Lower respiratory infections	78.7% (72.7% to 83.7%)	84.6% (80.2% to 88.5%)	78.7% (72.8% to 83.9%)	84.6% (80.2% to 88.4%)
Upper respiratory infections	1.3% (0.7% to 2.2%)	2.4% (1.2% to 4.2%)	0.0% (0.0% to 0.0%)	0.2% (0.1% to 0.3%)
Otitis media	2.2% (1.4% to 3.4%)	5.6% (3.7% to 7.7%)	3.3% (2.2% to 4.5%)	3.5% (2.3% to 4.7%)
Meningitis	2.0% (1.5% to 2.6%)	4.2% (3.2% to 5.2%)	0.0% (0.0% to 0.0%)	3.9% (3.0% to 4.9%)
Encephalitis	4.0% (2.6% to 5.8%)	5.1% (3.4% to 7.4%)	0.0% (0.0% to 0.0%)	3.2% (2.1% to 4.8%)
Measles	65.6% (52.5% to 82.1%)	66.6% (53.4% to 83.3%)	64.6% (51.7% to 81.0%)	66.6% (53.3% to 83.3%)
**Maternal disorders**	7.5% (3.8% to 11.6%)	7.5% (3.8% to 11.4%)	2.0% (1.0% to 3.2%)	7.1% (3.6% to 10.9%)
Maternal haemorrhage	17.3% (6.2% to 28.5%)	17.4% (6.2% to 28.5%)	17.3% (6.1% to 28.2%)	17.4% (6.2% to 28.5%)
Maternal sepsis and other pregnancy related infections	17.5% (6.3% to 28.7%)	17.6% (6.3% to 28.8%)	16.7% (5.9% to 27.5%)	17.6% (6.3% to 28.8%)
Maternal abortion, miscarriage, and ectopic pregnancy	19.6% (12.5% to 27.0%)	19.3% (12.2% to 26.5%)	19.5% (11.5% to 27.6%)	19.3% (12.2% to 26.5%)
**Neonatal disorders**	78.3% (75.9% to 80.9%)	78.4% (76.0% to 80.9%)	0.3% (0.2% to 0.6%)	74.2% (70.7% to 77.4%)
Neonatal preterm birth complications	94.7% (92.8% to 96.4%)	94.8% (92.8% to 96.4%)	1.0% (0.8% to 1.3%)	89.3% (86.5% to 91.9%)
Neonatal encephalopathy due to birth asphyxia and trauma	77.1% (74.1% to 80.3%)	77.2% (74.2% to 80.3%)	0.0% (0.0% to 0.0%)	73.8% (68.1% to 78.4%)
Neonatal sepsis and other neonatal infections	65.0% (61.2% to 68.9%)	65.1% (61.2% to 68.9%)	0.0% (0.0% to 0.0%)	59.5% (50.9% to 66.0%)
Hemolytic disease and other neonatal jaundice	70.7% (66.6% to 74.5%)	70.7% (66.7% to 74.6%)	0.0% (0.0% to 0.0%)	63.7% (59.1% to 68.2%)

**Supplementary Results Table 1. Age-standardised PAFs attributable to the joint distribution of all risk factors for all ages, both sexes combined for each GBD level 1 and level 2 cause and all causes in 2016 for Eastern Sub-Saharan Africa. Results are shown for deaths, YLLs, YLDs, and** Data in parenthesis are 95% uncertainty intervals. PAFs=population attributable fractions. GBD=Global Burden of Disease. YLLs=years of life lost. YLDs=years live with disability. DALYs=disability-adjusted life-years	**Deaths 2016**	**YLLs 2016**	**YLDs 2016**	**DALYs 2016**
Other neonatal disorders	70.1% (66.3% to 73.9%)	70.1% (66.3% to 74.0%)	0.0% (0.0% to 0.0%)	68.7% (65.2% to 72.3%)
**Nutritional deficiencies**	23.0% (19.8% to 27.0%)	56.4% (51.8% to 61.8%)	87.3% (85.1% to 89.2%)	66.7% (62.8% to 71.2%)
Protein-energy malnutrition	27.2% (23.1% to 33.4%)	62.3% (57.2% to 68.5%)	97.9% (97.7% to 98.2%)	63.4% (58.3% to 69.4%)
Vitamin A deficiency	-	-	99.9% (99.9% to 99.9%)	99.9% (99.9% to 99.9%)
Iron-deficiency anaemia	100.0% (100.0% to 100.0%)	100.0% (100.0% to 100.0%)	99.9% (99.9% to 99.9%)	99.9% (99.9% to 99.9%)
**Other communicable, maternal, neonatal, and nutritional diseases**	5.6% (4.2% to 7.0%)	3.4% (2.3% to 4.6%)	73.0% (68.2% to 77.4%)	11.0% (7.4% to 16.0%)
Sexually transmitted diseases excluding HIV	12.8% (7.7% to 20.6%)	5.6% (3.2% to 9.4%)	99.9% (99.9% to 99.9%)	18.0% (10.5% to 29.4%)
Hepatitis	0.6% (0.5% to 0.8%)	0.7% (0.5% to 1.0%)	0.6% (0.4% to 1.0%)	0.7% (0.5% to 1.0%)
**Neoplasms**	30.5% (27.4% to 34.5%)	30.2% (27.1% to 34.2%)	32.3% (28.8% to 37.2%)	30.2% (27.1% to 34.2%)
Oesophageal cancer	55.7% (43.6% to 66.2%)	57.7% (45.9% to 67.8%)	56.3% (44.2% to 66.7%)	57.7% (45.9% to 67.8%)
Stomach cancer	4.5% (1.1% to 12.3%)	4.4% (1.2% to 11.5%)	4.3% (1.1% to 11.8%)	4.4% (1.2% to 11.5%)
Liver cancer	27.4% (22.3% to 33.9%)	27.8% (22.8% to 34.1%)	27.6% (22.5% to 34.1%)	27.8% (22.8% to 34.1%)
Larynx cancer	47.7% (36.3% to 58.4%)	50.1% (38.7% to 61.0%)	47.6% (36.6% to 58.2%)	50.1% (38.7% to 61.0%)
Tracheal, bronchus, and lung cancer	78.3% (72.5% to 83.6%)	78.0% (72.1% to 83.4%)	77.7% (71.9% to 83.0%)	78.0% (72.0% to 83.4%)
Breast cancer	18.3% (13.0% to 25.1%)	17.5% (12.5% to 23.6%)	17.8% (12.7% to 24.1%)	17.6% (12.5% to 23.6%)
Cervical cancer	100.0% (100.0% to 100.0%)	100.0% (100.0% to 100.0%)	99.9% (99.9% to 99.9%)	100.0% (100.0% to 100.0%)
Uterine cancer	22.5% (13.3% to 33.0%)	23.8% (14.5% to 34.3%)	23.6% (14.3% to 34.1%)	23.8% (14.5% to 34.3%)
Prostate cancer	0.7% (0.4% to 1.1%)	0.8% (0.5% to 1.2%)	0.8% (0.5% to 1.1%)	0.8% (0.5% to 1.2%)
Colon and rectum cancer	45.0% (36.2% to 54.1%)	45.0% (36.7% to 53.5%)	45.0% (36.5% to 54.0%)	45.0% (36.7% to 53.5%)
Lip and oral cavity cancer	52.7% (43.9% to 61.0%)	54.1% (45.2% to 62.0%)	52.9% (44.2% to 60.6%)	54.0% (45.2% to 61.9%)
Nasopharynx cancer	49.2% (41.4% to 57.1%)	50.6% (43.3% to 57.8%)	50.0% (42.8% to 56.9%)	50.6% (43.2% to 57.8%)

Supplementary Results Table 1. Age-standardised PAFs attributable to the joint distribution of all risk factors for all ages, both sexes combined for each GBD level 1 and level 2 cause and all causes in 2016 for Eastern Sub-Saharan Africa. Results are shown for deaths, YLLs, YLDs, and
Data in parenthesis are 95% uncertainty intervals. PAFs=population attributable fractions. GBD=Global Burden of Disease. YLLs=years of life lost. YLDs=years live with disability. DALYs=disability-adjusted life-years

	Deaths 2016	YLLs 2016	YLDs 2016	DALYs 2016
Other pharynx cancer	43.1% (31.5% to 53.9%)	45.1% (33.7% to 55.7%)	43.7% (32.4% to 54.6%)	45.1% (33.7% to 55.7%)
Gallbladder and biliary tract cancer	9.3% (4.3% to 16.4%)	10.1% (4.9% to 17.6%)	9.7% (4.6% to 16.9%)	10.1% (4.9% to 17.6%)
Pancreatic cancer	10.7% (5.6% to 17.7%)	10.5% (5.7% to 17.0%)	10.5% (5.5% to 17.3%)	10.5% (5.7% to 17.0%)
Ovarian cancer	7.4% (3.0% to 13.8%)	6.5% (2.6% to 12.0%)	6.5% (2.6% to 12.0%)	6.5% (2.6% to 12.0%)
Kidney cancer	10.4% (6.1% to 16.3%)	9.0% (5.3% to 14.1%)	9.7% (5.7% to 15.3%)	9.1% (5.3% to 14.1%)
Bladder cancer	9.9% (5.1% to 17.0%)	9.5% (4.8% to 16.0%)	9.5% (4.8% to 16.3%)	9.5% (4.8% to 16.0%)
Thyroid cancer	5.5% (2.5% to 9.9%)	5.7% (2.7% to 10.0%)	5.7% (2.7% to 10.2%)	5.7% (2.7% to 10.0%)
Mesothelioma	85.7% (79.0% to 89.8%)	76.9% (68.2% to 82.6%)	82.2% (75.0% to 86.9%)	76.9% (68.4% to 82.7%)
Non-Hodgkin's lymphoma	2.2% (0.7% to 4.4%)	1.8% (0.6% to 3.6%)	2.1% (0.7% to 4.2%)	1.8% (0.6% to 3.6%)
Multiple myeloma	3.2% (1.2% to 6.4%)	3.3% (1.2% to 6.5%)	3.3% (1.2% to 6.5%)	3.3% (1.2% to 6.5%)
Leukaemia	5.8% (3.3% to 8.9%)	5.6% (3.3% to 8.4%)	5.7% (3.3% to 8.8%)	5.6% (3.3% to 8.4%)
**Cardiovascular diseases**	82.5% (80.0% to 84.7%)	83.5% (81.3% to 85.3%)	61.9% (57.8% to 65.9%)	82.0% (79.9% to 83.8%)
Rheumatic heart disease	29.4% (20.2% to 44.7%)	28.2% (19.4% to 42.4%)	23.0% (14.8% to 32.8%)	26.1% (18.1% to 37.5%)
Ischaemic heart disease	92.7% (89.6% to 95.2%)	93.8% (91.9% to 95.5%)	89.0% (86.2% to 91.8%)	93.7% (91.7% to 95.3%)
Cerebrovascular disease	87.9% (84.6% to 90.8%)	90.2% (87.9% to 92.2%)	87.0% (84.0% to 89.9%)	90.1% (87.8% to 92.1%)
Hypertensive heart disease	100.0% (100.0% to 100.0%)	100.0% (100.0% to 100.0%)	99.9% (99.9% to 99.9%)	100.0% (100.0% to 100.0%)
Cardiomyopathy and myocarditis	41.2% (33.6% to 49.9%)	39.5% (32.5% to 46.9%)	41.4% (35.0% to 48.0%)	39.7% (32.8% to 47.0%)
Atrial fibrillation and flutter	45.1% (37.4% to 52.2%)	49.4% (42.6% to 55.4%)	55.7% (50.8% to 60.3%)	52.5% (46.4% to 57.8%)
Aortic aneurysm	43.8% (36.8% to 50.6%)	48.2% (42.2% to 53.7%)	-	48.2% (42.2% to 53.7%)
Peripheral vascular disease	51.7% (45.1% to 58.8%)	58.7% (53.4% to 64.1%)	59.2% (53.8% to 64.7%)	59.1% (54.0% to 64.1%)
Endocarditis	39.1% (30.7% to 48.9%)	37.5% (29.7% to 45.3%)	33.2% (25.2% to 42.6%)	37.5% (29.7% to 45.3%)

Supplementary Results Table 1. Age-standardised PAFs attributable to the joint distribution of all risk factors for all ages, both sexes combined for each GBD level 1 and level 2 cause and all causes in 2016 for Eastern Sub-Saharan Africa. Results are shown for deaths, YLLs, YLDs, and
Data in parenthesis are 95% uncertainty intervals. PAFs=population attributable fractions. GBD=Global Burden of Disease. YLLs=years of life lost. YLDs=years live with disability. DALYs=disability-adjusted life-years

	Deaths 2016	YLLs 2016	YLDs 2016	DALYs 2016
Other cardiovascular and circulatory diseases	45.3% (40.2% to 50.5%)	48.0% (43.8% to 52.1%)	37.1% (31.4% to 42.9%)	46.7% (42.4% to 50.7%)
**Chronic respiratory diseases**	60.7% (52.0% to 68.5%)	56.9% (49.2% to 64.1%)	38.6% (32.8% to 45.4%)	51.4% (45.0% to 57.7%)
Chronic obstructive pulmonary disease	74.2% (65.8% to 83.9%)	73.3% (64.9% to 82.7%)	62.6% (51.9% to 73.6%)	71.1% (62.6% to 80.3%)
Pneumoconiosis	100.0% (100.0% to 100.0%)	100.0% (100.0% to 100.0%)	99.9% (99.9% to 99.9%)	100.0% (100.0% to 100.0%)
Asthma	26.8% (21.1% to 33.9%)	29.6% (24.1% to 35.8%)	23.7% (18.9% to 29.6%)	27.0% (22.1% to 33.0%)
Other chronic respiratory diseases	2.9% (1.5% to 4.9%)	2.8% (1.0% to 5.7%)	1.5% (0.4% to 3.1%)	2.4% (0.9% to 4.9%)
**Cirrhosis and other chronic liver diseases**	31.6% (28.4% to 34.7%)	31.4% (28.3% to 34.6%)	25.2% (22.7% to 27.8%)	31.2% (28.1% to 34.4%)
Cirrhosis and other chronic liver diseases due to hepatitis B	0.2% (0.2% to 0.4%)	0.3% (0.2% to 0.4%)	0.3% (0.2% to 0.4%)	0.3% (0.2% to 0.4%)
Cirrhosis and other chronic liver diseases due to hepatitis C	10.0% (6.7% to 14.1%)	15.2% (10.6% to 20.8%)	17.8% (13.0% to 23.4%)	15.3% (10.7% to 20.9%)
Cirrhosis and other chronic liver diseases due to alcohol use	100.0% (100.0% to 100.0%)	100.0% (100.0% to 100.0%)	99.9% (99.9% to 99.9%)	100.0% (100.0% to 100.0%)
**Digestive diseases**	3.7% (2.7% to 5.0%)	3.8% (2.9% to 5.0%)	1.6% (1.1% to 2.1%)	3.7% (2.8% to 4.8%)
Peptic ulcer disease	6.6% (4.5% to 9.1%)	7.5% (5.1% to 10.3%)	6.1% (4.2% to 8.4%)	7.4% (5.1% to 10.1%)
Gallbladder and biliary diseases	18.9% (10.2% to 30.1%)	20.3% (11.6% to 31.2%)	22.1% (13.3% to 33.1%)	20.3% (11.7% to 31.3%)
Pancreatitis	24.3% (14.0% to 34.9%)	27.8% (17.4% to 38.3%)	25.1% (15.9% to 35.0%)	27.8% (17.4% to 38.2%)
**Neurological disorders**	12.2% (5.9% to 20.5%)	13.1% (7.9% to 19.6%)	3.9% (2.5% to 5.4%)	7.5% (4.8% to 11.0%)
Alzheimer's disease and other dementias	13.0% (5.9% to 22.6%)	13.8% (6.4% to 23.6%)	13.8% (6.3% to 23.4%)	13.8% (6.4% to 23.5%)
Parkinson's disease	-5.9% (-7.1% to -4.6%)	-6.9% (-8.4% to -5.4%)	-7.4% (-8.9% to -5.8%)	-7.0% (-8.5% to -5.5%)
Epilepsy	17.4% (11.8% to 22.8%)	17.6% (12.3% to 22.9%)	14.4% (9.7% to 19.6%)	15.9% (10.9% to 20.8%)
Multiple sclerosis	4.6% (2.2% to 7.4%)	4.6% (2.1% to 7.6%)	3.4% (2.0% to 4.9%)	4.2% (2.1% to 6.6%)
**Mental and substance use disorders**	99.9% (99.7% to 99.9%)	99.7% (99.5% to 99.9%)	19.7% (17.4% to 22.0%)	22.9% (20.5% to 25.4%)
Alcohol use disorders	100.0% (100.0% to 100.0%)	100.0% (100.0% to 100.0%)	99.9% (99.9% to 99.9%)	100.0% (99.9% to 100.0%)

Supplementary Results Table 1. Age-standardised PAFs attributable to the joint distribution of all risk factors for all ages, both sexes combined for each GBD level 1 and level 2 cause and all causes in 2016 for Eastern Sub-Saharan Africa. Results are shown for deaths, YLLs, YLDs, and
Data in parenthesis are 95% uncertainty intervals. PAFs=population attributable fractions. GBD=Global Burden of Disease. YLLs=years of life lost. YLDs=years live with disability. DALYs=disability-adjusted life-years

	Deaths 2016	YLLs 2016	YLDs 2016	DALYs 2016
Drug use disorders	100.0% (100.0% to 100.0%)	100.0% (100.0% to 100.0%)	99.9% (99.9% to 99.9%)	99.9% (99.9% to 99.9%)
Depressive disorders	-	-	10.4% (8.7% to 12.3%)	10.4% (8.7% to 12.3%)
Idiopathic developmental intellectual disability	-	-	60.7% (30.5% to 80.2%)	60.7% (30.5% to 80.2%)
**Diabetes, urogenital, blood, and endocrine diseases**	79.8% (78.2% to 81.3%)	72.6% (69.6% to 74.6%)	61.7% (57.3% to 65.7%)	68.4% (65.6% to 70.6%)
Diabetes mellitus	100.0% (100.0% to 100.0%)	100.0% (100.0% to 100.0%)	99.9% (99.9% to 99.9%)	100.0% (100.0% to 100.0%)
Chronic kidney disease	100.0% (100.0% to 100.0%)	100.0% (100.0% to 100.0%)	99.9% (99.9% to 99.9%)	100.0% (100.0% to 100.0%)
**Musculoskeletal disorders**	0.9% (0.3% to 1.6%)	0.7% (0.3% to 1.2%)	27.9% (25.0% to 31.0%)	27.3% (24.6% to 30.4%)
Rheumatoid arthritis	2.1% (0.8% to 3.7%)	2.2% (0.8% to 3.8%)	2.3% (0.9% to 3.9%)	2.3% (0.9% to 3.9%)
Osteoarthritis	-	-	11.7% (5.3% to 20.1%)	11.7% (5.3% to 20.1%)
Low back and neck pain	-	-	36.2% (33.2% to 39.4%)	36.2% (33.2% to 39.4%)
Gout	-	-	36.4% (29.3% to 46.0%)	36.4% (29.3% to 46.0%)
**Other non-communicable diseases**	0.3% (0.1% to 0.6%)	0.6% (0.2% to 1.0%)	10.1% (8.6% to 11.6%)	8.5% (7.3% to 9.8%)
Sense organ diseases	-	-	19.8% (17.9% to 21.8%)	19.8% (17.9% to 21.8%)
Sudden infant death syndrome	4.7% (2.8% to 6.7%)	4.7% (2.8% to 6.7%)	-	4.7% (2.8% to 6.7%)
**Transport injuries**	35.4% (31.1% to 40.5%)	33.1% (28.7% to 38.0%)	39.9% (35.7% to 44.8%)	34.0% (29.6% to 38.9%)
Road injuries	35.3% (31.0% to 40.4%)	33.0% (28.6% to 37.9%)	41.3% (37.0% to 46.2%)	34.0% (29.6% to 38.9%)
Other transport injuries	37.6% (32.7% to 43.1%)	34.7% (29.8% to 40.3%)	32.0% (28.0% to 36.6%)	33.9% (29.4% to 39.0%)
**Unintentional injuries**	33.9% (29.7% to 36.4%)	21.3% (19.5% to 23.4%)	26.1% (24.3% to 28.2%)	22.6% (20.9% to 24.6%)
Falls	65.4% (55.2% to 68.1%)	45.0% (40.4% to 47.4%)	33.6% (31.7% to 35.6%)	40.1% (37.3% to 42.3%)
Drowning	15.1% (12.4% to 18.6%)	16.3% (13.8% to 19.2%)	19.1% (15.8% to 23.1%)	16.3% (13.8% to 19.3%)
Fire, heat, and hot substances	11.3% (8.6% to 15.0%)	13.4% (10.9% to 16.6%)	23.3% (20.0% to 27.2%)	15.4% (12.6% to 18.8%)

Supplementary Results Table 1. Age-standardised PAFs attributable to the joint distribution of all risk factors for all ages, both sexes combined for each GBD level 1 and level 2 cause and all causes in 2016 for Eastern Sub-Saharan Africa. Results are shown for deaths, YLLs, YLDs, and
Data in parenthesis are 95% uncertainty intervals. PAFs=population attributable fractions. GBD=Global Burden of Disease. YLLs=years of life lost. YLDs=years live with disability. DALYs=disability-adjusted life-years

	**Deaths 2016**	**YLLs 2016**	**YLDs 2016**	**DALYs 2016**
Poisonings	14.6% (11.6% to 18.8%)	14.5% (11.0% to 18.5%)	23.0% (19.8% to 26.7%)	14.8% (11.3% to 18.8%)
Exposure to mechanical forces	25.4% (23.4% to 27.7%)	23.3% (20.8% to 26.2%)	26.7% (24.4% to 29.3%)	24.3% (21.9% to 27.0%)
Animal contact	9.9% (8.7% to 11.3%)	11.8% (10.5% to 13.6%)	17.6% (15.7% to 20.0%)	12.8% (11.3% to 14.6%)
Foreign body	9.3% (6.9% to 11.7%)	9.3% (5.7% to 12.4%)	15.9% (13.8% to 18.3%)	11.1% (8.0% to 13.9%)
Other unintentional injuries	18.3% (15.0% to 22.2%)	21.0% (17.9% to 24.7%)	20.0% (17.0% to 23.6%)	20.6% (17.6% to 24.2%)
**Self-harm and interpersonal violence**	18.0% (12.4% to 24.0%)	18.0% (12.9% to 23.3%)	24.1% (21.5% to 27.0%)	18.5% (13.5% to 23.5%)
Self-harm	16.7% (8.1% to 25.6%)	18.1% (9.3% to 26.9%)	16.3% (8.0% to 25.1%)	18.1% (9.3% to 26.8%)
Interpersonal violence	19.9% (14.3% to 25.2%)	17.9% (12.4% to 23.2%)	24.8% (22.1% to 27.8%)	18.9% (13.7% to 23.8%)
**Forces of nature, conflict and terrorism, and executions and police conflict**	0.2% (0.2% to 0.3%)	0.1% (0.1% to 0.1%)	0.1% (0.0% to 0.1%)	0.1% (0.1% to 0.1%)
Forces of nature, conflict and terrorism, and state actor violence	6.9% (6.4% to 7.4%)	2.8% (2.5% to 3.1%)	6.8% (4.6% to 9.9%)	3.5% (2.9% to 4.7%)

Supplementary Results Table 1. Age-standardised PAFs attributable to the joint distribution of all risk factors for all ages, both sexes combined for each GBD level 1 and level 2 cause and all causes in 2016 for High-income Asia Pacific. Results are shown for deaths, YLLs, YLDs, and DALYs
Data in parenthesis are 95% uncertainty intervals. PAFs=population attributable fractions. GBD=Global Burden of Disease. YLLs=years of life lost. YLDs=years live with disability. DALYs=disability-adjusted life-years

	Deaths 2016	YLLs 2016	YLDs 2016	DALYs 2016
**All causes**	44.3% (41.8% to 46.9%)	42.0% (39.5% to 44.3%)	21.6% (20.4% to 22.9%)	30.3% (28.2% to 32.5%)
**HIV/AIDS and tuberculosis**	41.8% (28.9% to 53.7%)	49.2% (37.9% to 58.9%)	67.7% (56.9% to 76.3%)	53.2% (42.5% to 62.3%)
Tuberculosis	38.5% (25.0% to 50.8%)	43.7% (30.1% to 55.2%)	45.6% (33.3% to 56.5%)	44.0% (31.1% to 55.3%)
HIV/AIDS	89.1% (87.3% to 91.9%)	81.0% (78.6% to 85.6%)	95.1% (92.7% to 96.8%)	87.3% (84.2% to 90.1%)
**Diarrhoea, lower respiratory infections, and other common infectious diseases**	40.4% (33.2% to 48.4%)	45.4% (39.1% to 52.4%)	5.4% (4.0% to 7.2%)	31.9% (26.1% to 38.6%)
Diarrhoeal diseases	30.3% (16.7% to 49.8%)	45.2% (32.8% to 60.8%)	41.0% (28.3% to 58.4%)	43.6% (31.7% to 59.6%)
Lower respiratory infections	41.7% (34.1% to 50.2%)	48.7% (41.5% to 56.8%)	46.0% (38.1% to 54.7%)	48.6% (41.5% to 56.7%)
Upper respiratory infections	0.3% (0.2% to 0.6%)	0.9% (0.5% to 1.9%)	0.0% (0.0% to 0.0%)	0.0% (0.0% to 0.0%)
Otitis media	1.2% (0.9% to 1.6%)	3.6% (2.6% to 4.7%)	7.5% (5.1% to 9.7%)	7.5% (5.1% to 9.7%)
Meningitis	1.5% (1.1% to 1.9%)	3.4% (2.7% to 4.1%)	0.0% (0.0% to 0.0%)	3.0% (2.0% to 3.7%)
Encephalitis	0.4% (0.2% to 0.7%)	0.9% (0.4% to 1.4%)	0.0% (0.0% to 0.0%)	0.5% (0.2% to 0.8%)
Measles	15.7% (3.3% to 49.6%)	16.4% (3.4% to 51.6%)	19.6% (5.0% to 59.1%)	16.4% (3.4% to 51.6%)
**Maternal disorders**	3.8% (1.6% to 6.2%)	3.7% (1.6% to 6.2%)	3.0% (1.5% to 4.6%)	3.4% (1.6% to 5.4%)
Maternal haemorrhage	12.9% (4.6% to 21.0%)	12.9% (4.6% to 21.0%)	12.3% (4.4% to 19.9%)	12.8% (4.6% to 20.7%)
Maternal sepsis and other pregnancy related infections	13.1% (4.8% to 21.6%)	13.1% (4.7% to 21.6%)	11.7% (4.3% to 18.9%)	12.3% (4.5% to 20.1%)
Maternal abortion, miscarriage, and ectopic pregnancy	3.3% (1.9% to 4.9%)	3.4% (1.9% to 5.1%)	3.6% (1.8% to 6.1%)	3.5% (1.8% to 5.6%)
**Neonatal disorders**	69.1% (65.4% to 71.8%)	69.2% (65.6% to 71.9%)	0.1% (0.1% to 0.1%)	38.7% (32.0% to 44.7%)
Neonatal preterm birth complications	80.6% (75.0% to 84.2%)	80.8% (75.2% to 84.3%)	0.2% (0.1% to 0.2%)	42.9% (33.6% to 52.0%)
Neonatal encephalopathy due to birth asphyxia and trauma	57.1% (53.8% to 60.2%)	57.2% (53.9% to 60.3%)	0.0% (0.0% to 0.0%)	28.6% (21.4% to 36.3%)
Neonatal sepsis and other neonatal infections	54.7% (48.2% to 61.2%)	54.8% (48.3% to 61.2%)	0.0% (0.0% to 0.0%)	23.9% (12.1% to 40.0%)
Hemolytic disease and other neonatal jaundice	50.8% (41.2% to 56.8%)	51.0% (41.4% to 57.0%)	0.0% (0.0% to 0.0%)	12.0% (8.2% to 16.1%)

Supplementary Results Table 1. Age-standardised PAFs attributable to the joint distribution of all risk factors for all ages, both sexes combined for each GBD level 1 and level 2 cause and all causes in 2016 for High-income Asia Pacific. Results are shown for deaths, YLLs, YLDs, and DALYs
Data in parenthesis are 95% uncertainty intervals. PAFs=population attributable fractions. GBD=Global Burden of Disease. YLLs=years of life lost. YLDs=years live with disability. DALYs=disability-adjusted life-years

	Deaths 2016	YLLs 2016	YLDs 2016	DALYs 2016
Other neonatal disorders	61.5% (58.1% to 64.7%)	61.6% (58.3% to 64.8%)	0.0% (0.0% to 0.0%)	56.6% (52.6% to 60.0%)
**Nutritional deficiencies**	1.9% (1.6% to 2.7%)	7.1% (6.3% to 8.7%)	91.8% (90.2% to 93.3%)	86.3% (82.9% to 89.1%)
Protein-energy malnutrition	0.9% (0.7% to 1.2%)	4.8% (4.1% to 6.6%)	98.3% (98.1% to 98.5%)	52.7% (42.7% to 63.8%)
Vitamin A deficiency	-	-	0.0% (0.0% to 0.0%)	0.0% (0.0% to 0.0%)
Iron-deficiency anaemia	100.0% (100.0% to 100.0%)	100.0% (100.0% to 100.0%)	100.0% (100.0% to 100.0%)	100.0% (100.0% to 100.0%)
**Other communicable, maternal, neonatal, and nutritional diseases**	4.4% (4.0% to 4.9%)	3.5% (3.1% to 4.1%)	58.2% (49.4% to 66.6%)	27.6% (18.8% to 39.6%)
Sexually transmitted diseases excluding HIV	82.1% (55.8% to 99.0%)	57.8% (23.0% to 96.1%)	100.0% (100.0% to 100.0%)	93.7% (80.7% to 99.8%)
Hepatitis	1.1% (0.8% to 1.4%)	1.3% (1.0% to 1.8%)	0.7% (0.5% to 1.2%)	1.3% (1.0% to 1.7%)
**Neoplasms**	35.1% (31.5% to 38.8%)	33.2% (29.8% to 36.8%)	29.5% (26.3% to 33.0%)	33.1% (29.7% to 36.7%)
Oesophageal cancer	63.3% (50.9% to 74.0%)	63.0% (50.6% to 73.8%)	62.4% (50.1% to 73.2%)	63.0% (50.6% to 73.8%)
Stomach cancer	18.8% (9.9% to 29.8%)	16.6% (8.6% to 26.2%)	17.8% (9.3% to 28.4%)	16.6% (8.6% to 26.2%)
Liver cancer	34.0% (26.5% to 42.4%)	36.5% (29.1% to 45.0%)	34.6% (27.5% to 42.8%)	36.5% (29.1% to 44.9%)
Larynx cancer	70.2% (58.7% to 79.5%)	66.4% (54.2% to 77.1%)	66.7% (54.6% to 76.5%)	66.4% (54.2% to 77.0%)
Tracheal, bronchus, and lung cancer	74.6% (69.3% to 79.4%)	71.4% (65.7% to 76.7%)	72.1% (66.6% to 76.9%)	71.4% (65.7% to 76.7%)
Breast cancer	27.8% (22.0% to 34.3%)	27.9% (22.6% to 34.0%)	27.7% (22.3% to 33.9%)	27.9% (22.6% to 34.0%)
Cervical cancer	100.0% (100.0% to 100.0%)	100.0% (100.0% to 100.0%)	100.0% (100.0% to 100.0%)	100.0% (100.0% to 100.0%)
Uterine cancer	19.3% (8.7% to 32.3%)	19.5% (8.7% to 32.7%)	19.3% (8.7% to 32.3%)	19.4% (8.7% to 32.6%)
Prostate cancer	3.4% (2.3% to 4.6%)	2.9% (1.9% to 3.9%)	2.7% (1.9% to 3.7%)	2.8% (1.9% to 3.9%)
Colon and rectum cancer	47.4% (38.1% to 56.2%)	47.8% (38.7% to 56.5%)	47.4% (38.2% to 56.2%)	47.8% (38.7% to 56.4%)
Lip and oral cavity cancer	56.8% (46.4% to 65.9%)	56.4% (45.8% to 65.6%)	55.2% (44.9% to 64.2%)	56.3% (45.7% to 65.5%)
Nasopharynx cancer	59.4% (52.5% to 66.0%)	56.7% (50.1% to 63.3%)	56.7% (50.1% to 63.1%)	56.7% (50.1% to 63.3%)

Supplementary Results Table 1. Age-standardised PAFs attributable to the joint distribution of all risk factors for all ages, both sexes combined for each GBD level 1 and level 2 cause and all causes in 2016 for High-income Asia Pacific. Results are shown for deaths, YLLs, YLDs, and DALYs
Data in parenthesis are 95% uncertainty intervals. PAFs=population attributable fractions. GBD=Global Burden of Disease. YLLs=years of life lost. YLDs=years live with disability. DALYs=disability-adjusted life-years

	**Deaths 2016**	**YLLs 2016**	**YLDs 2016**	**DALYs 2016**
Other pharynx cancer	43.8% (29.8% to 56.1%)	45.8% (31.9% to 58.3%)	45.2% (31.3% to 57.7%)	45.8% (31.9% to 58.2%)
Gallbladder and biliary tract cancer	8.1% (2.9% to 15.9%)	8.3% (3.0% to 16.2%)	8.2% (3.0% to 16.1%)	8.3% (3.0% to 16.2%)
Pancreatic cancer	17.6% (11.1% to 25.4%)	16.0% (9.8% to 23.7%)	16.9% (10.7% to 24.6%)	16.1% (9.8% to 23.7%)
Ovarian cancer	8.4% (3.7% to 15.2%)	6.6% (2.5% to 12.5%)	6.7% (2.7% to 12.4%)	6.6% (2.5% to 12.5%)
Kidney cancer	17.5% (10.7% to 26.3%)	16.2% (9.6% to 24.5%)	15.8% (9.3% to 24.2%)	16.2% (9.6% to 24.5%)
Bladder cancer	24.6% (16.8% to 33.9%)	21.6% (14.0% to 30.6%)	21.6% (14.4% to 30.4%)	21.6% (14.1% to 30.5%)
Thyroid cancer	6.0% (2.1% to 12.1%)	6.4% (2.3% to 13.2%)	5.8% (2.1% to 11.3%)	6.3% (2.3% to 12.6%)
Mesothelioma	92.8% (90.6% to 94.5%)	86.3% (82.9% to 89.2%)	91.0% (88.6% to 93.0%)	86.4% (83.0% to 89.3%)
Non-Hodgkin's lymphoma	2.9% (0.9% to 6.2%)	3.0% (0.9% to 6.3%)	3.0% (0.9% to 6.2%)	3.0% (0.9% to 6.3%)
Multiple myeloma	3.3% (1.1% to 6.9%)	3.5% (1.1% to 7.2%)	3.5% (1.1% to 7.2%)	3.5% (1.1% to 7.3%)
Leukaemia	8.8% (5.0% to 13.5%)	7.0% (3.9% to 10.9%)	6.4% (3.5% to 9.8%)	7.0% (3.9% to 10.9%)
**Cardiovascular diseases**	77.3% (74.1% to 80.3%)	80.6% (78.4% to 82.6%)	73.5% (70.5% to 76.2%)	79.0% (76.7% to 81.1%)
Rheumatic heart disease	24.9% (14.1% to 47.8%)	24.3% (15.9% to 39.0%)	23.3% (15.8% to 35.3%)	24.3% (15.9% to 38.7%)
Ischaemic heart disease	88.8% (83.7% to 93.1%)	91.3% (88.1% to 94.0%)	88.4% (85.1% to 91.5%)	91.0% (87.8% to 93.6%)
Cerebrovascular disease	79.3% (73.4% to 84.8%)	85.1% (81.6% to 88.4%)	81.9% (77.5% to 86.0%)	84.3% (80.6% to 87.8%)
Hypertensive heart disease	100.0% (100.0% to 100.0%)	100.0% (100.0% to 100.0%)	100.0% (100.0% to 100.0%)	100.0% (100.0% to 100.0%)
Cardiomyopathy and myocarditis	34.0% (25.7% to 43.4%)	31.7% (25.3% to 38.2%)	34.3% (28.3% to 40.8%)	32.2% (26.2% to 38.6%)
Atrial fibrillation and flutter	46.3% (39.1% to 53.7%)	52.1% (45.9% to 58.1%)	57.6% (52.5% to 62.4%)	55.4% (49.9% to 60.6%)
Aortic aneurysm	42.0% (34.6% to 49.7%)	49.1% (42.8% to 54.8%)	-	49.1% (42.8% to 54.8%)
Peripheral vascular disease	48.9% (41.7% to 56.4%)	55.8% (50.2% to 61.3%)	56.3% (51.3% to 61.2%)	56.1% (51.0% to 61.1%)
Endocarditis	38.9% (28.7% to 52.0%)	38.4% (30.4% to 47.3%)	41.3% (31.6% to 51.9%)	38.6% (30.4% to 47.6%)

Supplementary Results Table 1. Age-standardised PAFs attributable to the joint distribution of all risk factors for all ages, both sexes combined for each GBD level 1 and level 2 cause and all causes in 2016 for High-income Asia Pacific. Results are shown for deaths, YLLs, YLDs, and DALYs
Data in parenthesis are 95% uncertainty intervals. PAFs=population attributable fractions. GBD=Global Burden of Disease. YLLs=years of life lost. YLDs=years live with disability. DALYs=disability-adjusted life-years

	Deaths 2016	YLLs 2016	YLDs 2016	DALYs 2016
Other cardiovascular and circulatory diseases	41.3% (35.8% to 47.0%)	44.7% (40.6% to 48.9%)	48.9% (44.3% to 53.5%)	47.0% (42.5% to 51.2%)
**Chronic respiratory diseases**	49.7% (44.5% to 56.4%)	44.1% (38.9% to 50.6%)	35.9% (30.5% to 41.9%)	38.6% (33.8% to 44.0%)
Chronic obstructive pulmonary disease	70.2% (63.2% to 76.3%)	66.4% (59.3% to 73.0%)	57.6% (49.5% to 65.3%)	61.5% (53.7% to 68.8%)
Pneumoconiosis	100.0% (100.0% to 100.0%)	100.0% (100.0% to 100.0%)	100.0% (100.0% to 100.0%)	100.0% (100.0% to 100.0%)
Asthma	26.4% (19.3% to 35.6%)	30.9% (24.2% to 39.0%)	24.4% (18.4% to 32.1%)	24.9% (18.9% to 32.4%)
Other chronic respiratory diseases	6.3% (4.2% to 8.9%)	3.5% (1.6% to 6.6%)	3.5% (1.4% to 6.9%)	3.5% (1.5% to 6.9%)
**Cirrhosis and other chronic liver diseases**	37.3% (34.8% to 39.8%)	43.4% (40.9% to 46.3%)	37.4% (35.1% to 39.6%)	43.1% (40.5% to 45.9%)
Cirrhosis and other chronic liver diseases due to hepatitis B	0.2% (0.1% to 0.3%)	0.2% (0.1% to 0.4%)	0.2% (0.1% to 0.3%)	0.2% (0.1% to 0.4%)
Cirrhosis and other chronic liver diseases due to hepatitis C	34.4% (30.6% to 38.3%)	51.3% (47.0% to 55.4%)	52.9% (49.0% to 56.6%)	51.4% (47.1% to 55.4%)
Cirrhosis and other chronic liver diseases due to alcohol use	100.0% (100.0% to 100.0%)	100.0% (100.0% to 100.0%)	100.0% (100.0% to 100.0%)	100.0% (100.0% to 100.0%)
**Digestive diseases**	7.3% (4.8% to 10.3%)	8.2% (5.3% to 11.1%)	2.1% (1.5% to 2.9%)	5.9% (3.9% to 8.0%)
Peptic ulcer disease	14.5% (10.3% to 19.1%)	18.8% (13.7% to 24.5%)	12.0% (8.6% to 15.6%)	16.9% (12.2% to 22.3%)
Gallbladder and biliary diseases	19.1% (9.6% to 32.2%)	20.2% (10.5% to 33.0%)	21.3% (10.9% to 35.0%)	20.3% (10.6% to 33.3%)
Pancreatitis	15.5% (-1.1% to 30.4%)	18.5% (1.4% to 34.5%)	17.4% (0.9% to 32.9%)	18.4% (1.4% to 34.5%)
**Neurological disorders**	14.0% (6.5% to 23.8%)	13.8% (7.0% to 22.6%)	3.3% (1.9% to 5.1%)	7.2% (3.8% to 11.8%)
Alzheimer's disease and other dementias	15.7% (7.4% to 26.5%)	16.9% (8.3% to 27.5%)	16.6% (8.0% to 27.3%)	16.8% (8.2% to 27.4%)
Parkinson's disease	-11.9% (-14.1% to -9.4%)	-14.3% (-16.9% to -11.2%)	-14.7% (-17.4% to -11.4%)	-14.4% (-16.9% to -11.3%)
Epilepsy	22.3% (12.2% to 31.3%)	21.1% (11.9% to 29.4%)	17.6% (10.0% to 25.0%)	18.8% (10.7% to 26.2%)
Multiple sclerosis	10.4% (6.4% to 14.5%)	10.8% (6.7% to 15.1%)	8.5% (5.5% to 11.6%)	9.1% (5.8% to 12.5%)
**Mental and substance use disorders**	95.7% (94.7% to 97.0%)	93.3% (91.7% to 95.6%)	16.9% (15.2% to 18.9%)	18.3% (16.5% to 20.3%)
Alcohol use disorders	100.0% (100.0% to 100.0%)	100.0% (100.0% to 100.0%)	100.0% (100.0% to 100.0%)	100.0% (100.0% to 100.0%)

Supplementary Results Table 1. Age-standardised PAFs attributable to the joint distribution of all risk factors for all ages, both sexes combined for each GBD level 1 and level 2 cause and all causes in 2016 for High-income Asia Pacific. Results are shown for deaths, YLLs, YLDs, and DALYs
Data in parenthesis are 95% uncertainty intervals. PAFs=population attributable fractions. GBD=Global Burden of Disease. YLLs=years of life lost. YLDs=years live with disability. DALYs=disability-adjusted life-years

	**Deaths 2016**	**YLLs 2016**	**YLDs 2016**	**DALYs 2016**
Drug use disorders	100.0% (100.0% to 100.0%)	100.0% (100.0% to 100.0%)	100.0% (100.0% to 100.0%)	100.0% (100.0% to 100.0%)
Depressive disorders	-	-	8.0% (6.7% to 9.5%)	8.0% (6.7% to 9.5%)
Idiopathic developmental intellectual disability	-	-	41.8% (20.2% to 59.1%)	41.8% (20.2% to 59.1%)
**Diabetes, urogenital, blood, and endocrine diseases**	80.2% (77.9% to 81.6%)	78.8% (76.2% to 80.7%)	67.6% (63.2% to 71.8%)	70.9% (67.4% to 74.1%)
Diabetes mellitus	100.0% (100.0% to 100.0%)	100.0% (100.0% to 100.0%)	100.0% (100.0% to 100.0%)	100.0% (100.0% to 100.0%)
Chronic kidney disease	100.0% (100.0% to 100.0%)	100.0% (100.0% to 100.0%)	100.0% (100.0% to 100.0%)	100.0% (100.0% to 100.0%)
**Musculoskeletal disorders**	1.4% (0.6% to 2.4%)	1.3% (0.5% to 2.3%)	17.1% (14.8% to 19.9%)	16.9% (14.6% to 19.7%)
Rheumatoid arthritis	4.1% (1.7% to 6.9%)	4.8% (1.9% to 8.1%)	5.1% (2.1% to 8.4%)	5.1% (2.1% to 8.4%)
Osteoarthritis	-	-	12.1% (4.7% to 22.4%)	12.1% (4.7% to 22.4%)
Low back and neck pain	-	-	22.4% (19.7% to 25.2%)	22.4% (19.7% to 25.2%)
Gout	-	-	29.5% (18.2% to 44.4%)	29.5% (18.2% to 44.4%)
**Other non-communicable diseases**	0.2% (0.1% to 0.2%)	0.2% (0.2% to 0.3%)	2.2% (1.9% to 2.5%)	2.0% (1.7% to 2.3%)
Sense organ diseases	-	-	6.5% (5.8% to 7.3%)	6.5% (5.8% to 7.3%)
Sudden infant death syndrome	2.3% (1.9% to 2.9%)	2.3% (1.9% to 2.9%)	-	2.3% (1.9% to 2.9%)
**Transport injuries**	39.4% (32.5% to 46.4%)	37.3% (30.0% to 44.5%)	44.9% (39.1% to 50.6%)	39.9% (33.1% to 46.7%)
Road injuries	39.0% (32.1% to 46.1%)	37.0% (29.6% to 44.3%)	45.9% (40.1% to 51.6%)	39.9% (33.0% to 46.7%)
Other transport injuries	42.8% (35.7% to 49.7%)	40.7% (33.5% to 47.7%)	40.6% (35.0% to 46.4%)	40.6% (34.3% to 47.1%)
**Unintentional injuries**	28.3% (25.7% to 31.4%)	23.3% (20.6% to 26.2%)	27.7% (25.6% to 30.1%)	26.2% (23.9% to 28.8%)
Falls	56.6% (52.2% to 60.2%)	39.7% (35.5% to 44.0%)	30.9% (29.0% to 33.2%)	32.7% (30.6% to 35.5%)
Drowning	16.5% (11.8% to 22.9%)	20.0% (15.7% to 25.5%)	16.3% (11.3% to 23.0%)	19.8% (15.5% to 25.3%)
Fire, heat, and hot substances	18.3% (13.4% to 24.2%)	21.9% (17.4% to 26.7%)	27.4% (22.5% to 33.9%)	25.4% (20.6% to 31.2%)

Supplementary Results Table 1. Age-standardised PAFs attributable to the joint distribution of all risk factors for all ages, both sexes combined for each GBD level 1 and level 2 cause and all causes in 2016 for High-income Asia Pacific. Results are shown for deaths, YLLs, YLDs, and DALYs
Data in parenthesis are 95% uncertainty intervals. PAFs=population attributable fractions. GBD=Global Burden of Disease. YLLs=years of life lost. YLDs=years live with disability. DALYs=disability-adjusted life-years

	**Deaths 2016**	**YLLs 2016**	**YLDs 2016**	**DALYs 2016**
Poisonings	30.2% (24.2% to 37.0%)	34.9% (28.7% to 41.5%)	25.6% (20.9% to 31.5%)	30.6% (25.4% to 37.1%)
Exposure to mechanical forces	24.0% (20.2% to 28.6%)	21.8% (17.8% to 26.6%)	30.4% (27.9% to 33.4%)	27.8% (24.9% to 31.5%)
Animal contact	15.0% (12.6% to 18.1%)	16.0% (12.9% to 19.8%)	19.5% (17.5% to 21.9%)	19.1% (17.0% to 21.6%)
Foreign body	6.7% (5.9% to 7.7%)	10.7% (9.0% to 12.1%)	13.2% (11.4% to 15.0%)	11.5% (10.0% to 12.9%)
Other unintentional injuries	30.4% (23.3% to 38.4%)	32.9% (25.6% to 40.7%)	24.9% (20.2% to 31.1%)	25.7% (20.8% to 31.9%)
**Self-harm and interpersonal violence**	21.5% (6.8% to 34.1%)	22.9% (8.0% to 35.4%)	22.1% (16.0% to 28.3%)	22.8% (8.4% to 34.8%)
Self-harm	21.4% (6.0% to 34.4%)	23.0% (7.5% to 36.1%)	20.0% (5.7% to 32.5%)	22.9% (7.4% to 36.0%)
Interpersonal violence	23.9% (15.8% to 31.6%)	19.9% (12.7% to 26.9%)	23.4% (19.0% to 28.0%)	21.4% (15.5% to 27.0%)
**Forces of nature, conflict and terrorism, and executions and police conflict**	0.0% (0.0% to 0.0%)	0.0% (0.0% to 0.0%)	7.7% (5.0% to 11.1%)	6.7% (3.4% to 10.2%)
Forces of nature, conflict and terrorism, and state actor violence	0.0% (0.0% to 0.0%)	0.0% (0.0% to 0.0%)	8.3% (5.6% to 12.0%)	8.3% (5.6% to 12.0%)

Supplementary Results Table 1. Age-standardised PAFs attributable to the joint distribution of all risk factors for all ages, both sexes combined for each GBD level 1 and level 2 cause and all causes in 2016 for High-income North America. Results are shown for deaths, YLLs, YLDs, and
Data in parenthesis are 95% uncertainty intervals. PAFs=population attributable fractions. GBD=Global Burden of Disease. YLLs=years of life lost. YLDs=years live with disability. DALYs=disability-adjusted life-years

	Deaths 2016	YLLs 2016	YLDs 2016	DALYs 2016
**All causes**	59.6% (57.6% to 61.7%)	57.1% (55.5% to 58.6%)	26.1% (24.3% to 27.8%)	41.8% (39.1% to 44.6%)
**HIV/AIDS and tuberculosis**	86.3% (85.0% to 87.6%)	86.7% (85.7% to 87.7%)	91.0% (89.9% to 91.9%)	88.1% (86.9% to 89.2%)
Tuberculosis	46.5% (36.8% to 55.8%)	49.9% (40.8% to 58.4%)	49.4% (40.6% to 57.0%)	49.9% (40.7% to 58.2%)
HIV/AIDS	91.0% (90.1% to 91.7%)	89.1% (88.2% to 90.0%)	91.6% (90.6% to 92.5%)	90.0% (89.0% to 90.9%)
**Diarrhoea, lower respiratory infections, and other common infectious diseases**	28.4% (21.6% to 36.2%)	36.0% (29.6% to 43.1%)	6.7% (4.9% to 8.6%)	26.8% (21.9% to 32.6%)
Diarrhoeal diseases	21.3% (15.8% to 28.8%)	37.9% (32.2% to 44.5%)	29.0% (22.2% to 36.6%)	34.4% (28.3% to 41.3%)
Lower respiratory infections	30.2% (22.7% to 39.1%)	39.0% (31.4% to 47.7%)	38.5% (30.7% to 47.3%)	39.0% (31.4% to 47.7%)
Upper respiratory infections	0.7% (0.5% to 1.1%)	2.0% (1.3% to 2.9%)	0.0% (0.0% to 0.0%)	0.0% (0.0% to 0.0%)
Otitis media	2.7% (1.9% to 3.5%)	5.7% (4.1% to 7.3%)	5.7% (3.8% to 7.5%)	5.7% (3.8% to 7.5%)
Meningitis	1.5% (1.2% to 1.7%)	2.7% (2.3% to 3.0%)	0.0% (0.0% to 0.0%)	2.3% (2.0% to 2.6%)
Encephalitis	0.6% (0.5% to 0.7%)	1.5% (1.2% to 1.7%)	0.0% (0.0% to 0.0%)	1.2% (1.0% to 1.3%)
Measles	12.3% (2.7% to 44.1%)	12.8% (2.7% to 46.1%)	13.2% (2.9% to 48.2%)	12.8% (2.7% to 46.1%)
**Maternal disorders**	1.3% (0.6% to 1.9%)	1.3% (0.6% to 1.9%)	2.2% (0.9% to 3.8%)	1.5% (0.7% to 2.2%)
Maternal haemorrhage	9.7% (3.5% to 15.7%)	9.7% (3.5% to 15.8%)	9.8% (3.5% to 15.9%)	9.8% (3.5% to 15.8%)
Maternal sepsis and other pregnancy related infections	9.7% (3.5% to 15.8%)	9.8% (3.5% to 15.9%)	9.4% (3.4% to 15.3%)	9.7% (3.5% to 15.8%)
Maternal abortion, miscarriage, and ectopic pregnancy	1.9% (1.1% to 2.8%)	2.0% (1.2% to 3.0%)	2.4% (1.2% to 4.0%)	2.1% (1.2% to 3.1%)
**Neonatal disorders**	81.2% (80.0% to 82.2%)	81.2% (80.1% to 82.3%)	0.1% (0.1% to 0.2%)	62.6% (57.5% to 67.1%)
Neonatal preterm birth complications	93.4% (92.5% to 93.9%)	93.4% (92.6% to 94.0%)	0.2% (0.1% to 0.2%)	66.9% (59.1% to 73.4%)
Neonatal encephalopathy due to birth asphyxia and trauma	64.6% (62.8% to 66.7%)	64.7% (62.8% to 66.7%)	0.0% (0.0% to 0.0%)	57.8% (53.7% to 61.3%)
Neonatal sepsis and other neonatal infections	59.1% (56.8% to 61.5%)	59.1% (56.8% to 61.6%)	0.0% (0.0% to 0.0%)	36.9% (24.5% to 46.8%)
Hemolytic disease and other neonatal jaundice	52.0% (48.7% to 54.6%)	52.2% (48.8% to 54.7%)	0.0% (0.0% to 0.0%)	15.2% (11.8% to 19.2%)

Supplementary Results Table 1. Age-standardised PAFs attributable to the joint distribution of all risk factors for all ages, both sexes combined for each GBD level 1 and level 2 cause and all causes in 2016 for High-income North America. Results are shown for deaths, YLLs, YLDs, and
Data in parenthesis are 95% uncertainty intervals. PAFs=population attributable fractions. GBD=Global Burden of Disease. YLLs=years of life lost. YLDs=years live with disability. DALYs=disability-adjusted life-years

	Deaths 2016	YLLs 2016	YLDs 2016	DALYs 2016
Other neonatal disorders	66.5% (64.5% to 68.5%)	66.5% (64.6% to 68.5%)	0.0% (0.0% to 0.0%)	64.0% (62.0% to 66.1%)
**Nutritional deficiencies**	1.6% (1.2% to 2.0%)	6.6% (5.7% to 7.8%)	77.7% (74.0% to 81.2%)	64.6% (58.1% to 70.3%)
Protein-energy malnutrition	0.6% (0.6% to 0.7%)	4.1% (3.7% to 4.9%)	98.4% (98.3% to 98.5%)	49.2% (39.7% to 58.3%)
Vitamin A deficiency	-	-	0.0% (0.0% to 0.0%)	0.0% (0.0% to 0.0%)
Iron-deficiency anaemia	100.0% (100.0% to 100.0%)	100.0% (100.0% to 100.0%)	100.0% (100.0% to 100.0%)	100.0% (100.0% to 100.0%)
**Other communicable, maternal, neonatal, and nutritional diseases**	6.9% (5.9% to 7.9%)	6.6% (5.5% to 7.6%)	77.8% (72.8% to 82.8%)	30.4% (22.7% to 41.5%)
Sexually transmitted diseases excluding HIV	88.2% (80.6% to 93.8%)	72.1% (58.9% to 83.5%)	100.0% (100.0% to 100.0%)	96.6% (93.6% to 98.5%)
Hepatitis	13.3% (10.4% to 16.8%)	14.0% (10.8% to 17.8%)	5.6% (4.2% to 7.2%)	13.2% (10.2% to 17.0%)
**Neoplasms**	52.3% (49.6% to 55.3%)	51.0% (48.4% to 53.7%)	35.5% (32.0% to 39.0%)	50.3% (47.6% to 53.0%)
Oesophageal cancer	86.9% (79.4% to 92.7%)	87.4% (80.1% to 93.2%)	87.0% (79.7% to 92.6%)	87.4% (80.1% to 93.2%)
Stomach cancer	27.2% (17.7% to 36.8%)	25.6% (16.8% to 34.4%)	26.7% (17.4% to 36.2%)	25.6% (16.8% to 34.5%)
Liver cancer	78.5% (71.9% to 84.1%)	78.0% (71.1% to 83.7%)	77.7% (70.6% to 83.6%)	78.0% (71.1% to 83.7%)
Larynx cancer	89.6% (84.3% to 93.7%)	89.1% (83.5% to 93.2%)	89.2% (83.7% to 93.3%)	89.1% (83.6% to 93.2%)
Tracheal, bronchus, and lung cancer	91.6% (90.2% to 92.9%)	90.7% (89.3% to 92.1%)	91.1% (89.7% to 92.5%)	90.7% (89.3% to 92.1%)
Breast cancer	37.1% (28.7% to 46.2%)	35.4% (27.3% to 43.7%)	36.0% (27.7% to 44.7%)	35.5% (27.4% to 43.8%)
Cervical cancer	100.0% (100.0% to 100.0%)	100.0% (100.0% to 100.0%)	100.0% (100.0% to 100.0%)	100.0% (100.0% to 100.0%)
Uterine cancer	53.3% (39.0% to 66.2%)	55.0% (40.6% to 67.5%)	55.4% (41.0% to 67.7%)	55.0% (40.7% to 67.5%)
Prostate cancer	7.5% (5.5% to 9.7%)	7.5% (5.6% to 9.5%)	7.5% (5.6% to 9.4%)	7.5% (5.6% to 9.5%)
Colon and rectum cancer	56.9% (47.9% to 65.7%)	57.6% (49.0% to 65.7%)	57.2% (48.4% to 65.8%)	57.5% (49.0% to 65.7%)
Lip and oral cavity cancer	82.7% (77.1% to 87.5%)	83.2% (77.6% to 87.9%)	82.5% (76.9% to 87.3%)	83.2% (77.6% to 87.8%)
Nasopharynx cancer	82.3% (78.3% to 86.1%)	80.5% (76.4% to 84.2%)	79.0% (74.4% to 82.7%)	80.5% (76.4% to 84.2%)

Supplementary Results Table 1. Age-standardised PAFs attributable to the joint distribution of all risk factors for all ages, both sexes combined for each GBD level 1 and level 2 cause and all causes in 2016 for High-income North America. Results are shown for deaths, YLLs, YLDs, and
Data in parenthesis are 95% uncertainty intervals. PAFs=population attributable fractions. GBD=Global Burden of Disease. YLLs=years of life lost. YLDs=years live with disability. DALYs=disability-adjusted life-years

	Deaths 2016	YLLs 2016	YLDs 2016	DALYs 2016
Other pharynx cancer	51.1% (38.0% to 62.4%)	54.5% (41.5% to 65.4%)	54.3% (41.1% to 65.2%)	54.5% (41.4% to 65.4%)
Gallbladder and biliary tract cancer	26.1% (14.9% to 38.4%)	27.1% (15.7% to 39.6%)	25.8% (14.8% to 38.3%)	27.1% (15.6% to 39.6%)
Pancreatic cancer	44.3% (36.1% to 52.9%)	43.6% (35.4% to 52.2%)	44.0% (35.8% to 52.6%)	43.6% (35.4% to 52.2%)
Ovarian cancer	16.3% (7.6% to 26.7%)	14.2% (6.0% to 23.9%)	13.9% (6.0% to 23.2%)	14.2% (6.0% to 23.8%)
Kidney cancer	45.4% (34.9% to 55.8%)	44.9% (34.6% to 55.0%)	44.8% (34.9% to 54.8%)	44.9% (34.6% to 55.0%)
Bladder cancer	47.7% (36.9% to 57.9%)	46.8% (36.3% to 56.6%)	46.3% (35.9% to 56.0%)	46.8% (36.3% to 56.5%)
Thyroid cancer	18.6% (9.7% to 29.0%)	19.6% (10.2% to 30.6%)	19.9% (10.8% to 30.4%)	19.7% (10.5% to 30.6%)
Mesothelioma	95.8% (94.7% to 96.7%)	91.0% (89.1% to 92.7%)	94.4% (93.1% to 95.6%)	91.0% (89.2% to 92.8%)
Non-Hodgkin's lymphoma	8.9% (4.0% to 14.7%)	9.3% (4.1% to 15.1%)	9.3% (4.2% to 15.2%)	9.3% (4.2% to 15.1%)
Multiple myeloma	10.1% (4.6% to 17.2%)	10.7% (4.9% to 17.9%)	10.6% (4.9% to 18.0%)	10.7% (4.9% to 17.9%)
Leukaemia	25.2% (16.2% to 33.9%)	21.9% (14.5% to 29.0%)	22.9% (14.8% to 30.7%)	21.9% (14.5% to 29.0%)
**Cardiovascular diseases**	82.3% (79.6% to 84.6%)	83.2% (81.2% to 84.8%)	70.6% (67.4% to 73.5%)	81.3% (79.3% to 83.1%)
Rheumatic heart disease	22.5% (13.5% to 40.4%)	20.3% (13.7% to 32.4%)	20.8% (13.6% to 33.8%)	20.3% (13.8% to 32.4%)
Ischaemic heart disease	92.3% (88.8% to 95.2%)	93.9% (91.7% to 95.9%)	89.7% (86.7% to 92.5%)	93.8% (91.5% to 95.7%)
Cerebrovascular disease	80.2% (74.1% to 86.2%)	84.1% (80.5% to 87.7%)	82.9% (78.2% to 87.3%)	83.8% (79.9% to 87.5%)
Hypertensive heart disease	100.0% (100.0% to 100.0%)	100.0% (100.0% to 100.0%)	100.0% (100.0% to 100.0%)	100.0% (100.0% to 100.0%)
Cardiomyopathy and myocarditis	45.4% (38.3% to 53.4%)	42.1% (36.2% to 48.4%)	42.2% (36.3% to 48.8%)	42.1% (36.2% to 48.4%)
Atrial fibrillation and flutter	50.7% (43.1% to 58.1%)	53.8% (47.5% to 60.2%)	57.1% (51.4% to 62.8%)	56.1% (50.3% to 62.0%)
Aortic aneurysm	37.0% (30.7% to 43.8%)	40.6% (35.2% to 46.1%)	-	40.6% (35.2% to 46.1%)
Peripheral vascular disease	55.2% (49.7% to 61.2%)	60.9% (56.8% to 65.0%)	60.9% (56.7% to 65.2%)	60.9% (56.7% to 65.0%)
Endocarditis	35.4% (26.1% to 46.4%)	33.6% (25.4% to 42.8%)	35.2% (26.1% to 45.2%)	33.6% (25.4% to 42.8%)

Supplementary Results Table 1. Age-standardised PAFs attributable to the joint distribution of all risk factors for all ages, both sexes combined for each GBD level 1 and level 2 cause and all causes in 2016 for High-income North America. Results are shown for deaths, YLLs, YLDs, and
Data in parenthesis are 95% uncertainty intervals. PAFs=population attributable fractions. GBD=Global Burden of Disease. YLLs=years of life lost. YLDs=years live with disability. DALYs=disability-adjusted life-years

	Deaths 2016	YLLs 2016	YLDs 2016	DALYs 2016
Other cardiovascular and circulatory diseases	39.5% (34.2% to 44.7%)	41.3% (36.5% to 46.0%)	41.2% (36.3% to 46.2%)	41.3% (36.4% to 46.1%)
**Chronic respiratory diseases**	75.4% (72.6% to 79.6%)	72.7% (70.0% to 76.5%)	60.6% (54.8% to 66.1%)	67.3% (63.6% to 71.0%)
Chronic obstructive pulmonary disease	86.1% (83.4% to 88.5%)	85.5% (83.0% to 87.9%)	80.6% (77.5% to 83.4%)	83.8% (81.2% to 86.4%)
Pneumoconiosis	100.0% (100.0% to 100.0%)	100.0% (100.0% to 100.0%)	100.0% (100.0% to 100.0%)	100.0% (100.0% to 100.0%)
Asthma	48.3% (38.4% to 58.7%)	49.0% (39.8% to 58.4%)	39.3% (30.3% to 49.5%)	40.6% (31.7% to 50.7%)
Other chronic respiratory diseases	24.1% (19.9% to 28.1%)	17.7% (14.3% to 20.9%)	13.3% (11.0% to 16.0%)	15.9% (13.1% to 19.1%)
**Cirrhosis and other chronic liver diseases**	67.8% (65.0% to 70.2%)	68.7% (65.9% to 71.3%)	60.5% (58.0% to 62.8%)	68.4% (65.6% to 70.9%)
Cirrhosis and other chronic liver diseases due to hepatitis B	11.5% (8.2% to 15.4%)	13.2% (9.3% to 17.9%)	10.6% (7.6% to 14.5%)	13.1% (9.3% to 17.8%)
Cirrhosis and other chronic liver diseases due to hepatitis C	88.3% (87.8% to 88.8%)	89.5% (89.2% to 89.7%)	88.6% (88.1% to 89.2%)	89.4% (89.2% to 89.7%)
Cirrhosis and other chronic liver diseases due to alcohol use	100.0% (100.0% to 100.0%)	100.0% (100.0% to 100.0%)	100.0% (100.0% to 100.0%)	100.0% (100.0% to 100.0%)
**Digestive diseases**	8.1% (6.1% to 10.2%)	9.8% (7.5% to 12.4%)	3.9% (3.1% to 4.8%)	7.6% (5.8% to 9.4%)
Peptic ulcer disease	8.7% (6.0% to 11.8%)	11.6% (8.1% to 15.6%)	10.2% (7.1% to 13.8%)	11.0% (7.7% to 14.9%)
Gallbladder and biliary diseases	48.2% (33.7% to 62.7%)	50.1% (35.8% to 63.9%)	53.4% (39.5% to 66.3%)	50.8% (36.6% to 64.4%)
Pancreatitis	26.4% (13.1% to 40.1%)	33.6% (19.2% to 47.7%)	29.4% (15.8% to 42.6%)	33.0% (18.6% to 47.0%)
**Neurological disorders**	23.2% (11.5% to 36.4%)	20.6% (11.1% to 30.9%)	5.9% (3.8% to 8.3%)	11.2% (6.6% to 16.8%)
Alzheimer's disease and other dementias	28.8% (14.3% to 45.1%)	30.3% (15.9% to 46.3%)	30.5% (16.1% to 46.5%)	30.3% (15.9% to 46.3%)
Parkinson's disease	-4.9% (-5.5% to -4.2%)	-6.4% (-7.2% to -5.4%)	-7.5% (-8.5% to -6.4%)	-6.6% (-7.5% to -5.6%)
Epilepsy	30.4% (21.6% to 38.5%)	31.4% (22.9% to 39.0%)	24.6% (17.3% to 31.3%)	26.1% (18.3% to 33.0%)
Multiple sclerosis	7.3% (4.7% to 10.1%)	7.9% (5.1% to 11.0%)	7.1% (4.5% to 10.0%)	7.4% (4.8% to 10.3%)
**Mental and substance use disorders**	99.8% (99.8% to 99.8%)	99.8% (99.8% to 99.8%)	32.3% (29.4% to 35.5%)	42.4% (38.6% to 46.3%)
Alcohol use disorders	100.0% (100.0% to 100.0%)	100.0% (100.0% to 100.0%)	100.0% (100.0% to 100.0%)	100.0% (100.0% to 100.0%)

Supplementary Results Table 1. Age-standardised PAFs attributable to the joint distribution of all risk factors for all ages, both sexes combined for each GBD level 1 and level 2 cause and all causes in 2016 for High-income North America. Results are shown for deaths, YLLs, YLDs, and
Data in parenthesis are 95% uncertainty intervals. PAFs=population attributable fractions. GBD=Global Burden of Disease. YLLs=years of life lost. YLDs=years live with disability. DALYs=disability-adjusted life-years

	Deaths 2016	YLLs 2016	YLDs 2016	DALYs 2016
Drug use disorders	100.0% (100.0% to 100.0%)	100.0% (100.0% to 100.0%)	100.0% (100.0% to 100.0%)	100.0% (100.0% to 100.0%)
Depressive disorders	-	-	10.1% (8.6% to 12.0%)	10.1% (8.6% to 12.0%)
Idiopathic developmental intellectual disability	-	-	53.9% (28.1% to 71.0%)	53.9% (28.1% to 71.0%)
**Diabetes, urogenital, blood, and endocrine diseases**	75.3% (74.4% to 78.4%)	71.1% (69.8% to 74.4%)	72.6% (68.9% to 75.8%)	71.9% (69.8% to 74.0%)
Diabetes mellitus	100.0% (100.0% to 100.0%)	100.0% (100.0% to 100.0%)	100.0% (100.0% to 100.0%)	100.0% (100.0% to 100.0%)
Chronic kidney disease	100.0% (100.0% to 100.0%)	100.0% (100.0% to 100.0%)	100.0% (100.0% to 100.0%)	100.0% (100.0% to 100.0%)
**Musculoskeletal disorders**	0.9% (0.3% to 1.5%)	0.8% (0.3% to 1.4%)	14.8% (12.2% to 17.7%)	14.5% (11.9% to 17.4%)
Rheumatoid arthritis	3.2% (1.3% to 5.6%)	4.1% (1.6% to 6.9%)	4.5% (1.8% to 7.7%)	4.5% (1.8% to 7.6%)
Osteoarthritis	-	-	33.9% (19.7% to 47.3%)	33.9% (19.7% to 47.3%)
Low back and neck pain	-	-	18.4% (16.0% to 20.8%)	18.4% (16.0% to 20.8%)
Gout	-	-	58.8% (40.9% to 75.2%)	58.8% (40.9% to 75.2%)
**Other non-communicable diseases**	0.5% (0.4% to 0.6%)	0.7% (0.5% to 0.8%)	2.1% (1.9% to 2.4%)	1.9% (1.7% to 2.1%)
Sense organ diseases	-	-	7.7% (7.0% to 8.5%)	7.7% (7.0% to 8.5%)
Sudden infant death syndrome	3.7% (3.3% to 4.1%)	3.7% (3.3% to 4.1%)	-	3.7% (3.3% to 4.1%)
**Transport injuries**	33.4% (25.8% to 42.6%)	31.3% (23.3% to 40.9%)	36.8% (29.9% to 44.8%)	32.5% (24.8% to 41.7%)
Road injuries	33.2% (25.6% to 42.4%)	31.1% (23.1% to 40.8%)	37.3% (30.4% to 45.4%)	32.4% (24.6% to 41.7%)
Other transport injuries	36.1% (28.4% to 45.1%)	33.5% (25.5% to 42.6%)	33.8% (27.2% to 41.4%)	33.6% (26.5% to 42.0%)
**Unintentional injuries**	33.7% (27.5% to 36.3%)	18.5% (16.0% to 20.6%)	19.6% (18.5% to 21.0%)	19.1% (17.6% to 20.6%)
Falls	61.4% (57.3% to 63.5%)	40.7% (35.8% to 42.6%)	22.9% (22.0% to 23.8%)	27.4% (25.4% to 29.2%)
Drowning	16.5% (11.9% to 22.5%)	14.7% (10.7% to 19.8%)	16.7% (11.8% to 23.7%)	14.8% (10.7% to 20.0%)
Fire, heat, and hot substances	12.1% (7.8% to 17.8%)	13.1% (9.1% to 17.9%)	19.0% (13.8% to 26.0%)	15.7% (11.1% to 21.6%)

Supplementary Results Table 1. Age-standardised PAFs attributable to the joint distribution of all risk factors for all ages, both sexes combined for each GBD level 1 and level 2 cause and all causes in 2016 for High-income North America. Results are shown for deaths, YLLs, YLDs, and
Data in parenthesis are 95% uncertainty intervals. PAFs=population attributable fractions. GBD=Global Burden of Disease. YLLs=years of life lost. YLDs=years live with disability. DALYs=disability-adjusted life-years

	Deaths 2016	YLLs 2016	YLDs 2016	DALYs 2016
Poisonings	19.3% (13.7% to 26.2%)	19.2% (13.8% to 25.9%)	16.7% (11.9% to 22.9%)	18.6% (13.4% to 25.1%)
Exposure to mechanical forces	11.7% (10.4% to 14.2%)	9.9% (8.4% to 13.1%)	18.2% (17.3% to 19.0%)	14.4% (12.9% to 16.2%)
Animal contact	11.4% (10.7% to 12.5%)	8.7% (8.0% to 9.7%)	9.3% (8.7% to 9.8%)	9.2% (8.6% to 9.7%)
Foreign body	4.1% (3.8% to 4.7%)	5.5% (5.2% to 6.3%)	6.0% (5.4% to 6.6%)	5.7% (5.3% to 6.2%)
Other unintentional injuries	22.8% (16.9% to 30.5%)	23.7% (17.6% to 31.2%)	17.0% (11.8% to 24.0%)	18.1% (12.9% to 25.1%)
**Self-harm and interpersonal violence**	36.3% (27.2% to 44.6%)	37.5% (29.0% to 45.3%)	32.0% (26.5% to 37.1%)	37.0% (28.9% to 44.5%)
Self-harm	39.1% (26.1% to 50.3%)	42.4% (30.0% to 53.2%)	33.3% (21.1% to 44.2%)	42.1% (29.7% to 52.9%)
Interpersonal violence	29.7% (22.0% to 37.8%)	28.4% (21.1% to 36.1%)	31.6% (25.6% to 37.0%)	28.9% (21.9% to 36.4%)
**Forces of nature, conflict and terrorism, and executions and police conflict**	0.0% (0.0% to 0.0%)	0.0% (0.0% to 0.0%)	1.4% (0.9% to 2.2%)	0.6% (0.2% to 1.2%)
Forces of nature, conflict and terrorism, and state actor violence	0.0% (0.0% to 0.0%)	0.0% (0.0% to 0.0%)	6.5% (4.2% to 9.9%)	6.5% (4.2% to 9.9%)

Supplementary Results Table 1. Age-standardised PAFs attributable to the joint distribution of all risk factors for all ages, both sexes combined for each GBD level 1 and level 2 cause and all causes in 2016 for North Africa and Middle East. Results are shown for deaths, YLLs, YLDs, and
Data in parenthesis are 95% uncertainty intervals. PAFs=population attributable fractions. GBD=Global Burden of Disease. YLLs=years of life lost. YLDs=years live with disability. DALYs=disability-adjusted life-years

	Deaths 2016	YLLs 2016	YLDs 2016	DALYs 2016
**All causes**	61.1% (59.3% to 63.0%)	52.8% (51.2% to 54.6%)	26.2% (24.8% to 27.7%)	42.8% (40.7% to 45.1%)
**HIV/AIDS and tuberculosis**	27.4% (21.5% to 34.2%)	29.7% (24.4% to 36.2%)	37.5% (30.2% to 46.9%)	30.0% (24.8% to 36.4%)
Tuberculosis	22.8% (16.5% to 29.4%)	22.2% (16.9% to 27.6%)	21.2% (16.3% to 26.0%)	22.1% (16.9% to 27.6%)
HIV/AIDS	80.6% (77.1% to 84.4%)	78.2% (74.3% to 82.5%)	81.6% (78.5% to 85.1%)	78.4% (74.6% to 82.6%)
**Diarrhoea, lower respiratory infections, and other common infectious diseases**	61.5% (57.0% to 66.2%)	68.1% (63.5% to 72.5%)	35.5% (28.2% to 42.0%)	62.9% (58.6% to 67.3%)
Diarrhoeal diseases	90.0% (80.8% to 96.2%)	95.7% (91.3% to 98.4%)	80.8% (65.8% to 91.9%)	92.1% (85.0% to 96.9%)
Lower respiratory infections	65.0% (59.3% to 70.6%)	78.1% (73.1% to 82.6%)	62.0% (56.2% to 67.8%)	77.9% (72.9% to 82.4%)
Upper respiratory infections	2.2% (0.9% to 4.3%)	4.3% (1.7% to 8.7%)	0.0% (0.0% to 0.0%)	0.1% (0.0% to 0.1%)
Otitis media	3.4% (1.2% to 6.9%)	7.3% (3.9% to 11.1%)	7.3% (4.9% to 9.6%)	7.3% (5.0% to 9.6%)
Meningitis	4.5% (2.8% to 6.8%)	6.3% (4.2% to 9.1%)	0.0% (0.0% to 0.0%)	5.6% (3.7% to 8.2%)
Encephalitis	1.9% (0.9% to 3.5%)	3.2% (1.6% to 5.6%)	0.0% (0.0% to 0.0%)	1.7% (0.8% to 3.3%)
Measles	62.4% (49.0% to 79.2%)	63.7% (50.1% to 80.6%)	43.4% (25.4% to 73.5%)	63.5% (49.8% to 80.5%)
**Maternal disorders**	9.4% (4.1% to 14.9%)	9.2% (3.9% to 14.9%)	3.1% (1.4% to 5.2%)	8.7% (3.7% to 13.9%)
Maternal haemorrhage	18.5% (6.7% to 30.2%)	18.5% (6.7% to 30.2%)	15.9% (5.7% to 26.0%)	18.5% (6.7% to 30.1%)
Maternal sepsis and other pregnancy related infections	18.8% (6.8% to 30.5%)	18.8% (6.8% to 30.4%)	16.1% (5.8% to 26.2%)	18.7% (6.8% to 30.3%)
Maternal abortion, miscarriage, and ectopic pregnancy	18.2% (11.7% to 25.8%)	17.7% (11.5% to 25.1%)	13.9% (8.0% to 20.1%)	17.4% (11.3% to 24.7%)
**Neonatal disorders**	77.9% (75.4% to 80.1%)	77.9% (75.5% to 80.1%)	0.1% (0.1% to 0.2%)	70.3% (64.3% to 74.1%)
Neonatal preterm birth complications	89.8% (87.3% to 91.9%)	89.9% (87.4% to 91.9%)	0.3% (0.2% to 0.3%)	83.1% (79.0% to 86.6%)
Neonatal encephalopathy due to birth asphyxia and trauma	63.8% (59.4% to 67.9%)	63.9% (59.5% to 68.0%)	0.0% (0.0% to 0.0%)	59.9% (53.2% to 65.4%)
Neonatal sepsis and other neonatal infections	57.6% (52.5% to 62.4%)	57.7% (52.6% to 62.4%)	0.0% (0.0% to 0.0%)	45.5% (28.4% to 54.8%)
Hemolytic disease and other neonatal jaundice	67.8% (63.5% to 71.9%)	67.9% (63.5% to 71.9%)	0.0% (0.0% to 0.0%)	39.1% (31.0% to 48.0%)

Supplementary Results Table 1. Age-standardised PAFs attributable to the joint distribution of all risk factors for all ages, both sexes combined for each GBD level 1 and level 2 cause and all causes in 2016 for North Africa and Middle East. Results are shown for deaths, YLLs, YLDs, and Data in parenthesis are 95% uncertainty intervals. PAFs=population attributable fractions. GBD=Global Burden of Disease. YLLs=years of life lost. YLDs=years live with disability. DALYs=disability-adjusted life-years				
	**Deaths 2016**	**YLLs 2016**	**YLDs 2016**	**DALYs 2016**
Other neonatal disorders	61.7% (57.4% to 65.5%)	61.8% (57.5% to 65.6%)	0.0% (0.0% to 0.0%)	60.0% (55.5% to 63.8%)
**Nutritional deficiencies**	16.2% (12.8% to 20.6%)	47.2% (40.6% to 54.3%)	87.9% (85.5% to 90.0%)	85.2% (82.5% to 87.6%)
Protein-energy malnutrition	17.0% (12.8% to 22.3%)	52.2% (44.6% to 59.9%)	97.3% (96.9% to 97.5%)	74.6% (68.7% to 79.3%)
Vitamin A deficiency	-	-	99.9% (99.9% to 99.9%)	99.9% (99.9% to 99.9%)
Iron-deficiency anaemia	100.0% (100.0% to 100.0%)	100.0% (100.0% to 100.0%)	99.9% (99.9% to 99.9%)	99.9% (99.9% to 99.9%)
**Other communicable, maternal, neonatal, and nutritional diseases**	1.6% (1.1% to 2.2%)	1.0% (0.6% to 1.6%)	78.3% (73.1% to 83.0%)	26.5% (16.6% to 37.8%)
Sexually transmitted diseases excluding HIV	3.2% (1.6% to 6.0%)	1.3% (0.6% to 2.5%)	99.9% (99.9% to 99.9%)	38.6% (22.9% to 57.6%)
Hepatitis	1.3% (0.9% to 1.7%)	1.3% (1.0% to 1.8%)	1.0% (0.6% to 1.5%)	1.3% (1.0% to 1.8%)
**Neoplasms**	35.4% (31.9% to 39.3%)	33.9% (30.7% to 37.5%)	29.4% (25.9% to 33.4%)	33.8% (30.6% to 37.4%)
Oesophageal cancer	60.2% (46.7% to 72.9%)	61.7% (48.5% to 74.5%)	60.1% (46.4% to 72.9%)	61.7% (48.4% to 74.5%)
Stomach cancer	14.4% (7.2% to 22.6%)	15.2% (8.1% to 23.1%)	14.4% (7.3% to 22.2%)	15.2% (8.1% to 23.1%)
Liver cancer	44.7% (34.3% to 55.8%)	47.6% (37.1% to 58.4%)	45.4% (35.0% to 56.6%)	47.6% (37.1% to 58.4%)
Larynx cancer	68.7% (59.3% to 76.6%)	70.0% (61.1% to 77.5%)	69.0% (60.4% to 76.6%)	69.9% (61.1% to 77.5%)
Tracheal, bronchus, and lung cancer	84.1% (80.5% to 87.3%)	84.6% (81.3% to 87.6%)	83.4% (80.0% to 86.6%)	84.6% (81.3% to 87.6%)
Breast cancer	19.1% (10.6% to 29.0%)	15.5% (7.6% to 24.4%)	16.7% (8.5% to 26.0%)	15.5% (7.6% to 24.4%)
Cervical cancer	100.0% (100.0% to 100.0%)	100.0% (100.0% to 100.0%)	99.9% (99.9% to 99.9%)	100.0% (100.0% to 100.0%)
Uterine cancer	50.1% (37.2% to 63.0%)	50.6% (37.7% to 63.4%)	53.1% (39.5% to 66.1%)	50.7% (37.8% to 63.5%)
Prostate cancer	3.6% (2.4% to 4.9%)	3.9% (2.7% to 5.3%)	4.1% (3.0% to 5.5%)	3.9% (2.8% to 5.3%)
Colon and rectum cancer	46.8% (36.7% to 57.0%)	46.8% (37.3% to 56.4%)	46.5% (36.6% to 56.6%)	46.8% (37.3% to 56.4%)
Lip and oral cavity cancer	47.0% (40.4% to 53.5%)	49.2% (43.0% to 55.5%)	44.2% (38.4% to 50.7%)	49.0% (42.9% to 55.3%)
Nasopharynx cancer	51.0% (44.0% to 57.4%)	50.7% (44.2% to 56.4%)	49.2% (43.1% to 55.2%)	50.7% (44.1% to 56.4%)

Supplementary Results Table 1. Age-standardised PAFs attributable to the joint distribution of all risk factors for all ages, both sexes combined for each GBD level 1 and level 2 cause and all causes in 2016 for North Africa and Middle East. Results are shown for deaths, YLLs, YLDs, and
Data in parenthesis are 95% uncertainty intervals. PAFs=population attributable fractions. GBD=Global Burden of Disease. YLLs=years of life lost. YLDs=years live with disability. DALYs=disability-adjusted life-years

	Deaths 2016	YLLs 2016	YLDs 2016	DALYs 2016
Other pharynx cancer	16.2% (10.0% to 23.4%)	17.7% (11.2% to 24.4%)	16.6% (10.5% to 23.6%)	17.6% (11.2% to 24.4%)
Gallbladder and biliary tract cancer	24.9% (14.9% to 36.7%)	25.8% (15.6% to 37.8%)	25.4% (15.3% to 37.2%)	25.8% (15.6% to 37.8%)
Pancreatic cancer	28.5% (19.3% to 39.0%)	29.8% (21.1% to 39.9%)	28.6% (19.6% to 38.9%)	29.8% (21.1% to 39.9%)
Ovarian cancer	15.7% (6.5% to 26.9%)	14.1% (5.6% to 24.6%)	14.0% (5.6% to 24.1%)	14.1% (5.6% to 24.6%)
Kidney cancer	33.6% (24.4% to 43.9%)	32.6% (23.9% to 42.3%)	33.4% (24.8% to 43.3%)	32.7% (23.9% to 42.3%)
Bladder cancer	28.0% (18.5% to 38.7%)	28.7% (19.4% to 39.2%)	28.7% (19.5% to 39.0%)	28.7% (19.4% to 39.2%)
Thyroid cancer	15.4% (8.7% to 24.3%)	15.7% (8.8% to 24.9%)	16.6% (9.6% to 25.1%)	15.7% (8.8% to 24.9%)
Mesothelioma	91.3% (88.8% to 93.2%)	85.0% (81.1% to 88.2%)	89.5% (86.8% to 91.8%)	85.0% (81.2% to 88.2%)
Non-Hodgkin's lymphoma	7.6% (3.3% to 13.0%)	7.1% (3.0% to 12.2%)	7.8% (3.3% to 13.2%)	7.1% (3.1% to 12.2%)
Multiple myeloma	9.2% (4.2% to 15.6%)	9.5% (4.3% to 16.0%)	9.6% (4.4% to 16.3%)	9.5% (4.3% to 16.0%)
Leukaemia	16.2% (10.5% to 22.2%)	13.4% (8.9% to 18.4%)	15.7% (10.2% to 21.4%)	13.4% (8.9% to 18.5%)
**Cardiovascular diseases**	87.8% (85.3% to 89.9%)	88.5% (86.9% to 90.0%)	74.5% (71.7% to 77.3%)	87.3% (85.7% to 88.8%)
Rheumatic heart disease	26.3% (18.4% to 39.8%)	23.4% (16.2% to 33.9%)	21.4% (13.7% to 30.7%)	22.9% (15.5% to 32.9%)
Ischaemic heart disease	93.8% (91.0% to 96.1%)	95.1% (93.4% to 96.5%)	92.0% (89.7% to 94.2%)	95.0% (93.3% to 96.4%)
Cerebrovascular disease	84.2% (79.7% to 89.0%)	86.3% (83.5% to 89.0%)	86.3% (82.4% to 90.3%)	86.3% (83.5% to 89.1%)
Hypertensive heart disease	100.0% (100.0% to 100.0%)	100.0% (100.0% to 100.0%)	99.9% (99.9% to 99.9%)	100.0% (100.0% to 100.0%)
Cardiomyopathy and myocarditis	38.2% (31.6% to 45.8%)	31.8% (26.4% to 37.2%)	31.7% (26.0% to 37.9%)	31.8% (26.4% to 37.2%)
Atrial fibrillation and flutter	47.0% (39.7% to 54.3%)	51.6% (44.8% to 57.8%)	58.4% (53.5% to 63.6%)	55.5% (49.6% to 60.9%)
Aortic aneurysm	44.7% (38.0% to 51.4%)	50.9% (45.4% to 56.1%)	-	50.9% (45.4% to 56.1%)
Peripheral vascular disease	55.7% (47.6% to 62.6%)	63.9% (55.8% to 69.5%)	65.9% (61.0% to 70.7%)	65.1% (59.6% to 70.0%)
Endocarditis	38.9% (29.8% to 50.2%)	36.2% (28.8% to 44.0%)	39.5% (30.7% to 48.9%)	36.3% (28.9% to 44.0%)

Supplementary Results Table 1. Age-standardised PAFs attributable to the joint distribution of all risk factors for all ages, both sexes combined for each GBD level 1 and level 2 cause and all causes in 2016 for North Africa and Middle East. Results are shown for deaths, YLLs, YLDs, and
Data in parenthesis are 95% uncertainty intervals. PAFs=population attributable fractions. GBD=Global Burden of Disease. YLLs=years of life lost. YLDs=years live with disability. DALYs=disability-adjusted life-years

	Deaths 2016	YLLs 2016	YLDs 2016	DALYs 2016
Other cardiovascular and circulatory diseases	43.4% (38.6% to 48.3%)	44.0% (40.0% to 48.0%)	45.8% (41.1% to 50.4%)	44.5% (40.5% to 48.5%)
**Chronic respiratory diseases**	59.3% (53.4% to 65.2%)	57.9% (52.8% to 63.3%)	45.4% (38.9% to 52.0%)	53.1% (48.1% to 58.8%)
Chronic obstructive pulmonary disease	70.1% (63.0% to 77.4%)	71.1% (64.7% to 77.8%)	65.7% (59.1% to 72.8%)	69.9% (63.5% to 76.6%)
Pneumoconiosis	100.0% (100.0% to 100.0%)	100.0% (100.0% to 100.0%)	99.9% (99.9% to 99.9%)	100.0% (100.0% to 100.0%)
Asthma	41.4% (32.1% to 51.9%)	42.2% (33.6% to 51.8%)	36.0% (27.2% to 45.7%)	38.3% (29.9% to 48.0%)
Other chronic respiratory diseases	10.4% (7.4% to 13.7%)	9.4% (6.6% to 12.2%)	6.1% (4.2% to 8.3%)	8.7% (6.1% to 11.4%)
**Cirrhosis and other chronic liver diseases**	17.6% (15.0% to 20.6%)	22.9% (19.5% to 26.6%)	20.9% (18.3% to 23.6%)	22.8% (19.5% to 26.5%)
Cirrhosis and other chronic liver diseases due to hepatitis B	0.3% (0.2% to 0.4%)	0.3% (0.2% to 0.5%)	0.3% (0.2% to 0.5%)	0.3% (0.2% to 0.5%)
Cirrhosis and other chronic liver diseases due to hepatitis C	20.7% (15.7% to 27.0%)	33.3% (26.2% to 41.1%)	48.0% (41.6% to 54.0%)	33.7% (26.6% to 41.5%)
Cirrhosis and other chronic liver diseases due to alcohol use	100.0% (100.0% to 100.0%)	100.0% (100.0% to 100.0%)	99.9% (99.9% to 99.9%)	100.0% (100.0% to 100.0%)
**Digestive diseases**	11.2% (8.0% to 14.3%)	11.0% (7.8% to 14.1%)	2.9% (2.2% to 3.6%)	8.3% (6.0% to 10.4%)
Peptic ulcer disease	11.7% (8.1% to 15.5%)	13.5% (9.5% to 17.8%)	7.6% (5.4% to 10.1%)	11.6% (8.1% to 15.3%)
Gallbladder and biliary diseases	46.2% (33.2% to 59.4%)	47.0% (33.7% to 59.8%)	49.0% (35.8% to 62.1%)	47.1% (34.0% to 60.0%)
Pancreatitis	9.1% (4.6% to 15.4%)	11.3% (6.4% to 18.1%)	10.2% (5.9% to 15.9%)	11.2% (6.4% to 18.0%)
**Neurological disorders**	25.2% (12.8% to 39.2%)	22.7% (12.1% to 34.9%)	3.7% (2.0% to 5.8%)	10.3% (5.3% to 16.3%)
Alzheimer's disease and other dementias	28.9% (15.0% to 44.7%)	30.0% (16.1% to 45.6%)	30.1% (15.7% to 46.0%)	30.0% (16.1% to 45.7%)
Parkinson's disease	-9.4% (-11.4% to -7.4%)	-11.5% (-13.8% to -9.0%)	-12.1% (-14.4% to -9.4%)	-11.6% (-13.9% to -9.1%)
Epilepsy	4.8% (3.0% to 6.7%)	4.0% (2.6% to 5.6%)	3.5% (2.1% to 5.4%)	3.7% (2.3% to 5.4%)
Multiple sclerosis	8.1% (4.8% to 11.7%)	8.3% (4.9% to 11.9%)	6.4% (4.0% to 9.0%)	7.1% (4.3% to 10.2%)
**Mental and substance use disorders**	99.8% (99.7% to 99.8%)	99.7% (99.6% to 99.8%)	20.3% (17.7% to 23.1%)	22.5% (19.7% to 25.5%)
Alcohol use disorders	100.0% (100.0% to 100.0%)	100.0% (100.0% to 100.0%)	99.9% (99.9% to 99.9%)	99.9% (99.9% to 99.9%)

Supplementary Results Table 1. Age-standardised PAFs attributable to the joint distribution of all risk factors for all ages, both sexes combined for each GBD level 1 and level 2 cause and all causes in 2016 for North Africa and Middle East. Results are shown for deaths, YLLs, YLDs, and Data in parenthesis are 95% uncertainty intervals. PAFs=population attributable fractions. GBD=Global Burden of Disease. YLLs=years of life lost. YLDs=years live with disability. DALYs=disability-adjusted life-years	Deaths 2016	YLLs 2016	YLDs 2016	DALYs 2016
Drug use disorders	100.0% (100.0% to 100.0%)	100.0% (100.0% to 100.0%)	99.9% (99.9% to 99.9%)	99.9% (99.9% to 99.9%)
Depressive disorders	-	-	8.2% (6.6% to 9.9%)	8.2% (6.6% to 9.9%)
Idiopathic developmental intellectual disability	-	-	66.8% (35.5% to 84.9%)	66.8% (35.5% to 84.9%)
**Diabetes, urogenital, blood, and endocrine diseases**	89.5% (88.5% to 90.4%)	85.2% (83.3% to 86.5%)	70.5% (66.2% to 74.3%)	78.2% (75.5% to 80.5%)
Diabetes mellitus	100.0% (100.0% to 100.0%)	100.0% (100.0% to 100.0%)	99.9% (99.9% to 99.9%)	99.9% (99.9% to 100.0%)
Chronic kidney disease	100.0% (100.0% to 100.0%)	100.0% (100.0% to 100.0%)	99.9% (99.9% to 99.9%)	100.0% (100.0% to 100.0%)
**Musculoskeletal disorders**	0.7% (0.3% to 1.2%)	0.5% (0.2% to 0.9%)	16.8% (13.7% to 20.2%)	16.6% (13.6% to 20.0%)
Rheumatoid arthritis	3.3% (1.3% to 5.6%)	3.4% (1.4% to 5.7%)	3.4% (1.4% to 5.6%)	3.4% (1.4% to 5.6%)
Osteoarthritis	-	-	31.9% (18.3% to 45.2%)	31.9% (18.3% to 45.2%)
Low back and neck pain	-	-	19.7% (16.8% to 22.9%)	19.7% (16.8% to 22.9%)
Gout	-	-	55.6% (41.8% to 69.9%)	55.6% (41.8% to 69.9%)
**Other non-communicable diseases**	0.1% (0.1% to 0.2%)	0.2% (0.1% to 0.2%)	5.5% (4.6% to 6.7%)	3.8% (3.1% to 4.6%)
Sense organ diseases	-	-	11.5% (9.8% to 13.6%)	11.5% (9.8% to 13.6%)
Sudden infant death syndrome	4.9% (3.5% to 6.5%)	4.9% (3.5% to 6.5%)	-	4.9% (3.5% to 6.5%)
**Transport injuries**	22.5% (21.0% to 24.0%)	19.1% (17.6% to 20.6%)	27.6% (25.9% to 29.4%)	19.9% (18.5% to 21.5%)
Road injuries	22.3% (20.9% to 23.9%)	19.0% (17.6% to 20.6%)	29.9% (28.2% to 31.8%)	19.9% (18.4% to 21.5%)
Other transport injuries	27.7% (25.9% to 29.6%)	21.0% (19.1% to 22.9%)	20.1% (18.5% to 21.8%)	20.5% (19.0% to 22.1%)
**Unintentional injuries**	22.3% (19.3% to 24.2%)	13.3% (12.2% to 14.4%)	17.3% (16.3% to 18.5%)	14.8% (13.8% to 15.9%)
Falls	45.7% (37.3% to 49.3%)	24.3% (21.2% to 27.0%)	25.2% (24.0% to 26.4%)	24.7% (22.9% to 26.4%)
Drowning	12.0% (10.7% to 13.4%)	10.2% (9.0% to 11.6%)	12.5% (11.2% to 13.9%)	10.3% (9.0% to 11.6%)
Fire, heat, and hot substances	8.3% (7.0% to 9.5%)	8.9% (7.8% to 10.1%)	11.5% (10.3% to 12.9%)	9.6% (8.6% to 10.9%)

Supplementary Results Table 1. Age-standardised PAFs attributable to the joint distribution of all risk factors for all ages, both sexes combined for each GBD level 1 and level 2 cause and all causes in 2016 for North Africa and Middle East. Results are shown for deaths, YLLs, YLDs, and
Data in parenthesis are 95% uncertainty intervals. PAFs=population attributable fractions. GBD=Global Burden of Disease. YLLs=years of life lost. YLDs=years live with disability. DALYs=disability-adjusted life-years

	**Deaths 2016**	**YLLs 2016**	**YLDs 2016**	**DALYs 2016**
Poisonings	9.4% (7.8% to 10.9%)	8.8% (7.1% to 10.3%)	11.8% (10.7% to 13.2%)	9.1% (7.6% to 10.6%)
Exposure to mechanical forces	19.1% (16.4% to 21.1%)	16.3% (13.1% to 18.5%)	22.2% (20.8% to 23.8%)	18.4% (16.2% to 20.3%)
Animal contact	11.3% (9.6% to 13.0%)	7.7% (6.3% to 9.6%)	11.2% (10.2% to 12.3%)	9.8% (8.7% to 11.0%)
Foreign body	6.2% (5.2% to 7.1%)	5.6% (4.6% to 6.5%)	9.1% (8.1% to 10.2%)	7.0% (6.1% to 7.9%)
Other unintentional injuries	16.0% (13.9% to 18.4%)	15.5% (13.5% to 17.9%)	11.6% (10.5% to 13.0%)	13.2% (11.8% to 14.8%)
**Self-harm and interpersonal violence**	10.4% (8.1% to 13.0%)	10.3% (8.0% to 13.0%)	22.1% (20.5% to 23.8%)	11.3% (9.1% to 13.8%)
Self-harm	10.9% (7.6% to 14.9%)	11.7% (8.1% to 15.8%)	9.7% (6.6% to 13.3%)	11.6% (8.1% to 15.7%)
Interpersonal violence	9.6% (7.0% to 12.8%)	8.5% (6.0% to 11.6%)	23.7% (22.2% to 25.5%)	11.1% (8.8% to 14.7%)
**Forces of nature, conflict and terrorism, and executions and police conflict**	0.0% (0.0% to 0.0%)	0.0% (0.0% to 0.0%)	0.2% (0.1% to 0.3%)	0.0% (0.0% to 0.0%)
Forces of nature, conflict and terrorism, and state actor violence	4.8% (4.4% to 5.1%)	1.8% (1.6% to 2.0%)	6.6% (4.6% to 9.5%)	5.3% (3.3% to 7.6%)

Supplementary Results Table 1. Age-standardised PAFs attributable to the joint distribution of all risk factors for all ages, both sexes combined for each GBD level 1 and level 2 cause and all causes in 2016 for Oceania. Results are shown for deaths, YLLs, YLDs, and DALYs
Data in parenthesis are 95% uncertainty intervals. PAFs=population attributable fractions. GBD=Global Burden of Disease. YLLs=years of life lost. YLDs=years live with disability. DALYs=disability-adjusted life-years

	Deaths 2016	YLLs 2016	YLDs 2016	DALYs 2016
**All causes**	66.9% (65.0% to 68.6%)	60.9% (58.8% to 62.9%)	29.3% (28.1% to 30.7%)	53.3% (51.0% to 55.7%)
**HIV/AIDS and tuberculosis**	60.7% (54.1% to 67.2%)	63.8% (58.4% to 69.0%)	61.3% (55.4% to 67.1%)	63.5% (58.5% to 68.6%)
Tuberculosis	47.9% (38.9% to 57.0%)	50.4% (42.3% to 58.3%)	44.7% (37.6% to 51.7%)	49.8% (41.7% to 57.4%)
HIV/AIDS	82.4% (76.2% to 88.5%)	76.4% (69.3% to 84.1%)	79.2% (74.8% to 82.8%)	76.6% (69.9% to 83.8%)
**Diarrhoea, lower respiratory infections, and other common infectious diseases**	72.5% (66.5% to 78.3%)	70.7% (64.3% to 76.3%)	44.5% (37.7% to 49.6%)	68.7% (62.8% to 74.4%)
Diarrhoeal diseases	91.7% (79.1% to 97.7%)	93.2% (82.1% to 98.2%)	83.7% (71.9% to 89.8%)	91.5% (80.3% to 96.8%)
Lower respiratory infections	73.7% (67.1% to 79.7%)	82.7% (76.6% to 87.9%)	65.8% (60.3% to 70.9%)	82.6% (76.5% to 87.8%)
Upper respiratory infections	1.5% (0.2% to 4.9%)	3.0% (0.4% to 9.7%)	0.0% (0.0% to 0.0%)	0.1% (0.0% to 0.5%)
Otitis media	4.1% (0.9% to 9.7%)	7.8% (2.2% to 15.5%)	8.5% (5.8% to 11.0%)	8.5% (5.8% to 11.0%)
Meningitis	2.4% (1.2% to 4.1%)	3.5% (1.8% to 5.8%)	0.0% (0.0% to 0.0%)	3.1% (1.7% to 5.2%)
Encephalitis	1.4% (0.7% to 2.6%)	2.4% (1.2% to 4.2%)	0.0% (0.0% to 0.0%)	1.8% (0.9% to 3.2%)
Measles	63.2% (47.4% to 81.9%)	64.3% (48.2% to 83.1%)	57.1% (42.4% to 76.4%)	64.3% (48.2% to 83.1%)
**Maternal disorders**	8.6% (3.5% to 14.0%)	8.5% (3.5% to 13.9%)	3.7% (1.8% to 6.1%)	8.3% (3.4% to 13.7%)
Maternal haemorrhage	20.1% (7.3% to 32.7%)	20.1% (7.2% to 32.6%)	18.7% (6.7% to 30.4%)	20.0% (7.2% to 32.6%)
Maternal sepsis and other pregnancy related infections	20.2% (7.3% to 32.7%)	20.1% (7.3% to 32.6%)	18.5% (6.7% to 30.2%)	20.1% (7.3% to 32.6%)
Maternal abortion, miscarriage, and ectopic pregnancy	15.7% (8.9% to 23.1%)	15.5% (8.7% to 22.7%)	13.9% (7.9% to 19.8%)	15.4% (8.7% to 22.4%)
**Neonatal disorders**	76.4% (68.7% to 81.9%)	76.5% (68.8% to 82.0%)	0.3% (0.2% to 0.4%)	71.5% (63.9% to 77.7%)
Neonatal preterm birth complications	92.3% (85.3% to 96.8%)	92.3% (85.4% to 96.8%)	0.4% (0.3% to 0.5%)	82.7% (74.5% to 89.8%)
Neonatal encephalopathy due to birth asphyxia and trauma	68.2% (59.4% to 74.5%)	68.2% (59.6% to 74.6%)	0.0% (0.0% to 0.0%)	66.5% (57.6% to 73.4%)
Neonatal sepsis and other neonatal infections	64.2% (54.0% to 71.0%)	64.2% (54.1% to 71.0%)	0.0% (0.0% to 0.0%)	60.7% (50.1% to 68.6%)
Hemolytic disease and other neonatal jaundice	68.7% (61.1% to 74.6%)	68.7% (61.1% to 74.7%)	0.0% (0.0% to 0.0%)	55.6% (45.1% to 65.0%)

Supplementary Results Table 1. Age-standardised PAFs attributable to the joint distribution of all risk factors for all ages, both sexes combined for each GBD level 1 and level 2 cause and all causes in 2016 for Oceania. Results are shown for deaths, YLLs, YLDs, and DALYs
Data in parenthesis are 95% uncertainty intervals. PAFs=population attributable fractions. GBD=Global Burden of Disease. YLLs=years of life lost. YLDs=years live with disability. DALYs=disability-adjusted life-years

	Deaths 2016	YLLs 2016	YLDs 2016	DALYs 2016
Other neonatal disorders	58.7% (45.9% to 68.3%)	58.8% (46.1% to 68.3%)	0.0% (0.0% to 0.0%)	58.1% (45.3% to 67.7%)
**Nutritional deficiencies**	39.1% (28.2% to 51.3%)	75.2% (64.0% to 85.1%)	93.0% (92.8% to 93.2%)	87.7% (85.0% to 90.1%)
Protein-energy malnutrition	27.1% (15.3% to 42.1%)	71.5% (56.8% to 83.8%)	95.1% (94.3% to 95.7%)	75.9% (65.6% to 85.2%)
Vitamin A deficiency	-	-	94.4% (93.8% to 94.8%)	94.4% (93.8% to 94.8%)
Iron-deficiency anaemia	100.0% (100.0% to 100.0%)	100.0% (100.0% to 100.0%)	93.5% (93.4% to 93.7%)	94.0% (93.8% to 94.3%)
**Other communicable, maternal, neonatal, and nutritional diseases**	3.9% (1.9% to 6.1%)	2.7% (1.1% to 4.9%)	56.9% (52.0% to 62.2%)	6.9% (3.2% to 11.9%)
Sexually transmitted diseases excluding HIV	7.6% (2.7% to 16.4%)	3.9% (1.3% to 8.7%)	93.1% (92.8% to 93.3%)	10.1% (3.9% to 21.1%)
Hepatitis	0.3% (0.2% to 0.5%)	0.4% (0.2% to 0.5%)	0.3% (0.2% to 0.5%)	0.4% (0.2% to 0.5%)
**Neoplasms**	43.1% (38.3% to 48.1%)	43.2% (38.5% to 48.0%)	38.5% (33.6% to 43.0%)	43.1% (38.5% to 47.9%)
Oesophageal cancer	72.9% (62.7% to 81.9%)	75.4% (65.7% to 83.6%)	63.4% (54.7% to 70.9%)	75.3% (65.5% to 83.5%)
Stomach cancer	12.5% (6.8% to 18.7%)	14.0% (7.9% to 20.9%)	11.2% (6.2% to 16.7%)	14.0% (7.8% to 20.8%)
Liver cancer	38.7% (28.2% to 48.8%)	40.3% (29.2% to 50.6%)	33.8% (24.6% to 42.7%)	40.3% (29.1% to 50.5%)
Larynx cancer	75.9% (66.5% to 83.3%)	77.5% (69.2% to 84.1%)	65.4% (57.5% to 71.9%)	77.2% (69.0% to 83.9%)
Tracheal, bronchus, and lung cancer	87.8% (83.8% to 91.2%)	87.3% (83.5% to 90.6%)	75.9% (72.3% to 79.0%)	87.2% (83.3% to 90.5%)
Breast cancer	43.1% (27.9% to 58.3%)	41.0% (27.4% to 55.0%)	38.2% (25.0% to 51.3%)	40.9% (27.3% to 54.9%)
Cervical cancer	100.0% (100.0% to 100.0%)	100.0% (100.0% to 100.0%)	90.9% (90.4% to 91.3%)	99.8% (99.7% to 99.8%)
Uterine cancer	36.1% (22.2% to 50.7%)	36.9% (22.8% to 51.5%)	36.2% (24.6% to 48.5%)	36.9% (22.9% to 51.4%)
Prostate cancer	3.7% (2.5% to 5.2%)	3.8% (2.6% to 5.6%)	3.2% (2.2% to 4.4%)	3.8% (2.6% to 5.6%)
Colon and rectum cancer	54.8% (43.7% to 66.2%)	54.8% (44.2% to 65.5%)	48.0% (38.6% to 57.9%)	54.6% (44.1% to 65.4%)
Lip and oral cavity cancer	65.8% (57.8% to 73.6%)	69.6% (62.5% to 76.7%)	57.5% (51.0% to 64.3%)	69.2% (62.1% to 76.3%)
Nasopharynx cancer	72.1% (63.8% to 79.5%)	73.2% (65.7% to 80.0%)	64.5% (57.7% to 70.8%)	73.0% (65.5% to 79.8%)

Supplementary Results Table 1. Age-standardised PAFs attributable to the joint distribution of all risk factors for all ages, both sexes combined for each GBD level 1 and level 2 cause and all causes in 2016 for Oceania. Results are shown for deaths, YLLs, YLDs, and DALYs
Data in parenthesis are 95% uncertainty intervals. PAFs=population attributable fractions. GBD=Global Burden of Disease. YLLs=years of life lost. YLDs=years live with disability. DALYs=disability-adjusted life-years

	Deaths 2016	YLLs 2016	YLDs 2016	DALYs 2016
Other pharynx cancer	34.3% (20.8% to 47.7%)	37.0% (23.5% to 50.0%)	30.7% (19.3% to 41.9%)	36.9% (23.4% to 49.9%)
Gallbladder and biliary tract cancer	17.9% (9.2% to 28.8%)	18.8% (9.6% to 30.1%)	15.7% (8.0% to 25.1%)	18.7% (9.6% to 30.0%)
Pancreatic cancer	34.2% (22.4% to 48.3%)	35.2% (23.8% to 48.6%)	29.5% (19.5% to 41.4%)	35.1% (23.8% to 48.5%)
Ovarian cancer	20.9% (7.3% to 38.5%)	19.3% (6.8% to 35.6%)	17.1% (6.0% to 31.6%)	19.2% (6.8% to 35.5%)
Kidney cancer	31.1% (22.3% to 41.0%)	30.7% (21.7% to 40.0%)	26.9% (19.3% to 34.8%)	30.6% (21.6% to 39.8%)
Bladder cancer	34.9% (22.8% to 48.8%)	36.1% (23.9% to 49.5%)	31.1% (20.5% to 43.0%)	36.0% (23.8% to 49.3%)
Thyroid cancer	11.3% (6.0% to 18.3%)	11.6% (6.1% to 18.8%)	12.1% (6.8% to 18.4%)	11.6% (6.1% to 18.8%)
Mesothelioma	86.5% (81.5% to 90.8%)	78.7% (71.5% to 85.1%)	74.0% (68.8% to 78.3%)	78.7% (71.4% to 85.0%)
Non-Hodgkin's lymphoma	5.5% (2.2% to 9.9%)	5.1% (2.0% to 9.0%)	4.9% (2.0% to 8.7%)	5.1% (2.0% to 9.0%)
Multiple myeloma	6.9% (2.8% to 12.6%)	7.2% (2.9% to 13.0%)	6.3% (2.6% to 11.3%)	7.1% (2.9% to 13.0%)
Leukaemia	13.4% (8.3% to 19.0%)	11.0% (6.9% to 15.6%)	11.3% (7.1% to 15.8%)	11.0% (6.9% to 15.7%)
**Cardiovascular diseases**	86.9% (85.1% to 88.8%)	85.1% (83.1% to 87.0%)	62.9% (58.8% to 66.6%)	84.0% (82.0% to 86.0%)
Rheumatic heart disease	22.9% (16.0% to 35.5%)	19.2% (13.0% to 28.3%)	16.6% (10.2% to 24.8%)	18.9% (12.7% to 27.5%)
Ischaemic heart disease	96.1% (94.1% to 97.7%)	96.8% (95.6% to 97.7%)	82.2% (80.3% to 83.7%)	96.5% (95.4% to 97.5%)
Cerebrovascular disease	89.5% (86.8% to 92.3%)	88.6% (85.7% to 91.0%)	79.2% (76.9% to 81.4%)	88.0% (85.2% to 90.3%)
Hypertensive heart disease	100.0% (100.0% to 100.0%)	100.0% (100.0% to 100.0%)	86.6% (86.1% to 87.0%)	99.6% (99.4% to 99.7%)
Cardiomyopathy and myocarditis	28.0% (22.8% to 34.2%)	25.2% (20.1% to 30.8%)	21.0% (17.1% to 25.2%)	25.1% (20.0% to 30.6%)
Atrial fibrillation and flutter	48.0% (40.6% to 55.1%)	52.8% (46.3% to 59.1%)	51.2% (46.6% to 56.2%)	51.9% (46.7% to 57.4%)
Aortic aneurysm	46.1% (39.5% to 52.6%)	52.5% (46.7% to 58.0%)	-	52.5% (46.7% to 58.0%)
Peripheral vascular disease	63.5% (57.6% to 69.5%)	73.6% (69.2% to 77.9%)	61.8% (58.0% to 65.5%)	65.9% (61.6% to 70.6%)
Endocarditis	35.5% (27.8% to 44.2%)	32.7% (26.0% to 39.7%)	29.8% (23.2% to 36.7%)	32.7% (26.0% to 39.7%)

Supplementary Results Table 1. Age-standardised PAFs attributable to the joint distribution of all risk factors for all ages, both sexes combined for each GBD level 1 and level 2 cause and all causes in 2016 for Oceania. Results are shown for deaths, YLLs, YLDs, and DALYs
Data in parenthesis are 95% uncertainty intervals. PAFs=population attributable fractions. GBD=Global Burden of Disease. YLLs=years of life lost. YLDs=years live with disability. DALYs=disability-adjusted life-years

	Deaths 2016	YLLs 2016	YLDs 2016	DALYs 2016
Other cardiovascular and circulatory diseases	47.3% (42.4% to 51.9%)	46.2% (40.9% to 51.2%)	36.7% (32.0% to 41.4%)	45.2% (40.3% to 50.1%)
**Chronic respiratory diseases**	61.0% (54.6% to 66.7%)	60.0% (54.0% to 65.3%)	46.4% (41.1% to 52.0%)	57.9% (52.3% to 63.1%)
Chronic obstructive pulmonary disease	79.7% (72.8% to 85.9%)	79.3% (73.1% to 85.2%)	64.2% (58.4% to 69.5%)	77.3% (71.0% to 83.1%)
Pneumoconiosis	100.0% (100.0% to 100.0%)	100.0% (100.0% to 100.0%)	86.1% (85.9% to 86.4%)	97.7% (96.5% to 98.6%)
Asthma	35.6% (26.7% to 45.8%)	39.9% (31.6% to 49.2%)	32.1% (25.1% to 40.2%)	38.3% (30.5% to 47.1%)
Other chronic respiratory diseases	16.2% (10.1% to 24.0%)	16.7% (10.4% to 24.6%)	7.6% (4.9% to 10.8%)	16.0% (10.1% to 23.5%)
**Cirrhosis and other chronic liver diseases**	17.4% (15.9% to 19.0%)	17.6% (16.0% to 19.5%)	11.3% (9.9% to 12.9%)	17.4% (15.8% to 19.3%)
Cirrhosis and other chronic liver diseases due to hepatitis B	0.1% (0.0% to 0.1%)	0.1% (0.0% to 0.1%)	0.1% (0.0% to 0.1%)	0.1% (0.0% to 0.1%)
Cirrhosis and other chronic liver diseases due to hepatitis C	16.8% (12.1% to 22.0%)	24.4% (17.9% to 31.1%)	18.3% (13.7% to 23.9%)	24.3% (17.9% to 31.0%)
Cirrhosis and other chronic liver diseases due to alcohol use	100.0% (100.0% to 100.0%)	100.0% (100.0% to 100.0%)	89.4% (89.0% to 89.9%)	99.8% (99.7% to 99.9%)
**Digestive diseases**	12.0% (9.2% to 14.8%)	12.6% (9.7% to 15.7%)	5.1% (3.8% to 6.6%)	12.0% (9.2% to 14.9%)
Peptic ulcer disease	17.0% (12.3% to 22.2%)	18.9% (13.7% to 24.9%)	14.9% (10.6% to 19.8%)	18.7% (13.5% to 24.6%)
Gallbladder and biliary diseases	34.4% (21.8% to 48.8%)	34.7% (22.6% to 48.6%)	34.7% (22.7% to 48.1%)	34.7% (22.6% to 48.6%)
Pancreatitis	29.2% (13.7% to 47.8%)	36.3% (19.6% to 53.5%)	26.6% (13.4% to 41.3%)	36.2% (19.6% to 53.4%)
**Neurological disorders**	26.9% (13.6% to 42.8%)	25.6% (14.6% to 38.4%)	5.5% (3.1% to 8.2%)	13.4% (7.6% to 20.5%)
Alzheimer's disease and other dementias	29.8% (14.7% to 47.3%)	31.3% (15.8% to 48.9%)	25.8% (13.0% to 40.5%)	30.0% (15.1% to 47.0%)
Parkinson's disease	-10.2% (-12.3% to -8.1%)	-13.3% (-16.0% to -10.4%)	-13.3% (-16.4% to -10.3%)	-13.3% (-16.1% to -10.4%)
Epilepsy	22.9% (16.0% to 30.2%)	22.5% (15.6% to 29.7%)	14.9% (10.0% to 20.9%)	18.6% (12.9% to 25.0%)
Multiple sclerosis	12.1% (7.3% to 18.3%)	12.8% (7.6% to 19.5%)	8.6% (5.5% to 11.8%)	11.9% (7.3% to 17.9%)
**Mental and substance use disorders**	99.7% (99.5% to 99.9%)	99.6% (99.3% to 99.9%)	15.3% (13.6% to 17.0%)	17.9% (15.7% to 21.4%)
Alcohol use disorders	100.0% (100.0% to 100.0%)	100.0% (100.0% to 100.0%)	93.0% (92.7% to 93.3%)	94.8% (94.0% to 96.9%)

Supplementary Results Table 1. Age-standardised PAFs attributable to the joint distribution of all risk factors for all ages, both sexes combined for each GBD level 1 and level 2 cause and all causes in 2016 for Oceania. Results are shown for deaths, YLLs, YLDs, and DALYs
Data in parenthesis are 95% uncertainty intervals. PAFs=population attributable fractions. GBD=Global Burden of Disease. YLLs=years of life lost. YLDs=years live with disability. DALYs=disability-adjusted life-years

	Deaths 2016	YLLs 2016	YLDs 2016	DALYs 2016
Drug use disorders	100.0% (100.0% to 100.0%)	100.0% (100.0% to 100.0%)	93.3% (93.1% to 93.6%)	94.5% (93.9% to 95.2%)
Depressive disorders	-	-	10.7% (9.0% to 12.4%)	10.7% (9.0% to 12.4%)
Idiopathic developmental intellectual disability	-	-	56.1% (27.5% to 74.6%)	56.1% (27.5% to 74.6%)
**Diabetes, urogenital, blood, and endocrine diseases**	94.1% (93.1% to 94.8%)	92.4% (90.9% to 93.4%)	70.0% (67.7% to 72.1%)	86.3% (84.2% to 88.1%)
Diabetes mellitus	100.0% (100.0% to 100.0%)	100.0% (100.0% to 100.0%)	89.4% (89.2% to 89.6%)	97.0% (96.3% to 97.8%)
Chronic kidney disease	100.0% (100.0% to 100.0%)	100.0% (100.0% to 100.0%)	88.4% (88.0% to 88.8%)	98.4% (97.9% to 98.8%)
**Musculoskeletal disorders**	1.0% (0.4% to 1.9%)	0.7% (0.3% to 1.4%)	16.5% (13.3% to 20.0%)	16.1% (12.9% to 19.5%)
Rheumatoid arthritis	5.7% (2.4% to 9.4%)	6.3% (2.6% to 10.6%)	5.9% (2.5% to 9.9%)	6.0% (2.5% to 10.0%)
Osteoarthritis	-	-	20.3% (10.4% to 31.5%)	20.3% (10.4% to 31.5%)
Low back and neck pain	-	-	21.1% (17.5% to 25.0%)	21.1% (17.5% to 25.0%)
Gout	-	-	38.2% (27.0% to 51.1%)	38.2% (27.0% to 51.1%)
**Other non-communicable diseases**	0.3% (0.2% to 0.6%)	0.5% (0.2% to 1.0%)	6.8% (5.8% to 7.9%)	4.8% (3.7% to 6.0%)
Sense organ diseases	-	-	12.9% (11.4% to 14.5%)	12.9% (11.4% to 14.5%)
Sudden infant death syndrome	14.0% (7.5% to 21.7%)	14.1% (7.6% to 21.8%)	-	14.1% (7.6% to 21.8%)
**Transport injuries**	28.5% (22.0% to 37.0%)	25.7% (18.9% to 34.4%)	28.4% (23.8% to 34.7%)	26.1% (19.5% to 34.4%)
Road injuries	28.3% (21.8% to 37.0%)	25.8% (19.0% to 34.5%)	31.3% (26.6% to 37.6%)	26.3% (19.7% to 34.8%)
Other transport injuries	30.1% (23.4% to 38.2%)	25.2% (18.6% to 33.3%)	22.8% (18.2% to 29.0%)	24.4% (18.6% to 32.2%)
**Unintentional injuries**	14.6% (11.2% to 17.7%)	10.8% (8.1% to 13.9%)	11.4% (10.0% to 12.9%)	10.9% (8.4% to 13.7%)
Falls	48.1% (26.3% to 56.4%)	24.1% (17.9% to 28.8%)	20.1% (18.9% to 21.5%)	22.3% (18.7% to 25.0%)
Drowning	13.8% (9.5% to 19.3%)	12.9% (8.8% to 18.1%)	11.8% (8.2% to 16.3%)	12.9% (8.8% to 18.1%)
Fire, heat, and hot substances	8.6% (5.8% to 12.5%)	8.8% (5.9% to 12.6%)	10.5% (7.4% to 14.5%)	9.1% (6.1% to 12.9%)

Supplementary Results Table 1. Age-standardised PAFs attributable to the joint distribution of all risk factors for all ages, both sexes combined for each GBD level 1 and level 2 cause and all causes in 2016 for Oceania. Results are shown for deaths, YLLs, YLDs, and DALYs
Data in parenthesis are 95% uncertainty intervals. PAFs=population attributable fractions. GBD=Global Burden of Disease. YLLs=years of life lost. YLDs=years live with disability. DALYs=disability-adjusted life-years

	**Deaths 2016**	**YLLs 2016**	**YLDs 2016**	**DALYs 2016**
Poisonings	11.6% (7.5% to 16.7%)	12.0% (7.6% to 17.4%)	11.6% (8.2% to 15.6%)	11.9% (7.6% to 17.3%)
Exposure to mechanical forces	13.5% (11.4% to 15.7%)	10.3% (8.2% to 12.6%)	14.9% (13.6% to 16.6%)	11.2% (9.2% to 13.4%)
Animal contact	7.8% (5.8% to 10.6%)	7.2% (4.9% to 10.5%)	7.4% (6.1% to 9.0%)	7.2% (5.3% to 9.9%)
Foreign body	4.8% (3.6% to 5.9%)	4.9% (3.3% to 6.6%)	5.2% (4.2% to 6.5%)	4.9% (3.6% to 6.4%)
Other unintentional injuries	14.8% (10.0% to 20.8%)	15.7% (10.8% to 22.1%)	10.9% (7.6% to 15.0%)	13.9% (9.7% to 19.5%)
**Self-harm and interpersonal violence**	23.6% (16.5% to 32.1%)	24.3% (16.8% to 32.8%)	27.5% (24.1% to 32.0%)	24.5% (17.4% to 32.6%)
Self-harm	22.7% (12.7% to 33.6%)	24.0% (13.8% to 35.3%)	17.9% (9.9% to 26.1%)	24.0% (13.7% to 35.2%)
Interpersonal violence	25.5% (18.4% to 34.3%)	24.6% (17.4% to 33.5%)	28.4% (25.0% to 33.0%)	25.3% (18.8% to 33.4%)
**Forces of nature, conflict and terrorism, and executions and police conflict**	0.0% (0.0% to 0.0%)	0.0% (0.0% to 0.0%)	4.4% (3.0% to 6.3%)	4.2% (2.9% to 6.1%)
Forces of nature, conflict and terrorism, and state actor violence	0.0% (0.0% to 0.0%)	0.0% (0.0% to 0.0%)	5.8% (4.0% to 8.3%)	5.8% (4.0% to 8.3%)

Supplementary Results Table 1. Age-standardised PAFs attributable to the joint distribution of all risk factors for all ages, both sexes combined for each GBD level 1 and level 2 cause and all causes in 2016 for South Asia. Results are shown for deaths, YLLs, YLDs, and DALYs
Data in parenthesis are 95% uncertainty intervals. PAFs=population attributable fractions. GBD=Global Burden of Disease. YLLs=years of life lost. YLDs=years live with disability. DALYs=disability-adjusted life-years

	Deaths 2016	YLLs 2016	YLDs 2016	DALYs 2016
**All causes**	63.7% (61.8% to 65.6%)	58.6% (57.2% to 60.1%)	30.0% (28.7% to 31.2%)	49.8% (47.8% to 51.9%)
**HIV/AIDS and tuberculosis**	35.9% (30.3% to 41.3%)	39.3% (34.5% to 43.8%)	41.2% (35.8% to 46.1%)	39.4% (34.7% to 43.9%)
Tuberculosis	30.3% (23.9% to 36.2%)	30.5% (24.7% to 36.0%)	32.2% (26.3% to 38.2%)	30.6% (24.8% to 36.1%)
HIV/AIDS	78.1% (75.4% to 80.5%)	74.7% (71.8% to 77.2%)	78.6% (75.6% to 81.2%)	74.9% (72.1% to 77.5%)
**Diarrhoea, lower respiratory infections, and other common infectious diseases**	76.2% (68.7% to 82.4%)	70.9% (64.9% to 76.6%)	43.2% (36.4% to 48.7%)	68.9% (63.0% to 74.5%)
Diarrhoeal diseases	91.2% (78.8% to 97.4%)	93.3% (83.9% to 98.1%)	90.4% (77.7% to 97.1%)	93.0% (83.4% to 98.0%)
Lower respiratory infections	77.3% (72.9% to 81.9%)	86.6% (83.7% to 89.5%)	74.2% (69.3% to 79.1%)	86.5% (83.6% to 89.4%)
Upper respiratory infections	2.4% (1.3% to 3.9%)	4.9% (2.8% to 8.0%)	0.0% (0.0% to 0.0%)	0.1% (0.1% to 0.3%)
Otitis media	2.4% (1.3% to 4.0%)	5.8% (3.5% to 8.4%)	7.2% (4.8% to 9.3%)	7.2% (4.8% to 9.3%)
Meningitis	2.8% (1.5% to 4.2%)	4.7% (2.9% to 6.8%)	0.0% (0.0% to 0.0%)	4.3% (2.7% to 6.2%)
Encephalitis	0.6% (0.5% to 0.7%)	1.4% (1.2% to 1.6%)	0.0% (0.0% to 0.0%)	1.2% (1.0% to 1.4%)
Measles	75.4% (66.6% to 85.2%)	76.6% (67.7% to 86.5%)	72.2% (63.1% to 82.4%)	76.5% (67.7% to 86.4%)
**Maternal disorders**	13.7% (5.9% to 21.6%)	13.7% (5.8% to 21.7%)	2.6% (1.3% to 4.1%)	13.0% (5.6% to 20.7%)
Maternal haemorrhage	22.5% (8.5% to 35.8%)	22.6% (8.6% to 36.0%)	24.0% (8.9% to 38.0%)	22.7% (8.6% to 36.0%)
Maternal sepsis and other pregnancy related infections	24.3% (9.0% to 38.6%)	24.4% (9.1% to 38.7%)	22.7% (8.5% to 36.0%)	24.4% (9.1% to 38.7%)
Maternal abortion, miscarriage, and ectopic pregnancy	16.1% (10.5% to 22.0%)	16.1% (10.2% to 22.1%)	16.6% (8.9% to 24.8%)	16.2% (10.2% to 22.0%)
**Neonatal disorders**	82.6% (80.5% to 84.9%)	82.6% (80.5% to 84.9%)	0.3% (0.2% to 0.3%)	75.6% (72.5% to 78.5%)
Neonatal preterm birth complications	97.1% (95.9% to 98.1%)	97.1% (95.9% to 98.2%)	0.4% (0.3% to 0.4%)	80.7% (75.3% to 85.2%)
Neonatal encephalopathy due to birth asphyxia and trauma	76.7% (73.6% to 80.1%)	76.7% (73.7% to 80.1%)	0.0% (0.0% to 0.0%)	75.4% (71.5% to 79.1%)
Neonatal sepsis and other neonatal infections	71.4% (68.3% to 74.3%)	71.4% (68.3% to 74.3%)	0.0% (0.0% to 0.0%)	60.4% (47.6% to 68.8%)
Hemolytic disease and other neonatal jaundice	70.4% (67.0% to 73.4%)	70.4% (67.0% to 73.4%)	0.0% (0.0% to 0.0%)	65.0% (61.7% to 68.1%)

Supplementary Results Table 1. Age-standardised PAFs attributable to the joint distribution of all risk factors for all ages, both sexes combined for each GBD level 1 and level 2 cause and all causes in 2016 for South Asia. Results are shown for deaths, YLLs, YLDs, and DALYs
Data in parenthesis are 95% uncertainty intervals. PAFs=population attributable fractions. GBD=Global Burden of Disease. YLLs=years of life lost. YLDs=years live with disability. DALYs=disability-adjusted life-years

	Deaths 2016	YLLs 2016	YLDs 2016	DALYs 2016
Other neonatal disorders	76.1% (73.0% to 78.9%)	76.1% (73.0% to 78.9%)	0.0% (0.0% to 0.0%)	75.0% (71.9% to 78.0%)
**Nutritional deficiencies**	25.9% (23.1% to 29.5%)	55.6% (52.2% to 59.5%)	94.1% (93.1% to 94.9%)	88.4% (86.0% to 90.3%)
Protein-energy malnutrition	46.6% (42.3% to 51.0%)	81.0% (78.3% to 83.5%)	97.7% (97.6% to 97.8%)	88.3% (86.1% to 90.3%)
Vitamin A deficiency	-	-	100.0% (100.0% to 100.0%)	100.0% (100.0% to 100.0%)
Iron-deficiency anaemia	100.0% (100.0% to 100.0%)	100.0% (100.0% to 100.0%)	100.0% (100.0% to 100.0%)	100.0% (100.0% to 100.0%)
**Other communicable, maternal, neonatal, and nutritional diseases**	2.7% (2.2% to 3.2%)	2.3% (1.8% to 2.8%)	38.6% (33.4% to 44.3%)	8.5% (6.2% to 11.3%)
Sexually transmitted diseases excluding HIV	22.7% (12.8% to 37.0%)	10.3% (5.4% to 18.6%)	100.0% (100.0% to 100.0%)	34.3% (20.0% to 51.6%)
Hepatitis	0.1% (0.1% to 0.2%)	0.1% (0.1% to 0.2%)	0.1% (0.1% to 0.2%)	0.1% (0.1% to 0.2%)
**Neoplasms**	32.8% (30.2% to 35.6%)	31.9% (29.3% to 34.4%)	33.1% (30.4% to 35.8%)	31.9% (29.4% to 34.4%)
Oesophageal cancer	60.1% (49.4% to 70.0%)	61.8% (51.3% to 71.2%)	61.0% (50.2% to 70.6%)	61.7% (51.3% to 71.2%)
Stomach cancer	8.8% (3.6% to 16.7%)	9.0% (3.9% to 15.9%)	9.3% (4.0% to 16.9%)	9.0% (3.9% to 15.9%)
Liver cancer	26.6% (20.4% to 33.3%)	27.3% (21.1% to 34.1%)	27.1% (20.7% to 34.0%)	27.3% (21.1% to 34.1%)
Larynx cancer	53.6% (43.8% to 63.1%)	57.2% (47.5% to 66.4%)	55.3% (45.7% to 64.3%)	57.1% (47.5% to 66.4%)
Tracheal, bronchus, and lung cancer	82.5% (78.5% to 86.4%)	82.7% (78.8% to 86.4%)	83.0% (79.2% to 86.7%)	82.7% (78.8% to 86.4%)
Breast cancer	13.8% (7.4% to 22.2%)	12.3% (6.4% to 20.0%)	12.8% (6.7% to 20.9%)	12.3% (6.4% to 20.0%)
Cervical cancer	100.0% (100.0% to 100.0%)	100.0% (100.0% to 100.0%)	100.0% (100.0% to 100.0%)	100.0% (100.0% to 100.0%)
Uterine cancer	22.0% (13.1% to 32.2%)	23.6% (14.4% to 34.2%)	23.5% (14.4% to 33.6%)	23.6% (14.4% to 34.2%)
Prostate cancer	1.4% (1.0% to 1.9%)	1.6% (1.1% to 2.1%)	1.6% (1.1% to 2.2%)	1.6% (1.1% to 2.1%)
Colon and rectum cancer	45.4% (36.2% to 54.9%)	44.8% (36.0% to 54.0%)	45.5% (36.4% to 54.9%)	44.8% (36.0% to 54.0%)
Lip and oral cavity cancer	63.4% (56.7% to 69.5%)	64.4% (57.7% to 70.4%)	62.1% (55.8% to 68.2%)	64.3% (57.7% to 70.3%)
Nasopharynx cancer	44.6% (37.2% to 52.0%)	45.3% (38.0% to 52.1%)	45.4% (38.2% to 51.9%)	45.3% (38.0% to 52.1%)

Supplementary Results Table 1. Age-standardised PAFs attributable to the joint distribution of all risk factors for all ages, both sexes combined for each GBD level 1 and level 2 cause and all causes in 2016 for South Asia. Results are shown for deaths, YLLs, YLDs, and DALYs
Data in parenthesis are 95% uncertainty intervals. PAFs=population attributable fractions. GBD=Global Burden of Disease. YLLs=years of life lost. YLDs=years live with disability. DALYs=disability-adjusted life-years

	Deaths 2016	YLLs 2016	YLDs 2016	DALYs 2016
Other pharynx cancer	29.9% (21.1% to 38.8%)	31.3% (22.6% to 39.9%)	30.1% (21.5% to 38.7%)	31.3% (22.6% to 39.9%)
Gallbladder and biliary tract cancer	9.3% (4.4% to 16.4%)	10.0% (4.8% to 17.4%)	9.4% (4.4% to 16.4%)	10.0% (4.8% to 17.4%)
Pancreatic cancer	15.1% (8.6% to 23.8%)	15.7% (9.6% to 23.9%)	15.7% (9.2% to 24.4%)	15.7% (9.6% to 23.9%)
Ovarian cancer	9.3% (3.2% to 18.8%)	8.6% (2.9% to 17.2%)	8.4% (2.9% to 16.9%)	8.5% (2.9% to 17.2%)
Kidney cancer	14.0% (9.1% to 20.1%)	14.6% (9.7% to 20.8%)	14.9% (9.8% to 21.0%)	14.6% (9.7% to 20.8%)
Bladder cancer	18.1% (11.0% to 27.5%)	19.5% (12.4% to 28.5%)	19.4% (12.2% to 28.7%)	19.5% (12.4% to 28.5%)
Thyroid cancer	5.4% (2.5% to 9.7%)	5.6% (2.6% to 10.1%)	5.8% (2.8% to 10.1%)	5.6% (2.6% to 10.1%)
Mesothelioma	85.3% (80.0% to 89.2%)	76.1% (68.9% to 81.7%)	82.4% (76.3% to 86.9%)	76.2% (69.0% to 81.7%)
Non-Hodgkin's lymphoma	2.6% (1.0% to 4.9%)	2.5% (0.9% to 4.8%)	2.7% (1.0% to 5.0%)	2.5% (0.9% to 4.8%)
Multiple myeloma	3.1% (1.2% to 5.9%)	3.4% (1.4% to 6.3%)	3.2% (1.3% to 6.1%)	3.4% (1.4% to 6.3%)
Leukaemia	7.1% (4.4% to 10.4%)	6.4% (4.0% to 9.4%)	7.0% (4.4% to 10.2%)	6.4% (4.0% to 9.4%)
**Cardiovascular diseases**	87.7% (85.5% to 89.6%)	88.7% (87.2% to 90.1%)	74.8% (71.6% to 77.6%)	88.0% (86.4% to 89.5%)
Rheumatic heart disease	25.2% (17.2% to 39.4%)	23.6% (16.5% to 34.9%)	20.8% (13.4% to 30.2%)	23.3% (16.2% to 34.3%)
Ischaemic heart disease	92.9% (89.9% to 95.2%)	94.2% (92.4% to 95.7%)	89.4% (86.5% to 92.2%)	94.1% (92.2% to 95.6%)
Cerebrovascular disease	86.3% (82.9% to 89.6%)	89.7% (87.3% to 92.0%)	85.9% (82.5% to 89.1%)	89.5% (87.1% to 91.8%)
Hypertensive heart disease	100.0% (100.0% to 100.0%)	100.0% (100.0% to 100.0%)	100.0% (100.0% to 100.0%)	100.0% (100.0% to 100.0%)
Cardiomyopathy and myocarditis	33.2% (26.5% to 41.0%)	26.9% (20.5% to 35.1%)	17.8% (13.3% to 23.0%)	26.2% (20.0% to 34.0%)
Atrial fibrillation and flutter	41.1% (34.3% to 47.7%)	45.6% (39.5% to 51.1%)	52.4% (47.8% to 57.1%)	49.5% (44.4% to 54.1%)
Aortic aneurysm	41.5% (35.1% to 47.9%)	46.1% (40.6% to 51.3%)	-	46.1% (40.6% to 51.3%)
Peripheral vascular disease	52.3% (46.2% to 58.9%)	59.2% (54.1% to 64.5%)	59.4% (54.6% to 64.2%)	59.4% (54.6% to 64.1%)
Endocarditis	38.6% (29.9% to 48.3%)	37.1% (29.0% to 45.2%)	35.7% (27.4% to 45.0%)	37.1% (29.0% to 45.2%)

Supplementary Results Table 1. Age-standardised PAFs attributable to the joint distribution of all risk factors for all ages, both sexes combined for each GBD level 1 and level 2 cause and all causes in 2016 for South Asia. Results are shown for deaths, YLLs, YLDs, and DALYs
Data in parenthesis are 95% uncertainty intervals. PAFs=population attributable fractions. GBD=Global Burden of Disease. YLLs=years of life lost. YLDs=years live with disability. DALYs=disability-adjusted life-years

	**Deaths 2016**	**YLLs 2016**	**YLDs 2016**	**DALYs 2016**
Other cardiovascular and circulatory diseases	42.8% (37.7% to 47.9%)	47.7% (43.2% to 52.0%)	43.5% (39.1% to 48.1%)	46.9% (42.4% to 51.2%)
**Chronic respiratory diseases**	64.5% (57.4% to 71.6%)	63.8% (57.1% to 70.3%)	55.4% (47.8% to 62.5%)	61.6% (55.3% to 68.1%)
Chronic obstructive pulmonary disease	73.8% (65.8% to 82.0%)	74.8% (67.1% to 82.5%)	64.8% (56.0% to 73.5%)	72.3% (64.3% to 79.9%)
Pneumoconiosis	100.0% (100.0% to 100.0%)	100.0% (100.0% to 100.0%)	100.0% (100.0% to 100.0%)	100.0% (100.0% to 100.0%)
Asthma	27.2% (21.2% to 34.5%)	30.5% (24.5% to 37.5%)	27.4% (22.5% to 33.1%)	29.7% (24.1% to 36.4%)
Other chronic respiratory diseases	5.3% (3.6% to 7.3%)	5.5% (3.7% to 8.0%)	3.1% (1.9% to 4.8%)	4.3% (2.8% to 6.3%)
**Cirrhosis and other chronic liver diseases**	29.1% (26.9% to 31.3%)	28.1% (25.9% to 30.5%)	22.5% (20.6% to 24.5%)	27.9% (25.6% to 30.2%)
Cirrhosis and other chronic liver diseases due to hepatitis B	0.1% (0.1% to 0.1%)	0.1% (0.1% to 0.2%)	0.1% (0.1% to 0.2%)	0.1% (0.1% to 0.2%)
Cirrhosis and other chronic liver diseases due to hepatitis C	10.3% (7.0% to 14.3%)	14.7% (9.9% to 20.5%)	14.9% (10.0% to 20.7%)	14.7% (10.0% to 20.5%)
Cirrhosis and other chronic liver diseases due to alcohol use	100.0% (100.0% to 100.0%)	100.0% (100.0% to 100.0%)	100.0% (100.0% to 100.0%)	100.0% (100.0% to 100.0%)
**Digestive diseases**	5.7% (4.5% to 7.1%)	5.7% (4.5% to 7.1%)	2.2% (1.6% to 2.8%)	5.3% (4.2% to 6.7%)
Peptic ulcer disease	12.6% (9.0% to 16.7%)	12.8% (9.1% to 17.0%)	7.6% (5.4% to 10.1%)	12.3% (8.8% to 16.2%)
Gallbladder and biliary diseases	19.7% (11.6% to 30.1%)	20.2% (12.2% to 30.4%)	21.3% (12.7% to 31.6%)	20.3% (12.2% to 30.5%)
Pancreatitis	14.8% (9.5% to 21.0%)	16.9% (11.7% to 23.4%)	15.0% (10.7% to 20.6%)	16.9% (11.7% to 23.4%)
**Neurological disorders**	12.6% (6.1% to 20.8%)	10.6% (5.8% to 16.8%)	1.5% (0.9% to 2.4%)	4.2% (2.4% to 7.0%)
Alzheimer's disease and other dementias	15.8% (7.7% to 26.2%)	16.7% (8.4% to 27.3%)	16.8% (8.5% to 27.3%)	16.8% (8.4% to 27.4%)
Parkinson's disease	-10.0% (-12.1% to -7.8%)	-11.3% (-13.5% to -8.8%)	-11.8% (-14.1% to -9.1%)	-11.4% (-13.6% to -8.8%)
Epilepsy	7.8% (5.5% to 10.4%)	6.6% (4.7% to 8.6%)	5.9% (4.1% to 8.1%)	6.3% (4.4% to 8.2%)
Multiple sclerosis	6.6% (3.6% to 10.2%)	6.4% (3.4% to 10.0%)	4.7% (2.8% to 6.7%)	5.6% (3.2% to 8.4%)
**Mental and substance use disorders**	99.9% (99.8% to 99.9%)	99.8% (99.7% to 99.9%)	20.7% (18.4% to 23.0%)	24.8% (22.2% to 27.5%)
Alcohol use disorders	100.0% (100.0% to 100.0%)	100.0% (100.0% to 100.0%)	100.0% (100.0% to 100.0%)	100.0% (100.0% to 100.0%)

Supplementary Results Table 1. Age-standardised PAFs attributable to the joint distribution of all risk factors for all ages, both sexes combined for each GBD level 1 and level 2 cause and all causes in 2016 for South Asia. Results are shown for deaths, YLLs, YLDs, and DALYs
Data in parenthesis are 95% uncertainty intervals. PAFs=population attributable fractions. GBD=Global Burden of Disease. YLLs=years of life lost. YLDs=years live with disability. DALYs=disability-adjusted life-years

	**Deaths 2016**	**YLLs 2016**	**YLDs 2016**	**DALYs 2016**
Drug use disorders	100.0% (100.0% to 100.0%)	100.0% (100.0% to 100.0%)	100.0% (100.0% to 100.0%)	100.0% (100.0% to 100.0%)
Depressive disorders	-	-	11.3% (9.7% to 13.1%)	11.3% (9.7% to 13.1%)
Idiopathic developmental intellectual disability	-	-	65.3% (33.8% to 84.2%)	65.3% (33.8% to 84.2%)
**Diabetes, urogenital, blood, and endocrine diseases**	84.5% (83.6% to 85.2%)	82.1% (80.8% to 83.0%)	59.0% (55.2% to 62.6%)	73.2% (70.6% to 75.8%)
Diabetes mellitus	100.0% (100.0% to 100.0%)	100.0% (100.0% to 100.0%)	100.0% (100.0% to 100.0%)	100.0% (100.0% to 100.0%)
Chronic kidney disease	100.0% (100.0% to 100.0%)	100.0% (100.0% to 100.0%)	100.0% (100.0% to 100.0%)	100.0% (100.0% to 100.0%)
**Musculoskeletal disorders**	1.7% (0.6% to 2.9%)	1.5% (0.6% to 2.6%)	15.2% (13.0% to 17.6%)	15.0% (12.9% to 17.5%)
Rheumatoid arthritis	3.3% (1.3% to 5.6%)	3.4% (1.3% to 5.8%)	3.2% (1.3% to 5.3%)	3.2% (1.3% to 5.4%)
Osteoarthritis	-	-	11.0% (5.1% to 18.5%)	11.0% (5.1% to 18.5%)
Low back and neck pain	-	-	26.6% (23.9% to 29.7%)	26.6% (23.9% to 29.7%)
Gout	-	-	31.7% (23.5% to 42.5%)	31.7% (23.5% to 42.5%)
**Other non-communicable diseases**	0.4% (0.2% to 0.5%)	0.5% (0.3% to 0.7%)	9.0% (7.7% to 10.6%)	7.2% (6.1% to 8.3%)
Sense organ diseases	-	-	16.4% (14.4% to 18.5%)	16.4% (14.4% to 18.5%)
Sudden infant death syndrome	6.5% (4.9% to 8.3%)	6.6% (4.9% to 8.4%)	-	6.6% (4.9% to 8.4%)
**Transport injuries**	25.0% (22.5% to 28.0%)	21.3% (18.8% to 24.2%)	28.1% (25.7% to 30.9%)	22.4% (20.0% to 25.3%)
Road injuries	24.6% (22.0% to 27.5%)	21.1% (18.6% to 24.1%)	30.7% (28.2% to 33.6%)	22.5% (20.0% to 25.6%)
Other transport injuries	28.7% (26.2% to 31.6%)	22.4% (19.8% to 25.5%)	20.9% (19.0% to 23.3%)	21.9% (19.6% to 24.7%)
**Unintentional injuries**	34.2% (29.5% to 37.6%)	17.1% (15.7% to 19.2%)	17.2% (16.3% to 18.2%)	17.1% (16.0% to 18.7%)
Falls	60.5% (52.1% to 62.8%)	38.4% (35.5% to 40.2%)	27.4% (26.4% to 28.4%)	34.1% (31.8% to 35.8%)
Drowning	7.7% (6.4% to 9.3%)	6.9% (5.8% to 8.1%)	9.8% (8.3% to 11.7%)	6.9% (5.8% to 8.2%)
Fire, heat, and hot substances	5.3% (4.3% to 7.0%)	5.7% (4.7% to 7.4%)	8.5% (7.3% to 9.9%)	6.4% (5.3% to 8.2%)

Supplementary Results Table 1. Age-standardised PAFs attributable to the joint distribution of all risk factors for all ages, both sexes combined for each GBD level 1 and level 2 cause and all causes in 2016 for South Asia. Results are shown for deaths, YLLs, YLDs, and DALYs

Data in parenthesis are 95% uncertainty intervals. PAFs=population attributable fractions. GBD=Global Burden of Disease. YLLs=years of life lost. YLDs=years live with disability. DALYs=disability-adjusted life-years

	**Deaths 2016**	**YLLs 2016**	**YLDs 2016**	**DALYs 2016**
Poisonings	7.7% (6.3% to 9.5%)	6.9% (5.5% to 8.5%)	10.3% (8.9% to 12.0%)	7.3% (5.9% to 8.9%)
Exposure to mechanical forces	15.9% (15.2% to 16.6%)	11.1% (10.4% to 12.1%)	16.9% (16.0% to 17.8%)	13.2% (12.3% to 14.2%)
Animal contact	5.7% (4.8% to 7.9%)	6.0% (5.0% to 8.4%)	8.4% (7.7% to 9.2%)	6.7% (5.7% to 8.5%)
Foreign body	2.9% (2.4% to 3.7%)	3.3% (2.8% to 4.2%)	6.0% (5.5% to 6.6%)	4.8% (4.2% to 5.5%)
Other unintentional injuries	12.4% (10.4% to 15.0%)	14.1% (12.1% to 16.8%)	10.6% (9.0% to 12.6%)	12.3% (10.6% to 14.7%)
**Self-harm and interpersonal violence**	11.5% (8.3% to 15.0%)	11.0% (7.8% to 14.2%)	19.4% (17.9% to 20.8%)	11.4% (8.4% to 14.6%)
Self-harm	10.7% (6.6% to 14.9%)	10.4% (6.6% to 14.3%)	10.2% (6.3% to 14.3%)	10.4% (6.6% to 14.3%)
Interpersonal violence	14.7% (12.0% to 18.1%)	13.0% (10.4% to 16.1%)	21.5% (20.1% to 23.0%)	14.7% (12.4% to 17.5%)
**Forces of nature, conflict and terrorism, and executions and police conflict**	4.0% (3.2% to 4.7%)	1.7% (1.6% to 1.9%)	3.9% (2.7% to 5.6%)	2.7% (2.1% to 3.9%)
Forces of nature, conflict and terrorism, and state actor violence	6.8% (6.4% to 7.1%)	2.7% (2.5% to 2.9%)	5.7% (4.0% to 8.1%)	4.1% (3.2% to 5.9%)

Supplementary Results Table 1. Age-standardised PAFs attributable to the joint distribution of all risk factors for all ages, both sexes combined for each GBD level 1 and level 2 cause and all causes in 2016 for Southeast Asia. Results are shown for deaths, YLLs, YLDs, and DALYs
Data in parenthesis are 95% uncertainty intervals. PAFs=population attributable fractions. GBD=Global Burden of Disease. YLLs=years of life lost. YLDs=years live with disability. DALYs=disability-adjusted life-years

	Deaths 2016	YLLs 2016	YLDs 2016	DALYs 2016
**All causes**	61.5% (60.0% to 62.9%)	57.3% (56.2% to 58.4%)	26.9% (25.8% to 28.1%)	47.2% (45.0% to 49.4%)
**HIV/AIDS and tuberculosis**	42.2% (36.0% to 48.1%)	45.2% (40.0% to 50.1%)	51.3% (45.8% to 57.1%)	45.8% (40.7% to 50.5%)
Tuberculosis	36.1% (29.1% to 43.1%)	34.9% (28.4% to 41.1%)	36.4% (30.0% to 42.5%)	35.0% (28.6% to 41.1%)
HIV/AIDS	79.8% (78.1% to 81.5%)	77.7% (75.9% to 79.5%)	82.5% (81.1% to 83.9%)	78.3% (76.7% to 80.0%)
**Diarrhoea, lower respiratory infections, and other common infectious diseases**	62.9% (55.6% to 69.0%)	61.9% (56.1% to 67.3%)	36.2% (26.2% to 43.4%)	58.8% (52.9% to 64.0%)
Diarrhoeal diseases	81.0% (55.3% to 94.7%)	86.7% (68.5% to 96.3%)	81.7% (58.6% to 94.2%)	85.6% (66.4% to 95.8%)
Lower respiratory infections	67.6% (61.3% to 73.7%)	77.3% (72.4% to 82.1%)	68.0% (61.9% to 74.0%)	77.2% (72.3% to 82.0%)
Upper respiratory infections	1.4% (0.8% to 2.4%)	3.2% (1.8% to 5.3%)	0.0% (0.0% to 0.0%)	0.0% (0.0% to 0.1%)
Otitis media	2.6% (0.8% to 7.3%)	6.7% (2.4% to 14.5%)	8.6% (5.8% to 11.0%)	8.5% (5.8% to 11.1%)
Meningitis	1.6% (1.3% to 2.0%)	2.3% (1.9% to 2.9%)	0.0% (0.0% to 0.0%)	2.0% (1.6% to 2.4%)
Encephalitis	0.5% (0.3% to 0.7%)	0.9% (0.6% to 1.2%)	0.0% (0.0% to 0.0%)	0.6% (0.4% to 0.8%)
Measles	64.8% (50.2% to 82.3%)	65.8% (51.1% to 83.6%)	63.4% (49.1% to 81.3%)	65.8% (51.1% to 83.6%)
**Maternal disorders**	6.9% (3.2% to 10.9%)	6.9% (3.1% to 10.8%)	2.7% (1.2% to 4.4%)	6.6% (3.0% to 10.4%)
Maternal haemorrhage	15.1% (5.5% to 24.6%)	15.1% (5.5% to 24.5%)	14.8% (5.3% to 24.0%)	15.1% (5.5% to 24.4%)
Maternal sepsis and other pregnancy related infections	14.9% (5.4% to 24.1%)	14.8% (5.4% to 24.0%)	14.3% (5.2% to 23.3%)	14.7% (5.4% to 24.0%)
Maternal abortion, miscarriage, and ectopic pregnancy	9.6% (5.6% to 14.3%)	9.3% (5.3% to 13.8%)	8.7% (4.8% to 13.1%)	9.3% (5.3% to 13.7%)
**Neonatal disorders**	79.0% (77.2% to 80.9%)	79.0% (77.2% to 81.0%)	0.2% (0.1% to 0.2%)	71.5% (68.4% to 74.4%)
Neonatal preterm birth complications	95.5% (94.5% to 96.3%)	95.5% (94.5% to 96.3%)	0.3% (0.2% to 0.3%)	81.5% (76.1% to 85.8%)
Neonatal encephalopathy due to birth asphyxia and trauma	69.8% (66.9% to 72.9%)	69.8% (67.0% to 73.0%)	0.0% (0.0% to 0.0%)	68.5% (65.5% to 71.9%)
Neonatal sepsis and other neonatal infections	61.1% (57.4% to 64.4%)	61.1% (57.4% to 64.5%)	0.0% (0.0% to 0.0%)	55.9% (48.7% to 60.8%)
Hemolytic disease and other neonatal jaundice	64.5% (61.1% to 67.8%)	64.5% (61.1% to 67.9%)	0.0% (0.0% to 0.0%)	34.7% (29.0% to 40.6%)

Supplementary Results Table 1. Age-standardised PAFs attributable to the joint distribution of all risk factors for all ages, both sexes combined for each GBD level 1 and level 2 cause and all causes in 2016 for Southeast Asia. Results are shown for deaths, YLLs, YLDs, and DALYs
Data in parenthesis are 95% uncertainty intervals. PAFs=population attributable fractions. GBD=Global Burden of Disease. YLLs=years of life lost. YLDs=years live with disability. DALYs=disability-adjusted life-years

	Deaths 2016	YLLs 2016	YLDs 2016	DALYs 2016
Other neonatal disorders	60.8% (57.7% to 64.2%)	60.8% (57.8% to 64.2%)	0.0% (0.0% to 0.0%)	58.5% (55.1% to 62.0%)
**Nutritional deficiencies**	5.9% (5.0% to 7.1%)	26.3% (22.9% to 30.2%)	94.8% (94.0% to 95.5%)	79.7% (74.4% to 84.3%)
Protein-energy malnutrition	4.8% (4.0% to 5.9%)	26.1% (22.4% to 30.3%)	96.6% (96.0% to 97.1%)	46.6% (40.3% to 53.2%)
Vitamin A deficiency	-	-	99.9% (99.9% to 99.9%)	99.9% (99.9% to 99.9%)
Iron-deficiency anaemia	100.0% (100.0% to 100.0%)	100.0% (100.0% to 100.0%)	99.9% (99.9% to 99.9%)	99.9% (99.9% to 99.9%)
**Other communicable, maternal, neonatal, and nutritional diseases**	4.4% (3.7% to 5.2%)	3.4% (2.7% to 4.2%)	68.1% (64.0% to 71.8%)	17.2% (12.4% to 23.7%)
Sexually transmitted diseases excluding HIV	26.1% (15.0% to 40.5%)	11.6% (6.0% to 19.8%)	99.9% (99.9% to 99.9%)	46.6% (30.3% to 63.9%)
Hepatitis	0.7% (0.5% to 0.9%)	0.7% (0.6% to 1.0%)	0.5% (0.3% to 0.8%)	0.7% (0.6% to 1.0%)
**Neoplasms**	45.3% (42.4% to 48.2%)	42.1% (39.3% to 44.8%)	39.9% (36.7% to 43.3%)	42.1% (39.3% to 44.8%)
Oesophageal cancer	75.3% (68.5% to 81.8%)	74.8% (67.8% to 81.6%)	74.1% (66.9% to 80.9%)	74.8% (67.8% to 81.6%)
Stomach cancer	16.2% (9.8% to 23.4%)	16.0% (9.6% to 22.9%)	15.7% (9.5% to 22.7%)	16.0% (9.6% to 22.9%)
Liver cancer	49.5% (41.2% to 57.0%)	49.8% (41.4% to 57.4%)	49.5% (41.4% to 56.8%)	49.7% (41.4% to 57.4%)
Larynx cancer	73.4% (66.3% to 79.1%)	73.2% (66.0% to 79.0%)	71.4% (63.9% to 77.2%)	73.2% (66.0% to 78.9%)
Tracheal, bronchus, and lung cancer	88.0% (85.9% to 90.0%)	86.4% (84.0% to 88.5%)	86.6% (84.2% to 88.6%)	86.4% (84.0% to 88.5%)
Breast cancer	29.5% (19.1% to 40.7%)	28.6% (18.7% to 39.3%)	29.2% (19.1% to 40.1%)	28.6% (18.7% to 39.3%)
Cervical cancer	100.0% (100.0% to 100.0%)	100.0% (100.0% to 100.0%)	99.8% (99.8% to 99.8%)	100.0% (100.0% to 100.0%)
Uterine cancer	23.6% (14.2% to 34.7%)	25.9% (16.1% to 37.1%)	27.6% (18.0% to 38.8%)	26.0% (16.2% to 37.2%)
Prostate cancer	4.4% (3.2% to 5.7%)	4.4% (3.3% to 5.6%)	4.2% (3.1% to 5.3%)	4.4% (3.3% to 5.6%)
Colon and rectum cancer	56.1% (47.6% to 64.4%)	54.8% (46.4% to 62.9%)	55.5% (47.1% to 63.7%)	54.8% (46.4% to 62.9%)
Lip and oral cavity cancer	75.3% (70.9% to 79.3%)	72.0% (67.6% to 76.2%)	71.6% (67.0% to 75.8%)	72.0% (67.6% to 76.2%)
Nasopharynx cancer	62.1% (55.4% to 67.8%)	61.6% (55.2% to 67.1%)	60.7% (54.2% to 66.1%)	61.6% (55.2% to 67.1%)

Supplementary Results Table 1. Age-standardised PAFs attributable to the joint distribution of all risk factors for all ages, both sexes combined for each GBD level 1 and level 2 cause and all causes in 2016 for Southeast Asia. Results are shown for deaths, YLLs, YLDs, and DALYs
Data in parenthesis are 95% uncertainty intervals. PAFs=population attributable fractions. GBD=Global Burden of Disease. YLLs=years of life lost. YLDs=years live with disability. DALYs=disability-adjusted life-years

	Deaths 2016	YLLs 2016	YLDs 2016	DALYs 2016
Other pharynx cancer	48.7% (40.1% to 57.4%)	50.7% (42.4% to 59.1%)	48.2% (39.9% to 56.7%)	50.7% (42.4% to 59.1%)
Gallbladder and biliary tract cancer	10.5% (5.0% to 18.6%)	11.5% (5.7% to 20.0%)	10.8% (5.2% to 18.8%)	11.5% (5.7% to 20.0%)
Pancreatic cancer	26.4% (19.8% to 34.6%)	25.7% (19.0% to 33.8%)	25.8% (19.1% to 34.1%)	25.7% (19.0% to 33.8%)
Ovarian cancer	10.9% (4.3% to 20.5%)	9.3% (3.4% to 17.9%)	8.9% (3.2% to 17.0%)	9.3% (3.4% to 17.9%)
Kidney cancer	21.7% (15.6% to 28.8%)	20.9% (15.0% to 27.7%)	20.7% (14.9% to 27.6%)	20.9% (15.0% to 27.7%)
Bladder cancer	31.5% (23.3% to 40.3%)	30.6% (22.6% to 39.2%)	30.1% (22.3% to 38.5%)	30.6% (22.6% to 39.2%)
Thyroid cancer	5.8% (2.7% to 10.4%)	6.2% (3.0% to 11.0%)	7.2% (3.7% to 12.0%)	6.3% (3.0% to 11.0%)
Mesothelioma	83.9% (79.8% to 87.8%)	73.9% (68.0% to 79.8%)	79.5% (74.7% to 84.4%)	74.0% (68.1% to 79.8%)
Non-Hodgkin's lymphoma	2.8% (1.0% to 5.3%)	2.7% (1.1% to 5.1%)	2.9% (1.2% to 5.5%)	2.7% (1.1% to 5.1%)
Multiple myeloma	3.6% (1.4% to 6.8%)	3.8% (1.6% to 7.2%)	3.8% (1.5% to 7.3%)	3.8% (1.6% to 7.2%)
Leukaemia	13.2% (8.1% to 18.5%)	10.6% (6.8% to 14.9%)	12.5% (7.9% to 17.6%)	10.6% (6.8% to 14.9%)
**Cardiovascular diseases**	88.2% (86.3% to 90.1%)	88.9% (87.6% to 90.1%)	78.4% (75.8% to 80.8%)	88.1% (86.7% to 89.3%)
Rheumatic heart disease	28.4% (19.9% to 41.8%)	23.9% (16.8% to 34.3%)	21.3% (13.7% to 30.7%)	23.6% (16.3% to 33.6%)
Ischaemic heart disease	94.4% (91.7% to 96.5%)	95.5% (93.9% to 96.7%)	92.3% (90.2% to 94.3%)	95.3% (93.8% to 96.6%)
Cerebrovascular disease	88.0% (84.3% to 91.2%)	90.6% (88.3% to 92.6%)	88.4% (85.4% to 91.1%)	90.4% (88.2% to 92.4%)
Hypertensive heart disease	100.0% (100.0% to 100.0%)	100.0% (100.0% to 100.0%)	99.8% (99.8% to 99.8%)	100.0% (100.0% to 100.0%)
Cardiomyopathy and myocarditis	42.8% (36.1% to 50.4%)	40.9% (34.7% to 46.4%)	35.6% (30.5% to 40.8%)	40.6% (34.6% to 46.1%)
Atrial fibrillation and flutter	51.8% (44.3% to 58.2%)	55.8% (49.3% to 61.3%)	61.6% (57.0% to 65.9%)	59.0% (53.6% to 63.5%)
Aortic aneurysm	48.8% (41.6% to 55.8%)	53.4% (47.1% to 58.6%)	-	53.4% (47.1% to 58.6%)
Peripheral vascular disease	55.2% (48.7% to 62.2%)	62.9% (58.0% to 67.8%)	62.8% (57.4% to 67.8%)	62.8% (57.6% to 67.7%)
Endocarditis	39.1% (31.1% to 47.6%)	36.3% (28.8% to 43.9%)	36.8% (29.2% to 45.1%)	36.3% (28.9% to 43.9%)

Supplementary Results Table 1. Age-standardised PAFs attributable to the joint distribution of all risk factors for all ages, both sexes combined for each GBD level 1 and level 2 cause and all causes in 2016 for Southeast Asia. Results are shown for deaths, YLLs, YLDs, and DALYs
Data in parenthesis are 95% uncertainty intervals. PAFs=population attributable fractions. GBD=Global Burden of Disease. YLLs=years of life lost. YLDs=years live with disability. DALYs=disability-adjusted life-years

	Deaths 2016	YLLs 2016	YLDs 2016	DALYs 2016
Other cardiovascular and circulatory diseases	51.6% (46.7% to 56.2%)	51.9% (47.8% to 56.0%)	46.5% (42.0% to 51.2%)	50.4% (46.4% to 54.5%)
**Chronic respiratory diseases**	62.4% (58.0% to 66.7%)	59.0% (54.7% to 63.0%)	51.4% (46.6% to 56.5%)	55.9% (51.9% to 60.1%)
Chronic obstructive pulmonary disease	77.3% (72.5% to 82.2%)	75.5% (70.5% to 80.6%)	70.1% (65.1% to 75.3%)	73.4% (68.5% to 78.4%)
Pneumoconiosis	100.0% (100.0% to 100.0%)	100.0% (100.0% to 100.0%)	99.8% (99.8% to 99.8%)	99.9% (99.8% to 99.9%)
Asthma	33.9% (27.7% to 41.0%)	36.4% (30.5% to 42.9%)	26.7% (21.6% to 32.9%)	32.5% (27.2% to 38.7%)
Other chronic respiratory diseases	12.3% (8.9% to 17.0%)	8.5% (6.3% to 10.9%)	8.0% (6.0% to 10.1%)	8.3% (6.3% to 10.3%)
**Cirrhosis and other chronic liver diseases**	30.0% (27.2% to 32.9%)	31.3% (28.4% to 34.3%)	24.2% (21.8% to 26.8%)	31.1% (28.2% to 34.2%)
Cirrhosis and other chronic liver diseases due to hepatitis B	0.4% (0.3% to 0.5%)	0.5% (0.3% to 0.6%)	0.4% (0.3% to 0.6%)	0.5% (0.3% to 0.6%)
Cirrhosis and other chronic liver diseases due to hepatitis C	39.7% (35.1% to 44.6%)	48.9% (43.5% to 54.1%)	46.2% (41.3% to 50.9%)	48.9% (43.5% to 54.0%)
Cirrhosis and other chronic liver diseases due to alcohol use	100.0% (100.0% to 100.0%)	100.0% (100.0% to 100.0%)	99.8% (99.8% to 99.8%)	100.0% (100.0% to 100.0%)
**Digestive diseases**	10.5% (8.4% to 12.7%)	10.8% (8.7% to 12.9%)	5.7% (4.5% to 7.0%)	10.4% (8.5% to 12.4%)
Peptic ulcer disease	19.1% (14.0% to 24.4%)	20.6% (15.3% to 26.4%)	15.9% (11.8% to 20.4%)	20.4% (15.1% to 26.0%)
Gallbladder and biliary diseases	22.1% (13.6% to 32.9%)	24.9% (16.2% to 35.5%)	28.1% (18.6% to 39.7%)	25.0% (16.3% to 35.7%)
Pancreatitis	23.9% (17.3% to 31.3%)	24.0% (18.2% to 30.7%)	25.6% (19.7% to 32.7%)	24.0% (18.3% to 30.7%)
**Neurological disorders**	15.3% (7.7% to 24.8%)	15.3% (8.3% to 24.1%)	4.0% (2.4% to 5.8%)	8.6% (4.9% to 13.5%)
Alzheimer's disease and other dementias	17.7% (9.3% to 28.2%)	19.2% (10.3% to 30.2%)	19.2% (10.2% to 30.1%)	19.2% (10.3% to 30.2%)
Parkinson's disease	-20.5% (-24.5% to -15.8%)	-23.5% (-28.0% to -17.9%)	-24.1% (-28.6% to -18.2%)	-23.6% (-28.2% to -17.9%)
Epilepsy	16.6% (11.7% to 21.5%)	14.8% (10.4% to 19.1%)	9.9% (6.6% to 13.8%)	11.5% (8.0% to 15.4%)
Multiple sclerosis	10.4% (6.3% to 14.8%)	10.2% (6.2% to 14.5%)	6.7% (4.2% to 9.3%)	9.4% (5.6% to 13.2%)
**Mental and substance use disorders**	99.5% (99.2% to 99.6%)	99.2% (98.8% to 99.4%)	15.5% (13.7% to 17.4%)	19.1% (17.0% to 21.3%)
Alcohol use disorders	100.0% (100.0% to 100.0%)	100.0% (100.0% to 100.0%)	99.9% (99.9% to 99.9%)	99.9% (99.9% to 99.9%)

Supplementary Results Table 1. Age-standardised PAFs attributable to the joint distribution of all risk factors for all ages, both sexes combined for each GBD level 1 and level 2 cause and all causes in 2016 for Southeast Asia. Results are shown for deaths, YLLs, YLDs, and DALYs
Data in parenthesis are 95% uncertainty intervals. PAFs=population attributable fractions. GBD=Global Burden of Disease. YLLs=years of life lost. YLDs=years live with disability. DALYs=disability-adjusted life-years

	Deaths 2016	YLLs 2016	YLDs 2016	DALYs 2016
Drug use disorders	100.0% (100.0% to 100.0%)	100.0% (100.0% to 100.0%)	99.9% (99.9% to 99.9%)	99.9% (99.9% to 99.9%)
Depressive disorders	-	-	4.8% (3.9% to 5.9%)	4.8% (3.9% to 5.9%)
Idiopathic developmental intellectual disability	-	-	65.2% (34.0% to 84.4%)	65.2% (34.0% to 84.4%)
**Diabetes, urogenital, blood, and endocrine diseases**	87.2% (86.3% to 87.7%)	86.1% (85.4% to 86.7%)	68.6% (64.6% to 72.4%)	80.4% (78.2% to 82.2%)
Diabetes mellitus	100.0% (100.0% to 100.0%)	100.0% (100.0% to 100.0%)	99.8% (99.8% to 99.8%)	99.9% (99.9% to 100.0%)
Chronic kidney disease	100.0% (100.0% to 100.0%)	100.0% (100.0% to 100.0%)	99.8% (99.8% to 99.8%)	100.0% (100.0% to 100.0%)
**Musculoskeletal disorders**	1.3% (0.5% to 2.2%)	0.8% (0.3% to 1.4%)	19.7% (17.2% to 22.5%)	19.4% (16.9% to 22.1%)
Rheumatoid arthritis	6.4% (2.6% to 10.7%)	6.5% (2.7% to 10.9%)	4.8% (2.0% to 7.9%)	5.0% (2.1% to 8.2%)
Osteoarthritis	-	-	13.6% (6.7% to 22.4%)	13.6% (6.7% to 22.4%)
Low back and neck pain	-	-	28.1% (25.1% to 31.2%)	28.1% (25.1% to 31.2%)
Gout	-	-	29.2% (20.0% to 41.3%)	29.2% (20.0% to 41.3%)
**Other non-communicable diseases**	0.2% (0.1% to 0.2%)	0.3% (0.2% to 0.3%)	7.8% (6.5% to 9.2%)	6.2% (5.2% to 7.1%)
Sense organ diseases	-	-	15.8% (14.1% to 17.6%)	15.8% (14.1% to 17.6%)
Sudden infant death syndrome	6.6% (4.7% to 8.6%)	6.6% (4.7% to 8.7%)	-	6.6% (4.7% to 8.7%)
**Transport injuries**	36.4% (32.4% to 41.1%)	32.9% (28.8% to 37.6%)	40.5% (37.0% to 44.2%)	34.1% (30.2% to 38.6%)
Road injuries	36.1% (32.1% to 40.8%)	32.8% (28.7% to 37.5%)	43.6% (40.0% to 47.6%)	34.2% (30.3% to 38.7%)
Other transport injuries	40.3% (36.3% to 45.0%)	33.9% (29.7% to 38.9%)	31.8% (28.6% to 35.2%)	33.0% (29.3% to 37.4%)
**Unintentional injuries**	36.4% (25.1% to 41.2%)	22.1% (19.3% to 24.4%)	25.1% (23.5% to 26.8%)	22.9% (20.6% to 24.9%)
Falls	60.4% (36.3% to 67.4%)	40.8% (32.7% to 44.5%)	34.8% (33.2% to 36.4%)	38.7% (33.4% to 41.5%)
Drowning	15.4% (12.5% to 18.7%)	13.8% (11.4% to 16.6%)	22.1% (19.3% to 25.4%)	13.9% (11.6% to 16.7%)
Fire, heat, and hot substances	14.8% (12.5% to 17.6%)	16.6% (14.3% to 19.1%)	24.1% (21.3% to 27.1%)	19.2% (16.8% to 21.8%)

Supplementary Results Table 1. Age-standardised PAFs attributable to the joint distribution of all risk factors for all ages, both sexes combined for each GBD level 1 and level 2 cause and all causes in 2016 for Southeast Asia. Results are shown for deaths, YLLs, YLDs, and DALYs
Data in parenthesis are 95% uncertainty intervals. PAFs=population attributable fractions. GBD=Global Burden of Disease. YLLs=years of life lost. YLDs=years live with disability. DALYs=disability-adjusted life-years

	**Deaths 2016**	**YLLs 2016**	**YLDs 2016**	**DALYs 2016**
Poisonings	22.5% (16.2% to 28.7%)	24.0% (18.1% to 30.2%)	24.4% (21.5% to 27.6%)	24.1% (18.6% to 29.9%)
Exposure to mechanical forces	26.1% (22.0% to 28.6%)	22.3% (16.8% to 25.3%)	29.2% (27.3% to 31.0%)	25.1% (21.4% to 27.5%)
Animal contact	18.5% (16.9% to 20.5%)	18.3% (16.3% to 20.2%)	21.3% (19.3% to 23.2%)	19.1% (17.3% to 20.8%)
Foreign body	9.8% (8.4% to 11.6%)	9.6% (8.2% to 11.3%)	17.4% (15.8% to 19.0%)	11.6% (10.1% to 13.3%)
Other unintentional injuries	27.6% (21.5% to 33.0%)	28.4% (22.4% to 33.7%)	23.4% (20.4% to 26.7%)	26.1% (21.6% to 30.4%)
**Self-harm and interpersonal violence**	20.9% (16.3% to 25.6%)	20.6% (16.1% to 25.2%)	25.1% (21.7% to 28.8%)	21.0% (16.7% to 25.3%)
Self-harm	21.5% (14.6% to 28.6%)	22.1% (15.3% to 29.0%)	16.4% (10.7% to 21.9%)	22.0% (15.2% to 28.9%)
Interpersonal violence	20.0% (14.2% to 26.0%)	18.5% (13.0% to 24.3%)	25.8% (22.3% to 29.8%)	20.0% (15.0% to 25.3%)
**Forces of nature, conflict and terrorism, and executions and police conflict**	0.8% (0.6% to 1.1%)	0.3% (0.2% to 0.4%)	2.9% (2.1% to 3.7%)	1.7% (1.0% to 2.4%)
Forces of nature, conflict and terrorism, and state actor violence	6.9% (6.4% to 7.4%)	2.9% (2.6% to 3.1%)	6.9% (5.1% to 9.1%)	6.2% (4.6% to 8.2%)

**Supplementary Results Table 1. Age-standardised PAFs attributable to the joint distribution of all risk factors for all ages, both sexes combined for each GBD level 1 and level 2 cause and all causes in 2016 for Southern Latin America. Results are shown for deaths, YLLs, YLDs, and DALYs** Data in parenthesis are 95% uncertainty intervals. PAFs=population attributable fractions. GBD=Global Burden of Disease. YLLs=years of life lost. YLDs=years live with disability. DALYs=disability-adjusted life-years				
	**Deaths 2016**	**YLLs 2016**	**YLDs 2016**	**DALYs 2016**
**All causes**	56.1% (54.3% to 58.0%)	52.8% (51.3% to 54.4%)	22.0% (20.7% to 23.3%)	39.3% (36.8% to 42.0%)
**HIV/AIDS and tuberculosis**	77.1% (72.8% to 80.7%)	79.3% (76.3% to 81.7%)	87.7% (84.7% to 90.1%)	80.5% (77.7% to 83.0%)
Tuberculosis	51.9% (39.5% to 62.3%)	53.4% (42.7% to 62.1%)	49.8% (39.3% to 58.2%)	53.2% (42.6% to 61.9%)
HIV/AIDS	89.6% (88.2% to 90.8%)	87.3% (85.9% to 88.6%)	91.2% (89.6% to 92.5%)	88.0% (86.5% to 89.3%)
**Diarrhoea, lower respiratory infections, and other common infectious diseases**	39.7% (31.9% to 48.2%)	46.2% (39.7% to 53.4%)	17.5% (12.2% to 23.7%)	41.3% (35.5% to 48.1%)
Diarrhoeal diseases	46.9% (28.6% to 68.7%)	63.5% (49.9% to 79.2%)	58.5% (41.7% to 77.0%)	61.0% (45.9% to 78.3%)
Lower respiratory infections	40.8% (32.2% to 49.9%)	49.7% (42.1% to 58.2%)	49.0% (41.0% to 57.8%)	49.7% (42.1% to 58.2%)
Upper respiratory infections	1.1% (0.2% to 3.2%)	2.7% (0.5% to 7.9%)	0.0% (0.0% to 0.0%)	0.0% (0.0% to 0.0%)
Otitis media	3.0% (2.0% to 4.4%)	6.4% (4.3% to 8.8%)	7.8% (5.3% to 10.3%)	7.8% (5.3% to 10.3%)
Meningitis	1.5% (1.2% to 2.0%)	2.9% (2.2% to 3.6%)	0.0% (0.0% to 0.0%)	2.7% (2.1% to 3.4%)
Encephalitis	0.5% (0.4% to 0.6%)	0.9% (0.7% to 1.1%)	0.0% (0.0% to 0.0%)	0.7% (0.6% to 0.9%)
Measles	21.0% (5.8% to 57.1%)	22.3% (6.2% to 60.3%)	12.2% (2.4% to 44.4%)	22.3% (6.2% to 60.3%)
**Maternal disorders**	5.1% (2.3% to 8.1%)	5.0% (2.3% to 7.9%)	3.5% (1.9% to 5.3%)	4.7% (2.2% to 7.3%)
Maternal haemorrhage	11.7% (4.2% to 19.0%)	11.6% (4.2% to 18.9%)	11.5% (4.1% to 18.6%)	11.6% (4.2% to 18.9%)
Maternal sepsis and other pregnancy related infections	11.7% (4.2% to 19.1%)	11.6% (4.2% to 18.9%)	11.2% (4.0% to 18.2%)	11.6% (4.2% to 18.8%)
Maternal abortion, miscarriage, and ectopic pregnancy	6.0% (3.5% to 9.0%)	6.2% (3.5% to 9.3%)	6.6% (3.4% to 10.2%)	6.3% (3.5% to 9.5%)
**Neonatal disorders**	79.4% (77.4% to 81.2%)	79.4% (77.4% to 81.2%)	0.1% (0.1% to 0.1%)	68.2% (64.3% to 71.7%)
Neonatal preterm birth complications	92.6% (90.8% to 94.1%)	92.6% (90.9% to 94.2%)	0.2% (0.1% to 0.2%)	79.3% (74.0% to 83.9%)
Neonatal encephalopathy due to birth asphyxia and trauma	61.5% (57.8% to 64.9%)	61.6% (57.8% to 65.0%)	0.0% (0.0% to 0.0%)	53.0% (44.6% to 59.6%)
Neonatal sepsis and other neonatal infections	57.9% (54.2% to 61.2%)	57.9% (54.2% to 61.3%)	0.0% (0.0% to 0.0%)	49.6% (40.6% to 55.9%)
Hemolytic disease and other neonatal jaundice	64.9% (61.0% to 68.3%)	64.9% (61.0% to 68.3%)	0.0% (0.0% to 0.0%)	12.4% (7.8% to 18.4%)

Supplementary Results Table 1. Age-standardised PAFs attributable to the joint distribution of all risk factors for all ages, both sexes combined for each GBD level 1 and level 2 cause and all causes in 2016 for Southern Latin America. Results are shown for deaths, YLLs, YLDs, and DALYs
Data in parenthesis are 95% uncertainty intervals. PAFs=population attributable fractions. GBD=Global Burden of Disease. YLLs=years of life lost. YLDs=years live with disability. DALYs=disability-adjusted life-years

	Deaths 2016	YLLs 2016	YLDs 2016	DALYs 2016
Other neonatal disorders	59.5% (55.8% to 63.1%)	59.6% (55.9% to 63.2%)	0.0% (0.0% to 0.0%)	57.8% (54.1% to 61.5%)
**Nutritional deficiencies**	5.3% (4.2% to 6.9%)	25.5% (20.9% to 31.0%)	91.2% (89.2% to 93.0%)	73.4% (66.4% to 80.0%)
Protein-energy malnutrition	5.5% (4.2% to 7.2%)	27.3% (22.0% to 33.6%)	98.3% (97.2% to 99.0%)	31.4% (25.8% to 37.7%)
Vitamin A deficiency	-	-	0.0% (0.0% to 0.0%)	0.0% (0.0% to 0.0%)
Iron-deficiency anaemia	100.0% (100.0% to 100.0%)	100.0% (100.0% to 100.0%)	100.0% (100.0% to 100.0%)	100.0% (100.0% to 100.0%)
**Other communicable, maternal, neonatal, and nutritional diseases**	7.8% (4.9% to 11.2%)	6.1% (2.8% to 11.2%)	65.1% (59.2% to 71.4%)	19.4% (9.7% to 32.7%)
Sexually transmitted diseases excluding HIV	28.6% (7.7% to 82.0%)	16.3% (3.2% to 64.2%)	100.0% (100.0% to 100.0%)	39.1% (12.8% to 90.0%)
Hepatitis	4.5% (3.4% to 6.0%)	4.5% (3.4% to 6.1%)	1.5% (1.0% to 2.4%)	4.2% (3.1% to 5.6%)
**Neoplasms**	41.4% (38.3% to 44.5%)	41.4% (38.7% to 44.3%)	36.8% (33.9% to 40.0%)	41.3% (38.5% to 44.1%)
Oesophageal cancer	79.4% (69.9% to 87.4%)	81.4% (72.7% to 88.8%)	79.9% (70.6% to 87.6%)	81.4% (72.6% to 88.8%)
Stomach cancer	17.8% (9.4% to 27.3%)	18.1% (9.9% to 26.9%)	17.6% (9.5% to 27.1%)	18.1% (9.9% to 26.9%)
Liver cancer	66.9% (60.2% to 73.6%)	70.4% (64.3% to 76.4%)	67.7% (61.4% to 74.1%)	70.4% (64.3% to 76.3%)
Larynx cancer	85.2% (77.2% to 90.9%)	85.9% (78.6% to 91.3%)	85.4% (77.5% to 91.0%)	85.9% (78.6% to 91.3%)
Tracheal, bronchus, and lung cancer	84.2% (81.7% to 86.7%)	84.6% (82.1% to 86.9%)	83.9% (81.0% to 86.5%)	84.6% (82.1% to 86.9%)
Breast cancer	34.3% (27.7% to 41.8%)	33.4% (27.0% to 40.3%)	33.6% (27.2% to 40.5%)	33.4% (27.0% to 40.3%)
Cervical cancer	100.0% (100.0% to 100.0%)	100.0% (100.0% to 100.0%)	100.0% (100.0% to 100.0%)	100.0% (100.0% to 100.0%)
Uterine cancer	45.8% (31.7% to 60.3%)	46.7% (32.2% to 60.9%)	47.0% (32.4% to 61.3%)	46.7% (32.2% to 60.9%)
Prostate cancer	4.5% (3.2% to 5.9%)	5.0% (3.6% to 6.5%)	4.9% (3.6% to 6.5%)	5.0% (3.6% to 6.5%)
Colon and rectum cancer	58.1% (49.3% to 65.6%)	59.0% (50.6% to 66.2%)	58.7% (50.0% to 66.1%)	59.0% (50.5% to 66.2%)
Lip and oral cavity cancer	76.8% (68.9% to 83.7%)	79.4% (72.1% to 85.7%)	76.7% (68.5% to 83.3%)	79.3% (72.1% to 85.6%)
Nasopharynx cancer	78.0% (71.9% to 82.9%)	76.9% (71.7% to 81.3%)	75.3% (70.2% to 80.0%)	76.8% (71.7% to 81.3%)

Supplementary Results Table 1. Age-standardised PAFs attributable to the joint distribution of all risk factors for all ages, both sexes combined for each GBD level 1 and level 2 cause and all causes in 2016 for Southern Latin America. Results are shown for deaths, YLLs, YLDs, and DALYs
Data in parenthesis are 95% uncertainty intervals. PAFs=population attributable fractions. GBD=Global Burden of Disease. YLLs=years of life lost. YLDs=years live with disability. DALYs=disability-adjusted life-years

	Deaths 2016	YLLs 2016	YLDs 2016	DALYs 2016
Other pharynx cancer	55.1% (39.7% to 68.0%)	57.2% (41.9% to 69.6%)	56.3% (40.8% to 69.0%)	57.2% (41.9% to 69.5%)
Gallbladder and biliary tract cancer	24.9% (13.7% to 38.0%)	25.5% (14.2% to 38.5%)	25.1% (14.2% to 37.9%)	25.5% (14.2% to 38.5%)
Pancreatic cancer	30.1% (22.8% to 38.4%)	31.4% (24.1% to 39.4%)	30.4% (23.0% to 38.6%)	31.4% (24.1% to 39.4%)
Ovarian cancer	12.4% (5.4% to 20.5%)	10.8% (4.5% to 18.1%)	10.5% (4.5% to 17.7%)	10.8% (4.5% to 18.1%)
Kidney cancer	37.3% (27.1% to 48.2%)	37.4% (27.3% to 47.7%)	36.8% (26.9% to 47.2%)	37.4% (27.3% to 47.7%)
Bladder cancer	32.7% (24.0% to 41.7%)	34.6% (25.9% to 43.8%)	33.5% (25.1% to 42.6%)	34.5% (25.8% to 43.7%)
Thyroid cancer	15.9% (8.3% to 25.7%)	16.5% (8.4% to 26.4%)	15.4% (8.4% to 24.2%)	16.4% (8.5% to 26.3%)
Mesothelioma	91.0% (88.2% to 93.1%)	84.4% (80.3% to 87.7%)	88.9% (85.8% to 91.3%)	84.5% (80.4% to 87.8%)
Non-Hodgkin's lymphoma	8.0% (3.4% to 13.7%)	7.6% (3.3% to 13.0%)	7.9% (3.4% to 13.2%)	7.6% (3.3% to 13.0%)
Multiple myeloma	9.3% (4.0% to 15.8%)	9.5% (4.2% to 16.1%)	9.4% (4.1% to 16.3%)	9.5% (4.2% to 16.1%)
Leukaemia	18.6% (12.2% to 25.2%)	14.9% (10.0% to 20.0%)	17.5% (11.5% to 23.6%)	14.9% (10.0% to 20.1%)
**Cardiovascular diseases**	82.1% (79.7% to 84.2%)	82.7% (80.8% to 84.3%)	76.5% (73.6% to 79.2%)	82.0% (80.1% to 83.6%)
Rheumatic heart disease	29.8% (17.9% to 52.3%)	28.1% (18.9% to 43.8%)	27.7% (18.9% to 42.5%)	28.1% (18.9% to 43.8%)
Ischaemic heart disease	93.1% (89.6% to 95.7%)	94.2% (92.1% to 96.0%)	91.5% (89.0% to 93.9%)	94.1% (91.9% to 95.9%)
Cerebrovascular disease	84.7% (79.8% to 89.2%)	87.1% (84.1% to 89.8%)	85.9% (81.6% to 89.6%)	86.9% (83.8% to 89.7%)
Hypertensive heart disease	100.0% (100.0% to 100.0%)	100.0% (100.0% to 100.0%)	100.0% (100.0% to 100.0%)	100.0% (100.0% to 100.0%)
Cardiomyopathy and myocarditis	42.5% (34.1% to 52.3%)	40.1% (33.3% to 47.1%)	41.1% (34.1% to 48.7%)	40.1% (33.3% to 47.1%)
Atrial fibrillation and flutter	57.2% (49.4% to 65.3%)	60.6% (53.7% to 67.5%)	65.8% (60.5% to 71.2%)	63.7% (57.8% to 69.9%)
Aortic aneurysm	46.6% (38.9% to 54.1%)	51.4% (45.0% to 57.2%)	-	51.4% (45.0% to 57.2%)
Peripheral vascular disease	53.0% (46.0% to 60.7%)	58.9% (53.3% to 64.5%)	60.9% (55.2% to 66.3%)	59.9% (54.4% to 65.3%)
Endocarditis	41.7% (32.1% to 54.1%)	38.8% (31.3% to 47.2%)	41.9% (32.6% to 52.4%)	38.9% (31.3% to 47.2%)

Supplementary Results Table 1. Age-standardised PAFs attributable to the joint distribution of all risk factors for all ages, both sexes combined for each GBD level 1 and level 2 cause and all causes in 2016 for Southern Latin America. Results are shown for deaths, YLLs, YLDs, and DALYs
Data in parenthesis are 95% uncertainty intervals. PAFs=population attributable fractions. GBD=Global Burden of Disease. YLLs=years of life lost. YLDs=years live with disability. DALYs=disability-adjusted life-years

	Deaths 2016	YLLs 2016	YLDs 2016	DALYs 2016
Other cardiovascular and circulatory diseases	48.8% (43.1% to 54.4%)	50.5% (45.7% to 54.8%)	51.8% (46.9% to 56.6%)	50.8% (46.1% to 55.1%)
**Chronic respiratory diseases**	62.7% (58.0% to 67.1%)	61.1% (57.1% to 64.9%)	40.2% (32.8% to 48.3%)	53.2% (48.7% to 58.0%)
Chronic obstructive pulmonary disease	73.8% (68.4% to 78.5%)	74.6% (70.1% to 78.9%)	71.3% (66.6% to 76.0%)	74.2% (69.7% to 78.5%)
Pneumoconiosis	100.0% (100.0% to 100.0%)	100.0% (100.0% to 100.0%)	100.0% (100.0% to 100.0%)	100.0% (100.0% to 100.0%)
Asthma	43.0% (32.6% to 54.7%)	46.2% (37.0% to 56.7%)	34.6% (26.3% to 44.2%)	35.6% (27.3% to 45.2%)
Other chronic respiratory diseases	17.4% (12.7% to 22.4%)	15.4% (11.2% to 20.1%)	6.8% (5.0% to 9.2%)	14.8% (10.7% to 19.3%)
**Cirrhosis and other chronic liver diseases**	61.2% (58.2% to 63.8%)	63.9% (61.2% to 66.5%)	52.5% (49.4% to 55.7%)	63.5% (60.7% to 66.1%)
Cirrhosis and other chronic liver diseases due to hepatitis B	1.8% (0.9% to 2.9%)	2.3% (1.2% to 3.9%)	2.0% (1.1% to 3.3%)	2.3% (1.2% to 3.9%)
Cirrhosis and other chronic liver diseases due to hepatitis C	74.5% (69.8% to 78.9%)	81.8% (78.6% to 84.6%)	78.2% (74.8% to 81.2%)	81.7% (78.5% to 84.4%)
Cirrhosis and other chronic liver diseases due to alcohol use	100.0% (100.0% to 100.0%)	100.0% (100.0% to 100.0%)	100.0% (100.0% to 100.0%)	100.0% (100.0% to 100.0%)
**Digestive diseases**	10.8% (7.7% to 14.0%)	12.5% (9.0% to 16.1%)	5.0% (3.7% to 6.3%)	11.2% (8.2% to 14.3%)
Peptic ulcer disease	11.6% (8.2% to 15.5%)	15.4% (11.0% to 20.4%)	14.9% (10.6% to 19.8%)	15.3% (11.0% to 20.2%)
Gallbladder and biliary diseases	45.4% (31.0% to 60.4%)	46.1% (31.9% to 60.5%)	49.9% (35.7% to 64.8%)	46.5% (32.2% to 60.8%)
Pancreatitis	28.8% (10.9% to 45.8%)	32.6% (15.2% to 48.8%)	32.4% (15.9% to 48.4%)	32.6% (15.2% to 48.8%)
**Neurological disorders**	19.7% (9.5% to 31.9%)	18.1% (9.9% to 27.6%)	4.2% (2.4% to 6.2%)	8.4% (5.0% to 12.6%)
Alzheimer's disease and other dementias	25.2% (12.3% to 40.4%)	26.6% (13.7% to 41.4%)	26.6% (13.4% to 41.6%)	26.6% (13.6% to 41.4%)
Parkinson's disease	-6.1% (-7.3% to -5.0%)	-8.4% (-9.9% to -6.8%)	-10.4% (-12.4% to -8.4%)	-8.8% (-10.5% to -7.1%)
Epilepsy	31.8% (22.4% to 40.5%)	30.8% (22.4% to 38.7%)	26.1% (18.2% to 33.4%)	27.7% (19.7% to 35.3%)
Multiple sclerosis	10.0% (6.5% to 13.8%)	10.8% (7.1% to 14.8%)	10.2% (6.7% to 14.0%)	10.4% (6.8% to 14.3%)
**Mental and substance use disorders**	99.7% (99.7% to 99.8%)	99.5% (99.4% to 99.6%)	19.9% (17.8% to 22.3%)	22.9% (20.6% to 25.6%)
Alcohol use disorders	100.0% (100.0% to 100.0%)	100.0% (100.0% to 100.0%)	100.0% (100.0% to 100.0%)	100.0% (100.0% to 100.0%)

Supplementary Results Table 1. Age-standardised PAFs attributable to the joint distribution of all risk factors for all ages, both sexes combined for each GBD level 1 and level 2 cause and all causes in 2016 for Southern Latin America. Results are shown for deaths, YLLs, YLDs, and DALYs
Data in parenthesis are 95% uncertainty intervals. PAFs=population attributable fractions. GBD=Global Burden of Disease. YLLs=years of life lost. YLDs=years live with disability. DALYs=disability-adjusted life-years

	Deaths 2016	YLLs 2016	YLDs 2016	DALYs 2016
Drug use disorders	100.0% (100.0% to 100.0%)	100.0% (100.0% to 100.0%)	100.0% (100.0% to 100.0%)	100.0% (100.0% to 100.0%)
Depressive disorders	-	-	6.9% (5.7% to 8.3%)	6.9% (5.7% to 8.3%)
Idiopathic developmental intellectual disability	-	-	62.0% (31.8% to 81.9%)	62.0% (31.8% to 81.9%)
**Diabetes, urogenital, blood, and endocrine diseases**	77.9% (75.7% to 79.0%)	76.7% (73.2% to 77.9%)	65.2% (60.9% to 69.2%)	71.7% (69.2% to 73.8%)
Diabetes mellitus	100.0% (100.0% to 100.0%)	100.0% (100.0% to 100.0%)	100.0% (100.0% to 100.0%)	100.0% (100.0% to 100.0%)
Chronic kidney disease	100.0% (100.0% to 100.0%)	100.0% (100.0% to 100.0%)	100.0% (100.0% to 100.0%)	100.0% (100.0% to 100.0%)
**Musculoskeletal disorders**	1.6% (0.6% to 2.8%)	1.5% (0.6% to 2.6%)	17.1% (14.3% to 20.1%)	16.9% (14.1% to 19.8%)
Rheumatoid arthritis	4.9% (2.0% to 8.3%)	6.0% (2.4% to 10.0%)	6.4% (2.6% to 10.7%)	6.4% (2.6% to 10.7%)
Osteoarthritis	-	-	29.3% (16.3% to 42.4%)	29.3% (16.3% to 42.4%)
Low back and neck pain	-	-	22.8% (19.7% to 26.1%)	22.8% (19.7% to 26.1%)
Gout	-	-	52.7% (35.2% to 70.4%)	52.7% (35.2% to 70.4%)
**Other non-communicable diseases**	0.3% (0.2% to 0.5%)	0.4% (0.3% to 0.6%)	3.5% (3.0% to 4.0%)	2.7% (2.3% to 3.1%)
Sense organ diseases	-	-	9.5% (8.5% to 10.7%)	9.5% (8.5% to 10.7%)
Sudden infant death syndrome	5.9% (4.4% to 7.8%)	5.9% (4.4% to 7.8%)	-	5.9% (4.4% to 7.8%)
**Transport injuries**	44.8% (36.5% to 53.5%)	42.2% (33.6% to 51.1%)	48.4% (40.9% to 56.2%)	43.0% (34.6% to 51.7%)
Road injuries	44.6% (36.3% to 53.5%)	42.0% (33.4% to 51.1%)	49.8% (42.2% to 57.8%)	43.0% (34.5% to 51.7%)
Other transport injuries	47.4% (39.1% to 55.9%)	44.8% (35.9% to 53.8%)	39.2% (32.2% to 46.6%)	43.5% (35.2% to 51.8%)
**Unintentional injuries**	28.8% (25.0% to 33.6%)	22.7% (18.5% to 28.2%)	28.7% (24.8% to 33.9%)	25.3% (21.3% to 30.5%)
Falls	63.2% (58.3% to 67.5%)	43.8% (38.3% to 50.1%)	31.7% (28.3% to 36.1%)	35.0% (31.3% to 39.7%)
Drowning	25.9% (19.8% to 32.9%)	23.3% (17.8% to 29.6%)	26.4% (20.1% to 34.4%)	23.4% (17.9% to 29.7%)
Fire, heat, and hot substances	20.5% (15.1% to 27.6%)	22.8% (17.5% to 29.2%)	26.7% (20.6% to 34.4%)	24.3% (18.7% to 30.9%)

Supplementary Results Table 1. Age-standardised PAFs attributable to the joint distribution of all risk factors for all ages, both sexes combined for each GBD level 1 and level 2 cause and all causes in 2016 for Southern Latin America. Results are shown for deaths, YLLs, YLDs, and DALYs
Data in parenthesis are 95% uncertainty intervals. PAFs=population attributable fractions. GBD=Global Burden of Disease. YLLs=years of life lost. YLDs=years live with disability. DALYs=disability-adjusted life-years

	**Deaths 2016**	**YLLs 2016**	**YLDs 2016**	**DALYs 2016**
Poisonings	24.7% (18.7% to 32.7%)	23.2% (17.4% to 31.1%)	26.9% (20.6% to 34.3%)	23.9% (18.3% to 31.3%)
Exposure to mechanical forces	29.2% (23.2% to 37.5%)	27.3% (21.3% to 35.3%)	32.9% (27.3% to 40.7%)	30.2% (24.6% to 38.1%)
Animal contact	20.3% (16.1% to 26.2%)	17.9% (13.8% to 23.8%)	19.5% (15.2% to 24.9%)	19.0% (14.8% to 24.4%)
Foreign body	15.1% (11.1% to 20.6%)	15.3% (11.2% to 20.9%)	15.6% (12.0% to 20.5%)	15.3% (11.4% to 20.8%)
Other unintentional injuries	33.9% (26.3% to 43.8%)	32.2% (24.9% to 41.7%)	27.5% (21.3% to 35.4%)	29.6% (23.1% to 37.8%)
**Self-harm and interpersonal violence**	29.8% (18.8% to 39.3%)	30.5% (19.7% to 39.8%)	30.2% (25.0% to 35.6%)	30.5% (20.0% to 39.6%)
Self-harm	31.1% (15.9% to 43.5%)	32.9% (17.3% to 46.0%)	29.4% (14.7% to 41.4%)	32.8% (17.3% to 45.9%)
Interpersonal violence	27.0% (17.1% to 36.2%)	26.0% (16.2% to 35.2%)	30.4% (24.4% to 36.2%)	26.6% (17.2% to 35.3%)
**Forces of nature, conflict and terrorism, and executions and police conflict**	0.0% (0.0% to 0.0%)	0.0% (0.0% to 0.0%)	2.0% (1.2% to 3.1%)	1.7% (0.8% to 2.8%)
Forces of nature, conflict and terrorism, and state actor violence	0.0% (0.0% to 0.0%)	0.0% (0.0% to 0.0%)	8.2% (5.3% to 12.2%)	8.2% (5.3% to 12.2%)

Supplementary Results Table 1. Age-standardised PAFs attributable to the joint distribution of all risk factors for all ages, both sexes combined for each GBD level 1 and level 2 cause and all causes in 2016 for Southern Sub-Saharan Africa. Results are shown for deaths, YLLs, YLDs, and
Data in parenthesis are 95% uncertainty intervals. PAFs=population attributable fractions. GBD=Global Burden of Disease. YLLs=years of life lost. YLDs=years live with disability. DALYs=disability-adjusted life-years

	Deaths 2016	YLLs 2016	YLDs 2016	DALYs 2016
**All causes**	67.9% (66.4% to 69.5%)	65.8% (64.1% to 67.8%)	34.3% (32.6% to 36.0%)	58.9% (56.8% to 61.1%)
**HIV/AIDS and tuberculosis**	82.9% (80.5% to 85.3%)	79.6% (76.6% to 82.5%)	88.9% (87.0% to 90.5%)	80.3% (77.6% to 83.0%)
Tuberculosis	50.3% (42.6% to 58.5%)	49.1% (42.0% to 57.1%)	36.6% (28.7% to 45.3%)	48.7% (41.4% to 56.6%)
HIV/AIDS	90.2% (88.4% to 91.8%)	84.2% (81.2% to 87.0%)	92.0% (90.7% to 93.1%)	84.9% (82.1% to 87.4%)
**Diarrhoea, lower respiratory infections, and other common infectious diseases**	69.6% (64.3% to 74.4%)	73.4% (69.2% to 77.2%)	46.4% (37.9% to 53.5%)	71.6% (67.4% to 75.5%)
Diarrhoeal diseases	91.1% (81.0% to 97.2%)	94.1% (87.1% to 98.3%)	85.3% (71.4% to 95.0%)	93.3% (85.7% to 97.9%)
Lower respiratory infections	67.1% (61.3% to 73.0%)	73.3% (68.1% to 78.3%)	64.4% (58.4% to 70.5%)	73.3% (68.1% to 78.3%)
Upper respiratory infections	1.9% (0.6% to 5.1%)	3.7% (1.1% to 10.1%)	0.0% (0.0% to 0.0%)	0.1% (0.0% to 0.3%)
Otitis media	3.4% (2.0% to 5.0%)	5.3% (3.4% to 7.5%)	5.7% (3.8% to 7.5%)	5.7% (3.8% to 7.4%)
Meningitis	1.5% (1.0% to 2.2%)	2.7% (1.8% to 4.0%)	0.0% (0.0% to 0.0%)	2.6% (1.7% to 3.8%)
Encephalitis	0.6% (0.4% to 0.9%)	1.1% (0.7% to 1.6%)	0.0% (0.0% to 0.0%)	0.7% (0.4% to 1.2%)
Measles	48.4% (30.8% to 73.7%)	49.4% (31.4% to 75.3%)	49.5% (30.7% to 77.7%)	49.4% (31.4% to 75.3%)
**Maternal disorders**	3.8% (1.8% to 6.2%)	3.8% (1.7% to 6.1%)	1.9% (0.9% to 3.2%)	3.7% (1.7% to 5.9%)
Maternal haemorrhage	16.7% (5.9% to 27.2%)	16.7% (5.9% to 27.1%)	16.2% (5.8% to 26.5%)	16.7% (5.9% to 27.1%)
Maternal sepsis and other pregnancy related infections	16.3% (5.8% to 26.7%)	16.3% (5.8% to 26.6%)	15.9% (5.7% to 26.0%)	16.3% (5.8% to 26.6%)
Maternal abortion, miscarriage, and ectopic pregnancy	9.4% (5.4% to 14.2%)	9.3% (5.3% to 14.1%)	9.6% (5.5% to 14.4%)	9.4% (5.3% to 14.1%)
**Neonatal disorders**	80.2% (77.8% to 82.4%)	80.3% (77.8% to 82.4%)	0.3% (0.2% to 0.5%)	75.4% (71.9% to 78.4%)
Neonatal preterm birth complications	96.4% (94.3% to 97.9%)	96.4% (94.3% to 97.9%)	0.6% (0.5% to 0.8%)	87.9% (82.9% to 91.4%)
Neonatal encephalopathy due to birth asphyxia and trauma	73.9% (70.3% to 77.3%)	73.9% (70.3% to 77.3%)	0.0% (0.0% to 0.0%)	72.6% (68.8% to 76.3%)
Neonatal sepsis and other neonatal infections	66.8% (62.9% to 70.2%)	66.8% (63.0% to 70.3%)	0.0% (0.0% to 0.0%)	59.4% (47.8% to 66.6%)
Hemolytic disease and other neonatal jaundice	68.5% (64.4% to 71.9%)	68.5% (64.4% to 71.9%)	0.0% (0.0% to 0.0%)	48.5% (43.1% to 54.4%)

Supplementary Results Table 1. Age-standardised PAFs attributable to the joint distribution of all risk factors for all ages, both sexes combined for each GBD level 1 and level 2 cause and all causes in 2016 for Southern Sub-Saharan Africa. Results are shown for deaths, YLLs, YLDs, and
Data in parenthesis are 95% uncertainty intervals. PAFs=population attributable fractions. GBD=Global Burden of Disease. YLLs=years of life lost. YLDs=years live with disability. DALYs=disability-adjusted life-years

	**Deaths 2016**	**YLLs 2016**	**YLDs 2016**	**DALYs 2016**
Other neonatal disorders	71.0% (68.1% to 74.1%)	71.0% (68.1% to 74.1%)	0.0% (0.0% to 0.0%)	69.8% (66.9% to 73.0%)
**Nutritional deficiencies**	35.4% (28.5% to 43.1%)	65.7% (59.0% to 72.7%)	74.7% (71.3% to 78.3%)	70.5% (66.6% to 74.4%)
Protein-energy malnutrition	40.1% (32.6% to 48.3%)	70.2% (63.7% to 76.9%)	97.7% (97.1% to 98.2%)	71.8% (65.6% to 77.9%)
Vitamin A deficiency	-	-	100.0% (100.0% to 100.0%)	100.0% (100.0% to 100.0%)
Iron-deficiency anaemia	100.0% (100.0% to 100.0%)	100.0% (100.0% to 100.0%)	100.0% (100.0% to 100.0%)	100.0% (100.0% to 100.0%)
**Other communicable, maternal, neonatal, and nutritional diseases**	9.8% (7.6% to 16.5%)	6.7% (4.8% to 13.1%)	85.3% (82.1% to 88.0%)	28.7% (20.8% to 39.7%)
Sexually transmitted diseases excluding HIV	44.7% (29.4% to 61.9%)	22.7% (12.6% to 37.9%)	100.0% (100.0% to 100.0%)	63.2% (44.8% to 78.6%)
Hepatitis	1.9% (1.5% to 2.5%)	1.6% (1.2% to 2.1%)	1.3% (0.8% to 2.1%)	1.6% (1.2% to 2.1%)
**Neoplasms**	45.8% (43.1% to 49.2%)	46.2% (43.7% to 49.1%)	40.9% (37.9% to 44.4%)	46.1% (43.6% to 49.0%)
Oesophageal cancer	75.8% (65.3% to 84.4%)	78.6% (68.8% to 86.4%)	77.2% (67.0% to 85.7%)	78.6% (68.8% to 86.4%)
Stomach cancer	15.4% (8.4% to 25.6%)	16.5% (9.2% to 26.4%)	16.3% (9.3% to 26.2%)	16.5% (9.2% to 26.3%)
Liver cancer	53.4% (45.6% to 60.7%)	52.0% (44.2% to 59.4%)	54.1% (46.3% to 61.7%)	52.0% (44.2% to 59.5%)
Larynx cancer	80.6% (73.2% to 86.6%)	82.5% (75.4% to 87.8%)	82.2% (75.2% to 87.6%)	82.5% (75.4% to 87.8%)
Tracheal, bronchus, and lung cancer	88.3% (85.8% to 90.7%)	88.7% (86.3% to 90.9%)	88.1% (85.5% to 90.5%)	88.7% (86.3% to 90.9%)
Breast cancer	28.4% (18.8% to 39.1%)	24.8% (15.4% to 35.5%)	27.2% (17.1% to 38.9%)	24.9% (15.5% to 35.6%)
Cervical cancer	100.0% (100.0% to 100.0%)	100.0% (100.0% to 100.0%)	100.0% (100.0% to 100.0%)	100.0% (100.0% to 100.0%)
Uterine cancer	49.5% (37.6% to 61.3%)	51.5% (39.5% to 63.2%)	55.8% (43.3% to 67.3%)	51.7% (39.6% to 63.4%)
Prostate cancer	3.7% (2.7% to 4.9%)	4.4% (3.2% to 5.6%)	4.8% (3.5% to 6.3%)	4.4% (3.2% to 5.6%)
Colon and rectum cancer	55.8% (47.2% to 64.4%)	56.4% (48.3% to 64.5%)	56.5% (48.2% to 64.9%)	56.4% (48.3% to 64.5%)
Lip and oral cavity cancer	76.1% (69.4% to 81.9%)	79.2% (73.3% to 84.3%)	75.9% (69.4% to 81.3%)	79.1% (73.2% to 84.2%)
Nasopharynx cancer	73.1% (66.9% to 78.1%)	72.4% (66.9% to 76.7%)	71.7% (66.4% to 76.1%)	72.3% (66.9% to 76.7%)

Supplementary Results Table 1. Age-standardised PAFs attributable to the joint distribution of all risk factors for all ages, both sexes combined for each GBD level 1 and level 2 cause and all causes in 2016 for Southern Sub-Saharan Africa. Results are shown for deaths, YLLs, YLDs, and
Data in parenthesis are 95% uncertainty intervals. PAFs=population attributable fractions. GBD=Global Burden of Disease. YLLs=years of life lost. YLDs=years live with disability. DALYs=disability-adjusted life-years

	Deaths 2016	YLLs 2016	YLDs 2016	DALYs 2016
Other pharynx cancer	55.0% (43.4% to 65.7%)	57.7% (46.3% to 68.1%)	56.5% (44.6% to 67.4%)	57.6% (46.3% to 68.1%)
Gallbladder and biliary tract cancer	27.3% (16.5% to 39.5%)	27.8% (17.0% to 39.6%)	28.2% (17.1% to 40.6%)	27.8% (17.0% to 39.6%)
Pancreatic cancer	31.0% (22.4% to 41.2%)	32.4% (24.0% to 41.9%)	31.9% (23.4% to 41.9%)	32.4% (24.0% to 41.9%)
Ovarian cancer	15.9% (6.8% to 26.8%)	13.9% (5.6% to 23.7%)	14.1% (5.7% to 23.8%)	13.9% (5.6% to 23.7%)
Kidney cancer	34.0% (25.7% to 42.9%)	31.0% (23.6% to 39.0%)	32.1% (24.7% to 40.2%)	31.1% (23.7% to 39.1%)
Bladder cancer	28.9% (20.4% to 38.6%)	29.9% (21.6% to 38.9%)	31.5% (22.9% to 40.9%)	29.9% (21.7% to 38.9%)
Thyroid cancer	12.7% (7.4% to 19.2%)	13.1% (7.8% to 19.7%)	15.7% (9.4% to 22.8%)	13.2% (7.8% to 19.9%)
Mesothelioma	95.6% (94.4% to 96.5%)	92.2% (90.4% to 93.7%)	94.7% (93.3% to 95.7%)	92.2% (90.4% to 93.7%)
Non-Hodgkin's lymphoma	6.2% (2.5% to 10.7%)	5.5% (2.3% to 9.5%)	6.7% (2.7% to 11.3%)	5.6% (2.3% to 9.5%)
Multiple myeloma	8.3% (3.8% to 14.2%)	8.6% (3.9% to 14.5%)	9.1% (4.2% to 15.5%)	8.6% (3.9% to 14.6%)
Leukaemia	17.4% (11.3% to 24.2%)	14.5% (9.6% to 19.9%)	17.2% (11.1% to 23.8%)	14.5% (9.6% to 19.9%)
**Cardiovascular diseases**	87.7% (85.9% to 89.3%)	86.2% (84.9% to 87.4%)	70.5% (66.6% to 74.1%)	84.9% (83.6% to 86.2%)
Rheumatic heart disease	29.7% (20.7% to 44.4%)	25.9% (18.2% to 36.6%)	27.1% (18.3% to 38.0%)	26.3% (18.3% to 37.1%)
Ischaemic heart disease	95.5% (93.2% to 97.5%)	96.6% (95.2% to 97.8%)	93.7% (91.7% to 95.5%)	96.5% (95.0% to 97.7%)
Cerebrovascular disease	89.0% (85.5% to 92.3%)	88.8% (86.6% to 90.9%)	90.4% (87.1% to 93.5%)	89.0% (86.8% to 91.0%)
Hypertensive heart disease	100.0% (100.0% to 100.0%)	100.0% (100.0% to 100.0%)	100.0% (100.0% to 100.0%)	100.0% (100.0% to 100.0%)
Cardiomyopathy and myocarditis	46.3% (38.4% to 55.5%)	41.6% (35.1% to 48.0%)	49.4% (42.4% to 56.4%)	42.0% (35.5% to 48.5%)
Atrial fibrillation and flutter	56.1% (48.5% to 63.3%)	60.6% (54.1% to 66.5%)	67.5% (62.6% to 72.0%)	64.5% (59.1% to 69.6%)
Aortic aneurysm	49.2% (42.2% to 56.4%)	54.5% (48.7% to 60.1%)	-	54.5% (48.7% to 60.1%)
Peripheral vascular disease	66.3% (61.2% to 71.5%)	74.0% (70.1% to 77.7%)	68.7% (63.7% to 73.5%)	72.5% (68.7% to 76.5%)
Endocarditis	42.4% (34.0% to 51.5%)	37.7% (29.3% to 46.7%)	40.2% (31.2% to 51.0%)	37.8% (29.4% to 46.6%)

Supplementary Results Table 1. Age-standardised PAFs attributable to the joint distribution of all risk factors for all ages, both sexes combined for each GBD level 1 and level 2 cause and all causes in 2016 for Southern Sub-Saharan Africa. Results are shown for deaths, YLLs, YLDs, and
Data in parenthesis are 95% uncertainty intervals. PAFs=population attributable fractions. GBD=Global Burden of Disease. YLLs=years of life lost. YLDs=years live with disability. DALYs=disability-adjusted life-years

	Deaths 2016	YLLs 2016	YLDs 2016	DALYs 2016
Other cardiovascular and circulatory diseases	49.7% (44.8% to 54.7%)	47.1% (42.4% to 52.3%)	46.4% (41.2% to 51.4%)	46.9% (42.5% to 51.8%)
**Chronic respiratory diseases**	62.3% (57.7% to 67.0%)	60.5% (56.3% to 64.7%)	51.1% (45.4% to 56.9%)	57.3% (52.9% to 61.7%)
Chronic obstructive pulmonary disease	73.2% (67.4% to 79.1%)	74.7% (69.3% to 79.9%)	70.5% (65.1% to 76.1%)	73.5% (68.1% to 78.7%)
Pneumoconiosis	100.0% (100.0% to 100.0%)	100.0% (100.0% to 100.0%)	100.0% (100.0% to 100.0%)	100.0% (100.0% to 100.0%)
Asthma	41.5% (32.6% to 51.4%)	42.5% (34.7% to 51.4%)	33.0% (25.5% to 41.7%)	38.1% (30.7% to 46.9%)
Other chronic respiratory diseases	6.3% (4.7% to 8.3%)	3.6% (2.4% to 4.9%)	8.3% (6.2% to 10.5%)	5.2% (3.8% to 6.8%)
**Cirrhosis and other chronic liver diseases**	49.4% (47.3% to 51.4%)	48.1% (46.0% to 50.2%)	35.1% (33.0% to 37.1%)	47.6% (45.6% to 49.6%)
Cirrhosis and other chronic liver diseases due to hepatitis B	0.5% (0.3% to 0.6%)	0.5% (0.3% to 0.6%)	0.4% (0.3% to 0.6%)	0.5% (0.3% to 0.6%)
Cirrhosis and other chronic liver diseases due to hepatitis C	81.2% (76.8% to 84.9%)	85.7% (83.1% to 87.8%)	83.6% (80.8% to 85.8%)	85.6% (83.0% to 87.8%)
Cirrhosis and other chronic liver diseases due to alcohol use	100.0% (100.0% to 100.0%)	100.0% (100.0% to 100.0%)	100.0% (100.0% to 100.0%)	100.0% (100.0% to 100.0%)
**Digestive diseases**	8.3% (6.7% to 10.1%)	8.4% (6.8% to 10.0%)	4.1% (3.2% to 5.0%)	8.1% (6.5% to 9.6%)
Peptic ulcer disease	11.2% (7.9% to 15.0%)	12.9% (9.1% to 17.0%)	9.0% (6.3% to 12.1%)	12.6% (8.9% to 16.6%)
Gallbladder and biliary diseases	41.5% (30.1% to 53.6%)	41.2% (30.1% to 53.1%)	47.8% (36.5% to 59.8%)	41.5% (30.5% to 53.2%)
Pancreatitis	43.1% (30.2% to 56.3%)	50.5% (38.0% to 63.4%)	46.9% (34.2% to 60.0%)	50.5% (38.0% to 63.4%)
**Neurological disorders**	25.2% (14.8% to 36.5%)	24.6% (17.7% to 31.9%)	7.0% (4.8% to 9.6%)	15.1% (10.5% to 20.1%)
Alzheimer's disease and other dementias	27.9% (13.5% to 44.0%)	29.0% (14.3% to 45.1%)	29.7% (14.5% to 46.1%)	29.2% (14.3% to 45.3%)
Parkinson's disease	-8.2% (-9.5% to -6.7%)	-9.7% (-11.3% to -7.8%)	-10.3% (-12.0% to -8.3%)	-9.8% (-11.4% to -7.9%)
Epilepsy	27.5% (19.4% to 36.0%)	26.6% (19.0% to 34.4%)	21.8% (15.4% to 28.7%)	25.0% (17.7% to 32.4%)
Multiple sclerosis	7.4% (4.8% to 10.6%)	7.6% (4.8% to 10.9%)	6.1% (4.0% to 8.4%)	6.9% (4.5% to 9.6%)
**Mental and substance use disorders**	99.8% (99.6% to 99.9%)	99.7% (99.5% to 99.9%)	20.6% (18.4% to 23.0%)	26.4% (23.3% to 30.8%)
Alcohol use disorders	100.0% (100.0% to 100.0%)	100.0% (100.0% to 100.0%)	100.0% (100.0% to 100.0%)	100.0% (100.0% to 100.0%)

Supplementary Results Table 1. Age-standardised PAFs attributable to the joint distribution of all risk factors for all ages, both sexes combined for each GBD level 1 and level 2 cause and all causes in 2016 for Southern Sub-Saharan Africa. Results are shown for deaths, YLLs, YLDs, and
Data in parenthesis are 95% uncertainty intervals. PAFs=population attributable fractions. GBD=Global Burden of Disease. YLLs=years of life lost. YLDs=years live with disability. DALYs=disability-adjusted life-years

	Deaths 2016	YLLs 2016	YLDs 2016	DALYs 2016
Drug use disorders	100.0% (100.0% to 100.0%)	100.0% (100.0% to 100.0%)	100.0% (100.0% to 100.0%)	100.0% (100.0% to 100.0%)
Depressive disorders	-	-	7.9% (6.5% to 9.5%)	7.9% (6.5% to 9.5%)
Idiopathic developmental intellectual disability	-	-	62.5% (31.4% to 81.4%)	62.5% (31.4% to 81.4%)
**Diabetes, urogenital, blood, and endocrine diseases**	86.1% (85.3% to 88.1%)	79.5% (78.2% to 82.7%)	73.9% (70.4% to 77.0%)	78.0% (76.5% to 80.6%)
Diabetes mellitus	100.0% (100.0% to 100.0%)	100.0% (100.0% to 100.0%)	100.0% (100.0% to 100.0%)	100.0% (100.0% to 100.0%)
Chronic kidney disease	100.0% (100.0% to 100.0%)	100.0% (100.0% to 100.0%)	100.0% (100.0% to 100.0%)	100.0% (100.0% to 100.0%)
**Musculoskeletal disorders**	1.6% (0.6% to 2.7%)	1.4% (0.6% to 2.3%)	18.8% (15.7% to 22.2%)	18.2% (15.1% to 21.6%)
Rheumatoid arthritis	4.2% (1.7% to 7.0%)	4.7% (1.9% to 7.8%)	4.3% (1.7% to 7.2%)	4.4% (1.7% to 7.3%)
Osteoarthritis	-	-	29.3% (17.6% to 41.6%)	29.3% (17.6% to 41.6%)
Low back and neck pain	-	-	21.3% (18.5% to 24.2%)	21.3% (18.5% to 24.2%)
Gout	-	-	55.4% (44.8% to 67.3%)	55.4% (44.8% to 67.3%)
**Other non-communicable diseases**	0.2% (0.2% to 0.3%)	0.4% (0.3% to 0.5%)	6.0% (5.2% to 6.9%)	5.0% (4.3% to 5.6%)
Sense organ diseases	-	-	12.9% (11.4% to 14.5%)	12.9% (11.4% to 14.5%)
Sudden infant death syndrome	12.4% (9.4% to 15.7%)	12.4% (9.5% to 15.7%)	-	12.4% (9.5% to 15.7%)
**Transport injuries**	29.7% (23.8% to 36.5%)	24.7% (19.0% to 31.3%)	37.6% (31.9% to 44.5%)	25.8% (20.1% to 32.5%)
Road injuries	29.4% (23.4% to 36.3%)	24.5% (18.9% to 31.1%)	39.1% (33.3% to 46.1%)	25.7% (20.0% to 32.4%)
Other transport injuries	34.4% (28.7% to 41.1%)	27.3% (21.6% to 34.4%)	29.4% (24.1% to 35.8%)	27.8% (22.2% to 34.7%)
**Unintentional injuries**	17.7% (15.6% to 19.9%)	11.3% (9.6% to 13.3%)	17.1% (15.6% to 19.0%)	12.9% (11.1% to 14.8%)
Falls	56.8% (54.3% to 59.2%)	35.3% (33.4% to 37.5%)	26.7% (25.2% to 28.3%)	29.9% (28.4% to 31.5%)
Drowning	13.0% (9.4% to 17.3%)	11.1% (8.1% to 14.6%)	15.2% (11.0% to 20.3%)	11.2% (8.1% to 14.7%)
Fire, heat, and hot substances	12.5% (8.9% to 17.3%)	13.3% (9.9% to 17.6%)	17.0% (12.8% to 22.1%)	14.0% (10.5% to 18.4%)

**Supplementary Results Table 1. Age-standardised PAFs attributable to the joint distribution of all risk factors for all ages, both sexes combined for each GBD level 1 and level 2 cause and all causes in 2016 for Southern Sub-Saharan Africa. Results are shown for deaths, YLLs, YLDs, and** Data in parenthesis are 95% uncertainty intervals. PAFs=population attributable fractions. GBD=Global Burden of Disease. YLLs=years of life lost. YLDs=years live with disability. DALYs=disability-adjusted life-years				
	**Deaths 2016**	**YLLs 2016**	**YLDs 2016**	**DALYs 2016**
Poisonings	13.1% (9.5% to 18.1%)	11.8% (8.3% to 16.2%)	16.1% (12.1% to 20.6%)	12.0% (8.6% to 16.4%)
Exposure to mechanical forces	13.2% (11.9% to 14.5%)	10.1% (8.7% to 11.3%)	17.9% (16.3% to 19.9%)	12.0% (10.6% to 13.4%)
Animal contact	10.5% (9.5% to 11.5%)	9.0% (8.1% to 10.1%)	10.9% (9.5% to 12.7%)	9.5% (8.5% to 10.7%)
Foreign body	5.6% (4.8% to 6.7%)	4.3% (3.7% to 5.4%)	8.9% (7.6% to 10.6%)	5.5% (4.7% to 6.6%)
Other unintentional injuries	12.6% (9.2% to 17.0%)	12.5% (9.2% to 16.4%)	14.8% (11.0% to 19.3%)	13.8% (10.4% to 17.9%)
**Self-harm and interpersonal violence**	25.5% (19.7% to 31.8%)	25.0% (19.4% to 31.4%)	28.7% (24.5% to 33.6%)	25.2% (19.7% to 31.5%)
Self-harm	27.6% (17.9% to 37.3%)	29.7% (19.6% to 39.3%)	25.7% (16.2% to 35.5%)	29.7% (19.5% to 39.2%)
Interpersonal violence	24.3% (17.6% to 31.8%)	22.9% (16.5% to 30.5%)	29.0% (24.6% to 34.0%)	23.2% (16.9% to 30.6%)
**Forces of nature, conflict and terrorism, and executions and police conflict**	0.0% (0.0% to 0.0%)	0.0% (0.0% to 0.0%)	0.9% (0.6% to 1.3%)	0.5% (0.2% to 0.8%)
Forces of nature, conflict and terrorism, and state actor violence	0.0% (0.0% to 0.0%)	0.0% (0.0% to 0.0%)	6.8% (4.5% to 10.1%)	6.8% (4.5% to 10.1%)

Supplementary Results Table 1. Age-standardised PAFs attributable to the joint distribution of all risk factors for all ages, both sexes combined for each GBD level 1 and level 2 cause and all causes in 2016 for Tropical Latin America. Results are shown for deaths, YLLs, YLDs, and DALYs
Data in parenthesis are 95% uncertainty intervals. PAFs=population attributable fractions. GBD=Global Burden of Disease. YLLs=years of life lost. YLDs=years live with disability. DALYs=disability-adjusted life-years

	Deaths 2016	YLLs 2016	YLDs 2016	DALYs 2016
**All causes**	54.3% (52.5% to 56.1%)	50.3% (48.8% to 51.7%)	23.9% (22.8% to 25.2%)	40.1% (38.1% to 42.2%)
**HIV/AIDS and tuberculosis**	79.2% (76.6% to 81.9%)	80.6% (78.6% to 82.7%)	87.0% (83.6% to 89.9%)	81.1% (79.1% to 83.1%)
Tuberculosis	49.4% (40.5% to 57.7%)	52.2% (44.3% to 60.4%)	49.4% (41.5% to 57.6%)	52.1% (44.2% to 60.3%)
HIV/AIDS	91.4% (90.2% to 92.3%)	88.6% (87.3% to 89.5%)	93.7% (92.5% to 94.6%)	88.9% (87.7% to 89.9%)
**Diarrhoea, lower respiratory infections, and other common infectious diseases**	46.0% (40.4% to 53.3%)	54.1% (49.1% to 59.7%)	48.3% (39.3% to 56.9%)	52.7% (48.2% to 57.7%)
Diarrhoeal diseases	76.7% (62.7% to 89.0%)	86.7% (77.9% to 93.8%)	79.0% (66.1% to 90.2%)	82.1% (70.9% to 91.7%)
Lower respiratory infections	45.3% (39.0% to 53.5%)	56.0% (49.6% to 63.1%)	50.9% (44.5% to 58.5%)	55.9% (49.5% to 63.1%)
Upper respiratory infections	1.2% (0.7% to 1.8%)	2.6% (1.5% to 3.9%)	0.0% (0.0% to 0.0%)	0.0% (0.0% to 0.0%)
Otitis media	4.5% (3.5% to 5.6%)	7.8% (6.1% to 9.6%)	5.4% (3.5% to 7.1%)	5.4% (3.6% to 7.1%)
Meningitis	0.8% (0.7% to 0.9%)	1.2% (1.0% to 1.3%)	0.0% (0.0% to 0.0%)	1.1% (1.0% to 1.2%)
Encephalitis	0.9% (0.7% to 1.3%)	1.5% (1.2% to 1.9%)	0.0% (0.0% to 0.0%)	1.1% (0.8% to 1.5%)
Measles	23.8% (8.5% to 59.8%)	24.8% (8.9% to 62.2%)	27.4% (10.6% to 65.2%)	24.8% (8.9% to 62.2%)
**Maternal disorders**	5.5% (2.9% to 8.2%)	5.4% (2.8% to 8.0%)	3.8% (2.1% to 5.7%)	5.1% (2.7% to 7.6%)
Maternal haemorrhage	15.1% (5.4% to 24.6%)	15.0% (5.4% to 24.5%)	14.8% (5.3% to 24.1%)	15.0% (5.4% to 24.5%)
Maternal sepsis and other pregnancy related infections	14.9% (5.3% to 24.3%)	14.8% (5.3% to 24.1%)	14.5% (5.2% to 23.6%)	14.8% (5.3% to 24.1%)
Maternal abortion, miscarriage, and ectopic pregnancy	13.1% (8.5% to 18.2%)	12.8% (8.2% to 17.9%)	13.0% (7.4% to 18.9%)	12.9% (8.1% to 17.9%)
**Neonatal disorders**	71.8% (70.4% to 73.3%)	71.8% (70.5% to 73.3%)	0.1% (0.1% to 0.2%)	63.9% (60.9% to 66.5%)
Neonatal preterm birth complications	91.1% (90.1% to 92.0%)	91.2% (90.1% to 92.0%)	0.2% (0.2% to 0.3%)	76.8% (71.9% to 80.9%)
Neonatal encephalopathy due to birth asphyxia and trauma	61.7% (59.4% to 64.3%)	61.8% (59.5% to 64.4%)	0.0% (0.0% to 0.0%)	57.6% (52.8% to 61.4%)
Neonatal sepsis and other neonatal infections	54.2% (51.9% to 58.0%)	54.3% (51.9% to 58.1%)	0.0% (0.0% to 0.0%)	48.2% (40.9% to 53.5%)
Hemolytic disease and other neonatal jaundice	61.7% (59.3% to 64.2%)	61.7% (59.4% to 64.2%)	0.0% (0.0% to 0.0%)	34.9% (29.9% to 40.1%)

Supplementary Results Table 1. Age-standardised PAFs attributable to the joint distribution of all risk factors for all ages, both sexes combined for each GBD level 1 and level 2 cause and all causes in 2016 for Tropical Latin America. Results are shown for deaths, YLLs, YLDs, and DALYs
Data in parenthesis are 95% uncertainty intervals. PAFs=population attributable fractions. GBD=Global Burden of Disease. YLLs=years of life lost. YLDs=years live with disability. DALYs=disability-adjusted life-years

	Deaths 2016	YLLs 2016	YLDs 2016	DALYs 2016
Other neonatal disorders	59.6% (57.3% to 62.2%)	59.6% (57.3% to 62.2%)	0.0% (0.0% to 0.0%)	58.4% (56.2% to 60.9%)
**Nutritional deficiencies**	9.1% (8.2% to 10.1%)	37.9% (35.2% to 40.6%)	97.1% (96.6% to 97.6%)	83.7% (79.0% to 87.7%)
Protein-energy malnutrition	9.5% (8.5% to 10.8%)	41.0% (37.8% to 44.8%)	96.0% (95.4% to 96.6%)	53.0% (48.7% to 58.1%)
Vitamin A deficiency	-	-	100.0% (100.0% to 100.0%)	100.0% (100.0% to 100.0%)
Iron-deficiency anaemia	100.0% (100.0% to 100.0%)	100.0% (100.0% to 100.0%)	100.0% (100.0% to 100.0%)	100.0% (100.0% to 100.0%)
**Other communicable, maternal, neonatal, and nutritional diseases**	6.9% (6.0% to 7.7%)	5.2% (4.0% to 6.3%)	61.0% (54.4% to 67.8%)	23.6% (17.1% to 33.0%)
Sexually transmitted diseases excluding HIV	32.2% (19.3% to 48.3%)	15.6% (8.4% to 26.2%)	100.0% (100.0% to 100.0%)	54.4% (36.3% to 74.1%)
Hepatitis	0.4% (0.2% to 0.5%)	0.4% (0.3% to 0.6%)	0.3% (0.2% to 0.6%)	0.4% (0.3% to 0.6%)
**Neoplasms**	36.2% (33.5% to 38.7%)	35.9% (33.6% to 38.4%)	31.4% (28.7% to 34.3%)	35.8% (33.5% to 38.3%)
Oesophageal cancer	73.1% (63.7% to 81.8%)	75.1% (65.8% to 83.5%)	73.5% (64.2% to 82.1%)	75.1% (65.8% to 83.5%)
Stomach cancer	13.7% (6.6% to 22.5%)	13.2% (6.4% to 21.4%)	13.7% (6.6% to 22.3%)	13.2% (6.4% to 21.4%)
Liver cancer	47.4% (38.7% to 56.5%)	47.8% (39.0% to 57.2%)	47.5% (38.9% to 56.6%)	47.8% (39.1% to 57.2%)
Larynx cancer	78.0% (68.5% to 85.0%)	78.5% (69.4% to 85.4%)	78.7% (69.8% to 85.5%)	78.5% (69.4% to 85.4%)
Tracheal, bronchus, and lung cancer	79.7% (76.6% to 82.5%)	79.1% (76.1% to 82.0%)	79.2% (76.0% to 82.1%)	79.1% (76.1% to 82.0%)
Breast cancer	23.6% (16.5% to 31.4%)	22.1% (15.1% to 30.3%)	22.8% (15.6% to 30.9%)	22.1% (15.1% to 30.4%)
Cervical cancer	100.0% (100.0% to 100.0%)	100.0% (100.0% to 100.0%)	100.0% (100.0% to 100.0%)	100.0% (100.0% to 100.0%)
Uterine cancer	45.4% (32.8% to 58.7%)	46.2% (33.4% to 59.9%)	47.2% (34.2% to 61.1%)	46.3% (33.4% to 60.0%)
Prostate cancer	3.0% (2.1% to 4.0%)	2.9% (2.1% to 3.9%)	3.1% (2.2% to 4.1%)	3.0% (2.1% to 3.9%)
Colon and rectum cancer	51.8% (43.1% to 60.0%)	53.0% (44.9% to 60.7%)	52.3% (43.9% to 60.5%)	53.0% (44.9% to 60.7%)
Lip and oral cavity cancer	70.8% (62.8% to 77.6%)	74.6% (67.1% to 81.1%)	70.7% (63.0% to 77.3%)	74.5% (67.0% to 81.0%)
Nasopharynx cancer	70.4% (64.8% to 75.2%)	68.8% (63.5% to 73.3%)	68.0% (62.7% to 72.6%)	68.8% (63.5% to 73.3%)

Supplementary Results Table 1. Age-standardised PAFs attributable to the joint distribution of all risk factors for all ages, both sexes combined for each GBD level 1 and level 2 cause and all causes in 2016 for Tropical Latin America. Results are shown for deaths, YLLs, YLDs, and DALYs
Data in parenthesis are 95% uncertainty intervals. PAFs=population attributable fractions. GBD=Global Burden of Disease. YLLs=years of life lost. YLDs=years live with disability. DALYs=disability-adjusted life-years

	Deaths 2016	YLLs 2016	YLDs 2016	DALYs 2016
Other pharynx cancer	56.0% (44.0% to 66.4%)	60.4% (48.1% to 70.4%)	58.1% (45.9% to 68.2%)	60.4% (48.0% to 70.3%)
Gallbladder and biliary tract cancer	22.9% (13.2% to 34.8%)	23.5% (13.7% to 35.5%)	23.0% (13.3% to 35.1%)	23.5% (13.7% to 35.5%)
Pancreatic cancer	24.1% (17.7% to 31.8%)	23.9% (17.7% to 31.4%)	24.0% (17.6% to 31.6%)	23.9% (17.7% to 31.4%)
Ovarian cancer	11.7% (5.2% to 19.1%)	9.7% (4.0% to 16.5%)	9.8% (4.2% to 16.4%)	9.7% (4.0% to 16.5%)
Kidney cancer	29.3% (20.9% to 39.0%)	27.1% (19.4% to 36.0%)	27.9% (19.9% to 37.1%)	27.1% (19.4% to 36.0%)
Bladder cancer	25.3% (17.7% to 33.5%)	24.5% (17.2% to 32.2%)	24.7% (17.2% to 32.3%)	24.5% (17.2% to 32.2%)
Thyroid cancer	13.8% (7.3% to 22.7%)	14.3% (7.4% to 23.5%)	14.4% (7.9% to 22.8%)	14.3% (7.5% to 23.5%)
Mesothelioma	90.1% (87.5% to 92.3%)	82.8% (78.9% to 86.2%)	87.7% (84.7% to 90.3%)	82.8% (79.0% to 86.2%)
Non-Hodgkin's lymphoma	6.6% (2.6% to 11.4%)	6.1% (2.5% to 10.5%)	6.6% (2.7% to 11.4%)	6.1% (2.5% to 10.5%)
Multiple myeloma	7.9% (3.5% to 13.9%)	8.2% (3.7% to 14.3%)	8.1% (3.6% to 14.2%)	8.2% (3.7% to 14.3%)
Leukaemia	13.8% (8.9% to 19.0%)	10.5% (7.0% to 14.3%)	13.0% (8.5% to 18.0%)	10.5% (7.0% to 14.4%)
**Cardiovascular diseases**	81.6% (78.9% to 84.0%)	82.5% (80.6% to 84.1%)	74.5% (71.7% to 76.9%)	81.8% (79.9% to 83.4%)
Rheumatic heart disease	26.0% (17.9% to 39.9%)	22.3% (15.6% to 32.7%)	17.7% (11.3% to 25.8%)	21.4% (14.7% to 31.2%)
Ischaemic heart disease	91.5% (87.8% to 94.6%)	93.2% (90.9% to 95.2%)	90.3% (87.6% to 92.8%)	93.0% (90.8% to 95.0%)
Cerebrovascular disease	81.3% (76.0% to 86.5%)	84.7% (81.3% to 87.7%)	83.2% (78.4% to 87.4%)	84.6% (81.1% to 87.7%)
Hypertensive heart disease	100.0% (100.0% to 100.0%)	100.0% (100.0% to 100.0%)	100.0% (100.0% to 100.0%)	100.0% (100.0% to 100.0%)
Cardiomyopathy and myocarditis	43.0% (34.8% to 52.3%)	42.5% (35.9% to 49.6%)	41.0% (34.3% to 48.2%)	42.5% (35.9% to 49.6%)
Atrial fibrillation and flutter	50.8% (43.6% to 57.9%)	56.0% (49.4% to 61.9%)	63.1% (58.1% to 68.1%)	60.4% (55.1% to 65.6%)
Aortic aneurysm	44.4% (37.6% to 50.9%)	48.3% (42.5% to 53.7%)	-	48.3% (42.5% to 53.7%)
Peripheral vascular disease	49.8% (43.7% to 56.5%)	55.7% (50.5% to 60.7%)	56.1% (50.6% to 61.4%)	55.8% (50.5% to 60.8%)
Endocarditis	37.7% (29.3% to 47.4%)	31.3% (23.6% to 38.9%)	36.5% (28.6% to 44.8%)	31.3% (23.7% to 39.0%)

Supplementary Results Table 1. Age-standardised PAFs attributable to the joint distribution of all risk factors for all ages, both sexes combined for each GBD level 1 and level 2 cause and all causes in 2016 for Tropical Latin America. Results are shown for deaths, YLLs, YLDs, and DALYs
Data in parenthesis are 95% uncertainty intervals. PAFs=population attributable fractions. GBD=Global Burden of Disease. YLLs=years of life lost. YLDs=years live with disability. DALYs=disability-adjusted life-years

	Deaths 2016	YLLs 2016	YLDs 2016	DALYs 2016
Other cardiovascular and circulatory diseases	45.0% (40.1% to 49.9%)	45.2% (40.8% to 49.3%)	45.3% (40.4% to 50.1%)	45.2% (40.9% to 49.3%)
**Chronic respiratory diseases**	65.9% (60.9% to 70.6%)	63.4% (59.0% to 68.0%)	38.4% (30.5% to 46.6%)	56.7% (51.8% to 61.4%)
Chronic obstructive pulmonary disease	70.7% (65.2% to 75.7%)	70.0% (64.9% to 74.9%)	66.0% (60.9% to 71.4%)	69.5% (64.6% to 74.6%)
Pneumoconiosis	100.0% (100.0% to 100.0%)	100.0% (100.0% to 100.0%)	100.0% (100.0% to 100.0%)	100.0% (100.0% to 100.0%)
Asthma	36.9% (27.7% to 47.4%)	38.1% (29.8% to 48.1%)	27.9% (19.0% to 39.0%)	29.8% (21.1% to 40.1%)
Other chronic respiratory diseases	8.4% (6.5% to 10.6%)	5.1% (3.5% to 7.1%)	4.1% (2.7% to 6.1%)	4.9% (3.4% to 6.8%)
**Cirrhosis and other chronic liver diseases**	39.9% (37.7% to 42.3%)	41.3% (38.4% to 44.3%)	33.8% (31.3% to 36.2%)	41.1% (38.2% to 44.1%)
Cirrhosis and other chronic liver diseases due to hepatitis B	0.0% (0.0% to 0.1%)	0.0% (0.0% to 0.1%)	0.0% (0.0% to 0.1%)	0.0% (0.0% to 0.1%)
Cirrhosis and other chronic liver diseases due to hepatitis C	13.8% (9.7% to 18.3%)	19.4% (13.4% to 25.9%)	19.2% (13.4% to 25.0%)	19.4% (13.4% to 25.9%)
Cirrhosis and other chronic liver diseases due to alcohol use	100.0% (100.0% to 100.0%)	100.0% (100.0% to 100.0%)	100.0% (100.0% to 100.0%)	100.0% (100.0% to 100.0%)
**Digestive diseases**	13.7% (10.7% to 16.8%)	15.6% (12.4% to 18.7%)	3.7% (2.8% to 4.6%)	12.7% (10.2% to 15.4%)
Peptic ulcer disease	9.3% (6.4% to 12.6%)	10.9% (7.5% to 14.7%)	10.0% (6.9% to 13.6%)	10.6% (7.4% to 14.4%)
Gallbladder and biliary diseases	42.5% (29.9% to 56.2%)	43.2% (30.6% to 56.8%)	47.2% (34.4% to 61.4%)	43.5% (30.9% to 57.2%)
Pancreatitis	40.2% (27.1% to 54.6%)	49.0% (35.7% to 62.8%)	38.1% (25.2% to 51.8%)	48.9% (35.5% to 62.7%)
**Neurological disorders**	21.5% (10.8% to 34.1%)	21.0% (11.5% to 31.9%)	5.7% (3.7% to 8.1%)	11.5% (6.8% to 17.3%)
Alzheimer's disease and other dementias	23.3% (11.4% to 36.9%)	24.1% (12.3% to 37.6%)	24.1% (12.2% to 37.6%)	24.1% (12.3% to 37.6%)
Parkinson's disease	-5.7% (-6.5% to -4.8%)	-7.1% (-8.1% to -6.0%)	-7.9% (-9.1% to -6.7%)	-7.2% (-8.3% to -6.1%)
Epilepsy	28.0% (21.1% to 35.3%)	27.0% (20.6% to 33.8%)	23.0% (17.1% to 29.5%)	24.3% (18.3% to 30.7%)
Multiple sclerosis	5.9% (3.8% to 8.4%)	6.5% (4.2% to 9.1%)	5.9% (3.8% to 8.2%)	6.3% (4.0% to 8.7%)
**Mental and substance use disorders**	99.7% (99.7% to 99.8%)	99.6% (99.6% to 99.6%)	21.2% (19.1% to 23.6%)	26.7% (24.0% to 29.6%)
Alcohol use disorders	100.0% (100.0% to 100.0%)	100.0% (100.0% to 100.0%)	100.0% (100.0% to 100.0%)	100.0% (100.0% to 100.0%)

Supplementary Results Table 1. Age-standardised PAFs attributable to the joint distribution of all risk factors for all ages, both sexes combined for each GBD level 1 and level 2 cause and all causes in 2016 for Tropical Latin America. Results are shown for deaths, YLLs, YLDs, and DALYs
Data in parenthesis are 95% uncertainty intervals. PAFs=population attributable fractions. GBD=Global Burden of Disease. YLLs=years of life lost. YLDs=years live with disability. DALYs=disability-adjusted life-years

	Deaths 2016	YLLs 2016	YLDs 2016	DALYs 2016
Drug use disorders	100.0% (100.0% to 100.0%)	100.0% (100.0% to 100.0%)	100.0% (100.0% to 100.0%)	100.0% (100.0% to 100.0%)
Depressive disorders	-	-	5.0% (4.2% to 6.1%)	5.0% (4.2% to 6.1%)
Idiopathic developmental intellectual disability	-	-	67.9% (37.5% to 86.6%)	67.9% (37.5% to 86.6%)
**Diabetes, urogenital, blood, and endocrine diseases**	76.7% (75.9% to 78.5%)	75.0% (74.2% to 77.7%)	66.1% (61.5% to 70.1%)	72.0% (70.0% to 74.0%)
Diabetes mellitus	100.0% (100.0% to 100.0%)	100.0% (100.0% to 100.0%)	100.0% (100.0% to 100.0%)	100.0% (100.0% to 100.0%)
Chronic kidney disease	100.0% (100.0% to 100.0%)	100.0% (100.0% to 100.0%)	100.0% (100.0% to 100.0%)	100.0% (100.0% to 100.0%)
**Musculoskeletal disorders**	0.6% (0.2% to 1.1%)	0.5% (0.2% to 0.9%)	18.4% (15.6% to 21.5%)	18.0% (15.3% to 21.0%)
Rheumatoid arthritis	3.4% (1.4% to 5.9%)	3.8% (1.5% to 6.5%)	3.9% (1.6% to 6.7%)	3.9% (1.6% to 6.7%)
Osteoarthritis	-	-	26.5% (14.7% to 39.2%)	26.5% (14.7% to 39.2%)
Low back and neck pain	-	-	23.8% (21.0% to 26.9%)	23.8% (21.0% to 26.9%)
Gout	-	-	47.0% (32.6% to 62.7%)	47.0% (32.6% to 62.7%)
**Other non-communicable diseases**	0.0% (0.0% to 0.1%)	0.1% (0.1% to 0.1%)	4.7% (4.1% to 5.3%)	3.6% (3.1% to 4.0%)
Sense organ diseases	-	-	11.0% (9.8% to 12.3%)	11.0% (9.8% to 12.3%)
Sudden infant death syndrome	5.0% (4.3% to 5.8%)	5.0% (4.3% to 5.8%)	-	5.0% (4.3% to 5.8%)
**Transport injuries**	35.8% (29.0% to 44.1%)	33.3% (26.5% to 41.5%)	40.3% (33.9% to 48.1%)	33.9% (27.2% to 42.1%)
Road injuries	35.8% (29.0% to 44.1%)	33.3% (26.5% to 41.5%)	40.5% (34.1% to 48.3%)	34.0% (27.2% to 42.2%)
Other transport injuries	37.4% (30.8% to 45.2%)	33.5% (26.8% to 41.2%)	34.2% (27.8% to 42.0%)	33.5% (26.9% to 41.2%)
**Unintentional injuries**	33.6% (28.6% to 36.0%)	19.4% (16.8% to 21.7%)	21.1% (20.0% to 22.3%)	20.2% (18.4% to 21.7%)
Falls	59.7% (56.0% to 61.8%)	37.3% (33.4% to 39.1%)	26.4% (25.4% to 27.4%)	30.1% (28.3% to 31.7%)
Drowning	18.4% (14.3% to 23.6%)	16.7% (13.1% to 21.3%)	19.3% (14.5% to 25.2%)	16.8% (13.1% to 21.3%)
Fire, heat, and hot substances	12.5% (9.0% to 16.9%)	13.9% (10.5% to 18.0%)	18.2% (14.0% to 23.5%)	15.5% (12.0% to 19.9%)

Supplementary Results Table 1. Age-standardised PAFs attributable to the joint distribution of all risk factors for all ages, both sexes combined for each GBD level 1 and level 2 cause and all causes in 2016 for Tropical Latin America. Results are shown for deaths, YLLs, YLDs, and DALYs
Data in parenthesis are 95% uncertainty intervals. PAFs=population attributable fractions. GBD=Global Burden of Disease. YLLs=years of life lost. YLDs=years live with disability. DALYs=disability-adjusted life-years

	Deaths 2016	YLLs 2016	YLDs 2016	DALYs 2016
Poisonings	13.7% (10.2% to 18.2%)	14.0% (10.8% to 18.2%)	16.4% (12.5% to 21.2%)	14.3% (11.0% to 18.6%)
Exposure to mechanical forces	18.7% (16.8% to 20.6%)	16.0% (14.0% to 18.0%)	20.5% (19.3% to 21.7%)	17.5% (15.6% to 19.1%)
Animal contact	11.0% (9.8% to 12.1%)	10.2% (9.4% to 11.7%)	11.9% (10.6% to 12.7%)	10.7% (9.8% to 11.8%)
Foreign body	4.3% (3.8% to 5.4%)	4.4% (3.9% to 5.7%)	6.0% (4.9% to 7.0%)	4.7% (4.2% to 5.8%)
Other unintentional injuries	20.9% (16.3% to 26.9%)	22.2% (17.5% to 28.1%)	16.8% (12.6% to 21.9%)	19.7% (15.3% to 25.3%)
**Self-harm and interpersonal violence**	24.4% (18.2% to 31.6%)	23.9% (17.4% to 31.2%)	22.0% (18.0% to 26.4%)	23.8% (17.4% to 31.0%)
Self-harm	32.6% (21.2% to 43.1%)	34.3% (23.0% to 44.5%)	29.4% (18.8% to 39.7%)	34.3% (23.0% to 44.5%)
Interpersonal violence	22.4% (15.3% to 30.7%)	21.8% (14.6% to 30.0%)	21.6% (17.4% to 26.3%)	21.8% (14.8% to 29.9%)
**Forces of nature, conflict and terrorism, and executions and police conflict**	0.0% (0.0% to 0.0%)	0.0% (0.0% to 0.0%)	3.0% (2.0% to 4.3%)	0.2% (0.0% to 1.4%)
Forces of nature, conflict and terrorism, and state actor violence	0.0% (0.0% to 0.0%)	0.0% (0.0% to 0.0%)	4.5% (3.0% to 6.6%)	4.5% (3.0% to 6.6%)

Supplementary Results Table 1. Age-standardised PAFs attributable to the joint distribution of all risk factors for all ages, both sexes combined for each GBD level 1 and level 2 cause and all causes in 2016 for Western Europe. Results are shown for deaths, YLLs, YLDs, and DALYs
Data in parenthesis are 95% uncertainty intervals. PAFs=population attributable fractions. GBD=Global Burden of Disease. YLLs=years of life lost. YLDs=years live with disability. DALYs=disability-adjusted life-years

	Deaths 2016	YLLs 2016	YLDs 2016	DALYs 2016
**All causes**	55.8% (53.6% to 57.9%)	53.7% (52.0% to 55.3%)	19.6% (18.3% to 20.8%)	34.8% (32.1% to 37.6%)
**HIV/AIDS and tuberculosis**	77.8% (74.2% to 80.9%)	81.5% (79.7% to 83.1%)	88.5% (86.1% to 90.1%)	84.3% (82.2% to 86.1%)
Tuberculosis	59.4% (50.8% to 66.7%)	60.3% (53.1% to 66.8%)	51.5% (42.8% to 59.0%)	58.7% (51.4% to 65.2%)
HIV/AIDS	90.1% (89.2% to 90.9%)	87.6% (86.6% to 88.4%)	91.5% (90.3% to 92.4%)	89.3% (88.2% to 90.3%)
**Diarrhoea, lower respiratory infections, and other common infectious diseases**	35.5% (27.8% to 43.3%)	40.5% (34.3% to 47.0%)	6.6% (4.8% to 8.9%)	24.9% (19.8% to 30.4%)
Diarrhoeal diseases	14.5% (8.5% to 24.3%)	26.3% (20.3% to 35.0%)	31.2% (23.0% to 40.6%)	29.1% (22.2% to 38.1%)
Lower respiratory infections	39.2% (30.3% to 47.9%)	47.4% (39.5% to 55.7%)	45.6% (37.7% to 54.3%)	47.3% (39.5% to 55.7%)
Upper respiratory infections	0.5% (0.3% to 0.8%)	1.6% (1.0% to 2.6%)	0.0% (0.0% to 0.0%)	0.0% (0.0% to 0.0%)
Otitis media	5.0% (3.3% to 7.0%)	10.1% (7.1% to 12.9%)	7.8% (5.4% to 10.3%)	7.9% (5.4% to 10.3%)
Meningitis	2.4% (2.0% to 2.8%)	4.4% (3.7% to 4.9%)	0.0% (0.0% to 0.0%)	3.8% (3.2% to 4.3%)
Encephalitis	0.4% (0.4% to 0.5%)	1.0% (0.8% to 1.2%)	0.0% (0.0% to 0.0%)	0.6% (0.5% to 0.8%)
Measles	6.5% (1.3% to 23.7%)	7.4% (1.5% to 27.1%)	11.7% (2.6% to 42.6%)	7.5% (1.6% to 27.6%)
**Maternal disorders**	2.6% (1.4% to 3.8%)	2.5% (1.4% to 3.8%)	2.6% (1.3% to 4.3%)	2.6% (1.3% to 4.0%)
Maternal haemorrhage	8.9% (3.2% to 14.4%)	8.9% (3.2% to 14.4%)	9.1% (3.3% to 14.8%)	9.0% (3.3% to 14.6%)
Maternal sepsis and other pregnancy related infections	8.9% (3.2% to 14.4%)	8.9% (3.3% to 14.5%)	9.0% (3.3% to 14.5%)	8.9% (3.3% to 14.5%)
Maternal abortion, miscarriage, and ectopic pregnancy	4.8% (2.9% to 7.1%)	4.9% (2.9% to 7.2%)	5.4% (2.7% to 8.7%)	5.1% (2.9% to 7.8%)
**Neonatal disorders**	74.3% (72.5% to 75.8%)	74.4% (72.6% to 75.9%)	0.1% (0.1% to 0.1%)	47.7% (41.7% to 53.1%)
Neonatal preterm birth complications	86.4% (85.3% to 87.5%)	86.5% (85.4% to 87.6%)	0.2% (0.1% to 0.2%)	56.9% (49.0% to 64.0%)
Neonatal encephalopathy due to birth asphyxia and trauma	64.1% (61.8% to 66.7%)	64.2% (61.8% to 66.8%)	0.0% (0.0% to 0.0%)	36.8% (28.9% to 44.6%)
Neonatal sepsis and other neonatal infections	55.3% (52.3% to 58.4%)	55.3% (52.4% to 58.4%)	0.0% (0.0% to 0.0%)	23.2% (11.8% to 38.7%)
Hemolytic disease and other neonatal jaundice	58.0% (54.1% to 61.8%)	58.1% (54.2% to 62.0%)	0.0% (0.0% to 0.0%)	17.8% (13.3% to 23.1%)

Supplementary Results Table 1. Age-standardised PAFs attributable to the joint distribution of all risk factors for all ages, both sexes combined for each GBD level 1 and level 2 cause and all causes in 2016 for Western Europe. Results are shown for deaths, YLLs, YLDs, and DALYs
Data in parenthesis are 95% uncertainty intervals. PAFs=population attributable fractions. GBD=Global Burden of Disease. YLLs=years of life lost. YLDs=years live with disability. DALYs=disability-adjusted life-years

	Deaths 2016	YLLs 2016	YLDs 2016	DALYs 2016
Other neonatal disorders	60.0% (57.6% to 62.6%)	60.1% (57.7% to 62.7%)	0.0% (0.0% to 0.0%)	55.7% (52.9% to 58.6%)
**Nutritional deficiencies**	4.1% (1.3% to 5.5%)	9.6% (6.7% to 11.0%)	65.4% (59.1% to 71.5%)	61.4% (55.2% to 67.8%)
Protein-energy malnutrition	0.5% (0.4% to 0.7%)	4.6% (3.8% to 6.1%)	98.2% (98.2% to 98.3%)	66.4% (56.6% to 74.6%)
Vitamin A deficiency	-	-	0.0% (0.0% to 0.0%)	0.0% (0.0% to 0.0%)
Iron-deficiency anaemia	100.0% (100.0% to 100.0%)	100.0% (100.0% to 100.0%)	99.9% (99.9% to 99.9%)	99.9% (99.9% to 99.9%)
**Other communicable, maternal, neonatal, and nutritional diseases**	5.1% (4.6% to 6.1%)	4.5% (4.0% to 5.2%)	67.4% (61.2% to 73.5%)	30.9% (23.2% to 41.3%)
Sexually transmitted diseases excluding HIV	95.3% (90.6% to 97.8%)	85.3% (73.3% to 92.5%)	99.9% (99.9% to 99.9%)	98.8% (97.6% to 99.5%)
Hepatitis	5.1% (3.9% to 6.5%)	5.7% (4.4% to 7.3%)	2.3% (1.6% to 3.3%)	5.4% (4.2% to 6.9%)
**Neoplasms**	46.9% (44.4% to 49.5%)	46.9% (44.8% to 49.2%)	34.8% (32.1% to 37.8%)	46.5% (44.2% to 48.8%)
Oesophageal cancer	87.4% (81.6% to 92.2%)	88.2% (82.6% to 92.9%)	87.4% (81.6% to 92.2%)	88.2% (82.6% to 92.9%)
Stomach cancer	21.0% (12.8% to 30.0%)	21.6% (13.4% to 30.3%)	21.1% (12.9% to 30.0%)	21.6% (13.3% to 30.3%)
Liver cancer	75.2% (69.3% to 80.3%)	77.3% (71.4% to 82.0%)	76.2% (70.4% to 81.1%)	77.3% (71.4% to 82.0%)
Larynx cancer	92.0% (87.2% to 95.1%)	91.9% (87.2% to 94.9%)	91.6% (86.6% to 94.7%)	91.9% (87.2% to 94.8%)
Tracheal, bronchus, and lung cancer	89.9% (88.3% to 91.4%)	89.6% (88.0% to 91.0%)	89.4% (87.8% to 90.9%)	89.6% (88.0% to 91.0%)
Breast cancer	36.2% (29.9% to 42.7%)	35.3% (29.5% to 41.2%)	35.4% (29.5% to 41.6%)	35.3% (29.5% to 41.2%)
Cervical cancer	100.0% (100.0% to 100.0%)	100.0% (100.0% to 100.0%)	99.9% (99.9% to 99.9%)	100.0% (100.0% to 100.0%)
Uterine cancer	42.1% (29.6% to 55.4%)	42.7% (30.1% to 56.3%)	42.5% (29.8% to 56.0%)	42.7% (30.1% to 56.3%)
Prostate cancer	6.4% (4.7% to 8.3%)	6.6% (4.9% to 8.4%)	6.7% (4.9% to 8.6%)	6.6% (4.9% to 8.5%)
Colon and rectum cancer	58.7% (50.9% to 66.3%)	59.6% (52.2% to 66.7%)	59.1% (51.4% to 66.5%)	59.6% (52.1% to 66.7%)
Lip and oral cavity cancer	86.7% (82.6% to 90.1%)	87.6% (83.7% to 90.7%)	85.7% (81.7% to 89.1%)	87.5% (83.7% to 90.6%)
Nasopharynx cancer	87.8% (84.8% to 90.3%)	86.1% (83.4% to 88.4%)	83.1% (80.1% to 85.7%)	86.1% (83.4% to 88.4%)

Supplementary Results Table 1. Age-standardised PAFs attributable to the joint distribution of all risk factors for all ages, both sexes combined for each GBD level 1 and level 2 cause and all causes in 2016 for Western Europe. Results are shown for deaths, YLLs, YLDs, and DALYs
Data in parenthesis are 95% uncertainty intervals. PAFs=population attributable fractions. GBD=Global Burden of Disease. YLLs=years of life lost. YLDs=years live with disability. DALYs=disability-adjusted life-years

	Deaths 2016	YLLs 2016	YLDs 2016	DALYs 2016
Other pharynx cancer	72.4% (63.1% to 79.2%)	73.0% (63.8% to 79.8%)	72.4% (63.2% to 79.4%)	73.0% (63.8% to 79.8%)
Gallbladder and biliary tract cancer	19.9% (10.7% to 31.5%)	19.9% (10.7% to 31.6%)	20.6% (11.3% to 32.2%)	19.9% (10.7% to 31.6%)
Pancreatic cancer	33.6% (26.3% to 41.1%)	34.5% (27.5% to 41.8%)	33.3% (26.4% to 41.0%)	34.5% (27.5% to 41.8%)
Ovarian cancer	15.9% (8.7% to 23.8%)	12.6% (6.6% to 19.5%)	12.7% (6.8% to 19.5%)	12.6% (6.6% to 19.5%)
Kidney cancer	37.8% (28.0% to 48.4%)	38.0% (28.2% to 48.4%)	37.3% (27.9% to 47.6%)	38.0% (28.2% to 48.4%)
Bladder cancer	39.7% (29.7% to 49.2%)	40.7% (31.3% to 49.6%)	40.3% (30.8% to 49.3%)	40.7% (31.2% to 49.6%)
Thyroid cancer	14.2% (7.1% to 23.9%)	14.7% (7.0% to 24.9%)	13.2% (6.8% to 21.9%)	14.5% (7.1% to 24.3%)
Mesothelioma	97.5% (96.8% to 98.1%)	94.9% (93.7% to 96.1%)	96.6% (95.8% to 97.4%)	95.0% (93.8% to 96.1%)
Non-Hodgkin's lymphoma	6.9% (2.7% to 12.4%)	6.8% (2.7% to 12.0%)	6.9% (2.7% to 12.2%)	6.8% (2.7% to 12.0%)
Multiple myeloma	7.8% (3.4% to 14.0%)	8.0% (3.5% to 14.4%)	8.1% (3.5% to 14.4%)	8.0% (3.5% to 14.4%)
Leukaemia	21.1% (13.2% to 28.8%)	18.6% (12.0% to 25.2%)	19.0% (12.1% to 25.8%)	18.6% (12.0% to 25.2%)
**Cardiovascular diseases**	80.3% (77.5% to 82.6%)	82.8% (80.9% to 84.4%)	73.5% (70.5% to 76.1%)	81.0% (79.0% to 82.7%)
Rheumatic heart disease	26.7% (15.8% to 48.3%)	27.0% (18.0% to 42.6%)	25.9% (17.4% to 39.1%)	27.0% (18.0% to 42.5%)
Ischaemic heart disease	92.3% (88.4% to 95.6%)	94.2% (91.7% to 96.3%)	91.2% (88.6% to 93.6%)	94.0% (91.5% to 96.1%)
Cerebrovascular disease	81.8% (75.8% to 87.0%)	86.0% (82.3% to 89.4%)	84.8% (80.7% to 88.4%)	85.7% (82.0% to 89.0%)
Hypertensive heart disease	100.0% (100.0% to 100.0%)	100.0% (100.0% to 100.0%)	99.9% (99.9% to 99.9%)	100.0% (100.0% to 100.0%)
Cardiomyopathy and myocarditis	41.3% (34.3% to 50.1%)	47.6% (41.0% to 53.6%)	44.8% (39.5% to 50.6%)	47.2% (41.1% to 53.0%)
Atrial fibrillation and flutter	56.0% (48.8% to 63.3%)	60.3% (53.8% to 66.3%)	67.0% (62.0% to 71.7%)	64.1% (58.7% to 69.2%)
Aortic aneurysm	42.8% (35.6% to 50.2%)	49.0% (42.8% to 54.6%)	-	49.0% (42.8% to 54.6%)
Peripheral vascular disease	48.9% (42.4% to 56.1%)	54.9% (49.6% to 60.4%)	56.1% (50.7% to 61.3%)	55.3% (50.1% to 60.4%)
Endocarditis	38.1% (28.8% to 50.1%)	39.1% (31.3% to 47.6%)	39.3% (30.7% to 48.8%)	39.1% (31.3% to 47.6%)

Supplementary Results Table 1. Age-standardised PAFs attributable to the joint distribution of all risk factors for all ages, both sexes combined for each GBD level 1 and level 2 cause and all causes in 2016 for Western Europe. Results are shown for deaths, YLLs, YLDs, and DALYs
Data in parenthesis are 95% uncertainty intervals. PAFs=population attributable fractions. GBD=Global Burden of Disease. YLLs=years of life lost. YLDs=years live with disability. DALYs=disability-adjusted life-years

	Deaths 2016	YLLs 2016	YLDs 2016	DALYs 2016
Other cardiovascular and circulatory diseases	43.5% (37.9% to 49.6%)	49.9% (45.1% to 54.4%)	51.7% (47.0% to 56.7%)	50.7% (45.9% to 55.3%)
**Chronic respiratory diseases**	69.5% (65.6% to 74.0%)	68.2% (64.5% to 72.6%)	43.2% (36.6% to 50.3%)	55.1% (50.0% to 60.3%)
Chronic obstructive pulmonary disease	78.1% (74.1% to 81.9%)	79.4% (75.8% to 82.7%)	73.0% (69.1% to 77.0%)	77.8% (74.2% to 81.3%)
Pneumoconiosis	100.0% (100.0% to 100.0%)	100.0% (100.0% to 100.0%)	99.9% (99.9% to 99.9%)	100.0% (100.0% to 100.0%)
Asthma	38.8% (29.0% to 49.8%)	42.3% (33.8% to 52.0%)	34.5% (26.7% to 43.5%)	35.0% (27.1% to 43.9%)
Other chronic respiratory diseases	18.0% (14.1% to 21.9%)	14.1% (10.3% to 17.6%)	8.5% (6.1% to 11.5%)	11.6% (8.8% to 14.7%)
**Cirrhosis and other chronic liver diseases**	71.3% (68.3% to 74.3%)	74.2% (71.2% to 77.1%)	60.1% (57.4% to 62.9%)	73.2% (70.3% to 76.1%)
Cirrhosis and other chronic liver diseases due to hepatitis B	1.8% (1.2% to 2.6%)	2.3% (1.5% to 3.2%)	2.4% (1.5% to 3.5%)	2.3% (1.5% to 3.2%)
Cirrhosis and other chronic liver diseases due to hepatitis C	75.4% (71.2% to 78.8%)	82.9% (80.5% to 84.8%)	81.0% (78.7% to 83.1%)	82.8% (80.4% to 84.7%)
Cirrhosis and other chronic liver diseases due to alcohol use	100.0% (100.0% to 100.0%)	100.0% (100.0% to 100.0%)	99.9% (99.9% to 99.9%)	100.0% (100.0% to 100.0%)
**Digestive diseases**	11.0% (8.8% to 13.4%)	13.1% (10.8% to 15.8%)	3.1% (2.4% to 3.9%)	10.0% (8.2% to 12.2%)
Peptic ulcer disease	10.4% (7.3% to 14.0%)	14.6% (10.4% to 19.1%)	14.9% (10.6% to 19.8%)	14.6% (10.5% to 19.2%)
Gallbladder and biliary diseases	40.5% (27.2% to 54.7%)	41.0% (27.6% to 55.2%)	44.0% (31.0% to 58.3%)	41.4% (28.2% to 55.7%)
Pancreatitis	48.4% (37.5% to 59.2%)	50.5% (39.7% to 60.8%)	43.3% (30.8% to 55.3%)	50.3% (39.4% to 60.7%)
**Neurological disorders**	19.7% (10.0% to 31.5%)	18.2% (10.4% to 27.5%)	4.3% (2.8% to 6.2%)	8.9% (5.3% to 13.3%)
Alzheimer's disease and other dementias	23.2% (11.4% to 37.5%)	24.4% (12.6% to 38.2%)	24.4% (12.6% to 38.4%)	24.4% (12.6% to 38.2%)
Parkinson's disease	-6.6% (-7.7% to -5.5%)	-8.8% (-10.2% to -7.3%)	-10.6% (-12.4% to -8.7%)	-9.2% (-10.6% to -7.5%)
Epilepsy	37.2% (28.1% to 44.7%)	33.0% (24.4% to 39.9%)	27.7% (20.0% to 34.4%)	29.6% (21.8% to 36.4%)
Multiple sclerosis	10.1% (6.6% to 13.9%)	11.2% (7.3% to 15.3%)	10.1% (6.6% to 13.9%)	10.6% (7.0% to 14.5%)
**Mental and substance use disorders**	99.5% (99.4% to 99.6%)	99.3% (99.2% to 99.4%)	16.7% (15.1% to 18.5%)	22.6% (20.3% to 25.3%)
Alcohol use disorders	100.0% (100.0% to 100.0%)	100.0% (100.0% to 100.0%)	99.9% (99.9% to 99.9%)	99.9% (99.9% to 99.9%)

Supplementary Results Table 1. Age-standardised PAFs attributable to the joint distribution of all risk factors for all ages, both sexes combined for each GBD level 1 and level 2 cause and all causes in 2016 for Western Europe. Results are shown for deaths, YLLs, YLDs, and DALYs
Data in parenthesis are 95% uncertainty intervals. PAFs=population attributable fractions. GBD=Global Burden of Disease. YLLs=years of life lost. YLDs=years live with disability. DALYs=disability-adjusted life-years

	Deaths 2016	YLLs 2016	YLDs 2016	DALYs 2016
Drug use disorders	100.0% (100.0% to 100.0%)	100.0% (100.0% to 100.0%)	99.9% (99.9% to 99.9%)	99.9% (99.9% to 99.9%)
Depressive disorders	-	-	7.7% (6.4% to 9.2%)	7.7% (6.4% to 9.2%)
Idiopathic developmental intellectual disability	-	-	57.9% (29.7% to 77.1%)	57.9% (29.7% to 77.1%)
**Diabetes, urogenital, blood, and endocrine diseases**	73.7% (72.7% to 76.7%)	65.7% (64.1% to 69.6%)	55.4% (51.2% to 59.6%)	59.5% (56.5% to 62.4%)
Diabetes mellitus	100.0% (100.0% to 100.0%)	100.0% (100.0% to 100.0%)	99.9% (99.9% to 99.9%)	99.9% (99.9% to 99.9%)
Chronic kidney disease	100.0% (100.0% to 100.0%)	100.0% (100.0% to 100.0%)	99.9% (99.9% to 99.9%)	100.0% (100.0% to 100.0%)
**Musculoskeletal disorders**	1.1% (0.4% to 1.8%)	1.3% (0.5% to 2.3%)	17.5% (14.7% to 20.6%)	17.3% (14.5% to 20.4%)
Rheumatoid arthritis	3.9% (1.5% to 6.6%)	5.0% (2.0% to 8.4%)	6.3% (2.6% to 10.6%)	6.2% (2.5% to 10.4%)
Osteoarthritis	-	-	24.0% (13.2% to 35.7%)	24.0% (13.2% to 35.7%)
Low back and neck pain	-	-	20.0% (17.1% to 23.0%)	20.0% (17.1% to 23.0%)
Gout	-	-	45.8% (28.4% to 63.5%)	45.8% (28.4% to 63.5%)
**Other non-communicable diseases**	0.3% (0.2% to 0.4%)	0.4% (0.4% to 0.5%)	2.5% (2.1% to 2.8%)	2.2% (1.9% to 2.6%)
Sense organ diseases	-	-	7.6% (6.8% to 8.5%)	7.6% (6.8% to 8.5%)
Sudden infant death syndrome	4.1% (3.5% to 4.8%)	4.1% (3.5% to 4.8%)	-	4.1% (3.5% to 4.8%)
**Transport injuries**	40.7% (32.5% to 49.1%)	36.6% (28.4% to 44.9%)	43.5% (36.1% to 51.0%)	39.1% (31.1% to 47.4%)
Road injuries	40.5% (32.3% to 48.8%)	36.3% (28.1% to 44.6%)	43.6% (36.1% to 51.0%)	39.0% (31.0% to 47.3%)
Other transport injuries	43.8% (35.8% to 52.2%)	39.8% (31.6% to 48.1%)	42.9% (35.2% to 50.8%)	40.9% (33.0% to 49.0%)
**Unintentional injuries**	40.7% (37.6% to 43.2%)	26.0% (23.5% to 28.6%)	22.8% (20.8% to 25.2%)	23.8% (21.7% to 26.1%)
Falls	64.4% (60.9% to 66.8%)	43.8% (41.2% to 46.0%)	24.4% (22.7% to 26.3%)	28.7% (26.5% to 31.1%)
Drowning	22.3% (16.0% to 29.6%)	20.9% (15.6% to 26.9%)	20.7% (14.5% to 28.5%)	20.9% (15.6% to 26.9%)
Fire, heat, and hot substances	17.7% (11.1% to 25.9%)	20.1% (14.8% to 26.3%)	23.3% (17.1% to 31.0%)	22.1% (16.3% to 29.3%)

Supplementary Results Table 1. Age-standardised PAFs attributable to the joint distribution of all risk factors for all ages, both sexes combined for each GBD level 1 and level 2 cause and all causes in 2016 for Western Europe. Results are shown for deaths, YLLs, YLDs, and DALYs
Data in parenthesis are 95% uncertainty intervals. PAFs=population attributable fractions. GBD=Global Burden of Disease. YLLs=years of life lost. YLDs=years live with disability. DALYs=disability-adjusted life-years

	Deaths 2016	YLLs 2016	YLDs 2016	DALYs 2016
Poisonings	22.3% (15.6% to 29.5%)	22.9% (17.2% to 29.3%)	20.4% (15.2% to 27.0%)	21.5% (16.1% to 27.8%)
Exposure to mechanical forces	25.5% (23.2% to 28.3%)	20.1% (17.7% to 23.0%)	22.9% (20.7% to 25.5%)	22.2% (20.0% to 24.9%)
Animal contact	17.1% (15.4% to 18.9%)	14.5% (12.5% to 17.0%)	12.4% (10.6% to 14.5%)	13.0% (11.1% to 15.1%)
Foreign body	6.5% (4.6% to 10.0%)	10.0% (8.0% to 13.2%)	8.7% (7.1% to 10.5%)	9.6% (7.8% to 12.1%)
Other unintentional injuries	28.2% (21.1% to 36.2%)	28.9% (22.6% to 36.2%)	22.4% (16.0% to 30.2%)	22.9% (16.5% to 30.5%)
**Self-harm and interpersonal violence**	35.8% (23.1% to 47.1%)	34.4% (21.9% to 45.9%)	35.1% (30.4% to 39.9%)	34.5% (22.8% to 45.3%)
Self-harm	35.9% (21.8% to 48.2%)	34.8% (21.0% to 47.3%)	30.0% (16.4% to 42.4%)	34.7% (20.9% to 47.2%)
Interpersonal violence	35.0% (27.2% to 42.3%)	31.1% (23.7% to 37.7%)	36.6% (32.5% to 41.1%)	33.4% (27.6% to 39.0%)
**Forces of nature, conflict and terrorism, and executions and police conflict**	5.3% (3.1% to 6.4%)	1.9% (1.0% to 2.4%)	2.1% (1.3% to 3.1%)	2.0% (1.4% to 2.6%)
Forces of nature, conflict and terrorism, and state actor violence	7.6% (7.2% to 8.0%)	3.0% (2.7% to 3.2%)	7.9% (4.9% to 12.5%)	4.1% (3.2% to 6.3%)

**Supplementary Results Table 1. Age-standardised PAFs attributable to the joint distribution of all risk factors for all ages, both sexes combined for each GBD level 1 and level 2 cause and all causes in 2016 for Western Sub-Saharan Africa. Results are shown for deaths, YLLs, YLDs, and** Data in parenthesis are 95% uncertainty intervals. PAFs=population attributable fractions. GBD=Global Burden of Disease. YLLs=years of life lost. YLDs=years live with disability. DALYs=disability-adjusted life-years				
	**Deaths 2016**	**YLLs 2016**	**YLDs 2016**	**DALYs 2016**
**All causes**	55.7% (54.0% to 57.7%)	51.2% (49.1% to 53.3%)	28.9% (27.8% to 30.1%)	46.2% (44.3% to 48.2%)
**HIV/AIDS and tuberculosis**	67.1% (62.6% to 71.3%)	72.7% (69.4% to 75.8%)	82.0% (78.7% to 85.3%)	73.2% (70.0% to 76.2%)
Tuberculosis	35.7% (26.9% to 44.1%)	37.2% (29.2% to 45.2%)	39.8% (31.0% to 48.5%)	37.3% (29.3% to 45.2%)
HIV/AIDS	89.5% (88.2% to 90.6%)	86.2% (84.6% to 87.5%)	89.8% (88.1% to 91.4%)	86.4% (84.9% to 87.7%)
**Diarrhoea, lower respiratory infections, and other common infectious diseases**	76.0% (71.5% to 80.8%)	74.8% (70.1% to 79.2%)	44.3% (39.3% to 48.9%)	73.6% (69.1% to 78.0%)
Diarrhoeal diseases	95.6% (88.7% to 98.9%)	97.4% (93.3% to 99.4%)	95.3% (87.9% to 98.7%)	97.3% (93.1% to 99.3%)
Lower respiratory infections	80.2% (74.9% to 85.0%)	86.3% (82.2% to 89.8%)	80.0% (74.6% to 84.9%)	86.3% (82.2% to 89.8%)
Upper respiratory infections	1.7% (0.8% to 3.5%)	3.0% (1.3% to 6.1%)	0.0% (0.0% to 0.0%)	0.2% (0.1% to 0.5%)
Otitis media	1.0% (0.5% to 2.1%)	4.1% (2.4% to 6.7%)	2.7% (1.8% to 3.6%)	2.7% (1.8% to 3.6%)
Meningitis	2.8% (1.8% to 4.0%)	4.9% (3.4% to 6.7%)	0.0% (0.0% to 0.0%)	4.7% (3.2% to 6.4%)
Encephalitis	0.9% (0.7% to 1.2%)	1.6% (1.2% to 2.0%)	0.0% (0.0% to 0.0%)	1.0% (0.7% to 1.3%)
Measles	66.4% (52.3% to 82.9%)	67.4% (53.2% to 84.1%)	65.1% (50.5% to 81.8%)	67.4% (53.2% to 84.1%)
**Maternal disorders**	7.9% (3.6% to 12.7%)	7.8% (3.6% to 12.6%)	1.6% (0.7% to 2.8%)	7.6% (3.5% to 12.1%)
Maternal haemorrhage	18.7% (6.8% to 30.2%)	18.7% (6.8% to 30.3%)	18.8% (6.8% to 30.5%)	18.7% (6.8% to 30.3%)
Maternal sepsis and other pregnancy related infections	18.3% (6.6% to 29.8%)	18.4% (6.6% to 29.9%)	18.1% (6.5% to 29.3%)	18.4% (6.6% to 29.8%)
Maternal abortion, miscarriage, and ectopic pregnancy	12.8% (8.1% to 17.9%)	12.7% (7.9% to 17.9%)	13.4% (8.0% to 19.2%)	12.7% (7.9% to 17.9%)
**Neonatal disorders**	82.2% (79.8% to 84.3%)	82.2% (79.8% to 84.3%)	0.6% (0.3% to 1.0%)	80.0% (77.2% to 82.5%)
Neonatal preterm birth complications	98.3% (97.7% to 99.0%)	98.3% (97.7% to 99.0%)	1.6% (1.1% to 2.2%)	95.6% (94.0% to 96.8%)
Neonatal encephalopathy due to birth asphyxia and trauma	79.0% (75.8% to 82.5%)	79.1% (75.8% to 82.5%)	0.0% (0.0% to 0.0%)	78.0% (74.4% to 81.6%)
Neonatal sepsis and other neonatal infections	69.3% (65.8% to 72.7%)	69.4% (65.9% to 72.7%)	0.0% (0.0% to 0.0%)	65.9% (59.2% to 70.5%)
Hemolytic disease and other neonatal jaundice	69.7% (65.5% to 74.0%)	69.7% (65.5% to 74.0%)	0.0% (0.0% to 0.0%)	66.3% (61.9% to 70.7%)

Supplementary Results Table 1. Age-standardised PAFs attributable to the joint distribution of all risk factors for all ages, both sexes combined for each GBD level 1 and level 2 cause and all causes in 2016 for Western Sub-Saharan Africa. Results are shown for deaths, YLLs, YLDs, and Data in parenthesis are 95% uncertainty intervals. PAFs=population attributable fractions. GBD=Global Burden of Disease. YLLs=years of life lost. YLDs=years live with disability. DALYs=disability-adjusted life-years				
	**Deaths 2016**	**YLLs 2016**	**YLDs 2016**	**DALYs 2016**
Other neonatal disorders	70.7% (66.4% to 75.0%)	70.7% (66.4% to 75.0%)	0.0% (0.0% to 0.0%)	68.9% (64.7% to 73.4%)
**Nutritional deficiencies**	40.9% (34.3% to 48.7%)	74.1% (68.5% to 79.3%)	94.6% (93.6% to 95.4%)	84.8% (82.0% to 87.3%)
Protein-energy malnutrition	44.8% (37.8% to 53.3%)	78.6% (73.5% to 83.7%)	97.7% (97.4% to 98.0%)	79.4% (74.6% to 84.2%)
Vitamin A deficiency	-	-	100.0% (100.0% to 100.0%)	100.0% (100.0% to 100.0%)
Iron-deficiency anaemia	100.0% (100.0% to 100.0%)	100.0% (100.0% to 100.0%)	100.0% (100.0% to 100.0%)	100.0% (100.0% to 100.0%)
**Other communicable, maternal, neonatal, and nutritional diseases**	2.8% (2.1% to 3.8%)	2.1% (1.4% to 2.9%)	59.8% (54.9% to 65.2%)	11.2% (7.2% to 16.4%)
Sexually transmitted diseases excluding HIV	7.6% (4.3% to 12.9%)	3.8% (2.1% to 6.6%)	100.0% (100.0% to 100.0%)	20.1% (11.6% to 32.1%)
Hepatitis	0.0% (0.0% to 0.1%)	0.1% (0.0% to 0.1%)	0.1% (0.0% to 0.1%)	0.1% (0.0% to 0.1%)
**Neoplasms**	28.1% (25.3% to 31.9%)	29.0% (26.4% to 32.4%)	27.3% (24.3% to 31.5%)	29.0% (26.4% to 32.4%)
Oesophageal cancer	63.0% (52.4% to 73.2%)	65.0% (54.3% to 75.0%)	63.8% (53.0% to 73.8%)	65.0% (54.3% to 75.0%)
Stomach cancer	5.6% (2.0% to 13.7%)	5.9% (2.3% to 13.2%)	5.5% (1.9% to 13.1%)	5.9% (2.3% to 13.2%)
Liver cancer	30.1% (23.5% to 37.9%)	29.8% (22.8% to 37.8%)	30.3% (23.7% to 38.0%)	29.8% (22.9% to 37.8%)
Larynx cancer	54.9% (44.9% to 65.0%)	57.3% (47.5% to 67.1%)	54.8% (44.2% to 65.2%)	57.3% (47.4% to 67.1%)
Tracheal, bronchus, and lung cancer	80.7% (75.8% to 85.3%)	80.9% (76.0% to 85.4%)	79.9% (74.7% to 84.6%)	80.8% (76.0% to 85.4%)
Breast cancer	19.2% (13.8% to 25.7%)	17.7% (12.4% to 23.9%)	17.5% (12.2% to 23.6%)	17.7% (12.4% to 23.9%)
Cervical cancer	100.0% (100.0% to 100.0%)	100.0% (100.0% to 100.0%)	100.0% (100.0% to 100.0%)	100.0% (100.0% to 100.0%)
Uterine cancer	31.5% (20.6% to 43.8%)	33.1% (22.0% to 45.5%)	32.3% (21.2% to 44.6%)	33.1% (22.0% to 45.5%)
Prostate cancer	0.8% (0.5% to 1.2%)	0.9% (0.5% to 1.4%)	0.8% (0.4% to 1.2%)	0.9% (0.5% to 1.4%)
Colon and rectum cancer	45.9% (37.4% to 54.4%)	46.3% (37.9% to 54.5%)	46.4% (37.8% to 54.7%)	46.3% (37.9% to 54.5%)
Lip and oral cavity cancer	57.7% (49.6% to 65.3%)	59.0% (51.6% to 66.3%)	57.0% (49.0% to 64.5%)	59.0% (51.6% to 66.2%)
Nasopharynx cancer	56.5% (48.7% to 63.3%)	56.3% (49.0% to 62.9%)	58.0% (49.8% to 65.2%)	56.3% (49.0% to 63.0%)

Supplementary Results Table 1. Age-standardised PAFs attributable to the joint distribution of all risk factors for all ages, both sexes combined for each GBD level 1 and level 2 cause and all causes in 2016 for Western Sub-Saharan Africa. Results are shown for deaths, YLLs, YLDs, and
Data in parenthesis are 95% uncertainty intervals. PAFs=population attributable fractions. GBD=Global Burden of Disease. YLLs=years of life lost. YLDs=years live with disability. DALYs=disability-adjusted life-years

	Deaths 2016	YLLs 2016	YLDs 2016	DALYs 2016
Other pharynx cancer	39.2% (28.4% to 49.8%)	41.1% (31.1% to 51.5%)	40.4% (29.7% to 51.3%)	41.1% (31.1% to 51.5%)
Gallbladder and biliary tract cancer	15.3% (7.8% to 25.2%)	16.3% (8.5% to 26.2%)	15.3% (7.9% to 24.8%)	16.3% (8.5% to 26.2%)
Pancreatic cancer	13.1% (7.5% to 20.7%)	13.3% (7.9% to 20.4%)	12.7% (7.2% to 20.0%)	13.3% (7.9% to 20.4%)
Ovarian cancer	8.8% (3.8% to 15.4%)	7.6% (2.9% to 13.5%)	7.4% (3.0% to 13.3%)	7.6% (2.9% to 13.5%)
Kidney cancer	16.4% (10.5% to 23.8%)	15.7% (10.2% to 22.5%)	15.4% (9.9% to 22.5%)	15.7% (10.2% to 22.5%)
Bladder cancer	12.2% (7.0% to 18.8%)	12.8% (7.6% to 19.2%)	11.8% (6.8% to 18.2%)	12.8% (7.6% to 19.2%)
Thyroid cancer	8.3% (4.0% to 14.6%)	8.7% (4.2% to 15.1%)	8.5% (4.1% to 14.8%)	8.7% (4.2% to 15.1%)
Mesothelioma	82.4% (77.6% to 86.6%)	72.0% (65.5% to 78.0%)	76.6% (70.7% to 82.2%)	72.0% (65.6% to 78.1%)
Non-Hodgkin's lymphoma	3.7% (1.4% to 6.8%)	3.1% (1.2% to 5.8%)	3.4% (1.4% to 6.5%)	3.1% (1.2% to 5.8%)
Multiple myeloma	4.9% (1.9% to 9.0%)	5.2% (2.1% to 9.5%)	5.0% (2.0% to 9.2%)	5.2% (2.1% to 9.5%)
Leukaemia	7.9% (4.8% to 11.9%)	7.4% (4.6% to 10.7%)	7.7% (4.7% to 11.5%)	7.4% (4.6% to 10.7%)
**Cardiovascular diseases**	84.6% (82.2% to 86.6%)	84.6% (82.7% to 86.2%)	63.4% (59.7% to 66.8%)	82.6% (80.7% to 84.3%)
Rheumatic heart disease	27.9% (18.2% to 45.4%)	25.5% (17.4% to 37.7%)	23.8% (15.7% to 33.6%)	25.0% (17.4% to 36.3%)
Ischaemic heart disease	93.0% (90.0% to 95.5%)	94.5% (92.5% to 96.1%)	90.2% (87.5% to 92.6%)	94.3% (92.4% to 95.9%)
Cerebrovascular disease	86.5% (83.0% to 89.8%)	88.3% (86.0% to 90.4%)	87.1% (83.5% to 90.2%)	88.2% (85.9% to 90.4%)
Hypertensive heart disease	100.0% (100.0% to 100.0%)	100.0% (100.0% to 100.0%)	100.0% (100.0% to 100.0%)	100.0% (100.0% to 100.0%)
Cardiomyopathy and myocarditis	39.0% (32.1% to 47.0%)	38.3% (31.6% to 45.5%)	46.6% (40.2% to 52.9%)	39.1% (32.6% to 46.0%)
Atrial fibrillation and flutter	46.9% (39.2% to 54.5%)	51.2% (44.2% to 57.5%)	58.2% (53.1% to 63.2%)	55.1% (49.2% to 60.4%)
Aortic aneurysm	42.0% (34.8% to 49.1%)	46.1% (39.9% to 51.8%)	-	46.1% (39.9% to 51.8%)
Peripheral vascular disease	50.1% (43.3% to 57.7%)	56.4% (51.0% to 62.1%)	59.6% (54.3% to 65.3%)	59.0% (53.8% to 64.4%)
Endocarditis	38.6% (30.1% to 48.0%)	34.5% (27.3% to 41.8%)	36.8% (27.8% to 47.9%)	34.5% (27.4% to 41.9%)

Supplementary Results Table 1. Age-standardised PAFs attributable to the joint distribution of all risk factors for all ages, both sexes combined for each GBD level 1 and level 2 cause and all causes in 2016 for Western Sub-Saharan Africa. Results are shown for deaths, YLLs, YLDs, and Data in parenthesis are 95% uncertainty intervals. PAFs=population attributable fractions. GBD=Global Burden of Disease. YLLs=years of life lost. YLDs=years live with disability. DALYs=disability-adjusted life-years	Deaths 2016	YLLs 2016	YLDs 2016	DALYs 2016
Other cardiovascular and circulatory diseases	44.8% (39.8% to 49.7%)	45.3% (41.0% to 49.6%)	41.1% (36.3% to 46.1%)	44.1% (39.6% to 48.3%)
**Chronic respiratory diseases**	59.8% (53.5% to 66.4%)	56.3% (50.5% to 62.2%)	40.8% (34.6% to 47.8%)	51.4% (45.9% to 57.7%)
Chronic obstructive pulmonary disease	77.4% (69.3% to 85.6%)	76.7% (69.2% to 84.5%)	67.2% (57.3% to 76.9%)	74.5% (66.5% to 82.4%)
Pneumoconiosis	100.0% (100.0% to 100.0%)	100.0% (100.0% to 100.0%)	100.0% (100.0% to 100.0%)	100.0% (100.0% to 100.0%)
Asthma	27.1% (19.9% to 36.2%)	30.9% (24.2% to 39.1%)	23.4% (17.8% to 30.4%)	27.3% (21.2% to 34.6%)
Other chronic respiratory diseases	4.3% (2.8% to 6.0%)	4.1% (2.6% to 6.1%)	2.1% (0.9% to 4.0%)	4.0% (2.4% to 5.9%)
**Cirrhosis and other chronic liver diseases**	25.7% (23.3% to 28.3%)	24.6% (22.0% to 27.3%)	19.6% (17.4% to 21.9%)	24.5% (21.9% to 27.1%)
Cirrhosis and other chronic liver diseases due to hepatitis B	0.0% (0.0% to 0.0%)	0.0% (0.0% to 0.0%)	0.0% (0.0% to 0.0%)	0.0% (0.0% to 0.0%)
Cirrhosis and other chronic liver diseases due to hepatitis C	1.5% (0.6% to 2.9%)	2.2% (0.9% to 4.5%)	2.5% (1.0% to 5.2%)	2.2% (0.9% to 4.5%)
Cirrhosis and other chronic liver diseases due to alcohol use	100.0% (100.0% to 100.0%)	100.0% (100.0% to 100.0%)	100.0% (100.0% to 100.0%)	100.0% (100.0% to 100.0%)
**Digestive diseases**	5.3% (4.0% to 6.7%)	6.2% (4.6% to 8.1%)	1.7% (1.2% to 2.2%)	5.9% (4.4% to 7.6%)
Peptic ulcer disease	4.6% (3.1% to 6.3%)	5.0% (3.4% to 6.9%)	4.2% (2.9% to 5.7%)	5.0% (3.4% to 6.8%)
Gallbladder and biliary diseases	25.3% (15.2% to 37.5%)	24.7% (15.2% to 36.1%)	29.0% (18.6% to 41.2%)	24.9% (15.5% to 36.4%)
Pancreatitis	33.0% (22.7% to 43.7%)	38.4% (27.6% to 49.2%)	34.9% (24.0% to 46.0%)	38.4% (27.6% to 49.2%)
**Neurological disorders**	13.9% (7.0% to 22.7%)	14.7% (8.9% to 21.7%)	3.5% (2.0% to 5.4%)	7.5% (4.8% to 10.9%)
Alzheimer's disease and other dementias	14.7% (6.7% to 25.2%)	15.6% (7.2% to 26.3%)	15.4% (7.1% to 25.9%)	15.5% (7.2% to 26.3%)
Parkinson's disease	-3.2% (-3.8% to -2.5%)	-3.7% (-4.5% to -2.9%)	-4.0% (-4.7% to -3.1%)	-3.8% (-4.5% to -3.0%)
Epilepsy	20.6% (14.6% to 28.4%)	19.1% (13.1% to 27.2%)	15.7% (10.3% to 21.3%)	17.5% (12.1% to 23.6%)
Multiple sclerosis	2.9% (1.4% to 4.7%)	3.0% (1.4% to 5.0%)	2.2% (1.3% to 3.3%)	2.7% (1.4% to 4.3%)
**Mental and substance use disorders**	99.9% (99.8% to 99.9%)	99.8% (99.7% to 99.9%)	19.7% (17.7% to 21.9%)	23.4% (21.0% to 26.1%)
Alcohol use disorders	100.0% (100.0% to 100.0%)	100.0% (100.0% to 100.0%)	100.0% (100.0% to 100.0%)	100.0% (100.0% to 100.0%)

Supplementary Results Table 1. Age-standardised PAFs attributable to the joint distribution of all risk factors for all ages, both sexes combined for each GBD level 1 and level 2 cause and all causes in 2016 for Western Sub-Saharan Africa. Results are shown for deaths, YLLs, YLDs, and
Data in parenthesis are 95% uncertainty intervals. PAFs=population attributable fractions. GBD=Global Burden of Disease. YLLs=years of life lost. YLDs=years live with disability. DALYs=disability-adjusted life-years

	Deaths 2016	YLLs 2016	YLDs 2016	DALYs 2016
Drug use disorders	100.0% (100.0% to 100.0%)	100.0% (100.0% to 100.0%)	100.0% (100.0% to 100.0%)	100.0% (100.0% to 100.0%)
Depressive disorders	-	-	12.2% (10.5% to 14.0%)	12.2% (10.5% to 14.0%)
Idiopathic developmental intellectual disability	-	-	61.2% (30.9% to 80.7%)	61.2% (30.9% to 80.7%)
**Diabetes, urogenital, blood, and endocrine diseases**	72.1% (69.9% to 74.3%)	58.3% (55.5% to 61.7%)	52.6% (48.6% to 56.1%)	56.1% (53.5% to 58.7%)
Diabetes mellitus	100.0% (100.0% to 100.0%)	100.0% (100.0% to 100.0%)	100.0% (100.0% to 100.0%)	100.0% (100.0% to 100.0%)
Chronic kidney disease	100.0% (100.0% to 100.0%)	100.0% (100.0% to 100.0%)	100.0% (100.0% to 100.0%)	100.0% (100.0% to 100.0%)
**Musculoskeletal disorders**	0.2% (0.1% to 0.4%)	0.1% (0.1% to 0.3%)	23.2% (20.3% to 26.5%)	22.9% (20.0% to 26.1%)
Rheumatoid arthritis	1.0% (0.4% to 1.9%)	1.1% (0.4% to 2.0%)	1.5% (0.6% to 2.6%)	1.5% (0.6% to 2.6%)
Osteoarthritis	-	-	17.2% (8.6% to 27.5%)	17.2% (8.6% to 27.5%)
Low back and neck pain	-	-	28.2% (25.2% to 31.5%)	28.2% (25.2% to 31.5%)
Gout	-	-	47.0% (38.0% to 58.3%)	47.0% (38.0% to 58.3%)
**Other non-communicable diseases**	0.1% (0.1% to 0.3%)	0.2% (0.1% to 0.4%)	9.3% (7.9% to 10.7%)	6.1% (4.7% to 7.8%)
Sense organ diseases	-	-	17.6% (15.7% to 19.6%)	17.6% (15.7% to 19.6%)
Sudden infant death syndrome	4.6% (2.2% to 7.4%)	4.6% (2.2% to 7.4%)	-	4.6% (2.2% to 7.4%)
**Transport injuries**	32.1% (27.6% to 37.8%)	25.8% (21.8% to 31.1%)	38.1% (33.1% to 44.0%)	27.6% (23.4% to 32.9%)
Road injuries	32.1% (27.6% to 37.8%)	25.9% (21.9% to 31.1%)	40.2% (34.9% to 46.2%)	27.7% (23.4% to 33.0%)
Other transport injuries	32.3% (27.0% to 39.0%)	25.3% (19.8% to 32.9%)	29.3% (24.7% to 34.4%)	26.8% (22.1% to 32.6%)
**Unintentional injuries**	24.7% (18.0% to 32.8%)	13.9% (11.4% to 17.0%)	21.6% (19.8% to 23.5%)	16.0% (13.9% to 18.4%)
Falls	50.6% (34.0% to 68.6%)	34.6% (27.2% to 43.5%)	30.4% (28.7% to 32.1%)	32.7% (28.4% to 37.9%)
Drowning	10.5% (7.8% to 13.7%)	9.6% (7.5% to 12.1%)	16.1% (12.2% to 20.6%)	9.7% (7.6% to 12.2%)
Fire, heat, and hot substances	9.2% (6.3% to 12.8%)	9.7% (7.3% to 13.1%)	18.8% (14.7% to 23.3%)	11.6% (8.8% to 15.1%)

Supplementary Results Table 1. Age-standardised PAFs attributable to the joint distribution of all risk factors for all ages, both sexes combined for each GBD level 1 and level 2 cause and all causes in 2016 for Western Sub-Saharan Africa. Results are shown for deaths, YLLs, YLDs, and
Data in parenthesis are 95% uncertainty intervals. PAFs=population attributable fractions. GBD=Global Burden of Disease. YLLs=years of life lost. YLDs=years live with disability. DALYs=disability-adjusted life-years

	**Deaths 2016**	**YLLs 2016**	**YLDs 2016**	**DALYs 2016**
Poisonings	12.6% (9.0% to 17.1%)	12.2% (8.0% to 16.8%)	18.8% (14.7% to 22.9%)	12.5% (8.4% to 17.2%)
Exposure to mechanical forces	18.4% (15.8% to 21.3%)	12.8% (10.3% to 16.4%)	21.2% (19.2% to 23.7%)	15.0% (12.7% to 17.9%)
Animal contact	7.4% (6.5% to 8.3%)	8.1% (7.1% to 9.1%)	13.0% (11.4% to 15.1%)	9.1% (8.0% to 10.4%)
Foreign body	5.3% (3.7% to 6.8%)	6.0% (3.4% to 8.3%)	10.8% (9.3% to 12.7%)	7.4% (4.9% to 9.5%)
Other unintentional injuries	13.1% (10.1% to 16.8%)	13.2% (10.3% to 17.2%)	16.7% (12.8% to 20.9%)	14.7% (11.5% to 18.1%)
**Self-harm and interpersonal violence**	18.5% (12.0% to 25.8%)	19.0% (13.2% to 25.6%)	19.9% (17.3% to 23.0%)	19.1% (13.7% to 25.3%)
Self-harm	18.9% (11.2% to 27.5%)	20.5% (12.8% to 28.8%)	22.9% (13.6% to 32.6%)	20.5% (12.9% to 28.8%)
Interpersonal violence	16.7% (12.3% to 22.0%)	14.7% (10.6% to 19.8%)	19.5% (17.0% to 22.4%)	15.9% (12.3% to 20.3%)
**Forces of nature, conflict and terrorism, and executions and police conflict**	0.0% (0.0% to 0.0%)	0.0% (0.0% to 0.0%)	0.2% (0.1% to 0.2%)	0.0% (0.0% to 0.1%)
Forces of nature, conflict and terrorism, and state actor violence	0.0% (0.0% to 0.0%)	0.0% (0.0% to 0.0%)	7.7% (5.3% to 11.2%)	7.7% (5.3% to 11.2%)

Supplementary Results Table 1. Age-standardised PAFs attributable to the joint distribution of all risk factors for all ages, both sexes combined for each GBD level 1 and level 2 cause and all causes in 2016 for Afghanistan. Results are shown for deaths, YLLs, YLDs, and DALYs
Data in parenthesis are 95% uncertainty intervals. PAFs=population attributable fractions. GBD=Global Burden of Disease. YLLs=years of life lost. YLDs=years live with disability. DALYs=disability-adjusted life-years

	Deaths 2016	YLLs 2016	YLDs 2016	DALYs 2016
**All causes**	64.0% (61.0% to 67.0%)	54.8% (51.2% to 58.7%)	25.8% (23.8% to 27.6%)	48.8% (45.8% to 51.9%)
**HIV/AIDS and tuberculosis**	20.1% (13.2% to 27.4%)	20.5% (14.1% to 26.9%)	21.0% (13.0% to 31.5%)	20.5% (14.1% to 26.9%)
Tuberculosis	19.7% (13.0% to 26.9%)	19.8% (13.6% to 26.1%)	16.8% (11.2% to 22.3%)	19.8% (13.5% to 26.0%)
HIV/AIDS	80.0% (74.6% to 86.0%)	77.9% (69.9% to 85.3%)	82.1% (77.9% to 86.8%)	78.5% (71.7% to 85.5%)
**Diarrhoea, lower respiratory infections, and other common infectious diseases**	65.9% (58.8% to 71.6%)	65.0% (55.6% to 72.0%)	44.9% (38.9% to 50.1%)	63.7% (54.8% to 70.3%)
Diarrhoeal diseases	97.1% (92.2% to 99.3%)	98.7% (96.5% to 99.7%)	96.1% (89.3% to 99.0%)	98.2% (95.2% to 99.6%)
Lower respiratory infections	83.3% (77.6% to 88.0%)	90.1% (86.0% to 93.1%)	81.6% (76.1% to 86.3%)	90.0% (86.0% to 93.1%)
Upper respiratory infections	1.3% (0.1% to 4.3%)	2.7% (0.2% to 9.5%)	0.0% (0.0% to 0.0%)	0.2% (0.0% to 0.8%)
Otitis media	1.6% (0.3% to 4.6%)	4.6% (1.4% to 9.1%)	5.8% (3.8% to 7.7%)	5.7% (3.8% to 7.6%)
Meningitis	4.4% (2.2% to 7.5%)	6.0% (3.4% to 10.0%)	0.0% (0.0% to 0.0%)	5.9% (3.3% to 9.7%)
Encephalitis	0.7% (0.4% to 1.1%)	1.3% (0.8% to 2.1%)	0.0% (0.0% to 0.0%)	0.7% (0.4% to 1.2%)
Measles	67.6% (55.7% to 80.7%)	69.1% (57.2% to 82.2%)	72.3% (61.7% to 82.6%)	69.1% (57.2% to 82.2%)
**Maternal disorders**	9.7% (4.2% to 15.7%)	9.6% (4.1% to 15.5%)	4.8% (2.4% to 8.1%)	9.5% (4.1% to 15.4%)
Maternal haemorrhage	17.5% (6.2% to 28.8%)	17.5% (6.2% to 28.7%)	17.2% (6.1% to 28.2%)	17.5% (6.2% to 28.7%)
Maternal sepsis and other pregnancy related infections	17.5% (6.2% to 28.6%)	17.4% (6.2% to 28.5%)	17.2% (6.1% to 28.1%)	17.4% (6.2% to 28.5%)
Maternal abortion, miscarriage, and ectopic pregnancy	28.9% (19.0% to 38.7%)	28.5% (18.9% to 38.1%)	28.1% (17.7% to 38.4%)	28.5% (18.9% to 38.2%)
**Neonatal disorders**	74.3% (68.0% to 79.8%)	74.4% (68.1% to 79.8%)	0.2% (0.1% to 0.4%)	70.7% (64.4% to 76.4%)
Neonatal preterm birth complications	91.0% (83.3% to 95.8%)	91.0% (83.4% to 95.9%)	0.5% (0.4% to 0.7%)	86.4% (78.7% to 92.0%)
Neonatal encephalopathy due to birth asphyxia and trauma	66.7% (57.8% to 73.8%)	66.8% (57.9% to 73.9%)	0.0% (0.0% to 0.0%)	64.4% (55.5% to 72.2%)
Neonatal sepsis and other neonatal infections	57.9% (46.6% to 66.0%)	57.9% (46.7% to 66.1%)	0.0% (0.0% to 0.0%)	53.7% (41.1% to 63.0%)
Hemolytic disease and other neonatal jaundice	66.8% (58.0% to 73.2%)	66.8% (58.0% to 73.2%)	0.0% (0.0% to 0.0%)	51.7% (40.8% to 61.5%)

Supplementary Results Table 1. Age-standardised PAFs attributable to the joint distribution of all risk factors for all ages, both sexes combined for each GBD level 1 and level 2 cause and all causes in 2016 for Afghanistan. Results are shown for deaths, YLLs, YLDs, and DALYs
Data in parenthesis are 95% uncertainty intervals. PAFs=population attributable fractions. GBD=Global Burden of Disease. YLLs=years of life lost. YLDs=years live with disability. DALYs=disability-adjusted life-years

	Deaths 2016	YLLs 2016	YLDs 2016	DALYs 2016
Other neonatal disorders	61.5% (51.4% to 69.0%)	61.6% (51.6% to 69.1%)	0.0% (0.0% to 0.0%)	60.9% (50.9% to 68.6%)
**Nutritional deficiencies**	15.8% (9.2% to 24.8%)	41.3% (27.7% to 55.4%)	78.6% (74.0% to 83.1%)	71.5% (66.3% to 76.2%)
Protein-energy malnutrition	20.3% (11.4% to 32.1%)	46.1% (31.7% to 61.2%)	97.9% (97.2% to 98.6%)	61.3% (51.5% to 70.6%)
Vitamin A deficiency	-	-	100.0% (100.0% to 100.0%)	100.0% (100.0% to 100.0%)
Iron-deficiency anaemia	100.0% (100.0% to 100.0%)	100.0% (100.0% to 100.0%)	100.0% (100.0% to 100.0%)	100.0% (100.0% to 100.0%)
**Other communicable, maternal, neonatal, and nutritional diseases**	2.3% (1.0% to 3.7%)	1.5% (0.5% to 2.8%)	79.4% (74.1% to 84.3%)	14.3% (7.8% to 23.5%)
Sexually transmitted diseases excluding HIV	4.4% (1.2% to 9.2%)	2.0% (0.5% to 4.5%)	100.0% (100.0% to 100.0%)	20.6% (9.9% to 36.4%)
Hepatitis	1.4% (0.9% to 2.0%)	1.3% (0.9% to 1.9%)	1.1% (0.7% to 1.7%)	1.3% (0.9% to 1.9%)
**Neoplasms**	37.2% (30.4% to 44.6%)	36.5% (29.5% to 43.8%)	34.3% (28.1% to 40.7%)	36.5% (29.5% to 43.8%)
Oesophageal cancer	69.1% (55.9% to 79.8%)	71.1% (58.0% to 81.5%)	69.5% (56.4% to 80.1%)	71.1% (58.0% to 81.5%)
Stomach cancer	17.6% (8.1% to 28.2%)	19.1% (9.3% to 29.6%)	17.8% (8.3% to 28.4%)	19.1% (9.3% to 29.6%)
Liver cancer	40.1% (29.4% to 51.0%)	43.6% (32.4% to 55.1%)	40.3% (29.7% to 51.7%)	43.5% (32.4% to 55.1%)
Larynx cancer	78.0% (63.5% to 89.0%)	80.6% (66.4% to 90.4%)	78.2% (64.0% to 89.1%)	80.5% (66.4% to 90.4%)
Tracheal, bronchus, and lung cancer	93.2% (88.9% to 96.2%)	93.5% (89.3% to 96.4%)	93.1% (88.9% to 96.1%)	93.5% (89.3% to 96.4%)
Breast cancer	21.2% (11.1% to 33.8%)	19.3% (9.3% to 32.1%)	20.7% (10.8% to 33.1%)	19.3% (9.3% to 32.1%)
Cervical cancer	100.0% (100.0% to 100.0%)	100.0% (100.0% to 100.0%)	100.0% (100.0% to 100.0%)	100.0% (100.0% to 100.0%)
Uterine cancer	35.5% (22.8% to 48.3%)	36.1% (23.4% to 48.8%)	35.7% (22.9% to 48.5%)	36.1% (23.4% to 48.8%)
Prostate cancer	3.5% (1.6% to 6.7%)	4.2% (1.9% to 8.2%)	3.7% (1.7% to 7.0%)	4.2% (1.9% to 8.2%)
Colon and rectum cancer	52.8% (42.8% to 63.3%)	52.8% (43.0% to 63.0%)	52.8% (42.7% to 63.2%)	52.8% (43.0% to 63.0%)
Lip and oral cavity cancer	64.1% (51.4% to 75.4%)	66.4% (53.4% to 77.6%)	62.5% (50.4% to 73.4%)	66.3% (53.4% to 77.6%)
Nasopharynx cancer	57.6% (42.5% to 70.0%)	57.7% (43.1% to 69.6%)	55.4% (40.3% to 68.3%)	57.7% (43.1% to 69.7%)

Supplementary Results Table 1. Age-standardised PAFs attributable to the joint distribution of all risk factors for all ages, both sexes combined for each GBD level 1 and level 2 cause and all causes in 2016 for Afghanistan. Results are shown for deaths, YLLs, YLDs, and DALYs
Data in parenthesis are 95% uncertainty intervals. PAFs=population attributable fractions. GBD=Global Burden of Disease. YLLs=years of life lost. YLDs=years live with disability. DALYs=disability-adjusted life-years

	Deaths 2016	YLLs 2016	YLDs 2016	DALYs 2016
Other pharynx cancer	13.0% (4.3% to 22.7%)	13.5% (4.7% to 23.1%)	13.1% (4.3% to 22.8%)	13.5% (4.7% to 23.1%)
Gallbladder and biliary tract cancer	17.0% (8.7% to 28.3%)	17.9% (9.4% to 29.2%)	17.3% (8.9% to 28.5%)	17.9% (9.4% to 29.1%)
Pancreatic cancer	29.8% (19.0% to 42.5%)	32.1% (20.6% to 44.9%)	30.0% (19.0% to 42.8%)	32.1% (20.5% to 44.9%)
Ovarian cancer	21.5% (10.2% to 36.7%)	19.0% (8.7% to 33.4%)	20.1% (9.5% to 35.2%)	19.0% (8.7% to 33.4%)
Kidney cancer	28.0% (18.0% to 39.0%)	28.3% (18.2% to 39.6%)	27.5% (17.8% to 38.2%)	28.3% (18.2% to 39.5%)
Bladder cancer	31.4% (18.2% to 46.1%)	34.3% (20.1% to 49.3%)	32.2% (18.8% to 47.0%)	34.3% (20.1% to 49.3%)
Thyroid cancer	9.4% (4.8% to 15.5%)	9.8% (5.0% to 16.2%)	9.4% (4.7% to 15.3%)	9.7% (5.0% to 16.2%)
Mesothelioma	93.1% (89.6% to 96.4%)	89.1% (83.2% to 94.3%)	92.3% (88.7% to 96.0%)	89.1% (83.2% to 94.4%)
Non-Hodgkin's lymphoma	4.8% (1.9% to 8.7%)	4.7% (1.9% to 8.5%)	4.7% (1.9% to 8.6%)	4.7% (1.9% to 8.5%)
Multiple myeloma	5.7% (2.5% to 10.3%)	5.9% (2.6% to 10.8%)	5.7% (2.5% to 10.3%)	5.9% (2.6% to 10.8%)
Leukaemia	14.1% (7.9% to 21.8%)	12.7% (7.2% to 20.2%)	13.9% (7.7% to 21.9%)	12.7% (7.2% to 20.2%)
**Cardiovascular diseases**	91.1% (89.1% to 93.2%)	92.4% (91.0% to 94.2%)	76.0% (73.1% to 78.9%)	91.9% (90.5% to 93.7%)
Rheumatic heart disease	26.5% (18.4% to 40.1%)	25.4% (16.9% to 36.1%)	21.0% (12.8% to 30.7%)	25.0% (16.6% to 35.4%)
Ischaemic heart disease	96.2% (94.2% to 97.7%)	97.1% (95.9% to 98.1%)	93.3% (90.8% to 95.4%)	97.0% (95.8% to 98.0%)
Cerebrovascular disease	88.9% (85.5% to 92.4%)	91.7% (89.7% to 93.7%)	89.6% (86.5% to 92.9%)	91.7% (89.5% to 93.7%)
Hypertensive heart disease	100.0% (100.0% to 100.0%)	100.0% (100.0% to 100.0%)	100.0% (100.0% to 100.0%)	100.0% (100.0% to 100.0%)
Cardiomyopathy and myocarditis	46.5% (37.0% to 55.8%)	43.3% (34.2% to 52.1%)	30.6% (24.6% to 37.0%)	42.7% (33.7% to 51.2%)
Atrial fibrillation and flutter	40.2% (32.6% to 47.6%)	45.4% (38.5% to 51.8%)	52.4% (47.1% to 57.9%)	49.5% (43.5% to 55.1%)
Aortic aneurysm	40.8% (33.7% to 47.2%)	45.8% (39.7% to 51.5%)	-	45.8% (39.7% to 51.5%)
Peripheral vascular disease	53.0% (46.0% to 60.5%)	62.7% (56.5% to 68.8%)	67.8% (63.1% to 72.4%)	67.3% (62.7% to 71.9%)
Endocarditis	40.9% (31.2% to 52.8%)	41.5% (32.6% to 50.7%)	38.2% (28.7% to 48.6%)	41.4% (32.6% to 50.7%)

Supplementary Results Table 1. Age-standardised PAFs attributable to the joint distribution of all risk factors for all ages, both sexes combined for each GBD level 1 and level 2 cause and all causes in 2016 for Afghanistan. Results are shown for deaths, YLLs, YLDs, and DALYs
Data in parenthesis are 95% uncertainty intervals. PAFs=population attributable fractions. GBD=Global Burden of Disease. YLLs=years of life lost. YLDs=years live with disability. DALYs=disability-adjusted life-years

	Deaths 2016	YLLs 2016	YLDs 2016	DALYs 2016
Other cardiovascular and circulatory diseases	43.4% (37.8% to 49.1%)	47.0% (42.3% to 51.8%)	40.7% (35.6% to 46.1%)	45.9% (41.2% to 50.7%)
**Chronic respiratory diseases**	70.0% (61.6% to 76.5%)	68.2% (61.1% to 74.3%)	47.5% (40.5% to 54.5%)	64.6% (58.1% to 70.4%)
Chronic obstructive pulmonary disease	87.3% (81.0% to 92.7%)	87.2% (81.7% to 92.1%)	81.6% (73.9% to 87.7%)	86.6% (80.9% to 91.5%)
Pneumoconiosis	100.0% (100.0% to 100.0%)	100.0% (100.0% to 100.0%)	100.0% (100.0% to 100.0%)	100.0% (100.0% to 100.0%)
Asthma	32.5% (23.7% to 42.6%)	35.5% (27.0% to 45.1%)	28.8% (21.6% to 37.2%)	33.6% (25.7% to 42.5%)
Other chronic respiratory diseases	15.3% (7.6% to 27.6%)	16.4% (7.4% to 29.8%)	10.8% (5.1% to 17.1%)	15.3% (7.0% to 26.9%)
**Cirrhosis and other chronic liver diseases**	24.0% (18.3% to 30.1%)	27.8% (21.8% to 34.4%)	20.9% (17.0% to 24.9%)	27.7% (21.7% to 34.1%)
Cirrhosis and other chronic liver diseases due to hepatitis B	0.4% (0.2% to 0.8%)	0.5% (0.2% to 0.9%)	0.5% (0.2% to 0.8%)	0.5% (0.2% to 0.9%)
Cirrhosis and other chronic liver diseases due to hepatitis C	47.0% (32.6% to 58.7%)	60.0% (46.0% to 70.7%)	55.8% (46.5% to 65.0%)	60.0% (46.1% to 70.5%)
Cirrhosis and other chronic liver diseases due to alcohol use	100.0% (100.0% to 100.0%)	100.0% (100.0% to 100.0%)	100.0% (100.0% to 100.0%)	100.0% (100.0% to 100.0%)
**Digestive diseases**	6.8% (4.6% to 9.7%)	7.1% (4.9% to 9.8%)	1.7% (1.1% to 2.5%)	6.1% (4.2% to 8.6%)
Peptic ulcer disease	7.7% (4.9% to 10.9%)	9.1% (5.9% to 12.6%)	6.3% (3.8% to 9.8%)	8.8% (5.7% to 12.4%)
Gallbladder and biliary diseases	32.2% (18.9% to 46.7%)	33.6% (21.1% to 47.0%)	34.3% (21.8% to 47.0%)	33.6% (21.1% to 46.8%)
Pancreatitis	0.9% (-2.3% to 4.8%)	1.1% (-2.3% to 5.2%)	1.0% (-2.1% to 4.9%)	1.1% (-2.3% to 5.2%)
**Neurological disorders**	19.1% (8.8% to 31.2%)	16.8% (8.0% to 27.4%)	2.9% (1.4% to 4.9%)	8.8% (4.3% to 14.8%)
Alzheimer's disease and other dementias	21.8% (10.1% to 35.4%)	23.5% (11.1% to 37.7%)	23.5% (11.1% to 37.9%)	23.5% (11.1% to 37.8%)
Parkinson's disease	-4.3% (-6.0% to -2.9%)	-5.5% (-7.6% to -3.9%)	-6.3% (-8.8% to -4.4%)	-5.7% (-7.8% to -4.0%)
Epilepsy	1.8% (0.5% to 3.6%)	1.5% (0.4% to 3.2%)	1.3% (0.4% to 2.7%)	1.5% (0.4% to 3.0%)
Multiple sclerosis	6.4% (2.8% to 11.8%)	6.5% (2.7% to 12.3%)	4.7% (2.7% to 7.1%)	5.8% (2.7% to 10.2%)
**Mental and substance use disorders**	99.8% (99.5% to 99.9%)	99.7% (99.2% to 99.9%)	24.4% (20.9% to 28.3%)	26.8% (22.8% to 31.7%)
Alcohol use disorders	100.0% (100.0% to 100.0%)	100.0% (100.0% to 100.0%)	100.0% (100.0% to 100.0%)	100.0% (100.0% to 100.0%)

Supplementary Results Table 1. Age-standardised PAFs attributable to the joint distribution of all risk factors for all ages, both sexes combined for each GBD level 1 and level 2 cause and all causes in 2016 for Afghanistan. Results are shown for deaths, YLLs, YLDs, and DALYs
Data in parenthesis are 95% uncertainty intervals. PAFs=population attributable fractions. GBD=Global Burden of Disease. YLLs=years of life lost. YLDs=years live with disability. DALYs=disability-adjusted life-years

	Deaths 2016	YLLs 2016	YLDs 2016	DALYs 2016
Drug use disorders	100.0% (100.0% to 100.0%)	100.0% (100.0% to 100.0%)	100.0% (100.0% to 100.0%)	100.0% (100.0% to 100.0%)
Depressive disorders	-	-	7.7% (6.2% to 9.5%)	7.7% (6.2% to 9.5%)
Idiopathic developmental intellectual disability	-	-	70.0% (36.9% to 88.2%)	70.0% (36.9% to 88.2%)
**Diabetes, urogenital, blood, and endocrine diseases**	93.8% (92.3% to 94.9%)	90.5% (87.4% to 92.9%)	71.9% (67.0% to 75.9%)	85.4% (82.4% to 88.2%)
Diabetes mellitus	100.0% (100.0% to 100.0%)	100.0% (100.0% to 100.0%)	100.0% (100.0% to 100.0%)	100.0% (100.0% to 100.0%)
Chronic kidney disease	100.0% (100.0% to 100.0%)	100.0% (100.0% to 100.0%)	100.0% (100.0% to 100.0%)	100.0% (100.0% to 100.0%)
**Musculoskeletal disorders**	0.7% (0.2% to 1.5%)	0.5% (0.1% to 1.2%)	18.2% (14.9% to 21.9%)	17.8% (14.7% to 21.5%)
Rheumatoid arthritis	2.3% (0.8% to 4.4%)	2.2% (0.8% to 4.2%)	2.7% (1.0% to 5.1%)	2.7% (0.9% to 5.1%)
Osteoarthritis	-	-	20.8% (10.6% to 32.5%)	20.8% (10.6% to 32.5%)
Low back and neck pain	-	-	22.6% (19.2% to 26.6%)	22.6% (19.2% to 26.6%)
Gout	-	-	48.8% (37.4% to 61.9%)	48.8% (37.4% to 61.9%)
**Other non-communicable diseases**	0.2% (0.1% to 0.5%)	0.2% (0.1% to 0.6%)	8.5% (7.0% to 10.1%)	4.9% (3.5% to 6.7%)
Sense organ diseases	-	-	15.8% (13.2% to 18.3%)	15.8% (13.2% to 18.3%)
Sudden infant death syndrome	4.8% (2.2% to 9.1%)	4.8% (2.2% to 9.1%)	-	4.8% (2.2% to 9.1%)
**Transport injuries**	27.6% (23.2% to 33.0%)	25.4% (20.8% to 31.2%)	28.0% (24.0% to 32.5%)	25.5% (21.0% to 31.3%)
Road injuries	27.6% (23.1% to 33.0%)	25.4% (20.8% to 31.2%)	31.3% (26.9% to 36.2%)	25.6% (21.0% to 31.4%)
Other transport injuries	29.5% (23.6% to 34.5%)	24.8% (18.7% to 30.6%)	19.4% (16.3% to 23.0%)	22.0% (17.8% to 26.7%)
**Unintentional injuries**	21.1% (17.2% to 25.7%)	14.5% (11.2% to 18.2%)	18.9% (16.0% to 22.1%)	15.3% (12.2% to 18.6%)
Falls	36.7% (28.2% to 44.2%)	18.5% (11.9% to 28.0%)	24.8% (21.7% to 28.3%)	19.9% (14.5% to 27.2%)
Drowning	16.9% (13.0% to 21.9%)	14.1% (10.4% to 18.8%)	18.3% (14.2% to 23.3%)	14.1% (10.4% to 18.9%)
Fire, heat, and hot substances	6.9% (4.7% to 11.3%)	7.0% (4.5% to 11.9%)	14.4% (11.2% to 18.1%)	8.6% (6.0% to 13.0%)

Supplementary Results Table 1. Age-standardised PAFs attributable to the joint distribution of all risk factors for all ages, both sexes combined for each GBD level 1 and level 2 cause and all causes in 2016 for Afghanistan. Results are shown for deaths, YLLs, YLDs, and DALYs
Data in parenthesis are 95% uncertainty intervals. PAFs=population attributable fractions. GBD=Global Burden of Disease. YLLs=years of life lost. YLDs=years live with disability. DALYs=disability-adjusted life-years

	**Deaths 2016**	**YLLs 2016**	**YLDs 2016**	**DALYs 2016**
Poisonings	7.2% (4.6% to 9.9%)	6.5% (4.2% to 9.2%)	14.5% (11.2% to 18.4%)	7.0% (4.6% to 9.7%)
Exposure to mechanical forces	23.7% (18.8% to 29.2%)	21.2% (16.0% to 26.9%)	25.5% (21.2% to 30.4%)	21.8% (16.9% to 27.1%)
Animal contact	14.9% (10.2% to 19.0%)	11.2% (6.6% to 16.1%)	14.5% (11.6% to 18.1%)	12.8% (9.3% to 16.5%)
Foreign body	8.1% (5.4% to 11.0%)	7.4% (4.8% to 10.5%)	11.6% (8.9% to 14.7%)	8.7% (6.4% to 11.7%)
Other unintentional injuries	21.0% (14.4% to 27.7%)	20.4% (14.6% to 26.9%)	15.4% (12.1% to 19.4%)	18.5% (14.0% to 23.5%)
**Self-harm and interpersonal violence**	6.1% (4.0% to 9.2%)	5.8% (3.6% to 9.2%)	17.2% (15.3% to 19.3%)	6.2% (4.1% to 9.6%)
Self-harm	9.0% (5.1% to 13.5%)	10.3% (6.0% to 15.3%)	8.1% (4.5% to 12.4%)	10.3% (6.0% to 15.2%)
Interpersonal violence	4.9% (2.6% to 8.4%)	4.2% (2.0% to 7.8%)	18.0% (16.0% to 20.0%)	4.8% (2.6% to 8.6%)
**Forces of nature, conflict and terrorism, and executions and police conflict**	0.0% (0.0% to 0.0%)	0.0% (0.0% to 0.0%)	0.1% (0.0% to 0.2%)	0.0% (0.0% to 0.1%)
Forces of nature, conflict and terrorism, and state actor violence	0.0% (0.0% to 0.0%)	0.0% (0.0% to 0.0%)	5.7% (3.5% to 8.7%)	5.7% (3.5% to 8.7%)

Supplementary Results Table 1. Age-standardised PAFs attributable to the joint distribution of all risk factors for all ages, both sexes combined for each GBD level 1 and level 2 cause and all causes in 2016 for Albania. Results are shown for deaths, YLLs, YLDs, and DALYs
Data in parenthesis are 95% uncertainty intervals. PAFs=population attributable fractions. GBD=Global Burden of Disease. YLLs=years of life lost. YLDs=years live with disability. DALYs=disability-adjusted life-years

	Deaths 2016	YLLs 2016	YLDs 2016	DALYs 2016
**All causes**	65.2% (62.4% to 67.8%)	57.1% (54.4% to 59.6%)	23.9% (22.5% to 25.4%)	42.7% (39.7% to 45.8%)
**HIV/AIDS and tuberculosis**	56.2% (47.4% to 65.5%)	62.0% (54.6% to 69.0%)	48.5% (38.8% to 57.6%)	59.3% (51.8% to 66.6%)
Tuberculosis	49.2% (39.2% to 59.8%)	50.2% (40.8% to 59.8%)	44.2% (34.0% to 53.9%)	48.8% (39.1% to 58.2%)
HIV/AIDS	96.1% (95.1% to 96.8%)	94.7% (92.8% to 95.9%)	95.9% (95.1% to 96.6%)	94.8% (93.1% to 95.9%)
**Diarrhoea, lower respiratory infections, and other common infectious diseases**	58.8% (52.7% to 65.2%)	63.6% (56.8% to 70.2%)	34.5% (27.4% to 41.3%)	56.8% (50.3% to 63.4%)
Diarrhoeal diseases	75.9% (54.8% to 91.3%)	85.1% (69.9% to 94.8%)	80.1% (63.5% to 92.7%)	80.8% (64.6% to 93.0%)
Lower respiratory infections	67.2% (60.2% to 74.2%)	77.8% (71.0% to 83.7%)	64.4% (57.4% to 71.4%)	77.5% (70.7% to 83.5%)
Upper respiratory infections	1.2% (0.1% to 4.1%)	2.9% (0.2% to 10.0%)	0.0% (0.0% to 0.0%)	0.0% (0.0% to 0.1%)
Otitis media	4.4% (1.7% to 9.4%)	14.6% (8.5% to 23.7%)	7.9% (5.3% to 10.4%)	8.0% (5.3% to 10.4%)
Meningitis	3.8% (2.0% to 6.2%)	5.7% (3.3% to 9.2%)	0.0% (0.0% to 0.0%)	4.9% (2.9% to 7.6%)
Encephalitis	1.6% (0.5% to 3.1%)	2.4% (0.8% to 4.6%)	0.0% (0.0% to 0.0%)	2.1% (0.7% to 3.9%)
Measles	46.6% (26.5% to 78.9%)	47.4% (27.0% to 79.8%)	47.2% (26.9% to 78.1%)	47.4% (26.9% to 79.7%)
**Maternal disorders**	6.5% (4.0% to 9.5%)	6.5% (3.9% to 9.5%)	4.2% (1.9% to 7.0%)	5.4% (3.0% to 8.0%)
Maternal haemorrhage	11.7% (4.3% to 19.1%)	11.7% (4.3% to 19.1%)	11.7% (4.3% to 19.1%)	11.7% (4.3% to 19.1%)
Maternal sepsis and other pregnancy related infections	11.6% (4.3% to 18.9%)	11.6% (4.2% to 18.9%)	11.6% (4.2% to 18.9%)	11.6% (4.2% to 19.0%)
Maternal abortion, miscarriage, and ectopic pregnancy	7.4% (4.2% to 11.6%)	7.3% (4.0% to 11.6%)	6.8% (3.0% to 11.9%)	7.2% (3.9% to 11.4%)
**Neonatal disorders**	71.6% (66.7% to 76.1%)	71.6% (66.8% to 76.2%)	0.1% (0.0% to 0.1%)	58.4% (49.9% to 66.1%)
Neonatal preterm birth complications	94.4% (87.9% to 97.9%)	94.5% (88.0% to 97.9%)	0.2% (0.2% to 0.3%)	73.0% (59.7% to 83.9%)
Neonatal encephalopathy due to birth asphyxia and trauma	64.3% (56.3% to 70.2%)	64.3% (56.4% to 70.2%)	0.0% (0.0% to 0.0%)	36.3% (18.9% to 54.5%)
Neonatal sepsis and other neonatal infections	52.4% (41.7% to 60.2%)	52.4% (41.7% to 60.2%)	0.0% (0.0% to 0.0%)	30.9% (16.6% to 45.3%)
Hemolytic disease and other neonatal jaundice	66.8% (60.3% to 71.6%)	66.8% (60.4% to 71.6%)	0.0% (0.0% to 0.0%)	25.6% (14.0% to 39.1%)

Supplementary Results Table 1. Age-standardised PAFs attributable to the joint distribution of all risk factors for all ages, both sexes combined for each GBD level 1 and level 2 cause and all causes in 2016 for Albania. Results are shown for deaths, YLLs, YLDs, and DALYs
Data in parenthesis are 95% uncertainty intervals. PAFs=population attributable fractions. GBD=Global Burden of Disease. YLLs=years of life lost. YLDs=years live with disability. DALYs=disability-adjusted life-years

	Deaths 2016	YLLs 2016	YLDs 2016	DALYs 2016
Other neonatal disorders	62.0% (57.0% to 66.7%)	62.1% (57.1% to 66.7%)	0.0% (0.0% to 0.0%)	61.3% (56.3% to 66.0%)
**Nutritional deficiencies**	45.8% (32.9% to 59.7%)	72.7% (56.8% to 82.4%)	89.9% (86.2% to 92.7%)	89.3% (85.7% to 92.1%)
Protein-energy malnutrition	54.6% (38.3% to 70.4%)	83.8% (73.7% to 91.1%)	98.1% (96.8% to 98.9%)	95.8% (93.7% to 97.3%)
Vitamin A deficiency	-	-	0.0% (0.0% to 0.0%)	0.0% (0.0% to 0.0%)
Iron-deficiency anaemia	100.0% (100.0% to 100.0%)	100.0% (100.0% to 100.0%)	100.0% (100.0% to 100.0%)	100.0% (100.0% to 100.0%)
**Other communicable, maternal, neonatal, and nutritional diseases**	2.2% (1.8% to 2.7%)	2.0% (1.4% to 2.5%)	76.8% (72.5% to 80.5%)	37.0% (26.7% to 48.2%)
Sexually transmitted diseases excluding HIV	88.3% (74.0% to 95.8%)	74.4% (50.9% to 89.5%)	100.0% (100.0% to 100.0%)	99.0% (97.4% to 99.7%)
Hepatitis	3.0% (2.2% to 4.0%)	2.5% (1.7% to 3.4%)	1.2% (0.7% to 1.8%)	2.4% (1.7% to 3.2%)
**Neoplasms**	41.5% (36.9% to 45.6%)	38.6% (34.6% to 42.2%)	34.1% (29.5% to 37.6%)	38.5% (34.5% to 42.2%)
Oesophageal cancer	79.4% (71.0% to 86.9%)	80.7% (72.9% to 87.4%)	79.7% (71.7% to 87.0%)	80.7% (72.8% to 87.4%)
Stomach cancer	24.1% (14.3% to 34.4%)	24.3% (14.6% to 34.3%)	24.0% (14.5% to 34.2%)	24.3% (14.6% to 34.3%)
Liver cancer	62.6% (53.1% to 71.1%)	62.5% (52.7% to 70.9%)	62.8% (53.6% to 71.0%)	62.5% (52.7% to 70.9%)
Larynx cancer	87.5% (80.4% to 92.4%)	88.0% (81.3% to 92.6%)	87.9% (81.1% to 92.7%)	88.0% (81.3% to 92.6%)
Tracheal, bronchus, and lung cancer	88.0% (84.8% to 90.6%)	87.6% (84.6% to 90.2%)	87.8% (84.7% to 90.3%)	87.6% (84.6% to 90.2%)
Breast cancer	22.0% (16.4% to 28.5%)	20.6% (15.1% to 26.6%)	21.2% (15.7% to 27.4%)	20.6% (15.1% to 26.7%)
Cervical cancer	100.0% (100.0% to 100.0%)	100.0% (100.0% to 100.0%)	100.0% (100.0% to 100.0%)	100.0% (100.0% to 100.0%)
Uterine cancer	39.2% (24.3% to 54.4%)	39.1% (24.3% to 54.1%)	39.4% (24.5% to 54.3%)	39.1% (24.3% to 54.1%)
Prostate cancer	5.9% (4.1% to 8.3%)	6.5% (4.6% to 9.0%)	6.6% (4.6% to 9.0%)	6.5% (4.6% to 9.0%)
Colon and rectum cancer	44.9% (36.9% to 52.6%)	45.3% (37.5% to 53.1%)	45.4% (37.6% to 53.0%)	45.3% (37.5% to 53.1%)
Lip and oral cavity cancer	76.2% (67.8% to 83.4%)	77.9% (70.3% to 84.4%)	74.7% (66.6% to 82.0%)	77.8% (70.3% to 84.3%)
Nasopharynx cancer	74.5% (68.3% to 80.3%)	72.2% (65.6% to 78.3%)	73.1% (66.3% to 79.1%)	72.2% (65.8% to 78.3%)

Supplementary Results Table 1. Age-standardised PAFs attributable to the joint distribution of all risk factors for all ages, both sexes combined for each GBD level 1 and level 2 cause and all causes in 2016 for Albania. Results are shown for deaths, YLLs, YLDs, and DALYs
Data in parenthesis are 95% uncertainty intervals. PAFs=population attributable fractions. GBD=Global Burden of Disease. YLLs=years of life lost. YLDs=years live with disability. DALYs=disability-adjusted life-years

	Deaths 2016	YLLs 2016	YLDs 2016	DALYs 2016
Other pharynx cancer	58.8% (45.5% to 70.0%)	61.3% (48.3% to 71.9%)	59.3% (46.3% to 70.2%)	61.3% (48.3% to 71.9%)
Gallbladder and biliary tract cancer	18.2% (8.6% to 29.7%)	18.0% (8.7% to 29.3%)	18.3% (8.7% to 29.6%)	18.0% (8.7% to 29.3%)
Pancreatic cancer	30.3% (22.6% to 38.0%)	31.8% (24.3% to 39.6%)	30.5% (23.0% to 37.8%)	31.8% (24.3% to 39.6%)
Ovarian cancer	7.2% (2.7% to 12.9%)	6.1% (2.0% to 11.4%)	6.1% (1.9% to 11.2%)	6.1% (2.0% to 11.4%)
Kidney cancer	35.1% (24.1% to 46.3%)	33.0% (22.7% to 43.8%)	33.6% (23.6% to 43.9%)	33.0% (22.7% to 43.8%)
Bladder cancer	31.8% (23.1% to 41.4%)	34.4% (25.5% to 44.4%)	32.8% (24.0% to 41.5%)	34.3% (25.4% to 44.3%)
Thyroid cancer	13.1% (5.6% to 23.4%)	13.1% (5.5% to 23.7%)	12.3% (5.8% to 20.9%)	13.0% (5.6% to 23.5%)
Mesothelioma	84.1% (75.2% to 90.4%)	75.2% (62.4% to 84.6%)	81.9% (72.1% to 88.6%)	75.3% (62.6% to 84.6%)
Non-Hodgkin's lymphoma	5.8% (2.1% to 10.6%)	5.1% (1.9% to 9.2%)	5.7% (2.1% to 10.5%)	5.1% (1.9% to 9.2%)
Multiple myeloma	7.1% (2.7% to 13.3%)	7.1% (2.7% to 13.1%)	7.1% (2.7% to 13.3%)	7.1% (2.7% to 13.1%)
Leukaemia	16.9% (10.2% to 24.2%)	12.2% (7.4% to 17.7%)	16.4% (10.0% to 23.3%)	12.2% (7.4% to 17.8%)
**Cardiovascular diseases**	82.6% (79.4% to 85.6%)	82.2% (79.7% to 84.5%)	73.1% (70.0% to 75.8%)	81.2% (78.8% to 83.5%)
Rheumatic heart disease	30.0% (20.1% to 47.7%)	27.9% (20.0% to 41.4%)	28.8% (19.1% to 40.3%)	28.3% (19.8% to 40.4%)
Ischaemic heart disease	92.6% (89.3% to 95.3%)	92.7% (90.5% to 94.6%)	91.9% (89.5% to 94.2%)	92.6% (90.5% to 94.5%)
Cerebrovascular disease	82.9% (76.7% to 89.1%)	84.0% (79.5% to 88.1%)	86.3% (82.8% to 89.4%)	84.3% (80.0% to 88.1%)
Hypertensive heart disease	100.0% (100.0% to 100.0%)	100.0% (100.0% to 100.0%)	100.0% (100.0% to 100.0%)	100.0% (100.0% to 100.0%)
Cardiomyopathy and myocarditis	20.7% (15.6% to 27.4%)	20.9% (16.0% to 27.3%)	27.4% (22.9% to 32.3%)	21.4% (16.8% to 27.5%)
Atrial fibrillation and flutter	55.6% (47.6% to 63.8%)	60.2% (53.0% to 66.8%)	66.8% (61.3% to 72.3%)	64.0% (57.8% to 69.9%)
Aortic aneurysm	50.1% (43.0% to 56.6%)	54.2% (48.4% to 59.3%)	-	54.2% (48.4% to 59.3%)
Peripheral vascular disease	47.2% (39.1% to 55.6%)	53.5% (46.5% to 60.2%)	55.7% (49.4% to 61.6%)	55.3% (49.3% to 61.0%)
Endocarditis	42.1% (31.7% to 52.6%)	36.7% (25.9% to 48.0%)	45.8% (37.1% to 54.5%)	36.9% (26.1% to 48.1%)

Supplementary Results Table 1. Age-standardised PAFs attributable to the joint distribution of all risk factors for all ages, both sexes combined for each GBD level 1 and level 2 cause and all causes in 2016 for Albania. Results are shown for deaths, YLLs, YLDs, and DALYs
Data in parenthesis are 95% uncertainty intervals. PAFs=population attributable fractions. GBD=Global Burden of Disease. YLLs=years of life lost. YLDs=years live with disability. DALYs=disability-adjusted life-years

	Deaths 2016	YLLs 2016	YLDs 2016	DALYs 2016
Other cardiovascular and circulatory diseases	50.8% (45.4% to 56.0%)	51.6% (46.3% to 57.8%)	58.1% (53.7% to 62.4%)	53.6% (48.9% to 58.7%)
**Chronic respiratory diseases**	63.6% (58.6% to 68.8%)	61.0% (55.9% to 65.9%)	43.9% (37.4% to 50.5%)	54.1% (49.0% to 59.3%)
Chronic obstructive pulmonary disease	78.2% (71.7% to 83.6%)	78.2% (72.4% to 83.3%)	75.4% (70.2% to 80.4%)	77.6% (72.1% to 82.4%)
Pneumoconiosis	100.0% (100.0% to 100.0%)	100.0% (100.0% to 100.0%)	100.0% (100.0% to 100.0%)	100.0% (100.0% to 100.0%)
Asthma	40.9% (30.0% to 52.2%)	41.9% (31.9% to 52.6%)	31.4% (23.2% to 41.3%)	34.7% (26.5% to 44.3%)
Other chronic respiratory diseases	14.1% (9.5% to 19.7%)	12.1% (7.4% to 17.8%)	7.9% (5.4% to 10.9%)	11.4% (7.2% to 16.5%)
**Cirrhosis and other chronic liver diseases**	55.4% (50.2% to 60.3%)	55.7% (51.2% to 59.9%)	44.5% (39.7% to 49.0%)	54.7% (50.3% to 58.9%)
Cirrhosis and other chronic liver diseases due to hepatitis B	0.8% (0.3% to 1.4%)	1.0% (0.4% to 1.8%)	1.0% (0.4% to 1.8%)	1.0% (0.4% to 1.8%)
Cirrhosis and other chronic liver diseases due to hepatitis C	76.7% (69.3% to 83.0%)	83.6% (78.8% to 87.1%)	83.4% (78.9% to 86.9%)	83.6% (78.8% to 87.1%)
Cirrhosis and other chronic liver diseases due to alcohol use	100.0% (100.0% to 100.0%)	100.0% (100.0% to 100.0%)	100.0% (100.0% to 100.0%)	100.0% (100.0% to 100.0%)
**Digestive diseases**	12.1% (8.6% to 15.9%)	11.4% (7.6% to 15.9%)	2.8% (2.0% to 3.6%)	6.9% (4.7% to 9.6%)
Peptic ulcer disease	17.4% (12.2% to 23.1%)	18.2% (13.0% to 24.1%)	8.9% (6.2% to 11.8%)	14.1% (9.9% to 18.9%)
Gallbladder and biliary diseases	36.8% (21.9% to 52.5%)	35.2% (21.3% to 50.0%)	39.3% (24.8% to 55.1%)	37.0% (23.0% to 52.1%)
Pancreatitis	41.3% (22.0% to 62.4%)	43.4% (24.3% to 62.7%)	38.1% (21.1% to 56.0%)	43.1% (24.0% to 62.4%)
**Neurological disorders**	17.1% (8.7% to 27.6%)	14.1% (8.2% to 21.1%)	3.5% (1.6% to 5.9%)	7.0% (4.1% to 10.6%)
Alzheimer's disease and other dementias	23.5% (12.4% to 37.1%)	23.9% (12.9% to 37.2%)	23.8% (12.8% to 37.2%)	23.9% (12.9% to 37.2%)
Parkinson's disease	-18.2% (-23.1% to -13.4%)	-19.6% (-24.8% to -14.3%)	-18.6% (-23.2% to -13.7%)	-19.4% (-24.3% to -14.2%)
Epilepsy	22.0% (14.8% to 29.9%)	18.0% (11.3% to 24.7%)	20.2% (13.7% to 26.7%)	19.0% (12.6% to 25.5%)
Multiple sclerosis	9.2% (5.1% to 13.7%)	9.3% (5.1% to 14.1%)	6.4% (3.8% to 9.1%)	8.2% (4.7% to 12.1%)
**Mental and substance use disorders**	99.4% (99.1% to 99.7%)	99.1% (98.6% to 99.6%)	18.8% (16.7% to 21.2%)	21.3% (19.0% to 23.6%)
Alcohol use disorders	100.0% (100.0% to 100.0%)	100.0% (100.0% to 100.0%)	100.0% (100.0% to 100.0%)	100.0% (100.0% to 100.0%)

Supplementary Results Table 1. Age-standardised PAFs attributable to the joint distribution of all risk factors for all ages, both sexes combined for each GBD level 1 and level 2 cause and all causes in 2016 for Albania. Results are shown for deaths, YLLs, YLDs, and DALYs
Data in parenthesis are 95% uncertainty intervals. PAFs=population attributable fractions. GBD=Global Burden of Disease. YLLs=years of life lost. YLDs=years live with disability. DALYs=disability-adjusted life-years

	Deaths 2016	YLLs 2016	YLDs 2016	DALYs 2016
Drug use disorders	100.0% (100.0% to 100.0%)	100.0% (100.0% to 100.0%)	100.0% (100.0% to 100.0%)	100.0% (100.0% to 100.0%)
Depressive disorders	-	-	5.4% (4.3% to 6.7%)	5.4% (4.3% to 6.7%)
Idiopathic developmental intellectual disability	-	-	61.1% (31.3% to 81.4%)	61.1% (31.3% to 81.4%)
**Diabetes, urogenital, blood, and endocrine diseases**	77.3% (73.7% to 79.9%)	70.8% (60.5% to 74.5%)	42.4% (38.1% to 46.6%)	54.8% (50.0% to 59.0%)
Diabetes mellitus	100.0% (100.0% to 100.0%)	100.0% (100.0% to 100.0%)	100.0% (100.0% to 100.0%)	100.0% (100.0% to 100.0%)
Chronic kidney disease	100.0% (100.0% to 100.0%)	100.0% (100.0% to 100.0%)	100.0% (100.0% to 100.0%)	100.0% (100.0% to 100.0%)
**Musculoskeletal disorders**	2.0% (0.8% to 3.5%)	1.8% (0.8% to 3.2%)	28.0% (23.7% to 32.8%)	27.9% (23.6% to 32.7%)
Rheumatoid arthritis	4.7% (1.9% to 7.8%)	4.5% (1.9% to 7.6%)	3.8% (1.5% to 6.2%)	3.8% (1.5% to 6.2%)
Osteoarthritis	-	-	23.5% (11.3% to 36.9%)	23.5% (11.3% to 36.9%)
Low back and neck pain	-	-	30.8% (26.4% to 35.4%)	30.8% (26.4% to 35.4%)
Gout	-	-	41.3% (25.5% to 58.3%)	41.3% (25.5% to 58.3%)
**Other non-communicable diseases**	0.6% (0.3% to 1.1%)	0.6% (0.3% to 1.1%)	4.2% (3.7% to 4.7%)	3.3% (2.8% to 3.8%)
Sense organ diseases	-	-	9.0% (8.1% to 10.1%)	9.0% (8.1% to 10.1%)
Sudden infant death syndrome	6.5% (3.3% to 11.7%)	6.5% (3.4% to 11.8%)	-	6.5% (3.4% to 11.8%)
**Transport injuries**	33.3% (25.4% to 42.1%)	29.3% (21.3% to 38.4%)	37.4% (30.2% to 45.8%)	31.9% (24.2% to 40.5%)
Road injuries	33.0% (25.1% to 42.0%)	29.4% (21.3% to 38.4%)	37.7% (30.5% to 46.2%)	32.1% (24.3% to 40.8%)
Other transport injuries	34.9% (27.3% to 43.9%)	28.8% (20.9% to 38.4%)	30.4% (23.6% to 38.2%)	29.0% (21.6% to 37.6%)
**Unintentional injuries**	19.8% (15.8% to 24.7%)	14.2% (10.6% to 19.0%)	21.8% (18.3% to 25.8%)	19.5% (15.9% to 23.8%)
Falls	39.7% (33.6% to 51.7%)	24.6% (19.2% to 32.1%)	24.3% (20.8% to 28.2%)	24.3% (20.9% to 28.4%)
Drowning	14.6% (9.9% to 20.3%)	12.3% (8.1% to 17.4%)	16.8% (11.6% to 23.1%)	12.4% (8.1% to 17.4%)
Fire, heat, and hot substances	13.7% (8.9% to 19.6%)	13.8% (9.0% to 20.0%)	18.5% (13.0% to 25.0%)	16.7% (11.4% to 22.3%)

Supplementary Results Table 1. Age-standardised PAFs attributable to the joint distribution of all risk factors for all ages, both sexes combined for each GBD level 1 and level 2 cause and all causes in 2016 for Albania. Results are shown for deaths, YLLs, YLDs, and DALYs
Data in parenthesis are 95% uncertainty intervals. PAFs=population attributable fractions. GBD=Global Burden of Disease. YLLs=years of life lost. YLDs=years live with disability. DALYs=disability-adjusted life-years

	**Deaths 2016**	**YLLs 2016**	**YLDs 2016**	**DALYs 2016**
Poisonings	15.0% (9.7% to 22.0%)	14.5% (9.0% to 21.4%)	17.4% (12.1% to 23.5%)	15.3% (9.8% to 21.8%)
Exposure to mechanical forces	16.8% (12.5% to 22.6%)	13.1% (9.0% to 19.2%)	21.3% (16.7% to 27.1%)	17.8% (13.2% to 23.5%)
Animal contact	15.0% (11.8% to 18.7%)	11.4% (8.3% to 15.5%)	10.9% (7.6% to 15.1%)	10.9% (7.7% to 15.1%)
Foreign body	6.8% (4.2% to 11.4%)	6.2% (3.6% to 11.0%)	8.4% (5.7% to 11.8%)	7.2% (4.8% to 11.0%)
Other unintentional injuries	23.1% (15.5% to 31.5%)	22.2% (14.8% to 30.5%)	16.8% (11.7% to 22.8%)	19.2% (13.2% to 26.0%)
**Self-harm and interpersonal violence**	25.1% (15.5% to 34.6%)	24.0% (14.6% to 33.4%)	25.7% (20.5% to 31.1%)	24.2% (15.5% to 32.7%)
Self-harm	25.4% (12.8% to 37.7%)	24.7% (11.8% to 36.9%)	20.9% (10.5% to 31.6%)	24.6% (11.7% to 36.7%)
Interpersonal violence	24.3% (16.8% to 32.0%)	22.7% (15.3% to 30.4%)	26.5% (21.1% to 32.1%)	23.7% (16.9% to 30.8%)
**Forces of nature, conflict and terrorism, and executions and police conflict**	0.0% (0.0% to 0.0%)	0.0% (0.0% to 0.0%)	0.6% (0.3% to 1.0%)	0.5% (0.3% to 0.8%)
Forces of nature, conflict and terrorism, and state actor violence	0.0% (0.0% to 0.0%)	0.0% (0.0% to 0.0%)	4.8% (3.1% to 7.1%)	4.8% (3.1% to 7.1%)

**Supplementary Results Table 1. Age-standardised PAFs attributable to the joint distribution of all risk factors for all ages, both sexes combined for each GBD level 1 and level 2 cause and all causes in 2016 for Algeria. Results are shown for deaths, YLLs, YLDs, and DALYs**<br>Data in parenthesis are 95% uncertainty intervals. PAFs=population attributable fractions. GBD=Global Burden of Disease. YLLs=years of life lost. YLDs=years live with disability. DALYs=disability-adjusted life-years				
	**Deaths 2016**	**YLLs 2016**	**YLDs 2016**	**DALYs 2016**
**All causes**	61.3% (58.2% to 64.1%)	54.2% (51.6% to 57.2%)	24.7% (23.1% to 26.3%)	40.6% (38.0% to 43.5%)
**HIV/AIDS and tuberculosis**	25.9% (18.1% to 34.7%)	29.0% (22.1% to 36.8%)	34.0% (26.7% to 41.2%)	29.4% (22.5% to 36.7%)
Tuberculosis	23.7% (15.9% to 32.5%)	24.6% (17.9% to 32.3%)	21.4% (14.9% to 28.1%)	24.4% (17.8% to 31.8%)
HIV/AIDS	82.4% (78.3% to 86.9%)	81.9% (77.0% to 87.0%)	82.4% (78.6% to 86.6%)	82.0% (77.5% to 86.7%)
**Diarrhoea, lower respiratory infections, and other common infectious diseases**	46.7% (39.8% to 53.4%)	51.2% (44.5% to 57.6%)	28.0% (20.0% to 35.4%)	44.5% (38.4% to 50.7%)
Diarrhoeal diseases	69.2% (45.7% to 87.6%)	80.9% (63.9% to 92.8%)	71.4% (49.8% to 87.8%)	75.4% (56.5% to 90.1%)
Lower respiratory infections	48.4% (41.1% to 56.3%)	56.9% (48.5% to 65.6%)	51.7% (44.7% to 58.8%)	56.8% (48.5% to 65.4%)
Upper respiratory infections	2.1% (0.2% to 7.3%)	5.0% (0.4% to 17.0%)	0.0% (0.0% to 0.0%)	0.0% (0.0% to 0.1%)
Otitis media	1.6% (0.4% to 3.8%)	5.3% (1.9% to 9.6%)	6.3% (4.2% to 8.3%)	6.3% (4.2% to 8.3%)
Meningitis	1.8% (0.9% to 3.0%)	3.5% (1.8% to 5.6%)	0.0% (0.0% to 0.0%)	2.4% (1.2% to 3.8%)
Encephalitis	0.8% (0.5% to 1.3%)	1.6% (0.9% to 2.5%)	0.0% (0.0% to 0.0%)	0.6% (0.3% to 1.0%)
Measles	36.2% (16.1% to 73.7%)	36.8% (16.4% to 74.8%)	36.7% (16.9% to 73.7%)	36.8% (16.4% to 74.8%)
**Maternal disorders**	7.7% (3.0% to 13.0%)	7.6% (2.9% to 12.7%)	3.0% (1.2% to 5.4%)	7.0% (2.7% to 11.8%)
Maternal haemorrhage	15.0% (5.5% to 25.2%)	14.8% (5.4% to 24.7%)	15.1% (5.5% to 25.1%)	14.9% (5.4% to 24.7%)
Maternal sepsis and other pregnancy related infections	14.7% (5.5% to 24.7%)	14.6% (5.4% to 24.5%)	15.1% (5.4% to 25.1%)	14.6% (5.4% to 24.6%)
Maternal abortion, miscarriage, and ectopic pregnancy	11.1% (6.4% to 16.9%)	10.7% (6.1% to 16.6%)	12.2% (6.7% to 19.3%)	11.2% (6.3% to 17.0%)
**Neonatal disorders**	79.3% (73.8% to 84.3%)	79.3% (73.8% to 84.4%)	0.1% (0.0% to 0.2%)	68.7% (59.2% to 75.5%)
Neonatal preterm birth complications	89.3% (83.1% to 94.0%)	89.3% (83.1% to 94.0%)	0.2% (0.2% to 0.3%)	81.9% (73.9% to 88.0%)
Neonatal encephalopathy due to birth asphyxia and trauma	59.7% (48.7% to 69.0%)	59.8% (48.8% to 69.1%)	0.0% (0.0% to 0.0%)	54.6% (41.7% to 65.1%)
Neonatal sepsis and other neonatal infections	53.1% (42.7% to 62.3%)	53.2% (42.8% to 62.3%)	0.0% (0.0% to 0.0%)	35.4% (17.4% to 48.7%)
Hemolytic disease and other neonatal jaundice	67.5% (60.7% to 73.1%)	67.5% (60.8% to 73.2%)	0.0% (0.0% to 0.0%)	16.1% (8.7% to 25.8%)

Supplementary Results Table 1. Age-standardised PAFs attributable to the joint distribution of all risk factors for all ages, both sexes combined for each GBD level 1 and level 2 cause and all causes in 2016 for Algeria. Results are shown for deaths, YLLs, YLDs, and DALYs
Data in parenthesis are 95% uncertainty intervals. PAFs=population attributable fractions. GBD=Global Burden of Disease. YLLs=years of life lost. YLDs=years live with disability. DALYs=disability-adjusted life-years

	**Deaths 2016**	**YLLs 2016**	**YLDs 2016**	**DALYs 2016**
Other neonatal disorders	58.6% (47.6% to 67.1%)	58.7% (47.7% to 67.2%)	0.0% (0.0% to 0.0%)	54.9% (44.1% to 63.5%)
**Nutritional deficiencies**	12.0% (7.7% to 18.0%)	41.0% (28.6% to 54.3%)	87.6% (83.1% to 91.3%)	86.3% (81.9% to 90.1%)
Protein-energy malnutrition	10.9% (5.8% to 18.4%)	44.0% (29.1% to 59.7%)	93.5% (90.7% to 95.5%)	80.5% (74.3% to 85.9%)
Vitamin A deficiency	-	-	100.0% (100.0% to 100.0%)	100.0% (100.0% to 100.0%)
Iron-deficiency anaemia	100.0% (100.0% to 100.0%)	100.0% (100.0% to 100.0%)	100.0% (100.0% to 100.0%)	100.0% (100.0% to 100.0%)
**Other communicable, maternal, neonatal, and nutritional diseases**	2.1% (1.5% to 2.8%)	2.2% (1.3% to 3.0%)	80.5% (73.9% to 85.7%)	47.1% (34.9% to 58.3%)
Sexually transmitted diseases excluding HIV	24.5% (10.2% to 49.5%)	12.8% (4.7% to 29.3%)	100.0% (100.0% to 100.0%)	88.5% (75.0% to 96.6%)
Hepatitis	0.9% (0.6% to 1.3%)	1.0% (0.6% to 1.4%)	0.8% (0.4% to 1.3%)	1.0% (0.6% to 1.4%)
**Neoplasms**	26.9% (22.6% to 31.5%)	26.0% (22.1% to 30.3%)	24.4% (20.5% to 29.1%)	26.0% (22.0% to 30.3%)
Oesophageal cancer	51.1% (34.1% to 66.2%)	54.8% (38.7% to 69.5%)	51.7% (35.0% to 66.7%)	54.8% (38.7% to 69.4%)
Stomach cancer	8.6% (2.0% to 17.7%)	8.9% (2.5% to 17.1%)	8.6% (2.1% to 17.7%)	8.9% (2.5% to 17.1%)
Liver cancer	37.3% (27.1% to 48.2%)	40.6% (30.5% to 51.5%)	37.8% (27.7% to 48.6%)	40.6% (30.5% to 51.5%)
Larynx cancer	42.9% (30.0% to 56.3%)	47.9% (34.5% to 61.0%)	44.1% (31.5% to 57.0%)	47.8% (34.5% to 60.9%)
Tracheal, bronchus, and lung cancer	64.7% (55.3% to 72.6%)	67.8% (58.8% to 75.5%)	64.9% (56.0% to 72.9%)	67.8% (58.7% to 75.4%)
Breast cancer	17.5% (9.3% to 27.6%)	13.8% (6.0% to 23.1%)	15.1% (6.7% to 24.9%)	13.8% (6.0% to 23.1%)
Cervical cancer	100.0% (100.0% to 100.0%)	100.0% (100.0% to 100.0%)	100.0% (100.0% to 100.0%)	100.0% (100.0% to 100.0%)
Uterine cancer	53.5% (40.2% to 65.4%)	54.8% (42.4% to 66.1%)	54.6% (41.8% to 66.0%)	54.7% (42.4% to 66.1%)
Prostate cancer	0.7% (0.3% to 1.2%)	1.0% (0.5% to 1.6%)	1.0% (0.5% to 1.5%)	1.0% (0.5% to 1.6%)
Colon and rectum cancer	42.9% (32.3% to 53.9%)	42.3% (31.8% to 52.5%)	42.8% (32.2% to 53.5%)	42.3% (31.8% to 52.5%)
Lip and oral cavity cancer	30.2% (18.9% to 41.7%)	35.0% (23.3% to 46.4%)	29.4% (18.5% to 40.3%)	34.9% (23.1% to 46.2%)
Nasopharynx cancer	37.8% (27.3% to 48.2%)	38.2% (28.1% to 48.1%)	37.1% (27.5% to 46.5%)	38.2% (28.1% to 48.0%)

Supplementary Results Table 1. Age-standardised PAFs attributable to the joint distribution of all risk factors for all ages, both sexes combined for each GBD level 1 and level 2 cause and all causes in 2016 for Algeria. Results are shown for deaths, YLLs, YLDs, and DALYs
Data in parenthesis are 95% uncertainty intervals. PAFs=population attributable fractions. GBD=Global Burden of Disease. YLLs=years of life lost. YLDs=years live with disability. DALYs=disability-adjusted life-years

	Deaths 2016	YLLs 2016	YLDs 2016	DALYs 2016
Other pharynx cancer	19.4% (9.2% to 31.9%)	20.7% (10.1% to 32.9%)	19.8% (9.6% to 31.8%)	20.7% (10.1% to 32.9%)
Gallbladder and biliary tract cancer	28.1% (17.2% to 41.2%)	30.0% (18.6% to 43.4%)	28.6% (17.5% to 41.6%)	30.0% (18.6% to 43.4%)
Pancreatic cancer	20.0% (11.0% to 32.1%)	20.7% (11.8% to 32.2%)	20.1% (11.2% to 31.8%)	20.7% (11.8% to 32.2%)
Ovarian cancer	14.2% (5.2% to 26.1%)	13.0% (4.5% to 23.7%)	12.9% (4.6% to 23.4%)	13.0% (4.5% to 23.7%)
Kidney cancer	25.6% (17.5% to 35.4%)	25.7% (17.8% to 34.9%)	26.3% (18.4% to 35.5%)	25.7% (17.8% to 34.9%)
Bladder cancer	16.7% (7.7% to 28.3%)	18.2% (9.0% to 29.3%)	17.5% (8.6% to 28.5%)	18.2% (9.0% to 29.3%)
Thyroid cancer	15.1% (9.1% to 22.5%)	15.4% (9.1% to 22.7%)	15.2% (9.3% to 22.2%)	15.4% (9.1% to 22.7%)
Mesothelioma	79.4% (71.4% to 86.5%)	68.7% (59.2% to 77.7%)	76.2% (68.1% to 83.9%)	68.8% (59.3% to 77.8%)
Non-Hodgkin's lymphoma	6.8% (3.0% to 11.6%)	6.4% (2.7% to 11.0%)	6.7% (2.8% to 11.5%)	6.4% (2.7% to 11.0%)
Multiple myeloma	8.6% (3.9% to 14.7%)	8.9% (4.0% to 15.2%)	8.8% (4.0% to 15.0%)	8.9% (4.0% to 15.2%)
Leukaemia	11.9% (7.3% to 17.0%)	10.8% (6.7% to 15.6%)	11.9% (7.4% to 16.8%)	10.8% (6.8% to 15.6%)
**Cardiovascular diseases**	85.7% (82.7% to 88.5%)	86.0% (83.6% to 88.1%)	72.6% (69.6% to 75.6%)	84.6% (82.3% to 86.9%)
Rheumatic heart disease	23.9% (15.7% to 39.6%)	19.9% (14.0% to 30.3%)	19.6% (12.4% to 29.5%)	19.8% (13.4% to 29.9%)
Ischaemic heart disease	92.8% (89.3% to 95.7%)	93.8% (91.5% to 95.7%)	90.8% (88.0% to 93.5%)	93.6% (91.3% to 95.6%)
Cerebrovascular disease	81.8% (76.2% to 87.0%)	84.0% (80.4% to 87.3%)	85.0% (80.8% to 89.1%)	84.1% (80.4% to 87.4%)
Hypertensive heart disease	100.0% (100.0% to 100.0%)	100.0% (100.0% to 100.0%)	100.0% (100.0% to 100.0%)	100.0% (100.0% to 100.0%)
Cardiomyopathy and myocarditis	40.1% (31.8% to 50.5%)	33.1% (26.1% to 41.0%)	33.4% (27.1% to 40.0%)	33.1% (26.4% to 40.8%)
Atrial fibrillation and flutter	47.3% (39.2% to 55.1%)	50.4% (43.2% to 57.0%)	54.7% (48.9% to 60.2%)	52.7% (46.3% to 58.6%)
Aortic aneurysm	39.7% (32.6% to 47.3%)	43.2% (37.2% to 48.8%)	-	43.2% (37.2% to 48.8%)
Peripheral vascular disease	54.4% (47.6% to 62.5%)	60.7% (55.4% to 66.4%)	64.7% (60.0% to 69.5%)	63.9% (59.2% to 68.8%)
Endocarditis	37.7% (28.0% to 50.0%)	33.6% (25.5% to 42.3%)	37.9% (29.0% to 47.2%)	33.7% (25.5% to 42.4%)

Supplementary Results Table 1. Age-standardised PAFs attributable to the joint distribution of all risk factors for all ages, both sexes combined for each GBD level 1 and level 2 cause and all causes in 2016 for Algeria. Results are shown for deaths, YLLs, YLDs, and DALYs
Data in parenthesis are 95% uncertainty intervals. PAFs=population attributable fractions. GBD=Global Burden of Disease. YLLs=years of life lost. YLDs=years live with disability. DALYs=disability-adjusted life-years

	Deaths 2016	YLLs 2016	YLDs 2016	DALYs 2016
Other cardiovascular and circulatory diseases	41.0% (35.3% to 47.0%)	39.9% (35.1% to 44.6%)	42.3% (37.5% to 47.2%)	40.6% (35.9% to 45.1%)
**Chronic respiratory diseases**	42.1% (34.0% to 50.3%)	42.7% (35.3% to 49.8%)	38.0% (31.4% to 45.0%)	40.2% (34.0% to 47.0%)
Chronic obstructive pulmonary disease	50.5% (40.0% to 62.1%)	52.6% (43.0% to 63.2%)	49.6% (40.3% to 59.8%)	51.6% (42.2% to 61.5%)
Pneumoconiosis	100.0% (100.0% to 100.0%)	100.0% (100.0% to 100.0%)	100.0% (100.0% to 100.0%)	100.0% (100.0% to 100.0%)
Asthma	38.6% (27.9% to 49.8%)	40.7% (30.9% to 50.9%)	34.0% (25.1% to 43.9%)	35.6% (26.7% to 45.5%)
Other chronic respiratory diseases	3.4% (1.7% to 5.7%)	4.3% (2.2% to 7.1%)	2.7% (1.0% to 5.3%)	3.8% (1.8% to 6.4%)
**Cirrhosis and other chronic liver diseases**	20.2% (16.5% to 24.9%)	25.1% (20.3% to 30.6%)	20.8% (17.1% to 25.2%)	24.7% (20.2% to 30.2%)
Cirrhosis and other chronic liver diseases due to hepatitis B	0.2% (0.1% to 0.4%)	0.3% (0.1% to 0.5%)	0.2% (0.1% to 0.4%)	0.3% (0.1% to 0.5%)
Cirrhosis and other chronic liver diseases due to hepatitis C	35.3% (25.4% to 46.6%)	50.6% (38.6% to 61.7%)	52.6% (42.1% to 62.6%)	50.7% (39.1% to 61.7%)
Cirrhosis and other chronic liver diseases due to alcohol use	100.0% (100.0% to 100.0%)	100.0% (100.0% to 100.0%)	100.0% (100.0% to 100.0%)	100.0% (100.0% to 100.0%)
**Digestive diseases**	8.7% (5.5% to 12.0%)	8.4% (5.6% to 11.3%)	2.7% (1.9% to 3.7%)	6.0% (4.2% to 8.3%)
Peptic ulcer disease	8.6% (5.5% to 12.5%)	10.4% (6.9% to 14.8%)	6.8% (4.2% to 10.4%)	8.7% (5.8% to 12.4%)
Gallbladder and biliary diseases	43.8% (28.9% to 59.0%)	45.8% (32.5% to 59.3%)	48.8% (36.9% to 61.1%)	46.2% (33.3% to 59.2%)
Pancreatitis	5.8% (0.2% to 17.3%)	7.4% (0.5% to 20.9%)	7.1% (0.5% to 19.0%)	7.4% (0.5% to 20.8%)
**Neurological disorders**	23.9% (11.3% to 38.2%)	22.5% (11.3% to 35.2%)	3.4% (1.8% to 5.4%)	10.1% (5.1% to 16.4%)
Alzheimer's disease and other dementias	27.1% (13.0% to 43.2%)	28.5% (14.5% to 44.3%)	28.5% (14.3% to 44.7%)	28.5% (14.5% to 44.4%)
Parkinson's disease	-6.8% (-9.0% to -4.9%)	-8.7% (-11.3% to -6.2%)	-9.1% (-11.6% to -6.7%)	-8.7% (-11.3% to -6.3%)
Epilepsy	4.9% (2.0% to 8.5%)	4.2% (1.8% to 7.0%)	3.7% (1.6% to 6.3%)	3.9% (1.6% to 6.5%)
Multiple sclerosis	6.2% (3.3% to 9.3%)	6.1% (3.2% to 9.2%)	4.3% (2.4% to 6.8%)	5.0% (2.8% to 7.6%)
**Mental and substance use disorders**	99.8% (99.6% to 99.9%)	99.7% (99.5% to 99.8%)	18.8% (16.2% to 21.6%)	20.5% (17.9% to 23.4%)
Alcohol use disorders	100.0% (100.0% to 100.0%)	100.0% (100.0% to 100.0%)	100.0% (100.0% to 100.0%)	100.0% (100.0% to 100.0%)

Supplementary Results Table 1. Age-standardised PAFs attributable to the joint distribution of all risk factors for all ages, both sexes combined for each GBD level 1 and level 2 cause and all causes in 2016 for Algeria. Results are shown for deaths, YLLs, YLDs, and DALYs
Data in parenthesis are 95% uncertainty intervals. PAFs=population attributable fractions. GBD=Global Burden of Disease. YLLs=years of life lost. YLDs=years live with disability. DALYs=disability-adjusted life-years

	Deaths 2016	YLLs 2016	YLDs 2016	DALYs 2016
Drug use disorders	100.0% (100.0% to 100.0%)	100.0% (100.0% to 100.0%)	100.0% (100.0% to 100.0%)	100.0% (100.0% to 100.0%)
Depressive disorders	-	-	7.9% (6.3% to 9.9%)	7.9% (6.3% to 9.9%)
Idiopathic developmental intellectual disability	-	-	69.0% (36.6% to 87.9%)	69.0% (36.6% to 87.9%)
**Diabetes, urogenital, blood, and endocrine diseases**	89.4% (86.3% to 91.2%)	85.5% (81.6% to 88.1%)	71.9% (67.4% to 76.0%)	78.5% (75.1% to 81.4%)
Diabetes mellitus	100.0% (100.0% to 100.0%)	100.0% (100.0% to 100.0%)	100.0% (100.0% to 100.0%)	100.0% (100.0% to 100.0%)
Chronic kidney disease	100.0% (100.0% to 100.0%)	100.0% (100.0% to 100.0%)	100.0% (100.0% to 100.0%)	100.0% (100.0% to 100.0%)
**Musculoskeletal disorders**	0.3% (0.1% to 0.7%)	0.2% (0.1% to 0.5%)	13.7% (10.8% to 16.9%)	13.6% (10.7% to 16.8%)
Rheumatoid arthritis	2.2% (0.8% to 4.1%)	2.1% (0.8% to 3.9%)	2.6% (1.0% to 4.5%)	2.6% (1.0% to 4.5%)
Osteoarthritis	-	-	30.5% (17.8% to 44.1%)	30.5% (17.8% to 44.1%)
Low back and neck pain	-	-	15.4% (12.7% to 18.2%)	15.4% (12.7% to 18.2%)
Gout	-	-	53.8% (40.5% to 67.9%)	53.8% (40.5% to 67.9%)
**Other non-communicable diseases**	0.1% (0.0% to 0.2%)	0.1% (0.1% to 0.2%)	4.9% (4.0% to 6.1%)	3.4% (2.6% to 4.3%)
Sense organ diseases	-	-	10.1% (8.4% to 12.2%)	10.1% (8.4% to 12.2%)
Sudden infant death syndrome	3.9% (2.0% to 7.3%)	3.9% (2.0% to 7.4%)	-	3.9% (2.0% to 7.4%)
**Transport injuries**	31.4% (26.7% to 36.7%)	28.5% (23.3% to 34.6%)	34.1% (29.9% to 39.3%)	29.2% (23.9% to 35.1%)
Road injuries	31.2% (26.4% to 36.5%)	28.5% (23.3% to 34.6%)	37.2% (32.8% to 42.6%)	29.3% (24.0% to 35.4%)
Other transport injuries	37.2% (33.2% to 41.3%)	28.1% (23.6% to 33.5%)	25.0% (21.5% to 29.2%)	26.4% (22.5% to 30.9%)
**Unintentional injuries**	25.7% (21.4% to 30.1%)	19.3% (15.5% to 23.6%)	21.2% (18.2% to 25.0%)	20.1% (16.9% to 24.2%)
Falls	51.1% (43.1% to 56.0%)	35.3% (29.9% to 41.3%)	29.0% (26.0% to 32.8%)	31.5% (27.8% to 36.1%)
Drowning	19.4% (14.8% to 24.9%)	17.3% (13.0% to 22.5%)	16.4% (12.8% to 20.9%)	17.3% (13.0% to 22.4%)
Fire, heat, and hot substances	13.3% (9.2% to 17.8%)	15.9% (11.3% to 20.9%)	16.3% (12.7% to 20.6%)	16.0% (12.0% to 20.4%)

Supplementary Results Table 1. Age-standardised PAFs attributable to the joint distribution of all risk factors for all ages, both sexes combined for each GBD level 1 and level 2 cause and all causes in 2016 for Algeria. Results are shown for deaths, YLLs, YLDs, and DALYs
Data in parenthesis are 95% uncertainty intervals. PAFs=population attributable fractions. GBD=Global Burden of Disease. YLLs=years of life lost. YLDs=years live with disability. DALYs=disability-adjusted life-years

	**Deaths 2016**	**YLLs 2016**	**YLDs 2016**	**DALYs 2016**
Poisonings	17.1% (11.2% to 22.9%)	16.6% (10.6% to 22.9%)	16.1% (12.4% to 20.4%)	16.5% (11.4% to 22.1%)
Exposure to mechanical forces	26.1% (21.1% to 32.2%)	23.1% (17.5% to 29.5%)	27.2% (22.8% to 32.3%)	24.8% (19.7% to 30.6%)
Animal contact	14.9% (12.1% to 17.9%)	11.2% (8.0% to 15.3%)	15.3% (11.9% to 19.4%)	14.2% (11.2% to 17.8%)
Foreign body	7.8% (5.5% to 10.5%)	7.2% (4.8% to 10.0%)	13.1% (9.8% to 16.7%)	9.6% (6.9% to 12.8%)
Other unintentional injuries	27.0% (19.7% to 35.7%)	27.8% (20.6% to 36.5%)	15.8% (12.3% to 20.2%)	18.1% (14.1% to 23.2%)
**Self-harm and interpersonal violence**	10.8% (7.4% to 14.8%)	10.9% (7.6% to 14.6%)	23.5% (21.6% to 25.7%)	12.5% (9.4% to 15.9%)
Self-harm	9.8% (5.5% to 14.5%)	10.2% (6.0% to 14.9%)	9.0% (4.9% to 13.6%)	10.2% (6.0% to 14.9%)
Interpersonal violence	14.4% (10.3% to 19.8%)	13.0% (9.2% to 18.5%)	25.5% (23.6% to 27.5%)	17.0% (13.8% to 21.7%)
**Forces of nature, conflict and terrorism, and executions and police conflict**	0.0% (0.0% to 0.0%)	0.0% (0.0% to 0.0%)	1.1% (0.7% to 1.5%)	0.7% (0.3% to 1.1%)
Forces of nature, conflict and terrorism, and state actor violence	0.0% (0.0% to 0.0%)	0.0% (0.0% to 0.0%)	7.8% (5.4% to 11.2%)	7.8% (5.4% to 11.2%)

Supplementary Results Table 1. Age-standardised PAFs attributable to the joint distribution of all risk factors for all ages, both sexes combined for each GBD level 1 and level 2 cause and all causes in 2016 for American Samoa. Results are shown for deaths, YLLs, YLDs, and DALYs
Data in parenthesis are 95% uncertainty intervals. PAFs=population attributable fractions. GBD=Global Burden of Disease. YLLs=years of life lost. YLDs=years live with disability. DALYs=disability-adjusted life-years

	Deaths 2016	YLLs 2016	YLDs 2016	DALYs 2016
**All causes**	64.5% (62.3% to 66.6%)	59.4% (57.3% to 61.5%)	36.0% (34.1% to 37.9%)	50.9% (48.5% to 53.4%)
**HIV/AIDS and tuberculosis**	70.1% (49.6% to 89.1%)	73.6% (54.5% to 88.6%)	57.2% (40.3% to 81.6%)	72.5% (54.0% to 87.9%)
Tuberculosis	45.2% (34.4% to 57.7%)	47.1% (37.1% to 58.3%)	41.7% (32.0% to 52.7%)	45.7% (36.0% to 56.8%)
HIV/AIDS	89.8% (83.8% to 93.7%)	87.3% (80.3% to 92.1%)	87.5% (79.9% to 92.6%)	87.5% (80.6% to 92.2%)
**Diarrhoea, lower respiratory infections, and other common infectious diseases**	37.7% (30.8% to 45.6%)	41.2% (34.8% to 48.0%)	36.6% (23.0% to 47.1%)	40.0% (33.7% to 46.4%)
Diarrhoeal diseases	69.0% (39.7% to 89.7%)	73.3% (48.9% to 91.3%)	71.1% (42.6% to 90.1%)	71.8% (45.1% to 90.6%)
Lower respiratory infections	36.4% (29.8% to 45.0%)	44.9% (37.7% to 52.5%)	41.8% (35.4% to 50.0%)	44.8% (37.7% to 52.5%)
Upper respiratory infections	0.4% (0.0% to 1.3%)	1.1% (0.1% to 3.6%)	0.0% (0.0% to 0.0%)	0.0% (0.0% to 0.0%)
Otitis media	1.5% (0.5% to 4.4%)	3.5% (1.3% to 8.4%)	7.6% (5.1% to 9.9%)	7.6% (5.1% to 9.8%)
Meningitis	1.2% (0.8% to 2.0%)	2.3% (1.5% to 3.5%)	0.0% (0.0% to 0.0%)	1.5% (1.0% to 2.3%)
Encephalitis	0.5% (0.2% to 0.7%)	1.1% (0.6% to 1.7%)	0.0% (0.0% to 0.0%)	0.4% (0.2% to 0.6%)
Measles	29.8% (9.0% to 69.9%)	30.4% (9.2% to 71.1%)	29.8% (8.8% to 71.1%)	30.4% (9.2% to 71.1%)
**Maternal disorders**	7.7% (3.2% to 12.5%)	7.6% (3.2% to 12.6%)	5.2% (2.4% to 8.6%)	7.5% (3.1% to 12.2%)
Maternal haemorrhage	16.3% (5.8% to 27.0%)	16.3% (5.8% to 26.9%)	16.3% (5.8% to 26.9%)	16.3% (5.8% to 26.9%)
Maternal sepsis and other pregnancy related infections	16.2% (5.8% to 26.8%)	16.2% (5.8% to 26.8%)	16.2% (5.7% to 26.7%)	16.2% (5.8% to 26.7%)
Maternal abortion, miscarriage, and ectopic pregnancy	10.4% (5.7% to 16.1%)	10.3% (5.6% to 15.9%)	9.4% (5.1% to 15.1%)	10.2% (5.5% to 15.7%)
**Neonatal disorders**	74.4% (70.6% to 77.6%)	74.5% (70.7% to 77.7%)	0.2% (0.1% to 0.2%)	54.8% (47.4% to 61.0%)
Neonatal preterm birth complications	92.2% (88.5% to 94.9%)	92.3% (88.5% to 94.9%)	0.2% (0.2% to 0.3%)	57.1% (45.7% to 67.2%)
Neonatal encephalopathy due to birth asphyxia and trauma	64.9% (59.8% to 69.5%)	65.0% (59.9% to 69.6%)	0.0% (0.0% to 0.0%)	57.9% (51.1% to 64.1%)
Neonatal sepsis and other neonatal infections	63.2% (52.8% to 70.1%)	63.3% (52.9% to 70.1%)	0.0% (0.0% to 0.0%)	42.8% (27.0% to 56.2%)
Hemolytic disease and other neonatal jaundice	56.5% (41.4% to 65.9%)	56.7% (41.6% to 65.9%)	0.0% (0.0% to 0.0%)	24.9% (16.3% to 34.3%)

Supplementary Results Table 1. Age-standardised PAFs attributable to the joint distribution of all risk factors for all ages, both sexes combined for each GBD level 1 and level 2 cause and all causes in 2016 for American Samoa. Results are shown for deaths, YLLs, YLDs, and DALYs
Data in parenthesis are 95% uncertainty intervals. PAFs=population attributable fractions. GBD=Global Burden of Disease. YLLs=years of life lost. YLDs=years live with disability. DALYs=disability-adjusted life-years

	Deaths 2016	YLLs 2016	YLDs 2016	DALYs 2016
Other neonatal disorders	53.6% (44.9% to 62.2%)	53.7% (45.0% to 62.2%)	0.0% (0.0% to 0.0%)	50.9% (42.1% to 59.5%)
**Nutritional deficiencies**	6.1% (3.7% to 9.6%)	32.6% (22.1% to 44.9%)	99.3% (99.1% to 99.5%)	91.3% (87.6% to 94.1%)
Protein-energy malnutrition	6.2% (3.6% to 10.1%)	35.5% (24.3% to 48.7%)	97.1% (95.0% to 98.4%)	54.2% (43.8% to 64.5%)
Vitamin A deficiency	-	-	0.0% (0.0% to 0.0%)	0.0% (0.0% to 0.0%)
Iron-deficiency anaemia	100.0% (100.0% to 100.0%)	100.0% (100.0% to 100.0%)	100.0% (100.0% to 100.0%)	100.0% (100.0% to 100.0%)
**Other communicable, maternal, neonatal, and nutritional diseases**	2.1% (1.2% to 3.6%)	1.6% (0.7% to 3.4%)	67.7% (61.8% to 73.8%)	12.0% (5.7% to 23.2%)
Sexually transmitted diseases excluding HIV	7.3% (1.8% to 29.7%)	4.4% (0.8% to 16.1%)	100.0% (100.0% to 100.0%)	20.6% (7.1% to 66.6%)
Hepatitis	0.6% (0.4% to 0.9%)	0.7% (0.5% to 1.0%)	0.4% (0.2% to 0.7%)	0.7% (0.5% to 1.0%)
**Neoplasms**	44.7% (39.8% to 49.5%)	44.2% (39.6% to 48.6%)	40.2% (34.9% to 45.6%)	44.1% (39.5% to 48.5%)
Oesophageal cancer	79.9% (68.3% to 88.8%)	80.9% (69.7% to 89.2%)	80.1% (69.3% to 88.8%)	80.9% (69.7% to 89.1%)
Stomach cancer	15.1% (9.1% to 21.7%)	15.4% (9.2% to 22.4%)	15.2% (9.1% to 21.8%)	15.4% (9.2% to 22.4%)
Liver cancer	56.2% (43.9% to 67.7%)	57.5% (45.4% to 68.7%)	56.6% (44.6% to 67.9%)	57.5% (45.4% to 68.7%)
Larynx cancer	81.7% (73.6% to 88.2%)	79.7% (72.0% to 86.3%)	81.1% (73.1% to 87.6%)	79.8% (72.0% to 86.3%)
Tracheal, bronchus, and lung cancer	87.0% (82.8% to 90.6%)	85.4% (80.9% to 89.3%)	86.2% (81.7% to 90.0%)	85.5% (80.9% to 89.3%)
Breast cancer	59.2% (39.0% to 74.1%)	56.2% (39.3% to 69.9%)	56.6% (39.4% to 70.2%)	56.2% (39.3% to 69.9%)
Cervical cancer	100.0% (100.0% to 100.0%)	100.0% (100.0% to 100.0%)	100.0% (100.0% to 100.0%)	100.0% (100.0% to 100.0%)
Uterine cancer	67.4% (54.0% to 77.1%)	68.7% (55.6% to 77.9%)	69.0% (55.9% to 78.1%)	68.7% (55.6% to 77.9%)
Prostate cancer	6.2% (4.2% to 8.3%)	6.1% (4.2% to 8.3%)	6.2% (4.2% to 8.4%)	6.1% (4.2% to 8.3%)
Colon and rectum cancer	60.0% (48.5% to 71.8%)	60.0% (49.0% to 71.0%)	60.3% (48.8% to 71.9%)	60.0% (49.0% to 71.1%)
Lip and oral cavity cancer	57.8% (47.4% to 68.6%)	59.5% (49.3% to 69.8%)	57.2% (46.4% to 68.0%)	59.4% (49.3% to 69.8%)
Nasopharynx cancer	67.3% (58.4% to 75.7%)	67.2% (58.3% to 75.9%)	65.8% (56.8% to 74.5%)	67.2% (58.3% to 75.9%)

Supplementary Results Table 1. Age-standardised PAFs attributable to the joint distribution of all risk factors for all ages, both sexes combined for each GBD level 1 and level 2 cause and all causes in 2016 for American Samoa. Results are shown for deaths, YLLs, YLDs, and DALYs
Data in parenthesis are 95% uncertainty intervals. PAFs=population attributable fractions. GBD=Global Burden of Disease. YLLs=years of life lost. YLDs=years live with disability. DALYs=disability-adjusted life-years

	Deaths 2016	YLLs 2016	YLDs 2016	DALYs 2016
Other pharynx cancer	20.5% (9.3% to 35.6%)	22.4% (10.9% to 37.0%)	21.6% (10.2% to 36.4%)	22.4% (10.9% to 37.0%)
Gallbladder and biliary tract cancer	42.3% (28.4% to 57.1%)	43.7% (29.3% to 58.0%)	42.8% (28.8% to 57.5%)	43.6% (29.3% to 58.0%)
Pancreatic cancer	47.0% (33.6% to 61.7%)	47.2% (33.7% to 61.7%)	46.9% (33.6% to 61.7%)	47.2% (33.7% to 61.7%)
Ovarian cancer	29.9% (12.8% to 49.7%)	28.4% (12.0% to 47.6%)	27.6% (11.5% to 46.2%)	28.3% (12.0% to 47.5%)
Kidney cancer	49.0% (38.7% to 59.2%)	47.8% (37.7% to 58.0%)	47.2% (37.3% to 56.9%)	47.8% (37.7% to 57.9%)
Bladder cancer	45.6% (31.7% to 60.2%)	45.6% (31.6% to 60.1%)	45.2% (31.7% to 59.7%)	45.6% (31.6% to 60.1%)
Thyroid cancer	23.8% (15.1% to 33.0%)	25.2% (15.9% to 34.4%)	25.5% (16.7% to 34.3%)	25.2% (16.0% to 34.4%)
Mesothelioma	90.1% (87.0% to 92.7%)	83.0% (78.3% to 87.1%)	87.7% (84.3% to 90.7%)	83.1% (78.4% to 87.1%)
Non-Hodgkin's lymphoma	12.3% (4.9% to 20.5%)	12.0% (5.1% to 19.3%)	12.3% (4.9% to 20.2%)	12.0% (5.1% to 19.3%)
Multiple myeloma	16.1% (7.2% to 26.6%)	16.8% (7.5% to 27.9%)	16.4% (7.2% to 27.0%)	16.8% (7.5% to 27.8%)
Leukaemia	25.0% (16.6% to 33.5%)	21.6% (14.6% to 28.7%)	22.8% (15.2% to 30.4%)	21.6% (14.6% to 28.7%)
**Cardiovascular diseases**	85.8% (83.7% to 87.6%)	86.3% (84.7% to 87.8%)	74.0% (69.7% to 77.8%)	85.1% (83.4% to 86.7%)
Rheumatic heart disease	31.3% (21.7% to 48.2%)	29.3% (20.7% to 41.1%)	26.7% (17.8% to 37.2%)	28.4% (19.9% to 39.7%)
Ischaemic heart disease	95.4% (92.8% to 97.3%)	96.5% (95.1% to 97.6%)	93.9% (92.0% to 95.5%)	96.5% (95.0% to 97.6%)
Cerebrovascular disease	87.6% (83.5% to 91.4%)	90.1% (87.7% to 92.3%)	89.1% (85.9% to 91.9%)	89.9% (87.6% to 92.1%)
Hypertensive heart disease	100.0% (100.0% to 100.0%)	100.0% (100.0% to 100.0%)	100.0% (100.0% to 100.0%)	100.0% (100.0% to 100.0%)
Cardiomyopathy and myocarditis	43.6% (35.7% to 51.6%)	43.8% (36.4% to 50.4%)	38.3% (31.4% to 44.9%)	43.6% (36.3% to 50.1%)
Atrial fibrillation and flutter	57.5% (49.1% to 65.0%)	61.7% (54.4% to 67.9%)	68.2% (63.1% to 73.1%)	65.3% (59.3% to 70.9%)
Aortic aneurysm	49.0% (41.3% to 56.7%)	56.3% (50.3% to 62.0%)	-	56.3% (50.3% to 62.0%)
Peripheral vascular disease	72.9% (67.3% to 78.0%)	81.8% (78.2% to 85.2%)	77.2% (72.9% to 81.1%)	80.2% (76.3% to 83.7%)
Endocarditis	42.5% (33.3% to 52.3%)	42.4% (34.1% to 49.5%)	42.0% (33.6% to 50.3%)	42.4% (34.1% to 49.5%)

Supplementary Results Table 1. Age-standardised PAFs attributable to the joint distribution of all risk factors for all ages, both sexes combined for each GBD level 1 and level 2 cause and all causes in 2016 for American Samoa. Results are shown for deaths, YLLs, YLDs, and DALYs
Data in parenthesis are 95% uncertainty intervals. PAFs=population attributable fractions. GBD=Global Burden of Disease. YLLs=years of life lost. YLDs=years live with disability. DALYs=disability-adjusted life-years

	Deaths 2016	YLLs 2016	YLDs 2016	DALYs 2016
Other cardiovascular and circulatory diseases	53.1% (47.8% to 58.1%)	55.7% (51.1% to 60.2%)	50.7% (45.4% to 56.0%)	54.7% (50.3% to 59.1%)
**Chronic respiratory diseases**	66.9% (62.2% to 71.4%)	63.0% (58.2% to 67.5%)	60.0% (54.0% to 66.2%)	61.8% (57.2% to 66.6%)
Chronic obstructive pulmonary disease	75.6% (70.4% to 80.1%)	72.8% (67.4% to 77.5%)	70.5% (64.8% to 75.5%)	72.1% (66.5% to 76.9%)
Pneumoconiosis	100.0% (100.0% to 100.0%)	100.0% (100.0% to 100.0%)	100.0% (100.0% to 100.0%)	100.0% (100.0% to 100.0%)
Asthma	57.6% (45.5% to 69.9%)	61.7% (50.8% to 72.1%)	55.4% (44.9% to 66.2%)	57.6% (47.9% to 67.6%)
Other chronic respiratory diseases	19.7% (13.9% to 26.7%)	18.6% (12.6% to 26.0%)	7.6% (5.0% to 10.5%)	16.8% (11.1% to 23.7%)
**Cirrhosis and other chronic liver diseases**	25.2% (23.2% to 27.4%)	26.1% (24.0% to 28.5%)	17.9% (15.3% to 20.5%)	25.6% (23.6% to 28.0%)
Cirrhosis and other chronic liver diseases due to hepatitis B	0.1% (0.0% to 0.1%)	0.1% (0.0% to 0.1%)	0.1% (0.0% to 0.1%)	0.1% (0.0% to 0.1%)
Cirrhosis and other chronic liver diseases due to hepatitis C	62.4% (54.2% to 69.2%)	72.4% (65.4% to 77.8%)	65.6% (56.9% to 73.0%)	72.1% (65.3% to 77.5%)
Cirrhosis and other chronic liver diseases due to alcohol use	100.0% (100.0% to 100.0%)	100.0% (100.0% to 100.0%)	100.0% (100.0% to 100.0%)	100.0% (100.0% to 100.0%)
**Digestive diseases**	17.8% (13.9% to 21.3%)	18.2% (14.4% to 21.8%)	7.6% (6.0% to 9.3%)	16.7% (13.2% to 19.9%)
Peptic ulcer disease	13.0% (8.9% to 17.5%)	16.0% (11.2% to 21.3%)	15.3% (10.5% to 20.4%)	15.9% (11.2% to 21.2%)
Gallbladder and biliary diseases	64.1% (51.1% to 75.3%)	64.0% (51.7% to 74.9%)	68.2% (56.0% to 78.3%)	64.2% (51.9% to 75.0%)
Pancreatitis	11.6% (1.3% to 28.5%)	16.1% (3.0% to 35.2%)	12.5% (1.7% to 29.8%)	16.0% (3.0% to 35.1%)
**Neurological disorders**	39.4% (20.8% to 57.0%)	34.3% (19.9% to 48.1%)	8.1% (4.8% to 11.7%)	19.5% (10.9% to 28.2%)
Alzheimer's disease and other dementias	44.9% (23.8% to 65.0%)	46.6% (25.6% to 66.5%)	46.6% (25.3% to 66.6%)	46.6% (25.7% to 66.6%)
Parkinson's disease	-7.7% (-9.9% to -5.8%)	-10.5% (-13.4% to -7.9%)	-12.5% (-15.9% to -9.5%)	-10.9% (-13.8% to -8.3%)
Epilepsy	15.7% (7.0% to 25.1%)	16.2% (7.1% to 25.9%)	11.3% (4.8% to 18.5%)	13.5% (5.9% to 22.1%)
Multiple sclerosis	10.3% (6.4% to 14.6%)	11.0% (6.8% to 15.4%)	8.9% (5.7% to 12.7%)	10.4% (6.7% to 14.6%)
**Mental and substance use disorders**	99.1% (98.6% to 99.4%)	98.7% (98.1% to 99.2%)	15.5% (13.9% to 17.3%)	18.2% (16.4% to 20.3%)
Alcohol use disorders	100.0% (100.0% to 100.0%)	100.0% (100.0% to 100.0%)	100.0% (100.0% to 100.0%)	100.0% (100.0% to 100.0%)

Supplementary Results Table 1. Age-standardised PAFs attributable to the joint distribution of all risk factors for all ages, both sexes combined for each GBD level 1 and level 2 cause and all causes in 2016 for American Samoa. Results are shown for deaths, YLLs, YLDs, and DALYs
Data in parenthesis are 95% uncertainty intervals. PAFs=population attributable fractions. GBD=Global Burden of Disease. YLLs=years of life lost. YLDs=years live with disability. DALYs=disability-adjusted life-years

	Deaths 2016	YLLs 2016	YLDs 2016	DALYs 2016
Drug use disorders	100.0% (100.0% to 100.0%)	100.0% (100.0% to 100.0%)	100.0% (100.0% to 100.0%)	100.0% (100.0% to 100.0%)
Depressive disorders	-	-	10.4% (8.4% to 12.4%)	10.4% (8.4% to 12.4%)
Idiopathic developmental intellectual disability	-	-	57.4% (27.5% to 78.1%)	57.4% (27.5% to 78.1%)
**Diabetes, urogenital, blood, and endocrine diseases**	87.8% (86.5% to 89.4%)	85.8% (84.3% to 87.6%)	84.3% (82.1% to 86.2%)	85.3% (84.0% to 86.7%)
Diabetes mellitus	100.0% (100.0% to 100.0%)	100.0% (100.0% to 100.0%)	100.0% (100.0% to 100.0%)	100.0% (100.0% to 100.0%)
Chronic kidney disease	100.0% (100.0% to 100.0%)	100.0% (100.0% to 100.0%)	100.0% (100.0% to 100.0%)	100.0% (100.0% to 100.0%)
**Musculoskeletal disorders**	0.3% (0.1% to 0.5%)	0.2% (0.1% to 0.4%)	23.4% (18.5% to 28.1%)	22.6% (17.9% to 27.2%)
Rheumatoid arthritis	4.3% (1.7% to 7.6%)	5.0% (2.0% to 8.9%)	5.6% (2.3% to 9.5%)	5.6% (2.2% to 9.4%)
Osteoarthritis	-	-	46.3% (30.3% to 60.7%)	46.3% (30.3% to 60.7%)
Low back and neck pain	-	-	23.6% (19.3% to 28.0%)	23.6% (19.3% to 28.0%)
Gout	-	-	69.4% (54.1% to 82.4%)	69.4% (54.1% to 82.4%)
**Other non-communicable diseases**	0.3% (0.1% to 0.5%)	0.9% (0.5% to 1.5%)	6.9% (5.9% to 8.1%)	5.3% (4.5% to 6.2%)
Sense organ diseases	-	-	14.4% (12.6% to 16.4%)	14.4% (12.6% to 16.4%)
Sudden infant death syndrome	16.5% (9.5% to 25.5%)	16.6% (9.5% to 25.6%)	-	16.6% (9.5% to 25.6%)
**Transport injuries**	27.5% (23.0% to 33.0%)	21.6% (16.7% to 27.8%)	26.9% (23.3% to 31.7%)	22.8% (18.2% to 28.7%)
Road injuries	26.4% (21.9% to 31.9%)	21.2% (16.3% to 27.3%)	30.2% (26.4% to 35.2%)	22.9% (18.1% to 28.7%)
Other transport injuries	32.3% (27.3% to 38.5%)	23.5% (18.6% to 29.9%)	21.6% (18.0% to 26.4%)	22.7% (18.3% to 28.5%)
**Unintentional injuries**	15.5% (11.5% to 18.3%)	11.0% (8.9% to 13.6%)	12.3% (10.8% to 14.1%)	11.3% (9.4% to 13.7%)
Falls	51.5% (27.7% to 59.5%)	28.2% (20.8% to 33.1%)	23.5% (21.9% to 25.6%)	26.1% (21.9% to 29.2%)
Drowning	13.1% (9.6% to 17.9%)	13.5% (9.9% to 18.2%)	10.4% (7.5% to 14.6%)	13.4% (9.9% to 18.1%)
Fire, heat, and hot substances	8.6% (6.1% to 12.1%)	10.6% (7.7% to 14.7%)	10.8% (7.8% to 14.8%)	10.6% (7.8% to 14.6%)

Supplementary Results Table 1. Age-standardised PAFs attributable to the joint distribution of all risk factors for all ages, both sexes combined for each GBD level 1 and level 2 cause and all causes in 2016 for American Samoa. Results are shown for deaths, YLLs, YLDs, and DALYs
Data in parenthesis are 95% uncertainty intervals. PAFs=population attributable fractions. GBD=Global Burden of Disease. YLLs=years of life lost. YLDs=years live with disability. DALYs=disability-adjusted life-years

	**Deaths 2016**	**YLLs 2016**	**YLDs 2016**	**DALYs 2016**
Poisonings	10.6% (7.6% to 14.4%)	12.8% (9.4% to 17.0%)	11.0% (8.0% to 14.8%)	12.6% (9.3% to 16.9%)
Exposure to mechanical forces	16.3% (14.0% to 19.2%)	12.4% (10.1% to 15.4%)	16.7% (14.8% to 19.2%)	13.7% (11.4% to 16.7%)
Animal contact	18.1% (15.5% to 20.6%)	12.3% (10.2% to 15.1%)	8.3% (6.5% to 10.7%)	10.0% (8.2% to 12.5%)
Foreign body	5.2% (3.9% to 6.8%)	6.0% (4.5% to 8.1%)	6.0% (4.4% to 7.8%)	6.0% (4.5% to 8.1%)
Other unintentional injuries	13.0% (9.4% to 17.7%)	15.0% (10.9% to 20.4%)	10.1% (7.3% to 13.7%)	11.8% (8.9% to 16.0%)
**Self-harm and interpersonal violence**	15.6% (9.4% to 22.6%)	15.9% (9.4% to 23.3%)	26.4% (22.7% to 30.1%)	17.0% (10.8% to 23.9%)
Self-harm	13.7% (5.3% to 23.7%)	15.0% (6.0% to 25.6%)	11.6% (4.2% to 20.9%)	15.0% (5.9% to 25.5%)
Interpersonal violence	17.9% (11.7% to 24.2%)	16.9% (10.5% to 23.6%)	27.7% (24.1% to 31.6%)	18.9% (12.9% to 25.1%)
**Forces of nature, conflict and terrorism, and executions and police conflict**	0.0% (0.0% to 0.0%)	0.0% (0.0% to 0.0%)	2.9% (2.0% to 4.1%)	2.7% (1.9% to 4.0%)
Forces of nature, conflict and terrorism, and state actor violence	0.0% (0.0% to 0.0%)	0.0% (0.0% to 0.0%)	5.4% (3.7% to 7.8%)	5.4% (3.7% to 7.8%)

Supplementary Results Table 1. Age-standardised PAFs attributable to the joint distribution of all risk factors for all ages, both sexes combined for each GBD level 1 and level 2 cause and all causes in 2016 for Andorra. Results are shown for deaths, YLLs, YLDs, and DALYs
Data in parenthesis are 95% uncertainty intervals. PAFs=population attributable fractions. GBD=Global Burden of Disease. YLLs=years of life lost. YLDs=years live with disability. DALYs=disability-adjusted life-years

	Deaths 2016	YLLs 2016	YLDs 2016	DALYs 2016
**All causes**	51.2% (48.1% to 54.2%)	50.0% (47.0% to 52.7%)	19.1% (17.6% to 20.7%)	31.9% (29.0% to 35.3%)
**HIV/AIDS and tuberculosis**	89.4% (75.7% to 96.0%)	91.1% (80.8% to 96.0%)	89.0% (75.9% to 95.6%)	91.4% (83.3% to 95.7%)
Tuberculosis	62.7% (50.4% to 73.1%)	60.8% (50.3% to 70.3%)	56.2% (43.7% to 65.8%)	59.5% (49.2% to 68.4%)
HIV/AIDS	97.1% (95.9% to 98.1%)	96.2% (93.9% to 97.7%)	97.2% (96.0% to 98.2%)	96.5% (94.6% to 97.7%)
**Diarrhoea, lower respiratory infections, and other common infectious diseases**	38.1% (27.0% to 50.3%)	40.8% (31.6% to 49.9%)	6.6% (4.8% to 8.9%)	26.8% (19.3% to 35.2%)
Diarrhoeal diseases	15.9% (8.8% to 26.2%)	32.7% (19.9% to 48.8%)	29.4% (20.9% to 38.6%)	30.3% (21.8% to 39.2%)
Lower respiratory infections	40.5% (28.3% to 53.5%)	47.5% (36.7% to 58.3%)	46.8% (37.4% to 56.9%)	47.5% (36.7% to 58.3%)
Upper respiratory infections	0.3% (0.0% to 1.1%)	0.9% (0.1% to 3.3%)	0.0% (0.0% to 0.0%)	0.0% (0.0% to 0.0%)
Otitis media	3.1% (1.1% to 6.6%)	7.2% (3.2% to 12.1%)	8.2% (5.5% to 10.9%)	8.2% (5.5% to 10.9%)
Meningitis	1.1% (0.5% to 1.9%)	1.7% (0.8% to 3.0%)	0.0% (0.0% to 0.0%)	1.5% (0.7% to 2.8%)
Encephalitis	0.3% (0.1% to 0.5%)	0.6% (0.3% to 1.0%)	0.0% (0.0% to 0.0%)	0.3% (0.2% to 0.6%)
Measles	7.5% (0.0% to 40.5%)	7.6% (0.0% to 41.0%)	7.9% (0.0% to 39.7%)	9.7% (0.0% to 42.2%)
**Maternal disorders**	1.6% (0.6% to 2.7%)	1.5% (0.6% to 2.6%)	1.8% (0.7% to 3.3%)	1.7% (0.7% to 2.9%)
Maternal haemorrhage	8.4% (3.0% to 13.9%)	8.4% (3.0% to 13.8%)	8.6% (3.1% to 14.1%)	8.5% (3.1% to 13.9%)
Maternal sepsis and other pregnancy related infections	8.5% (3.1% to 13.9%)	8.4% (3.1% to 13.8%)	8.4% (3.1% to 13.8%)	8.4% (3.1% to 13.8%)
Maternal abortion, miscarriage, and ectopic pregnancy	4.4% (2.5% to 7.0%)	4.4% (2.5% to 7.1%)	4.7% (2.2% to 8.2%)	4.7% (2.3% to 8.0%)
**Neonatal disorders**	72.4% (65.1% to 78.0%)	72.5% (65.2% to 78.1%)	0.1% (0.1% to 0.1%)	38.3% (28.7% to 46.6%)
Neonatal preterm birth complications	83.1% (74.1% to 89.7%)	83.2% (74.2% to 89.8%)	0.2% (0.1% to 0.2%)	47.8% (34.5% to 59.2%)
Neonatal encephalopathy due to birth asphyxia and trauma	59.0% (49.1% to 66.7%)	59.1% (49.3% to 66.8%)	0.0% (0.0% to 0.0%)	26.8% (16.5% to 38.9%)
Neonatal sepsis and other neonatal infections	53.2% (41.2% to 61.6%)	53.2% (41.2% to 61.6%)	0.0% (0.0% to 0.0%)	16.3% (5.9% to 35.1%)
Hemolytic disease and other neonatal jaundice	39.2% (24.8% to 52.7%)	39.6% (25.2% to 53.1%)	0.0% (0.0% to 0.0%)	4.7% (2.4% to 7.9%)

Supplementary Results Table 1. Age-standardised PAFs attributable to the joint distribution of all risk factors for all ages, both sexes combined for each GBD level 1 and level 2 cause and all causes in 2016 for Andorra. Results are shown for deaths, YLLs, YLDs, and DALYs
Data in parenthesis are 95% uncertainty intervals. PAFs=population attributable fractions. GBD=Global Burden of Disease. YLLs=years of life lost. YLDs=years live with disability. DALYs=disability-adjusted life-years

	Deaths 2016	YLLs 2016	YLDs 2016	DALYs 2016
Other neonatal disorders	50.4% (39.4% to 61.0%)	50.5% (39.5% to 61.1%)	0.0% (0.0% to 0.0%)	41.1% (30.6% to 52.5%)
**Nutritional deficiencies**	7.3% (5.8% to 9.5%)	19.6% (16.5% to 23.5%)	48.8% (38.4% to 58.7%)	48.1% (38.0% to 57.8%)
Protein-energy malnutrition	1.3% (0.7% to 2.5%)	7.9% (4.3% to 13.8%)	98.3% (98.1% to 98.5%)	90.1% (85.9% to 93.2%)
Vitamin A deficiency	-	-	0.0% (0.0% to 0.0%)	0.0% (0.0% to 0.0%)
Iron-deficiency anaemia	100.0% (100.0% to 100.0%)	100.0% (100.0% to 100.0%)	100.0% (100.0% to 100.0%)	100.0% (100.0% to 100.0%)
**Other communicable, maternal, neonatal, and nutritional diseases**	5.6% (3.5% to 8.8%)	4.0% (1.8% to 6.5%)	68.5% (59.0% to 77.0%)	25.1% (12.3% to 39.6%)
Sexually transmitted diseases excluding HIV	98.3% (95.3% to 99.5%)	94.5% (86.0% to 98.4%)	100.0% (100.0% to 100.0%)	99.4% (98.4% to 99.9%)
Hepatitis	5.8% (4.2% to 7.9%)	5.4% (3.8% to 7.6%)	2.0% (1.3% to 3.0%)	5.1% (3.6% to 7.1%)
**Neoplasms**	41.4% (37.4% to 45.1%)	40.0% (36.0% to 43.8%)	28.4% (25.2% to 32.3%)	39.5% (35.4% to 43.2%)
Oesophageal cancer	84.0% (76.4% to 90.3%)	84.7% (76.9% to 90.9%)	83.6% (76.3% to 89.7%)	84.7% (76.9% to 90.9%)
Stomach cancer	20.3% (10.8% to 30.9%)	19.7% (9.9% to 29.9%)	19.9% (10.3% to 30.0%)	19.7% (9.9% to 29.8%)
Liver cancer	78.8% (72.6% to 84.1%)	77.1% (70.4% to 82.9%)	77.3% (71.1% to 82.7%)	77.1% (70.4% to 82.9%)
Larynx cancer	90.6% (84.0% to 94.7%)	89.6% (82.5% to 94.0%)	90.0% (83.2% to 94.4%)	89.6% (82.6% to 94.0%)
Tracheal, bronchus, and lung cancer	86.9% (82.7% to 89.8%)	85.7% (80.9% to 89.1%)	85.2% (80.4% to 89.3%)	85.7% (80.9% to 89.1%)
Breast cancer	33.9% (25.8% to 45.2%)	33.3% (25.3% to 44.2%)	33.4% (25.3% to 44.5%)	33.3% (25.3% to 44.2%)
Cervical cancer	100.0% (100.0% to 100.0%)	100.0% (100.0% to 100.0%)	100.0% (100.0% to 100.0%)	100.0% (100.0% to 100.0%)
Uterine cancer	33.0% (18.8% to 48.0%)	33.7% (19.3% to 48.6%)	34.1% (19.6% to 49.0%)	33.7% (19.5% to 48.7%)
Prostate cancer	6.0% (3.8% to 8.4%)	5.7% (3.6% to 8.0%)	5.6% (3.5% to 8.0%)	5.7% (3.6% to 8.0%)
Colon and rectum cancer	57.2% (48.5% to 65.3%)	58.1% (49.8% to 65.6%)	57.7% (49.1% to 65.7%)	58.1% (49.8% to 65.6%)
Lip and oral cavity cancer	85.1% (77.3% to 90.7%)	85.5% (78.6% to 90.7%)	83.5% (75.4% to 89.2%)	85.4% (78.5% to 90.6%)
Nasopharynx cancer	87.2% (81.5% to 91.6%)	84.8% (78.9% to 89.3%)	81.3% (74.0% to 86.9%)	84.7% (78.8% to 89.3%)

Supplementary Results Table 1. Age-standardised PAFs attributable to the joint distribution of all risk factors for all ages, both sexes combined for each GBD level 1 and level 2 cause and all causes in 2016 for Andorra. Results are shown for deaths, YLLs, YLDs, and DALYs
Data in parenthesis are 95% uncertainty intervals. PAFs=population attributable fractions. GBD=Global Burden of Disease. YLLs=years of life lost. YLDs=years live with disability. DALYs=disability-adjusted life-years

	Deaths 2016	YLLs 2016	YLDs 2016	DALYs 2016
Other pharynx cancer	72.2% (59.0% to 81.3%)	73.1% (60.6% to 81.7%)	73.1% (60.5% to 81.7%)	73.1% (60.6% to 81.7%)
Gallbladder and biliary tract cancer	15.4% (7.2% to 26.3%)	15.6% (7.3% to 26.5%)	15.9% (7.3% to 26.9%)	15.6% (7.3% to 26.5%)
Pancreatic cancer	27.8% (19.9% to 36.1%)	27.5% (19.6% to 36.2%)	27.1% (19.1% to 34.9%)	27.5% (19.6% to 36.2%)
Ovarian cancer	10.4% (5.0% to 17.2%)	8.3% (3.7% to 14.2%)	8.3% (3.7% to 14.0%)	8.3% (3.7% to 14.2%)
Kidney cancer	32.3% (21.7% to 43.3%)	31.3% (21.0% to 42.6%)	30.4% (20.5% to 40.9%)	31.3% (21.0% to 42.5%)
Bladder cancer	35.5% (24.9% to 45.7%)	34.5% (24.0% to 44.3%)	34.0% (23.9% to 44.0%)	34.4% (24.0% to 44.2%)
Thyroid cancer	12.2% (5.4% to 21.7%)	12.8% (5.7% to 22.7%)	11.8% (5.7% to 19.9%)	12.6% (5.7% to 22.3%)
Mesothelioma	98.4% (97.4% to 99.1%)	96.7% (94.7% to 98.0%)	97.6% (95.9% to 98.6%)	96.7% (94.7% to 98.1%)
Non-Hodgkin's lymphoma	5.8% (2.3% to 10.6%)	5.8% (2.3% to 10.5%)	5.8% (2.3% to 10.4%)	5.8% (2.3% to 10.5%)
Multiple myeloma	6.5% (2.5% to 12.0%)	6.7% (2.7% to 12.5%)	6.7% (2.7% to 12.5%)	6.7% (2.7% to 12.5%)
Leukaemia	18.5% (10.6% to 27.7%)	15.3% (9.0% to 22.7%)	15.0% (8.7% to 22.4%)	15.3% (9.0% to 22.7%)
**Cardiovascular diseases**	77.8% (73.9% to 81.0%)	80.9% (77.9% to 83.5%)	71.0% (67.4% to 74.3%)	78.7% (75.8% to 81.4%)
Rheumatic heart disease	25.7% (15.4% to 45.7%)	25.7% (17.4% to 40.7%)	24.8% (16.4% to 38.2%)	25.7% (17.4% to 40.6%)
Ischaemic heart disease	91.0% (86.7% to 94.4%)	93.4% (90.8% to 95.5%)	89.8% (86.8% to 92.5%)	93.1% (90.4% to 95.2%)
Cerebrovascular disease	80.8% (74.2% to 86.4%)	84.9% (80.3% to 88.8%)	83.7% (79.2% to 87.6%)	84.5% (80.2% to 88.2%)
Hypertensive heart disease	100.0% (100.0% to 100.0%)	100.0% (100.0% to 100.0%)	100.0% (100.0% to 100.0%)	100.0% (100.0% to 100.0%)
Cardiomyopathy and myocarditis	39.7% (30.8% to 48.9%)	42.1% (34.0% to 49.5%)	42.9% (37.0% to 48.9%)	42.3% (34.6% to 49.3%)
Atrial fibrillation and flutter	55.7% (46.4% to 66.4%)	60.1% (51.9% to 69.7%)	67.2% (60.4% to 74.3%)	64.0% (56.6% to 72.2%)
Aortic aneurysm	40.9% (33.4% to 49.0%)	46.6% (39.6% to 53.4%)	-	46.6% (39.6% to 53.4%)
Peripheral vascular disease	43.8% (36.8% to 52.1%)	49.6% (43.5% to 56.4%)	53.2% (47.6% to 59.2%)	50.7% (44.9% to 57.0%)
Endocarditis	37.9% (28.5% to 49.9%)	38.9% (30.8% to 47.8%)	38.9% (30.1% to 49.2%)	39.0% (30.8% to 47.8%)

Supplementary Results Table 1. Age-standardised PAFs attributable to the joint distribution of all risk factors for all ages, both sexes combined for each GBD level 1 and level 2 cause and all causes in 2016 for Andorra. Results are shown for deaths, YLLs, YLDs, and DALYs
Data in parenthesis are 95% uncertainty intervals. PAFs=population attributable fractions. GBD=Global Burden of Disease. YLLs=years of life lost. YLDs=years live with disability. DALYs=disability-adjusted life-years

	Deaths 2016	YLLs 2016	YLDs 2016	DALYs 2016
Other cardiovascular and circulatory diseases	43.4% (37.2% to 49.5%)	49.8% (44.4% to 55.1%)	51.0% (45.8% to 56.1%)	50.4% (45.2% to 55.3%)
**Chronic respiratory diseases**	67.1% (59.9% to 73.9%)	63.1% (56.0% to 69.9%)	40.4% (34.0% to 47.5%)	50.9% (44.3% to 57.1%)
Chronic obstructive pulmonary disease	76.1% (69.5% to 81.8%)	75.0% (68.3% to 80.7%)	66.4% (58.1% to 73.8%)	72.7% (65.6% to 78.9%)
Pneumoconiosis	100.0% (100.0% to 100.0%)	100.0% (100.0% to 100.0%)	100.0% (100.0% to 100.0%)	100.0% (100.0% to 100.0%)
Asthma	34.5% (25.0% to 44.8%)	35.8% (27.5% to 44.6%)	32.3% (25.0% to 41.1%)	32.6% (25.2% to 41.3%)
Other chronic respiratory diseases	11.1% (7.3% to 15.9%)	5.6% (3.0% to 9.3%)	6.1% (3.3% to 9.9%)	5.8% (3.3% to 9.0%)
**Cirrhosis and other chronic liver diseases**	76.7% (73.1% to 80.1%)	76.6% (72.7% to 80.3%)	62.8% (58.5% to 67.1%)	75.4% (71.5% to 79.0%)
Cirrhosis and other chronic liver diseases due to hepatitis B	1.7% (0.7% to 3.2%)	2.1% (0.8% to 4.0%)	1.9% (0.7% to 3.6%)	2.0% (0.8% to 3.9%)
Cirrhosis and other chronic liver diseases due to hepatitis C	89.5% (88.3% to 90.0%)	89.9% (89.7% to 90.0%)	88.1% (86.6% to 89.1%)	89.7% (89.5% to 89.9%)
Cirrhosis and other chronic liver diseases due to alcohol use	100.0% (100.0% to 100.0%)	100.0% (100.0% to 100.0%)	100.0% (100.0% to 100.0%)	100.0% (100.0% to 100.0%)
**Digestive diseases**	9.6% (6.9% to 12.3%)	11.9% (8.9% to 15.2%)	2.7% (1.9% to 3.6%)	9.3% (6.8% to 12.1%)
Peptic ulcer disease	10.9% (7.4% to 15.0%)	15.1% (10.5% to 20.5%)	13.3% (9.0% to 18.3%)	14.9% (10.4% to 20.1%)
Gallbladder and biliary diseases	33.3% (19.9% to 47.9%)	34.3% (21.2% to 48.6%)	37.0% (23.8% to 51.9%)	34.8% (21.7% to 49.1%)
Pancreatitis	56.7% (33.8% to 76.1%)	58.2% (37.2% to 76.6%)	55.5% (34.9% to 73.1%)	58.1% (37.1% to 76.6%)
**Neurological disorders**	17.0% (8.3% to 28.0%)	16.3% (9.3% to 24.9%)	3.8% (2.0% to 6.3%)	8.1% (4.7% to 12.5%)
Alzheimer's disease and other dementias	19.9% (9.5% to 32.8%)	21.0% (10.7% to 33.7%)	21.0% (10.5% to 33.6%)	21.0% (10.7% to 33.7%)
Parkinson's disease	-6.9% (-8.5% to -5.3%)	-8.8% (-11.1% to -6.9%)	-10.4% (-12.8% to -8.2%)	-9.2% (-11.3% to -7.2%)
Epilepsy	39.7% (29.0% to 49.4%)	35.4% (24.9% to 45.2%)	31.7% (22.1% to 40.5%)	33.5% (23.3% to 42.2%)
Multiple sclerosis	10.0% (6.4% to 14.0%)	11.2% (7.1% to 15.6%)	10.2% (6.6% to 14.2%)	10.6% (6.9% to 14.7%)
**Mental and substance use disorders**	99.8% (99.4% to 99.9%)	99.7% (99.3% to 99.9%)	15.3% (13.6% to 17.4%)	20.8% (18.0% to 24.1%)
Alcohol use disorders	100.0% (100.0% to 100.0%)	100.0% (100.0% to 100.0%)	100.0% (100.0% to 100.0%)	100.0% (100.0% to 100.0%)

Supplementary Results Table 1. Age-standardised PAFs attributable to the joint distribution of all risk factors for all ages, both sexes combined for each GBD level 1 and level 2 cause and all causes in 2016 for Andorra. Results are shown for deaths, YLLs, YLDs, and DALYs
Data in parenthesis are 95% uncertainty intervals. PAFs=population attributable fractions. GBD=Global Burden of Disease. YLLs=years of life lost. YLDs=years live with disability. DALYs=disability-adjusted life-years

	Deaths 2016	YLLs 2016	YLDs 2016	DALYs 2016
Drug use disorders	100.0% (100.0% to 100.0%)	100.0% (100.0% to 100.0%)	100.0% (100.0% to 100.0%)	100.0% (100.0% to 100.0%)
Depressive disorders	-	-	7.0% (5.8% to 8.5%)	7.0% (5.8% to 8.5%)
Idiopathic developmental intellectual disability	-	-	63.0% (33.6% to 82.7%)	63.0% (33.6% to 82.7%)
**Diabetes, urogenital, blood, and endocrine diseases**	64.5% (59.5% to 68.9%)	58.5% (53.1% to 64.9%)	51.1% (44.9% to 56.6%)	53.4% (48.5% to 57.9%)
Diabetes mellitus	100.0% (100.0% to 100.0%)	100.0% (100.0% to 100.0%)	100.0% (100.0% to 100.0%)	100.0% (100.0% to 100.0%)
Chronic kidney disease	100.0% (100.0% to 100.0%)	100.0% (100.0% to 100.0%)	100.0% (100.0% to 100.0%)	100.0% (100.0% to 100.0%)
**Musculoskeletal disorders**	1.1% (0.4% to 2.1%)	1.5% (0.6% to 2.7%)	19.5% (15.6% to 23.8%)	19.3% (15.5% to 23.6%)
Rheumatoid arthritis	3.7% (1.5% to 6.4%)	4.8% (1.9% to 8.2%)	5.9% (2.3% to 10.0%)	5.8% (2.3% to 9.8%)
Osteoarthritis	-	-	20.0% (10.3% to 31.0%)	20.0% (10.3% to 31.0%)
Low back and neck pain	-	-	22.1% (18.0% to 26.5%)	22.1% (18.0% to 26.5%)
Gout	-	-	42.9% (26.5% to 60.7%)	42.9% (26.5% to 60.7%)
**Other non-communicable diseases**	0.2% (0.1% to 0.3%)	0.3% (0.1% to 0.4%)	2.1% (1.8% to 2.5%)	1.9% (1.6% to 2.3%)
Sense organ diseases	-	-	7.3% (6.3% to 8.3%)	7.3% (6.3% to 8.3%)
Sudden infant death syndrome	4.2% (2.1% to 7.5%)	4.2% (2.1% to 7.5%)	-	4.2% (2.1% to 7.5%)
**Transport injuries**	39.3% (29.5% to 49.8%)	34.6% (24.8% to 45.1%)	45.3% (36.1% to 55.3%)	39.0% (29.5% to 49.3%)
Road injuries	39.1% (29.2% to 49.6%)	34.3% (24.5% to 44.9%)	45.4% (36.1% to 55.3%)	38.8% (29.3% to 49.1%)
Other transport injuries	43.5% (33.0% to 54.9%)	38.6% (28.2% to 49.3%)	44.9% (35.3% to 55.1%)	41.5% (31.4% to 52.0%)
**Unintentional injuries**	42.4% (37.5% to 47.2%)	25.2% (20.8% to 30.6%)	23.7% (19.0% to 30.7%)	24.1% (19.6% to 30.6%)
Falls	62.6% (55.9% to 68.3%)	41.0% (34.1% to 48.6%)	25.2% (20.5% to 32.0%)	28.5% (23.9% to 35.0%)
Drowning	21.0% (13.9% to 28.9%)	17.9% (11.7% to 24.8%)	22.9% (14.9% to 32.6%)	18.1% (11.9% to 25.0%)
Fire, heat, and hot substances	17.7% (10.6% to 26.8%)	17.0% (10.9% to 23.8%)	24.6% (15.9% to 34.4%)	21.7% (14.1% to 30.7%)

Supplementary Results Table 1. Age-standardised PAFs attributable to the joint distribution of all risk factors for all ages, both sexes combined for each GBD level 1 and level 2 cause and all causes in 2016 for Andorra. Results are shown for deaths, YLLs, YLDs, and DALYs
Data in parenthesis are 95% uncertainty intervals. PAFs=population attributable fractions. GBD=Global Burden of Disease. YLLs=years of life lost. YLDs=years live with disability. DALYs=disability-adjusted life-years

	Deaths 2016	YLLs 2016	YLDs 2016	DALYs 2016
Poisonings	24.9% (16.4% to 34.6%)	24.5% (16.1% to 34.5%)	22.0% (14.5% to 31.2%)	23.1% (15.4% to 32.0%)
Exposure to mechanical forces	24.5% (19.2% to 30.9%)	17.8% (12.4% to 25.4%)	23.7% (17.6% to 32.8%)	22.2% (16.5% to 30.8%)
Animal contact	18.0% (13.9% to 24.2%)	15.4% (10.5% to 22.7%)	13.4% (8.0% to 22.2%)	14.0% (8.9% to 22.3%)
Foreign body	8.3% (5.2% to 13.1%)	10.6% (6.5% to 16.7%)	9.5% (5.5% to 15.7%)	10.2% (6.4% to 16.1%)
Other unintentional injuries	26.5% (18.1% to 36.3%)	26.1% (18.2% to 35.6%)	23.7% (15.3% to 33.3%)	23.8% (15.5% to 33.5%)
**Self-harm and interpersonal violence**	38.2% (23.3% to 51.6%)	36.9% (22.3% to 50.1%)	37.5% (32.1% to 43.5%)	36.9% (23.4% to 49.3%)
Self-harm	38.5% (22.7% to 52.5%)	37.6% (21.8% to 51.7%)	35.5% (20.0% to 48.9%)	37.5% (21.6% to 51.7%)
Interpersonal violence	34.0% (25.5% to 43.3%)	29.4% (21.5% to 38.0%)	38.1% (32.8% to 43.7%)	33.5% (27.2% to 40.3%)
**Forces of nature, conflict and terrorism, and executions and police conflict**	0.0% (0.0% to 0.0%)	0.0% (0.0% to 0.0%)	0.0% (0.0% to 0.0%)	0.0% (0.0% to 0.0%)
Forces of nature, conflict and terrorism, and state actor violence	0.0% (0.0% to 0.0%)	0.0% (0.0% to 0.0%)	0.0% (0.0% to 0.0%)	0.0% (0.0% to 0.0%)

Supplementary Results Table 1. Age-standardised PAFs attributable to the joint distribution of all risk factors for all ages, both sexes combined for each GBD level 1 and level 2 cause and all causes in 2016 for Angola. Results are shown for deaths, YLLs, YLDs, and DALYs
Data in parenthesis are 95% uncertainty intervals. PAFs=population attributable fractions. GBD=Global Burden of Disease. YLLs=years of life lost. YLDs=years live with disability. DALYs=disability-adjusted life-years

	Deaths 2016	YLLs 2016	YLDs 2016	DALYs 2016
**All causes**	57.0% (53.4% to 60.5%)	54.7% (51.6% to 57.8%)	26.7% (25.4% to 28.1%)	47.8% (44.8% to 50.8%)
**HIV/AIDS and tuberculosis**	58.1% (45.1% to 68.5%)	65.2% (54.2% to 73.8%)	78.6% (69.5% to 86.1%)	65.7% (55.5% to 74.1%)
Tuberculosis	40.5% (26.3% to 54.0%)	43.6% (30.4% to 56.2%)	41.5% (28.5% to 54.1%)	43.5% (30.4% to 56.1%)
HIV/AIDS	93.7% (91.7% to 95.3%)	90.4% (86.7% to 93.4%)	94.1% (92.3% to 95.4%)	90.7% (87.2% to 93.4%)
**Diarrhoea, lower respiratory infections, and other common infectious diseases**	75.9% (69.0% to 82.5%)	75.9% (70.2% to 81.3%)	49.4% (43.4% to 54.9%)	74.3% (68.7% to 79.7%)
Diarrhoeal diseases	95.0% (86.3% to 98.8%)	96.4% (89.9% to 99.2%)	95.4% (87.4% to 98.9%)	96.3% (89.8% to 99.1%)
Lower respiratory infections	69.2% (59.3% to 77.3%)	77.9% (70.1% to 84.6%)	70.7% (61.7% to 78.6%)	77.9% (70.0% to 84.5%)
Upper respiratory infections	1.2% (0.1% to 4.0%)	2.4% (0.2% to 7.9%)	0.0% (0.0% to 0.0%)	0.1% (0.0% to 0.2%)
Otitis media	1.1% (0.3% to 3.0%)	3.6% (1.5% to 6.6%)	3.7% (2.3% to 5.0%)	3.7% (2.3% to 5.0%)
Meningitis	2.5% (1.2% to 4.6%)	4.4% (2.3% to 7.6%)	0.0% (0.0% to 0.0%)	4.1% (2.2% to 7.2%)
Encephalitis	0.8% (0.5% to 1.3%)	1.4% (0.8% to 2.1%)	0.0% (0.0% to 0.0%)	0.9% (0.5% to 1.4%)
Measles	56.8% (35.2% to 82.5%)	57.6% (35.8% to 83.6%)	55.7% (35.0% to 79.8%)	57.6% (35.8% to 83.6%)
**Maternal disorders**	5.2% (2.2% to 8.4%)	5.2% (2.2% to 8.6%)	2.8% (1.3% to 4.7%)	5.1% (2.2% to 8.2%)
Maternal haemorrhage	15.4% (5.6% to 25.5%)	15.6% (5.7% to 25.7%)	16.1% (5.8% to 26.4%)	15.6% (5.7% to 25.7%)
Maternal sepsis and other pregnancy related infections	14.9% (5.3% to 24.6%)	15.3% (5.5% to 25.1%)	15.6% (5.6% to 25.6%)	15.3% (5.5% to 25.1%)
Maternal abortion, miscarriage, and ectopic pregnancy	27.9% (18.0% to 37.2%)	27.6% (17.9% to 36.9%)	27.4% (16.2% to 37.3%)	27.6% (17.8% to 36.8%)
**Neonatal disorders**	78.2% (74.3% to 82.0%)	78.2% (74.3% to 82.0%)	0.4% (0.2% to 0.8%)	74.1% (69.3% to 78.5%)
Neonatal preterm birth complications	95.5% (91.6% to 98.4%)	95.5% (91.6% to 98.4%)	1.6% (1.0% to 2.3%)	91.4% (86.8% to 94.9%)
Neonatal encephalopathy due to birth asphyxia and trauma	72.2% (66.4% to 76.8%)	72.2% (66.4% to 76.8%)	0.0% (0.0% to 0.0%)	68.3% (59.7% to 74.7%)
Neonatal sepsis and other neonatal infections	68.4% (62.9% to 72.6%)	68.4% (63.0% to 72.6%)	0.0% (0.0% to 0.0%)	63.5% (53.2% to 69.9%)
Hemolytic disease and other neonatal jaundice	70.0% (62.8% to 74.7%)	70.0% (62.8% to 74.8%)	0.0% (0.0% to 0.0%)	57.0% (47.0% to 66.5%)

Supplementary Results Table 1. Age-standardised PAFs attributable to the joint distribution of all risk factors for all ages, both sexes combined for each GBD level 1 and level 2 cause and all causes in 2016 for Angola. Results are shown for deaths, YLLs, YLDs, and DALYs
Data in parenthesis are 95% uncertainty intervals. PAFs=population attributable fractions. GBD=Global Burden of Disease. YLLs=years of life lost. YLDs=years live with disability. DALYs=disability-adjusted life-years

	Deaths 2016	YLLs 2016	YLDs 2016	DALYs 2016
Other neonatal disorders	69.0% (63.2% to 74.1%)	69.0% (63.3% to 74.2%)	0.0% (0.0% to 0.0%)	67.5% (61.4% to 72.9%)
**Nutritional deficiencies**	19.5% (8.9% to 30.8%)	52.0% (31.5% to 67.8%)	81.5% (76.7% to 86.0%)	63.1% (44.4% to 73.4%)
Protein-energy malnutrition	23.9% (11.1% to 36.8%)	58.9% (37.8% to 74.0%)	98.2% (97.5% to 98.7%)	60.4% (39.0% to 74.9%)
Vitamin A deficiency	-	-	100.0% (100.0% to 100.0%)	100.0% (100.0% to 100.0%)
Iron-deficiency anaemia	100.0% (100.0% to 100.0%)	100.0% (100.0% to 100.0%)	100.0% (100.0% to 100.0%)	100.0% (100.0% to 100.0%)
**Other communicable, maternal, neonatal, and nutritional diseases**	3.4% (1.8% to 6.0%)	2.1% (0.9% to 3.8%)	69.9% (65.6% to 74.4%)	9.0% (4.5% to 15.2%)
Sexually transmitted diseases excluding HIV	9.4% (3.7% to 18.5%)	3.9% (1.4% to 8.1%)	100.0% (100.0% to 100.0%)	16.1% (6.6% to 30.7%)
Hepatitis	0.0% (0.0% to 0.0%)	0.0% (0.0% to 0.0%)	0.0% (0.0% to 0.1%)	0.0% (0.0% to 0.0%)
**Neoplasms**	36.8% (32.5% to 41.3%)	38.6% (34.2% to 42.9%)	38.3% (33.6% to 43.1%)	38.6% (34.2% to 42.9%)
Oesophageal cancer	67.0% (53.6% to 78.5%)	70.0% (57.3% to 80.5%)	68.0% (54.8% to 79.1%)	70.0% (57.3% to 80.5%)
Stomach cancer	9.3% (2.6% to 20.5%)	9.6% (3.1% to 20.1%)	9.4% (2.8% to 20.3%)	9.6% (3.1% to 20.1%)
Liver cancer	35.0% (26.2% to 44.5%)	35.8% (27.1% to 45.3%)	35.4% (26.8% to 45.1%)	35.8% (27.1% to 45.3%)
Larynx cancer	64.4% (48.9% to 77.5%)	68.6% (54.2% to 80.2%)	65.7% (50.9% to 78.2%)	68.6% (54.2% to 80.2%)
Tracheal, bronchus, and lung cancer	78.0% (68.8% to 85.3%)	79.4% (71.0% to 86.3%)	78.5% (69.7% to 85.4%)	79.4% (71.0% to 86.3%)
Breast cancer	22.7% (15.7% to 31.7%)	21.1% (14.1% to 30.2%)	21.7% (14.7% to 30.2%)	21.2% (14.1% to 30.2%)
Cervical cancer	100.0% (100.0% to 100.0%)	100.0% (100.0% to 100.0%)	100.0% (100.0% to 100.0%)	100.0% (100.0% to 100.0%)
Uterine cancer	42.8% (29.3% to 56.2%)	43.5% (30.0% to 56.6%)	43.1% (29.7% to 56.4%)	43.5% (30.0% to 56.6%)
Prostate cancer	1.1% (0.5% to 2.1%)	1.5% (0.7% to 2.5%)	1.3% (0.7% to 2.2%)	1.5% (0.7% to 2.5%)
Colon and rectum cancer	50.2% (41.0% to 59.7%)	50.7% (41.9% to 59.8%)	50.4% (41.3% to 59.7%)	50.7% (41.9% to 59.8%)
Lip and oral cavity cancer	64.2% (49.7% to 77.0%)	68.5% (54.7% to 79.9%)	65.4% (51.3% to 77.3%)	68.4% (54.6% to 79.9%)
Nasopharynx cancer	61.3% (47.6% to 73.2%)	62.6% (49.2% to 73.9%)	61.0% (48.1% to 72.0%)	62.6% (49.2% to 73.8%)

Supplementary Results Table 1. Age-standardised PAFs attributable to the joint distribution of all risk factors for all ages, both sexes combined for each GBD level 1 and level 2 cause and all causes in 2016 for Angola. Results are shown for deaths, YLLs, YLDs, and DALYs
Data in parenthesis are 95% uncertainty intervals. PAFs=population attributable fractions. GBD=Global Burden of Disease. YLLs=years of life lost. YLDs=years live with disability. DALYs=disability-adjusted life-years

	Deaths 2016	YLLs 2016	YLDs 2016	DALYs 2016
Other pharynx cancer	45.7% (26.9% to 61.7%)	48.6% (30.1% to 64.2%)	46.7% (28.0% to 62.7%)	48.6% (30.0% to 64.2%)
Gallbladder and biliary tract cancer	21.4% (12.0% to 33.2%)	22.1% (12.5% to 34.0%)	21.5% (12.1% to 33.5%)	22.1% (12.5% to 34.0%)
Pancreatic cancer	18.7% (11.6% to 27.6%)	19.6% (12.4% to 28.3%)	18.9% (11.6% to 27.9%)	19.6% (12.4% to 28.2%)
Ovarian cancer	10.7% (4.5% to 18.8%)	9.7% (3.8% to 17.1%)	9.7% (3.8% to 17.2%)	9.7% (3.8% to 17.1%)
Kidney cancer	23.2% (15.4% to 32.8%)	22.4% (14.8% to 31.7%)	22.6% (15.1% to 31.7%)	22.4% (14.8% to 31.7%)
Bladder cancer	15.2% (7.9% to 24.3%)	17.1% (9.4% to 26.0%)	16.2% (8.7% to 25.1%)	17.1% (9.4% to 26.0%)
Thyroid cancer	12.1% (6.5% to 19.1%)	12.3% (6.7% to 19.5%)	12.1% (6.5% to 19.1%)	12.3% (6.7% to 19.5%)
Mesothelioma	87.2% (81.2% to 91.0%)	79.3% (71.7% to 84.7%)	84.3% (77.2% to 88.9%)	79.4% (71.8% to 84.7%)
Non-Hodgkin's lymphoma	5.6% (2.2% to 10.0%)	5.2% (2.1% to 9.1%)	5.5% (2.2% to 9.8%)	5.2% (2.1% to 9.2%)
Multiple myeloma	7.2% (3.0% to 12.6%)	7.4% (3.1% to 13.0%)	7.2% (3.0% to 12.6%)	7.4% (3.1% to 13.0%)
Leukaemia	10.3% (6.1% to 15.6%)	8.5% (5.0% to 13.0%)	9.9% (5.9% to 14.7%)	8.5% (5.0% to 13.0%)
**Cardiovascular diseases**	84.0% (81.3% to 86.4%)	85.1% (82.9% to 87.2%)	61.9% (57.5% to 66.1%)	83.4% (81.1% to 85.6%)
Rheumatic heart disease	29.4% (19.9% to 45.4%)	29.2% (20.3% to 43.5%)	25.2% (16.3% to 35.8%)	27.7% (19.0% to 40.0%)
Ischaemic heart disease	92.6% (89.4% to 95.2%)	94.1% (91.9% to 96.1%)	89.8% (86.9% to 92.5%)	94.0% (91.7% to 95.9%)
Cerebrovascular disease	85.5% (81.4% to 89.2%)	88.8% (85.8% to 91.5%)	86.1% (82.1% to 89.6%)	88.6% (85.6% to 91.3%)
Hypertensive heart disease	100.0% (100.0% to 100.0%)	100.0% (100.0% to 100.0%)	100.0% (100.0% to 100.0%)	100.0% (100.0% to 100.0%)
Cardiomyopathy and myocarditis	42.8% (34.9% to 51.6%)	41.9% (34.3% to 49.8%)	44.7% (37.7% to 51.9%)	42.1% (34.7% to 49.9%)
Atrial fibrillation and flutter	47.8% (39.7% to 55.7%)	52.4% (45.3% to 59.6%)	58.7% (52.7% to 64.7%)	55.5% (49.2% to 62.0%)
Aortic aneurysm	42.4% (35.5% to 49.2%)	47.4% (40.9% to 53.1%)	-	47.4% (40.9% to 53.1%)
Peripheral vascular disease	52.4% (46.0% to 59.3%)	59.5% (54.4% to 64.7%)	59.6% (54.3% to 65.2%)	59.6% (54.4% to 64.9%)
Endocarditis	41.3% (32.0% to 51.5%)	41.7% (33.5% to 50.8%)	37.0% (27.6% to 48.3%)	41.6% (33.5% to 50.7%)

Supplementary Results Table 1. Age-standardised PAFs attributable to the joint distribution of all risk factors for all ages, both sexes combined for each GBD level 1 and level 2 cause and all causes in 2016 for Angola. Results are shown for deaths, YLLs, YLDs, and DALYs
Data in parenthesis are 95% uncertainty intervals. PAFs=population attributable fractions. GBD=Global Burden of Disease. YLLs=years of life lost. YLDs=years live with disability. DALYs=disability-adjusted life-years

	Deaths 2016	YLLs 2016	YLDs 2016	DALYs 2016
Other cardiovascular and circulatory diseases	45.0% (40.0% to 50.2%)	48.4% (43.9% to 52.9%)	40.6% (35.4% to 46.0%)	46.9% (42.2% to 51.4%)
**Chronic respiratory diseases**	61.6% (51.9% to 70.3%)	60.6% (52.3% to 68.2%)	42.8% (36.0% to 50.8%)	54.4% (46.5% to 62.2%)
Chronic obstructive pulmonary disease	71.4% (59.1% to 82.3%)	73.5% (63.0% to 82.9%)	65.0% (54.0% to 75.4%)	71.5% (61.2% to 81.1%)
Pneumoconiosis	100.0% (100.0% to 100.0%)	100.0% (100.0% to 100.0%)	100.0% (100.0% to 100.0%)	100.0% (100.0% to 100.0%)
Asthma	33.7% (23.8% to 45.1%)	35.0% (26.1% to 45.3%)	27.9% (20.1% to 37.5%)	31.3% (23.2% to 40.6%)
Other chronic respiratory diseases	5.3% (2.6% to 9.2%)	5.2% (2.1% to 9.3%)	3.5% (1.5% to 6.3%)	4.6% (2.0% to 8.3%)
**Cirrhosis and other chronic liver diseases**	26.0% (24.2% to 27.7%)	24.9% (23.0% to 26.6%)	19.9% (17.4% to 22.5%)	24.7% (23.0% to 26.5%)
Cirrhosis and other chronic liver diseases due to hepatitis B	0.0% (0.0% to 0.0%)	0.0% (0.0% to 0.0%)	0.0% (0.0% to 0.0%)	0.0% (0.0% to 0.0%)
Cirrhosis and other chronic liver diseases due to hepatitis C	0.6% (0.3% to 1.0%)	0.8% (0.4% to 1.4%)	1.1% (0.5% to 2.0%)	0.8% (0.4% to 1.4%)
Cirrhosis and other chronic liver diseases due to alcohol use	100.0% (100.0% to 100.0%)	100.0% (100.0% to 100.0%)	100.0% (100.0% to 100.0%)	100.0% (100.0% to 100.0%)
**Digestive diseases**	6.2% (4.4% to 8.4%)	6.8% (4.9% to 9.2%)	3.2% (2.4% to 4.2%)	6.6% (4.7% to 8.9%)
Peptic ulcer disease	5.5% (3.4% to 8.0%)	6.8% (4.3% to 9.7%)	6.5% (4.4% to 9.2%)	6.8% (4.3% to 9.7%)
Gallbladder and biliary diseases	39.6% (25.9% to 53.7%)	40.1% (27.3% to 53.3%)	40.6% (28.0% to 54.1%)	40.1% (27.4% to 53.4%)
Pancreatitis	33.5% (10.6% to 63.0%)	38.7% (13.3% to 67.6%)	35.1% (11.6% to 63.4%)	38.7% (13.3% to 67.6%)
**Neurological disorders**	19.7% (9.5% to 32.3%)	19.5% (10.8% to 30.0%)	5.0% (2.6% to 7.9%)	11.3% (6.5% to 17.3%)
Alzheimer's disease and other dementias	21.0% (9.6% to 34.9%)	21.8% (10.3% to 35.6%)	21.9% (10.1% to 35.7%)	21.8% (10.3% to 35.6%)
Parkinson's disease	-4.2% (-6.0% to -2.7%)	-5.1% (-7.1% to -3.4%)	-5.6% (-7.4% to -3.9%)	-5.2% (-7.2% to -3.6%)
Epilepsy	20.7% (11.5% to 30.2%)	19.2% (10.8% to 28.2%)	17.3% (9.5% to 25.8%)	18.4% (10.3% to 27.1%)
Multiple sclerosis	3.9% (1.7% to 6.4%)	4.1% (1.8% to 7.0%)	3.4% (1.9% to 5.1%)	3.9% (1.9% to 6.2%)
**Mental and substance use disorders**	99.8% (99.6% to 99.9%)	99.7% (99.4% to 99.8%)	18.2% (16.1% to 20.3%)	21.7% (19.2% to 24.6%)
Alcohol use disorders	100.0% (100.0% to 100.0%)	100.0% (100.0% to 100.0%)	100.0% (100.0% to 100.0%)	100.0% (100.0% to 100.0%)

Supplementary Results Table 1. Age-standardised PAFs attributable to the joint distribution of all risk factors for all ages, both sexes combined for each GBD level 1 and level 2 cause and all causes in 2016 for Angola. Results are shown for deaths, YLLs, YLDs, and DALYs
Data in parenthesis are 95% uncertainty intervals. PAFs=population attributable fractions. GBD=Global Burden of Disease. YLLs=years of life lost. YLDs=years live with disability. DALYs=disability-adjusted life-years

	Deaths 2016	YLLs 2016	YLDs 2016	DALYs 2016
Drug use disorders	100.0% (100.0% to 100.0%)	100.0% (100.0% to 100.0%)	100.0% (100.0% to 100.0%)	100.0% (100.0% to 100.0%)
Depressive disorders	-	-	9.5% (7.9% to 11.4%)	9.5% (7.9% to 11.4%)
Idiopathic developmental intellectual disability	-	-	65.3% (33.9% to 84.3%)	65.3% (33.9% to 84.3%)
**Diabetes, urogenital, blood, and endocrine diseases**	80.8% (77.8% to 83.6%)	74.2% (69.4% to 78.1%)	60.7% (56.3% to 64.7%)	68.5% (64.4% to 71.9%)
Diabetes mellitus	100.0% (100.0% to 100.0%)	100.0% (100.0% to 100.0%)	100.0% (100.0% to 100.0%)	100.0% (100.0% to 100.0%)
Chronic kidney disease	100.0% (100.0% to 100.0%)	100.0% (100.0% to 100.0%)	100.0% (100.0% to 100.0%)	100.0% (100.0% to 100.0%)
**Musculoskeletal disorders**	0.5% (0.2% to 0.9%)	0.4% (0.1% to 0.8%)	19.3% (16.2% to 22.7%)	18.9% (15.9% to 22.3%)
Rheumatoid arthritis	1.3% (0.5% to 2.5%)	1.5% (0.6% to 2.9%)	2.1% (0.8% to 3.5%)	2.0% (0.8% to 3.4%)
Osteoarthritis	-	-	24.2% (13.2% to 36.3%)	24.2% (13.2% to 36.3%)
Low back and neck pain	-	-	21.7% (18.8% to 24.7%)	21.7% (18.8% to 24.7%)
Gout	-	-	50.5% (38.8% to 63.7%)	50.5% (38.8% to 63.7%)
**Other non-communicable diseases**	0.2% (0.1% to 0.5%)	0.4% (0.1% to 0.7%)	6.1% (5.0% to 7.2%)	4.8% (3.9% to 5.7%)
Sense organ diseases	-	-	11.5% (9.5% to 13.3%)	11.5% (9.5% to 13.3%)
Sudden infant death syndrome	6.6% (2.9% to 12.9%)	6.6% (3.0% to 12.9%)	-	6.6% (3.0% to 12.9%)
**Transport injuries**	35.9% (28.8% to 43.8%)	30.6% (24.0% to 38.2%)	42.8% (35.3% to 51.6%)	32.2% (25.3% to 40.1%)
Road injuries	35.8% (28.7% to 43.8%)	30.4% (23.8% to 37.9%)	44.9% (37.3% to 53.7%)	32.0% (25.2% to 39.9%)
Other transport injuries	40.7% (32.3% to 50.0%)	34.7% (26.4% to 44.4%)	34.6% (27.5% to 42.9%)	34.7% (27.4% to 42.8%)
**Unintentional injuries**	33.0% (24.6% to 38.3%)	19.8% (15.8% to 24.3%)	28.6% (23.0% to 34.9%)	22.2% (18.1% to 27.1%)
Falls	67.6% (45.2% to 74.4%)	46.7% (36.8% to 53.9%)	36.5% (31.0% to 42.7%)	41.8% (35.7% to 47.4%)
Drowning	15.4% (10.8% to 21.4%)	12.1% (8.1% to 17.4%)	21.2% (15.0% to 28.1%)	12.2% (8.2% to 17.6%)
Fire, heat, and hot substances	13.4% (9.6% to 18.7%)	14.8% (10.5% to 19.8%)	25.1% (17.8% to 33.0%)	16.9% (11.9% to 22.5%)

Supplementary Results Table 1. Age-standardised PAFs attributable to the joint distribution of all risk factors for all ages, both sexes combined for each GBD level 1 and level 2 cause and all causes in 2016 for Angola. Results are shown for deaths, YLLs, YLDs, and DALYs

Data in parenthesis are 95% uncertainty intervals. PAFs=population attributable fractions. GBD=Global Burden of Disease. YLLs=years of life lost. YLDs=years live with disability. DALYs=disability-adjusted life-years

	**Deaths 2016**	**YLLs 2016**	**YLDs 2016**	**DALYs 2016**
Poisonings	17.9% (12.2% to 24.6%)	17.6% (10.2% to 25.0%)	23.5% (16.5% to 31.3%)	17.9% (10.5% to 25.4%)
Exposure to mechanical forces	27.5% (21.7% to 34.5%)	21.6% (15.5% to 29.7%)	31.6% (24.0% to 40.9%)	24.7% (18.0% to 32.8%)
Animal contact	14.5% (10.4% to 19.1%)	15.6% (11.0% to 21.0%)	19.6% (13.3% to 26.8%)	16.5% (11.6% to 22.4%)
Foreign body	11.7% (7.4% to 16.7%)	10.7% (5.4% to 16.4%)	17.1% (11.4% to 23.9%)	12.4% (7.3% to 18.3%)
Other unintentional injuries	20.6% (15.1% to 27.2%)	22.3% (16.4% to 29.1%)	22.6% (16.1% to 29.8%)	22.4% (16.5% to 29.2%)
**Self-harm and interpersonal violence**	19.0% (9.9% to 29.6%)	19.3% (11.1% to 28.3%)	22.2% (18.5% to 26.8%)	19.6% (12.0% to 28.1%)
Self-harm	19.0% (7.4% to 32.6%)	20.8% (8.8% to 34.5%)	20.0% (7.9% to 33.9%)	20.8% (8.7% to 34.5%)
Interpersonal violence	18.9% (12.5% to 26.5%)	17.4% (11.2% to 24.9%)	22.5% (19.1% to 26.7%)	18.4% (12.6% to 25.0%)
**Forces of nature, conflict and terrorism, and executions and police conflict**	4.5% (3.8% to 4.9%)	1.8% (1.5% to 2.0%)	0.0% (0.0% to 0.0%)	0.4% (0.1% to 0.9%)
Forces of nature, conflict and terrorism, and state actor violence	7.2% (6.7% to 7.6%)	2.9% (2.6% to 3.2%)	5.6% (3.5% to 8.4%)	3.0% (2.7% to 3.4%)

Supplementary Results Table 1. Age-standardised PAFs attributable to the joint distribution of all risk factors for all ages, both sexes combined for each GBD level 1 and level 2 cause and all causes in 2016 for Antigua and Barbuda. Results are shown for deaths, YLLs, YLDs, and DALYs
Data in parenthesis are 95% uncertainty intervals. PAFs=population attributable fractions. GBD=Global Burden of Disease. YLLs=years of life lost. YLDs=years live with disability. DALYs=disability-adjusted life-years

	Deaths 2016	YLLs 2016	YLDs 2016	DALYs 2016
**All causes**	55.9% (53.9% to 57.9%)	52.2% (50.6% to 54.0%)	25.7% (24.2% to 27.2%)	40.8% (38.5% to 43.1%)
**HIV/AIDS and tuberculosis**	78.2% (71.6% to 83.1%)	75.2% (67.4% to 80.3%)	74.7% (65.9% to 82.1%)	75.2% (67.5% to 80.4%)
Tuberculosis	32.5% (18.7% to 44.4%)	34.6% (20.5% to 46.8%)	28.8% (15.6% to 40.0%)	34.1% (20.2% to 46.2%)
HIV/AIDS	81.3% (74.4% to 86.2%)	76.8% (69.0% to 82.1%)	79.3% (71.4% to 85.1%)	77.0% (69.3% to 82.4%)
**Diarrhoea, lower respiratory infections, and other common infectious diseases**	34.3% (26.1% to 43.9%)	44.7% (36.5% to 52.9%)	39.6% (28.1% to 48.9%)	43.5% (36.7% to 50.7%)
Diarrhoeal diseases	80.7% (58.5% to 93.7%)	87.5% (72.4% to 96.0%)	79.7% (56.0% to 93.3%)	82.3% (61.9% to 94.2%)
Lower respiratory infections	33.8% (24.7% to 44.3%)	45.8% (36.5% to 55.5%)	40.5% (31.5% to 50.6%)	45.8% (36.4% to 55.4%)
Upper respiratory infections	0.9% (0.1% to 3.2%)	2.2% (0.2% to 8.4%)	0.0% (0.0% to 0.0%)	0.0% (0.0% to 0.0%)
Otitis media	1.1% (0.6% to 1.9%)	3.0% (1.7% to 5.0%)	3.6% (2.4% to 4.9%)	3.6% (2.4% to 4.9%)
Meningitis	2.1% (1.3% to 3.2%)	4.2% (2.7% to 6.0%)	0.0% (0.0% to 0.0%)	3.7% (2.3% to 5.3%)
Encephalitis	1.0% (0.6% to 1.5%)	1.7% (1.1% to 2.4%)	0.0% (0.0% to 0.0%)	1.3% (0.8% to 1.9%)
Measles	17.3% (5.1% to 47.7%)	19.1% (5.7% to 51.9%)	12.5% (0.0% to 59.9%)	19.1% (5.7% to 51.9%)
**Maternal disorders**	4.0% (1.7% to 6.9%)	3.9% (1.7% to 6.7%)	3.1% (1.4% to 5.3%)	3.7% (1.6% to 6.2%)
Maternal haemorrhage	15.6% (5.7% to 25.6%)	15.6% (5.7% to 25.5%)	15.8% (5.7% to 26.1%)	15.6% (5.7% to 25.6%)
Maternal sepsis and other pregnancy related infections	15.5% (5.6% to 25.2%)	15.5% (5.6% to 25.0%)	15.6% (5.6% to 25.7%)	15.5% (5.6% to 25.3%)
Maternal abortion, miscarriage, and ectopic pregnancy	5.0% (2.7% to 8.1%)	5.1% (2.7% to 8.2%)	5.4% (2.5% to 9.0%)	5.2% (2.8% to 8.1%)
**Neonatal disorders**	76.9% (73.3% to 79.8%)	76.9% (73.4% to 79.8%)	0.1% (0.1% to 0.2%)	58.1% (46.4% to 66.7%)
Neonatal preterm birth complications	92.6% (90.0% to 94.6%)	92.7% (90.1% to 94.6%)	0.2% (0.2% to 0.2%)	67.7% (53.4% to 79.0%)
Neonatal encephalopathy due to birth asphyxia and trauma	60.9% (56.1% to 65.5%)	61.0% (56.2% to 65.5%)	0.0% (0.0% to 0.0%)	56.9% (49.1% to 63.0%)
Neonatal sepsis and other neonatal infections	53.3% (46.9% to 60.7%)	53.4% (46.9% to 60.7%)	0.0% (0.0% to 0.0%)	29.8% (14.8% to 45.1%)
Hemolytic disease and other neonatal jaundice	54.7% (46.4% to 62.5%)	54.7% (46.4% to 62.5%)	0.0% (0.0% to 0.0%)	45.6% (36.2% to 54.4%)

Supplementary Results Table 1. Age-standardised PAFs attributable to the joint distribution of all risk factors for all ages, both sexes combined for each GBD level 1 and level 2 cause and all causes in 2016 for Antigua and Barbuda. Results are shown for deaths, YLLs, YLDs, and DALYs
Data in parenthesis are 95% uncertainty intervals. PAFs=population attributable fractions. GBD=Global Burden of Disease. YLLs=years of life lost. YLDs=years live with disability. DALYs=disability-adjusted life-years

	Deaths 2016	YLLs 2016	YLDs 2016	DALYs 2016
Other neonatal disorders	55.6% (50.3% to 60.7%)	55.7% (50.4% to 60.8%)	0.0% (0.0% to 0.0%)	51.9% (45.7% to 57.6%)
**Nutritional deficiencies**	13.1% (8.1% to 19.8%)	45.4% (33.6% to 58.0%)	98.4% (97.9% to 98.9%)	91.8% (88.6% to 94.2%)
Protein-energy malnutrition	13.1% (8.0% to 20.1%)	46.4% (34.1% to 59.5%)	96.3% (94.8% to 97.6%)	51.8% (41.6% to 62.8%)
Vitamin A deficiency	-	-	100.0% (100.0% to 100.0%)	100.0% (100.0% to 100.0%)
Iron-deficiency anaemia	100.0% (100.0% to 100.0%)	100.0% (100.0% to 100.0%)	100.0% (100.0% to 100.0%)	100.0% (100.0% to 100.0%)
**Other communicable, maternal, neonatal, and nutritional diseases**	7.7% (6.2% to 9.3%)	6.2% (4.9% to 7.7%)	50.1% (42.4% to 58.3%)	23.5% (17.8% to 31.4%)
Sexually transmitted diseases excluding HIV	80.0% (61.3% to 95.4%)	59.2% (35.4% to 87.6%)	100.0% (100.0% to 100.0%)	90.3% (78.1% to 98.2%)
Hepatitis	8.7% (5.8% to 12.7%)	9.1% (5.7% to 13.6%)	6.0% (3.6% to 9.2%)	8.9% (5.6% to 13.2%)
**Neoplasms**	24.3% (21.0% to 27.9%)	25.2% (22.0% to 28.6%)	19.1% (16.2% to 22.4%)	25.0% (21.8% to 28.4%)
Oesophageal cancer	57.5% (41.7% to 71.4%)	60.9% (45.0% to 74.1%)	58.3% (42.1% to 71.8%)	60.9% (45.0% to 74.0%)
Stomach cancer	6.4% (1.9% to 13.8%)	6.9% (2.4% to 13.9%)	6.7% (2.0% to 14.0%)	6.9% (2.4% to 13.9%)
Liver cancer	56.5% (48.8% to 65.0%)	56.6% (49.1% to 65.0%)	56.5% (48.7% to 65.0%)	56.6% (49.1% to 65.0%)
Larynx cancer	52.5% (37.9% to 66.2%)	56.4% (41.7% to 69.4%)	55.1% (40.3% to 68.1%)	56.4% (41.6% to 69.4%)
Tracheal, bronchus, and lung cancer	60.8% (51.5% to 69.1%)	63.4% (55.1% to 71.0%)	61.3% (52.3% to 69.2%)	63.4% (55.1% to 70.9%)
Breast cancer	22.3% (13.8% to 32.7%)	20.0% (11.9% to 29.5%)	20.7% (12.4% to 30.4%)	20.1% (12.0% to 29.6%)
Cervical cancer	100.0% (100.0% to 100.0%)	100.0% (100.0% to 100.0%)	100.0% (100.0% to 100.0%)	100.0% (100.0% to 100.0%)
Uterine cancer	47.0% (31.1% to 62.1%)	49.1% (33.4% to 64.2%)	49.7% (34.0% to 64.6%)	49.2% (33.5% to 64.2%)
Prostate cancer	0.9% (0.4% to 1.4%)	1.1% (0.6% to 1.7%)	1.1% (0.6% to 1.7%)	1.1% (0.6% to 1.7%)
Colon and rectum cancer	52.2% (41.9% to 62.2%)	52.6% (42.6% to 62.1%)	52.5% (42.5% to 62.4%)	52.6% (42.6% to 62.1%)
Lip and oral cavity cancer	47.0% (32.1% to 61.5%)	51.5% (36.3% to 65.6%)	46.7% (32.3% to 60.4%)	51.3% (36.2% to 65.5%)
Nasopharynx cancer	48.0% (35.9% to 59.2%)	48.0% (36.2% to 59.1%)	47.1% (35.5% to 57.8%)	48.0% (36.2% to 59.1%)

Supplementary Results Table 1. Age-standardised PAFs attributable to the joint distribution of all risk factors for all ages, both sexes combined for each GBD level 1 and level 2 cause and all causes in 2016 for Antigua and Barbuda. Results are shown for deaths, YLLs, YLDs, and DALYs
Data in parenthesis are 95% uncertainty intervals. PAFs=population attributable fractions. GBD=Global Burden of Disease. YLLs=years of life lost. YLDs=years live with disability. DALYs=disability-adjusted life-years

	Deaths 2016	YLLs 2016	YLDs 2016	DALYs 2016
Other pharynx cancer	37.4% (20.7% to 53.7%)	40.2% (22.9% to 56.8%)	38.8% (21.6% to 55.3%)	40.2% (22.9% to 56.8%)
Gallbladder and biliary tract cancer	24.2% (13.5% to 36.2%)	25.3% (14.2% to 37.9%)	24.3% (13.6% to 36.5%)	25.3% (14.2% to 37.8%)
Pancreatic cancer	20.5% (11.5% to 32.2%)	21.4% (12.4% to 32.8%)	20.8% (11.7% to 32.4%)	21.4% (12.4% to 32.8%)
Ovarian cancer	13.2% (4.2% to 24.9%)	11.9% (3.7% to 22.5%)	10.7% (3.3% to 20.6%)	11.9% (3.7% to 22.4%)
Kidney cancer	26.0% (17.2% to 36.3%)	25.3% (17.0% to 34.8%)	26.0% (17.3% to 35.9%)	25.3% (17.0% to 34.9%)
Bladder cancer	15.6% (7.3% to 26.7%)	16.8% (8.5% to 27.8%)	16.4% (8.5% to 27.2%)	16.8% (8.5% to 27.8%)
Thyroid cancer	14.4% (7.8% to 21.9%)	15.0% (8.2% to 22.6%)	14.8% (8.1% to 22.0%)	15.0% (8.2% to 22.5%)
Mesothelioma	70.0% (62.7% to 76.3%)	54.9% (46.5% to 62.6%)	65.0% (57.9% to 71.7%)	55.1% (46.7% to 62.8%)
Non-Hodgkin's lymphoma	7.4% (3.2% to 12.7%)	7.1% (3.1% to 12.1%)	7.4% (3.2% to 12.6%)	7.2% (3.1% to 12.1%)
Multiple myeloma	8.8% (3.9% to 15.3%)	9.1% (4.1% to 15.8%)	9.0% (4.1% to 15.4%)	9.1% (4.1% to 15.8%)
Leukaemia	12.3% (7.6% to 17.5%)	10.9% (6.9% to 15.3%)	11.7% (7.3% to 16.7%)	10.9% (6.9% to 15.3%)
**Cardiovascular diseases**	80.5% (77.7% to 83.0%)	80.2% (78.0% to 82.3%)	69.1% (66.1% to 72.1%)	79.1% (76.9% to 81.2%)
Rheumatic heart disease	24.7% (15.7% to 42.2%)	21.0% (14.5% to 32.1%)	25.3% (17.1% to 38.4%)	21.0% (14.6% to 32.1%)
Ischaemic heart disease	92.0% (88.1% to 95.2%)	93.7% (91.2% to 95.8%)	90.6% (87.7% to 93.2%)	93.5% (90.9% to 95.6%)
Cerebrovascular disease	81.5% (76.0% to 86.3%)	84.1% (80.2% to 87.5%)	82.3% (77.4% to 87.2%)	83.9% (79.9% to 87.4%)
Hypertensive heart disease	100.0% (100.0% to 100.0%)	100.0% (100.0% to 100.0%)	100.0% (100.0% to 100.0%)	100.0% (100.0% to 100.0%)
Cardiomyopathy and myocarditis	30.1% (24.5% to 36.3%)	31.1% (25.6% to 36.7%)	28.1% (23.3% to 33.4%)	30.9% (25.5% to 36.5%)
Atrial fibrillation and flutter	45.9% (37.6% to 53.6%)	49.8% (42.6% to 56.4%)	54.9% (49.3% to 60.6%)	52.8% (46.6% to 58.7%)
Aortic aneurysm	37.8% (30.3% to 44.9%)	40.7% (34.1% to 46.6%)	-	40.7% (34.1% to 46.6%)
Peripheral vascular disease	48.6% (41.4% to 56.5%)	55.2% (49.3% to 61.2%)	59.6% (54.5% to 64.8%)	56.7% (51.2% to 62.0%)
Endocarditis	37.3% (29.2% to 46.7%)	34.9% (27.7% to 42.3%)	35.6% (27.9% to 43.9%)	34.9% (27.7% to 42.3%)

Supplementary Results Table 1. Age-standardised PAFs attributable to the joint distribution of all risk factors for all ages, both sexes combined for each GBD level 1 and level 2 cause and all causes in 2016 for Antigua and Barbuda. Results are shown for deaths, YLLs, YLDs, and DALYs
Data in parenthesis are 95% uncertainty intervals. PAFs=population attributable fractions. GBD=Global Burden of Disease. YLLs=years of life lost. YLDs=years live with disability. DALYs=disability-adjusted life-years

	Deaths 2016	YLLs 2016	YLDs 2016	DALYs 2016
Other cardiovascular and circulatory diseases	40.6% (35.7% to 45.8%)	40.7% (36.5% to 45.3%)	38.7% (34.0% to 43.7%)	40.3% (36.2% to 44.8%)
**Chronic respiratory diseases**	36.2% (28.8% to 43.2%)	36.1% (29.8% to 42.1%)	31.4% (23.3% to 41.0%)	33.0% (26.4% to 40.2%)
Chronic obstructive pulmonary disease	46.8% (36.0% to 57.5%)	50.0% (40.3% to 59.5%)	48.3% (39.0% to 58.1%)	49.5% (39.9% to 58.9%)
Pneumoconiosis	100.0% (100.0% to 100.0%)	100.0% (100.0% to 100.0%)	100.0% (100.0% to 100.0%)	100.0% (100.0% to 100.0%)
Asthma	36.9% (26.4% to 48.5%)	38.2% (28.9% to 48.6%)	29.6% (20.3% to 40.8%)	30.6% (21.5% to 41.2%)
Other chronic respiratory diseases	3.9% (2.0% to 6.2%)	4.4% (2.4% to 7.0%)	1.6% (0.5% to 3.2%)	4.2% (2.2% to 6.7%)
**Cirrhosis and other chronic liver diseases**	66.9% (64.2% to 69.5%)	66.8% (64.3% to 69.2%)	54.4% (50.7% to 58.2%)	66.2% (63.7% to 68.5%)
Cirrhosis and other chronic liver diseases due to hepatitis B	5.8% (2.6% to 10.1%)	7.5% (3.3% to 13.0%)	5.9% (2.6% to 10.7%)	7.4% (3.3% to 12.9%)
Cirrhosis and other chronic liver diseases due to hepatitis C	84.3% (80.4% to 87.7%)	88.1% (86.5% to 89.4%)	84.4% (80.6% to 87.4%)	88.0% (86.2% to 89.4%)
Cirrhosis and other chronic liver diseases due to alcohol use	100.0% (100.0% to 100.0%)	100.0% (100.0% to 100.0%)	100.0% (100.0% to 100.0%)	100.0% (100.0% to 100.0%)
**Digestive diseases**	6.2% (4.3% to 8.7%)	6.9% (4.6% to 10.0%)	2.4% (1.6% to 3.4%)	5.5% (3.8% to 7.8%)
Peptic ulcer disease	2.0% (1.3% to 3.0%)	2.6% (1.7% to 3.8%)	1.7% (1.1% to 2.6%)	2.3% (1.5% to 3.3%)
Gallbladder and biliary diseases	44.5% (29.7% to 59.6%)	45.7% (31.1% to 59.8%)	49.8% (34.4% to 64.5%)	46.4% (31.8% to 60.4%)
Pancreatitis	17.5% (2.8% to 38.3%)	20.4% (3.7% to 42.5%)	13.2% (1.4% to 28.2%)	20.2% (3.7% to 42.2%)
**Neurological disorders**	20.9% (10.2% to 32.8%)	19.0% (10.7% to 28.5%)	3.6% (1.8% to 5.7%)	8.6% (4.7% to 13.0%)
Alzheimer's disease and other dementias	24.4% (11.0% to 39.1%)	25.7% (11.9% to 40.7%)	25.8% (12.0% to 40.7%)	25.8% (11.9% to 40.7%)
Parkinson's disease	-0.8% (-1.0% to -0.6%)	-1.0% (-1.3% to -0.8%)	-1.2% (-1.5% to -0.9%)	-1.1% (-1.3% to -0.8%)
Epilepsy	16.3% (6.7% to 26.4%)	16.8% (7.2% to 26.7%)	13.5% (5.6% to 22.1%)	15.2% (6.6% to 24.7%)
Multiple sclerosis	1.0% (0.6% to 1.6%)	1.0% (0.6% to 1.6%)	1.0% (0.6% to 1.6%)	1.0% (0.6% to 1.6%)
**Mental and substance use disorders**	99.9% (99.9% to 99.9%)	99.8% (99.8% to 99.9%)	19.1% (17.0% to 21.3%)	25.7% (22.7% to 28.9%)
Alcohol use disorders	100.0% (100.0% to 100.0%)	100.0% (100.0% to 100.0%)	100.0% (100.0% to 100.0%)	100.0% (100.0% to 100.0%)

**Supplementary Results Table 1. Age-standardised PAFs attributable to the joint distribution of all risk factors for all ages, both sexes combined for each GBD level 1 and level 2 cause and all causes in 2016 for Antigua and Barbuda. Results are shown for deaths, YLLs, YLDs, and DALYs** Data in parenthesis are 95% uncertainty intervals. PAFs=population attributable fractions. GBD=Global Burden of Disease. YLLs=years of life lost. YLDs=years live with disability. DALYs=disability-adjusted life-years				
	**Deaths 2016**	**YLLs 2016**	**YLDs 2016**	**DALYs 2016**
Drug use disorders	100.0% (100.0% to 100.0%)	100.0% (100.0% to 100.0%)	100.0% (100.0% to 100.0%)	100.0% (100.0% to 100.0%)
Depressive disorders	-	-	6.5% (5.2% to 8.1%)	6.5% (5.2% to 8.1%)
Idiopathic developmental intellectual disability	-	-	64.0% (32.9% to 84.8%)	64.0% (32.9% to 84.8%)
**Diabetes, urogenital, blood, and endocrine diseases**	86.6% (84.4% to 87.6%)	83.3% (80.1% to 84.6%)	73.8% (69.8% to 77.1%)	79.9% (77.1% to 81.6%)
Diabetes mellitus	100.0% (100.0% to 100.0%)	100.0% (100.0% to 100.0%)	100.0% (100.0% to 100.0%)	100.0% (100.0% to 100.0%)
Chronic kidney disease	100.0% (100.0% to 100.0%)	100.0% (100.0% to 100.0%)	100.0% (100.0% to 100.0%)	100.0% (100.0% to 100.0%)
**Musculoskeletal disorders**	0.1% (0.0% to 0.2%)	0.1% (0.0% to 0.1%)	12.9% (10.0% to 16.0%)	12.4% (9.6% to 15.4%)
Rheumatoid arthritis	0.5% (0.2% to 0.9%)	0.6% (0.2% to 1.1%)	0.7% (0.2% to 1.2%)	0.7% (0.2% to 1.2%)
Osteoarthritis	-	-	29.6% (16.5% to 42.4%)	29.6% (16.5% to 42.4%)
Low back and neck pain	-	-	15.7% (13.1% to 18.5%)	15.7% (13.1% to 18.5%)
Gout	-	-	52.7% (37.4% to 68.3%)	52.7% (37.4% to 68.3%)
**Other non-communicable diseases**	0.2% (0.1% to 0.3%)	0.3% (0.2% to 0.5%)	5.0% (4.2% to 6.0%)	4.1% (3.4% to 4.8%)
Sense organ diseases	-	-	12.3% (10.7% to 14.2%)	12.3% (10.7% to 14.2%)
Sudden infant death syndrome	7.2% (4.3% to 11.0%)	7.2% (4.3% to 11.0%)	-	7.2% (4.3% to 11.0%)
**Transport injuries**	24.7% (18.6% to 32.1%)	19.6% (13.3% to 27.4%)	28.4% (22.4% to 35.5%)	21.1% (15.0% to 28.8%)
Road injuries	23.7% (17.6% to 31.3%)	19.3% (12.9% to 27.2%)	28.7% (22.7% to 36.0%)	21.0% (14.8% to 28.9%)
Other transport injuries	31.7% (26.9% to 37.4%)	21.8% (16.3% to 28.4%)	23.6% (18.4% to 29.9%)	22.0% (16.6% to 28.6%)
**Unintentional injuries**	12.5% (9.9% to 16.2%)	9.3% (6.5% to 13.4%)	16.0% (13.6% to 19.0%)	11.8% (9.1% to 15.4%)
Falls	50.1% (46.1% to 54.3%)	26.1% (23.0% to 30.2%)	23.4% (21.1% to 26.5%)	23.9% (21.6% to 26.9%)
Drowning	9.9% (5.9% to 16.0%)	9.3% (5.5% to 14.9%)	11.5% (7.3% to 16.6%)	9.4% (5.5% to 15.0%)
Fire, heat, and hot substances	7.2% (4.6% to 10.7%)	8.4% (5.6% to 12.4%)	14.1% (9.6% to 19.7%)	10.3% (6.9% to 14.7%)

Supplementary Results Table 1. Age-standardised PAFs attributable to the joint distribution of all risk factors for all ages, both sexes combined for each GBD level 1 and level 2 cause and all causes in 2016 for Antigua and Barbuda. Results are shown for deaths, YLLs, YLDs, and DALYs
Data in parenthesis are 95% uncertainty intervals. PAFs=population attributable fractions. GBD=Global Burden of Disease. YLLs=years of life lost. YLDs=years live with disability. DALYs=disability-adjusted life-years

	Deaths 2016	YLLs 2016	YLDs 2016	DALYs 2016
Poisonings	11.5% (8.0% to 16.1%)	11.5% (7.8% to 16.1%)	14.0% (9.5% to 19.3%)	12.0% (8.2% to 16.5%)
Exposure to mechanical forces	12.7% (8.9% to 17.9%)	10.9% (7.0% to 16.5%)	14.5% (11.9% to 18.6%)	11.9% (8.4% to 16.7%)
Animal contact	11.7% (9.3% to 14.9%)	9.9% (7.3% to 13.5%)	9.8% (6.8% to 13.8%)	9.9% (7.1% to 13.4%)
Foreign body	3.7% (2.5% to 5.7%)	3.9% (2.4% to 5.9%)	6.4% (4.2% to 9.4%)	4.6% (3.0% to 6.8%)
Other unintentional injuries	10.7% (6.5% to 16.8%)	10.9% (6.8% to 17.0%)	12.6% (8.5% to 17.7%)	12.2% (8.2% to 17.3%)
**Self-harm and interpersonal violence**	21.4% (14.7% to 28.4%)	20.9% (14.0% to 28.3%)	27.1% (23.4% to 30.4%)	21.6% (15.0% to 28.4%)
Self-harm	17.7% (6.2% to 30.4%)	18.6% (6.8% to 31.6%)	14.0% (4.6% to 24.3%)	18.4% (6.7% to 31.5%)
Interpersonal violence	23.2% (15.5% to 31.7%)	22.0% (14.3% to 30.6%)	28.0% (24.5% to 31.2%)	22.8% (15.6% to 30.5%)
**Forces of nature, conflict and terrorism, and executions and police conflict**	0.0% (0.0% to 0.0%)	0.0% (0.0% to 0.0%)	0.0% (0.0% to 0.0%)	0.0% (0.0% to 0.0%)
Forces of nature, conflict and terrorism, and state actor violence	0.0% (0.0% to 0.0%)	0.0% (0.0% to 0.0%)	0.0% (0.0% to 0.0%)	0.0% (0.0% to 0.0%)

Supplementary Results Table 1. Age-standardised PAFs attributable to the joint distribution of all risk factors for all ages, both sexes combined for each GBD level 1 and level 2 cause and all causes in 2016 for Argentina. Results are shown for deaths, YLLs, YLDs, and DALYs
Data in parenthesis are 95% uncertainty intervals. PAFs=population attributable fractions. GBD=Global Burden of Disease. YLLs=years of life lost. YLDs=years live with disability. DALYs=disability-adjusted life-years

	Deaths 2016	YLLs 2016	YLDs 2016	DALYs 2016
**All causes**	57.4% (55.5% to 59.3%)	54.1% (52.5% to 55.9%)	22.4% (21.0% to 23.8%)	41.0% (38.3% to 43.7%)
**HIV/AIDS and tuberculosis**	78.4% (74.2% to 82.0%)	79.0% (75.9% to 81.8%)	88.0% (84.9% to 90.5%)	80.5% (77.5% to 83.1%)
Tuberculosis	52.6% (38.4% to 63.9%)	52.9% (40.7% to 62.9%)	48.0% (35.1% to 58.3%)	52.6% (40.5% to 62.7%)
HIV/AIDS	88.6% (86.9% to 89.9%)	86.3% (84.5% to 87.7%)	90.8% (89.1% to 92.3%)	87.1% (85.3% to 88.7%)
**Diarrhoea, lower respiratory infections, and other common infectious diseases**	40.0% (31.8% to 48.7%)	46.5% (39.9% to 53.9%)	19.3% (13.5% to 26.0%)	42.6% (36.6% to 49.5%)
Diarrhoeal diseases	52.4% (32.8% to 75.3%)	68.4% (54.2% to 84.3%)	60.9% (43.5% to 80.3%)	64.6% (49.3% to 82.0%)
Lower respiratory infections	40.9% (32.2% to 50.2%)	49.5% (41.7% to 58.0%)	49.0% (41.0% to 57.8%)	49.5% (41.7% to 58.0%)
Upper respiratory infections	1.1% (0.1% to 3.7%)	2.7% (0.2% to 9.0%)	0.0% (0.0% to 0.0%)	0.0% (0.0% to 0.0%)
Otitis media	5.7% (3.6% to 8.1%)	10.1% (7.2% to 13.3%)	7.4% (4.9% to 9.8%)	7.4% (4.9% to 9.8%)
Meningitis	1.4% (1.1% to 1.9%)	2.7% (2.0% to 3.5%)	0.0% (0.0% to 0.0%)	2.5% (1.9% to 3.3%)
Encephalitis	0.5% (0.4% to 0.6%)	0.9% (0.7% to 1.2%)	0.0% (0.0% to 0.0%)	0.8% (0.6% to 1.0%)
Measles	21.8% (6.0% to 58.4%)	23.0% (6.4% to 61.9%)	12.0% (0.0% to 58.6%)	23.0% (6.4% to 61.9%)
**Maternal disorders**	5.4% (2.4% to 8.6%)	5.3% (2.4% to 8.4%)	3.6% (2.0% to 5.4%)	5.0% (2.3% to 7.8%)
Maternal haemorrhage	11.7% (4.2% to 19.1%)	11.7% (4.2% to 18.9%)	11.5% (4.2% to 18.7%)	11.7% (4.2% to 18.9%)
Maternal sepsis and other pregnancy related infections	11.8% (4.2% to 19.1%)	11.7% (4.2% to 18.9%)	11.2% (4.0% to 18.3%)	11.6% (4.2% to 18.9%)
Maternal abortion, miscarriage, and ectopic pregnancy	6.0% (3.5% to 9.0%)	6.2% (3.5% to 9.4%)	6.7% (3.4% to 10.3%)	6.4% (3.5% to 9.5%)
**Neonatal disorders**	79.1% (76.7% to 81.3%)	79.1% (76.8% to 81.3%)	0.1% (0.1% to 0.1%)	68.9% (64.8% to 72.4%)
Neonatal preterm birth complications	92.5% (90.3% to 94.4%)	92.5% (90.4% to 94.4%)	0.2% (0.1% to 0.2%)	81.0% (75.5% to 85.4%)
Neonatal encephalopathy due to birth asphyxia and trauma	60.7% (56.4% to 64.7%)	60.8% (56.5% to 64.7%)	0.0% (0.0% to 0.0%)	52.7% (43.9% to 59.9%)
Neonatal sepsis and other neonatal infections	57.4% (53.1% to 61.2%)	57.4% (53.1% to 61.3%)	0.0% (0.0% to 0.0%)	49.9% (41.5% to 56.2%)
Hemolytic disease and other neonatal jaundice	65.5% (61.0% to 69.0%)	65.6% (61.1% to 69.0%)	0.0% (0.0% to 0.0%)	11.2% (6.6% to 17.0%)

Supplementary Results Table 1. Age-standardised PAFs attributable to the joint distribution of all risk factors for all ages, both sexes combined for each GBD level 1 and level 2 cause and all causes in 2016 for Argentina. Results are shown for deaths, YLLs, YLDs, and DALYs
Data in parenthesis are 95% uncertainty intervals. PAFs=population attributable fractions. GBD=Global Burden of Disease. YLLs=years of life lost. YLDs=years live with disability. DALYs=disability-adjusted life-years

	Deaths 2016	YLLs 2016	YLDs 2016	DALYs 2016
Other neonatal disorders	59.0% (54.5% to 63.3%)	59.0% (54.5% to 63.3%)	0.0% (0.0% to 0.0%)	57.3% (52.8% to 61.7%)
**Nutritional deficiencies**	6.4% (4.8% to 8.5%)	28.5% (22.9% to 34.9%)	91.4% (89.2% to 93.2%)	73.8% (66.7% to 80.2%)
Protein-energy malnutrition	6.6% (4.8% to 8.9%)	30.4% (24.3% to 37.9%)	98.3% (96.8% to 99.2%)	33.8% (27.2% to 40.8%)
Vitamin A deficiency	-	-	0.0% (0.0% to 0.0%)	0.0% (0.0% to 0.0%)
Iron-deficiency anaemia	100.0% (100.0% to 100.0%)	100.0% (100.0% to 100.0%)	100.0% (100.0% to 100.0%)	100.0% (100.0% to 100.0%)
**Other communicable, maternal, neonatal, and nutritional diseases**	8.2% (4.7% to 12.7%)	6.6% (2.6% to 13.2%)	64.0% (57.8% to 70.6%)	18.5% (8.4% to 33.1%)
Sexually transmitted diseases excluding HIV	29.9% (6.6% to 95.9%)	20.0% (2.8% to 90.8%)	100.0% (100.0% to 100.0%)	37.6% (10.1% to 97.6%)
Hepatitis	4.8% (3.5% to 6.5%)	4.9% (3.6% to 6.7%)	1.6% (1.0% to 2.4%)	4.5% (3.3% to 6.2%)
**Neoplasms**	43.3% (40.1% to 46.6%)	43.6% (40.7% to 46.5%)	39.1% (35.9% to 42.5%)	43.5% (40.6% to 46.4%)
Oesophageal cancer	80.0% (70.6% to 88.0%)	82.2% (73.4% to 89.3%)	80.6% (71.3% to 88.3%)	82.1% (73.4% to 89.3%)
Stomach cancer	19.3% (10.6% to 29.0%)	20.2% (11.5% to 29.5%)	19.5% (11.0% to 28.9%)	20.1% (11.5% to 29.5%)
Liver cancer	67.0% (60.0% to 73.8%)	70.9% (64.5% to 77.1%)	68.0% (61.1% to 74.6%)	70.9% (64.4% to 77.0%)
Larynx cancer	86.0% (78.5% to 91.8%)	86.9% (79.7% to 92.3%)	86.7% (79.4% to 92.2%)	86.8% (79.7% to 92.3%)
Tracheal, bronchus, and lung cancer	85.3% (82.8% to 87.6%)	85.9% (83.5% to 88.0%)	85.1% (82.5% to 87.6%)	85.9% (83.5% to 88.0%)
Breast cancer	35.1% (28.2% to 42.7%)	34.3% (27.8% to 41.4%)	34.4% (27.8% to 41.7%)	34.3% (27.8% to 41.4%)
Cervical cancer	100.0% (100.0% to 100.0%)	100.0% (100.0% to 100.0%)	100.0% (100.0% to 100.0%)	100.0% (100.0% to 100.0%)
Uterine cancer	44.9% (30.8% to 59.5%)	45.7% (31.3% to 60.4%)	45.8% (31.3% to 60.5%)	45.7% (31.3% to 60.4%)
Prostate cancer	4.9% (3.5% to 6.5%)	5.4% (3.9% to 7.1%)	5.5% (4.0% to 7.3%)	5.4% (3.9% to 7.1%)
Colon and rectum cancer	58.2% (48.9% to 66.1%)	59.1% (50.3% to 66.7%)	58.8% (49.6% to 66.4%)	59.1% (50.3% to 66.7%)
Lip and oral cavity cancer	77.8% (69.2% to 85.3%)	80.5% (72.7% to 87.3%)	77.8% (69.5% to 85.1%)	80.4% (72.5% to 87.2%)
Nasopharynx cancer	78.9% (72.4% to 84.3%)	78.1% (72.3% to 83.1%)	76.5% (70.7% to 81.9%)	78.0% (72.3% to 83.1%)

Supplementary Results Table 1. Age-standardised PAFs attributable to the joint distribution of all risk factors for all ages, both sexes combined for each GBD level 1 and level 2 cause and all causes in 2016 for Argentina. Results are shown for deaths, YLLs, YLDs, and DALYs
Data in parenthesis are 95% uncertainty intervals. PAFs=population attributable fractions. GBD=Global Burden of Disease. YLLs=years of life lost. YLDs=years live with disability. DALYs=disability-adjusted life-years

	Deaths 2016	YLLs 2016	YLDs 2016	DALYs 2016
Other pharynx cancer	55.5% (38.7% to 69.5%)	57.4% (40.7% to 71.2%)	56.5% (39.8% to 70.3%)	57.4% (40.7% to 71.1%)
Gallbladder and biliary tract cancer	23.6% (13.3% to 36.3%)	23.8% (13.5% to 36.6%)	23.7% (13.3% to 36.3%)	23.8% (13.5% to 36.6%)
Pancreatic cancer	31.1% (23.5% to 39.6%)	32.8% (25.2% to 41.0%)	31.6% (24.1% to 39.9%)	32.8% (25.2% to 41.0%)
Ovarian cancer	13.1% (6.1% to 21.6%)	11.4% (4.9% to 18.8%)	11.1% (4.9% to 18.4%)	11.3% (4.9% to 18.8%)
Kidney cancer	38.4% (27.9% to 49.2%)	38.7% (28.3% to 49.0%)	38.1% (28.0% to 48.3%)	38.7% (28.3% to 48.9%)
Bladder cancer	34.2% (25.1% to 43.8%)	36.7% (27.4% to 46.6%)	36.1% (26.9% to 45.5%)	36.7% (27.4% to 46.5%)
Thyroid cancer	15.7% (8.1% to 25.5%)	16.2% (8.2% to 26.2%)	14.8% (8.0% to 23.4%)	16.1% (8.2% to 26.0%)
Mesothelioma	92.0% (89.6% to 93.8%)	85.8% (82.2% to 88.7%)	90.0% (87.4% to 92.2%)	85.9% (82.3% to 88.8%)
Non-Hodgkin's lymphoma	7.8% (3.3% to 13.5%)	7.5% (3.2% to 12.7%)	7.7% (3.3% to 13.0%)	7.5% (3.2% to 12.7%)
Multiple myeloma	9.0% (4.0% to 15.6%)	9.2% (4.1% to 15.7%)	9.1% (4.0% to 15.5%)	9.2% (4.1% to 15.7%)
Leukaemia	19.2% (12.4% to 26.2%)	15.5% (10.3% to 21.1%)	18.3% (11.9% to 24.8%)	15.6% (10.3% to 21.1%)
**Cardiovascular diseases**	81.7% (79.2% to 83.8%)	82.3% (80.4% to 83.9%)	76.0% (72.9% to 78.7%)	81.6% (79.7% to 83.3%)
Rheumatic heart disease	30.0% (17.9% to 53.0%)	28.7% (19.2% to 44.3%)	28.7% (19.5% to 44.1%)	28.7% (19.2% to 44.4%)
Ischaemic heart disease	92.9% (89.3% to 95.7%)	94.0% (91.8% to 95.8%)	91.4% (88.9% to 93.8%)	93.9% (91.7% to 95.8%)
Cerebrovascular disease	84.8% (80.0% to 89.2%)	86.8% (83.8% to 89.6%)	85.7% (81.2% to 89.5%)	86.6% (83.4% to 89.5%)
Hypertensive heart disease	100.0% (100.0% to 100.0%)	100.0% (100.0% to 100.0%)	100.0% (100.0% to 100.0%)	100.0% (100.0% to 100.0%)
Cardiomyopathy and myocarditis	41.9% (33.3% to 52.2%)	39.7% (32.9% to 46.9%)	41.6% (34.6% to 49.5%)	39.8% (33.1% to 47.1%)
Atrial fibrillation and flutter	57.2% (49.2% to 65.6%)	60.7% (53.5% to 67.7%)	66.0% (60.5% to 71.5%)	63.8% (57.9% to 70.0%)
Aortic aneurysm	46.4% (38.6% to 54.1%)	51.4% (44.9% to 57.3%)	-	51.4% (44.9% to 57.3%)
Peripheral vascular disease	54.2% (47.3% to 62.0%)	60.3% (54.7% to 66.1%)	61.6% (56.0% to 66.9%)	61.1% (55.5% to 66.5%)
Endocarditis	41.9% (32.0% to 54.7%)	39.2% (31.5% to 47.7%)	42.4% (32.9% to 52.7%)	39.3% (31.6% to 47.7%)

Supplementary Results Table 1. Age-standardised PAFs attributable to the joint distribution of all risk factors for all ages, both sexes combined for each GBD level 1 and level 2 cause and all causes in 2016 for Argentina. Results are shown for deaths, YLLs, YLDs, and DALYs
Data in parenthesis are 95% uncertainty intervals. PAFs=population attributable fractions. GBD=Global Burden of Disease. YLLs=years of life lost. YLDs=years live with disability. DALYs=disability-adjusted life-years

	Deaths 2016	YLLs 2016	YLDs 2016	DALYs 2016
Other cardiovascular and circulatory diseases	48.9% (43.2% to 54.5%)	50.6% (45.9% to 54.8%)	51.9% (46.8% to 56.7%)	50.8% (46.1% to 55.3%)
**Chronic respiratory diseases**	64.8% (59.5% to 69.1%)	63.6% (59.2% to 67.3%)	39.4% (32.5% to 47.3%)	55.2% (50.5% to 59.8%)
Chronic obstructive pulmonary disease	73.9% (68.6% to 78.6%)	75.2% (70.8% to 79.3%)	73.1% (68.6% to 77.5%)	75.0% (70.5% to 79.0%)
Pneumoconiosis	100.0% (100.0% to 100.0%)	100.0% (100.0% to 100.0%)	100.0% (100.0% to 100.0%)	100.0% (100.0% to 100.0%)
Asthma	43.3% (33.0% to 55.0%)	45.8% (36.7% to 56.2%)	32.5% (24.8% to 41.5%)	33.8% (26.0% to 43.1%)
Other chronic respiratory diseases	17.8% (12.9% to 23.0%)	16.1% (11.7% to 21.0%)	7.8% (5.7% to 10.7%)	15.7% (11.4% to 20.4%)
**Cirrhosis and other chronic liver diseases**	60.4% (57.1% to 63.4%)	63.2% (60.3% to 66.0%)	51.6% (47.5% to 55.8%)	62.7% (59.8% to 65.5%)
Cirrhosis and other chronic liver diseases due to hepatitis B	1.6% (0.7% to 2.9%)	2.1% (1.0% to 3.8%)	1.9% (0.8% to 3.4%)	2.1% (1.0% to 3.8%)
Cirrhosis and other chronic liver diseases due to hepatitis C	72.3% (66.0% to 77.9%)	80.7% (76.4% to 84.2%)	77.4% (72.5% to 81.3%)	80.5% (76.3% to 84.0%)
Cirrhosis and other chronic liver diseases due to alcohol use	100.0% (100.0% to 100.0%)	100.0% (100.0% to 100.0%)	100.0% (100.0% to 100.0%)	100.0% (100.0% to 100.0%)
**Digestive diseases**	10.3% (7.1% to 13.7%)	12.0% (8.1% to 15.9%)	4.8% (3.6% to 6.2%)	10.8% (7.5% to 14.3%)
Peptic ulcer disease	11.6% (8.2% to 15.5%)	15.0% (10.7% to 19.9%)	13.4% (9.4% to 17.9%)	14.8% (10.5% to 19.6%)
Gallbladder and biliary diseases	44.9% (30.3% to 60.0%)	45.4% (31.2% to 59.7%)	48.6% (34.3% to 63.9%)	45.7% (31.6% to 60.1%)
Pancreatitis	28.3% (7.7% to 47.6%)	31.8% (11.8% to 50.5%)	31.6% (12.1% to 50.3%)	31.8% (11.8% to 50.5%)
**Neurological disorders**	19.7% (9.5% to 32.2%)	17.9% (9.7% to 27.5%)	3.9% (2.0% to 6.3%)	8.1% (4.7% to 12.3%)
Alzheimer's disease and other dementias	25.4% (12.4% to 40.5%)	26.7% (13.5% to 41.5%)	26.7% (13.5% to 41.5%)	26.7% (13.5% to 41.5%)
Parkinson's disease	-6.2% (-7.4% to -5.0%)	-8.2% (-9.9% to -6.6%)	-10.0% (-11.9% to -7.9%)	-8.5% (-10.3% to -6.9%)
Epilepsy	31.4% (20.1% to 41.6%)	29.7% (19.3% to 38.8%)	25.6% (16.2% to 34.3%)	26.9% (17.3% to 35.9%)
Multiple sclerosis	9.6% (6.2% to 13.5%)	10.2% (6.6% to 14.2%)	9.3% (6.0% to 12.9%)	9.6% (6.3% to 13.3%)
**Mental and substance use disorders**	99.7% (99.6% to 99.7%)	99.4% (99.2% to 99.5%)	20.4% (18.2% to 22.9%)	23.5% (21.1% to 26.1%)
Alcohol use disorders	100.0% (100.0% to 100.0%)	100.0% (100.0% to 100.0%)	100.0% (100.0% to 100.0%)	100.0% (100.0% to 100.0%)

Supplementary Results Table 1. Age-standardised PAFs attributable to the joint distribution of all risk factors for all ages, both sexes combined for each GBD level 1 and level 2 cause and all causes in 2016 for Argentina. Results are shown for deaths, YLLs, YLDs, and DALYs
Data in parenthesis are 95% uncertainty intervals. PAFs=population attributable fractions. GBD=Global Burden of Disease. YLLs=years of life lost. YLDs=years live with disability. DALYs=disability-adjusted life-years

	Deaths 2016	YLLs 2016	YLDs 2016	DALYs 2016
Drug use disorders	100.0% (100.0% to 100.0%)	100.0% (100.0% to 100.0%)	100.0% (100.0% to 100.0%)	100.0% (100.0% to 100.0%)
Depressive disorders	-	-	7.0% (5.8% to 8.4%)	7.0% (5.8% to 8.4%)
Idiopathic developmental intellectual disability	-	-	63.7% (32.8% to 83.5%)	63.7% (32.8% to 83.5%)
**Diabetes, urogenital, blood, and endocrine diseases**	78.6% (76.0% to 79.9%)	77.6% (73.5% to 79.0%)	66.4% (62.2% to 70.3%)	72.9% (70.2% to 75.0%)
Diabetes mellitus	100.0% (100.0% to 100.0%)	100.0% (100.0% to 100.0%)	100.0% (100.0% to 100.0%)	100.0% (100.0% to 100.0%)
Chronic kidney disease	100.0% (100.0% to 100.0%)	100.0% (100.0% to 100.0%)	100.0% (100.0% to 100.0%)	100.0% (100.0% to 100.0%)
**Musculoskeletal disorders**	1.5% (0.6% to 2.5%)	1.3% (0.5% to 2.3%)	16.3% (13.4% to 19.3%)	16.1% (13.2% to 19.0%)
Rheumatoid arthritis	4.9% (2.0% to 8.3%)	5.8% (2.3% to 9.5%)	5.8% (2.4% to 9.6%)	5.8% (2.4% to 9.6%)
Osteoarthritis	-	-	28.7% (16.0% to 41.6%)	28.7% (16.0% to 41.6%)
Low back and neck pain	-	-	21.7% (18.5% to 25.0%)	21.7% (18.5% to 25.0%)
Gout	-	-	52.8% (35.5% to 70.4%)	52.8% (35.5% to 70.4%)
**Other non-communicable diseases**	0.3% (0.2% to 0.6%)	0.5% (0.3% to 0.7%)	3.6% (3.1% to 4.1%)	2.8% (2.4% to 3.2%)
Sense organ diseases	-	-	9.7% (8.7% to 10.9%)	9.7% (8.7% to 10.9%)
Sudden infant death syndrome	6.2% (4.2% to 8.7%)	6.2% (4.2% to 8.7%)	-	6.2% (4.2% to 8.7%)
**Transport injuries**	47.3% (37.7% to 57.9%)	44.5% (34.3% to 55.5%)	50.6% (42.2% to 59.7%)	45.3% (35.3% to 56.2%)
Road injuries	47.1% (37.5% to 57.7%)	44.3% (34.1% to 55.3%)	52.1% (43.5% to 61.5%)	45.3% (35.2% to 56.3%)
Other transport injuries	50.0% (40.1% to 60.5%)	47.4% (36.5% to 58.8%)	40.8% (33.0% to 49.1%)	45.9% (36.1% to 56.2%)
**Unintentional injuries**	27.3% (22.6% to 33.7%)	23.0% (17.6% to 30.1%)	30.8% (25.6% to 38.1%)	26.3% (20.8% to 33.2%)
Falls	64.4% (59.4% to 69.8%)	46.5% (39.3% to 55.7%)	33.5% (28.7% to 39.8%)	36.7% (31.4% to 43.5%)
Drowning	27.7% (20.4% to 37.1%)	24.0% (17.7% to 32.4%)	29.1% (21.6% to 38.2%)	24.1% (17.8% to 32.5%)
Fire, heat, and hot substances	22.0% (16.0% to 30.3%)	24.7% (18.5% to 32.8%)	29.2% (21.9% to 37.9%)	26.4% (19.6% to 34.8%)

Supplementary Results Table 1. Age-standardised PAFs attributable to the joint distribution of all risk factors for all ages, both sexes combined for each GBD level 1 and level 2 cause and all causes in 2016 for Argentina. Results are shown for deaths, YLLs, YLDs, and DALYs
Data in parenthesis are 95% uncertainty intervals. PAFs=population attributable fractions. GBD=Global Burden of Disease. YLLs=years of life lost. YLDs=years live with disability. DALYs=disability-adjusted life-years

	Deaths 2016	YLLs 2016	YLDs 2016	DALYs 2016
Poisonings	25.3% (18.6% to 34.4%)	23.6% (17.2% to 32.6%)	29.1% (21.8% to 37.8%)	24.5% (18.0% to 33.3%)
Exposure to mechanical forces	31.4% (23.7% to 41.8%)	28.9% (21.3% to 39.1%)	36.2% (28.2% to 46.7%)	32.5% (24.6% to 42.6%)
Animal contact	22.1% (16.1% to 30.5%)	19.5% (13.8% to 27.5%)	21.9% (15.9% to 29.7%)	21.1% (15.4% to 28.8%)
Foreign body	17.3% (11.8% to 24.7%)	16.8% (11.6% to 23.9%)	17.5% (12.5% to 23.8%)	16.9% (11.8% to 23.9%)
Other unintentional injuries	35.6% (26.9% to 47.3%)	33.4% (25.1% to 44.2%)	29.8% (22.3% to 38.7%)	31.6% (23.9% to 41.3%)
**Self-harm and interpersonal violence**	30.2% (18.3% to 40.9%)	30.8% (19.0% to 41.2%)	31.4% (25.4% to 37.1%)	30.9% (19.8% to 40.9%)
Self-harm	31.6% (15.2% to 45.4%)	33.4% (16.8% to 47.8%)	29.7% (13.7% to 43.2%)	33.3% (16.8% to 47.8%)
Interpersonal violence	27.7% (17.2% to 37.0%)	26.6% (16.1% to 36.1%)	31.6% (25.5% to 37.7%)	27.3% (17.1% to 36.2%)
**Forces of nature, conflict and terrorism, and executions and police conflict**	0.0% (0.0% to 0.0%)	0.0% (0.0% to 0.0%)	0.4% (0.2% to 0.6%)	0.3% (0.1% to 0.5%)
Forces of nature, conflict and terrorism, and state actor violence	0.0% (0.0% to 0.0%)	0.0% (0.0% to 0.0%)	8.1% (5.2% to 12.4%)	8.1% (5.2% to 12.4%)

Supplementary Results Table 1. Age-standardised PAFs attributable to the joint distribution of all risk factors for all ages, both sexes combined for each GBD level 1 and level 2 cause and all causes in 2016 for Armenia. Results are shown for deaths, YLLs, YLDs, and DALYs
Data in parenthesis are 95% uncertainty intervals. PAFs=population attributable fractions. GBD=Global Burden of Disease. YLLs=years of life lost. YLDs=years live with disability. DALYs=disability-adjusted life-years

	Deaths 2016	YLLs 2016	YLDs 2016	DALYs 2016
**All causes**	68.3% (66.6% to 69.9%)	62.1% (60.4% to 63.7%)	25.4% (24.0% to 26.9%)	47.4% (44.5% to 50.4%)
**HIV/AIDS and tuberculosis**	58.6% (49.6% to 66.3%)	57.5% (49.6% to 64.3%)	54.1% (45.9% to 61.7%)	57.3% (49.4% to 64.0%)
Tuberculosis	55.9% (43.6% to 65.9%)	56.2% (44.2% to 65.9%)	44.9% (34.5% to 54.4%)	55.4% (43.7% to 65.2%)
HIV/AIDS	67.0% (64.5% to 69.2%)	60.3% (56.7% to 63.5%)	77.1% (76.2% to 78.0%)	61.2% (57.6% to 64.2%)
**Diarrhoea, lower respiratory infections, and other common infectious diseases**	65.8% (59.1% to 72.3%)	73.8% (67.5% to 79.4%)	31.6% (24.1% to 39.7%)	63.4% (56.9% to 69.5%)
Diarrhoeal diseases	80.9% (68.5% to 92.2%)	87.3% (78.4% to 94.9%)	70.9% (53.2% to 87.7%)	75.8% (61.0% to 89.8%)
Lower respiratory infections	69.1% (61.8% to 76.0%)	78.1% (71.0% to 84.2%)	59.5% (51.3% to 68.2%)	77.9% (70.7% to 84.0%)
Upper respiratory infections	0.9% (0.1% to 3.1%)	2.3% (0.2% to 7.4%)	0.0% (0.0% to 0.0%)	0.0% (0.0% to 0.1%)
Otitis media	2.9% (1.3% to 5.1%)	7.8% (4.2% to 12.3%)	10.2% (7.1% to 13.2%)	10.2% (7.1% to 13.2%)
Meningitis	0.9% (0.6% to 1.3%)	1.5% (1.0% to 2.1%)	0.0% (0.0% to 0.0%)	0.7% (0.4% to 1.0%)
Encephalitis	0.8% (0.4% to 1.4%)	1.2% (0.7% to 1.9%)	0.0% (0.0% to 0.0%)	0.9% (0.5% to 1.5%)
Measles	28.8% (11.7% to 61.3%)	30.4% (12.3% to 64.1%)	35.7% (14.7% to 72.2%)	30.6% (12.4% to 64.4%)
**Maternal disorders**	7.1% (3.4% to 11.1%)	6.8% (3.3% to 10.8%)	4.5% (2.1% to 7.6%)	6.1% (3.0% to 9.7%)
Maternal haemorrhage	13.7% (4.9% to 22.4%)	13.6% (4.9% to 22.2%)	13.4% (4.8% to 22.0%)	13.6% (4.9% to 22.2%)
Maternal sepsis and other pregnancy related infections	13.6% (4.9% to 22.2%)	13.5% (4.9% to 22.1%)	13.2% (4.7% to 21.6%)	13.2% (4.8% to 21.7%)
Maternal abortion, miscarriage, and ectopic pregnancy	8.6% (5.1% to 12.7%)	8.2% (5.0% to 12.3%)	7.9% (3.6% to 13.6%)	8.2% (4.7% to 12.2%)
**Neonatal disorders**	73.7% (70.5% to 77.4%)	73.7% (70.5% to 77.4%)	0.1% (0.1% to 0.1%)	56.3% (48.3% to 64.0%)
Neonatal preterm birth complications	91.9% (88.6% to 95.0%)	92.0% (88.6% to 95.0%)	0.2% (0.2% to 0.3%)	75.3% (66.7% to 82.9%)
Neonatal encephalopathy due to birth asphyxia and trauma	61.8% (56.6% to 66.7%)	61.8% (56.6% to 66.8%)	0.0% (0.0% to 0.0%)	24.9% (11.8% to 46.8%)
Neonatal sepsis and other neonatal infections	52.2% (46.3% to 56.8%)	52.3% (46.4% to 56.8%)	0.0% (0.0% to 0.0%)	40.0% (27.8% to 48.8%)
Hemolytic disease and other neonatal jaundice	59.2% (52.3% to 64.3%)	59.3% (52.4% to 64.3%)	0.0% (0.0% to 0.0%)	17.0% (10.4% to 25.0%)

Supplementary Results Table 1. Age-standardised PAFs attributable to the joint distribution of all risk factors for all ages, both sexes combined for each GBD level 1 and level 2 cause and all causes in 2016 for Armenia. Results are shown for deaths, YLLs, YLDs, and DALYs
Data in parenthesis are 95% uncertainty intervals. PAFs=population attributable fractions. GBD=Global Burden of Disease. YLLs=years of life lost. YLDs=years live with disability. DALYs=disability-adjusted life-years

	Deaths 2016	YLLs 2016	YLDs 2016	DALYs 2016
Other neonatal disorders	58.5% (53.0% to 63.7%)	58.5% (53.1% to 63.7%)	0.0% (0.0% to 0.0%)	55.1% (49.5% to 60.7%)
**Nutritional deficiencies**	22.4% (19.9% to 24.3%)	28.3% (25.3% to 30.5%)	77.3% (73.4% to 81.0%)	76.4% (72.5% to 80.3%)
Protein-energy malnutrition	2.5% (1.3% to 4.5%)	5.6% (2.9% to 9.2%)	97.9% (96.3% to 98.9%)	90.9% (85.2% to 95.0%)
Vitamin A deficiency	-	-	0.0% (0.0% to 0.0%)	0.0% (0.0% to 0.0%)
Iron-deficiency anaemia	100.0% (100.0% to 100.0%)	100.0% (100.0% to 100.0%)	100.0% (100.0% to 100.0%)	100.0% (100.0% to 100.0%)
**Other communicable, maternal, neonatal, and nutritional diseases**	12.4% (6.5% to 18.9%)	6.8% (2.7% to 11.7%)	77.9% (73.6% to 82.0%)	51.1% (32.8% to 62.7%)
Sexually transmitted diseases excluding HIV	96.6% (91.0% to 99.8%)	90.0% (75.9% to 99.3%)	100.0% (100.0% to 100.0%)	99.5% (98.7% to 100.0%)
Hepatitis	2.7% (2.0% to 3.6%)	2.2% (1.6% to 3.0%)	1.1% (0.8% to 1.7%)	2.1% (1.5% to 2.8%)
**Neoplasms**	46.7% (43.4% to 50.1%)	45.5% (42.3% to 48.5%)	39.6% (35.9% to 43.5%)	45.4% (42.2% to 48.4%)
Oesophageal cancer	79.2% (71.1% to 86.7%)	82.0% (75.0% to 88.7%)	79.9% (71.8% to 87.3%)	82.0% (74.9% to 88.7%)
Stomach cancer	27.4% (17.1% to 37.6%)	28.8% (18.2% to 38.9%)	27.6% (17.4% to 37.5%)	28.8% (18.2% to 38.9%)
Liver cancer	65.0% (57.3% to 71.9%)	66.9% (59.5% to 73.8%)	65.5% (57.7% to 72.5%)	66.9% (59.5% to 73.7%)
Larynx cancer	89.4% (83.8% to 93.7%)	90.2% (85.0% to 94.1%)	89.9% (85.0% to 93.9%)	90.2% (85.0% to 94.1%)
Tracheal, bronchus, and lung cancer	91.1% (88.8% to 92.9%)	91.1% (89.0% to 92.9%)	90.7% (88.4% to 92.7%)	91.1% (89.0% to 92.9%)
Breast cancer	26.3% (17.0% to 36.9%)	25.0% (16.1% to 35.5%)	25.0% (15.9% to 35.5%)	25.0% (16.1% to 35.5%)
Cervical cancer	100.0% (100.0% to 100.0%)	100.0% (100.0% to 100.0%)	100.0% (100.0% to 100.0%)	100.0% (100.0% to 100.0%)
Uterine cancer	45.0% (29.7% to 59.7%)	46.0% (30.1% to 60.9%)	46.2% (30.2% to 61.2%)	46.0% (30.1% to 60.9%)
Prostate cancer	8.1% (5.9% to 10.5%)	9.6% (7.0% to 12.3%)	9.6% (7.0% to 12.4%)	9.6% (7.0% to 12.3%)
Colon and rectum cancer	48.2% (38.9% to 57.4%)	48.8% (39.8% to 57.9%)	48.7% (39.6% to 57.8%)	48.8% (39.8% to 57.8%)
Lip and oral cavity cancer	79.4% (72.5% to 84.6%)	81.0% (74.8% to 85.7%)	78.2% (71.4% to 83.5%)	80.9% (74.7% to 85.6%)
Nasopharynx cancer	76.3% (70.7% to 80.9%)	72.0% (66.5% to 77.1%)	72.0% (65.4% to 77.8%)	72.0% (66.5% to 77.0%)

Supplementary Results Table 1. Age-standardised PAFs attributable to the joint distribution of all risk factors for all ages, both sexes combined for each GBD level 1 and level 2 cause and all causes in 2016 for Armenia. Results are shown for deaths, YLLs, YLDs, and DALYs

Data in parenthesis are 95% uncertainty intervals. PAFs=population attributable fractions. GBD=Global Burden of Disease. YLLs=years of life lost. YLDs=years live with disability. DALYs=disability-adjusted life-years

	Deaths 2016	YLLs 2016	YLDs 2016	DALYs 2016
Other pharynx cancer	51.1% (36.9% to 63.8%)	53.2% (38.9% to 65.7%)	52.0% (37.9% to 64.6%)	53.1% (38.8% to 65.7%)
Gallbladder and biliary tract cancer	22.6% (12.2% to 35.1%)	22.8% (12.4% to 35.4%)	22.8% (12.4% to 35.4%)	22.8% (12.5% to 35.4%)
Pancreatic cancer	33.8% (25.8% to 41.8%)	37.0% (29.0% to 45.0%)	34.9% (26.7% to 42.7%)	37.0% (29.0% to 45.0%)
Ovarian cancer	14.0% (7.0% to 22.4%)	12.3% (5.6% to 20.0%)	12.1% (5.6% to 20.0%)	12.3% (5.6% to 20.0%)
Kidney cancer	38.7% (28.3% to 49.1%)	39.3% (29.3% to 49.7%)	38.5% (28.4% to 48.5%)	39.3% (29.3% to 49.6%)
Bladder cancer	44.8% (34.1% to 55.0%)	49.2% (38.0% to 59.3%)	47.4% (36.9% to 57.2%)	49.1% (37.9% to 59.2%)
Thyroid cancer	13.0% (6.4% to 22.0%)	13.3% (6.4% to 22.4%)	13.2% (6.7% to 21.6%)	13.3% (6.4% to 22.3%)
Mesothelioma	92.9% (90.7% to 94.5%)	87.8% (84.4% to 90.5%)	91.3% (88.8% to 93.3%)	87.8% (84.5% to 90.6%)
Non-Hodgkin's lymphoma	6.2% (2.4% to 11.2%)	5.8% (2.2% to 10.4%)	6.2% (2.3% to 11.0%)	5.8% (2.2% to 10.4%)
Multiple myeloma	7.9% (3.2% to 14.5%)	8.0% (3.3% to 14.5%)	8.0% (3.2% to 14.6%)	8.0% (3.2% to 14.5%)
Leukaemia	22.2% (14.0% to 30.4%)	18.0% (11.5% to 24.7%)	21.2% (13.6% to 29.0%)	18.1% (11.5% to 24.8%)
**Cardiovascular diseases**	90.8% (88.7% to 92.7%)	91.7% (90.3% to 93.0%)	85.4% (83.4% to 87.1%)	91.2% (89.8% to 92.4%)
Rheumatic heart disease	35.8% (25.2% to 52.9%)	34.9% (25.1% to 49.1%)	36.4% (25.2% to 51.2%)	34.9% (25.1% to 49.2%)
Ischaemic heart disease	94.5% (91.7% to 96.7%)	95.6% (93.9% to 97.0%)	93.3% (91.1% to 95.2%)	95.5% (93.8% to 97.0%)
Cerebrovascular disease	88.1% (84.1% to 91.7%)	91.0% (88.3% to 93.4%)	89.2% (86.0% to 92.0%)	90.7% (88.0% to 93.2%)
Hypertensive heart disease	100.0% (100.0% to 100.0%)	100.0% (100.0% to 100.0%)	100.0% (100.0% to 100.0%)	100.0% (100.0% to 100.0%)
Cardiomyopathy and myocarditis	55.8% (47.7% to 64.0%)	53.9% (46.7% to 61.4%)	47.7% (40.9% to 54.2%)	53.2% (46.5% to 60.4%)
Atrial fibrillation and flutter	58.3% (50.0% to 65.8%)	62.6% (55.2% to 68.6%)	69.2% (63.9% to 74.0%)	66.5% (60.1% to 71.7%)
Aortic aneurysm	56.8% (49.6% to 63.4%)	62.5% (56.1% to 67.8%)	-	62.5% (56.1% to 67.8%)
Peripheral vascular disease	55.3% (46.9% to 64.1%)	62.2% (54.6% to 69.4%)	65.7% (60.0% to 71.3%)	65.3% (59.7% to 70.9%)
Endocarditis	48.6% (39.2% to 58.8%)	45.3% (35.8% to 54.2%)	45.7% (37.4% to 54.2%)	45.3% (35.8% to 54.1%)

Supplementary Results Table 1. Age-standardised PAFs attributable to the joint distribution of all risk factors for all ages, both sexes combined for each GBD level 1 and level 2 cause and all causes in 2016 for Armenia. Results are shown for deaths, YLLs, YLDs, and DALYs
Data in parenthesis are 95% uncertainty intervals. PAFs=population attributable fractions. GBD=Global Burden of Disease. YLLs=years of life lost. YLDs=years live with disability. DALYs=disability-adjusted life-years

	Deaths 2016	YLLs 2016	YLDs 2016	DALYs 2016
Other cardiovascular and circulatory diseases	55.6% (50.3% to 60.9%)	60.1% (55.7% to 64.3%)	54.6% (49.6% to 59.7%)	58.4% (54.1% to 62.5%)
**Chronic respiratory diseases**	67.7% (61.4% to 74.4%)	70.2% (65.3% to 75.4%)	42.8% (36.2% to 49.6%)	59.8% (54.9% to 65.1%)
Chronic obstructive pulmonary disease	76.1% (69.4% to 82.5%)	80.0% (74.7% to 84.9%)	74.6% (68.9% to 79.7%)	79.2% (74.0% to 83.9%)
Pneumoconiosis	100.0% (100.0% to 100.0%)	100.0% (100.0% to 100.0%)	100.0% (100.0% to 100.0%)	100.0% (100.0% to 100.0%)
Asthma	42.2% (31.6% to 53.9%)	44.8% (34.9% to 55.5%)	34.4% (25.9% to 43.7%)	35.0% (26.5% to 44.2%)
Other chronic respiratory diseases	20.5% (14.7% to 27.8%)	18.2% (12.5% to 25.6%)	7.2% (4.7% to 10.1%)	10.1% (6.8% to 14.1%)
**Cirrhosis and other chronic liver diseases**	42.6% (40.1% to 45.1%)	44.7% (42.5% to 47.1%)	31.0% (28.0% to 34.0%)	44.0% (41.8% to 46.4%)
Cirrhosis and other chronic liver diseases due to hepatitis B	0.4% (0.2% to 0.7%)	0.6% (0.3% to 1.0%)	0.5% (0.2% to 0.8%)	0.6% (0.3% to 1.0%)
Cirrhosis and other chronic liver diseases due to hepatitis C	75.2% (68.9% to 80.9%)	82.8% (78.9% to 86.2%)	80.7% (75.9% to 84.8%)	82.7% (78.9% to 86.1%)
Cirrhosis and other chronic liver diseases due to alcohol use	100.0% (100.0% to 100.0%)	100.0% (100.0% to 100.0%)	100.0% (100.0% to 100.0%)	100.0% (100.0% to 100.0%)
**Digestive diseases**	14.7% (11.5% to 18.4%)	15.5% (12.2% to 19.0%)	3.5% (2.6% to 4.4%)	12.0% (9.4% to 14.9%)
Peptic ulcer disease	19.9% (14.6% to 26.0%)	23.7% (17.7% to 30.1%)	11.4% (8.0% to 15.1%)	21.8% (16.2% to 27.5%)
Gallbladder and biliary diseases	42.5% (27.9% to 58.0%)	42.6% (28.3% to 57.7%)	45.7% (30.8% to 61.5%)	42.9% (28.6% to 58.1%)
Pancreatitis	33.7% (18.7% to 50.2%)	33.4% (18.1% to 49.8%)	37.8% (22.8% to 55.2%)	33.5% (18.2% to 49.8%)
**Neurological disorders**	18.2% (8.2% to 30.6%)	16.5% (8.2% to 26.9%)	3.3% (1.6% to 5.6%)	6.9% (3.7% to 11.1%)
Alzheimer's disease and other dementias	23.0% (11.2% to 37.1%)	24.5% (12.6% to 38.7%)	24.4% (12.5% to 39.0%)	24.5% (12.6% to 38.8%)
Parkinson's disease	-14.9% (-20.4% to -10.3%)	-19.5% (-26.2% to -13.5%)	-20.7% (-27.0% to -14.4%)	-19.8% (-26.2% to -13.8%)
Epilepsy	22.9% (14.9% to 30.1%)	20.3% (12.8% to 27.0%)	18.5% (12.0% to 24.4%)	18.9% (12.4% to 25.1%)
Multiple sclerosis	9.6% (5.3% to 14.1%)	10.5% (5.8% to 15.2%)	7.3% (4.3% to 10.3%)	8.9% (5.2% to 12.7%)
**Mental and substance use disorders**	100.0% (100.0% to 100.0%)	100.0% (99.9% to 100.0%)	20.1% (17.9% to 22.5%)	24.1% (21.5% to 26.9%)
Alcohol use disorders	100.0% (100.0% to 100.0%)	100.0% (100.0% to 100.0%)	100.0% (100.0% to 100.0%)	100.0% (100.0% to 100.0%)

Supplementary Results Table 1. Age-standardised PAFs attributable to the joint distribution of all risk factors for all ages, both sexes combined for each GBD level 1 and level 2 cause and all causes in 2016 for Armenia. Results are shown for deaths, YLLs, YLDs, and DALYs
Data in parenthesis are 95% uncertainty intervals. PAFs=population attributable fractions. GBD=Global Burden of Disease. YLLs=years of life lost. YLDs=years live with disability. DALYs=disability-adjusted life-years

	**Deaths 2016**	**YLLs 2016**	**YLDs 2016**	**DALYs 2016**
Drug use disorders	100.0% (100.0% to 100.0%)	100.0% (100.0% to 100.0%)	100.0% (100.0% to 100.0%)	100.0% (100.0% to 100.0%)
Depressive disorders	-	-	3.3% (2.6% to 4.2%)	3.3% (2.6% to 4.2%)
Idiopathic developmental intellectual disability	-	-	55.5% (26.9% to 75.9%)	55.5% (26.9% to 75.9%)
**Diabetes, urogenital, blood, and endocrine diseases**	75.5% (74.1% to 77.0%)	76.3% (75.0% to 77.8%)	61.9% (57.5% to 65.8%)	70.3% (67.8% to 72.5%)
Diabetes mellitus	100.0% (100.0% to 100.0%)	100.0% (100.0% to 100.0%)	100.0% (100.0% to 100.0%)	100.0% (100.0% to 100.0%)
Chronic kidney disease	100.0% (100.0% to 100.0%)	100.0% (100.0% to 100.0%)	100.0% (100.0% to 100.0%)	100.0% (100.0% to 100.0%)
**Musculoskeletal disorders**	1.4% (0.6% to 2.5%)	1.3% (0.5% to 2.3%)	26.3% (21.8% to 30.9%)	26.1% (21.6% to 30.7%)
Rheumatoid arthritis	3.7% (1.5% to 6.7%)	4.4% (1.8% to 7.8%)	4.4% (1.8% to 7.1%)	4.4% (1.8% to 7.1%)
Osteoarthritis	-	-	26.0% (13.0% to 39.3%)	26.0% (13.0% to 39.3%)
Low back and neck pain	-	-	28.6% (24.4% to 33.0%)	28.6% (24.4% to 33.0%)
Gout	-	-	47.0% (32.8% to 62.0%)	47.0% (32.8% to 62.0%)
**Other non-communicable diseases**	0.1% (0.1% to 0.2%)	0.1% (0.1% to 0.2%)	5.2% (4.5% to 6.0%)	4.0% (3.4% to 4.8%)
Sense organ diseases	-	-	11.1% (9.7% to 12.7%)	11.1% (9.7% to 12.7%)
Sudden infant death syndrome	6.1% (3.6% to 9.1%)	6.1% (3.6% to 9.1%)	-	6.1% (3.6% to 9.1%)
**Transport injuries**	32.5% (25.7% to 39.7%)	29.3% (22.5% to 36.6%)	37.6% (31.3% to 44.2%)	31.2% (24.5% to 38.3%)
Road injuries	32.5% (25.7% to 39.7%)	29.4% (22.6% to 36.7%)	38.5% (32.1% to 45.1%)	31.4% (24.7% to 38.5%)
Other transport injuries	32.4% (25.4% to 39.4%)	28.3% (21.4% to 35.4%)	28.7% (22.9% to 34.8%)	28.4% (22.0% to 35.2%)
**Unintentional injuries**	19.0% (15.8% to 22.1%)	13.9% (11.1% to 16.9%)	21.2% (18.6% to 24.2%)	18.1% (15.3% to 21.1%)
Falls	45.5% (31.1% to 51.3%)	24.9% (17.6% to 29.3%)	25.8% (23.1% to 28.8%)	25.6% (22.2% to 28.8%)
Drowning	17.0% (12.7% to 22.4%)	15.1% (11.3% to 19.9%)	18.2% (13.5% to 24.0%)	15.2% (11.4% to 20.0%)
Fire, heat, and hot substances	14.4% (10.5% to 19.5%)	13.9% (10.2% to 18.4%)	17.4% (12.8% to 22.8%)	15.8% (11.7% to 20.7%)

**Supplementary Results Table 1. Age-standardised PAFs attributable to the joint distribution of all risk factors for all ages, both sexes combined for each GBD level 1 and level 2 cause and all causes in 2016 for Armenia. Results are shown for deaths, YLLs, YLDs, and DALYs**<br>Data in parenthesis are 95% uncertainty intervals. PAFs=population attributable fractions. GBD=Global Burden of Disease. YLLs=years of life lost. YLDs=years live with disability. DALYs=disability-adjusted life-years				
	**Deaths 2016**	**YLLs 2016**	**YLDs 2016**	**DALYs 2016**
Poisonings	19.1% (14.7% to 25.0%)	17.8% (13.3% to 23.5%)	16.5% (12.2% to 21.9%)	17.6% (13.4% to 23.1%)
Exposure to mechanical forces	16.9% (13.5% to 20.5%)	15.7% (12.1% to 19.6%)	20.4% (17.1% to 24.3%)	18.6% (15.5% to 22.4%)
Animal contact	14.4% (11.6% to 17.6%)	11.0% (8.4% to 14.0%)	11.1% (8.6% to 14.0%)	11.0% (8.7% to 13.8%)
Foreign body	8.4% (6.6% to 10.6%)	7.2% (5.4% to 9.3%)	9.1% (7.0% to 11.6%)	7.9% (6.2% to 10.1%)
Other unintentional injuries	20.5% (15.5% to 26.5%)	19.2% (14.6% to 25.0%)	17.7% (13.2% to 23.2%)	18.4% (14.0% to 23.9%)
**Self-harm and interpersonal violence**	22.3% (13.4% to 31.2%)	22.0% (13.2% to 31.0%)	23.7% (18.6% to 28.8%)	22.2% (13.8% to 30.7%)
Self-harm	22.4% (10.0% to 34.4%)	22.8% (9.8% to 35.4%)	22.2% (11.4% to 33.2%)	22.8% (9.9% to 35.3%)
Interpersonal violence	22.2% (14.6% to 29.7%)	20.7% (12.7% to 28.3%)	23.9% (18.5% to 29.1%)	21.4% (14.1% to 28.4%)
**Forces of nature, conflict and terrorism, and executions and police conflict**	0.0% (0.0% to 0.0%)	0.0% (0.0% to 0.0%)	6.8% (4.9% to 9.3%)	6.8% (4.8% to 9.2%)
Forces of nature, conflict and terrorism, and state actor violence	0.0% (0.0% to 0.0%)	0.0% (0.0% to 0.0%)	7.1% (5.1% to 9.7%)	7.1% (5.1% to 9.7%)

Supplementary Results Table 1. Age-standardised PAFs attributable to the joint distribution of all risk factors for all ages, both sexes combined for each GBD level 1 and level 2 cause and all causes in 2016 for Australia. Results are shown for deaths, YLLs, YLDs, and DALYs
Data in parenthesis are 95% uncertainty intervals. PAFs=population attributable fractions. GBD=Global Burden of Disease. YLLs=years of life lost. YLDs=years live with disability. DALYs=disability-adjusted life-years

	Deaths 2016	YLLs 2016	YLDs 2016	DALYs 2016
**All causes**	54.1% (51.9% to 56.3%)	51.3% (49.5% to 53.0%)	20.2% (18.7% to 21.7%)	33.3% (30.9% to 36.1%)
**HIV/AIDS and tuberculosis**	77.3% (71.9% to 82.0%)	83.1% (80.4% to 85.4%)	91.9% (88.5% to 94.1%)	87.1% (83.9% to 89.7%)
Tuberculosis	53.9% (41.9% to 64.4%)	55.3% (44.8% to 64.6%)	54.1% (42.6% to 63.9%)	55.0% (44.5% to 64.2%)
HIV/AIDS	94.3% (93.8% to 94.8%)	91.6% (90.8% to 92.3%)	96.2% (95.6% to 96.7%)	93.8% (92.8% to 94.6%)
**Diarrhoea, lower respiratory infections, and other common infectious diseases**	27.0% (19.5% to 35.5%)	36.2% (29.7% to 43.6%)	4.9% (3.5% to 7.1%)	21.1% (16.2% to 26.8%)
Diarrhoeal diseases	29.3% (15.6% to 50.3%)	44.7% (31.9% to 62.1%)	32.9% (19.2% to 53.1%)	39.0% (26.3% to 58.1%)
Lower respiratory infections	28.0% (19.9% to 37.0%)	40.0% (32.0% to 48.7%)	40.7% (33.3% to 49.4%)	40.0% (32.1% to 48.8%)
Upper respiratory infections	0.6% (0.0% to 2.4%)	1.9% (0.1% to 6.9%)	0.0% (0.0% to 0.0%)	0.0% (0.0% to 0.0%)
Otitis media	0.6% (0.3% to 1.0%)	3.0% (1.7% to 4.6%)	6.6% (4.3% to 8.9%)	6.6% (4.3% to 8.9%)
Meningitis	3.3% (2.5% to 4.2%)	5.7% (4.4% to 7.1%)	0.0% (0.0% to 0.0%)	4.7% (3.4% to 6.1%)
Encephalitis	0.5% (0.3% to 0.6%)	1.1% (0.8% to 1.5%)	0.0% (0.0% to 0.0%)	0.6% (0.4% to 0.9%)
Measles	4.7% (0.4% to 19.3%)	5.6% (0.5% to 22.4%)	12.6% (1.1% to 50.9%)	5.6% (0.5% to 22.6%)
**Maternal disorders**	2.8% (1.2% to 4.6%)	2.8% (1.2% to 4.6%)	3.3% (1.5% to 5.5%)	3.1% (1.3% to 4.9%)
Maternal haemorrhage	9.8% (3.5% to 15.9%)	9.7% (3.5% to 15.8%)	9.8% (3.5% to 15.9%)	9.8% (3.5% to 15.9%)
Maternal sepsis and other pregnancy related infections	9.8% (3.6% to 16.1%)	9.8% (3.5% to 16.0%)	9.7% (3.5% to 15.8%)	9.7% (3.5% to 15.9%)
Maternal abortion, miscarriage, and ectopic pregnancy	3.4% (1.8% to 5.2%)	3.4% (1.9% to 5.3%)	3.8% (1.8% to 6.1%)	3.6% (1.9% to 5.8%)
**Neonatal disorders**	74.7% (72.2% to 77.4%)	74.8% (72.2% to 77.5%)	0.1% (0.1% to 0.1%)	51.8% (44.9% to 57.8%)
Neonatal preterm birth complications	87.4% (83.9% to 90.4%)	87.4% (84.0% to 90.5%)	0.2% (0.1% to 0.2%)	51.3% (41.4% to 60.5%)
Neonatal encephalopathy due to birth asphyxia and trauma	66.4% (62.8% to 70.1%)	66.5% (62.9% to 70.1%)	0.0% (0.0% to 0.0%)	57.4% (51.6% to 62.9%)
Neonatal sepsis and other neonatal infections	61.2% (56.1% to 66.4%)	61.2% (56.1% to 66.5%)	0.0% (0.0% to 0.0%)	20.0% (8.3% to 41.1%)
Hemolytic disease and other neonatal jaundice	60.6% (53.6% to 66.7%)	60.8% (53.8% to 66.8%)	0.0% (0.0% to 0.0%)	18.7% (12.4% to 27.2%)

Supplementary Results Table 1. Age-standardised PAFs attributable to the joint distribution of all risk factors for all ages, both sexes combined for each GBD level 1 and level 2 cause and all causes in 2016 for Australia. Results are shown for deaths, YLLs, YLDs, and DALYs
Data in parenthesis are 95% uncertainty intervals. PAFs=population attributable fractions. GBD=Global Burden of Disease. YLLs=years of life lost. YLDs=years live with disability. DALYs=disability-adjusted life-years

	Deaths 2016	YLLs 2016	YLDs 2016	DALYs 2016
Other neonatal disorders	66.5% (62.5% to 70.2%)	66.6% (62.6% to 70.3%)	0.0% (0.0% to 0.0%)	63.2% (58.9% to 67.3%)
**Nutritional deficiencies**	2.9% (1.6% to 3.6%)	10.5% (8.0% to 12.5%)	76.9% (71.8% to 81.5%)	71.0% (65.2% to 76.6%)
Protein-energy malnutrition	0.8% (0.5% to 1.1%)	5.8% (3.5% to 8.1%)	98.0% (97.3% to 98.6%)	70.7% (60.6% to 79.5%)
Vitamin A deficiency	-	-	0.0% (0.0% to 0.0%)	0.0% (0.0% to 0.0%)
Iron-deficiency anaemia	100.0% (100.0% to 100.0%)	100.0% (100.0% to 100.0%)	100.0% (100.0% to 100.0%)	100.0% (100.0% to 100.0%)
**Other communicable, maternal, neonatal, and nutritional diseases**	4.5% (3.7% to 5.9%)	4.1% (3.2% to 5.4%)	75.2% (68.8% to 81.4%)	29.4% (20.4% to 41.7%)
Sexually transmitted diseases excluding HIV	90.6% (83.0% to 96.5%)	71.7% (55.4% to 87.5%)	100.0% (100.0% to 100.0%)	98.4% (96.6% to 99.5%)
Hepatitis	6.1% (4.2% to 8.4%)	6.4% (4.4% to 8.9%)	4.0% (2.5% to 5.7%)	6.2% (4.2% to 8.7%)
**Neoplasms**	43.6% (41.1% to 46.4%)	42.8% (40.4% to 45.3%)	27.6% (24.9% to 30.4%)	41.9% (39.5% to 44.5%)
Oesophageal cancer	85.9% (79.0% to 91.6%)	86.7% (80.2% to 92.2%)	85.6% (78.7% to 91.5%)	86.7% (80.1% to 92.2%)
Stomach cancer	16.8% (10.0% to 25.0%)	15.7% (9.3% to 23.1%)	16.4% (9.8% to 24.4%)	15.7% (9.4% to 23.1%)
Liver cancer	77.0% (70.5% to 82.7%)	76.7% (70.7% to 82.0%)	77.3% (70.9% to 82.9%)	76.7% (70.7% to 82.0%)
Larynx cancer	90.5% (85.1% to 94.4%)	89.8% (84.0% to 93.7%)	90.1% (84.4% to 94.0%)	89.8% (84.1% to 93.7%)
Tracheal, bronchus, and lung cancer	88.2% (85.9% to 90.2%)	86.6% (84.2% to 88.7%)	87.5% (85.1% to 89.6%)	86.7% (84.2% to 88.7%)
Breast cancer	36.4% (29.2% to 43.8%)	35.1% (28.1% to 41.8%)	35.6% (28.3% to 42.5%)	35.2% (28.1% to 41.9%)
Cervical cancer	100.0% (100.0% to 100.0%)	100.0% (100.0% to 100.0%)	100.0% (100.0% to 100.0%)	100.0% (100.0% to 100.0%)
Uterine cancer	50.9% (36.4% to 63.5%)	52.4% (38.3% to 64.6%)	52.8% (39.0% to 65.0%)	52.4% (38.3% to 64.6%)
Prostate cancer	5.1% (3.6% to 6.9%)	4.9% (3.5% to 6.4%)	4.8% (3.4% to 6.2%)	4.9% (3.5% to 6.4%)
Colon and rectum cancer	59.2% (50.9% to 67.0%)	60.7% (52.7% to 68.2%)	60.1% (51.9% to 67.8%)	60.7% (52.6% to 68.2%)
Lip and oral cavity cancer	83.5% (77.2% to 88.0%)	84.5% (78.8% to 88.8%)	84.6% (78.8% to 88.9%)	84.5% (78.8% to 88.8%)
Nasopharynx cancer	86.6% (82.0% to 90.2%)	85.5% (81.2% to 88.9%)	84.7% (80.2% to 88.3%)	85.5% (81.2% to 88.8%)

Supplementary Results Table 1. Age-standardised PAFs attributable to the joint distribution of all risk factors for all ages, both sexes combined for each GBD level 1 and level 2 cause and all causes in 2016 for Australia. Results are shown for deaths, YLLs, YLDs, and DALYs
Data in parenthesis are 95% uncertainty intervals. PAFs=population attributable fractions. GBD=Global Burden of Disease. YLLs=years of life lost. YLDs=years live with disability. DALYs=disability-adjusted life-years

	Deaths 2016	YLLs 2016	YLDs 2016	DALYs 2016
Other pharynx cancer	66.2% (55.1% to 75.1%)	70.0% (59.5% to 78.1%)	69.2% (58.6% to 77.6%)	70.0% (59.6% to 78.1%)
Gallbladder and biliary tract cancer	23.9% (13.6% to 36.1%)	24.5% (14.1% to 36.8%)	25.4% (14.7% to 37.8%)	24.5% (14.1% to 36.8%)
Pancreatic cancer	31.8% (24.7% to 39.6%)	31.0% (24.0% to 38.8%)	31.7% (24.6% to 39.4%)	31.1% (24.0% to 38.8%)
Ovarian cancer	19.8% (11.6% to 28.3%)	15.6% (8.6% to 23.3%)	15.9% (9.1% to 23.7%)	15.6% (8.6% to 23.3%)
Kidney cancer	36.9% (27.3% to 47.3%)	36.3% (26.7% to 46.4%)	36.4% (26.7% to 46.6%)	36.3% (26.7% to 46.4%)
Bladder cancer	35.2% (25.5% to 44.7%)	34.0% (24.9% to 43.2%)	33.9% (24.9% to 42.9%)	34.0% (24.9% to 43.2%)
Thyroid cancer	16.4% (8.6% to 26.3%)	17.2% (9.1% to 27.5%)	16.9% (9.6% to 26.0%)	17.1% (9.3% to 27.2%)
Mesothelioma	98.4% (98.0% to 98.8%)	96.4% (95.5% to 97.2%)	97.8% (97.1% to 98.4%)	96.5% (95.6% to 97.3%)
Non-Hodgkin's lymphoma	7.8% (3.3% to 13.0%)	8.0% (3.4% to 13.5%)	8.1% (3.5% to 13.6%)	8.0% (3.4% to 13.5%)
Multiple myeloma	8.8% (4.0% to 15.1%)	9.3% (4.3% to 15.9%)	9.3% (4.2% to 15.9%)	9.3% (4.3% to 15.9%)
Leukaemia	20.4% (12.8% to 28.0%)	17.7% (11.3% to 24.0%)	18.5% (11.7% to 25.4%)	17.7% (11.4% to 24.1%)
**Cardiovascular diseases**	79.2% (75.6% to 82.1%)	81.8% (79.4% to 83.9%)	73.0% (70.0% to 75.9%)	80.2% (77.9% to 82.3%)
Rheumatic heart disease	24.8% (15.2% to 44.0%)	24.2% (16.6% to 37.7%)	21.5% (14.6% to 32.1%)	24.1% (16.6% to 37.6%)
Ischaemic heart disease	90.7% (86.1% to 94.5%)	93.3% (90.3% to 95.6%)	90.7% (87.9% to 93.3%)	93.1% (90.1% to 95.4%)
Cerebrovascular disease	77.1% (69.6% to 83.9%)	83.2% (78.1% to 87.5%)	83.2% (78.5% to 87.5%)	83.2% (78.3% to 87.4%)
Hypertensive heart disease	100.0% (100.0% to 100.0%)	100.0% (100.0% to 100.0%)	100.0% (100.0% to 100.0%)	100.0% (100.0% to 100.0%)
Cardiomyopathy and myocarditis	52.1% (45.3% to 59.8%)	53.8% (46.7% to 59.6%)	49.7% (44.8% to 54.8%)	53.3% (46.8% to 58.9%)
Atrial fibrillation and flutter	52.0% (44.2% to 59.5%)	56.9% (49.9% to 63.3%)	64.7% (59.4% to 69.9%)	61.7% (55.9% to 67.3%)
Aortic aneurysm	37.2% (29.9% to 44.6%)	42.8% (36.4% to 48.7%)	-	42.8% (36.4% to 48.7%)
Peripheral vascular disease	43.4% (36.3% to 51.5%)	48.7% (42.7% to 54.8%)	54.0% (48.8% to 59.3%)	50.0% (44.4% to 55.6%)
Endocarditis	35.8% (27.3% to 46.5%)	36.3% (28.8% to 44.5%)	37.3% (29.1% to 46.4%)	36.4% (28.8% to 44.5%)

Supplementary Results Table 1. Age-standardised PAFs attributable to the joint distribution of all risk factors for all ages, both sexes combined for each GBD level 1 and level 2 cause and all causes in 2016 for Australia. Results are shown for deaths, YLLs, YLDs, and DALYs
Data in parenthesis are 95% uncertainty intervals. PAFs=population attributable fractions. GBD=Global Burden of Disease. YLLs=years of life lost. YLDs=years live with disability. DALYs=disability-adjusted life-years

	Deaths 2016	YLLs 2016	YLDs 2016	DALYs 2016
Other cardiovascular and circulatory diseases	39.9% (34.5% to 45.6%)	45.7% (41.1% to 50.3%)	48.1% (43.0% to 53.0%)	46.8% (41.9% to 51.4%)
**Chronic respiratory diseases**	66.2% (61.9% to 73.0%)	63.9% (59.7% to 70.4%)	42.1% (34.9% to 50.3%)	50.9% (45.0% to 57.2%)
Chronic obstructive pulmonary disease	76.6% (72.5% to 80.6%)	76.1% (72.2% to 80.0%)	71.2% (66.9% to 75.7%)	74.9% (70.9% to 79.0%)
Pneumoconiosis	100.0% (100.0% to 100.0%)	100.0% (100.0% to 100.0%)	100.0% (100.0% to 100.0%)	100.0% (100.0% to 100.0%)
Asthma	41.7% (31.1% to 52.9%)	43.9% (35.2% to 53.8%)	37.0% (28.6% to 46.8%)	37.5% (29.1% to 47.2%)
Other chronic respiratory diseases	15.6% (12.4% to 19.1%)	10.8% (8.4% to 13.8%)	7.0% (4.7% to 10.3%)	9.3% (7.0% to 12.3%)
**Cirrhosis and other chronic liver diseases**	51.7% (48.8% to 54.6%)	54.0% (51.2% to 56.9%)	43.4% (38.9% to 48.2%)	53.4% (50.6% to 56.4%)
Cirrhosis and other chronic liver diseases due to hepatitis B	2.4% (1.0% to 4.1%)	2.9% (1.3% to 4.9%)	2.8% (1.2% to 4.7%)	2.9% (1.3% to 4.9%)
Cirrhosis and other chronic liver diseases due to hepatitis C	81.7% (76.4% to 85.8%)	87.4% (85.0% to 89.0%)	85.5% (82.2% to 87.8%)	87.3% (84.9% to 89.0%)
Cirrhosis and other chronic liver diseases due to alcohol use	100.0% (100.0% to 100.0%)	100.0% (100.0% to 100.0%)	100.0% (100.0% to 100.0%)	100.0% (100.0% to 100.0%)
**Digestive diseases**	9.2% (7.0% to 11.6%)	11.6% (9.2% to 14.3%)	5.4% (4.1% to 6.8%)	9.7% (7.8% to 12.0%)
Peptic ulcer disease	6.5% (4.3% to 9.0%)	9.4% (6.4% to 12.9%)	10.1% (6.8% to 14.1%)	9.7% (6.7% to 13.4%)
Gallbladder and biliary diseases	45.5% (30.9% to 59.6%)	46.3% (32.5% to 59.9%)	53.2% (39.4% to 66.3%)	47.7% (33.8% to 61.3%)
Pancreatitis	40.9% (25.0% to 56.5%)	50.0% (33.5% to 64.5%)	40.1% (24.2% to 54.4%)	49.4% (32.8% to 64.0%)
**Neurological disorders**	19.6% (9.7% to 31.1%)	17.6% (10.3% to 26.4%)	3.6% (1.9% to 5.8%)	8.2% (4.7% to 12.6%)
Alzheimer's disease and other dementias	24.5% (12.0% to 38.9%)	25.4% (12.8% to 40.0%)	25.5% (12.9% to 40.2%)	25.4% (12.8% to 40.0%)
Parkinson's disease	-4.5% (-5.4% to -3.7%)	-5.8% (-6.9% to -4.8%)	-7.0% (-8.3% to -5.8%)	-6.0% (-7.2% to -5.0%)
Epilepsy	38.0% (28.6% to 46.4%)	36.1% (27.1% to 44.3%)	29.2% (20.7% to 37.2%)	32.6% (23.8% to 41.0%)
Multiple sclerosis	6.8% (4.4% to 9.5%)	7.6% (4.9% to 10.7%)	7.3% (4.6% to 10.2%)	7.4% (4.7% to 10.3%)
**Mental and substance use disorders**	99.6% (99.5% to 99.7%)	99.5% (99.4% to 99.6%)	21.2% (18.9% to 23.7%)	27.1% (24.3% to 30.2%)
Alcohol use disorders	100.0% (100.0% to 100.0%)	100.0% (100.0% to 100.0%)	100.0% (100.0% to 100.0%)	100.0% (100.0% to 100.0%)

Supplementary Results Table 1. Age-standardised PAFs attributable to the joint distribution of all risk factors for all ages, both sexes combined for each GBD level 1 and level 2 cause and all causes in 2016 for Australia. Results are shown for deaths, YLLs, YLDs, and DALYs
Data in parenthesis are 95% uncertainty intervals. PAFs=population attributable fractions. GBD=Global Burden of Disease. YLLs=years of life lost. YLDs=years live with disability. DALYs=disability-adjusted life-years

	Deaths 2016	YLLs 2016	YLDs 2016	DALYs 2016
Drug use disorders	100.0% (100.0% to 100.0%)	100.0% (100.0% to 100.0%)	100.0% (100.0% to 100.0%)	100.0% (100.0% to 100.0%)
Depressive disorders	-	-	7.7% (6.4% to 9.1%)	7.7% (6.4% to 9.1%)
Idiopathic developmental intellectual disability	-	-	64.5% (34.5% to 83.4%)	64.5% (34.5% to 83.4%)
**Diabetes, urogenital, blood, and endocrine diseases**	73.9% (72.3% to 77.3%)	65.9% (63.4% to 69.4%)	63.2% (59.4% to 66.9%)	64.4% (61.9% to 66.7%)
Diabetes mellitus	100.0% (100.0% to 100.0%)	100.0% (100.0% to 100.0%)	100.0% (100.0% to 100.0%)	100.0% (100.0% to 100.0%)
Chronic kidney disease	100.0% (100.0% to 100.0%)	100.0% (100.0% to 100.0%)	100.0% (100.0% to 100.0%)	100.0% (100.0% to 100.0%)
**Musculoskeletal disorders**	0.7% (0.3% to 1.2%)	0.9% (0.3% to 1.5%)	16.5% (13.8% to 19.5%)	16.3% (13.6% to 19.2%)
Rheumatoid arthritis	2.7% (1.0% to 4.7%)	3.4% (1.3% to 5.9%)	4.5% (1.8% to 7.7%)	4.4% (1.7% to 7.6%)
Osteoarthritis	-	-	30.7% (17.5% to 43.8%)	30.7% (17.5% to 43.8%)
Low back and neck pain	-	-	22.0% (19.0% to 25.0%)	22.0% (19.0% to 25.0%)
Gout	-	-	52.3% (34.1% to 70.1%)	52.3% (34.1% to 70.1%)
**Other non-communicable diseases**	0.4% (0.2% to 0.5%)	0.6% (0.4% to 0.8%)	2.0% (1.8% to 2.3%)	1.9% (1.6% to 2.1%)
Sense organ diseases	-	-	6.7% (6.0% to 7.5%)	6.7% (6.0% to 7.5%)
Sudden infant death syndrome	3.7% (2.6% to 5.2%)	3.7% (2.6% to 5.3%)	-	3.7% (2.6% to 5.3%)
**Transport injuries**	37.8% (28.6% to 47.9%)	33.4% (24.3% to 43.6%)	42.3% (33.9% to 51.6%)	36.5% (27.6% to 46.4%)
Road injuries	37.4% (28.2% to 47.5%)	33.0% (24.1% to 43.2%)	42.9% (34.3% to 52.1%)	36.2% (27.4% to 46.2%)
Other transport injuries	41.7% (31.6% to 52.2%)	37.4% (27.5% to 48.3%)	39.3% (30.9% to 48.4%)	38.2% (29.1% to 48.0%)
**Unintentional injuries**	46.3% (41.2% to 49.7%)	24.6% (20.3% to 28.1%)	19.6% (16.9% to 22.9%)	21.2% (18.2% to 24.3%)
Falls	71.1% (67.6% to 74.9%)	48.7% (44.6% to 52.2%)	22.1% (19.8% to 24.8%)	28.1% (25.0% to 31.4%)
Drowning	18.8% (12.0% to 26.2%)	15.9% (10.1% to 22.1%)	18.7% (11.8% to 26.9%)	16.0% (10.3% to 22.2%)
Fire, heat, and hot substances	14.9% (8.8% to 22.6%)	16.2% (10.5% to 23.1%)	21.2% (13.6% to 29.7%)	19.7% (12.7% to 27.7%)

Supplementary Results Table 1. Age-standardised PAFs attributable to the joint distribution of all risk factors for all ages, both sexes combined for each GBD level 1 and level 2 cause and all causes in 2016 for Australia. Results are shown for deaths, YLLs, YLDs, and DALYs
Data in parenthesis are 95% uncertainty intervals. PAFs=population attributable fractions. GBD=Global Burden of Disease. YLLs=years of life lost. YLDs=years live with disability. DALYs=disability-adjusted life-years

	Deaths 2016	YLLs 2016	YLDs 2016	DALYs 2016
Poisonings	20.4% (13.1% to 28.7%)	20.0% (13.2% to 28.0%)	18.0% (11.5% to 25.2%)	18.9% (12.2% to 26.4%)
Exposure to mechanical forces	16.1% (12.8% to 20.2%)	12.0% (8.6% to 16.4%)	19.0% (15.9% to 23.0%)	17.1% (13.8% to 21.2%)
Animal contact	12.3% (9.4% to 16.0%)	9.6% (6.6% to 13.6%)	9.2% (6.6% to 12.5%)	9.3% (6.6% to 12.7%)
Foreign body	4.2% (2.7% to 6.5%)	6.2% (4.1% to 9.1%)	5.7% (3.9% to 8.2%)	6.0% (4.2% to 8.5%)
Other unintentional injuries	24.2% (15.9% to 33.7%)	24.3% (16.1% to 33.3%)	20.1% (12.9% to 28.5%)	20.5% (13.2% to 28.8%)
**Self-harm and interpersonal violence**	43.1% (30.3% to 54.4%)	43.1% (30.7% to 54.2%)	36.8% (31.2% to 42.5%)	42.6% (30.8% to 53.1%)
Self-harm	43.8% (29.3% to 55.8%)	44.4% (30.0% to 56.2%)	35.0% (20.5% to 47.5%)	44.1% (29.9% to 56.0%)
Interpersonal violence	37.1% (28.9% to 44.5%)	34.0% (26.1% to 41.0%)	37.4% (32.5% to 42.3%)	35.1% (28.3% to 41.0%)
**Forces of nature, conflict and terrorism, and executions and police conflict**	0.0% (0.0% to 0.0%)	0.0% (0.0% to 0.0%)	1.5% (0.9% to 2.3%)	0.6% (0.2% to 1.2%)
Forces of nature, conflict and terrorism, and state actor violence	0.0% (0.0% to 0.0%)	0.0% (0.0% to 0.0%)	7.4% (4.9% to 11.1%)	7.4% (4.9% to 11.1%)

Supplementary Results Table 1. Age-standardised PAFs attributable to the joint distribution of all risk factors for all ages, both sexes combined for each GBD level 1 and level 2 cause and all causes in 2016 for Austria. Results are shown for deaths, YLLs, YLDs, and DALYs
Data in parenthesis are 95% uncertainty intervals. PAFs=population attributable fractions. GBD=Global Burden of Disease. YLLs=years of life lost. YLDs=years live with disability. DALYs=disability-adjusted life-years

	Deaths 2016	YLLs 2016	YLDs 2016	DALYs 2016
**All causes**	59.5% (57.0% to 61.8%)	56.1% (54.0% to 58.1%)	20.3% (19.0% to 21.7%)	36.4% (33.4% to 39.5%)
**HIV/AIDS and tuberculosis**	76.1% (71.0% to 80.4%)	78.3% (75.2% to 81.1%)	84.7% (81.2% to 86.8%)	81.4% (78.3% to 83.8%)
Tuberculosis	65.1% (54.7% to 74.1%)	65.7% (56.3% to 73.9%)	55.7% (44.0% to 66.4%)	63.7% (54.4% to 72.3%)
HIV/AIDS	86.1% (85.2% to 87.1%)	83.8% (82.8% to 84.9%)	87.5% (86.3% to 88.7%)	85.8% (84.5% to 87.0%)
**Diarrhoea, lower respiratory infections, and other common infectious diseases**	37.8% (29.1% to 46.4%)	39.9% (33.4% to 45.9%)	8.7% (6.2% to 11.8%)	18.5% (14.4% to 23.3%)
Diarrhoeal diseases	21.9% (13.9% to 32.5%)	39.2% (29.7% to 49.0%)	34.1% (25.1% to 44.5%)	35.0% (25.9% to 45.1%)
Lower respiratory infections	43.9% (33.3% to 53.9%)	52.4% (43.1% to 61.1%)	49.0% (39.8% to 57.8%)	52.3% (43.1% to 61.0%)
Upper respiratory infections	0.5% (0.0% to 1.9%)	1.7% (0.2% to 5.9%)	0.0% (0.0% to 0.0%)	0.0% (0.0% to 0.0%)
Otitis media	0.3% (0.2% to 0.6%)	2.0% (1.0% to 3.3%)	8.8% (6.0% to 11.7%)	8.8% (6.0% to 11.7%)
Meningitis	2.6% (1.8% to 3.6%)	4.9% (3.6% to 6.5%)	0.0% (0.0% to 0.0%)	4.3% (3.1% to 5.8%)
Encephalitis	0.4% (0.3% to 0.6%)	0.9% (0.7% to 1.3%)	0.0% (0.0% to 0.0%)	0.6% (0.4% to 0.8%)
Measles	5.3% (1.0% to 20.3%)	6.4% (1.2% to 24.3%)	11.4% (2.2% to 42.3%)	9.3% (1.8% to 34.8%)
**Maternal disorders**	2.4% (1.1% to 3.7%)	2.3% (1.1% to 3.7%)	3.8% (1.9% to 6.0%)	3.2% (1.6% to 5.0%)
Maternal haemorrhage	8.5% (3.1% to 14.0%)	8.5% (3.1% to 14.0%)	8.7% (3.2% to 14.4%)	8.6% (3.2% to 14.2%)
Maternal sepsis and other pregnancy related infections	8.6% (3.1% to 14.1%)	8.6% (3.1% to 14.0%)	8.5% (3.1% to 14.0%)	8.5% (3.1% to 14.0%)
Maternal abortion, miscarriage, and ectopic pregnancy	3.5% (2.0% to 5.2%)	3.5% (2.1% to 5.2%)	3.8% (1.9% to 6.2%)	3.8% (2.0% to 5.9%)
**Neonatal disorders**	78.2% (75.3% to 80.8%)	78.3% (75.4% to 80.8%)	0.1% (0.1% to 0.1%)	46.2% (38.0% to 54.8%)
Neonatal preterm birth complications	89.3% (86.3% to 91.9%)	89.4% (86.4% to 92.0%)	0.2% (0.1% to 0.2%)	56.1% (45.3% to 66.4%)
Neonatal encephalopathy due to birth asphyxia and trauma	67.4% (63.5% to 71.5%)	67.5% (63.5% to 71.6%)	0.0% (0.0% to 0.0%)	32.6% (21.4% to 44.0%)
Neonatal sepsis and other neonatal infections	56.7% (49.8% to 62.8%)	56.8% (49.8% to 62.8%)	0.0% (0.0% to 0.0%)	17.3% (6.4% to 33.5%)
Hemolytic disease and other neonatal jaundice	64.0% (58.4% to 69.2%)	64.1% (58.6% to 69.4%)	0.0% (0.0% to 0.0%)	17.6% (10.5% to 27.1%)

Supplementary Results Table 1. Age-standardised PAFs attributable to the joint distribution of all risk factors for all ages, both sexes combined for each GBD level 1 and level 2 cause and all causes in 2016 for Austria. Results are shown for deaths, YLLs, YLDs, and DALYs
Data in parenthesis are 95% uncertainty intervals. PAFs=population attributable fractions. GBD=Global Burden of Disease. YLLs=years of life lost. YLDs=years live with disability. DALYs=disability-adjusted life-years

	**Deaths 2016**	**YLLs 2016**	**YLDs 2016**	**DALYs 2016**
Other neonatal disorders	58.9% (53.0% to 64.5%)	59.0% (53.1% to 64.5%)	0.0% (0.0% to 0.0%)	53.1% (46.6% to 58.8%)
**Nutritional deficiencies**	20.4% (6.6% to 29.0%)	25.4% (17.9% to 30.1%)	66.0% (55.8% to 75.7%)	65.2% (55.2% to 74.7%)
Protein-energy malnutrition	7.7% (3.2% to 12.6%)	25.2% (14.0% to 36.7%)	98.3% (98.1% to 98.5%)	95.1% (92.3% to 96.3%)
Vitamin A deficiency	-	-	0.0% (0.0% to 0.0%)	0.0% (0.0% to 0.0%)
Iron-deficiency anaemia	100.0% (100.0% to 100.0%)	100.0% (100.0% to 100.0%)	100.0% (100.0% to 100.0%)	100.0% (100.0% to 100.0%)
**Other communicable, maternal, neonatal, and nutritional diseases**	3.9% (3.2% to 5.5%)	3.1% (2.5% to 4.4%)	70.7% (63.0% to 77.6%)	36.6% (26.9% to 48.7%)
Sexually transmitted diseases excluding HIV	92.9% (82.1% to 97.8%)	81.1% (59.3% to 93.3%)	100.0% (100.0% to 100.0%)	99.1% (97.3% to 99.8%)
Hepatitis	5.5% (4.2% to 7.2%)	5.0% (3.8% to 6.5%)	1.5% (1.0% to 2.2%)	4.6% (3.4% to 6.0%)
**Neoplasms**	44.7% (41.9% to 47.5%)	45.8% (43.2% to 48.4%)	33.8% (30.8% to 36.9%)	45.3% (42.6% to 47.9%)
Oesophageal cancer	87.0% (80.4% to 92.0%)	88.0% (81.8% to 92.9%)	87.2% (80.7% to 92.3%)	88.0% (81.8% to 92.9%)
Stomach cancer	19.5% (10.7% to 29.4%)	20.5% (12.0% to 30.1%)	19.8% (11.2% to 29.5%)	20.4% (12.0% to 30.1%)
Liver cancer	81.8% (76.5% to 86.3%)	81.7% (76.6% to 86.2%)	81.3% (76.0% to 85.8%)	81.7% (76.6% to 86.2%)
Larynx cancer	90.8% (85.0% to 94.8%)	90.8% (85.3% to 94.6%)	90.9% (85.4% to 94.7%)	90.8% (85.3% to 94.6%)
Tracheal, bronchus, and lung cancer	88.1% (86.0% to 90.0%)	88.4% (86.5% to 90.1%)	87.6% (85.4% to 89.6%)	88.4% (86.4% to 90.1%)
Breast cancer	37.2% (30.7% to 44.1%)	36.9% (30.5% to 43.5%)	36.7% (30.5% to 43.2%)	36.8% (30.5% to 43.5%)
Cervical cancer	100.0% (100.0% to 100.0%)	100.0% (100.0% to 100.0%)	100.0% (100.0% to 100.0%)	100.0% (100.0% to 100.0%)
Uterine cancer	41.2% (27.8% to 54.6%)	41.9% (28.6% to 55.5%)	41.9% (28.6% to 55.8%)	41.9% (28.5% to 55.6%)
Prostate cancer	4.6% (3.2% to 6.1%)	5.1% (3.7% to 6.7%)	5.7% (4.1% to 7.4%)	5.2% (3.7% to 6.7%)
Colon and rectum cancer	60.2% (51.7% to 67.8%)	61.5% (53.3% to 68.8%)	60.8% (52.6% to 68.1%)	61.5% (53.3% to 68.8%)
Lip and oral cavity cancer	87.4% (81.7% to 91.8%)	88.4% (83.1% to 92.3%)	86.7% (80.8% to 91.1%)	88.4% (83.0% to 92.3%)
Nasopharynx cancer	88.3% (83.9% to 91.7%)	86.8% (82.8% to 90.1%)	82.8% (77.3% to 87.8%)	86.7% (82.6% to 89.9%)

Supplementary Results Table 1. Age-standardised PAFs attributable to the joint distribution of all risk factors for all ages, both sexes combined for each GBD level 1 and level 2 cause and all causes in 2016 for Austria. Results are shown for deaths, YLLs, YLDs, and DALYs
Data in parenthesis are 95% uncertainty intervals. PAFs=population attributable fractions. GBD=Global Burden of Disease. YLLs=years of life lost. YLDs=years live with disability. DALYs=disability-adjusted life-years

	**Deaths 2016**	**YLLs 2016**	**YLDs 2016**	**DALYs 2016**
Other pharynx cancer	74.2% (63.0% to 82.3%)	74.7% (63.9% to 82.7%)	75.0% (64.2% to 82.8%)	74.7% (63.9% to 82.7%)
Gallbladder and biliary tract cancer	19.7% (10.3% to 31.5%)	20.0% (10.6% to 31.9%)	20.6% (11.0% to 32.6%)	20.0% (10.6% to 31.9%)
Pancreatic cancer	30.5% (23.8% to 38.2%)	32.2% (25.5% to 39.6%)	30.5% (23.8% to 38.1%)	32.1% (25.5% to 39.6%)
Ovarian cancer	13.4% (7.1% to 20.7%)	11.0% (5.5% to 17.4%)	10.9% (5.6% to 17.2%)	11.0% (5.5% to 17.4%)
Kidney cancer	34.2% (24.4% to 44.9%)	35.1% (25.5% to 45.3%)	34.3% (25.0% to 44.5%)	35.1% (25.5% to 45.3%)
Bladder cancer	32.7% (24.0% to 41.5%)	35.2% (26.3% to 44.0%)	34.8% (26.2% to 43.6%)	35.1% (26.2% to 44.0%)
Thyroid cancer	14.1% (6.7% to 23.9%)	14.6% (6.8% to 25.1%)	13.4% (6.7% to 22.5%)	14.4% (6.8% to 24.6%)
Mesothelioma	95.9% (94.7% to 97.2%)	92.3% (90.3% to 94.6%)	94.7% (93.3% to 96.4%)	92.3% (90.4% to 94.6%)
Non-Hodgkin's lymphoma	6.8% (2.6% to 12.2%)	6.7% (2.7% to 11.9%)	6.8% (2.6% to 12.0%)	6.7% (2.7% to 11.9%)
Multiple myeloma	7.8% (3.3% to 14.1%)	8.0% (3.5% to 14.5%)	8.0% (3.5% to 14.4%)	8.0% (3.5% to 14.5%)
Leukaemia	18.8% (11.7% to 26.0%)	17.2% (10.9% to 23.4%)	17.2% (10.7% to 23.8%)	17.2% (10.9% to 23.4%)
**Cardiovascular diseases**	82.4% (79.3% to 85.0%)	84.9% (82.6% to 86.7%)	75.6% (72.6% to 78.4%)	83.0% (80.7% to 85.0%)
Rheumatic heart disease	27.8% (16.1% to 51.4%)	28.7% (19.1% to 45.0%)	27.6% (18.9% to 40.5%)	28.7% (19.0% to 44.8%)
Ischaemic heart disease	92.0% (87.7% to 95.5%)	94.0% (91.2% to 96.3%)	91.5% (88.8% to 93.8%)	93.8% (91.0% to 96.1%)
Cerebrovascular disease	83.4% (77.0% to 88.7%)	87.6% (83.5% to 90.9%)	85.8% (81.2% to 89.6%)	87.0% (82.8% to 90.3%)
Hypertensive heart disease	100.0% (100.0% to 100.0%)	100.0% (100.0% to 100.0%)	100.0% (100.0% to 100.0%)	100.0% (100.0% to 100.0%)
Cardiomyopathy and myocarditis	41.7% (31.5% to 54.6%)	48.3% (40.9% to 57.4%)	44.1% (37.7% to 51.7%)	47.7% (40.7% to 56.5%)
Atrial fibrillation and flutter	58.0% (49.7% to 66.7%)	62.8% (55.3% to 70.2%)	70.0% (64.4% to 75.9%)	67.0% (60.5% to 73.4%)
Aortic aneurysm	45.8% (38.5% to 53.3%)	52.6% (46.7% to 58.4%)	-	52.6% (46.7% to 58.4%)
Peripheral vascular disease	47.6% (40.0% to 55.8%)	54.4% (48.1% to 60.5%)	57.7% (52.1% to 63.4%)	55.1% (49.0% to 60.8%)
Endocarditis	40.5% (30.6% to 52.7%)	41.5% (33.1% to 49.8%)	41.8% (32.9% to 51.6%)	41.5% (33.0% to 49.9%)

Supplementary Results Table 1. Age-standardised PAFs attributable to the joint distribution of all risk factors for all ages, both sexes combined for each GBD level 1 and level 2 cause and all causes in 2016 for Austria. Results are shown for deaths, YLLs, YLDs, and DALYs
Data in parenthesis are 95% uncertainty intervals. PAFs=population attributable fractions. GBD=Global Burden of Disease. YLLs=years of life lost. YLDs=years live with disability. DALYs=disability-adjusted life-years

	Deaths 2016	YLLs 2016	YLDs 2016	DALYs 2016
Other cardiovascular and circulatory diseases	44.2% (37.9% to 51.0%)	51.2% (46.2% to 56.1%)	54.6% (49.6% to 59.2%)	52.8% (47.9% to 57.3%)
**Chronic respiratory diseases**	70.7% (65.9% to 74.9%)	70.9% (66.7% to 74.5%)	41.7% (35.4% to 48.4%)	54.4% (48.9% to 59.9%)
Chronic obstructive pulmonary disease	76.1% (71.1% to 80.5%)	78.0% (73.7% to 82.0%)	72.3% (67.4% to 76.7%)	76.6% (72.4% to 80.6%)
Pneumoconiosis	100.0% (100.0% to 100.0%)	100.0% (100.0% to 100.0%)	100.0% (100.0% to 100.0%)	100.0% (100.0% to 100.0%)
Asthma	38.9% (28.9% to 50.3%)	42.4% (33.5% to 52.1%)	33.6% (26.7% to 41.7%)	34.0% (27.1% to 42.0%)
Other chronic respiratory diseases	15.2% (11.5% to 19.1%)	11.7% (8.1% to 15.6%)	7.5% (4.8% to 11.2%)	9.4% (6.5% to 12.8%)
**Cirrhosis and other chronic liver diseases**	77.5% (73.7% to 81.0%)	78.1% (74.3% to 81.8%)	64.9% (60.6% to 69.1%)	77.4% (73.6% to 80.9%)
Cirrhosis and other chronic liver diseases due to hepatitis B	1.2% (0.5% to 2.0%)	1.5% (0.6% to 2.4%)	1.7% (0.8% to 3.0%)	1.5% (0.6% to 2.4%)
Cirrhosis and other chronic liver diseases due to hepatitis C	88.4% (86.2% to 89.8%)	89.7% (89.1% to 90.0%)	87.8% (86.3% to 88.9%)	89.6% (89.0% to 89.9%)
Cirrhosis and other chronic liver diseases due to alcohol use	100.0% (100.0% to 100.0%)	100.0% (100.0% to 100.0%)	100.0% (100.0% to 100.0%)	100.0% (100.0% to 100.0%)
**Digestive diseases**	11.7% (8.6% to 15.1%)	15.2% (11.3% to 19.7%)	4.0% (2.9% to 5.1%)	10.8% (8.1% to 14.2%)
Peptic ulcer disease	11.5% (8.0% to 15.6%)	16.6% (11.7% to 21.9%)	17.3% (12.0% to 23.4%)	16.8% (12.0% to 22.1%)
Gallbladder and biliary diseases	39.2% (25.1% to 54.3%)	40.2% (26.5% to 54.8%)	40.8% (27.7% to 54.6%)	40.4% (27.1% to 54.8%)
Pancreatitis	54.1% (34.3% to 72.2%)	56.6% (37.3% to 73.8%)	48.8% (30.7% to 65.6%)	56.2% (37.0% to 73.4%)
**Neurological disorders**	18.7% (9.2% to 30.3%)	17.6% (9.8% to 27.0%)	3.8% (2.0% to 6.1%)	8.1% (4.6% to 12.4%)
Alzheimer's disease and other dementias	22.0% (10.8% to 35.9%)	23.5% (12.3% to 37.4%)	23.4% (12.2% to 37.4%)	23.5% (12.3% to 37.4%)
Parkinson's disease	-7.4% (-9.0% to -5.9%)	-10.2% (-12.3% to -8.1%)	-12.5% (-14.9% to -10.1%)	-10.6% (-12.8% to -8.5%)
Epilepsy	40.3% (29.7% to 49.9%)	35.3% (25.1% to 44.1%)	29.9% (20.9% to 38.7%)	31.9% (22.6% to 40.9%)
Multiple sclerosis	11.5% (7.5% to 15.8%)	12.7% (8.4% to 17.3%)	11.7% (7.7% to 16.2%)	12.2% (8.0% to 16.6%)
**Mental and substance use disorders**	99.3% (99.0% to 99.5%)	99.0% (98.6% to 99.2%)	19.3% (17.3% to 21.5%)	26.5% (23.3% to 30.0%)
Alcohol use disorders	100.0% (100.0% to 100.0%)	100.0% (100.0% to 100.0%)	100.0% (100.0% to 100.0%)	100.0% (100.0% to 100.0%)

Supplementary Results Table 1. Age-standardised PAFs attributable to the joint distribution of all risk factors for all ages, both sexes combined for each GBD level 1 and level 2 cause and all causes in 2016 for Austria. Results are shown for deaths, YLLs, YLDs, and DALYs
Data in parenthesis are 95% uncertainty intervals. PAFs=population attributable fractions. GBD=Global Burden of Disease. YLLs=years of life lost. YLDs=years live with disability. DALYs=disability-adjusted life-years

	Deaths 2016	YLLs 2016	YLDs 2016	DALYs 2016
Drug use disorders	100.0% (100.0% to 100.0%)	100.0% (100.0% to 100.0%)	100.0% (100.0% to 100.0%)	100.0% (100.0% to 100.0%)
Depressive disorders	-	-	6.1% (5.0% to 7.4%)	6.1% (5.0% to 7.4%)
Idiopathic developmental intellectual disability	-	-	58.0% (30.1% to 78.3%)	58.0% (30.1% to 78.3%)
**Diabetes, urogenital, blood, and endocrine diseases**	76.5% (73.7% to 87.4%)	63.6% (59.4% to 80.9%)	46.8% (43.0% to 50.8%)	54.7% (51.2% to 61.9%)
Diabetes mellitus	100.0% (100.0% to 100.0%)	100.0% (100.0% to 100.0%)	100.0% (100.0% to 100.0%)	100.0% (100.0% to 100.0%)
Chronic kidney disease	100.0% (100.0% to 100.0%)	100.0% (100.0% to 100.0%)	100.0% (100.0% to 100.0%)	100.0% (100.0% to 100.0%)
**Musculoskeletal disorders**	1.1% (0.4% to 1.9%)	1.4% (0.6% to 2.4%)	18.7% (15.7% to 21.9%)	18.5% (15.6% to 21.8%)
Rheumatoid arthritis	4.6% (1.8% to 7.7%)	6.0% (2.4% to 10.0%)	7.1% (2.9% to 11.7%)	7.0% (2.9% to 11.6%)
Osteoarthritis	-	-	23.2% (12.1% to 35.0%)	23.2% (12.1% to 35.0%)
Low back and neck pain	-	-	21.5% (18.4% to 24.8%)	21.5% (18.4% to 24.8%)
Gout	-	-	45.1% (27.6% to 62.7%)	45.1% (27.6% to 62.7%)
**Other non-communicable diseases**	0.3% (0.2% to 0.4%)	0.3% (0.2% to 0.5%)	2.3% (1.9% to 2.7%)	2.1% (1.7% to 2.4%)
Sense organ diseases	-	-	7.3% (6.4% to 8.4%)	7.3% (6.4% to 8.4%)
Sudden infant death syndrome	2.9% (1.9% to 4.4%)	3.0% (1.9% to 4.4%)	-	3.0% (1.9% to 4.4%)
**Transport injuries**	42.8% (32.6% to 53.2%)	39.4% (29.2% to 49.7%)	45.0% (36.1% to 54.9%)	41.6% (31.8% to 51.7%)
Road injuries	42.4% (32.2% to 52.8%)	39.0% (28.9% to 49.6%)	45.0% (36.1% to 54.8%)	41.4% (31.6% to 51.5%)
Other transport injuries	46.0% (35.5% to 56.5%)	42.2% (31.5% to 52.5%)	45.3% (35.8% to 55.5%)	43.1% (33.0% to 53.1%)
**Unintentional injuries**	43.5% (39.7% to 49.8%)	27.7% (23.3% to 33.9%)	24.3% (20.1% to 29.9%)	25.4% (21.2% to 31.0%)
Falls	61.5% (57.7% to 65.8%)	42.1% (37.5% to 48.0%)	25.9% (21.9% to 31.2%)	30.0% (25.8% to 35.2%)
Drowning	21.8% (13.8% to 30.6%)	20.4% (13.5% to 28.0%)	21.6% (14.1% to 30.5%)	20.5% (13.6% to 28.2%)
Fire, heat, and hot substances	19.3% (11.2% to 29.2%)	21.7% (14.2% to 30.1%)	24.4% (16.5% to 33.8%)	23.6% (15.7% to 32.5%)

Supplementary Results Table 1. Age-standardised PAFs attributable to the joint distribution of all risk factors for all ages, both sexes combined for each GBD level 1 and level 2 cause and all causes in 2016 for Austria. Results are shown for deaths, YLLs, YLDs, and DALYs
Data in parenthesis are 95% uncertainty intervals. PAFs=population attributable fractions. GBD=Global Burden of Disease. YLLs=years of life lost. YLDs=years live with disability. DALYs=disability-adjusted life-years

	**Deaths 2016**	**YLLs 2016**	**YLDs 2016**	**DALYs 2016**
Poisonings	26.9% (18.0% to 36.7%)	28.4% (19.5% to 38.3%)	22.1% (14.9% to 30.5%)	25.1% (17.2% to 33.8%)
Exposure to mechanical forces	25.1% (19.3% to 32.6%)	21.6% (15.2% to 30.5%)	24.2% (18.9% to 31.6%)	23.5% (17.7% to 31.3%)
Animal contact	15.5% (12.1% to 20.0%)	14.7% (10.5% to 20.1%)	13.4% (9.0% to 19.2%)	13.8% (9.5% to 19.4%)
Foreign body	8.9% (5.7% to 13.0%)	11.0% (7.1% to 16.2%)	9.2% (5.9% to 13.7%)	10.4% (6.7% to 15.3%)
Other unintentional injuries	30.7% (21.1% to 41.5%)	30.5% (21.1% to 41.5%)	23.9% (15.8% to 33.2%)	24.4% (16.2% to 33.9%)
**Self-harm and interpersonal violence**	39.1% (23.7% to 52.8%)	37.8% (22.3% to 50.6%)	38.9% (32.5% to 45.0%)	37.9% (23.1% to 50.1%)
Self-harm	38.9% (22.6% to 53.1%)	37.9% (21.3% to 51.5%)	32.6% (16.7% to 46.9%)	37.7% (21.2% to 51.5%)
Interpersonal violence	41.8% (32.9% to 49.3%)	37.0% (28.8% to 44.3%)	40.9% (35.5% to 46.7%)	38.8% (32.0% to 45.2%)
**Forces of nature, conflict and terrorism, and executions and police conflict**	0.0% (0.0% to 0.0%)	0.0% (0.0% to 0.0%)	5.8% (3.8% to 8.9%)	4.2% (2.1% to 7.0%)
Forces of nature, conflict and terrorism, and state actor violence	0.0% (0.0% to 0.0%)	0.0% (0.0% to 0.0%)	7.1% (4.7% to 10.8%)	7.1% (4.7% to 10.8%)

Supplementary Results Table 1. Age-standardised PAFs attributable to the joint distribution of all risk factors for all ages, both sexes combined for each GBD level 1 and level 2 cause and all causes in 2016 for Azerbaijan. Results are shown for deaths, YLLs, YLDs, and DALYs
Data in parenthesis are 95% uncertainty intervals. PAFs=population attributable fractions. GBD=Global Burden of Disease. YLLs=years of life lost. YLDs=years live with disability. DALYs=disability-adjusted life-years

	Deaths 2016	YLLs 2016	YLDs 2016	DALYs 2016
**All causes**	72.6% (70.7% to 74.3%)	66.3% (64.5% to 68.0%)	27.9% (26.5% to 29.3%)	54.1% (50.9% to 57.2%)
**HIV/AIDS and tuberculosis**	59.5% (48.5% to 68.7%)	58.6% (48.3% to 68.0%)	51.3% (41.6% to 60.2%)	58.2% (48.0% to 67.5%)
Tuberculosis	58.7% (47.0% to 68.4%)	57.8% (46.8% to 67.7%)	47.7% (36.2% to 58.2%)	57.2% (46.0% to 67.2%)
HIV/AIDS	72.2% (69.4% to 74.3%)	71.0% (67.6% to 73.6%)	72.2% (69.3% to 74.1%)	71.1% (68.0% to 73.6%)
**Diarrhoea, lower respiratory infections, and other common infectious diseases**	69.5% (62.5% to 76.6%)	74.6% (67.5% to 81.0%)	41.0% (32.4% to 48.8%)	71.6% (64.9% to 78.2%)
Diarrhoeal diseases	92.8% (84.6% to 97.6%)	94.6% (88.7% to 98.2%)	79.9% (62.6% to 92.5%)	88.8% (77.9% to 96.1%)
Lower respiratory infections	74.9% (67.7% to 82.2%)	80.9% (74.5% to 87.0%)	61.4% (53.4% to 70.7%)	80.8% (74.4% to 86.8%)
Upper respiratory infections	2.0% (0.1% to 7.3%)	4.4% (0.3% to 16.0%)	0.0% (0.0% to 0.0%)	0.1% (0.0% to 0.3%)
Otitis media	4.0% (1.3% to 8.9%)	9.0% (4.1% to 15.4%)	10.0% (6.9% to 13.0%)	10.0% (6.9% to 13.0%)
Meningitis	3.8% (2.2% to 5.8%)	5.2% (3.1% to 7.6%)	0.0% (0.0% to 0.0%)	4.1% (2.5% to 6.2%)
Encephalitis	3.6% (1.1% to 7.1%)	4.3% (1.5% to 8.2%)	0.0% (0.0% to 0.0%)	3.9% (1.3% to 7.6%)
Measles	17.3% (0.0% to 73.5%)	17.6% (0.0% to 74.7%)	19.0% (0.0% to 72.8%)	27.9% (0.0% to 75.1%)
**Maternal disorders**	9.0% (4.1% to 14.2%)	8.8% (3.9% to 14.0%)	3.0% (1.3% to 5.5%)	6.8% (3.1% to 11.0%)
Maternal haemorrhage	16.3% (5.9% to 26.4%)	16.2% (5.9% to 26.4%)	16.1% (5.8% to 26.1%)	16.2% (5.9% to 26.4%)
Maternal sepsis and other pregnancy related infections	16.2% (5.9% to 26.4%)	16.2% (5.9% to 26.3%)	15.9% (5.7% to 25.8%)	16.0% (5.8% to 26.1%)
Maternal abortion, miscarriage, and ectopic pregnancy	7.4% (4.3% to 11.1%)	7.2% (4.1% to 11.0%)	5.7% (2.5% to 10.2%)	6.9% (3.9% to 10.3%)
**Neonatal disorders**	79.2% (74.6% to 83.9%)	79.2% (74.6% to 83.9%)	0.1% (0.1% to 0.2%)	74.2% (69.2% to 79.1%)
Neonatal preterm birth complications	96.5% (93.0% to 98.7%)	96.5% (93.0% to 98.7%)	0.3% (0.3% to 0.4%)	89.5% (82.6% to 93.9%)
Neonatal encephalopathy due to birth asphyxia and trauma	70.6% (65.8% to 75.5%)	70.6% (65.8% to 75.5%)	0.0% (0.0% to 0.0%)	68.2% (62.5% to 73.6%)
Neonatal sepsis and other neonatal infections	54.3% (45.5% to 62.4%)	54.3% (45.5% to 62.4%)	0.0% (0.0% to 0.0%)	46.8% (34.5% to 56.9%)
Hemolytic disease and other neonatal jaundice	74.9% (69.8% to 79.2%)	74.9% (69.8% to 79.2%)	0.0% (0.0% to 0.0%)	33.8% (20.8% to 47.8%)

Supplementary Results Table 1. Age-standardised PAFs attributable to the joint distribution of all risk factors for all ages, both sexes combined for each GBD level 1 and level 2 cause and all causes in 2016 for Azerbaijan. Results are shown for deaths, YLLs, YLDs, and DALYs
Data in parenthesis are 95% uncertainty intervals. PAFs=population attributable fractions. GBD=Global Burden of Disease. YLLs=years of life lost. YLDs=years live with disability. DALYs=disability-adjusted life-years

	Deaths 2016	YLLs 2016	YLDs 2016	DALYs 2016
Other neonatal disorders	64.3% (53.2% to 73.1%)	64.3% (53.2% to 73.1%)	0.0% (0.0% to 0.0%)	58.4% (47.1% to 67.7%)
**Nutritional deficiencies**	40.5% (26.0% to 54.9%)	56.6% (36.6% to 70.6%)	94.4% (92.8% to 95.8%)	92.3% (90.1% to 94.1%)
Protein-energy malnutrition	49.0% (29.8% to 66.8%)	70.6% (51.3% to 84.1%)	98.3% (97.0% to 99.2%)	87.5% (80.9% to 92.3%)
Vitamin A deficiency	-	-	0.0% (0.0% to 0.0%)	0.0% (0.0% to 0.0%)
Iron-deficiency anaemia	100.0% (100.0% to 100.0%)	100.0% (100.0% to 100.0%)	100.0% (100.0% to 100.0%)	100.0% (100.0% to 100.0%)
**Other communicable, maternal, neonatal, and nutritional diseases**	4.3% (1.4% to 10.7%)	2.3% (0.6% to 6.9%)	73.7% (69.5% to 78.0%)	28.4% (11.8% to 42.4%)
Sexually transmitted diseases excluding HIV	63.9% (31.8% to 98.6%)	41.8% (14.2% to 95.9%)	100.0% (100.0% to 100.0%)	94.2% (84.3% to 99.9%)
Hepatitis	2.4% (1.7% to 3.2%)	1.9% (1.3% to 2.6%)	1.0% (0.6% to 1.5%)	1.8% (1.3% to 2.5%)
**Neoplasms**	41.8% (36.9% to 46.5%)	40.3% (35.7% to 44.7%)	36.2% (32.3% to 40.0%)	40.3% (35.6% to 44.7%)
Oesophageal cancer	73.3% (62.2% to 82.8%)	77.4% (67.6% to 85.7%)	74.5% (63.8% to 83.8%)	77.3% (67.6% to 85.7%)
Stomach cancer	20.6% (11.3% to 31.6%)	22.8% (13.0% to 34.2%)	21.3% (12.0% to 31.9%)	22.8% (13.0% to 34.2%)
Liver cancer	61.1% (53.3% to 68.3%)	62.7% (55.0% to 69.9%)	61.8% (54.2% to 69.0%)	62.7% (55.0% to 69.9%)
Larynx cancer	80.4% (70.9% to 87.3%)	84.0% (75.8% to 89.9%)	82.5% (73.8% to 88.7%)	84.0% (75.7% to 89.9%)
Tracheal, bronchus, and lung cancer	84.1% (77.8% to 88.6%)	85.7% (80.6% to 89.7%)	84.5% (78.7% to 88.8%)	85.7% (80.5% to 89.7%)
Breast cancer	28.5% (21.5% to 35.2%)	26.4% (19.6% to 33.1%)	26.9% (20.1% to 33.7%)	26.5% (19.7% to 33.1%)
Cervical cancer	100.0% (100.0% to 100.0%)	100.0% (100.0% to 100.0%)	100.0% (100.0% to 100.0%)	100.0% (100.0% to 100.0%)
Uterine cancer	48.6% (33.6% to 62.8%)	49.3% (33.9% to 63.4%)	49.8% (34.2% to 64.1%)	49.3% (33.9% to 63.5%)
Prostate cancer	3.4% (2.0% to 5.3%)	4.8% (2.9% to 7.3%)	4.9% (3.0% to 7.4%)	4.8% (2.9% to 7.3%)
Colon and rectum cancer	50.1% (41.2% to 58.2%)	51.2% (42.7% to 59.1%)	51.0% (42.3% to 59.1%)	51.2% (42.7% to 59.1%)
Lip and oral cavity cancer	68.8% (57.2% to 78.4%)	72.5% (62.4% to 80.5%)	67.5% (55.8% to 77.0%)	72.4% (62.2% to 80.5%)
Nasopharynx cancer	66.9% (59.0% to 73.9%)	60.8% (52.7% to 67.9%)	62.5% (53.7% to 70.3%)	60.8% (52.8% to 67.8%)

Supplementary Results Table 1. Age-standardised PAFs attributable to the joint distribution of all risk factors for all ages, both sexes combined for each GBD level 1 and level 2 cause and all causes in 2016 for Azerbaijan. Results are shown for deaths, YLLs, YLDs, and DALYs
Data in parenthesis are 95% uncertainty intervals. PAFs=population attributable fractions. GBD=Global Burden of Disease. YLLs=years of life lost. YLDs=years live with disability. DALYs=disability-adjusted life-years

	Deaths 2016	YLLs 2016	YLDs 2016	DALYs 2016
Other pharynx cancer	61.4% (46.5% to 72.3%)	63.7% (50.0% to 73.6%)	61.9% (48.0% to 72.6%)	63.7% (50.0% to 73.6%)
Gallbladder and biliary tract cancer	22.6% (12.2% to 34.8%)	23.5% (13.0% to 35.6%)	23.0% (12.6% to 34.6%)	23.5% (13.0% to 35.6%)
Pancreatic cancer	25.6% (17.5% to 34.4%)	29.4% (20.7% to 38.2%)	26.6% (18.6% to 35.0%)	29.4% (20.7% to 38.2%)
Ovarian cancer	9.3% (3.0% to 16.8%)	8.5% (2.4% to 15.5%)	8.1% (2.3% to 14.7%)	8.5% (2.4% to 15.5%)
Kidney cancer	34.0% (23.4% to 45.2%)	33.7% (23.1% to 44.6%)	34.3% (24.4% to 44.6%)	33.7% (23.1% to 44.6%)
Bladder cancer	29.2% (19.0% to 39.4%)	35.2% (23.6% to 46.3%)	33.7% (23.0% to 43.8%)	35.2% (23.6% to 46.2%)
Thyroid cancer	13.5% (6.9% to 22.1%)	13.6% (6.9% to 22.3%)	13.8% (7.5% to 21.6%)	13.6% (6.9% to 22.2%)
Mesothelioma	72.2% (64.4% to 79.2%)	59.3% (50.1% to 68.4%)	68.4% (60.2% to 76.1%)	59.5% (50.3% to 68.5%)
Non-Hodgkin's lymphoma	5.8% (2.1% to 10.2%)	4.9% (1.9% to 9.0%)	5.7% (2.1% to 10.3%)	4.9% (1.9% to 9.0%)
Multiple myeloma	7.6% (3.2% to 13.8%)	7.8% (3.3% to 14.0%)	7.8% (3.3% to 14.0%)	7.8% (3.3% to 14.0%)
Leukaemia	13.7% (8.6% to 19.5%)	9.6% (5.9% to 13.9%)	12.4% (7.8% to 17.7%)	9.6% (5.9% to 14.0%)
**Cardiovascular diseases**	90.2% (87.6% to 92.4%)	90.7% (88.9% to 92.2%)	79.5% (76.3% to 82.0%)	90.0% (88.1% to 91.5%)
Rheumatic heart disease	27.9% (19.4% to 43.3%)	24.4% (16.9% to 36.2%)	23.7% (15.5% to 34.0%)	24.2% (16.6% to 35.7%)
Ischaemic heart disease	93.5% (90.4% to 96.0%)	94.7% (92.7% to 96.2%)	92.1% (89.6% to 94.4%)	94.6% (92.5% to 96.1%)
Cerebrovascular disease	86.2% (81.7% to 90.3%)	89.7% (86.6% to 92.5%)	87.3% (83.6% to 90.6%)	89.5% (86.3% to 92.2%)
Hypertensive heart disease	100.0% (100.0% to 100.0%)	100.0% (100.0% to 100.0%)	100.0% (100.0% to 100.0%)	100.0% (100.0% to 100.0%)
Cardiomyopathy and myocarditis	49.2% (42.4% to 56.6%)	42.4% (35.3% to 52.7%)	47.8% (42.5% to 53.4%)	42.7% (35.7% to 52.5%)
Atrial fibrillation and flutter	53.4% (45.3% to 61.5%)	57.7% (50.4% to 64.5%)	64.4% (58.7% to 70.1%)	61.7% (55.7% to 67.9%)
Aortic aneurysm	50.5% (43.2% to 57.3%)	55.9% (49.9% to 61.5%)	-	55.9% (49.9% to 61.5%)
Peripheral vascular disease	50.7% (42.7% to 59.3%)	57.9% (50.9% to 64.5%)	61.5% (55.8% to 66.9%)	61.0% (55.5% to 66.4%)
Endocarditis	40.4% (31.4% to 49.6%)	33.3% (24.1% to 42.2%)	41.1% (32.5% to 49.7%)	33.4% (24.2% to 42.3%)

Supplementary Results Table 1. Age-standardised PAFs attributable to the joint distribution of all risk factors for all ages, both sexes combined for each GBD level 1 and level 2 cause and all causes in 2016 for Azerbaijan. Results are shown for deaths, YLLs, YLDs, and DALYs
Data in parenthesis are 95% uncertainty intervals. PAFs=population attributable fractions. GBD=Global Burden of Disease. YLLs=years of life lost. YLDs=years live with disability. DALYs=disability-adjusted life-years

	Deaths 2016	YLLs 2016	YLDs 2016	DALYs 2016
Other cardiovascular and circulatory diseases	51.5% (45.9% to 56.4%)	52.8% (47.1% to 57.8%)	52.0% (47.3% to 56.7%)	52.6% (47.7% to 57.3%)
**Chronic respiratory diseases**	57.3% (49.3% to 65.1%)	59.2% (52.4% to 65.4%)	42.9% (36.4% to 50.2%)	53.6% (47.9% to 59.3%)
Chronic obstructive pulmonary disease	64.7% (55.4% to 74.0%)	68.7% (60.7% to 76.1%)	69.2% (61.2% to 76.1%)	68.8% (61.1% to 76.1%)
Pneumoconiosis	100.0% (100.0% to 100.0%)	100.0% (100.0% to 100.0%)	100.0% (100.0% to 100.0%)	100.0% (100.0% to 100.0%)
Asthma	39.8% (29.7% to 51.3%)	43.6% (33.8% to 54.6%)	35.1% (26.5% to 44.8%)	36.8% (28.4% to 46.9%)
Other chronic respiratory diseases	14.1% (8.4% to 20.7%)	16.1% (9.6% to 23.8%)	7.2% (4.3% to 10.5%)	15.0% (8.9% to 22.1%)
**Cirrhosis and other chronic liver diseases**	41.3% (38.4% to 44.0%)	42.8% (40.2% to 45.3%)	32.1% (29.3% to 34.9%)	42.4% (39.8% to 44.9%)
Cirrhosis and other chronic liver diseases due to hepatitis B	0.7% (0.4% to 1.2%)	1.0% (0.5% to 1.7%)	0.8% (0.4% to 1.4%)	1.0% (0.5% to 1.6%)
Cirrhosis and other chronic liver diseases due to hepatitis C	70.9% (62.8% to 78.3%)	80.5% (75.5% to 84.8%)	79.2% (74.0% to 83.4%)	80.5% (75.4% to 84.7%)
Cirrhosis and other chronic liver diseases due to alcohol use	100.0% (100.0% to 100.0%)	100.0% (100.0% to 100.0%)	100.0% (100.0% to 100.0%)	100.0% (100.0% to 100.0%)
**Digestive diseases**	11.9% (9.0% to 15.2%)	12.3% (9.3% to 15.5%)	3.3% (2.5% to 4.3%)	9.6% (7.2% to 12.1%)
Peptic ulcer disease	21.5% (15.4% to 28.2%)	22.8% (16.5% to 29.5%)	9.9% (7.1% to 13.0%)	20.7% (14.9% to 26.8%)
Gallbladder and biliary diseases	42.1% (28.9% to 56.4%)	42.5% (29.1% to 56.3%)	47.5% (33.4% to 62.1%)	43.7% (30.0% to 57.6%)
Pancreatitis	40.3% (20.3% to 59.5%)	46.6% (26.2% to 65.0%)	33.1% (17.0% to 50.1%)	46.1% (25.9% to 64.4%)
**Neurological disorders**	18.5% (9.1% to 30.0%)	17.1% (10.8% to 24.5%)	4.1% (2.0% to 6.6%)	8.3% (5.2% to 12.2%)
Alzheimer's disease and other dementias	22.8% (10.8% to 36.8%)	23.7% (11.8% to 37.7%)	23.8% (11.6% to 37.8%)	23.7% (11.7% to 37.8%)
Parkinson's disease	-12.0% (-17.3% to -7.9%)	-14.7% (-20.6% to -10.0%)	-15.6% (-21.1% to -10.9%)	-14.9% (-20.4% to -10.2%)
Epilepsy	23.1% (15.5% to 30.7%)	19.1% (12.4% to 25.9%)	20.7% (13.6% to 27.9%)	19.8% (13.2% to 26.4%)
Multiple sclerosis	9.7% (4.9% to 14.9%)	10.2% (5.2% to 15.5%)	6.4% (3.5% to 9.3%)	7.8% (4.3% to 11.4%)
**Mental and substance use disorders**	100.0% (100.0% to 100.0%)	100.0% (99.9% to 100.0%)	24.8% (22.2% to 27.6%)	37.1% (32.2% to 46.6%)
Alcohol use disorders	100.0% (100.0% to 100.0%)	100.0% (100.0% to 100.0%)	100.0% (100.0% to 100.0%)	100.0% (100.0% to 100.0%)

Supplementary Results Table 1. Age-standardised PAFs attributable to the joint distribution of all risk factors for all ages, both sexes combined for each GBD level 1 and level 2 cause and all causes in 2016 for Azerbaijan. Results are shown for deaths, YLLs, YLDs, and DALYs
Data in parenthesis are 95% uncertainty intervals. PAFs=population attributable fractions. GBD=Global Burden of Disease. YLLs=years of life lost. YLDs=years live with disability. DALYs=disability-adjusted life-years

	Deaths 2016	YLLs 2016	YLDs 2016	DALYs 2016
Drug use disorders	100.0% (100.0% to 100.0%)	100.0% (100.0% to 100.0%)	100.0% (100.0% to 100.0%)	100.0% (100.0% to 100.0%)
Depressive disorders	-	-	4.1% (3.1% to 5.2%)	4.1% (3.1% to 5.2%)
Idiopathic developmental intellectual disability	-	-	56.5% (27.3% to 77.1%)	56.5% (27.3% to 77.1%)
**Diabetes, urogenital, blood, and endocrine diseases**	82.5% (80.6% to 84.2%)	74.2% (70.0% to 77.9%)	59.1% (54.5% to 63.1%)	68.0% (64.4% to 71.3%)
Diabetes mellitus	100.0% (100.0% to 100.0%)	100.0% (100.0% to 100.0%)	100.0% (100.0% to 100.0%)	100.0% (100.0% to 100.0%)
Chronic kidney disease	100.0% (100.0% to 100.0%)	100.0% (100.0% to 100.0%)	100.0% (100.0% to 100.0%)	100.0% (100.0% to 100.0%)
**Musculoskeletal disorders**	0.7% (0.3% to 1.3%)	0.6% (0.2% to 1.1%)	25.7% (21.7% to 29.9%)	25.6% (21.7% to 29.8%)
Rheumatoid arthritis	2.7% (1.1% to 5.0%)	2.8% (1.1% to 5.3%)	3.7% (1.6% to 6.2%)	3.7% (1.6% to 6.1%)
Osteoarthritis	-	-	27.0% (14.2% to 39.9%)	27.0% (14.2% to 39.9%)
Low back and neck pain	-	-	27.9% (24.2% to 31.7%)	27.9% (24.2% to 31.7%)
Gout	-	-	48.0% (34.4% to 62.6%)	48.0% (34.4% to 62.6%)
**Other non-communicable diseases**	0.2% (0.1% to 0.4%)	0.2% (0.1% to 0.4%)	5.5% (4.7% to 6.3%)	4.2% (3.4% to 5.0%)
Sense organ diseases	-	-	11.6% (10.2% to 13.2%)	11.6% (10.2% to 13.2%)
Sudden infant death syndrome	6.8% (3.2% to 12.7%)	6.8% (3.2% to 12.8%)	-	6.8% (3.2% to 12.8%)
**Transport injuries**	34.3% (26.4% to 43.6%)	30.4% (22.8% to 39.6%)	38.7% (31.4% to 47.1%)	32.4% (24.8% to 41.2%)
Road injuries	34.2% (26.3% to 43.6%)	30.6% (22.9% to 39.8%)	39.8% (32.4% to 48.1%)	32.7% (25.0% to 41.5%)
Other transport injuries	35.6% (27.9% to 43.7%)	27.7% (20.4% to 35.8%)	28.7% (22.0% to 36.3%)	28.1% (21.3% to 35.5%)
**Unintentional injuries**	15.9% (12.7% to 19.7%)	12.1% (9.3% to 15.5%)	19.2% (16.5% to 22.4%)	15.5% (12.7% to 18.8%)
Falls	31.5% (26.6% to 36.7%)	18.2% (14.6% to 22.6%)	23.3% (20.8% to 26.6%)	22.2% (19.3% to 25.7%)
Drowning	16.1% (11.6% to 21.8%)	13.7% (9.8% to 18.6%)	18.2% (12.8% to 25.4%)	13.8% (9.8% to 18.6%)
Fire, heat, and hot substances	15.4% (10.9% to 20.8%)	13.4% (9.4% to 18.2%)	17.7% (12.5% to 24.5%)	14.7% (10.3% to 20.0%)

**Supplementary Results Table 1. Age-standardised PAFs attributable to the joint distribution of all risk factors for all ages, both sexes combined for each GBD level 1 and level 2 cause and all causes in 2016 for Azerbaijan. Results are shown for deaths, YLLs, YLDs, and DALYs** Data in parenthesis are 95% uncertainty intervals. PAFs=population attributable fractions. GBD=Global Burden of Disease. YLLs=years of life lost. YLDs=years live with disability. DALYs=disability-adjusted life-years				
	**Deaths 2016**	**YLLs 2016**	**YLDs 2016**	**DALYs 2016**
Poisonings	19.2% (13.2% to 26.0%)	16.5% (11.3% to 22.9%)	16.8% (11.8% to 23.3%)	16.6% (11.6% to 22.6%)
Exposure to mechanical forces	15.6% (11.5% to 21.0%)	13.0% (9.1% to 18.4%)	18.1% (14.9% to 22.4%)	15.7% (12.3% to 19.9%)
Animal contact	9.7% (7.4% to 13.1%)	6.7% (4.6% to 9.5%)	9.2% (6.7% to 12.4%)	8.2% (6.0% to 11.0%)
Foreign body	4.6% (3.2% to 6.4%)	3.8% (2.6% to 5.4%)	8.0% (5.8% to 11.0%)	5.5% (3.9% to 7.6%)
Other unintentional injuries	20.9% (14.9% to 28.4%)	18.4% (13.0% to 25.2%)	16.8% (11.8% to 23.5%)	17.6% (12.6% to 24.0%)
**Self-harm and interpersonal violence**	27.4% (19.3% to 35.6%)	26.2% (18.6% to 34.5%)	26.2% (20.8% to 32.6%)	26.2% (19.0% to 34.0%)
Self-harm	30.9% (18.3% to 43.2%)	30.9% (18.6% to 42.7%)	26.2% (13.6% to 38.8%)	30.8% (18.5% to 42.5%)
Interpersonal violence	23.6% (15.8% to 32.7%)	21.8% (14.1% to 30.9%)	26.2% (20.5% to 32.8%)	22.6% (15.5% to 31.3%)
**Forces of nature, conflict and terrorism, and executions and police conflict**	0.0% (0.0% to 0.0%)	0.0% (0.0% to 0.0%)	0.1% (0.1% to 0.1%)	0.1% (0.1% to 0.1%)
Forces of nature, conflict and terrorism, and state actor violence	0.0% (0.0% to 0.0%)	0.0% (0.0% to 0.0%)	4.6% (3.1% to 6.6%)	4.6% (3.1% to 6.6%)

Supplementary Results Table 1. Age-standardised PAFs attributable to the joint distribution of all risk factors for all ages, both sexes combined for each GBD level 1 and level 2 cause and all causes in 2016 for Bahrain. Results are shown for deaths, YLLs, YLDs, and DALYs
Data in parenthesis are 95% uncertainty intervals. PAFs=population attributable fractions. GBD=Global Burden of Disease. YLLs=years of life lost. YLDs=years live with disability. DALYs=disability-adjusted life-years

	Deaths 2016	YLLs 2016	YLDs 2016	DALYs 2016
**All causes**	64.8% (61.9% to 67.4%)	59.6% (57.5% to 61.7%)	30.2% (28.4% to 32.2%)	45.5% (42.7% to 48.6%)
**HIV/AIDS and tuberculosis**	50.5% (43.0% to 57.9%)	56.2% (51.2% to 61.4%)	48.8% (38.3% to 59.0%)	55.7% (50.9% to 60.8%)
Tuberculosis	36.9% (25.2% to 48.6%)	37.7% (27.2% to 47.7%)	31.1% (21.3% to 40.8%)	37.1% (27.1% to 47.0%)
HIV/AIDS	73.7% (71.9% to 75.0%)	71.3% (68.0% to 73.7%)	74.0% (73.1% to 74.7%)	71.5% (68.2% to 73.7%)
**Diarrhoea, lower respiratory infections, and other common infectious diseases**	54.4% (47.3% to 61.7%)	54.2% (47.8% to 60.5%)	28.4% (19.7% to 36.3%)	45.2% (38.8% to 51.5%)
Diarrhoeal diseases	62.4% (36.3% to 84.1%)	67.1% (43.6% to 86.6%)	68.7% (47.1% to 86.4%)	68.2% (46.5% to 86.4%)
Lower respiratory infections	58.4% (50.5% to 66.2%)	63.0% (55.9% to 70.3%)	59.0% (51.4% to 66.5%)	62.9% (55.8% to 70.2%)
Upper respiratory infections	0.5% (0.0% to 1.5%)	1.4% (0.1% to 4.6%)	0.0% (0.0% to 0.0%)	0.0% (0.0% to 0.0%)
Otitis media	0.6% (0.3% to 1.2%)	2.4% (1.3% to 4.2%)	5.3% (3.5% to 7.0%)	5.3% (3.4% to 7.0%)
Meningitis	0.7% (0.4% to 1.1%)	1.8% (1.2% to 2.6%)	0.0% (0.0% to 0.0%)	1.1% (0.7% to 1.6%)
Encephalitis	0.5% (0.3% to 0.8%)	1.2% (0.8% to 1.7%)	0.0% (0.0% to 0.0%)	0.6% (0.4% to 0.9%)
Measles	27.7% (8.6% to 67.9%)	28.2% (8.8% to 69.1%)	27.5% (8.6% to 66.9%)	28.1% (8.8% to 69.0%)
**Maternal disorders**	8.6% (5.5% to 12.1%)	8.3% (5.3% to 11.8%)	4.3% (2.0% to 6.9%)	7.2% (4.6% to 10.3%)
Maternal haemorrhage	15.3% (5.6% to 24.6%)	15.3% (5.6% to 24.6%)	15.4% (5.6% to 24.9%)	15.3% (5.6% to 24.6%)
Maternal sepsis and other pregnancy related infections	15.1% (5.6% to 24.3%)	15.1% (5.6% to 24.3%)	15.3% (5.6% to 24.6%)	15.2% (5.6% to 24.5%)
Maternal abortion, miscarriage, and ectopic pregnancy	11.1% (6.5% to 16.4%)	10.8% (6.3% to 15.8%)	11.8% (6.4% to 17.9%)	10.8% (6.3% to 16.1%)
**Neonatal disorders**	64.5% (59.3% to 69.0%)	64.6% (59.4% to 69.1%)	0.1% (0.1% to 0.2%)	46.7% (37.7% to 53.3%)
Neonatal preterm birth complications	75.1% (68.5% to 80.4%)	75.2% (68.6% to 80.5%)	0.2% (0.1% to 0.2%)	55.6% (45.9% to 63.7%)
Neonatal encephalopathy due to birth asphyxia and trauma	48.8% (41.2% to 56.1%)	48.9% (41.4% to 56.2%)	0.0% (0.0% to 0.0%)	38.1% (29.4% to 46.9%)
Neonatal sepsis and other neonatal infections	45.3% (33.0% to 55.9%)	45.4% (33.1% to 56.0%)	0.0% (0.0% to 0.0%)	23.5% (8.7% to 39.6%)
Hemolytic disease and other neonatal jaundice	49.1% (37.4% to 58.4%)	49.2% (37.6% to 58.5%)	0.0% (0.0% to 0.0%)	23.3% (16.3% to 31.8%)

Supplementary Results Table 1. Age-standardised PAFs attributable to the joint distribution of all risk factors for all ages, both sexes combined for each GBD level 1 and level 2 cause and all causes in 2016 for Bahrain. Results are shown for deaths, YLLs, YLDs, and DALYs
Data in parenthesis are 95% uncertainty intervals. PAFs=population attributable fractions. GBD=Global Burden of Disease. YLLs=years of life lost. YLDs=years live with disability. DALYs=disability-adjusted life-years

	Deaths 2016	YLLs 2016	YLDs 2016	DALYs 2016
Other neonatal disorders	49.9% (42.2% to 57.2%)	50.0% (42.3% to 57.3%)	0.0% (0.0% to 0.0%)	46.0% (38.8% to 53.3%)
**Nutritional deficiencies**	4.9% (3.0% to 7.3%)	25.0% (16.7% to 34.1%)	89.3% (85.8% to 92.3%)	86.6% (82.8% to 89.5%)
Protein-energy malnutrition	6.0% (3.4% to 9.5%)	32.4% (21.1% to 44.0%)	98.0% (97.2% to 98.7%)	77.1% (68.9% to 84.0%)
Vitamin A deficiency	-	-	0.0% (0.0% to 0.0%)	0.0% (0.0% to 0.0%)
Iron-deficiency anaemia	100.0% (100.0% to 100.0%)	100.0% (100.0% to 100.0%)	100.0% (100.0% to 100.0%)	100.0% (100.0% to 100.0%)
**Other communicable, maternal, neonatal, and nutritional diseases**	4.2% (2.4% to 5.3%)	4.0% (3.0% to 4.9%)	82.1% (76.5% to 87.1%)	48.3% (37.5% to 59.1%)
Sexually transmitted diseases excluding HIV	87.5% (72.3% to 97.5%)	64.9% (40.0% to 90.3%)	100.0% (100.0% to 100.0%)	98.3% (95.6% to 99.7%)
Hepatitis	1.0% (0.6% to 1.4%)	1.2% (0.8% to 1.8%)	1.0% (0.6% to 1.6%)	1.2% (0.8% to 1.8%)
**Neoplasms**	38.9% (33.7% to 44.4%)	36.6% (31.5% to 42.1%)	32.1% (26.7% to 38.2%)	36.5% (31.4% to 42.1%)
Oesophageal cancer	68.5% (54.4% to 81.1%)	67.9% (53.9% to 81.2%)	68.1% (54.4% to 81.1%)	67.9% (53.9% to 81.2%)
Stomach cancer	14.9% (6.9% to 24.7%)	13.6% (6.2% to 22.7%)	14.2% (6.5% to 23.6%)	13.6% (6.2% to 22.7%)
Liver cancer	45.8% (34.1% to 57.9%)	47.4% (36.0% to 59.5%)	46.1% (34.8% to 58.3%)	47.4% (36.0% to 59.5%)
Larynx cancer	72.0% (60.6% to 81.6%)	68.0% (56.4% to 78.5%)	70.4% (58.6% to 81.0%)	68.1% (56.5% to 78.5%)
Tracheal, bronchus, and lung cancer	86.4% (81.2% to 90.7%)	84.1% (78.6% to 89.0%)	85.2% (79.8% to 89.7%)	84.2% (78.6% to 89.0%)
Breast cancer	30.0% (18.4% to 44.2%)	26.1% (15.1% to 39.3%)	26.5% (15.3% to 40.1%)	26.1% (15.1% to 39.3%)
Cervical cancer	100.0% (100.0% to 100.0%)	100.0% (100.0% to 100.0%)	100.0% (100.0% to 100.0%)	100.0% (100.0% to 100.0%)
Uterine cancer	54.5% (38.4% to 68.4%)	55.9% (40.7% to 68.9%)	56.2% (40.8% to 69.4%)	55.9% (40.7% to 68.9%)
Prostate cancer	3.7% (2.2% to 5.5%)	3.5% (2.1% to 5.3%)	3.5% (2.1% to 5.3%)	3.5% (2.1% to 5.3%)
Colon and rectum cancer	51.7% (39.5% to 64.6%)	51.3% (39.7% to 63.5%)	51.7% (39.8% to 64.4%)	51.3% (39.7% to 63.6%)
Lip and oral cavity cancer	57.7% (46.6% to 69.4%)	55.3% (44.3% to 67.2%)	55.3% (44.6% to 67.2%)	55.3% (44.4% to 67.1%)
Nasopharynx cancer	53.5% (42.4% to 64.1%)	49.0% (38.4% to 59.6%)	47.7% (37.6% to 57.4%)	49.0% (38.4% to 59.6%)

Supplementary Results Table 1. Age-standardised PAFs attributable to the joint distribution of all risk factors for all ages, both sexes combined for each GBD level 1 and level 2 cause and all causes in 2016 for Bahrain. Results are shown for deaths, YLLs, YLDs, and DALYs
Data in parenthesis are 95% uncertainty intervals. PAFs=population attributable fractions. GBD=Global Burden of Disease. YLLs=years of life lost. YLDs=years live with disability. DALYs=disability-adjusted life-years

	Deaths 2016	YLLs 2016	YLDs 2016	DALYs 2016
Other pharynx cancer	24.6% (11.3% to 40.6%)	27.2% (13.5% to 42.1%)	26.0% (12.6% to 41.6%)	27.2% (13.5% to 42.1%)
Gallbladder and biliary tract cancer	25.8% (14.7% to 38.8%)	26.8% (15.4% to 39.9%)	26.2% (15.0% to 38.9%)	26.7% (15.4% to 39.9%)
Pancreatic cancer	35.0% (22.0% to 49.8%)	33.6% (21.2% to 48.1%)	34.6% (21.8% to 49.4%)	33.6% (21.2% to 48.1%)
Ovarian cancer	21.7% (8.5% to 38.7%)	20.3% (7.6% to 36.4%)	19.6% (7.5% to 35.1%)	20.2% (7.6% to 36.3%)
Kidney cancer	34.6% (24.6% to 45.9%)	32.9% (23.1% to 43.8%)	33.4% (23.6% to 44.1%)	32.9% (23.2% to 43.8%)
Bladder cancer	35.5% (22.2% to 50.8%)	33.2% (20.4% to 48.5%)	32.8% (20.1% to 47.8%)	33.2% (20.3% to 48.5%)
Thyroid cancer	17.6% (9.9% to 26.6%)	18.4% (10.4% to 27.8%)	18.3% (10.6% to 26.6%)	18.4% (10.4% to 27.7%)
Mesothelioma	92.9% (89.2% to 95.1%)	86.7% (81.4% to 90.4%)	91.0% (86.9% to 93.6%)	86.8% (81.5% to 90.5%)
Non-Hodgkin's lymphoma	8.9% (4.0% to 14.9%)	8.8% (3.9% to 14.5%)	8.9% (4.0% to 14.8%)	8.8% (3.9% to 14.5%)
Multiple myeloma	10.3% (4.6% to 17.5%)	10.7% (4.8% to 17.9%)	10.6% (4.7% to 18.0%)	10.7% (4.8% to 17.9%)
Leukaemia	18.6% (11.6% to 25.7%)	16.0% (10.3% to 22.0%)	18.0% (11.4% to 24.9%)	16.0% (10.3% to 22.1%)
**Cardiovascular diseases**	86.4% (83.3% to 88.9%)	87.4% (85.3% to 89.2%)	76.4% (73.7% to 79.0%)	86.1% (83.9% to 87.9%)
Rheumatic heart disease	24.7% (15.9% to 41.1%)	22.9% (15.5% to 35.2%)	25.5% (17.2% to 38.2%)	22.9% (15.6% to 35.3%)
Ischaemic heart disease	92.9% (89.2% to 95.9%)	94.2% (91.9% to 96.1%)	91.8% (89.2% to 94.3%)	94.0% (91.8% to 95.9%)
Cerebrovascular disease	82.7% (77.1% to 88.1%)	85.2% (81.5% to 88.5%)	86.0% (81.6% to 90.4%)	85.4% (81.6% to 88.9%)
Hypertensive heart disease	100.0% (100.0% to 100.0%)	100.0% (100.0% to 100.0%)	100.0% (100.0% to 100.0%)	100.0% (100.0% to 100.0%)
Cardiomyopathy and myocarditis	39.0% (30.7% to 48.4%)	37.6% (30.3% to 45.3%)	38.3% (31.2% to 45.4%)	37.6% (30.5% to 45.3%)
Atrial fibrillation and flutter	46.4% (38.2% to 54.3%)	50.8% (43.3% to 57.8%)	57.6% (51.8% to 63.2%)	54.6% (47.9% to 60.8%)
Aortic aneurysm	38.9% (32.0% to 45.8%)	43.6% (37.3% to 49.3%)	-	43.6% (37.3% to 49.3%)
Peripheral vascular disease	62.3% (55.3% to 68.3%)	69.4% (62.9% to 74.1%)	71.1% (66.7% to 75.4%)	69.8% (64.0% to 74.1%)
Endocarditis	35.9% (26.4% to 47.3%)	34.4% (26.6% to 42.5%)	37.0% (28.1% to 46.3%)	34.5% (26.7% to 42.5%)

Supplementary Results Table 1. Age-standardised PAFs attributable to the joint distribution of all risk factors for all ages, both sexes combined for each GBD level 1 and level 2 cause and all causes in 2016 for Bahrain. Results are shown for deaths, YLLs, YLDs, and DALYs
Data in parenthesis are 95% uncertainty intervals. PAFs=population attributable fractions. GBD=Global Burden of Disease. YLLs=years of life lost. YLDs=years live with disability. DALYs=disability-adjusted life-years

	Deaths 2016	YLLs 2016	YLDs 2016	DALYs 2016
Other cardiovascular and circulatory diseases	39.9% (34.7% to 45.4%)	41.3% (36.7% to 46.3%)	43.7% (38.4% to 48.7%)	42.2% (37.7% to 46.8%)
**Chronic respiratory diseases**	65.3% (59.8% to 70.1%)	61.2% (55.9% to 66.2%)	49.3% (42.5% to 56.5%)	55.8% (50.4% to 61.2%)
Chronic obstructive pulmonary disease	81.2% (74.3% to 86.8%)	79.1% (72.3% to 84.9%)	69.9% (61.2% to 77.9%)	76.1% (69.0% to 82.7%)
Pneumoconiosis	100.0% (100.0% to 100.0%)	100.0% (100.0% to 100.0%)	100.0% (100.0% to 100.0%)	100.0% (100.0% to 100.0%)
Asthma	43.2% (31.0% to 55.7%)	45.5% (33.6% to 57.3%)	39.1% (29.4% to 49.5%)	40.6% (31.0% to 51.3%)
Other chronic respiratory diseases	11.7% (7.8% to 17.1%)	8.5% (5.0% to 13.1%)	3.7% (1.6% to 7.3%)	7.1% (4.2% to 11.2%)
**Cirrhosis and other chronic liver diseases**	16.4% (13.8% to 19.4%)	21.9% (17.8% to 26.3%)	20.3% (16.1% to 24.7%)	21.7% (17.8% to 26.2%)
Cirrhosis and other chronic liver diseases due to hepatitis B	0.2% (0.1% to 0.4%)	0.3% (0.1% to 0.5%)	0.3% (0.1% to 0.6%)	0.3% (0.1% to 0.5%)
Cirrhosis and other chronic liver diseases due to hepatitis C	23.2% (15.6% to 32.6%)	38.9% (28.6% to 50.3%)	51.1% (39.5% to 60.9%)	39.7% (29.7% to 50.8%)
Cirrhosis and other chronic liver diseases due to alcohol use	100.0% (100.0% to 100.0%)	100.0% (100.0% to 100.0%)	100.0% (100.0% to 100.0%)	100.0% (100.0% to 100.0%)
**Digestive diseases**	9.3% (6.5% to 12.4%)	9.2% (6.4% to 12.6%)	2.9% (2.1% to 3.8%)	7.4% (5.3% to 10.0%)
Peptic ulcer disease	8.4% (5.1% to 12.6%)	8.9% (5.5% to 12.6%)	6.6% (4.1% to 9.7%)	8.2% (5.1% to 11.7%)
Gallbladder and biliary diseases	46.5% (31.2% to 61.6%)	47.8% (32.8% to 62.3%)	50.7% (36.1% to 64.4%)	48.1% (33.1% to 62.4%)
Pancreatitis	13.8% (2.0% to 37.7%)	17.5% (3.6% to 40.5%)	17.8% (4.0% to 39.9%)	17.5% (3.6% to 40.5%)
**Neurological disorders**	30.8% (16.2% to 47.5%)	28.9% (15.4% to 43.7%)	4.8% (2.8% to 7.4%)	13.4% (7.2% to 21.0%)
Alzheimer's disease and other dementias	35.2% (18.7% to 53.1%)	36.8% (19.8% to 55.1%)	36.9% (19.7% to 55.3%)	36.8% (19.8% to 55.1%)
Parkinson's disease	-7.5% (-10.8% to -5.2%)	-8.1% (-11.2% to -5.8%)	-8.0% (-10.8% to -5.9%)	-8.1% (-11.1% to -5.9%)
Epilepsy	8.9% (4.1% to 15.2%)	7.8% (3.8% to 12.6%)	7.0% (3.4% to 11.6%)	7.3% (3.6% to 11.9%)
Multiple sclerosis	4.8% (2.7% to 7.2%)	4.6% (2.6% to 7.0%)	4.1% (2.4% to 6.2%)	4.2% (2.5% to 6.3%)
**Mental and substance use disorders**	99.4% (99.3% to 99.6%)	99.1% (98.8% to 99.4%)	18.9% (16.3% to 21.8%)	21.3% (18.4% to 24.6%)
Alcohol use disorders	100.0% (100.0% to 100.0%)	100.0% (100.0% to 100.0%)	100.0% (100.0% to 100.0%)	100.0% (100.0% to 100.0%)

Supplementary Results Table 1. Age-standardised PAFs attributable to the joint distribution of all risk factors for all ages, both sexes combined for each GBD level 1 and level 2 cause and all causes in 2016 for Bahrain. Results are shown for deaths, YLLs, YLDs, and DALYs
Data in parenthesis are 95% uncertainty intervals. PAFs=population attributable fractions. GBD=Global Burden of Disease. YLLs=years of life lost. YLDs=years live with disability. DALYs=disability-adjusted life-years

	Deaths 2016	YLLs 2016	YLDs 2016	DALYs 2016
Drug use disorders	100.0% (100.0% to 100.0%)	100.0% (100.0% to 100.0%)	100.0% (100.0% to 100.0%)	100.0% (100.0% to 100.0%)
Depressive disorders	-	-	6.5% (5.3% to 7.8%)	6.5% (5.3% to 7.8%)
Idiopathic developmental intellectual disability	-	-	66.3% (34.4% to 85.4%)	66.3% (34.4% to 85.4%)
**Diabetes, urogenital, blood, and endocrine diseases**	86.8% (85.7% to 88.1%)	84.0% (82.3% to 85.3%)	78.9% (75.2% to 82.2%)	82.1% (80.2% to 83.6%)
Diabetes mellitus	100.0% (100.0% to 100.0%)	100.0% (100.0% to 100.0%)	100.0% (100.0% to 100.0%)	100.0% (100.0% to 100.0%)
Chronic kidney disease	100.0% (100.0% to 100.0%)	100.0% (100.0% to 100.0%)	100.0% (100.0% to 100.0%)	100.0% (100.0% to 100.0%)
**Musculoskeletal disorders**	0.9% (0.3% to 1.7%)	0.8% (0.3% to 1.5%)	15.2% (11.6% to 18.8%)	15.0% (11.5% to 18.5%)
Rheumatoid arthritis	2.8% (1.0% to 5.4%)	2.7% (0.9% to 5.2%)	2.6% (0.9% to 5.0%)	2.6% (0.9% to 5.0%)
Osteoarthritis	-	-	34.8% (19.7% to 48.8%)	34.8% (19.7% to 48.8%)
Low back and neck pain	-	-	17.4% (14.2% to 20.7%)	17.4% (14.2% to 20.7%)
Gout	-	-	59.4% (42.3% to 75.3%)	59.4% (42.3% to 75.3%)
**Other non-communicable diseases**	0.0% (0.0% to 0.0%)	0.1% (0.0% to 0.1%)	6.2% (4.9% to 7.7%)	4.8% (3.8% to 6.0%)
Sense organ diseases	-	-	13.3% (10.9% to 16.3%)	13.3% (10.9% to 16.3%)
Sudden infant death syndrome	5.8% (3.3% to 9.6%)	5.8% (3.3% to 9.7%)	-	5.8% (3.3% to 9.7%)
**Transport injuries**	34.7% (29.1% to 41.2%)	32.2% (25.5% to 39.8%)	39.2% (32.5% to 47.0%)	33.5% (26.9% to 41.5%)
Road injuries	34.4% (28.7% to 41.1%)	32.2% (25.5% to 39.8%)	42.0% (35.0% to 50.2%)	33.7% (26.8% to 41.8%)
Other transport injuries	38.0% (32.9% to 44.1%)	32.1% (26.1% to 39.3%)	31.0% (25.1% to 38.0%)	31.6% (26.0% to 38.7%)
**Unintentional injuries**	30.2% (25.5% to 35.0%)	23.9% (18.9% to 29.6%)	24.9% (19.3% to 31.5%)	24.5% (19.1% to 30.6%)
Falls	57.6% (49.8% to 63.7%)	42.9% (35.5% to 50.8%)	32.9% (27.1% to 40.0%)	35.7% (29.8% to 42.6%)
Drowning	19.8% (14.6% to 25.5%)	19.2% (14.1% to 25.2%)	20.0% (14.3% to 26.6%)	19.2% (14.2% to 25.1%)
Fire, heat, and hot substances	17.1% (12.9% to 22.0%)	22.5% (17.2% to 28.6%)	23.0% (16.4% to 30.6%)	22.8% (17.1% to 29.5%)

Supplementary Results Table 1. Age-standardised PAFs attributable to the joint distribution of all risk factors for all ages, both sexes combined for each GBD level 1 and level 2 cause and all causes in 2016 for Bahrain. Results are shown for deaths, YLLs, YLDs, and DALYs
Data in parenthesis are 95% uncertainty intervals. PAFs=population attributable fractions. GBD=Global Burden of Disease. YLLs=years of life lost. YLDs=years live with disability. DALYs=disability-adjusted life-years

	**Deaths 2016**	**YLLs 2016**	**YLDs 2016**	**DALYs 2016**
Poisonings	22.7% (17.0% to 30.2%)	25.7% (19.1% to 33.9%)	22.6% (16.2% to 30.5%)	24.6% (18.5% to 32.2%)
Exposure to mechanical forces	26.0% (17.4% to 34.3%)	23.5% (12.5% to 33.1%)	32.8% (25.0% to 42.3%)	28.8% (20.2% to 38.4%)
Animal contact	16.9% (14.2% to 19.6%)	12.3% (9.5% to 15.6%)	21.1% (14.7% to 29.0%)	18.3% (13.0% to 24.7%)
Foreign body	15.2% (11.3% to 20.2%)	16.5% (12.1% to 21.5%)	17.7% (12.0% to 24.5%)	17.1% (12.2% to 22.5%)
Other unintentional injuries	24.9% (18.3% to 33.1%)	28.8% (20.8% to 38.6%)	21.7% (15.3% to 29.0%)	23.6% (17.4% to 31.5%)
**Self-harm and interpersonal violence**	14.6% (9.8% to 19.9%)	14.4% (9.7% to 19.4%)	20.1% (18.2% to 22.4%)	14.9% (10.4% to 19.5%)
Self-harm	13.2% (7.8% to 19.3%)	13.8% (8.3% to 20.1%)	13.4% (7.7% to 19.8%)	13.8% (8.3% to 20.1%)
Interpersonal violence	19.8% (13.6% to 25.6%)	16.4% (10.9% to 22.2%)	21.5% (19.8% to 23.5%)	17.7% (13.3% to 22.1%)
**Forces of nature, conflict and terrorism, and executions and police conflict**	0.0% (0.0% to 0.0%)	0.0% (0.0% to 0.0%)	0.0% (0.0% to 0.0%)	0.0% (0.0% to 0.0%)
Forces of nature, conflict and terrorism, and state actor violence	0.0% (0.0% to 0.0%)	0.0% (0.0% to 0.0%)	0.0% (0.0% to 0.0%)	0.0% (0.0% to 0.0%)

Supplementary Results Table 1. Age-standardised PAFs attributable to the joint distribution of all risk factors for all ages, both sexes combined for each GBD level 1 and level 2 cause and all causes in 2016 for Bangladesh. Results are shown for deaths, YLLs, YLDs, and DALYs
Data in parenthesis are 95% uncertainty intervals. PAFs=population attributable fractions. GBD=Global Burden of Disease. YLLs=years of life lost. YLDs=years live with disability. DALYs=disability-adjusted life-years

	Deaths 2016	YLLs 2016	YLDs 2016	DALYs 2016
**All causes**	66.6% (64.5% to 68.3%)	61.4% (59.6% to 63.0%)	25.3% (24.3% to 26.4%)	48.8% (46.1% to 51.6%)
**HIV/AIDS and tuberculosis**	25.9% (17.4% to 34.6%)	27.8% (18.8% to 37.1%)	26.7% (18.5% to 35.3%)	27.7% (18.9% to 36.8%)
Tuberculosis	25.4% (17.1% to 34.0%)	26.7% (18.3% to 35.2%)	25.6% (17.2% to 34.0%)	26.7% (18.2% to 35.0%)
HIV/AIDS	78.7% (70.2% to 84.9%)	75.2% (64.9% to 82.7%)	79.8% (75.1% to 83.9%)	76.4% (67.9% to 82.9%)
**Diarrhoea, lower respiratory infections, and other common infectious diseases**	77.4% (70.7% to 84.2%)	70.1% (61.7% to 78.0%)	38.6% (33.0% to 43.7%)	66.0% (58.5% to 73.3%)
Diarrhoeal diseases	93.7% (84.1% to 98.2%)	95.1% (87.7% to 98.7%)	94.3% (85.6% to 98.4%)	94.9% (87.3% to 98.6%)
Lower respiratory infections	86.6% (82.6% to 90.3%)	92.2% (89.4% to 94.6%)	85.6% (81.4% to 89.4%)	92.2% (89.4% to 94.5%)
Upper respiratory infections	3.3% (0.2% to 12.0%)	6.4% (0.5% to 22.1%)	0.0% (0.0% to 0.0%)	0.1% (0.0% to 0.4%)
Otitis media	0.5% (0.1% to 2.1%)	3.0% (1.0% to 8.7%)	9.0% (6.1% to 11.7%)	9.0% (6.1% to 11.7%)
Meningitis	2.4% (1.3% to 3.8%)	3.5% (1.9% to 5.6%)	0.0% (0.0% to 0.0%)	2.0% (1.2% to 3.4%)
Encephalitis	0.7% (0.4% to 1.0%)	2.1% (1.3% to 3.2%)	0.0% (0.0% to 0.0%)	1.2% (0.8% to 1.9%)
Measles	66.6% (47.3% to 85.2%)	67.5% (48.0% to 86.3%)	64.9% (46.8% to 81.9%)	67.4% (47.9% to 86.1%)
**Maternal disorders**	6.2% (2.5% to 10.4%)	6.1% (2.5% to 10.3%)	1.6% (0.6% to 3.0%)	5.9% (2.4% to 10.0%)
Maternal haemorrhage	14.5% (5.3% to 23.6%)	14.6% (5.3% to 23.8%)	14.8% (5.4% to 24.1%)	14.6% (5.3% to 23.8%)
Maternal sepsis and other pregnancy related infections	14.5% (5.2% to 23.5%)	14.5% (5.3% to 23.7%)	14.4% (5.3% to 23.4%)	14.5% (5.2% to 23.7%)
Maternal abortion, miscarriage, and ectopic pregnancy	14.6% (8.9% to 21.3%)	14.6% (8.8% to 21.1%)	14.3% (8.1% to 20.5%)	14.6% (8.8% to 21.1%)
**Neonatal disorders**	82.0% (78.7% to 86.5%)	82.0% (78.7% to 86.5%)	0.3% (0.2% to 0.4%)	76.2% (72.3% to 81.4%)
Neonatal preterm birth complications	97.7% (95.2% to 99.3%)	97.7% (95.2% to 99.3%)	0.5% (0.4% to 0.6%)	79.3% (69.8% to 90.0%)
Neonatal encephalopathy due to birth asphyxia and trauma	79.2% (75.4% to 82.9%)	79.2% (75.4% to 82.9%)	0.0% (0.0% to 0.0%)	77.9% (73.1% to 82.0%)
Neonatal sepsis and other neonatal infections	74.2% (70.3% to 77.8%)	74.2% (70.3% to 77.8%)	0.0% (0.0% to 0.0%)	59.1% (41.3% to 70.1%)
Hemolytic disease and other neonatal jaundice	74.4% (70.6% to 78.1%)	74.5% (70.6% to 78.1%)	0.0% (0.0% to 0.0%)	69.0% (64.1% to 73.4%)

Supplementary Results Table 1. Age-standardised PAFs attributable to the joint distribution of all risk factors for all ages, both sexes combined for each GBD level 1 and level 2 cause and all causes in 2016 for Bangladesh. Results are shown for deaths, YLLs, YLDs, and DALYs
Data in parenthesis are 95% uncertainty intervals. PAFs=population attributable fractions. GBD=Global Burden of Disease. YLLs=years of life lost. YLDs=years live with disability. DALYs=disability-adjusted life-years

	Deaths 2016	YLLs 2016	YLDs 2016	DALYs 2016
Other neonatal disorders	77.5% (73.6% to 81.1%)	77.5% (73.6% to 81.2%)	0.0% (0.0% to 0.0%)	76.6% (72.7% to 80.2%)
**Nutritional deficiencies**	14.9% (10.4% to 20.3%)	50.0% (40.2% to 59.6%)	95.3% (94.2% to 96.3%)	78.2% (72.5% to 82.8%)
Protein-energy malnutrition	16.4% (11.2% to 22.8%)	57.4% (46.6% to 67.7%)	97.7% (96.1% to 98.7%)	62.9% (54.1% to 71.0%)
Vitamin A deficiency	-	-	100.0% (100.0% to 100.0%)	100.0% (100.0% to 100.0%)
Iron-deficiency anaemia	100.0% (100.0% to 100.0%)	100.0% (100.0% to 100.0%)	100.0% (100.0% to 100.0%)	100.0% (100.0% to 100.0%)
**Other communicable, maternal, neonatal, and nutritional diseases**	1.9% (1.0% to 6.9%)	1.6% (0.8% to 5.6%)	56.7% (50.2% to 63.6%)	8.5% (5.0% to 13.7%)
Sexually transmitted diseases excluding HIV	9.5% (3.8% to 24.3%)	3.3% (1.2% to 10.8%)	100.0% (100.0% to 100.0%)	17.3% (7.7% to 32.3%)
Hepatitis	0.1% (0.0% to 0.1%)	0.1% (0.1% to 0.2%)	0.1% (0.0% to 0.1%)	0.1% (0.1% to 0.2%)
**Neoplasms**	52.2% (49.2% to 55.1%)	47.9% (45.0% to 50.7%)	48.5% (45.3% to 51.6%)	47.9% (45.0% to 50.7%)
Oesophageal cancer	77.2% (68.1% to 85.3%)	76.9% (67.6% to 84.8%)	77.3% (68.3% to 85.3%)	76.9% (67.6% to 84.8%)
Stomach cancer	22.4% (13.5% to 31.8%)	21.4% (13.3% to 30.3%)	22.0% (13.4% to 31.4%)	21.4% (13.3% to 30.3%)
Liver cancer	31.9% (22.4% to 41.8%)	31.9% (22.5% to 41.4%)	32.0% (22.4% to 41.6%)	31.9% (22.5% to 41.4%)
Larynx cancer	79.1% (69.9% to 86.2%)	79.4% (70.6% to 86.4%)	78.9% (69.8% to 85.8%)	79.4% (70.5% to 86.3%)
Tracheal, bronchus, and lung cancer	94.0% (92.0% to 95.7%)	93.1% (91.0% to 95.0%)	93.7% (91.7% to 95.4%)	93.1% (91.0% to 95.0%)
Breast cancer	14.2% (7.8% to 22.2%)	14.5% (7.7% to 22.5%)	14.4% (7.9% to 22.6%)	14.5% (7.7% to 22.5%)
Cervical cancer	100.0% (100.0% to 100.0%)	100.0% (100.0% to 100.0%)	100.0% (100.0% to 100.0%)	100.0% (100.0% to 100.0%)
Uterine cancer	14.7% (6.3% to 25.2%)	16.4% (7.5% to 27.0%)	15.5% (6.8% to 26.0%)	16.4% (7.5% to 26.9%)
Prostate cancer	7.0% (5.0% to 9.3%)	6.2% (4.4% to 8.2%)	6.6% (4.7% to 8.8%)	6.2% (4.4% to 8.2%)
Colon and rectum cancer	51.0% (42.0% to 60.1%)	49.3% (40.9% to 57.8%)	50.9% (41.8% to 60.0%)	49.3% (40.9% to 57.9%)
Lip and oral cavity cancer	80.7% (73.4% to 86.1%)	79.3% (73.0% to 84.1%)	79.8% (73.2% to 85.2%)	79.3% (72.9% to 84.2%)
Nasopharynx cancer	58.1% (49.4% to 65.6%)	56.2% (47.4% to 63.6%)	56.2% (47.3% to 64.2%)	56.2% (47.4% to 63.6%)

Supplementary Results Table 1. Age-standardised PAFs attributable to the joint distribution of all risk factors for all ages, both sexes combined for each GBD level 1 and level 2 cause and all causes in 2016 for Bangladesh. Results are shown for deaths, YLLs, YLDs, and DALYs
Data in parenthesis are 95% uncertainty intervals. PAFs=population attributable fractions. GBD=Global Burden of Disease. YLLs=years of life lost. YLDs=years live with disability. DALYs=disability-adjusted life-years

	Deaths 2016	YLLs 2016	YLDs 2016	DALYs 2016
Other pharynx cancer	10.3% (2.1% to 20.0%)	10.4% (2.3% to 20.1%)	10.4% (2.2% to 20.2%)	10.4% (2.3% to 20.1%)
Gallbladder and biliary tract cancer	6.8% (2.6% to 13.1%)	7.8% (3.1% to 14.7%)	6.9% (2.7% to 13.4%)	7.8% (3.1% to 14.7%)
Pancreatic cancer	28.7% (20.9% to 37.0%)	28.4% (20.9% to 36.6%)	28.6% (20.8% to 36.9%)	28.4% (20.9% to 36.6%)
Ovarian cancer	8.1% (2.4% to 16.8%)	7.3% (2.2% to 15.1%)	7.4% (2.3% to 15.4%)	7.3% (2.2% to 15.1%)
Kidney cancer	24.6% (16.1% to 33.4%)	23.0% (15.2% to 31.2%)	23.4% (15.6% to 31.5%)	23.0% (15.2% to 31.2%)
Bladder cancer	38.5% (27.6% to 49.3%)	36.5% (26.5% to 47.0%)	37.6% (27.3% to 48.2%)	36.5% (26.6% to 47.1%)
Thyroid cancer	4.1% (1.5% to 8.3%)	4.5% (1.7% to 9.0%)	4.4% (1.7% to 8.6%)	4.5% (1.7% to 9.0%)
Mesothelioma	80.4% (69.7% to 87.7%)	69.3% (55.7% to 79.4%)	78.3% (67.0% to 86.1%)	69.5% (55.8% to 79.5%)
Non-Hodgkin's lymphoma	1.8% (0.6% to 3.9%)	1.9% (0.6% to 3.8%)	1.9% (0.6% to 3.9%)	1.9% (0.6% to 3.8%)
Multiple myeloma	2.1% (0.7% to 4.4%)	2.4% (0.9% to 5.0%)	2.2% (0.7% to 4.5%)	2.4% (0.9% to 5.0%)
Leukaemia	13.4% (7.5% to 20.2%)	11.0% (6.1% to 17.2%)	13.3% (7.4% to 20.0%)	11.1% (6.1% to 17.2%)
**Cardiovascular diseases**	88.7% (86.8% to 90.6%)	90.3% (88.9% to 91.6%)	77.5% (74.4% to 80.0%)	89.4% (88.0% to 90.8%)
Rheumatic heart disease	23.1% (15.7% to 37.2%)	20.1% (13.8% to 30.6%)	18.1% (11.5% to 26.9%)	19.8% (13.5% to 29.8%)
Ischaemic heart disease	94.6% (92.2% to 96.3%)	95.7% (94.4% to 96.8%)	91.1% (88.6% to 93.4%)	95.5% (94.0% to 96.6%)
Cerebrovascular disease	87.5% (84.4% to 90.7%)	91.2% (89.1% to 93.1%)	88.1% (85.1% to 90.9%)	91.0% (89.0% to 92.9%)
Hypertensive heart disease	100.0% (100.0% to 100.0%)	100.0% (100.0% to 100.0%)	100.0% (100.0% to 100.0%)	100.0% (100.0% to 100.0%)
Cardiomyopathy and myocarditis	31.1% (23.7% to 40.2%)	23.8% (16.6% to 31.9%)	17.7% (12.7% to 23.4%)	23.0% (16.4% to 30.7%)
Atrial fibrillation and flutter	40.0% (33.3% to 46.6%)	44.8% (38.7% to 50.3%)	52.4% (47.6% to 57.0%)	49.6% (44.5% to 54.6%)
Aortic aneurysm	43.7% (37.0% to 50.2%)	48.6% (42.6% to 54.2%)	-	48.6% (42.6% to 54.2%)
Peripheral vascular disease	53.1% (47.1% to 59.5%)	60.5% (54.9% to 65.8%)	61.7% (56.6% to 66.6%)	61.6% (56.7% to 66.3%)
Endocarditis	38.8% (29.7% to 49.4%)	35.7% (27.5% to 44.6%)	35.8% (27.4% to 45.3%)	35.7% (27.6% to 44.6%)

Supplementary Results Table 1. Age-standardised PAFs attributable to the joint distribution of all risk factors for all ages, both sexes combined for each GBD level 1 and level 2 cause and all causes in 2016 for Bangladesh. Results are shown for deaths, YLLs, YLDs, and DALYs
Data in parenthesis are 95% uncertainty intervals. PAFs=population attributable fractions. GBD=Global Burden of Disease. YLLs=years of life lost. YLDs=years live with disability. DALYs=disability-adjusted life-years

	Deaths 2016	YLLs 2016	YLDs 2016	DALYs 2016
Other cardiovascular and circulatory diseases	44.4% (38.9% to 49.8%)	49.0% (43.5% to 54.8%)	45.3% (40.4% to 50.4%)	47.7% (42.7% to 52.8%)
**Chronic respiratory diseases**	77.6% (70.0% to 82.6%)	74.7% (66.9% to 79.7%)	54.6% (47.9% to 61.2%)	71.0% (64.3% to 76.0%)
Chronic obstructive pulmonary disease	89.2% (85.0% to 93.4%)	89.1% (85.2% to 93.0%)	82.8% (77.6% to 87.8%)	88.3% (84.3% to 92.3%)
Pneumoconiosis	100.0% (100.0% to 100.0%)	100.0% (100.0% to 100.0%)	100.0% (100.0% to 100.0%)	100.0% (100.0% to 100.0%)
Asthma	31.1% (23.2% to 40.1%)	34.4% (26.6% to 43.8%)	27.4% (22.7% to 33.1%)	32.1% (25.6% to 40.2%)
Other chronic respiratory diseases	18.6% (13.4% to 24.4%)	15.0% (10.5% to 20.4%)	9.4% (6.6% to 12.6%)	13.1% (9.3% to 17.8%)
**Cirrhosis and other chronic liver diseases**	27.6% (23.6% to 31.7%)	25.5% (21.7% to 29.6%)	20.3% (16.7% to 24.3%)	25.2% (21.5% to 29.3%)
Cirrhosis and other chronic liver diseases due to hepatitis B	0.1% (0.0% to 0.1%)	0.1% (0.0% to 0.1%)	0.1% (0.0% to 0.1%)	0.1% (0.0% to 0.1%)
Cirrhosis and other chronic liver diseases due to hepatitis C	5.5% (3.7% to 7.7%)	8.9% (6.1% to 12.3%)	7.7% (5.3% to 10.7%)	8.9% (6.1% to 12.2%)
Cirrhosis and other chronic liver diseases due to alcohol use	100.0% (100.0% to 100.0%)	100.0% (100.0% to 100.0%)	100.0% (100.0% to 100.0%)	100.0% (100.0% to 100.0%)
**Digestive diseases**	7.4% (5.0% to 10.5%)	7.0% (4.6% to 10.0%)	2.8% (2.0% to 3.8%)	6.4% (4.3% to 9.2%)
Peptic ulcer disease	22.1% (15.8% to 28.4%)	22.3% (16.2% to 28.4%)	13.4% (9.8% to 17.0%)	21.5% (15.5% to 27.6%)
Gallbladder and biliary diseases	16.5% (8.7% to 26.7%)	18.1% (9.8% to 28.4%)	19.6% (10.8% to 30.2%)	18.2% (9.9% to 28.4%)
Pancreatitis	2.4% (1.0% to 5.0%)	2.3% (1.0% to 4.9%)	2.5% (1.1% to 5.0%)	2.3% (1.0% to 4.9%)
**Neurological disorders**	12.0% (5.5% to 20.3%)	8.5% (3.9% to 14.7%)	1.1% (0.5% to 2.0%)	3.2% (1.4% to 5.8%)
Alzheimer's disease and other dementias	16.4% (8.2% to 26.5%)	17.5% (9.0% to 27.9%)	17.5% (9.1% to 27.8%)	17.5% (9.0% to 27.8%)
Parkinson's disease	-20.7% (-26.7% to -14.9%)	-24.1% (-30.5% to -17.3%)	-25.1% (-31.9% to -18.0%)	-24.3% (-30.7% to -17.5%)
Epilepsy	0.7% (0.4% to 1.1%)	0.6% (0.3% to 1.0%)	0.5% (0.3% to 0.9%)	0.6% (0.3% to 0.9%)
Multiple sclerosis	10.7% (5.6% to 16.5%)	10.2% (5.0% to 16.1%)	7.6% (4.5% to 10.7%)	8.9% (5.0% to 13.1%)
**Mental and substance use disorders**	99.8% (99.7% to 99.9%)	99.6% (99.5% to 99.8%)	20.6% (18.5% to 22.8%)	22.0% (19.9% to 24.3%)
Alcohol use disorders	100.0% (100.0% to 100.0%)	100.0% (100.0% to 100.0%)	100.0% (100.0% to 100.0%)	100.0% (100.0% to 100.0%)

Supplementary Results Table 1. Age-standardised PAFs attributable to the joint distribution of all risk factors for all ages, both sexes combined for each GBD level 1 and level 2 cause and all causes in 2016 for Bangladesh. Results are shown for deaths, YLLs, YLDs, and DALYs
Data in parenthesis are 95% uncertainty intervals. PAFs=population attributable fractions. GBD=Global Burden of Disease. YLLs=years of life lost. YLDs=years live with disability. DALYs=disability-adjusted life-years

	**Deaths 2016**	**YLLs 2016**	**YLDs 2016**	**DALYs 2016**
Drug use disorders	100.0% (100.0% to 100.0%)	100.0% (100.0% to 100.0%)	100.0% (100.0% to 100.0%)	100.0% (100.0% to 100.0%)
Depressive disorders	-	-	14.9% (12.6% to 17.4%)	14.9% (12.6% to 17.4%)
Idiopathic developmental intellectual disability	-	-	70.5% (38.8% to 88.6%)	70.5% (38.8% to 88.6%)
**Diabetes, urogenital, blood, and endocrine diseases**	82.9% (81.5% to 84.8%)	80.7% (78.2% to 82.1%)	62.2% (55.8% to 67.2%)	72.9% (69.0% to 75.9%)
Diabetes mellitus	100.0% (100.0% to 100.0%)	100.0% (100.0% to 100.0%)	100.0% (100.0% to 100.0%)	100.0% (100.0% to 100.0%)
Chronic kidney disease	100.0% (100.0% to 100.0%)	100.0% (100.0% to 100.0%)	100.0% (100.0% to 100.0%)	100.0% (100.0% to 100.0%)
**Musculoskeletal disorders**	3.1% (1.1% to 5.4%)	2.6% (1.0% to 4.7%)	16.9% (14.5% to 19.6%)	16.8% (14.4% to 19.5%)
Rheumatoid arthritis	5.5% (2.2% to 9.7%)	5.6% (2.3% to 9.7%)	5.0% (2.1% to 8.2%)	5.1% (2.1% to 8.4%)
Osteoarthritis	-	-	8.7% (3.4% to 16.0%)	8.7% (3.4% to 16.0%)
Low back and neck pain	-	-	28.5% (25.4% to 31.9%)	28.5% (25.4% to 31.9%)
Gout	-	-	31.1% (23.3% to 41.8%)	31.1% (23.3% to 41.8%)
**Other non-communicable diseases**	0.5% (0.2% to 0.9%)	0.7% (0.3% to 1.4%)	12.0% (10.1% to 13.7%)	10.0% (8.5% to 11.6%)
Sense organ diseases	-	-	22.1% (19.4% to 24.9%)	22.1% (19.4% to 24.9%)
Sudden infant death syndrome	6.8% (2.8% to 12.8%)	6.8% (2.8% to 12.9%)	-	6.8% (2.8% to 12.9%)
**Transport injuries**	26.9% (22.8% to 31.4%)	23.7% (19.2% to 29.0%)	30.3% (26.5% to 34.3%)	24.9% (20.4% to 29.9%)
Road injuries	26.1% (22.1% to 30.6%)	23.2% (18.8% to 28.6%)	33.7% (29.7% to 38.0%)	24.6% (20.2% to 29.8%)
Other transport injuries	30.8% (25.9% to 36.2%)	26.3% (21.0% to 32.5%)	24.8% (21.5% to 28.6%)	25.8% (21.3% to 30.8%)
**Unintentional injuries**	20.1% (15.5% to 24.9%)	10.3% (8.0% to 14.1%)	20.0% (17.3% to 22.9%)	13.7% (11.2% to 17.0%)
Falls	60.7% (44.7% to 65.6%)	36.4% (30.0% to 41.4%)	31.5% (28.9% to 34.5%)	33.4% (30.2% to 36.5%)
Drowning	6.8% (4.9% to 9.4%)	5.3% (3.8% to 7.6%)	13.0% (9.8% to 16.6%)	5.4% (3.8% to 7.8%)
Fire, heat, and hot substances	7.1% (5.1% to 10.0%)	9.7% (6.9% to 12.9%)	13.2% (9.9% to 17.0%)	11.0% (8.0% to 14.4%)

Supplementary Results Table 1. Age-standardised PAFs attributable to the joint distribution of all risk factors for all ages, both sexes combined for each GBD level 1 and level 2 cause and all causes in 2016 for Bangladesh. Results are shown for deaths, YLLs, YLDs, and DALYs
Data in parenthesis are 95% uncertainty intervals. PAFs=population attributable fractions. GBD=Global Burden of Disease. YLLs=years of life lost. YLDs=years live with disability. DALYs=disability-adjusted life-years

	**Deaths 2016**	**YLLs 2016**	**YLDs 2016**	**DALYs 2016**
Poisonings	6.0% (3.5% to 11.3%)	5.4% (2.8% to 10.5%)	14.4% (10.8% to 18.4%)	6.6% (3.9% to 11.6%)
Exposure to mechanical forces	17.7% (14.3% to 21.1%)	13.1% (9.5% to 16.7%)	23.2% (19.8% to 26.9%)	18.0% (14.5% to 21.7%)
Animal contact	14.0% (10.6% to 18.0%)	14.2% (10.6% to 18.5%)	13.9% (10.5% to 17.6%)	13.9% (10.7% to 17.6%)
Foreign body	5.9% (4.2% to 7.9%)	5.9% (4.1% to 8.2%)	10.9% (8.2% to 14.1%)	8.8% (6.5% to 11.5%)
Other unintentional injuries	6.3% (4.1% to 20.3%)	14.0% (10.1% to 20.8%)	13.6% (10.3% to 17.2%)	13.9% (10.5% to 18.4%)
**Self-harm and interpersonal violence**	4.5% (3.4% to 6.3%)	4.5% (3.4% to 6.4%)	17.3% (16.0% to 18.7%)	5.8% (4.6% to 7.5%)
Self-harm	3.1% (2.0% to 4.6%)	3.5% (2.2% to 5.2%)	2.9% (1.9% to 4.2%)	3.5% (2.2% to 5.2%)
Interpersonal violence	8.7% (6.4% to 13.5%)	7.2% (5.1% to 11.8%)	19.9% (18.5% to 21.2%)	10.3% (8.0% to 13.4%)
**Forces of nature, conflict and terrorism, and executions and police conflict**	0.0% (0.0% to 0.0%)	0.0% (0.0% to 0.0%)	7.1% (5.1% to 9.9%)	5.6% (3.4% to 8.6%)
Forces of nature, conflict and terrorism, and state actor violence	0.0% (0.0% to 0.0%)	0.0% (0.0% to 0.0%)	7.4% (5.2% to 10.2%)	7.4% (5.2% to 10.2%)

Supplementary Results Table 1. Age-standardised PAFs attributable to the joint distribution of all risk factors for all ages, both sexes combined for each GBD level 1 and level 2 cause and all causes in 2016 for Barbados. Results are shown for deaths, YLLs, YLDs, and DALYs
Data in parenthesis are 95% uncertainty intervals. PAFs=population attributable fractions. GBD=Global Burden of Disease. YLLs=years of life lost. YLDs=years live with disability. DALYs=disability-adjusted life-years

	Deaths 2016	YLLs 2016	YLDs 2016	DALYs 2016
**All causes**	51.5% (49.5% to 53.7%)	49.0% (47.4% to 50.8%)	24.5% (22.9% to 26.0%)	38.9% (36.9% to 41.2%)
**HIV/AIDS and tuberculosis**	77.6% (71.5% to 82.0%)	73.6% (66.5% to 78.6%)	79.8% (72.9% to 84.5%)	74.1% (67.0% to 79.1%)
Tuberculosis	47.5% (37.7% to 57.0%)	50.9% (41.7% to 60.0%)	44.2% (35.3% to 52.4%)	50.4% (41.2% to 59.4%)
HIV/AIDS	80.2% (73.7% to 84.9%)	74.7% (67.4% to 79.9%)	81.8% (74.5% to 86.8%)	75.3% (67.9% to 80.6%)
**Diarrhoea, lower respiratory infections, and other common infectious diseases**	33.9% (23.9% to 43.9%)	44.3% (34.8% to 53.6%)	38.7% (25.8% to 49.0%)	43.0% (35.1% to 51.3%)
Diarrhoeal diseases	76.1% (50.7% to 91.7%)	85.0% (67.9% to 95.2%)	75.4% (50.9% to 91.1%)	77.8% (55.0% to 92.2%)
Lower respiratory infections	34.5% (24.0% to 45.5%)	47.7% (37.2% to 58.4%)	41.5% (31.7% to 51.9%)	47.6% (37.1% to 58.3%)
Upper respiratory infections	1.4% (0.1% to 5.5%)	3.6% (0.2% to 12.6%)	0.0% (0.0% to 0.0%)	0.0% (0.0% to 0.1%)
Otitis media	1.1% (0.5% to 2.3%)	3.3% (1.5% to 6.4%)	2.6% (1.7% to 3.6%)	2.6% (1.7% to 3.6%)
Meningitis	2.4% (1.1% to 4.4%)	4.9% (2.6% to 7.8%)	0.0% (0.0% to 0.0%)	4.3% (2.2% to 7.0%)
Encephalitis	1.2% (0.5% to 2.2%)	2.0% (1.0% to 3.7%)	0.0% (0.0% to 0.0%)	1.7% (0.8% to 3.2%)
Measles	19.0% (6.5% to 48.1%)	22.0% (7.8% to 53.8%)	16.7% (0.0% to 68.7%)	22.0% (7.8% to 53.8%)
**Maternal disorders**	5.0% (2.4% to 7.9%)	4.9% (2.4% to 7.8%)	2.6% (1.4% to 4.1%)	4.4% (2.2% to 6.8%)
Maternal haemorrhage	15.0% (5.4% to 24.4%)	14.9% (5.4% to 24.3%)	15.1% (5.5% to 24.7%)	14.9% (5.4% to 24.3%)
Maternal sepsis and other pregnancy related infections	15.0% (5.4% to 24.4%)	14.9% (5.4% to 24.3%)	14.8% (5.4% to 24.1%)	14.9% (5.4% to 24.3%)
Maternal abortion, miscarriage, and ectopic pregnancy	5.7% (3.2% to 8.8%)	5.7% (3.2% to 8.9%)	5.9% (3.0% to 9.3%)	5.8% (3.2% to 8.9%)
**Neonatal disorders**	73.8% (71.0% to 76.5%)	73.8% (71.1% to 76.6%)	0.1% (0.1% to 0.2%)	57.8% (43.5% to 66.9%)
Neonatal preterm birth complications	94.6% (92.3% to 96.4%)	94.7% (92.3% to 96.5%)	0.2% (0.2% to 0.3%)	69.6% (51.5% to 82.8%)
Neonatal encephalopathy due to birth asphyxia and trauma	65.7% (62.4% to 69.6%)	65.8% (62.4% to 69.6%)	0.0% (0.0% to 0.0%)	64.7% (60.5% to 68.8%)
Neonatal sepsis and other neonatal infections	57.3% (52.7% to 61.8%)	57.3% (52.7% to 61.8%)	0.0% (0.0% to 0.0%)	36.1% (17.8% to 50.6%)
Hemolytic disease and other neonatal jaundice	64.9% (59.2% to 70.1%)	65.0% (59.2% to 70.1%)	0.0% (0.0% to 0.0%)	35.4% (20.1% to 51.2%)

Supplementary Results Table 1. Age-standardised PAFs attributable to the joint distribution of all risk factors for all ages, both sexes combined for each GBD level 1 and level 2 cause and all causes in 2016 for Barbados. Results are shown for deaths, YLLs, YLDs, and DALYs
Data in parenthesis are 95% uncertainty intervals. PAFs=population attributable fractions. GBD=Global Burden of Disease. YLLs=years of life lost. YLDs=years live with disability. DALYs=disability-adjusted life-years

	**Deaths 2016**	**YLLs 2016**	**YLDs 2016**	**DALYs 2016**
Other neonatal disorders	62.8% (58.6% to 67.2%)	62.9% (58.6% to 67.3%)	0.0% (0.0% to 0.0%)	58.5% (52.0% to 64.3%)
**Nutritional deficiencies**	10.8% (5.1% to 19.6%)	38.8% (22.9% to 56.5%)	97.6% (96.8% to 98.2%)	91.2% (88.0% to 93.6%)
Protein-energy malnutrition	13.1% (5.9% to 24.6%)	47.2% (28.2% to 67.1%)	97.1% (95.9% to 98.1%)	64.0% (51.5% to 75.4%)
Vitamin A deficiency	-	-	100.0% (100.0% to 100.0%)	100.0% (100.0% to 100.0%)
Iron-deficiency anaemia	100.0% (100.0% to 100.0%)	100.0% (100.0% to 100.0%)	100.0% (100.0% to 100.0%)	100.0% (100.0% to 100.0%)
**Other communicable, maternal, neonatal, and nutritional diseases**	5.5% (4.6% to 6.8%)	4.9% (3.9% to 6.1%)	63.5% (55.3% to 71.2%)	18.9% (13.6% to 26.6%)
Sexually transmitted diseases excluding HIV	91.5% (77.9% to 98.1%)	80.6% (57.0% to 95.0%)	100.0% (100.0% to 100.0%)	95.7% (88.3% to 99.1%)
Hepatitis	9.1% (5.7% to 13.2%)	9.7% (5.9% to 14.8%)	6.5% (3.7% to 9.9%)	9.5% (5.8% to 14.5%)
**Neoplasms**	27.8% (24.1% to 31.8%)	28.7% (25.3% to 32.4%)	23.5% (20.0% to 27.3%)	28.5% (25.0% to 32.2%)
Oesophageal cancer	63.0% (50.8% to 74.9%)	67.1% (55.5% to 77.7%)	64.1% (52.3% to 75.4%)	67.1% (55.5% to 77.7%)
Stomach cancer	6.9% (2.4% to 14.6%)	7.7% (3.1% to 14.4%)	7.4% (2.7% to 14.5%)	7.7% (3.1% to 14.4%)
Liver cancer	55.9% (48.4% to 64.0%)	56.5% (49.3% to 64.7%)	55.9% (48.4% to 64.1%)	56.5% (49.3% to 64.7%)
Larynx cancer	64.2% (52.0% to 74.6%)	68.3% (56.8% to 77.8%)	66.6% (54.5% to 76.3%)	68.3% (56.7% to 77.8%)
Tracheal, bronchus, and lung cancer	64.4% (56.6% to 71.9%)	66.6% (59.9% to 73.3%)	64.8% (56.8% to 72.4%)	66.6% (59.9% to 73.3%)
Breast cancer	24.0% (15.3% to 34.8%)	21.4% (13.3% to 31.0%)	21.9% (13.7% to 31.5%)	21.5% (13.4% to 31.0%)
Cervical cancer	100.0% (100.0% to 100.0%)	100.0% (100.0% to 100.0%)	100.0% (100.0% to 100.0%)	100.0% (100.0% to 100.0%)
Uterine cancer	47.7% (32.1% to 61.7%)	50.3% (34.8% to 64.1%)	51.2% (35.6% to 65.2%)	50.4% (34.9% to 64.2%)
Prostate cancer	1.1% (0.6% to 1.7%)	1.3% (0.8% to 1.9%)	1.4% (0.9% to 1.9%)	1.3% (0.8% to 1.9%)
Colon and rectum cancer	52.8% (42.7% to 62.8%)	53.7% (44.1% to 63.3%)	53.2% (43.1% to 63.0%)	53.7% (44.1% to 63.3%)
Lip and oral cavity cancer	61.9% (51.2% to 71.0%)	67.3% (57.8% to 75.5%)	61.4% (51.0% to 70.4%)	67.1% (57.6% to 75.2%)
Nasopharynx cancer	63.9% (55.6% to 70.8%)	64.7% (57.4% to 71.1%)	63.8% (56.9% to 69.8%)	64.7% (57.3% to 71.1%)

Supplementary Results Table 1. Age-standardised PAFs attributable to the joint distribution of all risk factors for all ages, both sexes combined for each GBD level 1 and level 2 cause and all causes in 2016 for Barbados. Results are shown for deaths, YLLs, YLDs, and DALYs
Data in parenthesis are 95% uncertainty intervals. PAFs=population attributable fractions. GBD=Global Burden of Disease. YLLs=years of life lost. YLDs=years live with disability. DALYs=disability-adjusted life-years

	Deaths 2016	YLLs 2016	YLDs 2016	DALYs 2016
Other pharynx cancer	54.3% (41.6% to 65.4%)	58.2% (45.8% to 68.9%)	56.2% (43.7% to 66.8%)	58.1% (45.7% to 68.8%)
Gallbladder and biliary tract cancer	24.1% (13.5% to 36.6%)	25.9% (14.7% to 38.8%)	24.4% (13.8% to 36.7%)	25.9% (14.6% to 38.8%)
Pancreatic cancer	22.0% (12.5% to 34.1%)	23.1% (14.0% to 34.6%)	22.3% (13.0% to 34.2%)	23.1% (14.0% to 34.6%)
Ovarian cancer	15.4% (5.5% to 28.6%)	14.0% (4.8% to 25.7%)	13.1% (4.6% to 24.3%)	14.0% (4.8% to 25.7%)
Kidney cancer	25.1% (16.4% to 35.2%)	25.6% (17.1% to 35.2%)	26.3% (17.7% to 36.1%)	25.6% (17.1% to 35.2%)
Bladder cancer	18.4% (9.5% to 30.0%)	19.5% (10.7% to 30.7%)	19.2% (10.5% to 30.3%)	19.5% (10.7% to 30.7%)
Thyroid cancer	14.0% (7.5% to 22.2%)	15.0% (8.1% to 23.5%)	15.1% (8.4% to 22.8%)	15.0% (8.2% to 23.5%)
Mesothelioma	82.0% (77.6% to 86.1%)	70.7% (64.5% to 76.7%)	77.5% (72.6% to 82.3%)	70.8% (64.6% to 76.8%)
Non-Hodgkin's lymphoma	6.9% (2.7% to 12.1%)	6.9% (2.7% to 11.7%)	7.0% (2.7% to 12.2%)	6.9% (2.7% to 11.8%)
Multiple myeloma	8.3% (3.5% to 14.6%)	8.8% (3.8% to 15.2%)	8.7% (3.7% to 15.1%)	8.8% (3.8% to 15.2%)
Leukaemia	12.8% (7.9% to 18.4%)	12.1% (7.6% to 17.2%)	12.1% (7.5% to 17.0%)	12.1% (7.6% to 17.2%)
**Cardiovascular diseases**	79.3% (76.6% to 81.8%)	79.4% (77.2% to 81.5%)	70.8% (67.9% to 73.7%)	78.4% (76.2% to 80.5%)
Rheumatic heart disease	25.5% (16.3% to 42.5%)	21.8% (15.3% to 32.9%)	25.7% (17.4% to 38.5%)	21.9% (15.3% to 33.0%)
Ischaemic heart disease	92.3% (88.5% to 95.4%)	94.0% (91.6% to 96.0%)	90.8% (87.9% to 93.2%)	93.7% (91.3% to 95.8%)
Cerebrovascular disease	82.0% (76.5% to 87.1%)	84.6% (80.9% to 88.1%)	83.7% (79.2% to 88.1%)	84.6% (80.8% to 88.0%)
Hypertensive heart disease	100.0% (100.0% to 100.0%)	100.0% (100.0% to 100.0%)	100.0% (100.0% to 100.0%)	100.0% (100.0% to 100.0%)
Cardiomyopathy and myocarditis	42.0% (35.3% to 48.8%)	42.6% (36.2% to 48.9%)	41.1% (35.3% to 46.8%)	42.5% (36.2% to 48.8%)
Atrial fibrillation and flutter	48.9% (40.8% to 56.5%)	53.2% (46.0% to 59.8%)	58.8% (52.8% to 64.4%)	56.5% (50.0% to 62.4%)
Aortic aneurysm	39.9% (32.4% to 47.1%)	43.0% (36.7% to 48.6%)	-	43.0% (36.7% to 48.6%)
Peripheral vascular disease	50.5% (43.2% to 59.1%)	56.8% (50.9% to 63.0%)	62.1% (57.1% to 67.1%)	57.7% (52.2% to 63.6%)
Endocarditis	38.2% (29.7% to 48.0%)	36.1% (28.7% to 44.0%)	38.1% (30.0% to 47.1%)	36.1% (28.7% to 44.0%)

Supplementary Results Table 1. Age-standardised PAFs attributable to the joint distribution of all risk factors for all ages, both sexes combined for each GBD level 1 and level 2 cause and all causes in 2016 for Barbados. Results are shown for deaths, YLLs, YLDs, and DALYs
Data in parenthesis are 95% uncertainty intervals. PAFs=population attributable fractions. GBD=Global Burden of Disease. YLLs=years of life lost. YLDs=years live with disability. DALYs=disability-adjusted life-years

	Deaths 2016	YLLs 2016	YLDs 2016	DALYs 2016
Other cardiovascular and circulatory diseases	41.7% (36.4% to 46.8%)	41.7% (37.0% to 46.2%)	41.9% (37.1% to 46.8%)	41.8% (37.2% to 46.2%)
**Chronic respiratory diseases**	36.4% (29.2% to 43.6%)	36.5% (30.8% to 42.7%)	30.2% (22.3% to 39.7%)	32.6% (26.1% to 40.0%)
Chronic obstructive pulmonary disease	48.4% (36.7% to 59.4%)	51.1% (41.2% to 60.9%)	50.2% (40.5% to 60.2%)	50.9% (41.1% to 60.6%)
Pneumoconiosis	100.0% (100.0% to 100.0%)	100.0% (100.0% to 100.0%)	100.0% (100.0% to 100.0%)	100.0% (100.0% to 100.0%)
Asthma	33.8% (23.6% to 45.2%)	37.6% (28.0% to 48.0%)	28.2% (19.3% to 39.1%)	29.9% (21.0% to 40.5%)
Other chronic respiratory diseases	5.2% (3.1% to 7.7%)	6.1% (3.7% to 9.1%)	2.3% (1.1% to 4.3%)	5.5% (3.4% to 8.3%)
**Cirrhosis and other chronic liver diseases**	67.8% (65.4% to 70.2%)	68.2% (65.7% to 70.6%)	53.4% (49.0% to 57.4%)	67.5% (64.9% to 69.9%)
Cirrhosis and other chronic liver diseases due to hepatitis B	6.5% (2.6% to 11.5%)	8.4% (3.5% to 14.9%)	6.4% (2.6% to 11.3%)	8.3% (3.5% to 14.7%)
Cirrhosis and other chronic liver diseases due to hepatitis C	83.9% (80.4% to 87.1%)	87.9% (86.3% to 89.2%)	83.4% (79.5% to 86.6%)	87.7% (86.1% to 89.1%)
Cirrhosis and other chronic liver diseases due to alcohol use	100.0% (100.0% to 100.0%)	100.0% (100.0% to 100.0%)	100.0% (100.0% to 100.0%)	100.0% (100.0% to 100.0%)
**Digestive diseases**	6.9% (5.2% to 8.9%)	9.6% (7.2% to 12.2%)	2.3% (1.7% to 3.2%)	7.6% (5.8% to 9.5%)
Peptic ulcer disease	3.1% (2.0% to 4.5%)	4.3% (2.8% to 6.1%)	2.1% (1.3% to 3.0%)	3.7% (2.4% to 5.2%)
Gallbladder and biliary diseases	41.9% (28.1% to 57.1%)	44.6% (30.8% to 59.1%)	49.3% (34.5% to 63.9%)	45.5% (31.6% to 60.1%)
Pancreatitis	45.6% (29.7% to 62.3%)	51.9% (35.8% to 67.9%)	36.7% (23.5% to 51.9%)	51.6% (35.6% to 67.6%)
**Neurological disorders**	20.9% (9.9% to 33.8%)	19.9% (11.4% to 29.7%)	4.2% (2.1% to 6.7%)	9.4% (5.5% to 14.2%)
Alzheimer's disease and other dementias	24.2% (11.0% to 40.2%)	25.7% (12.1% to 42.1%)	25.8% (12.0% to 42.2%)	25.7% (12.1% to 42.1%)
Parkinson's disease	-1.5% (-2.1% to -1.1%)	-1.9% (-2.6% to -1.3%)	-1.9% (-2.5% to -1.4%)	-1.9% (-2.6% to -1.3%)
Epilepsy	27.8% (20.2% to 35.9%)	29.0% (20.9% to 37.3%)	21.7% (15.4% to 28.2%)	25.0% (18.2% to 32.7%)
Multiple sclerosis	1.4% (0.8% to 2.1%)	1.5% (0.9% to 2.3%)	1.5% (0.8% to 2.2%)	1.5% (0.9% to 2.2%)
**Mental and substance use disorders**	99.4% (99.3% to 99.5%)	99.0% (98.8% to 99.2%)	16.0% (14.2% to 18.0%)	18.2% (16.3% to 20.3%)
Alcohol use disorders	100.0% (100.0% to 100.0%)	100.0% (100.0% to 100.0%)	100.0% (100.0% to 100.0%)	100.0% (100.0% to 100.0%)

Supplementary Results Table 1. Age-standardised PAFs attributable to the joint distribution of all risk factors for all ages, both sexes combined for each GBD level 1 and level 2 cause and all causes in 2016 for Barbados. Results are shown for deaths, YLLs, YLDs, and DALYs
Data in parenthesis are 95% uncertainty intervals. PAFs=population attributable fractions. GBD=Global Burden of Disease. YLLs=years of life lost. YLDs=years live with disability. DALYs=disability-adjusted life-years

	Deaths 2016	YLLs 2016	YLDs 2016	DALYs 2016
Drug use disorders	100.0% (100.0% to 100.0%)	100.0% (100.0% to 100.0%)	100.0% (100.0% to 100.0%)	100.0% (100.0% to 100.0%)
Depressive disorders	-	-	6.3% (5.1% to 7.8%)	6.3% (5.1% to 7.8%)
Idiopathic developmental intellectual disability	-	-	59.6% (30.5% to 80.2%)	59.6% (30.5% to 80.2%)
**Diabetes, urogenital, blood, and endocrine diseases**	79.6% (77.8% to 81.1%)	76.6% (73.5% to 78.6%)	76.0% (72.4% to 79.2%)	76.3% (74.0% to 78.1%)
Diabetes mellitus	100.0% (100.0% to 100.0%)	100.0% (100.0% to 100.0%)	100.0% (100.0% to 100.0%)	100.0% (100.0% to 100.0%)
Chronic kidney disease	100.0% (100.0% to 100.0%)	100.0% (100.0% to 100.0%)	100.0% (100.0% to 100.0%)	100.0% (100.0% to 100.0%)
**Musculoskeletal disorders**	0.1% (0.0% to 0.1%)	0.0% (0.0% to 0.1%)	12.5% (9.8% to 15.6%)	11.7% (9.1% to 14.6%)
Rheumatoid arthritis	0.3% (0.1% to 0.6%)	0.5% (0.2% to 0.9%)	0.8% (0.3% to 1.4%)	0.8% (0.3% to 1.3%)
Osteoarthritis	-	-	28.1% (15.0% to 41.2%)	28.1% (15.0% to 41.2%)
Low back and neck pain	-	-	16.5% (13.8% to 19.5%)	16.5% (13.8% to 19.5%)
Gout	-	-	48.4% (33.7% to 64.2%)	48.4% (33.7% to 64.2%)
**Other non-communicable diseases**	0.1% (0.0% to 0.1%)	0.2% (0.1% to 0.2%)	4.7% (3.9% to 5.6%)	3.4% (2.7% to 4.1%)
Sense organ diseases	-	-	12.0% (10.5% to 13.9%)	12.0% (10.5% to 13.9%)
Sudden infant death syndrome	6.1% (3.9% to 8.9%)	6.1% (3.9% to 9.0%)	-	6.1% (3.9% to 9.0%)
**Transport injuries**	33.7% (25.9% to 43.3%)	30.2% (21.3% to 40.5%)	36.4% (28.8% to 45.7%)	31.2% (22.5% to 41.1%)
Road injuries	33.3% (25.2% to 43.0%)	30.2% (21.2% to 40.5%)	36.8% (29.2% to 46.2%)	31.3% (22.5% to 41.2%)
Other transport injuries	39.4% (33.3% to 46.6%)	30.4% (23.1% to 39.9%)	29.4% (23.0% to 37.5%)	30.3% (23.2% to 39.0%)
**Unintentional injuries**	17.4% (13.9% to 21.7%)	13.2% (8.9% to 18.7%)	19.2% (15.7% to 24.8%)	15.8% (12.1% to 21.2%)
Falls	56.1% (51.6% to 60.9%)	32.3% (27.2% to 39.6%)	25.7% (22.2% to 31.5%)	27.0% (23.4% to 32.9%)
Drowning	15.9% (9.3% to 24.1%)	15.6% (9.1% to 23.7%)	15.3% (9.7% to 22.2%)	15.6% (9.1% to 23.6%)
Fire, heat, and hot substances	10.2% (6.5% to 15.4%)	13.4% (8.7% to 19.3%)	18.7% (13.0% to 25.6%)	15.6% (10.6% to 22.0%)

Supplementary Results Table 1. Age-standardised PAFs attributable to the joint distribution of all risk factors for all ages, both sexes combined for each GBD level 1 and level 2 cause and all causes in 2016 for Barbados. Results are shown for deaths, YLLs, YLDs, and DALYs
Data in parenthesis are 95% uncertainty intervals. PAFs=population attributable fractions. GBD=Global Burden of Disease. YLLs=years of life lost. YLDs=years live with disability. DALYs=disability-adjusted life-years

	Deaths 2016	YLLs 2016	YLDs 2016	DALYs 2016
Poisonings	16.6% (10.7% to 23.8%)	17.3% (11.1% to 25.1%)	18.2% (12.3% to 25.1%)	17.5% (11.6% to 25.0%)
Exposure to mechanical forces	18.0% (12.7% to 25.6%)	15.2% (9.7% to 23.6%)	18.0% (13.6% to 25.6%)	16.5% (11.4% to 24.1%)
Animal contact	13.7% (10.5% to 18.2%)	13.4% (9.3% to 19.8%)	12.3% (8.2% to 18.5%)	12.6% (8.6% to 18.8%)
Foreign body	5.5% (3.0% to 10.1%)	6.2% (3.2% to 11.5%)	8.4% (5.3% to 13.3%)	6.9% (3.9% to 11.8%)
Other unintentional injuries	17.6% (10.3% to 26.8%)	18.5% (10.7% to 28.0%)	16.7% (11.6% to 23.3%)	17.0% (11.5% to 24.2%)
**Self-harm and interpersonal violence**	29.4% (22.6% to 36.8%)	29.4% (22.4% to 37.0%)	29.9% (26.3% to 34.6%)	29.4% (22.6% to 36.8%)
Self-harm	29.1% (18.0% to 40.4%)	30.5% (19.0% to 42.4%)	24.9% (14.7% to 35.2%)	30.4% (18.9% to 42.2%)
Interpersonal violence	29.7% (21.2% to 38.9%)	29.0% (20.6% to 38.3%)	30.2% (26.4% to 35.0%)	29.1% (21.1% to 37.8%)
**Forces of nature, conflict and terrorism, and executions and police conflict**	0.0% (0.0% to 0.0%)	0.0% (0.0% to 0.0%)	0.8% (0.3% to 1.3%)	0.0% (0.0% to 0.1%)
Forces of nature, conflict and terrorism, and state actor violence	0.0% (0.0% to 0.0%)	0.0% (0.0% to 0.0%)	4.8% (3.2% to 7.0%)	4.8% (3.2% to 7.0%)

Supplementary Results Table 1. Age-standardised PAFs attributable to the joint distribution of all risk factors for all ages, both sexes combined for each GBD level 1 and level 2 cause and all causes in 2016 for Belarus. Results are shown for deaths, YLLs, YLDs, and DALYs
Data in parenthesis are 95% uncertainty intervals. PAFs=population attributable fractions. GBD=Global Burden of Disease. YLLs=years of life lost. YLDs=years live with disability. DALYs=disability-adjusted life-years

	Deaths 2016	YLLs 2016	YLDs 2016	DALYs 2016
**All causes**	72.9% (70.8% to 74.7%)	66.6% (64.8% to 68.4%)	25.1% (23.3% to 26.9%)	51.1% (47.4% to 54.7%)
**HIV/AIDS and tuberculosis**	76.9% (72.2% to 80.9%)	77.3% (73.2% to 80.6%)	75.1% (72.1% to 77.9%)	77.2% (73.2% to 80.4%)
Tuberculosis	74.1% (65.2% to 82.2%)	75.4% (66.6% to 83.5%)	67.3% (58.7% to 75.1%)	75.1% (66.2% to 83.2%)
HIV/AIDS	79.7% (78.2% to 81.0%)	78.7% (77.2% to 80.1%)	78.3% (76.0% to 80.6%)	78.7% (77.2% to 80.1%)
**Diarrhoea, lower respiratory infections, and other common infectious diseases**	56.7% (49.9% to 62.9%)	56.7% (50.7% to 62.2%)	23.4% (17.4% to 29.7%)	42.2% (36.1% to 47.9%)
Diarrhoeal diseases	66.3% (48.5% to 83.2%)	76.1% (62.2% to 88.3%)	68.9% (51.4% to 84.5%)	69.4% (52.2% to 84.8%)
Lower respiratory infections	66.4% (59.0% to 73.2%)	71.6% (64.9% to 77.5%)	54.4% (45.8% to 63.3%)	70.9% (64.2% to 76.9%)
Upper respiratory infections	0.3% (0.0% to 1.2%)	0.9% (0.1% to 3.4%)	0.0% (0.0% to 0.0%)	0.0% (0.0% to 0.0%)
Otitis media	0.6% (0.3% to 1.0%)	2.2% (1.2% to 3.4%)	9.9% (6.8% to 12.6%)	9.9% (6.8% to 12.6%)
Meningitis	2.4% (1.6% to 3.3%)	3.9% (2.8% to 5.0%)	0.0% (0.0% to 0.0%)	2.7% (1.9% to 3.6%)
Encephalitis	1.6% (0.5% to 3.0%)	2.4% (1.0% to 4.3%)	0.0% (0.0% to 0.0%)	1.8% (0.7% to 3.2%)
Measles	6.4% (1.2% to 21.5%)	8.2% (1.6% to 27.5%)	17.0% (0.0% to 58.7%)	8.3% (1.6% to 27.8%)
**Maternal disorders**	4.8% (2.7% to 7.1%)	4.6% (2.7% to 6.9%)	4.8% (2.0% to 8.2%)	4.7% (2.4% to 7.4%)
Maternal haemorrhage	12.0% (4.3% to 19.5%)	11.9% (4.3% to 19.3%)	11.7% (4.2% to 19.0%)	11.7% (4.2% to 19.1%)
Maternal sepsis and other pregnancy related infections	11.9% (4.3% to 19.1%)	11.8% (4.3% to 19.0%)	11.6% (4.2% to 18.8%)	11.6% (4.2% to 18.8%)
Maternal abortion, miscarriage, and ectopic pregnancy	6.9% (4.0% to 10.4%)	6.7% (3.8% to 10.2%)	6.2% (3.0% to 10.4%)	6.6% (3.8% to 10.0%)
**Neonatal disorders**	72.9% (69.7% to 76.1%)	73.0% (69.7% to 76.1%)	0.1% (0.0% to 0.1%)	43.5% (34.9% to 50.9%)
Neonatal preterm birth complications	88.2% (83.9% to 91.6%)	88.2% (84.0% to 91.7%)	0.2% (0.2% to 0.3%)	55.6% (43.9% to 66.6%)
Neonatal encephalopathy due to birth asphyxia and trauma	67.2% (62.9% to 71.6%)	67.2% (62.9% to 71.7%)	0.0% (0.0% to 0.0%)	34.6% (24.3% to 44.0%)
Neonatal sepsis and other neonatal infections	61.7% (55.6% to 67.4%)	61.7% (55.7% to 67.4%)	0.0% (0.0% to 0.0%)	33.7% (19.7% to 46.7%)
Hemolytic disease and other neonatal jaundice	67.7% (62.8% to 72.9%)	67.8% (62.9% to 73.0%)	0.0% (0.0% to 0.0%)	21.4% (13.8% to 33.5%)

**Supplementary Results Table 1. Age-standardised PAFs attributable to the joint distribution of all risk factors for all ages, both sexes combined for each GBD level 1 and level 2 cause and all causes in 2016 for Belarus. Results are shown for deaths, YLLs, YLDs, and DALYs**<br>Data in parenthesis are 95% uncertainty intervals. PAFs=population attributable fractions. GBD=Global Burden of Disease. YLLs=years of life lost. YLDs=years live with disability. DALYs=disability-adjusted life-years	**Deaths 2016**	**YLLs 2016**	**YLDs 2016**	**DALYs 2016**
Other neonatal disorders	62.8% (57.1% to 68.0%)	62.8% (57.2% to 68.1%)	0.0% (0.0% to 0.0%)	55.8% (49.7% to 61.9%)
**Nutritional deficiencies**	22.3% (17.5% to 28.5%)	37.0% (29.1% to 45.9%)	78.2% (72.7% to 83.7%)	76.1% (70.6% to 81.4%)
Protein-energy malnutrition	24.2% (14.7% to 35.6%)	43.3% (30.3% to 57.4%)	96.3% (94.4% to 97.7%)	80.7% (72.2% to 87.4%)
Vitamin A deficiency	-	-	0.0% (0.0% to 0.0%)	0.0% (0.0% to 0.0%)
Iron-deficiency anaemia	100.0% (100.0% to 100.0%)	100.0% (100.0% to 100.0%)	100.0% (100.0% to 100.0%)	100.0% (100.0% to 100.0%)
**Other communicable, maternal, neonatal, and nutritional diseases**	3.4% (2.2% to 5.2%)	2.1% (1.2% to 3.6%)	79.8% (75.9% to 83.4%)	33.4% (21.9% to 45.5%)
Sexually transmitted diseases excluding HIV	76.8% (57.4% to 93.1%)	56.6% (33.5% to 83.1%)	100.0% (100.0% to 100.0%)	98.2% (95.8% to 99.6%)
Hepatitis	4.8% (3.2% to 6.8%)	4.1% (2.6% to 5.9%)	2.7% (1.6% to 3.9%)	4.0% (2.5% to 5.8%)
**Neoplasms**	45.2% (41.0% to 49.3%)	45.8% (41.9% to 49.8%)	38.2% (34.6% to 41.8%)	45.6% (41.7% to 49.6%)
Oesophageal cancer	89.3% (83.3% to 93.9%)	91.2% (85.7% to 95.2%)	89.7% (83.9% to 94.1%)	91.2% (85.7% to 95.2%)
Stomach cancer	21.2% (11.7% to 32.3%)	23.1% (13.3% to 34.3%)	20.6% (11.8% to 30.6%)	23.1% (13.2% to 34.3%)
Liver cancer	70.3% (62.6% to 77.0%)	69.6% (61.8% to 76.6%)	69.8% (62.6% to 76.1%)	69.6% (61.8% to 76.6%)
Larynx cancer	92.3% (87.6% to 95.6%)	93.3% (89.0% to 96.2%)	92.2% (87.8% to 95.5%)	93.3% (88.9% to 96.2%)
Tracheal, bronchus, and lung cancer	86.4% (82.7% to 89.5%)	88.0% (84.7% to 90.8%)	85.4% (82.0% to 88.3%)	88.0% (84.7% to 90.7%)
Breast cancer	31.8% (22.4% to 44.2%)	31.6% (21.7% to 45.1%)	31.4% (21.6% to 44.4%)	31.6% (21.7% to 45.1%)
Cervical cancer	100.0% (100.0% to 100.0%)	100.0% (100.0% to 100.0%)	100.0% (100.0% to 100.0%)	100.0% (100.0% to 100.0%)
Uterine cancer	50.8% (35.5% to 65.4%)	51.5% (36.2% to 65.7%)	51.7% (36.5% to 66.0%)	51.5% (36.2% to 65.7%)
Prostate cancer	7.5% (5.1% to 10.2%)	8.9% (6.1% to 12.2%)	9.0% (6.2% to 12.3%)	8.9% (6.1% to 12.2%)
Colon and rectum cancer	54.3% (45.4% to 61.9%)	56.6% (47.7% to 64.3%)	55.5% (47.0% to 63.0%)	56.6% (47.7% to 64.2%)
Lip and oral cavity cancer	88.7% (83.5% to 92.6%)	91.3% (87.2% to 94.4%)	86.3% (80.3% to 90.8%)	91.2% (87.1% to 94.3%)
Nasopharynx cancer	90.4% (86.8% to 93.2%)	90.5% (87.3% to 93.1%)	90.3% (87.2% to 92.8%)	90.5% (87.3% to 93.1%)

Supplementary Results Table 1. Age-standardised PAFs attributable to the joint distribution of all risk factors for all ages, both sexes combined for each GBD level 1 and level 2 cause and all causes in 2016 for Belarus. Results are shown for deaths, YLLs, YLDs, and DALYs
Data in parenthesis are 95% uncertainty intervals. PAFs=population attributable fractions. GBD=Global Burden of Disease. YLLs=years of life lost. YLDs=years live with disability. DALYs=disability-adjusted life-years

	Deaths 2016	YLLs 2016	YLDs 2016	DALYs 2016
Other pharynx cancer	77.5% (69.3% to 84.5%)	78.7% (70.7% to 85.5%)	77.8% (69.4% to 84.6%)	78.7% (70.7% to 85.5%)
Gallbladder and biliary tract cancer	25.1% (14.2% to 37.4%)	24.8% (14.0% to 36.9%)	25.7% (14.6% to 38.2%)	24.8% (14.0% to 36.9%)
Pancreatic cancer	31.2% (23.0% to 39.7%)	34.6% (26.1% to 43.4%)	31.6% (24.0% to 39.6%)	34.6% (26.1% to 43.4%)
Ovarian cancer	11.0% (4.6% to 18.1%)	9.9% (3.9% to 16.6%)	9.7% (3.8% to 16.3%)	9.9% (3.9% to 16.6%)
Kidney cancer	41.3% (30.0% to 52.7%)	42.3% (31.1% to 53.3%)	40.7% (30.0% to 51.6%)	42.3% (31.0% to 53.3%)
Bladder cancer	37.8% (27.3% to 48.6%)	43.6% (31.8% to 54.7%)	40.1% (30.3% to 50.1%)	43.4% (31.7% to 54.6%)
Thyroid cancer	15.7% (8.0% to 25.5%)	16.0% (7.9% to 26.2%)	15.2% (8.2% to 23.9%)	15.9% (8.0% to 25.9%)
Mesothelioma	87.4% (83.6% to 90.5%)	80.3% (75.0% to 84.9%)	85.0% (80.8% to 88.3%)	80.4% (75.1% to 84.9%)
Non-Hodgkin's lymphoma	7.3% (2.7% to 13.0%)	6.8% (2.7% to 11.9%)	7.2% (2.8% to 12.9%)	6.8% (2.7% to 11.9%)
Multiple myeloma	9.5% (3.9% to 16.9%)	9.6% (4.0% to 16.9%)	9.6% (4.0% to 17.1%)	9.6% (4.0% to 16.9%)
Leukaemia	22.7% (13.9% to 31.8%)	19.8% (12.2% to 28.1%)	19.3% (11.7% to 27.0%)	19.8% (12.2% to 28.1%)
**Cardiovascular diseases**	91.5% (88.8% to 93.6%)	93.1% (91.5% to 94.4%)	86.5% (83.9% to 88.7%)	92.6% (91.0% to 93.9%)
Rheumatic heart disease	33.8% (23.0% to 49.8%)	33.6% (22.8% to 46.9%)	32.6% (21.7% to 47.1%)	33.6% (22.8% to 46.9%)
Ischaemic heart disease	93.9% (90.8% to 96.5%)	95.6% (93.7% to 97.2%)	92.8% (90.5% to 94.9%)	95.5% (93.5% to 97.1%)
Cerebrovascular disease	87.0% (82.5% to 91.4%)	91.5% (88.7% to 93.9%)	88.5% (84.6% to 91.9%)	91.0% (88.1% to 93.6%)
Hypertensive heart disease	100.0% (100.0% to 100.0%)	100.0% (100.0% to 100.0%)	100.0% (100.0% to 100.0%)	100.0% (100.0% to 100.0%)
Cardiomyopathy and myocarditis	69.5% (63.5% to 78.3%)	69.0% (62.1% to 78.3%)	51.3% (44.5% to 57.9%)	68.0% (61.1% to 77.3%)
Atrial fibrillation and flutter	57.1% (48.2% to 66.1%)	62.1% (53.9% to 69.8%)	69.7% (63.5% to 75.5%)	66.6% (60.0% to 73.3%)
Aortic aneurysm	54.3% (47.5% to 60.2%)	59.9% (54.0% to 65.3%)	-	59.9% (54.0% to 65.3%)
Peripheral vascular disease	52.9% (45.7% to 60.2%)	61.1% (54.9% to 67.3%)	58.3% (52.7% to 64.3%)	60.0% (54.2% to 65.8%)
Endocarditis	47.7% (39.0% to 56.6%)	47.5% (37.4% to 57.3%)	43.3% (34.8% to 52.0%)	47.5% (37.5% to 57.2%)

Supplementary Results Table 1. Age-standardised PAFs attributable to the joint distribution of all risk factors for all ages, both sexes combined for each GBD level 1 and level 2 cause and all causes in 2016 for Belarus. Results are shown for deaths, YLLs, YLDs, and DALYs
Data in parenthesis are 95% uncertainty intervals. PAFs=population attributable fractions. GBD=Global Burden of Disease. YLLs=years of life lost. YLDs=years live with disability. DALYs=disability-adjusted life-years

	**Deaths 2016**	**YLLs 2016**	**YLDs 2016**	**DALYs 2016**
Other cardiovascular and circulatory diseases	53.9% (48.6% to 58.9%)	61.1% (56.2% to 65.7%)	54.7% (49.4% to 59.6%)	59.3% (54.6% to 63.8%)
**Chronic respiratory diseases**	66.7% (60.7% to 72.7%)	70.8% (65.5% to 75.9%)	38.3% (32.2% to 45.1%)	54.4% (48.4% to 60.5%)
Chronic obstructive pulmonary disease	70.8% (64.1% to 77.4%)	76.7% (70.7% to 82.2%)	61.6% (53.5% to 69.7%)	73.8% (67.7% to 79.7%)
Pneumoconiosis	100.0% (100.0% to 100.0%)	100.0% (100.0% to 100.0%)	100.0% (100.0% to 100.0%)	100.0% (100.0% to 100.0%)
Asthma	46.6% (35.9% to 58.1%)	50.4% (40.5% to 61.0%)	34.0% (26.6% to 42.5%)	35.9% (28.3% to 44.4%)
Other chronic respiratory diseases	22.1% (14.8% to 29.8%)	22.4% (14.9% to 30.5%)	5.8% (3.5% to 8.6%)	13.9% (8.7% to 20.1%)
**Cirrhosis and other chronic liver diseases**	59.2% (56.6% to 61.8%)	58.5% (55.9% to 61.3%)	45.0% (41.7% to 48.5%)	58.0% (55.4% to 60.7%)
Cirrhosis and other chronic liver diseases due to hepatitis B	3.3% (1.4% to 5.5%)	3.8% (1.6% to 6.2%)	3.3% (1.4% to 5.5%)	3.8% (1.6% to 6.2%)
Cirrhosis and other chronic liver diseases due to hepatitis C	89.9% (89.7% to 90.0%)	90.0% (89.9% to 90.0%)	89.1% (88.7% to 89.3%)	89.9% (89.9% to 90.0%)
Cirrhosis and other chronic liver diseases due to alcohol use	100.0% (100.0% to 100.0%)	100.0% (100.0% to 100.0%)	100.0% (100.0% to 100.0%)	100.0% (100.0% to 100.0%)
**Digestive diseases**	26.2% (20.8% to 31.2%)	35.8% (29.0% to 42.0%)	5.4% (4.3% to 6.6%)	26.7% (21.2% to 32.3%)
Peptic ulcer disease	19.6% (14.1% to 25.5%)	24.3% (17.7% to 30.9%)	14.7% (10.3% to 19.8%)	21.3% (15.4% to 27.5%)
Gallbladder and biliary diseases	46.2% (31.7% to 61.6%)	46.2% (32.4% to 60.8%)	48.7% (35.1% to 62.8%)	46.9% (33.2% to 61.2%)
Pancreatitis	71.8% (57.5% to 84.3%)	76.1% (62.9% to 86.8%)	60.2% (45.3% to 74.4%)	75.8% (62.4% to 86.5%)
**Neurological disorders**	20.5% (9.3% to 33.5%)	21.0% (12.6% to 30.7%)	3.7% (1.7% to 6.4%)	8.7% (4.9% to 13.0%)
Alzheimer's disease and other dementias	24.2% (10.5% to 40.2%)	25.3% (11.8% to 40.8%)	25.3% (11.6% to 41.0%)	25.3% (11.8% to 40.8%)
Parkinson's disease	-8.3% (-11.2% to -5.6%)	-11.4% (-15.1% to -7.9%)	-12.9% (-16.6% to -9.4%)	-11.7% (-15.2% to -8.3%)
Epilepsy	47.4% (36.2% to 57.9%)	45.4% (35.0% to 55.5%)	35.4% (27.0% to 44.0%)	40.7% (30.8% to 50.4%)
Multiple sclerosis	11.6% (7.3% to 16.2%)	12.6% (8.2% to 17.4%)	10.3% (6.7% to 14.0%)	11.6% (7.5% to 15.8%)
**Mental and substance use disorders**	100.0% (100.0% to 100.0%)	100.0% (100.0% to 100.0%)	31.2% (28.4% to 34.3%)	47.8% (41.9% to 54.3%)
Alcohol use disorders	100.0% (100.0% to 100.0%)	100.0% (100.0% to 100.0%)	100.0% (100.0% to 100.0%)	100.0% (100.0% to 100.0%)

Supplementary Results Table 1. Age-standardised PAFs attributable to the joint distribution of all risk factors for all ages, both sexes combined for each GBD level 1 and level 2 cause and all causes in 2016 for Belarus. Results are shown for deaths, YLLs, YLDs, and DALYs
Data in parenthesis are 95% uncertainty intervals. PAFs=population attributable fractions. GBD=Global Burden of Disease. YLLs=years of life lost. YLDs=years live with disability. DALYs=disability-adjusted life-years

	Deaths 2016	YLLs 2016	YLDs 2016	DALYs 2016
Drug use disorders	100.0% (100.0% to 100.0%)	100.0% (100.0% to 100.0%)	100.0% (100.0% to 100.0%)	100.0% (100.0% to 100.0%)
Depressive disorders	-	-	10.5% (9.0% to 12.2%)	10.5% (9.0% to 12.2%)
Idiopathic developmental intellectual disability	-	-	60.9% (29.8% to 81.9%)	60.9% (29.8% to 81.9%)
**Diabetes, urogenital, blood, and endocrine diseases**	66.3% (64.7% to 67.9%)	61.5% (59.5% to 64.6%)	48.7% (44.5% to 52.7%)	52.3% (48.8% to 55.5%)
Diabetes mellitus	100.0% (100.0% to 100.0%)	100.0% (100.0% to 100.0%)	100.0% (100.0% to 100.0%)	100.0% (100.0% to 100.0%)
Chronic kidney disease	100.0% (100.0% to 100.0%)	100.0% (100.0% to 100.0%)	100.0% (100.0% to 100.0%)	100.0% (100.0% to 100.0%)
**Musculoskeletal disorders**	1.1% (0.4% to 2.1%)	1.4% (0.6% to 2.5%)	23.6% (19.6% to 28.1%)	23.6% (19.5% to 28.0%)
Rheumatoid arthritis	2.5% (1.0% to 4.8%)	3.6% (1.4% to 6.5%)	5.5% (2.2% to 9.0%)	5.4% (2.2% to 8.9%)
Osteoarthritis	-	-	30.0% (16.3% to 43.9%)	30.0% (16.3% to 43.9%)
Low back and neck pain	-	-	24.1% (20.7% to 28.1%)	24.1% (20.7% to 28.1%)
Gout	-	-	48.9% (33.5% to 64.4%)	48.9% (33.5% to 64.4%)
**Other non-communicable diseases**	0.3% (0.1% to 0.5%)	0.4% (0.2% to 0.6%)	3.8% (3.3% to 4.3%)	3.2% (2.8% to 3.7%)
Sense organ diseases	-	-	9.1% (8.2% to 10.2%)	9.1% (8.2% to 10.2%)
Sudden infant death syndrome	5.2% (3.0% to 8.1%)	5.2% (3.0% to 8.1%)	-	5.2% (3.0% to 8.1%)
**Transport injuries**	40.2% (28.5% to 53.3%)	37.9% (26.4% to 51.5%)	41.4% (31.1% to 53.2%)	38.7% (27.6% to 51.5%)
Road injuries	40.0% (28.4% to 53.1%)	37.7% (26.3% to 51.3%)	41.8% (31.5% to 53.7%)	38.7% (27.6% to 51.5%)
Other transport injuries	42.3% (30.4% to 55.6%)	39.6% (27.4% to 53.5%)	34.7% (24.5% to 45.9%)	38.6% (26.8% to 52.0%)
**Unintentional injuries**	18.0% (13.1% to 23.8%)	15.1% (10.2% to 20.8%)	16.5% (14.6% to 18.9%)	15.7% (12.2% to 19.9%)
Falls	26.8% (23.1% to 31.7%)	16.0% (13.2% to 19.7%)	19.2% (17.5% to 21.2%)	18.1% (16.1% to 20.8%)
Drowning	21.3% (11.5% to 32.9%)	20.4% (11.4% to 31.6%)	19.7% (10.3% to 30.7%)	20.4% (11.4% to 31.6%)
Fire, heat, and hot substances	19.9% (10.2% to 31.3%)	20.5% (10.8% to 31.6%)	18.8% (10.1% to 28.9%)	20.1% (10.7% to 31.0%)

Supplementary Results Table 1. Age-standardised PAFs attributable to the joint distribution of all risk factors for all ages, both sexes combined for each GBD level 1 and level 2 cause and all causes in 2016 for Belarus. Results are shown for deaths, YLLs, YLDs, and DALYs

Data in parenthesis are 95% uncertainty intervals. PAFs=population attributable fractions. GBD=Global Burden of Disease. YLLs=years of life lost. YLDs=years live with disability. DALYs=disability-adjusted life-years

	**Deaths 2016**	**YLLs 2016**	**YLDs 2016**	**DALYs 2016**
Poisonings	18.6% (9.6% to 28.9%)	16.8% (9.1% to 25.5%)	17.4% (9.9% to 26.7%)	16.9% (9.1% to 25.5%)
Exposure to mechanical forces	12.0% (9.2% to 15.7%)	10.4% (7.8% to 14.2%)	12.0% (10.1% to 14.6%)	11.4% (9.2% to 14.3%)
Animal contact	8.0% (6.2% to 10.6%)	7.2% (5.3% to 9.9%)	4.9% (3.4% to 7.0%)	6.0% (4.4% to 8.3%)
Foreign body	5.0% (3.2% to 7.8%)	5.3% (3.4% to 8.1%)	3.8% (2.5% to 5.6%)	5.0% (3.2% to 7.6%)
Other unintentional injuries	24.3% (12.7% to 37.8%)	24.0% (13.0% to 37.0%)	17.1% (9.1% to 26.6%)	20.0% (10.8% to 30.8%)
**Self-harm and interpersonal violence**	45.9% (34.5% to 56.7%)	46.2% (35.1% to 57.0%)	34.1% (26.3% to 42.3%)	45.6% (34.8% to 56.0%)
Self-harm	48.7% (34.8% to 61.5%)	49.7% (35.7% to 62.4%)	43.1% (29.8% to 55.7%)	49.6% (35.7% to 62.4%)
Interpersonal violence	35.7% (23.4% to 49.6%)	34.4% (22.3% to 47.8%)	32.1% (23.7% to 41.4%)	34.0% (22.5% to 46.7%)
**Forces of nature, conflict and terrorism, and executions and police conflict**	0.0% (0.0% to 0.0%)	0.0% (0.0% to 0.0%)	1.4% (0.9% to 2.1%)	0.4% (0.1% to 0.9%)
Forces of nature, conflict and terrorism, and state actor violence	0.0% (0.0% to 0.0%)	0.0% (0.0% to 0.0%)	3.6% (2.3% to 5.3%)	3.6% (2.3% to 5.3%)

Supplementary Results Table 1. Age-standardised PAFs attributable to the joint distribution of all risk factors for all ages, both sexes combined for each GBD level 1 and level 2 cause and all causes in 2016 for Belgium. Results are shown for deaths, YLLs, YLDs, and DALYs
Data in parenthesis are 95% uncertainty intervals. PAFs=population attributable fractions. GBD=Global Burden of Disease. YLLs=years of life lost. YLDs=years live with disability. DALYs=disability-adjusted life-years

	Deaths 2016	YLLs 2016	YLDs 2016	DALYs 2016
**All causes**	54.7% (52.3% to 57.3%)	53.0% (50.9% to 55.2%)	19.1% (17.9% to 20.4%)	34.8% (32.1% to 38.0%)
**HIV/AIDS and tuberculosis**	75.1% (68.9% to 80.2%)	78.6% (75.1% to 81.4%)	91.0% (86.4% to 93.8%)	83.4% (79.4% to 86.8%)
Tuberculosis	58.7% (46.2% to 68.9%)	59.4% (48.5% to 68.1%)	51.0% (38.8% to 60.7%)	57.9% (47.1% to 66.6%)
HIV/AIDS	91.6% (90.9% to 92.2%)	87.0% (86.0% to 87.9%)	96.2% (95.5% to 96.8%)	91.0% (89.3% to 92.7%)
**Diarrhoea, lower respiratory infections, and other common infectious diseases**	36.1% (27.9% to 44.7%)	41.3% (34.4% to 48.6%)	7.5% (5.4% to 10.1%)	28.3% (22.5% to 34.8%)
Diarrhoeal diseases	13.6% (7.4% to 23.2%)	23.7% (17.0% to 33.1%)	30.1% (21.6% to 39.8%)	27.0% (19.7% to 36.3%)
Lower respiratory infections	40.2% (30.7% to 50.2%)	48.0% (39.3% to 56.9%)	47.5% (39.3% to 56.2%)	48.0% (39.3% to 56.9%)
Upper respiratory infections	0.5% (0.0% to 1.9%)	1.6% (0.2% to 5.4%)	0.0% (0.0% to 0.0%)	0.0% (0.0% to 0.0%)
Otitis media	1.6% (0.9% to 2.5%)	4.1% (2.5% to 5.9%)	7.2% (4.8% to 9.7%)	7.2% (4.8% to 9.7%)
Meningitis	1.8% (1.3% to 2.4%)	3.1% (2.4% to 4.0%)	0.0% (0.0% to 0.0%)	2.8% (2.1% to 3.6%)
Encephalitis	0.4% (0.3% to 0.6%)	1.0% (0.8% to 1.3%)	0.0% (0.0% to 0.0%)	0.7% (0.5% to 0.9%)
Measles	5.7% (0.9% to 23.0%)	6.4% (1.0% to 25.7%)	10.9% (1.9% to 42.0%)	6.5% (1.0% to 25.9%)
**Maternal disorders**	2.6% (1.5% to 3.7%)	2.5% (1.5% to 3.6%)	1.3% (0.5% to 2.3%)	1.8% (0.9% to 2.7%)
Maternal haemorrhage	8.4% (3.0% to 13.9%)	8.5% (3.0% to 13.9%)	8.6% (3.1% to 14.2%)	8.6% (3.1% to 14.1%)
Maternal sepsis and other pregnancy related infections	8.5% (3.1% to 13.9%)	8.5% (3.1% to 14.0%)	8.4% (3.0% to 13.7%)	8.5% (3.0% to 13.9%)
Maternal abortion, miscarriage, and ectopic pregnancy	5.7% (3.4% to 8.4%)	5.8% (3.5% to 8.6%)	6.4% (3.3% to 10.3%)	5.9% (3.6% to 8.8%)
**Neonatal disorders**	71.5% (68.6% to 74.5%)	71.6% (68.6% to 74.5%)	0.1% (0.1% to 0.1%)	49.1% (42.7% to 55.4%)
Neonatal preterm birth complications	87.0% (82.8% to 90.8%)	87.1% (82.9% to 90.9%)	0.2% (0.1% to 0.2%)	49.1% (39.2% to 59.6%)
Neonatal encephalopathy due to birth asphyxia and trauma	66.5% (63.1% to 69.9%)	66.6% (63.1% to 70.0%)	0.0% (0.0% to 0.0%)	53.5% (46.7% to 59.4%)
Neonatal sepsis and other neonatal infections	54.3% (47.1% to 59.9%)	54.3% (47.2% to 59.9%)	0.0% (0.0% to 0.0%)	25.9% (13.1% to 44.7%)
Hemolytic disease and other neonatal jaundice	62.8% (57.5% to 67.1%)	62.9% (57.7% to 67.2%)	0.0% (0.0% to 0.0%)	17.8% (10.6% to 25.9%)

Supplementary Results Table 1. Age-standardised PAFs attributable to the joint distribution of all risk factors for all ages, both sexes combined for each GBD level 1 and level 2 cause and all causes in 2016 for Belgium. Results are shown for deaths, YLLs, YLDs, and DALYs
Data in parenthesis are 95% uncertainty intervals. PAFs=population attributable fractions. GBD=Global Burden of Disease. YLLs=years of life lost. YLDs=years live with disability. DALYs=disability-adjusted life-years

	Deaths 2016	YLLs 2016	YLDs 2016	DALYs 2016
Other neonatal disorders	60.5% (55.7% to 65.2%)	60.5% (55.8% to 65.2%)	0.0% (0.0% to 0.0%)	57.7% (53.1% to 62.5%)
**Nutritional deficiencies**	1.0% (0.8% to 1.5%)	4.9% (3.7% to 6.1%)	69.7% (59.8% to 78.2%)	62.6% (53.0% to 71.9%)
Protein-energy malnutrition	0.2% (0.1% to 0.4%)	2.2% (1.2% to 3.8%)	98.3% (98.1% to 98.5%)	53.1% (41.2% to 66.6%)
Vitamin A deficiency	-	-	0.0% (0.0% to 0.0%)	0.0% (0.0% to 0.0%)
Iron-deficiency anaemia	100.0% (100.0% to 100.0%)	100.0% (100.0% to 100.0%)	100.0% (100.0% to 100.0%)	100.0% (100.0% to 100.0%)
**Other communicable, maternal, neonatal, and nutritional diseases**	3.1% (2.6% to 4.4%)	2.8% (2.3% to 3.8%)	66.2% (59.1% to 72.4%)	23.4% (17.0% to 32.8%)
Sexually transmitted diseases excluding HIV	93.9% (88.5% to 97.2%)	81.1% (67.5% to 90.6%)	100.0% (100.0% to 100.0%)	98.7% (97.3% to 99.5%)
Hepatitis	4.8% (3.4% to 6.5%)	5.9% (4.2% to 7.8%)	2.5% (1.6% to 3.7%)	5.7% (4.0% to 7.5%)
**Neoplasms**	50.4% (47.4% to 53.4%)	51.2% (48.4% to 53.8%)	36.6% (33.2% to 39.7%)	50.6% (47.7% to 53.2%)
Oesophageal cancer	88.8% (83.6% to 93.3%)	90.0% (85.1% to 94.1%)	88.8% (83.6% to 93.3%)	89.9% (85.1% to 94.1%)
Stomach cancer	23.9% (14.5% to 34.0%)	25.2% (15.6% to 34.5%)	24.7% (15.3% to 34.5%)	25.2% (15.7% to 34.5%)
Liver cancer	76.8% (70.3% to 82.1%)	78.0% (71.8% to 83.1%)	76.6% (70.4% to 82.3%)	78.0% (71.8% to 83.1%)
Larynx cancer	93.7% (89.5% to 96.4%)	93.4% (89.2% to 96.1%)	93.6% (89.3% to 96.2%)	93.4% (89.2% to 96.1%)
Tracheal, bronchus, and lung cancer	92.2% (90.5% to 93.5%)	91.9% (90.2% to 93.3%)	91.7% (89.9% to 93.3%)	91.9% (90.2% to 93.4%)
Breast cancer	37.2% (30.9% to 43.9%)	37.0% (30.8% to 43.2%)	36.9% (30.7% to 43.4%)	36.9% (30.8% to 43.2%)
Cervical cancer	100.0% (100.0% to 100.0%)	100.0% (100.0% to 100.0%)	100.0% (100.0% to 100.0%)	100.0% (100.0% to 100.0%)
Uterine cancer	40.4% (27.2% to 53.5%)	40.6% (27.9% to 53.9%)	40.8% (28.0% to 54.1%)	40.6% (28.0% to 53.9%)
Prostate cancer	8.9% (6.3% to 11.9%)	9.1% (6.5% to 12.0%)	9.3% (6.7% to 12.1%)	9.1% (6.5% to 12.0%)
Colon and rectum cancer	57.1% (48.5% to 65.1%)	58.5% (50.3% to 66.1%)	58.0% (49.8% to 65.7%)	58.5% (50.3% to 66.1%)
Lip and oral cavity cancer	89.0% (84.6% to 92.8%)	89.9% (85.7% to 93.3%)	87.8% (83.0% to 91.9%)	89.8% (85.6% to 93.3%)
Nasopharynx cancer	89.7% (86.1% to 92.6%)	88.4% (85.0% to 91.2%)	85.2% (80.6% to 89.1%)	88.3% (85.0% to 91.0%)

Supplementary Results Table 1. Age-standardised PAFs attributable to the joint distribution of all risk factors for all ages, both sexes combined for each GBD level 1 and level 2 cause and all causes in 2016 for Belgium. Results are shown for deaths, YLLs, YLDs, and DALYs
Data in parenthesis are 95% uncertainty intervals. PAFs=population attributable fractions. GBD=Global Burden of Disease. YLLs=years of life lost. YLDs=years live with disability. DALYs=disability-adjusted life-years

	Deaths 2016	YLLs 2016	YLDs 2016	DALYs 2016
Other pharynx cancer	71.0% (58.7% to 80.0%)	72.0% (59.8% to 80.6%)	72.0% (60.1% to 80.6%)	72.0% (59.8% to 80.7%)
Gallbladder and biliary tract cancer	18.7% (10.0% to 29.5%)	18.6% (9.8% to 29.3%)	19.0% (10.2% to 30.0%)	18.6% (9.8% to 29.3%)
Pancreatic cancer	36.2% (28.8% to 43.4%)	37.9% (30.5% to 45.0%)	36.3% (29.1% to 43.5%)	37.9% (30.4% to 45.0%)
Ovarian cancer	14.7% (7.9% to 22.2%)	11.9% (6.2% to 18.5%)	11.9% (6.4% to 18.4%)	11.9% (6.2% to 18.5%)
Kidney cancer	40.1% (29.7% to 50.6%)	40.7% (30.3% to 51.2%)	39.8% (29.8% to 49.8%)	40.6% (30.3% to 51.1%)
Bladder cancer	44.5% (34.0% to 54.1%)	46.1% (35.7% to 55.4%)	45.9% (35.7% to 55.5%)	46.1% (35.7% to 55.4%)
Thyroid cancer	13.9% (6.5% to 23.8%)	14.6% (6.3% to 25.6%)	13.7% (6.6% to 23.4%)	14.4% (6.4% to 25.2%)
Mesothelioma	97.7% (97.0% to 98.3%)	95.3% (94.0% to 96.4%)	96.8% (95.8% to 97.7%)	95.3% (94.1% to 96.4%)
Non-Hodgkin's lymphoma	6.5% (2.6% to 11.7%)	6.4% (2.6% to 11.4%)	6.4% (2.6% to 11.4%)	6.4% (2.6% to 11.4%)
Multiple myeloma	7.3% (3.2% to 13.2%)	7.6% (3.2% to 13.7%)	7.5% (3.3% to 13.7%)	7.6% (3.2% to 13.7%)
Leukaemia	23.6% (14.6% to 32.4%)	20.9% (13.2% to 28.5%)	21.0% (12.9% to 28.9%)	20.9% (13.2% to 28.5%)
**Cardiovascular diseases**	79.0% (76.0% to 81.8%)	82.6% (80.6% to 84.6%)	73.6% (70.5% to 76.5%)	80.6% (78.4% to 82.7%)
Rheumatic heart disease	28.6% (16.2% to 53.0%)	29.6% (19.4% to 48.0%)	28.8% (19.8% to 43.1%)	29.5% (19.4% to 48.0%)
Ischaemic heart disease	92.2% (88.3% to 95.4%)	94.2% (91.6% to 96.2%)	91.3% (88.4% to 93.8%)	93.9% (91.3% to 96.0%)
Cerebrovascular disease	82.7% (76.8% to 88.2%)	86.7% (82.8% to 90.0%)	85.4% (81.2% to 89.2%)	86.3% (82.6% to 89.7%)
Hypertensive heart disease	100.0% (100.0% to 100.0%)	100.0% (100.0% to 100.0%)	100.0% (100.0% to 100.0%)	100.0% (100.0% to 100.0%)
Cardiomyopathy and myocarditis	41.4% (32.2% to 53.2%)	48.3% (42.0% to 55.7%)	47.6% (41.9% to 54.0%)	48.2% (42.1% to 55.1%)
Atrial fibrillation and flutter	56.4% (48.4% to 63.9%)	60.9% (53.9% to 67.3%)	68.1% (62.8% to 73.0%)	64.7% (58.9% to 70.4%)
Aortic aneurysm	43.7% (35.8% to 51.7%)	50.0% (43.3% to 56.2%)	-	50.0% (43.3% to 56.2%)
Peripheral vascular disease	48.2% (41.0% to 56.4%)	54.4% (48.3% to 60.3%)	57.5% (51.5% to 63.2%)	55.8% (49.7% to 61.3%)
Endocarditis	40.8% (30.0% to 53.6%)	42.6% (34.2% to 51.5%)	42.7% (33.5% to 52.3%)	42.6% (34.2% to 51.6%)

Supplementary Results Table 1. Age-standardised PAFs attributable to the joint distribution of all risk factors for all ages, both sexes combined for each GBD level 1 and level 2 cause and all causes in 2016 for Belgium. Results are shown for deaths, YLLs, YLDs, and DALYs
Data in parenthesis are 95% uncertainty intervals. PAFs=population attributable fractions. GBD=Global Burden of Disease. YLLs=years of life lost. YLDs=years live with disability. DALYs=disability-adjusted life-years

	Deaths 2016	YLLs 2016	YLDs 2016	DALYs 2016
Other cardiovascular and circulatory diseases	45.4% (39.3% to 51.6%)	52.5% (47.4% to 57.1%)	54.8% (49.9% to 59.7%)	53.6% (48.6% to 58.4%)
**Chronic respiratory diseases**	75.3% (71.1% to 79.0%)	75.0% (71.5% to 78.3%)	47.2% (40.8% to 53.7%)	62.5% (57.5% to 67.3%)
Chronic obstructive pulmonary disease	81.2% (77.0% to 84.7%)	83.0% (79.4% to 86.1%)	77.4% (72.8% to 81.3%)	81.6% (78.1% to 84.8%)
Pneumoconiosis	100.0% (100.0% to 100.0%)	100.0% (100.0% to 100.0%)	100.0% (100.0% to 100.0%)	100.0% (100.0% to 100.0%)
Asthma	39.1% (29.9% to 49.8%)	41.6% (33.2% to 51.0%)	31.7% (24.5% to 40.2%)	32.4% (25.3% to 41.0%)
Other chronic respiratory diseases	21.3% (16.4% to 26.6%)	16.6% (11.6% to 21.8%)	10.8% (7.6% to 14.6%)	13.9% (10.1% to 18.0%)
**Cirrhosis and other chronic liver diseases**	77.7% (74.3% to 80.6%)	79.6% (76.4% to 82.5%)	63.1% (59.1% to 67.0%)	78.3% (75.2% to 81.1%)
Cirrhosis and other chronic liver diseases due to hepatitis B	2.3% (1.1% to 3.8%)	2.9% (1.3% to 4.8%)	3.1% (1.4% to 5.2%)	2.9% (1.4% to 4.8%)
Cirrhosis and other chronic liver diseases due to hepatitis C	78.1% (73.0% to 82.7%)	85.2% (82.3% to 87.4%)	83.2% (79.9% to 85.8%)	85.0% (82.2% to 87.2%)
Cirrhosis and other chronic liver diseases due to alcohol use	100.0% (100.0% to 100.0%)	100.0% (100.0% to 100.0%)	100.0% (100.0% to 100.0%)	100.0% (100.0% to 100.0%)
**Digestive diseases**	8.9% (6.7% to 11.6%)	10.6% (7.8% to 13.9%)	3.1% (2.2% to 4.1%)	8.2% (6.2% to 10.8%)
Peptic ulcer disease	9.9% (6.9% to 13.4%)	14.2% (10.0% to 18.9%)	14.9% (10.2% to 20.2%)	14.4% (10.2% to 19.0%)
Gallbladder and biliary diseases	40.3% (25.9% to 54.3%)	39.7% (25.9% to 53.4%)	42.0% (29.2% to 55.7%)	40.1% (26.6% to 53.7%)
Pancreatitis	41.9% (24.2% to 59.9%)	45.8% (27.7% to 63.6%)	40.1% (22.5% to 57.5%)	45.6% (27.5% to 63.4%)
**Neurological disorders**	19.3% (9.7% to 30.6%)	18.0% (10.4% to 26.8%)	4.3% (2.3% to 6.9%)	9.0% (5.3% to 13.5%)
Alzheimer's disease and other dementias	22.4% (10.8% to 36.3%)	23.4% (12.1% to 36.9%)	23.4% (11.8% to 36.9%)	23.4% (11.9% to 36.8%)
Parkinson's disease	-6.9% (-8.2% to -5.7%)	-9.0% (-10.7% to -7.3%)	-10.7% (-12.6% to -8.7%)	-9.4% (-11.1% to -7.6%)
Epilepsy	37.0% (25.7% to 47.1%)	33.4% (23.4% to 42.3%)	28.2% (18.5% to 37.0%)	30.4% (20.7% to 39.3%)
Multiple sclerosis	9.9% (6.5% to 13.6%)	10.9% (7.1% to 15.0%)	9.6% (6.1% to 13.4%)	10.1% (6.6% to 14.1%)
**Mental and substance use disorders**	99.4% (99.2% to 99.5%)	99.0% (98.7% to 99.3%)	15.3% (13.6% to 17.2%)	21.3% (18.8% to 24.1%)
Alcohol use disorders	100.0% (100.0% to 100.0%)	100.0% (100.0% to 100.0%)	100.0% (100.0% to 100.0%)	100.0% (100.0% to 100.0%)

Supplementary Results Table 1. Age-standardised PAFs attributable to the joint distribution of all risk factors for all ages, both sexes combined for each GBD level 1 and level 2 cause and all causes in 2016 for Belgium. Results are shown for deaths, YLLs, YLDs, and DALYs
Data in parenthesis are 95% uncertainty intervals. PAFs=population attributable fractions. GBD=Global Burden of Disease. YLLs=years of life lost. YLDs=years live with disability. DALYs=disability-adjusted life-years

	Deaths 2016	YLLs 2016	YLDs 2016	DALYs 2016
Drug use disorders	100.0% (100.0% to 100.0%)	100.0% (100.0% to 100.0%)	100.0% (100.0% to 100.0%)	100.0% (100.0% to 100.0%)
Depressive disorders	-	-	8.0% (6.6% to 9.6%)	8.0% (6.6% to 9.6%)
Idiopathic developmental intellectual disability	-	-	66.7% (36.2% to 85.2%)	66.7% (36.2% to 85.2%)
**Diabetes, urogenital, blood, and endocrine diseases**	68.2% (66.3% to 71.6%)	60.9% (57.8% to 63.9%)	51.5% (47.0% to 56.1%)	55.0% (51.7% to 58.2%)
Diabetes mellitus	100.0% (100.0% to 100.0%)	100.0% (100.0% to 100.0%)	100.0% (100.0% to 100.0%)	100.0% (100.0% to 100.0%)
Chronic kidney disease	100.0% (100.0% to 100.0%)	100.0% (100.0% to 100.0%)	100.0% (100.0% to 100.0%)	100.0% (100.0% to 100.0%)
**Musculoskeletal disorders**	0.9% (0.3% to 1.6%)	1.2% (0.4% to 2.0%)	16.4% (13.6% to 19.5%)	16.2% (13.5% to 19.3%)
Rheumatoid arthritis	4.1% (1.6% to 6.9%)	5.2% (2.1% to 8.7%)	6.1% (2.5% to 10.3%)	6.0% (2.4% to 10.2%)
Osteoarthritis	-	-	22.6% (11.9% to 34.0%)	22.6% (11.9% to 34.0%)
Low back and neck pain	-	-	18.3% (15.4% to 21.3%)	18.3% (15.4% to 21.3%)
Gout	-	-	44.0% (26.9% to 61.5%)	44.0% (26.9% to 61.5%)
**Other non-communicable diseases**	0.4% (0.2% to 0.6%)	0.6% (0.4% to 0.9%)	2.1% (1.7% to 2.5%)	1.9% (1.6% to 2.3%)
Sense organ diseases	-	-	6.5% (5.6% to 7.5%)	6.5% (5.6% to 7.5%)
Sudden infant death syndrome	3.2% (2.2% to 4.6%)	3.2% (2.2% to 4.6%)	-	3.2% (2.2% to 4.6%)
**Transport injuries**	37.7% (28.1% to 48.0%)	33.2% (23.6% to 43.4%)	40.8% (31.9% to 50.2%)	35.7% (26.5% to 45.5%)
Road injuries	37.7% (28.1% to 48.0%)	33.2% (23.6% to 43.3%)	40.8% (31.9% to 50.1%)	35.7% (26.6% to 45.6%)
Other transport injuries	38.2% (28.2% to 48.5%)	33.7% (24.0% to 44.3%)	40.8% (31.2% to 50.4%)	35.8% (26.1% to 46.1%)
**Unintentional injuries**	38.9% (33.6% to 44.5%)	23.2% (20.2% to 27.0%)	20.7% (17.7% to 24.5%)	21.6% (18.7% to 25.2%)
Falls	63.0% (55.3% to 69.3%)	41.2% (37.3% to 45.9%)	22.4% (20.0% to 25.9%)	27.2% (24.4% to 30.7%)
Drowning	18.3% (11.4% to 28.1%)	16.2% (10.7% to 24.0%)	17.5% (10.6% to 27.6%)	16.3% (10.6% to 24.2%)
Fire, heat, and hot substances	14.9% (8.3% to 24.3%)	15.5% (9.8% to 22.9%)	20.0% (12.7% to 30.3%)	17.7% (11.3% to 26.4%)

Supplementary Results Table 1. Age-standardised PAFs attributable to the joint distribution of all risk factors for all ages, both sexes combined for each GBD level 1 and level 2 cause and all causes in 2016 for Belgium. Results are shown for deaths, YLLs, YLDs, and DALYs
Data in parenthesis are 95% uncertainty intervals. PAFs=population attributable fractions. GBD=Global Burden of Disease. YLLs=years of life lost. YLDs=years live with disability. DALYs=disability-adjusted life-years

	**Deaths 2016**	**YLLs 2016**	**YLDs 2016**	**DALYs 2016**
Poisonings	18.3% (11.2% to 27.6%)	18.2% (11.7% to 26.9%)	17.8% (11.4% to 26.4%)	18.0% (11.7% to 26.3%)
Exposure to mechanical forces	21.6% (18.0% to 26.2%)	14.9% (11.1% to 19.8%)	19.3% (15.4% to 24.6%)	18.0% (14.1% to 23.2%)
Animal contact	13.8% (11.4% to 16.8%)	10.8% (7.7% to 14.6%)	9.2% (6.2% to 13.5%)	9.7% (6.8% to 13.6%)
Foreign body	3.6% (2.0% to 6.6%)	5.7% (3.5% to 8.9%)	5.1% (3.2% to 7.6%)	5.6% (3.5% to 8.7%)
Other unintentional injuries	22.1% (14.3% to 32.6%)	23.0% (15.2% to 33.2%)	19.6% (12.1% to 30.1%)	20.0% (12.6% to 30.2%)
**Self-harm and interpersonal violence**	34.3% (18.5% to 47.9%)	33.3% (17.6% to 46.7%)	35.6% (29.3% to 41.5%)	33.5% (18.5% to 46.3%)
Self-harm	34.1% (17.7% to 48.7%)	33.4% (16.4% to 47.9%)	28.7% (12.0% to 42.6%)	33.3% (16.4% to 47.8%)
Interpersonal violence	37.3% (28.5% to 45.1%)	33.1% (24.8% to 40.8%)	38.3% (32.5% to 44.1%)	34.9% (27.8% to 41.6%)
**Forces of nature, conflict and terrorism, and executions and police conflict**	0.0% (0.0% to 0.0%)	0.0% (0.0% to 0.0%)	0.0% (0.0% to 0.0%)	0.0% (0.0% to 0.0%)
Forces of nature, conflict and terrorism, and state actor violence	0.0% (0.0% to 0.0%)	0.0% (0.0% to 0.0%)	0.0% (0.0% to 0.0%)	0.0% (0.0% to 0.0%)

Supplementary Results Table 1. Age-standardised PAFs attributable to the joint distribution of all risk factors for all ages, both sexes combined for each GBD level 1 and level 2 cause and all causes in 2016 for Belize. Results are shown for deaths, YLLs, YLDs, and DALYs
Data in parenthesis are 95% uncertainty intervals. PAFs=population attributable fractions. GBD=Global Burden of Disease. YLLs=years of life lost. YLDs=years live with disability. DALYs=disability-adjusted life-years

	Deaths 2016	YLLs 2016	YLDs 2016	DALYs 2016
**All causes**	61.0% (59.2% to 62.8%)	57.7% (55.7% to 59.8%)	26.4% (24.9% to 27.9%)	47.4% (44.9% to 50.1%)
**HIV/AIDS and tuberculosis**	72.2% (66.2% to 77.3%)	70.9% (64.2% to 76.0%)	78.1% (71.1% to 83.2%)	71.3% (64.7% to 76.3%)
Tuberculosis	53.0% (42.9% to 63.8%)	56.3% (47.0% to 66.3%)	50.1% (41.4% to 59.7%)	56.1% (46.8% to 66.1%)
HIV/AIDS	78.8% (71.2% to 84.1%)	73.7% (65.7% to 79.3%)	80.4% (72.8% to 85.8%)	74.1% (66.1% to 79.6%)
**Diarrhoea, lower respiratory infections, and other common infectious diseases**	48.1% (40.4% to 56.0%)	55.5% (48.3% to 62.8%)	42.9% (33.3% to 50.5%)	53.5% (47.1% to 60.0%)
Diarrhoeal diseases	88.2% (70.6% to 96.7%)	92.1% (80.2% to 97.8%)	88.3% (70.9% to 96.7%)	90.2% (76.2% to 97.3%)
Lower respiratory infections	47.7% (39.4% to 56.2%)	56.6% (48.3% to 64.9%)	52.6% (45.0% to 60.6%)	56.6% (48.3% to 64.9%)
Upper respiratory infections	0.9% (0.1% to 3.3%)	2.4% (0.2% to 8.3%)	0.0% (0.0% to 0.0%)	0.0% (0.0% to 0.1%)
Otitis media	0.8% (0.3% to 1.7%)	2.5% (1.1% to 4.8%)	3.5% (2.2% to 4.6%)	3.4% (2.2% to 4.6%)
Meningitis	1.8% (0.9% to 3.2%)	3.9% (2.1% to 6.0%)	0.0% (0.0% to 0.0%)	3.4% (1.8% to 5.3%)
Encephalitis	1.0% (0.5% to 1.6%)	1.7% (0.9% to 2.7%)	0.0% (0.0% to 0.0%)	1.5% (0.7% to 2.3%)
Measles	17.7% (5.8% to 44.0%)	20.2% (6.9% to 49.5%)	16.9% (0.0% to 67.7%)	20.2% (6.9% to 49.5%)
**Maternal disorders**	5.2% (2.5% to 8.2%)	5.1% (2.4% to 8.0%)	4.7% (2.5% to 7.1%)	5.0% (2.5% to 7.5%)
Maternal haemorrhage	15.9% (5.7% to 26.0%)	15.8% (5.7% to 25.9%)	15.8% (5.8% to 25.9%)	15.8% (5.7% to 25.9%)
Maternal sepsis and other pregnancy related infections	15.8% (5.7% to 25.8%)	15.8% (5.7% to 25.9%)	15.6% (5.6% to 25.6%)	15.7% (5.7% to 25.7%)
Maternal abortion, miscarriage, and ectopic pregnancy	7.7% (4.5% to 11.7%)	7.8% (4.4% to 11.9%)	7.8% (4.0% to 12.2%)	7.8% (4.2% to 11.8%)
**Neonatal disorders**	75.7% (72.7% to 78.3%)	75.7% (72.7% to 78.3%)	0.1% (0.1% to 0.2%)	59.3% (46.3% to 68.4%)
Neonatal preterm birth complications	94.7% (92.6% to 96.5%)	94.8% (92.7% to 96.5%)	0.3% (0.2% to 0.3%)	72.0% (56.2% to 83.8%)
Neonatal encephalopathy due to birth asphyxia and trauma	65.2% (61.6% to 69.1%)	65.3% (61.7% to 69.1%)	0.0% (0.0% to 0.0%)	62.6% (56.9% to 67.3%)
Neonatal sepsis and other neonatal infections	56.6% (51.3% to 62.6%)	56.7% (51.4% to 62.6%)	0.0% (0.0% to 0.0%)	36.2% (17.4% to 51.9%)
Hemolytic disease and other neonatal jaundice	61.5% (54.0% to 67.1%)	61.5% (54.1% to 67.2%)	0.0% (0.0% to 0.0%)	39.9% (25.6% to 52.5%)

Supplementary Results Table 1. Age-standardised PAFs attributable to the joint distribution of all risk factors for all ages, both sexes combined for each GBD level 1 and level 2 cause and all causes in 2016 for Belize. Results are shown for deaths, YLLs, YLDs, and DALYs
Data in parenthesis are 95% uncertainty intervals. PAFs=population attributable fractions. GBD=Global Burden of Disease. YLLs=years of life lost. YLDs=years live with disability. DALYs=disability-adjusted life-years

	Deaths 2016	YLLs 2016	YLDs 2016	DALYs 2016
Other neonatal disorders	61.0% (56.4% to 65.7%)	61.1% (56.5% to 65.8%)	0.0% (0.0% to 0.0%)	55.9% (49.2% to 61.9%)
**Nutritional deficiencies**	9.2% (4.0% to 17.7%)	34.3% (18.4% to 52.9%)	97.4% (96.8% to 98.1%)	81.4% (75.0% to 86.6%)
Protein-energy malnutrition	10.7% (4.5% to 20.6%)	39.4% (21.3% to 59.8%)	95.5% (93.9% to 96.7%)	45.1% (29.9% to 62.2%)
Vitamin A deficiency	-	-	100.0% (100.0% to 100.0%)	100.0% (100.0% to 100.0%)
Iron-deficiency anaemia	100.0% (100.0% to 100.0%)	100.0% (100.0% to 100.0%)	100.0% (100.0% to 100.0%)	100.0% (100.0% to 100.0%)
**Other communicable, maternal, neonatal, and nutritional diseases**	8.4% (6.1% to 11.4%)	6.9% (4.1% to 10.0%)	65.0% (55.2% to 74.7%)	30.3% (19.9% to 42.8%)
Sexually transmitted diseases excluding HIV	33.3% (13.4% to 61.2%)	16.8% (5.5% to 37.7%)	100.0% (100.0% to 100.0%)	61.9% (34.1% to 86.6%)
Hepatitis	6.7% (4.4% to 9.4%)	7.3% (4.6% to 10.2%)	4.9% (3.0% to 7.4%)	7.1% (4.5% to 10.1%)
**Neoplasms**	39.0% (35.7% to 42.3%)	41.1% (37.8% to 44.3%)	36.5% (33.1% to 40.2%)	41.0% (37.7% to 44.3%)
Oesophageal cancer	75.1% (65.3% to 84.1%)	78.1% (69.0% to 86.4%)	75.8% (66.3% to 84.6%)	78.1% (69.0% to 86.4%)
Stomach cancer	12.9% (6.6% to 21.0%)	14.2% (7.5% to 22.2%)	13.2% (6.9% to 21.2%)	14.2% (7.5% to 22.2%)
Liver cancer	58.1% (50.4% to 66.1%)	59.4% (51.6% to 67.4%)	58.2% (50.5% to 66.3%)	59.4% (51.6% to 67.3%)
Larynx cancer	82.2% (74.0% to 88.8%)	83.9% (76.1% to 89.7%)	83.0% (75.1% to 89.2%)	83.9% (76.0% to 89.7%)
Tracheal, bronchus, and lung cancer	84.1% (79.6% to 87.6%)	83.9% (79.7% to 87.7%)	83.4% (79.0% to 87.0%)	83.9% (79.7% to 87.7%)
Breast cancer	25.0% (16.0% to 35.8%)	22.8% (13.9% to 33.2%)	23.5% (14.8% to 34.5%)	22.8% (13.9% to 33.2%)
Cervical cancer	100.0% (100.0% to 100.0%)	100.0% (100.0% to 100.0%)	100.0% (100.0% to 100.0%)	100.0% (100.0% to 100.0%)
Uterine cancer	49.4% (33.6% to 64.0%)	52.3% (36.7% to 66.1%)	52.6% (36.8% to 66.5%)	52.3% (36.7% to 66.2%)
Prostate cancer	3.1% (2.0% to 4.3%)	3.6% (2.4% to 5.0%)	3.5% (2.3% to 4.9%)	3.6% (2.4% to 5.0%)
Colon and rectum cancer	55.3% (45.5% to 65.1%)	56.7% (47.1% to 65.9%)	55.8% (46.1% to 65.2%)	56.6% (47.1% to 65.9%)
Lip and oral cavity cancer	71.4% (62.8% to 79.3%)	75.1% (67.0% to 82.1%)	69.9% (61.3% to 77.5%)	75.0% (66.9% to 82.0%)
Nasopharynx cancer	74.2% (67.2% to 80.0%)	73.2% (66.7% to 78.9%)	71.8% (65.6% to 76.8%)	73.2% (66.7% to 78.8%)

**Supplementary Results Table 1. Age-standardised PAFs attributable to the joint distribution of all risk factors for all ages, both sexes combined for each GBD level 1 and level 2 cause and all causes in 2016 for Belize. Results are shown for deaths, YLLs, YLDs, and DALYs** Data in parenthesis are 95% uncertainty intervals. PAFs=population attributable fractions. GBD=Global Burden of Disease. YLLs=years of life lost. YLDs=years live with disability. DALYs=disability-adjusted life-years				
	**Deaths 2016**	**YLLs 2016**	**YLDs 2016**	**DALYs 2016**
Other pharynx cancer	56.5% (43.9% to 67.5%)	60.4% (48.0% to 70.8%)	57.9% (45.6% to 68.3%)	60.3% (47.9% to 70.7%)
Gallbladder and biliary tract cancer	24.8% (13.6% to 37.1%)	26.6% (15.1% to 39.0%)	25.5% (14.1% to 37.6%)	26.6% (15.1% to 39.0%)
Pancreatic cancer	28.8% (19.6% to 40.2%)	30.5% (21.1% to 41.5%)	29.3% (20.1% to 40.3%)	30.5% (21.1% to 41.5%)
Ovarian cancer	16.4% (6.7% to 28.9%)	14.8% (5.4% to 26.0%)	14.4% (5.3% to 25.2%)	14.8% (5.4% to 25.9%)
Kidney cancer	31.6% (22.0% to 41.6%)	31.5% (22.2% to 41.1%)	31.5% (22.3% to 41.0%)	31.5% (22.3% to 41.1%)
Bladder cancer	28.5% (18.5% to 39.7%)	30.6% (20.3% to 42.0%)	29.6% (19.6% to 40.7%)	30.6% (20.3% to 41.9%)
Thyroid cancer	14.7% (7.5% to 23.9%)	15.7% (8.1% to 25.0%)	15.6% (8.4% to 24.3%)	15.7% (8.1% to 25.0%)
Mesothelioma	93.6% (91.2% to 95.4%)	88.1% (84.1% to 91.5%)	90.9% (88.0% to 93.3%)	88.1% (84.1% to 91.5%)
Non-Hodgkin's lymphoma	7.2% (2.9% to 12.4%)	7.2% (3.0% to 12.1%)	7.2% (2.9% to 12.3%)	7.2% (3.0% to 12.1%)
Multiple myeloma	8.4% (3.5% to 14.8%)	9.0% (3.9% to 15.6%)	8.7% (3.6% to 15.3%)	9.0% (3.8% to 15.6%)
Leukaemia	16.8% (10.6% to 23.6%)	15.1% (9.7% to 21.1%)	16.4% (10.5% to 22.7%)	15.1% (9.8% to 21.1%)
**Cardiovascular diseases**	82.3% (79.7% to 84.8%)	82.5% (80.4% to 84.4%)	66.2% (62.9% to 69.4%)	81.2% (79.2% to 83.2%)
Rheumatic heart disease	22.1% (13.9% to 38.4%)	18.4% (12.6% to 29.2%)	16.0% (10.1% to 23.7%)	17.6% (11.8% to 27.3%)
Ischaemic heart disease	92.6% (88.9% to 95.6%)	94.3% (91.9% to 96.4%)	91.2% (88.4% to 93.8%)	94.2% (91.7% to 96.3%)
Cerebrovascular disease	83.2% (78.3% to 88.1%)	86.0% (82.6% to 89.4%)	84.4% (79.9% to 89.3%)	85.9% (82.5% to 89.4%)
Hypertensive heart disease	100.0% (100.0% to 100.0%)	100.0% (100.0% to 100.0%)	100.0% (100.0% to 100.0%)	100.0% (100.0% to 100.0%)
Cardiomyopathy and myocarditis	31.2% (25.3% to 37.9%)	31.8% (26.2% to 38.0%)	29.1% (23.9% to 34.7%)	31.7% (26.1% to 37.9%)
Atrial fibrillation and flutter	47.6% (39.2% to 55.8%)	52.2% (44.8% to 59.5%)	58.8% (52.6% to 64.9%)	55.9% (49.4% to 62.2%)
Aortic aneurysm	37.8% (30.5% to 45.0%)	41.5% (35.0% to 47.2%)	-	41.5% (35.0% to 47.2%)
Peripheral vascular disease	49.5% (42.4% to 57.0%)	57.0% (51.2% to 62.8%)	62.0% (57.2% to 66.8%)	59.4% (54.2% to 64.2%)
Endocarditis	33.5% (25.4% to 43.6%)	31.0% (24.4% to 38.4%)	31.8% (24.7% to 39.9%)	31.1% (24.5% to 38.4%)

**Supplementary Results Table 1. Age-standardised PAFs attributable to the joint distribution of all risk factors for all ages, both sexes combined for each GBD level 1 and level 2 cause and all causes in 2016 for Belize. Results are shown for deaths, YLLs, YLDs, and DALYs**<br>Data in parenthesis are 95% uncertainty intervals. PAFs=population attributable fractions. GBD=Global Burden of Disease. YLLs=years of life lost. YLDs=years live with disability. DALYs=disability-adjusted life-years				
	**Deaths 2016**	**YLLs 2016**	**YLDs 2016**	**DALYs 2016**
Other cardiovascular and circulatory diseases	39.8% (34.9% to 44.6%)	40.4% (35.9% to 45.0%)	38.4% (33.7% to 43.0%)	40.1% (35.8% to 44.6%)
**Chronic respiratory diseases**	55.4% (49.4% to 60.9%)	51.8% (46.3% to 56.7%)	33.6% (25.9% to 42.3%)	45.1% (39.3% to 50.7%)
Chronic obstructive pulmonary disease	71.5% (64.7% to 77.8%)	71.6% (65.3% to 77.5%)	68.1% (62.0% to 74.3%)	71.2% (65.0% to 77.1%)
Pneumoconiosis	100.0% (100.0% to 100.0%)	100.0% (100.0% to 100.0%)	100.0% (100.0% to 100.0%)	100.0% (100.0% to 100.0%)
Asthma	37.0% (26.7% to 48.5%)	38.2% (29.0% to 48.2%)	28.5% (19.9% to 38.7%)	30.3% (21.8% to 40.3%)
Other chronic respiratory diseases	12.5% (8.3% to 17.8%)	13.0% (8.8% to 18.8%)	5.0% (3.0% to 7.5%)	12.4% (8.4% to 17.7%)
**Cirrhosis and other chronic liver diseases**	66.6% (63.8% to 69.2%)	67.0% (64.1% to 69.6%)	55.5% (52.1% to 59.1%)	66.7% (63.7% to 69.3%)
Cirrhosis and other chronic liver diseases due to hepatitis B	5.2% (2.1% to 8.7%)	6.6% (2.7% to 11.1%)	5.2% (2.1% to 8.7%)	6.6% (2.7% to 11.0%)
Cirrhosis and other chronic liver diseases due to hepatitis C	79.0% (74.6% to 82.7%)	84.2% (81.3% to 86.6%)	78.3% (73.0% to 82.4%)	84.0% (81.2% to 86.5%)
Cirrhosis and other chronic liver diseases due to alcohol use	100.0% (100.0% to 100.0%)	100.0% (100.0% to 100.0%)	100.0% (100.0% to 100.0%)	100.0% (100.0% to 100.0%)
**Digestive diseases**	9.8% (7.4% to 12.1%)	11.8% (9.2% to 14.5%)	2.7% (2.0% to 3.6%)	9.7% (7.5% to 11.9%)
Peptic ulcer disease	7.1% (4.7% to 10.2%)	8.6% (5.8% to 12.2%)	4.1% (2.7% to 5.9%)	7.6% (5.1% to 10.7%)
Gallbladder and biliary diseases	42.8% (27.7% to 58.9%)	45.2% (30.3% to 60.0%)	51.5% (36.4% to 65.9%)	45.7% (30.7% to 60.5%)
Pancreatitis	53.0% (33.2% to 70.6%)	59.1% (40.3% to 75.0%)	43.8% (27.2% to 60.3%)	58.8% (40.0% to 74.7%)
**Neurological disorders**	21.8% (11.1% to 34.4%)	22.1% (13.2% to 32.5%)	4.6% (2.5% to 7.4%)	10.6% (6.4% to 15.7%)
Alzheimer's disease and other dementias	24.5% (11.6% to 39.4%)	26.2% (12.9% to 41.3%)	26.3% (12.9% to 41.5%)	26.2% (12.9% to 41.3%)
Parkinson's disease	-3.7% (-4.9% to -2.7%)	-4.7% (-6.3% to -3.5%)	-5.1% (-6.6% to -3.8%)	-4.8% (-6.3% to -3.6%)
Epilepsy	29.0% (20.3% to 38.0%)	30.0% (21.2% to 39.0%)	23.8% (16.8% to 31.6%)	26.9% (18.9% to 35.5%)
Multiple sclerosis	4.9% (2.6% to 7.6%)	4.7% (2.5% to 7.3%)	2.7% (1.6% to 3.9%)	3.9% (2.1% to 6.0%)
**Mental and substance use disorders**	99.9% (99.9% to 99.9%)	99.9% (99.8% to 99.9%)	17.5% (15.6% to 19.7%)	22.7% (20.0% to 25.6%)
Alcohol use disorders	100.0% (100.0% to 100.0%)	100.0% (100.0% to 100.0%)	100.0% (100.0% to 100.0%)	100.0% (100.0% to 100.0%)

Supplementary Results Table 1. Age-standardised PAFs attributable to the joint distribution of all risk factors for all ages, both sexes combined for each GBD level 1 and level 2 cause and all causes in 2016 for Belize. Results are shown for deaths, YLLs, YLDs, and DALYs
Data in parenthesis are 95% uncertainty intervals. PAFs=population attributable fractions. GBD=Global Burden of Disease. YLLs=years of life lost. YLDs=years live with disability. DALYs=disability-adjusted life-years

	**Deaths 2016**	**YLLs 2016**	**YLDs 2016**	**DALYs 2016**
Drug use disorders	100.0% (100.0% to 100.0%)	100.0% (100.0% to 100.0%)	100.0% (100.0% to 100.0%)	100.0% (100.0% to 100.0%)
Depressive disorders	-	-	5.1% (4.2% to 6.2%)	5.1% (4.2% to 6.2%)
Idiopathic developmental intellectual disability	-	-	65.0% (33.3% to 85.1%)	65.0% (33.3% to 85.1%)
**Diabetes, urogenital, blood, and endocrine diseases**	85.8% (84.2% to 86.7%)	84.5% (82.0% to 85.8%)	75.7% (71.9% to 78.9%)	82.1% (79.9% to 83.6%)
Diabetes mellitus	100.0% (100.0% to 100.0%)	100.0% (100.0% to 100.0%)	100.0% (100.0% to 100.0%)	100.0% (100.0% to 100.0%)
Chronic kidney disease	100.0% (100.0% to 100.0%)	100.0% (100.0% to 100.0%)	100.0% (100.0% to 100.0%)	100.0% (100.0% to 100.0%)
**Musculoskeletal disorders**	0.3% (0.1% to 0.5%)	0.2% (0.1% to 0.3%)	14.2% (11.2% to 17.5%)	13.6% (10.6% to 16.7%)
Rheumatoid arthritis	1.1% (0.4% to 2.1%)	1.2% (0.5% to 2.3%)	1.8% (0.7% to 3.2%)	1.7% (0.7% to 3.1%)
Osteoarthritis	-	-	29.0% (15.9% to 42.1%)	29.0% (15.9% to 42.1%)
Low back and neck pain	-	-	18.6% (15.7% to 21.6%)	18.6% (15.7% to 21.6%)
Gout	-	-	51.7% (36.2% to 67.6%)	51.7% (36.2% to 67.6%)
**Other non-communicable diseases**	0.4% (0.2% to 0.6%)	0.6% (0.4% to 0.9%)	5.6% (4.7% to 6.7%)	4.6% (3.8% to 5.5%)
Sense organ diseases	-	-	12.7% (11.1% to 14.7%)	12.7% (11.1% to 14.7%)
Sudden infant death syndrome	8.8% (5.5% to 13.1%)	8.8% (5.5% to 13.1%)	-	8.8% (5.5% to 13.1%)
**Transport injuries**	38.0% (28.5% to 49.8%)	34.6% (24.4% to 46.8%)	41.2% (31.5% to 52.4%)	35.3% (25.3% to 47.3%)
Road injuries	37.6% (27.9% to 49.6%)	34.6% (24.3% to 46.9%)	41.7% (32.0% to 53.1%)	35.4% (25.3% to 47.5%)
Other transport injuries	41.5% (33.1% to 51.2%)	34.2% (24.9% to 45.3%)	34.0% (25.5% to 44.1%)	34.2% (25.1% to 45.0%)
**Unintentional injuries**	19.0% (14.5% to 25.7%)	16.2% (10.5% to 25.3%)	20.5% (16.0% to 28.3%)	17.4% (12.1% to 25.8%)
Falls	53.3% (48.1% to 59.1%)	33.2% (26.7% to 42.7%)	27.1% (22.6% to 35.2%)	29.2% (24.2% to 37.7%)
Drowning	19.3% (11.2% to 30.3%)	19.3% (11.4% to 31.0%)	17.4% (10.1% to 26.7%)	19.3% (11.4% to 30.9%)
Fire, heat, and hot substances	11.3% (6.6% to 17.6%)	14.0% (8.5% to 21.8%)	18.7% (12.0% to 27.4%)	15.7% (9.5% to 23.7%)

Supplementary Results Table 1. Age-standardised PAFs attributable to the joint distribution of all risk factors for all ages, both sexes combined for each GBD level 1 and level 2 cause and all causes in 2016 for Belize. Results are shown for deaths, YLLs, YLDs, and DALYs
Data in parenthesis are 95% uncertainty intervals. PAFs=population attributable fractions. GBD=Global Burden of Disease. YLLs=years of life lost. YLDs=years live with disability. DALYs=disability-adjusted life-years

	**Deaths 2016**	**YLLs 2016**	**YLDs 2016**	**DALYs 2016**
Poisonings	18.1% (10.9% to 27.8%)	18.7% (11.4% to 29.0%)	18.1% (11.6% to 26.8%)	18.7% (11.4% to 28.8%)
Exposure to mechanical forces	23.1% (14.7% to 35.8%)	22.9% (13.7% to 37.0%)	21.1% (14.4% to 32.9%)	22.4% (14.0% to 35.9%)
Animal contact	12.5% (8.0% to 20.6%)	12.8% (6.9% to 23.1%)	11.4% (6.4% to 20.3%)	12.4% (6.8% to 22.1%)
Foreign body	5.7% (2.8% to 11.0%)	6.6% (3.0% to 13.2%)	8.1% (4.4% to 14.9%)	6.9% (3.3% to 13.4%)
Other unintentional injuries	21.7% (12.8% to 34.2%)	23.5% (13.8% to 37.4%)	16.7% (10.6% to 24.8%)	19.3% (12.1% to 29.5%)
**Self-harm and interpersonal violence**	30.3% (22.0% to 39.5%)	30.2% (21.8% to 39.8%)	30.7% (25.7% to 37.1%)	30.3% (22.1% to 39.6%)
Self-harm	34.1% (20.9% to 45.6%)	35.6% (22.4% to 46.7%)	30.3% (17.8% to 41.4%)	35.6% (22.4% to 46.6%)
Interpersonal violence	28.9% (18.9% to 40.1%)	28.5% (18.5% to 39.5%)	30.8% (25.2% to 37.1%)	28.6% (18.9% to 39.4%)
**Forces of nature, conflict and terrorism, and executions and police conflict**	0.0% (0.0% to 0.0%)	0.0% (0.0% to 0.0%)	1.8% (1.2% to 2.6%)	1.0% (0.3% to 1.7%)
Forces of nature, conflict and terrorism, and state actor violence	0.0% (0.0% to 0.0%)	0.0% (0.0% to 0.0%)	4.6% (3.1% to 6.7%)	4.6% (3.1% to 6.7%)

Supplementary Results Table 1. Age-standardised PAFs attributable to the joint distribution of all risk factors for all ages, both sexes combined for each GBD level 1 and level 2 cause and all causes in 2016 for Benin. Results are shown for deaths, YLLs, YLDs, and DALYs
Data in parenthesis are 95% uncertainty intervals. PAFs=population attributable fractions. GBD=Global Burden of Disease. YLLs=years of life lost. YLDs=years live with disability. DALYs=disability-adjusted life-years

	Deaths 2016	YLLs 2016	YLDs 2016	DALYs 2016
**All causes**	55.8% (53.6% to 58.3%)	52.0% (49.7% to 54.5%)	29.1% (27.9% to 30.5%)	46.4% (44.2% to 48.8%)
**HIV/AIDS and tuberculosis**	43.7% (33.5% to 54.2%)	53.0% (43.4% to 62.0%)	74.3% (68.8% to 79.3%)	54.0% (44.6% to 62.7%)
Tuberculosis	24.7% (12.8% to 38.2%)	25.8% (14.6% to 38.3%)	24.7% (14.1% to 37.5%)	25.8% (14.6% to 38.3%)
HIV/AIDS	90.6% (88.2% to 92.5%)	87.0% (84.0% to 89.6%)	91.7% (89.6% to 93.4%)	87.3% (84.6% to 89.8%)
**Diarrhoea, lower respiratory infections, and other common infectious diseases**	77.8% (73.1% to 82.9%)	77.4% (71.9% to 82.1%)	42.2% (37.2% to 46.6%)	76.1% (70.6% to 80.7%)
Diarrhoeal diseases	96.2% (89.8% to 99.1%)	97.7% (93.9% to 99.5%)	96.1% (89.8% to 99.0%)	97.6% (93.7% to 99.5%)
Lower respiratory infections	81.1% (75.4% to 86.0%)	85.9% (81.0% to 89.7%)	81.0% (74.9% to 86.0%)	85.9% (81.0% to 89.7%)
Upper respiratory infections	1.5% (0.1% to 5.3%)	3.0% (0.2% to 10.6%)	0.0% (0.0% to 0.0%)	0.2% (0.0% to 0.8%)
Otitis media	0.4% (0.1% to 1.0%)	1.7% (0.6% to 3.4%)	2.4% (1.5% to 3.3%)	2.4% (1.5% to 3.3%)
Meningitis	1.9% (1.0% to 3.2%)	3.7% (2.0% to 5.8%)	0.0% (0.0% to 0.0%)	3.5% (1.9% to 5.6%)
Encephalitis	0.6% (0.4% to 0.9%)	1.2% (0.8% to 1.7%)	0.0% (0.0% to 0.0%)	0.7% (0.5% to 1.1%)
Measles	61.8% (46.4% to 80.9%)	63.0% (47.4% to 82.3%)	62.0% (45.5% to 81.6%)	63.0% (47.4% to 82.3%)
**Maternal disorders**	9.8% (5.2% to 14.7%)	9.8% (5.2% to 14.7%)	1.3% (0.6% to 2.2%)	9.2% (4.9% to 13.9%)
Maternal haemorrhage	18.2% (6.5% to 29.8%)	18.3% (6.6% to 29.8%)	18.5% (6.7% to 30.0%)	18.3% (6.6% to 29.8%)
Maternal sepsis and other pregnancy related infections	18.1% (6.6% to 29.5%)	18.2% (6.6% to 29.6%)	18.1% (6.5% to 29.4%)	18.2% (6.6% to 29.6%)
Maternal abortion, miscarriage, and ectopic pregnancy	15.5% (9.1% to 22.7%)	15.4% (9.0% to 22.4%)	15.7% (8.5% to 23.6%)	15.4% (9.0% to 22.4%)
**Neonatal disorders**	83.7% (80.0% to 86.6%)	83.7% (80.0% to 86.6%)	0.5% (0.2% to 1.0%)	80.3% (76.2% to 84.0%)
Neonatal preterm birth complications	97.7% (95.3% to 99.0%)	97.7% (95.4% to 99.0%)	1.6% (1.1% to 2.3%)	94.4% (91.2% to 96.6%)
Neonatal encephalopathy due to birth asphyxia and trauma	79.0% (75.3% to 82.9%)	79.0% (75.3% to 82.9%)	0.0% (0.0% to 0.0%)	76.9% (71.5% to 81.3%)
Neonatal sepsis and other neonatal infections	70.6% (64.4% to 75.4%)	70.7% (64.5% to 75.4%)	0.0% (0.0% to 0.0%)	65.0% (54.2% to 72.8%)
Hemolytic disease and other neonatal jaundice	70.4% (59.2% to 77.6%)	70.4% (59.3% to 77.6%)	0.0% (0.0% to 0.0%)	65.1% (54.7% to 72.9%)

Supplementary Results Table 1. Age-standardised PAFs attributable to the joint distribution of all risk factors for all ages, both sexes combined for each GBD level 1 and level 2 cause and all causes in 2016 for Benin. Results are shown for deaths, YLLs, YLDs, and DALYs
Data in parenthesis are 95% uncertainty intervals. PAFs=population attributable fractions. GBD=Global Burden of Disease. YLLs=years of life lost. YLDs=years live with disability. DALYs=disability-adjusted life-years

	Deaths 2016	YLLs 2016	YLDs 2016	DALYs 2016
Other neonatal disorders	70.2% (61.7% to 76.1%)	70.3% (61.8% to 76.2%)	0.0% (0.0% to 0.0%)	67.6% (59.2% to 73.8%)
**Nutritional deficiencies**	26.9% (17.5% to 38.2%)	60.8% (48.2% to 73.2%)	94.2% (92.5% to 95.5%)	81.7% (77.0% to 85.7%)
Protein-energy malnutrition	30.4% (20.0% to 42.6%)	66.3% (53.6% to 77.9%)	98.8% (98.4% to 99.1%)	70.1% (59.7% to 79.7%)
Vitamin A deficiency	-	-	100.0% (100.0% to 100.0%)	100.0% (100.0% to 100.0%)
Iron-deficiency anaemia	100.0% (100.0% to 100.0%)	100.0% (100.0% to 100.0%)	100.0% (100.0% to 100.0%)	100.0% (100.0% to 100.0%)
**Other communicable, maternal, neonatal, and nutritional diseases**	3.4% (2.2% to 4.5%)	3.3% (2.0% to 4.6%)	55.9% (50.9% to 60.7%)	16.2% (11.0% to 21.9%)
Sexually transmitted diseases excluding HIV	32.1% (17.3% to 51.6%)	19.1% (9.3% to 34.8%)	100.0% (100.0% to 100.0%)	58.9% (38.1% to 78.2%)
Hepatitis	0.0% (0.0% to 0.1%)	0.1% (0.0% to 0.1%)	0.1% (0.0% to 0.1%)	0.1% (0.0% to 0.1%)
**Neoplasms**	34.6% (31.1% to 38.5%)	35.4% (31.7% to 39.3%)	35.7% (31.6% to 39.9%)	35.4% (31.7% to 39.3%)
Oesophageal cancer	69.6% (56.5% to 80.3%)	71.3% (58.4% to 81.9%)	70.2% (57.4% to 80.8%)	71.3% (58.4% to 81.9%)
Stomach cancer	7.3% (2.9% to 15.7%)	7.8% (3.1% to 15.5%)	7.4% (2.9% to 15.6%)	7.8% (3.1% to 15.5%)
Liver cancer	34.0% (25.1% to 44.3%)	33.8% (24.8% to 44.6%)	33.8% (25.0% to 44.2%)	33.8% (24.8% to 44.6%)
Larynx cancer	61.9% (49.5% to 72.5%)	63.6% (51.4% to 74.2%)	62.3% (49.7% to 72.8%)	63.6% (51.4% to 74.2%)
Tracheal, bronchus, and lung cancer	87.6% (83.5% to 91.0%)	87.8% (83.9% to 91.1%)	87.4% (83.3% to 90.9%)	87.8% (83.9% to 91.1%)
Breast cancer	25.7% (17.8% to 33.7%)	24.4% (16.8% to 31.8%)	24.3% (16.7% to 31.8%)	24.4% (16.8% to 31.8%)
Cervical cancer	100.0% (100.0% to 100.0%)	100.0% (100.0% to 100.0%)	100.0% (100.0% to 100.0%)	100.0% (100.0% to 100.0%)
Uterine cancer	44.1% (29.8% to 58.8%)	45.9% (31.5% to 60.2%)	44.8% (30.6% to 59.2%)	45.9% (31.5% to 60.1%)
Prostate cancer	1.9% (1.2% to 2.6%)	2.1% (1.4% to 3.0%)	2.0% (1.3% to 2.8%)	2.1% (1.4% to 3.0%)
Colon and rectum cancer	45.0% (35.7% to 53.7%)	45.1% (36.3% to 53.4%)	45.1% (35.7% to 53.5%)	45.1% (36.3% to 53.4%)
Lip and oral cavity cancer	63.1% (51.2% to 73.3%)	63.8% (52.4% to 73.9%)	62.8% (50.9% to 73.2%)	63.7% (52.4% to 73.9%)
Nasopharynx cancer	52.2% (41.0% to 62.6%)	51.2% (39.7% to 61.3%)	49.2% (37.8% to 59.3%)	51.1% (39.7% to 61.3%)

Supplementary Results Table 1. Age-standardised PAFs attributable to the joint distribution of all risk factors for all ages, both sexes combined for each GBD level 1 and level 2 cause and all causes in 2016 for Benin. Results are shown for deaths, YLLs, YLDs, and DALYs
Data in parenthesis are 95% uncertainty intervals. PAFs=population attributable fractions. GBD=Global Burden of Disease. YLLs=years of life lost. YLDs=years live with disability. DALYs=disability-adjusted life-years

	Deaths 2016	YLLs 2016	YLDs 2016	DALYs 2016
Other pharynx cancer	35.5% (20.2% to 53.3%)	37.1% (21.7% to 54.0%)	36.1% (20.9% to 53.3%)	37.0% (21.7% to 54.0%)
Gallbladder and biliary tract cancer	23.3% (12.7% to 35.7%)	24.2% (13.5% to 36.7%)	23.6% (12.8% to 35.8%)	24.2% (13.5% to 36.7%)
Pancreatic cancer	17.9% (11.5% to 25.8%)	18.5% (12.2% to 26.2%)	18.0% (11.7% to 25.8%)	18.5% (12.2% to 26.2%)
Ovarian cancer	10.6% (4.4% to 17.8%)	9.2% (3.5% to 15.8%)	9.3% (3.7% to 16.0%)	9.2% (3.5% to 15.8%)
Kidney cancer	25.0% (17.0% to 34.4%)	23.9% (16.4% to 32.8%)	23.2% (15.9% to 31.7%)	23.9% (16.4% to 32.7%)
Bladder cancer	16.9% (10.3% to 24.7%)	18.1% (11.2% to 25.7%)	17.3% (10.7% to 24.8%)	18.1% (11.2% to 25.7%)
Thyroid cancer	12.9% (6.9% to 20.7%)	13.3% (7.2% to 21.7%)	12.8% (6.9% to 20.5%)	13.3% (7.2% to 21.7%)
Mesothelioma	84.1% (79.1% to 88.3%)	74.3% (67.7% to 80.6%)	80.2% (74.6% to 85.0%)	74.4% (67.8% to 80.7%)
Non-Hodgkin's lymphoma	5.6% (2.3% to 9.9%)	4.8% (2.0% to 8.5%)	5.2% (2.1% to 9.1%)	4.8% (2.0% to 8.5%)
Multiple myeloma	7.7% (3.3% to 13.5%)	7.9% (3.4% to 13.7%)	7.8% (3.4% to 13.6%)	7.9% (3.4% to 13.7%)
Leukaemia	12.2% (7.6% to 17.6%)	10.7% (6.8% to 15.4%)	11.7% (7.2% to 16.8%)	10.8% (6.8% to 15.4%)
**Cardiovascular diseases**	83.1% (80.5% to 85.5%)	83.7% (81.3% to 85.7%)	59.9% (55.9% to 64.0%)	81.7% (79.4% to 83.8%)
Rheumatic heart disease	25.4% (16.6% to 41.4%)	24.0% (16.6% to 36.2%)	20.9% (13.4% to 29.9%)	23.2% (16.0% to 34.8%)
Ischaemic heart disease	92.3% (89.1% to 94.8%)	93.9% (91.8% to 95.6%)	88.7% (85.9% to 91.5%)	93.7% (91.7% to 95.5%)
Cerebrovascular disease	86.1% (82.3% to 89.6%)	88.7% (86.1% to 90.9%)	86.0% (82.3% to 89.2%)	88.5% (85.9% to 90.8%)
Hypertensive heart disease	100.0% (100.0% to 100.0%)	100.0% (100.0% to 100.0%)	100.0% (100.0% to 100.0%)	100.0% (100.0% to 100.0%)
Cardiomyopathy and myocarditis	35.4% (28.5% to 43.7%)	34.8% (28.3% to 41.8%)	40.4% (33.9% to 47.2%)	35.3% (28.8% to 42.3%)
Atrial fibrillation and flutter	43.9% (35.4% to 52.0%)	47.8% (40.1% to 55.1%)	53.4% (47.4% to 59.0%)	50.7% (44.2% to 56.9%)
Aortic aneurysm	38.0% (31.0% to 44.8%)	41.7% (35.2% to 47.6%)	-	41.7% (35.2% to 47.6%)
Peripheral vascular disease	48.4% (41.8% to 55.9%)	55.2% (49.8% to 60.7%)	58.1% (52.7% to 63.4%)	57.5% (52.3% to 62.8%)
Endocarditis	35.5% (27.0% to 45.5%)	32.3% (25.1% to 40.1%)	33.3% (24.3% to 44.4%)	32.3% (25.1% to 40.2%)

Supplementary Results Table 1. Age-standardised PAFs attributable to the joint distribution of all risk factors for all ages, both sexes combined for each GBD level 1 and level 2 cause and all causes in 2016 for Benin. Results are shown for deaths, YLLs, YLDs, and DALYs
Data in parenthesis are 95% uncertainty intervals. PAFs=population attributable fractions. GBD=Global Burden of Disease. YLLs=years of life lost. YLDs=years live with disability. DALYs=disability-adjusted life-years

	Deaths 2016	YLLs 2016	YLDs 2016	DALYs 2016
Other cardiovascular and circulatory diseases	41.4% (36.2% to 46.6%)	42.3% (37.7% to 47.1%)	36.8% (31.6% to 42.0%)	40.9% (36.2% to 45.7%)
**Chronic respiratory diseases**	67.5% (61.5% to 73.5%)	64.6% (59.4% to 69.9%)	49.1% (42.7% to 56.1%)	60.7% (55.3% to 66.6%)
Chronic obstructive pulmonary disease	83.9% (76.7% to 90.5%)	83.5% (76.9% to 89.8%)	76.2% (68.0% to 84.0%)	82.2% (75.5% to 88.7%)
Pneumoconiosis	100.0% (100.0% to 100.0%)	100.0% (100.0% to 100.0%)	100.0% (100.0% to 100.0%)	100.0% (100.0% to 100.0%)
Asthma	36.6% (27.0% to 47.9%)	40.3% (31.8% to 50.5%)	29.6% (22.4% to 37.9%)	36.1% (28.4% to 45.5%)
Other chronic respiratory diseases	7.6% (4.9% to 11.1%)	7.3% (4.5% to 11.0%)	3.5% (1.8% to 6.0%)	7.0% (4.3% to 10.6%)
**Cirrhosis and other chronic liver diseases**	23.9% (21.4% to 26.8%)	23.1% (20.6% to 25.9%)	18.2% (15.6% to 20.9%)	23.0% (20.5% to 25.8%)
Cirrhosis and other chronic liver diseases due to hepatitis B	0.0% (0.0% to 0.0%)	0.0% (0.0% to 0.0%)	0.0% (0.0% to 0.0%)	0.0% (0.0% to 0.0%)
Cirrhosis and other chronic liver diseases due to hepatitis C	1.1% (0.5% to 2.2%)	1.7% (0.6% to 3.5%)	1.8% (0.6% to 3.9%)	1.7% (0.6% to 3.5%)
Cirrhosis and other chronic liver diseases due to alcohol use	100.0% (100.0% to 100.0%)	100.0% (100.0% to 100.0%)	100.0% (100.0% to 100.0%)	100.0% (100.0% to 100.0%)
**Digestive diseases**	4.3% (2.7% to 6.3%)	4.4% (2.6% to 7.0%)	1.7% (1.2% to 2.4%)	4.3% (2.6% to 6.6%)
Peptic ulcer disease	4.3% (2.7% to 6.2%)	4.9% (3.1% to 6.9%)	3.8% (2.5% to 5.4%)	4.9% (3.1% to 6.9%)
Gallbladder and biliary diseases	37.3% (24.5% to 52.2%)	35.9% (23.9% to 49.7%)	39.6% (27.3% to 53.0%)	36.1% (24.2% to 49.7%)
Pancreatitis	12.3% (0.4% to 29.7%)	13.6% (1.5% to 32.6%)	13.3% (0.6% to 30.9%)	13.6% (1.5% to 32.6%)
**Neurological disorders**	16.9% (8.0% to 28.1%)	15.2% (8.2% to 23.7%)	3.2% (1.6% to 5.1%)	7.6% (4.2% to 11.9%)
Alzheimer's disease and other dementias	19.1% (8.5% to 31.7%)	20.3% (9.5% to 33.5%)	20.3% (9.6% to 33.6%)	20.3% (9.5% to 33.5%)
Parkinson's disease	-2.6% (-3.5% to -1.8%)	-3.3% (-4.4% to -2.3%)	-3.7% (-4.8% to -2.6%)	-3.3% (-4.5% to -2.4%)
Epilepsy	10.6% (4.5% to 18.1%)	9.6% (4.0% to 16.2%)	9.7% (3.9% to 16.6%)	9.7% (4.1% to 16.2%)
Multiple sclerosis	2.9% (1.3% to 4.8%)	2.9% (1.3% to 5.0%)	2.1% (1.2% to 3.1%)	2.7% (1.3% to 4.4%)
**Mental and substance use disorders**	99.9% (99.8% to 100.0%)	99.8% (99.7% to 99.9%)	19.9% (17.7% to 22.3%)	24.2% (21.4% to 27.9%)
Alcohol use disorders	100.0% (100.0% to 100.0%)	100.0% (100.0% to 100.0%)	100.0% (100.0% to 100.0%)	100.0% (100.0% to 100.0%)

Supplementary Results Table 1. Age-standardised PAFs attributable to the joint distribution of all risk factors for all ages, both sexes combined for each GBD level 1 and level 2 cause and all causes in 2016 for Benin. Results are shown for deaths, YLLs, YLDs, and DALYs
Data in parenthesis are 95% uncertainty intervals. PAFs=population attributable fractions. GBD=Global Burden of Disease. YLLs=years of life lost. YLDs=years live with disability. DALYs=disability-adjusted life-years

	Deaths 2016	YLLs 2016	YLDs 2016	DALYs 2016
Drug use disorders	100.0% (100.0% to 100.0%)	100.0% (100.0% to 100.0%)	100.0% (100.0% to 100.0%)	100.0% (100.0% to 100.0%)
Depressive disorders	-	-	11.4% (9.8% to 13.1%)	11.4% (9.8% to 13.1%)
Idiopathic developmental intellectual disability	-	-	63.9% (32.4% to 83.2%)	63.9% (32.4% to 83.2%)
**Diabetes, urogenital, blood, and endocrine diseases**	72.2% (69.7% to 75.3%)	59.6% (56.1% to 64.0%)	52.5% (48.5% to 56.5%)	57.2% (54.2% to 60.6%)
Diabetes mellitus	100.0% (100.0% to 100.0%)	100.0% (100.0% to 100.0%)	100.0% (100.0% to 100.0%)	100.0% (100.0% to 100.0%)
Chronic kidney disease	100.0% (100.0% to 100.0%)	100.0% (100.0% to 100.0%)	100.0% (100.0% to 100.0%)	100.0% (100.0% to 100.0%)
**Musculoskeletal disorders**	0.2% (0.1% to 0.5%)	0.2% (0.1% to 0.3%)	28.1% (24.2% to 32.2%)	27.7% (23.9% to 31.7%)
Rheumatoid arthritis	0.9% (0.3% to 2.0%)	1.0% (0.4% to 2.1%)	1.5% (0.6% to 2.6%)	1.4% (0.5% to 2.5%)
Osteoarthritis	-	-	25.7% (13.9% to 38.8%)	25.7% (13.9% to 38.8%)
Low back and neck pain	-	-	33.6% (29.7% to 38.0%)	33.6% (29.7% to 38.0%)
Gout	-	-	55.8% (44.5% to 67.7%)	55.8% (44.5% to 67.7%)
**Other non-communicable diseases**	0.1% (0.0% to 0.2%)	0.1% (0.0% to 0.3%)	10.8% (9.0% to 12.7%)	7.5% (5.9% to 9.1%)
Sense organ diseases	-	-	19.9% (17.4% to 22.2%)	19.9% (17.4% to 22.2%)
Sudden infant death syndrome	3.7% (1.2% to 8.1%)	3.8% (1.2% to 8.1%)	-	3.8% (1.2% to 8.1%)
**Transport injuries**	27.9% (23.7% to 32.2%)	22.4% (18.6% to 26.8%)	32.3% (28.3% to 36.7%)	23.8% (19.8% to 28.3%)
Road injuries	27.9% (23.7% to 32.3%)	22.5% (18.6% to 26.9%)	33.7% (29.7% to 38.0%)	23.9% (19.8% to 28.3%)
Other transport injuries	26.4% (22.0% to 30.6%)	21.1% (16.9% to 25.4%)	25.2% (20.9% to 30.2%)	23.1% (19.0% to 27.2%)
**Unintentional injuries**	23.7% (16.5% to 32.8%)	14.5% (11.4% to 18.0%)	19.8% (18.0% to 22.1%)	16.0% (13.4% to 19.0%)
Falls	49.9% (31.8% to 70.4%)	36.6% (27.7% to 47.3%)	29.6% (27.8% to 31.6%)	33.5% (28.3% to 40.1%)
Drowning	9.6% (7.1% to 12.9%)	9.7% (7.4% to 12.5%)	12.8% (9.5% to 16.9%)	9.7% (7.4% to 12.5%)
Fire, heat, and hot substances	8.8% (6.0% to 13.0%)	10.1% (7.5% to 13.3%)	15.6% (12.1% to 20.1%)	11.2% (8.4% to 14.5%)

Supplementary Results Table 1. Age-standardised PAFs attributable to the joint distribution of all risk factors for all ages, both sexes combined for each GBD level 1 and level 2 cause and all causes in 2016 for Benin. Results are shown for deaths, YLLs, YLDs, and DALYs
Data in parenthesis are 95% uncertainty intervals. PAFs=population attributable fractions. GBD=Global Burden of Disease. YLLs=years of life lost. YLDs=years live with disability. DALYs=disability-adjusted life-years

	**Deaths 2016**	**YLLs 2016**	**YLDs 2016**	**DALYs 2016**
Poisonings	11.4% (8.0% to 15.2%)	11.9% (7.6% to 16.0%)	15.0% (11.6% to 19.1%)	12.1% (7.8% to 16.0%)
Exposure to mechanical forces	18.2% (14.9% to 20.9%)	13.1% (10.2% to 15.9%)	19.7% (17.3% to 22.6%)	14.8% (12.1% to 17.6%)
Animal contact	7.3% (5.9% to 9.0%)	8.2% (6.4% to 10.3%)	11.6% (9.5% to 14.1%)	9.0% (7.2% to 11.1%)
Foreign body	5.6% (4.2% to 7.3%)	6.7% (4.7% to 9.1%)	9.4% (7.5% to 11.7%)	7.5% (5.6% to 9.5%)
Other unintentional injuries	11.9% (8.8% to 15.5%)	12.8% (9.7% to 16.4%)	13.3% (10.1% to 17.1%)	13.0% (9.9% to 16.6%)
**Self-harm and interpersonal violence**	10.2% (4.2% to 17.4%)	10.1% (4.9% to 16.5%)	15.9% (13.7% to 18.6%)	10.5% (5.5% to 16.7%)
Self-harm	10.0% (2.5% to 18.8%)	10.5% (3.4% to 18.6%)	9.8% (2.6% to 18.1%)	10.5% (3.4% to 18.6%)
Interpersonal violence	10.7% (7.0% to 15.2%)	9.2% (5.8% to 13.3%)	16.8% (14.8% to 19.1%)	10.6% (7.4% to 14.2%)
**Forces of nature, conflict and terrorism, and executions and police conflict**	0.0% (0.0% to 0.0%)	0.0% (0.0% to 0.0%)	3.8% (2.5% to 5.6%)	1.0% (0.3% to 2.1%)
Forces of nature, conflict and terrorism, and state actor violence	0.0% (0.0% to 0.0%)	0.0% (0.0% to 0.0%)	7.3% (4.9% to 10.6%)	7.3% (4.9% to 10.6%)

Supplementary Results Table 1. Age-standardised PAFs attributable to the joint distribution of all risk factors for all ages, both sexes combined for each GBD level 1 and level 2 cause and all causes in 2016 for Bermuda. Results are shown for deaths, YLLs, YLDs, and DALYs
Data in parenthesis are 95% uncertainty intervals. PAFs=population attributable fractions. GBD=Global Burden of Disease. YLLs=years of life lost. YLDs=years live with disability. DALYs=disability-adjusted life-years

	Deaths 2016	YLLs 2016	YLDs 2016	DALYs 2016
**All causes**	57.7% (55.3% to 59.9%)	56.5% (54.4% to 58.6%)	24.6% (23.2% to 26.2%)	41.4% (38.5% to 44.3%)
**HIV/AIDS and tuberculosis**	82.4% (75.5% to 87.4%)	79.2% (71.3% to 84.6%)	78.9% (71.6% to 85.0%)	79.2% (71.5% to 84.6%)
Tuberculosis	48.1% (36.6% to 62.3%)	50.5% (38.4% to 64.2%)	50.6% (37.2% to 65.5%)	50.5% (38.1% to 64.8%)
HIV/AIDS	82.7% (75.9% to 87.7%)	79.3% (71.4% to 84.8%)	80.0% (72.3% to 85.7%)	79.4% (71.5% to 84.8%)
**Diarrhoea, lower respiratory infections, and other common infectious diseases**	41.2% (32.8% to 51.0%)	46.5% (38.5% to 54.7%)	40.5% (27.6% to 50.8%)	43.9% (36.7% to 50.6%)
Diarrhoeal diseases	73.7% (47.5% to 90.5%)	80.2% (58.7% to 92.7%)	76.3% (51.8% to 91.5%)	76.7% (52.9% to 91.6%)
Lower respiratory infections	42.3% (33.5% to 52.7%)	50.8% (42.4% to 59.8%)	47.0% (38.8% to 56.2%)	50.7% (42.3% to 59.7%)
Upper respiratory infections	0.3% (0.0% to 1.1%)	1.0% (0.1% to 3.5%)	0.0% (0.0% to 0.0%)	0.0% (0.0% to 0.0%)
Otitis media	0.4% (0.2% to 0.7%)	1.4% (0.7% to 2.5%)	4.9% (3.2% to 6.5%)	4.9% (3.2% to 6.5%)
Meningitis	1.0% (0.5% to 1.7%)	2.7% (1.6% to 4.2%)	0.0% (0.0% to 0.0%)	1.8% (1.1% to 2.8%)
Encephalitis	0.7% (0.3% to 1.3%)	1.2% (0.7% to 2.2%)	0.0% (0.0% to 0.0%)	1.0% (0.5% to 1.8%)
Measles	13.3% (3.2% to 39.5%)	15.8% (3.9% to 46.6%)	23.5% (6.1% to 63.8%)	22.7% (6.0% to 62.0%)
**Maternal disorders**	3.4% (1.6% to 5.6%)	3.4% (1.6% to 5.6%)	3.3% (1.7% to 5.4%)	3.3% (1.7% to 5.4%)
Maternal haemorrhage	13.9% (5.0% to 23.1%)	14.1% (5.0% to 23.2%)	14.9% (5.3% to 24.2%)	14.6% (5.3% to 23.9%)
Maternal sepsis and other pregnancy related infections	13.3% (4.7% to 22.3%)	13.6% (4.8% to 22.5%)	14.6% (5.3% to 23.9%)	14.6% (5.3% to 23.9%)
Maternal abortion, miscarriage, and ectopic pregnancy	4.4% (2.3% to 7.5%)	4.4% (2.2% to 7.7%)	5.1% (2.4% to 8.5%)	5.0% (2.4% to 8.5%)
**Neonatal disorders**	64.2% (59.3% to 68.8%)	64.3% (59.5% to 68.9%)	0.1% (0.1% to 0.2%)	37.5% (28.0% to 45.7%)
Neonatal preterm birth complications	89.2% (83.8% to 93.5%)	89.3% (83.9% to 93.6%)	0.2% (0.2% to 0.2%)	27.3% (16.2% to 40.2%)
Neonatal encephalopathy due to birth asphyxia and trauma	62.0% (55.9% to 67.5%)	62.1% (56.1% to 67.6%)	0.0% (0.0% to 0.0%)	53.0% (45.7% to 59.5%)
Neonatal sepsis and other neonatal infections	51.1% (42.9% to 61.0%)	51.2% (43.0% to 61.0%)	0.0% (0.0% to 0.0%)	19.2% (8.7% to 34.8%)
Hemolytic disease and other neonatal jaundice	54.9% (44.6% to 63.8%)	55.0% (44.7% to 63.9%)	0.0% (0.0% to 0.0%)	22.9% (14.9% to 33.8%)

Supplementary Results Table 1. Age-standardised PAFs attributable to the joint distribution of all risk factors for all ages, both sexes combined for each GBD level 1 and level 2 cause and all causes in 2016 for Bermuda. Results are shown for deaths, YLLs, YLDs, and DALYs
Data in parenthesis are 95% uncertainty intervals. PAFs=population attributable fractions. GBD=Global Burden of Disease. YLLs=years of life lost. YLDs=years live with disability. DALYs=disability-adjusted life-years

	Deaths 2016	YLLs 2016	YLDs 2016	DALYs 2016
Other neonatal disorders	54.8% (48.9% to 60.5%)	55.0% (49.0% to 60.6%)	0.0% (0.0% to 0.0%)	52.0% (46.3% to 57.6%)
**Nutritional deficiencies**	5.7% (3.4% to 9.1%)	26.9% (17.4% to 38.3%)	96.9% (96.0% to 97.6%)	95.1% (93.6% to 96.3%)
Protein-energy malnutrition	6.5% (3.1% to 11.4%)	34.1% (19.6% to 49.7%)	96.3% (94.5% to 97.6%)	71.1% (60.1% to 80.0%)
Vitamin A deficiency	-	-	0.0% (0.0% to 0.0%)	0.0% (0.0% to 0.0%)
Iron-deficiency anaemia	100.0% (100.0% to 100.0%)	100.0% (100.0% to 100.0%)	100.0% (100.0% to 100.0%)	100.0% (100.0% to 100.0%)
**Other communicable, maternal, neonatal, and nutritional diseases**	9.1% (6.6% to 11.2%)	6.8% (4.7% to 8.9%)	70.4% (61.4% to 80.1%)	51.3% (39.6% to 63.6%)
Sexually transmitted diseases excluding HIV	60.4% (33.1% to 88.5%)	32.3% (12.4% to 68.0%)	100.0% (100.0% to 100.0%)	91.0% (77.4% to 98.5%)
Hepatitis	7.5% (5.2% to 10.3%)	7.7% (5.0% to 10.9%)	3.4% (2.2% to 4.8%)	7.3% (4.8% to 10.4%)
**Neoplasms**	42.8% (39.5% to 46.4%)	43.4% (40.0% to 46.9%)	32.7% (28.9% to 36.7%)	43.0% (39.6% to 46.5%)
Oesophageal cancer	82.4% (73.9% to 89.8%)	84.0% (75.6% to 90.9%)	82.9% (74.6% to 89.9%)	84.0% (75.6% to 90.8%)
Stomach cancer	21.4% (12.7% to 30.9%)	22.0% (13.0% to 31.3%)	21.7% (12.7% to 31.0%)	22.0% (13.0% to 31.3%)
Liver cancer	73.7% (67.2% to 79.6%)	73.2% (66.2% to 79.3%)	73.4% (66.5% to 79.4%)	73.2% (66.2% to 79.3%)
Larynx cancer	88.8% (82.1% to 93.4%)	89.1% (82.8% to 93.4%)	89.1% (83.0% to 93.5%)	89.1% (82.9% to 93.4%)
Tracheal, bronchus, and lung cancer	87.9% (85.4% to 90.2%)	87.1% (84.4% to 89.3%)	87.4% (84.4% to 89.7%)	87.1% (84.4% to 89.3%)
Breast cancer	28.8% (17.6% to 42.8%)	26.9% (14.3% to 42.5%)	27.1% (15.2% to 42.4%)	26.9% (14.4% to 42.5%)
Cervical cancer	100.0% (100.0% to 100.0%)	100.0% (100.0% to 100.0%)	100.0% (100.0% to 100.0%)	100.0% (100.0% to 100.0%)
Uterine cancer	50.5% (35.1% to 64.8%)	52.8% (37.7% to 66.7%)	54.0% (39.0% to 67.7%)	52.9% (37.7% to 66.8%)
Prostate cancer	6.1% (4.2% to 8.0%)	6.2% (4.4% to 8.1%)	6.4% (4.5% to 8.4%)	6.3% (4.4% to 8.2%)
Colon and rectum cancer	58.5% (49.0% to 67.3%)	59.6% (50.1% to 68.2%)	58.8% (49.2% to 67.4%)	59.6% (50.1% to 68.2%)
Lip and oral cavity cancer	84.2% (78.9% to 89.2%)	85.7% (80.8% to 90.4%)	83.0% (77.5% to 87.9%)	85.6% (80.7% to 90.3%)
Nasopharynx cancer	85.8% (81.8% to 89.2%)	85.5% (81.7% to 88.6%)	84.1% (80.7% to 86.9%)	85.5% (81.7% to 88.5%)

Supplementary Results Table 1. Age-standardised PAFs attributable to the joint distribution of all risk factors for all ages, both sexes combined for each GBD level 1 and level 2 cause and all causes in 2016 for Bermuda. Results are shown for deaths, YLLs, YLDs, and DALYs
Data in parenthesis are 95% uncertainty intervals. PAFs=population attributable fractions. GBD=Global Burden of Disease. YLLs=years of life lost. YLDs=years live with disability. DALYs=disability-adjusted life-years

	Deaths 2016	YLLs 2016	YLDs 2016	DALYs 2016
Other pharynx cancer	61.2% (49.3% to 71.9%)	64.4% (52.5% to 74.6%)	63.0% (50.9% to 73.5%)	64.4% (52.4% to 74.6%)
Gallbladder and biliary tract cancer	25.7% (14.4% to 38.8%)	27.1% (15.4% to 40.4%)	26.3% (15.1% to 39.6%)	27.1% (15.4% to 40.4%)
Pancreatic cancer	36.8% (28.4% to 45.6%)	37.1% (28.6% to 45.9%)	36.6% (28.2% to 45.2%)	37.1% (28.6% to 45.9%)
Ovarian cancer	15.0% (6.8% to 24.4%)	13.5% (5.8% to 22.7%)	13.4% (5.8% to 22.2%)	13.5% (5.8% to 22.7%)
Kidney cancer	40.5% (29.8% to 51.3%)	40.2% (29.5% to 50.8%)	40.0% (29.6% to 50.0%)	40.2% (29.5% to 50.8%)
Bladder cancer	41.5% (30.8% to 51.8%)	41.6% (31.3% to 52.0%)	41.1% (31.1% to 51.5%)	41.6% (31.3% to 52.0%)
Thyroid cancer	16.7% (8.9% to 26.7%)	17.9% (9.5% to 28.2%)	17.6% (9.9% to 26.5%)	17.9% (9.5% to 28.1%)
Mesothelioma	94.0% (92.0% to 95.7%)	88.4% (84.9% to 91.6%)	91.6% (89.1% to 93.8%)	88.4% (85.0% to 91.6%)
Non-Hodgkin's lymphoma	8.6% (3.6% to 14.8%)	8.9% (3.7% to 14.8%)	8.9% (3.7% to 14.9%)	8.9% (3.7% to 14.8%)
Multiple myeloma	9.8% (4.3% to 16.8%)	10.4% (4.6% to 17.6%)	10.3% (4.5% to 17.6%)	10.4% (4.6% to 17.6%)
Leukaemia	24.0% (15.5% to 32.9%)	22.3% (14.6% to 30.5%)	22.4% (14.5% to 30.6%)	22.3% (14.6% to 30.5%)
**Cardiovascular diseases**	78.5% (75.6% to 81.1%)	80.7% (78.4% to 82.7%)	69.5% (66.4% to 72.3%)	79.3% (77.1% to 81.6%)
Rheumatic heart disease	23.0% (13.7% to 42.1%)	20.5% (13.9% to 32.0%)	22.7% (14.8% to 35.6%)	20.6% (14.0% to 32.1%)
Ischaemic heart disease	90.9% (87.0% to 94.4%)	93.1% (90.6% to 95.4%)	90.5% (87.9% to 93.1%)	93.0% (90.5% to 95.2%)
Cerebrovascular disease	79.3% (73.5% to 84.6%)	83.7% (79.6% to 87.4%)	81.0% (76.2% to 85.5%)	83.4% (79.2% to 87.1%)
Hypertensive heart disease	100.0% (100.0% to 100.0%)	100.0% (100.0% to 100.0%)	100.0% (100.0% to 100.0%)	100.0% (100.0% to 100.0%)
Cardiomyopathy and myocarditis	37.1% (29.8% to 45.7%)	38.4% (31.2% to 45.2%)	31.9% (26.1% to 38.1%)	38.0% (31.0% to 44.7%)
Atrial fibrillation and flutter	53.0% (44.3% to 61.1%)	57.3% (49.3% to 64.1%)	63.9% (57.8% to 70.0%)	61.4% (54.6% to 67.7%)
Aortic aneurysm	39.3% (32.0% to 46.5%)	44.5% (38.1% to 50.2%)	-	44.5% (38.1% to 50.2%)
Peripheral vascular disease	43.7% (36.1% to 52.1%)	50.3% (43.6% to 57.2%)	56.0% (50.7% to 61.2%)	51.4% (44.9% to 57.5%)
Endocarditis	35.9% (27.5% to 46.4%)	35.0% (26.7% to 44.1%)	31.8% (24.4% to 39.8%)	35.0% (26.7% to 44.0%)

Supplementary Results Table 1. Age-standardised PAFs attributable to the joint distribution of all risk factors for all ages, both sexes combined for each GBD level 1 and level 2 cause and all causes in 2016 for Bermuda. Results are shown for deaths, YLLs, YLDs, and DALYs
Data in parenthesis are 95% uncertainty intervals. PAFs=population attributable fractions. GBD=Global Burden of Disease. YLLs=years of life lost. YLDs=years live with disability. DALYs=disability-adjusted life-years

	Deaths 2016	YLLs 2016	YLDs 2016	DALYs 2016
Other cardiovascular and circulatory diseases	40.5% (35.2% to 45.7%)	44.4% (39.8% to 48.7%)	38.8% (33.9% to 43.8%)	42.9% (38.4% to 47.2%)
**Chronic respiratory diseases**	55.7% (51.4% to 60.8%)	49.4% (44.8% to 54.9%)	38.7% (29.8% to 48.5%)	42.6% (35.8% to 49.3%)
Chronic obstructive pulmonary disease	84.1% (79.6% to 87.9%)	82.3% (77.6% to 86.1%)	73.4% (67.8% to 78.3%)	79.5% (74.9% to 83.6%)
Pneumoconiosis	100.0% (100.0% to 100.0%)	100.0% (100.0% to 100.0%)	100.0% (100.0% to 100.0%)	100.0% (100.0% to 100.0%)
Asthma	40.5% (29.6% to 52.9%)	44.0% (33.9% to 55.2%)	33.7% (23.8% to 45.3%)	34.3% (24.4% to 45.7%)
Other chronic respiratory diseases	22.6% (16.3% to 29.5%)	20.6% (14.7% to 27.4%)	6.5% (4.2% to 9.4%)	17.5% (12.5% to 23.5%)
**Cirrhosis and other chronic liver diseases**	71.1% (68.7% to 73.5%)	71.2% (68.7% to 73.6%)	55.1% (51.1% to 58.7%)	70.1% (67.6% to 72.5%)
Cirrhosis and other chronic liver diseases due to hepatitis B	6.1% (2.5% to 10.3%)	8.1% (3.3% to 13.8%)	6.4% (2.8% to 10.7%)	8.0% (3.3% to 13.6%)
Cirrhosis and other chronic liver diseases due to hepatitis C	88.4% (86.5% to 89.8%)	89.6% (88.9% to 90.0%)	87.8% (86.1% to 88.9%)	89.5% (88.8% to 89.9%)
Cirrhosis and other chronic liver diseases due to alcohol use	100.0% (100.0% to 100.0%)	100.0% (100.0% to 100.0%)	100.0% (100.0% to 100.0%)	100.0% (100.0% to 100.0%)
**Digestive diseases**	10.2% (8.2% to 12.5%)	14.2% (11.5% to 17.1%)	4.2% (3.2% to 5.3%)	11.0% (8.8% to 13.3%)
Peptic ulcer disease	7.9% (5.3% to 10.8%)	9.8% (6.6% to 13.6%)	7.3% (4.8% to 10.4%)	8.9% (6.0% to 12.3%)
Gallbladder and biliary diseases	44.3% (29.1% to 59.7%)	46.1% (31.3% to 60.4%)	53.8% (39.3% to 67.8%)	48.3% (34.2% to 62.6%)
Pancreatitis	63.6% (47.5% to 80.3%)	69.5% (54.7% to 83.8%)	60.6% (46.7% to 74.8%)	69.3% (54.6% to 83.7%)
**Neurological disorders**	22.0% (10.9% to 35.0%)	21.6% (12.0% to 33.2%)	4.5% (2.5% to 7.5%)	9.6% (5.5% to 14.6%)
Alzheimer's disease and other dementias	25.2% (12.2% to 40.1%)	26.7% (13.4% to 41.6%)	26.8% (13.5% to 41.5%)	26.7% (13.4% to 41.6%)
Parkinson's disease	-4.2% (-5.6% to -3.1%)	-5.2% (-6.8% to -3.9%)	-5.8% (-7.2% to -4.5%)	-5.4% (-6.8% to -4.1%)
Epilepsy	31.6% (18.1% to 45.1%)	33.6% (18.8% to 47.2%)	26.3% (15.1% to 37.2%)	28.5% (16.2% to 40.4%)
Multiple sclerosis	5.1% (3.1% to 7.4%)	5.2% (3.1% to 7.4%)	4.2% (2.6% to 6.1%)	4.7% (2.9% to 6.6%)
**Mental and substance use disorders**	100.0% (100.0% to 100.0%)	99.9% (99.9% to 100.0%)	18.4% (16.4% to 20.8%)	21.2% (18.9% to 23.8%)
Alcohol use disorders	100.0% (100.0% to 100.0%)	100.0% (100.0% to 100.0%)	100.0% (100.0% to 100.0%)	100.0% (100.0% to 100.0%)

Supplementary Results Table 1. Age-standardised PAFs attributable to the joint distribution of all risk factors for all ages, both sexes combined for each GBD level 1 and level 2 cause and all causes in 2016 for Bermuda. Results are shown for deaths, YLLs, YLDs, and DALYs
Data in parenthesis are 95% uncertainty intervals. PAFs=population attributable fractions. GBD=Global Burden of Disease. YLLs=years of life lost. YLDs=years live with disability. DALYs=disability-adjusted life-years

	Deaths 2016	YLLs 2016	YLDs 2016	DALYs 2016
Drug use disorders	100.0% (100.0% to 100.0%)	100.0% (100.0% to 100.0%)	100.0% (100.0% to 100.0%)	100.0% (100.0% to 100.0%)
Depressive disorders	-	-	6.2% (5.1% to 7.6%)	6.2% (5.1% to 7.6%)
Idiopathic developmental intellectual disability	-	-	61.1% (32.9% to 80.8%)	61.1% (32.9% to 80.8%)
**Diabetes, urogenital, blood, and endocrine diseases**	81.2% (77.8% to 82.8%)	79.0% (73.1% to 81.0%)	70.7% (66.3% to 74.5%)	75.1% (71.8% to 77.4%)
Diabetes mellitus	100.0% (100.0% to 100.0%)	100.0% (100.0% to 100.0%)	100.0% (100.0% to 100.0%)	100.0% (100.0% to 100.0%)
Chronic kidney disease	100.0% (100.0% to 100.0%)	100.0% (100.0% to 100.0%)	100.0% (100.0% to 100.0%)	100.0% (100.0% to 100.0%)
**Musculoskeletal disorders**	0.4% (0.2% to 0.8%)	0.3% (0.1% to 0.5%)	13.6% (10.5% to 16.9%)	13.1% (10.1% to 16.3%)
Rheumatoid arthritis	1.9% (0.7% to 3.2%)	2.3% (0.9% to 3.9%)	2.8% (1.1% to 4.8%)	2.8% (1.1% to 4.8%)
Osteoarthritis	-	-	32.1% (17.9% to 45.8%)	32.1% (17.9% to 45.8%)
Low back and neck pain	-	-	16.9% (14.0% to 19.9%)	16.9% (14.0% to 19.9%)
Gout	-	-	54.1% (37.4% to 70.2%)	54.1% (37.4% to 70.2%)
**Other non-communicable diseases**	1.1% (0.5% to 2.2%)	1.9% (1.2% to 3.1%)	4.9% (4.2% to 5.7%)	4.6% (4.0% to 5.3%)
Sense organ diseases	-	-	12.5% (11.0% to 14.3%)	12.5% (11.0% to 14.3%)
Sudden infant death syndrome	16.7% (10.8% to 23.1%)	16.8% (10.9% to 23.1%)	-	16.8% (10.9% to 23.1%)
**Transport injuries**	41.3% (29.4% to 54.0%)	39.9% (26.0% to 54.1%)	44.7% (33.2% to 56.9%)	40.9% (27.7% to 54.8%)
Road injuries	40.8% (28.8% to 53.8%)	39.8% (25.8% to 54.2%)	44.9% (33.4% to 57.2%)	41.0% (27.6% to 54.8%)
Other transport injuries	46.1% (35.8% to 57.1%)	40.5% (28.2% to 53.4%)	39.5% (29.3% to 50.3%)	40.3% (28.5% to 53.0%)
**Unintentional injuries**	26.4% (21.5% to 31.5%)	18.8% (13.2% to 26.1%)	24.8% (20.0% to 31.0%)	22.2% (17.2% to 28.9%)
Falls	55.2% (49.4% to 60.2%)	34.9% (28.5% to 42.9%)	29.5% (25.2% to 35.9%)	30.8% (26.7% to 37.4%)
Drowning	19.0% (9.8% to 31.3%)	19.7% (10.2% to 32.3%)	22.8% (14.3% to 33.0%)	19.8% (10.4% to 32.4%)
Fire, heat, and hot substances	12.2% (6.7% to 19.9%)	16.7% (9.8% to 25.5%)	29.0% (19.3% to 38.6%)	26.7% (17.6% to 36.5%)

Supplementary Results Table 1. Age-standardised PAFs attributable to the joint distribution of all risk factors for all ages, both sexes combined for each GBD level 1 and level 2 cause and all causes in 2016 for Bermuda. Results are shown for deaths, YLLs, YLDs, and DALYs
Data in parenthesis are 95% uncertainty intervals. PAFs=population attributable fractions. GBD=Global Burden of Disease. YLLs=years of life lost. YLDs=years live with disability. DALYs=disability-adjusted life-years

	**Deaths 2016**	**YLLs 2016**	**YLDs 2016**	**DALYs 2016**
Poisonings	19.7% (10.6% to 32.1%)	21.7% (11.6% to 34.5%)	28.1% (18.8% to 38.2%)	24.1% (14.5% to 35.5%)
Exposure to mechanical forces	22.6% (17.7% to 29.6%)	18.7% (12.5% to 28.0%)	22.4% (16.7% to 31.0%)	21.0% (15.3% to 29.8%)
Animal contact	15.5% (11.6% to 20.9%)	16.1% (10.3% to 24.2%)	20.2% (12.9% to 27.7%)	18.8% (12.3% to 26.4%)
Foreign body	5.3% (2.8% to 9.6%)	7.4% (3.9% to 13.2%)	13.5% (8.3% to 19.1%)	9.9% (5.7% to 15.8%)
Other unintentional injuries	20.6% (10.3% to 34.2%)	22.7% (11.5% to 37.5%)	24.9% (16.6% to 34.6%)	24.7% (16.3% to 34.9%)
**Self-harm and interpersonal violence**	36.6% (26.4% to 45.7%)	37.5% (27.0% to 47.0%)	32.0% (25.2% to 38.4%)	36.8% (27.0% to 45.9%)
Self-harm	39.0% (25.2% to 51.4%)	41.6% (27.3% to 54.0%)	35.9% (22.9% to 47.7%)	41.4% (27.2% to 53.9%)
Interpersonal violence	33.5% (18.0% to 47.8%)	32.9% (17.2% to 47.7%)	31.6% (24.2% to 38.6%)	32.6% (18.9% to 45.6%)
**Forces of nature, conflict and terrorism, and executions and police conflict**	0.0% (0.0% to 0.0%)	0.0% (0.0% to 0.0%)	4.5% (3.0% to 6.6%)	2.3% (0.5% to 4.3%)
Forces of nature, conflict and terrorism, and state actor violence	0.0% (0.0% to 0.0%)	0.0% (0.0% to 0.0%)	4.7% (3.2% to 6.9%)	4.7% (3.2% to 6.9%)

Supplementary Results Table 1. Age-standardised PAFs attributable to the joint distribution of all risk factors for all ages, both sexes combined for each GBD level 1 and level 2 cause and all causes in 2016 for Bhutan. Results are shown for deaths, YLLs, YLDs, and DALYs
Data in parenthesis are 95% uncertainty intervals. PAFs=population attributable fractions. GBD=Global Burden of Disease. YLLs=years of life lost. YLDs=years live with disability. DALYs=disability-adjusted life-years

	Deaths 2016	YLLs 2016	YLDs 2016	DALYs 2016
**All causes**	61.1% (58.7% to 63.3%)	56.4% (54.0% to 58.7%)	33.5% (31.8% to 35.1%)	47.6% (45.5% to 50.0%)
**HIV/AIDS and tuberculosis**	49.6% (30.0% to 73.6%)	54.1% (32.2% to 77.3%)	43.0% (26.2% to 65.4%)	53.5% (33.6% to 76.2%)
Tuberculosis	34.1% (20.8% to 49.6%)	33.0% (21.2% to 46.5%)	30.2% (19.5% to 41.1%)	32.7% (21.1% to 45.8%)
HIV/AIDS	91.1% (86.7% to 94.7%)	87.7% (81.4% to 92.8%)	91.8% (87.9% to 94.7%)	88.1% (82.3% to 92.8%)
**Diarrhoea, lower respiratory infections, and other common infectious diseases**	54.7% (47.0% to 63.8%)	54.0% (46.5% to 61.0%)	55.5% (42.7% to 65.6%)	54.3% (47.5% to 61.2%)
Diarrhoeal diseases	78.6% (59.1% to 93.2%)	85.4% (68.4% to 96.9%)	78.3% (60.2% to 91.8%)	81.1% (65.7% to 93.2%)
Lower respiratory infections	65.9% (58.8% to 73.0%)	78.6% (72.6% to 84.1%)	67.9% (61.9% to 74.0%)	78.6% (72.6% to 84.0%)
Upper respiratory infections	2.8% (0.2% to 9.5%)	5.8% (0.4% to 20.0%)	0.0% (0.0% to 0.0%)	0.1% (0.0% to 0.4%)
Otitis media	1.4% (0.3% to 3.4%)	3.2% (0.8% to 6.8%)	3.3% (2.2% to 4.7%)	3.3% (2.2% to 4.7%)
Meningitis	2.2% (1.0% to 3.6%)	3.6% (1.9% to 6.0%)	0.0% (0.0% to 0.0%)	3.1% (1.7% to 5.2%)
Encephalitis	0.7% (0.4% to 1.0%)	1.4% (1.0% to 2.3%)	0.0% (0.0% to 0.0%)	1.2% (0.8% to 2.0%)
Measles	55.4% (34.1% to 82.5%)	56.1% (34.6% to 83.4%)	52.6% (31.1% to 79.7%)	56.1% (34.5% to 83.3%)
**Maternal disorders**	12.1% (4.9% to 19.5%)	12.1% (4.8% to 19.6%)	3.1% (1.3% to 5.6%)	11.7% (4.7% to 18.9%)
Maternal haemorrhage	22.6% (8.4% to 35.8%)	22.7% (8.5% to 36.0%)	22.7% (8.5% to 36.2%)	22.7% (8.5% to 36.0%)
Maternal sepsis and other pregnancy related infections	22.4% (8.4% to 35.8%)	22.5% (8.4% to 36.0%)	22.0% (8.3% to 35.0%)	22.5% (8.4% to 36.0%)
Maternal abortion, miscarriage, and ectopic pregnancy	12.2% (6.9% to 18.4%)	12.1% (6.8% to 18.2%)	12.9% (6.9% to 20.5%)	12.2% (6.8% to 18.2%)
**Neonatal disorders**	84.3% (80.9% to 87.5%)	84.3% (80.9% to 87.5%)	0.3% (0.2% to 0.4%)	78.7% (74.7% to 82.2%)
Neonatal preterm birth complications	97.2% (94.9% to 98.7%)	97.2% (94.9% to 98.8%)	0.5% (0.4% to 0.7%)	86.9% (80.8% to 91.3%)
Neonatal encephalopathy due to birth asphyxia and trauma	77.9% (73.8% to 81.8%)	77.9% (73.8% to 81.9%)	0.0% (0.0% to 0.0%)	76.8% (72.3% to 81.0%)
Neonatal sepsis and other neonatal infections	73.0% (67.7% to 77.5%)	73.1% (67.7% to 77.5%)	0.0% (0.0% to 0.0%)	61.2% (44.3% to 71.1%)
Hemolytic disease and other neonatal jaundice	72.5% (66.8% to 77.4%)	72.6% (66.8% to 77.4%)	0.0% (0.0% to 0.0%)	65.6% (58.5% to 71.4%)

Supplementary Results Table 1. Age-standardised PAFs attributable to the joint distribution of all risk factors for all ages, both sexes combined for each GBD level 1 and level 2 cause and all causes in 2016 for Bhutan. Results are shown for deaths, YLLs, YLDs, and DALYs
Data in parenthesis are 95% uncertainty intervals. PAFs=population attributable fractions. GBD=Global Burden of Disease. YLLs=years of life lost. YLDs=years live with disability. DALYs=disability-adjusted life-years

	Deaths 2016	YLLs 2016	YLDs 2016	DALYs 2016
Other neonatal disorders	77.1% (73.0% to 80.8%)	77.2% (73.0% to 80.8%)	0.0% (0.0% to 0.0%)	76.2% (71.8% to 79.9%)
**Nutritional deficiencies**	26.1% (17.6% to 36.0%)	48.3% (34.4% to 62.3%)	98.8% (98.5% to 99.0%)	95.2% (93.0% to 96.8%)
Protein-energy malnutrition	60.7% (44.5% to 75.0%)	87.5% (78.8% to 93.5%)	96.2% (92.1% to 98.4%)	90.6% (85.4% to 94.4%)
Vitamin A deficiency	-	-	100.0% (100.0% to 100.0%)	100.0% (100.0% to 100.0%)
Iron-deficiency anaemia	100.0% (100.0% to 100.0%)	100.0% (100.0% to 100.0%)	100.0% (100.0% to 100.0%)	100.0% (100.0% to 100.0%)
**Other communicable, maternal, neonatal, and nutritional diseases**	3.4% (1.9% to 5.8%)	2.4% (1.1% to 4.4%)	38.8% (32.6% to 46.5%)	11.2% (6.7% to 17.1%)
Sexually transmitted diseases excluding HIV	14.6% (4.7% to 34.4%)	6.3% (1.8% to 16.7%)	100.0% (100.0% to 100.0%)	29.0% (11.4% to 54.9%)
Hepatitis	0.1% (0.1% to 0.2%)	0.2% (0.1% to 0.3%)	0.1% (0.1% to 0.2%)	0.2% (0.1% to 0.3%)
**Neoplasms**	29.9% (25.1% to 34.9%)	28.9% (24.3% to 33.9%)	30.9% (25.5% to 36.3%)	29.0% (24.4% to 33.9%)
Oesophageal cancer	59.7% (46.1% to 72.1%)	62.6% (49.3% to 74.4%)	60.8% (47.2% to 73.2%)	62.6% (49.3% to 74.4%)
Stomach cancer	10.8% (2.9% to 21.5%)	10.0% (3.0% to 19.0%)	10.6% (3.0% to 20.4%)	10.0% (3.0% to 19.0%)
Liver cancer	22.5% (15.1% to 32.9%)	23.1% (15.4% to 33.6%)	22.7% (15.1% to 32.9%)	23.1% (15.4% to 33.5%)
Larynx cancer	43.6% (26.6% to 59.9%)	46.0% (28.9% to 62.2%)	44.7% (27.4% to 60.9%)	46.0% (28.9% to 62.2%)
Tracheal, bronchus, and lung cancer	68.5% (58.9% to 76.9%)	68.6% (59.0% to 76.6%)	68.5% (59.2% to 76.7%)	68.6% (59.0% to 76.6%)
Breast cancer	14.4% (7.6% to 23.5%)	12.0% (5.7% to 20.1%)	13.0% (6.5% to 21.4%)	12.1% (5.7% to 20.2%)
Cervical cancer	100.0% (100.0% to 100.0%)	100.0% (100.0% to 100.0%)	100.0% (100.0% to 100.0%)	100.0% (100.0% to 100.0%)
Uterine cancer	33.1% (19.0% to 48.7%)	34.8% (20.7% to 50.1%)	34.5% (20.3% to 49.8%)	34.8% (20.7% to 50.1%)
Prostate cancer	0.5% (0.1% to 0.9%)	0.5% (0.1% to 1.0%)	0.5% (0.1% to 1.0%)	0.5% (0.1% to 1.0%)
Colon and rectum cancer	48.8% (38.6% to 58.8%)	48.2% (38.8% to 57.7%)	48.8% (38.9% to 58.5%)	48.3% (38.8% to 57.7%)
Lip and oral cavity cancer	60.0% (45.2% to 72.9%)	63.3% (50.3% to 74.9%)	61.2% (48.4% to 72.6%)	63.2% (50.3% to 74.8%)
Nasopharynx cancer	44.2% (26.6% to 59.6%)	43.4% (26.6% to 58.5%)	43.4% (27.1% to 57.5%)	43.4% (26.6% to 58.5%)

Supplementary Results Table 1. Age-standardised PAFs attributable to the joint distribution of all risk factors for all ages, both sexes combined for each GBD level 1 and level 2 cause and all causes in 2016 for Bhutan. Results are shown for deaths, YLLs, YLDs, and DALYs
Data in parenthesis are 95% uncertainty intervals. PAFs=population attributable fractions. GBD=Global Burden of Disease. YLLs=years of life lost. YLDs=years live with disability. DALYs=disability-adjusted life-years

	Deaths 2016	YLLs 2016	YLDs 2016	DALYs 2016
Other pharynx cancer	38.6% (20.0% to 55.7%)	39.1% (21.2% to 55.0%)	38.8% (20.2% to 55.4%)	39.1% (21.2% to 55.0%)
Gallbladder and biliary tract cancer	14.5% (7.0% to 24.9%)	15.5% (7.6% to 26.1%)	14.8% (7.2% to 25.1%)	15.5% (7.6% to 26.1%)
Pancreatic cancer	14.1% (6.2% to 25.0%)	14.1% (6.7% to 24.6%)	14.1% (6.2% to 24.9%)	14.1% (6.7% to 24.6%)
Ovarian cancer	10.7% (3.5% to 21.1%)	9.8% (3.1% to 19.3%)	9.7% (3.2% to 19.0%)	9.8% (3.1% to 19.3%)
Kidney cancer	13.4% (6.7% to 22.2%)	13.6% (7.1% to 22.1%)	14.1% (7.6% to 22.8%)	13.6% (7.1% to 22.2%)
Bladder cancer	11.8% (4.3% to 22.2%)	11.8% (4.6% to 21.7%)	11.9% (4.5% to 22.0%)	11.8% (4.6% to 21.7%)
Thyroid cancer	8.7% (3.7% to 16.4%)	8.9% (3.9% to 16.6%)	8.8% (4.0% to 15.9%)	8.9% (3.9% to 16.5%)
Mesothelioma	85.9% (79.5% to 90.3%)	76.3% (67.1% to 83.0%)	83.5% (76.6% to 88.3%)	76.4% (67.3% to 83.1%)
Non-Hodgkin's lymphoma	3.7% (1.3% to 7.4%)	3.5% (1.2% to 6.9%)	3.8% (1.3% to 7.6%)	3.5% (1.3% to 6.9%)
Multiple myeloma	4.7% (1.7% to 9.2%)	5.0% (1.9% to 9.6%)	4.9% (1.7% to 9.4%)	5.0% (1.9% to 9.6%)
Leukaemia	6.7% (3.6% to 10.4%)	5.9% (3.2% to 9.2%)	6.6% (3.6% to 10.4%)	5.9% (3.3% to 9.2%)
**Cardiovascular diseases**	86.7% (84.2% to 88.9%)	88.1% (86.3% to 89.7%)	75.0% (71.9% to 77.9%)	87.2% (85.3% to 88.8%)
Rheumatic heart disease	24.5% (16.2% to 40.5%)	22.0% (15.3% to 32.8%)	19.9% (12.7% to 28.9%)	21.7% (15.1% to 31.8%)
Ischaemic heart disease	92.6% (89.4% to 95.2%)	94.1% (92.1% to 95.7%)	90.6% (87.9% to 93.2%)	93.9% (92.0% to 95.6%)
Cerebrovascular disease	84.1% (79.9% to 88.2%)	88.1% (85.3% to 90.8%)	85.9% (82.2% to 89.3%)	87.9% (85.0% to 90.6%)
Hypertensive heart disease	100.0% (100.0% to 100.0%)	100.0% (100.0% to 100.0%)	100.0% (100.0% to 100.0%)	100.0% (100.0% to 100.0%)
Cardiomyopathy and myocarditis	34.2% (26.7% to 43.3%)	26.7% (19.3% to 35.0%)	20.7% (15.2% to 26.7%)	26.2% (18.9% to 34.1%)
Atrial fibrillation and flutter	42.7% (34.1% to 51.8%)	46.3% (38.7% to 54.0%)	52.1% (45.6% to 58.6%)	49.4% (42.3% to 56.3%)
Aortic aneurysm	35.3% (28.5% to 42.6%)	39.4% (33.6% to 45.3%)	-	39.4% (33.6% to 45.3%)
Peripheral vascular disease	49.8% (43.5% to 56.9%)	56.3% (51.1% to 61.6%)	58.8% (53.7% to 63.9%)	57.9% (52.7% to 62.7%)
Endocarditis	39.8% (30.2% to 49.9%)	37.0% (28.7% to 45.3%)	38.9% (29.8% to 48.5%)	37.0% (28.7% to 45.4%)

Supplementary Results Table 1. Age-standardised PAFs attributable to the joint distribution of all risk factors for all ages, both sexes combined for each GBD level 1 and level 2 cause and all causes in 2016 for Bhutan. Results are shown for deaths, YLLs, YLDs, and DALYs
Data in parenthesis are 95% uncertainty intervals. PAFs=population attributable fractions. GBD=Global Burden of Disease. YLLs=years of life lost. YLDs=years live with disability. DALYs=disability-adjusted life-years

	Deaths 2016	YLLs 2016	YLDs 2016	DALYs 2016
Other cardiovascular and circulatory diseases	38.6% (33.1% to 43.9%)	42.6% (37.9% to 47.3%)	41.3% (36.7% to 46.3%)	42.3% (37.9% to 46.9%)
**Chronic respiratory diseases**	57.4% (48.9% to 66.2%)	56.2% (48.2% to 64.5%)	48.4% (41.0% to 55.5%)	54.0% (46.3% to 61.6%)
Chronic obstructive pulmonary disease	63.7% (54.0% to 73.2%)	64.3% (54.6% to 73.3%)	57.9% (47.9% to 67.7%)	62.6% (53.0% to 71.9%)
Pneumoconiosis	100.0% (100.0% to 100.0%)	100.0% (100.0% to 100.0%)	100.0% (100.0% to 100.0%)	100.0% (100.0% to 100.0%)
Asthma	29.7% (21.5% to 39.6%)	34.0% (26.3% to 43.1%)	32.5% (25.5% to 40.6%)	33.4% (26.1% to 42.0%)
Other chronic respiratory diseases	1.9% (0.5% to 3.7%)	1.9% (0.4% to 4.1%)	1.6% (0.3% to 3.6%)	1.7% (0.4% to 3.8%)
**Cirrhosis and other chronic liver diseases**	23.1% (19.5% to 27.1%)	21.7% (18.3% to 25.6%)	17.8% (14.5% to 21.5%)	21.5% (18.1% to 25.5%)
Cirrhosis and other chronic liver diseases due to hepatitis B	0.1% (0.0% to 0.2%)	0.1% (0.1% to 0.2%)	0.1% (0.0% to 0.2%)	0.1% (0.1% to 0.2%)
Cirrhosis and other chronic liver diseases due to hepatitis C	7.1% (4.4% to 10.9%)	10.2% (6.3% to 15.5%)	9.2% (5.6% to 14.2%)	10.1% (6.3% to 15.5%)
Cirrhosis and other chronic liver diseases due to alcohol use	100.0% (100.0% to 100.0%)	100.0% (100.0% to 100.0%)	100.0% (100.0% to 100.0%)	100.0% (100.0% to 100.0%)
**Digestive diseases**	5.2% (2.8% to 8.4%)	5.2% (2.7% to 8.4%)	1.5% (1.1% to 2.1%)	4.7% (2.5% to 7.6%)
Peptic ulcer disease	4.3% (2.7% to 6.3%)	4.7% (3.0% to 6.7%)	3.7% (2.4% to 5.3%)	4.5% (2.9% to 6.5%)
Gallbladder and biliary diseases	26.4% (13.9% to 41.5%)	25.9% (14.2% to 39.4%)	28.5% (16.6% to 42.4%)	26.1% (14.5% to 39.5%)
Pancreatitis	23.5% (5.1% to 46.3%)	23.7% (6.7% to 44.3%)	21.9% (6.5% to 40.5%)	23.7% (6.7% to 44.3%)
**Neurological disorders**	14.8% (6.3% to 25.5%)	12.5% (6.3% to 20.3%)	1.9% (1.0% to 3.1%)	5.1% (2.6% to 8.7%)
Alzheimer's disease and other dementias	17.6% (7.2% to 30.7%)	18.4% (7.8% to 31.5%)	18.5% (7.8% to 31.6%)	18.4% (7.8% to 31.5%)
Parkinson's disease	-2.7% (-3.7% to -1.9%)	-3.0% (-3.9% to -2.2%)	-3.1% (-3.9% to -2.4%)	-3.0% (-3.9% to -2.3%)
Epilepsy	10.3% (4.8% to 16.7%)	7.5% (3.7% to 12.1%)	8.1% (3.9% to 12.6%)	7.8% (3.8% to 12.2%)
Multiple sclerosis	2.7% (1.6% to 4.1%)	2.8% (1.7% to 4.3%)	2.4% (1.4% to 3.6%)	2.6% (1.6% to 4.0%)
**Mental and substance use disorders**	99.9% (99.8% to 100.0%)	99.8% (99.7% to 99.9%)	23.8% (21.3% to 26.6%)	27.3% (24.3% to 31.4%)
Alcohol use disorders	100.0% (100.0% to 100.0%)	100.0% (100.0% to 100.0%)	100.0% (100.0% to 100.0%)	100.0% (100.0% to 100.0%)

Supplementary Results Table 1. Age-standardised PAFs attributable to the joint distribution of all risk factors for all ages, both sexes combined for each GBD level 1 and level 2 cause and all causes in 2016 for Bhutan. Results are shown for deaths, YLLs, YLDs, and DALYs
Data in parenthesis are 95% uncertainty intervals. PAFs=population attributable fractions. GBD=Global Burden of Disease. YLLs=years of life lost. YLDs=years live with disability. DALYs=disability-adjusted life-years

	Deaths 2016	YLLs 2016	YLDs 2016	DALYs 2016
Drug use disorders	100.0% (100.0% to 100.0%)	100.0% (100.0% to 100.0%)	100.0% (100.0% to 100.0%)	100.0% (100.0% to 100.0%)
Depressive disorders	-	-	9.6% (8.0% to 11.4%)	9.6% (8.0% to 11.4%)
Idiopathic developmental intellectual disability	-	-	66.3% (34.7% to 85.2%)	66.3% (34.7% to 85.2%)
**Diabetes, urogenital, blood, and endocrine diseases**	85.4% (82.9% to 87.6%)	82.6% (79.4% to 85.3%)	61.0% (54.5% to 66.4%)	73.6% (69.0% to 77.2%)
Diabetes mellitus	100.0% (100.0% to 100.0%)	100.0% (100.0% to 100.0%)	100.0% (100.0% to 100.0%)	100.0% (100.0% to 100.0%)
Chronic kidney disease	100.0% (100.0% to 100.0%)	100.0% (100.0% to 100.0%)	100.0% (100.0% to 100.0%)	100.0% (100.0% to 100.0%)
**Musculoskeletal disorders**	0.6% (0.2% to 1.2%)	0.6% (0.2% to 1.1%)	23.6% (20.4% to 27.3%)	23.4% (20.3% to 27.1%)
Rheumatoid arthritis	1.3% (0.5% to 2.4%)	1.4% (0.5% to 2.4%)	1.5% (0.6% to 2.7%)	1.5% (0.6% to 2.7%)
Osteoarthritis	-	-	17.0% (7.7% to 28.3%)	17.0% (7.7% to 28.3%)
Low back and neck pain	-	-	38.1% (34.7% to 42.2%)	38.1% (34.7% to 42.2%)
Gout	-	-	37.7% (26.8% to 51.4%)	37.7% (26.8% to 51.4%)
**Other non-communicable diseases**	0.3% (0.2% to 0.6%)	0.4% (0.2% to 0.7%)	8.5% (7.4% to 9.8%)	6.4% (5.3% to 7.5%)
Sense organ diseases	-	-	17.0% (15.2% to 19.0%)	17.0% (15.2% to 19.0%)
Sudden infant death syndrome	6.3% (3.0% to 11.3%)	6.3% (3.0% to 11.4%)	-	6.3% (3.0% to 11.4%)
**Transport injuries**	28.3% (23.1% to 34.5%)	23.7% (18.7% to 29.6%)	33.6% (28.6% to 39.9%)	26.2% (21.3% to 32.2%)
Road injuries	28.1% (22.9% to 34.4%)	23.7% (18.7% to 29.6%)	36.1% (30.9% to 42.8%)	26.4% (21.3% to 32.6%)
Other transport injuries	29.8% (24.3% to 36.4%)	23.2% (17.7% to 29.9%)	26.3% (21.7% to 31.9%)	24.7% (20.1% to 30.4%)
**Unintentional injuries**	39.8% (32.3% to 44.9%)	21.0% (17.7% to 24.6%)	22.8% (19.7% to 26.8%)	21.8% (19.0% to 25.0%)
Falls	61.3% (49.9% to 67.0%)	38.3% (31.6% to 43.9%)	31.2% (28.3% to 34.8%)	34.9% (30.5% to 39.0%)
Drowning	13.3% (9.6% to 17.1%)	11.1% (7.7% to 14.6%)	16.7% (12.3% to 22.6%)	11.2% (7.8% to 14.8%)
Fire, heat, and hot substances	9.6% (7.0% to 13.5%)	10.0% (7.3% to 14.2%)	15.0% (11.3% to 19.9%)	11.9% (8.9% to 16.0%)

Supplementary Results Table 1. Age-standardised PAFs attributable to the joint distribution of all risk factors for all ages, both sexes combined for each GBD level 1 and level 2 cause and all causes in 2016 for Bhutan. Results are shown for deaths, YLLs, YLDs, and DALYs

Data in parenthesis are 95% uncertainty intervals. PAFs=population attributable fractions. GBD=Global Burden of Disease. YLLs=years of life lost. YLDs=years live with disability. DALYs=disability-adjusted life-years

	**Deaths 2016**	**YLLs 2016**	**YLDs 2016**	**DALYs 2016**
Poisonings	11.1% (6.8% to 16.8%)	9.3% (4.8% to 15.4%)	16.4% (12.2% to 22.1%)	10.3% (5.7% to 15.8%)
Exposure to mechanical forces	20.5% (17.0% to 24.5%)	15.9% (12.3% to 20.7%)	23.2% (19.2% to 28.5%)	19.0% (15.2% to 23.6%)
Animal contact	10.3% (7.4% to 14.6%)	8.2% (5.3% to 13.2%)	13.9% (10.1% to 19.2%)	10.5% (7.5% to 15.0%)
Foreign body	5.6% (3.9% to 7.4%)	5.9% (4.0% to 8.1%)	10.5% (7.5% to 14.5%)	8.3% (6.0% to 11.5%)
Other unintentional injuries	21.5% (15.5% to 28.1%)	21.7% (15.6% to 29.0%)	17.2% (12.7% to 23.0%)	19.1% (14.2% to 24.9%)
**Self-harm and interpersonal violence**	13.6% (6.7% to 21.7%)	12.6% (6.9% to 19.6%)	19.9% (17.3% to 22.8%)	13.4% (7.8% to 19.8%)
Self-harm	13.6% (5.8% to 22.9%)	12.8% (6.1% to 21.4%)	13.7% (5.3% to 23.8%)	12.9% (6.1% to 21.5%)
Interpersonal violence	13.6% (8.9% to 19.1%)	11.5% (7.2% to 16.4%)	21.1% (19.0% to 23.4%)	14.9% (11.3% to 18.9%)
**Forces of nature, conflict and terrorism, and executions and police conflict**	0.0% (0.0% to 0.0%)	0.0% (0.0% to 0.0%)	5.5% (3.8% to 7.9%)	4.9% (3.2% to 7.1%)
Forces of nature, conflict and terrorism, and state actor violence	0.0% (0.0% to 0.0%)	0.0% (0.0% to 0.0%)	5.6% (3.8% to 8.0%)	5.6% (3.8% to 8.0%)

Supplementary Results Table 1. Age-standardised PAFs attributable to the joint distribution of all risk factors for all ages, both sexes combined for each GBD level 1 and level 2 cause and all causes in 2016 for Bolivia. Results are shown for deaths, YLLs, YLDs, and DALYs
Data in parenthesis are 95% uncertainty intervals. PAFs=population attributable fractions. GBD=Global Burden of Disease. YLLs=years of life lost. YLDs=years live with disability. DALYs=disability-adjusted life-years

	Deaths 2016	YLLs 2016	YLDs 2016	DALYs 2016
**All causes**	53.7% (51.3% to 56.3%)	50.3% (47.9% to 53.3%)	26.2% (24.9% to 27.7%)	42.0% (39.6% to 44.7%)
**HIV/AIDS and tuberculosis**	48.8% (32.2% to 72.9%)	53.3% (35.3% to 78.3%)	63.6% (45.4% to 83.1%)	54.0% (36.8% to 77.9%)
Tuberculosis	36.7% (25.6% to 47.8%)	37.2% (27.1% to 47.7%)	33.0% (22.7% to 43.0%)	37.1% (27.1% to 47.7%)
HIV/AIDS	95.9% (92.3% to 97.6%)	94.8% (89.1% to 97.3%)	95.9% (93.6% to 97.0%)	94.8% (89.4% to 97.1%)
**Diarrhoea, lower respiratory infections, and other common infectious diseases**	51.1% (43.7% to 58.7%)	61.9% (53.9% to 69.1%)	41.8% (29.2% to 52.9%)	59.7% (52.3% to 66.6%)
Diarrhoeal diseases	73.7% (51.3% to 90.6%)	82.5% (66.4% to 94.1%)	72.6% (51.1% to 90.0%)	78.8% (61.0% to 92.2%)
Lower respiratory infections	51.5% (43.1% to 60.1%)	65.9% (57.3% to 73.9%)	52.8% (44.7% to 61.3%)	65.8% (57.3% to 73.8%)
Upper respiratory infections	1.7% (0.2% to 6.2%)	3.5% (0.3% to 12.5%)	0.0% (0.0% to 0.0%)	0.1% (0.0% to 0.2%)
Otitis media	3.8% (0.9% to 8.7%)	6.2% (2.3% to 10.9%)	3.7% (2.3% to 5.1%)	3.7% (2.3% to 5.1%)
Meningitis	2.7% (1.6% to 4.4%)	3.9% (2.3% to 6.3%)	0.0% (0.0% to 0.0%)	3.7% (2.2% to 5.9%)
Encephalitis	1.5% (1.0% to 2.2%)	2.2% (1.4% to 3.3%)	0.0% (0.0% to 0.0%)	2.0% (1.3% to 3.0%)
Measles	16.7% (0.0% to 69.9%)	17.0% (0.0% to 70.9%)	16.3% (0.0% to 68.7%)	25.5% (0.0% to 72.7%)
**Maternal disorders**	10.8% (5.2% to 16.3%)	10.6% (5.0% to 16.2%)	4.4% (2.3% to 7.0%)	10.0% (4.9% to 15.4%)
Maternal haemorrhage	17.9% (6.4% to 29.3%)	17.9% (6.3% to 29.2%)	17.5% (6.2% to 28.6%)	17.9% (6.3% to 29.2%)
Maternal sepsis and other pregnancy related infections	17.9% (6.4% to 29.3%)	17.8% (6.4% to 29.2%)	17.4% (6.2% to 28.3%)	17.8% (6.3% to 29.1%)
Maternal abortion, miscarriage, and ectopic pregnancy	16.6% (10.3% to 23.3%)	16.5% (10.3% to 23.3%)	16.8% (9.7% to 24.3%)	16.6% (10.3% to 23.3%)
**Neonatal disorders**	77.6% (72.5% to 81.8%)	77.6% (72.6% to 81.8%)	0.2% (0.1% to 0.3%)	71.4% (66.0% to 76.2%)
Neonatal preterm birth complications	92.5% (86.1% to 96.6%)	92.6% (86.1% to 96.6%)	0.4% (0.3% to 0.5%)	86.2% (80.2% to 91.0%)
Neonatal encephalopathy due to birth asphyxia and trauma	65.6% (59.0% to 71.0%)	65.7% (59.1% to 71.0%)	0.0% (0.0% to 0.0%)	63.9% (56.7% to 69.8%)
Neonatal sepsis and other neonatal infections	60.4% (53.2% to 66.7%)	60.5% (53.3% to 66.7%)	0.0% (0.0% to 0.0%)	51.1% (37.9% to 60.9%)
Hemolytic disease and other neonatal jaundice	65.1% (59.2% to 70.5%)	65.1% (59.2% to 70.5%)	0.0% (0.0% to 0.0%)	28.9% (19.9% to 39.6%)

Supplementary Results Table 1. Age-standardised PAFs attributable to the joint distribution of all risk factors for all ages, both sexes combined for each GBD level 1 and level 2 cause and all causes in 2016 for Bolivia. Results are shown for deaths, YLLs, YLDs, and DALYs
Data in parenthesis are 95% uncertainty intervals. PAFs=population attributable fractions. GBD=Global Burden of Disease. YLLs=years of life lost. YLDs=years live with disability. DALYs=disability-adjusted life-years

	Deaths 2016	YLLs 2016	YLDs 2016	DALYs 2016
Other neonatal disorders	58.4% (48.6% to 66.4%)	58.4% (48.7% to 66.4%)	0.0% (0.0% to 0.0%)	55.8% (46.3% to 63.6%)
**Nutritional deficiencies**	15.7% (9.8% to 23.1%)	52.1% (39.7% to 64.0%)	99.4% (99.2% to 99.5%)	81.6% (75.5% to 86.5%)
Protein-energy malnutrition	17.5% (11.0% to 25.7%)	57.5% (44.7% to 69.6%)	96.2% (92.6% to 98.2%)	58.4% (46.3% to 70.0%)
Vitamin A deficiency	-	-	100.0% (100.0% to 100.0%)	100.0% (100.0% to 100.0%)
Iron-deficiency anaemia	100.0% (100.0% to 100.0%)	100.0% (100.0% to 100.0%)	100.0% (100.0% to 100.0%)	100.0% (100.0% to 100.0%)
**Other communicable, maternal, neonatal, and nutritional diseases**	4.9% (2.9% to 7.2%)	2.8% (1.4% to 4.5%)	49.1% (42.5% to 55.9%)	12.4% (7.1% to 20.0%)
Sexually transmitted diseases excluding HIV	9.9% (4.2% to 19.7%)	4.0% (1.6% to 8.5%)	100.0% (100.0% to 100.0%)	20.1% (9.7% to 35.2%)
Hepatitis	1.6% (1.1% to 2.3%)	1.6% (1.1% to 2.2%)	1.3% (0.7% to 2.2%)	1.6% (1.1% to 2.2%)
**Neoplasms**	30.5% (26.6% to 34.7%)	30.1% (26.4% to 34.1%)	31.6% (27.6% to 35.7%)	30.1% (26.4% to 34.1%)
Oesophageal cancer	66.1% (53.0% to 77.7%)	67.5% (54.8% to 78.6%)	66.4% (53.2% to 78.1%)	67.5% (54.8% to 78.6%)
Stomach cancer	10.2% (3.8% to 18.9%)	9.4% (3.5% to 17.5%)	9.9% (3.7% to 18.4%)	9.4% (3.5% to 17.5%)
Liver cancer	38.7% (29.3% to 49.4%)	38.6% (29.3% to 49.3%)	38.7% (29.4% to 49.5%)	38.6% (29.3% to 49.3%)
Larynx cancer	69.4% (57.4% to 79.3%)	68.9% (56.0% to 79.2%)	69.1% (56.8% to 79.0%)	68.9% (55.9% to 79.2%)
Tracheal, bronchus, and lung cancer	77.0% (70.2% to 82.9%)	75.2% (68.0% to 81.6%)	76.1% (68.8% to 82.3%)	75.2% (68.0% to 81.6%)
Breast cancer	26.0% (18.7% to 34.0%)	24.5% (17.5% to 32.2%)	24.8% (17.9% to 32.9%)	24.5% (17.5% to 32.2%)
Cervical cancer	100.0% (100.0% to 100.0%)	100.0% (100.0% to 100.0%)	100.0% (100.0% to 100.0%)	100.0% (100.0% to 100.0%)
Uterine cancer	48.5% (33.2% to 63.1%)	50.0% (34.6% to 64.3%)	50.1% (34.7% to 64.1%)	50.0% (34.7% to 64.3%)
Prostate cancer	1.9% (1.1% to 2.8%)	1.7% (1.0% to 2.6%)	1.8% (1.0% to 2.7%)	1.7% (1.0% to 2.6%)
Colon and rectum cancer	53.5% (44.3% to 62.2%)	53.9% (45.2% to 62.3%)	53.3% (44.4% to 61.9%)	53.9% (45.2% to 62.2%)
Lip and oral cavity cancer	54.7% (42.3% to 66.3%)	56.2% (44.2% to 67.6%)	53.7% (41.1% to 65.7%)	56.1% (44.1% to 67.5%)
Nasopharynx cancer	56.8% (46.9% to 66.5%)	54.6% (45.3% to 63.8%)	53.6% (44.6% to 62.2%)	54.6% (45.3% to 63.8%)

**Supplementary Results Table 1. Age-standardised PAFs attributable to the joint distribution of all risk factors for all ages, both sexes combined for each GBD level 1 and level 2 cause and all causes in 2016 for Bolivia. Results are shown for deaths, YLLs, YLDs, and DALYs**<br>Data in parenthesis are 95% uncertainty intervals. PAFs=population attributable fractions. GBD=Global Burden of Disease. YLLs=years of life lost. YLDs=years live with disability. DALYs=disability-adjusted life-years				
	**Deaths 2016**	**YLLs 2016**	**YLDs 2016**	**DALYs 2016**
Other pharynx cancer	34.0% (21.1% to 48.1%)	37.9% (24.5% to 52.4%)	35.6% (22.2% to 49.7%)	37.9% (24.5% to 52.3%)
Gallbladder and biliary tract cancer	25.5% (14.1% to 38.1%)	26.7% (14.5% to 39.7%)	25.7% (14.0% to 38.3%)	26.6% (14.5% to 39.7%)
Pancreatic cancer	21.9% (14.0% to 31.2%)	21.3% (13.4% to 30.8%)	21.6% (13.9% to 31.1%)	21.3% (13.5% to 30.8%)
Ovarian cancer	13.8% (6.6% to 22.5%)	11.9% (5.1% to 20.1%)	11.6% (5.0% to 19.6%)	11.9% (5.1% to 20.0%)
Kidney cancer	27.5% (18.0% to 37.8%)	26.0% (16.8% to 35.8%)	27.1% (17.7% to 37.2%)	26.0% (16.8% to 35.9%)
Bladder cancer	20.0% (12.2% to 29.0%)	18.6% (11.1% to 27.4%)	18.9% (11.4% to 27.7%)	18.6% (11.1% to 27.4%)
Thyroid cancer	14.6% (7.7% to 23.2%)	15.1% (8.0% to 23.9%)	14.8% (8.0% to 22.9%)	15.1% (8.0% to 23.9%)
Mesothelioma	89.2% (85.1% to 92.3%)	80.2% (73.9% to 85.4%)	86.2% (81.3% to 90.1%)	80.3% (74.0% to 85.5%)
Non-Hodgkin's lymphoma	6.9% (2.7% to 12.3%)	6.5% (2.5% to 11.3%)	6.9% (2.7% to 12.2%)	6.5% (2.5% to 11.3%)
Multiple myeloma	8.8% (3.5% to 15.7%)	9.1% (3.7% to 16.1%)	8.9% (3.6% to 16.0%)	9.1% (3.7% to 16.1%)
Leukaemia	11.7% (7.2% to 17.1%)	8.6% (5.4% to 12.5%)	11.3% (7.0% to 16.3%)	8.6% (5.4% to 12.6%)
**Cardiovascular diseases**	82.0% (78.7% to 84.8%)	80.3% (77.7% to 82.7%)	63.7% (59.6% to 67.3%)	78.9% (76.3% to 81.3%)
Rheumatic heart disease	19.6% (11.9% to 34.4%)	15.4% (10.0% to 25.1%)	11.6% (6.9% to 17.5%)	13.9% (9.1% to 21.7%)
Ischaemic heart disease	89.7% (85.4% to 93.3%)	90.9% (87.9% to 93.5%)	87.9% (84.3% to 91.1%)	90.8% (87.8% to 93.5%)
Cerebrovascular disease	77.6% (72.3% to 82.5%)	78.0% (74.2% to 81.7%)	79.5% (74.3% to 84.2%)	78.1% (74.3% to 81.7%)
Hypertensive heart disease	100.0% (100.0% to 100.0%)	100.0% (100.0% to 100.0%)	100.0% (100.0% to 100.0%)	100.0% (100.0% to 100.0%)
Cardiomyopathy and myocarditis	25.8% (19.9% to 32.5%)	17.3% (12.5% to 22.4%)	20.3% (15.0% to 26.3%)	17.4% (12.7% to 22.4%)
Atrial fibrillation and flutter	43.3% (35.7% to 51.0%)	46.5% (39.6% to 53.2%)	51.1% (45.3% to 57.4%)	49.2% (43.2% to 55.7%)
Aortic aneurysm	32.7% (26.6% to 39.2%)	35.3% (29.6% to 40.8%)	-	35.3% (29.6% to 40.8%)
Peripheral vascular disease	43.9% (37.5% to 51.3%)	49.2% (43.8% to 54.6%)	52.3% (47.5% to 57.4%)	51.9% (47.2% to 57.1%)
Endocarditis	26.5% (19.1% to 36.1%)	16.5% (11.3% to 22.7%)	27.3% (19.9% to 36.5%)	16.6% (11.5% to 22.8%)

Supplementary Results Table 1. Age-standardised PAFs attributable to the joint distribution of all risk factors for all ages, both sexes combined for each GBD level 1 and level 2 cause and all causes in 2016 for Bolivia. Results are shown for deaths, YLLs, YLDs, and DALYs
Data in parenthesis are 95% uncertainty intervals. PAFs=population attributable fractions. GBD=Global Burden of Disease. YLLs=years of life lost. YLDs=years live with disability. DALYs=disability-adjusted life-years

	Deaths 2016	YLLs 2016	YLDs 2016	DALYs 2016
Other cardiovascular and circulatory diseases	32.5% (27.8% to 37.4%)	26.1% (21.6% to 30.8%)	29.6% (23.9% to 35.3%)	26.6% (22.5% to 31.1%)
**Chronic respiratory diseases**	57.5% (50.4% to 63.7%)	51.1% (44.0% to 57.8%)	34.7% (26.9% to 43.8%)	45.9% (39.2% to 52.3%)
Chronic obstructive pulmonary disease	72.6% (64.7% to 79.5%)	68.4% (60.2% to 75.8%)	60.5% (51.1% to 68.8%)	67.4% (59.1% to 74.8%)
Pneumoconiosis	100.0% (100.0% to 100.0%)	100.0% (100.0% to 100.0%)	100.0% (100.0% to 100.0%)	100.0% (100.0% to 100.0%)
Asthma	37.3% (27.1% to 48.4%)	35.7% (25.1% to 46.6%)	29.2% (19.8% to 40.9%)	30.3% (21.0% to 41.5%)
Other chronic respiratory diseases	6.6% (4.0% to 10.0%)	3.6% (1.7% to 6.3%)	2.1% (0.8% to 3.9%)	3.4% (1.6% to 5.8%)
**Cirrhosis and other chronic liver diseases**	46.7% (43.4% to 50.2%)	47.6% (44.3% to 51.2%)	38.9% (35.4% to 42.6%)	47.4% (44.1% to 51.0%)
Cirrhosis and other chronic liver diseases due to hepatitis B	0.5% (0.2% to 0.8%)	0.6% (0.2% to 1.0%)	0.5% (0.2% to 0.8%)	0.6% (0.2% to 1.0%)
Cirrhosis and other chronic liver diseases due to hepatitis C	39.8% (29.2% to 51.2%)	51.6% (40.4% to 62.5%)	43.5% (33.8% to 52.7%)	51.4% (40.4% to 62.2%)
Cirrhosis and other chronic liver diseases due to alcohol use	100.0% (100.0% to 100.0%)	100.0% (100.0% to 100.0%)	100.0% (100.0% to 100.0%)	100.0% (100.0% to 100.0%)
**Digestive diseases**	10.0% (6.9% to 13.5%)	9.5% (6.5% to 13.0%)	3.4% (2.5% to 4.3%)	8.3% (5.7% to 11.3%)
Peptic ulcer disease	6.4% (4.2% to 9.2%)	8.1% (5.3% to 11.4%)	7.0% (4.7% to 9.6%)	7.9% (5.2% to 11.0%)
Gallbladder and biliary diseases	44.2% (28.9% to 59.0%)	45.3% (29.9% to 59.7%)	47.4% (32.4% to 62.0%)	45.4% (30.1% to 60.0%)
Pancreatitis	17.1% (4.7% to 32.0%)	19.9% (6.5% to 35.5%)	16.4% (4.1% to 30.4%)	19.9% (6.4% to 35.4%)
**Neurological disorders**	20.4% (10.2% to 32.5%)	19.2% (10.5% to 28.8%)	3.5% (1.9% to 5.5%)	8.6% (4.8% to 13.3%)
Alzheimer's disease and other dementias	23.2% (11.3% to 37.6%)	24.3% (12.0% to 39.1%)	24.4% (12.1% to 39.0%)	24.3% (12.0% to 39.0%)
Parkinson's disease	-4.0% (-5.0% to -3.0%)	-5.0% (-6.3% to -3.9%)	-5.6% (-6.9% to -4.4%)	-5.1% (-6.4% to -4.0%)
Epilepsy	18.6% (10.3% to 26.5%)	16.7% (9.2% to 23.7%)	15.8% (8.6% to 23.0%)	16.3% (8.9% to 23.2%)
Multiple sclerosis	5.0% (3.0% to 7.4%)	5.4% (3.3% to 8.0%)	4.4% (2.7% to 6.3%)	5.1% (3.1% to 7.4%)
**Mental and substance use disorders**	99.7% (99.6% to 99.8%)	99.5% (99.3% to 99.7%)	21.8% (19.7% to 24.2%)	26.5% (23.7% to 30.4%)
Alcohol use disorders	100.0% (100.0% to 100.0%)	100.0% (100.0% to 100.0%)	100.0% (100.0% to 100.0%)	100.0% (100.0% to 100.0%)

Supplementary Results Table 1. Age-standardised PAFs attributable to the joint distribution of all risk factors for all ages, both sexes combined for each GBD level 1 and level 2 cause and all causes in 2016 for Bolivia. Results are shown for deaths, YLLs, YLDs, and DALYs
Data in parenthesis are 95% uncertainty intervals. PAFs=population attributable fractions. GBD=Global Burden of Disease. YLLs=years of life lost. YLDs=years live with disability. DALYs=disability-adjusted life-years

	**Deaths 2016**	**YLLs 2016**	**YLDs 2016**	**DALYs 2016**
Drug use disorders	100.0% (100.0% to 100.0%)	100.0% (100.0% to 100.0%)	100.0% (100.0% to 100.0%)	100.0% (100.0% to 100.0%)
Depressive disorders	-	-	11.6% (9.8% to 13.5%)	11.6% (9.8% to 13.5%)
Idiopathic developmental intellectual disability	-	-	69.8% (39.4% to 88.6%)	69.8% (39.4% to 88.6%)
**Diabetes, urogenital, blood, and endocrine diseases**	90.7% (88.7% to 92.0%)	88.7% (85.9% to 90.4%)	67.5% (62.9% to 71.6%)	81.7% (78.5% to 84.4%)
Diabetes mellitus	100.0% (100.0% to 100.0%)	100.0% (100.0% to 100.0%)	100.0% (100.0% to 100.0%)	100.0% (100.0% to 100.0%)
Chronic kidney disease	100.0% (100.0% to 100.0%)	100.0% (100.0% to 100.0%)	100.0% (100.0% to 100.0%)	100.0% (100.0% to 100.0%)
**Musculoskeletal disorders**	0.9% (0.4% to 1.7%)	0.8% (0.3% to 1.4%)	26.0% (22.3% to 30.0%)	25.5% (21.8% to 29.5%)
Rheumatoid arthritis	2.0% (0.8% to 3.5%)	2.4% (0.9% to 4.1%)	2.8% (1.1% to 4.8%)	2.7% (1.0% to 4.7%)
Osteoarthritis	-	-	28.3% (14.9% to 41.5%)	28.3% (14.9% to 41.5%)
Low back and neck pain	-	-	32.6% (28.7% to 36.7%)	32.6% (28.7% to 36.7%)
Gout	-	-	48.3% (32.8% to 64.4%)	48.3% (32.8% to 64.4%)
**Other non-communicable diseases**	0.1% (0.1% to 0.2%)	0.1% (0.1% to 0.3%)	6.3% (5.3% to 7.5%)	4.7% (3.9% to 5.6%)
Sense organ diseases	-	-	13.4% (11.6% to 15.3%)	13.4% (11.6% to 15.3%)
Sudden infant death syndrome	6.5% (3.2% to 11.4%)	6.5% (3.2% to 11.4%)	-	6.5% (3.2% to 11.4%)
**Transport injuries**	27.6% (21.7% to 34.1%)	22.4% (16.9% to 28.7%)	33.4% (27.0% to 40.4%)	23.4% (17.7% to 29.7%)
Road injuries	27.4% (21.6% to 33.9%)	22.3% (16.7% to 28.5%)	33.6% (27.2% to 40.7%)	23.3% (17.7% to 29.6%)
Other transport injuries	30.2% (23.8% to 37.2%)	24.7% (18.7% to 32.0%)	27.2% (20.9% to 33.8%)	24.9% (18.9% to 32.1%)
**Unintentional injuries**	18.7% (15.4% to 22.2%)	9.8% (7.5% to 12.4%)	18.0% (15.6% to 21.4%)	12.1% (9.7% to 14.9%)
Falls	48.6% (43.3% to 54.4%)	28.2% (24.0% to 33.1%)	25.4% (23.0% to 28.7%)	26.6% (23.8% to 30.3%)
Drowning	11.1% (7.3% to 15.8%)	9.3% (6.1% to 13.3%)	13.6% (8.8% to 19.3%)	9.3% (6.1% to 13.4%)
Fire, heat, and hot substances	8.8% (5.5% to 13.2%)	8.2% (5.4% to 11.9%)	14.8% (10.0% to 20.4%)	10.0% (6.6% to 14.1%)

Supplementary Results Table 1. Age-standardised PAFs attributable to the joint distribution of all risk factors for all ages, both sexes combined for each GBD level 1 and level 2 cause and all causes in 2016 for Bolivia. Results are shown for deaths, YLLs, YLDs, and DALYs
Data in parenthesis are 95% uncertainty intervals. PAFs=population attributable fractions. GBD=Global Burden of Disease. YLLs=years of life lost. YLDs=years live with disability. DALYs=disability-adjusted life-years

	**Deaths 2016**	**YLLs 2016**	**YLDs 2016**	**DALYs 2016**
Poisonings	11.8% (7.4% to 17.5%)	10.2% (5.7% to 15.3%)	13.7% (9.3% to 19.2%)	10.4% (5.9% to 15.5%)
Exposure to mechanical forces	13.7% (10.6% to 17.3%)	10.6% (7.5% to 14.6%)	16.8% (13.7% to 21.6%)	12.4% (9.2% to 16.4%)
Animal contact	7.9% (6.0% to 10.3%)	6.8% (4.8% to 9.4%)	9.0% (6.3% to 13.3%)	7.7% (5.5% to 10.6%)
Foreign body	3.8% (2.4% to 5.6%)	2.8% (1.7% to 4.4%)	6.6% (4.5% to 9.8%)	3.4% (2.1% to 5.1%)
Other unintentional injuries	16.0% (10.8% to 22.1%)	16.4% (11.3% to 22.4%)	13.3% (8.7% to 18.9%)	14.9% (10.2% to 20.5%)
**Self-harm and interpersonal violence**	21.6% (14.3% to 28.8%)	21.2% (14.4% to 28.1%)	32.9% (28.3% to 37.6%)	21.9% (15.4% to 28.5%)
Self-harm	20.4% (9.2% to 31.3%)	21.1% (9.9% to 31.4%)	19.8% (8.5% to 30.3%)	21.0% (9.9% to 31.4%)
Interpersonal violence	23.0% (15.6% to 30.3%)	21.4% (14.2% to 28.5%)	33.8% (29.2% to 38.7%)	22.7% (15.7% to 29.4%)
**Forces of nature, conflict and terrorism, and executions and police conflict**	0.0% (0.0% to 0.0%)	0.0% (0.0% to 0.0%)	4.9% (3.3% to 7.1%)	3.2% (1.1% to 5.4%)
Forces of nature, conflict and terrorism, and state actor violence	0.0% (0.0% to 0.0%)	0.0% (0.0% to 0.0%)	5.3% (3.5% to 7.6%)	5.3% (3.5% to 7.6%)

Supplementary Results Table 1. Age-standardised PAFs attributable to the joint distribution of all risk factors for all ages, both sexes combined for each GBD level 1 and level 2 cause and all causes in 2016 for Bosnia and Herzegovina. Results are shown for deaths, YLLs, YLDs, and DALYs
Data in parenthesis are 95% uncertainty intervals. PAFs=population attributable fractions. GBD=Global Burden of Disease. YLLs=years of life lost. YLDs=years live with disability. DALYs=disability-adjusted life-years

	Deaths 2016	YLLs 2016	YLDs 2016	DALYs 2016
**All causes**	70.1% (68.1% to 71.9%)	66.0% (64.5% to 67.6%)	26.2% (24.6% to 27.7%)	47.7% (44.4% to 51.2%)
**HIV/AIDS and tuberculosis**	58.5% (45.0% to 72.8%)	63.4% (50.4% to 76.6%)	53.5% (40.4% to 67.2%)	62.6% (49.7% to 75.8%)
Tuberculosis	57.3% (43.3% to 72.1%)	61.4% (47.5% to 75.5%)	51.0% (37.6% to 65.5%)	60.5% (46.8% to 74.7%)
HIV/AIDS	94.5% (92.9% to 95.8%)	93.2% (91.0% to 95.0%)	94.4% (92.7% to 95.6%)	93.3% (91.2% to 95.1%)
**Diarrhoea, lower respiratory infections, and other common infectious diseases**	64.1% (57.7% to 70.4%)	59.4% (50.0% to 66.8%)	30.7% (23.5% to 38.3%)	46.1% (39.7% to 52.4%)
Diarrhoeal diseases	78.0% (61.3% to 91.0%)	88.3% (79.3% to 95.2%)	69.3% (51.5% to 85.1%)	73.1% (57.6% to 87.3%)
Lower respiratory infections	72.8% (66.3% to 78.6%)	76.3% (70.9% to 81.5%)	72.5% (65.7% to 78.4%)	76.1% (70.7% to 81.3%)
Upper respiratory infections	0.6% (0.0% to 2.0%)	1.8% (0.1% to 5.8%)	0.0% (0.0% to 0.0%)	0.0% (0.0% to 0.0%)
Otitis media	0.7% (0.1% to 2.1%)	3.3% (1.0% to 8.6%)	11.0% (7.5% to 14.1%)	11.0% (7.5% to 14.1%)
Meningitis	6.5% (3.4% to 10.6%)	9.8% (5.5% to 15.3%)	0.0% (0.0% to 0.0%)	6.8% (3.8% to 10.7%)
Encephalitis	0.6% (0.3% to 1.0%)	1.3% (0.8% to 2.0%)	0.0% (0.0% to 0.0%)	0.8% (0.4% to 1.2%)
Measles	40.8% (21.8% to 74.4%)	41.6% (22.3% to 75.8%)	41.2% (21.6% to 76.0%)	41.6% (22.1% to 75.7%)
**Maternal disorders**	4.3% (2.3% to 6.6%)	4.3% (2.2% to 6.5%)	5.5% (2.4% to 9.2%)	5.0% (2.5% to 8.0%)
Maternal haemorrhage	13.3% (4.8% to 22.2%)	13.3% (4.8% to 22.2%)	13.4% (4.7% to 22.7%)	13.3% (4.7% to 22.5%)
Maternal sepsis and other pregnancy related infections	13.1% (4.7% to 22.1%)	13.1% (4.7% to 21.9%)	13.2% (4.7% to 22.0%)	13.2% (4.7% to 22.0%)
Maternal abortion, miscarriage, and ectopic pregnancy	6.7% (3.7% to 10.5%)	6.5% (3.6% to 10.3%)	7.1% (3.1% to 12.5%)	6.8% (3.4% to 11.0%)
**Neonatal disorders**	79.0% (74.8% to 82.9%)	79.0% (74.9% to 82.9%)	0.1% (0.0% to 0.1%)	51.9% (43.8% to 60.2%)
Neonatal preterm birth complications	93.6% (89.2% to 96.7%)	93.6% (89.3% to 96.7%)	0.2% (0.1% to 0.2%)	72.7% (64.0% to 80.7%)
Neonatal encephalopathy due to birth asphyxia and trauma	59.7% (53.4% to 65.3%)	59.7% (53.5% to 65.3%)	0.0% (0.0% to 0.0%)	25.5% (16.2% to 36.2%)
Neonatal sepsis and other neonatal infections	55.4% (48.7% to 61.6%)	55.4% (48.8% to 61.6%)	0.0% (0.0% to 0.0%)	26.6% (13.6% to 39.1%)
Hemolytic disease and other neonatal jaundice	53.3% (47.0% to 59.5%)	53.4% (47.2% to 59.7%)	0.0% (0.0% to 0.0%)	4.2% (2.5% to 6.6%)

Supplementary Results Table 1. Age-standardised PAFs attributable to the joint distribution of all risk factors for all ages, both sexes combined for each GBD level 1 and level 2 cause and all causes in 2016 for Bosnia and Herzegovina. Results are shown for deaths, YLLs, YLDs, and DALYs
Data in parenthesis are 95% uncertainty intervals. PAFs=population attributable fractions. GBD=Global Burden of Disease. YLLs=years of life lost. YLDs=years live with disability. DALYs=disability-adjusted life-years

	Deaths 2016	YLLs 2016	YLDs 2016	DALYs 2016
Other neonatal disorders	50.8% (45.0% to 56.6%)	50.8% (45.0% to 56.6%)	0.0% (0.0% to 0.0%)	46.2% (40.0% to 51.9%)
**Nutritional deficiencies**	7.4% (6.3% to 9.8%)	16.0% (14.1% to 18.6%)	84.7% (81.0% to 87.6%)	84.2% (80.4% to 87.1%)
Protein-energy malnutrition	1.1% (0.7% to 1.7%)	5.0% (3.1% to 7.4%)	96.7% (94.6% to 98.1%)	93.5% (90.3% to 96.0%)
Vitamin A deficiency	-	-	0.0% (0.0% to 0.0%)	0.0% (0.0% to 0.0%)
Iron-deficiency anaemia	100.0% (100.0% to 100.0%)	100.0% (100.0% to 100.0%)	100.0% (100.0% to 100.0%)	100.0% (100.0% to 100.0%)
**Other communicable, maternal, neonatal, and nutritional diseases**	3.3% (2.7% to 4.9%)	3.9% (3.0% to 4.8%)	67.1% (60.4% to 73.4%)	46.9% (38.3% to 55.9%)
Sexually transmitted diseases excluding HIV	81.2% (59.1% to 93.9%)	69.1% (41.4% to 88.3%)	100.0% (100.0% to 100.0%)	99.0% (97.2% to 99.8%)
Hepatitis	3.7% (2.7% to 4.9%)	3.2% (2.4% to 4.3%)	1.3% (0.8% to 2.1%)	3.1% (2.3% to 4.1%)
**Neoplasms**	49.8% (45.9% to 53.6%)	49.5% (45.5% to 53.2%)	42.0% (38.1% to 46.2%)	49.3% (45.3% to 53.0%)
Oesophageal cancer	85.6% (78.9% to 91.4%)	88.0% (82.1% to 92.8%)	86.2% (79.6% to 91.7%)	88.0% (82.1% to 92.8%)
Stomach cancer	29.5% (18.7% to 40.2%)	30.8% (20.1% to 41.3%)	29.8% (19.3% to 40.1%)	30.7% (20.1% to 41.3%)
Liver cancer	70.4% (62.3% to 77.6%)	71.3% (63.3% to 78.3%)	70.6% (62.5% to 77.6%)	71.3% (63.3% to 78.3%)
Larynx cancer	92.4% (87.4% to 95.8%)	93.2% (88.6% to 96.2%)	92.8% (88.0% to 95.9%)	93.2% (88.6% to 96.2%)
Tracheal, bronchus, and lung cancer	92.9% (91.0% to 94.6%)	93.2% (91.3% to 94.8%)	92.8% (90.8% to 94.5%)	93.2% (91.3% to 94.8%)
Breast cancer	24.2% (13.1% to 37.7%)	22.7% (11.3% to 37.2%)	23.0% (11.7% to 37.1%)	22.7% (11.3% to 37.2%)
Cervical cancer	100.0% (100.0% to 100.0%)	100.0% (100.0% to 100.0%)	100.0% (100.0% to 100.0%)	100.0% (100.0% to 100.0%)
Uterine cancer	45.8% (31.5% to 59.6%)	45.5% (31.0% to 59.8%)	45.8% (31.0% to 60.0%)	45.5% (31.0% to 59.8%)
Prostate cancer	8.6% (5.9% to 11.6%)	10.0% (7.1% to 13.5%)	10.0% (7.1% to 13.4%)	10.0% (7.1% to 13.5%)
Colon and rectum cancer	50.2% (39.3% to 60.1%)	51.5% (40.7% to 61.4%)	51.0% (40.0% to 61.2%)	51.4% (40.7% to 61.3%)
Lip and oral cavity cancer	86.8% (82.4% to 90.7%)	89.7% (85.7% to 92.8%)	85.9% (81.4% to 89.9%)	89.6% (85.7% to 92.7%)
Nasopharynx cancer	81.7% (77.6% to 85.5%)	81.3% (77.5% to 84.8%)	81.2% (77.4% to 84.9%)	81.3% (77.5% to 84.8%)

Supplementary Results Table 1. Age-standardised PAFs attributable to the joint distribution of all risk factors for all ages, both sexes combined for each GBD level 1 and level 2 cause and all causes in 2016 for Bosnia and Herzegovina. Results are shown for deaths, YLLs, YLDs, and DALYs
Data in parenthesis are 95% uncertainty intervals. PAFs=population attributable fractions. GBD=Global Burden of Disease. YLLs=years of life lost. YLDs=years live with disability. DALYs=disability-adjusted life-years

	Deaths 2016	YLLs 2016	YLDs 2016	DALYs 2016
Other pharynx cancer	66.3% (54.6% to 75.9%)	67.6% (56.4% to 77.1%)	66.3% (54.8% to 76.1%)	67.6% (56.4% to 77.0%)
Gallbladder and biliary tract cancer	21.7% (11.5% to 34.0%)	22.2% (11.5% to 34.5%)	22.0% (11.4% to 34.0%)	22.2% (11.5% to 34.5%)
Pancreatic cancer	38.4% (30.1% to 47.5%)	41.5% (33.1% to 50.3%)	39.3% (31.2% to 47.8%)	41.5% (33.1% to 50.3%)
Ovarian cancer	9.9% (3.2% to 18.5%)	9.0% (2.8% to 16.7%)	8.7% (2.8% to 16.3%)	9.0% (2.8% to 16.7%)
Kidney cancer	43.9% (33.0% to 54.9%)	44.5% (33.6% to 55.3%)	43.6% (33.2% to 54.1%)	44.5% (33.6% to 55.3%)
Bladder cancer	44.6% (34.6% to 54.6%)	49.0% (38.4% to 58.8%)	46.8% (36.9% to 56.0%)	49.0% (38.3% to 58.6%)
Thyroid cancer	13.5% (6.9% to 22.1%)	13.8% (6.9% to 22.8%)	12.8% (6.6% to 20.4%)	13.7% (6.9% to 22.6%)
Mesothelioma	80.0% (74.7% to 85.9%)	67.3% (59.5% to 78.3%)	76.8% (70.9% to 83.8%)	67.4% (59.7% to 78.4%)
Non-Hodgkin's lymphoma	6.2% (2.4% to 11.0%)	5.5% (2.2% to 9.9%)	6.0% (2.4% to 11.0%)	5.5% (2.2% to 10.0%)
Multiple myeloma	7.8% (3.3% to 13.9%)	8.0% (3.3% to 14.2%)	8.0% (3.4% to 14.1%)	8.0% (3.3% to 14.2%)
Leukaemia	24.4% (14.9% to 33.7%)	20.1% (12.4% to 28.0%)	23.9% (14.6% to 32.9%)	20.2% (12.4% to 28.1%)
**Cardiovascular diseases**	87.2% (84.7% to 89.4%)	89.1% (87.4% to 90.7%)	82.4% (80.0% to 84.6%)	88.2% (86.5% to 89.7%)
Rheumatic heart disease	33.2% (21.5% to 51.7%)	32.4% (22.5% to 46.6%)	33.7% (23.0% to 48.6%)	32.4% (22.5% to 46.7%)
Ischaemic heart disease	94.5% (91.6% to 96.8%)	95.9% (94.2% to 97.4%)	93.2% (91.1% to 95.0%)	95.8% (94.0% to 97.2%)
Cerebrovascular disease	87.3% (82.7% to 91.5%)	89.8% (86.7% to 92.5%)	88.9% (85.3% to 91.9%)	89.7% (86.5% to 92.4%)
Hypertensive heart disease	100.0% (100.0% to 100.0%)	100.0% (100.0% to 100.0%)	100.0% (100.0% to 100.0%)	100.0% (100.0% to 100.0%)
Cardiomyopathy and myocarditis	43.2% (35.2% to 54.3%)	48.7% (41.5% to 57.0%)	46.9% (41.0% to 53.0%)	48.6% (41.6% to 56.7%)
Atrial fibrillation and flutter	60.0% (51.9% to 67.5%)	64.9% (57.7% to 71.4%)	72.9% (67.8% to 77.5%)	69.2% (63.3% to 74.4%)
Aortic aneurysm	55.4% (48.2% to 62.0%)	61.7% (55.8% to 67.2%)	-	61.7% (55.8% to 67.2%)
Peripheral vascular disease	54.2% (47.0% to 61.9%)	62.9% (57.5% to 68.4%)	63.7% (57.8% to 69.3%)	63.5% (58.0% to 68.8%)
Endocarditis	47.4% (37.9% to 57.3%)	45.4% (37.0% to 53.5%)	45.2% (36.7% to 53.7%)	45.4% (37.1% to 53.4%)

**Supplementary Results Table 1. Age-standardised PAFs attributable to the joint distribution of all risk factors for all ages, both sexes combined for each GBD level 1 and level 2 cause and all causes in 2016 for Bosnia and Herzegovina. Results are shown for deaths, YLLs, YLDs, and DALYs** Data in parenthesis are 95% uncertainty intervals. PAFs=population attributable fractions. GBD=Global Burden of Disease. YLLs=years of life lost. YLDs=years live with disability. DALYs=disability-adjusted life-years				
	**Deaths 2016**	**YLLs 2016**	**YLDs 2016**	**DALYs 2016**
Other cardiovascular and circulatory diseases	57.0% (51.9% to 62.0%)	63.0% (58.5% to 67.0%)	62.7% (58.1% to 66.9%)	62.9% (58.6% to 66.8%)
**Chronic respiratory diseases**	78.4% (73.1% to 82.7%)	79.7% (75.6% to 83.1%)	49.2% (42.6% to 55.9%)	66.9% (61.4% to 72.0%)
Chronic obstructive pulmonary disease	83.9% (78.5% to 88.5%)	86.2% (82.0% to 89.9%)	82.1% (77.8% to 86.1%)	85.4% (81.2% to 89.1%)
Pneumoconiosis	100.0% (100.0% to 100.0%)	100.0% (100.0% to 100.0%)	100.0% (100.0% to 100.0%)	100.0% (100.0% to 100.0%)
Asthma	42.5% (32.5% to 53.2%)	45.2% (35.7% to 55.2%)	34.6% (26.8% to 43.5%)	35.8% (27.9% to 44.7%)
Other chronic respiratory diseases	19.1% (12.7% to 26.2%)	17.4% (10.0% to 25.8%)	11.0% (7.8% to 14.7%)	12.9% (8.9% to 17.7%)
**Cirrhosis and other chronic liver diseases**	62.1% (57.1% to 66.8%)	62.1% (56.9% to 66.6%)	47.1% (41.7% to 51.8%)	61.0% (55.9% to 65.7%)
Cirrhosis and other chronic liver diseases due to hepatitis B	1.0% (0.4% to 1.7%)	1.2% (0.5% to 2.1%)	1.1% (0.5% to 1.9%)	1.2% (0.5% to 2.0%)
Cirrhosis and other chronic liver diseases due to hepatitis C	86.0% (81.1% to 89.1%)	88.1% (85.2% to 89.8%)	87.1% (83.7% to 89.1%)	88.1% (85.2% to 89.7%)
Cirrhosis and other chronic liver diseases due to alcohol use	100.0% (100.0% to 100.0%)	100.0% (100.0% to 100.0%)	100.0% (100.0% to 100.0%)	100.0% (100.0% to 100.0%)
**Digestive diseases**	18.9% (13.9% to 24.1%)	21.1% (16.6% to 26.0%)	6.5% (4.8% to 8.6%)	14.5% (11.6% to 18.0%)
Peptic ulcer disease	20.9% (14.9% to 27.7%)	24.6% (18.0% to 31.8%)	24.2% (17.7% to 31.4%)	24.4% (18.0% to 31.5%)
Gallbladder and biliary diseases	40.8% (27.2% to 54.9%)	41.7% (28.0% to 55.5%)	44.2% (30.7% to 59.0%)	42.3% (28.6% to 56.3%)
Pancreatitis	65.7% (47.9% to 83.3%)	69.3% (51.7% to 85.7%)	62.8% (45.9% to 78.9%)	69.1% (51.4% to 85.3%)
**Neurological disorders**	19.3% (10.0% to 31.2%)	18.5% (11.2% to 27.7%)	3.5% (1.8% to 5.8%)	8.1% (4.7% to 12.0%)
Alzheimer's disease and other dementias	24.7% (13.2% to 38.9%)	26.5% (14.9% to 40.7%)	26.5% (14.8% to 40.6%)	26.5% (14.9% to 40.7%)
Parkinson's disease	-17.1% (-21.1% to -12.9%)	-20.9% (-26.1% to -15.9%)	-23.4% (-28.5% to -17.9%)	-21.4% (-26.7% to -16.3%)
Epilepsy	26.6% (14.2% to 39.3%)	24.1% (13.2% to 35.5%)	20.5% (10.7% to 31.1%)	22.2% (11.9% to 33.3%)
Multiple sclerosis	17.0% (11.3% to 22.9%)	17.7% (11.8% to 23.6%)	15.1% (10.0% to 20.4%)	16.7% (11.3% to 22.5%)
**Mental and substance use disorders**	100.0% (100.0% to 100.0%)	100.0% (100.0% to 100.0%)	23.8% (21.1% to 26.7%)	28.5% (25.4% to 32.5%)
Alcohol use disorders	100.0% (100.0% to 100.0%)	100.0% (100.0% to 100.0%)	100.0% (100.0% to 100.0%)	100.0% (100.0% to 100.0%)

Supplementary Results Table 1. Age-standardised PAFs attributable to the joint distribution of all risk factors for all ages, both sexes combined for each GBD level 1 and level 2 cause and all causes in 2016 for Bosnia and Herzegovina. Results are shown for deaths, YLLs, YLDs, and DALYs
Data in parenthesis are 95% uncertainty intervals. PAFs=population attributable fractions. GBD=Global Burden of Disease. YLLs=years of life lost. YLDs=years live with disability. DALYs=disability-adjusted life-years

	Deaths 2016	YLLs 2016	YLDs 2016	DALYs 2016
Drug use disorders	100.0% (100.0% to 100.0%)	100.0% (100.0% to 100.0%)	100.0% (100.0% to 100.0%)	100.0% (100.0% to 100.0%)
Depressive disorders	-	-	5.9% (4.8% to 7.2%)	5.9% (4.8% to 7.2%)
Idiopathic developmental intellectual disability	-	-	61.4% (31.1% to 81.3%)	61.4% (31.1% to 81.3%)
**Diabetes, urogenital, blood, and endocrine diseases**	95.9% (95.2% to 96.3%)	94.4% (92.9% to 95.0%)	59.2% (55.0% to 63.1%)	78.5% (74.4% to 82.0%)
Diabetes mellitus	100.0% (100.0% to 100.0%)	100.0% (100.0% to 100.0%)	100.0% (100.0% to 100.0%)	100.0% (100.0% to 100.0%)
Chronic kidney disease	100.0% (100.0% to 100.0%)	100.0% (100.0% to 100.0%)	100.0% (100.0% to 100.0%)	100.0% (100.0% to 100.0%)
**Musculoskeletal disorders**	2.8% (1.0% to 5.9%)	2.5% (0.9% to 5.6%)	22.7% (18.5% to 27.1%)	22.5% (18.4% to 26.9%)
Rheumatoid arthritis	7.1% (2.9% to 12.1%)	8.5% (3.5% to 14.2%)	9.7% (4.0% to 16.0%)	9.6% (4.0% to 15.9%)
Osteoarthritis	-	-	26.2% (13.8% to 39.5%)	26.2% (13.8% to 39.5%)
Low back and neck pain	-	-	23.5% (19.5% to 27.5%)	23.5% (19.5% to 27.5%)
Gout	-	-	42.9% (27.0% to 59.3%)	42.9% (27.0% to 59.3%)
**Other non-communicable diseases**	0.3% (0.2% to 0.7%)	0.4% (0.2% to 0.7%)	3.3% (2.9% to 3.8%)	3.0% (2.6% to 3.4%)
Sense organ diseases	-	-	7.3% (6.3% to 8.2%)	7.3% (6.3% to 8.2%)
Sudden infant death syndrome	12.5% (5.9% to 21.7%)	12.5% (6.0% to 21.7%)	-	12.5% (6.0% to 21.7%)
**Transport injuries**	39.3% (28.3% to 51.1%)	36.5% (25.3% to 48.9%)	39.8% (29.7% to 50.3%)	37.7% (27.0% to 49.4%)
Road injuries	39.1% (28.1% to 51.0%)	36.4% (25.2% to 48.8%)	40.2% (30.0% to 50.7%)	37.8% (27.1% to 49.4%)
Other transport injuries	41.4% (30.3% to 52.7%)	37.9% (26.3% to 50.0%)	33.0% (23.3% to 43.3%)	36.8% (25.6% to 48.2%)
**Unintentional injuries**	21.7% (16.5% to 27.6%)	17.3% (12.9% to 23.4%)	22.2% (18.4% to 27.4%)	21.1% (17.2% to 26.3%)
Falls	45.0% (39.3% to 50.7%)	31.5% (25.8% to 38.7%)	24.5% (20.8% to 29.3%)	25.0% (21.3% to 30.1%)
Drowning	17.8% (10.7% to 26.9%)	17.9% (11.4% to 26.8%)	17.1% (10.3% to 26.2%)	17.9% (11.4% to 26.6%)
Fire, heat, and hot substances	16.5% (9.2% to 27.1%)	18.8% (11.6% to 28.7%)	19.4% (12.1% to 28.5%)	19.1% (11.9% to 28.5%)

Supplementary Results Table 1. Age-standardised PAFs attributable to the joint distribution of all risk factors for all ages, both sexes combined for each GBD level 1 and level 2 cause and all causes in 2016 for Bosnia and Herzegovina. Results are shown for deaths, YLLs, YLDs, and DALYs
Data in parenthesis are 95% uncertainty intervals. PAFs=population attributable fractions. GBD=Global Burden of Disease. YLLs=years of life lost. YLDs=years live with disability. DALYs=disability-adjusted life-years

	**Deaths 2016**	**YLLs 2016**	**YLDs 2016**	**DALYs 2016**
Poisonings	18.7% (11.6% to 28.4%)	19.7% (12.6% to 29.0%)	18.8% (11.7% to 27.8%)	19.4% (12.5% to 28.2%)
Exposure to mechanical forces	20.2% (14.9% to 27.6%)	18.5% (12.9% to 26.4%)	22.1% (17.0% to 29.5%)	21.4% (16.3% to 29.0%)
Animal contact	12.9% (9.7% to 16.7%)	12.6% (8.8% to 17.4%)	11.5% (8.0% to 16.6%)	11.8% (8.4% to 16.9%)
Foreign body	9.6% (6.4% to 13.9%)	10.5% (7.0% to 15.5%)	9.3% (6.3% to 13.7%)	9.9% (6.7% to 14.5%)
Other unintentional injuries	20.5% (12.6% to 30.6%)	23.7% (15.1% to 34.4%)	18.2% (11.1% to 27.1%)	19.7% (12.3% to 28.8%)
**Self-harm and interpersonal violence**	33.6% (22.9% to 43.4%)	33.0% (22.7% to 42.8%)	27.1% (18.4% to 36.1%)	32.4% (22.7% to 41.5%)
Self-harm	35.7% (22.4% to 48.6%)	35.7% (22.8% to 48.6%)	27.2% (16.4% to 37.9%)	35.6% (22.7% to 48.4%)
Interpersonal violence	27.2% (14.5% to 41.0%)	25.3% (13.0% to 38.6%)	27.1% (17.4% to 37.3%)	25.8% (14.2% to 38.5%)
**Forces of nature, conflict and terrorism, and executions and police conflict**	0.0% (0.0% to 0.0%)	0.0% (0.0% to 0.0%)	0.0% (0.0% to 0.0%)	0.0% (0.0% to 0.0%)
Forces of nature, conflict and terrorism, and state actor violence	0.0% (0.0% to 0.0%)	0.0% (0.0% to 0.0%)	4.0% (2.6% to 6.1%)	4.0% (2.6% to 6.1%)

Supplementary Results Table 1. Age-standardised PAFs attributable to the joint distribution of all risk factors for all ages, both sexes combined for each GBD level 1 and level 2 cause and all causes in 2016 for Botswana. Results are shown for deaths, YLLs, YLDs, and DALYs
Data in parenthesis are 95% uncertainty intervals. PAFs=population attributable fractions. GBD=Global Burden of Disease. YLLs=years of life lost. YLDs=years live with disability. DALYs=disability-adjusted life-years

	Deaths 2016	YLLs 2016	YLDs 2016	DALYs 2016
**All causes**	71.9% (68.9% to 75.5%)	72.7% (68.4% to 78.8%)	35.9% (34.4% to 37.5%)	62.9% (59.6% to 66.2%)
**HIV/AIDS and tuberculosis**	89.3% (83.3% to 93.9%)	91.2% (86.4% to 94.8%)	94.3% (93.2% to 95.3%)	91.6% (87.3% to 94.8%)
Tuberculosis	56.5% (44.5% to 68.9%)	57.9% (46.8% to 69.9%)	46.2% (34.2% to 59.2%)	57.4% (46.4% to 69.4%)
HIV/AIDS	97.6% (96.8% to 98.4%)	97.1% (95.8% to 98.2%)	96.6% (96.1% to 97.0%)	97.0% (95.8% to 98.0%)
**Diarrhoea, lower respiratory infections, and other common infectious diseases**	68.7% (60.4% to 76.6%)	72.7% (65.5% to 79.3%)	40.2% (33.7% to 46.2%)	70.2% (63.3% to 76.6%)
Diarrhoeal diseases	91.0% (78.6% to 97.6%)	93.2% (82.5% to 98.3%)	91.4% (80.2% to 97.7%)	93.0% (82.5% to 98.3%)
Lower respiratory infections	62.5% (54.0% to 71.2%)	67.4% (59.9% to 75.0%)	64.2% (56.4% to 72.4%)	67.4% (59.9% to 75.0%)
Upper respiratory infections	0.7% (0.0% to 2.7%)	1.7% (0.1% to 6.6%)	0.0% (0.0% to 0.0%)	0.0% (0.0% to 0.1%)
Otitis media	1.5% (0.3% to 4.4%)	2.9% (0.7% to 7.4%)	6.9% (4.7% to 9.1%)	6.9% (4.6% to 9.0%)
Meningitis	0.8% (0.2% to 1.9%)	1.6% (0.5% to 3.6%)	0.0% (0.0% to 0.0%)	1.4% (0.5% to 3.1%)
Encephalitis	0.3% (0.1% to 0.8%)	0.7% (0.3% to 1.5%)	0.0% (0.0% to 0.0%)	0.4% (0.1% to 0.8%)
Measles	47.3% (27.3% to 76.7%)	48.4% (28.1% to 78.1%)	49.4% (29.0% to 77.9%)	48.4% (28.1% to 78.1%)
**Maternal disorders**	3.1% (1.2% to 5.4%)	3.0% (1.2% to 5.3%)	2.5% (1.1% to 4.4%)	3.0% (1.2% to 5.1%)
Maternal haemorrhage	15.9% (5.7% to 25.9%)	15.9% (5.7% to 25.9%)	16.0% (5.7% to 26.2%)	15.9% (5.7% to 25.9%)
Maternal sepsis and other pregnancy related infections	15.8% (5.7% to 25.9%)	15.8% (5.7% to 25.9%)	15.5% (5.6% to 25.4%)	15.8% (5.6% to 25.8%)
Maternal abortion, miscarriage, and ectopic pregnancy	10.1% (5.7% to 15.7%)	9.9% (5.6% to 15.3%)	10.7% (5.7% to 16.2%)	10.0% (5.6% to 15.2%)
**Neonatal disorders**	78.2% (73.1% to 83.2%)	78.2% (73.2% to 83.2%)	0.3% (0.2% to 0.4%)	67.0% (59.4% to 73.7%)
Neonatal preterm birth complications	93.1% (84.9% to 98.9%)	93.1% (85.0% to 98.9%)	0.5% (0.4% to 0.6%)	70.4% (55.7% to 81.9%)
Neonatal encephalopathy due to birth asphyxia and trauma	73.7% (66.9% to 79.1%)	73.7% (67.0% to 79.1%)	0.0% (0.0% to 0.0%)	67.7% (56.5% to 75.9%)
Neonatal sepsis and other neonatal infections	70.0% (63.3% to 74.5%)	70.0% (63.3% to 74.5%)	0.0% (0.0% to 0.0%)	56.0% (39.4% to 68.7%)
Hemolytic disease and other neonatal jaundice	72.2% (67.1% to 76.4%)	72.3% (67.2% to 76.4%)	0.0% (0.0% to 0.0%)	27.0% (14.8% to 42.5%)

Supplementary Results Table 1. Age-standardised PAFs attributable to the joint distribution of all risk factors for all ages, both sexes combined for each GBD level 1 and level 2 cause and all causes in 2016 for Botswana. Results are shown for deaths, YLLs, YLDs, and DALYs
Data in parenthesis are 95% uncertainty intervals. PAFs=population attributable fractions. GBD=Global Burden of Disease. YLLs=years of life lost. YLDs=years live with disability. DALYs=disability-adjusted life-years

	Deaths 2016	YLLs 2016	YLDs 2016	DALYs 2016
Other neonatal disorders	69.5% (61.7% to 77.1%)	69.6% (61.8% to 77.1%)	0.0% (0.0% to 0.0%)	67.7% (60.1% to 74.8%)
**Nutritional deficiencies**	18.9% (8.1% to 37.4%)	46.6% (25.6% to 70.3%)	85.3% (82.1% to 88.3%)	73.6% (65.2% to 80.9%)
Protein-energy malnutrition	23.8% (10.5% to 45.5%)	53.3% (31.2% to 77.3%)	97.7% (95.7% to 98.9%)	60.3% (41.4% to 79.5%)
Vitamin A deficiency	-	-	100.0% (100.0% to 100.0%)	100.0% (100.0% to 100.0%)
Iron-deficiency anaemia	100.0% (100.0% to 100.0%)	100.0% (100.0% to 100.0%)	100.0% (100.0% to 100.0%)	100.0% (100.0% to 100.0%)
**Other communicable, maternal, neonatal, and nutritional diseases**	9.7% (4.1% to 24.0%)	7.3% (2.2% to 18.8%)	80.1% (75.5% to 84.1%)	26.9% (14.9% to 41.5%)
Sexually transmitted diseases excluding HIV	40.5% (13.0% to 79.6%)	22.6% (5.3% to 61.0%)	100.0% (100.0% to 100.0%)	57.7% (30.6% to 89.3%)
Hepatitis	2.6% (1.8% to 3.6%)	2.6% (1.9% to 3.6%)	2.0% (1.3% to 3.3%)	2.6% (1.9% to 3.5%)
**Neoplasms**	46.2% (40.2% to 51.7%)	48.1% (42.0% to 53.3%)	41.4% (36.4% to 46.9%)	48.0% (41.8% to 53.2%)
Oesophageal cancer	80.3% (68.9% to 88.6%)	83.5% (72.8% to 90.9%)	81.3% (70.6% to 89.4%)	83.5% (72.8% to 90.9%)
Stomach cancer	14.8% (5.5% to 25.5%)	17.2% (6.7% to 28.4%)	15.2% (5.8% to 25.8%)	17.2% (6.7% to 28.4%)
Liver cancer	53.1% (42.9% to 62.6%)	53.8% (43.4% to 63.6%)	53.2% (43.3% to 62.3%)	53.8% (43.4% to 63.6%)
Larynx cancer	83.9% (70.2% to 91.8%)	86.2% (72.8% to 93.0%)	84.7% (70.6% to 92.1%)	86.1% (72.8% to 92.9%)
Tracheal, bronchus, and lung cancer	86.7% (79.1% to 91.5%)	87.6% (79.4% to 92.4%)	86.1% (75.7% to 91.7%)	87.6% (79.4% to 92.4%)
Breast cancer	28.3% (18.2% to 41.3%)	26.2% (16.0% to 39.6%)	26.4% (16.2% to 39.7%)	26.2% (16.0% to 39.6%)
Cervical cancer	100.0% (100.0% to 100.0%)	100.0% (100.0% to 100.0%)	100.0% (100.0% to 100.0%)	100.0% (100.0% to 100.0%)
Uterine cancer	52.7% (39.0% to 64.7%)	56.0% (44.4% to 66.8%)	56.1% (44.5% to 66.9%)	56.0% (44.4% to 66.8%)
Prostate cancer	3.6% (1.6% to 5.6%)	4.4% (1.9% to 6.9%)	4.4% (2.0% to 6.6%)	4.4% (1.9% to 6.9%)
Colon and rectum cancer	54.5% (44.1% to 64.5%)	56.1% (46.3% to 65.5%)	55.2% (45.0% to 65.0%)	56.1% (46.3% to 65.5%)
Lip and oral cavity cancer	84.1% (74.0% to 90.9%)	86.8% (77.8% to 92.5%)	84.1% (75.2% to 90.7%)	86.8% (77.7% to 92.5%)
Nasopharynx cancer	77.0% (65.6% to 85.1%)	78.6% (67.4% to 85.7%)	76.2% (66.3% to 83.2%)	78.5% (67.4% to 85.6%)

Supplementary Results Table 1. Age-standardised PAFs attributable to the joint distribution of all risk factors for all ages, both sexes combined for each GBD level 1 and level 2 cause and all causes in 2016 for Botswana. Results are shown for deaths, YLLs, YLDs, and DALYs<br>Data in parenthesis are 95% uncertainty intervals. PAFs=population attributable fractions. GBD=Global Burden of Disease. YLLs=years of life lost. YLDs=years live with disability. DALYs=disability-adjusted life-years				
	**Deaths 2016**	**YLLs 2016**	**YLDs 2016**	**DALYs 2016**
Other pharynx cancer	60.9% (44.3% to 73.2%)	63.9% (48.5% to 75.4%)	62.1% (45.7% to 74.0%)	63.9% (48.4% to 75.3%)
Gallbladder and biliary tract cancer	24.5% (13.6% to 36.5%)	25.5% (14.6% to 37.9%)	25.2% (15.1% to 37.0%)	25.5% (14.7% to 37.8%)
Pancreatic cancer	28.8% (17.5% to 40.8%)	31.8% (19.4% to 44.0%)	29.3% (18.0% to 41.1%)	31.8% (19.4% to 44.0%)
Ovarian cancer	13.6% (4.9% to 24.9%)	12.6% (4.4% to 22.8%)	11.9% (4.0% to 21.7%)	12.6% (4.3% to 22.7%)
Kidney cancer	30.9% (20.0% to 41.9%)	31.6% (19.4% to 43.2%)	30.4% (20.6% to 40.9%)	31.5% (19.6% to 43.1%)
Bladder cancer	28.6% (15.7% to 40.8%)	32.0% (17.8% to 44.2%)	30.3% (16.4% to 42.4%)	31.9% (17.7% to 44.2%)
Thyroid cancer	13.2% (7.6% to 20.0%)	14.0% (8.2% to 20.9%)	14.7% (8.9% to 21.1%)	14.1% (8.3% to 21.0%)
Mesothelioma	95.0% (89.8% to 97.5%)	91.6% (83.5% to 95.4%)	93.8% (87.6% to 96.8%)	91.6% (83.6% to 95.5%)
Non-Hodgkin's lymphoma	5.6% (2.2% to 9.9%)	5.5% (2.2% to 9.6%)	5.7% (2.3% to 10.0%)	5.5% (2.2% to 9.7%)
Multiple myeloma	7.1% (3.2% to 12.1%)	7.5% (3.4% to 12.9%)	7.5% (3.4% to 12.6%)	7.5% (3.4% to 12.9%)
Leukaemia	17.0% (9.5% to 25.4%)	16.7% (9.2% to 25.6%)	16.5% (9.7% to 24.8%)	16.7% (9.2% to 25.6%)
**Cardiovascular diseases**	88.6% (86.1% to 90.7%)	89.5% (87.3% to 91.3%)	78.3% (75.5% to 81.2%)	88.8% (86.5% to 90.6%)
Rheumatic heart disease	31.4% (21.6% to 48.3%)	31.3% (21.9% to 46.2%)	32.1% (21.8% to 46.4%)	31.3% (21.9% to 46.2%)
Ischaemic heart disease	95.4% (92.6% to 97.4%)	96.6% (94.8% to 98.0%)	93.2% (90.8% to 95.2%)	96.4% (94.6% to 97.9%)
Cerebrovascular disease	89.6% (85.5% to 93.2%)	92.7% (90.1% to 95.0%)	90.1% (86.6% to 93.5%)	92.5% (89.9% to 94.8%)
Hypertensive heart disease	100.0% (100.0% to 100.0%)	100.0% (100.0% to 100.0%)	100.0% (100.0% to 100.0%)	100.0% (100.0% to 100.0%)
Cardiomyopathy and myocarditis	47.5% (38.8% to 57.2%)	47.3% (39.0% to 55.6%)	47.4% (40.3% to 54.5%)	47.4% (39.3% to 55.5%)
Atrial fibrillation and flutter	54.7% (45.4% to 64.1%)	59.4% (51.1% to 67.4%)	66.5% (60.5% to 72.6%)	63.4% (56.8% to 70.1%)
Aortic aneurysm	46.0% (38.1% to 53.5%)	51.5% (44.9% to 57.5%)	-	51.5% (44.9% to 57.5%)
Peripheral vascular disease	64.9% (58.9% to 70.8%)	72.6% (67.9% to 77.3%)	68.5% (63.3% to 73.4%)	71.2% (66.3% to 76.0%)
Endocarditis	43.4% (34.2% to 53.1%)	40.7% (30.3% to 50.4%)	34.9% (26.2% to 45.6%)	40.6% (30.3% to 50.4%)

Supplementary Results Table 1. Age-standardised PAFs attributable to the joint distribution of all risk factors for all ages, both sexes combined for each GBD level 1 and level 2 cause and all causes in 2016 for Botswana. Results are shown for deaths, YLLs, YLDs, and DALYs
Data in parenthesis are 95% uncertainty intervals. PAFs=population attributable fractions. GBD=Global Burden of Disease. YLLs=years of life lost. YLDs=years live with disability. DALYs=disability-adjusted life-years

	Deaths 2016	YLLs 2016	YLDs 2016	DALYs 2016
Other cardiovascular and circulatory diseases	51.1% (45.4% to 56.4%)	54.8% (49.6% to 59.8%)	39.1% (33.5% to 44.7%)	51.1% (45.1% to 56.3%)
**Chronic respiratory diseases**	63.7% (55.9% to 70.6%)	64.2% (57.1% to 70.2%)	51.1% (43.0% to 58.9%)	59.8% (51.3% to 66.6%)
Chronic obstructive pulmonary disease	74.0% (64.0% to 81.9%)	76.8% (67.1% to 84.0%)	71.6% (57.1% to 80.3%)	75.4% (64.3% to 83.2%)
Pneumoconiosis	100.0% (100.0% to 100.0%)	100.0% (100.0% to 100.0%)	100.0% (100.0% to 100.0%)	100.0% (100.0% to 100.0%)
Asthma	39.1% (29.6% to 49.2%)	43.4% (35.0% to 52.4%)	33.0% (26.0% to 41.3%)	38.4% (30.7% to 47.0%)
Other chronic respiratory diseases	8.8% (3.4% to 14.7%)	6.4% (1.9% to 12.0%)	9.7% (4.0% to 15.3%)	7.7% (3.0% to 13.0%)
**Cirrhosis and other chronic liver diseases**	48.7% (45.7% to 51.8%)	49.1% (46.1% to 52.4%)	35.1% (31.1% to 39.1%)	48.6% (45.6% to 51.9%)
Cirrhosis and other chronic liver diseases due to hepatitis B	0.5% (0.2% to 0.8%)	0.5% (0.2% to 0.8%)	0.5% (0.2% to 0.8%)	0.5% (0.2% to 0.8%)
Cirrhosis and other chronic liver diseases due to hepatitis C	72.5% (59.7% to 84.2%)	80.3% (69.8% to 87.9%)	78.0% (68.3% to 85.5%)	80.2% (69.8% to 87.9%)
Cirrhosis and other chronic liver diseases due to alcohol use	100.0% (100.0% to 100.0%)	100.0% (100.0% to 100.0%)	100.0% (100.0% to 100.0%)	100.0% (100.0% to 100.0%)
**Digestive diseases**	8.5% (6.3% to 10.9%)	9.4% (7.2% to 11.8%)	4.2% (3.2% to 5.4%)	8.9% (6.8% to 11.2%)
Peptic ulcer disease	9.3% (6.1% to 13.1%)	12.1% (8.2% to 16.8%)	8.5% (5.7% to 11.5%)	11.8% (8.0% to 16.3%)
Gallbladder and biliary diseases	40.8% (26.7% to 55.7%)	41.8% (28.6% to 55.9%)	47.3% (36.2% to 58.9%)	42.2% (29.3% to 56.0%)
Pancreatitis	53.5% (27.6% to 77.2%)	62.0% (37.9% to 81.4%)	54.0% (29.3% to 74.4%)	61.9% (37.8% to 81.4%)
**Neurological disorders**	24.0% (13.6% to 35.1%)	27.0% (18.6% to 35.8%)	8.2% (4.6% to 12.6%)	16.6% (10.8% to 23.0%)
Alzheimer's disease and other dementias	24.3% (10.7% to 40.2%)	26.0% (12.0% to 41.8%)	26.2% (11.9% to 42.2%)	26.0% (12.0% to 41.8%)
Parkinson's disease	-5.3% (-6.9% to -3.9%)	-6.9% (-8.8% to -5.1%)	-7.8% (-9.7% to -5.9%)	-7.1% (-9.0% to -5.3%)
Epilepsy	33.5% (21.6% to 46.5%)	34.2% (22.9% to 47.6%)	25.5% (16.7% to 35.5%)	30.9% (20.2% to 43.2%)
Multiple sclerosis	6.8% (4.1% to 10.2%)	7.2% (4.2% to 11.1%)	5.3% (3.3% to 7.7%)	6.3% (3.9% to 9.1%)
**Mental and substance use disorders**	99.9% (99.8% to 99.9%)	99.8% (99.7% to 99.9%)	22.3% (19.9% to 24.9%)	29.4% (25.0% to 34.2%)
Alcohol use disorders	100.0% (100.0% to 100.0%)	100.0% (100.0% to 100.0%)	100.0% (100.0% to 100.0%)	100.0% (100.0% to 100.0%)

Supplementary Results Table 1. Age-standardised PAFs attributable to the joint distribution of all risk factors for all ages, both sexes combined for each GBD level 1 and level 2 cause and all causes in 2016 for Botswana. Results are shown for deaths, YLLs, YLDs, and DALYs
Data in parenthesis are 95% uncertainty intervals. PAFs=population attributable fractions. GBD=Global Burden of Disease. YLLs=years of life lost. YLDs=years live with disability. DALYs=disability-adjusted life-years

	Deaths 2016	YLLs 2016	YLDs 2016	DALYs 2016
Drug use disorders	100.0% (100.0% to 100.0%)	100.0% (100.0% to 100.0%)	100.0% (100.0% to 100.0%)	100.0% (100.0% to 100.0%)
Depressive disorders	-	-	10.0% (8.1% to 12.1%)	10.0% (8.1% to 12.1%)
Idiopathic developmental intellectual disability	-	-	62.0% (31.4% to 81.7%)	62.0% (31.4% to 81.7%)
**Diabetes, urogenital, blood, and endocrine diseases**	89.8% (87.8% to 91.7%)	85.5% (81.3% to 89.3%)	74.8% (71.5% to 77.7%)	82.5% (78.8% to 85.8%)
Diabetes mellitus	100.0% (100.0% to 100.0%)	100.0% (100.0% to 100.0%)	100.0% (100.0% to 100.0%)	100.0% (100.0% to 100.0%)
Chronic kidney disease	100.0% (100.0% to 100.0%)	100.0% (100.0% to 100.0%)	100.0% (100.0% to 100.0%)	100.0% (100.0% to 100.0%)
**Musculoskeletal disorders**	1.6% (0.6% to 2.7%)	1.6% (0.6% to 2.8%)	20.6% (17.6% to 23.8%)	20.0% (17.1% to 23.3%)
Rheumatoid arthritis	3.6% (1.4% to 6.1%)	4.4% (1.7% to 7.4%)	3.9% (1.5% to 6.6%)	3.9% (1.5% to 6.7%)
Osteoarthritis	-	-	27.6% (15.8% to 39.5%)	27.6% (15.8% to 39.5%)
Low back and neck pain	-	-	24.5% (21.7% to 27.7%)	24.5% (21.7% to 27.7%)
Gout	-	-	53.3% (43.1% to 65.3%)	53.3% (43.1% to 65.3%)
**Other non-communicable diseases**	0.2% (0.0% to 0.5%)	0.5% (0.1% to 1.1%)	6.0% (5.1% to 7.2%)	5.4% (4.5% to 6.4%)
Sense organ diseases	-	-	13.1% (11.3% to 15.0%)	13.1% (11.3% to 15.0%)
Sudden infant death syndrome	12.1% (4.8% to 25.4%)	12.1% (4.8% to 25.5%)	-	12.1% (4.8% to 25.5%)
**Transport injuries**	34.0% (25.0% to 46.0%)	30.4% (21.5% to 42.5%)	39.1% (30.5% to 51.6%)	31.9% (22.9% to 43.7%)
Road injuries	33.8% (24.8% to 45.9%)	30.3% (21.4% to 42.4%)	40.3% (31.6% to 52.9%)	31.8% (22.8% to 43.8%)
Other transport injuries	38.2% (28.7% to 50.9%)	34.2% (24.7% to 47.4%)	31.2% (23.2% to 43.1%)	32.9% (24.2% to 45.0%)
**Unintentional injuries**	20.6% (16.1% to 27.5%)	15.6% (11.1% to 22.3%)	17.8% (13.9% to 27.9%)	16.4% (12.0% to 24.0%)
Falls	57.0% (51.4% to 64.6%)	39.0% (33.1% to 48.7%)	27.2% (23.6% to 37.6%)	31.3% (27.7% to 39.3%)
Drowning	18.8% (11.6% to 27.6%)	18.4% (11.4% to 26.7%)	16.8% (9.4% to 27.6%)	18.4% (11.4% to 26.7%)
Fire, heat, and hot substances	15.6% (9.4% to 23.7%)	18.1% (11.1% to 27.4%)	18.1% (10.3% to 31.1%)	18.2% (11.0% to 28.4%)

Supplementary Results Table 1. Age-standardised PAFs attributable to the joint distribution of all risk factors for all ages, both sexes combined for each GBD level 1 and level 2 cause and all causes in 2016 for Botswana. Results are shown for deaths, YLLs, YLDs, and DALYs
Data in parenthesis are 95% uncertainty intervals. PAFs=population attributable fractions. GBD=Global Burden of Disease. YLLs=years of life lost. YLDs=years live with disability. DALYs=disability-adjusted life-years

	**Deaths 2016**	**YLLs 2016**	**YLDs 2016**	**DALYs 2016**
Poisonings	18.2% (10.7% to 28.3%)	17.7% (9.8% to 27.7%)	16.4% (9.4% to 29.2%)	17.6% (9.8% to 27.5%)
Exposure to mechanical forces	18.2% (13.7% to 27.2%)	15.2% (10.7% to 23.5%)	18.5% (14.0% to 31.1%)	16.3% (11.6% to 27.0%)
Animal contact	13.4% (9.0% to 21.4%)	12.4% (8.1% to 20.8%)	11.3% (6.7% to 24.8%)	12.2% (8.1% to 21.5%)
Foreign body	7.4% (4.6% to 12.7%)	6.7% (3.9% to 12.0%)	8.7% (4.8% to 20.3%)	7.3% (4.3% to 14.8%)
Other unintentional injuries	18.2% (11.3% to 27.3%)	19.6% (12.2% to 28.7%)	15.5% (8.8% to 26.4%)	16.9% (10.2% to 27.0%)
**Self-harm and interpersonal violence**	29.7% (20.0% to 39.8%)	29.8% (20.5% to 39.9%)	26.3% (20.1% to 33.5%)	29.6% (20.5% to 39.5%)
Self-harm	33.7% (20.7% to 46.6%)	35.5% (22.2% to 48.2%)	30.9% (17.8% to 43.8%)	35.4% (22.1% to 48.0%)
Interpersonal violence	23.8% (13.7% to 36.5%)	22.9% (12.9% to 35.4%)	25.7% (19.3% to 33.4%)	23.2% (13.6% to 35.1%)
**Forces of nature, conflict and terrorism, and executions and police conflict**	0.0% (0.0% to 0.0%)	0.0% (0.0% to 0.0%)	3.0% (2.0% to 4.5%)	0.9% (0.2% to 1.9%)
Forces of nature, conflict and terrorism, and state actor violence	0.0% (0.0% to 0.0%)	0.0% (0.0% to 0.0%)	6.3% (4.2% to 9.3%)	6.3% (4.2% to 9.3%)

Supplementary Results Table 1. Age-standardised PAFs attributable to the joint distribution of all risk factors for all ages, both sexes combined for each GBD level 1 and level 2 cause and all causes in 2016 for Brazil. Results are shown for deaths, YLLs, YLDs, and DALYs
Data in parenthesis are 95% uncertainty intervals. PAFs=population attributable fractions. GBD=Global Burden of Disease. YLLs=years of life lost. YLDs=years live with disability. DALYs=disability-adjusted life-years

	Deaths 2016	YLLs 2016	YLDs 2016	DALYs 2016
**All causes**	54.1% (52.3% to 55.9%)	50.1% (48.6% to 51.5%)	23.9% (22.7% to 25.1%)	40.0% (38.0% to 42.1%)
**HIV/AIDS and tuberculosis**	79.5% (76.8% to 82.1%)	80.8% (78.8% to 82.8%)	87.2% (83.8% to 90.0%)	81.2% (79.3% to 83.2%)
Tuberculosis	49.2% (40.2% to 57.9%)	52.1% (43.9% to 60.6%)	49.4% (41.4% to 57.8%)	52.0% (43.8% to 60.5%)
HIV/AIDS	91.4% (90.2% to 92.3%)	88.4% (87.2% to 89.4%)	93.7% (92.5% to 94.6%)	88.8% (87.6% to 89.8%)
**Diarrhoea, lower respiratory infections, and other common infectious diseases**	45.7% (40.2% to 53.1%)	54.0% (48.9% to 59.7%)	48.2% (39.3% to 56.7%)	52.7% (48.1% to 57.6%)
Diarrhoeal diseases	76.7% (62.6% to 89.0%)	86.8% (78.3% to 93.9%)	79.2% (66.5% to 90.3%)	82.2% (71.3% to 91.8%)
Lower respiratory infections	45.0% (38.7% to 53.2%)	55.7% (49.2% to 62.8%)	50.3% (43.8% to 57.9%)	55.7% (49.2% to 62.7%)
Upper respiratory infections	1.2% (0.7% to 1.9%)	2.6% (1.5% to 3.9%)	0.0% (0.0% to 0.0%)	0.0% (0.0% to 0.0%)
Otitis media	4.5% (3.5% to 5.6%)	7.8% (6.1% to 9.6%)	5.4% (3.5% to 7.1%)	5.4% (3.6% to 7.2%)
Meningitis	0.8% (0.7% to 0.9%)	1.1% (1.0% to 1.3%)	0.0% (0.0% to 0.0%)	1.0% (0.9% to 1.2%)
Encephalitis	0.9% (0.7% to 1.3%)	1.5% (1.2% to 2.0%)	0.0% (0.0% to 0.0%)	1.1% (0.8% to 1.5%)
Measles	23.8% (8.5% to 59.8%)	24.8% (8.9% to 62.2%)	27.4% (10.6% to 65.2%)	24.8% (8.9% to 62.2%)
**Maternal disorders**	5.6% (2.9% to 8.2%)	5.4% (2.8% to 8.0%)	3.8% (2.1% to 5.8%)	5.2% (2.7% to 7.7%)
Maternal haemorrhage	15.1% (5.4% to 24.6%)	15.0% (5.3% to 24.5%)	14.8% (5.3% to 24.0%)	15.0% (5.3% to 24.4%)
Maternal sepsis and other pregnancy related infections	14.9% (5.3% to 24.3%)	14.8% (5.3% to 24.1%)	14.5% (5.2% to 23.6%)	14.8% (5.3% to 24.0%)
Maternal abortion, miscarriage, and ectopic pregnancy	13.2% (8.5% to 18.2%)	12.9% (8.3% to 18.0%)	13.1% (7.4% to 18.9%)	12.9% (8.2% to 18.1%)
**Neonatal disorders**	71.4% (70.0% to 73.0%)	71.5% (70.0% to 73.0%)	0.1% (0.1% to 0.2%)	63.6% (60.6% to 66.2%)
Neonatal preterm birth complications	90.9% (89.7% to 91.7%)	90.9% (89.7% to 91.8%)	0.2% (0.2% to 0.3%)	76.3% (71.2% to 80.5%)
Neonatal encephalopathy due to birth asphyxia and trauma	61.7% (59.4% to 64.3%)	61.7% (59.5% to 64.3%)	0.0% (0.0% to 0.0%)	57.5% (52.8% to 61.3%)
Neonatal sepsis and other neonatal infections	54.2% (51.8% to 58.1%)	54.2% (51.8% to 58.1%)	0.0% (0.0% to 0.0%)	48.3% (41.2% to 53.6%)
Hemolytic disease and other neonatal jaundice	61.7% (59.3% to 64.3%)	61.7% (59.3% to 64.3%)	0.0% (0.0% to 0.0%)	35.4% (30.5% to 40.8%)

Supplementary Results Table 1. Age-standardised PAFs attributable to the joint distribution of all risk factors for all ages, both sexes combined for each GBD level 1 and level 2 cause and all causes in 2016 for Brazil. Results are shown for deaths, YLLs, YLDs, and DALYs

Data in parenthesis are 95% uncertainty intervals. PAFs=population attributable fractions. GBD=Global Burden of Disease. YLLs=years of life lost. YLDs=years live with disability. DALYs=disability-adjusted life-years

	**Deaths 2016**	**YLLs 2016**	**YLDs 2016**	**DALYs 2016**
Other neonatal disorders	59.6% (57.2% to 62.1%)	59.6% (57.3% to 62.2%)	0.0% (0.0% to 0.0%)	58.4% (56.2% to 60.9%)
**Nutritional deficiencies**	8.8% (7.9% to 9.8%)	36.9% (34.2% to 39.9%)	97.1% (96.6% to 97.6%)	83.7% (79.0% to 87.7%)
Protein-energy malnutrition	9.2% (8.1% to 10.5%)	40.0% (36.9% to 44.3%)	96.0% (95.4% to 96.6%)	52.8% (48.3% to 58.2%)
Vitamin A deficiency	-	-	100.0% (100.0% to 100.0%)	100.0% (100.0% to 100.0%)
Iron-deficiency anaemia	100.0% (100.0% to 100.0%)	100.0% (100.0% to 100.0%)	100.0% (100.0% to 100.0%)	100.0% (100.0% to 100.0%)
**Other communicable, maternal, neonatal, and nutritional diseases**	7.2% (6.3% to 7.9%)	5.7% (4.6% to 6.6%)	60.9% (54.3% to 67.8%)	24.8% (18.2% to 33.9%)
Sexually transmitted diseases excluding HIV	39.5% (24.8% to 56.5%)	20.3% (11.5% to 32.9%)	100.0% (100.0% to 100.0%)	62.0% (43.0% to 79.9%)
Hepatitis	0.3% (0.2% to 0.5%)	0.4% (0.3% to 0.6%)	0.3% (0.2% to 0.5%)	0.4% (0.3% to 0.6%)
**Neoplasms**	36.1% (33.4% to 38.7%)	35.8% (33.5% to 38.3%)	31.3% (28.6% to 34.1%)	35.7% (33.4% to 38.2%)
Oesophageal cancer	73.1% (63.6% to 81.8%)	75.0% (65.7% to 83.5%)	73.5% (64.1% to 82.1%)	75.0% (65.7% to 83.4%)
Stomach cancer	13.7% (6.6% to 22.5%)	13.1% (6.4% to 21.4%)	13.6% (6.5% to 22.3%)	13.1% (6.4% to 21.4%)
Liver cancer	47.3% (38.6% to 56.5%)	47.8% (39.0% to 57.1%)	47.5% (38.9% to 56.6%)	47.8% (39.0% to 57.1%)
Larynx cancer	77.9% (68.5% to 84.9%)	78.4% (69.3% to 85.3%)	78.6% (69.7% to 85.5%)	78.4% (69.3% to 85.3%)
Tracheal, bronchus, and lung cancer	79.5% (76.4% to 82.4%)	79.0% (76.0% to 81.9%)	79.1% (75.9% to 82.0%)	79.0% (76.0% to 81.9%)
Breast cancer	23.6% (16.4% to 31.4%)	22.1% (15.0% to 30.4%)	22.8% (15.6% to 31.1%)	22.1% (15.0% to 30.5%)
Cervical cancer	100.0% (100.0% to 100.0%)	100.0% (100.0% to 100.0%)	100.0% (100.0% to 100.0%)	100.0% (100.0% to 100.0%)
Uterine cancer	45.6% (33.0% to 58.9%)	46.4% (33.6% to 60.1%)	47.3% (34.3% to 61.1%)	46.5% (33.6% to 60.1%)
Prostate cancer	3.0% (2.1% to 4.0%)	2.9% (2.1% to 3.9%)	3.1% (2.2% to 4.1%)	2.9% (2.1% to 3.9%)
Colon and rectum cancer	51.8% (43.1% to 60.0%)	53.0% (44.8% to 60.7%)	52.3% (43.8% to 60.5%)	53.0% (44.8% to 60.7%)
Lip and oral cavity cancer	70.7% (62.7% to 77.6%)	74.5% (67.0% to 81.0%)	70.6% (62.9% to 77.3%)	74.4% (66.9% to 81.0%)
Nasopharynx cancer	70.3% (64.8% to 75.2%)	68.7% (63.3% to 73.2%)	67.9% (62.5% to 72.6%)	68.7% (63.3% to 73.2%)

Supplementary Results Table 1. Age-standardised PAFs attributable to the joint distribution of all risk factors for all ages, both sexes combined for each GBD level 1 and level 2 cause and all causes in 2016 for Brazil. Results are shown for deaths, YLLs, YLDs, and DALYs
Data in parenthesis are 95% uncertainty intervals. PAFs=population attributable fractions. GBD=Global Burden of Disease. YLLs=years of life lost. YLDs=years live with disability. DALYs=disability-adjusted life-years

	Deaths 2016	YLLs 2016	YLDs 2016	DALYs 2016
Other pharynx cancer	55.9% (43.7% to 66.4%)	60.3% (48.0% to 70.2%)	58.1% (45.8% to 68.2%)	60.3% (47.9% to 70.2%)
Gallbladder and biliary tract cancer	23.0% (13.2% to 34.9%)	23.5% (13.7% to 35.6%)	23.1% (13.3% to 35.1%)	23.5% (13.7% to 35.6%)
Pancreatic cancer	24.1% (17.7% to 31.7%)	23.9% (17.7% to 31.3%)	23.9% (17.6% to 31.5%)	23.9% (17.7% to 31.3%)
Ovarian cancer	11.6% (5.2% to 19.0%)	9.6% (4.0% to 16.3%)	9.8% (4.2% to 16.4%)	9.6% (4.0% to 16.3%)
Kidney cancer	29.3% (21.0% to 39.0%)	27.1% (19.4% to 36.0%)	27.9% (19.9% to 37.1%)	27.1% (19.4% to 36.0%)
Bladder cancer	25.2% (17.6% to 33.4%)	24.4% (17.1% to 32.0%)	24.6% (17.2% to 32.2%)	24.4% (17.1% to 32.0%)
Thyroid cancer	13.8% (7.3% to 22.8%)	14.4% (7.5% to 23.6%)	14.4% (8.0% to 22.9%)	14.4% (7.5% to 23.6%)
Mesothelioma	90.1% (87.6% to 92.3%)	82.8% (79.1% to 86.3%)	87.8% (84.8% to 90.3%)	82.9% (79.2% to 86.3%)
Non-Hodgkin's lymphoma	6.6% (2.7% to 11.4%)	6.1% (2.5% to 10.5%)	6.6% (2.7% to 11.4%)	6.1% (2.5% to 10.5%)
Multiple myeloma	7.9% (3.5% to 13.9%)	8.2% (3.7% to 14.3%)	8.2% (3.6% to 14.2%)	8.2% (3.7% to 14.3%)
Leukaemia	13.8% (9.0% to 19.0%)	10.5% (7.0% to 14.3%)	13.0% (8.6% to 18.0%)	10.5% (7.0% to 14.3%)
**Cardiovascular diseases**	81.5% (78.8% to 83.8%)	82.3% (80.5% to 84.0%)	74.4% (71.6% to 76.9%)	81.6% (79.7% to 83.3%)
Rheumatic heart disease	26.0% (17.9% to 39.9%)	22.3% (15.6% to 32.6%)	17.6% (11.2% to 25.7%)	21.3% (14.6% to 31.2%)
Ischaemic heart disease	91.4% (87.7% to 94.6%)	93.2% (90.9% to 95.1%)	90.3% (87.6% to 92.8%)	93.0% (90.7% to 95.0%)
Cerebrovascular disease	81.2% (75.8% to 86.4%)	84.6% (81.2% to 87.7%)	83.1% (78.3% to 87.4%)	84.5% (81.0% to 87.7%)
Hypertensive heart disease	100.0% (100.0% to 100.0%)	100.0% (100.0% to 100.0%)	100.0% (100.0% to 100.0%)	100.0% (100.0% to 100.0%)
Cardiomyopathy and myocarditis	43.0% (34.8% to 52.3%)	42.5% (35.9% to 49.5%)	41.1% (34.4% to 48.3%)	42.4% (35.9% to 49.5%)
Atrial fibrillation and flutter	50.9% (43.5% to 58.0%)	56.1% (49.4% to 61.9%)	63.1% (58.1% to 68.2%)	60.4% (55.1% to 65.7%)
Aortic aneurysm	44.4% (37.6% to 50.9%)	48.3% (42.4% to 53.7%)	-	48.3% (42.4% to 53.7%)
Peripheral vascular disease	49.8% (43.7% to 56.5%)	55.7% (50.4% to 60.7%)	56.0% (50.5% to 61.3%)	55.8% (50.4% to 60.8%)
Endocarditis	37.7% (29.3% to 47.4%)	31.2% (23.6% to 38.9%)	36.6% (28.7% to 44.9%)	31.3% (23.6% to 38.9%)

Supplementary Results Table 1. Age-standardised PAFs attributable to the joint distribution of all risk factors for all ages, both sexes combined for each GBD level 1 and level 2 cause and all causes in 2016 for Brazil. Results are shown for deaths, YLLs, YLDs, and DALYs
Data in parenthesis are 95% uncertainty intervals. PAFs=population attributable fractions. GBD=Global Burden of Disease. YLLs=years of life lost. YLDs=years live with disability. DALYs=disability-adjusted life-years

	Deaths 2016	YLLs 2016	YLDs 2016	DALYs 2016
Other cardiovascular and circulatory diseases	45.0% (40.1% to 49.9%)	45.1% (40.7% to 49.3%)	45.4% (40.5% to 50.2%)	45.2% (40.8% to 49.3%)
**Chronic respiratory diseases**	65.8% (60.8% to 70.5%)	63.3% (58.8% to 67.8%)	38.7% (30.7% to 46.8%)	56.7% (52.0% to 61.5%)
Chronic obstructive pulmonary disease	70.6% (65.1% to 75.6%)	69.9% (64.8% to 74.8%)	66.0% (60.9% to 71.3%)	69.4% (64.4% to 74.5%)
Pneumoconiosis	100.0% (100.0% to 100.0%)	100.0% (100.0% to 100.0%)	100.0% (100.0% to 100.0%)	100.0% (100.0% to 100.0%)
Asthma	36.9% (27.8% to 47.5%)	38.1% (29.7% to 48.1%)	28.1% (19.1% to 39.1%)	29.9% (21.2% to 40.3%)
Other chronic respiratory diseases	8.4% (6.5% to 10.6%)	5.1% (3.5% to 7.1%)	4.1% (2.6% to 6.1%)	4.9% (3.4% to 6.9%)
**Cirrhosis and other chronic liver diseases**	39.8% (37.7% to 42.2%)	41.1% (38.3% to 44.3%)	33.5% (31.1% to 35.9%)	40.9% (38.1% to 44.0%)
Cirrhosis and other chronic liver diseases due to hepatitis B	0.0% (0.0% to 0.1%)	0.0% (0.0% to 0.1%)	0.0% (0.0% to 0.1%)	0.0% (0.0% to 0.1%)
Cirrhosis and other chronic liver diseases due to hepatitis C	13.5% (9.4% to 18.0%)	19.0% (13.2% to 25.5%)	18.5% (12.8% to 24.3%)	19.0% (13.2% to 25.5%)
Cirrhosis and other chronic liver diseases due to alcohol use	100.0% (100.0% to 100.0%)	100.0% (100.0% to 100.0%)	100.0% (100.0% to 100.0%)	100.0% (100.0% to 100.0%)
**Digestive diseases**	13.8% (10.8% to 17.0%)	15.7% (12.5% to 18.9%)	3.7% (2.9% to 4.6%)	12.8% (10.3% to 15.6%)
Peptic ulcer disease	9.2% (6.3% to 12.5%)	10.8% (7.5% to 14.7%)	10.0% (7.0% to 13.7%)	10.6% (7.3% to 14.4%)
Gallbladder and biliary diseases	42.6% (30.1% to 56.3%)	43.3% (30.8% to 56.9%)	47.3% (34.5% to 61.6%)	43.6% (31.0% to 57.3%)
Pancreatitis	40.4% (26.9% to 55.2%)	49.2% (35.7% to 63.2%)	38.3% (25.0% to 52.2%)	49.1% (35.5% to 63.1%)
**Neurological disorders**	21.5% (10.8% to 34.2%)	21.0% (11.5% to 31.8%)	5.7% (3.7% to 8.2%)	11.5% (6.7% to 17.3%)
Alzheimer's disease and other dementias	23.3% (11.4% to 36.8%)	24.1% (12.3% to 37.6%)	24.1% (12.2% to 37.7%)	24.1% (12.2% to 37.6%)
Parkinson's disease	-5.6% (-6.5% to -4.8%)	-7.0% (-8.0% to -5.9%)	-7.9% (-9.0% to -6.6%)	-7.2% (-8.2% to -6.0%)
Epilepsy	27.9% (21.0% to 35.2%)	26.9% (20.4% to 33.8%)	23.0% (17.1% to 29.5%)	24.3% (18.3% to 30.6%)
Multiple sclerosis	5.9% (3.8% to 8.3%)	6.5% (4.2% to 9.2%)	5.9% (3.8% to 8.3%)	6.3% (4.0% to 8.8%)
**Mental and substance use disorders**	99.7% (99.7% to 99.8%)	99.6% (99.6% to 99.6%)	21.3% (19.2% to 23.7%)	26.8% (24.1% to 29.7%)
Alcohol use disorders	100.0% (100.0% to 100.0%)	100.0% (100.0% to 100.0%)	100.0% (100.0% to 100.0%)	100.0% (100.0% to 100.0%)

Supplementary Results Table 1. Age-standardised PAFs attributable to the joint distribution of all risk factors for all ages, both sexes combined for each GBD level 1 and level 2 cause and all causes in 2016 for Brazil. Results are shown for deaths, YLLs, YLDs, and DALYs
Data in parenthesis are 95% uncertainty intervals. PAFs=population attributable fractions. GBD=Global Burden of Disease. YLLs=years of life lost. YLDs=years live with disability. DALYs=disability-adjusted life-years

	**Deaths 2016**	**YLLs 2016**	**YLDs 2016**	**DALYs 2016**
Drug use disorders	100.0% (100.0% to 100.0%)	100.0% (100.0% to 100.0%)	100.0% (100.0% to 100.0%)	100.0% (100.0% to 100.0%)
Depressive disorders	-	-	5.1% (4.2% to 6.1%)	5.1% (4.2% to 6.1%)
Idiopathic developmental intellectual disability	-	-	67.8% (37.4% to 86.6%)	67.8% (37.4% to 86.6%)
**Diabetes, urogenital, blood, and endocrine diseases**	76.1% (75.4% to 78.1%)	74.5% (73.6% to 77.3%)	65.8% (61.2% to 69.9%)	71.6% (69.5% to 73.7%)
Diabetes mellitus	100.0% (100.0% to 100.0%)	100.0% (100.0% to 100.0%)	100.0% (100.0% to 100.0%)	100.0% (100.0% to 100.0%)
Chronic kidney disease	100.0% (100.0% to 100.0%)	100.0% (100.0% to 100.0%)	100.0% (100.0% to 100.0%)	100.0% (100.0% to 100.0%)
**Musculoskeletal disorders**	0.6% (0.2% to 1.0%)	0.5% (0.2% to 0.8%)	18.4% (15.5% to 21.4%)	18.0% (15.2% to 21.0%)
Rheumatoid arthritis	3.4% (1.4% to 5.9%)	3.8% (1.5% to 6.5%)	4.0% (1.6% to 6.7%)	3.9% (1.6% to 6.7%)
Osteoarthritis	-	-	26.6% (14.8% to 39.2%)	26.6% (14.8% to 39.2%)
Low back and neck pain	-	-	23.7% (20.9% to 26.8%)	23.7% (20.9% to 26.8%)
Gout	-	-	47.1% (32.7% to 62.8%)	47.1% (32.7% to 62.8%)
**Other non-communicable diseases**	0.0% (0.0% to 0.1%)	0.1% (0.1% to 0.1%)	4.7% (4.1% to 5.3%)	3.5% (3.1% to 4.0%)
Sense organ diseases	-	-	11.0% (9.8% to 12.2%)	11.0% (9.8% to 12.2%)
Sudden infant death syndrome	5.2% (4.6% to 6.1%)	5.2% (4.6% to 6.1%)	-	5.2% (4.6% to 6.1%)
**Transport injuries**	35.7% (28.8% to 44.0%)	33.2% (26.3% to 41.5%)	40.2% (33.9% to 48.0%)	33.8% (27.0% to 41.9%)
Road injuries	35.7% (28.7% to 43.9%)	33.2% (26.3% to 41.5%)	40.4% (34.1% to 48.1%)	33.9% (27.0% to 42.0%)
Other transport injuries	37.3% (30.6% to 45.1%)	33.4% (26.7% to 41.2%)	34.2% (27.8% to 42.0%)	33.4% (26.7% to 41.3%)
**Unintentional injuries**	33.6% (28.6% to 36.0%)	19.4% (16.8% to 21.6%)	21.1% (20.0% to 22.3%)	20.2% (18.3% to 21.7%)
Falls	59.5% (56.0% to 61.7%)	37.2% (33.3% to 39.0%)	26.4% (25.4% to 27.4%)	30.1% (28.3% to 31.6%)
Drowning	18.4% (14.2% to 23.6%)	16.7% (13.0% to 21.1%)	19.2% (14.4% to 25.1%)	16.7% (13.0% to 21.1%)
Fire, heat, and hot substances	12.4% (9.0% to 17.0%)	13.9% (10.6% to 18.0%)	18.2% (13.9% to 23.4%)	15.4% (11.9% to 19.9%)

Supplementary Results Table 1. Age-standardised PAFs attributable to the joint distribution of all risk factors for all ages, both sexes combined for each GBD level 1 and level 2 cause and all causes in 2016 for Brazil. Results are shown for deaths, YLLs, YLDs, and DALYs
Data in parenthesis are 95% uncertainty intervals. PAFs=population attributable fractions. GBD=Global Burden of Disease. YLLs=years of life lost. YLDs=years live with disability. DALYs=disability-adjusted life-years

	Deaths 2016	YLLs 2016	YLDs 2016	DALYs 2016
Poisonings	13.7% (10.1% to 18.3%)	14.2% (10.9% to 18.4%)	16.4% (12.5% to 21.1%)	14.5% (11.2% to 18.7%)
Exposure to mechanical forces	18.7% (16.7% to 20.6%)	16.0% (13.9% to 18.0%)	20.4% (19.2% to 21.7%)	17.4% (15.6% to 19.1%)
Animal contact	10.9% (9.7% to 12.0%)	10.2% (9.3% to 11.6%)	11.8% (10.6% to 12.7%)	10.6% (9.7% to 11.8%)
Foreign body	4.2% (3.8% to 5.4%)	4.4% (3.8% to 5.8%)	6.0% (4.8% to 7.0%)	4.7% (4.2% to 5.8%)
Other unintentional injuries	20.8% (16.2% to 26.8%)	22.1% (17.4% to 28.1%)	16.7% (12.6% to 21.8%)	19.7% (15.3% to 25.3%)
**Self-harm and interpersonal violence**	24.3% (18.1% to 31.6%)	23.8% (17.3% to 31.2%)	22.0% (17.9% to 26.4%)	23.8% (17.3% to 31.0%)
Self-harm	32.7% (21.3% to 43.2%)	34.5% (23.1% to 44.9%)	29.4% (18.7% to 39.8%)	34.4% (23.1% to 44.9%)
Interpersonal violence	22.3% (15.2% to 30.7%)	21.7% (14.6% to 30.0%)	21.6% (17.4% to 26.3%)	21.7% (14.7% to 29.9%)
**Forces of nature, conflict and terrorism, and executions and police conflict**	0.0% (0.0% to 0.0%)	0.0% (0.0% to 0.0%)	2.9% (1.9% to 4.3%)	0.2% (0.0% to 1.4%)
Forces of nature, conflict and terrorism, and state actor violence	0.0% (0.0% to 0.0%)	0.0% (0.0% to 0.0%)	4.6% (3.0% to 6.7%)	4.6% (3.0% to 6.7%)

Supplementary Results Table 1. Age-standardised PAFs attributable to the joint distribution of all risk factors for all ages, both sexes combined for each GBD level 1 and level 2 cause and all causes in 2016 for Brunei. Results are shown for deaths, YLLs, YLDs, and DALYs
Data in parenthesis are 95% uncertainty intervals. PAFs=population attributable fractions. GBD=Global Burden of Disease. YLLs=years of life lost. YLDs=years live with disability. DALYs=disability-adjusted life-years

	Deaths 2016	YLLs 2016	YLDs 2016	DALYs 2016
**All causes**	57.0% (54.9% to 59.3%)	52.0% (49.9% to 54.1%)	25.9% (24.0% to 27.6%)	41.0% (38.0% to 43.7%)
**HIV/AIDS and tuberculosis**	27.8% (20.6% to 35.4%)	33.3% (27.3% to 39.7%)	64.1% (52.9% to 72.3%)	38.4% (31.6% to 45.1%)
Tuberculosis	20.8% (12.4% to 28.8%)	20.9% (13.6% to 28.2%)	18.7% (11.9% to 25.5%)	20.7% (13.5% to 28.0%)
HIV/AIDS	83.9% (80.2% to 87.0%)	79.5% (74.5% to 83.6%)	82.3% (77.3% to 86.1%)	80.6% (75.7% to 84.5%)
**Diarrhoea, lower respiratory infections, and other common infectious diseases**	29.3% (24.2% to 36.3%)	37.3% (32.2% to 43.4%)	5.2% (3.8% to 7.1%)	30.9% (26.5% to 36.6%)
Diarrhoeal diseases	32.7% (17.6% to 53.9%)	50.9% (35.4% to 67.9%)	44.6% (31.6% to 61.7%)	49.1% (35.2% to 66.1%)
Lower respiratory infections	30.5% (24.8% to 38.0%)	41.0% (35.1% to 48.1%)	37.6% (31.4% to 45.4%)	41.0% (35.0% to 48.1%)
Upper respiratory infections	0.7% (0.1% to 2.4%)	1.9% (0.2% to 6.6%)	0.0% (0.0% to 0.0%)	0.0% (0.0% to 0.0%)
Otitis media	0.8% (0.4% to 1.8%)	2.2% (1.1% to 4.8%)	6.7% (4.5% to 8.8%)	6.7% (4.5% to 8.8%)
Meningitis	3.0% (1.9% to 4.1%)	5.8% (4.0% to 7.7%)	0.0% (0.0% to 0.0%)	5.6% (3.9% to 7.5%)
Encephalitis	0.7% (0.2% to 1.3%)	1.4% (0.4% to 2.5%)	0.0% (0.0% to 0.0%)	1.1% (0.3% to 1.9%)
Measles	31.8% (0.0% to 69.4%)	32.3% (0.0% to 70.5%)	31.9% (0.0% to 69.5%)	33.2% (13.6% to 70.6%)
**Maternal disorders**	2.9% (1.1% to 5.3%)	2.9% (1.1% to 5.3%)	2.8% (1.3% to 4.8%)	2.9% (1.1% to 5.1%)
Maternal haemorrhage	12.7% (4.6% to 21.1%)	12.6% (4.6% to 21.0%)	12.7% (4.6% to 21.3%)	12.6% (4.6% to 21.1%)
Maternal sepsis and other pregnancy related infections	12.8% (4.7% to 21.2%)	12.7% (4.6% to 21.1%)	12.2% (4.4% to 20.3%)	12.5% (4.5% to 20.8%)
Maternal abortion, miscarriage, and ectopic pregnancy	3.7% (2.0% to 5.8%)	3.8% (2.0% to 6.0%)	4.2% (2.0% to 7.1%)	3.9% (2.1% to 6.3%)
**Neonatal disorders**	72.0% (68.1% to 75.7%)	72.1% (68.2% to 75.8%)	0.1% (0.1% to 0.2%)	59.3% (54.1% to 64.4%)
Neonatal preterm birth complications	86.8% (80.9% to 91.9%)	86.9% (81.0% to 91.9%)	0.2% (0.1% to 0.2%)	66.1% (57.7% to 74.0%)
Neonatal encephalopathy due to birth asphyxia and trauma	60.9% (51.9% to 67.6%)	61.0% (52.1% to 67.7%)	0.0% (0.0% to 0.0%)	55.8% (44.3% to 64.5%)
Neonatal sepsis and other neonatal infections	56.1% (47.2% to 64.1%)	56.2% (47.3% to 64.2%)	0.0% (0.0% to 0.0%)	41.9% (28.1% to 53.6%)
Hemolytic disease and other neonatal jaundice	48.0% (33.7% to 60.2%)	48.1% (33.8% to 60.4%)	0.0% (0.0% to 0.0%)	23.7% (17.5% to 31.7%)

Supplementary Results Table 1. Age-standardised PAFs attributable to the joint distribution of all risk factors for all ages, both sexes combined for each GBD level 1 and level 2 cause and all causes in 2016 for Brunei. Results are shown for deaths, YLLs, YLDs, and DALYs
Data in parenthesis are 95% uncertainty intervals. PAFs=population attributable fractions. GBD=Global Burden of Disease. YLLs=years of life lost. YLDs=years live with disability. DALYs=disability-adjusted life-years

	Deaths 2016	YLLs 2016	YLDs 2016	DALYs 2016
Other neonatal disorders	64.6% (58.6% to 70.0%)	64.7% (58.7% to 70.0%)	0.0% (0.0% to 0.0%)	62.8% (56.8% to 68.3%)
**Nutritional deficiencies**	2.9% (2.3% to 3.6%)	8.9% (7.3% to 10.9%)	89.2% (86.3% to 91.5%)	86.0% (82.3% to 89.0%)
Protein-energy malnutrition	1.2% (0.6% to 2.1%)	6.3% (3.2% to 10.4%)	98.3% (97.7% to 98.9%)	82.8% (74.9% to 89.0%)
Vitamin A deficiency	-	-	0.0% (0.0% to 0.0%)	0.0% (0.0% to 0.0%)
Iron-deficiency anaemia	100.0% (100.0% to 100.0%)	100.0% (100.0% to 100.0%)	100.0% (100.0% to 100.0%)	100.0% (100.0% to 100.0%)
**Other communicable, maternal, neonatal, and nutritional diseases**	3.6% (2.9% to 4.6%)	2.9% (2.2% to 3.7%)	53.9% (43.2% to 64.6%)	11.5% (7.3% to 17.9%)
Sexually transmitted diseases excluding HIV	91.3% (76.2% to 100.0%)	76.4% (47.2% to 100.0%)	100.0% (100.0% to 100.0%)	93.4% (80.9% to 100.0%)
Hepatitis	0.9% (0.6% to 1.3%)	1.1% (0.8% to 1.7%)	0.7% (0.4% to 1.2%)	1.1% (0.7% to 1.6%)
**Neoplasms**	38.0% (33.7% to 42.4%)	34.4% (30.5% to 38.5%)	29.1% (24.9% to 33.6%)	34.2% (30.3% to 38.3%)
Oesophageal cancer	57.6% (44.2% to 69.7%)	56.2% (42.6% to 68.5%)	57.3% (43.9% to 69.3%)	56.2% (42.6% to 68.5%)
Stomach cancer	19.3% (10.3% to 30.1%)	17.3% (9.2% to 26.8%)	18.6% (9.9% to 28.8%)	17.3% (9.3% to 26.9%)
Liver cancer	30.8% (22.0% to 40.9%)	31.7% (22.7% to 42.0%)	31.1% (22.4% to 41.1%)	31.7% (22.7% to 42.0%)
Larynx cancer	70.0% (59.5% to 79.2%)	66.3% (55.1% to 76.1%)	67.5% (55.7% to 77.7%)	66.4% (55.1% to 76.2%)
Tracheal, bronchus, and lung cancer	79.5% (74.6% to 84.1%)	76.9% (71.3% to 82.2%)	78.6% (73.0% to 83.7%)	77.0% (71.3% to 82.2%)
Breast cancer	18.2% (9.7% to 29.0%)	16.2% (8.1% to 26.2%)	17.1% (8.7% to 27.7%)	16.3% (8.2% to 26.3%)
Cervical cancer	100.0% (100.0% to 100.0%)	100.0% (100.0% to 100.0%)	100.0% (100.0% to 100.0%)	100.0% (100.0% to 100.0%)
Uterine cancer	25.7% (13.7% to 38.9%)	26.5% (14.4% to 39.6%)	26.6% (14.5% to 39.7%)	26.5% (14.4% to 39.6%)
Prostate cancer	3.2% (2.1% to 4.7%)	3.0% (1.9% to 4.3%)	3.0% (1.9% to 4.3%)	3.0% (1.9% to 4.3%)
Colon and rectum cancer	48.7% (37.3% to 60.2%)	47.9% (36.8% to 59.1%)	48.6% (37.1% to 60.1%)	47.9% (36.8% to 59.2%)
Lip and oral cavity cancer	44.8% (35.7% to 53.9%)	40.6% (30.9% to 50.3%)	42.1% (32.6% to 51.5%)	40.6% (30.8% to 50.3%)
Nasopharynx cancer	42.3% (32.0% to 51.7%)	37.5% (27.1% to 47.4%)	37.2% (26.0% to 48.1%)	37.5% (27.1% to 47.4%)

Supplementary Results Table 1. Age-standardised PAFs attributable to the joint distribution of all risk factors for all ages, both sexes combined for each GBD level 1 and level 2 cause and all causes in 2016 for Brunei. Results are shown for deaths, YLLs, YLDs, and DALYs
Data in parenthesis are 95% uncertainty intervals. PAFs=population attributable fractions. GBD=Global Burden of Disease. YLLs=years of life lost. YLDs=years live with disability. DALYs=disability-adjusted life-years

	Deaths 2016	YLLs 2016	YLDs 2016	DALYs 2016
Other pharynx cancer	6.9% (0.2% to 14.9%)	7.1% (0.3% to 15.2%)	7.0% (0.3% to 15.1%)	7.1% (0.3% to 15.2%)
Gallbladder and biliary tract cancer	11.0% (4.6% to 20.0%)	11.6% (5.1% to 20.5%)	11.1% (4.7% to 20.2%)	11.6% (5.0% to 20.5%)
Pancreatic cancer	27.7% (17.6% to 39.8%)	26.2% (16.4% to 38.1%)	27.5% (17.5% to 39.5%)	26.2% (16.4% to 38.1%)
Ovarian cancer	15.7% (6.9% to 28.1%)	12.4% (5.2% to 23.0%)	12.3% (5.4% to 22.5%)	12.4% (5.2% to 23.0%)
Kidney cancer	21.0% (13.6% to 30.2%)	19.6% (12.5% to 28.4%)	19.7% (12.6% to 28.8%)	19.6% (12.5% to 28.4%)
Bladder cancer	32.0% (21.1% to 44.7%)	30.0% (19.4% to 42.3%)	30.2% (19.6% to 42.7%)	30.0% (19.4% to 42.3%)
Thyroid cancer	7.5% (3.1% to 13.7%)	8.0% (3.4% to 14.6%)	7.5% (3.4% to 13.2%)	7.9% (3.4% to 14.2%)
Mesothelioma	91.3% (88.4% to 93.5%)	84.0% (79.5% to 87.5%)	90.0% (87.3% to 92.3%)	84.1% (79.7% to 87.6%)
Non-Hodgkin's lymphoma	3.8% (1.3% to 7.3%)	3.6% (1.3% to 7.0%)	3.7% (1.3% to 7.1%)	3.6% (1.3% to 7.0%)
Multiple myeloma	4.3% (1.5% to 8.6%)	4.6% (1.6% to 9.0%)	4.5% (1.6% to 8.9%)	4.6% (1.6% to 9.0%)
Leukaemia	9.8% (5.6% to 14.6%)	7.8% (4.4% to 11.8%)	6.5% (3.5% to 10.2%)	7.7% (4.4% to 11.7%)
**Cardiovascular diseases**	83.8% (81.0% to 86.2%)	84.3% (82.3% to 86.1%)	76.4% (73.5% to 79.2%)	83.5% (81.5% to 85.3%)
Rheumatic heart disease	26.5% (16.7% to 45.6%)	25.4% (17.3% to 39.3%)	25.7% (17.1% to 38.5%)	25.5% (17.3% to 39.3%)
Ischaemic heart disease	93.5% (90.2% to 96.3%)	94.5% (92.4% to 96.2%)	92.5% (89.8% to 94.9%)	94.4% (92.3% to 96.1%)
Cerebrovascular disease	83.5% (77.9% to 89.0%)	87.4% (83.8% to 90.7%)	85.4% (81.0% to 89.8%)	87.0% (83.2% to 90.4%)
Hypertensive heart disease	100.0% (100.0% to 100.0%)	100.0% (100.0% to 100.0%)	100.0% (100.0% to 100.0%)	100.0% (100.0% to 100.0%)
Cardiomyopathy and myocarditis	49.6% (43.3% to 56.6%)	46.4% (41.1% to 51.9%)	55.7% (50.4% to 61.3%)	47.1% (41.8% to 52.2%)
Atrial fibrillation and flutter	42.9% (35.1% to 50.1%)	47.9% (40.9% to 53.7%)	53.4% (48.1% to 58.4%)	51.2% (45.2% to 56.5%)
Aortic aneurysm	41.8% (34.5% to 48.9%)	46.6% (40.4% to 52.4%)	-	46.6% (40.4% to 52.4%)
Peripheral vascular disease	59.2% (53.2% to 65.5%)	65.6% (60.8% to 70.2%)	66.4% (61.5% to 71.0%)	66.1% (61.4% to 70.5%)
Endocarditis	40.8% (30.4% to 52.3%)	40.0% (31.5% to 48.7%)	43.0% (33.0% to 53.3%)	40.1% (31.6% to 48.9%)

Supplementary Results Table 1. Age-standardised PAFs attributable to the joint distribution of all risk factors for all ages, both sexes combined for each GBD level 1 and level 2 cause and all causes in 2016 for Brunei. Results are shown for deaths, YLLs, YLDs, and DALYs
Data in parenthesis are 95% uncertainty intervals. PAFs=population attributable fractions. GBD=Global Burden of Disease. YLLs=years of life lost. YLDs=years live with disability. DALYs=disability-adjusted life-years

	Deaths 2016	YLLs 2016	YLDs 2016	DALYs 2016
Other cardiovascular and circulatory diseases	43.0% (37.7% to 48.3%)	43.3% (38.9% to 47.7%)	47.8% (43.2% to 52.4%)	44.7% (40.3% to 49.0%)
**Chronic respiratory diseases**	57.9% (52.4% to 62.9%)	53.3% (48.1% to 58.2%)	41.9% (36.0% to 48.1%)	49.0% (44.0% to 54.0%)
Chronic obstructive pulmonary disease	68.4% (61.5% to 74.3%)	64.9% (58.2% to 70.9%)	60.4% (51.5% to 68.5%)	63.7% (56.4% to 70.3%)
Pneumoconiosis	100.0% (100.0% to 100.0%)	100.0% (100.0% to 100.0%)	100.0% (100.0% to 100.0%)	100.0% (100.0% to 100.0%)
Asthma	29.8% (22.1% to 39.3%)	34.0% (26.8% to 42.9%)	27.0% (20.4% to 35.1%)	29.7% (23.2% to 37.6%)
Other chronic respiratory diseases	8.9% (5.8% to 12.2%)	5.1% (2.6% to 8.3%)	5.6% (2.9% to 9.2%)	5.3% (3.1% to 8.1%)
**Cirrhosis and other chronic liver diseases**	26.4% (22.0% to 31.7%)	29.8% (24.7% to 35.5%)	26.1% (21.2% to 31.3%)	29.5% (24.5% to 35.0%)
Cirrhosis and other chronic liver diseases due to hepatitis B	0.2% (0.1% to 0.3%)	0.2% (0.1% to 0.4%)	0.2% (0.1% to 0.4%)	0.2% (0.1% to 0.4%)
Cirrhosis and other chronic liver diseases due to hepatitis C	24.5% (16.1% to 34.7%)	37.9% (26.3% to 49.0%)	45.0% (33.6% to 56.3%)	38.4% (27.0% to 49.4%)
Cirrhosis and other chronic liver diseases due to alcohol use	100.0% (100.0% to 100.0%)	100.0% (100.0% to 100.0%)	100.0% (100.0% to 100.0%)	100.0% (100.0% to 100.0%)
**Digestive diseases**	7.2% (4.5% to 10.3%)	6.8% (4.6% to 9.5%)	2.6% (1.8% to 3.6%)	5.9% (4.0% to 8.2%)
Peptic ulcer disease	10.4% (7.1% to 14.3%)	13.2% (9.1% to 18.0%)	8.8% (6.1% to 12.0%)	12.5% (8.7% to 17.1%)
Gallbladder and biliary diseases	22.3% (11.2% to 35.9%)	23.8% (13.0% to 37.3%)	26.7% (15.2% to 40.5%)	24.0% (13.3% to 37.4%)
Pancreatitis	0.2% (-2.2% to 3.1%)	0.2% (-2.6% to 3.8%)	0.2% (-2.5% to 3.6%)	0.2% (-2.6% to 3.8%)
**Neurological disorders**	18.1% (7.9% to 30.9%)	15.2% (6.8% to 26.0%)	2.7% (1.2% to 5.0%)	7.6% (3.3% to 13.5%)
Alzheimer's disease and other dementias	21.4% (9.6% to 35.9%)	22.9% (10.7% to 37.8%)	22.7% (10.5% to 37.5%)	22.9% (10.6% to 37.7%)
Parkinson's disease	-6.9% (-8.5% to -5.3%)	-8.3% (-10.2% to -6.4%)	-8.8% (-10.7% to -6.8%)	-8.4% (-10.2% to -6.5%)
Epilepsy	0.7% (-0.0% to 2.6%)	0.6% (-0.0% to 2.4%)	0.6% (-0.0% to 2.2%)	0.6% (-0.0% to 2.3%)
Multiple sclerosis	6.8% (3.9% to 10.3%)	6.8% (3.8% to 10.4%)	5.1% (3.1% to 7.2%)	5.8% (3.5% to 8.4%)
**Mental and substance use disorders**	99.3% (99.2% to 99.5%)	99.0% (98.8% to 99.3%)	16.9% (14.9% to 19.2%)	17.7% (15.6% to 20.0%)
Alcohol use disorders	100.0% (100.0% to 100.0%)	100.0% (100.0% to 100.0%)	100.0% (100.0% to 100.0%)	100.0% (100.0% to 100.0%)

Supplementary Results Table 1. Age-standardised PAFs attributable to the joint distribution of all risk factors for all ages, both sexes combined for each GBD level 1 and level 2 cause and all causes in 2016 for Brunei. Results are shown for deaths, YLLs, YLDs, and DALYs
Data in parenthesis are 95% uncertainty intervals. PAFs=population attributable fractions. GBD=Global Burden of Disease. YLLs=years of life lost. YLDs=years live with disability. DALYs=disability-adjusted life-years

	Deaths 2016	YLLs 2016	YLDs 2016	DALYs 2016
Drug use disorders	100.0% (100.0% to 100.0%)	100.0% (100.0% to 100.0%)	100.0% (100.0% to 100.0%)	100.0% (100.0% to 100.0%)
Depressive disorders	-	-	7.1% (5.8% to 8.5%)	7.1% (5.8% to 8.5%)
Idiopathic developmental intellectual disability	-	-	52.3% (26.4% to 71.4%)	52.3% (26.4% to 71.4%)
**Diabetes, urogenital, blood, and endocrine diseases**	81.7% (80.2% to 83.2%)	80.6% (78.9% to 82.1%)	79.9% (77.0% to 82.5%)	80.3% (79.0% to 81.7%)
Diabetes mellitus	100.0% (100.0% to 100.0%)	100.0% (100.0% to 100.0%)	100.0% (100.0% to 100.0%)	100.0% (100.0% to 100.0%)
Chronic kidney disease	100.0% (100.0% to 100.0%)	100.0% (100.0% to 100.0%)	100.0% (100.0% to 100.0%)	100.0% (100.0% to 100.0%)
**Musculoskeletal disorders**	0.7% (0.3% to 1.2%)	0.6% (0.2% to 1.0%)	13.6% (11.3% to 16.3%)	13.4% (11.1% to 16.0%)
Rheumatoid arthritis	3.1% (1.2% to 5.4%)	3.4% (1.4% to 5.9%)	3.2% (1.2% to 5.4%)	3.2% (1.3% to 5.4%)
Osteoarthritis	-	-	15.9% (6.9% to 27.1%)	15.9% (6.9% to 27.1%)
Low back and neck pain	-	-	17.6% (15.2% to 20.2%)	17.6% (15.2% to 20.2%)
Gout	-	-	35.8% (23.1% to 51.2%)	35.8% (23.1% to 51.2%)
**Other non-communicable diseases**	0.1% (0.1% to 0.2%)	0.2% (0.1% to 0.3%)	2.0% (1.7% to 2.4%)	1.6% (1.3% to 1.8%)
Sense organ diseases	-	-	6.0% (5.1% to 7.1%)	6.0% (5.1% to 7.1%)
Sudden infant death syndrome	3.6% (1.8% to 6.4%)	3.6% (1.8% to 6.4%)	-	3.6% (1.8% to 6.4%)
**Transport injuries**	38.6% (27.5% to 48.3%)	35.6% (23.3% to 46.4%)	44.2% (31.5% to 54.1%)	37.7% (25.4% to 48.5%)
Road injuries	37.6% (26.9% to 47.2%)	34.9% (23.0% to 45.6%)	45.1% (32.0% to 55.3%)	37.4% (25.6% to 47.8%)
Other transport injuries	44.1% (29.6% to 55.7%)	40.2% (22.6% to 54.0%)	39.2% (28.7% to 47.7%)	39.9% (24.5% to 51.9%)
**Unintentional injuries**	35.5% (26.8% to 42.7%)	30.0% (17.9% to 39.3%)	35.0% (23.2% to 44.2%)	32.6% (20.9% to 41.8%)
Falls	61.2% (51.9% to 68.2%)	47.4% (32.4% to 59.0%)	39.1% (27.6% to 48.2%)	41.4% (28.7% to 51.1%)
Drowning	23.4% (14.4% to 31.8%)	23.8% (13.8% to 32.5%)	17.1% (11.0% to 22.7%)	23.6% (13.8% to 32.1%)
Fire, heat, and hot substances	21.0% (12.3% to 29.1%)	25.1% (13.1% to 35.1%)	29.9% (15.5% to 41.2%)	27.2% (14.4% to 37.4%)

Supplementary Results Table 1. Age-standardised PAFs attributable to the joint distribution of all risk factors for all ages, both sexes combined for each GBD level 1 and level 2 cause and all causes in 2016 for Brunei. Results are shown for deaths, YLLs, YLDs, and DALYs
Data in parenthesis are 95% uncertainty intervals. PAFs=population attributable fractions. GBD=Global Burden of Disease. YLLs=years of life lost. YLDs=years live with disability. DALYs=disability-adjusted life-years

	**Deaths 2016**	**YLLs 2016**	**YLDs 2016**	**DALYs 2016**
Poisonings	30.9% (15.9% to 44.3%)	34.1% (17.3% to 48.6%)	27.1% (15.7% to 37.1%)	31.6% (16.7% to 44.1%)
Exposure to mechanical forces	38.4% (19.8% to 51.6%)	36.0% (16.2% to 50.4%)	41.5% (24.5% to 54.3%)	38.8% (20.7% to 52.4%)
Animal contact	28.4% (18.8% to 37.2%)	28.2% (15.4% to 38.7%)	27.8% (16.1% to 37.3%)	28.0% (16.1% to 37.1%)
Foreign body	11.6% (7.6% to 16.2%)	13.0% (7.2% to 19.6%)	20.8% (12.3% to 28.3%)	15.6% (9.0% to 21.7%)
Other unintentional injuries	36.8% (15.2% to 52.8%)	37.6% (15.2% to 54.2%)	27.3% (15.1% to 36.8%)	29.5% (15.3% to 40.7%)
**Self-harm and interpersonal violence**	6.2% (4.4% to 9.0%)	6.6% (4.6% to 9.5%)	13.2% (12.2% to 14.5%)	7.4% (5.5% to 10.1%)
Self-harm	5.2% (3.1% to 8.5%)	6.2% (3.7% to 9.8%)	4.3% (2.4% to 7.1%)	6.1% (3.6% to 9.7%)
Interpersonal violence	10.0% (6.5% to 13.2%)	7.8% (4.7% to 11.0%)	15.9% (15.0% to 17.3%)	10.3% (7.9% to 12.9%)
**Forces of nature, conflict and terrorism, and executions and police conflict**	0.0% (0.0% to 0.0%)	0.0% (0.0% to 0.0%)	1.4% (0.7% to 2.3%)	0.6% (0.1% to 1.4%)
Forces of nature, conflict and terrorism, and state actor violence	0.0% (0.0% to 0.0%)	0.0% (0.0% to 0.0%)	6.5% (4.2% to 9.8%)	6.5% (4.2% to 9.8%)

Supplementary Results Table 1. Age-standardised PAFs attributable to the joint distribution of all risk factors for all ages, both sexes combined for each GBD level 1 and level 2 cause and all causes in 2016 for Bulgaria. Results are shown for deaths, YLLs, YLDs, and DALYs
Data in parenthesis are 95% uncertainty intervals. PAFs=population attributable fractions. GBD=Global Burden of Disease. YLLs=years of life lost. YLDs=years live with disability. DALYs=disability-adjusted life-years

	Deaths 2016	YLLs 2016	YLDs 2016	DALYs 2016
**All causes**	71.8% (69.9% to 73.7%)	66.6% (64.7% to 68.4%)	25.2% (23.8% to 26.7%)	50.3% (47.0% to 53.7%)
**HIV/AIDS and tuberculosis**	69.7% (64.4% to 74.4%)	71.1% (67.2% to 74.5%)	62.4% (54.9% to 70.2%)	70.3% (66.5% to 73.8%)
Tuberculosis	64.9% (56.0% to 72.5%)	67.9% (59.9% to 75.0%)	51.2% (42.0% to 60.3%)	65.9% (58.1% to 73.1%)
HIV/AIDS	77.0% (76.0% to 78.1%)	73.8% (72.4% to 75.3%)	80.3% (79.4% to 81.2%)	74.3% (72.8% to 75.8%)
**Diarrhoea, lower respiratory infections, and other common infectious diseases**	56.9% (50.9% to 63.2%)	62.7% (57.1% to 68.4%)	32.1% (23.5% to 41.0%)	54.8% (49.2% to 60.8%)
Diarrhoeal diseases	71.1% (53.4% to 85.7%)	81.3% (68.4% to 90.7%)	65.6% (47.2% to 82.0%)	69.1% (51.9% to 84.0%)
Lower respiratory infections	61.4% (54.8% to 68.6%)	71.3% (64.8% to 78.0%)	62.4% (55.2% to 69.5%)	71.2% (64.6% to 77.9%)
Upper respiratory infections	0.6% (0.0% to 1.9%)	1.6% (0.1% to 5.2%)	0.0% (0.0% to 0.0%)	0.0% (0.0% to 0.0%)
Otitis media	2.0% (0.8% to 4.4%)	7.3% (3.9% to 11.9%)	10.8% (7.4% to 13.9%)	10.8% (7.4% to 13.9%)
Meningitis	3.3% (2.1% to 5.0%)	4.9% (3.3% to 6.9%)	0.0% (0.0% to 0.0%)	3.9% (2.5% to 5.7%)
Encephalitis	1.2% (0.5% to 2.3%)	1.9% (0.8% to 3.4%)	0.0% (0.0% to 0.0%)	1.6% (0.6% to 2.9%)
Measles	26.4% (8.8% to 63.9%)	27.7% (9.4% to 66.5%)	14.6% (0.0% to 66.2%)	27.7% (9.4% to 66.5%)
**Maternal disorders**	6.1% (3.4% to 8.9%)	6.1% (3.4% to 9.0%)	5.5% (2.4% to 9.3%)	5.9% (3.1% to 9.0%)
Maternal haemorrhage	12.4% (4.5% to 20.8%)	12.6% (4.5% to 21.1%)	12.8% (4.5% to 21.4%)	12.7% (4.5% to 21.1%)
Maternal sepsis and other pregnancy related infections	12.2% (4.4% to 20.1%)	12.4% (4.4% to 20.4%)	12.7% (4.5% to 21.1%)	12.5% (4.4% to 20.6%)
Maternal abortion, miscarriage, and ectopic pregnancy	7.8% (4.7% to 11.3%)	7.6% (4.6% to 11.0%)	7.6% (3.8% to 12.4%)	7.6% (4.7% to 10.9%)
**Neonatal disorders**	82.6% (80.2% to 84.5%)	82.6% (80.2% to 84.6%)	0.1% (0.0% to 0.1%)	59.2% (47.9% to 69.0%)
Neonatal preterm birth complications	93.7% (91.0% to 95.6%)	93.7% (91.1% to 95.6%)	0.2% (0.1% to 0.2%)	76.0% (64.5% to 84.7%)
Neonatal encephalopathy due to birth asphyxia and trauma	69.2% (66.4% to 72.2%)	69.2% (66.4% to 72.2%)	0.0% (0.0% to 0.0%)	39.5% (24.9% to 53.0%)
Neonatal sepsis and other neonatal infections	56.9% (50.4% to 61.5%)	57.0% (50.4% to 61.5%)	0.0% (0.0% to 0.0%)	27.3% (13.1% to 41.1%)
Hemolytic disease and other neonatal jaundice	61.6% (55.2% to 66.7%)	61.8% (55.5% to 66.9%)	0.0% (0.0% to 0.0%)	16.0% (8.1% to 26.3%)

Supplementary Results Table 1. Age-standardised PAFs attributable to the joint distribution of all risk factors for all ages, both sexes combined for each GBD level 1 and level 2 cause and all causes in 2016 for Bulgaria. Results are shown for deaths, YLLs, YLDs, and DALYs
Data in parenthesis are 95% uncertainty intervals. PAFs=population attributable fractions. GBD=Global Burden of Disease. YLLs=years of life lost. YLDs=years live with disability. DALYs=disability-adjusted life-years

	Deaths 2016	YLLs 2016	YLDs 2016	DALYs 2016
Other neonatal disorders	65.9% (62.0% to 69.4%)	65.9% (62.1% to 69.4%)	0.0% (0.0% to 0.0%)	60.6% (55.2% to 65.2%)
**Nutritional deficiencies**	15.8% (11.5% to 21.7%)	32.1% (24.4% to 43.7%)	90.6% (88.1% to 92.7%)	89.3% (86.5% to 91.7%)
Protein-energy malnutrition	24.8% (13.3% to 40.9%)	56.2% (39.0% to 71.9%)	97.9% (96.3% to 98.9%)	93.5% (89.8% to 96.4%)
Vitamin A deficiency	-	-	0.0% (0.0% to 0.0%)	0.0% (0.0% to 0.0%)
Iron-deficiency anaemia	100.0% (100.0% to 100.0%)	100.0% (100.0% to 100.0%)	100.0% (100.0% to 100.0%)	100.0% (100.0% to 100.0%)
**Other communicable, maternal, neonatal, and nutritional diseases**	4.8% (3.8% to 5.8%)	4.1% (3.1% to 5.2%)	72.4% (66.6% to 77.9%)	45.6% (36.2% to 56.2%)
Sexually transmitted diseases excluding HIV	69.1% (48.8% to 87.7%)	51.5% (29.6% to 76.4%)	100.0% (100.0% to 100.0%)	97.1% (93.2% to 99.3%)
Hepatitis	2.2% (1.6% to 3.0%)	1.8% (1.3% to 2.5%)	1.0% (0.6% to 1.6%)	1.7% (1.2% to 2.4%)
**Neoplasms**	46.8% (43.3% to 50.4%)	48.1% (44.7% to 51.5%)	40.2% (36.7% to 44.1%)	47.9% (44.5% to 51.3%)
Oesophageal cancer	86.1% (79.2% to 91.3%)	88.8% (82.9% to 93.1%)	86.9% (80.5% to 91.9%)	88.8% (82.9% to 93.1%)
Stomach cancer	23.8% (14.3% to 34.0%)	27.1% (17.6% to 37.1%)	25.2% (15.5% to 35.2%)	27.1% (17.5% to 37.1%)
Liver cancer	67.2% (59.3% to 74.8%)	68.9% (60.5% to 76.4%)	67.7% (59.7% to 75.0%)	68.9% (60.5% to 76.4%)
Larynx cancer	90.9% (85.7% to 94.6%)	92.8% (88.4% to 95.8%)	91.9% (87.4% to 95.2%)	92.8% (88.4% to 95.8%)
Tracheal, bronchus, and lung cancer	88.2% (85.1% to 90.8%)	90.0% (87.4% to 92.3%)	88.5% (85.5% to 91.0%)	90.0% (87.4% to 92.2%)
Breast cancer	30.8% (24.5% to 37.7%)	30.8% (24.5% to 37.2%)	30.9% (24.5% to 37.6%)	30.8% (24.5% to 37.2%)
Cervical cancer	100.0% (100.0% to 100.0%)	100.0% (100.0% to 100.0%)	100.0% (100.0% to 100.0%)	100.0% (100.0% to 100.0%)
Uterine cancer	44.8% (29.8% to 59.9%)	45.7% (30.7% to 60.4%)	46.5% (31.2% to 61.1%)	45.8% (30.7% to 60.4%)
Prostate cancer	5.1% (3.4% to 7.0%)	7.0% (4.8% to 9.5%)	6.7% (4.6% to 9.1%)	7.0% (4.8% to 9.5%)
Colon and rectum cancer	57.8% (49.4% to 65.5%)	59.7% (51.7% to 66.7%)	59.0% (51.1% to 66.4%)	59.7% (51.7% to 66.7%)
Lip and oral cavity cancer	84.6% (78.9% to 88.9%)	88.5% (83.7% to 91.8%)	84.9% (79.4% to 89.3%)	88.3% (83.6% to 91.7%)
Nasopharynx cancer	82.2% (77.6% to 86.1%)	82.2% (78.1% to 86.1%)	81.2% (76.1% to 85.9%)	82.1% (78.0% to 86.1%)

Supplementary Results Table 1. Age-standardised PAFs attributable to the joint distribution of all risk factors for all ages, both sexes combined for each GBD level 1 and level 2 cause and all causes in 2016 for Bulgaria. Results are shown for deaths, YLLs, YLDs, and DALYs
Data in parenthesis are 95% uncertainty intervals. PAFs=population attributable fractions. GBD=Global Burden of Disease. YLLs=years of life lost. YLDs=years live with disability. DALYs=disability-adjusted life-years

	Deaths 2016	YLLs 2016	YLDs 2016	DALYs 2016
Other pharynx cancer	72.6% (62.6% to 79.8%)	73.5% (63.7% to 80.7%)	73.2% (63.6% to 80.5%)	73.5% (63.7% to 80.7%)
Gallbladder and biliary tract cancer	22.8% (12.5% to 35.7%)	23.4% (13.1% to 36.1%)	23.7% (12.8% to 36.9%)	23.4% (13.1% to 36.1%)
Pancreatic cancer	32.9% (25.1% to 41.4%)	37.7% (29.6% to 46.3%)	33.9% (26.1% to 42.2%)	37.7% (29.6% to 46.2%)
Ovarian cancer	8.6% (2.7% to 16.0%)	7.8% (2.2% to 14.6%)	7.7% (2.2% to 14.5%)	7.8% (2.2% to 14.6%)
Kidney cancer	40.0% (29.4% to 50.8%)	41.1% (30.4% to 51.6%)	40.2% (29.8% to 50.2%)	41.1% (30.4% to 51.5%)
Bladder cancer	35.0% (25.4% to 44.5%)	41.8% (31.3% to 51.3%)	40.2% (30.4% to 49.4%)	41.7% (31.2% to 51.1%)
Thyroid cancer	14.5% (7.0% to 24.2%)	14.8% (7.2% to 24.7%)	13.5% (6.8% to 22.1%)	14.6% (7.1% to 24.4%)
Mesothelioma	66.7% (57.7% to 74.8%)	57.0% (47.5% to 65.9%)	63.5% (54.4% to 72.0%)	57.1% (47.6% to 66.0%)
Non-Hodgkin's lymphoma	6.6% (2.6% to 11.9%)	5.9% (2.3% to 10.4%)	6.7% (2.6% to 11.8%)	5.9% (2.3% to 10.5%)
Multiple myeloma	8.4% (3.5% to 15.1%)	8.4% (3.6% to 15.2%)	8.7% (3.6% to 15.4%)	8.4% (3.6% to 15.2%)
Leukaemia	20.6% (12.9% to 28.7%)	17.3% (11.1% to 24.1%)	19.9% (12.3% to 27.5%)	17.4% (11.2% to 24.1%)
**Cardiovascular diseases**	88.2% (85.6% to 90.4%)	89.7% (88.0% to 91.2%)	78.2% (75.4% to 81.0%)	88.6% (86.9% to 90.0%)
Rheumatic heart disease	33.6% (23.3% to 48.3%)	32.6% (22.7% to 45.8%)	33.8% (22.9% to 49.3%)	32.6% (22.7% to 45.9%)
Ischaemic heart disease	93.3% (89.7% to 96.2%)	95.2% (93.1% to 96.9%)	92.5% (90.1% to 94.6%)	95.1% (93.0% to 96.8%)
Cerebrovascular disease	85.4% (80.2% to 89.8%)	89.2% (86.1% to 92.2%)	87.8% (84.0% to 91.3%)	89.1% (85.9% to 92.0%)
Hypertensive heart disease	100.0% (100.0% to 100.0%)	100.0% (100.0% to 100.0%)	100.0% (100.0% to 100.0%)	100.0% (100.0% to 100.0%)
Cardiomyopathy and myocarditis	28.9% (23.0% to 35.2%)	30.5% (25.1% to 37.0%)	28.2% (23.5% to 33.4%)	30.3% (25.0% to 36.5%)
Atrial fibrillation and flutter	54.8% (47.2% to 62.8%)	60.5% (53.5% to 67.1%)	69.1% (64.2% to 74.0%)	65.5% (59.7% to 71.2%)
Aortic aneurysm	58.3% (52.4% to 63.8%)	62.2% (56.7% to 67.0%)	-	62.2% (56.7% to 67.0%)
Peripheral vascular disease	53.4% (45.7% to 60.9%)	62.3% (55.2% to 68.0%)	59.8% (53.7% to 66.0%)	60.7% (54.9% to 66.4%)
Endocarditis	45.9% (36.6% to 54.8%)	41.7% (32.1% to 51.1%)	42.0% (32.7% to 50.6%)	41.7% (32.2% to 51.1%)

Supplementary Results Table 1. Age-standardised PAFs attributable to the joint distribution of all risk factors for all ages, both sexes combined for each GBD level 1 and level 2 cause and all causes in 2016 for Bulgaria. Results are shown for deaths, YLLs, YLDs, and DALYs
Data in parenthesis are 95% uncertainty intervals. PAFs=population attributable fractions. GBD=Global Burden of Disease. YLLs=years of life lost. YLDs=years live with disability. DALYs=disability-adjusted life-years

	Deaths 2016	YLLs 2016	YLDs 2016	DALYs 2016
Other cardiovascular and circulatory diseases	56.2% (51.3% to 60.7%)	61.2% (56.7% to 65.3%)	54.6% (49.7% to 59.6%)	59.0% (54.8% to 63.3%)
**Chronic respiratory diseases**	65.8% (58.1% to 72.9%)	72.0% (66.3% to 77.3%)	49.8% (43.3% to 56.0%)	63.3% (58.2% to 68.6%)
Chronic obstructive pulmonary disease	67.5% (59.5% to 75.0%)	75.1% (69.1% to 80.7%)	75.1% (69.7% to 80.2%)	75.1% (69.4% to 80.4%)
Pneumoconiosis	100.0% (100.0% to 100.0%)	100.0% (100.0% to 100.0%)	100.0% (100.0% to 100.0%)	100.0% (100.0% to 100.0%)
Asthma	40.9% (30.7% to 52.2%)	46.5% (37.3% to 56.7%)	36.4% (27.9% to 46.0%)	36.9% (28.6% to 46.5%)
Other chronic respiratory diseases	18.1% (11.8% to 25.0%)	18.9% (12.7% to 26.6%)	11.6% (8.3% to 15.2%)	16.5% (11.4% to 22.8%)
**Cirrhosis and other chronic liver diseases**	57.9% (53.0% to 62.7%)	58.1% (53.1% to 62.9%)	44.5% (39.4% to 49.4%)	57.6% (52.6% to 62.3%)
Cirrhosis and other chronic liver diseases due to hepatitis B	0.9% (0.4% to 1.7%)	1.1% (0.4% to 1.9%)	0.9% (0.4% to 1.7%)	1.1% (0.4% to 1.9%)
Cirrhosis and other chronic liver diseases due to hepatitis C	82.1% (76.6% to 86.1%)	85.8% (81.9% to 88.2%)	82.3% (76.8% to 86.3%)	85.7% (81.9% to 88.1%)
Cirrhosis and other chronic liver diseases due to alcohol use	100.0% (100.0% to 100.0%)	100.0% (100.0% to 100.0%)	100.0% (100.0% to 100.0%)	100.0% (100.0% to 100.0%)
**Digestive diseases**	13.9% (10.9% to 16.9%)	17.3% (13.7% to 21.3%)	6.0% (4.6% to 7.8%)	12.8% (10.0% to 15.9%)
Peptic ulcer disease	17.0% (12.3% to 22.3%)	22.8% (16.9% to 29.4%)	21.7% (15.7% to 28.4%)	22.4% (16.5% to 28.7%)
Gallbladder and biliary diseases	38.8% (24.6% to 54.5%)	41.7% (27.5% to 56.5%)	45.7% (31.5% to 60.2%)	43.1% (29.1% to 57.9%)
Pancreatitis	46.2% (32.2% to 60.5%)	50.8% (36.0% to 64.7%)	39.8% (27.1% to 52.7%)	50.5% (35.7% to 64.5%)
**Neurological disorders**	17.6% (8.5% to 29.1%)	18.1% (10.7% to 27.3%)	3.7% (1.9% to 6.4%)	8.1% (4.8% to 12.2%)
Alzheimer's disease and other dementias	21.2% (10.0% to 35.4%)	23.3% (11.8% to 37.5%)	23.3% (11.7% to 37.4%)	23.3% (11.7% to 37.5%)
Parkinson's disease	-8.3% (-10.4% to -6.3%)	-12.0% (-14.9% to -9.1%)	-15.4% (-18.9% to -11.8%)	-12.7% (-15.7% to -9.8%)
Epilepsy	36.1% (27.7% to 44.2%)	32.1% (24.1% to 39.9%)	25.3% (18.1% to 32.7%)	28.2% (20.6% to 35.8%)
Multiple sclerosis	13.7% (8.9% to 19.0%)	16.5% (10.9% to 22.0%)	14.9% (10.0% to 20.2%)	15.9% (10.6% to 21.4%)
**Mental and substance use disorders**	99.9% (99.9% to 99.9%)	99.9% (99.9% to 99.9%)	18.9% (16.8% to 21.3%)	22.3% (20.0% to 25.0%)
Alcohol use disorders	100.0% (100.0% to 100.0%)	100.0% (100.0% to 100.0%)	100.0% (100.0% to 100.0%)	100.0% (100.0% to 100.0%)

Supplementary Results Table 1. Age-standardised PAFs attributable to the joint distribution of all risk factors for all ages, both sexes combined for each GBD level 1 and level 2 cause and all causes in 2016 for Bulgaria. Results are shown for deaths, YLLs, YLDs, and DALYs
Data in parenthesis are 95% uncertainty intervals. PAFs=population attributable fractions. GBD=Global Burden of Disease. YLLs=years of life lost. YLDs=years live with disability. DALYs=disability-adjusted life-years

	Deaths 2016	YLLs 2016	YLDs 2016	DALYs 2016
Drug use disorders	100.0% (100.0% to 100.0%)	100.0% (100.0% to 100.0%)	100.0% (100.0% to 100.0%)	100.0% (100.0% to 100.0%)
Depressive disorders	-	-	6.2% (5.1% to 7.4%)	6.2% (5.1% to 7.4%)
Idiopathic developmental intellectual disability	-	-	54.3% (27.3% to 73.7%)	54.3% (27.3% to 73.7%)
**Diabetes, urogenital, blood, and endocrine diseases**	84.8% (83.6% to 87.4%)	80.2% (78.1% to 84.0%)	54.7% (50.6% to 58.9%)	66.6% (63.0% to 70.0%)
Diabetes mellitus	100.0% (100.0% to 100.0%)	100.0% (100.0% to 100.0%)	100.0% (100.0% to 100.0%)	100.0% (100.0% to 100.0%)
Chronic kidney disease	100.0% (100.0% to 100.0%)	100.0% (100.0% to 100.0%)	100.0% (100.0% to 100.0%)	100.0% (100.0% to 100.0%)
**Musculoskeletal disorders**	1.6% (0.6% to 2.9%)	1.6% (0.7% to 2.8%)	22.9% (18.8% to 27.2%)	22.8% (18.7% to 27.1%)
Rheumatoid arthritis	5.6% (2.3% to 9.3%)	7.4% (3.0% to 12.4%)	8.8% (3.7% to 14.5%)	8.7% (3.6% to 14.4%)
Osteoarthritis	-	-	27.0% (13.8% to 41.1%)	27.0% (13.8% to 41.1%)
Low back and neck pain	-	-	23.7% (20.1% to 27.7%)	23.7% (20.1% to 27.7%)
Gout	-	-	45.0% (28.7% to 62.1%)	45.0% (28.7% to 62.1%)
**Other non-communicable diseases**	0.1% (0.1% to 0.1%)	0.1% (0.1% to 0.2%)	3.6% (3.1% to 4.1%)	2.9% (2.4% to 3.3%)
Sense organ diseases	-	-	8.2% (7.3% to 9.2%)	8.2% (7.3% to 9.2%)
Sudden infant death syndrome	5.3% (3.3% to 8.0%)	5.4% (3.3% to 8.0%)	-	5.4% (3.3% to 8.0%)
**Transport injuries**	35.8% (26.6% to 45.4%)	32.1% (22.9% to 41.9%)	38.8% (30.2% to 47.5%)	34.3% (25.4% to 43.6%)
Road injuries	35.4% (26.1% to 45.0%)	31.8% (22.6% to 41.7%)	39.1% (30.4% to 47.9%)	34.4% (25.4% to 43.6%)
Other transport injuries	39.6% (30.4% to 49.1%)	34.3% (25.1% to 44.1%)	32.1% (23.9% to 40.8%)	33.9% (24.9% to 43.4%)
**Unintentional injuries**	22.0% (17.2% to 32.2%)	15.2% (11.5% to 20.5%)	20.3% (17.7% to 23.8%)	18.6% (15.6% to 22.5%)
Falls	39.1% (32.8% to 54.6%)	23.8% (18.7% to 34.2%)	22.6% (20.1% to 26.0%)	22.8% (20.0% to 27.0%)
Drowning	17.4% (11.2% to 25.1%)	15.1% (9.8% to 21.8%)	17.8% (11.7% to 26.1%)	15.1% (9.8% to 21.9%)
Fire, heat, and hot substances	16.0% (10.1% to 23.6%)	15.4% (10.1% to 22.7%)	19.1% (12.9% to 27.8%)	17.2% (11.6% to 24.9%)

Supplementary Results Table 1. Age-standardised PAFs attributable to the joint distribution of all risk factors for all ages, both sexes combined for each GBD level 1 and level 2 cause and all causes in 2016 for Bulgaria. Results are shown for deaths, YLLs, YLDs, and DALYs
Data in parenthesis are 95% uncertainty intervals. PAFs=population attributable fractions. GBD=Global Burden of Disease. YLLs=years of life lost. YLDs=years live with disability. DALYs=disability-adjusted life-years

	Deaths 2016	YLLs 2016	YLDs 2016	DALYs 2016
Poisonings	17.8% (11.4% to 25.3%)	17.1% (11.3% to 24.3%)	18.3% (12.3% to 26.5%)	17.4% (11.7% to 24.8%)
Exposure to mechanical forces	16.6% (13.1% to 21.2%)	13.6% (9.9% to 18.3%)	17.9% (14.8% to 22.5%)	16.4% (13.1% to 21.0%)
Animal contact	12.4% (9.8% to 15.8%)	10.6% (7.8% to 14.4%)	8.9% (6.3% to 12.7%)	9.5% (6.9% to 13.2%)
Foreign body	7.9% (5.2% to 11.6%)	6.9% (4.0% to 10.8%)	7.0% (4.8% to 10.2%)	7.0% (4.4% to 10.5%)
Other unintentional injuries	22.2% (14.4% to 31.8%)	21.7% (14.4% to 30.9%)	17.6% (11.8% to 25.7%)	19.2% (13.0% to 27.7%)
**Self-harm and interpersonal violence**	33.6% (21.4% to 44.4%)	32.9% (21.0% to 43.5%)	23.5% (18.0% to 29.0%)	32.1% (20.9% to 41.9%)
Self-harm	35.2% (20.5% to 48.0%)	35.4% (21.0% to 48.3%)	26.8% (13.8% to 38.8%)	35.2% (20.9% to 48.2%)
Interpersonal violence	26.1% (17.7% to 34.1%)	23.2% (15.1% to 31.1%)	22.9% (17.2% to 28.9%)	23.2% (15.7% to 30.4%)
**Forces of nature, conflict and terrorism, and executions and police conflict**	0.0% (0.0% to 0.0%)	0.0% (0.0% to 0.0%)	3.7% (2.5% to 5.4%)	2.6% (1.4% to 4.2%)
Forces of nature, conflict and terrorism, and state actor violence	0.0% (0.0% to 0.0%)	0.0% (0.0% to 0.0%)	4.3% (2.9% to 6.4%)	4.3% (2.9% to 6.4%)

Supplementary Results Table 1. Age-standardised PAFs attributable to the joint distribution of all risk factors for all ages, both sexes combined for each GBD level 1 and level 2 cause and all causes in 2016 for Burkina Faso. Results are shown for deaths, YLLs, YLDs, and DALYs
Data in parenthesis are 95% uncertainty intervals. PAFs=population attributable fractions. GBD=Global Burden of Disease. YLLs=years of life lost. YLDs=years live with disability. DALYs=disability-adjusted life-years

	Deaths 2016	YLLs 2016	YLDs 2016	DALYs 2016
**All causes**	52.3% (49.5% to 55.1%)	44.7% (42.0% to 47.7%)	31.6% (30.5% to 32.8%)	42.0% (39.8% to 44.4%)
**HIV/AIDS and tuberculosis**	55.5% (41.0% to 67.6%)	59.8% (48.3% to 69.3%)	76.5% (69.8% to 82.4%)	60.5% (49.3% to 69.7%)
Tuberculosis	47.1% (30.3% to 61.5%)	47.6% (32.2% to 60.8%)	46.6% (31.2% to 59.9%)	47.6% (32.2% to 60.8%)
HIV/AIDS	90.8% (88.6% to 93.2%)	87.4% (84.8% to 90.0%)	91.4% (88.9% to 93.7%)	87.7% (85.3% to 90.2%)
**Diarrhoea, lower respiratory infections, and other common infectious diseases**	78.0% (73.2% to 82.6%)	73.6% (68.4% to 78.4%)	45.7% (40.7% to 50.3%)	72.5% (67.4% to 77.1%)
Diarrhoeal diseases	95.7% (88.9% to 98.9%)	96.7% (91.5% to 99.2%)	96.0% (89.6% to 98.9%)	96.7% (91.4% to 99.2%)
Lower respiratory infections	86.2% (81.3% to 90.3%)	90.6% (86.7% to 93.6%)	84.7% (79.7% to 89.2%)	90.6% (86.7% to 93.6%)
Upper respiratory infections	1.6% (0.1% to 5.6%)	2.8% (0.2% to 9.7%)	0.0% (0.0% to 0.0%)	0.3% (0.0% to 1.0%)
Otitis media	1.9% (0.5% to 4.6%)	6.4% (3.1% to 10.6%)	4.4% (2.8% to 6.1%)	4.5% (2.9% to 6.2%)
Meningitis	2.6% (1.4% to 4.2%)	4.5% (2.6% to 7.2%)	0.0% (0.0% to 0.0%)	4.3% (2.5% to 7.0%)
Encephalitis	2.6% (0.7% to 5.3%)	3.6% (1.3% to 7.0%)	0.0% (0.0% to 0.0%)	2.0% (0.5% to 4.3%)
Measles	70.9% (58.3% to 84.7%)	72.1% (59.5% to 86.0%)	70.5% (58.9% to 82.9%)	72.1% (59.5% to 86.0%)
**Maternal disorders**	9.6% (4.0% to 15.7%)	9.5% (3.9% to 15.8%)	2.4% (0.9% to 4.3%)	9.2% (3.8% to 15.3%)
Maternal haemorrhage	21.8% (8.0% to 35.0%)	21.9% (8.0% to 35.1%)	22.0% (8.0% to 35.3%)	21.9% (8.0% to 35.1%)
Maternal sepsis and other pregnancy related infections	21.7% (7.9% to 34.8%)	21.8% (7.9% to 35.0%)	21.5% (7.9% to 34.6%)	21.8% (7.9% to 35.0%)
Maternal abortion, miscarriage, and ectopic pregnancy	7.3% (4.3% to 10.8%)	7.2% (4.1% to 10.7%)	7.3% (4.0% to 11.1%)	7.2% (4.1% to 10.7%)
**Neonatal disorders**	81.3% (77.9% to 84.5%)	81.3% (77.9% to 84.5%)	0.7% (0.3% to 1.2%)	78.9% (75.2% to 82.4%)
Neonatal preterm birth complications	97.9% (95.8% to 99.1%)	97.9% (95.8% to 99.1%)	1.9% (1.2% to 2.7%)	95.1% (92.3% to 97.1%)
Neonatal encephalopathy due to birth asphyxia and trauma	76.6% (71.8% to 81.0%)	76.6% (71.9% to 81.0%)	0.0% (0.0% to 0.0%)	75.0% (69.9% to 79.7%)
Neonatal sepsis and other neonatal infections	69.4% (64.0% to 74.3%)	69.5% (64.1% to 74.3%)	0.0% (0.0% to 0.0%)	66.5% (59.6% to 72.4%)
Hemolytic disease and other neonatal jaundice	65.6% (53.7% to 74.2%)	65.6% (53.7% to 74.2%)	0.0% (0.0% to 0.0%)	62.1% (50.9% to 70.7%)

Supplementary Results Table 1. Age-standardised PAFs attributable to the joint distribution of all risk factors for all ages, both sexes combined for each GBD level 1 and level 2 cause and all causes in 2016 for Burkina Faso. Results are shown for deaths, YLLs, YLDs, and DALYs
Data in parenthesis are 95% uncertainty intervals. PAFs=population attributable fractions. GBD=Global Burden of Disease. YLLs=years of life lost. YLDs=years live with disability. DALYs=disability-adjusted life-years

	Deaths 2016	YLLs 2016	YLDs 2016	DALYs 2016
Other neonatal disorders	68.4% (59.5% to 75.2%)	68.5% (59.6% to 75.2%)	0.0% (0.0% to 0.0%)	66.9% (58.1% to 73.7%)
**Nutritional deficiencies**	26.5% (16.5% to 37.4%)	59.5% (45.6% to 71.2%)	96.5% (95.9% to 97.1%)	78.6% (73.0% to 83.5%)
Protein-energy malnutrition	33.2% (21.3% to 45.9%)	69.2% (55.5% to 80.0%)	97.1% (96.0% to 97.9%)	70.5% (58.2% to 80.4%)
Vitamin A deficiency	-	-	100.0% (100.0% to 100.0%)	100.0% (100.0% to 100.0%)
Iron-deficiency anaemia	100.0% (100.0% to 100.0%)	100.0% (100.0% to 100.0%)	100.0% (100.0% to 100.0%)	100.0% (100.0% to 100.0%)
**Other communicable, maternal, neonatal, and nutritional diseases**	6.2% (3.9% to 8.8%)	4.9% (2.5% to 8.2%)	50.0% (45.1% to 55.6%)	12.5% (6.9% to 18.7%)
Sexually transmitted diseases excluding HIV	26.3% (13.5% to 46.1%)	14.0% (6.7% to 27.2%)	100.0% (100.0% to 100.0%)	32.6% (17.0% to 54.6%)
Hepatitis	0.0% (0.0% to 0.1%)	0.1% (0.0% to 0.1%)	0.1% (0.0% to 0.1%)	0.1% (0.0% to 0.1%)
**Neoplasms**	33.3% (29.3% to 37.1%)	34.0% (29.9% to 37.7%)	36.0% (30.7% to 40.8%)	34.0% (30.0% to 37.7%)
Oesophageal cancer	69.4% (55.2% to 80.1%)	70.6% (57.2% to 80.6%)	70.0% (56.3% to 80.5%)	70.6% (57.2% to 80.6%)
Stomach cancer	4.3% (0.9% to 12.6%)	4.3% (0.9% to 12.0%)	4.3% (0.9% to 12.4%)	4.3% (0.9% to 12.0%)
Liver cancer	28.3% (22.2% to 35.9%)	27.5% (21.1% to 35.5%)	28.3% (22.0% to 36.0%)	27.5% (21.1% to 35.5%)
Larynx cancer	57.2% (41.5% to 71.5%)	59.8% (44.2% to 73.6%)	58.1% (42.1% to 72.3%)	59.7% (44.1% to 73.6%)
Tracheal, bronchus, and lung cancer	83.6% (77.6% to 88.5%)	83.5% (77.7% to 88.2%)	83.6% (77.8% to 88.4%)	83.5% (77.7% to 88.2%)
Breast cancer	26.8% (20.0% to 36.5%)	25.4% (18.7% to 35.4%)	25.9% (19.3% to 35.3%)	25.4% (18.7% to 35.4%)
Cervical cancer	100.0% (100.0% to 100.0%)	100.0% (100.0% to 100.0%)	100.0% (100.0% to 100.0%)	100.0% (100.0% to 100.0%)
Uterine cancer	31.2% (18.2% to 46.8%)	30.8% (18.1% to 45.9%)	31.1% (18.0% to 46.1%)	30.8% (18.1% to 45.9%)
Prostate cancer	0.7% (0.3% to 1.1%)	0.8% (0.4% to 1.3%)	0.7% (0.4% to 1.2%)	0.8% (0.4% to 1.3%)
Colon and rectum cancer	47.7% (38.0% to 57.0%)	48.3% (38.7% to 57.5%)	48.0% (38.4% to 57.4%)	48.3% (38.7% to 57.5%)
Lip and oral cavity cancer	68.2% (53.6% to 81.0%)	72.0% (58.4% to 83.1%)	70.2% (56.4% to 82.1%)	72.0% (58.4% to 83.0%)
Nasopharynx cancer	65.4% (50.3% to 77.0%)	64.8% (50.4% to 75.7%)	64.3% (50.4% to 75.4%)	64.8% (50.4% to 75.7%)

Supplementary Results Table 1. Age-standardised PAFs attributable to the joint distribution of all risk factors for all ages, both sexes combined for each GBD level 1 and level 2 cause and all causes in 2016 for Burkina Faso. Results are shown for deaths, YLLs, YLDs, and DALYs
Data in parenthesis are 95% uncertainty intervals. PAFs=population attributable fractions. GBD=Global Burden of Disease. YLLs=years of life lost. YLDs=years live with disability. DALYs=disability-adjusted life-years

	Deaths 2016	YLLs 2016	YLDs 2016	DALYs 2016
Other pharynx cancer	55.5% (35.5% to 72.1%)	58.0% (39.0% to 74.3%)	56.3% (36.5% to 72.7%)	58.0% (39.0% to 74.3%)
Gallbladder and biliary tract cancer	16.3% (7.3% to 27.7%)	16.1% (7.3% to 27.6%)	16.1% (7.2% to 27.5%)	16.1% (7.3% to 27.6%)
Pancreatic cancer	11.6% (6.1% to 19.1%)	11.4% (6.1% to 18.4%)	11.5% (6.1% to 18.8%)	11.4% (6.1% to 18.4%)
Ovarian cancer	8.7% (3.8% to 15.5%)	7.4% (3.0% to 13.3%)	7.8% (3.3% to 13.8%)	7.4% (3.0% to 13.3%)
Kidney cancer	13.7% (7.7% to 21.8%)	12.3% (6.9% to 19.8%)	12.4% (6.9% to 19.9%)	12.3% (6.9% to 19.8%)
Bladder cancer	9.5% (4.6% to 16.1%)	9.7% (4.7% to 16.1%)	9.7% (4.8% to 16.3%)	9.7% (4.7% to 16.1%)
Thyroid cancer	7.8% (3.5% to 13.9%)	7.6% (3.4% to 13.6%)	7.6% (3.5% to 13.7%)	7.6% (3.4% to 13.5%)
Mesothelioma	83.4% (77.7% to 88.7%)	74.4% (66.9% to 82.3%)	80.2% (73.7% to 86.4%)	74.5% (67.0% to 82.3%)
Non-Hodgkin's lymphoma	3.1% (1.0% to 6.2%)	2.7% (0.9% to 5.4%)	2.9% (1.0% to 5.7%)	2.7% (0.9% to 5.4%)
Multiple myeloma	4.7% (1.6% to 9.2%)	4.7% (1.6% to 9.2%)	4.7% (1.6% to 9.3%)	4.7% (1.6% to 9.2%)
Leukaemia	7.0% (3.9% to 10.9%)	6.4% (3.6% to 10.2%)	6.8% (3.8% to 10.6%)	6.4% (3.6% to 10.2%)
**Cardiovascular diseases**	81.3% (76.0% to 84.3%)	81.4% (77.0% to 84.2%)	60.3% (56.4% to 64.2%)	79.9% (75.5% to 82.7%)
Rheumatic heart disease	25.6% (16.5% to 41.7%)	23.8% (15.9% to 35.4%)	19.3% (12.2% to 28.4%)	23.0% (15.6% to 34.2%)
Ischaemic heart disease	92.6% (89.7% to 95.1%)	93.9% (91.7% to 95.7%)	88.5% (84.9% to 91.5%)	93.8% (91.5% to 95.6%)
Cerebrovascular disease	89.2% (86.0% to 92.1%)	91.3% (89.1% to 93.2%)	88.1% (84.5% to 91.2%)	91.1% (88.9% to 93.1%)
Hypertensive heart disease	100.0% (100.0% to 100.0%)	100.0% (100.0% to 100.0%)	100.0% (100.0% to 100.0%)	100.0% (100.0% to 100.0%)
Cardiomyopathy and myocarditis	37.5% (30.3% to 45.7%)	37.1% (30.0% to 45.2%)	42.1% (35.9% to 48.6%)	37.4% (30.5% to 45.4%)
Atrial fibrillation and flutter	48.2% (40.3% to 57.2%)	52.3% (44.9% to 60.7%)	58.0% (51.4% to 66.0%)	54.8% (47.5% to 62.6%)
Aortic aneurysm	41.8% (34.9% to 48.3%)	45.0% (38.7% to 51.1%)	-	45.0% (38.7% to 51.1%)
Peripheral vascular disease	51.3% (45.0% to 58.0%)	57.1% (51.8% to 62.7%)	59.1% (53.7% to 64.2%)	58.6% (53.6% to 63.7%)
Endocarditis	36.3% (28.0% to 45.8%)	33.4% (25.9% to 41.6%)	33.6% (25.1% to 44.4%)	33.4% (25.9% to 41.7%)

Supplementary Results Table 1. Age-standardised PAFs attributable to the joint distribution of all risk factors for all ages, both sexes combined for each GBD level 1 and level 2 cause and all causes in 2016 for Burkina Faso. Results are shown for deaths, YLLs, YLDs, and DALYs
Data in parenthesis are 95% uncertainty intervals. PAFs=population attributable fractions. GBD=Global Burden of Disease. YLLs=years of life lost. YLDs=years live with disability. DALYs=disability-adjusted life-years

	**Deaths 2016**	**YLLs 2016**	**YLDs 2016**	**DALYs 2016**
Other cardiovascular and circulatory diseases	43.5% (38.2% to 48.7%)	44.9% (40.0% to 49.8%)	39.8% (34.8% to 44.7%)	44.0% (39.1% to 48.8%)
**Chronic respiratory diseases**	61.9% (54.3% to 68.9%)	57.0% (49.9% to 63.9%)	40.9% (34.5% to 48.4%)	51.8% (45.3% to 58.9%)
Chronic obstructive pulmonary disease	80.2% (72.5% to 89.0%)	78.8% (71.1% to 87.5%)	71.0% (61.1% to 81.2%)	77.0% (69.1% to 85.7%)
Pneumoconiosis	100.0% (100.0% to 100.0%)	100.0% (100.0% to 100.0%)	100.0% (100.0% to 100.0%)	100.0% (100.0% to 100.0%)
Asthma	29.8% (21.7% to 39.7%)	31.1% (23.8% to 40.1%)	23.9% (17.8% to 31.2%)	27.8% (21.3% to 35.6%)
Other chronic respiratory diseases	2.8% (1.4% to 4.7%)	2.8% (1.2% to 5.1%)	1.6% (0.3% to 4.0%)	2.6% (1.1% to 5.0%)
**Cirrhosis and other chronic liver diseases**	24.3% (21.7% to 27.2%)	23.3% (20.6% to 26.2%)	18.5% (15.8% to 21.3%)	23.1% (20.5% to 26.0%)
Cirrhosis and other chronic liver diseases due to hepatitis B	0.0% (0.0% to 0.0%)	0.0% (0.0% to 0.0%)	0.0% (0.0% to 0.0%)	0.0% (0.0% to 0.0%)
Cirrhosis and other chronic liver diseases due to hepatitis C	1.1% (0.4% to 2.3%)	1.7% (0.5% to 4.0%)	2.3% (0.8% to 5.1%)	1.7% (0.5% to 4.0%)
Cirrhosis and other chronic liver diseases due to alcohol use	100.0% (100.0% to 100.0%)	100.0% (100.0% to 100.0%)	100.0% (100.0% to 100.0%)	100.0% (100.0% to 100.0%)
**Digestive diseases**	5.7% (3.5% to 8.5%)	6.5% (3.8% to 9.7%)	1.8% (1.3% to 2.4%)	6.2% (3.6% to 9.3%)
Peptic ulcer disease	6.5% (4.1% to 9.4%)	6.5% (4.2% to 9.3%)	7.0% (4.9% to 9.4%)	6.5% (4.2% to 9.3%)
Gallbladder and biliary diseases	27.1% (15.2% to 41.4%)	25.1% (14.4% to 38.2%)	26.0% (15.0% to 39.2%)	25.2% (14.4% to 38.3%)
Pancreatitis	42.6% (16.0% to 71.2%)	48.8% (19.3% to 76.1%)	43.2% (16.8% to 71.1%)	48.8% (19.3% to 76.1%)
**Neurological disorders**	16.2% (8.0% to 26.9%)	16.8% (10.3% to 25.0%)	3.8% (1.6% to 7.3%)	8.8% (5.1% to 13.4%)
Alzheimer's disease and other dementias	17.3% (8.0% to 29.7%)	17.9% (8.4% to 30.1%)	17.8% (8.4% to 30.1%)	17.8% (8.4% to 30.1%)
Parkinson's disease	-9.4% (-12.5% to -6.4%)	-9.3% (-12.1% to -6.6%)	-8.8% (-11.2% to -6.2%)	-9.3% (-11.9% to -6.6%)
Epilepsy	25.1% (14.2% to 36.2%)	22.4% (12.9% to 32.7%)	20.9% (11.6% to 30.7%)	21.7% (12.3% to 31.7%)
Multiple sclerosis	4.7% (2.3% to 7.7%)	4.8% (2.3% to 8.1%)	3.9% (2.3% to 5.6%)	4.6% (2.4% to 7.2%)
**Mental and substance use disorders**	99.9% (99.7% to 99.9%)	99.8% (99.5% to 99.9%)	18.4% (16.0% to 20.8%)	21.5% (19.0% to 24.1%)
Alcohol use disorders	100.0% (100.0% to 100.0%)	100.0% (100.0% to 100.0%)	100.0% (100.0% to 100.0%)	100.0% (100.0% to 100.0%)

Supplementary Results Table 1. Age-standardised PAFs attributable to the joint distribution of all risk factors for all ages, both sexes combined for each GBD level 1 and level 2 cause and all causes in 2016 for Burkina Faso. Results are shown for deaths, YLLs, YLDs, and DALYs
Data in parenthesis are 95% uncertainty intervals. PAFs=population attributable fractions. GBD=Global Burden of Disease. YLLs=years of life lost. YLDs=years live with disability. DALYs=disability-adjusted life-years

	Deaths 2016	YLLs 2016	YLDs 2016	DALYs 2016
Drug use disorders	100.0% (100.0% to 100.0%)	100.0% (100.0% to 100.0%)	100.0% (100.0% to 100.0%)	100.0% (100.0% to 100.0%)
Depressive disorders	-	-	9.4% (8.1% to 10.8%)	9.4% (8.1% to 10.8%)
Idiopathic developmental intellectual disability	-	-	63.8% (32.3% to 82.8%)	63.8% (32.3% to 82.8%)
**Diabetes, urogenital, blood, and endocrine diseases**	65.3% (62.2% to 68.3%)	47.4% (43.3% to 51.5%)	48.7% (44.8% to 52.1%)	47.8% (44.8% to 50.9%)
Diabetes mellitus	100.0% (100.0% to 100.0%)	100.0% (100.0% to 100.0%)	100.0% (100.0% to 100.0%)	100.0% (100.0% to 100.0%)
Chronic kidney disease	100.0% (100.0% to 100.0%)	100.0% (100.0% to 100.0%)	100.0% (100.0% to 100.0%)	100.0% (100.0% to 100.0%)
**Musculoskeletal disorders**	0.5% (0.2% to 1.1%)	0.3% (0.1% to 0.6%)	27.4% (23.7% to 31.0%)	27.0% (23.3% to 30.6%)
Rheumatoid arthritis	2.1% (0.7% to 4.2%)	1.9% (0.7% to 3.7%)	2.6% (1.0% to 4.4%)	2.5% (1.0% to 4.3%)
Osteoarthritis	-	-	15.5% (7.1% to 26.2%)	15.5% (7.1% to 26.2%)
Low back and neck pain	-	-	34.4% (30.0% to 38.3%)	34.4% (30.0% to 38.3%)
Gout	-	-	43.1% (34.7% to 53.7%)	43.1% (34.7% to 53.7%)
**Other non-communicable diseases**	0.1% (0.0% to 0.3%)	0.2% (0.1% to 0.5%)	11.5% (9.6% to 13.3%)	6.7% (4.9% to 9.1%)
Sense organ diseases	-	-	21.9% (19.8% to 24.0%)	21.9% (19.8% to 24.0%)
Sudden infant death syndrome	5.3% (2.0% to 11.3%)	5.3% (2.0% to 11.3%)	-	5.3% (2.0% to 11.3%)
**Transport injuries**	31.1% (23.6% to 41.5%)	23.5% (16.7% to 33.1%)	35.1% (27.6% to 45.9%)	24.9% (18.0% to 34.6%)
Road injuries	31.1% (23.7% to 41.5%)	23.6% (16.8% to 33.2%)	36.8% (29.2% to 47.8%)	24.9% (18.1% to 34.6%)
Other transport injuries	28.3% (20.5% to 38.4%)	20.9% (14.0% to 30.2%)	26.5% (19.3% to 36.9%)	23.7% (16.8% to 33.2%)
**Unintentional injuries**	24.8% (16.8% to 34.1%)	12.5% (9.4% to 16.1%)	16.3% (14.9% to 17.9%)	13.3% (10.7% to 16.3%)
Falls	49.9% (31.2% to 69.0%)	33.2% (24.3% to 43.6%)	25.0% (23.9% to 26.0%)	30.2% (24.5% to 36.8%)
Drowning	9.2% (5.3% to 14.5%)	7.5% (4.7% to 11.5%)	11.9% (7.1% to 18.7%)	7.6% (4.8% to 11.5%)
Fire, heat, and hot substances	9.2% (4.9% to 15.6%)	8.1% (4.7% to 13.0%)	12.6% (7.8% to 19.2%)	8.7% (5.1% to 13.8%)

Supplementary Results Table 1. Age-standardised PAFs attributable to the joint distribution of all risk factors for all ages, both sexes combined for each GBD level 1 and level 2 cause and all causes in 2016 for Burkina Faso. Results are shown for deaths, YLLs, YLDs, and DALYs
Data in parenthesis are 95% uncertainty intervals. PAFs=population attributable fractions. GBD=Global Burden of Disease. YLLs=years of life lost. YLDs=years live with disability. DALYs=disability-adjusted life-years

	**Deaths 2016**	**YLLs 2016**	**YLDs 2016**	**DALYs 2016**
Poisonings	10.0% (5.2% to 16.5%)	8.4% (3.8% to 15.0%)	12.1% (7.8% to 18.2%)	8.5% (3.9% to 15.1%)
Exposure to mechanical forces	15.8% (10.8% to 18.9%)	8.7% (4.7% to 11.3%)	13.4% (12.3% to 14.5%)	9.8% (6.1% to 12.1%)
Animal contact	4.0% (3.3% to 5.3%)	3.6% (2.9% to 4.6%)	5.5% (4.8% to 6.3%)	3.9% (3.2% to 4.8%)
Foreign body	2.5% (1.7% to 3.4%)	2.8% (1.5% to 4.1%)	4.0% (3.4% to 4.8%)	3.2% (2.0% to 4.2%)
Other unintentional injuries	10.9% (6.5% to 16.9%)	10.1% (6.4% to 15.1%)	11.7% (7.0% to 18.1%)	10.7% (6.6% to 16.0%)
**Self-harm and interpersonal violence**	25.1% (10.9% to 40.1%)	25.3% (11.5% to 39.5%)	19.7% (15.9% to 23.9%)	24.9% (11.8% to 38.4%)
Self-harm	25.3% (10.2% to 41.1%)	26.1% (10.9% to 41.3%)	25.4% (10.3% to 40.4%)	26.0% (10.9% to 41.3%)
Interpersonal violence	22.2% (14.9% to 30.8%)	19.1% (12.4% to 27.3%)	18.8% (16.1% to 22.4%)	19.0% (13.9% to 25.4%)
**Forces of nature, conflict and terrorism, and executions and police conflict**	0.0% (0.0% to 0.0%)	0.0% (0.0% to 0.0%)	0.4% (0.2% to 0.6%)	0.0% (0.0% to 0.1%)
Forces of nature, conflict and terrorism, and state actor violence	0.0% (0.0% to 0.0%)	0.0% (0.0% to 0.0%)	6.6% (4.4% to 9.7%)	6.6% (4.4% to 9.7%)

Supplementary Results Table 1. Age-standardised PAFs attributable to the joint distribution of all risk factors for all ages, both sexes combined for each GBD level 1 and level 2 cause and all causes in 2016 for Burundi. Results are shown for deaths, YLLs, YLDs, and DALYs
Data in parenthesis are 95% uncertainty intervals. PAFs=population attributable fractions. GBD=Global Burden of Disease. YLLs=years of life lost. YLDs=years live with disability. DALYs=disability-adjusted life-years

	Deaths 2016	YLLs 2016	YLDs 2016	DALYs 2016
**All causes**	59.8% (56.9% to 63.4%)	58.3% (55.3% to 61.8%)	27.3% (25.8% to 28.7%)	52.5% (49.5% to 56.1%)
**HIV/AIDS and tuberculosis**	60.5% (49.7% to 73.0%)	62.2% (52.0% to 73.0%)	75.1% (68.1% to 82.3%)	62.5% (52.5% to 73.2%)
Tuberculosis	56.1% (43.6% to 70.6%)	56.2% (43.4% to 70.2%)	54.8% (42.4% to 69.1%)	56.1% (43.4% to 70.2%)
HIV/AIDS	89.4% (86.1% to 92.7%)	85.2% (81.1% to 89.4%)	90.5% (87.4% to 93.0%)	85.6% (81.7% to 89.5%)
**Diarrhoea, lower respiratory infections, and other common infectious diseases**	81.5% (76.6% to 86.4%)	80.8% (75.9% to 85.1%)	50.4% (45.6% to 54.7%)	79.7% (75.0% to 83.9%)
Diarrhoeal diseases	96.2% (89.9% to 99.0%)	97.4% (92.7% to 99.3%)	96.3% (90.0% to 99.0%)	97.3% (92.6% to 99.3%)
Lower respiratory infections	83.6% (77.8% to 88.1%)	88.4% (83.8% to 91.7%)	84.2% (79.0% to 88.5%)	88.3% (83.8% to 91.7%)
Upper respiratory infections	1.5% (0.1% to 5.1%)	2.7% (0.2% to 10.1%)	0.0% (0.0% to 0.0%)	0.4% (0.0% to 1.5%)
Otitis media	1.9% (0.6% to 4.3%)	4.9% (2.1% to 9.5%)	2.8% (1.8% to 3.8%)	2.9% (1.9% to 4.0%)
Meningitis	2.2% (1.2% to 3.9%)	4.5% (2.6% to 7.6%)	0.0% (0.0% to 0.0%)	4.3% (2.4% to 7.2%)
Encephalitis	3.7% (2.4% to 5.8%)	4.7% (3.2% to 7.2%)	0.0% (0.0% to 0.0%)	2.9% (1.8% to 4.6%)
Measles	65.5% (50.5% to 82.4%)	66.6% (51.3% to 83.5%)	65.6% (50.3% to 81.3%)	66.5% (51.3% to 83.5%)
**Maternal disorders**	6.5% (3.1% to 10.3%)	6.4% (3.1% to 10.2%)	1.8% (0.9% to 3.0%)	6.1% (2.9% to 9.6%)
Maternal haemorrhage	14.6% (5.2% to 23.9%)	14.6% (5.2% to 24.0%)	14.5% (5.2% to 23.9%)	14.6% (5.2% to 24.0%)
Maternal sepsis and other pregnancy related infections	14.5% (5.2% to 23.9%)	14.5% (5.2% to 23.9%)	14.1% (5.0% to 23.1%)	14.5% (5.2% to 23.8%)
Maternal abortion, miscarriage, and ectopic pregnancy	16.2% (10.0% to 23.5%)	16.1% (9.7% to 23.0%)	17.0% (9.1% to 26.2%)	16.1% (9.7% to 23.1%)
**Neonatal disorders**	78.3% (73.9% to 81.9%)	78.3% (73.9% to 81.9%)	0.7% (0.3% to 1.2%)	76.5% (72.2% to 80.5%)
Neonatal preterm birth complications	94.5% (89.3% to 97.4%)	94.6% (89.3% to 97.4%)	1.7% (1.1% to 2.4%)	91.3% (85.7% to 94.9%)
Neonatal encephalopathy due to birth asphyxia and trauma	76.5% (72.2% to 80.4%)	76.5% (72.2% to 80.4%)	0.0% (0.0% to 0.0%)	75.4% (70.7% to 79.6%)
Neonatal sepsis and other neonatal infections	65.1% (56.6% to 71.1%)	65.2% (56.7% to 71.1%)	0.0% (0.0% to 0.0%)	63.5% (53.8% to 70.3%)
Hemolytic disease and other neonatal jaundice	69.5% (60.1% to 75.7%)	69.5% (60.1% to 75.8%)	0.0% (0.0% to 0.0%)	64.1% (53.8% to 71.4%)

Supplementary Results Table 1. Age-standardised PAFs attributable to the joint distribution of all risk factors for all ages, both sexes combined for each GBD level 1 and level 2 cause and all causes in 2016 for Burundi. Results are shown for deaths, YLLs, YLDs, and DALYs
Data in parenthesis are 95% uncertainty intervals. PAFs=population attributable fractions. GBD=Global Burden of Disease. YLLs=years of life lost. YLDs=years live with disability. DALYs=disability-adjusted life-years

	Deaths 2016	YLLs 2016	YLDs 2016	DALYs 2016
Other neonatal disorders	69.4% (61.7% to 75.3%)	69.5% (61.8% to 75.4%)	0.0% (0.0% to 0.0%)	68.6% (60.9% to 74.6%)
**Nutritional deficiencies**	27.0% (17.2% to 37.7%)	60.5% (47.2% to 71.8%)	84.0% (81.3% to 86.5%)	63.9% (52.9% to 73.1%)
Protein-energy malnutrition	31.1% (20.2% to 43.2%)	65.5% (51.8% to 76.4%)	98.2% (97.2% to 98.8%)	65.8% (52.6% to 76.7%)
Vitamin A deficiency	-	-	100.0% (100.0% to 100.0%)	100.0% (100.0% to 100.0%)
Iron-deficiency anaemia	100.0% (100.0% to 100.0%)	100.0% (100.0% to 100.0%)	100.0% (100.0% to 100.0%)	100.0% (100.0% to 100.0%)
**Other communicable, maternal, neonatal, and nutritional diseases**	6.9% (3.5% to 10.3%)	5.7% (2.2% to 9.7%)	80.9% (74.6% to 86.6%)	20.1% (8.8% to 31.0%)
Sexually transmitted diseases excluding HIV	54.6% (30.9% to 77.1%)	34.2% (15.7% to 58.1%)	100.0% (100.0% to 100.0%)	68.5% (47.2% to 85.7%)
Hepatitis	0.3% (0.2% to 0.4%)	0.3% (0.2% to 0.5%)	0.3% (0.2% to 0.5%)	0.3% (0.2% to 0.5%)
**Neoplasms**	33.3% (29.1% to 38.5%)	33.3% (29.1% to 38.1%)	35.5% (30.6% to 42.0%)	33.3% (29.1% to 38.2%)
Oesophageal cancer	70.0% (59.3% to 79.4%)	71.2% (60.8% to 80.1%)	70.4% (60.0% to 79.7%)	71.2% (60.8% to 80.1%)
Stomach cancer	3.9% (0.6% to 11.9%)	3.8% (0.6% to 11.1%)	3.9% (0.6% to 11.6%)	3.8% (0.6% to 11.2%)
Liver cancer	29.9% (24.7% to 36.7%)	29.9% (24.4% to 36.6%)	29.9% (24.8% to 36.5%)	29.9% (24.4% to 36.6%)
Larynx cancer	59.0% (44.2% to 71.6%)	61.1% (45.4% to 73.7%)	59.7% (44.7% to 72.3%)	61.1% (45.4% to 73.6%)
Tracheal, bronchus, and lung cancer	79.3% (71.9% to 85.1%)	78.9% (71.4% to 84.9%)	79.1% (71.8% to 84.9%)	78.9% (71.4% to 84.9%)
Breast cancer	22.6% (6.8% to 45.7%)	22.5% (5.9% to 46.4%)	22.6% (6.4% to 45.3%)	22.5% (5.9% to 46.4%)
Cervical cancer	100.0% (100.0% to 100.0%)	100.0% (100.0% to 100.0%)	100.0% (100.0% to 100.0%)	100.0% (100.0% to 100.0%)
Uterine cancer	8.9% (2.6% to 18.5%)	9.3% (2.7% to 19.3%)	9.1% (2.6% to 18.8%)	9.3% (2.7% to 19.2%)
Prostate cancer	0.5% (0.2% to 1.0%)	0.5% (0.2% to 1.0%)	0.5% (0.2% to 1.0%)	0.5% (0.2% to 1.0%)
Colon and rectum cancer	51.7% (41.9% to 61.3%)	52.2% (42.5% to 62.0%)	52.0% (42.2% to 61.7%)	52.2% (42.5% to 62.0%)
Lip and oral cavity cancer	81.5% (73.2% to 88.3%)	82.5% (75.3% to 88.6%)	82.0% (74.3% to 88.3%)	82.5% (75.3% to 88.6%)
Nasopharynx cancer	71.1% (62.5% to 77.6%)	72.1% (64.6% to 77.9%)	71.8% (64.4% to 77.5%)	72.1% (64.7% to 77.9%)

Supplementary Results Table 1. Age-standardised PAFs attributable to the joint distribution of all risk factors for all ages, both sexes combined for each GBD level 1 and level 2 cause and all causes in 2016 for Burundi. Results are shown for deaths, YLLs, YLDs, and DALYs
Data in parenthesis are 95% uncertainty intervals. PAFs=population attributable fractions. GBD=Global Burden of Disease. YLLs=years of life lost. YLDs=years live with disability. DALYs=disability-adjusted life-years

	Deaths 2016	YLLs 2016	YLDs 2016	DALYs 2016
Other pharynx cancer	66.8% (54.5% to 76.7%)	68.0% (56.6% to 77.5%)	67.2% (55.5% to 77.0%)	68.0% (56.6% to 77.5%)
Gallbladder and biliary tract cancer	4.7% (1.3% to 10.3%)	5.0% (1.4% to 10.8%)	4.8% (1.3% to 10.5%)	5.0% (1.4% to 10.8%)
Pancreatic cancer	8.5% (3.7% to 15.4%)	8.2% (3.6% to 14.6%)	8.3% (3.7% to 15.2%)	8.2% (3.6% to 14.6%)
Ovarian cancer	6.3% (2.4% to 12.3%)	5.4% (2.0% to 10.4%)	5.5% (2.1% to 10.6%)	5.4% (2.0% to 10.4%)
Kidney cancer	6.8% (3.2% to 12.3%)	5.9% (2.6% to 10.9%)	6.1% (2.8% to 11.2%)	5.9% (2.6% to 10.9%)
Bladder cancer	8.1% (3.2% to 14.7%)	7.8% (3.1% to 14.3%)	7.9% (3.2% to 14.5%)	7.8% (3.1% to 14.3%)
Thyroid cancer	3.3% (0.9% to 7.8%)	3.4% (1.0% to 7.6%)	3.3% (1.0% to 7.6%)	3.4% (1.0% to 7.6%)
Mesothelioma	86.0% (78.7% to 90.8%)	77.6% (68.2% to 84.2%)	82.9% (74.4% to 88.4%)	77.6% (68.3% to 84.3%)
Non-Hodgkin's lymphoma	1.6% (0.5% to 3.4%)	1.3% (0.4% to 2.8%)	1.5% (0.5% to 3.2%)	1.3% (0.4% to 2.8%)
Multiple myeloma	2.0% (0.6% to 4.3%)	2.0% (0.6% to 4.5%)	2.0% (0.6% to 4.4%)	2.0% (0.6% to 4.5%)
Leukaemia	3.8% (1.9% to 6.5%)	3.7% (1.9% to 6.2%)	3.9% (2.0% to 6.5%)	3.7% (1.9% to 6.2%)
**Cardiovascular diseases**	84.2% (81.5% to 86.6%)	84.7% (82.4% to 86.8%)	61.8% (57.2% to 66.1%)	83.3% (81.0% to 85.5%)
Rheumatic heart disease	30.4% (20.7% to 46.3%)	28.8% (19.9% to 42.2%)	23.1% (14.8% to 33.5%)	26.8% (18.4% to 38.5%)
Ischaemic heart disease	94.0% (91.5% to 96.1%)	94.8% (93.1% to 96.3%)	90.6% (87.8% to 93.1%)	94.7% (93.0% to 96.2%)
Cerebrovascular disease	90.3% (87.3% to 92.9%)	92.1% (90.0% to 93.8%)	89.4% (86.7% to 91.9%)	92.0% (90.0% to 93.7%)
Hypertensive heart disease	100.0% (100.0% to 100.0%)	100.0% (100.0% to 100.0%)	100.0% (100.0% to 100.0%)	100.0% (100.0% to 100.0%)
Cardiomyopathy and myocarditis	43.9% (35.7% to 53.1%)	41.3% (33.5% to 50.2%)	47.2% (40.2% to 54.3%)	41.7% (34.1% to 50.2%)
Atrial fibrillation and flutter	52.8% (43.1% to 62.3%)	57.1% (48.3% to 65.4%)	63.5% (56.5% to 70.5%)	60.3% (52.3% to 67.9%)
Aortic aneurysm	42.6% (35.6% to 49.6%)	46.3% (39.9% to 52.3%)	-	46.3% (39.9% to 52.3%)
Peripheral vascular disease	51.7% (44.6% to 59.3%)	58.9% (52.9% to 64.6%)	59.0% (53.6% to 64.6%)	58.9% (53.7% to 64.6%)
Endocarditis	39.8% (31.1% to 49.7%)	37.4% (29.8% to 45.3%)	32.7% (24.1% to 42.1%)	37.4% (29.8% to 45.2%)

Supplementary Results Table 1. Age-standardised PAFs attributable to the joint distribution of all risk factors for all ages, both sexes combined for each GBD level 1 and level 2 cause and all causes in 2016 for Burundi. Results are shown for deaths, YLLs, YLDs, and DALYs
Data in parenthesis are 95% uncertainty intervals. PAFs=population attributable fractions. GBD=Global Burden of Disease. YLLs=years of life lost. YLDs=years live with disability. DALYs=disability-adjusted life-years

	Deaths 2016	YLLs 2016	YLDs 2016	DALYs 2016
Other cardiovascular and circulatory diseases	45.6% (40.2% to 50.7%)	47.6% (43.4% to 52.0%)	36.0% (28.9% to 42.9%)	46.3% (41.9% to 50.7%)
**Chronic respiratory diseases**	60.7% (51.8% to 68.8%)	56.5% (48.7% to 64.1%)	36.1% (29.6% to 43.4%)	51.4% (43.9% to 58.7%)
Chronic obstructive pulmonary disease	75.9% (67.3% to 86.3%)	74.9% (66.5% to 84.9%)	63.9% (52.2% to 76.3%)	73.1% (64.3% to 83.5%)
Pneumoconiosis	100.0% (100.0% to 100.0%)	100.0% (100.0% to 100.0%)	100.0% (100.0% to 100.0%)	100.0% (100.0% to 100.0%)
Asthma	21.0% (15.6% to 28.1%)	23.9% (18.7% to 30.5%)	18.6% (14.6% to 23.7%)	21.9% (17.5% to 28.0%)
Other chronic respiratory diseases	2.0% (0.6% to 4.2%)	1.9% (0.3% to 5.0%)	1.1% (0.1% to 2.7%)	1.7% (0.3% to 4.3%)
**Cirrhosis and other chronic liver diseases**	36.9% (32.5% to 41.5%)	36.0% (31.2% to 40.7%)	28.4% (24.1% to 32.9%)	35.8% (31.1% to 40.5%)
Cirrhosis and other chronic liver diseases due to hepatitis B	0.1% (0.0% to 0.2%)	0.1% (0.1% to 0.3%)	0.1% (0.1% to 0.2%)	0.1% (0.1% to 0.3%)
Cirrhosis and other chronic liver diseases due to hepatitis C	4.9% (2.4% to 8.5%)	7.8% (3.8% to 13.7%)	9.3% (5.0% to 16.4%)	7.9% (3.9% to 13.8%)
Cirrhosis and other chronic liver diseases due to alcohol use	100.0% (100.0% to 100.0%)	100.0% (100.0% to 100.0%)	100.0% (100.0% to 100.0%)	100.0% (100.0% to 100.0%)
**Digestive diseases**	3.7% (2.5% to 5.3%)	4.3% (3.0% to 6.0%)	1.1% (0.8% to 1.5%)	4.1% (2.9% to 5.7%)
Peptic ulcer disease	4.1% (2.3% to 6.2%)	4.8% (2.8% to 7.3%)	4.4% (2.9% to 6.2%)	4.8% (2.8% to 7.2%)
Gallbladder and biliary diseases	10.6% (4.1% to 20.9%)	11.3% (4.5% to 21.3%)	11.5% (4.7% to 21.6%)	11.3% (4.5% to 21.4%)
Pancreatitis	70.0% (51.7% to 85.3%)	73.5% (58.1% to 87.0%)	69.0% (54.3% to 82.6%)	73.5% (58.1% to 87.0%)
**Neurological disorders**	10.8% (5.4% to 17.9%)	15.4% (9.8% to 21.7%)	5.4% (2.0% to 9.7%)	9.3% (5.6% to 13.4%)
Alzheimer's disease and other dementias	9.5% (3.7% to 17.6%)	10.2% (4.1% to 18.4%)	10.1% (4.1% to 18.3%)	10.2% (4.2% to 18.4%)
Parkinson's disease	-3.4% (-5.2% to -2.1%)	-4.1% (-6.1% to -2.7%)	-4.5% (-6.5% to -3.1%)	-4.2% (-6.2% to -2.7%)
Epilepsy	33.8% (20.8% to 46.2%)	32.6% (19.6% to 44.7%)	26.4% (15.8% to 36.8%)	29.7% (17.8% to 40.8%)
Multiple sclerosis	3.4% (1.4% to 6.2%)	3.5% (1.4% to 6.6%)	2.6% (1.5% to 3.8%)	3.2% (1.5% to 5.7%)
**Mental and substance use disorders**	99.9% (99.8% to 99.9%)	99.8% (99.5% to 99.9%)	19.9% (17.1% to 22.7%)	23.6% (20.2% to 26.9%)
Alcohol use disorders	100.0% (100.0% to 100.0%)	100.0% (100.0% to 100.0%)	100.0% (100.0% to 100.0%)	100.0% (100.0% to 100.0%)

Supplementary Results Table 1. Age-standardised PAFs attributable to the joint distribution of all risk factors for all ages, both sexes combined for each GBD level 1 and level 2 cause and all causes in 2016 for Burundi. Results are shown for deaths, YLLs, YLDs, and DALYs
Data in parenthesis are 95% uncertainty intervals. PAFs=population attributable fractions. GBD=Global Burden of Disease. YLLs=years of life lost. YLDs=years live with disability. DALYs=disability-adjusted life-years

	Deaths 2016	YLLs 2016	YLDs 2016	DALYs 2016
Drug use disorders	100.0% (100.0% to 100.0%)	100.0% (100.0% to 100.0%)	100.0% (100.0% to 100.0%)	100.0% (100.0% to 100.0%)
Depressive disorders	-	-	8.9% (7.4% to 10.7%)	8.9% (7.4% to 10.7%)
Idiopathic developmental intellectual disability	-	-	59.8% (29.1% to 79.6%)	59.8% (29.1% to 79.6%)
**Diabetes, urogenital, blood, and endocrine diseases**	79.7% (77.2% to 81.9%)	71.4% (67.4% to 74.6%)	64.3% (59.7% to 68.7%)	68.8% (65.6% to 71.6%)
Diabetes mellitus	100.0% (100.0% to 100.0%)	100.0% (100.0% to 100.0%)	100.0% (100.0% to 100.0%)	100.0% (100.0% to 100.0%)
Chronic kidney disease	100.0% (100.0% to 100.0%)	100.0% (100.0% to 100.0%)	100.0% (100.0% to 100.0%)	100.0% (100.0% to 100.0%)
**Musculoskeletal disorders**	0.6% (0.2% to 1.3%)	0.5% (0.2% to 1.0%)	28.8% (25.4% to 32.3%)	28.3% (24.7% to 31.8%)
Rheumatoid arthritis	1.4% (0.5% to 2.8%)	1.6% (0.5% to 3.1%)	1.6% (0.6% to 2.9%)	1.6% (0.6% to 2.9%)
Osteoarthritis	-	-	6.9% (2.2% to 14.0%)	6.9% (2.2% to 14.0%)
Low back and neck pain	-	-	37.5% (33.5% to 41.5%)	37.5% (33.5% to 41.5%)
Gout	-	-	36.1% (28.7% to 46.3%)	36.1% (28.7% to 46.3%)
**Other non-communicable diseases**	0.4% (0.1% to 0.8%)	0.7% (0.2% to 1.4%)	9.0% (7.4% to 10.5%)	7.3% (6.1% to 8.5%)
Sense organ diseases	-	-	18.2% (16.0% to 20.4%)	18.2% (16.0% to 20.4%)
Sudden infant death syndrome	6.0% (2.4% to 11.7%)	6.0% (2.4% to 11.7%)	-	6.0% (2.4% to 11.7%)
**Transport injuries**	43.8% (34.2% to 55.3%)	40.5% (30.5% to 51.1%)	48.5% (38.6% to 58.7%)	41.4% (31.5% to 51.9%)
Road injuries	43.7% (34.1% to 55.3%)	40.4% (30.5% to 51.1%)	49.7% (39.7% to 60.0%)	41.4% (31.5% to 52.0%)
Other transport injuries	45.2% (34.3% to 56.3%)	41.2% (30.2% to 52.8%)	40.8% (31.4% to 51.2%)	41.2% (30.9% to 51.9%)
**Unintentional injuries**	33.1% (27.4% to 38.4%)	21.7% (17.2% to 26.7%)	27.1% (23.1% to 32.4%)	23.1% (18.7% to 28.2%)
Falls	63.9% (49.6% to 69.1%)	43.4% (36.9% to 48.9%)	33.5% (30.0% to 38.2%)	39.0% (34.6% to 43.7%)
Drowning	19.4% (12.5% to 27.4%)	19.1% (12.4% to 26.2%)	23.3% (14.9% to 33.3%)	19.1% (12.4% to 26.2%)
Fire, heat, and hot substances	15.7% (9.3% to 24.4%)	16.6% (10.8% to 23.9%)	26.9% (18.4% to 37.0%)	18.4% (12.2% to 26.0%)

Supplementary Results Table 1. Age-standardised PAFs attributable to the joint distribution of all risk factors for all ages, both sexes combined for each GBD level 1 and level 2 cause and all causes in 2016 for Burundi. Results are shown for deaths, YLLs, YLDs, and DALYs
Data in parenthesis are 95% uncertainty intervals. PAFs=population attributable fractions. GBD=Global Burden of Disease. YLLs=years of life lost. YLDs=years live with disability. DALYs=disability-adjusted life-years

	**Deaths 2016**	**YLLs 2016**	**YLDs 2016**	**DALYs 2016**
Poisonings	18.4% (10.5% to 27.7%)	17.0% (7.7% to 26.5%)	25.9% (17.7% to 35.7%)	17.3% (8.0% to 26.8%)
Exposure to mechanical forces	26.0% (21.0% to 32.4%)	23.5% (17.5% to 31.4%)	26.7% (21.6% to 34.0%)	24.4% (18.7% to 31.9%)
Animal contact	10.1% (7.4% to 14.4%)	12.1% (8.8% to 17.5%)	17.2% (13.3% to 22.8%)	13.0% (9.6% to 18.3%)
Foreign body	9.8% (5.7% to 15.3%)	9.9% (4.3% to 16.1%)	15.6% (11.4% to 21.6%)	11.5% (6.5% to 17.4%)
Other unintentional injuries	22.3% (14.8% to 31.8%)	23.5% (16.1% to 32.7%)	23.9% (15.8% to 33.5%)	23.6% (16.2% to 32.1%)
**Self-harm and interpersonal violence**	34.6% (22.0% to 46.9%)	35.3% (23.4% to 47.2%)	28.0% (23.6% to 32.8%)	34.5% (23.7% to 45.2%)
Self-harm	35.1% (21.4% to 48.9%)	36.6% (23.2% to 50.1%)	35.4% (21.8% to 48.7%)	36.5% (23.2% to 50.1%)
Interpersonal violence	27.9% (15.1% to 40.9%)	25.7% (13.4% to 38.5%)	27.0% (22.6% to 31.9%)	26.4% (18.1% to 34.7%)
**Forces of nature, conflict and terrorism, and executions and police conflict**	2.0% (1.8% to 2.2%)	0.8% (0.7% to 0.8%)	0.0% (0.0% to 0.0%)	0.4% (0.2% to 0.6%)
Forces of nature, conflict and terrorism, and state actor violence	7.2% (6.7% to 7.6%)	2.9% (2.6% to 3.1%)	5.2% (3.3% to 7.7%)	2.9% (2.6% to 3.2%)

Supplementary Results Table 1. Age-standardised PAFs attributable to the joint distribution of all risk factors for all ages, both sexes combined for each GBD level 1 and level 2 cause and all causes in 2016 for Cambodia. Results are shown for deaths, YLLs, YLDs, and DALYs
Data in parenthesis are 95% uncertainty intervals. PAFs=population attributable fractions. GBD=Global Burden of Disease. YLLs=years of life lost. YLDs=years live with disability. DALYs=disability-adjusted life-years

	Deaths 2016	YLLs 2016	YLDs 2016	DALYs 2016
**All causes**	58.6% (57.0% to 60.2%)	55.6% (53.9% to 57.2%)	30.8% (29.6% to 32.0%)	48.5% (46.5% to 50.4%)
**HIV/AIDS and tuberculosis**	59.1% (48.0% to 70.1%)	62.8% (53.3% to 71.6%)	61.3% (52.6% to 70.7%)	62.6% (53.6% to 71.6%)
Tuberculosis	52.1% (39.7% to 65.9%)	53.7% (41.5% to 66.6%)	50.2% (38.4% to 63.3%)	53.1% (41.0% to 66.1%)
HIV/AIDS	82.3% (79.3% to 85.8%)	79.3% (75.2% to 84.5%)	84.5% (82.5% to 86.2%)	80.0% (76.2% to 84.6%)
**Diarrhoea, lower respiratory infections, and other common infectious diseases**	75.4% (70.1% to 80.2%)	74.2% (69.0% to 79.2%)	44.2% (35.9% to 50.3%)	72.0% (67.2% to 76.7%)
Diarrhoeal diseases	87.0% (67.2% to 96.6%)	90.3% (75.6% to 97.5%)	88.7% (72.0% to 96.9%)	89.8% (74.8% to 97.3%)
Lower respiratory infections	81.3% (75.2% to 86.1%)	86.3% (81.9% to 89.8%)	81.1% (75.4% to 85.7%)	86.3% (81.9% to 89.8%)
Upper respiratory infections	1.3% (0.1% to 4.9%)	3.1% (0.2% to 10.9%)	0.0% (0.0% to 0.0%)	0.1% (0.0% to 0.4%)
Otitis media	0.8% (0.2% to 2.3%)	2.7% (0.8% to 6.6%)	7.1% (4.8% to 9.4%)	7.1% (4.8% to 9.3%)
Meningitis	1.9% (1.1% to 3.2%)	3.0% (1.7% to 5.0%)	0.0% (0.0% to 0.0%)	2.6% (1.5% to 4.3%)
Encephalitis	0.4% (0.3% to 0.7%)	0.8% (0.5% to 1.2%)	0.0% (0.0% to 0.0%)	0.5% (0.3% to 0.8%)
Measles	61.0% (43.0% to 80.8%)	62.1% (43.6% to 82.2%)	62.3% (43.9% to 81.3%)	62.1% (43.6% to 82.2%)
**Maternal disorders**	8.6% (5.6% to 12.2%)	8.6% (5.5% to 12.1%)	2.9% (1.4% to 4.7%)	8.0% (5.2% to 11.5%)
Maternal haemorrhage	17.5% (6.3% to 28.1%)	17.4% (6.3% to 27.9%)	16.9% (6.1% to 27.2%)	17.3% (6.3% to 27.9%)
Maternal sepsis and other pregnancy related infections	17.5% (6.4% to 28.2%)	17.5% (6.4% to 28.1%)	16.9% (6.2% to 27.2%)	17.4% (6.3% to 27.8%)
Maternal abortion, miscarriage, and ectopic pregnancy	8.4% (5.0% to 12.4%)	8.3% (5.0% to 12.3%)	7.1% (3.6% to 10.9%)	8.3% (5.0% to 12.3%)
**Neonatal disorders**	79.4% (74.5% to 83.5%)	79.4% (74.6% to 83.6%)	0.2% (0.2% to 0.3%)	73.9% (68.7% to 78.4%)
Neonatal preterm birth complications	95.2% (90.9% to 97.9%)	95.3% (90.9% to 97.9%)	0.3% (0.2% to 0.4%)	85.2% (78.0% to 90.3%)
Neonatal encephalopathy due to birth asphyxia and trauma	68.9% (63.4% to 73.4%)	68.9% (63.5% to 73.4%)	0.0% (0.0% to 0.0%)	68.0% (62.4% to 72.8%)
Neonatal sepsis and other neonatal infections	61.5% (54.7% to 67.2%)	61.5% (54.7% to 67.2%)	0.0% (0.0% to 0.0%)	56.4% (46.4% to 63.9%)
Hemolytic disease and other neonatal jaundice	64.1% (55.5% to 70.4%)	64.1% (55.5% to 70.4%)	0.0% (0.0% to 0.0%)	36.5% (24.2% to 47.8%)

Supplementary Results Table 1. Age-standardised PAFs attributable to the joint distribution of all risk factors for all ages, both sexes combined for each GBD level 1 and level 2 cause and all causes in 2016 for Cambodia. Results are shown for deaths, YLLs, YLDs, and DALYs
Data in parenthesis are 95% uncertainty intervals. PAFs=population attributable fractions. GBD=Global Burden of Disease. YLLs=years of life lost. YLDs=years live with disability. DALYs=disability-adjusted life-years

	Deaths 2016	YLLs 2016	YLDs 2016	DALYs 2016
Other neonatal disorders	60.5% (52.0% to 67.4%)	60.6% (52.0% to 67.4%)	0.0% (0.0% to 0.0%)	59.1% (50.9% to 65.8%)
**Nutritional deficiencies**	3.6% (2.0% to 5.9%)	18.7% (11.0% to 28.3%)	97.9% (97.4% to 98.2%)	87.0% (82.6% to 90.6%)
Protein-energy malnutrition	3.7% (1.9% to 6.3%)	20.5% (11.8% to 31.3%)	97.8% (95.3% to 99.1%)	37.8% (27.2% to 48.6%)
Vitamin A deficiency	-	-	100.0% (100.0% to 100.0%)	100.0% (100.0% to 100.0%)
Iron-deficiency anaemia	100.0% (100.0% to 100.0%)	100.0% (100.0% to 100.0%)	100.0% (100.0% to 100.0%)	100.0% (100.0% to 100.0%)
**Other communicable, maternal, neonatal, and nutritional diseases**	17.5% (12.4% to 22.2%)	13.4% (6.8% to 19.2%)	49.5% (44.8% to 54.1%)	18.4% (10.0% to 24.5%)
Sexually transmitted diseases excluding HIV	98.7% (97.2% to 99.5%)	95.8% (91.4% to 98.4%)	100.0% (100.0% to 100.0%)	97.3% (94.4% to 99.0%)
Hepatitis	0.1% (0.1% to 0.2%)	0.2% (0.1% to 0.2%)	0.1% (0.1% to 0.2%)	0.2% (0.1% to 0.2%)
**Neoplasms**	45.7% (41.7% to 49.7%)	44.5% (40.7% to 48.1%)	43.9% (40.0% to 47.8%)	44.5% (40.7% to 48.1%)
Oesophageal cancer	78.2% (69.9% to 85.4%)	79.3% (71.6% to 85.9%)	78.3% (70.2% to 85.4%)	79.3% (71.6% to 85.9%)
Stomach cancer	16.7% (9.2% to 25.2%)	17.1% (9.7% to 24.8%)	16.6% (9.4% to 24.7%)	17.1% (9.7% to 24.8%)
Liver cancer	37.5% (28.0% to 46.9%)	38.8% (28.9% to 48.1%)	37.6% (28.3% to 46.9%)	38.8% (28.9% to 48.1%)
Larynx cancer	88.5% (81.8% to 93.2%)	88.9% (82.8% to 93.2%)	88.7% (82.2% to 93.2%)	88.9% (82.8% to 93.2%)
Tracheal, bronchus, and lung cancer	92.1% (89.2% to 94.4%)	91.8% (89.2% to 94.1%)	91.8% (88.9% to 94.2%)	91.8% (89.2% to 94.1%)
Breast cancer	27.6% (16.4% to 41.4%)	27.0% (16.2% to 40.0%)	27.2% (16.1% to 40.3%)	27.0% (16.2% to 40.0%)
Cervical cancer	100.0% (100.0% to 100.0%)	100.0% (100.0% to 100.0%)	100.0% (100.0% to 100.0%)	100.0% (100.0% to 100.0%)
Uterine cancer	17.0% (7.7% to 28.8%)	17.8% (8.3% to 29.5%)	17.4% (8.0% to 29.1%)	17.8% (8.3% to 29.4%)
Prostate cancer	6.4% (3.9% to 9.5%)	6.9% (4.4% to 10.0%)	6.7% (4.2% to 9.9%)	6.9% (4.4% to 10.0%)
Colon and rectum cancer	57.6% (48.6% to 66.2%)	57.7% (48.9% to 66.3%)	57.6% (48.7% to 66.2%)	57.7% (48.9% to 66.3%)
Lip and oral cavity cancer	86.7% (81.3% to 91.1%)	87.3% (82.7% to 91.1%)	86.0% (80.8% to 90.3%)	87.2% (82.7% to 91.1%)
Nasopharynx cancer	81.5% (75.8% to 86.3%)	81.0% (76.0% to 85.4%)	79.9% (74.8% to 84.4%)	81.0% (75.9% to 85.4%)

Supplementary Results Table 1. Age-standardised PAFs attributable to the joint distribution of all risk factors for all ages, both sexes combined for each GBD level 1 and level 2 cause and all causes in 2016 for Cambodia. Results are shown for deaths, YLLs, YLDs, and DALYs
Data in parenthesis are 95% uncertainty intervals. PAFs=population attributable fractions. GBD=Global Burden of Disease. YLLs=years of life lost. YLDs=years live with disability. DALYs=disability-adjusted life-years

	Deaths 2016	YLLs 2016	YLDs 2016	DALYs 2016
Other pharynx cancer	53.2% (40.9% to 65.1%)	54.9% (42.9% to 66.4%)	53.4% (41.2% to 65.2%)	54.8% (42.9% to 66.4%)
Gallbladder and biliary tract cancer	7.0% (2.6% to 13.5%)	7.5% (2.9% to 14.3%)	7.2% (2.7% to 13.8%)	7.5% (2.9% to 14.3%)
Pancreatic cancer	28.4% (20.7% to 37.1%)	29.5% (22.2% to 37.8%)	28.4% (21.0% to 37.0%)	29.5% (22.1% to 37.8%)
Ovarian cancer	8.8% (3.3% to 17.2%)	7.6% (2.6% to 15.3%)	7.6% (2.7% to 15.2%)	7.6% (2.6% to 15.3%)
Kidney cancer	24.5% (15.9% to 33.9%)	24.2% (15.9% to 33.3%)	22.9% (15.1% to 31.6%)	24.2% (15.9% to 33.2%)
Bladder cancer	35.8% (25.3% to 47.3%)	37.3% (27.0% to 48.3%)	36.3% (26.1% to 47.3%)	37.3% (27.0% to 48.3%)
Thyroid cancer	4.3% (1.7% to 8.5%)	4.5% (1.7% to 8.8%)	4.3% (1.7% to 8.5%)	4.5% (1.7% to 8.8%)
Mesothelioma	82.4% (76.4% to 87.6%)	71.5% (63.0% to 79.7%)	78.5% (71.7% to 84.7%)	71.6% (63.1% to 79.7%)
Non-Hodgkin's lymphoma	2.0% (0.6% to 4.3%)	1.9% (0.6% to 4.1%)	1.9% (0.6% to 4.2%)	1.9% (0.6% to 4.1%)
Multiple myeloma	2.5% (0.8% to 5.3%)	2.6% (0.9% to 5.4%)	2.5% (0.8% to 5.3%)	2.6% (0.9% to 5.4%)
Leukaemia	13.6% (7.6% to 20.6%)	11.1% (6.6% to 16.6%)	12.6% (7.3% to 18.9%)	11.1% (6.6% to 16.7%)
**Cardiovascular diseases**	85.5% (83.1% to 87.9%)	87.0% (85.1% to 88.7%)	70.3% (66.3% to 73.8%)	85.9% (84.0% to 87.6%)
Rheumatic heart disease	17.4% (11.4% to 29.0%)	15.6% (10.4% to 24.7%)	12.2% (7.3% to 19.2%)	14.7% (9.6% to 22.9%)
Ischaemic heart disease	93.0% (90.0% to 95.4%)	94.6% (92.8% to 96.2%)	90.0% (87.2% to 92.6%)	94.5% (92.6% to 96.0%)
Cerebrovascular disease	85.8% (82.2% to 89.2%)	89.2% (86.7% to 91.6%)	86.7% (83.4% to 89.7%)	89.1% (86.6% to 91.4%)
Hypertensive heart disease	100.0% (100.0% to 100.0%)	100.0% (100.0% to 100.0%)	100.0% (100.0% to 100.0%)	100.0% (100.0% to 100.0%)
Cardiomyopathy and myocarditis	34.0% (27.3% to 41.6%)	32.0% (25.4% to 38.9%)	29.3% (24.9% to 34.0%)	31.9% (25.5% to 38.7%)
Atrial fibrillation and flutter	45.1% (37.6% to 52.7%)	48.7% (42.0% to 55.4%)	54.7% (48.9% to 60.8%)	52.0% (45.8% to 58.4%)
Aortic aneurysm	39.6% (33.3% to 45.9%)	43.0% (37.1% to 48.6%)	-	43.0% (37.1% to 48.6%)
Peripheral vascular disease	48.8% (42.7% to 55.4%)	56.5% (50.8% to 62.0%)	54.7% (49.9% to 59.6%)	55.0% (50.3% to 59.8%)
Endocarditis	26.3% (19.4% to 34.5%)	24.0% (17.7% to 31.1%)	23.4% (16.8% to 31.0%)	24.0% (17.7% to 31.1%)

Supplementary Results Table 1. Age-standardised PAFs attributable to the joint distribution of all risk factors for all ages, both sexes combined for each GBD level 1 and level 2 cause and all causes in 2016 for Cambodia. Results are shown for deaths, YLLs, YLDs, and DALYs
Data in parenthesis are 95% uncertainty intervals. PAFs=population attributable fractions. GBD=Global Burden of Disease. YLLs=years of life lost. YLDs=years live with disability. DALYs=disability-adjusted life-years

	Deaths 2016	YLLs 2016	YLDs 2016	DALYs 2016
Other cardiovascular and circulatory diseases	40.1% (34.4% to 45.7%)	40.5% (34.7% to 46.6%)	34.1% (29.2% to 39.4%)	39.2% (34.0% to 44.8%)
**Chronic respiratory diseases**	67.6% (59.4% to 73.5%)	65.7% (58.3% to 70.9%)	57.0% (51.4% to 62.7%)	62.5% (57.4% to 67.1%)
Chronic obstructive pulmonary disease	87.7% (82.8% to 91.8%)	87.6% (83.6% to 91.3%)	82.6% (77.6% to 87.1%)	85.8% (81.5% to 89.7%)
Pneumoconiosis	100.0% (100.0% to 100.0%)	100.0% (100.0% to 100.0%)	100.0% (100.0% to 100.0%)	100.0% (100.0% to 100.0%)
Asthma	37.0% (29.8% to 45.3%)	39.2% (32.6% to 46.7%)	25.4% (21.5% to 30.1%)	34.3% (29.0% to 40.8%)
Other chronic respiratory diseases	10.1% (6.9% to 13.7%)	10.2% (6.9% to 13.8%)	9.6% (6.8% to 13.1%)	9.8% (7.1% to 13.1%)
**Cirrhosis and other chronic liver diseases**	24.2% (20.8% to 27.7%)	23.9% (20.6% to 27.9%)	19.6% (16.1% to 23.3%)	23.9% (20.5% to 27.8%)
Cirrhosis and other chronic liver diseases due to hepatitis B	0.1% (0.0% to 0.1%)	0.1% (0.0% to 0.1%)	0.1% (0.0% to 0.1%)	0.1% (0.0% to 0.1%)
Cirrhosis and other chronic liver diseases due to hepatitis C	3.5% (2.2% to 5.4%)	5.8% (3.5% to 8.8%)	3.9% (2.3% to 6.1%)	5.8% (3.5% to 8.7%)
Cirrhosis and other chronic liver diseases due to alcohol use	100.0% (100.0% to 100.0%)	100.0% (100.0% to 100.0%)	100.0% (100.0% to 100.0%)	100.0% (100.0% to 100.0%)
**Digestive diseases**	14.3% (11.3% to 17.6%)	15.4% (12.2% to 19.0%)	6.6% (5.1% to 8.4%)	15.1% (12.0% to 18.5%)
Peptic ulcer disease	18.9% (13.8% to 24.9%)	19.2% (14.2% to 24.8%)	18.1% (13.2% to 23.0%)	19.2% (14.2% to 24.8%)
Gallbladder and biliary diseases	18.1% (9.0% to 30.5%)	18.9% (9.9% to 30.3%)	19.8% (10.4% to 31.8%)	18.9% (9.9% to 30.4%)
Pancreatitis	61.8% (45.8% to 79.3%)	65.5% (50.3% to 80.7%)	59.9% (44.0% to 76.4%)	65.5% (50.3% to 80.7%)
**Neurological disorders**	14.9% (7.4% to 24.2%)	15.5% (8.7% to 23.7%)	5.0% (2.6% to 7.9%)	9.6% (5.6% to 14.6%)
Alzheimer's disease and other dementias	17.6% (9.2% to 27.6%)	18.6% (10.0% to 28.6%)	18.3% (9.7% to 28.5%)	18.6% (9.9% to 28.6%)
Parkinson's disease	-35.1% (-47.0% to -24.0%)	-35.9% (-47.2% to -25.0%)	-31.7% (-40.9% to -22.4%)	-35.2% (-46.2% to -24.5%)
Epilepsy	25.7% (14.3% to 37.3%)	23.9% (13.3% to 34.4%)	17.7% (10.0% to 26.3%)	20.2% (11.4% to 29.8%)
Multiple sclerosis	11.5% (6.2% to 17.4%)	10.9% (5.8% to 16.9%)	6.7% (4.2% to 9.2%)	10.1% (5.6% to 15.2%)
**Mental and substance use disorders**	99.8% (99.7% to 99.9%)	99.7% (99.6% to 99.9%)	15.6% (13.6% to 17.8%)	20.5% (17.5% to 24.3%)
Alcohol use disorders	100.0% (100.0% to 100.0%)	100.0% (100.0% to 100.0%)	100.0% (100.0% to 100.0%)	100.0% (100.0% to 100.0%)

Supplementary Results Table 1. Age-standardised PAFs attributable to the joint distribution of all risk factors for all ages, both sexes combined for each GBD level 1 and level 2 cause and all causes in 2016 for Cambodia. Results are shown for deaths, YLLs, YLDs, and DALYs
Data in parenthesis are 95% uncertainty intervals. PAFs=population attributable fractions. GBD=Global Burden of Disease. YLLs=years of life lost. YLDs=years live with disability. DALYs=disability-adjusted life-years

	Deaths 2016	YLLs 2016	YLDs 2016	DALYs 2016
Drug use disorders	100.0% (100.0% to 100.0%)	100.0% (100.0% to 100.0%)	100.0% (100.0% to 100.0%)	100.0% (100.0% to 100.0%)
Depressive disorders	-	-	4.9% (4.1% to 5.8%)	4.9% (4.1% to 5.8%)
Idiopathic developmental intellectual disability	-	-	66.9% (35.5% to 86.1%)	66.9% (35.5% to 86.1%)
**Diabetes, urogenital, blood, and endocrine diseases**	84.5% (82.8% to 86.2%)	82.7% (80.5% to 84.4%)	57.3% (52.7% to 61.8%)	73.3% (70.1% to 76.3%)
Diabetes mellitus	100.0% (100.0% to 100.0%)	100.0% (100.0% to 100.0%)	100.0% (100.0% to 100.0%)	100.0% (100.0% to 100.0%)
Chronic kidney disease	100.0% (100.0% to 100.0%)	100.0% (100.0% to 100.0%)	100.0% (100.0% to 100.0%)	100.0% (100.0% to 100.0%)
**Musculoskeletal disorders**	1.8% (0.7% to 3.2%)	1.2% (0.5% to 2.1%)	29.2% (26.1% to 32.5%)	28.8% (25.8% to 32.2%)
Rheumatoid arthritis	6.5% (2.6% to 10.9%)	6.2% (2.5% to 10.5%)	4.7% (2.0% to 7.7%)	4.9% (2.0% to 8.0%)
Osteoarthritis	-	-	9.3% (3.6% to 17.5%)	9.3% (3.6% to 17.5%)
Low back and neck pain	-	-	40.2% (36.7% to 43.8%)	40.2% (36.7% to 43.8%)
Gout	-	-	23.1% (15.2% to 34.9%)	23.1% (15.2% to 34.9%)
**Other non-communicable diseases**	0.3% (0.1% to 0.6%)	0.5% (0.2% to 0.8%)	11.7% (9.8% to 13.6%)	10.4% (8.7% to 12.0%)
Sense organ diseases	-	-	21.3% (18.8% to 23.6%)	21.3% (18.8% to 23.6%)
Sudden infant death syndrome	5.9% (2.4% to 11.2%)	5.9% (2.4% to 11.2%)	-	5.9% (2.4% to 11.2%)
**Transport injuries**	35.3% (26.6% to 45.6%)	30.4% (21.7% to 41.2%)	35.7% (28.1% to 44.7%)	31.0% (22.3% to 41.8%)
Road injuries	34.9% (26.2% to 45.3%)	30.3% (21.6% to 41.1%)	39.3% (31.3% to 48.5%)	31.1% (22.3% to 41.8%)
Other transport injuries	41.4% (32.5% to 52.1%)	32.7% (23.2% to 44.4%)	27.1% (20.0% to 35.3%)	30.3% (22.1% to 40.6%)
**Unintentional injuries**	32.5% (22.3% to 37.7%)	17.8% (14.4% to 21.6%)	18.3% (16.2% to 20.8%)	17.9% (14.8% to 21.3%)
Falls	54.4% (34.5% to 61.4%)	33.6% (26.4% to 38.6%)	27.7% (25.5% to 30.0%)	32.3% (26.5% to 36.3%)
Drowning	11.6% (7.6% to 17.2%)	10.4% (6.9% to 15.1%)	15.6% (10.3% to 23.0%)	10.5% (6.9% to 15.1%)
Fire, heat, and hot substances	12.7% (7.9% to 19.2%)	14.5% (9.8% to 21.0%)	16.0% (10.9% to 22.9%)	14.8% (10.0% to 21.3%)

Supplementary Results Table 1. Age-standardised PAFs attributable to the joint distribution of all risk factors for all ages, both sexes combined for each GBD level 1 and level 2 cause and all causes in 2016 for Cambodia. Results are shown for deaths, YLLs, YLDs, and DALYs
Data in parenthesis are 95% uncertainty intervals. PAFs=population attributable fractions. GBD=Global Burden of Disease. YLLs=years of life lost. YLDs=years live with disability. DALYs=disability-adjusted life-years

	**Deaths 2016**	**YLLs 2016**	**YLDs 2016**	**DALYs 2016**
Poisonings	16.1% (10.7% to 23.8%)	17.3% (11.7% to 24.8%)	16.0% (11.0% to 23.2%)	17.2% (11.7% to 24.7%)
Exposure to mechanical forces	16.6% (13.5% to 20.2%)	13.2% (10.2% to 17.0%)	18.2% (16.0% to 20.8%)	14.8% (11.9% to 18.1%)
Animal contact	11.3% (8.8% to 13.9%)	10.6% (8.3% to 13.7%)	10.5% (8.5% to 13.0%)	10.6% (8.5% to 13.3%)
Foreign body	5.8% (4.3% to 7.7%)	5.8% (3.8% to 8.1%)	7.7% (6.1% to 9.7%)	6.2% (4.4% to 8.4%)
Other unintentional injuries	20.2% (13.1% to 29.4%)	20.6% (13.4% to 29.9%)	15.1% (10.0% to 22.1%)	18.5% (12.1% to 26.6%)
**Self-harm and interpersonal violence**	29.0% (20.1% to 37.5%)	28.7% (20.1% to 37.4%)	28.4% (21.0% to 35.7%)	28.7% (20.4% to 37.1%)
Self-harm	31.6% (20.1% to 43.1%)	32.8% (21.5% to 44.9%)	27.8% (17.4% to 38.4%)	32.7% (21.5% to 44.8%)
Interpersonal violence	24.6% (13.0% to 37.2%)	23.2% (11.7% to 36.0%)	28.5% (21.1% to 36.4%)	24.2% (13.4% to 36.0%)
**Forces of nature, conflict and terrorism, and executions and police conflict**	0.0% (0.0% to 0.0%)	0.0% (0.0% to 0.0%)	0.1% (0.0% to 0.1%)	0.1% (0.0% to 0.1%)
Forces of nature, conflict and terrorism, and state actor violence	0.0% (0.0% to 0.0%)	0.0% (0.0% to 0.0%)	6.2% (4.0% to 9.2%)	6.2% (4.0% to 9.2%)

Supplementary Results Table 1. Age-standardised PAFs attributable to the joint distribution of all risk factors for all ages, both sexes combined for each GBD level 1 and level 2 cause and all causes in 2016 for Cameroon. Results are shown for deaths, YLLs, YLDs, and DALYs
Data in parenthesis are 95% uncertainty intervals. PAFs=population attributable fractions. GBD=Global Burden of Disease. YLLs=years of life lost. YLDs=years live with disability. DALYs=disability-adjusted life-years

	Deaths 2016	YLLs 2016	YLDs 2016	DALYs 2016
**All causes**	58.4% (56.0% to 60.6%)	53.5% (50.5% to 56.4%)	29.1% (27.4% to 30.8%)	48.6% (46.1% to 51.2%)
**HIV/AIDS and tuberculosis**	82.5% (76.4% to 87.9%)	85.2% (80.2% to 89.1%)	91.9% (89.4% to 94.2%)	85.5% (80.8% to 89.3%)
Tuberculosis	43.5% (32.6% to 57.7%)	43.9% (33.5% to 57.3%)	40.7% (30.5% to 53.5%)	43.9% (33.5% to 57.1%)
HIV/AIDS	95.5% (94.5% to 96.4%)	93.0% (91.1% to 94.4%)	95.4% (93.9% to 96.8%)	93.1% (91.3% to 94.5%)
**Diarrhoea, lower respiratory infections, and other common infectious diseases**	76.8% (71.9% to 81.7%)	74.1% (68.6% to 79.2%)	42.7% (37.2% to 47.6%)	72.6% (67.4% to 77.5%)
Diarrhoeal diseases	95.2% (87.7% to 98.8%)	96.5% (90.6% to 99.1%)	95.6% (88.3% to 98.8%)	96.5% (90.6% to 99.1%)
Lower respiratory infections	83.2% (77.6% to 88.0%)	88.0% (83.6% to 91.9%)	83.0% (77.4% to 87.8%)	88.0% (83.6% to 91.9%)
Upper respiratory infections	1.5% (0.1% to 5.7%)	2.8% (0.2% to 10.9%)	0.0% (0.0% to 0.0%)	0.2% (0.0% to 0.6%)
Otitis media	0.7% (0.2% to 1.8%)	2.9% (1.1% to 5.4%)	3.2% (2.1% to 4.4%)	3.2% (2.1% to 4.4%)
Meningitis	2.0% (1.1% to 3.4%)	3.6% (2.2% to 6.2%)	0.0% (0.0% to 0.0%)	3.5% (2.1% to 5.9%)
Encephalitis	0.7% (0.5% to 1.0%)	1.3% (0.9% to 1.8%)	0.0% (0.0% to 0.0%)	0.9% (0.6% to 1.3%)
Measles	57.5% (39.7% to 80.6%)	58.4% (40.4% to 81.8%)	56.9% (40.1% to 79.1%)	58.4% (40.4% to 81.8%)
**Maternal disorders**	7.7% (3.5% to 12.2%)	7.7% (3.5% to 12.0%)	1.6% (0.7% to 2.7%)	7.4% (3.4% to 11.7%)
Maternal haemorrhage	17.4% (6.2% to 28.3%)	17.4% (6.2% to 28.4%)	17.4% (6.2% to 28.5%)	17.4% (6.2% to 28.4%)
Maternal sepsis and other pregnancy related infections	17.2% (6.1% to 28.0%)	17.3% (6.1% to 28.2%)	17.1% (6.1% to 28.0%)	17.3% (6.1% to 28.1%)
Maternal abortion, miscarriage, and ectopic pregnancy	18.6% (11.9% to 25.5%)	18.5% (11.9% to 25.5%)	19.4% (11.1% to 27.7%)	18.5% (11.9% to 25.5%)
**Neonatal disorders**	81.2% (77.5% to 84.5%)	81.2% (77.6% to 84.6%)	0.6% (0.3% to 1.1%)	78.7% (75.1% to 82.4%)
Neonatal preterm birth complications	97.2% (94.8% to 98.7%)	97.2% (94.8% to 98.7%)	1.5% (1.0% to 2.0%)	94.0% (91.0% to 96.3%)
Neonatal encephalopathy due to birth asphyxia and trauma	76.4% (72.5% to 80.1%)	76.4% (72.5% to 80.1%)	0.0% (0.0% to 0.0%)	75.4% (71.2% to 79.2%)
Neonatal sepsis and other neonatal infections	66.5% (60.9% to 71.4%)	66.6% (61.0% to 71.4%)	0.0% (0.0% to 0.0%)	63.1% (55.5% to 69.3%)
Hemolytic disease and other neonatal jaundice	64.6% (52.6% to 72.7%)	64.7% (52.7% to 72.8%)	0.0% (0.0% to 0.0%)	60.3% (49.1% to 68.3%)

Supplementary Results Table 1. Age-standardised PAFs attributable to the joint distribution of all risk factors for all ages, both sexes combined for each GBD level 1 and level 2 cause and all causes in 2016 for Cameroon. Results are shown for deaths, YLLs, YLDs, and DALYs
Data in parenthesis are 95% uncertainty intervals. PAFs=population attributable fractions. GBD=Global Burden of Disease. YLLs=years of life lost. YLDs=years live with disability. DALYs=disability-adjusted life-years

	Deaths 2016	YLLs 2016	YLDs 2016	DALYs 2016
Other neonatal disorders	66.1% (57.4% to 73.3%)	66.2% (57.5% to 73.4%)	0.0% (0.0% to 0.0%)	64.0% (55.2% to 71.2%)
**Nutritional deficiencies**	36.6% (23.9% to 50.8%)	69.8% (56.8% to 80.3%)	91.5% (89.1% to 93.6%)	80.2% (74.2% to 85.0%)
Protein-energy malnutrition	41.4% (28.0% to 55.8%)	75.7% (63.8% to 85.4%)	96.5% (95.4% to 97.5%)	76.4% (65.3% to 85.7%)
Vitamin A deficiency	-	-	100.0% (100.0% to 100.0%)	100.0% (100.0% to 100.0%)
Iron-deficiency anaemia	100.0% (100.0% to 100.0%)	100.0% (100.0% to 100.0%)	100.0% (100.0% to 100.0%)	100.0% (100.0% to 100.0%)
**Other communicable, maternal, neonatal, and nutritional diseases**	2.5% (1.3% to 4.0%)	1.7% (0.8% to 3.1%)	68.8% (63.8% to 73.6%)	9.3% (5.0% to 15.6%)
Sexually transmitted diseases excluding HIV	5.2% (2.0% to 11.3%)	2.7% (1.0% to 5.7%)	100.0% (100.0% to 100.0%)	14.2% (6.4% to 27.6%)
Hepatitis	0.1% (0.0% to 0.1%)	0.1% (0.0% to 0.1%)	0.1% (0.0% to 0.1%)	0.1% (0.0% to 0.1%)
**Neoplasms**	36.5% (32.0% to 41.3%)	36.8% (32.3% to 41.4%)	36.6% (31.5% to 41.3%)	36.8% (32.3% to 41.4%)
Oesophageal cancer	70.6% (59.7% to 80.4%)	71.8% (61.2% to 82.0%)	71.0% (59.9% to 81.4%)	71.8% (61.2% to 82.0%)
Stomach cancer	8.5% (3.5% to 16.6%)	8.8% (3.7% to 16.6%)	8.5% (3.5% to 16.6%)	8.8% (3.7% to 16.6%)
Liver cancer	41.0% (31.7% to 50.7%)	40.2% (30.4% to 50.2%)	40.8% (31.5% to 50.5%)	40.2% (30.4% to 50.2%)
Larynx cancer	71.6% (59.8% to 81.0%)	72.6% (61.1% to 82.0%)	71.9% (60.0% to 81.4%)	72.6% (61.1% to 82.0%)
Tracheal, bronchus, and lung cancer	88.2% (84.0% to 91.8%)	88.1% (83.7% to 91.6%)	88.0% (83.5% to 91.7%)	88.1% (83.7% to 91.6%)
Breast cancer	25.0% (13.4% to 39.9%)	22.8% (11.3% to 38.5%)	22.9% (11.5% to 38.0%)	22.8% (11.3% to 38.5%)
Cervical cancer	100.0% (100.0% to 100.0%)	100.0% (100.0% to 100.0%)	100.0% (100.0% to 100.0%)	100.0% (100.0% to 100.0%)
Uterine cancer	47.2% (33.7% to 61.1%)	48.5% (35.1% to 61.7%)	48.0% (34.4% to 61.4%)	48.5% (35.1% to 61.7%)
Prostate cancer	2.3% (1.4% to 3.4%)	2.5% (1.5% to 3.7%)	2.4% (1.5% to 3.5%)	2.5% (1.5% to 3.7%)
Colon and rectum cancer	50.0% (40.7% to 59.1%)	50.1% (41.1% to 58.8%)	50.1% (41.0% to 58.9%)	50.1% (41.1% to 58.8%)
Lip and oral cavity cancer	74.7% (65.0% to 83.1%)	75.5% (66.5% to 83.1%)	74.6% (65.4% to 82.4%)	75.5% (66.5% to 83.1%)
Nasopharynx cancer	68.5% (59.9% to 76.2%)	67.0% (58.7% to 74.3%)	65.9% (57.6% to 73.2%)	67.0% (58.7% to 74.3%)

Supplementary Results Table 1. Age-standardised PAFs attributable to the joint distribution of all risk factors for all ages, both sexes combined for each GBD level 1 and level 2 cause and all causes in 2016 for Cameroon. Results are shown for deaths, YLLs, YLDs, and DALYs
Data in parenthesis are 95% uncertainty intervals. PAFs=population attributable fractions. GBD=Global Burden of Disease. YLLs=years of life lost. YLDs=years live with disability. DALYs=disability-adjusted life-years

	Deaths 2016	YLLs 2016	YLDs 2016	DALYs 2016
Other pharynx cancer	50.0% (35.0% to 63.1%)	51.8% (38.3% to 64.6%)	50.8% (36.7% to 63.8%)	51.8% (38.2% to 64.6%)
Gallbladder and biliary tract cancer	25.0% (14.0% to 38.4%)	25.7% (14.6% to 39.1%)	25.3% (14.5% to 38.3%)	25.7% (14.6% to 39.1%)
Pancreatic cancer	21.0% (13.4% to 30.0%)	21.2% (13.6% to 29.9%)	20.9% (13.5% to 29.9%)	21.2% (13.6% to 29.9%)
Ovarian cancer	12.5% (5.5% to 21.4%)	10.8% (4.1% to 18.9%)	10.8% (4.4% to 18.6%)	10.8% (4.1% to 18.9%)
Kidney cancer	26.2% (17.4% to 36.1%)	24.0% (15.8% to 33.3%)	24.5% (16.5% to 33.7%)	24.0% (15.8% to 33.3%)
Bladder cancer	20.6% (12.2% to 29.9%)	21.4% (12.6% to 30.7%)	20.8% (12.2% to 30.2%)	21.3% (12.6% to 30.6%)
Thyroid cancer	13.6% (7.1% to 22.1%)	13.8% (7.2% to 22.1%)	13.5% (7.2% to 21.7%)	13.8% (7.2% to 22.1%)
Mesothelioma	86.6% (82.1% to 90.1%)	77.6% (70.7% to 83.4%)	82.9% (77.6% to 87.2%)	77.7% (70.8% to 83.4%)
Non-Hodgkin's lymphoma	5.7% (2.3% to 10.0%)	4.8% (1.9% to 8.6%)	5.4% (2.2% to 9.4%)	4.8% (1.9% to 8.6%)
Multiple myeloma	7.9% (3.3% to 13.8%)	8.1% (3.3% to 14.2%)	8.0% (3.4% to 14.2%)	8.1% (3.3% to 14.2%)
Leukaemia	13.0% (7.9% to 18.9%)	11.0% (6.7% to 16.2%)	12.8% (7.8% to 18.5%)	11.0% (6.7% to 16.2%)
**Cardiovascular diseases**	82.8% (79.9% to 85.4%)	83.2% (80.2% to 85.3%)	60.3% (56.1% to 64.3%)	81.3% (78.6% to 83.8%)
Rheumatic heart disease	24.6% (16.1% to 40.5%)	23.5% (16.2% to 34.7%)	23.2% (14.8% to 32.9%)	23.4% (16.4% to 34.2%)
Ischaemic heart disease	92.5% (89.6% to 95.3%)	94.2% (92.2% to 96.0%)	89.5% (86.8% to 92.4%)	94.1% (92.1% to 95.9%)
Cerebrovascular disease	86.3% (82.7% to 89.9%)	88.1% (85.5% to 90.6%)	86.6% (83.1% to 90.6%)	88.0% (85.4% to 90.5%)
Hypertensive heart disease	100.0% (100.0% to 100.0%)	100.0% (100.0% to 100.0%)	100.0% (100.0% to 100.0%)	100.0% (100.0% to 100.0%)
Cardiomyopathy and myocarditis	37.1% (30.6% to 44.7%)	37.3% (30.8% to 44.1%)	44.8% (38.2% to 51.4%)	37.9% (31.5% to 44.3%)
Atrial fibrillation and flutter	47.9% (39.0% to 56.7%)	52.1% (43.6% to 60.0%)	59.2% (52.2% to 65.6%)	55.7% (47.8% to 62.5%)
Aortic aneurysm	38.2% (31.3% to 45.0%)	43.8% (37.6% to 50.3%)	-	43.8% (37.6% to 50.3%)
Peripheral vascular disease	48.4% (42.0% to 55.6%)	55.3% (49.9% to 61.1%)	58.6% (53.4% to 63.8%)	57.7% (52.6% to 62.9%)
Endocarditis	34.2% (26.0% to 43.5%)	31.1% (23.7% to 38.5%)	32.7% (23.8% to 43.5%)	31.1% (23.7% to 38.6%)

Supplementary Results Table 1. Age-standardised PAFs attributable to the joint distribution of all risk factors for all ages, both sexes combined for each GBD level 1 and level 2 cause and all causes in 2016 for Cameroon. Results are shown for deaths, YLLs, YLDs, and DALYs
Data in parenthesis are 95% uncertainty intervals. PAFs=population attributable fractions. GBD=Global Burden of Disease. YLLs=years of life lost. YLDs=years live with disability. DALYs=disability-adjusted life-years

	Deaths 2016	YLLs 2016	YLDs 2016	DALYs 2016
Other cardiovascular and circulatory diseases	40.7% (35.9% to 45.7%)	42.9% (38.1% to 47.6%)	39.9% (34.8% to 45.0%)	42.1% (37.5% to 46.7%)
**Chronic respiratory diseases**	69.9% (64.3% to 75.7%)	65.6% (59.8% to 71.2%)	52.9% (45.8% to 60.3%)	62.4% (56.5% to 68.5%)
Chronic obstructive pulmonary disease	85.5% (79.1% to 91.5%)	84.4% (78.2% to 90.2%)	78.8% (71.6% to 85.9%)	83.4% (77.1% to 89.2%)
Pneumoconiosis	100.0% (100.0% to 100.0%)	100.0% (100.0% to 100.0%)	100.0% (100.0% to 100.0%)	100.0% (100.0% to 100.0%)
Asthma	37.9% (28.2% to 49.4%)	40.2% (31.6% to 50.4%)	31.0% (23.1% to 40.2%)	36.5% (28.2% to 46.1%)
Other chronic respiratory diseases	9.0% (5.4% to 13.5%)	8.2% (4.4% to 12.9%)	4.2% (2.1% to 7.1%)	7.9% (4.3% to 12.4%)
**Cirrhosis and other chronic liver diseases**	28.5% (25.3% to 31.8%)	27.4% (24.0% to 30.9%)	21.5% (18.5% to 24.9%)	27.3% (23.9% to 30.7%)
Cirrhosis and other chronic liver diseases due to hepatitis B	0.0% (0.0% to 0.0%)	0.0% (0.0% to 0.0%)	0.0% (0.0% to 0.0%)	0.0% (0.0% to 0.0%)
Cirrhosis and other chronic liver diseases due to hepatitis C	1.3% (0.5% to 2.6%)	1.9% (0.6% to 4.0%)	1.9% (0.7% to 4.2%)	1.9% (0.7% to 4.0%)
Cirrhosis and other chronic liver diseases due to alcohol use	100.0% (100.0% to 100.0%)	100.0% (100.0% to 100.0%)	100.0% (100.0% to 100.0%)	100.0% (100.0% to 100.0%)
**Digestive diseases**	9.4% (6.6% to 12.2%)	10.7% (7.5% to 13.9%)	2.3% (1.8% to 3.0%)	10.1% (7.2% to 13.1%)
Peptic ulcer disease	4.6% (2.9% to 6.9%)	5.2% (3.2% to 7.7%)	4.9% (3.3% to 6.8%)	5.2% (3.2% to 7.6%)
Gallbladder and biliary diseases	38.3% (25.3% to 52.7%)	36.0% (24.1% to 49.5%)	41.5% (29.1% to 55.0%)	36.2% (24.3% to 49.7%)
Pancreatitis	55.1% (35.0% to 75.7%)	58.6% (40.5% to 77.0%)	55.9% (38.1% to 74.1%)	58.6% (40.5% to 77.0%)
**Neurological disorders**	19.7% (10.1% to 31.5%)	18.9% (11.2% to 28.0%)	4.6% (2.1% to 8.2%)	10.2% (6.0% to 14.9%)
Alzheimer's disease and other dementias	21.6% (10.0% to 35.4%)	22.7% (10.8% to 36.5%)	22.7% (10.8% to 36.7%)	22.7% (10.8% to 36.5%)
Parkinson's disease	-3.0% (-4.1% to -2.1%)	-4.0% (-5.4% to -2.8%)	-4.8% (-6.3% to -3.5%)	-4.1% (-5.4% to -2.9%)
Epilepsy	21.9% (12.9% to 32.3%)	19.4% (11.0% to 28.9%)	17.0% (10.0% to 25.0%)	18.2% (10.5% to 27.2%)
Multiple sclerosis	3.7% (1.8% to 6.1%)	3.9% (1.8% to 6.4%)	2.8% (1.5% to 4.1%)	3.5% (1.7% to 5.7%)
**Mental and substance use disorders**	99.9% (99.8% to 99.9%)	99.8% (99.6% to 99.9%)	21.1% (18.9% to 23.4%)	26.8% (23.7% to 30.7%)
Alcohol use disorders	100.0% (100.0% to 100.0%)	100.0% (100.0% to 100.0%)	100.0% (100.0% to 100.0%)	100.0% (100.0% to 100.0%)

Supplementary Results Table 1. Age-standardised PAFs attributable to the joint distribution of all risk factors for all ages, both sexes combined for each GBD level 1 and level 2 cause and all causes in 2016 for Cameroon. Results are shown for deaths, YLLs, YLDs, and DALYs
Data in parenthesis are 95% uncertainty intervals. PAFs=population attributable fractions. GBD=Global Burden of Disease. YLLs=years of life lost. YLDs=years live with disability. DALYs=disability-adjusted life-years

	**Deaths 2016**	**YLLs 2016**	**YLDs 2016**	**DALYs 2016**
Drug use disorders	100.0% (100.0% to 100.0%)	100.0% (100.0% to 100.0%)	100.0% (100.0% to 100.0%)	100.0% (100.0% to 100.0%)
Depressive disorders	-	-	13.4% (11.5% to 15.6%)	13.4% (11.5% to 15.6%)
Idiopathic developmental intellectual disability	-	-	60.9% (29.8% to 80.3%)	60.9% (29.8% to 80.3%)
**Diabetes, urogenital, blood, and endocrine diseases**	74.9% (72.1% to 78.2%)	64.9% (60.4% to 70.0%)	58.0% (53.5% to 62.0%)	62.9% (59.3% to 66.6%)
Diabetes mellitus	100.0% (100.0% to 100.0%)	100.0% (100.0% to 100.0%)	100.0% (100.0% to 100.0%)	100.0% (100.0% to 100.0%)
Chronic kidney disease	100.0% (100.0% to 100.0%)	100.0% (100.0% to 100.0%)	100.0% (100.0% to 100.0%)	100.0% (100.0% to 100.0%)
**Musculoskeletal disorders**	0.2% (0.1% to 0.4%)	0.1% (0.0% to 0.2%)	27.2% (23.2% to 31.1%)	26.7% (22.7% to 30.6%)
Rheumatoid arthritis	1.0% (0.3% to 2.0%)	1.1% (0.4% to 2.3%)	1.9% (0.7% to 3.3%)	1.9% (0.7% to 3.2%)
Osteoarthritis	-	-	26.7% (14.4% to 39.2%)	26.7% (14.4% to 39.2%)
Low back and neck pain	-	-	32.6% (28.5% to 36.7%)	32.6% (28.5% to 36.7%)
Gout	-	-	55.1% (43.8% to 68.2%)	55.1% (43.8% to 68.2%)
**Other non-communicable diseases**	0.1% (0.0% to 0.2%)	0.1% (0.1% to 0.3%)	9.5% (8.0% to 11.0%)	5.7% (4.2% to 7.4%)
Sense organ diseases	-	-	19.3% (17.1% to 21.4%)	19.3% (17.1% to 21.4%)
Sudden infant death syndrome	3.8% (1.5% to 8.4%)	3.9% (1.5% to 8.5%)	-	3.9% (1.5% to 8.5%)
**Transport injuries**	29.4% (23.6% to 37.0%)	23.2% (17.8% to 30.1%)	34.0% (27.9% to 41.8%)	24.4% (18.8% to 31.5%)
Road injuries	29.5% (23.6% to 37.1%)	23.3% (17.9% to 30.2%)	35.8% (29.6% to 43.7%)	24.5% (18.9% to 31.6%)
Other transport injuries	27.4% (21.2% to 34.5%)	22.1% (16.0% to 28.6%)	26.2% (20.3% to 33.5%)	23.2% (17.3% to 29.8%)
**Unintentional injuries**	23.1% (16.3% to 31.8%)	12.4% (9.6% to 15.8%)	18.1% (16.4% to 20.3%)	13.7% (11.2% to 16.5%)
Falls	48.8% (32.4% to 67.2%)	32.8% (24.8% to 43.3%)	27.0% (25.3% to 28.9%)	30.5% (25.5% to 36.7%)
Drowning	9.2% (5.9% to 13.9%)	8.4% (5.6% to 12.0%)	11.9% (7.9% to 17.6%)	8.4% (5.6% to 12.1%)
Fire, heat, and hot substances	8.5% (4.9% to 13.9%)	8.5% (5.5% to 12.9%)	13.5% (9.2% to 19.1%)	9.4% (6.2% to 13.8%)

Supplementary Results Table 1. Age-standardised PAFs attributable to the joint distribution of all risk factors for all ages, both sexes combined for each GBD level 1 and level 2 cause and all causes in 2016 for Cameroon. Results are shown for deaths, YLLs, YLDs, and DALYs
Data in parenthesis are 95% uncertainty intervals. PAFs=population attributable fractions. GBD=Global Burden of Disease. YLLs=years of life lost. YLDs=years live with disability. DALYs=disability-adjusted life-years

	**Deaths 2016**	**YLLs 2016**	**YLDs 2016**	**DALYs 2016**
Poisonings	10.1% (5.9% to 15.6%)	9.5% (4.8% to 14.5%)	13.0% (9.1% to 18.1%)	9.7% (5.0% to 14.6%)
Exposure to mechanical forces	15.3% (12.5% to 18.5%)	9.7% (7.5% to 12.2%)	16.0% (14.1% to 18.3%)	11.2% (9.0% to 13.5%)
Animal contact	5.6% (4.6% to 7.0%)	5.6% (4.4% to 7.1%)	8.5% (6.8% to 10.8%)	6.2% (4.8% to 7.8%)
Foreign body	3.6% (2.7% to 4.6%)	4.0% (2.5% to 5.5%)	6.7% (5.1% to 8.7%)	4.7% (3.2% to 6.3%)
Other unintentional injuries	10.7% (7.1% to 15.5%)	10.6% (7.3% to 14.7%)	12.0% (8.1% to 17.3%)	11.1% (7.7% to 15.5%)
**Self-harm and interpersonal violence**	21.5% (13.5% to 30.1%)	20.8% (13.4% to 28.4%)	20.0% (16.9% to 23.7%)	20.7% (13.7% to 28.1%)
Self-harm	23.0% (13.2% to 33.5%)	23.5% (13.9% to 33.5%)	22.8% (13.2% to 33.1%)	23.5% (13.8% to 33.5%)
Interpersonal violence	15.0% (8.4% to 22.6%)	13.5% (7.3% to 20.8%)	19.5% (16.5% to 23.0%)	14.5% (8.9% to 21.1%)
**Forces of nature, conflict and terrorism, and executions and police conflict**	0.0% (0.0% to 0.0%)	0.0% (0.0% to 0.0%)	2.5% (1.5% to 3.7%)	0.2% (0.0% to 0.7%)
Forces of nature, conflict and terrorism, and state actor violence	0.0% (0.0% to 0.0%)	0.0% (0.0% to 0.0%)	8.5% (5.8% to 12.5%)	8.5% (5.8% to 12.5%)

Supplementary Results Table 1. Age-standardised PAFs attributable to the joint distribution of all risk factors for all ages, both sexes combined for each GBD level 1 and level 2 cause and all causes in 2016 for Canada. Results are shown for deaths, YLLs, YLDs, and DALYs
Data in parenthesis are 95% uncertainty intervals. PAFs=population attributable fractions. GBD=Global Burden of Disease. YLLs=years of life lost. YLDs=years live with disability. DALYs=disability-adjusted life-years

	Deaths 2016	YLLs 2016	YLDs 2016	DALYs 2016
**All causes**	55.7% (53.5% to 57.8%)	53.3% (51.5% to 54.9%)	22.3% (20.9% to 23.8%)	36.4% (33.9% to 39.1%)
**HIV/AIDS and tuberculosis**	76.3% (72.6% to 79.8%)	79.7% (77.4% to 82.2%)	85.2% (82.8% to 87.8%)	81.9% (79.4% to 84.6%)
Tuberculosis	44.6% (31.2% to 56.5%)	47.4% (35.8% to 57.4%)	50.7% (41.4% to 58.9%)	48.0% (36.8% to 57.6%)
HIV/AIDS	85.8% (83.8% to 88.2%)	83.9% (81.8% to 86.3%)	86.6% (84.4% to 89.0%)	85.1% (82.8% to 87.5%)
**Diarrhoea, lower respiratory infections, and other common infectious diseases**	25.9% (19.7% to 33.6%)	33.0% (27.5% to 39.4%)	5.1% (3.7% to 6.8%)	21.4% (16.8% to 26.3%)
Diarrhoeal diseases	17.7% (10.1% to 28.3%)	25.7% (17.6% to 35.7%)	29.6% (21.4% to 39.0%)	27.1% (19.1% to 36.9%)
Lower respiratory infections	28.3% (20.5% to 37.2%)	38.3% (31.2% to 46.3%)	41.4% (34.5% to 49.7%)	38.3% (31.3% to 46.3%)
Upper respiratory infections	0.8% (0.0% to 3.0%)	2.3% (0.1% to 8.7%)	0.0% (0.0% to 0.0%)	0.0% (0.0% to 0.0%)
Otitis media	3.5% (1.9% to 6.2%)	7.6% (5.0% to 10.7%)	5.9% (3.9% to 7.9%)	5.9% (3.9% to 7.9%)
Meningitis	2.2% (1.5% to 2.9%)	3.6% (2.7% to 4.6%)	0.0% (0.0% to 0.0%)	3.0% (2.2% to 3.9%)
Encephalitis	0.6% (0.4% to 0.8%)	1.3% (1.0% to 1.7%)	0.0% (0.0% to 0.0%)	0.8% (0.6% to 1.1%)
Measles	5.4% (0.9% to 20.9%)	6.4% (1.1% to 24.6%)	12.8% (2.6% to 48.0%)	6.6% (1.2% to 25.3%)
**Maternal disorders**	1.1% (0.5% to 1.7%)	1.1% (0.5% to 1.7%)	1.9% (0.8% to 3.3%)	1.3% (0.6% to 2.1%)
Maternal haemorrhage	9.7% (3.5% to 15.8%)	9.7% (3.5% to 15.7%)	9.9% (3.6% to 16.1%)	9.8% (3.5% to 15.9%)
Maternal sepsis and other pregnancy related infections	9.8% (3.6% to 15.8%)	9.8% (3.6% to 15.8%)	9.4% (3.4% to 15.5%)	9.6% (3.5% to 15.6%)
Maternal abortion, miscarriage, and ectopic pregnancy	1.5% (0.9% to 2.3%)	1.6% (0.9% to 2.4%)	1.9% (0.9% to 3.2%)	1.7% (0.9% to 2.5%)
**Neonatal disorders**	75.9% (73.2% to 78.2%)	76.0% (73.3% to 78.3%)	0.1% (0.1% to 0.1%)	57.6% (51.4% to 63.0%)
Neonatal preterm birth complications	90.7% (87.7% to 93.1%)	90.8% (87.8% to 93.1%)	0.2% (0.1% to 0.2%)	62.3% (52.8% to 70.2%)
Neonatal encephalopathy due to birth asphyxia and trauma	63.0% (59.3% to 66.5%)	63.1% (59.4% to 66.6%)	0.0% (0.0% to 0.0%)	52.6% (45.7% to 58.2%)
Neonatal sepsis and other neonatal infections	54.7% (49.4% to 59.4%)	54.7% (49.4% to 59.4%)	0.0% (0.0% to 0.0%)	31.6% (18.5% to 43.9%)
Hemolytic disease and other neonatal jaundice	51.7% (39.8% to 59.1%)	51.9% (40.0% to 59.2%)	0.0% (0.0% to 0.0%)	14.5% (7.1% to 22.4%)

Supplementary Results Table 1. Age-standardised PAFs attributable to the joint distribution of all risk factors for all ages, both sexes combined for each GBD level 1 and level 2 cause and all causes in 2016 for Canada. Results are shown for deaths, YLLs, YLDs, and DALYs
Data in parenthesis are 95% uncertainty intervals. PAFs=population attributable fractions. GBD=Global Burden of Disease. YLLs=years of life lost. YLDs=years live with disability. DALYs=disability-adjusted life-years

	Deaths 2016	YLLs 2016	YLDs 2016	DALYs 2016
Other neonatal disorders	61.3% (57.5% to 64.9%)	61.3% (57.5% to 64.9%)	0.0% (0.0% to 0.0%)	59.2% (55.3% to 63.0%)
**Nutritional deficiencies**	1.8% (1.2% to 2.2%)	7.8% (5.9% to 9.6%)	79.5% (75.2% to 83.4%)	72.3% (66.6% to 77.6%)
Protein-energy malnutrition	0.5% (0.3% to 0.8%)	4.1% (2.5% to 6.0%)	98.3% (97.8% to 98.7%)	62.6% (51.7% to 73.0%)
Vitamin A deficiency	-	-	0.0% (0.0% to 0.0%)	0.0% (0.0% to 0.0%)
Iron-deficiency anaemia	100.0% (100.0% to 100.0%)	100.0% (100.0% to 100.0%)	100.0% (100.0% to 100.0%)	100.0% (100.0% to 100.0%)
**Other communicable, maternal, neonatal, and nutritional diseases**	4.9% (4.1% to 6.0%)	3.9% (3.1% to 5.0%)	71.2% (64.9% to 77.3%)	27.6% (19.9% to 38.9%)
Sexually transmitted diseases excluding HIV	59.4% (41.5% to 78.4%)	28.9% (15.9% to 49.1%)	100.0% (100.0% to 100.0%)	85.6% (73.5% to 94.7%)
Hepatitis	4.9% (3.0% to 7.6%)	5.2% (3.0% to 8.2%)	2.5% (1.4% to 4.0%)	5.0% (2.9% to 7.9%)
**Neoplasms**	49.7% (46.8% to 52.6%)	48.5% (45.8% to 51.2%)	35.5% (32.1% to 38.7%)	48.0% (45.2% to 50.7%)
Oesophageal cancer	84.9% (77.0% to 91.2%)	85.3% (77.4% to 91.7%)	85.0% (77.4% to 91.2%)	85.3% (77.4% to 91.6%)
Stomach cancer	26.1% (16.4% to 36.4%)	24.6% (15.5% to 33.9%)	25.6% (16.1% to 35.4%)	24.6% (15.5% to 33.9%)
Liver cancer	71.1% (62.5% to 78.7%)	70.5% (62.0% to 77.9%)	70.6% (61.9% to 77.9%)	70.5% (62.0% to 77.9%)
Larynx cancer	88.9% (82.8% to 93.4%)	87.9% (81.7% to 92.7%)	88.3% (82.1% to 93.0%)	87.9% (81.7% to 92.7%)
Tracheal, bronchus, and lung cancer	91.2% (89.7% to 92.6%)	90.4% (88.8% to 91.8%)	90.8% (89.3% to 92.2%)	90.4% (88.8% to 91.8%)
Breast cancer	39.0% (30.9% to 46.9%)	38.0% (30.6% to 45.4%)	38.2% (30.4% to 45.8%)	38.0% (30.6% to 45.4%)
Cervical cancer	100.0% (100.0% to 100.0%)	100.0% (100.0% to 100.0%)	100.0% (100.0% to 100.0%)	100.0% (100.0% to 100.0%)
Uterine cancer	49.0% (34.2% to 63.1%)	50.1% (35.6% to 63.9%)	50.3% (35.8% to 64.2%)	50.1% (35.6% to 64.0%)
Prostate cancer	6.9% (4.9% to 9.2%)	6.8% (5.0% to 8.8%)	6.8% (5.0% to 8.9%)	6.8% (5.0% to 8.8%)
Colon and rectum cancer	57.1% (47.8% to 65.4%)	57.9% (49.0% to 65.8%)	57.7% (48.7% to 65.8%)	57.9% (48.9% to 65.8%)
Lip and oral cavity cancer	82.1% (75.5% to 87.4%)	82.4% (75.8% to 87.6%)	81.9% (75.5% to 87.1%)	82.4% (75.7% to 87.6%)
Nasopharynx cancer	82.2% (77.7% to 86.0%)	80.2% (75.8% to 84.1%)	78.5% (71.6% to 83.9%)	80.2% (75.7% to 84.0%)

Supplementary Results Table 1. Age-standardised PAFs attributable to the joint distribution of all risk factors for all ages, both sexes combined for each GBD level 1 and level 2 cause and all causes in 2016 for Canada. Results are shown for deaths, YLLs, YLDs, and DALYs
Data in parenthesis are 95% uncertainty intervals. PAFs=population attributable fractions. GBD=Global Burden of Disease. YLLs=years of life lost. YLDs=years live with disability. DALYs=disability-adjusted life-years

	Deaths 2016	YLLs 2016	YLDs 2016	DALYs 2016
Other pharynx cancer	52.3% (37.2% to 65.1%)	55.3% (39.9% to 67.4%)	54.4% (38.9% to 66.9%)	55.3% (39.9% to 67.4%)
Gallbladder and biliary tract cancer	23.5% (12.9% to 36.2%)	23.9% (13.1% to 36.3%)	23.4% (12.9% to 35.8%)	23.9% (13.1% to 36.3%)
Pancreatic cancer	41.0% (33.2% to 48.8%)	40.1% (32.3% to 47.6%)	40.8% (33.0% to 48.4%)	40.1% (32.3% to 47.6%)
Ovarian cancer	13.8% (6.5% to 22.5%)	11.9% (5.2% to 19.8%)	11.7% (5.1% to 19.4%)	11.9% (5.2% to 19.8%)
Kidney cancer	41.9% (31.8% to 52.3%)	41.1% (31.1% to 51.3%)	41.0% (31.2% to 51.1%)	41.1% (31.0% to 51.3%)
Bladder cancer	45.0% (34.9% to 55.2%)	44.0% (34.2% to 53.6%)	43.8% (34.1% to 53.2%)	44.0% (34.2% to 53.6%)
Thyroid cancer	16.4% (8.2% to 26.7%)	17.1% (8.5% to 27.6%)	16.3% (8.9% to 25.5%)	16.9% (8.6% to 27.2%)
Mesothelioma	97.2% (96.3% to 97.9%)	94.0% (92.4% to 95.4%)	96.2% (95.1% to 97.2%)	94.0% (92.4% to 95.4%)
Non-Hodgkin's lymphoma	7.9% (3.5% to 13.5%)	8.1% (3.6% to 13.6%)	8.1% (3.6% to 13.5%)	8.1% (3.6% to 13.5%)
Multiple myeloma	9.0% (3.9% to 15.4%)	9.4% (4.1% to 16.0%)	9.3% (4.1% to 16.0%)	9.4% (4.1% to 16.0%)
Leukaemia	23.1% (14.7% to 31.7%)	19.4% (12.6% to 26.4%)	20.6% (13.1% to 28.3%)	19.5% (12.7% to 26.5%)
**Cardiovascular diseases**	79.4% (75.8% to 82.3%)	81.7% (79.3% to 83.7%)	69.0% (65.3% to 72.3%)	79.1% (76.7% to 81.3%)
Rheumatic heart disease	22.9% (13.7% to 41.1%)	22.4% (14.7% to 36.5%)	22.0% (14.4% to 35.7%)	22.3% (14.7% to 36.5%)
Ischaemic heart disease	89.9% (85.4% to 93.7%)	92.2% (89.4% to 94.8%)	89.1% (85.7% to 92.5%)	92.0% (89.1% to 94.7%)
Cerebrovascular disease	75.7% (67.9% to 83.2%)	81.0% (76.1% to 86.0%)	81.4% (76.5% to 86.4%)	81.1% (76.3% to 86.0%)
Hypertensive heart disease	100.0% (100.0% to 100.0%)	100.0% (100.0% to 100.0%)	100.0% (100.0% to 100.0%)	100.0% (100.0% to 100.0%)
Cardiomyopathy and myocarditis	50.0% (42.9% to 57.1%)	46.2% (40.5% to 53.6%)	45.2% (39.0% to 51.7%)	46.1% (40.6% to 53.1%)
Atrial fibrillation and flutter	49.1% (41.1% to 56.5%)	53.3% (46.4% to 60.0%)	58.2% (52.6% to 64.0%)	56.7% (50.8% to 62.7%)
Aortic aneurysm	36.3% (29.2% to 43.5%)	40.8% (34.6% to 47.0%)	-	40.8% (34.6% to 47.0%)
Peripheral vascular disease	48.2% (42.0% to 55.2%)	54.0% (49.0% to 59.1%)	55.4% (50.3% to 60.5%)	54.4% (49.6% to 59.3%)
Endocarditis	35.2% (25.8% to 46.1%)	34.7% (26.3% to 43.5%)	35.9% (26.9% to 45.6%)	34.8% (26.3% to 43.6%)

Supplementary Results Table 1. Age-standardised PAFs attributable to the joint distribution of all risk factors for all ages, both sexes combined for each GBD level 1 and level 2 cause and all causes in 2016 for Canada. Results are shown for deaths, YLLs, YLDs, and DALYs
Data in parenthesis are 95% uncertainty intervals. PAFs=population attributable fractions. GBD=Global Burden of Disease. YLLs=years of life lost. YLDs=years live with disability. DALYs=disability-adjusted life-years

	Deaths 2016	YLLs 2016	YLDs 2016	DALYs 2016
Other cardiovascular and circulatory diseases	38.5% (33.3% to 44.1%)	42.5% (37.5% to 47.3%)	44.2% (38.9% to 49.6%)	43.4% (38.3% to 48.6%)
**Chronic respiratory diseases**	72.2% (68.3% to 78.2%)	69.1% (65.5% to 74.7%)	44.9% (37.5% to 53.1%)	56.3% (50.4% to 62.4%)
Chronic obstructive pulmonary disease	84.6% (81.5% to 87.4%)	84.2% (81.3% to 87.1%)	80.0% (76.4% to 83.4%)	83.3% (80.3% to 86.2%)
Pneumoconiosis	100.0% (100.0% to 100.0%)	100.0% (100.0% to 100.0%)	100.0% (100.0% to 100.0%)	100.0% (100.0% to 100.0%)
Asthma	41.4% (30.9% to 53.1%)	43.6% (34.8% to 53.5%)	37.2% (28.7% to 47.0%)	37.4% (29.0% to 47.2%)
Other chronic respiratory diseases	22.8% (18.4% to 26.9%)	16.2% (13.0% to 19.8%)	10.8% (8.2% to 14.0%)	14.5% (11.6% to 17.7%)
**Cirrhosis and other chronic liver diseases**	63.6% (60.8% to 66.6%)	65.9% (63.3% to 68.6%)	57.5% (53.6% to 61.3%)	65.4% (62.8% to 67.9%)
Cirrhosis and other chronic liver diseases due to hepatitis B	3.3% (1.3% to 6.0%)	3.9% (1.6% to 7.2%)	3.3% (1.3% to 6.1%)	3.9% (1.6% to 7.1%)
Cirrhosis and other chronic liver diseases due to hepatitis C	77.9% (72.6% to 83.6%)	84.0% (80.0% to 87.8%)	81.7% (76.4% to 86.8%)	83.8% (79.9% to 87.7%)
Cirrhosis and other chronic liver diseases due to alcohol use	100.0% (100.0% to 100.0%)	100.0% (100.0% to 100.0%)	100.0% (100.0% to 100.0%)	100.0% (100.0% to 100.0%)
**Digestive diseases**	7.4% (5.3% to 9.7%)	8.5% (6.2% to 11.0%)	7.0% (5.4% to 8.9%)	8.1% (6.1% to 10.2%)
Peptic ulcer disease	7.9% (5.4% to 10.9%)	10.7% (7.5% to 14.6%)	9.9% (6.7% to 13.7%)	10.4% (7.3% to 14.2%)
Gallbladder and biliary diseases	44.3% (29.3% to 60.1%)	45.2% (30.5% to 60.4%)	47.8% (34.1% to 61.7%)	45.8% (31.9% to 60.7%)
Pancreatitis	23.9% (7.6% to 40.1%)	29.4% (14.3% to 45.8%)	31.3% (16.9% to 45.7%)	29.8% (14.8% to 45.9%)
**Neurological disorders**	18.3% (8.8% to 29.3%)	15.7% (8.7% to 23.8%)	3.7% (2.0% to 5.8%)	7.7% (4.4% to 11.9%)
Alzheimer's disease and other dementias	25.8% (12.3% to 41.1%)	27.0% (13.8% to 42.0%)	27.1% (13.5% to 42.1%)	27.0% (13.7% to 42.0%)
Parkinson's disease	-5.0% (-5.8% to -4.1%)	-6.2% (-7.2% to -5.2%)	-7.3% (-8.5% to -6.1%)	-6.5% (-7.5% to -5.4%)
Epilepsy	31.9% (21.8% to 40.9%)	33.0% (23.4% to 41.2%)	27.3% (19.0% to 35.1%)	29.5% (20.5% to 37.0%)
Multiple sclerosis	7.3% (4.7% to 10.0%)	8.0% (5.1% to 11.1%)	7.2% (4.5% to 10.1%)	7.5% (4.8% to 10.4%)
**Mental and substance use disorders**	99.7% (99.6% to 99.8%)	99.6% (99.4% to 99.7%)	24.5% (22.0% to 27.2%)	31.4% (28.3% to 34.7%)
Alcohol use disorders	100.0% (100.0% to 100.0%)	100.0% (100.0% to 100.0%)	100.0% (100.0% to 100.0%)	100.0% (100.0% to 100.0%)

Supplementary Results Table 1. Age-standardised PAFs attributable to the joint distribution of all risk factors for all ages, both sexes combined for each GBD level 1 and level 2 cause and all causes in 2016 for Canada. Results are shown for deaths, YLLs, YLDs, and DALYs
Data in parenthesis are 95% uncertainty intervals. PAFs=population attributable fractions. GBD=Global Burden of Disease. YLLs=years of life lost. YLDs=years live with disability. DALYs=disability-adjusted life-years

	Deaths 2016	YLLs 2016	YLDs 2016	DALYs 2016
Drug use disorders	100.0% (100.0% to 100.0%)	100.0% (100.0% to 100.0%)	100.0% (100.0% to 100.0%)	100.0% (100.0% to 100.0%)
Depressive disorders	-	-	7.8% (6.6% to 9.1%)	7.8% (6.6% to 9.1%)
Idiopathic developmental intellectual disability	-	-	52.4% (26.5% to 70.6%)	52.4% (26.5% to 70.6%)
**Diabetes, urogenital, blood, and endocrine diseases**	74.9% (73.3% to 78.1%)	68.2% (65.9% to 72.2%)	66.9% (63.0% to 70.6%)	67.4% (64.8% to 70.0%)
Diabetes mellitus	100.0% (100.0% to 100.0%)	100.0% (100.0% to 100.0%)	100.0% (100.0% to 100.0%)	100.0% (100.0% to 100.0%)
Chronic kidney disease	100.0% (100.0% to 100.0%)	100.0% (100.0% to 100.0%)	100.0% (100.0% to 100.0%)	100.0% (100.0% to 100.0%)
**Musculoskeletal disorders**	0.9% (0.3% to 1.6%)	1.0% (0.4% to 1.7%)	17.1% (14.1% to 20.5%)	16.8% (13.9% to 20.2%)
Rheumatoid arthritis	3.0% (1.1% to 5.1%)	3.8% (1.5% to 6.5%)	4.6% (1.8% to 7.9%)	4.5% (1.8% to 7.8%)
Osteoarthritis	-	-	30.2% (16.7% to 43.5%)	30.2% (16.7% to 43.5%)
Low back and neck pain	-	-	22.4% (19.3% to 25.7%)	22.4% (19.3% to 25.7%)
Gout	-	-	52.0% (33.6% to 69.7%)	52.0% (33.6% to 69.7%)
**Other non-communicable diseases**	0.3% (0.2% to 0.4%)	0.4% (0.2% to 0.5%)	2.2% (1.9% to 2.5%)	1.9% (1.7% to 2.2%)
Sense organ diseases	-	-	7.8% (7.0% to 8.6%)	7.8% (7.0% to 8.6%)
Sudden infant death syndrome	2.7% (1.7% to 3.9%)	2.7% (1.7% to 3.9%)	-	2.7% (1.7% to 3.9%)
**Transport injuries**	41.6% (34.5% to 49.1%)	39.2% (31.9% to 46.9%)	44.4% (37.6% to 51.6%)	40.7% (33.6% to 48.1%)
Road injuries	40.8% (33.7% to 48.2%)	38.4% (31.1% to 46.1%)	45.0% (38.1% to 52.3%)	40.2% (33.0% to 47.6%)
Other transport injuries	46.3% (38.9% to 54.3%)	44.0% (36.5% to 52.0%)	42.4% (35.5% to 49.7%)	43.4% (36.1% to 51.1%)
**Unintentional injuries**	47.4% (37.0% to 51.8%)	30.1% (24.6% to 33.7%)	26.9% (24.8% to 29.2%)	28.0% (25.1% to 30.5%)
Falls	70.2% (62.2% to 73.6%)	52.5% (45.5% to 55.8%)	29.4% (27.7% to 31.2%)	35.2% (31.8% to 38.1%)
Drowning	25.7% (21.0% to 31.6%)	23.4% (19.1% to 28.5%)	24.4% (18.8% to 31.8%)	23.4% (19.1% to 28.7%)
Fire, heat, and hot substances	16.6% (11.7% to 23.0%)	19.8% (14.7% to 25.2%)	27.8% (22.0% to 35.1%)	24.4% (19.0% to 31.0%)

Supplementary Results Table 1. Age-standardised PAFs attributable to the joint distribution of all risk factors for all ages, both sexes combined for each GBD level 1 and level 2 cause and all causes in 2016 for Canada. Results are shown for deaths, YLLs, YLDs, and DALYs

Data in parenthesis are 95% uncertainty intervals. PAFs=population attributable fractions. GBD=Global Burden of Disease. YLLs=years of life lost. YLDs=years live with disability. DALYs=disability-adjusted life-years

	**Deaths 2016**	**YLLs 2016**	**YLDs 2016**	**DALYs 2016**
Poisonings	29.1% (22.8% to 36.6%)	30.0% (23.5% to 37.3%)	23.7% (18.4% to 30.6%)	27.4% (21.6% to 34.5%)
Exposure to mechanical forces	20.2% (17.3% to 23.1%)	20.2% (16.5% to 23.7%)	29.2% (26.3% to 32.3%)	26.6% (23.6% to 29.8%)
Animal contact	19.7% (17.2% to 22.5%)	17.0% (14.0% to 20.0%)	17.4% (15.1% to 20.0%)	17.4% (15.1% to 19.9%)
Foreign body	8.5% (6.9% to 10.1%)	11.9% (9.7% to 14.3%)	11.7% (10.0% to 13.8%)	11.9% (10.0% to 13.8%)
Other unintentional injuries	33.2% (27.5% to 40.0%)	35.3% (29.4% to 42.3%)	25.1% (19.3% to 32.3%)	26.3% (20.5% to 33.4%)
**Self-harm and interpersonal violence**	34.1% (21.3% to 45.5%)	35.5% (22.8% to 46.4%)	32.6% (27.4% to 38.2%)	35.2% (23.7% to 45.6%)
Self-harm	34.6% (20.2% to 47.4%)	36.7% (22.0% to 49.4%)	31.4% (17.2% to 44.1%)	36.5% (21.8% to 49.2%)
Interpersonal violence	30.1% (21.2% to 38.5%)	28.3% (19.7% to 36.4%)	33.0% (27.5% to 38.5%)	30.0% (22.5% to 36.9%)
**Forces of nature, conflict and terrorism, and executions and police conflict**	0.0% (0.0% to 0.0%)	0.0% (0.0% to 0.0%)	2.2% (1.3% to 3.6%)	0.8% (0.2% to 1.8%)
Forces of nature, conflict and terrorism, and state actor violence	0.0% (0.0% to 0.0%)	0.0% (0.0% to 0.0%)	9.9% (6.4% to 15.1%)	9.9% (6.4% to 15.1%)

Supplementary Results Table 1. Age-standardised PAFs attributable to the joint distribution of all risk factors for all ages, both sexes combined for each GBD level 1 and level 2 cause and all causes in 2016 for Cape Verde. Results are shown for deaths, YLLs, YLDs, and DALYs
Data in parenthesis are 95% uncertainty intervals. PAFs=population attributable fractions. GBD=Global Burden of Disease. YLLs=years of life lost. YLDs=years live with disability. DALYs=disability-adjusted life-years

	Deaths 2016	YLLs 2016	YLDs 2016	DALYs 2016
**All causes**	60.0% (58.0% to 62.1%)	55.1% (53.1% to 57.1%)	28.8% (27.4% to 30.1%)	45.6% (43.3% to 48.0%)
**HIV/AIDS and tuberculosis**	76.2% (67.5% to 83.1%)	81.0% (74.7% to 85.6%)	83.3% (78.4% to 87.9%)	81.2% (75.6% to 85.6%)
Tuberculosis	40.2% (29.8% to 53.1%)	41.4% (31.1% to 53.8%)	39.1% (29.4% to 51.6%)	41.3% (31.0% to 53.7%)
HIV/AIDS	92.9% (90.6% to 94.9%)	90.4% (87.2% to 93.2%)	92.2% (90.0% to 94.2%)	90.5% (87.6% to 93.2%)
**Diarrhoea, lower respiratory infections, and other common infectious diseases**	61.9% (53.6% to 70.4%)	60.5% (53.0% to 67.7%)	36.4% (29.8% to 41.7%)	57.3% (50.7% to 64.0%)
Diarrhoeal diseases	88.1% (73.6% to 96.6%)	90.7% (79.0% to 97.4%)	88.7% (74.1% to 96.8%)	90.0% (77.5% to 97.2%)
Lower respiratory infections	65.3% (55.9% to 74.9%)	69.7% (61.6% to 78.4%)	66.7% (57.5% to 76.2%)	69.7% (61.5% to 78.4%)
Upper respiratory infections	1.0% (0.1% to 3.5%)	2.3% (0.1% to 8.1%)	0.0% (0.0% to 0.0%)	0.0% (0.0% to 0.1%)
Otitis media	0.2% (0.1% to 0.9%)	1.2% (0.3% to 3.6%)	3.4% (2.1% to 4.8%)	3.4% (2.1% to 4.8%)
Meningitis	1.1% (0.6% to 1.8%)	2.6% (1.4% to 4.1%)	0.0% (0.0% to 0.0%)	2.0% (1.2% to 3.2%)
Encephalitis	0.3% (0.1% to 0.6%)	0.5% (0.3% to 1.0%)	0.0% (0.0% to 0.0%)	0.3% (0.2% to 0.7%)
Measles	34.6% (12.3% to 74.7%)	34.9% (12.5% to 75.6%)	33.0% (11.9% to 70.8%)	34.9% (12.5% to 75.5%)
**Maternal disorders**	8.6% (4.8% to 12.8%)	8.5% (4.6% to 12.7%)	2.9% (1.3% to 5.1%)	7.0% (3.8% to 10.5%)
Maternal haemorrhage	17.7% (6.4% to 28.7%)	17.7% (6.4% to 28.7%)	18.0% (6.5% to 29.0%)	17.7% (6.4% to 28.8%)
Maternal sepsis and other pregnancy related infections	17.5% (6.4% to 28.5%)	17.6% (6.4% to 28.6%)	17.4% (6.3% to 28.1%)	17.5% (6.3% to 28.3%)
Maternal abortion, miscarriage, and ectopic pregnancy	13.7% (7.8% to 20.5%)	13.4% (7.6% to 20.0%)	13.2% (7.4% to 20.7%)	13.4% (7.6% to 19.9%)
**Neonatal disorders**	78.1% (72.7% to 83.1%)	78.2% (72.8% to 83.1%)	0.2% (0.1% to 0.4%)	66.7% (59.1% to 73.3%)
Neonatal preterm birth complications	91.9% (85.6% to 96.6%)	92.0% (85.7% to 96.6%)	0.7% (0.5% to 0.9%)	81.4% (73.6% to 87.9%)
Neonatal encephalopathy due to birth asphyxia and trauma	71.1% (64.4% to 77.2%)	71.2% (64.5% to 77.3%)	0.0% (0.0% to 0.0%)	64.5% (53.9% to 73.2%)
Neonatal sepsis and other neonatal infections	62.7% (54.9% to 68.7%)	62.8% (54.9% to 68.8%)	0.0% (0.0% to 0.0%)	45.3% (29.5% to 57.1%)
Hemolytic disease and other neonatal jaundice	61.4% (45.2% to 71.2%)	61.4% (45.3% to 71.3%)	0.0% (0.0% to 0.0%)	45.5% (33.6% to 55.7%)

Supplementary Results Table 1. Age-standardised PAFs attributable to the joint distribution of all risk factors for all ages, both sexes combined for each GBD level 1 and level 2 cause and all causes in 2016 for Cape Verde. Results are shown for deaths, YLLs, YLDs, and DALYs
Data in parenthesis are 95% uncertainty intervals. PAFs=population attributable fractions. GBD=Global Burden of Disease. YLLs=years of life lost. YLDs=years live with disability. DALYs=disability-adjusted life-years

	**Deaths 2016**	**YLLs 2016**	**YLDs 2016**	**DALYs 2016**
Other neonatal disorders	57.4% (44.2% to 68.3%)	57.5% (44.4% to 68.4%)	0.0% (0.0% to 0.0%)	48.8% (37.2% to 59.2%)
**Nutritional deficiencies**	15.0% (9.9% to 22.1%)	44.2% (33.5% to 55.9%)	84.4% (81.6% to 87.1%)	81.2% (78.0% to 84.1%)
Protein-energy malnutrition	20.2% (13.3% to 29.3%)	56.0% (44.2% to 68.0%)	97.4% (96.1% to 98.3%)	66.0% (57.2% to 74.3%)
Vitamin A deficiency	-	-	100.0% (100.0% to 100.0%)	100.0% (100.0% to 100.0%)
Iron-deficiency anaemia	100.0% (100.0% to 100.0%)	100.0% (100.0% to 100.0%)	100.0% (100.0% to 100.0%)	100.0% (100.0% to 100.0%)
**Other communicable, maternal, neonatal, and nutritional diseases**	1.8% (1.4% to 2.4%)	1.9% (1.4% to 2.5%)	64.5% (58.6% to 70.3%)	32.8% (24.7% to 42.8%)
Sexually transmitted diseases excluding HIV	27.0% (11.3% to 55.8%)	14.0% (5.0% to 34.3%)	100.0% (100.0% to 100.0%)	82.4% (63.8% to 95.5%)
Hepatitis	0.1% (0.0% to 0.2%)	0.1% (0.0% to 0.3%)	0.1% (0.0% to 0.3%)	0.1% (0.0% to 0.3%)
**Neoplasms**	36.7% (33.2% to 40.7%)	36.5% (33.1% to 40.1%)	34.4% (30.8% to 39.2%)	36.5% (33.1% to 40.0%)
Oesophageal cancer	75.4% (65.6% to 84.1%)	74.7% (64.1% to 83.8%)	75.1% (65.3% to 83.9%)	74.7% (64.2% to 83.8%)
Stomach cancer	13.0% (6.4% to 22.3%)	12.4% (6.1% to 21.7%)	12.6% (6.2% to 21.5%)	12.4% (6.1% to 21.7%)
Liver cancer	39.0% (30.7% to 47.6%)	37.5% (29.4% to 46.6%)	38.3% (30.1% to 47.0%)	37.5% (29.4% to 46.6%)
Larynx cancer	76.3% (66.1% to 84.6%)	74.2% (63.8% to 83.0%)	75.9% (66.0% to 84.3%)	74.2% (63.9% to 83.0%)
Tracheal, bronchus, and lung cancer	84.9% (79.8% to 89.2%)	83.3% (78.0% to 88.1%)	83.7% (77.9% to 88.5%)	83.3% (78.0% to 88.1%)
Breast cancer	22.9% (12.6% to 37.2%)	20.9% (10.4% to 35.2%)	21.6% (11.2% to 35.7%)	20.9% (10.4% to 35.2%)
Cervical cancer	100.0% (100.0% to 100.0%)	100.0% (100.0% to 100.0%)	100.0% (100.0% to 100.0%)	100.0% (100.0% to 100.0%)
Uterine cancer	37.0% (23.2% to 52.2%)	39.9% (26.3% to 54.5%)	38.9% (25.3% to 53.5%)	39.8% (26.3% to 54.5%)
Prostate cancer	4.2% (2.8% to 5.8%)	4.2% (2.8% to 5.9%)	4.3% (3.0% to 6.0%)	4.2% (2.8% to 5.9%)
Colon and rectum cancer	48.1% (38.7% to 56.7%)	47.9% (38.6% to 56.3%)	48.1% (39.0% to 56.8%)	47.9% (38.6% to 56.3%)
Lip and oral cavity cancer	78.7% (69.9% to 85.3%)	77.5% (69.3% to 84.3%)	78.2% (69.9% to 84.9%)	77.6% (69.3% to 84.2%)
Nasopharynx cancer	68.5% (60.7% to 76.5%)	65.9% (57.9% to 74.0%)	64.3% (56.2% to 72.6%)	65.8% (57.9% to 74.0%)

Supplementary Results Table 1. Age-standardised PAFs attributable to the joint distribution of all risk factors for all ages, both sexes combined for each GBD level 1 and level 2 cause and all causes in 2016 for Cape Verde. Results are shown for deaths, YLLs, YLDs, and DALYs
Data in parenthesis are 95% uncertainty intervals. PAFs=population attributable fractions. GBD=Global Burden of Disease. YLLs=years of life lost. YLDs=years live with disability. DALYs=disability-adjusted life-years

	Deaths 2016	YLLs 2016	YLDs 2016	DALYs 2016
Other pharynx cancer	46.4% (31.1% to 59.9%)	49.1% (35.9% to 61.4%)	47.4% (32.9% to 60.7%)	49.0% (35.8% to 61.4%)
Gallbladder and biliary tract cancer	19.6% (10.1% to 31.4%)	20.5% (11.2% to 32.2%)	20.3% (10.8% to 31.7%)	20.5% (11.2% to 32.2%)
Pancreatic cancer	22.3% (15.4% to 30.5%)	21.3% (14.6% to 29.3%)	21.9% (15.2% to 29.9%)	21.3% (14.6% to 29.3%)
Ovarian cancer	9.4% (3.4% to 17.2%)	8.2% (2.6% to 15.5%)	8.4% (2.8% to 15.7%)	8.2% (2.6% to 15.5%)
Kidney cancer	23.7% (16.0% to 33.0%)	20.3% (13.4% to 28.8%)	21.9% (14.7% to 30.4%)	20.3% (13.4% to 28.8%)
Bladder cancer	27.7% (18.8% to 37.7%)	25.5% (17.0% to 35.2%)	25.5% (17.2% to 35.0%)	25.5% (17.0% to 35.2%)
Thyroid cancer	10.6% (4.8% to 18.5%)	10.9% (5.0% to 19.4%)	10.8% (5.3% to 18.2%)	10.9% (5.0% to 19.4%)
Mesothelioma	70.0% (62.4% to 76.4%)	53.1% (43.7% to 61.6%)	64.2% (55.9% to 71.4%)	53.2% (43.8% to 61.8%)
Non-Hodgkin's lymphoma	4.5% (1.7% to 8.3%)	3.8% (1.4% to 7.1%)	4.4% (1.7% to 8.1%)	3.8% (1.4% to 7.1%)
Multiple myeloma	6.2% (2.5% to 11.7%)	6.5% (2.6% to 12.2%)	6.5% (2.6% to 12.2%)	6.5% (2.6% to 12.2%)
Leukaemia	12.3% (7.2% to 18.6%)	9.2% (5.4% to 14.7%)	12.1% (7.2% to 17.9%)	9.3% (5.4% to 14.8%)
**Cardiovascular diseases**	86.1% (83.4% to 88.2%)	86.5% (84.1% to 88.2%)	65.5% (61.7% to 69.0%)	83.9% (81.6% to 85.9%)
Rheumatic heart disease	33.3% (21.8% to 53.3%)	33.0% (23.7% to 47.3%)	30.7% (21.1% to 42.0%)	31.9% (23.0% to 44.5%)
Ischaemic heart disease	93.4% (90.2% to 96.0%)	94.7% (92.7% to 96.4%)	91.9% (89.7% to 93.9%)	94.6% (92.6% to 96.3%)
Cerebrovascular disease	87.1% (83.0% to 90.8%)	89.5% (86.8% to 91.7%)	88.0% (84.4% to 91.1%)	89.3% (86.6% to 91.6%)
Hypertensive heart disease	100.0% (100.0% to 100.0%)	100.0% (100.0% to 100.0%)	100.0% (100.0% to 100.0%)	100.0% (100.0% to 100.0%)
Cardiomyopathy and myocarditis	44.2% (36.4% to 52.4%)	43.4% (36.2% to 49.8%)	47.6% (40.7% to 54.2%)	44.1% (37.1% to 50.2%)
Atrial fibrillation and flutter	53.9% (44.4% to 62.1%)	58.2% (50.1% to 65.4%)	65.4% (59.5% to 70.9%)	61.9% (55.2% to 68.0%)
Aortic aneurysm	48.5% (40.0% to 56.2%)	53.8% (46.7% to 59.9%)	-	53.8% (46.7% to 59.9%)
Peripheral vascular disease	54.8% (47.2% to 62.8%)	61.1% (55.0% to 67.1%)	64.0% (58.6% to 69.7%)	62.9% (57.3% to 68.5%)
Endocarditis	42.8% (34.0% to 53.0%)	36.6% (28.5% to 44.5%)	41.9% (32.4% to 53.1%)	36.8% (28.7% to 44.6%)

Supplementary Results Table 1. Age-standardised PAFs attributable to the joint distribution of all risk factors for all ages, both sexes combined for each GBD level 1 and level 2 cause and all causes in 2016 for Cape Verde. Results are shown for deaths, YLLs, YLDs, and DALYs
Data in parenthesis are 95% uncertainty intervals. PAFs=population attributable fractions. GBD=Global Burden of Disease. YLLs=years of life lost. YLDs=years live with disability. DALYs=disability-adjusted life-years

	Deaths 2016	YLLs 2016	YLDs 2016	DALYs 2016
Other cardiovascular and circulatory diseases	50.4% (44.7% to 55.6%)	52.0% (47.5% to 56.1%)	46.7% (41.4% to 52.1%)	50.2% (45.7% to 54.6%)
**Chronic respiratory diseases**	65.2% (59.3% to 71.3%)	59.9% (54.2% to 65.6%)	46.0% (39.7% to 53.2%)	54.6% (48.9% to 60.8%)
Chronic obstructive pulmonary disease	79.0% (71.6% to 86.2%)	76.6% (69.2% to 84.0%)	71.4% (63.6% to 78.8%)	75.1% (67.7% to 82.6%)
Pneumoconiosis	100.0% (100.0% to 100.0%)	100.0% (100.0% to 100.0%)	100.0% (100.0% to 100.0%)	100.0% (100.0% to 100.0%)
Asthma	31.5% (22.2% to 41.9%)	35.7% (27.2% to 45.4%)	27.1% (19.9% to 35.9%)	30.4% (22.8% to 39.5%)
Other chronic respiratory diseases	13.3% (9.0% to 18.4%)	10.1% (6.3% to 14.8%)	4.1% (2.0% to 7.2%)	9.5% (5.8% to 14.0%)
**Cirrhosis and other chronic liver diseases**	28.3% (25.3% to 31.5%)	27.8% (24.7% to 31.2%)	21.1% (18.3% to 24.2%)	27.5% (24.5% to 30.9%)
Cirrhosis and other chronic liver diseases due to hepatitis B	0.0% (0.0% to 0.0%)	0.0% (0.0% to 0.0%)	0.0% (0.0% to 0.0%)	0.0% (0.0% to 0.0%)
Cirrhosis and other chronic liver diseases due to hepatitis C	2.9% (1.1% to 5.9%)	4.3% (1.5% to 9.0%)	4.6% (1.6% to 10.3%)	4.4% (1.5% to 9.0%)
Cirrhosis and other chronic liver diseases due to alcohol use	100.0% (100.0% to 100.0%)	100.0% (100.0% to 100.0%)	100.0% (100.0% to 100.0%)	100.0% (100.0% to 100.0%)
**Digestive diseases**	11.1% (7.6% to 15.0%)	14.2% (10.1% to 19.0%)	2.2% (1.6% to 2.8%)	12.3% (8.8% to 16.5%)
Peptic ulcer disease	4.6% (2.9% to 6.7%)	5.6% (3.6% to 8.1%)	4.8% (3.1% to 6.8%)	5.5% (3.6% to 7.8%)
Gallbladder and biliary diseases	28.6% (16.9% to 42.3%)	27.5% (16.6% to 40.6%)	36.8% (24.6% to 50.1%)	28.3% (17.5% to 41.4%)
Pancreatitis	51.5% (32.8% to 72.6%)	55.7% (37.9% to 75.4%)	52.6% (35.6% to 71.5%)	55.7% (37.9% to 75.4%)
**Neurological disorders**	16.9% (8.6% to 27.1%)	17.2% (10.6% to 24.8%)	4.6% (2.2% to 7.7%)	8.8% (5.4% to 12.6%)
Alzheimer's disease and other dementias	18.0% (8.2% to 30.6%)	19.1% (8.9% to 31.3%)	19.3% (8.7% to 31.8%)	19.1% (8.9% to 31.4%)
Parkinson's disease	-3.1% (-4.3% to -2.1%)	-4.0% (-5.3% to -2.8%)	-4.3% (-5.7% to -3.1%)	-4.0% (-5.4% to -2.8%)
Epilepsy	21.4% (12.7% to 32.5%)	20.2% (11.3% to 31.2%)	16.1% (9.3% to 24.3%)	18.0% (10.3% to 27.7%)
Multiple sclerosis	3.5% (1.9% to 5.5%)	3.6% (1.9% to 5.7%)	3.0% (1.8% to 4.5%)	3.4% (1.9% to 5.1%)
**Mental and substance use disorders**	99.9% (99.9% to 100.0%)	99.9% (99.9% to 100.0%)	22.2% (19.9% to 24.7%)	29.1% (25.4% to 34.2%)
Alcohol use disorders	100.0% (100.0% to 100.0%)	100.0% (100.0% to 100.0%)	100.0% (100.0% to 100.0%)	100.0% (100.0% to 100.0%)

Supplementary Results Table 1. Age-standardised PAFs attributable to the joint distribution of all risk factors for all ages, both sexes combined for each GBD level 1 and level 2 cause and all causes in 2016 for Cape Verde. Results are shown for deaths, YLLs, YLDs, and DALYs
Data in parenthesis are 95% uncertainty intervals. PAFs=population attributable fractions. GBD=Global Burden of Disease. YLLs=years of life lost. YLDs=years live with disability. DALYs=disability-adjusted life-years

	Deaths 2016	YLLs 2016	YLDs 2016	DALYs 2016
Drug use disorders	100.0% (100.0% to 100.0%)	100.0% (100.0% to 100.0%)	100.0% (100.0% to 100.0%)	100.0% (100.0% to 100.0%)
Depressive disorders	-	-	11.4% (9.7% to 13.1%)	11.4% (9.7% to 13.1%)
Idiopathic developmental intellectual disability	-	-	61.1% (30.2% to 80.9%)	61.1% (30.2% to 80.9%)
**Diabetes, urogenital, blood, and endocrine diseases**	87.5% (84.0% to 88.8%)	84.1% (80.1% to 85.7%)	61.3% (56.9% to 65.3%)	74.2% (70.5% to 77.1%)
Diabetes mellitus	100.0% (100.0% to 100.0%)	100.0% (100.0% to 100.0%)	100.0% (100.0% to 100.0%)	100.0% (100.0% to 100.0%)
Chronic kidney disease	100.0% (100.0% to 100.0%)	100.0% (100.0% to 100.0%)	100.0% (100.0% to 100.0%)	100.0% (100.0% to 100.0%)
**Musculoskeletal disorders**	0.2% (0.1% to 0.5%)	0.2% (0.1% to 0.4%)	20.6% (17.2% to 24.1%)	20.3% (17.0% to 23.9%)
Rheumatoid arthritis	1.4% (0.5% to 2.8%)	1.6% (0.6% to 2.9%)	2.1% (0.8% to 3.5%)	2.0% (0.8% to 3.5%)
Osteoarthritis	-	-	22.3% (11.4% to 34.3%)	22.3% (11.4% to 34.3%)
Low back and neck pain	-	-	23.8% (20.4% to 27.3%)	23.8% (20.4% to 27.3%)
Gout	-	-	50.9% (40.8% to 62.4%)	50.9% (40.8% to 62.4%)
**Other non-communicable diseases**	0.1% (0.0% to 0.2%)	0.2% (0.1% to 0.3%)	6.4% (5.1% to 7.8%)	5.1% (4.1% to 6.2%)
Sense organ diseases	-	-	13.7% (11.5% to 16.0%)	13.7% (11.5% to 16.0%)
Sudden infant death syndrome	2.6% (1.2% to 4.7%)	2.6% (1.2% to 4.7%)	-	2.6% (1.2% to 4.7%)
**Transport injuries**	29.3% (23.9% to 36.8%)	22.8% (17.4% to 30.1%)	33.1% (27.7% to 40.2%)	24.8% (19.3% to 31.8%)
Road injuries	29.5% (24.1% to 37.0%)	22.9% (17.6% to 30.0%)	34.8% (29.3% to 42.0%)	25.0% (19.5% to 31.9%)
Other transport injuries	27.0% (21.2% to 34.7%)	21.6% (16.1% to 29.4%)	26.4% (21.3% to 33.2%)	23.3% (18.0% to 30.5%)
**Unintentional injuries**	13.9% (10.2% to 18.8%)	9.7% (7.4% to 12.4%)	16.4% (14.7% to 18.4%)	12.0% (9.8% to 14.5%)
Falls	47.1% (31.4% to 65.4%)	31.2% (24.2% to 41.5%)	25.8% (24.2% to 27.7%)	27.2% (24.6% to 30.9%)
Drowning	10.9% (7.3% to 15.7%)	10.3% (6.8% to 14.4%)	11.9% (8.2% to 16.8%)	10.4% (6.8% to 14.4%)
Fire, heat, and hot substances	8.4% (5.1% to 13.4%)	9.6% (6.2% to 14.0%)	13.5% (9.6% to 18.3%)	11.3% (7.7% to 16.1%)

Supplementary Results Table 1. Age-standardised PAFs attributable to the joint distribution of all risk factors for all ages, both sexes combined for each GBD level 1 and level 2 cause and all causes in 2016 for Cape Verde. Results are shown for deaths, YLLs, YLDs, and DALYs
Data in parenthesis are 95% uncertainty intervals. PAFs=population attributable fractions. GBD=Global Burden of Disease. YLLs=years of life lost. YLDs=years live with disability. DALYs=disability-adjusted life-years

	**Deaths 2016**	**YLLs 2016**	**YLDs 2016**	**DALYs 2016**
Poisonings	10.7% (7.2% to 15.7%)	11.4% (7.5% to 16.6%)	12.8% (9.2% to 17.3%)	11.5% (7.7% to 16.7%)
Exposure to mechanical forces	13.3% (10.3% to 17.0%)	9.1% (6.7% to 12.4%)	16.0% (14.3% to 18.1%)	11.6% (9.3% to 14.5%)
Animal contact	8.8% (7.0% to 11.2%)	6.6% (5.3% to 8.3%)	9.1% (7.4% to 11.2%)	7.8% (6.4% to 9.7%)
Foreign body	3.8% (3.0% to 4.7%)	4.2% (3.0% to 5.5%)	6.8% (5.4% to 8.6%)	5.1% (3.9% to 6.7%)
Other unintentional injuries	10.0% (6.4% to 14.9%)	11.5% (7.7% to 16.2%)	11.5% (8.0% to 16.1%)	11.5% (7.9% to 16.1%)
**Self-harm and interpersonal violence**	19.1% (13.2% to 25.6%)	18.0% (12.6% to 24.2%)	19.9% (15.9% to 24.8%)	18.1% (12.8% to 24.1%)
Self-harm	23.6% (14.7% to 32.8%)	24.6% (15.6% to 33.9%)	22.9% (13.9% to 32.0%)	24.5% (15.6% to 33.8%)
Interpersonal violence	12.9% (7.1% to 19.7%)	11.3% (6.0% to 18.1%)	19.5% (15.1% to 24.5%)	11.9% (6.7% to 18.6%)
**Forces of nature, conflict and terrorism, and executions and police conflict**	0.0% (0.0% to 0.0%)	0.0% (0.0% to 0.0%)	7.5% (4.8% to 11.2%)	2.8% (0.7% to 5.9%)
Forces of nature, conflict and terrorism, and state actor violence	0.0% (0.0% to 0.0%)	0.0% (0.0% to 0.0%)	9.4% (6.5% to 13.5%)	9.4% (6.5% to 13.5%)

Supplementary Results Table 1. Age-standardised PAFs attributable to the joint distribution of all risk factors for all ages, both sexes combined for each GBD level 1 and level 2 cause and all causes in 2016 for Central African Republic. Results are shown for deaths, YLLs, YLDs, and
Data in parenthesis are 95% uncertainty intervals. PAFs=population attributable fractions. GBD=Global Burden of Disease. YLLs=years of life lost. YLDs=years live with disability. DALYs=disability-adjusted life-years

	Deaths 2016	YLLs 2016	YLDs 2016	DALYs 2016
**All causes**	58.6% (54.1% to 62.8%)	56.8% (52.9% to 60.5%)	27.4% (26.0% to 29.3%)	52.5% (48.8% to 56.0%)
**HIV/AIDS and tuberculosis**	56.8% (45.0% to 67.3%)	62.5% (52.6% to 71.2%)	83.3% (77.6% to 88.7%)	63.0% (53.4% to 71.5%)
Tuberculosis	41.6% (26.5% to 55.4%)	43.6% (29.2% to 56.0%)	40.2% (26.5% to 52.4%)	43.6% (29.2% to 56.0%)
HIV/AIDS	94.8% (93.9% to 95.6%)	92.1% (90.5% to 93.4%)	93.7% (91.8% to 95.6%)	92.2% (90.7% to 93.4%)
**Diarrhoea, lower respiratory infections, and other common infectious diseases**	80.4% (74.2% to 85.8%)	77.5% (69.7% to 83.2%)	46.8% (40.7% to 52.0%)	76.6% (69.1% to 82.2%)
Diarrhoeal diseases	96.2% (89.8% to 99.1%)	97.3% (92.8% to 99.4%)	96.5% (90.5% to 99.1%)	97.3% (92.7% to 99.4%)
Lower respiratory infections	83.5% (77.7% to 88.1%)	89.3% (84.7% to 92.9%)	82.9% (77.6% to 87.2%)	89.3% (84.6% to 92.9%)
Upper respiratory infections	1.5% (0.1% to 4.9%)	2.8% (0.2% to 10.1%)	0.0% (0.0% to 0.0%)	0.3% (0.0% to 0.9%)
Otitis media	1.6% (0.4% to 3.7%)	4.0% (1.6% to 7.1%)	3.4% (2.1% to 4.7%)	3.4% (2.2% to 4.7%)
Meningitis	3.2% (1.5% to 6.4%)	5.2% (2.6% to 10.1%)	0.0% (0.0% to 0.0%)	4.9% (2.5% to 9.5%)
Encephalitis	1.0% (0.6% to 1.6%)	1.6% (1.0% to 2.6%)	0.0% (0.0% to 0.0%)	1.0% (0.6% to 1.5%)
Measles	68.5% (55.4% to 83.7%)	69.6% (56.3% to 84.9%)	68.2% (55.0% to 82.3%)	69.6% (56.3% to 84.9%)
**Maternal disorders**	6.1% (2.8% to 10.0%)	6.2% (2.8% to 10.0%)	1.8% (0.9% to 2.9%)	6.1% (2.8% to 9.8%)
Maternal haemorrhage	16.5% (6.0% to 27.3%)	16.9% (6.1% to 27.9%)	17.8% (6.5% to 29.0%)	16.9% (6.1% to 27.9%)
Maternal sepsis and other pregnancy related infections	15.7% (5.7% to 26.2%)	16.3% (5.9% to 26.9%)	17.4% (6.3% to 28.4%)	16.3% (5.9% to 26.9%)
Maternal abortion, miscarriage, and ectopic pregnancy	32.1% (22.1% to 42.1%)	31.9% (22.1% to 41.8%)	31.8% (20.5% to 42.2%)	31.9% (22.2% to 41.7%)
**Neonatal disorders**	77.7% (74.0% to 81.2%)	77.7% (74.0% to 81.2%)	0.9% (0.4% to 1.5%)	76.0% (72.4% to 79.8%)
Neonatal preterm birth complications	96.8% (93.8% to 98.7%)	96.8% (93.8% to 98.7%)	2.4% (1.5% to 3.4%)	94.5% (90.8% to 96.7%)
Neonatal encephalopathy due to birth asphyxia and trauma	70.9% (67.4% to 74.4%)	70.9% (67.4% to 74.4%)	0.0% (0.0% to 0.0%)	69.4% (65.0% to 73.3%)
Neonatal sepsis and other neonatal infections	67.0% (62.6% to 70.5%)	67.0% (62.6% to 70.5%)	0.0% (0.0% to 0.0%)	65.4% (60.5% to 69.6%)
Hemolytic disease and other neonatal jaundice	68.5% (63.2% to 72.9%)	68.5% (63.2% to 72.9%)	0.0% (0.0% to 0.0%)	64.5% (58.7% to 69.6%)

Supplementary Results Table 1. Age-standardised PAFs attributable to the joint distribution of all risk factors for all ages, both sexes combined for each GBD level 1 and level 2 cause and all causes in 2016 for Central African Republic. Results are shown for deaths, YLLs, YLDs, and
Data in parenthesis are 95% uncertainty intervals. PAFs=population attributable fractions. GBD=Global Burden of Disease. YLLs=years of life lost. YLDs=years live with disability. DALYs=disability-adjusted life-years

	Deaths 2016	YLLs 2016	YLDs 2016	DALYs 2016
Other neonatal disorders	68.3% (63.2% to 72.6%)	68.3% (63.3% to 72.6%)	0.0% (0.0% to 0.0%)	67.6% (62.6% to 72.0%)
**Nutritional deficiencies**	24.6% (10.3% to 37.0%)	55.3% (33.1% to 69.4%)	79.8% (77.0% to 82.6%)	62.5% (43.3% to 72.3%)
Protein-energy malnutrition	29.4% (13.4% to 42.8%)	61.7% (39.3% to 75.1%)	97.9% (97.1% to 98.5%)	62.2% (40.0% to 75.6%)
Vitamin A deficiency	-	-	100.0% (100.0% to 100.0%)	100.0% (100.0% to 100.0%)
Iron-deficiency anaemia	100.0% (100.0% to 100.0%)	100.0% (100.0% to 100.0%)	100.0% (100.0% to 100.0%)	100.0% (100.0% to 100.0%)
**Other communicable, maternal, neonatal, and nutritional diseases**	2.6% (1.2% to 5.4%)	1.4% (0.6% to 2.9%)	61.1% (55.6% to 66.6%)	4.4% (2.3% to 7.5%)
Sexually transmitted diseases excluding HIV	4.9% (2.1% to 10.0%)	2.0% (0.7% to 4.1%)	100.0% (100.0% to 100.0%)	6.3% (3.1% to 11.6%)
Hepatitis	0.0% (0.0% to 0.0%)	0.0% (0.0% to 0.0%)	0.0% (0.0% to 0.0%)	0.0% (0.0% to 0.0%)
**Neoplasms**	37.5% (31.2% to 43.2%)	39.6% (32.6% to 45.5%)	39.4% (31.7% to 45.3%)	39.6% (32.6% to 45.5%)
Oesophageal cancer	67.5% (54.3% to 79.0%)	71.1% (57.7% to 82.3%)	68.3% (54.6% to 79.5%)	71.1% (57.6% to 82.3%)
Stomach cancer	11.7% (3.7% to 22.8%)	13.1% (4.4% to 24.2%)	12.0% (3.8% to 23.0%)	13.1% (4.4% to 24.2%)
Liver cancer	27.2% (19.5% to 37.0%)	29.0% (20.4% to 40.1%)	27.4% (19.6% to 37.1%)	29.0% (20.4% to 40.1%)
Larynx cancer	70.7% (55.4% to 83.0%)	75.1% (59.8% to 86.8%)	71.4% (56.2% to 83.4%)	75.0% (59.8% to 86.7%)
Tracheal, bronchus, and lung cancer	88.4% (82.0% to 92.9%)	89.5% (83.2% to 93.7%)	88.5% (82.0% to 93.1%)	89.5% (83.2% to 93.7%)
Breast cancer	19.2% (12.7% to 28.2%)	19.0% (12.5% to 28.2%)	19.0% (12.5% to 28.0%)	19.0% (12.5% to 28.2%)
Cervical cancer	100.0% (100.0% to 100.0%)	100.0% (100.0% to 100.0%)	100.0% (100.0% to 100.0%)	100.0% (100.0% to 100.0%)
Uterine cancer	10.8% (3.3% to 20.6%)	11.3% (3.4% to 21.4%)	10.9% (3.4% to 20.8%)	11.3% (3.4% to 21.4%)
Prostate cancer	1.6% (0.6% to 3.0%)	2.3% (0.9% to 4.3%)	1.8% (0.7% to 3.4%)	2.3% (0.8% to 4.2%)
Colon and rectum cancer	50.3% (40.9% to 59.5%)	51.0% (41.6% to 60.0%)	50.5% (41.2% to 59.6%)	51.0% (41.6% to 60.0%)
Lip and oral cavity cancer	67.9% (51.9% to 80.6%)	72.6% (57.4% to 84.3%)	68.2% (52.3% to 80.4%)	72.5% (57.2% to 84.2%)
Nasopharynx cancer	66.1% (49.8% to 78.6%)	67.2% (52.1% to 78.7%)	65.5% (50.2% to 76.9%)	67.2% (52.1% to 78.7%)

Supplementary Results Table 1. Age-standardised PAFs attributable to the joint distribution of all risk factors for all ages, both sexes combined for each GBD level 1 and level 2 cause and all causes in 2016 for Central African Republic. Results are shown for deaths, YLLs, YLDs, and
Data in parenthesis are 95% uncertainty intervals. PAFs=population attributable fractions. GBD=Global Burden of Disease. YLLs=years of life lost. YLDs=years live with disability. DALYs=disability-adjusted life-years

	Deaths 2016	YLLs 2016	YLDs 2016	DALYs 2016
Other pharynx cancer	47.9% (28.2% to 63.5%)	50.5% (30.4% to 65.8%)	48.6% (28.9% to 64.3%)	50.5% (30.4% to 65.8%)
Gallbladder and biliary tract cancer	5.9% (2.0% to 12.5%)	6.3% (2.2% to 12.9%)	6.0% (2.0% to 12.4%)	6.3% (2.2% to 12.9%)
Pancreatic cancer	16.1% (8.4% to 25.0%)	18.1% (9.6% to 27.4%)	16.3% (8.7% to 25.0%)	18.1% (9.6% to 27.4%)
Ovarian cancer	8.2% (3.3% to 15.6%)	7.3% (2.8% to 14.0%)	7.2% (2.8% to 13.9%)	7.3% (2.8% to 14.0%)
Kidney cancer	15.9% (8.3% to 25.1%)	16.6% (8.8% to 26.4%)	15.3% (8.2% to 23.8%)	16.6% (8.8% to 26.3%)
Bladder cancer	17.5% (8.4% to 28.6%)	21.0% (10.1% to 33.4%)	18.8% (8.9% to 30.0%)	20.9% (10.0% to 33.4%)
Thyroid cancer	4.0% (1.3% to 8.3%)	4.2% (1.4% to 8.7%)	4.0% (1.4% to 8.3%)	4.2% (1.4% to 8.7%)
Mesothelioma	87.9% (82.7% to 92.2%)	80.9% (73.3% to 87.4%)	84.6% (78.3% to 89.8%)	81.0% (73.4% to 87.4%)
Non-Hodgkin's lymphoma	2.3% (0.7% to 4.6%)	2.2% (0.7% to 4.5%)	2.3% (0.7% to 4.6%)	2.2% (0.7% to 4.5%)
Multiple myeloma	2.5% (0.8% to 5.3%)	2.6% (0.8% to 5.4%)	2.5% (0.8% to 5.3%)	2.6% (0.8% to 5.4%)
Leukaemia	7.5% (3.3% to 13.2%)	6.4% (3.0% to 11.2%)	6.9% (3.2% to 11.7%)	6.4% (3.0% to 11.1%)
**Cardiovascular diseases**	86.3% (83.9% to 88.5%)	86.9% (84.9% to 88.9%)	59.7% (55.0% to 64.2%)	85.7% (83.6% to 87.8%)
Rheumatic heart disease	27.7% (19.0% to 41.6%)	26.7% (18.6% to 39.0%)	22.9% (14.5% to 33.2%)	25.9% (17.8% to 36.6%)
Ischaemic heart disease	94.3% (91.8% to 96.3%)	95.5% (93.8% to 96.8%)	90.1% (87.2% to 92.7%)	95.4% (93.7% to 96.8%)
Cerebrovascular disease	88.7% (85.4% to 91.5%)	90.8% (88.4% to 92.8%)	88.0% (84.7% to 91.2%)	90.7% (88.3% to 92.7%)
Hypertensive heart disease	100.0% (100.0% to 100.0%)	100.0% (100.0% to 100.0%)	100.0% (100.0% to 100.0%)	100.0% (100.0% to 100.0%)
Cardiomyopathy and myocarditis	41.5% (33.7% to 50.3%)	38.6% (30.7% to 46.6%)	43.8% (37.1% to 51.1%)	38.9% (31.1% to 46.9%)
Atrial fibrillation and flutter	42.1% (34.4% to 49.7%)	47.0% (39.8% to 54.1%)	53.2% (47.2% to 59.4%)	50.2% (43.7% to 56.7%)
Aortic aneurysm	41.8% (34.6% to 48.3%)	46.1% (39.5% to 52.0%)	-	46.1% (39.5% to 52.0%)
Peripheral vascular disease	52.8% (45.8% to 59.6%)	60.1% (54.0% to 65.8%)	58.6% (53.4% to 64.0%)	59.1% (54.2% to 64.0%)
Endocarditis	39.7% (30.6% to 50.4%)	39.2% (30.2% to 49.0%)	34.9% (25.3% to 45.7%)	39.1% (30.2% to 48.9%)

Supplementary Results Table 1. Age-standardised PAFs attributable to the joint distribution of all risk factors for all ages, both sexes combined for each GBD level 1 and level 2 cause and all causes in 2016 for Central African Republic. Results are shown for deaths, YLLs, YLDs, and
Data in parenthesis are 95% uncertainty intervals. PAFs=population attributable fractions. GBD=Global Burden of Disease. YLLs=years of life lost. YLDs=years live with disability. DALYs=disability-adjusted life-years

	Deaths 2016	YLLs 2016	YLDs 2016	DALYs 2016
Other cardiovascular and circulatory diseases	44.1% (39.2% to 49.5%)	46.6% (42.1% to 51.2%)	38.2% (32.8% to 43.8%)	45.3% (40.7% to 50.1%)
**Chronic respiratory diseases**	63.1% (54.3% to 70.0%)	60.0% (53.2% to 66.4%)	37.8% (31.3% to 45.0%)	54.9% (48.1% to 61.4%)
Chronic obstructive pulmonary disease	81.6% (73.6% to 89.3%)	82.5% (74.8% to 89.6%)	74.0% (63.1% to 83.2%)	81.2% (73.0% to 88.4%)
Pneumoconiosis	100.0% (100.0% to 100.0%)	100.0% (100.0% to 100.0%)	100.0% (100.0% to 100.0%)	100.0% (100.0% to 100.0%)
Asthma	18.5% (11.8% to 27.4%)	20.8% (14.2% to 29.2%)	14.9% (10.5% to 20.8%)	18.8% (13.3% to 26.1%)
Other chronic respiratory diseases	9.0% (3.4% to 17.2%)	9.5% (3.1% to 18.3%)	4.9% (1.8% to 8.8%)	8.1% (2.7% to 15.8%)
**Cirrhosis and other chronic liver diseases**	24.2% (22.5% to 25.9%)	23.1% (21.4% to 24.9%)	18.2% (15.7% to 20.7%)	23.0% (21.3% to 24.8%)
Cirrhosis and other chronic liver diseases due to hepatitis B	0.0% (0.0% to 0.0%)	0.0% (0.0% to 0.0%)	0.0% (0.0% to 0.0%)	0.0% (0.0% to 0.0%)
Cirrhosis and other chronic liver diseases due to hepatitis C	0.6% (0.3% to 1.1%)	0.9% (0.3% to 1.6%)	1.1% (0.5% to 2.0%)	0.9% (0.3% to 1.6%)
Cirrhosis and other chronic liver diseases due to alcohol use	100.0% (100.0% to 100.0%)	100.0% (100.0% to 100.0%)	100.0% (100.0% to 100.0%)	100.0% (100.0% to 100.0%)
**Digestive diseases**	4.7% (3.1% to 6.4%)	5.6% (3.6% to 7.6%)	1.3% (0.9% to 1.9%)	5.4% (3.5% to 7.3%)
Peptic ulcer disease	6.8% (4.1% to 9.9%)	8.1% (4.9% to 11.9%)	6.0% (4.0% to 8.6%)	8.1% (4.9% to 11.9%)
Gallbladder and biliary diseases	14.0% (5.8% to 24.4%)	14.7% (6.4% to 25.0%)	14.8% (6.8% to 25.5%)	14.7% (6.5% to 25.0%)
Pancreatitis	36.5% (11.5% to 65.4%)	40.9% (13.6% to 69.3%)	36.2% (11.8% to 64.2%)	40.9% (13.6% to 69.3%)
**Neurological disorders**	10.8% (4.8% to 19.4%)	12.5% (7.1% to 19.3%)	2.9% (1.2% to 5.2%)	7.2% (3.9% to 11.4%)
Alzheimer's disease and other dementias	10.8% (4.2% to 20.2%)	11.4% (4.5% to 21.0%)	11.3% (4.4% to 20.9%)	11.4% (4.5% to 20.9%)
Parkinson's disease	-3.5% (-4.9% to -2.3%)	-4.3% (-5.9% to -3.0%)	-4.6% (-6.3% to -3.2%)	-4.4% (-6.0% to -3.0%)
Epilepsy	21.2% (11.8% to 30.7%)	19.4% (10.7% to 28.7%)	17.2% (9.0% to 25.6%)	18.8% (10.3% to 27.5%)
Multiple sclerosis	4.3% (1.7% to 8.4%)	4.6% (1.7% to 9.0%)	3.0% (1.7% to 4.6%)	4.1% (1.8% to 7.8%)
**Mental and substance use disorders**	99.8% (99.6% to 99.9%)	99.7% (99.4% to 99.9%)	18.9% (16.4% to 21.3%)	22.8% (19.5% to 26.7%)
Alcohol use disorders	100.0% (100.0% to 100.0%)	100.0% (100.0% to 100.0%)	100.0% (100.0% to 100.0%)	100.0% (100.0% to 100.0%)

**Supplementary Results Table 1. Age-standardised PAFs attributable to the joint distribution of all risk factors for all ages, both sexes combined for each GBD level 1 and level 2 cause and all causes in 2016 for Central African Republic. Results are shown for deaths, YLLs, YLDs, and** Data in parenthesis are 95% uncertainty intervals. PAFs=population attributable fractions. GBD=Global Burden of Disease. YLLs=years of life lost. YLDs=years live with disability. DALYs=disability-adjusted life-years				
	**Deaths 2016**	**YLLs 2016**	**YLDs 2016**	**DALYs 2016**
Drug use disorders	100.0% (100.0% to 100.0%)	100.0% (100.0% to 100.0%)	100.0% (100.0% to 100.0%)	100.0% (100.0% to 100.0%)
Depressive disorders	-	-	8.5% (7.1% to 10.0%)	8.5% (7.1% to 10.0%)
Idiopathic developmental intellectual disability	-	-	62.8% (31.3% to 82.1%)	62.8% (31.3% to 82.1%)
**Diabetes, urogenital, blood, and endocrine diseases**	80.8% (77.9% to 83.4%)	73.0% (65.4% to 77.0%)	58.6% (53.8% to 62.7%)	68.0% (63.0% to 71.4%)
Diabetes mellitus	100.0% (100.0% to 100.0%)	100.0% (100.0% to 100.0%)	100.0% (100.0% to 100.0%)	100.0% (100.0% to 100.0%)
Chronic kidney disease	100.0% (100.0% to 100.0%)	100.0% (100.0% to 100.0%)	100.0% (100.0% to 100.0%)	100.0% (100.0% to 100.0%)
**Musculoskeletal disorders**	0.6% (0.2% to 1.3%)	0.5% (0.2% to 1.1%)	17.0% (14.7% to 19.7%)	16.6% (14.4% to 19.4%)
Rheumatoid arthritis	1.4% (0.5% to 2.7%)	1.6% (0.6% to 3.1%)	1.8% (0.7% to 3.2%)	1.8% (0.7% to 3.1%)
Osteoarthritis	-	-	8.6% (3.3% to 15.9%)	8.6% (3.3% to 15.9%)
Low back and neck pain	-	-	21.5% (18.9% to 24.4%)	21.5% (18.9% to 24.4%)
Gout	-	-	38.0% (29.6% to 49.0%)	38.0% (29.6% to 49.0%)
**Other non-communicable diseases**	0.3% (0.1% to 0.6%)	0.4% (0.1% to 0.9%)	6.8% (5.6% to 8.0%)	4.8% (3.8% to 6.0%)
Sense organ diseases	-	-	12.7% (10.6% to 14.7%)	12.7% (10.6% to 14.7%)
Sudden infant death syndrome	7.5% (3.0% to 14.2%)	7.5% (3.0% to 14.3%)	-	7.5% (3.0% to 14.3%)
**Transport injuries**	34.8% (28.3% to 43.1%)	29.5% (23.4% to 37.4%)	40.8% (33.8% to 49.3%)	30.1% (23.9% to 37.9%)
Road injuries	34.7% (28.3% to 42.9%)	29.4% (23.3% to 37.3%)	44.0% (37.0% to 52.7%)	30.0% (23.8% to 37.9%)
Other transport injuries	39.2% (30.8% to 49.2%)	33.4% (25.3% to 43.2%)	32.9% (26.6% to 41.0%)	33.1% (26.1% to 41.9%)
**Unintentional injuries**	26.8% (21.7% to 32.0%)	17.6% (13.6% to 22.1%)	26.0% (21.3% to 32.0%)	19.1% (14.8% to 23.9%)
Falls	61.8% (47.1% to 68.9%)	40.7% (32.2% to 48.4%)	34.5% (30.0% to 40.3%)	38.1% (32.2% to 44.0%)
Drowning	15.6% (10.7% to 21.9%)	11.9% (7.8% to 18.1%)	21.8% (15.3% to 29.1%)	12.0% (7.8% to 18.1%)
Fire, heat, and hot substances	15.3% (10.4% to 21.1%)	15.6% (10.9% to 21.4%)	23.3% (16.7% to 30.7%)	16.5% (11.6% to 22.2%)

Supplementary Results Table 1. Age-standardised PAFs attributable to the joint distribution of all risk factors for all ages, both sexes combined for each GBD level 1 and level 2 cause and all causes in 2016 for Central African Republic. Results are shown for deaths, YLLs, YLDs, and
Data in parenthesis are 95% uncertainty intervals. PAFs=population attributable fractions. GBD=Global Burden of Disease. YLLs=years of life lost. YLDs=years live with disability. DALYs=disability-adjusted life-years

	**Deaths 2016**	**YLLs 2016**	**YLDs 2016**	**DALYs 2016**
Poisonings	19.2% (12.2% to 27.3%)	18.0% (10.2% to 26.7%)	22.0% (15.7% to 29.3%)	18.1% (10.2% to 26.6%)
Exposure to mechanical forces	26.8% (19.9% to 35.0%)	21.8% (15.1% to 30.7%)	29.8% (22.9% to 38.7%)	23.2% (16.4% to 32.0%)
Animal contact	15.5% (11.3% to 21.2%)	16.0% (11.2% to 22.7%)	18.5% (13.5% to 25.3%)	16.6% (12.0% to 22.9%)
Foreign body	11.8% (7.5% to 17.0%)	10.8% (5.5% to 16.9%)	15.8% (10.9% to 22.0%)	12.2% (7.1% to 18.2%)
Other unintentional injuries	22.8% (15.8% to 30.6%)	22.6% (15.6% to 30.1%)	22.6% (16.1% to 30.1%)	22.6% (15.8% to 29.8%)
**Self-harm and interpersonal violence**	19.8% (10.2% to 31.1%)	19.7% (10.8% to 29.9%)	21.0% (17.3% to 25.2%)	19.8% (11.2% to 29.6%)
Self-harm	20.5% (7.7% to 35.7%)	21.9% (8.9% to 36.6%)	20.1% (7.9% to 34.9%)	21.9% (8.9% to 36.6%)
Interpersonal violence	18.1% (11.2% to 25.9%)	16.8% (10.1% to 24.3%)	21.1% (17.9% to 24.9%)	17.3% (11.1% to 24.3%)
**Forces of nature, conflict and terrorism, and executions and police conflict**	0.0% (0.0% to 0.0%)	0.0% (0.0% to 0.0%)	0.0% (0.0% to 0.0%)	0.0% (0.0% to 0.0%)
Forces of nature, conflict and terrorism, and state actor violence	0.0% (0.0% to 0.0%)	0.0% (0.0% to 0.0%)	0.0% (0.0% to 0.0%)	0.0% (0.0% to 0.0%)

Supplementary Results Table 1. Age-standardised PAFs attributable to the joint distribution of all risk factors for all ages, both sexes combined for each GBD level 1 and level 2 cause and all causes in 2016 for Chad. Results are shown for deaths, YLLs, YLDs, and DALYs
Data in parenthesis are 95% uncertainty intervals. PAFs=population attributable fractions. GBD=Global Burden of Disease. YLLs=years of life lost. YLDs=years live with disability. DALYs=disability-adjusted life-years

	Deaths 2016	YLLs 2016	YLDs 2016	DALYs 2016
**All causes**	58.9% (56.5% to 61.7%)	56.7% (53.9% to 59.8%)	30.9% (29.5% to 32.3%)	51.5% (48.8% to 54.2%)
**HIV/AIDS and tuberculosis**	63.7% (55.9% to 70.2%)	70.4% (64.4% to 75.4%)	83.7% (79.5% to 87.9%)	71.0% (65.0% to 75.8%)
Tuberculosis	30.8% (21.6% to 39.1%)	33.6% (24.7% to 41.8%)	32.7% (24.3% to 41.0%)	33.6% (24.6% to 41.8%)
HIV/AIDS	90.8% (88.7% to 92.4%)	86.9% (84.0% to 89.3%)	91.4% (88.8% to 93.7%)	87.2% (84.4% to 89.4%)
**Diarrhoea, lower respiratory infections, and other common infectious diseases**	78.4% (73.3% to 83.7%)	77.0% (69.7% to 82.1%)	49.4% (44.3% to 54.1%)	76.1% (69.2% to 81.1%)
Diarrhoeal diseases	96.9% (92.0% to 99.3%)	98.3% (95.2% to 99.6%)	96.7% (91.2% to 99.2%)	98.2% (95.2% to 99.6%)
Lower respiratory infections	84.3% (79.4% to 88.5%)	90.1% (86.4% to 93.1%)	82.9% (77.8% to 87.4%)	90.1% (86.4% to 93.1%)
Upper respiratory infections	1.3% (0.1% to 4.7%)	2.3% (0.2% to 9.0%)	0.0% (0.0% to 0.0%)	0.2% (0.0% to 0.6%)
Otitis media	0.9% (0.2% to 2.4%)	3.3% (1.3% to 5.6%)	3.1% (1.9% to 4.2%)	3.1% (1.9% to 4.1%)
Meningitis	2.2% (1.2% to 3.7%)	3.7% (2.0% to 6.0%)	0.0% (0.0% to 0.0%)	3.6% (2.0% to 5.8%)
Encephalitis	0.7% (0.4% to 0.9%)	1.1% (0.7% to 1.6%)	0.0% (0.0% to 0.0%)	0.8% (0.5% to 1.1%)
Measles	72.8% (62.2% to 84.5%)	73.9% (63.3% to 85.6%)	71.6% (60.0% to 83.9%)	73.9% (63.3% to 85.6%)
**Maternal disorders**	8.9% (3.9% to 14.5%)	8.8% (3.9% to 14.2%)	2.7% (1.2% to 4.7%)	8.7% (3.9% to 14.0%)
Maternal haemorrhage	20.4% (7.4% to 32.9%)	20.5% (7.5% to 32.8%)	21.1% (7.6% to 34.1%)	20.5% (7.5% to 32.8%)
Maternal sepsis and other pregnancy related infections	20.2% (7.4% to 33.0%)	20.4% (7.5% to 33.1%)	20.6% (7.4% to 33.2%)	20.4% (7.5% to 33.1%)
Maternal abortion, miscarriage, and ectopic pregnancy	12.7% (8.0% to 18.1%)	12.5% (7.8% to 17.7%)	13.3% (7.3% to 19.5%)	12.5% (7.8% to 17.7%)
**Neonatal disorders**	80.7% (76.8% to 83.9%)	80.7% (76.9% to 83.9%)	0.8% (0.4% to 1.3%)	78.9% (75.0% to 82.1%)
Neonatal preterm birth complications	96.6% (93.3% to 98.3%)	96.6% (93.4% to 98.3%)	2.0% (1.3% to 2.9%)	94.2% (90.8% to 96.4%)
Neonatal encephalopathy due to birth asphyxia and trauma	76.9% (72.6% to 81.1%)	76.9% (72.7% to 81.1%)	0.0% (0.0% to 0.0%)	75.6% (70.5% to 80.0%)
Neonatal sepsis and other neonatal infections	67.7% (61.8% to 72.3%)	67.8% (61.8% to 72.3%)	0.0% (0.0% to 0.0%)	66.1% (59.4% to 71.3%)
Hemolytic disease and other neonatal jaundice	63.2% (50.2% to 71.7%)	63.2% (50.3% to 71.7%)	0.0% (0.0% to 0.0%)	60.1% (47.6% to 68.6%)

Supplementary Results Table 1. Age-standardised PAFs attributable to the joint distribution of all risk factors for all ages, both sexes combined for each GBD level 1 and level 2 cause and all causes in 2016 for Chad. Results are shown for deaths, YLLs, YLDs, and DALYs
Data in parenthesis are 95% uncertainty intervals. PAFs=population attributable fractions. GBD=Global Burden of Disease. YLLs=years of life lost. YLDs=years live with disability. DALYs=disability-adjusted life-years

	Deaths 2016	YLLs 2016	YLDs 2016	DALYs 2016
Other neonatal disorders	66.0% (55.2% to 73.9%)	66.0% (55.3% to 74.0%)	0.0% (0.0% to 0.0%)	64.6% (54.0% to 72.8%)
**Nutritional deficiencies**	45.0% (31.6% to 57.4%)	76.3% (65.8% to 84.1%)	96.7% (96.0% to 97.3%)	86.1% (82.2% to 89.6%)
Protein-energy malnutrition	48.6% (35.1% to 60.8%)	79.6% (70.1% to 86.8%)	97.1% (96.1% to 97.9%)	80.4% (71.6% to 87.1%)
Vitamin A deficiency	-	-	100.0% (100.0% to 100.0%)	100.0% (100.0% to 100.0%)
Iron-deficiency anaemia	100.0% (100.0% to 100.0%)	100.0% (100.0% to 100.0%)	100.0% (100.0% to 100.0%)	100.0% (100.0% to 100.0%)
**Other communicable, maternal, neonatal, and nutritional diseases**	1.6% (0.8% to 2.6%)	1.0% (0.5% to 1.8%)	54.8% (48.9% to 61.1%)	5.0% (2.6% to 8.4%)
Sexually transmitted diseases excluding HIV	2.6% (1.1% to 5.2%)	1.3% (0.5% to 2.6%)	100.0% (100.0% to 100.0%)	6.6% (3.1% to 12.5%)
Hepatitis	0.1% (0.0% to 0.1%)	0.1% (0.0% to 0.1%)	0.1% (0.0% to 0.1%)	0.1% (0.0% to 0.1%)
**Neoplasms**	31.3% (28.2% to 34.5%)	31.8% (28.9% to 35.0%)	33.0% (29.2% to 36.7%)	31.8% (28.9% to 35.0%)
Oesophageal cancer	63.8% (51.1% to 75.2%)	66.0% (53.7% to 76.9%)	64.4% (52.0% to 75.5%)	66.0% (53.7% to 76.9%)
Stomach cancer	6.7% (2.4% to 15.1%)	6.9% (2.6% to 14.5%)	6.7% (2.5% to 14.5%)	6.9% (2.6% to 14.5%)
Liver cancer	27.0% (20.6% to 34.2%)	26.2% (19.5% to 33.8%)	26.7% (20.4% to 33.9%)	26.2% (19.5% to 33.8%)
Larynx cancer	61.7% (48.6% to 73.1%)	63.8% (51.1% to 74.8%)	62.1% (48.9% to 73.3%)	63.8% (51.1% to 74.7%)
Tracheal, bronchus, and lung cancer	87.4% (82.8% to 91.1%)	87.3% (82.8% to 91.0%)	87.2% (82.6% to 91.0%)	87.3% (82.8% to 91.0%)
Breast cancer	16.2% (11.3% to 22.3%)	15.6% (11.1% to 20.9%)	15.4% (10.9% to 20.7%)	15.5% (11.1% to 20.9%)
Cervical cancer	100.0% (100.0% to 100.0%)	100.0% (100.0% to 100.0%)	100.0% (100.0% to 100.0%)	100.0% (100.0% to 100.0%)
Uterine cancer	14.6% (5.9% to 25.6%)	15.3% (6.3% to 26.2%)	14.7% (6.0% to 25.7%)	15.3% (6.3% to 26.2%)
Prostate cancer	1.6% (1.0% to 2.4%)	1.8% (1.1% to 2.7%)	1.7% (1.0% to 2.5%)	1.8% (1.1% to 2.7%)
Colon and rectum cancer	44.8% (36.3% to 53.9%)	45.2% (36.9% to 53.7%)	45.0% (36.6% to 53.8%)	45.2% (36.9% to 53.7%)
Lip and oral cavity cancer	60.0% (48.1% to 70.2%)	62.2% (51.4% to 72.1%)	59.2% (48.2% to 69.6%)	62.1% (51.3% to 72.0%)
Nasopharynx cancer	56.6% (46.6% to 66.2%)	56.4% (46.7% to 65.7%)	55.9% (46.6% to 64.8%)	56.4% (46.7% to 65.7%)

Supplementary Results Table 1. Age-standardised PAFs attributable to the joint distribution of all risk factors for all ages, both sexes combined for each GBD level 1 and level 2 cause and all causes in 2016 for Chad. Results are shown for deaths, YLLs, YLDs, and DALYs
Data in parenthesis are 95% uncertainty intervals. PAFs=population attributable fractions. GBD=Global Burden of Disease. YLLs=years of life lost. YLDs=years live with disability. DALYs=disability-adjusted life-years

	Deaths 2016	YLLs 2016	YLDs 2016	DALYs 2016
Other pharynx cancer	34.8% (21.8% to 47.7%)	38.1% (24.7% to 50.3%)	35.8% (22.6% to 48.5%)	38.1% (24.7% to 50.2%)
Gallbladder and biliary tract cancer	7.6% (2.8% to 14.6%)	8.0% (3.0% to 15.2%)	7.7% (2.8% to 14.8%)	8.0% (3.0% to 15.2%)
Pancreatic cancer	13.0% (7.6% to 20.1%)	13.2% (8.0% to 19.8%)	13.0% (7.7% to 19.9%)	13.2% (8.0% to 19.8%)
Ovarian cancer	7.9% (3.4% to 14.4%)	6.5% (2.6% to 12.4%)	6.6% (2.8% to 12.4%)	6.5% (2.6% to 12.4%)
Kidney cancer	12.5% (7.3% to 18.9%)	11.2% (6.4% to 17.0%)	11.1% (6.5% to 17.3%)	11.2% (6.4% to 17.0%)
Bladder cancer	15.0% (8.9% to 22.7%)	15.6% (9.4% to 23.3%)	15.2% (9.1% to 22.6%)	15.6% (9.5% to 23.3%)
Thyroid cancer	4.9% (1.9% to 9.5%)	5.1% (2.0% to 9.8%)	4.8% (1.9% to 9.3%)	5.1% (2.0% to 9.8%)
Mesothelioma	83.3% (77.1% to 88.1%)	72.6% (64.5% to 80.0%)	78.3% (71.4% to 84.5%)	72.6% (64.6% to 80.0%)
Non-Hodgkin's lymphoma	2.2% (0.8% to 4.4%)	1.8% (0.6% to 3.7%)	2.1% (0.7% to 4.1%)	1.8% (0.6% to 3.7%)
Multiple myeloma	2.7% (0.9% to 5.5%)	2.8% (1.0% to 5.6%)	2.7% (0.9% to 5.5%)	2.8% (1.0% to 5.6%)
Leukaemia	6.3% (3.6% to 9.6%)	5.3% (3.1% to 8.3%)	6.2% (3.5% to 9.5%)	5.4% (3.1% to 8.3%)
**Cardiovascular diseases**	84.3% (80.9% to 86.8%)	83.6% (80.6% to 85.6%)	61.4% (57.3% to 65.3%)	81.7% (78.8% to 83.7%)
Rheumatic heart disease	25.4% (16.5% to 40.5%)	22.0% (15.0% to 32.1%)	21.0% (13.4% to 30.6%)	21.7% (15.1% to 31.9%)
Ischaemic heart disease	93.1% (90.3% to 95.4%)	94.5% (92.6% to 96.0%)	89.9% (87.2% to 92.4%)	94.3% (92.4% to 95.9%)
Cerebrovascular disease	87.0% (83.7% to 90.1%)	87.6% (85.1% to 89.8%)	87.6% (84.3% to 90.6%)	87.6% (85.1% to 89.8%)
Hypertensive heart disease	100.0% (100.0% to 100.0%)	100.0% (100.0% to 100.0%)	100.0% (100.0% to 100.0%)	100.0% (100.0% to 100.0%)
Cardiomyopathy and myocarditis	36.6% (29.3% to 44.8%)	34.3% (27.3% to 42.2%)	42.4% (35.9% to 49.1%)	35.1% (28.3% to 42.4%)
Atrial fibrillation and flutter	40.9% (33.3% to 48.6%)	45.4% (38.5% to 51.8%)	52.2% (47.0% to 57.1%)	49.2% (43.3% to 54.6%)
Aortic aneurysm	41.4% (34.4% to 48.1%)	45.3% (39.0% to 51.0%)	-	45.3% (39.0% to 51.0%)
Peripheral vascular disease	49.9% (43.1% to 57.1%)	56.7% (50.9% to 62.3%)	59.5% (53.7% to 64.6%)	59.1% (53.7% to 64.2%)
Endocarditis	35.7% (27.2% to 46.2%)	30.4% (22.5% to 38.2%)	34.1% (25.1% to 44.8%)	30.4% (22.5% to 38.2%)

Supplementary Results Table 1. Age-standardised PAFs attributable to the joint distribution of all risk factors for all ages, both sexes combined for each GBD level 1 and level 2 cause and all causes in 2016 for Chad. Results are shown for deaths, YLLs, YLDs, and DALYs
Data in parenthesis are 95% uncertainty intervals. PAFs=population attributable fractions. GBD=Global Burden of Disease. YLLs=years of life lost. YLDs=years live with disability. DALYs=disability-adjusted life-years

	Deaths 2016	YLLs 2016	YLDs 2016	DALYs 2016
Other cardiovascular and circulatory diseases	43.0% (37.7% to 48.3%)	42.6% (37.8% to 47.5%)	38.7% (33.6% to 43.7%)	41.6% (37.0% to 46.3%)
**Chronic respiratory diseases**	61.7% (54.8% to 68.2%)	57.2% (50.8% to 63.1%)	42.4% (36.2% to 49.5%)	53.3% (47.8% to 59.2%)
Chronic obstructive pulmonary disease	82.3% (75.4% to 89.9%)	81.3% (74.7% to 88.4%)	73.9% (65.3% to 83.0%)	79.8% (73.1% to 87.1%)
Pneumoconiosis	100.0% (100.0% to 100.0%)	100.0% (100.0% to 100.0%)	100.0% (100.0% to 100.0%)	100.0% (100.0% to 100.0%)
Asthma	22.9% (16.8% to 30.3%)	26.0% (20.1% to 32.9%)	18.8% (14.6% to 23.7%)	23.3% (18.2% to 29.4%)
Other chronic respiratory diseases	6.1% (3.4% to 9.4%)	5.4% (2.8% to 8.6%)	2.6% (1.1% to 5.0%)	5.2% (2.7% to 8.3%)
**Cirrhosis and other chronic liver diseases**	26.5% (23.6% to 29.7%)	25.3% (22.4% to 28.4%)	19.9% (17.0% to 23.1%)	25.2% (22.3% to 28.2%)
Cirrhosis and other chronic liver diseases due to hepatitis B	0.0% (0.0% to 0.0%)	0.0% (0.0% to 0.0%)	0.0% (0.0% to 0.0%)	0.0% (0.0% to 0.0%)
Cirrhosis and other chronic liver diseases due to hepatitis C	1.4% (0.5% to 2.8%)	2.1% (0.7% to 4.5%)	2.0% (0.7% to 4.5%)	2.1% (0.7% to 4.5%)
Cirrhosis and other chronic liver diseases due to alcohol use	100.0% (100.0% to 100.0%)	100.0% (100.0% to 100.0%)	100.0% (100.0% to 100.0%)	100.0% (100.0% to 100.0%)
**Digestive diseases**	4.5% (2.9% to 6.4%)	5.4% (3.2% to 8.2%)	1.3% (0.9% to 1.8%)	5.2% (3.1% to 7.9%)
Peptic ulcer disease	6.6% (4.1% to 9.5%)	6.4% (4.0% to 9.1%)	5.8% (3.8% to 8.2%)	6.4% (4.1% to 9.0%)
Gallbladder and biliary diseases	15.3% (7.3% to 26.0%)	14.9% (7.3% to 25.1%)	16.1% (7.8% to 27.0%)	14.9% (7.4% to 25.1%)
Pancreatitis	26.0% (11.8% to 42.0%)	33.4% (15.7% to 53.0%)	25.6% (11.5% to 41.5%)	33.4% (15.7% to 53.0%)
**Neurological disorders**	11.1% (5.6% to 18.3%)	12.3% (7.6% to 18.0%)	2.7% (1.1% to 5.0%)	6.3% (3.9% to 9.5%)
Alzheimer's disease and other dementias	11.7% (5.2% to 20.1%)	12.5% (5.8% to 21.1%)	12.5% (5.7% to 21.2%)	12.5% (5.8% to 21.1%)
Parkinson's disease	-5.6% (-8.0% to -3.9%)	-6.3% (-8.7% to -4.4%)	-6.3% (-8.5% to -4.5%)	-6.3% (-8.6% to -4.5%)
Epilepsy	17.7% (11.0% to 24.8%)	16.8% (10.8% to 23.9%)	12.6% (7.8% to 17.9%)	15.0% (9.4% to 21.4%)
Multiple sclerosis	4.1% (1.9% to 6.9%)	4.1% (1.9% to 7.1%)	3.1% (1.7% to 4.6%)	3.8% (2.0% to 6.2%)
**Mental and substance use disorders**	99.9% (99.8% to 100.0%)	99.8% (99.7% to 99.9%)	19.8% (17.5% to 22.1%)	23.2% (20.5% to 26.1%)
Alcohol use disorders	100.0% (100.0% to 100.0%)	100.0% (100.0% to 100.0%)	100.0% (100.0% to 100.0%)	100.0% (100.0% to 100.0%)

Supplementary Results Table 1. Age-standardised PAFs attributable to the joint distribution of all risk factors for all ages, both sexes combined for each GBD level 1 and level 2 cause and all causes in 2016 for Chad. Results are shown for deaths, YLLs, YLDs, and DALYs
Data in parenthesis are 95% uncertainty intervals. PAFs=population attributable fractions. GBD=Global Burden of Disease. YLLs=years of life lost. YLDs=years live with disability. DALYs=disability-adjusted life-years

	Deaths 2016	YLLs 2016	YLDs 2016	DALYs 2016
Drug use disorders	100.0% (100.0% to 100.0%)	100.0% (100.0% to 100.0%)	100.0% (100.0% to 100.0%)	100.0% (100.0% to 100.0%)
Depressive disorders	-	-	12.3% (10.6% to 14.3%)	12.3% (10.6% to 14.3%)
Idiopathic developmental intellectual disability	-	-	63.1% (31.8% to 82.6%)	63.1% (31.8% to 82.6%)
**Diabetes, urogenital, blood, and endocrine diseases**	75.1% (71.9% to 78.6%)	63.8% (58.9% to 69.3%)	55.7% (51.4% to 59.7%)	61.0% (57.4% to 64.8%)
Diabetes mellitus	100.0% (100.0% to 100.0%)	100.0% (100.0% to 100.0%)	100.0% (100.0% to 100.0%)	100.0% (100.0% to 100.0%)
Chronic kidney disease	100.0% (100.0% to 100.0%)	100.0% (100.0% to 100.0%)	100.0% (100.0% to 100.0%)	100.0% (100.0% to 100.0%)
**Musculoskeletal disorders**	0.5% (0.2% to 1.0%)	0.3% (0.1% to 0.6%)	25.2% (22.0% to 28.7%)	24.9% (21.8% to 28.3%)
Rheumatoid arthritis	1.8% (0.6% to 3.6%)	1.8% (0.6% to 3.4%)	2.2% (0.8% to 3.8%)	2.1% (0.8% to 3.8%)
Osteoarthritis	-	-	9.8% (3.8% to 17.7%)	9.8% (3.8% to 17.7%)
Low back and neck pain	-	-	31.6% (27.8% to 35.5%)	31.6% (27.8% to 35.5%)
Gout	-	-	42.6% (34.7% to 52.6%)	42.6% (34.7% to 52.6%)
**Other non-communicable diseases**	0.2% (0.1% to 0.5%)	0.3% (0.1% to 0.7%)	8.8% (7.3% to 10.3%)	6.1% (4.6% to 7.8%)
Sense organ diseases	-	-	17.1% (14.8% to 19.4%)	17.1% (14.8% to 19.4%)
Sudden infant death syndrome	4.5% (1.6% to 9.6%)	4.5% (1.6% to 9.6%)	-	4.5% (1.6% to 9.6%)
**Transport injuries**	29.0% (25.1% to 34.2%)	23.9% (19.7% to 29.4%)	34.6% (30.7% to 39.9%)	25.2% (20.9% to 30.7%)
Road injuries	28.9% (25.1% to 34.1%)	23.8% (19.7% to 29.4%)	36.8% (32.7% to 42.1%)	25.2% (20.9% to 30.7%)
Other transport injuries	30.3% (24.8% to 36.4%)	24.4% (18.5% to 30.9%)	26.1% (22.2% to 30.8%)	25.0% (20.1% to 30.6%)
**Unintentional injuries**	21.6% (15.4% to 28.5%)	12.7% (10.1% to 15.6%)	20.8% (18.6% to 23.1%)	14.6% (12.1% to 17.2%)
Falls	50.2% (32.2% to 67.5%)	33.5% (24.6% to 42.9%)	30.9% (28.8% to 33.1%)	32.3% (27.1% to 37.9%)
Drowning	9.9% (7.5% to 13.1%)	9.2% (6.8% to 12.2%)	14.3% (11.1% to 18.2%)	9.3% (6.8% to 12.3%)
Fire, heat, and hot substances	8.0% (5.8% to 10.8%)	8.7% (6.4% to 11.8%)	16.7% (13.1% to 20.6%)	10.1% (7.5% to 13.3%)

**Supplementary Results Table 1. Age-standardised PAFs attributable to the joint distribution of all risk factors for all ages, both sexes combined for each GBD level 1 and level 2 cause and all causes in 2016 for Chad. Results are shown for deaths, YLLs, YLDs, and DALYs**<br>Data in parenthesis are 95% uncertainty intervals. PAFs=population attributable fractions. GBD=Global Burden of Disease. YLLs=years of life lost. YLDs=years live with disability. DALYs=disability-adjusted life-years				
	**Deaths 2016**	**YLLs 2016**	**YLDs 2016**	**DALYs 2016**
Poisonings	11.8% (7.7% to 16.1%)	11.7% (5.9% to 16.9%)	16.8% (13.1% to 20.7%)	11.9% (6.1% to 17.0%)
Exposure to mechanical forces	17.4% (14.3% to 20.7%)	13.1% (9.9% to 17.0%)	21.2% (18.5% to 24.4%)	14.8% (11.8% to 18.2%)
Animal contact	8.0% (6.4% to 9.9%)	8.6% (6.7% to 10.8%)	13.0% (10.6% to 15.6%)	9.3% (7.3% to 11.5%)
Foreign body	6.0% (4.2% to 8.0%)	6.6% (3.6% to 9.6%)	11.0% (8.8% to 13.6%)	7.9% (5.2% to 10.5%)
Other unintentional injuries	12.7% (9.5% to 16.4%)	12.5% (9.1% to 16.7%)	14.8% (11.5% to 18.4%)	13.3% (10.1% to 16.8%)
**Self-harm and interpersonal violence**	13.6% (8.0% to 20.0%)	14.5% (9.3% to 20.8%)	18.4% (16.1% to 20.9%)	14.9% (9.9% to 20.7%)
Self-harm	13.3% (6.2% to 21.5%)	15.6% (8.0% to 24.3%)	15.3% (7.6% to 23.9%)	15.6% (8.0% to 24.2%)
Interpersonal violence	14.5% (10.3% to 19.8%)	12.8% (8.9% to 18.1%)	18.7% (16.6% to 21.1%)	14.0% (10.3% to 18.5%)
**Forces of nature, conflict and terrorism, and executions and police conflict**	0.0% (0.0% to 0.0%)	0.0% (0.0% to 0.0%)	0.0% (0.0% to 0.1%)	0.0% (0.0% to 0.1%)
Forces of nature, conflict and terrorism, and state actor violence	0.0% (0.0% to 0.0%)	0.0% (0.0% to 0.0%)	7.5% (5.0% to 10.8%)	7.5% (5.0% to 10.8%)

Supplementary Results Table 1. Age-standardised PAFs attributable to the joint distribution of all risk factors for all ages, both sexes combined for each GBD level 1 and level 2 cause and all causes in 2016 for Chile. Results are shown for deaths, YLLs, YLDs, and DALYs
Data in parenthesis are 95% uncertainty intervals. PAFs=population attributable fractions. GBD=Global Burden of Disease. YLLs=years of life lost. YLDs=years live with disability. DALYs=disability-adjusted life-years

	Deaths 2016	YLLs 2016	YLDs 2016	DALYs 2016
**All causes**	53.0% (50.9% to 55.1%)	49.5% (47.7% to 51.4%)	21.3% (19.9% to 22.8%)	35.4% (32.6% to 38.6%)
**HIV/AIDS and tuberculosis**	74.3% (67.0% to 80.7%)	81.5% (76.9% to 85.2%)	89.8% (85.3% to 93.1%)	82.2% (78.0% to 85.8%)
Tuberculosis	51.1% (37.3% to 63.1%)	55.0% (43.4% to 65.2%)	53.9% (42.6% to 63.2%)	55.0% (43.4% to 65.0%)
HIV/AIDS	96.1% (95.6% to 96.5%)	94.1% (93.5% to 94.7%)	97.4% (96.8% to 97.8%)	94.5% (93.9% to 95.1%)
**Diarrhoea, lower respiratory infections, and other common infectious diseases**	39.3% (31.4% to 48.5%)	43.6% (37.3% to 51.2%)	13.4% (9.2% to 18.7%)	34.6% (28.8% to 41.5%)
Diarrhoeal diseases	38.4% (19.8% to 61.7%)	45.3% (28.2% to 66.2%)	49.2% (32.9% to 68.9%)	47.5% (31.2% to 67.8%)
Lower respiratory infections	41.1% (32.0% to 51.4%)	50.1% (41.9% to 59.2%)	49.2% (40.9% to 58.5%)	50.1% (41.9% to 59.2%)
Upper respiratory infections	1.1% (0.1% to 4.2%)	3.0% (0.2% to 10.8%)	0.0% (0.0% to 0.0%)	0.0% (0.0% to 0.0%)
Otitis media	2.8% (1.6% to 4.5%)	6.1% (3.5% to 9.4%)	9.2% (6.4% to 12.1%)	9.2% (6.4% to 12.1%)
Meningitis	2.0% (1.3% to 2.7%)	3.7% (2.6% to 4.8%)	0.0% (0.0% to 0.0%)	3.4% (2.4% to 4.4%)
Encephalitis	0.4% (0.2% to 0.6%)	0.7% (0.4% to 1.0%)	0.0% (0.0% to 0.0%)	0.5% (0.3% to 0.8%)
Measles	7.0% (1.2% to 28.8%)	7.7% (1.3% to 31.8%)	10.6% (2.1% to 42.4%)	7.7% (1.3% to 31.9%)
**Maternal disorders**	3.2% (1.4% to 5.3%)	3.1% (1.4% to 5.2%)	3.0% (1.5% to 4.9%)	3.1% (1.5% to 4.9%)
Maternal haemorrhage	11.4% (4.1% to 18.5%)	11.3% (4.1% to 18.4%)	11.2% (4.1% to 18.2%)	11.3% (4.1% to 18.3%)
Maternal sepsis and other pregnancy related infections	11.4% (4.1% to 18.6%)	11.3% (4.1% to 18.4%)	10.9% (4.0% to 17.8%)	11.2% (4.1% to 18.2%)
Maternal abortion, miscarriage, and ectopic pregnancy	5.5% (3.0% to 8.4%)	5.7% (3.1% to 8.7%)	6.0% (3.1% to 9.6%)	5.9% (3.1% to 9.3%)
**Neonatal disorders**	81.5% (78.7% to 84.3%)	81.6% (78.8% to 84.3%)	0.1% (0.1% to 0.2%)	66.0% (60.5% to 71.3%)
Neonatal preterm birth complications	93.5% (91.0% to 95.9%)	93.6% (91.0% to 95.9%)	0.2% (0.1% to 0.2%)	73.7% (66.2% to 80.0%)
Neonatal encephalopathy due to birth asphyxia and trauma	64.0% (59.8% to 68.3%)	64.1% (59.9% to 68.4%)	0.0% (0.0% to 0.0%)	53.2% (44.4% to 61.1%)
Neonatal sepsis and other neonatal infections	60.0% (56.0% to 63.8%)	60.0% (56.0% to 63.8%)	0.0% (0.0% to 0.0%)	45.0% (31.6% to 55.3%)
Hemolytic disease and other neonatal jaundice	60.4% (53.2% to 66.6%)	60.5% (53.3% to 66.7%)	0.0% (0.0% to 0.0%)	20.6% (12.3% to 36.3%)

Supplementary Results Table 1. Age-standardised PAFs attributable to the joint distribution of all risk factors for all ages, both sexes combined for each GBD level 1 and level 2 cause and all causes in 2016 for Chile. Results are shown for deaths, YLLs, YLDs, and DALYs
Data in parenthesis are 95% uncertainty intervals. PAFs=population attributable fractions. GBD=Global Burden of Disease. YLLs=years of life lost. YLDs=years live with disability. DALYs=disability-adjusted life-years

	Deaths 2016	YLLs 2016	YLDs 2016	DALYs 2016
Other neonatal disorders	61.6% (57.2% to 66.0%)	61.7% (57.2% to 66.1%)	0.0% (0.0% to 0.0%)	59.3% (54.7% to 63.7%)
**Nutritional deficiencies**	1.6% (1.2% to 2.3%)	9.5% (6.6% to 13.1%)	90.0% (87.5% to 91.9%)	70.7% (62.2% to 77.9%)
Protein-energy malnutrition	1.3% (0.8% to 1.9%)	8.8% (5.8% to 12.8%)	98.2% (96.5% to 99.2%)	15.7% (10.8% to 22.4%)
Vitamin A deficiency	-	-	0.0% (0.0% to 0.0%)	0.0% (0.0% to 0.0%)
Iron-deficiency anaemia	100.0% (100.0% to 100.0%)	100.0% (100.0% to 100.0%)	100.0% (100.0% to 100.0%)	100.0% (100.0% to 100.0%)
**Other communicable, maternal, neonatal, and nutritional diseases**	6.6% (5.0% to 8.3%)	5.0% (3.0% to 7.0%)	68.6% (62.8% to 74.1%)	24.7% (15.9% to 36.3%)
Sexually transmitted diseases excluding HIV	47.6% (15.4% to 98.2%)	28.7% (5.4% to 94.6%)	100.0% (100.0% to 100.0%)	65.5% (31.8% to 99.3%)
Hepatitis	3.9% (2.7% to 5.3%)	3.5% (2.4% to 4.8%)	1.5% (1.0% to 2.3%)	3.3% (2.3% to 4.5%)
**Neoplasms**	35.3% (31.5% to 39.0%)	34.4% (30.8% to 37.9%)	30.4% (27.2% to 33.7%)	34.3% (30.7% to 37.8%)
Oesophageal cancer	76.3% (64.8% to 85.7%)	77.3% (65.9% to 86.4%)	76.5% (64.8% to 85.9%)	77.3% (65.9% to 86.4%)
Stomach cancer	14.6% (6.5% to 24.7%)	13.8% (6.2% to 22.7%)	14.2% (6.4% to 24.0%)	13.8% (6.2% to 22.7%)
Liver cancer	66.9% (59.3% to 74.4%)	69.9% (62.8% to 76.6%)	67.5% (60.7% to 74.5%)	69.9% (62.8% to 76.6%)
Larynx cancer	77.0% (65.1% to 86.5%)	76.7% (64.0% to 86.0%)	77.0% (64.9% to 86.6%)	76.7% (64.0% to 86.0%)
Tracheal, bronchus, and lung cancer	78.8% (72.9% to 83.8%)	77.6% (71.5% to 83.0%)	77.9% (71.5% to 83.3%)	77.6% (71.5% to 83.0%)
Breast cancer	33.1% (26.4% to 40.7%)	31.3% (25.2% to 38.4%)	31.9% (25.6% to 39.1%)	31.4% (25.3% to 38.4%)
Cervical cancer	100.0% (100.0% to 100.0%)	100.0% (100.0% to 100.0%)	100.0% (100.0% to 100.0%)	100.0% (100.0% to 100.0%)
Uterine cancer	47.8% (32.7% to 62.3%)	49.2% (33.9% to 63.6%)	49.7% (34.1% to 63.8%)	49.3% (33.9% to 63.6%)
Prostate cancer	2.7% (1.6% to 4.2%)	2.8% (1.7% to 4.5%)	2.9% (1.7% to 4.5%)	2.8% (1.7% to 4.5%)
Colon and rectum cancer	59.6% (51.1% to 67.6%)	60.1% (51.9% to 68.0%)	59.9% (51.4% to 67.6%)	60.1% (51.9% to 68.0%)
Lip and oral cavity cancer	71.7% (60.1% to 80.9%)	72.8% (61.4% to 82.0%)	71.5% (60.7% to 80.5%)	72.7% (61.3% to 81.9%)
Nasopharynx cancer	72.2% (63.6% to 79.8%)	69.9% (61.9% to 77.3%)	68.9% (61.6% to 75.7%)	69.9% (61.8% to 77.3%)

Supplementary Results Table 1. Age-standardised PAFs attributable to the joint distribution of all risk factors for all ages, both sexes combined for each GBD level 1 and level 2 cause and all causes in 2016 for Chile. Results are shown for deaths, YLLs, YLDs, and DALYs
Data in parenthesis are 95% uncertainty intervals. PAFs=population attributable fractions. GBD=Global Burden of Disease. YLLs=years of life lost. YLDs=years live with disability. DALYs=disability-adjusted life-years

	Deaths 2016	YLLs 2016	YLDs 2016	DALYs 2016
Other pharynx cancer	55.8% (40.1% to 70.0%)	58.2% (43.1% to 71.3%)	57.2% (41.9% to 70.9%)	58.2% (43.0% to 71.3%)
Gallbladder and biliary tract cancer	26.2% (14.5% to 39.9%)	27.3% (15.1% to 41.1%)	26.6% (15.0% to 40.0%)	27.3% (15.1% to 41.1%)
Pancreatic cancer	25.1% (17.4% to 33.9%)	24.5% (16.9% to 33.2%)	24.8% (17.2% to 33.3%)	24.5% (16.9% to 33.2%)
Ovarian cancer	10.7% (4.1% to 18.5%)	9.6% (3.4% to 16.9%)	9.3% (3.2% to 16.3%)	9.6% (3.4% to 16.9%)
Kidney cancer	32.2% (21.9% to 43.6%)	31.6% (21.5% to 42.8%)	31.7% (21.5% to 42.8%)	31.6% (21.5% to 42.8%)
Bladder cancer	24.1% (15.8% to 33.7%)	23.6% (15.4% to 33.2%)	23.4% (15.2% to 32.6%)	23.6% (15.4% to 33.0%)
Thyroid cancer	15.9% (8.4% to 25.6%)	16.8% (8.7% to 26.7%)	16.2% (8.9% to 25.2%)	16.7% (8.7% to 26.5%)
Mesothelioma	88.7% (84.9% to 91.9%)	81.0% (75.2% to 86.0%)	86.2% (81.8% to 89.7%)	81.1% (75.3% to 86.0%)
Non-Hodgkin's lymphoma	8.0% (3.3% to 13.7%)	7.9% (3.3% to 13.3%)	8.0% (3.3% to 13.6%)	7.9% (3.3% to 13.3%)
Multiple myeloma	9.4% (4.1% to 16.4%)	9.7% (4.2% to 16.9%)	9.7% (4.2% to 16.8%)	9.7% (4.2% to 16.9%)
Leukaemia	15.0% (9.3% to 20.9%)	11.6% (7.4% to 16.7%)	13.3% (8.4% to 18.6%)	11.7% (7.5% to 16.7%)
**Cardiovascular diseases**	84.2% (81.7% to 86.5%)	85.0% (83.2% to 86.7%)	78.0% (75.1% to 80.5%)	83.9% (81.9% to 85.7%)
Rheumatic heart disease	28.8% (18.4% to 47.9%)	25.2% (17.3% to 38.8%)	24.5% (16.6% to 36.6%)	25.2% (17.2% to 38.8%)
Ischaemic heart disease	93.8% (90.8% to 96.2%)	95.0% (93.0% to 96.7%)	91.8% (89.1% to 94.0%)	94.8% (92.7% to 96.5%)
Cerebrovascular disease	85.2% (80.3% to 89.8%)	88.4% (85.3% to 91.2%)	86.5% (82.3% to 90.0%)	88.1% (84.9% to 91.1%)
Hypertensive heart disease	100.0% (100.0% to 100.0%)	100.0% (100.0% to 100.0%)	100.0% (100.0% to 100.0%)	100.0% (100.0% to 100.0%)
Cardiomyopathy and myocarditis	43.6% (35.1% to 52.3%)	40.5% (33.3% to 47.9%)	38.7% (31.5% to 46.4%)	40.4% (33.3% to 47.8%)
Atrial fibrillation and flutter	57.8% (49.6% to 66.2%)	61.0% (53.6% to 68.1%)	65.9% (60.3% to 71.4%)	63.9% (57.4% to 69.8%)
Aortic aneurysm	47.4% (40.1% to 54.6%)	51.3% (45.2% to 57.2%)	-	51.3% (45.2% to 57.2%)
Peripheral vascular disease	51.9% (44.5% to 60.0%)	57.5% (51.4% to 63.3%)	60.1% (54.4% to 65.4%)	58.4% (52.4% to 63.9%)
Endocarditis	41.3% (31.9% to 51.5%)	37.4% (29.8% to 46.1%)	40.7% (31.7% to 50.8%)	37.5% (29.9% to 46.1%)

Supplementary Results Table 1. Age-standardised PAFs attributable to the joint distribution of all risk factors for all ages, both sexes combined for each GBD level 1 and level 2 cause and all causes in 2016 for Chile. Results are shown for deaths, YLLs, YLDs, and DALYs
Data in parenthesis are 95% uncertainty intervals. PAFs=population attributable fractions. GBD=Global Burden of Disease. YLLs=years of life lost. YLDs=years live with disability. DALYs=disability-adjusted life-years

	Deaths 2016	YLLs 2016	YLDs 2016	DALYs 2016
Other cardiovascular and circulatory diseases	50.5% (44.7% to 55.7%)	52.6% (47.7% to 57.5%)	51.8% (46.5% to 57.2%)	52.3% (47.3% to 57.3%)
**Chronic respiratory diseases**	53.2% (46.9% to 61.8%)	49.2% (43.6% to 57.7%)	41.7% (33.0% to 51.3%)	45.7% (39.6% to 52.0%)
Chronic obstructive pulmonary disease	71.6% (64.1% to 78.3%)	69.7% (62.1% to 76.3%)	65.5% (56.3% to 73.8%)	69.1% (60.9% to 75.9%)
Pneumoconiosis	100.0% (100.0% to 100.0%)	100.0% (100.0% to 100.0%)	100.0% (100.0% to 100.0%)	100.0% (100.0% to 100.0%)
Asthma	41.7% (30.3% to 54.0%)	46.8% (36.4% to 57.9%)	39.0% (29.1% to 50.3%)	39.5% (29.7% to 50.6%)
Other chronic respiratory diseases	9.9% (6.0% to 14.5%)	7.6% (4.2% to 11.7%)	3.7% (1.9% to 6.2%)	6.7% (3.8% to 10.5%)
**Cirrhosis and other chronic liver diseases**	63.4% (59.9% to 66.6%)	65.6% (62.4% to 68.7%)	55.3% (51.6% to 59.0%)	65.3% (62.1% to 68.3%)
Cirrhosis and other chronic liver diseases due to hepatitis B	2.0% (0.9% to 3.7%)	2.7% (1.2% to 5.0%)	2.4% (1.0% to 4.3%)	2.7% (1.2% to 5.0%)
Cirrhosis and other chronic liver diseases due to hepatitis C	80.7% (75.5% to 84.8%)	85.3% (82.0% to 87.7%)	83.0% (79.4% to 85.8%)	85.2% (81.9% to 87.6%)
Cirrhosis and other chronic liver diseases due to alcohol use	100.0% (100.0% to 100.0%)	100.0% (100.0% to 100.0%)	100.0% (100.0% to 100.0%)	100.0% (100.0% to 100.0%)
**Digestive diseases**	12.4% (8.9% to 15.9%)	14.3% (10.4% to 18.3%)	5.2% (3.9% to 6.6%)	12.4% (9.1% to 15.7%)
Peptic ulcer disease	11.8% (8.2% to 15.8%)	16.4% (11.7% to 21.5%)	17.7% (12.7% to 23.5%)	16.7% (12.1% to 21.9%)
Gallbladder and biliary diseases	45.5% (30.4% to 60.5%)	46.9% (31.9% to 61.9%)	51.8% (37.1% to 66.5%)	47.3% (32.5% to 62.1%)
Pancreatitis	30.6% (11.8% to 50.2%)	35.0% (16.7% to 53.5%)	34.5% (16.6% to 53.3%)	34.9% (16.7% to 53.5%)
**Neurological disorders**	19.8% (9.8% to 32.4%)	18.7% (11.0% to 28.0%)	4.8% (2.4% to 7.9%)	9.1% (5.3% to 13.5%)
Alzheimer's disease and other dementias	24.8% (11.9% to 40.7%)	26.3% (13.8% to 41.9%)	26.3% (13.6% to 42.0%)	26.3% (13.8% to 42.1%)
Parkinson's disease	-6.0% (-7.5% to -4.7%)	-8.7% (-10.7% to -6.8%)	-11.4% (-13.8% to -9.0%)	-9.3% (-11.3% to -7.3%)
Epilepsy	33.2% (22.7% to 43.4%)	33.1% (22.8% to 42.3%)	27.0% (18.5% to 34.7%)	29.2% (20.0% to 37.6%)
Multiple sclerosis	11.6% (7.4% to 15.8%)	12.9% (8.3% to 17.6%)	12.2% (8.0% to 17.0%)	12.4% (8.0% to 17.0%)
**Mental and substance use disorders**	99.9% (99.8% to 99.9%)	99.8% (99.7% to 99.9%)	19.5% (17.3% to 21.9%)	22.4% (20.0% to 25.3%)
Alcohol use disorders	100.0% (100.0% to 100.0%)	100.0% (100.0% to 100.0%)	100.0% (100.0% to 100.0%)	100.0% (100.0% to 100.0%)

Supplementary Results Table 1. Age-standardised PAFs attributable to the joint distribution of all risk factors for all ages, both sexes combined for each GBD level 1 and level 2 cause and all causes in 2016 for Chile. Results are shown for deaths, YLLs, YLDs, and DALYs
Data in parenthesis are 95% uncertainty intervals. PAFs=population attributable fractions. GBD=Global Burden of Disease. YLLs=years of life lost. YLDs=years live with disability. DALYs=disability-adjusted life-years

	Deaths 2016	YLLs 2016	YLDs 2016	DALYs 2016
Drug use disorders	100.0% (100.0% to 100.0%)	100.0% (100.0% to 100.0%)	100.0% (100.0% to 100.0%)	100.0% (100.0% to 100.0%)
Depressive disorders	-	-	6.7% (5.5% to 8.2%)	6.7% (5.5% to 8.2%)
Idiopathic developmental intellectual disability	-	-	56.7% (27.9% to 77.1%)	56.7% (27.9% to 77.1%)
**Diabetes, urogenital, blood, and endocrine diseases**	77.4% (75.9% to 78.7%)	75.9% (74.0% to 77.5%)	63.4% (59.1% to 67.5%)	70.0% (67.2% to 72.6%)
Diabetes mellitus	100.0% (100.0% to 100.0%)	100.0% (100.0% to 100.0%)	100.0% (100.0% to 100.0%)	100.0% (100.0% to 100.0%)
Chronic kidney disease	100.0% (100.0% to 100.0%)	100.0% (100.0% to 100.0%)	100.0% (100.0% to 100.0%)	100.0% (100.0% to 100.0%)
**Musculoskeletal disorders**	2.0% (0.8% to 3.6%)	1.9% (0.7% to 3.6%)	18.7% (15.9% to 21.8%)	18.4% (15.7% to 21.5%)
Rheumatoid arthritis	5.0% (2.0% to 8.5%)	6.7% (2.7% to 11.2%)	7.6% (3.1% to 12.7%)	7.6% (3.1% to 12.6%)
Osteoarthritis	-	-	30.3% (16.6% to 43.6%)	30.3% (16.6% to 43.6%)
Low back and neck pain	-	-	24.8% (21.5% to 28.4%)	24.8% (21.5% to 28.4%)
Gout	-	-	52.3% (34.3% to 69.9%)	52.3% (34.3% to 69.9%)
**Other non-communicable diseases**	0.2% (0.1% to 0.3%)	0.3% (0.2% to 0.4%)	3.2% (2.8% to 3.8%)	2.6% (2.2% to 3.0%)
Sense organ diseases	-	-	9.0% (8.1% to 10.1%)	9.0% (8.1% to 10.1%)
Sudden infant death syndrome	4.9% (3.2% to 7.1%)	4.9% (3.3% to 7.1%)	-	4.9% (3.3% to 7.1%)
**Transport injuries**	40.5% (30.6% to 50.8%)	38.2% (28.0% to 48.6%)	44.5% (35.8% to 53.7%)	39.0% (28.9% to 49.3%)
Road injuries	40.3% (30.5% to 50.4%)	37.9% (27.8% to 48.2%)	45.8% (36.8% to 55.2%)	38.9% (28.9% to 49.3%)
Other transport injuries	43.3% (32.4% to 54.4%)	41.1% (29.7% to 52.5%)	36.5% (28.1% to 44.9%)	40.1% (29.5% to 50.7%)
**Unintentional injuries**	35.5% (28.9% to 42.1%)	24.5% (18.8% to 31.5%)	25.2% (20.5% to 31.5%)	24.9% (19.6% to 31.1%)
Falls	62.4% (54.0% to 69.0%)	40.9% (32.9% to 49.5%)	28.7% (24.8% to 34.1%)	32.6% (27.9% to 38.5%)
Drowning	24.7% (16.6% to 34.3%)	23.3% (16.0% to 31.9%)	23.2% (15.5% to 32.1%)	23.3% (16.0% to 31.9%)
Fire, heat, and hot substances	18.7% (12.2% to 26.6%)	20.2% (14.0% to 27.4%)	22.9% (15.2% to 31.6%)	21.2% (14.7% to 28.8%)

Supplementary Results Table 1. Age-standardised PAFs attributable to the joint distribution of all risk factors for all ages, both sexes combined for each GBD level 1 and level 2 cause and all causes in 2016 for Chile. Results are shown for deaths, YLLs, YLDs, and DALYs
Data in parenthesis are 95% uncertainty intervals. PAFs=population attributable fractions. GBD=Global Burden of Disease. YLLs=years of life lost. YLDs=years live with disability. DALYs=disability-adjusted life-years

	Deaths 2016	YLLs 2016	YLDs 2016	DALYs 2016
Poisonings	25.3% (17.3% to 34.4%)	26.3% (18.3% to 35.5%)	23.2% (15.6% to 31.8%)	25.2% (17.4% to 33.9%)
Exposure to mechanical forces	25.4% (18.2% to 35.6%)	24.1% (16.5% to 34.4%)	26.7% (20.3% to 36.4%)	25.8% (19.0% to 35.7%)
Animal contact	17.1% (12.3% to 23.4%)	15.4% (10.4% to 22.0%)	15.2% (10.0% to 22.2%)	15.3% (10.3% to 21.9%)
Foreign body	12.2% (7.6% to 18.5%)	13.4% (8.2% to 20.3%)	11.8% (7.4% to 17.9%)	12.9% (8.1% to 19.2%)
Other unintentional injuries	30.5% (20.4% to 42.2%)	30.6% (20.4% to 42.0%)	23.1% (15.7% to 32.4%)	25.6% (17.4% to 34.8%)
**Self-harm and interpersonal violence**	29.7% (17.8% to 40.9%)	30.3% (18.7% to 41.0%)	27.9% (22.2% to 33.4%)	30.2% (19.3% to 40.5%)
Self-harm	31.3% (15.4% to 45.2%)	32.8% (16.7% to 47.0%)	29.3% (14.8% to 42.7%)	32.8% (16.6% to 47.0%)
Interpersonal violence	25.6% (15.7% to 35.1%)	24.8% (15.0% to 34.2%)	27.6% (21.6% to 33.7%)	25.2% (16.1% to 33.9%)
**Forces of nature, conflict and terrorism, and executions and police conflict**	0.0% (0.0% to 0.0%)	0.0% (0.0% to 0.0%)	7.1% (4.5% to 10.7%)	6.0% (2.9% to 9.6%)
Forces of nature, conflict and terrorism, and state actor violence	0.0% (0.0% to 0.0%)	0.0% (0.0% to 0.0%)	7.7% (5.1% to 11.6%)	7.7% (5.1% to 11.6%)

Supplementary Results Table 1. Age-standardised PAFs attributable to the joint distribution of all risk factors for all ages, both sexes combined for each GBD level 1 and level 2 cause and all causes in 2016 for China. Results are shown for deaths, YLLs, YLDs, and DALYs
Data in parenthesis are 95% uncertainty intervals. PAFs=population attributable fractions. GBD=Global Burden of Disease. YLLs=years of life lost. YLDs=years live with disability. DALYs=disability-adjusted life-years

	Deaths 2016	YLLs 2016	YLDs 2016	DALYs 2016
**All causes**	63.8% (60.7% to 66.9%)	56.6% (54.3% to 59.2%)	23.2% (21.8% to 24.5%)	43.9% (41.1% to 46.9%)
**HIV/AIDS and tuberculosis**	61.5% (57.5% to 65.6%)	62.9% (59.8% to 65.9%)	43.8% (38.1% to 49.5%)	57.8% (54.5% to 61.5%)
Tuberculosis	49.1% (42.3% to 55.7%)	47.8% (41.7% to 53.9%)	38.5% (33.3% to 43.4%)	44.0% (38.3% to 49.7%)
HIV/AIDS	79.1% (77.1% to 81.1%)	75.3% (72.5% to 77.7%)	81.3% (79.6% to 83.1%)	75.7% (73.1% to 78.1%)
**Diarrhoea, lower respiratory infections, and other common infectious diseases**	66.6% (61.7% to 71.6%)	67.6% (62.9% to 71.9%)	17.4% (12.9% to 21.7%)	53.3% (48.0% to 58.5%)
Diarrhoeal diseases	81.8% (65.0% to 93.2%)	89.0% (78.4% to 96.0%)	74.6% (52.9% to 89.7%)	80.7% (64.0% to 92.3%)
Lower respiratory infections	72.1% (66.9% to 77.5%)	80.3% (75.4% to 84.5%)	71.6% (65.5% to 77.3%)	80.2% (75.3% to 84.4%)
Upper respiratory infections	0.9% (0.5% to 1.4%)	2.3% (1.4% to 3.6%)	0.0% (0.0% to 0.0%)	0.0% (0.0% to 0.0%)
Otitis media	2.6% (1.8% to 3.5%)	6.2% (4.4% to 8.0%)	9.1% (6.2% to 11.7%)	9.1% (6.3% to 11.8%)
Meningitis	1.2% (0.8% to 1.6%)	2.0% (1.5% to 2.4%)	0.0% (0.0% to 0.0%)	1.7% (1.2% to 2.1%)
Encephalitis	0.9% (0.8% to 1.1%)	1.2% (1.0% to 1.4%)	0.0% (0.0% to 0.0%)	0.5% (0.4% to 0.7%)
Measles	36.5% (16.4% to 72.0%)	37.1% (16.7% to 73.1%)	33.1% (13.3% to 69.4%)	36.7% (16.4% to 72.6%)
**Maternal disorders**	6.0% (2.8% to 9.1%)	5.8% (2.7% to 8.9%)	3.2% (1.6% to 4.9%)	5.1% (2.4% to 7.9%)
Maternal haemorrhage	14.3% (5.1% to 23.2%)	14.2% (5.1% to 23.1%)	13.9% (5.0% to 22.7%)	14.2% (5.0% to 23.1%)
Maternal sepsis and other pregnancy related infections	13.5% (4.8% to 22.1%)	13.6% (4.9% to 22.3%)	13.8% (4.9% to 22.5%)	13.7% (4.9% to 22.3%)
Maternal abortion, miscarriage, and ectopic pregnancy	9.4% (5.6% to 13.8%)	9.2% (5.3% to 13.4%)	8.1% (3.9% to 12.7%)	8.9% (5.0% to 13.2%)
**Neonatal disorders**	75.6% (74.1% to 77.3%)	75.7% (74.2% to 77.4%)	0.1% (0.1% to 0.2%)	65.4% (62.0% to 68.4%)
Neonatal preterm birth complications	91.7% (90.7% to 93.4%)	91.7% (90.8% to 93.4%)	0.2% (0.1% to 0.2%)	75.4% (69.8% to 80.0%)
Neonatal encephalopathy due to birth asphyxia and trauma	64.3% (61.7% to 67.7%)	64.3% (61.8% to 67.7%)	0.0% (0.0% to 0.0%)	61.1% (57.5% to 64.8%)
Neonatal sepsis and other neonatal infections	49.3% (46.5% to 53.5%)	49.4% (46.5% to 53.5%)	0.0% (0.0% to 0.0%)	34.8% (24.8% to 43.4%)
Hemolytic disease and other neonatal jaundice	46.0% (43.2% to 48.7%)	46.1% (43.3% to 48.8%)	0.0% (0.0% to 0.0%)	30.0% (26.7% to 33.2%)

Supplementary Results Table 1. Age-standardised PAFs attributable to the joint distribution of all risk factors for all ages, both sexes combined for each GBD level 1 and level 2 cause and all causes in 2016 for China. Results are shown for deaths, YLLs, YLDs, and DALYs
Data in parenthesis are 95% uncertainty intervals. PAFs=population attributable fractions. GBD=Global Burden of Disease. YLLs=years of life lost. YLDs=years live with disability. DALYs=disability-adjusted life-years

	Deaths 2016	YLLs 2016	YLDs 2016	DALYs 2016
Other neonatal disorders	48.0% (45.2% to 50.7%)	48.1% (45.3% to 50.8%)	0.0% (0.0% to 0.0%)	46.3% (43.6% to 48.9%)
**Nutritional deficiencies**	5.3% (4.6% to 6.4%)	26.6% (23.4% to 32.6%)	75.6% (72.3% to 78.4%)	68.4% (64.0% to 72.5%)
Protein-energy malnutrition	4.9% (4.1% to 6.2%)	32.4% (28.5% to 37.7%)	97.6% (97.4% to 97.9%)	53.9% (47.7% to 59.7%)
Vitamin A deficiency	-	-	99.8% (99.8% to 99.9%)	100.0% (100.0% to 100.0%)
Iron-deficiency anaemia	100.0% (100.0% to 100.0%)	100.0% (100.0% to 100.0%)	99.8% (99.8% to 99.9%)	100.0% (100.0% to 100.0%)
**Other communicable, maternal, neonatal, and nutritional diseases**	4.5% (3.6% to 4.9%)	4.0% (3.3% to 4.4%)	86.0% (83.5% to 88.2%)	48.9% (39.9% to 58.7%)
Sexually transmitted diseases excluding HIV	77.6% (60.5% to 89.0%)	55.4% (34.9% to 73.6%)	99.7% (99.7% to 99.8%)	97.4% (94.3% to 99.0%)
Hepatitis	1.1% (0.9% to 1.4%)	1.2% (0.9% to 1.5%)	0.9% (0.7% to 1.2%)	1.2% (0.9% to 1.5%)
**Neoplasms**	44.4% (37.0% to 52.4%)	40.9% (34.3% to 48.5%)	36.9% (31.1% to 43.2%)	40.8% (34.2% to 48.3%)
Oesophageal cancer	66.2% (52.4% to 80.1%)	66.5% (53.4% to 80.2%)	64.4% (50.4% to 78.9%)	66.5% (53.4% to 80.1%)
Stomach cancer	21.5% (10.6% to 33.6%)	20.0% (10.5% to 31.5%)	20.6% (10.5% to 32.5%)	20.0% (10.5% to 31.5%)
Liver cancer	35.0% (25.4% to 48.1%)	33.1% (23.5% to 46.2%)	34.4% (24.9% to 47.3%)	33.2% (23.5% to 46.2%)
Larynx cancer	73.0% (53.1% to 88.3%)	71.5% (52.7% to 86.9%)	72.5% (53.8% to 87.6%)	71.6% (52.8% to 87.0%)
Tracheal, bronchus, and lung cancer	79.7% (66.4% to 90.2%)	78.0% (64.8% to 89.0%)	78.9% (65.8% to 89.7%)	78.0% (64.8% to 89.0%)
Breast cancer	26.5% (16.5% to 38.6%)	25.3% (16.1% to 36.2%)	25.7% (16.2% to 36.8%)	25.4% (16.2% to 36.2%)
Cervical cancer	100.0% (100.0% to 100.0%)	100.0% (100.0% to 100.0%)	100.0% (100.0% to 100.0%)	100.0% (100.0% to 100.0%)
Uterine cancer	23.0% (11.7% to 36.6%)	23.8% (12.4% to 37.6%)	24.7% (13.0% to 38.5%)	23.9% (12.4% to 37.6%)
Prostate cancer	3.2% (0.8% to 7.4%)	2.7% (0.7% to 6.3%)	2.7% (0.7% to 6.3%)	2.7% (0.7% to 6.3%)
Colon and rectum cancer	52.3% (43.7% to 60.7%)	52.2% (44.1% to 60.1%)	52.5% (44.3% to 60.7%)	52.2% (44.1% to 60.2%)
Lip and oral cavity cancer	69.1% (57.9% to 80.2%)	69.0% (59.2% to 79.2%)	67.0% (56.4% to 78.0%)	68.9% (59.1% to 79.2%)
Nasopharynx cancer	65.5% (56.5% to 76.4%)	64.6% (56.5% to 74.4%)	62.0% (54.1% to 72.1%)	64.5% (56.4% to 74.4%)

Supplementary Results Table 1. Age-standardised PAFs attributable to the joint distribution of all risk factors for all ages, both sexes combined for each GBD level 1 and level 2 cause and all causes in 2016 for China. Results are shown for deaths, YLLs, YLDs, and DALYs
Data in parenthesis are 95% uncertainty intervals. PAFs=population attributable fractions. GBD=Global Burden of Disease. YLLs=years of life lost. YLDs=years live with disability. DALYs=disability-adjusted life-years

	Deaths 2016	YLLs 2016	YLDs 2016	DALYs 2016
Other pharynx cancer	57.1% (48.4% to 64.9%)	59.5% (51.3% to 66.9%)	61.3% (52.8% to 68.8%)	59.6% (51.3% to 67.0%)
Gallbladder and biliary tract cancer	9.8% (3.9% to 18.1%)	10.4% (4.3% to 19.0%)	10.2% (4.2% to 18.7%)	10.4% (4.3% to 19.0%)
Pancreatic cancer	19.2% (8.8% to 32.2%)	17.7% (8.4% to 30.2%)	18.7% (8.8% to 31.7%)	17.7% (8.4% to 30.2%)
Ovarian cancer	9.3% (3.7% to 17.3%)	8.0% (2.9% to 15.1%)	7.9% (3.0% to 14.9%)	8.0% (2.9% to 15.1%)
Kidney cancer	17.7% (8.2% to 30.3%)	15.2% (7.1% to 25.9%)	15.7% (7.5% to 26.4%)	15.2% (7.1% to 25.9%)
Bladder cancer	24.6% (9.9% to 42.4%)	21.7% (8.4% to 38.0%)	22.1% (8.7% to 38.4%)	21.8% (8.4% to 38.0%)
Thyroid cancer	6.7% (2.3% to 13.8%)	7.1% (2.5% to 14.3%)	7.0% (2.8% to 13.6%)	7.1% (2.5% to 14.2%)
Mesothelioma	80.4% (75.5% to 84.4%)	69.2% (63.0% to 74.6%)	76.7% (71.4% to 81.1%)	69.3% (63.1% to 74.8%)
Non-Hodgkin's lymphoma	3.1% (1.0% to 6.5%)	2.9% (0.9% to 6.0%)	3.2% (1.0% to 6.7%)	2.9% (0.9% to 6.0%)
Multiple myeloma	3.6% (1.2% to 7.4%)	3.7% (1.2% to 7.5%)	3.7% (1.3% to 7.6%)	3.7% (1.2% to 7.5%)
Leukaemia	8.0% (3.9% to 14.5%)	5.7% (3.0% to 9.9%)	6.4% (3.2% to 11.6%)	5.7% (3.0% to 10.0%)
**Cardiovascular diseases**	86.2% (83.7% to 88.6%)	87.5% (85.9% to 89.2%)	79.7% (77.0% to 82.3%)	86.6% (84.9% to 88.3%)
Rheumatic heart disease	25.4% (16.0% to 43.0%)	24.5% (16.4% to 37.9%)	20.5% (13.6% to 29.9%)	24.2% (16.4% to 37.6%)
Ischaemic heart disease	91.8% (88.0% to 94.9%)	93.3% (90.9% to 95.2%)	89.6% (86.9% to 92.2%)	93.2% (90.8% to 95.1%)
Cerebrovascular disease	84.9% (81.0% to 88.7%)	88.4% (85.7% to 90.8%)	86.1% (82.8% to 89.2%)	88.1% (85.5% to 90.5%)
Hypertensive heart disease	100.0% (100.0% to 100.0%)	100.0% (100.0% to 100.0%)	100.0% (100.0% to 100.0%)	100.0% (100.0% to 100.0%)
Cardiomyopathy and myocarditis	23.7% (18.9% to 30.4%)	21.3% (17.8% to 25.9%)	18.6% (15.1% to 22.6%)	21.1% (17.7% to 25.5%)
Atrial fibrillation and flutter	46.9% (39.8% to 53.2%)	51.5% (45.4% to 56.9%)	58.4% (53.9% to 62.8%)	55.5% (50.3% to 60.2%)
Aortic aneurysm	46.6% (40.2% to 53.0%)	52.6% (46.8% to 57.7%)	-	52.6% (46.8% to 57.7%)
Peripheral vascular disease	51.4% (45.4% to 57.9%)	58.4% (53.6% to 63.3%)	56.7% (51.5% to 61.6%)	57.1% (52.0% to 61.9%)
Endocarditis	39.9% (30.1% to 51.6%)	33.2% (25.1% to 41.0%)	39.9% (31.3% to 49.0%)	33.3% (25.2% to 41.2%)

Supplementary Results Table 1. Age-standardised PAFs attributable to the joint distribution of all risk factors for all ages, both sexes combined for each GBD level 1 and level 2 cause and all causes in 2016 for China. Results are shown for deaths, YLLs, YLDs, and DALYs
Data in parenthesis are 95% uncertainty intervals. PAFs=population attributable fractions. GBD=Global Burden of Disease. YLLs=years of life lost. YLDs=years live with disability. DALYs=disability-adjusted life-years

	Deaths 2016	YLLs 2016	YLDs 2016	DALYs 2016
Other cardiovascular and circulatory diseases	44.3% (39.2% to 49.5%)	41.5% (37.6% to 45.6%)	42.8% (38.2% to 47.4%)	42.0% (38.0% to 46.1%)
**Chronic respiratory diseases**	77.8% (63.5% to 87.8%)	75.7% (62.7% to 85.6%)	48.7% (39.8% to 58.1%)	68.7% (57.0% to 78.0%)
Chronic obstructive pulmonary disease	79.5% (64.7% to 89.9%)	77.9% (64.0% to 88.5%)	68.9% (54.2% to 81.4%)	76.4% (62.3% to 87.1%)
Pneumoconiosis	100.0% (100.0% to 100.0%)	100.0% (100.0% to 100.0%)	100.0% (100.0% to 100.0%)	100.0% (100.0% to 100.0%)
Asthma	29.7% (22.8% to 38.0%)	33.7% (26.7% to 41.7%)	23.6% (17.7% to 30.9%)	25.4% (19.6% to 32.6%)
Other chronic respiratory diseases	8.7% (2.1% to 19.7%)	4.9% (1.2% to 11.6%)	3.1% (0.6% to 7.5%)	3.9% (0.9% to 9.2%)
**Cirrhosis and other chronic liver diseases**	22.5% (21.2% to 23.9%)	24.5% (23.0% to 26.1%)	21.4% (20.0% to 22.8%)	24.3% (22.8% to 25.9%)
Cirrhosis and other chronic liver diseases due to hepatitis B	0.6% (0.5% to 0.8%)	0.7% (0.5% to 0.9%)	0.7% (0.5% to 0.8%)	0.7% (0.5% to 0.9%)
Cirrhosis and other chronic liver diseases due to hepatitis C	59.5% (54.8% to 64.3%)	70.9% (66.8% to 75.1%)	71.3% (67.2% to 75.2%)	70.9% (66.8% to 75.1%)
Cirrhosis and other chronic liver diseases due to alcohol use	100.0% (100.0% to 100.0%)	100.0% (100.0% to 100.0%)	100.0% (100.0% to 100.0%)	100.0% (100.0% to 100.0%)
**Digestive diseases**	11.7% (9.4% to 14.2%)	12.4% (10.3% to 14.9%)	2.9% (2.2% to 3.6%)	7.9% (6.2% to 9.6%)
Peptic ulcer disease	20.2% (14.8% to 25.9%)	22.7% (16.9% to 28.7%)	9.4% (6.8% to 12.2%)	17.3% (12.6% to 22.3%)
Gallbladder and biliary diseases	20.6% (10.8% to 33.6%)	21.5% (11.8% to 34.4%)	25.6% (14.5% to 39.7%)	22.4% (12.4% to 35.6%)
Pancreatitis	35.9% (27.2% to 45.0%)	39.3% (31.2% to 48.0%)	24.0% (18.9% to 29.5%)	38.1% (30.2% to 46.3%)
**Neurological disorders**	15.3% (7.5% to 25.4%)	14.8% (7.7% to 24.0%)	4.6% (2.8% to 7.0%)	9.1% (5.1% to 14.5%)
Alzheimer's disease and other dementias	18.1% (9.2% to 29.2%)	19.3% (10.1% to 30.4%)	19.2% (9.9% to 30.6%)	19.3% (10.0% to 30.5%)
Parkinson's disease	-19.9% (-24.0% to -15.4%)	-23.6% (-28.7% to -18.0%)	-24.6% (-29.9% to -18.5%)	-23.9% (-29.0% to -18.1%)
Epilepsy	19.9% (15.1% to 24.3%)	16.5% (12.3% to 20.2%)	14.0% (10.4% to 17.6%)	14.9% (11.3% to 18.4%)
Multiple sclerosis	11.8% (7.1% to 16.6%)	11.4% (6.8% to 16.1%)	8.1% (5.0% to 11.3%)	10.0% (6.1% to 13.8%)
**Mental and substance use disorders**	99.9% (99.9% to 99.9%)	99.8% (99.8% to 99.9%)	17.7% (15.9% to 19.7%)	22.7% (20.3% to 25.1%)
Alcohol use disorders	100.0% (100.0% to 100.0%)	100.0% (100.0% to 100.0%)	100.0% (100.0% to 100.0%)	100.0% (100.0% to 100.0%)

Supplementary Results Table 1. Age-standardised PAFs attributable to the joint distribution of all risk factors for all ages, both sexes combined for each GBD level 1 and level 2 cause and all causes in 2016 for China. Results are shown for deaths, YLLs, YLDs, and DALYs
Data in parenthesis are 95% uncertainty intervals. PAFs=population attributable fractions. GBD=Global Burden of Disease. YLLs=years of life lost. YLDs=years live with disability. DALYs=disability-adjusted life-years

	Deaths 2016	YLLs 2016	YLDs 2016	DALYs 2016
Drug use disorders	100.0% (100.0% to 100.0%)	100.0% (100.0% to 100.0%)	100.0% (100.0% to 100.0%)	100.0% (100.0% to 100.0%)
Depressive disorders	-	-	4.6% (3.8% to 5.7%)	4.6% (3.8% to 5.7%)
Idiopathic developmental intellectual disability	-	-	64.9% (33.2% to 84.0%)	64.9% (33.2% to 84.0%)
**Diabetes, urogenital, blood, and endocrine diseases**	86.3% (85.7% to 87.3%)	81.8% (80.6% to 83.2%)	52.4% (49.0% to 55.7%)	63.7% (60.1% to 67.1%)
Diabetes mellitus	100.0% (100.0% to 100.0%)	100.0% (100.0% to 100.0%)	100.0% (100.0% to 100.0%)	100.0% (100.0% to 100.0%)
Chronic kidney disease	100.0% (100.0% to 100.0%)	100.0% (100.0% to 100.0%)	100.0% (100.0% to 100.0%)	100.0% (100.0% to 100.0%)
**Musculoskeletal disorders**	3.2% (1.4% to 5.3%)	2.3% (1.0% to 3.9%)	13.2% (10.6% to 16.2%)	13.0% (10.5% to 15.9%)
Rheumatoid arthritis	6.2% (2.6% to 10.1%)	6.5% (2.8% to 10.7%)	4.2% (1.8% to 7.0%)	4.6% (1.9% to 7.6%)
Osteoarthritis	-	-	14.1% (5.7% to 25.1%)	14.1% (5.7% to 25.1%)
Low back and neck pain	-	-	17.1% (14.6% to 20.0%)	17.1% (14.6% to 20.0%)
Gout	-	-	27.6% (16.9% to 41.8%)	27.6% (16.9% to 41.8%)
**Other non-communicable diseases**	0.1% (0.1% to 0.1%)	0.1% (0.1% to 0.1%)	6.7% (6.0% to 7.5%)	5.3% (4.7% to 5.9%)
Sense organ diseases	-	-	14.2% (12.9% to 15.5%)	14.2% (12.9% to 15.5%)
Sudden infant death syndrome	7.2% (6.2% to 8.2%)	7.2% (6.2% to 8.3%)	-	7.2% (6.2% to 8.3%)
**Transport injuries**	34.2% (28.9% to 40.2%)	30.2% (25.5% to 35.6%)	38.4% (34.1% to 43.3%)	31.5% (26.8% to 36.9%)
Road injuries	34.1% (28.7% to 40.1%)	30.1% (25.4% to 35.6%)	40.7% (36.2% to 45.7%)	31.6% (26.8% to 37.1%)
Other transport injuries	37.3% (32.1% to 43.3%)	31.8% (26.8% to 37.5%)	29.7% (25.7% to 33.9%)	30.7% (26.3% to 35.6%)
**Unintentional injuries**	28.5% (20.7% to 31.7%)	15.6% (14.0% to 17.1%)	21.5% (20.3% to 22.9%)	17.2% (15.8% to 18.6%)
Falls	52.1% (33.5% to 58.9%)	30.3% (25.0% to 33.0%)	30.1% (29.0% to 31.4%)	30.2% (26.8% to 32.1%)
Drowning	9.6% (7.6% to 12.2%)	7.7% (6.4% to 9.4%)	15.0% (12.3% to 18.1%)	7.8% (6.5% to 9.5%)
Fire, heat, and hot substances	12.3% (8.8% to 16.7%)	13.8% (10.9% to 17.3%)	21.3% (18.0% to 25.0%)	17.2% (14.1% to 20.8%)

Supplementary Results Table 1. Age-standardised PAFs attributable to the joint distribution of all risk factors for all ages, both sexes combined for each GBD level 1 and level 2 cause and all causes in 2016 for China. Results are shown for deaths, YLLs, YLDs, and DALYs
Data in parenthesis are 95% uncertainty intervals. PAFs=population attributable fractions. GBD=Global Burden of Disease. YLLs=years of life lost. YLDs=years live with disability. DALYs=disability-adjusted life-years

	**Deaths 2016**	**YLLs 2016**	**YLDs 2016**	**DALYs 2016**
Poisonings	14.0% (10.9% to 18.0%)	13.5% (11.0% to 17.0%)	20.0% (16.9% to 23.7%)	13.8% (11.2% to 17.3%)
Exposure to mechanical forces	16.5% (15.4% to 18.8%)	12.8% (11.6% to 15.5%)	24.0% (22.7% to 25.7%)	15.7% (14.1% to 18.6%)
Animal contact	12.4% (11.4% to 14.7%)	11.3% (10.4% to 13.2%)	16.2% (14.9% to 17.9%)	13.5% (12.4% to 15.1%)
Foreign body	3.5% (3.2% to 4.3%)	3.0% (2.6% to 3.7%)	11.1% (10.3% to 12.3%)	5.5% (4.5% to 6.7%)
Other unintentional injuries	23.9% (19.1% to 29.4%)	23.8% (19.4% to 28.7%)	18.9% (15.2% to 23.5%)	22.0% (17.8% to 26.6%)
**Self-harm and interpersonal violence**	22.1% (15.5% to 28.6%)	21.1% (15.1% to 26.6%)	29.5% (25.9% to 33.1%)	22.0% (16.6% to 27.1%)
Self-harm	21.8% (14.3% to 29.2%)	21.3% (14.1% to 28.2%)	19.0% (12.7% to 25.2%)	21.2% (14.0% to 28.1%)
Interpersonal violence	24.0% (19.2% to 28.9%)	20.2% (16.1% to 24.6%)	30.4% (26.7% to 34.3%)	24.1% (19.9% to 28.2%)
**Forces of nature, conflict and terrorism, and executions and police conflict**	3.6% (1.1% to 4.8%)	1.3% (0.4% to 1.9%)	1.0% (0.6% to 1.5%)	1.2% (0.7% to 1.6%)
Forces of nature, conflict and terrorism, and state actor violence	6.8% (6.3% to 7.1%)	2.6% (2.4% to 2.8%)	5.4% (3.7% to 7.8%)	3.5% (2.7% to 5.0%)

Supplementary Results Table 1. Age-standardised PAFs attributable to the joint distribution of all risk factors for all ages, both sexes combined for each GBD level 1 and level 2 cause and all causes in 2016 for Colombia. Results are shown for deaths, YLLs, YLDs, and DALYs
Data in parenthesis are 95% uncertainty intervals. PAFs=population attributable fractions. GBD=Global Burden of Disease. YLLs=years of life lost. YLDs=years live with disability. DALYs=disability-adjusted life-years

	Deaths 2016	YLLs 2016	YLDs 2016	DALYs 2016
**All causes**	54.8% (52.8% to 56.7%)	47.4% (45.4% to 49.3%)	21.5% (20.1% to 22.8%)	36.7% (34.4% to 39.0%)
**HIV/AIDS and tuberculosis**	80.9% (77.1% to 84.2%)	83.8% (81.5% to 85.8%)	87.7% (82.6% to 91.9%)	84.0% (81.9% to 86.0%)
Tuberculosis	46.9% (35.4% to 57.2%)	47.5% (37.7% to 56.1%)	44.5% (34.3% to 53.9%)	47.3% (37.6% to 55.9%)
HIV/AIDS	95.6% (95.0% to 96.1%)	92.8% (92.0% to 93.5%)	97.8% (96.9% to 98.4%)	93.1% (92.2% to 93.8%)
**Diarrhoea, lower respiratory infections, and other common infectious diseases**	47.8% (41.1% to 55.6%)	54.8% (48.8% to 60.8%)	29.6% (19.8% to 39.4%)	47.3% (40.9% to 53.4%)
Diarrhoeal diseases	70.0% (49.0% to 86.9%)	81.6% (67.6% to 92.2%)	68.9% (46.9% to 86.3%)	74.0% (55.6% to 88.8%)
Lower respiratory infections	50.7% (42.5% to 59.6%)	62.1% (54.3% to 69.7%)	53.8% (45.4% to 62.6%)	62.1% (54.2% to 69.6%)
Upper respiratory infections	1.6% (0.1% to 5.4%)	3.5% (0.3% to 12.4%)	0.0% (0.0% to 0.0%)	0.0% (0.0% to 0.1%)
Otitis media	2.7% (1.6% to 4.5%)	5.9% (3.9% to 8.4%)	5.2% (3.4% to 6.8%)	5.2% (3.4% to 6.8%)
Meningitis	2.5% (1.8% to 3.3%)	4.2% (3.2% to 5.5%)	0.0% (0.0% to 0.0%)	3.7% (2.8% to 4.8%)
Encephalitis	1.2% (0.8% to 1.7%)	1.9% (1.4% to 2.5%)	0.0% (0.0% to 0.0%)	1.6% (1.1% to 2.2%)
Measles	22.8% (6.8% to 57.9%)	23.8% (7.1% to 60.1%)	24.4% (0.0% to 69.2%)	23.8% (7.1% to 60.1%)
**Maternal disorders**	6.2% (3.7% to 8.9%)	6.0% (3.6% to 8.6%)	3.8% (2.2% to 5.8%)	5.6% (3.4% to 7.9%)
Maternal haemorrhage	12.4% (4.5% to 20.5%)	12.4% (4.4% to 20.4%)	12.4% (4.4% to 20.5%)	12.4% (4.4% to 20.4%)
Maternal sepsis and other pregnancy related infections	12.3% (4.4% to 20.5%)	12.2% (4.4% to 20.3%)	12.2% (4.3% to 20.1%)	12.2% (4.4% to 20.3%)
Maternal abortion, miscarriage, and ectopic pregnancy	11.8% (7.4% to 16.9%)	11.6% (7.1% to 16.7%)	12.0% (6.5% to 18.3%)	11.6% (7.1% to 16.8%)
**Neonatal disorders**	74.2% (71.4% to 76.8%)	74.3% (71.4% to 76.8%)	0.1% (0.1% to 0.2%)	67.0% (63.3% to 70.4%)
Neonatal preterm birth complications	93.9% (91.7% to 96.0%)	94.0% (91.7% to 96.1%)	0.2% (0.2% to 0.3%)	83.8% (78.8% to 88.1%)
Neonatal encephalopathy due to birth asphyxia and trauma	61.6% (58.4% to 64.5%)	61.6% (58.4% to 64.6%)	0.0% (0.0% to 0.0%)	55.4% (49.5% to 61.1%)
Neonatal sepsis and other neonatal infections	55.0% (51.0% to 58.5%)	55.0% (51.0% to 58.6%)	0.0% (0.0% to 0.0%)	50.2% (43.2% to 55.5%)
Hemolytic disease and other neonatal jaundice	57.6% (52.2% to 62.1%)	57.6% (52.3% to 62.1%)	0.0% (0.0% to 0.0%)	22.2% (13.9% to 35.6%)

Supplementary Results Table 1. Age-standardised PAFs attributable to the joint distribution of all risk factors for all ages, both sexes combined for each GBD level 1 and level 2 cause and all causes in 2016 for Colombia. Results are shown for deaths, YLLs, YLDs, and DALYs
Data in parenthesis are 95% uncertainty intervals. PAFs=population attributable fractions. GBD=Global Burden of Disease. YLLs=years of life lost. YLDs=years live with disability. DALYs=disability-adjusted life-years

	Deaths 2016	YLLs 2016	YLDs 2016	DALYs 2016
Other neonatal disorders	58.7% (54.9% to 62.1%)	58.7% (54.9% to 62.2%)	0.0% (0.0% to 0.0%)	57.5% (53.5% to 60.9%)
**Nutritional deficiencies**	17.3% (12.6% to 22.6%)	54.3% (45.5% to 62.8%)	72.9% (67.0% to 78.1%)	62.7% (56.8% to 68.4%)
Protein-energy malnutrition	17.7% (12.8% to 23.2%)	55.9% (46.7% to 64.8%)	93.3% (89.4% to 96.2%)	56.7% (47.8% to 65.4%)
Vitamin A deficiency	-	-	100.0% (100.0% to 100.0%)	100.0% (100.0% to 100.0%)
Iron-deficiency anaemia	100.0% (100.0% to 100.0%)	100.0% (100.0% to 100.0%)	100.0% (100.0% to 100.0%)	100.0% (100.0% to 100.0%)
**Other communicable, maternal, neonatal, and nutritional diseases**	8.7% (6.8% to 10.4%)	6.7% (4.9% to 8.5%)	73.1% (66.5% to 78.9%)	33.7% (24.3% to 45.2%)
Sexually transmitted diseases excluding HIV	48.2% (29.2% to 68.5%)	27.7% (14.2% to 46.2%)	100.0% (100.0% to 100.0%)	75.7% (56.8% to 89.9%)
Hepatitis	2.5% (1.7% to 3.4%)	2.3% (1.6% to 3.2%)	1.3% (0.8% to 2.1%)	2.3% (1.6% to 3.1%)
**Neoplasms**	31.7% (28.6% to 34.8%)	29.3% (26.4% to 32.1%)	27.0% (24.2% to 29.8%)	29.2% (26.4% to 32.0%)
Oesophageal cancer	70.1% (58.9% to 79.8%)	70.9% (60.4% to 80.5%)	70.3% (59.8% to 79.9%)	70.9% (60.4% to 80.5%)
Stomach cancer	12.0% (4.9% to 21.5%)	10.7% (4.3% to 19.1%)	11.6% (4.7% to 20.7%)	10.7% (4.3% to 19.1%)
Liver cancer	53.3% (45.4% to 61.7%)	54.1% (46.2% to 62.5%)	53.5% (45.2% to 61.9%)	54.1% (46.3% to 62.5%)
Larynx cancer	73.9% (63.1% to 82.8%)	72.9% (62.3% to 81.6%)	73.9% (63.2% to 82.6%)	73.0% (62.3% to 81.7%)
Tracheal, bronchus, and lung cancer	74.7% (69.6% to 79.4%)	71.8% (66.1% to 77.0%)	73.8% (68.5% to 78.5%)	71.9% (66.1% to 77.0%)
Breast cancer	26.3% (19.9% to 33.6%)	24.5% (18.6% to 31.2%)	25.2% (19.1% to 32.0%)	24.5% (18.6% to 31.2%)
Cervical cancer	100.0% (100.0% to 100.0%)	100.0% (100.0% to 100.0%)	100.0% (100.0% to 100.0%)	100.0% (100.0% to 100.0%)
Uterine cancer	43.8% (29.5% to 58.6%)	44.9% (30.1% to 59.4%)	45.2% (30.5% to 59.7%)	44.9% (30.1% to 59.4%)
Prostate cancer	2.2% (1.4% to 3.2%)	2.0% (1.3% to 2.9%)	2.0% (1.3% to 2.9%)	2.0% (1.3% to 2.9%)
Colon and rectum cancer	52.5% (43.6% to 61.0%)	53.1% (44.2% to 61.1%)	52.5% (43.8% to 61.0%)	53.1% (44.2% to 61.1%)
Lip and oral cavity cancer	63.6% (52.7% to 73.2%)	64.7% (54.1% to 73.6%)	61.7% (50.7% to 71.3%)	64.6% (54.0% to 73.5%)
Nasopharynx cancer	68.7% (60.5% to 76.5%)	66.3% (59.3% to 73.0%)	66.1% (58.7% to 72.7%)	66.3% (59.3% to 73.0%)

Supplementary Results Table 1. Age-standardised PAFs attributable to the joint distribution of all risk factors for all ages, both sexes combined for each GBD level 1 and level 2 cause and all causes in 2016 for Colombia. Results are shown for deaths, YLLs, YLDs, and DALYs
Data in parenthesis are 95% uncertainty intervals. PAFs=population attributable fractions. GBD=Global Burden of Disease. YLLs=years of life lost. YLDs=years live with disability. DALYs=disability-adjusted life-years

	**Deaths 2016**	**YLLs 2016**	**YLDs 2016**	**DALYs 2016**
Other pharynx cancer	51.4% (36.3% to 64.0%)	54.7% (40.5% to 66.5%)	52.4% (37.5% to 64.7%)	54.6% (40.5% to 66.5%)
Gallbladder and biliary tract cancer	22.5% (12.4% to 34.5%)	23.3% (12.8% to 35.5%)	22.7% (12.4% to 34.8%)	23.3% (12.8% to 35.5%)
Pancreatic cancer	20.6% (13.9% to 28.9%)	19.2% (12.7% to 27.1%)	20.3% (13.7% to 28.5%)	19.2% (12.7% to 27.2%)
Ovarian cancer	9.9% (3.7% to 17.6%)	8.8% (3.0% to 15.8%)	8.6% (3.0% to 15.5%)	8.8% (3.0% to 15.8%)
Kidney cancer	24.4% (16.5% to 34.1%)	21.0% (13.9% to 29.6%)	22.4% (14.9% to 31.5%)	21.1% (13.9% to 29.7%)
Bladder cancer	20.6% (13.7% to 29.1%)	18.4% (11.9% to 26.3%)	18.9% (12.5% to 26.8%)	18.4% (11.9% to 26.3%)
Thyroid cancer	12.8% (6.8% to 20.9%)	13.4% (7.0% to 21.7%)	13.0% (6.9% to 20.7%)	13.3% (7.0% to 21.7%)
Mesothelioma	85.4% (81.5% to 88.7%)	76.6% (71.2% to 81.3%)	82.8% (78.6% to 86.4%)	76.7% (71.4% to 81.4%)
Non-Hodgkin's lymphoma	6.1% (2.4% to 10.7%)	5.5% (2.2% to 9.6%)	6.0% (2.4% to 10.6%)	5.5% (2.2% to 9.6%)
Multiple myeloma	7.7% (3.2% to 13.7%)	7.9% (3.4% to 14.2%)	7.8% (3.2% to 14.0%)	7.9% (3.4% to 14.2%)
Leukaemia	10.9% (6.9% to 15.6%)	7.7% (4.8% to 11.2%)	10.2% (6.4% to 14.6%)	7.7% (4.8% to 11.2%)
**Cardiovascular diseases**	85.3% (82.3% to 87.7%)	84.5% (82.4% to 86.3%)	74.2% (71.4% to 76.5%)	83.4% (81.4% to 85.2%)
Rheumatic heart disease	27.6% (18.3% to 43.5%)	23.0% (16.0% to 34.3%)	26.2% (17.2% to 38.9%)	23.1% (16.0% to 34.4%)
Ischaemic heart disease	91.7% (87.6% to 95.0%)	93.0% (90.2% to 95.2%)	90.5% (87.7% to 93.1%)	92.8% (90.1% to 95.0%)
Cerebrovascular disease	82.1% (77.1% to 87.1%)	83.1% (79.4% to 86.2%)	83.3% (79.1% to 87.2%)	83.1% (79.5% to 86.3%)
Hypertensive heart disease	100.0% (100.0% to 100.0%)	100.0% (100.0% to 100.0%)	100.0% (100.0% to 100.0%)	100.0% (100.0% to 100.0%)
Cardiomyopathy and myocarditis	38.2% (29.8% to 49.1%)	30.2% (23.5% to 37.5%)	30.1% (23.7% to 36.9%)	30.2% (23.7% to 37.3%)
Atrial fibrillation and flutter	52.8% (44.7% to 60.7%)	57.2% (49.9% to 64.0%)	63.2% (57.8% to 68.5%)	60.9% (54.7% to 66.6%)
Aortic aneurysm	45.2% (37.1% to 52.6%)	48.6% (41.6% to 54.7%)	-	48.6% (41.6% to 54.7%)
Peripheral vascular disease	52.0% (44.6% to 59.8%)	57.4% (51.2% to 63.5%)	60.5% (54.9% to 65.9%)	59.1% (53.1% to 64.8%)
Endocarditis	39.9% (30.9% to 49.9%)	32.4% (23.4% to 41.8%)	38.9% (30.4% to 47.2%)	32.4% (23.6% to 41.9%)

Supplementary Results Table 1. Age-standardised PAFs attributable to the joint distribution of all risk factors for all ages, both sexes combined for each GBD level 1 and level 2 cause and all causes in 2016 for Colombia. Results are shown for deaths, YLLs, YLDs, and DALYs
Data in parenthesis are 95% uncertainty intervals. PAFs=population attributable fractions. GBD=Global Burden of Disease. YLLs=years of life lost. YLDs=years live with disability. DALYs=disability-adjusted life-years

	Deaths 2016	YLLs 2016	YLDs 2016	DALYs 2016
Other cardiovascular and circulatory diseases	46.4% (41.0% to 51.8%)	46.4% (41.8% to 50.6%)	47.3% (42.3% to 52.1%)	46.6% (42.2% to 50.8%)
**Chronic respiratory diseases**	66.3% (60.1% to 72.1%)	60.8% (54.6% to 66.5%)	33.7% (27.3% to 40.7%)	50.0% (44.2% to 55.5%)
Chronic obstructive pulmonary disease	70.1% (62.9% to 76.1%)	67.5% (60.6% to 74.0%)	58.0% (50.3% to 65.3%)	66.1% (59.0% to 72.7%)
Pneumoconiosis	100.0% (100.0% to 100.0%)	100.0% (100.0% to 100.0%)	100.0% (100.0% to 100.0%)	100.0% (100.0% to 100.0%)
Asthma	34.5% (24.8% to 45.4%)	32.8% (24.6% to 42.5%)	26.3% (18.6% to 35.5%)	26.8% (19.0% to 35.7%)
Other chronic respiratory diseases	6.2% (3.4% to 8.9%)	2.7% (1.1% to 4.2%)	1.7% (0.7% to 3.4%)	2.5% (1.1% to 4.0%)
**Cirrhosis and other chronic liver diseases**	58.1% (53.2% to 63.1%)	60.7% (56.0% to 65.2%)	51.8% (47.4% to 56.0%)	60.0% (55.4% to 64.4%)
Cirrhosis and other chronic liver diseases due to hepatitis B	0.4% (0.2% to 0.7%)	0.5% (0.2% to 0.9%)	0.4% (0.2% to 0.8%)	0.5% (0.2% to 0.9%)
Cirrhosis and other chronic liver diseases due to hepatitis C	50.5% (38.4% to 63.9%)	64.4% (53.2% to 75.0%)	61.4% (51.5% to 70.8%)	64.2% (53.2% to 74.6%)
Cirrhosis and other chronic liver diseases due to alcohol use	100.0% (100.0% to 100.0%)	100.0% (100.0% to 100.0%)	100.0% (100.0% to 100.0%)	100.0% (100.0% to 100.0%)
**Digestive diseases**	10.2% (7.5% to 13.1%)	10.7% (8.0% to 13.8%)	3.6% (2.6% to 4.7%)	8.6% (6.5% to 11.0%)
Peptic ulcer disease	7.6% (5.0% to 10.7%)	9.5% (6.4% to 13.1%)	6.3% (4.1% to 9.1%)	8.1% (5.5% to 11.2%)
Gallbladder and biliary diseases	39.3% (26.0% to 53.8%)	40.1% (26.9% to 54.5%)	45.5% (31.4% to 60.5%)	40.6% (27.5% to 54.8%)
Pancreatitis	30.5% (15.0% to 47.9%)	36.1% (21.1% to 52.9%)	29.5% (17.0% to 43.3%)	36.0% (21.0% to 52.6%)
**Neurological disorders**	20.2% (10.0% to 32.7%)	19.6% (11.0% to 29.7%)	5.1% (2.6% to 8.1%)	9.7% (5.7% to 14.5%)
Alzheimer's disease and other dementias	22.3% (10.8% to 35.9%)	23.1% (11.7% to 36.9%)	23.1% (11.5% to 37.1%)	23.1% (11.6% to 36.9%)
Parkinson's disease	-5.3% (-7.0% to -3.8%)	-6.6% (-8.5% to -5.0%)	-7.2% (-9.0% to -5.6%)	-6.7% (-8.5% to -5.1%)
Epilepsy	26.5% (18.1% to 34.6%)	24.1% (16.0% to 31.1%)	21.0% (13.9% to 28.0%)	22.0% (14.4% to 28.9%)
Multiple sclerosis	5.6% (3.5% to 8.2%)	5.6% (3.5% to 8.1%)	4.3% (2.7% to 6.3%)	5.2% (3.3% to 7.6%)
**Mental and substance use disorders**	99.4% (99.3% to 99.5%)	99.0% (98.8% to 99.2%)	22.8% (20.6% to 25.4%)	24.0% (21.7% to 26.6%)
Alcohol use disorders	100.0% (100.0% to 100.0%)	100.0% (100.0% to 100.0%)	100.0% (100.0% to 100.0%)	100.0% (100.0% to 100.0%)

Supplementary Results Table 1. Age-standardised PAFs attributable to the joint distribution of all risk factors for all ages, both sexes combined for each GBD level 1 and level 2 cause and all causes in 2016 for Colombia. Results are shown for deaths, YLLs, YLDs, and DALYs
Data in parenthesis are 95% uncertainty intervals. PAFs=population attributable fractions. GBD=Global Burden of Disease. YLLs=years of life lost. YLDs=years live with disability. DALYs=disability-adjusted life-years

	Deaths 2016	YLLs 2016	YLDs 2016	DALYs 2016
Drug use disorders	100.0% (100.0% to 100.0%)	100.0% (100.0% to 100.0%)	100.0% (100.0% to 100.0%)	100.0% (100.0% to 100.0%)
Depressive disorders	-	-	9.5% (7.8% to 11.4%)	9.5% (7.8% to 11.4%)
Idiopathic developmental intellectual disability	-	-	67.4% (37.2% to 87.0%)	67.4% (37.2% to 87.0%)
**Diabetes, urogenital, blood, and endocrine diseases**	81.7% (80.1% to 82.9%)	79.8% (77.9% to 81.2%)	72.8% (68.6% to 76.6%)	76.6% (74.3% to 78.5%)
Diabetes mellitus	100.0% (100.0% to 100.0%)	100.0% (100.0% to 100.0%)	100.0% (100.0% to 100.0%)	100.0% (100.0% to 100.0%)
Chronic kidney disease	100.0% (100.0% to 100.0%)	100.0% (100.0% to 100.0%)	100.0% (100.0% to 100.0%)	100.0% (100.0% to 100.0%)
**Musculoskeletal disorders**	0.7% (0.2% to 1.2%)	0.5% (0.2% to 0.8%)	14.0% (11.1% to 17.2%)	13.5% (10.8% to 16.6%)
Rheumatoid arthritis	2.7% (1.0% to 4.6%)	3.0% (1.2% to 5.3%)	3.1% (1.3% to 5.4%)	3.1% (1.2% to 5.3%)
Osteoarthritis	-	-	25.8% (13.9% to 38.6%)	25.8% (13.9% to 38.6%)
Low back and neck pain	-	-	16.8% (14.3% to 19.7%)	16.8% (14.3% to 19.7%)
Gout	-	-	48.2% (34.6% to 62.6%)	48.2% (34.6% to 62.6%)
**Other non-communicable diseases**	0.1% (0.0% to 0.2%)	0.1% (0.1% to 0.2%)	5.7% (4.8% to 6.8%)	4.4% (3.7% to 5.3%)
Sense organ diseases	-	-	12.0% (10.3% to 13.8%)	12.0% (10.3% to 13.8%)
Sudden infant death syndrome	8.0% (5.0% to 12.3%)	8.1% (5.0% to 12.3%)	-	8.1% (5.0% to 12.3%)
**Transport injuries**	33.5% (26.0% to 42.2%)	29.5% (22.0% to 38.1%)	38.8% (31.6% to 47.3%)	30.7% (23.3% to 39.4%)
Road injuries	33.4% (25.9% to 42.0%)	29.4% (21.9% to 38.0%)	39.0% (31.8% to 47.5%)	30.7% (23.3% to 39.3%)
Other transport injuries	35.7% (27.6% to 44.9%)	31.3% (23.3% to 40.5%)	32.2% (25.2% to 40.3%)	31.4% (23.5% to 40.4%)
**Unintentional injuries**	24.2% (21.3% to 28.6%)	13.4% (11.0% to 15.9%)	20.3% (18.6% to 22.2%)	16.1% (14.0% to 18.3%)
Falls	49.6% (45.8% to 56.7%)	28.4% (25.2% to 32.9%)	24.6% (23.2% to 26.2%)	25.9% (24.1% to 27.9%)
Drowning	12.5% (8.4% to 17.8%)	10.5% (7.2% to 14.8%)	14.5% (9.6% to 20.9%)	10.6% (7.2% to 14.9%)
Fire, heat, and hot substances	10.8% (6.8% to 16.1%)	10.5% (7.0% to 15.0%)	16.2% (11.4% to 22.2%)	13.3% (9.4% to 18.5%)

Supplementary Results Table 1. Age-standardised PAFs attributable to the joint distribution of all risk factors for all ages, both sexes combined for each GBD level 1 and level 2 cause and all causes in 2016 for Colombia. Results are shown for deaths, YLLs, YLDs, and DALYs
Data in parenthesis are 95% uncertainty intervals. PAFs=population attributable fractions. GBD=Global Burden of Disease. YLLs=years of life lost. YLDs=years live with disability. DALYs=disability-adjusted life-years

	**Deaths 2016**	**YLLs 2016**	**YLDs 2016**	**DALYs 2016**
Poisonings	15.0% (10.3% to 20.7%)	14.3% (10.0% to 19.7%)	15.6% (10.9% to 21.5%)	14.6% (10.3% to 20.0%)
Exposure to mechanical forces	14.7% (12.5% to 17.3%)	12.3% (10.1% to 14.9%)	17.2% (15.2% to 19.7%)	14.3% (12.1% to 17.0%)
Animal contact	8.8% (6.7% to 10.4%)	7.5% (5.1% to 9.3%)	8.6% (7.0% to 10.5%)	7.9% (5.9% to 9.8%)
Foreign body	3.4% (2.3% to 5.6%)	3.0% (1.9% to 5.1%)	5.8% (4.7% to 7.2%)	3.6% (2.5% to 5.3%)
Other unintentional injuries	20.1% (14.2% to 28.0%)	19.7% (13.9% to 27.0%)	16.7% (11.5% to 23.3%)	18.5% (13.2% to 25.6%)
**Self-harm and interpersonal violence**	24.0% (16.5% to 32.0%)	23.2% (15.3% to 31.2%)	21.9% (16.0% to 28.5%)	23.2% (15.4% to 31.1%)
Self-harm	31.4% (17.6% to 43.7%)	31.3% (17.7% to 43.4%)	25.7% (14.0% to 37.2%)	31.2% (17.7% to 43.3%)
Interpersonal violence	22.6% (14.1% to 31.7%)	21.9% (13.3% to 30.9%)	21.7% (15.5% to 28.5%)	21.9% (13.5% to 30.7%)
**Forces of nature, conflict and terrorism, and executions and police conflict**	0.0% (0.0% to 0.0%)	0.0% (0.0% to 0.0%)	3.4% (2.3% to 4.7%)	1.2% (0.4% to 2.9%)
Forces of nature, conflict and terrorism, and state actor violence	0.0% (0.0% to 0.0%)	0.0% (0.0% to 0.0%)	5.9% (4.1% to 8.4%)	5.9% (4.1% to 8.4%)

Supplementary Results Table 1. Age-standardised PAFs attributable to the joint distribution of all risk factors for all ages, both sexes combined for each GBD level 1 and level 2 cause and all causes in 2016 for Comoros. Results are shown for deaths, YLLs, YLDs, and DALYs
Data in parenthesis are 95% uncertainty intervals. PAFs=population attributable fractions. GBD=Global Burden of Disease. YLLs=years of life lost. YLDs=years live with disability. DALYs=disability-adjusted life-years

	Deaths 2016	YLLs 2016	YLDs 2016	DALYs 2016
**All causes**	54.7% (51.8% to 58.0%)	53.0% (49.5% to 56.4%)	26.1% (24.8% to 27.5%)	45.9% (43.0% to 49.2%)
**HIV/AIDS and tuberculosis**	17.0% (9.4% to 29.6%)	17.8% (9.1% to 34.7%)	22.0% (11.5% to 43.1%)	18.0% (9.4% to 35.0%)
Tuberculosis	13.2% (7.6% to 19.4%)	12.0% (6.8% to 17.6%)	11.0% (6.0% to 16.4%)	12.0% (6.8% to 17.5%)
HIV/AIDS	93.3% (88.7% to 96.8%)	91.2% (84.7% to 96.0%)	93.1% (89.3% to 95.8%)	91.4% (85.5% to 95.9%)
**Diarrhoea, lower respiratory infections, and other common infectious diseases**	73.8% (67.0% to 80.7%)	71.2% (63.2% to 78.3%)	47.8% (42.2% to 52.9%)	69.8% (62.4% to 76.8%)
Diarrhoeal diseases	93.7% (84.1% to 98.3%)	94.6% (86.3% to 98.6%)	94.4% (86.0% to 98.5%)	94.5% (86.2% to 98.6%)
Lower respiratory infections	72.2% (63.7% to 79.3%)	79.5% (72.4% to 85.3%)	70.9% (62.3% to 78.1%)	79.5% (72.3% to 85.3%)
Upper respiratory infections	2.6% (0.2% to 10.1%)	5.2% (0.3% to 19.2%)	0.0% (0.0% to 0.0%)	0.3% (0.0% to 1.2%)
Otitis media	2.4% (0.9% to 4.7%)	6.4% (3.3% to 9.9%)	4.3% (2.8% to 5.7%)	4.4% (2.8% to 5.8%)
Meningitis	2.8% (1.5% to 4.7%)	5.9% (3.3% to 9.8%)	0.0% (0.0% to 0.0%)	5.5% (3.1% to 9.1%)
Encephalitis	4.3% (2.5% to 7.6%)	5.7% (3.1% to 10.4%)	0.0% (0.0% to 0.0%)	3.2% (2.0% to 5.3%)
Measles	64.3% (49.3% to 83.4%)	65.4% (50.3% to 84.5%)	64.3% (48.7% to 82.2%)	65.4% (50.3% to 84.5%)
**Maternal disorders**	6.5% (2.6% to 11.3%)	6.4% (2.5% to 11.3%)	0.4% (0.2% to 0.8%)	4.9% (1.9% to 8.6%)
Maternal haemorrhage	17.4% (6.3% to 29.1%)	17.4% (6.3% to 29.1%)	17.6% (6.4% to 29.4%)	17.4% (6.3% to 29.0%)
Maternal sepsis and other pregnancy related infections	17.3% (6.3% to 29.1%)	17.3% (6.3% to 29.1%)	16.6% (6.1% to 27.8%)	17.3% (6.3% to 29.0%)
Maternal abortion, miscarriage, and ectopic pregnancy	4.4% (2.2% to 7.5%)	4.4% (2.2% to 7.4%)	4.1% (2.2% to 6.2%)	4.3% (2.2% to 7.3%)
**Neonatal disorders**	81.2% (76.9% to 85.1%)	81.3% (77.0% to 85.1%)	0.3% (0.1% to 0.6%)	77.5% (72.8% to 82.0%)
Neonatal preterm birth complications	96.2% (92.2% to 98.5%)	96.2% (92.2% to 98.6%)	0.8% (0.6% to 1.1%)	90.5% (85.1% to 94.6%)
Neonatal encephalopathy due to birth asphyxia and trauma	78.9% (74.5% to 82.8%)	78.9% (74.5% to 82.8%)	0.0% (0.0% to 0.0%)	77.4% (72.2% to 81.6%)
Neonatal sepsis and other neonatal infections	68.5% (60.9% to 74.9%)	68.5% (61.0% to 74.9%)	0.0% (0.0% to 0.0%)	62.6% (51.2% to 71.0%)
Hemolytic disease and other neonatal jaundice	76.0% (68.8% to 81.2%)	76.1% (68.9% to 81.2%)	0.0% (0.0% to 0.0%)	69.9% (61.8% to 76.2%)

Supplementary Results Table 1. Age-standardised PAFs attributable to the joint distribution of all risk factors for all ages, both sexes combined for each GBD level 1 and level 2 cause and all causes in 2016 for Comoros. Results are shown for deaths, YLLs, YLDs, and DALYs
Data in parenthesis are 95% uncertainty intervals. PAFs=population attributable fractions. GBD=Global Burden of Disease. YLLs=years of life lost. YLDs=years live with disability. DALYs=disability-adjusted life-years

	Deaths 2016	YLLs 2016	YLDs 2016	DALYs 2016
Other neonatal disorders	73.3% (65.4% to 78.9%)	73.3% (65.5% to 78.9%)	0.0% (0.0% to 0.0%)	71.9% (63.9% to 77.6%)
**Nutritional deficiencies**	19.4% (12.2% to 28.3%)	51.4% (38.3% to 64.1%)	90.1% (87.9% to 92.0%)	65.4% (57.5% to 73.1%)
Protein-energy malnutrition	23.4% (14.3% to 35.0%)	57.4% (43.0% to 70.9%)	97.3% (96.1% to 98.1%)	59.1% (46.2% to 71.7%)
Vitamin A deficiency	-	-	100.0% (100.0% to 100.0%)	100.0% (100.0% to 100.0%)
Iron-deficiency anaemia	100.0% (100.0% to 100.0%)	100.0% (100.0% to 100.0%)	100.0% (100.0% to 100.0%)	100.0% (100.0% to 100.0%)
**Other communicable, maternal, neonatal, and nutritional diseases**	4.7% (2.5% to 8.2%)	2.7% (1.2% to 5.2%)	77.7% (70.8% to 83.9%)	11.8% (5.8% to 20.6%)
Sexually transmitted diseases excluding HIV	9.8% (4.2% to 19.5%)	4.3% (1.7% to 9.0%)	100.0% (100.0% to 100.0%)	18.2% (8.1% to 34.7%)
Hepatitis	0.3% (0.2% to 0.5%)	0.5% (0.3% to 0.7%)	0.5% (0.2% to 0.8%)	0.5% (0.3% to 0.7%)
**Neoplasms**	25.9% (21.9% to 30.3%)	24.9% (20.8% to 29.4%)	27.1% (22.3% to 32.5%)	24.9% (20.9% to 29.5%)
Oesophageal cancer	53.1% (38.5% to 66.5%)	52.8% (37.8% to 65.8%)	53.1% (38.0% to 66.3%)	52.8% (37.8% to 65.8%)
Stomach cancer	4.0% (0.6% to 12.1%)	3.8% (0.5% to 11.2%)	3.9% (0.6% to 11.9%)	3.8% (0.5% to 11.2%)
Liver cancer	18.7% (13.8% to 25.6%)	19.3% (14.1% to 26.3%)	19.0% (14.2% to 25.8%)	19.3% (14.1% to 26.3%)
Larynx cancer	28.2% (14.9% to 42.4%)	28.2% (14.4% to 43.2%)	28.5% (14.8% to 43.2%)	28.2% (14.4% to 43.2%)
Tracheal, bronchus, and lung cancer	69.4% (59.3% to 77.7%)	68.5% (58.1% to 77.1%)	69.1% (59.1% to 77.4%)	68.5% (58.2% to 77.1%)
Breast cancer	12.2% (7.1% to 18.7%)	10.7% (6.0% to 16.3%)	11.4% (6.3% to 17.4%)	10.7% (6.0% to 16.3%)
Cervical cancer	100.0% (100.0% to 100.0%)	100.0% (100.0% to 100.0%)	100.0% (100.0% to 100.0%)	100.0% (100.0% to 100.0%)
Uterine cancer	18.2% (7.9% to 30.3%)	19.7% (8.8% to 32.0%)	19.0% (8.2% to 30.9%)	19.7% (8.8% to 32.0%)
Prostate cancer	0.5% (0.1% to 1.0%)	0.6% (0.1% to 1.0%)	0.6% (0.2% to 1.0%)	0.6% (0.1% to 1.0%)
Colon and rectum cancer	43.5% (34.2% to 53.1%)	42.6% (33.7% to 51.8%)	43.3% (33.9% to 52.6%)	42.6% (33.7% to 51.8%)
Lip and oral cavity cancer	52.8% (41.7% to 62.7%)	50.3% (40.0% to 60.0%)	52.7% (41.8% to 62.2%)	50.4% (40.1% to 60.0%)
Nasopharynx cancer	18.7% (8.5% to 29.1%)	18.3% (8.9% to 28.4%)	18.8% (8.9% to 29.0%)	18.3% (8.9% to 28.5%)

Supplementary Results Table 1. Age-standardised PAFs attributable to the joint distribution of all risk factors for all ages, both sexes combined for each GBD level 1 and level 2 cause and all causes in 2016 for Comoros. Results are shown for deaths, YLLs, YLDs, and DALYs

Data in parenthesis are 95% uncertainty intervals. PAFs=population attributable fractions. GBD=Global Burden of Disease. YLLs=years of life lost. YLDs=years live with disability. DALYs=disability-adjusted life-years

	Deaths 2016	YLLs 2016	YLDs 2016	DALYs 2016
Other pharynx cancer	12.7% (4.3% to 22.2%)	12.9% (4.4% to 22.2%)	12.8% (4.4% to 22.3%)	12.9% (4.4% to 22.2%)
Gallbladder and biliary tract cancer	7.2% (2.5% to 14.4%)	7.8% (2.8% to 15.3%)	7.4% (2.6% to 14.6%)	7.8% (2.8% to 15.3%)
Pancreatic cancer	9.3% (4.0% to 16.8%)	8.9% (3.9% to 15.8%)	9.2% (4.0% to 16.4%)	8.9% (3.9% to 15.8%)
Ovarian cancer	7.1% (2.6% to 13.8%)	6.1% (2.3% to 11.8%)	6.2% (2.3% to 12.0%)	6.1% (2.3% to 11.8%)
Kidney cancer	7.6% (3.4% to 13.7%)	6.9% (3.0% to 12.1%)	7.4% (3.3% to 13.1%)	6.9% (3.0% to 12.1%)
Bladder cancer	8.6% (3.2% to 16.0%)	8.2% (3.2% to 15.2%)	8.5% (3.3% to 15.7%)	8.2% (3.2% to 15.2%)
Thyroid cancer	4.5% (1.6% to 9.3%)	4.8% (1.8% to 9.7%)	4.8% (1.8% to 9.6%)	4.8% (1.8% to 9.7%)
Mesothelioma	84.6% (77.4% to 89.5%)	75.2% (65.6% to 82.0%)	82.1% (74.6% to 87.3%)	75.2% (65.7% to 82.1%)
Non-Hodgkin's lymphoma	1.7% (0.4% to 3.9%)	1.5% (0.4% to 3.3%)	1.7% (0.4% to 3.8%)	1.5% (0.4% to 3.3%)
Multiple myeloma	2.5% (0.7% to 5.6%)	2.7% (0.8% to 5.8%)	2.6% (0.7% to 5.7%)	2.7% (0.8% to 5.8%)
Leukaemia	4.4% (2.2% to 7.3%)	4.3% (2.2% to 7.1%)	4.4% (2.2% to 7.4%)	4.3% (2.2% to 7.1%)
**Cardiovascular diseases**	83.9% (81.1% to 86.2%)	84.5% (82.2% to 86.3%)	62.6% (58.2% to 66.8%)	82.7% (80.5% to 84.6%)
Rheumatic heart disease	31.7% (21.7% to 48.8%)	30.5% (21.0% to 44.3%)	25.2% (16.3% to 36.1%)	27.9% (19.3% to 39.7%)
Ischaemic heart disease	93.9% (90.9% to 96.4%)	94.8% (92.8% to 96.5%)	91.3% (88.8% to 93.6%)	94.7% (92.7% to 96.4%)
Cerebrovascular disease	87.5% (83.5% to 91.0%)	89.4% (86.6% to 91.7%)	88.1% (84.7% to 91.0%)	89.4% (86.6% to 91.7%)
Hypertensive heart disease	100.0% (100.0% to 100.0%)	100.0% (100.0% to 100.0%)	100.0% (100.0% to 100.0%)	100.0% (100.0% to 100.0%)
Cardiomyopathy and myocarditis	41.9% (34.3% to 50.8%)	39.4% (32.1% to 47.5%)	43.1% (36.0% to 50.6%)	39.7% (32.5% to 47.6%)
Atrial fibrillation and flutter	44.7% (36.4% to 52.5%)	48.8% (41.7% to 55.1%)	55.0% (50.2% to 59.9%)	51.9% (45.7% to 57.1%)
Aortic aneurysm	47.0% (39.6% to 53.7%)	50.8% (44.5% to 56.7%)	-	50.8% (44.5% to 56.7%)
Peripheral vascular disease	53.8% (46.8% to 61.2%)	60.0% (54.2% to 65.7%)	61.2% (55.6% to 66.6%)	60.8% (55.4% to 66.0%)
Endocarditis	41.0% (32.3% to 51.1%)	38.9% (31.2% to 47.2%)	34.4% (26.0% to 43.9%)	38.9% (31.2% to 47.1%)

Supplementary Results Table 1. Age-standardised PAFs attributable to the joint distribution of all risk factors for all ages, both sexes combined for each GBD level 1 and level 2 cause and all causes in 2016 for Comoros. Results are shown for deaths, YLLs, YLDs, and DALYs
Data in parenthesis are 95% uncertainty intervals. PAFs=population attributable fractions. GBD=Global Burden of Disease. YLLs=years of life lost. YLDs=years live with disability. DALYs=disability-adjusted life-years

	**Deaths 2016**	**YLLs 2016**	**YLDs 2016**	**DALYs 2016**
Other cardiovascular and circulatory diseases	48.1% (42.7% to 53.5%)	50.2% (45.7% to 54.5%)	38.5% (31.5% to 45.6%)	48.4% (43.8% to 53.2%)
**Chronic respiratory diseases**	51.9% (42.7% to 61.0%)	48.8% (40.6% to 57.0%)	31.0% (24.6% to 37.9%)	41.9% (34.7% to 49.4%)
Chronic obstructive pulmonary disease	63.8% (52.1% to 76.0%)	63.1% (51.9% to 75.2%)	52.2% (39.4% to 64.3%)	60.5% (48.8% to 72.1%)
Pneumoconiosis	100.0% (100.0% to 100.0%)	100.0% (100.0% to 100.0%)	100.0% (100.0% to 100.0%)	100.0% (100.0% to 100.0%)
Asthma	22.5% (15.9% to 30.9%)	25.2% (18.7% to 32.9%)	21.1% (15.1% to 28.6%)	22.9% (17.1% to 30.3%)
Other chronic respiratory diseases	2.0% (0.5% to 4.1%)	2.0% (0.3% to 5.4%)	1.0% (0.1% to 3.0%)	1.7% (0.2% to 4.5%)
**Cirrhosis and other chronic liver diseases**	28.1% (24.7% to 32.0%)	28.2% (24.7% to 32.2%)	23.5% (20.1% to 27.1%)	28.0% (24.5% to 32.1%)
Cirrhosis and other chronic liver diseases due to hepatitis B	0.1% (0.0% to 0.2%)	0.1% (0.0% to 0.2%)	0.1% (0.1% to 0.2%)	0.1% (0.1% to 0.2%)
Cirrhosis and other chronic liver diseases due to hepatitis C	8.1% (4.1% to 13.6%)	14.0% (7.4% to 23.1%)	17.5% (9.9% to 28.2%)	14.2% (7.5% to 23.2%)
Cirrhosis and other chronic liver diseases due to alcohol use	100.0% (100.0% to 100.0%)	100.0% (100.0% to 100.0%)	100.0% (100.0% to 100.0%)	100.0% (100.0% to 100.0%)
**Digestive diseases**	2.7% (1.7% to 4.0%)	2.4% (1.5% to 3.4%)	1.6% (1.1% to 2.3%)	2.3% (1.5% to 3.3%)
Peptic ulcer disease	8.0% (4.9% to 12.0%)	8.4% (5.3% to 12.3%)	8.0% (5.4% to 11.1%)	8.4% (5.4% to 12.2%)
Gallbladder and biliary diseases	16.3% (7.0% to 28.3%)	17.5% (7.9% to 29.7%)	19.1% (9.0% to 31.1%)	17.5% (8.0% to 29.7%)
Pancreatitis	0.7% (-2.4% to 4.6%)	0.7% (-2.3% to 4.5%)	0.8% (-2.7% to 5.0%)	0.7% (-2.3% to 4.5%)
**Neurological disorders**	10.8% (4.6% to 19.5%)	8.8% (3.8% to 15.7%)	1.5% (0.7% to 2.5%)	4.4% (1.9% to 8.0%)
Alzheimer's disease and other dementias	13.0% (5.8% to 22.8%)	13.5% (6.2% to 23.5%)	13.5% (6.1% to 23.2%)	13.5% (6.2% to 23.5%)
Parkinson's disease	-10.2% (-15.9% to -6.1%)	-10.7% (-16.4% to -6.7%)	-9.7% (-13.9% to -6.4%)	-10.5% (-16.1% to -6.7%)
Epilepsy	1.9% (0.5% to 4.4%)	1.4% (0.4% to 3.2%)	1.6% (0.5% to 3.3%)	1.5% (0.4% to 3.3%)
Multiple sclerosis	4.4% (2.0% to 7.3%)	4.4% (2.0% to 7.2%)	3.5% (2.1% to 5.2%)	4.1% (2.1% to 6.5%)
**Mental and substance use disorders**	99.8% (99.6% to 99.9%)	99.6% (99.2% to 99.8%)	17.5% (15.1% to 19.9%)	20.2% (17.5% to 23.0%)
Alcohol use disorders	100.0% (100.0% to 100.0%)	100.0% (100.0% to 100.0%)	100.0% (100.0% to 100.0%)	100.0% (100.0% to 100.0%)

Supplementary Results Table 1. Age-standardised PAFs attributable to the joint distribution of all risk factors for all ages, both sexes combined for each GBD level 1 and level 2 cause and all causes in 2016 for Comoros. Results are shown for deaths, YLLs, YLDs, and DALYs
Data in parenthesis are 95% uncertainty intervals. PAFs=population attributable fractions. GBD=Global Burden of Disease. YLLs=years of life lost. YLDs=years live with disability. DALYs=disability-adjusted life-years

	Deaths 2016	YLLs 2016	YLDs 2016	DALYs 2016
Drug use disorders	100.0% (100.0% to 100.0%)	100.0% (100.0% to 100.0%)	100.0% (100.0% to 100.0%)	100.0% (100.0% to 100.0%)
Depressive disorders	-	-	4.8% (4.0% to 5.7%)	4.8% (4.0% to 5.7%)
Idiopathic developmental intellectual disability	-	-	57.8% (28.1% to 77.4%)	57.8% (28.1% to 77.4%)
**Diabetes, urogenital, blood, and endocrine diseases**	79.6% (77.0% to 82.3%)	72.5% (67.7% to 76.2%)	65.4% (60.9% to 69.4%)	69.6% (65.9% to 72.6%)
Diabetes mellitus	100.0% (100.0% to 100.0%)	100.0% (100.0% to 100.0%)	100.0% (100.0% to 100.0%)	100.0% (100.0% to 100.0%)
Chronic kidney disease	100.0% (100.0% to 100.0%)	100.0% (100.0% to 100.0%)	100.0% (100.0% to 100.0%)	100.0% (100.0% to 100.0%)
**Musculoskeletal disorders**	0.9% (0.3% to 1.7%)	0.6% (0.2% to 1.2%)	22.8% (19.5% to 26.5%)	22.4% (19.1% to 26.1%)
Rheumatoid arthritis	2.0% (0.7% to 3.9%)	2.0% (0.7% to 3.8%)	2.3% (0.9% to 4.0%)	2.3% (0.9% to 3.9%)
Osteoarthritis	-	-	9.1% (3.2% to 17.6%)	9.1% (3.2% to 17.6%)
Low back and neck pain	-	-	30.0% (25.9% to 34.2%)	30.0% (25.9% to 34.2%)
Gout	-	-	33.1% (26.1% to 42.8%)	33.1% (26.1% to 42.8%)
**Other non-communicable diseases**	0.3% (0.1% to 0.6%)	0.5% (0.2% to 1.1%)	8.1% (6.6% to 9.6%)	6.7% (5.6% to 7.9%)
Sense organ diseases	-	-	15.5% (13.3% to 17.6%)	15.5% (13.3% to 17.6%)
Sudden infant death syndrome	5.0% (1.8% to 10.5%)	5.1% (1.8% to 10.5%)	-	5.1% (1.8% to 10.5%)
**Transport injuries**	26.4% (22.8% to 30.8%)	22.7% (17.9% to 28.7%)	31.6% (27.0% to 37.5%)	24.3% (19.4% to 30.4%)
Road injuries	26.3% (22.8% to 30.6%)	22.6% (18.0% to 28.5%)	32.9% (28.2% to 38.9%)	24.3% (19.4% to 30.3%)
Other transport injuries	27.9% (21.0% to 36.9%)	24.5% (17.5% to 34.7%)	24.0% (20.0% to 29.2%)	24.3% (18.3% to 32.6%)
**Unintentional injuries**	33.2% (25.1% to 37.9%)	18.4% (14.9% to 22.1%)	21.7% (18.0% to 26.5%)	19.4% (16.0% to 23.4%)
Falls	66.6% (47.9% to 72.1%)	45.0% (37.3% to 50.4%)	30.0% (26.4% to 34.9%)	38.4% (33.4% to 43.1%)
Drowning	9.0% (6.4% to 12.7%)	10.4% (7.0% to 14.9%)	12.9% (9.1% to 18.3%)	10.5% (7.1% to 15.0%)
Fire, heat, and hot substances	6.1% (4.3% to 8.5%)	8.4% (5.9% to 11.5%)	15.6% (11.0% to 22.0%)	10.1% (7.2% to 14.1%)

Supplementary Results Table 1. Age-standardised PAFs attributable to the joint distribution of all risk factors for all ages, both sexes combined for each GBD level 1 and level 2 cause and all causes in 2016 for Comoros. Results are shown for deaths, YLLs, YLDs, and DALYs
Data in parenthesis are 95% uncertainty intervals. PAFs=population attributable fractions. GBD=Global Burden of Disease. YLLs=years of life lost. YLDs=years live with disability. DALYs=disability-adjusted life-years

	**Deaths 2016**	**YLLs 2016**	**YLDs 2016**	**DALYs 2016**
Poisonings	8.8% (5.6% to 12.7%)	9.5% (4.8% to 14.3%)	15.4% (10.6% to 21.9%)	9.8% (5.0% to 14.8%)
Exposure to mechanical forces	23.0% (18.8% to 27.8%)	18.7% (13.6% to 24.8%)	23.6% (18.7% to 30.4%)	20.4% (15.4% to 26.8%)
Animal contact	7.6% (5.3% to 10.5%)	9.0% (6.1% to 12.9%)	14.1% (10.1% to 19.5%)	10.1% (7.0% to 14.1%)
Foreign body	7.7% (4.6% to 11.9%)	7.7% (3.9% to 12.9%)	12.8% (8.6% to 18.4%)	9.2% (5.4% to 14.2%)
Other unintentional injuries	11.0% (7.3% to 16.4%)	13.9% (9.2% to 20.4%)	12.8% (9.1% to 17.9%)	13.4% (9.2% to 19.0%)
**Self-harm and interpersonal violence**	6.4% (3.8% to 9.2%)	6.1% (3.8% to 8.9%)	17.0% (15.6% to 18.6%)	7.1% (4.9% to 9.8%)
Self-harm	2.5% (0.6% to 5.0%)	2.8% (0.8% to 5.4%)	2.5% (0.6% to 4.8%)	2.8% (0.8% to 5.4%)
Interpersonal violence	10.5% (6.2% to 14.6%)	8.5% (4.7% to 12.6%)	18.2% (16.7% to 19.9%)	9.9% (6.5% to 13.7%)
**Forces of nature, conflict and terrorism, and executions and police conflict**	0.0% (0.0% to 0.0%)	0.0% (0.0% to 0.0%)	1.4% (0.9% to 2.1%)	0.9% (0.4% to 1.4%)
Forces of nature, conflict and terrorism, and state actor violence	0.0% (0.0% to 0.0%)	0.0% (0.0% to 0.0%)	8.8% (6.0% to 13.0%)	8.8% (6.0% to 13.0%)

Supplementary Results Table 1. Age-standardised PAFs attributable to the joint distribution of all risk factors for all ages, both sexes combined for each GBD level 1 and level 2 cause and all causes in 2016 for Congo (Brazzaville). Results are shown for deaths, YLLs, YLDs, and DALYs
Data in parenthesis are 95% uncertainty intervals. PAFs=population attributable fractions. GBD=Global Burden of Disease. YLLs=years of life lost. YLDs=years live with disability. DALYs=disability-adjusted life-years

	Deaths 2016	YLLs 2016	YLDs 2016	DALYs 2016
**All causes**	59.3% (56.5% to 62.1%)	56.4% (53.6% to 59.5%)	28.2% (26.8% to 29.6%)	49.8% (47.3% to 52.7%)
**HIV/AIDS and tuberculosis**	71.6% (64.2% to 78.1%)	75.6% (69.9% to 80.7%)	84.4% (79.9% to 88.7%)	76.0% (70.4% to 80.8%)
Tuberculosis	42.9% (30.1% to 55.1%)	46.1% (33.8% to 57.1%)	42.7% (30.5% to 53.6%)	46.0% (33.7% to 57.1%)
HIV/AIDS	92.6% (91.4% to 93.7%)	88.0% (85.7% to 90.1%)	92.5% (90.0% to 94.8%)	88.3% (86.1% to 90.3%)
**Diarrhoea, lower respiratory infections, and other common infectious diseases**	78.1% (70.6% to 84.6%)	75.8% (69.3% to 81.8%)	46.8% (41.2% to 52.1%)	74.1% (67.7% to 80.1%)
Diarrhoeal diseases	94.3% (86.1% to 98.4%)	95.2% (88.3% to 98.7%)	95.1% (87.9% to 98.7%)	95.2% (88.3% to 98.7%)
Lower respiratory infections	72.5% (63.4% to 80.0%)	79.3% (71.8% to 85.9%)	74.4% (66.5% to 81.4%)	79.3% (71.8% to 85.9%)
Upper respiratory infections	1.4% (0.1% to 5.7%)	3.0% (0.2% to 11.3%)	0.0% (0.0% to 0.0%)	0.1% (0.0% to 0.3%)
Otitis media	1.0% (0.3% to 2.6%)	3.3% (1.3% to 6.1%)	4.0% (2.6% to 5.4%)	4.0% (2.6% to 5.4%)
Meningitis	2.1% (1.0% to 4.1%)	3.8% (1.9% to 7.1%)	0.0% (0.0% to 0.0%)	3.5% (1.8% to 6.6%)
Encephalitis	0.8% (0.5% to 1.3%)	1.3% (0.8% to 2.2%)	0.0% (0.0% to 0.0%)	0.8% (0.5% to 1.3%)
Measles	53.2% (33.4% to 80.9%)	54.0% (34.0% to 82.1%)	52.0% (31.9% to 79.4%)	54.0% (34.0% to 82.1%)
**Maternal disorders**	5.7% (2.5% to 9.4%)	5.8% (2.5% to 9.4%)	2.6% (1.2% to 4.3%)	5.7% (2.4% to 9.2%)
Maternal haemorrhage	15.7% (5.7% to 25.7%)	16.2% (5.8% to 26.3%)	17.1% (6.2% to 27.5%)	16.2% (5.8% to 26.3%)
Maternal sepsis and other pregnancy related infections	14.7% (5.4% to 23.9%)	15.4% (5.6% to 25.3%)	16.7% (6.1% to 27.0%)	15.4% (5.6% to 25.3%)
Maternal abortion, miscarriage, and ectopic pregnancy	25.6% (16.9% to 34.7%)	25.5% (16.9% to 34.5%)	25.1% (14.8% to 34.9%)	25.5% (16.9% to 34.2%)
**Neonatal disorders**	79.8% (75.7% to 83.5%)	79.8% (75.8% to 83.5%)	0.5% (0.2% to 0.8%)	75.3% (70.3% to 79.5%)
Neonatal preterm birth complications	97.1% (93.8% to 98.9%)	97.1% (93.9% to 98.9%)	1.2% (0.8% to 1.6%)	91.1% (86.1% to 94.8%)
Neonatal encephalopathy due to birth asphyxia and trauma	72.9% (68.6% to 77.1%)	73.0% (68.6% to 77.2%)	0.0% (0.0% to 0.0%)	70.6% (65.0% to 75.6%)
Neonatal sepsis and other neonatal infections	69.2% (64.2% to 73.4%)	69.3% (64.2% to 73.4%)	0.0% (0.0% to 0.0%)	63.2% (51.4% to 71.0%)
Hemolytic disease and other neonatal jaundice	72.4% (67.2% to 76.7%)	72.5% (67.3% to 76.7%)	0.0% (0.0% to 0.0%)	60.5% (50.3% to 67.8%)

Supplementary Results Table 1. Age-standardised PAFs attributable to the joint distribution of all risk factors for all ages, both sexes combined for each GBD level 1 and level 2 cause and all causes in 2016 for Congo (Brazzaville). Results are shown for deaths, YLLs, YLDs, and DALYs
Data in parenthesis are 95% uncertainty intervals. PAFs=population attributable fractions. GBD=Global Burden of Disease. YLLs=years of life lost. YLDs=years live with disability. DALYs=disability-adjusted life-years

	**Deaths 2016**	**YLLs 2016**	**YLDs 2016**	**DALYs 2016**
Other neonatal disorders	70.7% (66.2% to 75.3%)	70.8% (66.2% to 75.3%)	0.0% (0.0% to 0.0%)	69.0% (64.0% to 73.8%)
**Nutritional deficiencies**	15.9% (7.1% to 26.6%)	45.6% (26.6% to 62.5%)	74.2% (70.7% to 77.8%)	59.8% (44.3% to 68.7%)
Protein-energy malnutrition	20.0% (8.7% to 32.8%)	53.4% (31.5% to 70.7%)	95.4% (94.2% to 96.5%)	54.7% (32.9% to 71.1%)
Vitamin A deficiency	-	-	100.0% (100.0% to 100.0%)	100.0% (100.0% to 100.0%)
Iron-deficiency anaemia	100.0% (100.0% to 100.0%)	100.0% (100.0% to 100.0%)	100.0% (100.0% to 100.0%)	100.0% (100.0% to 100.0%)
**Other communicable, maternal, neonatal, and nutritional diseases**	4.9% (2.4% to 9.8%)	4.2% (1.7% to 8.7%)	64.2% (59.4% to 68.8%)	15.8% (8.1% to 24.6%)
Sexually transmitted diseases excluding HIV	40.6% (20.1% to 64.0%)	22.0% (8.9% to 41.9%)	100.0% (100.0% to 100.0%)	56.1% (35.3% to 75.5%)
Hepatitis	0.0% (0.0% to 0.0%)	0.0% (0.0% to 0.1%)	0.0% (0.0% to 0.1%)	0.0% (0.0% to 0.1%)
**Neoplasms**	36.8% (32.5% to 41.1%)	38.4% (34.0% to 42.7%)	37.4% (32.1% to 42.2%)	38.4% (34.0% to 42.7%)
Oesophageal cancer	69.2% (57.6% to 79.3%)	72.1% (61.1% to 81.4%)	70.1% (58.5% to 80.0%)	72.1% (61.1% to 81.4%)
Stomach cancer	9.8% (3.1% to 20.5%)	10.3% (3.8% to 20.3%)	9.9% (3.3% to 20.3%)	10.3% (3.8% to 20.3%)
Liver cancer	29.4% (21.8% to 38.3%)	29.9% (22.3% to 39.1%)	29.6% (22.0% to 38.6%)	29.9% (22.3% to 39.1%)
Larynx cancer	65.9% (52.8% to 77.7%)	70.2% (58.0% to 80.7%)	66.4% (53.4% to 78.3%)	70.1% (57.9% to 80.7%)
Tracheal, bronchus, and lung cancer	82.1% (75.0% to 88.0%)	83.2% (76.5% to 88.8%)	82.2% (74.8% to 88.2%)	83.2% (76.5% to 88.8%)
Breast cancer	24.9% (17.4% to 33.6%)	23.8% (16.4% to 33.1%)	24.0% (16.4% to 32.8%)	23.8% (16.4% to 33.0%)
Cervical cancer	100.0% (100.0% to 100.0%)	100.0% (100.0% to 100.0%)	100.0% (100.0% to 100.0%)	100.0% (100.0% to 100.0%)
Uterine cancer	38.4% (25.0% to 51.7%)	38.6% (25.7% to 52.1%)	38.5% (25.3% to 52.0%)	38.6% (25.7% to 52.1%)
Prostate cancer	0.8% (0.3% to 1.6%)	1.1% (0.5% to 2.0%)	1.0% (0.4% to 1.7%)	1.1% (0.5% to 2.0%)
Colon and rectum cancer	54.4% (45.3% to 63.5%)	55.0% (46.0% to 63.7%)	54.7% (45.7% to 63.7%)	55.0% (46.0% to 63.7%)
Lip and oral cavity cancer	68.7% (57.0% to 79.0%)	72.7% (61.8% to 81.9%)	69.8% (58.5% to 79.7%)	72.6% (61.7% to 81.9%)
Nasopharynx cancer	64.1% (52.5% to 74.1%)	65.6% (54.1% to 75.0%)	63.2% (52.1% to 72.7%)	65.6% (54.1% to 75.0%)

Supplementary Results Table 1. Age-standardised PAFs attributable to the joint distribution of all risk factors for all ages, both sexes combined for each GBD level 1 and level 2 cause and all causes in 2016 for Congo (Brazzaville). Results are shown for deaths, YLLs, YLDs, and DALYs
Data in parenthesis are 95% uncertainty intervals. PAFs=population attributable fractions. GBD=Global Burden of Disease. YLLs=years of life lost. YLDs=years live with disability. DALYs=disability-adjusted life-years

	Deaths 2016	YLLs 2016	YLDs 2016	DALYs 2016
Other pharynx cancer	48.5% (31.4% to 63.1%)	51.3% (34.5% to 65.7%)	49.7% (32.8% to 64.4%)	51.3% (34.4% to 65.7%)
Gallbladder and biliary tract cancer	18.5% (9.1% to 29.8%)	19.0% (9.4% to 30.2%)	18.2% (9.0% to 29.4%)	19.0% (9.4% to 30.2%)
Pancreatic cancer	19.6% (11.9% to 28.8%)	20.5% (13.0% to 29.5%)	19.5% (12.1% to 28.6%)	20.5% (13.0% to 29.5%)
Ovarian cancer	11.9% (5.1% to 21.0%)	10.6% (4.4% to 19.0%)	10.6% (4.3% to 18.9%)	10.6% (4.4% to 19.0%)
Kidney cancer	19.5% (12.0% to 28.2%)	19.1% (11.7% to 27.7%)	18.9% (11.9% to 27.4%)	19.1% (11.8% to 27.7%)
Bladder cancer	16.6% (9.0% to 26.3%)	18.5% (10.5% to 28.3%)	17.4% (9.8% to 27.2%)	18.5% (10.5% to 28.2%)
Thyroid cancer	9.6% (4.9% to 16.1%)	9.7% (5.0% to 16.0%)	9.5% (4.9% to 15.8%)	9.7% (5.0% to 16.0%)
Mesothelioma	88.7% (84.8% to 92.2%)	81.7% (76.2% to 86.9%)	86.1% (81.4% to 90.2%)	81.8% (76.3% to 86.9%)
Non-Hodgkin's lymphoma	4.3% (1.5% to 8.2%)	3.9% (1.4% to 7.4%)	4.1% (1.5% to 7.8%)	3.9% (1.4% to 7.4%)
Multiple myeloma	5.7% (2.2% to 10.4%)	5.8% (2.3% to 10.5%)	5.7% (2.2% to 10.2%)	5.8% (2.3% to 10.5%)
Leukaemia	8.8% (4.9% to 13.5%)	7.5% (4.2% to 11.2%)	8.3% (4.7% to 12.5%)	7.5% (4.2% to 11.3%)
**Cardiovascular diseases**	86.0% (83.6% to 88.1%)	87.0% (84.9% to 88.6%)	63.2% (58.8% to 67.5%)	85.4% (83.1% to 87.0%)
Rheumatic heart disease	30.5% (20.7% to 46.4%)	30.5% (21.2% to 45.1%)	26.2% (17.3% to 37.3%)	28.8% (20.3% to 41.8%)
Ischaemic heart disease	94.3% (91.3% to 96.5%)	95.5% (93.5% to 97.1%)	91.0% (88.3% to 93.6%)	95.4% (93.4% to 97.0%)
Cerebrovascular disease	87.7% (83.6% to 91.1%)	90.6% (87.8% to 92.8%)	87.8% (84.2% to 91.1%)	90.4% (87.7% to 92.7%)
Hypertensive heart disease	100.0% (100.0% to 100.0%)	100.0% (100.0% to 100.0%)	100.0% (100.0% to 100.0%)	100.0% (100.0% to 100.0%)
Cardiomyopathy and myocarditis	43.7% (35.9% to 52.0%)	43.1% (35.7% to 50.2%)	46.0% (39.0% to 53.1%)	43.3% (36.0% to 50.3%)
Atrial fibrillation and flutter	47.1% (38.5% to 54.8%)	52.0% (44.2% to 59.0%)	58.3% (52.4% to 64.0%)	55.1% (48.3% to 61.2%)
Aortic aneurysm	42.4% (35.3% to 49.2%)	47.4% (40.8% to 53.4%)	-	47.4% (40.8% to 53.4%)
Peripheral vascular disease	53.5% (47.0% to 60.5%)	60.4% (55.1% to 65.8%)	61.3% (56.1% to 66.5%)	61.0% (55.8% to 66.1%)
Endocarditis	42.3% (32.9% to 52.5%)	42.9% (34.5% to 51.8%)	37.5% (28.4% to 48.3%)	42.8% (34.4% to 51.7%)

Supplementary Results Table 1. Age-standardised PAFs attributable to the joint distribution of all risk factors for all ages, both sexes combined for each GBD level 1 and level 2 cause and all causes in 2016 for Congo (Brazzaville). Results are shown for deaths, YLLs, YLDs, and DALYs
Data in parenthesis are 95% uncertainty intervals. PAFs=population attributable fractions. GBD=Global Burden of Disease. YLLs=years of life lost. YLDs=years live with disability. DALYs=disability-adjusted life-years

	Deaths 2016	YLLs 2016	YLDs 2016	DALYs 2016
Other cardiovascular and circulatory diseases	45.6% (40.5% to 50.5%)	49.0% (44.7% to 53.6%)	40.9% (35.5% to 46.4%)	47.5% (42.9% to 52.1%)
**Chronic respiratory diseases**	65.4% (57.4% to 72.4%)	63.6% (56.7% to 69.6%)	44.3% (37.5% to 51.4%)	57.6% (50.9% to 63.9%)
Chronic obstructive pulmonary disease	77.4% (68.0% to 85.8%)	79.1% (70.6% to 86.4%)	70.7% (62.3% to 78.9%)	77.2% (68.9% to 84.6%)
Pneumoconiosis	100.0% (100.0% to 100.0%)	100.0% (100.0% to 100.0%)	100.0% (100.0% to 100.0%)	100.0% (100.0% to 100.0%)
Asthma	28.6% (18.6% to 39.8%)	29.4% (20.7% to 39.4%)	22.4% (15.8% to 30.2%)	26.1% (18.6% to 34.8%)
Other chronic respiratory diseases	5.2% (2.8% to 8.2%)	5.0% (2.3% to 8.5%)	4.1% (2.1% to 6.6%)	4.7% (2.3% to 7.8%)
**Cirrhosis and other chronic liver diseases**	23.7% (22.0% to 25.3%)	22.7% (21.0% to 24.5%)	18.1% (15.8% to 20.7%)	22.6% (21.0% to 24.3%)
Cirrhosis and other chronic liver diseases due to hepatitis B	0.0% (0.0% to 0.0%)	0.0% (0.0% to 0.0%)	0.0% (0.0% to 0.0%)	0.0% (0.0% to 0.0%)
Cirrhosis and other chronic liver diseases due to hepatitis C	0.7% (0.3% to 1.1%)	0.9% (0.4% to 1.8%)	1.2% (0.5% to 2.1%)	0.9% (0.4% to 1.8%)
Cirrhosis and other chronic liver diseases due to alcohol use	100.0% (100.0% to 100.0%)	100.0% (100.0% to 100.0%)	100.0% (100.0% to 100.0%)	100.0% (100.0% to 100.0%)
**Digestive diseases**	5.9% (4.0% to 7.9%)	6.6% (4.7% to 8.8%)	2.7% (1.9% to 3.6%)	6.4% (4.5% to 8.5%)
Peptic ulcer disease	4.4% (2.5% to 6.7%)	5.5% (3.1% to 8.4%)	5.7% (3.8% to 7.9%)	5.5% (3.2% to 8.3%)
Gallbladder and biliary diseases	34.4% (19.8% to 49.0%)	34.8% (21.5% to 49.2%)	34.2% (21.8% to 48.1%)	34.8% (21.5% to 49.2%)
Pancreatitis	34.9% (13.5% to 59.5%)	40.9% (17.9% to 65.9%)	36.9% (15.2% to 61.6%)	40.9% (17.8% to 65.9%)
**Neurological disorders**	18.0% (8.1% to 29.7%)	18.4% (10.0% to 28.6%)	5.2% (2.7% to 8.1%)	11.0% (6.3% to 16.9%)
Alzheimer's disease and other dementias	19.0% (7.9% to 31.9%)	19.9% (8.5% to 33.1%)	19.9% (8.5% to 32.9%)	19.9% (8.5% to 33.1%)
Parkinson's disease	-3.5% (-5.0% to -2.3%)	-4.2% (-5.9% to -2.8%)	-4.7% (-6.3% to -3.3%)	-4.3% (-6.0% to -2.9%)
Epilepsy	23.0% (13.5% to 33.4%)	21.9% (12.7% to 31.5%)	19.2% (11.6% to 27.6%)	20.6% (12.2% to 29.5%)
Multiple sclerosis	3.2% (1.4% to 5.7%)	3.5% (1.5% to 6.2%)	3.2% (1.8% to 4.8%)	3.4% (1.6% to 5.7%)
**Mental and substance use disorders**	99.7% (99.5% to 99.9%)	99.6% (99.2% to 99.8%)	18.6% (16.5% to 20.8%)	22.6% (19.9% to 25.6%)
Alcohol use disorders	100.0% (100.0% to 100.0%)	100.0% (100.0% to 100.0%)	100.0% (100.0% to 100.0%)	100.0% (100.0% to 100.0%)

Supplementary Results Table 1. Age-standardised PAFs attributable to the joint distribution of all risk factors for all ages, both sexes combined for each GBD level 1 and level 2 cause and all causes in 2016 for Congo (Brazzaville). Results are shown for deaths, YLLs, YLDs, and DALYs
Data in parenthesis are 95% uncertainty intervals. PAFs=population attributable fractions. GBD=Global Burden of Disease. YLLs=years of life lost. YLDs=years live with disability. DALYs=disability-adjusted life-years

	Deaths 2016	YLLs 2016	YLDs 2016	DALYs 2016
Drug use disorders	100.0% (100.0% to 100.0%)	100.0% (100.0% to 100.0%)	100.0% (100.0% to 100.0%)	100.0% (100.0% to 100.0%)
Depressive disorders	-	-	9.4% (7.9% to 11.2%)	9.4% (7.9% to 11.2%)
Idiopathic developmental intellectual disability	-	-	63.0% (31.8% to 82.7%)	63.0% (31.8% to 82.7%)
**Diabetes, urogenital, blood, and endocrine diseases**	82.4% (80.0% to 84.9%)	77.5% (74.1% to 80.9%)	63.4% (59.0% to 67.1%)	71.8% (68.5% to 74.8%)
Diabetes mellitus	100.0% (100.0% to 100.0%)	100.0% (100.0% to 100.0%)	100.0% (100.0% to 100.0%)	100.0% (100.0% to 100.0%)
Chronic kidney disease	100.0% (100.0% to 100.0%)	100.0% (100.0% to 100.0%)	100.0% (100.0% to 100.0%)	100.0% (100.0% to 100.0%)
**Musculoskeletal disorders**	0.3% (0.1% to 0.6%)	0.2% (0.1% to 0.5%)	18.0% (15.4% to 21.3%)	17.6% (14.9% to 20.8%)
Rheumatoid arthritis	1.0% (0.4% to 1.9%)	1.2% (0.4% to 2.3%)	1.9% (0.7% to 3.2%)	1.8% (0.7% to 3.1%)
Osteoarthritis	-	-	19.4% (9.5% to 30.9%)	19.4% (9.5% to 30.9%)
Low back and neck pain	-	-	21.3% (18.6% to 24.2%)	21.3% (18.6% to 24.2%)
Gout	-	-	43.9% (33.7% to 55.8%)	43.9% (33.7% to 55.8%)
**Other non-communicable diseases**	0.1% (0.0% to 0.2%)	0.2% (0.1% to 0.4%)	6.9% (5.7% to 8.0%)	5.3% (4.3% to 6.2%)
Sense organ diseases	-	-	13.3% (11.4% to 15.2%)	13.3% (11.4% to 15.2%)
Sudden infant death syndrome	5.6% (2.3% to 10.9%)	5.7% (2.3% to 11.0%)	-	5.7% (2.3% to 11.0%)
**Transport injuries**	36.0% (29.4% to 43.5%)	30.9% (24.4% to 38.4%)	43.2% (36.0% to 51.2%)	32.5% (26.0% to 39.9%)
Road injuries	35.7% (29.2% to 43.1%)	30.7% (24.3% to 38.1%)	45.6% (38.2% to 53.8%)	32.3% (25.8% to 39.7%)
Other transport injuries	41.2% (33.0% to 50.6%)	35.5% (27.2% to 45.4%)	35.0% (28.2% to 42.3%)	35.3% (28.1% to 43.1%)
**Unintentional injuries**	34.7% (27.2% to 40.3%)	21.0% (16.8% to 25.3%)	28.4% (23.3% to 35.0%)	23.1% (18.9% to 27.7%)
Falls	67.4% (48.3% to 73.8%)	46.9% (38.7% to 53.5%)	36.3% (31.4% to 42.8%)	42.0% (36.3% to 47.7%)
Drowning	16.6% (12.2% to 22.0%)	13.3% (9.3% to 18.4%)	23.2% (16.4% to 31.4%)	13.4% (9.4% to 18.5%)
Fire, heat, and hot substances	14.1% (10.2% to 19.4%)	15.7% (11.4% to 20.9%)	25.7% (18.3% to 34.3%)	17.8% (13.1% to 23.5%)

Supplementary Results Table 1. Age-standardised PAFs attributable to the joint distribution of all risk factors for all ages, both sexes combined for each GBD level 1 and level 2 cause and all causes in 2016 for Congo (Brazzaville). Results are shown for deaths, YLLs, YLDs, and DALYs
Data in parenthesis are 95% uncertainty intervals. PAFs=population attributable fractions. GBD=Global Burden of Disease. YLLs=years of life lost. YLDs=years live with disability. DALYs=disability-adjusted life-years

	Deaths 2016	YLLs 2016	YLDs 2016	DALYs 2016
Poisonings	18.3% (12.6% to 24.6%)	18.2% (11.1% to 25.7%)	24.0% (17.2% to 32.0%)	18.6% (11.7% to 25.9%)
Exposure to mechanical forces	27.1% (21.5% to 34.3%)	21.3% (15.4% to 29.7%)	31.2% (23.7% to 41.1%)	24.6% (18.3% to 32.2%)
Animal contact	15.5% (11.8% to 20.8%)	16.3% (12.0% to 22.3%)	19.7% (14.0% to 27.2%)	17.2% (12.7% to 23.3%)
Foreign body	11.5% (7.7% to 16.2%)	10.7% (6.1% to 16.7%)	16.8% (11.5% to 23.9%)	12.4% (7.8% to 18.3%)
Other unintentional injuries	20.7% (15.0% to 27.2%)	22.7% (16.5% to 30.0%)	22.7% (16.3% to 30.3%)	22.7% (16.8% to 29.6%)
**Self-harm and interpersonal violence**	21.4% (12.3% to 31.2%)	21.8% (13.7% to 30.9%)	22.9% (19.1% to 27.7%)	22.0% (14.3% to 30.5%)
Self-harm	21.3% (9.1% to 34.0%)	23.2% (10.9% to 36.1%)	22.1% (9.9% to 34.8%)	23.2% (10.9% to 36.1%)
Interpersonal violence	21.6% (14.6% to 29.8%)	20.1% (13.4% to 27.7%)	23.0% (19.4% to 27.7%)	20.6% (14.4% to 27.7%)
**Forces of nature, conflict and terrorism, and executions and police conflict**	0.0% (0.0% to 0.0%)	0.0% (0.0% to 0.0%)	0.0% (0.0% to 0.0%)	0.0% (0.0% to 0.0%)
Forces of nature, conflict and terrorism, and state actor violence	0.0% (0.0% to 0.0%)	0.0% (0.0% to 0.0%)	5.8% (3.9% to 8.6%)	5.8% (3.9% to 8.6%)

Supplementary Results Table 1. Age-standardised PAFs attributable to the joint distribution of all risk factors for all ages, both sexes combined for each GBD level 1 and level 2 cause and all causes in 2016 for Costa Rica. Results are shown for deaths, YLLs, YLDs, and DALYs
Data in parenthesis are 95% uncertainty intervals. PAFs=population attributable fractions. GBD=Global Burden of Disease. YLLs=years of life lost. YLDs=years live with disability. DALYs=disability-adjusted life-years

	Deaths 2016	YLLs 2016	YLDs 2016	DALYs 2016
**All causes**	50.7% (48.3% to 52.9%)	46.5% (44.7% to 48.6%)	22.2% (20.8% to 23.7%)	35.1% (32.9% to 37.3%)
**HIV/AIDS and tuberculosis**	82.7% (79.5% to 85.5%)	84.4% (82.5% to 86.2%)	90.3% (86.6% to 93.0%)	84.8% (83.0% to 86.6%)
Tuberculosis	48.7% (37.6% to 59.2%)	50.0% (40.3% to 58.9%)	43.3% (33.8% to 52.4%)	49.6% (39.9% to 58.5%)
HIV/AIDS	94.3% (93.6% to 94.9%)	90.8% (89.7% to 91.7%)	97.4% (96.4% to 98.1%)	91.2% (90.3% to 92.1%)
**Diarrhoea, lower respiratory infections, and other common infectious diseases**	43.0% (36.3% to 50.7%)	48.3% (41.9% to 54.8%)	32.6% (21.0% to 43.8%)	41.3% (34.0% to 48.9%)
Diarrhoeal diseases	56.8% (35.2% to 77.9%)	69.4% (52.9% to 84.6%)	60.0% (39.7% to 79.5%)	62.8% (43.8% to 80.9%)
Lower respiratory infections	46.0% (38.1% to 55.3%)	57.1% (49.0% to 65.4%)	48.5% (40.1% to 57.4%)	56.9% (48.8% to 65.3%)
Upper respiratory infections	1.5% (0.1% to 5.4%)	3.6% (0.2% to 11.6%)	0.0% (0.0% to 0.0%)	0.0% (0.0% to 0.0%)
Otitis media	2.4% (1.4% to 3.6%)	3.0% (1.8% to 4.5%)	3.9% (2.6% to 5.3%)	3.9% (2.6% to 5.3%)
Meningitis	2.7% (1.7% to 3.9%)	4.8% (3.4% to 6.6%)	0.0% (0.0% to 0.0%)	4.1% (2.8% to 5.7%)
Encephalitis	0.8% (0.3% to 1.7%)	1.2% (0.5% to 2.4%)	0.0% (0.0% to 0.0%)	1.0% (0.4% to 2.1%)
Measles	11.0% (2.4% to 34.6%)	12.6% (2.7% to 39.0%)	11.4% (0.0% to 60.8%)	12.6% (2.7% to 39.0%)
**Maternal disorders**	2.9% (1.2% to 4.8%)	2.8% (1.1% to 4.7%)	2.8% (1.4% to 4.4%)	2.8% (1.3% to 4.5%)
Maternal haemorrhage	12.7% (4.5% to 20.8%)	12.6% (4.5% to 20.6%)	12.5% (4.5% to 20.5%)	12.6% (4.5% to 20.5%)
Maternal sepsis and other pregnancy related infections	12.7% (4.6% to 20.7%)	12.6% (4.5% to 20.5%)	12.3% (4.3% to 20.2%)	12.4% (4.4% to 20.3%)
Maternal abortion, miscarriage, and ectopic pregnancy	6.5% (3.6% to 10.2%)	6.4% (3.6% to 10.0%)	6.2% (3.3% to 10.0%)	6.3% (3.4% to 9.7%)
**Neonatal disorders**	79.6% (77.3% to 82.1%)	79.6% (77.4% to 82.1%)	0.1% (0.1% to 0.2%)	69.6% (63.8% to 74.4%)
Neonatal preterm birth complications	94.3% (92.2% to 96.0%)	94.3% (92.2% to 96.0%)	0.2% (0.2% to 0.2%)	81.2% (73.9% to 87.1%)
Neonatal encephalopathy due to birth asphyxia and trauma	63.1% (59.5% to 67.0%)	63.2% (59.6% to 67.0%)	0.0% (0.0% to 0.0%)	56.4% (48.7% to 62.6%)
Neonatal sepsis and other neonatal infections	55.4% (50.4% to 60.0%)	55.4% (50.4% to 60.0%)	0.0% (0.0% to 0.0%)	42.0% (28.6% to 51.8%)
Hemolytic disease and other neonatal jaundice	58.7% (54.1% to 63.0%)	58.8% (54.1% to 63.1%)	0.0% (0.0% to 0.0%)	13.2% (6.8% to 20.9%)

Supplementary Results Table 1. Age-standardised PAFs attributable to the joint distribution of all risk factors for all ages, both sexes combined for each GBD level 1 and level 2 cause and all causes in 2016 for Costa Rica. Results are shown for deaths, YLLs, YLDs, and DALYs
Data in parenthesis are 95% uncertainty intervals. PAFs=population attributable fractions. GBD=Global Burden of Disease. YLLs=years of life lost. YLDs=years live with disability. DALYs=disability-adjusted life-years

	Deaths 2016	YLLs 2016	YLDs 2016	DALYs 2016
Other neonatal disorders	58.7% (54.3% to 63.0%)	58.7% (54.3% to 63.1%)	0.0% (0.0% to 0.0%)	57.5% (53.0% to 62.0%)
**Nutritional deficiencies**	11.7% (7.4% to 17.5%)	46.3% (34.4% to 58.2%)	77.9% (73.0% to 82.1%)	75.0% (69.9% to 79.6%)
Protein-energy malnutrition	11.2% (6.4% to 17.6%)	48.3% (34.9% to 61.7%)	95.8% (93.4% to 97.8%)	62.3% (52.6% to 71.4%)
Vitamin A deficiency	-	-	100.0% (100.0% to 100.0%)	100.0% (100.0% to 100.0%)
Iron-deficiency anaemia	100.0% (100.0% to 100.0%)	100.0% (100.0% to 100.0%)	100.0% (100.0% to 100.0%)	100.0% (100.0% to 100.0%)
**Other communicable, maternal, neonatal, and nutritional diseases**	7.5% (5.2% to 9.0%)	5.9% (4.1% to 7.3%)	71.6% (65.1% to 77.7%)	33.5% (24.5% to 45.2%)
Sexually transmitted diseases excluding HIV	58.6% (28.3% to 83.7%)	35.2% (12.4% to 64.0%)	100.0% (100.0% to 100.0%)	84.5% (63.5% to 95.9%)
Hepatitis	3.3% (2.3% to 4.4%)	3.5% (2.6% to 4.7%)	1.6% (1.0% to 2.7%)	3.4% (2.5% to 4.5%)
**Neoplasms**	25.8% (22.5% to 29.2%)	25.1% (22.1% to 28.1%)	23.5% (20.4% to 26.6%)	25.1% (22.1% to 28.1%)
Oesophageal cancer	63.7% (51.1% to 75.8%)	65.7% (53.2% to 77.4%)	64.0% (51.6% to 76.0%)	65.7% (53.2% to 77.4%)
Stomach cancer	7.5% (2.0% to 16.2%)	6.8% (1.8% to 14.2%)	7.3% (1.9% to 15.4%)	6.8% (1.8% to 14.2%)
Liver cancer	51.8% (43.4% to 61.0%)	53.4% (45.2% to 62.6%)	52.1% (43.7% to 60.9%)	53.4% (45.2% to 62.6%)
Larynx cancer	59.8% (45.9% to 71.9%)	59.8% (46.0% to 71.0%)	60.1% (46.5% to 71.6%)	59.8% (46.1% to 71.1%)
Tracheal, bronchus, and lung cancer	60.3% (51.9% to 67.5%)	58.3% (50.2% to 65.2%)	59.3% (50.5% to 66.8%)	58.3% (50.2% to 65.2%)
Breast cancer	25.3% (18.9% to 32.5%)	23.4% (17.3% to 29.9%)	24.1% (17.7% to 30.9%)	23.5% (17.3% to 29.9%)
Cervical cancer	100.0% (100.0% to 100.0%)	100.0% (100.0% to 100.0%)	100.0% (100.0% to 100.0%)	100.0% (100.0% to 100.0%)
Uterine cancer	47.9% (33.1% to 62.1%)	49.2% (34.2% to 63.2%)	49.7% (34.7% to 63.6%)	49.2% (34.2% to 63.2%)
Prostate cancer	1.2% (0.7% to 1.9%)	1.1% (0.7% to 1.7%)	1.2% (0.7% to 1.7%)	1.1% (0.7% to 1.7%)
Colon and rectum cancer	51.3% (42.1% to 60.2%)	51.8% (43.2% to 60.2%)	51.3% (42.5% to 60.1%)	51.8% (43.2% to 60.2%)
Lip and oral cavity cancer	59.3% (47.4% to 70.1%)	61.0% (49.3% to 70.6%)	57.3% (45.7% to 68.1%)	60.9% (49.2% to 70.5%)
Nasopharynx cancer	61.4% (52.5% to 69.3%)	56.8% (49.1% to 63.5%)	56.2% (48.6% to 63.3%)	56.8% (49.1% to 63.4%)

Supplementary Results Table 1. Age-standardised PAFs attributable to the joint distribution of all risk factors for all ages, both sexes combined for each GBD level 1 and level 2 cause and all causes in 2016 for Costa Rica. Results are shown for deaths, YLLs, YLDs, and DALYs
Data in parenthesis are 95% uncertainty intervals. PAFs=population attributable fractions. GBD=Global Burden of Disease. YLLs=years of life lost. YLDs=years live with disability. DALYs=disability-adjusted life-years

	Deaths 2016	YLLs 2016	YLDs 2016	DALYs 2016
Other pharynx cancer	55.2% (41.7% to 67.0%)	58.1% (45.1% to 69.4%)	56.4% (43.2% to 68.0%)	58.1% (45.1% to 69.4%)
Gallbladder and biliary tract cancer	24.0% (13.2% to 36.5%)	25.0% (14.0% to 37.7%)	24.2% (13.5% to 36.4%)	25.0% (13.9% to 37.7%)
Pancreatic cancer	16.7% (9.6% to 25.9%)	16.1% (9.1% to 25.1%)	16.6% (9.5% to 25.7%)	16.1% (9.1% to 25.1%)
Ovarian cancer	9.9% (3.4% to 17.8%)	8.8% (2.7% to 16.0%)	8.6% (2.7% to 15.7%)	8.8% (2.7% to 16.0%)
Kidney cancer	24.4% (15.7% to 35.2%)	23.4% (15.0% to 33.8%)	24.1% (15.5% to 34.6%)	23.4% (15.0% to 33.8%)
Bladder cancer	14.5% (7.7% to 22.8%)	12.9% (6.8% to 20.6%)	13.2% (7.1% to 21.1%)	12.9% (6.9% to 20.6%)
Thyroid cancer	14.7% (7.7% to 23.8%)	15.4% (7.9% to 24.8%)	15.0% (8.0% to 23.8%)	15.4% (7.9% to 24.8%)
Mesothelioma	77.3% (71.6% to 82.7%)	62.6% (55.4% to 70.5%)	72.8% (66.6% to 78.9%)	62.7% (55.6% to 70.6%)
Non-Hodgkin's lymphoma	7.1% (2.9% to 12.4%)	6.8% (2.8% to 11.9%)	7.1% (2.9% to 12.3%)	6.8% (2.8% to 11.9%)
Multiple myeloma	8.5% (3.7% to 15.1%)	8.9% (3.8% to 15.5%)	8.7% (3.8% to 15.4%)	8.9% (3.8% to 15.5%)
Leukaemia	11.4% (7.0% to 16.5%)	9.6% (6.0% to 14.0%)	11.1% (6.9% to 15.8%)	9.7% (6.0% to 14.0%)
**Cardiovascular diseases**	84.3% (81.5% to 86.5%)	83.9% (81.8% to 85.6%)	75.9% (73.1% to 78.2%)	82.8% (80.8% to 84.5%)
Rheumatic heart disease	28.9% (20.0% to 43.7%)	26.7% (18.1% to 38.0%)	28.6% (19.2% to 41.4%)	26.8% (18.1% to 38.0%)
Ischaemic heart disease	92.5% (88.9% to 95.5%)	94.2% (91.9% to 96.1%)	91.0% (88.3% to 93.5%)	93.9% (91.6% to 95.9%)
Cerebrovascular disease	81.6% (75.8% to 86.6%)	84.7% (80.9% to 87.9%)	83.1% (78.5% to 87.1%)	84.5% (80.7% to 87.6%)
Hypertensive heart disease	100.0% (100.0% to 100.0%)	100.0% (100.0% to 100.0%)	100.0% (100.0% to 100.0%)	100.0% (100.0% to 100.0%)
Cardiomyopathy and myocarditis	42.6% (35.2% to 51.3%)	38.1% (31.4% to 44.8%)	39.3% (32.6% to 46.3%)	38.2% (31.5% to 44.8%)
Atrial fibrillation and flutter	52.9% (44.8% to 60.8%)	57.6% (50.2% to 64.7%)	63.7% (57.8% to 69.7%)	61.3% (55.2% to 67.4%)
Aortic aneurysm	43.6% (36.4% to 50.7%)	46.7% (40.6% to 52.4%)	-	46.7% (40.6% to 52.4%)
Peripheral vascular disease	51.3% (44.0% to 59.1%)	57.1% (51.1% to 62.9%)	60.1% (54.3% to 65.7%)	58.9% (53.2% to 64.3%)
Endocarditis	41.9% (32.5% to 51.2%)	37.8% (27.3% to 47.9%)	42.2% (33.9% to 50.4%)	37.9% (27.5% to 48.0%)

Supplementary Results Table 1. Age-standardised PAFs attributable to the joint distribution of all risk factors for all ages, both sexes combined for each GBD level 1 and level 2 cause and all causes in 2016 for Costa Rica. Results are shown for deaths, YLLs, YLDs, and DALYs
Data in parenthesis are 95% uncertainty intervals. PAFs=population attributable fractions. GBD=Global Burden of Disease. YLLs=years of life lost. YLDs=years live with disability. DALYs=disability-adjusted life-years

	Deaths 2016	YLLs 2016	YLDs 2016	DALYs 2016
Other cardiovascular and circulatory diseases	45.8% (40.2% to 51.0%)	47.6% (43.2% to 52.1%)	49.0% (44.3% to 53.9%)	48.1% (43.6% to 52.5%)
**Chronic respiratory diseases**	46.1% (37.6% to 53.9%)	41.4% (33.8% to 48.5%)	33.7% (25.8% to 43.1%)	37.4% (31.0% to 43.8%)
Chronic obstructive pulmonary disease	52.9% (43.0% to 62.1%)	50.9% (41.7% to 60.2%)	44.7% (35.4% to 53.8%)	49.6% (40.3% to 58.7%)
Pneumoconiosis	100.0% (100.0% to 100.0%)	100.0% (100.0% to 100.0%)	100.0% (100.0% to 100.0%)	100.0% (100.0% to 100.0%)
Asthma	40.1% (29.0% to 52.4%)	41.8% (31.3% to 53.3%)	31.7% (21.8% to 43.6%)	32.5% (22.8% to 44.2%)
Other chronic respiratory diseases	2.3% (1.0% to 4.4%)	1.4% (0.4% to 3.8%)	1.1% (0.2% to 2.9%)	1.3% (0.3% to 3.3%)
**Cirrhosis and other chronic liver diseases**	62.0% (57.4% to 66.3%)	66.0% (61.7% to 69.6%)	54.5% (50.4% to 58.5%)	65.4% (61.1% to 69.0%)
Cirrhosis and other chronic liver diseases due to hepatitis B	0.7% (0.3% to 1.3%)	0.8% (0.3% to 1.6%)	0.7% (0.3% to 1.3%)	0.8% (0.3% to 1.5%)
Cirrhosis and other chronic liver diseases due to hepatitis C	58.0% (46.4% to 67.4%)	69.5% (59.5% to 77.1%)	59.5% (49.5% to 68.1%)	69.1% (59.0% to 76.7%)
Cirrhosis and other chronic liver diseases due to alcohol use	100.0% (100.0% to 100.0%)	100.0% (100.0% to 100.0%)	100.0% (100.0% to 100.0%)	100.0% (100.0% to 100.0%)
**Digestive diseases**	10.8% (7.9% to 14.0%)	12.4% (9.1% to 15.8%)	3.5% (2.6% to 4.6%)	9.8% (7.3% to 12.6%)
Peptic ulcer disease	6.2% (4.1% to 8.7%)	7.9% (5.2% to 10.9%)	5.8% (3.6% to 8.3%)	7.1% (4.7% to 9.9%)
Gallbladder and biliary diseases	41.9% (27.3% to 56.2%)	43.4% (29.0% to 57.4%)	49.6% (35.1% to 65.1%)	44.2% (29.8% to 58.6%)
Pancreatitis	36.3% (19.4% to 55.2%)	40.2% (23.8% to 56.4%)	33.2% (18.2% to 48.3%)	40.1% (23.7% to 56.2%)
**Neurological disorders**	21.0% (10.4% to 33.4%)	19.0% (10.5% to 29.0%)	4.8% (2.7% to 7.5%)	9.6% (5.6% to 14.4%)
Alzheimer's disease and other dementias	23.6% (11.7% to 37.8%)	24.3% (12.2% to 38.6%)	24.3% (12.2% to 38.6%)	24.3% (12.2% to 38.7%)
Parkinson's disease	-3.6% (-4.6% to -2.8%)	-4.5% (-5.7% to -3.5%)	-4.9% (-6.1% to -3.8%)	-4.6% (-5.7% to -3.5%)
Epilepsy	28.4% (20.2% to 36.4%)	26.7% (18.6% to 34.3%)	19.4% (12.9% to 25.9%)	21.4% (14.4% to 28.4%)
Multiple sclerosis	3.9% (2.4% to 5.7%)	4.1% (2.5% to 6.1%)	3.4% (2.1% to 4.8%)	3.9% (2.4% to 5.7%)
**Mental and substance use disorders**	99.9% (99.9% to 99.9%)	99.9% (99.8% to 99.9%)	20.2% (18.2% to 22.5%)	22.6% (20.5% to 25.0%)
Alcohol use disorders	100.0% (100.0% to 100.0%)	100.0% (100.0% to 100.0%)	100.0% (100.0% to 100.0%)	100.0% (100.0% to 100.0%)

**Supplementary Results Table 1. Age-standardised PAFs attributable to the joint distribution of all risk factors for all ages, both sexes combined for each GBD level 1 and level 2 cause and all causes in 2016 for Costa Rica. Results are shown for deaths, YLLs, YLDs, and DALYs** Data in parenthesis are 95% uncertainty intervals. PAFs=population attributable fractions. GBD=Global Burden of Disease. YLLs=years of life lost. YLDs=years live with disability. DALYs=disability-adjusted life-years				
	**Deaths 2016**	**YLLs 2016**	**YLDs 2016**	**DALYs 2016**
Drug use disorders	100.0% (100.0% to 100.0%)	100.0% (100.0% to 100.0%)	100.0% (100.0% to 100.0%)	100.0% (100.0% to 100.0%)
Depressive disorders	-	-	14.1% (12.1% to 16.2%)	14.1% (12.1% to 16.2%)
Idiopathic developmental intellectual disability	-	-	70.0% (38.1% to 89.4%)	70.0% (38.1% to 89.4%)
**Diabetes, urogenital, blood, and endocrine diseases**	84.6% (82.0% to 85.6%)	82.3% (79.1% to 83.7%)	74.4% (70.5% to 77.8%)	78.4% (75.8% to 80.4%)
Diabetes mellitus	100.0% (100.0% to 100.0%)	100.0% (100.0% to 100.0%)	100.0% (100.0% to 100.0%)	100.0% (100.0% to 100.0%)
Chronic kidney disease	100.0% (100.0% to 100.0%)	100.0% (100.0% to 100.0%)	100.0% (100.0% to 100.0%)	100.0% (100.0% to 100.0%)
**Musculoskeletal disorders**	0.5% (0.2% to 0.9%)	0.4% (0.1% to 0.6%)	14.3% (11.1% to 17.8%)	13.7% (10.7% to 17.1%)
Rheumatoid arthritis	1.7% (0.7% to 3.0%)	1.9% (0.8% to 3.4%)	2.3% (0.9% to 3.9%)	2.2% (0.9% to 3.8%)
Osteoarthritis	-	-	28.6% (15.4% to 41.9%)	28.6% (15.4% to 41.9%)
Low back and neck pain	-	-	16.8% (14.2% to 19.7%)	16.8% (14.2% to 19.7%)
Gout	-	-	51.7% (37.5% to 66.6%)	51.7% (37.5% to 66.6%)
**Other non-communicable diseases**	0.1% (0.0% to 0.1%)	0.1% (0.0% to 0.2%)	5.6% (4.8% to 6.6%)	4.4% (3.6% to 5.2%)
Sense organ diseases	-	-	12.0% (10.6% to 13.7%)	12.0% (10.6% to 13.7%)
Sudden infant death syndrome	6.4% (3.9% to 10.0%)	6.4% (3.9% to 10.0%)	-	6.4% (3.9% to 10.0%)
**Transport injuries**	37.7% (30.9% to 45.6%)	34.1% (27.2% to 42.3%)	41.5% (34.9% to 49.4%)	35.2% (28.4% to 43.0%)
Road injuries	37.6% (30.8% to 45.5%)	34.0% (27.0% to 42.1%)	41.7% (35.1% to 49.6%)	35.2% (28.4% to 42.9%)
Other transport injuries	39.3% (32.2% to 47.7%)	35.5% (28.2% to 44.0%)	36.2% (29.6% to 43.7%)	35.5% (28.4% to 44.1%)
**Unintentional injuries**	33.0% (23.9% to 37.2%)	20.9% (16.3% to 24.7%)	24.5% (22.6% to 26.5%)	22.5% (19.4% to 25.3%)
Falls	61.8% (50.8% to 66.4%)	41.7% (32.0% to 46.3%)	28.3% (26.5% to 30.2%)	32.4% (28.8% to 35.2%)
Drowning	20.8% (15.4% to 27.1%)	19.0% (13.9% to 24.8%)	17.1% (12.2% to 23.3%)	18.9% (13.8% to 24.7%)
Fire, heat, and hot substances	11.3% (7.4% to 16.9%)	14.1% (10.4% to 18.7%)	20.3% (16.0% to 25.3%)	17.2% (13.3% to 21.8%)

Supplementary Results Table 1. Age-standardised PAFs attributable to the joint distribution of all risk factors for all ages, both sexes combined for each GBD level 1 and level 2 cause and all causes in 2016 for Costa Rica. Results are shown for deaths, YLLs, YLDs, and DALYs
Data in parenthesis are 95% uncertainty intervals. PAFs=population attributable fractions. GBD=Global Burden of Disease. YLLs=years of life lost. YLDs=years live with disability. DALYs=disability-adjusted life-years

	Deaths 2016	YLLs 2016	YLDs 2016	DALYs 2016
Poisonings	22.8% (17.1% to 29.5%)	22.8% (16.7% to 29.0%)	19.6% (15.5% to 24.6%)	22.1% (16.6% to 27.7%)
Exposure to mechanical forces	20.8% (18.6% to 23.2%)	17.8% (15.2% to 20.6%)	22.8% (20.2% to 25.7%)	20.8% (18.3% to 23.4%)
Animal contact	14.8% (12.0% to 17.4%)	14.2% (9.0% to 18.2%)	13.7% (11.4% to 16.1%)	14.0% (10.6% to 16.8%)
Foreign body	6.9% (5.1% to 10.0%)	6.9% (5.0% to 9.2%)	9.4% (7.8% to 11.3%)	7.7% (6.0% to 9.7%)
Other unintentional injuries	27.4% (21.5% to 34.1%)	26.5% (20.8% to 33.2%)	20.6% (15.9% to 26.2%)	23.9% (18.7% to 29.8%)
**Self-harm and interpersonal violence**	26.2% (18.7% to 33.7%)	25.0% (17.4% to 32.5%)	27.5% (22.5% to 32.4%)	25.2% (17.9% to 32.3%)
Self-harm	28.8% (16.4% to 41.4%)	27.7% (14.8% to 40.6%)	25.7% (14.3% to 37.6%)	27.7% (14.8% to 40.5%)
Interpersonal violence	23.9% (15.8% to 31.2%)	22.9% (14.7% to 30.0%)	27.7% (22.6% to 32.7%)	23.4% (15.5% to 30.3%)
**Forces of nature, conflict and terrorism, and executions and police conflict**	6.2% (5.8% to 6.6%)	2.5% (2.2% to 2.7%)	4.3% (2.9% to 6.3%)	2.6% (2.3% to 2.9%)
Forces of nature, conflict and terrorism, and state actor violence	6.6% (6.2% to 7.0%)	2.6% (2.4% to 2.9%)	4.5% (3.0% to 6.6%)	2.8% (2.5% to 3.1%)

Supplementary Results Table 1. Age-standardised PAFs attributable to the joint distribution of all risk factors for all ages, both sexes combined for each GBD level 1 and level 2 cause and all causes in 2016 for Cote d'Ivoire. Results are shown for deaths, YLLs, YLDs, and DALYs
Data in parenthesis are 95% uncertainty intervals. PAFs=population attributable fractions. GBD=Global Burden of Disease. YLLs=years of life lost. YLDs=years live with disability. DALYs=disability-adjusted life-years

	Deaths 2016	YLLs 2016	YLDs 2016	DALYs 2016
**All causes**	57.3% (55.1% to 59.6%)	54.5% (51.8% to 57.0%)	29.8% (28.4% to 31.3%)	49.5% (47.1% to 52.0%)
**HIV/AIDS and tuberculosis**	72.6% (65.0% to 79.4%)	77.9% (72.3% to 82.9%)	87.9% (84.8% to 90.8%)	78.4% (73.2% to 83.2%)
Tuberculosis	38.8% (24.0% to 53.4%)	42.3% (27.5% to 55.9%)	40.1% (26.1% to 53.7%)	42.3% (27.5% to 55.9%)
HIV/AIDS	92.8% (90.6% to 94.3%)	90.1% (87.8% to 92.2%)	92.8% (90.7% to 94.7%)	90.3% (87.9% to 92.2%)
**Diarrhoea, lower respiratory infections, and other common infectious diseases**	74.3% (69.0% to 79.9%)	76.3% (71.2% to 81.1%)	44.5% (38.9% to 49.5%)	75.0% (70.0% to 79.7%)
Diarrhoeal diseases	95.2% (88.0% to 98.7%)	97.2% (92.5% to 99.3%)	94.7% (87.0% to 98.5%)	97.1% (92.4% to 99.3%)
Lower respiratory infections	77.8% (71.0% to 84.0%)	84.1% (78.7% to 88.9%)	78.3% (71.9% to 84.3%)	84.0% (78.7% to 88.9%)
Upper respiratory infections	1.8% (0.1% to 6.8%)	3.4% (0.2% to 12.8%)	0.0% (0.0% to 0.0%)	0.2% (0.0% to 0.8%)
Otitis media	0.9% (0.2% to 2.5%)	3.8% (1.4% to 7.4%)	4.6% (2.9% to 6.2%)	4.6% (2.9% to 6.2%)
Meningitis	2.2% (1.1% to 3.9%)	4.2% (2.3% to 7.4%)	0.0% (0.0% to 0.0%)	4.0% (2.2% to 7.1%)
Encephalitis	0.7% (0.5% to 1.1%)	1.4% (0.9% to 2.0%)	0.0% (0.0% to 0.0%)	0.9% (0.6% to 1.4%)
Measles	56.5% (38.1% to 79.6%)	57.7% (38.9% to 81.0%)	58.6% (40.0% to 80.4%)	57.7% (38.9% to 81.0%)
**Maternal disorders**	8.0% (3.2% to 13.4%)	8.0% (3.2% to 13.3%)	1.5% (0.6% to 2.7%)	7.8% (3.0% to 12.9%)
Maternal haemorrhage	18.1% (6.5% to 29.4%)	18.2% (6.5% to 29.5%)	18.5% (6.6% to 29.9%)	18.2% (6.5% to 29.5%)
Maternal sepsis and other pregnancy related infections	18.0% (6.5% to 29.2%)	18.1% (6.5% to 29.3%)	18.1% (6.5% to 29.4%)	18.1% (6.5% to 29.3%)
Maternal abortion, miscarriage, and ectopic pregnancy	15.6% (9.7% to 22.1%)	15.4% (9.4% to 21.8%)	15.1% (8.8% to 22.0%)	15.4% (9.4% to 21.8%)
**Neonatal disorders**	83.9% (80.8% to 86.6%)	83.9% (80.8% to 86.6%)	0.6% (0.3% to 1.1%)	81.5% (78.2% to 84.6%)
Neonatal preterm birth complications	97.9% (96.2% to 99.0%)	98.0% (96.2% to 99.1%)	1.6% (1.0% to 2.1%)	95.2% (92.5% to 97.1%)
Neonatal encephalopathy due to birth asphyxia and trauma	78.5% (74.5% to 82.4%)	78.5% (74.5% to 82.4%)	0.0% (0.0% to 0.0%)	77.2% (72.4% to 81.5%)
Neonatal sepsis and other neonatal infections	70.9% (65.7% to 75.3%)	71.0% (65.8% to 75.3%)	0.0% (0.0% to 0.0%)	67.7% (59.9% to 73.6%)
Hemolytic disease and other neonatal jaundice	70.4% (60.6% to 76.8%)	70.4% (60.7% to 76.8%)	0.0% (0.0% to 0.0%)	66.5% (56.9% to 73.1%)

Supplementary Results Table 1. Age-standardised PAFs attributable to the joint distribution of all risk factors for all ages, both sexes combined for each GBD level 1 and level 2 cause and all causes in 2016 for Cote d'Ivoire. Results are shown for deaths, YLLs, YLDs, and DALYs
Data in parenthesis are 95% uncertainty intervals. PAFs=population attributable fractions. GBD=Global Burden of Disease. YLLs=years of life lost. YLDs=years live with disability. DALYs=disability-adjusted life-years

	Deaths 2016	YLLs 2016	YLDs 2016	DALYs 2016
Other neonatal disorders	70.4% (62.1% to 76.6%)	70.4% (62.2% to 76.7%)	0.0% (0.0% to 0.0%)	68.4% (60.3% to 75.0%)
**Nutritional deficiencies**	27.1% (17.3% to 38.4%)	59.5% (46.4% to 71.6%)	94.6% (93.3% to 95.7%)	80.0% (74.9% to 84.3%)
Protein-energy malnutrition	30.6% (19.7% to 43.2%)	64.9% (51.5% to 76.6%)	97.8% (96.8% to 98.5%)	66.4% (53.8% to 77.0%)
Vitamin A deficiency	-	-	100.0% (100.0% to 100.0%)	100.0% (100.0% to 100.0%)
Iron-deficiency anaemia	100.0% (100.0% to 100.0%)	100.0% (100.0% to 100.0%)	100.0% (100.0% to 100.0%)	100.0% (100.0% to 100.0%)
**Other communicable, maternal, neonatal, and nutritional diseases**	3.0% (1.7% to 4.7%)	2.6% (1.3% to 4.3%)	56.8% (51.6% to 62.1%)	11.3% (6.5% to 16.9%)
Sexually transmitted diseases excluding HIV	10.9% (4.0% to 23.0%)	6.0% (2.0% to 13.4%)	100.0% (100.0% to 100.0%)	24.1% (10.5% to 44.7%)
Hepatitis	0.0% (0.0% to 0.0%)	0.0% (0.0% to 0.0%)	0.0% (0.0% to 0.0%)	0.0% (0.0% to 0.0%)
**Neoplasms**	24.0% (20.6% to 28.3%)	26.1% (22.9% to 29.7%)	23.7% (19.9% to 29.0%)	26.1% (22.8% to 29.6%)
Oesophageal cancer	57.7% (44.3% to 69.6%)	61.3% (47.6% to 72.9%)	58.5% (45.1% to 70.5%)	61.2% (47.6% to 72.8%)
Stomach cancer	4.7% (1.1% to 12.9%)	4.9% (1.3% to 13.0%)	4.8% (1.2% to 13.0%)	4.9% (1.3% to 13.0%)
Liver cancer	26.1% (19.6% to 34.0%)	27.1% (20.2% to 35.8%)	26.6% (19.9% to 34.7%)	27.1% (20.2% to 35.7%)
Larynx cancer	51.1% (35.2% to 64.9%)	55.0% (38.9% to 68.5%)	52.0% (36.8% to 65.7%)	54.9% (38.9% to 68.4%)
Tracheal, bronchus, and lung cancer	77.6% (70.9% to 83.6%)	78.1% (71.4% to 83.9%)	77.7% (71.1% to 83.9%)	78.1% (71.4% to 83.9%)
Breast cancer	19.2% (13.3% to 26.9%)	18.0% (12.2% to 25.4%)	18.0% (12.2% to 25.2%)	18.0% (12.2% to 25.4%)
Cervical cancer	100.0% (100.0% to 100.0%)	100.0% (100.0% to 100.0%)	100.0% (100.0% to 100.0%)	100.0% (100.0% to 100.0%)
Uterine cancer	23.6% (12.2% to 36.7%)	26.3% (14.2% to 39.8%)	24.8% (12.8% to 38.2%)	26.3% (14.2% to 39.8%)
Prostate cancer	0.7% (0.4% to 1.2%)	0.9% (0.5% to 1.4%)	0.8% (0.4% to 1.2%)	0.9% (0.5% to 1.4%)
Colon and rectum cancer	45.0% (35.8% to 54.5%)	45.7% (36.6% to 54.8%)	45.3% (36.0% to 54.5%)	45.7% (36.6% to 54.8%)
Lip and oral cavity cancer	52.8% (36.7% to 68.8%)	57.5% (41.9% to 72.3%)	54.5% (38.5% to 70.2%)	57.5% (41.8% to 72.2%)
Nasopharynx cancer	58.8% (43.4% to 71.7%)	58.0% (42.3% to 70.9%)	57.3% (42.1% to 70.1%)	58.0% (42.3% to 70.8%)

Supplementary Results Table 1. Age-standardised PAFs attributable to the joint distribution of all risk factors for all ages, both sexes combined for each GBD level 1 and level 2 cause and all causes in 2016 for Cote d'Ivoire. Results are shown for deaths, YLLs, YLDs, and DALYs
Data in parenthesis are 95% uncertainty intervals. PAFs=population attributable fractions. GBD=Global Burden of Disease. YLLs=years of life lost. YLDs=years live with disability. DALYs=disability-adjusted life-years

	Deaths 2016	YLLs 2016	YLDs 2016	DALYs 2016
Other pharynx cancer	41.0% (23.7% to 58.8%)	43.8% (26.6% to 61.2%)	41.9% (24.6% to 59.7%)	43.8% (26.6% to 61.2%)
Gallbladder and biliary tract cancer	12.2% (5.5% to 21.5%)	13.8% (6.5% to 23.5%)	12.5% (5.7% to 21.8%)	13.8% (6.5% to 23.5%)
Pancreatic cancer	12.2% (6.3% to 20.2%)	12.8% (6.9% to 20.5%)	12.4% (6.6% to 20.2%)	12.8% (6.9% to 20.5%)
Ovarian cancer	10.4% (4.4% to 18.2%)	9.2% (3.8% to 16.4%)	9.2% (3.8% to 16.4%)	9.2% (3.8% to 16.4%)
Kidney cancer	13.1% (7.5% to 20.8%)	14.9% (8.8% to 22.7%)	14.4% (8.5% to 22.2%)	14.9% (8.8% to 22.7%)
Bladder cancer	10.4% (5.2% to 17.9%)	11.4% (6.0% to 18.5%)	11.0% (5.7% to 18.3%)	11.4% (6.0% to 18.5%)
Thyroid cancer	7.0% (3.3% to 12.5%)	7.8% (3.8% to 13.5%)	7.4% (3.5% to 12.9%)	7.8% (3.8% to 13.5%)
Mesothelioma	86.1% (81.2% to 90.2%)	78.2% (70.6% to 84.4%)	83.5% (77.6% to 88.3%)	78.3% (70.7% to 84.4%)
Non-Hodgkin's lymphoma	3.6% (1.4% to 6.8%)	3.3% (1.3% to 6.3%)	3.4% (1.3% to 6.5%)	3.3% (1.3% to 6.3%)
Multiple myeloma	3.5% (1.3% to 7.1%)	4.3% (1.6% to 8.2%)	3.9% (1.4% to 7.6%)	4.3% (1.6% to 8.2%)
Leukaemia	7.0% (3.9% to 11.3%)	7.3% (4.2% to 11.2%)	7.1% (3.9% to 11.4%)	7.3% (4.2% to 11.2%)
**Cardiovascular diseases**	86.8% (84.5% to 88.9%)	87.3% (85.5% to 89.0%)	64.9% (61.0% to 68.5%)	85.9% (83.9% to 87.7%)
Rheumatic heart disease	31.0% (21.0% to 48.6%)	29.5% (20.9% to 43.8%)	25.3% (16.9% to 35.9%)	28.5% (20.0% to 41.7%)
Ischaemic heart disease	94.0% (91.2% to 96.2%)	95.4% (93.7% to 96.8%)	91.3% (88.9% to 93.5%)	95.3% (93.5% to 96.7%)
Cerebrovascular disease	88.4% (84.9% to 91.5%)	90.3% (87.9% to 92.4%)	88.1% (84.5% to 91.2%)	90.2% (87.8% to 92.3%)
Hypertensive heart disease	100.0% (100.0% to 100.0%)	100.0% (100.0% to 100.0%)	100.0% (100.0% to 100.0%)	100.0% (100.0% to 100.0%)
Cardiomyopathy and myocarditis	42.8% (35.5% to 50.7%)	41.1% (34.2% to 47.9%)	47.8% (41.0% to 54.5%)	41.6% (34.8% to 48.3%)
Atrial fibrillation and flutter	46.3% (38.1% to 55.5%)	51.5% (44.2% to 59.6%)	59.3% (53.5% to 65.4%)	55.6% (49.1% to 62.5%)
Aortic aneurysm	46.9% (39.2% to 54.2%)	51.9% (45.3% to 57.8%)	-	51.9% (45.3% to 57.8%)
Peripheral vascular disease	53.3% (46.4% to 60.9%)	60.7% (55.1% to 66.3%)	63.3% (57.6% to 68.8%)	62.8% (57.3% to 68.3%)
Endocarditis	41.8% (32.8% to 51.3%)	37.6% (29.7% to 45.6%)	39.8% (30.2% to 51.0%)	37.6% (29.7% to 45.5%)

Supplementary Results Table 1. Age-standardised PAFs attributable to the joint distribution of all risk factors for all ages, both sexes combined for each GBD level 1 and level 2 cause and all causes in 2016 for Cote d'Ivoire. Results are shown for deaths, YLLs, YLDs, and DALYs
Data in parenthesis are 95% uncertainty intervals. PAFs=population attributable fractions. GBD=Global Burden of Disease. YLLs=years of life lost. YLDs=years live with disability. DALYs=disability-adjusted life-years

	Deaths 2016	YLLs 2016	YLDs 2016	DALYs 2016
Other cardiovascular and circulatory diseases	49.3% (44.1% to 54.4%)	50.6% (45.9% to 55.3%)	45.1% (39.8% to 50.1%)	49.2% (44.6% to 53.9%)
**Chronic respiratory diseases**	57.4% (49.9% to 64.5%)	55.2% (48.5% to 61.6%)	42.2% (35.5% to 49.2%)	51.9% (45.8% to 58.3%)
Chronic obstructive pulmonary disease	73.8% (64.6% to 83.3%)	73.5% (64.7% to 82.7%)	65.1% (53.8% to 75.3%)	71.9% (63.1% to 80.9%)
Pneumoconiosis	100.0% (100.0% to 100.0%)	100.0% (100.0% to 100.0%)	100.0% (100.0% to 100.0%)	100.0% (100.0% to 100.0%)
Asthma	27.7% (20.6% to 36.8%)	33.5% (26.6% to 41.8%)	25.8% (19.4% to 33.2%)	30.4% (24.1% to 38.4%)
Other chronic respiratory diseases	3.2% (1.7% to 5.3%)	3.4% (1.5% to 6.4%)	1.7% (0.4% to 4.2%)	3.3% (1.4% to 6.2%)
**Cirrhosis and other chronic liver diseases**	23.7% (21.1% to 26.5%)	22.8% (20.2% to 25.6%)	17.7% (15.1% to 20.6%)	22.7% (20.1% to 25.5%)
Cirrhosis and other chronic liver diseases due to hepatitis B	0.0% (0.0% to 0.0%)	0.0% (0.0% to 0.0%)	0.0% (0.0% to 0.0%)	0.0% (0.0% to 0.0%)
Cirrhosis and other chronic liver diseases due to hepatitis C	0.3% (0.1% to 0.6%)	0.5% (0.2% to 0.8%)	0.5% (0.2% to 1.0%)	0.5% (0.2% to 0.8%)
Cirrhosis and other chronic liver diseases due to alcohol use	100.0% (100.0% to 100.0%)	100.0% (100.0% to 100.0%)	100.0% (100.0% to 100.0%)	100.0% (100.0% to 100.0%)
**Digestive diseases**	6.2% (3.4% to 9.4%)	8.1% (4.3% to 12.1%)	1.9% (1.3% to 2.5%)	7.7% (4.1% to 11.6%)
Peptic ulcer disease	6.2% (4.0% to 8.8%)	7.6% (5.0% to 10.5%)	6.0% (4.1% to 8.4%)	7.5% (4.9% to 10.5%)
Gallbladder and biliary diseases	20.7% (10.8% to 32.6%)	22.6% (12.8% to 34.2%)	28.2% (17.0% to 40.9%)	22.8% (13.1% to 34.3%)
Pancreatitis	34.5% (10.4% to 60.2%)	41.3% (12.8% to 68.6%)	34.1% (10.5% to 61.1%)	41.3% (12.8% to 68.6%)
**Neurological disorders**	13.0% (6.4% to 21.6%)	15.2% (9.1% to 21.6%)	3.7% (1.5% to 7.0%)	8.4% (4.9% to 11.9%)
Alzheimer's disease and other dementias	13.0% (5.4% to 23.3%)	14.1% (6.1% to 24.5%)	14.1% (6.2% to 24.8%)	14.1% (6.1% to 24.6%)
Parkinson's disease	-2.4% (-3.5% to -1.7%)	-3.4% (-4.7% to -2.4%)	-4.5% (-6.1% to -3.3%)	-3.6% (-4.9% to -2.6%)
Epilepsy	24.2% (12.4% to 35.8%)	23.4% (12.2% to 34.4%)	17.0% (8.2% to 25.5%)	20.7% (10.3% to 30.7%)
Multiple sclerosis	4.7% (2.4% to 7.7%)	5.2% (2.6% to 8.5%)	4.0% (2.3% to 5.8%)	4.9% (2.5% to 7.7%)
**Mental and substance use disorders**	99.9% (99.7% to 99.9%)	99.8% (99.6% to 99.9%)	20.1% (17.9% to 22.5%)	25.2% (22.1% to 28.6%)
Alcohol use disorders	100.0% (100.0% to 100.0%)	100.0% (100.0% to 100.0%)	100.0% (100.0% to 100.0%)	100.0% (100.0% to 100.0%)

Supplementary Results Table 1. Age-standardised PAFs attributable to the joint distribution of all risk factors for all ages, both sexes combined for each GBD level 1 and level 2 cause and all causes in 2016 for Cote d'Ivoire. Results are shown for deaths, YLLs, YLDs, and DALYs
Data in parenthesis are 95% uncertainty intervals. PAFs=population attributable fractions. GBD=Global Burden of Disease. YLLs=years of life lost. YLDs=years live with disability. DALYs=disability-adjusted life-years

	Deaths 2016	YLLs 2016	YLDs 2016	DALYs 2016
Drug use disorders	100.0% (100.0% to 100.0%)	100.0% (100.0% to 100.0%)	100.0% (100.0% to 100.0%)	100.0% (100.0% to 100.0%)
Depressive disorders	-	-	10.3% (8.8% to 12.0%)	10.3% (8.8% to 12.0%)
Idiopathic developmental intellectual disability	-	-	62.1% (30.9% to 81.3%)	62.1% (30.9% to 81.3%)
**Diabetes, urogenital, blood, and endocrine diseases**	77.8% (75.4% to 80.0%)	69.8% (66.6% to 73.3%)	57.0% (53.2% to 60.7%)	65.9% (63.1% to 69.1%)
Diabetes mellitus	100.0% (100.0% to 100.0%)	100.0% (100.0% to 100.0%)	100.0% (100.0% to 100.0%)	100.0% (100.0% to 100.0%)
Chronic kidney disease	100.0% (100.0% to 100.0%)	100.0% (100.0% to 100.0%)	100.0% (100.0% to 100.0%)	100.0% (100.0% to 100.0%)
**Musculoskeletal disorders**	0.3% (0.1% to 0.7%)	0.2% (0.1% to 0.4%)	24.4% (21.0% to 28.0%)	23.9% (20.6% to 27.6%)
Rheumatoid arthritis	1.5% (0.5% to 3.4%)	1.8% (0.7% to 3.5%)	2.6% (1.0% to 4.4%)	2.5% (1.0% to 4.3%)
Osteoarthritis	-	-	16.0% (7.6% to 26.4%)	16.0% (7.6% to 26.4%)
Low back and neck pain	-	-	30.0% (26.4% to 33.7%)	30.0% (26.4% to 33.7%)
Gout	-	-	49.3% (40.0% to 60.7%)	49.3% (40.0% to 60.7%)
**Other non-communicable diseases**	0.1% (0.0% to 0.3%)	0.2% (0.1% to 0.4%)	8.6% (7.1% to 10.2%)	5.3% (3.9% to 6.9%)
Sense organ diseases	-	-	16.8% (14.7% to 19.0%)	16.8% (14.7% to 19.0%)
Sudden infant death syndrome	5.0% (1.8% to 10.8%)	5.0% (1.8% to 10.8%)	-	5.0% (1.8% to 10.8%)
**Transport injuries**	29.2% (22.9% to 37.6%)	24.1% (17.9% to 32.1%)	32.7% (26.8% to 40.7%)	25.0% (18.8% to 33.1%)
Road injuries	29.2% (22.8% to 37.7%)	24.1% (17.8% to 32.1%)	34.6% (28.5% to 42.6%)	25.1% (18.9% to 33.2%)
Other transport injuries	29.7% (22.8% to 38.1%)	23.9% (17.3% to 32.3%)	23.8% (18.5% to 30.9%)	23.9% (17.7% to 31.8%)
**Unintentional injuries**	21.2% (15.6% to 28.8%)	12.3% (9.8% to 15.6%)	17.2% (15.9% to 18.9%)	13.4% (11.2% to 16.2%)
Falls	47.8% (34.0% to 64.6%)	32.3% (25.6% to 40.7%)	26.0% (24.6% to 27.4%)	29.7% (25.4% to 34.8%)
Drowning	9.4% (6.0% to 14.4%)	9.1% (6.2% to 13.3%)	11.0% (7.2% to 16.5%)	9.1% (6.2% to 13.3%)
Fire, heat, and hot substances	8.3% (5.0% to 13.4%)	8.9% (5.8% to 13.5%)	12.1% (8.4% to 17.5%)	9.3% (6.1% to 14.1%)

Supplementary Results Table 1. Age-standardised PAFs attributable to the joint distribution of all risk factors for all ages, both sexes combined for each GBD level 1 and level 2 cause and all causes in 2016 for Cote d'Ivoire. Results are shown for deaths, YLLs, YLDs, and DALYs
Data in parenthesis are 95% uncertainty intervals. PAFs=population attributable fractions. GBD=Global Burden of Disease. YLLs=years of life lost. YLDs=years live with disability. DALYs=disability-adjusted life-years

	Deaths 2016	YLLs 2016	YLDs 2016	DALYs 2016
Poisonings	10.6% (6.5% to 16.7%)	10.6% (6.0% to 16.5%)	11.9% (8.3% to 17.1%)	10.6% (6.1% to 16.5%)
Exposure to mechanical forces	15.0% (12.4% to 17.5%)	10.5% (8.5% to 12.9%)	15.0% (13.6% to 16.7%)	11.5% (9.6% to 13.6%)
Animal contact	4.7% (3.8% to 5.7%)	5.1% (4.1% to 6.4%)	7.1% (5.8% to 8.6%)	5.4% (4.4% to 6.7%)
Foreign body	3.4% (2.7% to 4.3%)	3.9% (2.8% to 5.2%)	5.5% (4.4% to 6.9%)	4.4% (3.3% to 5.6%)
Other unintentional injuries	11.4% (7.4% to 17.0%)	11.7% (7.9% to 17.1%)	11.1% (7.5% to 16.4%)	11.5% (7.8% to 16.9%)
**Self-harm and interpersonal violence**	20.4% (9.8% to 33.3%)	21.7% (11.2% to 34.9%)	19.0% (15.6% to 23.6%)	21.6% (11.5% to 34.0%)
Self-harm	20.3% (8.4% to 34.3%)	22.2% (9.8% to 36.6%)	19.7% (8.0% to 33.6%)	22.2% (9.8% to 36.6%)
Interpersonal violence	22.6% (13.6% to 29.7%)	20.7% (12.4% to 27.5%)	18.9% (15.8% to 22.5%)	20.0% (12.9% to 26.0%)
**Forces of nature, conflict and terrorism, and executions and police conflict**	0.0% (0.0% to 0.0%)	0.0% (0.0% to 0.0%)	0.1% (0.1% to 0.1%)	0.0% (0.0% to 0.1%)
Forces of nature, conflict and terrorism, and state actor violence	0.0% (0.0% to 0.0%)	0.0% (0.0% to 0.0%)	6.1% (4.1% to 8.7%)	6.1% (4.1% to 8.7%)

Supplementary Results Table 1. Age-standardised PAFs attributable to the joint distribution of all risk factors for all ages, both sexes combined for each GBD level 1 and level 2 cause and all causes in 2016 for Croatia. Results are shown for deaths, YLLs, YLDs, and DALYs
Data in parenthesis are 95% uncertainty intervals. PAFs=population attributable fractions. GBD=Global Burden of Disease. YLLs=years of life lost. YLDs=years live with disability. DALYs=disability-adjusted life-years

	Deaths 2016	YLLs 2016	YLDs 2016	DALYs 2016
**All causes**	66.5% (64.6% to 68.5%)	62.2% (60.5% to 63.9%)	23.6% (22.1% to 25.1%)	44.9% (41.3% to 48.3%)
**HIV/AIDS and tuberculosis**	65.6% (58.5% to 73.2%)	69.8% (63.6% to 76.1%)	62.5% (54.6% to 69.9%)	69.0% (63.1% to 75.2%)
Tuberculosis	62.4% (53.8% to 71.3%)	66.1% (58.3% to 74.3%)	52.6% (44.5% to 60.7%)	64.7% (57.0% to 72.8%)
HIV/AIDS	88.0% (86.6% to 89.4%)	82.4% (79.9% to 84.5%)	94.2% (93.4% to 95.0%)	83.7% (81.4% to 85.8%)
**Diarrhoea, lower respiratory infections, and other common infectious diseases**	54.2% (46.2% to 61.1%)	56.0% (49.8% to 61.4%)	34.7% (25.6% to 44.3%)	44.8% (38.2% to 51.5%)
Diarrhoeal diseases	53.7% (30.2% to 76.7%)	66.4% (48.3% to 83.2%)	66.5% (49.1% to 83.1%)	66.5% (49.3% to 82.8%)
Lower respiratory infections	57.8% (49.0% to 65.6%)	64.6% (57.1% to 71.2%)	61.8% (54.0% to 69.5%)	64.5% (57.0% to 71.2%)
Upper respiratory infections	0.5% (0.0% to 1.7%)	1.5% (0.1% to 5.4%)	0.0% (0.0% to 0.0%)	0.0% (0.0% to 0.0%)
Otitis media	1.6% (0.7% to 3.2%)	3.3% (1.5% to 6.1%)	9.9% (6.7% to 12.7%)	9.9% (6.7% to 12.7%)
Meningitis	1.8% (1.2% to 2.5%)	3.6% (2.6% to 4.9%)	0.0% (0.0% to 0.0%)	2.1% (1.5% to 2.9%)
Encephalitis	0.5% (0.2% to 0.8%)	0.9% (0.5% to 1.3%)	0.0% (0.0% to 0.0%)	0.5% (0.3% to 0.9%)
Measles	13.5% (3.9% to 37.4%)	16.4% (4.7% to 44.7%)	26.7% (8.1% to 68.9%)	22.5% (6.9% to 57.3%)
**Maternal disorders**	4.4% (1.9% to 7.0%)	4.3% (1.9% to 6.9%)	5.0% (2.0% to 8.4%)	4.6% (1.9% to 7.4%)
Maternal haemorrhage	11.6% (4.2% to 19.0%)	11.6% (4.2% to 19.0%)	11.7% (4.2% to 19.2%)	11.7% (4.1% to 19.1%)
Maternal sepsis and other pregnancy related infections	11.5% (4.1% to 18.7%)	11.5% (4.1% to 18.8%)	11.6% (4.0% to 19.0%)	11.5% (4.0% to 18.8%)
Maternal abortion, miscarriage, and ectopic pregnancy	3.5% (2.1% to 5.3%)	3.5% (2.1% to 5.2%)	3.6% (1.7% to 6.2%)	3.5% (2.0% to 5.4%)
**Neonatal disorders**	73.3% (70.7% to 76.0%)	73.4% (70.7% to 76.1%)	0.1% (0.0% to 0.1%)	50.4% (43.5% to 56.5%)
Neonatal preterm birth complications	94.8% (92.9% to 96.5%)	94.9% (92.9% to 96.5%)	0.2% (0.1% to 0.2%)	59.1% (48.8% to 69.0%)
Neonatal encephalopathy due to birth asphyxia and trauma	65.4% (61.8% to 69.1%)	65.4% (61.9% to 69.1%)	0.0% (0.0% to 0.0%)	45.5% (37.4% to 52.8%)
Neonatal sepsis and other neonatal infections	53.4% (48.8% to 57.3%)	53.5% (48.9% to 57.3%)	0.0% (0.0% to 0.0%)	26.5% (14.4% to 37.8%)
Hemolytic disease and other neonatal jaundice	66.3% (62.5% to 69.9%)	66.4% (62.5% to 69.9%)	0.0% (0.0% to 0.0%)	40.6% (30.4% to 49.5%)

Supplementary Results Table 1. Age-standardised PAFs attributable to the joint distribution of all risk factors for all ages, both sexes combined for each GBD level 1 and level 2 cause and all causes in 2016 for Croatia. Results are shown for deaths, YLLs, YLDs, and DALYs
Data in parenthesis are 95% uncertainty intervals. PAFs=population attributable fractions. GBD=Global Burden of Disease. YLLs=years of life lost. YLDs=years live with disability. DALYs=disability-adjusted life-years

	Deaths 2016	YLLs 2016	YLDs 2016	DALYs 2016
Other neonatal disorders	62.8% (58.7% to 66.8%)	62.8% (58.7% to 66.8%)	0.0% (0.0% to 0.0%)	60.0% (55.5% to 64.2%)
**Nutritional deficiencies**	13.4% (9.9% to 16.3%)	25.9% (21.5% to 32.9%)	78.4% (71.1% to 84.2%)	77.4% (70.3% to 83.4%)
Protein-energy malnutrition	13.4% (7.7% to 21.5%)	37.1% (25.7% to 48.8%)	96.3% (93.7% to 98.1%)	94.1% (90.6% to 96.6%)
Vitamin A deficiency	-	-	0.0% (0.0% to 0.0%)	0.0% (0.0% to 0.0%)
Iron-deficiency anaemia	100.0% (100.0% to 100.0%)	100.0% (100.0% to 100.0%)	100.0% (100.0% to 100.0%)	100.0% (100.0% to 100.0%)
**Other communicable, maternal, neonatal, and nutritional diseases**	5.1% (4.2% to 6.2%)	4.6% (3.8% to 5.7%)	82.6% (78.7% to 86.0%)	64.8% (55.7% to 73.5%)
Sexually transmitted diseases excluding HIV	88.4% (72.8% to 97.5%)	73.8% (48.1% to 93.0%)	100.0% (100.0% to 100.0%)	99.4% (98.4% to 99.9%)
Hepatitis	2.3% (1.7% to 3.0%)	2.0% (1.5% to 2.7%)	0.8% (0.5% to 1.4%)	1.9% (1.4% to 2.5%)
**Neoplasms**	49.0% (45.6% to 52.6%)	50.2% (46.9% to 53.5%)	39.5% (35.9% to 43.1%)	49.9% (46.5% to 53.3%)
Oesophageal cancer	89.8% (84.8% to 94.0%)	91.5% (86.7% to 95.1%)	90.3% (85.4% to 94.3%)	91.4% (86.7% to 95.1%)
Stomach cancer	28.2% (17.8% to 38.4%)	30.4% (20.1% to 40.7%)	27.9% (18.0% to 38.5%)	30.4% (20.1% to 40.6%)
Liver cancer	68.1% (58.5% to 76.3%)	69.7% (60.4% to 77.4%)	68.2% (59.1% to 76.3%)	69.6% (60.3% to 77.4%)
Larynx cancer	94.3% (90.7% to 96.8%)	94.7% (91.2% to 97.1%)	94.5% (91.0% to 96.8%)	94.7% (91.2% to 97.1%)
Tracheal, bronchus, and lung cancer	91.9% (90.1% to 93.5%)	92.5% (90.8% to 94.0%)	91.3% (89.3% to 93.1%)	92.4% (90.8% to 93.9%)
Breast cancer	31.4% (25.0% to 38.9%)	31.3% (25.2% to 38.0%)	31.2% (25.1% to 38.3%)	31.3% (25.2% to 38.0%)
Cervical cancer	100.0% (100.0% to 100.0%)	100.0% (100.0% to 100.0%)	100.0% (100.0% to 100.0%)	100.0% (100.0% to 100.0%)
Uterine cancer	43.0% (27.2% to 58.7%)	44.6% (29.0% to 60.3%)	44.8% (29.1% to 60.4%)	44.6% (29.0% to 60.3%)
Prostate cancer	7.8% (5.5% to 10.6%)	9.1% (6.5% to 12.2%)	9.5% (6.7% to 12.7%)	9.1% (6.5% to 12.2%)
Colon and rectum cancer	59.0% (50.6% to 67.0%)	60.4% (52.2% to 68.2%)	59.5% (51.2% to 67.6%)	60.4% (52.1% to 68.2%)
Lip and oral cavity cancer	90.2% (86.9% to 93.0%)	92.3% (89.4% to 94.6%)	89.4% (85.6% to 92.4%)	92.3% (89.3% to 94.5%)
Nasopharynx cancer	88.5% (85.3% to 91.0%)	87.2% (84.1% to 89.8%)	87.3% (83.3% to 90.5%)	87.2% (84.1% to 89.8%)

Supplementary Results Table 1. Age-standardised PAFs attributable to the joint distribution of all risk factors for all ages, both sexes combined for each GBD level 1 and level 2 cause and all causes in 2016 for Croatia. Results are shown for deaths, YLLs, YLDs, and DALYs
Data in parenthesis are 95% uncertainty intervals. PAFs=population attributable fractions. GBD=Global Burden of Disease. YLLs=years of life lost. YLDs=years live with disability. DALYs=disability-adjusted life-years

	Deaths 2016	YLLs 2016	YLDs 2016	DALYs 2016
Other pharynx cancer	76.2% (68.2% to 83.1%)	77.0% (69.1% to 83.5%)	76.2% (68.3% to 83.0%)	77.0% (69.1% to 83.5%)
Gallbladder and biliary tract cancer	22.2% (11.5% to 35.3%)	22.8% (12.1% to 35.9%)	23.3% (12.0% to 36.0%)	22.8% (12.1% to 35.9%)
Pancreatic cancer	37.4% (29.7% to 45.3%)	40.6% (32.7% to 48.6%)	36.9% (29.4% to 44.6%)	40.6% (32.7% to 48.6%)
Ovarian cancer	12.1% (5.7% to 19.5%)	10.3% (4.5% to 17.1%)	10.0% (4.2% to 16.5%)	10.3% (4.5% to 17.1%)
Kidney cancer	43.1% (31.8% to 54.4%)	45.1% (33.8% to 56.3%)	41.9% (31.5% to 52.5%)	44.9% (33.7% to 56.1%)
Bladder cancer	41.3% (31.2% to 51.0%)	45.9% (35.5% to 55.4%)	45.4% (35.6% to 54.8%)	45.8% (35.5% to 55.4%)
Thyroid cancer	14.4% (7.0% to 23.9%)	15.0% (7.3% to 24.8%)	13.3% (6.8% to 21.3%)	14.7% (7.2% to 24.4%)
Mesothelioma	96.3% (93.7% to 97.3%)	93.8% (89.8% to 95.4%)	95.5% (92.3% to 96.7%)	93.9% (89.9% to 95.4%)
Non-Hodgkin's lymphoma	7.0% (2.7% to 12.6%)	6.6% (2.6% to 11.8%)	6.9% (2.7% to 12.4%)	6.6% (2.6% to 11.8%)
Multiple myeloma	8.4% (3.5% to 15.4%)	8.6% (3.6% to 15.6%)	8.6% (3.5% to 15.7%)	8.6% (3.6% to 15.6%)
Leukaemia	23.9% (14.6% to 32.5%)	21.9% (13.7% to 29.6%)	22.4% (13.9% to 30.7%)	21.9% (13.8% to 29.6%)
**Cardiovascular diseases**	86.9% (84.5% to 89.1%)	89.2% (87.6% to 90.7%)	81.9% (79.6% to 84.0%)	88.3% (86.7% to 89.9%)
Rheumatic heart disease	31.9% (20.7% to 50.5%)	32.3% (22.1% to 47.0%)	32.3% (21.7% to 47.2%)	32.3% (22.1% to 47.0%)
Ischaemic heart disease	93.6% (90.1% to 96.4%)	95.3% (93.1% to 97.2%)	92.8% (90.7% to 94.7%)	95.2% (92.9% to 97.0%)
Cerebrovascular disease	86.0% (81.2% to 90.3%)	89.5% (86.3% to 92.2%)	87.7% (83.9% to 90.9%)	89.2% (85.9% to 91.9%)
Hypertensive heart disease	100.0% (100.0% to 100.0%)	100.0% (100.0% to 100.0%)	100.0% (100.0% to 100.0%)	100.0% (100.0% to 100.0%)
Cardiomyopathy and myocarditis	36.8% (30.2% to 48.3%)	50.2% (44.8% to 57.9%)	51.6% (46.9% to 56.3%)	50.3% (45.4% to 57.5%)
Atrial fibrillation and flutter	57.0% (48.8% to 64.3%)	62.6% (55.6% to 69.0%)	71.1% (65.9% to 75.8%)	67.5% (61.6% to 72.7%)
Aortic aneurysm	53.9% (47.1% to 60.0%)	59.5% (53.5% to 64.9%)	-	59.5% (53.5% to 64.9%)
Peripheral vascular disease	50.7% (43.1% to 58.7%)	57.7% (51.2% to 64.5%)	59.5% (53.2% to 65.4%)	58.0% (51.5% to 64.2%)
Endocarditis	46.5% (37.7% to 56.0%)	45.3% (36.9% to 53.4%)	44.2% (35.4% to 53.1%)	45.2% (37.0% to 53.4%)

Supplementary Results Table 1. Age-standardised PAFs attributable to the joint distribution of all risk factors for all ages, both sexes combined for each GBD level 1 and level 2 cause and all causes in 2016 for Croatia. Results are shown for deaths, YLLs, YLDs, and DALYs
Data in parenthesis are 95% uncertainty intervals. PAFs=population attributable fractions. GBD=Global Burden of Disease. YLLs=years of life lost. YLDs=years live with disability. DALYs=disability-adjusted life-years

	Deaths 2016	YLLs 2016	YLDs 2016	DALYs 2016
Other cardiovascular and circulatory diseases	52.8% (47.5% to 57.9%)	59.0% (54.2% to 63.3%)	58.7% (54.0% to 63.3%)	58.9% (54.2% to 63.3%)
**Chronic respiratory diseases**	77.0% (71.8% to 81.5%)	79.0% (75.0% to 82.6%)	49.0% (42.4% to 55.6%)	65.7% (60.1% to 70.7%)
Chronic obstructive pulmonary disease	79.8% (74.4% to 84.4%)	82.9% (78.6% to 86.5%)	80.4% (75.9% to 84.0%)	82.3% (78.1% to 85.9%)
Pneumoconiosis	100.0% (100.0% to 100.0%)	100.0% (100.0% to 100.0%)	100.0% (100.0% to 100.0%)	100.0% (100.0% to 100.0%)
Asthma	38.8% (27.5% to 51.2%)	43.5% (33.2% to 54.5%)	35.4% (27.1% to 44.6%)	36.0% (27.6% to 45.1%)
Other chronic respiratory diseases	20.3% (15.0% to 27.3%)	17.6% (12.7% to 24.4%)	10.7% (7.8% to 14.1%)	12.5% (9.2% to 17.0%)
**Cirrhosis and other chronic liver diseases**	58.3% (53.2% to 63.2%)	59.0% (53.9% to 63.9%)	46.6% (41.4% to 51.4%)	58.5% (53.5% to 63.4%)
Cirrhosis and other chronic liver diseases due to hepatitis B	0.2% (0.1% to 0.4%)	0.3% (0.1% to 0.5%)	0.3% (0.1% to 0.4%)	0.3% (0.1% to 0.5%)
Cirrhosis and other chronic liver diseases due to hepatitis C	82.6% (77.4% to 86.5%)	86.0% (82.7% to 88.5%)	83.8% (79.5% to 87.3%)	86.0% (82.7% to 88.4%)
Cirrhosis and other chronic liver diseases due to alcohol use	100.0% (100.0% to 100.0%)	100.0% (100.0% to 100.0%)	100.0% (100.0% to 100.0%)	100.0% (100.0% to 100.0%)
**Digestive diseases**	15.4% (12.4% to 18.7%)	19.4% (15.8% to 23.6%)	5.3% (4.0% to 6.9%)	13.5% (11.0% to 16.5%)
Peptic ulcer disease	14.9% (10.5% to 20.0%)	19.6% (14.0% to 25.7%)	21.3% (15.4% to 27.7%)	20.2% (14.6% to 26.4%)
Gallbladder and biliary diseases	39.2% (24.2% to 54.9%)	41.0% (26.4% to 56.2%)	44.1% (29.9% to 59.4%)	41.6% (27.3% to 56.8%)
Pancreatitis	53.0% (41.2% to 66.0%)	58.6% (46.5% to 70.3%)	47.7% (36.3% to 58.0%)	58.2% (46.2% to 70.0%)
**Neurological disorders**	18.8% (9.6% to 30.1%)	18.7% (11.5% to 27.4%)	3.6% (1.8% to 6.1%)	8.5% (5.1% to 12.5%)
Alzheimer's disease and other dementias	23.0% (11.4% to 37.0%)	25.0% (13.3% to 39.3%)	25.0% (13.3% to 39.2%)	25.0% (13.3% to 39.2%)
Parkinson's disease	-9.8% (-12.1% to -7.7%)	-13.0% (-15.9% to -10.3%)	-15.7% (-18.9% to -12.4%)	-13.5% (-16.4% to -10.7%)
Epilepsy	39.2% (30.3% to 48.1%)	35.2% (27.2% to 43.3%)	26.9% (20.0% to 33.5%)	31.2% (23.6% to 38.5%)
Multiple sclerosis	14.4% (9.5% to 19.5%)	15.6% (10.3% to 21.0%)	13.8% (9.0% to 18.9%)	15.0% (9.9% to 20.3%)
**Mental and substance use disorders**	99.8% (99.7% to 99.9%)	99.7% (99.6% to 99.8%)	20.0% (18.0% to 22.3%)	28.5% (25.3% to 32.0%)
Alcohol use disorders	100.0% (100.0% to 100.0%)	100.0% (100.0% to 100.0%)	100.0% (100.0% to 100.0%)	100.0% (100.0% to 100.0%)

Supplementary Results Table 1. Age-standardised PAFs attributable to the joint distribution of all risk factors for all ages, both sexes combined for each GBD level 1 and level 2 cause and all causes in 2016 for Croatia. Results are shown for deaths, YLLs, YLDs, and DALYs
Data in parenthesis are 95% uncertainty intervals. PAFs=population attributable fractions. GBD=Global Burden of Disease. YLLs=years of life lost. YLDs=years live with disability. DALYs=disability-adjusted life-years

	Deaths 2016	YLLs 2016	YLDs 2016	DALYs 2016
Drug use disorders	100.0% (100.0% to 100.0%)	100.0% (100.0% to 100.0%)	100.0% (100.0% to 100.0%)	100.0% (100.0% to 100.0%)
Depressive disorders	-	-	6.3% (5.2% to 7.4%)	6.3% (5.2% to 7.4%)
Idiopathic developmental intellectual disability	-	-	55.1% (25.7% to 74.5%)	55.1% (25.7% to 74.5%)
**Diabetes, urogenital, blood, and endocrine diseases**	79.0% (77.2% to 80.6%)	77.4% (74.4% to 79.1%)	56.6% (52.5% to 60.8%)	65.3% (62.0% to 68.4%)
Diabetes mellitus	100.0% (100.0% to 100.0%)	100.0% (100.0% to 100.0%)	100.0% (100.0% to 100.0%)	100.0% (100.0% to 100.0%)
Chronic kidney disease	100.0% (100.0% to 100.0%)	100.0% (100.0% to 100.0%)	100.0% (100.0% to 100.0%)	100.0% (100.0% to 100.0%)
**Musculoskeletal disorders**	2.2% (0.8% to 3.7%)	2.1% (0.8% to 3.6%)	22.9% (18.9% to 27.1%)	22.6% (18.7% to 26.7%)
Rheumatoid arthritis	5.6% (2.2% to 9.3%)	7.4% (3.0% to 12.2%)	8.7% (3.6% to 14.3%)	8.6% (3.6% to 14.0%)
Osteoarthritis	-	-	27.0% (13.8% to 40.5%)	27.0% (13.8% to 40.5%)
Low back and neck pain	-	-	24.1% (20.3% to 27.9%)	24.1% (20.3% to 27.9%)
Gout	-	-	45.3% (28.6% to 62.6%)	45.3% (28.6% to 62.6%)
**Other non-communicable diseases**	0.5% (0.3% to 0.7%)	0.6% (0.4% to 0.8%)	3.3% (2.8% to 3.7%)	2.9% (2.5% to 3.3%)
Sense organ diseases	-	-	8.0% (7.2% to 9.0%)	8.0% (7.2% to 9.0%)
Sudden infant death syndrome	5.6% (3.7% to 7.9%)	5.6% (3.7% to 7.9%)	-	5.6% (3.7% to 7.9%)
**Transport injuries**	35.3% (26.4% to 45.7%)	31.0% (22.1% to 41.7%)	37.1% (28.5% to 46.8%)	33.1% (24.8% to 43.6%)
Road injuries	34.7% (25.9% to 45.2%)	30.6% (21.8% to 41.2%)	37.2% (28.6% to 46.9%)	33.0% (24.7% to 43.5%)
Other transport injuries	39.5% (29.3% to 51.0%)	34.3% (25.0% to 45.2%)	32.8% (23.9% to 42.6%)	34.1% (24.8% to 45.0%)
**Unintentional injuries**	46.0% (29.8% to 52.6%)	25.5% (16.7% to 30.2%)	19.1% (17.1% to 21.4%)	21.1% (17.8% to 23.8%)
Falls	63.9% (50.1% to 70.1%)	42.6% (29.6% to 48.7%)	21.5% (19.3% to 23.6%)	26.0% (21.9% to 29.1%)
Drowning	17.7% (10.0% to 26.4%)	16.3% (9.9% to 23.9%)	15.3% (9.3% to 22.6%)	16.2% (9.9% to 23.9%)
Fire, heat, and hot substances	13.7% (7.1% to 21.9%)	15.0% (8.6% to 22.3%)	16.7% (10.1% to 24.6%)	16.0% (9.5% to 23.5%)

Supplementary Results Table 1. Age-standardised PAFs attributable to the joint distribution of all risk factors for all ages, both sexes combined for each GBD level 1 and level 2 cause and all causes in 2016 for Croatia. Results are shown for deaths, YLLs, YLDs, and DALYs
Data in parenthesis are 95% uncertainty intervals. PAFs=population attributable fractions. GBD=Global Burden of Disease. YLLs=years of life lost. YLDs=years live with disability. DALYs=disability-adjusted life-years

	**Deaths 2016**	**YLLs 2016**	**YLDs 2016**	**DALYs 2016**
Poisonings	14.8% (8.4% to 22.3%)	14.4% (8.7% to 21.3%)	15.6% (9.9% to 22.8%)	15.1% (9.4% to 21.6%)
Exposure to mechanical forces	15.4% (12.3% to 18.6%)	11.8% (9.0% to 15.2%)	13.5% (11.2% to 16.3%)	13.1% (10.6% to 15.8%)
Animal contact	10.6% (8.7% to 12.8%)	8.7% (6.7% to 11.2%)	5.4% (3.9% to 7.3%)	6.4% (4.8% to 8.5%)
Foreign body	4.0% (2.7% to 5.8%)	4.6% (3.2% to 6.5%)	3.7% (2.6% to 5.2%)	4.3% (3.0% to 6.0%)
Other unintentional injuries	19.4% (11.2% to 29.2%)	19.4% (12.0% to 28.5%)	15.4% (9.3% to 22.8%)	16.2% (9.8% to 23.8%)
**Self-harm and interpersonal violence**	38.5% (26.3% to 49.7%)	37.0% (25.4% to 48.1%)	36.3% (30.7% to 42.5%)	36.9% (25.9% to 47.4%)
Self-harm	38.2% (25.1% to 50.6%)	36.8% (23.9% to 49.2%)	30.0% (19.0% to 41.0%)	36.7% (23.8% to 49.1%)
Interpersonal violence	42.1% (32.8% to 51.7%)	38.9% (30.4% to 48.2%)	37.8% (31.8% to 44.7%)	38.5% (31.0% to 46.7%)
**Forces of nature, conflict and terrorism, and executions and police conflict**	0.0% (0.0% to 0.0%)	0.0% (0.0% to 0.0%)	0.0% (0.0% to 0.0%)	0.0% (0.0% to 0.0%)
Forces of nature, conflict and terrorism, and state actor violence	0.0% (0.0% to 0.0%)	0.0% (0.0% to 0.0%)	4.8% (3.1% to 7.1%)	4.8% (3.1% to 7.1%)

Supplementary Results Table 1. Age-standardised PAFs attributable to the joint distribution of all risk factors for all ages, both sexes combined for each GBD level 1 and level 2 cause and all causes in 2016 for Cuba. Results are shown for deaths, YLLs, YLDs, and DALYs

Data in parenthesis are 95% uncertainty intervals. PAFs=population attributable fractions. GBD=Global Burden of Disease. YLLs=years of life lost. YLDs=years live with disability. DALYs=disability-adjusted life-years

	Deaths 2016	YLLs 2016	YLDs 2016	DALYs 2016
**All causes**	60.0% (57.9% to 62.0%)	57.3% (55.1% to 59.4%)	23.4% (22.0% to 25.0%)	41.6% (38.7% to 44.7%)
**HIV/AIDS and tuberculosis**	83.1% (80.7% to 85.2%)	81.1% (78.2% to 83.3%)	88.3% (85.2% to 90.9%)	82.1% (79.2% to 84.5%)
Tuberculosis	46.7% (37.6% to 56.1%)	50.1% (41.2% to 58.9%)	45.7% (36.8% to 53.7%)	49.4% (40.6% to 57.9%)
HIV/AIDS	86.5% (83.8% to 88.5%)	82.5% (79.4% to 84.7%)	90.7% (87.9% to 92.8%)	83.6% (80.5% to 86.0%)
**Diarrhoea, lower respiratory infections, and other common infectious diseases**	48.4% (41.9% to 56.2%)	52.7% (47.1% to 58.9%)	34.1% (23.8% to 42.8%)	47.5% (42.0% to 53.1%)
Diarrhoeal diseases	76.1% (50.4% to 91.4%)	81.3% (61.1% to 93.3%)	78.0% (54.4% to 92.2%)	79.0% (56.5% to 92.6%)
Lower respiratory infections	48.8% (42.0% to 57.1%)	56.5% (50.1% to 63.9%)	52.6% (45.6% to 60.6%)	56.5% (50.1% to 63.8%)
Upper respiratory infections	0.5% (0.0% to 1.6%)	1.4% (0.1% to 4.3%)	0.0% (0.0% to 0.0%)	0.0% (0.0% to 0.0%)
Otitis media	2.1% (1.0% to 3.7%)	4.2% (2.3% to 6.7%)	6.4% (4.3% to 8.4%)	6.4% (4.3% to 8.4%)
Meningitis	2.0% (1.2% to 2.8%)	3.5% (2.5% to 4.8%)	0.0% (0.0% to 0.0%)	3.0% (2.1% to 4.1%)
Encephalitis	0.9% (0.6% to 1.3%)	1.4% (0.9% to 2.1%)	0.0% (0.0% to 0.0%)	1.1% (0.7% to 1.7%)
Measles	20.1% (6.9% to 51.1%)	21.1% (7.2% to 53.2%)	13.1% (0.0% to 60.1%)	21.1% (7.2% to 53.2%)
**Maternal disorders**	3.8% (1.8% to 5.9%)	3.7% (1.8% to 5.8%)	3.4% (1.7% to 5.4%)	3.6% (1.8% to 5.5%)
Maternal haemorrhage	14.7% (5.3% to 24.1%)	14.7% (5.3% to 24.1%)	14.7% (5.3% to 24.3%)	14.7% (5.3% to 24.1%)
Maternal sepsis and other pregnancy related infections	14.7% (5.3% to 24.0%)	14.7% (5.3% to 24.0%)	14.5% (5.2% to 23.7%)	14.6% (5.3% to 24.0%)
Maternal abortion, miscarriage, and ectopic pregnancy	5.5% (2.9% to 8.4%)	5.4% (2.9% to 8.4%)	5.4% (2.6% to 8.8%)	5.4% (2.8% to 8.4%)
**Neonatal disorders**	67.5% (63.7% to 71.2%)	67.6% (63.8% to 71.2%)	0.1% (0.1% to 0.2%)	37.8% (28.5% to 46.4%)
Neonatal preterm birth complications	88.3% (83.4% to 92.6%)	88.4% (83.5% to 92.7%)	0.2% (0.2% to 0.2%)	32.4% (21.2% to 44.9%)
Neonatal encephalopathy due to birth asphyxia and trauma	59.7% (54.7% to 64.2%)	59.8% (54.9% to 64.3%)	0.0% (0.0% to 0.0%)	49.1% (41.0% to 56.2%)
Neonatal sepsis and other neonatal infections	60.7% (56.5% to 64.5%)	60.7% (56.5% to 64.5%)	0.0% (0.0% to 0.0%)	25.9% (11.1% to 40.4%)
Hemolytic disease and other neonatal jaundice	61.2% (53.9% to 67.1%)	61.2% (54.1% to 67.1%)	0.0% (0.0% to 0.0%)	40.3% (30.0% to 49.9%)

Supplementary Results Table 1. Age-standardised PAFs attributable to the joint distribution of all risk factors for all ages, both sexes combined for each GBD level 1 and level 2 cause and all causes in 2016 for Cuba. Results are shown for deaths, YLLs, YLDs, and DALYs
Data in parenthesis are 95% uncertainty intervals. PAFs=population attributable fractions. GBD=Global Burden of Disease. YLLs=years of life lost. YLDs=years live with disability. DALYs=disability-adjusted life-years

	Deaths 2016	YLLs 2016	YLDs 2016	DALYs 2016
Other neonatal disorders	58.4% (53.9% to 63.0%)	58.5% (53.9% to 63.1%)	0.0% (0.0% to 0.0%)	54.1% (48.8% to 59.2%)
**Nutritional deficiencies**	11.3% (8.5% to 14.8%)	34.0% (27.6% to 40.9%)	97.5% (96.7% to 98.3%)	95.5% (93.9% to 96.7%)
Protein-energy malnutrition	13.6% (9.4% to 18.5%)	45.3% (35.4% to 55.4%)	98.1% (97.1% to 98.8%)	68.7% (59.9% to 77.6%)
Vitamin A deficiency	-	-	0.0% (0.0% to 0.0%)	0.0% (0.0% to 0.0%)
Iron-deficiency anaemia	100.0% (100.0% to 100.0%)	100.0% (100.0% to 100.0%)	100.0% (100.0% to 100.0%)	100.0% (100.0% to 100.0%)
**Other communicable, maternal, neonatal, and nutritional diseases**	16.1% (13.0% to 18.8%)	16.0% (12.6% to 19.6%)	67.3% (57.2% to 77.8%)	44.6% (35.2% to 56.0%)
Sexually transmitted diseases excluding HIV	84.2% (67.7% to 94.9%)	73.0% (48.1% to 90.2%)	100.0% (100.0% to 100.0%)	94.4% (87.0% to 98.6%)
Hepatitis	7.7% (5.2% to 11.1%)	6.8% (4.4% to 9.9%)	4.3% (2.6% to 6.4%)	6.6% (4.3% to 9.6%)
**Neoplasms**	46.8% (44.3% to 49.6%)	46.6% (44.2% to 49.3%)	36.1% (33.1% to 39.5%)	46.3% (43.9% to 49.0%)
Oesophageal cancer	81.2% (73.0% to 88.1%)	82.5% (74.7% to 89.1%)	81.5% (73.4% to 88.3%)	82.5% (74.7% to 89.1%)
Stomach cancer	22.1% (13.7% to 30.6%)	22.3% (14.2% to 30.6%)	22.4% (14.2% to 30.9%)	22.3% (14.2% to 30.6%)
Liver cancer	64.1% (56.7% to 71.1%)	64.0% (56.6% to 70.9%)	64.2% (56.6% to 71.2%)	64.0% (56.6% to 70.9%)
Larynx cancer	88.0% (81.2% to 92.7%)	88.5% (82.1% to 92.9%)	88.5% (82.0% to 92.8%)	88.5% (82.1% to 92.9%)
Tracheal, bronchus, and lung cancer	88.7% (86.7% to 90.6%)	88.8% (86.9% to 90.6%)	88.7% (86.7% to 90.6%)	88.8% (86.9% to 90.6%)
Breast cancer	26.6% (19.4% to 34.3%)	25.6% (18.1% to 33.0%)	26.0% (18.5% to 33.5%)	25.6% (18.2% to 33.0%)
Cervical cancer	100.0% (100.0% to 100.0%)	100.0% (100.0% to 100.0%)	100.0% (100.0% to 100.0%)	100.0% (100.0% to 100.0%)
Uterine cancer	43.2% (29.9% to 57.3%)	45.8% (31.7% to 59.5%)	46.9% (32.3% to 60.9%)	45.9% (31.7% to 59.6%)
Prostate cancer	7.1% (5.0% to 9.3%)	7.1% (5.1% to 9.3%)	7.3% (5.3% to 9.5%)	7.2% (5.1% to 9.3%)
Colon and rectum cancer	48.0% (39.1% to 56.8%)	49.0% (40.3% to 57.1%)	48.5% (39.7% to 57.0%)	48.9% (40.3% to 57.1%)
Lip and oral cavity cancer	80.4% (74.1% to 86.0%)	82.2% (76.5% to 87.5%)	79.5% (73.7% to 85.3%)	82.1% (76.5% to 87.4%)
Nasopharynx cancer	80.9% (75.1% to 85.5%)	79.8% (74.5% to 84.0%)	78.5% (73.2% to 82.9%)	79.8% (74.5% to 84.0%)

Supplementary Results Table 1. Age-standardised PAFs attributable to the joint distribution of all risk factors for all ages, both sexes combined for each GBD level 1 and level 2 cause and all causes in 2016 for Cuba. Results are shown for deaths, YLLs, YLDs, and DALYs
Data in parenthesis are 95% uncertainty intervals. PAFs=population attributable fractions. GBD=Global Burden of Disease. YLLs=years of life lost. YLDs=years live with disability. DALYs=disability-adjusted life-years

	Deaths 2016	YLLs 2016	YLDs 2016	DALYs 2016
Other pharynx cancer	48.9% (35.1% to 62.0%)	52.5% (38.9% to 65.6%)	50.6% (37.0% to 63.6%)	52.5% (38.8% to 65.6%)
Gallbladder and biliary tract cancer	19.9% (10.7% to 31.0%)	21.3% (11.6% to 32.9%)	20.1% (11.0% to 31.7%)	21.3% (11.6% to 32.9%)
Pancreatic cancer	36.7% (29.5% to 43.7%)	37.2% (30.1% to 44.2%)	36.8% (29.6% to 43.8%)	37.2% (30.1% to 44.2%)
Ovarian cancer	9.1% (3.1% to 16.6%)	8.6% (2.7% to 15.6%)	8.3% (2.7% to 15.0%)	8.6% (2.7% to 15.6%)
Kidney cancer	36.3% (27.1% to 45.9%)	34.1% (25.4% to 43.2%)	34.7% (25.8% to 44.0%)	34.1% (25.4% to 43.2%)
Bladder cancer	42.8% (33.1% to 52.8%)	43.0% (33.3% to 52.6%)	42.8% (33.3% to 52.4%)	43.0% (33.3% to 52.6%)
Thyroid cancer	11.9% (6.2% to 19.7%)	12.8% (6.6% to 21.2%)	13.2% (7.2% to 21.2%)	12.8% (6.7% to 21.2%)
Mesothelioma	73.7% (67.8% to 79.7%)	61.3% (54.5% to 68.9%)	69.4% (63.2% to 75.8%)	61.4% (54.6% to 69.0%)
Non-Hodgkin's lymphoma	5.5% (2.2% to 9.9%)	5.3% (2.1% to 9.5%)	5.6% (2.2% to 10.0%)	5.3% (2.1% to 9.5%)
Multiple myeloma	6.7% (2.9% to 12.1%)	7.2% (3.1% to 12.8%)	7.1% (3.1% to 12.9%)	7.2% (3.1% to 12.8%)
Leukaemia	20.1% (12.8% to 27.7%)	16.1% (10.5% to 22.0%)	18.9% (12.0% to 25.9%)	16.1% (10.5% to 22.0%)
**Cardiovascular diseases**	82.0% (78.8% to 84.7%)	84.4% (82.1% to 86.4%)	74.2% (71.3% to 76.7%)	83.5% (81.1% to 85.4%)
Rheumatic heart disease	22.8% (15.6% to 36.3%)	21.2% (14.4% to 31.7%)	23.0% (15.5% to 34.9%)	21.2% (14.4% to 31.8%)
Ischaemic heart disease	89.8% (85.5% to 93.3%)	92.2% (89.4% to 94.6%)	88.1% (85.0% to 91.0%)	92.0% (89.2% to 94.5%)
Cerebrovascular disease	79.0% (73.6% to 84.1%)	83.8% (80.1% to 87.3%)	80.0% (75.1% to 84.5%)	83.4% (79.7% to 87.0%)
Hypertensive heart disease	100.0% (100.0% to 100.0%)	100.0% (100.0% to 100.0%)	100.0% (100.0% to 100.0%)	100.0% (100.0% to 100.0%)
Cardiomyopathy and myocarditis	54.9% (43.9% to 62.0%)	59.1% (47.2% to 65.4%)	43.5% (38.1% to 49.2%)	58.1% (46.9% to 64.3%)
Atrial fibrillation and flutter	45.8% (38.4% to 53.3%)	50.9% (44.2% to 57.4%)	58.4% (53.0% to 63.6%)	55.5% (49.7% to 61.1%)
Aortic aneurysm	42.6% (36.0% to 49.2%)	48.0% (41.7% to 54.1%)	-	48.0% (41.7% to 54.1%)
Peripheral vascular disease	48.8% (40.1% to 56.1%)	55.6% (47.7% to 61.7%)	55.5% (50.1% to 60.6%)	55.6% (48.5% to 61.4%)
Endocarditis	33.7% (25.9% to 42.7%)	32.0% (24.8% to 39.8%)	31.4% (24.1% to 39.5%)	32.0% (24.8% to 39.8%)

Supplementary Results Table 1. Age-standardised PAFs attributable to the joint distribution of all risk factors for all ages, both sexes combined for each GBD level 1 and level 2 cause and all causes in 2016 for Cuba. Results are shown for deaths, YLLs, YLDs, and DALYs
Data in parenthesis are 95% uncertainty intervals. PAFs=population attributable fractions. GBD=Global Burden of Disease. YLLs=years of life lost. YLDs=years live with disability. DALYs=disability-adjusted life-years

	Deaths 2016	YLLs 2016	YLDs 2016	DALYs 2016
Other cardiovascular and circulatory diseases	44.6% (39.6% to 49.5%)	48.6% (44.0% to 53.2%)	46.8% (41.8% to 51.8%)	48.1% (43.5% to 52.9%)
**Chronic respiratory diseases**	79.0% (75.9% to 82.1%)	76.4% (73.5% to 79.3%)	36.3% (28.8% to 45.0%)	56.3% (49.6% to 62.8%)
Chronic obstructive pulmonary disease	85.0% (81.6% to 88.0%)	85.1% (82.0% to 87.9%)	78.2% (74.1% to 81.9%)	84.0% (80.8% to 86.9%)
Pneumoconiosis	100.0% (100.0% to 100.0%)	100.0% (100.0% to 100.0%)	100.0% (100.0% to 100.0%)	100.0% (100.0% to 100.0%)
Asthma	42.9% (34.5% to 52.3%)	44.1% (36.1% to 53.2%)	28.9% (20.9% to 38.9%)	30.8% (22.7% to 40.3%)
Other chronic respiratory diseases	24.9% (18.1% to 31.7%)	20.9% (15.1% to 27.0%)	6.9% (4.7% to 9.8%)	17.3% (12.5% to 22.2%)
**Cirrhosis and other chronic liver diseases**	69.4% (66.6% to 71.9%)	70.0% (67.3% to 72.4%)	54.7% (50.7% to 58.6%)	69.1% (66.6% to 71.5%)
Cirrhosis and other chronic liver diseases due to hepatitis B	6.4% (2.7% to 11.3%)	8.2% (3.5% to 14.4%)	6.4% (2.7% to 11.2%)	8.1% (3.5% to 14.3%)
Cirrhosis and other chronic liver diseases due to hepatitis C	87.0% (84.1% to 89.5%)	88.7% (87.2% to 90.0%)	86.0% (82.8% to 88.7%)	88.6% (87.0% to 89.9%)
Cirrhosis and other chronic liver diseases due to alcohol use	100.0% (100.0% to 100.0%)	100.0% (100.0% to 100.0%)	100.0% (100.0% to 100.0%)	100.0% (100.0% to 100.0%)
**Digestive diseases**	9.7% (7.5% to 12.1%)	11.9% (9.2% to 14.7%)	4.1% (3.0% to 5.4%)	9.3% (7.2% to 11.5%)
Peptic ulcer disease	15.4% (10.7% to 20.7%)	17.8% (12.5% to 23.7%)	12.9% (8.5% to 17.9%)	16.1% (11.2% to 21.6%)
Gallbladder and biliary diseases	37.7% (25.3% to 51.3%)	40.5% (28.3% to 54.1%)	45.3% (32.0% to 59.1%)	41.3% (28.9% to 54.9%)
Pancreatitis	31.8% (16.6% to 49.9%)	38.2% (22.7% to 56.7%)	28.3% (15.7% to 43.8%)	38.0% (22.6% to 56.4%)
**Neurological disorders**	18.0% (9.4% to 28.8%)	16.6% (9.4% to 25.2%)	3.5% (1.8% to 5.5%)	7.7% (4.3% to 11.7%)
Alzheimer's disease and other dementias	21.4% (11.2% to 33.8%)	22.8% (12.3% to 35.1%)	22.8% (12.3% to 35.1%)	22.8% (12.3% to 35.1%)
Parkinson's disease	-11.3% (-14.5% to -8.6%)	-13.9% (-17.4% to -10.4%)	-15.1% (-19.0% to -11.5%)	-14.1% (-17.8% to -10.7%)
Epilepsy	26.2% (18.7% to 33.6%)	25.4% (18.3% to 32.5%)	19.2% (12.9% to 25.5%)	21.3% (14.8% to 27.9%)
Multiple sclerosis	9.2% (5.9% to 12.9%)	8.9% (5.7% to 12.5%)	7.8% (5.0% to 10.9%)	8.6% (5.5% to 12.0%)
**Mental and substance use disorders**	100.0% (100.0% to 100.0%)	99.9% (99.9% to 99.9%)	17.4% (15.5% to 19.5%)	21.8% (19.4% to 24.3%)
Alcohol use disorders	100.0% (100.0% to 100.0%)	100.0% (100.0% to 100.0%)	100.0% (100.0% to 100.0%)	100.0% (100.0% to 100.0%)

Supplementary Results Table 1. Age-standardised PAFs attributable to the joint distribution of all risk factors for all ages, both sexes combined for each GBD level 1 and level 2 cause and all causes in 2016 for Cuba. Results are shown for deaths, YLLs, YLDs, and DALYs
Data in parenthesis are 95% uncertainty intervals. PAFs=population attributable fractions. GBD=Global Burden of Disease. YLLs=years of life lost. YLDs=years live with disability. DALYs=disability-adjusted life-years

	Deaths 2016	YLLs 2016	YLDs 2016	DALYs 2016
Drug use disorders	100.0% (100.0% to 100.0%)	100.0% (100.0% to 100.0%)	100.0% (100.0% to 100.0%)	100.0% (100.0% to 100.0%)
Depressive disorders	-	-	6.4% (5.3% to 7.8%)	6.4% (5.3% to 7.8%)
Idiopathic developmental intellectual disability	-	-	69.4% (37.4% to 89.1%)	69.4% (37.4% to 89.1%)
**Diabetes, urogenital, blood, and endocrine diseases**	84.2% (81.9% to 85.2%)	79.5% (77.6% to 81.0%)	68.0% (63.5% to 72.2%)	73.3% (70.5% to 75.8%)
Diabetes mellitus	100.0% (100.0% to 100.0%)	100.0% (100.0% to 100.0%)	100.0% (100.0% to 100.0%)	100.0% (100.0% to 100.0%)
Chronic kidney disease	100.0% (100.0% to 100.0%)	100.0% (100.0% to 100.0%)	100.0% (100.0% to 100.0%)	100.0% (100.0% to 100.0%)
**Musculoskeletal disorders**	1.2% (0.5% to 2.1%)	0.8% (0.3% to 1.5%)	13.0% (10.3% to 16.1%)	12.7% (10.0% to 15.7%)
Rheumatoid arthritis	4.9% (1.9% to 8.4%)	5.3% (2.0% to 9.2%)	5.6% (2.2% to 9.5%)	5.6% (2.2% to 9.5%)
Osteoarthritis	-	-	23.9% (12.9% to 36.0%)	23.9% (12.9% to 36.0%)
Low back and neck pain	-	-	16.8% (14.2% to 19.8%)	16.8% (14.2% to 19.8%)
Gout	-	-	41.7% (28.2% to 56.9%)	41.7% (28.2% to 56.9%)
**Other non-communicable diseases**	0.1% (0.0% to 0.1%)	0.1% (0.1% to 0.2%)	5.8% (5.0% to 6.7%)	5.1% (4.3% to 5.9%)
Sense organ diseases	-	-	13.8% (12.3% to 15.4%)	13.8% (12.3% to 15.4%)
Sudden infant death syndrome	6.1% (3.8% to 9.1%)	6.2% (3.8% to 9.2%)	-	6.2% (3.8% to 9.2%)
**Transport injuries**	46.7% (28.5% to 63.6%)	44.9% (23.6% to 64.8%)	49.6% (33.4% to 64.2%)	45.6% (25.2% to 64.6%)
Road injuries	45.8% (28.6% to 61.7%)	43.9% (23.9% to 63.2%)	50.1% (33.9% to 64.8%)	45.0% (25.5% to 63.5%)
Other transport injuries	51.8% (26.4% to 77.7%)	49.4% (22.0% to 79.1%)	42.5% (27.8% to 55.5%)	48.8% (22.7% to 77.5%)
**Unintentional injuries**	48.2% (32.4% to 56.5%)	35.6% (20.7% to 49.9%)	33.9% (21.6% to 46.2%)	34.8% (21.9% to 47.6%)
Falls	69.6% (59.7% to 74.4%)	55.9% (38.3% to 66.4%)	39.4% (27.4% to 51.1%)	45.4% (33.2% to 56.7%)
Drowning	30.9% (10.8% to 48.8%)	30.7% (8.6% to 51.1%)	31.6% (16.4% to 45.0%)	30.7% (8.7% to 51.0%)
Fire, heat, and hot substances	17.2% (9.8% to 24.1%)	24.4% (12.9% to 36.0%)	34.1% (17.7% to 50.4%)	30.7% (15.8% to 45.5%)

Supplementary Results Table 1. Age-standardised PAFs attributable to the joint distribution of all risk factors for all ages, both sexes combined for each GBD level 1 and level 2 cause and all causes in 2016 for Cuba. Results are shown for deaths, YLLs, YLDs, and DALYs
Data in parenthesis are 95% uncertainty intervals. PAFs=population attributable fractions. GBD=Global Burden of Disease. YLLs=years of life lost. YLDs=years live with disability. DALYs=disability-adjusted life-years

	Deaths 2016	YLLs 2016	YLDs 2016	DALYs 2016
Poisonings	19.0% (10.1% to 33.1%)	21.5% (10.3% to 35.6%)	32.7% (16.0% to 49.1%)	25.2% (12.1% to 38.6%)
Exposure to mechanical forces	27.0% (14.4% to 43.3%)	27.4% (9.7% to 47.2%)	35.3% (16.3% to 54.1%)	31.5% (13.3% to 50.3%)
Animal contact	18.4% (12.2% to 29.8%)	24.7% (12.0% to 37.5%)	27.6% (10.8% to 44.4%)	26.0% (11.3% to 40.1%)
Foreign body	11.9% (5.2% to 20.7%)	15.1% (5.4% to 25.2%)	19.4% (7.2% to 31.6%)	16.6% (6.1% to 27.2%)
Other unintentional injuries	35.7% (10.1% to 60.8%)	35.5% (8.3% to 62.5%)	31.1% (16.3% to 45.7%)	33.1% (13.0% to 53.0%)
**Self-harm and interpersonal violence**	25.6% (17.8% to 33.7%)	27.2% (19.3% to 34.6%)	27.9% (24.9% to 31.3%)	27.3% (19.7% to 34.4%)
Self-harm	24.7% (14.5% to 35.7%)	27.2% (16.5% to 38.4%)	22.3% (12.4% to 32.8%)	27.1% (16.4% to 38.3%)
Interpersonal violence	27.7% (20.3% to 35.0%)	27.4% (19.8% to 35.0%)	28.6% (25.6% to 32.0%)	27.6% (20.5% to 34.4%)
**Forces of nature, conflict and terrorism, and executions and police conflict**	0.0% (0.0% to 0.0%)	0.0% (0.0% to 0.0%)	4.0% (2.6% to 5.8%)	2.5% (0.8% to 4.3%)
Forces of nature, conflict and terrorism, and state actor violence	0.0% (0.0% to 0.0%)	0.0% (0.0% to 0.0%)	4.6% (3.1% to 6.7%)	4.6% (3.1% to 6.7%)

Supplementary Results Table 1. Age-standardised PAFs attributable to the joint distribution of all risk factors for all ages, both sexes combined for each GBD level 1 and level 2 cause and all causes in 2016 for Cyprus. Results are shown for deaths, YLLs, YLDs, and DALYs
Data in parenthesis are 95% uncertainty intervals. PAFs=population attributable fractions. GBD=Global Burden of Disease. YLLs=years of life lost. YLDs=years live with disability. DALYs=disability-adjusted life-years

	Deaths 2016	YLLs 2016	YLDs 2016	DALYs 2016
**All causes**	57.7% (55.4% to 60.1%)	54.5% (52.6% to 56.6%)	19.2% (18.0% to 20.5%)	35.9% (33.2% to 39.0%)
**HIV/AIDS and tuberculosis**	76.6% (70.9% to 81.7%)	84.8% (81.3% to 87.8%)	87.3% (80.9% to 91.1%)	85.7% (82.4% to 88.4%)
Tuberculosis	52.8% (41.5% to 63.1%)	55.4% (45.4% to 64.3%)	44.0% (33.0% to 54.1%)	52.8% (43.1% to 61.6%)
HIV/AIDS	94.9% (93.0% to 96.2%)	94.0% (90.9% to 95.9%)	94.9% (93.4% to 96.1%)	94.3% (92.0% to 95.9%)
**Diarrhoea, lower respiratory infections, and other common infectious diseases**	36.7% (29.4% to 44.7%)	41.1% (34.9% to 47.9%)	8.3% (5.8% to 11.3%)	24.4% (19.6% to 30.2%)
Diarrhoeal diseases	18.7% (10.8% to 30.1%)	35.6% (24.6% to 49.0%)	32.3% (23.1% to 42.9%)	33.8% (24.3% to 44.7%)
Lower respiratory infections	42.8% (33.3% to 51.9%)	50.7% (42.2% to 59.0%)	51.1% (43.4% to 59.6%)	50.7% (42.2% to 58.9%)
Upper respiratory infections	0.4% (0.0% to 1.4%)	1.3% (0.1% to 4.7%)	0.0% (0.0% to 0.0%)	0.0% (0.0% to 0.0%)
Otitis media	8.5% (4.7% to 13.6%)	14.7% (10.0% to 19.6%)	9.6% (6.6% to 12.7%)	9.6% (6.6% to 12.7%)
Meningitis	1.9% (1.2% to 2.9%)	3.8% (2.4% to 5.4%)	0.0% (0.0% to 0.0%)	3.4% (2.1% to 4.8%)
Encephalitis	0.4% (0.2% to 0.6%)	0.9% (0.6% to 1.3%)	0.0% (0.0% to 0.0%)	0.7% (0.4% to 1.0%)
Measles	6.1% (0.0% to 37.9%)	6.2% (0.0% to 38.5%)	6.0% (0.0% to 39.6%)	9.1% (0.0% to 41.8%)
**Maternal disorders**	1.7% (0.7% to 2.7%)	1.6% (0.7% to 2.8%)	2.8% (1.1% to 4.8%)	2.1% (0.9% to 3.4%)
Maternal haemorrhage	8.4% (3.0% to 13.8%)	8.4% (3.0% to 13.8%)	8.5% (3.1% to 14.0%)	8.5% (3.0% to 13.9%)
Maternal sepsis and other pregnancy related infections	8.5% (3.1% to 14.0%)	8.4% (3.1% to 14.0%)	8.4% (3.0% to 13.7%)	8.4% (3.0% to 13.8%)
Maternal abortion, miscarriage, and ectopic pregnancy	3.3% (1.9% to 5.0%)	3.3% (2.0% to 5.1%)	3.6% (1.7% to 6.4%)	3.4% (1.9% to 5.5%)
**Neonatal disorders**	73.1% (69.6% to 76.2%)	73.1% (69.6% to 76.3%)	0.1% (0.1% to 0.1%)	40.2% (31.6% to 48.7%)
Neonatal preterm birth complications	89.6% (85.4% to 93.2%)	89.6% (85.4% to 93.2%)	0.2% (0.1% to 0.2%)	43.6% (31.5% to 55.3%)
Neonatal encephalopathy due to birth asphyxia and trauma	68.6% (64.8% to 72.4%)	68.7% (64.8% to 72.5%)	0.0% (0.0% to 0.0%)	27.4% (17.2% to 38.7%)
Neonatal sepsis and other neonatal infections	44.5% (30.9% to 63.1%)	44.5% (31.0% to 63.2%)	0.0% (0.0% to 0.0%)	21.5% (9.5% to 51.1%)
Hemolytic disease and other neonatal jaundice	62.9% (53.4% to 69.2%)	63.0% (53.5% to 69.3%)	0.0% (0.0% to 0.0%)	36.9% (25.9% to 47.1%)

Supplementary Results Table 1. Age-standardised PAFs attributable to the joint distribution of all risk factors for all ages, both sexes combined for each GBD level 1 and level 2 cause and all causes in 2016 for Cyprus. Results are shown for deaths, YLLs, YLDs, and DALYs
Data in parenthesis are 95% uncertainty intervals. PAFs=population attributable fractions. GBD=Global Burden of Disease. YLLs=years of life lost. YLDs=years live with disability. DALYs=disability-adjusted life-years

	Deaths 2016	YLLs 2016	YLDs 2016	DALYs 2016
Other neonatal disorders	64.2% (59.0% to 68.9%)	64.3% (59.1% to 68.9%)	0.0% (0.0% to 0.0%)	60.1% (53.6% to 65.3%)
**Nutritional deficiencies**	5.2% (2.7% to 7.5%)	13.9% (10.5% to 18.1%)	48.2% (38.0% to 58.3%)	45.4% (36.3% to 55.0%)
Protein-energy malnutrition	1.4% (0.7% to 2.4%)	11.7% (6.6% to 18.9%)	98.3% (98.1% to 98.5%)	80.3% (72.8% to 85.7%)
Vitamin A deficiency	-	-	0.0% (0.0% to 0.0%)	0.0% (0.0% to 0.0%)
Iron-deficiency anaemia	100.0% (100.0% to 100.0%)	100.0% (100.0% to 100.0%)	100.0% (100.0% to 100.0%)	100.0% (100.0% to 100.0%)
**Other communicable, maternal, neonatal, and nutritional diseases**	7.4% (6.0% to 10.6%)	6.2% (5.0% to 8.0%)	70.3% (61.7% to 78.3%)	30.7% (21.3% to 42.5%)
Sexually transmitted diseases excluding HIV	98.3% (96.6% to 99.3%)	94.1% (88.3% to 97.4%)	100.0% (100.0% to 100.0%)	99.2% (98.2% to 99.7%)
Hepatitis	2.2% (1.6% to 3.1%)	2.7% (2.0% to 3.7%)	1.3% (0.8% to 2.0%)	2.6% (1.9% to 3.6%)
**Neoplasms**	39.2% (35.9% to 42.7%)	38.9% (35.7% to 42.0%)	29.1% (25.8% to 32.9%)	38.4% (35.3% to 41.5%)
Oesophageal cancer	80.1% (71.8% to 87.0%)	82.6% (75.2% to 89.0%)	80.4% (72.4% to 87.4%)	82.5% (75.2% to 89.0%)
Stomach cancer	18.2% (9.5% to 28.4%)	18.8% (10.2% to 27.8%)	18.5% (10.0% to 28.4%)	18.8% (10.2% to 27.9%)
Liver cancer	63.5% (55.2% to 71.9%)	66.5% (58.4% to 74.4%)	64.1% (55.8% to 72.2%)	66.5% (58.4% to 74.4%)
Larynx cancer	88.9% (82.4% to 93.3%)	88.9% (82.1% to 93.2%)	89.1% (82.6% to 93.4%)	88.9% (82.1% to 93.2%)
Tracheal, bronchus, and lung cancer	87.2% (84.4% to 89.7%)	86.6% (84.0% to 89.0%)	87.4% (84.4% to 90.0%)	86.7% (84.0% to 89.0%)
Breast cancer	28.6% (20.8% to 37.3%)	27.2% (19.9% to 35.2%)	27.2% (19.7% to 35.3%)	27.2% (19.9% to 35.2%)
Cervical cancer	100.0% (100.0% to 100.0%)	100.0% (100.0% to 100.0%)	100.0% (100.0% to 100.0%)	100.0% (100.0% to 100.0%)
Uterine cancer	40.1% (26.5% to 53.7%)	41.5% (28.3% to 55.3%)	41.9% (28.4% to 55.5%)	41.6% (28.3% to 55.3%)
Prostate cancer	5.3% (3.7% to 7.0%)	5.7% (4.1% to 7.5%)	5.9% (4.3% to 7.7%)	5.8% (4.1% to 7.5%)
Colon and rectum cancer	57.5% (48.0% to 66.6%)	58.3% (49.1% to 66.9%)	57.8% (48.4% to 66.6%)	58.3% (49.0% to 66.9%)
Lip and oral cavity cancer	73.1% (65.1% to 79.8%)	76.1% (68.6% to 82.1%)	70.7% (62.4% to 78.1%)	75.9% (68.4% to 82.0%)
Nasopharynx cancer	81.9% (76.9% to 86.4%)	80.1% (75.2% to 84.5%)	75.9% (69.4% to 81.4%)	80.0% (75.2% to 84.4%)

Supplementary Results Table 1. Age-standardised PAFs attributable to the joint distribution of all risk factors for all ages, both sexes combined for each GBD level 1 and level 2 cause and all causes in 2016 for Cyprus. Results are shown for deaths, YLLs, YLDs, and DALYs
Data in parenthesis are 95% uncertainty intervals. PAFs=population attributable fractions. GBD=Global Burden of Disease. YLLs=years of life lost. YLDs=years live with disability. DALYs=disability-adjusted life-years

	Deaths 2016	YLLs 2016	YLDs 2016	DALYs 2016
Other pharynx cancer	58.7% (46.3% to 69.0%)	60.3% (47.9% to 70.6%)	60.6% (48.3% to 70.5%)	60.3% (48.0% to 70.5%)
Gallbladder and biliary tract cancer	18.4% (9.8% to 29.2%)	19.1% (10.1% to 30.5%)	18.9% (10.0% to 30.4%)	19.1% (10.1% to 30.5%)
Pancreatic cancer	30.1% (21.6% to 39.4%)	31.0% (22.7% to 39.8%)	30.1% (21.6% to 39.2%)	31.0% (22.7% to 39.8%)
Ovarian cancer	15.0% (7.7% to 24.4%)	12.2% (5.7% to 20.3%)	11.9% (5.7% to 19.9%)	12.2% (5.7% to 20.3%)
Kidney cancer	34.0% (24.1% to 44.1%)	34.8% (24.7% to 45.1%)	34.0% (24.2% to 44.0%)	34.8% (24.7% to 45.1%)
Bladder cancer	36.5% (26.2% to 47.6%)	38.1% (27.8% to 48.7%)	37.7% (27.5% to 48.4%)	38.1% (27.7% to 48.7%)
Thyroid cancer	13.5% (6.4% to 23.1%)	14.5% (6.8% to 25.0%)	13.9% (7.1% to 23.0%)	14.4% (6.9% to 24.7%)
Mesothelioma	96.7% (95.7% to 97.7%)	93.4% (91.7% to 95.4%)	95.7% (94.4% to 97.0%)	93.5% (91.8% to 95.4%)
Non-Hodgkin's lymphoma	6.5% (2.7% to 11.8%)	6.7% (2.7% to 11.7%)	6.6% (2.7% to 11.6%)	6.6% (2.7% to 11.7%)
Multiple myeloma	7.3% (3.0% to 13.2%)	7.8% (3.3% to 13.8%)	7.7% (3.2% to 13.8%)	7.8% (3.3% to 13.8%)
Leukaemia	18.4% (11.5% to 25.9%)	16.5% (10.3% to 23.1%)	17.4% (10.4% to 24.9%)	16.5% (10.3% to 23.0%)
**Cardiovascular diseases**	82.8% (79.9% to 85.3%)	85.4% (83.3% to 87.2%)	73.8% (70.8% to 76.4%)	83.6% (81.3% to 85.4%)
Rheumatic heart disease	28.1% (17.6% to 48.3%)	28.8% (19.5% to 44.4%)	28.5% (19.1% to 42.5%)	28.8% (19.5% to 44.4%)
Ischaemic heart disease	93.2% (89.4% to 96.2%)	95.1% (92.7% to 97.0%)	92.1% (89.4% to 94.4%)	94.9% (92.5% to 96.8%)
Cerebrovascular disease	82.2% (75.8% to 87.6%)	86.0% (81.8% to 89.9%)	85.5% (81.3% to 89.5%)	85.9% (81.7% to 89.7%)
Hypertensive heart disease	100.0% (100.0% to 100.0%)	100.0% (100.0% to 100.0%)	100.0% (100.0% to 100.0%)	100.0% (100.0% to 100.0%)
Cardiomyopathy and myocarditis	29.3% (23.0% to 37.2%)	33.3% (27.4% to 39.6%)	31.1% (25.7% to 36.6%)	33.0% (27.3% to 39.2%)
Atrial fibrillation and flutter	52.6% (44.8% to 60.7%)	57.8% (50.6% to 64.9%)	66.0% (61.0% to 71.4%)	62.4% (56.7% to 68.4%)
Aortic aneurysm	47.0% (39.5% to 54.7%)	53.6% (47.2% to 59.3%)	-	53.6% (47.2% to 59.3%)
Peripheral vascular disease	51.2% (44.4% to 58.9%)	57.9% (52.4% to 63.7%)	62.3% (57.0% to 67.1%)	60.0% (54.8% to 65.1%)
Endocarditis	40.1% (30.3% to 52.1%)	41.6% (32.8% to 50.6%)	40.7% (31.7% to 50.3%)	41.5% (32.7% to 50.5%)

Supplementary Results Table 1. Age-standardised PAFs attributable to the joint distribution of all risk factors for all ages, both sexes combined for each GBD level 1 and level 2 cause and all causes in 2016 for Cyprus. Results are shown for deaths, YLLs, YLDs, and DALYs
Data in parenthesis are 95% uncertainty intervals. PAFs=population attributable fractions. GBD=Global Burden of Disease. YLLs=years of life lost. YLDs=years live with disability. DALYs=disability-adjusted life-years

	Deaths 2016	YLLs 2016	YLDs 2016	DALYs 2016
Other cardiovascular and circulatory diseases	45.6% (39.2% to 52.1%)	52.1% (46.9% to 57.4%)	52.6% (47.7% to 57.5%)	52.4% (47.5% to 57.1%)
**Chronic respiratory diseases**	56.6% (49.7% to 62.8%)	55.9% (49.5% to 60.9%)	41.8% (35.3% to 49.3%)	49.3% (44.1% to 54.4%)
Chronic obstructive pulmonary disease	68.8% (60.6% to 76.6%)	72.0% (65.2% to 78.3%)	67.5% (61.2% to 73.4%)	71.0% (64.8% to 76.8%)
Pneumoconiosis	100.0% (100.0% to 100.0%)	100.0% (100.0% to 100.0%)	100.0% (100.0% to 100.0%)	100.0% (100.0% to 100.0%)
Asthma	37.5% (27.9% to 48.5%)	42.3% (33.6% to 51.9%)	35.2% (27.2% to 44.6%)	36.2% (28.2% to 45.5%)
Other chronic respiratory diseases	11.6% (8.0% to 15.7%)	10.2% (5.8% to 14.5%)	6.3% (4.0% to 9.3%)	8.7% (5.4% to 12.3%)
**Cirrhosis and other chronic liver diseases**	65.5% (60.7% to 70.5%)	69.4% (65.2% to 73.7%)	56.3% (51.4% to 61.3%)	68.3% (64.2% to 72.6%)
Cirrhosis and other chronic liver diseases due to hepatitis B	0.3% (0.1% to 0.5%)	0.4% (0.2% to 0.6%)	0.4% (0.2% to 0.6%)	0.4% (0.2% to 0.6%)
Cirrhosis and other chronic liver diseases due to hepatitis C	55.4% (45.0% to 65.3%)	68.9% (60.1% to 76.6%)	70.7% (62.0% to 77.6%)	69.1% (60.3% to 76.7%)
Cirrhosis and other chronic liver diseases due to alcohol use	100.0% (100.0% to 100.0%)	100.0% (100.0% to 100.0%)	100.0% (100.0% to 100.0%)	100.0% (100.0% to 100.0%)
**Digestive diseases**	13.4% (9.6% to 17.4%)	15.4% (11.3% to 19.6%)	2.7% (1.9% to 3.7%)	11.5% (8.5% to 14.7%)
Peptic ulcer disease	11.6% (8.2% to 15.7%)	16.8% (12.1% to 22.1%)	14.8% (10.4% to 19.8%)	16.5% (12.0% to 21.8%)
Gallbladder and biliary diseases	37.7% (23.5% to 52.3%)	39.6% (25.9% to 54.1%)	44.1% (31.0% to 58.6%)	40.0% (26.5% to 54.3%)
Pancreatitis	32.7% (15.3% to 49.0%)	37.8% (19.6% to 53.7%)	31.2% (14.6% to 46.4%)	37.6% (19.5% to 53.5%)
**Neurological disorders**	19.3% (9.5% to 31.2%)	18.2% (9.7% to 28.5%)	3.5% (1.8% to 5.6%)	8.3% (4.5% to 13.1%)
Alzheimer's disease and other dementias	23.0% (11.4% to 36.9%)	25.0% (13.1% to 39.2%)	24.9% (12.9% to 39.2%)	25.0% (13.1% to 39.2%)
Parkinson's disease	-9.9% (-12.6% to -7.3%)	-13.5% (-16.9% to -10.1%)	-15.9% (-19.9% to -11.9%)	-14.0% (-17.5% to -10.4%)
Epilepsy	29.6% (20.3% to 37.8%)	28.9% (19.3% to 36.6%)	22.0% (14.7% to 29.1%)	24.3% (16.5% to 32.1%)
Multiple sclerosis	13.1% (8.3% to 18.1%)	14.4% (9.2% to 19.7%)	11.5% (7.6% to 15.8%)	12.8% (8.5% to 17.5%)
**Mental and substance use disorders**	98.3% (97.5% to 99.0%)	97.9% (97.0% to 98.9%)	13.0% (11.5% to 14.6%)	17.0% (14.7% to 19.5%)
Alcohol use disorders	100.0% (100.0% to 100.0%)	100.0% (100.0% to 100.0%)	100.0% (100.0% to 100.0%)	100.0% (100.0% to 100.0%)

Supplementary Results Table 1. Age-standardised PAFs attributable to the joint distribution of all risk factors for all ages, both sexes combined for each GBD level 1 and level 2 cause and all causes in 2016 for Cyprus. Results are shown for deaths, YLLs, YLDs, and DALYs
Data in parenthesis are 95% uncertainty intervals. PAFs=population attributable fractions. GBD=Global Burden of Disease. YLLs=years of life lost. YLDs=years live with disability. DALYs=disability-adjusted life-years

	**Deaths 2016**	**YLLs 2016**	**YLDs 2016**	**DALYs 2016**
Drug use disorders	100.0% (100.0% to 100.0%)	100.0% (100.0% to 100.0%)	100.0% (100.0% to 100.0%)	100.0% (100.0% to 100.0%)
Depressive disorders	-	-	5.8% (4.8% to 7.0%)	5.8% (4.8% to 7.0%)
Idiopathic developmental intellectual disability	-	-	61.3% (32.0% to 80.9%)	61.3% (32.0% to 80.9%)
**Diabetes, urogenital, blood, and endocrine diseases**	81.5% (80.0% to 83.2%)	77.2% (75.0% to 78.8%)	59.9% (55.6% to 64.1%)	69.8% (66.7% to 72.4%)
Diabetes mellitus	100.0% (100.0% to 100.0%)	100.0% (100.0% to 100.0%)	100.0% (100.0% to 100.0%)	100.0% (100.0% to 100.0%)
Chronic kidney disease	100.0% (100.0% to 100.0%)	100.0% (100.0% to 100.0%)	100.0% (100.0% to 100.0%)	100.0% (100.0% to 100.0%)
**Musculoskeletal disorders**	1.5% (0.6% to 2.6%)	2.0% (0.8% to 3.4%)	17.8% (14.9% to 20.8%)	17.5% (14.7% to 20.5%)
Rheumatoid arthritis	3.7% (1.5% to 6.3%)	5.1% (2.0% to 8.5%)	6.2% (2.6% to 10.5%)	6.1% (2.5% to 10.2%)
Osteoarthritis	-	-	23.3% (12.6% to 34.9%)	23.3% (12.6% to 34.9%)
Low back and neck pain	-	-	20.2% (17.3% to 23.4%)	20.2% (17.3% to 23.4%)
Gout	-	-	46.7% (29.8% to 64.8%)	46.7% (29.8% to 64.8%)
**Other non-communicable diseases**	0.1% (0.1% to 0.2%)	0.3% (0.2% to 0.5%)	2.3% (1.9% to 2.7%)	2.1% (1.7% to 2.5%)
Sense organ diseases	-	-	7.1% (6.2% to 8.2%)	7.1% (6.2% to 8.2%)
Sudden infant death syndrome	4.4% (2.5% to 7.4%)	4.5% (2.6% to 7.4%)	-	4.5% (2.6% to 7.4%)
**Transport injuries**	36.8% (28.6% to 46.8%)	32.5% (23.8% to 43.2%)	42.5% (34.4% to 52.3%)	35.1% (26.6% to 45.8%)
Road injuries	36.5% (28.2% to 46.5%)	32.3% (23.6% to 42.9%)	42.7% (34.7% to 52.5%)	35.1% (26.6% to 45.8%)
Other transport injuries	38.7% (30.3% to 48.5%)	33.8% (25.1% to 44.1%)	40.8% (32.6% to 50.8%)	35.2% (26.7% to 45.4%)
**Unintentional injuries**	33.1% (30.0% to 36.7%)	21.1% (17.4% to 25.6%)	22.2% (17.5% to 29.2%)	21.8% (17.6% to 27.5%)
Falls	63.3% (59.9% to 66.5%)	41.6% (38.0% to 45.9%)	24.2% (19.7% to 31.2%)	28.2% (23.8% to 34.3%)
Drowning	18.0% (11.4% to 26.5%)	17.4% (11.1% to 25.0%)	19.8% (12.9% to 28.9%)	17.4% (11.1% to 25.1%)
Fire, heat, and hot substances	14.9% (9.0% to 23.1%)	18.0% (11.8% to 26.0%)	22.2% (14.4% to 32.6%)	20.3% (13.4% to 29.2%)

Supplementary Results Table 1. Age-standardised PAFs attributable to the joint distribution of all risk factors for all ages, both sexes combined for each GBD level 1 and level 2 cause and all causes in 2016 for Cyprus. Results are shown for deaths, YLLs, YLDs, and DALYs
Data in parenthesis are 95% uncertainty intervals. PAFs=population attributable fractions. GBD=Global Burden of Disease. YLLs=years of life lost. YLDs=years live with disability. DALYs=disability-adjusted life-years

	**Deaths 2016**	**YLLs 2016**	**YLDs 2016**	**DALYs 2016**
Poisonings	20.6% (13.5% to 30.3%)	22.1% (14.9% to 31.8%)	19.9% (12.7% to 29.4%)	20.7% (13.6% to 29.6%)
Exposure to mechanical forces	23.1% (19.2% to 27.4%)	16.6% (12.4% to 22.0%)	21.8% (17.0% to 28.4%)	19.8% (15.1% to 25.5%)
Animal contact	17.1% (13.0% to 22.9%)	14.8% (10.0% to 22.5%)	13.7% (8.0% to 22.9%)	14.2% (8.8% to 22.6%)
Foreign body	5.7% (3.3% to 9.7%)	8.2% (4.9% to 12.8%)	9.1% (5.1% to 15.1%)	8.5% (5.1% to 13.4%)
Other unintentional injuries	22.2% (14.1% to 32.0%)	22.7% (14.8% to 32.2%)	20.9% (13.9% to 29.9%)	21.1% (14.1% to 30.1%)
**Self-harm and interpersonal violence**	32.4% (20.5% to 42.8%)	32.1% (20.8% to 42.3%)	34.7% (29.4% to 40.1%)	32.5% (21.9% to 41.8%)
Self-harm	32.3% (16.9% to 46.1%)	33.1% (17.6% to 46.9%)	19.8% (6.9% to 30.9%)	32.7% (17.2% to 46.2%)
Interpersonal violence	32.8% (24.5% to 40.8%)	30.1% (22.1% to 37.9%)	37.4% (32.1% to 42.9%)	32.1% (24.9% to 39.0%)
**Forces of nature, conflict and terrorism, and executions and police conflict**	0.0% (0.0% to 0.0%)	0.0% (0.0% to 0.0%)	0.9% (0.5% to 1.4%)	0.3% (0.1% to 0.6%)
Forces of nature, conflict and terrorism, and state actor violence	0.0% (0.0% to 0.0%)	0.0% (0.0% to 0.0%)	6.4% (4.1% to 9.7%)	6.4% (4.1% to 9.7%)

Supplementary Results Table 1. Age-standardised PAFs attributable to the joint distribution of all risk factors for all ages, both sexes combined for each GBD level 1 and level 2 cause and all causes in 2016 for Czech Republic. Results are shown for deaths, YLLs, YLDs, and DALYs
Data in parenthesis are 95% uncertainty intervals. PAFs=population attributable fractions. GBD=Global Burden of Disease. YLLs=years of life lost. YLDs=years live with disability. DALYs=disability-adjusted life-years

	Deaths 2016	YLLs 2016	YLDs 2016	DALYs 2016
**All causes**	65.3% (63.0% to 67.4%)	60.4% (58.5% to 62.2%)	25.7% (24.3% to 27.3%)	42.8% (39.9% to 45.8%)
**HIV/AIDS and tuberculosis**	72.4% (67.2% to 76.7%)	74.4% (71.2% to 77.5%)	72.6% (65.0% to 78.7%)	74.1% (70.9% to 77.3%)
Tuberculosis	64.4% (56.1% to 71.5%)	67.5% (60.4% to 73.5%)	56.6% (48.2% to 63.9%)	65.5% (58.3% to 71.7%)
HIV/AIDS	86.1% (84.3% to 87.7%)	79.7% (76.9% to 82.1%)	93.9% (93.0% to 94.8%)	81.4% (78.9% to 83.6%)
**Diarrhoea, lower respiratory infections, and other common infectious diseases**	49.6% (41.3% to 57.3%)	54.9% (48.2% to 61.6%)	31.8% (21.7% to 42.2%)	45.9% (39.2% to 52.9%)
Diarrhoeal diseases	45.3% (21.3% to 70.6%)	53.1% (31.6% to 75.5%)	58.6% (39.0% to 78.2%)	57.7% (38.1% to 77.3%)
Lower respiratory infections	51.3% (42.7% to 59.8%)	59.2% (51.5% to 66.8%)	54.1% (46.1% to 62.8%)	59.1% (51.4% to 66.7%)
Upper respiratory infections	0.3% (0.0% to 0.9%)	0.9% (0.1% to 2.9%)	0.0% (0.0% to 0.0%)	0.0% (0.0% to 0.0%)
Otitis media	5.0% (2.8% to 8.4%)	10.5% (6.9% to 14.9%)	9.2% (6.2% to 11.9%)	9.2% (6.3% to 11.9%)
Meningitis	2.1% (1.4% to 2.9%)	4.2% (2.9% to 5.5%)	0.0% (0.0% to 0.0%)	2.5% (1.7% to 3.6%)
Encephalitis	0.6% (0.4% to 0.9%)	1.2% (0.8% to 1.6%)	0.0% (0.0% to 0.0%)	0.6% (0.4% to 0.8%)
Measles	4.8% (0.9% to 18.2%)	7.0% (1.4% to 26.1%)	19.1% (4.9% to 58.7%)	7.2% (1.5% to 26.4%)
**Maternal disorders**	3.5% (1.8% to 5.4%)	3.4% (1.8% to 5.3%)	4.4% (2.1% to 7.2%)	4.0% (2.0% to 6.3%)
Maternal haemorrhage	12.6% (4.6% to 20.9%)	12.6% (4.6% to 20.9%)	12.9% (4.6% to 21.5%)	12.8% (4.6% to 21.3%)
Maternal sepsis and other pregnancy related infections	12.4% (4.5% to 20.3%)	12.4% (4.5% to 20.2%)	12.6% (4.5% to 21.0%)	12.6% (4.5% to 20.9%)
Maternal abortion, miscarriage, and ectopic pregnancy	5.1% (3.1% to 7.7%)	5.0% (3.0% to 7.5%)	5.3% (2.6% to 8.7%)	5.2% (2.9% to 8.1%)
**Neonatal disorders**	65.0% (62.0% to 68.1%)	65.1% (62.0% to 68.1%)	0.1% (0.1% to 0.1%)	36.9% (29.1% to 44.5%)
Neonatal preterm birth complications	82.2% (77.1% to 86.4%)	82.3% (77.2% to 86.5%)	0.2% (0.1% to 0.2%)	43.0% (32.2% to 54.3%)
Neonatal encephalopathy due to birth asphyxia and trauma	57.3% (53.0% to 61.4%)	57.4% (53.1% to 61.4%)	0.0% (0.0% to 0.0%)	29.9% (22.1% to 38.4%)
Neonatal sepsis and other neonatal infections	51.9% (47.7% to 56.4%)	52.0% (47.8% to 56.4%)	0.0% (0.0% to 0.0%)	20.1% (9.7% to 32.0%)
Hemolytic disease and other neonatal jaundice	60.4% (56.4% to 64.0%)	60.5% (56.6% to 64.1%)	0.0% (0.0% to 0.0%)	21.0% (12.6% to 31.0%)

Supplementary Results Table 1. Age-standardised PAFs attributable to the joint distribution of all risk factors for all ages, both sexes combined for each GBD level 1 and level 2 cause and all causes in 2016 for Czech Republic. Results are shown for deaths, YLLs, YLDs, and DALYs
Data in parenthesis are 95% uncertainty intervals. PAFs=population attributable fractions. GBD=Global Burden of Disease. YLLs=years of life lost. YLDs=years live with disability. DALYs=disability-adjusted life-years

	Deaths 2016	YLLs 2016	YLDs 2016	DALYs 2016
Other neonatal disorders	52.6% (48.0% to 57.2%)	52.7% (48.1% to 57.3%)	0.0% (0.0% to 0.0%)	49.0% (44.2% to 53.8%)
**Nutritional deficiencies**	3.1% (2.1% to 6.2%)	8.6% (6.1% to 15.0%)	89.9% (86.9% to 92.3%)	85.4% (81.0% to 89.0%)
Protein-energy malnutrition	0.5% (0.3% to 1.0%)	3.0% (1.7% to 5.4%)	97.9% (96.2% to 99.0%)	45.1% (29.4% to 64.8%)
Vitamin A deficiency	-	-	0.0% (0.0% to 0.0%)	0.0% (0.0% to 0.0%)
Iron-deficiency anaemia	100.0% (100.0% to 100.0%)	100.0% (100.0% to 100.0%)	100.0% (100.0% to 100.0%)	100.0% (100.0% to 100.0%)
**Other communicable, maternal, neonatal, and nutritional diseases**	8.9% (7.5% to 10.8%)	7.1% (5.7% to 8.9%)	78.0% (72.8% to 82.5%)	57.6% (50.0% to 66.1%)
Sexually transmitted diseases excluding HIV	95.6% (90.5% to 98.6%)	87.9% (75.7% to 95.9%)	100.0% (100.0% to 100.0%)	99.6% (99.0% to 99.9%)
Hepatitis	5.4% (3.9% to 7.3%)	5.4% (3.7% to 7.3%)	2.7% (1.8% to 3.9%)	5.2% (3.6% to 7.0%)
**Neoplasms**	47.2% (43.7% to 50.6%)	47.8% (44.6% to 50.9%)	36.7% (33.2% to 40.3%)	47.4% (44.3% to 50.6%)
Oesophageal cancer	89.2% (83.5% to 93.5%)	90.0% (84.8% to 94.3%)	89.5% (84.2% to 93.8%)	90.0% (84.8% to 94.3%)
Stomach cancer	23.9% (15.1% to 34.0%)	24.5% (15.8% to 33.7%)	23.8% (15.0% to 33.6%)	24.5% (15.7% to 33.6%)
Liver cancer	69.7% (60.8% to 77.2%)	69.0% (60.1% to 76.6%)	69.6% (60.4% to 77.0%)	69.0% (60.1% to 76.6%)
Larynx cancer	92.2% (87.6% to 95.3%)	92.1% (87.5% to 95.2%)	92.2% (87.7% to 95.3%)	92.1% (87.5% to 95.2%)
Tracheal, bronchus, and lung cancer	89.6% (87.5% to 91.5%)	90.0% (88.1% to 91.7%)	89.3% (87.2% to 91.4%)	89.9% (88.1% to 91.7%)
Breast cancer	35.6% (28.6% to 43.3%)	35.2% (28.6% to 42.1%)	35.1% (28.6% to 42.0%)	35.2% (28.6% to 42.1%)
Cervical cancer	100.0% (100.0% to 100.0%)	100.0% (100.0% to 100.0%)	100.0% (100.0% to 100.0%)	100.0% (100.0% to 100.0%)
Uterine cancer	46.0% (29.6% to 60.8%)	46.0% (29.8% to 60.9%)	45.9% (29.9% to 60.8%)	46.0% (29.8% to 60.9%)
Prostate cancer	6.1% (4.4% to 8.0%)	6.9% (5.0% to 9.0%)	7.2% (5.3% to 9.4%)	7.0% (5.1% to 9.1%)
Colon and rectum cancer	60.6% (52.5% to 68.3%)	62.2% (54.6% to 69.4%)	61.6% (53.8% to 68.9%)	62.2% (54.5% to 69.4%)
Lip and oral cavity cancer	88.5% (84.4% to 91.8%)	89.8% (86.1% to 92.7%)	87.0% (82.7% to 90.6%)	89.7% (86.0% to 92.7%)
Nasopharynx cancer	86.9% (83.9% to 89.8%)	85.5% (82.5% to 88.3%)	85.1% (81.5% to 88.5%)	85.5% (82.5% to 88.3%)

Supplementary Results Table 1. Age-standardised PAFs attributable to the joint distribution of all risk factors for all ages, both sexes combined for each GBD level 1 and level 2 cause and all causes in 2016 for Czech Republic. Results are shown for deaths, YLLs, YLDs, and DALYs
Data in parenthesis are 95% uncertainty intervals. PAFs=population attributable fractions. GBD=Global Burden of Disease. YLLs=years of life lost. YLDs=years live with disability. DALYs=disability-adjusted life-years

	Deaths 2016	YLLs 2016	YLDs 2016	DALYs 2016
Other pharynx cancer	77.2% (69.0% to 83.7%)	78.2% (70.1% to 84.6%)	77.0% (68.4% to 83.5%)	78.2% (70.1% to 84.6%)
Gallbladder and biliary tract cancer	24.0% (12.9% to 36.8%)	24.0% (13.0% to 36.7%)	24.1% (13.0% to 36.9%)	24.0% (13.0% to 36.7%)
Pancreatic cancer	34.7% (26.6% to 43.3%)	35.9% (27.6% to 44.3%)	34.6% (26.7% to 43.0%)	35.9% (27.6% to 44.2%)
Ovarian cancer	12.0% (5.0% to 20.6%)	10.5% (4.1% to 18.4%)	10.5% (4.2% to 18.2%)	10.5% (4.1% to 18.4%)
Kidney cancer	39.0% (28.3% to 50.0%)	40.1% (29.3% to 51.0%)	39.1% (28.7% to 50.0%)	40.1% (29.2% to 50.9%)
Bladder cancer	38.1% (28.8% to 47.7%)	40.6% (31.2% to 50.0%)	40.0% (30.9% to 49.3%)	40.5% (31.2% to 49.9%)
Thyroid cancer	15.1% (7.4% to 24.7%)	15.3% (7.4% to 25.3%)	13.9% (7.0% to 22.6%)	15.1% (7.4% to 24.8%)
Mesothelioma	89.1% (86.1% to 91.8%)	82.2% (77.7% to 86.2%)	86.5% (83.0% to 89.6%)	82.3% (77.8% to 86.3%)
Non-Hodgkin's lymphoma	7.3% (2.8% to 12.8%)	6.9% (2.6% to 12.0%)	7.2% (2.8% to 12.7%)	6.9% (2.7% to 12.0%)
Multiple myeloma	8.8% (3.6% to 15.7%)	8.9% (3.6% to 15.7%)	8.9% (3.6% to 15.6%)	8.9% (3.6% to 15.7%)
Leukaemia	21.7% (13.3% to 29.8%)	19.5% (12.2% to 26.7%)	20.8% (12.9% to 28.6%)	19.6% (12.2% to 26.8%)
**Cardiovascular diseases**	86.7% (83.8% to 89.1%)	88.1% (86.3% to 89.7%)	78.9% (76.3% to 81.3%)	86.6% (84.6% to 88.2%)
Rheumatic heart disease	30.8% (20.2% to 50.0%)	31.5% (21.4% to 47.0%)	31.2% (20.8% to 45.6%)	31.5% (21.3% to 47.0%)
Ischaemic heart disease	93.5% (89.8% to 96.4%)	95.2% (92.9% to 97.1%)	93.0% (90.8% to 95.0%)	95.1% (92.7% to 96.9%)
Cerebrovascular disease	84.3% (78.2% to 89.9%)	88.8% (85.2% to 92.2%)	87.7% (84.0% to 91.2%)	88.5% (85.0% to 91.9%)
Hypertensive heart disease	100.0% (100.0% to 100.0%)	100.0% (100.0% to 100.0%)	100.0% (100.0% to 100.0%)	100.0% (100.0% to 100.0%)
Cardiomyopathy and myocarditis	39.8% (31.0% to 47.7%)	45.3% (38.7% to 51.1%)	40.9% (34.9% to 47.2%)	44.7% (38.2% to 50.5%)
Atrial fibrillation and flutter	57.9% (50.0% to 65.4%)	62.8% (55.7% to 69.3%)	70.5% (65.5% to 75.4%)	67.4% (61.6% to 72.8%)
Aortic aneurysm	50.8% (43.7% to 57.6%)	56.5% (50.6% to 62.1%)	-	56.5% (50.6% to 62.1%)
Peripheral vascular disease	53.2% (46.6% to 60.7%)	60.8% (55.3% to 66.2%)	60.7% (55.0% to 66.2%)	60.8% (55.3% to 66.1%)
Endocarditis	46.4% (37.5% to 56.0%)	46.5% (37.3% to 55.3%)	44.7% (36.1% to 54.0%)	46.4% (37.4% to 55.1%)

Supplementary Results Table 1. Age-standardised PAFs attributable to the joint distribution of all risk factors for all ages, both sexes combined for each GBD level 1 and level 2 cause and all causes in 2016 for Czech Republic. Results are shown for deaths, YLLs, YLDs, and DALYs
Data in parenthesis are 95% uncertainty intervals. PAFs=population attributable fractions. GBD=Global Burden of Disease. YLLs=years of life lost. YLDs=years live with disability. DALYs=disability-adjusted life-years

	Deaths 2016	YLLs 2016	YLDs 2016	DALYs 2016
Other cardiovascular and circulatory diseases	50.7% (44.9% to 55.9%)	57.8% (53.1% to 62.0%)	58.3% (53.5% to 63.0%)	58.0% (53.2% to 62.3%)
**Chronic respiratory diseases**	73.4% (69.3% to 78.1%)	72.9% (69.0% to 77.7%)	48.6% (42.3% to 55.0%)	62.4% (57.5% to 67.2%)
Chronic obstructive pulmonary disease	80.0% (75.5% to 83.9%)	81.6% (77.8% to 85.0%)	77.1% (72.9% to 80.9%)	80.5% (76.8% to 83.9%)
Pneumoconiosis	100.0% (100.0% to 100.0%)	100.0% (100.0% to 100.0%)	100.0% (100.0% to 100.0%)	100.0% (100.0% to 100.0%)
Asthma	43.6% (32.7% to 54.8%)	45.3% (36.0% to 55.4%)	34.4% (26.5% to 43.3%)	35.5% (27.6% to 44.4%)
Other chronic respiratory diseases	15.8% (12.1% to 20.3%)	13.1% (9.4% to 18.4%)	7.4% (5.2% to 10.3%)	9.4% (6.7% to 13.1%)
**Cirrhosis and other chronic liver diseases**	47.9% (43.5% to 52.2%)	48.1% (43.6% to 52.5%)	36.8% (32.6% to 41.3%)	47.5% (43.0% to 51.9%)
Cirrhosis and other chronic liver diseases due to hepatitis B	2.8% (1.3% to 4.8%)	3.2% (1.5% to 5.4%)	3.1% (1.3% to 5.1%)	3.2% (1.5% to 5.4%)
Cirrhosis and other chronic liver diseases due to hepatitis C	89.3% (88.5% to 89.8%)	89.8% (89.6% to 90.0%)	89.2% (88.6% to 89.6%)	89.8% (89.6% to 90.0%)
Cirrhosis and other chronic liver diseases due to alcohol use	100.0% (100.0% to 100.0%)	100.0% (100.0% to 100.0%)	100.0% (100.0% to 100.0%)	100.0% (100.0% to 100.0%)
**Digestive diseases**	18.3% (15.1% to 21.9%)	22.3% (18.4% to 26.1%)	5.3% (4.1% to 6.9%)	15.0% (12.1% to 18.2%)
Peptic ulcer disease	14.6% (10.4% to 19.4%)	19.6% (14.3% to 25.5%)	17.9% (12.6% to 23.7%)	18.9% (13.6% to 24.8%)
Gallbladder and biliary diseases	43.1% (27.0% to 58.4%)	43.0% (27.6% to 58.0%)	43.3% (28.6% to 58.2%)	43.0% (27.7% to 58.1%)
Pancreatitis	55.3% (42.3% to 67.3%)	59.9% (47.4% to 71.2%)	48.4% (35.7% to 59.6%)	59.6% (47.1% to 70.8%)
**Neurological disorders**	21.4% (10.6% to 33.9%)	20.0% (11.6% to 29.7%)	4.1% (2.1% to 6.7%)	8.9% (5.1% to 13.4%)
Alzheimer's disease and other dementias	26.5% (13.1% to 42.5%)	27.8% (14.7% to 43.1%)	27.8% (14.6% to 43.5%)	27.8% (14.7% to 43.2%)
Parkinson's disease	-8.9% (-10.9% to -7.2%)	-12.2% (-14.6% to -9.8%)	-14.7% (-17.5% to -12.0%)	-12.7% (-15.2% to -10.2%)
Epilepsy	40.9% (32.3% to 49.0%)	37.4% (28.7% to 44.8%)	28.6% (21.0% to 35.4%)	32.1% (23.5% to 39.8%)
Multiple sclerosis	12.5% (8.2% to 17.0%)	13.1% (8.6% to 17.8%)	11.2% (7.2% to 15.3%)	12.3% (8.1% to 16.7%)
**Mental and substance use disorders**	99.7% (99.6% to 99.8%)	99.5% (99.4% to 99.7%)	19.9% (17.8% to 22.2%)	25.7% (23.2% to 28.6%)
Alcohol use disorders	100.0% (100.0% to 100.0%)	100.0% (100.0% to 100.0%)	100.0% (100.0% to 100.0%)	100.0% (100.0% to 100.0%)

Supplementary Results Table 1. Age-standardised PAFs attributable to the joint distribution of all risk factors for all ages, both sexes combined for each GBD level 1 and level 2 cause and all causes in 2016 for Czech Republic. Results are shown for deaths, YLLs, YLDs, and DALYs
Data in parenthesis are 95% uncertainty intervals. PAFs=population attributable fractions. GBD=Global Burden of Disease. YLLs=years of life lost. YLDs=years live with disability. DALYs=disability-adjusted life-years

	Deaths 2016	YLLs 2016	YLDs 2016	DALYs 2016
Drug use disorders	100.0% (100.0% to 100.0%)	100.0% (100.0% to 100.0%)	100.0% (100.0% to 100.0%)	100.0% (100.0% to 100.0%)
Depressive disorders	-	-	8.3% (7.0% to 9.7%)	8.3% (7.0% to 9.7%)
Idiopathic developmental intellectual disability	-	-	41.1% (18.0% to 59.2%)	41.1% (18.0% to 59.2%)
**Diabetes, urogenital, blood, and endocrine diseases**	82.3% (80.7% to 84.5%)	77.7% (75.5% to 81.7%)	57.1% (52.8% to 61.5%)	64.0% (60.2% to 67.4%)
Diabetes mellitus	100.0% (100.0% to 100.0%)	100.0% (100.0% to 100.0%)	100.0% (100.0% to 100.0%)	100.0% (100.0% to 100.0%)
Chronic kidney disease	100.0% (100.0% to 100.0%)	100.0% (100.0% to 100.0%)	100.0% (100.0% to 100.0%)	100.0% (100.0% to 100.0%)
**Musculoskeletal disorders**	1.6% (0.6% to 2.8%)	1.5% (0.6% to 2.7%)	22.7% (18.6% to 26.9%)	22.5% (18.5% to 26.7%)
Rheumatoid arthritis	6.0% (2.4% to 10.1%)	7.0% (2.8% to 11.7%)	7.4% (2.9% to 12.5%)	7.4% (2.9% to 12.4%)
Osteoarthritis	-	-	27.7% (14.5% to 41.1%)	27.7% (14.5% to 41.1%)
Low back and neck pain	-	-	23.2% (19.7% to 27.1%)	23.2% (19.7% to 27.1%)
Gout	-	-	44.8% (27.7% to 62.3%)	44.8% (27.7% to 62.3%)
**Other non-communicable diseases**	0.4% (0.2% to 0.6%)	0.5% (0.3% to 0.7%)	3.8% (3.3% to 4.3%)	3.5% (3.1% to 4.0%)
Sense organ diseases	-	-	9.3% (8.3% to 10.3%)	9.3% (8.3% to 10.3%)
Sudden infant death syndrome	4.5% (2.9% to 6.5%)	4.5% (2.9% to 6.6%)	-	4.5% (2.9% to 6.6%)
**Transport injuries**	42.7% (33.9% to 52.1%)	39.0% (30.0% to 48.4%)	43.4% (35.0% to 52.5%)	40.9% (32.1% to 50.1%)
Road injuries	42.3% (33.4% to 51.6%)	38.6% (29.6% to 47.7%)	43.6% (35.2% to 52.6%)	40.8% (32.1% to 50.0%)
Other transport injuries	45.0% (35.9% to 54.4%)	41.5% (32.0% to 51.0%)	39.6% (30.9% to 48.9%)	41.2% (31.8% to 50.6%)
**Unintentional injuries**	39.9% (35.0% to 44.5%)	25.9% (21.5% to 30.9%)	23.9% (20.4% to 28.3%)	24.3% (20.8% to 28.8%)
Falls	62.4% (52.0% to 67.4%)	43.4% (34.2% to 49.3%)	25.6% (22.0% to 29.9%)	27.9% (24.0% to 32.4%)
Drowning	24.8% (16.9% to 33.7%)	23.6% (16.5% to 32.1%)	22.1% (15.2% to 30.3%)	23.6% (16.5% to 32.0%)
Fire, heat, and hot substances	18.3% (11.3% to 27.1%)	22.7% (15.5% to 31.1%)	23.5% (16.6% to 31.7%)	23.2% (16.3% to 31.4%)

Supplementary Results Table 1. Age-standardised PAFs attributable to the joint distribution of all risk factors for all ages, both sexes combined for each GBD level 1 and level 2 cause and all causes in 2016 for Czech Republic. Results are shown for deaths, YLLs, YLDs, and DALYs
Data in parenthesis are 95% uncertainty intervals. PAFs=population attributable fractions. GBD=Global Burden of Disease. YLLs=years of life lost. YLDs=years live with disability. DALYs=disability-adjusted life-years

	Deaths 2016	YLLs 2016	YLDs 2016	DALYs 2016
Poisonings	24.6% (16.6% to 33.4%)	27.2% (18.7% to 36.6%)	23.2% (16.4% to 31.2%)	25.3% (17.7% to 34.1%)
Exposure to mechanical forces	25.7% (18.9% to 31.8%)	22.2% (16.4% to 29.3%)	22.1% (17.3% to 28.3%)	22.1% (17.2% to 28.6%)
Animal contact	15.8% (11.6% to 21.2%)	16.4% (11.4% to 22.7%)	12.6% (8.7% to 17.6%)	13.4% (9.4% to 18.5%)
Foreign body	10.3% (7.0% to 15.1%)	11.9% (8.0% to 17.2%)	9.6% (6.5% to 13.4%)	11.2% (7.7% to 16.0%)
Other unintentional injuries	29.2% (20.2% to 39.3%)	31.0% (22.0% to 41.8%)	22.5% (15.6% to 30.4%)	24.4% (17.2% to 32.8%)
**Self-harm and interpersonal violence**	41.5% (28.6% to 53.3%)	40.5% (27.8% to 52.4%)	41.3% (35.9% to 46.7%)	40.6% (29.1% to 51.6%)
Self-harm	41.1% (27.0% to 54.2%)	40.3% (26.4% to 53.6%)	33.9% (20.8% to 46.6%)	40.2% (26.3% to 53.5%)
Interpersonal violence	46.2% (37.9% to 54.8%)	42.6% (34.7% to 51.7%)	42.9% (37.4% to 48.7%)	42.7% (36.4% to 49.2%)
**Forces of nature, conflict and terrorism, and executions and police conflict**	0.0% (0.0% to 0.0%)	0.0% (0.0% to 0.0%)	1.1% (0.6% to 1.8%)	0.3% (0.1% to 0.7%)
Forces of nature, conflict and terrorism, and state actor violence	0.0% (0.0% to 0.0%)	0.0% (0.0% to 0.0%)	3.9% (2.5% to 5.9%)	3.9% (2.5% to 5.9%)

Supplementary Results Table 1. Age-standardised PAFs attributable to the joint distribution of all risk factors for all ages, both sexes combined for each GBD level 1 and level 2 cause and all causes in 2016 for Democratic Republic of the Congo. Results are shown for deaths, YLLs, YLDs,
Data in parenthesis are 95% uncertainty intervals. PAFs=population attributable fractions. GBD=Global Burden of Disease. YLLs=years of life lost. YLDs=years live with disability. DALYs=disability-adjusted life-years

	**Deaths 2016**	**YLLs 2016**	**YLDs 2016**	**DALYs 2016**
**All causes**	52.0% (47.3% to 55.7%)	48.3% (44.9% to 51.2%)	24.6% (23.2% to 25.9%)	42.9% (40.1% to 45.6%)
**HIV/AIDS and tuberculosis**	43.4% (30.8% to 55.6%)	50.2% (38.8% to 60.9%)	64.9% (55.1% to 73.9%)	50.5% (39.3% to 61.3%)
Tuberculosis	33.2% (19.7% to 47.6%)	35.9% (21.7% to 49.3%)	34.3% (20.7% to 48.0%)	35.9% (21.7% to 49.3%)
HIV/AIDS	93.0% (91.0% to 94.5%)	89.4% (86.1% to 91.7%)	92.1% (89.2% to 94.6%)	89.6% (86.5% to 91.7%)
**Diarrhoea, lower respiratory infections, and other common infectious diseases**	78.9% (73.4% to 84.2%)	75.2% (67.0% to 81.0%)	44.6% (39.2% to 49.6%)	73.5% (65.9% to 79.3%)
Diarrhoeal diseases	95.9% (88.9% to 99.0%)	96.9% (91.2% to 99.3%)	96.4% (90.2% to 99.1%)	96.9% (91.1% to 99.3%)
Lower respiratory infections	81.1% (75.2% to 86.2%)	86.7% (81.9% to 90.8%)	81.3% (75.4% to 86.2%)	86.7% (81.8% to 90.8%)
Upper respiratory infections	1.3% (0.1% to 4.6%)	2.5% (0.2% to 9.6%)	0.0% (0.0% to 0.0%)	0.2% (0.0% to 0.8%)
Otitis media	1.7% (0.5% to 3.9%)	4.5% (2.0% to 7.8%)	4.0% (2.6% to 5.3%)	4.0% (2.6% to 5.4%)
Meningitis	2.1% (1.0% to 4.0%)	3.6% (1.8% to 6.4%)	0.0% (0.0% to 0.0%)	3.4% (1.7% to 6.0%)
Encephalitis	0.7% (0.4% to 1.1%)	1.2% (0.7% to 1.9%)	0.0% (0.0% to 0.0%)	0.7% (0.4% to 1.0%)
Measles	66.3% (52.3% to 83.0%)	67.5% (53.4% to 84.0%)	67.1% (54.2% to 81.7%)	67.5% (53.4% to 84.0%)
**Maternal disorders**	6.1% (2.9% to 9.8%)	6.3% (2.9% to 10.2%)	3.2% (1.6% to 5.1%)	6.2% (2.8% to 10.0%)
Maternal haemorrhage	16.0% (5.9% to 26.4%)	16.5% (6.0% to 27.2%)	17.9% (6.4% to 29.1%)	16.6% (6.1% to 27.2%)
Maternal sepsis and other pregnancy related infections	14.8% (5.3% to 25.0%)	15.6% (5.7% to 26.0%)	17.5% (6.2% to 28.6%)	15.6% (5.7% to 26.1%)
Maternal abortion, miscarriage, and ectopic pregnancy	31.1% (21.4% to 40.9%)	30.9% (21.8% to 40.5%)	31.0% (19.8% to 41.5%)	30.9% (21.8% to 40.4%)
**Neonatal disorders**	76.0% (71.7% to 80.0%)	76.1% (71.7% to 80.0%)	0.7% (0.3% to 1.2%)	73.5% (68.7% to 77.7%)
Neonatal preterm birth complications	95.6% (90.5% to 98.4%)	95.6% (90.6% to 98.4%)	1.9% (1.3% to 2.6%)	91.8% (86.2% to 95.5%)
Neonatal encephalopathy due to birth asphyxia and trauma	70.0% (65.1% to 74.0%)	70.0% (65.1% to 74.1%)	0.0% (0.0% to 0.0%)	68.5% (62.4% to 72.9%)
Neonatal sepsis and other neonatal infections	65.0% (59.2% to 69.4%)	65.0% (59.3% to 69.4%)	0.0% (0.0% to 0.0%)	61.8% (53.3% to 68.0%)
Hemolytic disease and other neonatal jaundice	67.9% (60.1% to 72.9%)	67.9% (60.2% to 72.9%)	0.0% (0.0% to 0.0%)	60.6% (50.7% to 67.6%)

**Supplementary Results Table 1. Age-standardised PAFs attributable to the joint distribution of all risk factors for all ages, both sexes combined for each GBD level 1 and level 2 cause and all causes in 2016 for Democratic Republic of the Congo. Results are shown for deaths, YLLs, YLDs,** Data in parenthesis are 95% uncertainty intervals. PAFs=population attributable fractions. GBD=Global Burden of Disease. YLLs=years of life lost. YLDs=years live with disability. DALYs=disability-adjusted life-years	**Deaths 2016**	**YLLs 2016**	**YLDs 2016**	**DALYs 2016**
Other neonatal disorders	66.6% (60.4% to 71.3%)	66.7% (60.4% to 71.3%)	0.0% (0.0% to 0.0%)	65.6% (59.6% to 70.5%)
**Nutritional deficiencies**	20.4% (8.1% to 33.4%)	52.1% (28.2% to 69.5%)	80.2% (77.6% to 82.8%)	60.9% (39.4% to 72.6%)
Protein-energy malnutrition	25.2% (10.2% to 40.2%)	59.1% (34.3% to 75.7%)	97.7% (96.8% to 98.4%)	60.0% (35.3% to 76.1%)
Vitamin A deficiency	-	-	100.0% (100.0% to 100.0%)	100.0% (100.0% to 100.0%)
Iron-deficiency anaemia	100.0% (100.0% to 100.0%)	100.0% (100.0% to 100.0%)	100.0% (100.0% to 100.0%)	100.0% (100.0% to 100.0%)
**Other communicable, maternal, neonatal, and nutritional diseases**	2.8% (1.6% to 5.6%)	1.7% (0.8% to 3.5%)	65.1% (60.1% to 69.8%)	7.3% (3.9% to 12.4%)
Sexually transmitted diseases excluding HIV	8.4% (3.7% to 16.5%)	3.3% (1.4% to 6.9%)	100.0% (100.0% to 100.0%)	13.9% (6.7% to 25.3%)
Hepatitis	0.0% (0.0% to 0.0%)	0.0% (0.0% to 0.1%)	0.0% (0.0% to 0.1%)	0.0% (0.0% to 0.1%)
**Neoplasms**	32.3% (28.6% to 36.7%)	34.0% (29.9% to 38.4%)	34.3% (29.3% to 39.5%)	34.0% (29.9% to 38.4%)
Oesophageal cancer	61.0% (47.5% to 72.7%)	63.8% (50.0% to 74.9%)	61.8% (48.1% to 73.3%)	63.8% (49.9% to 74.9%)
Stomach cancer	7.6% (1.8% to 18.0%)	7.8% (2.2% to 17.6%)	7.6% (1.9% to 17.7%)	7.8% (2.2% to 17.6%)
Liver cancer	22.9% (16.9% to 30.5%)	23.5% (17.3% to 31.2%)	23.1% (17.0% to 30.5%)	23.5% (17.3% to 31.2%)
Larynx cancer	54.4% (40.6% to 68.0%)	58.6% (45.4% to 72.0%)	55.1% (41.8% to 68.5%)	58.5% (45.4% to 72.0%)
Tracheal, bronchus, and lung cancer	83.2% (77.5% to 87.9%)	83.9% (78.3% to 88.5%)	83.3% (77.7% to 88.1%)	83.9% (78.3% to 88.5%)
Breast cancer	18.8% (13.0% to 25.9%)	18.4% (12.8% to 25.0%)	18.3% (12.8% to 25.2%)	18.4% (12.8% to 25.0%)
Cervical cancer	100.0% (100.0% to 100.0%)	100.0% (100.0% to 100.0%)	100.0% (100.0% to 100.0%)	100.0% (100.0% to 100.0%)
Uterine cancer	18.4% (7.9% to 31.9%)	19.1% (8.5% to 32.4%)	18.5% (8.0% to 31.6%)	19.1% (8.4% to 32.3%)
Prostate cancer	0.7% (0.3% to 1.2%)	0.9% (0.5% to 1.6%)	0.8% (0.4% to 1.3%)	0.9% (0.5% to 1.6%)
Colon and rectum cancer	50.2% (40.8% to 59.2%)	50.3% (41.1% to 59.2%)	50.3% (41.0% to 59.3%)	50.3% (41.1% to 59.2%)
Lip and oral cavity cancer	58.2% (44.5% to 71.5%)	61.9% (47.8% to 75.1%)	58.8% (45.1% to 72.5%)	61.8% (47.8% to 75.0%)
Nasopharynx cancer	51.1% (38.0% to 64.6%)	52.3% (38.6% to 65.3%)	50.8% (37.7% to 63.7%)	52.3% (38.6% to 65.3%)

Supplementary Results Table 1. Age-standardised PAFs attributable to the joint distribution of all risk factors for all ages, both sexes combined for each GBD level 1 and level 2 cause and all causes in 2016 for Democratic Republic of the Congo. Results are shown for deaths, YLLs, YLDs,
Data in parenthesis are 95% uncertainty intervals. PAFs=population attributable fractions. GBD=Global Burden of Disease. YLLs=years of life lost. YLDs=years live with disability. DALYs=disability-adjusted life-years

	**Deaths 2016**	**YLLs 2016**	**YLDs 2016**	**DALYs 2016**
Other pharynx cancer	39.0% (21.7% to 56.9%)	41.6% (23.7% to 59.7%)	39.9% (22.6% to 57.7%)	41.6% (23.7% to 59.7%)
Gallbladder and biliary tract cancer	8.7% (3.4% to 16.7%)	9.1% (3.5% to 17.2%)	8.7% (3.4% to 16.7%)	9.1% (3.5% to 17.2%)
Pancreatic cancer	13.0% (6.9% to 21.4%)	13.5% (7.8% to 21.3%)	13.0% (7.2% to 21.2%)	13.5% (7.8% to 21.3%)
Ovarian cancer	8.4% (3.2% to 16.2%)	7.4% (2.8% to 14.3%)	7.4% (2.8% to 14.2%)	7.4% (2.8% to 14.4%)
Kidney cancer	11.6% (6.4% to 18.2%)	11.4% (6.2% to 18.0%)	11.0% (6.0% to 17.5%)	11.4% (6.2% to 18.0%)
Bladder cancer	12.1% (6.0% to 20.5%)	13.4% (7.3% to 21.8%)	12.6% (6.4% to 21.0%)	13.4% (7.3% to 21.8%)
Thyroid cancer	4.9% (1.9% to 9.4%)	5.1% (1.9% to 9.7%)	4.9% (1.8% to 9.5%)	5.1% (1.9% to 9.7%)
Mesothelioma	85.0% (77.6% to 89.5%)	76.3% (66.8% to 82.5%)	81.0% (72.6% to 86.5%)	76.4% (66.9% to 82.6%)
Non-Hodgkin's lymphoma	2.3% (0.8% to 4.8%)	2.2% (0.7% to 4.5%)	2.3% (0.7% to 4.7%)	2.2% (0.7% to 4.5%)
Multiple myeloma	2.9% (1.0% to 6.0%)	3.0% (1.0% to 6.0%)	2.9% (1.0% to 6.0%)	3.0% (1.0% to 6.0%)
Leukaemia	5.1% (2.7% to 8.2%)	4.3% (2.2% to 7.1%)	4.8% (2.5% to 7.7%)	4.3% (2.2% to 7.1%)
**Cardiovascular diseases**	84.2% (81.8% to 86.5%)	85.4% (83.4% to 87.1%)	58.9% (54.4% to 63.5%)	83.6% (81.5% to 85.6%)
Rheumatic heart disease	26.9% (18.2% to 41.4%)	26.9% (18.4% to 40.1%)	24.0% (15.2% to 33.9%)	25.9% (17.8% to 37.0%)
Ischaemic heart disease	93.9% (91.0% to 96.2%)	95.2% (93.3% to 96.8%)	90.2% (87.2% to 92.9%)	95.0% (93.1% to 96.7%)
Cerebrovascular disease	87.1% (83.5% to 90.8%)	89.8% (87.2% to 92.2%)	87.5% (84.0% to 90.9%)	89.7% (87.1% to 92.1%)
Hypertensive heart disease	100.0% (100.0% to 100.0%)	100.0% (100.0% to 100.0%)	100.0% (100.0% to 100.0%)	100.0% (100.0% to 100.0%)
Cardiomyopathy and myocarditis	38.6% (30.8% to 48.0%)	37.6% (30.0% to 46.0%)	41.8% (34.8% to 49.1%)	37.9% (30.5% to 46.3%)
Atrial fibrillation and flutter	38.7% (31.5% to 45.9%)	43.7% (37.2% to 50.0%)	50.9% (45.4% to 56.3%)	47.2% (41.1% to 53.0%)
Aortic aneurysm	40.2% (33.3% to 46.4%)	45.9% (39.6% to 51.5%)	-	45.9% (39.6% to 51.5%)
Peripheral vascular disease	51.2% (45.3% to 57.0%)	58.7% (53.4% to 63.8%)	58.0% (53.0% to 62.9%)	58.2% (53.4% to 63.0%)
Endocarditis	37.9% (29.1% to 48.4%)	39.0% (30.5% to 48.1%)	34.1% (25.3% to 44.3%)	38.9% (30.4% to 48.0%)

**Supplementary Results Table 1. Age-standardised PAFs attributable to the joint distribution of all risk factors for all ages, both sexes combined for each GBD level 1 and level 2 cause and all causes in 2016 for Democratic Republic of the Congo. Results are shown for deaths, YLLs, YLDs,** Data in parenthesis are 95% uncertainty intervals. PAFs=population attributable fractions. GBD=Global Burden of Disease. YLLs=years of life lost. YLDs=years live with disability. DALYs=disability-adjusted life-years	**Deaths 2016**	**YLLs 2016**	**YLDs 2016**	**DALYs 2016**
Other cardiovascular and circulatory diseases	41.5% (36.5% to 46.4%)	45.6% (41.3% to 49.9%)	38.7% (33.6% to 43.9%)	44.3% (40.1% to 48.8%)
**Chronic respiratory diseases**	60.9% (49.6% to 68.9%)	57.7% (48.0% to 64.8%)	34.2% (27.7% to 41.4%)	50.6% (43.4% to 57.5%)
Chronic obstructive pulmonary disease	77.7% (69.0% to 87.5%)	78.2% (70.0% to 87.2%)	68.1% (57.9% to 79.0%)	76.3% (68.1% to 85.5%)
Pneumoconiosis	100.0% (100.0% to 100.0%)	100.0% (100.0% to 100.0%)	100.0% (100.0% to 100.0%)	100.0% (100.0% to 100.0%)
Asthma	18.1% (10.9% to 27.2%)	20.2% (13.5% to 28.3%)	15.9% (11.2% to 22.0%)	18.3% (12.7% to 25.0%)
Other chronic respiratory diseases	3.5% (1.7% to 6.3%)	3.6% (1.6% to 7.0%)	2.0% (0.8% to 3.8%)	3.1% (1.4% to 5.8%)
**Cirrhosis and other chronic liver diseases**	23.3% (21.5% to 24.9%)	22.2% (20.5% to 23.9%)	17.9% (15.6% to 20.4%)	22.1% (20.4% to 23.8%)
Cirrhosis and other chronic liver diseases due to hepatitis B	0.0% (0.0% to 0.0%)	0.0% (0.0% to 0.0%)	0.0% (0.0% to 0.0%)	0.0% (0.0% to 0.0%)
Cirrhosis and other chronic liver diseases due to hepatitis C	1.1% (0.5% to 1.8%)	1.5% (0.7% to 2.5%)	2.1% (0.9% to 3.7%)	1.5% (0.7% to 2.6%)
Cirrhosis and other chronic liver diseases due to alcohol use	100.0% (100.0% to 100.0%)	100.0% (100.0% to 100.0%)	100.0% (100.0% to 100.0%)	100.0% (100.0% to 100.0%)
**Digestive diseases**	4.0% (2.6% to 5.8%)	4.6% (3.0% to 6.5%)	1.7% (1.1% to 2.3%)	4.4% (2.9% to 6.2%)
Peptic ulcer disease	6.4% (3.7% to 9.4%)	8.1% (4.9% to 11.6%)	7.6% (5.2% to 10.3%)	8.1% (4.9% to 11.6%)
Gallbladder and biliary diseases	18.0% (8.3% to 30.5%)	18.5% (9.1% to 30.1%)	18.4% (9.2% to 30.4%)	18.5% (9.1% to 30.2%)
Pancreatitis	20.5% (4.2% to 46.4%)	24.0% (5.8% to 52.5%)	21.1% (4.5% to 46.5%)	23.9% (5.8% to 52.5%)
**Neurological disorders**	12.2% (5.2% to 22.2%)	12.7% (6.5% to 21.1%)	3.0% (1.4% to 5.0%)	7.2% (3.7% to 11.9%)
Alzheimer's disease and other dementias	12.8% (5.2% to 23.6%)	13.6% (5.7% to 24.4%)	13.6% (5.7% to 24.2%)	13.6% (5.7% to 24.4%)
Parkinson's disease	-4.1% (-5.8% to -2.7%)	-5.3% (-7.2% to -3.7%)	-5.9% (-8.1% to -4.2%)	-5.4% (-7.3% to -3.8%)
Epilepsy	15.8% (6.5% to 25.4%)	14.6% (6.0% to 23.6%)	12.8% (5.1% to 20.6%)	13.9% (5.8% to 22.3%)
Multiple sclerosis	4.5% (1.7% to 8.4%)	4.8% (1.8% to 9.1%)	3.5% (1.9% to 5.2%)	4.4% (2.0% to 7.8%)
**Mental and substance use disorders**	99.8% (99.6% to 99.9%)	99.8% (99.5% to 99.9%)	19.6% (17.2% to 22.1%)	23.1% (20.2% to 27.0%)
Alcohol use disorders	100.0% (100.0% to 100.0%)	100.0% (100.0% to 100.0%)	100.0% (100.0% to 100.0%)	100.0% (100.0% to 100.0%)

Supplementary Results Table 1. Age-standardised PAFs attributable to the joint distribution of all risk factors for all ages, both sexes combined for each GBD level 1 and level 2 cause and all causes in 2016 for Democratic Republic of the Congo. Results are shown for deaths, YLLs, YLDs,
Data in parenthesis are 95% uncertainty intervals. PAFs=population attributable fractions. GBD=Global Burden of Disease. YLLs=years of life lost. YLDs=years live with disability. DALYs=disability-adjusted life-years

	Deaths 2016	YLLs 2016	YLDs 2016	DALYs 2016
Drug use disorders	100.0% (100.0% to 100.0%)	100.0% (100.0% to 100.0%)	100.0% (100.0% to 100.0%)	100.0% (100.0% to 100.0%)
Depressive disorders	-	-	10.3% (8.6% to 12.4%)	10.3% (8.6% to 12.4%)
Idiopathic developmental intellectual disability	-	-	62.3% (31.0% to 81.9%)	62.3% (31.0% to 81.9%)
**Diabetes, urogenital, blood, and endocrine diseases**	81.7% (78.1% to 84.5%)	73.5% (66.3% to 77.8%)	58.8% (54.5% to 62.7%)	66.9% (62.5% to 70.4%)
Diabetes mellitus	100.0% (100.0% to 100.0%)	100.0% (100.0% to 100.0%)	100.0% (100.0% to 100.0%)	100.0% (100.0% to 100.0%)
Chronic kidney disease	100.0% (100.0% to 100.0%)	100.0% (100.0% to 100.0%)	100.0% (100.0% to 100.0%)	100.0% (100.0% to 100.0%)
**Musculoskeletal disorders**	0.7% (0.3% to 1.3%)	0.6% (0.2% to 1.1%)	17.5% (15.1% to 20.3%)	17.2% (14.8% to 20.0%)
Rheumatoid arthritis	1.6% (0.5% to 3.1%)	1.9% (0.7% to 3.7%)	2.2% (0.9% to 3.8%)	2.1% (0.9% to 3.7%)
Osteoarthritis	-	-	10.5% (4.3% to 19.2%)	10.5% (4.3% to 19.2%)
Low back and neck pain	-	-	21.8% (19.2% to 24.7%)	21.8% (19.2% to 24.7%)
Gout	-	-	36.4% (28.6% to 47.0%)	36.4% (28.6% to 47.0%)
**Other non-communicable diseases**	0.1% (0.0% to 0.3%)	0.2% (0.1% to 0.5%)	7.0% (5.8% to 8.2%)	5.3% (4.2% to 6.4%)
Sense organ diseases	-	-	12.9% (10.7% to 14.8%)	12.9% (10.7% to 14.8%)
Sudden infant death syndrome	4.8% (1.9% to 9.6%)	4.8% (1.9% to 9.7%)	-	4.8% (1.9% to 9.7%)
**Transport injuries**	42.5% (32.4% to 49.5%)	37.2% (26.8% to 45.0%)	50.2% (40.7% to 57.2%)	38.6% (28.5% to 45.9%)
Road injuries	42.2% (32.3% to 49.2%)	36.9% (26.6% to 44.7%)	53.8% (43.9% to 60.7%)	38.3% (28.4% to 45.7%)
Other transport injuries	49.3% (35.7% to 59.0%)	43.5% (28.0% to 54.1%)	40.5% (32.9% to 46.9%)	42.2% (30.6% to 50.4%)
**Unintentional injuries**	35.0% (28.8% to 40.6%)	24.3% (18.0% to 30.4%)	36.2% (28.8% to 41.1%)	27.2% (21.0% to 32.7%)
Falls	70.0% (53.5% to 75.3%)	51.0% (42.0% to 58.1%)	44.6% (37.5% to 49.4%)	47.8% (40.8% to 53.2%)
Drowning	21.9% (15.6% to 29.5%)	17.1% (11.0% to 25.2%)	32.4% (26.3% to 38.6%)	17.3% (11.2% to 25.3%)
Fire, heat, and hot substances	18.3% (12.7% to 24.3%)	20.3% (13.5% to 26.9%)	34.9% (25.9% to 41.2%)	22.9% (16.3% to 29.5%)

Supplementary Results Table 1. Age-standardised PAFs attributable to the joint distribution of all risk factors for all ages, both sexes combined for each GBD level 1 and level 2 cause and all causes in 2016 for Democratic Republic of the Congo. Results are shown for deaths, YLLs, YLDs,
Data in parenthesis are 95% uncertainty intervals. PAFs=population attributable fractions. GBD=Global Burden of Disease. YLLs=years of life lost. YLDs=years live with disability. DALYs=disability-adjusted life-years

	**Deaths 2016**	**YLLs 2016**	**YLDs 2016**	**DALYs 2016**
Poisonings	25.1% (15.8% to 33.6%)	24.5% (13.5% to 35.4%)	31.6% (23.3% to 37.5%)	24.8% (13.9% to 35.4%)
Exposure to mechanical forces	36.3% (25.4% to 44.5%)	30.4% (18.4% to 41.0%)	43.2% (30.9% to 50.5%)	33.5% (22.2% to 42.7%)
Animal contact	23.9% (18.2% to 30.9%)	25.5% (17.9% to 33.4%)	29.9% (22.0% to 35.5%)	26.5% (19.0% to 33.4%)
Foreign body	18.7% (10.4% to 26.2%)	17.3% (7.4% to 27.4%)	25.6% (18.0% to 30.9%)	19.8% (10.9% to 27.8%)
Other unintentional injuries	29.5% (17.6% to 39.3%)	30.4% (17.8% to 41.5%)	31.5% (23.4% to 37.2%)	31.0% (21.4% to 38.1%)
**Self-harm and interpersonal violence**	15.0% (6.8% to 24.9%)	15.4% (7.6% to 24.2%)	20.6% (17.6% to 23.9%)	16.0% (9.0% to 24.2%)
Self-harm	14.6% (4.0% to 26.8%)	16.0% (4.6% to 28.6%)	15.0% (4.3% to 27.8%)	15.9% (4.6% to 28.6%)
Interpersonal violence	15.9% (9.9% to 22.1%)	14.6% (9.0% to 20.6%)	21.2% (18.3% to 24.2%)	16.0% (11.2% to 21.3%)
**Forces of nature, conflict and terrorism, and executions and police conflict**	0.0% (0.0% to 0.0%)	0.0% (0.0% to 0.0%)	0.0% (0.0% to 0.0%)	0.0% (0.0% to 0.0%)
Forces of nature, conflict and terrorism, and state actor violence	0.0% (0.0% to 0.0%)	0.0% (0.0% to 0.0%)	6.4% (4.3% to 9.4%)	6.4% (4.3% to 9.4%)

Supplementary Results Table 1. Age-standardised PAFs attributable to the joint distribution of all risk factors for all ages, both sexes combined for each GBD level 1 and level 2 cause and all causes in 2016 for Denmark. Results are shown for deaths, YLLs, YLDs, and DALYs
Data in parenthesis are 95% uncertainty intervals. PAFs=population attributable fractions. GBD=Global Burden of Disease. YLLs=years of life lost. YLDs=years live with disability. DALYs=disability-adjusted life-years

	Deaths 2016	YLLs 2016	YLDs 2016	DALYs 2016
**All causes**	58.7% (56.4% to 60.9%)	57.5% (55.4% to 59.5%)	20.6% (19.1% to 22.1%)	37.3% (34.3% to 40.6%)
**HIV/AIDS and tuberculosis**	81.1% (76.4% to 85.1%)	83.3% (80.5% to 85.7%)	90.1% (86.7% to 92.3%)	86.4% (83.5% to 88.9%)
Tuberculosis	66.0% (55.2% to 74.9%)	64.5% (54.6% to 72.6%)	56.0% (44.4% to 65.9%)	62.6% (52.9% to 70.9%)
HIV/AIDS	92.4% (91.7% to 93.1%)	90.2% (89.3% to 91.1%)	94.0% (93.1% to 94.8%)	92.1% (91.0% to 93.1%)
**Diarrhoea, lower respiratory infections, and other common infectious diseases**	39.1% (29.7% to 48.3%)	41.9% (34.1% to 49.8%)	6.9% (4.9% to 9.4%)	27.5% (21.3% to 34.2%)
Diarrhoeal diseases	12.6% (6.8% to 21.9%)	22.5% (16.0% to 31.9%)	28.4% (20.4% to 37.6%)	25.5% (18.6% to 34.6%)
Lower respiratory infections	43.9% (33.0% to 54.8%)	49.2% (39.4% to 59.4%)	46.0% (36.6% to 55.4%)	49.1% (39.4% to 59.3%)
Upper respiratory infections	0.6% (0.0% to 2.1%)	2.0% (0.1% to 7.1%)	0.0% (0.0% to 0.0%)	0.0% (0.0% to 0.0%)
Otitis media	2.2% (1.2% to 3.9%)	5.4% (3.1% to 8.3%)	6.4% (4.3% to 8.6%)	6.4% (4.3% to 8.6%)
Meningitis	1.9% (1.3% to 2.5%)	4.1% (2.8% to 5.4%)	0.0% (0.0% to 0.0%)	3.6% (2.6% to 4.8%)
Encephalitis	0.7% (0.3% to 1.1%)	1.4% (0.7% to 2.4%)	0.0% (0.0% to 0.0%)	0.8% (0.3% to 1.3%)
Measles	4.2% (0.7% to 17.1%)	5.6% (1.0% to 23.2%)	10.4% (2.0% to 40.8%)	5.7% (1.0% to 23.6%)
**Maternal disorders**	1.8% (1.1% to 2.6%)	1.7% (1.0% to 2.5%)	2.0% (0.9% to 3.5%)	1.9% (1.0% to 3.0%)
Maternal haemorrhage	8.2% (3.0% to 13.4%)	8.2% (3.0% to 13.6%)	8.5% (3.0% to 14.2%)	8.4% (3.0% to 13.9%)
Maternal sepsis and other pregnancy related infections	8.3% (3.0% to 13.7%)	8.3% (3.0% to 13.7%)	8.3% (3.0% to 13.8%)	8.3% (3.0% to 13.8%)
Maternal abortion, miscarriage, and ectopic pregnancy	4.7% (2.9% to 6.9%)	4.8% (2.9% to 7.0%)	5.2% (2.5% to 8.4%)	4.9% (2.8% to 7.5%)
**Neonatal disorders**	83.0% (80.5% to 85.3%)	83.1% (80.5% to 85.4%)	0.1% (0.1% to 0.1%)	58.7% (51.1% to 65.3%)
Neonatal preterm birth complications	91.2% (88.5% to 93.2%)	91.2% (88.6% to 93.2%)	0.2% (0.1% to 0.2%)	69.6% (61.4% to 76.8%)
Neonatal encephalopathy due to birth asphyxia and trauma	65.0% (60.7% to 68.9%)	65.1% (60.8% to 68.9%)	0.0% (0.0% to 0.0%)	40.4% (31.1% to 48.8%)
Neonatal sepsis and other neonatal infections	58.3% (50.9% to 64.6%)	58.3% (50.9% to 64.6%)	0.0% (0.0% to 0.0%)	14.4% (6.3% to 25.4%)
Hemolytic disease and other neonatal jaundice	62.9% (57.0% to 68.5%)	63.0% (57.1% to 68.5%)	0.0% (0.0% to 0.0%)	22.3% (13.8% to 31.1%)

Supplementary Results Table 1. Age-standardised PAFs attributable to the joint distribution of all risk factors for all ages, both sexes combined for each GBD level 1 and level 2 cause and all causes in 2016 for Denmark. Results are shown for deaths, YLLs, YLDs, and DALYs
Data in parenthesis are 95% uncertainty intervals. PAFs=population attributable fractions. GBD=Global Burden of Disease. YLLs=years of life lost. YLDs=years live with disability. DALYs=disability-adjusted life-years

	Deaths 2016	YLLs 2016	YLDs 2016	DALYs 2016
Other neonatal disorders	62.6% (57.5% to 67.6%)	62.6% (57.6% to 67.7%)	0.0% (0.0% to 0.0%)	56.1% (50.5% to 61.6%)
**Nutritional deficiencies**	2.4% (1.2% to 3.4%)	8.4% (6.1% to 10.4%)	48.4% (39.9% to 57.4%)	45.1% (36.9% to 53.8%)
Protein-energy malnutrition	0.6% (0.3% to 1.2%)	5.1% (2.5% to 8.2%)	98.3% (98.1% to 98.5%)	72.6% (62.6% to 81.7%)
Vitamin A deficiency	-	-	0.0% (0.0% to 0.0%)	0.0% (0.0% to 0.0%)
Iron-deficiency anaemia	100.0% (100.0% to 100.0%)	100.0% (100.0% to 100.0%)	100.0% (100.0% to 100.0%)	100.0% (100.0% to 100.0%)
**Other communicable, maternal, neonatal, and nutritional diseases**	4.4% (3.6% to 6.3%)	2.8% (2.2% to 4.1%)	63.6% (53.6% to 72.6%)	23.9% (15.3% to 35.8%)
Sexually transmitted diseases excluding HIV	96.0% (92.6% to 98.3%)	82.2% (70.2% to 91.4%)	100.0% (100.0% to 100.0%)	98.7% (97.2% to 99.5%)
Hepatitis	6.2% (4.4% to 8.2%)	5.9% (4.1% to 8.3%)	3.0% (1.8% to 4.4%)	5.7% (3.9% to 8.0%)
**Neoplasms**	49.8% (46.9% to 52.8%)	48.9% (46.2% to 51.7%)	36.1% (33.1% to 39.4%)	48.4% (45.7% to 51.2%)
Oesophageal cancer	88.9% (83.4% to 93.2%)	88.8% (83.0% to 93.2%)	88.8% (83.2% to 93.2%)	88.8% (83.0% to 93.2%)
Stomach cancer	24.3% (15.1% to 33.5%)	23.2% (14.5% to 32.4%)	24.0% (15.1% to 33.2%)	23.2% (14.5% to 32.4%)
Liver cancer	81.8% (75.7% to 86.5%)	80.5% (74.5% to 85.6%)	80.7% (74.6% to 85.6%)	80.5% (74.5% to 85.6%)
Larynx cancer	92.5% (87.6% to 95.8%)	91.5% (86.2% to 95.2%)	92.1% (87.1% to 95.7%)	91.5% (86.3% to 95.2%)
Tracheal, bronchus, and lung cancer	92.3% (90.7% to 93.6%)	91.3% (89.5% to 92.7%)	91.8% (90.1% to 93.2%)	91.3% (89.5% to 92.8%)
Breast cancer	42.8% (35.3% to 50.6%)	41.8% (34.7% to 49.4%)	41.8% (34.5% to 49.2%)	41.8% (34.7% to 49.4%)
Cervical cancer	100.0% (100.0% to 100.0%)	100.0% (100.0% to 100.0%)	100.0% (100.0% to 100.0%)	100.0% (100.0% to 100.0%)
Uterine cancer	36.3% (23.6% to 48.9%)	37.9% (25.4% to 50.7%)	38.5% (25.9% to 51.3%)	38.0% (25.4% to 50.8%)
Prostate cancer	6.9% (4.8% to 9.3%)	6.8% (4.7% to 9.1%)	6.7% (4.7% to 9.0%)	6.8% (4.7% to 9.1%)
Colon and rectum cancer	60.6% (51.8% to 68.4%)	61.0% (52.6% to 68.6%)	60.9% (52.2% to 68.7%)	61.0% (52.6% to 68.6%)
Lip and oral cavity cancer	90.2% (85.0% to 93.8%)	90.0% (84.6% to 93.7%)	89.4% (84.2% to 93.4%)	89.9% (84.5% to 93.7%)
Nasopharynx cancer	90.1% (86.2% to 93.1%)	88.0% (84.1% to 91.0%)	84.5% (79.6% to 88.5%)	87.9% (84.0% to 91.0%)

Supplementary Results Table 1. Age-standardised PAFs attributable to the joint distribution of all risk factors for all ages, both sexes combined for each GBD level 1 and level 2 cause and all causes in 2016 for Denmark. Results are shown for deaths, YLLs, YLDs, and DALYs
Data in parenthesis are 95% uncertainty intervals. PAFs=population attributable fractions. GBD=Global Burden of Disease. YLLs=years of life lost. YLDs=years live with disability. DALYs=disability-adjusted life-years

	Deaths 2016	YLLs 2016	YLDs 2016	DALYs 2016
Other pharynx cancer	73.9% (62.2% to 82.4%)	74.5% (63.1% to 82.7%)	74.2% (62.6% to 82.7%)	74.5% (63.1% to 82.7%)
Gallbladder and biliary tract cancer	16.8% (8.5% to 27.5%)	17.4% (9.1% to 28.5%)	17.8% (9.3% to 29.0%)	17.4% (9.1% to 28.5%)
Pancreatic cancer	39.9% (32.7% to 47.7%)	38.9% (31.6% to 46.5%)	39.6% (32.3% to 47.3%)	38.9% (31.6% to 46.5%)
Ovarian cancer	16.3% (9.0% to 24.6%)	12.9% (6.9% to 19.8%)	13.2% (7.0% to 20.4%)	12.9% (6.9% to 19.8%)
Kidney cancer	38.1% (27.9% to 48.8%)	37.6% (27.4% to 48.3%)	37.4% (27.5% to 47.7%)	37.6% (27.3% to 48.3%)
Bladder cancer	45.1% (35.2% to 54.6%)	44.2% (34.5% to 53.6%)	43.8% (33.9% to 53.0%)	44.1% (34.5% to 53.6%)
Thyroid cancer	12.0% (5.6% to 20.3%)	12.8% (5.9% to 21.7%)	12.4% (6.3% to 20.6%)	12.7% (6.0% to 21.4%)
Mesothelioma	97.5% (96.8% to 98.1%)	95.1% (93.9% to 96.2%)	96.7% (95.8% to 97.5%)	95.2% (94.0% to 96.2%)
Non-Hodgkin's lymphoma	6.0% (2.4% to 11.1%)	6.1% (2.4% to 11.0%)	6.2% (2.5% to 11.3%)	6.1% (2.4% to 11.0%)
Multiple myeloma	6.7% (2.8% to 12.2%)	7.1% (2.9% to 12.8%)	7.0% (3.0% to 12.9%)	7.1% (2.9% to 12.8%)
Leukaemia	21.5% (12.6% to 30.8%)	18.7% (11.4% to 26.4%)	19.4% (11.6% to 27.2%)	18.8% (11.4% to 26.4%)
**Cardiovascular diseases**	82.1% (79.0% to 84.7%)	84.6% (82.4% to 86.5%)	77.2% (74.2% to 79.9%)	83.0% (80.7% to 85.0%)
Rheumatic heart disease	30.4% (18.2% to 54.9%)	31.8% (21.7% to 49.5%)	30.4% (21.1% to 45.6%)	31.8% (21.7% to 49.4%)
Ischaemic heart disease	93.0% (89.0% to 96.2%)	94.8% (92.1% to 96.8%)	92.3% (89.7% to 94.6%)	94.5% (91.8% to 96.6%)
Cerebrovascular disease	84.8% (78.7% to 90.2%)	88.8% (84.7% to 92.2%)	86.8% (82.6% to 90.6%)	88.3% (84.2% to 91.8%)
Hypertensive heart disease	100.0% (100.0% to 100.0%)	100.0% (100.0% to 100.0%)	100.0% (100.0% to 100.0%)	100.0% (100.0% to 100.0%)
Cardiomyopathy and myocarditis	42.0% (34.9% to 48.2%)	48.8% (42.0% to 54.2%)	46.0% (40.6% to 51.4%)	48.4% (42.3% to 53.6%)
Atrial fibrillation and flutter	61.0% (52.7% to 68.5%)	65.4% (58.2% to 71.7%)	71.8% (66.2% to 76.9%)	68.9% (62.7% to 74.4%)
Aortic aneurysm	48.8% (40.6% to 56.6%)	53.5% (46.2% to 59.9%)	-	53.5% (46.2% to 59.9%)
Peripheral vascular disease	52.6% (45.2% to 60.5%)	58.4% (52.2% to 64.1%)	61.7% (55.8% to 66.9%)	59.6% (53.6% to 64.8%)
Endocarditis	43.4% (33.3% to 55.3%)	45.9% (37.2% to 54.6%)	44.3% (35.6% to 53.4%)	45.8% (37.2% to 54.5%)

Supplementary Results Table 1. Age-standardised PAFs attributable to the joint distribution of all risk factors for all ages, both sexes combined for each GBD level 1 and level 2 cause and all causes in 2016 for Denmark. Results are shown for deaths, YLLs, YLDs, and DALYs
Data in parenthesis are 95% uncertainty intervals. PAFs=population attributable fractions. GBD=Global Burden of Disease. YLLs=years of life lost. YLDs=years live with disability. DALYs=disability-adjusted life-years

	Deaths 2016	YLLs 2016	YLDs 2016	DALYs 2016
Other cardiovascular and circulatory diseases	49.3% (43.1% to 55.6%)	55.8% (50.8% to 60.3%)	58.2% (53.3% to 62.7%)	57.0% (52.0% to 61.4%)
**Chronic respiratory diseases**	80.7% (77.4% to 83.4%)	78.5% (75.2% to 81.3%)	47.8% (40.8% to 54.8%)	66.5% (61.6% to 71.2%)
Chronic obstructive pulmonary disease	85.9% (82.8% to 88.6%)	85.5% (82.5% to 88.3%)	78.7% (74.7% to 82.5%)	84.2% (81.0% to 87.2%)
Pneumoconiosis	100.0% (100.0% to 100.0%)	100.0% (100.0% to 100.0%)	100.0% (100.0% to 100.0%)	100.0% (100.0% to 100.0%)
Asthma	38.7% (29.7% to 48.5%)	41.8% (33.8% to 51.2%)	33.9% (26.0% to 43.4%)	34.4% (26.5% to 43.8%)
Other chronic respiratory diseases	21.6% (15.9% to 26.9%)	13.5% (8.0% to 18.9%)	9.8% (7.2% to 13.3%)	11.5% (7.9% to 15.5%)
**Cirrhosis and other chronic liver diseases**	77.0% (73.0% to 80.7%)	77.5% (73.6% to 81.3%)	63.1% (58.8% to 67.7%)	76.6% (72.6% to 80.2%)
Cirrhosis and other chronic liver diseases due to hepatitis B	3.0% (1.3% to 5.2%)	3.6% (1.6% to 6.2%)	3.8% (1.7% to 6.7%)	3.6% (1.6% to 6.3%)
Cirrhosis and other chronic liver diseases due to hepatitis C	89.0% (87.8% to 89.8%)	89.8% (89.5% to 90.0%)	88.0% (86.5% to 89.0%)	89.7% (89.4% to 89.9%)
Cirrhosis and other chronic liver diseases due to alcohol use	100.0% (100.0% to 100.0%)	100.0% (100.0% to 100.0%)	100.0% (100.0% to 100.0%)	100.0% (100.0% to 100.0%)
**Digestive diseases**	12.4% (9.7% to 15.2%)	15.1% (11.6% to 18.7%)	3.7% (2.7% to 4.8%)	12.9% (9.8% to 16.0%)
Peptic ulcer disease	13.8% (9.5% to 18.5%)	16.1% (11.2% to 21.5%)	15.5% (11.0% to 20.8%)	16.1% (11.2% to 21.5%)
Gallbladder and biliary diseases	35.7% (22.3% to 50.0%)	37.1% (23.9% to 51.4%)	41.9% (29.0% to 56.1%)	37.8% (24.7% to 51.9%)
Pancreatitis	52.6% (32.9% to 70.8%)	54.2% (35.2% to 71.5%)	49.8% (31.3% to 67.5%)	54.2% (35.1% to 71.4%)
**Neurological disorders**	18.5% (10.0% to 29.0%)	17.5% (10.9% to 25.4%)	3.7% (2.1% to 5.9%)	8.1% (5.0% to 11.9%)
Alzheimer's disease and other dementias	22.7% (12.0% to 35.9%)	23.9% (13.0% to 36.9%)	23.9% (13.1% to 37.1%)	23.9% (13.1% to 36.9%)
Parkinson's disease	-11.6% (-13.5% to -9.7%)	-13.2% (-15.3% to -11.0%)	-14.4% (-16.6% to -12.0%)	-13.4% (-15.5% to -11.2%)
Epilepsy	43.6% (31.2% to 53.5%)	39.7% (27.6% to 49.1%)	32.8% (22.1% to 41.8%)	36.0% (24.4% to 45.1%)
Multiple sclerosis	10.8% (7.0% to 15.0%)	11.1% (7.3% to 15.4%)	9.5% (6.0% to 13.2%)	10.2% (6.6% to 14.0%)
**Mental and substance use disorders**	99.8% (99.7% to 99.8%)	99.7% (99.6% to 99.8%)	18.7% (16.8% to 20.9%)	32.2% (28.0% to 36.8%)
Alcohol use disorders	100.0% (100.0% to 100.0%)	100.0% (100.0% to 100.0%)	100.0% (100.0% to 100.0%)	100.0% (100.0% to 100.0%)

Supplementary Results Table 1. Age-standardised PAFs attributable to the joint distribution of all risk factors for all ages, both sexes combined for each GBD level 1 and level 2 cause and all causes in 2016 for Denmark. Results are shown for deaths, YLLs, YLDs, and DALYs
Data in parenthesis are 95% uncertainty intervals. PAFs=population attributable fractions. GBD=Global Burden of Disease. YLLs=years of life lost. YLDs=years live with disability. DALYs=disability-adjusted life-years

	Deaths 2016	YLLs 2016	YLDs 2016	DALYs 2016
Drug use disorders	100.0% (100.0% to 100.0%)	100.0% (100.0% to 100.0%)	100.0% (100.0% to 100.0%)	100.0% (100.0% to 100.0%)
Depressive disorders	-	-	7.8% (6.3% to 9.6%)	7.8% (6.3% to 9.6%)
Idiopathic developmental intellectual disability	-	-	63.4% (33.0% to 82.7%)	63.4% (33.0% to 82.7%)
**Diabetes, urogenital, blood, and endocrine diseases**	73.2% (71.1% to 78.9%)	70.1% (66.9% to 79.0%)	52.5% (47.1% to 57.4%)	60.3% (55.9% to 64.7%)
Diabetes mellitus	100.0% (100.0% to 100.0%)	100.0% (100.0% to 100.0%)	100.0% (100.0% to 100.0%)	100.0% (100.0% to 100.0%)
Chronic kidney disease	100.0% (100.0% to 100.0%)	100.0% (100.0% to 100.0%)	100.0% (100.0% to 100.0%)	100.0% (100.0% to 100.0%)
**Musculoskeletal disorders**	1.6% (0.6% to 2.9%)	1.8% (0.7% to 3.1%)	19.9% (17.1% to 23.1%)	19.7% (16.9% to 22.8%)
Rheumatoid arthritis	5.6% (2.2% to 9.4%)	6.3% (2.5% to 10.4%)	6.6% (2.6% to 11.1%)	6.6% (2.6% to 11.0%)
Osteoarthritis	-	-	21.5% (11.5% to 32.6%)	21.5% (11.5% to 32.6%)
Low back and neck pain	-	-	22.4% (19.5% to 25.6%)	22.4% (19.5% to 25.6%)
Gout	-	-	43.5% (26.5% to 61.4%)	43.5% (26.5% to 61.4%)
**Other non-communicable diseases**	0.2% (0.1% to 0.2%)	0.2% (0.1% to 0.3%)	1.5% (1.3% to 1.7%)	1.3% (1.2% to 1.5%)
Sense organ diseases	-	-	6.6% (5.9% to 7.4%)	6.6% (5.9% to 7.4%)
Sudden infant death syndrome	4.5% (2.8% to 6.7%)	4.5% (2.8% to 6.7%)	-	4.5% (2.8% to 6.7%)
**Transport injuries**	43.4% (32.9% to 54.5%)	39.5% (28.7% to 50.6%)	46.9% (36.8% to 58.3%)	42.7% (32.2% to 54.2%)
Road injuries	43.0% (32.5% to 54.1%)	39.2% (28.4% to 50.4%)	47.0% (36.9% to 58.4%)	42.5% (32.0% to 54.1%)
Other transport injuries	47.4% (36.5% to 58.5%)	43.4% (32.3% to 55.3%)	46.6% (36.4% to 57.9%)	44.7% (34.2% to 56.2%)
**Unintentional injuries**	48.5% (40.7% to 53.2%)	31.2% (25.6% to 36.4%)	25.2% (20.1% to 32.9%)	26.8% (22.0% to 33.3%)
Falls	69.7% (60.4% to 74.3%)	52.1% (41.3% to 58.1%)	26.4% (21.7% to 33.9%)	31.2% (26.0% to 37.9%)
Drowning	23.0% (15.3% to 31.9%)	20.3% (14.1% to 28.0%)	24.9% (16.1% to 35.8%)	20.7% (14.2% to 28.6%)
Fire, heat, and hot substances	20.5% (12.2% to 30.6%)	24.1% (16.2% to 33.1%)	27.2% (18.1% to 37.9%)	25.9% (17.5% to 35.8%)

Supplementary Results Table 1. Age-standardised PAFs attributable to the joint distribution of all risk factors for all ages, both sexes combined for each GBD level 1 and level 2 cause and all causes in 2016 for Denmark. Results are shown for deaths, YLLs, YLDs, and DALYs
Data in parenthesis are 95% uncertainty intervals. PAFs=population attributable fractions. GBD=Global Burden of Disease. YLLs=years of life lost. YLDs=years live with disability. DALYs=disability-adjusted life-years

	**Deaths 2016**	**YLLs 2016**	**YLDs 2016**	**DALYs 2016**
Poisonings	28.1% (19.2% to 38.0%)	29.7% (20.9% to 39.4%)	25.0% (17.0% to 34.8%)	27.0% (18.8% to 36.6%)
Exposure to mechanical forces	26.4% (21.6% to 32.5%)	20.7% (15.2% to 28.5%)	25.1% (19.2% to 33.5%)	24.1% (18.4% to 32.4%)
Animal contact	17.3% (13.6% to 23.3%)	15.4% (10.7% to 22.9%)	16.0% (10.2% to 26.1%)	15.9% (10.3% to 25.2%)
Foreign body	7.9% (5.0% to 11.9%)	12.1% (7.6% to 18.4%)	11.0% (6.7% to 17.6%)	11.7% (7.4% to 17.8%)
Other unintentional injuries	26.2% (17.6% to 36.2%)	26.1% (18.4% to 34.9%)	26.0% (17.3% to 36.3%)	26.0% (17.4% to 36.2%)
**Self-harm and interpersonal violence**	38.9% (22.1% to 53.5%)	37.8% (21.5% to 52.5%)	39.0% (32.6% to 44.9%)	37.9% (22.7% to 51.6%)
Self-harm	38.8% (20.9% to 54.7%)	38.1% (20.0% to 54.0%)	33.2% (15.8% to 49.0%)	38.0% (20.0% to 53.8%)
Interpersonal violence	40.3% (31.4% to 48.9%)	35.7% (27.2% to 44.3%)	40.8% (35.0% to 46.3%)	37.9% (31.0% to 44.3%)
**Forces of nature, conflict and terrorism, and executions and police conflict**	0.0% (0.0% to 0.0%)	0.0% (0.0% to 0.0%)	0.0% (0.0% to 0.0%)	0.0% (0.0% to 0.0%)
Forces of nature, conflict and terrorism, and state actor violence	0.0% (0.0% to 0.0%)	0.0% (0.0% to 0.0%)	0.0% (0.0% to 0.0%)	0.0% (0.0% to 0.0%)

Supplementary Results Table 1. Age-standardised PAFs attributable to the joint distribution of all risk factors for all ages, both sexes combined for each GBD level 1 and level 2 cause and all causes in 2016 for Djibouti. Results are shown for deaths, YLLs, YLDs, and DALYs
Data in parenthesis are 95% uncertainty intervals. PAFs=population attributable fractions. GBD=Global Burden of Disease. YLLs=years of life lost. YLDs=years live with disability. DALYs=disability-adjusted life-years

	Deaths 2016	YLLs 2016	YLDs 2016	DALYs 2016
**All causes**	55.7% (52.7% to 59.2%)	54.9% (51.4% to 59.2%)	29.1% (27.5% to 30.9%)	48.4% (45.3% to 51.7%)
**HIV/AIDS and tuberculosis**	57.2% (43.6% to 70.9%)	62.7% (50.9% to 74.7%)	71.8% (58.4% to 82.9%)	63.2% (51.6% to 74.6%)
Tuberculosis	20.8% (9.1% to 32.7%)	20.1% (8.2% to 32.3%)	17.7% (6.0% to 29.4%)	20.0% (8.1% to 32.1%)
HIV/AIDS	93.4% (91.3% to 96.1%)	88.7% (84.6% to 93.3%)	93.7% (91.1% to 96.0%)	89.0% (85.1% to 93.4%)
**Diarrhoea, lower respiratory infections, and other common infectious diseases**	68.4% (60.9% to 76.5%)	66.4% (58.1% to 74.2%)	39.5% (34.2% to 44.3%)	64.9% (57.0% to 72.5%)
Diarrhoeal diseases	92.8% (81.0% to 98.1%)	94.0% (83.9% to 98.5%)	93.6% (83.6% to 98.3%)	93.9% (83.7% to 98.5%)
Lower respiratory infections	73.2% (64.1% to 82.2%)	81.2% (73.8% to 88.0%)	71.5% (62.0% to 81.2%)	81.2% (73.7% to 88.0%)
Upper respiratory infections	1.3% (0.1% to 4.7%)	2.5% (0.2% to 8.9%)	0.0% (0.0% to 0.0%)	0.1% (0.0% to 0.4%)
Otitis media	5.5% (1.8% to 11.7%)	11.0% (6.1% to 16.3%)	7.4% (4.9% to 9.7%)	8.0% (5.3% to 10.8%)
Meningitis	1.8% (0.9% to 3.5%)	3.7% (2.0% to 6.6%)	0.0% (0.0% to 0.0%)	3.5% (1.9% to 6.2%)
Encephalitis	2.8% (1.8% to 4.6%)	3.6% (2.4% to 5.7%)	0.0% (0.0% to 0.0%)	2.3% (1.3% to 3.8%)
Measles	69.8% (55.4% to 86.4%)	70.7% (56.2% to 87.4%)	67.9% (54.3% to 82.8%)	70.7% (56.2% to 87.4%)
**Maternal disorders**	6.7% (2.7% to 11.4%)	6.6% (2.6% to 11.2%)	2.1% (1.0% to 3.6%)	6.3% (2.5% to 10.7%)
Maternal haemorrhage	17.5% (6.3% to 28.8%)	17.5% (6.3% to 28.8%)	17.6% (6.3% to 29.1%)	17.5% (6.3% to 28.8%)
Maternal sepsis and other pregnancy related infections	17.4% (6.3% to 28.5%)	17.3% (6.2% to 28.4%)	17.0% (6.1% to 28.0%)	17.3% (6.2% to 28.4%)
Maternal abortion, miscarriage, and ectopic pregnancy	14.4% (8.4% to 21.1%)	14.0% (8.1% to 20.9%)	16.0% (8.7% to 24.6%)	14.2% (8.2% to 21.2%)
**Neonatal disorders**	78.3% (73.3% to 82.7%)	78.3% (73.3% to 82.8%)	0.3% (0.1% to 0.6%)	73.3% (67.2% to 78.6%)
Neonatal preterm birth complications	93.2% (86.9% to 96.9%)	93.2% (86.9% to 96.9%)	0.9% (0.7% to 1.2%)	87.4% (80.4% to 92.5%)
Neonatal encephalopathy due to birth asphyxia and trauma	75.8% (70.3% to 80.2%)	75.8% (70.4% to 80.2%)	0.0% (0.0% to 0.0%)	72.5% (63.8% to 78.3%)
Neonatal sepsis and other neonatal infections	63.5% (53.9% to 70.4%)	63.6% (54.0% to 70.4%)	0.0% (0.0% to 0.0%)	57.0% (43.0% to 66.9%)
Hemolytic disease and other neonatal jaundice	69.5% (58.0% to 76.7%)	69.5% (58.1% to 76.7%)	0.0% (0.0% to 0.0%)	59.3% (47.0% to 70.0%)

Supplementary Results Table 1. Age-standardised PAFs attributable to the joint distribution of all risk factors for all ages, both sexes combined for each GBD level 1 and level 2 cause and all causes in 2016 for Djibouti. Results are shown for deaths, YLLs, YLDs, and DALYs
Data in parenthesis are 95% uncertainty intervals. PAFs=population attributable fractions. GBD=Global Burden of Disease. YLLs=years of life lost. YLDs=years live with disability. DALYs=disability-adjusted life-years

	Deaths 2016	YLLs 2016	YLDs 2016	DALYs 2016
Other neonatal disorders	68.2% (58.6% to 75.6%)	68.3% (58.7% to 75.7%)	0.0% (0.0% to 0.0%)	66.1% (56.4% to 74.0%)
**Nutritional deficiencies**	25.0% (16.0% to 36.7%)	59.5% (46.8% to 72.4%)	89.3% (86.4% to 91.7%)	70.0% (61.6% to 78.0%)
Protein-energy malnutrition	28.0% (17.5% to 42.0%)	63.3% (49.3% to 76.4%)	97.7% (96.8% to 98.5%)	65.3% (52.4% to 77.5%)
Vitamin A deficiency	-	-	100.0% (100.0% to 100.0%)	100.0% (100.0% to 100.0%)
Iron-deficiency anaemia	100.0% (100.0% to 100.0%)	100.0% (100.0% to 100.0%)	100.0% (100.0% to 100.0%)	100.0% (100.0% to 100.0%)
**Other communicable, maternal, neonatal, and nutritional diseases**	5.3% (2.4% to 9.0%)	3.3% (1.3% to 6.3%)	75.3% (67.9% to 82.1%)	15.7% (7.5% to 27.4%)
Sexually transmitted diseases excluding HIV	15.0% (4.5% to 34.8%)	6.5% (1.8% to 17.4%)	100.0% (100.0% to 100.0%)	28.2% (10.4% to 55.7%)
Hepatitis	0.5% (0.3% to 0.7%)	0.6% (0.4% to 0.9%)	0.6% (0.3% to 1.0%)	0.6% (0.4% to 0.9%)
**Neoplasms**	28.7% (23.5% to 33.6%)	28.0% (22.7% to 33.2%)	29.8% (23.7% to 36.0%)	28.0% (22.7% to 33.3%)
Oesophageal cancer	58.1% (41.4% to 72.3%)	59.8% (43.1% to 73.8%)	58.8% (41.9% to 72.5%)	59.8% (43.1% to 73.8%)
Stomach cancer	4.4% (0.7% to 12.5%)	4.6% (0.8% to 12.2%)	4.5% (0.7% to 12.5%)	4.6% (0.8% to 12.2%)
Liver cancer	28.4% (20.8% to 37.7%)	29.5% (21.8% to 38.9%)	28.9% (21.2% to 38.2%)	29.5% (21.8% to 38.9%)
Larynx cancer	36.4% (18.1% to 54.8%)	38.8% (18.8% to 58.2%)	37.2% (18.4% to 56.1%)	38.8% (18.8% to 58.2%)
Tracheal, bronchus, and lung cancer	69.0% (55.4% to 80.8%)	69.5% (55.3% to 81.2%)	68.9% (54.9% to 80.5%)	69.5% (55.3% to 81.2%)
Breast cancer	17.6% (10.8% to 25.2%)	15.5% (9.0% to 22.3%)	16.2% (9.2% to 23.3%)	15.5% (9.0% to 22.3%)
Cervical cancer	100.0% (100.0% to 100.0%)	100.0% (100.0% to 100.0%)	100.0% (100.0% to 100.0%)	100.0% (100.0% to 100.0%)
Uterine cancer	43.3% (29.9% to 56.2%)	44.6% (31.4% to 57.4%)	44.0% (30.5% to 56.6%)	44.6% (31.4% to 57.5%)
Prostate cancer	0.6% (0.2% to 1.3%)	0.7% (0.2% to 1.4%)	0.7% (0.2% to 1.4%)	0.7% (0.2% to 1.4%)
Colon and rectum cancer	47.0% (37.8% to 56.4%)	46.6% (37.5% to 55.7%)	47.0% (37.8% to 56.3%)	46.6% (37.5% to 55.7%)
Lip and oral cavity cancer	45.6% (32.4% to 57.7%)	45.7% (32.6% to 57.7%)	45.5% (32.7% to 57.5%)	45.7% (32.6% to 57.7%)
Nasopharynx cancer	31.3% (17.8% to 44.8%)	32.6% (18.9% to 46.3%)	32.2% (18.7% to 45.2%)	32.6% (18.9% to 46.3%)

Supplementary Results Table 1. Age-standardised PAFs attributable to the joint distribution of all risk factors for all ages, both sexes combined for each GBD level 1 and level 2 cause and all causes in 2016 for Djibouti. Results are shown for deaths, YLLs, YLDs, and DALYs
Data in parenthesis are 95% uncertainty intervals. PAFs=population attributable fractions. GBD=Global Burden of Disease. YLLs=years of life lost. YLDs=years live with disability. DALYs=disability-adjusted life-years

	Deaths 2016	YLLs 2016	YLDs 2016	DALYs 2016
Other pharynx cancer	22.7% (10.6% to 36.3%)	23.7% (11.3% to 37.3%)	23.0% (10.9% to 36.5%)	23.7% (11.3% to 37.2%)
Gallbladder and biliary tract cancer	17.4% (9.5% to 27.1%)	18.2% (10.0% to 28.2%)	17.9% (9.8% to 28.1%)	18.2% (10.0% to 28.2%)
Pancreatic cancer	13.4% (6.9% to 22.2%)	13.5% (7.1% to 21.9%)	13.4% (7.0% to 22.0%)	13.5% (7.1% to 21.9%)
Ovarian cancer	9.1% (3.4% to 16.4%)	8.2% (2.8% to 14.8%)	8.1% (2.8% to 14.5%)	8.2% (2.8% to 14.8%)
Kidney cancer	16.8% (10.4% to 24.3%)	14.4% (8.9% to 21.4%)	16.3% (10.5% to 23.1%)	14.4% (8.9% to 21.4%)
Bladder cancer	10.0% (3.8% to 18.2%)	10.1% (3.9% to 18.2%)	10.0% (4.0% to 18.2%)	10.1% (3.9% to 18.2%)
Thyroid cancer	11.0% (5.7% to 18.1%)	11.3% (6.0% to 18.5%)	11.2% (5.9% to 17.9%)	11.3% (6.0% to 18.5%)
Mesothelioma	85.0% (76.3% to 90.7%)	75.8% (64.4% to 83.7%)	81.8% (72.2% to 88.1%)	75.9% (64.6% to 83.8%)
Non-Hodgkin's lymphoma	4.1% (1.6% to 7.4%)	3.3% (1.3% to 5.8%)	4.0% (1.6% to 7.2%)	3.3% (1.3% to 5.9%)
Multiple myeloma	6.4% (2.7% to 11.4%)	6.5% (2.8% to 11.6%)	6.5% (2.7% to 11.7%)	6.5% (2.8% to 11.6%)
Leukaemia	8.3% (4.6% to 13.1%)	7.4% (4.3% to 11.6%)	8.4% (4.8% to 13.2%)	7.4% (4.3% to 11.7%)
**Cardiovascular diseases**	84.3% (81.9% to 86.6%)	85.1% (83.2% to 86.8%)	63.7% (59.3% to 68.0%)	83.3% (81.1% to 85.3%)
Rheumatic heart disease	30.7% (21.0% to 47.4%)	29.3% (20.2% to 43.7%)	24.0% (15.4% to 34.1%)	26.6% (18.0% to 37.8%)
Ischaemic heart disease	93.7% (90.5% to 96.1%)	94.6% (92.5% to 96.3%)	91.7% (89.3% to 93.9%)	94.5% (92.4% to 96.1%)
Cerebrovascular disease	87.5% (83.6% to 90.9%)	89.9% (87.3% to 92.1%)	88.2% (84.7% to 91.2%)	89.8% (87.1% to 92.1%)
Hypertensive heart disease	100.0% (100.0% to 100.0%)	100.0% (100.0% to 100.0%)	100.0% (100.0% to 100.0%)	100.0% (100.0% to 100.0%)
Cardiomyopathy and myocarditis	41.2% (33.6% to 50.5%)	38.6% (30.9% to 47.3%)	41.9% (34.9% to 48.9%)	38.9% (31.5% to 47.3%)
Atrial fibrillation and flutter	49.6% (41.1% to 56.9%)	53.9% (46.9% to 59.9%)	60.7% (55.7% to 65.5%)	57.4% (51.6% to 62.9%)
Aortic aneurysm	50.5% (43.0% to 57.0%)	55.8% (49.2% to 61.4%)	-	55.8% (49.2% to 61.4%)
Peripheral vascular disease	56.7% (49.8% to 63.7%)	63.7% (57.8% to 69.4%)	63.8% (58.6% to 69.0%)	63.8% (58.8% to 68.8%)
Endocarditis	40.4% (31.6% to 50.4%)	38.1% (29.9% to 46.7%)	33.7% (25.2% to 43.1%)	38.1% (29.8% to 46.6%)

Supplementary Results Table 1. Age-standardised PAFs attributable to the joint distribution of all risk factors for all ages, both sexes combined for each GBD level 1 and level 2 cause and all causes in 2016 for Djibouti. Results are shown for deaths, YLLs, YLDs, and DALYs
Data in parenthesis are 95% uncertainty intervals. PAFs=population attributable fractions. GBD=Global Burden of Disease. YLLs=years of life lost. YLDs=years live with disability. DALYs=disability-adjusted life-years

	Deaths 2016	YLLs 2016	YLDs 2016	DALYs 2016
Other cardiovascular and circulatory diseases	50.1% (44.5% to 55.2%)	52.9% (48.3% to 57.6%)	40.0% (32.5% to 47.4%)	50.8% (46.2% to 55.4%)
**Chronic respiratory diseases**	52.8% (42.1% to 64.3%)	50.6% (41.2% to 60.6%)	38.2% (31.3% to 45.6%)	45.8% (37.7% to 54.6%)
Chronic obstructive pulmonary disease	59.5% (45.3% to 74.1%)	59.3% (45.4% to 73.6%)	54.2% (41.8% to 67.4%)	57.9% (44.2% to 71.9%)
Pneumoconiosis	100.0% (100.0% to 100.0%)	100.0% (100.0% to 100.0%)	100.0% (100.0% to 100.0%)	100.0% (100.0% to 100.0%)
Asthma	39.5% (31.4% to 48.3%)	40.1% (32.8% to 48.3%)	28.6% (21.8% to 36.8%)	33.8% (26.8% to 42.0%)
Other chronic respiratory diseases	2.9% (0.7% to 6.1%)	3.0% (0.4% to 6.9%)	1.6% (0.2% to 3.7%)	2.6% (0.3% to 6.0%)
**Cirrhosis and other chronic liver diseases**	29.1% (25.6% to 32.9%)	29.7% (26.4% to 33.6%)	24.0% (20.4% to 28.1%)	29.5% (26.1% to 33.4%)
Cirrhosis and other chronic liver diseases due to hepatitis B	0.1% (0.1% to 0.2%)	0.2% (0.1% to 0.3%)	0.1% (0.1% to 0.2%)	0.2% (0.1% to 0.3%)
Cirrhosis and other chronic liver diseases due to hepatitis C	11.5% (6.8% to 18.1%)	19.2% (11.2% to 29.7%)	21.2% (12.3% to 33.4%)	19.3% (11.2% to 29.7%)
Cirrhosis and other chronic liver diseases due to alcohol use	100.0% (100.0% to 100.0%)	100.0% (100.0% to 100.0%)	100.0% (100.0% to 100.0%)	100.0% (100.0% to 100.0%)
**Digestive diseases**	5.7% (4.3% to 7.6%)	5.1% (3.7% to 6.6%)	3.3% (2.4% to 4.3%)	4.9% (3.7% to 6.4%)
Peptic ulcer disease	15.2% (10.2% to 21.0%)	17.0% (11.8% to 22.9%)	14.1% (9.9% to 19.0%)	16.8% (11.7% to 22.6%)
Gallbladder and biliary diseases	36.7% (23.9% to 50.1%)	37.3% (25.8% to 50.1%)	40.0% (28.5% to 52.5%)	37.5% (26.0% to 50.1%)
Pancreatitis	2.5% (-8.2% to 14.8%)	2.9% (-9.0% to 15.5%)	2.7% (-8.2% to 14.4%)	2.9% (-9.0% to 15.4%)
**Neurological disorders**	18.6% (8.9% to 30.2%)	15.8% (8.1% to 24.5%)	2.9% (1.5% to 4.5%)	8.0% (4.0% to 12.9%)
Alzheimer's disease and other dementias	22.0% (10.7% to 35.0%)	22.9% (11.5% to 35.8%)	23.0% (11.6% to 35.9%)	23.0% (11.5% to 35.8%)
Parkinson's disease	-16.7% (-23.7% to -11.0%)	-18.8% (-25.9% to -12.5%)	-19.6% (-26.6% to -13.9%)	-18.9% (-25.7% to -12.9%)
Epilepsy	5.7% (1.7% to 11.5%)	5.6% (1.7% to 11.3%)	4.6% (1.4% to 9.7%)	5.1% (1.6% to 10.4%)
Multiple sclerosis	9.4% (4.8% to 14.9%)	9.5% (4.8% to 15.0%)	7.0% (4.3% to 10.0%)	8.6% (4.8% to 13.2%)
**Mental and substance use disorders**	99.8% (99.7% to 99.9%)	99.7% (99.4% to 99.8%)	18.3% (16.1% to 20.7%)	20.7% (18.1% to 23.3%)
Alcohol use disorders	100.0% (100.0% to 100.0%)	100.0% (100.0% to 100.0%)	100.0% (100.0% to 100.0%)	100.0% (100.0% to 100.0%)

Supplementary Results Table 1. Age-standardised PAFs attributable to the joint distribution of all risk factors for all ages, both sexes combined for each GBD level 1 and level 2 cause and all causes in 2016 for Djibouti. Results are shown for deaths, YLLs, YLDs, and DALYs
Data in parenthesis are 95% uncertainty intervals. PAFs=population attributable fractions. GBD=Global Burden of Disease. YLLs=years of life lost. YLDs=years live with disability. DALYs=disability-adjusted life-years

	Deaths 2016	YLLs 2016	YLDs 2016	DALYs 2016
Drug use disorders	100.0% (100.0% to 100.0%)	100.0% (100.0% to 100.0%)	100.0% (100.0% to 100.0%)	100.0% (100.0% to 100.0%)
Depressive disorders	-	-	8.8% (7.2% to 10.5%)	8.8% (7.2% to 10.5%)
Idiopathic developmental intellectual disability	-	-	61.5% (30.6% to 80.9%)	61.5% (30.6% to 80.9%)
**Diabetes, urogenital, blood, and endocrine diseases**	79.1% (76.3% to 81.9%)	71.7% (65.1% to 76.0%)	64.3% (59.3% to 68.6%)	68.6% (64.2% to 72.0%)
Diabetes mellitus	100.0% (100.0% to 100.0%)	100.0% (100.0% to 100.0%)	100.0% (100.0% to 100.0%)	100.0% (100.0% to 100.0%)
Chronic kidney disease	100.0% (100.0% to 100.0%)	100.0% (100.0% to 100.0%)	100.0% (100.0% to 100.0%)	100.0% (100.0% to 100.0%)
**Musculoskeletal disorders**	1.8% (0.7% to 3.3%)	1.3% (0.5% to 2.4%)	24.1% (20.3% to 28.2%)	23.7% (20.0% to 27.8%)
Rheumatoid arthritis	4.3% (1.7% to 7.6%)	4.4% (1.7% to 7.7%)	4.9% (2.0% to 8.1%)	4.8% (1.9% to 8.0%)
Osteoarthritis	-	-	22.3% (12.0% to 33.4%)	22.3% (12.0% to 33.4%)
Low back and neck pain	-	-	29.5% (24.7% to 33.8%)	29.5% (24.7% to 33.8%)
Gout	-	-	47.3% (37.1% to 59.7%)	47.3% (37.1% to 59.7%)
**Other non-communicable diseases**	0.3% (0.1% to 0.6%)	0.5% (0.1% to 1.1%)	6.8% (5.5% to 8.2%)	5.7% (4.6% to 6.8%)
Sense organ diseases	-	-	13.4% (11.4% to 15.7%)	13.4% (11.4% to 15.7%)
Sudden infant death syndrome	4.4% (1.7% to 9.0%)	4.4% (1.7% to 9.0%)	-	4.4% (1.7% to 9.0%)
**Transport injuries**	25.4% (20.6% to 30.3%)	20.1% (15.0% to 25.4%)	31.2% (26.3% to 36.4%)	22.2% (17.0% to 27.9%)
Road injuries	25.5% (20.7% to 30.4%)	20.1% (15.0% to 25.3%)	32.3% (27.4% to 37.6%)	22.3% (17.0% to 27.9%)
Other transport injuries	23.8% (17.2% to 30.8%)	20.0% (13.5% to 27.0%)	23.3% (18.5% to 28.4%)	21.2% (15.2% to 27.4%)
**Unintentional injuries**	30.7% (23.3% to 35.7%)	15.1% (12.3% to 18.3%)	18.4% (15.9% to 21.8%)	16.1% (13.7% to 18.9%)
Falls	64.6% (47.7% to 69.8%)	41.4% (34.8% to 46.3%)	26.8% (24.3% to 30.1%)	34.7% (30.8% to 38.4%)
Drowning	7.5% (4.8% to 10.9%)	7.5% (4.9% to 10.6%)	11.0% (7.4% to 15.4%)	7.5% (5.0% to 10.7%)
Fire, heat, and hot substances	6.0% (3.6% to 9.6%)	6.9% (4.5% to 10.2%)	13.2% (9.0% to 18.2%)	8.5% (5.6% to 12.3%)

Supplementary Results Table 1. Age-standardised PAFs attributable to the joint distribution of all risk factors for all ages, both sexes combined for each GBD level 1 and level 2 cause and all causes in 2016 for Djibouti. Results are shown for deaths, YLLs, YLDs, and DALYs
Data in parenthesis are 95% uncertainty intervals. PAFs=population attributable fractions. GBD=Global Burden of Disease. YLLs=years of life lost. YLDs=years live with disability. DALYs=disability-adjusted life-years

	**Deaths 2016**	**YLLs 2016**	**YLDs 2016**	**DALYs 2016**
Poisonings	8.1% (5.0% to 12.3%)	7.9% (3.9% to 12.4%)	12.7% (8.7% to 17.7%)	8.1% (4.1% to 12.7%)
Exposure to mechanical forces	18.6% (15.4% to 22.4%)	13.4% (10.2% to 18.0%)	19.2% (16.0% to 23.5%)	15.4% (12.1% to 20.0%)
Animal contact	5.9% (4.4% to 7.9%)	6.4% (4.6% to 8.9%)	10.4% (7.6% to 14.3%)	7.2% (5.2% to 10.0%)
Foreign body	4.8% (3.0% to 7.2%)	4.4% (2.3% to 7.3%)	8.8% (6.0% to 12.5%)	5.6% (3.3% to 8.5%)
Other unintentional injuries	9.2% (6.1% to 13.4%)	10.1% (6.7% to 14.5%)	11.3% (7.8% to 15.9%)	10.7% (7.4% to 14.9%)
**Self-harm and interpersonal violence**	9.0% (4.0% to 15.5%)	8.4% (3.4% to 14.6%)	19.9% (16.5% to 24.1%)	9.8% (5.0% to 15.6%)
Self-harm	5.3% (-1.7% to 13.4%)	5.8% (-1.6% to 14.1%)	5.5% (-1.7% to 13.6%)	5.8% (-1.6% to 14.1%)
Interpersonal violence	12.0% (5.7% to 19.1%)	9.9% (3.7% to 16.8%)	21.2% (17.5% to 25.3%)	11.7% (6.0% to 18.2%)
**Forces of nature, conflict and terrorism, and executions and police conflict**	0.0% (0.0% to 0.0%)	0.0% (0.0% to 0.0%)	1.7% (1.1% to 2.5%)	1.3% (0.8% to 1.9%)
Forces of nature, conflict and terrorism, and state actor violence	0.0% (0.0% to 0.0%)	0.0% (0.0% to 0.0%)	7.9% (5.4% to 11.6%)	7.9% (5.4% to 11.6%)

Supplementary Results Table 1. Age-standardised PAFs attributable to the joint distribution of all risk factors for all ages, both sexes combined for each GBD level 1 and level 2 cause and all causes in 2016 for Dominica. Results are shown for deaths, YLLs, YLDs, and DALYs
Data in parenthesis are 95% uncertainty intervals. PAFs=population attributable fractions. GBD=Global Burden of Disease. YLLs=years of life lost. YLDs=years live with disability. DALYs=disability-adjusted life-years

	Deaths 2016	YLLs 2016	YLDs 2016	DALYs 2016
**All causes**	56.2% (54.5% to 58.2%)	52.9% (51.4% to 54.7%)	24.5% (23.0% to 26.1%)	43.3% (41.0% to 45.8%)
**HIV/AIDS and tuberculosis**	69.4% (63.4% to 74.3%)	69.0% (62.7% to 73.7%)	72.6% (64.2% to 80.5%)	69.2% (62.8% to 74.0%)
Tuberculosis	51.7% (41.5% to 61.4%)	55.0% (45.8% to 63.5%)	49.5% (40.5% to 58.1%)	54.8% (45.6% to 63.2%)
HIV/AIDS	79.0% (71.8% to 84.2%)	73.4% (65.6% to 78.8%)	80.0% (71.9% to 86.0%)	73.8% (65.8% to 79.2%)
**Diarrhoea, lower respiratory infections, and other common infectious diseases**	40.2% (31.9% to 48.9%)	50.4% (43.0% to 57.5%)	41.5% (31.0% to 49.9%)	48.6% (42.0% to 55.0%)
Diarrhoeal diseases	85.2% (66.7% to 95.4%)	90.8% (78.6% to 97.2%)	84.1% (64.4% to 95.1%)	86.6% (69.5% to 95.9%)
Lower respiratory infections	40.8% (31.7% to 50.6%)	53.8% (45.2% to 62.5%)	45.7% (37.0% to 55.3%)	53.7% (45.1% to 62.4%)
Upper respiratory infections	2.4% (0.2% to 8.2%)	5.7% (0.4% to 18.2%)	0.0% (0.0% to 0.0%)	0.0% (0.0% to 0.2%)
Otitis media	1.7% (0.9% to 3.1%)	4.8% (2.6% to 8.1%)	3.7% (2.4% to 5.0%)	3.7% (2.4% to 5.0%)
Meningitis	4.2% (2.4% to 6.2%)	7.9% (5.2% to 11.0%)	0.0% (0.0% to 0.0%)	7.0% (4.4% to 9.9%)
Encephalitis	1.5% (0.7% to 2.5%)	2.5% (1.2% to 4.1%)	0.0% (0.0% to 0.0%)	2.2% (1.0% to 3.5%)
Measles	15.0% (4.0% to 42.8%)	16.7% (4.5% to 46.8%)	14.7% (0.0% to 65.6%)	16.7% (4.5% to 46.8%)
**Maternal disorders**	6.3% (2.6% to 10.6%)	6.3% (2.6% to 10.4%)	1.8% (0.8% to 3.1%)	3.9% (1.7% to 6.7%)
Maternal haemorrhage	14.1% (5.0% to 23.1%)	14.1% (5.0% to 23.0%)	14.2% (5.0% to 23.5%)	14.1% (5.0% to 23.1%)
Maternal sepsis and other pregnancy related infections	13.8% (4.9% to 22.8%)	13.9% (5.0% to 22.8%)	14.0% (5.0% to 22.9%)	14.0% (5.0% to 22.8%)
Maternal abortion, miscarriage, and ectopic pregnancy	5.5% (3.2% to 8.4%)	5.6% (3.2% to 8.6%)	6.3% (3.1% to 10.6%)	6.0% (3.3% to 9.3%)
**Neonatal disorders**	72.6% (70.1% to 75.4%)	72.7% (70.1% to 75.5%)	0.1% (0.1% to 0.2%)	63.3% (55.5% to 68.6%)
Neonatal preterm birth complications	96.1% (94.4% to 97.4%)	96.1% (94.4% to 97.5%)	0.2% (0.2% to 0.3%)	76.6% (65.4% to 85.9%)
Neonatal encephalopathy due to birth asphyxia and trauma	67.9% (64.6% to 71.3%)	67.9% (64.7% to 71.3%)	0.0% (0.0% to 0.0%)	67.7% (64.5% to 71.1%)
Neonatal sepsis and other neonatal infections	57.1% (53.0% to 61.3%)	57.1% (53.0% to 61.3%)	0.0% (0.0% to 0.0%)	44.9% (30.3% to 56.0%)
Hemolytic disease and other neonatal jaundice	63.2% (58.7% to 67.3%)	63.2% (58.7% to 67.4%)	0.0% (0.0% to 0.0%)	50.9% (42.1% to 58.6%)

Supplementary Results Table 1. Age-standardised PAFs attributable to the joint distribution of all risk factors for all ages, both sexes combined for each GBD level 1 and level 2 cause and all causes in 2016 for Dominica. Results are shown for deaths, YLLs, YLDs, and DALYs
Data in parenthesis are 95% uncertainty intervals. PAFs=population attributable fractions. GBD=Global Burden of Disease. YLLs=years of life lost. YLDs=years live with disability. DALYs=disability-adjusted life-years

	**Deaths 2016**	**YLLs 2016**	**YLDs 2016**	**DALYs 2016**
Other neonatal disorders	64.0% (59.8% to 68.3%)	64.1% (59.8% to 68.3%)	0.0% (0.0% to 0.0%)	60.3% (54.9% to 65.3%)
**Nutritional deficiencies**	10.1% (5.7% to 16.4%)	36.1% (24.4% to 48.3%)	96.7% (95.6% to 97.7%)	83.0% (77.5% to 87.7%)
Protein-energy malnutrition	16.4% (8.6% to 27.6%)	54.7% (37.2% to 70.9%)	96.3% (94.7% to 97.6%)	62.2% (49.3% to 74.9%)
Vitamin A deficiency	-	-	100.0% (100.0% to 100.0%)	100.0% (100.0% to 100.0%)
Iron-deficiency anaemia	100.0% (100.0% to 100.0%)	100.0% (100.0% to 100.0%)	100.0% (100.0% to 100.0%)	100.0% (100.0% to 100.0%)
**Other communicable, maternal, neonatal, and nutritional diseases**	9.8% (7.3% to 15.0%)	7.0% (4.9% to 10.6%)	72.2% (63.6% to 81.0%)	27.3% (18.5% to 38.4%)
Sexually transmitted diseases excluding HIV	65.3% (39.6% to 87.4%)	38.9% (17.2% to 67.8%)	100.0% (100.0% to 100.0%)	77.1% (53.9% to 93.0%)
Hepatitis	7.1% (4.8% to 10.1%)	7.6% (4.8% to 11.0%)	5.0% (3.1% to 7.5%)	7.4% (4.7% to 10.7%)
**Neoplasms**	28.3% (25.2% to 31.5%)	30.3% (27.4% to 33.3%)	22.8% (20.0% to 26.2%)	30.1% (27.2% to 33.1%)
Oesophageal cancer	72.1% (62.1% to 81.6%)	74.9% (65.5% to 83.7%)	72.8% (63.1% to 82.0%)	74.9% (65.5% to 83.7%)
Stomach cancer	12.4% (6.4% to 20.5%)	13.7% (7.2% to 21.7%)	12.9% (6.7% to 21.0%)	13.7% (7.2% to 21.7%)
Liver cancer	59.3% (51.9% to 66.6%)	59.4% (52.0% to 66.6%)	59.4% (51.7% to 66.5%)	59.4% (52.0% to 66.6%)
Larynx cancer	80.3% (70.9% to 87.4%)	82.0% (73.4% to 88.3%)	81.3% (72.3% to 88.0%)	82.0% (73.4% to 88.3%)
Tracheal, bronchus, and lung cancer	80.4% (75.8% to 84.5%)	80.6% (76.3% to 84.5%)	80.2% (75.5% to 84.4%)	80.6% (76.3% to 84.5%)
Breast cancer	26.7% (17.7% to 37.2%)	23.9% (15.4% to 33.6%)	24.9% (16.6% to 35.1%)	24.0% (15.5% to 33.7%)
Cervical cancer	100.0% (100.0% to 100.0%)	100.0% (100.0% to 100.0%)	100.0% (100.0% to 100.0%)	100.0% (100.0% to 100.0%)
Uterine cancer	48.4% (32.9% to 62.9%)	50.0% (34.8% to 64.2%)	50.5% (35.3% to 64.7%)	50.0% (34.9% to 64.3%)
Prostate cancer	2.9% (1.9% to 4.1%)	3.3% (2.1% to 4.5%)	3.3% (2.2% to 4.5%)	3.3% (2.1% to 4.5%)
Colon and rectum cancer	53.4% (43.1% to 64.0%)	54.4% (44.7% to 64.3%)	53.8% (43.9% to 64.2%)	54.3% (44.7% to 64.3%)
Lip and oral cavity cancer	71.6% (62.1% to 80.0%)	75.4% (66.9% to 82.7%)	70.4% (61.0% to 78.6%)	75.3% (66.7% to 82.6%)
Nasopharynx cancer	73.6% (66.6% to 80.0%)	72.1% (65.7% to 78.1%)	71.2% (65.3% to 76.9%)	72.1% (65.7% to 78.1%)

Supplementary Results Table 1. Age-standardised PAFs attributable to the joint distribution of all risk factors for all ages, both sexes combined for each GBD level 1 and level 2 cause and all causes in 2016 for Dominica. Results are shown for deaths, YLLs, YLDs, and DALYs
Data in parenthesis are 95% uncertainty intervals. PAFs=population attributable fractions. GBD=Global Burden of Disease. YLLs=years of life lost. YLDs=years live with disability. DALYs=disability-adjusted life-years

	**Deaths 2016**	**YLLs 2016**	**YLDs 2016**	**DALYs 2016**
Other pharynx cancer	56.7% (42.1% to 68.3%)	60.4% (47.2% to 71.2%)	58.3% (43.7% to 69.5%)	60.4% (47.1% to 71.2%)
Gallbladder and biliary tract cancer	21.7% (11.6% to 33.2%)	22.4% (12.5% to 33.8%)	21.8% (11.9% to 33.3%)	22.4% (12.5% to 33.8%)
Pancreatic cancer	29.0% (19.9% to 40.4%)	30.7% (21.7% to 41.3%)	29.4% (20.4% to 40.6%)	30.7% (21.7% to 41.3%)
Ovarian cancer	15.1% (4.9% to 28.1%)	14.1% (4.4% to 26.1%)	13.9% (4.3% to 25.9%)	14.1% (4.4% to 26.1%)
Kidney cancer	27.8% (19.4% to 37.2%)	27.5% (19.5% to 36.4%)	28.2% (20.1% to 37.3%)	27.5% (19.5% to 36.4%)
Bladder cancer	27.9% (18.1% to 39.2%)	29.3% (19.4% to 40.4%)	28.8% (19.0% to 39.9%)	29.3% (19.4% to 40.4%)
Thyroid cancer	12.3% (6.0% to 21.0%)	12.7% (6.3% to 21.7%)	13.2% (7.0% to 21.7%)	12.7% (6.4% to 21.7%)
Mesothelioma	86.3% (81.9% to 89.8%)	76.9% (69.9% to 82.6%)	83.3% (78.6% to 87.6%)	77.0% (70.0% to 82.7%)
Non-Hodgkin's lymphoma	5.6% (2.1% to 10.2%)	5.4% (2.1% to 9.8%)	5.7% (2.1% to 10.2%)	5.4% (2.1% to 9.8%)
Multiple myeloma	7.2% (3.0% to 12.7%)	7.4% (3.1% to 13.2%)	7.4% (3.0% to 13.2%)	7.4% (3.1% to 13.2%)
Leukaemia	15.2% (9.4% to 21.7%)	13.1% (8.2% to 18.8%)	14.8% (9.3% to 21.2%)	13.1% (8.3% to 18.8%)
**Cardiovascular diseases**	80.0% (77.2% to 82.7%)	78.9% (76.4% to 81.3%)	69.4% (66.1% to 72.6%)	78.1% (75.8% to 80.5%)
Rheumatic heart disease	25.9% (16.6% to 44.3%)	22.9% (16.3% to 34.1%)	21.9% (14.2% to 31.5%)	22.5% (15.2% to 33.1%)
Ischaemic heart disease	92.6% (88.8% to 95.9%)	94.2% (91.6% to 96.4%)	91.2% (88.4% to 93.8%)	94.1% (91.5% to 96.2%)
Cerebrovascular disease	83.4% (78.1% to 88.7%)	85.1% (81.4% to 88.6%)	84.8% (80.1% to 89.5%)	85.1% (81.4% to 88.5%)
Hypertensive heart disease	100.0% (100.0% to 100.0%)	100.0% (100.0% to 100.0%)	100.0% (100.0% to 100.0%)	100.0% (100.0% to 100.0%)
Cardiomyopathy and myocarditis	39.7% (32.1% to 47.6%)	40.1% (33.4% to 47.3%)	37.6% (30.8% to 44.4%)	40.1% (33.3% to 47.3%)
Atrial fibrillation and flutter	48.9% (40.6% to 56.7%)	53.1% (45.7% to 60.2%)	59.2% (53.3% to 65.4%)	56.7% (50.0% to 63.1%)
Aortic aneurysm	39.2% (31.5% to 47.0%)	43.5% (36.6% to 49.5%)	-	43.5% (36.6% to 49.5%)
Peripheral vascular disease	51.6% (44.3% to 59.3%)	58.4% (52.7% to 64.6%)	63.1% (58.1% to 68.0%)	60.0% (54.7% to 65.5%)
Endocarditis	38.0% (29.5% to 48.8%)	36.7% (29.6% to 44.5%)	36.7% (28.7% to 45.3%)	36.7% (29.6% to 44.5%)

Supplementary Results Table 1. Age-standardised PAFs attributable to the joint distribution of all risk factors for all ages, both sexes combined for each GBD level 1 and level 2 cause and all causes in 2016 for Dominica. Results are shown for deaths, YLLs, YLDs, and DALYs
Data in parenthesis are 95% uncertainty intervals. PAFs=population attributable fractions. GBD=Global Burden of Disease. YLLs=years of life lost. YLDs=years live with disability. DALYs=disability-adjusted life-years

	Deaths 2016	YLLs 2016	YLDs 2016	DALYs 2016
Other cardiovascular and circulatory diseases	42.0% (37.0% to 47.3%)	43.1% (38.9% to 47.5%)	43.1% (38.6% to 47.8%)	43.1% (38.9% to 47.4%)
**Chronic respiratory diseases**	54.8% (49.6% to 60.0%)	51.2% (46.3% to 55.7%)	31.7% (24.0% to 40.8%)	42.3% (36.2% to 48.7%)
Chronic obstructive pulmonary disease	70.0% (63.3% to 76.1%)	69.5% (63.6% to 75.2%)	66.2% (59.4% to 72.0%)	69.1% (63.0% to 74.6%)
Pneumoconiosis	100.0% (100.0% to 100.0%)	100.0% (100.0% to 100.0%)	100.0% (100.0% to 100.0%)	100.0% (100.0% to 100.0%)
Asthma	30.4% (21.4% to 40.7%)	33.0% (24.9% to 42.1%)	27.0% (18.0% to 37.5%)	28.5% (19.9% to 38.3%)
Other chronic respiratory diseases	12.5% (8.6% to 16.9%)	13.2% (8.7% to 17.9%)	5.2% (3.1% to 7.8%)	12.6% (8.4% to 17.2%)
**Cirrhosis and other chronic liver diseases**	66.5% (63.8% to 69.0%)	66.6% (64.1% to 69.0%)	54.3% (50.4% to 58.2%)	66.1% (63.6% to 68.6%)
Cirrhosis and other chronic liver diseases due to hepatitis B	5.6% (2.4% to 9.4%)	7.4% (3.2% to 12.6%)	5.8% (2.5% to 10.2%)	7.4% (3.2% to 12.5%)
Cirrhosis and other chronic liver diseases due to hepatitis C	82.1% (78.1% to 86.1%)	86.8% (84.7% to 88.8%)	83.0% (79.1% to 86.7%)	86.7% (84.5% to 88.7%)
Cirrhosis and other chronic liver diseases due to alcohol use	100.0% (100.0% to 100.0%)	100.0% (100.0% to 100.0%)	100.0% (100.0% to 100.0%)	100.0% (100.0% to 100.0%)
**Digestive diseases**	6.9% (5.2% to 8.8%)	9.4% (7.2% to 11.8%)	2.0% (1.4% to 2.7%)	7.4% (5.7% to 9.3%)
Peptic ulcer disease	4.1% (2.7% to 5.8%)	5.9% (4.0% to 8.3%)	2.1% (1.3% to 3.1%)	4.7% (3.1% to 6.7%)
Gallbladder and biliary diseases	40.8% (26.9% to 55.1%)	41.6% (28.1% to 55.5%)	46.5% (32.4% to 60.5%)	42.4% (28.9% to 56.2%)
Pancreatitis	47.4% (29.2% to 66.0%)	53.7% (35.9% to 70.4%)	39.8% (24.0% to 57.2%)	53.5% (35.7% to 70.2%)
**Neurological disorders**	21.4% (11.1% to 33.3%)	21.9% (14.5% to 30.6%)	5.0% (2.2% to 8.0%)	11.0% (6.8% to 15.5%)
Alzheimer's disease and other dementias	23.7% (10.4% to 39.2%)	24.9% (11.1% to 40.4%)	25.0% (11.2% to 40.5%)	24.9% (11.1% to 40.6%)
Parkinson's disease	-1.3% (-1.7% to -0.9%)	-1.8% (-2.3% to -1.2%)	-2.2% (-2.8% to -1.6%)	-1.9% (-2.4% to -1.3%)
Epilepsy	30.0% (21.4% to 39.8%)	30.5% (21.8% to 40.0%)	25.1% (17.8% to 33.3%)	28.3% (20.3% to 37.1%)
Multiple sclerosis	2.5% (1.3% to 3.8%)	2.7% (1.4% to 4.2%)	1.9% (1.1% to 2.9%)	2.3% (1.3% to 3.5%)
**Mental and substance use disorders**	99.9% (99.9% to 99.9%)	99.8% (99.8% to 99.9%)	18.6% (16.6% to 20.9%)	24.6% (22.0% to 27.6%)
Alcohol use disorders	100.0% (100.0% to 100.0%)	100.0% (100.0% to 100.0%)	100.0% (100.0% to 100.0%)	100.0% (100.0% to 100.0%)

Supplementary Results Table 1. Age-standardised PAFs attributable to the joint distribution of all risk factors for all ages, both sexes combined for each GBD level 1 and level 2 cause and all causes in 2016 for Dominica. Results are shown for deaths, YLLs, YLDs, and DALYs
Data in parenthesis are 95% uncertainty intervals. PAFs=population attributable fractions. GBD=Global Burden of Disease. YLLs=years of life lost. YLDs=years live with disability. DALYs=disability-adjusted life-years

	Deaths 2016	YLLs 2016	YLDs 2016	DALYs 2016
Drug use disorders	100.0% (100.0% to 100.0%)	100.0% (100.0% to 100.0%)	100.0% (100.0% to 100.0%)	100.0% (100.0% to 100.0%)
Depressive disorders	-	-	6.2% (5.0% to 7.7%)	6.2% (5.0% to 7.7%)
Idiopathic developmental intellectual disability	-	-	65.1% (34.7% to 85.7%)	65.1% (34.7% to 85.7%)
**Diabetes, urogenital, blood, and endocrine diseases**	89.5% (88.0% to 90.3%)	86.2% (83.7% to 87.5%)	77.9% (74.1% to 80.9%)	83.6% (81.4% to 85.1%)
Diabetes mellitus	100.0% (100.0% to 100.0%)	100.0% (100.0% to 100.0%)	100.0% (100.0% to 100.0%)	100.0% (100.0% to 100.0%)
Chronic kidney disease	100.0% (100.0% to 100.0%)	100.0% (100.0% to 100.0%)	100.0% (100.0% to 100.0%)	100.0% (100.0% to 100.0%)
**Musculoskeletal disorders**	0.2% (0.1% to 0.4%)	0.2% (0.1% to 0.3%)	14.0% (11.1% to 17.4%)	13.5% (10.7% to 16.8%)
Rheumatoid arthritis	1.0% (0.4% to 1.7%)	1.3% (0.5% to 2.3%)	1.2% (0.5% to 2.1%)	1.2% (0.5% to 2.1%)
Osteoarthritis	-	-	25.4% (13.5% to 37.7%)	25.4% (13.5% to 37.7%)
Low back and neck pain	-	-	19.0% (15.9% to 23.0%)	19.0% (15.9% to 23.0%)
Gout	-	-	44.4% (32.6% to 58.9%)	44.4% (32.6% to 58.9%)
**Other non-communicable diseases**	0.5% (0.3% to 0.9%)	1.0% (0.6% to 1.6%)	4.9% (4.0% to 5.9%)	3.6% (2.9% to 4.3%)
Sense organ diseases	-	-	11.7% (10.0% to 13.6%)	11.7% (10.0% to 13.6%)
Sudden infant death syndrome	9.7% (6.6% to 13.3%)	9.7% (6.6% to 13.3%)	-	9.7% (6.6% to 13.3%)
**Transport injuries**	30.4% (22.8% to 39.6%)	25.6% (17.5% to 35.1%)	34.0% (26.4% to 43.6%)	26.6% (18.7% to 36.0%)
Road injuries	30.0% (22.2% to 39.4%)	25.6% (17.4% to 35.2%)	34.4% (26.7% to 44.0%)	26.6% (18.5% to 36.2%)
Other transport injuries	36.4% (30.3% to 43.7%)	25.8% (19.2% to 34.0%)	27.6% (21.2% to 35.8%)	26.0% (19.4% to 34.1%)
**Unintentional injuries**	13.1% (9.9% to 16.8%)	9.5% (5.9% to 13.7%)	15.5% (13.4% to 18.0%)	11.6% (8.5% to 15.0%)
Falls	42.9% (39.1% to 46.7%)	21.2% (18.3% to 24.7%)	21.4% (19.7% to 23.9%)	21.3% (19.5% to 23.9%)
Drowning	12.5% (5.8% to 20.1%)	11.8% (5.6% to 19.1%)	12.9% (6.9% to 20.1%)	11.8% (5.6% to 19.1%)
Fire, heat, and hot substances	8.1% (4.3% to 13.1%)	9.2% (5.2% to 14.3%)	14.3% (9.0% to 20.5%)	11.0% (6.5% to 16.4%)

Supplementary Results Table 1. Age-standardised PAFs attributable to the joint distribution of all risk factors for all ages, both sexes combined for each GBD level 1 and level 2 cause and all causes in 2016 for Dominica. Results are shown for deaths, YLLs, YLDs, and DALYs
Data in parenthesis are 95% uncertainty intervals. PAFs=population attributable fractions. GBD=Global Burden of Disease. YLLs=years of life lost. YLDs=years live with disability. DALYs=disability-adjusted life-years

	Deaths 2016	YLLs 2016	YLDs 2016	DALYs 2016
Poisonings	12.5% (6.5% to 19.2%)	12.2% (6.4% to 18.9%)	13.8% (8.5% to 19.8%)	12.3% (6.5% to 19.0%)
Exposure to mechanical forces	12.7% (9.7% to 16.4%)	9.3% (6.0% to 13.4%)	12.7% (10.6% to 15.5%)	10.7% (8.0% to 13.8%)
Animal contact	9.6% (7.7% to 11.9%)	6.9% (5.2% to 9.6%)	6.9% (4.7% to 9.9%)	6.9% (5.0% to 9.7%)
Foreign body	2.3% (1.3% to 3.9%)	2.4% (1.3% to 4.2%)	4.6% (3.0% to 6.8%)	3.1% (1.8% to 4.9%)
Other unintentional injuries	13.2% (6.4% to 21.1%)	13.2% (6.3% to 21.0%)	13.0% (8.0% to 18.7%)	13.1% (7.5% to 19.3%)
**Self-harm and interpersonal violence**	29.2% (21.5% to 38.1%)	28.9% (21.3% to 38.1%)	29.8% (25.9% to 35.1%)	29.0% (21.7% to 37.7%)
Self-harm	32.8% (20.7% to 45.3%)	34.2% (21.8% to 47.2%)	28.0% (16.9% to 39.3%)	34.1% (21.8% to 47.1%)
Interpersonal violence	27.5% (18.8% to 37.8%)	26.7% (18.2% to 37.2%)	29.9% (26.0% to 35.2%)	27.0% (18.8% to 37.0%)
**Forces of nature, conflict and terrorism, and executions and police conflict**	0.0% (0.0% to 0.0%)	0.0% (0.0% to 0.0%)	5.4% (3.5% to 7.9%)	2.7% (0.6% to 5.2%)
Forces of nature, conflict and terrorism, and state actor violence	0.0% (0.0% to 0.0%)	0.0% (0.0% to 0.0%)	5.6% (3.7% to 8.2%)	5.6% (3.7% to 8.2%)

Supplementary Results Table 1. Age-standardised PAFs attributable to the joint distribution of all risk factors for all ages, both sexes combined for each GBD level 1 and level 2 cause and all causes in 2016 for Dominican Republic. Results are shown for deaths, YLLs, YLDs, and DALYs
Data in parenthesis are 95% uncertainty intervals. PAFs=population attributable fractions. GBD=Global Burden of Disease. YLLs=years of life lost. YLDs=years live with disability. DALYs=disability-adjusted life-years

	Deaths 2016	YLLs 2016	YLDs 2016	DALYs 2016
**All causes**	62.5% (60.6% to 64.3%)	59.2% (57.2% to 61.2%)	22.8% (21.4% to 24.3%)	45.8% (42.8% to 48.8%)
**HIV/AIDS and tuberculosis**	80.0% (72.9% to 85.1%)	78.4% (70.6% to 83.9%)	80.3% (72.4% to 86.3%)	78.6% (70.8% to 84.0%)
Tuberculosis	43.9% (34.2% to 53.1%)	43.4% (34.8% to 51.8%)	41.5% (32.0% to 50.0%)	43.4% (34.7% to 51.7%)
HIV/AIDS	83.7% (75.9% to 89.3%)	81.1% (72.6% to 87.3%)	82.2% (73.8% to 88.1%)	81.2% (72.7% to 87.3%)
**Diarrhoea, lower respiratory infections, and other common infectious diseases**	57.1% (51.2% to 63.5%)	60.7% (53.1% to 67.7%)	47.3% (37.8% to 55.1%)	58.2% (51.8% to 64.4%)
Diarrhoeal diseases	91.1% (78.2% to 97.5%)	94.7% (86.7% to 98.6%)	89.6% (75.3% to 97.0%)	92.8% (82.8% to 98.0%)
Lower respiratory infections	58.9% (52.0% to 66.3%)	68.5% (61.6% to 75.8%)	57.2% (50.0% to 64.6%)	68.4% (61.5% to 75.6%)
Upper respiratory infections	3.3% (0.2% to 11.3%)	6.8% (0.5% to 23.9%)	0.0% (0.0% to 0.0%)	0.1% (0.0% to 0.2%)
Otitis media	3.2% (1.6% to 5.2%)	6.1% (4.1% to 8.5%)	3.5% (2.3% to 4.6%)	3.5% (2.3% to 4.6%)
Meningitis	5.7% (3.3% to 8.6%)	8.2% (5.0% to 12.3%)	0.0% (0.0% to 0.0%)	7.6% (4.6% to 11.4%)
Encephalitis	1.8% (1.0% to 2.9%)	2.8% (1.6% to 4.6%)	0.0% (0.0% to 0.0%)	2.1% (1.3% to 3.5%)
Measles	14.6% (0.0% to 64.9%)	14.8% (0.0% to 66.0%)	13.5% (0.0% to 64.3%)	21.0% (0.0% to 67.8%)
**Maternal disorders**	4.0% (1.5% to 6.9%)	3.9% (1.5% to 6.7%)	1.9% (0.8% to 3.4%)	3.5% (1.3% to 6.0%)
Maternal haemorrhage	14.8% (5.2% to 24.1%)	14.7% (5.2% to 24.0%)	14.7% (5.2% to 24.1%)	14.7% (5.2% to 24.0%)
Maternal sepsis and other pregnancy related infections	14.7% (5.2% to 24.2%)	14.7% (5.2% to 24.1%)	14.5% (5.2% to 23.8%)	14.7% (5.2% to 24.0%)
Maternal abortion, miscarriage, and ectopic pregnancy	7.9% (4.6% to 12.0%)	7.8% (4.5% to 11.9%)	7.7% (4.1% to 12.4%)	7.8% (4.4% to 11.7%)
**Neonatal disorders**	81.1% (78.0% to 83.8%)	81.2% (78.1% to 83.9%)	0.1% (0.0% to 0.2%)	70.0% (60.8% to 76.0%)
Neonatal preterm birth complications	97.7% (96.0% to 98.9%)	97.7% (96.0% to 98.9%)	0.3% (0.2% to 0.3%)	87.6% (81.6% to 92.0%)
Neonatal encephalopathy due to birth asphyxia and trauma	70.3% (66.3% to 74.4%)	70.3% (66.3% to 74.4%)	0.0% (0.0% to 0.0%)	69.1% (64.4% to 73.6%)
Neonatal sepsis and other neonatal infections	63.6% (59.1% to 67.8%)	63.6% (59.1% to 67.8%)	0.0% (0.0% to 0.0%)	48.7% (34.4% to 59.5%)
Hemolytic disease and other neonatal jaundice	67.4% (61.7% to 72.4%)	67.4% (61.7% to 72.4%)	0.0% (0.0% to 0.0%)	40.2% (29.6% to 51.1%)

Supplementary Results Table 1. Age-standardised PAFs attributable to the joint distribution of all risk factors for all ages, both sexes combined for each GBD level 1 and level 2 cause and all causes in 2016 for Dominican Republic. Results are shown for deaths, YLLs, YLDs, and DALYs
Data in parenthesis are 95% uncertainty intervals. PAFs=population attributable fractions. GBD=Global Burden of Disease. YLLs=years of life lost. YLDs=years live with disability. DALYs=disability-adjusted life-years

	Deaths 2016	YLLs 2016	YLDs 2016	DALYs 2016
Other neonatal disorders	66.9% (61.5% to 71.7%)	66.9% (61.5% to 71.7%)	0.0% (0.0% to 0.0%)	61.9% (55.2% to 67.8%)
**Nutritional deficiencies**	25.7% (17.0% to 36.0%)	62.1% (50.2% to 73.2%)	97.9% (97.3% to 98.5%)	89.4% (85.9% to 92.3%)
Protein-energy malnutrition	27.2% (17.9% to 38.5%)	65.5% (53.7% to 76.5%)	95.7% (93.9% to 97.1%)	68.6% (58.6% to 77.9%)
Vitamin A deficiency	-	-	100.0% (100.0% to 100.0%)	100.0% (100.0% to 100.0%)
Iron-deficiency anaemia	100.0% (100.0% to 100.0%)	100.0% (100.0% to 100.0%)	100.0% (100.0% to 100.0%)	100.0% (100.0% to 100.0%)
**Other communicable, maternal, neonatal, and nutritional diseases**	8.7% (6.7% to 15.9%)	8.6% (6.5% to 13.1%)	75.0% (67.3% to 82.6%)	34.3% (24.8% to 45.7%)
Sexually transmitted diseases excluding HIV	55.3% (33.9% to 77.7%)	33.2% (16.7% to 56.3%)	100.0% (100.0% to 100.0%)	80.2% (63.5% to 91.9%)
Hepatitis	6.1% (3.9% to 9.0%)	6.8% (4.3% to 10.0%)	4.8% (2.9% to 7.2%)	6.7% (4.2% to 9.9%)
**Neoplasms**	29.4% (26.0% to 33.0%)	29.2% (25.9% to 32.4%)	24.8% (21.7% to 28.0%)	29.1% (25.8% to 32.3%)
Oesophageal cancer	58.4% (47.9% to 69.3%)	59.4% (48.4% to 70.6%)	58.5% (47.8% to 69.5%)	59.4% (48.4% to 70.6%)
Stomach cancer	8.7% (3.5% to 16.0%)	7.4% (2.9% to 13.8%)	8.2% (3.3% to 15.3%)	7.4% (3.0% to 13.8%)
Liver cancer	47.4% (40.3% to 54.9%)	49.1% (42.0% to 57.1%)	47.8% (40.8% to 55.5%)	49.1% (42.0% to 57.1%)
Larynx cancer	67.2% (54.8% to 77.6%)	64.6% (51.9% to 75.4%)	66.3% (53.8% to 76.9%)	64.7% (52.0% to 75.4%)
Tracheal, bronchus, and lung cancer	69.4% (61.7% to 75.5%)	66.2% (58.0% to 72.8%)	68.0% (59.8% to 74.6%)	66.2% (58.0% to 72.8%)
Breast cancer	20.5% (14.7% to 27.3%)	19.1% (13.5% to 25.3%)	19.7% (14.0% to 26.6%)	19.2% (13.5% to 25.3%)
Cervical cancer	100.0% (100.0% to 100.0%)	100.0% (100.0% to 100.0%)	100.0% (100.0% to 100.0%)	100.0% (100.0% to 100.0%)
Uterine cancer	29.2% (16.3% to 44.1%)	35.1% (21.2% to 50.2%)	36.2% (21.9% to 51.6%)	35.1% (21.2% to 50.3%)
Prostate cancer	2.2% (1.3% to 3.1%)	1.9% (1.1% to 2.7%)	1.9% (1.2% to 2.8%)	1.9% (1.1% to 2.7%)
Colon and rectum cancer	50.3% (41.0% to 59.5%)	51.0% (41.9% to 59.8%)	50.6% (41.3% to 59.6%)	51.0% (41.9% to 59.8%)
Lip and oral cavity cancer	58.2% (46.7% to 68.4%)	58.8% (47.0% to 69.0%)	57.1% (45.6% to 67.2%)	58.7% (46.9% to 68.9%)
Nasopharynx cancer	58.2% (49.1% to 66.2%)	56.6% (48.1% to 64.5%)	57.3% (49.4% to 64.7%)	56.6% (48.1% to 64.4%)

Supplementary Results Table 1. Age-standardised PAFs attributable to the joint distribution of all risk factors for all ages, both sexes combined for each GBD level 1 and level 2 cause and all causes in 2016 for Dominican Republic. Results are shown for deaths, YLLs, YLDs, and DALYs
Data in parenthesis are 95% uncertainty intervals. PAFs=population attributable fractions. GBD=Global Burden of Disease. YLLs=years of life lost. YLDs=years live with disability. DALYs=disability-adjusted life-years

	Deaths 2016	YLLs 2016	YLDs 2016	DALYs 2016
Other pharynx cancer	41.8% (28.2% to 54.8%)	46.2% (32.4% to 59.4%)	43.7% (29.9% to 56.8%)	46.2% (32.3% to 59.4%)
Gallbladder and biliary tract cancer	13.0% (5.4% to 22.7%)	15.4% (6.8% to 26.1%)	13.5% (5.8% to 23.3%)	15.4% (6.8% to 26.0%)
Pancreatic cancer	18.5% (11.7% to 26.9%)	17.6% (10.8% to 25.9%)	18.2% (11.4% to 26.6%)	17.6% (10.8% to 25.9%)
Ovarian cancer	8.1% (2.6% to 15.6%)	7.8% (2.3% to 14.8%)	7.6% (2.3% to 14.5%)	7.8% (2.3% to 14.8%)
Kidney cancer	19.5% (12.0% to 28.6%)	17.8% (10.7% to 26.2%)	19.3% (12.0% to 28.1%)	17.9% (10.7% to 26.3%)
Bladder cancer	20.5% (12.8% to 29.2%)	18.1% (11.2% to 26.3%)	18.6% (11.5% to 26.9%)	18.1% (11.3% to 26.3%)
Thyroid cancer	9.2% (3.7% to 17.3%)	10.9% (4.8% to 20.0%)	11.0% (5.1% to 19.4%)	10.9% (4.8% to 20.0%)
Mesothelioma	63.2% (52.5% to 72.7%)	46.1% (34.1% to 57.9%)	56.6% (45.0% to 66.9%)	46.2% (34.4% to 58.1%)
Non-Hodgkin's lymphoma	4.7% (1.7% to 8.8%)	4.6% (1.7% to 8.5%)	4.8% (1.7% to 9.0%)	4.6% (1.7% to 8.5%)
Multiple myeloma	5.3% (2.0% to 10.1%)	6.1% (2.4% to 11.4%)	5.6% (2.1% to 10.6%)	6.1% (2.4% to 11.4%)
Leukaemia	9.4% (5.5% to 14.0%)	7.2% (4.2% to 10.7%)	9.3% (5.6% to 13.6%)	7.2% (4.3% to 10.8%)
**Cardiovascular diseases**	84.3% (81.5% to 86.8%)	84.6% (82.5% to 86.6%)	68.0% (64.7% to 71.2%)	83.1% (80.8% to 85.2%)
Rheumatic heart disease	24.8% (15.1% to 44.1%)	20.2% (13.8% to 31.2%)	19.7% (12.8% to 28.8%)	20.0% (13.8% to 30.6%)
Ischaemic heart disease	90.5% (86.4% to 94.0%)	92.2% (89.5% to 94.5%)	89.3% (86.3% to 92.2%)	92.0% (89.4% to 94.4%)
Cerebrovascular disease	79.8% (74.8% to 84.6%)	81.5% (77.6% to 85.0%)	81.5% (76.8% to 85.9%)	81.5% (77.7% to 85.0%)
Hypertensive heart disease	100.0% (100.0% to 100.0%)	100.0% (100.0% to 100.0%)	100.0% (100.0% to 100.0%)	100.0% (100.0% to 100.0%)
Cardiomyopathy and myocarditis	27.0% (21.8% to 32.9%)	26.5% (21.4% to 31.7%)	25.1% (20.6% to 30.4%)	26.4% (21.5% to 31.4%)
Atrial fibrillation and flutter	47.7% (40.4% to 54.8%)	52.5% (45.7% to 58.5%)	58.9% (53.6% to 64.0%)	56.3% (50.6% to 61.8%)
Aortic aneurysm	42.7% (35.8% to 50.1%)	46.9% (40.9% to 52.5%)	-	46.9% (40.9% to 52.5%)
Peripheral vascular disease	48.3% (41.1% to 56.0%)	54.4% (48.3% to 60.0%)	58.0% (52.4% to 63.3%)	57.1% (51.7% to 62.4%)
Endocarditis	34.7% (26.0% to 45.3%)	26.9% (19.4% to 34.5%)	34.8% (27.4% to 43.0%)	27.0% (19.6% to 34.6%)

Supplementary Results Table 1. Age-standardised PAFs attributable to the joint distribution of all risk factors for all ages, both sexes combined for each GBD level 1 and level 2 cause and all causes in 2016 for Dominican Republic. Results are shown for deaths, YLLs, YLDs, and DALYs
Data in parenthesis are 95% uncertainty intervals. PAFs=population attributable fractions. GBD=Global Burden of Disease. YLLs=years of life lost. YLDs=years live with disability. DALYs=disability-adjusted life-years

	Deaths 2016	YLLs 2016	YLDs 2016	DALYs 2016
Other cardiovascular and circulatory diseases	43.5% (38.1% to 48.6%)	39.7% (34.8% to 44.6%)	41.8% (36.9% to 46.8%)	40.3% (35.9% to 44.6%)
**Chronic respiratory diseases**	55.3% (49.6% to 61.2%)	48.1% (42.3% to 53.6%)	29.6% (22.4% to 37.4%)	38.6% (32.3% to 45.3%)
Chronic obstructive pulmonary disease	69.0% (61.3% to 75.8%)	64.3% (56.0% to 71.2%)	57.0% (48.2% to 65.0%)	62.8% (54.7% to 70.0%)
Pneumoconiosis	100.0% (100.0% to 100.0%)	100.0% (100.0% to 100.0%)	100.0% (100.0% to 100.0%)	100.0% (100.0% to 100.0%)
Asthma	30.3% (22.1% to 40.2%)	31.7% (23.7% to 41.3%)	25.2% (17.2% to 34.4%)	26.6% (18.7% to 35.8%)
Other chronic respiratory diseases	8.2% (5.2% to 11.7%)	5.2% (3.0% to 7.9%)	1.9% (0.7% to 3.7%)	4.9% (2.8% to 7.5%)
**Cirrhosis and other chronic liver diseases**	59.2% (55.2% to 63.5%)	61.8% (58.2% to 65.5%)	51.6% (47.7% to 55.4%)	61.3% (57.7% to 65.0%)
Cirrhosis and other chronic liver diseases due to hepatitis B	4.3% (1.8% to 7.4%)	5.9% (2.5% to 10.3%)	5.2% (2.2% to 9.1%)	5.9% (2.5% to 10.3%)
Cirrhosis and other chronic liver diseases due to hepatitis C	60.9% (51.2% to 70.6%)	73.5% (66.3% to 80.2%)	68.4% (60.9% to 75.0%)	73.3% (66.1% to 80.0%)
Cirrhosis and other chronic liver diseases due to alcohol use	100.0% (100.0% to 100.0%)	100.0% (100.0% to 100.0%)	100.0% (100.0% to 100.0%)	100.0% (100.0% to 100.0%)
**Digestive diseases**	7.5% (5.5% to 9.5%)	6.8% (4.8% to 9.0%)	3.3% (2.3% to 4.3%)	5.6% (4.0% to 7.3%)
Peptic ulcer disease	12.9% (8.7% to 17.6%)	11.8% (8.1% to 16.1%)	9.2% (6.0% to 13.0%)	11.2% (7.6% to 15.1%)
Gallbladder and biliary diseases	25.3% (13.8% to 39.9%)	29.7% (17.3% to 44.0%)	40.2% (25.3% to 56.3%)	33.2% (20.1% to 48.4%)
Pancreatitis	24.8% (11.3% to 39.1%)	30.5% (15.9% to 45.3%)	24.5% (12.4% to 37.3%)	30.3% (15.8% to 45.1%)
**Neurological disorders**	15.3% (7.6% to 24.8%)	15.7% (9.1% to 24.4%)	3.3% (1.7% to 5.3%)	6.8% (4.0% to 10.4%)
Alzheimer's disease and other dementias	17.7% (9.0% to 28.6%)	19.3% (10.2% to 30.5%)	19.3% (10.1% to 30.4%)	19.3% (10.2% to 30.4%)
Parkinson's disease	-11.8% (-15.2% to -8.8%)	-12.3% (-15.7% to -9.5%)	-11.8% (-14.7% to -9.3%)	-12.2% (-15.5% to -9.4%)
Epilepsy	22.8% (14.8% to 30.1%)	21.5% (14.6% to 28.4%)	20.2% (13.4% to 26.8%)	20.7% (13.7% to 27.3%)
Multiple sclerosis	6.7% (4.1% to 9.7%)	5.9% (3.7% to 8.6%)	4.8% (3.0% to 6.9%)	5.4% (3.4% to 7.7%)
**Mental and substance use disorders**	99.8% (99.7% to 99.9%)	99.6% (99.4% to 99.8%)	16.9% (15.0% to 19.0%)	19.5% (17.2% to 22.4%)
Alcohol use disorders	100.0% (100.0% to 100.0%)	100.0% (100.0% to 100.0%)	100.0% (100.0% to 100.0%)	100.0% (100.0% to 100.0%)

Supplementary Results Table 1. Age-standardised PAFs attributable to the joint distribution of all risk factors for all ages, both sexes combined for each GBD level 1 and level 2 cause and all causes in 2016 for Dominican Republic. Results are shown for deaths, YLLs, YLDs, and DALYs
Data in parenthesis are 95% uncertainty intervals. PAFs=population attributable fractions. GBD=Global Burden of Disease. YLLs=years of life lost. YLDs=years live with disability. DALYs=disability-adjusted life-years

	Deaths 2016	YLLs 2016	YLDs 2016	DALYs 2016
Drug use disorders	100.0% (100.0% to 100.0%)	100.0% (100.0% to 100.0%)	100.0% (100.0% to 100.0%)	100.0% (100.0% to 100.0%)
Depressive disorders	-	-	5.6% (4.5% to 6.7%)	5.6% (4.5% to 6.7%)
Idiopathic developmental intellectual disability	-	-	72.2% (40.9% to 90.7%)	72.2% (40.9% to 90.7%)
**Diabetes, urogenital, blood, and endocrine diseases**	91.8% (88.0% to 93.0%)	87.0% (80.5% to 89.5%)	68.0% (63.7% to 72.1%)	79.3% (74.8% to 82.3%)
Diabetes mellitus	100.0% (100.0% to 100.0%)	100.0% (100.0% to 100.0%)	100.0% (100.0% to 100.0%)	100.0% (100.0% to 100.0%)
Chronic kidney disease	100.0% (100.0% to 100.0%)	100.0% (100.0% to 100.0%)	100.0% (100.0% to 100.0%)	100.0% (100.0% to 100.0%)
**Musculoskeletal disorders**	1.0% (0.4% to 2.0%)	0.5% (0.2% to 0.9%)	13.8% (11.2% to 16.9%)	13.6% (11.0% to 16.7%)
Rheumatoid arthritis	4.6% (1.7% to 7.9%)	4.3% (1.6% to 7.3%)	3.8% (1.5% to 6.6%)	3.8% (1.5% to 6.6%)
Osteoarthritis	-	-	20.3% (9.8% to 32.4%)	20.3% (9.8% to 32.4%)
Low back and neck pain	-	-	18.4% (15.8% to 21.2%)	18.4% (15.8% to 21.2%)
Gout	-	-	41.9% (27.8% to 57.6%)	41.9% (27.8% to 57.6%)
**Other non-communicable diseases**	0.1% (0.0% to 0.1%)	0.1% (0.0% to 0.1%)	5.1% (4.3% to 6.0%)	3.8% (3.2% to 4.5%)
Sense organ diseases	-	-	11.6% (10.2% to 13.3%)	11.6% (10.2% to 13.3%)
Sudden infant death syndrome	4.9% (2.5% to 8.2%)	4.9% (2.5% to 8.2%)	-	4.9% (2.5% to 8.2%)
**Transport injuries**	39.4% (28.6% to 53.2%)	36.1% (24.7% to 50.9%)	46.1% (35.0% to 60.2%)	37.4% (26.0% to 51.8%)
Road injuries	39.4% (28.5% to 53.2%)	36.1% (24.6% to 51.0%)	46.3% (35.0% to 60.5%)	37.4% (25.9% to 51.9%)
Other transport injuries	43.6% (33.4% to 56.7%)	37.2% (25.8% to 52.7%)	39.7% (29.7% to 51.1%)	37.8% (26.9% to 51.7%)
**Unintentional injuries**	17.8% (12.9% to 25.2%)	14.7% (9.2% to 23.3%)	29.8% (21.6% to 41.6%)	21.0% (14.1% to 31.0%)
Falls	57.2% (50.7% to 65.0%)	38.0% (28.4% to 51.9%)	37.2% (28.7% to 49.9%)	37.3% (28.9% to 49.8%)
Drowning	17.9% (10.3% to 28.7%)	16.0% (9.1% to 26.3%)	26.4% (16.9% to 38.2%)	16.2% (9.2% to 26.5%)
Fire, heat, and hot substances	13.8% (8.8% to 20.7%)	13.7% (8.4% to 21.8%)	33.0% (21.2% to 45.9%)	21.5% (13.3% to 31.3%)

Supplementary Results Table 1. Age-standardised PAFs attributable to the joint distribution of all risk factors for all ages, both sexes combined for each GBD level 1 and level 2 cause and all causes in 2016 for Dominican Republic. Results are shown for deaths, YLLs, YLDs, and DALYs
Data in parenthesis are 95% uncertainty intervals. PAFs=population attributable fractions. GBD=Global Burden of Disease. YLLs=years of life lost. YLDs=years live with disability. DALYs=disability-adjusted life-years

	**Deaths 2016**	**YLLs 2016**	**YLDs 2016**	**DALYs 2016**
Poisonings	16.1% (8.8% to 27.8%)	13.8% (6.2% to 26.8%)	31.8% (20.8% to 44.4%)	17.3% (9.0% to 29.0%)
Exposure to mechanical forces	26.3% (16.5% to 41.4%)	24.5% (14.3% to 40.2%)	31.4% (20.2% to 49.5%)	27.4% (16.7% to 43.9%)
Animal contact	18.1% (12.4% to 26.0%)	20.6% (12.5% to 30.8%)	26.3% (15.4% to 40.1%)	23.4% (14.0% to 35.3%)
Foreign body	8.4% (4.1% to 16.4%)	7.4% (3.1% to 16.2%)	18.5% (10.5% to 28.5%)	9.7% (4.7% to 18.9%)
Other unintentional injuries	24.8% (14.2% to 39.4%)	23.0% (13.2% to 36.7%)	28.5% (18.3% to 40.7%)	25.0% (15.3% to 38.1%)
**Self-harm and interpersonal violence**	23.3% (16.5% to 30.0%)	23.0% (16.4% to 29.4%)	27.3% (23.3% to 31.3%)	23.2% (16.9% to 29.5%)
Self-harm	24.3% (12.3% to 35.5%)	26.3% (13.9% to 38.2%)	21.7% (10.5% to 32.1%)	26.3% (13.8% to 38.1%)
Interpersonal violence	22.8% (15.0% to 30.1%)	21.9% (14.4% to 29.0%)	27.7% (23.6% to 31.9%)	22.3% (14.8% to 29.1%)
**Forces of nature, conflict and terrorism, and executions and police conflict**	0.0% (0.0% to 0.0%)	0.0% (0.0% to 0.0%)	4.1% (2.7% to 5.9%)	2.8% (1.0% to 4.6%)
Forces of nature, conflict and terrorism, and state actor violence	0.0% (0.0% to 0.0%)	0.0% (0.0% to 0.0%)	4.8% (3.2% to 6.9%)	4.8% (3.2% to 6.9%)

Supplementary Results Table 1. Age-standardised PAFs attributable to the joint distribution of all risk factors for all ages, both sexes combined for each GBD level 1 and level 2 cause and all causes in 2016 for Ecuador. Results are shown for deaths, YLLs, YLDs, and DALYs
Data in parenthesis are 95% uncertainty intervals. PAFs=population attributable fractions. GBD=Global Burden of Disease. YLLs=years of life lost. YLDs=years live with disability. DALYs=disability-adjusted life-years

	Deaths 2016	YLLs 2016	YLDs 2016	DALYs 2016
**All causes**	53.0% (51.2% to 54.7%)	48.1% (46.2% to 50.0%)	21.8% (20.2% to 23.2%)	37.5% (35.3% to 39.8%)
**HIV/AIDS and tuberculosis**	72.3% (66.3% to 78.6%)	75.9% (70.9% to 80.8%)	83.4% (77.0% to 88.7%)	76.2% (71.3% to 81.2%)
Tuberculosis	41.8% (28.8% to 57.4%)	42.9% (28.5% to 58.9%)	40.1% (26.7% to 56.5%)	42.8% (28.4% to 58.8%)
HIV/AIDS	93.8% (93.2% to 94.3%)	90.6% (89.7% to 91.3%)	95.7% (94.9% to 96.3%)	90.8% (90.0% to 91.6%)
**Diarrhoea, lower respiratory infections, and other common infectious diseases**	44.9% (38.4% to 52.4%)	55.8% (48.9% to 62.1%)	37.8% (28.2% to 47.5%)	52.1% (45.7% to 58.2%)
Diarrhoeal diseases	74.8% (57.7% to 89.0%)	83.5% (71.3% to 92.8%)	73.2% (55.7% to 88.3%)	77.6% (62.7% to 90.3%)
Lower respiratory infections	45.1% (37.6% to 53.2%)	58.1% (49.9% to 65.7%)	47.5% (40.2% to 55.5%)	58.0% (49.9% to 65.7%)
Upper respiratory infections	1.4% (0.1% to 5.0%)	2.9% (0.2% to 10.5%)	0.0% (0.0% to 0.0%)	0.0% (0.0% to 0.1%)
Otitis media	1.8% (0.9% to 2.8%)	3.5% (2.2% to 4.9%)	2.4% (1.5% to 3.4%)	2.4% (1.5% to 3.4%)
Meningitis	3.0% (2.3% to 3.9%)	5.0% (3.9% to 6.2%)	0.0% (0.0% to 0.0%)	4.4% (3.4% to 5.5%)
Encephalitis	1.1% (0.8% to 1.6%)	1.8% (1.3% to 2.3%)	0.0% (0.0% to 0.0%)	1.5% (1.1% to 2.0%)
Measles	26.1% (10.4% to 57.9%)	27.3% (10.9% to 60.4%)	17.7% (0.0% to 67.1%)	27.3% (10.9% to 60.4%)
**Maternal disorders**	5.9% (2.8% to 9.0%)	5.8% (2.7% to 8.8%)	5.6% (3.0% to 8.5%)	5.8% (2.8% to 8.7%)
Maternal haemorrhage	12.8% (4.6% to 20.9%)	12.7% (4.6% to 20.7%)	12.6% (4.5% to 20.5%)	12.7% (4.6% to 20.7%)
Maternal sepsis and other pregnancy related infections	12.7% (4.6% to 20.7%)	12.7% (4.5% to 20.6%)	12.5% (4.5% to 20.3%)	12.6% (4.5% to 20.5%)
Maternal abortion, miscarriage, and ectopic pregnancy	13.4% (8.2% to 19.1%)	13.2% (7.9% to 18.8%)	13.4% (7.3% to 19.6%)	13.2% (7.9% to 18.9%)
**Neonatal disorders**	77.9% (74.4% to 81.2%)	78.0% (74.4% to 81.2%)	0.2% (0.1% to 0.2%)	68.7% (64.7% to 72.7%)
Neonatal preterm birth complications	89.8% (86.6% to 92.7%)	89.9% (86.7% to 92.7%)	0.3% (0.2% to 0.3%)	79.7% (74.5% to 84.0%)
Neonatal encephalopathy due to birth asphyxia and trauma	65.1% (60.4% to 69.7%)	65.2% (60.5% to 69.8%)	0.0% (0.0% to 0.0%)	58.9% (52.1% to 65.1%)
Neonatal sepsis and other neonatal infections	58.2% (51.2% to 64.3%)	58.3% (51.2% to 64.4%)	0.0% (0.0% to 0.0%)	48.9% (38.0% to 57.7%)
Hemolytic disease and other neonatal jaundice	70.6% (67.1% to 74.1%)	70.7% (67.1% to 74.1%)	0.0% (0.0% to 0.0%)	25.3% (16.8% to 35.3%)

Supplementary Results Table 1. Age-standardised PAFs attributable to the joint distribution of all risk factors for all ages, both sexes combined for each GBD level 1 and level 2 cause and all causes in 2016 for Ecuador. Results are shown for deaths, YLLs, YLDs, and DALYs<br>Data in parenthesis are 95% uncertainty intervals. PAFs=population attributable fractions. GBD=Global Burden of Disease. YLLs=years of life lost. YLDs=years live with disability. DALYs=disability-adjusted life-years	Deaths<br>2016	YLLs<br>2016	YLDs<br>2016	DALYs<br>2016
Other neonatal disorders	59.5%<br>(52.8% to 65.3%)	59.5%<br>(52.9% to 65.4%)	0.0%<br>(0.0% to 0.0%)	57.1%<br>(50.8% to 62.7%)
**Nutritional deficiencies**	9.9%<br>(6.7% to 13.4%)	42.3%<br>(33.8% to 50.4%)	95.3%<br>(94.1% to 96.4%)	59.9%<br>(53.0% to 66.1%)
Protein-energy malnutrition	11.2%<br>(7.6% to 15.3%)	48.2%<br>(38.5% to 57.1%)	96.3%<br>(93.0% to 98.2%)	50.4%<br>(41.1% to 58.9%)
Vitamin A deficiency	-	-	100.0%<br>(100.0% to 100.0%)	100.0%<br>(100.0% to 100.0%)
Iron-deficiency anaemia	100.0%<br>(100.0% to 100.0%)	100.0%<br>(100.0% to 100.0%)	100.0%<br>(100.0% to 100.0%)	100.0%<br>(100.0% to 100.0%)
**Other communicable, maternal, neonatal, and nutritional diseases**	4.9%<br>(3.6% to 6.3%)	3.6%<br>(2.1% to 5.0%)	77.6%<br>(72.9% to 82.5%)	25.2%<br>(15.4% to 37.5%)
Sexually transmitted diseases excluding HIV	13.9%<br>(6.6% to 24.6%)	6.3%<br>(2.8% to 11.8%)	100.0%<br>(100.0% to 100.0%)	41.6%<br>(22.0% to 63.2%)
Hepatitis	2.2%<br>(1.6% to 3.0%)	2.2%<br>(1.7% to 3.0%)	1.7%<br>(1.0% to 2.7%)	2.2%<br>(1.7% to 2.9%)
**Neoplasms**	25.8%<br>(22.5% to 29.2%)	25.0%<br>(22.1% to 28.0%)	26.3%<br>(22.8% to 29.5%)	25.0%<br>(22.1% to 28.0%)
Oesophageal cancer	57.3%<br>(43.3% to 71.0%)	58.8%<br>(43.9% to 72.6%)	57.7%<br>(43.5% to 71.6%)	58.8%<br>(43.9% to 72.6%)
Stomach cancer	7.8%<br>(2.2% to 16.9%)	6.6%<br>(1.6% to 14.2%)	7.4%<br>(2.0% to 16.0%)	6.7%<br>(1.6% to 14.2%)
Liver cancer	39.5%<br>(30.2% to 50.3%)	40.5%<br>(30.8% to 51.5%)	39.8%<br>(30.3% to 50.8%)	40.4%<br>(30.8% to 51.5%)
Larynx cancer	58.0%<br>(44.7% to 69.2%)	56.2%<br>(42.7% to 67.7%)	57.6%<br>(44.4% to 69.0%)	56.2%<br>(42.8% to 67.7%)
Tracheal, bronchus, and lung cancer	60.0%<br>(52.2% to 67.1%)	56.3%<br>(48.5% to 63.5%)	58.6%<br>(51.0% to 65.8%)	56.3%<br>(48.5% to 63.5%)
Breast cancer	18.9%<br>(9.4% to 30.4%)	16.4%<br>(6.0% to 29.0%)	17.3%<br>(7.5% to 29.5%)	16.4%<br>(6.1% to 29.0%)
Cervical cancer	100.0%<br>(100.0% to 100.0%)	100.0%<br>(100.0% to 100.0%)	100.0%<br>(100.0% to 100.0%)	100.0%<br>(100.0% to 100.0%)
Uterine cancer	49.0%<br>(33.4% to 63.4%)	53.1%<br>(38.2% to 66.4%)	54.1%<br>(38.9% to 67.3%)	53.1%<br>(38.3% to 66.4%)
Prostate cancer	1.2%<br>(0.7% to 1.8%)	0.9%<br>(0.5% to 1.4%)	1.0%<br>(0.6% to 1.5%)	0.9%<br>(0.5% to 1.4%)
Colon and rectum cancer	52.8%<br>(43.3% to 61.8%)	53.4%<br>(43.9% to 62.0%)	52.4%<br>(42.8% to 61.2%)	53.4%<br>(43.8% to 62.0%)
Lip and oral cavity cancer	53.2%<br>(44.0% to 62.9%)	55.2%<br>(45.7% to 64.5%)	52.5%<br>(43.0% to 62.1%)	55.1%<br>(45.6% to 64.4%)
Nasopharynx cancer	55.2%<br>(49.3% to 60.4%)	52.7%<br>(47.5% to 57.5%)	52.9%<br>(48.0% to 57.4%)	52.7%<br>(47.5% to 57.5%)

Supplementary Results Table 1. Age-standardised PAFs attributable to the joint distribution of all risk factors for all ages, both sexes combined for each GBD level 1 and level 2 cause and all causes in 2016 for Ecuador. Results are shown for deaths, YLLs, YLDs, and DALYs
Data in parenthesis are 95% uncertainty intervals. PAFs=population attributable fractions. GBD=Global Burden of Disease. YLLs=years of life lost. YLDs=years live with disability. DALYs=disability-adjusted life-years

	Deaths 2016	YLLs 2016	YLDs 2016	DALYs 2016
Other pharynx cancer	36.3% (26.2% to 47.8%)	40.7% (30.6% to 52.3%)	38.2% (28.1% to 49.7%)	40.7% (30.6% to 52.2%)
Gallbladder and biliary tract cancer	25.0% (14.3% to 37.1%)	27.4% (16.1% to 40.2%)	25.6% (14.6% to 38.3%)	27.4% (16.1% to 40.2%)
Pancreatic cancer	17.7% (10.5% to 27.2%)	17.1% (9.9% to 26.4%)	17.5% (10.4% to 26.9%)	17.1% (9.9% to 26.4%)
Ovarian cancer	11.9% (5.0% to 20.3%)	10.5% (3.6% to 18.3%)	10.2% (3.7% to 17.9%)	10.5% (3.6% to 18.3%)
Kidney cancer	26.2% (17.2% to 36.8%)	25.2% (16.8% to 34.9%)	26.5% (17.8% to 36.6%)	25.2% (16.8% to 35.0%)
Bladder cancer	14.3% (7.9% to 22.3%)	12.0% (6.2% to 19.8%)	12.8% (6.8% to 20.6%)	12.0% (6.2% to 19.8%)
Thyroid cancer	14.7% (7.8% to 22.8%)	16.2% (9.0% to 24.9%)	16.3% (9.3% to 24.3%)	16.2% (9.0% to 24.8%)
Mesothelioma	81.4% (77.5% to 85.3%)	67.7% (62.3% to 73.4%)	77.0% (72.5% to 81.5%)	67.9% (62.5% to 73.6%)
Non-Hodgkin's lymphoma	7.5% (3.1% to 12.7%)	7.1% (3.0% to 11.9%)	7.5% (3.3% to 12.7%)	7.1% (3.0% to 11.9%)
Multiple myeloma	9.2% (4.0% to 15.6%)	10.0% (4.3% to 16.7%)	9.5% (4.2% to 16.2%)	10.0% (4.3% to 16.7%)
Leukaemia	10.6% (6.5% to 15.3%)	8.2% (5.1% to 11.8%)	10.2% (6.3% to 14.6%)	8.2% (5.1% to 11.9%)
**Cardiovascular diseases**	82.1% (79.2% to 84.4%)	80.8% (78.7% to 82.6%)	63.7% (59.9% to 67.2%)	78.5% (76.3% to 80.6%)
Rheumatic heart disease	20.9% (12.8% to 36.3%)	17.6% (12.0% to 28.0%)	15.5% (9.5% to 23.1%)	16.6% (11.0% to 25.9%)
Ischaemic heart disease	90.6% (86.7% to 94.0%)	91.8% (89.4% to 93.9%)	89.6% (86.6% to 92.4%)	91.7% (89.2% to 93.8%)
Cerebrovascular disease	77.1% (72.1% to 82.0%)	78.8% (75.3% to 82.1%)	78.9% (74.0% to 83.5%)	78.8% (75.3% to 82.2%)
Hypertensive heart disease	100.0% (100.0% to 100.0%)	100.0% (100.0% to 100.0%)	100.0% (100.0% to 100.0%)	100.0% (100.0% to 100.0%)
Cardiomyopathy and myocarditis	30.5% (24.0% to 38.6%)	24.6% (19.2% to 30.8%)	22.9% (17.9% to 28.8%)	24.5% (19.2% to 30.6%)
Atrial fibrillation and flutter	47.1% (39.4% to 54.7%)	51.0% (43.6% to 57.6%)	56.6% (50.3% to 62.7%)	54.3% (47.6% to 60.7%)
Aortic aneurysm	33.8% (27.5% to 40.6%)	35.8% (30.1% to 41.3%)	-	35.8% (30.1% to 41.3%)
Peripheral vascular disease	44.4% (37.9% to 51.7%)	49.9% (44.7% to 55.2%)	52.6% (47.7% to 57.7%)	52.2% (47.4% to 57.3%)
Endocarditis	29.0% (21.4% to 38.5%)	21.4% (15.7% to 27.8%)	29.7% (22.2% to 38.1%)	21.5% (15.8% to 27.9%)

Supplementary Results Table 1. Age-standardised PAFs attributable to the joint distribution of all risk factors for all ages, both sexes combined for each GBD level 1 and level 2 cause and all causes in 2016 for Ecuador. Results are shown for deaths, YLLs, YLDs, and DALYs
Data in parenthesis are 95% uncertainty intervals. PAFs=population attributable fractions. GBD=Global Burden of Disease. YLLs=years of life lost. YLDs=years live with disability. DALYs=disability-adjusted life-years

	Deaths 2016	YLLs 2016	YLDs 2016	DALYs 2016
Other cardiovascular and circulatory diseases	34.6% (29.7% to 39.3%)	30.6% (26.4% to 34.8%)	31.7% (26.8% to 36.4%)	30.9% (26.8% to 35.1%)
**Chronic respiratory diseases**	45.5% (38.3% to 52.7%)	37.5% (31.1% to 44.1%)	30.3% (23.1% to 38.6%)	34.5% (28.6% to 40.6%)
Chronic obstructive pulmonary disease	57.1% (48.0% to 65.5%)	51.0% (41.9% to 59.5%)	44.4% (35.4% to 53.3%)	49.7% (40.8% to 58.3%)
Pneumoconiosis	100.0% (100.0% to 100.0%)	100.0% (100.0% to 100.0%)	100.0% (100.0% to 100.0%)	100.0% (100.0% to 100.0%)
Asthma	35.4% (24.9% to 47.6%)	33.7% (24.7% to 43.6%)	28.1% (18.9% to 39.6%)	28.6% (19.4% to 39.7%)
Other chronic respiratory diseases	4.3% (2.7% to 6.3%)	2.3% (1.2% to 3.7%)	1.0% (0.3% to 2.5%)	2.0% (1.0% to 3.4%)
**Cirrhosis and other chronic liver diseases**	53.7% (50.7% to 56.6%)	54.2% (51.3% to 57.0%)	44.0% (40.3% to 47.7%)	53.8% (51.0% to 56.7%)
Cirrhosis and other chronic liver diseases due to hepatitis B	0.5% (0.2% to 0.9%)	0.6% (0.3% to 1.1%)	0.5% (0.2% to 0.9%)	0.6% (0.3% to 1.1%)
Cirrhosis and other chronic liver diseases due to hepatitis C	56.8% (46.0% to 66.9%)	68.7% (59.5% to 76.4%)	57.9% (47.8% to 67.7%)	68.4% (59.3% to 76.2%)
Cirrhosis and other chronic liver diseases due to alcohol use	100.0% (100.0% to 100.0%)	100.0% (100.0% to 100.0%)	100.0% (100.0% to 100.0%)	100.0% (100.0% to 100.0%)
**Digestive diseases**	16.2% (12.4% to 20.4%)	17.6% (13.6% to 22.0%)	2.9% (2.2% to 3.8%)	13.3% (10.3% to 16.7%)
Peptic ulcer disease	6.2% (4.0% to 8.5%)	6.7% (4.4% to 9.1%)	2.1% (1.4% to 3.1%)	4.7% (3.1% to 6.5%)
Gallbladder and biliary diseases	43.7% (28.4% to 59.4%)	47.2% (32.2% to 61.9%)	52.3% (37.8% to 66.1%)	47.7% (32.8% to 62.4%)
Pancreatitis	54.4% (37.8% to 71.8%)	59.2% (42.3% to 75.3%)	51.2% (35.5% to 68.0%)	59.1% (42.2% to 75.1%)
**Neurological disorders**	19.8% (9.9% to 31.7%)	19.2% (11.1% to 28.7%)	4.4% (2.2% to 7.6%)	8.6% (5.0% to 12.8%)
Alzheimer's disease and other dementias	22.8% (11.0% to 36.9%)	24.3% (12.2% to 38.7%)	24.3% (12.3% to 38.9%)	24.3% (12.3% to 38.7%)
Parkinson's disease	-4.9% (-6.3% to -3.8%)	-5.6% (-7.0% to -4.2%)	-5.3% (-6.6% to -4.1%)	-5.5% (-6.9% to -4.2%)
Epilepsy	21.3% (10.7% to 32.7%)	19.9% (10.0% to 30.7%)	17.1% (8.7% to 26.5%)	18.1% (9.2% to 28.0%)
Multiple sclerosis	3.4% (2.0% to 5.2%)	3.1% (1.8% to 4.7%)	2.1% (1.3% to 3.0%)	2.8% (1.6% to 4.2%)
**Mental and substance use disorders**	99.8% (99.7% to 99.8%)	99.6% (99.4% to 99.6%)	20.4% (18.4% to 22.5%)	25.6% (23.1% to 28.4%)
Alcohol use disorders	100.0% (100.0% to 100.0%)	100.0% (100.0% to 100.0%)	100.0% (100.0% to 100.0%)	100.0% (100.0% to 100.0%)

Supplementary Results Table 1. Age-standardised PAFs attributable to the joint distribution of all risk factors for all ages, both sexes combined for each GBD level 1 and level 2 cause and all causes in 2016 for Ecuador. Results are shown for deaths, YLLs, YLDs, and DALYs
Data in parenthesis are 95% uncertainty intervals. PAFs=population attributable fractions. GBD=Global Burden of Disease. YLLs=years of life lost. YLDs=years live with disability. DALYs=disability-adjusted life-years

	Deaths 2016	YLLs 2016	YLDs 2016	DALYs 2016
Drug use disorders	100.0% (100.0% to 100.0%)	100.0% (100.0% to 100.0%)	100.0% (100.0% to 100.0%)	100.0% (100.0% to 100.0%)
Depressive disorders	-	-	11.6% (9.8% to 13.4%)	11.6% (9.8% to 13.4%)
Idiopathic developmental intellectual disability	-	-	67.9% (37.5% to 87.1%)	67.9% (37.5% to 87.1%)
**Diabetes, urogenital, blood, and endocrine diseases**	93.3% (92.3% to 93.7%)	91.3% (89.4% to 92.0%)	70.3% (65.7% to 74.4%)	84.4% (81.9% to 86.4%)
Diabetes mellitus	100.0% (100.0% to 100.0%)	100.0% (100.0% to 100.0%)	100.0% (100.0% to 100.0%)	100.0% (100.0% to 100.0%)
Chronic kidney disease	100.0% (100.0% to 100.0%)	100.0% (100.0% to 100.0%)	100.0% (100.0% to 100.0%)	100.0% (100.0% to 100.0%)
**Musculoskeletal disorders**	0.6% (0.2% to 1.1%)	0.4% (0.1% to 0.7%)	21.5% (17.8% to 25.1%)	21.0% (17.4% to 24.5%)
Rheumatoid arthritis	1.4% (0.5% to 2.5%)	1.5% (0.6% to 2.6%)	1.5% (0.6% to 2.7%)	1.5% (0.6% to 2.7%)
Osteoarthritis	-	-	31.6% (17.6% to 45.0%)	31.6% (17.6% to 45.0%)
Low back and neck pain	-	-	25.7% (22.4% to 29.3%)	25.7% (22.4% to 29.3%)
Gout	-	-	53.8% (37.9% to 69.9%)	53.8% (37.9% to 69.9%)
**Other non-communicable diseases**	0.1% (0.1% to 0.2%)	0.1% (0.1% to 0.2%)	5.4% (4.5% to 6.4%)	4.3% (3.6% to 5.1%)
Sense organ diseases	-	-	11.9% (10.2% to 13.8%)	11.9% (10.2% to 13.8%)
Sudden infant death syndrome	6.5% (4.0% to 10.3%)	6.5% (4.1% to 10.3%)	-	6.5% (4.1% to 10.3%)
**Transport injuries**	36.2% (27.2% to 45.8%)	32.6% (23.8% to 41.9%)	41.4% (32.8% to 50.6%)	33.4% (24.5% to 42.8%)
Road injuries	36.1% (27.1% to 45.7%)	32.5% (23.7% to 41.8%)	41.6% (33.1% to 50.9%)	33.3% (24.4% to 42.8%)
Other transport injuries	39.1% (29.8% to 48.8%)	35.2% (25.5% to 44.9%)	34.5% (26.4% to 43.5%)	35.2% (25.4% to 44.6%)
**Unintentional injuries**	25.6% (21.6% to 30.0%)	17.1% (13.1% to 21.8%)	23.9% (20.6% to 28.3%)	19.6% (15.8% to 24.2%)
Falls	49.7% (45.2% to 54.2%)	32.4% (27.3% to 38.7%)	31.2% (27.7% to 35.8%)	31.7% (27.8% to 36.9%)
Drowning	18.2% (12.2% to 25.1%)	16.1% (10.9% to 22.2%)	18.2% (12.3% to 25.4%)	16.1% (10.9% to 22.2%)
Fire, heat, and hot substances	13.6% (9.1% to 19.0%)	14.7% (10.2% to 20.2%)	20.5% (14.3% to 27.6%)	16.9% (11.6% to 23.0%)

Supplementary Results Table 1. Age-standardised PAFs attributable to the joint distribution of all risk factors for all ages, both sexes combined for each GBD level 1 and level 2 cause and all causes in 2016 for Ecuador. Results are shown for deaths, YLLs, YLDs, and DALYs
Data in parenthesis are 95% uncertainty intervals. PAFs=population attributable fractions. GBD=Global Burden of Disease. YLLs=years of life lost. YLDs=years live with disability. DALYs=disability-adjusted life-years

	**Deaths 2016**	**YLLs 2016**	**YLDs 2016**	**DALYs 2016**
Poisonings	19.6% (13.5% to 27.0%)	18.3% (12.7% to 25.0%)	19.3% (13.4% to 26.2%)	18.4% (12.9% to 25.2%)
Exposure to mechanical forces	20.8% (16.3% to 26.8%)	17.6% (13.1% to 23.6%)	23.4% (19.0% to 29.3%)	19.8% (15.3% to 25.5%)
Animal contact	13.4% (9.9% to 17.8%)	12.6% (8.9% to 17.3%)	15.4% (11.1% to 20.9%)	13.7% (9.9% to 18.5%)
Foreign body	9.8% (6.9% to 13.9%)	7.7% (5.3% to 11.3%)	11.7% (8.5% to 16.1%)	8.6% (6.0% to 12.3%)
Other unintentional injuries	26.5% (18.0% to 36.6%)	26.9% (18.5% to 37.1%)	18.7% (12.9% to 25.3%)	22.7% (15.7% to 30.8%)
**Self-harm and interpersonal violence**	24.3% (14.9% to 33.6%)	23.7% (14.4% to 32.9%)	27.9% (21.6% to 34.7%)	23.8% (14.7% to 32.9%)
Self-harm	28.0% (17.6% to 38.5%)	27.4% (17.4% to 37.6%)	26.0% (16.2% to 36.4%)	27.4% (17.4% to 37.6%)
Interpersonal violence	22.1% (10.3% to 35.0%)	21.5% (9.9% to 34.2%)	28.0% (21.5% to 35.2%)	21.9% (10.4% to 34.3%)
**Forces of nature, conflict and terrorism, and executions and police conflict**	6.6% (6.1% to 7.0%)	2.7% (2.4% to 2.9%)	4.3% (2.7% to 6.6%)	2.7% (2.5% to 3.0%)
Forces of nature, conflict and terrorism, and state actor violence	6.6% (6.1% to 7.0%)	2.7% (2.4% to 2.9%)	4.4% (2.7% to 6.7%)	2.8% (2.5% to 3.0%)

**Supplementary Results Table 1. Age-standardised PAFs attributable to the joint distribution of all risk factors for all ages, both sexes combined for each GBD level 1 and level 2 cause and all causes in 2016 for Egypt. Results are shown for deaths, YLLs, YLDs, and DALYs**<br>Data in parenthesis are 95% uncertainty intervals. PAFs=population attributable fractions. GBD=Global Burden of Disease. YLLs=years of life lost. YLDs=years live with disability. DALYs=disability-adjusted life-years				
	**Deaths 2016**	**YLLs 2016**	**YLDs 2016**	**DALYs 2016**
**All causes**	62.2% (59.9% to 64.7%)	59.6% (57.2% to 61.8%)	26.9% (25.2% to 28.6%)	47.7% (45.0% to 50.6%)
**HIV/AIDS and tuberculosis**	32.6% (25.9% to 39.6%)	32.4% (26.4% to 39.0%)	32.3% (24.5% to 41.6%)	32.4% (26.4% to 38.7%)
Tuberculosis	28.1% (21.2% to 35.9%)	26.2% (19.9% to 33.1%)	21.2% (15.2% to 28.0%)	25.9% (19.7% to 32.7%)
HIV/AIDS	78.3% (74.0% to 83.2%)	75.0% (69.8% to 80.4%)	81.4% (77.7% to 85.8%)	75.5% (70.4% to 80.9%)
**Diarrhoea, lower respiratory infections, and other common infectious diseases**	69.8% (63.4% to 75.7%)	76.5% (70.3% to 82.1%)	37.4% (30.7% to 43.6%)	71.7% (65.1% to 77.9%)
Diarrhoeal diseases	89.8% (80.1% to 96.2%)	94.6% (89.8% to 98.0%)	81.3% (67.9% to 91.9%)	92.7% (86.5% to 97.2%)
Lower respiratory infections	70.0% (63.0% to 76.5%)	76.5% (70.1% to 82.2%)	66.0% (58.9% to 72.8%)	76.4% (70.0% to 82.2%)
Upper respiratory infections	1.4% (0.1% to 4.6%)	3.5% (0.3% to 10.9%)	0.0% (0.0% to 0.0%)	0.0% (0.0% to 0.1%)
Otitis media	4.6% (1.2% to 10.8%)	9.8% (3.5% to 19.4%)	8.0% (5.5% to 10.4%)	8.0% (5.5% to 10.4%)
Meningitis	4.1% (1.9% to 7.8%)	6.7% (3.5% to 11.4%)	0.0% (0.0% to 0.0%)	5.1% (2.6% to 9.1%)
Encephalitis	2.1% (0.9% to 4.5%)	3.3% (1.7% to 6.4%)	0.0% (0.0% to 0.0%)	2.2% (0.9% to 4.6%)
Measles	41.4% (22.1% to 74.4%)	42.3% (22.6% to 75.9%)	43.5% (23.5% to 76.5%)	42.4% (22.7% to 76.0%)
**Maternal disorders**	8.7% (3.3% to 14.2%)	8.6% (3.3% to 14.1%)	2.2% (0.9% to 3.9%)	7.4% (2.9% to 12.2%)
Maternal haemorrhage	13.2% (4.8% to 21.4%)	13.2% (4.8% to 21.4%)	13.2% (4.8% to 21.5%)	13.2% (4.8% to 21.4%)
Maternal sepsis and other pregnancy related infections	13.0% (4.7% to 21.3%)	13.0% (4.7% to 21.2%)	13.2% (4.8% to 21.4%)	13.0% (4.7% to 21.2%)
Maternal abortion, miscarriage, and ectopic pregnancy	10.4% (6.1% to 15.8%)	10.1% (5.8% to 15.6%)	10.7% (5.7% to 16.3%)	10.2% (5.8% to 15.3%)
**Neonatal disorders**	80.9% (76.1% to 84.7%)	80.9% (76.2% to 84.7%)	0.1% (0.0% to 0.2%)	65.2% (52.1% to 73.1%)
Neonatal preterm birth complications	96.5% (92.0% to 99.2%)	96.5% (92.1% to 99.2%)	0.3% (0.2% to 0.3%)	78.2% (66.1% to 87.3%)
Neonatal encephalopathy due to birth asphyxia and trauma	72.2% (63.7% to 78.4%)	72.2% (63.8% to 78.4%)	0.0% (0.0% to 0.0%)	65.4% (51.9% to 74.8%)
Neonatal sepsis and other neonatal infections	66.4% (58.4% to 72.5%)	66.4% (58.4% to 72.5%)	0.0% (0.0% to 0.0%)	45.6% (22.9% to 60.3%)
Hemolytic disease and other neonatal jaundice	71.8% (66.4% to 76.9%)	71.8% (66.5% to 76.9%)	0.0% (0.0% to 0.0%)	27.8% (16.8% to 41.3%)

Supplementary Results Table 1. Age-standardised PAFs attributable to the joint distribution of all risk factors for all ages, both sexes combined for each GBD level 1 and level 2 cause and all causes in 2016 for Egypt. Results are shown for deaths, YLLs, YLDs, and DALYs
Data in parenthesis are 95% uncertainty intervals. PAFs=population attributable fractions. GBD=Global Burden of Disease. YLLs=years of life lost. YLDs=years live with disability. DALYs=disability-adjusted life-years

	Deaths 2016	YLLs 2016	YLDs 2016	DALYs 2016
Other neonatal disorders	70.6% (62.7% to 76.8%)	70.6% (62.8% to 76.8%)	0.0% (0.0% to 0.0%)	66.7% (57.6% to 73.7%)
**Nutritional deficiencies**	8.2% (4.8% to 15.2%)	31.7% (19.5% to 50.0%)	90.3% (88.2% to 92.5%)	86.0% (82.3% to 89.1%)
Protein-energy malnutrition	7.2% (3.3% to 15.1%)	32.5% (18.3% to 53.8%)	98.0% (97.3% to 98.5%)	70.0% (57.4% to 80.4%)
Vitamin A deficiency	-	-	100.0% (100.0% to 100.0%)	100.0% (100.0% to 100.0%)
Iron-deficiency anaemia	100.0% (100.0% to 100.0%)	100.0% (100.0% to 100.0%)	100.0% (100.0% to 100.0%)	100.0% (100.0% to 100.0%)
**Other communicable, maternal, neonatal, and nutritional diseases**	1.2% (0.8% to 1.7%)	1.4% (0.9% to 2.0%)	83.4% (79.2% to 87.4%)	44.6% (33.9% to 56.0%)
Sexually transmitted diseases excluding HIV	18.7% (7.4% to 36.4%)	10.4% (3.8% to 22.2%)	100.0% (100.0% to 100.0%)	90.0% (79.1% to 96.5%)
Hepatitis	0.7% (0.4% to 1.1%)	0.8% (0.5% to 1.2%)	0.6% (0.3% to 0.9%)	0.8% (0.5% to 1.2%)
**Neoplasms**	31.0% (25.0% to 37.2%)	30.6% (25.0% to 36.0%)	26.1% (20.8% to 31.7%)	30.5% (24.9% to 35.9%)
Oesophageal cancer	55.1% (36.3% to 71.7%)	57.0% (39.1% to 72.8%)	55.2% (35.9% to 72.1%)	57.0% (39.1% to 72.8%)
Stomach cancer	8.8% (2.4% to 17.9%)	9.4% (3.3% to 17.4%)	8.8% (2.6% to 17.4%)	9.4% (3.3% to 17.4%)
Liver cancer	46.7% (34.2% to 59.5%)	50.8% (38.6% to 63.0%)	47.6% (35.7% to 59.6%)	50.8% (38.6% to 62.9%)
Larynx cancer	44.6% (31.9% to 57.3%)	50.0% (36.4% to 62.5%)	45.8% (32.6% to 58.2%)	49.9% (36.4% to 62.5%)
Tracheal, bronchus, and lung cancer	71.0% (62.5% to 78.1%)	73.1% (65.5% to 79.8%)	70.6% (62.4% to 77.4%)	73.1% (65.4% to 79.7%)
Breast cancer	18.2% (8.3% to 29.4%)	13.5% (3.6% to 23.8%)	15.6% (5.7% to 26.9%)	13.6% (3.7% to 23.9%)
Cervical cancer	100.0% (100.0% to 100.0%)	100.0% (100.0% to 100.0%)	100.0% (100.0% to 100.0%)	100.0% (100.0% to 100.0%)
Uterine cancer	62.2% (48.1% to 73.6%)	62.0% (48.0% to 73.5%)	62.4% (48.2% to 73.9%)	62.0% (48.1% to 73.5%)
Prostate cancer	0.7% (0.3% to 1.2%)	1.0% (0.5% to 1.6%)	1.0% (0.5% to 1.6%)	1.0% (0.5% to 1.6%)
Colon and rectum cancer	45.7% (35.0% to 56.1%)	45.0% (34.9% to 54.7%)	45.5% (34.9% to 55.7%)	45.0% (34.9% to 54.7%)
Lip and oral cavity cancer	30.6% (22.7% to 39.2%)	34.2% (25.6% to 43.3%)	29.3% (22.0% to 37.0%)	34.1% (25.4% to 43.1%)
Nasopharynx cancer	31.5% (22.8% to 40.5%)	31.2% (22.4% to 40.5%)	29.4% (21.2% to 38.7%)	31.2% (22.3% to 40.5%)

Supplementary Results Table 1. Age-standardised PAFs attributable to the joint distribution of all risk factors for all ages, both sexes combined for each GBD level 1 and level 2 cause and all causes in 2016 for Egypt. Results are shown for deaths, YLLs, YLDs, and DALYs
Data in parenthesis are 95% uncertainty intervals. PAFs=population attributable fractions. GBD=Global Burden of Disease. YLLs=years of life lost. YLDs=years live with disability. DALYs=disability-adjusted life-years

	Deaths 2016	YLLs 2016	YLDs 2016	DALYs 2016
Other pharynx cancer	10.3% (5.9% to 16.5%)	10.9% (6.4% to 17.3%)	10.6% (6.1% to 16.7%)	10.9% (6.4% to 17.3%)
Gallbladder and biliary tract cancer	28.8% (16.8% to 42.2%)	28.7% (16.8% to 41.6%)	29.8% (17.7% to 43.0%)	28.7% (16.8% to 41.6%)
Pancreatic cancer	23.1% (13.2% to 35.6%)	24.0% (14.3% to 35.9%)	23.3% (13.2% to 35.6%)	23.9% (14.3% to 35.9%)
Ovarian cancer	15.8% (5.7% to 27.7%)	14.7% (5.1% to 26.0%)	14.5% (5.0% to 25.7%)	14.7% (5.1% to 25.9%)
Kidney cancer	32.2% (22.3% to 42.8%)	30.0% (20.5% to 40.5%)	31.5% (22.1% to 42.1%)	30.1% (20.6% to 40.6%)
Bladder cancer	18.9% (9.9% to 30.5%)	20.8% (11.3% to 32.4%)	19.8% (10.8% to 31.2%)	20.7% (11.3% to 32.4%)
Thyroid cancer	21.0% (11.7% to 31.9%)	20.8% (11.4% to 31.9%)	20.6% (12.0% to 30.7%)	20.8% (11.5% to 31.9%)
Mesothelioma	83.2% (76.0% to 88.8%)	75.2% (64.3% to 82.5%)	80.9% (72.7% to 87.1%)	75.2% (64.5% to 82.6%)
Non-Hodgkin's lymphoma	9.2% (4.1% to 15.1%)	8.0% (3.5% to 13.3%)	9.0% (4.0% to 14.6%)	8.0% (3.5% to 13.3%)
Multiple myeloma	11.7% (5.4% to 19.5%)	11.7% (5.4% to 19.2%)	11.9% (5.5% to 19.7%)	11.7% (5.4% to 19.2%)
Leukaemia	14.9% (9.5% to 21.0%)	12.7% (8.0% to 18.2%)	14.7% (9.1% to 20.8%)	12.8% (8.0% to 18.2%)
**Cardiovascular diseases**	89.2% (87.0% to 91.3%)	91.0% (89.4% to 92.5%)	75.6% (72.6% to 78.6%)	90.0% (88.4% to 91.6%)
Rheumatic heart disease	27.4% (18.9% to 42.6%)	26.4% (17.9% to 39.2%)	23.8% (15.1% to 34.6%)	25.4% (16.9% to 37.3%)
Ischaemic heart disease	95.1% (92.5% to 97.2%)	96.2% (94.8% to 97.5%)	93.2% (90.9% to 95.4%)	96.2% (94.7% to 97.5%)
Cerebrovascular disease	85.2% (80.8% to 90.2%)	88.3% (85.4% to 91.3%)	87.1% (83.1% to 91.5%)	88.2% (85.3% to 91.3%)
Hypertensive heart disease	100.0% (100.0% to 100.0%)	100.0% (100.0% to 100.0%)	100.0% (100.0% to 100.0%)	100.0% (100.0% to 100.0%)
Cardiomyopathy and myocarditis	42.7% (34.4% to 51.5%)	39.1% (31.6% to 46.6%)	34.5% (27.8% to 41.0%)	38.8% (31.5% to 46.0%)
Atrial fibrillation and flutter	48.9% (40.8% to 56.5%)	53.4% (46.3% to 60.1%)	61.1% (55.8% to 66.4%)	57.6% (51.1% to 63.2%)
Aortic aneurysm	45.2% (37.7% to 51.7%)	52.2% (45.2% to 58.0%)	-	52.2% (45.2% to 58.0%)
Peripheral vascular disease	55.2% (48.2% to 62.0%)	63.1% (56.6% to 69.4%)	66.7% (61.6% to 71.6%)	65.9% (61.0% to 70.7%)
Endocarditis	37.8% (28.3% to 49.1%)	38.6% (30.2% to 47.0%)	38.3% (29.2% to 47.8%)	38.6% (30.2% to 47.0%)

Supplementary Results Table 1. Age-standardised PAFs attributable to the joint distribution of all risk factors for all ages, both sexes combined for each GBD level 1 and level 2 cause and all causes in 2016 for Egypt. Results are shown for deaths, YLLs, YLDs, and DALYs
Data in parenthesis are 95% uncertainty intervals. PAFs=population attributable fractions. GBD=Global Burden of Disease. YLLs=years of life lost. YLDs=years live with disability. DALYs=disability-adjusted life-years

	Deaths 2016	YLLs 2016	YLDs 2016	DALYs 2016
Other cardiovascular and circulatory diseases	42.6% (36.9% to 48.6%)	46.5% (41.8% to 51.6%)	47.3% (42.6% to 52.3%)	46.7% (42.4% to 51.4%)
**Chronic respiratory diseases**	54.0% (45.5% to 62.5%)	54.5% (46.8% to 62.1%)	46.6% (39.2% to 54.4%)	51.8% (44.6% to 58.7%)
Chronic obstructive pulmonary disease	62.0% (51.2% to 73.1%)	64.2% (54.3% to 74.6%)	61.5% (51.7% to 72.2%)	63.7% (53.8% to 74.3%)
Pneumoconiosis	100.0% (100.0% to 100.0%)	100.0% (100.0% to 100.0%)	100.0% (100.0% to 100.0%)	100.0% (100.0% to 100.0%)
Asthma	52.8% (42.0% to 64.2%)	51.9% (41.2% to 62.5%)	41.2% (31.2% to 51.8%)	45.8% (36.0% to 56.1%)
Other chronic respiratory diseases	4.2% (2.1% to 7.4%)	4.7% (2.2% to 8.5%)	3.9% (1.8% to 6.6%)	4.6% (2.2% to 7.9%)
**Cirrhosis and other chronic liver diseases**	15.3% (12.5% to 18.9%)	21.4% (17.3% to 26.5%)	20.9% (17.2% to 24.6%)	21.4% (17.3% to 26.5%)
Cirrhosis and other chronic liver diseases due to hepatitis B	0.2% (0.1% to 0.4%)	0.3% (0.1% to 0.5%)	0.2% (0.1% to 0.4%)	0.3% (0.1% to 0.5%)
Cirrhosis and other chronic liver diseases due to hepatitis C	13.7% (9.1% to 19.7%)	24.6% (17.2% to 33.1%)	30.9% (23.5% to 38.4%)	24.7% (17.4% to 33.2%)
Cirrhosis and other chronic liver diseases due to alcohol use	100.0% (100.0% to 100.0%)	100.0% (100.0% to 100.0%)	100.0% (100.0% to 100.0%)	100.0% (100.0% to 100.0%)
**Digestive diseases**	18.8% (13.2% to 24.7%)	18.5% (12.8% to 24.4%)	3.6% (2.7% to 4.5%)	15.2% (10.4% to 20.1%)
Peptic ulcer disease	21.2% (14.7% to 28.0%)	22.2% (15.7% to 28.9%)	7.4% (5.3% to 9.8%)	18.4% (12.7% to 24.4%)
Gallbladder and biliary diseases	52.8% (38.5% to 66.4%)	51.8% (37.3% to 65.3%)	56.0% (41.7% to 68.6%)	52.0% (37.7% to 65.3%)
Pancreatitis	11.3% (5.0% to 21.9%)	12.5% (5.9% to 24.2%)	12.3% (5.9% to 23.5%)	12.5% (5.9% to 24.2%)
**Neurological disorders**	31.0% (16.4% to 45.9%)	27.7% (14.7% to 41.1%)	3.6% (1.8% to 5.5%)	10.8% (5.6% to 16.8%)
Alzheimer's disease and other dementias	36.3% (19.7% to 53.4%)	36.7% (20.3% to 53.8%)	36.6% (19.6% to 53.9%)	36.7% (20.2% to 53.8%)
Parkinson's disease	-15.7% (-20.0% to -11.2%)	-18.6% (-23.5% to -13.4%)	-17.4% (-21.9% to -12.8%)	-18.3% (-23.0% to -13.3%)
Epilepsy	2.2% (1.0% to 3.8%)	2.0% (0.9% to 3.5%)	1.7% (0.8% to 2.9%)	1.7% (0.8% to 3.0%)
Multiple sclerosis	10.3% (5.1% to 16.6%)	10.3% (5.1% to 16.7%)	6.0% (3.2% to 8.7%)	7.2% (4.0% to 10.6%)
**Mental and substance use disorders**	99.1% (98.5% to 99.5%)	98.8% (97.8% to 99.3%)	19.6% (16.7% to 22.6%)	20.0% (17.2% to 23.2%)
Alcohol use disorders	100.0% (100.0% to 100.0%)	100.0% (100.0% to 100.0%)	100.0% (100.0% to 100.0%)	100.0% (100.0% to 100.0%)

Supplementary Results Table 1. Age-standardised PAFs attributable to the joint distribution of all risk factors for all ages, both sexes combined for each GBD level 1 and level 2 cause and all causes in 2016 for Egypt. Results are shown for deaths, YLLs, YLDs, and DALYs
Data in parenthesis are 95% uncertainty intervals. PAFs=population attributable fractions. GBD=Global Burden of Disease. YLLs=years of life lost. YLDs=years live with disability. DALYs=disability-adjusted life-years

	Deaths 2016	YLLs 2016	YLDs 2016	DALYs 2016
Drug use disorders	100.0% (100.0% to 100.0%)	100.0% (100.0% to 100.0%)	100.0% (100.0% to 100.0%)	100.0% (100.0% to 100.0%)
Depressive disorders	-	-	6.9% (5.5% to 8.5%)	6.9% (5.5% to 8.5%)
Idiopathic developmental intellectual disability	-	-	68.1% (36.4% to 87.0%)	68.1% (36.4% to 87.0%)
**Diabetes, urogenital, blood, and endocrine diseases**	88.9% (86.6% to 91.5%)	84.5% (81.4% to 87.6%)	74.5% (70.2% to 78.2%)	79.6% (76.7% to 82.2%)
Diabetes mellitus	100.0% (100.0% to 100.0%)	100.0% (100.0% to 100.0%)	100.0% (100.0% to 100.0%)	100.0% (100.0% to 100.0%)
Chronic kidney disease	100.0% (100.0% to 100.0%)	100.0% (100.0% to 100.0%)	100.0% (100.0% to 100.0%)	100.0% (100.0% to 100.0%)
**Musculoskeletal disorders**	2.1% (0.8% to 3.6%)	1.5% (0.6% to 2.8%)	18.9% (15.2% to 22.7%)	18.9% (15.2% to 22.6%)
Rheumatoid arthritis	4.5% (1.9% to 7.8%)	4.7% (2.0% to 8.1%)	3.1% (1.3% to 4.9%)	3.1% (1.3% to 5.0%)
Osteoarthritis	-	-	38.5% (23.2% to 52.7%)	38.5% (23.2% to 52.7%)
Low back and neck pain	-	-	21.9% (18.4% to 25.7%)	21.9% (18.4% to 25.7%)
Gout	-	-	62.0% (47.1% to 76.0%)	62.0% (47.1% to 76.0%)
**Other non-communicable diseases**	0.3% (0.1% to 0.7%)	0.3% (0.1% to 0.8%)	5.6% (4.5% to 6.8%)	4.3% (3.3% to 5.5%)
Sense organ diseases	-	-	11.0% (9.1% to 13.3%)	11.0% (9.1% to 13.3%)
Sudden infant death syndrome	8.2% (4.0% to 14.0%)	8.2% (4.0% to 14.0%)	-	8.2% (4.0% to 14.0%)
**Transport injuries**	18.4% (16.3% to 20.7%)	15.2% (13.0% to 17.7%)	21.6% (19.7% to 23.8%)	15.6% (13.4% to 18.2%)
Road injuries	18.4% (16.3% to 20.8%)	15.1% (13.0% to 17.7%)	24.6% (22.6% to 26.9%)	15.6% (13.5% to 18.2%)
Other transport injuries	18.3% (15.9% to 21.1%)	15.2% (12.6% to 18.1%)	14.6% (13.0% to 16.7%)	14.9% (12.8% to 17.4%)
**Unintentional injuries**	20.5% (17.3% to 23.6%)	11.7% (9.8% to 13.7%)	13.2% (11.8% to 15.1%)	12.4% (11.0% to 14.1%)
Falls	38.9% (32.5% to 46.5%)	23.1% (20.1% to 27.1%)	21.3% (19.7% to 23.1%)	22.1% (20.1% to 24.6%)
Drowning	7.8% (5.6% to 10.4%)	7.4% (5.1% to 10.0%)	7.0% (5.3% to 9.2%)	7.4% (5.2% to 10.0%)
Fire, heat, and hot substances	5.7% (4.1% to 7.7%)	6.3% (4.6% to 8.2%)	6.4% (4.9% to 8.3%)	6.3% (4.8% to 8.1%)

Supplementary Results Table 1. Age-standardised PAFs attributable to the joint distribution of all risk factors for all ages, both sexes combined for each GBD level 1 and level 2 cause and all causes in 2016 for Egypt. Results are shown for deaths, YLLs, YLDs, and DALYs

Data in parenthesis are 95% uncertainty intervals. PAFs=population attributable fractions. GBD=Global Burden of Disease. YLLs=years of life lost. YLDs=years live with disability. DALYs=disability-adjusted life-years

	**Deaths 2016**	**YLLs 2016**	**YLDs 2016**	**DALYs 2016**
Poisonings	6.3% (4.2% to 8.9%)	6.4% (4.1% to 9.4%)	6.6% (5.0% to 8.6%)	6.5% (4.6% to 8.8%)
Exposure to mechanical forces	12.0% (9.4% to 14.9%)	9.7% (6.9% to 12.8%)	16.8% (14.7% to 19.5%)	13.9% (11.6% to 16.5%)
Animal contact	3.0% (1.4% to 7.7%)	1.4% (0.6% to 3.5%)	6.7% (5.2% to 8.5%)	4.8% (3.4% to 6.8%)
Foreign body	3.5% (2.4% to 4.7%)	3.4% (2.4% to 4.7%)	5.2% (3.9% to 6.8%)	4.2% (3.1% to 5.5%)
Other unintentional injuries	11.7% (8.8% to 15.2%)	11.8% (8.8% to 15.5%)	5.9% (4.5% to 7.7%)	7.2% (5.5% to 9.3%)
**Self-harm and interpersonal violence**	8.2% (5.9% to 10.9%)	8.6% (6.1% to 11.6%)	21.2% (19.8% to 22.8%)	10.2% (7.8% to 12.9%)
Self-harm	7.5% (5.1% to 10.7%)	8.2% (5.5% to 11.6%)	6.9% (4.6% to 9.7%)	8.2% (5.5% to 11.6%)
Interpersonal violence	12.0% (7.9% to 17.0%)	10.7% (6.8% to 15.8%)	23.2% (21.7% to 24.7%)	15.9% (12.7% to 19.8%)
**Forces of nature, conflict and terrorism, and executions and police conflict**	0.0% (0.0% to 0.0%)	0.0% (0.0% to 0.0%)	0.2% (0.1% to 0.3%)	0.0% (0.0% to 0.1%)
Forces of nature, conflict and terrorism, and state actor violence	0.0% (0.0% to 0.0%)	0.0% (0.0% to 0.0%)	6.6% (4.6% to 9.6%)	6.6% (4.6% to 9.6%)

Supplementary Results Table 1. Age-standardised PAFs attributable to the joint distribution of all risk factors for all ages, both sexes combined for each GBD level 1 and level 2 cause and all causes in 2016 for El Salvador. Results are shown for deaths, YLLs, YLDs, and DALYs
Data in parenthesis are 95% uncertainty intervals. PAFs=population attributable fractions. GBD=Global Burden of Disease. YLLs=years of life lost. YLDs=years live with disability. DALYs=disability-adjusted life-years

	Deaths 2016	YLLs 2016	YLDs 2016	DALYs 2016
**All causes**	58.9% (56.7% to 60.8%)	51.4% (49.3% to 53.5%)	24.1% (22.6% to 25.6%)	41.6% (39.2% to 44.1%)
**HIV/AIDS and tuberculosis**	79.6% (71.6% to 88.9%)	85.5% (79.9% to 91.7%)	89.9% (86.4% to 92.6%)	86.1% (81.3% to 91.7%)
Tuberculosis	47.9% (36.9% to 58.4%)	48.3% (39.0% to 57.2%)	39.0% (30.1% to 47.5%)	47.6% (38.7% to 56.5%)
HIV/AIDS	98.0% (97.2% to 98.6%)	97.6% (96.4% to 98.4%)	97.7% (96.6% to 98.3%)	97.6% (96.6% to 98.3%)
**Diarrhoea, lower respiratory infections, and other common infectious diseases**	54.4% (46.9% to 61.9%)	59.1% (52.1% to 66.2%)	46.5% (37.4% to 54.5%)	56.5% (50.1% to 62.9%)
Diarrhoeal diseases	85.6% (71.5% to 94.7%)	90.3% (79.8% to 96.6%)	86.0% (72.4% to 94.8%)	88.3% (77.4% to 95.7%)
Lower respiratory infections	54.1% (46.1% to 63.1%)	62.4% (54.9% to 70.1%)	58.7% (51.1% to 66.6%)	62.4% (54.9% to 70.0%)
Upper respiratory infections	0.7% (0.1% to 2.7%)	1.6% (0.1% to 6.3%)	0.0% (0.0% to 0.0%)	0.0% (0.0% to 0.1%)
Otitis media	2.8% (1.4% to 4.5%)	5.3% (3.1% to 7.4%)	3.9% (2.6% to 5.3%)	3.9% (2.6% to 5.3%)
Meningitis	0.8% (0.5% to 1.3%)	1.6% (0.9% to 2.4%)	0.0% (0.0% to 0.0%)	1.4% (0.9% to 2.2%)
Encephalitis	0.7% (0.3% to 1.6%)	1.3% (0.6% to 2.2%)	0.0% (0.0% to 0.0%)	1.0% (0.4% to 1.9%)
Measles	19.5% (0.0% to 70.8%)	19.8% (0.0% to 71.8%)	18.5% (0.0% to 69.6%)	27.5% (0.0% to 72.4%)
**Maternal disorders**	3.5% (1.4% to 5.9%)	3.5% (1.4% to 5.8%)	2.8% (1.4% to 4.6%)	3.3% (1.4% to 5.6%)
Maternal haemorrhage	11.9% (4.2% to 19.2%)	11.8% (4.2% to 19.1%)	11.6% (4.1% to 18.8%)	11.8% (4.2% to 19.1%)
Maternal sepsis and other pregnancy related infections	11.8% (4.3% to 19.3%)	11.8% (4.2% to 19.1%)	11.4% (4.1% to 18.6%)	11.6% (4.2% to 18.9%)
Maternal abortion, miscarriage, and ectopic pregnancy	9.8% (5.4% to 15.2%)	9.7% (5.3% to 15.1%)	9.5% (4.7% to 15.4%)	9.6% (5.2% to 14.7%)
**Neonatal disorders**	73.5% (69.9% to 76.9%)	73.6% (70.0% to 77.0%)	0.1% (0.1% to 0.2%)	63.7% (58.0% to 68.5%)
Neonatal preterm birth complications	87.5% (83.1% to 93.0%)	87.5% (83.2% to 93.1%)	0.2% (0.2% to 0.3%)	77.1% (71.1% to 82.8%)
Neonatal encephalopathy due to birth asphyxia and trauma	60.6% (55.6% to 65.8%)	60.7% (55.7% to 65.8%)	0.0% (0.0% to 0.0%)	51.1% (39.9% to 59.9%)
Neonatal sepsis and other neonatal infections	55.2% (48.3% to 61.6%)	55.2% (48.4% to 61.6%)	0.0% (0.0% to 0.0%)	48.0% (36.9% to 58.3%)
Hemolytic disease and other neonatal jaundice	57.6% (51.1% to 63.9%)	57.7% (51.3% to 63.9%)	0.0% (0.0% to 0.0%)	4.0% (2.0% to 6.9%)

Supplementary Results Table 1. Age-standardised PAFs attributable to the joint distribution of all risk factors for all ages, both sexes combined for each GBD level 1 and level 2 cause and all causes in 2016 for El Salvador. Results are shown for deaths, YLLs, YLDs, and DALYs
Data in parenthesis are 95% uncertainty intervals. PAFs=population attributable fractions. GBD=Global Burden of Disease. YLLs=years of life lost. YLDs=years live with disability. DALYs=disability-adjusted life-years

	**Deaths 2016**	**YLLs 2016**	**YLDs 2016**	**DALYs 2016**
Other neonatal disorders	41.3% (33.2% to 49.6%)	41.3% (33.3% to 49.7%)	0.0% (0.0% to 0.0%)	39.2% (31.5% to 47.1%)
**Nutritional deficiencies**	7.2% (4.2% to 11.5%)	28.4% (18.8% to 40.6%)	72.8% (67.9% to 77.0%)	48.4% (41.3% to 56.1%)
Protein-energy malnutrition	7.0% (4.1% to 11.4%)	28.4% (18.6% to 40.6%)	96.1% (93.4% to 97.8%)	31.2% (22.0% to 42.5%)
Vitamin A deficiency	-	-	100.0% (100.0% to 100.0%)	100.0% (100.0% to 100.0%)
Iron-deficiency anaemia	100.0% (100.0% to 100.0%)	100.0% (100.0% to 100.0%)	100.0% (100.0% to 100.0%)	100.0% (100.0% to 100.0%)
**Other communicable, maternal, neonatal, and nutritional diseases**	6.3% (4.9% to 7.9%)	6.9% (4.4% to 8.9%)	79.2% (74.2% to 83.8%)	36.6% (26.1% to 49.8%)
Sexually transmitted diseases excluding HIV	65.6% (46.3% to 82.6%)	48.4% (29.0% to 69.7%)	100.0% (100.0% to 100.0%)	89.3% (78.6% to 96.2%)
Hepatitis	4.7% (3.5% to 6.3%)	4.4% (3.2% to 5.8%)	2.5% (1.5% to 3.9%)	4.3% (3.1% to 5.7%)
**Neoplasms**	32.6% (29.0% to 36.2%)	32.6% (29.1% to 35.9%)	32.5% (28.7% to 36.4%)	32.6% (29.1% to 35.9%)
Oesophageal cancer	65.3% (51.9% to 77.0%)	67.1% (54.2% to 78.2%)	65.7% (52.2% to 77.2%)	67.1% (54.1% to 78.2%)
Stomach cancer	6.7% (1.6% to 15.2%)	6.2% (1.6% to 13.7%)	6.5% (1.6% to 14.6%)	6.2% (1.6% to 13.7%)
Liver cancer	62.6% (54.9% to 70.3%)	62.4% (55.2% to 70.6%)	62.6% (55.1% to 70.4%)	62.4% (55.1% to 70.6%)
Larynx cancer	59.2% (45.6% to 71.1%)	60.7% (48.5% to 71.6%)	59.9% (47.3% to 71.4%)	60.6% (48.5% to 71.6%)
Tracheal, bronchus, and lung cancer	65.6% (57.1% to 73.3%)	65.2% (56.9% to 72.7%)	65.3% (56.6% to 73.0%)	65.2% (56.9% to 72.7%)
Breast cancer	23.8% (16.2% to 32.8%)	20.9% (13.4% to 29.0%)	22.2% (14.5% to 30.7%)	21.0% (13.5% to 29.1%)
Cervical cancer	100.0% (100.0% to 100.0%)	100.0% (100.0% to 100.0%)	100.0% (100.0% to 100.0%)	100.0% (100.0% to 100.0%)
Uterine cancer	48.6% (34.6% to 62.9%)	50.5% (36.1% to 64.6%)	50.8% (36.5% to 65.1%)	50.5% (36.1% to 64.6%)
Prostate cancer	0.8% (0.3% to 1.3%)	0.7% (0.3% to 1.2%)	0.8% (0.4% to 1.3%)	0.7% (0.3% to 1.2%)
Colon and rectum cancer	50.4% (40.8% to 59.6%)	51.0% (41.8% to 59.6%)	50.6% (41.2% to 59.6%)	50.9% (41.8% to 59.6%)
Lip and oral cavity cancer	57.9% (45.2% to 69.5%)	58.8% (47.2% to 69.2%)	55.6% (43.1% to 67.1%)	58.7% (47.1% to 69.0%)
Nasopharynx cancer	61.5% (50.6% to 70.6%)	59.6% (51.3% to 67.1%)	59.0% (50.3% to 66.6%)	59.6% (51.3% to 67.0%)

Supplementary Results Table 1. Age-standardised PAFs attributable to the joint distribution of all risk factors for all ages, both sexes combined for each GBD level 1 and level 2 cause and all causes in 2016 for El Salvador. Results are shown for deaths, YLLs, YLDs, and DALYs
Data in parenthesis are 95% uncertainty intervals. PAFs=population attributable fractions. GBD=Global Burden of Disease. YLLs=years of life lost. YLDs=years live with disability. DALYs=disability-adjusted life-years

	Deaths 2016	YLLs 2016	YLDs 2016	DALYs 2016
Other pharynx cancer	51.7% (36.6% to 64.5%)	52.6% (38.1% to 64.4%)	51.9% (36.9% to 64.8%)	52.6% (38.1% to 64.4%)
Gallbladder and biliary tract cancer	26.2% (14.9% to 39.3%)	28.0% (16.1% to 41.5%)	26.6% (15.2% to 40.1%)	27.9% (16.1% to 41.5%)
Pancreatic cancer	18.6% (10.7% to 29.1%)	18.5% (10.7% to 28.4%)	18.5% (10.6% to 29.0%)	18.5% (10.7% to 28.4%)
Ovarian cancer	11.1% (3.5% to 20.8%)	10.4% (3.2% to 19.4%)	10.0% (3.2% to 18.5%)	10.4% (3.2% to 19.4%)
Kidney cancer	25.1% (16.1% to 35.8%)	24.2% (15.4% to 34.2%)	25.5% (16.4% to 35.8%)	24.2% (15.4% to 34.3%)
Bladder cancer	13.7% (6.5% to 23.2%)	12.8% (6.2% to 21.7%)	13.0% (6.4% to 22.0%)	12.8% (6.2% to 21.7%)
Thyroid cancer	14.7% (7.9% to 23.5%)	15.5% (8.3% to 24.5%)	15.2% (8.3% to 23.6%)	15.5% (8.3% to 24.5%)
Mesothelioma	74.9% (67.9% to 81.7%)	65.0% (56.8% to 72.8%)	70.8% (63.3% to 77.9%)	65.1% (56.9% to 72.9%)
Non-Hodgkin's lymphoma	7.1% (2.8% to 12.3%)	6.7% (2.7% to 11.5%)	7.1% (2.9% to 12.3%)	6.7% (2.7% to 11.5%)
Multiple myeloma	9.3% (3.8% to 16.6%)	9.7% (4.1% to 17.2%)	9.5% (3.9% to 16.9%)	9.7% (4.1% to 17.2%)
Leukaemia	11.3% (6.9% to 16.7%)	9.8% (6.0% to 14.4%)	11.4% (7.0% to 16.6%)	9.9% (6.0% to 14.5%)
**Cardiovascular diseases**	86.4% (83.3% to 89.0%)	85.3% (83.3% to 87.2%)	70.8% (67.7% to 73.6%)	83.9% (81.7% to 85.7%)
Rheumatic heart disease	23.6% (15.9% to 38.5%)	19.7% (13.5% to 30.4%)	23.9% (16.1% to 35.8%)	19.7% (13.6% to 30.5%)
Ischaemic heart disease	91.8% (87.9% to 94.9%)	92.7% (90.3% to 94.6%)	90.6% (87.7% to 93.4%)	92.6% (90.2% to 94.6%)
Cerebrovascular disease	81.7% (76.7% to 86.0%)	81.4% (78.1% to 84.2%)	83.6% (79.5% to 87.3%)	81.6% (78.4% to 84.4%)
Hypertensive heart disease	100.0% (100.0% to 100.0%)	100.0% (100.0% to 100.0%)	100.0% (100.0% to 100.0%)	100.0% (100.0% to 100.0%)
Cardiomyopathy and myocarditis	25.8% (20.1% to 33.1%)	21.3% (16.5% to 28.9%)	18.7% (14.8% to 23.0%)	21.0% (16.5% to 28.1%)
Atrial fibrillation and flutter	50.0% (41.7% to 58.2%)	54.1% (46.6% to 61.5%)	60.0% (53.7% to 66.2%)	57.6% (51.0% to 64.1%)
Aortic aneurysm	37.0% (29.9% to 44.0%)	41.3% (35.4% to 47.0%)	-	41.3% (35.4% to 47.0%)
Peripheral vascular disease	51.2% (44.2% to 58.9%)	57.7% (52.1% to 63.7%)	61.9% (56.8% to 66.8%)	61.4% (56.4% to 66.3%)
Endocarditis	34.8% (26.6% to 44.7%)	27.7% (20.0% to 35.4%)	35.2% (27.0% to 43.3%)	27.8% (20.2% to 35.6%)

Supplementary Results Table 1. Age-standardised PAFs attributable to the joint distribution of all risk factors for all ages, both sexes combined for each GBD level 1 and level 2 cause and all causes in 2016 for El Salvador. Results are shown for deaths, YLLs, YLDs, and DALYs
Data in parenthesis are 95% uncertainty intervals. PAFs=population attributable fractions. GBD=Global Burden of Disease. YLLs=years of life lost. YLDs=years live with disability. DALYs=disability-adjusted life-years

	Deaths 2016	YLLs 2016	YLDs 2016	DALYs 2016
Other cardiovascular and circulatory diseases	39.0% (33.9% to 44.2%)	36.7% (31.9% to 41.6%)	36.2% (31.2% to 40.9%)	36.6% (32.1% to 41.1%)
**Chronic respiratory diseases**	51.7% (43.2% to 59.7%)	47.5% (39.9% to 54.3%)	33.3% (26.4% to 41.6%)	40.2% (33.5% to 46.7%)
Chronic obstructive pulmonary disease	60.7% (49.7% to 70.3%)	58.6% (48.4% to 68.1%)	52.6% (42.4% to 62.3%)	57.3% (47.2% to 66.5%)
Pneumoconiosis	100.0% (100.0% to 100.0%)	100.0% (100.0% to 100.0%)	100.0% (100.0% to 100.0%)	100.0% (100.0% to 100.0%)
Asthma	36.0% (25.1% to 48.6%)	38.4% (28.2% to 49.5%)	29.2% (20.5% to 39.3%)	30.7% (21.7% to 40.9%)
Other chronic respiratory diseases	2.5% (1.2% to 4.2%)	1.3% (0.5% to 2.6%)	1.2% (0.4% to 2.4%)	1.2% (0.5% to 2.4%)
**Cirrhosis and other chronic liver diseases**	72.0% (69.6% to 74.4%)	72.8% (70.9% to 74.7%)	63.0% (59.9% to 65.9%)	72.4% (70.5% to 74.3%)
Cirrhosis and other chronic liver diseases due to hepatitis B	0.7% (0.3% to 1.2%)	0.9% (0.3% to 1.5%)	0.6% (0.3% to 1.1%)	0.8% (0.3% to 1.5%)
Cirrhosis and other chronic liver diseases due to hepatitis C	84.1% (79.5% to 87.8%)	87.7% (85.1% to 89.5%)	83.8% (78.8% to 87.8%)	87.5% (85.0% to 89.4%)
Cirrhosis and other chronic liver diseases due to alcohol use	100.0% (100.0% to 100.0%)	100.0% (100.0% to 100.0%)	100.0% (100.0% to 100.0%)	100.0% (100.0% to 100.0%)
**Digestive diseases**	10.3% (7.7% to 13.0%)	11.3% (8.7% to 14.2%)	3.0% (2.2% to 4.0%)	9.1% (6.8% to 11.8%)
Peptic ulcer disease	4.8% (3.1% to 6.8%)	6.6% (4.3% to 9.2%)	3.4% (2.0% to 5.1%)	5.9% (3.8% to 8.2%)
Gallbladder and biliary diseases	44.9% (30.5% to 60.4%)	47.0% (32.4% to 62.1%)	51.7% (36.3% to 66.8%)	47.7% (33.2% to 62.9%)
Pancreatitis	39.3% (21.2% to 60.0%)	41.1% (25.3% to 58.2%)	33.3% (18.5% to 50.1%)	41.0% (25.2% to 58.0%)
**Neurological disorders**	22.3% (10.8% to 35.9%)	21.6% (11.7% to 33.5%)	4.6% (2.5% to 6.9%)	9.9% (5.5% to 15.1%)
Alzheimer's disease and other dementias	24.1% (11.5% to 39.1%)	25.2% (12.4% to 40.1%)	25.2% (12.3% to 40.0%)	25.2% (12.3% to 40.1%)
Parkinson's disease	-3.3% (-4.4% to -2.3%)	-4.1% (-5.6% to -2.9%)	-4.5% (-5.8% to -3.2%)	-4.2% (-5.6% to -3.0%)
Epilepsy	23.9% (16.7% to 31.5%)	20.8% (14.2% to 27.6%)	16.8% (11.2% to 22.4%)	18.0% (12.3% to 24.1%)
Multiple sclerosis	3.9% (2.2% to 6.1%)	4.2% (2.3% to 6.4%)	2.7% (1.6% to 4.0%)	3.6% (2.1% to 5.5%)
**Mental and substance use disorders**	99.9% (99.8% to 99.9%)	99.8% (99.7% to 99.9%)	23.4% (20.9% to 26.0%)	41.7% (36.5% to 48.5%)
Alcohol use disorders	100.0% (100.0% to 100.0%)	100.0% (100.0% to 100.0%)	100.0% (100.0% to 100.0%)	100.0% (100.0% to 100.0%)

Supplementary Results Table 1. Age-standardised PAFs attributable to the joint distribution of all risk factors for all ages, both sexes combined for each GBD level 1 and level 2 cause and all causes in 2016 for El Salvador. Results are shown for deaths, YLLs, YLDs, and DALYs
Data in parenthesis are 95% uncertainty intervals. PAFs=population attributable fractions. GBD=Global Burden of Disease. YLLs=years of life lost. YLDs=years live with disability. DALYs=disability-adjusted life-years

	Deaths 2016	YLLs 2016	YLDs 2016	DALYs 2016
Drug use disorders	100.0% (100.0% to 100.0%)	100.0% (100.0% to 100.0%)	100.0% (100.0% to 100.0%)	100.0% (100.0% to 100.0%)
Depressive disorders	-	-	9.5% (7.8% to 11.6%)	9.5% (7.8% to 11.6%)
Idiopathic developmental intellectual disability	-	-	71.0% (39.9% to 89.9%)	71.0% (39.9% to 89.9%)
**Diabetes, urogenital, blood, and endocrine diseases**	93.9% (91.5% to 94.6%)	93.5% (90.6% to 94.2%)	79.5% (75.8% to 82.7%)	89.6% (86.8% to 91.1%)
Diabetes mellitus	100.0% (100.0% to 100.0%)	100.0% (100.0% to 100.0%)	100.0% (100.0% to 100.0%)	100.0% (100.0% to 100.0%)
Chronic kidney disease	100.0% (100.0% to 100.0%)	100.0% (100.0% to 100.0%)	100.0% (100.0% to 100.0%)	100.0% (100.0% to 100.0%)
**Musculoskeletal disorders**	0.3% (0.1% to 0.5%)	0.2% (0.1% to 0.4%)	16.2% (13.0% to 20.0%)	15.7% (12.5% to 19.3%)
Rheumatoid arthritis	1.5% (0.6% to 2.7%)	1.9% (0.7% to 3.4%)	1.9% (0.7% to 3.4%)	1.9% (0.7% to 3.4%)
Osteoarthritis	-	-	29.6% (15.8% to 43.3%)	29.6% (15.8% to 43.3%)
Low back and neck pain	-	-	19.0% (16.2% to 21.9%)	19.0% (16.2% to 21.9%)
Gout	-	-	55.9% (42.8% to 69.3%)	55.9% (42.8% to 69.3%)
**Other non-communicable diseases**	0.3% (0.1% to 0.6%)	0.3% (0.2% to 0.8%)	5.8% (4.8% to 6.8%)	4.6% (3.8% to 5.5%)
Sense organ diseases	-	-	11.8% (10.1% to 13.6%)	11.8% (10.1% to 13.6%)
Sudden infant death syndrome	6.9% (4.2% to 11.1%)	7.0% (4.2% to 11.1%)	-	7.0% (4.2% to 11.1%)
**Transport injuries**	27.0% (19.7% to 35.7%)	21.4% (14.2% to 29.8%)	31.0% (24.0% to 39.2%)	22.3% (15.2% to 30.9%)
Road injuries	26.5% (19.2% to 35.2%)	21.2% (13.9% to 29.5%)	31.2% (24.2% to 39.4%)	22.2% (15.2% to 30.7%)
Other transport injuries	30.3% (22.4% to 39.0%)	22.8% (15.2% to 31.3%)	27.2% (19.8% to 35.6%)	23.0% (15.5% to 31.5%)
**Unintentional injuries**	30.8% (24.4% to 34.6%)	13.1% (10.1% to 16.1%)	16.2% (14.9% to 17.7%)	14.3% (12.1% to 16.4%)
Falls	53.8% (46.3% to 57.9%)	27.5% (20.7% to 30.8%)	21.1% (19.9% to 22.4%)	23.9% (20.9% to 25.8%)
Drowning	9.4% (4.7% to 15.8%)	8.4% (4.5% to 14.3%)	9.3% (4.2% to 15.9%)	8.5% (4.5% to 14.3%)
Fire, heat, and hot substances	7.6% (3.5% to 13.3%)	7.1% (3.5% to 12.3%)	8.0% (4.1% to 12.9%)	7.5% (3.8% to 12.5%)

Supplementary Results Table 1. Age-standardised PAFs attributable to the joint distribution of all risk factors for all ages, both sexes combined for each GBD level 1 and level 2 cause and all causes in 2016 for El Salvador. Results are shown for deaths, YLLs, YLDs, and DALYs
Data in parenthesis are 95% uncertainty intervals. PAFs=population attributable fractions. GBD=Global Burden of Disease. YLLs=years of life lost. YLDs=years live with disability. DALYs=disability-adjusted life-years

	Deaths 2016	YLLs 2016	YLDs 2016	DALYs 2016
Poisonings	9.3% (4.6% to 15.1%)	8.9% (4.5% to 14.4%)	7.7% (4.0% to 12.5%)	8.6% (4.4% to 13.8%)
Exposure to mechanical forces	10.8% (8.4% to 12.6%)	6.0% (4.7% to 7.6%)	9.5% (8.4% to 10.6%)	8.3% (7.0% to 9.4%)
Animal contact	4.7% (2.8% to 6.4%)	2.5% (1.7% to 3.2%)	2.1% (1.7% to 2.5%)	2.2% (1.8% to 2.6%)
Foreign body	1.1% (0.7% to 1.4%)	1.0% (0.6% to 1.4%)	1.1% (0.8% to 1.3%)	1.0% (0.7% to 1.4%)
Other unintentional injuries	10.8% (5.4% to 17.6%)	10.3% (5.3% to 17.1%)	9.0% (4.3% to 14.7%)	9.8% (5.0% to 16.2%)
**Self-harm and interpersonal violence**	19.3% (13.3% to 26.6%)	18.0% (11.9% to 25.2%)	20.8% (15.7% to 27.1%)	18.1% (12.0% to 25.2%)
Self-harm	25.5% (15.1% to 36.2%)	24.0% (14.1% to 35.2%)	22.8% (12.8% to 33.0%)	24.0% (14.1% to 35.2%)
Interpersonal violence	18.1% (12.0% to 26.2%)	17.0% (10.7% to 24.8%)	20.7% (15.6% to 27.2%)	17.1% (10.9% to 24.9%)
**Forces of nature, conflict and terrorism, and executions and police conflict**	0.0% (0.0% to 0.0%)	0.0% (0.0% to 0.0%)	0.6% (0.4% to 0.8%)	0.6% (0.4% to 0.8%)
Forces of nature, conflict and terrorism, and state actor violence	0.0% (0.0% to 0.0%)	0.0% (0.0% to 0.0%)	5.2% (3.5% to 7.5%)	5.2% (3.5% to 7.5%)

Supplementary Results Table 1. Age-standardised PAFs attributable to the joint distribution of all risk factors for all ages, both sexes combined for each GBD level 1 and level 2 cause and all causes in 2016 for Equatorial Guinea. Results are shown for deaths, YLLs, YLDs, and DALYs
Data in parenthesis are 95% uncertainty intervals. PAFs=population attributable fractions. GBD=Global Burden of Disease. YLLs=years of life lost. YLDs=years live with disability. DALYs=disability-adjusted life-years

	Deaths 2016	YLLs 2016	YLDs 2016	DALYs 2016
**All causes**	57.6% (53.7% to 61.1%)	53.8% (49.0% to 58.6%)	28.9% (26.9% to 31.2%)	47.4% (43.7% to 51.1%)
**HIV/AIDS and tuberculosis**	85.9% (81.1% to 89.7%)	86.1% (82.2% to 89.1%)	89.4% (86.1% to 92.6%)	86.3% (82.6% to 89.2%)
Tuberculosis	50.6% (36.7% to 63.2%)	53.6% (41.1% to 65.9%)	48.7% (37.2% to 60.9%)	53.3% (41.0% to 65.6%)
HIV/AIDS	93.7% (92.5% to 94.7%)	90.2% (87.9% to 92.0%)	93.0% (90.3% to 95.1%)	90.4% (88.3% to 92.1%)
**Diarrhoea, lower respiratory infections, and other common infectious diseases**	67.8% (57.2% to 77.0%)	64.4% (54.8% to 73.1%)	47.9% (39.5% to 54.1%)	62.5% (54.1% to 70.6%)
Diarrhoeal diseases	90.8% (76.0% to 97.7%)	92.2% (78.4% to 98.1%)	91.8% (78.4% to 98.0%)	92.0% (78.6% to 98.0%)
Lower respiratory infections	72.6% (60.5% to 81.0%)	78.8% (68.9% to 85.7%)	74.7% (63.2% to 82.5%)	78.8% (68.8% to 85.7%)
Upper respiratory infections	2.7% (0.2% to 10.5%)	5.8% (0.4% to 20.2%)	0.0% (0.0% to 0.0%)	0.0% (0.0% to 0.1%)
Otitis media	0.6% (0.1% to 1.8%)	2.1% (0.7% to 4.4%)	2.3% (1.4% to 3.1%)	2.3% (1.4% to 3.1%)
Meningitis	3.8% (1.5% to 7.3%)	7.0% (3.1% to 13.1%)	0.0% (0.0% to 0.0%)	6.3% (2.8% to 11.7%)
Encephalitis	1.2% (0.6% to 2.1%)	2.3% (1.3% to 3.6%)	0.0% (0.0% to 0.0%)	1.2% (0.7% to 1.9%)
Measles	32.9% (13.0% to 69.9%)	33.8% (13.5% to 71.4%)	35.7% (14.0% to 73.5%)	33.8% (13.5% to 71.4%)
**Maternal disorders**	4.6% (2.1% to 7.5%)	4.7% (2.2% to 7.6%)	1.9% (0.9% to 3.0%)	4.5% (2.1% to 7.2%)
Maternal haemorrhage	13.4% (4.8% to 22.6%)	13.8% (5.0% to 23.1%)	14.8% (5.4% to 24.6%)	13.8% (5.0% to 23.2%)
Maternal sepsis and other pregnancy related infections	12.5% (4.4% to 21.3%)	13.2% (4.6% to 22.1%)	14.4% (5.2% to 23.6%)	13.2% (4.6% to 22.1%)
Maternal abortion, miscarriage, and ectopic pregnancy	21.7% (14.3% to 29.9%)	21.6% (14.1% to 29.3%)	21.7% (12.7% to 31.0%)	21.6% (14.1% to 29.4%)
**Neonatal disorders**	81.7% (77.9% to 85.4%)	81.7% (77.9% to 85.4%)	0.5% (0.2% to 1.0%)	78.6% (74.3% to 82.6%)
Neonatal preterm birth complications	98.4% (96.4% to 99.4%)	98.4% (96.4% to 99.4%)	1.6% (1.0% to 2.4%)	94.9% (91.7% to 97.1%)
Neonatal encephalopathy due to birth asphyxia and trauma	74.7% (71.4% to 78.5%)	74.7% (71.4% to 78.5%)	0.0% (0.0% to 0.0%)	73.3% (68.7% to 77.6%)
Neonatal sepsis and other neonatal infections	71.5% (67.4% to 75.2%)	71.5% (67.4% to 75.2%)	0.0% (0.0% to 0.0%)	66.6% (57.8% to 72.6%)
Hemolytic disease and other neonatal jaundice	73.7% (69.6% to 77.7%)	73.7% (69.7% to 77.8%)	0.0% (0.0% to 0.0%)	62.3% (53.4% to 69.5%)

**Supplementary Results Table 1. Age-standardised PAFs attributable to the joint distribution of all risk factors for all ages, both sexes combined for each GBD level 1 and level 2 cause and all causes in 2016 for Equatorial Guinea. Results are shown for deaths, YLLs, YLDs, and DALYs** Data in parenthesis are 95% uncertainty intervals. PAFs=population attributable fractions. GBD=Global Burden of Disease. YLLs=years of life lost. YLDs=years live with disability. DALYs=disability-adjusted life-years				
	**Deaths 2016**	**YLLs 2016**	**YLDs 2016**	**DALYs 2016**
Other neonatal disorders	73.1% (69.2% to 76.9%)	73.1% (69.3% to 76.9%)	0.0% (0.0% to 0.0%)	71.9% (67.9% to 75.8%)
**Nutritional deficiencies**	12.6% (4.4% to 23.0%)	41.7% (18.0% to 61.7%)	73.2% (66.9% to 79.1%)	57.9% (36.7% to 69.3%)
Protein-energy malnutrition	15.9% (5.4% to 28.0%)	49.1% (22.6% to 68.8%)	97.1% (96.1% to 97.9%)	53.1% (25.8% to 71.9%)
Vitamin A deficiency	-	-	100.0% (100.0% to 100.0%)	100.0% (100.0% to 100.0%)
Iron-deficiency anaemia	100.0% (100.0% to 100.0%)	100.0% (100.0% to 100.0%)	100.0% (100.0% to 100.0%)	100.0% (100.0% to 100.0%)
**Other communicable, maternal, neonatal, and nutritional diseases**	4.2% (2.1% to 7.6%)	3.3% (1.2% to 6.5%)	74.9% (70.3% to 79.1%)	20.1% (10.1% to 32.6%)
Sexually transmitted diseases excluding HIV	25.8% (8.5% to 51.5%)	11.6% (3.2% to 27.9%)	100.0% (100.0% to 100.0%)	49.2% (23.9% to 73.3%)
Hepatitis	0.0% (0.0% to 0.0%)	0.0% (0.0% to 0.1%)	0.0% (0.0% to 0.1%)	0.0% (0.0% to 0.1%)
**Neoplasms**	38.7% (31.7% to 45.2%)	39.7% (33.3% to 46.2%)	35.7% (29.5% to 41.8%)	39.6% (33.2% to 46.1%)
Oesophageal cancer	72.4% (58.1% to 83.2%)	74.4% (60.6% to 84.6%)	73.2% (59.6% to 83.9%)	74.4% (60.6% to 84.6%)
Stomach cancer	10.3% (2.5% to 22.0%)	10.3% (2.7% to 21.1%)	10.3% (2.6% to 21.8%)	10.3% (2.7% to 21.1%)
Liver cancer	45.1% (34.2% to 57.4%)	45.6% (34.4% to 58.0%)	45.5% (34.5% to 57.5%)	45.6% (34.4% to 58.0%)
Larynx cancer	67.7% (48.6% to 82.4%)	70.0% (51.7% to 84.0%)	69.2% (51.0% to 83.3%)	70.0% (51.7% to 84.0%)
Tracheal, bronchus, and lung cancer	82.3% (70.1% to 90.2%)	82.4% (70.0% to 90.3%)	82.2% (70.3% to 90.3%)	82.4% (70.0% to 90.3%)
Breast cancer	27.7% (16.9% to 41.8%)	25.0% (12.9% to 40.4%)	25.1% (13.6% to 40.3%)	25.0% (12.9% to 40.4%)
Cervical cancer	100.0% (100.0% to 100.0%)	100.0% (100.0% to 100.0%)	100.0% (100.0% to 100.0%)	100.0% (100.0% to 100.0%)
Uterine cancer	51.9% (37.1% to 66.1%)	52.5% (37.4% to 66.4%)	52.5% (37.4% to 66.4%)	52.5% (37.4% to 66.4%)
Prostate cancer	1.5% (0.3% to 2.9%)	1.7% (0.5% to 3.4%)	1.7% (0.5% to 3.4%)	1.7% (0.5% to 3.4%)
Colon and rectum cancer	53.5% (43.7% to 62.4%)	54.3% (44.9% to 63.3%)	54.0% (44.7% to 63.0%)	54.3% (44.9% to 63.3%)
Lip and oral cavity cancer	73.5% (60.0% to 84.2%)	76.5% (64.1% to 86.1%)	74.8% (62.6% to 84.6%)	76.5% (64.1% to 86.1%)
Nasopharynx cancer	70.2% (55.7% to 80.6%)	70.8% (57.6% to 80.6%)	68.5% (56.9% to 78.0%)	70.8% (57.5% to 80.5%)

Supplementary Results Table 1. Age-standardised PAFs attributable to the joint distribution of all risk factors for all ages, both sexes combined for each GBD level 1 and level 2 cause and all causes in 2016 for Equatorial Guinea. Results are shown for deaths, YLLs, YLDs, and DALYs
Data in parenthesis are 95% uncertainty intervals. PAFs=population attributable fractions. GBD=Global Burden of Disease. YLLs=years of life lost. YLDs=years live with disability. DALYs=disability-adjusted life-years

	Deaths 2016	YLLs 2016	YLDs 2016	DALYs 2016
Other pharynx cancer	57.1% (40.2% to 70.0%)	60.0% (43.8% to 72.1%)	58.6% (42.0% to 70.9%)	60.0% (43.8% to 72.1%)
Gallbladder and biliary tract cancer	27.5% (15.7% to 40.6%)	28.2% (16.1% to 41.8%)	27.7% (15.8% to 41.1%)	28.2% (16.1% to 41.8%)
Pancreatic cancer	24.9% (14.1% to 36.9%)	25.0% (14.3% to 36.8%)	25.0% (14.2% to 36.8%)	25.0% (14.3% to 36.8%)
Ovarian cancer	13.2% (5.0% to 23.7%)	12.0% (4.3% to 21.6%)	11.6% (4.2% to 21.1%)	12.0% (4.3% to 21.6%)
Kidney cancer	30.3% (20.4% to 42.1%)	29.6% (19.6% to 41.2%)	28.3% (18.7% to 39.8%)	29.5% (19.6% to 41.1%)
Bladder cancer	20.2% (8.9% to 33.2%)	21.2% (9.5% to 34.1%)	20.9% (9.5% to 33.9%)	21.2% (9.5% to 34.1%)
Thyroid cancer	17.8% (9.4% to 28.2%)	18.1% (9.6% to 28.7%)	17.4% (9.8% to 26.8%)	18.1% (9.6% to 28.5%)
Mesothelioma	86.3% (79.3% to 91.8%)	77.3% (67.6% to 85.5%)	82.2% (73.6% to 89.1%)	77.4% (67.7% to 85.6%)
Non-Hodgkin's lymphoma	8.6% (3.7% to 14.3%)	8.2% (3.5% to 13.7%)	8.2% (3.4% to 13.9%)	8.2% (3.5% to 13.7%)
Multiple myeloma	10.2% (4.6% to 17.6%)	10.5% (4.7% to 17.9%)	10.4% (4.6% to 18.0%)	10.5% (4.7% to 17.9%)
Leukaemia	14.2% (8.5% to 21.4%)	12.1% (7.0% to 18.3%)	12.7% (7.7% to 18.9%)	12.1% (7.0% to 18.3%)
**Cardiovascular diseases**	82.2% (78.8% to 85.1%)	83.2% (80.5% to 85.6%)	68.8% (65.0% to 72.4%)	81.7% (78.7% to 84.3%)
Rheumatic heart disease	26.6% (18.0% to 42.1%)	26.7% (18.8% to 39.6%)	27.0% (18.2% to 39.9%)	26.7% (18.7% to 39.6%)
Ischaemic heart disease	92.4% (88.8% to 95.3%)	94.1% (91.5% to 96.1%)	90.1% (87.1% to 93.1%)	93.9% (91.3% to 96.0%)
Cerebrovascular disease	85.1% (80.2% to 89.3%)	88.5% (84.8% to 91.4%)	86.0% (81.8% to 90.2%)	88.2% (84.6% to 91.2%)
Hypertensive heart disease	100.0% (100.0% to 100.0%)	100.0% (100.0% to 100.0%)	100.0% (100.0% to 100.0%)	100.0% (100.0% to 100.0%)
Cardiomyopathy and myocarditis	42.7% (34.1% to 52.5%)	43.5% (35.5% to 51.9%)	45.6% (38.5% to 52.7%)	43.7% (35.8% to 51.6%)
Atrial fibrillation and flutter	50.3% (41.4% to 59.6%)	54.4% (45.8% to 62.5%)	60.8% (53.7% to 68.1%)	57.8% (50.5% to 65.2%)
Aortic aneurysm	35.9% (28.7% to 43.0%)	40.1% (33.5% to 46.3%)	-	40.1% (33.5% to 46.3%)
Peripheral vascular disease	51.8% (45.2% to 58.6%)	58.3% (52.8% to 63.9%)	60.1% (54.8% to 65.3%)	59.3% (54.2% to 64.3%)
Endocarditis	38.1% (28.7% to 49.1%)	39.4% (30.8% to 48.4%)	34.6% (25.5% to 44.9%)	39.4% (30.8% to 48.4%)

Supplementary Results Table 1. Age-standardised PAFs attributable to the joint distribution of all risk factors for all ages, both sexes combined for each GBD level 1 and level 2 cause and all causes in 2016 for Equatorial Guinea. Results are shown for deaths, YLLs, YLDs, and DALYs
Data in parenthesis are 95% uncertainty intervals. PAFs=population attributable fractions. GBD=Global Burden of Disease. YLLs=years of life lost. YLDs=years live with disability. DALYs=disability-adjusted life-years

	Deaths 2016	YLLs 2016	YLDs 2016	DALYs 2016
Other cardiovascular and circulatory diseases	39.4% (34.2% to 44.7%)	42.9% (38.3% to 47.7%)	36.1% (31.0% to 41.4%)	41.2% (36.6% to 46.1%)
**Chronic respiratory diseases**	67.2% (55.6% to 75.7%)	65.1% (55.0% to 73.2%)	50.5% (41.4% to 59.8%)	58.0% (48.2% to 66.9%)
Chronic obstructive pulmonary disease	77.2% (62.8% to 87.3%)	77.8% (63.5% to 87.4%)	71.1% (56.9% to 81.8%)	75.4% (61.0% to 85.7%)
Pneumoconiosis	100.0% (100.0% to 100.0%)	100.0% (100.0% to 100.0%)	100.0% (100.0% to 100.0%)	100.0% (100.0% to 100.0%)
Asthma	39.3% (26.9% to 52.8%)	40.6% (29.1% to 53.3%)	35.8% (24.6% to 48.2%)	37.3% (25.9% to 49.5%)
Other chronic respiratory diseases	6.5% (2.4% to 12.7%)	6.0% (1.9% to 12.1%)	3.8% (1.1% to 7.7%)	5.1% (1.6% to 10.6%)
**Cirrhosis and other chronic liver diseases**	28.7% (26.8% to 30.6%)	27.6% (25.6% to 29.6%)	22.0% (19.4% to 24.7%)	27.4% (25.5% to 29.3%)
Cirrhosis and other chronic liver diseases due to hepatitis B	0.0% (0.0% to 0.0%)	0.0% (0.0% to 0.0%)	0.0% (0.0% to 0.0%)	0.0% (0.0% to 0.0%)
Cirrhosis and other chronic liver diseases due to hepatitis C	0.6% (0.3% to 1.1%)	0.9% (0.4% to 1.8%)	1.3% (0.5% to 2.5%)	0.9% (0.4% to 1.8%)
Cirrhosis and other chronic liver diseases due to alcohol use	100.0% (100.0% to 100.0%)	100.0% (100.0% to 100.0%)	100.0% (100.0% to 100.0%)	100.0% (100.0% to 100.0%)
**Digestive diseases**	7.0% (4.9% to 9.8%)	8.0% (5.6% to 11.4%)	3.1% (2.3% to 4.2%)	7.5% (5.4% to 10.7%)
Peptic ulcer disease	1.6% (1.0% to 2.5%)	2.2% (1.3% to 3.3%)	2.1% (1.3% to 3.0%)	2.2% (1.3% to 3.2%)
Gallbladder and biliary diseases	47.2% (32.7% to 61.9%)	47.4% (33.1% to 62.2%)	48.7% (34.9% to 62.8%)	47.5% (33.1% to 62.3%)
Pancreatitis	55.0% (29.4% to 77.4%)	61.4% (36.9% to 80.9%)	57.5% (33.5% to 77.5%)	61.4% (36.9% to 80.9%)
**Neurological disorders**	26.2% (12.8% to 41.5%)	25.6% (13.9% to 38.8%)	6.7% (3.7% to 10.6%)	14.5% (8.3% to 22.0%)
Alzheimer's disease and other dementias	27.9% (13.4% to 44.8%)	28.6% (13.7% to 45.7%)	28.7% (13.7% to 45.6%)	28.6% (13.7% to 45.6%)
Parkinson's disease	-0.9% (-1.3% to -0.6%)	-1.2% (-1.7% to -0.9%)	-1.4% (-1.9% to -1.0%)	-1.2% (-1.7% to -0.9%)
Epilepsy	28.6% (19.0% to 39.7%)	27.2% (18.2% to 38.0%)	23.6% (15.3% to 33.6%)	25.2% (16.7% to 35.5%)
Multiple sclerosis	1.3% (0.7% to 2.0%)	1.4% (0.7% to 2.2%)	1.2% (0.7% to 1.9%)	1.3% (0.7% to 2.0%)
**Mental and substance use disorders**	99.8% (99.6% to 99.9%)	99.7% (99.5% to 99.8%)	18.1% (16.1% to 20.2%)	21.8% (18.8% to 25.3%)
Alcohol use disorders	100.0% (100.0% to 100.0%)	100.0% (100.0% to 100.0%)	100.0% (100.0% to 100.0%)	100.0% (100.0% to 100.0%)

Supplementary Results Table 1. Age-standardised PAFs attributable to the joint distribution of all risk factors for all ages, both sexes combined for each GBD level 1 and level 2 cause and all causes in 2016 for Equatorial Guinea. Results are shown for deaths, YLLs, YLDs, and DALYs
Data in parenthesis are 95% uncertainty intervals. PAFs=population attributable fractions. GBD=Global Burden of Disease. YLLs=years of life lost. YLDs=years live with disability. DALYs=disability-adjusted life-years

	Deaths 2016	YLLs 2016	YLDs 2016	DALYs 2016
Drug use disorders	100.0% (100.0% to 100.0%)	100.0% (100.0% to 100.0%)	100.0% (100.0% to 100.0%)	100.0% (100.0% to 100.0%)
Depressive disorders	-	-	9.2% (7.6% to 10.9%)	9.2% (7.6% to 10.9%)
Idiopathic developmental intellectual disability	-	-	62.1% (30.8% to 81.4%)	62.1% (30.8% to 81.4%)
**Diabetes, urogenital, blood, and endocrine diseases**	84.3% (81.0% to 87.0%)	79.6% (74.7% to 83.6%)	67.0% (62.9% to 70.6%)	73.5% (69.3% to 77.5%)
Diabetes mellitus	100.0% (100.0% to 100.0%)	100.0% (100.0% to 100.0%)	100.0% (100.0% to 100.0%)	100.0% (100.0% to 100.0%)
Chronic kidney disease	100.0% (100.0% to 100.0%)	100.0% (100.0% to 100.0%)	100.0% (100.0% to 100.0%)	100.0% (100.0% to 100.0%)
**Musculoskeletal disorders**	0.1% (0.0% to 0.2%)	0.1% (0.0% to 0.2%)	19.2% (15.6% to 23.2%)	18.9% (15.3% to 22.8%)
Rheumatoid arthritis	0.5% (0.2% to 0.9%)	0.6% (0.2% to 1.1%)	0.8% (0.3% to 1.3%)	0.7% (0.3% to 1.3%)
Osteoarthritis	-	-	32.0% (18.6% to 45.2%)	32.0% (18.6% to 45.2%)
Low back and neck pain	-	-	20.5% (17.5% to 23.8%)	20.5% (17.5% to 23.8%)
Gout	-	-	59.2% (45.1% to 73.4%)	59.2% (45.1% to 73.4%)
**Other non-communicable diseases**	0.2% (0.1% to 0.3%)	0.3% (0.1% to 0.5%)	5.6% (4.5% to 6.6%)	4.3% (3.4% to 5.2%)
Sense organ diseases	-	-	11.9% (9.9% to 13.9%)	11.9% (9.9% to 13.9%)
Sudden infant death syndrome	8.3% (3.2% to 16.2%)	8.3% (3.3% to 16.2%)	-	8.3% (3.3% to 16.2%)
**Transport injuries**	38.8% (29.9% to 48.9%)	33.7% (25.3% to 43.1%)	43.4% (34.7% to 53.2%)	35.4% (26.6% to 45.2%)
Road injuries	38.6% (29.8% to 48.5%)	33.5% (25.2% to 42.9%)	45.3% (36.4% to 55.3%)	35.3% (26.6% to 45.1%)
Other transport injuries	43.3% (33.1% to 54.0%)	37.8% (27.8% to 48.6%)	35.9% (27.5% to 45.7%)	36.8% (27.8% to 46.7%)
**Unintentional injuries**	36.6% (28.0% to 43.6%)	22.3% (17.8% to 28.0%)	25.1% (19.7% to 32.6%)	23.4% (18.8% to 29.7%)
Falls	68.3% (48.5% to 76.1%)	46.2% (37.4% to 53.6%)	32.0% (26.5% to 39.5%)	38.4% (33.1% to 44.5%)
Drowning	19.0% (13.3% to 26.0%)	16.3% (11.3% to 22.8%)	21.6% (14.4% to 30.4%)	16.4% (11.4% to 23.0%)
Fire, heat, and hot substances	14.7% (9.1% to 21.8%)	17.0% (10.7% to 24.2%)	22.9% (15.3% to 33.1%)	18.8% (12.2% to 26.2%)

Supplementary Results Table 1. Age-standardised PAFs attributable to the joint distribution of all risk factors for all ages, both sexes combined for each GBD level 1 and level 2 cause and all causes in 2016 for Equatorial Guinea. Results are shown for deaths, YLLs, YLDs, and DALYs
Data in parenthesis are 95% uncertainty intervals. PAFs=population attributable fractions. GBD=Global Burden of Disease. YLLs=years of life lost. YLDs=years live with disability. DALYs=disability-adjusted life-years

	Deaths 2016	YLLs 2016	YLDs 2016	DALYs 2016
Poisonings	18.2% (11.3% to 26.9%)	18.5% (10.7% to 28.2%)	21.7% (14.4% to 31.5%)	19.0% (11.4% to 28.2%)
Exposure to mechanical forces	26.3% (20.3% to 34.5%)	20.6% (14.3% to 30.1%)	26.9% (19.2% to 37.3%)	23.4% (16.6% to 32.9%)
Animal contact	13.6% (9.0% to 20.1%)	15.2% (9.8% to 23.1%)	15.4% (9.2% to 24.0%)	15.3% (9.9% to 23.3%)
Foreign body	10.1% (5.4% to 16.3%)	10.5% (4.4% to 17.8%)	13.1% (7.4% to 20.9%)	11.3% (5.7% to 18.6%)
Other unintentional injuries	20.1% (13.3% to 28.3%)	22.1% (15.1% to 30.7%)	21.0% (14.0% to 29.8%)	21.5% (14.6% to 30.3%)
**Self-harm and interpersonal violence**	27.0% (16.9% to 37.3%)	27.1% (17.7% to 36.4%)	25.8% (20.6% to 32.0%)	27.0% (18.0% to 35.7%)
Self-harm	28.4% (15.5% to 42.0%)	30.6% (17.7% to 43.2%)	29.6% (16.4% to 43.1%)	30.6% (17.7% to 43.2%)
Interpersonal violence	23.6% (14.8% to 34.4%)	22.1% (13.5% to 32.8%)	25.1% (20.1% to 31.3%)	22.8% (14.9% to 32.4%)
**Forces of nature, conflict and terrorism, and executions and police conflict**	0.0% (0.0% to 0.0%)	0.0% (0.0% to 0.0%)	0.0% (0.0% to 0.0%)	0.0% (0.0% to 0.0%)
Forces of nature, conflict and terrorism, and state actor violence	0.0% (0.0% to 0.0%)	0.0% (0.0% to 0.0%)	0.0% (0.0% to 0.0%)	0.0% (0.0% to 0.0%)

Supplementary Results Table 1. Age-standardised PAFs attributable to the joint distribution of all risk factors for all ages, both sexes combined for each GBD level 1 and level 2 cause and all causes in 2016 for Eritrea. Results are shown for deaths, YLLs, YLDs, and DALYs

Data in parenthesis are 95% uncertainty intervals. PAFs=population attributable fractions. GBD=Global Burden of Disease. YLLs=years of life lost. YLDs=years live with disability. DALYs=disability-adjusted life-years

	Deaths 2016	YLLs 2016	YLDs 2016	DALYs 2016
**All causes**	51.8% (48.2% to 55.7%)	50.5% (47.2% to 54.3%)	26.4% (25.0% to 27.7%)	45.2% (42.1% to 48.8%)
**HIV/AIDS and tuberculosis**	30.1% (18.6% to 42.4%)	35.2% (23.9% to 47.2%)	56.8% (45.2% to 69.1%)	35.8% (24.5% to 47.7%)
Tuberculosis	21.8% (9.8% to 35.1%)	23.0% (10.7% to 36.8%)	22.0% (9.8% to 36.2%)	23.0% (10.6% to 36.7%)
HIV/AIDS	87.3% (83.8% to 90.7%)	81.5% (76.3% to 86.5%)	90.7% (87.8% to 93.4%)	82.1% (77.2% to 86.9%)
**Diarrhoea, lower respiratory infections, and other common infectious diseases**	72.3% (63.3% to 79.7%)	72.6% (65.6% to 78.8%)	46.5% (41.0% to 51.2%)	71.3% (64.3% to 77.4%)
Diarrhoeal diseases	95.0% (86.4% to 98.8%)	96.1% (89.3% to 99.1%)	95.3% (87.6% to 98.9%)	96.1% (89.2% to 99.1%)
Lower respiratory infections	69.8% (55.7% to 80.3%)	78.2% (67.1% to 86.2%)	70.9% (57.5% to 81.1%)	78.2% (67.1% to 86.2%)
Upper respiratory infections	0.9% (0.1% to 3.2%)	1.8% (0.1% to 6.6%)	0.0% (0.0% to 0.0%)	0.2% (0.0% to 0.6%)
Otitis media	1.6% (0.5% to 3.6%)	4.2% (1.8% to 7.6%)	3.1% (2.0% to 4.3%)	3.2% (2.0% to 4.5%)
Meningitis	1.5% (0.7% to 3.0%)	3.2% (1.7% to 5.9%)	0.0% (0.0% to 0.0%)	3.0% (1.6% to 5.6%)
Encephalitis	2.7% (1.7% to 4.0%)	3.6% (2.5% to 5.4%)	0.0% (0.0% to 0.0%)	2.1% (1.3% to 3.4%)
Measles	68.7% (53.4% to 84.8%)	69.8% (54.2% to 86.0%)	68.0% (52.8% to 82.7%)	69.8% (54.2% to 86.0%)
**Maternal disorders**	10.4% (6.2% to 14.8%)	10.3% (6.1% to 14.7%)	2.7% (1.4% to 4.4%)	10.1% (6.0% to 14.3%)
Maternal haemorrhage	17.7% (6.4% to 29.5%)	17.7% (6.4% to 29.5%)	17.9% (6.4% to 30.0%)	17.7% (6.4% to 29.6%)
Maternal sepsis and other pregnancy related infections	17.7% (6.3% to 29.6%)	17.7% (6.3% to 29.6%)	17.1% (6.2% to 28.7%)	17.7% (6.3% to 29.6%)
Maternal abortion, miscarriage, and ectopic pregnancy	18.4% (11.0% to 26.7%)	18.0% (10.5% to 26.0%)	18.7% (10.3% to 27.9%)	18.0% (10.5% to 26.0%)
**Neonatal disorders**	78.1% (73.2% to 82.2%)	78.1% (73.3% to 82.2%)	0.3% (0.1% to 0.5%)	71.0% (64.9% to 76.9%)
Neonatal preterm birth complications	93.9% (88.0% to 97.4%)	93.9% (88.1% to 97.5%)	1.0% (0.7% to 1.3%)	86.5% (78.6% to 91.6%)
Neonatal encephalopathy due to birth asphyxia and trauma	76.6% (71.4% to 80.8%)	76.6% (71.5% to 80.9%)	0.0% (0.0% to 0.0%)	68.0% (55.4% to 77.5%)
Neonatal sepsis and other neonatal infections	65.4% (55.5% to 72.1%)	65.5% (55.6% to 72.1%)	0.0% (0.0% to 0.0%)	58.3% (45.6% to 67.9%)
Hemolytic disease and other neonatal jaundice	71.0% (61.3% to 77.1%)	71.0% (61.4% to 77.1%)	0.0% (0.0% to 0.0%)	60.3% (49.3% to 69.6%)

Supplementary Results Table 1. Age-standardised PAFs attributable to the joint distribution of all risk factors for all ages, both sexes combined for each GBD level 1 and level 2 cause and all causes in 2016 for Eritrea. Results are shown for deaths, YLLs, YLDs, and DALYs
Data in parenthesis are 95% uncertainty intervals. PAFs=population attributable fractions. GBD=Global Burden of Disease. YLLs=years of life lost. YLDs=years live with disability. DALYs=disability-adjusted life-years

	Deaths 2016	YLLs 2016	YLDs 2016	DALYs 2016
Other neonatal disorders	69.5% (60.9% to 75.8%)	69.6% (61.0% to 75.8%)	0.0% (0.0% to 0.0%)	67.0% (58.4% to 73.4%)
**Nutritional deficiencies**	19.3% (12.3% to 28.2%)	50.7% (37.9% to 63.3%)	82.6% (79.0% to 85.6%)	61.6% (53.6% to 69.0%)
Protein-energy malnutrition	23.5% (14.7% to 34.7%)	57.0% (43.6% to 69.9%)	98.1% (97.2% to 98.8%)	58.2% (45.5% to 70.4%)
Vitamin A deficiency	-	-	100.0% (100.0% to 100.0%)	100.0% (100.0% to 100.0%)
Iron-deficiency anaemia	100.0% (100.0% to 100.0%)	100.0% (100.0% to 100.0%)	100.0% (100.0% to 100.0%)	100.0% (100.0% to 100.0%)
**Other communicable, maternal, neonatal, and nutritional diseases**	7.5% (4.6% to 10.5%)	5.2% (2.5% to 8.4%)	73.0% (65.2% to 80.3%)	15.7% (7.9% to 25.1%)
Sexually transmitted diseases excluding HIV	30.9% (14.7% to 54.0%)	15.5% (6.3% to 31.3%)	100.0% (100.0% to 100.0%)	38.6% (19.0% to 63.5%)
Hepatitis	0.3% (0.2% to 0.4%)	0.4% (0.2% to 0.5%)	0.3% (0.2% to 0.5%)	0.4% (0.2% to 0.5%)
**Neoplasms**	30.2% (25.9% to 34.8%)	29.7% (25.1% to 34.0%)	32.3% (27.2% to 37.6%)	29.7% (25.1% to 34.1%)
Oesophageal cancer	50.0% (33.7% to 64.2%)	52.4% (36.9% to 66.3%)	50.9% (35.2% to 64.9%)	52.4% (36.9% to 66.3%)
Stomach cancer	4.6% (1.0% to 12.5%)	4.5% (1.1% to 12.0%)	4.6% (1.1% to 12.2%)	4.5% (1.1% to 12.0%)
Liver cancer	20.8% (16.1% to 26.8%)	20.9% (16.1% to 26.9%)	20.9% (16.2% to 27.0%)	20.9% (16.1% to 26.9%)
Larynx cancer	43.3% (29.2% to 57.2%)	45.3% (29.9% to 60.0%)	43.9% (29.5% to 58.2%)	45.3% (29.8% to 60.0%)
Tracheal, bronchus, and lung cancer	73.4% (61.8% to 82.4%)	73.5% (62.1% to 82.3%)	73.3% (61.5% to 82.2%)	73.5% (62.1% to 82.3%)
Breast cancer	15.3% (9.7% to 21.6%)	14.5% (9.3% to 20.2%)	14.8% (9.4% to 20.9%)	14.5% (9.3% to 20.2%)
Cervical cancer	100.0% (100.0% to 100.0%)	100.0% (100.0% to 100.0%)	100.0% (100.0% to 100.0%)	100.0% (100.0% to 100.0%)
Uterine cancer	19.4% (9.3% to 31.1%)	20.0% (9.7% to 31.7%)	19.9% (9.5% to 31.6%)	20.0% (9.7% to 31.7%)
Prostate cancer	0.8% (0.4% to 1.3%)	0.9% (0.4% to 1.4%)	0.8% (0.4% to 1.4%)	0.9% (0.4% to 1.4%)
Colon and rectum cancer	44.5% (35.1% to 54.2%)	44.0% (34.7% to 53.3%)	44.4% (35.0% to 54.0%)	44.0% (34.7% to 53.3%)
Lip and oral cavity cancer	39.2% (25.3% to 53.6%)	41.1% (26.4% to 56.3%)	38.6% (24.5% to 53.5%)	41.0% (26.4% to 56.3%)
Nasopharynx cancer	38.4% (25.6% to 51.7%)	39.6% (26.6% to 53.3%)	39.2% (26.3% to 52.7%)	39.6% (26.6% to 53.3%)

**Supplementary Results Table 1. Age-standardised PAFs attributable to the joint distribution of all risk factors for all ages, both sexes combined for each GBD level 1 and level 2 cause and all causes in 2016 for Eritrea. Results are shown for deaths, YLLs, YLDs, and DALYs** Data in parenthesis are 95% uncertainty intervals. PAFs=population attributable fractions. GBD=Global Burden of Disease. YLLs=years of life lost. YLDs=years live with disability. DALYs=disability-adjusted life-years				
	**Deaths 2016**	**YLLs 2016**	**YLDs 2016**	**DALYs 2016**
Other pharynx cancer	30.0% (15.5% to 46.2%)	31.5% (16.5% to 48.1%)	30.5% (15.9% to 46.9%)	31.5% (16.5% to 48.1%)
Gallbladder and biliary tract cancer	8.3% (3.4% to 15.5%)	8.6% (3.5% to 15.8%)	8.4% (3.5% to 15.5%)	8.6% (3.5% to 15.8%)
Pancreatic cancer	10.1% (4.9% to 17.3%)	9.8% (4.9% to 16.3%)	10.0% (4.9% to 16.9%)	9.8% (4.9% to 16.3%)
Ovarian cancer	7.8% (3.3% to 14.5%)	6.7% (2.8% to 12.5%)	6.7% (2.8% to 12.6%)	6.7% (2.8% to 12.5%)
Kidney cancer	8.8% (4.8% to 14.3%)	7.6% (4.0% to 12.3%)	7.9% (4.3% to 12.9%)	7.6% (4.0% to 12.3%)
Bladder cancer	9.6% (4.5% to 16.7%)	9.5% (4.4% to 16.0%)	9.6% (4.6% to 16.5%)	9.5% (4.4% to 16.0%)
Thyroid cancer	4.3% (1.8% to 7.7%)	4.3% (1.8% to 7.7%)	4.2% (1.8% to 7.7%)	4.3% (1.8% to 7.7%)
Mesothelioma	86.8% (81.1% to 90.7%)	79.4% (72.0% to 84.7%)	84.1% (77.6% to 88.5%)	79.4% (72.1% to 84.7%)
Non-Hodgkin's lymphoma	1.6% (0.5% to 3.3%)	1.3% (0.4% to 2.6%)	1.5% (0.5% to 3.1%)	1.3% (0.4% to 2.6%)
Multiple myeloma	2.6% (0.9% to 5.3%)	2.6% (0.9% to 5.3%)	2.6% (0.9% to 5.4%)	2.6% (0.9% to 5.3%)
Leukaemia	4.9% (2.8% to 7.8%)	4.7% (2.6% to 7.5%)	4.9% (2.7% to 7.7%)	4.7% (2.6% to 7.5%)
**Cardiovascular diseases**	79.5% (75.6% to 82.8%)	80.6% (77.2% to 83.5%)	51.5% (46.3% to 56.6%)	78.7% (75.4% to 81.7%)
Rheumatic heart disease	24.9% (16.3% to 39.4%)	23.5% (15.7% to 36.5%)	16.9% (10.5% to 24.8%)	20.3% (13.5% to 30.1%)
Ischaemic heart disease	91.6% (87.8% to 94.6%)	92.7% (89.9% to 94.9%)	88.0% (84.5% to 91.0%)	92.5% (89.7% to 94.8%)
Cerebrovascular disease	84.4% (79.8% to 88.8%)	87.4% (83.6% to 90.5%)	85.0% (80.9% to 88.8%)	87.3% (83.5% to 90.4%)
Hypertensive heart disease	100.0% (100.0% to 100.0%)	100.0% (100.0% to 100.0%)	100.0% (100.0% to 100.0%)	100.0% (100.0% to 100.0%)
Cardiomyopathy and myocarditis	36.6% (29.2% to 44.7%)	34.8% (28.1% to 42.3%)	36.4% (29.7% to 43.9%)	34.9% (28.3% to 42.4%)
Atrial fibrillation and flutter	36.9% (29.4% to 44.2%)	40.6% (34.1% to 46.8%)	46.1% (40.8% to 51.3%)	43.2% (37.2% to 48.7%)
Aortic aneurysm	36.3% (29.6% to 42.8%)	39.5% (33.4% to 45.1%)	-	39.5% (33.4% to 45.1%)
Peripheral vascular disease	47.7% (40.9% to 54.8%)	54.4% (48.5% to 60.3%)	55.3% (50.3% to 60.5%)	55.1% (50.2% to 60.2%)
Endocarditis	34.1% (25.9% to 44.3%)	31.9% (24.6% to 40.0%)	27.1% (19.5% to 36.8%)	31.9% (24.6% to 40.0%)

Supplementary Results Table 1. Age-standardised PAFs attributable to the joint distribution of all risk factors for all ages, both sexes combined for each GBD level 1 and level 2 cause and all causes in 2016 for Eritrea. Results are shown for deaths, YLLs, YLDs, and DALYs
Data in parenthesis are 95% uncertainty intervals. PAFs=population attributable fractions. GBD=Global Burden of Disease. YLLs=years of life lost. YLDs=years live with disability. DALYs=disability-adjusted life-years

	Deaths 2016	YLLs 2016	YLDs 2016	DALYs 2016
Other cardiovascular and circulatory diseases	38.0% (33.0% to 43.1%)	40.2% (35.7% to 44.6%)	28.5% (22.3% to 35.1%)	38.9% (34.3% to 43.4%)
**Chronic respiratory diseases**	52.9% (41.2% to 63.7%)	49.5% (39.0% to 59.1%)	30.6% (24.2% to 37.5%)	44.3% (34.9% to 52.8%)
Chronic obstructive pulmonary disease	66.7% (50.6% to 80.2%)	66.6% (51.0% to 79.4%)	58.9% (45.4% to 71.4%)	65.2% (50.1% to 77.8%)
Pneumoconiosis	100.0% (100.0% to 100.0%)	100.0% (100.0% to 100.0%)	100.0% (100.0% to 100.0%)	100.0% (100.0% to 100.0%)
Asthma	16.0% (10.5% to 23.5%)	17.4% (12.3% to 24.1%)	12.3% (8.7% to 17.1%)	15.3% (10.9% to 21.2%)
Other chronic respiratory diseases	3.0% (1.1% to 5.5%)	2.9% (0.8% to 6.7%)	1.5% (0.3% to 3.4%)	2.5% (0.6% to 5.6%)
**Cirrhosis and other chronic liver diseases**	28.6% (25.1% to 32.6%)	28.3% (24.7% to 32.0%)	22.6% (18.9% to 26.5%)	28.1% (24.6% to 31.8%)
Cirrhosis and other chronic liver diseases due to hepatitis B	0.1% (0.0% to 0.2%)	0.1% (0.1% to 0.2%)	0.1% (0.1% to 0.2%)	0.1% (0.1% to 0.2%)
Cirrhosis and other chronic liver diseases due to hepatitis C	4.8% (2.7% to 8.1%)	8.1% (4.6% to 13.6%)	9.9% (5.5% to 17.8%)	8.1% (4.6% to 13.7%)
Cirrhosis and other chronic liver diseases due to alcohol use	100.0% (100.0% to 100.0%)	100.0% (100.0% to 100.0%)	100.0% (100.0% to 100.0%)	100.0% (100.0% to 100.0%)
**Digestive diseases**	2.2% (1.3% to 3.5%)	2.3% (1.4% to 3.4%)	0.9% (0.6% to 1.3%)	2.2% (1.4% to 3.3%)
Peptic ulcer disease	2.6% (1.5% to 3.9%)	3.6% (2.2% to 5.4%)	2.7% (1.7% to 4.0%)	3.6% (2.2% to 5.3%)
Gallbladder and biliary diseases	17.2% (8.0% to 29.6%)	17.8% (8.7% to 29.6%)	16.8% (8.1% to 27.1%)	17.8% (8.7% to 29.5%)
Pancreatitis	8.8% (-2.0% to 25.1%)	10.2% (-1.9% to 28.0%)	9.0% (-1.8% to 25.0%)	10.2% (-1.9% to 28.0%)
**Neurological disorders**	10.0% (4.1% to 18.1%)	9.9% (4.8% to 16.6%)	2.5% (1.1% to 4.4%)	5.6% (2.8% to 9.3%)
Alzheimer's disease and other dementias	10.7% (3.9% to 20.2%)	11.4% (4.3% to 20.8%)	11.4% (4.4% to 21.0%)	11.4% (4.3% to 20.8%)
Parkinson's disease	-1.4% (-2.1% to -0.9%)	-1.9% (-2.7% to -1.3%)	-2.3% (-3.1% to -1.7%)	-2.0% (-2.7% to -1.4%)
Epilepsy	9.8% (3.1% to 19.2%)	9.9% (3.2% to 18.7%)	7.8% (2.5% to 14.8%)	8.9% (2.9% to 16.4%)
Multiple sclerosis	2.1% (0.8% to 3.8%)	2.4% (0.8% to 4.3%)	1.8% (0.9% to 2.8%)	2.2% (0.9% to 3.8%)
**Mental and substance use disorders**	99.8% (99.7% to 99.9%)	99.7% (99.5% to 99.9%)	18.8% (16.4% to 21.2%)	22.1% (19.3% to 25.9%)
Alcohol use disorders	100.0% (100.0% to 100.0%)	100.0% (100.0% to 100.0%)	100.0% (100.0% to 100.0%)	100.0% (100.0% to 100.0%)

Supplementary Results Table 1. Age-standardised PAFs attributable to the joint distribution of all risk factors for all ages, both sexes combined for each GBD level 1 and level 2 cause and all causes in 2016 for Eritrea. Results are shown for deaths, YLLs, YLDs, and DALYs
Data in parenthesis are 95% uncertainty intervals. PAFs=population attributable fractions. GBD=Global Burden of Disease. YLLs=years of life lost. YLDs=years live with disability. DALYs=disability-adjusted life-years

	Deaths 2016	YLLs 2016	YLDs 2016	DALYs 2016
Drug use disorders	100.0% (100.0% to 100.0%)	100.0% (100.0% to 100.0%)	100.0% (100.0% to 100.0%)	100.0% (100.0% to 100.0%)
Depressive disorders	-	-	9.0% (7.4% to 10.8%)	9.0% (7.4% to 10.8%)
Idiopathic developmental intellectual disability	-	-	59.0% (29.2% to 79.0%)	59.0% (29.2% to 79.0%)
**Diabetes, urogenital, blood, and endocrine diseases**	82.1% (79.4% to 84.5%)	75.4% (71.2% to 78.6%)	62.2% (57.1% to 66.7%)	70.5% (67.0% to 73.5%)
Diabetes mellitus	100.0% (100.0% to 100.0%)	100.0% (100.0% to 100.0%)	100.0% (100.0% to 100.0%)	100.0% (100.0% to 100.0%)
Chronic kidney disease	100.0% (100.0% to 100.0%)	100.0% (100.0% to 100.0%)	100.0% (100.0% to 100.0%)	100.0% (100.0% to 100.0%)
**Musculoskeletal disorders**	0.3% (0.1% to 0.6%)	0.3% (0.1% to 0.5%)	16.6% (14.3% to 19.3%)	16.2% (13.9% to 18.9%)
Rheumatoid arthritis	0.6% (0.2% to 1.3%)	0.8% (0.3% to 1.6%)	1.1% (0.4% to 1.8%)	1.0% (0.4% to 1.7%)
Osteoarthritis	-	-	9.2% (3.8% to 16.8%)	9.2% (3.8% to 16.8%)
Low back and neck pain	-	-	21.2% (18.6% to 23.9%)	21.2% (18.6% to 23.9%)
Gout	-	-	34.3% (27.9% to 42.8%)	34.3% (27.9% to 42.8%)
**Other non-communicable diseases**	0.3% (0.1% to 0.8%)	0.7% (0.2% to 1.5%)	8.5% (6.9% to 10.2%)	7.4% (6.0% to 8.9%)
Sense organ diseases	-	-	16.2% (13.6% to 18.7%)	16.2% (13.6% to 18.7%)
Sudden infant death syndrome	4.8% (2.0% to 9.1%)	4.8% (2.1% to 9.1%)	-	4.8% (2.1% to 9.1%)
**Transport injuries**	29.6% (25.2% to 35.4%)	26.6% (21.4% to 33.6%)	33.5% (28.7% to 39.8%)	27.6% (22.4% to 34.6%)
Road injuries	29.6% (25.2% to 35.2%)	26.5% (21.4% to 33.4%)	34.9% (30.0% to 41.4%)	27.6% (22.4% to 34.7%)
Other transport injuries	31.1% (24.4% to 40.4%)	28.7% (21.8% to 38.6%)	25.2% (20.9% to 31.0%)	27.6% (21.6% to 36.0%)
**Unintentional injuries**	31.8% (24.2% to 36.5%)	18.7% (15.4% to 22.5%)	22.0% (19.1% to 26.0%)	19.6% (16.4% to 23.4%)
Falls	67.1% (47.4% to 72.7%)	45.8% (37.9% to 51.2%)	30.4% (27.5% to 34.3%)	39.2% (34.4% to 43.3%)
Drowning	11.3% (7.9% to 15.3%)	12.6% (8.8% to 17.2%)	14.3% (10.2% to 19.3%)	12.6% (8.8% to 17.2%)
Fire, heat, and hot substances	7.9% (5.3% to 11.5%)	9.9% (7.0% to 13.6%)	16.9% (12.3% to 22.6%)	11.3% (8.1% to 15.4%)

Supplementary Results Table 1. Age-standardised PAFs attributable to the joint distribution of all risk factors for all ages, both sexes combined for each GBD level 1 and level 2 cause and all causes in 2016 for Eritrea. Results are shown for deaths, YLLs, YLDs, and DALYs

Data in parenthesis are 95% uncertainty intervals. PAFs=population attributable fractions. GBD=Global Burden of Disease. YLLs=years of life lost. YLDs=years live with disability. DALYs=disability-adjusted life-years

	Deaths 2016	YLLs 2016	YLDs 2016	DALYs 2016
Poisonings	10.9% (6.9% to 16.0%)	11.4% (6.3% to 17.4%)	16.6% (12.2% to 22.5%)	11.6% (6.4% to 17.5%)
Exposure to mechanical forces	23.1% (19.2% to 27.8%)	19.8% (14.7% to 26.4%)	22.6% (18.4% to 28.2%)	20.5% (15.8% to 26.9%)
Animal contact	8.0% (5.8% to 10.9%)	9.1% (6.5% to 12.7%)	13.1% (9.8% to 17.5%)	9.7% (6.8% to 13.3%)
Foreign body	7.2% (4.7% to 10.3%)	7.4% (4.2% to 11.3%)	11.8% (8.4% to 16.7%)	8.6% (5.5% to 12.5%)
Other unintentional injuries	13.2% (9.2% to 18.2%)	15.8% (10.9% to 21.8%)	14.2% (10.4% to 19.1%)	15.2% (10.8% to 20.3%)
**Self-harm and interpersonal violence**	12.1% (6.1% to 18.9%)	12.0% (6.5% to 18.3%)	21.6% (18.4% to 25.1%)	12.8% (7.5% to 18.9%)
Self-harm	8.9% (1.2% to 18.6%)	9.7% (1.5% to 19.6%)	9.1% (1.3% to 19.0%)	9.7% (1.5% to 19.6%)
Interpersonal violence	16.4% (9.7% to 22.5%)	14.1% (7.8% to 20.1%)	22.8% (19.5% to 26.1%)	15.3% (9.4% to 20.9%)
**Forces of nature, conflict and terrorism, and executions and police conflict**	0.0% (0.0% to 0.0%)	0.0% (0.0% to 0.0%)	0.0% (0.0% to 0.0%)	0.0% (0.0% to 0.0%)
Forces of nature, conflict and terrorism, and state actor violence	0.0% (0.0% to 0.0%)	0.0% (0.0% to 0.0%)	0.0% (0.0% to 0.0%)	0.0% (0.0% to 0.0%)

Supplementary Results Table 1. Age-standardised PAFs attributable to the joint distribution of all risk factors for all ages, both sexes combined for each GBD level 1 and level 2 cause and all causes in 2016 for Estonia. Results are shown for deaths, YLLs, YLDs, and DALYs
Data in parenthesis are 95% uncertainty intervals. PAFs=population attributable fractions. GBD=Global Burden of Disease. YLLs=years of life lost. YLDs=years live with disability. DALYs=disability-adjusted life-years

	Deaths 2016	YLLs 2016	YLDs 2016	DALYs 2016
**All causes**	68.3% (66.2% to 70.1%)	62.9% (60.9% to 64.8%)	26.2% (24.3% to 27.7%)	46.2% (43.0% to 49.5%)
**HIV/AIDS and tuberculosis**	74.3% (71.1% to 77.5%)	74.7% (72.0% to 77.3%)	72.4% (69.3% to 75.3%)	74.6% (72.0% to 77.1%)
Tuberculosis	70.9% (63.3% to 78.9%)	72.4% (64.4% to 80.1%)	61.4% (52.7% to 69.7%)	71.7% (63.7% to 79.4%)
HIV/AIDS	76.3% (74.4% to 78.2%)	75.6% (73.5% to 77.5%)	76.5% (74.2% to 78.5%)	75.7% (73.5% to 77.6%)
**Diarrhoea, lower respiratory infections, and other common infectious diseases**	56.0% (48.7% to 63.0%)	58.4% (51.7% to 64.7%)	35.2% (25.3% to 45.0%)	46.1% (39.0% to 52.6%)
Diarrhoeal diseases	65.6% (44.3% to 84.0%)	75.2% (58.5% to 88.9%)	68.4% (48.6% to 85.3%)	68.6% (48.8% to 85.4%)
Lower respiratory infections	61.5% (53.8% to 69.2%)	66.2% (59.1% to 73.3%)	50.2% (41.4% to 59.6%)	65.6% (58.5% to 72.7%)
Upper respiratory infections	0.2% (0.0% to 1.0%)	0.7% (0.0% to 2.8%)	0.0% (0.0% to 0.0%)	0.0% (0.0% to 0.0%)
Otitis media	0.2% (0.0% to 0.4%)	0.9% (0.4% to 1.9%)	8.7% (6.0% to 11.2%)	8.7% (6.0% to 11.2%)
Meningitis	1.3% (0.7% to 2.1%)	2.4% (1.4% to 3.8%)	0.0% (0.0% to 0.0%)	1.5% (0.8% to 2.4%)
Encephalitis	0.8% (0.5% to 1.2%)	1.6% (0.9% to 2.5%)	0.0% (0.0% to 0.0%)	0.9% (0.5% to 1.4%)
Measles	10.0% (1.8% to 34.3%)	11.8% (2.2% to 40.1%)	18.2% (0.9% to 58.0%)	12.5% (2.3% to 41.5%)
**Maternal disorders**	3.1% (1.4% to 5.0%)	3.0% (1.3% to 5.0%)	5.2% (2.0% to 8.8%)	4.4% (1.8% to 7.4%)
Maternal haemorrhage	11.6% (4.2% to 19.1%)	11.6% (4.2% to 19.0%)	11.5% (4.2% to 18.8%)	11.5% (4.2% to 18.8%)
Maternal sepsis and other pregnancy related infections	11.6% (4.2% to 18.8%)	11.5% (4.2% to 18.7%)	11.4% (4.1% to 18.5%)	11.4% (4.1% to 18.5%)
Maternal abortion, miscarriage, and ectopic pregnancy	3.4% (2.0% to 5.3%)	3.2% (1.8% to 5.0%)	3.7% (1.8% to 6.1%)	3.5% (1.8% to 5.4%)
**Neonatal disorders**	69.6% (66.1% to 72.8%)	69.6% (66.1% to 72.9%)	0.1% (0.0% to 0.1%)	30.8% (22.0% to 38.8%)
Neonatal preterm birth complications	83.2% (78.2% to 87.7%)	83.2% (78.3% to 87.8%)	0.2% (0.2% to 0.3%)	42.7% (30.4% to 54.1%)
Neonatal encephalopathy due to birth asphyxia and trauma	63.6% (59.2% to 68.0%)	63.6% (59.3% to 68.1%)	0.0% (0.0% to 0.0%)	22.2% (13.9% to 30.9%)
Neonatal sepsis and other neonatal infections	55.6% (48.6% to 61.9%)	55.6% (48.7% to 62.0%)	0.0% (0.0% to 0.0%)	21.4% (9.8% to 38.6%)
Hemolytic disease and other neonatal jaundice	61.4% (54.7% to 68.0%)	61.5% (54.8% to 68.1%)	0.0% (0.0% to 0.0%)	10.7% (5.7% to 18.3%)

Supplementary Results Table 1. Age-standardised PAFs attributable to the joint distribution of all risk factors for all ages, both sexes combined for each GBD level 1 and level 2 cause and all causes in 2016 for Estonia. Results are shown for deaths, YLLs, YLDs, and DALYs
Data in parenthesis are 95% uncertainty intervals. PAFs=population attributable fractions. GBD=Global Burden of Disease. YLLs=years of life lost. YLDs=years live with disability. DALYs=disability-adjusted life-years

	Deaths 2016	YLLs 2016	YLDs 2016	DALYs 2016
Other neonatal disorders	60.4% (55.1% to 65.3%)	60.5% (55.1% to 65.4%)	0.0% (0.0% to 0.0%)	48.7% (41.4% to 55.3%)
**Nutritional deficiencies**	15.5% (10.1% to 19.2%)	29.7% (22.0% to 36.7%)	77.6% (71.6% to 82.9%)	76.0% (69.9% to 81.2%)
Protein-energy malnutrition	10.9% (5.9% to 17.0%)	28.6% (16.9% to 40.5%)	97.7% (96.5% to 98.6%)	86.6% (79.6% to 91.5%)
Vitamin A deficiency	-	-	0.0% (0.0% to 0.0%)	0.0% (0.0% to 0.0%)
Iron-deficiency anaemia	100.0% (100.0% to 100.0%)	100.0% (100.0% to 100.0%)	100.0% (100.0% to 100.0%)	100.0% (100.0% to 100.0%)
**Other communicable, maternal, neonatal, and nutritional diseases**	4.9% (3.7% to 8.2%)	3.6% (2.4% to 6.0%)	83.0% (79.1% to 86.4%)	60.6% (50.4% to 69.9%)
Sexually transmitted diseases excluding HIV	83.2% (66.9% to 97.9%)	64.1% (40.1% to 94.2%)	100.0% (100.0% to 100.0%)	99.3% (98.1% to 99.9%)
Hepatitis	6.2% (4.1% to 8.7%)	5.8% (3.6% to 8.4%)	2.8% (1.7% to 4.0%)	5.5% (3.4% to 8.1%)
**Neoplasms**	43.7% (39.8% to 47.5%)	44.0% (40.5% to 47.4%)	34.3% (30.8% to 38.6%)	43.7% (40.1% to 47.2%)
Oesophageal cancer	88.7% (83.0% to 93.3%)	90.2% (84.7% to 94.4%)	89.1% (83.5% to 93.6%)	90.2% (84.7% to 94.4%)
Stomach cancer	21.1% (12.1% to 30.6%)	21.7% (12.7% to 30.7%)	19.8% (11.3% to 29.1%)	21.6% (12.7% to 30.6%)
Liver cancer	72.0% (65.1% to 78.3%)	71.4% (64.1% to 77.6%)	71.2% (64.3% to 77.2%)	71.4% (64.1% to 77.6%)
Larynx cancer	92.6% (87.8% to 95.8%)	92.7% (88.0% to 95.7%)	92.5% (87.8% to 95.6%)	92.7% (88.1% to 95.7%)
Tracheal, bronchus, and lung cancer	87.0% (84.1% to 89.6%)	87.7% (84.8% to 90.3%)	84.5% (80.9% to 87.8%)	87.7% (84.8% to 90.3%)
Breast cancer	32.7% (26.2% to 40.0%)	32.3% (26.1% to 39.4%)	32.0% (25.7% to 39.1%)	32.3% (26.0% to 39.4%)
Cervical cancer	100.0% (100.0% to 100.0%)	100.0% (100.0% to 100.0%)	100.0% (100.0% to 100.0%)	100.0% (100.0% to 100.0%)
Uterine cancer	48.9% (34.6% to 63.0%)	50.0% (35.8% to 63.8%)	50.1% (35.8% to 63.9%)	50.0% (35.8% to 63.8%)
Prostate cancer	7.2% (5.0% to 9.7%)	8.0% (5.7% to 10.6%)	8.2% (5.8% to 10.8%)	8.0% (5.8% to 10.6%)
Colon and rectum cancer	52.9% (44.0% to 61.2%)	54.8% (46.0% to 62.6%)	53.5% (44.7% to 61.5%)	54.8% (46.0% to 62.6%)
Lip and oral cavity cancer	86.7% (82.0% to 90.9%)	89.4% (85.3% to 92.7%)	83.9% (78.2% to 89.0%)	89.2% (85.1% to 92.6%)
Nasopharynx cancer	85.6% (81.4% to 89.3%)	83.8% (79.8% to 87.6%)	82.9% (78.9% to 86.7%)	83.8% (79.8% to 87.6%)

Supplementary Results Table 1. Age-standardised PAFs attributable to the joint distribution of all risk factors for all ages, both sexes combined for each GBD level 1 and level 2 cause and all causes in 2016 for Estonia. Results are shown for deaths, YLLs, YLDs, and DALYs
Data in parenthesis are 95% uncertainty intervals. PAFs=population attributable fractions. GBD=Global Burden of Disease. YLLs=years of life lost. YLDs=years live with disability. DALYs=disability-adjusted life-years

	Deaths 2016	YLLs 2016	YLDs 2016	DALYs 2016
Other pharynx cancer	78.7% (70.1% to 85.3%)	79.7% (71.4% to 86.0%)	78.8% (70.2% to 85.4%)	79.7% (71.4% to 86.0%)
Gallbladder and biliary tract cancer	25.3% (14.4% to 38.3%)	25.2% (14.3% to 38.1%)	26.1% (14.9% to 39.2%)	25.2% (14.3% to 38.1%)
Pancreatic cancer	32.3% (24.5% to 40.5%)	34.1% (26.1% to 41.9%)	32.4% (24.8% to 40.7%)	34.1% (26.1% to 41.9%)
Ovarian cancer	11.7% (4.8% to 20.1%)	10.6% (4.1% to 18.5%)	10.2% (3.8% to 17.7%)	10.6% (4.1% to 18.4%)
Kidney cancer	40.0% (29.6% to 50.6%)	41.1% (30.6% to 51.7%)	40.7% (30.4% to 51.4%)	41.1% (30.6% to 51.7%)
Bladder cancer	38.1% (28.0% to 47.9%)	41.3% (30.9% to 50.9%)	39.0% (29.2% to 48.0%)	41.1% (30.9% to 50.7%)
Thyroid cancer	15.4% (8.0% to 24.4%)	15.9% (8.4% to 25.7%)	15.0% (8.2% to 23.2%)	15.8% (8.3% to 25.2%)
Mesothelioma	86.4% (82.0% to 89.7%)	77.6% (70.9% to 82.8%)	83.1% (78.0% to 87.1%)	77.7% (71.0% to 82.8%)
Non-Hodgkin's lymphoma	7.5% (3.0% to 12.8%)	7.1% (3.0% to 12.1%)	7.4% (2.9% to 12.8%)	7.1% (3.0% to 12.2%)
Multiple myeloma	9.3% (4.0% to 16.4%)	9.5% (4.1% to 16.3%)	9.4% (4.0% to 16.1%)	9.5% (4.1% to 16.3%)
Leukaemia	21.8% (13.4% to 30.1%)	18.7% (11.7% to 25.6%)	18.4% (11.5% to 25.3%)	18.6% (11.7% to 25.6%)
**Cardiovascular diseases**	91.2% (88.4% to 93.3%)	91.4% (89.5% to 92.8%)	86.0% (83.7% to 87.9%)	90.8% (89.0% to 92.2%)
Rheumatic heart disease	35.8% (24.1% to 51.6%)	35.0% (23.8% to 49.1%)	33.5% (22.7% to 48.1%)	34.9% (23.8% to 49.0%)
Ischaemic heart disease	94.6% (91.3% to 97.2%)	96.1% (94.0% to 97.7%)	93.9% (91.9% to 95.7%)	95.9% (93.9% to 97.6%)
Cerebrovascular disease	88.1% (83.2% to 92.6%)	92.1% (89.2% to 94.6%)	89.9% (86.3% to 93.1%)	91.5% (88.4% to 94.2%)
Hypertensive heart disease	100.0% (100.0% to 100.0%)	100.0% (100.0% to 100.0%)	100.0% (100.0% to 100.0%)	100.0% (100.0% to 100.0%)
Cardiomyopathy and myocarditis	73.2% (62.0% to 79.8%)	73.2% (62.3% to 79.7%)	52.2% (44.7% to 59.2%)	72.3% (61.7% to 78.8%)
Atrial fibrillation and flutter	59.9% (51.5% to 67.9%)	64.8% (57.6% to 71.2%)	72.3% (67.3% to 77.1%)	69.0% (63.1% to 74.5%)
Aortic aneurysm	57.0% (49.9% to 63.6%)	62.0% (55.8% to 67.6%)	-	62.0% (55.8% to 67.6%)
Peripheral vascular disease	56.9% (48.7% to 65.1%)	64.1% (57.5% to 70.8%)	65.2% (59.2% to 71.1%)	64.5% (58.5% to 70.4%)
Endocarditis	50.6% (41.9% to 59.4%)	48.9% (39.0% to 57.7%)	44.7% (36.6% to 53.0%)	48.8% (39.0% to 57.7%)

Supplementary Results Table 1. Age-standardised PAFs attributable to the joint distribution of all risk factors for all ages, both sexes combined for each GBD level 1 and level 2 cause and all causes in 2016 for Estonia. Results are shown for deaths, YLLs, YLDs, and DALYs
Data in parenthesis are 95% uncertainty intervals. PAFs=population attributable fractions. GBD=Global Burden of Disease. YLLs=years of life lost. YLDs=years live with disability. DALYs=disability-adjusted life-years

	Deaths 2016	YLLs 2016	YLDs 2016	DALYs 2016
Other cardiovascular and circulatory diseases	57.1% (51.9% to 62.2%)	62.5% (58.0% to 66.9%)	56.5% (51.7% to 61.3%)	61.3% (56.9% to 65.7%)
**Chronic respiratory diseases**	67.0% (62.1% to 71.6%)	66.6% (62.2% to 70.8%)	40.7% (34.2% to 47.8%)	51.4% (45.6% to 57.1%)
Chronic obstructive pulmonary disease	75.8% (70.2% to 81.2%)	78.2% (73.3% to 82.9%)	65.7% (59.1% to 71.7%)	75.0% (70.0% to 79.8%)
Pneumoconiosis	100.0% (100.0% to 100.0%)	100.0% (100.0% to 100.0%)	100.0% (100.0% to 100.0%)	100.0% (100.0% to 100.0%)
Asthma	45.3% (35.0% to 56.5%)	49.1% (39.8% to 59.6%)	37.1% (29.1% to 45.8%)	38.5% (30.7% to 47.4%)
Other chronic respiratory diseases	19.5% (13.2% to 26.1%)	17.1% (10.2% to 25.2%)	4.4% (2.6% to 7.0%)	10.3% (5.5% to 16.1%)
**Cirrhosis and other chronic liver diseases**	60.5% (57.7% to 63.3%)	60.1% (57.5% to 62.9%)	44.2% (40.5% to 47.7%)	59.3% (56.7% to 62.1%)
Cirrhosis and other chronic liver diseases due to hepatitis B	4.9% (2.2% to 8.1%)	5.5% (2.5% to 9.0%)	4.8% (2.1% to 8.0%)	5.4% (2.5% to 9.0%)
Cirrhosis and other chronic liver diseases due to hepatitis C	89.9% (89.7% to 90.0%)	90.0% (89.9% to 90.0%)	88.9% (88.6% to 89.2%)	89.9% (89.9% to 90.0%)
Cirrhosis and other chronic liver diseases due to alcohol use	100.0% (100.0% to 100.0%)	100.0% (100.0% to 100.0%)	100.0% (100.0% to 100.0%)	100.0% (100.0% to 100.0%)
**Digestive diseases**	18.1% (14.7% to 21.8%)	25.6% (21.1% to 30.2%)	5.0% (3.9% to 6.2%)	18.8% (15.1% to 23.1%)
Peptic ulcer disease	17.7% (12.5% to 23.2%)	21.9% (15.8% to 28.1%)	15.1% (10.6% to 20.2%)	20.2% (14.6% to 26.4%)
Gallbladder and biliary diseases	45.4% (30.7% to 60.3%)	45.9% (32.1% to 60.1%)	49.4% (35.8% to 63.3%)	47.0% (33.4% to 61.2%)
Pancreatitis	64.9% (51.3% to 77.3%)	70.7% (58.7% to 81.7%)	47.2% (33.0% to 61.2%)	70.1% (57.9% to 80.9%)
**Neurological disorders**	21.6% (11.2% to 33.9%)	22.6% (15.3% to 31.0%)	4.0% (1.9% to 6.9%)	9.7% (6.2% to 14.0%)
Alzheimer's disease and other dementias	25.5% (12.2% to 41.2%)	26.9% (13.7% to 42.4%)	26.9% (13.6% to 42.5%)	26.9% (13.7% to 42.4%)
Parkinson's disease	-8.4% (-10.4% to -6.5%)	-11.7% (-14.3% to -9.1%)	-13.7% (-16.6% to -10.8%)	-12.1% (-14.8% to -9.5%)
Epilepsy	48.5% (36.8% to 58.8%)	46.2% (34.9% to 55.9%)	35.7% (27.1% to 43.8%)	42.0% (32.0% to 51.8%)
Multiple sclerosis	11.5% (7.5% to 15.7%)	11.4% (7.5% to 15.7%)	9.8% (6.5% to 13.4%)	10.8% (7.1% to 14.8%)
**Mental and substance use disorders**	100.0% (100.0% to 100.0%)	100.0% (100.0% to 100.0%)	33.3% (30.5% to 36.6%)	52.2% (47.3% to 57.7%)
Alcohol use disorders	100.0% (100.0% to 100.0%)	100.0% (100.0% to 100.0%)	100.0% (100.0% to 100.0%)	100.0% (100.0% to 100.0%)

Supplementary Results Table 1. Age-standardised PAFs attributable to the joint distribution of all risk factors for all ages, both sexes combined for each GBD level 1 and level 2 cause and all causes in 2016 for Estonia. Results are shown for deaths, YLLs, YLDs, and DALYs
Data in parenthesis are 95% uncertainty intervals. PAFs=population attributable fractions. GBD=Global Burden of Disease. YLLs=years of life lost. YLDs=years live with disability. DALYs=disability-adjusted life-years

	Deaths 2016	YLLs 2016	YLDs 2016	DALYs 2016
Drug use disorders	100.0% (100.0% to 100.0%)	100.0% (100.0% to 100.0%)	100.0% (100.0% to 100.0%)	100.0% (100.0% to 100.0%)
Depressive disorders	-	-	9.9% (8.5% to 11.4%)	9.9% (8.5% to 11.4%)
Idiopathic developmental intellectual disability	-	-	59.1% (28.6% to 79.6%)	59.1% (28.6% to 79.6%)
**Diabetes, urogenital, blood, and endocrine diseases**	75.1% (73.6% to 76.8%)	73.5% (71.7% to 75.6%)	55.1% (50.7% to 59.3%)	61.1% (57.6% to 64.3%)
Diabetes mellitus	100.0% (100.0% to 100.0%)	100.0% (100.0% to 100.0%)	100.0% (100.0% to 100.0%)	100.0% (100.0% to 100.0%)
Chronic kidney disease	100.0% (100.0% to 100.0%)	100.0% (100.0% to 100.0%)	100.0% (100.0% to 100.0%)	100.0% (100.0% to 100.0%)
**Musculoskeletal disorders**	2.3% (0.9% to 3.8%)	2.3% (0.9% to 3.8%)	22.6% (18.7% to 26.7%)	22.3% (18.4% to 26.3%)
Rheumatoid arthritis	5.3% (2.1% to 9.0%)	6.2% (2.6% to 10.4%)	6.4% (2.6% to 10.9%)	6.4% (2.6% to 10.8%)
Osteoarthritis	-	-	29.8% (16.0% to 43.5%)	29.8% (16.0% to 43.5%)
Low back and neck pain	-	-	23.2% (19.8% to 26.8%)	23.2% (19.8% to 26.8%)
Gout	-	-	51.6% (36.3% to 67.3%)	51.6% (36.3% to 67.3%)
**Other non-communicable diseases**	0.1% (0.1% to 0.2%)	0.2% (0.1% to 0.3%)	3.6% (3.2% to 4.1%)	3.3% (2.9% to 3.7%)
Sense organ diseases	-	-	9.8% (8.9% to 10.8%)	9.8% (8.9% to 10.8%)
Sudden infant death syndrome	3.5% (2.2% to 5.2%)	3.5% (2.2% to 5.2%)	-	3.5% (2.2% to 5.2%)
**Transport injuries**	39.5% (28.7% to 51.1%)	36.8% (26.0% to 49.0%)	40.2% (30.6% to 50.1%)	37.9% (27.4% to 49.1%)
Road injuries	39.2% (28.5% to 50.8%)	36.5% (25.6% to 48.7%)	40.8% (31.2% to 50.8%)	37.9% (27.5% to 49.2%)
Other transport injuries	41.5% (30.1% to 54.1%)	39.0% (27.7% to 51.2%)	33.5% (24.4% to 43.1%)	37.7% (27.0% to 49.2%)
**Unintentional injuries**	17.9% (13.8% to 22.3%)	14.0% (9.9% to 18.2%)	16.9% (14.7% to 19.3%)	15.5% (12.7% to 18.7%)
Falls	33.1% (27.4% to 37.3%)	19.6% (16.2% to 23.6%)	20.0% (18.0% to 22.1%)	19.9% (17.7% to 22.3%)
Drowning	20.5% (11.1% to 30.1%)	19.1% (10.8% to 27.5%)	18.0% (10.0% to 27.0%)	19.1% (10.8% to 27.5%)
Fire, heat, and hot substances	21.1% (11.2% to 31.4%)	21.3% (11.7% to 31.3%)	19.2% (11.3% to 28.0%)	20.5% (11.7% to 29.7%)

Supplementary Results Table 1. Age-standardised PAFs attributable to the joint distribution of all risk factors for all ages, both sexes combined for each GBD level 1 and level 2 cause and all causes in 2016 for Estonia. Results are shown for deaths, YLLs, YLDs, and DALYs
Data in parenthesis are 95% uncertainty intervals. PAFs=population attributable fractions. GBD=Global Burden of Disease. YLLs=years of life lost. YLDs=years live with disability. DALYs=disability-adjusted life-years

	**Deaths 2016**	**YLLs 2016**	**YLDs 2016**	**DALYs 2016**
Poisonings	22.5% (12.5% to 32.7%)	21.8% (12.1% to 31.4%)	17.8% (10.8% to 25.8%)	20.5% (11.8% to 29.0%)
Exposure to mechanical forces	13.0% (10.0% to 16.6%)	11.2% (8.3% to 15.0%)	13.7% (11.4% to 16.3%)	12.9% (10.4% to 15.7%)
Animal contact	9.5% (7.4% to 12.1%)	8.5% (6.3% to 11.4%)	6.2% (4.4% to 8.5%)	6.6% (4.8% to 9.1%)
Foreign body	6.6% (4.2% to 9.9%)	7.0% (4.5% to 10.5%)	5.0% (3.4% to 7.1%)	6.5% (4.3% to 9.6%)
Other unintentional injuries	24.7% (13.7% to 36.3%)	24.1% (14.0% to 34.8%)	17.2% (10.2% to 25.2%)	18.5% (11.1% to 26.5%)
**Self-harm and interpersonal violence**	45.1% (33.7% to 54.1%)	45.2% (34.0% to 54.2%)	31.7% (24.3% to 38.8%)	44.2% (33.6% to 52.7%)
Self-harm	48.6% (34.5% to 60.1%)	49.6% (35.7% to 61.2%)	40.9% (27.7% to 52.8%)	49.5% (35.5% to 61.0%)
Interpersonal violence	33.6% (22.4% to 45.6%)	32.0% (21.4% to 43.6%)	30.0% (22.3% to 38.3%)	31.6% (21.7% to 42.7%)
**Forces of nature, conflict and terrorism, and executions and police conflict**	0.0% (0.0% to 0.0%)	0.0% (0.0% to 0.0%)	0.3% (0.1% to 0.4%)	0.1% (0.0% to 0.2%)
Forces of nature, conflict and terrorism, and state actor violence	0.0% (0.0% to 0.0%)	0.0% (0.0% to 0.0%)	4.1% (2.7% to 6.0%)	4.1% (2.7% to 6.0%)

Supplementary Results Table 1. Age-standardised PAFs attributable to the joint distribution of all risk factors for all ages, both sexes combined for each GBD level 1 and level 2 cause and all causes in 2016 for Ethiopia. Results are shown for deaths, YLLs, YLDs, and DALYs
Data in parenthesis are 95% uncertainty intervals. PAFs=population attributable fractions. GBD=Global Burden of Disease. YLLs=years of life lost. YLDs=years live with disability. DALYs=disability-adjusted life-years

	Deaths 2016	YLLs 2016	YLDs 2016	DALYs 2016
**All causes**	55.4% (53.2% to 57.8%)	53.8% (51.4% to 56.6%)	27.4% (26.1% to 28.7%)	47.4% (45.0% to 50.1%)
**HIV/AIDS and tuberculosis**	40.2% (30.7% to 49.9%)	47.0% (37.7% to 56.2%)	65.0% (58.1% to 72.3%)	47.7% (38.6% to 56.8%)
Tuberculosis	27.9% (16.4% to 39.9%)	29.9% (18.1% to 42.3%)	26.6% (15.1% to 38.7%)	29.9% (18.0% to 42.2%)
HIV/AIDS	90.4% (87.2% to 93.2%)	86.7% (82.8% to 90.1%)	91.4% (88.8% to 93.6%)	87.1% (83.3% to 90.2%)
**Diarrhoea, lower respiratory infections, and other common infectious diseases**	74.8% (67.8% to 80.7%)	70.0% (60.7% to 76.9%)	47.9% (43.1% to 52.3%)	68.8% (60.1% to 75.6%)
Diarrhoeal diseases	95.8% (88.9% to 99.0%)	96.5% (90.8% to 99.2%)	96.2% (90.1% to 99.1%)	96.5% (90.7% to 99.2%)
Lower respiratory infections	77.8% (71.4% to 83.3%)	83.1% (77.8% to 87.4%)	79.1% (73.5% to 84.3%)	83.1% (77.8% to 87.4%)
Upper respiratory infections	1.1% (0.1% to 3.9%)	2.5% (0.2% to 8.6%)	0.0% (0.0% to 0.0%)	0.2% (0.0% to 0.7%)
Otitis media	0.3% (0.1% to 1.1%)	1.3% (0.4% to 3.1%)	1.9% (1.2% to 2.7%)	1.9% (1.2% to 2.7%)
Meningitis	1.6% (1.0% to 2.7%)	3.7% (2.2% to 5.9%)	0.0% (0.0% to 0.0%)	3.4% (2.1% to 5.4%)
Encephalitis	5.7% (2.4% to 11.7%)	7.3% (3.2% to 14.4%)	0.0% (0.0% to 0.0%)	4.5% (1.8% to 9.7%)
Measles	65.0% (52.0% to 81.5%)	66.2% (53.0% to 82.8%)	65.4% (51.9% to 81.4%)	66.2% (53.0% to 82.8%)
**Maternal disorders**	7.5% (4.1% to 11.1%)	7.5% (4.1% to 11.1%)	2.5% (1.3% to 4.1%)	7.2% (4.0% to 10.6%)
Maternal haemorrhage	16.6% (5.9% to 27.3%)	16.6% (5.9% to 27.4%)	16.8% (5.9% to 27.8%)	16.6% (5.9% to 27.4%)
Maternal sepsis and other pregnancy related infections	16.4% (5.9% to 27.0%)	16.5% (5.9% to 27.3%)	16.1% (5.7% to 26.6%)	16.5% (5.9% to 27.2%)
Maternal abortion, miscarriage, and ectopic pregnancy	20.2% (12.7% to 28.4%)	19.9% (12.4% to 27.9%)	20.3% (11.6% to 29.0%)	19.9% (12.4% to 27.8%)
**Neonatal disorders**	79.3% (76.2% to 82.4%)	79.3% (76.3% to 82.4%)	0.3% (0.1% to 0.6%)	73.8% (68.9% to 78.0%)
Neonatal preterm birth complications	96.8% (93.6% to 98.5%)	96.9% (93.7% to 98.5%)	1.2% (0.9% to 1.7%)	90.3% (84.9% to 94.0%)
Neonatal encephalopathy due to birth asphyxia and trauma	79.3% (75.8% to 82.6%)	79.4% (75.8% to 82.6%)	0.0% (0.0% to 0.0%)	73.0% (63.3% to 79.8%)
Neonatal sepsis and other neonatal infections	67.6% (62.0% to 72.4%)	67.6% (62.0% to 72.4%)	0.0% (0.0% to 0.0%)	61.5% (50.4% to 69.0%)
Hemolytic disease and other neonatal jaundice	74.3% (68.6% to 78.6%)	74.4% (68.7% to 78.6%)	0.0% (0.0% to 0.0%)	66.4% (59.4% to 72.3%)

Supplementary Results Table 1. Age-standardised PAFs attributable to the joint distribution of all risk factors for all ages, both sexes combined for each GBD level 1 and level 2 cause and all causes in 2016 for Ethiopia. Results are shown for deaths, YLLs, YLDs, and DALYs
Data in parenthesis are 95% uncertainty intervals. PAFs=population attributable fractions. GBD=Global Burden of Disease. YLLs=years of life lost. YLDs=years live with disability. DALYs=disability-adjusted life-years

	Deaths 2016	YLLs 2016	YLDs 2016	DALYs 2016
Other neonatal disorders	72.6% (66.5% to 77.7%)	72.7% (66.6% to 77.7%)	0.0% (0.0% to 0.0%)	71.0% (65.0% to 76.1%)
**Nutritional deficiencies**	18.2% (12.2% to 25.2%)	49.7% (38.8% to 60.4%)	90.7% (88.7% to 92.5%)	62.9% (55.9% to 69.7%)
Protein-energy malnutrition	22.2% (15.0% to 31.8%)	56.5% (44.6% to 67.5%)	98.6% (98.0% to 99.1%)	58.3% (47.9% to 68.6%)
Vitamin A deficiency	-	-	100.0% (100.0% to 100.0%)	100.0% (100.0% to 100.0%)
Iron-deficiency anaemia	100.0% (100.0% to 100.0%)	100.0% (100.0% to 100.0%)	100.0% (100.0% to 100.0%)	100.0% (100.0% to 100.0%)
**Other communicable, maternal, neonatal, and nutritional diseases**	6.0% (4.1% to 8.3%)	4.2% (2.4% to 6.6%)	76.0% (71.2% to 80.3%)	14.3% (8.7% to 21.4%)
Sexually transmitted diseases excluding HIV	15.8% (7.6% to 28.7%)	7.2% (3.2% to 14.1%)	100.0% (100.0% to 100.0%)	23.4% (12.0% to 40.2%)
Hepatitis	0.2% (0.1% to 0.4%)	0.3% (0.2% to 0.5%)	0.3% (0.2% to 0.5%)	0.3% (0.2% to 0.5%)
**Neoplasms**	30.8% (26.7% to 36.9%)	30.6% (26.2% to 37.5%)	32.5% (27.4% to 40.7%)	30.6% (26.2% to 37.5%)
Oesophageal cancer	54.4% (37.6% to 67.6%)	55.6% (38.7% to 68.7%)	55.1% (38.3% to 68.3%)	55.6% (38.7% to 68.7%)
Stomach cancer	4.8% (1.2% to 12.8%)	4.5% (1.1% to 12.0%)	4.7% (1.2% to 12.4%)	4.5% (1.1% to 12.0%)
Liver cancer	25.3% (20.2% to 31.4%)	25.0% (20.0% to 30.9%)	25.4% (20.4% to 31.2%)	25.0% (20.0% to 30.9%)
Larynx cancer	48.0% (33.0% to 61.4%)	49.4% (33.7% to 63.6%)	48.7% (33.4% to 62.4%)	49.4% (33.7% to 63.6%)
Tracheal, bronchus, and lung cancer	81.9% (75.7% to 87.0%)	81.1% (74.8% to 86.3%)	81.6% (75.4% to 86.7%)	81.2% (74.8% to 86.3%)
Breast cancer	16.7% (11.3% to 23.1%)	16.0% (10.9% to 22.0%)	16.3% (11.0% to 22.5%)	16.0% (10.9% to 22.0%)
Cervical cancer	100.0% (100.0% to 100.0%)	100.0% (100.0% to 100.0%)	100.0% (100.0% to 100.0%)	100.0% (100.0% to 100.0%)
Uterine cancer	16.8% (7.6% to 27.6%)	16.7% (7.7% to 27.3%)	16.8% (7.6% to 27.6%)	16.7% (7.7% to 27.3%)
Prostate cancer	0.9% (0.5% to 1.5%)	0.9% (0.5% to 1.5%)	0.9% (0.5% to 1.5%)	0.9% (0.5% to 1.5%)
Colon and rectum cancer	42.9% (33.4% to 52.0%)	42.3% (33.2% to 51.2%)	42.8% (33.5% to 51.9%)	42.3% (33.2% to 51.2%)
Lip and oral cavity cancer	43.3% (30.3% to 55.6%)	43.0% (30.1% to 55.3%)	42.0% (29.2% to 54.2%)	43.0% (30.0% to 55.3%)
Nasopharynx cancer	45.6% (34.7% to 57.5%)	46.2% (34.9% to 57.9%)	46.7% (36.3% to 57.5%)	46.2% (34.9% to 57.9%)

Supplementary Results Table 1. Age-standardised PAFs attributable to the joint distribution of all risk factors for all ages, both sexes combined for each GBD level 1 and level 2 cause and all causes in 2016 for Ethiopia. Results are shown for deaths, YLLs, YLDs, and DALYs
Data in parenthesis are 95% uncertainty intervals. PAFs=population attributable fractions. GBD=Global Burden of Disease. YLLs=years of life lost. YLDs=years live with disability. DALYs=disability-adjusted life-years

	**Deaths 2016**	**YLLs 2016**	**YLDs 2016**	**DALYs 2016**
Other pharynx cancer	38.5% (24.6% to 53.0%)	40.1% (26.1% to 54.7%)	39.3% (25.3% to 53.6%)	40.1% (26.1% to 54.7%)
Gallbladder and biliary tract cancer	7.2% (2.9% to 13.5%)	7.4% (2.8% to 13.6%)	7.2% (2.9% to 13.3%)	7.4% (2.8% to 13.6%)
Pancreatic cancer	10.5% (5.5% to 17.4%)	9.8% (5.0% to 16.1%)	10.4% (5.5% to 17.0%)	9.8% (5.0% to 16.1%)
Ovarian cancer	7.3% (3.0% to 13.8%)	6.2% (2.5% to 11.8%)	6.3% (2.6% to 12.1%)	6.2% (2.5% to 11.8%)
Kidney cancer	9.2% (5.1% to 14.5%)	7.7% (4.0% to 12.7%)	7.9% (4.3% to 13.0%)	7.7% (4.0% to 12.7%)
Bladder cancer	10.8% (5.4% to 18.3%)	9.8% (4.7% to 16.8%)	10.4% (5.2% to 17.7%)	9.8% (4.7% to 16.9%)
Thyroid cancer	4.1% (1.6% to 7.9%)	4.0% (1.5% to 7.6%)	4.1% (1.6% to 7.8%)	4.0% (1.5% to 7.6%)
Mesothelioma	87.0% (80.1% to 91.3%)	78.7% (69.9% to 84.8%)	84.3% (76.7% to 89.3%)	78.7% (70.1% to 84.8%)
Non-Hodgkin's lymphoma	1.7% (0.6% to 3.4%)	1.3% (0.4% to 2.7%)	1.5% (0.5% to 3.1%)	1.3% (0.4% to 2.7%)
Multiple myeloma	2.4% (0.9% to 4.9%)	2.4% (0.8% to 4.8%)	2.4% (0.9% to 4.8%)	2.4% (0.8% to 4.8%)
Leukaemia	5.4% (3.2% to 8.6%)	5.1% (2.9% to 8.0%)	5.4% (3.2% to 8.4%)	5.1% (2.9% to 8.0%)
**Cardiovascular diseases**	80.9% (78.0% to 83.5%)	82.1% (79.5% to 84.3%)	61.1% (56.8% to 65.1%)	80.8% (78.3% to 83.0%)
Rheumatic heart disease	25.0% (16.4% to 40.3%)	23.4% (15.7% to 35.7%)	16.9% (10.3% to 24.9%)	21.1% (14.2% to 31.1%)
Ischaemic heart disease	91.5% (88.1% to 94.2%)	92.7% (90.2% to 94.6%)	86.2% (82.5% to 89.7%)	92.5% (90.0% to 94.4%)
Cerebrovascular disease	86.1% (82.5% to 89.4%)	89.0% (86.2% to 91.4%)	85.0% (81.2% to 88.3%)	88.7% (86.0% to 91.1%)
Hypertensive heart disease	100.0% (100.0% to 100.0%)	100.0% (100.0% to 100.0%)	100.0% (100.0% to 100.0%)	100.0% (100.0% to 100.0%)
Cardiomyopathy and myocarditis	36.9% (29.2% to 45.8%)	35.3% (28.2% to 43.2%)	38.8% (32.5% to 45.9%)	35.8% (28.9% to 43.4%)
Atrial fibrillation and flutter	39.7% (32.2% to 47.0%)	43.2% (36.5% to 49.3%)	48.1% (43.0% to 53.3%)	45.7% (39.7% to 51.2%)
Aortic aneurysm	37.2% (30.2% to 44.1%)	39.4% (33.5% to 45.0%)	-	39.4% (33.5% to 45.0%)
Peripheral vascular disease	49.1% (42.7% to 56.3%)	55.6% (50.1% to 60.9%)	56.2% (50.9% to 61.7%)	56.1% (50.8% to 61.3%)
Endocarditis	34.8% (26.7% to 44.9%)	32.3% (25.0% to 40.4%)	29.0% (21.0% to 38.7%)	32.3% (25.0% to 40.4%)

Supplementary Results Table 1. Age-standardised PAFs attributable to the joint distribution of all risk factors for all ages, both sexes combined for each GBD level 1 and level 2 cause and all causes in 2016 for Ethiopia. Results are shown for deaths, YLLs, YLDs, and DALYs
Data in parenthesis are 95% uncertainty intervals. PAFs=population attributable fractions. GBD=Global Burden of Disease. YLLs=years of life lost. YLDs=years live with disability. DALYs=disability-adjusted life-years

	Deaths 2016	YLLs 2016	YLDs 2016	DALYs 2016
Other cardiovascular and circulatory diseases	39.6% (34.2% to 44.9%)	41.1% (36.4% to 45.6%)	32.1% (26.4% to 38.2%)	40.2% (35.6% to 44.7%)
**Chronic respiratory diseases**	62.2% (52.9% to 70.4%)	58.1% (49.6% to 65.9%)	38.4% (32.0% to 45.4%)	52.5% (45.3% to 59.1%)
Chronic obstructive pulmonary disease	77.1% (68.7% to 86.6%)	76.1% (67.6% to 85.5%)	65.1% (54.3% to 76.6%)	73.9% (65.4% to 83.6%)
Pneumoconiosis	100.0% (100.0% to 100.0%)	100.0% (100.0% to 100.0%)	100.0% (100.0% to 100.0%)	100.0% (100.0% to 100.0%)
Asthma	21.4% (15.6% to 28.6%)	24.0% (18.6% to 30.6%)	19.0% (14.8% to 24.1%)	21.9% (17.2% to 27.9%)
Other chronic respiratory diseases	3.5% (1.7% to 6.1%)	3.1% (0.9% to 6.7%)	1.5% (0.3% to 3.4%)	2.6% (0.7% to 5.7%)
**Cirrhosis and other chronic liver diseases**	27.7% (24.9% to 30.6%)	27.4% (24.6% to 30.3%)	21.9% (18.7% to 25.4%)	27.3% (24.5% to 30.2%)
Cirrhosis and other chronic liver diseases due to hepatitis B	0.1% (0.0% to 0.2%)	0.1% (0.1% to 0.2%)	0.1% (0.1% to 0.2%)	0.1% (0.1% to 0.2%)
Cirrhosis and other chronic liver diseases due to hepatitis C	6.1% (3.8% to 9.4%)	10.0% (6.2% to 15.6%)	11.5% (6.8% to 17.8%)	10.0% (6.2% to 15.6%)
Cirrhosis and other chronic liver diseases due to alcohol use	100.0% (100.0% to 100.0%)	100.0% (100.0% to 100.0%)	100.0% (100.0% to 100.0%)	100.0% (100.0% to 100.0%)
**Digestive diseases**	2.4% (1.5% to 3.7%)	2.4% (1.5% to 3.5%)	1.0% (0.7% to 1.4%)	2.3% (1.5% to 3.4%)
Peptic ulcer disease	3.5% (2.0% to 5.3%)	4.0% (2.4% to 6.0%)	3.5% (2.2% to 5.0%)	4.0% (2.4% to 6.0%)
Gallbladder and biliary diseases	16.5% (7.7% to 27.7%)	16.5% (7.7% to 27.3%)	15.4% (7.3% to 25.3%)	16.5% (7.7% to 27.2%)
Pancreatitis	12.7% (-0.1% to 27.5%)	14.9% (0.7% to 30.7%)	12.5% (-0.3% to 26.2%)	14.9% (0.6% to 30.7%)
**Neurological disorders**	11.0% (5.0% to 18.8%)	11.7% (6.4% to 18.1%)	3.1% (1.5% to 5.2%)	6.7% (3.7% to 10.4%)
Alzheimer's disease and other dementias	11.7% (4.8% to 21.0%)	12.3% (5.1% to 21.5%)	12.3% (5.2% to 21.6%)	12.3% (5.2% to 21.5%)
Parkinson's disease	-2.6% (-4.0% to -1.6%)	-3.2% (-4.7% to -2.0%)	-3.4% (-4.8% to -2.3%)	-3.2% (-4.8% to -2.1%)
Epilepsy	13.6% (6.1% to 22.2%)	14.5% (6.6% to 23.3%)	11.0% (5.1% to 18.1%)	12.8% (5.9% to 20.8%)
Multiple sclerosis	2.3% (0.8% to 4.2%)	2.3% (0.8% to 4.2%)	1.7% (0.9% to 2.6%)	2.1% (0.9% to 3.7%)
**Mental and substance use disorders**	99.9% (99.8% to 100.0%)	99.8% (99.7% to 99.9%)	21.8% (19.2% to 24.3%)	25.5% (22.7% to 28.4%)
Alcohol use disorders	100.0% (100.0% to 100.0%)	100.0% (100.0% to 100.0%)	100.0% (100.0% to 100.0%)	100.0% (100.0% to 100.0%)

Supplementary Results Table 1. Age-standardised PAFs attributable to the joint distribution of all risk factors for all ages, both sexes combined for each GBD level 1 and level 2 cause and all causes in 2016 for Ethiopia. Results are shown for deaths, YLLs, YLDs, and DALYs
Data in parenthesis are 95% uncertainty intervals. PAFs=population attributable fractions. GBD=Global Burden of Disease. YLLs=years of life lost. YLDs=years live with disability. DALYs=disability-adjusted life-years

	Deaths 2016	YLLs 2016	YLDs 2016	DALYs 2016
Drug use disorders	100.0% (100.0% to 100.0%)	100.0% (100.0% to 100.0%)	100.0% (100.0% to 100.0%)	100.0% (100.0% to 100.0%)
Depressive disorders	-	-	10.4% (8.5% to 12.6%)	10.4% (8.5% to 12.6%)
Idiopathic developmental intellectual disability	-	-	60.7% (30.0% to 80.4%)	60.7% (30.0% to 80.4%)
**Diabetes, urogenital, blood, and endocrine diseases**	81.3% (79.4% to 82.9%)	75.4% (71.9% to 77.7%)	63.3% (59.0% to 67.2%)	70.9% (67.9% to 73.3%)
Diabetes mellitus	100.0% (100.0% to 100.0%)	100.0% (100.0% to 100.0%)	100.0% (100.0% to 100.0%)	100.0% (100.0% to 100.0%)
Chronic kidney disease	100.0% (100.0% to 100.0%)	100.0% (100.0% to 100.0%)	100.0% (100.0% to 100.0%)	100.0% (100.0% to 100.0%)
**Musculoskeletal disorders**	0.5% (0.2% to 0.9%)	0.4% (0.1% to 0.7%)	23.1% (20.2% to 26.4%)	22.7% (19.9% to 25.9%)
Rheumatoid arthritis	1.0% (0.3% to 2.0%)	1.0% (0.4% to 2.0%)	1.1% (0.4% to 2.0%)	1.1% (0.4% to 2.0%)
Osteoarthritis	-	-	8.6% (3.4% to 15.7%)	8.6% (3.4% to 15.7%)
Low back and neck pain	-	-	30.1% (26.7% to 33.9%)	30.1% (26.7% to 33.9%)
Gout	-	-	35.0% (28.3% to 43.7%)	35.0% (28.3% to 43.7%)
**Other non-communicable diseases**	0.4% (0.1% to 1.0%)	0.9% (0.2% to 2.1%)	10.4% (8.8% to 12.0%)	9.3% (7.8% to 10.7%)
Sense organ diseases	-	-	20.6% (18.5% to 22.6%)	20.6% (18.5% to 22.6%)
Sudden infant death syndrome	5.2% (2.3% to 9.7%)	5.3% (2.3% to 9.8%)	-	5.3% (2.3% to 9.8%)
**Transport injuries**	36.5% (30.6% to 43.4%)	36.3% (29.0% to 44.5%)	40.3% (33.9% to 47.5%)	36.8% (29.6% to 44.7%)
Road injuries	36.3% (30.5% to 43.0%)	36.1% (28.8% to 44.0%)	41.8% (35.3% to 49.2%)	36.7% (29.6% to 44.5%)
Other transport injuries	42.7% (33.4% to 53.8%)	41.7% (31.6% to 53.9%)	31.8% (26.0% to 38.1%)	38.2% (29.7% to 47.9%)
**Unintentional injuries**	37.2% (32.2% to 41.7%)	25.4% (21.4% to 30.0%)	28.1% (23.6% to 34.0%)	26.2% (22.1% to 31.0%)
Falls	66.6% (56.8% to 70.7%)	48.4% (42.8% to 53.2%)	35.7% (31.3% to 41.4%)	43.0% (38.7% to 48.1%)
Drowning	17.0% (12.4% to 22.0%)	19.9% (14.3% to 26.1%)	20.3% (14.8% to 26.9%)	19.9% (14.3% to 26.1%)
Fire, heat, and hot substances	11.7% (8.1% to 16.4%)	15.3% (11.2% to 20.0%)	25.1% (18.9% to 32.9%)	17.6% (13.0% to 23.2%)

Supplementary Results Table 1. Age-standardised PAFs attributable to the joint distribution of all risk factors for all ages, both sexes combined for each GBD level 1 and level 2 cause and all causes in 2016 for Ethiopia. Results are shown for deaths, YLLs, YLDs, and DALYs
Data in parenthesis are 95% uncertainty intervals. PAFs=population attributable fractions. GBD=Global Burden of Disease. YLLs=years of life lost. YLDs=years live with disability. DALYs=disability-adjusted life-years

	Deaths 2016	YLLs 2016	YLDs 2016	DALYs 2016
Poisonings	15.8% (11.3% to 21.3%)	17.8% (12.3% to 24.5%)	25.4% (19.2% to 33.4%)	18.1% (12.6% to 24.8%)
Exposure to mechanical forces	28.2% (22.9% to 34.6%)	28.5% (21.3% to 37.9%)	29.3% (23.0% to 37.7%)	28.7% (21.7% to 37.4%)
Animal contact	10.8% (8.2% to 14.1%)	13.5% (10.0% to 18.0%)	19.5% (14.6% to 26.3%)	14.6% (10.8% to 19.5%)
Foreign body	12.0% (8.2% to 16.9%)	13.0% (8.1% to 19.0%)	18.3% (12.9% to 25.6%)	14.6% (10.0% to 20.7%)
Other unintentional injuries	20.2% (14.7% to 26.8%)	24.9% (18.1% to 33.2%)	21.3% (16.0% to 27.5%)	23.3% (17.4% to 30.3%)
**Self-harm and interpersonal violence**	16.5% (10.0% to 23.6%)	16.5% (10.1% to 23.2%)	23.7% (20.1% to 27.6%)	17.1% (10.9% to 23.4%)
Self-harm	12.8% (2.2% to 23.4%)	13.8% (3.1% to 24.6%)	13.0% (2.4% to 23.7%)	13.8% (3.1% to 24.6%)
Interpersonal violence	20.5% (13.5% to 27.2%)	18.5% (11.4% to 25.3%)	24.7% (21.0% to 28.4%)	19.2% (12.6% to 25.6%)
**Forces of nature, conflict and terrorism, and executions and police conflict**	0.0% (0.0% to 0.0%)	0.0% (0.0% to 0.0%)	0.0% (0.0% to 0.0%)	0.0% (0.0% to 0.0%)
Forces of nature, conflict and terrorism, and state actor violence	0.0% (0.0% to 0.0%)	0.0% (0.0% to 0.0%)	6.6% (4.4% to 9.7%)	6.6% (4.4% to 9.7%)

Supplementary Results Table 1. Age-standardised PAFs attributable to the joint distribution of all risk factors for all ages, both sexes combined for each GBD level 1 and level 2 cause and all causes in 2016 for Federated States of Micronesia. Results are shown for deaths, YLLs, YLDs, and
Data in parenthesis are 95% uncertainty intervals. PAFs=population attributable fractions. GBD=Global Burden of Disease. YLLs=years of life lost. YLDs=years live with disability. DALYs=disability-adjusted life-years

	Deaths 2016	YLLs 2016	YLDs 2016	DALYs 2016
**All causes**	68.2% (65.9% to 70.9%)	63.5% (60.7% to 67.4%)	34.0% (32.2% to 36.1%)	56.1% (53.0% to 59.6%)
**HIV/AIDS and tuberculosis**	65.5% (40.8% to 87.2%)	70.8% (45.4% to 88.8%)	63.4% (42.8% to 87.0%)	71.5% (47.6% to 88.3%)
Tuberculosis	37.8% (27.8% to 47.5%)	39.0% (30.0% to 47.7%)	35.8% (27.7% to 44.0%)	38.7% (30.0% to 47.0%)
HIV/AIDS	91.0% (85.9% to 95.1%)	90.1% (84.7% to 94.6%)	90.5% (85.5% to 94.7%)	90.2% (84.9% to 94.6%)
**Diarrhoea, lower respiratory infections, and other common infectious diseases**	48.9% (41.4% to 57.2%)	49.0% (41.6% to 56.7%)	37.0% (26.1% to 44.8%)	47.3% (40.4% to 54.1%)
Diarrhoeal diseases	77.6% (51.1% to 93.0%)	80.5% (56.2% to 94.2%)	79.3% (55.2% to 93.6%)	79.8% (55.5% to 93.8%)
Lower respiratory infections	50.1% (41.3% to 59.3%)	57.3% (48.6% to 66.2%)	54.1% (46.3% to 62.5%)	57.3% (48.6% to 66.1%)
Upper respiratory infections	0.6% (0.0% to 2.2%)	1.6% (0.1% to 5.8%)	0.0% (0.0% to 0.0%)	0.1% (0.0% to 0.2%)
Otitis media	1.3% (0.3% to 3.7%)	3.1% (0.8% to 7.7%)	5.9% (3.9% to 7.8%)	5.9% (3.9% to 7.8%)
Meningitis	1.7% (0.8% to 3.2%)	2.9% (1.5% to 5.3%)	0.0% (0.0% to 0.0%)	2.3% (1.2% to 4.1%)
Encephalitis	0.8% (0.4% to 1.4%)	1.7% (0.9% to 2.8%)	0.0% (0.0% to 0.0%)	0.7% (0.3% to 1.2%)
Measles	36.3% (14.2% to 73.5%)	37.0% (14.4% to 74.8%)	37.1% (14.7% to 73.9%)	37.0% (14.4% to 74.8%)
**Maternal disorders**	7.6% (3.0% to 12.7%)	7.6% (3.0% to 12.7%)	3.6% (1.7% to 6.1%)	7.4% (3.0% to 12.3%)
Maternal haemorrhage	16.9% (6.1% to 27.8%)	16.9% (6.1% to 27.8%)	16.9% (6.1% to 28.1%)	16.9% (6.1% to 27.8%)
Maternal sepsis and other pregnancy related infections	16.7% (6.1% to 27.7%)	16.8% (6.1% to 27.7%)	17.0% (6.1% to 28.2%)	16.8% (6.1% to 27.7%)
Maternal abortion, miscarriage, and ectopic pregnancy	12.2% (6.7% to 18.7%)	12.0% (6.5% to 18.5%)	12.1% (6.3% to 19.0%)	12.1% (6.8% to 18.2%)
**Neonatal disorders**	77.4% (69.8% to 82.9%)	77.4% (69.9% to 82.9%)	0.2% (0.1% to 0.3%)	67.2% (60.2% to 73.5%)
Neonatal preterm birth complications	95.0% (89.9% to 98.0%)	95.0% (90.0% to 98.0%)	0.3% (0.2% to 0.3%)	75.3% (64.2% to 83.9%)
Neonatal encephalopathy due to birth asphyxia and trauma	66.4% (57.0% to 72.9%)	66.4% (57.1% to 73.0%)	0.0% (0.0% to 0.0%)	63.4% (54.7% to 70.9%)
Neonatal sepsis and other neonatal infections	63.9% (54.2% to 70.5%)	64.0% (54.2% to 70.5%)	0.0% (0.0% to 0.0%)	56.5% (43.5% to 65.2%)
Hemolytic disease and other neonatal jaundice	66.5% (60.3% to 72.2%)	66.5% (60.3% to 72.2%)	0.0% (0.0% to 0.0%)	42.2% (30.4% to 53.3%)

Supplementary Results Table 1. Age-standardised PAFs attributable to the joint distribution of all risk factors for all ages, both sexes combined for each GBD level 1 and level 2 cause and all causes in 2016 for Federated States of Micronesia. Results are shown for deaths, YLLs, YLDs, and
Data in parenthesis are 95% uncertainty intervals. PAFs=population attributable fractions. GBD=Global Burden of Disease. YLLs=years of life lost. YLDs=years live with disability. DALYs=disability-adjusted life-years

	Deaths 2016	YLLs 2016	YLDs 2016	DALYs 2016
Other neonatal disorders	59.4% (45.6% to 68.4%)	59.4% (45.7% to 68.5%)	0.0% (0.0% to 0.0%)	57.8% (44.6% to 66.6%)
**Nutritional deficiencies**	7.7% (3.8% to 13.7%)	35.5% (20.6% to 52.3%)	99.4% (99.2% to 99.5%)	84.8% (78.7% to 89.7%)
Protein-energy malnutrition	7.7% (3.7% to 13.8%)	36.3% (21.1% to 53.4%)	97.1% (95.1% to 98.4%)	45.8% (33.6% to 59.3%)
Vitamin A deficiency	-	-	100.0% (100.0% to 100.0%)	100.0% (100.0% to 100.0%)
Iron-deficiency anaemia	100.0% (100.0% to 100.0%)	100.0% (100.0% to 100.0%)	100.0% (100.0% to 100.0%)	100.0% (100.0% to 100.0%)
**Other communicable, maternal, neonatal, and nutritional diseases**	5.6% (2.8% to 9.0%)	4.9% (1.9% to 9.7%)	61.2% (55.5% to 67.2%)	13.4% (6.9% to 22.9%)
Sexually transmitted diseases excluding HIV	22.8% (5.5% to 80.4%)	13.9% (2.6% to 66.5%)	100.0% (100.0% to 100.0%)	29.8% (9.0% to 86.5%)
Hepatitis	0.3% (0.2% to 0.5%)	0.4% (0.3% to 0.7%)	0.3% (0.2% to 0.6%)	0.4% (0.3% to 0.6%)
**Neoplasms**	45.0% (39.0% to 51.3%)	45.2% (38.9% to 51.5%)	44.2% (37.1% to 50.9%)	45.2% (38.9% to 51.5%)
Oesophageal cancer	75.6% (63.0% to 85.5%)	77.6% (65.3% to 86.8%)	76.0% (63.7% to 85.8%)	77.6% (65.2% to 86.8%)
Stomach cancer	14.8% (8.1% to 22.5%)	16.2% (9.0% to 24.2%)	15.2% (8.3% to 22.9%)	16.2% (9.0% to 24.2%)
Liver cancer	45.8% (33.5% to 57.6%)	47.7% (35.3% to 59.7%)	46.3% (34.0% to 58.1%)	47.7% (35.3% to 59.6%)
Larynx cancer	78.9% (68.7% to 87.2%)	78.8% (68.6% to 86.5%)	78.8% (68.3% to 86.8%)	78.8% (68.6% to 86.5%)
Tracheal, bronchus, and lung cancer	87.5% (82.3% to 91.8%)	86.9% (81.4% to 91.2%)	87.0% (81.4% to 91.3%)	86.9% (81.4% to 91.2%)
Breast cancer	49.8% (30.8% to 66.4%)	47.9% (30.7% to 63.5%)	48.1% (30.6% to 63.4%)	47.9% (30.7% to 63.4%)
Cervical cancer	100.0% (100.0% to 100.0%)	100.0% (100.0% to 100.0%)	100.0% (100.0% to 100.0%)	100.0% (100.0% to 100.0%)
Uterine cancer	56.1% (40.3% to 69.4%)	57.9% (42.7% to 70.8%)	57.6% (42.2% to 70.4%)	57.9% (42.7% to 70.8%)
Prostate cancer	4.9% (3.1% to 7.6%)	5.3% (3.3% to 8.3%)	5.2% (3.2% to 8.1%)	5.3% (3.3% to 8.3%)
Colon and rectum cancer	58.0% (46.6% to 69.8%)	57.9% (47.3% to 69.1%)	58.1% (47.1% to 69.7%)	57.9% (47.3% to 69.1%)
Lip and oral cavity cancer	57.4% (47.3% to 67.5%)	60.4% (50.1% to 70.2%)	56.1% (45.7% to 66.3%)	60.3% (50.0% to 70.1%)
Nasopharynx cancer	65.9% (54.1% to 75.7%)	66.4% (54.8% to 76.3%)	64.8% (53.4% to 75.1%)	66.4% (54.8% to 76.2%)

Supplementary Results Table 1. Age-standardised PAFs attributable to the joint distribution of all risk factors for all ages, both sexes combined for each GBD level 1 and level 2 cause and all causes in 2016 for Federated States of Micronesia. Results are shown for deaths, YLLs, YLDs, and
Data in parenthesis are 95% uncertainty intervals. PAFs=population attributable fractions. GBD=Global Burden of Disease. YLLs=years of life lost. YLDs=years live with disability. DALYs=disability-adjusted life-years

	Deaths 2016	YLLs 2016	YLDs 2016	DALYs 2016
Other pharynx cancer	15.3% (6.8% to 24.7%)	16.7% (7.7% to 26.4%)	15.9% (7.3% to 25.3%)	16.7% (7.7% to 26.3%)
Gallbladder and biliary tract cancer	28.6% (16.3% to 42.1%)	30.5% (17.8% to 44.2%)	28.7% (16.6% to 42.2%)	30.5% (17.8% to 44.2%)
Pancreatic cancer	40.4% (27.3% to 55.5%)	41.5% (28.9% to 56.1%)	40.5% (27.5% to 55.5%)	41.5% (28.9% to 56.1%)
Ovarian cancer	24.3% (9.3% to 42.8%)	22.9% (8.8% to 40.1%)	22.3% (8.4% to 39.2%)	22.8% (8.8% to 40.1%)
Kidney cancer	39.8% (28.7% to 51.4%)	40.6% (29.5% to 51.9%)	39.7% (29.0% to 50.8%)	40.6% (29.4% to 51.9%)
Bladder cancer	40.5% (26.8% to 55.3%)	42.0% (27.8% to 56.5%)	41.7% (27.5% to 56.3%)	42.0% (27.8% to 56.5%)
Thyroid cancer	17.4% (9.9% to 26.2%)	18.4% (10.5% to 27.3%)	18.3% (10.7% to 27.0%)	18.4% (10.5% to 27.3%)
Mesothelioma	88.2% (82.4% to 92.1%)	80.6% (71.6% to 87.0%)	85.8% (79.4% to 90.2%)	80.6% (71.7% to 87.0%)
Non-Hodgkin's lymphoma	8.4% (3.5% to 14.1%)	8.1% (3.4% to 13.5%)	8.3% (3.5% to 14.1%)	8.1% (3.4% to 13.5%)
Multiple myeloma	10.7% (4.6% to 18.0%)	11.5% (5.0% to 19.1%)	10.9% (4.8% to 18.3%)	11.4% (5.0% to 19.1%)
Leukaemia	20.3% (12.6% to 28.7%)	17.6% (10.7% to 25.2%)	19.2% (11.8% to 27.3%)	17.6% (10.7% to 25.2%)
**Cardiovascular diseases**	86.1% (84.0% to 88.1%)	86.2% (84.5% to 87.9%)	70.4% (65.4% to 74.4%)	85.4% (83.7% to 87.2%)
Rheumatic heart disease	24.6% (16.9% to 38.8%)	21.4% (14.6% to 31.4%)	18.2% (11.4% to 26.7%)	20.8% (14.2% to 30.5%)
Ischaemic heart disease	95.4% (93.0% to 97.3%)	96.4% (95.0% to 97.5%)	93.3% (91.2% to 95.0%)	96.4% (94.9% to 97.5%)
Cerebrovascular disease	87.4% (83.8% to 90.8%)	89.2% (86.7% to 91.5%)	88.9% (85.8% to 91.7%)	89.2% (86.7% to 91.5%)
Hypertensive heart disease	100.0% (100.0% to 100.0%)	100.0% (100.0% to 100.0%)	100.0% (100.0% to 100.0%)	100.0% (100.0% to 100.0%)
Cardiomyopathy and myocarditis	30.7% (24.4% to 37.2%)	29.2% (23.0% to 35.3%)	25.5% (20.7% to 30.7%)	29.1% (23.0% to 35.3%)
Atrial fibrillation and flutter	49.1% (41.0% to 56.7%)	53.5% (46.3% to 60.0%)	59.5% (54.2% to 64.9%)	56.3% (50.0% to 62.3%)
Aortic aneurysm	45.2% (38.3% to 52.1%)	51.1% (45.1% to 57.0%)	-	51.1% (45.1% to 57.0%)
Peripheral vascular disease	66.2% (60.0% to 72.3%)	75.7% (71.2% to 79.9%)	73.9% (69.7% to 77.9%)	74.8% (70.8% to 78.7%)
Endocarditis	35.5% (27.5% to 44.9%)	32.9% (25.8% to 40.3%)	34.0% (26.5% to 42.5%)	32.9% (25.8% to 40.3%)

Supplementary Results Table 1. Age-standardised PAFs attributable to the joint distribution of all risk factors for all ages, both sexes combined for each GBD level 1 and level 2 cause and all causes in 2016 for Federated States of Micronesia. Results are shown for deaths, YLLs, YLDs, and
Data in parenthesis are 95% uncertainty intervals. PAFs=population attributable fractions. GBD=Global Burden of Disease. YLLs=years of life lost. YLDs=years live with disability. DALYs=disability-adjusted life-years

	Deaths 2016	YLLs 2016	YLDs 2016	DALYs 2016
Other cardiovascular and circulatory diseases	47.4% (42.5% to 52.4%)	48.0% (42.7% to 53.2%)	42.2% (36.7% to 47.3%)	47.4% (42.3% to 52.6%)
**Chronic respiratory diseases**	62.9% (57.1% to 68.8%)	60.5% (54.6% to 66.0%)	54.7% (48.5% to 61.4%)	59.1% (53.2% to 64.7%)
Chronic obstructive pulmonary disease	74.8% (67.6% to 81.4%)	73.4% (65.8% to 80.0%)	70.5% (62.1% to 77.6%)	72.9% (65.1% to 79.8%)
Pneumoconiosis	100.0% (100.0% to 100.0%)	100.0% (100.0% to 100.0%)	100.0% (100.0% to 100.0%)	100.0% (100.0% to 100.0%)
Asthma	48.3% (36.9% to 60.8%)	52.6% (42.3% to 63.3%)	45.1% (35.9% to 55.2%)	49.7% (40.1% to 60.2%)
Other chronic respiratory diseases	22.4% (13.5% to 32.7%)	22.0% (12.7% to 32.8%)	8.8% (4.9% to 13.7%)	20.9% (12.0% to 31.6%)
**Cirrhosis and other chronic liver diseases**	18.9% (17.0% to 20.9%)	19.9% (17.5% to 22.3%)	14.0% (11.8% to 16.1%)	19.8% (17.3% to 22.1%)
Cirrhosis and other chronic liver diseases due to hepatitis B	0.1% (0.0% to 0.1%)	0.1% (0.0% to 0.1%)	0.1% (0.0% to 0.1%)	0.1% (0.0% to 0.1%)
Cirrhosis and other chronic liver diseases due to hepatitis C	24.4% (17.0% to 31.7%)	35.7% (26.3% to 44.2%)	29.3% (22.5% to 36.9%)	35.6% (26.4% to 44.1%)
Cirrhosis and other chronic liver diseases due to alcohol use	100.0% (100.0% to 100.0%)	100.0% (100.0% to 100.0%)	100.0% (100.0% to 100.0%)	100.0% (100.0% to 100.0%)
**Digestive diseases**	12.6% (9.7% to 15.6%)	12.9% (10.1% to 15.7%)	6.0% (4.6% to 7.6%)	12.3% (9.6% to 14.9%)
Peptic ulcer disease	14.5% (10.1% to 19.5%)	16.7% (11.7% to 22.2%)	14.8% (10.1% to 20.1%)	16.5% (11.6% to 22.0%)
Gallbladder and biliary diseases	51.6% (35.8% to 66.4%)	52.9% (38.3% to 66.1%)	56.5% (42.6% to 69.5%)	53.1% (38.5% to 66.2%)
Pancreatitis	4.8% (-1.1% to 11.8%)	6.9% (-0.4% to 15.9%)	5.2% (-0.8% to 12.2%)	6.9% (-0.4% to 15.8%)
**Neurological disorders**	31.4% (16.1% to 48.0%)	28.7% (15.4% to 43.3%)	6.0% (3.3% to 9.4%)	16.7% (8.8% to 26.1%)
Alzheimer's disease and other dementias	35.7% (18.6% to 54.1%)	37.9% (20.4% to 56.8%)	37.8% (20.3% to 56.8%)	37.9% (20.4% to 56.7%)
Parkinson's disease	-9.9% (-12.5% to -7.4%)	-12.7% (-16.0% to -9.6%)	-14.6% (-18.4% to -11.1%)	-13.0% (-16.4% to -9.9%)
Epilepsy	9.5% (4.2% to 16.1%)	10.0% (4.5% to 16.8%)	6.3% (2.7% to 10.8%)	8.0% (3.5% to 13.5%)
Multiple sclerosis	11.0% (6.6% to 16.8%)	11.5% (6.9% to 17.5%)	8.7% (5.6% to 12.3%)	11.0% (6.7% to 16.6%)
**Mental and substance use disorders**	99.6% (99.3% to 99.8%)	99.5% (99.1% to 99.7%)	16.3% (14.6% to 18.2%)	20.1% (17.4% to 23.3%)
Alcohol use disorders	100.0% (100.0% to 100.0%)	100.0% (100.0% to 100.0%)	100.0% (100.0% to 100.0%)	100.0% (100.0% to 100.0%)

Supplementary Results Table 1. Age-standardised PAFs attributable to the joint distribution of all risk factors for all ages, both sexes combined for each GBD level 1 and level 2 cause and all causes in 2016 for Federated States of Micronesia. Results are shown for deaths, YLLs, YLDs, and
Data in parenthesis are 95% uncertainty intervals. PAFs=population attributable fractions. GBD=Global Burden of Disease. YLLs=years of life lost. YLDs=years live with disability. DALYs=disability-adjusted life-years

	Deaths 2016	YLLs 2016	YLDs 2016	DALYs 2016
Drug use disorders	100.0% (100.0% to 100.0%)	100.0% (100.0% to 100.0%)	100.0% (100.0% to 100.0%)	100.0% (100.0% to 100.0%)
Depressive disorders	-	-	11.0% (9.1% to 13.1%)	11.0% (9.1% to 13.1%)
Idiopathic developmental intellectual disability	-	-	58.6% (28.5% to 78.4%)	58.6% (28.5% to 78.4%)
**Diabetes, urogenital, blood, and endocrine diseases**	93.0% (91.8% to 93.9%)	91.8% (90.2% to 92.9%)	82.8% (80.2% to 84.9%)	89.6% (88.0% to 90.8%)
Diabetes mellitus	100.0% (100.0% to 100.0%)	100.0% (100.0% to 100.0%)	100.0% (100.0% to 100.0%)	100.0% (100.0% to 100.0%)
Chronic kidney disease	100.0% (100.0% to 100.0%)	100.0% (100.0% to 100.0%)	100.0% (100.0% to 100.0%)	100.0% (100.0% to 100.0%)
**Musculoskeletal disorders**	0.6% (0.2% to 1.2%)	0.4% (0.2% to 0.8%)	19.1% (15.0% to 23.5%)	18.4% (14.4% to 22.8%)
Rheumatoid arthritis	4.7% (1.8% to 8.1%)	5.3% (2.1% to 9.0%)	5.6% (2.2% to 9.4%)	5.5% (2.2% to 9.3%)
Osteoarthritis	-	-	34.6% (19.7% to 49.0%)	34.6% (19.7% to 49.0%)
Low back and neck pain	-	-	21.9% (17.8% to 26.2%)	21.9% (17.8% to 26.2%)
Gout	-	-	57.0% (41.4% to 72.5%)	57.0% (41.4% to 72.5%)
**Other non-communicable diseases**	0.2% (0.1% to 0.3%)	0.4% (0.2% to 0.6%)	6.9% (5.8% to 8.0%)	5.2% (4.3% to 6.1%)
Sense organ diseases	-	-	13.6% (11.8% to 15.4%)	13.6% (11.8% to 15.4%)
Sudden infant death syndrome	15.5% (8.0% to 24.9%)	15.6% (8.0% to 25.0%)	-	15.6% (8.0% to 25.0%)
**Transport injuries**	21.6% (17.7% to 26.1%)	17.0% (12.8% to 21.7%)	25.1% (21.4% to 30.9%)	18.1% (13.9% to 22.8%)
Road injuries	21.3% (17.4% to 25.7%)	16.9% (12.7% to 21.6%)	28.4% (24.5% to 34.3%)	18.1% (13.8% to 22.9%)
Other transport injuries	24.2% (19.3% to 29.5%)	17.9% (13.5% to 23.3%)	19.0% (15.4% to 24.5%)	18.3% (14.2% to 23.0%)
**Unintentional injuries**	15.1% (10.7% to 18.5%)	9.8% (7.6% to 12.6%)	11.5% (9.6% to 16.0%)	10.2% (8.0% to 12.9%)
Falls	51.3% (26.1% to 60.4%)	27.3% (18.9% to 33.0%)	22.3% (20.2% to 27.0%)	25.3% (20.0% to 29.6%)
Drowning	10.0% (7.1% to 13.9%)	10.0% (7.1% to 13.9%)	9.0% (5.8% to 15.3%)	10.0% (7.1% to 13.9%)
Fire, heat, and hot substances	6.4% (4.4% to 9.5%)	7.5% (5.1% to 10.6%)	8.9% (5.8% to 16.2%)	7.8% (5.3% to 11.7%)

Supplementary Results Table 1. Age-standardised PAFs attributable to the joint distribution of all risk factors for all ages, both sexes combined for each GBD level 1 and level 2 cause and all causes in 2016 for Federated States of Micronesia. Results are shown for deaths, YLLs, YLDs, and
Data in parenthesis are 95% uncertainty intervals. PAFs=population attributable fractions. GBD=Global Burden of Disease. YLLs=years of life lost. YLDs=years live with disability. DALYs=disability-adjusted life-years

	Deaths 2016	YLLs 2016	YLDs 2016	DALYs 2016
Poisonings	7.6% (5.2% to 10.8%)	8.9% (6.2% to 12.6%)	9.1% (6.0% to 14.9%)	8.9% (6.2% to 12.6%)
Exposure to mechanical forces	13.4% (11.3% to 16.2%)	9.6% (7.6% to 12.3%)	15.8% (13.3% to 21.8%)	11.0% (8.7% to 14.6%)
Animal contact	14.9% (12.3% to 18.0%)	10.0% (7.8% to 12.9%)	7.2% (4.9% to 13.0%)	8.2% (6.0% to 12.8%)
Foreign body	4.0% (2.8% to 5.4%)	4.5% (3.1% to 6.4%)	5.0% (3.2% to 9.4%)	4.7% (3.2% to 6.9%)
Other unintentional injuries	10.4% (7.2% to 14.5%)	11.9% (8.2% to 16.8%)	8.5% (5.6% to 13.9%)	10.5% (7.3% to 15.2%)
**Self-harm and interpersonal violence**	12.4% (6.9% to 18.2%)	12.9% (7.2% to 19.4%)	25.1% (22.3% to 28.0%)	13.6% (8.2% to 19.7%)
Self-harm	10.0% (3.2% to 17.2%)	10.9% (3.5% to 19.0%)	8.0% (2.2% to 14.0%)	10.9% (3.5% to 18.9%)
Interpersonal violence	19.1% (13.0% to 25.4%)	18.2% (11.9% to 24.6%)	27.0% (24.1% to 30.0%)	19.6% (14.2% to 25.5%)
**Forces of nature, conflict and terrorism, and executions and police conflict**	0.0% (0.0% to 0.0%)	0.0% (0.0% to 0.0%)	5.4% (3.7% to 8.1%)	4.8% (3.2% to 7.2%)
Forces of nature, conflict and terrorism, and state actor violence	0.0% (0.0% to 0.0%)	0.0% (0.0% to 0.0%)	5.7% (3.8% to 8.4%)	5.7% (3.8% to 8.4%)

Supplementary Results Table 1. Age-standardised PAFs attributable to the joint distribution of all risk factors for all ages, both sexes combined for each GBD level 1 and level 2 cause and all causes in 2016 for Fiji. Results are shown for deaths, YLLs, YLDs, and DALYs
Data in parenthesis are 95% uncertainty intervals. PAFs=population attributable fractions. GBD=Global Burden of Disease. YLLs=years of life lost. YLDs=years live with disability. DALYs=disability-adjusted life-years

	Deaths 2016	YLLs 2016	YLDs 2016	DALYs 2016
**All causes**	72.2% (70.4% to 74.0%)	65.6% (63.1% to 67.8%)	35.2% (33.4% to 37.0%)	58.0% (55.1% to 60.8%)
**HIV/AIDS and tuberculosis**	56.1% (46.1% to 65.2%)	59.1% (50.1% to 67.5%)	56.8% (47.9% to 66.0%)	58.9% (50.6% to 66.7%)
Tuberculosis	48.2% (36.8% to 58.0%)	46.9% (37.7% to 55.5%)	46.7% (38.5% to 54.4%)	46.9% (37.7% to 55.2%)
HIV/AIDS	93.9% (91.4% to 95.8%)	92.4% (89.4% to 94.9%)	92.7% (90.4% to 94.3%)	92.5% (89.6% to 94.8%)
**Diarrhoea, lower respiratory infections, and other common infectious diseases**	48.6% (41.2% to 56.7%)	53.5% (45.7% to 61.8%)	35.1% (23.3% to 44.4%)	51.6% (44.3% to 59.8%)
Diarrhoeal diseases	72.6% (47.2% to 91.3%)	81.0% (61.6% to 94.0%)	72.5% (45.5% to 91.1%)	78.7% (58.2% to 93.3%)
Lower respiratory infections	48.2% (40.1% to 57.1%)	60.1% (50.6% to 69.5%)	49.0% (41.6% to 56.7%)	60.1% (50.6% to 69.5%)
Upper respiratory infections	1.5% (0.1% to 5.6%)	3.2% (0.3% to 11.8%)	0.0% (0.0% to 0.0%)	0.1% (0.0% to 0.3%)
Otitis media	2.6% (0.8% to 5.2%)	5.5% (2.1% to 9.5%)	6.2% (4.2% to 8.2%)	6.2% (4.2% to 8.2%)
Meningitis	2.7% (1.6% to 4.5%)	3.8% (2.3% to 6.1%)	0.0% (0.0% to 0.0%)	3.3% (2.0% to 5.3%)
Encephalitis	1.9% (0.8% to 3.6%)	3.3% (1.7% to 6.2%)	0.0% (0.0% to 0.0%)	1.9% (0.9% to 3.5%)
Measles	34.6% (15.1% to 73.4%)	35.2% (15.4% to 74.5%)	34.7% (14.8% to 72.1%)	35.2% (15.4% to 74.5%)
**Maternal disorders**	8.8% (5.5% to 12.4%)	8.6% (5.5% to 12.3%)	1.8% (0.9% to 2.9%)	7.8% (5.0% to 11.2%)
Maternal haemorrhage	15.0% (5.4% to 24.3%)	15.0% (5.4% to 24.2%)	14.9% (5.4% to 24.2%)	14.9% (5.4% to 24.2%)
Maternal sepsis and other pregnancy related infections	15.0% (5.5% to 24.2%)	15.0% (5.4% to 24.2%)	14.8% (5.4% to 24.1%)	15.0% (5.4% to 24.2%)
Maternal abortion, miscarriage, and ectopic pregnancy	10.4% (6.0% to 15.7%)	10.2% (5.9% to 15.4%)	9.5% (5.0% to 15.0%)	10.2% (5.9% to 15.4%)
**Neonatal disorders**	80.4% (74.6% to 84.2%)	80.4% (74.7% to 84.2%)	0.2% (0.2% to 0.3%)	75.0% (69.6% to 79.3%)
Neonatal preterm birth complications	94.8% (89.8% to 97.4%)	94.8% (89.9% to 97.4%)	0.3% (0.2% to 0.4%)	85.3% (78.7% to 90.5%)
Neonatal encephalopathy due to birth asphyxia and trauma	68.1% (61.8% to 73.1%)	68.2% (61.9% to 73.2%)	0.0% (0.0% to 0.0%)	67.5% (61.0% to 72.4%)
Neonatal sepsis and other neonatal infections	64.4% (55.5% to 70.6%)	64.4% (55.6% to 70.7%)	0.0% (0.0% to 0.0%)	60.6% (51.3% to 67.9%)
Hemolytic disease and other neonatal jaundice	69.6% (62.6% to 75.6%)	69.6% (62.6% to 75.6%)	0.0% (0.0% to 0.0%)	50.1% (39.7% to 59.8%)

Supplementary Results Table 1. Age-standardised PAFs attributable to the joint distribution of all risk factors for all ages, both sexes combined for each GBD level 1 and level 2 cause and all causes in 2016 for Fiji. Results are shown for deaths, YLLs, YLDs, and DALYs
Data in parenthesis are 95% uncertainty intervals. PAFs=population attributable fractions. GBD=Global Burden of Disease. YLLs=years of life lost. YLDs=years live with disability. DALYs=disability-adjusted life-years

	Deaths 2016	YLLs 2016	YLDs 2016	DALYs 2016
Other neonatal disorders	59.0% (48.0% to 67.6%)	59.1% (48.1% to 67.7%)	0.0% (0.0% to 0.0%)	57.4% (46.8% to 65.7%)
**Nutritional deficiencies**	24.7% (13.3% to 40.8%)	66.5% (49.7% to 81.3%)	99.5% (99.4% to 99.6%)	93.0% (89.9% to 95.4%)
Protein-energy malnutrition	25.5% (13.6% to 42.3%)	68.9% (52.1% to 83.6%)	98.0% (96.6% to 99.0%)	73.0% (60.3% to 84.7%)
Vitamin A deficiency	-	-	100.0% (100.0% to 100.0%)	100.0% (100.0% to 100.0%)
Iron-deficiency anaemia	100.0% (100.0% to 100.0%)	100.0% (100.0% to 100.0%)	100.0% (100.0% to 100.0%)	100.0% (100.0% to 100.0%)
**Other communicable, maternal, neonatal, and nutritional diseases**	4.5% (3.1% to 6.7%)	4.7% (2.9% to 7.2%)	63.5% (58.4% to 68.5%)	12.6% (8.4% to 18.2%)
Sexually transmitted diseases excluding HIV	51.3% (21.7% to 92.9%)	36.2% (11.7% to 86.2%)	100.0% (100.0% to 100.0%)	60.2% (28.7% to 95.2%)
Hepatitis	0.5% (0.3% to 0.7%)	0.5% (0.3% to 0.8%)	0.4% (0.2% to 0.7%)	0.5% (0.3% to 0.8%)
**Neoplasms**	39.0% (32.4% to 46.4%)	38.1% (31.8% to 44.7%)	41.1% (33.9% to 48.7%)	38.1% (31.9% to 44.7%)
Oesophageal cancer	63.6% (47.5% to 77.2%)	66.2% (50.8% to 78.4%)	64.3% (48.5% to 77.5%)	66.2% (50.7% to 78.4%)
Stomach cancer	4.5% (1.2% to 10.5%)	4.7% (1.3% to 10.1%)	4.5% (1.3% to 10.1%)	4.7% (1.3% to 10.1%)
Liver cancer	38.1% (26.4% to 51.4%)	38.9% (27.0% to 52.3%)	38.5% (27.0% to 51.5%)	38.9% (27.0% to 52.3%)
Larynx cancer	50.3% (33.2% to 64.6%)	54.0% (36.8% to 67.4%)	52.6% (35.8% to 66.7%)	53.9% (36.8% to 67.4%)
Tracheal, bronchus, and lung cancer	68.3% (54.7% to 78.9%)	68.0% (54.6% to 78.3%)	67.9% (54.6% to 78.3%)	68.0% (54.6% to 78.4%)
Breast cancer	49.4% (29.7% to 67.2%)	46.1% (29.0% to 62.8%)	47.1% (29.7% to 63.8%)	46.1% (29.1% to 62.8%)
Cervical cancer	100.0% (100.0% to 100.0%)	100.0% (100.0% to 100.0%)	100.0% (100.0% to 100.0%)	100.0% (100.0% to 100.0%)
Uterine cancer	53.3% (36.9% to 66.9%)	54.3% (38.7% to 67.1%)	54.6% (38.8% to 67.2%)	54.3% (38.7% to 67.1%)
Prostate cancer	0.8% (0.3% to 1.5%)	0.9% (0.3% to 1.6%)	0.9% (0.3% to 1.6%)	0.9% (0.3% to 1.6%)
Colon and rectum cancer	58.6% (45.5% to 71.9%)	58.0% (45.9% to 70.6%)	58.8% (46.0% to 71.9%)	58.1% (45.9% to 70.6%)
Lip and oral cavity cancer	42.0% (29.4% to 53.8%)	47.2% (34.9% to 58.5%)	42.4% (30.5% to 53.4%)	47.0% (34.7% to 58.3%)
Nasopharynx cancer	47.8% (34.6% to 59.5%)	49.5% (36.8% to 60.8%)	50.1% (38.4% to 59.8%)	49.5% (36.9% to 60.8%)

Supplementary Results Table 1. Age-standardised PAFs attributable to the joint distribution of all risk factors for all ages, both sexes combined for each GBD level 1 and level 2 cause and all causes in 2016 for Fiji. Results are shown for deaths, YLLs, YLDs, and DALYs
Data in parenthesis are 95% uncertainty intervals. PAFs=population attributable fractions. GBD=Global Burden of Disease. YLLs=years of life lost. YLDs=years live with disability. DALYs=disability-adjusted life-years

	Deaths 2016	YLLs 2016	YLDs 2016	DALYs 2016
Other pharynx cancer	23.9% (13.3% to 35.4%)	26.1% (15.6% to 37.1%)	24.8% (14.1% to 35.9%)	26.1% (15.6% to 37.0%)
Gallbladder and biliary tract cancer	25.7% (14.6% to 38.5%)	27.2% (16.0% to 40.3%)	26.3% (15.5% to 38.6%)	27.2% (16.0% to 40.3%)
Pancreatic cancer	34.1% (17.4% to 54.2%)	33.9% (17.8% to 53.2%)	34.1% (17.4% to 53.9%)	33.9% (17.8% to 53.2%)
Ovarian cancer	27.0% (9.4% to 47.6%)	24.3% (8.5% to 43.2%)	23.0% (7.9% to 41.6%)	24.3% (8.5% to 43.1%)
Kidney cancer	26.7% (17.8% to 36.9%)	24.3% (16.0% to 33.7%)	25.7% (17.5% to 35.1%)	24.4% (16.1% to 33.7%)
Bladder cancer	29.6% (12.0% to 49.9%)	29.7% (12.7% to 49.5%)	29.4% (12.5% to 48.8%)	29.7% (12.7% to 49.4%)
Thyroid cancer	15.9% (9.0% to 23.9%)	16.3% (9.3% to 24.3%)	16.3% (9.6% to 23.7%)	16.3% (9.4% to 24.3%)
Mesothelioma	88.6% (83.8% to 92.2%)	81.0% (73.8% to 86.9%)	86.9% (81.7% to 90.9%)	81.1% (73.9% to 87.0%)
Non-Hodgkin's lymphoma	7.5% (3.1% to 12.4%)	6.6% (2.7% to 11.0%)	7.5% (3.3% to 12.6%)	6.6% (2.7% to 11.0%)
Multiple myeloma	9.6% (4.2% to 16.4%)	10.0% (4.5% to 16.9%)	9.9% (4.4% to 16.7%)	10.0% (4.5% to 16.9%)
Leukaemia	11.2% (6.8% to 16.0%)	8.8% (5.2% to 12.7%)	10.1% (6.3% to 14.3%)	8.8% (5.2% to 12.7%)
**Cardiovascular diseases**	84.7% (82.6% to 86.5%)	82.8% (80.4% to 85.0%)	75.5% (71.9% to 78.6%)	82.4% (80.0% to 84.6%)
Rheumatic heart disease	27.6% (19.3% to 42.2%)	23.7% (16.6% to 34.0%)	24.2% (15.5% to 34.4%)	23.8% (16.5% to 34.0%)
Ischaemic heart disease	95.5% (93.5% to 97.2%)	96.2% (94.9% to 97.3%)	93.6% (91.7% to 95.3%)	96.2% (94.9% to 97.2%)
Cerebrovascular disease	87.8% (84.3% to 91.1%)	86.8% (83.6% to 89.5%)	89.9% (87.0% to 92.5%)	87.2% (84.1% to 89.8%)
Hypertensive heart disease	100.0% (100.0% to 100.0%)	100.0% (100.0% to 100.0%)	100.0% (100.0% to 100.0%)	100.0% (100.0% to 100.0%)
Cardiomyopathy and myocarditis	22.8% (18.2% to 27.7%)	20.7% (16.2% to 25.5%)	21.0% (17.0% to 25.3%)	20.7% (16.2% to 25.4%)
Atrial fibrillation and flutter	51.0% (42.2% to 59.4%)	55.3% (47.8% to 62.4%)	62.0% (56.5% to 67.4%)	59.2% (52.8% to 65.1%)
Aortic aneurysm	45.9% (38.6% to 53.2%)	51.4% (45.2% to 57.0%)	-	51.4% (45.2% to 57.0%)
Peripheral vascular disease	71.4% (65.9% to 77.1%)	77.9% (73.9% to 81.9%)	79.4% (75.6% to 82.8%)	79.0% (75.3% to 82.2%)
Endocarditis	39.6% (31.1% to 48.5%)	36.6% (29.2% to 43.7%)	38.9% (30.8% to 47.7%)	36.6% (29.3% to 43.7%)

Supplementary Results Table 1. Age-standardised PAFs attributable to the joint distribution of all risk factors for all ages, both sexes combined for each GBD level 1 and level 2 cause and all causes in 2016 for Fiji. Results are shown for deaths, YLLs, YLDs, and DALYs
Data in parenthesis are 95% uncertainty intervals. PAFs=population attributable fractions. GBD=Global Burden of Disease. YLLs=years of life lost. YLDs=years live with disability. DALYs=disability-adjusted life-years

	Deaths 2016	YLLs 2016	YLDs 2016	DALYs 2016
Other cardiovascular and circulatory diseases	47.5% (42.7% to 52.5%)	44.6% (38.9% to 49.7%)	43.7% (38.5% to 48.8%)	44.5% (39.2% to 49.5%)
**Chronic respiratory diseases**	40.4% (33.1% to 47.8%)	39.5% (32.9% to 46.4%)	40.2% (33.2% to 47.1%)	39.7% (33.3% to 46.4%)
Chronic obstructive pulmonary disease	46.9% (34.8% to 57.4%)	46.3% (34.5% to 56.8%)	44.0% (32.0% to 54.6%)	45.5% (33.6% to 56.1%)
Pneumoconiosis	100.0% (100.0% to 100.0%)	100.0% (100.0% to 100.0%)	100.0% (100.0% to 100.0%)	100.0% (100.0% to 100.0%)
Asthma	44.8% (33.6% to 56.2%)	47.5% (37.7% to 57.7%)	40.1% (31.1% to 50.4%)	45.1% (35.9% to 55.4%)
Other chronic respiratory diseases	4.4% (1.4% to 8.2%)	4.6% (1.3% to 8.9%)	2.2% (0.5% to 4.6%)	4.5% (1.3% to 8.7%)
**Cirrhosis and other chronic liver diseases**	20.0% (18.1% to 22.1%)	20.1% (17.9% to 22.7%)	14.1% (11.9% to 16.7%)	19.8% (17.6% to 22.3%)
Cirrhosis and other chronic liver diseases due to hepatitis B	0.1% (0.0% to 0.1%)	0.1% (0.0% to 0.1%)	0.1% (0.0% to 0.1%)	0.1% (0.0% to 0.1%)
Cirrhosis and other chronic liver diseases due to hepatitis C	22.7% (16.1% to 30.2%)	33.6% (24.6% to 42.9%)	29.4% (21.6% to 39.2%)	33.5% (24.5% to 42.5%)
Cirrhosis and other chronic liver diseases due to alcohol use	100.0% (100.0% to 100.0%)	100.0% (100.0% to 100.0%)	100.0% (100.0% to 100.0%)	100.0% (100.0% to 100.0%)
**Digestive diseases**	9.3% (7.0% to 11.6%)	9.3% (7.2% to 11.5%)	4.8% (3.7% to 6.0%)	8.9% (6.9% to 10.9%)
Peptic ulcer disease	11.5% (7.7% to 15.6%)	12.6% (8.5% to 17.0%)	9.8% (7.0% to 13.2%)	12.4% (8.4% to 16.6%)
Gallbladder and biliary diseases	44.9% (30.0% to 59.9%)	44.3% (30.4% to 58.1%)	52.5% (38.0% to 65.5%)	44.7% (31.1% to 58.6%)
Pancreatitis	23.8% (11.2% to 39.5%)	33.2% (17.4% to 50.0%)	22.2% (11.5% to 34.4%)	33.1% (17.3% to 49.7%)
**Neurological disorders**	35.1% (17.3% to 53.3%)	31.2% (17.3% to 45.2%)	7.8% (4.5% to 11.2%)	17.8% (9.9% to 26.8%)
Alzheimer's disease and other dementias	39.6% (19.1% to 60.6%)	41.4% (20.8% to 62.2%)	41.4% (20.8% to 62.1%)	41.4% (20.8% to 62.2%)
Parkinson's disease	-6.0% (-8.0% to -4.5%)	-7.8% (-10.2% to -5.7%)	-8.8% (-10.8% to -6.7%)	-8.0% (-10.2% to -6.0%)
Epilepsy	19.7% (13.0% to 27.2%)	19.2% (12.6% to 26.8%)	14.1% (9.6% to 19.3%)	16.7% (11.1% to 22.6%)
Multiple sclerosis	7.1% (3.9% to 11.5%)	7.4% (4.0% to 11.9%)	5.3% (3.3% to 7.5%)	6.9% (3.9% to 10.8%)
**Mental and substance use disorders**	99.1% (98.6% to 99.3%)	98.7% (98.1% to 99.1%)	15.4% (13.7% to 17.2%)	17.0% (15.2% to 18.9%)
Alcohol use disorders	100.0% (100.0% to 100.0%)	100.0% (100.0% to 100.0%)	100.0% (100.0% to 100.0%)	100.0% (100.0% to 100.0%)

Supplementary Results Table 1. Age-standardised PAFs attributable to the joint distribution of all risk factors for all ages, both sexes combined for each GBD level 1 and level 2 cause and all causes in 2016 for Fiji. Results are shown for deaths, YLLs, YLDs, and DALYs
Data in parenthesis are 95% uncertainty intervals. PAFs=population attributable fractions. GBD=Global Burden of Disease. YLLs=years of life lost. YLDs=years live with disability. DALYs=disability-adjusted life-years

	Deaths 2016	YLLs 2016	YLDs 2016	DALYs 2016
Drug use disorders	100.0% (100.0% to 100.0%)	100.0% (100.0% to 100.0%)	100.0% (100.0% to 100.0%)	100.0% (100.0% to 100.0%)
Depressive disorders	-	-	11.0% (9.1% to 13.0%)	11.0% (9.1% to 13.0%)
Idiopathic developmental intellectual disability	-	-	55.4% (26.1% to 75.8%)	55.4% (26.1% to 75.8%)
**Diabetes, urogenital, blood, and endocrine diseases**	97.3% (97.0% to 97.5%)	96.3% (95.8% to 96.7%)	88.4% (86.9% to 89.8%)	94.7% (93.9% to 95.4%)
Diabetes mellitus	100.0% (100.0% to 100.0%)	100.0% (100.0% to 100.0%)	100.0% (100.0% to 100.0%)	100.0% (100.0% to 100.0%)
Chronic kidney disease	100.0% (100.0% to 100.0%)	100.0% (100.0% to 100.0%)	100.0% (100.0% to 100.0%)	100.0% (100.0% to 100.0%)
**Musculoskeletal disorders**	0.3% (0.1% to 0.6%)	0.2% (0.1% to 0.4%)	14.9% (11.3% to 19.0%)	14.6% (11.0% to 18.6%)
Rheumatoid arthritis	3.6% (1.4% to 6.2%)	3.7% (1.4% to 6.3%)	3.3% (1.3% to 5.6%)	3.3% (1.3% to 5.7%)
Osteoarthritis	-	-	32.7% (18.6% to 46.7%)	32.7% (18.6% to 46.7%)
Low back and neck pain	-	-	14.9% (11.9% to 18.3%)	14.9% (11.9% to 18.3%)
Gout	-	-	57.2% (42.0% to 72.3%)	57.2% (42.0% to 72.3%)
**Other non-communicable diseases**	0.1% (0.1% to 0.2%)	0.1% (0.1% to 0.2%)	6.6% (5.6% to 7.8%)	4.2% (3.3% to 5.2%)
Sense organ diseases	-	-	13.8% (12.0% to 15.7%)	13.8% (12.0% to 15.7%)
Sudden infant death syndrome	12.4% (6.3% to 21.3%)	12.4% (6.4% to 21.4%)	-	12.4% (6.4% to 21.4%)
**Transport injuries**	25.5% (20.2% to 32.7%)	20.3% (14.8% to 28.1%)	27.6% (23.4% to 32.8%)	21.7% (16.5% to 28.8%)
Road injuries	25.0% (19.7% to 32.2%)	20.4% (14.8% to 28.3%)	31.1% (26.7% to 36.6%)	21.9% (16.6% to 29.3%)
Other transport injuries	29.3% (24.2% to 35.3%)	19.5% (14.3% to 26.6%)	21.2% (17.5% to 26.1%)	20.2% (15.8% to 26.1%)
**Unintentional injuries**	13.3% (9.9% to 16.3%)	8.7% (6.4% to 11.4%)	12.0% (10.1% to 14.5%)	9.3% (7.1% to 11.8%)
Falls	47.8% (26.7% to 57.1%)	23.0% (16.2% to 28.8%)	22.5% (20.6% to 25.3%)	22.7% (18.6% to 26.6%)
Drowning	11.3% (7.7% to 15.6%)	10.4% (6.8% to 14.6%)	11.1% (7.4% to 15.7%)	10.4% (6.8% to 14.6%)
Fire, heat, and hot substances	5.9% (3.9% to 8.5%)	6.1% (3.9% to 8.9%)	9.1% (6.3% to 12.5%)	6.6% (4.3% to 9.4%)

Supplementary Results Table 1. Age-standardised PAFs attributable to the joint distribution of all risk factors for all ages, both sexes combined for each GBD level 1 and level 2 cause and all causes in 2016 for Fiji. Results are shown for deaths, YLLs, YLDs, and DALYs
Data in parenthesis are 95% uncertainty intervals. PAFs=population attributable fractions. GBD=Global Burden of Disease. YLLs=years of life lost. YLDs=years live with disability. DALYs=disability-adjusted life-years

	**Deaths 2016**	**YLLs 2016**	**YLDs 2016**	**DALYs 2016**
Poisonings	9.4% (6.4% to 13.4%)	9.9% (6.3% to 14.8%)	11.1% (7.5% to 15.7%)	10.0% (6.4% to 14.8%)
Exposure to mechanical forces	11.6% (9.4% to 14.4%)	7.5% (5.7% to 10.1%)	14.9% (12.7% to 18.3%)	9.5% (7.3% to 12.5%)
Animal contact	15.1% (12.7% to 17.8%)	9.9% (7.7% to 12.5%)	8.0% (5.6% to 11.8%)	8.8% (6.7% to 11.8%)
Foreign body	3.9% (2.7% to 5.4%)	3.5% (2.1% to 5.5%)	5.6% (3.8% to 8.3%)	3.9% (2.4% to 5.8%)
Other unintentional injuries	12.0% (8.1% to 16.8%)	12.9% (8.7% to 18.1%)	10.1% (6.8% to 14.1%)	11.6% (8.0% to 15.9%)
**Self-harm and interpersonal violence**	20.7% (13.8% to 28.3%)	21.4% (14.7% to 29.5%)	28.8% (25.6% to 33.0%)	22.0% (15.6% to 29.8%)
Self-harm	19.7% (11.2% to 29.4%)	21.5% (12.6% to 32.1%)	16.1% (8.8% to 23.8%)	21.4% (12.5% to 31.9%)
Interpersonal violence	23.0% (16.7% to 30.6%)	21.4% (15.0% to 29.1%)	30.4% (26.9% to 34.8%)	23.0% (17.4% to 30.1%)
**Forces of nature, conflict and terrorism, and executions and police conflict**	0.0% (0.0% to 0.0%)	0.0% (0.0% to 0.0%)	6.4% (4.4% to 9.3%)	5.3% (3.4% to 7.9%)
Forces of nature, conflict and terrorism, and state actor violence	0.0% (0.0% to 0.0%)	0.0% (0.0% to 0.0%)	6.8% (4.7% to 9.9%)	6.8% (4.7% to 9.9%)

Supplementary Results Table 1. Age-standardised PAFs attributable to the joint distribution of all risk factors for all ages, both sexes combined for each GBD level 1 and level 2 cause and all causes in 2016 for Finland. Results are shown for deaths, YLLs, YLDs, and DALYs
Data in parenthesis are 95% uncertainty intervals. PAFs=population attributable fractions. GBD=Global Burden of Disease. YLLs=years of life lost. YLDs=years live with disability. DALYs=disability-adjusted life-years

	Deaths 2016	YLLs 2016	YLDs 2016	DALYs 2016
**All causes**	57.0% (54.2% to 59.8%)	55.0% (52.4% to 57.3%)	21.2% (19.7% to 22.7%)	35.9% (33.0% to 38.9%)
**HIV/AIDS and tuberculosis**	58.0% (47.2% to 67.2%)	65.1% (58.1% to 71.0%)	82.7% (75.3% to 87.1%)	70.7% (64.0% to 75.8%)
Tuberculosis	51.9% (38.4% to 63.0%)	54.9% (43.5% to 64.5%)	55.2% (44.7% to 63.4%)	55.0% (44.0% to 64.1%)
HIV/AIDS	87.1% (85.8% to 88.2%)	81.6% (80.1% to 83.1%)	92.4% (90.9% to 93.5%)	86.7% (84.3% to 88.8%)
**Diarrhoea, lower respiratory infections, and other common infectious diseases**	24.0% (16.1% to 33.3%)	29.9% (23.1% to 37.5%)	8.1% (5.7% to 11.0%)	15.4% (11.7% to 19.9%)
Diarrhoeal diseases	12.8% (7.1% to 21.9%)	22.4% (15.4% to 31.7%)	29.2% (20.9% to 38.9%)	28.4% (20.6% to 37.8%)
Lower respiratory infections	25.9% (17.2% to 36.5%)	35.7% (27.1% to 45.2%)	36.8% (29.1% to 46.1%)	35.7% (27.2% to 45.3%)
Upper respiratory infections	0.3% (0.0% to 1.1%)	1.0% (0.1% to 3.6%)	0.0% (0.0% to 0.0%)	0.0% (0.0% to 0.0%)
Otitis media	0.1% (0.0% to 0.1%)	0.5% (0.3% to 1.0%)	6.6% (4.3% to 8.7%)	6.6% (4.3% to 8.7%)
Meningitis	0.8% (0.5% to 1.2%)	1.8% (1.2% to 2.5%)	0.0% (0.0% to 0.0%)	1.5% (1.0% to 2.1%)
Encephalitis	0.4% (0.2% to 0.5%)	0.8% (0.6% to 1.1%)	0.0% (0.0% to 0.0%)	0.5% (0.3% to 0.7%)
Measles	4.1% (0.9% to 15.9%)	5.3% (1.2% to 20.5%)	11.0% (0.0% to 41.2%)	5.3% (1.2% to 20.6%)
**Maternal disorders**	2.5% (1.3% to 3.7%)	2.4% (1.3% to 3.6%)	1.7% (0.8% to 2.9%)	2.0% (1.0% to 3.0%)
Maternal haemorrhage	8.3% (3.0% to 13.7%)	8.3% (3.0% to 13.7%)	8.6% (3.1% to 14.2%)	8.4% (3.1% to 13.8%)
Maternal sepsis and other pregnancy related infections	8.5% (3.1% to 13.9%)	8.4% (3.1% to 13.8%)	8.3% (3.0% to 13.8%)	8.4% (3.0% to 13.7%)
Maternal abortion, miscarriage, and ectopic pregnancy	5.4% (3.2% to 7.9%)	5.5% (3.3% to 8.0%)	6.0% (3.2% to 9.8%)	5.7% (3.3% to 8.4%)
**Neonatal disorders**	72.5% (69.0% to 76.1%)	72.6% (69.1% to 76.2%)	0.1% (0.0% to 0.1%)	31.4% (23.4% to 39.9%)
Neonatal preterm birth complications	86.3% (81.4% to 91.0%)	86.4% (81.5% to 91.0%)	0.2% (0.1% to 0.2%)	40.4% (28.9% to 51.9%)
Neonatal encephalopathy due to birth asphyxia and trauma	59.6% (54.9% to 64.6%)	59.7% (55.1% to 64.7%)	0.0% (0.0% to 0.0%)	17.2% (10.9% to 24.4%)
Neonatal sepsis and other neonatal infections	59.5% (52.6% to 64.6%)	59.5% (52.6% to 64.6%)	0.0% (0.0% to 0.0%)	17.8% (7.4% to 30.7%)
Hemolytic disease and other neonatal jaundice	58.9% (51.2% to 65.5%)	59.0% (51.4% to 65.6%)	0.0% (0.0% to 0.0%)	17.4% (10.2% to 29.1%)

Supplementary Results Table 1. Age-standardised PAFs attributable to the joint distribution of all risk factors for all ages, both sexes combined for each GBD level 1 and level 2 cause and all causes in 2016 for Finland. Results are shown for deaths, YLLs, YLDs, and DALYs
Data in parenthesis are 95% uncertainty intervals. PAFs=population attributable fractions. GBD=Global Burden of Disease. YLLs=years of life lost. YLDs=years live with disability. DALYs=disability-adjusted life-years

	Deaths 2016	YLLs 2016	YLDs 2016	DALYs 2016
Other neonatal disorders	62.9% (58.7% to 66.7%)	62.9% (58.8% to 66.8%)	0.0% (0.0% to 0.0%)	55.0% (49.4% to 60.0%)
**Nutritional deficiencies**	6.8% (5.5% to 8.2%)	17.3% (14.0% to 20.5%)	58.6% (48.5% to 67.9%)	57.1% (47.4% to 66.4%)
Protein-energy malnutrition	3.3% (1.5% to 5.4%)	15.3% (7.5% to 22.9%)	98.3% (98.1% to 98.5%)	92.9% (89.7% to 94.9%)
Vitamin A deficiency	-	-	0.0% (0.0% to 0.0%)	0.0% (0.0% to 0.0%)
Iron-deficiency anaemia	100.0% (100.0% to 100.0%)	100.0% (100.0% to 100.0%)	100.0% (100.0% to 100.0%)	100.0% (100.0% to 100.0%)
**Other communicable, maternal, neonatal, and nutritional diseases**	5.7% (4.7% to 6.9%)	3.6% (2.8% to 4.3%)	64.1% (54.8% to 72.7%)	31.3% (20.6% to 43.6%)
Sexually transmitted diseases excluding HIV	95.5% (91.2% to 98.1%)	80.1% (65.6% to 90.5%)	100.0% (100.0% to 100.0%)	98.6% (96.9% to 99.5%)
Hepatitis	6.1% (4.6% to 8.0%)	5.5% (4.0% to 7.4%)	1.5% (1.0% to 2.2%)	5.0% (3.6% to 6.7%)
**Neoplasms**	39.1% (35.9% to 42.6%)	37.8% (34.7% to 41.0%)	27.2% (24.1% to 30.7%)	37.3% (34.2% to 40.5%)
Oesophageal cancer	82.6% (74.7% to 89.3%)	83.7% (76.1% to 90.0%)	83.0% (75.2% to 89.6%)	83.7% (76.1% to 90.0%)
Stomach cancer	16.4% (8.6% to 26.0%)	15.4% (8.1% to 24.0%)	15.8% (8.3% to 24.6%)	15.4% (8.1% to 24.1%)
Liver cancer	76.6% (69.7% to 82.5%)	75.5% (68.4% to 81.5%)	75.2% (68.6% to 81.3%)	75.5% (68.5% to 81.5%)
Larynx cancer	87.3% (80.4% to 92.0%)	85.9% (79.2% to 90.9%)	86.7% (79.8% to 91.5%)	86.0% (79.3% to 90.9%)
Tracheal, bronchus, and lung cancer	85.3% (82.3% to 87.9%)	83.7% (80.7% to 86.4%)	84.2% (80.9% to 87.2%)	83.7% (80.7% to 86.4%)
Breast cancer	34.2% (27.5% to 41.8%)	33.3% (27.2% to 39.9%)	33.5% (27.3% to 40.3%)	33.3% (27.2% to 39.9%)
Cervical cancer	100.0% (100.0% to 100.0%)	100.0% (100.0% to 100.0%)	100.0% (100.0% to 100.0%)	100.0% (100.0% to 100.0%)
Uterine cancer	43.3% (29.1% to 57.5%)	44.4% (29.8% to 58.4%)	44.6% (30.2% to 58.6%)	44.4% (29.9% to 58.4%)
Prostate cancer	4.7% (3.3% to 6.3%)	4.6% (3.3% to 6.1%)	4.6% (3.3% to 6.0%)	4.6% (3.3% to 6.1%)
Colon and rectum cancer	51.9% (42.8% to 60.7%)	53.0% (44.4% to 61.3%)	52.6% (43.8% to 61.0%)	53.0% (44.3% to 61.3%)
Lip and oral cavity cancer	78.1% (70.1% to 84.3%)	79.8% (72.5% to 85.2%)	76.9% (68.7% to 83.3%)	79.7% (72.4% to 85.1%)
Nasopharynx cancer	82.5% (77.2% to 86.9%)	80.0% (75.5% to 84.0%)	77.1% (72.3% to 81.2%)	79.9% (75.4% to 83.9%)

Supplementary Results Table 1. Age-standardised PAFs attributable to the joint distribution of all risk factors for all ages, both sexes combined for each GBD level 1 and level 2 cause and all causes in 2016 for Finland. Results are shown for deaths, YLLs, YLDs, and DALYs
Data in parenthesis are 95% uncertainty intervals. PAFs=population attributable fractions. GBD=Global Burden of Disease. YLLs=years of life lost. YLDs=years live with disability. DALYs=disability-adjusted life-years

	Deaths 2016	YLLs 2016	YLDs 2016	DALYs 2016
Other pharynx cancer	67.7% (56.1% to 77.0%)	70.4% (59.4% to 78.8%)	70.1% (58.9% to 78.9%)	70.4% (59.4% to 78.9%)
Gallbladder and biliary tract cancer	21.7% (11.6% to 34.1%)	21.9% (11.8% to 34.1%)	22.1% (11.9% to 34.3%)	21.9% (11.8% to 34.1%)
Pancreatic cancer	28.4% (21.0% to 37.0%)	27.6% (20.3% to 36.1%)	28.1% (20.8% to 36.7%)	27.6% (20.3% to 36.1%)
Ovarian cancer	15.2% (7.9% to 23.6%)	12.5% (6.0% to 19.8%)	12.4% (6.1% to 19.6%)	12.5% (6.0% to 19.8%)
Kidney cancer	33.6% (23.9% to 44.6%)	33.1% (23.1% to 44.0%)	32.8% (23.0% to 43.7%)	33.1% (23.1% to 44.0%)
Bladder cancer	32.8% (22.8% to 42.3%)	31.8% (22.4% to 41.3%)	31.6% (22.4% to 40.9%)	31.8% (22.4% to 41.3%)
Thyroid cancer	14.8% (7.4% to 24.0%)	15.2% (7.5% to 24.9%)	14.7% (7.6% to 24.1%)	15.1% (7.5% to 24.7%)
Mesothelioma	97.0% (96.1% to 97.7%)	94.2% (92.6% to 95.5%)	96.0% (94.8% to 97.0%)	94.2% (92.7% to 95.6%)
Non-Hodgkin's lymphoma	7.4% (3.1% to 12.8%)	7.4% (3.0% to 12.8%)	7.6% (3.1% to 13.1%)	7.4% (3.0% to 12.8%)
Multiple myeloma	8.4% (3.6% to 14.7%)	8.7% (3.7% to 15.3%)	8.7% (3.7% to 15.4%)	8.7% (3.7% to 15.4%)
Leukaemia	17.7% (11.1% to 24.8%)	14.7% (9.3% to 20.5%)	15.6% (9.6% to 21.9%)	14.8% (9.3% to 20.5%)
**Cardiovascular diseases**	84.8% (81.7% to 87.4%)	86.7% (84.7% to 88.4%)	79.7% (76.9% to 82.1%)	85.4% (83.3% to 87.1%)
Rheumatic heart disease	32.3% (20.6% to 54.2%)	33.2% (22.8% to 49.8%)	31.1% (21.4% to 45.5%)	33.2% (22.8% to 49.5%)
Ischaemic heart disease	93.6% (89.7% to 96.5%)	94.9% (92.4% to 96.8%)	92.3% (89.7% to 94.5%)	94.7% (92.2% to 96.6%)
Cerebrovascular disease	83.0% (75.2% to 89.6%)	87.2% (82.3% to 91.3%)	86.0% (81.3% to 89.8%)	86.9% (82.2% to 90.8%)
Hypertensive heart disease	100.0% (100.0% to 100.0%)	100.0% (100.0% to 100.0%)	100.0% (100.0% to 100.0%)	100.0% (100.0% to 100.0%)
Cardiomyopathy and myocarditis	62.4% (43.7% to 71.9%)	67.1% (53.3% to 73.4%)	56.2% (51.5% to 60.8%)	66.1% (53.6% to 72.1%)
Atrial fibrillation and flutter	58.0% (49.6% to 66.0%)	62.2% (55.1% to 68.8%)	69.1% (64.0% to 74.0%)	66.3% (60.5% to 72.0%)
Aortic aneurysm	48.6% (40.5% to 56.2%)	53.8% (46.9% to 60.0%)	-	53.8% (46.9% to 60.0%)
Peripheral vascular disease	51.0% (42.6% to 60.0%)	55.9% (48.6% to 63.2%)	59.6% (53.7% to 65.3%)	56.9% (49.9% to 63.5%)
Endocarditis	45.6% (36.1% to 56.3%)	46.9% (38.5% to 55.4%)	45.3% (36.3% to 55.2%)	46.8% (38.4% to 55.3%)

Supplementary Results Table 1. Age-standardised PAFs attributable to the joint distribution of all risk factors for all ages, both sexes combined for each GBD level 1 and level 2 cause and all causes in 2016 for Finland. Results are shown for deaths, YLLs, YLDs, and DALYs
Data in parenthesis are 95% uncertainty intervals. PAFs=population attributable fractions. GBD=Global Burden of Disease. YLLs=years of life lost. YLDs=years live with disability. DALYs=disability-adjusted life-years

	Deaths 2016	YLLs 2016	YLDs 2016	DALYs 2016
Other cardiovascular and circulatory diseases	49.2% (42.8% to 55.6%)	55.5% (50.7% to 60.2%)	56.4% (51.4% to 61.1%)	55.8% (51.0% to 60.4%)
**Chronic respiratory diseases**	59.3% (54.5% to 66.2%)	58.3% (53.7% to 65.2%)	40.2% (33.5% to 47.4%)	46.3% (40.6% to 52.1%)
Chronic obstructive pulmonary disease	72.2% (67.2% to 77.2%)	71.7% (67.0% to 76.6%)	61.3% (55.5% to 67.3%)	67.8% (62.6% to 72.8%)
Pneumoconiosis	100.0% (100.0% to 100.0%)	100.0% (100.0% to 100.0%)	100.0% (100.0% to 100.0%)	100.0% (100.0% to 100.0%)
Asthma	36.1% (25.1% to 48.6%)	40.5% (30.6% to 51.3%)	34.6% (26.3% to 44.1%)	34.8% (26.5% to 44.3%)
Other chronic respiratory diseases	12.7% (9.6% to 16.2%)	9.2% (5.8% to 13.1%)	4.7% (2.4% to 8.5%)	6.2% (3.7% to 9.7%)
**Cirrhosis and other chronic liver diseases**	77.2% (73.2% to 81.0%)	77.5% (73.4% to 81.5%)	64.8% (60.1% to 69.5%)	76.8% (72.8% to 80.7%)
Cirrhosis and other chronic liver diseases due to hepatitis B	2.8% (1.2% to 4.9%)	3.3% (1.4% to 5.7%)	3.2% (1.3% to 5.5%)	3.3% (1.4% to 5.6%)
Cirrhosis and other chronic liver diseases due to hepatitis C	89.4% (88.7% to 89.9%)	89.9% (89.7% to 90.0%)	88.3% (87.1% to 89.1%)	89.8% (89.7% to 89.9%)
Cirrhosis and other chronic liver diseases due to alcohol use	100.0% (100.0% to 100.0%)	100.0% (100.0% to 100.0%)	100.0% (100.0% to 100.0%)	100.0% (100.0% to 100.0%)
**Digestive diseases**	15.3% (11.5% to 19.1%)	21.2% (15.3% to 26.3%)	4.6% (3.5% to 5.8%)	17.5% (12.6% to 22.0%)
Peptic ulcer disease	10.4% (7.2% to 14.1%)	14.2% (9.8% to 19.0%)	11.7% (8.0% to 15.8%)	13.9% (9.6% to 18.6%)
Gallbladder and biliary diseases	42.0% (27.9% to 57.2%)	42.1% (28.2% to 57.4%)	45.7% (32.3% to 60.4%)	42.7% (28.8% to 57.7%)
Pancreatitis	52.4% (35.5% to 68.1%)	58.5% (42.2% to 73.2%)	49.4% (34.1% to 63.8%)	58.3% (41.9% to 73.1%)
**Neurological disorders**	21.4% (10.6% to 34.2%)	20.1% (11.6% to 30.3%)	4.2% (2.3% to 6.7%)	10.1% (5.7% to 15.4%)
Alzheimer's disease and other dementias	24.8% (12.1% to 39.4%)	25.7% (13.0% to 40.4%)	25.7% (13.2% to 40.5%)	25.7% (13.1% to 40.5%)
Parkinson's disease	-5.3% (-6.4% to -4.2%)	-7.1% (-8.6% to -5.7%)	-8.6% (-10.1% to -6.8%)	-7.4% (-8.8% to -5.9%)
Epilepsy	41.5% (32.0% to 50.4%)	41.1% (31.9% to 49.7%)	32.2% (23.0% to 39.9%)	36.4% (27.1% to 44.8%)
Multiple sclerosis	8.7% (5.6% to 12.1%)	9.4% (6.1% to 13.1%)	8.0% (5.2% to 11.1%)	8.6% (5.5% to 11.9%)
**Mental and substance use disorders**	99.9% (99.9% to 99.9%)	99.9% (99.8% to 99.9%)	22.5% (20.4% to 24.8%)	35.0% (31.2% to 39.7%)
Alcohol use disorders	100.0% (100.0% to 100.0%)	100.0% (100.0% to 100.0%)	100.0% (100.0% to 100.0%)	100.0% (100.0% to 100.0%)

Supplementary Results Table 1. Age-standardised PAFs attributable to the joint distribution of all risk factors for all ages, both sexes combined for each GBD level 1 and level 2 cause and all causes in 2016 for Finland. Results are shown for deaths, YLLs, YLDs, and DALYs
Data in parenthesis are 95% uncertainty intervals. PAFs=population attributable fractions. GBD=Global Burden of Disease. YLLs=years of life lost. YLDs=years live with disability. DALYs=disability-adjusted life-years

	Deaths 2016	YLLs 2016	YLDs 2016	DALYs 2016
Drug use disorders	100.0% (100.0% to 100.0%)	100.0% (100.0% to 100.0%)	100.0% (100.0% to 100.0%)	100.0% (100.0% to 100.0%)
Depressive disorders	-	-	7.3% (5.9% to 8.8%)	7.3% (5.9% to 8.8%)
Idiopathic developmental intellectual disability	-	-	53.5% (26.1% to 73.7%)	53.5% (26.1% to 73.7%)
**Diabetes, urogenital, blood, and endocrine diseases**	68.7% (66.9% to 70.7%)	64.2% (60.6% to 70.4%)	59.3% (53.3% to 64.5%)	60.5% (55.6% to 64.6%)
Diabetes mellitus	100.0% (100.0% to 100.0%)	100.0% (100.0% to 100.0%)	100.0% (100.0% to 100.0%)	100.0% (100.0% to 100.0%)
Chronic kidney disease	100.0% (100.0% to 100.0%)	100.0% (100.0% to 100.0%)	100.0% (100.0% to 100.0%)	100.0% (100.0% to 100.0%)
**Musculoskeletal disorders**	1.8% (0.7% to 3.0%)	2.3% (0.9% to 3.9%)	18.2% (15.2% to 21.3%)	18.0% (15.1% to 21.1%)
Rheumatoid arthritis	3.3% (1.3% to 5.7%)	4.3% (1.7% to 7.4%)	5.0% (2.0% to 8.6%)	4.9% (1.9% to 8.4%)
Osteoarthritis	-	-	26.1% (14.0% to 38.4%)	26.1% (14.0% to 38.4%)
Low back and neck pain	-	-	20.2% (17.3% to 23.3%)	20.2% (17.3% to 23.3%)
Gout	-	-	47.1% (28.7% to 65.5%)	47.1% (28.7% to 65.5%)
**Other non-communicable diseases**	0.3% (0.2% to 0.5%)	0.4% (0.2% to 0.6%)	1.7% (1.4% to 1.9%)	1.6% (1.3% to 1.8%)
Sense organ diseases	-	-	6.5% (5.7% to 7.3%)	6.5% (5.7% to 7.3%)
Sudden infant death syndrome	3.6% (2.4% to 5.1%)	3.6% (2.4% to 5.2%)	-	3.6% (2.4% to 5.2%)
**Transport injuries**	37.7% (27.8% to 47.4%)	34.3% (24.3% to 44.4%)	39.5% (30.5% to 48.4%)	36.4% (26.9% to 45.9%)
Road injuries	36.4% (26.7% to 46.0%)	32.9% (23.1% to 42.9%)	39.2% (30.4% to 48.1%)	35.7% (26.0% to 45.1%)
Other transport injuries	42.4% (31.4% to 53.4%)	40.3% (28.9% to 51.7%)	42.7% (33.0% to 52.3%)	40.8% (29.9% to 51.8%)
**Unintentional injuries**	43.8% (38.6% to 47.3%)	25.6% (21.7% to 29.2%)	19.2% (16.7% to 22.4%)	21.4% (18.7% to 24.6%)
Falls	60.9% (56.7% to 65.6%)	39.0% (35.3% to 42.4%)	21.1% (19.0% to 23.9%)	25.9% (23.2% to 28.7%)
Drowning	19.4% (11.3% to 28.9%)	19.5% (12.0% to 28.2%)	17.9% (10.6% to 26.8%)	19.4% (11.9% to 28.1%)
Fire, heat, and hot substances	19.1% (11.1% to 28.5%)	20.9% (12.8% to 30.2%)	19.1% (11.8% to 27.8%)	19.8% (12.4% to 29.0%)

Supplementary Results Table 1. Age-standardised PAFs attributable to the joint distribution of all risk factors for all ages, both sexes combined for each GBD level 1 and level 2 cause and all causes in 2016 for Finland. Results are shown for deaths, YLLs, YLDs, and DALYs
Data in parenthesis are 95% uncertainty intervals. PAFs=population attributable fractions. GBD=Global Burden of Disease. YLLs=years of life lost. YLDs=years live with disability. DALYs=disability-adjusted life-years

	**Deaths 2016**	**YLLs 2016**	**YLDs 2016**	**DALYs 2016**
Poisonings	21.4% (13.0% to 31.1%)	23.0% (14.2% to 32.8%)	16.9% (10.4% to 24.9%)	20.0% (12.3% to 28.7%)
Exposure to mechanical forces	18.2% (14.4% to 23.8%)	14.4% (10.6% to 19.5%)	16.6% (13.7% to 20.6%)	16.1% (13.1% to 20.4%)
Animal contact	11.7% (9.3% to 14.9%)	10.0% (7.5% to 13.6%)	7.1% (4.8% to 10.5%)	7.9% (5.6% to 11.3%)
Foreign body	3.9% (2.5% to 5.8%)	6.0% (3.9% to 9.1%)	5.2% (3.4% to 8.0%)	5.7% (3.7% to 8.6%)
Other unintentional injuries	21.3% (12.8% to 31.2%)	22.7% (14.2% to 32.3%)	18.1% (10.8% to 26.5%)	18.3% (11.2% to 26.8%)
**Self-harm and interpersonal violence**	40.6% (27.7% to 52.6%)	41.1% (27.9% to 53.2%)	39.4% (33.1% to 45.0%)	41.0% (28.6% to 52.5%)
Self-harm	40.6% (26.3% to 53.8%)	41.4% (26.9% to 54.7%)	30.8% (16.4% to 44.1%)	41.2% (26.6% to 54.5%)
Interpersonal violence	41.3% (32.0% to 49.6%)	38.9% (29.5% to 47.1%)	42.8% (37.4% to 48.1%)	40.2% (32.3% to 47.1%)
**Forces of nature, conflict and terrorism, and executions and police conflict**	0.0% (0.0% to 0.0%)	0.0% (0.0% to 0.0%)	4.5% (2.9% to 6.9%)	2.6% (1.2% to 4.7%)
Forces of nature, conflict and terrorism, and state actor violence	0.0% (0.0% to 0.0%)	0.0% (0.0% to 0.0%)	7.1% (4.6% to 10.8%)	7.1% (4.6% to 10.8%)

Supplementary Results Table 1. Age-standardised PAFs attributable to the joint distribution of all risk factors for all ages, both sexes combined for each GBD level 1 and level 2 cause and all causes in 2016 for France. Results are shown for deaths, YLLs, YLDs, and DALYs
Data in parenthesis are 95% uncertainty intervals. PAFs=population attributable fractions. GBD=Global Burden of Disease. YLLs=years of life lost. YLDs=years live with disability. DALYs=disability-adjusted life-years

	Deaths 2016	YLLs 2016	YLDs 2016	DALYs 2016
**All causes**	52.3% (50.0% to 54.6%)	52.3% (50.1% to 54.5%)	18.6% (17.3% to 19.9%)	33.8% (31.0% to 36.7%)
**HIV/AIDS and tuberculosis**	76.9% (69.8% to 82.5%)	84.8% (81.1% to 87.6%)	87.5% (78.3% to 93.2%)	85.5% (81.9% to 88.5%)
Tuberculosis	60.6% (48.5% to 70.4%)	62.8% (51.9% to 71.6%)	56.8% (44.4% to 66.7%)	61.9% (51.0% to 70.7%)
HIV/AIDS	95.8% (95.3% to 96.2%)	93.6% (93.0% to 94.2%)	97.2% (96.6% to 97.7%)	94.4% (93.6% to 95.2%)
**Diarrhoea, lower respiratory infections, and other common infectious diseases**	33.9% (24.3% to 43.2%)	40.2% (32.0% to 47.8%)	6.9% (5.2% to 9.2%)	24.3% (18.5% to 30.3%)
Diarrhoeal diseases	15.6% (9.2% to 25.7%)	28.6% (20.8% to 38.2%)	31.7% (23.1% to 41.5%)	30.1% (22.7% to 39.4%)
Lower respiratory infections	38.4% (26.5% to 49.6%)	48.0% (37.4% to 58.5%)	44.8% (35.4% to 55.1%)	47.9% (37.4% to 58.4%)
Upper respiratory infections	0.6% (0.0% to 2.0%)	1.8% (0.2% to 6.0%)	0.0% (0.0% to 0.0%)	0.0% (0.0% to 0.0%)
Otitis media	2.9% (1.3% to 5.8%)	7.5% (4.0% to 12.3%)	8.7% (5.8% to 11.4%)	8.7% (5.8% to 11.4%)
Meningitis	3.5% (2.4% to 4.7%)	6.5% (4.7% to 8.5%)	0.0% (0.0% to 0.0%)	5.7% (3.9% to 7.6%)
Encephalitis	0.6% (0.3% to 0.8%)	1.3% (0.8% to 2.1%)	0.0% (0.0% to 0.0%)	0.9% (0.5% to 1.3%)
Measles	5.4% (1.0% to 20.3%)	6.2% (1.2% to 23.5%)	12.0% (2.5% to 44.1%)	6.2% (1.2% to 23.6%)
**Maternal disorders**	2.6% (1.3% to 4.1%)	2.6% (1.3% to 4.1%)	2.5% (1.2% to 4.1%)	2.5% (1.3% to 3.9%)
Maternal haemorrhage	9.0% (3.2% to 14.5%)	9.0% (3.2% to 14.5%)	9.0% (3.2% to 14.6%)	9.0% (3.2% to 14.6%)
Maternal sepsis and other pregnancy related infections	9.1% (3.3% to 14.7%)	9.0% (3.3% to 14.6%)	8.9% (3.2% to 14.4%)	8.9% (3.2% to 14.5%)
Maternal abortion, miscarriage, and ectopic pregnancy	5.7% (3.3% to 8.6%)	5.8% (3.4% to 8.7%)	6.2% (3.0% to 10.1%)	6.0% (3.3% to 9.4%)
**Neonatal disorders**	68.4% (65.2% to 71.6%)	68.4% (65.3% to 71.6%)	0.1% (0.1% to 0.1%)	47.3% (40.6% to 54.0%)
Neonatal preterm birth complications	83.6% (78.8% to 88.3%)	83.7% (78.9% to 88.4%)	0.2% (0.1% to 0.2%)	44.1% (33.3% to 54.7%)
Neonatal encephalopathy due to birth asphyxia and trauma	65.5% (61.1% to 69.4%)	65.5% (61.2% to 69.4%)	0.0% (0.0% to 0.0%)	55.8% (48.8% to 61.5%)
Neonatal sepsis and other neonatal infections	59.8% (54.3% to 64.8%)	59.9% (54.3% to 64.9%)	0.0% (0.0% to 0.0%)	27.9% (13.1% to 45.4%)
Hemolytic disease and other neonatal jaundice	56.2% (46.0% to 63.9%)	56.3% (46.2% to 64.0%)	0.0% (0.0% to 0.0%)	19.1% (11.6% to 27.8%)

Supplementary Results Table 1. Age-standardised PAFs attributable to the joint distribution of all risk factors for all ages, both sexes combined for each GBD level 1 and level 2 cause and all causes in 2016 for France. Results are shown for deaths, YLLs, YLDs, and DALYs
Data in parenthesis are 95% uncertainty intervals. PAFs=population attributable fractions. GBD=Global Burden of Disease. YLLs=years of life lost. YLDs=years live with disability. DALYs=disability-adjusted life-years

	Deaths 2016	YLLs 2016	YLDs 2016	DALYs 2016
Other neonatal disorders	59.6% (54.8% to 64.6%)	59.6% (54.8% to 64.6%)	0.0% (0.0% to 0.0%)	57.3% (52.6% to 62.2%)
**Nutritional deficiencies**	0.9% (0.3% to 1.2%)	3.5% (2.1% to 4.5%)	50.6% (42.7% to 58.7%)	37.8% (31.1% to 44.6%)
Protein-energy malnutrition	0.1% (0.1% to 0.2%)	1.4% (0.9% to 2.2%)	98.3% (98.1% to 98.5%)	36.1% (27.2% to 45.5%)
Vitamin A deficiency	-	-	0.0% (0.0% to 0.0%)	0.0% (0.0% to 0.0%)
Iron-deficiency anaemia	100.0% (100.0% to 100.0%)	100.0% (100.0% to 100.0%)	100.0% (100.0% to 100.0%)	100.0% (100.0% to 100.0%)
**Other communicable, maternal, neonatal, and nutritional diseases**	3.4% (2.9% to 4.3%)	2.8% (2.3% to 3.4%)	70.2% (62.2% to 78.0%)	27.7% (18.2% to 41.2%)
Sexually transmitted diseases excluding HIV	94.3% (85.7% to 98.2%)	82.3% (60.5% to 93.7%)	100.0% (100.0% to 100.0%)	98.7% (96.2% to 99.7%)
Hepatitis	3.7% (2.7% to 4.9%)	3.8% (2.8% to 5.0%)	1.4% (0.9% to 2.2%)	3.6% (2.6% to 4.8%)
**Neoplasms**	48.5% (45.7% to 51.4%)	50.7% (48.3% to 53.3%)	36.7% (33.7% to 39.9%)	50.2% (47.8% to 52.8%)
Oesophageal cancer	89.5% (84.4% to 93.7%)	91.1% (86.6% to 94.7%)	89.7% (84.8% to 93.7%)	91.1% (86.5% to 94.7%)
Stomach cancer	21.5% (12.6% to 31.2%)	24.6% (15.2% to 33.8%)	22.7% (13.8% to 32.0%)	24.5% (15.1% to 33.7%)
Liver cancer	76.9% (69.6% to 83.0%)	79.2% (72.1% to 84.5%)	77.5% (70.5% to 83.2%)	79.1% (72.1% to 84.5%)
Larynx cancer	94.0% (90.0% to 96.7%)	94.4% (90.7% to 96.8%)	94.2% (90.4% to 96.6%)	94.4% (90.7% to 96.8%)
Tracheal, bronchus, and lung cancer	90.4% (88.6% to 91.9%)	91.0% (89.4% to 92.5%)	90.1% (88.1% to 91.9%)	91.0% (89.4% to 92.5%)
Breast cancer	36.9% (30.7% to 43.2%)	36.7% (30.6% to 43.1%)	36.7% (30.7% to 43.2%)	36.7% (30.6% to 43.2%)
Cervical cancer	100.0% (100.0% to 100.0%)	100.0% (100.0% to 100.0%)	100.0% (100.0% to 100.0%)	100.0% (100.0% to 100.0%)
Uterine cancer	39.0% (26.3% to 51.7%)	39.7% (27.2% to 52.5%)	39.9% (27.4% to 53.0%)	39.8% (27.3% to 52.6%)
Prostate cancer	6.2% (4.4% to 8.0%)	7.1% (5.2% to 9.0%)	7.5% (5.5% to 9.6%)	7.1% (5.2% to 9.1%)
Colon and rectum cancer	59.9% (51.6% to 67.7%)	61.6% (53.6% to 68.9%)	60.9% (53.0% to 68.4%)	61.5% (53.6% to 68.9%)
Lip and oral cavity cancer	90.2% (85.9% to 93.5%)	91.8% (88.0% to 94.7%)	89.5% (84.8% to 93.2%)	91.7% (87.9% to 94.7%)
Nasopharynx cancer	91.3% (87.9% to 93.9%)	91.1% (87.9% to 93.4%)	89.0% (84.9% to 92.1%)	91.0% (87.9% to 93.3%)

**Supplementary Results Table 1. Age-standardised PAFs attributable to the joint distribution of all risk factors for all ages, both sexes combined for each GBD level 1 and level 2 cause and all causes in 2016 for France. Results are shown for deaths, YLLs, YLDs, and DALYs** Data in parenthesis are 95% uncertainty intervals. PAFs=population attributable fractions. GBD=Global Burden of Disease. YLLs=years of life lost. YLDs=years live with disability. DALYs=disability-adjusted life-years	**Deaths 2016**	**YLLs 2016**	**YLDs 2016**	**DALYs 2016**
Other pharynx cancer	77.0% (66.8% to 84.6%)	77.4% (67.3% to 84.7%)	77.4% (67.4% to 84.8%)	77.4% (67.3% to 84.7%)
Gallbladder and biliary tract cancer	17.7% (9.2% to 28.5%)	17.7% (9.2% to 28.6%)	17.9% (9.4% to 28.9%)	17.7% (9.2% to 28.6%)
Pancreatic cancer	31.8% (25.2% to 38.9%)	35.5% (28.7% to 42.3%)	32.6% (25.8% to 39.5%)	35.5% (28.6% to 42.3%)
Ovarian cancer	14.0% (7.9% to 20.7%)	11.0% (5.9% to 16.9%)	11.1% (6.0% to 17.1%)	11.0% (5.9% to 16.9%)
Kidney cancer	37.1% (27.1% to 47.1%)	39.5% (29.2% to 49.4%)	38.7% (28.7% to 48.5%)	39.5% (29.2% to 49.4%)
Bladder cancer	37.7% (28.2% to 47.0%)	42.0% (31.9% to 51.0%)	41.6% (31.7% to 50.5%)	42.0% (31.9% to 51.0%)
Thyroid cancer	13.1% (6.2% to 22.6%)	13.6% (6.1% to 23.8%)	12.6% (6.4% to 21.4%)	13.4% (6.2% to 23.3%)
Mesothelioma	97.5% (96.8% to 98.2%)	95.2% (93.9% to 96.4%)	96.8% (95.8% to 97.6%)	95.2% (93.9% to 96.4%)
Non-Hodgkin's lymphoma	6.1% (2.5% to 11.2%)	6.1% (2.4% to 10.9%)	6.1% (2.4% to 11.0%)	6.1% (2.4% to 10.9%)
Multiple myeloma	6.9% (2.9% to 12.5%)	7.1% (3.0% to 13.1%)	7.1% (3.0% to 12.9%)	7.1% (3.0% to 13.1%)
Leukaemia	21.1% (13.2% to 28.9%)	20.3% (13.0% to 27.5%)	19.2% (12.0% to 26.6%)	20.2% (13.0% to 27.4%)
**Cardiovascular diseases**	76.9% (74.1% to 79.7%)	80.1% (78.0% to 82.1%)	71.1% (67.7% to 74.1%)	77.9% (75.4% to 80.1%)
Rheumatic heart disease	26.3% (15.5% to 48.2%)	26.4% (17.7% to 42.4%)	25.6% (17.3% to 39.7%)	26.4% (17.7% to 42.3%)
Ischaemic heart disease	92.4% (88.4% to 95.7%)	94.5% (92.0% to 96.6%)	91.3% (88.6% to 93.7%)	94.2% (91.7% to 96.3%)
Cerebrovascular disease	82.7% (76.5% to 88.2%)	87.0% (83.0% to 90.4%)	85.2% (80.6% to 89.0%)	86.5% (82.7% to 90.0%)
Hypertensive heart disease	100.0% (100.0% to 100.0%)	100.0% (100.0% to 100.0%)	100.0% (100.0% to 100.0%)	100.0% (100.0% to 100.0%)
Cardiomyopathy and myocarditis	40.4% (31.9% to 51.3%)	44.3% (37.9% to 51.3%)	44.0% (37.6% to 50.6%)	44.2% (38.0% to 51.2%)
Atrial fibrillation and flutter	56.0% (48.1% to 64.2%)	60.5% (53.3% to 67.8%)	67.8% (61.6% to 73.7%)	64.8% (58.1% to 71.2%)
Aortic aneurysm	42.8% (35.3% to 50.4%)	50.1% (43.9% to 55.8%)	-	50.1% (43.9% to 55.8%)
Peripheral vascular disease	45.4% (38.1% to 53.1%)	52.7% (45.8% to 58.4%)	52.9% (47.3% to 58.5%)	52.8% (47.2% to 58.2%)
Endocarditis	38.0% (28.4% to 49.4%)	39.2% (31.1% to 47.8%)	39.5% (30.5% to 49.3%)	39.2% (31.1% to 47.9%)

Supplementary Results Table 1. Age-standardised PAFs attributable to the joint distribution of all risk factors for all ages, both sexes combined for each GBD level 1 and level 2 cause and all causes in 2016 for France. Results are shown for deaths, YLLs, YLDs, and DALYs
Data in parenthesis are 95% uncertainty intervals. PAFs=population attributable fractions. GBD=Global Burden of Disease. YLLs=years of life lost. YLDs=years live with disability. DALYs=disability-adjusted life-years

	Deaths 2016	YLLs 2016	YLDs 2016	DALYs 2016
Other cardiovascular and circulatory diseases	42.4% (36.3% to 48.9%)	49.9% (45.0% to 54.8%)	52.3% (47.4% to 57.3%)	51.1% (46.3% to 55.8%)
**Chronic respiratory diseases**	62.9% (57.7% to 68.0%)	62.2% (57.6% to 66.7%)	34.0% (27.2% to 41.9%)	45.6% (40.1% to 51.8%)
Chronic obstructive pulmonary disease	73.0% (67.2% to 78.4%)	76.2% (71.4% to 80.4%)	69.2% (62.2% to 75.5%)	75.5% (70.9% to 79.7%)
Pneumoconiosis	100.0% (100.0% to 100.0%)	100.0% (100.0% to 100.0%)	100.0% (100.0% to 100.0%)	100.0% (100.0% to 100.0%)
Asthma	36.7% (27.3% to 47.8%)	41.9% (33.8% to 51.2%)	32.6% (25.4% to 41.0%)	33.1% (26.0% to 41.6%)
Other chronic respiratory diseases	19.0% (14.2% to 24.0%)	18.8% (13.1% to 24.3%)	8.7% (5.0% to 14.1%)	17.4% (12.0% to 22.5%)
**Cirrhosis and other chronic liver diseases**	75.1% (71.1% to 78.8%)	76.7% (72.7% to 80.4%)	62.1% (57.8% to 66.4%)	75.7% (71.9% to 79.3%)
Cirrhosis and other chronic liver diseases due to hepatitis B	1.1% (0.4% to 1.9%)	1.4% (0.5% to 2.3%)	1.4% (0.5% to 2.4%)	1.4% (0.5% to 2.3%)
Cirrhosis and other chronic liver diseases due to hepatitis C	82.3% (77.6% to 85.9%)	87.4% (85.5% to 88.8%)	84.7% (81.8% to 87.0%)	87.3% (85.3% to 88.7%)
Cirrhosis and other chronic liver diseases due to alcohol use	100.0% (100.0% to 100.0%)	100.0% (100.0% to 100.0%)	100.0% (100.0% to 100.0%)	100.0% (100.0% to 100.0%)
**Digestive diseases**	10.3% (7.6% to 13.6%)	13.3% (9.9% to 17.2%)	3.5% (2.6% to 4.5%)	10.4% (7.8% to 13.6%)
Peptic ulcer disease	9.8% (6.8% to 13.4%)	15.1% (10.8% to 20.1%)	15.8% (10.9% to 21.4%)	15.3% (11.0% to 20.3%)
Gallbladder and biliary diseases	36.8% (22.3% to 51.7%)	37.1% (23.6% to 51.2%)	41.3% (28.2% to 55.3%)	37.8% (24.7% to 51.8%)
Pancreatitis	54.5% (34.9% to 72.5%)	57.8% (37.6% to 74.8%)	49.7% (30.7% to 66.7%)	57.6% (37.4% to 74.6%)
**Neurological disorders**	17.4% (8.9% to 27.7%)	17.0% (10.5% to 24.8%)	4.5% (2.1% to 7.8%)	8.3% (5.1% to 12.3%)
Alzheimer's disease and other dementias	20.1% (9.6% to 33.2%)	21.3% (10.8% to 34.1%)	21.3% (10.9% to 34.2%)	21.3% (10.8% to 34.1%)
Parkinson's disease	-5.4% (-6.5% to -4.5%)	-7.5% (-8.9% to -6.0%)	-9.6% (-11.6% to -7.6%)	-7.9% (-9.5% to -6.4%)
Epilepsy	43.6% (32.7% to 53.7%)	41.0% (30.5% to 50.0%)	31.4% (22.1% to 39.3%)	35.1% (25.5% to 44.0%)
Multiple sclerosis	10.7% (7.0% to 14.7%)	12.3% (8.1% to 16.8%)	11.4% (7.3% to 15.7%)	11.8% (7.7% to 16.0%)
**Mental and substance use disorders**	99.5% (99.4% to 99.6%)	99.2% (99.0% to 99.4%)	14.9% (13.2% to 16.8%)	21.6% (18.9% to 24.5%)
Alcohol use disorders	100.0% (100.0% to 100.0%)	100.0% (100.0% to 100.0%)	100.0% (100.0% to 100.0%)	100.0% (100.0% to 100.0%)

Supplementary Results Table 1. Age-standardised PAFs attributable to the joint distribution of all risk factors for all ages, both sexes combined for each GBD level 1 and level 2 cause and all causes in 2016 for France. Results are shown for deaths, YLLs, YLDs, and DALYs
Data in parenthesis are 95% uncertainty intervals. PAFs=population attributable fractions. GBD=Global Burden of Disease. YLLs=years of life lost. YLDs=years live with disability. DALYs=disability-adjusted life-years

	Deaths 2016	YLLs 2016	YLDs 2016	DALYs 2016
Drug use disorders	100.0% (100.0% to 100.0%)	100.0% (100.0% to 100.0%)	100.0% (100.0% to 100.0%)	100.0% (100.0% to 100.0%)
Depressive disorders	-	-	4.8% (3.7% to 6.3%)	4.8% (3.7% to 6.3%)
Idiopathic developmental intellectual disability	-	-	56.3% (27.8% to 76.3%)	56.3% (27.8% to 76.3%)
**Diabetes, urogenital, blood, and endocrine diseases**	70.0% (67.9% to 74.0%)	60.7% (57.0% to 63.8%)	50.2% (45.6% to 54.7%)	54.3% (50.7% to 57.5%)
Diabetes mellitus	100.0% (100.0% to 100.0%)	100.0% (100.0% to 100.0%)	100.0% (100.0% to 100.0%)	100.0% (100.0% to 100.0%)
Chronic kidney disease	100.0% (100.0% to 100.0%)	100.0% (100.0% to 100.0%)	100.0% (100.0% to 100.0%)	100.0% (100.0% to 100.0%)
**Musculoskeletal disorders**	0.5% (0.2% to 1.0%)	0.8% (0.3% to 1.3%)	17.6% (14.7% to 20.6%)	17.3% (14.5% to 20.4%)
Rheumatoid arthritis	3.2% (1.2% to 5.5%)	4.4% (1.7% to 7.5%)	6.7% (2.7% to 11.2%)	6.5% (2.7% to 11.0%)
Osteoarthritis	-	-	21.7% (11.7% to 32.7%)	21.7% (11.7% to 32.7%)
Low back and neck pain	-	-	20.6% (17.7% to 23.7%)	20.6% (17.7% to 23.7%)
Gout	-	-	41.9% (25.3% to 59.7%)	41.9% (25.3% to 59.7%)
**Other non-communicable diseases**	0.4% (0.3% to 0.6%)	0.7% (0.5% to 0.9%)	2.2% (1.9% to 2.6%)	2.0% (1.8% to 2.3%)
Sense organ diseases	-	-	7.2% (6.5% to 8.0%)	7.2% (6.5% to 8.0%)
Sudden infant death syndrome	3.8% (2.6% to 5.6%)	3.9% (2.6% to 5.6%)	-	3.9% (2.6% to 5.6%)
**Transport injuries**	49.4% (38.2% to 60.4%)	46.6% (35.2% to 58.1%)	51.0% (41.4% to 61.5%)	48.0% (37.3% to 58.6%)
Road injuries	49.1% (38.0% to 60.2%)	46.4% (34.8% to 57.9%)	51.0% (41.5% to 61.5%)	47.9% (37.2% to 58.4%)
Other transport injuries	51.7% (40.3% to 62.6%)	48.8% (37.5% to 60.0%)	50.6% (40.7% to 60.9%)	49.3% (38.3% to 59.9%)
**Unintentional injuries**	39.6% (35.0% to 44.0%)	30.2% (24.5% to 37.1%)	29.5% (23.6% to 37.5%)	29.8% (24.3% to 37.3%)
Falls	65.5% (60.8% to 69.3%)	48.5% (42.6% to 55.4%)	30.7% (24.9% to 38.2%)	35.4% (29.4% to 42.8%)
Drowning	28.7% (19.7% to 38.9%)	27.8% (19.4% to 37.4%)	28.1% (19.0% to 38.5%)	27.8% (19.4% to 37.4%)
Fire, heat, and hot substances	23.7% (15.2% to 33.9%)	29.4% (20.8% to 38.7%)	32.1% (22.3% to 43.4%)	30.9% (22.0% to 41.0%)

Supplementary Results Table 1. Age-standardised PAFs attributable to the joint distribution of all risk factors for all ages, both sexes combined for each GBD level 1 and level 2 cause and all causes in 2016 for France. Results are shown for deaths, YLLs, YLDs, and DALYs
Data in parenthesis are 95% uncertainty intervals. PAFs=population attributable fractions. GBD=Global Burden of Disease. YLLs=years of life lost. YLDs=years live with disability. DALYs=disability-adjusted life-years

	**Deaths 2016**	**YLLs 2016**	**YLDs 2016**	**DALYs 2016**
Poisonings	28.5% (19.1% to 40.3%)	30.9% (21.7% to 42.7%)	28.6% (19.9% to 39.4%)	29.7% (20.9% to 40.6%)
Exposure to mechanical forces	32.9% (26.8% to 40.7%)	30.3% (22.3% to 40.1%)	31.1% (23.3% to 41.5%)	30.8% (23.1% to 41.0%)
Animal contact	22.2% (16.3% to 30.2%)	22.4% (15.5% to 32.6%)	20.6% (13.3% to 30.7%)	21.2% (14.1% to 31.0%)
Foreign body	9.1% (5.2% to 16.1%)	15.7% (10.1% to 23.8%)	14.3% (9.2% to 21.7%)	15.5% (10.0% to 23.2%)
Other unintentional injuries	35.9% (24.8% to 48.3%)	39.9% (28.3% to 52.8%)	30.1% (20.3% to 40.5%)	31.6% (21.7% to 42.2%)
**Self-harm and interpersonal violence**	40.1% (24.0% to 54.1%)	38.8% (23.0% to 52.7%)	37.2% (30.8% to 43.6%)	38.7% (23.6% to 52.2%)
Self-harm	40.2% (23.4% to 54.8%)	39.2% (22.4% to 53.9%)	32.5% (16.5% to 47.1%)	39.1% (22.3% to 53.9%)
Interpersonal violence	37.5% (28.5% to 46.1%)	33.1% (24.5% to 41.2%)	38.8% (32.8% to 44.7%)	35.4% (28.3% to 42.1%)
**Forces of nature, conflict and terrorism, and executions and police conflict**	0.0% (0.0% to 0.0%)	0.0% (0.0% to 0.0%)	1.3% (0.8% to 2.0%)	0.7% (0.3% to 1.2%)
Forces of nature, conflict and terrorism, and state actor violence	0.0% (0.0% to 0.0%)	0.0% (0.0% to 0.0%)	6.4% (4.1% to 9.8%)	6.4% (4.1% to 9.8%)

Supplementary Results Table 1. Age-standardised PAFs attributable to the joint distribution of all risk factors for all ages, both sexes combined for each GBD level 1 and level 2 cause and all causes in 2016 for Gabon. Results are shown for deaths, YLLs, YLDs, and DALYs
Data in parenthesis are 95% uncertainty intervals. PAFs=population attributable fractions. GBD=Global Burden of Disease. YLLs=years of life lost. YLDs=years live with disability. DALYs=disability-adjusted life-years

	Deaths 2016	YLLs 2016	YLDs 2016	DALYs 2016
**All causes**	55.4% (52.7% to 58.1%)	53.1% (50.5% to 55.8%)	29.4% (28.0% to 31.0%)	46.6% (44.3% to 49.1%)
**HIV/AIDS and tuberculosis**	79.5% (73.5% to 84.8%)	84.0% (79.3% to 88.1%)	89.6% (86.7% to 92.2%)	84.4% (80.0% to 88.2%)
Tuberculosis	48.7% (36.8% to 60.2%)	51.9% (41.1% to 63.3%)	48.4% (38.4% to 58.8%)	51.8% (41.0% to 63.0%)
HIV/AIDS	96.4% (95.4% to 97.4%)	94.9% (93.0% to 96.6%)	95.9% (95.0% to 96.7%)	94.9% (93.2% to 96.6%)
**Diarrhoea, lower respiratory infections, and other common infectious diseases**	63.7% (55.1% to 72.9%)	64.2% (57.0% to 71.4%)	42.1% (35.4% to 47.7%)	62.4% (55.6% to 69.3%)
Diarrhoeal diseases	89.7% (77.3% to 96.9%)	91.7% (81.1% to 97.6%)	90.9% (80.3% to 97.3%)	91.6% (81.1% to 97.6%)
Lower respiratory infections	58.8% (50.4% to 68.3%)	67.8% (60.3% to 75.6%)	62.0% (53.8% to 69.9%)	67.7% (60.3% to 75.5%)
Upper respiratory infections	1.9% (0.1% to 6.9%)	4.1% (0.3% to 14.2%)	0.0% (0.0% to 0.0%)	0.0% (0.0% to 0.2%)
Otitis media	1.3% (0.3% to 3.3%)	4.2% (1.7% to 7.5%)	4.9% (3.2% to 6.6%)	4.9% (3.2% to 6.6%)
Meningitis	2.9% (1.3% to 5.5%)	5.3% (2.6% to 9.5%)	0.0% (0.0% to 0.0%)	4.8% (2.4% to 8.6%)
Encephalitis	1.0% (0.6% to 1.7%)	1.7% (1.1% to 2.8%)	0.0% (0.0% to 0.0%)	1.1% (0.7% to 1.7%)
Measles	39.6% (18.9% to 74.9%)	40.3% (19.4% to 76.1%)	39.0% (18.5% to 73.6%)	40.3% (19.4% to 76.1%)
**Maternal disorders**	5.7% (2.4% to 9.4%)	5.8% (2.5% to 9.6%)	2.3% (1.1% to 3.9%)	5.6% (2.4% to 9.2%)
Maternal haemorrhage	17.3% (6.4% to 28.8%)	17.8% (6.6% to 29.2%)	19.1% (7.0% to 30.8%)	17.8% (6.6% to 29.2%)
Maternal sepsis and other pregnancy related infections	16.1% (5.9% to 26.7%)	16.9% (6.1% to 28.1%)	18.5% (6.9% to 30.0%)	17.0% (6.1% to 28.1%)
Maternal abortion, miscarriage, and ectopic pregnancy	19.9% (13.2% to 27.2%)	19.8% (13.2% to 27.0%)	19.4% (11.9% to 27.9%)	19.8% (13.3% to 27.0%)
**Neonatal disorders**	79.0% (75.4% to 82.7%)	79.0% (75.5% to 82.7%)	0.3% (0.1% to 0.5%)	73.2% (66.5% to 78.2%)
Neonatal preterm birth complications	97.2% (94.2% to 98.9%)	97.2% (94.2% to 98.9%)	0.9% (0.7% to 1.3%)	91.3% (85.6% to 95.1%)
Neonatal encephalopathy due to birth asphyxia and trauma	72.2% (67.6% to 76.2%)	72.2% (67.6% to 76.2%)	0.0% (0.0% to 0.0%)	69.7% (62.7% to 74.5%)
Neonatal sepsis and other neonatal infections	66.9% (62.0% to 70.9%)	66.9% (62.0% to 70.9%)	0.0% (0.0% to 0.0%)	58.2% (45.7% to 67.0%)
Hemolytic disease and other neonatal jaundice	71.1% (66.4% to 75.1%)	71.1% (66.4% to 75.1%)	0.0% (0.0% to 0.0%)	58.4% (49.7% to 65.6%)

Supplementary Results Table 1. Age-standardised PAFs attributable to the joint distribution of all risk factors for all ages, both sexes combined for each GBD level 1 and level 2 cause and all causes in 2016 for Gabon. Results are shown for deaths, YLLs, YLDs, and DALYs
Data in parenthesis are 95% uncertainty intervals. PAFs=population attributable fractions. GBD=Global Burden of Disease. YLLs=years of life lost. YLDs=years live with disability. DALYs=disability-adjusted life-years

	Deaths 2016	YLLs 2016	YLDs 2016	DALYs 2016
Other neonatal disorders	69.5% (64.1% to 74.1%)	69.5% (64.1% to 74.1%)	0.0% (0.0% to 0.0%)	67.3% (61.6% to 72.0%)
**Nutritional deficiencies**	14.8% (7.1% to 23.8%)	44.9% (26.9% to 60.7%)	95.2% (94.1% to 96.3%)	72.5% (52.6% to 81.7%)
Protein-energy malnutrition	18.2% (8.7% to 28.9%)	52.0% (31.7% to 68.1%)	97.7% (96.8% to 98.4%)	53.2% (33.0% to 69.1%)
Vitamin A deficiency	-	-	100.0% (100.0% to 100.0%)	100.0% (100.0% to 100.0%)
Iron-deficiency anaemia	100.0% (100.0% to 100.0%)	100.0% (100.0% to 100.0%)	100.0% (100.0% to 100.0%)	100.0% (100.0% to 100.0%)
**Other communicable, maternal, neonatal, and nutritional diseases**	4.4% (2.1% to 9.6%)	3.6% (1.4% to 8.1%)	63.8% (59.2% to 68.4%)	17.1% (7.8% to 28.0%)
Sexually transmitted diseases excluding HIV	42.1% (22.4% to 64.5%)	22.2% (9.5% to 40.7%)	100.0% (100.0% to 100.0%)	62.6% (42.5% to 80.9%)
Hepatitis	0.0% (0.0% to 0.1%)	0.1% (0.0% to 0.1%)	0.1% (0.0% to 0.1%)	0.1% (0.0% to 0.1%)
**Neoplasms**	34.3% (29.4% to 39.6%)	35.8% (31.0% to 40.6%)	34.4% (29.5% to 39.9%)	35.7% (30.9% to 40.6%)
Oesophageal cancer	62.5% (49.3% to 74.8%)	66.0% (53.0% to 77.5%)	63.8% (50.8% to 75.6%)	66.0% (53.0% to 77.5%)
Stomach cancer	8.2% (1.8% to 19.4%)	8.5% (2.2% to 18.3%)	8.3% (2.0% to 18.8%)	8.5% (2.2% to 18.3%)
Liver cancer	34.9% (26.0% to 45.6%)	35.9% (26.9% to 47.0%)	35.4% (26.6% to 46.2%)	35.9% (26.9% to 47.0%)
Larynx cancer	61.4% (45.3% to 76.4%)	66.0% (51.0% to 79.9%)	63.1% (46.8% to 77.7%)	66.0% (50.9% to 79.9%)
Tracheal, bronchus, and lung cancer	68.1% (56.4% to 77.3%)	69.6% (58.0% to 78.4%)	68.4% (56.9% to 77.2%)	69.6% (58.0% to 78.4%)
Breast cancer	26.7% (17.1% to 38.4%)	24.6% (14.7% to 37.1%)	25.0% (15.1% to 37.4%)	24.6% (14.7% to 37.1%)
Cervical cancer	100.0% (100.0% to 100.0%)	100.0% (100.0% to 100.0%)	100.0% (100.0% to 100.0%)	100.0% (100.0% to 100.0%)
Uterine cancer	50.5% (35.6% to 64.1%)	51.5% (37.2% to 65.1%)	51.3% (37.2% to 64.9%)	51.5% (37.2% to 65.1%)
Prostate cancer	0.8% (0.2% to 1.7%)	1.1% (0.3% to 2.0%)	1.0% (0.3% to 1.9%)	1.1% (0.3% to 2.0%)
Colon and rectum cancer	53.5% (43.6% to 63.0%)	54.3% (44.7% to 63.4%)	54.0% (44.1% to 63.3%)	54.3% (44.6% to 63.4%)
Lip and oral cavity cancer	63.5% (48.2% to 75.6%)	68.4% (54.7% to 78.9%)	65.1% (51.0% to 76.3%)	68.3% (54.6% to 78.9%)
Nasopharynx cancer	62.8% (49.9% to 73.7%)	64.6% (52.3% to 74.4%)	63.0% (51.8% to 72.3%)	64.6% (52.3% to 74.3%)

**Supplementary Results Table 1. Age-standardised PAFs attributable to the joint distribution of all risk factors for all ages, both sexes combined for each GBD level 1 and level 2 cause and all causes in 2016 for Gabon. Results are shown for deaths, YLLs, YLDs, and DALYs** Data in parenthesis are 95% uncertainty intervals. PAFs=population attributable fractions. GBD=Global Burden of Disease. YLLs=years of life lost. YLDs=years live with disability. DALYs=disability-adjusted life-years				
	**Deaths 2016**	**YLLs 2016**	**YLDs 2016**	**DALYs 2016**
Other pharynx cancer	51.9% (34.4% to 66.1%)	55.1% (38.0% to 68.2%)	53.4% (36.0% to 67.0%)	55.1% (38.0% to 68.1%)
Gallbladder and biliary tract cancer	25.4% (14.1% to 38.3%)	26.3% (15.2% to 39.3%)	25.1% (14.1% to 37.5%)	26.3% (15.2% to 39.2%)
Pancreatic cancer	20.1% (11.3% to 31.0%)	20.7% (12.0% to 31.0%)	20.2% (11.4% to 30.8%)	20.7% (12.0% to 31.0%)
Ovarian cancer	13.8% (5.4% to 24.4%)	12.5% (4.6% to 22.0%)	12.2% (4.6% to 21.6%)	12.5% (4.6% to 22.0%)
Kidney cancer	24.2% (16.0% to 34.1%)	24.0% (15.8% to 33.4%)	23.9% (15.9% to 33.6%)	24.0% (15.7% to 33.4%)
Bladder cancer	15.4% (6.7% to 26.2%)	16.7% (7.6% to 27.3%)	16.2% (7.5% to 26.8%)	16.7% (7.6% to 27.3%)
Thyroid cancer	14.2% (7.8% to 22.4%)	14.5% (8.1% to 23.0%)	14.3% (8.0% to 22.1%)	14.5% (8.1% to 23.0%)
Mesothelioma	88.2% (84.2% to 91.8%)	80.5% (74.6% to 85.7%)	85.4% (80.7% to 89.4%)	80.5% (74.7% to 85.7%)
Non-Hodgkin's lymphoma	6.4% (2.5% to 11.6%)	6.0% (2.4% to 10.7%)	6.2% (2.5% to 11.0%)	6.0% (2.4% to 10.7%)
Multiple myeloma	8.4% (3.7% to 14.5%)	8.6% (3.7% to 15.0%)	8.4% (3.6% to 14.5%)	8.6% (3.7% to 15.0%)
Leukaemia	10.9% (6.4% to 16.6%)	9.3% (5.3% to 14.3%)	10.2% (6.0% to 15.5%)	9.3% (5.3% to 14.3%)
**Cardiovascular diseases**	84.1% (81.4% to 86.3%)	84.9% (82.7% to 86.7%)	70.2% (66.7% to 73.5%)	83.8% (81.5% to 85.8%)
Rheumatic heart disease	30.4% (20.8% to 46.9%)	30.0% (21.1% to 45.1%)	30.3% (20.7% to 44.7%)	30.0% (21.1% to 45.1%)
Ischaemic heart disease	93.3% (89.9% to 96.2%)	94.7% (92.4% to 96.6%)	91.2% (88.4% to 93.8%)	94.5% (92.3% to 96.5%)
Cerebrovascular disease	86.0% (81.6% to 90.0%)	89.2% (86.1% to 91.8%)	86.9% (82.8% to 90.9%)	89.0% (85.9% to 91.7%)
Hypertensive heart disease	100.0% (100.0% to 100.0%)	100.0% (100.0% to 100.0%)	100.0% (100.0% to 100.0%)	100.0% (100.0% to 100.0%)
Cardiomyopathy and myocarditis	44.7% (37.1% to 53.6%)	44.1% (36.9% to 51.6%)	47.1% (40.4% to 54.4%)	44.3% (37.2% to 51.8%)
Atrial fibrillation and flutter	51.7% (43.0% to 60.2%)	56.3% (48.8% to 63.9%)	62.6% (56.2% to 68.9%)	59.3% (52.5% to 66.0%)
Aortic aneurysm	43.9% (37.0% to 51.0%)	48.8% (42.5% to 54.5%)	-	48.8% (42.5% to 54.5%)
Peripheral vascular disease	55.9% (49.3% to 63.3%)	62.5% (57.1% to 67.7%)	63.7% (58.5% to 68.9%)	63.1% (57.9% to 68.0%)
Endocarditis	42.6% (33.4% to 53.4%)	42.7% (34.5% to 51.7%)	38.1% (28.8% to 49.3%)	42.7% (34.5% to 51.6%)

Supplementary Results Table 1. Age-standardised PAFs attributable to the joint distribution of all risk factors for all ages, both sexes combined for each GBD level 1 and level 2 cause and all causes in 2016 for Gabon. Results are shown for deaths, YLLs, YLDs, and DALYs
Data in parenthesis are 95% uncertainty intervals. PAFs=population attributable fractions. GBD=Global Burden of Disease. YLLs=years of life lost. YLDs=years live with disability. DALYs=disability-adjusted life-years

	Deaths 2016	YLLs 2016	YLDs 2016	DALYs 2016
Other cardiovascular and circulatory diseases	46.6% (41.3% to 51.9%)	49.9% (45.5% to 54.4%)	41.9% (36.6% to 47.3%)	48.3% (43.7% to 52.9%)
**Chronic respiratory diseases**	51.6% (42.5% to 61.0%)	52.2% (43.9% to 60.4%)	39.7% (32.4% to 47.1%)	46.7% (39.1% to 54.3%)
Chronic obstructive pulmonary disease	58.0% (46.5% to 70.2%)	60.9% (49.8% to 72.0%)	57.9% (47.0% to 68.4%)	60.0% (48.8% to 70.9%)
Pneumoconiosis	100.0% (100.0% to 100.0%)	100.0% (100.0% to 100.0%)	100.0% (100.0% to 100.0%)	100.0% (100.0% to 100.0%)
Asthma	36.5% (25.2% to 49.4%)	37.7% (27.5% to 49.1%)	28.3% (19.9% to 37.9%)	31.5% (22.6% to 41.4%)
Other chronic respiratory diseases	4.3% (1.7% to 8.2%)	4.4% (1.4% to 8.9%)	2.9% (1.0% to 5.2%)	3.9% (1.3% to 7.7%)
**Cirrhosis and other chronic liver diseases**	25.5% (23.8% to 27.2%)	24.5% (22.8% to 26.3%)	20.0% (17.5% to 22.5%)	24.5% (22.7% to 26.2%)
Cirrhosis and other chronic liver diseases due to hepatitis B	0.0% (0.0% to 0.0%)	0.0% (0.0% to 0.0%)	0.0% (0.0% to 0.0%)	0.0% (0.0% to 0.0%)
Cirrhosis and other chronic liver diseases due to hepatitis C	1.1% (0.5% to 2.0%)	1.7% (0.7% to 2.9%)	2.3% (0.9% to 4.2%)	1.7% (0.7% to 2.9%)
Cirrhosis and other chronic liver diseases due to alcohol use	100.0% (100.0% to 100.0%)	100.0% (100.0% to 100.0%)	100.0% (100.0% to 100.0%)	100.0% (100.0% to 100.0%)
**Digestive diseases**	7.8% (5.7% to 10.1%)	8.9% (6.6% to 11.4%)	3.9% (2.9% to 5.1%)	8.5% (6.4% to 11.0%)
Peptic ulcer disease	5.3% (3.2% to 7.6%)	6.9% (4.2% to 9.9%)	6.3% (4.3% to 8.7%)	6.9% (4.3% to 9.8%)
Gallbladder and biliary diseases	43.9% (29.3% to 58.4%)	44.7% (30.9% to 58.7%)	45.7% (32.7% to 59.3%)	44.7% (30.9% to 58.8%)
Pancreatitis	45.7% (21.6% to 70.1%)	52.3% (26.2% to 74.3%)	48.3% (23.7% to 70.8%)	52.2% (26.2% to 74.3%)
**Neurological disorders**	23.2% (10.8% to 37.4%)	23.0% (12.2% to 35.1%)	6.5% (3.6% to 9.9%)	13.9% (8.0% to 21.2%)
Alzheimer's disease and other dementias	24.8% (10.9% to 40.6%)	26.0% (11.7% to 41.7%)	26.0% (11.9% to 41.8%)	26.0% (11.7% to 41.8%)
Parkinson's disease	-3.6% (-5.1% to -2.4%)	-4.6% (-6.5% to -3.2%)	-5.5% (-7.3% to -3.9%)	-4.8% (-6.5% to -3.3%)
Epilepsy	25.7% (17.4% to 35.5%)	24.3% (16.3% to 34.0%)	21.6% (14.3% to 30.0%)	22.9% (15.2% to 31.7%)
Multiple sclerosis	4.0% (2.1% to 6.5%)	4.4% (2.2% to 7.1%)	3.9% (2.3% to 5.8%)	4.2% (2.3% to 6.5%)
**Mental and substance use disorders**	99.7% (99.5% to 99.9%)	99.6% (99.4% to 99.8%)	18.3% (16.3% to 20.5%)	22.4% (19.7% to 25.2%)
Alcohol use disorders	100.0% (100.0% to 100.0%)	100.0% (100.0% to 100.0%)	100.0% (100.0% to 100.0%)	100.0% (100.0% to 100.0%)

Supplementary Results Table 1. Age-standardised PAFs attributable to the joint distribution of all risk factors for all ages, both sexes combined for each GBD level 1 and level 2 cause and all causes in 2016 for Gabon. Results are shown for deaths, YLLs, YLDs, and DALYs
Data in parenthesis are 95% uncertainty intervals. PAFs=population attributable fractions. GBD=Global Burden of Disease. YLLs=years of life lost. YLDs=years live with disability. DALYs=disability-adjusted life-years

	Deaths 2016	YLLs 2016	YLDs 2016	DALYs 2016
Drug use disorders	100.0% (100.0% to 100.0%)	100.0% (100.0% to 100.0%)	100.0% (100.0% to 100.0%)	100.0% (100.0% to 100.0%)
Depressive disorders	-	-	9.1% (7.6% to 10.8%)	9.1% (7.6% to 10.8%)
Idiopathic developmental intellectual disability	-	-	60.5% (30.6% to 80.1%)	60.5% (30.6% to 80.1%)
**Diabetes, urogenital, blood, and endocrine diseases**	82.4% (79.7% to 85.2%)	78.3% (74.5% to 82.0%)	66.2% (62.2% to 69.9%)	73.3% (69.9% to 76.3%)
Diabetes mellitus	100.0% (100.0% to 100.0%)	100.0% (100.0% to 100.0%)	100.0% (100.0% to 100.0%)	100.0% (100.0% to 100.0%)
Chronic kidney disease	100.0% (100.0% to 100.0%)	100.0% (100.0% to 100.0%)	100.0% (100.0% to 100.0%)	100.0% (100.0% to 100.0%)
**Musculoskeletal disorders**	0.3% (0.1% to 0.6%)	0.3% (0.1% to 0.5%)	17.8% (14.5% to 21.5%)	17.4% (14.2% to 20.9%)
Rheumatoid arthritis	1.3% (0.5% to 2.4%)	1.5% (0.6% to 3.0%)	2.3% (0.9% to 4.0%)	2.2% (0.9% to 3.9%)
Osteoarthritis	-	-	27.6% (15.3% to 40.8%)	27.6% (15.3% to 40.8%)
Low back and neck pain	-	-	19.4% (16.6% to 22.6%)	19.4% (16.6% to 22.6%)
Gout	-	-	51.2% (39.2% to 64.5%)	51.2% (39.2% to 64.5%)
**Other non-communicable diseases**	0.1% (0.1% to 0.3%)	0.2% (0.1% to 0.4%)	5.4% (4.5% to 6.4%)	4.1% (3.3% to 4.9%)
Sense organ diseases	-	-	11.0% (9.3% to 12.8%)	11.0% (9.3% to 12.8%)
Sudden infant death syndrome	6.1% (2.6% to 11.8%)	6.2% (2.6% to 11.8%)	-	6.2% (2.6% to 11.8%)
**Transport injuries**	35.7% (28.9% to 43.8%)	30.7% (24.1% to 38.6%)	40.7% (33.8% to 49.1%)	32.0% (25.3% to 39.8%)
Road injuries	35.5% (28.8% to 43.6%)	30.6% (24.0% to 38.5%)	42.6% (35.6% to 51.0%)	31.9% (25.2% to 39.7%)
Other transport injuries	39.5% (31.5% to 48.6%)	34.0% (26.3% to 43.1%)	33.2% (26.7% to 41.3%)	33.6% (26.7% to 41.6%)
**Unintentional injuries**	35.7% (27.2% to 42.6%)	20.7% (16.8% to 25.1%)	24.6% (20.2% to 29.9%)	21.9% (18.2% to 26.4%)
Falls	67.6% (47.6% to 74.7%)	45.9% (37.5% to 53.0%)	32.4% (28.4% to 37.3%)	39.5% (34.4% to 44.9%)
Drowning	16.5% (12.0% to 21.8%)	13.6% (9.7% to 18.4%)	19.6% (13.5% to 27.2%)	13.7% (9.8% to 18.5%)
Fire, heat, and hot substances	13.3% (9.0% to 18.7%)	15.1% (10.8% to 19.9%)	22.3% (15.6% to 30.1%)	16.9% (12.0% to 22.2%)

Supplementary Results Table 1. Age-standardised PAFs attributable to the joint distribution of all risk factors for all ages, both sexes combined for each GBD level 1 and level 2 cause and all causes in 2016 for Gabon. Results are shown for deaths, YLLs, YLDs, and DALYs

Data in parenthesis are 95% uncertainty intervals. PAFs=population attributable fractions. GBD=Global Burden of Disease. YLLs=years of life lost. YLDs=years live with disability. DALYs=disability-adjusted life-years

	Deaths 2016	YLLs 2016	YLDs 2016	DALYs 2016
Poisonings	16.7% (11.2% to 23.4%)	16.6% (9.7% to 24.1%)	21.1% (14.6% to 28.9%)	17.1% (10.4% to 24.3%)
Exposure to mechanical forces	25.0% (20.1% to 31.1%)	19.0% (14.0% to 26.1%)	25.6% (19.7% to 33.9%)	21.2% (16.1% to 28.6%)
Animal contact	13.0% (9.9% to 17.2%)	13.8% (10.2% to 19.1%)	16.0% (11.2% to 22.4%)	14.3% (10.5% to 19.3%)
Foreign body	9.2% (6.3% to 12.8%)	8.7% (5.0% to 13.1%)	13.6% (9.2% to 19.6%)	10.0% (6.3% to 14.5%)
Other unintentional injuries	19.4% (13.8% to 26.2%)	21.2% (15.5% to 28.0%)	19.9% (13.8% to 27.0%)	20.5% (14.7% to 27.0%)
**Self-harm and interpersonal violence**	23.9% (14.5% to 34.4%)	24.0% (15.5% to 33.6%)	23.9% (19.7% to 29.2%)	24.0% (15.9% to 33.1%)
Self-harm	24.9% (12.5% to 38.4%)	26.9% (14.3% to 39.9%)	25.3% (12.8% to 38.5%)	26.9% (14.3% to 39.8%)
Interpersonal violence	21.8% (14.5% to 30.7%)	20.2% (13.1% to 28.8%)	23.7% (19.9% to 28.7%)	20.8% (14.3% to 28.5%)
**Forces of nature, conflict and terrorism, and executions and police conflict**	0.0% (0.0% to 0.0%)	0.0% (0.0% to 0.0%)	0.0% (0.0% to 0.0%)	0.0% (0.0% to 0.0%)
Forces of nature, conflict and terrorism, and state actor violence	0.0% (0.0% to 0.0%)	0.0% (0.0% to 0.0%)	0.0% (0.0% to 0.0%)	0.0% (0.0% to 0.0%)

Supplementary Results Table 1. Age-standardised PAFs attributable to the joint distribution of all risk factors for all ages, both sexes combined for each GBD level 1 and level 2 cause and all causes in 2016 for Georgia. Results are shown for deaths, YLLs, YLDs, and DALYs
Data in parenthesis are 95% uncertainty intervals. PAFs=population attributable fractions. GBD=Global Burden of Disease. YLLs=years of life lost. YLDs=years live with disability. DALYs=disability-adjusted life-years

	Deaths 2016	YLLs 2016	YLDs 2016	DALYs 2016
**All causes**	71.5% (69.8% to 73.2%)	63.6% (61.9% to 65.2%)	27.6% (26.3% to 29.0%)	50.9% (47.9% to 53.8%)
**HIV/AIDS and tuberculosis**	61.4% (53.8% to 68.5%)	59.1% (51.6% to 66.2%)	49.1% (41.5% to 56.1%)	58.5% (51.3% to 65.5%)
Tuberculosis	61.9% (52.5% to 70.4%)	61.5% (52.0% to 70.2%)	43.5% (34.2% to 52.3%)	60.4% (51.0% to 69.0%)
HIV/AIDS	58.9% (56.1% to 61.5%)	50.6% (46.9% to 54.0%)	69.6% (66.9% to 71.6%)	51.7% (48.1% to 54.9%)
**Diarrhoea, lower respiratory infections, and other common infectious diseases**	58.6% (50.4% to 65.8%)	60.0% (53.0% to 66.5%)	33.6% (26.8% to 39.9%)	52.8% (46.6% to 58.6%)
Diarrhoeal diseases	87.2% (76.9% to 95.4%)	91.1% (83.6% to 96.8%)	81.8% (67.5% to 93.1%)	84.9% (72.7% to 94.3%)
Lower respiratory infections	66.3% (56.3% to 75.0%)	71.1% (62.0% to 79.2%)	62.2% (51.4% to 72.1%)	70.9% (61.9% to 79.1%)
Upper respiratory infections	1.1% (0.1% to 4.3%)	2.7% (0.2% to 10.5%)	0.0% (0.0% to 0.0%)	0.0% (0.0% to 0.2%)
Otitis media	13.2% (8.9% to 17.8%)	15.8% (10.8% to 20.4%)	9.8% (6.7% to 12.6%)	9.8% (6.7% to 12.6%)
Meningitis	2.1% (1.4% to 2.9%)	3.2% (2.3% to 4.3%)	0.0% (0.0% to 0.0%)	2.3% (1.6% to 3.1%)
Encephalitis	1.6% (0.8% to 2.8%)	2.6% (1.1% to 4.6%)	0.0% (0.0% to 0.0%)	2.2% (0.9% to 3.7%)
Measles	14.0% (4.5% to 35.8%)	17.7% (6.0% to 44.5%)	29.2% (11.7% to 67.0%)	20.8% (7.8% to 49.8%)
**Maternal disorders**	6.4% (3.2% to 10.0%)	6.3% (3.1% to 9.7%)	4.9% (2.2% to 8.1%)	6.0% (3.0% to 9.2%)
Maternal haemorrhage	15.8% (5.6% to 25.7%)	15.8% (5.6% to 25.7%)	15.6% (5.6% to 25.6%)	15.7% (5.6% to 25.7%)
Maternal sepsis and other pregnancy related infections	15.7% (5.7% to 25.7%)	15.7% (5.6% to 25.6%)	15.3% (5.5% to 25.2%)	15.6% (5.6% to 25.5%)
Maternal abortion, miscarriage, and ectopic pregnancy	8.5% (5.2% to 12.5%)	8.2% (4.9% to 12.1%)	8.1% (4.0% to 13.1%)	8.2% (4.9% to 12.1%)
**Neonatal disorders**	66.7% (63.7% to 69.8%)	66.8% (63.8% to 69.8%)	0.1% (0.1% to 0.1%)	59.2% (54.5% to 63.3%)
Neonatal preterm birth complications	88.8% (84.7% to 92.4%)	88.9% (84.8% to 92.4%)	0.2% (0.2% to 0.3%)	77.5% (70.8% to 82.9%)
Neonatal encephalopathy due to birth asphyxia and trauma	60.3% (55.5% to 64.4%)	60.3% (55.6% to 64.5%)	0.0% (0.0% to 0.0%)	53.2% (44.7% to 60.3%)
Neonatal sepsis and other neonatal infections	42.7% (35.1% to 49.7%)	42.8% (35.2% to 49.8%)	0.0% (0.0% to 0.0%)	36.1% (26.9% to 44.5%)
Hemolytic disease and other neonatal jaundice	60.4% (51.6% to 65.5%)	60.4% (51.7% to 65.5%)	0.0% (0.0% to 0.0%)	24.7% (17.1% to 33.6%)

Supplementary Results Table 1. Age-standardised PAFs attributable to the joint distribution of all risk factors for all ages, both sexes combined for each GBD level 1 and level 2 cause and all causes in 2016 for Georgia. Results are shown for deaths, YLLs, YLDs, and DALYs
Data in parenthesis are 95% uncertainty intervals. PAFs=population attributable fractions. GBD=Global Burden of Disease. YLLs=years of life lost. YLDs=years live with disability. DALYs=disability-adjusted life-years

	Deaths 2016	YLLs 2016	YLDs 2016	DALYs 2016
Other neonatal disorders	57.4% (52.0% to 62.8%)	57.4% (52.1% to 62.9%)	0.0% (0.0% to 0.0%)	56.4% (50.9% to 61.9%)
**Nutritional deficiencies**	14.3% (12.0% to 17.5%)	23.4% (19.1% to 28.8%)	87.4% (84.6% to 89.9%)	86.4% (83.4% to 89.0%)
Protein-energy malnutrition	15.0% (7.5% to 27.3%)	32.0% (18.9% to 46.4%)	96.1% (92.8% to 98.0%)	89.0% (82.5% to 93.8%)
Vitamin A deficiency	-	-	0.0% (0.0% to 0.0%)	0.0% (0.0% to 0.0%)
Iron-deficiency anaemia	100.0% (100.0% to 100.0%)	100.0% (100.0% to 100.0%)	100.0% (100.0% to 100.0%)	100.0% (100.0% to 100.0%)
**Other communicable, maternal, neonatal, and nutritional diseases**	4.0% (2.9% to 4.8%)	3.1% (1.8% to 4.0%)	71.0% (66.0% to 75.6%)	34.2% (24.1% to 44.1%)
Sexually transmitted diseases excluding HIV	63.6% (38.4% to 91.7%)	40.7% (18.3% to 79.6%)	100.0% (100.0% to 100.0%)	95.3% (88.7% to 99.4%)
Hepatitis	3.5% (2.5% to 4.8%)	2.9% (1.9% to 4.0%)	1.7% (1.1% to 2.5%)	2.8% (1.9% to 3.9%)
**Neoplasms**	43.8% (39.8% to 47.9%)	42.8% (38.9% to 46.8%)	36.6% (33.1% to 40.3%)	42.7% (38.8% to 46.6%)
Oesophageal cancer	78.1% (67.5% to 86.4%)	80.4% (70.9% to 87.6%)	78.6% (69.0% to 86.3%)	80.3% (70.9% to 87.6%)
Stomach cancer	24.8% (14.6% to 36.1%)	26.5% (16.1% to 37.3%)	25.0% (15.5% to 35.4%)	26.5% (16.1% to 37.3%)
Liver cancer	70.6% (63.6% to 76.8%)	69.9% (62.5% to 76.2%)	70.4% (63.2% to 76.6%)	69.9% (62.5% to 76.2%)
Larynx cancer	86.7% (80.1% to 91.3%)	89.1% (83.5% to 93.1%)	87.5% (81.6% to 91.6%)	89.1% (83.4% to 93.0%)
Tracheal, bronchus, and lung cancer	89.1% (85.6% to 92.2%)	89.7% (86.4% to 92.7%)	88.8% (85.3% to 91.9%)	89.7% (86.4% to 92.6%)
Breast cancer	20.4% (14.1% to 27.3%)	18.5% (12.1% to 25.1%)	18.8% (12.3% to 25.4%)	18.5% (12.1% to 25.1%)
Cervical cancer	100.0% (100.0% to 100.0%)	100.0% (100.0% to 100.0%)	100.0% (100.0% to 100.0%)	100.0% (100.0% to 100.0%)
Uterine cancer	44.4% (31.2% to 57.8%)	43.9% (30.5% to 57.5%)	44.2% (30.6% to 58.0%)	43.9% (30.5% to 57.6%)
Prostate cancer	5.6% (3.7% to 7.9%)	7.2% (4.7% to 10.0%)	7.2% (4.8% to 10.0%)	7.2% (4.7% to 10.0%)
Colon and rectum cancer	51.5% (43.1% to 59.8%)	52.7% (44.6% to 61.1%)	52.3% (44.3% to 60.0%)	52.7% (44.6% to 61.1%)
Lip and oral cavity cancer	73.6% (66.0% to 79.9%)	76.0% (69.1% to 81.8%)	69.5% (62.2% to 76.1%)	75.8% (69.0% to 81.6%)
Nasopharynx cancer	68.4% (61.9% to 74.2%)	65.9% (59.3% to 72.0%)	68.4% (62.8% to 73.7%)	65.9% (59.5% to 72.0%)

**Supplementary Results Table 1. Age-standardised PAFs attributable to the joint distribution of all risk factors for all ages, both sexes combined for each GBD level 1 and level 2 cause and all causes in 2016 for Georgia. Results are shown for deaths, YLLs, YLDs, and DALYs** Data in parenthesis are 95% uncertainty intervals. PAFs=population attributable fractions. GBD=Global Burden of Disease. YLLs=years of life lost. YLDs=years live with disability. DALYs=disability-adjusted life-years				
	**Deaths 2016**	**YLLs 2016**	**YLDs 2016**	**DALYs 2016**
Other pharynx cancer	67.5% (55.9% to 76.2%)	69.1% (58.4% to 77.5%)	67.6% (56.7% to 76.3%)	69.1% (58.4% to 77.4%)
Gallbladder and biliary tract cancer	22.7% (12.8% to 34.9%)	22.7% (12.7% to 34.9%)	22.8% (12.9% to 34.7%)	22.7% (12.7% to 34.9%)
Pancreatic cancer	28.5% (20.5% to 37.0%)	31.6% (23.2% to 40.0%)	29.0% (21.9% to 36.8%)	31.6% (23.2% to 40.0%)
Ovarian cancer	9.9% (4.2% to 16.3%)	8.4% (3.2% to 14.6%)	8.3% (3.3% to 14.2%)	8.4% (3.2% to 14.6%)
Kidney cancer	39.7% (28.5% to 51.1%)	40.3% (29.3% to 51.7%)	39.3% (28.7% to 49.5%)	40.2% (29.3% to 51.6%)
Bladder cancer	36.7% (26.0% to 47.7%)	42.7% (30.9% to 53.8%)	40.5% (30.3% to 51.0%)	42.6% (30.9% to 53.8%)
Thyroid cancer	13.4% (7.0% to 22.3%)	13.4% (7.0% to 22.3%)	12.5% (6.6% to 20.1%)	13.3% (6.9% to 22.2%)
Mesothelioma	86.1% (80.5% to 90.1%)	77.0% (68.7% to 82.7%)	83.3% (77.3% to 87.7%)	77.1% (68.8% to 82.8%)
Non-Hodgkin's lymphoma	6.3% (2.5% to 11.1%)	5.7% (2.2% to 10.1%)	6.1% (2.4% to 11.0%)	5.7% (2.2% to 10.1%)
Multiple myeloma	7.6% (3.2% to 13.9%)	7.5% (3.2% to 13.6%)	7.6% (3.3% to 13.6%)	7.5% (3.2% to 13.6%)
Leukaemia	19.2% (11.5% to 27.0%)	14.9% (9.0% to 21.2%)	18.3% (11.3% to 25.2%)	14.9% (9.0% to 21.2%)
**Cardiovascular diseases**	89.8% (87.6% to 91.8%)	90.2% (88.8% to 91.6%)	79.8% (76.9% to 82.4%)	89.4% (88.0% to 90.8%)
Rheumatic heart disease	30.5% (20.3% to 49.0%)	26.3% (18.8% to 38.3%)	25.7% (17.1% to 36.1%)	26.2% (18.6% to 37.7%)
Ischaemic heart disease	94.3% (91.5% to 96.7%)	95.5% (93.7% to 96.9%)	92.9% (90.5% to 94.9%)	95.4% (93.6% to 96.8%)
Cerebrovascular disease	87.5% (83.1% to 91.4%)	90.9% (88.1% to 93.1%)	88.7% (85.2% to 91.6%)	90.6% (87.9% to 92.9%)
Hypertensive heart disease	100.0% (100.0% to 100.0%)	100.0% (100.0% to 100.0%)	100.0% (100.0% to 100.0%)	100.0% (100.0% to 100.0%)
Cardiomyopathy and myocarditis	36.2% (26.9% to 49.7%)	32.2% (22.8% to 46.7%)	27.5% (22.2% to 32.8%)	31.9% (22.8% to 45.7%)
Atrial fibrillation and flutter	54.8% (47.0% to 62.6%)	59.3% (52.2% to 65.7%)	66.2% (61.3% to 70.9%)	63.2% (57.3% to 68.5%)
Aortic aneurysm	56.3% (49.5% to 62.5%)	61.3% (55.4% to 66.5%)	-	61.3% (55.4% to 66.5%)
Peripheral vascular disease	57.1% (49.2% to 64.5%)	64.9% (58.7% to 70.7%)	62.4% (56.7% to 68.1%)	63.2% (57.6% to 69.0%)
Endocarditis	46.8% (38.2% to 56.4%)	42.8% (34.7% to 51.5%)	42.9% (34.3% to 51.6%)	42.8% (34.7% to 51.5%)

Supplementary Results Table 1. Age-standardised PAFs attributable to the joint distribution of all risk factors for all ages, both sexes combined for each GBD level 1 and level 2 cause and all causes in 2016 for Georgia. Results are shown for deaths, YLLs, YLDs, and DALYs
Data in parenthesis are 95% uncertainty intervals. PAFs=population attributable fractions. GBD=Global Burden of Disease. YLLs=years of life lost. YLDs=years live with disability. DALYs=disability-adjusted life-years

	Deaths 2016	YLLs 2016	YLDs 2016	DALYs 2016
Other cardiovascular and circulatory diseases	56.8% (52.0% to 61.4%)	58.1% (53.5% to 62.5%)	49.3% (44.0% to 54.5%)	56.3% (52.0% to 60.7%)
**Chronic respiratory diseases**	61.4% (54.2% to 68.7%)	61.6% (55.8% to 67.4%)	44.1% (37.8% to 51.0%)	56.0% (50.6% to 61.5%)
Chronic obstructive pulmonary disease	70.8% (62.3% to 79.3%)	73.5% (66.7% to 80.1%)	69.9% (62.5% to 76.6%)	73.0% (66.3% to 79.4%)
Pneumoconiosis	100.0% (100.0% to 100.0%)	100.0% (100.0% to 100.0%)	100.0% (100.0% to 100.0%)	100.0% (100.0% to 100.0%)
Asthma	42.6% (33.3% to 52.8%)	41.1% (32.1% to 51.8%)	36.8% (28.6% to 46.4%)	37.8% (29.6% to 47.3%)
Other chronic respiratory diseases	21.3% (14.5% to 29.0%)	23.9% (15.7% to 32.5%)	7.2% (4.5% to 10.5%)	21.0% (13.2% to 29.1%)
**Cirrhosis and other chronic liver diseases**	47.7% (45.4% to 50.1%)	48.0% (45.5% to 50.4%)	32.2% (29.2% to 35.4%)	47.3% (44.9% to 49.8%)
Cirrhosis and other chronic liver diseases due to hepatitis B	1.5% (0.6% to 2.6%)	1.8% (0.7% to 3.0%)	1.2% (0.5% to 2.1%)	1.7% (0.7% to 3.0%)
Cirrhosis and other chronic liver diseases due to hepatitis C	89.2% (87.9% to 89.9%)	89.8% (89.4% to 90.0%)	87.9% (87.0% to 88.6%)	89.7% (89.4% to 89.9%)
Cirrhosis and other chronic liver diseases due to alcohol use	100.0% (100.0% to 100.0%)	100.0% (100.0% to 100.0%)	100.0% (100.0% to 100.0%)	100.0% (100.0% to 100.0%)
**Digestive diseases**	11.8% (9.1% to 15.3%)	13.1% (10.0% to 16.7%)	3.6% (2.7% to 4.6%)	10.2% (7.7% to 13.2%)
Peptic ulcer disease	19.1% (13.5% to 24.9%)	22.3% (16.1% to 28.6%)	11.4% (8.2% to 15.1%)	20.5% (14.7% to 26.7%)
Gallbladder and biliary diseases	40.8% (28.2% to 54.4%)	39.1% (26.3% to 52.7%)	44.8% (31.5% to 59.5%)	40.7% (27.7% to 54.5%)
Pancreatitis	36.1% (21.1% to 51.2%)	46.0% (29.7% to 61.0%)	27.5% (16.2% to 39.0%)	45.5% (29.4% to 60.4%)
**Neurological disorders**	18.8% (8.8% to 30.6%)	17.4% (9.6% to 26.8%)	3.4% (1.6% to 5.4%)	7.3% (4.0% to 11.1%)
Alzheimer's disease and other dementias	22.9% (11.1% to 36.6%)	23.7% (11.9% to 37.3%)	23.7% (11.7% to 37.4%)	23.7% (11.8% to 37.4%)
Parkinson's disease	-10.3% (-13.9% to -7.2%)	-13.2% (-17.7% to -9.5%)	-14.6% (-18.6% to -10.5%)	-13.5% (-17.7% to -9.7%)
Epilepsy	26.3% (18.9% to 33.9%)	22.6% (15.8% to 29.5%)	17.0% (12.1% to 22.5%)	18.9% (13.5% to 25.0%)
Multiple sclerosis	8.2% (4.7% to 12.1%)	9.8% (5.7% to 14.3%)	8.0% (5.0% to 11.1%)	8.7% (5.3% to 12.2%)
**Mental and substance use disorders**	99.9% (99.9% to 100.0%)	99.9% (99.9% to 99.9%)	19.5% (17.3% to 21.9%)	23.5% (20.9% to 26.3%)
Alcohol use disorders	100.0% (100.0% to 100.0%)	100.0% (100.0% to 100.0%)	100.0% (100.0% to 100.0%)	100.0% (100.0% to 100.0%)

Supplementary Results Table 1. Age-standardised PAFs attributable to the joint distribution of all risk factors for all ages, both sexes combined for each GBD level 1 and level 2 cause and all causes in 2016 for Georgia. Results are shown for deaths, YLLs, YLDs, and DALYs
Data in parenthesis are 95% uncertainty intervals. PAFs=population attributable fractions. GBD=Global Burden of Disease. YLLs=years of life lost. YLDs=years live with disability. DALYs=disability-adjusted life-years

	Deaths 2016	YLLs 2016	YLDs 2016	DALYs 2016
Drug use disorders	100.0% (100.0% to 100.0%)	100.0% (100.0% to 100.0%)	100.0% (100.0% to 100.0%)	100.0% (100.0% to 100.0%)
Depressive disorders	-	-	1.9% (1.5% to 2.4%)	1.9% (1.5% to 2.4%)
Idiopathic developmental intellectual disability	-	-	53.5% (25.7% to 74.0%)	53.5% (25.7% to 74.0%)
**Diabetes, urogenital, blood, and endocrine diseases**	89.6% (88.6% to 92.1%)	86.3% (84.9% to 89.1%)	62.6% (58.3% to 66.5%)	75.1% (71.4% to 78.2%)
Diabetes mellitus	100.0% (100.0% to 100.0%)	100.0% (100.0% to 100.0%)	100.0% (100.0% to 100.0%)	100.0% (100.0% to 100.0%)
Chronic kidney disease	100.0% (100.0% to 100.0%)	100.0% (100.0% to 100.0%)	100.0% (100.0% to 100.0%)	100.0% (100.0% to 100.0%)
**Musculoskeletal disorders**	0.9% (0.4% to 1.6%)	0.9% (0.3% to 1.6%)	31.0% (27.1% to 35.2%)	30.8% (26.9% to 35.0%)
Rheumatoid arthritis	3.7% (1.4% to 6.5%)	4.5% (1.7% to 7.6%)	4.7% (1.9% to 7.8%)	4.7% (1.9% to 7.8%)
Osteoarthritis	-	-	26.0% (14.1% to 38.7%)	26.0% (14.1% to 38.7%)
Low back and neck pain	-	-	35.2% (31.5% to 39.3%)	35.2% (31.5% to 39.3%)
Gout	-	-	49.0% (35.1% to 64.0%)	49.0% (35.1% to 64.0%)
**Other non-communicable diseases**	0.1% (0.1% to 0.2%)	0.2% (0.1% to 0.3%)	6.3% (5.4% to 7.2%)	5.4% (4.5% to 6.2%)
Sense organ diseases	-	-	13.0% (11.3% to 14.7%)	13.0% (11.3% to 14.7%)
Sudden infant death syndrome	4.2% (2.5% to 6.6%)	4.3% (2.6% to 6.6%)	-	4.3% (2.6% to 6.6%)
**Transport injuries**	34.2% (24.8% to 44.7%)	31.2% (21.8% to 41.6%)	38.4% (30.2% to 46.5%)	32.3% (23.2% to 42.5%)
Road injuries	34.1% (24.6% to 44.6%)	31.2% (21.7% to 41.7%)	39.0% (30.8% to 47.2%)	32.4% (23.3% to 42.6%)
Other transport injuries	36.5% (27.2% to 46.8%)	31.6% (22.2% to 42.4%)	30.4% (22.9% to 38.0%)	31.4% (22.3% to 41.6%)
**Unintentional injuries**	17.4% (13.3% to 21.5%)	12.8% (9.4% to 16.6%)	17.9% (15.9% to 20.6%)	15.1% (12.3% to 18.2%)
Falls	36.1% (25.8% to 41.5%)	18.0% (13.2% to 21.4%)	22.0% (20.0% to 24.5%)	21.0% (18.7% to 23.6%)
Drowning	18.2% (11.9% to 26.4%)	16.4% (10.7% to 23.8%)	16.2% (10.9% to 22.9%)	16.4% (10.7% to 23.8%)
Fire, heat, and hot substances	17.0% (11.2% to 24.5%)	16.3% (10.3% to 23.4%)	16.2% (10.9% to 22.6%)	16.2% (10.6% to 23.0%)

**Supplementary Results Table 1. Age-standardised PAFs attributable to the joint distribution of all risk factors for all ages, both sexes combined for each GBD level 1 and level 2 cause and all causes in 2016 for Georgia. Results are shown for deaths, YLLs, YLDs, and DALYs** Data in parenthesis are 95% uncertainty intervals. PAFs=population attributable fractions. GBD=Global Burden of Disease. YLLs=years of life lost. YLDs=years live with disability. DALYs=disability-adjusted life-years				
	**Deaths 2016**	**YLLs 2016**	**YLDs 2016**	**DALYs 2016**
Poisonings	16.4% (10.7% to 23.5%)	14.1% (8.7% to 20.6%)	14.0% (9.6% to 19.7%)	14.1% (8.9% to 20.5%)
Exposure to mechanical forces	15.0% (11.8% to 18.6%)	12.7% (9.6% to 16.1%)	16.3% (13.6% to 19.7%)	14.4% (11.5% to 17.8%)
Animal contact	12.3% (9.6% to 14.8%)	7.3% (5.8% to 9.1%)	7.2% (5.4% to 9.5%)	7.2% (5.7% to 9.1%)
Foreign body	4.9% (3.7% to 6.4%)	4.1% (3.0% to 5.5%)	5.7% (4.2% to 7.6%)	4.6% (3.4% to 6.0%)
Other unintentional injuries	19.9% (12.8% to 28.6%)	19.1% (12.3% to 27.1%)	14.7% (9.9% to 20.9%)	16.8% (11.2% to 23.9%)
**Self-harm and interpersonal violence**	31.8% (22.2% to 41.3%)	31.4% (22.2% to 40.9%)	26.6% (21.0% to 33.3%)	31.0% (22.3% to 40.0%)
Self-harm	34.4% (21.2% to 46.8%)	35.1% (22.2% to 47.3%)	25.1% (15.1% to 34.8%)	34.9% (22.1% to 47.2%)
Interpersonal violence	26.9% (18.1% to 37.4%)	25.7% (17.2% to 35.9%)	26.8% (20.6% to 34.0%)	25.9% (17.7% to 35.7%)
**Forces of nature, conflict and terrorism, and executions and police conflict**	0.0% (0.0% to 0.0%)	0.0% (0.0% to 0.0%)	0.1% (0.0% to 0.1%)	0.1% (0.0% to 0.1%)
Forces of nature, conflict and terrorism, and state actor violence	0.0% (0.0% to 0.0%)	0.0% (0.0% to 0.0%)	4.2% (2.7% to 6.2%)	4.2% (2.7% to 6.2%)

Supplementary Results Table 1. Age-standardised PAFs attributable to the joint distribution of all risk factors for all ages, both sexes combined for each GBD level 1 and level 2 cause and all causes in 2016 for Germany. Results are shown for deaths, YLLs, YLDs, and DALYs
Data in parenthesis are 95% uncertainty intervals. PAFs=population attributable fractions. GBD=Global Burden of Disease. YLLs=years of life lost. YLDs=years live with disability. DALYs=disability-adjusted life-years

	Deaths 2016	YLLs 2016	YLDs 2016	DALYs 2016
**All causes**	59.5% (57.1% to 61.8%)	56.6% (54.6% to 58.6%)	20.8% (19.5% to 22.1%)	37.2% (34.3% to 40.3%)
**HIV/AIDS and tuberculosis**	79.9% (75.2% to 83.6%)	82.2% (79.6% to 84.2%)	89.0% (84.7% to 91.4%)	85.0% (82.0% to 87.4%)
Tuberculosis	61.7% (50.2% to 71.4%)	61.4% (51.0% to 69.9%)	50.6% (36.7% to 61.9%)	59.0% (48.3% to 68.1%)
HIV/AIDS	91.4% (90.7% to 92.0%)	88.3% (87.5% to 89.1%)	93.4% (92.6% to 94.0%)	90.5% (89.4% to 91.6%)
**Diarrhoea, lower respiratory infections, and other common infectious diseases**	34.8% (26.5% to 43.1%)	40.1% (32.8% to 47.3%)	7.3% (5.1% to 9.9%)	24.5% (19.1% to 30.5%)
Diarrhoeal diseases	12.0% (6.1% to 21.3%)	18.3% (12.2% to 27.3%)	28.6% (20.3% to 37.3%)	23.9% (16.9% to 32.8%)
Lower respiratory infections	40.9% (30.7% to 51.5%)	48.6% (39.3% to 57.8%)	46.0% (37.5% to 55.2%)	48.6% (39.3% to 57.7%)
Upper respiratory infections	0.5% (0.0% to 1.7%)	1.6% (0.1% to 5.3%)	0.0% (0.0% to 0.0%)	0.0% (0.0% to 0.0%)
Otitis media	9.4% (6.1% to 13.2%)	13.5% (9.5% to 17.0%)	7.8% (5.3% to 10.3%)	7.9% (5.3% to 10.3%)
Meningitis	1.7% (1.2% to 2.3%)	3.1% (2.3% to 4.0%)	0.0% (0.0% to 0.0%)	2.7% (1.8% to 3.6%)
Encephalitis	0.4% (0.2% to 0.5%)	0.8% (0.6% to 1.1%)	0.0% (0.0% to 0.0%)	0.5% (0.3% to 0.6%)
Measles	5.9% (1.2% to 22.6%)	6.7% (1.4% to 25.6%)	11.7% (2.7% to 42.1%)	7.1% (1.5% to 26.9%)
**Maternal disorders**	2.7% (1.3% to 4.1%)	2.7% (1.3% to 4.1%)	3.2% (1.4% to 5.3%)	3.0% (1.3% to 4.7%)
Maternal haemorrhage	8.8% (3.2% to 14.3%)	8.9% (3.2% to 14.4%)	9.1% (3.3% to 14.8%)	9.0% (3.3% to 14.6%)
Maternal sepsis and other pregnancy related infections	8.8% (3.2% to 14.3%)	8.9% (3.2% to 14.4%)	9.0% (3.2% to 14.6%)	9.0% (3.2% to 14.5%)
Maternal abortion, miscarriage, and ectopic pregnancy	4.1% (2.4% to 6.2%)	4.2% (2.5% to 6.2%)	4.6% (2.3% to 7.5%)	4.3% (2.5% to 6.6%)
**Neonatal disorders**	75.1% (70.8% to 77.9%)	75.1% (70.9% to 78.0%)	0.1% (0.0% to 0.1%)	44.0% (36.4% to 51.7%)
Neonatal preterm birth complications	85.5% (81.8% to 88.5%)	85.6% (81.9% to 88.6%)	0.2% (0.1% to 0.2%)	59.2% (49.3% to 67.5%)
Neonatal encephalopathy due to birth asphyxia and trauma	62.5% (58.0% to 66.6%)	62.6% (58.1% to 66.7%)	0.0% (0.0% to 0.0%)	27.4% (19.5% to 36.4%)
Neonatal sepsis and other neonatal infections	53.0% (45.2% to 59.9%)	53.1% (45.3% to 59.9%)	0.0% (0.0% to 0.0%)	16.2% (6.7% to 36.4%)
Hemolytic disease and other neonatal jaundice	56.6% (44.8% to 64.4%)	56.8% (45.0% to 64.5%)	0.0% (0.0% to 0.0%)	19.7% (12.5% to 30.7%)

Supplementary Results Table 1. Age-standardised PAFs attributable to the joint distribution of all risk factors for all ages, both sexes combined for each GBD level 1 and level 2 cause and all causes in 2016 for Germany. Results are shown for deaths, YLLs, YLDs, and DALYs
Data in parenthesis are 95% uncertainty intervals. PAFs=population attributable fractions. GBD=Global Burden of Disease. YLLs=years of life lost. YLDs=years live with disability. DALYs=disability-adjusted life-years

	Deaths 2016	YLLs 2016	YLDs 2016	DALYs 2016
Other neonatal disorders	57.1% (51.1% to 62.8%)	57.2% (51.2% to 62.9%)	0.0% (0.0% to 0.0%)	49.6% (43.6% to 55.7%)
**Nutritional deficiencies**	4.9% (2.1% to 6.7%)	11.9% (8.4% to 14.3%)	74.3% (67.2% to 80.5%)	72.0% (65.0% to 78.5%)
Protein-energy malnutrition	0.7% (0.4% to 1.3%)	6.2% (3.4% to 9.6%)	98.4% (98.2% to 98.6%)	82.8% (75.8% to 88.5%)
Vitamin A deficiency	-	-	0.0% (0.0% to 0.0%)	0.0% (0.0% to 0.0%)
Iron-deficiency anaemia	100.0% (100.0% to 100.0%)	100.0% (100.0% to 100.0%)	100.0% (100.0% to 100.0%)	100.0% (100.0% to 100.0%)
**Other communicable, maternal, neonatal, and nutritional diseases**	4.0% (3.4% to 5.3%)	3.9% (3.3% to 4.7%)	69.8% (63.5% to 75.7%)	36.7% (27.4% to 47.6%)
Sexually transmitted diseases excluding HIV	98.4% (96.9% to 99.3%)	95.1% (90.8% to 97.7%)	100.0% (100.0% to 100.0%)	99.8% (99.6% to 99.9%)
Hepatitis	4.0% (2.9% to 5.4%)	4.3% (3.1% to 5.7%)	1.4% (0.9% to 2.3%)	4.0% (2.9% to 5.3%)
**Neoplasms**	46.7% (43.6% to 49.8%)	47.5% (44.7% to 50.4%)	35.5% (32.5% to 39.0%)	47.0% (44.2% to 49.9%)
Oesophageal cancer	88.7% (82.5% to 93.4%)	89.6% (83.6% to 94.2%)	88.9% (83.0% to 93.6%)	89.6% (83.6% to 94.2%)
Stomach cancer	20.3% (11.6% to 29.7%)	21.4% (12.7% to 30.8%)	20.7% (12.1% to 30.0%)	21.4% (12.7% to 30.8%)
Liver cancer	78.5% (73.3% to 83.3%)	80.9% (76.1% to 85.2%)	79.5% (74.4% to 84.4%)	80.9% (76.0% to 85.1%)
Larynx cancer	91.9% (86.7% to 95.4%)	91.5% (86.4% to 95.1%)	91.6% (86.6% to 95.2%)	91.5% (86.4% to 95.1%)
Tracheal, bronchus, and lung cancer	89.7% (87.6% to 91.6%)	89.7% (87.7% to 91.5%)	89.2% (87.1% to 91.2%)	89.7% (87.7% to 91.5%)
Breast cancer	39.7% (33.0% to 46.7%)	39.1% (32.7% to 45.7%)	39.1% (32.7% to 45.8%)	39.1% (32.7% to 45.7%)
Cervical cancer	100.0% (100.0% to 100.0%)	100.0% (100.0% to 100.0%)	100.0% (100.0% to 100.0%)	100.0% (100.0% to 100.0%)
Uterine cancer	43.3% (29.5% to 57.4%)	44.4% (30.7% to 58.8%)	44.5% (31.0% to 59.0%)	44.4% (30.8% to 58.6%)
Prostate cancer	5.9% (4.1% to 7.7%)	6.3% (4.5% to 8.3%)	6.5% (4.7% to 8.5%)	6.3% (4.6% to 8.3%)
Colon and rectum cancer	58.9% (49.9% to 67.2%)	60.2% (51.4% to 68.0%)	59.5% (50.8% to 67.5%)	60.1% (51.3% to 68.0%)
Lip and oral cavity cancer	87.6% (82.2% to 91.7%)	88.3% (82.9% to 92.1%)	87.1% (81.6% to 91.4%)	88.2% (82.8% to 92.0%)
Nasopharynx cancer	88.4% (84.2% to 91.8%)	86.8% (82.8% to 90.0%)	82.2% (77.3% to 86.5%)	86.6% (82.6% to 89.9%)

Supplementary Results Table 1. Age-standardised PAFs attributable to the joint distribution of all risk factors for all ages, both sexes combined for each GBD level 1 and level 2 cause and all causes in 2016 for Germany. Results are shown for deaths, YLLs, YLDs, and DALYs
Data in parenthesis are 95% uncertainty intervals. PAFs=population attributable fractions. GBD=Global Burden of Disease. YLLs=years of life lost. YLDs=years live with disability. DALYs=disability-adjusted life-years

	Deaths 2016	YLLs 2016	YLDs 2016	DALYs 2016
Other pharynx cancer	72.7% (60.9% to 81.4%)	73.0% (61.5% to 81.4%)	73.1% (61.3% to 81.6%)	73.0% (61.5% to 81.4%)
Gallbladder and biliary tract cancer	21.2% (11.4% to 33.4%)	21.4% (11.7% to 33.5%)	21.6% (11.6% to 33.8%)	21.4% (11.7% to 33.5%)
Pancreatic cancer	34.0% (26.4% to 42.6%)	35.6% (28.0% to 43.7%)	34.0% (26.5% to 42.5%)	35.6% (28.0% to 43.7%)
Ovarian cancer	15.8% (8.4% to 24.3%)	12.9% (6.3% to 20.4%)	13.2% (6.9% to 20.9%)	12.9% (6.3% to 20.4%)
Kidney cancer	38.1% (27.9% to 48.9%)	39.2% (28.8% to 50.0%)	38.5% (28.5% to 49.2%)	39.2% (28.8% to 50.0%)
Bladder cancer	37.4% (27.5% to 47.3%)	39.4% (29.5% to 49.3%)	39.1% (29.3% to 48.9%)	39.4% (29.5% to 49.3%)
Thyroid cancer	15.4% (7.5% to 25.9%)	16.0% (7.6% to 26.7%)	14.7% (7.8% to 24.0%)	15.8% (7.7% to 26.4%)
Mesothelioma	96.9% (96.0% to 97.6%)	93.7% (92.1% to 95.2%)	95.9% (94.8% to 96.9%)	93.7% (92.1% to 95.2%)
Non-Hodgkin's lymphoma	7.5% (3.0% to 13.0%)	7.4% (3.1% to 12.8%)	7.4% (3.0% to 12.9%)	7.4% (3.1% to 12.8%)
Multiple myeloma	8.4% (3.6% to 15.2%)	8.7% (3.8% to 15.7%)	8.7% (3.7% to 15.5%)	8.7% (3.8% to 15.6%)
Leukaemia	21.1% (13.5% to 29.0%)	19.3% (12.6% to 26.2%)	19.5% (12.3% to 27.1%)	19.3% (12.6% to 26.2%)
**Cardiovascular diseases**	82.2% (79.2% to 84.6%)	84.3% (82.3% to 86.0%)	75.2% (72.2% to 78.0%)	82.7% (80.6% to 84.5%)
Rheumatic heart disease	26.7% (15.2% to 50.4%)	27.0% (17.4% to 44.8%)	26.0% (17.5% to 40.4%)	27.0% (17.5% to 44.8%)
Ischaemic heart disease	92.5% (88.4% to 95.7%)	94.3% (91.6% to 96.5%)	91.6% (88.9% to 94.0%)	94.1% (91.5% to 96.3%)
Cerebrovascular disease	82.9% (76.5% to 88.6%)	87.2% (83.2% to 90.8%)	85.4% (81.0% to 89.3%)	86.7% (82.6% to 90.3%)
Hypertensive heart disease	100.0% (100.0% to 100.0%)	100.0% (100.0% to 100.0%)	100.0% (100.0% to 100.0%)	100.0% (100.0% to 100.0%)
Cardiomyopathy and myocarditis	54.2% (43.7% to 62.9%)	62.6% (52.3% to 68.8%)	59.5% (54.8% to 64.4%)	62.3% (53.0% to 68.3%)
Atrial fibrillation and flutter	56.8% (48.9% to 64.8%)	61.1% (54.1% to 67.9%)	67.3% (61.7% to 72.9%)	64.5% (58.3% to 70.7%)
Aortic aneurysm	42.7% (35.3% to 50.3%)	49.2% (43.1% to 55.3%)	-	49.2% (43.1% to 55.3%)
Peripheral vascular disease	50.5% (43.8% to 57.6%)	56.8% (50.9% to 62.3%)	57.9% (52.6% to 63.2%)	57.0% (51.5% to 62.3%)
Endocarditis	37.9% (28.5% to 49.7%)	38.3% (30.5% to 47.1%)	38.3% (29.4% to 48.0%)	38.3% (30.5% to 47.1%)

Supplementary Results Table 1. Age-standardised PAFs attributable to the joint distribution of all risk factors for all ages, both sexes combined for each GBD level 1 and level 2 cause and all causes in 2016 for Germany. Results are shown for deaths, YLLs, YLDs, and DALYs
Data in parenthesis are 95% uncertainty intervals. PAFs=population attributable fractions. GBD=Global Burden of Disease. YLLs=years of life lost. YLDs=years live with disability. DALYs=disability-adjusted life-years

	Deaths 2016	YLLs 2016	YLDs 2016	DALYs 2016
Other cardiovascular and circulatory diseases	43.9% (38.2% to 50.3%)	50.1% (45.3% to 54.9%)	51.5% (46.3% to 56.4%)	50.6% (45.8% to 55.5%)
**Chronic respiratory diseases**	70.8% (66.4% to 74.9%)	70.6% (66.7% to 74.3%)	47.7% (41.3% to 54.4%)	59.1% (54.2% to 64.2%)
Chronic obstructive pulmonary disease	77.8% (73.4% to 82.1%)	79.8% (75.9% to 83.5%)	74.7% (70.3% to 79.0%)	78.4% (74.5% to 82.2%)
Pneumoconiosis	100.0% (100.0% to 100.0%)	100.0% (100.0% to 100.0%)	100.0% (100.0% to 100.0%)	100.0% (100.0% to 100.0%)
Asthma	42.2% (31.9% to 53.6%)	46.5% (37.4% to 56.7%)	36.4% (28.4% to 45.5%)	37.1% (29.1% to 46.2%)
Other chronic respiratory diseases	17.3% (13.1% to 21.6%)	13.8% (9.1% to 18.5%)	9.0% (6.2% to 12.6%)	11.0% (7.7% to 14.7%)
**Cirrhosis and other chronic liver diseases**	73.6% (69.3% to 77.6%)	75.9% (71.9% to 79.8%)	62.6% (58.1% to 67.0%)	75.2% (71.3% to 79.1%)
Cirrhosis and other chronic liver diseases due to hepatitis B	0.9% (0.4% to 1.6%)	1.1% (0.5% to 2.1%)	1.3% (0.5% to 2.3%)	1.1% (0.5% to 2.1%)
Cirrhosis and other chronic liver diseases due to hepatitis C	78.1% (70.8% to 83.8%)	85.6% (81.5% to 88.2%)	83.4% (79.2% to 86.5%)	85.5% (81.5% to 88.1%)
Cirrhosis and other chronic liver diseases due to alcohol use	100.0% (100.0% to 100.0%)	100.0% (100.0% to 100.0%)	100.0% (100.0% to 100.0%)	100.0% (100.0% to 100.0%)
**Digestive diseases**	9.9% (7.4% to 12.5%)	12.4% (9.1% to 15.5%)	3.4% (2.5% to 4.4%)	10.0% (7.4% to 12.5%)
Peptic ulcer disease	10.2% (7.0% to 13.7%)	15.0% (10.6% to 19.6%)	14.4% (10.1% to 19.1%)	14.9% (10.5% to 19.6%)
Gallbladder and biliary diseases	41.5% (26.9% to 56.4%)	42.4% (28.5% to 57.3%)	46.6% (33.1% to 61.0%)	43.1% (29.2% to 57.9%)
Pancreatitis	45.3% (27.2% to 62.9%)	46.0% (28.4% to 62.7%)	40.9% (23.6% to 57.0%)	45.8% (28.3% to 62.5%)
**Neurological disorders**	20.9% (10.8% to 33.2%)	19.3% (11.6% to 28.4%)	5.2% (2.6% to 8.7%)	9.7% (5.8% to 14.5%)
Alzheimer's disease and other dementias	24.5% (12.1% to 39.7%)	25.9% (13.3% to 40.8%)	25.8% (13.3% to 40.9%)	25.9% (13.4% to 40.9%)
Parkinson's disease	-6.5% (-7.7% to -5.2%)	-8.7% (-10.3% to -7.0%)	-10.6% (-12.7% to -8.6%)	-9.1% (-10.8% to -7.3%)
Epilepsy	37.8% (25.2% to 48.5%)	32.5% (21.3% to 41.6%)	27.4% (17.4% to 36.0%)	29.3% (18.6% to 38.0%)
Multiple sclerosis	10.5% (6.8% to 14.3%)	11.6% (7.6% to 15.8%)	10.6% (6.8% to 14.5%)	11.1% (7.3% to 15.1%)
**Mental and substance use disorders**	99.5% (99.3% to 99.6%)	99.2% (98.9% to 99.4%)	17.4% (15.6% to 19.4%)	24.8% (22.0% to 28.0%)
Alcohol use disorders	100.0% (100.0% to 100.0%)	100.0% (100.0% to 100.0%)	100.0% (100.0% to 100.0%)	100.0% (100.0% to 100.0%)

Supplementary Results Table 1. Age-standardised PAFs attributable to the joint distribution of all risk factors for all ages, both sexes combined for each GBD level 1 and level 2 cause and all causes in 2016 for Germany. Results are shown for deaths, YLLs, YLDs, and DALYs
Data in parenthesis are 95% uncertainty intervals. PAFs=population attributable fractions. GBD=Global Burden of Disease. YLLs=years of life lost. YLDs=years live with disability. DALYs=disability-adjusted life-years

	Deaths 2016	YLLs 2016	YLDs 2016	DALYs 2016
Drug use disorders	100.0% (100.0% to 100.0%)	100.0% (100.0% to 100.0%)	100.0% (100.0% to 100.0%)	100.0% (100.0% to 100.0%)
Depressive disorders	-	-	8.9% (7.4% to 10.6%)	8.9% (7.4% to 10.6%)
Idiopathic developmental intellectual disability	-	-	57.2% (28.9% to 76.9%)	57.2% (28.9% to 76.9%)
**Diabetes, urogenital, blood, and endocrine diseases**	77.3% (75.9% to 81.1%)	68.8% (66.9% to 73.5%)	58.0% (54.0% to 62.1%)	62.6% (59.5% to 65.5%)
Diabetes mellitus	100.0% (100.0% to 100.0%)	100.0% (100.0% to 100.0%)	100.0% (100.0% to 100.0%)	100.0% (100.0% to 100.0%)
Chronic kidney disease	100.0% (100.0% to 100.0%)	100.0% (100.0% to 100.0%)	100.0% (100.0% to 100.0%)	100.0% (100.0% to 100.0%)
**Musculoskeletal disorders**	1.1% (0.4% to 1.9%)	1.4% (0.5% to 2.5%)	18.3% (15.5% to 21.6%)	18.2% (15.3% to 21.4%)
Rheumatoid arthritis	4.0% (1.6% to 6.8%)	5.3% (2.1% to 8.9%)	6.4% (2.6% to 10.6%)	6.3% (2.5% to 10.5%)
Osteoarthritis	-	-	25.3% (14.0% to 37.4%)	25.3% (14.0% to 37.4%)
Low back and neck pain	-	-	20.5% (17.6% to 23.7%)	20.5% (17.6% to 23.7%)
Gout	-	-	48.5% (30.7% to 66.4%)	48.5% (30.7% to 66.4%)
**Other non-communicable diseases**	0.4% (0.3% to 0.6%)	0.5% (0.4% to 0.8%)	3.2% (2.8% to 3.7%)	2.9% (2.6% to 3.3%)
Sense organ diseases	-	-	9.3% (8.4% to 10.3%)	9.3% (8.4% to 10.3%)
Sudden infant death syndrome	3.7% (2.4% to 5.4%)	3.7% (2.4% to 5.4%)	-	3.7% (2.4% to 5.4%)
**Transport injuries**	39.7% (29.4% to 49.7%)	35.9% (25.0% to 46.0%)	43.3% (34.1% to 52.8%)	38.8% (28.7% to 48.6%)
Road injuries	39.6% (29.2% to 49.5%)	35.7% (24.9% to 45.7%)	43.3% (34.1% to 52.8%)	38.7% (28.7% to 48.4%)
Other transport injuries	41.7% (31.6% to 51.8%)	37.7% (26.9% to 48.0%)	43.2% (34.0% to 53.1%)	39.7% (29.8% to 49.7%)
**Unintentional injuries**	43.9% (40.6% to 47.5%)	27.3% (23.7% to 31.8%)	23.4% (19.2% to 28.9%)	24.5% (20.6% to 29.7%)
Falls	64.4% (60.6% to 67.8%)	44.8% (40.2% to 49.7%)	24.7% (21.0% to 30.0%)	29.2% (25.2% to 34.3%)
Drowning	20.5% (13.5% to 28.8%)	18.4% (12.6% to 25.0%)	22.7% (15.2% to 32.3%)	18.6% (12.8% to 25.4%)
Fire, heat, and hot substances	18.7% (11.3% to 27.8%)	20.1% (13.7% to 27.7%)	24.4% (16.3% to 33.8%)	22.9% (15.5% to 31.5%)

Supplementary Results Table 1. Age-standardised PAFs attributable to the joint distribution of all risk factors for all ages, both sexes combined for each GBD level 1 and level 2 cause and all causes in 2016 for Germany. Results are shown for deaths, YLLs, YLDs, and DALYs
Data in parenthesis are 95% uncertainty intervals. PAFs=population attributable fractions. GBD=Global Burden of Disease. YLLs=years of life lost. YLDs=years live with disability. DALYs=disability-adjusted life-years

	**Deaths 2016**	**YLLs 2016**	**YLDs 2016**	**DALYs 2016**
Poisonings	24.8% (16.8% to 34.3%)	26.7% (18.6% to 35.7%)	21.8% (15.0% to 30.1%)	23.3% (16.2% to 31.5%)
Exposure to mechanical forces	23.7% (18.9% to 30.1%)	19.7% (14.1% to 27.0%)	23.5% (18.2% to 30.8%)	22.6% (17.5% to 29.8%)
Animal contact	16.7% (13.3% to 21.1%)	14.2% (10.1% to 19.7%)	13.3% (8.9% to 19.4%)	13.5% (9.3% to 19.5%)
Foreign body	6.9% (4.2% to 11.3%)	10.0% (6.4% to 15.1%)	9.3% (6.0% to 13.9%)	9.8% (6.3% to 14.5%)
Other unintentional injuries	25.0% (16.8% to 34.4%)	25.1% (17.6% to 33.9%)	23.4% (15.4% to 33.0%)	23.6% (15.6% to 33.0%)
**Self-harm and interpersonal violence**	33.5% (17.7% to 47.1%)	31.6% (15.8% to 45.0%)	31.4% (25.3% to 37.6%)	31.6% (16.5% to 44.5%)
Self-harm	33.5% (16.7% to 48.0%)	31.9% (14.8% to 46.5%)	29.3% (11.9% to 43.6%)	31.9% (14.7% to 46.5%)
Interpersonal violence	32.9% (24.3% to 41.0%)	28.3% (20.2% to 35.9%)	32.0% (26.6% to 37.4%)	29.9% (23.1% to 36.3%)
**Forces of nature, conflict and terrorism, and executions and police conflict**	0.0% (0.0% to 0.0%)	0.0% (0.0% to 0.0%)	0.0% (0.0% to 0.0%)	0.0% (0.0% to 0.0%)
Forces of nature, conflict and terrorism, and state actor violence	0.0% (0.0% to 0.0%)	0.0% (0.0% to 0.0%)	0.0% (0.0% to 0.0%)	0.0% (0.0% to 0.0%)

Supplementary Results Table 1. Age-standardised PAFs attributable to the joint distribution of all risk factors for all ages, both sexes combined for each GBD level 1 and level 2 cause and all causes in 2016 for Ghana. Results are shown for deaths, YLLs, YLDs, and DALYs
Data in parenthesis are 95% uncertainty intervals. PAFs=population attributable fractions. GBD=Global Burden of Disease. YLLs=years of life lost. YLDs=years live with disability. DALYs=disability-adjusted life-years

	Deaths 2016	YLLs 2016	YLDs 2016	DALYs 2016
**All causes**	57.6% (55.4% to 59.8%)	52.5% (50.5% to 54.6%)	30.4% (29.2% to 31.8%)	47.0% (45.1% to 49.1%)
**HIV/AIDS and tuberculosis**	75.3% (68.5% to 82.1%)	79.0% (74.2% to 83.5%)	83.3% (78.6% to 87.5%)	79.2% (74.5% to 83.5%)
Tuberculosis	55.4% (41.8% to 69.3%)	56.7% (44.2% to 70.5%)	52.5% (40.6% to 65.9%)	56.5% (44.2% to 70.3%)
HIV/AIDS	91.8% (90.6% to 92.8%)	88.3% (86.4% to 90.0%)	91.6% (89.7% to 93.5%)	88.5% (86.8% to 90.1%)
**Diarrhoea, lower respiratory infections, and other common infectious diseases**	68.5% (63.0% to 74.5%)	63.8% (58.5% to 69.8%)	40.1% (35.1% to 44.8%)	62.3% (57.2% to 68.1%)
Diarrhoeal diseases	94.8% (86.6% to 98.6%)	96.0% (89.4% to 98.9%)	95.3% (87.9% to 98.8%)	95.9% (89.2% to 98.9%)
Lower respiratory infections	77.9% (71.3% to 83.6%)	81.0% (75.2% to 85.9%)	78.2% (71.9% to 83.7%)	81.0% (75.2% to 85.9%)
Upper respiratory infections	1.7% (0.1% to 6.0%)	3.7% (0.2% to 13.6%)	0.0% (0.0% to 0.0%)	0.1% (0.0% to 0.5%)
Otitis media	0.2% (0.1% to 0.6%)	1.0% (0.4% to 2.1%)	1.4% (0.9% to 2.0%)	1.4% (0.9% to 2.0%)
Meningitis	1.4% (0.8% to 2.5%)	3.2% (1.8% to 5.6%)	0.0% (0.0% to 0.0%)	3.1% (1.7% to 5.5%)
Encephalitis	0.9% (0.5% to 1.5%)	1.6% (1.0% to 2.6%)	0.0% (0.0% to 0.0%)	1.1% (0.7% to 1.7%)
Measles	49.1% (27.4% to 78.8%)	50.0% (27.9% to 80.1%)	49.8% (28.2% to 78.6%)	50.0% (27.9% to 80.1%)
**Maternal disorders**	8.4% (4.3% to 12.7%)	8.3% (4.2% to 12.5%)	1.9% (0.8% to 3.3%)	7.7% (3.9% to 11.7%)
Maternal haemorrhage	18.2% (6.5% to 29.5%)	18.2% (6.6% to 29.5%)	18.3% (6.6% to 29.7%)	18.2% (6.6% to 29.5%)
Maternal sepsis and other pregnancy related infections	18.1% (6.5% to 29.5%)	18.2% (6.6% to 29.6%)	18.0% (6.5% to 29.3%)	18.2% (6.6% to 29.5%)
Maternal abortion, miscarriage, and ectopic pregnancy	13.4% (8.1% to 19.6%)	13.2% (7.7% to 19.4%)	13.8% (7.6% to 20.6%)	13.2% (7.8% to 19.4%)
**Neonatal disorders**	80.3% (76.9% to 83.5%)	80.3% (77.0% to 83.5%)	0.4% (0.2% to 0.6%)	76.0% (71.5% to 80.0%)
Neonatal preterm birth complications	98.4% (96.4% to 99.4%)	98.4% (96.4% to 99.4%)	1.0% (0.7% to 1.4%)	89.9% (83.5% to 94.4%)
Neonatal encephalopathy due to birth asphyxia and trauma	80.1% (76.9% to 83.4%)	80.1% (76.9% to 83.4%)	0.0% (0.0% to 0.0%)	78.4% (73.4% to 82.4%)
Neonatal sepsis and other neonatal infections	69.5% (63.9% to 74.3%)	69.6% (64.0% to 74.3%)	0.0% (0.0% to 0.0%)	62.8% (51.4% to 70.4%)
Hemolytic disease and other neonatal jaundice	69.7% (59.1% to 76.6%)	69.7% (59.2% to 76.6%)	0.0% (0.0% to 0.0%)	63.3% (53.2% to 70.8%)

Supplementary Results Table 1. Age-standardised PAFs attributable to the joint distribution of all risk factors for all ages, both sexes combined for each GBD level 1 and level 2 cause and all causes in 2016 for Ghana. Results are shown for deaths, YLLs, YLDs, and DALYs
Data in parenthesis are 95% uncertainty intervals. PAFs=population attributable fractions. GBD=Global Burden of Disease. YLLs=years of life lost. YLDs=years live with disability. DALYs=disability-adjusted life-years

	Deaths 2016	YLLs 2016	YLDs 2016	DALYs 2016
Other neonatal disorders	71.0% (62.9% to 77.1%)	71.1% (63.0% to 77.1%)	0.0% (0.0% to 0.0%)	68.9% (60.3% to 75.3%)
**Nutritional deficiencies**	44.2% (32.9% to 56.2%)	76.4% (66.2% to 84.6%)	95.6% (94.3% to 96.7%)	88.6% (85.4% to 91.4%)
Protein-energy malnutrition	39.0% (26.4% to 52.8%)	76.7% (65.6% to 85.4%)	98.8% (98.2% to 99.2%)	78.1% (68.4% to 86.1%)
Vitamin A deficiency	-	-	100.0% (100.0% to 100.0%)	100.0% (100.0% to 100.0%)
Iron-deficiency anaemia	100.0% (100.0% to 100.0%)	100.0% (100.0% to 100.0%)	100.0% (100.0% to 100.0%)	100.0% (100.0% to 100.0%)
**Other communicable, maternal, neonatal, and nutritional diseases**	8.7% (6.6% to 11.4%)	8.6% (5.9% to 11.7%)	63.5% (58.4% to 68.7%)	25.2% (18.6% to 33.1%)
Sexually transmitted diseases excluding HIV	47.7% (29.5% to 67.9%)	32.2% (17.4% to 51.8%)	100.0% (100.0% to 100.0%)	65.5% (46.5% to 81.9%)
Hepatitis	0.0% (0.0% to 0.1%)	0.1% (0.0% to 0.1%)	0.0% (0.0% to 0.1%)	0.1% (0.0% to 0.1%)
**Neoplasms**	29.0% (25.1% to 33.2%)	29.7% (25.5% to 33.9%)	31.7% (26.3% to 36.8%)	29.8% (25.5% to 33.9%)
Oesophageal cancer	59.2% (47.8% to 70.1%)	61.5% (50.1% to 72.4%)	59.9% (48.7% to 70.7%)	61.5% (50.1% to 72.4%)
Stomach cancer	3.4% (0.6% to 10.9%)	3.4% (0.7% to 10.3%)	3.4% (0.7% to 10.6%)	3.4% (0.7% to 10.3%)
Liver cancer	28.9% (22.1% to 37.5%)	27.6% (20.6% to 36.1%)	28.7% (21.9% to 36.9%)	27.6% (20.6% to 36.1%)
Larynx cancer	60.2% (46.2% to 72.1%)	62.9% (49.1% to 74.5%)	61.1% (47.3% to 72.7%)	62.9% (49.0% to 74.5%)
Tracheal, bronchus, and lung cancer	73.9% (66.7% to 80.3%)	74.4% (67.1% to 80.6%)	73.8% (66.5% to 80.3%)	74.4% (67.1% to 80.6%)
Breast cancer	22.6% (13.1% to 36.3%)	20.7% (10.6% to 36.3%)	21.6% (11.6% to 36.4%)	20.7% (10.6% to 36.3%)
Cervical cancer	100.0% (100.0% to 100.0%)	100.0% (100.0% to 100.0%)	100.0% (100.0% to 100.0%)	100.0% (100.0% to 100.0%)
Uterine cancer	38.2% (25.8% to 51.0%)	40.9% (28.0% to 53.4%)	39.9% (27.2% to 52.5%)	40.8% (28.0% to 53.4%)
Prostate cancer	0.8% (0.4% to 1.3%)	1.0% (0.5% to 1.5%)	0.9% (0.5% to 1.4%)	1.0% (0.5% to 1.5%)
Colon and rectum cancer	48.0% (38.3% to 57.7%)	48.5% (39.3% to 57.8%)	48.3% (39.0% to 57.8%)	48.5% (39.3% to 57.8%)
Lip and oral cavity cancer	59.2% (42.6% to 72.6%)	61.7% (46.4% to 73.5%)	58.8% (42.6% to 72.1%)	61.6% (46.3% to 73.5%)
Nasopharynx cancer	68.5% (56.6% to 75.8%)	68.5% (58.3% to 75.5%)	67.5% (56.7% to 74.1%)	68.5% (58.3% to 75.5%)

Supplementary Results Table 1. Age-standardised PAFs attributable to the joint distribution of all risk factors for all ages, both sexes combined for each GBD level 1 and level 2 cause and all causes in 2016 for Ghana. Results are shown for deaths, YLLs, YLDs, and DALYs
Data in parenthesis are 95% uncertainty intervals. PAFs=population attributable fractions. GBD=Global Burden of Disease. YLLs=years of life lost. YLDs=years live with disability. DALYs=disability-adjusted life-years

	Deaths 2016	YLLs 2016	YLDs 2016	DALYs 2016
Other pharynx cancer	52.8% (35.1% to 66.4%)	55.6% (39.5% to 68.2%)	53.9% (36.7% to 67.0%)	55.5% (39.5% to 68.2%)
Gallbladder and biliary tract cancer	18.2% (9.7% to 29.1%)	19.8% (10.8% to 31.2%)	18.8% (10.0% to 29.7%)	19.8% (10.8% to 31.2%)
Pancreatic cancer	13.3% (6.9% to 22.1%)	13.4% (7.1% to 21.8%)	13.4% (7.0% to 22.0%)	13.4% (7.1% to 21.8%)
Ovarian cancer	10.0% (4.0% to 17.5%)	9.0% (3.4% to 15.9%)	9.1% (3.6% to 16.1%)	9.0% (3.4% to 15.9%)
Kidney cancer	16.4% (10.4% to 24.3%)	14.6% (9.1% to 21.4%)	14.7% (9.5% to 21.5%)	14.6% (9.1% to 21.3%)
Bladder cancer	10.9% (5.2% to 19.0%)	11.1% (5.4% to 18.7%)	11.1% (5.4% to 18.5%)	11.1% (5.4% to 18.7%)
Thyroid cancer	9.3% (3.3% to 18.1%)	9.3% (3.5% to 17.7%)	9.4% (3.6% to 17.6%)	9.3% (3.5% to 17.7%)
Mesothelioma	84.8% (80.7% to 88.6%)	78.0% (72.5% to 83.0%)	82.4% (77.9% to 86.6%)	78.1% (72.6% to 83.1%)
Non-Hodgkin's lymphoma	3.9% (1.5% to 7.3%)	3.4% (1.4% to 6.5%)	3.6% (1.4% to 6.8%)	3.4% (1.4% to 6.5%)
Multiple myeloma	5.7% (2.3% to 10.4%)	6.0% (2.4% to 11.0%)	5.9% (2.4% to 10.7%)	6.0% (2.4% to 10.9%)
Leukaemia	8.3% (5.1% to 12.2%)	7.6% (4.6% to 11.3%)	8.0% (4.9% to 11.9%)	7.6% (4.6% to 11.3%)
**Cardiovascular diseases**	85.0% (82.4% to 87.2%)	86.1% (84.2% to 87.7%)	61.5% (57.4% to 65.2%)	83.9% (81.9% to 85.8%)
Rheumatic heart disease	28.6% (18.9% to 46.9%)	28.2% (19.4% to 43.1%)	23.2% (15.0% to 33.2%)	26.3% (18.3% to 39.6%)
Ischaemic heart disease	93.0% (90.1% to 95.4%)	94.7% (92.8% to 96.3%)	90.3% (87.7% to 92.9%)	94.6% (92.6% to 96.2%)
Cerebrovascular disease	86.0% (82.1% to 89.6%)	89.5% (86.7% to 91.7%)	86.4% (82.5% to 89.6%)	89.3% (86.5% to 91.6%)
Hypertensive heart disease	100.0% (100.0% to 100.0%)	100.0% (100.0% to 100.0%)	100.0% (100.0% to 100.0%)	100.0% (100.0% to 100.0%)
Cardiomyopathy and myocarditis	39.6% (32.4% to 47.2%)	40.1% (33.7% to 46.7%)	47.4% (40.8% to 53.9%)	40.4% (34.0% to 46.9%)
Atrial fibrillation and flutter	50.9% (41.5% to 60.9%)	55.1% (46.6% to 63.7%)	62.7% (55.5% to 70.0%)	59.0% (51.1% to 67.0%)
Aortic aneurysm	41.2% (34.0% to 48.3%)	44.0% (37.5% to 49.9%)	-	44.0% (37.5% to 49.9%)
Peripheral vascular disease	50.3% (42.8% to 58.3%)	57.0% (51.0% to 62.8%)	61.4% (55.8% to 66.9%)	60.6% (55.3% to 65.9%)
Endocarditis	37.5% (29.0% to 46.6%)	33.2% (25.8% to 40.6%)	36.2% (27.2% to 46.9%)	33.3% (26.0% to 40.9%)

Supplementary Results Table 1. Age-standardised PAFs attributable to the joint distribution of all risk factors for all ages, both sexes combined for each GBD level 1 and level 2 cause and all causes in 2016 for Ghana. Results are shown for deaths, YLLs, YLDs, and DALYs
Data in parenthesis are 95% uncertainty intervals. PAFs=population attributable fractions. GBD=Global Burden of Disease. YLLs=years of life lost. YLDs=years live with disability. DALYs=disability-adjusted life-years

	Deaths 2016	YLLs 2016	YLDs 2016	DALYs 2016
Other cardiovascular and circulatory diseases	45.2% (40.0% to 50.3%)	45.5% (41.0% to 50.3%)	41.3% (36.4% to 46.3%)	43.9% (39.3% to 48.8%)
**Chronic respiratory diseases**	54.6% (48.0% to 61.4%)	52.6% (46.7% to 58.9%)	42.6% (36.3% to 49.2%)	49.7% (43.9% to 55.9%)
Chronic obstructive pulmonary disease	72.0% (63.3% to 80.8%)	71.9% (63.4% to 80.5%)	62.9% (52.6% to 72.5%)	69.7% (60.8% to 78.2%)
Pneumoconiosis	100.0% (100.0% to 100.0%)	100.0% (100.0% to 100.0%)	100.0% (100.0% to 100.0%)	100.0% (100.0% to 100.0%)
Asthma	30.4% (21.5% to 40.8%)	34.9% (26.8% to 44.5%)	26.2% (20.3% to 33.0%)	31.1% (24.4% to 39.4%)
Other chronic respiratory diseases	3.3% (1.7% to 5.4%)	3.3% (1.5% to 6.0%)	1.6% (0.4% to 4.0%)	3.2% (1.4% to 5.8%)
**Cirrhosis and other chronic liver diseases**	23.5% (21.0% to 26.1%)	22.6% (20.2% to 25.1%)	17.6% (15.0% to 20.5%)	22.5% (20.0% to 25.0%)
Cirrhosis and other chronic liver diseases due to hepatitis B	0.0% (0.0% to 0.0%)	0.0% (0.0% to 0.0%)	0.0% (0.0% to 0.0%)	0.0% (0.0% to 0.0%)
Cirrhosis and other chronic liver diseases due to hepatitis C	1.3% (0.5% to 2.7%)	1.9% (0.7% to 4.0%)	1.7% (0.7% to 3.6%)	1.9% (0.7% to 4.0%)
Cirrhosis and other chronic liver diseases due to alcohol use	100.0% (100.0% to 100.0%)	100.0% (100.0% to 100.0%)	100.0% (100.0% to 100.0%)	100.0% (100.0% to 100.0%)
**Digestive diseases**	6.8% (4.4% to 9.0%)	8.2% (5.6% to 10.6%)	2.1% (1.6% to 2.7%)	7.7% (5.3% to 10.0%)
Peptic ulcer disease	4.9% (3.0% to 7.2%)	4.5% (2.9% to 6.7%)	3.6% (2.3% to 5.2%)	4.5% (2.8% to 6.6%)
Gallbladder and biliary diseases	29.2% (17.3% to 41.5%)	28.6% (17.6% to 40.5%)	34.7% (23.6% to 47.4%)	29.0% (18.0% to 41.0%)
Pancreatitis	59.8% (32.7% to 81.1%)	66.2% (39.7% to 84.9%)	60.7% (34.5% to 80.6%)	66.2% (39.6% to 84.9%)
**Neurological disorders**	16.5% (8.3% to 26.7%)	17.4% (10.6% to 25.6%)	5.5% (2.5% to 9.0%)	9.5% (5.9% to 13.8%)
Alzheimer's disease and other dementias	17.4% (7.9% to 29.2%)	18.2% (8.5% to 30.3%)	18.2% (8.5% to 30.2%)	18.2% (8.5% to 30.3%)
Parkinson's disease	-3.9% (-5.5% to -2.6%)	-3.9% (-5.3% to -2.8%)	-3.8% (-5.1% to -2.8%)	-3.9% (-5.3% to -2.8%)
Epilepsy	31.9% (21.5% to 44.3%)	29.9% (19.9% to 41.8%)	22.8% (15.2% to 32.2%)	25.2% (16.7% to 35.2%)
Multiple sclerosis	2.1% (1.1% to 3.5%)	1.9% (0.9% to 3.2%)	1.4% (0.8% to 2.2%)	1.7% (0.9% to 2.8%)
**Mental and substance use disorders**	99.8% (99.7% to 99.9%)	99.7% (99.5% to 99.8%)	19.2% (17.0% to 21.5%)	23.3% (20.8% to 26.3%)
Alcohol use disorders	100.0% (100.0% to 100.0%)	100.0% (100.0% to 100.0%)	100.0% (100.0% to 100.0%)	100.0% (100.0% to 100.0%)

Supplementary Results Table 1. Age-standardised PAFs attributable to the joint distribution of all risk factors for all ages, both sexes combined for each GBD level 1 and level 2 cause and all causes in 2016 for Ghana. Results are shown for deaths, YLLs, YLDs, and DALYs
Data in parenthesis are 95% uncertainty intervals. PAFs=population attributable fractions. GBD=Global Burden of Disease. YLLs=years of life lost. YLDs=years live with disability. DALYs=disability-adjusted life-years

	Deaths 2016	YLLs 2016	YLDs 2016	DALYs 2016
Drug use disorders	100.0% (100.0% to 100.0%)	100.0% (100.0% to 100.0%)	100.0% (100.0% to 100.0%)	100.0% (100.0% to 100.0%)
Depressive disorders	-	-	9.7% (8.3% to 11.2%)	9.7% (8.3% to 11.2%)
Idiopathic developmental intellectual disability	-	-	60.4% (30.2% to 79.8%)	60.4% (30.2% to 79.8%)
**Diabetes, urogenital, blood, and endocrine diseases**	74.0% (70.9% to 76.2%)	64.5% (60.8% to 67.4%)	59.9% (56.1% to 63.2%)	63.0% (60.1% to 65.3%)
Diabetes mellitus	100.0% (100.0% to 100.0%)	100.0% (100.0% to 100.0%)	100.0% (100.0% to 100.0%)	100.0% (100.0% to 100.0%)
Chronic kidney disease	100.0% (100.0% to 100.0%)	100.0% (100.0% to 100.0%)	100.0% (100.0% to 100.0%)	100.0% (100.0% to 100.0%)
**Musculoskeletal disorders**	0.3% (0.1% to 0.5%)	0.1% (0.0% to 0.2%)	24.5% (21.2% to 28.5%)	24.1% (20.8% to 27.9%)
Rheumatoid arthritis	1.6% (0.5% to 3.2%)	1.2% (0.4% to 2.5%)	1.2% (0.4% to 2.1%)	1.2% (0.4% to 2.1%)
Osteoarthritis	-	-	20.1% (10.2% to 31.3%)	20.1% (10.2% to 31.3%)
Low back and neck pain	-	-	30.4% (26.8% to 34.3%)	30.4% (26.8% to 34.3%)
Gout	-	-	49.0% (40.0% to 59.8%)	49.0% (40.0% to 59.8%)
**Other non-communicable diseases**	0.1% (0.0% to 0.2%)	0.1% (0.0% to 0.2%)	8.8% (7.5% to 10.1%)	6.0% (5.0% to 7.1%)
Sense organ diseases	-	-	16.6% (14.8% to 18.6%)	16.6% (14.8% to 18.6%)
Sudden infant death syndrome	4.4% (1.8% to 9.0%)	4.5% (1.8% to 9.0%)	-	4.5% (1.8% to 9.0%)
**Transport injuries**	37.4% (28.3% to 48.8%)	31.4% (22.7% to 42.4%)	39.6% (30.8% to 50.8%)	32.7% (24.0% to 43.7%)
Road injuries	37.3% (28.2% to 48.7%)	31.4% (22.7% to 42.3%)	41.7% (32.6% to 53.1%)	32.7% (24.1% to 43.9%)
Other transport injuries	39.0% (29.7% to 50.6%)	31.9% (23.1% to 43.1%)	31.0% (23.1% to 41.5%)	31.5% (23.3% to 42.2%)
**Unintentional injuries**	27.0% (17.8% to 37.7%)	15.8% (11.9% to 20.1%)	18.3% (16.6% to 20.3%)	16.5% (13.2% to 20.0%)
Falls	50.8% (30.6% to 71.2%)	37.2% (27.2% to 48.3%)	27.0% (25.8% to 28.4%)	33.0% (26.9% to 40.3%)
Drowning	13.3% (7.3% to 21.2%)	12.8% (7.8% to 19.5%)	14.8% (8.6% to 22.8%)	12.8% (7.8% to 19.5%)
Fire, heat, and hot substances	11.8% (5.9% to 19.7%)	12.4% (7.1% to 19.6%)	15.9% (10.0% to 23.6%)	13.2% (7.7% to 20.3%)

**Supplementary Results Table 1. Age-standardised PAFs attributable to the joint distribution of all risk factors for all ages, both sexes combined for each GBD level 1 and level 2 cause and all causes in 2016 for Ghana. Results are shown for deaths, YLLs, YLDs, and DALYs** Data in parenthesis are 95% uncertainty intervals. PAFs=population attributable fractions. GBD=Global Burden of Disease. YLLs=years of life lost. YLDs=years live with disability. DALYs=disability-adjusted life-years				
	**Deaths 2016**	**YLLs 2016**	**YLDs 2016**	**DALYs 2016**
Poisonings	14.1% (7.9% to 22.2%)	13.8% (8.1% to 20.9%)	15.3% (9.8% to 22.5%)	13.9% (8.2% to 20.8%)
Exposure to mechanical forces	18.5% (15.5% to 22.1%)	12.6% (9.8% to 16.0%)	16.3% (14.7% to 18.0%)	13.7% (11.4% to 16.5%)
Animal contact	5.4% (4.5% to 6.9%)	5.6% (4.6% to 6.8%)	7.6% (6.3% to 8.9%)	6.0% (4.9% to 7.2%)
Foreign body	3.3% (2.7% to 4.0%)	4.0% (3.2% to 5.0%)	5.9% (4.8% to 7.2%)	4.5% (3.6% to 5.6%)
Other unintentional injuries	14.2% (8.3% to 21.8%)	13.9% (8.6% to 20.6%)	14.3% (8.7% to 21.6%)	14.1% (8.8% to 21.0%)
**Self-harm and interpersonal violence**	32.4% (21.2% to 43.5%)	30.8% (20.5% to 41.2%)	25.1% (20.3% to 31.0%)	30.3% (20.6% to 40.0%)
Self-harm	35.2% (20.5% to 48.9%)	35.7% (21.5% to 48.2%)	34.5% (20.3% to 48.6%)	35.6% (21.5% to 48.2%)
Interpersonal violence	25.8% (16.2% to 36.9%)	23.6% (14.4% to 34.0%)	23.9% (19.2% to 29.8%)	23.6% (15.3% to 33.1%)
**Forces of nature, conflict and terrorism, and executions and police conflict**	0.0% (0.0% to 0.0%)	0.0% (0.0% to 0.0%)	0.4% (0.3% to 0.6%)	0.3% (0.1% to 0.4%)
Forces of nature, conflict and terrorism, and state actor violence	0.0% (0.0% to 0.0%)	0.0% (0.0% to 0.0%)	6.8% (4.6% to 9.9%)	6.8% (4.6% to 9.9%)

Supplementary Results Table 1. Age-standardised PAFs attributable to the joint distribution of all risk factors for all ages, both sexes combined for each GBD level 1 and level 2 cause and all causes in 2016 for Greece. Results are shown for deaths, YLLs, YLDs, and DALYs
Data in parenthesis are 95% uncertainty intervals. PAFs=population attributable fractions. GBD=Global Burden of Disease. YLLs=years of life lost. YLDs=years live with disability. DALYs=disability-adjusted life-years

	Deaths 2016	YLLs 2016	YLDs 2016	DALYs 2016
**All causes**	61.5% (59.2% to 63.7%)	58.3% (56.6% to 60.2%)	19.8% (18.4% to 21.2%)	37.9% (34.7% to 41.2%)
**HIV/AIDS and tuberculosis**	61.5% (52.6% to 69.6%)	64.7% (58.6% to 70.1%)	72.1% (68.2% to 74.5%)	66.9% (61.8% to 71.1%)
Tuberculosis	58.4% (46.9% to 68.6%)	60.5% (50.3% to 69.4%)	56.3% (44.9% to 66.3%)	60.0% (49.7% to 68.9%)
HIV/AIDS	73.5% (72.6% to 74.3%)	70.9% (69.9% to 71.8%)	75.9% (75.0% to 76.8%)	73.1% (71.9% to 74.4%)
**Diarrhoea, lower respiratory infections, and other common infectious diseases**	43.3% (34.6% to 52.3%)	47.6% (40.7% to 54.8%)	7.2% (5.3% to 9.5%)	27.0% (21.3% to 33.3%)
Diarrhoeal diseases	23.1% (15.6% to 33.5%)	43.1% (33.8% to 53.2%)	32.3% (23.4% to 42.7%)	33.5% (24.8% to 43.4%)
Lower respiratory infections	45.6% (36.4% to 55.2%)	53.5% (45.6% to 61.7%)	51.4% (43.2% to 60.0%)	53.4% (45.5% to 61.7%)
Upper respiratory infections	0.6% (0.0% to 2.0%)	1.7% (0.1% to 5.3%)	0.0% (0.0% to 0.0%)	0.0% (0.0% to 0.0%)
Otitis media	0.7% (0.4% to 1.3%)	3.5% (2.1% to 5.7%)	10.5% (7.2% to 13.5%)	10.5% (7.2% to 13.5%)
Meningitis	3.3% (2.4% to 4.5%)	6.2% (4.4% to 8.2%)	0.0% (0.0% to 0.0%)	5.6% (4.1% to 7.4%)
Encephalitis	0.4% (0.3% to 0.6%)	1.0% (0.7% to 1.3%)	0.0% (0.0% to 0.0%)	0.5% (0.4% to 0.7%)
Measles	6.1% (0.8% to 25.9%)	7.1% (0.9% to 29.6%)	9.1% (0.0% to 43.1%)	7.1% (0.9% to 29.6%)
**Maternal disorders**	3.7% (2.2% to 5.4%)	3.7% (2.1% to 5.3%)	2.5% (1.2% to 4.0%)	3.0% (1.6% to 4.5%)
Maternal haemorrhage	8.4% (3.1% to 13.7%)	8.5% (3.1% to 13.8%)	8.8% (3.2% to 14.6%)	8.6% (3.2% to 14.2%)
Maternal sepsis and other pregnancy related infections	8.6% (3.1% to 14.0%)	8.6% (3.1% to 14.1%)	8.6% (3.1% to 14.2%)	8.6% (3.1% to 14.0%)
Maternal abortion, miscarriage, and ectopic pregnancy	6.3% (3.8% to 9.2%)	6.4% (3.9% to 9.2%)	6.9% (3.5% to 11.2%)	6.6% (4.0% to 9.9%)
**Neonatal disorders**	87.9% (85.1% to 89.7%)	87.9% (85.1% to 89.7%)	0.1% (0.1% to 0.1%)	53.3% (42.9% to 62.2%)
Neonatal preterm birth complications	96.9% (94.9% to 98.1%)	97.0% (94.9% to 98.1%)	0.2% (0.1% to 0.2%)	68.8% (58.5% to 77.7%)
Neonatal encephalopathy due to birth asphyxia and trauma	65.8% (62.4% to 69.0%)	65.8% (62.5% to 69.0%)	0.0% (0.0% to 0.0%)	24.6% (15.0% to 35.1%)
Neonatal sepsis and other neonatal infections	58.1% (50.2% to 62.9%)	58.2% (50.2% to 62.9%)	0.0% (0.0% to 0.0%)	18.8% (8.0% to 32.5%)
Hemolytic disease and other neonatal jaundice	64.1% (57.6% to 68.8%)	64.1% (57.7% to 68.8%)	0.0% (0.0% to 0.0%)	20.5% (12.0% to 30.4%)

Supplementary Results Table 1. Age-standardised PAFs attributable to the joint distribution of all risk factors for all ages, both sexes combined for each GBD level 1 and level 2 cause and all causes in 2016 for Greece. Results are shown for deaths, YLLs, YLDs, and DALYs
Data in parenthesis are 95% uncertainty intervals. PAFs=population attributable fractions. GBD=Global Burden of Disease. YLLs=years of life lost. YLDs=years live with disability. DALYs=disability-adjusted life-years

	Deaths 2016	YLLs 2016	YLDs 2016	DALYs 2016
Other neonatal disorders	59.9% (54.3% to 64.3%)	60.0% (54.3% to 64.3%)	0.0% (0.0% to 0.0%)	50.0% (44.0% to 55.9%)
**Nutritional deficiencies**	13.0% (10.5% to 15.1%)	29.0% (25.0% to 32.9%)	63.0% (55.2% to 71.1%)	62.6% (54.9% to 70.6%)
Protein-energy malnutrition	4.6% (2.5% to 7.5%)	18.5% (11.3% to 26.5%)	98.3% (98.1% to 98.5%)	95.7% (94.1% to 96.6%)
Vitamin A deficiency	-	-	0.0% (0.0% to 0.0%)	0.0% (0.0% to 0.0%)
Iron-deficiency anaemia	100.0% (100.0% to 100.0%)	100.0% (100.0% to 100.0%)	100.0% (100.0% to 100.0%)	100.0% (100.0% to 100.0%)
**Other communicable, maternal, neonatal, and nutritional diseases**	3.7% (3.2% to 4.4%)	3.9% (3.3% to 4.5%)	64.7% (55.3% to 72.7%)	29.8% (20.4% to 41.9%)
Sexually transmitted diseases excluding HIV	90.0% (80.0% to 95.6%)	72.6% (53.4% to 86.2%)	100.0% (100.0% to 100.0%)	98.0% (95.5% to 99.3%)
Hepatitis	1.7% (1.2% to 2.4%)	2.2% (1.6% to 3.0%)	1.1% (0.7% to 1.8%)	2.1% (1.5% to 2.9%)
**Neoplasms**	47.1% (44.2% to 50.0%)	46.9% (44.1% to 49.7%)	35.6% (32.3% to 38.5%)	46.5% (43.8% to 49.3%)
Oesophageal cancer	86.0% (80.2% to 91.2%)	87.6% (82.2% to 92.2%)	86.0% (80.2% to 91.2%)	87.5% (82.2% to 92.2%)
Stomach cancer	24.5% (14.8% to 34.7%)	25.4% (15.7% to 35.1%)	24.6% (15.0% to 34.4%)	25.4% (15.7% to 35.1%)
Liver cancer	64.2% (54.8% to 72.9%)	66.0% (56.9% to 74.4%)	64.3% (55.6% to 72.5%)	65.9% (56.9% to 74.3%)
Larynx cancer	92.7% (88.1% to 95.9%)	92.9% (88.4% to 95.9%)	92.9% (88.6% to 96.0%)	92.9% (88.4% to 95.9%)
Tracheal, bronchus, and lung cancer	88.9% (87.0% to 90.5%)	88.8% (87.0% to 90.5%)	88.4% (86.2% to 90.2%)	88.8% (87.0% to 90.5%)
Breast cancer	34.0% (27.7% to 40.5%)	33.3% (27.2% to 39.1%)	33.5% (27.3% to 39.3%)	33.3% (27.2% to 39.1%)
Cervical cancer	100.0% (100.0% to 100.0%)	100.0% (100.0% to 100.0%)	100.0% (100.0% to 100.0%)	100.0% (100.0% to 100.0%)
Uterine cancer	43.0% (29.4% to 56.8%)	43.7% (30.1% to 57.5%)	43.8% (30.1% to 57.7%)	43.7% (30.1% to 57.5%)
Prostate cancer	7.8% (5.6% to 10.3%)	8.6% (6.2% to 10.9%)	8.8% (6.4% to 11.1%)	8.6% (6.2% to 11.0%)
Colon and rectum cancer	55.9% (47.2% to 63.5%)	57.1% (48.6% to 64.7%)	56.5% (47.8% to 64.2%)	57.1% (48.5% to 64.7%)
Lip and oral cavity cancer	83.5% (77.2% to 88.6%)	85.3% (79.7% to 89.7%)	81.6% (75.2% to 87.2%)	85.1% (79.5% to 89.6%)
Nasopharynx cancer	88.7% (85.1% to 91.7%)	86.6% (82.7% to 89.8%)	83.3% (77.8% to 87.7%)	86.5% (82.6% to 89.8%)

Supplementary Results Table 1. Age-standardised PAFs attributable to the joint distribution of all risk factors for all ages, both sexes combined for each GBD level 1 and level 2 cause and all causes in 2016 for Greece. Results are shown for deaths, YLLs, YLDs, and DALYs
Data in parenthesis are 95% uncertainty intervals. PAFs=population attributable fractions. GBD=Global Burden of Disease. YLLs=years of life lost. YLDs=years live with disability. DALYs=disability-adjusted life-years

	Deaths 2016	YLLs 2016	YLDs 2016	DALYs 2016
Other pharynx cancer	66.5% (53.7% to 76.2%)	67.6% (54.8% to 77.0%)	67.3% (54.7% to 77.0%)	67.5% (54.9% to 77.0%)
Gallbladder and biliary tract cancer	19.9% (10.7% to 31.7%)	20.5% (10.8% to 32.5%)	20.5% (10.9% to 32.7%)	20.5% (10.8% to 32.5%)
Pancreatic cancer	34.3% (27.4% to 41.5%)	36.2% (29.2% to 43.3%)	34.5% (27.6% to 41.9%)	36.2% (29.1% to 43.2%)
Ovarian cancer	9.8% (4.5% to 16.2%)	8.3% (3.5% to 14.1%)	8.2% (3.4% to 13.9%)	8.3% (3.5% to 14.1%)
Kidney cancer	41.5% (30.8% to 52.7%)	42.5% (31.9% to 53.5%)	41.4% (30.9% to 52.2%)	42.4% (31.8% to 53.5%)
Bladder cancer	43.9% (33.3% to 53.5%)	46.2% (35.6% to 55.9%)	45.5% (35.0% to 55.0%)	46.1% (35.5% to 55.9%)
Thyroid cancer	14.5% (7.1% to 24.1%)	15.7% (7.4% to 26.1%)	14.8% (7.5% to 24.0%)	15.6% (7.5% to 25.8%)
Mesothelioma	92.3% (90.2% to 95.3%)	86.7% (83.7% to 91.5%)	90.8% (88.4% to 94.4%)	86.8% (83.7% to 91.5%)
Non-Hodgkin's lymphoma	7.3% (2.9% to 12.7%)	7.1% (2.9% to 12.4%)	7.2% (2.9% to 12.5%)	7.1% (2.9% to 12.4%)
Multiple myeloma	8.2% (3.5% to 14.8%)	8.6% (3.6% to 15.4%)	8.5% (3.7% to 15.1%)	8.6% (3.6% to 15.4%)
Leukaemia	23.6% (14.6% to 32.0%)	20.7% (13.0% to 27.9%)	21.4% (13.4% to 29.3%)	20.7% (13.0% to 27.9%)
**Cardiovascular diseases**	83.8% (80.7% to 86.6%)	87.2% (85.2% to 89.0%)	75.8% (72.7% to 78.4%)	85.6% (83.5% to 87.5%)
Rheumatic heart disease	25.3% (14.6% to 47.5%)	26.0% (17.3% to 41.6%)	24.6% (16.5% to 37.8%)	26.0% (17.3% to 41.5%)
Ischaemic heart disease	91.5% (87.2% to 94.9%)	94.0% (91.5% to 96.0%)	90.6% (87.6% to 93.2%)	93.8% (91.3% to 95.8%)
Cerebrovascular disease	80.2% (74.0% to 85.9%)	84.9% (80.7% to 88.7%)	83.9% (79.6% to 87.9%)	84.7% (80.6% to 88.6%)
Hypertensive heart disease	100.0% (100.0% to 100.0%)	100.0% (100.0% to 100.0%)	100.0% (100.0% to 100.0%)	100.0% (100.0% to 100.0%)
Cardiomyopathy and myocarditis	36.6% (27.9% to 48.6%)	39.7% (31.9% to 48.2%)	34.8% (27.9% to 42.6%)	39.0% (31.4% to 47.4%)
Atrial fibrillation and flutter	55.1% (47.2% to 62.2%)	60.7% (53.4% to 66.9%)	68.8% (63.3% to 73.8%)	65.1% (58.7% to 70.7%)
Aortic aneurysm	47.2% (40.5% to 54.1%)	54.6% (48.5% to 60.3%)	-	54.6% (48.5% to 60.3%)
Peripheral vascular disease	45.2% (38.0% to 53.1%)	52.7% (46.5% to 58.6%)	56.3% (50.3% to 61.8%)	55.2% (49.6% to 60.7%)
Endocarditis	37.8% (27.6% to 50.6%)	38.8% (30.4% to 48.1%)	38.8% (29.3% to 49.3%)	38.8% (30.4% to 48.1%)

Supplementary Results Table 1. Age-standardised PAFs attributable to the joint distribution of all risk factors for all ages, both sexes combined for each GBD level 1 and level 2 cause and all causes in 2016 for Greece. Results are shown for deaths, YLLs, YLDs, and DALYs
Data in parenthesis are 95% uncertainty intervals. PAFs=population attributable fractions. GBD=Global Burden of Disease. YLLs=years of life lost. YLDs=years live with disability. DALYs=disability-adjusted life-years

	Deaths 2016	YLLs 2016	YLDs 2016	DALYs 2016
Other cardiovascular and circulatory diseases	45.0% (39.2% to 50.8%)	52.6% (47.3% to 57.4%)	54.0% (48.5% to 59.2%)	53.4% (48.3% to 58.4%)
**Chronic respiratory diseases**	73.3% (68.8% to 77.5%)	71.5% (67.7% to 75.8%)	54.1% (47.7% to 60.0%)	60.8% (55.8% to 65.8%)
Chronic obstructive pulmonary disease	77.8% (73.1% to 81.9%)	78.5% (74.4% to 82.2%)	73.7% (69.7% to 77.6%)	76.3% (72.5% to 79.9%)
Pneumoconiosis	100.0% (100.0% to 100.0%)	100.0% (100.0% to 100.0%)	100.0% (100.0% to 100.0%)	100.0% (100.0% to 100.0%)
Asthma	41.6% (31.7% to 53.0%)	46.2% (37.5% to 55.9%)	37.9% (29.1% to 47.8%)	38.2% (29.4% to 48.0%)
Other chronic respiratory diseases	18.3% (13.9% to 23.5%)	13.1% (9.4% to 17.2%)	9.8% (7.0% to 13.1%)	10.4% (7.6% to 13.8%)
**Cirrhosis and other chronic liver diseases**	60.3% (55.1% to 65.3%)	63.8% (59.0% to 68.8%)	50.9% (46.0% to 56.2%)	62.4% (57.9% to 67.4%)
Cirrhosis and other chronic liver diseases due to hepatitis B	0.4% (0.1% to 0.6%)	0.5% (0.2% to 0.8%)	0.5% (0.2% to 0.8%)	0.5% (0.2% to 0.8%)
Cirrhosis and other chronic liver diseases due to hepatitis C	51.1% (40.6% to 61.6%)	66.7% (56.8% to 74.7%)	69.0% (60.9% to 76.5%)	67.0% (57.3% to 74.9%)
Cirrhosis and other chronic liver diseases due to alcohol use	100.0% (100.0% to 100.0%)	100.0% (100.0% to 100.0%)	100.0% (100.0% to 100.0%)	100.0% (100.0% to 100.0%)
**Digestive diseases**	15.3% (11.6% to 19.2%)	18.3% (13.9% to 23.0%)	3.8% (2.8% to 5.1%)	13.6% (10.3% to 16.9%)
Peptic ulcer disease	14.1% (9.9% to 19.1%)	20.2% (14.6% to 26.4%)	21.6% (15.3% to 28.3%)	20.4% (14.8% to 26.7%)
Gallbladder and biliary diseases	40.4% (26.0% to 54.8%)	42.0% (28.1% to 56.2%)	46.1% (32.8% to 60.7%)	42.7% (29.1% to 57.1%)
Pancreatitis	42.7% (21.8% to 61.4%)	47.7% (26.7% to 65.4%)	42.8% (23.5% to 59.8%)	47.6% (26.7% to 65.1%)
**Neurological disorders**	19.0% (9.3% to 30.3%)	18.1% (9.9% to 27.7%)	3.8% (2.1% to 5.9%)	8.2% (4.8% to 12.7%)
Alzheimer's disease and other dementias	22.4% (11.1% to 35.3%)	24.1% (12.7% to 37.1%)	24.0% (12.6% to 37.2%)	24.1% (12.7% to 37.1%)
Parkinson's disease	-11.6% (-14.4% to -9.1%)	-15.7% (-19.2% to -12.3%)	-18.9% (-23.0% to -14.7%)	-16.4% (-19.9% to -12.7%)
Epilepsy	36.2% (25.1% to 46.3%)	32.3% (21.8% to 41.8%)	26.5% (16.9% to 34.8%)	28.2% (18.1% to 36.8%)
Multiple sclerosis	16.7% (11.3% to 22.8%)	17.8% (12.1% to 24.0%)	15.5% (10.3% to 20.7%)	16.7% (11.3% to 22.4%)
**Mental and substance use disorders**	99.3% (99.0% to 99.5%)	99.3% (99.0% to 99.5%)	14.5% (12.9% to 16.2%)	19.2% (16.7% to 21.9%)
Alcohol use disorders	100.0% (100.0% to 100.0%)	100.0% (100.0% to 100.0%)	100.0% (100.0% to 100.0%)	100.0% (100.0% to 100.0%)

Supplementary Results Table 1. Age-standardised PAFs attributable to the joint distribution of all risk factors for all ages, both sexes combined for each GBD level 1 and level 2 cause and all causes in 2016 for Greece. Results are shown for deaths, YLLs, YLDs, and DALYs
Data in parenthesis are 95% uncertainty intervals. PAFs=population attributable fractions. GBD=Global Burden of Disease. YLLs=years of life lost. YLDs=years live with disability. DALYs=disability-adjusted life-years

	Deaths 2016	YLLs 2016	YLDs 2016	DALYs 2016
Drug use disorders	100.0% (100.0% to 100.0%)	100.0% (100.0% to 100.0%)	100.0% (100.0% to 100.0%)	100.0% (100.0% to 100.0%)
Depressive disorders	-	-	7.2% (6.0% to 8.6%)	7.2% (6.0% to 8.6%)
Idiopathic developmental intellectual disability	-	-	64.6% (34.3% to 84.4%)	64.6% (34.3% to 84.4%)
**Diabetes, urogenital, blood, and endocrine diseases**	80.0% (78.0% to 82.9%)	73.2% (70.7% to 76.6%)	51.0% (46.1% to 55.9%)	59.7% (55.6% to 63.4%)
Diabetes mellitus	100.0% (100.0% to 100.0%)	100.0% (100.0% to 100.0%)	100.0% (100.0% to 100.0%)	100.0% (100.0% to 100.0%)
Chronic kidney disease	100.0% (100.0% to 100.0%)	100.0% (100.0% to 100.0%)	100.0% (100.0% to 100.0%)	100.0% (100.0% to 100.0%)
**Musculoskeletal disorders**	1.7% (0.7% to 3.0%)	1.9% (0.7% to 3.4%)	19.5% (16.5% to 23.1%)	19.5% (16.4% to 23.0%)
Rheumatoid arthritis	5.8% (2.4% to 9.7%)	7.3% (3.0% to 12.1%)	9.5% (4.0% to 15.4%)	9.4% (3.9% to 15.3%)
Osteoarthritis	-	-	25.8% (13.9% to 37.9%)	25.8% (13.9% to 37.9%)
Low back and neck pain	-	-	21.9% (18.6% to 25.3%)	21.9% (18.6% to 25.3%)
Gout	-	-	48.2% (29.9% to 66.3%)	48.2% (29.9% to 66.3%)
**Other non-communicable diseases**	0.2% (0.1% to 0.3%)	0.2% (0.1% to 0.4%)	2.4% (2.0% to 2.8%)	2.1% (1.8% to 2.5%)
Sense organ diseases	-	-	7.3% (6.4% to 8.2%)	7.3% (6.4% to 8.2%)
Sudden infant death syndrome	6.1% (3.6% to 9.4%)	6.2% (3.6% to 9.5%)	-	6.2% (3.6% to 9.5%)
**Transport injuries**	35.6% (25.3% to 46.0%)	31.5% (20.9% to 41.9%)	42.3% (33.3% to 50.8%)	34.5% (24.1% to 44.5%)
Road injuries	35.6% (25.2% to 46.0%)	31.5% (20.8% to 41.8%)	42.4% (33.4% to 50.9%)	34.4% (24.1% to 44.5%)
Other transport injuries	37.5% (27.3% to 47.7%)	32.8% (22.3% to 42.9%)	40.4% (31.3% to 49.4%)	35.9% (26.0% to 45.8%)
**Unintentional injuries**	24.7% (18.9% to 35.4%)	17.8% (13.1% to 23.2%)	19.5% (16.4% to 23.6%)	18.9% (15.4% to 23.2%)
Falls	49.4% (43.2% to 64.8%)	29.7% (24.0% to 40.7%)	21.2% (18.4% to 25.0%)	22.8% (19.6% to 27.4%)
Drowning	19.8% (11.6% to 28.9%)	19.7% (12.3% to 28.3%)	18.9% (11.5% to 27.6%)	19.7% (12.3% to 28.2%)
Fire, heat, and hot substances	17.5% (10.2% to 26.2%)	19.2% (12.0% to 27.4%)	19.8% (12.4% to 28.4%)	19.6% (12.6% to 27.9%)

Supplementary Results Table 1. Age-standardised PAFs attributable to the joint distribution of all risk factors for all ages, both sexes combined for each GBD level 1 and level 2 cause and all causes in 2016 for Greece. Results are shown for deaths, YLLs, YLDs, and DALYs
Data in parenthesis are 95% uncertainty intervals. PAFs=population attributable fractions. GBD=Global Burden of Disease. YLLs=years of life lost. YLDs=years live with disability. DALYs=disability-adjusted life-years

	Deaths 2016	YLLs 2016	YLDs 2016	DALYs 2016
Poisonings	21.7% (13.5% to 30.8%)	21.8% (14.1% to 30.6%)	17.2% (10.9% to 24.6%)	19.5% (12.7% to 27.6%)
Exposure to mechanical forces	19.5% (15.2% to 24.8%)	16.2% (12.0% to 22.0%)	18.8% (15.4% to 23.3%)	18.0% (14.5% to 22.8%)
Animal contact	13.3% (10.3% to 17.3%)	10.9% (7.5% to 15.5%)	9.0% (6.0% to 13.3%)	9.4% (6.3% to 13.7%)
Foreign body	6.3% (4.0% to 9.8%)	6.3% (4.0% to 9.7%)	6.2% (4.0% to 9.3%)	6.3% (4.0% to 9.6%)
Other unintentional injuries	24.1% (15.5% to 34.1%)	23.0% (14.9% to 32.7%)	19.6% (12.2% to 28.2%)	20.0% (12.5% to 28.7%)
**Self-harm and interpersonal violence**	36.2% (23.5% to 48.2%)	34.4% (21.6% to 46.4%)	36.2% (30.6% to 41.5%)	34.7% (23.3% to 45.4%)
Self-harm	36.7% (21.9% to 52.0%)	35.3% (19.7% to 50.7%)	26.7% (12.1% to 41.2%)	35.0% (19.5% to 50.2%)
Interpersonal violence	34.6% (24.6% to 43.6%)	31.5% (21.4% to 40.2%)	38.0% (32.5% to 43.7%)	34.1% (25.9% to 41.4%)
**Forces of nature, conflict and terrorism, and executions and police conflict**	0.0% (0.0% to 0.0%)	0.0% (0.0% to 0.0%)	5.2% (3.4% to 7.9%)	3.0% (1.3% to 5.3%)
Forces of nature, conflict and terrorism, and state actor violence	0.0% (0.0% to 0.0%)	0.0% (0.0% to 0.0%)	7.5% (4.9% to 11.2%)	7.5% (4.9% to 11.2%)

Supplementary Results Table 1. Age-standardised PAFs attributable to the joint distribution of all risk factors for all ages, both sexes combined for each GBD level 1 and level 2 cause and all causes in 2016 for Greenland. Results are shown for deaths, YLLs, YLDs, and DALYs
Data in parenthesis are 95% uncertainty intervals. PAFs=population attributable fractions. GBD=Global Burden of Disease. YLLs=years of life lost. YLDs=years live with disability. DALYs=disability-adjusted life-years

	Deaths 2016	YLLs 2016	YLDs 2016	DALYs 2016
**All causes**	60.6% (58.1% to 64.1%)	55.0% (52.0% to 58.4%)	23.9% (22.3% to 25.4%)	45.3% (42.2% to 48.9%)
**HIV/AIDS and tuberculosis**	51.7% (37.1% to 64.8%)	61.3% (49.9% to 71.8%)	83.5% (79.6% to 86.6%)	66.9% (56.9% to 75.3%)
Tuberculosis	42.0% (24.4% to 58.5%)	46.2% (29.2% to 61.9%)	48.0% (32.9% to 61.5%)	46.3% (29.2% to 61.8%)
HIV/AIDS	88.4% (86.5% to 90.1%)	87.3% (85.1% to 89.2%)	87.5% (85.2% to 89.5%)	87.4% (85.2% to 89.3%)
**Diarrhoea, lower respiratory infections, and other common infectious diseases**	35.9% (29.5% to 43.5%)	39.6% (33.8% to 46.4%)	7.8% (5.7% to 10.2%)	34.8% (29.1% to 41.1%)
Diarrhoeal diseases	30.1% (18.3% to 44.3%)	45.4% (27.6% to 63.8%)	38.0% (27.8% to 49.6%)	42.8% (28.3% to 57.8%)
Lower respiratory infections	38.1% (30.8% to 46.2%)	46.4% (39.1% to 54.3%)	45.0% (37.6% to 53.2%)	46.4% (39.1% to 54.3%)
Upper respiratory infections	0.7% (0.0% to 2.6%)	1.8% (0.1% to 6.7%)	0.0% (0.0% to 0.0%)	0.0% (0.0% to 0.0%)
Otitis media	9.3% (3.9% to 16.5%)	13.0% (6.2% to 20.0%)	9.9% (6.8% to 12.9%)	10.2% (7.0% to 13.2%)
Meningitis	0.6% (0.3% to 1.3%)	1.2% (0.7% to 2.2%)	0.0% (0.0% to 0.0%)	1.2% (0.7% to 2.1%)
Encephalitis	1.8% (0.4% to 3.4%)	3.1% (0.9% to 5.9%)	0.0% (0.0% to 0.0%)	2.5% (0.6% to 4.8%)
Measles	14.6% (2.5% to 52.5%)	14.8% (2.5% to 53.2%)	14.4% (2.4% to 51.7%)	14.8% (2.5% to 53.2%)
**Maternal disorders**	0.9% (0.4% to 1.5%)	0.9% (0.4% to 1.5%)	1.7% (0.7% to 2.9%)	1.0% (0.4% to 1.6%)
Maternal haemorrhage	8.8% (3.2% to 14.6%)	8.7% (3.1% to 14.5%)	10.1% (3.7% to 16.4%)	9.0% (3.3% to 14.7%)
Maternal sepsis and other pregnancy related infections	8.9% (3.1% to 14.5%)	8.8% (3.1% to 14.5%)	9.7% (3.5% to 15.6%)	8.9% (3.2% to 14.6%)
Maternal abortion, miscarriage, and ectopic pregnancy	3.4% (1.4% to 6.1%)	3.6% (1.4% to 6.4%)	3.5% (1.6% to 6.1%)	3.6% (1.4% to 6.3%)
**Neonatal disorders**	76.6% (72.5% to 79.9%)	76.7% (72.6% to 80.0%)	0.2% (0.1% to 0.2%)	69.1% (64.8% to 73.0%)
Neonatal preterm birth complications	92.9% (88.6% to 95.7%)	93.0% (88.7% to 95.8%)	0.2% (0.2% to 0.3%)	81.5% (75.1% to 86.6%)
Neonatal encephalopathy due to birth asphyxia and trauma	56.3% (49.9% to 63.6%)	56.4% (50.1% to 63.7%)	0.0% (0.0% to 0.0%)	53.8% (47.2% to 61.4%)
Neonatal sepsis and other neonatal infections	56.1% (45.3% to 64.5%)	56.2% (45.4% to 64.6%)	0.0% (0.0% to 0.0%)	47.4% (35.6% to 58.3%)
Hemolytic disease and other neonatal jaundice	49.9% (35.1% to 60.9%)	50.0% (35.2% to 61.0%)	0.0% (0.0% to 0.0%)	24.0% (13.5% to 33.4%)

Supplementary Results Table 1. Age-standardised PAFs attributable to the joint distribution of all risk factors for all ages, both sexes combined for each GBD level 1 and level 2 cause and all causes in 2016 for Greenland. Results are shown for deaths, YLLs, YLDs, and DALYs
Data in parenthesis are 95% uncertainty intervals. PAFs=population attributable fractions. GBD=Global Burden of Disease. YLLs=years of life lost. YLDs=years live with disability. DALYs=disability-adjusted life-years

	Deaths 2016	YLLs 2016	YLDs 2016	DALYs 2016
Other neonatal disorders	56.7% (50.2% to 62.8%)	56.8% (50.3% to 62.9%)	0.0% (0.0% to 0.0%)	55.5% (49.0% to 61.5%)
**Nutritional deficiencies**	0.4% (0.3% to 0.5%)	1.4% (1.2% to 1.7%)	75.3% (70.4% to 79.7%)	50.8% (41.7% to 60.1%)
Protein-energy malnutrition	0.0% (0.0% to 0.0%)	0.2% (0.1% to 0.4%)	98.3% (97.8% to 98.7%)	23.5% (15.5% to 32.5%)
Vitamin A deficiency	-	-	0.0% (0.0% to 0.0%)	0.0% (0.0% to 0.0%)
Iron-deficiency anaemia	100.0% (100.0% to 100.0%)	100.0% (100.0% to 100.0%)	100.0% (100.0% to 100.0%)	100.0% (100.0% to 100.0%)
**Other communicable, maternal, neonatal, and nutritional diseases**	8.6% (6.2% to 11.9%)	10.7% (5.8% to 16.1%)	71.1% (64.6% to 77.5%)	30.3% (21.9% to 41.0%)
Sexually transmitted diseases excluding HIV	54.0% (28.3% to 77.1%)	36.8% (13.2% to 63.1%)	100.0% (100.0% to 100.0%)	70.4% (45.2% to 88.9%)
Hepatitis	3.9% (2.4% to 5.8%)	4.8% (2.8% to 7.2%)	2.1% (1.2% to 3.3%)	4.5% (2.7% to 6.8%)
**Neoplasms**	68.9% (66.4% to 71.4%)	67.2% (64.6% to 69.9%)	57.5% (54.1% to 60.8%)	67.0% (64.4% to 69.7%)
Oesophageal cancer	91.8% (87.1% to 95.3%)	91.7% (86.9% to 95.2%)	91.8% (87.1% to 95.2%)	91.7% (86.9% to 95.2%)
Stomach cancer	45.8% (33.3% to 57.2%)	43.6% (31.4% to 54.9%)	44.7% (32.5% to 55.7%)	43.6% (31.5% to 54.9%)
Liver cancer	78.4% (70.5% to 84.8%)	77.9% (70.0% to 84.3%)	78.3% (70.4% to 84.5%)	77.9% (70.0% to 84.4%)
Larynx cancer	95.0% (91.8% to 97.2%)	94.3% (90.8% to 96.7%)	94.6% (91.0% to 96.9%)	94.3% (90.8% to 96.7%)
Tracheal, bronchus, and lung cancer	96.2% (95.5% to 96.9%)	95.7% (94.9% to 96.5%)	95.9% (95.2% to 96.6%)	95.7% (94.9% to 96.5%)
Breast cancer	43.9% (32.7% to 53.9%)	42.2% (31.3% to 51.5%)	42.8% (31.9% to 52.7%)	42.3% (31.3% to 51.6%)
Cervical cancer	100.0% (100.0% to 100.0%)	100.0% (100.0% to 100.0%)	100.0% (100.0% to 100.0%)	100.0% (100.0% to 100.0%)
Uterine cancer	45.9% (31.0% to 60.2%)	47.4% (32.4% to 62.0%)	47.8% (32.9% to 62.5%)	47.4% (32.5% to 62.0%)
Prostate cancer	18.5% (14.2% to 22.8%)	18.0% (13.7% to 22.3%)	17.8% (13.5% to 22.2%)	18.0% (13.7% to 22.4%)
Colon and rectum cancer	59.1% (50.9% to 66.9%)	58.9% (50.6% to 66.6%)	59.1% (50.8% to 66.8%)	58.9% (50.6% to 66.6%)
Lip and oral cavity cancer	90.4% (86.6% to 93.8%)	90.2% (85.9% to 93.7%)	89.9% (85.7% to 93.3%)	90.2% (85.9% to 93.7%)
Nasopharynx cancer	90.4% (86.5% to 93.5%)	89.1% (84.5% to 92.6%)	88.3% (83.3% to 92.3%)	89.0% (84.5% to 92.6%)

Supplementary Results Table 1. Age-standardised PAFs attributable to the joint distribution of all risk factors for all ages, both sexes combined for each GBD level 1 and level 2 cause and all causes in 2016 for Greenland. Results are shown for deaths, YLLs, YLDs, and DALYs
Data in parenthesis are 95% uncertainty intervals. PAFs=population attributable fractions. GBD=Global Burden of Disease. YLLs=years of life lost. YLDs=years live with disability. DALYs=disability-adjusted life-years

	Deaths 2016	YLLs 2016	YLDs 2016	DALYs 2016
Other pharynx cancer	44.2% (27.2% to 62.3%)	46.6% (29.4% to 64.3%)	45.9% (28.6% to 63.8%)	46.6% (29.4% to 64.3%)
Gallbladder and biliary tract cancer	21.0% (11.1% to 32.8%)	21.2% (11.2% to 33.2%)	21.1% (11.3% to 32.9%)	21.2% (11.2% to 33.2%)
Pancreatic cancer	60.3% (53.9% to 66.4%)	59.2% (52.5% to 65.6%)	59.7% (53.1% to 66.1%)	59.2% (52.5% to 65.6%)
Ovarian cancer	13.1% (5.9% to 21.8%)	11.1% (4.5% to 18.7%)	10.9% (4.3% to 18.4%)	11.0% (4.5% to 18.6%)
Kidney cancer	57.1% (45.9% to 66.5%)	56.1% (45.2% to 65.8%)	55.6% (44.9% to 65.2%)	56.1% (45.2% to 65.8%)
Bladder cancer	67.8% (58.8% to 75.1%)	67.0% (57.5% to 75.0%)	66.7% (56.8% to 74.3%)	67.0% (57.4% to 74.9%)
Thyroid cancer	13.9% (7.3% to 22.7%)	14.8% (7.5% to 24.1%)	14.8% (7.9% to 23.9%)	14.8% (7.6% to 24.1%)
Mesothelioma	98.2% (97.0% to 98.8%)	95.5% (93.3% to 96.9%)	97.1% (95.3% to 98.1%)	95.6% (93.4% to 96.9%)
Non-Hodgkin's lymphoma	6.9% (2.8% to 12.3%)	7.0% (2.8% to 12.3%)	7.1% (2.8% to 12.4%)	7.0% (2.8% to 12.3%)
Multiple myeloma	7.9% (3.3% to 14.0%)	8.3% (3.5% to 14.6%)	8.2% (3.4% to 14.5%)	8.3% (3.5% to 14.6%)
Leukaemia	36.6% (23.2% to 48.5%)	29.0% (18.7% to 39.7%)	34.9% (22.4% to 45.8%)	29.1% (18.8% to 39.8%)
**Cardiovascular diseases**	76.7% (72.7% to 81.1%)	79.6% (76.4% to 83.3%)	70.9% (67.0% to 74.4%)	78.9% (75.6% to 82.5%)
Rheumatic heart disease	19.0% (10.9% to 35.8%)	18.3% (11.3% to 30.8%)	18.1% (11.1% to 30.2%)	18.3% (11.3% to 30.8%)
Ischaemic heart disease	90.0% (85.3% to 94.0%)	91.9% (88.4% to 94.7%)	89.7% (86.1% to 92.9%)	91.8% (88.4% to 94.7%)
Cerebrovascular disease	76.6% (69.2% to 83.9%)	81.3% (76.2% to 86.3%)	82.4% (77.2% to 87.9%)	81.4% (76.4% to 86.5%)
Hypertensive heart disease	100.0% (100.0% to 100.0%)	100.0% (100.0% to 100.0%)	100.0% (100.0% to 100.0%)	100.0% (100.0% to 100.0%)
Cardiomyopathy and myocarditis	36.9% (28.4% to 46.9%)	37.7% (30.0% to 45.9%)	36.1% (29.5% to 43.7%)	37.6% (30.0% to 45.7%)
Atrial fibrillation and flutter	47.4% (40.0% to 54.9%)	52.6% (45.8% to 59.2%)	59.0% (53.1% to 64.6%)	56.7% (50.7% to 62.5%)
Aortic aneurysm	38.5% (32.0% to 45.2%)	44.4% (38.1% to 50.6%)	-	44.4% (38.1% to 50.6%)
Peripheral vascular disease	48.7% (42.5% to 55.0%)	55.3% (49.6% to 60.6%)	59.0% (53.5% to 64.2%)	55.8% (50.2% to 60.8%)
Endocarditis	31.0% (21.8% to 42.7%)	31.0% (22.4% to 40.3%)	31.8% (22.9% to 41.8%)	31.0% (22.5% to 40.3%)

Supplementary Results Table 1. Age-standardised PAFs attributable to the joint distribution of all risk factors for all ages, both sexes combined for each GBD level 1 and level 2 cause and all causes in 2016 for Greenland. Results are shown for deaths, YLLs, YLDs, and DALYs
Data in parenthesis are 95% uncertainty intervals. PAFs=population attributable fractions. GBD=Global Burden of Disease. YLLs=years of life lost. YLDs=years live with disability. DALYs=disability-adjusted life-years

	Deaths 2016	YLLs 2016	YLDs 2016	DALYs 2016
Other cardiovascular and circulatory diseases	39.5% (34.0% to 45.2%)	45.8% (40.5% to 51.0%)	49.4% (44.0% to 54.8%)	46.6% (41.3% to 51.7%)
**Chronic respiratory diseases**	84.0% (81.1% to 88.5%)	82.4% (79.6% to 86.8%)	61.5% (55.0% to 68.0%)	75.5% (71.5% to 80.0%)
Chronic obstructive pulmonary disease	93.1% (91.7% to 94.0%)	92.5% (90.8% to 93.7%)	88.1% (85.9% to 90.2%)	91.6% (89.7% to 92.9%)
Pneumoconiosis	100.0% (100.0% to 100.0%)	100.0% (100.0% to 100.0%)	100.0% (100.0% to 100.0%)	100.0% (100.0% to 100.0%)
Asthma	48.4% (38.8% to 58.9%)	54.2% (45.3% to 63.8%)	38.3% (30.2% to 47.3%)	41.0% (33.3% to 49.9%)
Other chronic respiratory diseases	41.4% (33.9% to 49.3%)	28.7% (19.9% to 37.6%)	22.6% (18.3% to 27.3%)	25.5% (20.2% to 31.4%)
**Cirrhosis and other chronic liver diseases**	56.5% (51.7% to 60.7%)	58.7% (54.3% to 63.0%)	53.7% (49.5% to 57.7%)	58.4% (54.2% to 62.5%)
Cirrhosis and other chronic liver diseases due to hepatitis B	2.9% (1.3% to 4.9%)	3.9% (1.7% to 6.7%)	4.2% (1.9% to 7.0%)	3.9% (1.7% to 6.7%)
Cirrhosis and other chronic liver diseases due to hepatitis C	58.4% (49.7% to 65.7%)	65.2% (56.5% to 72.2%)	68.1% (62.4% to 73.6%)	65.3% (57.0% to 72.0%)
Cirrhosis and other chronic liver diseases due to alcohol use	100.0% (100.0% to 100.0%)	100.0% (100.0% to 100.0%)	100.0% (100.0% to 100.0%)	100.0% (100.0% to 100.0%)
**Digestive diseases**	11.3% (8.6% to 14.2%)	13.0% (10.1% to 16.2%)	11.6% (9.2% to 14.2%)	12.8% (10.0% to 16.0%)
Peptic ulcer disease	18.9% (13.7% to 24.7%)	23.5% (17.3% to 30.1%)	26.8% (20.1% to 34.2%)	23.8% (17.5% to 30.3%)
Gallbladder and biliary diseases	44.1% (29.8% to 58.7%)	45.7% (32.1% to 59.8%)	47.3% (34.7% to 61.1%)	45.9% (32.4% to 59.9%)
Pancreatitis	18.2% (-1.0% to 37.6%)	22.3% (2.0% to 43.4%)	22.7% (2.5% to 43.3%)	22.3% (2.1% to 43.3%)
**Neurological disorders**	20.7% (11.5% to 31.6%)	20.0% (12.8% to 28.1%)	5.8% (3.5% to 9.0%)	11.4% (7.4% to 16.2%)
Alzheimer's disease and other dementias	26.9% (14.6% to 40.7%)	28.6% (16.4% to 42.1%)	28.8% (16.5% to 42.5%)	28.6% (16.4% to 42.3%)
Parkinson's disease	-16.9% (-20.2% to -13.7%)	-22.0% (-26.4% to -17.6%)	-27.2% (-32.6% to -21.5%)	-23.0% (-27.6% to -18.4%)
Epilepsy	26.2% (12.5% to 40.4%)	27.8% (13.8% to 41.7%)	23.2% (11.4% to 34.8%)	25.5% (12.5% to 38.7%)
Multiple sclerosis	18.5% (12.3% to 24.8%)	19.9% (13.5% to 26.5%)	18.0% (12.1% to 23.7%)	18.9% (12.8% to 25.1%)
**Mental and substance use disorders**	99.9% (99.8% to 100.0%)	99.8% (99.6% to 99.9%)	24.9% (22.7% to 27.5%)	37.9% (33.2% to 44.1%)
Alcohol use disorders	100.0% (100.0% to 100.0%)	100.0% (100.0% to 100.0%)	100.0% (100.0% to 100.0%)	100.0% (100.0% to 100.0%)

Supplementary Results Table 1. Age-standardised PAFs attributable to the joint distribution of all risk factors for all ages, both sexes combined for each GBD level 1 and level 2 cause and all causes in 2016 for Greenland. Results are shown for deaths, YLLs, YLDs, and DALYs
Data in parenthesis are 95% uncertainty intervals. PAFs=population attributable fractions. GBD=Global Burden of Disease. YLLs=years of life lost. YLDs=years live with disability. DALYs=disability-adjusted life-years

	Deaths 2016	YLLs 2016	YLDs 2016	DALYs 2016
Drug use disorders	100.0% (100.0% to 100.0%)	100.0% (100.0% to 100.0%)	100.0% (100.0% to 100.0%)	100.0% (100.0% to 100.0%)
Depressive disorders	-	-	9.4% (7.8% to 11.2%)	9.4% (7.8% to 11.2%)
Idiopathic developmental intellectual disability	-	-	65.1% (34.9% to 84.4%)	65.1% (34.9% to 84.4%)
**Diabetes, urogenital, blood, and endocrine diseases**	54.7% (50.6% to 59.6%)	51.5% (47.8% to 56.6%)	63.1% (59.0% to 67.2%)	56.8% (54.2% to 60.0%)
Diabetes mellitus	100.0% (100.0% to 100.0%)	100.0% (100.0% to 100.0%)	100.0% (100.0% to 100.0%)	100.0% (100.0% to 100.0%)
Chronic kidney disease	100.0% (100.0% to 100.0%)	100.0% (100.0% to 100.0%)	100.0% (100.0% to 100.0%)	100.0% (100.0% to 100.0%)
**Musculoskeletal disorders**	1.4% (0.5% to 2.9%)	1.3% (0.5% to 2.6%)	16.5% (13.5% to 19.8%)	16.0% (13.1% to 19.3%)
Rheumatoid arthritis	8.9% (3.7% to 14.8%)	10.3% (4.4% to 16.9%)	12.5% (5.4% to 20.4%)	12.4% (5.3% to 20.1%)
Osteoarthritis	-	-	27.3% (14.6% to 40.3%)	27.3% (14.6% to 40.3%)
Low back and neck pain	-	-	22.7% (19.1% to 26.3%)	22.7% (19.1% to 26.3%)
Gout	-	-	47.8% (30.7% to 65.9%)	47.8% (30.7% to 65.9%)
**Other non-communicable diseases**	0.7% (0.4% to 1.2%)	1.0% (0.5% to 1.6%)	2.1% (1.9% to 2.4%)	1.8% (1.6% to 2.1%)
Sense organ diseases	-	-	7.4% (6.6% to 8.2%)	7.4% (6.6% to 8.2%)
Sudden infant death syndrome	3.0% (1.7% to 4.9%)	3.0% (1.7% to 4.9%)	-	3.0% (1.7% to 4.9%)
**Transport injuries**	30.0% (21.7% to 39.9%)	24.4% (16.6% to 34.4%)	34.4% (26.6% to 43.7%)	26.1% (18.2% to 36.0%)
Road injuries	28.1% (20.2% to 37.7%)	21.0% (13.8% to 29.5%)	34.3% (26.4% to 43.6%)	23.6% (16.2% to 33.0%)
Other transport injuries	31.8% (23.0% to 42.5%)	27.8% (19.0% to 38.9%)	34.6% (26.7% to 44.0%)	28.8% (20.0% to 39.9%)
**Unintentional injuries**	35.6% (30.6% to 42.1%)	19.2% (15.3% to 23.6%)	18.4% (16.7% to 20.4%)	19.0% (16.2% to 22.2%)
Falls	63.1% (58.2% to 68.7%)	40.9% (35.6% to 46.7%)	22.5% (21.0% to 24.1%)	31.7% (28.2% to 35.3%)
Drowning	15.4% (10.0% to 23.6%)	14.4% (9.2% to 21.6%)	15.1% (9.6% to 23.0%)	14.4% (9.3% to 21.7%)
Fire, heat, and hot substances	9.3% (4.8% to 15.3%)	8.7% (4.7% to 13.4%)	14.8% (9.3% to 22.6%)	10.0% (5.8% to 15.4%)

Supplementary Results Table 1. Age-standardised PAFs attributable to the joint distribution of all risk factors for all ages, both sexes combined for each GBD level 1 and level 2 cause and all causes in 2016 for Greenland. Results are shown for deaths, YLLs, YLDs, and DALYs

Data in parenthesis are 95% uncertainty intervals. PAFs=population attributable fractions. GBD=Global Burden of Disease. YLLs=years of life lost. YLDs=years live with disability. DALYs=disability-adjusted life-years

	**Deaths 2016**	**YLLs 2016**	**YLDs 2016**	**DALYs 2016**
Poisonings	14.5% (9.1% to 22.4%)	14.4% (9.3% to 21.6%)	14.0% (8.9% to 21.5%)	14.3% (9.2% to 21.5%)
Exposure to mechanical forces	15.3% (12.7% to 17.9%)	11.3% (9.0% to 14.2%)	15.8% (13.8% to 18.4%)	13.5% (11.3% to 16.0%)
Animal contact	6.2% (4.6% to 9.8%)	2.9% (2.0% to 4.3%)	6.8% (5.3% to 8.7%)	5.7% (4.4% to 7.6%)
Foreign body	2.5% (2.0% to 3.3%)	4.4% (3.0% to 5.9%)	4.1% (3.1% to 5.5%)	4.4% (3.1% to 5.7%)
Other unintentional injuries	17.2% (11.4% to 25.4%)	18.9% (12.9% to 27.4%)	14.9% (9.3% to 22.8%)	16.2% (10.7% to 24.5%)
**Self-harm and interpersonal violence**	29.0% (15.7% to 43.1%)	29.8% (16.5% to 43.6%)	30.4% (23.4% to 38.0%)	29.8% (16.7% to 43.5%)
Self-harm	28.6% (13.6% to 44.0%)	29.9% (14.8% to 45.3%)	25.9% (11.2% to 41.3%)	29.8% (14.8% to 45.3%)
Interpersonal violence	31.7% (22.7% to 40.2%)	29.4% (20.8% to 37.7%)	33.0% (26.5% to 39.4%)	29.8% (21.5% to 37.8%)
**Forces of nature, conflict and terrorism, and executions and police conflict**	0.0% (0.0% to 0.0%)	0.0% (0.0% to 0.0%)	4.9% (2.8% to 7.9%)	3.3% (1.0% to 6.3%)
Forces of nature, conflict and terrorism, and state actor violence	0.0% (0.0% to 0.0%)	0.0% (0.0% to 0.0%)	6.0% (3.7% to 9.6%)	6.0% (3.7% to 9.6%)

Supplementary Results Table 1. Age-standardised PAFs attributable to the joint distribution of all risk factors for all ages, both sexes combined for each GBD level 1 and level 2 cause and all causes in 2016 for Grenada. Results are shown for deaths, YLLs, YLDs, and DALYs
Data in parenthesis are 95% uncertainty intervals. PAFs=population attributable fractions. GBD=Global Burden of Disease. YLLs=years of life lost. YLDs=years live with disability. DALYs=disability-adjusted life-years

	Deaths 2016	YLLs 2016	YLDs 2016	DALYs 2016
**All causes**	58.2% (56.3% to 60.0%)	54.7% (53.1% to 56.6%)	25.2% (23.8% to 26.6%)	45.0% (42.5% to 47.5%)
**HIV/AIDS and tuberculosis**	78.1% (71.3% to 82.9%)	76.1% (68.6% to 81.3%)	76.2% (67.9% to 83.1%)	76.1% (68.6% to 81.5%)
Tuberculosis	52.8% (43.2% to 62.2%)	56.0% (47.6% to 64.9%)	48.3% (40.0% to 56.4%)	55.6% (47.2% to 64.5%)
HIV/AIDS	81.4% (73.6% to 86.5%)	77.7% (69.4% to 83.2%)	79.1% (70.9% to 85.1%)	77.8% (69.5% to 83.4%)
**Diarrhoea, lower respiratory infections, and other common infectious diseases**	39.8% (30.4% to 49.6%)	48.8% (40.4% to 57.2%)	48.1% (34.9% to 58.1%)	48.7% (40.9% to 56.4%)
Diarrhoeal diseases	81.5% (60.3% to 94.1%)	86.9% (70.7% to 95.9%)	81.5% (59.8% to 94.1%)	82.9% (62.5% to 94.5%)
Lower respiratory infections	40.0% (30.1% to 50.2%)	50.3% (41.0% to 59.9%)	45.6% (35.8% to 55.6%)	50.2% (40.9% to 59.9%)
Upper respiratory infections	1.1% (0.1% to 4.2%)	2.7% (0.2% to 9.9%)	0.0% (0.0% to 0.0%)	0.0% (0.0% to 0.1%)
Otitis media	1.0% (0.5% to 1.8%)	2.8% (1.4% to 5.2%)	4.1% (2.7% to 5.6%)	4.1% (2.7% to 5.5%)
Meningitis	1.8% (0.9% to 3.3%)	4.0% (2.1% to 6.4%)	0.0% (0.0% to 0.0%)	3.4% (1.8% to 5.5%)
Encephalitis	0.9% (0.5% to 1.5%)	1.6% (1.0% to 2.4%)	0.0% (0.0% to 0.0%)	1.3% (0.8% to 2.0%)
Measles	12.8% (3.9% to 34.3%)	15.0% (4.7% to 40.9%)	15.9% (0.0% to 66.9%)	15.0% (4.7% to 40.9%)
**Maternal disorders**	2.8% (1.2% to 4.7%)	2.7% (1.2% to 4.6%)	2.7% (1.4% to 4.4%)	2.7% (1.3% to 4.4%)
Maternal haemorrhage	14.7% (5.3% to 24.1%)	14.7% (5.3% to 24.1%)	14.8% (5.3% to 24.3%)	14.7% (5.3% to 24.1%)
Maternal sepsis and other pregnancy related infections	14.4% (5.2% to 23.6%)	14.6% (5.3% to 23.9%)	14.6% (5.3% to 23.9%)	14.6% (5.3% to 23.8%)
Maternal abortion, miscarriage, and ectopic pregnancy	5.8% (3.2% to 9.1%)	5.9% (3.3% to 9.2%)	6.3% (2.9% to 10.5%)	6.0% (3.3% to 9.4%)
**Neonatal disorders**	77.2% (74.6% to 79.9%)	77.2% (74.6% to 79.9%)	0.1% (0.1% to 0.2%)	62.0% (51.7% to 70.2%)
Neonatal preterm birth complications	95.9% (94.4% to 97.2%)	95.9% (94.5% to 97.2%)	0.2% (0.2% to 0.3%)	71.2% (56.3% to 82.9%)
Neonatal encephalopathy due to birth asphyxia and trauma	68.0% (64.6% to 71.8%)	68.1% (64.6% to 71.8%)	0.0% (0.0% to 0.0%)	67.1% (63.1% to 71.0%)
Neonatal sepsis and other neonatal infections	60.5% (55.5% to 65.5%)	60.6% (55.5% to 65.5%)	0.0% (0.0% to 0.0%)	39.9% (22.6% to 54.1%)
Hemolytic disease and other neonatal jaundice	63.0% (55.6% to 69.1%)	63.0% (55.7% to 69.1%)	0.0% (0.0% to 0.0%)	20.7% (10.9% to 33.9%)

Supplementary Results Table 1. Age-standardised PAFs attributable to the joint distribution of all risk factors for all ages, both sexes combined for each GBD level 1 and level 2 cause and all causes in 2016 for Grenada. Results are shown for deaths, YLLs, YLDs, and DALYs
Data in parenthesis are 95% uncertainty intervals. PAFs=population attributable fractions. GBD=Global Burden of Disease. YLLs=years of life lost. YLDs=years live with disability. DALYs=disability-adjusted life-years

	Deaths 2016	YLLs 2016	YLDs 2016	DALYs 2016
Other neonatal disorders	63.9% (59.4% to 68.3%)	64.0% (59.5% to 68.4%)	0.0% (0.0% to 0.0%)	58.8% (53.0% to 64.4%)
**Nutritional deficiencies**	9.5% (5.0% to 16.8%)	33.0% (20.2% to 49.4%)	97.2% (96.4% to 98.0%)	85.1% (79.8% to 89.2%)
Protein-energy malnutrition	11.1% (5.6% to 20.0%)	38.4% (23.4% to 56.2%)	96.3% (94.6% to 97.7%)	46.9% (34.6% to 61.2%)
Vitamin A deficiency	-	-	100.0% (100.0% to 100.0%)	100.0% (100.0% to 100.0%)
Iron-deficiency anaemia	100.0% (100.0% to 100.0%)	100.0% (100.0% to 100.0%)	100.0% (100.0% to 100.0%)	100.0% (100.0% to 100.0%)
**Other communicable, maternal, neonatal, and nutritional diseases**	9.3% (7.6% to 12.8%)	7.5% (5.7% to 10.3%)	73.4% (65.4% to 81.5%)	23.8% (16.4% to 33.5%)
Sexually transmitted diseases excluding HIV	66.2% (41.8% to 87.8%)	42.8% (20.3% to 72.1%)	100.0% (100.0% to 100.0%)	75.0% (51.8% to 91.9%)
Hepatitis	7.3% (4.8% to 10.6%)	8.0% (5.0% to 11.8%)	5.4% (3.2% to 8.3%)	7.9% (4.9% to 11.6%)
**Neoplasms**	32.7% (29.3% to 36.4%)	33.5% (30.4% to 37.1%)	28.3% (25.0% to 31.9%)	33.4% (30.3% to 37.0%)
Oesophageal cancer	72.9% (62.8% to 81.8%)	76.3% (66.6% to 84.5%)	73.9% (63.9% to 82.8%)	76.3% (66.6% to 84.5%)
Stomach cancer	11.3% (5.6% to 18.9%)	13.0% (6.7% to 20.5%)	11.9% (6.1% to 19.3%)	13.0% (6.7% to 20.5%)
Liver cancer	56.9% (49.6% to 64.3%)	58.0% (51.0% to 65.3%)	57.3% (50.0% to 64.6%)	58.0% (51.0% to 65.3%)
Larynx cancer	78.9% (69.5% to 86.3%)	81.1% (72.4% to 87.7%)	80.0% (71.0% to 87.1%)	81.1% (72.4% to 87.7%)
Tracheal, bronchus, and lung cancer	79.4% (73.6% to 84.1%)	80.4% (75.3% to 84.8%)	79.5% (74.0% to 84.3%)	80.4% (75.3% to 84.8%)
Breast cancer	26.7% (17.5% to 37.6%)	24.5% (15.7% to 34.6%)	25.4% (16.5% to 36.0%)	24.5% (15.7% to 34.7%)
Cervical cancer	100.0% (100.0% to 100.0%)	100.0% (100.0% to 100.0%)	100.0% (100.0% to 100.0%)	100.0% (100.0% to 100.0%)
Uterine cancer	46.2% (31.4% to 60.6%)	48.6% (33.9% to 63.0%)	49.4% (34.9% to 63.6%)	48.6% (33.9% to 63.0%)
Prostate cancer	2.3% (1.5% to 3.3%)	2.9% (1.9% to 4.0%)	2.9% (1.9% to 4.0%)	2.9% (1.9% to 4.0%)
Colon and rectum cancer	56.3% (46.6% to 66.6%)	57.3% (48.1% to 66.8%)	56.8% (47.1% to 66.7%)	57.3% (48.1% to 66.8%)
Lip and oral cavity cancer	72.2% (62.7% to 80.1%)	76.6% (68.4% to 83.5%)	71.5% (62.8% to 79.2%)	76.5% (68.2% to 83.3%)
Nasopharynx cancer	70.4% (63.2% to 77.0%)	69.9% (63.4% to 76.2%)	69.2% (63.1% to 75.0%)	69.9% (63.4% to 76.1%)

Supplementary Results Table 1. Age-standardised PAFs attributable to the joint distribution of all risk factors for all ages, both sexes combined for each GBD level 1 and level 2 cause and all causes in 2016 for Grenada. Results are shown for deaths, YLLs, YLDs, and DALYs
Data in parenthesis are 95% uncertainty intervals. PAFs=population attributable fractions. GBD=Global Burden of Disease. YLLs=years of life lost. YLDs=years live with disability. DALYs=disability-adjusted life-years

	Deaths 2016	YLLs 2016	YLDs 2016	DALYs 2016
Other pharynx cancer	54.7% (41.5% to 66.5%)	58.4% (45.5% to 69.4%)	56.1% (42.8% to 67.7%)	58.4% (45.5% to 69.4%)
Gallbladder and biliary tract cancer	23.4% (13.2% to 35.8%)	24.3% (13.9% to 36.9%)	23.5% (13.2% to 35.9%)	24.3% (13.9% to 36.9%)
Pancreatic cancer	28.4% (18.5% to 40.1%)	30.4% (20.6% to 41.7%)	29.0% (19.2% to 40.7%)	30.4% (20.6% to 41.7%)
Ovarian cancer	18.8% (8.1% to 32.5%)	16.9% (7.0% to 29.8%)	16.5% (6.9% to 28.8%)	16.9% (7.0% to 29.8%)
Kidney cancer	27.7% (19.2% to 37.2%)	27.9% (19.4% to 37.4%)	28.4% (20.0% to 37.6%)	27.9% (19.4% to 37.4%)
Bladder cancer	27.1% (17.1% to 39.3%)	30.0% (19.5% to 41.8%)	29.0% (18.9% to 40.9%)	29.9% (19.5% to 41.8%)
Thyroid cancer	12.5% (6.3% to 21.3%)	13.0% (6.5% to 22.2%)	13.5% (7.1% to 22.0%)	13.0% (6.5% to 22.2%)
Mesothelioma	90.4% (87.5% to 93.0%)	83.9% (79.2% to 88.3%)	88.4% (85.0% to 91.4%)	84.0% (79.3% to 88.3%)
Non-Hodgkin's lymphoma	5.9% (2.4% to 10.5%)	5.7% (2.3% to 10.2%)	5.9% (2.4% to 10.6%)	5.7% (2.3% to 10.2%)
Multiple myeloma	7.8% (3.2% to 14.1%)	8.1% (3.3% to 14.3%)	8.0% (3.2% to 14.2%)	8.1% (3.3% to 14.3%)
Leukaemia	15.0% (9.2% to 21.3%)	13.9% (8.7% to 19.9%)	14.9% (9.2% to 20.8%)	13.9% (8.7% to 19.9%)
**Cardiovascular diseases**	81.8% (79.3% to 84.0%)	81.8% (79.7% to 83.7%)	70.1% (66.9% to 73.2%)	81.0% (78.9% to 82.9%)
Rheumatic heart disease	25.3% (16.3% to 42.1%)	21.9% (15.2% to 33.1%)	19.9% (12.7% to 29.5%)	21.4% (14.5% to 32.1%)
Ischaemic heart disease	93.6% (90.1% to 96.3%)	95.0% (92.8% to 96.9%)	91.9% (89.1% to 94.2%)	94.9% (92.6% to 96.7%)
Cerebrovascular disease	84.4% (79.3% to 89.0%)	86.9% (83.5% to 89.9%)	85.4% (80.5% to 89.8%)	86.8% (83.4% to 89.9%)
Hypertensive heart disease	100.0% (100.0% to 100.0%)	100.0% (100.0% to 100.0%)	100.0% (100.0% to 100.0%)	100.0% (100.0% to 100.0%)
Cardiomyopathy and myocarditis	36.5% (30.2% to 42.9%)	37.9% (32.1% to 43.7%)	36.4% (30.9% to 42.1%)	37.9% (32.1% to 43.5%)
Atrial fibrillation and flutter	48.2% (40.6% to 55.9%)	52.6% (45.6% to 59.5%)	59.2% (53.7% to 64.8%)	56.5% (50.4% to 62.3%)
Aortic aneurysm	42.1% (35.1% to 48.7%)	46.5% (40.5% to 52.2%)	-	46.5% (40.5% to 52.2%)
Peripheral vascular disease	51.9% (44.9% to 59.6%)	59.4% (53.8% to 65.2%)	64.5% (59.6% to 69.4%)	60.7% (55.5% to 66.1%)
Endocarditis	37.7% (29.4% to 47.3%)	35.7% (28.6% to 43.2%)	37.3% (29.0% to 46.3%)	35.7% (28.6% to 43.2%)

Supplementary Results Table 1. Age-standardised PAFs attributable to the joint distribution of all risk factors for all ages, both sexes combined for each GBD level 1 and level 2 cause and all causes in 2016 for Grenada. Results are shown for deaths, YLLs, YLDs, and DALYs
Data in parenthesis are 95% uncertainty intervals. PAFs=population attributable fractions. GBD=Global Burden of Disease. YLLs=years of life lost. YLDs=years live with disability. DALYs=disability-adjusted life-years

	Deaths 2016	YLLs 2016	YLDs 2016	DALYs 2016
Other cardiovascular and circulatory diseases	43.7% (38.8% to 48.5%)	45.2% (40.9% to 49.9%)	44.5% (39.9% to 49.4%)	45.1% (40.9% to 49.7%)
**Chronic respiratory diseases**	50.3% (44.1% to 55.9%)	47.3% (42.0% to 52.5%)	31.3% (23.1% to 40.9%)	39.7% (33.8% to 45.8%)
Chronic obstructive pulmonary disease	65.3% (56.7% to 72.8%)	65.9% (58.7% to 72.7%)	65.5% (58.0% to 72.0%)	65.8% (58.8% to 72.6%)
Pneumoconiosis	100.0% (100.0% to 100.0%)	100.0% (100.0% to 100.0%)	100.0% (100.0% to 100.0%)	100.0% (100.0% to 100.0%)
Asthma	34.6% (25.5% to 45.1%)	35.7% (27.1% to 45.5%)	26.9% (18.0% to 37.6%)	28.3% (19.6% to 38.9%)
Other chronic respiratory diseases	11.2% (7.3% to 15.4%)	12.6% (8.0% to 18.2%)	5.4% (3.0% to 8.4%)	12.2% (7.8% to 17.7%)
**Cirrhosis and other chronic liver diseases**	67.7% (65.0% to 70.3%)	68.3% (65.6% to 71.0%)	54.0% (50.2% to 57.9%)	67.8% (65.2% to 70.5%)
Cirrhosis and other chronic liver diseases due to hepatitis B	6.3% (2.6% to 11.1%)	8.1% (3.4% to 14.2%)	5.9% (2.5% to 10.5%)	8.0% (3.4% to 14.1%)
Cirrhosis and other chronic liver diseases due to hepatitis C	82.5% (78.9% to 85.6%)	86.8% (84.9% to 88.4%)	80.8% (76.5% to 84.7%)	86.7% (84.7% to 88.3%)
Cirrhosis and other chronic liver diseases due to alcohol use	100.0% (100.0% to 100.0%)	100.0% (100.0% to 100.0%)	100.0% (100.0% to 100.0%)	100.0% (100.0% to 100.0%)
**Digestive diseases**	6.3% (4.9% to 7.8%)	8.1% (6.3% to 10.0%)	2.6% (1.9% to 3.4%)	7.0% (5.4% to 8.7%)
Peptic ulcer disease	8.3% (5.5% to 11.5%)	10.1% (6.8% to 13.9%)	4.6% (3.0% to 6.7%)	9.1% (6.2% to 12.7%)
Gallbladder and biliary diseases	35.7% (23.4% to 49.5%)	36.0% (23.7% to 49.3%)	46.4% (32.9% to 60.3%)	37.2% (24.8% to 50.4%)
Pancreatitis	45.1% (28.8% to 63.0%)	51.3% (34.3% to 68.0%)	36.1% (21.9% to 51.7%)	51.1% (34.1% to 67.7%)
**Neurological disorders**	22.0% (10.8% to 34.7%)	22.2% (13.7% to 31.8%)	4.8% (2.5% to 7.7%)	10.8% (6.7% to 15.7%)
Alzheimer's disease and other dementias	24.6% (10.7% to 40.6%)	26.2% (12.1% to 42.5%)	26.2% (12.0% to 42.5%)	26.2% (12.1% to 42.5%)
Parkinson's disease	-3.2% (-4.2% to -2.4%)	-4.3% (-5.5% to -3.1%)	-5.0% (-6.4% to -3.7%)	-4.4% (-5.6% to -3.3%)
Epilepsy	29.8% (21.7% to 38.3%)	30.9% (22.8% to 39.2%)	23.4% (17.0% to 30.2%)	27.4% (20.0% to 35.1%)
Multiple sclerosis	4.1% (2.5% to 6.3%)	4.1% (2.4% to 6.2%)	3.2% (1.9% to 4.6%)	3.9% (2.3% to 5.8%)
**Mental and substance use disorders**	99.9% (99.9% to 100.0%)	99.9% (99.9% to 99.9%)	18.4% (16.3% to 20.5%)	24.1% (21.3% to 27.1%)
Alcohol use disorders	100.0% (100.0% to 100.0%)	100.0% (100.0% to 100.0%)	100.0% (100.0% to 100.0%)	100.0% (100.0% to 100.0%)

Supplementary Results Table 1. Age-standardised PAFs attributable to the joint distribution of all risk factors for all ages, both sexes combined for each GBD level 1 and level 2 cause and all causes in 2016 for Grenada. Results are shown for deaths, YLLs, YLDs, and DALYs
Data in parenthesis are 95% uncertainty intervals. PAFs=population attributable fractions. GBD=Global Burden of Disease. YLLs=years of life lost. YLDs=years live with disability. DALYs=disability-adjusted life-years

	Deaths 2016	YLLs 2016	YLDs 2016	DALYs 2016
Drug use disorders	100.0% (100.0% to 100.0%)	100.0% (100.0% to 100.0%)	100.0% (100.0% to 100.0%)	100.0% (100.0% to 100.0%)
Depressive disorders	-	-	6.1% (5.0% to 7.5%)	6.1% (5.0% to 7.5%)
Idiopathic developmental intellectual disability	-	-	67.4% (35.8% to 87.5%)	67.4% (35.8% to 87.5%)
**Diabetes, urogenital, blood, and endocrine diseases**	86.3% (84.7% to 87.2%)	83.4% (81.0% to 84.6%)	78.8% (75.3% to 81.7%)	82.1% (80.3% to 83.3%)
Diabetes mellitus	100.0% (100.0% to 100.0%)	100.0% (100.0% to 100.0%)	100.0% (100.0% to 100.0%)	100.0% (100.0% to 100.0%)
Chronic kidney disease	100.0% (100.0% to 100.0%)	100.0% (100.0% to 100.0%)	100.0% (100.0% to 100.0%)	100.0% (100.0% to 100.0%)
**Musculoskeletal disorders**	0.1% (0.0% to 0.2%)	0.1% (0.0% to 0.1%)	12.0% (9.3% to 14.8%)	11.1% (8.6% to 13.8%)
Rheumatoid arthritis	1.3% (0.5% to 2.2%)	1.6% (0.6% to 2.8%)	2.1% (0.8% to 3.8%)	2.1% (0.8% to 3.7%)
Osteoarthritis	-	-	25.6% (14.2% to 38.5%)	25.6% (14.2% to 38.5%)
Low back and neck pain	-	-	17.0% (14.1% to 20.1%)	17.0% (14.1% to 20.1%)
Gout	-	-	45.1% (32.7% to 59.6%)	45.1% (32.7% to 59.6%)
**Other non-communicable diseases**	0.1% (0.0% to 0.2%)	0.2% (0.1% to 0.3%)	5.0% (4.1% to 6.0%)	3.9% (3.1% to 4.7%)
Sense organ diseases	-	-	11.7% (10.1% to 13.7%)	11.7% (10.1% to 13.7%)
Sudden infant death syndrome	5.9% (3.4% to 9.3%)	5.9% (3.4% to 9.3%)	-	5.9% (3.4% to 9.3%)
**Transport injuries**	29.5% (22.0% to 39.4%)	25.3% (17.4% to 35.5%)	33.0% (25.5% to 42.3%)	26.1% (18.3% to 36.1%)
Road injuries	28.8% (21.1% to 38.8%)	25.2% (17.2% to 35.5%)	33.5% (25.9% to 42.9%)	26.1% (18.2% to 36.2%)
Other transport injuries	34.5% (27.9% to 43.0%)	26.5% (19.2% to 35.7%)	27.3% (20.6% to 35.4%)	26.5% (19.5% to 35.6%)
**Unintentional injuries**	14.2% (11.3% to 17.6%)	9.5% (6.3% to 13.6%)	14.7% (13.0% to 16.7%)	11.0% (8.2% to 14.3%)
Falls	42.7% (38.7% to 47.4%)	20.7% (17.6% to 24.3%)	21.0% (19.4% to 22.9%)	20.8% (19.0% to 23.2%)
Drowning	12.2% (5.9% to 19.9%)	11.7% (5.7% to 19.4%)	11.8% (6.2% to 19.1%)	11.7% (5.7% to 19.4%)
Fire, heat, and hot substances	8.3% (4.5% to 13.8%)	9.7% (5.6% to 15.4%)	12.7% (7.8% to 18.9%)	10.7% (6.2% to 16.3%)

Supplementary Results Table 1. Age-standardised PAFs attributable to the joint distribution of all risk factors for all ages, both sexes combined for each GBD level 1 and level 2 cause and all causes in 2016 for Grenada. Results are shown for deaths, YLLs, YLDs, and DALYs
Data in parenthesis are 95% uncertainty intervals. PAFs=population attributable fractions. GBD=Global Burden of Disease. YLLs=years of life lost. YLDs=years live with disability. DALYs=disability-adjusted life-years

	**Deaths 2016**	**YLLs 2016**	**YLDs 2016**	**DALYs 2016**
Poisonings	11.7% (6.8% to 17.9%)	11.9% (7.0% to 18.1%)	12.2% (7.6% to 17.9%)	11.9% (7.1% to 18.0%)
Exposure to mechanical forces	12.1% (9.5% to 15.2%)	8.6% (5.9% to 11.8%)	12.1% (10.3% to 14.7%)	9.6% (7.1% to 12.5%)
Animal contact	9.8% (8.0% to 12.0%)	6.7% (5.0% to 8.9%)	5.6% (3.9% to 7.7%)	6.2% (4.6% to 8.3%)
Foreign body	2.3% (1.4% to 3.6%)	2.6% (1.5% to 4.1%)	3.8% (2.5% to 5.4%)	2.8% (1.7% to 4.3%)
Other unintentional injuries	12.6% (6.6% to 19.7%)	12.9% (6.8% to 20.0%)	11.4% (7.0% to 17.0%)	11.9% (6.9% to 17.9%)
**Self-harm and interpersonal violence**	30.3% (22.8% to 37.9%)	30.3% (22.7% to 38.3%)	30.0% (26.7% to 33.7%)	30.2% (23.0% to 38.0%)
Self-harm	31.4% (20.0% to 43.4%)	32.6% (21.2% to 44.7%)	27.4% (16.6% to 39.1%)	32.6% (21.1% to 44.6%)
Interpersonal violence	29.2% (21.2% to 38.1%)	28.1% (20.2% to 37.3%)	30.2% (27.0% to 34.1%)	28.3% (20.9% to 36.8%)
**Forces of nature, conflict and terrorism, and executions and police conflict**	0.0% (0.0% to 0.0%)	0.0% (0.0% to 0.0%)	3.7% (2.5% to 5.5%)	2.6% (1.0% to 4.4%)
Forces of nature, conflict and terrorism, and state actor violence	0.0% (0.0% to 0.0%)	0.0% (0.0% to 0.0%)	4.8% (3.2% to 7.1%)	4.8% (3.2% to 7.1%)

Supplementary Results Table 1. Age-standardised PAFs attributable to the joint distribution of all risk factors for all ages, both sexes combined for each GBD level 1 and level 2 cause and all causes in 2016 for Guam. Results are shown for deaths, YLLs, YLDs, and DALYs
Data in parenthesis are 95% uncertainty intervals. PAFs=population attributable fractions. GBD=Global Burden of Disease. YLLs=years of life lost. YLDs=years live with disability. DALYs=disability-adjusted life-years

	Deaths 2016	YLLs 2016	YLDs 2016	DALYs 2016
**All causes**	66.7% (64.7% to 68.7%)	60.6% (58.8% to 62.8%)	31.9% (30.3% to 33.7%)	50.9% (48.4% to 53.5%)
**HIV/AIDS and tuberculosis**	57.2% (35.5% to 84.8%)	62.8% (41.2% to 85.9%)	48.0% (31.7% to 75.6%)	61.8% (41.5% to 84.8%)
Tuberculosis	35.7% (23.6% to 48.1%)	38.0% (25.3% to 50.6%)	35.0% (22.5% to 47.4%)	37.4% (24.8% to 50.0%)
HIV/AIDS	89.9% (83.6% to 94.1%)	87.4% (80.2% to 92.6%)	87.3% (80.2% to 92.5%)	87.5% (80.3% to 92.7%)
**Diarrhoea, lower respiratory infections, and other common infectious diseases**	35.7% (28.3% to 45.6%)	43.4% (35.9% to 51.9%)	35.9% (22.0% to 47.6%)	41.5% (34.6% to 49.0%)
Diarrhoeal diseases	65.8% (36.1% to 88.9%)	72.2% (48.3% to 90.7%)	67.5% (38.9% to 89.3%)	68.0% (40.0% to 89.6%)
Lower respiratory infections	36.8% (28.9% to 47.4%)	48.7% (40.2% to 58.7%)	41.8% (33.5% to 52.1%)	48.7% (40.1% to 58.6%)
Upper respiratory infections	1.1% (0.1% to 3.9%)	2.8% (0.2% to 9.5%)	0.0% (0.0% to 0.0%)	0.0% (0.0% to 0.1%)
Otitis media	1.6% (0.4% to 4.7%)	3.9% (1.2% to 8.9%)	7.0% (4.7% to 9.2%)	7.0% (4.7% to 9.2%)
Meningitis	2.3% (1.2% to 3.8%)	3.9% (2.1% to 6.2%)	0.0% (0.0% to 0.0%)	2.5% (1.3% to 4.0%)
Encephalitis	1.2% (0.7% to 2.0%)	2.5% (1.5% to 4.1%)	0.0% (0.0% to 0.0%)	0.6% (0.3% to 1.0%)
Measles	26.0% (6.5% to 69.3%)	26.3% (6.6% to 70.2%)	25.5% (6.7% to 68.4%)	26.3% (6.6% to 70.2%)
**Maternal disorders**	7.8% (4.8% to 11.3%)	7.7% (4.8% to 11.0%)	4.5% (2.4% to 7.1%)	7.1% (4.3% to 10.2%)
Maternal haemorrhage	15.5% (5.5% to 25.6%)	15.4% (5.5% to 25.5%)	15.3% (5.4% to 25.4%)	15.4% (5.5% to 25.6%)
Maternal sepsis and other pregnancy related infections	15.5% (5.6% to 25.8%)	15.5% (5.6% to 25.7%)	15.2% (5.4% to 25.1%)	15.4% (5.6% to 25.5%)
Maternal abortion, miscarriage, and ectopic pregnancy	8.9% (5.1% to 13.6%)	8.8% (5.1% to 13.4%)	8.0% (4.0% to 12.5%)	8.7% (5.1% to 13.3%)
**Neonatal disorders**	76.9% (71.5% to 80.9%)	77.0% (71.6% to 80.9%)	0.2% (0.1% to 0.2%)	65.6% (58.9% to 71.2%)
Neonatal preterm birth complications	94.7% (90.1% to 97.4%)	94.8% (90.2% to 97.4%)	0.2% (0.2% to 0.3%)	71.9% (61.2% to 80.6%)
Neonatal encephalopathy due to birth asphyxia and trauma	67.1% (58.9% to 73.6%)	67.2% (58.9% to 73.6%)	0.0% (0.0% to 0.0%)	64.9% (55.9% to 71.8%)
Neonatal sepsis and other neonatal infections	65.4% (56.7% to 71.2%)	65.5% (56.8% to 71.2%)	0.0% (0.0% to 0.0%)	54.7% (40.9% to 64.3%)
Hemolytic disease and other neonatal jaundice	67.0% (58.2% to 73.1%)	67.1% (58.3% to 73.2%)	0.0% (0.0% to 0.0%)	48.2% (35.8% to 58.9%)

Supplementary Results Table 1. Age-standardised PAFs attributable to the joint distribution of all risk factors for all ages, both sexes combined for each GBD level 1 and level 2 cause and all causes in 2016 for Guam. Results are shown for deaths, YLLs, YLDs, and DALYs
Data in parenthesis are 95% uncertainty intervals. PAFs=population attributable fractions. GBD=Global Burden of Disease. YLLs=years of life lost. YLDs=years live with disability. DALYs=disability-adjusted life-years

	Deaths 2016	YLLs 2016	YLDs 2016	DALYs 2016
Other neonatal disorders	59.8% (49.9% to 68.3%)	59.9% (50.0% to 68.3%)	0.0% (0.0% to 0.0%)	58.5% (48.7% to 66.6%)
**Nutritional deficiencies**	10.0% (5.9% to 15.3%)	45.9% (33.0% to 58.8%)	99.2% (98.9% to 99.4%)	92.3% (89.2% to 94.8%)
Protein-energy malnutrition	9.9% (5.7% to 15.3%)	46.5% (33.2% to 59.6%)	97.1% (95.2% to 98.4%)	60.7% (50.9% to 69.7%)
Vitamin A deficiency	-	-	0.0% (0.0% to 0.0%)	0.0% (0.0% to 0.0%)
Iron-deficiency anaemia	100.0% (100.0% to 100.0%)	100.0% (100.0% to 100.0%)	100.0% (100.0% to 100.0%)	100.0% (100.0% to 100.0%)
**Other communicable, maternal, neonatal, and nutritional diseases**	2.3% (1.2% to 3.7%)	1.8% (0.7% to 3.8%)	68.4% (62.5% to 73.8%)	14.7% (6.8% to 28.0%)
Sexually transmitted diseases excluding HIV	10.8% (1.8% to 100.0%)	7.1% (0.8% to 100.0%)	100.0% (100.0% to 100.0%)	28.1% (8.7% to 100.0%)
Hepatitis	0.5% (0.3% to 0.7%)	0.6% (0.4% to 0.9%)	0.5% (0.3% to 0.8%)	0.6% (0.4% to 0.8%)
**Neoplasms**	55.6% (51.3% to 59.8%)	53.8% (49.6% to 57.9%)	43.2% (38.4% to 48.1%)	53.5% (49.2% to 57.7%)
Oesophageal cancer	82.9% (74.2% to 90.1%)	83.2% (74.3% to 90.4%)	82.9% (74.0% to 90.1%)	83.2% (74.3% to 90.4%)
Stomach cancer	25.2% (16.2% to 33.9%)	24.8% (16.1% to 34.0%)	24.8% (16.2% to 33.4%)	24.8% (16.1% to 33.9%)
Liver cancer	56.6% (43.5% to 67.5%)	57.9% (44.7% to 69.3%)	56.8% (43.9% to 67.6%)	57.9% (44.7% to 69.2%)
Larynx cancer	86.5% (79.6% to 91.7%)	84.6% (77.6% to 90.0%)	85.7% (78.8% to 90.9%)	84.6% (77.6% to 90.1%)
Tracheal, bronchus, and lung cancer	90.8% (88.4% to 93.0%)	89.2% (86.5% to 91.6%)	89.9% (87.3% to 92.2%)	89.2% (86.5% to 91.6%)
Breast cancer	49.2% (32.1% to 65.4%)	47.3% (31.7% to 62.2%)	47.3% (31.8% to 62.3%)	47.3% (31.7% to 62.2%)
Cervical cancer	100.0% (100.0% to 100.0%)	100.0% (100.0% to 100.0%)	100.0% (100.0% to 100.0%)	100.0% (100.0% to 100.0%)
Uterine cancer	50.9% (35.6% to 64.6%)	53.5% (39.1% to 66.1%)	54.1% (39.5% to 66.6%)	53.5% (39.2% to 66.1%)
Prostate cancer	9.3% (6.6% to 12.3%)	9.2% (6.5% to 12.1%)	9.2% (6.4% to 12.1%)	9.2% (6.5% to 12.1%)
Colon and rectum cancer	55.3% (44.7% to 66.1%)	55.1% (45.4% to 65.7%)	55.4% (45.1% to 66.0%)	55.1% (45.4% to 65.7%)
Lip and oral cavity cancer	76.6% (69.4% to 83.1%)	76.6% (69.1% to 83.2%)	74.4% (67.1% to 81.1%)	76.5% (69.1% to 83.2%)
Nasopharynx cancer	78.1% (70.2% to 84.7%)	77.1% (68.6% to 84.5%)	75.1% (65.2% to 83.0%)	77.0% (68.5% to 84.4%)

Supplementary Results Table 1. Age-standardised PAFs attributable to the joint distribution of all risk factors for all ages, both sexes combined for each GBD level 1 and level 2 cause and all causes in 2016 for Guam. Results are shown for deaths, YLLs, YLDs, and DALYs
Data in parenthesis are 95% uncertainty intervals. PAFs=population attributable fractions. GBD=Global Burden of Disease. YLLs=years of life lost. YLDs=years live with disability. DALYs=disability-adjusted life-years

	Deaths 2016	YLLs 2016	YLDs 2016	DALYs 2016
Other pharynx cancer	15.6% (6.0% to 28.1%)	17.8% (7.0% to 31.9%)	16.8% (6.6% to 30.3%)	17.8% (7.0% to 31.9%)
Gallbladder and biliary tract cancer	23.9% (13.2% to 36.2%)	25.7% (14.6% to 38.0%)	24.7% (13.7% to 36.6%)	25.7% (14.6% to 38.0%)
Pancreatic cancer	48.5% (38.1% to 59.9%)	48.5% (38.4% to 59.6%)	48.4% (38.1% to 59.7%)	48.5% (38.4% to 59.6%)
Ovarian cancer	19.7% (6.6% to 36.6%)	18.5% (6.3% to 34.2%)	17.6% (5.9% to 32.5%)	18.5% (6.2% to 34.2%)
Kidney cancer	47.1% (36.7% to 57.4%)	46.4% (35.7% to 56.5%)	46.1% (35.7% to 56.1%)	46.4% (35.7% to 56.5%)
Bladder cancer	55.0% (42.3% to 67.2%)	54.7% (42.0% to 66.7%)	54.3% (41.5% to 66.0%)	54.7% (42.0% to 66.7%)
Thyroid cancer	16.0% (8.6% to 25.4%)	17.4% (9.4% to 27.4%)	18.0% (10.3% to 27.2%)	17.5% (9.6% to 27.2%)
Mesothelioma	89.9% (86.1% to 92.9%)	82.9% (76.9% to 87.8%)	87.1% (82.5% to 90.6%)	83.0% (77.0% to 87.8%)
Non-Hodgkin's lymphoma	7.9% (3.5% to 13.3%)	7.9% (3.5% to 13.1%)	8.0% (3.6% to 13.4%)	7.9% (3.5% to 13.1%)
Multiple myeloma	9.1% (4.1% to 15.3%)	9.9% (4.5% to 16.7%)	9.7% (4.3% to 16.2%)	9.9% (4.5% to 16.7%)
Leukaemia	25.8% (16.8% to 34.6%)	20.7% (13.7% to 28.0%)	20.2% (12.7% to 28.3%)	20.7% (13.7% to 28.0%)
**Cardiovascular diseases**	87.9% (85.7% to 90.0%)	88.1% (86.7% to 89.6%)	73.6% (69.3% to 77.5%)	87.1% (85.5% to 88.7%)
Rheumatic heart disease	27.9% (19.3% to 43.8%)	24.7% (17.2% to 35.5%)	24.0% (15.7% to 34.6%)	24.4% (16.8% to 35.1%)
Ischaemic heart disease	94.5% (91.4% to 96.9%)	95.6% (94.0% to 97.0%)	93.1% (90.7% to 95.0%)	95.6% (93.9% to 97.0%)
Cerebrovascular disease	85.4% (80.9% to 89.7%)	86.7% (83.7% to 89.3%)	87.6% (83.8% to 91.2%)	86.8% (83.9% to 89.4%)
Hypertensive heart disease	100.0% (100.0% to 100.0%)	100.0% (100.0% to 100.0%)	100.0% (100.0% to 100.0%)	100.0% (100.0% to 100.0%)
Cardiomyopathy and myocarditis	36.7% (30.7% to 43.1%)	34.6% (29.2% to 40.5%)	30.5% (25.4% to 36.0%)	34.5% (29.1% to 40.3%)
Atrial fibrillation and flutter	49.1% (40.7% to 57.1%)	54.3% (46.9% to 61.0%)	62.3% (57.0% to 67.4%)	59.0% (52.7% to 64.8%)
Aortic aneurysm	47.7% (40.5% to 54.6%)	55.2% (49.2% to 60.6%)	-	55.2% (49.2% to 60.6%)
Peripheral vascular disease	64.0% (57.9% to 70.3%)	73.9% (69.5% to 78.1%)	71.3% (66.3% to 75.7%)	73.0% (68.7% to 77.3%)
Endocarditis	38.8% (30.3% to 48.2%)	37.0% (30.1% to 43.9%)	37.8% (29.9% to 46.5%)	37.0% (30.1% to 43.9%)

Supplementary Results Table 1. Age-standardised PAFs attributable to the joint distribution of all risk factors for all ages, both sexes combined for each GBD level 1 and level 2 cause and all causes in 2016 for Guam. Results are shown for deaths, YLLs, YLDs, and DALYs
Data in parenthesis are 95% uncertainty intervals. PAFs=population attributable fractions. GBD=Global Burden of Disease. YLLs=years of life lost. YLDs=years live with disability. DALYs=disability-adjusted life-years

	Deaths 2016	YLLs 2016	YLDs 2016	DALYs 2016
Other cardiovascular and circulatory diseases	48.4% (43.3% to 53.6%)	48.8% (43.9% to 53.5%)	44.8% (39.6% to 50.2%)	48.1% (43.4% to 52.5%)
**Chronic respiratory diseases**	65.7% (62.3% to 69.3%)	58.6% (55.0% to 62.4%)	59.7% (54.1% to 65.3%)	59.1% (55.1% to 62.8%)
Chronic obstructive pulmonary disease	84.9% (81.3% to 88.3%)	81.5% (77.4% to 85.2%)	79.6% (75.0% to 83.3%)	80.8% (76.5% to 84.5%)
Pneumoconiosis	100.0% (100.0% to 100.0%)	100.0% (100.0% to 100.0%)	100.0% (100.0% to 100.0%)	100.0% (100.0% to 100.0%)
Asthma	43.5% (32.4% to 55.5%)	49.9% (40.3% to 60.7%)	46.4% (37.1% to 56.7%)	47.3% (38.1% to 57.4%)
Other chronic respiratory diseases	29.6% (22.6% to 36.9%)	27.8% (20.4% to 35.2%)	12.8% (9.2% to 17.0%)	25.3% (18.4% to 32.3%)
**Cirrhosis and other chronic liver diseases**	20.1% (18.3% to 22.1%)	21.2% (19.1% to 23.6%)	15.1% (12.9% to 17.7%)	21.0% (19.0% to 23.3%)
Cirrhosis and other chronic liver diseases due to hepatitis B	0.1% (0.0% to 0.1%)	0.1% (0.0% to 0.2%)	0.1% (0.0% to 0.1%)	0.1% (0.0% to 0.2%)
Cirrhosis and other chronic liver diseases due to hepatitis C	26.7% (20.1% to 34.2%)	39.1% (30.6% to 47.9%)	32.1% (23.6% to 41.3%)	38.9% (30.5% to 47.7%)
Cirrhosis and other chronic liver diseases due to alcohol use	100.0% (100.0% to 100.0%)	100.0% (100.0% to 100.0%)	100.0% (100.0% to 100.0%)	100.0% (100.0% to 100.0%)
**Digestive diseases**	9.8% (7.2% to 12.4%)	10.5% (7.9% to 13.3%)	6.9% (5.4% to 8.7%)	9.8% (7.4% to 12.0%)
Peptic ulcer disease	12.5% (8.9% to 16.8%)	16.0% (11.5% to 21.2%)	16.7% (11.8% to 22.1%)	16.2% (11.6% to 21.2%)
Gallbladder and biliary diseases	46.2% (31.1% to 61.4%)	48.4% (34.2% to 62.1%)	55.1% (41.3% to 68.2%)	49.0% (34.9% to 62.5%)
Pancreatitis	8.1% (-3.1% to 24.5%)	11.8% (-3.0% to 34.3%)	8.0% (-3.0% to 23.3%)	11.8% (-3.0% to 34.2%)
**Neurological disorders**	27.7% (13.5% to 43.8%)	25.6% (13.0% to 39.2%)	5.9% (3.2% to 9.2%)	14.2% (7.5% to 22.4%)
Alzheimer's disease and other dementias	31.7% (15.7% to 50.0%)	33.7% (17.2% to 52.0%)	33.7% (17.2% to 52.2%)	33.7% (17.2% to 52.0%)
Parkinson's disease	-6.6% (-8.4% to -5.1%)	-9.8% (-12.1% to -7.6%)	-12.2% (-15.0% to -9.5%)	-10.3% (-12.6% to -8.0%)
Epilepsy	13.0% (3.4% to 24.7%)	13.8% (3.6% to 25.8%)	9.0% (2.2% to 17.8%)	10.3% (2.6% to 20.3%)
Multiple sclerosis	11.2% (7.2% to 15.6%)	12.1% (7.8% to 16.8%)	10.2% (6.6% to 14.1%)	11.6% (7.6% to 15.9%)
**Mental and substance use disorders**	99.9% (99.8% to 99.9%)	99.8% (99.7% to 99.8%)	15.7% (14.1% to 17.4%)	19.0% (17.1% to 21.1%)
Alcohol use disorders	100.0% (100.0% to 100.0%)	100.0% (100.0% to 100.0%)	100.0% (100.0% to 100.0%)	100.0% (100.0% to 100.0%)

Supplementary Results Table 1. Age-standardised PAFs attributable to the joint distribution of all risk factors for all ages, both sexes combined for each GBD level 1 and level 2 cause and all causes in 2016 for Guam. Results are shown for deaths, YLLs, YLDs, and DALYs
Data in parenthesis are 95% uncertainty intervals. PAFs=population attributable fractions. GBD=Global Burden of Disease. YLLs=years of life lost. YLDs=years live with disability. DALYs=disability-adjusted life-years

	Deaths 2016	YLLs 2016	YLDs 2016	DALYs 2016
Drug use disorders	100.0% (100.0% to 100.0%)	100.0% (100.0% to 100.0%)	100.0% (100.0% to 100.0%)	100.0% (100.0% to 100.0%)
Depressive disorders	-	-	11.4% (9.5% to 13.6%)	11.4% (9.5% to 13.6%)
Idiopathic developmental intellectual disability	-	-	52.0% (24.5% to 72.7%)	52.0% (24.5% to 72.7%)
**Diabetes, urogenital, blood, and endocrine diseases**	87.0% (85.8% to 88.3%)	84.5% (83.0% to 86.2%)	80.2% (77.6% to 82.6%)	82.8% (81.3% to 84.2%)
Diabetes mellitus	100.0% (100.0% to 100.0%)	100.0% (100.0% to 100.0%)	100.0% (100.0% to 100.0%)	100.0% (100.0% to 100.0%)
Chronic kidney disease	100.0% (100.0% to 100.0%)	100.0% (100.0% to 100.0%)	100.0% (100.0% to 100.0%)	100.0% (100.0% to 100.0%)
**Musculoskeletal disorders**	0.4% (0.1% to 0.7%)	0.3% (0.1% to 0.6%)	17.3% (13.4% to 21.5%)	16.8% (13.1% to 20.8%)
Rheumatoid arthritis	4.1% (1.6% to 7.0%)	5.1% (2.0% to 8.5%)	6.4% (2.6% to 10.7%)	6.3% (2.6% to 10.5%)
Osteoarthritis	-	-	32.7% (18.6% to 47.2%)	32.7% (18.6% to 47.2%)
Low back and neck pain	-	-	19.0% (15.8% to 22.8%)	19.0% (15.8% to 22.8%)
Gout	-	-	56.6% (40.2% to 72.2%)	56.6% (40.2% to 72.2%)
**Other non-communicable diseases**	0.6% (0.3% to 1.0%)	0.9% (0.5% to 1.6%)	5.9% (5.0% to 6.8%)	4.7% (4.0% to 5.4%)
Sense organ diseases	-	-	12.9% (11.4% to 14.6%)	12.9% (11.4% to 14.6%)
Sudden infant death syndrome	14.6% (7.1% to 24.6%)	14.6% (7.1% to 24.7%)	-	14.6% (7.1% to 24.7%)
**Transport injuries**	25.4% (20.0% to 32.2%)	20.5% (14.5% to 27.8%)	26.6% (21.8% to 32.1%)	21.7% (16.0% to 28.9%)
Road injuries	24.7% (19.4% to 31.5%)	20.3% (14.3% to 27.6%)	29.0% (24.3% to 34.5%)	21.7% (16.0% to 29.1%)
Other transport injuries	31.0% (25.4% to 37.8%)	22.5% (16.5% to 30.0%)	20.3% (15.7% to 25.6%)	21.6% (16.2% to 28.1%)
**Unintentional injuries**	21.3% (14.1% to 25.6%)	12.7% (10.2% to 15.7%)	12.1% (10.7% to 14.1%)	12.5% (10.4% to 15.1%)
Falls	53.5% (27.5% to 61.7%)	28.2% (20.0% to 33.4%)	22.8% (21.3% to 24.7%)	25.7% (21.2% to 28.9%)
Drowning	12.4% (8.6% to 17.5%)	12.9% (9.0% to 18.2%)	9.7% (6.6% to 14.1%)	12.8% (9.0% to 18.1%)
Fire, heat, and hot substances	7.7% (5.0% to 11.7%)	9.3% (6.5% to 13.1%)	10.0% (6.9% to 14.2%)	9.6% (6.7% to 13.6%)

Supplementary Results Table 1. Age-standardised PAFs attributable to the joint distribution of all risk factors for all ages, both sexes combined for each GBD level 1 and level 2 cause and all causes in 2016 for Guam. Results are shown for deaths, YLLs, YLDs, and DALYs
Data in parenthesis are 95% uncertainty intervals. PAFs=population attributable fractions. GBD=Global Burden of Disease. YLLs=years of life lost. YLDs=years live with disability. DALYs=disability-adjusted life-years

	Deaths 2016	YLLs 2016	YLDs 2016	DALYs 2016
Poisonings	9.2% (6.2% to 13.6%)	11.2% (7.9% to 15.5%)	10.4% (7.4% to 14.8%)	11.1% (7.8% to 15.2%)
Exposure to mechanical forces	14.7% (12.9% to 17.0%)	10.6% (8.8% to 13.0%)	15.2% (13.5% to 17.4%)	12.4% (10.6% to 14.7%)
Animal contact	17.8% (15.2% to 20.4%)	11.2% (9.4% to 13.4%)	7.3% (5.7% to 9.5%)	7.8% (6.2% to 9.9%)
Foreign body	4.1% (3.1% to 5.4%)	4.8% (3.4% to 6.5%)	5.4% (4.1% to 7.2%)	4.9% (3.6% to 6.5%)
Other unintentional injuries	12.6% (8.6% to 18.2%)	14.5% (10.3% to 20.4%)	9.3% (6.4% to 13.4%)	11.4% (8.1% to 16.2%)
**Self-harm and interpersonal violence**	14.4% (5.3% to 26.2%)	15.5% (5.7% to 28.3%)	25.7% (21.1% to 30.9%)	16.2% (6.8% to 28.3%)
Self-harm	13.1% (2.8% to 26.6%)	14.6% (3.4% to 29.6%)	10.6% (1.8% to 22.4%)	14.5% (3.4% to 29.5%)
Interpersonal violence	20.7% (13.1% to 28.3%)	19.6% (11.9% to 27.6%)	28.6% (24.1% to 33.4%)	21.7% (14.7% to 28.8%)
**Forces of nature, conflict and terrorism, and executions and police conflict**	0.0% (0.0% to 0.0%)	0.0% (0.0% to 0.0%)	0.0% (0.0% to 0.0%)	0.0% (0.0% to 0.0%)
Forces of nature, conflict and terrorism, and state actor violence	0.0% (0.0% to 0.0%)	0.0% (0.0% to 0.0%)	0.0% (0.0% to 0.0%)	0.0% (0.0% to 0.0%)

Supplementary Results Table 1. Age-standardised PAFs attributable to the joint distribution of all risk factors for all ages, both sexes combined for each GBD level 1 and level 2 cause and all causes in 2016 for Guatemala. Results are shown for deaths, YLLs, YLDs, and DALYs
Data in parenthesis are 95% uncertainty intervals. PAFs=population attributable fractions. GBD=Global Burden of Disease. YLLs=years of life lost. YLDs=years live with disability. DALYs=disability-adjusted life-years

	Deaths 2016	YLLs 2016	YLDs 2016	DALYs 2016
**All causes**	57.3% (55.4% to 59.2%)	55.0% (53.2% to 57.0%)	25.9% (24.3% to 27.4%)	45.7% (43.2% to 48.1%)
**HIV/AIDS and tuberculosis**	71.4% (65.3% to 77.8%)	72.4% (67.6% to 77.6%)	88.1% (83.0% to 92.4%)	73.4% (68.8% to 78.3%)
Tuberculosis	39.7% (28.2% to 54.4%)	39.3% (28.0% to 53.6%)	36.6% (26.4% to 50.8%)	39.2% (27.9% to 53.5%)
HIV/AIDS	92.6% (91.9% to 93.2%)	87.6% (86.4% to 88.6%)	97.2% (96.0% to 98.1%)	88.3% (87.1% to 89.4%)
**Diarrhoea, lower respiratory infections, and other common infectious diseases**	66.5% (59.8% to 73.1%)	73.8% (68.6% to 78.8%)	48.4% (39.8% to 56.2%)	71.7% (66.3% to 76.5%)
Diarrhoeal diseases	85.6% (70.3% to 95.4%)	89.8% (79.2% to 96.8%)	87.2% (73.4% to 95.8%)	89.4% (78.3% to 96.6%)
Lower respiratory infections	64.6% (56.5% to 72.6%)	74.3% (67.3% to 80.3%)	67.3% (59.4% to 74.5%)	74.2% (67.3% to 80.3%)
Upper respiratory infections	1.2% (0.1% to 4.1%)	2.3% (0.2% to 8.0%)	0.0% (0.0% to 0.0%)	0.0% (0.0% to 0.1%)
Otitis media	1.3% (0.7% to 2.3%)	3.2% (1.9% to 4.8%)	3.8% (2.5% to 5.0%)	3.8% (2.5% to 5.0%)
Meningitis	0.7% (0.5% to 1.0%)	1.3% (0.9% to 1.7%)	0.0% (0.0% to 0.0%)	1.2% (0.8% to 1.6%)
Encephalitis	1.1% (0.6% to 1.8%)	1.6% (1.0% to 2.5%)	0.0% (0.0% to 0.0%)	1.3% (0.7% to 2.1%)
Measles	34.1% (18.8% to 55.8%)	37.1% (20.9% to 59.8%)	25.1% (0.0% to 74.6%)	37.1% (20.9% to 59.8%)
**Maternal disorders**	7.0% (2.7% to 11.4%)	6.9% (2.7% to 11.3%)	3.5% (1.8% to 5.8%)	6.5% (2.7% to 10.7%)
Maternal haemorrhage	14.0% (5.0% to 22.9%)	13.9% (4.9% to 22.8%)	13.7% (4.9% to 22.4%)	13.9% (4.9% to 22.7%)
Maternal sepsis and other pregnancy related infections	13.8% (4.9% to 22.6%)	13.7% (4.9% to 22.5%)	13.5% (4.9% to 22.1%)	13.7% (4.9% to 22.4%)
Maternal abortion, miscarriage, and ectopic pregnancy	6.6% (4.1% to 9.6%)	6.5% (4.0% to 9.5%)	6.7% (3.7% to 10.2%)	6.6% (4.0% to 9.5%)
**Neonatal disorders**	75.9% (73.2% to 78.6%)	76.0% (73.2% to 78.6%)	0.1% (0.1% to 0.2%)	70.4% (66.5% to 73.9%)
Neonatal preterm birth complications	94.7% (92.7% to 96.4%)	94.8% (92.8% to 96.5%)	0.3% (0.2% to 0.4%)	87.8% (83.8% to 91.2%)
Neonatal encephalopathy due to birth asphyxia and trauma	64.7% (60.8% to 68.2%)	64.7% (60.9% to 68.3%)	0.0% (0.0% to 0.0%)	60.6% (53.8% to 65.7%)
Neonatal sepsis and other neonatal infections	56.3% (51.4% to 60.6%)	56.3% (51.5% to 60.7%)	0.0% (0.0% to 0.0%)	52.5% (46.0% to 58.0%)
Hemolytic disease and other neonatal jaundice	53.3% (44.5% to 60.9%)	53.4% (44.6% to 61.0%)	0.0% (0.0% to 0.0%)	18.4% (11.5% to 28.4%)

Supplementary Results Table 1. Age-standardised PAFs attributable to the joint distribution of all risk factors for all ages, both sexes combined for each GBD level 1 and level 2 cause and all causes in 2016 for Guatemala. Results are shown for deaths, YLLs, YLDs, and DALYs
Data in parenthesis are 95% uncertainty intervals. PAFs=population attributable fractions. GBD=Global Burden of Disease. YLLs=years of life lost. YLDs=years live with disability. DALYs=disability-adjusted life-years

	Deaths 2016	YLLs 2016	YLDs 2016	DALYs 2016
Other neonatal disorders	59.7% (54.8% to 64.1%)	59.7% (54.8% to 64.1%)	0.0% (0.0% to 0.0%)	58.5% (53.5% to 63.1%)
**Nutritional deficiencies**	6.5% (4.7% to 8.7%)	28.0% (21.9% to 35.2%)	78.7% (74.6% to 82.6%)	39.6% (32.7% to 47.0%)
Protein-energy malnutrition	6.8% (5.0% to 9.2%)	30.0% (23.5% to 37.8%)	96.0% (93.4% to 97.7%)	30.6% (24.1% to 38.4%)
Vitamin A deficiency	-	-	100.0% (100.0% to 100.0%)	100.0% (100.0% to 100.0%)
Iron-deficiency anaemia	100.0% (100.0% to 100.0%)	100.0% (100.0% to 100.0%)	100.0% (100.0% to 100.0%)	100.0% (100.0% to 100.0%)
**Other communicable, maternal, neonatal, and nutritional diseases**	8.4% (6.0% to 11.1%)	6.2% (3.9% to 9.0%)	69.1% (62.2% to 75.3%)	22.1% (14.8% to 31.5%)
Sexually transmitted diseases excluding HIV	24.0% (12.0% to 39.4%)	12.2% (5.4% to 22.4%)	100.0% (100.0% to 100.0%)	41.1% (23.2% to 60.8%)
Hepatitis	3.6% (2.6% to 4.9%)	3.4% (2.5% to 4.7%)	2.3% (1.3% to 3.9%)	3.4% (2.4% to 4.6%)
**Neoplasms**	29.0% (25.6% to 32.8%)	29.9% (26.4% to 33.7%)	30.2% (26.9% to 33.6%)	30.0% (26.4% to 33.7%)
Oesophageal cancer	53.3% (38.1% to 67.0%)	55.3% (39.9% to 68.9%)	53.7% (38.7% to 67.6%)	55.3% (39.9% to 68.9%)
Stomach cancer	2.5% (0.5% to 7.4%)	2.5% (0.5% to 6.5%)	2.4% (0.5% to 6.9%)	2.5% (0.5% to 6.5%)
Liver cancer	50.3% (41.8% to 58.8%)	50.6% (42.4% to 59.3%)	50.5% (42.1% to 59.2%)	50.6% (42.5% to 59.3%)
Larynx cancer	44.3% (27.4% to 59.6%)	46.0% (29.8% to 60.5%)	44.5% (28.3% to 59.1%)	46.0% (29.8% to 60.5%)
Tracheal, bronchus, and lung cancer	66.1% (56.2% to 75.7%)	65.5% (55.7% to 74.8%)	65.7% (55.7% to 75.1%)	65.5% (55.7% to 74.8%)
Breast cancer	20.7% (9.0% to 35.0%)	18.7% (6.6% to 33.3%)	19.6% (7.6% to 34.2%)	18.7% (6.6% to 33.3%)
Cervical cancer	100.0% (100.0% to 100.0%)	100.0% (100.0% to 100.0%)	100.0% (100.0% to 100.0%)	100.0% (100.0% to 100.0%)
Uterine cancer	44.8% (31.2% to 59.0%)	46.6% (32.7% to 61.0%)	46.9% (32.9% to 61.1%)	46.6% (32.7% to 61.0%)
Prostate cancer	0.6% (0.1% to 1.1%)	0.6% (0.1% to 1.1%)	0.6% (0.2% to 1.2%)	0.6% (0.1% to 1.1%)
Colon and rectum cancer	48.2% (38.1% to 58.4%)	48.4% (38.4% to 58.0%)	48.4% (38.3% to 58.2%)	48.4% (38.4% to 58.0%)
Lip and oral cavity cancer	48.2% (37.4% to 59.0%)	51.4% (40.5% to 62.0%)	48.9% (38.5% to 59.2%)	51.4% (40.4% to 62.0%)
Nasopharynx cancer	49.2% (41.0% to 57.3%)	49.9% (42.6% to 57.1%)	49.3% (42.5% to 56.2%)	49.9% (42.7% to 57.1%)

Supplementary Results Table 1. Age-standardised PAFs attributable to the joint distribution of all risk factors for all ages, both sexes combined for each GBD level 1 and level 2 cause and all causes in 2016 for Guatemala. Results are shown for deaths, YLLs, YLDs, and DALYs
Data in parenthesis are 95% uncertainty intervals. PAFs=population attributable fractions. GBD=Global Burden of Disease. YLLs=years of life lost. YLDs=years live with disability. DALYs=disability-adjusted life-years

	Deaths 2016	YLLs 2016	YLDs 2016	DALYs 2016
Other pharynx cancer	41.1% (29.9% to 52.8%)	43.9% (32.7% to 55.6%)	42.1% (30.9% to 53.8%)	43.8% (32.7% to 55.6%)
Gallbladder and biliary tract cancer	22.1% (12.3% to 34.0%)	23.7% (13.3% to 36.1%)	22.6% (12.7% to 34.1%)	23.7% (13.3% to 36.0%)
Pancreatic cancer	16.9% (8.7% to 28.2%)	17.0% (8.7% to 28.0%)	16.9% (8.7% to 28.1%)	17.0% (8.7% to 28.0%)
Ovarian cancer	11.6% (3.6% to 21.9%)	10.9% (3.3% to 20.4%)	10.6% (3.3% to 19.9%)	10.9% (3.3% to 20.4%)
Kidney cancer	21.2% (13.4% to 30.9%)	20.8% (13.1% to 30.0%)	22.2% (14.3% to 31.6%)	20.8% (13.2% to 30.0%)
Bladder cancer	12.6% (4.7% to 23.2%)	12.0% (4.4% to 22.0%)	12.2% (4.7% to 22.4%)	12.0% (4.4% to 22.0%)
Thyroid cancer	12.8% (6.9% to 20.5%)	13.5% (7.3% to 21.5%)	13.4% (7.4% to 21.0%)	13.5% (7.3% to 21.5%)
Mesothelioma	71.7% (63.0% to 79.6%)	60.0% (49.5% to 69.3%)	67.3% (58.4% to 76.0%)	60.1% (49.6% to 69.3%)
Non-Hodgkin's lymphoma	5.8% (2.3% to 10.4%)	5.3% (2.2% to 9.3%)	5.9% (2.3% to 10.4%)	5.3% (2.2% to 9.4%)
Multiple myeloma	7.7% (3.2% to 13.8%)	8.1% (3.5% to 14.3%)	7.9% (3.3% to 14.1%)	8.1% (3.5% to 14.3%)
Leukaemia	9.5% (5.5% to 14.2%)	8.2% (4.8% to 12.3%)	9.6% (5.6% to 14.3%)	8.2% (4.8% to 12.3%)
**Cardiovascular diseases**	85.0% (82.0% to 87.5%)	82.9% (80.7% to 84.7%)	62.4% (58.9% to 65.8%)	80.6% (78.1% to 82.8%)
Rheumatic heart disease	21.0% (13.5% to 34.7%)	17.3% (11.5% to 27.0%)	14.8% (9.1% to 21.9%)	15.6% (10.0% to 23.7%)
Ischaemic heart disease	91.6% (87.6% to 94.8%)	92.4% (89.9% to 94.5%)	89.8% (86.5% to 92.7%)	92.3% (89.8% to 94.4%)
Cerebrovascular disease	81.1% (76.4% to 85.9%)	80.4% (77.1% to 83.5%)	82.9% (78.4% to 87.4%)	80.6% (77.4% to 83.9%)
Hypertensive heart disease	100.0% (100.0% to 100.0%)	100.0% (100.0% to 100.0%)	100.0% (100.0% to 100.0%)	100.0% (100.0% to 100.0%)
Cardiomyopathy and myocarditis	28.0% (22.3% to 33.8%)	22.2% (16.0% to 27.6%)	20.0% (16.1% to 24.3%)	22.1% (16.2% to 27.1%)
Atrial fibrillation and flutter	45.7% (38.1% to 53.1%)	49.4% (42.2% to 56.0%)	54.4% (48.5% to 60.3%)	52.3% (46.3% to 58.5%)
Aortic aneurysm	32.7% (25.9% to 40.0%)	35.8% (29.9% to 41.6%)	-	35.8% (29.9% to 41.6%)
Peripheral vascular disease	51.4% (44.4% to 58.8%)	57.8% (51.9% to 63.7%)	61.7% (56.7% to 66.7%)	61.3% (56.5% to 66.1%)
Endocarditis	26.9% (20.1% to 34.2%)	18.1% (12.7% to 24.5%)	28.6% (22.3% to 35.5%)	18.2% (12.9% to 24.6%)

Supplementary Results Table 1. Age-standardised PAFs attributable to the joint distribution of all risk factors for all ages, both sexes combined for each GBD level 1 and level 2 cause and all causes in 2016 for Guatemala. Results are shown for deaths, YLLs, YLDs, and DALYs
Data in parenthesis are 95% uncertainty intervals. PAFs=population attributable fractions. GBD=Global Burden of Disease. YLLs=years of life lost. YLDs=years live with disability. DALYs=disability-adjusted life-years

	Deaths 2016	YLLs 2016	YLDs 2016	DALYs 2016
Other cardiovascular and circulatory diseases	34.7% (29.9% to 39.3%)	29.6% (25.1% to 34.1%)	30.6% (25.8% to 35.6%)	29.9% (25.7% to 34.5%)
**Chronic respiratory diseases**	51.6% (42.2% to 60.3%)	46.0% (37.9% to 53.6%)	33.8% (26.5% to 41.9%)	40.9% (33.6% to 48.4%)
Chronic obstructive pulmonary disease	61.5% (49.6% to 72.6%)	59.3% (48.0% to 70.2%)	50.8% (38.6% to 62.0%)	57.4% (46.2% to 68.4%)
Pneumoconiosis	100.0% (100.0% to 100.0%)	100.0% (100.0% to 100.0%)	100.0% (100.0% to 100.0%)	100.0% (100.0% to 100.0%)
Asthma	33.3% (23.4% to 44.5%)	33.9% (25.0% to 44.2%)	28.1% (19.4% to 38.3%)	29.7% (21.0% to 39.7%)
Other chronic respiratory diseases	1.5% (0.4% to 3.0%)	1.0% (0.2% to 2.2%)	0.8% (0.1% to 1.9%)	0.9% (0.1% to 2.1%)
**Cirrhosis and other chronic liver diseases**	70.2% (65.8% to 73.7%)	70.9% (67.1% to 74.0%)	60.6% (57.0% to 64.3%)	70.7% (66.9% to 73.7%)
Cirrhosis and other chronic liver diseases due to hepatitis B	0.7% (0.3% to 1.3%)	0.8% (0.3% to 1.5%)	0.6% (0.3% to 1.1%)	0.8% (0.3% to 1.5%)
Cirrhosis and other chronic liver diseases due to hepatitis C	74.4% (64.2% to 81.3%)	80.1% (72.9% to 84.6%)	69.5% (59.6% to 76.0%)	79.9% (72.6% to 84.4%)
Cirrhosis and other chronic liver diseases due to alcohol use	100.0% (100.0% to 100.0%)	100.0% (100.0% to 100.0%)	100.0% (100.0% to 100.0%)	100.0% (100.0% to 100.0%)
**Digestive diseases**	10.2% (7.6% to 13.1%)	11.7% (8.7% to 15.2%)	2.4% (1.7% to 3.1%)	9.8% (7.2% to 12.8%)
Peptic ulcer disease	4.5% (2.8% to 6.5%)	4.8% (3.0% to 7.1%)	2.5% (1.5% to 3.7%)	4.4% (2.8% to 6.3%)
Gallbladder and biliary diseases	41.0% (27.2% to 55.7%)	42.4% (28.8% to 57.1%)	48.0% (33.7% to 63.0%)	43.0% (29.5% to 57.9%)
Pancreatitis	54.2% (35.9% to 73.8%)	55.8% (37.8% to 74.4%)	51.1% (34.1% to 69.1%)	55.8% (37.8% to 74.3%)
**Neurological disorders**	20.9% (10.3% to 33.3%)	19.2% (10.8% to 29.5%)	4.0% (2.1% to 6.1%)	9.3% (5.0% to 14.3%)
Alzheimer's disease and other dementias	22.9% (10.8% to 36.9%)	23.7% (11.3% to 38.1%)	23.7% (11.4% to 38.4%)	23.7% (11.4% to 38.1%)
Parkinson's disease	-3.1% (-4.4% to -2.2%)	-3.4% (-4.7% to -2.4%)	-3.5% (-4.6% to -2.6%)	-3.5% (-4.6% to -2.5%)
Epilepsy	16.1% (8.0% to 25.6%)	14.8% (7.5% to 23.6%)	13.6% (6.9% to 21.8%)	14.2% (7.1% to 22.6%)
Multiple sclerosis	2.6% (1.5% to 4.2%)	2.7% (1.4% to 4.3%)	1.8% (1.1% to 2.7%)	2.4% (1.3% to 3.7%)
**Mental and substance use disorders**	99.8% (99.7% to 99.9%)	99.7% (99.6% to 99.8%)	24.5% (21.9% to 27.3%)	42.0% (36.2% to 48.9%)
Alcohol use disorders	100.0% (100.0% to 100.0%)	100.0% (100.0% to 100.0%)	100.0% (100.0% to 100.0%)	100.0% (100.0% to 100.0%)

**Supplementary Results Table 1. Age-standardised PAFs attributable to the joint distribution of all risk factors for all ages, both sexes combined for each GBD level 1 and level 2 cause and all causes in 2016 for Guatemala. Results are shown for deaths, YLLs, YLDs, and DALYs** Data in parenthesis are 95% uncertainty intervals. PAFs=population attributable fractions. GBD=Global Burden of Disease. YLLs=years of life lost. YLDs=years live with disability. DALYs=disability-adjusted life-years	**Deaths 2016**	**YLLs 2016**	**YLDs 2016**	**DALYs 2016**
Drug use disorders	100.0% (100.0% to 100.0%)	100.0% (100.0% to 100.0%)	100.0% (100.0% to 100.0%)	100.0% (100.0% to 100.0%)
Depressive disorders	-	-	7.4% (6.1% to 8.9%)	7.4% (6.1% to 8.9%)
Idiopathic developmental intellectual disability	-	-	70.9% (39.8% to 89.7%)	70.9% (39.8% to 89.7%)
**Diabetes, urogenital, blood, and endocrine diseases**	95.0% (92.6% to 95.5%)	93.6% (90.4% to 94.3%)	81.3% (77.8% to 84.1%)	89.9% (87.3% to 91.4%)
Diabetes mellitus	100.0% (100.0% to 100.0%)	100.0% (100.0% to 100.0%)	100.0% (100.0% to 100.0%)	100.0% (100.0% to 100.0%)
Chronic kidney disease	100.0% (100.0% to 100.0%)	100.0% (100.0% to 100.0%)	100.0% (100.0% to 100.0%)	100.0% (100.0% to 100.0%)
**Musculoskeletal disorders**	0.5% (0.2% to 1.1%)	0.4% (0.1% to 0.8%)	16.1% (13.3% to 19.3%)	15.7% (13.0% to 18.7%)
Rheumatoid arthritis	1.4% (0.5% to 2.6%)	1.4% (0.5% to 2.6%)	1.3% (0.5% to 2.3%)	1.3% (0.5% to 2.3%)
Osteoarthritis	-	-	26.0% (14.2% to 38.6%)	26.0% (14.2% to 38.6%)
Low back and neck pain	-	-	19.8% (17.0% to 22.7%)	19.8% (17.0% to 22.7%)
Gout	-	-	51.5% (38.9% to 64.5%)	51.5% (38.9% to 64.5%)
**Other non-communicable diseases**	0.5% (0.3% to 0.8%)	0.6% (0.4% to 1.0%)	7.4% (6.2% to 8.8%)	5.9% (5.0% to 7.0%)
Sense organ diseases	-	-	15.4% (13.3% to 17.7%)	15.4% (13.3% to 17.7%)
Sudden infant death syndrome	8.2% (5.3% to 11.8%)	8.2% (5.3% to 11.9%)	-	8.2% (5.3% to 11.9%)
**Transport injuries**	43.1% (34.0% to 53.4%)	42.1% (32.4% to 53.3%)	45.4% (37.0% to 55.1%)	42.4% (32.8% to 53.6%)
Road injuries	43.1% (33.8% to 53.5%)	42.2% (32.4% to 53.5%)	45.7% (37.2% to 55.4%)	42.5% (32.8% to 53.8%)
Other transport injuries	44.4% (35.9% to 54.0%)	41.2% (32.5% to 51.4%)	37.5% (30.0% to 46.3%)	40.9% (32.3% to 50.8%)
**Unintentional injuries**	32.0% (26.6% to 38.6%)	27.8% (22.2% to 34.9%)	31.7% (26.7% to 38.8%)	28.8% (23.4% to 36.0%)
Falls	56.3% (49.9% to 63.8%)	44.0% (36.4% to 54.0%)	35.8% (31.5% to 41.7%)	39.3% (34.2% to 46.5%)
Drowning	30.1% (20.5% to 40.9%)	31.0% (21.3% to 41.6%)	19.6% (13.4% to 27.5%)	30.8% (21.2% to 41.2%)
Fire, heat, and hot substances	18.5% (14.0% to 24.2%)	21.4% (16.6% to 27.3%)	25.3% (18.9% to 33.8%)	22.4% (17.1% to 28.6%)

Supplementary Results Table 1. Age-standardised PAFs attributable to the joint distribution of all risk factors for all ages, both sexes combined for each GBD level 1 and level 2 cause and all causes in 2016 for Guatemala. Results are shown for deaths, YLLs, YLDs, and DALYs
Data in parenthesis are 95% uncertainty intervals. PAFs=population attributable fractions. GBD=Global Burden of Disease. YLLs=years of life lost. YLDs=years live with disability. DALYs=disability-adjusted life-years

	**Deaths 2016**	**YLLs 2016**	**YLDs 2016**	**DALYs 2016**
Poisonings	29.2% (22.1% to 38.2%)	29.7% (21.7% to 38.9%)	25.3% (18.7% to 33.8%)	29.6% (21.7% to 38.6%)
Exposure to mechanical forces	33.3% (25.7% to 44.1%)	33.1% (24.8% to 44.2%)	34.4% (26.9% to 46.2%)	33.5% (25.8% to 44.5%)
Animal contact	19.3% (14.5% to 24.6%)	18.7% (12.5% to 24.6%)	22.8% (16.8% to 31.2%)	20.8% (15.7% to 27.4%)
Foreign body	18.4% (12.4% to 24.5%)	16.9% (10.7% to 22.9%)	18.1% (13.1% to 25.3%)	17.1% (11.3% to 22.9%)
Other unintentional injuries	31.0% (23.6% to 41.2%)	33.9% (25.7% to 44.9%)	24.7% (18.3% to 33.9%)	30.7% (23.4% to 40.4%)
**Self-harm and interpersonal violence**	13.4% (6.9% to 21.6%)	12.8% (6.5% to 20.7%)	14.4% (9.1% to 20.9%)	12.9% (6.6% to 20.7%)
Self-harm	22.0% (13.7% to 31.2%)	22.3% (14.1% to 31.2%)	19.6% (11.7% to 28.3%)	22.3% (14.1% to 31.1%)
Interpersonal violence	11.8% (4.2% to 21.0%)	11.1% (4.0% to 20.1%)	14.2% (8.7% to 20.9%)	11.2% (4.1% to 20.1%)
**Forces of nature, conflict and terrorism, and executions and police conflict**	0.0% (0.0% to 0.0%)	0.0% (0.0% to 0.0%)	0.4% (0.3% to 0.6%)	0.4% (0.3% to 0.6%)
Forces of nature, conflict and terrorism, and state actor violence	0.0% (0.0% to 0.0%)	0.0% (0.0% to 0.0%)	5.2% (3.5% to 7.4%)	5.2% (3.5% to 7.4%)

Supplementary Results Table 1. Age-standardised PAFs attributable to the joint distribution of all risk factors for all ages, both sexes combined for each GBD level 1 and level 2 cause and all causes in 2016 for Guinea. Results are shown for deaths, YLLs, YLDs, and DALYs
Data in parenthesis are 95% uncertainty intervals. PAFs=population attributable fractions. GBD=Global Burden of Disease. YLLs=years of life lost. YLDs=years live with disability. DALYs=disability-adjusted life-years

	Deaths 2016	YLLs 2016	YLDs 2016	DALYs 2016
**All causes**	53.4% (51.5% to 55.5%)	48.0% (45.8% to 50.3%)	27.6% (26.2% to 28.9%)	43.9% (41.7% to 46.1%)
**HIV/AIDS and tuberculosis**	39.9% (31.6% to 48.6%)	51.8% (42.9% to 60.7%)	75.3% (68.6% to 81.1%)	52.7% (44.3% to 61.2%)
Tuberculosis	10.4% (3.2% to 17.3%)	10.7% (3.4% to 17.8%)	10.2% (3.1% to 16.9%)	10.7% (3.4% to 17.8%)
HIV/AIDS	92.9% (90.6% to 94.5%)	90.5% (87.8% to 92.6%)	92.6% (90.5% to 94.4%)	90.6% (88.0% to 92.7%)
**Diarrhoea, lower respiratory infections, and other common infectious diseases**	72.6% (67.1% to 78.1%)	70.0% (62.3% to 76.0%)	43.5% (38.4% to 48.0%)	68.9% (61.6% to 74.7%)
Diarrhoeal diseases	95.0% (87.1% to 98.8%)	96.4% (90.7% to 99.1%)	95.4% (88.1% to 98.8%)	96.3% (90.6% to 99.1%)
Lower respiratory infections	79.6% (73.6% to 85.0%)	85.9% (81.1% to 90.0%)	79.0% (73.0% to 84.5%)	85.9% (81.1% to 89.9%)
Upper respiratory infections	1.7% (0.1% to 6.5%)	3.2% (0.2% to 12.0%)	0.0% (0.0% to 0.0%)	0.3% (0.0% to 1.3%)
Otitis media	0.7% (0.2% to 2.0%)	3.2% (1.1% to 6.2%)	4.0% (2.6% to 5.4%)	4.0% (2.6% to 5.4%)
Meningitis	2.4% (1.3% to 4.0%)	4.5% (2.5% to 7.0%)	0.0% (0.0% to 0.0%)	4.3% (2.4% to 6.7%)
Encephalitis	0.8% (0.5% to 1.1%)	1.4% (0.9% to 1.9%)	0.0% (0.0% to 0.0%)	0.9% (0.6% to 1.3%)
Measles	61.1% (44.3% to 81.6%)	62.1% (45.2% to 82.8%)	61.0% (42.9% to 81.4%)	62.1% (45.2% to 82.8%)
**Maternal disorders**	9.6% (4.9% to 14.3%)	9.5% (4.9% to 14.4%)	1.4% (0.6% to 2.4%)	9.2% (4.8% to 13.9%)
Maternal haemorrhage	18.7% (6.7% to 30.7%)	18.8% (6.7% to 30.7%)	18.8% (6.8% to 30.6%)	18.8% (6.7% to 30.7%)
Maternal sepsis and other pregnancy related infections	18.5% (6.7% to 30.2%)	18.6% (6.7% to 30.4%)	18.5% (6.6% to 30.2%)	18.6% (6.7% to 30.4%)
Maternal abortion, miscarriage, and ectopic pregnancy	16.1% (9.7% to 23.2%)	16.0% (9.5% to 23.3%)	16.2% (9.1% to 24.2%)	16.0% (9.5% to 23.3%)
**Neonatal disorders**	80.0% (75.9% to 83.7%)	80.1% (75.9% to 83.7%)	0.6% (0.3% to 1.2%)	77.9% (73.9% to 81.9%)
Neonatal preterm birth complications	96.4% (92.7% to 98.7%)	96.5% (92.8% to 98.7%)	2.0% (1.3% to 2.9%)	94.0% (90.1% to 96.7%)
Neonatal encephalopathy due to birth asphyxia and trauma	77.0% (72.8% to 80.9%)	77.0% (72.8% to 80.9%)	0.0% (0.0% to 0.0%)	75.6% (70.9% to 80.0%)
Neonatal sepsis and other neonatal infections	66.4% (59.9% to 71.7%)	66.4% (60.0% to 71.7%)	0.0% (0.0% to 0.0%)	63.3% (54.7% to 69.9%)
Hemolytic disease and other neonatal jaundice	63.9% (52.7% to 73.0%)	63.9% (52.8% to 73.0%)	0.0% (0.0% to 0.0%)	60.6% (49.8% to 69.7%)

Supplementary Results Table 1. Age-standardised PAFs attributable to the joint distribution of all risk factors for all ages, both sexes combined for each GBD level 1 and level 2 cause and all causes in 2016 for Guinea. Results are shown for deaths, YLLs, YLDs, and DALYs
Data in parenthesis are 95% uncertainty intervals. PAFs=population attributable fractions. GBD=Global Burden of Disease. YLLs=years of life lost. YLDs=years live with disability. DALYs=disability-adjusted life-years

	Deaths 2016	YLLs 2016	YLDs 2016	DALYs 2016
Other neonatal disorders	64.8% (54.6% to 72.9%)	64.8% (54.7% to 72.9%)	0.0% (0.0% to 0.0%)	63.2% (53.4% to 71.3%)
**Nutritional deficiencies**	31.9% (21.2% to 43.9%)	65.0% (51.7% to 75.8%)	83.8% (81.5% to 86.1%)	75.4% (70.1% to 79.9%)
Protein-energy malnutrition	36.4% (24.5% to 49.6%)	71.6% (59.5% to 81.6%)	98.4% (97.7% to 99.0%)	72.7% (61.7% to 82.0%)
Vitamin A deficiency	-	-	100.0% (100.0% to 100.0%)	100.0% (100.0% to 100.0%)
Iron-deficiency anaemia	100.0% (100.0% to 100.0%)	100.0% (100.0% to 100.0%)	100.0% (100.0% to 100.0%)	100.0% (100.0% to 100.0%)
**Other communicable, maternal, neonatal, and nutritional diseases**	2.8% (1.7% to 4.1%)	2.0% (1.1% to 3.2%)	61.5% (56.2% to 67.2%)	9.8% (6.0% to 15.1%)
Sexually transmitted diseases excluding HIV	6.8% (3.1% to 12.4%)	3.5% (1.5% to 6.3%)	100.0% (100.0% to 100.0%)	16.6% (8.1% to 28.2%)
Hepatitis	0.0% (0.0% to 0.1%)	0.1% (0.0% to 0.1%)	0.1% (0.0% to 0.1%)	0.1% (0.0% to 0.1%)
**Neoplasms**	32.7% (29.3% to 36.2%)	35.5% (31.7% to 39.4%)	37.1% (32.8% to 41.0%)	35.5% (31.7% to 39.3%)
Oesophageal cancer	49.3% (36.9% to 61.5%)	53.9% (42.1% to 66.1%)	51.0% (38.7% to 62.7%)	53.9% (42.0% to 66.1%)
Stomach cancer	9.0% (3.6% to 17.4%)	11.7% (5.1% to 20.2%)	10.0% (4.4% to 18.2%)	11.7% (5.1% to 20.2%)
Liver cancer	30.3% (21.9% to 39.0%)	32.8% (22.9% to 43.1%)	31.3% (22.7% to 40.1%)	32.8% (22.9% to 43.1%)
Larynx cancer	49.6% (36.4% to 62.5%)	57.0% (43.4% to 69.5%)	51.8% (39.1% to 64.0%)	56.9% (43.4% to 69.5%)
Tracheal, bronchus, and lung cancer	84.1% (78.8% to 88.7%)	86.2% (81.3% to 90.3%)	85.0% (79.9% to 89.3%)	86.2% (81.3% to 90.3%)
Breast cancer	13.4% (8.0% to 20.0%)	12.0% (6.8% to 18.0%)	11.9% (6.5% to 17.8%)	12.0% (6.8% to 18.0%)
Cervical cancer	100.0% (100.0% to 100.0%)	100.0% (100.0% to 100.0%)	100.0% (100.0% to 100.0%)	100.0% (100.0% to 100.0%)
Uterine cancer	28.8% (17.0% to 41.5%)	30.0% (18.1% to 42.5%)	29.1% (17.3% to 41.4%)	30.0% (18.1% to 42.5%)
Prostate cancer	1.2% (0.7% to 1.9%)	1.7% (1.0% to 2.5%)	1.4% (0.8% to 2.1%)	1.7% (1.0% to 2.5%)
Colon and rectum cancer	43.7% (35.0% to 52.7%)	43.8% (35.3% to 52.6%)	43.7% (35.1% to 52.7%)	43.8% (35.3% to 52.6%)
Lip and oral cavity cancer	46.8% (37.2% to 56.2%)	48.8% (39.2% to 58.6%)	44.6% (35.1% to 54.2%)	48.8% (39.1% to 58.6%)
Nasopharynx cancer	39.3% (29.7% to 49.3%)	42.3% (31.5% to 53.2%)	43.3% (30.8% to 54.9%)	42.3% (31.5% to 53.2%)

Supplementary Results Table 1. Age-standardised PAFs attributable to the joint distribution of all risk factors for all ages, both sexes combined for each GBD level 1 and level 2 cause and all causes in 2016 for Guinea. Results are shown for deaths, YLLs, YLDs, and DALYs
Data in parenthesis are 95% uncertainty intervals. PAFs=population attributable fractions. GBD=Global Burden of Disease. YLLs=years of life lost. YLDs=years live with disability. DALYs=disability-adjusted life-years

	Deaths 2016	YLLs 2016	YLDs 2016	DALYs 2016
Other pharynx cancer	12.1% (5.5% to 20.6%)	12.6% (5.9% to 21.2%)	12.3% (5.7% to 20.7%)	12.6% (5.9% to 21.2%)
Gallbladder and biliary tract cancer	13.7% (6.4% to 23.9%)	14.6% (7.2% to 24.7%)	13.8% (6.6% to 24.0%)	14.6% (7.2% to 24.7%)
Pancreatic cancer	13.6% (8.5% to 20.4%)	16.0% (10.4% to 22.6%)	14.3% (9.2% to 20.9%)	16.0% (10.4% to 22.6%)
Ovarian cancer	9.3% (4.3% to 16.1%)	7.6% (3.3% to 13.6%)	7.6% (3.4% to 13.4%)	7.6% (3.3% to 13.6%)
Kidney cancer	18.9% (12.0% to 26.9%)	21.3% (13.5% to 30.1%)	20.8% (13.3% to 29.5%)	21.3% (13.5% to 30.1%)
Bladder cancer	15.6% (9.5% to 23.4%)	20.0% (12.5% to 28.3%)	17.9% (11.4% to 25.6%)	20.0% (12.5% to 28.2%)
Thyroid cancer	6.4% (3.0% to 11.6%)	6.5% (3.0% to 11.5%)	6.4% (2.9% to 11.3%)	6.5% (3.0% to 11.5%)
Mesothelioma	86.3% (81.5% to 90.8%)	77.4% (70.5% to 84.6%)	82.1% (76.5% to 87.8%)	77.5% (70.6% to 84.7%)
Non-Hodgkin's lymphoma	2.5% (0.8% to 5.0%)	2.3% (0.8% to 4.6%)	2.4% (0.8% to 4.8%)	2.3% (0.8% to 4.6%)
Multiple myeloma	4.0% (1.4% to 8.0%)	4.1% (1.5% to 8.2%)	4.0% (1.4% to 8.0%)	4.1% (1.5% to 8.2%)
Leukaemia	9.9% (5.6% to 15.0%)	10.3% (5.9% to 16.1%)	9.9% (5.7% to 14.9%)	10.3% (5.9% to 16.1%)
**Cardiovascular diseases**	84.5% (82.1% to 86.8%)	84.9% (82.8% to 86.9%)	61.7% (57.9% to 65.3%)	83.2% (81.1% to 85.2%)
Rheumatic heart disease	27.4% (18.1% to 44.4%)	25.6% (17.7% to 37.8%)	24.6% (15.8% to 35.3%)	25.5% (17.9% to 38.2%)
Ischaemic heart disease	93.2% (90.2% to 95.4%)	94.5% (92.7% to 96.1%)	89.8% (87.1% to 92.4%)	94.4% (92.6% to 96.0%)
Cerebrovascular disease	85.2% (81.3% to 88.9%)	87.0% (84.2% to 89.4%)	85.7% (82.0% to 89.1%)	86.9% (84.1% to 89.4%)
Hypertensive heart disease	100.0% (100.0% to 100.0%)	100.0% (100.0% to 100.0%)	100.0% (100.0% to 100.0%)	100.0% (100.0% to 100.0%)
Cardiomyopathy and myocarditis	37.2% (30.1% to 45.0%)	35.9% (29.3% to 42.6%)	42.3% (35.4% to 48.7%)	36.3% (29.8% to 42.9%)
Atrial fibrillation and flutter	40.4% (32.5% to 48.0%)	44.7% (37.9% to 51.4%)	51.5% (46.6% to 56.7%)	48.2% (42.3% to 53.9%)
Aortic aneurysm	41.9% (34.6% to 48.9%)	46.7% (40.2% to 52.7%)	-	46.7% (40.2% to 52.7%)
Peripheral vascular disease	49.4% (42.4% to 57.3%)	56.3% (50.8% to 62.0%)	59.3% (53.9% to 64.9%)	58.9% (53.9% to 64.3%)
Endocarditis	37.7% (28.9% to 47.3%)	33.9% (26.5% to 41.9%)	36.7% (27.9% to 46.8%)	34.0% (26.6% to 41.9%)

Supplementary Results Table 1. Age-standardised PAFs attributable to the joint distribution of all risk factors for all ages, both sexes combined for each GBD level 1 and level 2 cause and all causes in 2016 for Guinea. Results are shown for deaths, YLLs, YLDs, and DALYs
Data in parenthesis are 95% uncertainty intervals. PAFs=population attributable fractions. GBD=Global Burden of Disease. YLLs=years of life lost. YLDs=years live with disability. DALYs=disability-adjusted life-years

	Deaths 2016	YLLs 2016	YLDs 2016	DALYs 2016
Other cardiovascular and circulatory diseases	44.4% (39.0% to 49.6%)	45.3% (40.7% to 50.2%)	41.7% (36.6% to 46.7%)	44.2% (39.8% to 48.8%)
**Chronic respiratory diseases**	59.4% (51.8% to 66.3%)	57.6% (50.9% to 64.1%)	40.9% (34.7% to 47.7%)	53.6% (47.5% to 59.8%)
Chronic obstructive pulmonary disease	77.2% (68.3% to 86.4%)	78.0% (69.8% to 86.4%)	71.3% (62.2% to 80.7%)	76.9% (68.9% to 85.4%)
Pneumoconiosis	100.0% (100.0% to 100.0%)	100.0% (100.0% to 100.0%)	100.0% (100.0% to 100.0%)	100.0% (100.0% to 100.0%)
Asthma	24.9% (17.3% to 33.7%)	28.6% (21.4% to 36.5%)	21.6% (16.0% to 28.3%)	25.9% (19.7% to 33.2%)
Other chronic respiratory diseases	7.3% (4.1% to 11.5%)	10.0% (5.6% to 15.4%)	7.0% (3.8% to 10.8%)	9.7% (5.6% to 15.1%)
**Cirrhosis and other chronic liver diseases**	22.0% (19.6% to 24.5%)	21.1% (18.8% to 23.6%)	16.8% (14.3% to 19.7%)	21.0% (18.7% to 23.4%)
Cirrhosis and other chronic liver diseases due to hepatitis B	0.0% (0.0% to 0.0%)	0.0% (0.0% to 0.0%)	0.0% (0.0% to 0.0%)	0.0% (0.0% to 0.0%)
Cirrhosis and other chronic liver diseases due to hepatitis C	1.2% (0.5% to 2.3%)	1.7% (0.7% to 3.7%)	1.9% (0.7% to 4.0%)	1.8% (0.7% to 3.7%)
Cirrhosis and other chronic liver diseases due to alcohol use	100.0% (100.0% to 100.0%)	100.0% (100.0% to 100.0%)	100.0% (100.0% to 100.0%)	100.0% (100.0% to 100.0%)
**Digestive diseases**	2.6% (1.6% to 3.7%)	2.5% (1.5% to 3.7%)	1.2% (0.8% to 1.7%)	2.4% (1.4% to 3.6%)
Peptic ulcer disease	5.3% (3.3% to 7.8%)	6.0% (3.7% to 8.9%)	5.5% (3.6% to 7.6%)	6.0% (3.7% to 8.8%)
Gallbladder and biliary diseases	22.1% (11.7% to 35.3%)	21.7% (11.9% to 33.5%)	24.0% (13.7% to 36.0%)	21.8% (12.0% to 33.6%)
Pancreatitis	1.2% (-5.1% to 8.3%)	1.4% (-5.4% to 9.4%)	1.2% (-5.1% to 8.8%)	1.4% (-5.4% to 9.4%)
**Neurological disorders**	10.6% (4.2% to 18.8%)	8.9% (4.0% to 15.1%)	1.3% (0.6% to 2.1%)	4.2% (1.9% to 7.3%)
Alzheimer's disease and other dementias	12.4% (5.0% to 22.2%)	13.5% (5.8% to 23.2%)	13.5% (5.9% to 23.1%)	13.5% (5.8% to 23.2%)
Parkinson's disease	-3.6% (-5.0% to -2.4%)	-4.6% (-6.4% to -3.2%)	-5.2% (-7.2% to -3.6%)	-4.7% (-6.5% to -3.3%)
Epilepsy	2.9% (0.8% to 5.8%)	2.6% (0.7% to 5.2%)	2.1% (0.6% to 4.3%)	2.4% (0.7% to 4.7%)
Multiple sclerosis	3.8% (1.5% to 6.8%)	4.1% (1.6% to 7.2%)	3.1% (1.7% to 4.7%)	3.8% (1.7% to 6.5%)
**Mental and substance use disorders**	99.9% (99.7% to 99.9%)	99.8% (99.6% to 99.9%)	19.7% (17.3% to 22.0%)	23.2% (20.5% to 26.1%)
Alcohol use disorders	100.0% (100.0% to 100.0%)	100.0% (100.0% to 100.0%)	100.0% (100.0% to 100.0%)	100.0% (100.0% to 100.0%)

Supplementary Results Table 1. Age-standardised PAFs attributable to the joint distribution of all risk factors for all ages, both sexes combined for each GBD level 1 and level 2 cause and all causes in 2016 for Guinea. Results are shown for deaths, YLLs, YLDs, and DALYs
Data in parenthesis are 95% uncertainty intervals. PAFs=population attributable fractions. GBD=Global Burden of Disease. YLLs=years of life lost. YLDs=years live with disability. DALYs=disability-adjusted life-years

	Deaths 2016	YLLs 2016	YLDs 2016	DALYs 2016
Drug use disorders	100.0% (100.0% to 100.0%)	100.0% (100.0% to 100.0%)	100.0% (100.0% to 100.0%)	100.0% (100.0% to 100.0%)
Depressive disorders	-	-	11.5% (9.9% to 13.4%)	11.5% (9.9% to 13.4%)
Idiopathic developmental intellectual disability	-	-	63.1% (31.5% to 82.5%)	63.1% (31.5% to 82.5%)
**Diabetes, urogenital, blood, and endocrine diseases**	75.6% (72.6% to 78.1%)	62.8% (59.0% to 67.3%)	53.0% (48.7% to 56.8%)	59.6% (56.3% to 62.9%)
Diabetes mellitus	100.0% (100.0% to 100.0%)	100.0% (100.0% to 100.0%)	100.0% (100.0% to 100.0%)	100.0% (100.0% to 100.0%)
Chronic kidney disease	100.0% (100.0% to 100.0%)	100.0% (100.0% to 100.0%)	100.0% (100.0% to 100.0%)	100.0% (100.0% to 100.0%)
**Musculoskeletal disorders**	0.3% (0.1% to 0.5%)	0.2% (0.1% to 0.4%)	23.8% (20.6% to 27.4%)	23.5% (20.3% to 27.0%)
Rheumatoid arthritis	1.0% (0.4% to 2.0%)	1.2% (0.4% to 2.4%)	2.1% (0.8% to 3.7%)	2.0% (0.8% to 3.5%)
Osteoarthritis	-	-	13.6% (5.9% to 23.0%)	13.6% (5.9% to 23.0%)
Low back and neck pain	-	-	29.8% (26.2% to 33.6%)	29.8% (26.2% to 33.6%)
Gout	-	-	42.3% (34.8% to 51.7%)	42.3% (34.8% to 51.7%)
**Other non-communicable diseases**	0.2% (0.1% to 0.3%)	0.2% (0.1% to 0.5%)	9.3% (7.6% to 10.9%)	5.9% (4.4% to 7.7%)
Sense organ diseases	-	-	17.6% (15.4% to 19.8%)	17.6% (15.4% to 19.8%)
Sudden infant death syndrome	4.9% (2.2% to 9.4%)	4.9% (2.2% to 9.5%)	-	4.9% (2.2% to 9.5%)
**Transport injuries**	31.0% (27.3% to 35.3%)	28.1% (23.6% to 33.5%)	36.9% (32.5% to 42.0%)	29.3% (24.9% to 34.7%)
Road injuries	30.9% (27.2% to 35.1%)	28.0% (23.5% to 33.4%)	39.2% (34.6% to 44.3%)	29.3% (24.9% to 34.6%)
Other transport injuries	34.5% (29.0% to 41.0%)	30.1% (23.8% to 37.4%)	28.0% (24.1% to 32.1%)	29.2% (24.3% to 35.0%)
**Unintentional injuries**	25.7% (19.1% to 33.3%)	17.8% (14.7% to 21.4%)	26.4% (23.2% to 30.6%)	19.9% (16.8% to 23.3%)
Falls	53.2% (35.7% to 70.8%)	40.6% (31.7% to 50.7%)	36.3% (33.2% to 40.2%)	38.8% (32.8% to 45.3%)
Drowning	12.3% (9.7% to 15.8%)	13.3% (10.1% to 17.4%)	18.4% (14.6% to 23.1%)	13.4% (10.2% to 17.5%)
Fire, heat, and hot substances	9.1% (7.1% to 11.7%)	12.2% (9.7% to 15.5%)	22.5% (17.9% to 27.9%)	14.1% (11.2% to 17.6%)

Supplementary Results Table 1. Age-standardised PAFs attributable to the joint distribution of all risk factors for all ages, both sexes combined for each GBD level 1 and level 2 cause and all causes in 2016 for Guinea. Results are shown for deaths, YLLs, YLDs, and DALYs
Data in parenthesis are 95% uncertainty intervals. PAFs=population attributable fractions. GBD=Global Burden of Disease. YLLs=years of life lost. YLDs=years live with disability. DALYs=disability-adjusted life-years

	**Deaths 2016**	**YLLs 2016**	**YLDs 2016**	**DALYs 2016**
Poisonings	15.5% (10.8% to 20.1%)	17.3% (10.6% to 23.3%)	22.7% (17.8% to 28.6%)	17.4% (10.8% to 23.4%)
Exposure to mechanical forces	23.4% (19.5% to 27.5%)	19.6% (15.2% to 25.0%)	29.4% (24.7% to 35.0%)	21.8% (17.5% to 26.7%)
Animal contact	11.4% (9.0% to 14.5%)	13.7% (10.6% to 17.8%)	20.3% (16.3% to 25.0%)	15.0% (11.8% to 19.2%)
Foreign body	9.6% (7.1% to 12.5%)	11.2% (7.4% to 15.6%)	17.6% (13.9% to 22.0%)	13.1% (9.4% to 17.3%)
Other unintentional injuries	16.8% (13.3% to 21.5%)	19.0% (14.6% to 24.4%)	19.2% (15.2% to 24.0%)	19.1% (15.1% to 24.0%)
**Self-harm and interpersonal violence**	4.9% (2.0% to 8.7%)	5.2% (2.5% to 8.5%)	13.4% (11.7% to 15.3%)	5.8% (3.3% to 9.0%)
Self-harm	3.7% (-0.0% to 8.5%)	4.2% (0.3% to 8.9%)	4.0% (0.1% to 8.7%)	4.2% (0.3% to 8.9%)
Interpersonal violence	8.1% (4.7% to 11.9%)	6.8% (3.6% to 10.2%)	14.5% (12.9% to 16.5%)	8.1% (5.3% to 11.3%)
**Forces of nature, conflict and terrorism, and executions and police conflict**	0.0% (0.0% to 0.0%)	0.0% (0.0% to 0.0%)	0.5% (0.3% to 0.8%)	0.4% (0.1% to 0.7%)
Forces of nature, conflict and terrorism, and state actor violence	0.0% (0.0% to 0.0%)	0.0% (0.0% to 0.0%)	8.6% (5.9% to 12.4%)	8.6% (5.9% to 12.4%)

Supplementary Results Table 1. Age-standardised PAFs attributable to the joint distribution of all risk factors for all ages, both sexes combined for each GBD level 1 and level 2 cause and all causes in 2016 for Guinea-Bissau. Results are shown for deaths, YLLs, YLDs, and DALYs
Data in parenthesis are 95% uncertainty intervals. PAFs=population attributable fractions. GBD=Global Burden of Disease. YLLs=years of life lost. YLDs=years live with disability. DALYs=disability-adjusted life-years

	Deaths 2016	YLLs 2016	YLDs 2016	DALYs 2016
**All causes**	60.9% (58.4% to 63.4%)	57.2% (54.7% to 59.7%)	31.2% (29.8% to 32.7%)	52.2% (49.9% to 54.8%)
**HIV/AIDS and tuberculosis**	62.6% (53.3% to 71.8%)	69.4% (61.8% to 76.2%)	84.0% (80.3% to 87.7%)	70.0% (62.5% to 76.5%)
Tuberculosis	36.4% (22.6% to 51.7%)	38.3% (24.8% to 51.4%)	35.0% (22.2% to 47.7%)	38.3% (24.8% to 51.4%)
HIV/AIDS	91.7% (89.7% to 93.2%)	88.6% (86.2% to 90.7%)	90.7% (88.2% to 93.1%)	88.7% (86.4% to 90.8%)
**Diarrhoea, lower respiratory infections, and other common infectious diseases**	72.9% (66.7% to 79.1%)	69.2% (60.7% to 76.2%)	40.1% (35.4% to 44.5%)	68.0% (59.7% to 74.8%)
Diarrhoeal diseases	96.1% (89.8% to 99.1%)	97.3% (92.4% to 99.4%)	96.3% (90.0% to 99.1%)	97.2% (92.4% to 99.4%)
Lower respiratory infections	79.6% (73.1% to 85.1%)	83.6% (78.2% to 88.2%)	80.0% (73.8% to 85.3%)	83.6% (78.2% to 88.2%)
Upper respiratory infections	1.2% (0.1% to 4.4%)	2.4% (0.2% to 8.5%)	0.0% (0.0% to 0.0%)	0.3% (0.0% to 0.9%)
Otitis media	0.8% (0.2% to 2.0%)	3.2% (1.3% to 6.1%)	4.0% (2.6% to 5.4%)	4.0% (2.5% to 5.4%)
Meningitis	1.9% (0.9% to 3.4%)	3.5% (1.8% to 6.0%)	0.0% (0.0% to 0.0%)	3.4% (1.7% to 5.8%)
Encephalitis	0.6% (0.4% to 0.8%)	1.1% (0.7% to 1.5%)	0.0% (0.0% to 0.0%)	0.7% (0.5% to 0.9%)
Measles	57.5% (38.2% to 81.3%)	58.4% (38.9% to 82.5%)	55.2% (32.9% to 80.5%)	58.4% (38.9% to 82.5%)
**Maternal disorders**	9.7% (3.7% to 16.3%)	9.7% (3.6% to 16.1%)	2.6% (1.0% to 4.7%)	9.4% (3.5% to 15.7%)
Maternal haemorrhage	19.8% (7.2% to 32.2%)	19.9% (7.3% to 32.3%)	20.3% (7.3% to 33.2%)	20.0% (7.3% to 32.3%)
Maternal sepsis and other pregnancy related infections	19.9% (7.2% to 32.5%)	20.0% (7.2% to 32.5%)	19.7% (7.2% to 32.2%)	20.0% (7.2% to 32.5%)
Maternal abortion, miscarriage, and ectopic pregnancy	17.9% (10.5% to 26.0%)	17.7% (10.1% to 25.9%)	18.1% (10.2% to 26.4%)	17.7% (10.3% to 25.6%)
**Neonatal disorders**	82.2% (78.8% to 85.4%)	82.3% (78.8% to 85.4%)	0.6% (0.3% to 1.1%)	79.6% (76.0% to 83.0%)
Neonatal preterm birth complications	97.3% (94.1% to 99.0%)	97.3% (94.2% to 99.0%)	1.9% (1.2% to 2.6%)	94.5% (91.0% to 96.7%)
Neonatal encephalopathy due to birth asphyxia and trauma	77.7% (73.5% to 81.7%)	77.7% (73.6% to 81.7%)	0.0% (0.0% to 0.0%)	75.6% (70.1% to 80.1%)
Neonatal sepsis and other neonatal infections	69.2% (63.7% to 73.9%)	69.2% (63.8% to 73.9%)	0.0% (0.0% to 0.0%)	66.1% (58.4% to 72.3%)
Hemolytic disease and other neonatal jaundice	70.1% (60.9% to 76.6%)	70.1% (61.0% to 76.6%)	0.0% (0.0% to 0.0%)	66.0% (57.4% to 72.6%)

Supplementary Results Table 1. Age-standardised PAFs attributable to the joint distribution of all risk factors for all ages, both sexes combined for each GBD level 1 and level 2 cause and all causes in 2016 for Guinea-Bissau. Results are shown for deaths, YLLs, YLDs, and DALYs
Data in parenthesis are 95% uncertainty intervals. PAFs=population attributable fractions. GBD=Global Burden of Disease. YLLs=years of life lost. YLDs=years live with disability. DALYs=disability-adjusted life-years

	Deaths 2016	YLLs 2016	YLDs 2016	DALYs 2016
Other neonatal disorders	68.4% (58.8% to 75.2%)	68.5% (58.9% to 75.2%)	0.0% (0.0% to 0.0%)	66.5% (57.1% to 73.6%)
**Nutritional deficiencies**	26.7% (17.1% to 38.4%)	58.8% (45.5% to 71.1%)	93.0% (91.6% to 94.3%)	79.1% (73.5% to 83.6%)
Protein-energy malnutrition	30.3% (19.6% to 42.8%)	64.4% (51.1% to 76.3%)	98.1% (97.2% to 98.7%)	65.5% (53.0% to 76.6%)
Vitamin A deficiency	-	-	100.0% (100.0% to 100.0%)	100.0% (100.0% to 100.0%)
Iron-deficiency anaemia	100.0% (100.0% to 100.0%)	100.0% (100.0% to 100.0%)	100.0% (100.0% to 100.0%)	100.0% (100.0% to 100.0%)
**Other communicable, maternal, neonatal, and nutritional diseases**	2.0% (1.1% to 3.6%)	1.3% (0.6% to 2.5%)	55.4% (49.7% to 61.5%)	4.9% (2.6% to 8.7%)
Sexually transmitted diseases excluding HIV	3.2% (1.5% to 6.8%)	1.6% (0.7% to 3.6%)	100.0% (100.0% to 100.0%)	6.5% (3.0% to 13.1%)
Hepatitis	0.0% (0.0% to 0.1%)	0.0% (0.0% to 0.1%)	0.0% (0.0% to 0.1%)	0.0% (0.0% to 0.1%)
**Neoplasms**	35.7% (30.7% to 40.5%)	36.7% (31.4% to 41.5%)	37.4% (31.3% to 42.4%)	36.7% (31.4% to 41.5%)
Oesophageal cancer	70.5% (57.9% to 81.7%)	72.8% (60.2% to 83.5%)	71.2% (58.9% to 82.2%)	72.8% (60.2% to 83.4%)
Stomach cancer	8.3% (3.1% to 16.7%)	9.0% (3.4% to 17.3%)	8.5% (3.2% to 16.7%)	9.0% (3.4% to 17.2%)
Liver cancer	34.6% (25.5% to 44.9%)	34.4% (24.9% to 45.3%)	34.6% (25.5% to 44.9%)	34.4% (24.9% to 45.3%)
Larynx cancer	68.4% (54.7% to 80.9%)	70.8% (56.9% to 82.7%)	69.0% (55.6% to 81.2%)	70.8% (56.8% to 82.7%)
Tracheal, bronchus, and lung cancer	87.3% (81.9% to 91.6%)	87.5% (82.0% to 91.7%)	87.2% (81.7% to 91.5%)	87.5% (82.0% to 91.7%)
Breast cancer	23.7% (16.4% to 31.8%)	21.7% (15.0% to 29.5%)	22.3% (15.5% to 30.7%)	21.7% (15.0% to 29.5%)
Cervical cancer	100.0% (100.0% to 100.0%)	100.0% (100.0% to 100.0%)	100.0% (100.0% to 100.0%)	100.0% (100.0% to 100.0%)
Uterine cancer	43.3% (30.2% to 55.7%)	44.9% (31.8% to 57.1%)	43.8% (30.9% to 56.1%)	44.9% (31.8% to 57.1%)
Prostate cancer	2.1% (1.1% to 3.4%)	2.4% (1.3% to 3.9%)	2.2% (1.1% to 3.5%)	2.4% (1.3% to 3.9%)
Colon and rectum cancer	52.8% (43.6% to 61.9%)	53.1% (44.1% to 61.8%)	52.9% (43.9% to 61.9%)	53.1% (44.1% to 61.8%)
Lip and oral cavity cancer	66.1% (51.6% to 79.0%)	68.1% (54.0% to 80.6%)	65.8% (51.7% to 78.7%)	68.1% (54.0% to 80.6%)
Nasopharynx cancer	63.5% (49.2% to 76.1%)	62.6% (48.5% to 74.6%)	61.8% (48.4% to 73.5%)	62.6% (48.5% to 74.6%)

Supplementary Results Table 1. Age-standardised PAFs attributable to the joint distribution of all risk factors for all ages, both sexes combined for each GBD level 1 and level 2 cause and all causes in 2016 for Guinea-Bissau. Results are shown for deaths, YLLs, YLDs, and DALYs
Data in parenthesis are 95% uncertainty intervals. PAFs=population attributable fractions. GBD=Global Burden of Disease. YLLs=years of life lost. YLDs=years live with disability. DALYs=disability-adjusted life-years

	Deaths 2016	YLLs 2016	YLDs 2016	DALYs 2016
Other pharynx cancer	42.0% (22.9% to 60.3%)	44.4% (24.9% to 62.0%)	42.7% (23.5% to 60.5%)	44.4% (24.9% to 62.0%)
Gallbladder and biliary tract cancer	22.0% (12.1% to 34.1%)	23.0% (12.9% to 35.5%)	22.3% (12.5% to 34.7%)	23.0% (12.9% to 35.4%)
Pancreatic cancer	18.8% (11.9% to 27.2%)	19.5% (12.6% to 27.6%)	18.9% (12.0% to 27.3%)	19.5% (12.6% to 27.6%)
Ovarian cancer	11.9% (5.6% to 20.5%)	10.4% (4.4% to 18.2%)	10.7% (4.8% to 18.6%)	10.4% (4.5% to 18.2%)
Kidney cancer	24.4% (16.6% to 33.8%)	23.0% (15.3% to 32.5%)	23.4% (15.8% to 32.5%)	23.0% (15.3% to 32.5%)
Bladder cancer	19.0% (10.5% to 28.8%)	20.6% (11.2% to 31.4%)	19.5% (10.8% to 29.6%)	20.6% (11.2% to 31.4%)
Thyroid cancer	12.0% (6.4% to 19.4%)	12.4% (6.7% to 19.8%)	12.0% (6.5% to 19.2%)	12.4% (6.7% to 19.7%)
Mesothelioma	88.0% (83.2% to 92.2%)	81.5% (73.7% to 87.7%)	86.0% (80.3% to 90.7%)	81.5% (73.8% to 87.8%)
Non-Hodgkin's lymphoma	5.1% (2.1% to 8.9%)	4.4% (1.9% to 7.6%)	4.9% (2.1% to 8.4%)	4.4% (1.9% to 7.6%)
Multiple myeloma	7.0% (2.9% to 12.3%)	7.2% (3.0% to 12.8%)	7.1% (3.0% to 12.6%)	7.2% (3.0% to 12.8%)
Leukaemia	12.1% (7.1% to 17.9%)	10.6% (6.1% to 16.0%)	11.9% (7.0% to 17.7%)	10.6% (6.1% to 16.0%)
**Cardiovascular diseases**	87.4% (85.3% to 89.6%)	87.9% (85.9% to 89.9%)	64.9% (61.1% to 68.7%)	86.6% (84.7% to 88.7%)
Rheumatic heart disease	28.5% (19.4% to 44.7%)	27.1% (18.9% to 40.3%)	24.1% (15.8% to 34.3%)	26.5% (18.6% to 39.4%)
Ischaemic heart disease	94.5% (92.0% to 96.4%)	95.7% (94.1% to 97.0%)	91.6% (89.2% to 93.7%)	95.6% (94.0% to 96.9%)
Cerebrovascular disease	88.8% (85.7% to 91.7%)	90.8% (88.7% to 92.7%)	88.4% (85.3% to 91.4%)	90.7% (88.6% to 92.6%)
Hypertensive heart disease	100.0% (100.0% to 100.0%)	100.0% (100.0% to 100.0%)	100.0% (100.0% to 100.0%)	100.0% (100.0% to 100.0%)
Cardiomyopathy and myocarditis	39.6% (32.9% to 46.7%)	38.1% (31.7% to 44.8%)	45.1% (38.4% to 51.7%)	38.6% (32.2% to 45.1%)
Atrial fibrillation and flutter	47.1% (38.6% to 55.4%)	51.8% (43.9% to 59.0%)	58.6% (52.5% to 64.9%)	55.4% (48.8% to 62.2%)
Aortic aneurysm	43.6% (36.7% to 50.5%)	47.7% (41.3% to 53.7%)	-	47.7% (41.3% to 53.7%)
Peripheral vascular disease	52.6% (46.2% to 59.8%)	60.1% (54.8% to 65.4%)	62.3% (57.0% to 67.7%)	62.0% (56.8% to 67.2%)
Endocarditis	38.6% (30.1% to 47.7%)	34.5% (26.7% to 42.4%)	36.1% (26.6% to 47.6%)	34.6% (26.9% to 42.3%)

Supplementary Results Table 1. Age-standardised PAFs attributable to the joint distribution of all risk factors for all ages, both sexes combined for each GBD level 1 and level 2 cause and all causes in 2016 for Guinea-Bissau. Results are shown for deaths, YLLs, YLDs, and DALYs
Data in parenthesis are 95% uncertainty intervals. PAFs=population attributable fractions. GBD=Global Burden of Disease. YLLs=years of life lost. YLDs=years live with disability. DALYs=disability-adjusted life-years

	**Deaths 2016**	**YLLs 2016**	**YLDs 2016**	**DALYs 2016**
Other cardiovascular and circulatory diseases	46.3% (41.2% to 51.3%)	47.3% (42.6% to 52.3%)	41.5% (36.3% to 46.8%)	46.1% (41.5% to 50.8%)
**Chronic respiratory diseases**	65.0% (59.0% to 71.3%)	62.3% (56.6% to 68.1%)	47.9% (41.1% to 55.0%)	59.5% (53.8% to 65.3%)
Chronic obstructive pulmonary disease	81.5% (74.0% to 89.3%)	81.3% (74.1% to 88.5%)	74.5% (64.9% to 83.0%)	80.3% (72.8% to 87.5%)
Pneumoconiosis	100.0% (100.0% to 100.0%)	100.0% (100.0% to 100.0%)	100.0% (100.0% to 100.0%)	100.0% (100.0% to 100.0%)
Asthma	35.3% (26.4% to 45.6%)	38.4% (30.3% to 47.9%)	28.1% (21.1% to 36.3%)	35.3% (27.7% to 43.9%)
Other chronic respiratory diseases	8.8% (4.5% to 14.2%)	8.7% (4.1% to 14.5%)	4.5% (1.9% to 8.0%)	8.4% (4.0% to 14.1%)
**Cirrhosis and other chronic liver diseases**	23.4% (20.9% to 26.2%)	22.7% (20.0% to 25.3%)	17.4% (14.8% to 20.2%)	22.6% (20.0% to 25.2%)
Cirrhosis and other chronic liver diseases due to hepatitis B	0.0% (0.0% to 0.0%)	0.0% (0.0% to 0.0%)	0.0% (0.0% to 0.0%)	0.0% (0.0% to 0.0%)
Cirrhosis and other chronic liver diseases due to hepatitis C	0.7% (0.3% to 1.5%)	1.1% (0.4% to 2.3%)	1.1% (0.4% to 2.4%)	1.1% (0.4% to 2.2%)
Cirrhosis and other chronic liver diseases due to alcohol use	100.0% (100.0% to 100.0%)	100.0% (100.0% to 100.0%)	100.0% (100.0% to 100.0%)	100.0% (100.0% to 100.0%)
**Digestive diseases**	6.3% (3.9% to 9.5%)	7.3% (4.2% to 11.3%)	1.8% (1.3% to 2.4%)	7.0% (4.0% to 10.7%)
Peptic ulcer disease	5.9% (3.6% to 8.6%)	6.6% (4.1% to 9.6%)	5.2% (3.5% to 7.3%)	6.6% (4.1% to 9.6%)
Gallbladder and biliary diseases	35.9% (23.2% to 48.8%)	35.0% (23.4% to 47.3%)	39.3% (27.3% to 51.8%)	35.1% (23.6% to 47.4%)
Pancreatitis	29.9% (6.4% to 57.9%)	33.7% (8.5% to 62.8%)	29.1% (7.0% to 57.3%)	33.7% (8.5% to 62.8%)
**Neurological disorders**	17.3% (9.0% to 27.8%)	17.2% (11.0% to 24.8%)	3.2% (1.5% to 6.0%)	9.0% (5.5% to 13.3%)
Alzheimer's disease and other dementias	18.8% (8.6% to 31.7%)	20.1% (9.5% to 33.1%)	20.1% (9.4% to 33.3%)	20.1% (9.5% to 33.1%)
Parkinson's disease	-3.5% (-4.9% to -2.3%)	-4.5% (-6.1% to -3.1%)	-4.9% (-6.6% to -3.5%)	-4.5% (-6.2% to -3.1%)
Epilepsy	19.4% (9.7% to 29.7%)	18.0% (9.1% to 26.8%)	14.3% (7.0% to 22.5%)	16.8% (8.4% to 25.0%)
Multiple sclerosis	4.0% (1.8% to 7.4%)	4.2% (1.9% to 7.8%)	2.9% (1.6% to 4.5%)	3.9% (1.9% to 6.9%)
**Mental and substance use disorders**	99.9% (99.7% to 99.9%)	99.8% (99.6% to 99.9%)	20.3% (18.0% to 22.7%)	24.9% (21.9% to 28.2%)
Alcohol use disorders	100.0% (100.0% to 100.0%)	100.0% (100.0% to 100.0%)	100.0% (100.0% to 100.0%)	100.0% (100.0% to 100.0%)

Supplementary Results Table 1. Age-standardised PAFs attributable to the joint distribution of all risk factors for all ages, both sexes combined for each GBD level 1 and level 2 cause and all causes in 2016 for Guinea-Bissau. Results are shown for deaths, YLLs, YLDs, and DALYs
Data in parenthesis are 95% uncertainty intervals. PAFs=population attributable fractions. GBD=Global Burden of Disease. YLLs=years of life lost. YLDs=years live with disability. DALYs=disability-adjusted life-years

	Deaths 2016	YLLs 2016	YLDs 2016	DALYs 2016
Drug use disorders	100.0% (100.0% to 100.0%)	100.0% (100.0% to 100.0%)	100.0% (100.0% to 100.0%)	100.0% (100.0% to 100.0%)
Depressive disorders	-	-	11.7% (10.0% to 13.5%)	11.7% (10.0% to 13.5%)
Idiopathic developmental intellectual disability	-	-	63.3% (31.6% to 82.8%)	63.3% (31.6% to 82.8%)
**Diabetes, urogenital, blood, and endocrine diseases**	79.6% (76.5% to 81.9%)	72.0% (66.6% to 75.5%)	61.4% (57.0% to 65.3%)	69.1% (65.1% to 72.1%)
Diabetes mellitus	100.0% (100.0% to 100.0%)	100.0% (100.0% to 100.0%)	100.0% (100.0% to 100.0%)	100.0% (100.0% to 100.0%)
Chronic kidney disease	100.0% (100.0% to 100.0%)	100.0% (100.0% to 100.0%)	100.0% (100.0% to 100.0%)	100.0% (100.0% to 100.0%)
**Musculoskeletal disorders**	0.2% (0.1% to 0.5%)	0.2% (0.1% to 0.4%)	24.3% (20.9% to 27.9%)	23.9% (20.5% to 27.4%)
Rheumatoid arthritis	1.1% (0.4% to 2.1%)	1.2% (0.5% to 2.4%)	2.0% (0.8% to 3.5%)	1.9% (0.8% to 3.4%)
Osteoarthritis	-	-	24.2% (13.8% to 36.1%)	24.2% (13.8% to 36.1%)
Low back and neck pain	-	-	28.7% (24.9% to 32.4%)	28.7% (24.9% to 32.4%)
Gout	-	-	56.3% (46.2% to 67.9%)	56.3% (46.2% to 67.9%)
**Other non-communicable diseases**	0.2% (0.1% to 0.4%)	0.2% (0.1% to 0.5%)	9.1% (7.3% to 10.7%)	6.2% (4.8% to 7.7%)
Sense organ diseases	-	-	17.4% (15.1% to 19.7%)	17.4% (15.1% to 19.7%)
Sudden infant death syndrome	5.0% (2.3% to 9.6%)	5.0% (2.3% to 9.7%)	-	5.0% (2.3% to 9.7%)
**Transport injuries**	26.1% (20.9% to 33.4%)	20.5% (15.4% to 27.1%)	30.3% (24.9% to 37.6%)	21.5% (16.5% to 27.9%)
Road injuries	26.2% (21.0% to 33.6%)	20.6% (15.6% to 27.2%)	32.1% (26.7% to 39.5%)	21.7% (16.6% to 28.2%)
Other transport injuries	23.7% (18.0% to 30.5%)	18.0% (12.8% to 24.0%)	21.9% (16.8% to 28.7%)	19.2% (14.1% to 25.3%)
**Unintentional injuries**	20.5% (14.6% to 27.3%)	11.2% (8.9% to 14.1%)	15.4% (14.2% to 17.0%)	12.1% (10.1% to 14.6%)
Falls	48.8% (32.2% to 65.7%)	32.6% (25.2% to 40.9%)	25.1% (23.9% to 26.4%)	29.7% (25.0% to 34.8%)
Drowning	8.1% (5.1% to 12.3%)	7.5% (4.9% to 11.0%)	9.5% (6.3% to 14.4%)	7.6% (5.0% to 11.0%)
Fire, heat, and hot substances	7.5% (4.3% to 12.3%)	7.6% (4.9% to 11.7%)	10.4% (7.2% to 15.2%)	8.0% (5.3% to 12.2%)

Supplementary Results Table 1. Age-standardised PAFs attributable to the joint distribution of all risk factors for all ages, both sexes combined for each GBD level 1 and level 2 cause and all causes in 2016 for Guinea-Bissau. Results are shown for deaths, YLLs, YLDs, and DALYs
Data in parenthesis are 95% uncertainty intervals. PAFs=population attributable fractions. GBD=Global Burden of Disease. YLLs=years of life lost. YLDs=years live with disability. DALYs=disability-adjusted life-years

	**Deaths 2016**	**YLLs 2016**	**YLDs 2016**	**DALYs 2016**
Poisonings	9.0% (5.6% to 13.9%)	8.7% (4.9% to 13.3%)	9.9% (7.0% to 14.1%)	8.7% (5.0% to 13.3%)
Exposure to mechanical forces	13.3% (10.9% to 15.6%)	8.9% (7.2% to 10.9%)	13.9% (12.6% to 15.3%)	9.9% (8.3% to 11.9%)
Animal contact	4.2% (3.3% to 5.4%)	3.9% (3.0% to 4.9%)	5.8% (4.9% to 6.9%)	4.2% (3.3% to 5.3%)
Foreign body	2.7% (2.1% to 3.3%)	3.1% (2.4% to 3.9%)	4.3% (3.4% to 5.3%)	3.5% (2.8% to 4.2%)
Other unintentional injuries	9.4% (6.2% to 14.0%)	9.4% (6.5% to 13.3%)	9.5% (6.4% to 14.3%)	9.5% (6.5% to 13.6%)
**Self-harm and interpersonal violence**	18.4% (10.6% to 27.0%)	18.2% (11.4% to 25.9%)	20.5% (17.5% to 24.1%)	18.3% (11.8% to 25.8%)
Self-harm	16.8% (6.4% to 29.4%)	17.7% (7.5% to 29.2%)	16.9% (6.6% to 29.2%)	17.7% (7.5% to 29.2%)
Interpersonal violence	21.2% (15.8% to 27.2%)	18.8% (13.7% to 24.6%)	20.9% (18.0% to 24.2%)	19.1% (14.3% to 24.5%)
**Forces of nature, conflict and terrorism, and executions and police conflict**	0.0% (0.0% to 0.0%)	0.0% (0.0% to 0.0%)	0.0% (0.0% to 0.0%)	0.0% (0.0% to 0.0%)
Forces of nature, conflict and terrorism, and state actor violence	0.0% (0.0% to 0.0%)	0.0% (0.0% to 0.0%)	0.0% (0.0% to 0.0%)	0.0% (0.0% to 0.0%)

Supplementary Results Table 1. Age-standardised PAFs attributable to the joint distribution of all risk factors for all ages, both sexes combined for each GBD level 1 and level 2 cause and all causes in 2016 for Guyana. Results are shown for deaths, YLLs, YLDs, and DALYs
Data in parenthesis are 95% uncertainty intervals. PAFs=population attributable fractions. GBD=Global Burden of Disease. YLLs=years of life lost. YLDs=years live with disability. DALYs=disability-adjusted life-years

	Deaths 2016	YLLs 2016	YLDs 2016	DALYs 2016
**All causes**	65.0% (63.5% to 66.5%)	60.9% (59.5% to 62.4%)	27.3% (25.9% to 29.0%)	51.5% (49.3% to 53.9%)
**HIV/AIDS and tuberculosis**	71.2% (65.0% to 76.0%)	71.2% (63.8% to 76.4%)	83.1% (76.1% to 88.3%)	72.6% (65.4% to 77.7%)
Tuberculosis	51.6% (43.3% to 59.7%)	54.1% (46.1% to 62.2%)	49.2% (40.7% to 57.5%)	54.0% (46.1% to 62.1%)
HIV/AIDS	79.9% (71.6% to 85.4%)	76.0% (66.8% to 81.8%)	84.4% (77.1% to 89.6%)	77.2% (68.6% to 82.8%)
**Diarrhoea, lower respiratory infections, and other common infectious diseases**	53.8% (47.3% to 60.2%)	62.1% (56.6% to 67.2%)	40.7% (32.3% to 48.3%)	59.3% (54.1% to 64.2%)
Diarrhoeal diseases	88.7% (73.1% to 96.6%)	92.6% (82.2% to 97.8%)	88.5% (72.6% to 96.6%)	91.7% (80.1% to 97.5%)
Lower respiratory infections	50.6% (43.4% to 58.3%)	60.1% (53.8% to 66.5%)	54.0% (46.8% to 61.4%)	60.1% (53.7% to 66.5%)
Upper respiratory infections	1.4% (0.1% to 5.0%)	3.2% (0.2% to 11.6%)	0.0% (0.0% to 0.0%)	0.1% (0.0% to 0.2%)
Otitis media	1.3% (0.7% to 2.2%)	3.3% (1.9% to 5.3%)	4.9% (3.2% to 6.5%)	4.9% (3.2% to 6.5%)
Meningitis	2.0% (1.3% to 2.8%)	3.9% (2.6% to 5.3%)	0.0% (0.0% to 0.0%)	3.4% (2.3% to 4.7%)
Encephalitis	1.0% (0.7% to 1.5%)	1.7% (1.3% to 2.4%)	0.0% (0.0% to 0.0%)	1.6% (1.1% to 2.1%)
Measles	20.9% (9.3% to 43.2%)	24.0% (10.6% to 48.4%)	20.9% (0.0% to 74.6%)	24.0% (10.6% to 48.4%)
**Maternal disorders**	7.1% (3.8% to 10.8%)	7.1% (3.7% to 10.7%)	2.9% (1.5% to 4.6%)	6.6% (3.5% to 9.8%)
Maternal haemorrhage	17.7% (6.4% to 28.9%)	17.7% (6.4% to 28.8%)	17.5% (6.3% to 28.6%)	17.7% (6.4% to 28.8%)
Maternal sepsis and other pregnancy related infections	17.7% (6.4% to 28.8%)	17.7% (6.4% to 28.8%)	17.3% (6.2% to 28.2%)	17.6% (6.4% to 28.7%)
Maternal abortion, miscarriage, and ectopic pregnancy	7.3% (4.1% to 11.0%)	7.3% (4.0% to 11.2%)	6.9% (3.5% to 10.9%)	7.3% (4.0% to 11.1%)
**Neonatal disorders**	79.4% (76.8% to 82.0%)	79.4% (76.8% to 82.1%)	0.1% (0.1% to 0.2%)	69.6% (63.2% to 74.2%)
Neonatal preterm birth complications	96.9% (95.4% to 98.0%)	96.9% (95.4% to 98.0%)	0.3% (0.2% to 0.3%)	83.7% (76.5% to 88.9%)
Neonatal encephalopathy due to birth asphyxia and trauma	70.5% (67.2% to 74.2%)	70.5% (67.2% to 74.2%)	0.0% (0.0% to 0.0%)	69.2% (65.3% to 73.1%)
Neonatal sepsis and other neonatal infections	63.5% (58.5% to 68.2%)	63.6% (58.6% to 68.3%)	0.0% (0.0% to 0.0%)	48.8% (34.6% to 59.9%)
Hemolytic disease and other neonatal jaundice	68.2% (61.8% to 73.6%)	68.2% (61.8% to 73.6%)	0.0% (0.0% to 0.0%)	41.6% (29.7% to 53.2%)

Supplementary Results Table 1. Age-standardised PAFs attributable to the joint distribution of all risk factors for all ages, both sexes combined for each GBD level 1 and level 2 cause and all causes in 2016 for Guyana. Results are shown for deaths, YLLs, YLDs, and DALYs
Data in parenthesis are 95% uncertainty intervals. PAFs=population attributable fractions. GBD=Global Burden of Disease. YLLs=years of life lost. YLDs=years live with disability. DALYs=disability-adjusted life-years

	Deaths 2016	YLLs 2016	YLDs 2016	DALYs 2016
Other neonatal disorders	67.4% (63.0% to 72.0%)	67.5% (63.1% to 72.0%)	0.0% (0.0% to 0.0%)	64.6% (59.5% to 69.5%)
**Nutritional deficiencies**	12.4% (8.4% to 17.7%)	39.4% (30.3% to 49.2%)	98.9% (98.6% to 99.1%)	78.5% (72.1% to 83.9%)
Protein-energy malnutrition	14.4% (9.7% to 20.7%)	45.3% (35.1% to 56.2%)	96.2% (94.7% to 97.4%)	48.9% (39.7% to 58.7%)
Vitamin A deficiency	-	-	100.0% (100.0% to 100.0%)	100.0% (100.0% to 100.0%)
Iron-deficiency anaemia	100.0% (100.0% to 100.0%)	100.0% (100.0% to 100.0%)	100.0% (100.0% to 100.0%)	100.0% (100.0% to 100.0%)
**Other communicable, maternal, neonatal, and nutritional diseases**	13.4% (11.2% to 17.2%)	11.8% (9.4% to 15.6%)	60.6% (51.8% to 70.5%)	26.5% (20.0% to 35.5%)
Sexually transmitted diseases excluding HIV	72.1% (51.6% to 89.0%)	51.8% (29.3% to 75.8%)	100.0% (100.0% to 100.0%)	79.1% (61.1% to 92.8%)
Hepatitis	8.2% (5.1% to 12.0%)	8.8% (5.3% to 13.0%)	6.5% (3.8% to 9.8%)	8.7% (5.2% to 12.8%)
**Neoplasms**	28.3% (24.7% to 32.1%)	30.1% (26.8% to 33.7%)	26.8% (23.2% to 30.8%)	30.0% (26.7% to 33.6%)
Oesophageal cancer	61.1% (47.9% to 73.2%)	64.7% (52.1% to 76.1%)	62.0% (49.0% to 73.9%)	64.7% (52.1% to 76.1%)
Stomach cancer	5.2% (1.3% to 12.4%)	5.7% (1.8% to 12.1%)	5.4% (1.5% to 12.4%)	5.7% (1.8% to 12.1%)
Liver cancer	48.0% (40.3% to 56.7%)	49.2% (41.7% to 57.6%)	48.3% (40.6% to 57.0%)	49.1% (41.7% to 57.6%)
Larynx cancer	58.3% (45.4% to 69.1%)	63.6% (50.7% to 73.8%)	60.2% (47.3% to 70.8%)	63.6% (50.6% to 73.8%)
Tracheal, bronchus, and lung cancer	61.9% (52.5% to 70.3%)	64.1% (54.9% to 71.8%)	62.5% (53.1% to 70.9%)	64.1% (54.9% to 71.8%)
Breast cancer	21.6% (12.8% to 32.8%)	19.0% (10.6% to 29.0%)	20.3% (11.7% to 31.3%)	19.0% (10.7% to 29.1%)
Cervical cancer	100.0% (100.0% to 100.0%)	100.0% (100.0% to 100.0%)	100.0% (100.0% to 100.0%)	100.0% (100.0% to 100.0%)
Uterine cancer	45.6% (30.8% to 60.1%)	47.7% (32.9% to 61.7%)	47.8% (33.0% to 61.9%)	47.7% (32.9% to 61.8%)
Prostate cancer	0.4% (0.2% to 0.8%)	0.6% (0.3% to 1.0%)	0.6% (0.3% to 1.0%)	0.6% (0.3% to 1.0%)
Colon and rectum cancer	51.0% (40.4% to 61.6%)	51.9% (41.4% to 62.0%)	51.3% (40.7% to 61.7%)	51.9% (41.4% to 62.0%)
Lip and oral cavity cancer	50.3% (40.6% to 58.9%)	55.2% (45.6% to 63.4%)	47.3% (37.9% to 55.8%)	55.0% (45.4% to 63.2%)
Nasopharynx cancer	53.8% (46.1% to 60.5%)	53.6% (46.5% to 59.9%)	55.3% (48.2% to 60.8%)	53.6% (46.6% to 59.9%)

Supplementary Results Table 1. Age-standardised PAFs attributable to the joint distribution of all risk factors for all ages, both sexes combined for each GBD level 1 and level 2 cause and all causes in 2016 for Guyana. Results are shown for deaths, YLLs, YLDs, and DALYs
Data in parenthesis are 95% uncertainty intervals. PAFs=population attributable fractions. GBD=Global Burden of Disease. YLLs=years of life lost. YLDs=years live with disability. DALYs=disability-adjusted life-years

	Deaths 2016	YLLs 2016	YLDs 2016	DALYs 2016
Other pharynx cancer	51.4% (40.0% to 61.3%)	54.8% (43.6% to 64.4%)	52.5% (41.6% to 62.4%)	54.8% (43.6% to 64.4%)
Gallbladder and biliary tract cancer	22.2% (12.0% to 33.6%)	23.1% (12.8% to 34.6%)	22.4% (12.3% to 34.0%)	23.1% (12.8% to 34.6%)
Pancreatic cancer	20.1% (10.6% to 32.5%)	20.9% (11.7% to 32.8%)	20.3% (10.9% to 32.6%)	20.9% (11.7% to 32.8%)
Ovarian cancer	16.4% (6.7% to 28.8%)	14.4% (5.8% to 25.4%)	13.4% (5.5% to 23.8%)	14.3% (5.8% to 25.4%)
Kidney cancer	21.1% (13.4% to 30.3%)	20.8% (13.4% to 29.5%)	21.7% (14.2% to 30.7%)	20.8% (13.4% to 29.5%)
Bladder cancer	15.2% (6.4% to 27.2%)	16.7% (7.6% to 28.3%)	16.1% (7.2% to 27.8%)	16.7% (7.6% to 28.3%)
Thyroid cancer	12.5% (6.2% to 21.3%)	12.9% (6.3% to 21.9%)	12.9% (6.7% to 21.3%)	12.9% (6.4% to 21.9%)
Mesothelioma	89.0% (85.7% to 91.9%)	82.6% (77.6% to 87.0%)	86.5% (82.6% to 90.0%)	82.7% (77.7% to 87.1%)
Non-Hodgkin's lymphoma	5.9% (2.2% to 10.5%)	5.6% (2.1% to 10.0%)	5.8% (2.2% to 10.3%)	5.6% (2.1% to 10.0%)
Multiple myeloma	7.8% (3.2% to 14.3%)	8.1% (3.4% to 14.5%)	7.9% (3.3% to 14.5%)	8.1% (3.4% to 14.5%)
Leukaemia	10.4% (6.1% to 15.4%)	9.4% (5.6% to 13.6%)	10.3% (6.1% to 15.2%)	9.4% (5.6% to 13.6%)
**Cardiovascular diseases**	86.7% (84.5% to 88.8%)	86.6% (84.9% to 88.1%)	72.6% (69.3% to 75.5%)	86.0% (84.2% to 87.6%)
Rheumatic heart disease	25.3% (17.1% to 40.8%)	21.7% (14.6% to 33.0%)	19.8% (12.5% to 28.9%)	21.2% (14.0% to 31.5%)
Ischaemic heart disease	93.9% (90.8% to 96.4%)	95.3% (93.3% to 97.1%)	92.1% (89.4% to 94.5%)	95.3% (93.2% to 97.0%)
Cerebrovascular disease	86.1% (82.0% to 90.0%)	87.6% (84.7% to 90.3%)	86.1% (82.0% to 90.3%)	87.6% (84.7% to 90.3%)
Hypertensive heart disease	100.0% (100.0% to 100.0%)	100.0% (100.0% to 100.0%)	100.0% (100.0% to 100.0%)	100.0% (100.0% to 100.0%)
Cardiomyopathy and myocarditis	32.8% (27.3% to 38.5%)	33.0% (27.8% to 38.8%)	32.5% (27.8% to 37.9%)	33.0% (27.8% to 38.7%)
Atrial fibrillation and flutter	47.9% (40.5% to 55.1%)	52.6% (45.8% to 58.8%)	59.5% (54.1% to 64.6%)	56.5% (50.7% to 62.0%)
Aortic aneurysm	44.4% (37.5% to 50.8%)	48.8% (43.0% to 54.4%)	-	48.8% (43.0% to 54.4%)
Peripheral vascular disease	52.9% (46.0% to 60.2%)	60.8% (55.3% to 66.2%)	64.3% (59.5% to 69.1%)	62.4% (57.5% to 67.1%)
Endocarditis	37.6% (29.3% to 46.5%)	34.5% (27.5% to 41.9%)	36.2% (28.6% to 44.8%)	34.5% (27.6% to 42.0%)

Supplementary Results Table 1. Age-standardised PAFs attributable to the joint distribution of all risk factors for all ages, both sexes combined for each GBD level 1 and level 2 cause and all causes in 2016 for Guyana. Results are shown for deaths, YLLs, YLDs, and DALYs
Data in parenthesis are 95% uncertainty intervals. PAFs=population attributable fractions. GBD=Global Burden of Disease. YLLs=years of life lost. YLDs=years live with disability. DALYs=disability-adjusted life-years

	Deaths 2016	YLLs 2016	YLDs 2016	DALYs 2016
Other cardiovascular and circulatory diseases	45.7% (41.2% to 50.1%)	45.9% (41.5% to 50.4%)	43.3% (38.4% to 48.2%)	45.6% (41.3% to 50.0%)
**Chronic respiratory diseases**	37.4% (30.7% to 44.3%)	37.8% (31.9% to 43.7%)	27.6% (21.0% to 35.5%)	33.3% (27.7% to 39.0%)
Chronic obstructive pulmonary disease	46.7% (36.7% to 57.1%)	49.7% (40.1% to 58.9%)	48.7% (39.2% to 57.9%)	49.5% (40.0% to 58.8%)
Pneumoconiosis	100.0% (100.0% to 100.0%)	100.0% (100.0% to 100.0%)	100.0% (100.0% to 100.0%)	100.0% (100.0% to 100.0%)
Asthma	37.4% (28.6% to 47.2%)	37.9% (29.9% to 46.9%)	25.0% (17.6% to 33.9%)	28.6% (21.4% to 37.4%)
Other chronic respiratory diseases	2.7% (1.3% to 4.5%)	3.6% (1.6% to 6.3%)	1.8% (0.7% to 3.7%)	3.5% (1.6% to 6.1%)
**Cirrhosis and other chronic liver diseases**	66.1% (63.2% to 69.1%)	67.2% (64.6% to 69.9%)	53.3% (49.6% to 57.1%)	67.0% (64.3% to 69.6%)
Cirrhosis and other chronic liver diseases due to hepatitis B	6.5% (2.8% to 11.3%)	8.2% (3.5% to 14.1%)	5.8% (2.5% to 10.0%)	8.1% (3.5% to 14.0%)
Cirrhosis and other chronic liver diseases due to hepatitis C	77.5% (71.6% to 82.5%)	83.9% (80.5% to 86.7%)	73.5% (67.0% to 79.2%)	83.7% (80.2% to 86.6%)
Cirrhosis and other chronic liver diseases due to alcohol use	100.0% (100.0% to 100.0%)	100.0% (100.0% to 100.0%)	100.0% (100.0% to 100.0%)	100.0% (100.0% to 100.0%)
**Digestive diseases**	9.9% (7.9% to 11.8%)	11.8% (9.6% to 13.9%)	2.7% (2.0% to 3.5%)	10.3% (8.3% to 12.2%)
Peptic ulcer disease	12.1% (8.3% to 16.3%)	14.1% (9.8% to 18.9%)	7.3% (4.9% to 10.1%)	13.4% (9.3% to 17.9%)
Gallbladder and biliary diseases	39.9% (26.6% to 54.2%)	40.8% (27.9% to 54.6%)	45.7% (32.2% to 59.4%)	41.3% (28.1% to 55.1%)
Pancreatitis	40.2% (26.8% to 53.1%)	46.7% (33.0% to 59.4%)	29.1% (19.3% to 38.0%)	46.6% (32.9% to 59.2%)
**Neurological disorders**	21.0% (10.8% to 33.0%)	21.3% (13.8% to 30.1%)	4.5% (2.3% to 6.7%)	10.4% (6.5% to 14.9%)
Alzheimer's disease and other dementias	23.7% (11.1% to 38.6%)	25.6% (12.5% to 41.0%)	25.7% (12.8% to 40.9%)	25.6% (12.5% to 41.0%)
Parkinson's disease	-6.3% (-8.3% to -4.5%)	-8.0% (-10.5% to -5.8%)	-8.8% (-11.4% to -6.3%)	-8.1% (-10.6% to -5.9%)
Epilepsy	28.4% (20.8% to 36.2%)	28.8% (21.0% to 36.9%)	20.0% (14.7% to 25.8%)	24.8% (18.2% to 31.9%)
Multiple sclerosis	6.4% (3.6% to 9.6%)	6.2% (3.5% to 9.2%)	4.4% (2.7% to 6.3%)	5.7% (3.3% to 8.3%)
**Mental and substance use disorders**	99.5% (99.3% to 99.6%)	99.2% (99.0% to 99.4%)	18.5% (16.5% to 20.8%)	24.5% (21.8% to 27.7%)
Alcohol use disorders	100.0% (100.0% to 100.0%)	100.0% (100.0% to 100.0%)	100.0% (100.0% to 100.0%)	100.0% (100.0% to 100.0%)

Supplementary Results Table 1. Age-standardised PAFs attributable to the joint distribution of all risk factors for all ages, both sexes combined for each GBD level 1 and level 2 cause and all causes in 2016 for Guyana. Results are shown for deaths, YLLs, YLDs, and DALYs
Data in parenthesis are 95% uncertainty intervals. PAFs=population attributable fractions. GBD=Global Burden of Disease. YLLs=years of life lost. YLDs=years live with disability. DALYs=disability-adjusted life-years

	**Deaths 2016**	**YLLs 2016**	**YLDs 2016**	**DALYs 2016**
Drug use disorders	100.0% (100.0% to 100.0%)	100.0% (100.0% to 100.0%)	100.0% (100.0% to 100.0%)	100.0% (100.0% to 100.0%)
Depressive disorders	-	-	6.7% (5.5% to 8.2%)	6.7% (5.5% to 8.2%)
Idiopathic developmental intellectual disability	-	-	65.7% (34.5% to 85.1%)	65.7% (34.5% to 85.1%)
**Diabetes, urogenital, blood, and endocrine diseases**	88.8% (87.2% to 89.7%)	85.7% (83.5% to 86.9%)	76.2% (72.4% to 79.5%)	83.2% (81.2% to 84.6%)
Diabetes mellitus	100.0% (100.0% to 100.0%)	100.0% (100.0% to 100.0%)	100.0% (100.0% to 100.0%)	100.0% (100.0% to 100.0%)
Chronic kidney disease	100.0% (100.0% to 100.0%)	100.0% (100.0% to 100.0%)	100.0% (100.0% to 100.0%)	100.0% (100.0% to 100.0%)
**Musculoskeletal disorders**	0.4% (0.1% to 0.7%)	0.2% (0.1% to 0.4%)	13.9% (11.0% to 17.0%)	13.5% (10.7% to 16.6%)
Rheumatoid arthritis	1.9% (0.7% to 3.4%)	2.2% (0.8% to 3.8%)	3.0% (1.2% to 5.1%)	3.0% (1.2% to 5.0%)
Osteoarthritis	-	-	24.9% (13.1% to 37.4%)	24.9% (13.1% to 37.4%)
Low back and neck pain	-	-	17.8% (15.0% to 20.9%)	17.8% (15.0% to 20.9%)
Gout	-	-	44.5% (31.8% to 59.2%)	44.5% (31.8% to 59.2%)
**Other non-communicable diseases**	0.0% (0.0% to 0.1%)	0.1% (0.0% to 0.1%)	5.4% (4.5% to 6.5%)	4.3% (3.6% to 5.0%)
Sense organ diseases	-	-	12.5% (10.8% to 14.4%)	12.5% (10.8% to 14.4%)
Sudden infant death syndrome	4.2% (2.6% to 6.4%)	4.3% (2.7% to 6.4%)	-	4.3% (2.7% to 6.4%)
**Transport injuries**	34.0% (25.1% to 44.6%)	30.6% (21.3% to 42.0%)	37.9% (29.3% to 48.1%)	31.2% (21.9% to 42.5%)
Road injuries	33.7% (24.5% to 44.6%)	30.8% (21.1% to 42.4%)	38.5% (29.8% to 48.9%)	31.4% (21.9% to 42.9%)
Other transport injuries	36.6% (29.0% to 46.0%)	29.6% (21.4% to 39.7%)	30.6% (23.5% to 39.2%)	29.7% (21.6% to 39.6%)
**Unintentional injuries**	17.9% (14.2% to 22.6%)	12.8% (8.5% to 18.4%)	17.2% (14.2% to 21.7%)	13.9% (9.9% to 19.3%)
Falls	48.5% (44.2% to 53.9%)	26.6% (22.2% to 33.5%)	24.7% (21.6% to 29.7%)	25.5% (22.0% to 31.3%)
Drowning	17.4% (9.2% to 27.3%)	16.8% (8.8% to 26.3%)	15.9% (8.8% to 24.5%)	16.8% (8.8% to 26.3%)
Fire, heat, and hot substances	10.1% (6.3% to 15.0%)	11.8% (7.4% to 17.7%)	15.5% (10.1% to 22.2%)	12.7% (8.0% to 18.7%)

Supplementary Results Table 1. Age-standardised PAFs attributable to the joint distribution of all risk factors for all ages, both sexes combined for each GBD level 1 and level 2 cause and all causes in 2016 for Guyana. Results are shown for deaths, YLLs, YLDs, and DALYs
Data in parenthesis are 95% uncertainty intervals. PAFs=population attributable fractions. GBD=Global Burden of Disease. YLLs=years of life lost. YLDs=years live with disability. DALYs=disability-adjusted life-years

	**Deaths 2016**	**YLLs 2016**	**YLDs 2016**	**DALYs 2016**
Poisonings	14.4% (9.1% to 21.3%)	14.2% (9.0% to 21.2%)	14.8% (9.9% to 21.1%)	14.3% (9.0% to 21.1%)
Exposure to mechanical forces	15.7% (11.5% to 22.2%)	12.7% (8.3% to 19.6%)	16.3% (12.3% to 23.1%)	13.6% (9.2% to 20.3%)
Animal contact	8.4% (5.5% to 13.5%)	8.5% (5.1% to 14.1%)	8.6% (5.2% to 13.9%)	8.5% (5.2% to 14.0%)
Foreign body	5.2% (2.7% to 10.0%)	5.1% (2.6% to 9.4%)	6.2% (3.6% to 10.7%)	5.3% (2.7% to 9.6%)
Other unintentional injuries	17.3% (9.6% to 27.1%)	17.7% (9.6% to 27.9%)	14.4% (9.0% to 21.2%)	16.4% (9.4% to 25.2%)
**Self-harm and interpersonal violence**	31.8% (23.3% to 40.4%)	32.4% (23.8% to 40.9%)	29.8% (25.4% to 34.8%)	32.3% (23.8% to 40.8%)
Self-harm	32.6% (20.3% to 43.4%)	33.9% (21.6% to 45.0%)	28.6% (17.9% to 38.6%)	33.9% (21.6% to 45.0%)
Interpersonal violence	30.5% (19.9% to 41.6%)	30.2% (19.6% to 41.8%)	30.0% (25.3% to 35.2%)	30.2% (19.8% to 41.5%)
**Forces of nature, conflict and terrorism, and executions and police conflict**	0.0% (0.0% to 0.0%)	0.0% (0.0% to 0.0%)	1.3% (0.7% to 2.0%)	0.3% (0.0% to 0.8%)
Forces of nature, conflict and terrorism, and state actor violence	0.0% (0.0% to 0.0%)	0.0% (0.0% to 0.0%)	4.6% (3.0% to 6.7%)	4.6% (3.0% to 6.7%)

Supplementary Results Table 1. Age-standardised PAFs attributable to the joint distribution of all risk factors for all ages, both sexes combined for each GBD level 1 and level 2 cause and all causes in 2016 for Haiti. Results are shown for deaths, YLLs, YLDs, and DALYs
Data in parenthesis are 95% uncertainty intervals. PAFs=population attributable fractions. GBD=Global Burden of Disease. YLLs=years of life lost. YLDs=years live with disability. DALYs=disability-adjusted life-years

	Deaths 2016	YLLs 2016	YLDs 2016	DALYs 2016
**All causes**	63.8% (61.8% to 65.5%)	57.8% (55.9% to 59.6%)	27.1% (25.8% to 28.5%)	50.6% (48.4% to 52.8%)
**HIV/AIDS and tuberculosis**	67.5% (60.8% to 73.9%)	66.2% (59.3% to 71.9%)	75.9% (67.8% to 82.5%)	66.7% (59.8% to 72.3%)
Tuberculosis	41.6% (32.0% to 51.3%)	43.7% (34.7% to 52.6%)	42.7% (33.7% to 51.2%)	43.7% (34.7% to 52.5%)
HIV/AIDS	76.3% (67.6% to 83.1%)	70.9% (62.5% to 77.6%)	77.8% (69.6% to 84.2%)	71.4% (63.0% to 77.8%)
**Diarrhoea, lower respiratory infections, and other common infectious diseases**	73.6% (67.4% to 79.6%)	73.7% (65.4% to 79.9%)	50.3% (42.6% to 56.9%)	72.1% (64.4% to 78.2%)
Diarrhoeal diseases	95.9% (88.7% to 99.1%)	97.6% (93.4% to 99.5%)	95.5% (88.1% to 98.9%)	97.4% (92.8% to 99.4%)
Lower respiratory infections	74.6% (67.4% to 81.2%)	82.8% (76.6% to 88.1%)	76.4% (69.8% to 82.4%)	82.8% (76.5% to 88.1%)
Upper respiratory infections	1.2% (0.1% to 4.6%)	2.5% (0.2% to 9.1%)	0.0% (0.0% to 0.0%)	0.1% (0.0% to 0.4%)
Otitis media	1.0% (0.3% to 2.7%)	2.5% (0.8% to 5.8%)	2.8% (1.8% to 3.7%)	2.7% (1.8% to 3.7%)
Meningitis	5.0% (2.9% to 8.6%)	6.3% (3.6% to 10.2%)	0.0% (0.0% to 0.0%)	6.1% (3.6% to 10.1%)
Encephalitis	0.9% (0.4% to 1.4%)	1.3% (0.7% to 2.2%)	0.0% (0.0% to 0.0%)	1.2% (0.6% to 1.9%)
Measles	26.7% (0.0% to 76.1%)	27.2% (0.0% to 77.4%)	27.2% (0.0% to 77.5%)	41.7% (0.0% to 78.9%)
**Maternal disorders**	5.8% (2.8% to 9.4%)	5.8% (2.7% to 9.3%)	4.4% (2.4% to 6.8%)	5.7% (2.7% to 9.2%)
Maternal haemorrhage	20.8% (7.7% to 33.5%)	20.8% (7.7% to 33.6%)	20.9% (7.6% to 33.7%)	20.8% (7.7% to 33.6%)
Maternal sepsis and other pregnancy related infections	20.6% (7.6% to 33.3%)	20.7% (7.7% to 33.4%)	20.7% (7.6% to 33.3%)	20.7% (7.7% to 33.4%)
Maternal abortion, miscarriage, and ectopic pregnancy	12.2% (7.3% to 17.7%)	12.4% (7.5% to 18.0%)	13.0% (7.3% to 19.5%)	12.4% (7.6% to 18.0%)
**Neonatal disorders**	67.5% (62.7% to 72.1%)	67.6% (62.8% to 72.2%)	0.3% (0.1% to 0.4%)	63.3% (58.2% to 68.2%)
Neonatal preterm birth complications	93.9% (87.2% to 98.0%)	94.0% (87.3% to 98.0%)	0.5% (0.4% to 0.6%)	80.2% (68.9% to 88.3%)
Neonatal encephalopathy due to birth asphyxia and trauma	63.3% (56.3% to 69.3%)	63.3% (56.3% to 69.3%)	0.0% (0.0% to 0.0%)	62.9% (55.8% to 69.0%)
Neonatal sepsis and other neonatal infections	57.2% (49.0% to 64.1%)	57.2% (49.1% to 64.1%)	0.0% (0.0% to 0.0%)	50.3% (36.3% to 59.9%)
Hemolytic disease and other neonatal jaundice	55.4% (40.9% to 65.6%)	55.5% (41.0% to 65.7%)	0.0% (0.0% to 0.0%)	42.4% (29.7% to 53.5%)

Supplementary Results Table 1. Age-standardised PAFs attributable to the joint distribution of all risk factors for all ages, both sexes combined for each GBD level 1 and level 2 cause and all causes in 2016 for Haiti. Results are shown for deaths, YLLs, YLDs, and DALYs

Data in parenthesis are 95% uncertainty intervals. PAFs=population attributable fractions. GBD=Global Burden of Disease. YLLs=years of life lost. YLDs=years live with disability. DALYs=disability-adjusted life-years

	Deaths 2016	YLLs 2016	YLDs 2016	DALYs 2016
Other neonatal disorders	59.4% (51.8% to 65.6%)	59.4% (51.9% to 65.7%)	0.0% (0.0% to 0.0%)	58.6% (51.3% to 64.9%)
**Nutritional deficiencies**	24.2% (9.9% to 40.2%)	57.2% (33.5% to 74.0%)	99.0% (98.7% to 99.2%)	85.0% (67.3% to 91.3%)
Protein-energy malnutrition	27.6% (11.8% to 44.6%)	63.1% (39.0% to 79.9%)	96.5% (95.0% to 97.5%)	65.1% (41.3% to 80.7%)
Vitamin A deficiency	-	-	100.0% (100.0% to 100.0%)	100.0% (100.0% to 100.0%)
Iron-deficiency anaemia	100.0% (100.0% to 100.0%)	100.0% (100.0% to 100.0%)	100.0% (100.0% to 100.0%)	100.0% (100.0% to 100.0%)
**Other communicable, maternal, neonatal, and nutritional diseases**	9.2% (4.5% to 15.7%)	5.2% (2.2% to 9.6%)	51.1% (44.1% to 57.6%)	11.2% (5.9% to 18.5%)
Sexually transmitted diseases excluding HIV	16.1% (5.9% to 32.7%)	6.9% (2.4% to 15.2%)	100.0% (100.0% to 100.0%)	16.6% (7.4% to 31.8%)
Hepatitis	6.2% (3.7% to 9.3%)	6.2% (3.6% to 9.6%)	4.9% (2.8% to 7.5%)	6.1% (3.6% to 9.4%)
**Neoplasms**	34.1% (30.2% to 38.4%)	35.6% (31.7% to 40.0%)	33.2% (28.8% to 37.8%)	35.6% (31.7% to 40.0%)
Oesophageal cancer	66.8% (55.2% to 76.5%)	70.1% (59.5% to 79.2%)	67.5% (56.3% to 77.0%)	70.1% (59.4% to 79.2%)
Stomach cancer	11.2% (5.3% to 18.9%)	12.3% (6.0% to 20.4%)	11.4% (5.6% to 19.0%)	12.3% (6.0% to 20.3%)
Liver cancer	46.7% (39.7% to 54.4%)	48.3% (41.2% to 56.1%)	47.0% (40.1% to 54.3%)	48.3% (41.2% to 56.1%)
Larynx cancer	77.5% (66.7% to 86.4%)	79.7% (69.8% to 87.7%)	78.1% (67.6% to 86.8%)	79.7% (69.8% to 87.7%)
Tracheal, bronchus, and lung cancer	87.8% (83.3% to 91.6%)	87.9% (83.8% to 91.6%)	87.8% (83.5% to 91.6%)	87.9% (83.8% to 91.6%)
Breast cancer	21.3% (13.9% to 30.7%)	20.3% (12.7% to 29.4%)	20.8% (13.1% to 29.9%)	20.3% (12.7% to 29.4%)
Cervical cancer	100.0% (100.0% to 100.0%)	100.0% (100.0% to 100.0%)	100.0% (100.0% to 100.0%)	100.0% (100.0% to 100.0%)
Uterine cancer	24.1% (13.3% to 37.6%)	26.2% (14.6% to 39.1%)	25.3% (14.0% to 38.7%)	26.2% (14.6% to 39.1%)
Prostate cancer	2.4% (1.3% to 4.3%)	2.8% (1.5% to 5.0%)	2.6% (1.4% to 4.6%)	2.8% (1.5% to 5.0%)
Colon and rectum cancer	52.5% (43.1% to 62.1%)	53.2% (44.0% to 62.4%)	52.7% (43.4% to 62.2%)	53.2% (44.0% to 62.4%)
Lip and oral cavity cancer	66.5% (55.1% to 76.0%)	70.9% (60.3% to 79.6%)	65.8% (55.3% to 75.3%)	70.8% (60.1% to 79.5%)
Nasopharynx cancer	67.0% (58.1% to 75.0%)	65.8% (57.5% to 73.0%)	65.5% (57.4% to 72.5%)	65.8% (57.5% to 73.0%)

Supplementary Results Table 1. Age-standardised PAFs attributable to the joint distribution of all risk factors for all ages, both sexes combined for each GBD level 1 and level 2 cause and all causes in 2016 for Haiti. Results are shown for deaths, YLLs, YLDs, and DALYs
Data in parenthesis are 95% uncertainty intervals. PAFs=population attributable fractions. GBD=Global Burden of Disease. YLLs=years of life lost. YLDs=years live with disability. DALYs=disability-adjusted life-years

	Deaths 2016	YLLs 2016	YLDs 2016	DALYs 2016
Other pharynx cancer	47.4% (33.3% to 59.7%)	51.2% (37.4% to 63.2%)	48.4% (34.7% to 60.6%)	51.2% (37.4% to 63.1%)
Gallbladder and biliary tract cancer	11.1% (5.0% to 19.5%)	12.2% (5.7% to 20.9%)	11.3% (5.1% to 20.0%)	12.2% (5.8% to 20.8%)
Pancreatic cancer	23.1% (14.7% to 33.2%)	25.0% (16.5% to 35.0%)	23.4% (15.3% to 33.4%)	25.0% (16.5% to 35.0%)
Ovarian cancer	13.2% (5.3% to 24.3%)	12.0% (4.8% to 22.5%)	12.0% (4.8% to 22.4%)	12.0% (4.8% to 22.5%)
Kidney cancer	19.1% (12.5% to 27.3%)	18.6% (12.1% to 27.0%)	18.8% (12.4% to 26.7%)	18.6% (12.2% to 27.0%)
Bladder cancer	24.3% (14.7% to 35.2%)	26.6% (16.7% to 37.5%)	25.2% (15.7% to 35.6%)	26.5% (16.7% to 37.5%)
Thyroid cancer	6.2% (2.8% to 11.1%)	6.7% (3.1% to 11.8%)	6.4% (3.0% to 11.2%)	6.7% (3.1% to 11.8%)
Mesothelioma	86.9% (81.2% to 91.8%)	79.0% (71.1% to 86.6%)	84.5% (77.9% to 90.2%)	79.0% (71.2% to 86.6%)
Non-Hodgkin's lymphoma	2.9% (1.0% to 5.8%)	2.8% (1.0% to 5.5%)	2.9% (1.0% to 5.8%)	2.8% (1.0% to 5.5%)
Multiple myeloma	3.6% (1.3% to 7.0%)	3.8% (1.5% to 7.3%)	3.6% (1.3% to 7.0%)	3.8% (1.5% to 7.2%)
Leukaemia	9.6% (5.3% to 14.9%)	8.2% (4.5% to 12.7%)	9.5% (5.3% to 14.3%)	8.2% (4.5% to 12.8%)
**Cardiovascular diseases**	85.3% (82.9% to 87.6%)	84.0% (81.7% to 86.1%)	68.2% (64.6% to 71.5%)	83.4% (81.1% to 85.5%)
Rheumatic heart disease	23.5% (15.9% to 36.8%)	19.1% (13.0% to 28.1%)	17.8% (11.1% to 26.4%)	18.9% (12.9% to 27.6%)
Ischaemic heart disease	93.2% (90.1% to 95.7%)	94.6% (92.7% to 96.3%)	89.5% (86.3% to 92.4%)	94.5% (92.5% to 96.2%)
Cerebrovascular disease	84.9% (80.7% to 88.7%)	84.7% (80.8% to 87.9%)	85.3% (81.2% to 89.2%)	84.7% (80.9% to 87.9%)
Hypertensive heart disease	100.0% (100.0% to 100.0%)	100.0% (100.0% to 100.0%)	100.0% (100.0% to 100.0%)	100.0% (100.0% to 100.0%)
Cardiomyopathy and myocarditis	35.0% (28.6% to 42.3%)	32.4% (25.3% to 40.0%)	33.6% (28.2% to 39.1%)	32.4% (25.4% to 39.8%)
Atrial fibrillation and flutter	42.6% (35.2% to 50.2%)	47.2% (40.5% to 53.5%)	53.1% (47.8% to 58.4%)	50.5% (44.5% to 56.1%)
Aortic aneurysm	38.5% (31.8% to 45.3%)	41.7% (35.7% to 47.4%)	-	41.7% (35.7% to 47.4%)
Peripheral vascular disease	47.6% (41.1% to 55.0%)	55.0% (49.7% to 60.5%)	58.8% (53.9% to 63.8%)	57.3% (52.3% to 62.3%)
Endocarditis	36.4% (28.1% to 46.3%)	32.3% (24.9% to 40.1%)	35.4% (27.5% to 44.0%)	32.3% (24.9% to 40.1%)

Supplementary Results Table 1. Age-standardised PAFs attributable to the joint distribution of all risk factors for all ages, both sexes combined for each GBD level 1 and level 2 cause and all causes in 2016 for Haiti. Results are shown for deaths, YLLs, YLDs, and DALYs
Data in parenthesis are 95% uncertainty intervals. PAFs=population attributable fractions. GBD=Global Burden of Disease. YLLs=years of life lost. YLDs=years live with disability. DALYs=disability-adjusted life-years

	Deaths 2016	YLLs 2016	YLDs 2016	DALYs 2016
Other cardiovascular and circulatory diseases	40.4% (35.6% to 45.2%)	38.4% (33.4% to 43.8%)	39.5% (34.6% to 44.5%)	38.5% (33.6% to 43.8%)
**Chronic respiratory diseases**	61.7% (54.7% to 68.3%)	54.4% (47.5% to 61.0%)	24.5% (18.8% to 31.0%)	46.4% (39.4% to 52.9%)
Chronic obstructive pulmonary disease	81.4% (74.4% to 88.1%)	80.3% (74.1% to 86.5%)	74.2% (66.1% to 81.7%)	79.9% (73.5% to 86.0%)
Pneumoconiosis	100.0% (100.0% to 100.0%)	100.0% (100.0% to 100.0%)	100.0% (100.0% to 100.0%)	100.0% (100.0% to 100.0%)
Asthma	24.0% (17.4% to 32.0%)	24.3% (17.8% to 31.8%)	17.1% (12.1% to 23.6%)	21.0% (15.3% to 27.7%)
Other chronic respiratory diseases	10.3% (5.6% to 17.0%)	10.5% (5.2% to 17.5%)	5.1% (2.7% to 8.4%)	10.1% (5.1% to 16.7%)
**Cirrhosis and other chronic liver diseases**	60.3% (56.3% to 64.5%)	60.7% (56.7% to 64.7%)	49.2% (45.3% to 53.4%)	60.4% (56.5% to 64.3%)
Cirrhosis and other chronic liver diseases due to hepatitis B	4.6% (1.9% to 8.0%)	5.8% (2.5% to 10.2%)	4.6% (2.0% to 7.8%)	5.8% (2.5% to 10.1%)
Cirrhosis and other chronic liver diseases due to hepatitis C	65.7% (56.6% to 74.1%)	73.7% (66.2% to 80.1%)	65.5% (57.4% to 72.4%)	73.6% (66.0% to 79.9%)
Cirrhosis and other chronic liver diseases due to alcohol use	100.0% (100.0% to 100.0%)	100.0% (100.0% to 100.0%)	100.0% (100.0% to 100.0%)	100.0% (100.0% to 100.0%)
**Digestive diseases**	5.3% (3.9% to 7.1%)	6.1% (4.3% to 8.6%)	1.4% (0.9% to 1.9%)	5.4% (3.8% to 7.6%)
Peptic ulcer disease	5.4% (3.6% to 7.7%)	6.4% (4.2% to 9.0%)	4.6% (3.0% to 6.4%)	6.3% (4.2% to 8.8%)
Gallbladder and biliary diseases	23.3% (12.5% to 36.5%)	25.0% (14.1% to 37.9%)	28.2% (16.8% to 41.5%)	25.2% (14.3% to 38.1%)
Pancreatitis	34.2% (18.9% to 51.7%)	39.7% (23.9% to 57.0%)	29.1% (17.2% to 43.3%)	39.6% (23.9% to 56.9%)
**Neurological disorders**	13.8% (7.0% to 23.2%)	15.8% (10.0% to 22.7%)	3.0% (1.3% to 5.4%)	7.7% (4.8% to 11.6%)
Alzheimer's disease and other dementias	14.4% (6.0% to 25.6%)	15.7% (6.8% to 27.5%)	15.7% (6.9% to 27.4%)	15.7% (6.8% to 27.5%)
Parkinson's disease	-2.5% (-3.2% to -1.9%)	-3.2% (-4.1% to -2.4%)	-3.8% (-4.7% to -2.8%)	-3.3% (-4.2% to -2.5%)
Epilepsy	24.5% (17.0% to 32.8%)	24.1% (16.8% to 33.0%)	20.0% (14.0% to 26.5%)	22.8% (16.1% to 30.7%)
Multiple sclerosis	3.4% (1.8% to 5.4%)	3.3% (1.7% to 5.4%)	2.7% (1.6% to 3.8%)	3.1% (1.7% to 5.0%)
**Mental and substance use disorders**	99.9% (99.8% to 99.9%)	99.8% (99.7% to 99.9%)	18.4% (16.3% to 20.5%)	24.1% (21.0% to 27.9%)
Alcohol use disorders	100.0% (100.0% to 100.0%)	100.0% (100.0% to 100.0%)	100.0% (100.0% to 100.0%)	100.0% (100.0% to 100.0%)

Supplementary Results Table 1. Age-standardised PAFs attributable to the joint distribution of all risk factors for all ages, both sexes combined for each GBD level 1 and level 2 cause and all causes in 2016 for Haiti. Results are shown for deaths, YLLs, YLDs, and DALYs
Data in parenthesis are 95% uncertainty intervals. PAFs=population attributable fractions. GBD=Global Burden of Disease. YLLs=years of life lost. YLDs=years live with disability. DALYs=disability-adjusted life-years

	Deaths 2016	YLLs 2016	YLDs 2016	DALYs 2016
Drug use disorders	100.0% (100.0% to 100.0%)	100.0% (100.0% to 100.0%)	100.0% (100.0% to 100.0%)	100.0% (100.0% to 100.0%)
Depressive disorders	-	-	5.8% (4.8% to 7.0%)	5.8% (4.8% to 7.0%)
Idiopathic developmental intellectual disability	-	-	69.2% (36.6% to 88.1%)	69.2% (36.6% to 88.1%)
**Diabetes, urogenital, blood, and endocrine diseases**	89.0% (87.4% to 90.4%)	83.7% (80.7% to 86.4%)	67.4% (63.2% to 71.3%)	79.4% (76.5% to 82.1%)
Diabetes mellitus	100.0% (100.0% to 100.0%)	100.0% (100.0% to 100.0%)	100.0% (100.0% to 100.0%)	100.0% (100.0% to 100.0%)
Chronic kidney disease	100.0% (100.0% to 100.0%)	100.0% (100.0% to 100.0%)	100.0% (100.0% to 100.0%)	100.0% (100.0% to 100.0%)
**Musculoskeletal disorders**	0.4% (0.1% to 0.8%)	0.3% (0.1% to 0.5%)	14.9% (12.3% to 17.9%)	14.2% (11.6% to 17.0%)
Rheumatoid arthritis	1.4% (0.5% to 2.5%)	1.6% (0.6% to 2.8%)	1.8% (0.7% to 3.2%)	1.8% (0.7% to 3.2%)
Osteoarthritis	-	-	13.4% (6.2% to 22.7%)	13.4% (6.2% to 22.7%)
Low back and neck pain	-	-	23.0% (19.6% to 26.9%)	23.0% (19.6% to 26.9%)
Gout	-	-	33.2% (24.0% to 45.5%)	33.2% (24.0% to 45.5%)
**Other non-communicable diseases**	0.2% (0.1% to 0.4%)	0.3% (0.1% to 0.5%)	5.8% (4.8% to 6.8%)	3.9% (3.0% to 4.9%)
Sense organ diseases	-	-	12.2% (10.5% to 14.0%)	12.2% (10.5% to 14.0%)
Sudden infant death syndrome	4.6% (1.9% to 8.9%)	4.7% (1.9% to 9.0%)	-	4.7% (1.9% to 9.0%)
**Transport injuries**	25.1% (18.4% to 33.7%)	20.0% (13.3% to 28.5%)	33.5% (26.2% to 42.8%)	20.8% (14.0% to 29.2%)
Road injuries	24.9% (18.2% to 33.6%)	20.0% (13.3% to 28.6%)	33.9% (26.6% to 43.3%)	20.8% (14.0% to 29.3%)
Other transport injuries	28.8% (21.8% to 36.9%)	19.7% (12.8% to 28.1%)	26.4% (19.8% to 34.6%)	20.3% (13.7% to 28.6%)
**Unintentional injuries**	12.5% (9.5% to 16.1%)	7.8% (4.9% to 11.2%)	15.3% (13.1% to 17.9%)	9.5% (6.8% to 12.6%)
Falls	46.3% (40.3% to 52.8%)	21.6% (16.8% to 27.5%)	23.0% (20.6% to 26.0%)	22.4% (19.5% to 25.7%)
Drowning	9.9% (5.2% to 15.2%)	8.5% (4.4% to 13.5%)	11.9% (6.2% to 18.4%)	8.6% (4.4% to 13.5%)
Fire, heat, and hot substances	6.6% (3.7% to 10.3%)	6.3% (3.5% to 10.4%)	11.5% (6.7% to 17.0%)	7.1% (4.0% to 11.3%)

Supplementary Results Table 1. Age-standardised PAFs attributable to the joint distribution of all risk factors for all ages, both sexes combined for each GBD level 1 and level 2 cause and all causes in 2016 for Haiti. Results are shown for deaths, YLLs, YLDs, and DALYs
Data in parenthesis are 95% uncertainty intervals. PAFs=population attributable fractions. GBD=Global Burden of Disease. YLLs=years of life lost. YLDs=years live with disability. DALYs=disability-adjusted life-years

	**Deaths 2016**	**YLLs 2016**	**YLDs 2016**	**DALYs 2016**
Poisonings	9.3% (5.0% to 14.9%)	8.4% (4.0% to 14.4%)	10.9% (6.5% to 16.2%)	8.5% (4.0% to 14.4%)
Exposure to mechanical forces	11.2% (7.4% to 15.6%)	8.7% (5.0% to 13.5%)	12.8% (10.3% to 16.6%)	9.5% (6.1% to 13.7%)
Animal contact	7.6% (5.4% to 10.5%)	5.6% (3.7% to 8.6%)	5.6% (3.7% to 8.7%)	5.6% (3.7% to 8.6%)
Foreign body	2.5% (1.2% to 4.7%)	2.2% (0.9% to 4.2%)	3.6% (2.1% to 6.0%)	2.5% (1.2% to 4.6%)
Other unintentional injuries	10.7% (5.7% to 16.5%)	9.9% (5.2% to 15.3%)	11.1% (6.4% to 16.6%)	10.3% (5.6% to 15.7%)
**Self-harm and interpersonal violence**	27.4% (19.9% to 35.4%)	27.7% (20.3% to 35.8%)	30.8% (27.9% to 34.2%)	27.9% (20.8% to 35.6%)
Self-harm	26.9% (15.3% to 39.4%)	28.1% (16.3% to 40.9%)	24.0% (13.7% to 35.0%)	28.1% (16.2% to 40.8%)
Interpersonal violence	28.0% (21.0% to 36.1%)	27.1% (20.1% to 35.1%)	31.4% (28.6% to 34.7%)	27.6% (21.1% to 34.9%)
**Forces of nature, conflict and terrorism, and executions and police conflict**	6.5% (6.0% to 6.9%)	2.6% (2.3% to 2.8%)	7.0% (5.1% to 9.1%)	3.8% (2.9% to 5.2%)
Forces of nature, conflict and terrorism, and state actor violence	6.5% (6.0% to 6.9%)	2.6% (2.4% to 2.9%)	7.1% (5.2% to 9.2%)	3.9% (2.9% to 5.2%)

Supplementary Results Table 1. Age-standardised PAFs attributable to the joint distribution of all risk factors for all ages, both sexes combined for each GBD level 1 and level 2 cause and all causes in 2016 for Honduras. Results are shown for deaths, YLLs, YLDs, and DALYs
Data in parenthesis are 95% uncertainty intervals. PAFs=population attributable fractions. GBD=Global Burden of Disease. YLLs=years of life lost. YLDs=years live with disability. DALYs=disability-adjusted life-years

	Deaths 2016	YLLs 2016	YLDs 2016	DALYs 2016
**All causes**	60.4% (58.2% to 62.4%)	55.3% (53.4% to 57.1%)	24.7% (23.3% to 26.1%)	45.3% (42.4% to 48.2%)
**HIV/AIDS and tuberculosis**	55.3% (47.1% to 64.7%)	64.6% (57.7% to 71.4%)	83.2% (78.5% to 87.1%)	65.7% (59.1% to 72.3%)
Tuberculosis	35.2% (26.4% to 47.1%)	37.0% (28.1% to 48.2%)	34.6% (26.9% to 44.6%)	37.0% (28.1% to 48.1%)
HIV/AIDS	94.0% (91.3% to 96.8%)	90.6% (86.0% to 95.4%)	95.3% (93.9% to 96.8%)	91.0% (86.5% to 95.4%)
**Diarrhoea, lower respiratory infections, and other common infectious diseases**	67.6% (58.0% to 77.0%)	68.8% (59.5% to 76.5%)	44.7% (35.9% to 52.8%)	63.9% (55.4% to 71.6%)
Diarrhoeal diseases	83.3% (65.0% to 94.3%)	87.8% (73.4% to 96.2%)	85.4% (69.1% to 95.1%)	87.2% (73.0% to 95.7%)
Lower respiratory infections	68.8% (60.7% to 76.0%)	77.5% (70.6% to 83.3%)	70.3% (63.2% to 77.0%)	77.5% (70.6% to 83.2%)
Upper respiratory infections	1.6% (0.1% to 5.5%)	3.7% (0.3% to 12.7%)	0.0% (0.0% to 0.0%)	0.1% (0.0% to 0.2%)
Otitis media	2.7% (0.8% to 5.8%)	6.1% (2.4% to 11.1%)	4.9% (3.2% to 6.5%)	4.9% (3.2% to 6.5%)
Meningitis	2.5% (1.2% to 4.8%)	5.0% (2.3% to 9.8%)	0.0% (0.0% to 0.0%)	4.6% (2.1% to 9.0%)
Encephalitis	2.2% (1.2% to 4.0%)	3.6% (1.9% to 6.3%)	0.0% (0.0% to 0.0%)	2.9% (1.6% to 5.1%)
Measles	19.3% (0.0% to 73.1%)	19.6% (0.0% to 74.2%)	20.5% (0.0% to 71.1%)	30.4% (0.0% to 74.5%)
**Maternal disorders**	4.9% (1.9% to 8.5%)	4.8% (1.8% to 8.2%)	3.0% (1.6% to 4.8%)	4.6% (1.8% to 8.0%)
Maternal haemorrhage	13.4% (4.8% to 21.8%)	13.3% (4.7% to 21.6%)	13.1% (4.7% to 21.4%)	13.3% (4.7% to 21.6%)
Maternal sepsis and other pregnancy related infections	13.3% (4.8% to 21.7%)	13.3% (4.8% to 21.5%)	12.9% (4.6% to 21.1%)	13.1% (4.7% to 21.3%)
Maternal abortion, miscarriage, and ectopic pregnancy	7.4% (4.3% to 11.1%)	7.4% (4.3% to 11.0%)	7.3% (4.2% to 10.9%)	7.4% (4.3% to 10.8%)
**Neonatal disorders**	77.4% (72.5% to 81.7%)	77.4% (72.5% to 81.7%)	0.1% (0.1% to 0.2%)	71.2% (66.2% to 76.0%)
Neonatal preterm birth complications	95.9% (91.6% to 98.5%)	95.9% (91.7% to 98.6%)	0.2% (0.2% to 0.3%)	87.3% (81.6% to 91.8%)
Neonatal encephalopathy due to birth asphyxia and trauma	64.4% (58.0% to 69.4%)	64.4% (58.0% to 69.4%)	0.0% (0.0% to 0.0%)	60.8% (53.3% to 66.9%)
Neonatal sepsis and other neonatal infections	57.3% (51.3% to 62.8%)	57.3% (51.3% to 62.9%)	0.0% (0.0% to 0.0%)	52.7% (45.0% to 59.2%)
Hemolytic disease and other neonatal jaundice	63.5% (58.2% to 68.6%)	63.6% (58.2% to 68.6%)	0.0% (0.0% to 0.0%)	26.9% (17.8% to 37.6%)

Supplementary Results Table 1. Age-standardised PAFs attributable to the joint distribution of all risk factors for all ages, both sexes combined for each GBD level 1 and level 2 cause and all causes in 2016 for Honduras. Results are shown for deaths, YLLs, YLDs, and DALYs
Data in parenthesis are 95% uncertainty intervals. PAFs=population attributable fractions. GBD=Global Burden of Disease. YLLs=years of life lost. YLDs=years live with disability. DALYs=disability-adjusted life-years

	Deaths 2016	YLLs 2016	YLDs 2016	DALYs 2016
Other neonatal disorders	58.7% (50.9% to 65.2%)	58.7% (50.9% to 65.2%)	0.0% (0.0% to 0.0%)	57.3% (49.5% to 63.7%)
**Nutritional deficiencies**	10.3% (6.1% to 17.0%)	31.6% (20.9% to 46.0%)	85.6% (82.7% to 88.1%)	62.5% (53.8% to 71.0%)
Protein-energy malnutrition	17.0% (9.7% to 28.0%)	45.8% (31.7% to 61.7%)	94.4% (90.6% to 96.8%)	47.6% (34.1% to 62.8%)
Vitamin A deficiency	-	-	100.0% (100.0% to 100.0%)	100.0% (100.0% to 100.0%)
Iron-deficiency anaemia	100.0% (100.0% to 100.0%)	100.0% (100.0% to 100.0%)	100.0% (100.0% to 100.0%)	100.0% (100.0% to 100.0%)
**Other communicable, maternal, neonatal, and nutritional diseases**	9.2% (5.9% to 13.7%)	6.8% (4.4% to 10.6%)	64.6% (57.6% to 71.2%)	22.6% (15.6% to 32.3%)
Sexually transmitted diseases excluding HIV	79.3% (62.1% to 92.3%)	54.6% (33.2% to 77.5%)	100.0% (100.0% to 100.0%)	85.0% (71.8% to 93.9%)
Hepatitis	3.6% (2.6% to 5.0%)	2.7% (1.9% to 3.9%)	1.9% (1.0% to 3.2%)	2.7% (1.9% to 3.8%)
**Neoplasms**	29.8% (25.9% to 33.8%)	29.8% (25.6% to 34.0%)	28.3% (24.7% to 32.4%)	29.8% (25.6% to 34.0%)
Oesophageal cancer	59.8% (44.8% to 73.4%)	63.0% (48.3% to 76.1%)	60.6% (45.8% to 73.6%)	63.0% (48.2% to 76.1%)
Stomach cancer	7.3% (2.0% to 15.5%)	7.7% (2.5% to 15.0%)	7.5% (2.2% to 15.4%)	7.7% (2.4% to 15.0%)
Liver cancer	53.9% (45.5% to 62.6%)	54.8% (46.3% to 63.6%)	54.5% (46.1% to 63.0%)	54.8% (46.3% to 63.6%)
Larynx cancer	52.6% (38.0% to 66.8%)	58.4% (43.4% to 71.5%)	54.9% (40.5% to 68.4%)	58.3% (43.4% to 71.4%)
Tracheal, bronchus, and lung cancer	77.0% (69.3% to 84.4%)	78.5% (71.1% to 85.6%)	77.2% (69.6% to 84.4%)	78.5% (71.1% to 85.6%)
Breast cancer	18.2% (10.4% to 27.4%)	15.3% (7.9% to 24.0%)	16.5% (8.8% to 25.7%)	15.3% (8.0% to 24.0%)
Cervical cancer	100.0% (100.0% to 100.0%)	100.0% (100.0% to 100.0%)	100.0% (100.0% to 100.0%)	100.0% (100.0% to 100.0%)
Uterine cancer	45.5% (30.4% to 60.3%)	47.6% (32.5% to 62.1%)	47.6% (32.5% to 62.2%)	47.6% (32.5% to 62.1%)
Prostate cancer	0.8% (0.3% to 1.7%)	1.1% (0.4% to 2.2%)	1.1% (0.4% to 2.1%)	1.1% (0.4% to 2.2%)
Colon and rectum cancer	47.8% (37.9% to 57.5%)	48.4% (38.8% to 57.7%)	48.3% (38.5% to 57.8%)	48.4% (38.8% to 57.7%)
Lip and oral cavity cancer	49.5% (39.5% to 59.0%)	50.1% (40.3% to 59.5%)	46.6% (36.5% to 56.4%)	50.0% (40.2% to 59.4%)
Nasopharynx cancer	55.7% (46.2% to 65.3%)	56.9% (48.0% to 65.9%)	55.9% (47.8% to 64.2%)	56.9% (47.9% to 65.8%)

**Supplementary Results Table 1. Age-standardised PAFs attributable to the joint distribution of all risk factors for all ages, both sexes combined for each GBD level 1 and level 2 cause and all causes in 2016 for Honduras. Results are shown for deaths, YLLs, YLDs, and DALYs** Data in parenthesis are 95% uncertainty intervals. PAFs=population attributable fractions. GBD=Global Burden of Disease. YLLs=years of life lost. YLDs=years live with disability. DALYs=disability-adjusted life-years				
	**Deaths 2016**	**YLLs 2016**	**YLDs 2016**	**DALYs 2016**
Other pharynx cancer	31.1% (21.8% to 42.1%)	31.8% (22.8% to 42.6%)	32.0% (22.4% to 43.3%)	31.8% (22.8% to 42.6%)
Gallbladder and biliary tract cancer	26.8% (14.3% to 40.7%)	28.4% (15.6% to 42.4%)	26.8% (14.9% to 40.7%)	28.3% (15.6% to 42.3%)
Pancreatic cancer	19.5% (11.2% to 29.9%)	20.3% (12.0% to 30.3%)	19.6% (11.2% to 29.6%)	20.3% (12.0% to 30.3%)
Ovarian cancer	11.8% (4.1% to 21.8%)	11.2% (3.7% to 20.8%)	11.0% (3.6% to 20.2%)	11.2% (3.7% to 20.7%)
Kidney cancer	23.8% (14.9% to 34.1%)	22.3% (13.9% to 31.8%)	23.9% (15.4% to 33.7%)	22.3% (14.0% to 31.8%)
Bladder cancer	14.1% (6.4% to 24.2%)	15.6% (7.7% to 25.5%)	14.8% (7.1% to 24.7%)	15.5% (7.7% to 25.5%)
Thyroid cancer	14.6% (6.8% to 25.3%)	15.2% (7.0% to 26.3%)	14.8% (6.8% to 25.8%)	15.2% (7.0% to 26.3%)
Mesothelioma	84.1% (77.5% to 89.3%)	76.1% (67.6% to 82.9%)	80.9% (73.7% to 86.7%)	76.2% (67.7% to 83.0%)
Non-Hodgkin's lymphoma	6.7% (2.2% to 12.4%)	6.3% (2.1% to 11.5%)	6.7% (2.4% to 12.0%)	6.3% (2.1% to 11.5%)
Multiple myeloma	8.4% (3.4% to 15.1%)	8.8% (3.6% to 15.4%)	8.4% (3.5% to 14.8%)	8.8% (3.6% to 15.4%)
Leukaemia	12.0% (6.9% to 18.2%)	10.7% (6.1% to 16.6%)	11.8% (6.9% to 17.7%)	10.7% (6.1% to 16.6%)
**Cardiovascular diseases**	89.0% (86.5% to 91.2%)	85.7% (83.3% to 88.1%)	69.0% (65.4% to 72.3%)	84.7% (82.1% to 87.1%)
Rheumatic heart disease	26.3% (17.7% to 40.5%)	24.5% (16.4% to 36.9%)	18.2% (11.3% to 26.9%)	21.3% (14.4% to 31.1%)
Ischaemic heart disease	93.5% (90.2% to 96.1%)	94.5% (92.6% to 96.2%)	91.6% (89.0% to 94.0%)	94.4% (92.5% to 96.2%)
Cerebrovascular disease	79.4% (74.7% to 84.0%)	67.3% (60.8% to 73.3%)	84.7% (80.7% to 88.5%)	68.4% (62.2% to 74.1%)
Hypertensive heart disease	100.0% (100.0% to 100.0%)	100.0% (100.0% to 100.0%)	100.0% (100.0% to 100.0%)	100.0% (100.0% to 100.0%)
Cardiomyopathy and myocarditis	36.4% (29.8% to 43.4%)	28.3% (22.1% to 34.7%)	24.1% (18.7% to 29.4%)	28.1% (22.0% to 34.3%)
Atrial fibrillation and flutter	48.1% (40.4% to 55.2%)	52.7% (45.6% to 58.9%)	58.7% (52.8% to 64.2%)	56.5% (50.1% to 62.2%)
Aortic aneurysm	39.6% (32.5% to 47.0%)	44.9% (38.1% to 51.1%)	-	44.9% (38.1% to 51.1%)
Peripheral vascular disease	52.5% (45.6% to 59.8%)	59.1% (53.6% to 64.5%)	62.0% (56.7% to 67.1%)	61.2% (56.1% to 66.0%)
Endocarditis	35.7% (27.4% to 44.3%)	27.4% (20.2% to 35.8%)	32.1% (24.3% to 40.4%)	27.4% (20.3% to 35.8%)

Supplementary Results Table 1. Age-standardised PAFs attributable to the joint distribution of all risk factors for all ages, both sexes combined for each GBD level 1 and level 2 cause and all causes in 2016 for Honduras. Results are shown for deaths, YLLs, YLDs, and DALYs
Data in parenthesis are 95% uncertainty intervals. PAFs=population attributable fractions. GBD=Global Burden of Disease. YLLs=years of life lost. YLDs=years live with disability. DALYs=disability-adjusted life-years

	Deaths 2016	YLLs 2016	YLDs 2016	DALYs 2016
Other cardiovascular and circulatory diseases	42.1% (36.8% to 47.1%)	40.8% (35.3% to 46.0%)	36.2% (30.9% to 41.6%)	39.6% (34.9% to 44.3%)
**Chronic respiratory diseases**	57.7% (49.1% to 66.3%)	55.3% (46.9% to 63.3%)	36.5% (29.7% to 43.9%)	50.3% (42.8% to 58.0%)
Chronic obstructive pulmonary disease	64.8% (54.4% to 74.7%)	67.0% (57.4% to 75.8%)	61.9% (52.2% to 70.9%)	66.4% (57.0% to 75.1%)
Pneumoconiosis	100.0% (100.0% to 100.0%)	100.0% (100.0% to 100.0%)	100.0% (100.0% to 100.0%)	100.0% (100.0% to 100.0%)
Asthma	34.9% (24.1% to 47.3%)	33.0% (23.5% to 44.6%)	28.1% (20.3% to 37.4%)	30.0% (21.7% to 40.2%)
Other chronic respiratory diseases	2.6% (1.1% to 4.6%)	1.5% (0.5% to 3.0%)	2.0% (0.6% to 3.7%)	1.6% (0.6% to 3.0%)
**Cirrhosis and other chronic liver diseases**	65.1% (60.2% to 69.6%)	66.6% (61.9% to 70.8%)	60.5% (57.0% to 63.5%)	66.4% (61.8% to 70.5%)
Cirrhosis and other chronic liver diseases due to hepatitis B	0.5% (0.2% to 1.0%)	0.7% (0.3% to 1.2%)	0.6% (0.3% to 1.1%)	0.7% (0.3% to 1.2%)
Cirrhosis and other chronic liver diseases due to hepatitis C	67.5% (57.8% to 76.2%)	74.0% (65.2% to 80.9%)	72.9% (66.9% to 77.9%)	74.0% (65.5% to 80.8%)
Cirrhosis and other chronic liver diseases due to alcohol use	100.0% (100.0% to 100.0%)	100.0% (100.0% to 100.0%)	100.0% (100.0% to 100.0%)	100.0% (100.0% to 100.0%)
**Digestive diseases**	14.4% (10.0% to 18.9%)	14.6% (10.5% to 18.8%)	3.9% (2.8% to 5.2%)	13.2% (9.4% to 17.0%)
Peptic ulcer disease	4.0% (2.4% to 6.3%)	5.3% (3.3% to 8.3%)	4.1% (2.6% to 6.0%)	5.1% (3.2% to 7.9%)
Gallbladder and biliary diseases	46.6% (31.3% to 63.1%)	48.7% (33.1% to 64.6%)	50.8% (35.1% to 66.5%)	48.8% (33.2% to 64.7%)
Pancreatitis	29.9% (16.8% to 48.8%)	31.2% (19.3% to 47.1%)	33.2% (22.4% to 48.3%)	31.2% (19.4% to 47.1%)
**Neurological disorders**	20.9% (10.3% to 33.8%)	19.0% (10.2% to 29.3%)	4.1% (2.4% to 6.2%)	9.7% (5.3% to 15.3%)
Alzheimer's disease and other dementias	23.3% (11.0% to 38.3%)	24.2% (11.8% to 39.1%)	24.2% (11.9% to 38.8%)	24.2% (11.8% to 39.1%)
Parkinson's disease	-3.8% (-5.4% to -2.6%)	-4.9% (-6.9% to -3.3%)	-5.8% (-7.8% to -4.3%)	-5.1% (-7.0% to -3.6%)
Epilepsy	13.8% (8.3% to 21.2%)	13.1% (7.8% to 19.6%)	11.6% (7.4% to 16.6%)	12.2% (7.9% to 17.6%)
Multiple sclerosis	3.8% (2.1% to 6.1%)	4.0% (2.2% to 6.5%)	3.3% (2.0% to 4.8%)	3.8% (2.1% to 6.0%)
**Mental and substance use disorders**	99.8% (99.6% to 99.9%)	99.6% (99.4% to 99.8%)	20.1% (17.9% to 22.4%)	28.3% (24.3% to 33.5%)
Alcohol use disorders	100.0% (100.0% to 100.0%)	100.0% (100.0% to 100.0%)	100.0% (100.0% to 100.0%)	100.0% (100.0% to 100.0%)

Supplementary Results Table 1. Age-standardised PAFs attributable to the joint distribution of all risk factors for all ages, both sexes combined for each GBD level 1 and level 2 cause and all causes in 2016 for Honduras. Results are shown for deaths, YLLs, YLDs, and DALYs
Data in parenthesis are 95% uncertainty intervals. PAFs=population attributable fractions. GBD=Global Burden of Disease. YLLs=years of life lost. YLDs=years live with disability. DALYs=disability-adjusted life-years

	Deaths 2016	YLLs 2016	YLDs 2016	DALYs 2016
Drug use disorders	100.0% (100.0% to 100.0%)	100.0% (100.0% to 100.0%)	100.0% (100.0% to 100.0%)	100.0% (100.0% to 100.0%)
Depressive disorders	-	-	7.8% (6.6% to 9.1%)	7.8% (6.6% to 9.1%)
Idiopathic developmental intellectual disability	-	-	71.0% (40.1% to 89.2%)	71.0% (40.1% to 89.2%)
**Diabetes, urogenital, blood, and endocrine diseases**	83.0% (80.3% to 85.2%)	81.5% (78.9% to 83.6%)	77.2% (73.4% to 80.4%)	80.1% (77.8% to 82.1%)
Diabetes mellitus	100.0% (100.0% to 100.0%)	100.0% (100.0% to 100.0%)	100.0% (100.0% to 100.0%)	100.0% (100.0% to 100.0%)
Chronic kidney disease	100.0% (100.0% to 100.0%)	100.0% (100.0% to 100.0%)	100.0% (100.0% to 100.0%)	100.0% (100.0% to 100.0%)
**Musculoskeletal disorders**	0.8% (0.3% to 1.6%)	0.7% (0.3% to 1.4%)	16.1% (13.1% to 19.7%)	15.4% (12.5% to 18.6%)
Rheumatoid arthritis	1.4% (0.5% to 2.8%)	1.7% (0.7% to 3.3%)	2.2% (0.8% to 3.8%)	2.0% (0.8% to 3.5%)
Osteoarthritis	-	-	27.3% (14.5% to 40.9%)	27.3% (14.5% to 40.9%)
Low back and neck pain	-	-	21.0% (18.1% to 24.0%)	21.0% (18.1% to 24.0%)
Gout	-	-	52.3% (38.8% to 66.3%)	52.3% (38.8% to 66.3%)
**Other non-communicable diseases**	0.5% (0.2% to 0.8%)	0.5% (0.3% to 0.9%)	6.8% (5.7% to 7.9%)	5.7% (4.8% to 6.6%)
Sense organ diseases	-	-	13.8% (12.1% to 15.7%)	13.8% (12.1% to 15.7%)
Sudden infant death syndrome	9.1% (4.6% to 15.7%)	9.2% (4.6% to 15.7%)	-	9.2% (4.6% to 15.7%)
**Transport injuries**	42.6% (33.7% to 50.9%)	40.9% (26.3% to 50.7%)	49.4% (40.3% to 57.6%)	41.9% (28.5% to 51.3%)
Road injuries	42.7% (33.8% to 50.9%)	41.2% (26.6% to 51.0%)	49.9% (40.7% to 58.1%)	42.2% (28.6% to 51.7%)
Other transport injuries	41.1% (31.0% to 49.9%)	34.1% (20.9% to 45.5%)	38.9% (31.2% to 46.1%)	34.7% (22.3% to 45.3%)
**Unintentional injuries**	28.7% (23.6% to 34.2%)	24.3% (15.4% to 32.0%)	35.1% (28.3% to 41.3%)	27.9% (19.7% to 35.6%)
Falls	60.1% (53.5% to 66.5%)	49.0% (41.4% to 55.6%)	38.2% (32.1% to 43.7%)	41.1% (34.9% to 46.7%)
Drowning	29.0% (18.2% to 37.9%)	29.4% (16.6% to 39.3%)	22.5% (14.9% to 29.0%)	29.3% (16.6% to 39.1%)
Fire, heat, and hot substances	13.7% (9.1% to 18.8%)	16.5% (9.3% to 23.9%)	29.5% (20.3% to 37.7%)	21.6% (13.6% to 29.9%)

Supplementary Results Table 1. Age-standardised PAFs attributable to the joint distribution of all risk factors for all ages, both sexes combined for each GBD level 1 and level 2 cause and all causes in 2016 for Honduras. Results are shown for deaths, YLLs, YLDs, and DALYs
Data in parenthesis are 95% uncertainty intervals. PAFs=population attributable fractions. GBD=Global Burden of Disease. YLLs=years of life lost. YLDs=years live with disability. DALYs=disability-adjusted life-years

	**Deaths 2016**	**YLLs 2016**	**YLDs 2016**	**DALYs 2016**
Poisonings	22.8% (13.9% to 37.5%)	24.4% (11.3% to 37.9%)	28.8% (18.9% to 37.2%)	24.8% (11.9% to 37.7%)
Exposure to mechanical forces	34.4% (25.4% to 42.6%)	32.0% (17.7% to 42.2%)	39.9% (29.3% to 49.5%)	34.8% (22.5% to 44.8%)
Animal contact	20.4% (15.3% to 26.1%)	24.9% (17.6% to 33.7%)	26.5% (16.9% to 34.8%)	25.9% (17.5% to 33.7%)
Foreign body	15.5% (9.2% to 21.3%)	13.1% (5.8% to 19.3%)	19.2% (13.0% to 24.9%)	13.9% (6.7% to 20.0%)
Other unintentional injuries	34.7% (21.8% to 46.1%)	39.8% (21.4% to 52.9%)	29.8% (20.6% to 37.6%)	37.8% (21.4% to 49.7%)
**Self-harm and interpersonal violence**	16.3% (10.2% to 23.2%)	15.5% (9.7% to 22.6%)	17.5% (12.6% to 23.2%)	15.6% (9.8% to 22.6%)
Self-harm	21.9% (13.9% to 30.9%)	23.8% (15.3% to 32.9%)	18.0% (11.2% to 25.1%)	23.6% (15.3% to 32.7%)
Interpersonal violence	15.5% (9.1% to 23.2%)	14.6% (8.5% to 22.0%)	17.5% (12.3% to 23.6%)	14.7% (8.6% to 22.0%)
**Forces of nature, conflict and terrorism, and executions and police conflict**	0.0% (0.0% to 0.0%)	0.0% (0.0% to 0.0%)	4.6% (3.2% to 6.5%)	4.5% (3.1% to 6.5%)
Forces of nature, conflict and terrorism, and state actor violence	0.0% (0.0% to 0.0%)	0.0% (0.0% to 0.0%)	4.7% (3.3% to 6.7%)	4.7% (3.3% to 6.7%)

Supplementary Results Table 1. Age-standardised PAFs attributable to the joint distribution of all risk factors for all ages, both sexes combined for each GBD level 1 and level 2 cause and all causes in 2016 for Hungary. Results are shown for deaths, YLLs, YLDs, and DALYs
Data in parenthesis are 95% uncertainty intervals. PAFs=population attributable fractions. GBD=Global Burden of Disease. YLLs=years of life lost. YLDs=years live with disability. DALYs=disability-adjusted life-years

	Deaths 2016	YLLs 2016	YLDs 2016	DALYs 2016
**All causes**	71.2% (69.1% to 72.9%)	67.3% (64.9% to 69.2%)	25.5% (24.0% to 27.0%)	49.9% (46.2% to 53.3%)
**HIV/AIDS and tuberculosis**	77.6% (69.6% to 84.8%)	83.3% (77.5% to 88.2%)	70.9% (58.7% to 80.7%)	81.8% (75.6% to 87.1%)
Tuberculosis	63.7% (49.3% to 76.6%)	66.9% (52.9% to 79.4%)	54.5% (38.9% to 66.7%)	64.8% (50.2% to 77.1%)
HIV/AIDS	96.6% (96.0% to 97.1%)	94.6% (93.8% to 95.4%)	97.9% (97.4% to 98.3%)	94.9% (94.1% to 95.6%)
**Diarrhoea, lower respiratory infections, and other common infectious diseases**	53.7% (47.2% to 60.2%)	57.0% (51.9% to 62.2%)	32.9% (22.3% to 43.2%)	45.5% (38.6% to 52.5%)
Diarrhoeal diseases	51.6% (25.9% to 76.1%)	59.9% (38.0% to 80.0%)	62.6% (41.7% to 81.1%)	62.1% (40.9% to 80.9%)
Lower respiratory infections	60.4% (52.5% to 68.1%)	68.3% (61.2% to 75.1%)	57.1% (48.6% to 65.6%)	67.9% (60.8% to 74.8%)
Upper respiratory infections	0.4% (0.0% to 1.5%)	1.3% (0.1% to 4.5%)	0.0% (0.0% to 0.0%)	0.0% (0.0% to 0.0%)
Otitis media	6.7% (3.8% to 10.8%)	13.2% (9.0% to 17.9%)	9.3% (6.4% to 12.1%)	9.4% (6.5% to 12.1%)
Meningitis	2.0% (1.2% to 3.1%)	3.8% (2.4% to 5.6%)	0.0% (0.0% to 0.0%)	2.8% (1.7% to 4.2%)
Encephalitis	0.8% (0.5% to 1.1%)	1.4% (0.9% to 2.1%)	0.0% (0.0% to 0.0%)	0.8% (0.5% to 1.1%)
Measles	9.4% (1.7% to 33.4%)	10.8% (2.0% to 37.9%)	8.2% (0.0% to 52.0%)	10.9% (2.0% to 38.0%)
**Maternal disorders**	6.0% (3.0% to 9.2%)	6.0% (3.0% to 9.2%)	6.5% (3.1% to 10.4%)	6.2% (3.1% to 9.5%)
Maternal haemorrhage	12.8% (4.7% to 21.6%)	12.9% (4.7% to 21.7%)	13.0% (4.6% to 22.0%)	12.9% (4.7% to 21.8%)
Maternal sepsis and other pregnancy related infections	12.7% (4.5% to 21.2%)	12.8% (4.5% to 21.4%)	12.9% (4.7% to 21.6%)	12.8% (4.6% to 21.4%)
Maternal abortion, miscarriage, and ectopic pregnancy	7.3% (4.4% to 10.8%)	7.2% (4.2% to 10.7%)	7.4% (3.9% to 12.1%)	7.3% (4.1% to 11.0%)
**Neonatal disorders**	72.4% (69.0% to 75.4%)	72.4% (69.1% to 75.5%)	0.1% (0.1% to 0.1%)	49.5% (40.4% to 57.0%)
Neonatal preterm birth complications	83.2% (78.4% to 87.3%)	83.3% (78.4% to 87.4%)	0.2% (0.1% to 0.2%)	61.4% (51.5% to 70.2%)
Neonatal encephalopathy due to birth asphyxia and trauma	63.0% (58.9% to 66.6%)	63.0% (59.0% to 66.7%)	0.0% (0.0% to 0.0%)	24.2% (14.2% to 36.5%)
Neonatal sepsis and other neonatal infections	50.9% (46.0% to 56.4%)	50.9% (46.1% to 56.5%)	0.0% (0.0% to 0.0%)	20.2% (9.7% to 31.6%)
Hemolytic disease and other neonatal jaundice	64.5% (60.4% to 67.8%)	64.5% (60.5% to 67.9%)	0.0% (0.0% to 0.0%)	27.9% (17.1% to 38.2%)

Supplementary Results Table 1. Age-standardised PAFs attributable to the joint distribution of all risk factors for all ages, both sexes combined for each GBD level 1 and level 2 cause and all causes in 2016 for Hungary. Results are shown for deaths, YLLs, YLDs, and DALYs
Data in parenthesis are 95% uncertainty intervals. PAFs=population attributable fractions. GBD=Global Burden of Disease. YLLs=years of life lost. YLDs=years live with disability. DALYs=disability-adjusted life-years

	**Deaths 2016**	**YLLs 2016**	**YLDs 2016**	**DALYs 2016**
Other neonatal disorders	58.2% (53.7% to 62.4%)	58.3% (53.8% to 62.5%)	0.0% (0.0% to 0.0%)	55.7% (51.2% to 60.2%)
**Nutritional deficiencies**	10.1% (7.1% to 13.3%)	31.3% (23.6% to 39.1%)	89.4% (86.2% to 91.9%)	87.6% (84.1% to 90.5%)
Protein-energy malnutrition	16.6% (9.6% to 27.4%)	47.9% (35.0% to 61.8%)	97.9% (96.1% to 99.0%)	72.8% (62.7% to 83.6%)
Vitamin A deficiency	-	-	0.0% (0.0% to 0.0%)	0.0% (0.0% to 0.0%)
Iron-deficiency anaemia	100.0% (100.0% to 100.0%)	100.0% (100.0% to 100.0%)	100.0% (100.0% to 100.0%)	100.0% (100.0% to 100.0%)
**Other communicable, maternal, neonatal, and nutritional diseases**	18.2% (15.1% to 22.3%)	13.7% (10.5% to 17.7%)	72.7% (66.8% to 78.6%)	50.7% (42.7% to 59.7%)
Sexually transmitted diseases excluding HIV	97.9% (95.3% to 99.4%)	93.9% (87.0% to 98.4%)	100.0% (100.0% to 100.0%)	99.4% (98.5% to 99.9%)
Hepatitis	2.9% (2.1% to 4.0%)	2.7% (2.0% to 3.6%)	1.2% (0.8% to 1.9%)	2.6% (1.9% to 3.5%)
**Neoplasms**	57.2% (54.0% to 59.9%)	59.4% (56.6% to 62.0%)	47.8% (44.1% to 51.2%)	59.2% (56.2% to 61.8%)
Oesophageal cancer	93.5% (89.3% to 96.4%)	94.5% (90.7% to 97.0%)	93.7% (89.7% to 96.6%)	94.5% (90.7% to 97.0%)
Stomach cancer	33.4% (22.7% to 43.9%)	36.9% (26.2% to 46.9%)	34.3% (23.9% to 44.6%)	36.9% (26.2% to 46.9%)
Liver cancer	77.8% (71.1% to 83.2%)	79.4% (72.6% to 84.7%)	78.1% (71.4% to 83.5%)	79.4% (72.5% to 84.7%)
Larynx cancer	95.9% (93.1% to 97.9%)	96.3% (93.6% to 98.0%)	96.1% (93.5% to 97.9%)	96.3% (93.6% to 98.0%)
Tracheal, bronchus, and lung cancer	93.7% (92.5% to 94.9%)	94.5% (93.3% to 95.5%)	93.8% (92.6% to 95.0%)	94.5% (93.3% to 95.5%)
Breast cancer	36.3% (27.7% to 45.4%)	37.1% (28.1% to 46.6%)	36.7% (27.9% to 46.1%)	37.1% (28.1% to 46.5%)
Cervical cancer	100.0% (100.0% to 100.0%)	100.0% (100.0% to 100.0%)	100.0% (100.0% to 100.0%)	100.0% (100.0% to 100.0%)
Uterine cancer	46.8% (31.8% to 61.4%)	48.7% (33.5% to 63.2%)	49.3% (33.9% to 63.7%)	48.8% (33.7% to 63.3%)
Prostate cancer	9.2% (6.7% to 12.0%)	11.2% (8.3% to 14.3%)	11.7% (8.5% to 15.0%)	11.2% (8.3% to 14.3%)
Colon and rectum cancer	64.7% (55.9% to 72.7%)	66.9% (58.1% to 74.7%)	66.4% (57.5% to 74.3%)	66.9% (58.0% to 74.7%)
Lip and oral cavity cancer	93.9% (91.2% to 95.9%)	95.0% (92.8% to 96.7%)	93.1% (90.0% to 95.3%)	95.0% (92.7% to 96.6%)
Nasopharynx cancer	92.5% (89.2% to 95.0%)	91.6% (88.5% to 94.1%)	91.1% (87.3% to 94.1%)	91.6% (88.5% to 94.1%)

Supplementary Results Table 1. Age-standardised PAFs attributable to the joint distribution of all risk factors for all ages, both sexes combined for each GBD level 1 and level 2 cause and all causes in 2016 for Hungary. Results are shown for deaths, YLLs, YLDs, and DALYs
Data in parenthesis are 95% uncertainty intervals. PAFs=population attributable fractions. GBD=Global Burden of Disease. YLLs=years of life lost. YLDs=years live with disability. DALYs=disability-adjusted life-years

	Deaths 2016	YLLs 2016	YLDs 2016	DALYs 2016
Other pharynx cancer	78.2% (68.8% to 85.5%)	78.7% (69.3% to 85.9%)	77.3% (67.6% to 84.9%)	78.7% (69.3% to 85.9%)
Gallbladder and biliary tract cancer	24.2% (13.1% to 37.4%)	25.0% (14.0% to 37.8%)	24.5% (13.5% to 37.7%)	25.0% (14.0% to 37.8%)
Pancreatic cancer	45.3% (37.7% to 52.6%)	49.6% (42.2% to 56.6%)	46.7% (39.3% to 54.1%)	49.6% (42.2% to 56.6%)
Ovarian cancer	10.9% (4.2% to 18.9%)	9.9% (3.5% to 17.3%)	9.7% (3.5% to 17.0%)	9.9% (3.5% to 17.2%)
Kidney cancer	47.3% (36.8% to 57.5%)	50.2% (39.6% to 60.1%)	48.9% (38.6% to 58.8%)	50.2% (39.6% to 60.1%)
Bladder cancer	48.8% (39.2% to 57.6%)	54.2% (44.7% to 62.6%)	52.9% (43.8% to 61.2%)	54.1% (44.6% to 62.5%)
Thyroid cancer	15.3% (8.0% to 24.4%)	16.2% (8.4% to 25.8%)	15.3% (8.4% to 23.7%)	16.1% (8.4% to 25.5%)
Mesothelioma	86.1% (82.4% to 89.8%)	79.0% (73.8% to 84.2%)	83.5% (79.3% to 87.5%)	79.1% (73.9% to 84.2%)
Non-Hodgkin's lymphoma	7.5% (3.0% to 13.0%)	7.3% (2.9% to 12.6%)	7.5% (3.0% to 12.9%)	7.3% (3.0% to 12.6%)
Multiple myeloma	9.1% (3.8% to 15.9%)	9.4% (4.0% to 16.3%)	9.4% (4.0% to 16.2%)	9.4% (4.0% to 16.3%)
Leukaemia	26.9% (17.4% to 36.2%)	25.0% (16.4% to 33.2%)	25.7% (16.2% to 34.2%)	25.0% (16.5% to 33.2%)
**Cardiovascular diseases**	90.8% (88.7% to 92.6%)	92.5% (91.2% to 93.6%)	85.5% (83.3% to 87.5%)	91.7% (90.3% to 92.8%)
Rheumatic heart disease	38.3% (25.6% to 59.3%)	38.1% (26.8% to 54.5%)	37.5% (25.7% to 53.8%)	38.1% (26.8% to 54.5%)
Ischaemic heart disease	95.7% (93.0% to 97.7%)	97.0% (95.3% to 98.2%)	94.4% (92.5% to 95.9%)	96.8% (95.2% to 98.1%)
Cerebrovascular disease	89.7% (84.7% to 94.0%)	92.9% (90.1% to 95.4%)	90.5% (86.9% to 93.6%)	92.5% (89.5% to 95.0%)
Hypertensive heart disease	100.0% (100.0% to 100.0%)	100.0% (100.0% to 100.0%)	100.0% (100.0% to 100.0%)	100.0% (100.0% to 100.0%)
Cardiomyopathy and myocarditis	70.5% (59.0% to 77.6%)	80.7% (71.4% to 85.0%)	68.9% (63.6% to 74.0%)	79.9% (71.1% to 84.2%)
Atrial fibrillation and flutter	64.5% (56.2% to 72.4%)	69.2% (62.3% to 75.8%)	76.3% (70.9% to 80.9%)	73.3% (67.5% to 78.7%)
Aortic aneurysm	58.8% (51.0% to 65.8%)	64.4% (58.4% to 69.7%)	-	64.4% (58.4% to 69.7%)
Peripheral vascular disease	60.6% (51.9% to 68.1%)	67.3% (60.2% to 73.1%)	64.8% (58.2% to 70.8%)	66.8% (60.2% to 72.3%)
Endocarditis	54.7% (45.1% to 64.1%)	53.5% (44.7% to 62.0%)	52.1% (42.8% to 60.9%)	53.5% (44.7% to 62.0%)

Supplementary Results Table 1. Age-standardised PAFs attributable to the joint distribution of all risk factors for all ages, both sexes combined for each GBD level 1 and level 2 cause and all causes in 2016 for Hungary. Results are shown for deaths, YLLs, YLDs, and DALYs
Data in parenthesis are 95% uncertainty intervals. PAFs=population attributable fractions. GBD=Global Burden of Disease. YLLs=years of life lost. YLDs=years live with disability. DALYs=disability-adjusted life-years

	**Deaths 2016**	**YLLs 2016**	**YLDs 2016**	**DALYs 2016**
Other cardiovascular and circulatory diseases	60.3% (54.7% to 65.5%)	66.1% (61.7% to 70.0%)	64.4% (59.9% to 68.8%)	65.5% (61.3% to 69.5%)
**Chronic respiratory diseases**	82.9% (80.0% to 85.7%)	83.8% (81.3% to 86.1%)	56.4% (50.2% to 62.3%)	75.2% (71.2% to 78.8%)
Chronic obstructive pulmonary disease	87.2% (84.3% to 90.0%)	89.0% (86.7% to 91.3%)	86.3% (83.5% to 88.7%)	88.5% (86.2% to 90.9%)
Pneumoconiosis	100.0% (100.0% to 100.0%)	100.0% (100.0% to 100.0%)	100.0% (100.0% to 100.0%)	100.0% (100.0% to 100.0%)
Asthma	44.7% (34.1% to 56.2%)	48.8% (39.6% to 58.8%)	36.8% (28.8% to 45.9%)	38.2% (30.1% to 47.3%)
Other chronic respiratory diseases	27.5% (21.9% to 33.0%)	24.4% (19.2% to 30.1%)	16.5% (13.0% to 20.0%)	19.7% (15.7% to 24.2%)
**Cirrhosis and other chronic liver diseases**	66.4% (63.2% to 69.4%)	67.0% (63.9% to 70.0%)	54.5% (50.7% to 58.4%)	66.6% (63.6% to 69.5%)
Cirrhosis and other chronic liver diseases due to hepatitis B	0.4% (0.2% to 0.7%)	0.5% (0.2% to 0.8%)	0.4% (0.2% to 0.7%)	0.5% (0.2% to 0.8%)
Cirrhosis and other chronic liver diseases due to hepatitis C	84.0% (79.8% to 86.9%)	86.8% (84.0% to 88.6%)	84.0% (80.2% to 86.8%)	86.7% (83.9% to 88.6%)
Cirrhosis and other chronic liver diseases due to alcohol use	100.0% (100.0% to 100.0%)	100.0% (100.0% to 100.0%)	100.0% (100.0% to 100.0%)	100.0% (100.0% to 100.0%)
**Digestive diseases**	19.0% (15.3% to 23.5%)	23.7% (19.2% to 29.1%)	7.3% (5.6% to 9.2%)	18.6% (15.0% to 22.7%)
Peptic ulcer disease	16.4% (11.8% to 21.5%)	21.6% (15.7% to 28.0%)	21.4% (15.3% to 28.1%)	21.5% (15.8% to 27.9%)
Gallbladder and biliary diseases	42.8% (27.7% to 58.5%)	45.0% (29.7% to 60.1%)	48.9% (34.8% to 63.4%)	45.7% (30.7% to 60.6%)
Pancreatitis	62.6% (45.6% to 78.2%)	66.4% (49.9% to 80.6%)	52.6% (35.5% to 67.9%)	66.1% (49.6% to 80.4%)
**Neurological disorders**	19.5% (9.3% to 32.2%)	18.6% (10.4% to 28.4%)	3.9% (1.9% to 6.8%)	8.3% (4.7% to 12.6%)
Alzheimer's disease and other dementias	24.0% (11.7% to 39.6%)	25.9% (13.3% to 41.5%)	26.0% (13.2% to 41.4%)	25.9% (13.3% to 41.5%)
Parkinson's disease	-8.8% (-10.7% to -7.0%)	-12.1% (-14.6% to -9.6%)	-14.8% (-18.0% to -11.8%)	-12.6% (-15.3% to -10.1%)
Epilepsy	39.4% (25.0% to 51.6%)	34.2% (21.6% to 45.5%)	26.9% (16.1% to 36.8%)	29.7% (18.4% to 40.0%)
Multiple sclerosis	14.2% (9.2% to 19.3%)	15.2% (9.9% to 20.6%)	13.2% (8.6% to 17.9%)	14.4% (9.4% to 19.5%)
**Mental and substance use disorders**	99.9% (99.8% to 99.9%)	99.8% (99.7% to 99.8%)	19.3% (17.1% to 21.7%)	25.0% (22.3% to 28.0%)
Alcohol use disorders	100.0% (100.0% to 100.0%)	100.0% (100.0% to 100.0%)	100.0% (100.0% to 100.0%)	100.0% (100.0% to 100.0%)

Supplementary Results Table 1. Age-standardised PAFs attributable to the joint distribution of all risk factors for all ages, both sexes combined for each GBD level 1 and level 2 cause and all causes in 2016 for Hungary. Results are shown for deaths, YLLs, YLDs, and DALYs
Data in parenthesis are 95% uncertainty intervals. PAFs=population attributable fractions. GBD=Global Burden of Disease. YLLs=years of life lost. YLDs=years live with disability. DALYs=disability-adjusted life-years

	Deaths 2016	YLLs 2016	YLDs 2016	DALYs 2016
Drug use disorders	100.0% (100.0% to 100.0%)	100.0% (100.0% to 100.0%)	100.0% (100.0% to 100.0%)	100.0% (100.0% to 100.0%)
Depressive disorders	-	-	5.4% (4.5% to 6.5%)	5.4% (4.5% to 6.5%)
Idiopathic developmental intellectual disability	-	-	58.4% (30.4% to 78.1%)	58.4% (30.4% to 78.1%)
**Diabetes, urogenital, blood, and endocrine diseases**	80.6% (79.0% to 83.6%)	74.3% (71.7% to 79.8%)	55.7% (51.6% to 59.6%)	63.3% (59.9% to 66.6%)
Diabetes mellitus	100.0% (100.0% to 100.0%)	100.0% (100.0% to 100.0%)	100.0% (100.0% to 100.0%)	100.0% (100.0% to 100.0%)
Chronic kidney disease	100.0% (100.0% to 100.0%)	100.0% (100.0% to 100.0%)	100.0% (100.0% to 100.0%)	100.0% (100.0% to 100.0%)
**Musculoskeletal disorders**	1.2% (0.5% to 2.2%)	1.4% (0.5% to 2.4%)	23.1% (19.0% to 27.4%)	22.8% (18.7% to 27.0%)
Rheumatoid arthritis	5.7% (2.2% to 9.7%)	7.3% (2.9% to 12.1%)	8.3% (3.3% to 13.7%)	8.2% (3.2% to 13.6%)
Osteoarthritis	-	-	29.5% (15.8% to 43.4%)	29.5% (15.8% to 43.4%)
Low back and neck pain	-	-	23.8% (20.1% to 27.6%)	23.8% (20.1% to 27.6%)
Gout	-	-	47.7% (30.9% to 64.3%)	47.7% (30.9% to 64.3%)
**Other non-communicable diseases**	0.2% (0.2% to 0.4%)	0.3% (0.2% to 0.5%)	3.5% (3.1% to 4.0%)	3.0% (2.6% to 3.4%)
Sense organ diseases	-	-	8.5% (7.6% to 9.4%)	8.5% (7.6% to 9.4%)
Sudden infant death syndrome	4.0% (2.3% to 6.3%)	4.0% (2.3% to 6.3%)	-	4.0% (2.3% to 6.3%)
**Transport injuries**	39.3% (24.5% to 52.7%)	35.6% (21.2% to 48.6%)	39.1% (25.5% to 51.4%)	36.9% (22.8% to 49.8%)
Road injuries	39.2% (24.3% to 52.5%)	35.5% (21.1% to 48.2%)	39.3% (25.7% to 51.7%)	36.9% (22.8% to 49.8%)
Other transport injuries	41.2% (26.3% to 55.6%)	37.1% (22.7% to 50.8%)	33.1% (19.5% to 45.3%)	36.2% (22.1% to 49.8%)
**Unintentional injuries**	40.2% (33.6% to 45.0%)	22.8% (19.5% to 26.4%)	19.6% (17.2% to 22.4%)	20.5% (18.1% to 23.3%)
Falls	59.4% (48.7% to 64.8%)	39.3% (29.5% to 44.4%)	21.5% (19.3% to 24.1%)	24.8% (21.8% to 27.5%)
Drowning	19.6% (8.3% to 32.1%)	17.6% (8.1% to 28.3%)	17.2% (7.2% to 28.9%)	17.6% (8.1% to 28.2%)
Fire, heat, and hot substances	17.6% (6.2% to 31.1%)	19.2% (8.1% to 31.6%)	18.6% (8.3% to 30.5%)	18.8% (8.1% to 30.8%)

Supplementary Results Table 1. Age-standardised PAFs attributable to the joint distribution of all risk factors for all ages, both sexes combined for each GBD level 1 and level 2 cause and all causes in 2016 for Hungary. Results are shown for deaths, YLLs, YLDs, and DALYs
Data in parenthesis are 95% uncertainty intervals. PAFs=population attributable fractions. GBD=Global Burden of Disease. YLLs=years of life lost. YLDs=years live with disability. DALYs=disability-adjusted life-years

	Deaths 2016	YLLs 2016	YLDs 2016	DALYs 2016
Poisonings	20.9% (8.9% to 34.1%)	21.2% (10.2% to 33.4%)	18.1% (8.5% to 28.8%)	19.8% (9.6% to 31.5%)
Exposure to mechanical forces	16.3% (13.0% to 20.2%)	13.6% (9.9% to 18.2%)	15.4% (12.4% to 19.6%)	15.1% (11.9% to 19.2%)
Animal contact	12.4% (10.3% to 15.1%)	10.2% (7.8% to 13.6%)	7.4% (5.2% to 10.5%)	8.3% (6.0% to 11.4%)
Foreign body	5.3% (3.5% to 7.6%)	6.2% (4.1% to 8.9%)	5.2% (3.6% to 7.6%)	6.0% (4.0% to 8.6%)
Other unintentional injuries	23.2% (10.7% to 36.7%)	22.4% (11.1% to 35.0%)	17.3% (7.6% to 28.5%)	18.5% (8.5% to 29.7%)
**Self-harm and interpersonal violence**	42.2% (25.0% to 58.2%)	41.0% (23.9% to 56.9%)	32.1% (20.4% to 42.9%)	40.5% (23.7% to 56.4%)
Self-harm	42.7% (25.2% to 59.1%)	41.9% (24.2% to 57.7%)	33.1% (16.7% to 48.2%)	41.8% (24.1% to 57.6%)
Interpersonal violence	36.9% (23.8% to 50.5%)	32.8% (20.6% to 46.4%)	31.8% (21.2% to 41.7%)	32.5% (20.9% to 44.9%)
**Forces of nature, conflict and terrorism, and executions and police conflict**	0.0% (0.0% to 0.0%)	0.0% (0.0% to 0.0%)	2.1% (1.3% to 3.1%)	0.7% (0.2% to 1.5%)
Forces of nature, conflict and terrorism, and state actor violence	0.0% (0.0% to 0.0%)	0.0% (0.0% to 0.0%)	4.4% (2.8% to 6.5%)	4.4% (2.8% to 6.5%)

Supplementary Results Table 1. Age-standardised PAFs attributable to the joint distribution of all risk factors for all ages, both sexes combined for each GBD level 1 and level 2 cause and all causes in 2016 for Iceland. Results are shown for deaths, YLLs, YLDs, and DALYs
Data in parenthesis are 95% uncertainty intervals. PAFs=population attributable fractions. GBD=Global Burden of Disease. YLLs=years of life lost. YLDs=years live with disability. DALYs=disability-adjusted life-years

	Deaths 2016	YLLs 2016	YLDs 2016	DALYs 2016
**All causes**	53.8% (51.3% to 56.4%)	50.4% (48.2% to 52.5%)	19.7% (18.3% to 21.2%)	32.6% (30.0% to 35.5%)
**HIV/AIDS and tuberculosis**	59.7% (51.2% to 67.3%)	65.1% (60.8% to 68.9%)	72.0% (67.9% to 74.5%)	68.3% (64.4% to 71.4%)
Tuberculosis	46.2% (30.5% to 61.3%)	46.7% (32.5% to 59.7%)	39.6% (24.2% to 53.3%)	45.0% (31.4% to 57.6%)
HIV/AIDS	73.9% (73.1% to 74.8%)	72.1% (71.2% to 73.0%)	76.1% (75.3% to 77.1%)	74.1% (73.0% to 75.1%)
**Diarrhoea, lower respiratory infections, and other common infectious diseases**	27.7% (19.0% to 36.5%)	31.5% (23.8% to 39.2%)	6.4% (4.4% to 8.7%)	20.2% (15.3% to 26.0%)
Diarrhoeal diseases	13.6% (7.8% to 23.1%)	26.4% (18.9% to 35.1%)	28.6% (20.7% to 38.0%)	28.0% (20.7% to 36.8%)
Lower respiratory infections	29.2% (19.8% to 38.9%)	34.8% (25.8% to 43.7%)	34.7% (26.4% to 44.3%)	34.8% (25.8% to 43.8%)
Upper respiratory infections	0.3% (0.0% to 0.9%)	0.9% (0.1% to 3.1%)	0.0% (0.0% to 0.0%)	0.0% (0.0% to 0.0%)
Otitis media	1.1% (0.4% to 2.1%)	2.6% (1.0% to 4.8%)	6.7% (4.4% to 9.0%)	6.7% (4.3% to 9.0%)
Meningitis	1.4% (1.0% to 2.0%)	3.1% (2.1% to 4.3%)	0.0% (0.0% to 0.0%)	2.8% (1.9% to 3.9%)
Encephalitis	0.4% (0.3% to 0.6%)	1.0% (0.7% to 1.3%)	0.0% (0.0% to 0.0%)	0.4% (0.3% to 0.6%)
Measles	5.6% (1.4% to 19.7%)	6.8% (1.8% to 24.4%)	6.0% (0.0% to 37.7%)	6.9% (1.8% to 24.4%)
**Maternal disorders**	1.0% (0.5% to 1.6%)	1.0% (0.5% to 1.5%)	2.0% (0.8% to 3.3%)	1.7% (0.7% to 2.7%)
Maternal haemorrhage	8.5% (3.1% to 13.8%)	8.4% (3.0% to 13.7%)	8.5% (3.1% to 14.0%)	8.5% (3.1% to 13.9%)
Maternal sepsis and other pregnancy related infections	8.6% (3.2% to 14.0%)	8.5% (3.2% to 13.9%)	8.3% (3.0% to 13.5%)	8.3% (3.0% to 13.6%)
Maternal abortion, miscarriage, and ectopic pregnancy	4.3% (2.1% to 7.6%)	4.3% (2.3% to 7.4%)	4.8% (2.3% to 8.3%)	4.7% (2.4% to 7.7%)
**Neonatal disorders**	74.4% (70.8% to 77.7%)	74.5% (70.9% to 77.8%)	0.1% (0.0% to 0.1%)	30.0% (22.4% to 38.4%)
Neonatal preterm birth complications	86.9% (82.3% to 90.6%)	87.0% (82.4% to 90.6%)	0.2% (0.1% to 0.2%)	39.5% (28.4% to 51.2%)
Neonatal encephalopathy due to birth asphyxia and trauma	63.6% (58.8% to 68.1%)	63.7% (58.9% to 68.2%)	0.0% (0.0% to 0.0%)	21.9% (14.2% to 29.8%)
Neonatal sepsis and other neonatal infections	59.3% (53.0% to 65.5%)	59.4% (53.0% to 65.5%)	0.0% (0.0% to 0.0%)	10.0% (4.1% to 21.6%)
Hemolytic disease and other neonatal jaundice	62.0% (55.1% to 68.1%)	62.2% (55.2% to 68.2%)	0.0% (0.0% to 0.0%)	15.2% (9.4% to 24.3%)
[truncated: 391,664 more chars]
