# Supplementary material for: Global, regional, and national comparative risk assessment of 84 behavioural, environmental and occupational, and metabolic risks or clusters of risks, 1990–2016: a systematic analysis for the Global Burden of Disease Study 2016
Source: Lancet. 2017 Sep 16;390(10100):1345–422. doi: 10.1016/S0140-6736(17)32366-8 (PMC5614451; doi:10.1016/S0140-6736(17)32366-8)

THE LANCET

Supplementary appendix 1

This appendix formed part of the original submission and has been peer reviewed. We post it as supplied by the authors.

Supplement to: GBD 2016 Risk Factors Collaborators. Global, regional, and national comparative risk assessment of 84 behavioural, environmental and occupational, and metabolic risks or clusters of risks, 1990–2016: a systematic analysis for the Global Burden of Disease Study 2016. *Lancet* 2017; **390:** 1345–422.

Methods appendix to Global, regional, and national comparative risk assessment of 84 behavioural, environmental and occupational, and metabolic risks or clusters of risks, 1990-2016: a systematic analysis for the Global Burden of Disease Study 2016

This appendix provides further methodological detail, supplemental figures, and more detailed results for risk factors. The appendix is organized into broad sections following the structure of the main paper.

Authors' Contributions

Managing the estimation process

Ashkan Afshin, Tahiya Alam, Kara Estep, Emmanuela Gakidou, Nick J Kassebaum, Darya Li Kappe, Steve S Lim, Christopher J L Murray, Helen E Olsen, Gregory A Roth, Joseph Salama, Chloe Shields, Mari Smith, Caitlyn Steiner, and Theo Vos.

Writing the first draft of the manuscript

Emmanuela Gakidou, Erin C Mullany, and Christopher J L Murray.

Providing data or critical feedback on data sources

Walid Ammar, Hossein Ansari, Palwasha Anwari, Nicholas Arian, Johan Ärnlöv, Al Artaman, Krishna Kumar Aryal, Hamid Asayesh, Solomon Weldegebreal Asgedom, Ashish Awasthi, Umar Bacha, Kalpana Balakrishnan, Shoshana H Ballew, Till Bärnighausen, Simon Barquera, Lars Barregard, Lope H Barrero, Carolina Batis, Katherine E Battle, Bernhard T Baune, Justin Beardsley, Neeraj Bedi, Ettore Beghi, Derrick A Bennett, Isabela M Bensenor, Eduardo Bernabé, Balem Demtsu Betsu, Addisu Shunu Beyene, Anil Bhansali, Boris Bikbov, Charles Birungi, Dube Jara Boneya, Ibrahim R Bou-Orm, Michael Brauer, Traolach S Brugha, Lemma Negesa Bulto Bulto, Blair R Bumgarner, Juan Jesus Carrero, Carlos A Castañeda-Orjuela, Ferrán Catalá-López, Fiona J Charlson, Abdulaal A Chitheer, Massimo Cirillo, Aaron J Cohen, Josef Coresh, Michael H Criqui, John A Crump, Lalit Dandona, Rakhi Dandona, José das Neves, Gail Davey, Louisa Degenhardt, Kebede Deribe, Samath D Dharmaratne, Huyen Phuc Do, Klara Dokova, E Ray Dorsey, Tim R Driscoll, Manisha Dubey, Bruce Bartholow Duncan, Aman Yesuf Endries, Sergey Petrovich Ermakov, Holly E Erskine, Sharareh Eskandarieh, Alireza Esteghamati, Kara Estep, Emerito Jose Aquino Faraon, Carla Sofia e Sa Farinha, André Faro, Farshad Farzadfar, Kairsten Fay, Valery L Feigin, Seyed-Mohammad Fereshtehnejad, João C Fernandes, Alize J Ferrari, Tesfaye Regassa Feyissa, Irina Filip, Florian Fischer, Christina Fitzmaurice, Kyle J Foreman, Thomas Fürst, Joao M Furtado, Emmanuela Gakidou, Johanna M Geleijnse, Ayele Geleto, Bikila Lencha Gemechu, Hailay Abrha Gesesew, Peter W Gething, Alireza Ghajar, Katherine B Gibney, Melkamu Dedefo Gishu, Philimon N Gona, Harish Chander Gugnani, Tanush Gupta, Nima Hafezi-Nejad, Gessessew Bugssa Hailu, Hilda L Harb, Habtamu Abera Hareri, Mohammad Sadegh Hassanvand, Rasmus Havmoeller, Simon I Hay, Mohammad T Hedayati, Delia Hendrie, Ileana Beatriz Heredia-Pi, Hans W Hoek, Nobuyuki Horita, H Dean Hosgood, Damian G Hoy, Mohamed Hsairi, Guoqing Hu, Kim Moesgaard Iburg, Chad Ikeda, Manami Inoue, Maria Delores Jackson, Kathryn H Jacobsen, Mihajlo (Michael) B Jakovljevic, Alejandra Jauregui, Mehdi Javanbakht, Panniyammakal Jeemon, Lars R K Johansson, Catherine O Johnson, Jost B Jonas, Mikk Jürisson, Zubair Kabir, Rajendra Kadel, Ritul Kamal, André Karch, Corine Kakizi Karema, Amir Kasaeian, Nicholas J Kassebaum, Srinivasa Vittal Katikireddi, Norito Kawakami, Sefonias Getachew Kelbore, Chandrasekharan Nair Kesavachandran, Yousef Saleh Khader, Ibrahim A Khalil, Ardeshir Khosravi, Jagdish Khubchandani, Christian Kieling, Jun Y Kim, Yun Jin Kim, Ruth W Kimokoti, Yohannes Kinfu, Mika Kivimaki, Luke D Knibbs, Ann Kristin Knudsen, Soewarta Kosen, Parvaiz A Koul, Michael Kravchenko, Hans Kromhout, Barthelemy Kuate Defo, Burcu Kucuk Bicer, G Anil Kumar, Michael Kutz, Dharmesh Kumar Lal, Qing Lan, Van C Lansingh, James Leigh, Janni Leung, Miriam Levi, Xiaofeng Liang, Misgan Legesse Liben, Stephen S Lim, Alan D Lopez, Stefan Lorkowski, Paulo A Lotufo, Rafael Lozano, Erlyn Rachelle King Macarayan, Marek Majdan, Azeem Majeed, Reza Malekzadeh, Abdullah A Mamun, Lorenzo G Mantovani, Randall V Martin, Jose Martinez-Raga, Manu Raj Mathur, Kunihiro Matsushita, Mohsen Mazidi, Colm McAlinden, John J McGrath, Suresh Mehata, Man Mohan Mehndiratta, Yohannes Adama Melaku, Ziad A Memish,

Walter Mendoza, Melkamu Merid Mengesha, Gert B M Mensink, Atte Meretoja, Tuomo J Meretoja, Renata Micha, Ted R Miller, Shiva Raj Mishra, NORLINAH MOHAMED IBRAHIM, Shafiu Mohammed, Ali H Mokdad, Lorenzo Monasta, Julio Cesar Montañez Hernandez, Marcella Montico, Maziar Moradi-Lakeh, Ulrich O Mueller, Christopher J L Murray, Gudlavalleti Venkata Satyanarayana Murthy, Kamarul Imran Musa, Mohsen Naghavi, Minh Nguyen, Quyen Le Nguyen, Marika Nomura, Vuong Minh Nong, Bo Norrving, Carla Makhlouf Obermeyer, Felix Akpojene Ogbo, Olanrewaju Oladimeji, Andrew Toyin Olagunju, Bolajoko Olubukunola Olusanya, Jacob Olusegun Olusanya, Alberto Ortiz, Erika Ota, Mayowa O Owolabi, Mahesh PA, Adrian Pana, Basant Kumar Panda, Songhomitra Panda-Jonas, Jeyaraj D Pandian, Christina Papachristou, Scott B Patten, George C Patton, Norberto Perico, Konrad Pesudovs, Max Petzold, Dietrich Plass, Suzanne Polinder, Farshad Pourmalek, Narayan Prasad, Mostafa Qorbani, Amir Radfar, Anwar Rafay, Afarin Rahimi-Movaghar, Vafa Rahimi-Movaghar, Mahfuzar Rahman, Rajesh Kumar Rai, Sasa Rajsic, Usha Ram, Salman Rawaf, Colin D Rehm, Jürgen Rehm, Robert C Reiner, Marissa B Reitsma, Giuseppe Remuzzi, Luz Myriam Reynales-Shigematsu, Antonio L Ribeiro, Juan A Rivera, David Rojas-Rueda, Yesenia Roman, Gholamreza Roshandel, Gregory A Roth, Enrico Rubagotti, Lesley Rushton, Nafis Sadat, Saeid Safiri, Joshua A Salomon, Abdallah M Samy, Tania G Sánchez-Pimienta, Damian Santomauro, Itamar S Santos, Milena M Santric Milicevic, Maheswar Satpathy, Monika Sawhney, Sonia Saxena, Maria Inês Schmidt, David C C Schwebel, Falk Schwendicke, Soraya Seedat, Sadaf G Sepanlou, Edson E Servan-Mori, Gavin Shaddick, Masood Ali Shaikh, Teresa Shamah Levy, Mansour Shamsipour, Morteza Shamsizadeh, Sheikh Mohammed Shariful Islam, Jayendra Sharma, Jiabin Shen, Peilin Shi, Reza Shirkoohi, Kawkab Shishani, Haitham Shoman, Mark G Shrime, Inga Dora Sigfusdottir, Diego Augusto Santos Silva, Dayane Gabriele Alves Silveira, Jasvinder A Singh, Virendra Singh, Eirini Skiadaresi, Erica Leigh Slepak, David L Smith, Eugene Sobngwi, Luciano A Sposato, Chandrashekhar T Sreeramareddy, Caitlyn Steiner, Mark Andrew Stokes, Muawiyyah Babale Sufiyan, Rizwan Abdulkader Suliankatchi, Patrick J Sur, Souyma Swaminathan, Bryan L Sykes, Cassandra E I Szoeke, Rafael Tabarés-Seisdedos, Jukka S Takala, Marcel Tanner, Mohammad Tavakkoli, Abdullah Sulieman Terkawi, Matthew Lloyd Thomas, Amanda G Thrift, Ruoyan Tobe-Gai, Myriam Tobollik, Mette C Tollanes, Marcello Tonelli, Bach Xuan Tran, Thomas Truelsen, Kingsley Nnanna Ukwaja, Chigozie Jesse Uneke, Olalekan A Uthman, Job F M van Boven, Aaron van Donkelaar, Tommi Vasankari, Narayanaswamy Venketasubramanian, Francesco S Violante, Vasiliy Victorovich Vlassov, Stein Emil Vollset, Theo Vos, Tolassa Wakayo, Yuan-Pang Wang, Elisabete Weiderpass, Robert G Weintraub, Daniel J Weiss, Ronny Westerman, Harvey A Whiteford, Charles Shey Wiysonge, Charles D A Wolfe, Rachel Woodbrook, Abdulhalik Workicho, Sarah Wulf Hanson, Denis Xavier, Gelin Xu, Bereket Yakob, Lijing L Yan, Hassen Hamid Yimam, Naohiro Yonemoto, Mustafa Z Younis, Zoubida Zaidi, Luis Zavala-Arciniega, Luis Zavala-Arciniega, and Xueying Zhang.

Developing methods or computational machinery

Cristiana Abbafati, Foad Abd-Allah, Semaw Ferede Abera, Laith J Abu-Raddad, Niveen M E Abu-Rmeileh, Isaac Akinkunmi Adedeji, Olatunji Adetokunboh, Ashkan Afshin, Aliasghar Ahmad Kiadaliri, Muktar Beshir Ahmed, Amani Nidhal Aichour, Ibtihel Aichour, Miloud Taki Eddine Aichour, Rufus Olusola Akinyemi, Nadia Akseer, Fares Alahdab, Khurshid Alam, Noore Alam, Deena Alasfoor, Kefyalew Addis Alene, Ala'a Alkerwi, François Alla, Peter Allebeck, Ubai Alsharif, Khalid A Altirkawi, Azmeraw T Amare, Erfan Amini, Walid Ammar, Hossein Ansari, Palwasha Anwari, Nicholas Arian, Johan Ärnlöv, Al Artaman, Krishna Kumar Aryal, Hamid Asayesh, Solomon Weldegebreal Asgedom, Ashish Awasthi, Umar Bacha, Kalpana Balakrishnan, Shoshana H Ballew, Ryan M Barber, Till Bärnighausen, Simon Barquera, Lars Barregard, Lope H Barrero, Carolina Batis, Katherine E Battle, Bernhard T Baune, Justin Beardsley, Neeraj Bedi, Ettore Beghi, Derrick A Bennett, Isabela M Bensenor, Eduardo Bernabé, Balem Demtsu Betsu,

Addisu Shunu Beyene, Anil Bhansali, Boris Bikbov, Charles Birungi, Stan Biryukov, Dube Jara Boneya, Ibrahim R Bou-Orm, Michael Brauer, Traolach S Brugha, Lemma Negesa Bulto Bulto, Juan Jesus Carrero, Carlos A Castañeda-Orjuela, Ferrán Catalá-López, Kelly Cercy, Fiona J Charlson, Abdulaal A Chitheer, Massimo Cirillo, Aaron J Cohen, Josef Coresh, Michael H Criqui, John A Crump, Lalit Dandona, Rakhi Dandona, José das Neves, Gail Davey, Louisa Degenhardt, Kebede Deribe, Aniruddha Deshpande, Samath D Dharmaratne, Huyen Phuc Do, Klara Dokova, E Ray Dorsey, Tim R Driscoll, Manisha Dubey, Bruce Bartholow Duncan, Aman Yesuf Endries, Sergey Petrovich Ermakov, Holly E Erskine, Sharareh Eskandarieh, Alireza Esteghamati, Emerito Jose Aquino Faraon, Carla Sofia e Sa Farinha, André Faro, Farshad Farzadfar, Valery L Feigin, Seyed-Mohammad Fereshtehnejad, João C Fernandes, Alize J Ferrari, Tesfaye Regassa Feyissa, Irina Filip, Florian Fischer, Christina Fitzmaurice, Abraham D Flaxman, Kyle J Foreman, Thomas Fürst, Joao M Furtado, Emmanuela Gakidou, Johanna M Geleijnse, Ayele Geleto, Bikila Lencha Gemechu, Hailay Abrha Gesesew, Peter W Gething, Alireza Ghajar, Katherine B Gibney, Melkamu Dedefo Gishu, William W Godwin, Philimon N Gona, Nicholas Graetz, Harish Chander Gugnani, Tanush Gupta, Nima Hafezi-Nejad, Gessessew Bugssa Hailu, Hilda L Harb, Habtamu Abera Hareri, Mohammad Sadegh Hassanvand, Rasmus Havmoeller, Simon I Hay, Mohammad T Hedayati, Delia Hendrie, Ileana Beatriz Heredia-Pi, Hans W Hoek, Nobuyuki Horita, H Dean Hosgood, Damian G Hoy, Mohamed Hsairi, Guoqing Hu, Kim Moesgaard Iburg, Manami Inoue, Maria Delores Jackson, Kathryn H Jacobsen, Mihajlo (Michael) B Jakovljevic, Alejandra Jauregui, Mehdi Javanbakht, Panniyammakal Jeemon, Lars R K Johansson, Catherine O Johnson, Jost B Jonas, Mikk Jürisson, Zubair Kabir, Rajendra Kadel, Ritul Kamal, André Karch, Corine Kakizi Karema, Amir Kasaeian, Nicholas J Kassebaum, Srinivasa Vittal Katikireddi, Norito Kawakami, Sefonias Getachew Kelbore, Chandrasekharan Nair Kesavachandran, Yousef Saleh Khader, Ibrahim A Khalil, Ardeshir Khosravi, Jagdish Khubchandani, Christian Kieling, Jun Y Kim, Yun Jin Kim, Ruth W Kimokoti, Yohannes Kinfu, Mika Kivimaki, Luke D Knibbs, Ann Kristin Knudsen, Soewarta Kosen, Parvaiz A Koul, Michael Kravchenko, Hans Kromhout, Barthelemy Kuate Defo, Burcu Kucuk Bicer, G Anil Kumar, Michael Kutz, Dharmesh Kumar Lal, Qing Lan, Van C Lansingh, Alexander Lee, James Leigh, Janni Leung, Miriam Levi, Xiaofeng Liang, Misgan Legesse Liben, Stephen S Lim, Patrick Liu, Alan D Lopez, Stefan Lorkowski, Paulo A Lotufo, Rafael Lozano, Erlyn Rachelle King Macarayan, Marek Majdan, Azeem Majeed, Reza Malekzadeh, Abdullah A Mamun, Helena Manguerra, Lorenzo G Mantovani, Randall V Martin, Jose Martinez-Raga, Manu Raj Mathur, Kunihiro Matsushita, Mohsen Mazidi, Colm McAlinden, John J McGrath, Suresh Mehata, Man Mohan Mehndiratta, Yohannes Adama Melaku, Ziad A Memish, Walter Mendoza, Melkamu Merid Mengesha, Gert B M Mensink, Atte Meretoja, Tuomo J Meretoja, Renata Micha, Anoushka Millear, Ted R Miller, Shiva Raj Mishra, NORLINAH MOHAMED IBRAHIM, Shafiu Mohammed, Ali H Mokdad, Lorenzo Monasta, Julio Cesar Montañez Hernandez, Marcella Montico, Maziar Moradi-Lakeh, Ulrich O Mueller, Christopher J L Murray, Gudlavalleti Venkata Satyanarayana Murthy, Kamarul Imran Musa, Mohsen Naghavi, Minh Nguyen, Quyen Le Nguyen, Emma Nichols, Marika Nomura, Vuong Minh Nong, Bo Norrving, Carla Makhlouf Obermeyer, Felix Akpojene Ogbo, Olanrewaju Oladimeji, Andrew Toyin Olagunju, Bolajoko Olubukunola Olusanya, Jacob Olusegun Olusanya, Alberto Ortiz, Erika Ota, Mayowa O Owolabi, Mahesh PA, Adrian Pana, Basant Kumar Panda, Songhomitra Panda-Jonas, Jeyaraj D Pandian, Christina Papachristou, Charles D Parry, Scott B Patten, George C Patton, Norberto Perico, Konrad Pesudovs, Max Petzold, Dietrich Plass, Suzanne Polinder, Farshad Pourmalek, Narayan Prasad, Mostafa Qorbani, Amir Radfar, Anwar Rafay, Afarin Rahimi-Movaghar, Vafa Rahimi-Movaghar, Mahfuzar Rahman, Rajesh Kumar Rai, Sasa Rajsic, Usha Ram, Salman Rawaf, Colin D Rehm, Jürgen Rehm, Robert C Reiner, Marissa B Reitsma, Giuseppe Remuzzi, Luz Myriam Reynales-Shigematsu, Antonio L Ribeiro, Juan A Rivera, David

Rojas-Rueda, Yesenia Roman, Gholamreza Roshandel, Gregory A Roth, Enrico Rubagotti, Lesley Rushton, Nafis Sadat, Saeid Safiri, Joshua A Salomon, Abdallah M Samy, Tania G Sánchez-Pimienta, Damian Santomauro, Itamar S Santos, Milena M Santric Milicevic, Maheswar Satpathy, Monika Sawhney, Sonia Saxena, Maria Inês Schmidt, David C C Schwebel, Falk Schwendicke, Soraya Seedat, Sadaf G Sepanlou, Edson E Servan-Mori, Gavin Shaddick, Masood Ali Shaikh, Teresa Shamah Levy, Mansour Shamsipour, Morteza Shamsizadeh, Sheikh Mohammed Shariful Islam, Jayendra Sharma, Jiabin Shen, Peilin Shi, Reza Shirkoohi, Kawkab Shishani, Haitham Shoman, Mark G Shrime, Inga Dora Sigfusdottir, Diego Augusto Santos Silva, Dayane Gabriele Alves Silveira, Jasvinder A Singh, Virendra Singh, Eirini Skiadaresi, Erica Leigh Slepak, David L Smith, Eugene Sobngwi, Luciano A Sposato, Chandrashekhar T Sreeramareddy, Vinay Srinivasan, Mark Andrew Stokes, Muawiyyah Babale Sufiyan, Rizwan Abdulkader Suliankatchi, Patrick J Sur, Souyma Swaminathan, Bryan L Sykes, Cassandra E I Szoeke, Rafael Tabarés-Seisdedos, Jukka S Takala, Marcel Tanner, Mohammad Tavakkoli, Abdullah Sulieman Terkawi, Ornwipa Thamsuwan, Matthew Lloyd Thomas, Amanda G Thrift, Ruoyan Tobe-Gai, Myriam Tobollik, Mette C Tollanes, Marcello Tonelli, Bach Xuan Tran, Thomas Truelsen, Kingsley Nnanna Ukwaja, Chigozie Jesse Uneke, Rachel L Updike, Olalekan A Uthman, Job F M van Boven, Aaron van Donkelaar, Tommi Vasankari, Narayanaswamy Venketasubramanian, Francesco S Violante, Vasiliy Victorovich Vlassov, Stein Emil Vollset, Theo Vos, Tolassa Wakayo, Yuan-Pang Wang, Elisabete Weiderpass, Robert G Weintraub, Daniel J Weiss, Ronny Westerman, Harvey A Whiteford, Charles Shey Wiysonge, Charles D A Wolfe, Rachel Woodbrook, Abdulhalik Workicho, Sarah Wulf Hanson, Denis Xavier, Gelin Xu, Simon Yadgir, Bereket Yakob, Lijing L Yan, Hassen Hamid Yimam, Naohiro Yonemoto, Mustafa Z Younis, Zoubida Zaidi, Maysaa El Sayed Zaki, Luis Zavala-Arciniega, and Xueying Zhang.

Applying analytical methods to produce estimates

Olatunji Adetokunboh, Ashkan Afshin, Muktar Beshir Ahmed, Fares Alahdab, Azmeraw T Amare, Shoshana H Ballew, Ryan M Barber, James R Bennett, Adugnaw Berhane, Charles Birungi, Stan Biryukov, Dube Jara Boneya, Michael Brauer, Lemma Negesa Bulto Bulto, Kelly Cercy, Aaron J Cohen, Josef Coresh, Aman Yesuf Endries, Christina Fitzmaurice, Kyle J Foreman, Emmanuela Gakidou, Ayele Geleto, William W Godwin, Gessessew Bugssa Hailu, Caleb Mackay Salpeter Irvine, Catherine O Johnson, Nicholas J Kassebaum, Jun Y Kim, Yohannes Kinfu, Hmwe H Kyu, Van C Lansingh, Alexander Lee, James Leigh, Janni Leung, Stephen S Lim, Rafael Lozano, Reza Malekzadeh, Helena Manguerra, Randall V Martin, Kunihiro Matsushita, Mohsen Mazidi, Anoushka Millear, Ali H Mokdad, Christopher J L Murray, Mohsen Naghavi, Grant Nguyen, Minh Nguyen, Emma Nichols, Carla Makhlouf Obermeyer, Bolajoko Olubukunola Olusanya, Jacob Olusegun Olusanya, Martin A Pletcher, Carrie Purcell, Robert C Reiner, Marissa B Reitsma, Gregory A Roth, Enrico Rubagotti, Joshua A Salomon, Damian Santomauro, Maheswar Satpathy, Monika Sawhney, Gavin Shaddick, Masood Ali Shaikh, David L Smith, Vinay Srinivasan, Patrick J Sur, Souyma Swaminathan, Ornwipa Thamsuwan, Matthew Lloyd Thomas, Rachel L Updike, Vidhya Venkateswaran, Theo Vos, Tolassa Wakayo, Ronny Westerman, Gelin Xu, Simon Yadgir, and Hassen Hamid Yimam.

Providing critical feedback on methods or results

Amanuel Alemu Abajobir, Cristiana Abbafati, Kaja M Abbas, Foad Abd-Allah, Abdishakur M Abdulle, Semaw Ferede Abera, Victor Aboyans, Laith J Abu-Raddad, Gebre Yitayih Abyu, Isaac Akinkunmi Adedeji, Olatunji Adetokunboh, Mohsen Afarideh , Ashkan Afshin, Anurag Agrawal, Aliasghar Ahmad Kiadaliri, Hamid Ahmadieh, Muktar Beshir Ahmed, Amani Nidhal Aichour, Ibtihel Aichour, Miloud Taki Eddine Aichour, Rufus Olusola Akinyemi, Nadia Akseer, Fares Alahdab, Ziyad Al-Aly, Khurshid Alam, Noore Alam,

Deena Alasfoor, Kefyalew Addis Alene, Reza Alizadeh-Navaei, Ala'a Alkerwi, Peter Allebeck, Rajaa Al-Raddadi, Ubai Alsharif, Khalid A Altirkawi, Nelson Alvis-Guzman, Azmeraw T Amare, Erfan Amini, Yaw Ampem Amoako, Hossein Ansari, Josep M Antó, Carl Abelardo T Antonio, Palwasha Anwari, Al Artaman, Krishna Kumar Aryal, Hamid Asayesh, Solomon Weldegebreal Asgedom, Tesfay Mehari Atey, Leticia Avila-Burgos, Euripide Frinel G Arthur Avokpaho, Ashish Awasthi, Peter Azzopardi, Umar Bacha, Alaa Badawi, Aleksandra Barac, Ryan M Barber, Suzanne L Barker-Collo, Till Bärnighausen, Simon Barquera, Lars Barregard, Lope H Barrero, Bernhard T Baune, Justin Beardsley, Neeraj Bedi, Ettore Beghi, Michelle L Bell, Derrick A Bennett, Isabela M Bensenor, Adugnaw Berhane, Derbew Fikadu Berhe, Eduardo Bernabé, Balem Demtsu Betsu, Mircea Beuran, Addisu Shunu Beyene, Zulfiqar A Bhutta, Boris Bikbov, Charles Birungi, Stan Biryukov, Christopher D Blosser, Dube Jara Boneya, Michael Brauer, Nicholas J K Breitborde, Hermann Brenner, Traolach S Brugha, Lemma Negesa Bulto Bulto, Blair R Bumgarner, Zahid A Butt, Lucero Cahuana-Hurtado, Rosario Cárdenas, Carlos A Castañeda-Orjuela, Ferrán Catalá-López, Kelly Cercy, Hsing-Yi Chang, Fiona J Charlson, Odgerel Chimed-Ochir, Vesper Hichilombwe Chisumpa, Devasahayam Jesudas Christopher, Massimo Cirillo, Aaron J Cohen, cyrus cooper, Michael H Criqui, John A Crump, Lalit Dandona, Rakhi Dandona, José das Neves, Kairat Davletov, Barbora de Courten, Louisa Degenhardt, Selina Deiparine, Robert P Dellavalle, Kebede Deribe, Aniruddha Deshpande, Samath D Dharmaratne, Eric L Ding, Huyen Phuc Do, Klara Dokova, David T Doku, Tim R Driscoll, Manisha Dubey, Bruce Bartholow Duncan, Ziad Ziad El-Khatib, Ahmadali Enayati, Aman Yesuf Endries, Holly E Erskine, Babak Eshrati, Sharareh Eskandarieh, Alireza Esteghamati, Kara Estep, Emerito Jose Aquino Faraon, Carla Sofia e Sa Farinha, André Faro, Farshad Farzadfar, Seyed-Mohammad Fereshtehnejad, João C Fernandes, Alize J Ferrari, Tesfaye Regassa Feyissa, Irina Filip, Florian Fischer, Christina Fitzmaurice, Nataliya Foigt, Kyle J Foreman, Nancy Fullman, Thomas Fürst, Joao M Furtado, Emmanuela Gakidou, Morsaleh Ganji, Tsegaye Tewelde Gebrehiwot, Ayele Geleto, Bikila Lencha Gemechu, Hailay Abrha Gesesew, Katherine B Gibney, Paramjit Singh Gill, Ababi Zergaw Giref, Melkamu Dedefo Gishu, Giorgia Giussani, Philimon N Gona, Amador Goodridge, Sameer Vali Gopalani, Yevgeniy Goryakin, Alessandra Carvalho Goulart, Harish Chander Gugnani, Rajeev Gupta, Tanush Gupta, Vipin Gupta, Reyna A Gutiérrez, Vladimir Hachinski, Nima Hafezi-Nejad, Randah Ribhi Hamadeh, Samer Hamidi, Mouhanad Hammami, Graeme J Hankey, Hilda L Harb, Habtamu Abera Hareri, Rasmus Havmoeller, Simon I Hay, Mohammad T Hedayati, Delia Hendrie, Ileana Beatriz Heredia-Pi, Nobuyuki Horita, H Dean Hosgood, Sorin Hostiuc, Damian G Hoy, Guoqing Hu, Hsiang Huang, John J Huang, Jon Huang, Kim Moesgaard Iburg, Manami Inoue, Kathryn H Jacobsen, Nader Jahanmehr, Mihajlo (Michael) B Jakovljevic, Alejandra Jauregui, Mehdi Javanbakht, Panniyammakal Jeemon, Lars R K Johansson, Catherine O Johnson, Jost B Jonas, Mikk Jürisson, Zubair Kabir, Rajendra Kadel, Amaha Kahsay, Ritul Kamal, André Karch, Amir Kasaeian, Nicholas J Kassebaum, Anshul Kastor, Srinivasa Vittal Katikireddi, Norito Kawakami, Peter Njenga Keiyoro, Sefonias Getachew Kelbore, Andre Pascal Kengne, Chandrasekharan Nair Kesavachandran, Yousef Saleh Khader, Ibrahim A Khalil, Young-Ho Khang, Christian Kieling, Daniel Kim, Yun Jin Kim, Ruth W Kimokoti, Yohannes Kinfu, Adnan Kisa, Katarzyna A Kissimova-Skarbek, Mika Kivimaki, Luke D Knibbs, Ann Kristin Knudsen, Jacek A Kopec, Parvaiz A Koul, Ai Koyanagi, Kristopher J Krohn, Hans Kromhout, Barthelemy Kuate Defo, Burcu Kucuk Bicer, G Anil Kumar, Dharmesh Kumar Lal, Ratilal Lalloo, Tea Lallukka, Qing Lan, Van C Lansingh, Anders Larsson, Alexander Lee, Paul H Lee, James Leigh, Janni Leung, Miriam Levi, Yongmei Li, Misgan Legesse Liben, Stephen S Lim, Shai Linn, Rakesh Lodha, Alan D Lopez, Stefan Lorkowski, Paulo A Lotufo, Rafael Lozano, Erlyn Rachelle King Macarayan, Hassan Magdy Abd El Razek, Mohammed Magdy Abd El Razek, Marek Majdan, Reza Majdzadeh, Azeem Majeed, Reza Malekzadeh, Rajesh Malhotra, Deborah Carvalho Malta, Abdullah A Mamun, Lorenzo G Mantovani, Chabila C

Mapoma, Jose Martinez-Raga, Francisco Rogerlândio Martins-Melo, Manu Raj Mathur, Richard Matzopoulos, Mohsen Mazidi, Colm McAlinden, John J McGrath, Toni Meier, Yohannes Adama Melaku, Peter Memiah, Ziad A Memish, Walter Mendoza, Melkamu Merid Mengesha, George A Mensah, Gert B M Mensink, Seid Tiku Mereta, Atte Meretoja, Tuomo J Meretoja, Haftay Berhane Mezgebe, Renata Micha, Ted R Miller, Mojde Mirarefin, Erkin M Mirrakhimov, Shiva Raj Mishra, NORLINAH MOHAMED IBRAHIM, Kedir Endris Mohammed, Shafiu Mohammed, Murali B V Mohan, Ali H Mokdad, Lorenzo Monasta, Maziar Moradi-Lakeh, Lidia Morawska, Shane D Morrison, Ulrich O Mueller, Erin C Mullany, Kate Muller, Christopher J L Murray, Gudlavalleti Venkata Satyanarayana Murthy, Kamarul Imran Musa, Mohsen Naghavi, Aliya Naheed, Vinay Nangia, Gopalakrishnan Natarajan, Ionut Negoi, Ruxandra Irina Negoi, Cuong Tat Nguyen, Grant Nguyen, Quyen Le Nguyen, Trang Huyen Nguyen, Dina Nur Anggraini Ningrum, Vuong Minh Nong, Bo Norrving, Jean Jacques N Noubiap, Carla Makhlouf Obermeyer, Felix Akpojene Ogbo, Olanrewaju Oladimeji, Andrew Toyin Olagunju, Tinuke Oluwasefunmi Olagunju, Bolajoko Olubukunola Olusanya, Jacob Olusegun Olusanya, John Nelson Opio, Eyal Oren, Mayowa O Owolabi, Mahesh PA, Rosana E Pacella, Adrian Pana, Basant Kumar Panda, Songhomitra Panda-Jonas, Jeyaraj D Pandian, Christina Papachristou, Eun-Kee Park, George C Patton, Norberto Perico, Konrad Pesudovs, Michael Robert Phillips, Julian David Pillay, Dietrich Plass, Svetlana Popova, Richie G Poulton, Farshad Pourmalek, Narayan Prasad, Mostafa Qorbani, Amir Radfar, Afarin Rahimi-Movaghar, Vafa Rahimi-Movaghar, Mahfuzar Rahman, Mohammad Hifz Ur Rahman, Muhammad Aziz Rahman, Rajesh Kumar Rai, Usha Ram, Salman Rawaf, Colin D Rehm, Jürgen Rehm, Robert C Reiner, Marissa B Reitsma, Giuseppe Remuzzi, Andre M N Renzaho, Serge Resnikoff, Satar Rezaei, Antonio L Ribeiro, Juan A Rivera, Kedir Teji Roba, Gholamreza Roshandel, Gregory A Roth, Dietrich Rothenbacher, Sare Safi, Saeid Safiri, Joshua A Salomon, Abdallah M Samy, Juan Ramon Sanabria, Maria Dolores Sanchez-Niño, Damian Santomauro, Itamar S Santos, Milena M Santric Milicevic, Benn Sartorius, Maheswar Satpathy, Monika Sawhney, Sonia Saxena, Maria Inês Schmidt, Ione J C Schneider, Aletta E Schutte, David C C Schwebel, Falk Schwendicke, Soraya Seedat, Sadaf G Sepanlou, Berrin Serdar, Amira Shaheen, Saeid Shahraz, Masood Ali Shaikh, Morteza Shamsizadeh, Sheikh Mohammed Shariful Islam, Rajesh Sharma, Jun She, Jiabin Shen, Kenji Shibuya, Chloe Shields, Mekonnen Sisay Shiferaw, Mika Shigematsu, Min-Jeong Shin, Rahman Shiri, Reza Shirkoohi, Haitham Shoman, Mark G Shrime, Inga Dora Sigfusdottir, Diego Augusto Santos Silva, João Pedro Silva, Dayane Gabriele Alves Silveira, Jasvinder A Singh, Dhirendra Narain Sinha, Eirini Skiadaresi, David L Smith, Mari Smith, Badr H A Sobaih, Eugene Sobngwi, Samir Soneji, Reed J D Sorensen, Luciano A Sposato, Chandrashekhar T Sreeramareddy, Nicholas Steel, Dan J Stein, Caitlyn Steiner, Sabine Steinke, Muawiyyah Babale Sufiyan, Rizwan Abdulkader Suliankatchi, Patrick J Sur, Souyma Swaminathan, Bryan L Sykes, Cassandra E I Szoeke, Rafael Tabarés-Seisdedos, Ken Takahashi, Jukka S Takala, Nikhil Tandon, Marcel Tanner, Yihunie L Tarekegn, Teketo Kassaw Tegegne, Abdullah Sulieman Terkawi, Belay Tesssema, JS Thakur, Kavumpurathu Raman Thankappan, Alan J Thomson, Amanda G Thrift, Taavi Tillmann, Ruoyan Tobe-Gai, Myriam Tobollik, Mette C Tollanes, Marcello Tonelli, Roman Topor-Madry, Miguel Tortajada, Mathilde Touvier, Bach Xuan Tran, Kald Beshir Tuem, Emin Murat Tuzcu, Stefanos Tyrovolas, Kingsley Nnanna Ukwaja, Chigozie Jesse Uneke, Olalekan A Uthman, Job F M van Boven, Santosh Varughese, Tommi Vasankari, Lennert J Veerman, Vidhya Venkateswaran, Narayanaswamy Venketasubramanian, Francesco S Violante, Sergey K Vladimirov, Vasiliy Victorovich Vlassov, Stein Emil Vollset, Theo Vos, Fiseha Wadilo, Tolassa Wakayo, Mitchell T Wallin, Scott Weichenthal, Elisabete Weiderpass, Robert G Weintraub, Andrea Werdecker, Ronny Westerman, Harvey A Whiteford, Charles Shey Wiysonge, Belete Getahun Woldeyes, Charles D A Wolfe, Abdulhalik Workicho, Denis Xavier, Gelin Xu, Bereket Yakob, Lijing L Yan, Hassen Hamid Yimam, Paul Yip, Naohiro

Yonemoto, Marcel Yotebieng, Mustafa Z Younis, Zoubida Zaidi, Maysaa El Sayed Zaki, Ben Zipkin, and Sanjay Zodpey.

Drafting the work or revising is critically for important intellectual content

Kalkidan Hassen Abate, Cristiana Abbafati, Foad Abd-Allah, Abdishakur M Abdulle, Semaw Ferede Abera, Gebre Yitayih Abyu, Isaac Akinkunmi Adedeji, Olatunji Adetokunboh, Mohsen Afarideh , Ashkan Afshin, Sutapa Agrawal, Aliasghar Ahmad Kiadaliri, Muktar Beshir Ahmed, Rufus Olusola Akinyemi, Nadia Akseer, Fares Alahdab, Khurshid Alam, Noore Alam, Ala'a Alkerwi, Peter Allebeck, Nelson Alvis-Guzman, Azmeraw T Amare, Erfan Amini, Walid Ammar, Yaw Ampem Amoako, Hossein Ansari, Josep M Antó, Carl Abelardo T Antonio, Johan Ärnlöv, Hamid Asayesh, Solomon Weldegebreal Asgedom, Tesfay Mehari Atey, Leticia Avila-Burgos, Euripide Frinel G Arthur Avokpaho, Ashish Awasthi, Peter Azzopardi, Alaa Badawi, Aleksandra Barac, Suzanne L Barker-Collo, Till Bärnighausen, Lars Barregard, Lope H Barrero, Carolina Batis, Bernhard T Baune, Neeraj Bedi, Michelle L Bell, Derrick A Bennett, Isabela M Bensenor, Adugnaw Berhane, Balem Demtsu Betsu, Mircea Beuran, Addisu Shunu Beyene, Christopher D Blosser, Michael Brauer, Nicholas J K Breitborde, Hermann Brenner, Traolach S Brugha, Lemma Negesa Bulto Bulto, Blair R Bumgarner, Zahid A Butt, Lucero Cahuana-Hurtado, Juan Jesus Carrero, Carlos A Castañeda-Orjuela, Ferrán Catalá-López, Hsing-Yi Chang, Fiona J Charlson, Hanne Christensen, Devasahayam Jesudas Christopher, Aaron J Cohen, cyrus cooper, Paolo Angelo Cortesi, Michael H Criqui, John A Crump, Lalit Dandona, Rakhi Dandona, José das Neves, Gail Davey, Dragos V Davitoiu, Barbora de Courten, Louisa Degenhardt, Selina Deiparine, Kebede Deribe, Samath D Dharmaratne, Huyen Phuc Do, E Ray Dorsey, Tim R Driscoll, Manisha Dubey, Bruce Bartholow Duncan, Hedyeh Ebrahimi, Aman Yesuf Endries, Holly E Erskine, Sharareh Eskandarieh, Alireza Esteghamati, Kara Estep, Emerito Jose Aquino Faraon, Carla Sofia e Sa Farinha, André Faro, Seyed-Mohammad Fereshtehnejad, João C Fernandes, Alize J Ferrari, Irina Filip, Florian Fischer, Christina Fitzmaurice, Nataliya Foigt, Kyle J Foreman, Nancy Fullman, Thomas Fürst, Joao M Furtado, Emmanuela Gakidou, Morsaleh Ganji, Alberto L Garcia-Basteiro, Tsegaye Tewelde Gebrehiwot, Johanna M Geleijnse, Ayele Geleto, Hailay Abrha Gesesew, Alireza Ghajar, Katherine B Gibney, Paramjit Singh Gill, Richard F Gillum, Melkamu Dedefo Gishu, Philimon N Gona, Amador Goodridge, Sameer Vali Gopalani, Alessandra Carvalho Goulart, Rajeev Gupta, Tanush Gupta, Reyna A Gutiérrez, Nima Hafezi-Nejad, Gessessew Bugssa Hailu, Randah Ribhi Hamadeh, Alexis J Handal, Hilda L Harb, Rasmus Havmoeller, Caitlin Hawley, Ileana Beatriz Heredia-Pi, Nobuyuki Horita, H Dean Hosgood, Sorin Hostiuc, Damian G Hoy, Guoqing Hu, Hsiang Huang, Kathryn H Jacobsen, Mihajlo (Michael) B Jakovljevic, Panniyammakal Jeemon, Jost B Jonas, Mikk Jürisson, Rajendra Kadel, Ritul Kamal, André Karch, Amir Kasaeian, Nicholas J Kassebaum, Srinivasa Vittal Katikireddi, Norito Kawakami, Peter Njenga Keiyoro, Andre Pascal Kengne, Chandrasekharan Nair Kesavachandran, Yousef Saleh Khader, Ejaz Ahmad Khan, Young-Ho Khang, Jagdish Khubchandani, Christian Kieling, Daniel Kim, Yun Jin Kim, Yohannes Kinfu, Mika Kivimaki, Ann Kristin Knudsen, Parvaiz A Koul, Ai Koyanagi, Kristopher J Krohn, Barthelemy Kuate Defo, G Anil Kumar, Dharmesh Kumar Lal, Tea Lallukka, Anders Larsson, Alexander Lee, James Leigh, Janni Leung, Miriam Levi, Misgan Legesse Liben, Stephen S Lim, Giancarlo Logroscino, Alan D Lopez, Paulo A Lotufo, Rafael Lozano, Hassan Magdy Abd El Razek, Mohammed Magdy Abd El Razek, Marek Majdan, Reza Majdzadeh, Azeem Majeed, Abdullah A Mamun, Jose Martinez-Raga, Francisco Rogerlândio Martins-Melo, Mohsen Mazidi, Colm McAlinden, Suresh Mehata, Toni Meier, Yohannes Adama Melaku, Walter Mendoza, Melkamu Merid Mengesha, George A Mensah, Atte Meretoja, Tuomo J Meretoja, Haftay Berhane Mezgebe, Ted R Miller, Shiva Raj Mishra, Karzan Abdulmuhsin Mohammad, Shafiu Mohammed, Ali H Mokdad, Lorenzo Monasta, Maziar Moradi-Lakeh, Paula Moraga, Shane D Morrison, Ulrich O Mueller, Erin C Mullany, Kate Muller, Christopher J L Murray,

Kamarul Imran Musa, Mohsen Naghavi, Ionut Negoi, Ruxandra Irina Negoi, Cuong Tat Nguyen, Quyen Le Nguyen, Trang Huyen Nguyen, Vuong Minh Nong, Ole F Norheim, Bo Norrving, Jean Jacques N Noubiap, Carla Makhlouf Obermeyer, Felix Akpojene Ogbo, In-Hwan Oh, Olanrewaju Oladimeji, Andrew Toyin Olagunju, Pedro R Olivares, Bolajoko Olubukunola Olusanya, Jacob Olusegun Olusanya, Eyal Oren, Alberto Ortiz, Erika Ota, Mayowa O Owolabi, Mahesh PA, Rosana E Pacella, Songhomitra Panda-Jonas, Konrad Pesudovs, Michael Robert Phillips, Michael A Piradov, Farhad Pishgar, Dietrich Plass, Richie G Poulton, Mostafa Qorbani, Amir Radfar, Anwar Rafay, Vafa Rahimi-Movaghar, Mahfuzar Rahman, Rajesh Kumar Rai, Salman Rawaf, Colin D Rehm, Robert C Reiner, Andre M N Renzaho, Satar Rezaei, Antonio L Ribeiro, David Rojas-Rueda, Robin Room, Gholamreza Roshandel, Dietrich Rothenbacher, Lesley Rushton, Mahdi Safdarian, Saeid Safiri, Ramesh Sahathevan, Joshua A Salomon, Abdallah M Samy, Juan Ramon Sanabria, Damian Santomauro, Itamar S Santos, Milena M Santric Milicevic, Maheswar Satpathy, Monika Sawhney, Sonia Saxena, Maria Inês Schmidt, Aletta E Schutte, David C C Schwebel, Falk Schwendicke, Sadaf G Sepanlou, Berrin Serdar, Masood Ali Shaikh, Morteza Shamsizadeh, Sheikh Mohammed Shariful Islam, Kenji Shibuya, Mika Shigematsu, Rahman Shiri, Reza Shirkoohi, Mark G Shrime, Inga Dora Sigfusdottir, Diego Augusto Santos Silva, Jasvinder A Singh, Dhirendra Narain Sinha, Eirini Skiadaresi, David L Smith, Luciano A Sposato, Chandrashekhar T Sreeramareddy, Dan J Stein, Caitlyn Steiner, Sabine Steinke, Mark Andrew Stokes, Muawiyyah Babale Sufiyan, Patrick J Sur, Souyma Swaminathan, Bryan L Sykes, Cassandra E I Szoeke, Santosh Kumar Tadakamadla, Marcel Tanner, Arash Tehrani-Banihashemi, Ornwipa Thamsuwan, Alan J Thomson, Amanda G Thrift, Taavi Tillmann, Mette C Tollanes, Marcello Tonelli, Roman Topor-Madry, Miguel Tortajada, Bach Xuan Tran, Thomas Truelsen, Kald Beshir Tuem, Stefanos Tyrovolas, Kingsley Nnanna Ukwaja, Olalekan A Uthman, Job F M van Boven, Tommi Vasankari, Narayanaswamy Venketasubramanian, Vasiliy Victorovich Vlassov, Stein Emil Vollset, Theo Vos, Tolassa Wakayo, Yuan-Pang Wang, Elisabete Weiderpass, Robert G Weintraub, Andrea Werdecker, Ronny Westerman, Harvey A Whiteford, Charles Shey Wiysonge, Denis Xavier, Gelin Xu, Bereket Yakob, Mehdi Yaseri, Hassen Hamid Yimam, Naohiro Yonemoto, Seok-Jun Yoon, Mustafa Z Younis, Zoubida Zaidi, and Maysaa El Sayed Zaki.

Extracting, cleaning, or cataloging data; designing or coding figures and tables

Kalkidan Hassen Abate, Isaac Akinkunmi Adedeji, Muktar Beshir Ahmed, Komal Ali, Nicholas Arian, Solomon Weldegebreal Asgedom, Aleksandra Barac, James R Bennett, Adugnaw Berhane, Dube Jara Boneya, Lemma Negesa Bulto Bulto, Haley Comfort, Leslie Cornaby, Rakhi Dandona, Sarah Duncan, Sergey Petrovich Ermakov, Kairsten Fay, Kyle J Foreman, Ayele Geleto, William W Godwin, Jingwen Guo, Chad Ikeda, Nicholas J Kassebaum, Laura Kemmer, Young-Ho Khang, Jun Y Kim, G Anil Kumar, Michael Kutz, Alexander Lee, Janni Leung, Yichong Li, Misgan Legesse Liben, Patrick Liu, Helena Manguerra, Manu Raj Mathur, Mohsen Mazidi, Melkamu Merid Mengesha, Haftay Berhane Mezgebe, Anoushka Millear, Shawn Minnig, Cliff Mountjoy-Venning, Ionut Negoi, Mahesh PA, Carrie Purcell, Colin D Rehm, Jürgen Rehm, Marissa B Reitsma, Yesenia Roman, Abdallah M Samy, Maheswar Satpathy, Peilin Shi, Erica Leigh Slepak, Bryan Strub, Michelle Subart, Patrick J Sur, Mohammad Tavakkoli, Andrew M Theis, Anna Torre, Rachel L Updike, Vidhya Venkateswaran, Rachel Woodbrook, Sarah Wulf Hanson, Simon Yadgir, Luis Zavala-Arciniega, Stephanie Raman M Zimsen, and Ben Zipkin.

Managing the overall research enterprise

Ashkan Afshin, Tahiya Alam, Blair R Bumgarner, Kara Estep, Christina Fitzmaurice, Kyle J Foreman, Emmanuela Gakidou, Caitlin Hawley, Simon I Hay, Nicholas J Kassebaum, Laura Kemmer, Yohannes Kinfu, Xiaofeng Liang, Stephen S Lim, Rafael Lozano, Ali H Mokdad, Erin C Mullany, Kate Muller,

Christopher J L Murray, Mohsen Naghavi, Carla Makhlouf Obermeyer, Helen E Olsen, Robert C Reiner, Joseph Salama, Joshua A Salomon, Chloe Shields, David L Smith, Caitlyn Steiner, Patrick J Sur, Souyma Swaminathan, and Theo Vos.

Contents

Authors' contributions i
Preamble 6
Section 1. GBD overview 7
GATHER statement 7
Location units of the analysis 7
GBD risk factor hierarchy 7
Time periods of the analysis 7
List of abbreviations 7
Section 2. Risk factor estimation 10
Overview 10
Step 1. Effect size estimation 11
1a. Collate relative risk data 11
1b. Determine relative risks 13
Step 2. Exposure estimation 14
2a. Collate exposure data 14
2b. Adjust exposure data 15
2c. Estimate exposure 15
Subnational Scaling and Aggregation 22
Step 4. TMREL 22
Step 5. Estimate population attributable fractions 22
Step 6. Mediation 23
Summary 23
Additional detail 23
Calculating burden of multiple risk factors 25
Adjusting for mediation 26
Calculating mediation factor 27
Piecewise aggregation (Pattern 3) 29
Uncertainty of aggregated and mediated PAFs 29
Important assumptions in aggregating risk factors and including mediation 29
Step 7. Estimate attributable burden 30

Other analysis: Decomposition of deaths and DALYs 30
Other analyses: SDI 32
Development of SDI 32
Updates to SDI Computation for GBD 2016 32
Additional Methods Information 33
Risk-specific comparisons to other estimates 33
References 39
Section 3. Risk-specific estimation 42
Unsafe water 43
Unsafe Sanitation 43
Unsafe Hygiene 43
Ambient Particulate Matter Pollution 43
Household Air Pollution 43
Ambient Ozone Pollution 43
Residual Radon 43
Environmental Lead 43
Occupational 43
Suboptimal Breastfeeding 43
Child Growth Failure 43
Low birth weight 43
Iron Deficiency 43
Vitamin A deficiency 43
Smoking 43
Second hand smoke 43
Smokeless tobacco 43
Alcohol use 43
Drug use 43
Diet 43
Childhood sexual assault 43
Unsafe sex 43
Low physical activity 43
Fasting plasma glucose 43
High cholesterol 43

High systolic blood pressure 43
High BMI 43
Bone mineral density 43
Impaired kidney function 43
Section 4: Tables and Figures 44
Appendix Table 1a. Relative risks used by age and sex for each outcome for all risk factors except for ambient air pollution and alcohol. 44
Appendix Table 1b. Relative risks used by age and sex for each outcome for the particulate matter integrated exposure response curve. 44
Appendix Table 1c. Relative risks used by age and sex for each outcome for alcohol use globally. 44
Appendix Table 1d. Relative risks used for IQ shifts by age and sex for lead exposure blood. 44
Appendix Table 2. Supplemental information for Figure 1: Epidemiological evidence supporting causality between risk-outcome pairs included in the Global Burden of Disease 2016 study including 2A. Citations and 2B. Additional information 44
Appendix Table 3. GBD 2016 risk factor hierarchy with levels, modeling strategies, and the main type of data sources used to estimate exposure levels 44
Appendix Table 4. Types of Comparative Risk Assessments (CRA) based on the time perspective and the nature of the counterfactual level or distribution of exposure. The shaded box represents the type of CRA currently undertaken in GBD 2016. GBD=Global Burden of Disease. 44
Appendix Table 5. Guidelines for Accurate and Transparent Health Estimates Reporting (GATHER) 18-items checklist with description of compliance and location of information for GBD 2016 risk factors capstone 44
Appendix Table 6: GBD 2016 location hierarchy with levels 44
Appendix Table 7. Mediation factors. For IHD, stroke, and diabetes we pooled all available cohorts and estimated relative risks with and without adjustment across all combinations of metabolic risk factors. We then computed the excess attenuated risk for each mediation-risk-cause set. 44
Appendix Table 8: Socio-demographic Index groupings by location, based on 2016 values 44
Appendix Table 9: Socio-demographic Index values for all estimated GBD 2016 locations, 1980–2016 44
Appendix Figure 1: A more general causal web of the causes of health outcomes with the categories of causes included in this analysis shown in blue. 44
Appendix Figure 2: Analytical flowchart of the comparative risk assessment for the estimation of population attributable fractions by geography, age, sex, and year for GBD 2016. Ovals represent data inputs, rectangular boxes represent analytical steps, cylinders represent databases, and parallelograms represent intermediate and final results. GBD=Global Burden of Disease. SEVs=Summary exposure values. TMREL=Theoretical minimum-risk exposure level. PAFs=Population attributable fractions. YLLs=years of life lost. YLDs=years lived with disability. DALYs=disability-adjusted life-years 45

Appendix Figure 3: GBD 2016 DisMod-MR 2.1 analytic cascade 45
Appendix Figure 4: ST-GPR flowchart 45

Preamble

This appendix provides further methodological detail and more detailed results for Global, regional, and national comparative risk assessment of 84 behavioural, environmental and occupational, and metabolic risks or clusters of risks: 1990-2016: a systematic analysis for the Global Burden of Disease Study 2016. This study complies with the Guidelines for Accurate and Transparent Health Estimates Reporting (GATHER) recommendations. It includes detailed tables and information on data in an effort to maximize transparency in our estimation processes and provide a comprehensive description of analytical steps. We intend this appendix to be a living document, to be updated with each iteration of the Global Burden of Disease Study.

Section 1. GBD overview

GATHER statement

This study complies with the Guidelines for Accurate and Transparent Health Estimates Reporting (GATHER) recommendations. We have documented the steps involved in our analytical procedures and detailed the data sources used in compliance with GATHER. For additional GATHER reporting, please refer to Appendix Table 5.

Location units of the analysis

The locations included in Global Burden of Diseases, Injuries, and Risk Factors Study 2016 (GBD 2016) have been arranged into a set of hierarchical categories composed of seven super-regions and a further nested set of 21 regions containing 195 countries and territories. The locations for which GBD estimated global, regional, and national risk exposure, relative risk, theoretical minimum-risk exposure level (TMREL), and population attributable fractions (PAFs), have not expanded following GBD 2015. New sub-national locations estimated for GBD 2016 are the local health authorities of England and provinces of Indonesia. Sub-national assessments for GBD 2016 include 26 states and one federal district for Brazil, 33 provinces and municipalities for China, nine regions and 150 local health authorities for England, 31 states and union territories by urbanicity for India, 34 provinces for Indonesia, 47 prefectures for Japan, 47 counties for Kenya, 31 states and one federal district for Mexico, two areas for Sweden, nine provinces for South Africa, 13 states for the Kingdom of Saudi Arabia, and 50 states and one federal district for the United States. Combined, there are a total of 335 locations at the first subnational unit Level. Included in subnational Level 1 locations are countries that have been subdivided into the first subnational Level, such as states or provinces, for the GBD analysis; subnational Level 2 only applies to India and England. For this paper we present data at the national and territory Level.

GBD risk factor hierarchy

In this analysis, we focus on three groups of risk factors: behavioural, environmental and occupational, and metabolic. The GBD 2016 risk factors hierarchy and Levels are summarized in Appendix Table 6.

The GBD risk list continues to evolve to reflect the policy relevance, public health, and medical care importance of major risk factors. The risk factors list expanded following feedback from GBD 2013 and input from GBD 2015 collaborators. Five risks added to the list for GBD 2016.

Time periods of the analysis

A complete set of risk-specific exposure, relative risk, TMREL, and PAFs were computed for the years 1990-2016.

All GBD 2016 results and online data visualizations are available at http://vizhub.healthdata.org/gbd-compare with access to results for all GBD metrics.

List of abbreviations

BMI: body-mass index

BMD: bone mineral density

CKD: chronic kidney disease

COD: causes of death

CODEm: cause of death ensemble modeling

COPD: chronic obstructive pulmonary disease

CSA: childhood sexual abuse

CSMR: cause-specific mortality rate

CRA: comparative risk assessment

CVD: cardiovascular disease

DALY: disability-adjusted life-year

DRI: data representativeness index

EMR: excess mortality rate

FAO: Food and Agriculture Organization

GATHER: Guidelines for Accurate and Transparent Health Estimates Reporting

GBD: Global Burden of Disease

IER: integrated exposure response

IHD: ischemic heart disease

ILO: International Labour Organization

IPV: intimate partner violence

LDI: lag distributed income per capita

LRI: lower respiratory infection

MDG: Millennium Development Goal

NCD: non-communicable disease

PAF: population attributable fraction

$PM_{2.5}$: particulate matter $<2.5\mu m$ in diameter

RCT: randomised controlled trial

RMSE: root mean square error

SBP: systolic blood pressure

SD: standard deviation

SDG: sustainable development goal

SDI: Socio-demographic Index

SEER: Surveillance, Epidemiology, and End Results Program

SEV: summary exposure value

SIR: smoking impact ratio

SSB: sugar-sweetened beverages

ST-GPR: spatiotemporal Gaussian process regression

TB: tuberculosis

TMREL: theoretical minimum-risk exposure level

UI: uncertainty interval

WHO: World Health Organization

YLD: years lived with disability

YLL: years of life lost

Section 2. Risk factor estimation

Overview

The comparative risk assessment (CRA) conceptual framework was developed by Murray and Lopez,[1] who established a causal web of hierarchically organised risks or causes that contribute to health outcomes (Appendix Figure 1), which allows for quantification of risks or causes at any Level in the framework. In GBD 2016, as in previous iterations of the GBD study, we evaluated a set of behavioural, environmental and occupational, and metabolic risks, where risk-outcome pairs were included based on evidence rules (see appendix pp 10-11). These risks were organised in four hierarchical Levels, where Level 1 represents the overarching categories (behavioural, environmental and occupational, and metabolic) nested within Level 1 risks; Level 2 contains both single risks and risk clusters (such as child and maternal malnutrition); Level 3 contains the disaggregated single risks from within Level 2 risk clusters (such as low birthweight and prematurity); and Level 4 details risks with the most granular disaggregation, such as for specific occupational carcinogens, the subcomponents of childhood undernutrition (stunting, wasting, underweight), and suboptimal breastfeeding (discontinued and non-exclusive breastfeeding). At each level of risk, we evaluated whether risk combinations were additive, multiplicative, or shared common pathways for intervention. This approach allows the quantification of the proportion of risk-attributable burden shared with another risk or combination of risks and the measurement of potential overlaps between behavioural, environmental and occupational, and metabolic risks. To date in the GBD we have not quantified the contribution of other classes of risk factors illustrated in Appendix Table 4. We do provide some insights into the potential magnitude of distal social, cultural, and economic factors through an analysis of the relationship between risk exposures and development measured using the Socio-demographic Index (SDI) (appendix pp 34-36)

Two types of risk assessments are possible within the CRA framework: attributable burden and avoidable burden. Attributable burden is the reduction in current disease burden that would have been possible if past population exposure had shifted to an alternative or counterfactual distribution of risk exposure. Avoidable burden is the potential reduction in future disease burden that could be achieved by changing the current distribution of exposure to a counterfactual distribution of exposure. Murray and Lopez identified four types of counterfactual exposure distributions: (1) theoretical minimum risk; (2) plausible minimum risk; (3) feasible minimum risk; and (4) cost-effective minimum risk.[2] The theoretical minimum risk level (TMREL) is the level of risk exposure that minimises risk at the population level, or the level of risk that captures the maximum attributable burden. Other possible forms of risk quantification include plausible minimum risk – which reflects the distribution of risk that is conceivably possible and would minimise population-level risk if achieved – while feasible minimum risk describes the lowest risk distribution that has been attained within a population, and the cost-effective minimum risk is the lowest risk distribution for a population that can be attained in a cost-effective manner. Because no robust set of forecasts for all components of GBD is available, in this study we focus on quantifying attributable burden using the theoretical minimum risk counterfactual distribution. Appendix Table 4 shows the eight possible types of risk quantification within the CRA framework, with the hatched box representing the type of CRA currently undertaken by the GBD study. As per the definition of avoidable burden, risk reversibility would be incorporated into this type of assessment, as it would involve reducing risk to the counterfactual for the index year, given a history of past risk exposure. Given the focus in this study on attributable burden, risk reversibility is not a criteria used in estimation here.

In general, this analysis follows the CRA methods used in GBD 2015.[3] The methods described here provide a high-level overview of the analytical logic with a focus on areas of notable change from the methods employed in GBD 2015. Here we aim to provide sufficient detail on the methodology and overall structure of the estimation process. This study complies with the GATHER recommendations proposed by the World Health Organization (WHO) and others, which include recommendations on documentation of data sources, estimation methods, and statistical analysis (Appendix Table 5).[4]

Step 1. Effect size estimation

1a. Collate relative risk data

Criteria for inclusion of risk-outcome pairs

In this study, as in GBD 2015, we have included risk-outcome pairs that we have assessed as meeting the World Cancer Research Fund (WCRF) grades of convincing or probable evidence.[5] In this framework, convincing evidence consists of biologically plausible associations between exposure and disease established from multiple epidemiological studies in different populations. Evidentiary studies must be substantial, include prospective observational studies, and where relevant, randomised controlled trials (RCTs) of sufficient size, duration, and quality, and showing consistent effects. Probable evidence is similarly based on epidemiological studies with consistent associations between exposure and disease, but for which shortcomings in the evidence exist, such as insufficient trials (or prospective observational studies) available.

The World Cancer Research Fund grading system

Convincing evidence

Evidence based on epidemiological studies showing consistent associations between exposure and disease, with little or no evidence to the contrary. The available evidence is based on a substantial number of studies including prospective observational studies and where relevant, randomized controlled trials of sufficient size, duration, and quality showing consistent effects. The association should be biologically plausible.

Probable evidence

Evidence based on epidemiological studies showing fairly consistent associations between exposure and disease, but for which there are perceived shortcomings in the available evidence or some evidence to the contrary, which precludes a more definite judgment. Shortcomings in the evidence may be any of the following: insufficient duration of trials (or studies); insufficient trials (or studies) available; inadequate sample sizes; or incomplete follow-up. Laboratory evidence is usually supportive. The association should be biologically plausible.

Possible evidence

Evidence based mainly on findings from case-control and cross-sectional studies. Insufficient randomized controlled trials, observational studies, or non-randomized controlled trials are available. Evidence based on non-epidemiological studies, such as clinical and laboratory investigations, is supportive. More trials are needed to support the tentative associations, which should be biologically plausible.

Insufficient evidence

Evidence based on findings of a few studies which are suggestive, but insufficient to establish an association between exposure and disease. Little or no evidence is available from randomized controlled trials. More well-designed research is needed to support the tentative association.

Causal criteria

As in GBD 2015, to be more objective, consistent, and transparent in our evaluation of the causal relationship, we summarized epidemiologic evidence supporting causality for each risk-outcome pair. For each pair, we collected data on the following domains:

Domains	*Description*
RCTs of disease endpoint	Number of independent RCTs evaluating the effect of the risk on the disease endpoint
	Percent of independent RCTs showing significant effect in the opposite direction
	Percent of independent RCTs showing no effect
Prospective observational studies of disease endpoint	Number of independent prospective observational studies evaluating the association of the risk with the disease endpoint
	Percent of independent prospective observational studies with significant association in the opposite direction
Strength	Lower Limit of RR in observational studies> 1.5 (Yes/No)
Dose response	Evidence of the dose-response relationship between the risk and the outcome (Yes/No)
Biologic plausibility	Potential biologic mechanism that could explain the effect of the risk on the disease endpoint (Yes/No)
Analogy	Evidence on the relationship between the risk factor and a disease endpoint from the same category (Yes/No)

In GBD 2016, for risk-outcome pairs with less than 5 prospective studies, we summarized evidence from case-control studies as well including (a) the number of independent case-control studies evaluating the association of the risk with the disease endpoint and (b) percent of independent case control studies with significant association in the opposite direction.

Fitting a distribution to exposure data

The most informative data describing the distribution of risk factors within a population come from individual-level data; additional sources of data include reported means and variances. In cases when a risk factor also defines a disease, such as haemoglobin level and anaemia, the prevalence of disease is also frequently reported. To model the distribution of any particular risk factor, we seek a family of probability density functions (PDFs), a fitting method, and a model selection criterion. To make use of the most data describing most populations, we used the method of moments (MoM); the first two empirical moments from a population, the mean and variance, were used to determine the PDF describing the distribution of risk within any population, where exceptions to this rule are justified by context. We used the Kolmogorov-Smirnov test to measure the goodness of fit (GoF), but in some cases, the GoF was based on the prediction error for the prevalence of disease.

We used an ensemble technique in which a model selection algorithm is used to choose the best model for each continuous risk factor.[6] We drew the initial set of candidate models from commonly used PDF families, raning from right skwewed to left skewed distributions. We fitted each PDF candidate family to each dataset using the MoM, and used the Kolmogorov-Smirnov test[7] as the measure of GoF. Preliminary analysis showed that the GoF ranking of PDF families varied across datasets for any particular risk factor and that combining the predictions of differently fitted PDF families could dramatically improve the GoF for each dataset. Therefore, we developed a new model for prediction using the ensemble of candidate models which is a weighted linear combination of all candidate models, {f}, where a set of weights {w} is chosen such that $\sum_i w_i = 1$, and the values of the weights were determined by a second GoF criterion with its own validation process. For each risk, we pooled all available microdata and performed Nelder-Mead numeric optimization across demographics subsets of data to derive a set of distribution specific weights such that the Kolmogorov–Smirnov (KS) statistic is minimized. The details can be summarised by 1) the summary statistics for each dataset; 2) a table showing the Kolmogorov-Smirnov statistic for each candidate model and URD; and 3) the weights defining the final ensemble model for each dataset.

1b. Determine relative risks

Effect size estimation

The relative risk by level of exposure, or by cause, for mortality or morbidity can be found in published and unpublished primary studies or in secondary studies that summarize relative risks. In Step 1a of the analytical process (Appendix Figure 2), we collated information from randomized controlled trials, cohort, pooled cohort, and case-control studies, and in Step 1b, used these data to determine the relative risk for the risk-outcome pairs included in GBD 2016. For most risks, data from pooled cohorts, or meta-analyses of cohorts, were used; in the case of the risk of cataracts from household air pollution cohort data were not available, and instead we used case-control data. We estimated relative risks of mortality and morbidity for 65 risk factors for which we determined attributable burden using relative risk and exposure. We incorporated relative risks from studies that controlled for confounding but not for factors along the causal pathway between exposure and outcome. For risk-outcome pairs with evidence available for only one of mortality or morbidity, we generally assumed that the estimated relative risks applied equally to both. Given evidence of statistically different relative risks for mortality and morbidity, we incorporated different relative risks for each. We did not find that relative risks were consistently higher or lower for mortality compared with morbidity. Details and citation information for the data sources used for relative risks are provided in searchable form through a new web-tool (http://ghdx.healthdata.org/). Available data sources for determining relative risks varied across risks. Details on how relative risks were calculated for each risk can be found in Appendix Section 3.

For all outcomes related to unsafe sex, the relative risk and exposure framework was not used to estimate attributable burden. For unsafe sex and HIV, we used a direct attribution approach to address the lack of data on unsafe sexual practices in most populations. The proportion of HIV attributable to unsafe sex was modelled directly using DisMod-MR 2.1 from data on the fraction of cases identified as being through sexual transmission, intravenous drug use, or blood transfusion.

For risks estimated from a continuous exposure distribution where the effect size was reported by categories in pooled or meta-analysis studies, we converted those categories to relative risk per unit increase in exposure. This implies a linear increase in the log of the relative risk and exposure; various

studies have suggested this is a reasonable approximation of the dose-response curve for many risks. An example of this is high systolic blood pressure, where data from the Prospective Cohort Study (PSC) and the Asia-Pacific Cohort Studies Collaboration (APCSC) were well-described by a linear increase in the logarithm of the relative risk by a 10-unit increase in high systolic blood pressure. This approximately log-linear relationship suggests that the proportional difference in the age-specific risk of stroke death associated with a given absolute difference in exposure is about the same at all levels of risk. Many meta-analyses convert relative risks to per unit increase for convenience, particularly when studies choose different categories that could not otherwise be compared. The log-linear approximation appears plausible[8] even where there is limited consensus on the appropriate TMREL. Where there were insufficient samples in the primary studies at high levels of exposure to inform the shape of the tail of the distribution, we applied a cap to the maximum relative risk using the midpoint of the last category for which a relative risk was reported.

Step 2. Exposure estimation

2a. Collate exposure data

Systematic reviews

For GBD 2016, we conducted systematic literature reviews for 23 risks. For other risk factors, only a small fraction of the existing data appears in the published literature and other sources predominate such as survey data and satellite data. Data were systematically screened from household surveys archived in the Global Health Data Exchange (ghdx.healthdata.org), including Demographic and Health Surveys, Multiple Indicator Cluster Surveys, Living Standards Measurement Surveys, and Reproductive Health Surveys. Other national health surveys were identified based on survey series that had yielded usable data for past rounds of GBD, sources suggested to us by in-country collaborators, and surveys identified in major multinational survey data catalogs, such as the International Household Survey Network and the WHO Central Data Catalog, as well as through country Ministry of Health and Central Statistical Office websites. Citations for all data sources used for risk factor estimation in GBD 2016 are provided in searchable form through a web-tool (http://ghdx.healthdata.org/). A description of the search terms employed for risk-specific systematic reviews are detailed by cause in Appendix Section 3.

Information on systematic reviews were managed using Research Electronic Data Capture (REDCap) electronic data capture tools hosted at the University of Washington.[9] REDCap is a secure, web-based application designed to support data capture for research studies, providing 1) an intuitive interface for validated data entry; 2) audit trails for tracking data manipulation and export procedures; 3) automated export procedures for seamless data downloads to common statistical packages; and 4) procedures for importing data from external sources

Search terms

Search terms for updates of systematic reviews for GBD 2016 are shown by risk in Appendix Section 3.

Survey data preparation

For GBD 2016, survey data constitutes a substantial part of the underlying data used in the estimation process. During extraction, we concentrate on demographic variables (such as location, gender, age), survey design variables (such as sampling strategy and sampling weights), and the variables used to define the population estimate (such a prevalence or a proportion) and a measure of uncertainty (standard error, confidence interval or sample size and number of cases).

2b. Adjust exposure data

A number of adjustments were applied to extracted exposure sources in order to make the data more consistent and suitable for modeling. Commonly applied adjustments included age-sex splitting, adding study-level covariates, and bias correction. Age-sex splitting was applied to literature data reported by age or sex but not by age and sex assuring that the total number of cases remained as reported. If a source did not report sample size by age or sex, we applied the age-sex distribution of the population for the same location and year to the reported total sample size. We relied on the metaregression component of DisMod-MR 2.1 for most of the bias correction of data for variations in study attributes such as case definitions and measurement method. DisMod-MR 2.1 calculates a single adjustment that is applied regardless of age, sex, or location. If enough data were available to differentiate these adjustments by age, sex, or location, or if detailed survey data were available to make more precise adjustments between different thresholds on a biochemical measure, we applied bias corrections to the data before entry into DisMod-MR 2.1.

2c. Estimate exposure

Mean exposure estimation

In Step 2a of the estimation process, we used systematic literature reviews to identify risk factor exposure studies published or identified since GBD 2015 and combined these with existing data from household and health examination surveys, census, morbidity, or satellite imagery and ground sensor data (used for PM2.5 estimation). Certain risks, such as diet and alcohol consumption, also incorporated administrative record systems. Data sources used in estimating risk factor exposure can be accessed through the data source tool at http://ghdx.healthdata.org/.

Once data were collected and compiled, step 2b of the analytical flowchart describes the adjustments applied, where necessary, to correct for bias. Examples of these adjustments include: use of urban studies for lead; crosswalks between different measurements, methods, and definitions, such as for self-report of obesity and glycated hemoglobin (HbA1C) for diabetes; and age-sex splitting of data, such as for fasting plasma glucose, cholesterol, and systolic blood pressure that may be reported from broad age-groups.

For the GBD, we developed two modeling approaches, a Bayesian meta-regression model (DisMod-MR 2.1) and a spatiotemporal Gaussian process regression model (ST-GPR), to pool data from different sources, control and adjust for bias in data, and incorporate other types of information such as country-level covariates. DisMod-MR 2.1 and ST-GPR are mixed effect models that borrow information across age, time, and locations to synthesise multiple data sources into unified estimates of levels and trends. A detailed description of the likelihood used for estimation, and a full description of improvements made for DisMod-MR 2.1, are detailed by Vos and colleagues[10] with additional detail in the appendix to that paper. The ST-GPR model has three main hyper-parameters that control for smoothing across time, age, and location. Values for these hyper-parameters were selected based on cross-validation. Cross-validation tests were conducted for different combinations of the hyper-parameters. In each test, 30% of the data were held out and the performance of each combination of hyper-parameters evaluated on the held out data. For each hyper-parameter combination, 25 cross-validation tests were conducted. The performance of each model in predicting the withheld 30% of the data was evaluated using a combined measure based on root mean square error (RMSE) and uncertainty interval coverage. A detailed description of the ST-GPR process regression can be found on appendix pp 17-21.

The main difference between these methods is their power to include unstructured types of data by sex and age group and in their degree of flexibility. Step 2c in Appendix Figure 2 outlines the use of DisMod-MR 2.1 for 23 risk factors where data were available by different age intervals or mixed sex groups; DisMod-MR 2.1 is the preferred tool in these cases because of its ability to integrate over age and adjust for different exposure definitions in the data; however, the use of Bayesian Markov Chain Monte Carlo (MCMC) simulations with large volumes of data renders the analysis computationally intensive and reduces the number of iterations that are possible. If large volumes of standard age-group data are available – as is generally the case for metabolic risks – using ST-GPR becomes the preferred approach.

In some cases, we adapted our methods of modeling exposure to risks where necessary to account for complexities in the risk-outcome relationship or the need for particular handling of data, for example, dietary risks and ambient air pollution (see Section 3 for more detail). A complete list of risks and the analytical method used is reported in Appendix Table 3. Additional details for adjustments or adaptations to particular risk models are located in Appendix Section 3.

DisMod-MR 2.1 Estimation

DisMod-MR 2.1 description

Until GBD 2010, nonfatal estimates in burden of disease assessments were based on a single data source on prevalence, incidence, remission or a mortality risk selected by the researcher as most relevant to a particular location and time. For GBD 2010, we set a more ambitious goal: to evaluate all available information on a disease that passes a minimum quality standard. That required a different analytical tool that would be able to pool disparate information presented in varying age groupings and from data sources using different methods. The DisMod-MR 1.0 tool used in GBD 2010 evaluated and pooled all available data, adjusted data for systematic bias associated with methods that varied from the reference and produced estimates by world regions with uncertainty intervals using Bayesian statistical methods. For GBD 2013, the improved DisMod-MR 2.0 had increased computational speed allowing computations that were consistent between all disease parameters at the country rather than region Level. The hundred-fold increase in speed of DisMod-MR 2.0 was partly due to a more efficient rewrite of the code in C++ but also by changing to a model specification using log rates rather than a negative binomial model used in DisMod-MR 1.0. In cross-validation tests, the log rates specification worked as well or better than the negative binomial specification.[11] For GBD 2015, we rewrote the 'wrapper' code that organizes the flow of data and settings at each level of the analytical cascade. The sequence of estimation occurs at five Levels: global, super-region, region, country and, where applicable, subnational location. The super-region priors are generated at the global Level with mixed-effects, nonlinear regression using all available data; the super-region fit, in turn, informs the region fit, and so on down the cascade. The wrapper gives analysts the choice to branch the cascade in terms of time and sex at different levels depending on data density. The default used in most models is to branch by sex after the global fit but to retain all years of data until the lowest Level in the cascade. Appendix Figure 3 summarizes the Dismod-MR process.

In updating the 'wrapper,' we consolidated the code base into a single language, Python, to make the code more transparent and efficient and to better deal with subnational estimation. The computational engine is limited to three levels of random effects; we differentiate estimates at the super-region, region and country Level. In GBD 2013, the subnational units of China, the UK and Mexico were treated as 'countries' such that a random effect was estimated for every location with contributing data. However,

the lack of a hierarchy between country and subnational units meant that the fit to country data contributed as much to the estimation of a subnational unit as the fits for all other countries in the region. We found inconsistency between the country fit and the aggregation of subnational estimates when the country's epidemiology varied from the average of the region. Adding an additional level of random effects required a prohibitively comprehensive rewrite of the underlying DisMod-MR engine. Instead, we added a fifth layer to the cascade, with subnational estimation informed by the country fit and country covariates, plus an adjustment based on the average of the residuals between the subnational location's available data and its prior. This mimicked the impact of a random effect on estimates between subnationals.

In GBD 2015, we also improved how country covariates differentiate nonfatal estimates for diseases with sparse data. The coefficients for country covariates are re-estimated at each Level of the cascade. For a given location, country coefficients are calculated using both data and prior information available for that location. In the absence of data, the coefficient of its parent location is used, in order to utilize the predictive power of our covariates in data sparse situations.

For GBD 2016, the computational engine (DisMod-MR 2.1) remained substantively unchanged from GBD 2015. We changed the prediction year set to generate fits for the years 1990, 1995, 2000, 2005, 2010, and 2016. We updated the age prediction sets to include age groups 80-84, 85-89, 90-94, and 95+, to comply with changes across all functional areas of the GBD. We also expanded the set of locations where subnational units are modeled; the set now includes: Brazil, China, England, India, Indonesia, Japan, Kenya, Mexico, Saudi Arabia, South Africa, Sweden, and the United States.

The flowchart for the DisMod-MR 2.1 process can be found in Appendix Figure 3.

DisMod-MR 2.1 likelihood estimation

Analysts have the choice of using a Gaussian, log-Gaussian, Laplace or Log-Laplace likelihood function in DisMod-MR 2.1. The default log-Gaussian equation for the data likelihood is:

$$-log\left[p\left(y_j|\Phi\right)\right] = \log\left(\sqrt{2\pi}\right) + \log\left(\delta_j + s_j\right) + \frac{1}{2}\left(\frac{\log\left(a_j + \eta_j\right) - \log\left(m_j + \eta_j\right)}{\delta_j + s_j}\right)^2$$

where, y_j is a 'measurement value' (i.e., data point); Φ denotes all model random variables; η_j is the offset value, eta, for a particular 'integrand' (prevalence, incidence, remission, excess mortality rate, with-condition mortality rate, cause-specific mortality rate, relative risk or standardized mortality ratio) and a_j is the adjusted measurement for data point j, defined by:

$$a_j = e^{(-u_j - c_j)} y_j$$

where u_j is the total 'area effect' (i.e., the sum of the random effects at three Levels of the cascade: super-region, region and country) and c_j is the total covariate effect (i.e., the mean combined fixed effects for sex, study level and country level covariates), defined by:

$$c_j = \sum_{k=0}^{K[I(j)]-1} \beta_{I(j),k} \hat{X}_{k,j}$$

with standard deviation

$$s_j = \sum_{l=0}^{L[I(j)]-1} \zeta_{I(j),l} \hat{Z}_{k,j}$$

where k denotes the mean value of each data point in relation to a covariate (also called x-covariate); I(j) denotes a data point for a particular integrand, j; $\beta_{I(j),k}$ is the multiplier of the kth x-covariate for the ith integrand; $\hat{X}_{k,j}$ is the covariate value corresponding to the data point j for covariate k; l denotes the standard deviation of each data point in relation to a covariate (also called z-covariate); $\zeta_{I(j),k}$ is the multiplier of the lth z-covariate for the ith integrand; and δ_j is the standard deviation for adjusted measurement j, defined by:

$$\delta_j = log\left[y_j + e^{(-u_j - c_j)}\eta_j + c_j\right] - log\left[y_j + e^{(-u_j - c_j)}\eta_j\right]$$

Where m_j denotes the model for the jth measurement, not counting effects or measurement noise and defined by:

$$m_j = \frac{1}{B(j) - A(j)} \int_{A(j)}^{B(j)} I_j(\text{a})\ \text{da}$$

where A(j) is the lower bound of the age range for a data point; B(j) is the upper bound of the age range for a data point; and I_j denotes the function of age corresponding to the integrand for data point j.

Spatiotemporal Gaussian process regression

Spatiotemporal Gaussian process regression (ST-GPR) has been used for risk factors where the data density is sufficient to estimate a very flexible time trend. The flowchart showing the analytic steps can be found in Appendix Figure 4. The approach is a stochastic modeling technique that is designed to detect signals amidst noisy data. It also serves as a powerful tool for interpolating non-linear trends.[12,13] Unlike classical linear models that assume that the trend underlying data follows a definitive functional form, GPR assumes that the specific trend of interest follows a Gaussian Process, which is defined by a mean function $m(\cdot)$ and a covariance function $Cov(\cdot)$. For example, let $p_{c,a,s,t}$ be the exposure, in normal, log, or logit space, observed in country c, for age group a, and sex s at time t:

$$(p_{c,a,s,t}) = g_{c,a,s}(t) + \epsilon_{c,a,s,t}$$

where

$$\epsilon_{c,a,s,t} \sim Normal(0, \sigma_p^2),$$

$$g_{c,a,s}(t) \sim GP\left(m_{c,a,s}(t), Cov\left(g_{c,a,s}(t)\right)\right).$$

The derivation of the mean and covariance functions, $m_{c,a,s}(t)$ and $Cov\left(g_{c,a,s}(t)\right)$, along with a more detailed description of the error variance (σ_p^2), is described below.

Estimating mean functions

We estimated mean functions using a two-step approach. To be more specific, $m_{c,a,s}(t)$ can be expressed, depending on the exposure transformation, as:

$$log(p_{c,a,s}(t)) = X_{c,a,s}\beta + h(r_{c,a,s,t})$$

$$logit(p_{c,a,s}(t)) = X_{c,a,s}\beta + h(r_{c,a,s,t})$$

$$p_{c,a,s}(t) = X_{c,a,s}\beta + h(r_{c,a,s,t})$$

where $X\beta$ is the summation of the components of linear regression, including the intercept and the product of covariates with their corresponding fixed effect coefficients. Some models were run as hierarchical mixed-effects linear regressions, with random effects on the levels of the geographic hierarchy. For most mixed-effects models, random effects were only used in the fit, not in the prediction. The second part of the equation, $h(r_{c,a,s,t})$, is a smoothing function for the residuals, $r_{c,a,s,t}$, derived from the linear model.[3] Descriptions of exposure transformations and which covariates were used in linear models can be found in Section 3 describing the risk-specific estimation approaches.

While the linear component captures the general trend in exposures over time, much of the data varaibility may still not be adequately accounted for. To address this, we fit a locally weighted polynomial regression (LOESS) function $h(r_{c,a,s,t})$ to systematically estimate this residual variability by borrowing strength across time, age, and space patterns (the spatiotemporal component of ST-GPR). The time adjustment parameter, defined by λ , aims to borrow strength from neighboring time points (i.e. the exposure in this year is highly correlated with exposure in the previous year but less so further back in time). The age adjustment parameter, defined by ω, borrows strength from data in neighboring age groups. The space adjustment parameter, defined by ξ, aims to borrow strength across the hierarchy of geographical locations.

Let $w_{c,a,s,t}$ be the final weight assigned to observation $r_{c,a,s,t}$ with reference to a focal observation r_{c_0,a_0,s_0,t_0}. We first generated a preliminary weight $w'_{c,a,s,t}$ for smoothing over time, which was based on the scaled distance along the time dimension of the two observations[14]:

$$w'_{c,a,s,t} = \left(1 - \left(\frac{|t - t_0|}{1 + \max|t - t_0|}\right)^{\lambda}\right)$$

Next, we calculated the weight $w''_{c,a,s,t}$ to smooth over age, which is based on a distance along the age dimension of two observations. For a point between the age a of the observation $r_{c,a,s,t}$ and a focal observation r_{c_0,a_0,s_0,t_0}, the weight is defined as follows:

$$w''_{c,a,s,t} = \frac{1}{e^{\omega|a-a_0|}}$$

Finally, these combined weights were multiplied and further adjusted to account for geographic patterns.

Specifically, we defined a geospatial relationship by categorizing data based on the GBD location hierarchy (Appendix Table 6). We adapted the weighting strategy used in previous studies estimating time series of global indicators to be more flexible with respect to estimating subnational locations and to borrow strength from all levels.[15,16] A vector of spatial weights corresponding to each level of the location hierarchy was derived as $[\xi, \xi * (1-\xi)^{n_1-1}, \ldots, \xi * (1-\xi)^{n_i-1}, (1-\xi)^{n_i}]$, where the vector is expanded to include the number, n_i, levels in the location hierarchy between the location being

estimated and global, which recieves a pre-rescaling weight of $(1-\xi)^{n_i}$. For example, estimating a country would use the following weighting scheme:

- Country data: ξ
- Regional data not from the country being estimated: $\xi * (1- \xi)$
- Data from other regions in the same super region: $\xi * (1-\xi)^2$
- Global data from other super regions: $(1-\xi)^3$

A full derivation of weights for each category follow, assuming the location being estimated was a country, follows:

1) If the observation $r_{c,t}$ belongs to the same country c_0 of the focal observation r_{c_0,t_0}:

$$w_{c,a,s,t} = \frac{\xi(w'_{c,a,s,t}w''_{c,a,s,t})}{\sum_{c=c_0}(w'_{c,a,s,t}w''_{c,a,s,t})} \qquad \forall c = c_0$$

2) If the observation $r_{c,t}$ belongs to a different country than the focal observation r_{c_0,t_0}, but both belong to the same region R:

$$w_{c,a,s,t} = \frac{\xi * (1-\xi)\,(w'_{c,a,s,t}w''_{c,a,s,t})}{\sum_{c\neq c_0}(w'_{c,a,s,t}w''_{c,a,s,t})} \qquad \forall c \neq c_0 \cap R[c] = R[c_0]$$

3) If the observation $r_{c,t}$ belongs to the same super region SR but to a both different country c_0 and region $R[c_0]$ than the focal observation r_{c_0,t_0}:

$$w_{c,a,s,t} = \frac{\xi * (1-\xi)^2\,(w'_{c,a,s,t}w''_{c,a,s,t})}{\sum_{c\neq c_0}(w'_{c,a,s,t}w''_{c,a,s,t})} \qquad \forall c \neq c_0 \cap R[c] \neq R[c_0] \cap SR[c] = SR[c_0]$$

4) If the observation $r_{c,t}$ is from a different super region than the focal observation r_{c_0,t_0}(ie. all other data currently not receiving a weight):

$$w_{c,a,s,t} = \frac{(1-\xi)^3\,(w'_{c,a,s,t}w''_{c,a,s,t})}{\sum_{c\neq c_0}(w'_{c,a,s,t}w''_{c,a,s,t})} \qquad \forall c \neq c_0 \cap R[c] \neq R[c_0] \cap SR[c] \neq SR[c_0]$$

To allow additional flexibility and specificity in weighting schemes, we allowed for two different ξ to be defined. The higher ξ was applied when at least one age-sex group in the country of estimation had at least five years in the time series covered by at least one data source. The lower ξ was applied when estimating data-scarce countries.

Observations could be downweighted by a factor of 0.1, usually because they were not geographically representative at the unit of estimation. Details of reasons for downweighting can be found in risk-specific modeling summaries. The final weights were then normalized such that the sum of weights across age, time, and geographic hierarchy for a reference group was 1.

Estimating error variance

σ_p^2 represents the error variance in normal or transformed space including sampling variance of the estimates and predication error from any crosswalks performed. First, variance was systematically imputed if the data extraction did not include any measure of uncertainty. When some sample sizes for data were available, missing sample sizes were imputed as the 5th percentile of available sample sizes. Missing variances were then calculated as $\sigma_p^2 = \frac{p*(1-p)}{n}$ for proportions or were predicted from the mean using a regression for continuous values. When sample sizes were entirely missing and could not be imputed, the 95th percentile of available variances at the most granular geographic level (ie. first country, then region, etc.) were used to impute missing variances. For proportions where *p*n* or *(1-p)*n* is < 20, variance was replaced using the Wilson Interval Score method.

Next, if the exposure was modeled as a log transformation, the error variance was transformed into log-space using the delta method approximation as follows,

$$\sigma_p^2 \cong \frac{\sigma_{p\prime}^2}{p_{c,a,s,t}^2}$$

where $\sigma_{p\prime}^2$ represents the error variance in normal space. If the exposure was modeled as a logit transformation, the error variance was transformed into logit-space using the delta method approximation as follows,

$$\sigma_p^2 \cong \frac{\sigma_{p\prime}^2}{(p_{c,a,s,t} * (1 - p_{c,a,s,t}))^2}$$

Finally, prior to GPR, an approximation of non-sampling variance was added to the error variance. Calculations of non-sampling variance were performed on normal-space variances. Non-sampling variance was calculated as the variance of inverse-variance weighted residuals from the spacetime estimate at a given location level hierarchy. If there were fewer than 5 data points at a given level of the location hierarchy the non-sampling variance was replaced with that of the next highest geography level with more than 5 data points.

Estimating the covariance function

The final input into GPR is the covariance function, which defines the shape and distribution of the trends. Here, we have chosen the Matern-Euclidian covariance function, which offers the flexibility to model a wide spectrum of trends with varying degrees of smoothness. The function is defined as follows:

$$M(t,t') = \sigma^2 \frac{2^{1-v}}{\Gamma(\mathrm{v})} \left(\frac{d(t,t')\sqrt{2v}}{l}\right)^v K_v\left(\frac{d(t,t')\sqrt{2v}}{l}\right)$$

where $d(\cdot)$is a distance function; σ^2, v, l, and K_v are hyperparameters of the covariance function—specifically σ^2 is the marginal variance, v is the smoothness parameter that defines the differentiability of the function, l is the length scale, which roughly defines the distance between which two points become uncorrelated, and K_v is the Bessel function. We approximated σ^2 by $MADN(r'_{c,t})$, which is the normalized absolute deviation of the difference of the first-stage linear regression estimate from the second-stage spatiotemporal smoothing step for each country, region, or super-region depending on the

data coverage at a given location hierarchy level. Here, we have used the parameter specifications $\upsilon = 2$. The scale parameter l and the level of the geographic hierarchy at which σ^2 was is calculated are reported in the risk-specific appendix sections.

Prediction using GPR

We integrated over $g_{c,t}(t_*)$ to predict a full time series for country c, age a, sex s, and the prediction time t_*:

$$p_{c,a,s}(t_*) \sim N\left(m_{c,a,s,t}(t_*), \sigma_p^2 I + Cov\left(g_{c,a,s,t}(t_*)\right)\right)$$

Random draws of 1000 samples were obtained from the distributions above for every country for a given indicator. The final estimated mean for each country was the mean of the draws. In addition, 95% uncertainty intervals were calculated by taking the 2.5 and 97.5 percentile of the sample distribution. The linear modeling process was implemented using the lmer4 package in R, and the ST-GPR analysis was implemented through the PyMC2 package in Python.

Subnational Scaling and Aggregation

To ensure internal consistency of the estimates between countries and their respective subnational locations, national estimates were either created by population-weighted aggregation or subnational estimates were adjusted by population-weighted scaling to the national estimates, depending on the data coverage of a given country compared to that of its subnational locations. For example, if there was better data coverage at the national level, relative to its corresponding subnational locations, for a given country and risk across age, sex, and time, estimates were rescaled to be consistent with the national level. Conversely, if there was better data coverage at the subnational level, estimates for its parent country were generated through population-weighted aggregation of subnational estimates.

Step 4. TMREL

In this and all previous GBD studies, the counterfactual level of risk exposure used is the risk exposure that is both theoretically possible and minimizes risk in the exposed population that consequently captures the maximum population attributable burden.[2] For each risk evaluated in GBD 2016, Step 4 of the analytical flowchart describes the use of the best available epidemiological evidence from published and unpublished relative risks by level of exposure and the lowest observed level of exposure from cohorts, used to select a single level of risk exposure that minimises risk from all causes of DALYs combined to establish the TMREL. In principle, the TMREL for a given risk may vary by age, sex, and location if supported by clear evidence. Based on the available evidence, the TMREL itself can be uncertain, which is reflected in the 95% uncertainty intervals (UIs) in Table X. An estimation of uncertainty was derived by resampling from a uniform distribution of TMRELs where evidence supporting the selection of the TMREL was uncertain (for example, elevated systolic blood pressure or cholesterol).

Step 5. Estimate population attributable fractions

Risks are categorised on the basis of how exposure was measured: dichotomous, polytomous, and continuous. High total cholesterol is an example of a risk measured on a continuous scale. The population attributable fraction (PAF), which represents the proportion of risk that would be reduced in a given year if the exposure to a risk factor in the past were reduced to an ideal exposure scenario, is defined for a continuous risk factor as:[17]

$$PAF_{joasgt} = \frac{\int_{x=l}^{u} RR_{joasg}(x)P_{jasgt}(x)dx - RR_{joasg}(TMREL_{jas})}{\int_{x=l}^{u} RR_{joasg}(x)P_{jasgt}(x)dx}$$

Where PAF_{joasgt} is the population attributable fraction for cause o due to risk factor j for age group a, sex s, location g, and year t. $RR_{joasg}(x)$ is the relative risk as a function of exposure level x for risk factor j for cause o, age group a, sex s, and location g with the lowest level of observed exposure as l and the highest as u; $P_{jasgt}(x)$ is the distribution of exposure at x for age group a, sex s, location g, and year t; $TMREL_{jas}$ is the TMREL for risk factor *j*, age group *a*, and sex *s*.

The PAF_{joasgt} for dichotomous and polytomous risk factors for every country is defined as:

$$PAF_{joasgt} = \frac{\sum_{x=1}^{u} RR_{joast}(x)P_{jasgt}(x) - RR_{joasg}(TMRE_{jas})}{\sum_{x=1}^{u} RR_{joas}(x)P_{jasgt}(x)}$$

Where PAF_{joasgt} is the population attributable fraction for cause o due to risk factor j for age group a, sex s, location g, and year t. $RR_{joasg}(x)$ is the relative risk as a function of exposure level x for risk factor j for cause o, age group a, sex s, and location g on a plausible range of exposure levels from l to u $P_{jasgt}(x)$ is the proportion of population in risk group (prevalence), for age group a, sex s, location g, and year t; $TMREL_{jas}$ is the TMREL for risk factor *j*, age group *a*, and sex *s*.

Step 6. Mediation

Summary

The portion of the burden of disease that is attributable to various combinations of risk factors or to all risk factors combined has been a topic of broad interest.[18] Assumptions about how one risk factor is mediated through other risk factors are needed in order to estimate the joint risk factor burden for combinations of metabolic risks and behavioural or environmental risks. To accomplish this, in Step 6 of the estimation process, for every two risk factors for an outcome, we estimated the fraction of risk that was mediated through the other risk. This resulted in a matrix of parameters containing each possible pairing of risk factors included in the GBD 2016. Using this matrix, we computed the aggregated burden of disease at each level of the GBD 2016 hierarchy and for all risk factors using the following formula:

$$PAF_{Joasgt} = 1 - \prod_{j=1}^{J} \left(1 - PAF_{joasgt} \prod_{i=1}^{J} (1 - MF_{jio})\right) \quad (5)$$

where J is a set of risk factors for the aggregation; PAF_{joasgt} is the PAF for risk j for age group a, sex s, location g, and year t; and MF_{jio} is the mediation factor for risk j mediated through i for cause o. Mediation factors can be found in Appendix Table 7.

Additional detail

In GBD 2010, we only aggregated the burden of risk factors for some clusters of risks including access to improved water and sanitation, child and maternal malnutrition, tobacco smoking, alcohol use, dietary risk factors, occupational risk factors, and sexual abuse and violence. We did not aggregate air pollution

and metabolic risk factors. In GBD 2013, GBD 2015, and GBD 2016, we aggregated all risk factors into three large categories: behavioral, environmental and occupational, and metabolic risks -- as well as aggregating all GBD risk factors into a single attributable fraction for each diseases and eventually for all-causes of burden.

Aggregating risk factors at different levels share three essential challenges:

1. Risk factor coexistence or aggregation: for example, metabolic risk factors often occur together or high-risk behaviors are related such as drug abuse and unsafe sex.
2. Mediation: a risk factor may effect another risk factor that lies in the physiological pathway to a disease outcome. It can be inside a cluster of risk factors such as the effect of obesity through an increase in fasting plasma glucose (FPG) and later cardiovascular disease outcomes, or between clusters of risk factors such as the effect of fiber on cholesterol.
3. The formula to calculate the aggregated PAF.

The aggregation method is conceptually applicable to other aggregations such as socioeconomic factors, education, homelessness and refugee status that are being considered for inclusion in future GBD iterations. In the next section, we explain our approach to deal with these challenges.

There are three patterns of associations between risk factors to take into consideration. The first concerns confounding; risk B affects risk A and outcome C (Pattern 1 in *Patterns of associations between risk factors*). In these cases, the relative risk (RR) for A should be adjusted for B, for example the fruit RR is adjusted for smoking. If part of the effect of A is through B, a mediator, we do not adjust the effect of A for B. For example, we do not adjust the RR of BMI for cholesterol as cholesterol lies in the biological pathway between BMI and cardiovascular outcomes (Pattern 2 in in *Patterns of associations between risk factors*). The third pattern occurs when risks A and B are proxies of a third variable Z and aggregation aims to estimate the total effect of a latent variable Z, on C. An example is childhood

undernutrition, which is measured by stunting, wasting, and underweight as proxies.

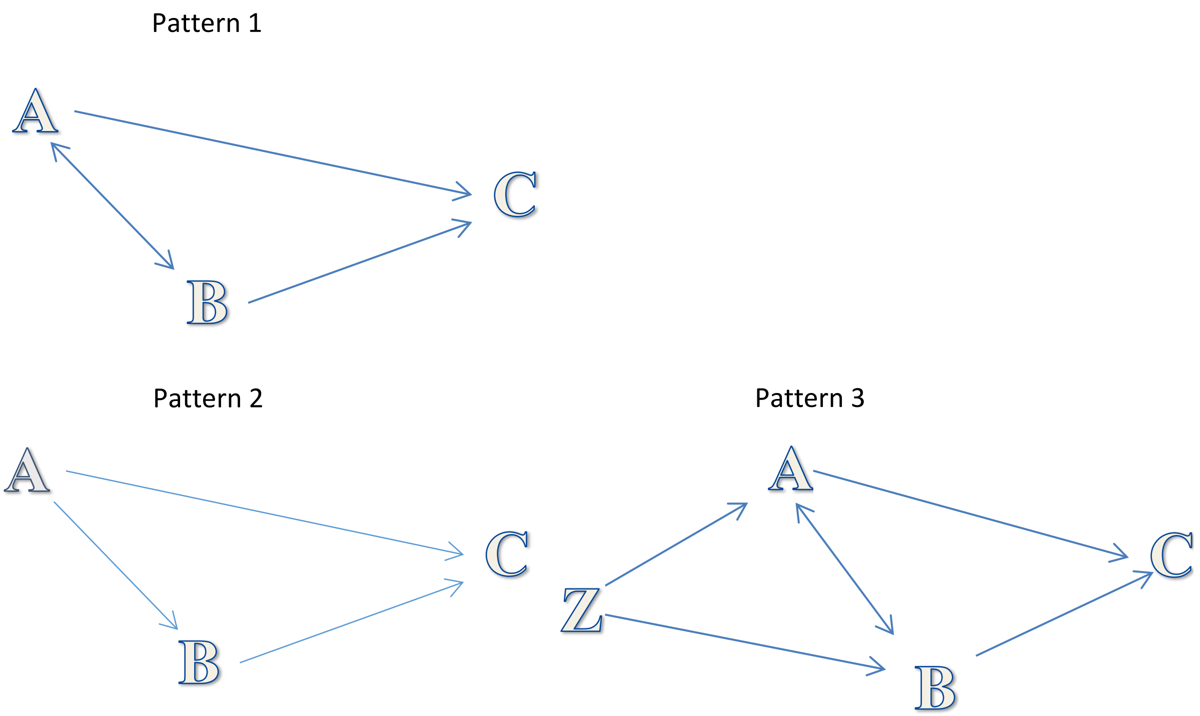

Patterns of associations between risk factors

Calculating burden of multiple risk factors

Validation studies have reported congruency between the true risk associated with multiple risk factors affecting the same outcome and a multiplicative aggregation of the population attributable fractions of the individual risk factors (formula below).[19]

$$PAF_{1..i} = 1 - \prod_{i=1}^{n}(1 - PAF_i)$$

Where *PAF* is the population attributable fraction and *i* is each individual risk factor. The same validation studies also found that the overestimation from ignoring the covariance between risk factors is small. This was important to note as there are few data sources from which we can draw information on covariance.

We endeavored to evaluate RRs that were controlled for confounders. However, as we had to rely on the literature for many RRs we did not always have full control over the choice of confounders controlled for in each study.

Adjusting for mediation

When aggregating the effects of multiple risk factors, we included a mediation factor if a part of the effect of one risk factor was included in the effect estimated for in the mediator. First we prepared a list of possible mediations especially between behavioural risks and metabolic risk factors with cardiometabolic outcomes. We did not assume any mediation effect between risk factors for cancers except for sugar sweetened beverages and BMI.

Danaei and colleagues assumed that part of the effect of BMI on ischemic heart disease (IHD) is through high SBP, cholesterol and FPG.[20] The proportion of the BMI effect that can be explained by other metabolic risk factors is the amount of mediation. The difference between the crude RR of BMI on IHD with the RR adjusted for SBP, FPG, and cholesterol reflects the amount of BMI effect on IHD that is mediated and already included in SBP, FPG, and cholesterol:

$$MF = \frac{RR_{crude} - RR_{adjusted}}{RR_{crude} - 1}$$

We used this approach for estimating mediation factors to adjust PAFs before aggregation.

$$MF = \frac{R_c^+ - R_a^+}{R_c^+ - R_c^-}$$

$$\text{So: } R_a^+ = R_c^+ - MF * (R_c^+ - R_c^-)$$

$$PAF_c = \frac{p * (R_c^+ - R_c^-)}{p * R_c^+ + (1-p) * R_c^-} = \frac{p * (R_c^+ - R_c^-)}{R_T}$$

If R_c^+: crude risk of outcome in exposed population

R_c^-: crude risk of outcome in non-exposed population

R_a^+: adjusted risk of outcome in exposed population

R_a^-: adjusted risk of outcome in non-exposed population

R_T is the overall rate of the outcome in the population. Since we are interested in the part which is from BMI but through cholesterol, the total risk in the population will be the same for the adjusted RR, so the unmediated part of the risk factor would be:

$$PAF_a = \frac{p*(R_a^+ - R_a^-)}{R_T} = \frac{p*(R_c^+ - MF*(R_c^+ - R_c^-) - R_c^-)}{R_T} = \frac{p*(R_c^+ - R_c^-)*(1-MF)}{R_T} = PAF_c * (1 - MF)$$

So for aggregating the PAF of multiple risk factors, we first calculated the part of the effect of every risk factor that is not mediated and then aggregated these assuming they are independent.

Therefore the aggregated PAF would be:

If MF is mediation factor of R2 through R1:

$$PAF_{1,2} = 1 - (1 - PAF_1) * \left(1 - PAF_2 * (1 - MF_{2/1})\right)$$

and a generalization for multiple pathways of R1 through other RFs:

$$PAF_{1..i} = 1 - \prod_{i=1}^{n}\left(1 - PAF_i * \left(1 - \prod_{j=1}^{n}(1 - MF_{i/j})\right)\right)$$

For every risk factor outcome pair, the matrix of possible mediations was calculated and used. For some risk factor aggregations, we simply added PAFs. For example, the total burden of smoking including smoking and secondhand smoke is the sum of the estimates of the individual risks because we estimate the burden of secondhand smoke in non-smokers only.

Calculating mediation factor

1 – Comparing crude RR versus mediator-adjusted RR

The best example is the mediation of BMI through SBP, FPG, and cholesterol reported by Danaei et al.[20] In their meta-analysis, they report the adjusted and unadjusted RR of BMI on IHD and stroke based on combined data from individual cohorts. They calculated the mediation factor using the equation below, and we used it directly as mediation factor in risk factor aggregation. Using individual level data from cohort studies, we estimated the mediation factor for other metabolic risk factors and some dietary risks.

$$MF = \frac{RR_{crude} - RR_{adjusted}}{RR_{crude} - 1}$$

2 – Estimating the mediation factor by pathway of the effect

For many other risk factors, there are no data available to use the first method. Instead, we searched studies to estimate the effect of the risk factor on the mediator and finally the expected increase in IHD risk. We pooled available studies to calculate the unit increase in the mediator per unit increase in the risk factor to calculate the size of the IHD RR.

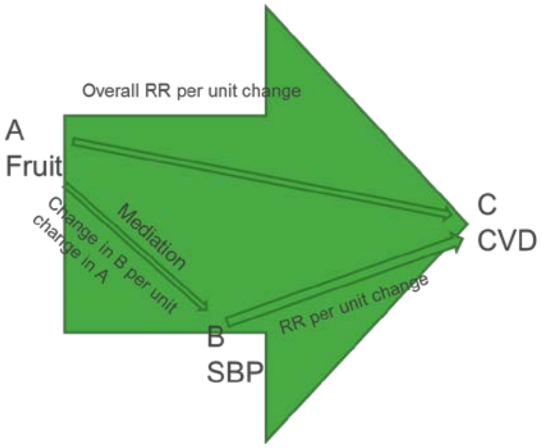

Example of pathway between fruit, high systolic blood pressure, and cardiovascular diseases

We have RRs for the effect of A on C and B on C in GBD from a meta-analysis of studies in the literature. The effect of A on B was estimated by analysis of diet trials.

$$RR_{ABC} = {RR_{BC}}^{\Delta_{AB}}$$

RR_{ABC} is expected effect of A through B on C

RR_{BC} is relative risk of each unit increase in mediator on outcome C

Δ_{AB} is change in mediator level B per each unit change in A

If RR_{AB} is the overall effect of A on B then:

The mediation factor would be

$$MF = \frac{RR_{ABC} - 1}{RR_{AB} - 1}$$

We kept uncertainty of each parameter by generating and following 1000 draws of the estimates to calculate 1000 draws of the posterior distribution of the mediation factor. We did not include risk-mediator pairs if the mediation factor was not significant at 5% level (more than 50 out of 1000 draws were negative). We truncated the mediation factor distribution at 1 where the whole effect of the risk factor on the outcome would be assumed to be through the mediator pathway.

Some mediation factors equal 1 where the whole effect was calculated through other risk factor, e.g. the effect of sugar-sweetened beverages through BMI or salt through SBP, or when we assumed other risk factors are sources of the exposure, for example, fiber is provided by consuming fruit, vegetable, and whole grains and all the beneficial effect of milk on colorectal cancer is mediated through calcium.

Dietary risk factors

For each dietary risk factor, we searched for randomized trials evaluating the effect of the diet component on metabolic risk factors and estimated the change in a given mediator per unit change in the diet component.

Physical activity

We found cohort studies on the effect of physical activity on FPG. The data was more on the effect of physical activity on diabetes incidence, so we calculated the shift in FPG using the provided RR value. We used this to calculate the mediated part of effect of physical activity on cardiovascular disease (CVD).[21–27]

Air pollution

We looked for cohort and time series studies but the data were limited. We found only one study with the effect of last year average of particle pollution PM 2.5 on SBP, FPG and cholesterol.[28] However, the effects through FPG and cholesterol were bigger than the effect expected for that level of PM2.5, indicating significant overestimation of the mediation. We found time series studies with different PM2.5 lag (by day) that show very short-term and confounded effects. So we decided to add this when stronger evidence is available.

Assumed mediations

For the risk factors with PAFs of 100% such as FPG and diabetes, low estimated glomerular filtration rate and chronic kidney disease, hypertension and hypertensive heart disease, alcohol and alcohol disorders, childhood underweight and protein-energy malnutrition, and childhood wasting and protein-energy malnutrition, and drug use and drug use disorders, no mediation is needed.

Piecewise aggregation (Pattern 3)

There are three anthropometric indicators that are highly correlated: childhood underweight, stunting, and wasting, as demonstrated in *Venn diagram demonstrating the correlation between childhood underweight, stunting, and wasting*. Available RRs for each indicator are not adjusted for the other two because there is a high correlation between these indicators and also interaction where the majority of the burden occurs. Estimating the total burden due to undernutrition, a latent variable, is difficult. The three anthropometric indicators are not independent, so the covariance between them should be considered. This was the main reason that GBD 2010 only included childhood underweight. If covariance between these indicators is significant (as is shown in the Figure below), aggregating these indicators assuming independence would overestimate the total burden significantly.

To use the best available data, we adjusted observed RRs reported by Olofin et al for underweight, stunting and wasting by simulating the joint distribution of the three indicators using the distribution of each indicator and covariance between indicators in the countries included in the meta-analysis (extracted from Demographic and Health Survey (DHS) micro-data).[29] Based on the analysis done by McDonald et al, we assumed there is an interaction between the three indicators, and extracted the interaction terms from the corresponding analysis.[30] We calculated the adjusted RRs by minimizing the error between observed crude RRs (from meta-analysis) and expected crude RRs derived from adjusted RRs.

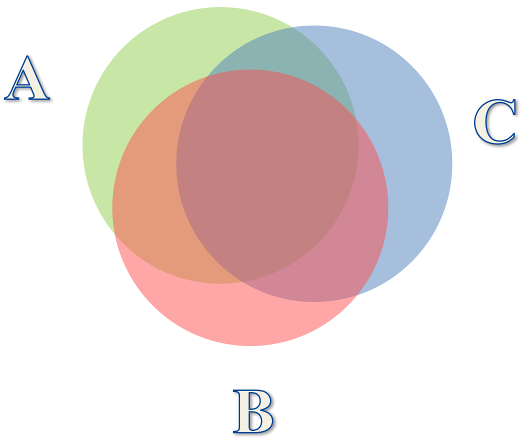

Venn diagram demonstrating the correlation between childhood underweight, stunting, and wasting

After adjusting for the three risk factors, we calculated the PAFs and aggregated underweight, stunting and wasting burden.

Uncertainty of aggregated and mediated PAFs

We generated 1000 draws of posterior distribution of mediation factor calculated by different methods to use beside draws of other inputs to the PAF aggregation.

Important assumptions in aggregating risk factors and including mediation

1 – The mediation factors or PAF adjustments are similar across countries, age, sex, and years. While it is quite likely that the size of mediation is different in different populations, there is little data to inform the covariance between different risk factors or the mediation factor amount by age and countries. For

example, in some countries, the size of the mediated BMI-IHD PAF through cholesterol, calculated by the mediation factor, was even bigger than the total burden of cholesterol, indicating that less effect of BMI is mediated through cholesterol and mediation factors are not similar across countries.

2 – For many risk-mediator-outcome pairs, there are no data available, so we assumed the mediation is zero.

3 – Since the covariance between undernutrition indicators is different by location (and across time, results were not reported), and there is an interaction between these indicators, the total burden might be underestimated.

4 – It is assumed that there is no significant covariance between PAFs, which might not be true between some risk factors such as between metabolic risk factors. While this overestimation is controlled by using adjusted RRs, using crude RRs for BMI and other metabolic risk factors may cause significant overestimation of aggregated metabolic risks burden.

Step 7. Estimate attributable burden

Four key components are included in estimation of the burden attributable to a given risk factor: the metric of burden being assessed (the number of deaths, years of life lost [YLLs], years lived with disability [YLDs], or DALYs [the sum of YLLs and YLDs]); the exposure levels for a risk factor; the relative risk of a given outcome due to exposure; and the counterfactual level of risk factor exposure. Estimates of attributable burden as DALYs for risk-outcome pairs were generated using the following model:

$$AB_{jasgt} = \sum_{o=1}^{w} DALY_{joasgt}\, PAF_{joasgt}$$

where AB_{jasgt} is the attributable burden for risk factor j for age group a, sex s, location g, and year t; $DALY_{joasgt}$ is total DALYs for cause o (of w relevant outcomes for risk factor j) for age group a, sex s, location g, and year t; PAF_{joasgt} is the population attributable fraction (PAF) for cause o due to risk factor j for age group a, sex s, location g, and year t. The proportion of deaths, YLLs, or YLDs attributable to a given risk factor or risk factor cluster were analogously computed by sequentially substituting each metric in place of DALYs in the equation above.

Other analysis: Decomposition of deaths and DALYs

We conducted two related decomposition analyses of changes in DALYs from 2006 to 2016: (1) decomposing changes in all-age cause-specific DALYs attributable to all risk factors due to changes in population growth, population age structure, exposure to all risks for a disease, and risk-deleted death and DALY rates; and (2) decomposing changes in age-specific all-cause DALYs attributable to all risk factors due to changes in population growth, population age structure, exposure to all risks for a disease, and risk-deleted DALY rates. In this case, risk-deleted rates are the rates after removing the effect of a risk factor or combination of risk factors; in other words, observed DALY rates multiplied by one minus the PAF for the risk or set of risks. Our decomposition analyses draw from methods developed by Das Gupta[31] to provide a computationally tractable solution to isolating drivers of burden changes whereby all combinations of possible pathways are averaged across factors. Attributable burden is determined, following the methods of Das Gupta, as a product of three factors such that:

$$T_{asgt} = (A_{asgt}\, B_{asgt}\, C_{asgt})$$

where T_{asgt} represents the attributable burden at year t; $A_{sgt}$is the age-specific population size for a given age group a, sex s and location g at year t; B_{asgt} is the underlying rate of the outcome unrelated to the risk factor or observed rate, multiplied by $1 - PAF$ for a given age group a, sex s and location g at year t; and where C_{asgt} is the ratio of attributable burden to the underlying rate, which reflects the risk effect for a given age group a, sex s, and location g at year t defined as $PAF/(1 - PAF)$ in the case of decomposing attributable burden to a risk. The contribution of each factor to total change in attributable burden was determined by changing the level of one factor from time t_0 to t_1 – here 2006 to 2016 – with all other factors held constant. Thus, the effect of any of the three factors, for example A_{asgt}on the change of attributable burden between 2006 (A_{06}) and 2016 (A_{16}) is calculated as:

$$E_A = (A_{16} - A_{06})\left(\frac{B_{06}C_{06} + B_{16}C_{16}}{3} + \frac{B_{06}C_{16} + B_{16}C_{06}}{6}\right)$$

Where E_A is the proportion of change due to factor A, and the subscripts for each factor in the equation denote the year for each estimate. Since the effect depends on the order of entry of the factor, we calculated the average of all combinations of the three factors.[31] The proportion of change due to factorA_{sgt}, the age-specific population size for a given age group a, sex s and location g at year t, is then further split, setting change in population growth equal to the percent change in all-age population from time t_0 to t_1 and change in population age structure to the residual, giving our final four factors.

This three factor decomposition method does not work for risks where the PAF, by definition, is 100% (such as high fasting plasma glucose and diabetes) or where the PAF is directly estimated (such as for unsafe sex and HIV). In the cases of child underweight and protein-energy malnutrition, child wasting and protein-energy malnutrition, short gestation for birth weight and neonatal preterm birth complications, low birth weight for gestation and neonatal preterm birth complications, iron deficiency and iron-deficiency anemia, vitamin A deficiency and vitamin A deficiency, alcohol use and liver cancer due to alcohol use, alcohol use and cirrhosis and other chronic liver diseases due to alcohol use, alcohol use and alcohol use disorders, alcohol use and alcoholic cardiomyopathy, drug use and drug use disorders, occupational particulate matter, gases, and fumes and other pneumoconiosis, occupational exposure to asbestos and asbestosis, and occupational exposure to silica and silicosis, we used a two factor decomposition method, which examines the contribution of population, ageing, and risk exposure. Effectively, we assume trends in these cases are driven by exposure, not change in the risk-deleted rates. Conversely for unsafe sex and sexually transmitted diseases excluding HIV, we used a two factor decomposition method, which examines the contribution of population, ageing, and risk-deleted death and DALY rates, assuming trends in these cases are driven by risk-deleted rates, not change in exposure. For high fasting plasma glucose and diabetes mellitus, high fasting plasma glucose and chronic kidney disease due to diabetes mellitus, high systolic blood pressure and hypertensive heart disease, high systolic blood pressure and chronic kidney disease due to hypertension, and impaired kidney function and chronic kidney disease, we used GBD estimates of SEVs for the given risk and the case-fatality rate decompose trends into the contribution of the three factors. Similarly for unsafe sex and cervical cancer, we used GBD estimates of the incidence of cervical cancer and the case-fatality rate to decompose trends into the contribution of the three factors. For unsafe sex and HIV we used spectrum counterfactual and CD4 risk-weighted prevalence.

Other analyses: SDI

Development of SDI

The SDI is a composite indicator of development status constructed for GBD-2015 whose components are strongly correlated with health outcomes. It is the geometric mean of 0 to 1 indices of total fertility rate, mean education for those aged 15 and older, and lag distributed income per capita.

SDI was calculated using the Human Development Index (HDI) methodology, wherein an index value was determined for each of the covariate inputs (log lag distributed income [LDI], mean educational attainment over age 15, and total fertility rate [TFR]). For GBD 2015, these indices were computed on the basis of a relative scale, in which the upper and lower bounds were established by the maximum and minimum observed values, respectively, for each input over the entire estimation period of 1980-2015.

Updates to SDI Computation for GBD 2016

Prompted by the observations that the scales (and by extension SDI) were sensitive to the addition of new subnational locations as GBD becomes more granular and to the length of the time period over which SDI is computed, for GBD 2016 we implemented fixed scales in determining individual indices. Thus, an index score of 0 now represents the minimum level of each covariate input past which selected summary health outcomes can get no worse. An index score of 1 represents the maximum level of each covariate input past which these selected health outcomes cease to improve. As a composite, a location with an SDI of 0 would have a theoretical minimum level of development relevant to these health outcomes, while a location with an SDI of 1 would have a theoretical maximum level of development relevant to these health ouctomes.

We selected the minima and maxima of the scales by examining the relationships each of the inputs had with life expectancy at birth and under-5 mortality and identifying points of limiting returns at both high and low values, if they occurred prior to theoretical limits (e.g., a TFR of 0). The final scales are summarised in the table below.

**Input**	**Lower Bound**	**Upper Bound**
TFR	1.5[a]	8
LDI per capita	250 USD (5.5 log USD)[b]	60,000 USD (11.0 log USD)
Mean educational attainment for ages 15 and older	0 years	17 years

[a] The low point of limiting returns for TFR was identified at 1 during GBD 2015; however, incorporating feedback with regard to accounting for a pattern of TFR rebound in highly developed countries, we instead set the lower limit of TFR at 1.5.
[b] The minimum for the LDI scale was originally set at the theoretical limit of 0 USD, as we did not observe an asymptotic relationship between log(LDI) and E_0 or 5q0 at lower values of log(LDI). Empirically, however, we also did not observe an LDI below 350 USD (5.86 log USD) for the estimation period 1970-2016. In log-space, this meant that approximately half of our scale was not being utilized, compressing the observed variation in LDI and diminishing its meaningful contribution to SDI. Accordingly, we set the lower limit on LDI to 250 USD (5.52 log USD) to ensure we were fully utilizing the range of the scale to capture its variation across space and time, as is the case with the other two inputs.

Using the limits on the scales described above, we computed the index scores underlying SDI analogously to GBD 2015 as follows:

$$I_{Cly} = {(C_{ly} - C_{low})}/{(C_{high} - C_{low})}$$

Where I_{cly} – the index for covariate *C*, location *l*, and year *y* – is equal to the difference between the

value of that covariate in that location-year and the lower bound of the covariate divided by the difference between the upper and lower bounds for that covariate. If the values of input covariates fell outside the upper or lower bounds (e.g. LDI per capita greater than 60,000 USD), they were mapped to the respective upper or lower bounds. We also note that the index value for TFR was computed as $1 - I_{TFRly}$, as lower TFRs correspond to higher levels of development, and thus higher index scores. For GBD 2016 we expanded the computation of SDI to 755 national and subnational locations spanning the time period 1970-2016.

The composite SDI is the geometric mean of these three indices for a given location-year. The cutoff values used to determine quintiles for analysis were then computed using country-level estimates of SDI for the year 2016, excluding countries with populations less than 1 million.

Example calculation

Below we present the calculation of SDI for Mexico in the year 2010:

$$TFR = 2.43;\ Mean\ educ\ yrs\ pc = 9.23;\ lnLDI = 9.58$$

$$I_{TFR} = 1 - \frac{2.43 - 1.5}{8 - 1.5} = .855$$

$$I_{Educ} = \frac{9.23 - 0}{17 - 0} = .543$$

$$I_{lnLDI} = \frac{9.58 - 5.52}{11.00 - 5.52} = .741$$

$$SDI = \sqrt[3]{I_{TFR} * I_{Educ} * I_{lnLDI}} = \sqrt[3]{.855 * .543 * .741} = .701$$

SDI values can be found in Appendix Table 8, and SDI by location can be found in Appendix Table 9.

Additional Methods Information

Risk-specific comparisons to other estimates

Low birth weight / Short Gestation:

GBD 2016 estimates of total preterm birth prevalence are generally in line with country-specific reports[30] as well as the most recent global analysis completed by Blencowe and colleagues.[31] GBD 2016 estimates of preterm birth rate are slightly higher, 12.52% (95% UI: 10.69% to 14.47%) versus 11.1%, than the estimates by Blencowe and colleagues. Close agreement is not surprising as most of the same data sources were used as data inputs to our modeling process, although the GBD analysis included almost eight times as many data points. Most reports, like GBD 2016, have assessed temporal trends in preterm birth in many locations to either be static or increasing. Compared to UNICEF estimates of low birth weight,[32] GBD 2016 estimates global birth prevalence of 13.83% (12.76% to 15.09%) are generally lower than the estimate of 15.5% birth prevalence globally, a difference which is most likely explained

by the fact that GBD 2016 has not implemented a uniform upward 24% correction factor for reported birth weights. The geographic variation in low birth weight largely mirrors that of the UNICEF report.

Smokeless Tobacco

We compared GBD estimates to recent research by Siddiqi et al.[33] on smokeless tobacco prevalence, risk, and burden in 2015. Our methods differed from Siddiqi on some key points:

- Prevalence estimation: We estimated both age-sex specific and aggregate current smokeless tobacco use prevalence for all countries and territories included in GBD from 1990-2016 using all available data. Siddiqi et al. used only the single most recent survey available containing data on smokeless tobacco use among adults.
- Relative risks and attributable burden: GBD excluded hospital-based case-control studies, while Siddiqi included them. GBD calculated separate relative risks by tobacco type (and by sex for oral cancer only), while Siddiqi calculated separate relative risks by geography, and then pooled these to produce global relative risks. In producing attributable burden, GBD classified countries as predominantly using snuff/snus or chew based on input from smokeless tobacco experts. Type-specific relative risks were then applied to countries based on their classification. Siddiqi used country- or region-specific relative risks where available, and in the absence of region-specific relative risks assigned global relative risks in countries predominantly using products with moderate to high pH and TSNAs levels.

The main differences in attributable burden come from the relative risk exclusion criteria. GBD's exclusion of hospital-based led to very different relative risk outputs: we found significant relative risks for oral cancer and oesophageal cancer, and only for users of chewing tobacco. Siddiqi found significant relative risks for oral, pharyngeal, and oesophageal cancers and ischemic heart disease, resulting in higher levels of global burden. Despite different methods in mapping tobacco types, both studies concluded that the causal evidence for applying risks in much of Europe and the Americas was too weak to estimate attributable burden.

Smoking

We compared GBD estimates to the most recent report on the global tobacco epidemic published by the WHO[32]. Overall, we found marked similarities in estimates. Among the 142 countries and territories included in the WHO report and estimated in GBD, the correlation coefficient for daily smoking prevalence estimates among females was 0.96 and among males was 0.90. In cases where estimates diverge, discordance can be attributed to differing modeling methods or data sources. GBD uses spatiotemporal Gaussian process regression to estimate smoking prevalence, whereas WHO uses Bayesian meta-regression (DisMod MR). Additionally, the WHO model was fit on 1,175 country-year data sources, whereas the GBD model was fit on 2,887 country-year data sources. There are no comparable global estimates of the burden of disease attributable to smoking, as GBD 2015 estimates of attributable burden were used in the most recent WHO report.

Ambient air pollution

In the past few years, other researchers have estimated the burden of disease due to air pollution using different data and methods. In recent estimates from WHO[33] of 3·0 million deaths in 2012 used the

same exposure estimates as presented here, but an earlier (GBD 2013) version of the integrated exposure response (IER) and somewhat different baseline disease burden estimates. Lelieveld and colleagues[34] analysed source sector contributions to air pollution and the resulting disease burden in 2010 and estimated the burden in 2050. These estimates used an older (GBD 2010) IER. Furthermore, the coarse spatial resolution (~100 × 100 km) of the exposure estimates introduced errors via spatial misalignment between exposure and population density compared with our estimates.

Occupational

Takala et al[35] reported 2.3 million deaths attributable to occupational injury/illness in 2011. In the comparison year 2010, GBD estimated 1.4 million deaths. This discrepancy is largely driven by the cause-outcome pairs that GBD currently has the evidence to include based on the criteria of the CRA framework. For example, 45% of Takala's reported burden is driven by occupational circulatory disease (35%) and occupational communicable disease (10%). Circulatory diseases are linked to occupational risks like shift work and lack of control but the GBD approach currently has insufficient evidence to estimate the variability in exposure to these factors on a global scale. Additionally, the use of a CRA approach in GBD estimates requires careful consideration of proposed counterfactual in order to derive the TMREL for a given risk. The TMREL for something like occupational lack of control is a challenging concept and as such these risks are still being reviewed for possible inclusion in future iterations of the GBD.

Takala also reports higher burden from occupational cancer based on the inclusion of carcinogens that are currently still out of the scope of GBD. For example, the authors use attributable fractions derived from Rushton et al to attribute pairs like breast cancer and shift work or skin cancer and solar radiation. These carcinogens, which form a large part of the cancer burden in Takala/Rushton are currently not included in the GBD based on limited exposure data across the time/space that GBD estimates for.[36]

In terms of fatal occupational injuries, Takala reported 353,000 deaths in 2011. The GBD 2016 estimate for deaths attributable to occupation was 335,000 deaths for 2016. The figures are similar but the GBD estimates are slightly lower, again due to the selection of risk-cause pairs. The ILO estimation strategy includes some kinds of injuries, such as deaths due to intentional violence that the GBD does not attribute to occupation.

Child growth failure (stunting, wasting, and underweight)

UNICEF et al.[37] estimate lower proportion of stunting (height-for-age z-score < -2 standard deviations below the reference median) in children under 5 in 2016 than GBD 2016, 22.9% (UNICEF et al) vs 25.9% [25.2-26.6%] (GBD 2016). The geographic patterns generally agree in identifying sub-Saharan Africa and South Asia as the regions with the largest burden of stunting (prevalence and magnitude, estimated as number of stunted children in UNICEF et al, and as DALYs in GBD 2016), with additional high prevalence in Oceania (excluding Australia and New Zealand) and moderate prevalence in Latin America and the Caribbean. While the UNICEF et al estimates highlight minimal or lack of progress in reducing stunting since 2000 in Africa and Oceania, GBD 2016 estimates show moderate decline in sub-Saharan Africa and North Africa and the Middle East, and a small decline in Oceania (compared to UNICEF et al's rise in stunting prevalence in Oceania).

There is generally high consistency between the UNICEF et al and GBD 2016 estimates for wasting (weight-for-height z-score < -2 standard deviations below the reference median) among children under 5 in 2016. Both sets of estimates identify the highest proportion of wasting in South Asia (15.4% UNICEF

et al, 15.5% [15.1-15.9%] GBD 2016), with the next highest in sub-Saharan Africa (UNICEF et al estimates range from 5.5-8.5% depending on the region in Africa, while GBD 2016 estimates 9.4% [9.1-9.8%]. There is a similar high burden identified in Oceania (excluding Australia and New Zealand). While both sets of estimate identify low prevalence in Latin America and the Caribbean (2.4% [2.2-2.5%] in GBD 2016), GBD 2016 does estimate a higher percentage of wasted children in high-income countries (1.0% [0.9-1.1%]) than does UNICEF et al (0.5% in North America).

GBD 2016 estimates show a downward trend in the prevalence of underweight (weight-for-age z-score < -2 standard deviations below the reference median) among children under 5 in 2016, driven largely by populations in sub-Saharan Africa and South Asia, a trend also reflected in the UNICEF et al estimates. For the year 2016, the two estimates are consistently somewhat higher in GBD compared with UNICEF, with the highest burdens in South Asia (28.1% in UNICEF et al, 34.0% [32.3-35.8%] in GBD 2016) and Africa (15.7% in UNICEF et al, 17.9% [17.0-18.8%] for sub-Saharan Africa in GBD 2016). Global prevalence in 2016 is estimated as 14.0% in UNICEF et al, while GBD 2016 estimates global prevalence at 16.2% [15.7-17.0%].

Impaired Kidney Function

Recently published estimates[38] from a meta-analysis of global data on exposure to impaired kidney function indicate prevalence of chronic kidney disease (CKD) stages 1-5 to be 13.4% (11.7-15.1%). These estimates are similar to the current GBD 2016 exposure estimates across all four levels of impaired kidney function, which indicate a prevalence for individuals over the age of 25 of 14.5% (13.5-15.5%).

Household Air Pollution

The WHO estimated 4.3 million deaths and 146.5 million DALYs attributable to exposure to household air pollution globally in 2012, as compared to GBD 2016 estimates for the year 2010 of 2.5 million deaths and 88 million DALYs. Differences in attributable burden arise between WHO estimates and GBD 2016 for a number of reasons. First, the IER curve was used for all outcomes (LRI, IHD, cerebrovascular stroke, COPD and lung cancer) except cataracts in our analysis, while WHO adapted relative risks for COPD based on epidemiological evidence. The resulting relative risks for COPD used by the WHO are stronger than the relative risks used in GBD, resulting in a larger PAF. Second, we have expanded the database that maps solid cooking fuel use to indoor PM 2.5 exposure, which has resulted in lower PM 2.5 exposure estimates globally. This also allows us to construct more granular relative risks using the IER curve, while the WHO relies on global relative risks. In addition to the differences in data sources, we estimated the burden of cataract attributable to household air pollution (HAP) only in women while WHO estimated for both sexes. One final reason GBD attributable burden estimates are lower than those produced by the WHO is that GBD adjusts for ambient air pollution exposure since some personal PM 2.5 exposure is due to ambient, not indoor, pollution. The WHO does not make any adjustment for ambient air pollution exposure.

Below is a comparison between the WHO and GBD 2016 of the percent of deaths attributed to HAP contributed by each cause (GBD 2016 on right):

- 12% -pneumonia/LRI 28%- pneumonia/LRI
- 34% -stroke 20%-stroke
- 26% -ischemic heart disease 24%-IHD

• 22% -COPD	23%-COPD
• 6% -lung cancer.	5%-lung cancer

Breastfeeding

CDC reports 18.8% of mothers in the United States in 2011 exclusively breastfeed their child up to 6 months of age, while GBD 2016 estimates that value to be 22.5%. Additionally, CDC reports 49.4% of mothers in the U.S. continue to breastfeed at 12 months of age. GBD estimates 24% of mothers in the U.S. continue to breastfeed from 6-24 months of age. In India, the WHO and UNICEF's estimate of exclusive breastfeeding prevalence in children under 6 months was 46% in 2011, while GBD estimates 40%. The main difference between the two estimation techniques is that GBD incorporates additional data sources beyond what the report by WHO and UNICEF used.

WaSH

Joint Monitoring Project[36] (JMP) lead by the WHO and UNICEF estimate water, sanitation, and handwashing access throughout the world. Globally, JMP estimates that 91% of population had access to an improved water source in 2015, while GBD estimates 88% of the population have access to improved water. Additionally, JMP reported the global prevalence of households with piped water connection to be 57% in 2015, while GBD reports piped prevalence of 51% for that year. JMP reported 68% of population had access to improved sanitation in 2015, whereas GBD estimates improved sanitation prevalence of 75%. The slight discrepancies in these estimates at the global Level can be largely attributed to differences in input data. The JMP relies almost exclusively on large-scale household surveys (DHS and Multiple Indicator Cluster Surveys [MICS]), while GBD estimates incorporate exposure data from smaller, yet still nationally representative, survey series such as Reproductive Health Survey and various country specific surveys. Due to the relative dearth of data regarding access to handwashing facility, the JMP only generates handwashing estimates for a select number of countries (mostly sub-Saharan Africa) where that data is actually collected. However, GBD models and predicts handwashing facility prevalence for all locations, even in the absence of data, and estimates that 67% of the globe has access to handwashing facility.

Lead

The most recent external estimates for the burden of lead exposure were conducted by the WHO in 2004 and provided disaggregated average exposures for children and adults in different regions of the world.[39] The GBD 2016 exposure estimates for the early 2000s match most of these regional estimates fairly well. The WHO study also calculated a global burden attributable to lead exposure of 13 million DALYs, which includes 229,000 deaths. This is very similar to our 2005 estimate of nearly 13.2 million DALYs attributable to lead exposure, including approximately 464,000 deaths.

While the overall estimates are similar, the breakdown of burden from intellectual disability and cardiovascular disease is very different. Both attribute 2-3% of global CVD to lead exposure, but our estimates of CVD burden in 2005 seem to be much higher than theirs (such that we estimate 9.7 million DALYs from CVD due to lead in 2005 compared to their estimate of 3.1 million). Additionally, our methodology for intellectual disability differs substantially from theirs. In the WHO study, they used a higher disability weight of 0.361 for intellectual disability whereas we currently use weights ranging from 0.01-0.2 (depending on the severity). However, their estimates of intelligence quotient (IQ) shift from lead exposure are much lower than ours, since recent studies have provided better evidence for

notable effects of lead on IQ at low levels of exposure. Still, due to differences in our estimates of the underlying burden of intellectual disability, our estimate of 2.9 million DALYs from intellectual disability attributable to lead exposure in 2005 is much smaller than their estimate of 9.8 million.

Intimate partner violence

The WHO reports a global lifetime prevalence of physical and/or sexual intimate partner violence among ever-partnered women of 30.0% (27.8-32.2%).[40] For GBD 2016, the estimated all-age global exposure for intimate partner violence (IPV) in 2016 is 17.2% (14.2-20.3%) among all women, which is a smaller estimate than the WHO estimate because the WHO estimates are among only ever-partnered women and the estimates used for GBD risk factor exposure are among all women. After making an adjustment using our model for the proportion of women who have ever been partnered, we estimate global lifetime IPV exposure as 29.3% (23.7-35.4%) among ever-partnered women – an estimate that agrees with the WHO report. The regional distribution reported by the WHO is in agreement with the distribution by GBD super-region; highest prevalence of IPV in North Africa and Middle East; South Asia; and sub-Saharan Africa and lower prevalence in Southeast Asia, East Asia, and Oceania; Central Europe, Eastern Europe, and Central Asia; High-income; and Latin American and Caribbean.

Iron deficiency

Iron deficiency (ID) was the 22nd ranked level 3 risk factor in 1990, increasing to 19th in 2016 after increasing 24.7% to 35.8 (24.1 - 50.7) million attributable DALYs, almost all of which was YLDs due to iron deficiency anaemia (IDA). We have not identified any other global, systematic analyses of ID as a risk factor for increased disease burden so we are not able to compare our estimates of ID-attributable health loss. There are a number of other studies that have evaluated the prevalence of ID and IDA, however.[37] The most comprehensive meta-analysis from LMIC estimated a much lower prevalence of ID/IDA than we have for GBD 2016. There are three aspects that make a direct comparison with GBD 2016 difficult. First, the study by Petry and colleagues likely underestimated ID/IDA somewhat by applying a single cutoff for diagnosing ID of <12 grams per deciliter of plasma ferritin concentration, especially with the acknowledged limitation of not being able to fully account for the effect of inflammation in many of its component studies. Second, Petry and colleagues did not distinguish etiologies of ID/IDA whereas GBD does distinguish many causes of anemia (e.g. hookworm, gastritis) that can manifest as ID. Third, whereas Petry and colleagues made direct estimation of ID/IDA from serum measurements, the GBD approach for estimating ID/IDA is indirect and therefore does not have a directly comparable case definition. We began by first estimating overall anemia then, after reassigning large portions to >25 other underlying causes, used fixed proportion redistribution methods to estimate IDA. The risk exposure for ID was then estimated as a counterfactual hemoglobin concentration in the absence of all the “other” causes rather than an explicit prevalence value. Unless all possible causes of anemia are included, the GBD approach has potential to overestimate the proportion of anemia to be redistributed to ID/IDA in places where other causes are important. We have begun work to address this in GBD 2016 by adding HIV as a cause of anemia, but there are still a number of others (e.g. cancers, alpha thalassemia, intestinal infections, cirrhosis, inflammatory bowel disease) that have yet to be included.

References

1 Murray CJ, Lopez AD. Global mortality, disability, and the contribution of risk factors: Global Burden of Disease Study. *Lancet* 1997; **349**: 1436–42.

2 Murray CJ, Lopez AD. On the comparable quantification of health risks: lessons from the Global Burden of Disease Study. *Epidemiology* 1999; **10**: 594–605.

3 Forouzanfar M, Afshin A, Alexander LT, Anderson H, Bhutta Z, Murray CJL. Global, regional, and national comparative risk assessment of 79 behavioural, environmental and occupational, and metabolic risks or clusters of risks, 1990-2015: a systematic analysis for the Global Burden of Disease Study 2015. *Lancet* 2016; **388**: 1659–724.

4 Stevens GA, Alkema L, Black RE, *et al.* Guidelines for Accurate and Transparent Health Estimates Reporting: the GATHER statement. *Lancet* 2016; published online June 28.

5 Food, nutrition, physical activity and the prevention of cancer: a global perspective. Washington, D.C: World Cancer Research Fund & American Institute for Cancer Research, 2007.

6 Džeroski S, Ženko B. Is Combining Classifiers with Stacking Better than Selecting the Best One? *Machine Learning* 2004; **54**: 255–73.

7 Massey FJ. The Kolmogorov-Smirnov Test for Goodness of Fit. *Journal of the American Statistical Association* 1951; **46**: 68–78.

8 Law MR, Morris JK, Wald NJ. Use of blood pressure lowering drugs in the prevention of cardiovascular disease: meta-analysis of 147 randomised trials in the context of expectations from prospective epidemiological studies. *BMJ* 2009; **338**. DOI:10.1136/bmj.b1665.

9 Harris PA, Taylor R, Thielke R, Payne J, Gonzalez N, Conde JG. Research Electronic Data Capture (REDCap) - A metadata-driven methodology and workflow process for providing translational research informatics support. *J Biomed Inform* 2009; **42**: 377–81.

10 GBD 2015 Diseases and Injury Incidence and prevalence Collaborators. Global, regional, and national incidence, prevalence, and years lived with disability (YLDs) for 310 acute and chronic diseases and injuries, 1990-2015: a systematic analysis for the Global Burden of Disease Study 2015. *The Lancet Under review*.

11 An Integrative Metaregression Framework for Descriptive Epidemiology (Publications on Global Health, Institute for Health Metrics and Evaluation): Abraham D. Flaxman, Theo Vos, Christopher J.L. Murray, Patricia Kiyono: 9780295991849: Amazon.com: Books. https://www.amazon.com/Integrative-Metaregression-Descriptive-Epidemiology-Publications/dp/0295991844 (accessed June 24, 2016).

12 Vasudevan S, Ramos F, Nettleton E, Durrant-Whyte H, Blair A. Gaussian Process modeling of large scale terrain. In: 2009 IEEE International Conference on Robotics and Automation. 2009: 1047–53.

13 Rasmussen CE, Williams CKI. Gaussian Processes for Machine Learning. Cambridge, Mass: The MIT Press, 2005.

14 Ng M, Freeman MK, Fleming TD, *et al.* Smoking Prevalence and Cigarette Consumption in 187 Countries, 1980-2012. *JAMA* 2014; **311**: 183–92.

15 Ng M, Freeman MK, Fleming TD, *et al.* Smoking prevalence and cigarette consumption in 187 countries, 1980-2012. *JAMA* 2014; **311**: 183–92.

16 Ng M, Fleming T, Robinson M, *et al.* Global, regional, and national prevalence of overweight and obesity in children and adults during 1980-2013: a systematic analysis for the Global Burden of Disease Study 2013. *Lancet* 2014; **384**: 766–81.

17 Vander Hoorn S, Ezzati M, Rodgers A, Lopez AD, Murray CJL. Estimating attributable burden of disease from exposure and hazard data. In: Comparative Quanitifcation of Health Risks: Global and regional burden of disease attribution to selected major risk factors. World Health Organisation, 2004: 2129–40.

18 Preston SH. Causes and Consequences of Mortality Declines in Less Developed Countries during the Twentieth Century. In: Population and economic change in developing countries. Chicago: Univ. of Chicago Pr, 1980: 289–360.

19 Carnahan E, Lim SS, Nelson EC, *et al.* Validation of a new predictive risk model: measuring the impact of major modifiable risks of death for patients and populations. *The Lancet* 2013; **381**: S26.

20 Danaei G, Singh GM, Paciorek CJ, *et al.* The global cardiovascular risk transition: associations of four metabolic risk factors with national income, urbanization, and Western diet in 1980 and 2008. *Circulation* 2013; **127**: 1493–502, 1502e1-8.

21 Nieman DC, Brock DW, Butterworth D, Utter AC, Nieman CC. Reducing diet and/or exercise training decreases the lipid and lipoprotein risk factors of moderately obese women. *J Am Coll Nutr* 2002; **21**: 344–50.

22 Tjønna AE, Lee SJ, Rognmo Ø, *et al.* Aerobic interval training versus continuous moderate exercise as a treatment for the metabolic syndrome: a pilot study. *Circulation* 2008; **118**: 346–54.

23 Snyder KA, Donnelly JE, Jabobsen DJ, Hertner G, Jakicic JM. The effects of long-term, moderate intensity, intermittent exercise on aerobic capacity, body composition, blood lipids, insulin and glucose in overweight females. *Int J Obes Relat Metab Disord* 1997; **21**: 1180–9.

24 Nordby P, Auerbach PL, Rosenkilde M, *et al.* Endurance training per se increases metabolic health in young, moderately overweight men. *Obesity (Silver Spring)* 2012; **20**: 2202–12.

25 Christiansen T, Paulsen SK, Bruun JM, Pedersen SB, Richelsen B. Exercise training versus diet-induced weight-loss on metabolic risk factors and inflammatory markers in obese subjects: a 12-week randomized intervention study. *Am J Physiol Endocrinol Metab* 2010; **298**: E824-831.

26 Coggan AR, Kohrt WM, Spina RJ, Bier DM, Holloszy JO. Endurance training decreases plasma glucose turnover and oxidation during moderate-intensity exercise in men. *J Appl Physiol* 1990; **68**: 990–6.

27 Arsenault BJ, Côté M, Cartier A, *et al.* Effect of exercise training on cardiometabolic risk markers among sedentary, but metabolically healthy overweight or obese post-menopausal women with elevated blood pressure. *Atherosclerosis* 2009; **207**: 530–3.

28 Chuang K-J, Yan Y-H, Chiu S-Y, Cheng T-J. Long-term air pollution exposure and risk factors for cardiovascular diseases among the elderly in Taiwan. *Occup Environ Med* 2011; **68**: 64–8.

29 Olofin I, McDonald CM, Ezzati M, *et al.* Associations of suboptimal growth with all-cause and cause-specific mortality in children under five years: a pooled analysis of ten prospective studies. *PLoS ONE* 2013; **8**: e64636.

30 McDonald CM, Olofin I, Flaxman S, *et al.* The effect of multiple anthropometric deficits on child mortality: meta-analysis of individual data in 10 prospective studies from developing countries. *Am J Clin Nutr* 2013; **97**: 896–901.

31 Das Gupta P. Standardization and Decomposition of Rates: A User's Manual. Washington D.C.: U.S. Bureau of the Census, 1993.

32 WHO report on the global tobacco epidemic, 2017: monitoring tobacco use and prevention policies. Geneva: World Health Organization, 2017.

33 Global Health Observatory data: Household air pollution. WHO http://www.who.int/gho/phe/indoor_air_pollution/en/.

34 Lelieveld J, Evans J, Fnais M, Giannadaki D, Pozzer A. The contribution of outdoor air pollution sources to premature mortality on a global scale. *Nature* 2015; : 367–71.

35 Takala J, Hamalainen P, Nenonen N, Takahashi K, Chimed-Ochir O, Rantanen J. Comparative Analysis of the Burden of Injury and Illness at Work in Selected Countries and Regions. *Central European Journal Occupational and Environmental Medicine* 2017; **23**: 6–32.

36 2016 ANNUAL REPORT WHO/UNICEF Joint Monitoring Programme for Water Supply, Sanitation and Hygiene (JMP). UNICEF.

37 Petry N, Olofin I, Hurrell RF, *et al.* The Proportion of Anemia Associated with Iron Deficiency in Low, Medium, and High Human Development Index Countries: A Systematic Analysis of National Surveys. *Nutrients* 2016; **8**. DOI:10.3390/nu8110693.

Section 3. Risk-specific estimation

The risk-specific modeling write-ups follow the order of the risk factor hierarchy for GBD 2016. In some cases, multiple risk factors are addressed in a single write-up, for example childhood underweight, wasting, and stunting are all included in a single detailed write-up.

Contents

Methods appendix to Global, regional, and national comparative risk assessment of 84 behavioural, environmental and occupational, and metabolic risks or clusters of risks, 1990-2016: a systematic analysis for the Global Burden of Disease Study 2016 1

Preamble 6

Section 1. GBD overview 7

GATHER statement 7

Location units of the analysis 7

GBD risk factor hierarchy 7

Time periods of the analysis 7

List of abbreviations 7

Section 2. Risk factor estimation 10

Overview 10

Step 1. Effect size estimation 11

1a. Collate relative risk data 11

1b. Determine relative risks 13

Step 2. Exposure estimation 14

2a. Collate exposure data 14

2b. Adjust exposure data 15

2c. Estimate exposure 15

Subnational Scaling and Aggregation 22

Step 4. TMREL 22

Step 5. Estimate population attributable fractions 22

Step 6. Mediation 23

Summary 23

Additional detail 23

Calculating burden of multiple risk factors 25

Adjusting for mediation 26

Calculating mediation factor 27

Piecewise aggregation (Pattern 3) 29

Uncertainty of aggregated and mediated PAFs 29

Important assumptions in aggregating risk factors and including mediation 29

Step 7. Estimate attributable burden 30

Other analysis: Decomposition of deaths and DALYs 30
Other analyses: SDI 32
Development of SDI 32
Updates to SDI Computation for GBD 2016........ 32
Additional Methods Information 33
Risk-specific comparisons to other estimates 33
References 39
Section 3. Risk-specific estimation........ 41
Unsafe water........ 43
Unsafe Sanitation........ 46
Unsafe Hygiene 49
Ambient Particulate Matter Pollution 51
Household Air Pollution 56
Ambient Ozone Pollution........ 58
Residual Radon........ 60
Environmental Lead 62
Occupational 65
Suboptimal Breastfeeding........ 71
Child Growth Failure 73
Low birth weight 77
Iron Deficiency 83
Vitamin A deficiency 85
Smoking........ 88
Second hand smoke 98
Smokeless tobacco........ 101
Alcohol use........ 109
Drug use 117
Diet........ 124
Childhood sexual assault........ 130
Unsafe sex........ 135
Low physical activity 138
Fasting plasma glucose 142
High cholesterol 147

High systolic blood pressure 154
High BMI........ 164
Bone mineral density 171
Impaired kidney function........ 177
Section 4: Tables and Figures........ 181
Appendix Table 1a. Relative risks used by age and sex for each outcome for all risk factors except for ambient air pollution and alcohol......... 181
Appendix Table 1b. Relative risks used by age and sex for each outcome for the particulate matter integrated exposure response curve. 333
Appendix Table 1c. Relative risks used by age and sex for each outcome for alcohol use globally....... 337
Appendix Table 1d. Relative risks used for IQ shifts by age and sex for lead exposure blood. 343
Appendix Table 2. Supplemental information for Figure 1: Epidemiological evidence supporting causality between risk-outcome pairs included in the Global Burden of Disease 2016 study including 2A. Citations and 2B. Additional information 344
Appendix Table 3. GBD 2016 risk factor hierarchy with levels, modeling strategies, and the main type of data sources used to estimate exposure levels........ 374
Appendix Table 4. Types of Comparative Risk Assessments (CRA) based on the time perspective and the nature of the counterfactual level or distribution of exposure. The shaded box represents the type of CRA currently undertaken in GBD 2016. GBD=Global Burden of Disease......... 376
Appendix Table 5. Guidelines for Accurate and Transparent Health Estimates Reporting (GATHER) 18-items checklist with description of compliance and location of information for GBD 2016 risk factors capstone........ 377
Appendix Table 6: GBD 2016 location hierarchy with levels 380
Appendix Table 7. Mediation factors. For IHD, stroke, and diabetes we pooled all available cohorts and estimated relative risks with and without adjustment across all combinations of metabolic risk factors. We then computed the excess attenuated risk for each mediation-risk-cause set. 397
Appendix Table 8: Socio-demographic Index groupings by location, based on 2016 values 402
Appendix Table 9: Socio-demographic Index values for all estimated GBD 2016 locations, 1980–2016 419
Appendix Figure 1: A more general causal web of the causes of health outcomes with the categories of causes included in this analysis shown in blue......... 432
Appendix Figure 2: Analytical flowchart of the comparative risk assessment for the estimation of population attributable fractions by geography, age, sex, and year for GBD 2016. Ovals represent data inputs, rectangular boxes represent analytical steps, cylinders represent databases, and parallelograms represent intermediate and final results. GBD=Global Burden of Disease. SEVs=Summary exposure values. TMREL=Theoretical minimum-risk exposure level. PAFs=Population attributable fractions. YLLs=years of life lost. YLDs=years lived with disability. DALYs=disability-adjusted life-years........ 433

Appendix Figure 3: GBD 2016 DisMod-MR 2.1 analytic cascade 434
Appendix Figure 4: ST-GPR flowchart 435

Unsafe Water Capstone Appendix

Flowchart

Unsafe Drinking Water

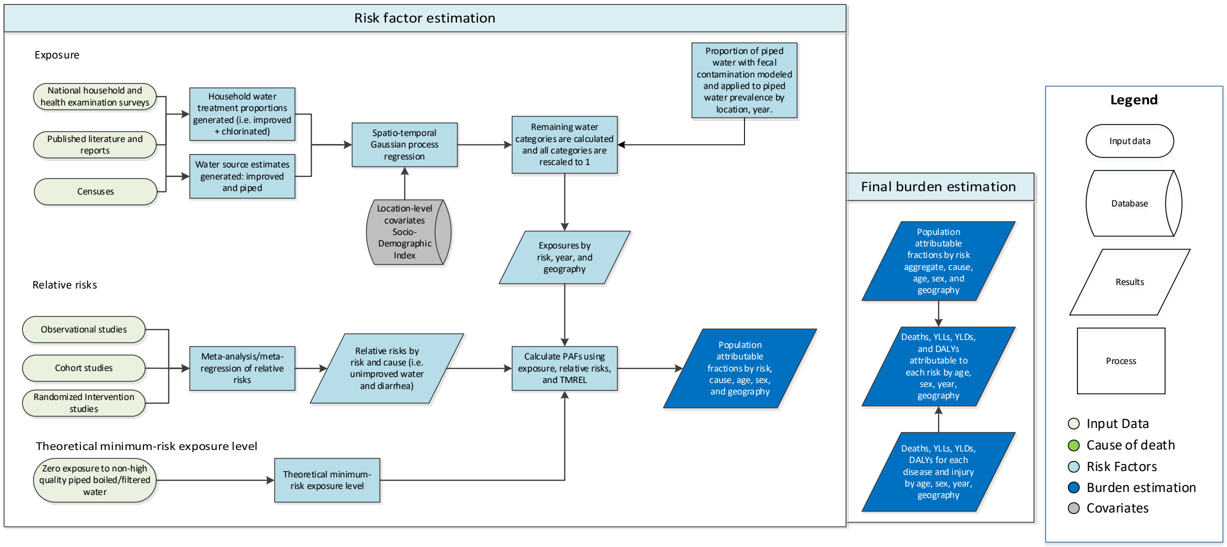

Input Data & Methodological Summary

Exposure

Case Definition

For GBD 2016, exposure to unsafe water is defined based on reported primary water source used by the household and use of household water treatment (HWT) to improve the quality of drinking water before consumption. Water sources were defined as “improved” based on the JMP designation (The WHO), which includes piped water as improved water, and households with access to piped water connection to the house, yard, or plot were defined as having access to piped water supply. One exception to this classification is that bottled water is considered “unimproved” by the JMP, however we treat it as an “improved” source. Solar treatment, chlorine treatment, boiling, or the use of filters were all assumed to be effective point-of-use household water treatments, and based on effect sizes published by Wolf et al. (2014) boiling or filtering was the most effective form of water treatment.

Input Data

The search for usable household surveys and censuses was conducted using the Global Health Data Exchange (GHDx) database. All surveys through December 2016 that provide household level micro-data on water source were added. Tabulated and report data was lower priority and was only updated when time permitted. HWT input data was limited to two large survey series (DHS and MICS) due to time constraints. An update to HWT input data is a top priority for estimating exposure to unsafe water in future iterations.

Modeling

Water source data is modeled in two distinct categories: household prevalence of improved water (excluding piped) and household prevalence of piped water. HWT is modeled in 6 distinct categories based on the 3 water treatment categories (filtered/boiled, solar/chlorine, or untreated) and 2 water

source categories (piped or improved). One modeling change made for GBD 2016 was to model prevalence of piped water independent of the improved water envelope, as was done in GBD 2015. By year and location, each of the above categories are modeled using a 3-step modeling scheme of mixed effect linear regression followed by spatio-temporal Gaussian process regression (ST-GPR), which outputs full time series estimates for each GBD 2016 location. Socio-demographic index (SDI), an index metric that includes a measure of education and income level, was used as a fixed effect in the linear regression since it proved to have significant coefficients. Random effects were placed at GBD 2016 region and super-region levels.

The process of vetting and validating models was accomplished primarily through an examination of ST-GPR scatter plots by GBD 2016 location from 1990-2016. Any unfitting data points were re-inspected for error at the level of extraction and survey implementation, and subsequently excluded from analysis if deemed appropriate. In addition to SDI, a number of different potential fixed effects were considered, including lag-distributed income and urbanicity, but SDI proved to be the strongest predictor of unsafe water. Uncertainty in the estimates was initially formed based on standard deviation by survey, then propagated through ST-GPR modeling by means of confidence intervals around each data point that reflect the point-estimate specific variance.

Once models are vetted, full time series outputs from ST-GPR modeling are then converted from proportion to prevalence by year and geography and then rescaled to form 9 mutually exclusive categories that sum up to 1. The table below provides the final result of this rescaling.

Category	*Definition*
Unimproved, no HWT	Proportion of households that use unimproved source, and *do not* use any HWT to purify their drinking water.
Unimproved, chlorine/solar	Proportion of households that use unimproved source, and solar or chlorine treatment to purify their drinking water.
Unimproved, boil/filter	Proportion of households that use unimproved source, and boil or filter to purify their drinking water.
Improved water except piped, no HWT	Proportion of households that use improved sources other than piped water supply, and *do not* use any HWT to purify their drinking water.
Improved water except piped, chlorine/solar	Proportion of households that use improved sources other than piped water supply, and use solar or chlorine treatment to purify their drinking water.
Improved water except piped, boil/filter	Proportion of households that use improved sources other than piped water supply, and boil/filter their drinking water.
Piped water, no boil/filter	Proportion of households that use piped water supply, and *do not* use any HWT to purify their drinking water
Piped water, chlorine/solar	Proportion of households that use piped water supply, and *use* solar or chlorine water treatment to purify their drinking water.

Piped water, boil/filter	Proportion of households that use piped water supply, and boil or filter to purify their drinking water

In previous GBD iterations, high income countries were assumed to have no risk of unsafe water. For GBD 2016, we estimated the risk of unsafe water in high income countries as well. Additionally, we modeled the microbiological quality of piped water sources primarily using data a review by Bain et al. (2014) that measured proportion of piped water sources contaminated with fecal indicators. We use the value generated from this model to split the prevalence of piped water into basic piped water and high quality piped water by location, year, age, and sex.

A substantial limitation in our analysis is the paucity of data on HWT and piped water quality. The inclusion of more location-specific data on water treatment utilization at the household level can greatly improve our estimates in future iterations.

Theoretical minimum-risk exposure level

The theoretical minimum-risk exposure level for unsafe water is defined as all households have access to high quality piped water that has been boiled or filtered before drinking.

Relative risks

Notable updates were made to the relative risks for unsafe water from GBD 2015. For GBD 2016, there is only 1 adverse health outcome paired with unsafe water, which is diarrheal disease. Note that previously typhoid fever and paratyphoid fever were also included as outcomes but were excluded this round due to the lack of direct evidence. A meta-analysis by Wolf et al. (2014) provided the bulk of the relative risk evidence for the relationship between unsafe water and diarrheal diseases. This meta-analysis was updated through a literature review that searched for related intervention studies post-2014 conducted in PubMed. Search terms used were identical to those provided by Wolf et al. (2014). Relative risk values for water-source interventions and point-of-use treatment interventions were calculated separately so the combined effect of a source intervention and point-of-use intervention was assumed to be multiplicative in order to match GBD 2016 exposure definitions. Please refer to appendix tables for more information on relative risk values and citations.

References

1. "Improved and Unimproved Water Sources and Sanitation Facilities."*WHO / UNICEF Joint Monitoring Programme: Wat/san Categories*. The WHO/UNICEF, n.d. Web. 08 June 2016
2. Wolf, Jennyfer, Annette Prüss-Ustün, Oliver Cumming, Jamie Bartram, Sophie Bonjour, Sandy Cairncross, Thomas Clasen, John M. Colford, Valerie Curtis, Jennifer De France, Lorna Fewtrell, Matthew C. Freeman, Bruce Gordon, Paul R. Hunter, Aurelie Jeandron, Richard B. Johnston, Daniel Mäusezahl, Colin Mathers, Maria Neira, and Julian P. T. Higgins. "Systematic Review: Assessing the Impact of Drinking Water and Sanitation on Diarrhoeal Disease in Low- and Middle-income Settings: Systematic Review and Meta-regression." Trop Med Int Health Tropical Medicine & International Health 19.8 (2014): 928-42. Web.
3. Bain, R., Cronk, R., Wright, J., Yang, H., Slaymaker, T., & Bartram, J. (2014). Fecal Contamination of Drinking-Water in Low- and Middle-Income Countries: A Systematic Review and Meta-Analysis. PLoS Medicine, 11(5). doi:10.1371/journal.pmed.1001644

Unsafe Sanitation Capstone Appendix

Flowchart

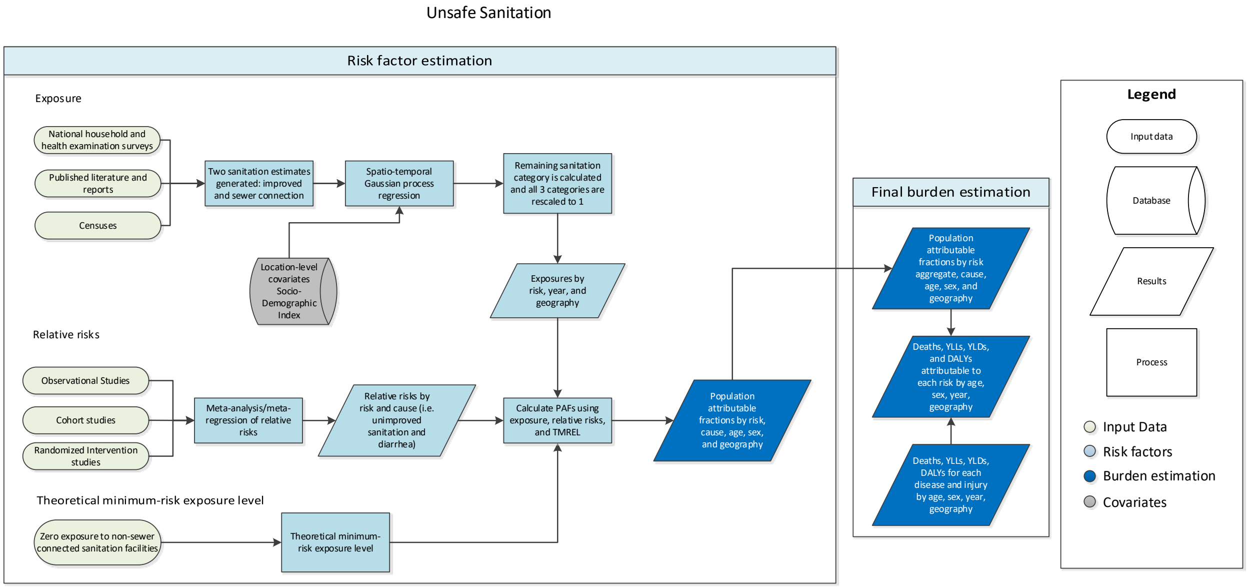

Input Data & Methodological Summary

Exposure

Case Definition

Exposure to unsafe sanitation were defined based on the primary toilet type used by households. Improved facilities are defined as such based on JMP designation (WHO). Sewer connection toilets included flush toilets or any toilet with connection to the sewer or septic tank.

Input Data

The search for usable household surveys and censuses was conducted using the Global Health Data Exchange (GHDx) database. Searches were conducted from October 2016 to December 2016, with the final search household level micro-data on toilet type conducted December 2016. Due to the organized nature of the GHDx, the only search term used was "unsafe sanitation", which yielded just under 1400 results, of which 795 were extracted and used as inputs for modeling. Tabulated and report data was lower priority and was only updated when time permitted.

Modeling

One modeling change made in GBD 2016 was that proportion of households with sewer connection is modeled independently, instead of within the "improved" sanitation envelope. Two distinct models were produced from sanitation data: prevalence of households with improved sanitation and the prevalence of households with a sewer connection. By each location-year, both models were generated using a 3-step modeling scheme of mixed effect linear regression followed by spatio-temporal Gaussian process regression (ST-GPR), which outputs full time series estimates for each GBD 2016 location. Socio-demographic index (SDI), an index metric that includes a measure of education and income level, was used as a fixed effect in the linear regression since it proved to have significant coefficients. Random effects were placed at GBD 2016 region and super-region levels.

The process of vetting and validating models was accomplished primarily through an examination of ST-GPR scatter plots by GBD 2016 location from 1990-2016. Any unfitting data points were re-inspected for error at the level of extraction and survey implementation, and subsequently excluded from analysis if deemed appropriate. In addition to SDI, a number of different potential fixed effects were considered, including lag-distributed income and urbanicity, but SDI proved to be the strongest predictor of unsafe sanitation. Uncertainty in the estimates was initially formed based on standard deviation by survey, then propagated through ST-GPR modeling by means of confidence intervals around each data point that reflect the point-estimate specific variance.

Once models were fully vetted, full time series outputs from ST-GPR modeling were rescaled to form 3 mutually exclusive categories that sum up to 1. The table below provides the final result of this rescaling.

Category	*Definition*
Unimproved sanitation	Proportion of households that use unimproved sanitation facilities.
Improved sanitation, excluding sewer	Proportion of households that use improved sanitation facilities except those with sewer connection.
Sanitation facilities with sewer connection	Proportion of households that use toilet facilities with sewer connection.

In previous GBD iterations, high income countries were assumed to have no risk of unsafe sanitation. For GBD 2016, we estimate the risk of unsafe sanitation in high income countries as well. One limitation that extends to the other two risk factors that comprise WaSH (unsafe water and unsafe hygiene) and can be improved upon in future iterations is taking into account covariance of access to water, sanitation and handwashing facilities. Currently, all 3 components of WaSH were modeled independently, which may lead to an overestimation of the burden of WaSH risk factors.

Theoretical minimum-risk exposure level

The theoretical minimum-risk exposure level for unsafe sanitation was defined as all households have access to a sanitation facility with sewer connection. Since it was assumed that all households in high-income countries have access to sewer-connected sanitation, this counterfactual exposure level is applied to all households in high-income countries.

Relative risks

Notable updates were made to the relative risks for unsafe sanitation from GBD 2015. For GBD 2016, there was only 1 adverse health outcome paired with unsafe sanitation, which was diarrheal disease. Note that previously typhoid fever and paratyphoid fever were also included as outcomes but were excluded this round due to the lack of direct evidence. A meta-analysis by Wolf et al. 2014 provides the bulk of the relative risk evidence for the relationship between unsafe sanitation and diarrheal diseases. This meta-analysis was updated through a literature review that searched for related intervention studies post-2014 conducted in PubMed. Search terms used were identical to those provided by Wolf et al. 2014. Please refer to appendix tables for more information on relative risk values and citations.

References

1. "Improved and Unimproved Water Sources and Sanitation Facilities."*WHO / UNICEF Joint Monitoring Programme: Wat/san Categories*. The WHO/UNICEF, n.d. Web. 08 June 2016
2. Wolf, Jennyfer, Annette Prüss-Ustün, Oliver Cumming, Jamie Bartram, Sophie Bonjour, Sandy Cairncross, Thomas Clasen, John M. Colford, Valerie Curtis, Jennifer De France, Lorna Fewtrell, Matthew C. Freeman, Bruce Gordon, Paul R. Hunter, Aurelie Jeandron, Richard B. Johnston, Daniel Mäusezahl, Colin Mathers, Maria Neira, and Julian P. T. Higgins. "Systematic Review: Assessing the Impact of Drinking Water and Sanitation on Diarrhoeal Disease in Low- and Middle-income Settings: Systematic Review and Meta-regression." Trop Med Int Health Tropical Medicine & International Health 19.8 (2014): 928-42. Web.

Unsafe Hygiene Capstone Appendix

Flowchart

Unsafe Handwashing

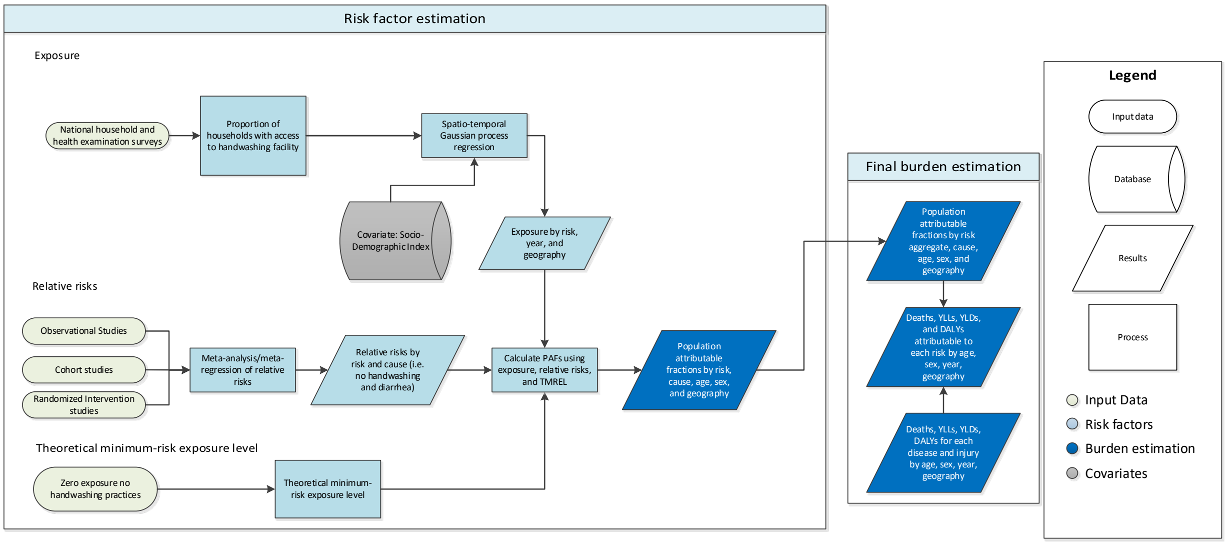

Input Data & Methodological Summary

Exposure

Case Definition

Lack of access to handwashing facility is defined access to a handwashing station with available soap and water. We estimated the burden of unsafe handwashing in both developed and developing settings.

Input Data

Since water and soap availability data were very limited, only country-specific Demographic Health Surveys (DHS) and Malaria Indicator Survey Series (MICS) conducted after 2006 were able to be used as input data.

Modeling Strategy

By year and location, proportion of households with handwashing facility is modeled using a 3-step modeling scheme of mixed effect linear regression followed by spatio-temporal Gaussian process regression (ST-GPR), which outputs full time series estimates for each GBD 2016 location. Socio-demographic index (SDI), an index metric that includes a measure of education and income level, was used as a fixed effect in the linear regression since it proved to have significant coefficients. Random effects were placed at GBD 2016 region and super-region levels.

The process of vetting and validating models was accomplished primarily through an examination of ST-GPR scatter plots by GBD 2016 location from 1990-2016. Any unreasonable data points were re-inspected for error at the level of extraction and survey implementation, and subsequently excluded from analysis if deemed appropriate. In addition to SDI, a number of different potential fixed effects

were considered, including lag-distributed income and urbanicity, however SDI proved to be the strongest predictor.

A considerable limitation for when estimating handwashing practices for over 190 independent locations around the world was data sparseness. Even when data were published on handwashing prevalence, the definition was often altered from the GBD 2016 standard definition or it may only have pertained to certain populations (such as hospital patients) and lacked representativeness at the geographic scale we required. The incorporation of questions about soap and water availability in DHS and MICS added much-needed information but there remains a large data gap that must be filled if we are to become more certain in handwashing access estimates.

Theoretical minimum-risk exposure level

The theoretical minimum-risk exposure level for unsafe hygiene is defined as all households engaging in handwashing with soap practices after any contact with excreta, including children's excreta.

Relative risks

Notable updates were made to the relative risks for unsafe water from GBD 2015. For GBD 2016, there were 2 adverse health outcome paired with unsafe water: diarrheal disease and lower respiratory infection. Note that previously typhoid fever and paratyphoid fever were also included as outcomes but were excluded this round due to the lack of direct evidence. A meta-analysis by Cairncross et al. (2010) provideed relative risk evidence for the relationship between lack of facility access and diarrheal diseases. A meta-analysis by Rabie and Curtis (2006) provided relative risk evidence for the relationship between lack of facility access and lower respiratory infection. Please refer to appendix tables for more information on relative risk values and citations.

References

1. Cairncross, S., Hunt, C., Boisson, S., Bostoen, K., Curtis, V., Fung, I. C., & Schmidt, W. P. (2010). Water, sanitation and hygiene for the prevention of diarrhoea. International Journal of Epidemiology, 39(Supplement 1), I193-I205. doi:10.1093/ije/dyq035
2. Rabie, T., & Curtis, V. (2006). Handwashing and risk of respiratory infections: a quantitative systematic review. Tropical Medicine and International Health, 11(3), 258-267. doi:10.1111/j.1365-3156.2006.01568.x

Ambient Particulate Matter Pollution

Flowchart

Ambient PM2.5

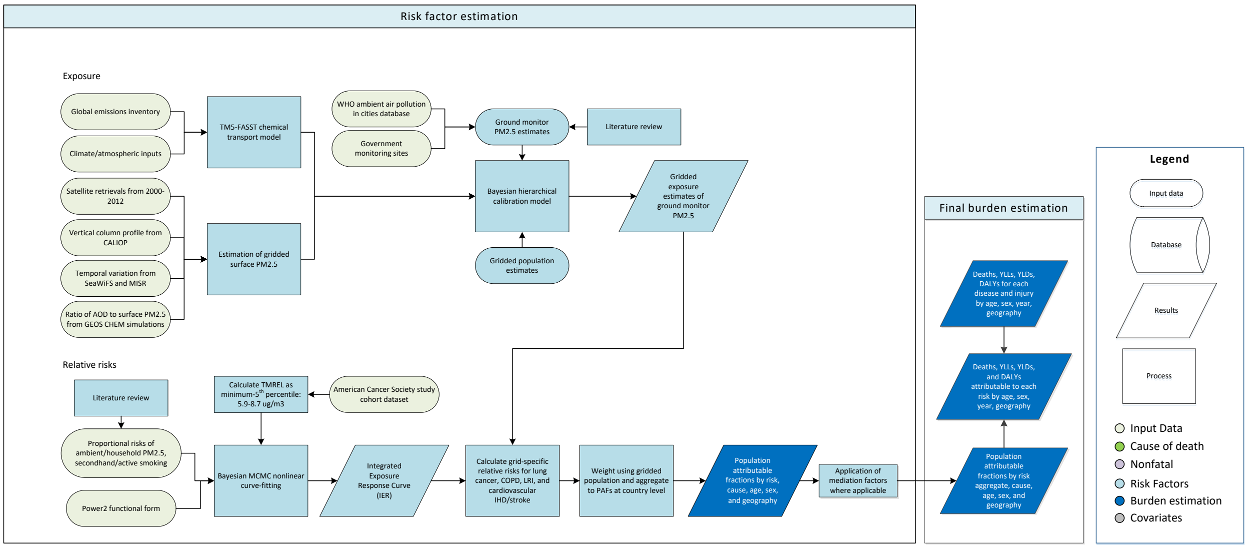

Input data and modeling strategy

Exposure

Definition

Exposure to ambient air pollution is defined as the population-weighted annual average mass concentration of particles with an aerodynamic diameter less than 2.5 micrometers ($PM_{2.5}$) in a cubic meter of air. This measurement is reported in $\mu g/m^3$.

Input Data

The data used to estimate exposure to ambient air pollution is drawn from multiple sources, including satellite observations of aerosols in the atmosphere, ground measurements, chemical transport model simulations, population estimates and land-use data.

The following details the updates in methodology and input data used in GBD2015 and GBD2016 from that used in GBD2013.

$PM_{2.5}$ ground measurement database

Updates of ground measurements used for GBD2015 and GBD2016 include using more recent data than that used in GBD2013 and the addition of data from locations where measurement data have become available. These updates were made in collaboration with the WHO and are included within the May 2016 update of the WHO Air Pollution in Cities database. Monitor-specific measurements (rather than city averages as reported in the WHO database) were used, resulting in measurements of concentrations of PM_{10} and $PM_{2.5}$ from 6,003 ground monitors from 117 countries. The majority of measurements were recorded in 2014 (as there is a lag in reporting measurements, little data

from 2015 were available). Where data were not available for 2014 (2760 monitors), data was used from 2015 (18 monitors), 2013 (2155), 2012 (564), 2011 (60), 2010 (375), 2009 (49), 2008 (21) and 2006 (1). For locations measuring only PM_{10}, $PM_{2.5}$ measurements were estimated from PM_{10}. This was performed using a locally derived conversion factor ($PM_{2.5}/PM_{10}$ ratio, for stations where measurements are available for the same year) that was estimated using population-weighted averages of location-specific conversion factors for the country. If country-level conversion factors were not available, the average of country-level conversion factors within a region were used. As in the GBD2013 database, additional information related to the ground measurements was also included where available, including monitor geo coordinates and monitor site type.

Satellite-based estimates

The updated satellite-based estimates for years 2000-2015 are described in detail in van Donkelaar et al. 2016[1]. These estimates were available at 0.1°×0.1° resolution (~11 x 11 km resolution at the equator) and combine aerosol optical depth retrievals from multiple satellites with the GEOS Chem chemical transport model and land use information.

Population data

A comprehensive set of population data on a high-resolution grid was obtained from the Gridded Population of the World (GPW) database. These data are provided on a 0.0417°×0.0417° resolution. Aggregation to each 0.1°×0.1° grid cell comprised of summing the central 3 × 3 population cells. As this resulted in a resolution higher than necessary, it was repeated four times, each offset by one cell in a North, South, East and West direction. The average of the resulting five quantities was used as the estimated population for each grid cell. Population estimates for 2000, 2005, 2010, 2015 and 2020 were availalble from GPW version 4. Populations for 2015 and 2016 were obtained by interpolation using natural splines with knots placed at 2000, 2005, 2010, 2015 and 2020. This was performed for each grid cell.

Chemical transport model simulations

Estimates of the sum of particulate sulfate, nitrate, ammonium and organic carbon and the compositional concentrations of mineral dust simulated using the GEOS Chem chemical transport model, and a measure combining elevation and the distance to the nearest urban land surface (as described in van Donkelaar et al. 2016[1]) were available for 2000 to 2015 for each 0.1°×0.1° grid cell. These were not included within the GBD2013 analysis.

Modelling Strategy

Significant advances have been made in the methodology used to estimate exposure to ambient particulate matter pollution since GBD2013. The following is a summary of the modelling approach, known as the Data Integration Model for Air Quality (DIMAQ) used in GBD2015 and 2016; further details can be found in Shaddick *et al.* (2017)[2]

In GBD2010 and GBD2013 exposure estimates were obtained using a single global function to calibrate available ground measurements to a ‘fused’ estimate of $PM_{2.5}$; the mean of satellite-based estimates and those from the TM5 chemical transport model, calculated for each 0.1°×0.1° grid cell. This was recognized to represent a trade-off between accuracy and computationally efficiency when utilising all the available data sources. In particular, the GBD2013 exposure estimates were known to underestimate ground measurements in specific locations (see discussion in Brauer et al., 2016[3]).

This underestimation was largely due to the use of a single, global, calibration function, whereas in reality the relationship between ground measurements and other variables will vary spatially.

In GBD2015 and GBD2016, coefficients in the calibration model were estimated for each country. Where data were insufficient within a country, information can be `borrowed' from a higher aggregation (region) and if enough information is still not available from an even higher level (super-region). Individual country level estimates were therefore based on a combination of information from the country, its region and super-region. This was implemented within a Bayesian Hierarchical modelling (BHM) framework. BHMs provide an extremely useful and flexible framework in which to model complex relationships and dependencies in data. Uncertainty can also be propagated through the model allowing uncertainty arising from different components, both data sources and models, to be incorporated within estimates of uncertainty associated with the final estimates. The results of the modelling comprise a posterior distribution for each grid cell, rather than just a single point estimate, allowing a variety of summaries to be calculated. The primary outputs here are the median and 95% credible intervals for each grid cell. Based on the availability of ground measurement data, modeling and evaluation was focused on the year 2014.

Due to both the complexity of the models and the size of the data, notably the number of spatial predictions that are required, recently developed techniques that perform 'approximate' Bayesian inference based on integrated nested Laplace approximations (INLA) were used[4]. Computation was performed using the R interface to the INLA computational engine (R-INLA). Fitting the models and performing predictions for each of the ca. 1.4 million grid cells required the use of a high performance computing cluster (HPC) making use of high memory nodes.

Model Evaluation

Model development and comparison was performed using within- and out-of-sample assessment. In the evaluation, cross validation was performed using 25 combinations of training (80%) and validation (20%) datasets. Validation sets were obtained by taking a stratified random sample, using sampling probabilities based on the cross-tabulation of $PM_{2.5}$ categories (0-24.9, 25-49.9, 50-74.9, 75-99.9, 100+ μg/m^3) and super-regions, resulting in them having the same distribution of $PM_{2.5}$ concentrations and super-regions as the overall set of sites. The following metrics were calculated for each training/evaluation set combination: for model fit - R^2 and deviance information criteria (DIC, a measure of model fit for Bayesian models); for predictive accuracy - root mean squared error (RMSE) and population weighted root mean squared error (PwRMSE).

All modelling was performed on the log-scale. The choice of which variables were included in the model was made based on their contribution to model fit and predictive ability. The following is a list variables and model structures that were considered in developing the model.

Continuous explanatory variables:

- (SAT) Estimate of $PM_{2.5}$ (in μgm^{-3}) for 2014 from satellite remote sensing on the log-scale.
- (CTM) Estimate of $PM_{2.5}$ (in μgm^{-3}) for 2010 from the TM5 chemical transport model on the log-scale.
- (POP) Estimate of population for 2014 on the log-scale.
- (SNAOC) Estimate of the sum of sulfate, nitrate, ammonium and organic carbon simulated using the GEOS Chem chemical transport model.

- (DST) Estimate of compositional concentrations of mineral dust simulated using the GEOS Chem chemical transport model.
- (EDxDU) The log of the elevation difference between the elevation at the ground measurement location and the mean elevation within the GEOS Chem simulation grid cell multiplied by the inverse distance to the nearest urban land surface.

Discrete explanatory variables:

- (LOC) Binary variable indicating whether exact location of ground measurement is known.
- (TYPE) Binary variable indicating whether exact type of ground monitor is known.
- (CONV) Binary variable indicating whether ground measurement is $PM_{2.5}$ or converted from PM_{10}.

Random Effects:

- Grid cell random effects on the intercept to allow for multiple ground monitors in a grid cell.
- Country-region-super-region hierarchical random effects for the intercept.
- Country-region-super-region hierarchical random effects for the coefficient associated with SAT .
- Country-region-super-region hierarchical random effects for the coefficient associated with the difference between estimates from CTM and SAT.
- Country-region-super-region hierarchical random effects for the coefficient associated with POP.
- Country level random effects for population uses a neighbourhood structure allowing specific borrowing of information from neighbouring countries.
- Within a region, country level effects of SAT and the difference between SAT AND CTM are assumed to be independent and identically distributed.
- Within a super-region, region level random effects are assumed to be independent and identically distributed.
- Super-region random effects are assumed to be independent and identically distributed.

Interactions*:*

- Interactions between the binary variables and the effects of SAT and CTM.

Results

The final model contained the following variables: SAT, POP, SNAOC, DST, EDxDU, LOC, TYPE, and CONV, together with interactions between SAT and each of LOC, TYPE and CONV. The model structure contained grid cell random effects on the intercept to allow for multiple ground monitors in a grid cell, country-region-super-region hierarchical random effects for intercepts and SAT and country level random effects for population using a neighbourhood structure allowing specific borrowing of information from neighbouring countries together with region-super-region hierarchical random effects for POP. Notably, based on the evaluation of candidate models, including estimates from the TM5 chemical transport model (CTM) used in GBD2013 did not improve the predictive ability of the model and was therefore not included.

Compared to the model used in GBD2013, DIMAQ showed improved predictions of ground measurements in all super regions (Table 1). Using this model resulted in an improvement in both within-sample fit; with an increase in R^2 from 0.64 (reported in GBD 2013[1]) to 0.91, and out-of-

sample predictive ability; with a global population-weighted RMSE of 12.1 µg/m^3 compared to 23.1 µg/m^3 when using the GBD 2013 approach.

	GBD2013	GBD2015/16
**Global**	23.1	12.1
High income	*6.4*	*2.7*
Central Europe, Eastern Europe and Central Asia	*9.7*	*6.0*
Latin America and Caribbean	*13.9*	*7.1*
Southeast Asia, East Asia and Oceania	*20.1*	*10.8*
North Africa / Middle East	*23.6*	*14.3*
Sub-Saharan Africa	*38.8*	*32.3*
South Asia	*44.8*	*22.0*

Table 1: Summary measures of predictive ability, globally and by super-region. Results are the median values of population weighted root mean squared error (µg/m^3), from 25 validation sets.

Estimates for other years

Satellite estimates, populations and quantities estimated using the GEOS-Chem model were available for 1990, 1995, 2000, 2005, 2010, 2011, 2012, 2013, 2014 and 2015. Population estimates for 2000, 2005, 2010, 2015 and 2020 were availalble from GPW version 4. For 1990 and 1995 data were extracted from GPW version 3, as in GBD2013[2]. As with populations for 2015, values for each cell for 2011, 2012, 2012, 2013 and 2014 were obtained by interpolation using natural splines with knots placed at 2000, 2005, 2010, 2015 and 2020.

These were used as inputs to DIMAQ, enabling estimates of exposures to be obtained for each of these years respectively. For 2016, estimates of exposures were obtained from predictions from locally-varying regression models[4]. For each cell a model was fit to the values within that cell over time, with a constraint placed on the rate of change between 2015 and 2016 to avoid unrealistic and/or unjustified extrapolation of trends. Measures of uncertainty were obtained by repeating the procedure for the limits of the 95% credible intervals, again on a cell-by-cell basis.

References

1. van Donkelaar, A.; Martin, R. V; Brauer, M.; Hsu, N. C.; Kahn, R. A.; Levy, R. C.; Lyapustin, A.; Sayer, A. M.; Winker, D. M. Global Estimates of Fine Particulate Matter using a Combined Geophysical-Statistical Method with Information from Satellites, Models, and Monitors. Environ. Sci. Technol. 2016, 50 (7), 3762–3772
2. Shaddick, G., Thomas, M.L., Jobling, A., Brauer, M., van Donkelaar, A., Burnett, R., Chang, H., Cohen, A., Van Dingenen, R., Dora, C. and Gumy, S., 2016. Data Integration Model for Air Quality: A Hierarchical Approach to the Global Estimation of Exposures to Ambient Air Pollution. Journal of Royal Statistical Society Series C (Applied Statistics).2017. DOI: 10.1111/rssc.12227
3. Brauer, M.; Freedman, G.; Frostad, J.; van Donkelaar, A.; Martin, R. V; Dentener, F.; Van Dingenen, R.; Estep, K.; Amini, H.; Apte, J. S.; et al. Ambient Air Pollution Exposure Estimation for the Global Burden of Disease 2013. Environ. Sci. Technol. 2015, 50 (1), 79–88.
4. Rue, H.; Martino, S.; Chopin, N.; Approximate Bayesian inference for latent Gaussian models by using integrated nested Laplace approximations. Journal of the royal statistical society: Series b (statistical methodology). 2009;71(2):319-92.
5. Cleveland, W.S. and Devlin, S.J., 1988. Locally weighted regression: an approach to regression analysis by local fitting. Journal of the American statistical association, 83(403), pp.596-610.

Household Air Pollution Capstone Appendix

Flowchart

Household Air Pollution from Solid Fuels

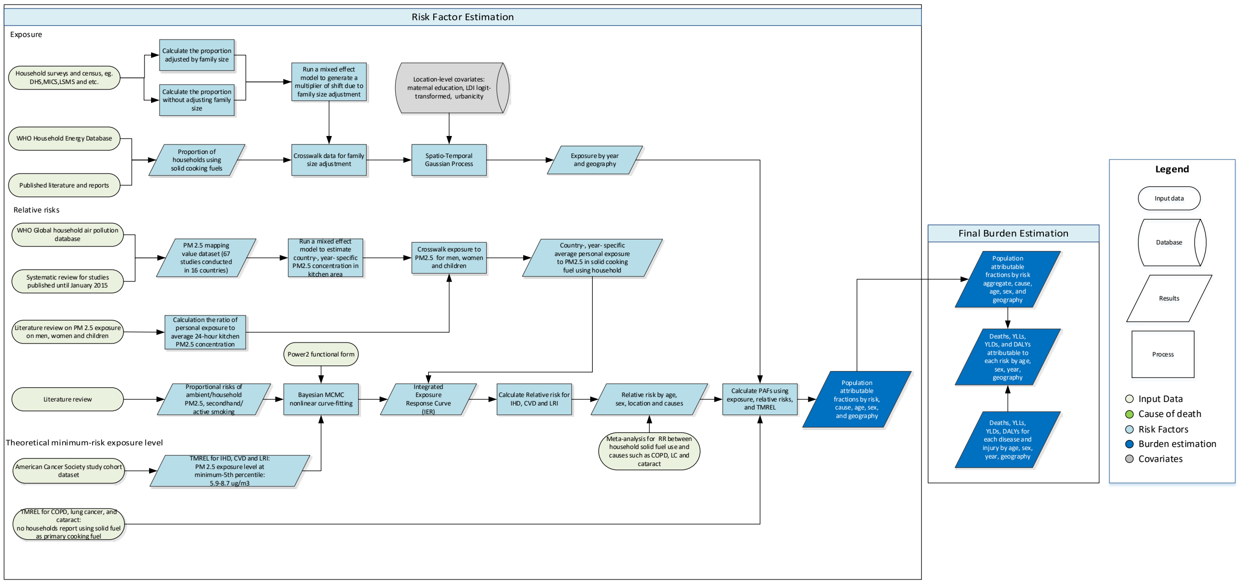

Input Data & Methodological Summary

Exposure

Case Definition

Exposure to household air pollution from solid fuels (HAP) is defined as the proportion of households using solid cooking fuels. The definition of solid fuel in our analysis includes coal, wood, charcoal, dung, and agricultural residues.

Input data

Data were extracted from the standard multi-country survey series such as Demographic and Health Surveys (DHS), Living Standards Measurement Surveys (LSMS), Multiple Indicator Cluster Surveys (MICS), and World Health Surveys (WHS), as well as country-specific survey series such as Kenya Welfare Monitoring Survey and South Africa General Household Survey. To fill the gaps of data in surveys and censuses, we also downloaded and updated HAP estimates from WHO Energy Database and extracted from literature through systematic review. Each nationally or sub-nationally representative data point provided an estimate for the percentage of households using solid cooking fuels. Estimates for the usage of solid fuels for non-cooking purpose were excluded, i.e. primary fuels for lighting. The database, with estimates from 1980 to 2016, contained about 680 studies from 150 countries. As updates to systematic reviews are performed on an ongoing schedule across all GBD causes and risk factors, an update for household air pollution will be performed in the next 1-2 iterations.

Modeling strategy

Household air pollution was modeled at household level using a three-step modeling strategy that uses linear regression, spatiotemporal regression and Gaussian Process Regression (GPR). The first step is a mixed-effect linear regression of logit-transformed proportion of households using solid cooking fuels.

The linear model contains maternal education, proportion of population living in urban areas, and lagged-distributed income as covariates and has nested random effect by GBD region, and GBD super region respectively. The full ST-GPR process is specified in Section 2 of this appendix.

No substantial modeling changes were in this round compared to GBD 2015. A variety of combinations of socioeconomic and environmental covariates in different transformation format were tested by running mixed-effect models with exposure data. The final list of covariates included in the exposure model are maternal education, proportion of population living in urban area, and lagged-distributed income since they proved to be the strongest predictors.

Theoretical minimum-risk exposure level

For outcomes where we extracted relative risks (RR) based on direct epidemiological evidence i.e. chronic obstructive pulmonary disease (COPD), lung cancer, and cataract, TMREL was defined such that no households would report using solid fuel as their primary cooking fuel. For outcomes that utilize evidence based on the Integrated Exposure Response (IER), the TMREL is defined as uniform distribution between 2.4 and 5.9 ug/m^3. TMREL for household air pollution.

Relative risks

The disease-outcomes paired with household air pollution have not changed since GBD 2015. These outcomes include lower respiratory infections (LRI), stroke, Ischemic Heart Disease (IHD), COPD, lung cancer and cataract. The relative risks of all outcomes, with the exception of cataracts, were generated by using the integrated exposure-response functions (IER). The relative risks for cataracts were extracted from a meta-analysis paper (1). The IER curves are updated to reflect the newly updated data and utilization of a new method that specified elsewhere.

PM2.5 mapping value

The relative risk estimates describing the association of HAP with outcomes including ischemic heart disease (IHD), cardiovascular disease (CVD), and lower respiratory infections (LRI) were derived from the IER curves. This is done by first estimating the crosswalk values that map household use of solid fuel to PM2.5 exposure because the IER curve measures exposure using PM2.5. For GBD 2015, this step of the analysis relied on 67 studies conducted in 16 countries to generate the PM2.5 mapping values. In this round, we have extracted PM2.5 data from about 20 additional studies to add to bring the total study sum of the database to almost 90 studies. The addition of more studies has provided more stability in the model and allowed us to use socio-demographic index as a covariate to predict exposure for all location-years. The PM2.5 exposures were then cross-walked to men, women and children by generating the ratio of personal exposure to average 24-hour kitchen PM2.5 concentration based on a study after the literature review in GBD 2013.

References

1. Smith KR, Bruce N, Balakrishnan K, Adair-Rohani H, Balmes J, Chafe Z, et al. Millions Dead: How Do We Know and What Does It Mean? Methods Used in the Comparative Risk Assessment of Household Air Pollution. Annu Rev Public Health. 2014;35(1):185–206.

Ambient Ozone Pollution

Flowchart

Ambient ozone

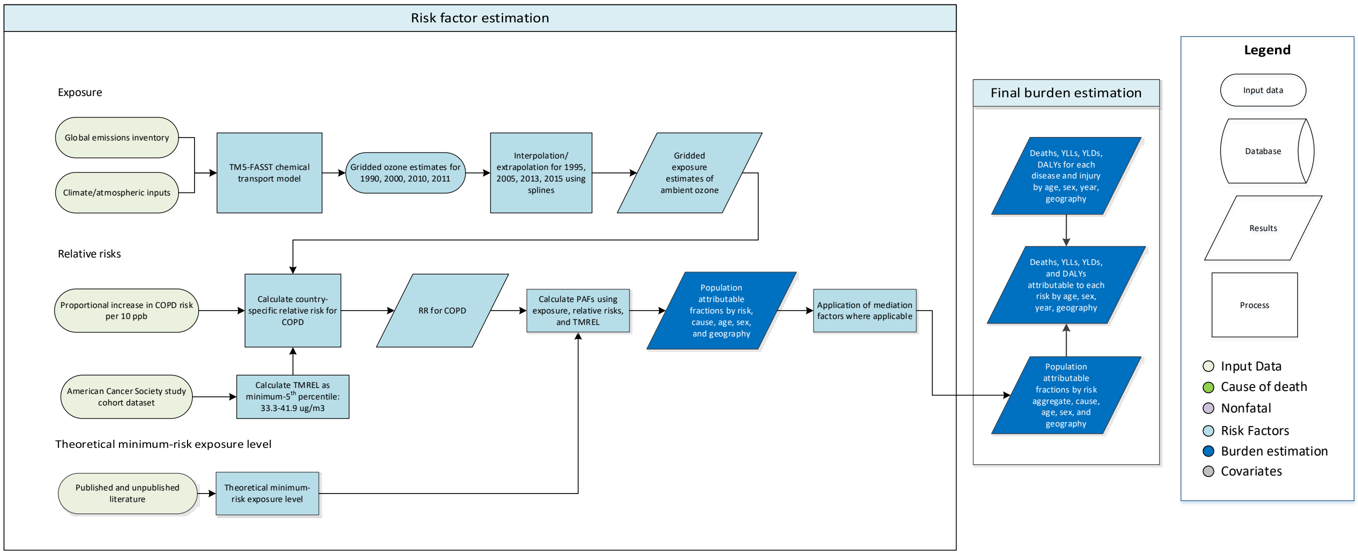

Input data and Methodological Summary

Exposure

Case Definition

For GBD 2016, exposure to ozone pollution is defined as the number of parts-per-billion (ppb) of ozone (O^3).

Input data

Data for estimating ozone exposure is derived from the TM5-FASST chemical transport model, which generates a 3-month running average of daily 1 hour maximum ozone values at the 0.1°×0.1° for the years 1990, 2000, and 2010.[1]

Modeling Strategy

The process for modeling ozone exposure has remained stable since GBD2010 and GBD2013. Natural cubic splines were used to interpolate for the years 1995, 2005, and 2011. Annualized rate of change was used to predict for the years 2013, 2015 and 2016. The uncertainty for exposure at the grid-level was assumed to be ±6% of the estimated concentration, in accordance with previous work. Uncertainty for ozone was calculated by assuming a +/- 6% uncertainty interval around the estimation concentration.

Theoretical minimum-risk exposure level

The TMREL of ozone was defined based on the exposure distribution from American Cancer Society CPS-II study, which was the source of the GBD 2016 ozone mortality RR estimate. As with PM2.5, a uniform distribution was drawn around the minimum and 5th percentile values experienced by the cohort. This value was not updated for GBD 2016, and continues to be defined as ~U(33.3, 41.9), in ppb.

No other significant changes were made from GBD 2013 to GBD 2016.

Relative Risks

The relative risk of ozone exposure for respiratory COPD was extracted from literature and was not updated for GBD 2016. The relative risk is applied linearly per 10 ppb of ozone exposure and is defined as 1.029 (1.010-1048).[2]

References

1. Brauer M, et al. Ambient Air Pollution Exposure Estimation for the Global Burden of Disease 2013. Environ Sci Technol 2016; 50: 79-88.
2. Jerrett M, Burnett RT, Pope CA, et al. Long-term ozone exposure and mortality. N Engl J Med 2009; 360: 1085–95.

Radon Exposure Capstone Appendix

Flowchart

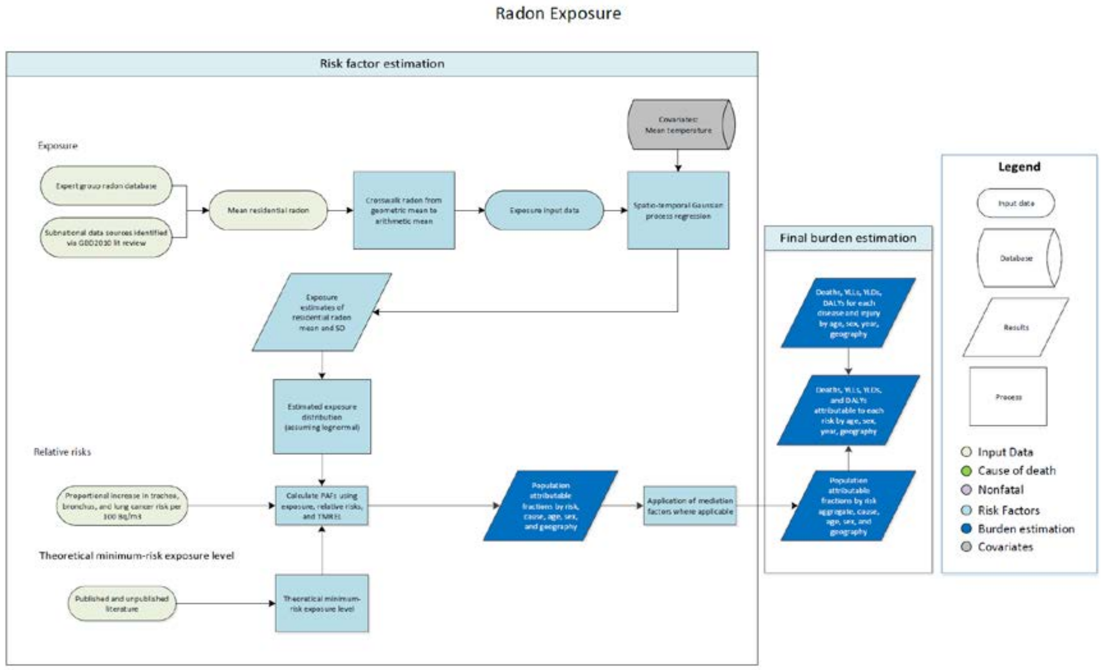

Input Data & Methodological Summary

Exposure

Case definition

Radon is a radioactive gas that is produced as a byproduct of the decay chain of uranium, occurring naturally within the Earth's crust. Some fraction of this natural radon production escapes into the atmosphere, where it forms at low concentration unless build-up is caused by enclosed spaces like homes, mines, or caves. Radon exposure is expressed as average daily exposure to indoor air radon gas levels measured in Becquerels (disintegrations per second) per cubic meter (Bq/m^3).

Input Data

Exposure to radon is determined using values curated by an expert group. These values are taken from a variety of sources including literature, government agencies, and monitoring stations. Their methodology is then inspected to determine if they are robust enough to be considered as country-level averages. This dataset was last updated for GBD 2013 by adding new data points across time and space. No new data points were added for GBD 2016.

Modeling Strategy

There have been minor changes to the methodology to estimate radon exposure. The modelling process was previously updated by shifting it from a nested random effects model to spatial-temporal GPR. For GBD 2016, the general spatial-temporal GPR modelling methodology was updated as detailed in the appendix specific to this analytical technique, which is common to a variety of risk factors. Radon is

naturally occurring, and is not considered to have much temporal fluctuation[1]. As such, we did not model radon over time, opting instead to assign all data points to a single year, predict across space using our radon database, and use the results for that year for the entire GBD time series. This eliminated any spurious time trends that might arise using the traditional ST-GPR approach. The only study-level covariate considered was whether a data point was reported as geometric or arithmetic mean. The only country-level covariate considered was a location's mean temperature, used as a proxy for the likelihood of adequate building ventilation.

We did not have the microdata necessary to use ensemble modeling to inform our radon exposure distribution, so for GBD 2016 we continued to assume a lognormal distribution. Arithmetic mean exposure estimates obtained from ST-GPR were used to fit the lognormal distribution before applying relative risks.

Theoretical minimum-risk exposure level

The TMREL was also taken directly from literature values that were not updated for GBD 2016. Given that radon is naturally occurring, zero exposure would be impossible. As such, we continue to use a TMREL of 10 Bq/m^3, which is equivalent to the outdoor concentration of radon[3].

Relative Risks

The relative risk for radon exposure was extracted from literature values – a 2005 meta-analysis of case-control studies showing the association of radon with lung cancer[2]. This value was used in GBD 2010 and has not been changed since.

References

1. Steck DJ. Annual average indoor radon variations over two decades. Health Phys. 2009;96(1):37-47.
2. Darby S, Hill D, Auvinen A, et al. Radon in homes and risk of lung cancer: collaborative analysis of individual data from 13 European case-control studies. BMJ. 2005;330(7485):223.
3. Menzler S, Piller G, Gruson M, Rosario AS, Wichmann HE, Kreienbrock L. Population attributable fraction for lung cancer due to residential radon in Switzerland and Germany. Health Phys. 2008;95(2):179-89.

Lead Exposure Capstone Appendix

Flowchart

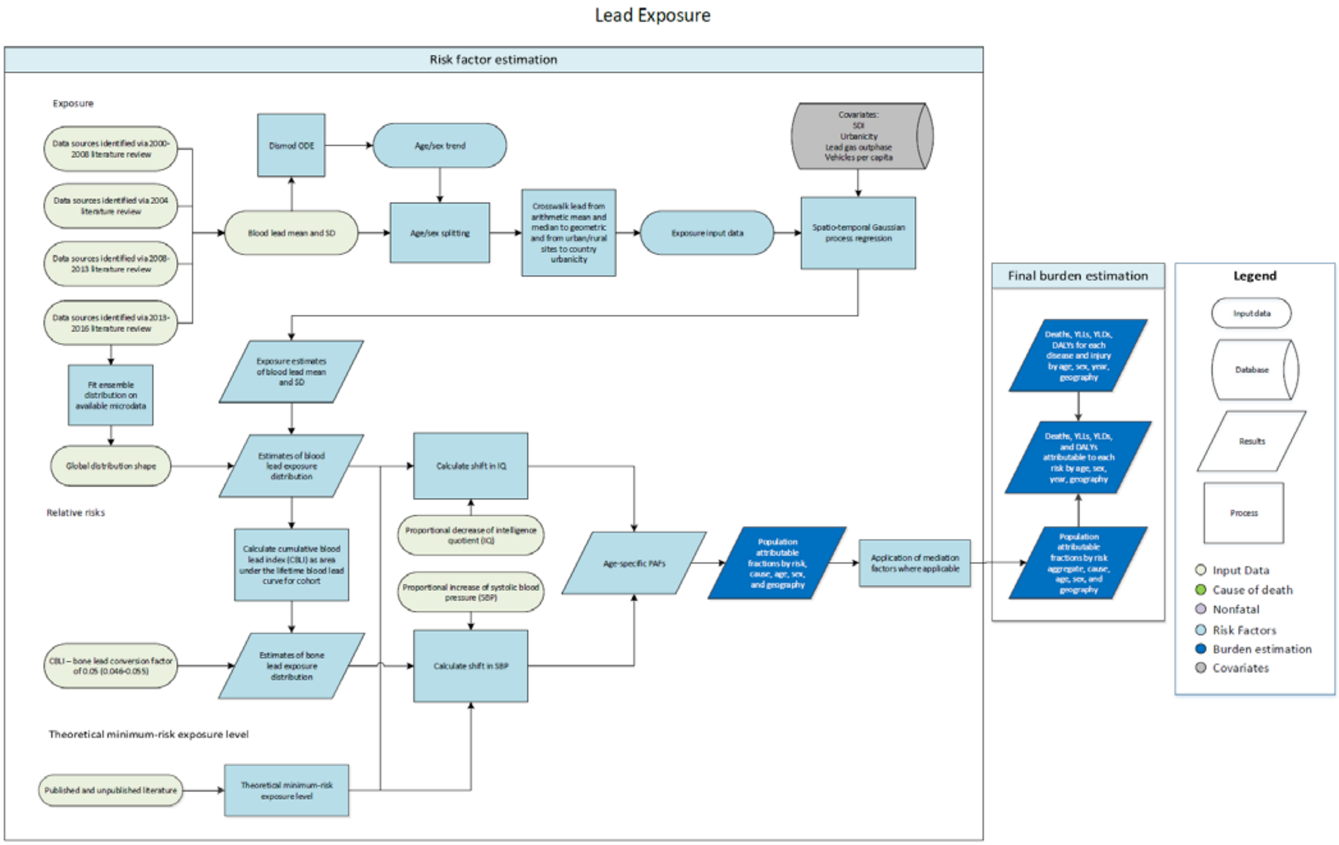

Input Data & Methodological Summary

Exposure

Case definition

Exposure to lead is defined in two different ways according to the currently known pathways of health loss. Acute lead exposure, relevant to disease burden through IQ loss in children, is measured as the micrograms of lead per deciliter of blood (µg/dL). Long-term lead exposure, relevant to disease burden in adults given the manifestation of health impact through increased systolic blood pressure and hence a decline of cardiovascular health, is measured as the accumulation of lead in the bone as micrograms of lead per gram of bone (µg/g).

Input data

The input data for lead exposure is primarily extracted from literature regarding blood lead, in addition to a few blood lead surveys. Blood lead values are derived from studies that take blood samples and analyze them using various techniques to determine the level of lead present. The blood lead database for GBD 2016 was augmented with an updated literature review for the years 2013-2016. In total, this approach yielded 3,151 usable data points from 563 different studies, which spanned the years 1970 to

2016. Nearly 2,000 new data points were added for GBD 2016, including 311 country-years and microdata from 11 blood lead surveys. The database of literature values was modelled for data-sparse countries using spatio-temporal GPR (ST-GPR). These values were used as blood lead exposure. The second pathway of burden is related to bone lead exposure, which was estimated by calculating a cumulative blood lead index for cohorts using estimated blood lead over their lifetime. The cumulative blood lead index is then used to estimate bone lead using a scalar defined in literature[1].

Modeling Strategy

The methodology to estimate lead exposure last underwent significant change in GBD 2013. Global exposure had been previously modelled using age-integrating Bayesian hierarchal modelling (DisMod-MR). The modelling process was updated for GBD 2013 by shifting to spatial-temporal GPR methodology. This allowed for estimates of all country-age-sex-year groups for single years instead of five year periods. This approach improved the granularity of estimates for bone lead, which requires back-estimation of previous blood lead to calculate a cumulative blood lead index.

For GBD 2016, the spatial-temporal GPR modelling methodology was updated as detailed in the appendix specific to this analytical technique, which is common to a variety of risk factors. In order to predict blood lead in country-years with insufficient data, covariates that have been produced across time and space relevant to this analysis were used. For blood lead exposure, the covariates determined to have predictive ability were the socio-demographic index (SDI), the proportion of a location's population living in urban settings (logit transformed), the combined number of 2 and 4-wheel vehicles per capita, and a covariate indicating whether leaded gasoline had been phased out in a given country-year (smoothed over the first 5 years of phase-out to reflect its gradual implementation). ST-GPR was used to produce estimates of mean and standard deviation of blood lead for all age groups, for both sexes, and for all GBD locations from 1970 to 2016.

In previous iterations of GBD, the distribution of lead exposure was assumed to be log-normal. For GBD 2016, ensemble modeling techniques were used to find an optimal global distribution by fitting a variety of distributions to the available blood lead microdata. This was a common update for all GBD 2016 continuous risk factors. The ST-GPR estimates of mean and standard deviation blood lead were used with the global distribution shape to determine distributions for blood lead exposure.

To calculate blood lead over the lifetime of a given cohort, blood lead was assumed to grow linearly from 2.0 ug/dL in 1920 (see TMREL) to the value for that cohort in 1970. Using the exposure distributions of blood lead over time and space, cohorts were constructed such that lifetime blood lead could be expressed as a curve over each year of life. The area under this curve was the cumulative blood lead index, which could be used to estimate bone lead in a given year with the aforementioned scalar.

Theoretical minimum-risk exposure level

In previous iterations of GBD, the TMREL was taken from literature estimates of pre-industrial blood lead in humans[4]. That value was estimated at 2.0 ug/dL. The decision was made that the TMREL of blood lead could not be 0 given the ambient sources of lead that would be impossible to eliminate[5].

However, average blood lead exposures in a number of countries have fallen below 2.0 ug/dL in the past few years, suggesting that the TMREL ought to be lowered. Unfortunately, we were not able to find literature with statistically significant estimates for relative risk at such low levels of blood lead exposure. As a result, we have continued to use a TMREL of 2.0 ug/dL for GBD 2016.

Relative Risks

Because the relative risk of IQ loss from lead exposure is specific to children, in GBD 2015, no burden of lead via IQ loss was estimated in the population aged 15 and above. To better account for the continued burden of past lead exposure on IQ in older age groups, for GBD 2016, cohorts were constructed from the entire population. Estimates of a cohort's lead exposure in early childhood (at 24 months of age) were used to determine past IQ loss, and thus calculate burden via the impact on concurrent IQ in the older population.

Blood lead relative risks were previously taken from a 2005 pooled analysis that was first incorporated in GBD 2010[2]. For GBD 2016, blood lead relative risks have been updated with a 2013 re-analysis of the findings of that 2005 paper, providing slightly adjusted relative risk estimates specific to exposure at 24 months of age[6]. The bone lead relative risks were taken from a 2008 meta-analysis that was updated for GBD 2010[3].

References

1. Hu H, Shih R, Rothenberg S, Schwartz BS. The epidemiology of lead toxicity in adults: measuring dose and consideration of other methodologic issues. Environ Health Perspect. 2007;115(3):455-62.
2. Lanphear BP, Hornung R, Khoury J, et al. Low-level environmental lead exposure and children's intellectual function: an international pooled analysis. Environ Health Perspect. 2005;113(7):894-9.
3. Navas-acien A, Schwartz BS, Rothenberg SJ, Hu H, Silbergeld EK, Guallar E. Bone lead levels and blood pressure endpoints: a meta-analysis. Epidemiology. 2008;19(3):496-504.
4. Flegal AR, Smith DR. Lead levels in preindustrial humans. N Engl J Med. 1992;326(19):1293-4.
5. Pruss-Astun A, Fewtrell L, Landrigan PJ, Ayuso-Mateos JL. Lead Exposure. In: Ezzati M, Lopez AD, Rodgers A, Murray CJ, eds. Comparative quantifications of health risks: Global and regional burden of disease attrituable to selected major risk factors. Geneva, World Health Organization, 2004: 1496-542
6. Crump K, Van Landingham C, Bowers T, Cahoy D, Chandalia J. A statistical reevaluation of the data used in the Lanphear et al. () pooled-analysis that related low levels of blood lead to intellectual deficits in children. Critical Reviews in Toxicology. 2013;43(9):785-799.

Occupational Risk Factors

Input Data and Methodological Summary

Exposure

Definition

The following definitions were used for occupational risk factor exposures. All exposures were estimated only for ages 15+

Occupational Asbestos	Cumulative exposure to occupational asbestos using mesothelioma death rate as an analogue.
Occupational Asthmagens	Proportion of working population exposed to asthmagens based on distribution of the population in nine occupational groups
Occupational Carcinogens (arsenic, acid, benzene, beryllium, cadmium, chromium, diesel, formaldehyde, nickel, polycyclic aromatic hydrocarbons, second-hand smoke, silica, trichloroethylene)	Proportion of working population ever exposed to carcinogens in high or low exposures groups, based on distribution of the population in seventeen economic activity groups
Occupational Injuries	Proportion of fatal injuries attributed to occupational work in seventeen economic activities, based on fatal injury rates in those economic activities.
Occupational Ergonomic Factors	Proportion of working population exposed to lower back pain, based on distribution of the population in nine occupational groups.
Occupational Noise	Proportion of working population exposed to 85+ decibels of noise, based on distribution in seventeen economic activities.
Occupational Particulates	Proportion of working population exposed based on distribution in seventeen economic activities

Estimates of the proportion of population involved in economic activities and occupations were coded into the following categories:

Economic Activities	Occupations
Agriculture, hunting, forestry	Legislators, senior officials, and managers
Fishing	Professionals
Mining and Quarrying	Technicians and associate professionals
Manufacturing	Clerks
Electricity, gas, and water	Service workers and shop/market sales workers
Construction	Skilled agricultural and fishery workers
Wholesale and retail trade/repair	Plant and machine operators and assemblers
Hospitality	Craft and related workers

Transport, storage, and communication	Elementary occupations
Financial intermediation	
Real estate/renting	
Public administration/defense; compulsory social security	
Education	
Health and social work	
Other community/social/personal service activities	
Private households	
Extra-territorial organizations/bodies	

Input data

Primary inputs were obtained from the ILO [1-4], using raw data on economic activity proportions, occupation proportions, fatal injury rates, and employment to population ratio estimates. A systematic web review was conducted in order to collect the underlying microdata from the ILO's estimates to aid in re-extraction at lower levels of granularity. Where freely available, survey datasets were downloaded from the survey organizations in question. Other datasets were obtained through submission of requests to the agencies and through the GBD collaborator network. Microdata was tabulated in order to create survey weighted estimates of economic activity and occupation for the GBD geographies and years. Various classification systems were crosswalked to ISIC Rev.3 (for economic activities) and ISCO 1988 (for occupations). Subnational estimates for UK and China were added to the datasets for economic activities and occupations [5-6].

For occupational asbestos, primary inputs were obtained through GBD 2016 cause of death estimates and published studies. [7, 13-14]

Uncertainty for inputs where microdata was not available was generated by fitting a Loess curve to the data and determining the standard deviation of the data from the fitted curve.

Modeling strategies

A spatial-temporal Gaussian process regression was used to generate estimates for all year/locations for the primary inputs (see app section 2). Study level covariates included for the prior model were education years per capita, geological covariates (for mining models), proportion of population living with access to coastline (for fishing models), the IHME socio-demographic index (SDI), mean temperature/latitude (for agriculture models), and proportion of population in urban areas. Space-time parameters were chosen by maximizing out-of-sample cross-validation and minimizing RMSE. For economic activity and occupation proportions, estimates from ST-GPR were then re-scaled to sum to 1 across categories by dividing each estimate by the sum of all the estimates.

The following sections describe the modeling approaches for each occupational risk's prevalence exposure.

Occupational carcinogens, occupational noise, occupational particulates

Prevalence of exposure to these risks was determined using the following equation:

$$Prevalence\ of\ Exposure_{c,y,s,a,r,l} = \sum_{EA} Proportion_{EA,c,y} * EAP_{c,y,s,a} * Exposure\ rate_{EA,r,l,d}$$

where:

EAP = Economically active population	c = country	r = risk
EA = economic activity	d = duration	s = sex
a = age	l = level of exposure	y =year

Exposure rate was provided by expert group recommendations and literature [8-11] (see table 1). The CAREX database was used in order to quantify the association between exposure by industry/carcinogen to SDI across all the countries in the database. This effect was used to predict exposure in countries that were not included in CAREX. Duration was considered for occupational carcinogens through application of occupational turnover factors [12] and for occupational noise and particulates by calculating cumulative exposure as the average exposure over the lifetime (past 50 years) for each age/sex cohort.

Occupational ergonomic factors and asthmagens

Prevalence of exposure to these risks was determined using the following equation:

$$Prevalence\ of\ Exposure_{c,y,s,a,r} = \sum_{EA} Proportion_{OCC,c,y} * EAP_{c,y,s,a}$$

where:

EAP = Economically active population	c = country	r = risk
OCC = occupation	a = age	s = sex
		y = year

Occupational injuries

Occupational injury counts were estimated using the following equation:

$$Occupational\ fatal\ injuries_{c,y,a,s} = \sum_{EA} Injury\ rate_{EA,c,y,s} * Population_{c,y,a,s} * EAP_{c,y,s,a} * Proportion_{EA,c,y}$$

where:

EAP = Economically active population	c = country	y = year
EA = economic activity	a = age	s = sex

Occupational asbestos

Prevalence of exposure to asbestos was estimated using the asbestos impact ratio (AIR), which is equivalent to the excess deaths due to mesothelioma observed in a population divided by excess deaths due to mesothelioma in a population heavily exposed to asbestos. Formally, this is defined using the following equation:

$$AIR = \frac{Mort_{c,y,s} - N_{c,y,s}}{Mort^{*}_{c,ys,} - N_{c,y,s}}$$

where:

Mort = Mortality rate due to mesothelioma	c = country
Mort* = Mortality rate due to mesothelioma in population highly exposed to asbestos	y = year
N = Mortality rate due to mesothelioma in population not exposed to asbestos	s = sex

Mortality rate due to mesothelioma was estimated from GBD 2015 causes of death [7]. Mortality rate due to mesothelioma in population not exposed to asbestos was calculated using the model in Lin et al. [13], while the mortality rate due to high exposure to asbestos was estimated in Goodman et al. [14] Asbestos exposure prevalence created using the AIR was used to estimate PAFs for all associated causes except for mesothelioma. Custom PAFs were calculated for mesothelioma by using the ratio of excess mortality compared to the unexposed population (Mort – N) to the mortality rate in the population in question (Mort). This calculation assumes that all mesothelioma is a product of occupational asbestos exposure and could potentially over-estimate burden due to occupational asbestos exposure in populations with high non-occupational asbestos exposure.

Theoretical minimum-risk exposure level

For all occupational risks, with the exception of occupational asbestos, the theoretical minimum-risk exposure level was assumed to be no exposure to that risk.

Relative risk

Relative risks were obtained for all occupational risks by conducting a systematic review of published meta-analysis. The estimates used, as well as the associated studies, are reported by category group in appendix table 1.

PAF

For all occupational risks, with the exception of injuries (outlined below) and mesothelioma (outlined above), PAFs were calculated using the prevalences estimated above, using the PAF formula in appendix section 2.

Occupational injuries PAF

The PAF for occupational injuries was calculated using the following formula:

$$PAF_{c,y,a,s} = \frac{Occupational\ fatal\ injuries_{c,y,a,s} - TMREL}{Fatal\ injuries_{c,y,a,s}}$$

where:

c = country
y = year
a = age
s = sex

Fatal injuries total was obtained from GBD 2016 causes of death [7].

Citations

[1] International Labour Organization (ILO). International Labour Organization Database (ILOSTAT) - Employment by Sex and Economic Activity. International Labour Organization (ILO).

[2] International Labour Organization (ILO). International Labour Organization Database (ILOSTAT) - Employment by Sex and Occupation. International Labour Organization (ILO).

[3] International Labour Organization (ILO). International Labour Organization Database (ILOSTAT) - Fatal Injuries by Sex and Economic Activity. International Labour Organization (ILO).

[4] International Labour Organization (ILO). International Labour Organization LABORSTA Economically Active Population, Estimates and Projections, October 2011. International Labour Organization (ILO), 2011.

[5] Office for National Statistics (United Kingdom). Nomis Official Labor Market Statistics - Annual Population Survey. Newport, United Kingdom: Office for National Statistics (United Kingdom).

[6] National Bureau of Statistics of China. China 1% National Population Sample Survey 1995. Ann Arbor, United States: China Data Center, University of Michigan.

[7] GBD 2015 Mortality and Causes of Death Collaborators. Global, regional, and national life expectancy, all-cause and cause-specific mortality for 249 causes of death, 1980–2015: a systematic analysis for the Global Burden of Disease Study 2015. *Lancet Rev*.

[8] Wilson DH, Walsh PG, Sanchez L, *et al.* The epidemiology of hearing impairment in an Australian adult population. *Int J Epidemiol* 1999; 28: 247–52

[9] Kauppinen T, Toikkanen J, Pederson D, Young R, Kogevinas M, Ahrens W, et al. Occupational Exposure to Carcinogens in the European Union in 1990-93. Helsinki, Finland: Finnish Institute of Occupational Health; 1998.

[10] Kauppinen T, Toikkanen J, Pedersen D, Young R, Ahrens W, Boffetta P, et al. Occupational exposure to carcinogens in the European Union. *Occup Environ Med* 2000; 57(1): 10–18.

[11] Driscoll T, et al. The global burden of non-malignant respiratory disease due to occupational airborne exposures. *American Journal of Industrial Medicine 2005; 48*(6): 432-445.

[12] Nelson, D. I., Concha-Barrientos, M., Driscoll, T., Steenland, K., Fingerhut, M., Punnett, L. & Corvalan, C. (2005). The global burden of selected occupational diseases and injury risks: Methodology and summary. *American journal of industrial medicine*, *48*(6), 400-418

[13] Lin R-T, Takahashi K, Karjalainen A, *et al.* Ecological association between asbestos-related diseases and historical asbestos consumption: an international analysis. *Lancet* 2007; **369**: 844–9.

[14] Goodman M, Morgan RW, Ray R, Malloy CD, Zhao K. Cancer in asbestos-exposed occupational cohorts: a meta-analysis. *Cancer Causes Control* 1999; **10**: 453–65.

Suboptimal Breastfeeding Capstone Appendix

Flowchart

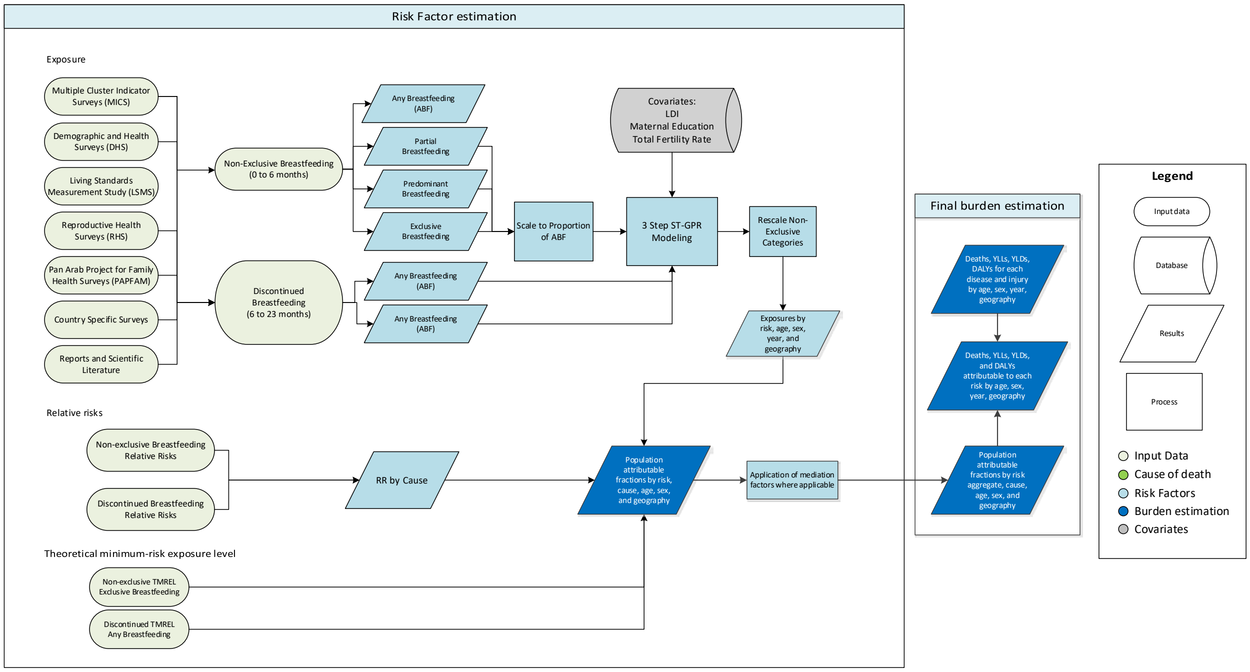

Input Data & Methodological Summary

Exposure

Definition

Exposure to suboptimal breastfeeding is composed of 2 distinct categories: nonexclusive breastfeeding and discontinued breastfeeding. Non-exclusive breastfeeding is defined as the proportion of children under 6 months who are not exclusively breastfed. Those not exclusively breastfed are then parsed into 3 categories – predominate, partial, and no breastfeeding. Discontinued breastfeeding is defined as the proportion of children between 6 to 23 months who receive no breast milk.

Input data

The data used in this analysis consists mostly of processed micro data from surveys and tabulated data from scientific literature and reports. The data was primarily sourced from the micro data of surveys. The data updates were focused on the extraction of the larger surveys at the subnational level, especially for those subnational locations added into GBD 2016. Tabulated data was only used when micro data was not available.

Modeling

A complete time series from 1980 to 2016 for the prevalence of breastfeeding patterns for children 0 to 6 months and 6 to 23 months were generated. This was accomplished by carrying the processed micro and tabulated data through a three-step modeling process. First, a robust linear regression incorporated the covariates of log-transformed lag-distributed income, total fertility rate, and the mean years of education of women of reproductive age. This was followed by a spatial-temporal regression that used the residuals of the predictions from the linear regression to perform a locally-weighted regression that

provided a greater weighting factor to those nearer in space and time. The predicted residuals from this step are added to those created in the linear regression. The final of the three steps was the Gaussian Process Regression. This step incorporated the variance of the input data as well as that of the model predictions. It used predictions from the spatial-temporal regression as the mean function and generated draws from a multinomial distribution, based on the data uncertainty in the prior, to generate the final prevalence estimates and their confidence intervals. One major change to our modeling process for this round was we now estimate exposure to suboptimal breastfeeding for high-income countries, whereas, exposure was assumed to be zero in GBD 2015.

Theoretical minimum-risk exposure level

For non-exclusive breastfeeding, those children that received no source of nourishment other than breastmilk were considered to be at the lowest risk of any of the disease outcomes. For discontinued breastfeeding, we assumed that children aged 6 to 23 months who received any breastmilk as a source of nourishment to be at the lowest risk of disease outcome.

Relative risks

Relative risks used for suboptimal breastfeeding were generated based on published review by the World Health Organization (Horta et al., 2013). New relative risks, for both non-exclusive and discontinued breastfeeding, were generated from the studies compiled by this review. Non-exclusive breastfeeding exposure was paired with diarrhea and LRI as disease outcomes. Discontinued breastfeeding was paired with diarrhea only. No new outcomes were added in GBD 2016.

We have also applied a novel adjustment to the existing relative risks in order to make them representative to their larger GBD age groups (post neo-natal in the case of nonexclusive breastfeeding and 1 to 4 years in the case of discontinued breastfeeding).

References

1. Horta, B., Voctora, C. (2013) Short-term effects of breastfeeding: a systematic review on the benefits of breastfeeding on diarrhoea and pneumonia mortality. The World Health Organization.

Child Growth Failure Capstone Appendix

Flowchart

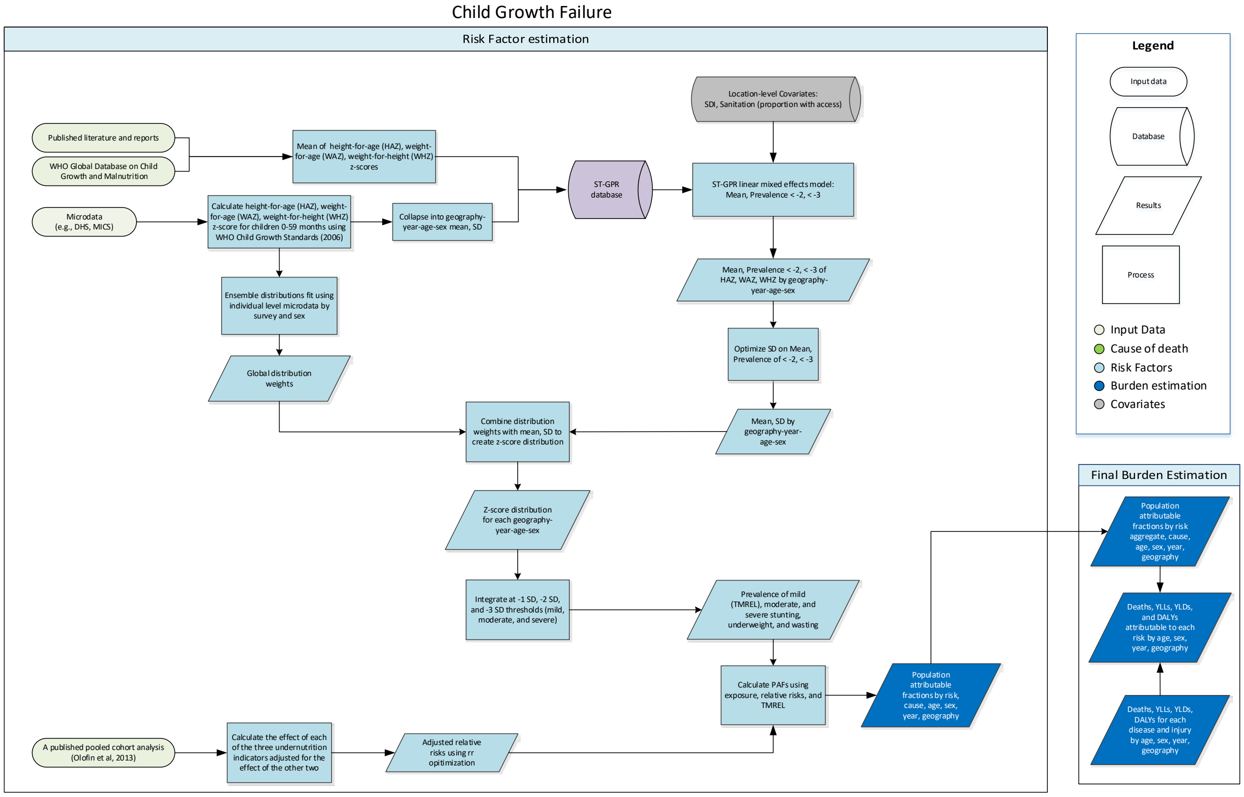

Input Data & Methodological Summary

Exposure

Case Definition

Child growth failure is estimated using three indicators, stunting, wasting, and underweight, all of which all of which are based on categorical definitions using the WHO 2006 growth standards for children 0-59 months.[1] Definitions are based on Z scores from the growth standards, which were derived from an international reference population. Mild, moderate, and severe categorical prevalences were estimated for each of the three indicators.

Input data

There are two main inputs for the GBD 2016 child growth failure models: microdata from population surveys and tabulated data from reports, literature, and the WHO Global Database on Child Growth and

[1] https://www.unicef.org/infobycountry/stats_popup2.html

Malnutrition.[2] Population surveys include a variety of multi-country and country-specific survey series such as Multiple Indicator Cluster Surveys (MICS), Demographic and Health Surveys (DHS), Living Standards Measurement Surveys (LSMS), and the China Health and Nutrition Survey (CHNS), as well as other one time country specific surveys such as the Indonesia Family Life Survey and the Brazil National Demographic and Health Survey of Children and Women. These microdata contain information about each individual child's age (from which age in weeks and age in months are calculated), as well as height and/or weight. From that information, a height-for-age z-score (HAZ), weight-for-age z-score (WAZ), and weight-for-height z-score (WHZ) are calculated using the WHO 2006 Child Growth Standards and the LMS method.[3,4]

The second source of data was tabulated data from survey reports, published literature, and the WHO Global Database on Child Growth and Malnutrition that contained the mean z-score and SD for stunting, wasting, and underweight (HAZ, WAZ, WHZ). Any data that was reported using the NCHS 1978 growth standards was given 10% weight in the regression; in future iterations of GBD, we will crosswalk this data to the WHO 2006 Child Growth Standards. All data used in this analysis is catalogued in the Global Health Data Exchange (http://ghdx.healthdata.org). A representative dataset coverage map for moderate stunting is shown below.

Figure 1: Number of data points in moderate stunting (<-2 HAZ) in males, 1990 to 2016

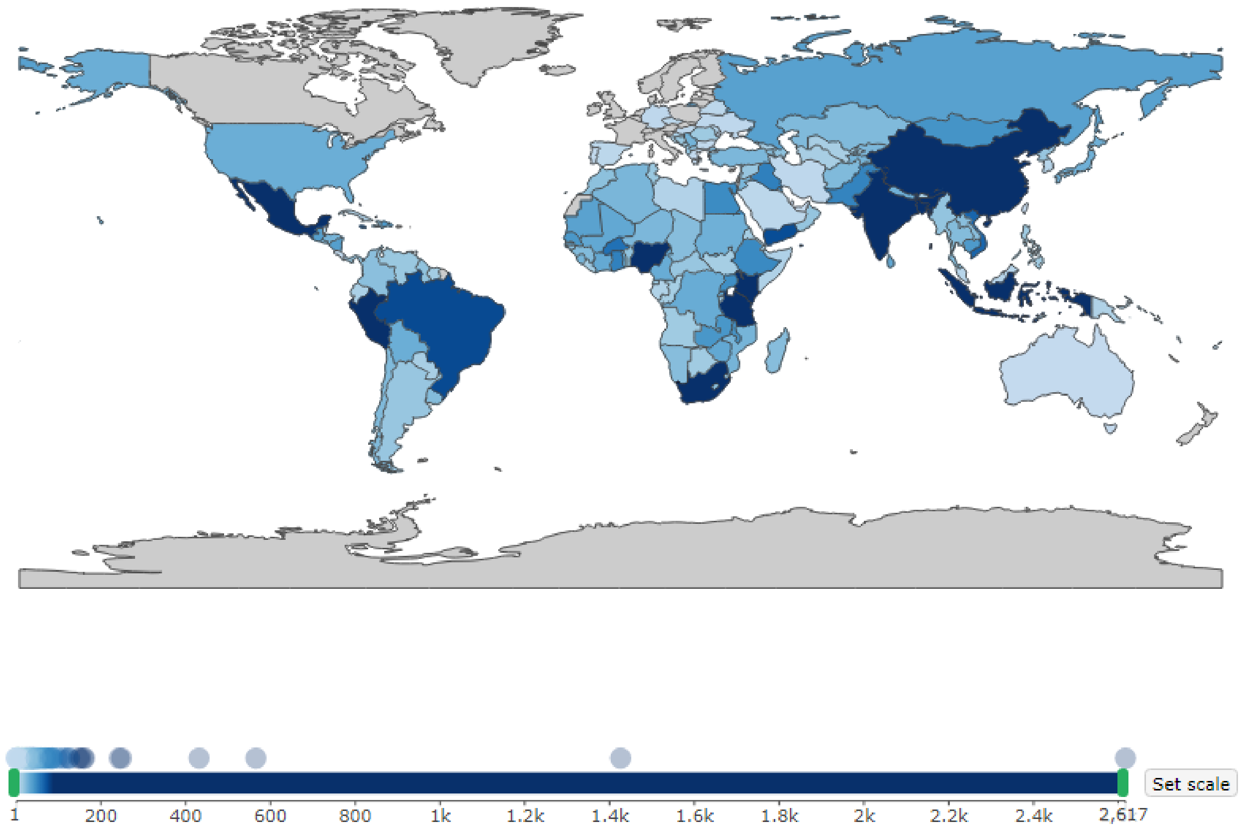

[2] http://www.who.int/nutgrowthdb/en/
[3] http://www.who.int/childgrowth/standards/en/
[4] http://webnt.calhoun.edu/distance/internet/Business/eco231/downloads/9781441917874-c1.pdf

Modeling strategy

Exposure Estimation

The following three-step modeling process was applied to each of stunting, wasting, and underweight.

First, all microdata was fit using an ensemble modeling process, a modeling framework developed for GBD 2016 that is described elsewhere in this appendix. A series of 12 individual distributions (normal, log normal, log logistic, exponential, gamma, mirror gamma, inverse gamma, gumbel, mirror gumbel, Weibull, inverse Weibull, and beta) were fit to the entire set of microdata (approximately 2.5 million individual z-scores) at the individual survey level. A weighting algorithm combined each distribution to find the optimal combination of these distributions for each survey, minimizing the absolute prediction error across the entire distribution. Ensemble weights for each survey were then averaged across all surveys to produce a single set of global weights of the ensemble distributions. Weights were different for each sex, but invariant across geography, time, and age group. All component distributions that were used to derive weights were parameterized using "method of moments," meaning that each corresponding probability density function (PDF) could be described as a function of the mean and variance of the quantity of interest.

Second, models were developed for mean Z scores and prevalence of moderate and severe growth failure. Individual level microdata were collapsed to calculate three metrics: mean z-score, moderate prevalence, and severe prevalence. These data were combined with that derived from literature, GHDx review, and the WHO Global Database on Child Growth and Malnutrition. For those sources where moderate prevalence was reported without a corresponding mean, we calculated a predicted mean using an ordinary-least square (OLS) regression from those sources where both metrics were present. Each of the three metrics was then modeled using spatiotemporal Gaussian process regression (ST-GPR), a common modeling framework used across GBD 2016 analyses, generating estimates for each age-group, sex, year, and location.

Third, we combined estimates of mean, prevalence (moderate and severe) with ensemble weights in an optimization framework in order to derive the variance that would best correspond to the predicted mean and prevalence. This variance was then paired with the mean and, using the method of moments equation for each of the component distributions of the ensemble, PDF of the distribution of Z-scores were calculated for each location, year, age-group, and sex. PDFs were integrated to determine the prevalence between -1 and -2 Z scores (mild), between -2 and -3 Z scores (moderate), and below -3 Z scores (severe). These were categorical exposures used for subsequent attributable risk analysis.

Differences from GBD 2015

There are several important differences from the GBD 2015 analysis. First, our systematic data searching efforts led to an approximately 30% increase in the number of data sources, including a significant increase in data sources for Oceania, Latin America, and South Asia. Most notable was the increase in data for India through our collaboration with the India Council for Medical Research (ICMR) and Public Health Foundation of India (PHFI). Second, while GBD 2015 also used ST-GPR to model growth failure, models were completed for a single 0-5 age group, followed by application of a pooled uniform age-sex split which resulted in the implicit assumption that the age pattern of growth failure is invariant over time and geography. GBD 2016 estimates, owing to smaller sample sizes in younger age groups, do have wider uncertainty in those age groups. Third, GBD 2015, like all analyses of growth failure before it, assumed

that high-income countries had zero prevalence of child growth failure. We have suspended this assumption for GBD 2016 as it is not accurate and instead made explicit estimates of growth failure in all locations. Fourth, GBD 2015 did not use an ensemble approach or estimate the entire distribution of Z scores. Fifth, we have changed the name of this risk factor category changed from childhood undernutrition to child growth failure to more explicitly identify the specific aspects of childhood undernutrition that are covered by the three component indicators.

Theoretical minimum-risk exposure level

Theoretical minimum risk exposure level (TMREL) for underweight, stunting, and wasting was assigned to be greater than or equal to -1 SD of the WHO 2006 standard weight-for-age, height-for-age, and weight-for-height curves respectively. This was unchanged from GBD 2015.

Relative risks

Relative risks (RRs) were derived from a pooled cohort analysis (source and risk-outcome pairs below), which remained the same as GBD 2013 & GBD 2015.[5] The final list of outcomes paired with child growth failure risks included lower respiratory infections (LRI), diarrhea, measles, and protein energy malnutrition (PEM). The RRs were adjusted using an optimization algorithm developed at IHME for GBD 2013 that takes into account covariance between the three child growth failure indicators.

Of historical note, URI and otitis media were included as outcomes in the GBD 2013 risk analysis, based on the "analogy" causal criterion, assuming there is similar pathway as LRI outcome. However, closer review for GBD 2015 did not find sufficient evidence to support their inclusion and they were excluded, a decision that was carried forward into GBD 2016. We also attributed 100% of PEM to childhood wasting and underweight but not stunting. To build on the existing literature base for GBD on risk-outcome pairs, a literature search was conducted for GBD 2016 searching for case-control studies published after January 1st, 1985; this search did not return any sources that were appropriate for this work.

Risk factor	Outcome
Child underweight	Diarrhoeal diseases
Child underweight	Lower respiratory infections
Child underweight	Measles
Child stunting	Diarrhoeal diseases
Child stunting	Lower respiratory infections
Child stunting	Measles
Child wasting	Diarrhoeal diseases
Child wasting	Lower respiratory infections
Child wasting	Measles

[5] Olofin I, McDonald CM, Ezzati M, et al. Associations of suboptimal growth with all-cause and cause-specific mortality in children under five years: a pooled analysis of ten prospective studies. PLoS ONE 2013; 8: e64636

Low Birth Weight and Short Gestation

Flowchart

Low birth weight and Short gestation Risk Factors

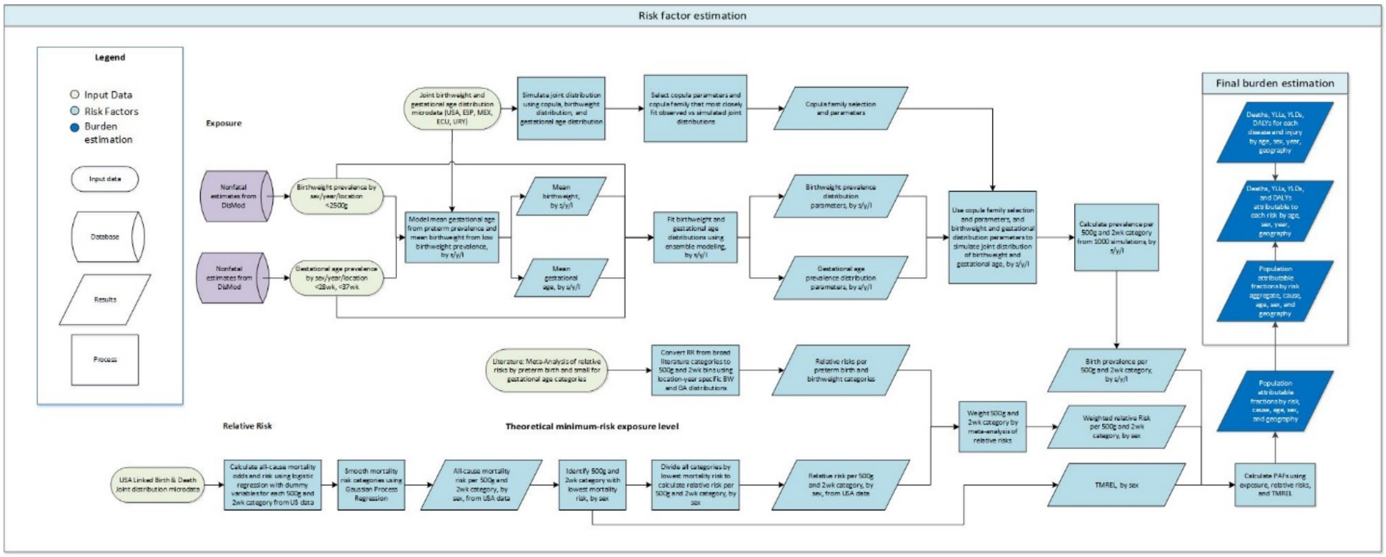

Input Data and Methodological Summary

The "Low Birth Weight and Short Gestation" risk factor and its child risks "Low Birth Weight for Gestation" and "Short Gestation for Birth Weight" are new risk factors for GBD 2016.

Although Low Birth Weight for Gestation and Short Gestation for Birth Weight are separate risk factors, the exposures and relative risks for both are estimated jointly through the Low Birth Weight and Short Gestation parent risk factor. Joint estimation of risk factors is a new approach in GBD 2016 and is thought to yield more accurate estimates of the PAF attributable to low birth weight and short gestation.

Case Definition

The term "low birth weight" is commonly used to refer to birth weight less than 2500 grams. Likewise, newborns are typically classified into gestational age categories of "extremely preterm" (<28 weeks of gestation), "very preterm" (28-<32 weeks of gestation), and "moderate to late preterm" (32-<37 weeks of gestation).

However, the use of "low birth weight" in these risk factors refers to birth weight below the Theoretical Minimum Risk Exposure Level (TMREL) for birth weight ([4000, 4500) grams). "Short gestation" refers to gestational age below the gestational age TMREL ([40, 42) weeks). Exposures and relative risks for the GBD Low birth weight and short gestation risk factors are divided into joint 500-gram birth weight and 2-week gestational age combinations.

Under this framework, the eight 500-gram birth weight categories less than the birth weight category associated with the Theoretical Minimum Risk Exposure Level ([4000-4500) grams – see below for

TMREL methods) refer to "low birth weight". "Short gestation" refers to the ten 2-wk gestational age categories less than the gestational age associated with the Theoretical Minimum Risk Exposure level ([40-42) weeks – see below for TMREL methods). Each combination of 500-grams and 2-wks is associated with a relative risk for mortality by neonatal period (early and late neonatal) and by the causes listed in Table 1, and relative to the joint TMREL [4000-4500) grams and [40-42) weeks.

Table 1: Cause list for low birth weight and short gestation

Cause ID	Cause name
302	Diarrheal diseases
322	Lower respiratory infections
328	Upper respiratory infections
329	Otitis media
333	Pneumococcal meningitis
334	H influenzae type B meningitis
335	Meningococcal meningitis
336	Other meningitis
337	Encephalitis
381	Neonatal preterm birth complications
382	Neonatal encephalopathy due to birth asphyxia and trauma
383	Neonatal sepsis and other neonatal infections
384	Hemolytic disease and other neonatal jaundice
385	Other neonatal disorders
686	Sudden infant death syndrome

Exposure

Input data

To model the joint distribution of exposure of low birth weight and short gestation for each location, year, and sex estimated in GBD 2016, three types of information are used:

- Distribution of gestational age for each location, year, and sex
- Distribution of birth weight for each location, year, and sex
- Copula family and parameters, specifying correlation between gestational age and birth weight distributions

Modeling strategy

<u>Distributions of Birth weight & Gestational Age</u>

To model the joint distribution of birth weight and gestational age for every sex/location/year, ensemble model methods standard to GBD risk factors (described elsewhere in the methods appendix), are first used to create separate distributions of birth weight and gestational age for every sex/location/year.

Microdata is the most ideal data source for modeling distributions; however, microdata is not widely available for birth weight and is even more scarce for gestational age. Much more readily available, and from a wider range of locations and years, is categorical prevalence data for low birth weight (<2500g), extremely preterm (<28 weeks of gestation), very preterm (28-32 weeks of gestation), moderate to late preterm (32-37 weeks of gestation), and preterm birth (<37 weeks of gestation).

Since GBD 2010, this categorical data has been used model birth prevalence of preterm birth by gestational age (<28 wks, 28-<32 wks, and 32-<37 wks) and low birth weight (<2500g) for every location, sex, and year estimated in GBD.

We use the ensemble model methods, with the categorical estimates of preterm birth and low birth weight, which are available for every sex/location/year, as inputs, to estimate gestational age and birth weight distributions for every sex/location/year. Mean birth weight and mean gestational age for every sex/location/year are also estimated from the categorical prevalence estimates, and also serve as inputs into the ensemble modeling methods.

Copula Optimization

Distributions of gestational age and birth weight are not independent; initial exploration of available joint microdata of births with birth weight and gestational age from five countries (the United States, Mexico, Spain, Uruguay, Ecuador) showed that the Spearman correlation for each country of data, pooling across all years of data available, ranged from 0.340-0.489 (Table 2). Because of the correlation between birth weight and gestational age, in order to model the joint distribution of gestational age and birth weight from the separate distributions, information is first needed about the correlation between the two distributions.

Table 2: Spearman Correlation and sample size of countries' microdata with joint birth weight and gestational age, combined sex

Country (years of data)	Spearman Correlation between birth weight & gestational age	Total births (all years)
USA (1990-2014)	0.400	81,929,879
URY (1996-2014)	0.489	698,622
ESP (1980-2014)	0.360	10,991,153
MEX (2008-2012)	0.354	10,253,571
ECU (2003-2015)	0.340	2,473,039

Copula modeling is used to model joint distributions between the birth weight and gestational age marginal distributions. The Copula and VineCopula packages in R were used to select the optimal copula family and copula parameters to model the joint distribution, using joint microdata from the country-years in Table 2. The copula family selected from the microdata was “Survival BB8”.

Using the Copula and VineCopula packages in R, the joint distribution could then be estimated from the

available information: uniform copula family and parameters for all sexes/location/years and separate distributions of gestational age and birth weight provided by the ensemble models for every sex/location/year. Each joint distribution was divided into 500g by 2wk bins to match the categorical bins of the relative risk surface.

Relative Risks & Theoretical minimum-risk exposure level

Input data

Data Source: National Center for Health Statistics, Linked Birth/Death Cohort Data (1996-2010)

In the US Linked Birth/Death Cohort datasets, live births are reported with gestational age, birth weight, and an indicator of death at 7 days and 28 days. For this analysis, gestational age was grouped into 2-week categories, and birth weight was grouped into 500-gram categories.

Modeling strategy

Using pooled US Linked Birth/Death Cohort Data from 1996-2010, the risk of all-cause mortality at the early neonatal period and late neonatal period at joint birth weight and gestational age combinations was calculated. Figures 1-4 display male and female relative risk surfaces in log space of all-cause mortality in the early neonatal period (0-6 days) and late neonatal period (7-27 days) at combinations of birth weight and gestational age. The relative risk of each joint gestational age and birth weight category are relative to the risk of mortality in the early neonatal period and late neonatal period at the joint gestational age and birth weight category with the lowest mortality risk, which was identified as [40-42) weeks and [4000-4500) grams.

To calculate relative risk at each 500g and 2wk combination, logistic regression was first used to calculate mortality odds for each joint 2-week gestational age and 500-gram birth weight category. A pooled country analysis[1] of mortality risk in the early neonatal period and late neonatal period by SGA category in developing countries in Asia, the Americas, and Sub-Saharan Africa was also used to weight the risk surfaces developed from the US Linked Birth/Death Cohort Data. The combined mortality odds for each gestational age and birth weight category were then smoothed with Gaussian Process Regression, with the independent distributions of mortality odds by birth weight and mortality odds by gestational age serving as priors in the regression.

The smoothed mortality odds were converted to mortality risk. Relative risks were calculated by dividing each the mortality risk in each joint gestational age and birth weight category by the risk at the TMREL. The relative risk surfaces, which were created using all-cause mortality, were then attributed to specific causes (Table 1) identified as closely linked to low birth weight and short gestation.

<u>Limitations</u>

[1] Katz et al. Mortality risk in preterm and small-for-gestational-age infants in low-income and middle-income countries: a pooled country analysis. 2013. Volume 382, Issue 9890. *The Lancet.*

A limitation of this approach is that the only linked birth and death data set available to create the relative risk surface is from the United States. Ideally, data from other locations would inform the relative risk surface. Additionally, relative risks were calculated from all-cause mortality, and then applied to specific causes. Cause-specific relative risks would be more ideal.

Figures

Early Neonatal

Late Neonatal

Figure 1: Female Log Relative Risk Surface

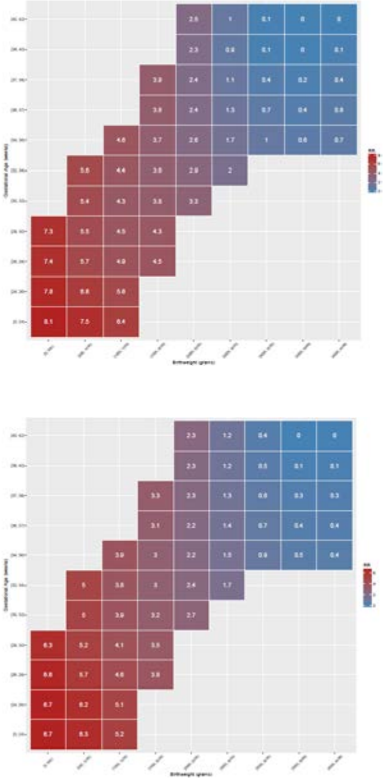

Figure 2: Male Log Relative Risk Surface

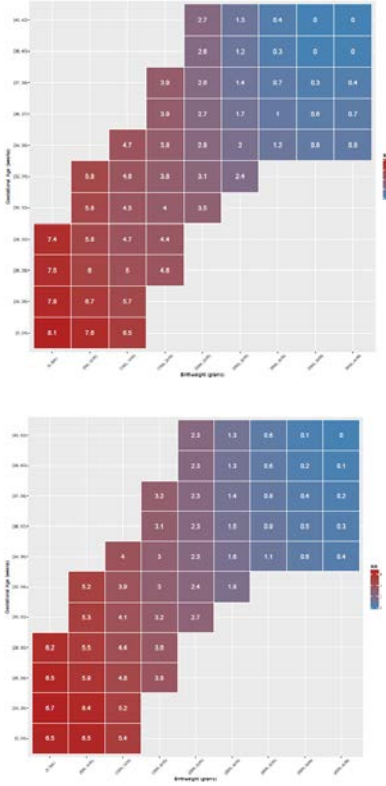

PAF Calculations

The total PAF for the Low Birth Weight and Short Gestation joint risk factor is calculated by summing the PAF calculated from each 500g x 2wk category, with the lowest risk category among all the 500g x 2wk categories serving as the TMREL. The equation for calculating PAF for each 500g x 2wk category is:

$$PAF_{joasgt} = \frac{\sum_{x=1}^{u} RR_{joast}(x)P_{jasgt}(x) - RR_{joasg}(TMRE_{jas})}{\sum_{x=1}^{u} RR_{joas}(x)P_{jasgt}(x)}$$

To calculate the overall PAF for the Short gestation for birth weight risk factor, PAF was once again calculated for each joint 500-gram and 2-week category. Unlike the joint PAF calculation, which used only one TMREL for all 500-gram and 2-week categories, the joint 500-gram and 2-week category with

the lowest risk for each 500-gram birth weight grouping served as the TMREL for that 500-gram birth weight grouping. For example, the [3000, 3500) gram birth weight grouping contains five joint categories: [34, 36) weeks and [3000, 3500) grams; [36, 37) weeks and [3000, 3500) grams; [37, 38) weeks and [3000, 3500) grams; [38, 40) weeks and [3000, 3500) grams; and [40, 42) weeks and [3000, 3500) grams. The [40, 42) weeks and [3000, 3500) grams joint category has the lowest risk, and so it serves as the TMREL for the [3000, 3500) gram birth weight grouping. In the Relative Risk surface figures, a birth weight grouping is one "column" of the birth weight and gestational age matrix.

The overall PAF for the Short gestation for birth weight risk factor was then calculated for all the joint 500-gram and 2-week categories using the formula below:

$$PAF_{1..i} = 1 - \prod_{i=1}^{n}(1 - PAF_i)$$

The same methodology was applied to calculate the total PAF for the Low birth weight for gestation risk factor, using 2-week gestational age categories (each "row" of the matrix) instead of 500-gram birth weight categories. For example, the [24, 26) weeks gestational age grouping contains three joint categories: [0, 500) grams and [24, 26) weeks; [500, 1000) grams and [24, 26) weeks; and [1000, 1500) grams and [24, 26) weeks. The [1000, 1500) grams and [24, 26) weeks joint category has the lowest risk, and so it serves as the TMREL for the [24, 26) weeks gestational age grouping.

After the short gestation for birth weight PAF and low birth weight for gestational age PAF were calculated, they were then scaled so that the sum of the short gestation for birth weight PAF and low birth weight for gestation PAF equal the low birth weight and short gestation parent PAF calculated for each location/year/sex/age group.

Iron Deficiency

Flowchart

Iron deficiency

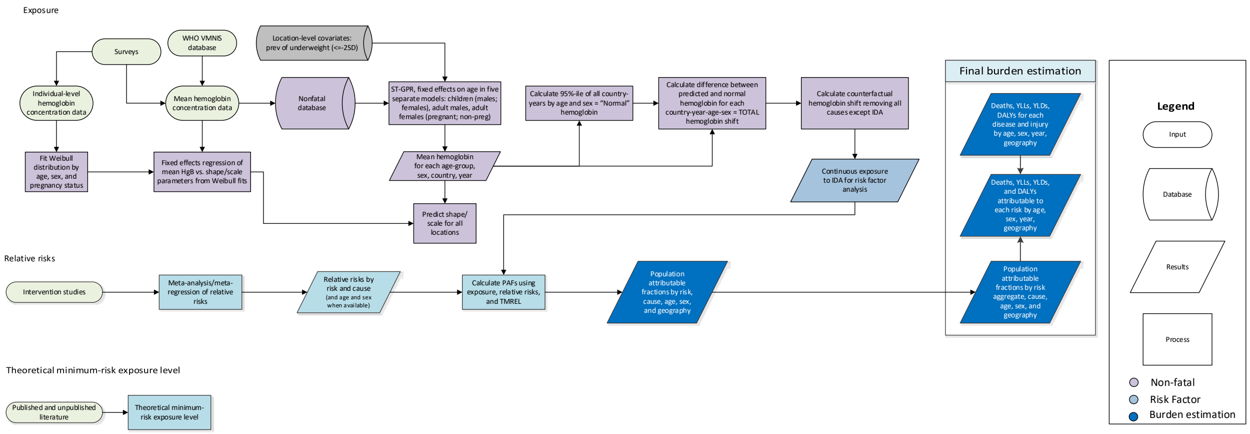

Input Data and Methodological Summary

Exposure

Case definition

For GBD 2016, as with GBD 2015, the anemia model has two main steps: estimation of the anemia envelope and causal attribution. Our analytic strategy began with calculation of an anemia envelope – a determination of mean hemoglobin, as well as a sum total of anemia prevalence, by severity for each country, age group, and both sexes for each year from 1990 through 2016. The envelope approach avoids double-counting while capturing potentially different disease profiles within each population group. We defined a population group as a specific geography, sex, age-group, and year.

Input data

Iron-deficiency anemia (IDA) estimates include acute and chronic hemorrhagic states for which supplementation may be helpful, but poor nutritional intake is not the only underlying problem. A few causes in this category – hookworm, schistosomiasis, upper gastrointestinal bleeding, and gynecologic diseases – were considered separately from IDA because there was enough data from GBD prevalence estimation processes to do so. Distribution of anemia burden to IDA only after assignment to "known" causes avoided double counting of these cases.

For our nonfatal anemia estimates, the envelope approach to the anemia impairment utilizes data from a variety of sources. Population-based surveys of hemoglobin concentration were the primary input to our analytic dataset. Examples include the Demographic and Health Survey (DHS) and Multiple Indicator Cluster Survey (MICS) series, along with other national and subnational surveys that completed hemoglobin testing. We supplemented with pertinent sources downloaded from the WHO Vitamin and Mineral Nutrition Information System (VMNIS) available at http://www.who.int/vmnis/database/anaemia/countries/en/. A full source list is available elsewhere in this appendix. Most used a HemoCue test, adjusted for altitude, and excluded those with terminal or

acute medical conditions. Inclusion, exclusion and diagnostic criteria for other studies were similar and can be found in each study.

Modeling strategy

For GBD 2016, we estimated the mean hemoglobin in g/dL among women aged 15 to 49 years of age and the implied mean hemoglobin among women in the absence of iron deficiency anemia, as the risk exposure for maternal iron deficiency anemia.

Theoretical minimum-risk exposure level

The population normal hemoglobin concentration is the theoretical minimum risk exposure level. This was used to calculate exposures for iron deficiency by subtracting the iron deficiency shift from the population normal hemoglobin concentration for each demographic. For example, if the normal hemoglobin concentration among 30-34 year old women in Ethiopia was 134.5 g/L, and the shift was 1.6 g/L in that demographic, then the exposure was 132.9 g/L. The GBD 2016 anemia modeling strategy provides details on how the iron deficiency shifts and population normal hemoglobin concentrations were calculated.

Relative risk

We attribute 100% of iron-deficiency anemia to iron deficiency. The other outcomes are maternal hemorrhage and maternal sepsis and other maternal infections. Sources of evidence for these relative risks are unchanged from GBD 2013.

References

1. Centers for Disease Control and Prevention (CDC). Iron deficiency--United States, 1999-2000. MMWR Morb Mortal Wkly Rep 2002; 51: 897–9.

2. Looker AC, Dallman PR, Carroll MD, Gunter EW, Johnson CL. Prevalence of iron deficiency in the united states. JAMA 1997; 277: 973–6.

Vitamin A Deficiency Capstone Appendix

Flowchart

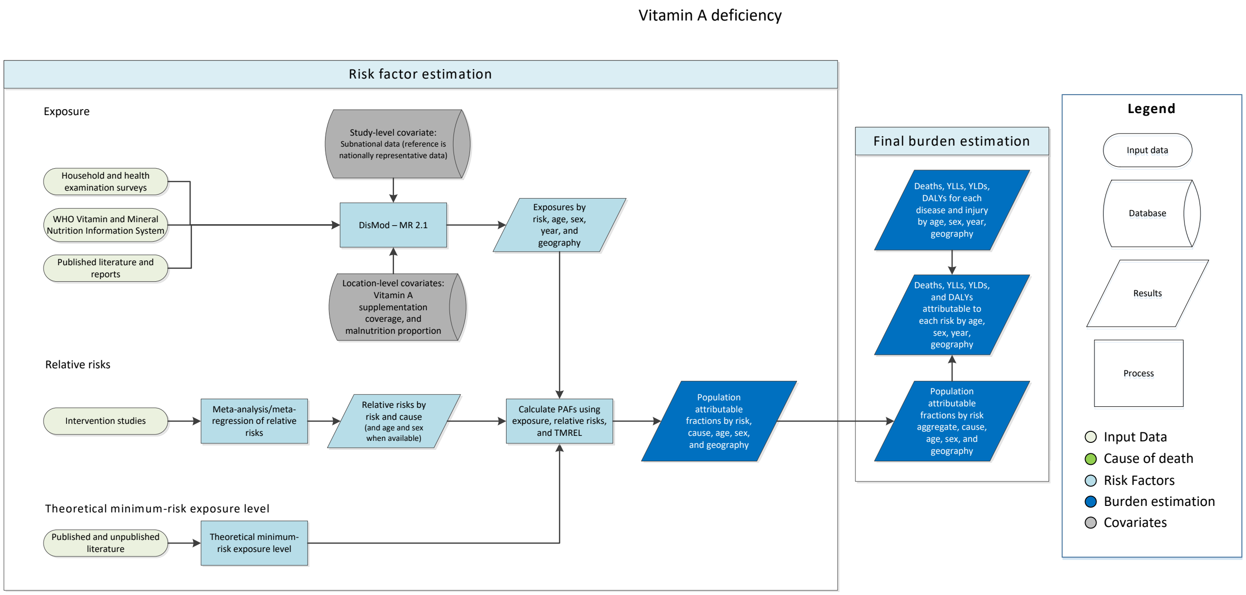

Input Data & Methodological Summary

Exposure

Definition

For GBD 2016, vitamin A deficiency is defined as serum retinol <70 µmol/L. We examined vitamin A deficiency as a risk factor in children aged 6 months to 5 years.

To ensure we were using as much information as possible, and therefore maximize the data basis of our estimates, we modeled Vitamin A deficiency sequentially. The first step was to estimate the coverage of Vitamin A supplementation. Although the typical metric on which supplementation is tracked is 2+ doses of Vitamin A in the previous 12 months for children under 5 years, most existing health surveys do not routinely provide sufficient information to calculate it. Our case definition for the supplementation model was therefore the proportion of children 6-59 months of age who received at least one dose of Vitamin A in the previous 6 months. Supplementation estimates were then used as a location-level covariate to guide exposure models of overall Vitamin A deficiency.

Input data

For GBD 2016, we used data from the WHO Vitamin and Mineral Nutrition Information System, health surveys such as DHS and MICS, and studies identified through literature review. A systematic review was last conducted for GBD 2013. The PubMed search terms were: ((vitamin A deficiency[Title/Abstract] AND prevalence[Title/Abstract]) AND ("2009"[Date – Publication] : "2013"[Date – Publication])). The table below shows the number of data points included in the final datasets**.** Exclusion criteria were:

1. Studies that were not population-based, e.g., hospital or clinic-based studies
2. Studies that did not provide primary data on epidemiological parameters, e.g., commentaries
3. Review articles
4. Case series
5. Self-reported cases

Table 1. Geographic representation of datasets used for three stages of Vitamin A deficiency risk factor burden estimation (number of data points per geography)

Geography	Supplementation (proportion)	Deficiency (prevalence)	Vision Loss (Relative risk)	Vision Loss (prevalence)
Global	900	365	1	81
East Asia	12	10		
Southeast Asia	102	45		21
Oceania	24	18		
Central Asia	51	31		
Central Europe	2	1		
Australasia		1		
Southern Latin America		1		
High-income North America		4		
Caribbean	17	12		1
Andean Latin America	25	10		
Central Latin America	33	54		1
Tropical Latin America	1	2		2
North Africa and Middle East	49	37		18
South Asia	61	21		21
Central Sub-Saharan Africa	60	9		1
Eastern Sub-Saharan Africa	182	63		10
Southern Sub-Saharan Africa	49	15		1
Western Sub-Saharan Africa	232	31		5

Modeling Strategy

All Vitamin A deficiency estimates were made using DisMod-MR 2.2. As described above, we first estimated Vitamin A supplementation coverage. Although all data was from ages 6-59 months, we assumed no difference in age pattern of supplementation coverage and used the natural log of lag-distributed income per capita (LN-LDI) as a location-level covariate to inform estimates where data was absent. DHS and MICS data was cross-walked to the reference data source, which came from UNICEF (http://data.worldbank.org/indicator/SN.ITK.VITA.ZS).

Table 2: Covariate effects for Vitamin A supplementation model

Measure	Covariate	Type	Value	Exponentiated
Prevalence	MICS	Study-level	-0.59 (-0.73 — -0.42)	0.56 (0.48 — 0.65)
Prevalence	DHS	Study-level	-0.092 (-0.22 — 0.038)	0.91 (0.80 — 1.04)
Prevalence	LDI (I$ per capita)	Country-level	0.0094 (0.00061 — 0.039)	1.01 (1.00 — 1.04)

Second, we estimated the age- and sex-specific prevalence of Vitamin A deficiency (serum retinol < 0.7 µmol/L). WHO VMNIS was the primary data source for this model and was supplemented with data from DHS and other health surveys where testing was performed. We assumed the following in our model: no excess mortality, birth prevalence is possible, and that incidence and remission are both decreasing after age 5. Data from subnational locations was crosswalked to the reference data sources of nationally-representative data. Females were found to have 1.09 times higher Vitamin A deficiency, although the uncertainty in that ratio ranged from 0.97 to 1.24. Location-level covariates were used for Vitamin A supplementation coverage from the above model as well as GBD 2016 Socio-demographic Index (SDI) numbers.

Table 3: Covariate effects for Vitamin A deficiency model

Measure	Covariate	Type	Value	Exponentiated
Prevalence	Sex	Study-level	0.086 (-0.027 — 0.21)	1.09 (0.97 — 1.24)
Prevalence	Subnational	Study-level	0.00074 (-0.15 — 0.17)	1.00 (0.86 — 1.19)
Prevalence	Vit A suppl. coverage	Country-level	-0.38 (-0.71 — -0.099)	0.68 (0.49 — 0.91)
Prevalence	SDI	Country-level	-2.25 (-2.87 — -1.36)	0.10 (0.057 — 0.26)

Theoretical minimum-risk exposure level

The theoretical minimum risk exposure is that the prevalence of vitamin A deficiency is zero.

Relative risks

The relative risks have not changed from GBD 2015.

Smoking Appendix

Flowchart

Smoking

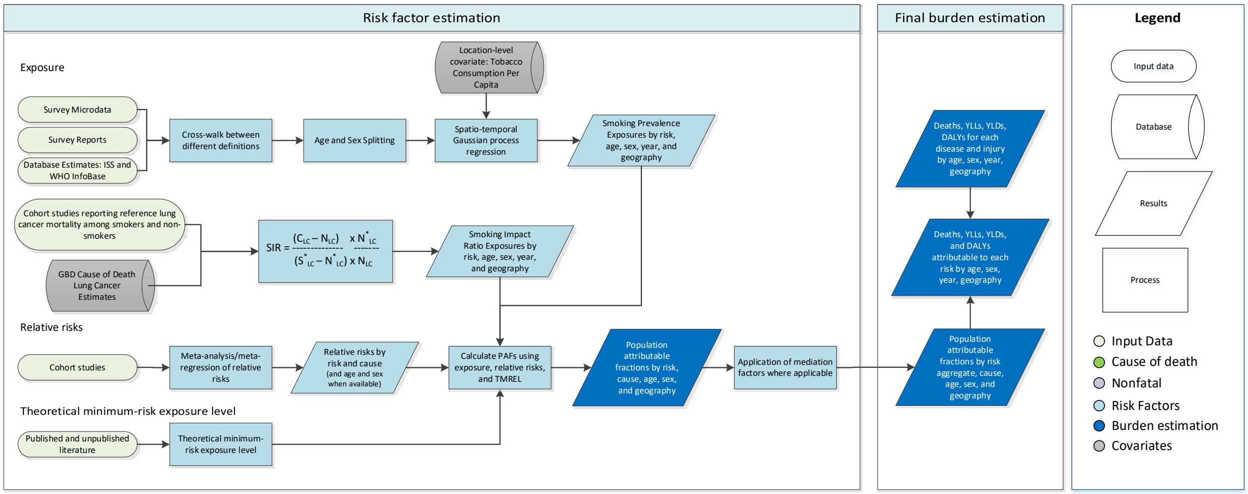

Inclusion criteria

We included nationally representative survey data sources that captured information on primary tobacco use among individuals over age 10. We included only self-reported smoking data and excluded data from questions asking about others' smoking behaviors. We included data that was collected between 1 January 1980 and 31 December 2016.

Data sources

A complete list of sources is available from the GBD 2016 Data Input Sources Tool.

Prevalence

We searched the Global Health Data Exchange (GHDx) database for primary data sources with the keyword "Tobacco Use" on 1 January 2017 to ensure all available data sources were captured. Together, these sources comprised 6,216 location-years of data and 130,308 location-year-age-sex data points.

In addition to the primary data sources identified through the GHDx, we supplemented with secondary database estimates from the WHO InfoBase Database and International Smoking Statistics Database for sources for which primary data are unavailable. We included 275 sources from the WHO InfoBase and 199 sources from the International Smoking Statistics Database.

Smoking Impact Ratio

The Smoking Impact Ratio (SIR) is computed using four estimates: 1) lung cancer mortality rates in a reference population of smokers, 2) lung cancer mortality rates in a reference population of never-smokers, 3) lung cancer mortality rates among never smokers in a population of interest, and 4) observed lung cancer mortality rates in a population of interest. We used available prospective cohort studies to estimate values 1, 2, and 3. A list of included prospective cohorts is available in the GBD 2016 Data Input Sources Tool. We used lung cancer mortality rate estimates from GBD 2016 for value 4.

Relative risk

Relative risk estimates were derived from prospective cohort studies. Sources used in relative risk estimation are reported in Appendix Table 1.

Smoking prevalence data preparation

Data extraction

We extracted primary data from individual-level microdata and survey report tabulations. We extracted data on current, former, and/or ever smoked tobacco use reported as any combination of frequency of use (daily, occasional, and unspecified, which includes both daily and occasional smokers) and type of smoked tobacco used (all smoked tobacco, cigarettes, hookah, and other smoked tobacco products such as cigars or pipes), resulting in 36 possible combinations. Other variants of tobacco products, for example hand-rolled cigarettes, were grouped into the four type categories listed above based on product similarities. Only smoked tobacco products are included, smoked drugs are estimated separately as part of the drug use risk factor.

For microdata, we extracted relevant demographic information, including age, sex, location, and year, as well as survey metadata, including survey weights, primary sampling units, and strata. This information allowed us to tabulate individual-level data in the standard GBD five-year age-sex groups and produce accurate estimates of uncertainty. For survey report tabulations, we extracted data at the most granular age-sex group provided.

Crosswalking

Our case-definition for smoking prevalence is daily use of any smoked tobacco products. All other data points were adjusted to be consistent with this definition. Some sources contained information on more than one indicator and these sources were used to develop the adjustment coefficient to transform that alternative definition to the GBD standard case-definition of daily use of smoked tobacco. The adjustment coefficient was the beta value derived from the following model:

$$\mathrm{p}_{\mathrm{daily-smoked,k}} = \beta \mathrm{p}_{\mathrm{i,k}} + \epsilon_{\mathrm{k}}$$

where $\mathrm{p}_{\mathrm{daily-smoked,k}}$ is the prevalence of daily smoking reported in survey k and $\mathrm{p}_{\mathrm{i,k}}$ is the prevalence of an alternative frequency-type combination i also reported in survey k. Models with adjusted R-squared values > 0.8 were used in order of their R-squared value. We fit the regression using the maximum of either 1) the 200 closest sources to the source to be crosswalked, or 2) all data from the same region as the source to be crosswalked.

For each source that needed adjusting, we assigned space weights based on GBD region and super-region to the sources containing more than one case definition. Data from the same region receiving a full weight of 1, and data from the same super-region received a weight of ½. We explored using a time weight, as we do in the age-sex splitting process, to control for possible changes in the relationship between smoking behaviors over time. We found incorporating temporal information did not significantly change the estimated coefficients but did undercut sample sizes, and chose to exclude the time weight. Crosswalk coefficients generated from fewer than 20 data sources were dropped.

We generated separate crosswalk coefficients for the 10-14 age group and the 15-19 age group, as we found the relationships between case definitions differed strongly in the younger age groups compared

to the 20+ age groups. Due to data limitations, we generated a global crosswalk coefficient for both the 10-14 and 15-19 age groups, using the same regression as above.

The estimated regression coefficients used for adjustment are reported in Tables 1 (a,b,c) below.

We propagated uncertainty at the survey (k) level from the crosswalk using the following equation:

$$PE_k = \sigma_\epsilon^2 + X_k^2 \mathrm{var}(\hat{\beta})$$

where PE_k is the crosswalk prediction error that is added to the sampling variance of the data point, σ_ϵ^2 is the variance of the error, X_k^2 is the squared value of the data being adjusted, and $\mathrm{var}(\hat{\beta})$ is the variance of the adjustment coefficient.

Age and sex splitting

We split data reported in broader age groups than the GBD 5-year age groups or as both sexes combined by adapting the method reported in Ng et al. (http://jamanetwork.com/journals/jama/fullarticle/1812960) to split using a sex- geography- time-specific reference age pattern. We separated the data into two sets: a training dataset, with data already falling into GBD sex-specific 5-year age groups, and a split dataset, which reported data in aggregated age or sex groups. Each source reporting aggregated data was temporarily duplicated into correct GBD categorizations covering their form of aggregation. These duplicated age groups were iteratively matched to the closest 200 sources of the same age and sex in the training dataset by geography and time. The mean of these 200 sources was used to generate an estimate for each duplicated estimate. Finally, we multiplied the original aggregated estimate by the population-weighted ratio of the mean estimates generated from the training data.

Similar to the crosswalk, we defined the “closest” sources in space by assigning space weights based on GBD regions. If a training source was from the same country or subnational unit as the source to be split, it received a full space weight of 1. If from a different country but the same region, it received a space weight of .66. If the sources only shared a super_region, it received a space weight of .33. The time weights were generated using the equation:

$$\text{Time weight} = 1 - \text{abs}\left(\text{year}_{\text{train}} - \text{year}_{\text{split}}\right) * .05$$

Essentially, sources from the training dataset published in the same year as the source to be split would receive a full time weight of 1, with diminishing weight as the difference in publication years increased. The time weight and space weight each made up 50% of a combined total weight. The 200 training sources with the highest total weights were then used to estimate the mean prevalence pattern for each source in need of splitting.

Smoking prevalence modeling

We used ST-GPR to model smoking prevalence given the abundance of age and sex-specific data. Full details on the ST-GPR method are available elsewhere. Briefly, the mean function input to GPR is a complete time series of estimates generated from a mixed effects hierarchical linear model plus

weighted residuals smoothed across time, space, and age. The linear model formula, fit separately by sex using restricted maximum likelihood in R, is:

$$\mathrm{logit}(p_{g,a,t}) = \beta_0 + \beta_1 \mathrm{CPC}_{g,t} + \sum_{k=2}^{19} \beta_k I_{A[a]} + \alpha_s + \alpha_r + \alpha_g + \epsilon_{g,a,t}$$

where $\mathrm{CPC}_{c,t}$ is the tobacco consumption covariate, by geography g and time t, described above, $I_{A[a]}$ is a dummy variable indicating specific age group A that the prevalence point $p_{g,a,t}$ captures, and α_s, α_r, and α_g are super region, region, and geography random intercepts, respectively. Random effects were used in model fitting but were not used in prediction.

We used out-of-sample cross validation for hyperparameter selection for the space (zeta), age (omega), and time (lambda) weights used in spatiotemporal smoothing along with the scale used in Gaussian process regression (details on the effects of different parameters have been previously published). We used a space weight of 0.95 in data-dense countries (at least five years covered in a geography-age-sex group) and space weight of 0.7 in data-sparse countries. The other parameters were consistent across data-density levels: age weight = 1, time weight = 1, and scale = 10.

Smoking Impact Ratio calculation

We calculated SIR for each geography, year, age group, and sex included in attributable burden analysis using the following formula:

$$SIR = \frac{C_{LC} - N_{LC}}{S_{LC}^{*} - N_{LC}^{*}} \times \frac{N_{LC}^{*}}{N_{LC}}$$

where C_{LC} is the lung cancer mortality rate specific to the age-sex-geography-year of interest, N_{LC} is the age- sex- geography- year-specific lung cancer mortality rate of never-smokers in the population of interest, S_{LC}^{*} is the lung cancer mortality rate in a reference population of smokers, N_{LC}^{*} is the lung cancer mortality rate in a reference population of never-smokers. Additional details on SIR calculation have been described elsewhere.

Estimating attributable burden

Assessment of risk-outcome pairs

We conducted a systematic literature review of meta-analyses, pooled analyses, and systematic reviews on the effects of smoking on health outcomes. We searched PubMed on December 14, 2016 using the following search string: ("Tobacco Smoke Pollution"[Mesh] OR "Smoking"[Mesh] OR "Smoking"[TiAb] OR"Tobacco Smoke"[tiab] OR "Passive Smoke"[tiab] OR "Secondhand Smoke"[tiab] OR "Second hand smoke"[tiab]) AND (Meta-Analysis[ptyp] OR "systematic review"[tiab] OR "pooled"[tiab]). The search yielded 4,506 sources which were reviewed. Sources were included if the outcome of interest was included in GBD, if at least one prospective cohort study was included, and if the summary effect size reported was statistically significant. Seven disease outcomes that were not included in GBD 2015 met inclusion criteria. For these outcomes, we completed causal criteria tables and evaluated the strength of evidence supporting a causal relationship. For GBD 2016 we also dropped 5 outcomes that were previously included in GBD 2015 (pneumoconiosis, coal worker's pneumoconiosis, asbestosis, silicosis, and interstitial lung disease and pulmonary sarcoidosis) for which there was insufficient evidence to support a causal effect, resulting in a total of 49 outcomes. Appendix Table 2 reports the strength of

evidence for included outcomes and the exposure metric (5-year lagged prevalence or SIR) used for each outcome. Appendix Table 2 reports the sources used in evaluating strength of included outcomes. New outcomes for GBD 2016 are: breast cancer, prostate cancer, Alzheimer disease, Parkinson disease, multiple sclerosis, gallbladder and biliary tract disease, and low back pain.

Relative risk

Appendix Table 1 reports relative risk estimates and uncertainty for the 49 outcomes included in analysis, by age and sex where applicable. Sources used in generating relative risk estimates are cited in the GBD 2016 Data Input Sources Tool.

Figures and Tables

Table 1a: Crosswalk coefficients for adult (20+ years) age group, by region

Crosswalk Coefficients, Ages 20+				
Indicator	Region	Crosswalk Coefficient	R-squared value	Number of sources used in regression
Current use of cigarettes	East Asia	0.931	0.991	199
Current use of cigarettes	Southeast Asia	0.967	0.994	127
Current use of cigarettes	Oceania	0.931	0.991	199
Current use of cigarettes	Central Asia	0.969	0.996	583
Current use of cigarettes	Central Europe	0.963	0.996	325
Current use of cigarettes	Eastern Europe	0.976	0.996	253
Current use of cigarettes	High-income Asia Pacific	0.881	0.954	6181
Current use of cigarettes	Australasia	0.881	0.954	6181
Current use of cigarettes	Western Europe	0.804	0.915	645
Current use of cigarettes	Southern Latin America	0.881	0.954	6181
Current use of cigarettes	High-income North America	0.894	0.959	5486
Current use of cigarettes	Caribbean	0.797	0.953	184
Current use of cigarettes	Andean Latin America	0.895	0.961	4189
Current use of cigarettes	Central Latin America	0.895	0.961	4189
Current use of cigarettes	Tropical Latin America	0.911	0.969	3918
Current use of cigarettes	North Africa and Middle East	0.927	0.979	96
Current use of cigarettes	South Asia	0.911	0.968	695
Current use of cigarettes	Central Sub-Saharan Africa	0.984	0.995	94
Current use of cigarettes	Eastern Sub-Saharan Africa	0.984	0.995	94
Current use of cigarettes	Southern Sub-Saharan Africa	0.984	0.995	94
Current use of cigarettes	Western Sub-Saharan Africa	0.984	0.995	94

Current use of cigarettes	East Asia	1.012	1	103
Current use of cigarettes	Southeast Asia	1.057	0.98	268
Current use of cigarettes	Oceania	1.01	0.983	131
Current use of cigarettes	Central Europe	1.002	0.999	498
Current use of cigarettes	High-income Asia Pacific	1.024	0.973	6647
Current use of cigarettes	Australasia	1.024	0.973	6647
Current use of cigarettes	Western Europe	1.008	0.994	1085
Current use of cigarettes	Southern Latin America	1.024	0.973	6647
Current use of cigarettes	High-income North America	1.028	0.968	5486
Current use of cigarettes	Caribbean	1.045	0.981	211
Current use of cigarettes	North Africa and Middle East	1.036	0.989	227
Current use of cigarettes	South Asia	1.045	0.963	993
Current use of cigarettes	Central Sub-Saharan Africa	1.026	0.903	746
Current use of cigarettes	Eastern Sub-Saharan Africa	1.008	0.919	184
Current use of cigarettes	Southern Sub-Saharan Africa	1.04	0.946	276
Occasional use of cigarettes	Central Sub-Saharan Africa	0.991	0.949	53
Occasional use of cigarettes	Eastern Sub-Saharan Africa	0.991	0.949	53
Occasional use of cigarettes	Southern Sub-Saharan Africa	0.991	0.949	53
Occasional use of cigarettes	Western Sub-Saharan Africa	0.991	0.949	53
Ever user of cigarettes	East Asia	0.809	0.984	58
Ever user of cigarettes	Southeast Asia	0.809	0.984	58
Ever user of cigarettes	Oceania	0.809	0.984	58
Ever user of cigarettes	Central Asia	0.498	0.819	165
Ever user of cigarettes	Central Europe	0.508	0.821	133
Ever user of cigarettes	Eastern Europe	0.498	0.819	165
Ever user of cigarettes	High-income Asia Pacific	0.474	0.871	4322
Ever user of cigarettes	Australasia	0.474	0.871	4322
Ever user of cigarettes	Western Europe	0.395	0.803	255
Ever user of cigarettes	Southern Latin America	0.474	0.871	4322
Ever user of cigarettes	High-income North America	0.479	0.875	4067
Ever user of cigarettes	Caribbean	0.374	0.856	151
Ever user of cigarettes	Central Sub-Saharan Africa	0.773	0.991	25
Ever user of cigarettes	Eastern Sub-Saharan Africa	0.773	0.991	25

Ever user of cigarettes	Southern Sub-Saharan Africa	0.773	0.991	25
Ever user of cigarettes	Western Sub-Saharan Africa	0.773	0.991	25
Ever used cigarettes daily	Central Asia	0.544	0.831	170
Ever used cigarettes daily	Central Europe	0.544	0.833	133
Ever used cigarettes daily	Eastern Europe	0.544	0.831	170
Ever used cigarettes daily	High-income Asia Pacific	0.447	0.823	255
Ever used cigarettes daily	Australasia	0.447	0.823	255
Ever used cigarettes daily	Western Europe	0.447	0.823	255
Ever used cigarettes daily	Southern Latin America	0.447	0.823	255
Ever used cigarettes daily	High-income North America	0.447	0.823	255
Current use of any smoked tobacco	East Asia	0.913	0.993	2633
Current use of any smoked tobacco	Southeast Asia	0.829	0.977	483
Current use of any smoked tobacco	Oceania	0.844	0.949	235
Current use of any smoked tobacco	Central Asia	0.865	0.981	123
Current use of any smoked tobacco	Central Europe	0.896	0.982	592
Current use of any smoked tobacco	Eastern Europe	0.896	0.983	563
Current use of any smoked tobacco	High-income Asia Pacific	0.851	0.983	7227
Current use of any smoked tobacco	Australasia	0.719	0.954	145
Current use of any smoked tobacco	Western Europe	0.849	0.971	1471
Current use of any smoked tobacco	Southern Latin America	0.851	0.983	7227
Current use of any smoked tobacco	High-income North America	0.855	0.987	5517
Current use of any smoked tobacco	Caribbean	0.787	0.96	263
Current use of any smoked tobacco	Andean Latin America	0.744	0.915	9048
Current use of any smoked tobacco	Central Latin America	0.615	0.837	1040
Current use of any smoked tobacco	Tropical Latin America	0.858	0.966	7703
Current use of any smoked tobacco	North Africa and Middle East	0.915	0.979	389

Current use of any smoked tobacco	South Asia	0.809	0.945	1719
Current use of any smoked tobacco	Central Sub-Saharan Africa	0.875	0.973	883
Current use of any smoked tobacco	Eastern Sub-Saharan Africa	0.918	0.975	300
Current use of any smoked tobacco	Southern Sub-Saharan Africa	0.817	0.976	213
Current use of any smoked tobacco	Western Sub-Saharan Africa	0.872	0.983	349
Occasional use of smoked tobacco	High-income Asia Pacific	1.254	0.812	6860
Occasional use of smoked tobacco	Southern Latin America	1.254	0.812	6860
Occasional use of smoked tobacco	High-income North America	1.283	0.838	5476
Occasional use of smoked tobacco	Central Latin America	0.844	0.818	258
Ever used any smoked tobacco	East Asia	0.676	0.886	180
Ever used any smoked tobacco	Southeast Asia	0.676	0.886	180
Ever used any smoked tobacco	Oceania	0.676	0.886	180
Ever used any smoked tobacco	Central Europe	0.468	0.825	193
Ever used any smoked tobacco	High-income Asia Pacific	0.467	0.867	5957
Ever used any smoked tobacco	Australasia	0.467	0.867	5957
Ever used any smoked tobacco	Southern Latin America	0.467	0.867	5957
Ever used any smoked tobacco	High-income North America	0.49	0.879	5397
Ever used any smoked tobacco	Caribbean	0.353	0.828	184
Ever used any smoked tobacco	South Asia	0.456	0.81	812
Ever used any smoked tobacco	Central Sub-Saharan Africa	0.878	0.956	42
Ever used any smoked tobacco	Eastern Sub-Saharan Africa	0.878	0.956	42
Ever used any smoked tobacco	Southern Sub-Saharan Africa	0.878	0.956	42
Ever used any smoked tobacco	Western Sub-Saharan Africa	0.878	0.956	42

Ever used any smoked tobacco daily	East Asia	0.687	0.94	81
Ever used any smoked tobacco daily	Southeast Asia	0.687	0.94	81
Ever used any smoked tobacco daily	Oceania	0.687	0.94	81
Ever used any smoked tobacco daily	Central Asia	0.589	0.86	230
Ever used any smoked tobacco daily	Central Europe	0.578	0.85	185
Ever used any smoked tobacco daily	Eastern Europe	0.589	0.86	230
Ever used any smoked tobacco daily	North Africa and Middle East	0.67	0.935	65
Ever used any smoked tobacco daily	South Asia	0.857	0.961	221
Ever used any smoked tobacco daily	Central Sub-Saharan Africa	0.668	0.96	213
Ever used any smoked tobacco daily	Eastern Sub-Saharan Africa	0.668	0.96	213
Ever used any smoked tobacco daily	Southern Sub-Saharan Africa	0.668	0.96	213
Ever used any smoked tobacco daily	Western Sub-Saharan Africa	0.645	0.954	104

Table 1b: Global crosswalk coefficients for 10-14 age group

**Crosswalk coefficients for 10-14 age group**			
Indicator	Crosswalk Coefficient	R-squared value	Number of sources used in regression
Current use of cigarettes	1.082	0.992	262
Current use of smoked tobacco other than cigarettes	1.005	0.891	113
Occasional use of smoked tobacco other than cigarettes	0.774	0.882	28

Table 1c: Global crosswalk coefficients for 15-19 age-group

**Crosswalk coefficients for 15-19 age group**			
Indicator	Crosswalk Coefficient	R-squared value	Number of sources used in regression
Ever user of cigarettes	0.604	0.898	357
Ever used cigarettes daily	0.942	0.986	22
Ever used any smoked tobacco daily	0.8	0.92	148

Secondhand Smoke Capstone Appendix

Flowchart

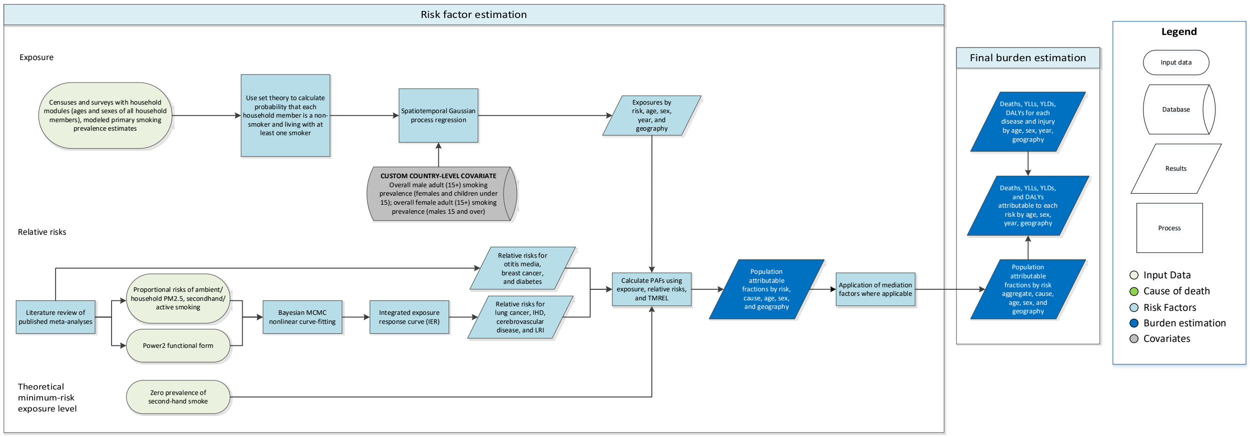

Exposure

Case Definition

We define secondhand smoke exposure as exposure to tobacco smoke among non-smokers by a household member. We use household composition as a proxy for exposure and make the assumption that all persons living with a daily smoker are exposed to tobacco smoke. Non-smokers are defined as all persons who are not daily smokers. Ex-smokers and occasional smokers are considered non-smokers in this analysis. Exposure was evaluated for both children and adults. This analysis does not include exposure to secondhand smoke through social networks or at a workplace.

Input data

To calculate the proportion of non-smokers who live with at least one smoker, we used unit record data on household composition, which included the ages and sexes of all persons living in the same household. Our sources included representative major survey series with a household composition module, including the Demographic Health Surveys (DHS), the Multiple Indicator Cluster Surveys (MICS), and the Living Standards Measurement Surveys (LSMS); and national and subnational censuses, which included those captured in the IPUMS project and identified using the Global Health Data Exchange catalog (GHDx).

Estimates of primary smoking prevalence in each location were also used in our calculations.

Modelling strategy

We made several substantial changes to our modelling strategy for secondhand smoke for GBD 2016. First, we ran a spatiotemporal Gaussian process regression (ST-GPR), whereas the model from GBD 2015 used DisMod-MR. Both methods modeled separately by sex, but the DisMod-MR method from GBD 2015 included children (under age 15) of both sexes in the female model and modeled adult males (age 15+) alone. Second, we used overall adult (age 15+) smoking prevalence in lieu of of age-standardized smoking prevalence as a covariate in the model (described in further detail below). Third, we used

mathematics (methods described below) to estimate the proportion of a population exposed, instead of using survey data asking more directly about exposure.

We estimated the probability that each person is living with a smoker and is also a non-smoker themselves using set theory. First, household composition data were used at the individual level to capture the ages and sexes of each person in the household. Second, we analyzed surveys with both household composition data and tobacco use questions and determined that the distribution of household size, mean age of the household members, and the age distribution were not significantly different between households with and without a self-reported smoker. Since we did not find that household composition varied between smokers and non-smokers, we then used the GBD 2016 primary smoking prevalence model to calculate the probability that each household member is a smoker. Next, we used the probability of the union of sets on each individual household member to calculate the overall probability that at least one of the other household members was a smoker. Finally, we multiplied this output by the probability that the respondent was not a smoker themselves (i.e. 1 minus primary smoking prevalence for that person's location, year, age, and sex). Once the microdata are collapsed into tabulations by location, year, age, sex, and survey iteration, these calculations give estimates for the proportion of each population who are both living with one or more smokers and are not smokers themselves.

These probabilities were modeled in the GBD ST-GPR framework, which generates exposure estimates from a mixed effects hierarchical linear model plus weighted residuals smoothed across time, space, and age. The linear model formula was fit separately by sex using restricted maximum likelihood in R.

We used the sex-specific overall smoking prevalence for adults (age 15 and older) as a country-level covariate in the model. The overall male adult daily smoking prevalence was used as the covariate for females of all ages and for males under age 15. The overall female adult daily smoking prevalence was used as the covariate for males age 15 and older. This was a modelling change from GBD 2015, in which we used the male age-standardized smoking prevalence for the adult female and children under 15 model, and the female age-standardized smoking prevalence for the adult male model.

All input data points from the probability calculation had a measure of uncertainty (variance and sample size) coming from the uncertainty of the primary smoking prevalence model and the sample size from the unit record data going into the modelling process. Geographic random effects were used in model fitting but were not used in prediction.

Theoretical minimum-risk exposure level

The theoretical minimum-risk exposure level for secondhand smoke is zero exposure among non-smokers, meaning that non-smokers would not live with any primary smokers.

Relative risks

For children ages 0-14, we estimated the burden of otitis media attributable to secondhand smoke exposure. For all ages we estimated the burden of lower respiratory infections (LRI), and for adults greater or equal to 25 years of age we estimated the burden of lung cancer, chronic obstructive pulmonary disease (COPD), ischemic heart disease, and cerebrovascular disease attributable to secondhand smoke exposure. This year, we found significant evidence for associations between secondhand smoke exposure and two additional outcomes: breast cancer and diabetes. These were added to the list of risk-outcome pairs for secondhand smoke exposure.

For lung cancer, ischemic heart disease, cerebrovascular disease, and LRI, we used country-specific relative risks created using integrated exposure response curves (IER) for PM2.5 air pollution. The relative risks for otitis media, breast cancer, and diabetes are derived from published meta-analyses.

We used the traditional GBD population attributable fraction (PAF) equation to estimate burden based on exposure and relative risks.

Smokeless Tobacco

Flowchart

Smokeless Tobacco: Data and Model Flow Chart

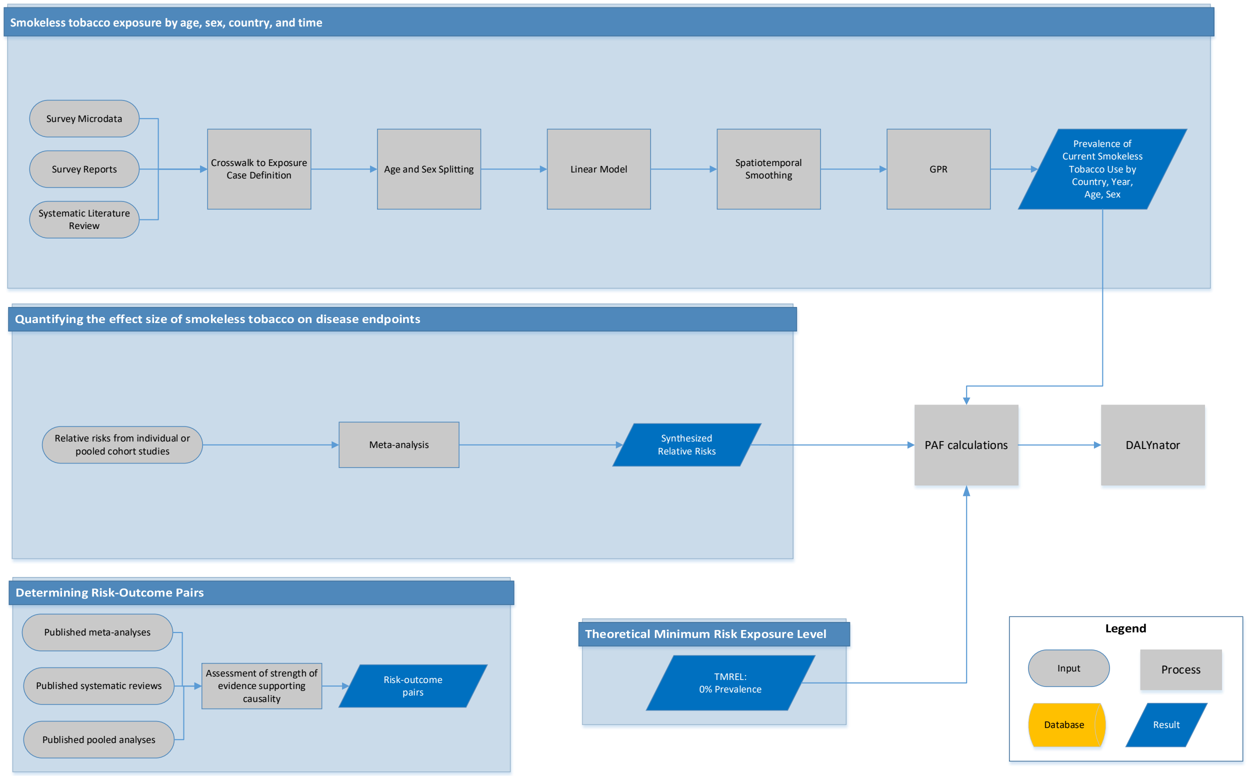

Input data and Methodological Summary

Input data

Inclusion criteria

We included sources that reported primary smokeless tobacco use among respondents over age 10. To be eligible for inclusion, sources had to be representative for their level of estimation (ie. national sources needed to be nationally representative, subnational sources subnationally representative). We included only self-reported smokeless tobacco use data and excluded data from questions asking about others' tobacco use behaviors. We included data collected between 1 January 1980 and 31 December 2016.

Data sources

A complete list of sources is available from the GBD 2016 Data Input Sources Tool (http://ghdx.healthdata.org/gbd-2016/data-input-sources).

Prevalence

We searched the Global Health Data Exchange (GHDx) database for primary data sources with the keyword "Tobacco Use" on January 1, 2017 to ensure all available data sources were captured. Of the 3,318 sources identified in the GHDx, 1,578 country-year sources met inclusion criteria and were included.

In addition to the primary data sources identified through the GHDx, we performed a systematic literature search on PubMed. The search was conducted on January 19, 2017 and returned 5982 hits, of which 267 were eligible for inclusion. Of these 267 sources, 200 had already been identified in the GHDx, so the Pubmed search yielded 67 additional sources overall. Figure 1 presents the PRISMA flowchart for the systematic literature review. The search string is shown below:

("smokeless tobacco"[tiab] OR "Tobacco, Smokeless"[Mesh] OR bajjar[tiab] OR ("betel quid"[tiab] AND tobacco[tiab]) OR "chewing tobacco"[tiab] OR chimó[tiab] OR snuff[tiab] OR snuif[tiab] OR dip[tiab] OR dohra[tiab] OR gudakhu[tiab] OR gul[tiab] OR gutka[tiab] OR gutkha[tiab] OR "hnat hsey"[tiab] OR iq'mik[tiab] OR khaini[tiab] OR kharra[tiab] OR khiwam[tiab] OR khimam[tiab] OR kiwam[tiab] OR kimam[tiab] OR "lal dant manjan"[tiab] OR ("loose leaf"[tiab] AND (chew[tiab] OR tobacco[tiab])) OR mainpuri[tiab] OR maras[tiab] OR mawa[tiab] OR mshri[tiab] OR naffa[tiab] OR nas[Supplementary Concept] OR ((nas[tiab] OR nass[tiab]) AND tobacco[tiab]) OR naswar[tiab] OR nasway[tiab] OR nasvay[tiab] OR neffa[tiab] OR ((pan[tiab] OR paan[tiab]) AND tobacco[tiab]) OR (plug[tiab] AND tobacco[tiab]) OR (rapé[tiab] AND tobacco[tiab]) OR ((red[tiab] OR tobacco[tiab]) AND (toothpowder[tiab] OR toothpaste[tiab])) OR shammah[tiab] OR snus[tiab] OR taaba[tiab] OR tapkeer[tiab] OR tawa[tiab] OR tombol[tiab] OR toombak[tiab] OR tuibur[tiab] OR "tobacco water"[tiab] OR (twist[tiab] AND tobacco[tiab]) OR zarda[tiab]) AND Humans[Mesh] AND English[Language] NOT Case Reports[ptyp]

Smokeless tobacco prevalence data preparation

Data extraction

We extracted primary data from individual-level microdata and survey report tabulations. We extracted data on current, former, and/or ever smokeless tobacco use as well as frequency of use (daily, occasional, and unspecified, which includes both daily and occasional smokers). We included all smokeless tobacco products in estimating exposure. Smokeless products that do not include tobacco, such as betel quid without tobacco, are excluded or estimated separately as part of the drug use risk factor, if applicable.

For microdata, we extracted relevant demographic information, including age, sex, location, and year, as well as survey metadata, including survey weights, primary sampling units, and strata. This information allowed us to tabulate individual-level data in the standard GBD five-year age-sex groups and produce accurate estimates of uncertainty. For survey report tabulations, we extracted data at the most granular age-sex group provided.

Crosswalking

Our GBD smokeless tobacco case definition is current use of any smokeless tobacco product. All other data points were adjusted to be consistent with this definition. Table 1 shows the number of data points extracted for each indicator. Some sources contained information on more than one case definition and these sources were used to develop the adjustment coefficient to transform alternative case definitions to the GBD case definition. The adjustment coefficient was the beta value derived from the following model:

$$p_{\text{current-all smokeless},k} = \beta p_{i,k} + \epsilon_k$$

where $p_{\text{current-all smokeless},k}$ is the prevalence of current smokeless tobacco use reported in survey k and $p_{i,k}$ is the prevalence of an alternative case definition i also reported in survey k. Models with

adjusted R-squared values > 0.8 were used in order of their R-squared value. We fit the regression using the maximum of either 1) the 200 closest sources to the source to be crosswalked, or 2) all data from the same region as the source to be crosswalked.

For each source that needed adjusting, we assigned space weights based on GBD region and super-region to the sources containing more than one case definition. Data from the same region receiving a full weight of 1, and data from the same super-region received a weight of ½. We explored using a time weight, as we do in the age-sex splitting process, to control for possible changes in the relationship between smokeless tobacco use behaviors over time. We found incorporating temporal information did not significantly change the estimated coefficients but did undercut sample sizes, and chose to exclude the time weight. Crosswalk coefficients generated from fewer than 20 data sources were dropped.

We generated separate crosswalk coefficients for the 10-14 age group and the 15-19 age group, as we found the relationships between case definitions differed strongly in the younger age groups compared to the 20+ age groups. To account for this, we attempted to generate a global crosswalk coefficient for both the 10-14 and 15-19 age groups, using the same regression as above. Due to data limitations, none of the crosswalk coefficients met the criteria outlined above, so no data covering youths under 20 years old were crosswalked. In other words, all data from these age groups that appear in the model were asked according to our case definition in the survey.

The estimated regression coefficients used for adjustment are reported in Table 2.

We propagated uncertainty at the survey (k) level from the crosswalk using the following equation:

$$\mathrm{PE_k} = \sigma_\epsilon^2 + \mathrm{X_k^2}\mathrm{var}(\hat{\beta})$$

where $\mathrm{PE_k}$ is the crosswalk prediction error that is added to the sampling variance of the data point, σ_ϵ^2 is the variance of the error, $\mathrm{X_k^2}$ is the squared value of the data being adjusted, and $\mathrm{var}(\hat{\beta})$ is the variance of the adjustment coefficient.

Age and sex splitting

We split data reported in broader age groups than the GBD 5-year age groups or as both sexes combined by adapting the method reported in Ng et al. (http://jamanetwork.com/journals/jama/fullarticle/1812960) to split using a sex- geography- time-specific reference age pattern. We separated the data into two sets: a training dataset, with data already falling into GBD sex-specific 5-year age groups, and a split dataset, which reported data in aggregated age or sex groups. Each source reporting aggregated data was temporarily duplicated into correct GBD categorizations covering their form of aggregation. These duplicated age groups were iteratively matched to the closest 200 sources of the same age and sex in the training dataset by geography and time. The mean of these 200 sources was used to generate an estimate for each duplicated estimate. Finally, we multiplied the original aggregated estimate by the population-weighted ratio of the mean estimates generated from the training data.

Similar to the crosswalk, we defined the "closest" sources in space by assigning space weights based on GBD regions. If a training source was from the same country or subnational unit as the source to be split, it received a full space weight of 1. If from a different country but the same region, it received a space weight of .66. If the sources only shared a super-region, it received a space weight of .33. The time weights were generated using the equation: $\mathrm{Time\ weight} = 1 - \mathrm{abs}(\mathrm{year_{train}} - \mathrm{year_{split}}) * .05$

Essentially, sources from the training dataset published in the same year as the source to be split would receive a full time weight of 1, with diminishing weight as the difference in publication years increased. The time weight and space weight each made up 50% of a combined total weight. The 200 training sources with the highest total weights were then used to estimate the mean prevalence pattern for each source in need of splitting.

Smokeless tobacco prevalence modeling

We used ST-GPR to model smokeless tobacco prevalence. Full details on the ST-GPR method are reported in Appendix Section 2. Briefly, the mean function input to GPR is a complete time series of estimates generated from a mixed effects hierarchical linear model plus weighted residuals smoothed across time, space, and age. The linear model formula, fit separately by sex using restricted maximum likelihood in R, is:

$$\text{logit}(p_{g,a,t}) = \beta_0$$

We chose this simple model after finding that all explored covariates and age-specific indicators were inconsistent across geographies, leading to artificial smokeless tobacco trends in certain locations.

We used a space weight of 0.95 in data-dense countries (at least five years covered in a geography-age-sex group) and space weight of 0.7 in data-sparse countries. The other parameters were consistent across data-density levels: age weight = 1, time weight = 1, and scale = 10. Amplitude was calculated at the region level.

Estimating attributable burden

Assessment of risk-outcome pairs

We included outcomes based on the strength of available evidence supporting a causal relationship. Table X reports the strength of evidence for included outcomes.

Relative risk

Relative risk estimates were derived from prospective cohort studies and population-based case-control studies. Sources used in relative risk estimation are reported in Table X.

Table X reports relative risk estimates and uncertainty for the two outcomes included in analysis, by sex. We extracted the underlying effect size estimates from prospective cohort studies and population-based case-control studies identified by performing a systematic literature review as well as by reviewing the underlying studies included in published meta-analyses. We did not include hospital-based case control studies due to concerns over representativeness. We only included sources that adequately adjusted for major confounders, especially smoking status. Summary effect size estimates were calculated in R, using the 'metafor' package. We performed a random effects meta-analysis using the DerSimonian and Laird method, which does not assume a true effect size but considers each input study as selected from a random sample of all possible sets of studies for the outcome of interest.[1] The random-effects method allows for more variation between the studies, and incorporates this variance into the estimation process. We used an inverse-variance weighting method to determine component study weights.

We found significantly different relative risks for oral cancer for males and females, and estimated relative risks separately by sex for oral cancer alone. The strength of evidence was only sufficient to attribute risk in countries who predominantly use chewing tobacco. As more evidence becomes available we will continue to re-evaluate risk-outcome pairs and the harm associated with additional smokeless tobacco products.

Theoretical minimum risk exposure level

The theoretical minimum risk exposure level is that everyone in the population has been a lifelong non-user of smokeless tobacco products.

Figures and Tables

Figure 1: PRISMA flowchart

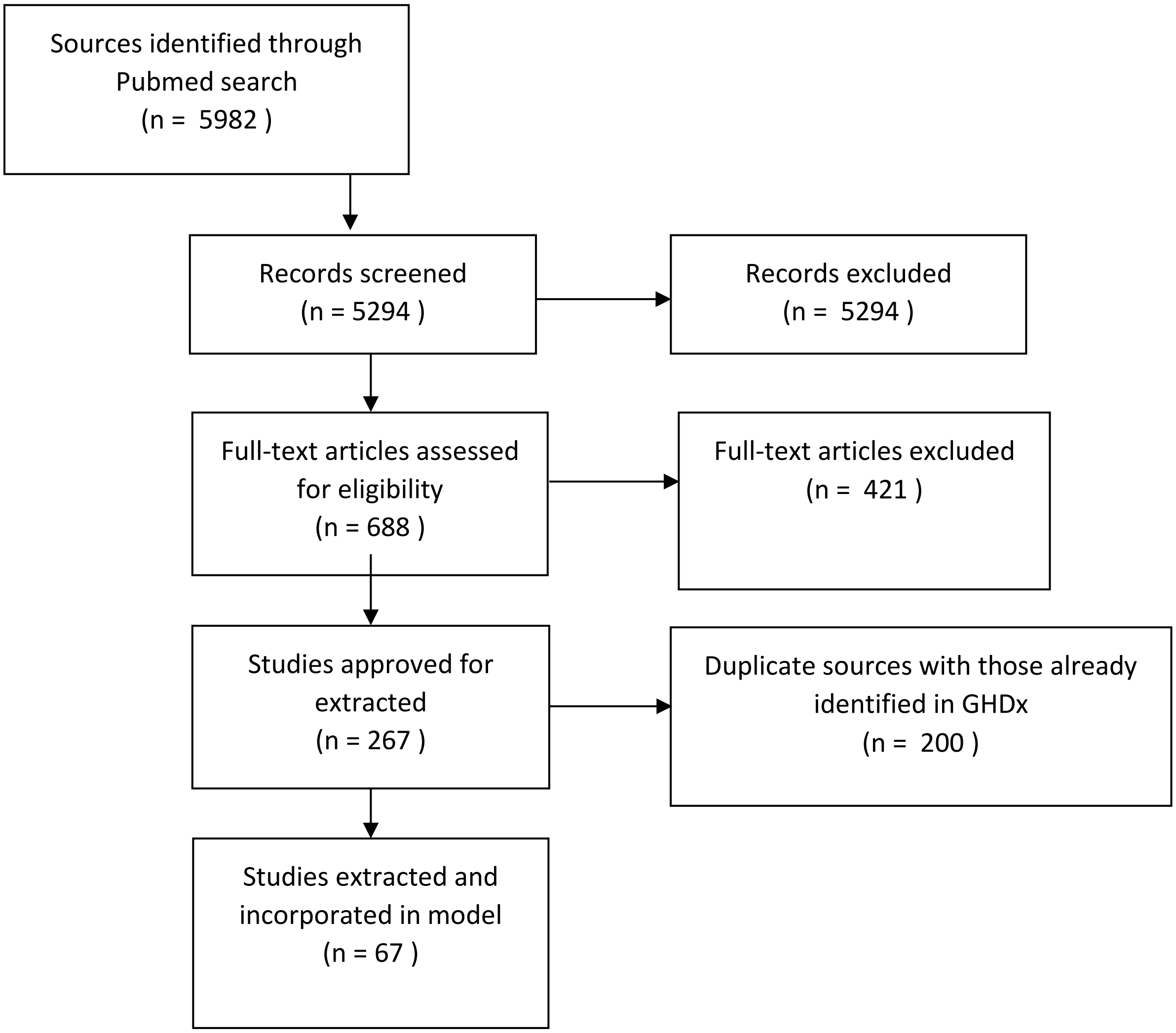

Table 1: Number of datapoints for each smokeless tobacco case definition (working definition in bold)

Case definition	Number of datapoints
**Current use of smokeless tobacco**	34036
Daily use of smokeless tobacco	16252
Occasional use of smokeless tobacco	15071
Ever user of smokeless tobacco	12186
Former-daily use of smokeless tobacco	2470
Ever daily use of smokeless tobacco	1463
Ever non-daily use of smokeless tobacco	441
Former use of smokeless tobacco	440
Former non-daily use of smokeless tobacco	144

Table 2: Regression coefficients used for crosswalk, by GBD super-region or (when possible) region

Case definition	Super-region	Region	Crosswalk Coefficient	R-squared value	Number of sources used in regression
Daily use of smokeless tobacco	Southeast Asia, East Asia, and Oceania	East Asia	1.152	0.832	351
Daily use of smokeless tobacco	Southeast Asia, East Asia, and Oceania	Southeast Asia	1.106	0.911	143
Daily use of smokeless tobacco	High-income		1.469	0.918	11403
Daily use of smokeless tobacco	Latin America and Caribbean	Tropical Latin America	1.002	0.819	566
Daily use of smokeless tobacco	North Africa and Middle East		1.074	0.925	106
Daily use of smokeless tobacco	South Asia		1.056	0.985	893
Daily use of smokeless tobacco	Sub-Saharan Africa	Central Sub-Saharan Africa	1.068	0.973	365
Daily use of smokeless tobacco	Sub-Saharan Africa	Eastern Sub-Saharan Africa	1.047	0.987	134
Daily use of smokeless tobacco	Sub-Saharan Africa	Southern Sub-Saharan Africa	1.068	0.973	365
Daily use of smokeless tobacco	Sub-Saharan Africa	Western Sub-Saharan Africa	1.15	0.978	173
Occasional use of smokeless tobacco	Southeast Asia, East Asia, and Oceania	Oceania	1.274	0.831	176
Occasional use of smokeless tobacco	Central Europe, Eastern Europe, and Central Asia		1.534	0.847	124
Occasional use of smokeless tobacco	High-income		2.02	0.822	11353

Occasional use of smokeless tobacco	Latin America and Caribbean	Caribbean	1.022	0.926	326
Ever used smokeless tobacco	South Asia		0.647	0.924	208
Ever used smokeless tobacco daily	South Asia		0.998	0.988	137
Ever used smokeless tobacco daily	Sub-Saharan Africa	Central Sub-Saharan Africa	0.949	0.962	185
Ever used smokeless tobacco daily	Sub-Saharan Africa	Eastern Sub-Saharan Africa	0.949	0.962	185
Ever used smokeless tobacco daily	Sub-Saharan Africa	Southern Sub-Saharan Africa	0.949	0.962	185
Ever used smokeless tobacco daily	Sub-Saharan Africa	Western Sub-Saharan Africa	0.882	0.968	104

Alcohol Use Capstone Write-up

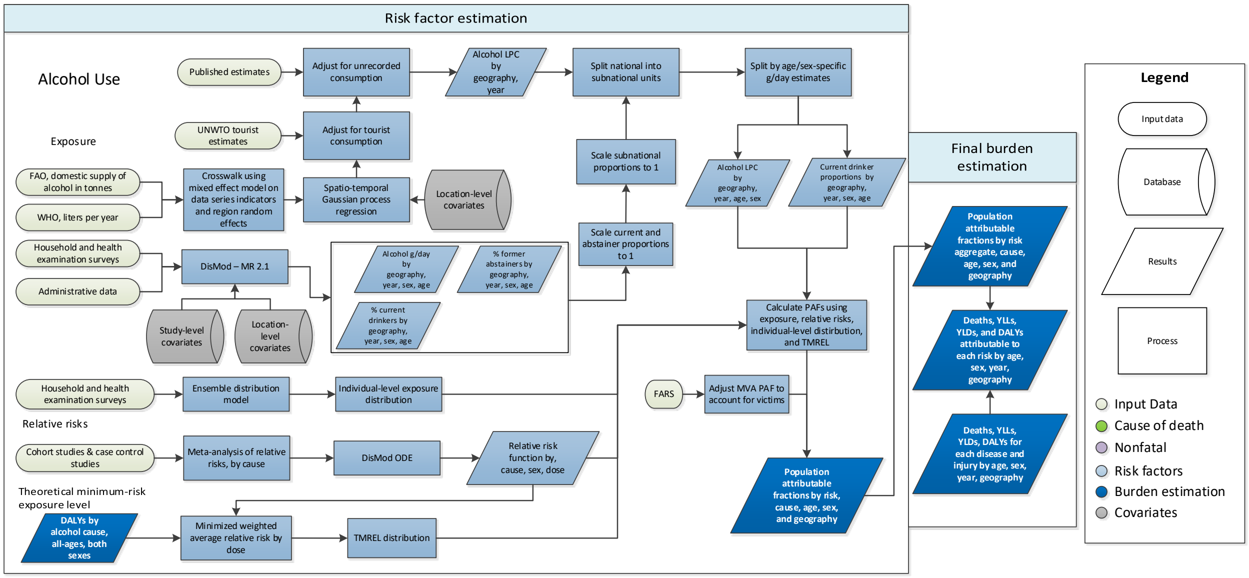

Exposure

Definitions

We defined exposure as the grams per day of pure alcohol consumed amongst drinkers. We constructed this exposure using the indicators outlined below:

1. Current drinkers, defined as the proportion of individuals who have consumed at least one alcoholic beverage (or some approximation) in a 12-month period.
2. Lifetime abstainers, defined as the proportion of individuals who have never consumed an alcoholic beverage.
3. Alcohol consumption (in grams per day), defined as grams of alcohol consumed by current drinkers, per day, over a 12-month period.
4. Alcohol liters per capita stock, defined in liters per capita of pure alcohol, over a 12-month period.

We also used three additional indicators to adjust alcohol exposure estimates to account for different types of bias:

1. Number of tourists within a location, defined as the total amount of visitors to a location within a 12 month period.
2. Tourists' duration of stay, defined as the number of days resided in a hosting country.
3. Unrecorded alcohol stock, defined as a percentage of the total alcohol stock produced outside established markets.

Input data

A systematic review of the literature was performed to extract data on our primary indicators. The Global Health Exchange (GHDx), IHME's online database of health-related data, was searched for population survey data containing participant-level information from which we could formulate the required alcohol use indicators on current drinkers, lifetime abstainers, alcohol consumption, and binge drinkers. Data-sources were included if they captured a sample representative of the geographic location under study. We documented relevant survey variables from each data-source in a spreadsheet and extracted using STATA 13.1 and R 3.3 . A total of 2,821 potential data-sources were available in the GHDx across countries with subnational locations, out of which 191 data-sources (corresponding 88,734 tabulated data-points by location/year/sex/age) were included across the four indicators mentioned above.

To generate estimates of alcohol consumption in liters per capita (LPC), we obtained data from FAOSTAT, and WHO GISAH database [1-2]. To provide more stable time trends in the model, we transformed FAO sales data (which calculates stock based on primary inputs) to a lagged five-year average. Given WHO uses FAO data in locations where WHO could not find data using their own methods, we removed FAO data in the locations where WHO used FAO data in place of their own. To correct for bias in the underlying data sources, we adjusted the input data (crosswalked), by running a mixed effect model on the log average of the data with dummy variables for the data series, as well as random effects on super region, region, country, and time. We adjusted the data points using the following equation:

$$Log\ Average\ Data = D + (Super\ Region\ |D, Region\ |\ D, Country\ |D, Year|\ D)$$

$$Transformed\ data = \ data\ *\ e^{\widehat{\beta_1} + \widehat{\beta_3}}$$

where:

$D\ is\ a\ dummy\ variable\ for\ a\ data\ source$

None of the data sources on liters per capita provided estimates of uncertainty, which is a component required for our eventual modeling strategy. To generate uncertainty, we ran a Loess model on the adjusted data points and the standard deviation between the difference of the Loess smoothed model and the adjusted data points across a five-year span was used as the standard deviation of the data. (i.e., if the total stock changes more variably in a narrow time frame, we believe the data to be more uncertain).

We obtained data on the number of tourists and their duration of stay from the UNWTO [4]. We applied a crosswalk across different tourist categories, similar to the one used for the liters per capita data, to arrive at a consistent definition (i.e. visitors to a country).

We obtained estimates on unrecorded alcohol stock from six published papers [4-9], consisting of 166 locations.

Modeling Strategy

While population-based surveys provide accurate estimates of the prevalence of lifetime abstainers and current drinkers, they typically underestimate real alcohol consumption levels [10-12]. As a result, we considered the liter per capita input to be a better estimate of overall volume of consumption. Per capita consumption, however, does not provide age- and sex-specific consumption estimates needed to compute alcohol-attributable burden of disease. Therefore, we use the age-sex pattern of consumption among drinkers modeled from the population survey data and the overall volume of consumption from FAO and GISAH to determine the total amount of alcohol consumed within a location. In the paragraphs we outline how we estimated each primary input in the alcohol exposure model, as well as how we combined these inputs to arrive at our final estimate of grams per day of pure alcohol. We estimated all models below using 1000 draws.

For data obtained through surveys, we used DisMod-MR 2.1 to construct estimates for each country/year/age/sex. We chose to use DisMod due to its ability to leverage information across the heterogeneous age groups reported in the surveys, through age-integration, as well as the model's ability to leverage information available from data in nearby locations or time-periods [13].

We modeled the alcohol liters per capita data, as well as the total number of tourists, using a spatio-temporal Gaussian process regression (ST-GPR). We chose parameters, as well as our final model, using out-of-sample 10-fold cross validation.

Given the heterogeneous nature of the estimates on unrecorded consumption, as well as the wide variation across countries and time-periods, we took 1000 draws from the uniform distribution of the lowest and highest estimates available for a given country. We did this to incorporate the diffuse uncertainty within the unrecorded estimates reported. We used these 1000 draws in the above equation. We adjusted LPC only for countries where estimates were available.

We adjusted the alcohol LPC for unrecorded consumption using the following equation:

$$Alcohol\ LPC = \frac{Alcohol\ LPC}{(1 - \%\ Unrecorded)}$$

We then adjusted the estimates for alcohol LPC for tourist consumption by adding in the per capita rate of consumption abroad and subtracting the per capita rate of tourist consumption domestically.

$$Alcohol\ LPC_d = Unadjusted\ Alcohol\ LPC_d + Alcohol\ LPC_{Domestic\ consumption\ abroad} - Alcohol\ LPC_{Tourist\ consumption\ domestically}$$

$$Alcohol\ LPC_i = \frac{\sum_l Tourist\ Population_l * Proportion\ of\ tourists_{i,l} * Unadjusted\ Alcohol\ LPC_l * \frac{Average\ length\ of\ stay_{i,l}}{365} *}{Population_d}$$

where:

l *is the set of all locations*, i *is either* Domestic consumption abroad *or* Tourist consumption domestically, *and* d *is a domestic location*

After adjusting alcohol LPC by tourist consumption and unrecorded consumption for all location/years reported, sex-specific and age-specific estimates were generated by incorporating estimates modeled in DisMod for percentage of current drinkers within a location/year/sex/age, as well as consumption trends modeled in the DisMod g/day model. We do this by first making sure the sum of percent current drinkers and percent abstainers sum to one for a given location/year/age/sex. We then calculate the proportion of total consumption for a given location/year by age and sex, using the estimates of alcohol consumed per day, the population size, and the percentage of current drinkers. Lastly, we then multiply this proportion of total stock for a given location/year/sex/age by the total stock for a given location/year to calculate the consumption in terms of liter per capita for a given location/year/sex/age. We then convert these estimates to be in terms of grams/per day. The following equations describe these calculations:

$$\%\, Current\ drinkers_{\,l,y,s,a} = \frac{\%\, Current\ drinkers_{\,l,y,s,a}}{\%\, Current\ drinkers_{\,l,y,s,a} + \%\, Abstainers_{l,y,s,a}}$$

$$Proportion\ of\ total\ consumption_{\,l,y,s,a} = \frac{Alcohol\ \ g/day_{\ l,y,s,a} \ *\ Population_{\,l,y,s,a} \ *\ \%\, Current\ drinkers_{\,l,y,s,a}}{\sum_{s,a} Alcohol\ \ g/day_{\,l,y,s,a} \ *\ Population_{\,l,y,s,a} \ *\ \%\, Current\ drinkers_{\,l,y,s,a}}$$

$$Alcohol\ LPC_{\,l,y,s,a} = \frac{Alcohol\ LPC_{\,l,y} \ *\ Population_{\,l,y} \ *\ Proportion\ of\ total\ consumption_{\,l,y,s,a}}{\%\, Current\ drinkers_{\,l,y,s,a} * Population_{\,l,y,s,a}}$$

$$Alcohol\ g/day_{\,l,y,s,a} = Alcohol\ LPC_{\,l,y,s,a} * \frac{1000}{365}$$

where:

$l\ is\ a\ location, y\ is\ a\ year, s\ is\ a\ sex, and\ a\ is\ an\ age\ group.$

We then used the gamma distribution to estimate individual level variation within location, year, sex, age drinking populations, following the recommendations of other published alcohol studies [7-8]. We chose parameters of the gamma distribution based on the mean and standard deviation of the 1000 draws of alcohol g/day exposure for a given population.

Theoretical minimum-risk exposure level

We calculated TMREL by first calculating the overall risk attributable to alcohol. We did this by weighting each relative risk curve by the share of overall DALYs for a given cause. We then took the minimum of this overall-risk curve as the TMREL of alcohol-use. More formally,

$$TMREL = argmin\ average\ overall\ risk_{\omega}(g/day)$$

$$Average\ overall\ risk_{\omega}(g/day) = \sum_{i}^{\omega} RR_i(g/day) * \frac{DALY_i}{\sum_i^{\omega} DALY_i}$$

Where:

ω *is the set of causes associated with alcohol*, i *is a given cause from that set*, *DALY is the global DALY rate in* 2010, *and RR is the dose response curve for a given cause and exposure level in grams per day.*

In other words, we chose TMREL as being the exposure that minimizes your risk of suffering burden from any given cause related to alcohol. We weight the risk for a particular cause in our aggregation by the proportion of DALYs due to that cause. (e.g. since more observed people die from IHD, we weight the risk for IHD more in the above calculation of average risk compared to, say, diabetes, even if both have the same relative risk for a given level of consumption)

Relative risks

For GBD2016, we performed a systematic literature review of all cohort and case-control studies reporting a relative risk, hazard ratio, or odds ratio for any risk-outcome pairs studied in GBD 2016. Studies were included if they reported a categorical or continuous dose for alcohol consumption, as well as uncertainty measures for their outcomes, and the population under study was representative. Relative risk estimates by dose can be found in Appendix Table 1.

We then used these studies to calculate a dose-response, modeled using DisMod ODE. We chose DisMod ODE rather than a conventional mixed effect meta-regression because of its ability to estimate nonparametric splines over doses (i.e. for most alcohol causes, there is a non-linear relationship with different doses) and incorporate heterogeneous doses through dose-integration (i.e. most studies report doses categorically in wide ranges. DisMod ODE estimates specific doses when categories overlap across studies, through an integration step.) We used the results of the meta-regression to estimate a non-parametric curve for all doses between 0-150 g/day and their corresponding relative risks. For all causes, we assumed the relative risk was the same for all-ages and sexes, with the exception of ischemic heart disease, ischemic stroke, hemorrhagic stroke, and diabetes, which we estimated by sex.

Regarding injuries outcomes, we constructed relative risks based on chronic exposure rather than acute, which has a weaker relationship to the outcome, though still significant [15-16, 18-21]. We decided to use chronic exposure given the lack of available data on acute exposure, as well as, the lack of cohort studies using acute exposure as a metric. Further, using chronic exposure allowed us to construct relative risks curves for unintentional injuries, interpersonal violence, motor vehicle accidents, and self-harm using the same method as reported above.

In the case of motor vehicle accidents, we adjusted the PAF to account for victims of drunk drivers that are involved in accidents. Using data from the Fatality Analysis Reporting System in the US [17], we calculated the average number of fatalities in a car crash involving alcohol, as well as the percentage of those fatalities distributed by age and sex (figures 1 and 2). We aggregated FARS data across the years 1985-2015, given there was little variation in the data temporally and the number of cases in old age groups had too much variance when constructing estimates by year. To adjust PAFs, we multiplied attributable deaths by the average number of fatalities from FARS and redistributed the PAF amongst each population, based on the probability of being a victim to a certain drunk driver by age and sex, based on the FARS data. The following equation describes this process:

$$Adjusted\ PAF_i = \frac{\sum_d PAF_d * DALY_d * Avg\ Fatalities_d * \ P(i\ is\ a\ victim)_d}{DALY_i}$$

where:

$i\ is\ a\ population\ by\ location, year, age, sex\ and$

$d\ is\ the\ set\ of\ all\ age\ and\ sex\ exposed\ groups\ within\ that\ location\ and\ year.$

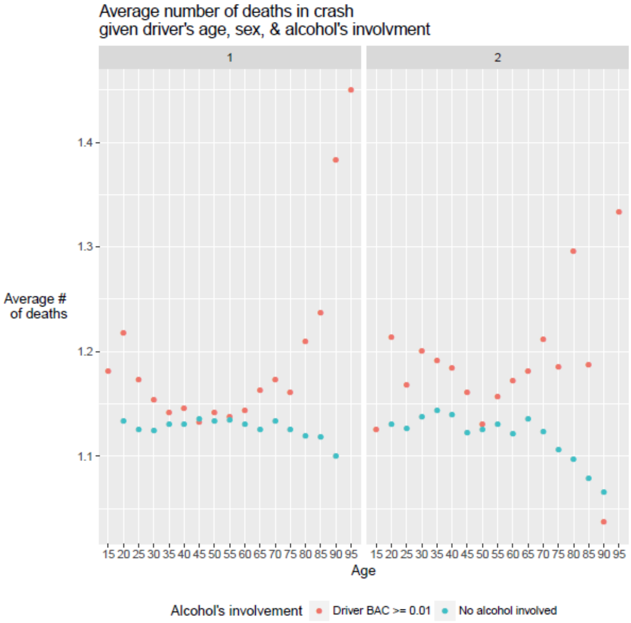

Figure 1

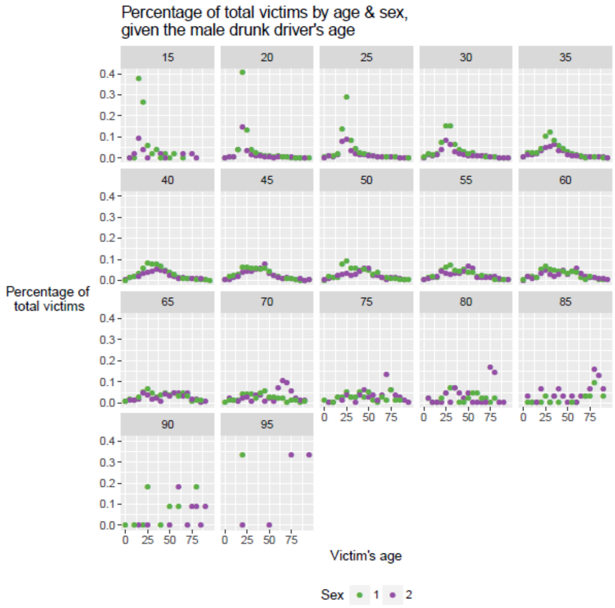

Figure 2

PAF

For all causes, we defined PAF as:

$$PAF(x) = \frac{P_A + \int_0^{150} P(x) * RR_C(x)\,dx - 1}{P_A + \int_0^{150} P(x) * RR_C(x)\,dx} \qquad P(x) = P_C * \Gamma(\boldsymbol{p})$$

where:

$P_C\ is\ the\ prevalence\ of\ current\ drinkers, P_a\ is\ the\ prevalence\ of\ abstainers, RR_c(x)\ is\ the\ relative$
$risk\ function\ for\ current\ drinkers, and\ \boldsymbol{p}\ are\ parameters\ determined\ by\ the\ mean\ and\ sd\ of\ exposure$

We performed the above equation for 1000 draws of the exposure and relative risk models. We then used the estimated PAF draws to calculate YLL, YLDs, and DALYs, as per the other risk factors.

References

1. Food and Agriculture Organization of the United Nations (FAO). FAOSTAT Food Balance Sheets, October 2014. Rome, Italy: Food and Agriculture Organization of the United Nations (FAO).
2. World Health Organization (WHO). WHO Global Health Observatory - Recorded adult per capita alcohol consumption, Total per country. Geneva, Switzerland: World Health Organization (WHO).
3. UN World Tourism Organization (UNWTO). UN World Tourism Organization Compendium of Tourism Statistics 2015 [Electronic]. Madrid, Spain: UN World Tourism Organization (UNWTO), 2016.
4. Norstrom, Thor. "Estimating changes in unrecorded alcohol consumption in Norway using indicators of harm." *Addiction* 93.10 (1998): 1531-1538.
5. Macdonald, Scott. "Unrecorded alcohol consumption in Ontario, Canada: estimation procedures and research implications." *Drug and Alcohol Review* 18.1 (1999): 21-29.
6. Meier, Petra Sylvia, et al. "Adjusting for unrecorded consumption in survey and per capita sales data: quantification of impact on gender-and age-specific alcohol-attributable fractions for oral and pharyngeal cancers in Great Britain." *Alcohol and Alcoholism* 48.2 (2013): 241-249.
7. Hao, Wei, Hanhui Chen, and Zhonghua Su. "China: alcohol today." *Addiction* 100.6 (2005): 737-741.
8. Rehm, Jürgen, and Vladimir Poznyak. "On monitoring unrecorded alcohol consumption." *Alcoholism and Drug Addiction* 28.2 (2015): 79-89.
9. Probst et al. "Unrecorded Alcohol Use: A global modeling study based on Delphi assessments and survey data". Toronto, Canada: CAMH.
10. Ramstedt, Mats. "How much alcohol do you buy? A comparison of self-reported alcohol purchases with actual sales." *Addiction* 105.4 (2010): 649-654.
11. Stockwell, Tim, et al. "Under-reporting of alcohol consumption in household surveys: a comparison of quantity–frequency, graduated–frequency and recent recall." *Addiction* 99.8 (2004): 1024-1033.
12. Kerr, William C., and Thomas K. Greenfield. "Distribution of alcohol consumption and expenditures and the impact of improved measurement on coverage of alcohol sales in the 2000 National Alcohol Survey." *Alcoholism: Clinical and Experimental Research* 31.10 (2007): 1714-1722.
13. An Integrative Metaregression Framework for Descriptive Epidemiology. Abraham D. Flaxman, Theo Vos, Christopher J. L. Murray. Seattle: University of Washington Press, [2015]
14. Taylor, Bruce, et al. "The more you drink, the harder you fall: a systematic review and meta-analysis of how acute alcohol consumption and injury or collision risk increase together." *Drug and alcohol dependence* 110.1 (2010): 108-116.
15. Vinson, Daniel C., Guilherme Borges, and Cheryl J. Cherpitel. "The risk of intentional injury with acute and chronic alcohol exposures: a case-control and case-crossover study." *Journal of studies on alcohol* 64.3 (2003): 350-357.
16. Vinson, Daniel C., et al. "A population-based case-crossover and case-control study of alcohol and the risk of injury." *Journal of studies on alcohol*64.3 (2003): 358-366.
17. Fatal Accident Reporting System (FARS). National Highway Traffic Safety Administration, National Center for Statistics and Analysis Data Reporting and Information Division (NVS-424); 1985, 1990, 1995, 2000, 2005, 2010, 2015

18. Chen, Li-Hui, Susan P. Baker, and Guohua Li. "Drinking history and risk of fatal injury: comparison among specific injury causes." *Accident Analysis & Prevention* 37.2 (2005): 245-251.
19. Bell, Nicole S., et al. "Self-reported risk-taking behaviors and hospitalization for motor vehicle injury among active duty army personnel." *American journal of preventive medicine* 18.3 (2000): 85-95.
20. Margolis, Karen L., et al. "Risk factors for motor vehicle crashes in older women." *The Journals of Gerontology Series A: Biological Sciences and Medical Sciences* 57.3 (2002): M186-M191.
21. Sorock, Gary S., et al. "Alcohol-drinking history and fatal injury in older adults." *Alcohol* 40.3 (2006): 193-199.

Injecting Drug Use Capstone Appendix

Flowchart

Drug Use

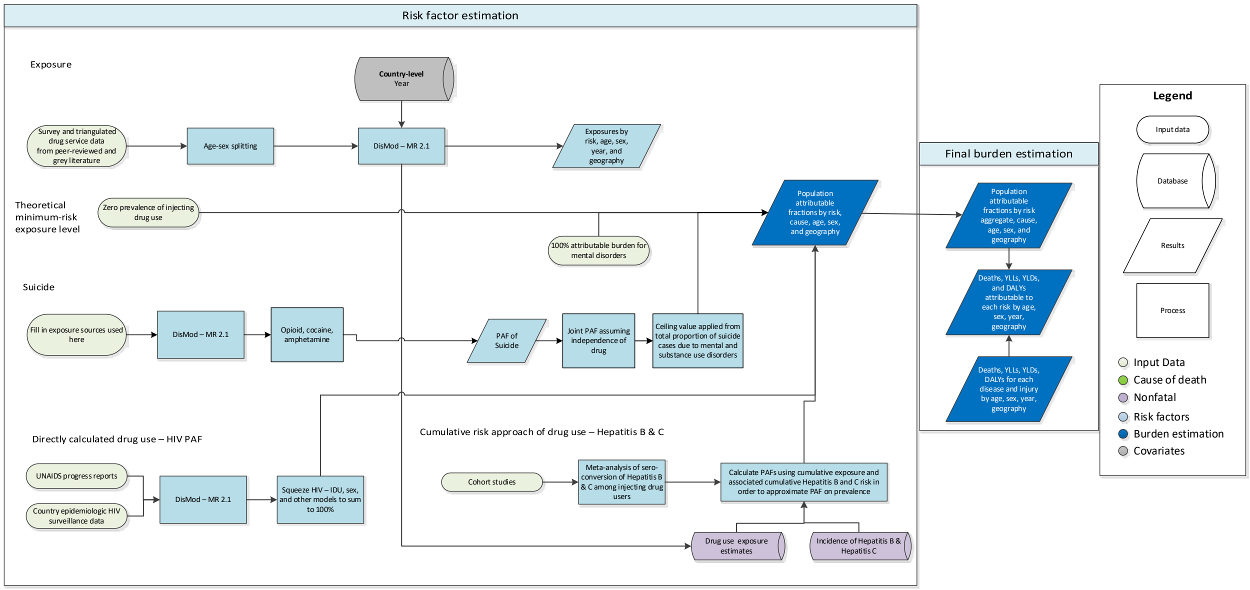

Input Data & Methodological Summary

Exposure

Case definition

Injecting drug users (IDU) are at high risk from blood-borne infections, including human immunodeficiency virus (HIV) and Hepatitis B and C viruses (HBV and HCV, respectively), through the use of shared needles and injection equipment. In GBD 2010, based on the available epidemiological literature and the availability of exposure estimates[2,3] we measure the burden of disease attributable to HIV, HBV and HCV due to injecting drug use. An injecting drug user was defined as a current or recent user aged 15-64 years old.

Input data

The major burden of mortality from viral hepatitis is due to cirrhosis and liver cancer resulting from chronic hepatitis infection. Cirrhosis mortality was modelled with vital registration data using CODEm. Etiologic proportion models, estimated using DisMod-MR 2.1, were used to split the overarching cirrhosis mortality estimates into cases of cirrhosis attributable to hepatitis B, hepatitis C, alcohol, and other causes.(1-4)

Liver cancer mortality was modelled using cancer registry data. The incidence numbers were transformed into mortality estimates using mortality to incidence ratios. The mortality estimates from cancer registries were then combined with vital registration system data as input data into CODEm, which produced the final mortality estimates for liver cancer. As with cirrhosis mortality, etiologic

proportions for liver cancer due to hepatitis B, and C, alcohol, and other causes were generated using DisMod-MR 2.1.

To estimate the burden of HIV cases attributable to IDU, we extracted data on the proportion of notified HIV cases by transmission route – sexual intercourse, injecting drug use, commercial sex work and other -- from a number of agencies that conduct surveillance of HIV across the globe.(6-13)

The prevalence of current injecting drug use was estimated using data from a 2008 review conducted by the Reference Group to the UN on HIV and injecting drug use (15), and a new review currently being conducted by international collaborations and experts. The reviews used a multistage process of systematic review adhering to international guidelines. It involved multiple stages of peer and expert review, with searches of the peer-reviewed literature in addition to an extensive review of online grey literature databases in the drug and alcohol and HIV fields. Additional data on the age and sex distribution of injecting drug use were sourced for this modelling exercise.

In order to generate a pooled incidence rate/absolute relative risk for viral hepatitis among people who inject drugs, we conducted a meta-analysis of longitudinal epidemiological studies that reported a hepatitis B (16-20) or hepatitis C (16-31) incidence rate among PWID. We calculated confidence intervals for the incidence rate (where no CI was reported) from a Poisson distribution around the number of cases.

We excluded studies that focused on non-representative subgroups, such as recent injectors or adolescents or because hepatitis incidence is far higher in those groups than for all people who inject drugs (e.g.(32)). We did not vary incidence among active injectors according the availability of blood borne virus prevention strategies (e.g. NSPs, opioid substitution therapy) because too few studies have examined different levels of incidence according to variable coverage, and we were not able to estimate coverage by country over time. In any case, in most countries, coverage of virus prevention strategies remains very low among people who inject drugs,(33) and would have been negligible in most countries until recent years.

Modeling strategy

As part of the GBD 2016 study, we measured the burden of hepatitis B and hepatitis C (including attributable cirrhosis and liver cancer) and HIV at the country, regional, and global level for each age-sex group for the years 1990 to 2016. For HIV, hepatitis B and hepatitis C, disease-specific natural history models were used to estimate deaths and YLDs, because the three-state model in DisMod-MR 2.01(susceptible, cases, dead) did not capture the complexity of the disease processes.

Mortality estimation

Mortality due to overall acute hepatitis was modelled with vital registration data using the Cause of Death Ensemble Modelling tool (CODEm), an analytical tool that tests the predictive power of hundreds of models to estimate trends in causes of death.(5) Due to poor coverage of cause of death data for each of the acute hepatitis varieties, four natural history models for hepatitis B and C were used to estimate mortality by deriving incidence from measurements of seroprevalence and then multiplying incidence by case fatality to estimate the number of deaths. These four models were then squeezed so as to fit the parent cause of death model.

We estimated HIV mortality using a modified UNAIDS Spectrum model.(2) This is a compartmental HIV progression model estimates age-specific incidence, prevalence and death rates using methods described elsewhere.(2) This modelling approach was adapted according to epidemic type, including concentrated and generalized epidemics. For concentrated epidemics, the Spectrum models were corrected for misclassification of HIV deaths and then calibrated to align with vital registration data. For generalised HIV epidemics, we minimised a loss function to select epidemic curves that were most consistent with the prevalence and all-cause mortality data.(2)

Estimation of Years Lived with Disability

For non-fatal estimation, we estimated the incidence of hepatitis B and C using seroprevalence data in DisMod-MR 2.1. For both hepatitis B and C, we use data on the seroprevalence of the hepatitis surface antigen (a marker of chronic infection in hepatitis B and a marker of ever-infection in hepatitis C), excess mortality, and remission, to estimate incidence of both hepatitis infections. Incidence of cirrhosis was also estimated in DisMod using cirrhosis hospital data and cause-specific mortality rate (CSMR) data.

Incidence of liver cancer was derived by dividing mortality by the mortality to incidence ratios, which were then used to predict liver cancer survival. Finally, we estimated prevalence as a function of incidence and survival by splitting prevalence into four phases. Each phase had different disability weights, which were used to generate YLDs for that phase.

Finally, incidence of HIV was also estimated using the UNAIDS Spectrum modelling approach described above in the mortality estimation section.

Burden of HIV attributable to injecting drug use

We then estimated the proportion of HIV cases attributable to three transmission categories (sex, IDU and other) for all country-time periods using DisMod-MR 2.1. The only covariate used in the model was one that added variance to the data points derived from data sources that attributed a portion of HIV cases to “unknown” transmission sources. We scaled the proportions from each of the three transmission models (sex, IDU and other) to ensure that they fit the total HIV transmission envelope by country, year, age and sex.

Burden of hepatitis B and hepatitis C attributable to injecting drug use

To estimate the relative contribution of IDU to hepatitis B and C disease burden at the country, regional and global level, we used a cohort method. We re-calibrated individuals according to history of injecting drug use, and their accumulated risk of incident hepatitis B and C due to IDU. We made use of data on prevalence of current injecting drug use, pooled in DisMod-MR 2.1; a meta-analysis of incidence rates of hepatitis B and hepatitis C among people who inject drugs; and estimates of population-level incidence of hepatitis B and C between 1990 and 2016. We used back extrapolations to estimate incidence before 1990. These steps are detailed below.

To estimate the lifetime risk of being infected with hepatitis B or C, we undertook a cohort analysis for each country, year, age, and sex category and estimated the probability of an individual having been infected in each preceding year. One of the main inputs to this cohort method was the probability of having injected drugs in a specific age cohort in a given calendar year. For example, for a cohort of 40-year-olds in 2015, the relevant probability in 2005 is the estimated prevalence of injecting drug use among 30-year-olds.

In addition to a global time series of estimated prevalence of injecting drug use, we also used the incidence of hepatitis B or C and the sero-conversion rate of hepatitis B and hepatitis C among people who inject drugs for each age-sex-country-year from 1960 to 2013 by 5-year age groups.

1. Incidence rate of Hepatitis B and C in the general population

We modelled the annual incidence rate of hepatitis B and hepatitis C using sero-prevalence data in DisMod-MR 2.1. We assumed a low remission (mean 0.015 and standard error 0.0075)(14) in the hepatitis B model to reflect the small proportion of cases who spontaneously clear the infection. We assumed zero remission for hepatitis C.

2. Prevalence of ever-injecting drug use

DisMod-MR 2.1 was used to estimate the prevalence of injecting drug use with year as a covariate to estimate the trends over time. DisMod makes an average estimate of the change in drug use over the time period from 1990-2016 and we took draws from a normal distribution of the coefficient to project IDU prevalence backward in time to 1960 from baseline level in 1990.

3. Pooled seroconversion hazard of hepatitis C and hepatitis B among people who ever injected drugs

This pooled sero-conversion hazard for both hepatitis C and hepatitis B was derived from a meta-analysis of longitudinal epidemiologic studies described above in the input data section.

Theoretical minimum-risk exposure level

The theoretical minimum-risk exposure level is defined as zero exposure to injecting drug use.

Relative risks

For drug use, there were not substantial changes made to the effect sizes from GBD 2015. We used a pooled absolute risk of Hepatitis C and Hepatitis B among those who have ever used injecting drugs.

In addition to assessing IDU as a risk factor for blood-borne infections, the broader category of mental and substance use disorders is assessed as risk factors for suicide. The suicide burden attributable to mental and substance use disorders is estimated by comparing the current health status with a theoretical-minimum-risk exposure defined as the counterfactual status of the absence of mental and substance use disorders (Ferrari, Norman et al 2014).

References

1. Fitzmaurice C, Dicker D, Pain A, Hamavid H, Moradi-Lakeh M, MacIntyre MF, et al. The Global Burden of Cancer 2013. JAMA oncology. 2015;1(4):505-27.
2. Murray CJ, Ortblad KF, Guinovart C, Lim SS, Wolock TM, Roberts DA, et al. Global, regional, and national incidence and mortality for HIV, tuberculosis, and malaria during 1990-2013: a systematic analysis for the Global Burden of Disease Study 2013. Lancet. 2014;384(9947):1005-70.

3. GBD 2013 YLDs Collaborators. Global, regional, and national incidence, prevalence and YLDs for 301 acute and chronic diseases and injuries for 188 countries, 1990-2013: A systematic analysis for the Global Burden of Disease Study 2013. The Lancet. 2015;386:743-800.
4. Naghavi M, Wang H, Lozano R, Davis A, Liang X, Zhou M, et al. Global, regional, and national age-sex specific all-cause and cause-specific mortality for 240 causes of death, 1990-2013: a systematic analysis for the Global Burden of Disease Study 2013. Lancet. 2015;385(9963):117-71.
5. Foreman KJ, Lozano R, Lopez AD, Murray C. Modeling causes of death: an integrated approach using CODEm. Population Health Metrics. 2012;10(1).
6. European Centre for Disease Prevention. HIV/AIDS surveillance in Europe 2014 Solna, Sweden. http://ecdc.europa.eu/en/publications/surveillance_reports/HIV_STI_and_blood_borne_viruses/Pages/HIV_STI_and_blood_borne_viruses.aspx: ECDC, 2014.
7. Family Health International, Bureau of AIDS TB and STIs Department of Disease Control. The Asian Epidemic Model (AEM) Projections for HIV/AIDS in Thailand:2005-2025. Bangkok: Family Health International (FHI) and Bureau of AIDS, TB and STIs, Department of Disease Control, Ministry of Public Health, Thailand, 2008.
8. Kirby Institute. 2015 Annual Surveillance Report of HIV, viral hepatitis, STIs. Sydney, New South Wales. https://kirby.unsw.edu.au/surveillance/2015-annual-surveillance-report-hiv-viral-hepatitis-stis: Kirby Institute, UNSW Australia, 2015.
9. Kirby Institute. Australian NSP survey national data report 2015. Sydney, New South Wales: Kirby Institute, University of New South Wales, 2015.
10. Country reports for Global AIDS Response Progress Reporting [Internet]. UNAIDS. 2014.
11. UNAIDS. UNAIDS Country reports. Geneva: Joint United Nations Programme on HIV/AIDS. http://www.unaids.org/en/regionscountries/countries, 2015.
12. United States Center for Disease Control and Prevention. HIV/AIDS Statistics. Atlanta, Georgia: US CDC. http://www.cdc.gov/hiv/statistics/index.html, 2015.
13. Gouws E, White PJ, Stover J, Brown T. Short term estimates of adult HIV incidence by mode of transmission: Kenya and Thailand as examples. Sex Transm Infect. 2006;82 Suppl 3:iii51-5.
14. McMahon B. The natural history of chronic hepatitis B virus infection. Hepatology. 2009;49(5 Suppl):S45-S55.
15. Mathers BM, Degenhardt L, Phillips B, Wiessing L, Hickman M, Strathdee SA, et al. Global epidemiology of injecting drug use and HIV among people who inject drugs: a systematic review. Lancet. 2008;372(9651):1733-45.
16. Jackson JB, Wei L, Liping F, Aramrattana A, Celentano DD, Walshe L, et al. Prevalence and Seroincidence of Hepatitis B and Hepatitis C Infection in High Risk People Who Inject Drugs in China and Thailand. Hepatitis research and treatment. 2014;2014.
17. Månsson A-S, Moestrup T, Nordenfelt E, Widell A. Continued transmission of hepatitis B and C viruses, but no transmission of human immunodeficiency virus among intravenous drug users participating in a syringe/needle exchange program. Scandinavian Journal of Infectious Diseases. 2000;32(3):253-8.
18. Blomé MA, Björkman P, Flamholc L, Jacobsson H, Molnegren V, Widell A. Minimal transmission of HIV despite persistently high transmission of hepatitis C virus in a Swedish needle exchange program. Journal of viral hepatitis. 2011;18(12):831-9.

19. Hagan H, McGough JP, Thiede H, Weiss NS, Hopkins S, Alexander ER. Syringe exchange and risk of infection with hepatitis B and C viruses. American journal of epidemiology. 1999;149(3):203-13.
20. Crofts N, Aitken CK. Incidence of bloodborne virus infection and risk behaviours in a cohort of injecting drug users in Victoria in 1990-1995. Medical Journal of Australia. 1997;167(1):17-20.
21. Roy K, Goldberg D, Taylor A, Hutchinson S, MacDonald L, Wilson K, et al. A method to detect the incidence of hepatitis C infection among injecting drug users in Glasgow 1993–98. Journal of Infection. 2001;43(3):200-5.
22. Abou-Saleh M, Davis P, Rice P, Checinski K, Drummond C, Maxwell D, et al. The effectiveness of behavioural interventions in the primary prevention of hepatitis C amongst injecting drug users: a randomised controlled trial and lessons learned. Harm reduction journal. 2008;5(1):1.
23. Turner KM, Hutchinson S, Vickerman P, Hope V, Craine N, Palmateer N, et al. The impact of needle and syringe provision and opiate substitution therapy on the incidence of hepatitis C virus in injecting drug users: pooling of UK evidence. Addiction. 2011;106(11):1978-88.
24. Grebely J, Lima VD, Marshall BD, Milloy M, DeBeck K, Montaner J, et al. Declining incidence of hepatitis C virus infection among people who inject drugs in a Canadian setting, 1996-2012. PloS one. 2014;9(6):e97726.
25. Foley S, Abou-Saleh MT. Risk behaviors and transmission of hepatitis C in injecting drug users. Addictive Disorders & Their Treatment. 2009;8(1):13-21.
26. Craine N, Hickman M, Parry J, Smith J, Walker A, Russell D, et al. Incidence of hepatitis C in drug injectors: the role of homelessness, opiate substitution treatment, equipment sharing, and community size. Epidemiology and Infection. 2009;137(09):1255-65.
27. Villano SA, Vlahov D, Nelson KE, Lyles CM, Cohn S, Thomas DL. Incidence and risk factors for hepatitis C among injection drug users in Baltimore, Maryland. Journal of clinical microbiology. 1997;35(12):3274-7.
28. Maher L, Jalaludin B, Chant KG, Jayasuriya R, Sladden T, Kaldor JM, et al. Incidence and risk factors for hepatitis C seroconversion in injecting drug users in Australia. Addiction. 2006;101(10):1499-508.
29. Lucidarme D, Bruandet A, Ilef D, Harbonnier J, Jacob C, Decoster A, et al. Incidence and risk factors of HCV and HIV infections in a cohort of intravenous drug users in the North and East of France. Epidemiology and infection. 2004;132(04):699-708.
30. Partanen A, Malin K, Perälä R, Harju O, Holopainen A, Holmström P, et al. Riski-tutkimus 2000-2003. Pistämällä huumeita käyttävien seurantatutkimus. A-Klinikkasäätiön Raporttisarja nro 52. Helsinki: A-Klinikkasäätiön, 2006.
31. Van Den Berg C, Smit C, Van Brussel G, Coutinho R, Prins M. Full participation in harm reduction programmes is associated with decreased risk for human immunodeficiency virus and hepatitis C virus: evidence from the Amsterdam Cohort Studies among drug users. Addiction. 2007;102(9):1454-62.
32. Larney S, Kopinski H, Beckwith CG, Zaller ND, Jarlais DD, Hagan H, et al. Incidence and prevalence of hepatitis C in prisons and other closed settings: results of a systematic review and meta-analysis. Hepatology. 2013;58(4):1215-24.

33. Degenhardt L, Mathers B, Vickerman P, Rhodes T, Latkin C, Hickman M. Prevention of HIV infection for people who inject drugs: Why individual, structural, and combination approaches are needed. The Lancet. 2010;376:285-301.

Dietary Risks Capstone Appendix

Flowchart

Dietary risks

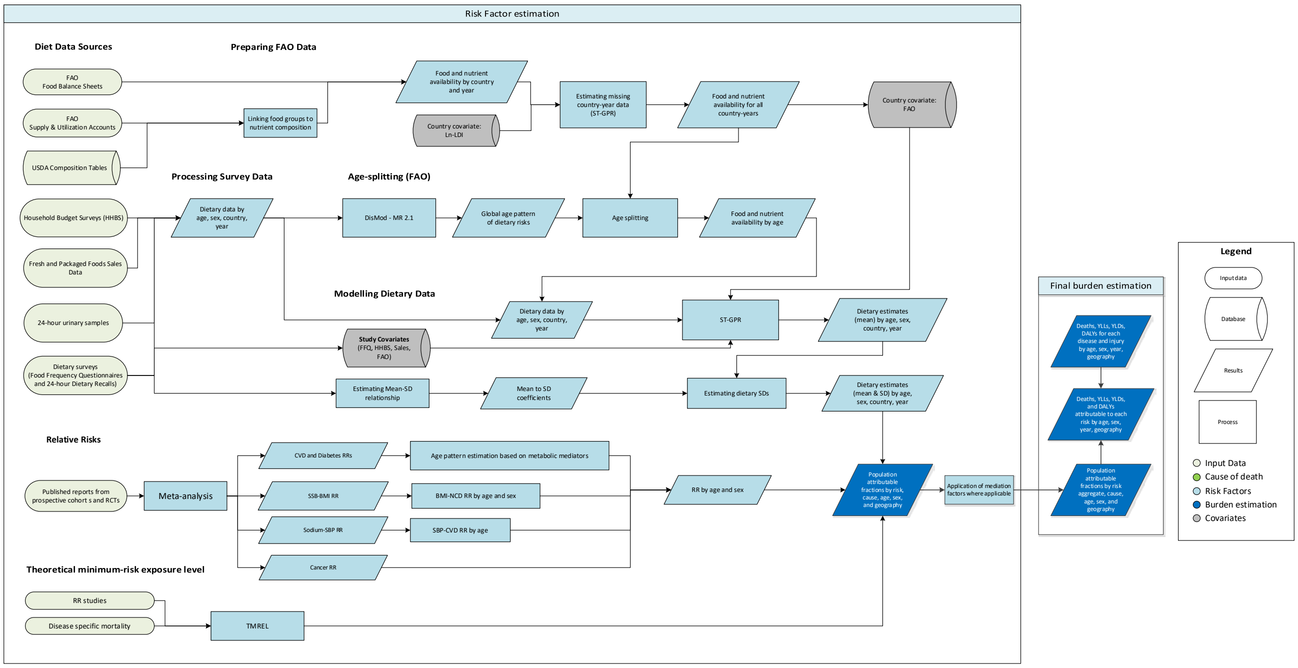

Input data & Methodological summary

Exposure

Case definition

For GBD 2016, risk factors associated with diet include: diet low in fruits, vegetables, legumes, whole grains, nuts and seeds, fiber, seafood omega-3 fatty acids, polyunsaturated fatty acids, calcium, milk; and diet high in red meat, processed meat, sugar sweetened beverages, trans fatty acids, and sodium. Exposure to a diet low in fruits is defined as average daily consumption of less than 250 grams per day of fruits (fresh, frozen, cooked, canned, or dried, excluding fruit juices and salted or pickled fruits). Exposure to diet low in vegetables is defined as average daily consumption of less than 360 grams per day of vegetables (fresh, frozen, cooked, canned or dried vegetables excluding legumes and salted or pickled vegetables, juices, nuts and seeds, and starchy vegetables such as potatoes or corn). Exposure to a diet low in legumes is defined as average daily consumption of less than 60 grams per day of legumes. Exposure to diet low in whole grains is defined as average daily consumption of less than 125 grams per day of whole grains (bran, germ, and endosperm in their natural proportion) from breakfast cereals, bread, rice, pasta, biscuits, muffins, tortillas, pancakes and other sources. Exposure to diet low in nuts and seeds is defined as average daily consumption of less than 20.5 grams per day of nuts and seeds. Exposure to diet low in milk is defined as average daily consumption of less than 435 grams per day of milk including non-fat, low-fat, and full-fat milk, excluding soy milk and other plant derivatives. Exposure to diet low in calcium is defined as average daily consumption of less than 1.15 grams per day of calcium from all sources, including milk, yogurt, and cheese. Exposure to diet low in fiber is defined as average daily consumption of less 23.5 grams per day of than fiber from all sources including fruits, vegetables,

grains, legumes and pulses. Exposure to diet low in seafood omega-3 fatty acids is defined as average daily consumption of less than 250 milligrams per day of eicosapentaenoic acid (EPA) and docosahexaenoic acid (DHA). Exposure to diet low in polyunsaturated fatty acids is defined as average daily consumption of less than 11% of total energy intake from polyunsaturated fatty acids as a replacement for high intake of saturated fatty acids (> 7% of total energy intake). Exposure to diet high in red meat is defined as average daily consumption of greater than 22.5 grams per day of red meat (beef, pork, lamb, and goat but excluding poultry, fish, eggs, and all processed meats). Exposure to diet high in processed meat is defined as average daily consumption of greater than 2 grams of meat preserved by smoking, curing, salting, or addition of chemical preservatives. Exposure to diet high in sugar sweetened beverages is defined as average daily consumption of greater than 2.5 grams per day of beverages with ≥50 kcal per 226.8 gram serving, including carbonated beverages, sodas, energy drinks, fruit drinks, but excluding 100% fruit and vegetable juices. Exposure to diet high in trans fatty acids is defined as average daily consumption of greater than 0.5% of trans fat from all sources, mainly partially hydrogenated vegetable oils and ruminant products. Exposure to diet high in sodium is defined as average 24 hour urinary sodium greater than 3 grams per day.

Input data

We used dietary data from multiple sources including nationally and sub-nationally representative nutrition surveys, household budget surveys, accounts of national sales, and United Nations FAO Food Balance Sheets and Supply and Utilization Accounts. Additionally, for sodium and trans fatty acids, we used data on 24-hour urinary sodium and availability of hydrogenated vegetable oil in packaged foods, respectively. Poly unsaturated and trans fatty acids were modeled as a percent of total dietary energy. We modelled missing country-year data from FAO using a space-time Gaussian process regression and lag-distributed country income as the covariate. For each dietary factor, we estimated the global age pattern of consumption based on nutrition surveys (i.e., 24-hour diet recall) and applied that age pattern to the FAO data. Substantive changes in input data compared to GBD 2015 are as follows: (a) re-extracting data from all nutrition surveys and standardizing the definition of dietary components across sources; (b) incorporating data gathered through a systematic review of literature for each of our dietary risk factors; (c) using sales data for fruit, vegetables, legumes, processed meats, red meats, sugar-sweetened beverages, and milk.

Modeling strategy

We used a spatio-temporal Gaussian process regression (ST-GPR) framework to estimate the intake of each dietary factor by age, sex, country, and year. In GBD 2016, for all dietary factors other than sodium, we considered data from 24-hour diet recall as the gold standard, and cross-walked other methods of assessment to the gold standard method. For sodium, the 24-hour urinary sodium was considered as the gold standard. To estimate the 24-hour urinary sodium based on dietary sodium, we performed a crosswalk adjustment between these two types of data.

Table 1 summarizes the study-level and country-level covariates used in modeling of each dietary factor.

Table 1. Types of data sources (other than 24-hour dietary recall) and covariates used in modeling of each dietary factor.

	Data Sources				Country level covariate
	Sales	FFQ[1]	HBS[2]	FAO	

Diet low in fruits	●	●	●	●	Lag-distributed income, total available kilocalories per person per day
Diet low in vegetables	●	●	●	●	Lag-distributed income, total available kilocalories per person per day
Diet low in legumes	●	-	●	●	Lag-distributed income, total available kilocalories per person per day
Diet low in whole grains	-	●	-	-	Proportion of wheat flour to wheat germ available per person per day
Diet low in nuts and seeds	-	-	●	●	Lag-distributed income, total available kilocalories per person per day
Diet low in milk	●	●	●	●	Lag-distributed income, total available kilocalories per person per day
Diet high in red meat	●	●	●	●	Lag-distributed income, total available kilocalories per person per day
Diet high in processed meat	●	●	●	-	National availability of red meat (grams/person/day), National availability of pig meat (% of energy/person/day), Lag-distributed income
Diet high in sugar-sweetened beverages	●	●	●	-	National availability of sugar (grams/person/day), Lag-distributed income, total available kilocalories per person per day
Diet low in fiber	-	●	-	●	Lag-distributed income, total available kilocalories per person per day
Diet suboptimal in calcium	-	●	-	●	Lag-distributed income, total available kilocalories per person per day
Diet low in seafood omega-3 fatty acids	-	-	-	●	Landlocked nation (Yes/No), Lag-distributed income
Diet low in polyunsaturated fatty acids	-	●	-	●	Lag-distributed income, total available kilocalories per person per day
Diet high in trans fatty acids	●	●	-	-	-
Diet high in sodium[3]	-	-	-	-	-

[1] Food Frequency Questionnaire
[2] Household Budge Survey
[3] For sodium, we used data from the 24-hour urinary sodium and 24-hour dietary recall.

To characterize the distribution of each dietary factor at population level, we use an ensemble approach that separately fit 12 distributions for individual level microdata to specific to each data source's sampled population. The respective goodness of fit of each family was assessed and a weighting scheme was determined to optimize overall fit to the unique distribution of each risk factor. A global mean of the weights for each risk factor's data sources was created. We then determined the standard deviation of each population's consumption through a linear regression that captured the relationship between the standard deviation and mean of intake in nationally representative nutrition surveys using 24-hour diet recalls:

$$ln\,(Standard\ deviation) = \beta_0 + \beta_1 \times ln\,(Mean_i)$$

Then we applied the coefficients of this regression to the outputs of our ST-GPR model to calculate the standard deviation of intake by age, sex, year, and country. We also quantified the within person variation in consumption of each dietary component and adjusted the standard deviations accordingly.

Theoretical minimum-risk exposure level

In GBD 2016, to estimate the TMREL for each dietary factor, we first calculated the level of intake associated with the lowest risk of mortality from each disease endpoint based on the studies included in the meta-analyses of the dietary relative risks. Then, we calculated the TMREL as the weighted average of these numbers using the global number of deaths from each of outcome as the weight (Table 2).

Table 2. Theoretical minimum-risk exposure level for dietary factors in GBD 2015 and GBD 2016.

Dietary Factor	GBD 2016	GBD 2015
Fruits	200-300 gr/day	200-300 gr/day
Vegetables	290-430 gr/day	340-500 gr/day
Legumes	50-70 gr/day	N/A
Whole grains	100-150 gr/day	100-150 gr/day
Nuts	16-25 gr/day	16-25 gr/day
Red meats	18-27 gr/day	18-27 gr/day
Processed meats	0-4 gr/day	0-4 gr/day
Milk	350-520 gr/day	350-520 gr/day
Sugar sweetened beverages	0-5 gr/day	0-5 gr/day
Polyunsaturated fatty acids	9-13% of total daily energy	9-13% of total daily energy
Seafood omega-3 fatty acids	200-300 mg/day	200-300 mg/day
Trans fatty acids	0-1% of total daily energy	0-1% of total daily energy
Dietary fiber	19-28 gr/day	19-28 gr/day
Dietary calcium	1.0-1.3 gr/day	1-1.3 gr/day

Relative Risk

We obtained the relative risk of each disease endpoint per serving of the dietary components from recent dose-response meta-analyses of prospective observational studies, and where available randomized controlled trials. In GBD 2016, we specifically updated the relative risks for the relationship between a diet low in legumes and ischemic heart disease, which is now being considered distinctly as opposed to being placed within the category of vegetables. Considering the well-established age trend of the relative risks of metabolic risk factors for cardiovascular disease and diabetes, we conducted a literature review to identify the most important metabolic mediators for each dietary factor and used the age trend of the relative risk of that mediator(s) and the disease endpoint to estimate the age-specific relative risk for each dietary factors (Table 3).

Table 3. Metabolic mediators used to determine the age trend of the effect of dietary factors on cardiometabolic outcomes.

	Body Mass Index	Total Serum Cholesterol	Fasting Plasma Glucose	Systolic Blood Pressure
Diet low in fruits	●	●	●	●
Diet low in vegetables	●	●	●	●
Diet low in legumes	●	●	●	●
Diet low in whole grains	●	●	●	-
Diet low in nuts and seeds	●	●	●	●
Diet high in red meats	●	-	●	-
Diet high in processed meats	●	-	●	●
Diet low in fiber	-	●	-	-
Diet low in seafood omega-3 fatty acids	●	-	-	●
Diet low in polyunsaturated fatty acids	-	●	●	-
Diet high in trans fatty acids	●	●	-	-

Zinc deficiency Capstone Appendix

Input data & Methodological summary

Exposure

Case definition

Exposure to zinc deficiency is defined as consumption of less than 2.5 milligrams of zinc per day among children between the ages of 1 and 4 years old.

Input data

We used dietary data from nationally and sub-nationally representative nutrition surveys and United Nations FAO Supply and Utilization Accounts to estimate the mean intake of zinc at the population level.

Modeling strategy

For GBD 2016, we first used a spatio-temporal Gaussian process regression (ST-GPR) framework to estimate the mean intake of zinc by age, sex, country, and year. We considered data from 24-hour diet recall as the gold standard, and adjusted data from other sources to the gold standard method. Using the method described in the dietary risks section, we characterized the distribution of zinc intake for children between ages of 1 and 4 years old and estimate the proportion of the children with intake of less than 2.5 milligrams of zinc per day.

Relative Risk

Relative risks used for zinc deficiency is based on the results of randomized trials that measured the effect of zinc supplementation.

Theoretical minimum-risk exposure level

The theoretical minimum-risk exposure level for proportion zinc deficient is zero percent deficient.

Childhood Sexual Abuse

Flowchart

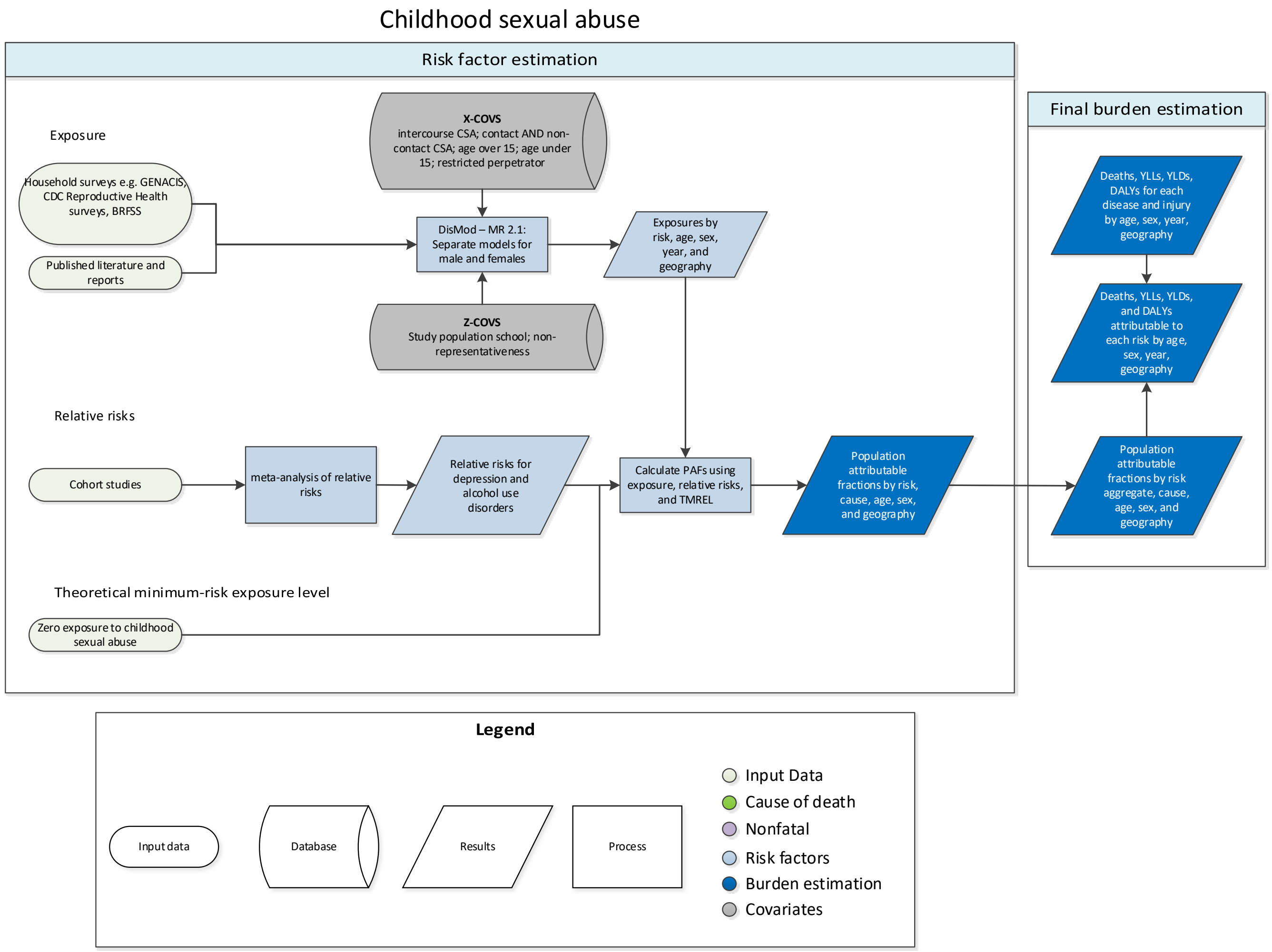

Input Data & Methodological Summary

Exposure

Case Definition

The case definition for childhood sexual abuse (CSA) is ever having had the experience of intercourse or other contact abuse (i.e. fondling and other sexual touching) when aged 15 years or younger, and the perpetrator or partner was greater than five years older than the victim.

Input data

Currently, we use self-reported survey data to measure CSA prevalence, not data from Child Protection Services (CPS) or other crime data. The reliability and comprehensiveness of CPS and crime statistics varies too much geographically to warrant including it.

An updated systematic review of CSA prevalence literature was conducted for sources published between August 2015 and January 2017. The following search terms were used:

(((("health surveys"[MeSH Terms] AND prevalence[Title/Abstract]) OR ("sentinel surveillance"[MeSH Terms] AND prevalence[Title/Abstract]) OR ("prevalence"[Title/Abstract] AND cross sectional studies[MeSH Terms])) AND (("child abuse"[MeSH Terms] OR "child abuse, sexual"[MeSH Terms]) OR ("sex offenses"[MeSH Terms] OR "child abuse, sexual"[MeSH Terms]) OR (child*[Title/Abstract] AND sexual[Title/Abstract] AND abuse[Title/Abstract]))) NOT ("comment"[Publication Type] OR "letter"[Publication Type] OR "editorial"[Publication Type]))

We supplemented with data from relevant national health surveys and violence-specific surveys. Several survey series used include the United States Behavioral Risk Factor Surveillance System, the CDC Reproductive Health Surveys, Brazil National Alcohol and Drug Survey, and the Gender, Alcohol, and Culture International Study (GENACIS).

A number of study level covariates were also extracted that were used in the modelling process to adjust for heterogeneous definitions across sources. All crosswalks and adjustments were done in DisMod-MR 2.1.

Modeling strategy

CSA prevalence was modeled as a single parameter prevalence model in DisMod-MR. CSA exposure is modeled separately for males and females because we observe little correlation between the prevalence of child abuse among females and males, and modeling both sexes together causes unreasonable estimates in countries where we only have data for one sex.

Three study-level covariates were used for alternate definitions of the violence.

- Study asked only about intercourse CSA
- Study asked about contact and non-contact CSA
- Study placed restrictions on the relationship between the perpetrator and the victim (e.g. only asked about CSA committed by a father)

We also included study-level fixed effects for varying age thresholds across studies.

- Study asked about recall for events before ages above 15 years (versus reference age threshold of 15)
- Study asked about recall for events before ages less than 15 years (versus reference age threshold of 15)

Two study-level covariate fixed effects on variance (z-cov) were also included in both the male and female models, including an indicator that the survey was not nationally representative, as well as

whether the survey was administered in schools. These study-level covariates were tested as x-covs first, but we did not find coefficients which would indicate systematic bias. We have not included any national-level covariates to date due to lack of knowledge about a covariate (for which we have a time series for all GBD locations) that predicts CSA prevalence.

Theoretical minimum-risk exposure level

The theoretical minimum risk exposure level is zero exposure to contact childhood sexual abuse.

Relative risks

We estimate burden attributable to CSA for the following health outcomes: unipolar depressive disorders (major depressive disorder and dysthymia) and alcohol use disorders.

In GBD 2015, we used one twin study that compared adverse outcome risks in same-sex discordant pairs.[1] This study was deemed reliable given that environmental and contextual factors are inherently controlled for when comparing between twins, avoiding potential confounding. However, to add to the strength of the evidence for GBD 2016, we performed a systematic review and a random effects meta-analysis to produce relative risks for depressive disorders and alcohol use disorders. In a departure from GBD 2015, suicide was not used as an outcome for CSA. This decision was based on the evidence available for the relative risk of suicide given exposure to CSA – not enough studies used suicide as an outcome, but instead used attempted suicide.

The pooled relative risk figures and 95% confidence intervals were 1.63 (1.41, 1.89) for depressive disorders and 1.54 (1.19, 1.99) for alcohol use disorders. The resulting forest plots are as follows:

CSA and depressive disorders meta-analysis

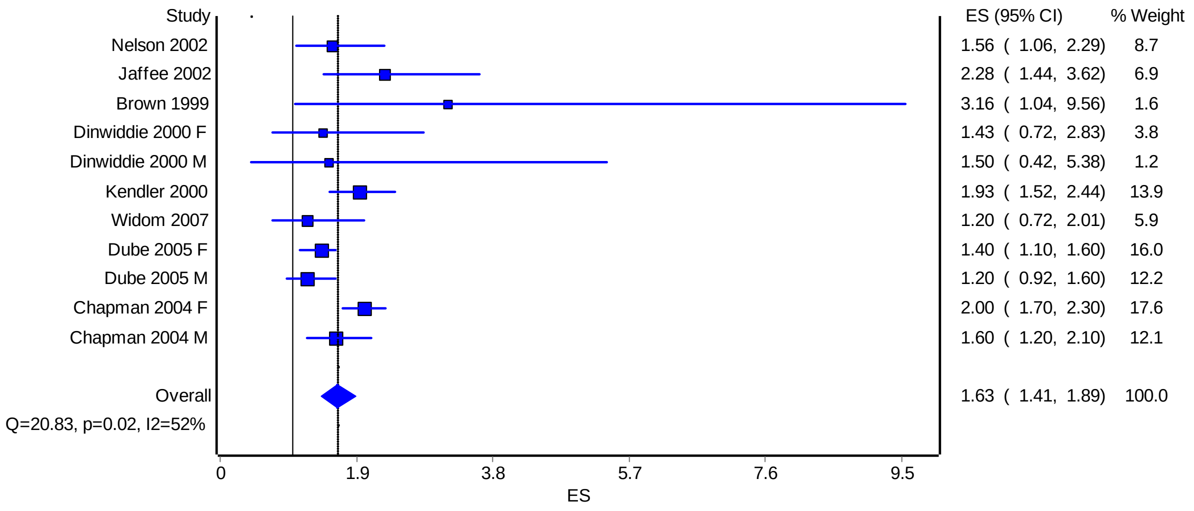

CSA and alcohol use disorders meta-analysis

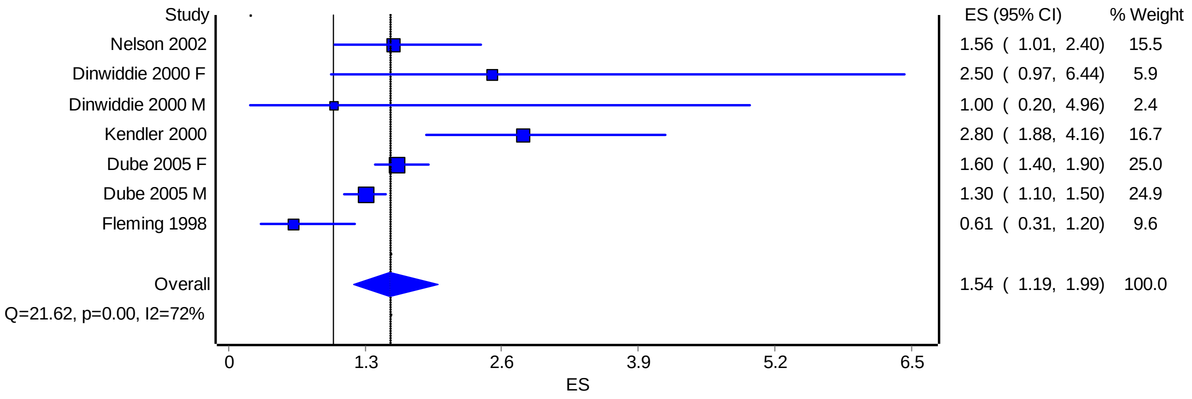

References

1. Brown J, Cohen P, Johnson JG., and Smailes EM. Childhood abuse and neglect: specificity of effects on adolescent and young adult depression and suicidality. Journal of the American Academy of Child & Adolescent Psychiatry. 1999; 38(12): 1490-1496.
2. Chapman DP, Whitfield CL, Felitti VJ, Dube SR, Edwards VJ and Anda RF. Adverse childhood experiences and the risk of depressive disorders in adulthood. Journal of affective disorders. 2004; 82(2): 217-225.
3. Cheasty M, Clare AW and Collins C. Relation between sexual abuse in childhood and adult depression: case-control study. Bmj. 1998; 316(7126): 198-201.
4. Dinwiddie S, Heath AC, Dunne MP, Bucholz KK, Madden PA, Slutske WS, Bierut LJ, Statham DB, Martin NG. Early sexual abuse and lifetime psychopathology: a co-twin-control study. *Psychol Med*. 2000; 30(1): 41–52.
5. Dube SR, Anda RF, Whitfield CL, Brown DW, Felitti VJ, Dong M and Giles WH. Long-term consequences of childhood sexual abuse by gender of victim. American journal of preventive medicine. 2005; 28(5): 430-438.
6. Ernst C, Angst J, Földényi M. The Zurich Study. XVII. Sexual abuse in childhood. Frequency and relevance for adult morbidity data of a longitudinal epidemiological study. *Eur Arch Psychiatry Clin Neurosci*. 1993; 242(5): 293–300.
7. Fleming J, Mullen PE, Sibthorpe B, Attewell R and Bammer G. The relationship between childhood sexual abuse and alcohol abuse in women-a case-control study. Addiction. 1998; 93(12): 1787-1798.
8. Jaffee SR, Moffitt TE, Caspi A, Fombonne E, Poulton R, Martin J. Differences in early childhood risk factors for juvenile-onset and adult-onset depression. *Arch Gen Psychiatry*. 2002; 59(3): 215-22.
9. Kendler KS, Bulik CM, Silberg J, Hettema JM, Myers J, Prescott CA. Childhood sexual abuse and adult psychiatric and substance use disorders in women: an epidemiological and cotwin control analysis. *Arch Gen Psychiatry*. 2000; 57(10): 953–9.
10. Molnar BE, Buka SL and Kessler, RC. Child sexual abuse and subsequent psychopathology: results from the National Comorbidity Survey. American journal of public health. 2001; 91(5): 753.
11. Nelson EC, Heath AC, Madden PA, Cooper ML, Dinwiddie SH, Bucholz KK, Glowinski A, McLaughlin T, Dunne MP, Statham DJ, Martin NG. Association between self-reported sexual abuse and adverse psychosocial outcomes: results from a twin study. Arch Gen Psychiatry. 2002; 59(2): 139-45.

12. Peleikis DE, Mykletun A and Dahl AA. The relative influence of childhood sexual abuse and other family background risk factors on adult adversities in female outpatients treated for anxiety disorders and depression. Child Abuse & Neglect. 2004; 28(1): 61-76.
13. Sartor CE, Lynskey MT, Bucholz KK, McCutcheon VV, Nelson EC, Waldron M, Heath AC. Childhood sexual abuse and the course of alcohol dependence development: findings from a female twin sample. *Drug Alcohol Depend*. 2007; 89(2-3): 139–44.
14. Silverman AB, Reinherz HZ and Giaconia RM. The long-term sequelae of child and adolescent abuse: A longitudinal community study. Child abuse & neglect. 1996; 20(8): 709-723.
15. Widom CS, DuMont K and Czaja SJ. A prospective investigation of major depressive disorder and comorbidity in abused and neglected children grown up. Archives of general psychiatry. 2007; 64(1): 49-56.

Unsafe Sex

Flowchart

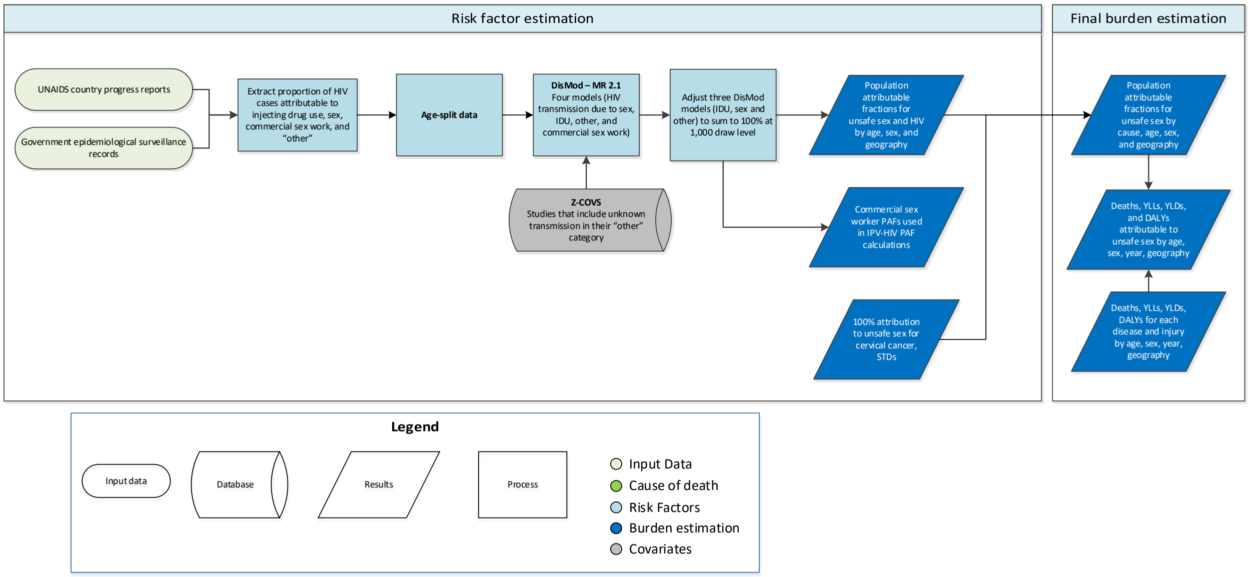

Input Data & Methodological Summary

Case definition and summary of GBD approach

Unsafe sex is defined as the risk of disease due to sexual transmission. The outcomes associated with unsafe sex that we estimate for GBD include HIV, cervical cancer, and all sexually transmitted diseases (STDs) except for those in neonates from vertical transmission, including HIV, opthalmia neonatorum and neonatal syphilis. We assume 100% of cervical cancer and STDs are attributable to unsafe sex and model the proportion of HIV incidence occurring through sexual transmission to estimate the attributable burden for HIV due to unsafe sex.

Input data

To be used in our models, sources must report HIV cases attributable to various modes of transmission. We screened all UNAIDS country progress reports and searched government epidemiological surveillance records for these data. The primary data sources we used were UNAIDS, the European CDC, and the US CDC.

For GBD 2016, we extracted all new European CDC, UNAIDS, and US CDC reports that had been published since the previous iteration of GBD. We also extracted US state-level HIV surveillance reports where available. These were found through the US CDC: National Center for HIV/AIDS, Viral Hepatitis, STD, and TB Prevention, Division of HIV/AIDS Prevention's website.

We excluded all extractions where the "other" category for HIV transmissions accounted for greater than 25 percent of all cases. We believe that such high proportions raise concerns about the quality of reporting .

Modeling strategy

We model the proportion of HIV cases attributable to unsafe sex. To do this we collect and clean data, run three DisMod models (HIV attributable to sex, HIV attributable to injection drug use, HIV attributable to other routes of transmission), adjust results of the three DisMod models to sum to one, and prepare PAFs.

All of the DisMod models included a study-level covariate fixed effect on integrand variance (z-cov) for sources that include cases of unknown transmission in their "other" category. We assumed that the inclusion of unknown cases in the other category would impact the uncertainty around the point estimates. No country level covariates were included in the models.

A new approach was introduced for GBD 2016 to inform an age-pattern in these HIV transmission models. All-age data points represent the majority of the available data, so we derived an age-pattern for the HIV-IDU transmission model from the age-pattern present in the GBD 2016 population attributable fraction for hepatitis B attributable to intravenous drug use. Assuming the proportion of HIV due to other is constant over time, the age-pattern for the proportion of HIV due to sex was set to be the complement to 1 of the age-pattern for the proportion of HIV due to IDU. The all-age data were split according to these age-patterns, and the three HIV transmission DisMod models were run on the age-split data. Additional priors were set to inform an age-pattern: zero proportion HIV transmission due to IDU before age 15, zero proportion HIV transmission due to sex before age 10, and 100% transmission due to other before age 10. The results from these HIV transmission models were adjusted to sum to 100% for a given country-year-age-sex group at each of 1,000 draws.

Squeezed global HIV transmission models by age (females, 2016):

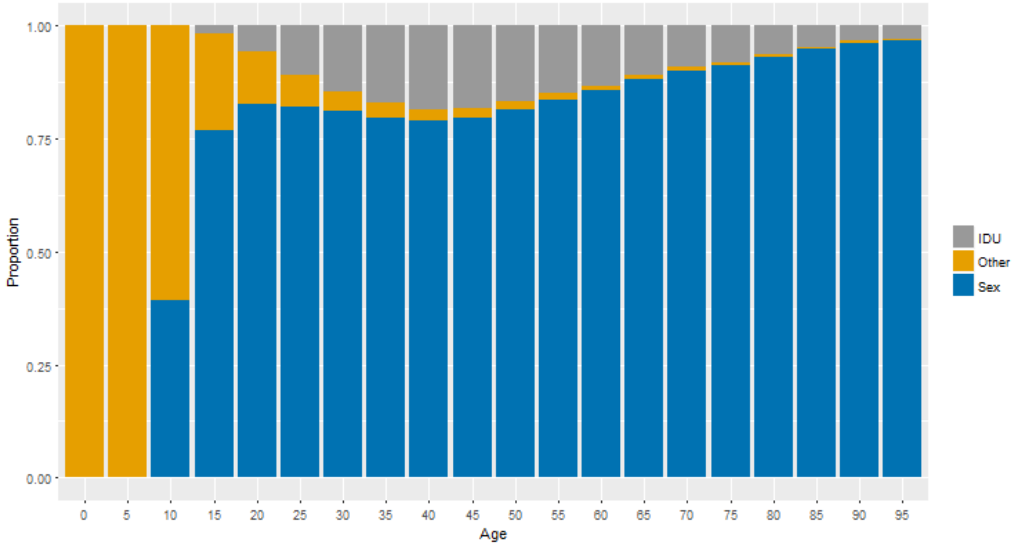

Theoretical minimum-risk exposure level

The theoretical minimum level used for unsafe sex is the absence of disease transmission due to sexual contact.

Population attributable fraction calculations

The outcomes associated with unsafe sex that we report on include HIV, cervical cancer, and all sexually transmitted diseases (STDs) except for those in neonates from vertical transmission, including HIV, opthalmia neonatorum and neonatal syphilis.

Based on evidence in the literature, we attribute 100% of cervical cancer to unsafe sex. These sources state that HPV infection is necessary for cervical cancer to develop and that HPV is only spread through sexual contact. The proportion of STDs attributable to unsafe sex is also 100%.

For HIV, the results from the single parameter proportion DisMod model for HIV transmission due to sex were used directly as the population attributable fraction.

Low Physical Activity Capstone Appendix

Flowchart

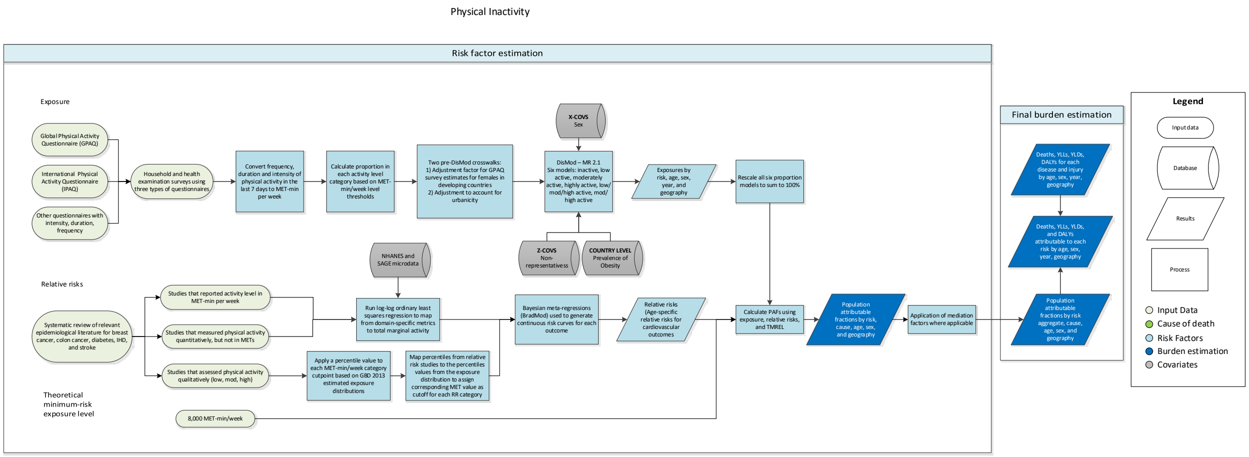

Input Data and Methodological Summary

Exposure

Case Definition

We measure physical activity performed by adults greater than or equal to 25 years of age, for durations of at least ten minutes at a time, across all domains of life (leisure/recreation, work/household and transport). We use frequency, duration and intensity of activity to calculate total metabolic equivalent-minutes per week. MET (Metabolic Equivalent) is the ratio of the working metabolic rate to the resting metabolic rate. One MET is equivalent to 1 kcal/kg/hour and is equal to the energy cost of sitting quietly. A MET is also defined as the oxygen uptake in ml/kg/min with one MET equal to the oxygen cost of sitting quietly, around 3.5 ml/kl/min.

Input data

We included surveys of the general adult population that captured self-reported physical activity in all domains of life (leisure/recreation, work/household and transport), where random sampling was used.

Data were primarily derived from two standardized questionnaires: The Global Physical Activity Questionnaire (GPAQ) and the International Physical Activity Questionnaire (IPAQ), although we included other survey instruments that asked about intensity, frequency and duration of physical activities performed across all activity domains.

Due to a lack of a consistent relationship on the individual level between activity performed in each domain and total activity, we were not able to use studies that included only recreational/leisure activities.

Physical activity level is categorized by total MET-minutes per week using four categories based on rounded values closest to the quartiles of the global distribution of total MET-minutes/week. The lower limit for the Level 1 category (600 MET-min/week) is the recommended minimum amount of physical

activity to get any health benefit. We used four categories with higher thresholds rather than the GPAQ and IPAQ recommended 3 categories to better capture any additional protective effects from higher activity levels.

- Level 0: < 600 MET-min/week (inactive)
- Level 1: 600-3999 MET-min/week (low-active)
- Level 2: 4000-7,999 MET-min/week (moderately-active)
- Level 3: ≤ 8,000 MET-min/week (highly active)

The GHDx was used to locate all surveys that use the GPAQ or IPAQ questionnaire. Although there were many other surveys that focused specifically on leisure activity, we were unable to use these sources because they did not comprise all three domains (work, transport and leisure). In addition, we excluded any surveys that did not report frequency, duration, and intensity of activity.

Modeling strategy

Pre-DisMod crosswalks

We conducted two crosswalks prior to DisMod to adjust the raw data to our "gold standard" definition. In GBD 2016, our gold standard definition was IPAQ due to concern that GPAQ was not accurately capturing "domestic" (house/yard) activities.

A sex-specific regression was fit on data from nationally representative surveys that used either GPAQ or IPAQ for each activity category, where the dependent variable was the logit of the proportion in the relevant activity level and the main independent variable was an survey instrument (1=GPAQ, 0=IPAQ), with fixed effects for age categories as well as a super-region, region, and country level random effects.

We also adjusted non-nationally-representative urban and rural data points. We constructed an urbanicity covariate that is equal to 1 for urban data points, 0 for rural data points and the proportion urban for the country for nationally representative data points. The dependent variable was the logit of the proportion in the relevant activity level and the main independent variable is urbanicity, with fixed effects for age categories and sex with super region, region and country level random effects.

DisMod modeling

Once the raw data had been adjusted to meet our gold standard definition of physical activity, we modeled activity as a single parameter proportion model in DisMod. We estimated the proportion of each country/year/age/sex subpopulation in each of the above four activity levels using six separate Dismod models. We use six models rather than four to accommodate the different MET-minute/week cutoffs presented in tabulated data sources where individual unit record data was not available. Since the accepted threshold/definition for inactivity is consistently <600 MET-minutes/week, the vast majority of tabulated data was broken down into proportion inactive (model A) and proportion low, moderate or highly active (model B).

	Label	MET-min/week	Name of sequelae in online visualization tool
A	inactive	<600	Physical inactivity and low physical activity, inactive

B	low/moderately/highly active	≥600	Physical inactivity and low physical activity, low/moderately/highly active
C	low active	600-3999	Physical inactivity and low physical activity, low active
D	moderately/highly active	>4000	Physical inactivity and low physical activity, moderately/highly active
E	moderately active	4000-7999	Physical inactivity and low physical activity, moderately active
F	highly active	≥8,000	Physical inactivity and low physical activity, highly active

These models have mesh points at 0 15 25 35 45 55 65 75 100, and a study-level fixed effect on integrand variance (Z-cov) for whether a study was nationally representative or not, to account for the heterogeneity introduced by studies that are not generalizable to the entire population. They also have national level fixed effects on prevalence of obesity.

After DisMod, we rescale these 6 models so that the proportions sum to one. Since we have the most data for models A and B, we rescale the sum of the proportion in each category to be equal to one. Next we rescale the sum of model C and D to be equal to the rescaled value from model B. Then we rescale the sum of models E and F to be equal to the rescaled value from model D. After these three rescales we are left with a proportion for each of the four categories that all sum to 1.

For the first time, we have directly estimated total MET-minutes per week globally through the use of a regression that estimated the relationship between total MET-mins/week and each of the categorical prevalences of physical activity. The resultant coefficients were then applied to country-year-age-sex specific estimates of categorical prevalence of physical activity. It takes the form:

$$\begin{aligned} log\ (Total\ MET - mins/week)_{cyas} &= \beta_{intercept} + \beta_{inact} \times Inactivity\ Prev_{cyas} + \beta_{lowact} \times Low\ Activity\ Prev_{cyas} \\ &+ \beta_{modact} \times Moderate\ Activity\ Prev_{cyas} + \beta_{highact} \times High\ Activity\ Prev_{cyas} \end{aligned}$$

Utilizing microdata on total MET-mins per week from individual-level surveys, we characterized the distribution of activity level at the population level. We then used an ensemble approach to distribution fitting, borrowing characteristics from individual distributions to tailor a unique distribution to fit the data using a weighting scheme. We characterized the standard deviation of each population's activity through a linear regression that captured the relationship between standard deviation and mean activity levels in nationally representative IPAQ surveys:

$$ln\ (Standard\ deviation) = \beta_0 + \beta_1 \times ln\ (Mean_i)$$

We then applied the coefficients of this regression to the outputs of our estimate of total MET-minutes per week regression outputs to calculate the standard deviation by country, year, age, and sex.

Theoretical minimum-risk exposure level

The theoretical minimum-risk exposure level for physical inactivity is 3000-4500 MET-min per week, which was calculated as the exposure at which minimal deaths across outcomes occurred.[4]

Relative risks

We used a recently published dose-response meta-analysis of prospective cohort studies to estimate the effect size of the change in physical activity level on breast cancer, colon cancer, diabetes, ischemic heart disease and ischemic stroke.[4]

References

1. Ainsworth BE, Haskell WL, Whitt MC, Irwin ML, Swartz AM, Strath SJ, O Brien WL, Bassett DR, Schmitz KH, Emplaincourt PO, Jacobs DR. Compendium of physical activities: an update of activity codes and MET intensities. Medicine and science in sports and exercise. 2000 Sep 1;32(9; SUPP/1):S498-504.

2. IPAQ Research Committee. Guidelines for data processing and analysis of the International Physical Activity Questionnaire (IPAQ)–short and long forms. Retrieved September. 2005;17:2008.

3. World Health Organization. Global Physical Activity Questionnaire (GPAQ) Analysis Guide. 2011. Geneva, Switzerland: WHO Google Scholar. 2013

4. Kyu HH, Bachman VF, Alexander LT, Mumford JE, Afshin A, Estep K, Veerman JL, Delwiche K, Iannarone ML, Moyer ML, Cercy K. Physical activity and risk of breast cancer, colon cancer, diabetes, ischemic heart disease, and ischemic stroke events: systematic review and dose-response meta-analysis for the Global Burden of Disease Study 2013. bmj. 2016 Aug 9;354:i3857.

High Fasting Plasma Glucose Capstone Appendix

Flowchart

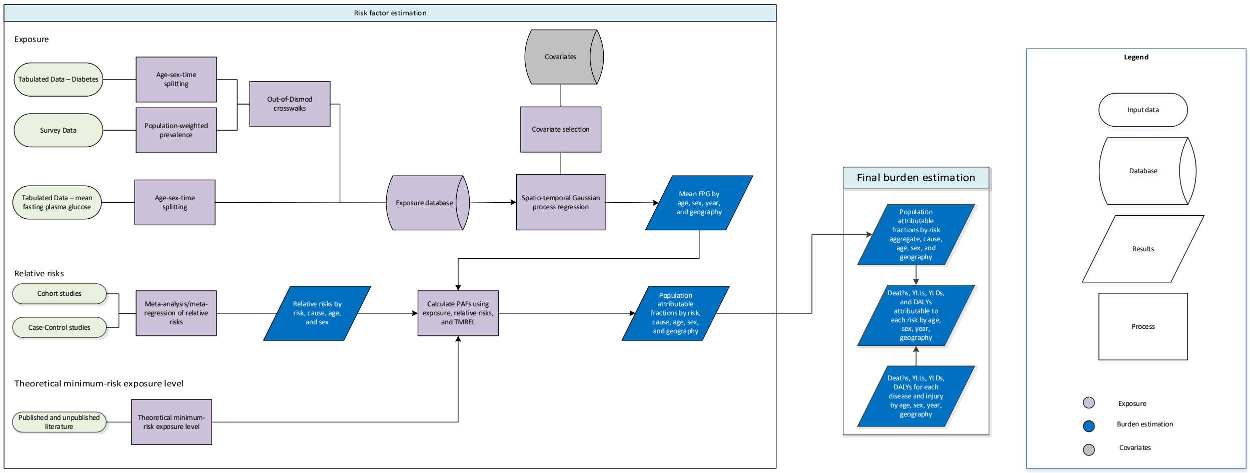

Case Definition

High fasting plasma glucose (FPG) is defined as FPF of greater than 5 mmol/L.

Data seeking

Exposure

1. A systematic review of the literature was done for GBD 2016 with the following search terms:

FPG search string: ((“glucose”[Mesh] OR “hyperglycemia”[Mesh] OR “prediabetic state”[Mesh]) AND "Geographic Locations"[Mesh] NOT "United States"[Mesh]) AND ("humans"[Mesh] AND "adult"[MeSH]) AND ("Data Collection"[Mesh] OR "Health Services Research"[Mesh] OR "Population Surveillance"[Mesh] OR "Vital statistics"[Mesh] OR "Population"[Mesh] OR "Epidemiology"[Mesh] OR "surve*"[TiAb]) NOT Comment[ptyp] NOT Case Reports[ptyp] AND ("2016/01/01"[PDAT] : "2016/12/31"[PDAT]) NOT "hospital"[TiAb]

And

Diabetes mellitus search string: (diabetes[TI] AND (prevalence[TIAB] OR incidence[TIAB])) OR ("diabetes mellitus"[MeSH Terms] AND "epidemiology"[MeSH Terms]) OR (diabetes[TI] AND "epidemiology"[MeSH Terms]) NOT gestational[All Fields] NOT ("neoplasms"[MeSH Terms] OR "neoplasms"[All Fields] OR "cancer"[All Fields]) NOT ("mice"[MeSH Terms] OR "mice"[All Fields]) NOT ("schizophrenia"[MeSH Terms] OR "schizophrenia"[All Fields]) NOT ("emigrants and immigrants"[MeSH Terms] OR ("emigrants"[All Fields] AND "immigrants"[All Fields]) OR "emigrants and immigrants"[All Fields] OR "immigrants"[All Fields]) NOT ("pregnancy"[MeSH Terms] OR "pregnancy"[All Fields] OR "gestation"[All Fields]) NOT ("rats"[MeSH Terms] OR "rats"[All Fields] OR "rat"[All Fields]) NOT ("kidney"[MeSH Terms] OR "kidney"[All Fields]) NOT renal[All Fields] NOT ("vitamins"[Pharmacological Action] OR

"vitamins"[MeSH Terms] OR "vitamins"[All Fields] OR "vitamin"[All Fields]) AND ("2016/01/01"[PDAT] : "2016/12/31"[PDAT])

Search date: January 5, 2017

The search took place for the following dates: 1/1/2016– 12/31/2016. The number of studies returned was 1,976, and the number of studies extracted was 26.

2. We systematically searched the Global Health Data Exchange (GHDx) for multi-country survey programs, national surveys, and longitudinal studies that was tagged with either fasting plasma glucose (FPG) or Diabetes mellitus. Each data source we found was tagged with whether the file contained microdata and whether the file contained data on FPG (biomarker). In the interest of time, we prioritized the data sources we reviewed and extracted based on whether the source was tagged with microdata and biomarker information.

Figure 1: PRISMA diagram of data sources used in GBD 2016 high fasting plasma glucose (FPG) model

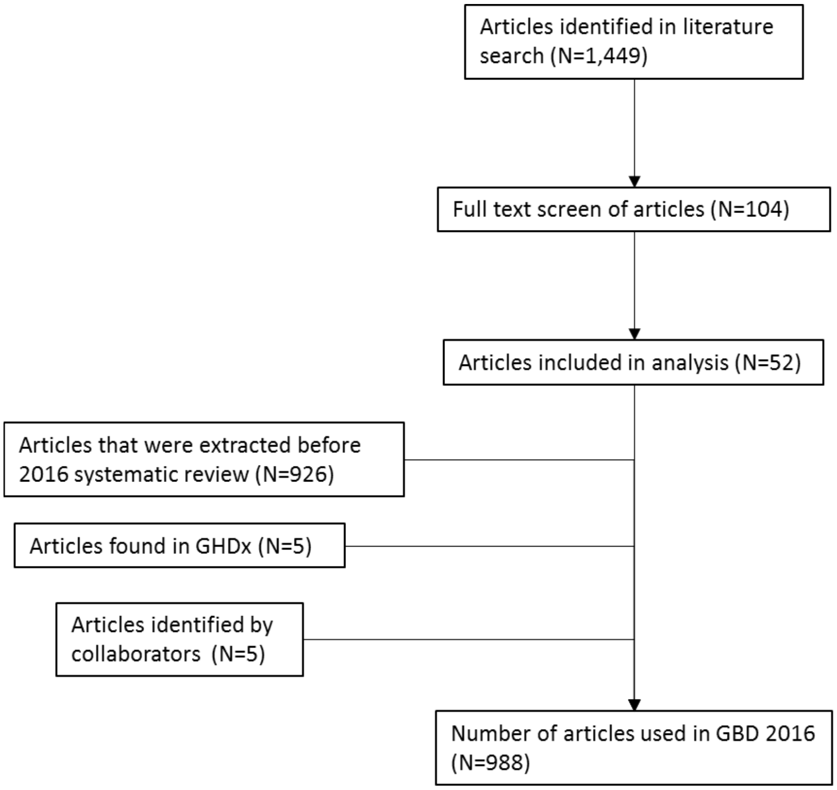

Data Inputs

Data:

Data inputs come from 3 sources:

- Estimates of mean FPG in a representative population
- Individual-level data of fasting plasma glucose measured from surveys

- Estimates of diabetes prevalence in a representative population

Data sources that did not report mean FPG or prevalence of diabetes were excluded from analysis. When a study reported both mean fasting plasma glucose (FPG) and prevalence of diabetes, we used the mean FPG for exposure estimates. Where possible, individual-level data superseded any data described in a study. Individual-level data was collapsed and aggregated to produce estimates for each age group, sex, location, and year a survey is conducted.

Data processing

We perform several processing steps to the data in order to address sampling and measurement inconsistencies that will ensure the data are comparable.

1. *Small sample size*
   Estimates in a sex and age group with a sample size <30 persons was considered a small sample size. In order to avoid small sample size problems that may bias estimates, data were collapsed into the next age group in the same study till the sample size reached at least 30 persons. The intent of collapsing the data is to preserve as much granularity between age groups as possible which determined whether the collapse occurred with a younger or older age group. If the entire study sample consisted of <30 persons and did not include a population-weight, the study was excluded from the modeling process. The estimates were re-calculated if case count and sample size were available or the population-weighted estimate was calculated when only sample size was available.

2. *Time, Age, and Sex Splitting*
   For more details on how datapoints on mean FPG was processed, please see the Diabetes mellitus capstone appendix in the GBD 2016 Non-fatal Paper.

3. *Crosswalks*
   We predicted mean FPG from diabetes prevalence using an ensemble distribution. We characterized the distribution of FPG using individual-level data. For more details on the ensemble distribution, please see the GBD 2016 Risk Factors Paper. Before predicting mean FPG from prevalence of diabetes, we ensured that the prevalence of diabetes was based on the reference case definition: fasting plasma glucose (FPG) >126 mg/dL (7 mmol/L) or on treatment. For more details on how the case-definition crosswalk was conducted, please see the Diabetes mellitus capstone appendix in the GBD 2016 Non-fatal Paper.

Exposure Modeling

Exposure estimates were produced from 1980 to 2016 for each national and subnational location, sex, and for each 5-year age group starting from 25+. As in GBD 2015, we used a Spatio-Temporal Gaussian Process Regression (ST-GPR) framework to model the mean fasting plasma glucose at the location-, year-, age-, sex- level. Updates to the ST-GR modeling framework for GBD 2016 are detailed in the appendix.

Fasting plasma glucose is frequently tested or reported in surveys aiming at assessing the prevalence of diabetes mellitus. In these surveys, the case definition of diabetes may include both a glucose test and questions about treatment for diabetes; people with positive history of diabetes treatment are generally excluded from the FPG test. Thus, the mean FPG in these surveys may not represent the mean FPG in the entire population. To address this limitation, using the data from the surveys reporting mean FPG in the entire population, we estimated a regression-based correction factor and adjusted the mean FPG to account for diabetics in the population. We also used an ensemble distribution to characterize the distribution of FPG in the population and developed an optimization function to estimate the standard deviation based on mean FPG and prevalence of diabetics.

To inform our estimates in data-sparse countries, we systematically tested a range of covariates and selected two covariates based on AIC and adjusted R^2. These included prevalence of obesity and lag-distributed income per capita (LDI).

Mean FPG was estimated using a mixed-effects linear regression, run separately by sex:

$$\text{logit}(\text{FPG}_{c,a,t}) = \beta_0 + \beta_1 \log(\text{LDI})_{c,t} + \beta_2 p_{\text{overweight}_{c,a,t}} + \sum_{k=2}^{16} \beta_k I_{A[a]} + \alpha_s + \alpha_r + \alpha_c + \epsilon_{c,a,t}$$

where $\log(\text{LDI})_{c,t}$ is the log of the lag-distributed income, $p_{\text{overweight}_{c,a,t}}$ is the prevalence of overweight, $I_{A[a]}$ is an indicator variable for a fixed effect on a given 5-year age group, and α_s α_r α_c are random effects at the super-region, region, and country level, respectively.

The estimates were then propagated through the ST-GPR framework to obtain 1000 draws for each location, year, age, and sex.

Theoretical minimum-risk exposure level

The theoretical minimum-risk exposure level (TMREL) for FPG is 4.5-5.4 mmol/L. This was calculated by taking the person-year weighted average of the levels of FPG that were associated with the lowest risk of mortality in the pooled analyses of prospective cohort studies.[1]

Relative risks

We estimate 15 outcomes due to high fasting plasma glucose (continuous risk) or diabetes (categorical risk).

**Risk**	**Outcome**
Ischemic heart disease	Fasting plasma glucose
Ischemic stroke	Fasting plasma glucose

Hemorrhagic stroke	Fasting plasma glucose
Peripheral vascular disease	Fasting plasma glucose
Tuberculosis	Diabetes mellitus
Liver cancer	Diabetes mellitus
Pancreatic cancer	Diabetes mellitus
Ovarian cancer	Diabetes mellitus
Colorectal cancer	Diabetes mellitus
Bladder cancer	Diabetes mellitus
Lung cancer	Diabetes mellitus
Breast cancer	Diabetes mellitus
Glaucoma	Diabetes mellitus
Cataracts	Diabetes mellitus
Dementia	Diabetes mellitus

Relative risks for High Fasting Plasma Glucose (continuous risk)

Relative risks (RR) were obtained from dose-response meta-analysis of prospective cohort studies. Please see the citation list for a full list of studies that are utilized. For cardiovascular outcomes, we estimated age-specific RRs using DisMod-MR 2.1 with log (RR) as the dependent variable and median age at event as the independent variable with an intercept at age 110. Morbidity and mortality directly caused by diabetes was considered directly attributable to FPG.

Relative risks for Diabetes mellitus (Categorical risk)

In GBD 2016, we added 10 additional outcomes for which we found sufficient evidence on their relationship with diabetes. These new outcomes included liver cancer, pancreas cancer, ovarian cancer, colorectal cancer, bladder cancer, lung cancer, breast cancer, glaucoma, cataracts, and dementia. In GBD 2016, tuberculosis was further split into drug-resistant tuberculosis, drug-susceptible tuberculosis, multi-drug resistant tuberculosis without extensive drug resistance, and extensively drug-resistant tuberculosis. Since studies of tuberculosis and diabetes did not differentiate types of tuberculosis, we assumed that the risk was the same. Relative risks were mostly obtained from meta-analysis of cohort studies. Please see the citation list for a full list of studies that are utilized.

References

1 Singh GM, Danaei G, Farzadfar F, *et al.* The age-specific quantitative effects of metabolic risk factors on cardiovascular diseases and diabetes: a pooled analysis. *PloS One* 2013; **8**: e65174.

High Total Blood Cholesterol Capstone Appendix

Flowchart

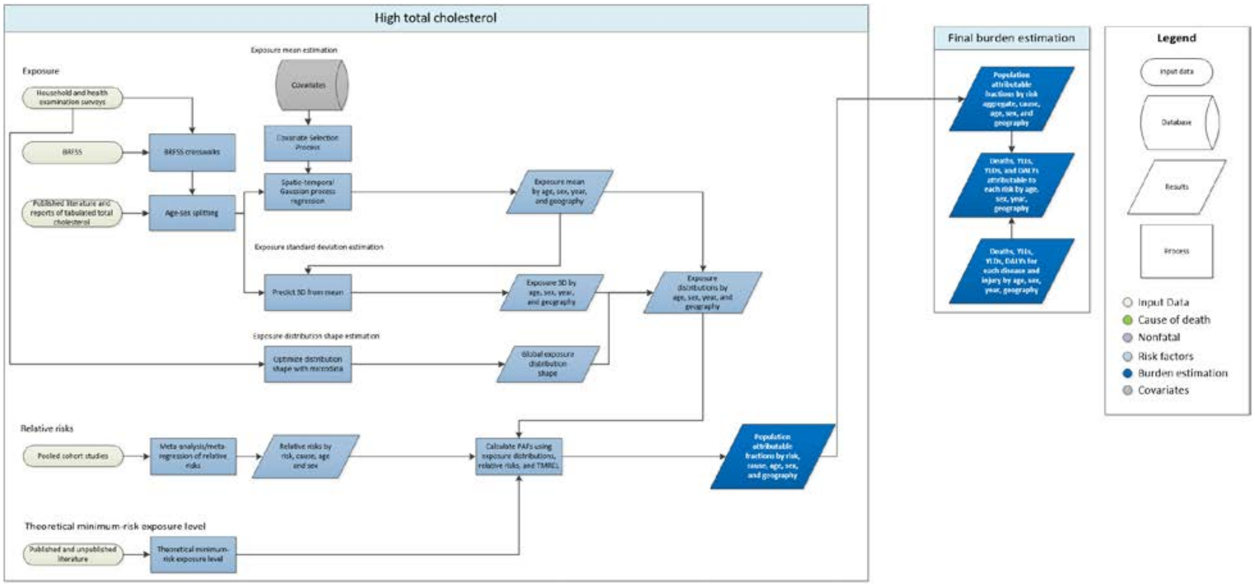

Input Data & Methodological Summary

Exposure

Case Definition

Blood total cholesterol in units of mmol/L.

Input Data

We utilized data on blood mean total cholesterol from literature and from household survey microdata and reports. Please see the appendix for a full list of included sources. For the GBD 2015 study, a systematic review of the literature was completed to capture population survey data on mean total blood cholesterol. For GBD 2016, we updated the systematic review using the same strategy, drawing from the GHDx and Medline via PubMed. In total, we have utilized 572 sources corresponding to 32,745 unique data points.

Literature Review

We systematically searched PubMed for articles published between 01 December 2015 and 31 December 2016 which provided national or subnational estimates of mean total blood cholesterol in the general population. The literature review was completed for systolic blood pressure, fasting plasma glucose, body mass index, and blood cholesterol simultaneously.

Search terms:

(("Hyperlipidemias"[Text Word] OR "Hypercholesterolemia"[Text Word] OR "Cholesterol"[Text Word]) AND "Geographic Locations"[Mesh] NOT "United States"[Mesh]) AND ("Data Collection"[Mesh] OR

"Health Services Research"[Mesh] OR "Population Surveillance"[Mesh] OR "Vital statistics"[Mesh] OR "Population"[Mesh] OR "Epidemiology"[Mesh] OR "surve*"[TiAb]) NOT Comment[ptyp] NOT Case Reports[ptyp] AND ("2015/12/01"[PDAT] : "2016/10/18"[PDAT]) NOT "hospital"[TiAb]

Inclusion Criteria

Studies were included if they were population-based and measured total blood cholesterol using a blood test. We assumed the data is representative of the location if the geography was not related to the diseases (a mining area) and if it is not an outlier compared to other data in the country or region.

Outliers

Data was utilized in the modeling process unless an assessment of data strongly suggested that the data was biased. A candidate source was excluded if the quality of study did not warrant a valid estimate because of selection (non-representative populations) or if the study did not provide methodological details for evaluation. In a small number of cases, data point was considered to be an outlier candidate if the level was implausibly low or high based on expert judgement and other country data.

Data Extraction

Where possible, individual level data on blood pressure estimates were extracted from survey microdata and these were collapsed across individuals and collapsed across demographic groupings to produce mean estimates in the standard GBD 5-year age-sex groups. If microdata were unavailable, information from survey reports or from literature were extracted along with any available measure of uncertainty including standard error, uncertainty intervals, and sample size. Standard deviations were also extracted.

Incorporating United States prevalence data

Survey reports and literature often report information only about the prevalence, but not the level, of hypercholesterolemia in the population studied. These sources were not used to model total cholesterol, with the exception of data from the Behavioral Risk Factors Surveillance System (BRFSS) because of the availability of a similarly structured exam survey covering the identical population (NHANES). BRFSS is a telephone survey conducted in the United States for all counties. It collects self-reported diagnosis of hypercholesterolemia. These self-reported values of prevalence of raised total cholesterol in each age group, sex, US state, and year were used to predict a mean total cholesterol for the same strata with a regression using data from the National Health and Nutrition Examination Survey, a nationally representative health examination survey of the US adult population. The regression was:

$$\mathrm{TC}_{l,a,t,s} = \beta_0 + \beta_1 \mathrm{prev}_{l,a,t,s}$$

where $\mathrm{TC}_{l,a,t,s}$ is the location, age, time, and sex specific mean total cholesterol and $\mathrm{prev}_{l,a,t,s}$ is the location, age, time, and sex specific prevalence of raised total cholesterol. The coefficients for both models are reported in Table 1.

Table 1. Coefficients in the sex-specific US states blood pressure prediction models

Term	Male model	Female model
Intercept	4.23	4.36
Prevalence	6.25	5.22

Out of sample RMSE was used to quantify the predictive validity of the model. The regression was repeated 10 times for each sex, each time randomly holding out 20% of the data. The RMSEs from each holdout analysis were averaged to get the average out of sample RMSE. The results of this holdout analysis are reported in Table 2.

Table 2. Out of sample RMSEs of the sex-specific US states blood pressure prediction models

	Male model	Female model
Out of sample RMSE	0.21 mmol/L	0.20 mmol/L

Age and Sex Splitting

Prior to modeling, data provided in age groups wider than the GBD 5-year age groups were processed using the approach outlined in Ng et al.[2] Briefly, age-sex patterns were identified using person-level microdata (58 sources), and estimate age-sex specific levels of total cholesterol from aggregated results reported in published literature or survey reports.

Modeling

Exposure estimates were produced from 1980 to 2016 for each national and subnational location, sex, and for each 5-year age group starting from 25+. As in GBD 2015, we used *a* Spatio-Temporal Gaussian Process Regression (ST-GPR) framework to model the mean total blood cholesterol at the location-, year-, age-, sex- level. Updates to the ST-GR modeling framework for GBD 2016 are detailed in the appendix.

Covariate selection

The first step of the ST-GPR framework requires the creation of a linear model for predicting total cholesterol at the location-, year-, age-, sex- level. Covariates for this model were selected in two stages. First a list of variables with an expected causal relationship with total cholesterol was created based on significant association found within high-quality prospective cohort studies reported in the published scientific literature. These variables were: dietary fiber availability, dietary fruit availability, dietary polyunsaturated fatty acid (PUFA) availability, dietary nuts and seeds availability, and the prevalence of overweight persons in a population. We also explored associations with the GBD study socio-demographic index (SDI) covariate, and the health access quality index (HAQI) covariate to represent the effect of proximal socioeconomic factors and access to health care on exposure levels. The second stage in covariate selection was to test the predictive validity of every possible combination of covariates in the linear model, given the covariates selected above. This was done separately for

each sex. Predictive validity was measured with out of sample root-mean-squared error. The linear model with the lowest root-mean squared error for each sex was then used in the ST-GPR model. For women, this linear model was:

$$\log(TC_{c,a,t}) = \beta_0 + \beta_1 SDI_{c,t} + \beta_2 HAQI_{c,a,t} + \beta_3 nuts_{c,a,t} + \beta_4 fiber_{c,a,t} + \sum_{k=2}^{16} \beta_k I_{A[a]} + \alpha_s + \alpha_r + \epsilon_{c,a,t}$$

For men, the linear model was:

$$\log(TC_{c,a,t}) = \beta_0 + \beta_1 SDI_{c,t} + \beta_2 HAQI_{c,a,t} + \beta_4 prev\_overweight_{c,a,t} + \beta_3 PUFA_{c,a,t} + \beta_4 fiber_{c,a,t} + \sum_{k=2}^{16} \beta_k I_{A[a]} + \alpha_s + \alpha_r + \epsilon_{c,a,t}$$

where $SDI_{c,t}$ is socio-demographic index (SDI), an index metric that includes a measure of education, fertility, and income, HAQI is the health access quality index, $prev\_overweight_{c,a,t}$ is the prevalence of overweight, $nuts_{c,a,t}$ is the calorie adjusted food availability of nuts and seeds, $PUFA_{c,a,t}$ is the calorie adjusted food availability of poly-unsaturated fatty acids per capita per day, $fiber_{c,a,t}$ is the calorie adjusted food availability of fiber, $I_{A[a]}$ is a dummy variable for a fixed effect on a given 5-year age group, and α_s and α_r are random effects at the super-region and region level, respectively. Table 3 contains the coefficients of the fixed effects used in the two regressions.

Table 3. Coefficients on covariates in sex-specific linear models

Covariate	Male	Female
Fiber	-0.00287<br>(-0.0034 to -0.0022)	-0.00196<br>(-0.0025 to -0.0014)
Nuts/seeds	NA	-0.00364<br>(-0.004 to -0.003)
SDI	0.317<br>(0.278 to 0.356)	0.307<br>(0.271 to 0.343)
HAQI	-0.0016<br>(-0.0021 to -0.0012)	-0.0008<br>(-0.0011 to -0.0004)
PUFA	-0.533<br>(-0.719 to -0.347)	NA

Prevalence of overweight	0.0364<br>(0.0138 to 0.0591)	NA

The estimates were then propagated through the ST-GPR framework to obtain 1000 draws for each location, year, age, and sex. Table 4 contains the out of sample root-mean-squared error (RMSE) of both the linear model and the final ST-GPR results for the male and female models.

Table 4. Out of sample RMSEs of the sex-specific linear and ST-GPR models

	**Out of sample RMSEs for male model**	**Out of sample RMSEs for female model**
Linear model	0.323 mmol/L	0.336 mmol/L
Final ST-GPR model	0.176 mmol/L	0.173 mmol/L

Estimate of standard deviation

The standard deviation of total cholesterol within a population was estimated for each national and subnational location, sex, and 5-year age group starting from age 25 using the standard deviation from person-level and some tabulated data sources. Person-level microdata accounted for 3009 of the total 4001 rows of data on standard deviation. The remaining 992 rows came from tabulated data. Tabulated data was only used to model standard deviation if it was sex and 5-year age group specific and reported a population standard deviation of total cholesterol. The total cholesterol standard deviation function was estimated using a linear regression:

$$\log(SD_{c,a,t,s}) = \beta_0 + \beta_1 TC_{c,a,t,s} + \beta_3 (TC_{c,a,t,s})^2 + \beta_4 sex + \sum_{k=2}^{16} \beta_k I_{A[a]} + \alpha_s + \epsilon_{c,a,t,s}$$

where $TC_{c,a,t,s}$ is the country, age, time, and sex specific mean total cholesterol estimate from ST-GPR, $I_{A[a]}$ is a dummy variable for a fixed effect on a given 5-year age group, and α_s is a random effect at the super-region level.

Distribution shape modelling

The shape of the distribution of total cholesterolwas estimated using all available person-level microdata sources, which was a subset of the input data into the modelling process. The distribution shape modelling framework for GBD 2016 is detailed in the appendix. Briefly, an ensemble distribution created from a weighted average of distribution families was fit for each individual microdata source,

separately by sex. The weights for the distribution families for each individual source were then averaged and weighted to create a global ensemble distribution for each sex.

Theoretical minimum-risk exposure level

The TMREL for total cholesterol was the same as that used in GBD2015. A Meta-analysis of randomized trials has shown that outcomes can be improved even at low levels of LDL-cholesterol, below 1.3 mmol/l.[3] Recent studies of PCSK-9 inhibitors support these results. We used the strong correlation between LDL-cholesterol and total cholesterol to map the proposed LDL-cholesterol TMREL of 0.7-1.3 mmol/l to a TMREL for total cholesterol of 2.8-3.4 mmol/l.

Relative Risks

We used Dismod-MR 2.1 to pool effect sizes from included studies and generate a dose-response curve for each of the outcomes associated with high total cholesterol. The tool enabled us to incorporate random effects across studies and include data with different age ranges. RRs were used universally for all countries and the meta-regression only helped to pool the three major sources and produce RRs with uncertainty and covariance across ages taking into account the uncertainty of the data points

As in GBD 2015, RRs for IHD and ischemic stroke are obtained from meta-regressions of pooled epidemiological studies: the Asia Pacific Cohort Studies Collaboration (APCSC) and the Prospective Studies Collaboration (PSC).[4] RRs for IHD were modeled with log (RR) as the dependent variable and median age at event as the independent variable with an age intercept (RR equals 1) at age 110. For total cholesterol and ischemic stroke, a similar approach was used, except that there was no age intercept at age 110, due to the fact that there was no statistically significant relationship between total cholesterol and stroke after age 70 with a mean RR less than one. We assumed that there is not a protective effect of high cholesterol and therefore did not include an RR for ages 80+.

References

1 Roth GA, Fihn SD, Mokdad AH, Aekplakorn W, Hasegawa T, Lim SS. High total serum cholesterol, medication coverage and therapeutic control: an analysis of national health examination survey data from eight countries. *Bull World Health Organ* 2011; **89**: 92–101.

2 Ng M, Fleming T, Robinson M, *et al.* Global, regional, and national prevalence of overweight and obesity in children and adults during 1980–2013: a systematic analysis for the Global Burden of Disease Study 2013. *The Lancet* 2014; **384**: 766–81.

3 Boekholdt SM, Hovingh GK, Mora S, *et al.* Very Low Levels of Atherogenic Lipoproteins and the Risk for Cardiovascular EventsA Meta-Analysis of Statin Trials. *J Am Coll Cardiol* 2014; **64**: 485–94.

4 Singh GM, Danaei G, Farzadfar F, *et al.* The age-specific quantitative effects of metabolic risk factors on cardiovascular diseases and diabetes: a pooled analysis. *PloS One* 2013; **8**: e65174.

High Systolic Blood Pressure Capstone Appendix

Flowchart

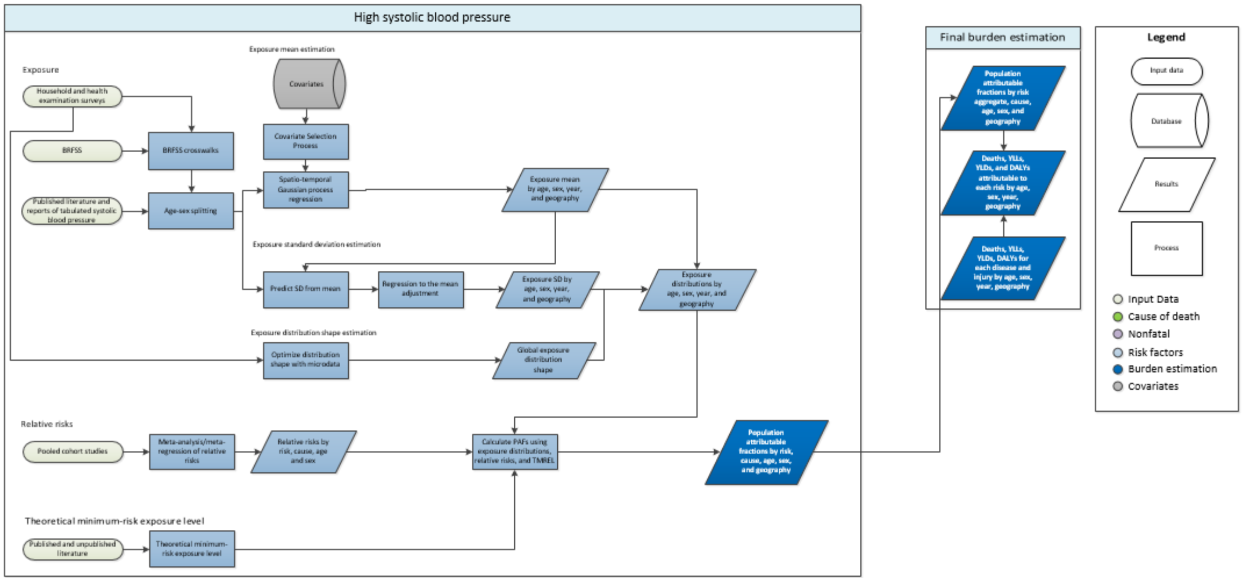

Input Data & Methodological Summary

Exposure

Case Definition

Brachial systolic blood pressure in mmHg.

Input Data

We utilized data on mean systolic blood pressure from literature and from household survey microdata and reports (e.g. STEPS, NHANES). Please see the appendix for a full list of included sources. In GBD 2015, a systematic review of the literature was completed to capture population survey data on mean systolic blood pressure. For GBD 2016, we updated the systematic review using the same strategy, drawing from the GHDx and Medline via PubMed. In total, we have utilized 934 sources corresponding to 49,690 unique data points.

Literature Review

We systematically searched PubMed for articles published between 01 December 2015 and 31 December 2016 which provided national or subnational estimates of mean systolic blood pressure. The literature review was completed for systolic blood pressure, fasting plasma glucose, body mass index, and blood cholesterol simultaneously.

Search terms:

(("Hyperlipidemias"[Text Word] OR "Hypercholesterolemia"[Text Word] OR "Cholesterol"[Text Word]) AND "Geographic Locations"[Mesh] NOT "United States"[Mesh]) AND ("Data Collection"[Mesh] OR "Health Services Research"[Mesh] OR "Population Surveillance"[Mesh] OR

"Vital statistics"[Mesh] OR "Population"[Mesh] OR "Epidemiology"[Mesh] OR "surve*"[TiAb]) NOT Comment[ptyp] NOT Case Reports[ptyp] AND ("2015/12/01"[PDAT] : "2016/12/31"[PDAT]) NOT "hospital"[TiAb]

Inclusion Criteria

Studies were included if they were population-based and measured systolic blood pressure using a blood test. We assumed the data is representative of the location if the geography was not selected because it was related to the diseases.

Outliers

Data was utilized in the modeling process unless an assessment of data strongly suggested that the data was biased. A candidate source was excluded if the quality of study did not warrant a valid estimate because of selection (non-representative populations) or if the study did not provide methodological details for evaluation. In a small number of cases, a data point was considered to be an outlier candidate if the level was implausibly low or high based on expert judgement and data from other country data.

Data Extraction

Where possible, individual level data on blood pressure estimates were extracted from survey microdata and these were collapsed across individuals and collapsed across demographic groupings to produce mean estimates in the standard GBD 5-year age-sex groups. If microdata were unavailable, information from survey reports or from literature were extracted along with any available measure of uncertainty including standard error, uncertainty intervals, and sample size. Standard deviations were also extracted.

Incorporating United States prevalence data

Survey reports and literature often report information only about the prevalence, but not the level, of hypertension in the population studied. These sources were not used to model systolic blood pressure, with the exception of data from the Behavioral Risk Factors Surveillance System (BRFSS) because of the availability of a similarly structured exam survey that is representative of the same population (NHANES). BRFSS is a telephone survey conducted in the United States for all US counties. It collects self-reported diagnosis of hypertension. These self-reported values of prevalence of raised blood pressure were adjusted for self-report bias and tabulated by age group, sex, US state, and year. These prevalences were used to predict a mean systolic blood pressure for the same strata with a regression using data from the National Health and Nutrition Examination Survey, a nationally representative health examination survey of the US adult population. The regression was run separately by sex, and was specified as:

$$\mathrm{SBP}_{l,a,t,s} = \beta_0 + \beta_1 \mathrm{prev}_{l,a,t,s}$$

where $\mathrm{SBP}_{l,a,t,s}$ is the location, age, time, and sex specific mean systolic blood pressure and $\mathrm{prev}_{l,a,t,s}$ is the location, age, time, and sex specific prevalence of raised blood pressure. The coefficients for both models are reported in Table 1.

Table 1. Coefficients in the sex-specific US states blood pressure prediction models

Term	Male model	Female model
Intercept	114.65	108.28
Prevalence	51.86	68.87

Out of sample RMSE was used to quantify the predictive validity of the model. The regression was repeated 10 times for each sex, each time randomly holding out 20% of the data. The RMSEs from each holdout analysis were averaged to get the average out of sample RMSE. The results of this holdout analysis are reported in Table 2.

Table 2. Out of sample RMSEs of the sex-specific US states blood pressure prediction models

	Male model	Female model
Out of sample RMSE	2.37 mmHg	3.27 mmHg

Age and Sex Splitting

Prior to modeling, data provided in age groups wider than the GBD 5-year age groups were processed using the approach outlined in Ng et al.[2] Briefly, age-sex patterns were identified using 115 sources of microdata with multiple age-sex groups, and these patterns were applied to estimate age-sex specific levels of mean systolic blood pressure from aggregated results reported in published literature or survey reports.

Modeling

Exposure estimates were produced from 1980 to 2016 for each national and subnational location, sex, and for each 5-year age group starting from 25+. As in GBD 2015, we used a Spatio-Temporal Gaussian Process Regression (ST-GPR) framework to model the mean systolic blood pressure at the location-, year-, age-, sex- level. Updates to the ST-GR modeling framework for GBD 2015 are detailed in the appendix.

Covariate selection

The first step of the ST-GPR framework requires the creation of a linear model for predicting systolic blood pressure at the location-, year-, age-, sex- level. Covariates for this model were selected in two stages. First a list of variables with an expected causal relationship with systolic blood pressure was created based on significant association found within high-quality prospective cohort studies reported in the published scientific literature. These variables were: urinary sodium, liters per capita of alcohol, dietary availability of vegetables, dietary availability of fruits, dietary availability of omega-3 fatty acids, the prevalence of smoking, dietary availability of nuts and seeds, and the prevalence of overweight. We also explored associations with the GBD study socio-demographic index (SDI) covariate, and the GBD sudy health access quality index (HAQI) covariate to represent the effect of proximal socioeconomic factors and access to health care on exposure levels. The second stage in covariate selection was to test the predictive validity of every possible combination of covariates in the linear model, given the

covariates selected above. This was done separately for each sex. Predictive validity was measured with out of sample root-mean-squared error. The linear model with the lowest root-mean squared error for each sex was then used in the ST-GPR model. For women, this linear model was:

$$\log(SBP_{l,a,t}) = \beta_0 + \beta_1 SDI_{l,t} + \beta_2 nuts + \beta_3 HAQI + \sum_{k=2}^{16} \beta_k I_{A[a]} + \alpha_s + \alpha_r + \alpha_c + \epsilon_{l,a,t}$$

For men, the linear model was:

$$\log(SBP_{l,a,t}) = \beta_0 + \beta_1 SDI_{l,t} + \beta_2 alcohol + \beta_3 HAQI + \sum_{k=2}^{16} \beta_k I_{A[a]} + \alpha_s + \alpha_r + \alpha_c + \epsilon_{l,a,t}$$

where $SDI_{c,t}$ is socio-demographic index (SDI), an index metric that includes a measure of education and income, $HAQI_{c,t}$ is the health access quality index, $nuts_{c,t}$ is the dietary availability of nuts and seeds, $alcohol_{c,t}$ is the liters per capita of alcohol consumed, $I_{A[a]}$ is a dummy variable for a fixed effect on a given 5-year age group, and α_s α_r α_c are random effects at the super-region, region, and country level, respectively. Table 3 contains the coefficients of the fixed effects used in the two regressions.

Table 3. Coefficients on covariates in the sex-specific linear models

Covariate	Coefficients from male model	Coefficients from female model
Alcohol LPC	0.0003<br>(0.0002 to -00.0004)	NA
Nuts/seeds	-0.0024<br>(-0.0027 to -0.0022)	-0.0028<br>(-0.0031 to -0.0025)
SDI	0.088<br>(0.074 to 0.102)	0.121<br>(0.100 to 0.142)
HAQI	NA	-0.0016<br>(-0.0019 to -0.0014)

The estimates were then propagated through the ST-GPR framework to obtain 1000 draws for each location, year, age, and sex. Table 4 contains the out of sample root-mean-squared error (RMSE) of both the linear model and the final ST-GPR results for the male and female models.

Table 4. Out of sample RMSEs of the sex-specific linear and ST-GPR models

	**Out of sample RMSEs for male model**	**Out of sample RMSEs for female model**
Linear model	6.469 mmHg	5.985 mmHg
Final ST-GPR model	3.56 mmHg	3.77 mmHg

Estimate of Standard Deviation

Currently, the ST-GPR model only produces an estimate of mean exposure level without standard deviation. Therefore, the standard deviation of systolic blood pressure within a population was estimated for each national and subnational location, sex, and 5-year age group starting from age 25 using the standard deviation from person-level and some tabulated data sources. Person-level microdata accounted for 10375 of the total 12570 rows of data on standard deviation. The remaining 2195 rows came from tabulated data. Tabulated data was only used to model standard deviation if it was sex and 5-year age group specific and reported a population standard deviation of systolic blood pressure. The systolic blood pressure standard deviation function was estimated using a linear regression:

$$\log(SD_{l,a,t,s}) = \beta_0 + \beta_1 SBP_{l,a,t,s} + \beta_3 (SBP_{l,a,t,s})^2 + \beta_4 sex + \sum_{k=2}^{16} \beta_k I_{A[a]} + \alpha_s + \epsilon_{l,a,t,s}$$

where $SBP_{l,a,t,s}$ is the location, age, time, and sex specific mean total cholesterol estimate from ST-GPR, $I_{A[a]}$ is a dummy variable for a fixed effect on a given 5-year age group, and α_s is a random effect at the super-region level.

Adjustment for Usual Levels of Blood Pressure

To account for in-person variation in systolic blood pressure, a ‘usual blood pressure’ adjustment was done. The need for this adjustment has been described elsewhere.[5] Briefly, measurements of a risk factor taken at a single time point may not accurately capture an individual’s true long-term exposure to that risk. Blood pressure readings are highly variable over time due to measurement error as well as diurnal, seasonal, or biological variation. These sources of variation result in an over-estimation of the variation in cross-sectional studies of the distribution of SBP.

To adjust for this overestimation, we applied a correction factor to each location-, age-, time-, and sex-specific standard deviation. These correction factors were age-specific, and represented the proportion of the variation in blood pressure within a population that would be observed if there were no within-person variation across time. Four longitudinal surveys were used to estimate these factors: the China Health and Retirement Longitudinal Survey (CHRLS), the Indonesia Family Life Survey (IFLS), the National Health and Nutrition Examination Survey I Epidemiological Follow-up Study (NHANES I/EFS), and the South Africa National Income Dynamics Survey (NIDS). The sample size and number of blood pressure measurements at each measurement period for each survey is reported in Table 5.

Table 5. Characteristics of longitudinal surveys used for the usual blood pressure adjustment

Source	Measurement period	Number of measurements	Sample size
CHRLS	2008	3	1967
	2012	3	1419
IFLS	1997	1	19418
	2000	1	16626
	2007	3	14136
NIDS	1997	2	14084
	2000	2	9612
	2007	2	9098
NHANES I/EFS	1971-1976	2	20716
	1982-1984	3	9932

For each survey, the following regression was created for each age group:

$$SBP_{i,a} = \beta_0 + \beta_1 sex + \beta_3 age + + \upsilon_i$$

where $SBP_{i,a}$ is the systolic blood pressure of an individual *i* at age *a,* sex is a dummy variable for the sex of an individual, age is a continuous variable for the age of an individual, and υ_i is a random intercept for each individual. Then, a blood pressure value $\widehat{SBP}_{i,b}$ was predicted for each individual *i* for his/her age at baseline *b*. The correction factor *cf* for each age group within each survey was calculated as variation in these predicted blood pressures was divided by the variation in the observed blood pressures at baseline, $SBP_{i,b}$:

$$cf = \frac{var(\widehat{SBP}_{b})}{var(SBP_{b})}$$

The average of the correction factors was taken over the three surveys to get one set of age-specific correction factors, which were then multiplied by the square of the modeled standard deviations to estimate standard deviation of the 'usual blood pressure' of each age, sex, location, and year. Because of low sample sizes, the correction factors for the 75-79 age group was used for all terminal age groups. The final correction factors for each age group are reported in Table 6. Figure 1 shows the correction factors by survey and age group ID.

s

Table 6. Age-specific usual blood pressure correction factors

Age group	Correction factor
25-29	.665
30-34	.713
35-39	.737
40-44	.733
45-49	.798
50-54	.771
55-59	.764
60-64	.753
65-69	.719
70-74	.689
75+	.678

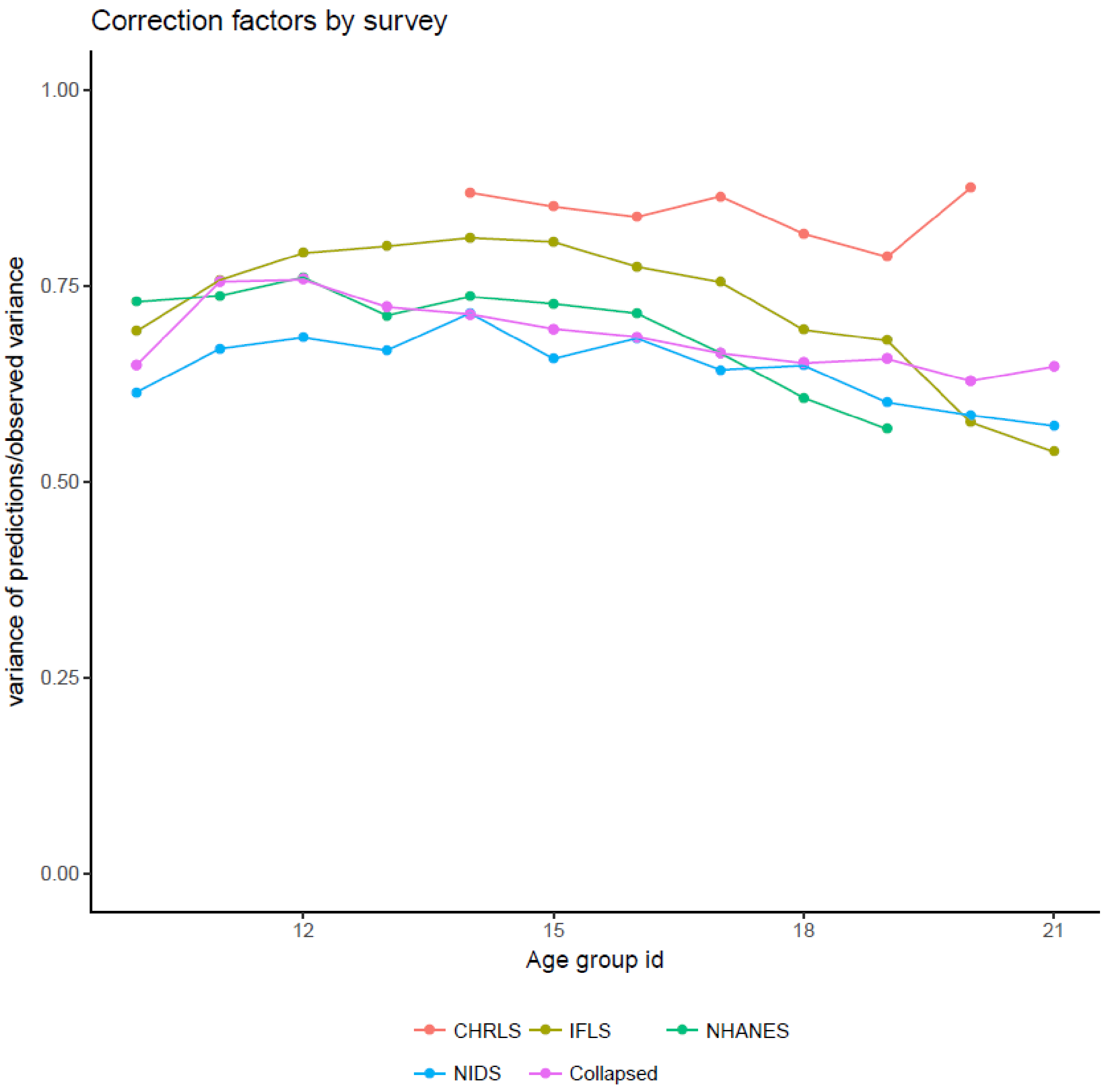

Figure 1: Correction factor by survey and age group id. The correction factor is equal to the variance of the predictions divided by the variance of the raw dataset. In pink is the average correction factor for each age group, summarized in Table 6.

A visualization of how the uncorrected blood pressure measurements overestimate the 'usual' blood pressure variation is shown in Figure 1. This image shows the density of the distribution of the observed blood pressure values $\mathrm{SBP}_{\mathrm{i,b}}$ in participants in the Indonesian Family Life Study survey in red, and the density of the predicted blood pressure values $\widehat{\mathrm{SBP}}_{\mathrm{i,b}}$ in blue. The ratio of the variance of the blue distribution to the variance of the red distribution is an example of the scalar adjustment factor being applied to the modeled standard deviations.

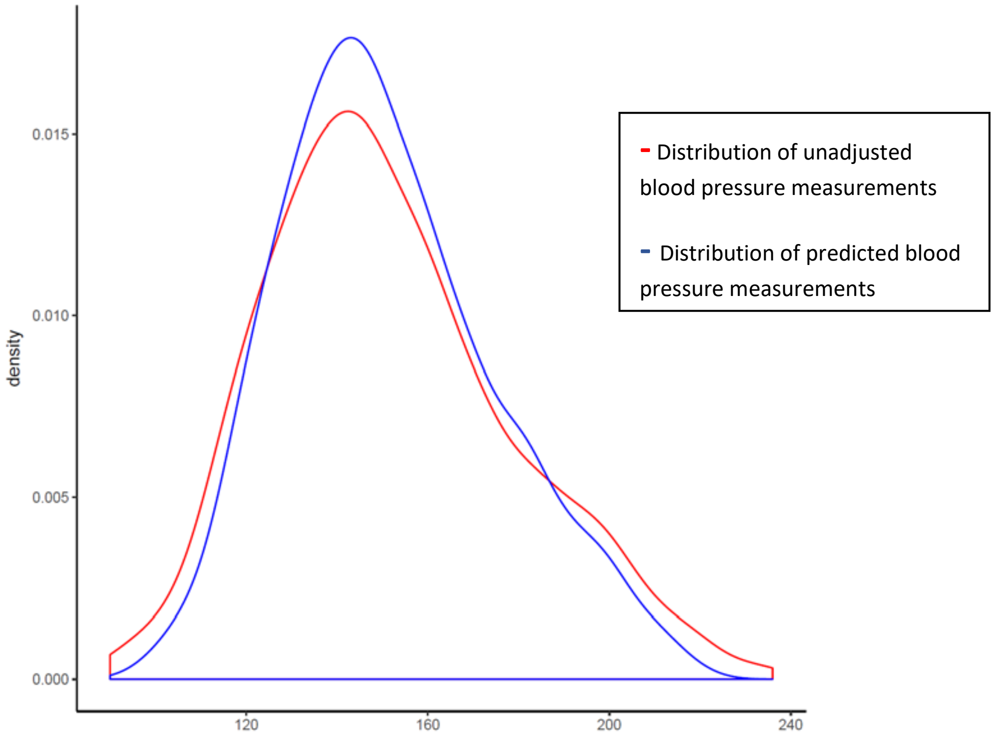

Figure 2: Raw and predicted distributions of blood pressure in the Indonesia Family Life Survey

Estimating the exposure distribution shape

The shape of the distribution of systolic blood pressure was estimated using all available person-level microdata sources, which was a subset of the input data into the modelling process. The distribution shape modelling framework for GBD 2016 is detailed in the appendix. Briefly, an ensemble distribution created from a weighted average of distribution families was fit for each individual microdata source, separately by sex. The weights for the distribution families for each individual source were then averaged and weighted to create a global ensemble distribution for each sex.

Theoretical minimum-risk exposure level

No changes were made to TMREL used in the GBD2015 study. We estimated that the TMREL of SBP ranges from 110 to 115 mm Hg based on pooled prospective cohort studies that show risk of mortality increases for SBP above that level.[3,4] Our selection of a TMREL of 110-115 mmHg is consistent with the GBD study approach of estimating all attributable health loss that could be prevented even if current interventions do not exist that can achieve such a change in exposure level, for example a tobacco smoking prevalence of zero percent. To include the uncertainty in the TMREL, we took a random draw from the uniform distribution of the interval between 110 mm and 115 mm Hg each time the population attributable burden was calculated.

Relative risks

No change was made to RR for blood pressure outcomes used in the GBD2015 study. RRs for chronic kidney disease are from the Renal Risk Collaboration meta-analysis of 2.7 million individuals in 106 cohorts. For other outcomes, we used data from two pooled epidemiological studies: the Asia Pacific Cohort Studies Collaboration (APCSC) and the Prospective Studies Collaboration (PSC).[4,7] In GBD 2015, we have added additional estimates of RR for cardiovascular outcomes from the CALIBER study, a health-record linkage cohort study from the UK.[8]

For cardiovascular disease, epidemiological studies have shown that the RR associated with SBP declines with age, with the log (RR) having an approximately linear relationship with age and reaching a value of 1 between the ages of 100 and 120. RRs were reported per 10 mm Hg increase in SBP above TMREL value (115 mm Hg) as in the equation below:

$$RR_x = RR^{(x-TMREL)}$$

We used Dismod-MR 2.1 to pool effect sizes from included studies and generate a dose-response curve for each of the outcomes associated with high SBP. The tool enabled us to incorporate random effects across studies and include data with different age ranges. RRs were used universally for all countries and the meta-regression only helped to pool the three major sources and produce RRs with uncertainty and covariance across ages taking into account the uncertainty of the data points

References

1 Bangalore S, Gong Y, Cooper-DeHoff RM, Pepine CJ, Messerli FH. 2014 Eighth Joint National Committee panel recommendation for blood pressure targets revisited: results from the INVEST study. *J Am Coll Cardiol* 2014; **64**: 784–93.

2 Ng M, Fleming T, Robinson M, *et al.* Global, regional, and national prevalence of overweight and obesity in children and adults during 1980–2013: a systematic analysis for the Global Burden of Disease Study 2013. *The Lancet* 2014; **384**: 766–81.

3 Singh GM, Danaei G, Farzadfar F, *et al.* The age-specific quantitative effects of metabolic risk factors on cardiovascular diseases and diabetes: a pooled analysis. *PloS One* 2013; **8**: e65174.

4 Collaboration APCS, others. Blood pressure and cardiovascular disease in the Asia Pacific region. *J Hypertens* 2003; **21**: 707–16.

5 Prospective Studies Collaboration. Age-specific relevance of usual blood pressure to vascular mortality: a meta-analysis of individual data for one million adults in 61 prospective studies. *The Lancet* 2002; **360**: 1903–13.

6 Rapsomaniki E, Timmis A, George J, *et al.* Blood pressure and incidence of twelve cardiovascular diseases: lifetime risks, healthy life-years lost, and age-specific associations in 1·25 million people. *Lancet Lond Engl* 2014; **383**: 1899–911.

High Body-Mass index

Flowchart

Adult (Ages 20+) High Body-Mass Index: Data and Model Flow Chart

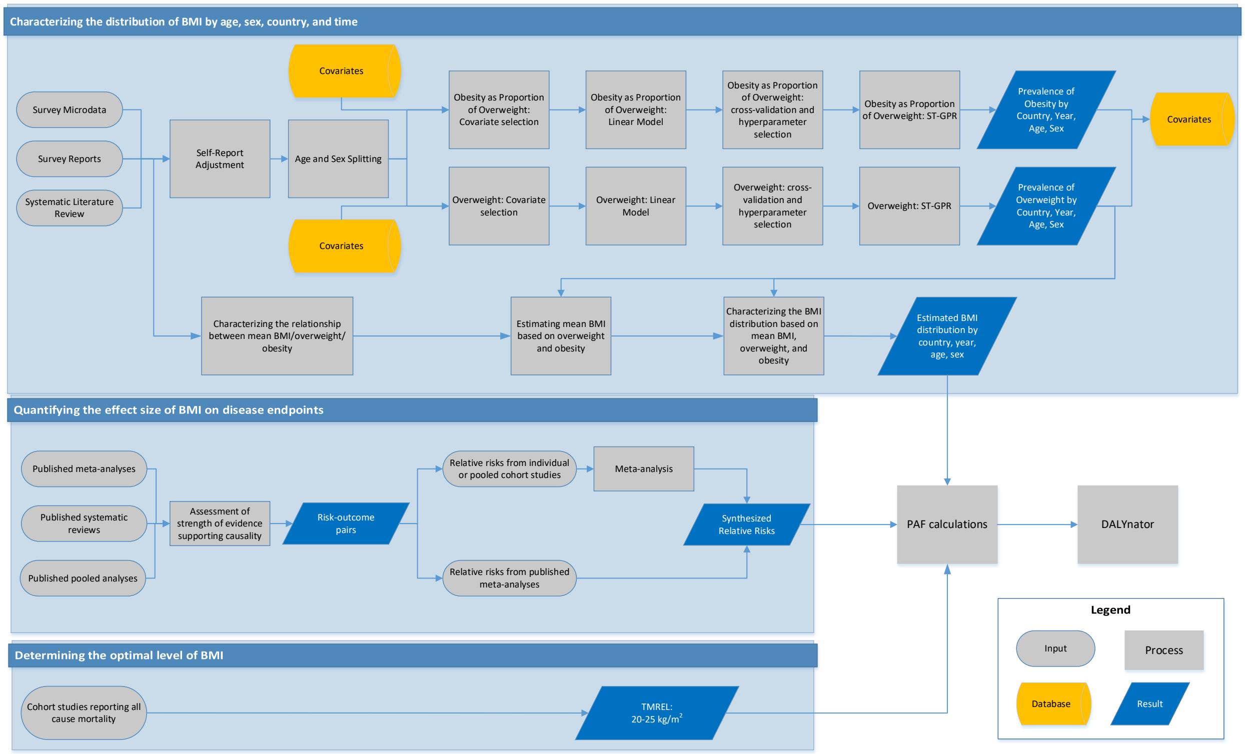

Childhood (Ages 2-19) High Body-Mass Index: Data and Model Flow Chart

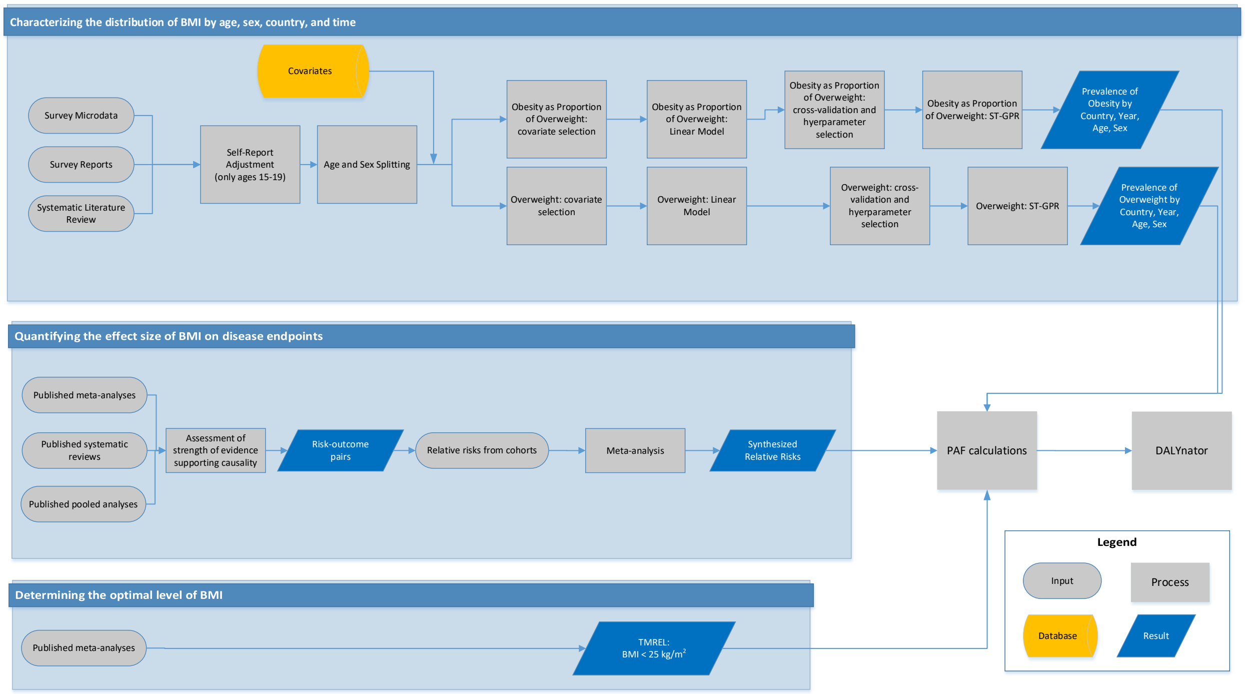

Case Definitions

High body-mass index (BMI) for adults (ages 20+) is defined as BMI greater than 22.5. High BMI for children (ages 1-19) is defined as being overweight or obese based on IOTF cutoffs.

Input data and Methodological Summary

Data Sources

We systematically searched Medline to identify studies providing nationally or subnationally representative estimates of overweight prevalence, obesity prevalence, or mean Body Mass Index (BMI) there were published between 1 January 2016 and 31 December 2016 to update the systematic literature search previously performed as part of GBD 2015.

The search for adults was conducted on 4 January 2017 using the following terms:

((("Body Mass Index"[Mesh] OR "Overweight"[Mesh] OR "Obesity"[Mesh]) AND ("Geographic Locations"[Mesh] NOT "United States"[Mesh]) AND ("humans"[Mesh] AND "adult"[MeSH]) AND ("Data Collection"[Mesh] OR "Health Services Research"[Mesh] OR "Population Surveillance"[Mesh] OR "Vital statistics"[Mesh] OR "Population"[Mesh] OR "Epidemiology"[Mesh] OR "surve*"[TiAb]) NOT (Comment[ptyp] OR Case Reports[ptyp] OR "hospital"[TiAb])) AND ("2016/01/01"[Date - Publication] : "2016/12/31"[Date - Publication]))

The search for children was conducted on 4 August 2016 using the following terms:
((("Body Mass Index"[Mesh] OR "Overweight"[Mesh] OR "Obesity"[Mesh]) AND ("Geographic Locations"[Mesh] NOT "United States"[Mesh]) AND ("humans"[Mesh] AND "child"[MeSH]) AND ("Data Collection"[Mesh] OR "Health Services Research"[Mesh] OR "Population Surveillance"[Mesh] OR "Vital statistics"[Mesh] OR "Population"[Mesh] OR "Epidemiology"[Mesh] OR "surve*"[TiAb]) NOT (Comment[ptyp] OR Case Reports[ptyp] OR "hospital"[TiAb])) AND ("2016/01/01"[Date - Publication] : "2016/12/31"[Date - Publication]))

Our search for adult estimates identified 456 abstracts, of which 25 met inclusion criteria and were extracted. The search for children estimates identified 137 articles, of which 4 were extracted. Including sources from the previous GBD systematic literature searches, a total of 11,220 articles were identified, of which 845 were included. Additionally, we searched the Global Health Data Exchange (GHDx) database for individual-level data from major multinational survey series or country-specific surveys and identified 5,385 location-year sources meeting the inclusion criteria.

Eligibility Criteria

We included representative studies providing data on mean BMI or prevalence of overweight or obesity among adults or children. For adults, studies were included if they defined overweight as BMI≥25 kg/m^2 and obesity as BMI≥30 kg/m^2, or if estimates using those cutoffs could be back-calculated from reported categories. For children (children ages 2-18), studies were included if they used International Obesity Task Force (IOTF) standards to define overweight and obesity thresholds. We only included studies reporting data collected between 1 January 1980 and 31 December 2016. Studies were excluded if using non-random samples (e.g., case-control studies or convenience samples); conducted among specific subpopulations (e.g., pregnant women, racial or ethnic minorities, immigrants, or individuals with specific diseases); using alternative methods to assess adiposity (e.g., waist-circumference, skin-fold thickness, or hydrodensitometry); having sample sizes of less than 20 per age-sex group; or providing inadequate

information on any of the inclusion criteria. We also excluded review articles and non-English language articles.

Data collection process

Where individual-level survey data were available, we computed mean BMI using weight and height and then used BMI to determine the prevalence of overweight and obesity. For individuals aged over 18 years, we considered them to be overweight if their BMI was greater than or equal to 25 kg/m^2, and obese if their BMI was greater than or equal to 30 kg/m^2. For individuals aged 2-18 years, we used monthly IOTF cutoffs[2] to determine overweight and obese status when age in months was available. When only age in years was available, we used the cutoff for the 6 month of that year. Individuals who were obese were also considered to be overweight. We excluded studies using the World Health Organization (WHO) standards or country-specific cutoffs to define childhood overweight and obesity. At the individual-level, we considered BMI<10 kg/m^2 and BMI>70 kg/m^2 to be biologically implausible and excluded those observations.

The rationale for choosing to use the IOTF cutoffs over the WHO standards has been described elsewhere. Briefly, the IOTF cutoffs provide consistent child-specific standards for ages 2-18 derived surveys covering multiple countries. On the other hand, the WHO growth standards apply to children under 5 and the WHO growth reference applies to children ages 5-19. The WHO growth reference for children ages 5-19 was derived from United States data which is less representative than the multinational data used by IOTF. Additionally, the switch between references at age 5 can produce artificial discontinuities. Given that we estimate global childhood overweight and obesity for ages 2-19 (with ages 19 using standard adult cutoffs), the IOTF cutoffs were preferable. Additionally, we found that IOTF cutoffs were more commonly used in scientific literature covering childhood obesity.

From report and literature data, we extracted data on mean BMI, prevalence of overweight, and prevalence of obesity, measures of uncertainty for each, and sample size, by the most granular age and sex groups available. Additionally, we extracted the same study-level covariates as were extracted from microdata (measurement, urbanicity, and representativeness), as well as location and year.

In addition to the primary indicators described above, we extracted relevant survey-design variables, including primary sampling unit, strata, and survey weights, which were used to tabulate individual-level microdata and produce accurate measures of uncertainty. We extracted three study-level covariates: 1) whether height and weight data were measured or self-reported, 2) whether the study was predominantly conducted in an urban area, rural area, or both, and 3) the level of representativeness of the study (national or subnational).

Finally, we extracted relevant demographic indicators, including location, year, age, and sex. We estimated the standard error of the mean from individual-level data where available and used the reported standard error of the mean for published data. When multiple data sources were available for the same country, we included all of them in our analysis. If data from the same data source were available in multiple formats such individual-level data and tabulated data, we used individual-level data.

Self-report bias adjustment

We included both measured and self-reported data. Of 72.6 million person-years of data, 18.8 million (26%) were self-reported. We tested for bias in self-report data compared to measured data, which is considered to be the gold-standard. There was no clear direction of bias for children ages 2-14, so for

these age groups we only included measured data. For individuals ages 15+, we adjusted self-reported data for overweight prevalence, obesity prevalence, and mean BMI using the following nested hierarchical mixed-effects regression models, fit using restricted maximum likelihood separately by sex:

$$\text{logit}(\text{overweight})_{c,a,t} = \beta_0 + \beta_1 m + \sum_{k=2}^{19} \beta_k I_{A[a]} + \sum_{l=20}^{55} \beta_l\, I_{A[a]} I_{M[m]} + \alpha_s + \alpha_s m + \alpha_r + \alpha_r m + \alpha_c + \alpha_c m + \alpha_t + \alpha_t m + \epsilon_{c,a,t}$$

$$\text{logit}(\text{obesity})_{c,a,t} = \beta_0 + \beta_1 m + \sum_{k=2}^{19} \beta_k I_{A[a]} + \sum_{l=20}^{55} \beta_l\, I_{A[a]} I_{M[m]} + \alpha_s + \alpha_s m + \alpha_r + \alpha_r m + \alpha_c + \alpha_c m + \alpha_t + \alpha_t m + \epsilon_{c,a,t}$$

$$\log(\text{BMI})_{c,a,t} = \beta_0 + \beta_1 m + \sum_{k=2}^{19} \beta_k I_{A[a]} + \sum_{l=20}^{55} \beta_l\, I_{A[a]} I_{M[m]} + \alpha_s + \alpha_s m + \alpha_r + \alpha_r m + \alpha_c + \alpha_c m + \alpha_t + \alpha_t m + \epsilon_{c,a,t}$$

Where m is a fixed effect on measurement (binary, either measured (1) or self-report (0)), $I_{A[a]}$ is an indicator variable for specific age group A, $I_{A[a]} I_{M[m]}$ is an interaction term between age and measurement, α_s, α_r, and α_c are random effects at the super region, region, and country, respectively, and α_t is a random effect by time-period (1980-1989, 1990-1999, 2000-2009, 2010-2016). Random effects at the country level and time-period level were used to fit the models, but were taken as noise and were not used in adjustment of self-reported data. We propagated the uncertainty in the self-report adjustment model by adding the variance of each of the regression coefficients used in adjustment to the data variance in delta-transformed space. After adjustment, regressions confirmed that self-reported data was no longer significantly different from measured data.

Age and sex splitting

Any report or literature data provided in age groups wider than the standard 5-year age groups or as both sexes combined were split using the approach used by Ng et al.[1] Briefly, age-sex patterns were identified using sources with data on multiple age-sex groups and these patterns were applied to split aggregated report and literature data. Uncertainty in the age-sex split was propagated by multiplying the standard error of the data by the square root of the number of splits performed. We did not propagate the uncertainty in the age pattern and sex pattern used to split the data as they seemed to have small effect.

Prevalence estimation for overweight and obesity

After adjusting for self-report bias and splitting aggregated data into 5-year age-sex groups, we used spatiotemporal Gaussian process regression (ST-GPR) to estimate the prevalence of overweight and obesity. This modeling approach has been described in detail elsewhere.

The linear model, which when added to the smoothed residuals forms the mean prior for GPR is as follows:

$$\text{logit}(\text{overweight})_{c,a,t} = \beta_0 + \beta_1 \text{energy}_{c,t} + \beta_2 \text{SDI}_{c,t} + \beta_3 \text{vehicles}_{c,t} + \beta_4 \text{agriculture}_{c,t} + \sum_{k=5}^{21} \beta_k I_{A[a]} + \alpha_s + \alpha_r + \alpha_c$$

$$\text{logit}(\text{obesity/overweight})_{c,a,t} = \beta_0 + \beta_1 \text{energy}_{c,t} + \beta_2 \text{SDI}_{c,t} + \beta_3 \text{vehicles}_{c,t} + \sum_{k=4}^{21} \beta_k I_{A[a]} + \alpha_s + \alpha_r + \alpha_c +$$

where energy is ten-year lag-distributed energy consumption per capita, SDI is a composite index of development including lag-distributed income per capita, education, and fertility, vehicles is is the number of two or four-wheel vehicles per capita, and agriculture is the proportion of the population working in agriculture. $I_{A[a]}$ is a dummy variable indicating specific age group A that the prevalence point captures, and α_s, α_r, and α_c are super region,

region, and country random intercepts, respectively. Random effects were used in model fitting but were not used in prediction.

We tested all combinations of the following covariates to see which performed best in terms of in-sample AIC for the overweight linear model and the obesity as a proportion of overweight linear model: ten-year lag distributed energy per capita, proportion of the population living in urban areas, SDI, lag-distributed income per capita, educational attainment (years) per capita, proportion of the population working in agriculture, grams of sugar adjusted for energy per capita, grams of sugar not adjusted for energy per capita, and the number of two or four-wheeled vehicles per capita. We selected these candidate covariates based on theory as well as reviewing covariates used in other publications. The final linear model was selected based on: 1) if the direction of covariates matched what is expected from theory, 2) all the included covariates were significant, and 3) minimizing in-sample AIC. The covariate selection process was performed using the dredge package in R.

We used different space weights by data density category: locations with 0-4 years covered by data used a space weight of 0.7, locations with 5-9 years covered by data used a space weight of 0.9, locations with 10-19 years covered by data used a space weight of 0.95, and locations with more than 20 years covered by data used a space weight of 0.99 . The other parameters were consistent across data-density levels: age weight = 1.2 for overweight and age weight = 1.4 for obesity, time weight = 1, and scale = 10. The GPR amplitude was calculated at the region level.

Estimating mean BMI

To estimate the mean BMI for adults in each country, age, sex, and time period 1980-2016, we first used the following nested hierarchical mixed-effects model, fit using restricted maximum likelihood on data from sources containing estimates of all three indicators (prevalence of overweight, prevalence of obesity, and mean BMI), in order to characterize the relationship between overweight, obesity, and mean BMI:

$$\log(\mathrm{BMI}_{c,a,s,t}) = \beta_0 + \beta_1 \mathrm{ow}_{c,a,s,t} + \beta_2 \mathrm{ob}_{c,a,s,t} + \beta_3 \mathrm{sex} + \sum_{k=4}^{20} \beta_k \mathrm{I}_{A[a]} + \alpha_s(1 + \mathrm{ow}_{c,a,s,t} + \mathrm{ob}_{c,a,s,t}) + \alpha_r(1 + \mathrm{ow}_{c,a,s,t} + \mathrm{ob}_{c,a,s,t}) + \alpha_c(1 + \mathrm{ow}_{c,a,s,t} + \mathrm{ob}_{c,a,s,t}) + \epsilon_{c,a,s,t}$$

where $\mathrm{ow}_{c,a,s,t}$ is the prevalence of overweight in country c, age a, sex s, and year t, $\mathrm{ob}_{c,a,s,t}$ is the prevalence of obesity in country c, age a, sex s, and year t, sex is a fixed effect on sex, $I_{A[a]}$ is an indicator variable for age, and α_s, α_r, and α_c are random effects at the super region, region, and country, respectively. The model was run in Stata 13.

We applied 1,000 draws of the regression coefficients to the 1,000 draws of overweight prevalence and obesity prevalence produced through ST-GPR to estimate 1,000 draws of mean BMI for each country, year, age, and sex. This approach ensured that overweight prevalence, obesity prevalence, and mean BMI were correlated at the draw level and uncertainty was propagated.

Estimating BMI distribution

We used the ensemble distribution approach described in the manuscript. We fit ensemble weights by source and sex, with source- and sex-specific weights averaged across all sources included to produce the final global weights. The ensemble weights were fit on measured microdata. The final ensemble weights were: exponential = 0.002, gamma = 0.028, inverse gamma = 0.085, log logistic = 0.187, gumbel = 0.220, inverse Weibull = 0.141, Weibull = 0.011, lognormal = 0.058, normal = 0.012, beta = 0.136, mirror gamma = 0.008, and mirror gumbel = 0.113.

One thousand draws of BMI distributions for each location, year, age group, and sex estimated were produced by fitting an ensemble distribution using 1,000 draws of estimated mean BMI, 1,000 draws of estimated standard deviation, and the ensemble weights. Estimated standard deviation was produced by optimizing a standard deviation to fit estimated overweight prevalence draws and estimated obesity prevalence draws.

Assessment of risk-outcome pairs

Risk-outcome pairs were defined based on strength of available evidence supporting a causal effect. We performed a systematic review of published meta-analyses, pooled analyses, and systematic reviews available through PubMed using the following search string: ("Body Mass Index"[Mesh] OR "Overweight"[Mesh] OR "Obesity"[Mesh]) AND (Meta-Analysis[ptyp] OR "systematic review"[tiab] OR "pooled analysis"[tiab]). Inclusion criteria are 1) the health outcome is included in GBD, 2) at least one prospective cohort is included, and 3) that the summary effect size is statistically significant. For outcomes meeting inclusion criteria we completed causal criteria tables to evaluate the strength of evidence supporting a causal relationship. Table X reports the results of our assessment for included risk-outcome pairs and Table X reports the supporting scientific literature. As a result of this effort we expanded the number of risk-outcome pairs to include a total of 38 outcomes. Gallbladder disease, cataract, multiple myeloma, gout, non-Hodgkin lymphoma, asthma, Alzheimer disease, and atrial fibrillation were added as new outcomes for GBD 2016.

Theoretical minimum risk exposure level

For adults (ages 20+), the theoretical minimum risk exposure level (TMREL) of BMI (20-25 kg/m2) was determined based on the BMI level that was associated with the lowest risk of all-cause mortality in prospective cohort studies.[2]

For children (ages 2-19), the TMREL is “normal weight”, that is, not overweight or obese, based on IOTF cutoffs.

Relative risk

The relative risk per 5-unit change in BMI for each disease endpoint was obtained from meta-analyses, and where available, pooled analyses of prospective observational studies. In cases where a relative risk per 5-unit change in BMI was not available we computed our own dose-response meta-analysis using two-step generalized least squares for time trends estimation methods.

For childhood outcomes (ages 2-19), we computed categorical relative risks for overweight and obesity using a random effects meta-analysis.

Relative risks for all 38 outcomes, by age and sex, are reported in Table X.

References

1.) Ng M, Fleming T, Robinson M, et al. Global, regional, and national prevalence of overweight and obesity in children and adults during 1980–2013: a systematic analysis for the Global Burden of Disease Study 2013. The Lancet 2014; 384: 766–81.

2.) Angelantonio ED, Bhupathiraju SN, Wormser D, et al. Body-mass index and all-cause mortality: individual-participant-data meta-analysis of 239 prospective studies in four continents. The Lancet 2016; 388: 776–86.

Bone Mineral Density Capstone Appendix

Flowchart

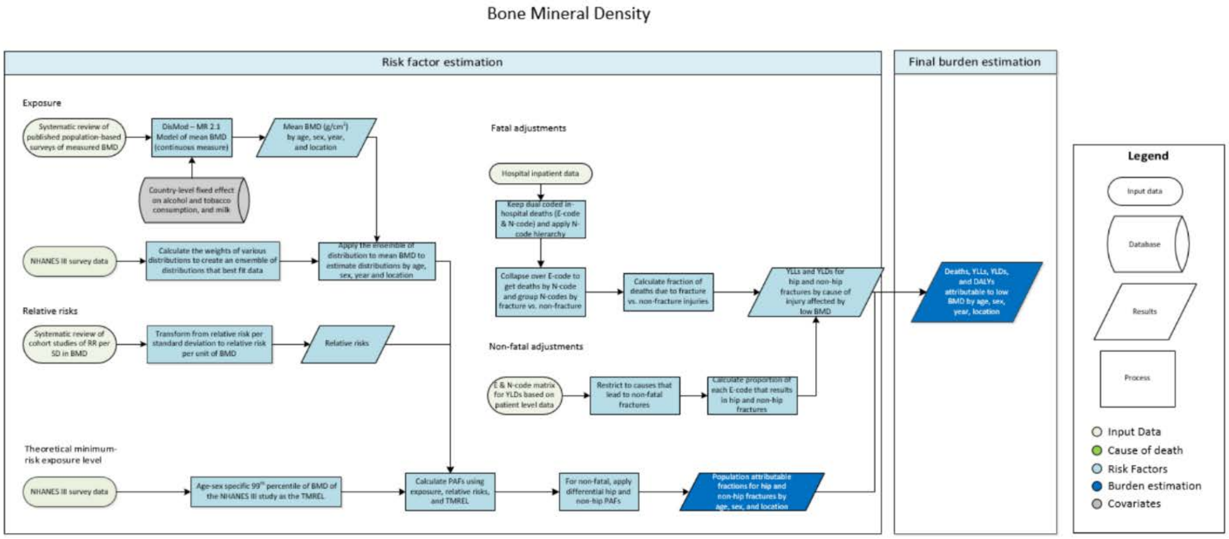

Input Data & Methodological Summary

Exposure

Case Definition

Bone mineral density (BMD) is a continuous variable measured by dual-x-ray-absorptiometry (DXA) at the femoral neck (FN) and is presented in g/cm^2 after standardizing for the brand of densitometer (sBMD). Low BMD is measured in terms of the difference between BMD of a population and the 99th percentile of a reference population at the same age and sex (theoretical minimum of risk exposure level, TMREL). The burden attributed to low bone mineral density is estimated for adults 20 years and older.

For estimating burden, we need to estimate:

Exposure: mean and standard deviation (SD) of BMD standardized for the brand of densitometer for each country and all subnational locations included in GBD.

Risk of osteoporotic outcomes, i.e. fractures, in people exposed to low BMD relative to people who have BMD equal or greater than the TMREL. We consider the risk of fatal and non-fatal outcomes for hip non-hip fractures, separately, as relative risk data provide different estimates. These osteoporotic non-hip fractures include fractures of vertebrae, clavicle, scapula, humerus, skull, sternum, rib, face bone, radius or ulna, femur, patella, tibia, fibula, ankle, pelvis and vertebra.

Input data

A systematic review (search string at the end of document) was conducted in GBD 2015 but it was not scheduled for systematic review in GBD 2016. Inclusion criteria that informed the search are:

- Representative, population-based surveys
- Reporting of quantitative BMD

- measured by DXA
- performed at the FN region
- measured in g/cm^2

Mean BMD was occasionally reported in stratified groups, e.g. by fracture status but not for total sample. In these cases, the stratified means were aggregated to obtain a total mean BMD at population level for an age or sex category.

The data availability by GBD super-region is shown in below table.

Super region	The number of data points
Southeast Asia, East Asia, and Oceania	314
Central Europe, Eastern Europe, and Central Asia	36
High-income	682
Latin America and Caribbean	97
North Africa and Middle East	110
South Asia	39
Sub-Saharan Africa	3

Modeling strategy

We modeled mean BMD in DisMod-MR 2.1 as a single 'continuous' parameter model by age and sex, and all GBD locations for years 1990 to 2016. The model had age mesh points at 0 10 20 25 30 40 50 60 70 80 90 & 100, a time window of 10 years for fitting data, and a minimum coefficient of variation of 0.1 for global, 0.06 super region and 0.04 for the region level.

The country covariates of alcohol consumption (litres per capita), tobacco consumption (cigarettes per capita) and adjusted calcium intake (g) were included in modelling. The country covariates of BMI and milk consumption did not have a significant effect on BMD so we excluded them from our final model.

The uncertainty of BMD was modeled using a new approach. Various distributions were tested for goodness-of-fit in NHANES III data, the only survey for which we had unit record data available. We applied a weighting ensemble on those distributions. The weights were calculated in an optimization model with an objective function that minimized Kolmogorov-Smirnov statistics. The weights of the distributions in the ensemble were calculated separately for males and females, and for ages below and above 70. Distribution weights are shown below.

sex	age (years)	gamma	gumbel	inverse weibull	weibull	log normal	normal	mirrored gamma	mirrored gumbel
male	< 70	0.03	0.01	0.28	0.00	0.20	0.35	0.06	0.07
male	>= 70	0.24	0.21	0.06	0.00	0.21	0.01	0.00	0.27
female	< 70	0.03	0.06	0.01	0.06	0.49	0.02	0.00	0.32
female	>= 70	0.00	0.00	0.42	0.00	0.12	0.44	0.00	0.01

There were various modelling steps after DisMod-MR 2.1 modelling of exposure to arrive at attributable fractions that can be applied to fatal and non-fatal fracture outcomes. First, we calculated the

proportion of injury deaths that are due to fractures. This proportion of death caused by fracture is the envelope that we use to attribute death to BMD. In order to do this, we assumed that hip fracture and some non-hip fractures (any fractures apart from fingers and toes) are potentially fatal fractures. As cause of death data from vital registrations and verbal autopsy attributed injury deaths to causes of death (e.g. fall or road injury) and not nature of injury (such as fractures), we turned to available hospital data to estimate the proportion of injury deaths during admission that could be ascribed to fractures. We restricted our analysis to cases that were dual-coded with both the cause of injury ("E-code") and nature of injury ("N-code"). As injury cases may have multiple forms of trauma, we applied a severity hierarchy to the fatal hospital data to determine the proportion of the deaths that could be attributed to the chosen fracture types but were not accompanied by more severe fatal trauma such as head trauma, spinal cord lesion, and intra-abdominal or thoracic organ damage. We collapsed all deaths over E-code to determine the ratio of deaths attributable to fracture versus non-fracture injuries. We applied this ratio to the YLL.

We restricted non-fatal estimates of low BMD to a list of causes that were deemed to cause osteoporotic fractures. Below is the list of injuries for which a PAF was calculated:

- Transport injuries
- Road injuries
- Pedestrian road injuries
- Cyclist road injuries
- Motorcyclist road injuries
- Motor vehicle road injuries
- Other road injuries
- Other transport injuries
- Unintentional injuries
- Falls
- Exposure to mechanical forces
- Other exposure to mechanical forces
- Non-venomous animal contact
- Interpersonal violence
- Assault by other means
- Exposure to forces of nature

We made use of the E to N-code matrix generated from dual-coded (E-code/N-code) patient level data in our injury analyses to determine the proportion of each E-code that results in a certain N-code. The hip and non-hip fracture population attributable fractions (as explained below) were applied to the appropriate combinations of external cause and fracture estimates of YLD.

Theoretical minimum-risk exposure level

The theoretical minimum of risk exposure level or TMREL was chosen as the age-sex specific 99th percentile of BMD of the NHANES III study as the reference population.

Relative risks

Relative risks must be reported per standard deviation or per unit bone mass density in order for us to use the data. Many studies report relative risk based on a z-score or the relative risks in the osteoporotic group versus the non-osteoporotic group; neither of these relative risks are usable.

For GBD 2016, we did not update the systematic review for the RR of BMD that was done in GBD 2013, from which twelve prospective observational studies were found but one meta-analysis of 12 studies (Johnell et al. 2005) reported the dose-response relationship between low BMD and high relative risk of hip and other fractures that are prone to osteoporosis, as shown in the below table.

BMD	*Any fracture*		*Osteoporotic fracture*		*Hip fracture*	
z score	*RR*	*95% CI*	*RR*	*95% CI*	*RR*	*95% CI*
−4	1.79	1.44–2.23	2.10	1.63–2.71	2.14	1.40–3.26
−3	1.71	1.44–2.02	1.96	1.61–2.39	2.12	1.54–2.92
−2	1.63	1.45–1.84	1.84	1.60–2.12	2.11	1.70–2.62
−1	1.56	1.45–1.69	1.73	1.59–1.89	2.11	1.86–2.39
0	1.50	1.44–1.56	1.62	1.54–1.71	2.08	1.91–2.26
1	1.39	1.32–1.46	1.42	1.34–1.51	2.04	1.78–2.34
2	1.32	1.21–1.45	1.33	1.19–1.48	2.03	1.60–2.56
3	1.26	1.10–1.45	1.25	1.06–1.47	2.01	1.44–2.81
4	1.21	1.00–1.45	1.17	0.93–1.46	1.99	1.28–3.10

The z score ranged from −5.1 to +5.8.

Reference

Johnell O, Kanis JA, Oden A, Johansson H, De Laet C, Delmas P, Eisman JA, Fujiwara S, Kroger H, Mellstrom D, Meunier PJ, Melton LJ, 3rd, O'Neill T, Pols H, Reeve J, Silman A, Tenenhouse A (2005) Predictive value of BMD for hip and other fractures. J Bone Miner Res 20 (7):1185-1194

Search string from GBD 2015 systematic review:

Search	Query	Items found	Time
#11	Search (#8 AND #10) Filters: Humans	326	12:37:09
#10	Search ("Cross-Sectional Studies"[Mesh] OR "cross-sectional"[title/abstract] OR "Health Surveys"[Mesh] OR Survey[title/abstract] OR cohort[title/abstract] OR "Diet Surveys"[Mesh] OR "Longitudinal Studies"[Mesh] OR "Nutrition Surveys"[Mesh] OR "Surveys and Questionnaires"[Mesh]) Filters: Humans	1324376	12:36:29
#8	Search (#7 AND #6) Filters: Humans	622	12:33:16
#7	Search ("Absorptiometry, Photon"[Mesh] OR "dual-energy x-ray absorptiometry" OR "dual energy x-ray absorptiometry") Filters: Humans	21368	12:32:34
#6	Search (#5 AND ("2010"[Date - Publication] : "3000"[Date - Publication])) Filters: Humans	1387	12:30:26
#5	Search ((#1 OR #2) AND #3) Filters: Humans	3702	12:29:47
#4	Search ((#1 OR #2) AND #3)	4015	12:29:33
#3	Search (((("bone mineral density"[title/abstract] OR "bone mineral densities"[title/abstract]) OR "Bone Density"[Mesh]) AND (mean[title/abstract] OR average[title/abstract])))	12892	12:29:00

#2	Search ((((multinational[TIAB] OR international[TIAB] OR national[TIAB] OR nationwide[TIAB] OR nation-wide[TIAB] OR equitorial[TIAB] OR equator[TIAB] OR global[TIAB] OR globe[TIAB] OR world[TIAB] OR worldwide[TIAB] OR world-wide[TIAB] OR countrywide[TIAB] OR countries[TIAB] OR continental[TIAB] OR continent[TIAB] OR continents[TIAB] OR global burden[TIAB] OR burden of disease[TIAB] OR disease burden[TIAB] OR tropic[TIAB] OR tropics[TIAB] OR tropical[TIAB] OR Oceania[TIAB] OR South America[TIAB] OR Central America[TIAB] OR Mesoamerica[TIAB] OR Americas[TIAB] OR Latin America[TIAB] OR paho[TIAB] OR pan-american[TIAB] OR panamerican[TIAB] OR pan-america[TIAB] OR Caribbean[TIAB] OR Indies[TIAB] OR Australasia[TIAB] OR Australasian[TIAB] OR developing countries[TIAB] OR developing nations[TIAB] OR developed countries[TIAB] OR developed nations[TIAB] OR commonwealth[TIAB] OR industrialized[TIAB] OR nonindustrialized[TIAB] OR non-industrialized[TIAB] OR underdeveloped countries[TIAB] OR underdeveloped nation[TIAB] OR underdeveloped nations[TIAB] OR under-developed country[TIAB] OR under-developed countries[TIAB] OR under-developed nation[TIAB] OR under-developed nations[TIAB] OR low-income country[TIAB] OR low-income countries[TIAB] OR low-income nation[TIAB] OR low-income nations[TIAB] OR nondeveloped country[TIAB] OR nondeveloped countries[TIAB] OR nondeveloped nation[TIAB] OR nondeveloped nations[TIAB] OR non-developed country[TIAB] OR non-developed countries[TIAB] OR non-developed nation[TIAB] OR non-developed nations[TIAB] OR International Cooperation[TIAB] OR World Health Organization[TIAB] OR Asia[TIAB] OR Far East[TIAB] OR Near East[TIAB] OR Middle East[TIAB] OR Scandinavia[TIAB] OR Europe[TIAB] OR European[TIAB] OR Eastern Hemisphere[TIAB] OR Western Hemisphere[TIAB] OR Northern Hemisphere[TIAB] OR Southern Hemisphere[TIAB] OR North America[TIAB] OR island[TIAB] OR islands[TIAB] OR United Nations[TIAB] OR unesco[TIAB] OR unicef[TIAB] OR Worldbank[TIAB] OR Benelux[TIAB] OR sub-Saharan[TIAB] OR subsaharan[TIAB] OR Sahara[TIAB] OR sub-Sahara[TIAB] OR Amazon[TIAB] OR Amazonian[TIAB] OR valley[TIAB] OR river[TIAB] OR mountain[TIAB] OR mountains[TIAB] OR forest[TIAB] OR forests[TIAB] OR rainforest[TIAB] OR rainforests[TIAB] OR jungle[TIAB] OR jungles[TIAB] OR archipelago[TIAB] OR archipelagos[TIAB] OR archipelagoes[TIAB] OR patagonia[TIAB] OR andes[TIAB] OR mediterranean region[TIAB] OR Africa[TIAB] OR registry[TIAB] OR North Korea[TIAB] OR Timor[TIAB] OR Palestine[TIAB] OR Syrian Arab Republic[TIAB] OR Baltic[TIAB] OR Atlantic Islands[TIAB] OR Indian Ocean[TIAB] OR Pacific[TIAB] OR multicenter[TIAB] OR multi-center[TIAB] OR registry[TIAB] OR registries[TIAB] OR Algeria[TIAB] OR Egypt[TIAB] OR Libya[TIAB] OR Morocco[TIAB] OR Tunisia[TIAB] OR Cameroon[TIAB] OR Central African Republic[TIAB] OR Chad[TIAB] OR Congo[TIAB] OR Congo[TIAB] OR Equatorial Guinea[TIAB] OR Gabon[TIAB] OR Burundi[TIAB] OR Djibouti[TIAB] OR Eritrea[TIAB] OR Ethiopia[TIAB] OR Kenya[TIAB] OR Rwanda[TIAB] OR Somalia[TIAB] OR Sudan[TIAB] OR Tanzania[TIAB] OR Uganda[TIAB] OR Angola[TIAB] OR Botswana[TIAB] OR Lesotho[TIAB] OR Malawi[TIAB] OR Mozambique[TIAB] OR Namibia[TIAB] OR South Africa[TIAB] OR Swaziland[TIAB] OR Zambia[TIAB] OR Zimbabwe[TIAB] OR Benin[TIAB] OR Burkina Faso[TIAB] OR Cote d'Ivoire[TIAB] OR Gambia[TIAB] OR Ghana[TIAB] OR Guinea[TIAB] OR Guinea-Bissau[TIAB] OR Liberia[TIAB] OR Mali[TIAB] OR Mauritania[TIAB] OR Niger[TIAB] OR Nigeria[TIAB] OR Senegal[TIAB] OR Sierra Leone[TIAB] OR Togo[TIAB] OR Antigua[TIAB] OR Bahamas[TIAB] OR Barbados[TIAB] OR Cuba[TIAB] OR Dominica[TIAB] OR Dominican Republic[TIAB] OR Grenada[TIAB] OR Guadeloupe[TIAB] OR Haiti[TIAB] OR Jamaica[TIAB] OR Martinique[TIAB] OR Netherlands Antilles[TIAB] OR Puerto Rico[TIAB] OR Saint Kitts and Nevis[TIAB] OR Saint Lucia[TIAB] OR Saint Vincent[TIAB] OR Grenadines[TIAB] OR Trinidad and Tobago[TIAB] OR Virgin Islands[TIAB] OR Belize[TIAB] OR Costa Rica[TIAB] OR El Salvador[TIAB] OR Guatemala[TIAB] OR Honduras[TIAB] OR Nicaragua[TIAB] OR Panama[TIAB] OR Mexico[TIAB] OR Argentina[TIAB] OR Bolivia[TIAB] OR Brazil[TIAB] OR Chile[TIAB] OR Colombia[TIAB] OR Ecuador[TIAB] OR French Guiana[TIAB] OR French Guiana[TIAB] OR Paraguay[TIAB] OR Peru[TIAB] OR Suriname[TIAB] OR Uruguay[TIAB] OR Venezuela[TIAB] OR Kazakhstan[TIAB] OR Kyrgyzstan[TIAB] OR Tajikistan[TIAB] OR Turkmenistan[TIAB] OR Uzbekistan[TIAB] OR Borneo[TIAB] OR Cambodia[TIAB] OR Timor[TIAB] OR Indonesia[TIAB] OR Laos[TIAB] OR Malaysia[TIAB] OR Mekong Valley[TIAB] OR Myanmar[TIAB] OR Philippines[TIAB] OR Thailand[TIAB] OR Vietnam[TIAB] OR Viet Nam[TIAB] OR Bangladesh[TIAB] OR Bhutan[TIAB] OR India[TIAB] OR Afghanistan[TIAB] OR Bahrain[TIAB] OR Iran[TIAB] OR Iraq[TIAB] OR Jordan[TIAB] OR Kuwait[TIAB] OR Lebanon[TIAB] OR Oman[TIAB] OR Qatar[TIAB] OR Saudi Arabia[TIAB] OR Syria[TIAB] OR Turkey[TIAB] OR United Arab Emirates[TIAB] OR Yemen[TIAB] OR Nepal[TIAB] OR Pakistan[TIAB] OR Sri Lanka[TIAB] OR China[TIAB] OR Macao[TIAB] OR Mongolia[TIAB] OR Taiwan[TIAB] OR Azores[TIAB] OR Bermuda[TIAB] OR Falkland Islands[TIAB] OR Albania[TIAB] OR Estonia[TIAB] OR Latvia[TIAB] OR Lithuania[TIAB] OR Bosnia-Herzegovina[TIAB] OR Bulgaria[TIAB] OR Byelarus[TIAB] OR Croatia[TIAB] OR Czech Republic[TIAB] OR Hungary[TIAB] OR Macedonia[TIAB] OR Moldova[TIAB] OR Montenegro[TIAB] OR Poland[TIAB] OR Romania[TIAB] OR Russia[TIAB] OR Slovakia[TIAB] OR Slovenia[TIAB] OR Ukraine[TIAB] OR Yugoslavia[TIAB] OR Armenia[TIAB] OR Azerbaijan[TIAB] OR Georgia[TIAB] OR Comoros[TIAB] OR Madagascar[TIAB] OR Mauritius[TIAB] OR Reunion[TIAB] OR Seychelles[TIAB] OR Fiji[TIAB] OR New Caledonia[TIAB] OR Papua New Guinea[TIAB] OR Vanuatu[TIAB] OR Guam[TIAB] OR Palau[TIAB] OR Pitcairn Island[TIAB] OR Samoa[TIAB] OR Tonga[TIAB] OR Czechoslovakia[TIAB] OR East Germany[TIAB] OR New Guinea[TIAB] OR USSR[TIAB] OR Yugoslavia[TIAB] OR Ivory Coast[TIAB] OR Hong Kong[TIAB] OR china[TIAB] OR North Korea[TIAB] OR Palestine[TIAB] OR Syrian Arab Republic[TIAB]) AND (hasabstract[text] AND Humans[Mesh] AND middle age[MeSH])) OR ((International Cooperation[Mesh:noexp] OR developing countries[Mesh] OR developed countries[Mesh] OR WORLD HEALTH[Mesh] OR WORLD HEALTH ORGANIZATION[Mesh] OR AFRICA[Mesh] OR	3585538	12:28:22

	Americas[Mesh:noexp] OR Caribbean Region[Mesh] OR West Indies[Mesh] OR Central America[Mesh] OR Latin America[Mesh:noexp] OR North America[Mesh:noexp] OR South America[Mesh] OR Antarctic Regions[Mesh:noexp] OR Arctic Regions[Mesh:noexp] OR Asia[Mesh:noexp] OR Asia, Central[Mesh] OR Asia, Southeastern[Mesh:noexp] OR Asia, Western[Mesh:noexp] OR Middle East[Mesh:noexp] OR Far East[Mesh:noexp] OR Atlantic Islands[Mesh] OR Europe[Mesh:noexp] OR Europe, Eastern[Mesh] OR Scandinavia[Mesh:noexp] OR Transcaucasia[Mesh] OR Indian Ocean Islands[Mesh] OR Oceania[Mesh:noexp] OR Australasia[Mesh:noexp] OR Pacific Islands[Mesh:noexp] OR Melanesia[Mesh:noexp] OR Micronesia[Mesh:noexp] OR Polynesia[Mesh:noexp] OR Mexico[Mesh] OR Borneo[Mesh] OR Cambodia[Mesh] OR East Timor[Mesh] OR Indonesia[Mesh] OR Laos[Mesh] OR Malaysia[Mesh] OR Mekong Valley[Mesh] OR Myanmar[Mesh] OR Philippines[Mesh] OR Thailand[Mesh] OR Vietnam[Mesh] OR Bangladesh[Mesh] OR Bhutan[Mesh] OR India[Mesh] OR Afghanistan[Mesh] OR Bahrain[Mesh] OR Iran[Mesh] OR Iraq[Mesh] OR Jordan[Mesh] OR Kuwait[Mesh] OR Lebanon[Mesh] OR Oman[Mesh] OR Qatar[Mesh] OR Saudi Arabia[Mesh] OR Syria[Mesh] OR Turkey[Mesh] OR United Arab Emirates[Mesh] OR Yemen[Mesh] OR Nepal[Mesh] OR Pakistan[Mesh] OR Sri Lanka[Mesh] OR China[Mesh] OR Macao[Mesh] OR Mongolia[Mesh] OR Taiwan[Mesh] OR Multicenter Studies As Topic[Mesh] OR Multicenter Study[PT] OR Algeria[PL] OR Egypt[PL] OR Libya[PL] OR Morocco[PL] OR Tunisia[PL] OR Cameroon[PL] OR Central African Republic[PL] OR Chad[PL] OR Congo[PL] OR Congo[PL] OR Equatorial Guinea[PL] OR Gabon[PL] OR Burundi[PL] OR Djibouti[PL] OR Eritrea[PL] OR Ethiopia[PL] OR Kenya[PL] OR Rwanda[PL] OR Somalia[PL] OR Sudan[PL] OR Tanzania[PL] OR Uganda[PL] OR Angola[PL] OR Botswana[PL] OR Lesotho[PL] OR Malawi[PL] OR Mozambique[PL] OR Namibia[PL] OR South Africa[PL] OR Swaziland[PL] OR Zambia[PL] OR Zimbabwe[PL] OR Benin[PL] OR Burkina Faso[PL] OR Cote d'Ivoire[PL] OR Gambia[PL] OR Ghana[PL] OR Guinea[PL] OR Guinea-Bissau[PL] OR Liberia[PL] OR Mali[PL] OR Mauritania[PL] OR Niger[PL] OR Nigeria[PL] OR Senegal[PL] OR Sierra Leone[PL] OR Togo[PL] OR Antigua[PL] OR Bahamas[PL] OR Barbados[PL] OR Cuba[PL] OR Dominica[PL] OR Dominican Republic[PL] OR Grenada[PL] OR Guadeloupe[PL] OR Haiti[PL] OR Jamaica[PL] OR Martinique[PL] OR Netherlands Antilles[PL] OR Puerto Rico[PL] OR Saint Kitts and Nevis[PL] OR Saint Lucia[PL] OR Saint Vincent[PL] OR Grenadines[PL] OR Trinidad and Tobago[PL] OR Virgin Islands[PL] OR Belize[PL] OR Costa Rica[PL] OR El Salvador[PL] OR Guatemala[PL] OR Honduras[PL] OR Nicaragua[PL] OR Panama[PL] OR Mexico[PL] OR Argentina[PL] OR Bolivia[PL] OR Brazil[PL] OR Chile[PL] OR Colombia[PL] OR Ecuador[PL] OR French Guiana[PL] OR French Guiana[PL] OR Paraguay[PL] OR Peru[PL] OR Suriname[PL] OR Uruguay[PL] OR Venezuela[PL] OR Kazakhstan[PL] OR Kyrgyzstan[PL] OR Tajikistan[PL] OR Turkmenistan[PL] OR Uzbekistan[PL] OR Borneo[PL] OR Cambodia[PL] OR East Timor[PL] OR Indonesia[PL] OR Laos[PL] OR Malaysia[PL] OR Mekong Valley[PL] OR Myanmar[PL] OR Philippines[PL] OR Thailand[PL] OR Vietnam[PL] OR Bangladesh[PL] OR Bhutan[PL] OR India[PL] OR Afghanistan[PL] OR Bahrain[PL] OR Iran[PL] OR Iraq[PL] OR Jordan[PL] OR Kuwait[PL] OR Lebanon[PL] OR Oman[PL] OR Qatar[PL] OR Saudi Arabia[PL] OR Syria[PL] OR Turkey[PL] OR United Arab Emirates[PL] OR Yemen[PL] OR Nepal[PL] OR Pakistan[PL] OR Sri Lanka[PL] OR China[PL] OR Macao[PL] OR Mongolia[PL] OR Taiwan[PL] OR Azores[PL] OR Bermuda[PL] OR Falkland Islands[PL] OR Albania[PL] OR Estonia[PL] OR Latvia[PL] OR Lithuania[PL] OR Bosnia-Herzegovina[PL] OR Bulgaria[PL] OR Byelarus[PL] OR Croatia[PL] OR Czech Republic[PL] OR Hungary[PL] OR Macedonia[PL] OR Moldova[PL] OR Montenegro[PL] OR Poland[PL] OR Romania[PL] OR Russia[PL] OR Slovakia[PL] OR Slovenia[PL] OR Ukraine[PL] OR Armenia[PL] OR Azerbaijan[PL] OR Georgia[PL] OR Comoros[PL] OR Madagascar[PL] OR Mauritius[PL] OR Reunion[PL] OR Seychelles[PL] OR Fiji[PL] OR New Caledonia[PL] OR Papua New Guinea[PL] OR Vanuatu[PL] OR Guam[PL] OR Palau[PL] OR Pitcairn Island[PL] OR Samoa[PL] OR Tonga[PL] OR Czechoslovakia[PL] OR Germany, East[PL] OR New Guinea[PL] OR USSR[PL] OR Yugoslavia[PL] OR Ivory Coast[PL] OR Hong Kong[PL] OR republic of china[PL])))		
#1	Search ((Canada[Mesh] OR Greenland[Mesh] OR United States[Mesh] OR Brunei[Mesh] OR Singapore[Mesh] OR Israel[Mesh] OR Japan[Mesh] OR Korea[Mesh] OR Australia[Mesh] OR Andorra[Mesh] OR Austria[Mesh] OR Belgium[Mesh] OR Finland[Mesh] OR France[Mesh] OR Germany[Mesh] OR Gibraltar[Mesh] OR Great Britain[Mesh] OR Greece[Mesh] OR Iceland[Mesh] OR Ireland[Mesh] OR Italy[Mesh] OR Liechtenstein[Mesh] OR Luxembourg[Mesh] OR Mediterranean Region[Mesh] OR Monaco[Mesh] OR Netherlands[Mesh] OR Portugal[Mesh] OR San Marino[Mesh] OR Scandinavia[Mesh] OR Spain[Mesh] OR Switzerland[Mesh] OR Vatican City[Mesh] OR Australia[Mesh] OR New Zealand[Mesh] OR Brunei[TIAB] OR Japan[TIAB] OR South Korea[TIAB] OR Singapore[TIAB] OR Andorra[TIAB] OR Austria[TIAB] OR Belgium[TIAB] OR Cyprus[TIAB] OR Denmark[TIAB] OR Finland[TIAB] OR France[TIAB] OR Germany[TIAB] OR Gibraltar[TIAB] OR Greece[TIAB] OR Greenland[TIAB] OR Vatican[TIAB] OR Iceland[TIAB] OR Ireland[TIAB] OR Israel[TIAB] OR Italy[TIAB] OR Liechtenstein[TIAB] OR Luxembourg[TIAB] OR Malta[TIAB] OR Monaco[TIAB] OR Netherlands[TIAB] OR Norway[TIAB] OR Portugal[TIAB] OR San Marino[TIAB] OR Spain[TIAB] OR Sweden[TIAB] OR Switzerland[TIAB] OR United Kingdom[TIAB] OR England[TIAB] OR Wales[TIAB] OR Scotland[TIAB] OR Canada[TIAB] OR United States[TIAB] OR Australia[TIAB] OR New Zealand[TIAB]))	3182449	12:27:20

Impaired Kidney Function Capstone Appendix

Flowchart

Impaired Kidney Function

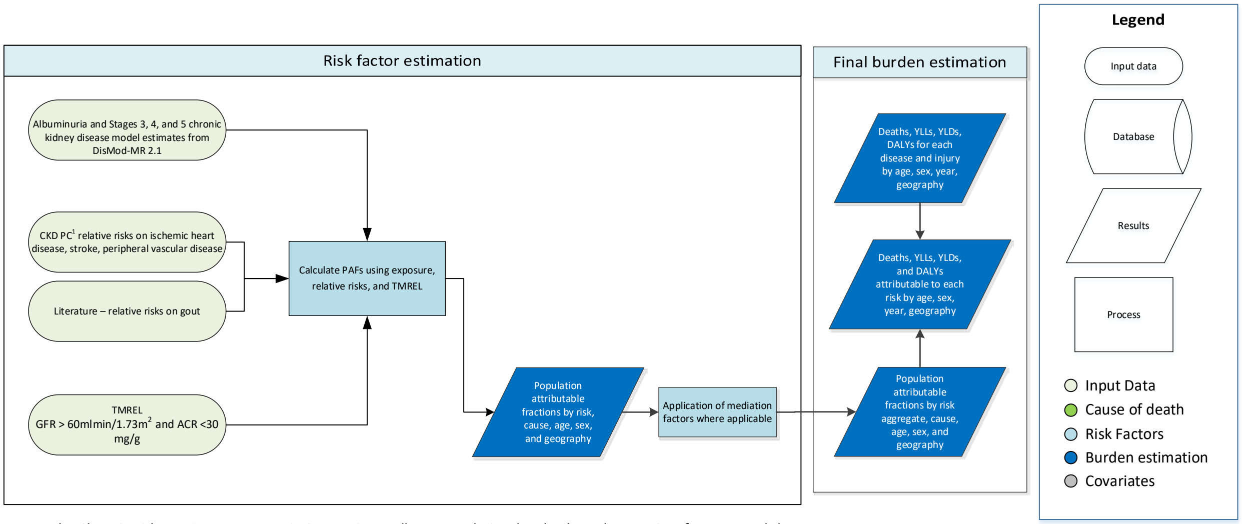

1. The Chronic Kidney Disease Prognosis Consortium collects population-level cohort data on CKD from around the world for the purpose of collective meta-analyses

Input Data & Methodological Summary

Exposure

Case Definition

For GBD 2016, the impaired kidney function risk factor exposure is divided into four categories of renal function defined by urinary albumin to creatinine ratio (ACR) and estimated glomerular filtration rate (GFR): albuminuria with preserved GFR (ACR >30 mg/g & GFR >=60 ml/min/1.73m^2), chronic kidney disease (CKD) stage 3 (GFR of 30-59 ml/min/1.73m^2), CKD stage 4 (GFR of 15-29 ml/min/1.73m^2), and CKD stage 5 (GFR <15ml/min/1.73m^2, not yet on renal replacement therapy). The modelling of CKD stages 3, 4, and 5 is described in detail in the appendix to the GBD 2016 non-fatal capstone paper as these are also disease sequelae.

This represents a change from GBD 2015 in which the “low glomerular filtration rate” risk factor was defined only as exposure to GFR <60 ml/min/1.73m^2 indicated by the prevalence of CKD stages 3, 4, and 5. For GBD 2016, albuminuria was added as an exposure category to capture the risk of cardiovascular outcomes due to impaired kidney function with preserved GFR.

Input data

For GBD 2010, a systematic review of the prevalence of low glomerular filtration rate throughout the world was conducted. This search was updated for GBD 2013 and GBD 2015. For GBD 2016 this literature search was repeated using PubMed search terms: ((((("chronic kidney disease"[Title/Abstract]) AND prevalen*[Title/Abstract]) AND ("2015/1/1"[Date - Publication] : "3000"[Date - Publication])) NOT ((animals[MeSH] NOT humans[MeSH])))). For GBD 2016, all previously extracted sources were reviewed for data pertaining to albuminuria.

Disease	Number of sources	Number of countries	Number of new sources for GBD 2016
Albuminuria	72	31	72
CKD Stage III	112	47	49
CKD Stage IV	94	40	45
CKD Stage V	92	38	49

Exclusion criteria included surveys that were not population-representative, studies not reporting on CKD by stage, and studies not reporting on albuminuria with preserved GFR (GFR >=60 ml/min/1.73m^2).

Modeling strategy

Estimates of exposure to CKD stages 3, 4, and 5 were obtained from the GBD 2016 non-fatal burden of disease analysis, which includes stage-specific prevalence estimates at the country level across twenty-three age-groups for both genders. The modeling strategy for these estimates is detailed in the appendix to the GBD 2016 non-fatal capstone paper.

Albuminuria exposure was modeled using DisMod-MR 2.1 to produce prevalence estimates by age, sex, year, and country. The albuminuria exposure model included country-level covariates indicating prevalence of diabetes mellitus and mean systolic blood pressure. As albuminuria classification is dependent on GFR, this model included a cross-walk adjusting data points obtained using estimating equations other than CKD-EPI to the CKD-EPI equation, which is our reference estimating equation for GBD 2016. We also applied a cross-walk to adjust alternate definitions of albuminuria to the reference definition of ACR > 30 mg/g & GFR >=60 ml/min/1.73m^2. This crosswalk was informed with priors obtained from a linear regression using NHANES data to compare age-standardized prevalence of the alternate definition to reference definition. Regression outputs were used to adjust prevalence from studies using lower cut points to the reference definition to those using the reference cut point.

Definition	Value
ACR > 17 mg/g	2.084 (1.530,2.639)
ACR > 20 mg/g	1.662 (1.220 2.103)
ACR > 25 mg/g	1.305 (1.112, 1.497)

The relative risks were calculated by the Chronic Kidney Disease Prognosis Consortium, a consortium composed of population-level cohorts with prospective data collection from several countries (details below). YLDs and YLLs for Cardiovascular and gout were obtained from the GBD 2016 Study for the same geographic, time-period, and age-groups as detailed above.

Theoretical minimum-risk exposure level

The theoretical minimum risk is a diagnosis of albuminuria or CKD stages 3, 4, or 5. An ACR above 30 mg/g and eGFR below 60ml/min/1.73m^2 have been demonstrated in the literature to be the thresholds at which increased cardiovascular and gout events occur secondary to impaired kidney function. (1-10)

Relative risk

A two-stage pooled meta-analysis was used to calculate relative risks for ischemic heart disease, stroke, and peripheral vascular disease. The relative risk of these conditions was first determined within each cohort, and then a pooled analysis of cohort-level relative risks was performed using a random effects meta-analysis approach. Uncertainty intervals largely overlapped for the relative risks of fatal and nonfatal cardiovascular events from impaired kidney function exposure. Thus, we decided to use the relative risks from the combined analysis for fatal and nonfatal cardiovascular outcomes. Gout relative risk was determined by meta-analysis of a literature review performed for GBD 2013. Search terms included "gout" and "chronic kidney disease". Exclusion criteria for search results included special populations, reversal of exposure and outcome categories, or unclear exposure category definition. This search resulted in four eligible studies. The literature review was repeated for GBD 2016, however, there were no new sources indicating increased risk of gout with albuminuria.

The relative risks were updated since the GBD 2015 analyses to account for a change in the reference group from those with GFR >=60 ml/min/1.73m^2 to those with GFR >=60 ml/min/1.73m^2 and ACR<=30 mg/g.

Population Attributable Fraction

We calculated the cardiovascular and gout fatal and nonfatal burden attributable to the categorical exposure to impaired kidney function using the following equation:

$$PAF = \frac{\sum_{i=1}^{n} P_i(RR_i - 1)}{\sum_{i=1}^{n} P_i(RR_i - 1) + 1}$$

Equation 1. PAF based on categorical exposure

where RR_i is the relative risk for exposure level *i*, P_i is the proportion of the population in that exposure category, and *n* is the number of exposure categories.(11)

References

1. Go AS, Chertow GM, Fan D, McCulloch CE, Hsu CY. Chronic kidney disease and the risks of death, cardiovascular events, and hospitalization. N Engl J Med. 2004;351(13):1296-305.
2. Ninomiya T, Kiyohara Y, Kubo M, Tanizaki Y, Doi Y, Okubo K, et al. Chronic kidney disease and cardiovascular disease in a general Japanese population: the Hisayama Study. Kidney international. 2005;68(1):228-36.
3. Shara NM, Wang H, Mete M, Al-Balha YR, Azalddin N, Lee ET, et al. Estimated GFR and incident cardiovascular disease events in American Indians: the Strong Heart Study. American journal of kidney diseases : the official journal of the National Kidney Foundation. 2012;60(5):795-803.

4. Mann JF, Gerstein HC, Pogue J, Bosch J, Yusuf S. Renal insufficiency as a predictor of cardiovascular outcomes and the impact of ramipril: the HOPE randomized trial. Annals of internal medicine. 2001;134(8):629-36.
5. Chronic Kidney Disease Prognosis C, Matsushita K, van der Velde M, Astor BC, Woodward M, Levey AS, et al. Association of estimated glomerular filtration rate and albuminuria with all-cause and cardiovascular mortality in general population cohorts: a collaborative meta-analysis. Lancet. 2010;375(9731):2073-81.
6. De Graauw J, Chonchol M, Poppert H, Etgen T, Sander D. Relationship between kidney function and risk of asymptomatic peripheral arterial disease in elderly subjects. Nephrology, dialysis, transplantation : official publication of the European Dialysis and Transplant Association - European Renal Association. 2011;26(3):927-32.
7. Wattanakit K, Folsom AR, Selvin E, Coresh J, Hirsch AT, Weatherley BD. Kidney function and risk of peripheral arterial disease: results from the Atherosclerosis Risk in Communities (ARIC) Study. Journal of the American Society of Nephrology : JASN. 2007;18(2):629-36.
8. O'Hare AM, Vittinghoff E, Hsia J, Shlipak MG. Renal insufficiency and the risk of lower extremity peripheral arterial disease: results from the Heart and Estrogen/Progestin Replacement Study (HERS). Journal of the American Society of Nephrology : JASN. 2004;15(4):1046-51.
9. Manjunath G, Tighiouart H, Coresh J, Macleod B, Salem DN, Griffith JL, et al. Level of kidney function as a risk factor for cardiovascular outcomes in the elderly. Kidney international. 2003;63(3):1121-9.
10. Manjunath G, Tighiouart H, Ibrahim H, MacLeod B, Salem DN, Griffith JL, et al. Level of kidney function as a risk factor for atherosclerotic cardiovascular outcomes in the community. Journal of the American College of Cardiology. 2003;41(1):47-55.
11. Miettinen OS. Proportion of disease caused or prevented by a given exposure, trait or intervention. American journal of epidemiology. 1974;99(5):325-32.

Appendix Table 1a. Relative risks used by age and sex for each outcome for all risk factors except for ambient air pollution and alcohol.

Risk - Outcome	Category / Units	Morbidity / Mortality	Sex	All-age	0-6 days	7-27 days	28-364 days	1-4 years	5-9 years	10-14 years	15-19 years	20-24 years	25-29 years
**Unsafe water source**													
Diarrhoeal diseases	Unimproved, untreated	Both	Both		11.501<br>(2.761 to 31.282)	11.501<br>(2.761 to 31.282)	11.501<br>(2.761 to 31.282)	11.501<br>(2.761 to 31.282)	11.501<br>(2.761 to 31.282)	11.501<br>(2.761 to 31.282)	11.501<br>(2.761 to 31.282)	11.501<br>(2.761 to 31.282)	11.501<br>(2.761 to 31.282)
Diarrhoeal diseases	Unimproved, chlorinated	Both	Both		7.914<br>(1.971 to 21.188)	7.914<br>(1.971 to 21.188)	7.914<br>(1.971 to 21.188)	7.914<br>(1.971 to 21.188)	7.914<br>(1.971 to 21.188)	7.914<br>(1.971 to 21.188)	7.914<br>(1.971 to 21.188)	7.914<br>(1.971 to 21.188)	7.914<br>(1.971 to 21.188)
Diarrhoeal diseases	Unimproved, filter	Both	Both		4.789<br>(1.204 to 12.752)	4.789<br>(1.204 to 12.752)	4.789<br>(1.204 to 12.752)	4.789<br>(1.204 to 12.752)	4.789<br>(1.204 to 12.752)	4.789<br>(1.204 to 12.752)	4.789<br>(1.204 to 12.752)	4.789<br>(1.204 to 12.752)	4.789<br>(1.204 to 12.752)
Diarrhoeal diseases	Improved, untreated	Both	Both		9.428<br>(2.782 to 23.028)	9.428<br>(2.782 to 23.028)	9.428<br>(2.782 to 23.028)	9.428<br>(2.782 to 23.028)	9.428<br>(2.782 to 23.028)	9.428<br>(2.782 to 23.028)	9.428<br>(2.782 to 23.028)	9.428<br>(2.782 to 23.028)	9.428<br>(2.782 to 23.028)
Diarrhoeal diseases	Improved, chlorinated	Both	Both		6.488<br>(1.997 to 15.677)	6.488<br>(1.997 to 15.677)	6.488<br>(1.997 to 15.677)	6.488<br>(1.997 to 15.677)	6.488<br>(1.997 to 15.677)	6.488<br>(1.997 to 15.677)	6.488<br>(1.997 to 15.677)	6.488<br>(1.997 to 15.677)	6.488<br>(1.997 to 15.677)
Diarrhoeal diseases	Improved, filtered	Both	Both		3.926<br>(1.22 to 9.401)	3.926<br>(1.22 to 9.401)	3.926<br>(1.22 to 9.401)	3.926<br>(1.22 to 9.401)	3.926<br>(1.22 to 9.401)	3.926<br>(1.22 to 9.401)	3.926<br>(1.22 to 9.401)	3.926<br>(1.22 to 9.401)	3.926<br>(1.22 to 9.401)
Diarrhoeal diseases	Piped, untreated	Both	Both		8.431<br>(2.533 to 20.446)	8.431<br>(2.533 to 20.446)	8.431<br>(2.533 to 20.446)	8.431<br>(2.533 to 20.446)	8.431<br>(2.533 to 20.446)	8.431<br>(2.533 to 20.446)	8.431<br>(2.533 to 20.446)	8.431<br>(2.533 to 20.446)	8.431<br>(2.533 to 20.446)
Diarrhoeal diseases	Piped, chlorinated	Both	Both		5.802<br>(1.807 to 13.843)	5.802<br>(1.807 to 13.843)	5.802<br>(1.807 to 13.843)	5.802<br>(1.807 to 13.843)	5.802<br>(1.807 to 13.843)	5.802<br>(1.807 to 13.843)	5.802<br>(1.807 to 13.843)	5.802<br>(1.807 to 13.843)	5.802<br>(1.807 to 13.843)
Diarrhoeal diseases	Piped, filtered	Both	Both		3.511<br>(1.107 to 8.331)	3.511<br>(1.107 to 8.331)	3.511<br>(1.107 to 8.331)	3.511<br>(1.107 to 8.331)	3.511<br>(1.107 to 8.331)	3.511<br>(1.107 to 8.331)	3.511<br>(1.107 to 8.331)	3.511<br>(1.107 to 8.331)	3.511<br>(1.107 to 8.331)
Diarrhoeal diseases	High quality (HQ) piped, untreated	Both	Both		2.401<br>(2.037 to 2.818)	2.401<br>(2.037 to 2.818)	2.401<br>(2.037 to 2.818)	2.401<br>(2.037 to 2.818)	2.401<br>(2.037 to 2.818)	2.401<br>(2.037 to 2.818)	2.401<br>(2.037 to 2.818)	2.401<br>(2.037 to 2.818)	2.401<br>(2.037 to 2.818)
Diarrhoeal diseases	HQ piped, chlorinated	Both	Both		1.653<br>(1.56 to 1.748)	1.653<br>(1.56 to 1.748)	1.653<br>(1.56 to 1.748)	1.653<br>(1.56 to 1.748)	1.653<br>(1.56 to 1.748)	1.653<br>(1.56 to 1.748)	1.653<br>(1.56 to 1.748)	1.653<br>(1.56 to 1.748)	1.653<br>(1.56 to 1.748)
Diarrhoeal diseases	HQ piped, filtered	Both	Both		1.0<br>(1.0 to 1.0)	1.0<br>(1.0 to 1.0)	1.0<br>(1.0 to 1.0)	1.0<br>(1.0 to 1.0)	1.0<br>(1.0 to 1.0)	1.0<br>(1.0 to 1.0)	1.0<br>(1.0 to 1.0)	1.0<br>(1.0 to 1.0)	1.0<br>(1.0 to 1.0)
**Unsafe sanitation**													
Diarrhoeal diseases	Unimproved & untreated	Both	Both		3.242<br>(2.528 to 4.067)	3.242<br>(2.528 to 4.067)	3.242<br>(2.528 to 4.067)	3.242<br>(2.528 to 4.067)	3.242<br>(2.528 to 4.067)	3.242<br>(2.528 to 4.067)	3.242<br>(2.528 to 4.067)	3.242<br>(2.528 to 4.067)	3.242<br>(2.528 to 4.067)
Diarrhoeal diseases	Improved	Both	Both		2.595<br>(2.044 to 3.285)	2.595<br>(2.044 to 3.285)	2.595<br>(2.044 to 3.285)	2.595<br>(2.044 to 3.285)	2.595<br>(2.044 to 3.285)	2.595<br>(2.044 to 3.285)	2.595<br>(2.044 to 3.285)	2.595<br>(2.044 to 3.285)	2.595<br>(2.044 to 3.285)
Diarrhoeal diseases	Sewer	Both	Both		1.0<br>(1.0 to 1.0)	1.0<br>(1.0 to 1.0)	1.0<br>(1.0 to 1.0)	1.0<br>(1.0 to 1.0)	1.0<br>(1.0 to 1.0)	1.0<br>(1.0 to 1.0)	1.0<br>(1.0 to 1.0)	1.0<br>(1.0 to 1.0)	1.0<br>(1.0 to 1.0)
**No access to handwashing facility**													
Diarrhoeal diseases	No handwashing w/soap & water	Both	Both		1.908<br>(1.32 to 2.668)	1.908<br>(1.32 to 2.668)	1.908<br>(1.32 to 2.668)	1.908<br>(1.32 to 2.668)	1.908<br>(1.32 to 2.668)	1.908<br>(1.32 to 2.668)	1.908<br>(1.32 to 2.668)	1.908<br>(1.32 to 2.668)	1.908<br>(1.32 to 2.668)
Diarrhoeal diseases	Handwashing w/soap & water	Both	Both		1.0<br>(1.0 to 1.0)	1.0<br>(1.0 to 1.0)	1.0<br>(1.0 to 1.0)	1.0<br>(1.0 to 1.0)	1.0<br>(1.0 to 1.0)	1.0<br>(1.0 to 1.0)	1.0<br>(1.0 to 1.0)	1.0<br>(1.0 to 1.0)	1.0<br>(1.0 to 1.0)
Lower respiratory infections	No handwashing w/soap & water	Both	Both		1.191<br>(1.119 to 1.266)	1.191<br>(1.119 to 1.266)	1.191<br>(1.119 to 1.266)	1.191<br>(1.119 to 1.266)	1.191<br>(1.119 to 1.266)	1.191<br>(1.119 to 1.266)	1.191<br>(1.119 to 1.266)	1.191<br>(1.119 to 1.266)	1.191<br>(1.119 to 1.266)
Lower respiratory infections	Handwashing w/soap & water	Both	Both		1.0<br>(1.0 to 1.0)	1.0<br>(1.0 to 1.0)	1.0<br>(1.0 to 1.0)	1.0<br>(1.0 to 1.0)	1.0<br>(1.0 to 1.0)	1.0<br>(1.0 to 1.0)	1.0<br>(1.0 to 1.0)	1.0<br>(1.0 to 1.0)	1.0<br>(1.0 to 1.0)
**Household air pollution from solid fuels**													
Cataract	Exposed	Morbidity	Both								2.526<br>(1.622 to 3.689)	2.534<br>(1.65 to 3.652)	2.545<br>(1.666 to 3.635)
Cataract	Not exposed	Morbidity	Both								1.0<br>(1.0 to 1.0)	1.0<br>(1.0 to 1.0)	1.0<br>(1.0 to 1.0)
**Ambient ozone pollution**													
Chronic obstructive pulmonary disease	10 ppb	Mortality	Both										1.029<br>(1.01 to 1.048)
**Residential radon**													
Tracheal, bronchus, and lung cancer	Bq/m3	Both	Both		1.002<br>(1.0 to 1.003)	1.002<br>(1.0 to 1.003)	1.002<br>(1.0 to 1.003)	1.002<br>(1.0 to 1.003)	1.002<br>(1.0 to 1.003)	1.002<br>(1.0 to 1.003)	1.002<br>(1.0 to 1.003)	1.002<br>(1.0 to 1.003)	1.002<br>(1.0 to 1.003)
**Lead exposure in blood**													

Ages													
**30-34 years**	**35-39 years**	**40-44 years**	**45-49 years**	**50-54 years**	**55-59 years**	**60-64 years**	**65-69 years**	**70-74 years**	**75-79 years**	**80-84 years**	**85-89 years**	**90-94 years**	**95+ years**
11.501 (2.761 to 31.282)	11.501 (2.761 to 31.282)	11.501 (2.761 to 31.282)	11.501 (2.761 to 31.282)	11.501 (2.761 to 31.282)	11.501 (2.761 to 31.282)	11.501 (2.761 to 31.282)	11.501 (2.761 to 31.282)	11.501 (2.761 to 31.282)	11.501 (2.761 to 31.282)	11.501 (2.761 to 31.282)	11.501 (2.761 to 31.282)	11.501 (2.761 to 31.282)	11.501 (2.761 to 31.282)
7.914 (1.971 to 21.188)	7.914 (1.971 to 21.188)	7.914 (1.971 to 21.188)	7.914 (1.971 to 21.188)	7.914 (1.971 to 21.188)	7.914 (1.971 to 21.188)	7.914 (1.971 to 21.188)	7.914 (1.971 to 21.188)	7.914 (1.971 to 21.188)	7.914 (1.971 to 21.188)	7.914 (1.971 to 21.188)	7.914 (1.971 to 21.188)	7.914 (1.971 to 21.188)	7.914 (1.971 to 21.188)
4.789 (1.204 to 12.752)	4.789 (1.204 to 12.752)	4.789 (1.204 to 12.752)	4.789 (1.204 to 12.752)	4.789 (1.204 to 12.752)	4.789 (1.204 to 12.752)	4.789 (1.204 to 12.752)	4.789 (1.204 to 12.752)	4.789 (1.204 to 12.752)	4.789 (1.204 to 12.752)	4.789 (1.204 to 12.752)	4.789 (1.204 to 12.752)	4.789 (1.204 to 12.752)	4.789 (1.204 to 12.752)
9.428 (2.782 to 23.028)	9.428 (2.782 to 23.028)	9.428 (2.782 to 23.028)	9.428 (2.782 to 23.028)	9.428 (2.782 to 23.028)	9.428 (2.782 to 23.028)	9.428 (2.782 to 23.028)	9.428 (2.782 to 23.028)	9.428 (2.782 to 23.028)	9.428 (2.782 to 23.028)	9.428 (2.782 to 23.028)	9.428 (2.782 to 23.028)	9.428 (2.782 to 23.028)	9.428 (2.782 to 23.028)
6.488 (1.997 to 15.677)	6.488 (1.997 to 15.677)	6.488 (1.997 to 15.677)	6.488 (1.997 to 15.677)	6.488 (1.997 to 15.677)	6.488 (1.997 to 15.677)	6.488 (1.997 to 15.677)	6.488 (1.997 to 15.677)	6.488 (1.997 to 15.677)	6.488 (1.997 to 15.677)	6.488 (1.997 to 15.677)	6.488 (1.997 to 15.677)	6.488 (1.997 to 15.677)	6.488 (1.997 to 15.677)
3.926 (1.22 to 9.401)	3.926 (1.22 to 9.401)	3.926 (1.22 to 9.401)	3.926 (1.22 to 9.401)	3.926 (1.22 to 9.401)	3.926 (1.22 to 9.401)	3.926 (1.22 to 9.401)	3.926 (1.22 to 9.401)	3.926 (1.22 to 9.401)	3.926 (1.22 to 9.401)	3.926 (1.22 to 9.401)	3.926 (1.22 to 9.401)	3.926 (1.22 to 9.401)	3.926 (1.22 to 9.401)
8.431 (2.533 to 20.446)	8.431 (2.533 to 20.446)	8.431 (2.533 to 20.446)	8.431 (2.533 to 20.446)	8.431 (2.533 to 20.446)	8.431 (2.533 to 20.446)	8.431 (2.533 to 20.446)	8.431 (2.533 to 20.446)	8.431 (2.533 to 20.446)	8.431 (2.533 to 20.446)	8.431 (2.533 to 20.446)	8.431 (2.533 to 20.446)	8.431 (2.533 to 20.446)	8.431 (2.533 to 20.446)
5.802 (1.807 to 13.843)	5.802 (1.807 to 13.843)	5.802 (1.807 to 13.843)	5.802 (1.807 to 13.843)	5.802 (1.807 to 13.843)	5.802 (1.807 to 13.843)	5.802 (1.807 to 13.843)	5.802 (1.807 to 13.843)	5.802 (1.807 to 13.843)	5.802 (1.807 to 13.843)	5.802 (1.807 to 13.843)	5.802 (1.807 to 13.843)	5.802 (1.807 to 13.843)	5.802 (1.807 to 13.843)
3.511 (1.107 to 8.331)	3.511 (1.107 to 8.331)	3.511 (1.107 to 8.331)	3.511 (1.107 to 8.331)	3.511 (1.107 to 8.331)	3.511 (1.107 to 8.331)	3.511 (1.107 to 8.331)	3.511 (1.107 to 8.331)	3.511 (1.107 to 8.331)	3.511 (1.107 to 8.331)	3.511 (1.107 to 8.331)	3.511 (1.107 to 8.331)	3.511 (1.107 to 8.331)	3.511 (1.107 to 8.331)
2.401 (2.037 to 2.818)	2.401 (2.037 to 2.818)	2.401 (2.037 to 2.818)	2.401 (2.037 to 2.818)	2.401 (2.037 to 2.818)	2.401 (2.037 to 2.818)	2.401 (2.037 to 2.818)	2.401 (2.037 to 2.818)	2.401 (2.037 to 2.818)	2.401 (2.037 to 2.818)	2.401 (2.037 to 2.818)	2.401 (2.037 to 2.818)	2.401 (2.037 to 2.818)	2.401 (2.037 to 2.818)
1.653 (1.56 to 1.748)	1.653 (1.56 to 1.748)	1.653 (1.56 to 1.748)	1.653 (1.56 to 1.748)	1.653 (1.56 to 1.748)	1.653 (1.56 to 1.748)	1.653 (1.56 to 1.748)	1.653 (1.56 to 1.748)	1.653 (1.56 to 1.748)	1.653 (1.56 to 1.748)	1.653 (1.56 to 1.748)	1.653 (1.56 to 1.748)	1.653 (1.56 to 1.748)	1.653 (1.56 to 1.748)
1.0 (1.0 to 1.0)	1.0 (1.0 to 1.0)	1.0 (1.0 to 1.0)	1.0 (1.0 to 1.0)	1.0 (1.0 to 1.0)	1.0 (1.0 to 1.0)	1.0 (1.0 to 1.0)	1.0 (1.0 to 1.0)	1.0 (1.0 to 1.0)	1.0 (1.0 to 1.0)	1.0 (1.0 to 1.0)	1.0 (1.0 to 1.0)	1.0 (1.0 to 1.0)	1.0 (1.0 to 1.0)
3.242 (2.528 to 4.067)	3.242 (2.528 to 4.067)	3.242 (2.528 to 4.067)	3.242 (2.528 to 4.067)	3.242 (2.528 to 4.067)	3.242 (2.528 to 4.067)	3.242 (2.528 to 4.067)	3.242 (2.528 to 4.067)	3.242 (2.528 to 4.067)	3.242 (2.528 to 4.067)	3.242 (2.528 to 4.067)	3.242 (2.528 to 4.067)	3.242 (2.528 to 4.067)	3.242 (2.528 to 4.067)
2.595 (2.044 to 3.285)	2.595 (2.044 to 3.285)	2.595 (2.044 to 3.285)	2.595 (2.044 to 3.285)	2.595 (2.044 to 3.285)	2.595 (2.044 to 3.285)	2.595 (2.044 to 3.285)	2.595 (2.044 to 3.285)	2.595 (2.044 to 3.285)	2.595 (2.044 to 3.285)	2.595 (2.044 to 3.285)	2.595 (2.044 to 3.285)	2.595 (2.044 to 3.285)	2.595 (2.044 to 3.285)
1.0 (1.0 to 1.0)	1.0 (1.0 to 1.0)	1.0 (1.0 to 1.0)	1.0 (1.0 to 1.0)	1.0 (1.0 to 1.0)	1.0 (1.0 to 1.0)	1.0 (1.0 to 1.0)	1.0 (1.0 to 1.0)	1.0 (1.0 to 1.0)	1.0 (1.0 to 1.0)	1.0 (1.0 to 1.0)	1.0 (1.0 to 1.0)	1.0 (1.0 to 1.0)	1.0 (1.0 to 1.0)
1.908 (1.32 to 2.668)	1.908 (1.32 to 2.668)	1.908 (1.32 to 2.668)	1.908 (1.32 to 2.668)	1.908 (1.32 to 2.668)	1.908 (1.32 to 2.668)	1.908 (1.32 to 2.668)	1.908 (1.32 to 2.668)	1.908 (1.32 to 2.668)	1.908 (1.32 to 2.668)	1.908 (1.32 to 2.668)	1.908 (1.32 to 2.668)	1.908 (1.32 to 2.668)	1.908 (1.32 to 2.668)
1.0 (1.0 to 1.0)	1.0 (1.0 to 1.0)	1.0 (1.0 to 1.0)	1.0 (1.0 to 1.0)	1.0 (1.0 to 1.0)	1.0 (1.0 to 1.0)	1.0 (1.0 to 1.0)	1.0 (1.0 to 1.0)	1.0 (1.0 to 1.0)	1.0 (1.0 to 1.0)	1.0 (1.0 to 1.0)	1.0 (1.0 to 1.0)	1.0 (1.0 to 1.0)	1.0 (1.0 to 1.0)
1.191 (1.119 to 1.266)	1.191 (1.119 to 1.266)	1.191 (1.119 to 1.266)	1.191 (1.119 to 1.266)	1.191 (1.119 to 1.266)	1.191 (1.119 to 1.266)	1.191 (1.119 to 1.266)	1.191 (1.119 to 1.266)	1.191 (1.119 to 1.266)	1.191 (1.119 to 1.266)	1.191 (1.119 to 1.266)	1.191 (1.119 to 1.266)	1.191 (1.119 to 1.266)	1.191 (1.119 to 1.266)
1.0 (1.0 to 1.0)	1.0 (1.0 to 1.0)	1.0 (1.0 to 1.0)	1.0 (1.0 to 1.0)	1.0 (1.0 to 1.0)	1.0 (1.0 to 1.0)	1.0 (1.0 to 1.0)	1.0 (1.0 to 1.0)	1.0 (1.0 to 1.0)	1.0 (1.0 to 1.0)	1.0 (1.0 to 1.0)	1.0 (1.0 to 1.0)	1.0 (1.0 to 1.0)	1.0 (1.0 to 1.0)
2.502 (1.639 to 3.667)	2.504 (1.611 to 3.764)	2.512 (1.691 to 3.656)	2.528 (1.608 to 3.672)	2.524 (1.598 to 3.703)	2.528 (1.69 to 3.701)	2.482 (1.586 to 3.635)	2.525 (1.679 to 3.6)	2.524 (1.621 to 3.688)	2.537 (1.646 to 3.716)	2.55 (1.62 to 3.824)	2.521 (1.587 to 3.763)	2.509 (1.612 to 3.748)	2.537 (1.606 to 3.856)
1.0 (1.0 to 1.0)	1.0 (1.0 to 1.0)	1.0 (1.0 to 1.0)	1.0 (1.0 to 1.0)	1.0 (1.0 to 1.0)	1.0 (1.0 to 1.0)	1.0 (1.0 to 1.0)	1.0 (1.0 to 1.0)	1.0 (1.0 to 1.0)	1.0 (1.0 to 1.0)	1.0 (1.0 to 1.0)	1.0 (1.0 to 1.0)	1.0 (1.0 to 1.0)	1.0 (1.0 to 1.0)
1.029 (1.01 to 1.048)	1.029 (1.01 to 1.048)	1.029 (1.01 to 1.048)	1.028 (1.008 to 1.047)	1.03 (1.011 to 1.049)	1.029 (1.011 to 1.047)	1.029 (1.01 to 1.048)	1.029 (1.011 to 1.048)	1.029 (1.012 to 1.048)	1.029 (1.01 to 1.049)	1.029 (1.01 to 1.049)	1.029 (1.01 to 1.049)	1.029 (1.01 to 1.049)	1.029 (1.01 to 1.049)
1.002 (1.0 to 1.003)	1.002 (1.0 to 1.003)	1.002 (1.0 to 1.003)	1.002 (1.0 to 1.003)	1.002 (1.0 to 1.003)	1.002 (1.0 to 1.003)	1.002 (1.0 to 1.003)	1.002 (1.0 to 1.003)	1.002 (1.0 to 1.003)	1.002 (1.0 to 1.003)	1.002 (1.0 to 1.003)	1.002 (1.0 to 1.003)	1.002 (1.0 to 1.003)	1.002 (1.0 to 1.003)

Appendix Table 1a. Relative risks used by age and sex for each outcome for all risk factors except for ambient air pollution and alcohol.

Risk - Outcome	Category / Units	Morbidity / Mortality	Sex	All-age	0-6 days	7-27 days	28-364 days	1-4 years	5-9 years	10-14 years	15-19 years	20-24 years	25-29 years
IQ shift	10 µg/dL	Morbidity	Both	4.688 (1.719 to 7.656)									
IQ shift	12 µg/dL	Morbidity	Both	5.014 (1.839 to 8.19)									
IQ shift	15 µg/dL	Morbidity	Both	5.42 (1.988 to 8.853)									
IQ shift	2 µg/dL	Morbidity	Both	0.0 (0.0 to 0.0)									
IQ shift	20 µg/dL	Morbidity	Both	5.952 (2.183 to 9.721)									
IQ shift	25 µg/dL	Morbidity	Both	6.37 (2.336 to 10.403)									
IQ shift	30 µg/dL	Morbidity	Both	6.713 (2.462 to 10.965)									
IQ shift	35 µg/dL	Morbidity	Both	7.006 (2.569 to 11.442)									
IQ shift	4 µg/dL	Morbidity	Both	3.146 (1.154 to 5.139)									
IQ shift	40 µg/dL	Morbidity	Both	7.26 (2.662 to 11.857)									
IQ shift	6 µg/dL	Morbidity	Both	3.804 (1.395 to 6.213)									
IQ shift	8 µg/dL	Morbidity	Both	4.296 (1.575 to 7.016)									
**Lead exposure in bone**													
Rheumatic heart disease	10 µg/g	Both	Both										1.03 (1.01 to 1.052)
Ischaemic heart disease	10 µg/g	Both	Both										1.042 (1.022 to 1.06)
Ischaemic stroke	10 µg/g	Both	Both										1.038 (1.021 to 1.06)
Hemorrhagic stroke	10 µg/g	Both	Both										1.047 (1.027 to 1.068)
Hypertensive heart disease	10 µg/g	Both	Both										1.066 (1.038 to 1.09)
Other cardiomyopathy	10 µg/g	Both	Both										1.035 (1.015 to 1.055)
Atrial fibrillation and flutter	10 µg/g	Both	Both										1.035 (1.018 to 1.056)
Aortic aneurysm	10 µg/g	Both	Both										1.027 (1.014 to 1.048)
Peripheral vascular disease	10 µg/g	Both	Both										1.034 (1.011 to 1.056)
Endocarditis	10 µg/g	Both	Both										1.035 (1.015 to 1.055)
Other cardiovascular and circulatory diseases	10 µg/g	Both	Both										1.034 (1.018 to 1.055)
Chronic kidney disease due to diabetes mellitus	10 µg/g	Both	Both										1.015 (1.01 to 1.021)
Chronic kidney disease due to hypertension	10 µg/g	Both	Both										1.015 (1.01 to 1.02)
Chronic kidney disease due to glomerulonephritis	10 µg/g	Both	Both										1.015 (1.01 to 1.02)
Chronic kidney disease due to other causes	10 µg/g	Both	Both										1.015 (1.01 to 1.021)
**Occupational exposure to asbestos**													
Larynx cancer	High exposure	Both	Males								1.38 (1.188 to 1.612)	1.38 (1.188 to 1.612)	1.38 (1.188 to 1.612)

Ages													
30-34 years	35-39 years	40-44 years	45-49 years	50-54 years	55-59 years	60-64 years	65-69 years	70-74 years	75-79 years	80-84 years	85-89 years	90-94 years	95+ years
1.024 (1.01 to 1.04)	1.017 (1.008 to 1.028)	1.013 (1.005 to 1.022)	1.012 (1.006 to 1.019)	1.011 (1.006 to 1.017)	1.01 (1.006 to 1.016)	1.009 (1.005 to 1.014)	1.008 (1.003 to 1.014)	1.007 (1.003 to 1.013)	1.007 (1.004 to 1.013)	1.006 (1.002 to 1.015)	1.006 (1.002 to 1.015)	1.006 (1.002 to 1.015)	1.006 (1.002 to 1.015)
1.037 (1.023 to 1.049)	1.032 (1.023 to 1.04)	1.028 (1.021 to 1.036)	1.026 (1.02 to 1.033)	1.024 (1.02 to 1.03)	1.023 (1.019 to 1.026)	1.021 (1.018 to 1.025)	1.019 (1.014 to 1.023)	1.018 (1.012 to 1.022)	1.016 (1.012 to 1.021)	1.014 (1.008 to 1.022)	1.014 (1.008 to 1.022)	1.014 (1.008 to 1.022)	1.014 (1.008 to 1.022)
1.036 (1.022 to 1.051)	1.033 (1.021 to 1.044)	1.03 (1.019 to 1.042)	1.028 (1.019 to 1.037)	1.026 (1.019 to 1.033)	1.024 (1.018 to 1.029)	1.021 (1.016 to 1.026)	1.019 (1.012 to 1.025)	1.017 (1.01 to 1.023)	1.015 (1.01 to 1.02)	1.011 (1.006 to 1.019)	1.011 (1.006 to 1.019)	1.011 (1.006 to 1.019)	1.011 (1.006 to 1.019)
1.045 (1.029 to 1.061)	1.042 (1.029 to 1.057)	1.039 (1.025 to 1.052)	1.036 (1.024 to 1.047)	1.032 (1.023 to 1.041)	1.028 (1.021 to 1.035)	1.024 (1.018 to 1.03)	1.02 (1.012 to 1.027)	1.017 (1.009 to 1.025)	1.017 (1.011 to 1.023)	1.015 (1.007 to 1.026)	1.015 (1.007 to 1.026)	1.015 (1.007 to 1.026)	1.015 (1.007 to 1.026)
1.066 (1.038 to 1.091)	1.065 (1.037 to 1.094)	1.063 (1.035 to 1.091)	1.058 (1.037 to 1.084)	1.052 (1.036 to 1.079)	1.046 (1.031 to 1.076)	1.04 (1.023 to 1.073)	1.033 (1.011 to 1.074)	1.03 (1.003 to 1.072)	1.031 (1.005 to 1.071)	1.033 (1.006 to 1.075)	1.033 (1.006 to 1.075)	1.033 (1.006 to 1.075)	1.033 (1.006 to 1.075)
1.029 (1.016 to 1.044)	1.023 (1.015 to 1.031)	1.019 (1.013 to 1.025)	1.018 (1.012 to 1.023)	1.016 (1.012 to 1.02)	1.015 (1.012 to 1.018)	1.014 (1.01 to 1.016)	1.012 (1.008 to 1.015)	1.011 (1.007 to 1.014)	1.01 (1.007 to 1.013)	1.007 (1.004 to 1.013)	1.007 (1.004 to 1.013)	1.007 (1.004 to 1.013)	1.007 (1.004 to 1.013)
1.03 (1.02 to 1.044)	1.025 (1.021 to 1.031)	1.022 (1.018 to 1.025)	1.02 (1.017 to 1.023)	1.019 (1.017 to 1.021)	1.018 (1.016 to 1.019)	1.016 (1.014 to 1.018)	1.015 (1.013 to 1.017)	1.013 (1.011 to 1.015)	1.012 (1.01 to 1.013)	1.008 (1.005 to 1.01)	1.008 (1.005 to 1.01)	1.008 (1.005 to 1.01)	1.008 (1.005 to 1.01)
1.024 (1.016 to 1.037)	1.02 (1.016 to 1.026)	1.018 (1.013 to 1.023)	1.017 (1.013 to 1.021)	1.016 (1.013 to 1.019)	1.015 (1.012 to 1.017)	1.014 (1.011 to 1.016)	1.012 (1.009 to 1.015)	1.011 (1.008 to 1.014)	1.01 (1.007 to 1.013)	1.007 (1.004 to 1.01)	1.007 (1.004 to 1.01)	1.007 (1.004 to 1.01)	1.007 (1.004 to 1.01)
1.025 (1.012 to 1.039)	1.014 (1.01 to 1.018)	1.008 (1.001 to 1.014)	1.008 (1.003 to 1.013)	1.008 (1.004 to 1.012)	1.009 (1.005 to 1.012)	1.009 (1.006 to 1.011)	1.009 (1.007 to 1.012)	1.009 (1.006 to 1.011)	1.008 (1.006 to 1.01)	1.006 (1.003 to 1.009)	1.006 (1.003 to 1.009)	1.006 (1.003 to 1.009)	1.006 (1.003 to 1.009)
1.029 (1.016 to 1.044)	1.023 (1.015 to 1.031)	1.019 (1.013 to 1.025)	1.018 (1.012 to 1.023)	1.016 (1.012 to 1.02)	1.015 (1.012 to 1.018)	1.014 (1.01 to 1.016)	1.012 (1.008 to 1.015)	1.011 (1.007 to 1.014)	1.01 (1.007 to 1.013)	1.007 (1.004 to 1.013)	1.007 (1.004 to 1.013)	1.007 (1.004 to 1.013)	1.007 (1.004 to 1.013)
1.03 (1.02 to 1.043)	1.025 (1.021 to 1.03)	1.022 (1.019 to 1.025)	1.021 (1.018 to 1.023)	1.019 (1.017 to 1.021)	1.018 (1.016 to 1.019)	1.016 (1.015 to 1.018)	1.014 (1.013 to 1.016)	1.013 (1.011 to 1.015)	1.012 (1.01 to 1.013)	1.008 (1.006 to 1.011)	1.008 (1.006 to 1.011)	1.008 (1.006 to 1.011)	1.008 (1.006 to 1.011)
1.015 (1.01 to 1.021)	1.015 (1.01 to 1.021)	1.015 (1.01 to 1.021)	1.015 (1.01 to 1.021)	1.015 (1.01 to 1.021)	1.015 (1.01 to 1.021)	1.015 (1.01 to 1.021)	1.015 (1.01 to 1.021)	1.015 (1.01 to 1.021)	1.015 (1.01 to 1.021)	1.015 (1.01 to 1.021)	1.015 (1.01 to 1.021)	1.015 (1.01 to 1.021)	1.015 (1.01 to 1.021)
1.015 (1.01 to 1.02)	1.015 (1.01 to 1.02)	1.015 (1.01 to 1.02)	1.015 (1.01 to 1.02)	1.015 (1.01 to 1.02)	1.015 (1.01 to 1.02)	1.015 (1.01 to 1.02)	1.015 (1.01 to 1.02)	1.015 (1.01 to 1.02)	1.015 (1.01 to 1.02)	1.015 (1.01 to 1.02)	1.015 (1.01 to 1.02)	1.015 (1.01 to 1.02)	1.015 (1.01 to 1.02)
1.015 (1.01 to 1.02)	1.015 (1.01 to 1.02)	1.015 (1.01 to 1.02)	1.015 (1.01 to 1.02)	1.015 (1.01 to 1.02)	1.015 (1.01 to 1.02)	1.015 (1.01 to 1.02)	1.015 (1.01 to 1.02)	1.015 (1.01 to 1.02)	1.015 (1.01 to 1.02)	1.015 (1.01 to 1.02)	1.015 (1.01 to 1.02)	1.015 (1.01 to 1.02)	1.015 (1.01 to 1.02)
1.015 (1.01 to 1.021)	1.015 (1.01 to 1.021)	1.015 (1.01 to 1.021)	1.015 (1.01 to 1.021)	1.015 (1.01 to 1.021)	1.015 (1.01 to 1.021)	1.015 (1.01 to 1.021)	1.015 (1.01 to 1.021)	1.015 (1.01 to 1.021)	1.015 (1.01 to 1.021)	1.015 (1.01 to 1.021)	1.015 (1.01 to 1.021)	1.015 (1.01 to 1.021)	1.015 (1.01 to 1.021)
1.38 (1.188 to 1.612)	1.38 (1.188 to 1.612)	1.38 (1.188 to 1.612)	1.38 (1.188 to 1.612)	1.38 (1.188 to 1.612)	1.38 (1.188 to 1.612)	1.38 (1.188 to 1.612)	1.38 (1.188 to 1.612)	1.38 (1.188 to 1.612)	1.38 (1.188 to 1.612)	1.38 (1.188 to 1.612)	1.38 (1.188 to 1.612)	1.38 (1.188 to 1.612)	1.38 (1.188 to 1.612)

Appendix Table 1a. Relative risks used by age and sex for each outcome for all risk factors except for ambient air pollution and alcohol.

Risk - Outcome	Category / Units	Morbidity / Mortality	Sex	All-age	0-6 days	7-27 days	28-364 days	1-4 years	5-9 years	10-14 years	15-19 years	20-24 years	25-29 years
Larynx cancer	High exposure	Both	Females								1.385 (1.187 to 1.598)	1.385 (1.187 to 1.598)	1.385 (1.187 to 1.598)
Larynx cancer	Low exposure	Both	Males								1.0 (1.0 to 1.0)	1.0 (1.0 to 1.0)	1.0 (1.0 to 1.0)
Larynx cancer	Low exposure	Both	Females								1.0 (1.0 to 1.0)	1.0 (1.0 to 1.0)	1.0 (1.0 to 1.0)
Tracheal, bronchus, and lung cancer	High exposure	Both	Males								2.279 (1.741 to 2.936)	2.279 (1.741 to 2.936)	2.279 (1.741 to 2.936)
Tracheal, bronchus, and lung cancer	High exposure	Both	Females								1.875 (1.589 to 2.208)	1.875 (1.589 to 2.208)	1.875 (1.589 to 2.208)
Tracheal, bronchus, and lung cancer	Low exposure	Both	Males								1.0 (1.0 to 1.0)	1.0 (1.0 to 1.0)	1.0 (1.0 to 1.0)
Tracheal, bronchus, and lung cancer	Low exposure	Both	Females								1.0 (1.0 to 1.0)	1.0 (1.0 to 1.0)	1.0 (1.0 to 1.0)
Ovarian cancer	High exposure	Both	Both								1.811 (1.385 to 2.306)	1.811 (1.385 to 2.306)	1.811 (1.385 to 2.306)
Ovarian cancer	Low exposure	Both	Both								1.0 (1.0 to 1.0)	1.0 (1.0 to 1.0)	1.0 (1.0 to 1.0)
**Occupational exposure to arsenic**													
Tracheal, bronchus, and lung cancer	High exposure	Both	Both								2.061 (1.521 to 2.553)	2.061 (1.521 to 2.553)	2.061 (1.521 to 2.553)
Tracheal, bronchus, and lung cancer	Low exposure	Both	Both								1.749 (0.698 to 2.775)	1.749 (0.698 to 2.775)	1.749 (0.698 to 2.775)
Tracheal, bronchus, and lung cancer	No exposure	Both	Both								1.0 (1.0 to 1.0)	1.0 (1.0 to 1.0)	1.0 (1.0 to 1.0)
**Occupational exposure to benzene**													
Leukaemia	High exposure	Both	Both								2.623 (1.222 to 3.975)	2.623 (1.222 to 3.975)	2.623 (1.222 to 3.975)
Leukaemia	Low exposure	Both	Both								1.626 (0.998 to 2.256)	1.626 (0.998 to 2.256)	1.626 (0.998 to 2.256)
Leukaemia	No exposure	Both	Both								1.0 (1.0 to 1.0)	1.0 (1.0 to 1.0)	1.0 (1.0 to 1.0)
**Occupational exposure to beryllium**													
Tracheal, bronchus, and lung cancer	High exposure	Both	Males								1.174 (1.086 to 1.269)	1.169 (1.065 to 1.276)	1.17 (1.073 to 1.274)
Tracheal, bronchus, and lung cancer	High exposure	Both	Females								1.17 (1.082 to 1.262)	1.17 (1.076 to 1.277)	1.169 (1.072 to 1.277)
Tracheal, bronchus, and lung cancer	Low exposure	Both	Males								1.0 (1.0 to 1.0)	1.0 (1.0 to 1.0)	1.0 (1.0 to 1.0)
Tracheal, bronchus, and lung cancer	Low exposure	Both	Females								1.0 (1.0 to 1.0)	1.0 (1.0 to 1.0)	1.0 (1.0 to 1.0)
Tracheal, bronchus, and lung cancer	No exposure	Both	Males								1.0 (1.0 to 1.0)	1.0 (1.0 to 1.0)	1.0 (1.0 to 1.0)
Tracheal, bronchus, and lung cancer	No exposure	Both	Females								1.0 (1.0 to 1.0)	1.0 (1.0 to 1.0)	1.0 (1.0 to 1.0)
**Occupational exposure to cadmium**													
Tracheal, bronchus, and lung cancer	High exposure	Both	Males								1.192 (1.097 to 1.292)	1.188 (1.083 to 1.287)	1.19 (1.102 to 1.295)
Tracheal, bronchus, and lung cancer	High exposure	Both	Females								1.191 (1.087 to 1.295)	1.191 (1.099 to 1.29)	1.188 (1.093 to 1.293)
Tracheal, bronchus, and lung cancer	Low exposure	Both	Males								1.0 (1.0 to 1.0)	1.0 (1.0 to 1.0)	1.0 (1.0 to 1.0)
Tracheal, bronchus, and lung cancer	Low exposure	Both	Females								1.0 (1.0 to 1.0)	1.0 (1.0 to 1.0)	1.0 (1.0 to 1.0)
Tracheal, bronchus, and lung cancer	No exposure	Both	Males								1.0 (1.0 to 1.0)	1.0 (1.0 to 1.0)	1.0 (1.0 to 1.0)

Ages													
**30-34 years**	**35-39 years**	**40-44 years**	**45-49 years**	**50-54 years**	**55-59 years**	**60-64 years**	**65-69 years**	**70-74 years**	**75-79 years**	**80-84 years**	**85-89 years**	**90-94 years**	**95+ years**
1.385 (1.187 to 1.598)	1.385 (1.187 to 1.598)	1.385 (1.187 to 1.598)	1.385 (1.187 to 1.598)	1.385 (1.187 to 1.598)	1.385 (1.187 to 1.598)	1.385 (1.187 to 1.598)	1.385 (1.187 to 1.598)	1.385 (1.187 to 1.598)	1.385 (1.187 to 1.598)	1.385 (1.187 to 1.598)	1.385 (1.187 to 1.598)	1.385 (1.187 to 1.598)	1.385 (1.187 to 1.598)
1.0 (1.0 to 1.0)	1.0 (1.0 to 1.0)	1.0 (1.0 to 1.0)	1.0 (1.0 to 1.0)	1.0 (1.0 to 1.0)	1.0 (1.0 to 1.0)	1.0 (1.0 to 1.0)	1.0 (1.0 to 1.0)	1.0 (1.0 to 1.0)	1.0 (1.0 to 1.0)	1.0 (1.0 to 1.0)	1.0 (1.0 to 1.0)	1.0 (1.0 to 1.0)	1.0 (1.0 to 1.0)
1.0 (1.0 to 1.0)	1.0 (1.0 to 1.0)	1.0 (1.0 to 1.0)	1.0 (1.0 to 1.0)	1.0 (1.0 to 1.0)	1.0 (1.0 to 1.0)	1.0 (1.0 to 1.0)	1.0 (1.0 to 1.0)	1.0 (1.0 to 1.0)	1.0 (1.0 to 1.0)	1.0 (1.0 to 1.0)	1.0 (1.0 to 1.0)	1.0 (1.0 to 1.0)	1.0 (1.0 to 1.0)
2.279 (1.741 to 2.936)	2.279 (1.741 to 2.936)	2.279 (1.741 to 2.936)	2.279 (1.741 to 2.936)	2.279 (1.741 to 2.936)	2.279 (1.741 to 2.936)	2.279 (1.741 to 2.936)	2.279 (1.741 to 2.936)	2.279 (1.741 to 2.936)	2.279 (1.741 to 2.936)	2.279 (1.741 to 2.936)	2.279 (1.741 to 2.936)	2.279 (1.741 to 2.936)	2.279 (1.741 to 2.936)
1.875 (1.589 to 2.208)	1.875 (1.589 to 2.208)	1.875 (1.589 to 2.208)	1.875 (1.589 to 2.208)	1.875 (1.589 to 2.208)	1.875 (1.589 to 2.208)	1.875 (1.589 to 2.208)	1.875 (1.589 to 2.208)	1.875 (1.589 to 2.208)	1.875 (1.589 to 2.208)	1.875 (1.589 to 2.208)	1.875 (1.589 to 2.208)	1.875 (1.589 to 2.208)	1.875 (1.589 to 2.208)
1.0 (1.0 to 1.0)	1.0 (1.0 to 1.0)	1.0 (1.0 to 1.0)	1.0 (1.0 to 1.0)	1.0 (1.0 to 1.0)	1.0 (1.0 to 1.0)	1.0 (1.0 to 1.0)	1.0 (1.0 to 1.0)	1.0 (1.0 to 1.0)	1.0 (1.0 to 1.0)	1.0 (1.0 to 1.0)	1.0 (1.0 to 1.0)	1.0 (1.0 to 1.0)	1.0 (1.0 to 1.0)
1.0 (1.0 to 1.0)	1.0 (1.0 to 1.0)	1.0 (1.0 to 1.0)	1.0 (1.0 to 1.0)	1.0 (1.0 to 1.0)	1.0 (1.0 to 1.0)	1.0 (1.0 to 1.0)	1.0 (1.0 to 1.0)	1.0 (1.0 to 1.0)	1.0 (1.0 to 1.0)	1.0 (1.0 to 1.0)	1.0 (1.0 to 1.0)	1.0 (1.0 to 1.0)	1.0 (1.0 to 1.0)
1.811 (1.385 to 2.306)	1.811 (1.385 to 2.306)	1.811 (1.385 to 2.306)	1.811 (1.385 to 2.306)	1.811 (1.385 to 2.306)	1.811 (1.385 to 2.306)	1.811 (1.385 to 2.306)	1.811 (1.385 to 2.306)	1.811 (1.385 to 2.306)	1.811 (1.385 to 2.306)	1.811 (1.385 to 2.306)	1.811 (1.385 to 2.306)	1.811 (1.385 to 2.306)	1.811 (1.385 to 2.306)
1.0 (1.0 to 1.0)	1.0 (1.0 to 1.0)	1.0 (1.0 to 1.0)	1.0 (1.0 to 1.0)	1.0 (1.0 to 1.0)	1.0 (1.0 to 1.0)	1.0 (1.0 to 1.0)	1.0 (1.0 to 1.0)	1.0 (1.0 to 1.0)	1.0 (1.0 to 1.0)	1.0 (1.0 to 1.0)	1.0 (1.0 to 1.0)	1.0 (1.0 to 1.0)	1.0 (1.0 to 1.0)
2.061 (1.521 to 2.553)	2.061 (1.521 to 2.553)	2.061 (1.521 to 2.553)	2.061 (1.521 to 2.553)	2.061 (1.521 to 2.553)	2.061 (1.521 to 2.553)	2.061 (1.521 to 2.553)	2.061 (1.521 to 2.553)	2.061 (1.521 to 2.553)	2.061 (1.521 to 2.553)	2.061 (1.521 to 2.553)	2.061 (1.521 to 2.553)	2.061 (1.521 to 2.553)	2.061 (1.521 to 2.553)
1.749 (0.698 to 2.775)	1.749 (0.698 to 2.775)	1.749 (0.698 to 2.775)	1.749 (0.698 to 2.775)	1.749 (0.698 to 2.775)	1.749 (0.698 to 2.775)	1.749 (0.698 to 2.775)	1.749 (0.698 to 2.775)	1.749 (0.698 to 2.775)	1.749 (0.698 to 2.775)	1.749 (0.698 to 2.775)	1.749 (0.698 to 2.775)	1.749 (0.698 to 2.775)	1.749 (0.698 to 2.775)
1.0 (1.0 to 1.0)	1.0 (1.0 to 1.0)	1.0 (1.0 to 1.0)	1.0 (1.0 to 1.0)	1.0 (1.0 to 1.0)	1.0 (1.0 to 1.0)	1.0 (1.0 to 1.0)	1.0 (1.0 to 1.0)	1.0 (1.0 to 1.0)	1.0 (1.0 to 1.0)	1.0 (1.0 to 1.0)	1.0 (1.0 to 1.0)	1.0 (1.0 to 1.0)	1.0 (1.0 to 1.0)
2.623 (1.222 to 3.975)	2.623 (1.222 to 3.975)	2.623 (1.222 to 3.975)	2.623 (1.222 to 3.975)	2.623 (1.222 to 3.975)	2.623 (1.222 to 3.975)	2.623 (1.222 to 3.975)	2.623 (1.222 to 3.975)	2.623 (1.222 to 3.975)	2.623 (1.222 to 3.975)	2.623 (1.222 to 3.975)	2.623 (1.222 to 3.975)	2.623 (1.222 to 3.975)	2.623 (1.222 to 3.975)
1.626 (0.998 to 2.256)	1.626 (0.998 to 2.256)	1.626 (0.998 to 2.256)	1.626 (0.998 to 2.256)	1.626 (0.998 to 2.256)	1.626 (0.998 to 2.256)	1.626 (0.998 to 2.256)	1.626 (0.998 to 2.256)	1.626 (0.998 to 2.256)	1.626 (0.998 to 2.256)	1.626 (0.998 to 2.256)	1.626 (0.998 to 2.256)	1.626 (0.998 to 2.256)	1.626 (0.998 to 2.256)
1.0 (1.0 to 1.0)	1.0 (1.0 to 1.0)	1.0 (1.0 to 1.0)	1.0 (1.0 to 1.0)	1.0 (1.0 to 1.0)	1.0 (1.0 to 1.0)	1.0 (1.0 to 1.0)	1.0 (1.0 to 1.0)	1.0 (1.0 to 1.0)	1.0 (1.0 to 1.0)	1.0 (1.0 to 1.0)	1.0 (1.0 to 1.0)	1.0 (1.0 to 1.0)	1.0 (1.0 to 1.0)
1.169 (1.073 to 1.269)	1.172 (1.081 to 1.271)	1.168 (1.07 to 1.276)	1.171 (1.071 to 1.274)	1.171 (1.075 to 1.273)	1.17 (1.073 to 1.271)	1.171 (1.072 to 1.271)	1.172 (1.081 to 1.276)	1.171 (1.076 to 1.27)	1.174 (1.079 to 1.276)	1.171 (1.072 to 1.273)	1.171 (1.072 to 1.273)	1.171 (1.072 to 1.273)	1.171 (1.072 to 1.273)
1.17 (1.077 to 1.275)	1.172 (1.075 to 1.274)	1.171 (1.078 to 1.276)	1.173 (1.074 to 1.276)	1.172 (1.074 to 1.273)	1.173 (1.081 to 1.277)	1.169 (1.074 to 1.269)	1.173 (1.077 to 1.282)	1.167 (1.069 to 1.269)	1.169 (1.073 to 1.274)	1.171 (1.068 to 1.279)	1.171 (1.068 to 1.279)	1.171 (1.068 to 1.279)	1.171 (1.068 to 1.279)
1.0 (1.0 to 1.0)	1.0 (1.0 to 1.0)	1.0 (1.0 to 1.0)	1.0 (1.0 to 1.0)	1.0 (1.0 to 1.0)	1.0 (1.0 to 1.0)	1.0 (1.0 to 1.0)	1.0 (1.0 to 1.0)	1.0 (1.0 to 1.0)	1.0 (1.0 to 1.0)	1.0 (1.0 to 1.0)	1.0 (1.0 to 1.0)	1.0 (1.0 to 1.0)	1.0 (1.0 to 1.0)
1.0 (1.0 to 1.0)	1.0 (1.0 to 1.0)	1.0 (1.0 to 1.0)	1.0 (1.0 to 1.0)	1.0 (1.0 to 1.0)	1.0 (1.0 to 1.0)	1.0 (1.0 to 1.0)	1.0 (1.0 to 1.0)	1.0 (1.0 to 1.0)	1.0 (1.0 to 1.0)	1.0 (1.0 to 1.0)	1.0 (1.0 to 1.0)	1.0 (1.0 to 1.0)	1.0 (1.0 to 1.0)
1.0 (1.0 to 1.0)	1.0 (1.0 to 1.0)	1.0 (1.0 to 1.0)	1.0 (1.0 to 1.0)	1.0 (1.0 to 1.0)	1.0 (1.0 to 1.0)	1.0 (1.0 to 1.0)	1.0 (1.0 to 1.0)	1.0 (1.0 to 1.0)	1.0 (1.0 to 1.0)	1.0 (1.0 to 1.0)	1.0 (1.0 to 1.0)	1.0 (1.0 to 1.0)	1.0 (1.0 to 1.0)
1.0 (1.0 to 1.0)	1.0 (1.0 to 1.0)	1.0 (1.0 to 1.0)	1.0 (1.0 to 1.0)	1.0 (1.0 to 1.0)	1.0 (1.0 to 1.0)	1.0 (1.0 to 1.0)	1.0 (1.0 to 1.0)	1.0 (1.0 to 1.0)	1.0 (1.0 to 1.0)	1.0 (1.0 to 1.0)	1.0 (1.0 to 1.0)	1.0 (1.0 to 1.0)	1.0 (1.0 to 1.0)
1.191 (1.092 to 1.304)	1.19 (1.091 to 1.298)	1.19 (1.092 to 1.297)	1.192 (1.096 to 1.298)	1.193 (1.096 to 1.296)	1.19 (1.089 to 1.293)	1.192 (1.089 to 1.297)	1.193 (1.088 to 1.3)	1.187 (1.095 to 1.29)	1.191 (1.095 to 1.289)	1.19 (1.088 to 1.296)	1.19 (1.088 to 1.296)	1.19 (1.088 to 1.296)	1.19 (1.088 to 1.296)
1.19 (1.092 to 1.302)	1.191 (1.089 to 1.292)	1.194 (1.098 to 1.297)	1.188 (1.091 to 1.3)	1.19 (1.095 to 1.291)	1.19 (1.096 to 1.294)	1.189 (1.095 to 1.29)	1.192 (1.095 to 1.296)	1.192 (1.09 to 1.298)	1.193 (1.097 to 1.297)	1.193 (1.1 to 1.301)	1.193 (1.1 to 1.301)	1.193 (1.1 to 1.301)	1.193 (1.1 to 1.301)
1.0 (1.0 to 1.0)	1.0 (1.0 to 1.0)	1.0 (1.0 to 1.0)	1.0 (1.0 to 1.0)	1.0 (1.0 to 1.0)	1.0 (1.0 to 1.0)	1.0 (1.0 to 1.0)	1.0 (1.0 to 1.0)	1.0 (1.0 to 1.0)	1.0 (1.0 to 1.0)	1.0 (1.0 to 1.0)	1.0 (1.0 to 1.0)	1.0 (1.0 to 1.0)	1.0 (1.0 to 1.0)
1.0 (1.0 to 1.0)	1.0 (1.0 to 1.0)	1.0 (1.0 to 1.0)	1.0 (1.0 to 1.0)	1.0 (1.0 to 1.0)	1.0 (1.0 to 1.0)	1.0 (1.0 to 1.0)	1.0 (1.0 to 1.0)	1.0 (1.0 to 1.0)	1.0 (1.0 to 1.0)	1.0 (1.0 to 1.0)	1.0 (1.0 to 1.0)	1.0 (1.0 to 1.0)	1.0 (1.0 to 1.0)
1.0 (1.0 to 1.0)	1.0 (1.0 to 1.0)	1.0 (1.0 to 1.0)	1.0 (1.0 to 1.0)	1.0 (1.0 to 1.0)	1.0 (1.0 to 1.0)	1.0 (1.0 to 1.0)	1.0 (1.0 to 1.0)	1.0 (1.0 to 1.0)	1.0 (1.0 to 1.0)	1.0 (1.0 to 1.0)	1.0 (1.0 to 1.0)	1.0 (1.0 to 1.0)	1.0 (1.0 to 1.0)

Appendix Table 1a. Relative risks used by age and sex for each outcome for all risk factors except for ambient air pollution and alcohol.

Risk - Outcome	Category / Units	Morbidity / Mortality	Sex	All-age	0-6 days	7-27 days	28-364 days	1-4 years	5-9 years	10-14 years	15-19 years	20-24 years	25-29 years
Tracheal, bronchus, and lung cancer	No exposure	Both	Females								1.0 (1.0 to 1.0)	1.0 (1.0 to 1.0)	1.0 (1.0 to 1.0)
**Occupational exposure to chromium**													
Tracheal, bronchus, and lung cancer	High exposure	Both	Males								1.179 (1.114 to 1.245)	1.181 (1.117 to 1.25)	1.181 (1.118 to 1.249)
Tracheal, bronchus, and lung cancer	High exposure	Both	Females								1.179 (1.116 to 1.247)	1.18 (1.115 to 1.248)	1.18 (1.115 to 1.244)
Tracheal, bronchus, and lung cancer	Low exposure	Both	Males								1.0 (1.0 to 1.0)	1.0 (1.0 to 1.0)	1.0 (1.0 to 1.0)
Tracheal, bronchus, and lung cancer	Low exposure	Both	Females								1.0 (1.0 to 1.0)	1.0 (1.0 to 1.0)	1.0 (1.0 to 1.0)
Tracheal, bronchus, and lung cancer	No exposure	Both	Males								1.0 (1.0 to 1.0)	1.0 (1.0 to 1.0)	1.0 (1.0 to 1.0)
Tracheal, bronchus, and lung cancer	No exposure	Both	Females								1.0 (1.0 to 1.0)	1.0 (1.0 to 1.0)	1.0 (1.0 to 1.0)
**Occupational exposure to diesel engine exhaust**													
Tracheal, bronchus, and lung cancer	High exposure	Both	Males								1.469 (1.294 to 1.658)	1.477 (1.301 to 1.665)	1.473 (1.29 to 1.668)
Tracheal, bronchus, and lung cancer	High exposure	Both	Females								1.473 (1.287 to 1.682)	1.476 (1.303 to 1.681)	1.469 (1.288 to 1.67)
Tracheal, bronchus, and lung cancer	Low exposure	Both	Males								1.0 (1.0 to 1.0)	1.0 (1.0 to 1.0)	1.0 (1.0 to 1.0)
Tracheal, bronchus, and lung cancer	Low exposure	Both	Females								1.0 (1.0 to 1.0)	1.0 (1.0 to 1.0)	1.0 (1.0 to 1.0)
Tracheal, bronchus, and lung cancer	No exposure	Both	Males								1.0 (1.0 to 1.0)	1.0 (1.0 to 1.0)	1.0 (1.0 to 1.0)
Tracheal, bronchus, and lung cancer	No exposure	Both	Females								1.0 (1.0 to 1.0)	1.0 (1.0 to 1.0)	1.0 (1.0 to 1.0)
**Occupational exposure to second-hand smoke**													
Tracheal, bronchus, and lung cancer	High exposure	Both	Males								1.241 (1.186 to 1.301)	1.24 (1.187 to 1.298)	1.242 (1.183 to 1.296)
Tracheal, bronchus, and lung cancer	High exposure	Both	Females								1.24 (1.184 to 1.299)	1.24 (1.189 to 1.294)	1.241 (1.19 to 1.296)
Tracheal, bronchus, and lung cancer	Low exposure	Both	Males								1.0 (1.0 to 1.0)	1.0 (1.0 to 1.0)	1.0 (1.0 to 1.0)
Tracheal, bronchus, and lung cancer	Low exposure	Both	Females								1.0 (1.0 to 1.0)	1.0 (1.0 to 1.0)	1.0 (1.0 to 1.0)
Tracheal, bronchus, and lung cancer	No exposure	Both	Males								1.0 (1.0 to 1.0)	1.0 (1.0 to 1.0)	1.0 (1.0 to 1.0)
Tracheal, bronchus, and lung cancer	No exposure	Both	Females								1.0 (1.0 to 1.0)	1.0 (1.0 to 1.0)	1.0 (1.0 to 1.0)
**Occupational exposure to formaldehyde**													
Nasopharynx cancer	High exposure	Both	Males								2.222 (1.026 to 4.233)	2.294 (1.056 to 4.409)	2.204 (1.023 to 4.036)
Nasopharynx cancer	High exposure	Both	Females								2.202 (1.04 to 4.059)	2.246 (1.039 to 4.184)	2.227 (1.05 to 4.207)
Nasopharynx cancer	Low exposure	Both	Males								1.0 (1.0 to 1.0)	1.0 (1.0 to 1.0)	1.0 (1.0 to 1.0)
Nasopharynx cancer	Low exposure	Both	Females								1.0 (1.0 to 1.0)	1.0 (1.0 to 1.0)	1.0 (1.0 to 1.0)
Nasopharynx cancer	No exposure	Both	Males								1.0 (1.0 to 1.0)	1.0 (1.0 to 1.0)	1.0 (1.0 to 1.0)
Nasopharynx cancer	No exposure	Both	Females								1.0 (1.0 to 1.0)	1.0 (1.0 to 1.0)	1.0 (1.0 to 1.0)
Acute lymphoid leukaemia	High exposure	Both	Males								1.483 (1.191 to 1.818)	1.479 (1.183 to 1.83)	1.474 (1.182 to 1.815)

Ages

30-34 years	35-39 years	40-44 years	45-49 years	50-54 years	55-59 years	60-64 years	65-69 years	70-74 years	75-79 years	80-84 years	85-89 years	90-94 years	95+ years
1.0 (1.0 to 1.0)	1.0 (1.0 to 1.0)	1.0 (1.0 to 1.0)	1.0 (1.0 to 1.0)	1.0 (1.0 to 1.0)	1.0 (1.0 to 1.0)	1.0 (1.0 to 1.0)	1.0 (1.0 to 1.0)	1.0 (1.0 to 1.0)	1.0 (1.0 to 1.0)	1.0 (1.0 to 1.0)	1.0 (1.0 to 1.0)	1.0 (1.0 to 1.0)	1.0 (1.0 to 1.0)
1.18 (1.117 to 1.244)	1.183 (1.117 to 1.249)	1.18 (1.117 to 1.239)	1.18 (1.117 to 1.249)	1.182 (1.12 to 1.246)	1.181 (1.119 to 1.246)	1.179 (1.115 to 1.242)	1.182 (1.121 to 1.247)	1.18 (1.118 to 1.248)	1.18 (1.113 to 1.245)	1.181 (1.117 to 1.244)	1.181 (1.117 to 1.244)	1.181 (1.117 to 1.244)	1.181 (1.117 to 1.244)
1.179 (1.118 to 1.243)	1.18 (1.116 to 1.248)	1.181 (1.115 to 1.245)	1.181 (1.119 to 1.25)	1.181 (1.118 to 1.245)	1.181 (1.117 to 1.248)	1.181 (1.117 to 1.244)	1.179 (1.121 to 1.245)	1.181 (1.116 to 1.254)	1.181 (1.116 to 1.245)	1.179 (1.113 to 1.247)	1.179 (1.113 to 1.247)	1.179 (1.113 to 1.247)	1.179 (1.113 to 1.247)
1.0 (1.0 to 1.0)	1.0 (1.0 to 1.0)	1.0 (1.0 to 1.0)	1.0 (1.0 to 1.0)	1.0 (1.0 to 1.0)	1.0 (1.0 to 1.0)	1.0 (1.0 to 1.0)	1.0 (1.0 to 1.0)	1.0 (1.0 to 1.0)	1.0 (1.0 to 1.0)	1.0 (1.0 to 1.0)	1.0 (1.0 to 1.0)	1.0 (1.0 to 1.0)	1.0 (1.0 to 1.0)
1.0 (1.0 to 1.0)	1.0 (1.0 to 1.0)	1.0 (1.0 to 1.0)	1.0 (1.0 to 1.0)	1.0 (1.0 to 1.0)	1.0 (1.0 to 1.0)	1.0 (1.0 to 1.0)	1.0 (1.0 to 1.0)	1.0 (1.0 to 1.0)	1.0 (1.0 to 1.0)	1.0 (1.0 to 1.0)	1.0 (1.0 to 1.0)	1.0 (1.0 to 1.0)	1.0 (1.0 to 1.0)
1.0 (1.0 to 1.0)	1.0 (1.0 to 1.0)	1.0 (1.0 to 1.0)	1.0 (1.0 to 1.0)	1.0 (1.0 to 1.0)	1.0 (1.0 to 1.0)	1.0 (1.0 to 1.0)	1.0 (1.0 to 1.0)	1.0 (1.0 to 1.0)	1.0 (1.0 to 1.0)	1.0 (1.0 to 1.0)	1.0 (1.0 to 1.0)	1.0 (1.0 to 1.0)	1.0 (1.0 to 1.0)
1.0 (1.0 to 1.0)	1.0 (1.0 to 1.0)	1.0 (1.0 to 1.0)	1.0 (1.0 to 1.0)	1.0 (1.0 to 1.0)	1.0 (1.0 to 1.0)	1.0 (1.0 to 1.0)	1.0 (1.0 to 1.0)	1.0 (1.0 to 1.0)	1.0 (1.0 to 1.0)	1.0 (1.0 to 1.0)	1.0 (1.0 to 1.0)	1.0 (1.0 to 1.0)	1.0 (1.0 to 1.0)
1.474 (1.293 to 1.675)	1.472 (1.301 to 1.669)	1.47 (1.294 to 1.662)	1.475 (1.3 to 1.667)	1.469 (1.301 to 1.644)	1.474 (1.295 to 1.676)	1.477 (1.292 to 1.671)	1.477 (1.311 to 1.68)	1.476 (1.302 to 1.669)	1.473 (1.287 to 1.669)	1.477 (1.302 to 1.666)	1.477 (1.302 to 1.666)	1.477 (1.302 to 1.666)	1.477 (1.302 to 1.666)
1.467 (1.282 to 1.67)	1.473 (1.288 to 1.66)	1.475 (1.294 to 1.68)	1.477 (1.3 to 1.679)	1.475 (1.302 to 1.674)	1.476 (1.292 to 1.68)	1.481 (1.309 to 1.681)	1.478 (1.295 to 1.683)	1.473 (1.291 to 1.661)	1.473 (1.29 to 1.676)	1.476 (1.289 to 1.662)	1.476 (1.289 to 1.662)	1.476 (1.289 to 1.662)	1.476 (1.289 to 1.662)
1.0 (1.0 to 1.0)	1.0 (1.0 to 1.0)	1.0 (1.0 to 1.0)	1.0 (1.0 to 1.0)	1.0 (1.0 to 1.0)	1.0 (1.0 to 1.0)	1.0 (1.0 to 1.0)	1.0 (1.0 to 1.0)	1.0 (1.0 to 1.0)	1.0 (1.0 to 1.0)	1.0 (1.0 to 1.0)	1.0 (1.0 to 1.0)	1.0 (1.0 to 1.0)	1.0 (1.0 to 1.0)
1.0 (1.0 to 1.0)	1.0 (1.0 to 1.0)	1.0 (1.0 to 1.0)	1.0 (1.0 to 1.0)	1.0 (1.0 to 1.0)	1.0 (1.0 to 1.0)	1.0 (1.0 to 1.0)	1.0 (1.0 to 1.0)	1.0 (1.0 to 1.0)	1.0 (1.0 to 1.0)	1.0 (1.0 to 1.0)	1.0 (1.0 to 1.0)	1.0 (1.0 to 1.0)	1.0 (1.0 to 1.0)
1.0 (1.0 to 1.0)	1.0 (1.0 to 1.0)	1.0 (1.0 to 1.0)	1.0 (1.0 to 1.0)	1.0 (1.0 to 1.0)	1.0 (1.0 to 1.0)	1.0 (1.0 to 1.0)	1.0 (1.0 to 1.0)	1.0 (1.0 to 1.0)	1.0 (1.0 to 1.0)	1.0 (1.0 to 1.0)	1.0 (1.0 to 1.0)	1.0 (1.0 to 1.0)	1.0 (1.0 to 1.0)
1.0 (1.0 to 1.0)	1.0 (1.0 to 1.0)	1.0 (1.0 to 1.0)	1.0 (1.0 to 1.0)	1.0 (1.0 to 1.0)	1.0 (1.0 to 1.0)	1.0 (1.0 to 1.0)	1.0 (1.0 to 1.0)	1.0 (1.0 to 1.0)	1.0 (1.0 to 1.0)	1.0 (1.0 to 1.0)	1.0 (1.0 to 1.0)	1.0 (1.0 to 1.0)	1.0 (1.0 to 1.0)
1.24 (1.185 to 1.292)	1.243 (1.19 to 1.296)	1.242 (1.187 to 1.298)	1.241 (1.185 to 1.293)	1.239 (1.187 to 1.298)	1.24 (1.188 to 1.292)	1.241 (1.187 to 1.298)	1.241 (1.19 to 1.299)	1.239 (1.186 to 1.298)	1.241 (1.187 to 1.297)	1.24 (1.187 to 1.295)	1.24 (1.187 to 1.295)	1.24 (1.187 to 1.295)	1.24 (1.187 to 1.295)
1.24 (1.183 to 1.298)	1.24 (1.189 to 1.296)	1.24 (1.186 to 1.298)	1.24 (1.186 to 1.294)	1.239 (1.185 to 1.295)	1.239 (1.188 to 1.295)	1.241 (1.188 to 1.298)	1.239 (1.189 to 1.293)	1.24 (1.187 to 1.294)	1.241 (1.187 to 1.293)	1.24 (1.184 to 1.297)	1.24 (1.184 to 1.297)	1.24 (1.184 to 1.297)	1.24 (1.184 to 1.297)
1.0 (1.0 to 1.0)	1.0 (1.0 to 1.0)	1.0 (1.0 to 1.0)	1.0 (1.0 to 1.0)	1.0 (1.0 to 1.0)	1.0 (1.0 to 1.0)	1.0 (1.0 to 1.0)	1.0 (1.0 to 1.0)	1.0 (1.0 to 1.0)	1.0 (1.0 to 1.0)	1.0 (1.0 to 1.0)	1.0 (1.0 to 1.0)	1.0 (1.0 to 1.0)	1.0 (1.0 to 1.0)
1.0 (1.0 to 1.0)	1.0 (1.0 to 1.0)	1.0 (1.0 to 1.0)	1.0 (1.0 to 1.0)	1.0 (1.0 to 1.0)	1.0 (1.0 to 1.0)	1.0 (1.0 to 1.0)	1.0 (1.0 to 1.0)	1.0 (1.0 to 1.0)	1.0 (1.0 to 1.0)	1.0 (1.0 to 1.0)	1.0 (1.0 to 1.0)	1.0 (1.0 to 1.0)	1.0 (1.0 to 1.0)
1.0 (1.0 to 1.0)	1.0 (1.0 to 1.0)	1.0 (1.0 to 1.0)	1.0 (1.0 to 1.0)	1.0 (1.0 to 1.0)	1.0 (1.0 to 1.0)	1.0 (1.0 to 1.0)	1.0 (1.0 to 1.0)	1.0 (1.0 to 1.0)	1.0 (1.0 to 1.0)	1.0 (1.0 to 1.0)	1.0 (1.0 to 1.0)	1.0 (1.0 to 1.0)	1.0 (1.0 to 1.0)
1.0 (1.0 to 1.0)	1.0 (1.0 to 1.0)	1.0 (1.0 to 1.0)	1.0 (1.0 to 1.0)	1.0 (1.0 to 1.0)	1.0 (1.0 to 1.0)	1.0 (1.0 to 1.0)	1.0 (1.0 to 1.0)	1.0 (1.0 to 1.0)	1.0 (1.0 to 1.0)	1.0 (1.0 to 1.0)	1.0 (1.0 to 1.0)	1.0 (1.0 to 1.0)	1.0 (1.0 to 1.0)
2.211 (0.994 to 4.221)	2.23 (1.042 to 4.13)	2.241 (1.077 to 4.068)	2.265 (1.056 to 4.33)	2.198 (1.041 to 4.081)	2.232 (1.104 to 4.091)	2.215 (1.022 to 4.191)	2.251 (1.056 to 4.236)	2.233 (1.022 to 4.329)	2.234 (1.02 to 4.325)	2.204 (1.064 to 4.14)	2.204 (1.064 to 4.14)	2.204 (1.064 to 4.14)	2.204 (1.064 to 4.14)
2.269 (1.084 to 4.182)	2.264 (1.069 to 4.385)	2.217 (1.041 to 4.21)	2.201 (1.035 to 4.162)	2.221 (1.035 to 4.234)	2.225 (1.025 to 4.212)	2.276 (1.072 to 4.204)	2.261 (1.04 to 4.208)	2.258 (1.042 to 4.371)	2.241 (1.031 to 4.156)	2.229 (1.025 to 4.237)	2.229 (1.025 to 4.237)	2.229 (1.025 to 4.237)	2.229 (1.025 to 4.237)
1.0 (1.0 to 1.0)	1.0 (1.0 to 1.0)	1.0 (1.0 to 1.0)	1.0 (1.0 to 1.0)	1.0 (1.0 to 1.0)	1.0 (1.0 to 1.0)	1.0 (1.0 to 1.0)	1.0 (1.0 to 1.0)	1.0 (1.0 to 1.0)	1.0 (1.0 to 1.0)	1.0 (1.0 to 1.0)	1.0 (1.0 to 1.0)	1.0 (1.0 to 1.0)	1.0 (1.0 to 1.0)
1.0 (1.0 to 1.0)	1.0 (1.0 to 1.0)	1.0 (1.0 to 1.0)	1.0 (1.0 to 1.0)	1.0 (1.0 to 1.0)	1.0 (1.0 to 1.0)	1.0 (1.0 to 1.0)	1.0 (1.0 to 1.0)	1.0 (1.0 to 1.0)	1.0 (1.0 to 1.0)	1.0 (1.0 to 1.0)	1.0 (1.0 to 1.0)	1.0 (1.0 to 1.0)	1.0 (1.0 to 1.0)
1.0 (1.0 to 1.0)	1.0 (1.0 to 1.0)	1.0 (1.0 to 1.0)	1.0 (1.0 to 1.0)	1.0 (1.0 to 1.0)	1.0 (1.0 to 1.0)	1.0 (1.0 to 1.0)	1.0 (1.0 to 1.0)	1.0 (1.0 to 1.0)	1.0 (1.0 to 1.0)	1.0 (1.0 to 1.0)	1.0 (1.0 to 1.0)	1.0 (1.0 to 1.0)	1.0 (1.0 to 1.0)
1.0 (1.0 to 1.0)	1.0 (1.0 to 1.0)	1.0 (1.0 to 1.0)	1.0 (1.0 to 1.0)	1.0 (1.0 to 1.0)	1.0 (1.0 to 1.0)	1.0 (1.0 to 1.0)	1.0 (1.0 to 1.0)	1.0 (1.0 to 1.0)	1.0 (1.0 to 1.0)	1.0 (1.0 to 1.0)	1.0 (1.0 to 1.0)	1.0 (1.0 to 1.0)	1.0 (1.0 to 1.0)
1.48 (1.193 to 1.827)	1.479 (1.197 to 1.804)	1.467 (1.174 to 1.843)	1.481 (1.178 to 1.839)	1.49 (1.199 to 1.831)	1.47 (1.197 to 1.842)	1.487 (1.2 to 1.846)	1.485 (1.188 to 1.803)	1.48 (1.18 to 1.836)	1.482 (1.202 to 1.839)	1.488 (1.198 to 1.827)	1.488 (1.198 to 1.827)	1.488 (1.198 to 1.827)	1.488 (1.198 to 1.827)

Appendix Table 1a. Relative risks used by age and sex for each outcome for all risk factors except for ambient air pollution and alcohol.

Risk - Outcome	Category / Units	Morbidity / Mortality	Sex	All-age	0-6 days	7-27 days	28-364 days	1-4 years	5-9 years	10-14 years	15-19 years	20-24 years	25-29 years
Acute lymphoid leukaemia	High exposure	Both	Females								1.485 (1.199 to 1.845)	1.485 (1.183 to 1.855)	1.479 (1.184 to 1.844)
Acute lymphoid leukaemia	Low exposure	Both	Males								1.0 (1.0 to 1.0)	1.0 (1.0 to 1.0)	1.0 (1.0 to 1.0)
Acute lymphoid leukaemia	Low exposure	Both	Females								1.0 (1.0 to 1.0)	1.0 (1.0 to 1.0)	1.0 (1.0 to 1.0)
Acute lymphoid leukaemia	No exposure	Both	Males								1.0 (1.0 to 1.0)	1.0 (1.0 to 1.0)	1.0 (1.0 to 1.0)
Acute lymphoid leukaemia	No exposure	Both	Females								1.0 (1.0 to 1.0)	1.0 (1.0 to 1.0)	1.0 (1.0 to 1.0)
Chronic lymphoid leukaemia	High exposure	Both	Males								1.483 (1.191 to 1.818)	1.479 (1.183 to 1.83)	1.474 (1.182 to 1.815)
Chronic lymphoid leukaemia	High exposure	Both	Females								1.485 (1.199 to 1.845)	1.485 (1.183 to 1.855)	1.479 (1.184 to 1.844)
Chronic lymphoid leukaemia	Low exposure	Both	Males								1.0 (1.0 to 1.0)	1.0 (1.0 to 1.0)	1.0 (1.0 to 1.0)
Chronic lymphoid leukaemia	Low exposure	Both	Females								1.0 (1.0 to 1.0)	1.0 (1.0 to 1.0)	1.0 (1.0 to 1.0)
Chronic lymphoid leukaemia	No exposure	Both	Males								1.0 (1.0 to 1.0)	1.0 (1.0 to 1.0)	1.0 (1.0 to 1.0)
Chronic lymphoid leukaemia	No exposure	Both	Females								1.0 (1.0 to 1.0)	1.0 (1.0 to 1.0)	1.0 (1.0 to 1.0)
Acute myeloid leukaemia	High exposure	Both	Males								1.483 (1.191 to 1.818)	1.479 (1.183 to 1.83)	1.474 (1.182 to 1.815)
Acute myeloid leukaemia	High exposure	Both	Females								1.485 (1.199 to 1.845)	1.485 (1.183 to 1.855)	1.479 (1.184 to 1.844)
Acute myeloid leukaemia	Low exposure	Both	Males								1.0 (1.0 to 1.0)	1.0 (1.0 to 1.0)	1.0 (1.0 to 1.0)
Acute myeloid leukaemia	Low exposure	Both	Females								1.0 (1.0 to 1.0)	1.0 (1.0 to 1.0)	1.0 (1.0 to 1.0)
Acute myeloid leukaemia	No exposure	Both	Males								1.0 (1.0 to 1.0)	1.0 (1.0 to 1.0)	1.0 (1.0 to 1.0)
Acute myeloid leukaemia	No exposure	Both	Females								1.0 (1.0 to 1.0)	1.0 (1.0 to 1.0)	1.0 (1.0 to 1.0)
Chronic myeloid leukaemia	High exposure	Both	Males								1.483 (1.191 to 1.818)	1.479 (1.183 to 1.83)	1.474 (1.182 to 1.815)
Chronic myeloid leukaemia	High exposure	Both	Females								1.485 (1.199 to 1.845)	1.485 (1.183 to 1.855)	1.479 (1.184 to 1.844)
Chronic myeloid leukaemia	Low exposure	Both	Males								1.0 (1.0 to 1.0)	1.0 (1.0 to 1.0)	1.0 (1.0 to 1.0)
Chronic myeloid leukaemia	Low exposure	Both	Females								1.0 (1.0 to 1.0)	1.0 (1.0 to 1.0)	1.0 (1.0 to 1.0)
Chronic myeloid leukaemia	No exposure	Both	Males								1.0 (1.0 to 1.0)	1.0 (1.0 to 1.0)	1.0 (1.0 to 1.0)
Chronic myeloid leukaemia	No exposure	Both	Females								1.0 (1.0 to 1.0)	1.0 (1.0 to 1.0)	1.0 (1.0 to 1.0)
Other leukaemia	High exposure	Both	Males								1.483 (1.191 to 1.818)	1.479 (1.183 to 1.83)	1.474 (1.182 to 1.815)
Other leukaemia	High exposure	Both	Females								1.485 (1.199 to 1.845)	1.485 (1.183 to 1.855)	1.479 (1.184 to 1.844)
Other leukaemia	Low exposure	Both	Males								1.0 (1.0 to 1.0)	1.0 (1.0 to 1.0)	1.0 (1.0 to 1.0)
Other leukaemia	Low exposure	Both	Females								1.0 (1.0 to 1.0)	1.0 (1.0 to 1.0)	1.0 (1.0 to 1.0)
Other leukaemia	No exposure	Both	Males								1.0 (1.0 to 1.0)	1.0 (1.0 to 1.0)	1.0 (1.0 to 1.0)
Other leukaemia	No exposure	Both	Females								1.0 (1.0 to 1.0)	1.0 (1.0 to 1.0)	1.0 (1.0 to 1.0)
**Occupational exposure to nickel**													

Ages													
**30-34 years**	**35-39 years**	**40-44 years**	**45-49 years**	**50-54 years**	**55-59 years**	**60-64 years**	**65-69 years**	**70-74 years**	**75-79 years**	**80-84 years**	**85-89 years**	**90-94 years**	**95+ years**
1.471 (1.181 to 1.774)	1.469 (1.183 to 1.819)	1.486 (1.196 to 1.823)	1.485 (1.19 to 1.848)	1.481 (1.191 to 1.856)	1.48 (1.199 to 1.814)	1.47 (1.184 to 1.812)	1.473 (1.19 to 1.814)	1.471 (1.194 to 1.789)	1.49 (1.209 to 1.859)	1.464 (1.18 to 1.788)	1.464 (1.18 to 1.788)	1.464 (1.18 to 1.788)	1.464 (1.18 to 1.788)
1.0 (1.0 to 1.0)	1.0 (1.0 to 1.0)	1.0 (1.0 to 1.0)	1.0 (1.0 to 1.0)	1.0 (1.0 to 1.0)	1.0 (1.0 to 1.0)	1.0 (1.0 to 1.0)	1.0 (1.0 to 1.0)	1.0 (1.0 to 1.0)	1.0 (1.0 to 1.0)	1.0 (1.0 to 1.0)	1.0 (1.0 to 1.0)	1.0 (1.0 to 1.0)	1.0 (1.0 to 1.0)
1.0 (1.0 to 1.0)	1.0 (1.0 to 1.0)	1.0 (1.0 to 1.0)	1.0 (1.0 to 1.0)	1.0 (1.0 to 1.0)	1.0 (1.0 to 1.0)	1.0 (1.0 to 1.0)	1.0 (1.0 to 1.0)	1.0 (1.0 to 1.0)	1.0 (1.0 to 1.0)	1.0 (1.0 to 1.0)	1.0 (1.0 to 1.0)	1.0 (1.0 to 1.0)	1.0 (1.0 to 1.0)
1.0 (1.0 to 1.0)	1.0 (1.0 to 1.0)	1.0 (1.0 to 1.0)	1.0 (1.0 to 1.0)	1.0 (1.0 to 1.0)	1.0 (1.0 to 1.0)	1.0 (1.0 to 1.0)	1.0 (1.0 to 1.0)	1.0 (1.0 to 1.0)	1.0 (1.0 to 1.0)	1.0 (1.0 to 1.0)	1.0 (1.0 to 1.0)	1.0 (1.0 to 1.0)	1.0 (1.0 to 1.0)
1.0 (1.0 to 1.0)	1.0 (1.0 to 1.0)	1.0 (1.0 to 1.0)	1.0 (1.0 to 1.0)	1.0 (1.0 to 1.0)	1.0 (1.0 to 1.0)	1.0 (1.0 to 1.0)	1.0 (1.0 to 1.0)	1.0 (1.0 to 1.0)	1.0 (1.0 to 1.0)	1.0 (1.0 to 1.0)	1.0 (1.0 to 1.0)	1.0 (1.0 to 1.0)	1.0 (1.0 to 1.0)
1.48 (1.193 to 1.827)	1.479 (1.197 to 1.804)	1.467 (1.174 to 1.843)	1.481 (1.178 to 1.839)	1.49 (1.199 to 1.831)	1.47 (1.197 to 1.842)	1.487 (1.2 to 1.846)	1.485 (1.188 to 1.803)	1.48 (1.18 to 1.836)	1.482 (1.202 to 1.839)	1.488 (1.198 to 1.827)	1.488 (1.198 to 1.827)	1.488 (1.198 to 1.827)	1.488 (1.198 to 1.827)
1.471 (1.181 to 1.774)	1.469 (1.183 to 1.819)	1.486 (1.196 to 1.823)	1.485 (1.19 to 1.848)	1.481 (1.191 to 1.856)	1.48 (1.199 to 1.814)	1.47 (1.184 to 1.812)	1.473 (1.19 to 1.814)	1.471 (1.194 to 1.789)	1.49 (1.209 to 1.859)	1.464 (1.18 to 1.788)	1.464 (1.18 to 1.788)	1.464 (1.18 to 1.788)	1.464 (1.18 to 1.788)
1.0 (1.0 to 1.0)	1.0 (1.0 to 1.0)	1.0 (1.0 to 1.0)	1.0 (1.0 to 1.0)	1.0 (1.0 to 1.0)	1.0 (1.0 to 1.0)	1.0 (1.0 to 1.0)	1.0 (1.0 to 1.0)	1.0 (1.0 to 1.0)	1.0 (1.0 to 1.0)	1.0 (1.0 to 1.0)	1.0 (1.0 to 1.0)	1.0 (1.0 to 1.0)	1.0 (1.0 to 1.0)
1.0 (1.0 to 1.0)	1.0 (1.0 to 1.0)	1.0 (1.0 to 1.0)	1.0 (1.0 to 1.0)	1.0 (1.0 to 1.0)	1.0 (1.0 to 1.0)	1.0 (1.0 to 1.0)	1.0 (1.0 to 1.0)	1.0 (1.0 to 1.0)	1.0 (1.0 to 1.0)	1.0 (1.0 to 1.0)	1.0 (1.0 to 1.0)	1.0 (1.0 to 1.0)	1.0 (1.0 to 1.0)
1.0 (1.0 to 1.0)	1.0 (1.0 to 1.0)	1.0 (1.0 to 1.0)	1.0 (1.0 to 1.0)	1.0 (1.0 to 1.0)	1.0 (1.0 to 1.0)	1.0 (1.0 to 1.0)	1.0 (1.0 to 1.0)	1.0 (1.0 to 1.0)	1.0 (1.0 to 1.0)	1.0 (1.0 to 1.0)	1.0 (1.0 to 1.0)	1.0 (1.0 to 1.0)	1.0 (1.0 to 1.0)
1.0 (1.0 to 1.0)	1.0 (1.0 to 1.0)	1.0 (1.0 to 1.0)	1.0 (1.0 to 1.0)	1.0 (1.0 to 1.0)	1.0 (1.0 to 1.0)	1.0 (1.0 to 1.0)	1.0 (1.0 to 1.0)	1.0 (1.0 to 1.0)	1.0 (1.0 to 1.0)	1.0 (1.0 to 1.0)	1.0 (1.0 to 1.0)	1.0 (1.0 to 1.0)	1.0 (1.0 to 1.0)
1.48 (1.193 to 1.827)	1.479 (1.197 to 1.804)	1.467 (1.174 to 1.843)	1.481 (1.178 to 1.839)	1.49 (1.199 to 1.831)	1.47 (1.197 to 1.842)	1.487 (1.2 to 1.846)	1.485 (1.188 to 1.803)	1.48 (1.18 to 1.836)	1.482 (1.202 to 1.839)	1.488 (1.198 to 1.827)	1.488 (1.198 to 1.827)	1.488 (1.198 to 1.827)	1.488 (1.198 to 1.827)
1.471 (1.181 to 1.774)	1.469 (1.183 to 1.819)	1.486 (1.196 to 1.823)	1.485 (1.19 to 1.848)	1.481 (1.191 to 1.856)	1.48 (1.199 to 1.814)	1.47 (1.184 to 1.812)	1.473 (1.19 to 1.814)	1.471 (1.194 to 1.789)	1.49 (1.209 to 1.859)	1.464 (1.18 to 1.788)	1.464 (1.18 to 1.788)	1.464 (1.18 to 1.788)	1.464 (1.18 to 1.788)
1.0 (1.0 to 1.0)	1.0 (1.0 to 1.0)	1.0 (1.0 to 1.0)	1.0 (1.0 to 1.0)	1.0 (1.0 to 1.0)	1.0 (1.0 to 1.0)	1.0 (1.0 to 1.0)	1.0 (1.0 to 1.0)	1.0 (1.0 to 1.0)	1.0 (1.0 to 1.0)	1.0 (1.0 to 1.0)	1.0 (1.0 to 1.0)	1.0 (1.0 to 1.0)	1.0 (1.0 to 1.0)
1.0 (1.0 to 1.0)	1.0 (1.0 to 1.0)	1.0 (1.0 to 1.0)	1.0 (1.0 to 1.0)	1.0 (1.0 to 1.0)	1.0 (1.0 to 1.0)	1.0 (1.0 to 1.0)	1.0 (1.0 to 1.0)	1.0 (1.0 to 1.0)	1.0 (1.0 to 1.0)	1.0 (1.0 to 1.0)	1.0 (1.0 to 1.0)	1.0 (1.0 to 1.0)	1.0 (1.0 to 1.0)
1.0 (1.0 to 1.0)	1.0 (1.0 to 1.0)	1.0 (1.0 to 1.0)	1.0 (1.0 to 1.0)	1.0 (1.0 to 1.0)	1.0 (1.0 to 1.0)	1.0 (1.0 to 1.0)	1.0 (1.0 to 1.0)	1.0 (1.0 to 1.0)	1.0 (1.0 to 1.0)	1.0 (1.0 to 1.0)	1.0 (1.0 to 1.0)	1.0 (1.0 to 1.0)	1.0 (1.0 to 1.0)
1.0 (1.0 to 1.0)	1.0 (1.0 to 1.0)	1.0 (1.0 to 1.0)	1.0 (1.0 to 1.0)	1.0 (1.0 to 1.0)	1.0 (1.0 to 1.0)	1.0 (1.0 to 1.0)	1.0 (1.0 to 1.0)	1.0 (1.0 to 1.0)	1.0 (1.0 to 1.0)	1.0 (1.0 to 1.0)	1.0 (1.0 to 1.0)	1.0 (1.0 to 1.0)	1.0 (1.0 to 1.0)
1.48 (1.193 to 1.827)	1.479 (1.197 to 1.804)	1.467 (1.174 to 1.843)	1.481 (1.178 to 1.839)	1.49 (1.199 to 1.831)	1.47 (1.197 to 1.842)	1.487 (1.2 to 1.846)	1.485 (1.188 to 1.803)	1.48 (1.18 to 1.836)	1.482 (1.202 to 1.839)	1.488 (1.198 to 1.827)	1.488 (1.198 to 1.827)	1.488 (1.198 to 1.827)	1.488 (1.198 to 1.827)
1.471 (1.181 to 1.774)	1.469 (1.183 to 1.819)	1.486 (1.196 to 1.823)	1.485 (1.19 to 1.848)	1.481 (1.191 to 1.856)	1.48 (1.199 to 1.814)	1.47 (1.184 to 1.812)	1.473 (1.19 to 1.814)	1.471 (1.194 to 1.789)	1.49 (1.209 to 1.859)	1.464 (1.18 to 1.788)	1.464 (1.18 to 1.788)	1.464 (1.18 to 1.788)	1.464 (1.18 to 1.788)
1.0 (1.0 to 1.0)	1.0 (1.0 to 1.0)	1.0 (1.0 to 1.0)	1.0 (1.0 to 1.0)	1.0 (1.0 to 1.0)	1.0 (1.0 to 1.0)	1.0 (1.0 to 1.0)	1.0 (1.0 to 1.0)	1.0 (1.0 to 1.0)	1.0 (1.0 to 1.0)	1.0 (1.0 to 1.0)	1.0 (1.0 to 1.0)	1.0 (1.0 to 1.0)	1.0 (1.0 to 1.0)
1.0 (1.0 to 1.0)	1.0 (1.0 to 1.0)	1.0 (1.0 to 1.0)	1.0 (1.0 to 1.0)	1.0 (1.0 to 1.0)	1.0 (1.0 to 1.0)	1.0 (1.0 to 1.0)	1.0 (1.0 to 1.0)	1.0 (1.0 to 1.0)	1.0 (1.0 to 1.0)	1.0 (1.0 to 1.0)	1.0 (1.0 to 1.0)	1.0 (1.0 to 1.0)	1.0 (1.0 to 1.0)
1.0 (1.0 to 1.0)	1.0 (1.0 to 1.0)	1.0 (1.0 to 1.0)	1.0 (1.0 to 1.0)	1.0 (1.0 to 1.0)	1.0 (1.0 to 1.0)	1.0 (1.0 to 1.0)	1.0 (1.0 to 1.0)	1.0 (1.0 to 1.0)	1.0 (1.0 to 1.0)	1.0 (1.0 to 1.0)	1.0 (1.0 to 1.0)	1.0 (1.0 to 1.0)	1.0 (1.0 to 1.0)
1.0 (1.0 to 1.0)	1.0 (1.0 to 1.0)	1.0 (1.0 to 1.0)	1.0 (1.0 to 1.0)	1.0 (1.0 to 1.0)	1.0 (1.0 to 1.0)	1.0 (1.0 to 1.0)	1.0 (1.0 to 1.0)	1.0 (1.0 to 1.0)	1.0 (1.0 to 1.0)	1.0 (1.0 to 1.0)	1.0 (1.0 to 1.0)	1.0 (1.0 to 1.0)	1.0 (1.0 to 1.0)
1.48 (1.193 to 1.827)	1.479 (1.197 to 1.804)	1.467 (1.174 to 1.843)	1.481 (1.178 to 1.839)	1.49 (1.199 to 1.831)	1.47 (1.197 to 1.842)	1.487 (1.2 to 1.846)	1.485 (1.188 to 1.803)	1.48 (1.18 to 1.836)	1.482 (1.202 to 1.839)	1.488 (1.198 to 1.827)	1.488 (1.198 to 1.827)	1.488 (1.198 to 1.827)	1.488 (1.198 to 1.827)
1.471 (1.181 to 1.774)	1.469 (1.183 to 1.819)	1.486 (1.196 to 1.823)	1.485 (1.19 to 1.848)	1.481 (1.191 to 1.856)	1.48 (1.199 to 1.814)	1.47 (1.184 to 1.812)	1.473 (1.19 to 1.814)	1.471 (1.194 to 1.789)	1.49 (1.209 to 1.859)	1.464 (1.18 to 1.788)	1.464 (1.18 to 1.788)	1.464 (1.18 to 1.788)	1.464 (1.18 to 1.788)
1.0 (1.0 to 1.0)	1.0 (1.0 to 1.0)	1.0 (1.0 to 1.0)	1.0 (1.0 to 1.0)	1.0 (1.0 to 1.0)	1.0 (1.0 to 1.0)	1.0 (1.0 to 1.0)	1.0 (1.0 to 1.0)	1.0 (1.0 to 1.0)	1.0 (1.0 to 1.0)	1.0 (1.0 to 1.0)	1.0 (1.0 to 1.0)	1.0 (1.0 to 1.0)	1.0 (1.0 to 1.0)
1.0 (1.0 to 1.0)	1.0 (1.0 to 1.0)	1.0 (1.0 to 1.0)	1.0 (1.0 to 1.0)	1.0 (1.0 to 1.0)	1.0 (1.0 to 1.0)	1.0 (1.0 to 1.0)	1.0 (1.0 to 1.0)	1.0 (1.0 to 1.0)	1.0 (1.0 to 1.0)	1.0 (1.0 to 1.0)	1.0 (1.0 to 1.0)	1.0 (1.0 to 1.0)	1.0 (1.0 to 1.0)
1.0 (1.0 to 1.0)	1.0 (1.0 to 1.0)	1.0 (1.0 to 1.0)	1.0 (1.0 to 1.0)	1.0 (1.0 to 1.0)	1.0 (1.0 to 1.0)	1.0 (1.0 to 1.0)	1.0 (1.0 to 1.0)	1.0 (1.0 to 1.0)	1.0 (1.0 to 1.0)	1.0 (1.0 to 1.0)	1.0 (1.0 to 1.0)	1.0 (1.0 to 1.0)	1.0 (1.0 to 1.0)
1.0 (1.0 to 1.0)	1.0 (1.0 to 1.0)	1.0 (1.0 to 1.0)	1.0 (1.0 to 1.0)	1.0 (1.0 to 1.0)	1.0 (1.0 to 1.0)	1.0 (1.0 to 1.0)	1.0 (1.0 to 1.0)	1.0 (1.0 to 1.0)	1.0 (1.0 to 1.0)	1.0 (1.0 to 1.0)	1.0 (1.0 to 1.0)	1.0 (1.0 to 1.0)	1.0 (1.0 to 1.0)

Appendix Table 1a. Relative risks used by age and sex for each outcome for all risk factors except for ambient air pollution and alcohol.

Risk - Outcome	Category / Units	Morbidity / Mortality	Sex	All-age	0-6 days	7-27 days	28-364 days	1-4 years	5-9 years	10-14 years	15-19 years	20-24 years	25-29 years
Tracheal, bronchus, and lung cancer	High exposure	Both	Both								2.148 (1.296 to 3.297)	2.148 (1.296 to 3.297)	2.148 (1.296 to 3.297)
Tracheal, bronchus, and lung cancer	Low exposure	Both	Both								1.538 (0.606 to 3.355)	1.538 (0.606 to 3.355)	1.538 (0.606 to 3.355)
Tracheal, bronchus, and lung cancer	No exposure	Both	Both								1.0 (1.0 to 1.0)	1.0 (1.0 to 1.0)	1.0 (1.0 to 1.0)
**Occupational exposure to polycyclic aromatic hydrocarbons**													
Tracheal, bronchus, and lung cancer	High exposure	Both	Males								1.31 (1.165 to 1.468)	1.304 (1.146 to 1.466)	1.313 (1.164 to 1.485)
Tracheal, bronchus, and lung cancer	High exposure	Both	Females								1.31 (1.154 to 1.486)	1.311 (1.157 to 1.469)	1.313 (1.154 to 1.472)
Tracheal, bronchus, and lung cancer	Low exposure	Both	Males								1.0 (1.0 to 1.0)	1.0 (1.0 to 1.0)	1.0 (1.0 to 1.0)
Tracheal, bronchus, and lung cancer	Low exposure	Both	Females								1.0 (1.0 to 1.0)	1.0 (1.0 to 1.0)	1.0 (1.0 to 1.0)
Tracheal, bronchus, and lung cancer	No exposure	Both	Males								1.0 (1.0 to 1.0)	1.0 (1.0 to 1.0)	1.0 (1.0 to 1.0)
Tracheal, bronchus, and lung cancer	No exposure	Both	Females								1.0 (1.0 to 1.0)	1.0 (1.0 to 1.0)	1.0 (1.0 to 1.0)
**Occupational exposure to silica**													
Tracheal, bronchus, and lung cancer	High exposure	Both	Both								1.698 (1.164 to 2.259)	1.698 (1.164 to 2.259)	1.698 (1.164 to 2.259)
Tracheal, bronchus, and lung cancer	Low exposure	Both	Both								1.537 (1.063 to 1.986)	1.537 (1.063 to 1.986)	1.537 (1.063 to 1.986)
Tracheal, bronchus, and lung cancer	No exposure	Both	Both								1.0 (1.0 to 1.0)	1.0 (1.0 to 1.0)	1.0 (1.0 to 1.0)
**Occupational exposure to sulfphuric acid**													
Larynx cancer	High exposure	Both	Both								4.566 (2.122 to 8.328)	4.566 (2.122 to 8.328)	4.566 (2.122 to 8.328)
Larynx cancer	Low exposure	Both	Both								2.024 (0.944 to 3.782)	2.024 (0.944 to 3.782)	2.024 (0.944 to 3.782)
Larynx cancer	No exposure	Both	Both								1.0 (1.0 to 1.0)	1.0 (1.0 to 1.0)	1.0 (1.0 to 1.0)
**Occupational exposure to trichloroethylene**													
Kidney cancer	High exposure	Both	Both								1.245 (1.054 to 1.456)	1.245 (1.054 to 1.456)	1.245 (1.054 to 1.456)
Kidney cancer	Low esposure	Both	Both								1.0 (1.0 to 1.0)	1.0 (1.0 to 1.0)	1.0 (1.0 to 1.0)
Kidney cancer	No exposure	Both	Both								1.0 (1.0 to 1.0)	1.0 (1.0 to 1.0)	1.0 (1.0 to 1.0)
**Occupational asthmagens**													
Asthma	Admin	Both	Males								1.0 (1.0 to 1.0)	1.0 (1.0 to 1.0)	1.0 (1.0 to 1.0)
Asthma	Admin	Both	Females								1.0 (1.0 to 1.0)	1.0 (1.0 to 1.0)	1.0 (1.0 to 1.0)
Asthma	Technical	Both	Males								1.05 (0.977 to 1.125)	1.051 (0.979 to 1.122)	1.051 (0.982 to 1.124)
Asthma	Technical	Both	Females								1.06 (1.028 to 1.095)	1.06 (1.024 to 1.099)	1.059 (1.025 to 1.095)
Asthma	Sales	Both	Males								1.14 (1.047 to 1.237)	1.14 (1.049 to 1.234)	1.144 (1.055 to 1.233)
Asthma	Sales	Both	Females								1.131 (1.083 to 1.182)	1.13 (1.083 to 1.178)	1.129 (1.083 to 1.178)
Asthma	Agriculture	Both	Males								1.519 (1.1 to 2.029)	1.527 (1.119 to 2.063)	1.524 (1.103 to 2.019)

Ages													
30-34 years	35-39 years	40-44 years	45-49 years	50-54 years	55-59 years	60-64 years	65-69 years	70-74 years	75-79 years	80-84 years	85-89 years	90-94 years	95+ years
2.148 (1.296 to 3.297)	2.148 (1.296 to 3.297)	2.148 (1.296 to 3.297)	2.148 (1.296 to 3.297)	2.148 (1.296 to 3.297)	2.148 (1.296 to 3.297)	2.148 (1.296 to 3.297)	2.148 (1.296 to 3.297)	2.148 (1.296 to 3.297)	2.148 (1.296 to 3.297)	2.148 (1.296 to 3.297)	2.148 (1.296 to 3.297)	2.148 (1.296 to 3.297)	2.148 (1.296 to 3.297)
1.538 (0.606 to 3.355)	1.538 (0.606 to 3.355)	1.538 (0.606 to 3.355)	1.538 (0.606 to 3.355)	1.538 (0.606 to 3.355)	1.538 (0.606 to 3.355)	1.538 (0.606 to 3.355)	1.538 (0.606 to 3.355)	1.538 (0.606 to 3.355)	1.538 (0.606 to 3.355)	1.538 (0.606 to 3.355)	1.538 (0.606 to 3.355)	1.538 (0.606 to 3.355)	1.538 (0.606 to 3.355)
1.0 (1.0 to 1.0)	1.0 (1.0 to 1.0)	1.0 (1.0 to 1.0)	1.0 (1.0 to 1.0)	1.0 (1.0 to 1.0)	1.0 (1.0 to 1.0)	1.0 (1.0 to 1.0)	1.0 (1.0 to 1.0)	1.0 (1.0 to 1.0)	1.0 (1.0 to 1.0)	1.0 (1.0 to 1.0)	1.0 (1.0 to 1.0)	1.0 (1.0 to 1.0)	1.0 (1.0 to 1.0)
1.314 (1.167 to 1.477)	1.315 (1.17 to 1.474)	1.314 (1.16 to 1.475)	1.315 (1.154 to 1.477)	1.312 (1.16 to 1.477)	1.318 (1.166 to 1.483)	1.311 (1.158 to 1.479)	1.313 (1.166 to 1.485)	1.314 (1.154 to 1.483)	1.313 (1.161 to 1.478)	1.309 (1.176 to 1.477)	1.309 (1.176 to 1.477)	1.309 (1.176 to 1.477)	1.309 (1.176 to 1.477)
1.313 (1.155 to 1.469)	1.316 (1.162 to 1.483)	1.313 (1.156 to 1.476)	1.314 (1.155 to 1.483)	1.314 (1.171 to 1.483)	1.311 (1.146 to 1.477)	1.312 (1.163 to 1.481)	1.309 (1.147 to 1.489)	1.316 (1.168 to 1.475)	1.315 (1.15 to 1.479)	1.315 (1.166 to 1.481)	1.315 (1.166 to 1.481)	1.315 (1.166 to 1.481)	1.315 (1.166 to 1.481)
1.0 (1.0 to 1.0)	1.0 (1.0 to 1.0)	1.0 (1.0 to 1.0)	1.0 (1.0 to 1.0)	1.0 (1.0 to 1.0)	1.0 (1.0 to 1.0)	1.0 (1.0 to 1.0)	1.0 (1.0 to 1.0)	1.0 (1.0 to 1.0)	1.0 (1.0 to 1.0)	1.0 (1.0 to 1.0)	1.0 (1.0 to 1.0)	1.0 (1.0 to 1.0)	1.0 (1.0 to 1.0)
1.0 (1.0 to 1.0)	1.0 (1.0 to 1.0)	1.0 (1.0 to 1.0)	1.0 (1.0 to 1.0)	1.0 (1.0 to 1.0)	1.0 (1.0 to 1.0)	1.0 (1.0 to 1.0)	1.0 (1.0 to 1.0)	1.0 (1.0 to 1.0)	1.0 (1.0 to 1.0)	1.0 (1.0 to 1.0)	1.0 (1.0 to 1.0)	1.0 (1.0 to 1.0)	1.0 (1.0 to 1.0)
1.0 (1.0 to 1.0)	1.0 (1.0 to 1.0)	1.0 (1.0 to 1.0)	1.0 (1.0 to 1.0)	1.0 (1.0 to 1.0)	1.0 (1.0 to 1.0)	1.0 (1.0 to 1.0)	1.0 (1.0 to 1.0)	1.0 (1.0 to 1.0)	1.0 (1.0 to 1.0)	1.0 (1.0 to 1.0)	1.0 (1.0 to 1.0)	1.0 (1.0 to 1.0)	1.0 (1.0 to 1.0)
1.0 (1.0 to 1.0)	1.0 (1.0 to 1.0)	1.0 (1.0 to 1.0)	1.0 (1.0 to 1.0)	1.0 (1.0 to 1.0)	1.0 (1.0 to 1.0)	1.0 (1.0 to 1.0)	1.0 (1.0 to 1.0)	1.0 (1.0 to 1.0)	1.0 (1.0 to 1.0)	1.0 (1.0 to 1.0)	1.0 (1.0 to 1.0)	1.0 (1.0 to 1.0)	1.0 (1.0 to 1.0)
1.698 (1.164 to 2.259)	1.698 (1.164 to 2.259)	1.698 (1.164 to 2.259)	1.698 (1.164 to 2.259)	1.698 (1.164 to 2.259)	1.698 (1.164 to 2.259)	1.698 (1.164 to 2.259)	1.698 (1.164 to 2.259)	1.698 (1.164 to 2.259)	1.698 (1.164 to 2.259)	1.698 (1.164 to 2.259)	1.698 (1.164 to 2.259)	1.698 (1.164 to 2.259)	1.698 (1.164 to 2.259)
1.537 (1.063 to 1.986)	1.537 (1.063 to 1.986)	1.537 (1.063 to 1.986)	1.537 (1.063 to 1.986)	1.537 (1.063 to 1.986)	1.537 (1.063 to 1.986)	1.537 (1.063 to 1.986)	1.537 (1.063 to 1.986)	1.537 (1.063 to 1.986)	1.537 (1.063 to 1.986)	1.537 (1.063 to 1.986)	1.537 (1.063 to 1.986)	1.537 (1.063 to 1.986)	1.537 (1.063 to 1.986)
1.0 (1.0 to 1.0)	1.0 (1.0 to 1.0)	1.0 (1.0 to 1.0)	1.0 (1.0 to 1.0)	1.0 (1.0 to 1.0)	1.0 (1.0 to 1.0)	1.0 (1.0 to 1.0)	1.0 (1.0 to 1.0)	1.0 (1.0 to 1.0)	1.0 (1.0 to 1.0)	1.0 (1.0 to 1.0)	1.0 (1.0 to 1.0)	1.0 (1.0 to 1.0)	1.0 (1.0 to 1.0)
4.566 (2.122 to 8.328)	4.566 (2.122 to 8.328)	4.566 (2.122 to 8.328)	4.566 (2.122 to 8.328)	4.566 (2.122 to 8.328)	4.566 (2.122 to 8.328)	4.566 (2.122 to 8.328)	4.566 (2.122 to 8.328)	4.566 (2.122 to 8.328)	4.566 (2.122 to 8.328)	4.566 (2.122 to 8.328)	4.566 (2.122 to 8.328)	4.566 (2.122 to 8.328)	4.566 (2.122 to 8.328)
2.024 (0.944 to 3.782)	2.024 (0.944 to 3.782)	2.024 (0.944 to 3.782)	2.024 (0.944 to 3.782)	2.024 (0.944 to 3.782)	2.024 (0.944 to 3.782)	2.024 (0.944 to 3.782)	2.024 (0.944 to 3.782)	2.024 (0.944 to 3.782)	2.024 (0.944 to 3.782)	2.024 (0.944 to 3.782)	2.024 (0.944 to 3.782)	2.024 (0.944 to 3.782)	2.024 (0.944 to 3.782)
1.0 (1.0 to 1.0)	1.0 (1.0 to 1.0)	1.0 (1.0 to 1.0)	1.0 (1.0 to 1.0)	1.0 (1.0 to 1.0)	1.0 (1.0 to 1.0)	1.0 (1.0 to 1.0)	1.0 (1.0 to 1.0)	1.0 (1.0 to 1.0)	1.0 (1.0 to 1.0)	1.0 (1.0 to 1.0)	1.0 (1.0 to 1.0)	1.0 (1.0 to 1.0)	1.0 (1.0 to 1.0)
1.245 (1.054 to 1.456)	1.245 (1.054 to 1.456)	1.245 (1.054 to 1.456)	1.245 (1.054 to 1.456)	1.245 (1.054 to 1.456)	1.245 (1.054 to 1.456)	1.245 (1.054 to 1.456)	1.245 (1.054 to 1.456)	1.245 (1.054 to 1.456)	1.245 (1.054 to 1.456)	1.245 (1.054 to 1.456)	1.245 (1.054 to 1.456)	1.245 (1.054 to 1.456)	1.245 (1.054 to 1.456)
1.0 (1.0 to 1.0)	1.0 (1.0 to 1.0)	1.0 (1.0 to 1.0)	1.0 (1.0 to 1.0)	1.0 (1.0 to 1.0)	1.0 (1.0 to 1.0)	1.0 (1.0 to 1.0)	1.0 (1.0 to 1.0)	1.0 (1.0 to 1.0)	1.0 (1.0 to 1.0)	1.0 (1.0 to 1.0)	1.0 (1.0 to 1.0)	1.0 (1.0 to 1.0)	1.0 (1.0 to 1.0)
1.0 (1.0 to 1.0)	1.0 (1.0 to 1.0)	1.0 (1.0 to 1.0)	1.0 (1.0 to 1.0)	1.0 (1.0 to 1.0)	1.0 (1.0 to 1.0)	1.0 (1.0 to 1.0)	1.0 (1.0 to 1.0)	1.0 (1.0 to 1.0)	1.0 (1.0 to 1.0)	1.0 (1.0 to 1.0)	1.0 (1.0 to 1.0)	1.0 (1.0 to 1.0)	1.0 (1.0 to 1.0)
1.0 (1.0 to 1.0)	1.0 (1.0 to 1.0)	1.0 (1.0 to 1.0)	1.0 (1.0 to 1.0)	1.0 (1.0 to 1.0)	1.0 (1.0 to 1.0)	1.0 (1.0 to 1.0)	1.0 (1.0 to 1.0)	1.0 (1.0 to 1.0)	1.0 (1.0 to 1.0)				
1.0 (1.0 to 1.0)	1.0 (1.0 to 1.0)	1.0 (1.0 to 1.0)	1.0 (1.0 to 1.0)	1.0 (1.0 to 1.0)	1.0 (1.0 to 1.0)	1.0 (1.0 to 1.0)	1.0 (1.0 to 1.0)	1.0 (1.0 to 1.0)	1.0 (1.0 to 1.0)				
1.051 (0.983 to 1.118)	1.05 (0.977 to 1.12)	1.05 (0.981 to 1.122)	1.051 (0.98 to 1.121)	1.051 (0.986 to 1.121)	1.05 (0.976 to 1.125)	1.05 (0.987 to 1.118)	1.051 (0.984 to 1.121)	1.051 (0.982 to 1.122)	1.051 (0.981 to 1.122)				
1.061 (1.026 to 1.099)	1.06 (1.027 to 1.093)	1.06 (1.026 to 1.094)	1.059 (1.024 to 1.093)	1.06 (1.028 to 1.094)	1.06 (1.027 to 1.092)	1.061 (1.026 to 1.095)	1.06 (1.027 to 1.096)	1.061 (1.023 to 1.096)	1.06 (1.027 to 1.096)				
1.142 (1.048 to 1.234)	1.14 (1.046 to 1.234)	1.141 (1.053 to 1.235)	1.143 (1.052 to 1.238)	1.14 (1.047 to 1.236)	1.141 (1.056 to 1.235)	1.138 (1.046 to 1.236)	1.139 (1.057 to 1.226)	1.141 (1.052 to 1.243)	1.14 (1.058 to 1.239)				
1.13 (1.081 to 1.182)	1.131 (1.084 to 1.18)	1.13 (1.08 to 1.181)	1.13 (1.083 to 1.181)	1.13 (1.079 to 1.178)	1.131 (1.077 to 1.184)	1.13 (1.081 to 1.182)	1.129 (1.082 to 1.181)	1.131 (1.083 to 1.184)	1.131 (1.083 to 1.183)				
1.513 (1.127 to 1.999)	1.523 (1.108 to 2.023)	1.516 (1.083 to 2.043)	1.522 (1.106 to 2.038)	1.52 (1.122 to 2.023)	1.519 (1.103 to 2.011)	1.531 (1.122 to 2.047)	1.52 (1.106 to 2.041)	1.508 (1.083 to 2.019)	1.498 (1.081 to 1.965)				

Appendix Table 1a. Relative risks used by age and sex for each outcome for all risk factors except for ambient air pollution and alcohol.

Risk - Outcome	Category / Units	Morbidity / Mortality	Sex	All-age	0-6 days	7-27 days	28-364 days	1-4 years	5-9 years	10-14 years	15-19 years	20-24 years	25-29 years
Asthma	Agriculture	Both	Females								1.52 (1.125 to 2.022)	1.506 (1.099 to 1.997)	1.52 (1.115 to 2.05)
Asthma	Mining	Both	Males								1.959 (1.576 to 2.413)	1.959 (1.568 to 2.381)	1.971 (1.602 to 2.414)
Asthma	Mining	Both	Females								1.956 (1.58 to 2.408)	1.961 (1.574 to 2.395)	1.967 (1.586 to 2.417)
Asthma	Transport	Both	Males								1.313 (1.22 to 1.402)	1.311 (1.222 to 1.406)	1.312 (1.225 to 1.399)
Asthma	Transport	Both	Females								1.221 (1.132 to 1.312)	1.22 (1.138 to 1.31)	1.217 (1.136 to 1.303)
Asthma	Manufact	Both	Males								1.559 (1.477 to 1.647)	1.561 (1.474 to 1.652)	1.56 (1.474 to 1.657)
Asthma	Manufact	Both	Females								1.33 (1.272 to 1.392)	1.331 (1.27 to 1.39)	1.33 (1.271 to 1.39)
Asthma	Services	Both	Males								1.531 (1.415 to 1.646)	1.531 (1.416 to 1.652)	1.529 (1.409 to 1.649)
Asthma	Services	Both	Females								1.41 (1.352 to 1.467)	1.41 (1.357 to 1.469)	1.412 (1.357 to 1.467)
Asthma	Other	Both	Males								1.0 (1.0 to 1.0)	1.0 (1.0 to 1.0)	1.0 (1.0 to 1.0)
Asthma	Other	Both	Females								1.0 (1.0 to 1.0)	1.0 (1.0 to 1.0)	1.0 (1.0 to 1.0)
**Occupational particulate matter, gases, and fumes**													
Chronic obstructive pulmonary disease	High	Both	Males								2.364 (1.463 to 3.64)	2.341 (1.426 to 3.555)	2.37 (1.41 to 3.596)
Chronic obstructive pulmonary disease	High	Both	Females								2.371 (1.459 to 3.719)	2.395 (1.483 to 3.669)	2.364 (1.473 to 3.694)
Chronic obstructive pulmonary disease	Low	Both	Males								1.462 (1.057 to 1.915)	1.457 (1.052 to 1.935)	1.464 (1.085 to 1.91)
Chronic obstructive pulmonary disease	Low	Both	Females								1.446 (1.063 to 1.904)	1.459 (1.09 to 1.954)	1.453 (1.055 to 2.003)
Chronic obstructive pulmonary disease	None	Both	Males								1.0 (1.0 to 1.0)	1.0 (1.0 to 1.0)	1.0 (1.0 to 1.0)
Chronic obstructive pulmonary disease	None	Both	Females								1.0 (1.0 to 1.0)	1.0 (1.0 to 1.0)	1.0 (1.0 to 1.0)
**Occupational ergonomic factors**													
Low back pain	Professional, technical and related workers	Morbidity	Both								1.173 (1.066 to 1.282)	1.172 (1.062 to 1.283)	1.169 (1.065 to 1.283)
Low back pain	Administrative and managerial workers	Morbidity	Both								1.211 (0.964 to 1.508)	1.21 (0.964 to 1.492)	1.209 (0.965 to 1.487)
Low back pain	Clerical and related workers	Morbidity	Both								1.0 (1.0 to 1.0)	1.0 (1.0 to 1.0)	1.0 (1.0 to 1.0)
Low back pain	Sales workers	Morbidity	Both								1.22 (1.029 to 1.434)	1.21 (1.018 to 1.418)	1.213 (1.028 to 1.434)
Low back pain	Service workers	Morbidity	Both								1.472 (1.385 to 1.568)	1.472 (1.383 to 1.569)	1.471 (1.372 to 1.563)
Low back pain	Agriculture, animal husbandry and forestry workers, fishermen and hunters	Morbidity	Both								3.789 (2.58 to 5.376)	3.762 (2.621 to 5.284)	3.869 (2.642 to 5.486)
Low back pain	Production and related workers, transport equipment operators and labourers	Morbidity	Both								1.543 (1.409 to 1.679)	1.54 (1.406 to 1.676)	1.542 (1.415 to 1.677)
Low back pain	Background	Morbidity	Both								1.0 (1.0 to 1.0)	1.0 (1.0 to 1.0)	1.0 (1.0 to 1.0)
**Non-exclusive breastfeeding**													
Diarrhoeal diseases	None	Both	Both			3.605 (2.716 to 4.703)	3.605 (2.716 to 4.703)						
Diarrhoeal diseases	Partial	Both	Both			2.633 (1.942 to 3.481)	2.633 (1.942 to 3.481)						

Ages													
30-34 years	35-39 years	40-44 years	45-49 years	50-54 years	55-59 years	60-64 years	65-69 years	70-74 years	75-79 years	80-84 years	85-89 years	90-94 years	95+ years
1.514 (1.109 to 2.048)	1.509 (1.101 to 1.969)	1.513 (1.128 to 2.026)	1.508 (1.108 to 2.0)	1.526 (1.108 to 2.07)	1.519 (1.117 to 1.981)	1.53 (1.105 to 2.017)	1.519 (1.107 to 2.024)	1.518 (1.115 to 2.018)	1.502 (1.094 to 2.03)				
1.966 (1.571 to 2.396)	1.963 (1.601 to 2.395)	1.959 (1.588 to 2.385)	1.956 (1.595 to 2.406)	1.964 (1.57 to 2.397)	1.969 (1.616 to 2.417)	1.954 (1.574 to 2.406)	1.965 (1.558 to 2.393)	1.955 (1.577 to 2.39)	1.953 (1.551 to 2.418)				
1.952 (1.567 to 2.371)	1.959 (1.575 to 2.416)	1.959 (1.567 to 2.417)	1.962 (1.585 to 2.386)	1.955 (1.591 to 2.393)	1.948 (1.57 to 2.381)	1.964 (1.597 to 2.409)	1.965 (1.59 to 2.414)	1.959 (1.588 to 2.39)	1.973 (1.586 to 2.392)				
1.31 (1.218 to 1.398)	1.312 (1.225 to 1.404)	1.311 (1.225 to 1.401)	1.313 (1.226 to 1.402)	1.31 (1.223 to 1.398)	1.314 (1.221 to 1.396)	1.313 (1.224 to 1.408)	1.311 (1.228 to 1.397)	1.312 (1.225 to 1.409)	1.311 (1.226 to 1.407)				
1.221 (1.138 to 1.313)	1.221 (1.137 to 1.317)	1.221 (1.132 to 1.314)	1.22 (1.132 to 1.312)	1.22 (1.134 to 1.313)	1.221 (1.14 to 1.315)	1.223 (1.139 to 1.309)	1.221 (1.133 to 1.312)	1.224 (1.135 to 1.316)	1.22 (1.133 to 1.313)				
1.562 (1.472 to 1.658)	1.561 (1.473 to 1.65)	1.561 (1.471 to 1.655)	1.56 (1.471 to 1.649)	1.562 (1.473 to 1.654)	1.558 (1.461 to 1.655)	1.562 (1.479 to 1.655)	1.559 (1.475 to 1.652)	1.562 (1.476 to 1.657)	1.561 (1.474 to 1.654)				
1.329 (1.271 to 1.388)	1.331 (1.268 to 1.392)	1.329 (1.269 to 1.39)	1.33 (1.269 to 1.391)	1.332 (1.272 to 1.394)	1.331 (1.273 to 1.389)	1.331 (1.272 to 1.391)	1.33 (1.268 to 1.392)	1.33 (1.268 to 1.394)	1.331 (1.272 to 1.39)				
1.532 (1.413 to 1.653)	1.529 (1.424 to 1.648)	1.529 (1.416 to 1.645)	1.533 (1.418 to 1.646)	1.535 (1.427 to 1.649)	1.531 (1.416 to 1.655)	1.528 (1.411 to 1.651)	1.53 (1.416 to 1.652)	1.53 (1.411 to 1.658)	1.531 (1.415 to 1.655)				
1.41 (1.356 to 1.464)	1.409 (1.353 to 1.463)	1.409 (1.354 to 1.467)	1.41 (1.354 to 1.467)	1.411 (1.357 to 1.464)	1.409 (1.357 to 1.46)	1.411 (1.355 to 1.466)	1.41 (1.354 to 1.465)	1.41 (1.357 to 1.467)	1.411 (1.357 to 1.464)				
1.0 (1.0 to 1.0)	1.0 (1.0 to 1.0)	1.0 (1.0 to 1.0)	1.0 (1.0 to 1.0)	1.0 (1.0 to 1.0)	1.0 (1.0 to 1.0)	1.0 (1.0 to 1.0)	1.0 (1.0 to 1.0)	1.0 (1.0 to 1.0)	1.0 (1.0 to 1.0)				
1.0 (1.0 to 1.0)	1.0 (1.0 to 1.0)	1.0 (1.0 to 1.0)	1.0 (1.0 to 1.0)	1.0 (1.0 to 1.0)	1.0 (1.0 to 1.0)	1.0 (1.0 to 1.0)	1.0 (1.0 to 1.0)	1.0 (1.0 to 1.0)	1.0 (1.0 to 1.0)				
2.391 (1.455 to 3.756)	2.387 (1.409 to 3.791)	2.379 (1.4 to 3.672)	2.409 (1.439 to 3.833)	2.361 (1.447 to 3.684)	2.367 (1.454 to 3.654)	2.373 (1.375 to 3.64)	2.357 (1.438 to 3.685)	2.402 (1.434 to 3.763)	2.398 (1.441 to 3.813)	2.359 (1.429 to 3.695)	2.359 (1.429 to 3.695)	2.359 (1.429 to 3.695)	2.359 (1.429 to 3.695)
2.377 (1.431 to 3.763)	2.375 (1.446 to 3.698)	2.326 (1.438 to 3.576)	2.35 (1.432 to 3.702)	2.35 (1.425 to 3.609)	2.364 (1.461 to 3.677)	2.364 (1.475 to 3.64)	2.395 (1.476 to 3.715)	2.336 (1.434 to 3.673)	2.363 (1.431 to 3.741)	2.404 (1.462 to 3.619)	2.404 (1.462 to 3.619)	2.404 (1.462 to 3.619)	2.404 (1.462 to 3.619)
1.45 (1.067 to 1.947)	1.452 (1.06 to 1.963)	1.454 (1.075 to 1.96)	1.446 (1.055 to 1.929)	1.457 (1.076 to 1.933)	1.453 (1.052 to 1.903)	1.46 (1.067 to 1.954)	1.443 (1.057 to 1.916)	1.462 (1.097 to 1.954)	1.464 (1.079 to 1.952)	1.462 (1.096 to 1.965)	1.462 (1.096 to 1.965)	1.462 (1.096 to 1.965)	1.462 (1.096 to 1.965)
1.459 (1.072 to 1.931)	1.456 (1.056 to 1.968)	1.47 (1.089 to 1.96)	1.44 (1.046 to 1.932)	1.457 (1.097 to 1.925)	1.455 (1.061 to 1.921)	1.451 (1.082 to 1.912)	1.459 (1.102 to 1.941)	1.448 (1.077 to 1.929)	1.467 (1.072 to 1.971)	1.456 (1.056 to 1.927)	1.456 (1.056 to 1.927)	1.456 (1.056 to 1.927)	1.456 (1.056 to 1.927)
1.0 (1.0 to 1.0)	1.0 (1.0 to 1.0)	1.0 (1.0 to 1.0)	1.0 (1.0 to 1.0)	1.0 (1.0 to 1.0)	1.0 (1.0 to 1.0)	1.0 (1.0 to 1.0)	1.0 (1.0 to 1.0)	1.0 (1.0 to 1.0)	1.0 (1.0 to 1.0)	1.0 (1.0 to 1.0)	1.0 (1.0 to 1.0)	1.0 (1.0 to 1.0)	1.0 (1.0 to 1.0)
1.0 (1.0 to 1.0)	1.0 (1.0 to 1.0)	1.0 (1.0 to 1.0)	1.0 (1.0 to 1.0)	1.0 (1.0 to 1.0)	1.0 (1.0 to 1.0)	1.0 (1.0 to 1.0)	1.0 (1.0 to 1.0)	1.0 (1.0 to 1.0)	1.0 (1.0 to 1.0)	1.0 (1.0 to 1.0)	1.0 (1.0 to 1.0)	1.0 (1.0 to 1.0)	1.0 (1.0 to 1.0)
1.17 (1.062 to 1.284)	1.17 (1.062 to 1.285)	1.172 (1.062 to 1.283)	1.171 (1.071 to 1.27)	1.169 (1.063 to 1.288)	1.171 (1.059 to 1.281)	1.17 (1.058 to 1.286)	1.17 (1.07 to 1.279)	1.172 (1.065 to 1.287)	1.172 (1.07 to 1.283)				
1.209 (0.963 to 1.524)	1.207 (0.976 to 1.496)	1.207 (0.965 to 1.5)	1.205 (0.946 to 1.489)	1.205 (0.967 to 1.472)	1.205 (0.961 to 1.509)	1.203 (0.948 to 1.515)	1.209 (0.976 to 1.479)	1.21 (0.964 to 1.49)	1.203 (0.961 to 1.501)				
1.0 (1.0 to 1.0)	1.0 (1.0 to 1.0)	1.0 (1.0 to 1.0)	1.0 (1.0 to 1.0)	1.0 (1.0 to 1.0)	1.0 (1.0 to 1.0)	1.0 (1.0 to 1.0)	1.0 (1.0 to 1.0)	1.0 (1.0 to 1.0)	1.0 (1.0 to 1.0)				
1.214 (1.004 to 1.448)	1.207 (1.017 to 1.445)	1.218 (1.016 to 1.455)	1.212 (1.012 to 1.444)	1.216 (1.01 to 1.448)	1.219 (1.019 to 1.45)	1.211 (1.014 to 1.444)	1.213 (1.015 to 1.455)	1.21 (1.007 to 1.423)	1.214 (1.017 to 1.446)				
1.472 (1.382 to 1.571)	1.469 (1.378 to 1.567)	1.472 (1.377 to 1.57)	1.469 (1.375 to 1.568)	1.47 (1.378 to 1.57)	1.472 (1.379 to 1.575)	1.472 (1.381 to 1.572)	1.474 (1.386 to 1.571)	1.47 (1.377 to 1.568)	1.472 (1.38 to 1.571)				
3.775 (2.569 to 5.369)	3.774 (2.606 to 5.314)	3.771 (2.532 to 5.317)	3.793 (2.632 to 5.361)	3.785 (2.556 to 5.333)	3.776 (2.645 to 5.157)	3.792 (2.536 to 5.421)	3.802 (2.684 to 5.428)	3.746 (2.609 to 5.175)	3.77 (2.635 to 5.151)				
1.543 (1.413 to 1.695)	1.542 (1.416 to 1.685)	1.543 (1.418 to 1.685)	1.541 (1.402 to 1.684)	1.542 (1.41 to 1.684)	1.541 (1.404 to 1.683)	1.54 (1.414 to 1.677)	1.54 (1.408 to 1.683)	1.538 (1.408 to 1.673)	1.541 (1.41 to 1.677)				
1.0 (1.0 to 1.0)	1.0 (1.0 to 1.0)	1.0 (1.0 to 1.0)	1.0 (1.0 to 1.0)	1.0 (1.0 to 1.0)	1.0 (1.0 to 1.0)	1.0 (1.0 to 1.0)	1.0 (1.0 to 1.0)	1.0 (1.0 to 1.0)	1.0 (1.0 to 1.0)				

Appendix Table 1a. Relative risks used by age and sex for each outcome for all risk factors except for ambient air pollution and alcohol.

Risk - Outcome	Category / Units	Morbidity / Mortality	Sex	All-age	0-6 days	7-27 days	28-364 days	1-4 years	5-9 years	10-14 years	15-19 years	20-24 years	25-29 years
Diarrhoeal diseases	Predominant	Both	Both			2.346 (1.667 to 3.234)	2.346 (1.667 to 3.234)						
Diarrhoeal diseases	Exclusive	Both	Both			1.0 (1.0 to 1.0)	1.0 (1.0 to 1.0)						
Lower respiratory infections	None	Both	Both			1.739 (1.493 to 2.025)	1.739 (1.493 to 2.025)						
Lower respiratory infections	Partial	Both	Both			1.483 (1.206 to 1.792)	1.483 (1.206 to 1.792)						
Lower respiratory infections	Predominant	Both	Both			1.369 (1.055 to 1.8)	1.369 (1.055 to 1.8)						
Lower respiratory infections	Exclusive	Both	Both			1.0 (1.0 to 1.0)	1.0 (1.0 to 1.0)						
**Discontinued breastfeeding**													
Diarrhoeal diseases	Continued	Both	Both				1.313 (1.111 to 1.549)	1.313 (1.111 to 1.549)					
Diarrhoeal diseases	Not continued	Both	Both				1.0 (1.0 to 1.0)	1.0 (1.0 to 1.0)					
**Child underweight**													
Diarrhoeal diseases	<-3 sd	Both	Both				2.332 (2.076 to 2.802)	2.332 (2.076 to 2.802)					
Diarrhoeal diseases	<-2 sd	Both	Both				1.23 (1.163 to 1.314)	1.23 (1.163 to 1.314)					
Diarrhoeal diseases	<-1 sd	Both	Both				1.088 (1.046 to 1.134)	1.088 (1.046 to 1.134)					
Diarrhoeal diseases	-1 sd and above	Both	Both				1.0 (1.0 to 1.0)	1.0 (1.0 to 1.0)					
Lower respiratory infections	<-3 sd	Both	Both				2.593 (1.908 to 4.39)	2.593 (1.908 to 4.39)					
Lower respiratory infections	<-2 sd	Both	Both				1.365 (1.215 to 1.755)	1.365 (1.215 to 1.755)					
Lower respiratory infections	<-1 sd	Both	Both				1.145 (1.044 to 1.364)	1.145 (1.044 to 1.364)					
Lower respiratory infections	-1 sd and above	Both	Both				1.0 (1.0 to 1.0)	1.0 (1.0 to 1.0)					
Measles	<-3 sd	Both	Both				5.668 (1.767 to 12.414)	5.668 (1.767 to 12.414)					
Measles	<-2 sd	Both	Both				2.458 (1.26 to 5.118)	2.458 (1.26 to 5.118)					
Measles	<-1 sd	Both	Both				0.995 (0.5 to 1.726)	0.995 (0.5 to 1.726)					
Measles	-1 sd and above	Both	Both				1.0 (1.0 to 1.0)	1.0 (1.0 to 1.0)					
**Child wasting**													
Diarrhoeal diseases	<-3 sd	Both	Both				105.759 (42.198 to 157.813)	105.759 (42.198 to 157.813)					
Diarrhoeal diseases	<-2 sd	Both	Both				23.261 (9.02 to 35.845)	23.261 (9.02 to 35.845)					
Diarrhoeal diseases	<-1 sd	Both	Both				6.601 (2.158 to 11.243)	6.601 (2.158 to 11.243)					
Diarrhoeal diseases	-1 sd and above	Both	Both				1.0 (1.0 to 1.0)	1.0 (1.0 to 1.0)					
Lower respiratory infections	<-3 sd	Both	Both				47.67 (15.923 to 94.874)	47.67 (15.923 to 94.874)					
Lower respiratory infections	<-2 sd	Both	Both				20.455 (7.084 to 37.929)	20.455 (7.084 to 37.929)					
Lower respiratory infections	<-1 sd	Both	Both				5.941 (1.972 to 11.992)	5.941 (1.972 to 11.992)					

Ages													
**30-34 years**	**35-39 years**	**40-44 years**	**45-49 years**	**50-54 years**	**55-59 years**	**60-64 years**	**65-69 years**	**70-74 years**	**75-79 years**	**80-84 years**	**85-89 years**	**90-94 years**	**95+ years**

Appendix Table 1a. Relative risks used by age and sex for each outcome for all risk factors except for ambient air pollution and alcohol.

Risk - Outcome	Category / Units	Morbidity / Mortality	Sex	All-age	0-6 days	7-27 days	28-364 days	1-4 years	5-9 years	10-14 years	15-19 years	20-24 years	25-29 years
Lower respiratory infections	-1 sd and above	Both	Both				1.0 (1.0 to 1.0)	1.0 (1.0 to 1.0)					
Measles	<-3 sd	Both	Both				37.936 (5.088 to 199.126)	37.936 (5.088 to 199.126)					
Measles	<-2 sd	Both	Both				8.477 (1.33 to 42.777)	8.477 (1.33 to 42.777)					
Measles	<-1 sd	Both	Both				1.833 (0.569 to 8.985)	1.833 (0.569 to 8.985)					
Measles	-1 sd and above	Both	Both				1.0 (1.0 to 1.0)	1.0 (1.0 to 1.0)					
**Child stunting**													
Diarrhoeal diseases	<-3 sd	Both	Both				1.851 (1.28 to 2.699)	1.851 (1.28 to 2.699)					
Diarrhoeal diseases	<-2 sd	Both	Both				1.222 (1.067 to 1.5)	1.222 (1.067 to 1.5)					
Diarrhoeal diseases	<-1 sd	Both	Both				1.111 (1.023 to 1.273)	1.111 (1.023 to 1.273)					
Diarrhoeal diseases	-1 sd and above	Both	Both				1.0 (1.0 to 1.0)	1.0 (1.0 to 1.0)					
Lower respiratory infections	<-3 sd	Both	Both				2.355 (1.15 to 5.114)	2.355 (1.15 to 5.114)					
Lower respiratory infections	<-2 sd	Both	Both				1.318 (1.014 to 2.165)	1.318 (1.014 to 2.165)					
Lower respiratory infections	<-1 sd	Both	Both				1.158 (0.998 to 1.655)	1.158 (0.998 to 1.655)					
Lower respiratory infections	-1 sd and above	Both	Both				1.0 (1.0 to 1.0)	1.0 (1.0 to 1.0)					
Measles	<-3 sd	Both	Both				2.487 (1.129 to 6.528)	2.487 (1.129 to 6.528)					
Measles	<-2 sd	Both	Both				1.54 (1.029 to 3.222)	1.54 (1.029 to 3.222)					
Measles	<-1 sd	Both	Both				1.103 (0.861 to 1.719)	1.103 (0.861 to 1.719)					
Measles	-1 sd and above	Both	Both				1.0 (1.0 to 1.0)	1.0 (1.0 to 1.0)					
**Low birth weight and short gestation**													
Diarrhoeal diseases	Birth prevalence - [22, 24) wks, [0, 500) g	Mortality	Males		3273.944 (2987.306 to 3600.961)	599.42 (512.777 to 692.914)							
Diarrhoeal diseases	Birth prevalence - [22, 24) wks, [0, 500) g	Mortality	Females		3353.667 (2964.862 to 3798.637)	771.075 (635.721 to 931.62)							
Diarrhoeal diseases	Birth prevalence - [24, 26) wks, [0, 500) g	Mortality	Males		2454.576 (2216.544 to 2713.168)	884.902 (766.03 to 1021.494)							
Diarrhoeal diseases	Birth prevalence - [24, 26) wks, [0, 500) g	Mortality	Females		2376.85 (2080.354 to 2699.254)	915.957 (765.805 to 1079.243)							
Diarrhoeal diseases	Birth prevalence - [26, 28) wks, [0, 500) g	Mortality	Males		1870.684 (1676.978 to 2075.277)	847.512 (727.279 to 983.252)							
Diarrhoeal diseases	Birth prevalence - [26, 28) wks, [0, 500) g	Mortality	Females		1708.132 (1477.512 to 1960.75)	822.906 (677.101 to 990.491)							
Diarrhoeal diseases	Birth prevalence - [28, 30) wks, [0, 500) g	Mortality	Males		1805.939 (1612.157 to 2006.088)	583.881 (485.096 to 690.434)							
Diarrhoeal diseases	Birth prevalence - [28, 30) wks, [0, 500) g	Mortality	Females		1585.788 (1377.653 to 1836.365)	636.164 (520.511 to 760.561)							
Diarrhoeal diseases	Birth prevalence - [22, 24) wks, [500, 1000) g	Mortality	Males		2037.627 (1843.599 to 2249.686)	720.117 (628.641 to 820.917)							
Diarrhoeal diseases	Birth prevalence - [22, 24) wks, [500, 1000) g	Mortality	Females		1833.266 (1596.246 to 2105.971)	610.368 (510.239 to 722.278)							
Diarrhoeal diseases	Birth prevalence - [24, 26) wks, [500, 1000) g	Mortality	Males		852.585 (753.604 to 959.026)	642.627 (558.091 to 730.626)							

Ages													
**30-34 years**	**35-39 years**	**40-44 years**	**45-49 years**	**50-54 years**	**55-59 years**	**60-64 years**	**65-69 years**	**70-74 years**	**75-79 years**	**80-84 years**	**85-89 years**	**90-94 years**	**95+ years**

Appendix Table 1a. Relative risks used by age and sex for each outcome for all risk factors except for ambient air pollution and alcohol.

Risk - Outcome	Category / Units	Morbidity / Mortality	Sex	All-age	0-6 days	7-27 days	28-364 days	1-4 years	5-9 years	10-14 years	15-19 years	20-24 years	25-29 years
Diarrhoeal diseases	Birth prevalence - [24, 26) wks, [500, 1000) g	Mortality	Females		715.379 (610.81 to 830.296)	546.057 (463.282 to 637.815)							
Diarrhoeal diseases	Birth prevalence - [26, 28) wks, [500, 1000) g	Mortality	Males		411.87 (365.037 to 467.411)	353.266 (305.647 to 405.904)							
Diarrhoeal diseases	Birth prevalence - [26, 28) wks, [500, 1000) g	Mortality	Females		308.092 (262.47 to 362.302)	292.936 (243.986 to 349.019)							
Diarrhoeal diseases	Birth prevalence - [32, 34) wks, [500, 1000) g	Mortality	Males		344.501 (301.197 to 397.343)	194.954 (163.844 to 231.771)							
Diarrhoeal diseases	Birth prevalence - [32, 34) wks, [500, 1000) g	Mortality	Females		299.264 (251.468 to 353.995)	161.548 (130.866 to 196.323)							
Diarrhoeal diseases	Birth prevalence - [22, 24) wks, [1000, 1500) g	Mortality	Males		673.302 (584.187 to 768.258)	213.181 (179.555 to 254.572)							
Diarrhoeal diseases	Birth prevalence - [22, 24) wks, [1000, 1500) g	Mortality	Females		620.446 (522.864 to 734.748)	186.215 (149.772 to 227.046)							
Diarrhoeal diseases	Birth prevalence - [30, 32) wks, [500, 1000) g	Mortality	Males		332.681 (293.52 to 377.86)	212.332 (181.213 to 245.271)							
Diarrhoeal diseases	Birth prevalence - [30, 32) wks, [500, 1000) g	Mortality	Females		239.082 (200.416 to 284.597)	163.95 (135.165 to 197.261)							
Diarrhoeal diseases	Birth prevalence - [28, 30) wks, [500, 1000) g	Mortality	Males		326.557 (286.01 to 366.811)	249.246 (216.248 to 289.232)							
Diarrhoeal diseases	Birth prevalence - [28, 30) wks, [500, 1000) g	Mortality	Females		231.533 (194.62 to 271.396)	187.47 (157.953 to 224.552)							
Diarrhoeal diseases	Birth prevalence - [24, 26) wks, [1000, 1500) g	Mortality	Males		273.536 (236.795 to 312.421)	182.743 (155.477 to 212.281)							
Diarrhoeal diseases	Birth prevalence - [24, 26) wks, [1000, 1500) g	Mortality	Females		268.033 (223.95 to 318.815)	157.928 (130.936 to 190.298)							
Diarrhoeal diseases	Birth prevalence - [26, 28) wks, [1000, 1500) g	Mortality	Males		164.685 (145.457 to 186.571)	120.878 (102.672 to 140.967)							
Diarrhoeal diseases	Birth prevalence - [26, 28) wks, [1000, 1500) g	Mortality	Females		142.274 (121.33 to 168.246)	97.496 (80.858 to 116.625)							
Diarrhoeal diseases	Birth prevalence - [26, 28) wks, [1500, 2000) g	Mortality	Males		113.21 (97.079 to 130.806)	49.449 (40.908 to 59.113)							
Diarrhoeal diseases	Birth prevalence - [26, 28) wks, [1500, 2000) g	Mortality	Females		108.393 (89.864 to 129.883)	49.979 (40.484 to 60.784)							
Diarrhoeal diseases	Birth prevalence - [34, 36) wks, [1000, 1500) g	Mortality	Males		119.602 (104.655 to 136.729)	56.519 (48.084 to 66.018)							
Diarrhoeal diseases	Birth prevalence - [34, 36) wks, [1000, 1500) g	Mortality	Females		109.231 (91.177 to 128.378)	52.834 (43.454 to 63.459)							
Diarrhoeal diseases	Birth prevalence - [28, 30) wks, [1500, 2000) g	Mortality	Males		87.285 (76.012 to 100.83)	37.15 (30.87 to 43.664)							
Diarrhoeal diseases	Birth prevalence - [28, 30) wks, [1500, 2000) g	Mortality	Females		86.639 (71.639 to 103.632)	38.3 (31.326 to 45.531)							
Diarrhoeal diseases	Birth prevalence - [28, 30) wks, [1000, 1500) g	Mortality	Males		107.077 (93.214 to 122.668)	81.407 (70.511 to 93.12)							
Diarrhoeal diseases	Birth prevalence - [28, 30) wks, [1000, 1500) g	Mortality	Females		87.409 (73.438 to 104.222)	64.981 (53.651 to 77.029)							
Diarrhoeal diseases	Birth prevalence - [32, 34) wks, [1000, 1500) g	Mortality	Males		98.982 (86.649 to 112.931)	51.159 (43.522 to 59.402)							
Diarrhoeal diseases	Birth prevalence - [32, 34) wks, [1000, 1500) g	Mortality	Females		84.081 (70.672 to 99.726)	44.695 (37.129 to 53.257)							
Diarrhoeal diseases	Birth prevalence - [30, 32) wks, [1000, 1500) g	Mortality	Males		90.239 (78.583 to 103.215)	59.239 (50.511 to 69.508)							
Diarrhoeal diseases	Birth prevalence - [30, 32) wks, [1000, 1500) g	Mortality	Females		70.119 (59.11 to 81.846)	47.493 (39.526 to 56.547)							
Diarrhoeal diseases	Birth prevalence - [37, 38) wks, [1500, 2000) g	Mortality	Males		55.659 (48.726 to 62.963)	25.45 (21.867 to 29.451)							
Diarrhoeal diseases	Birth prevalence - [37, 38) wks, [1500, 2000) g	Mortality	Females		54.453 (46.308 to 63.524)	29.339 (24.736 to 34.558)							
Diarrhoeal diseases	Birth prevalence - [36, 37) wks, [1500, 2000) g	Mortality	Males		53.885 (47.099 to 61.414)	23.622 (20.373 to 27.298)							

Ages													
30-34 years	35-39 years	40-44 years	45-49 years	50-54 years	55-59 years	60-64 years	65-69 years	70-74 years	75-79 years	80-84 years	85-89 years	90-94 years	95+ years

Appendix Table 1a. Relative risks used by age and sex for each outcome for all risk factors except for ambient air pollution and alcohol.

Risk - Outcome	Category / Units	Morbidity / Mortality	Sex	All-age	0-6 days	7-27 days	28-364 days	1-4 years	5-9 years	10-14 years	15-19 years	20-24 years	25-29 years
Diarrhoeal diseases	Birth prevalence - [36, 37) wks, [1500, 2000) g	Mortality	Females		47.995 (40.82 to 56.378)	23.905 (19.8 to 29.023)							
Diarrhoeal diseases	Birth prevalence - [30, 32) wks, [2000, 2500) g	Mortality	Males		39.701 (34.573 to 45.311)	16.391 (13.683 to 19.411)							
Diarrhoeal diseases	Birth prevalence - [30, 32) wks, [2000, 2500) g	Mortality	Females		34.894 (28.953 to 41.934)	16.549 (13.438 to 19.916)							
Diarrhoeal diseases	Birth prevalence - [30, 32) wks, [1500, 2000) g	Mortality	Males		55.142 (48.379 to 62.989)	26.391 (22.751 to 30.584)							
Diarrhoeal diseases	Birth prevalence - [30, 32) wks, [1500, 2000) g	Mortality	Females		48.414 (40.924 to 57.128)	26.986 (22.299 to 32.305)							
Diarrhoeal diseases	Birth prevalence - [34, 36) wks, [1500, 2000) g	Mortality	Males		45.579 (39.817 to 51.922)	19.836 (16.94 to 23.141)							
Diarrhoeal diseases	Birth prevalence - [34, 36) wks, [1500, 2000) g	Mortality	Females		38.405 (32.273 to 45.04)	18.409 (15.134 to 21.755)							
Diarrhoeal diseases	Birth prevalence - [32, 34) wks, [1500, 2000) g	Mortality	Males		41.734 (36.381 to 47.516)	19.083 (16.193 to 21.909)							
Diarrhoeal diseases	Birth prevalence - [32, 34) wks, [1500, 2000) g	Mortality	Females		36.162 (30.231 to 42.934)	19.528 (16.278 to 23.535)							
Diarrhoeal diseases	Birth prevalence - [32, 34) wks, [2000, 2500) g	Mortality	Males		22.379 (19.576 to 25.426)	11.555 (9.874 to 13.332)							
Diarrhoeal diseases	Birth prevalence - [32, 34) wks, [2000, 2500) g	Mortality	Females		20.326 (17.055 to 23.945)	12.376 (10.192 to 14.834)							
Diarrhoeal diseases	Birth prevalence - [40, 42) wks, [2000, 2500) g	Mortality	Males		17.218 (14.893 to 19.961)	11.519 (9.766 to 13.495)							
Diarrhoeal diseases	Birth prevalence - [40, 42) wks, [2000, 2500) g	Mortality	Females		13.555 (11.337 to 16.132)	11.838 (9.792 to 14.338)							
Diarrhoeal diseases	Birth prevalence - [38, 40) wks, [2000, 2500) g	Mortality	Males		14.227 (12.521 to 16.06)	10.475 (9.122 to 12.085)							
Diarrhoeal diseases	Birth prevalence - [38, 40) wks, [2000, 2500) g	Mortality	Females		10.867 (9.199 to 12.777)	10.493 (8.801 to 12.342)							
Diarrhoeal diseases	Birth prevalence - [32, 34) wks, [2500, 3000) g	Mortality	Males		12.775 (11.146 to 14.607)	6.296 (5.269 to 7.299)							
Diarrhoeal diseases	Birth prevalence - [32, 34) wks, [2500, 3000) g	Mortality	Females		9.041 (7.504 to 10.706)	6.205 (5.041 to 7.52)							
Diarrhoeal diseases	Birth prevalence - [34, 36) wks, [2000, 2500) g	Mortality	Males		16.472 (14.422 to 18.7)	9.777 (8.456 to 11.277)							
Diarrhoeal diseases	Birth prevalence - [34, 36) wks, [2000, 2500) g	Mortality	Females		12.895 (10.992 to 15.284)	9.44 (7.865 to 11.209)							
Diarrhoeal diseases	Birth prevalence - [37, 38) wks, [2000, 2500) g	Mortality	Males		14.003 (12.407 to 15.831)	9.977 (8.758 to 11.444)							
Diarrhoeal diseases	Birth prevalence - [37, 38) wks, [2000, 2500) g	Mortality	Females		10.456 (8.899 to 12.149)	9.83 (8.317 to 11.54)							
Diarrhoeal diseases	Birth prevalence - [36, 37) wks, [2000, 2500) g	Mortality	Males		14.873 (13.083 to 16.88)	9.756 (8.38 to 11.214)							
Diarrhoeal diseases	Birth prevalence - [36, 37) wks, [2000, 2500) g	Mortality	Females		10.517 (8.897 to 12.379)	9.233 (7.691 to 10.92)							
Diarrhoeal diseases	Birth prevalence - [34, 36) wks, [2500, 3000) g	Mortality	Males		8.216 (7.133 to 9.378)	5.419 (4.631 to 6.282)							
Diarrhoeal diseases	Birth prevalence - [34, 36) wks, [2500, 3000) g	Mortality	Females		6.037 (5.044 to 7.094)	5.198 (4.31 to 6.293)							
Diarrhoeal diseases	Birth prevalence - [34, 36) wks, [4000, 4500) g	Mortality	Males		2.68 (2.245 to 3.183)	1.532 (1.254 to 1.867)							
Diarrhoeal diseases	Birth prevalence - [34, 36) wks, [4000, 4500) g	Mortality	Females		2.458 (1.967 to 3.074)	1.673 (1.344 to 2.093)							
Diarrhoeal diseases	Birth prevalence - [34, 36) wks, [3000, 3500) g	Mortality	Males		3.961 (3.433 to 4.521)	3.194 (2.71 to 3.696)							
Diarrhoeal diseases	Birth prevalence - [34, 36) wks, [3000, 3500) g	Mortality	Females		3.267 (2.738 to 3.876)	2.783 (2.273 to 3.408)							
Diarrhoeal diseases	Birth prevalence - [36, 37) wks, [2500, 3000) g	Mortality	Males		5.363 (4.69 to 6.082)	4.559 (3.945 to 5.248)							

Ages													
30-34 years	35-39 years	40-44 years	45-49 years	50-54 years	55-59 years	60-64 years	65-69 years	70-74 years	75-79 years	80-84 years	85-89 years	90-94 years	95+ years

Appendix Table 1a. Relative risks used by age and sex for each outcome for all risk factors except for ambient air pollution and alcohol.

Risk - Outcome	Category / Units	Morbidity / Mortality	Sex	All-age	0-6 days	7-27 days	28-364 days	1-4 years	5-9 years	10-14 years	15-19 years	20-24 years	25-29 years
Diarrhoeal diseases	Birth prevalence - [36, 37) wks, [2500, 3000) g	Mortality	Females		3.969 (3.371 to 4.675)	4.138 (3.469 to 4.868)							
Diarrhoeal diseases	Birth prevalence - [34, 36) wks, [3500, 4000) g	Mortality	Males		2.678 (2.289 to 3.086)	2.014 (1.69 to 2.365)							
Diarrhoeal diseases	Birth prevalence - [34, 36) wks, [3500, 4000) g	Mortality	Females		2.342 (1.908 to 2.845)	1.756 (1.426 to 2.126)							
Diarrhoeal diseases	Birth prevalence - [37, 38) wks, [2500, 3000) g	Mortality	Males		4.093 (3.581 to 4.626)	4.139 (3.631 to 4.74)							
Diarrhoeal diseases	Birth prevalence - [37, 38) wks, [2500, 3000) g	Mortality	Females		3.07 (2.612 to 3.603)	3.618 (3.045 to 4.25)							
Diarrhoeal diseases	Birth prevalence - [40, 42) wks, [2500, 3000) g	Mortality	Males		3.833 (3.364 to 4.388)	3.828 (3.271 to 4.426)							
Diarrhoeal diseases	Birth prevalence - [40, 42) wks, [2500, 3000) g	Mortality	Females		2.699 (2.264 to 3.202)	3.379 (2.825 to 4.042)							
Diarrhoeal diseases	Birth prevalence - [38, 40) wks, [2500, 3000) g	Mortality	Males		3.396 (3.002 to 3.839)	3.858 (3.346 to 4.424)							
Diarrhoeal diseases	Birth prevalence - [38, 40) wks, [2500, 3000) g	Mortality	Females		2.382 (2.04 to 2.802)	3.27 (2.774 to 3.927)							
Diarrhoeal diseases	Birth prevalence - [36, 37) wks, [3000, 3500) g	Mortality	Males		2.819 (2.433 to 3.229)	2.683 (2.299 to 3.079)							
Diarrhoeal diseases	Birth prevalence - [36, 37) wks, [3000, 3500) g	Mortality	Females		2.335 (1.952 to 2.784)	2.434 (2.007 to 2.884)							
Diarrhoeal diseases	Birth prevalence - [36, 37) wks, [4000, 4500) g	Mortality	Males		2.331 (1.993 to 2.727)	1.471 (1.221 to 1.731)							
Diarrhoeal diseases	Birth prevalence - [36, 37) wks, [4000, 4500) g	Mortality	Females		2.141 (1.729 to 2.577)	1.601 (1.291 to 1.97)							
Diarrhoeal diseases	Birth prevalence - [36, 37) wks, [3500, 4000) g	Mortality	Males		2.084 (1.789 to 2.418)	1.923 (1.624 to 2.252)							
Diarrhoeal diseases	Birth prevalence - [36, 37) wks, [3500, 4000) g	Mortality	Females		1.845 (1.527 to 2.216)	1.731 (1.426 to 2.071)							
Diarrhoeal diseases	Birth prevalence - [37, 38) wks, [3000, 3500) g	Mortality	Males		2.027 (1.788 to 2.287)	2.236 (1.926 to 2.542)							
Diarrhoeal diseases	Birth prevalence - [37, 38) wks, [3000, 3500) g	Mortality	Females		1.667 (1.411 to 1.947)	1.989 (1.672 to 2.366)							
Diarrhoeal diseases	Birth prevalence - [37, 38) wks, [4000, 4500) g	Mortality	Males		1.572 (1.352 to 1.813)	1.28 (1.095 to 1.481)							
Diarrhoeal diseases	Birth prevalence - [37, 38) wks, [4000, 4500) g	Mortality	Females		1.647 (1.37 to 1.98)	1.396 (1.167 to 1.674)							
Diarrhoeal diseases	Birth prevalence - [37, 38) wks, [3500, 4000) g	Mortality	Males		1.502 (1.309 to 1.712)	1.563 (1.353 to 1.807)							
Diarrhoeal diseases	Birth prevalence - [37, 38) wks, [3500, 4000) g	Mortality	Females		1.275 (1.075 to 1.501)	1.444 (1.206 to 1.711)							
Diarrhoeal diseases	Birth prevalence - [40, 42) wks, [3000, 3500) g	Mortality	Males		1.446 (1.258 to 1.639)	1.79 (1.546 to 2.08)							
Diarrhoeal diseases	Birth prevalence - [40, 42) wks, [3000, 3500) g	Mortality	Females		1.103 (0.922 to 1.305)	1.491 (1.233 to 1.776)							
Diarrhoeal diseases	Birth prevalence - [38, 40) wks, [3000, 3500) g	Mortality	Males		1.414 (1.242 to 1.593)	1.874 (1.636 to 2.156)							
Diarrhoeal diseases	Birth prevalence - [38, 40) wks, [3000, 3500) g	Mortality	Females		1.096 (0.927 to 1.289)	1.617 (1.352 to 1.907)							
Diarrhoeal diseases	Birth prevalence - [38, 40) wks, [4000, 4500) g	Mortality	Males		1.052 (0.92 to 1.203)	1.093 (0.939 to 1.261)							
Diarrhoeal diseases	Birth prevalence - [38, 40) wks, [4000, 4500) g	Mortality	Females		1.121 (0.925 to 1.31)	1.149 (0.955 to 1.367)							
Diarrhoeal diseases	Birth prevalence - [38, 40) wks, [3500, 4000) g	Mortality	Males		1.009 (0.883 to 1.143)	1.243 (1.078 to 1.421)							
Diarrhoeal diseases	Birth prevalence - [38, 40) wks, [3500, 4000) g	Mortality	Females		0.828 (0.702 to 0.972)	1.155 (0.967 to 1.369)							
Diarrhoeal diseases	Birth prevalence - [40, 42) wks, [3500, 4000) g	Mortality	Males		0.98 (0.854 to 1.123)	1.155 (0.983 to 1.334)							

Ages													
30-34 years	35-39 years	40-44 years	45-49 years	50-54 years	55-59 years	60-64 years	65-69 years	70-74 years	75-79 years	80-84 years	85-89 years	90-94 years	95+ years

Appendix Table 1a. Relative risks used by age and sex for each outcome for all risk factors except for ambient air pollution and alcohol.

Risk - Outcome	Category / Units	Morbidity / Mortality	Sex	All-age	0-6 days	7-27 days	28-364 days	1-4 years	5-9 years	10-14 years	15-19 years	20-24 years	25-29 years
Diarrhoeal diseases	Birth prevalence - [40, 42) wks, [3500, 4000) g	Mortality	Females		0.819 (0.679 to 0.974)	1.045 (0.874 to 1.26)							
Lower respiratory infections	Birth prevalence - [22, 24) wks, [0, 500) g	Mortality	Males		3273.944 (2987.306 to 3600.961)	599.42 (512.777 to 692.914)							
Lower respiratory infections	Birth prevalence - [22, 24) wks, [0, 500) g	Mortality	Females		3353.667 (2964.862 to 3798.637)	771.075 (635.721 to 931.62)							
Lower respiratory infections	Birth prevalence - [24, 26) wks, [0, 500) g	Mortality	Males		2454.576 (2216.544 to 2713.168)	884.902 (766.03 to 1021.494)							
Lower respiratory infections	Birth prevalence - [24, 26) wks, [0, 500) g	Mortality	Females		2376.85 (2080.354 to 2699.254)	915.957 (765.805 to 1079.243)							
Lower respiratory infections	Birth prevalence - [26, 28) wks, [0, 500) g	Mortality	Males		1870.684 (1676.978 to 2075.277)	847.512 (727.279 to 983.252)							
Lower respiratory infections	Birth prevalence - [26, 28) wks, [0, 500) g	Mortality	Females		1708.132 (1477.512 to 1960.75)	822.906 (677.101 to 990.491)							
Lower respiratory infections	Birth prevalence - [28, 30) wks, [0, 500) g	Mortality	Males		1805.939 (1612.157 to 2006.088)	583.881 (485.096 to 690.434)							
Lower respiratory infections	Birth prevalence - [28, 30) wks, [0, 500) g	Mortality	Females		1585.788 (1377.653 to 1836.365)	636.164 (520.511 to 760.561)							
Lower respiratory infections	Birth prevalence - [22, 24) wks, [500, 1000) g	Mortality	Males		2037.627 (1843.599 to 2249.686)	720.117 (628.641 to 820.917)							
Lower respiratory infections	Birth prevalence - [22, 24) wks, [500, 1000) g	Mortality	Females		1833.266 (1596.246 to 2105.971)	610.368 (510.239 to 722.278)							
Lower respiratory infections	Birth prevalence - [24, 26) wks, [500, 1000) g	Mortality	Males		852.585 (753.604 to 959.026)	642.627 (558.091 to 730.626)							
Lower respiratory infections	Birth prevalence - [24, 26) wks, [500, 1000) g	Mortality	Females		715.379 (610.81 to 830.296)	546.057 (463.282 to 637.815)							
Lower respiratory infections	Birth prevalence - [26, 28) wks, [500, 1000) g	Mortality	Males		411.87 (365.037 to 467.411)	353.266 (305.647 to 405.904)							
Lower respiratory infections	Birth prevalence - [26, 28) wks, [500, 1000) g	Mortality	Females		308.092 (262.47 to 362.302)	292.936 (243.986 to 349.019)							
Lower respiratory infections	Birth prevalence - [32, 34) wks, [500, 1000) g	Mortality	Males		344.501 (301.197 to 397.343)	194.954 (163.844 to 231.771)							
Lower respiratory infections	Birth prevalence - [32, 34) wks, [500, 1000) g	Mortality	Females		299.264 (251.468 to 353.995)	161.548 (130.866 to 196.323)							
Lower respiratory infections	Birth prevalence - [22, 24) wks, [1000, 1500) g	Mortality	Males		673.302 (584.187 to 768.258)	213.181 (179.555 to 254.572)							
Lower respiratory infections	Birth prevalence - [22, 24) wks, [1000, 1500) g	Mortality	Females		620.446 (522.864 to 734.748)	186.215 (149.772 to 227.046)							
Lower respiratory infections	Birth prevalence - [30, 32) wks, [500, 1000) g	Mortality	Males		332.681 (293.52 to 377.86)	212.332 (181.213 to 245.271)							
Lower respiratory infections	Birth prevalence - [30, 32) wks, [500, 1000) g	Mortality	Females		239.082 (200.416 to 284.597)	163.95 (135.165 to 197.261)							
Lower respiratory infections	Birth prevalence - [28, 30) wks, [500, 1000) g	Mortality	Males		326.557 (286.01 to 366.811)	249.246 (216.248 to 289.232)							
Lower respiratory infections	Birth prevalence - [28, 30) wks, [500, 1000) g	Mortality	Females		231.533 (194.62 to 271.396)	187.47 (157.953 to 224.552)							
Lower respiratory infections	Birth prevalence - [24, 26) wks, [1000, 1500) g	Mortality	Males		273.536 (236.795 to 312.421)	182.743 (155.477 to 212.281)							
Lower respiratory infections	Birth prevalence - [24, 26) wks, [1000, 1500) g	Mortality	Females		268.033 (223.95 to 318.815)	157.928 (130.936 to 190.298)							
Lower respiratory infections	Birth prevalence - [26, 28) wks, [1000, 1500) g	Mortality	Males		164.685 (145.457 to 186.571)	120.878 (102.672 to 140.967)							
Lower respiratory infections	Birth prevalence - [26, 28) wks, [1000, 1500) g	Mortality	Females		142.274 (121.33 to 168.246)	97.496 (80.858 to 116.625)							
Lower respiratory infections	Birth prevalence - [26, 28) wks, [1500, 2000) g	Mortality	Males		113.21 (97.079 to 130.806)	49.449 (40.908 to 59.113)							
Lower respiratory infections	Birth prevalence - [26, 28) wks, [1500, 2000) g	Mortality	Females		108.393 (89.864 to 129.883)	49.979 (40.484 to 60.784)							
Lower respiratory infections	Birth prevalence - [34, 36) wks, [1000, 1500) g	Mortality	Males		119.602 (104.655 to 136.729)	56.519 (48.084 to 66.018)							

Ages													
30-34 years	35-39 years	40-44 years	45-49 years	50-54 years	55-59 years	60-64 years	65-69 years	70-74 years	75-79 years	80-84 years	85-89 years	90-94 years	95+ years

Appendix Table 1a. Relative risks used by age and sex for each outcome for all risk factors except for ambient air pollution and alcohol.

Risk - Outcome	Category / Units	Morbidity / Mortality	Sex	All-age	0-6 days	7-27 days	28-364 days	1-4 years	5-9 years	10-14 years	15-19 years	20-24 years	25-29 years
Lower respiratory infections	Birth prevalence - [34, 36) wks, [1000, 1500) g	Mortality	Females		109.231 (91.177 to 128.378)	52.834 (43.454 to 63.459)							
Lower respiratory infections	Birth prevalence - [28, 30) wks, [1500, 2000) g	Mortality	Males		87.285 (76.012 to 100.83)	37.15 (30.87 to 43.664)							
Lower respiratory infections	Birth prevalence - [28, 30) wks, [1500, 2000) g	Mortality	Females		86.639 (71.639 to 103.632)	38.3 (31.326 to 45.531)							
Lower respiratory infections	Birth prevalence - [28, 30) wks, [1000, 1500) g	Mortality	Males		107.077 (93.214 to 122.668)	81.407 (70.511 to 93.12)							
Lower respiratory infections	Birth prevalence - [28, 30) wks, [1000, 1500) g	Mortality	Females		87.409 (73.438 to 104.222)	64.981 (53.651 to 77.029)							
Lower respiratory infections	Birth prevalence - [32, 34) wks, [1000, 1500) g	Mortality	Males		98.982 (86.649 to 112.931)	51.159 (43.522 to 59.402)							
Lower respiratory infections	Birth prevalence - [32, 34) wks, [1000, 1500) g	Mortality	Females		84.081 (70.672 to 99.726)	44.695 (37.129 to 53.257)							
Lower respiratory infections	Birth prevalence - [30, 32) wks, [1000, 1500) g	Mortality	Males		90.239 (78.583 to 103.215)	59.239 (50.511 to 69.508)							
Lower respiratory infections	Birth prevalence - [30, 32) wks, [1000, 1500) g	Mortality	Females		70.119 (59.11 to 81.846)	47.493 (39.526 to 56.547)							
Lower respiratory infections	Birth prevalence - [37, 38) wks, [1500, 2000) g	Mortality	Males		55.659 (48.726 to 62.963)	25.45 (21.867 to 29.451)							
Lower respiratory infections	Birth prevalence - [37, 38) wks, [1500, 2000) g	Mortality	Females		54.453 (46.308 to 63.524)	29.339 (24.736 to 34.558)							
Lower respiratory infections	Birth prevalence - [36, 37) wks, [1500, 2000) g	Mortality	Males		53.885 (47.099 to 61.414)	23.622 (20.373 to 27.298)							
Lower respiratory infections	Birth prevalence - [36, 37) wks, [1500, 2000) g	Mortality	Females		47.995 (40.82 to 56.378)	23.905 (19.8 to 29.023)							
Lower respiratory infections	Birth prevalence - [30, 32) wks, [2000, 2500) g	Mortality	Males		39.701 (34.573 to 45.311)	16.391 (13.683 to 19.411)							
Lower respiratory infections	Birth prevalence - [30, 32) wks, [2000, 2500) g	Mortality	Females		34.894 (28.953 to 41.934)	16.549 (13.438 to 19.916)							
Lower respiratory infections	Birth prevalence - [30, 32) wks, [1500, 2000) g	Mortality	Males		55.142 (48.379 to 62.989)	26.391 (22.751 to 30.584)							
Lower respiratory infections	Birth prevalence - [30, 32) wks, [1500, 2000) g	Mortality	Females		48.414 (40.924 to 57.128)	26.986 (22.299 to 32.305)							
Lower respiratory infections	Birth prevalence - [34, 36) wks, [1500, 2000) g	Mortality	Males		45.579 (39.817 to 51.922)	19.836 (16.94 to 23.141)							
Lower respiratory infections	Birth prevalence - [34, 36) wks, [1500, 2000) g	Mortality	Females		38.405 (32.273 to 45.04)	18.409 (15.134 to 21.755)							
Lower respiratory infections	Birth prevalence - [32, 34) wks, [1500, 2000) g	Mortality	Males		41.734 (36.381 to 47.516)	19.083 (16.193 to 21.909)							
Lower respiratory infections	Birth prevalence - [32, 34) wks, [1500, 2000) g	Mortality	Females		36.162 (30.231 to 42.934)	19.528 (16.278 to 23.535)							
Lower respiratory infections	Birth prevalence - [32, 34) wks, [2000, 2500) g	Mortality	Males		22.379 (19.576 to 25.426)	11.555 (9.874 to 13.332)							
Lower respiratory infections	Birth prevalence - [32, 34) wks, [2000, 2500) g	Mortality	Females		20.326 (17.055 to 23.945)	12.376 (10.192 to 14.834)							
Lower respiratory infections	Birth prevalence - [40, 42) wks, [2000, 2500) g	Mortality	Males		17.218 (14.893 to 19.961)	11.519 (9.766 to 13.495)							
Lower respiratory infections	Birth prevalence - [40, 42) wks, [2000, 2500) g	Mortality	Females		13.555 (11.337 to 16.132)	11.838 (9.792 to 14.338)							
Lower respiratory infections	Birth prevalence - [38, 40) wks, [2000, 2500) g	Mortality	Males		14.227 (12.521 to 16.06)	10.475 (9.122 to 12.085)							
Lower respiratory infections	Birth prevalence - [38, 40) wks, [2000, 2500) g	Mortality	Females		10.867 (9.199 to 12.777)	10.493 (8.801 to 12.342)							
Lower respiratory infections	Birth prevalence - [32, 34) wks, [2500, 3000) g	Mortality	Males		12.775 (11.146 to 14.607)	6.296 (5.269 to 7.299)							
Lower respiratory infections	Birth prevalence - [32, 34) wks, [2500, 3000) g	Mortality	Females		9.041 (7.504 to 10.706)	6.205 (5.041 to 7.52)							
Lower respiratory infections	Birth prevalence - [34, 36) wks, [2000, 2500) g	Mortality	Males		16.472 (14.422 to 18.7)	9.777 (8.456 to 11.277)							

Ages													
30-34 years	35-39 years	40-44 years	45-49 years	50-54 years	55-59 years	60-64 years	65-69 years	70-74 years	75-79 years	80-84 years	85-89 years	90-94 years	95+ years

Appendix Table 1a. Relative risks used by age and sex for each outcome for all risk factors except for ambient air pollution and alcohol.

Risk - Outcome	Category / Units	Morbidity / Mortality	Sex	All-age	0-6 days	7-27 days	28-364 days	1-4 years	5-9 years	10-14 years	15-19 years	20-24 years	25-29 years
Lower respiratory infections	Birth prevalence - [34, 36) wks, [2000, 2500) g	Mortality	Females		12.895 (10.992 to 15.284)	9.44 (7.865 to 11.209)							
Lower respiratory infections	Birth prevalence - [37, 38) wks, [2000, 2500) g	Mortality	Males		14.003 (12.407 to 15.831)	9.977 (8.758 to 11.444)							
Lower respiratory infections	Birth prevalence - [37, 38) wks, [2000, 2500) g	Mortality	Females		10.456 (8.899 to 12.149)	9.83 (8.317 to 11.54)							
Lower respiratory infections	Birth prevalence - [36, 37) wks, [2000, 2500) g	Mortality	Males		14.873 (13.083 to 16.88)	9.756 (8.38 to 11.214)							
Lower respiratory infections	Birth prevalence - [36, 37) wks, [2000, 2500) g	Mortality	Females		10.517 (8.897 to 12.379)	9.233 (7.691 to 10.92)							
Lower respiratory infections	Birth prevalence - [34, 36) wks, [2500, 3000) g	Mortality	Males		8.216 (7.133 to 9.378)	5.419 (4.631 to 6.282)							
Lower respiratory infections	Birth prevalence - [34, 36) wks, [2500, 3000) g	Mortality	Females		6.037 (5.044 to 7.094)	5.198 (4.31 to 6.293)							
Lower respiratory infections	Birth prevalence - [34, 36) wks, [4000, 4500) g	Mortality	Males		2.68 (2.245 to 3.183)	1.532 (1.254 to 1.867)							
Lower respiratory infections	Birth prevalence - [34, 36) wks, [4000, 4500) g	Mortality	Females		2.458 (1.967 to 3.074)	1.673 (1.344 to 2.093)							
Lower respiratory infections	Birth prevalence - [34, 36) wks, [3000, 3500) g	Mortality	Males		3.961 (3.433 to 4.521)	3.194 (2.71 to 3.696)							
Lower respiratory infections	Birth prevalence - [34, 36) wks, [3000, 3500) g	Mortality	Females		3.267 (2.738 to 3.876)	2.783 (2.273 to 3.408)							
Lower respiratory infections	Birth prevalence - [36, 37) wks, [2500, 3000) g	Mortality	Males		5.363 (4.69 to 6.082)	4.559 (3.945 to 5.248)							
Lower respiratory infections	Birth prevalence - [36, 37) wks, [2500, 3000) g	Mortality	Females		3.969 (3.371 to 4.675)	4.138 (3.469 to 4.868)							
Lower respiratory infections	Birth prevalence - [34, 36) wks, [3500, 4000) g	Mortality	Males		2.678 (2.289 to 3.086)	2.014 (1.69 to 2.365)							
Lower respiratory infections	Birth prevalence - [34, 36) wks, [3500, 4000) g	Mortality	Females		2.342 (1.908 to 2.845)	1.756 (1.426 to 2.126)							
Lower respiratory infections	Birth prevalence - [37, 38) wks, [2500, 3000) g	Mortality	Males		4.093 (3.581 to 4.626)	4.139 (3.631 to 4.74)							
Lower respiratory infections	Birth prevalence - [37, 38) wks, [2500, 3000) g	Mortality	Females		3.07 (2.612 to 3.603)	3.618 (3.045 to 4.25)							
Lower respiratory infections	Birth prevalence - [40, 42) wks, [2500, 3000) g	Mortality	Males		3.833 (3.364 to 4.388)	3.828 (3.271 to 4.426)							
Lower respiratory infections	Birth prevalence - [40, 42) wks, [2500, 3000) g	Mortality	Females		2.699 (2.264 to 3.202)	3.379 (2.825 to 4.042)							
Lower respiratory infections	Birth prevalence - [38, 40) wks, [2500, 3000) g	Mortality	Males		3.396 (3.002 to 3.839)	3.858 (3.346 to 4.424)							
Lower respiratory infections	Birth prevalence - [38, 40) wks, [2500, 3000) g	Mortality	Females		2.382 (2.04 to 2.802)	3.27 (2.774 to 3.927)							
Lower respiratory infections	Birth prevalence - [36, 37) wks, [3000, 3500) g	Mortality	Males		2.819 (2.433 to 3.229)	2.683 (2.299 to 3.079)							
Lower respiratory infections	Birth prevalence - [36, 37) wks, [3000, 3500) g	Mortality	Females		2.335 (1.952 to 2.784)	2.434 (2.007 to 2.884)							
Lower respiratory infections	Birth prevalence - [36, 37) wks, [4000, 4500) g	Mortality	Males		2.331 (1.993 to 2.727)	1.471 (1.221 to 1.731)							
Lower respiratory infections	Birth prevalence - [36, 37) wks, [4000, 4500) g	Mortality	Females		2.141 (1.729 to 2.577)	1.601 (1.291 to 1.97)							
Lower respiratory infections	Birth prevalence - [36, 37) wks, [3500, 4000) g	Mortality	Males		2.084 (1.789 to 2.418)	1.923 (1.624 to 2.252)							
Lower respiratory infections	Birth prevalence - [36, 37) wks, [3500, 4000) g	Mortality	Females		1.845 (1.527 to 2.216)	1.731 (1.426 to 2.071)							
Lower respiratory infections	Birth prevalence - [37, 38) wks, [3000, 3500) g	Mortality	Males		2.027 (1.788 to 2.287)	2.236 (1.926 to 2.542)							
Lower respiratory infections	Birth prevalence - [37, 38) wks, [3000, 3500) g	Mortality	Females		1.667 (1.411 to 1.947)	1.989 (1.672 to 2.366)							
Lower respiratory infections	Birth prevalence - [37, 38) wks, [4000, 4500) g	Mortality	Males		1.572 (1.352 to 1.813)	1.28 (1.095 to 1.481)							

Ages													
30-34 years	35-39 years	40-44 years	45-49 years	50-54 years	55-59 years	60-64 years	65-69 years	70-74 years	75-79 years	80-84 years	85-89 years	90-94 years	95+ years

Appendix Table 1a. Relative risks used by age and sex for each outcome for all risk factors except for ambient air pollution and alcohol.

Risk - Outcome	Category / Units	Morbidity / Mortality	Sex	All-age	0-6 days	7-27 days	28-364 days	1-4 years	5-9 years	10-14 years	15-19 years	20-24 years	25-29 years
Lower respiratory infections	Birth prevalence - [37, 38) wks, [4000, 4500) g	Mortality	Females		1.647<br>(1.37 to 1.98)	1.396<br>(1.167 to 1.674)							
Lower respiratory infections	Birth prevalence - [37, 38) wks, [3500, 4000) g	Mortality	Males		1.502<br>(1.309 to 1.712)	1.563<br>(1.353 to 1.807)							
Lower respiratory infections	Birth prevalence - [37, 38) wks, [3500, 4000) g	Mortality	Females		1.275<br>(1.075 to 1.501)	1.444<br>(1.206 to 1.711)							
Lower respiratory infections	Birth prevalence - [40, 42) wks, [3000, 3500) g	Mortality	Males		1.446<br>(1.258 to 1.639)	1.79<br>(1.546 to 2.08)							
Lower respiratory infections	Birth prevalence - [40, 42) wks, [3000, 3500) g	Mortality	Females		1.103<br>(0.922 to 1.305)	1.491<br>(1.233 to 1.776)							
Lower respiratory infections	Birth prevalence - [38, 40) wks, [3000, 3500) g	Mortality	Males		1.414<br>(1.242 to 1.593)	1.874<br>(1.636 to 2.156)							
Lower respiratory infections	Birth prevalence - [38, 40) wks, [3000, 3500) g	Mortality	Females		1.096<br>(0.927 to 1.289)	1.617<br>(1.352 to 1.907)							
Lower respiratory infections	Birth prevalence - [38, 40) wks, [4000, 4500) g	Mortality	Males		1.052<br>(0.92 to 1.203)	1.093<br>(0.939 to 1.261)							
Lower respiratory infections	Birth prevalence - [38, 40) wks, [4000, 4500) g	Mortality	Females		1.121<br>(0.925 to 1.31)	1.149<br>(0.955 to 1.367)							
Lower respiratory infections	Birth prevalence - [38, 40) wks, [3500, 4000) g	Mortality	Males		1.009<br>(0.883 to 1.143)	1.243<br>(1.078 to 1.421)							
Lower respiratory infections	Birth prevalence - [38, 40) wks, [3500, 4000) g	Mortality	Females		0.828<br>(0.702 to 0.972)	1.155<br>(0.967 to 1.369)							
Lower respiratory infections	Birth prevalence - [40, 42) wks, [3500, 4000) g	Mortality	Males		0.98<br>(0.854 to 1.123)	1.155<br>(0.983 to 1.334)							
Lower respiratory infections	Birth prevalence - [40, 42) wks, [3500, 4000) g	Mortality	Females		0.819<br>(0.679 to 0.974)	1.045<br>(0.874 to 1.26)							
Upper respiratory infections	Birth prevalence - [22, 24) wks, [0, 500) g	Mortality	Males		3273.944<br>(2987.306 to 3600.961)	599.42<br>(512.777 to 692.914)							
Upper respiratory infections	Birth prevalence - [22, 24) wks, [0, 500) g	Mortality	Females		3353.667<br>(2964.862 to 3798.637)	771.075<br>(635.721 to 931.62)							
Upper respiratory infections	Birth prevalence - [24, 26) wks, [0, 500) g	Mortality	Males		2454.576<br>(2216.544 to 2713.168)	884.902<br>(766.03 to 1021.494)							
Upper respiratory infections	Birth prevalence - [24, 26) wks, [0, 500) g	Mortality	Females		2376.85<br>(2080.354 to 2699.254)	915.957<br>(765.805 to 1079.243)							
Upper respiratory infections	Birth prevalence - [26, 28) wks, [0, 500) g	Mortality	Males		1870.684<br>(1676.978 to 2075.277)	847.512<br>(727.279 to 983.252)							
Upper respiratory infections	Birth prevalence - [26, 28) wks, [0, 500) g	Mortality	Females		1708.132<br>(1477.512 to 1960.75)	822.906<br>(677.101 to 990.491)							
Upper respiratory infections	Birth prevalence - [28, 30) wks, [0, 500) g	Mortality	Males		1805.939<br>(1612.157 to 2006.088)	583.881<br>(485.096 to 690.434)							
Upper respiratory infections	Birth prevalence - [28, 30) wks, [0, 500) g	Mortality	Females		1585.788<br>(1377.653 to 1836.365)	636.164<br>(520.511 to 760.561)							
Upper respiratory infections	Birth prevalence - [22, 24) wks, [500, 1000) g	Mortality	Males		2037.627<br>(1843.599 to 2249.686)	720.117<br>(628.641 to 820.917)							
Upper respiratory infections	Birth prevalence - [22, 24) wks, [500, 1000) g	Mortality	Females		1833.266<br>(1596.246 to 2105.971)	610.368<br>(510.239 to 722.278)							
Upper respiratory infections	Birth prevalence - [24, 26) wks, [500, 1000) g	Mortality	Males		852.585<br>(753.604 to 959.026)	642.627<br>(558.091 to 730.626)							
Upper respiratory infections	Birth prevalence - [24, 26) wks, [500, 1000) g	Mortality	Females		715.379<br>(610.81 to 830.296)	546.057<br>(463.282 to 637.815)							
Upper respiratory infections	Birth prevalence - [26, 28) wks, [500, 1000) g	Mortality	Males		411.87<br>(365.037 to 467.411)	353.266<br>(305.647 to 405.904)							
Upper respiratory infections	Birth prevalence - [26, 28) wks, [500, 1000) g	Mortality	Females		308.092<br>(262.47 to 362.302)	292.936<br>(243.986 to 349.019)							
Upper respiratory infections	Birth prevalence - [32, 34) wks, [500, 1000) g	Mortality	Males		344.501<br>(301.197 to 397.343)	194.954<br>(163.844 to 231.771)							
Upper respiratory infections	Birth prevalence - [32, 34) wks, [500, 1000) g	Mortality	Females		299.264<br>(251.468 to 353.995)	161.548<br>(130.866 to 196.323)							
Upper respiratory infections	Birth prevalence - [22, 24) wks, [1000, 1500) g	Mortality	Males		673.302<br>(584.187 to 768.258)	213.181<br>(179.555 to 254.572)							

Ages													
30-34 years	35-39 years	40-44 years	45-49 years	50-54 years	55-59 years	60-64 years	65-69 years	70-74 years	75-79 years	80-84 years	85-89 years	90-94 years	95+ years

Appendix Table 1a. Relative risks used by age and sex for each outcome for all risk factors except for ambient air pollution and alcohol.

Risk - Outcome	Category / Units	Morbidity / Mortality	Sex	All-age	0-6 days	7-27 days	28-364 days	1-4 years	5-9 years	10-14 years	15-19 years	20-24 years	25-29 years
Upper respiratory infections	Birth prevalence - [22, 24) wks, [1000, 1500) g	Mortality	Females		620.446 (522.864 to 734.748)	186.215 (149.772 to 227.046)							
Upper respiratory infections	Birth prevalence - [30, 32) wks, [500, 1000) g	Mortality	Males		332.681 (293.52 to 377.86)	212.332 (181.213 to 245.271)							
Upper respiratory infections	Birth prevalence - [30, 32) wks, [500, 1000) g	Mortality	Females		239.082 (200.416 to 284.597)	163.95 (135.165 to 197.261)							
Upper respiratory infections	Birth prevalence - [28, 30) wks, [500, 1000) g	Mortality	Males		326.557 (286.01 to 366.811)	249.246 (216.248 to 289.232)							
Upper respiratory infections	Birth prevalence - [28, 30) wks, [500, 1000) g	Mortality	Females		231.533 (194.62 to 271.396)	187.47 (157.953 to 224.552)							
Upper respiratory infections	Birth prevalence - [24, 26) wks, [1000, 1500) g	Mortality	Males		273.536 (236.795 to 312.421)	182.743 (155.477 to 212.281)							
Upper respiratory infections	Birth prevalence - [24, 26) wks, [1000, 1500) g	Mortality	Females		268.033 (223.95 to 318.815)	157.928 (130.936 to 190.298)							
Upper respiratory infections	Birth prevalence - [26, 28) wks, [1000, 1500) g	Mortality	Males		164.685 (145.457 to 186.571)	120.878 (102.672 to 140.967)							
Upper respiratory infections	Birth prevalence - [26, 28) wks, [1000, 1500) g	Mortality	Females		142.274 (121.33 to 168.246)	97.496 (80.858 to 116.625)							
Upper respiratory infections	Birth prevalence - [26, 28) wks, [1500, 2000) g	Mortality	Males		113.21 (97.079 to 130.806)	49.449 (40.908 to 59.113)							
Upper respiratory infections	Birth prevalence - [26, 28) wks, [1500, 2000) g	Mortality	Females		108.393 (89.864 to 129.883)	49.979 (40.484 to 60.784)							
Upper respiratory infections	Birth prevalence - [34, 36) wks, [1000, 1500) g	Mortality	Males		119.602 (104.655 to 136.729)	56.519 (48.084 to 66.018)							
Upper respiratory infections	Birth prevalence - [34, 36) wks, [1000, 1500) g	Mortality	Females		109.231 (91.177 to 128.378)	52.834 (43.454 to 63.459)							
Upper respiratory infections	Birth prevalence - [28, 30) wks, [1500, 2000) g	Mortality	Males		87.285 (76.012 to 100.83)	37.15 (30.87 to 43.664)							
Upper respiratory infections	Birth prevalence - [28, 30) wks, [1500, 2000) g	Mortality	Females		86.639 (71.639 to 103.632)	38.3 (31.326 to 45.531)							
Upper respiratory infections	Birth prevalence - [28, 30) wks, [1000, 1500) g	Mortality	Males		107.077 (93.214 to 122.668)	81.407 (70.511 to 93.12)							
Upper respiratory infections	Birth prevalence - [28, 30) wks, [1000, 1500) g	Mortality	Females		87.409 (73.438 to 104.222)	64.981 (53.651 to 77.029)							
Upper respiratory infections	Birth prevalence - [32, 34) wks, [1000, 1500) g	Mortality	Males		98.982 (86.649 to 112.931)	51.159 (43.522 to 59.402)							
Upper respiratory infections	Birth prevalence - [32, 34) wks, [1000, 1500) g	Mortality	Females		84.081 (70.672 to 99.726)	44.695 (37.129 to 53.257)							
Upper respiratory infections	Birth prevalence - [30, 32) wks, [1000, 1500) g	Mortality	Males		90.239 (78.583 to 103.215)	59.239 (50.511 to 69.508)							
Upper respiratory infections	Birth prevalence - [30, 32) wks, [1000, 1500) g	Mortality	Females		70.119 (59.11 to 81.846)	47.493 (39.526 to 56.547)							
Upper respiratory infections	Birth prevalence - [37, 38) wks, [1500, 2000) g	Mortality	Males		55.659 (48.726 to 62.963)	25.45 (21.867 to 29.451)							
Upper respiratory infections	Birth prevalence - [37, 38) wks, [1500, 2000) g	Mortality	Females		54.453 (46.308 to 63.524)	29.339 (24.736 to 34.558)							
Upper respiratory infections	Birth prevalence - [36, 37) wks, [1500, 2000) g	Mortality	Males		53.885 (47.099 to 61.414)	23.622 (20.373 to 27.298)							
Upper respiratory infections	Birth prevalence - [36, 37) wks, [1500, 2000) g	Mortality	Females		47.995 (40.82 to 56.378)	23.905 (19.8 to 29.023)							
Upper respiratory infections	Birth prevalence - [30, 32) wks, [2000, 2500) g	Mortality	Males		39.701 (34.573 to 45.311)	16.391 (13.683 to 19.411)							
Upper respiratory infections	Birth prevalence - [30, 32) wks, [2000, 2500) g	Mortality	Females		34.894 (28.953 to 41.934)	16.549 (13.438 to 19.916)							
Upper respiratory infections	Birth prevalence - [30, 32) wks, [1500, 2000) g	Mortality	Males		55.142 (48.379 to 62.989)	26.391 (22.751 to 30.584)							
Upper respiratory infections	Birth prevalence - [30, 32) wks, [1500, 2000) g	Mortality	Females		48.414 (40.924 to 57.128)	26.986 (22.299 to 32.305)							
Upper respiratory infections	Birth prevalence - [34, 36) wks, [1500, 2000) g	Mortality	Males		45.579 (39.817 to 51.922)	19.836 (16.94 to 23.141)							

Ages													
30-34 years	35-39 years	40-44 years	45-49 years	50-54 years	55-59 years	60-64 years	65-69 years	70-74 years	75-79 years	80-84 years	85-89 years	90-94 years	95+ years

Appendix Table 1a. Relative risks used by age and sex for each outcome for all risk factors except for ambient air pollution and alcohol.

Risk - Outcome	Category / Units	Morbidity / Mortality	Sex	All-age	0-6 days	7-27 days	28-364 days	1-4 years	5-9 years	10-14 years	15-19 years	20-24 years	25-29 years
Upper respiratory infections	Birth prevalence - [34, 36) wks, [1500, 2000) g	Mortality	Females		38.405 (32.273 to 45.04)	18.409 (15.134 to 21.755)							
Upper respiratory infections	Birth prevalence - [32, 34) wks, [1500, 2000) g	Mortality	Males		41.734 (36.381 to 47.516)	19.083 (16.193 to 21.909)							
Upper respiratory infections	Birth prevalence - [32, 34) wks, [1500, 2000) g	Mortality	Females		36.162 (30.231 to 42.934)	19.528 (16.278 to 23.535)							
Upper respiratory infections	Birth prevalence - [32, 34) wks, [2000, 2500) g	Mortality	Males		22.379 (19.576 to 25.426)	11.555 (9.874 to 13.332)							
Upper respiratory infections	Birth prevalence - [32, 34) wks, [2000, 2500) g	Mortality	Females		20.326 (17.055 to 23.945)	12.376 (10.192 to 14.834)							
Upper respiratory infections	Birth prevalence - [40, 42) wks, [2000, 2500) g	Mortality	Males		17.218 (14.893 to 19.961)	11.519 (9.766 to 13.495)							
Upper respiratory infections	Birth prevalence - [40, 42) wks, [2000, 2500) g	Mortality	Females		13.555 (11.337 to 16.132)	11.838 (9.792 to 14.338)							
Upper respiratory infections	Birth prevalence - [38, 40) wks, [2000, 2500) g	Mortality	Males		14.227 (12.521 to 16.06)	10.475 (9.122 to 12.085)							
Upper respiratory infections	Birth prevalence - [38, 40) wks, [2000, 2500) g	Mortality	Females		10.867 (9.199 to 12.777)	10.493 (8.801 to 12.342)							
Upper respiratory infections	Birth prevalence - [32, 34) wks, [2500, 3000) g	Mortality	Males		12.775 (11.146 to 14.607)	6.296 (5.269 to 7.299)							
Upper respiratory infections	Birth prevalence - [32, 34) wks, [2500, 3000) g	Mortality	Females		9.041 (7.504 to 10.706)	6.205 (5.041 to 7.52)							
Upper respiratory infections	Birth prevalence - [34, 36) wks, [2000, 2500) g	Mortality	Males		16.472 (14.422 to 18.7)	9.777 (8.456 to 11.277)							
Upper respiratory infections	Birth prevalence - [34, 36) wks, [2000, 2500) g	Mortality	Females		12.895 (10.992 to 15.284)	9.44 (7.865 to 11.209)							
Upper respiratory infections	Birth prevalence - [37, 38) wks, [2000, 2500) g	Mortality	Males		14.003 (12.407 to 15.831)	9.977 (8.758 to 11.444)							
Upper respiratory infections	Birth prevalence - [37, 38) wks, [2000, 2500) g	Mortality	Females		10.456 (8.899 to 12.149)	9.83 (8.317 to 11.54)							
Upper respiratory infections	Birth prevalence - [36, 37) wks, [2000, 2500) g	Mortality	Males		14.873 (13.083 to 16.88)	9.756 (8.38 to 11.214)							
Upper respiratory infections	Birth prevalence - [36, 37) wks, [2000, 2500) g	Mortality	Females		10.517 (8.897 to 12.379)	9.233 (7.691 to 10.92)							
Upper respiratory infections	Birth prevalence - [34, 36) wks, [2500, 3000) g	Mortality	Males		8.216 (7.133 to 9.378)	5.419 (4.631 to 6.282)							
Upper respiratory infections	Birth prevalence - [34, 36) wks, [2500, 3000) g	Mortality	Females		6.037 (5.044 to 7.094)	5.198 (4.31 to 6.293)							
Upper respiratory infections	Birth prevalence - [34, 36) wks, [4000, 4500) g	Mortality	Males		2.68 (2.245 to 3.183)	1.532 (1.254 to 1.867)							
Upper respiratory infections	Birth prevalence - [34, 36) wks, [4000, 4500) g	Mortality	Females		2.458 (1.967 to 3.074)	1.673 (1.344 to 2.093)							
Upper respiratory infections	Birth prevalence - [34, 36) wks, [3000, 3500) g	Mortality	Males		3.961 (3.433 to 4.521)	3.194 (2.71 to 3.696)							
Upper respiratory infections	Birth prevalence - [34, 36) wks, [3000, 3500) g	Mortality	Females		3.267 (2.738 to 3.876)	2.783 (2.273 to 3.408)							
Upper respiratory infections	Birth prevalence - [36, 37) wks, [2500, 3000) g	Mortality	Males		5.363 (4.69 to 6.082)	4.559 (3.945 to 5.248)							
Upper respiratory infections	Birth prevalence - [36, 37) wks, [2500, 3000) g	Mortality	Females		3.969 (3.371 to 4.675)	4.138 (3.469 to 4.868)							
Upper respiratory infections	Birth prevalence - [34, 36) wks, [3500, 4000) g	Mortality	Males		2.678 (2.289 to 3.086)	2.014 (1.69 to 2.365)							
Upper respiratory infections	Birth prevalence - [34, 36) wks, [3500, 4000) g	Mortality	Females		2.342 (1.908 to 2.845)	1.756 (1.426 to 2.126)							
Upper respiratory infections	Birth prevalence - [37, 38) wks, [2500, 3000) g	Mortality	Males		4.093 (3.581 to 4.626)	4.139 (3.631 to 4.74)							
Upper respiratory infections	Birth prevalence - [37, 38) wks, [2500, 3000) g	Mortality	Females		3.07 (2.612 to 3.603)	3.618 (3.045 to 4.25)							
Upper respiratory infections	Birth prevalence - [40, 42) wks, [2500, 3000) g	Mortality	Males		3.833 (3.364 to 4.388)	3.828 (3.271 to 4.426)							

Ages													
30-34 years	35-39 years	40-44 years	45-49 years	50-54 years	55-59 years	60-64 years	65-69 years	70-74 years	75-79 years	80-84 years	85-89 years	90-94 years	95+ years

Appendix Table 1a. Relative risks used by age and sex for each outcome for all risk factors except for ambient air pollution and alcohol.

Risk - Outcome	Category / Units	Morbidity / Mortality	Sex	All-age	0-6 days	7-27 days	28-364 days	1-4 years	5-9 years	10-14 years	15-19 years	20-24 years	25-29 years
Upper respiratory infections	Birth prevalence - [40, 42) wks, [2500, 3000) g	Mortality	Females		2.699 (2.264 to 3.202)	3.379 (2.825 to 4.042)							
Upper respiratory infections	Birth prevalence - [38, 40) wks, [2500, 3000) g	Mortality	Males		3.396 (3.002 to 3.839)	3.858 (3.346 to 4.424)							
Upper respiratory infections	Birth prevalence - [38, 40) wks, [2500, 3000) g	Mortality	Females		2.382 (2.04 to 2.802)	3.27 (2.774 to 3.927)							
Upper respiratory infections	Birth prevalence - [36, 37) wks, [3000, 3500) g	Mortality	Males		2.819 (2.433 to 3.229)	2.683 (2.299 to 3.079)							
Upper respiratory infections	Birth prevalence - [36, 37) wks, [3000, 3500) g	Mortality	Females		2.335 (1.952 to 2.784)	2.434 (2.007 to 2.884)							
Upper respiratory infections	Birth prevalence - [36, 37) wks, [4000, 4500) g	Mortality	Males		2.331 (1.993 to 2.727)	1.471 (1.221 to 1.731)							
Upper respiratory infections	Birth prevalence - [36, 37) wks, [4000, 4500) g	Mortality	Females		2.141 (1.729 to 2.577)	1.601 (1.291 to 1.97)							
Upper respiratory infections	Birth prevalence - [36, 37) wks, [3500, 4000) g	Mortality	Males		2.084 (1.789 to 2.418)	1.923 (1.624 to 2.252)							
Upper respiratory infections	Birth prevalence - [36, 37) wks, [3500, 4000) g	Mortality	Females		1.845 (1.527 to 2.216)	1.731 (1.426 to 2.071)							
Upper respiratory infections	Birth prevalence - [37, 38) wks, [3000, 3500) g	Mortality	Males		2.027 (1.788 to 2.287)	2.236 (1.926 to 2.542)							
Upper respiratory infections	Birth prevalence - [37, 38) wks, [3000, 3500) g	Mortality	Females		1.667 (1.411 to 1.947)	1.989 (1.672 to 2.366)							
Upper respiratory infections	Birth prevalence - [37, 38) wks, [4000, 4500) g	Mortality	Males		1.572 (1.352 to 1.813)	1.28 (1.095 to 1.481)							
Upper respiratory infections	Birth prevalence - [37, 38) wks, [4000, 4500) g	Mortality	Females		1.647 (1.37 to 1.98)	1.396 (1.167 to 1.674)							
Upper respiratory infections	Birth prevalence - [37, 38) wks, [3500, 4000) g	Mortality	Males		1.502 (1.309 to 1.712)	1.563 (1.353 to 1.807)							
Upper respiratory infections	Birth prevalence - [37, 38) wks, [3500, 4000) g	Mortality	Females		1.275 (1.075 to 1.501)	1.444 (1.206 to 1.711)							
Upper respiratory infections	Birth prevalence - [40, 42) wks, [3000, 3500) g	Mortality	Males		1.446 (1.258 to 1.639)	1.79 (1.546 to 2.08)							
Upper respiratory infections	Birth prevalence - [40, 42) wks, [3000, 3500) g	Mortality	Females		1.103 (0.922 to 1.305)	1.491 (1.233 to 1.776)							
Upper respiratory infections	Birth prevalence - [38, 40) wks, [3000, 3500) g	Mortality	Males		1.414 (1.242 to 1.593)	1.874 (1.636 to 2.156)							
Upper respiratory infections	Birth prevalence - [38, 40) wks, [3000, 3500) g	Mortality	Females		1.096 (0.927 to 1.289)	1.617 (1.352 to 1.907)							
Upper respiratory infections	Birth prevalence - [38, 40) wks, [4000, 4500) g	Mortality	Males		1.052 (0.92 to 1.203)	1.093 (0.939 to 1.261)							
Upper respiratory infections	Birth prevalence - [38, 40) wks, [4000, 4500) g	Mortality	Females		1.121 (0.925 to 1.31)	1.149 (0.955 to 1.367)							
Upper respiratory infections	Birth prevalence - [38, 40) wks, [3500, 4000) g	Mortality	Males		1.009 (0.883 to 1.143)	1.243 (1.078 to 1.421)							
Upper respiratory infections	Birth prevalence - [38, 40) wks, [3500, 4000) g	Mortality	Females		0.828 (0.702 to 0.972)	1.155 (0.967 to 1.369)							
Upper respiratory infections	Birth prevalence - [40, 42) wks, [3500, 4000) g	Mortality	Males		0.98 (0.854 to 1.123)	1.155 (0.983 to 1.334)							
Upper respiratory infections	Birth prevalence - [40, 42) wks, [3500, 4000) g	Mortality	Females		0.819 (0.679 to 0.974)	1.045 (0.874 to 1.26)							
Otitis media	Birth prevalence - [22, 24) wks, [0, 500) g	Mortality	Males		3273.944 (2987.306 to 3600.961)	599.42 (512.777 to 692.914)							
Otitis media	Birth prevalence - [22, 24) wks, [0, 500) g	Mortality	Females		3353.667 (2964.862 to 3798.637)	771.075 (635.721 to 931.62)							
Otitis media	Birth prevalence - [24, 26) wks, [0, 500) g	Mortality	Males		2454.576 (2216.544 to 2713.168)	884.902 (766.03 to 1021.494)							
Otitis media	Birth prevalence - [24, 26) wks, [0, 500) g	Mortality	Females		2376.85 (2080.354 to 2699.254)	915.957 (765.805 to 1079.243)							
Otitis media	Birth prevalence - [26, 28) wks, [0, 500) g	Mortality	Males		1870.684 (1676.978 to 2075.277)	847.512 (727.279 to 983.252)							

Ages													
30-34 years	35-39 years	40-44 years	45-49 years	50-54 years	55-59 years	60-64 years	65-69 years	70-74 years	75-79 years	80-84 years	85-89 years	90-94 years	95+ years

Appendix Table 1a. Relative risks used by age and sex for each outcome for all risk factors except for ambient air pollution and alcohol.

Risk - Outcome	Category / Units	Morbidity / Mortality	Sex	All-age	0-6 days	7-27 days	28-364 days	1-4 years	5-9 years	10-14 years	15-19 years	20-24 years	25-29 years
Otitis media	Birth prevalence - [26, 28) wks, [0, 500) g	Mortality	Females		1708.132 (1477.512 to 1960.75)	822.906 (677.101 to 990.491)							
Otitis media	Birth prevalence - [28, 30) wks, [0, 500) g	Mortality	Males		1805.939 (1612.157 to 2006.088)	583.881 (485.096 to 690.434)							
Otitis media	Birth prevalence - [28, 30) wks, [0, 500) g	Mortality	Females		1585.788 (1377.653 to 1836.365)	636.164 (520.511 to 760.561)							
Otitis media	Birth prevalence - [22, 24) wks, [500, 1000) g	Mortality	Males		2037.627 (1843.599 to 2249.686)	720.117 (628.641 to 820.917)							
Otitis media	Birth prevalence - [22, 24) wks, [500, 1000) g	Mortality	Females		1833.266 (1596.246 to 2105.971)	610.368 (510.239 to 722.278)							
Otitis media	Birth prevalence - [24, 26) wks, [500, 1000) g	Mortality	Males		852.585 (753.604 to 959.026)	642.627 (558.091 to 730.626)							
Otitis media	Birth prevalence - [24, 26) wks, [500, 1000) g	Mortality	Females		715.379 (610.81 to 830.296)	546.057 (463.282 to 637.815)							
Otitis media	Birth prevalence - [26, 28) wks, [500, 1000) g	Mortality	Males		411.87 (365.037 to 467.411)	353.266 (305.647 to 405.904)							
Otitis media	Birth prevalence - [26, 28) wks, [500, 1000) g	Mortality	Females		308.092 (262.47 to 362.302)	292.936 (243.986 to 349.019)							
Otitis media	Birth prevalence - [32, 34) wks, [500, 1000) g	Mortality	Males		344.501 (301.197 to 397.343)	194.954 (163.844 to 231.771)							
Otitis media	Birth prevalence - [32, 34) wks, [500, 1000) g	Mortality	Females		299.264 (251.468 to 353.995)	161.548 (130.866 to 196.323)							
Otitis media	Birth prevalence - [22, 24) wks, [1000, 1500) g	Mortality	Males		673.302 (584.187 to 768.258)	213.181 (179.555 to 254.572)							
Otitis media	Birth prevalence - [22, 24) wks, [1000, 1500) g	Mortality	Females		620.446 (522.864 to 734.748)	186.215 (149.772 to 227.046)							
Otitis media	Birth prevalence - [30, 32) wks, [500, 1000) g	Mortality	Males		332.681 (293.52 to 377.86)	212.332 (181.213 to 245.271)							
Otitis media	Birth prevalence - [30, 32) wks, [500, 1000) g	Mortality	Females		239.082 (200.416 to 284.597)	163.95 (135.165 to 197.261)							
Otitis media	Birth prevalence - [28, 30) wks, [500, 1000) g	Mortality	Males		326.557 (286.01 to 366.811)	249.246 (216.248 to 289.232)							
Otitis media	Birth prevalence - [28, 30) wks, [500, 1000) g	Mortality	Females		231.533 (194.62 to 271.396)	187.47 (157.953 to 224.552)							
Otitis media	Birth prevalence - [24, 26) wks, [1000, 1500) g	Mortality	Males		273.536 (236.795 to 312.421)	182.743 (155.477 to 212.281)							
Otitis media	Birth prevalence - [24, 26) wks, [1000, 1500) g	Mortality	Females		268.033 (223.95 to 318.815)	157.928 (130.936 to 190.298)							
Otitis media	Birth prevalence - [26, 28) wks, [1000, 1500) g	Mortality	Males		164.685 (145.457 to 186.571)	120.878 (102.672 to 140.967)							
Otitis media	Birth prevalence - [26, 28) wks, [1000, 1500) g	Mortality	Females		142.274 (121.33 to 168.246)	97.496 (80.858 to 116.625)							
Otitis media	Birth prevalence - [26, 28) wks, [1500, 2000) g	Mortality	Males		113.21 (97.079 to 130.806)	49.449 (40.908 to 59.113)							
Otitis media	Birth prevalence - [26, 28) wks, [1500, 2000) g	Mortality	Females		108.393 (89.864 to 129.883)	49.979 (40.484 to 60.784)							
Otitis media	Birth prevalence - [34, 36) wks, [1000, 1500) g	Mortality	Males		119.602 (104.655 to 136.729)	56.519 (48.084 to 66.018)							
Otitis media	Birth prevalence - [34, 36) wks, [1000, 1500) g	Mortality	Females		109.231 (91.177 to 128.378)	52.834 (43.454 to 63.459)							
Otitis media	Birth prevalence - [28, 30) wks, [1500, 2000) g	Mortality	Males		87.285 (76.012 to 100.83)	37.15 (30.87 to 43.664)							
Otitis media	Birth prevalence - [28, 30) wks, [1500, 2000) g	Mortality	Females		86.639 (71.639 to 103.632)	38.3 (31.326 to 45.531)							
Otitis media	Birth prevalence - [28, 30) wks, [1000, 1500) g	Mortality	Males		107.077 (93.214 to 122.668)	81.407 (70.511 to 93.12)							
Otitis media	Birth prevalence - [28, 30) wks, [1000, 1500) g	Mortality	Females		87.409 (73.438 to 104.222)	64.981 (53.651 to 77.029)							
Otitis media	Birth prevalence - [32, 34) wks, [1000, 1500) g	Mortality	Males		98.982 (86.649 to 112.931)	51.159 (43.522 to 59.402)							

Ages													
30-34 years	35-39 years	40-44 years	45-49 years	50-54 years	55-59 years	60-64 years	65-69 years	70-74 years	75-79 years	80-84 years	85-89 years	90-94 years	95+ years

Appendix Table 1a. Relative risks used by age and sex for each outcome for all risk factors except for ambient air pollution and alcohol.

Risk - Outcome	Category / Units	Morbidity / Mortality	Sex	All-age	0-6 days	7-27 days	28-364 days	1-4 years	5-9 years	10-14 years	15-19 years	20-24 years	25-29 years
Otitis media	Birth prevalence - [32, 34) wks, [1000, 1500) g	Mortality	Females		84.081 (70.672 to 99.726)	44.695 (37.129 to 53.257)							
Otitis media	Birth prevalence - [30, 32) wks, [1000, 1500) g	Mortality	Males		90.239 (78.583 to 103.215)	59.239 (50.511 to 69.508)							
Otitis media	Birth prevalence - [30, 32) wks, [1000, 1500) g	Mortality	Females		70.119 (59.11 to 81.846)	47.493 (39.526 to 56.547)							
Otitis media	Birth prevalence - [37, 38) wks, [1500, 2000) g	Mortality	Males		55.659 (48.726 to 62.963)	25.45 (21.867 to 29.451)							
Otitis media	Birth prevalence - [37, 38) wks, [1500, 2000) g	Mortality	Females		54.453 (46.308 to 63.524)	29.339 (24.736 to 34.558)							
Otitis media	Birth prevalence - [36, 37) wks, [1500, 2000) g	Mortality	Males		53.885 (47.099 to 61.414)	23.622 (20.373 to 27.298)							
Otitis media	Birth prevalence - [36, 37) wks, [1500, 2000) g	Mortality	Females		47.995 (40.82 to 56.378)	23.905 (19.8 to 29.023)							
Otitis media	Birth prevalence - [30, 32) wks, [2000, 2500) g	Mortality	Males		39.701 (34.573 to 45.311)	16.391 (13.683 to 19.411)							
Otitis media	Birth prevalence - [30, 32) wks, [2000, 2500) g	Mortality	Females		34.894 (28.953 to 41.934)	16.549 (13.438 to 19.916)							
Otitis media	Birth prevalence - [30, 32) wks, [1500, 2000) g	Mortality	Males		55.142 (48.379 to 62.989)	26.391 (22.751 to 30.584)							
Otitis media	Birth prevalence - [30, 32) wks, [1500, 2000) g	Mortality	Females		48.414 (40.924 to 57.128)	26.986 (22.299 to 32.305)							
Otitis media	Birth prevalence - [34, 36) wks, [1500, 2000) g	Mortality	Males		45.579 (39.817 to 51.922)	19.836 (16.94 to 23.141)							
Otitis media	Birth prevalence - [34, 36) wks, [1500, 2000) g	Mortality	Females		38.405 (32.273 to 45.04)	18.409 (15.134 to 21.755)							
Otitis media	Birth prevalence - [32, 34) wks, [1500, 2000) g	Mortality	Males		41.734 (36.381 to 47.516)	19.083 (16.193 to 21.909)							
Otitis media	Birth prevalence - [32, 34) wks, [1500, 2000) g	Mortality	Females		36.162 (30.231 to 42.934)	19.528 (16.278 to 23.535)							
Otitis media	Birth prevalence - [32, 34) wks, [2000, 2500) g	Mortality	Males		22.379 (19.576 to 25.426)	11.555 (9.874 to 13.332)							
Otitis media	Birth prevalence - [32, 34) wks, [2000, 2500) g	Mortality	Females		20.326 (17.055 to 23.945)	12.376 (10.192 to 14.834)							
Otitis media	Birth prevalence - [40, 42) wks, [2000, 2500) g	Mortality	Males		17.218 (14.893 to 19.961)	11.519 (9.766 to 13.495)							
Otitis media	Birth prevalence - [40, 42) wks, [2000, 2500) g	Mortality	Females		13.555 (11.337 to 16.132)	11.838 (9.792 to 14.338)							
Otitis media	Birth prevalence - [38, 40) wks, [2000, 2500) g	Mortality	Males		14.227 (12.521 to 16.06)	10.475 (9.122 to 12.085)							
Otitis media	Birth prevalence - [38, 40) wks, [2000, 2500) g	Mortality	Females		10.867 (9.199 to 12.777)	10.493 (8.801 to 12.342)							
Otitis media	Birth prevalence - [32, 34) wks, [2500, 3000) g	Mortality	Males		12.775 (11.146 to 14.607)	6.296 (5.269 to 7.299)							
Otitis media	Birth prevalence - [32, 34) wks, [2500, 3000) g	Mortality	Females		9.041 (7.504 to 10.706)	6.205 (5.041 to 7.52)							
Otitis media	Birth prevalence - [34, 36) wks, [2000, 2500) g	Mortality	Males		16.472 (14.422 to 18.7)	9.777 (8.456 to 11.277)							
Otitis media	Birth prevalence - [34, 36) wks, [2000, 2500) g	Mortality	Females		12.895 (10.992 to 15.284)	9.44 (7.865 to 11.209)							
Otitis media	Birth prevalence - [37, 38) wks, [2000, 2500) g	Mortality	Males		14.003 (12.407 to 15.831)	9.977 (8.758 to 11.444)							
Otitis media	Birth prevalence - [37, 38) wks, [2000, 2500) g	Mortality	Females		10.456 (8.899 to 12.149)	9.83 (8.317 to 11.54)							
Otitis media	Birth prevalence - [36, 37) wks, [2000, 2500) g	Mortality	Males		14.873 (13.083 to 16.88)	9.756 (8.38 to 11.214)							
Otitis media	Birth prevalence - [36, 37) wks, [2000, 2500) g	Mortality	Females		10.517 (8.897 to 12.379)	9.233 (7.691 to 10.92)							
Otitis media	Birth prevalence - [34, 36) wks, [2500, 3000) g	Mortality	Males		8.216 (7.133 to 9.378)	5.419 (4.631 to 6.282)							

Ages													
30-34 years	35-39 years	40-44 years	45-49 years	50-54 years	55-59 years	60-64 years	65-69 years	70-74 years	75-79 years	80-84 years	85-89 years	90-94 years	95+ years

Appendix Table 1a. Relative risks used by age and sex for each outcome for all risk factors except for ambient air pollution and alcohol.

Risk - Outcome	Category / Units	Morbidity / Mortality	Sex	All-age	0-6 days	7-27 days	28-364 days	1-4 years	5-9 years	10-14 years	15-19 years	20-24 years	25-29 years
Otitis media	Birth prevalence - [34, 36) wks, [2500, 3000) g	Mortality	Females		6.037 (5.044 to 7.094)	5.198 (4.31 to 6.293)							
Otitis media	Birth prevalence - [34, 36) wks, [4000, 4500) g	Mortality	Males		2.68 (2.245 to 3.183)	1.532 (1.254 to 1.867)							
Otitis media	Birth prevalence - [34, 36) wks, [4000, 4500) g	Mortality	Females		2.458 (1.967 to 3.074)	1.673 (1.344 to 2.093)							
Otitis media	Birth prevalence - [34, 36) wks, [3000, 3500) g	Mortality	Males		3.961 (3.433 to 4.521)	3.194 (2.71 to 3.696)							
Otitis media	Birth prevalence - [34, 36) wks, [3000, 3500) g	Mortality	Females		3.267 (2.738 to 3.876)	2.783 (2.273 to 3.408)							
Otitis media	Birth prevalence - [36, 37) wks, [2500, 3000) g	Mortality	Males		5.363 (4.69 to 6.082)	4.559 (3.945 to 5.248)							
Otitis media	Birth prevalence - [36, 37) wks, [2500, 3000) g	Mortality	Females		3.969 (3.371 to 4.675)	4.138 (3.469 to 4.868)							
Otitis media	Birth prevalence - [34, 36) wks, [3500, 4000) g	Mortality	Males		2.678 (2.289 to 3.086)	2.014 (1.69 to 2.365)							
Otitis media	Birth prevalence - [34, 36) wks, [3500, 4000) g	Mortality	Females		2.342 (1.908 to 2.845)	1.756 (1.426 to 2.126)							
Otitis media	Birth prevalence - [37, 38) wks, [2500, 3000) g	Mortality	Males		4.093 (3.581 to 4.626)	4.139 (3.631 to 4.74)							
Otitis media	Birth prevalence - [37, 38) wks, [2500, 3000) g	Mortality	Females		3.07 (2.612 to 3.603)	3.618 (3.045 to 4.25)							
Otitis media	Birth prevalence - [40, 42) wks, [2500, 3000) g	Mortality	Males		3.833 (3.364 to 4.388)	3.828 (3.271 to 4.426)							
Otitis media	Birth prevalence - [40, 42) wks, [2500, 3000) g	Mortality	Females		2.699 (2.264 to 3.202)	3.379 (2.825 to 4.042)							
Otitis media	Birth prevalence - [38, 40) wks, [2500, 3000) g	Mortality	Males		3.396 (3.002 to 3.839)	3.858 (3.346 to 4.424)							
Otitis media	Birth prevalence - [38, 40) wks, [2500, 3000) g	Mortality	Females		2.382 (2.04 to 2.802)	3.27 (2.774 to 3.927)							
Otitis media	Birth prevalence - [36, 37) wks, [3000, 3500) g	Mortality	Males		2.819 (2.433 to 3.229)	2.683 (2.299 to 3.079)							
Otitis media	Birth prevalence - [36, 37) wks, [3000, 3500) g	Mortality	Females		2.335 (1.952 to 2.784)	2.434 (2.007 to 2.884)							
Otitis media	Birth prevalence - [36, 37) wks, [4000, 4500) g	Mortality	Males		2.331 (1.993 to 2.727)	1.471 (1.221 to 1.731)							
Otitis media	Birth prevalence - [36, 37) wks, [4000, 4500) g	Mortality	Females		2.141 (1.729 to 2.577)	1.601 (1.291 to 1.97)							
Otitis media	Birth prevalence - [36, 37) wks, [3500, 4000) g	Mortality	Males		2.084 (1.789 to 2.418)	1.923 (1.624 to 2.252)							
Otitis media	Birth prevalence - [36, 37) wks, [3500, 4000) g	Mortality	Females		1.845 (1.527 to 2.216)	1.731 (1.426 to 2.071)							
Otitis media	Birth prevalence - [37, 38) wks, [3000, 3500) g	Mortality	Males		2.027 (1.788 to 2.287)	2.236 (1.926 to 2.542)							
Otitis media	Birth prevalence - [37, 38) wks, [3000, 3500) g	Mortality	Females		1.667 (1.411 to 1.947)	1.989 (1.672 to 2.366)							
Otitis media	Birth prevalence - [37, 38) wks, [4000, 4500) g	Mortality	Males		1.572 (1.352 to 1.813)	1.28 (1.095 to 1.481)							
Otitis media	Birth prevalence - [37, 38) wks, [4000, 4500) g	Mortality	Females		1.647 (1.37 to 1.98)	1.396 (1.167 to 1.674)							
Otitis media	Birth prevalence - [37, 38) wks, [3500, 4000) g	Mortality	Males		1.502 (1.309 to 1.712)	1.563 (1.353 to 1.807)							
Otitis media	Birth prevalence - [37, 38) wks, [3500, 4000) g	Mortality	Females		1.275 (1.075 to 1.501)	1.444 (1.206 to 1.711)							
Otitis media	Birth prevalence - [40, 42) wks, [3000, 3500) g	Mortality	Males		1.446 (1.258 to 1.639)	1.79 (1.546 to 2.08)							
Otitis media	Birth prevalence - [40, 42) wks, [3000, 3500) g	Mortality	Females		1.103 (0.922 to 1.305)	1.491 (1.233 to 1.776)							
Otitis media	Birth prevalence - [38, 40) wks, [3000, 3500) g	Mortality	Males		1.414 (1.242 to 1.593)	1.874 (1.636 to 2.156)							

Ages													
30-34 years	35-39 years	40-44 years	45-49 years	50-54 years	55-59 years	60-64 years	65-69 years	70-74 years	75-79 years	80-84 years	85-89 years	90-94 years	95+ years

Appendix Table 1a. Relative risks used by age and sex for each outcome for all risk factors except for ambient air pollution and alcohol.

Risk - Outcome	Category / Units	Morbidity / Mortality	Sex	All-age	0-6 days	7-27 days	28-364 days	1-4 years	5-9 years	10-14 years	15-19 years	20-24 years	25-29 years
Otitis media	Birth prevalence - [38, 40) wks, [3000, 3500) g	Mortality	Females		1.096 (0.927 to 1.289)	1.617 (1.352 to 1.907)							
Otitis media	Birth prevalence - [38, 40) wks, [4000, 4500) g	Mortality	Males		1.052 (0.92 to 1.203)	1.093 (0.939 to 1.261)							
Otitis media	Birth prevalence - [38, 40) wks, [4000, 4500) g	Mortality	Females		1.121 (0.925 to 1.31)	1.149 (0.955 to 1.367)							
Otitis media	Birth prevalence - [38, 40) wks, [3500, 4000) g	Mortality	Males		1.009 (0.883 to 1.143)	1.243 (1.078 to 1.421)							
Otitis media	Birth prevalence - [38, 40) wks, [3500, 4000) g	Mortality	Females		0.828 (0.702 to 0.972)	1.155 (0.967 to 1.369)							
Otitis media	Birth prevalence - [40, 42) wks, [3500, 4000) g	Mortality	Males		0.98 (0.854 to 1.123)	1.155 (0.983 to 1.334)							
Otitis media	Birth prevalence - [40, 42) wks, [3500, 4000) g	Mortality	Females		0.819 (0.679 to 0.974)	1.045 (0.874 to 1.26)							
Pneumococcal meningitis	Birth prevalence - [22, 24) wks, [0, 500) g	Mortality	Males		3273.944 (2987.306 to 3600.961)	599.42 (512.777 to 692.914)							
Pneumococcal meningitis	Birth prevalence - [22, 24) wks, [0, 500) g	Mortality	Females		3353.667 (2964.862 to 3798.637)	771.075 (635.721 to 931.62)							
Pneumococcal meningitis	Birth prevalence - [24, 26) wks, [0, 500) g	Mortality	Males		2454.576 (2216.544 to 2713.168)	884.902 (766.03 to 1021.494)							
Pneumococcal meningitis	Birth prevalence - [24, 26) wks, [0, 500) g	Mortality	Females		2376.85 (2080.354 to 2699.254)	915.957 (765.805 to 1079.243)							
Pneumococcal meningitis	Birth prevalence - [26, 28) wks, [0, 500) g	Mortality	Males		1870.684 (1676.978 to 2075.277)	847.512 (727.279 to 983.252)							
Pneumococcal meningitis	Birth prevalence - [26, 28) wks, [0, 500) g	Mortality	Females		1708.132 (1477.512 to 1960.75)	822.906 (677.101 to 990.491)							
Pneumococcal meningitis	Birth prevalence - [28, 30) wks, [0, 500) g	Mortality	Males		1805.939 (1612.157 to 2006.088)	583.881 (485.096 to 690.434)							
Pneumococcal meningitis	Birth prevalence - [28, 30) wks, [0, 500) g	Mortality	Females		1585.788 (1377.653 to 1836.365)	636.164 (520.511 to 760.561)							
Pneumococcal meningitis	Birth prevalence - [22, 24) wks, [500, 1000) g	Mortality	Males		2037.627 (1843.599 to 2249.686)	720.117 (628.641 to 820.917)							
Pneumococcal meningitis	Birth prevalence - [22, 24) wks, [500, 1000) g	Mortality	Females		1833.266 (1596.246 to 2105.971)	610.368 (510.239 to 722.278)							
Pneumococcal meningitis	Birth prevalence - [24, 26) wks, [500, 1000) g	Mortality	Males		852.585 (753.604 to 959.026)	642.627 (558.091 to 730.626)							
Pneumococcal meningitis	Birth prevalence - [24, 26) wks, [500, 1000) g	Mortality	Females		715.379 (610.81 to 830.296)	546.057 (463.282 to 637.815)							
Pneumococcal meningitis	Birth prevalence - [26, 28) wks, [500, 1000) g	Mortality	Males		411.87 (365.037 to 467.411)	353.266 (305.647 to 405.904)							
Pneumococcal meningitis	Birth prevalence - [26, 28) wks, [500, 1000) g	Mortality	Females		308.092 (262.47 to 362.302)	292.936 (243.986 to 349.019)							
Pneumococcal meningitis	Birth prevalence - [32, 34) wks, [500, 1000) g	Mortality	Males		344.501 (301.197 to 397.343)	194.954 (163.844 to 231.771)							
Pneumococcal meningitis	Birth prevalence - [32, 34) wks, [500, 1000) g	Mortality	Females		299.264 (251.468 to 353.995)	161.548 (130.866 to 196.323)							
Pneumococcal meningitis	Birth prevalence - [22, 24) wks, [1000, 1500) g	Mortality	Males		673.302 (584.187 to 768.258)	213.181 (179.555 to 254.572)							
Pneumococcal meningitis	Birth prevalence - [22, 24) wks, [1000, 1500) g	Mortality	Females		620.446 (522.864 to 734.748)	186.215 (149.772 to 227.046)							
Pneumococcal meningitis	Birth prevalence - [30, 32) wks, [500, 1000) g	Mortality	Males		332.681 (293.52 to 377.86)	212.332 (181.213 to 245.271)							
Pneumococcal meningitis	Birth prevalence - [30, 32) wks, [500, 1000) g	Mortality	Females		239.082 (200.416 to 284.597)	163.95 (135.165 to 197.261)							
Pneumococcal meningitis	Birth prevalence - [28, 30) wks, [500, 1000) g	Mortality	Males		326.557 (286.01 to 366.811)	249.246 (216.248 to 289.232)							
Pneumococcal meningitis	Birth prevalence - [28, 30) wks, [500, 1000) g	Mortality	Females		231.533 (194.62 to 271.396)	187.47 (157.953 to 224.552)							
Pneumococcal meningitis	Birth prevalence - [24, 26) wks, [1000, 1500) g	Mortality	Males		273.536 (236.795 to 312.421)	182.743 (155.477 to 212.281)							

Ages													
30-34 years	35-39 years	40-44 years	45-49 years	50-54 years	55-59 years	60-64 years	65-69 years	70-74 years	75-79 years	80-84 years	85-89 years	90-94 years	95+ years

Appendix Table 1a. Relative risks used by age and sex for each outcome for all risk factors except for ambient air pollution and alcohol.

Risk - Outcome	Category / Units	Morbidity / Mortality	Sex	All-age	0-6 days	7-27 days	28-364 days	1-4 years	5-9 years	10-14 years	15-19 years	20-24 years	25-29 years
Pneumococcal meningitis	Birth prevalence - [24, 26) wks, [1000, 1500) g	Mortality	Females		268.033 (223.95 to 318.815)	157.928 (130.936 to 190.298)							
Pneumococcal meningitis	Birth prevalence - [26, 28) wks, [1000, 1500) g	Mortality	Males		164.685 (145.457 to 186.571)	120.878 (102.672 to 140.967)							
Pneumococcal meningitis	Birth prevalence - [26, 28) wks, [1000, 1500) g	Mortality	Females		142.274 (121.33 to 168.246)	97.496 (80.858 to 116.625)							
Pneumococcal meningitis	Birth prevalence - [26, 28) wks, [1500, 2000) g	Mortality	Males		113.21 (97.079 to 130.806)	49.449 (40.908 to 59.113)							
Pneumococcal meningitis	Birth prevalence - [26, 28) wks, [1500, 2000) g	Mortality	Females		108.393 (89.864 to 129.883)	49.979 (40.484 to 60.784)							
Pneumococcal meningitis	Birth prevalence - [34, 36) wks, [1000, 1500) g	Mortality	Males		119.602 (104.655 to 136.729)	56.519 (48.084 to 66.018)							
Pneumococcal meningitis	Birth prevalence - [34, 36) wks, [1000, 1500) g	Mortality	Females		109.231 (91.177 to 128.378)	52.834 (43.454 to 63.459)							
Pneumococcal meningitis	Birth prevalence - [28, 30) wks, [1500, 2000) g	Mortality	Males		87.285 (76.012 to 100.83)	37.15 (30.87 to 43.664)							
Pneumococcal meningitis	Birth prevalence - [28, 30) wks, [1500, 2000) g	Mortality	Females		86.639 (71.639 to 103.632)	38.3 (31.326 to 45.531)							
Pneumococcal meningitis	Birth prevalence - [28, 30) wks, [1000, 1500) g	Mortality	Males		107.077 (93.214 to 122.668)	81.407 (70.511 to 93.12)							
Pneumococcal meningitis	Birth prevalence - [28, 30) wks, [1000, 1500) g	Mortality	Females		87.409 (73.438 to 104.222)	64.981 (53.651 to 77.029)							
Pneumococcal meningitis	Birth prevalence - [32, 34) wks, [1000, 1500) g	Mortality	Males		98.982 (86.649 to 112.931)	51.159 (43.522 to 59.402)							
Pneumococcal meningitis	Birth prevalence - [32, 34) wks, [1000, 1500) g	Mortality	Females		84.081 (70.672 to 99.726)	44.695 (37.129 to 53.257)							
Pneumococcal meningitis	Birth prevalence - [30, 32) wks, [1000, 1500) g	Mortality	Males		90.239 (78.583 to 103.215)	59.239 (50.511 to 69.508)							
Pneumococcal meningitis	Birth prevalence - [30, 32) wks, [1000, 1500) g	Mortality	Females		70.119 (59.11 to 81.846)	47.493 (39.526 to 56.547)							
Pneumococcal meningitis	Birth prevalence - [37, 38) wks, [1500, 2000) g	Mortality	Males		55.659 (48.726 to 62.963)	25.45 (21.867 to 29.451)							
Pneumococcal meningitis	Birth prevalence - [37, 38) wks, [1500, 2000) g	Mortality	Females		54.453 (46.308 to 63.524)	29.339 (24.736 to 34.558)							
Pneumococcal meningitis	Birth prevalence - [36, 37) wks, [1500, 2000) g	Mortality	Males		53.885 (47.099 to 61.414)	23.622 (20.373 to 27.298)							
Pneumococcal meningitis	Birth prevalence - [36, 37) wks, [1500, 2000) g	Mortality	Females		47.995 (40.82 to 56.378)	23.905 (19.8 to 29.023)							
Pneumococcal meningitis	Birth prevalence - [30, 32) wks, [2000, 2500) g	Mortality	Males		39.701 (34.573 to 45.311)	16.391 (13.683 to 19.411)							
Pneumococcal meningitis	Birth prevalence - [30, 32) wks, [2000, 2500) g	Mortality	Females		34.894 (28.953 to 41.934)	16.549 (13.438 to 19.916)							
Pneumococcal meningitis	Birth prevalence - [30, 32) wks, [1500, 2000) g	Mortality	Males		55.142 (48.379 to 62.989)	26.391 (22.751 to 30.584)							
Pneumococcal meningitis	Birth prevalence - [30, 32) wks, [1500, 2000) g	Mortality	Females		48.414 (40.924 to 57.128)	26.986 (22.299 to 32.305)							
Pneumococcal meningitis	Birth prevalence - [34, 36) wks, [1500, 2000) g	Mortality	Males		45.579 (39.817 to 51.922)	19.836 (16.94 to 23.141)							
Pneumococcal meningitis	Birth prevalence - [34, 36) wks, [1500, 2000) g	Mortality	Females		38.405 (32.273 to 45.04)	18.409 (15.134 to 21.755)							
Pneumococcal meningitis	Birth prevalence - [32, 34) wks, [1500, 2000) g	Mortality	Males		41.734 (36.381 to 47.516)	19.083 (16.193 to 21.909)							
Pneumococcal meningitis	Birth prevalence - [32, 34) wks, [1500, 2000) g	Mortality	Females		36.162 (30.231 to 42.934)	19.528 (16.278 to 23.535)							
Pneumococcal meningitis	Birth prevalence - [32, 34) wks, [2000, 2500) g	Mortality	Males		22.379 (19.576 to 25.426)	11.555 (9.874 to 13.332)							
Pneumococcal meningitis	Birth prevalence - [32, 34) wks, [2000, 2500) g	Mortality	Females		20.326 (17.055 to 23.945)	12.376 (10.192 to 14.834)							
Pneumococcal meningitis	Birth prevalence - [40, 42) wks, [2000, 2500) g	Mortality	Males		17.218 (14.893 to 19.961)	11.519 (9.766 to 13.495)							

Ages													
30-34 years	35-39 years	40-44 years	45-49 years	50-54 years	55-59 years	60-64 years	65-69 years	70-74 years	75-79 years	80-84 years	85-89 years	90-94 years	95+ years

Appendix Table 1a. Relative risks used by age and sex for each outcome for all risk factors except for ambient air pollution and alcohol.

Risk - Outcome	Category / Units	Morbidity / Mortality	Sex	All-age	0-6 days	7-27 days	28-364 days	1-4 years	5-9 years	10-14 years	15-19 years	20-24 years	25-29 years
Pneumococcal meningitis	Birth prevalence - [40, 42) wks, [2000, 2500) g	Mortality	Females		13.555 (11.337 to 16.132)	11.838 (9.792 to 14.338)							
Pneumococcal meningitis	Birth prevalence - [38, 40) wks, [2000, 2500) g	Mortality	Males		14.227 (12.521 to 16.06)	10.475 (9.122 to 12.085)							
Pneumococcal meningitis	Birth prevalence - [38, 40) wks, [2000, 2500) g	Mortality	Females		10.867 (9.199 to 12.777)	10.493 (8.801 to 12.342)							
Pneumococcal meningitis	Birth prevalence - [32, 34) wks, [2500, 3000) g	Mortality	Males		12.775 (11.146 to 14.607)	6.296 (5.269 to 7.299)							
Pneumococcal meningitis	Birth prevalence - [32, 34) wks, [2500, 3000) g	Mortality	Females		9.041 (7.504 to 10.706)	6.205 (5.041 to 7.52)							
Pneumococcal meningitis	Birth prevalence - [34, 36) wks, [2000, 2500) g	Mortality	Males		16.472 (14.422 to 18.7)	9.777 (8.456 to 11.277)							
Pneumococcal meningitis	Birth prevalence - [34, 36) wks, [2000, 2500) g	Mortality	Females		12.895 (10.992 to 15.284)	9.44 (7.865 to 11.209)							
Pneumococcal meningitis	Birth prevalence - [37, 38) wks, [2000, 2500) g	Mortality	Males		14.003 (12.407 to 15.831)	9.977 (8.758 to 11.444)							
Pneumococcal meningitis	Birth prevalence - [37, 38) wks, [2000, 2500) g	Mortality	Females		10.456 (8.899 to 12.149)	9.83 (8.317 to 11.54)							
Pneumococcal meningitis	Birth prevalence - [36, 37) wks, [2000, 2500) g	Mortality	Males		14.873 (13.083 to 16.88)	9.756 (8.38 to 11.214)							
Pneumococcal meningitis	Birth prevalence - [36, 37) wks, [2000, 2500) g	Mortality	Females		10.517 (8.897 to 12.379)	9.233 (7.691 to 10.92)							
Pneumococcal meningitis	Birth prevalence - [34, 36) wks, [2500, 3000) g	Mortality	Males		8.216 (7.133 to 9.378)	5.419 (4.631 to 6.282)							
Pneumococcal meningitis	Birth prevalence - [34, 36) wks, [2500, 3000) g	Mortality	Females		6.037 (5.044 to 7.094)	5.198 (4.31 to 6.293)							
Pneumococcal meningitis	Birth prevalence - [34, 36) wks, [4000, 4500) g	Mortality	Males		2.68 (2.245 to 3.183)	1.532 (1.254 to 1.867)							
Pneumococcal meningitis	Birth prevalence - [34, 36) wks, [4000, 4500) g	Mortality	Females		2.458 (1.967 to 3.074)	1.673 (1.344 to 2.093)							
Pneumococcal meningitis	Birth prevalence - [34, 36) wks, [3000, 3500) g	Mortality	Males		3.961 (3.433 to 4.521)	3.194 (2.71 to 3.696)							
Pneumococcal meningitis	Birth prevalence - [34, 36) wks, [3000, 3500) g	Mortality	Females		3.267 (2.738 to 3.876)	2.783 (2.273 to 3.408)							
Pneumococcal meningitis	Birth prevalence - [36, 37) wks, [2500, 3000) g	Mortality	Males		5.363 (4.69 to 6.082)	4.559 (3.945 to 5.248)							
Pneumococcal meningitis	Birth prevalence - [36, 37) wks, [2500, 3000) g	Mortality	Females		3.969 (3.371 to 4.675)	4.138 (3.469 to 4.868)							
Pneumococcal meningitis	Birth prevalence - [34, 36) wks, [3500, 4000) g	Mortality	Males		2.678 (2.289 to 3.086)	2.014 (1.69 to 2.365)							
Pneumococcal meningitis	Birth prevalence - [34, 36) wks, [3500, 4000) g	Mortality	Females		2.342 (1.908 to 2.845)	1.756 (1.426 to 2.126)							
Pneumococcal meningitis	Birth prevalence - [37, 38) wks, [2500, 3000) g	Mortality	Males		4.093 (3.581 to 4.626)	4.139 (3.631 to 4.74)							
Pneumococcal meningitis	Birth prevalence - [37, 38) wks, [2500, 3000) g	Mortality	Females		3.07 (2.612 to 3.603)	3.618 (3.045 to 4.25)							
Pneumococcal meningitis	Birth prevalence - [40, 42) wks, [2500, 3000) g	Mortality	Males		3.833 (3.364 to 4.388)	3.828 (3.271 to 4.426)							
Pneumococcal meningitis	Birth prevalence - [40, 42) wks, [2500, 3000) g	Mortality	Females		2.699 (2.264 to 3.202)	3.379 (2.825 to 4.042)							
Pneumococcal meningitis	Birth prevalence - [38, 40) wks, [2500, 3000) g	Mortality	Males		3.396 (3.002 to 3.839)	3.858 (3.346 to 4.424)							
Pneumococcal meningitis	Birth prevalence - [38, 40) wks, [2500, 3000) g	Mortality	Females		2.382 (2.04 to 2.802)	3.27 (2.774 to 3.927)							
Pneumococcal meningitis	Birth prevalence - [36, 37) wks, [3000, 3500) g	Mortality	Males		2.819 (2.433 to 3.229)	2.683 (2.299 to 3.079)							
Pneumococcal meningitis	Birth prevalence - [36, 37) wks, [3000, 3500) g	Mortality	Females		2.335 (1.952 to 2.784)	2.434 (2.007 to 2.884)							
Pneumococcal meningitis	Birth prevalence - [36, 37) wks, [4000, 4500) g	Mortality	Males		2.331 (1.993 to 2.727)	1.471 (1.221 to 1.731)							

Ages													
30-34 years	35-39 years	40-44 years	45-49 years	50-54 years	55-59 years	60-64 years	65-69 years	70-74 years	75-79 years	80-84 years	85-89 years	90-94 years	95+ years

Appendix Table 1a. Relative risks used by age and sex for each outcome for all risk factors except for ambient air pollution and alcohol.

Risk - Outcome	Category / Units	Morbidity / Mortality	Sex	All-age	0-6 days	7-27 days	28-364 days	1-4 years	5-9 years	10-14 years	15-19 years	20-24 years	25-29 years
Pneumococcal meningitis	Birth prevalence - [36, 37) wks, [4000, 4500) g	Mortality	Females		2.141 (1.729 to 2.577)	1.601 (1.291 to 1.97)							
Pneumococcal meningitis	Birth prevalence - [36, 37) wks, [3500, 4000) g	Mortality	Males		2.084 (1.789 to 2.418)	1.923 (1.624 to 2.252)							
Pneumococcal meningitis	Birth prevalence - [36, 37) wks, [3500, 4000) g	Mortality	Females		1.845 (1.527 to 2.216)	1.731 (1.426 to 2.071)							
Pneumococcal meningitis	Birth prevalence - [37, 38) wks, [3000, 3500) g	Mortality	Males		2.027 (1.788 to 2.287)	2.236 (1.926 to 2.542)							
Pneumococcal meningitis	Birth prevalence - [37, 38) wks, [3000, 3500) g	Mortality	Females		1.667 (1.411 to 1.947)	1.989 (1.672 to 2.366)							
Pneumococcal meningitis	Birth prevalence - [37, 38) wks, [4000, 4500) g	Mortality	Males		1.572 (1.352 to 1.813)	1.28 (1.095 to 1.481)							
Pneumococcal meningitis	Birth prevalence - [37, 38) wks, [4000, 4500) g	Mortality	Females		1.647 (1.37 to 1.98)	1.396 (1.167 to 1.674)							
Pneumococcal meningitis	Birth prevalence - [37, 38) wks, [3500, 4000) g	Mortality	Males		1.502 (1.309 to 1.712)	1.563 (1.353 to 1.807)							
Pneumococcal meningitis	Birth prevalence - [37, 38) wks, [3500, 4000) g	Mortality	Females		1.275 (1.075 to 1.501)	1.444 (1.206 to 1.711)							
Pneumococcal meningitis	Birth prevalence - [40, 42) wks, [3000, 3500) g	Mortality	Males		1.446 (1.258 to 1.639)	1.79 (1.546 to 2.08)							
Pneumococcal meningitis	Birth prevalence - [40, 42) wks, [3000, 3500) g	Mortality	Females		1.103 (0.922 to 1.305)	1.491 (1.233 to 1.776)							
Pneumococcal meningitis	Birth prevalence - [38, 40) wks, [3000, 3500) g	Mortality	Males		1.414 (1.242 to 1.593)	1.874 (1.636 to 2.156)							
Pneumococcal meningitis	Birth prevalence - [38, 40) wks, [3000, 3500) g	Mortality	Females		1.096 (0.927 to 1.289)	1.617 (1.352 to 1.907)							
Pneumococcal meningitis	Birth prevalence - [38, 40) wks, [4000, 4500) g	Mortality	Males		1.052 (0.92 to 1.203)	1.093 (0.939 to 1.261)							
Pneumococcal meningitis	Birth prevalence - [38, 40) wks, [4000, 4500) g	Mortality	Females		1.121 (0.925 to 1.31)	1.149 (0.955 to 1.367)							
Pneumococcal meningitis	Birth prevalence - [38, 40) wks, [3500, 4000) g	Mortality	Males		1.009 (0.883 to 1.143)	1.243 (1.078 to 1.421)							
Pneumococcal meningitis	Birth prevalence - [38, 40) wks, [3500, 4000) g	Mortality	Females		0.828 (0.702 to 0.972)	1.155 (0.967 to 1.369)							
Pneumococcal meningitis	Birth prevalence - [40, 42) wks, [3500, 4000) g	Mortality	Males		0.98 (0.854 to 1.123)	1.155 (0.983 to 1.334)							
Pneumococcal meningitis	Birth prevalence - [40, 42) wks, [3500, 4000) g	Mortality	Females		0.819 (0.679 to 0.974)	1.045 (0.874 to 1.26)							
H influenzae type B meningitis	Birth prevalence - [22, 24) wks, [0, 500) g	Mortality	Males		3273.944 (2987.306 to 3600.961)	599.42 (512.777 to 692.914)							
H influenzae type B meningitis	Birth prevalence - [22, 24) wks, [0, 500) g	Mortality	Females		3353.667 (2964.862 to 3798.637)	771.075 (635.721 to 931.62)							
H influenzae type B meningitis	Birth prevalence - [24, 26) wks, [0, 500) g	Mortality	Males		2454.576 (2216.544 to 2713.168)	884.902 (766.03 to 1021.494)							
H influenzae type B meningitis	Birth prevalence - [24, 26) wks, [0, 500) g	Mortality	Females		2376.85 (2080.354 to 2699.254)	915.957 (765.805 to 1079.243)							
H influenzae type B meningitis	Birth prevalence - [26, 28) wks, [0, 500) g	Mortality	Males		1870.684 (1676.978 to 2075.277)	847.512 (727.279 to 983.252)							
H influenzae type B meningitis	Birth prevalence - [26, 28) wks, [0, 500) g	Mortality	Females		1708.132 (1477.512 to 1960.75)	822.906 (677.101 to 990.491)							
H influenzae type B meningitis	Birth prevalence - [28, 30) wks, [0, 500) g	Mortality	Males		1805.939 (1612.157 to 2006.088)	583.881 (485.096 to 690.434)							
H influenzae type B meningitis	Birth prevalence - [28, 30) wks, [0, 500) g	Mortality	Females		1585.788 (1377.653 to 1836.365)	636.164 (520.511 to 760.561)							
H influenzae type B meningitis	Birth prevalence - [22, 24) wks, [500, 1000) g	Mortality	Males		2037.627 (1843.599 to 2249.686)	720.117 (628.641 to 820.917)							
H influenzae type B meningitis	Birth prevalence - [22, 24) wks, [500, 1000) g	Mortality	Females		1833.266 (1596.246 to 2105.971)	610.368 (510.239 to 722.278)							
H influenzae type B meningitis	Birth prevalence - [24, 26) wks, [500, 1000) g	Mortality	Males		852.585 (753.604 to 959.026)	642.627 (558.091 to 730.626)							

Ages													
30-34 years	35-39 years	40-44 years	45-49 years	50-54 years	55-59 years	60-64 years	65-69 years	70-74 years	75-79 years	80-84 years	85-89 years	90-94 years	95+ years

Appendix Table 1a. Relative risks used by age and sex for each outcome for all risk factors except for ambient air pollution and alcohol.

Risk - Outcome	Category / Units	Morbidity / Mortality	Sex	All-age	0-6 days	7-27 days	28-364 days	1-4 years	5-9 years	10-14 years	15-19 years	20-24 years	25-29 years
H influenzae type B meningitis	Birth prevalence - [24, 26) wks, [500, 1000) g	Mortality	Females		715.379 (610.81 to 830.296)	546.057 (463.282 to 637.815)							
H influenzae type B meningitis	Birth prevalence - [26, 28) wks, [500, 1000) g	Mortality	Males		411.87 (365.037 to 467.411)	353.266 (305.647 to 405.904)							
H influenzae type B meningitis	Birth prevalence - [26, 28) wks, [500, 1000) g	Mortality	Females		308.092 (262.47 to 362.302)	292.936 (243.986 to 349.019)							
H influenzae type B meningitis	Birth prevalence - [32, 34) wks, [500, 1000) g	Mortality	Males		344.501 (301.197 to 397.343)	194.954 (163.844 to 231.771)							
H influenzae type B meningitis	Birth prevalence - [32, 34) wks, [500, 1000) g	Mortality	Females		299.264 (251.468 to 353.995)	161.548 (130.866 to 196.323)							
H influenzae type B meningitis	Birth prevalence - [22, 24) wks, [1000, 1500) g	Mortality	Males		673.302 (584.187 to 768.258)	213.181 (179.555 to 254.572)							
H influenzae type B meningitis	Birth prevalence - [22, 24) wks, [1000, 1500) g	Mortality	Females		620.446 (522.864 to 734.748)	186.215 (149.772 to 227.046)							
H influenzae type B meningitis	Birth prevalence - [30, 32) wks, [500, 1000) g	Mortality	Males		332.681 (293.52 to 377.86)	212.332 (181.213 to 245.271)							
H influenzae type B meningitis	Birth prevalence - [30, 32) wks, [500, 1000) g	Mortality	Females		239.082 (200.416 to 284.597)	163.95 (135.165 to 197.261)							
H influenzae type B meningitis	Birth prevalence - [28, 30) wks, [500, 1000) g	Mortality	Males		326.557 (286.01 to 366.811)	249.246 (216.248 to 289.232)							
H influenzae type B meningitis	Birth prevalence - [28, 30) wks, [500, 1000) g	Mortality	Females		231.533 (194.62 to 271.396)	187.47 (157.953 to 224.552)							
H influenzae type B meningitis	Birth prevalence - [24, 26) wks, [1000, 1500) g	Mortality	Males		273.536 (236.795 to 312.421)	182.743 (155.477 to 212.281)							
H influenzae type B meningitis	Birth prevalence - [24, 26) wks, [1000, 1500) g	Mortality	Females		268.033 (223.95 to 318.815)	157.928 (130.936 to 190.298)							
H influenzae type B meningitis	Birth prevalence - [26, 28) wks, [1000, 1500) g	Mortality	Males		164.685 (145.457 to 186.571)	120.878 (102.672 to 140.967)							
H influenzae type B meningitis	Birth prevalence - [26, 28) wks, [1000, 1500) g	Mortality	Females		142.274 (121.33 to 168.246)	97.496 (80.858 to 116.625)							
H influenzae type B meningitis	Birth prevalence - [26, 28) wks, [1500, 2000) g	Mortality	Males		113.21 (97.079 to 130.806)	49.449 (40.908 to 59.113)							
H influenzae type B meningitis	Birth prevalence - [26, 28) wks, [1500, 2000) g	Mortality	Females		108.393 (89.864 to 129.883)	49.979 (40.484 to 60.784)							
H influenzae type B meningitis	Birth prevalence - [34, 36) wks, [1000, 1500) g	Mortality	Males		119.602 (104.655 to 136.729)	56.519 (48.084 to 66.018)							
H influenzae type B meningitis	Birth prevalence - [34, 36) wks, [1000, 1500) g	Mortality	Females		109.231 (91.177 to 128.378)	52.834 (43.454 to 63.459)							
H influenzae type B meningitis	Birth prevalence - [28, 30) wks, [1500, 2000) g	Mortality	Males		87.285 (76.012 to 100.83)	37.15 (30.87 to 43.664)							
H influenzae type B meningitis	Birth prevalence - [28, 30) wks, [1500, 2000) g	Mortality	Females		86.639 (71.639 to 103.632)	38.3 (31.326 to 45.531)							
H influenzae type B meningitis	Birth prevalence - [28, 30) wks, [1000, 1500) g	Mortality	Males		107.077 (93.214 to 122.668)	81.407 (70.511 to 93.12)							
H influenzae type B meningitis	Birth prevalence - [28, 30) wks, [1000, 1500) g	Mortality	Females		87.409 (73.438 to 104.222)	64.981 (53.651 to 77.029)							
H influenzae type B meningitis	Birth prevalence - [32, 34) wks, [1000, 1500) g	Mortality	Males		98.982 (86.649 to 112.931)	51.159 (43.522 to 59.402)							
H influenzae type B meningitis	Birth prevalence - [32, 34) wks, [1000, 1500) g	Mortality	Females		84.081 (70.672 to 99.726)	44.695 (37.129 to 53.257)							
H influenzae type B meningitis	Birth prevalence - [30, 32) wks, [1000, 1500) g	Mortality	Males		90.239 (78.583 to 103.215)	59.239 (50.511 to 69.508)							
H influenzae type B meningitis	Birth prevalence - [30, 32) wks, [1000, 1500) g	Mortality	Females		70.119 (59.11 to 81.846)	47.493 (39.526 to 56.547)							
H influenzae type B meningitis	Birth prevalence - [37, 38) wks, [1500, 2000) g	Mortality	Males		55.659 (48.726 to 62.963)	25.45 (21.867 to 29.451)							
H influenzae type B meningitis	Birth prevalence - [37, 38) wks, [1500, 2000) g	Mortality	Females		54.453 (46.308 to 63.524)	29.339 (24.736 to 34.558)							
H influenzae type B meningitis	Birth prevalence - [36, 37) wks, [1500, 2000) g	Mortality	Males		53.885 (47.099 to 61.414)	23.622 (20.373 to 27.298)							

Ages													
30-34 years	35-39 years	40-44 years	45-49 years	50-54 years	55-59 years	60-64 years	65-69 years	70-74 years	75-79 years	80-84 years	85-89 years	90-94 years	95+ years

Appendix Table 1a. Relative risks used by age and sex for each outcome for all risk factors except for ambient air pollution and alcohol.													
**Risk - Outcome**	**Category / Units**	**Morbidity / Mortality**	**Sex**	**All-age**	**0-6 days**	**7-27 days**	**28-364 days**	**1-4 years**	**5-9 years**	**10-14 years**	**15-19 years**	**20-24 years**	**25-29 years**
H influenzae type B meningitis	Birth prevalence - [36, 37) wks, [1500, 2000) g	Mortality	Females		47.995 (40.82 to 56.378)	23.905 (19.8 to 29.023)							
H influenzae type B meningitis	Birth prevalence - [30, 32) wks, [2000, 2500) g	Mortality	Males		39.701 (34.573 to 45.311)	16.391 (13.683 to 19.411)							
H influenzae type B meningitis	Birth prevalence - [30, 32) wks, [2000, 2500) g	Mortality	Females		34.894 (28.953 to 41.934)	16.549 (13.438 to 19.916)							
H influenzae type B meningitis	Birth prevalence - [30, 32) wks, [1500, 2000) g	Mortality	Males		55.142 (48.379 to 62.989)	26.391 (22.751 to 30.584)							
H influenzae type B meningitis	Birth prevalence - [30, 32) wks, [1500, 2000) g	Mortality	Females		48.414 (40.924 to 57.128)	26.986 (22.299 to 32.305)							
H influenzae type B meningitis	Birth prevalence - [34, 36) wks, [1500, 2000) g	Mortality	Males		45.579 (39.817 to 51.922)	19.836 (16.94 to 23.141)							
H influenzae type B meningitis	Birth prevalence - [34, 36) wks, [1500, 2000) g	Mortality	Females		38.405 (32.273 to 45.04)	18.409 (15.134 to 21.755)							
H influenzae type B meningitis	Birth prevalence - [32, 34) wks, [1500, 2000) g	Mortality	Males		41.734 (36.381 to 47.516)	19.083 (16.193 to 21.909)							
H influenzae type B meningitis	Birth prevalence - [32, 34) wks, [1500, 2000) g	Mortality	Females		36.162 (30.231 to 42.934)	19.528 (16.278 to 23.535)							
H influenzae type B meningitis	Birth prevalence - [32, 34) wks, [2000, 2500) g	Mortality	Males		22.379 (19.576 to 25.426)	11.555 (9.874 to 13.332)							
H influenzae type B meningitis	Birth prevalence - [32, 34) wks, [2000, 2500) g	Mortality	Females		20.326 (17.055 to 23.945)	12.376 (10.192 to 14.834)							
H influenzae type B meningitis	Birth prevalence - [40, 42) wks, [2000, 2500) g	Mortality	Males		17.218 (14.893 to 19.961)	11.519 (9.766 to 13.495)							
H influenzae type B meningitis	Birth prevalence - [40, 42) wks, [2000, 2500) g	Mortality	Females		13.555 (11.337 to 16.132)	11.838 (9.792 to 14.338)							
H influenzae type B meningitis	Birth prevalence - [38, 40) wks, [2000, 2500) g	Mortality	Males		14.227 (12.521 to 16.06)	10.475 (9.122 to 12.085)							
H influenzae type B meningitis	Birth prevalence - [38, 40) wks, [2000, 2500) g	Mortality	Females		10.867 (9.199 to 12.777)	10.493 (8.801 to 12.342)							
H influenzae type B meningitis	Birth prevalence - [32, 34) wks, [2500, 3000) g	Mortality	Males		12.775 (11.146 to 14.607)	6.296 (5.269 to 7.299)							
H influenzae type B meningitis	Birth prevalence - [32, 34) wks, [2500, 3000) g	Mortality	Females		9.041 (7.504 to 10.706)	6.205 (5.041 to 7.52)							
H influenzae type B meningitis	Birth prevalence - [34, 36) wks, [2000, 2500) g	Mortality	Males		16.472 (14.422 to 18.7)	9.777 (8.456 to 11.277)							
H influenzae type B meningitis	Birth prevalence - [34, 36) wks, [2000, 2500) g	Mortality	Females		12.895 (10.992 to 15.284)	9.44 (7.865 to 11.209)							
H influenzae type B meningitis	Birth prevalence - [37, 38) wks, [2000, 2500) g	Mortality	Males		14.003 (12.407 to 15.831)	9.977 (8.758 to 11.444)							
H influenzae type B meningitis	Birth prevalence - [37, 38) wks, [2000, 2500) g	Mortality	Females		10.456 (8.899 to 12.149)	9.83 (8.317 to 11.54)							
H influenzae type B meningitis	Birth prevalence - [36, 37) wks, [2000, 2500) g	Mortality	Males		14.873 (13.083 to 16.88)	9.756 (8.38 to 11.214)							
H influenzae type B meningitis	Birth prevalence - [36, 37) wks, [2000, 2500) g	Mortality	Females		10.517 (8.897 to 12.379)	9.233 (7.691 to 10.92)							
H influenzae type B meningitis	Birth prevalence - [34, 36) wks, [2500, 3000) g	Mortality	Males		8.216 (7.133 to 9.378)	5.419 (4.631 to 6.282)							
H influenzae type B meningitis	Birth prevalence - [34, 36) wks, [2500, 3000) g	Mortality	Females		6.037 (5.044 to 7.094)	5.198 (4.31 to 6.293)							
H influenzae type B meningitis	Birth prevalence - [34, 36) wks, [4000, 4500) g	Mortality	Males		2.68 (2.245 to 3.183)	1.532 (1.254 to 1.867)							
H influenzae type B meningitis	Birth prevalence - [34, 36) wks, [4000, 4500) g	Mortality	Females		2.458 (1.967 to 3.074)	1.673 (1.344 to 2.093)							
H influenzae type B meningitis	Birth prevalence - [34, 36) wks, [3000, 3500) g	Mortality	Males		3.961 (3.433 to 4.521)	3.194 (2.71 to 3.696)							
H influenzae type B meningitis	Birth prevalence - [34, 36) wks, [3000, 3500) g	Mortality	Females		3.267 (2.738 to 3.876)	2.783 (2.273 to 3.408)							
H influenzae type B meningitis	Birth prevalence - [36, 37) wks, [2500, 3000) g	Mortality	Males		5.363 (4.69 to 6.082)	4.559 (3.945 to 5.248)							

Ages													
30-34 years	35-39 years	40-44 years	45-49 years	50-54 years	55-59 years	60-64 years	65-69 years	70-74 years	75-79 years	80-84 years	85-89 years	90-94 years	95+ years

Appendix Table 1a. Relative risks used by age and sex for each outcome for all risk factors except for ambient air pollution and alcohol.

Risk - Outcome	Category / Units	Morbidity / Mortality	Sex	All-age	0-6 days	7-27 days	28-364 days	1-4 years	5-9 years	10-14 years	15-19 years	20-24 years	25-29 years
H influenzae type B meningitis	Birth prevalence - [36, 37) wks, [2500, 3000) g	Mortality	Females		3.969 (3.371 to 4.675)	4.138 (3.469 to 4.868)							
H influenzae type B meningitis	Birth prevalence - [34, 36) wks, [3500, 4000) g	Mortality	Males		2.678 (2.289 to 3.086)	2.014 (1.69 to 2.365)							
H influenzae type B meningitis	Birth prevalence - [34, 36) wks, [3500, 4000) g	Mortality	Females		2.342 (1.908 to 2.845)	1.756 (1.426 to 2.126)							
H influenzae type B meningitis	Birth prevalence - [37, 38) wks, [2500, 3000) g	Mortality	Males		4.093 (3.581 to 4.626)	4.139 (3.631 to 4.74)							
H influenzae type B meningitis	Birth prevalence - [37, 38) wks, [2500, 3000) g	Mortality	Females		3.07 (2.612 to 3.603)	3.618 (3.045 to 4.25)							
H influenzae type B meningitis	Birth prevalence - [40, 42) wks, [2500, 3000) g	Mortality	Males		3.833 (3.364 to 4.388)	3.828 (3.271 to 4.426)							
H influenzae type B meningitis	Birth prevalence - [40, 42) wks, [2500, 3000) g	Mortality	Females		2.699 (2.264 to 3.202)	3.379 (2.825 to 4.042)							
H influenzae type B meningitis	Birth prevalence - [38, 40) wks, [2500, 3000) g	Mortality	Males		3.396 (3.002 to 3.839)	3.858 (3.346 to 4.424)							
H influenzae type B meningitis	Birth prevalence - [38, 40) wks, [2500, 3000) g	Mortality	Females		2.382 (2.04 to 2.802)	3.27 (2.774 to 3.927)							
H influenzae type B meningitis	Birth prevalence - [36, 37) wks, [3000, 3500) g	Mortality	Males		2.819 (2.433 to 3.229)	2.683 (2.299 to 3.079)							
H influenzae type B meningitis	Birth prevalence - [36, 37) wks, [3000, 3500) g	Mortality	Females		2.335 (1.952 to 2.784)	2.434 (2.007 to 2.884)							
H influenzae type B meningitis	Birth prevalence - [36, 37) wks, [4000, 4500) g	Mortality	Males		2.331 (1.993 to 2.727)	1.471 (1.221 to 1.731)							
H influenzae type B meningitis	Birth prevalence - [36, 37) wks, [4000, 4500) g	Mortality	Females		2.141 (1.729 to 2.577)	1.601 (1.291 to 1.97)							
H influenzae type B meningitis	Birth prevalence - [36, 37) wks, [3500, 4000) g	Mortality	Males		2.084 (1.789 to 2.418)	1.923 (1.624 to 2.252)							
H influenzae type B meningitis	Birth prevalence - [36, 37) wks, [3500, 4000) g	Mortality	Females		1.845 (1.527 to 2.216)	1.731 (1.426 to 2.071)							
H influenzae type B meningitis	Birth prevalence - [37, 38) wks, [3000, 3500) g	Mortality	Males		2.027 (1.788 to 2.287)	2.236 (1.926 to 2.542)							
H influenzae type B meningitis	Birth prevalence - [37, 38) wks, [3000, 3500) g	Mortality	Females		1.667 (1.411 to 1.947)	1.989 (1.672 to 2.366)							
H influenzae type B meningitis	Birth prevalence - [37, 38) wks, [4000, 4500) g	Mortality	Males		1.572 (1.352 to 1.813)	1.28 (1.095 to 1.481)							
H influenzae type B meningitis	Birth prevalence - [37, 38) wks, [4000, 4500) g	Mortality	Females		1.647 (1.37 to 1.98)	1.396 (1.167 to 1.674)							
H influenzae type B meningitis	Birth prevalence - [37, 38) wks, [3500, 4000) g	Mortality	Males		1.502 (1.309 to 1.712)	1.563 (1.353 to 1.807)							
H influenzae type B meningitis	Birth prevalence - [37, 38) wks, [3500, 4000) g	Mortality	Females		1.275 (1.075 to 1.501)	1.444 (1.206 to 1.711)							
H influenzae type B meningitis	Birth prevalence - [40, 42) wks, [3000, 3500) g	Mortality	Males		1.446 (1.258 to 1.639)	1.79 (1.546 to 2.08)							
H influenzae type B meningitis	Birth prevalence - [40, 42) wks, [3000, 3500) g	Mortality	Females		1.103 (0.922 to 1.305)	1.491 (1.233 to 1.776)							
H influenzae type B meningitis	Birth prevalence - [38, 40) wks, [3000, 3500) g	Mortality	Males		1.414 (1.242 to 1.593)	1.874 (1.636 to 2.156)							
H influenzae type B meningitis	Birth prevalence - [38, 40) wks, [3000, 3500) g	Mortality	Females		1.096 (0.927 to 1.289)	1.617 (1.352 to 1.907)							
H influenzae type B meningitis	Birth prevalence - [38, 40) wks, [4000, 4500) g	Mortality	Males		1.052 (0.92 to 1.203)	1.093 (0.939 to 1.261)							
H influenzae type B meningitis	Birth prevalence - [38, 40) wks, [4000, 4500) g	Mortality	Females		1.121 (0.925 to 1.31)	1.149 (0.955 to 1.367)							
H influenzae type B meningitis	Birth prevalence - [38, 40) wks, [3500, 4000) g	Mortality	Males		1.009 (0.883 to 1.143)	1.243 (1.078 to 1.421)							
H influenzae type B meningitis	Birth prevalence - [38, 40) wks, [3500, 4000) g	Mortality	Females		0.828 (0.702 to 0.972)	1.155 (0.967 to 1.369)							
H influenzae type B meningitis	Birth prevalence - [40, 42) wks, [3500, 4000) g	Mortality	Males		0.98 (0.854 to 1.123)	1.155 (0.983 to 1.334)							

Ages													
30-34 years	35-39 years	40-44 years	45-49 years	50-54 years	55-59 years	60-64 years	65-69 years	70-74 years	75-79 years	80-84 years	85-89 years	90-94 years	95+ years

Appendix Table 1a. Relative risks used by age and sex for each outcome for all risk factors except for ambient air pollution and alcohol.

Risk - Outcome	Category / Units	Morbidity / Mortality	Sex	All-age	0-6 days	7-27 days	28-364 days	1-4 years	5-9 years	10-14 years	15-19 years	20-24 years	25-29 years
H influenzae type B meningitis	Birth prevalence - [40, 42) wks, [3500, 4000) g	Mortality	Females		0.819<br>(0.679 to 0.974)	1.045<br>(0.874 to 1.26)							
Meningococcal infection	Birth prevalence - [22, 24) wks, [0, 500) g	Mortality	Males		3273.944<br>(2987.306 to 3600.961)	599.42<br>(512.777 to 692.914)							
Meningococcal infection	Birth prevalence - [22, 24) wks, [0, 500) g	Mortality	Females		3353.667<br>(2964.862 to 3798.637)	771.075<br>(635.721 to 931.62)							
Meningococcal infection	Birth prevalence - [24, 26) wks, [0, 500) g	Mortality	Males		2454.576<br>(2216.544 to 2713.168)	884.902<br>(766.03 to 1021.494)							
Meningococcal infection	Birth prevalence - [24, 26) wks, [0, 500) g	Mortality	Females		2376.85<br>(2080.354 to 2699.254)	915.957<br>(765.805 to 1079.243)							
Meningococcal infection	Birth prevalence - [26, 28) wks, [0, 500) g	Mortality	Males		1870.684<br>(1676.978 to 2075.277)	847.512<br>(727.279 to 983.252)							
Meningococcal infection	Birth prevalence - [26, 28) wks, [0, 500) g	Mortality	Females		1708.132<br>(1477.512 to 1960.75)	822.906<br>(677.101 to 990.491)							
Meningococcal infection	Birth prevalence - [28, 30) wks, [0, 500) g	Mortality	Males		1805.939<br>(1612.157 to 2006.088)	583.881<br>(485.096 to 690.434)							
Meningococcal infection	Birth prevalence - [28, 30) wks, [0, 500) g	Mortality	Females		1585.788<br>(1377.653 to 1836.365)	636.164<br>(520.511 to 760.561)							
Meningococcal infection	Birth prevalence - [22, 24) wks, [500, 1000) g	Mortality	Males		2037.627<br>(1843.599 to 2249.686)	720.117<br>(628.641 to 820.917)							
Meningococcal infection	Birth prevalence - [22, 24) wks, [500, 1000) g	Mortality	Females		1833.266<br>(1596.246 to 2105.971)	610.368<br>(510.239 to 722.278)							
Meningococcal infection	Birth prevalence - [24, 26) wks, [500, 1000) g	Mortality	Males		852.585<br>(753.604 to 959.026)	642.627<br>(558.091 to 730.626)							
Meningococcal infection	Birth prevalence - [24, 26) wks, [500, 1000) g	Mortality	Females		715.379<br>(610.81 to 830.296)	546.057<br>(463.282 to 637.815)							
Meningococcal infection	Birth prevalence - [26, 28) wks, [500, 1000) g	Mortality	Males		411.87<br>(365.037 to 467.411)	353.266<br>(305.647 to 405.904)							
Meningococcal infection	Birth prevalence - [26, 28) wks, [500, 1000) g	Mortality	Females		308.092<br>(262.47 to 362.302)	292.936<br>(243.986 to 349.019)							
Meningococcal infection	Birth prevalence - [32, 34) wks, [500, 1000) g	Mortality	Males		344.501<br>(301.197 to 397.343)	194.954<br>(163.844 to 231.771)							
Meningococcal infection	Birth prevalence - [32, 34) wks, [500, 1000) g	Mortality	Females		299.264<br>(251.468 to 353.995)	161.548<br>(130.866 to 196.323)							
Meningococcal infection	Birth prevalence - [22, 24) wks, [1000, 1500) g	Mortality	Males		673.302<br>(584.187 to 768.258)	213.181<br>(179.555 to 254.572)							
Meningococcal infection	Birth prevalence - [22, 24) wks, [1000, 1500) g	Mortality	Females		620.446<br>(522.864 to 734.748)	186.215<br>(149.772 to 227.046)							
Meningococcal infection	Birth prevalence - [30, 32) wks, [500, 1000) g	Mortality	Males		332.681<br>(293.52 to 377.86)	212.332<br>(181.213 to 245.271)							
Meningococcal infection	Birth prevalence - [30, 32) wks, [500, 1000) g	Mortality	Females		239.082<br>(200.416 to 284.597)	163.95<br>(135.165 to 197.261)							
Meningococcal infection	Birth prevalence - [28, 30) wks, [500, 1000) g	Mortality	Males		326.557<br>(286.01 to 366.811)	249.246<br>(216.248 to 289.232)							
Meningococcal infection	Birth prevalence - [28, 30) wks, [500, 1000) g	Mortality	Females		231.533<br>(194.62 to 271.396)	187.47<br>(157.953 to 224.552)							
Meningococcal infection	Birth prevalence - [24, 26) wks, [1000, 1500) g	Mortality	Males		273.536<br>(236.795 to 312.421)	182.743<br>(155.477 to 212.281)							
Meningococcal infection	Birth prevalence - [24, 26) wks, [1000, 1500) g	Mortality	Females		268.033<br>(223.95 to 318.815)	157.928<br>(130.936 to 190.298)							
Meningococcal infection	Birth prevalence - [26, 28) wks, [1000, 1500) g	Mortality	Males		164.685<br>(145.457 to 186.571)	120.878<br>(102.672 to 140.967)							
Meningococcal infection	Birth prevalence - [26, 28) wks, [1000, 1500) g	Mortality	Females		142.274<br>(121.33 to 168.246)	97.496<br>(80.858 to 116.625)							
Meningococcal infection	Birth prevalence - [26, 28) wks, [1500, 2000) g	Mortality	Males		113.21<br>(97.079 to 130.806)	49.449<br>(40.908 to 59.113)							
Meningococcal infection	Birth prevalence - [26, 28) wks, [1500, 2000) g	Mortality	Females		108.393<br>(89.864 to 129.883)	49.979<br>(40.484 to 60.784)							
Meningococcal infection	Birth prevalence - [34, 36) wks, [1000, 1500) g	Mortality	Males		119.602<br>(104.655 to 136.729)	56.519<br>(48.084 to 66.018)							

Ages													
30-34 years	35-39 years	40-44 years	45-49 years	50-54 years	55-59 years	60-64 years	65-69 years	70-74 years	75-79 years	80-84 years	85-89 years	90-94 years	95+ years

Appendix Table 1a. Relative risks used by age and sex for each outcome for all risk factors except for ambient air pollution and alcohol.

Risk - Outcome	Category / Units	Morbidity / Mortality	Sex	All-age	0-6 days	7-27 days	28-364 days	1-4 years	5-9 years	10-14 years	15-19 years	20-24 years	25-29 years
Meningococcal infection	Birth prevalence - [34, 36) wks, [1000, 1500) g	Mortality	Females		109.231 (91.177 to 128.378)	52.834 (43.454 to 63.459)							
Meningococcal infection	Birth prevalence - [28, 30) wks, [1500, 2000) g	Mortality	Males		87.285 (76.012 to 100.83)	37.15 (30.87 to 43.664)							
Meningococcal infection	Birth prevalence - [28, 30) wks, [1500, 2000) g	Mortality	Females		86.639 (71.639 to 103.632)	38.3 (31.326 to 45.531)							
Meningococcal infection	Birth prevalence - [28, 30) wks, [1000, 1500) g	Mortality	Males		107.077 (93.214 to 122.668)	81.407 (70.511 to 93.12)							
Meningococcal infection	Birth prevalence - [28, 30) wks, [1000, 1500) g	Mortality	Females		87.409 (73.438 to 104.222)	64.981 (53.651 to 77.029)							
Meningococcal infection	Birth prevalence - [32, 34) wks, [1000, 1500) g	Mortality	Males		98.982 (86.649 to 112.931)	51.159 (43.522 to 59.402)							
Meningococcal infection	Birth prevalence - [32, 34) wks, [1000, 1500) g	Mortality	Females		84.081 (70.672 to 99.726)	44.695 (37.129 to 53.257)							
Meningococcal infection	Birth prevalence - [30, 32) wks, [1000, 1500) g	Mortality	Males		90.239 (78.583 to 103.215)	59.239 (50.511 to 69.508)							
Meningococcal infection	Birth prevalence - [30, 32) wks, [1000, 1500) g	Mortality	Females		70.119 (59.11 to 81.846)	47.493 (39.526 to 56.547)							
Meningococcal infection	Birth prevalence - [37, 38) wks, [1500, 2000) g	Mortality	Males		55.659 (48.726 to 62.963)	25.45 (21.867 to 29.451)							
Meningococcal infection	Birth prevalence - [37, 38) wks, [1500, 2000) g	Mortality	Females		54.453 (46.308 to 63.524)	29.339 (24.736 to 34.558)							
Meningococcal infection	Birth prevalence - [36, 37) wks, [1500, 2000) g	Mortality	Males		53.885 (47.099 to 61.414)	23.622 (20.373 to 27.298)							
Meningococcal infection	Birth prevalence - [36, 37) wks, [1500, 2000) g	Mortality	Females		47.995 (40.82 to 56.378)	23.905 (19.8 to 29.023)							
Meningococcal infection	Birth prevalence - [30, 32) wks, [2000, 2500) g	Mortality	Males		39.701 (34.573 to 45.311)	16.391 (13.683 to 19.411)							
Meningococcal infection	Birth prevalence - [30, 32) wks, [2000, 2500) g	Mortality	Females		34.894 (28.953 to 41.934)	16.549 (13.438 to 19.916)							
Meningococcal infection	Birth prevalence - [30, 32) wks, [1500, 2000) g	Mortality	Males		55.142 (48.379 to 62.989)	26.391 (22.751 to 30.584)							
Meningococcal infection	Birth prevalence - [30, 32) wks, [1500, 2000) g	Mortality	Females		48.414 (40.924 to 57.128)	26.986 (22.299 to 32.305)							
Meningococcal infection	Birth prevalence - [34, 36) wks, [1500, 2000) g	Mortality	Males		45.579 (39.817 to 51.922)	19.836 (16.94 to 23.141)							
Meningococcal infection	Birth prevalence - [34, 36) wks, [1500, 2000) g	Mortality	Females		38.405 (32.273 to 45.04)	18.409 (15.134 to 21.755)							
Meningococcal infection	Birth prevalence - [32, 34) wks, [1500, 2000) g	Mortality	Males		41.734 (36.381 to 47.516)	19.083 (16.193 to 21.909)							
Meningococcal infection	Birth prevalence - [32, 34) wks, [1500, 2000) g	Mortality	Females		36.162 (30.231 to 42.934)	19.528 (16.278 to 23.535)							
Meningococcal infection	Birth prevalence - [32, 34) wks, [2000, 2500) g	Mortality	Males		22.379 (19.576 to 25.426)	11.555 (9.874 to 13.332)							
Meningococcal infection	Birth prevalence - [32, 34) wks, [2000, 2500) g	Mortality	Females		20.326 (17.055 to 23.945)	12.376 (10.192 to 14.834)							
Meningococcal infection	Birth prevalence - [40, 42) wks, [2000, 2500) g	Mortality	Males		17.218 (14.893 to 19.961)	11.519 (9.766 to 13.495)							
Meningococcal infection	Birth prevalence - [40, 42) wks, [2000, 2500) g	Mortality	Females		13.555 (11.337 to 16.132)	11.838 (9.792 to 14.338)							
Meningococcal infection	Birth prevalence - [38, 40) wks, [2000, 2500) g	Mortality	Males		14.227 (12.521 to 16.06)	10.475 (9.122 to 12.085)							
Meningococcal infection	Birth prevalence - [38, 40) wks, [2000, 2500) g	Mortality	Females		10.867 (9.199 to 12.777)	10.493 (8.801 to 12.342)							
Meningococcal infection	Birth prevalence - [32, 34) wks, [2500, 3000) g	Mortality	Males		12.775 (11.146 to 14.607)	6.296 (5.269 to 7.299)							
Meningococcal infection	Birth prevalence - [32, 34) wks, [2500, 3000) g	Mortality	Females		9.041 (7.504 to 10.706)	6.205 (5.041 to 7.52)							
Meningococcal infection	Birth prevalence - [34, 36) wks, [2000, 2500) g	Mortality	Males		16.472 (14.422 to 18.7)	9.777 (8.456 to 11.277)							

[redacted]

Ages													
30-34 years	35-39 years	40-44 years	45-49 years	50-54 years	55-59 years	60-64 years	65-69 years	70-74 years	75-79 years	80-84 years	85-89 years	90-94 years	95+ years

Appendix Table 1a. Relative risks used by age and sex for each outcome for all risk factors except for ambient air pollution and alcohol.

Risk - Outcome	Category / Units	Morbidity / Mortality	Sex	All-age	0-6 days	7-27 days	28-364 days	1-4 years	5-9 years	10-14 years	15-19 years	20-24 years	25-29 years
Meningococcal infection	Birth prevalence - [34, 36) wks, [2000, 2500) g	Mortality	Females		12.895 (10.992 to 15.284)	9.44 (7.865 to 11.209)							
Meningococcal infection	Birth prevalence - [37, 38) wks, [2000, 2500) g	Mortality	Males		14.003 (12.407 to 15.831)	9.977 (8.758 to 11.444)							
Meningococcal infection	Birth prevalence - [37, 38) wks, [2000, 2500) g	Mortality	Females		10.456 (8.899 to 12.149)	9.83 (8.317 to 11.54)							
Meningococcal infection	Birth prevalence - [36, 37) wks, [2000, 2500) g	Mortality	Males		14.873 (13.083 to 16.88)	9.756 (8.38 to 11.214)							
Meningococcal infection	Birth prevalence - [36, 37) wks, [2000, 2500) g	Mortality	Females		10.517 (8.897 to 12.379)	9.233 (7.691 to 10.92)							
Meningococcal infection	Birth prevalence - [34, 36) wks, [2500, 3000) g	Mortality	Males		8.216 (7.133 to 9.378)	5.419 (4.631 to 6.282)							
Meningococcal infection	Birth prevalence - [34, 36) wks, [2500, 3000) g	Mortality	Females		6.037 (5.044 to 7.094)	5.198 (4.31 to 6.293)							
Meningococcal infection	Birth prevalence - [34, 36) wks, [4000, 4500) g	Mortality	Males		2.68 (2.245 to 3.183)	1.532 (1.254 to 1.867)							
Meningococcal infection	Birth prevalence - [34, 36) wks, [4000, 4500) g	Mortality	Females		2.458 (1.967 to 3.074)	1.673 (1.344 to 2.093)							
Meningococcal infection	Birth prevalence - [34, 36) wks, [3000, 3500) g	Mortality	Males		3.961 (3.433 to 4.521)	3.194 (2.71 to 3.696)							
Meningococcal infection	Birth prevalence - [34, 36) wks, [3000, 3500) g	Mortality	Females		3.267 (2.738 to 3.876)	2.783 (2.273 to 3.408)							
Meningococcal infection	Birth prevalence - [36, 37) wks, [2500, 3000) g	Mortality	Males		5.363 (4.69 to 6.082)	4.559 (3.945 to 5.248)							
Meningococcal infection	Birth prevalence - [36, 37) wks, [2500, 3000) g	Mortality	Females		3.969 (3.371 to 4.675)	4.138 (3.469 to 4.868)							
Meningococcal infection	Birth prevalence - [34, 36) wks, [3500, 4000) g	Mortality	Males		2.678 (2.289 to 3.086)	2.014 (1.69 to 2.365)							
Meningococcal infection	Birth prevalence - [34, 36) wks, [3500, 4000) g	Mortality	Females		2.342 (1.908 to 2.845)	1.756 (1.426 to 2.126)							
Meningococcal infection	Birth prevalence - [37, 38) wks, [2500, 3000) g	Mortality	Males		4.093 (3.581 to 4.626)	4.139 (3.631 to 4.74)							
Meningococcal infection	Birth prevalence - [37, 38) wks, [2500, 3000) g	Mortality	Females		3.07 (2.612 to 3.603)	3.618 (3.045 to 4.25)							
Meningococcal infection	Birth prevalence - [40, 42) wks, [2500, 3000) g	Mortality	Males		3.833 (3.364 to 4.388)	3.828 (3.271 to 4.426)							
Meningococcal infection	Birth prevalence - [40, 42) wks, [2500, 3000) g	Mortality	Females		2.699 (2.264 to 3.202)	3.379 (2.825 to 4.042)							
Meningococcal infection	Birth prevalence - [38, 40) wks, [2500, 3000) g	Mortality	Males		3.396 (3.002 to 3.839)	3.858 (3.346 to 4.424)							
Meningococcal infection	Birth prevalence - [38, 40) wks, [2500, 3000) g	Mortality	Females		2.382 (2.04 to 2.802)	3.27 (2.774 to 3.927)							
Meningococcal infection	Birth prevalence - [36, 37) wks, [3000, 3500) g	Mortality	Males		2.819 (2.433 to 3.229)	2.683 (2.299 to 3.079)							
Meningococcal infection	Birth prevalence - [36, 37) wks, [3000, 3500) g	Mortality	Females		2.335 (1.952 to 2.784)	2.434 (2.007 to 2.884)							
Meningococcal infection	Birth prevalence - [36, 37) wks, [4000, 4500) g	Mortality	Males		2.331 (1.993 to 2.727)	1.471 (1.221 to 1.731)							
Meningococcal infection	Birth prevalence - [36, 37) wks, [4000, 4500) g	Mortality	Females		2.141 (1.729 to 2.577)	1.601 (1.291 to 1.97)							
Meningococcal infection	Birth prevalence - [36, 37) wks, [3500, 4000) g	Mortality	Males		2.084 (1.789 to 2.418)	1.923 (1.624 to 2.252)							
Meningococcal infection	Birth prevalence - [36, 37) wks, [3500, 4000) g	Mortality	Females		1.845 (1.527 to 2.216)	1.731 (1.426 to 2.071)							
Meningococcal infection	Birth prevalence - [37, 38) wks, [3000, 3500) g	Mortality	Males		2.027 (1.788 to 2.287)	2.236 (1.926 to 2.542)							
Meningococcal infection	Birth prevalence - [37, 38) wks, [3000, 3500) g	Mortality	Females		1.667 (1.411 to 1.947)	1.989 (1.672 to 2.366)							
Meningococcal infection	Birth prevalence - [37, 38) wks, [4000, 4500) g	Mortality	Males		1.572 (1.352 to 1.813)	1.28 (1.095 to 1.481)							

Ages													
30-34 years	35-39 years	40-44 years	45-49 years	50-54 years	55-59 years	60-64 years	65-69 years	70-74 years	75-79 years	80-84 years	85-89 years	90-94 years	95+ years

Appendix Table 1a. Relative risks used by age and sex for each outcome for all risk factors except for ambient air pollution and alcohol.

Risk - Outcome	Category / Units	Morbidity / Mortality	Sex	All-age	0-6 days	7-27 days	28-364 days	1-4 years	5-9 years	10-14 years	15-19 years	20-24 years	25-29 years
Meningococcal infection	Birth prevalence - [37, 38) wks, [4000, 4500) g	Mortality	Females		1.647 (1.37 to 1.98)	1.396 (1.167 to 1.674)							
Meningococcal infection	Birth prevalence - [37, 38) wks, [3500, 4000) g	Mortality	Males		1.502 (1.309 to 1.712)	1.563 (1.353 to 1.807)							
Meningococcal infection	Birth prevalence - [37, 38) wks, [3500, 4000) g	Mortality	Females		1.275 (1.075 to 1.501)	1.444 (1.206 to 1.711)							
Meningococcal infection	Birth prevalence - [40, 42) wks, [3000, 3500) g	Mortality	Males		1.446 (1.258 to 1.639)	1.79 (1.546 to 2.08)							
Meningococcal infection	Birth prevalence - [40, 42) wks, [3000, 3500) g	Mortality	Females		1.103 (0.922 to 1.305)	1.491 (1.233 to 1.776)							
Meningococcal infection	Birth prevalence - [38, 40) wks, [3000, 3500) g	Mortality	Males		1.414 (1.242 to 1.593)	1.874 (1.636 to 2.156)							
Meningococcal infection	Birth prevalence - [38, 40) wks, [3000, 3500) g	Mortality	Females		1.096 (0.927 to 1.289)	1.617 (1.352 to 1.907)							
Meningococcal infection	Birth prevalence - [38, 40) wks, [4000, 4500) g	Mortality	Males		1.052 (0.92 to 1.203)	1.093 (0.939 to 1.261)							
Meningococcal infection	Birth prevalence - [38, 40) wks, [4000, 4500) g	Mortality	Females		1.121 (0.925 to 1.31)	1.149 (0.955 to 1.367)							
Meningococcal infection	Birth prevalence - [38, 40) wks, [3500, 4000) g	Mortality	Males		1.009 (0.883 to 1.143)	1.243 (1.078 to 1.421)							
Meningococcal infection	Birth prevalence - [38, 40) wks, [3500, 4000) g	Mortality	Females		0.828 (0.702 to 0.972)	1.155 (0.967 to 1.369)							
Meningococcal infection	Birth prevalence - [40, 42) wks, [3500, 4000) g	Mortality	Males		0.98 (0.854 to 1.123)	1.155 (0.983 to 1.334)							
Meningococcal infection	Birth prevalence - [40, 42) wks, [3500, 4000) g	Mortality	Females		0.819 (0.679 to 0.974)	1.045 (0.874 to 1.26)							
Other meningitis	Birth prevalence - [22, 24) wks, [0, 500) g	Mortality	Males		3273.944 (2987.306 to 3600.961)	599.42 (512.777 to 692.914)							
Other meningitis	Birth prevalence - [22, 24) wks, [0, 500) g	Mortality	Females		3353.667 (2964.862 to 3798.637)	771.075 (635.721 to 931.62)							
Other meningitis	Birth prevalence - [24, 26) wks, [0, 500) g	Mortality	Males		2454.576 (2216.544 to 2713.168)	884.902 (766.03 to 1021.494)							
Other meningitis	Birth prevalence - [24, 26) wks, [0, 500) g	Mortality	Females		2376.85 (2080.354 to 2699.254)	915.957 (765.805 to 1079.243)							
Other meningitis	Birth prevalence - [26, 28) wks, [0, 500) g	Mortality	Males		1870.684 (1676.978 to 2075.277)	847.512 (727.279 to 983.252)							
Other meningitis	Birth prevalence - [26, 28) wks, [0, 500) g	Mortality	Females		1708.132 (1477.512 to 1960.75)	822.906 (677.101 to 990.491)							
Other meningitis	Birth prevalence - [28, 30) wks, [0, 500) g	Mortality	Males		1805.939 (1612.157 to 2006.088)	583.881 (485.096 to 690.434)							
Other meningitis	Birth prevalence - [28, 30) wks, [0, 500) g	Mortality	Females		1585.788 (1377.653 to 1836.365)	636.164 (520.511 to 760.561)							
Other meningitis	Birth prevalence - [22, 24) wks, [500, 1000) g	Mortality	Males		2037.627 (1843.599 to 2249.686)	720.117 (628.641 to 820.917)							
Other meningitis	Birth prevalence - [22, 24) wks, [500, 1000) g	Mortality	Females		1833.266 (1596.246 to 2105.971)	610.368 (510.239 to 722.278)							
Other meningitis	Birth prevalence - [24, 26) wks, [500, 1000) g	Mortality	Males		852.585 (753.604 to 959.026)	642.627 (558.091 to 730.626)							
Other meningitis	Birth prevalence - [24, 26) wks, [500, 1000) g	Mortality	Females		715.379 (610.81 to 830.296)	546.057 (463.282 to 637.815)							
Other meningitis	Birth prevalence - [26, 28) wks, [500, 1000) g	Mortality	Males		411.87 (365.037 to 467.411)	353.266 (305.647 to 405.904)							
Other meningitis	Birth prevalence - [26, 28) wks, [500, 1000) g	Mortality	Females		308.092 (262.47 to 362.302)	292.936 (243.986 to 349.019)							
Other meningitis	Birth prevalence - [32, 34) wks, [500, 1000) g	Mortality	Males		344.501 (301.197 to 397.343)	194.954 (163.844 to 231.771)							
Other meningitis	Birth prevalence - [32, 34) wks, [500, 1000) g	Mortality	Females		299.264 (251.468 to 353.995)	161.548 (130.866 to 196.323)							
Other meningitis	Birth prevalence - [22, 24) wks, [1000, 1500) g	Mortality	Males		673.302 (584.187 to 768.258)	213.181 (179.555 to 254.572)							

Ages													
30-34 years	35-39 years	40-44 years	45-49 years	50-54 years	55-59 years	60-64 years	65-69 years	70-74 years	75-79 years	80-84 years	85-89 years	90-94 years	95+ years

Appendix Table 1a. Relative risks used by age and sex for each outcome for all risk factors except for ambient air pollution and alcohol.

Risk - Outcome	Category / Units	Morbidity / Mortality	Sex	All-age	0-6 days	7-27 days	28-364 days	1-4 years	5-9 years	10-14 years	15-19 years	20-24 years	25-29 years
Other meningitis	Birth prevalence - [22, 24) wks, [1000, 1500) g	Mortality	Females		620.446 (522.864 to 734.748)	186.215 (149.772 to 227.046)							
Other meningitis	Birth prevalence - [30, 32) wks, [500, 1000) g	Mortality	Males		332.681 (293.52 to 377.86)	212.332 (181.213 to 245.271)							
Other meningitis	Birth prevalence - [30, 32) wks, [500, 1000) g	Mortality	Females		239.082 (200.416 to 284.597)	163.95 (135.165 to 197.261)							
Other meningitis	Birth prevalence - [28, 30) wks, [500, 1000) g	Mortality	Males		326.557 (286.01 to 366.811)	249.246 (216.248 to 289.232)							
Other meningitis	Birth prevalence - [28, 30) wks, [500, 1000) g	Mortality	Females		231.533 (194.62 to 271.396)	187.47 (157.953 to 224.552)							
Other meningitis	Birth prevalence - [24, 26) wks, [1000, 1500) g	Mortality	Males		273.536 (236.795 to 312.421)	182.743 (155.477 to 212.281)							
Other meningitis	Birth prevalence - [24, 26) wks, [1000, 1500) g	Mortality	Females		268.033 (223.95 to 318.815)	157.928 (130.936 to 190.298)							
Other meningitis	Birth prevalence - [26, 28) wks, [1000, 1500) g	Mortality	Males		164.685 (145.457 to 186.571)	120.878 (102.672 to 140.967)							
Other meningitis	Birth prevalence - [26, 28) wks, [1000, 1500) g	Mortality	Females		142.274 (121.33 to 168.246)	97.496 (80.858 to 116.625)							
Other meningitis	Birth prevalence - [26, 28) wks, [1500, 2000) g	Mortality	Males		113.21 (97.079 to 130.806)	49.449 (40.908 to 59.113)							
Other meningitis	Birth prevalence - [26, 28) wks, [1500, 2000) g	Mortality	Females		108.393 (89.864 to 129.883)	49.979 (40.484 to 60.784)							
Other meningitis	Birth prevalence - [34, 36) wks, [1000, 1500) g	Mortality	Males		119.602 (104.655 to 136.729)	56.519 (48.084 to 66.018)							
Other meningitis	Birth prevalence - [34, 36) wks, [1000, 1500) g	Mortality	Females		109.231 (91.177 to 128.378)	52.834 (43.454 to 63.459)							
Other meningitis	Birth prevalence - [28, 30) wks, [1500, 2000) g	Mortality	Males		87.285 (76.012 to 100.83)	37.15 (30.87 to 43.664)							
Other meningitis	Birth prevalence - [28, 30) wks, [1500, 2000) g	Mortality	Females		86.639 (71.639 to 103.632)	38.3 (31.326 to 45.531)							
Other meningitis	Birth prevalence - [28, 30) wks, [1000, 1500) g	Mortality	Males		107.077 (93.214 to 122.668)	81.407 (70.511 to 93.12)							
Other meningitis	Birth prevalence - [28, 30) wks, [1000, 1500) g	Mortality	Females		87.409 (73.438 to 104.222)	64.981 (53.651 to 77.029)							
Other meningitis	Birth prevalence - [32, 34) wks, [1000, 1500) g	Mortality	Males		98.982 (86.649 to 112.931)	51.159 (43.522 to 59.402)							
Other meningitis	Birth prevalence - [32, 34) wks, [1000, 1500) g	Mortality	Females		84.081 (70.672 to 99.726)	44.695 (37.129 to 53.257)							
Other meningitis	Birth prevalence - [30, 32) wks, [1000, 1500) g	Mortality	Males		90.239 (78.583 to 103.215)	59.239 (50.511 to 69.508)							
Other meningitis	Birth prevalence - [30, 32) wks, [1000, 1500) g	Mortality	Females		70.119 (59.11 to 81.846)	47.493 (39.526 to 56.547)							
Other meningitis	Birth prevalence - [37, 38) wks, [1500, 2000) g	Mortality	Males		55.659 (48.726 to 62.963)	25.45 (21.867 to 29.451)							
Other meningitis	Birth prevalence - [37, 38) wks, [1500, 2000) g	Mortality	Females		54.453 (46.308 to 63.524)	29.339 (24.736 to 34.558)							
Other meningitis	Birth prevalence - [36, 37) wks, [1500, 2000) g	Mortality	Males		53.885 (47.099 to 61.414)	23.622 (20.373 to 27.298)							
Other meningitis	Birth prevalence - [36, 37) wks, [1500, 2000) g	Mortality	Females		47.995 (40.82 to 56.378)	23.905 (19.8 to 29.023)							
Other meningitis	Birth prevalence - [30, 32) wks, [2000, 2500) g	Mortality	Males		39.701 (34.573 to 45.311)	16.391 (13.683 to 19.411)							
Other meningitis	Birth prevalence - [30, 32) wks, [2000, 2500) g	Mortality	Females		34.894 (28.953 to 41.934)	16.549 (13.438 to 19.916)							
Other meningitis	Birth prevalence - [30, 32) wks, [1500, 2000) g	Mortality	Males		55.142 (48.379 to 62.989)	26.391 (22.751 to 30.584)							
Other meningitis	Birth prevalence - [30, 32) wks, [1500, 2000) g	Mortality	Females		48.414 (40.924 to 57.128)	26.986 (22.299 to 32.305)							
Other meningitis	Birth prevalence - [34, 36) wks, [1500, 2000) g	Mortality	Males		45.579 (39.817 to 51.922)	19.836 (16.94 to 23.141)							

Ages													
30-34 years	35-39 years	40-44 years	45-49 years	50-54 years	55-59 years	60-64 years	65-69 years	70-74 years	75-79 years	80-84 years	85-89 years	90-94 years	95+ years

Appendix Table 1a. Relative risks used by age and sex for each outcome for all risk factors except for ambient air pollution and alcohol.

Risk - Outcome	Category / Units	Morbidity / Mortality	Sex	All-age	0-6 days	7-27 days	28-364 days	1-4 years	5-9 years	10-14 years	15-19 years	20-24 years	25-29 years
Other meningitis	Birth prevalence - [34, 36) wks, [1500, 2000) g	Mortality	Females		38.405 (32.273 to 45.04)	18.409 (15.134 to 21.755)							
Other meningitis	Birth prevalence - [32, 34) wks, [1500, 2000) g	Mortality	Males		41.734 (36.381 to 47.516)	19.083 (16.193 to 21.909)							
Other meningitis	Birth prevalence - [32, 34) wks, [1500, 2000) g	Mortality	Females		36.162 (30.231 to 42.934)	19.528 (16.278 to 23.535)							
Other meningitis	Birth prevalence - [32, 34) wks, [2000, 2500) g	Mortality	Males		22.379 (19.576 to 25.426)	11.555 (9.874 to 13.332)							
Other meningitis	Birth prevalence - [32, 34) wks, [2000, 2500) g	Mortality	Females		20.326 (17.055 to 23.945)	12.376 (10.192 to 14.834)							
Other meningitis	Birth prevalence - [40, 42) wks, [2000, 2500) g	Mortality	Males		17.218 (14.893 to 19.961)	11.519 (9.766 to 13.495)							
Other meningitis	Birth prevalence - [40, 42) wks, [2000, 2500) g	Mortality	Females		13.555 (11.337 to 16.132)	11.838 (9.792 to 14.338)							
Other meningitis	Birth prevalence - [38, 40) wks, [2000, 2500) g	Mortality	Males		14.227 (12.521 to 16.06)	10.475 (9.122 to 12.085)							
Other meningitis	Birth prevalence - [38, 40) wks, [2000, 2500) g	Mortality	Females		10.867 (9.199 to 12.777)	10.493 (8.801 to 12.342)							
Other meningitis	Birth prevalence - [32, 34) wks, [2500, 3000) g	Mortality	Males		12.775 (11.146 to 14.607)	6.296 (5.269 to 7.299)							
Other meningitis	Birth prevalence - [32, 34) wks, [2500, 3000) g	Mortality	Females		9.041 (7.504 to 10.706)	6.205 (5.041 to 7.52)							
Other meningitis	Birth prevalence - [34, 36) wks, [2000, 2500) g	Mortality	Males		16.472 (14.422 to 18.7)	9.777 (8.456 to 11.277)							
Other meningitis	Birth prevalence - [34, 36) wks, [2000, 2500) g	Mortality	Females		12.895 (10.992 to 15.284)	9.44 (7.865 to 11.209)							
Other meningitis	Birth prevalence - [37, 38) wks, [2000, 2500) g	Mortality	Males		14.003 (12.407 to 15.831)	9.977 (8.758 to 11.444)							
Other meningitis	Birth prevalence - [37, 38) wks, [2000, 2500) g	Mortality	Females		10.456 (8.899 to 12.149)	9.83 (8.317 to 11.54)							
Other meningitis	Birth prevalence - [36, 37) wks, [2000, 2500) g	Mortality	Males		14.873 (13.083 to 16.88)	9.756 (8.38 to 11.214)							
Other meningitis	Birth prevalence - [36, 37) wks, [2000, 2500) g	Mortality	Females		10.517 (8.897 to 12.379)	9.233 (7.691 to 10.92)							
Other meningitis	Birth prevalence - [34, 36) wks, [2500, 3000) g	Mortality	Males		8.216 (7.133 to 9.378)	5.419 (4.631 to 6.282)							
Other meningitis	Birth prevalence - [34, 36) wks, [2500, 3000) g	Mortality	Females		6.037 (5.044 to 7.094)	5.198 (4.31 to 6.293)							
Other meningitis	Birth prevalence - [34, 36) wks, [4000, 4500) g	Mortality	Males		2.68 (2.245 to 3.183)	1.532 (1.254 to 1.867)							
Other meningitis	Birth prevalence - [34, 36) wks, [4000, 4500) g	Mortality	Females		2.458 (1.967 to 3.074)	1.673 (1.344 to 2.093)							
Other meningitis	Birth prevalence - [34, 36) wks, [3000, 3500) g	Mortality	Males		3.961 (3.433 to 4.521)	3.194 (2.71 to 3.696)							
Other meningitis	Birth prevalence - [34, 36) wks, [3000, 3500) g	Mortality	Females		3.267 (2.738 to 3.876)	2.783 (2.273 to 3.408)							
Other meningitis	Birth prevalence - [36, 37) wks, [2500, 3000) g	Mortality	Males		5.363 (4.69 to 6.082)	4.559 (3.945 to 5.248)							
Other meningitis	Birth prevalence - [36, 37) wks, [2500, 3000) g	Mortality	Females		3.969 (3.371 to 4.675)	4.138 (3.469 to 4.868)							
Other meningitis	Birth prevalence - [34, 36) wks, [3500, 4000) g	Mortality	Males		2.678 (2.289 to 3.086)	2.014 (1.69 to 2.365)							
Other meningitis	Birth prevalence - [34, 36) wks, [3500, 4000) g	Mortality	Females		2.342 (1.908 to 2.845)	1.756 (1.426 to 2.126)							
Other meningitis	Birth prevalence - [37, 38) wks, [2500, 3000) g	Mortality	Males		4.093 (3.581 to 4.626)	4.139 (3.631 to 4.74)							
Other meningitis	Birth prevalence - [37, 38) wks, [2500, 3000) g	Mortality	Females		3.07 (2.612 to 3.603)	3.618 (3.045 to 4.25)							
Other meningitis	Birth prevalence - [40, 42) wks, [2500, 3000) g	Mortality	Males		3.833 (3.364 to 4.388)	3.828 (3.271 to 4.426)							

Ages													
30-34 years	35-39 years	40-44 years	45-49 years	50-54 years	55-59 years	60-64 years	65-69 years	70-74 years	75-79 years	80-84 years	85-89 years	90-94 years	95+ years

Appendix Table 1a. Relative risks used by age and sex for each outcome for all risk factors except for ambient air pollution and alcohol.

Risk - Outcome	Category / Units	Morbidity / Mortality	Sex	All-age	0-6 days	7-27 days	28-364 days	1-4 years	5-9 years	10-14 years	15-19 years	20-24 years	25-29 years
Other meningitis	Birth prevalence - [40, 42) wks, [2500, 3000) g	Mortality	Females		2.699 (2.264 to 3.202)	3.379 (2.825 to 4.042)							
Other meningitis	Birth prevalence - [38, 40) wks, [2500, 3000) g	Mortality	Males		3.396 (3.002 to 3.839)	3.858 (3.346 to 4.424)							
Other meningitis	Birth prevalence - [38, 40) wks, [2500, 3000) g	Mortality	Females		2.382 (2.04 to 2.802)	3.27 (2.774 to 3.927)							
Other meningitis	Birth prevalence - [36, 37) wks, [3000, 3500) g	Mortality	Males		2.819 (2.433 to 3.229)	2.683 (2.299 to 3.079)							
Other meningitis	Birth prevalence - [36, 37) wks, [3000, 3500) g	Mortality	Females		2.335 (1.952 to 2.784)	2.434 (2.007 to 2.884)							
Other meningitis	Birth prevalence - [36, 37) wks, [4000, 4500) g	Mortality	Males		2.331 (1.993 to 2.727)	1.471 (1.221 to 1.731)							
Other meningitis	Birth prevalence - [36, 37) wks, [4000, 4500) g	Mortality	Females		2.141 (1.729 to 2.577)	1.601 (1.291 to 1.97)							
Other meningitis	Birth prevalence - [36, 37) wks, [3500, 4000) g	Mortality	Males		2.084 (1.789 to 2.418)	1.923 (1.624 to 2.252)							
Other meningitis	Birth prevalence - [36, 37) wks, [3500, 4000) g	Mortality	Females		1.845 (1.527 to 2.216)	1.731 (1.426 to 2.071)							
Other meningitis	Birth prevalence - [37, 38) wks, [3000, 3500) g	Mortality	Males		2.027 (1.788 to 2.287)	2.236 (1.926 to 2.542)							
Other meningitis	Birth prevalence - [37, 38) wks, [3000, 3500) g	Mortality	Females		1.667 (1.411 to 1.947)	1.989 (1.672 to 2.366)							
Other meningitis	Birth prevalence - [37, 38) wks, [4000, 4500) g	Mortality	Males		1.572 (1.352 to 1.813)	1.28 (1.095 to 1.481)							
Other meningitis	Birth prevalence - [37, 38) wks, [4000, 4500) g	Mortality	Females		1.647 (1.37 to 1.98)	1.396 (1.167 to 1.674)							
Other meningitis	Birth prevalence - [37, 38) wks, [3500, 4000) g	Mortality	Males		1.502 (1.309 to 1.712)	1.563 (1.353 to 1.807)							
Other meningitis	Birth prevalence - [37, 38) wks, [3500, 4000) g	Mortality	Females		1.275 (1.075 to 1.501)	1.444 (1.206 to 1.711)							
Other meningitis	Birth prevalence - [40, 42) wks, [3000, 3500) g	Mortality	Males		1.446 (1.258 to 1.639)	1.79 (1.546 to 2.08)							
Other meningitis	Birth prevalence - [40, 42) wks, [3000, 3500) g	Mortality	Females		1.103 (0.922 to 1.305)	1.491 (1.233 to 1.776)							
Other meningitis	Birth prevalence - [38, 40) wks, [3000, 3500) g	Mortality	Males		1.414 (1.242 to 1.593)	1.874 (1.636 to 2.156)							
Other meningitis	Birth prevalence - [38, 40) wks, [3000, 3500) g	Mortality	Females		1.096 (0.927 to 1.289)	1.617 (1.352 to 1.907)							
Other meningitis	Birth prevalence - [38, 40) wks, [4000, 4500) g	Mortality	Males		1.052 (0.92 to 1.203)	1.093 (0.939 to 1.261)							
Other meningitis	Birth prevalence - [38, 40) wks, [4000, 4500) g	Mortality	Females		1.121 (0.925 to 1.31)	1.149 (0.955 to 1.367)							
Other meningitis	Birth prevalence - [38, 40) wks, [3500, 4000) g	Mortality	Males		1.009 (0.883 to 1.143)	1.243 (1.078 to 1.421)							
Other meningitis	Birth prevalence - [38, 40) wks, [3500, 4000) g	Mortality	Females		0.828 (0.702 to 0.972)	1.155 (0.967 to 1.369)							
Other meningitis	Birth prevalence - [40, 42) wks, [3500, 4000) g	Mortality	Males		0.98 (0.854 to 1.123)	1.155 (0.983 to 1.334)							
Other meningitis	Birth prevalence - [40, 42) wks, [3500, 4000) g	Mortality	Females		0.819 (0.679 to 0.974)	1.045 (0.874 to 1.26)							
Encephalitis	Birth prevalence - [22, 24) wks, [0, 500) g	Mortality	Males		3273.944 (2987.306 to 3600.961)	599.42 (512.777 to 692.914)							
Encephalitis	Birth prevalence - [22, 24) wks, [0, 500) g	Mortality	Females		3353.667 (2964.862 to 3798.637)	771.075 (635.721 to 931.62)							
Encephalitis	Birth prevalence - [24, 26) wks, [0, 500) g	Mortality	Males		2454.576 (2216.544 to 2713.168)	884.902 (766.03 to 1021.494)							
Encephalitis	Birth prevalence - [24, 26) wks, [0, 500) g	Mortality	Females		2376.85 (2080.354 to 2699.254)	915.957 (765.805 to 1079.243)							
Encephalitis	Birth prevalence - [26, 28) wks, [0, 500) g	Mortality	Males		1870.684 (1676.978 to 2075.277)	847.512 (727.279 to 983.252)							

Ages													
**30-34 years**	**35-39 years**	**40-44 years**	**45-49 years**	**50-54 years**	**55-59 years**	**60-64 years**	**65-69 years**	**70-74 years**	**75-79 years**	**80-84 years**	**85-89 years**	**90-94 years**	**95+ years**

Appendix Table 1a. Relative risks used by age and sex for each outcome for all risk factors except for ambient air pollution and alcohol.

Risk - Outcome	Category / Units	Morbidity / Mortality	Sex	All-age	0-6 days	7-27 days	28-364 days	1-4 years	5-9 years	10-14 years	15-19 years	20-24 years	25-29 years
Encephalitis	Birth prevalence - [26, 28) wks, [0, 500) g	Mortality	Females		1708.132 (1477.512 to 1960.75)	822.906 (677.101 to 990.491)							
Encephalitis	Birth prevalence - [28, 30) wks, [0, 500) g	Mortality	Males		1805.939 (1612.157 to 2006.088)	583.881 (485.096 to 690.434)							
Encephalitis	Birth prevalence - [28, 30) wks, [0, 500) g	Mortality	Females		1585.788 (1377.653 to 1836.365)	636.164 (520.511 to 760.561)							
Encephalitis	Birth prevalence - [22, 24) wks, [500, 1000) g	Mortality	Males		2037.627 (1843.599 to 2249.686)	720.117 (628.641 to 820.917)							
Encephalitis	Birth prevalence - [22, 24) wks, [500, 1000) g	Mortality	Females		1833.266 (1596.246 to 2105.971)	610.368 (510.239 to 722.278)							
Encephalitis	Birth prevalence - [24, 26) wks, [500, 1000) g	Mortality	Males		852.585 (753.604 to 959.026)	642.627 (558.091 to 730.626)							
Encephalitis	Birth prevalence - [24, 26) wks, [500, 1000) g	Mortality	Females		715.379 (610.81 to 830.296)	546.057 (463.282 to 637.815)							
Encephalitis	Birth prevalence - [26, 28) wks, [500, 1000) g	Mortality	Males		411.87 (365.037 to 467.411)	353.266 (305.647 to 405.904)							
Encephalitis	Birth prevalence - [26, 28) wks, [500, 1000) g	Mortality	Females		308.092 (262.47 to 362.302)	292.936 (243.986 to 349.019)							
Encephalitis	Birth prevalence - [32, 34) wks, [500, 1000) g	Mortality	Males		344.501 (301.197 to 397.343)	194.954 (163.844 to 231.771)							
Encephalitis	Birth prevalence - [32, 34) wks, [500, 1000) g	Mortality	Females		299.264 (251.468 to 353.995)	161.548 (130.866 to 196.323)							
Encephalitis	Birth prevalence - [22, 24) wks, [1000, 1500) g	Mortality	Males		673.302 (584.187 to 768.258)	213.181 (179.555 to 254.572)							
Encephalitis	Birth prevalence - [22, 24) wks, [1000, 1500) g	Mortality	Females		620.446 (522.864 to 734.748)	186.215 (149.772 to 227.046)							
Encephalitis	Birth prevalence - [30, 32) wks, [500, 1000) g	Mortality	Males		332.681 (293.52 to 377.86)	212.332 (181.213 to 245.271)							
Encephalitis	Birth prevalence - [30, 32) wks, [500, 1000) g	Mortality	Females		239.082 (200.416 to 284.597)	163.95 (135.165 to 197.261)							
Encephalitis	Birth prevalence - [28, 30) wks, [500, 1000) g	Mortality	Males		326.557 (286.01 to 366.811)	249.246 (216.248 to 289.232)							
Encephalitis	Birth prevalence - [28, 30) wks, [500, 1000) g	Mortality	Females		231.533 (194.62 to 271.396)	187.47 (157.953 to 224.552)							
Encephalitis	Birth prevalence - [24, 26) wks, [1000, 1500) g	Mortality	Males		273.536 (236.795 to 312.421)	182.743 (155.477 to 212.281)							
Encephalitis	Birth prevalence - [24, 26) wks, [1000, 1500) g	Mortality	Females		268.033 (223.95 to 318.815)	157.928 (130.936 to 190.298)							
Encephalitis	Birth prevalence - [26, 28) wks, [1000, 1500) g	Mortality	Males		164.685 (145.457 to 186.571)	120.878 (102.672 to 140.967)							
Encephalitis	Birth prevalence - [26, 28) wks, [1000, 1500) g	Mortality	Females		142.274 (121.33 to 168.246)	97.496 (80.858 to 116.625)							
Encephalitis	Birth prevalence - [26, 28) wks, [1500, 2000) g	Mortality	Males		113.21 (97.079 to 130.806)	49.449 (40.908 to 59.113)							
Encephalitis	Birth prevalence - [26, 28) wks, [1500, 2000) g	Mortality	Females		108.393 (89.864 to 129.883)	49.979 (40.484 to 60.784)							
Encephalitis	Birth prevalence - [34, 36) wks, [1000, 1500) g	Mortality	Males		119.602 (104.655 to 136.729)	56.519 (48.084 to 66.018)							
Encephalitis	Birth prevalence - [34, 36) wks, [1000, 1500) g	Mortality	Females		109.231 (91.177 to 128.378)	52.834 (43.454 to 63.459)							
Encephalitis	Birth prevalence - [28, 30) wks, [1500, 2000) g	Mortality	Males		87.285 (76.012 to 100.83)	37.15 (30.87 to 43.664)							
Encephalitis	Birth prevalence - [28, 30) wks, [1500, 2000) g	Mortality	Females		86.639 (71.639 to 103.632)	38.3 (31.326 to 45.531)							
Encephalitis	Birth prevalence - [28, 30) wks, [1000, 1500) g	Mortality	Males		107.077 (93.214 to 122.668)	81.407 (70.511 to 93.12)							
Encephalitis	Birth prevalence - [28, 30) wks, [1000, 1500) g	Mortality	Females		87.409 (73.438 to 104.222)	64.981 (53.651 to 77.029)							
Encephalitis	Birth prevalence - [32, 34) wks, [1000, 1500) g	Mortality	Males		98.982 (86.649 to 112.931)	51.159 (43.522 to 59.402)							

Ages													
30-34 years	35-39 years	40-44 years	45-49 years	50-54 years	55-59 years	60-64 years	65-69 years	70-74 years	75-79 years	80-84 years	85-89 years	90-94 years	95+ years

Appendix Table 1a. Relative risks used by age and sex for each outcome for all risk factors except for ambient air pollution and alcohol.

Risk - Outcome	Category / Units	Morbidity / Mortality	Sex	All-age	0-6 days	7-27 days	28-364 days	1-4 years	5-9 years	10-14 years	15-19 years	20-24 years	25-29 years
Encephalitis	Birth prevalence - [32, 34) wks, [1000, 1500) g	Mortality	Females		84.081 (70.672 to 99.726)	44.695 (37.129 to 53.257)							
Encephalitis	Birth prevalence - [30, 32) wks, [1000, 1500) g	Mortality	Males		90.239 (78.583 to 103.215)	59.239 (50.511 to 69.508)							
Encephalitis	Birth prevalence - [30, 32) wks, [1000, 1500) g	Mortality	Females		70.119 (59.11 to 81.846)	47.493 (39.526 to 56.547)							
Encephalitis	Birth prevalence - [37, 38) wks, [1500, 2000) g	Mortality	Males		55.659 (48.726 to 62.963)	25.45 (21.867 to 29.451)							
Encephalitis	Birth prevalence - [37, 38) wks, [1500, 2000) g	Mortality	Females		54.453 (46.308 to 63.524)	29.339 (24.736 to 34.558)							
Encephalitis	Birth prevalence - [36, 37) wks, [1500, 2000) g	Mortality	Males		53.885 (47.099 to 61.414)	23.622 (20.373 to 27.298)							
Encephalitis	Birth prevalence - [36, 37) wks, [1500, 2000) g	Mortality	Females		47.995 (40.82 to 56.378)	23.905 (19.8 to 29.023)							
Encephalitis	Birth prevalence - [30, 32) wks, [2000, 2500) g	Mortality	Males		39.701 (34.573 to 45.311)	16.391 (13.683 to 19.411)							
Encephalitis	Birth prevalence - [30, 32) wks, [2000, 2500) g	Mortality	Females		34.894 (28.953 to 41.934)	16.549 (13.438 to 19.916)							
Encephalitis	Birth prevalence - [30, 32) wks, [1500, 2000) g	Mortality	Males		55.142 (48.379 to 62.989)	26.391 (22.751 to 30.584)							
Encephalitis	Birth prevalence - [30, 32) wks, [1500, 2000) g	Mortality	Females		48.414 (40.924 to 57.128)	26.986 (22.299 to 32.305)							
Encephalitis	Birth prevalence - [34, 36) wks, [1500, 2000) g	Mortality	Males		45.579 (39.817 to 51.922)	19.836 (16.94 to 23.141)							
Encephalitis	Birth prevalence - [34, 36) wks, [1500, 2000) g	Mortality	Females		38.405 (32.273 to 45.04)	18.409 (15.134 to 21.755)							
Encephalitis	Birth prevalence - [32, 34) wks, [1500, 2000) g	Mortality	Males		41.734 (36.381 to 47.516)	19.083 (16.193 to 21.909)							
Encephalitis	Birth prevalence - [32, 34) wks, [1500, 2000) g	Mortality	Females		36.162 (30.231 to 42.934)	19.528 (16.278 to 23.535)							
Encephalitis	Birth prevalence - [32, 34) wks, [2000, 2500) g	Mortality	Males		22.379 (19.576 to 25.426)	11.555 (9.874 to 13.332)							
Encephalitis	Birth prevalence - [32, 34) wks, [2000, 2500) g	Mortality	Females		20.326 (17.055 to 23.945)	12.376 (10.192 to 14.834)							
Encephalitis	Birth prevalence - [40, 42) wks, [2000, 2500) g	Mortality	Males		17.218 (14.893 to 19.961)	11.519 (9.766 to 13.495)							
Encephalitis	Birth prevalence - [40, 42) wks, [2000, 2500) g	Mortality	Females		13.555 (11.337 to 16.132)	11.838 (9.792 to 14.338)							
Encephalitis	Birth prevalence - [38, 40) wks, [2000, 2500) g	Mortality	Males		14.227 (12.521 to 16.06)	10.475 (9.122 to 12.085)							
Encephalitis	Birth prevalence - [38, 40) wks, [2000, 2500) g	Mortality	Females		10.867 (9.199 to 12.777)	10.493 (8.801 to 12.342)							
Encephalitis	Birth prevalence - [32, 34) wks, [2500, 3000) g	Mortality	Males		12.775 (11.146 to 14.607)	6.296 (5.269 to 7.299)							
Encephalitis	Birth prevalence - [32, 34) wks, [2500, 3000) g	Mortality	Females		9.041 (7.504 to 10.706)	6.205 (5.041 to 7.52)							
Encephalitis	Birth prevalence - [34, 36) wks, [2000, 2500) g	Mortality	Males		16.472 (14.422 to 18.7)	9.777 (8.456 to 11.277)							
Encephalitis	Birth prevalence - [34, 36) wks, [2000, 2500) g	Mortality	Females		12.895 (10.992 to 15.284)	9.44 (7.865 to 11.209)							
Encephalitis	Birth prevalence - [37, 38) wks, [2000, 2500) g	Mortality	Males		14.003 (12.407 to 15.831)	9.977 (8.758 to 11.444)							
Encephalitis	Birth prevalence - [37, 38) wks, [2000, 2500) g	Mortality	Females		10.456 (8.899 to 12.149)	9.83 (8.317 to 11.54)							
Encephalitis	Birth prevalence - [36, 37) wks, [2000, 2500) g	Mortality	Males		14.873 (13.083 to 16.88)	9.756 (8.38 to 11.214)							
Encephalitis	Birth prevalence - [36, 37) wks, [2000, 2500) g	Mortality	Females		10.517 (8.897 to 12.379)	9.233 (7.691 to 10.92)							
Encephalitis	Birth prevalence - [34, 36) wks, [2500, 3000) g	Mortality	Males		8.216 (7.133 to 9.378)	5.419 (4.631 to 6.282)							

Ages													
**30-34 years**	**35-39 years**	**40-44 years**	**45-49 years**	**50-54 years**	**55-59 years**	**60-64 years**	**65-69 years**	**70-74 years**	**75-79 years**	**80-84 years**	**85-89 years**	**90-94 years**	**95+ years**

Appendix Table 1a. Relative risks used by age and sex for each outcome for all risk factors except for ambient air pollution and alcohol.

Risk - Outcome	Category / Units	Morbidity / Mortality	Sex	All-age	0-6 days	7-27 days	28-364 days	1-4 years	5-9 years	10-14 years	15-19 years	20-24 years	25-29 years
Encephalitis	Birth prevalence - [34, 36) wks, [2500, 3000) g	Mortality	Females		6.037 (5.044 to 7.094)	5.198 (4.31 to 6.293)							
Encephalitis	Birth prevalence - [34, 36) wks, [4000, 4500) g	Mortality	Males		2.68 (2.245 to 3.183)	1.532 (1.254 to 1.867)							
Encephalitis	Birth prevalence - [34, 36) wks, [4000, 4500) g	Mortality	Females		2.458 (1.967 to 3.074)	1.673 (1.344 to 2.093)							
Encephalitis	Birth prevalence - [34, 36) wks, [3000, 3500) g	Mortality	Males		3.961 (3.433 to 4.521)	3.194 (2.71 to 3.696)							
Encephalitis	Birth prevalence - [34, 36) wks, [3000, 3500) g	Mortality	Females		3.267 (2.738 to 3.876)	2.783 (2.273 to 3.408)							
Encephalitis	Birth prevalence - [36, 37) wks, [2500, 3000) g	Mortality	Males		5.363 (4.69 to 6.082)	4.559 (3.945 to 5.248)							
Encephalitis	Birth prevalence - [36, 37) wks, [2500, 3000) g	Mortality	Females		3.969 (3.371 to 4.675)	4.138 (3.469 to 4.868)							
Encephalitis	Birth prevalence - [34, 36) wks, [3500, 4000) g	Mortality	Males		2.678 (2.289 to 3.086)	2.014 (1.69 to 2.365)							
Encephalitis	Birth prevalence - [34, 36) wks, [3500, 4000) g	Mortality	Females		2.342 (1.908 to 2.845)	1.756 (1.426 to 2.126)							
Encephalitis	Birth prevalence - [37, 38) wks, [2500, 3000) g	Mortality	Males		4.093 (3.581 to 4.626)	4.139 (3.631 to 4.74)							
Encephalitis	Birth prevalence - [37, 38) wks, [2500, 3000) g	Mortality	Females		3.07 (2.612 to 3.603)	3.618 (3.045 to 4.25)							
Encephalitis	Birth prevalence - [40, 42) wks, [2500, 3000) g	Mortality	Males		3.833 (3.364 to 4.388)	3.828 (3.271 to 4.426)							
Encephalitis	Birth prevalence - [40, 42) wks, [2500, 3000) g	Mortality	Females		2.699 (2.264 to 3.202)	3.379 (2.825 to 4.042)							
Encephalitis	Birth prevalence - [38, 40) wks, [2500, 3000) g	Mortality	Males		3.396 (3.002 to 3.839)	3.858 (3.346 to 4.424)							
Encephalitis	Birth prevalence - [38, 40) wks, [2500, 3000) g	Mortality	Females		2.382 (2.04 to 2.802)	3.27 (2.774 to 3.927)							
Encephalitis	Birth prevalence - [36, 37) wks, [3000, 3500) g	Mortality	Males		2.819 (2.433 to 3.229)	2.683 (2.299 to 3.079)							
Encephalitis	Birth prevalence - [36, 37) wks, [3000, 3500) g	Mortality	Females		2.335 (1.952 to 2.784)	2.434 (2.007 to 2.884)							
Encephalitis	Birth prevalence - [36, 37) wks, [4000, 4500) g	Mortality	Males		2.331 (1.993 to 2.727)	1.471 (1.221 to 1.731)							
Encephalitis	Birth prevalence - [36, 37) wks, [4000, 4500) g	Mortality	Females		2.141 (1.729 to 2.577)	1.601 (1.291 to 1.97)							
Encephalitis	Birth prevalence - [36, 37) wks, [3500, 4000) g	Mortality	Males		2.084 (1.789 to 2.418)	1.923 (1.624 to 2.252)							
Encephalitis	Birth prevalence - [36, 37) wks, [3500, 4000) g	Mortality	Females		1.845 (1.527 to 2.216)	1.731 (1.426 to 2.071)							
Encephalitis	Birth prevalence - [37, 38) wks, [3000, 3500) g	Mortality	Males		2.027 (1.788 to 2.287)	2.236 (1.926 to 2.542)							
Encephalitis	Birth prevalence - [37, 38) wks, [3000, 3500) g	Mortality	Females		1.667 (1.411 to 1.947)	1.989 (1.672 to 2.366)							
Encephalitis	Birth prevalence - [37, 38) wks, [4000, 4500) g	Mortality	Males		1.572 (1.352 to 1.813)	1.28 (1.095 to 1.481)							
Encephalitis	Birth prevalence - [37, 38) wks, [4000, 4500) g	Mortality	Females		1.647 (1.37 to 1.98)	1.396 (1.167 to 1.674)							
Encephalitis	Birth prevalence - [37, 38) wks, [3500, 4000) g	Mortality	Males		1.502 (1.309 to 1.712)	1.563 (1.353 to 1.807)							
Encephalitis	Birth prevalence - [37, 38) wks, [3500, 4000) g	Mortality	Females		1.275 (1.075 to 1.501)	1.444 (1.206 to 1.711)							
Encephalitis	Birth prevalence - [40, 42) wks, [3000, 3500) g	Mortality	Males		1.446 (1.258 to 1.639)	1.79 (1.546 to 2.08)							
Encephalitis	Birth prevalence - [40, 42) wks, [3000, 3500) g	Mortality	Females		1.103 (0.922 to 1.305)	1.491 (1.233 to 1.776)							
Encephalitis	Birth prevalence - [38, 40) wks, [3000, 3500) g	Mortality	Males		1.414 (1.242 to 1.593)	1.874 (1.636 to 2.156)							

Ages													
30-34 years	35-39 years	40-44 years	45-49 years	50-54 years	55-59 years	60-64 years	65-69 years	70-74 years	75-79 years	80-84 years	85-89 years	90-94 years	95+ years

Appendix Table 1a. Relative risks used by age and sex for each outcome for all risk factors except for ambient air pollution and alcohol.

Risk - Outcome	Category / Units	Morbidity / Mortality	Sex	All-age	0-6 days	7-27 days	28-364 days	1-4 years	5-9 years	10-14 years	15-19 years	20-24 years	25-29 years
Encephalitis	Birth prevalence - [38, 40) wks, [3000, 3500) g	Mortality	Females		1.096 (0.927 to 1.289)	1.617 (1.352 to 1.907)							
Encephalitis	Birth prevalence - [38, 40) wks, [4000, 4500) g	Mortality	Males		1.052 (0.92 to 1.203)	1.093 (0.939 to 1.261)							
Encephalitis	Birth prevalence - [38, 40) wks, [4000, 4500) g	Mortality	Females		1.121 (0.925 to 1.31)	1.149 (0.955 to 1.367)							
Encephalitis	Birth prevalence - [38, 40) wks, [3500, 4000) g	Mortality	Males		1.009 (0.883 to 1.143)	1.243 (1.078 to 1.421)							
Encephalitis	Birth prevalence - [38, 40) wks, [3500, 4000) g	Mortality	Females		0.828 (0.702 to 0.972)	1.155 (0.967 to 1.369)							
Encephalitis	Birth prevalence - [40, 42) wks, [3500, 4000) g	Mortality	Males		0.98 (0.854 to 1.123)	1.155 (0.983 to 1.334)							
Encephalitis	Birth prevalence - [40, 42) wks, [3500, 4000) g	Mortality	Females		0.819 (0.679 to 0.974)	1.045 (0.874 to 1.26)							
Neonatal preterm birth complications	Birth prevalence - [22, 24) wks, [0, 500) g	Mortality	Males		3273.944 (2987.306 to 3600.961)	599.42 (512.777 to 692.914)							
Neonatal preterm birth complications	Birth prevalence - [22, 24) wks, [0, 500) g	Mortality	Females		3353.667 (2964.862 to 3798.637)	771.075 (635.721 to 931.62)							
Neonatal preterm birth complications	Birth prevalence - [24, 26) wks, [0, 500) g	Mortality	Males		2454.576 (2216.544 to 2713.168)	884.902 (766.03 to 1021.494)							
Neonatal preterm birth complications	Birth prevalence - [24, 26) wks, [0, 500) g	Mortality	Females		2376.85 (2080.354 to 2699.254)	915.957 (765.805 to 1079.243)							
Neonatal preterm birth complications	Birth prevalence - [26, 28) wks, [0, 500) g	Mortality	Males		1870.684 (1676.978 to 2075.277)	847.512 (727.279 to 983.252)							
Neonatal preterm birth complications	Birth prevalence - [26, 28) wks, [0, 500) g	Mortality	Females		1708.132 (1477.512 to 1960.75)	822.906 (677.101 to 990.491)							
Neonatal preterm birth complications	Birth prevalence - [28, 30) wks, [0, 500) g	Mortality	Males		1805.939 (1612.157 to 2006.088)	583.881 (485.096 to 690.434)							
Neonatal preterm birth complications	Birth prevalence - [28, 30) wks, [0, 500) g	Mortality	Females		1585.788 (1377.653 to 1836.365)	636.164 (520.511 to 760.561)							
Neonatal preterm birth complications	Birth prevalence - [22, 24) wks, [500, 1000) g	Mortality	Males		2037.627 (1843.599 to 2249.686)	720.117 (628.641 to 820.917)							
Neonatal preterm birth complications	Birth prevalence - [22, 24) wks, [500, 1000) g	Mortality	Females		1833.266 (1596.246 to 2105.971)	610.368 (510.239 to 722.278)							
Neonatal preterm birth complications	Birth prevalence - [24, 26) wks, [500, 1000) g	Mortality	Males		852.585 (753.604 to 959.026)	642.627 (558.091 to 730.626)							
Neonatal preterm birth complications	Birth prevalence - [24, 26) wks, [500, 1000) g	Mortality	Females		715.379 (610.81 to 830.296)	546.057 (463.282 to 637.815)							
Neonatal preterm birth complications	Birth prevalence - [26, 28) wks, [500, 1000) g	Mortality	Males		411.87 (365.037 to 467.411)	353.266 (305.647 to 405.904)							
Neonatal preterm birth complications	Birth prevalence - [26, 28) wks, [500, 1000) g	Mortality	Females		308.092 (262.47 to 362.302)	292.936 (243.986 to 349.019)							
Neonatal preterm birth complications	Birth prevalence - [32, 34) wks, [500, 1000) g	Mortality	Males		344.501 (301.197 to 397.343)	194.954 (163.844 to 231.771)							
Neonatal preterm birth complications	Birth prevalence - [32, 34) wks, [500, 1000) g	Mortality	Females		299.264 (251.468 to 353.995)	161.548 (130.866 to 196.323)							
Neonatal preterm birth complications	Birth prevalence - [22, 24) wks, [1000, 1500) g	Mortality	Males		673.302 (584.187 to 768.258)	213.181 (179.555 to 254.572)							
Neonatal preterm birth complications	Birth prevalence - [22, 24) wks, [1000, 1500) g	Mortality	Females		620.446 (522.864 to 734.748)	186.215 (149.772 to 227.046)							
Neonatal preterm birth complications	Birth prevalence - [30, 32) wks, [500, 1000) g	Mortality	Males		332.681 (293.52 to 377.86)	212.332 (181.213 to 245.271)							
Neonatal preterm birth complications	Birth prevalence - [30, 32) wks, [500, 1000) g	Mortality	Females		239.082 (200.416 to 284.597)	163.95 (135.165 to 197.261)							
Neonatal preterm birth complications	Birth prevalence - [28, 30) wks, [500, 1000) g	Mortality	Males		326.557 (286.01 to 366.811)	249.246 (216.248 to 289.232)							
Neonatal preterm birth complications	Birth prevalence - [28, 30) wks, [500, 1000) g	Mortality	Females		231.533 (194.62 to 271.396)	187.47 (157.953 to 224.552)							
Neonatal preterm birth complications	Birth prevalence - [24, 26) wks, [1000, 1500) g	Mortality	Males		273.536 (236.795 to 312.421)	182.743 (155.477 to 212.281)							

Ages													
30-34 years	35-39 years	40-44 years	45-49 years	50-54 years	55-59 years	60-64 years	65-69 years	70-74 years	75-79 years	80-84 years	85-89 years	90-94 years	95+ years

Appendix Table 1a. Relative risks used by age and sex for each outcome for all risk factors except for ambient air pollution and alcohol.

Risk - Outcome	Category / Units	Morbidity / Mortality	Sex	All-age	0-6 days	7-27 days	28-364 days	1-4 years	5-9 years	10-14 years	15-19 years	20-24 years	25-29 years
Neonatal preterm birth complications	Birth prevalence - [24, 26) wks, [1000, 1500) g	Mortality	Females		268.033<br>(223.95 to 318.815)	157.928<br>(130.936 to 190.298)							
Neonatal preterm birth complications	Birth prevalence - [26, 28) wks, [1000, 1500) g	Mortality	Males		164.685<br>(145.457 to 186.571)	120.878<br>(102.672 to 140.967)							
Neonatal preterm birth complications	Birth prevalence - [26, 28) wks, [1000, 1500) g	Mortality	Females		142.274<br>(121.33 to 168.246)	97.496<br>(80.858 to 116.625)							
Neonatal preterm birth complications	Birth prevalence - [26, 28) wks, [1500, 2000) g	Mortality	Males		113.21<br>(97.079 to 130.806)	49.449<br>(40.908 to 59.113)							
Neonatal preterm birth complications	Birth prevalence - [26, 28) wks, [1500, 2000) g	Mortality	Females		108.393<br>(89.864 to 129.883)	49.979<br>(40.484 to 60.784)							
Neonatal preterm birth complications	Birth prevalence - [34, 36) wks, [1000, 1500) g	Mortality	Males		119.602<br>(104.655 to 136.729)	56.519<br>(48.084 to 66.018)							
Neonatal preterm birth complications	Birth prevalence - [34, 36) wks, [1000, 1500) g	Mortality	Females		109.231<br>(91.177 to 128.378)	52.834<br>(43.454 to 63.459)							
Neonatal preterm birth complications	Birth prevalence - [28, 30) wks, [1500, 2000) g	Mortality	Males		87.285<br>(76.012 to 100.83)	37.15<br>(30.87 to 43.664)							
Neonatal preterm birth complications	Birth prevalence - [28, 30) wks, [1500, 2000) g	Mortality	Females		86.639<br>(71.639 to 103.632)	38.3<br>(31.326 to 45.531)							
Neonatal preterm birth complications	Birth prevalence - [28, 30) wks, [1000, 1500) g	Mortality	Males		107.077<br>(93.214 to 122.668)	81.407<br>(70.511 to 93.12)							
Neonatal preterm birth complications	Birth prevalence - [28, 30) wks, [1000, 1500) g	Mortality	Females		87.409<br>(73.438 to 104.222)	64.981<br>(53.651 to 77.029)							
Neonatal preterm birth complications	Birth prevalence - [32, 34) wks, [1000, 1500) g	Mortality	Males		98.982<br>(86.649 to 112.931)	51.159<br>(43.522 to 59.402)							
Neonatal preterm birth complications	Birth prevalence - [32, 34) wks, [1000, 1500) g	Mortality	Females		84.081<br>(70.672 to 99.726)	44.695<br>(37.129 to 53.257)							
Neonatal preterm birth complications	Birth prevalence - [30, 32) wks, [1000, 1500) g	Mortality	Males		90.239<br>(78.583 to 103.215)	59.239<br>(50.511 to 69.508)							
Neonatal preterm birth complications	Birth prevalence - [30, 32) wks, [1000, 1500) g	Mortality	Females		70.119<br>(59.11 to 81.846)	47.493<br>(39.526 to 56.547)							
Neonatal preterm birth complications	Birth prevalence - [37, 38) wks, [1500, 2000) g	Mortality	Males		55.659<br>(48.726 to 62.963)	25.45<br>(21.867 to 29.451)							
Neonatal preterm birth complications	Birth prevalence - [37, 38) wks, [1500, 2000) g	Mortality	Females		54.453<br>(46.308 to 63.524)	29.339<br>(24.736 to 34.558)							
Neonatal preterm birth complications	Birth prevalence - [36, 37) wks, [1500, 2000) g	Mortality	Males		53.885<br>(47.099 to 61.414)	23.622<br>(20.373 to 27.298)							
Neonatal preterm birth complications	Birth prevalence - [36, 37) wks, [1500, 2000) g	Mortality	Females		47.995<br>(40.82 to 56.378)	23.905<br>(19.8 to 29.023)							
Neonatal preterm birth complications	Birth prevalence - [30, 32) wks, [2000, 2500) g	Mortality	Males		39.701<br>(34.573 to 45.311)	16.391<br>(13.683 to 19.411)							
Neonatal preterm birth complications	Birth prevalence - [30, 32) wks, [2000, 2500) g	Mortality	Females		34.894<br>(28.953 to 41.934)	16.549<br>(13.438 to 19.916)							
Neonatal preterm birth complications	Birth prevalence - [30, 32) wks, [1500, 2000) g	Mortality	Males		55.142<br>(48.379 to 62.989)	26.391<br>(22.751 to 30.584)							
Neonatal preterm birth complications	Birth prevalence - [30, 32) wks, [1500, 2000) g	Mortality	Females		48.414<br>(40.924 to 57.128)	26.986<br>(22.299 to 32.305)							
Neonatal preterm birth complications	Birth prevalence - [34, 36) wks, [1500, 2000) g	Mortality	Males		45.579<br>(39.817 to 51.922)	19.836<br>(16.94 to 23.141)							
Neonatal preterm birth complications	Birth prevalence - [34, 36) wks, [1500, 2000) g	Mortality	Females		38.405<br>(32.273 to 45.04)	18.409<br>(15.134 to 21.755)							
Neonatal preterm birth complications	Birth prevalence - [32, 34) wks, [1500, 2000) g	Mortality	Males		41.734<br>(36.381 to 47.516)	19.083<br>(16.193 to 21.909)							
Neonatal preterm birth complications	Birth prevalence - [32, 34) wks, [1500, 2000) g	Mortality	Females		36.162<br>(30.231 to 42.934)	19.528<br>(16.278 to 23.535)							
Neonatal preterm birth complications	Birth prevalence - [32, 34) wks, [2000, 2500) g	Mortality	Males		22.379<br>(19.576 to 25.426)	11.555<br>(9.874 to 13.332)							
Neonatal preterm birth complications	Birth prevalence - [32, 34) wks, [2000, 2500) g	Mortality	Females		20.326<br>(17.055 to 23.945)	12.376<br>(10.192 to 14.834)							
Neonatal preterm birth complications	Birth prevalence - [40, 42) wks, [2000, 2500) g	Mortality	Males		17.218<br>(14.893 to 19.961)	11.519<br>(9.766 to 13.495)							

Ages													
30-34 years	35-39 years	40-44 years	45-49 years	50-54 years	55-59 years	60-64 years	65-69 years	70-74 years	75-79 years	80-84 years	85-89 years	90-94 years	95+ years

Appendix Table 1a. Relative risks used by age and sex for each outcome for all risk factors except for ambient air pollution and alcohol.

Risk - Outcome	Category / Units	Morbidity / Mortality	Sex	All-age	0-6 days	7-27 days	28-364 days	1-4 years	5-9 years	10-14 years	15-19 years	20-24 years	25-29 years
Neonatal preterm birth complications	Birth prevalence - [40, 42) wks, [2000, 2500) g	Mortality	Females		13.555 (11.337 to 16.132)	11.838 (9.792 to 14.338)							
Neonatal preterm birth complications	Birth prevalence - [38, 40) wks, [2000, 2500) g	Mortality	Males		14.227 (12.521 to 16.06)	10.475 (9.122 to 12.085)							
Neonatal preterm birth complications	Birth prevalence - [38, 40) wks, [2000, 2500) g	Mortality	Females		10.867 (9.199 to 12.777)	10.493 (8.801 to 12.342)							
Neonatal preterm birth complications	Birth prevalence - [32, 34) wks, [2500, 3000) g	Mortality	Males		12.775 (11.146 to 14.607)	6.296 (5.269 to 7.299)							
Neonatal preterm birth complications	Birth prevalence - [32, 34) wks, [2500, 3000) g	Mortality	Females		9.041 (7.504 to 10.706)	6.205 (5.041 to 7.52)							
Neonatal preterm birth complications	Birth prevalence - [34, 36) wks, [2000, 2500) g	Mortality	Males		16.472 (14.422 to 18.7)	9.777 (8.456 to 11.277)							
Neonatal preterm birth complications	Birth prevalence - [34, 36) wks, [2000, 2500) g	Mortality	Females		12.895 (10.992 to 15.284)	9.44 (7.865 to 11.209)							
Neonatal preterm birth complications	Birth prevalence - [37, 38) wks, [2000, 2500) g	Mortality	Males		14.003 (12.407 to 15.831)	9.977 (8.758 to 11.444)							
Neonatal preterm birth complications	Birth prevalence - [37, 38) wks, [2000, 2500) g	Mortality	Females		10.456 (8.899 to 12.149)	9.83 (8.317 to 11.54)							
Neonatal preterm birth complications	Birth prevalence - [36, 37) wks, [2000, 2500) g	Mortality	Males		14.873 (13.083 to 16.88)	9.756 (8.38 to 11.214)							
Neonatal preterm birth complications	Birth prevalence - [36, 37) wks, [2000, 2500) g	Mortality	Females		10.517 (8.897 to 12.379)	9.233 (7.691 to 10.92)							
Neonatal preterm birth complications	Birth prevalence - [34, 36) wks, [2500, 3000) g	Mortality	Males		8.216 (7.133 to 9.378)	5.419 (4.631 to 6.282)							
Neonatal preterm birth complications	Birth prevalence - [34, 36) wks, [2500, 3000) g	Mortality	Females		6.037 (5.044 to 7.094)	5.198 (4.31 to 6.293)							
Neonatal preterm birth complications	Birth prevalence - [34, 36) wks, [4000, 4500) g	Mortality	Males		2.68 (2.245 to 3.183)	1.532 (1.254 to 1.867)							
Neonatal preterm birth complications	Birth prevalence - [34, 36) wks, [4000, 4500) g	Mortality	Females		2.458 (1.967 to 3.074)	1.673 (1.344 to 2.093)							
Neonatal preterm birth complications	Birth prevalence - [34, 36) wks, [3000, 3500) g	Mortality	Males		3.961 (3.433 to 4.521)	3.194 (2.71 to 3.696)							
Neonatal preterm birth complications	Birth prevalence - [34, 36) wks, [3000, 3500) g	Mortality	Females		3.267 (2.738 to 3.876)	2.783 (2.273 to 3.408)							
Neonatal preterm birth complications	Birth prevalence - [36, 37) wks, [2500, 3000) g	Mortality	Males		5.363 (4.69 to 6.082)	4.559 (3.945 to 5.248)							
Neonatal preterm birth complications	Birth prevalence - [36, 37) wks, [2500, 3000) g	Mortality	Females		3.969 (3.371 to 4.675)	4.138 (3.469 to 4.868)							
Neonatal preterm birth complications	Birth prevalence - [34, 36) wks, [3500, 4000) g	Mortality	Males		2.678 (2.289 to 3.086)	2.014 (1.69 to 2.365)							
Neonatal preterm birth complications	Birth prevalence - [34, 36) wks, [3500, 4000) g	Mortality	Females		2.342 (1.908 to 2.845)	1.756 (1.426 to 2.126)							
Neonatal preterm birth complications	Birth prevalence - [37, 38) wks, [2500, 3000) g	Mortality	Males		4.093 (3.581 to 4.626)	4.139 (3.631 to 4.74)							
Neonatal preterm birth complications	Birth prevalence - [37, 38) wks, [2500, 3000) g	Mortality	Females		3.07 (2.612 to 3.603)	3.618 (3.045 to 4.25)							
Neonatal preterm birth complications	Birth prevalence - [40, 42) wks, [2500, 3000) g	Mortality	Males		3.833 (3.364 to 4.388)	3.828 (3.271 to 4.426)							
Neonatal preterm birth complications	Birth prevalence - [40, 42) wks, [2500, 3000) g	Mortality	Females		2.699 (2.264 to 3.202)	3.379 (2.825 to 4.042)							
Neonatal preterm birth complications	Birth prevalence - [38, 40) wks, [2500, 3000) g	Mortality	Males		3.396 (3.002 to 3.839)	3.858 (3.346 to 4.424)							
Neonatal preterm birth complications	Birth prevalence - [38, 40) wks, [2500, 3000) g	Mortality	Females		2.382 (2.04 to 2.802)	3.27 (2.774 to 3.927)							
Neonatal preterm birth complications	Birth prevalence - [36, 37) wks, [3000, 3500) g	Mortality	Males		2.819 (2.433 to 3.229)	2.683 (2.299 to 3.079)							
Neonatal preterm birth complications	Birth prevalence - [36, 37) wks, [3000, 3500) g	Mortality	Females		2.335 (1.952 to 2.784)	2.434 (2.007 to 2.884)							
Neonatal preterm birth complications	Birth prevalence - [36, 37) wks, [4000, 4500) g	Mortality	Males		2.331 (1.993 to 2.727)	1.471 (1.221 to 1.731)							

Ages													
**30-34 years**	**35-39 years**	**40-44 years**	**45-49 years**	**50-54 years**	**55-59 years**	**60-64 years**	**65-69 years**	**70-74 years**	**75-79 years**	**80-84 years**	**85-89 years**	**90-94 years**	**95+ years**

Appendix Table 1a. Relative risks used by age and sex for each outcome for all risk factors except for ambient air pollution and alcohol.

Risk - Outcome	Category / Units	Morbidity / Mortality	Sex	All-age	0-6 days	7-27 days	28-364 days	1-4 years	5-9 years	10-14 years	15-19 years	20-24 years	25-29 years
Neonatal preterm birth complications	Birth prevalence - [36, 37) wks, [4000, 4500) g	Mortality	Females		2.141 (1.729 to 2.577)	1.601 (1.291 to 1.97)							
Neonatal preterm birth complications	Birth prevalence - [36, 37) wks, [3500, 4000) g	Mortality	Males		2.084 (1.789 to 2.418)	1.923 (1.624 to 2.252)							
Neonatal preterm birth complications	Birth prevalence - [36, 37) wks, [3500, 4000) g	Mortality	Females		1.845 (1.527 to 2.216)	1.731 (1.426 to 2.071)							
Neonatal preterm birth complications	Birth prevalence - [37, 38) wks, [3000, 3500) g	Mortality	Males		2.027 (1.788 to 2.287)	2.236 (1.926 to 2.542)							
Neonatal preterm birth complications	Birth prevalence - [37, 38) wks, [3000, 3500) g	Mortality	Females		1.667 (1.411 to 1.947)	1.989 (1.672 to 2.366)							
Neonatal preterm birth complications	Birth prevalence - [37, 38) wks, [4000, 4500) g	Mortality	Males		1.572 (1.352 to 1.813)	1.28 (1.095 to 1.481)							
Neonatal preterm birth complications	Birth prevalence - [37, 38) wks, [4000, 4500) g	Mortality	Females		1.647 (1.37 to 1.98)	1.396 (1.167 to 1.674)							
Neonatal preterm birth complications	Birth prevalence - [37, 38) wks, [3500, 4000) g	Mortality	Males		1.502 (1.309 to 1.712)	1.563 (1.353 to 1.807)							
Neonatal preterm birth complications	Birth prevalence - [37, 38) wks, [3500, 4000) g	Mortality	Females		1.275 (1.075 to 1.501)	1.444 (1.206 to 1.711)							
Neonatal preterm birth complications	Birth prevalence - [40, 42) wks, [3000, 3500) g	Mortality	Males		1.446 (1.258 to 1.639)	1.79 (1.546 to 2.08)							
Neonatal preterm birth complications	Birth prevalence - [40, 42) wks, [3000, 3500) g	Mortality	Females		1.103 (0.922 to 1.305)	1.491 (1.233 to 1.776)							
Neonatal preterm birth complications	Birth prevalence - [38, 40) wks, [3000, 3500) g	Mortality	Males		1.414 (1.242 to 1.593)	1.874 (1.636 to 2.156)							
Neonatal preterm birth complications	Birth prevalence - [38, 40) wks, [3000, 3500) g	Mortality	Females		1.096 (0.927 to 1.289)	1.617 (1.352 to 1.907)							
Neonatal preterm birth complications	Birth prevalence - [38, 40) wks, [4000, 4500) g	Mortality	Males		1.052 (0.92 to 1.203)	1.093 (0.939 to 1.261)							
Neonatal preterm birth complications	Birth prevalence - [38, 40) wks, [4000, 4500) g	Mortality	Females		1.121 (0.925 to 1.31)	1.149 (0.955 to 1.367)							
Neonatal preterm birth complications	Birth prevalence - [38, 40) wks, [3500, 4000) g	Mortality	Males		1.009 (0.883 to 1.143)	1.243 (1.078 to 1.421)							
Neonatal preterm birth complications	Birth prevalence - [38, 40) wks, [3500, 4000) g	Mortality	Females		0.828 (0.702 to 0.972)	1.155 (0.967 to 1.369)							
Neonatal preterm birth complications	Birth prevalence - [40, 42) wks, [3500, 4000) g	Mortality	Males		0.98 (0.854 to 1.123)	1.155 (0.983 to 1.334)							
Neonatal preterm birth complications	Birth prevalence - [40, 42) wks, [3500, 4000) g	Mortality	Females		0.819 (0.679 to 0.974)	1.045 (0.874 to 1.26)							
Neonatal encephalopathy due to birth asphyxia and trauma	Birth prevalence - [22, 24) wks, [0, 500) g	Mortality	Males		3273.944 (2987.306 to 3600.961)	599.42 (512.777 to 692.914)							
Neonatal encephalopathy due to birth asphyxia and trauma	Birth prevalence - [22, 24) wks, [0, 500) g	Mortality	Females		3353.667 (2964.862 to 3798.637)	771.075 (635.721 to 931.62)							
Neonatal encephalopathy due to birth asphyxia and trauma	Birth prevalence - [24, 26) wks, [0, 500) g	Mortality	Males		2454.576 (2216.544 to 2713.168)	884.902 (766.03 to 1021.494)							
Neonatal encephalopathy due to birth asphyxia and trauma	Birth prevalence - [24, 26) wks, [0, 500) g	Mortality	Females		2376.85 (2080.354 to 2699.254)	915.957 (765.805 to 1079.243)							
Neonatal encephalopathy due to birth asphyxia and trauma	Birth prevalence - [26, 28) wks, [0, 500) g	Mortality	Males		1870.684 (1676.978 to 2075.277)	847.512 (727.279 to 983.252)							
Neonatal encephalopathy due to birth asphyxia and trauma	Birth prevalence - [26, 28) wks, [0, 500) g	Mortality	Females		1708.132 (1477.512 to 1960.75)	822.906 (677.101 to 990.491)							
Neonatal encephalopathy due to birth asphyxia and trauma	Birth prevalence - [28, 30) wks, [0, 500) g	Mortality	Males		1805.939 (1612.157 to 2006.088)	583.881 (485.096 to 690.434)							
Neonatal encephalopathy due to birth asphyxia and trauma	Birth prevalence - [28, 30) wks, [0, 500) g	Mortality	Females		1585.788 (1377.653 to 1836.365)	636.164 (520.511 to 760.561)							
Neonatal encephalopathy due to birth asphyxia and trauma	Birth prevalence - [22, 24) wks, [500, 1000) g	Mortality	Males		2037.627 (1843.599 to 2249.686)	720.117 (628.641 to 820.917)							
Neonatal encephalopathy due to birth asphyxia and trauma	Birth prevalence - [22, 24) wks, [500, 1000) g	Mortality	Females		1833.266 (1596.246 to 2105.971)	610.368 (510.239 to 722.278)							
Neonatal encephalopathy due to birth asphyxia and trauma	Birth prevalence - [24, 26) wks, [500, 1000) g	Mortality	Males		852.585 (753.604 to 959.026)	642.627 (558.091 to 730.626)							

Ages													
30-34 years	35-39 years	40-44 years	45-49 years	50-54 years	55-59 years	60-64 years	65-69 years	70-74 years	75-79 years	80-84 years	85-89 years	90-94 years	95+ years

Appendix Table 1a. Relative risks used by age and sex for each outcome for all risk factors except for ambient air pollution and alcohol.

Risk - Outcome	Category / Units	Morbidity / Mortality	Sex	All-age	0-6 days	7-27 days	28-364 days	1-4 years	5-9 years	10-14 years	15-19 years	20-24 years	25-29 years
Neonatal encephalopathy due to birth asphyxia and trauma	Birth prevalence - [24, 26) wks, [500, 1000) g	Mortality	Females		715.379 (610.81 to 830.296)	546.057 (463.282 to 637.815)							
Neonatal encephalopathy due to birth asphyxia and trauma	Birth prevalence - [26, 28) wks, [500, 1000) g	Mortality	Males		411.87 (365.037 to 467.411)	353.266 (305.647 to 405.904)							
Neonatal encephalopathy due to birth asphyxia and trauma	Birth prevalence - [26, 28) wks, [500, 1000) g	Mortality	Females		308.092 (262.47 to 362.302)	292.936 (243.986 to 349.019)							
Neonatal encephalopathy due to birth asphyxia and trauma	Birth prevalence - [32, 34) wks, [500, 1000) g	Mortality	Males		344.501 (301.197 to 397.343)	194.954 (163.844 to 231.771)							
Neonatal encephalopathy due to birth asphyxia and trauma	Birth prevalence - [32, 34) wks, [500, 1000) g	Mortality	Females		299.264 (251.468 to 353.995)	161.548 (130.866 to 196.323)							
Neonatal encephalopathy due to birth asphyxia and trauma	Birth prevalence - [22, 24) wks, [1000, 1500) g	Mortality	Males		673.302 (584.187 to 768.258)	213.181 (179.555 to 254.572)							
Neonatal encephalopathy due to birth asphyxia and trauma	Birth prevalence - [22, 24) wks, [1000, 1500) g	Mortality	Females		620.446 (522.864 to 734.748)	186.215 (149.772 to 227.046)							
Neonatal encephalopathy due to birth asphyxia and trauma	Birth prevalence - [30, 32) wks, [500, 1000) g	Mortality	Males		332.681 (293.52 to 377.86)	212.332 (181.213 to 245.271)							
Neonatal encephalopathy due to birth asphyxia and trauma	Birth prevalence - [30, 32) wks, [500, 1000) g	Mortality	Females		239.082 (200.416 to 284.597)	163.95 (135.165 to 197.261)							
Neonatal encephalopathy due to birth asphyxia and trauma	Birth prevalence - [28, 30) wks, [500, 1000) g	Mortality	Males		326.557 (286.01 to 366.811)	249.246 (216.248 to 289.232)							
Neonatal encephalopathy due to birth asphyxia and trauma	Birth prevalence - [28, 30) wks, [500, 1000) g	Mortality	Females		231.533 (194.62 to 271.396)	187.47 (157.953 to 224.552)							
Neonatal encephalopathy due to birth asphyxia and trauma	Birth prevalence - [24, 26) wks, [1000, 1500) g	Mortality	Males		273.536 (236.795 to 312.421)	182.743 (155.477 to 212.281)							
Neonatal encephalopathy due to birth asphyxia and trauma	Birth prevalence - [24, 26) wks, [1000, 1500) g	Mortality	Females		268.033 (223.95 to 318.815)	157.928 (130.936 to 190.298)							
Neonatal encephalopathy due to birth asphyxia and trauma	Birth prevalence - [26, 28) wks, [1000, 1500) g	Mortality	Males		164.685 (145.457 to 186.571)	120.878 (102.672 to 140.967)							
Neonatal encephalopathy due to birth asphyxia and trauma	Birth prevalence - [26, 28) wks, [1000, 1500) g	Mortality	Females		142.274 (121.33 to 168.246)	97.496 (80.858 to 116.625)							
Neonatal encephalopathy due to birth asphyxia and trauma	Birth prevalence - [26, 28) wks, [1500, 2000) g	Mortality	Males		113.21 (97.079 to 130.806)	49.449 (40.908 to 59.113)							
Neonatal encephalopathy due to birth asphyxia and trauma	Birth prevalence - [26, 28) wks, [1500, 2000) g	Mortality	Females		108.393 (89.864 to 129.883)	49.979 (40.484 to 60.784)							
Neonatal encephalopathy due to birth asphyxia and trauma	Birth prevalence - [34, 36) wks, [1000, 1500) g	Mortality	Males		119.602 (104.655 to 136.729)	56.519 (48.084 to 66.018)							
Neonatal encephalopathy due to birth asphyxia and trauma	Birth prevalence - [34, 36) wks, [1000, 1500) g	Mortality	Females		109.231 (91.177 to 128.378)	52.834 (43.454 to 63.459)							
Neonatal encephalopathy due to birth asphyxia and trauma	Birth prevalence - [28, 30) wks, [1500, 2000) g	Mortality	Males		87.285 (76.012 to 100.83)	37.15 (30.87 to 43.664)							
Neonatal encephalopathy due to birth asphyxia and trauma	Birth prevalence - [28, 30) wks, [1500, 2000) g	Mortality	Females		86.639 (71.639 to 103.632)	38.3 (31.326 to 45.531)							
Neonatal encephalopathy due to birth asphyxia and trauma	Birth prevalence - [28, 30) wks, [1000, 1500) g	Mortality	Males		107.077 (93.214 to 122.668)	81.407 (70.511 to 93.12)							
Neonatal encephalopathy due to birth asphyxia and trauma	Birth prevalence - [28, 30) wks, [1000, 1500) g	Mortality	Females		87.409 (73.438 to 104.222)	64.981 (53.651 to 77.029)							
Neonatal encephalopathy due to birth asphyxia and trauma	Birth prevalence - [32, 34) wks, [1000, 1500) g	Mortality	Males		98.982 (86.649 to 112.931)	51.159 (43.522 to 59.402)							
Neonatal encephalopathy due to birth asphyxia and trauma	Birth prevalence - [32, 34) wks, [1000, 1500) g	Mortality	Females		84.081 (70.672 to 99.726)	44.695 (37.129 to 53.257)							
Neonatal encephalopathy due to birth asphyxia and trauma	Birth prevalence - [30, 32) wks, [1000, 1500) g	Mortality	Males		90.239 (78.583 to 103.215)	59.239 (50.511 to 69.508)							
Neonatal encephalopathy due to birth asphyxia and trauma	Birth prevalence - [30, 32) wks, [1000, 1500) g	Mortality	Females		70.119 (59.11 to 81.846)	47.493 (39.526 to 56.547)							
Neonatal encephalopathy due to birth asphyxia and trauma	Birth prevalence - [37, 38) wks, [1500, 2000) g	Mortality	Males		55.659 (48.726 to 62.963)	25.45 (21.867 to 29.451)							
Neonatal encephalopathy due to birth asphyxia and trauma	Birth prevalence - [37, 38) wks, [1500, 2000) g	Mortality	Females		54.453 (46.308 to 63.524)	29.339 (24.736 to 34.558)							
Neonatal encephalopathy due to birth asphyxia and trauma	Birth prevalence - [36, 37) wks, [1500, 2000) g	Mortality	Males		53.885 (47.099 to 61.414)	23.622 (20.373 to 27.298)							

Ages													
**30-34 years**	**35-39 years**	**40-44 years**	**45-49 years**	**50-54 years**	**55-59 years**	**60-64 years**	**65-69 years**	**70-74 years**	**75-79 years**	**80-84 years**	**85-89 years**	**90-94 years**	**95+ years**

Appendix Table 1a. Relative risks used by age and sex for each outcome for all risk factors except for ambient air pollution and alcohol.

Risk - Outcome	Category / Units	Morbidity / Mortality	Sex	All-age	0-6 days	7-27 days	28-364 days	1-4 years	5-9 years	10-14 years	15-19 years	20-24 years	25-29 years
Neonatal encephalopathy due to birth asphyxia and trauma	Birth prevalence - [36, 37) wks, [1500, 2000) g	Mortality	Females		47.995 (40.82 to 56.378)	23.905 (19.8 to 29.023)							
Neonatal encephalopathy due to birth asphyxia and trauma	Birth prevalence - [30, 32) wks, [2000, 2500) g	Mortality	Males		39.701 (34.573 to 45.311)	16.391 (13.683 to 19.411)							
Neonatal encephalopathy due to birth asphyxia and trauma	Birth prevalence - [30, 32) wks, [2000, 2500) g	Mortality	Females		34.894 (28.953 to 41.934)	16.549 (13.438 to 19.916)							
Neonatal encephalopathy due to birth asphyxia and trauma	Birth prevalence - [30, 32) wks, [1500, 2000) g	Mortality	Males		55.142 (48.379 to 62.989)	26.391 (22.751 to 30.584)							
Neonatal encephalopathy due to birth asphyxia and trauma	Birth prevalence - [30, 32) wks, [1500, 2000) g	Mortality	Females		48.414 (40.924 to 57.128)	26.986 (22.299 to 32.305)							
Neonatal encephalopathy due to birth asphyxia and trauma	Birth prevalence - [34, 36) wks, [1500, 2000) g	Mortality	Males		45.579 (39.817 to 51.922)	19.836 (16.94 to 23.141)							
Neonatal encephalopathy due to birth asphyxia and trauma	Birth prevalence - [34, 36) wks, [1500, 2000) g	Mortality	Females		38.405 (32.273 to 45.04)	18.409 (15.134 to 21.755)							
Neonatal encephalopathy due to birth asphyxia and trauma	Birth prevalence - [32, 34) wks, [1500, 2000) g	Mortality	Males		41.734 (36.381 to 47.516)	19.083 (16.193 to 21.909)							
Neonatal encephalopathy due to birth asphyxia and trauma	Birth prevalence - [32, 34) wks, [1500, 2000) g	Mortality	Females		36.162 (30.231 to 42.934)	19.528 (16.278 to 23.535)							
Neonatal encephalopathy due to birth asphyxia and trauma	Birth prevalence - [32, 34) wks, [2000, 2500) g	Mortality	Males		22.379 (19.576 to 25.426)	11.555 (9.874 to 13.332)							
Neonatal encephalopathy due to birth asphyxia and trauma	Birth prevalence - [32, 34) wks, [2000, 2500) g	Mortality	Females		20.326 (17.055 to 23.945)	12.376 (10.192 to 14.834)							
Neonatal encephalopathy due to birth asphyxia and trauma	Birth prevalence - [40, 42) wks, [2000, 2500) g	Mortality	Males		17.218 (14.893 to 19.961)	11.519 (9.766 to 13.495)							
Neonatal encephalopathy due to birth asphyxia and trauma	Birth prevalence - [40, 42) wks, [2000, 2500) g	Mortality	Females		13.555 (11.337 to 16.132)	11.838 (9.792 to 14.338)							
Neonatal encephalopathy due to birth asphyxia and trauma	Birth prevalence - [38, 40) wks, [2000, 2500) g	Mortality	Males		14.227 (12.521 to 16.06)	10.475 (9.122 to 12.085)							
Neonatal encephalopathy due to birth asphyxia and trauma	Birth prevalence - [38, 40) wks, [2000, 2500) g	Mortality	Females		10.867 (9.199 to 12.777)	10.493 (8.801 to 12.342)							
Neonatal encephalopathy due to birth asphyxia and trauma	Birth prevalence - [32, 34) wks, [2500, 3000) g	Mortality	Males		12.775 (11.146 to 14.607)	6.296 (5.269 to 7.299)							
Neonatal encephalopathy due to birth asphyxia and trauma	Birth prevalence - [32, 34) wks, [2500, 3000) g	Mortality	Females		9.041 (7.504 to 10.706)	6.205 (5.041 to 7.52)							
Neonatal encephalopathy due to birth asphyxia and trauma	Birth prevalence - [34, 36) wks, [2000, 2500) g	Mortality	Males		16.472 (14.422 to 18.7)	9.777 (8.456 to 11.277)							
Neonatal encephalopathy due to birth asphyxia and trauma	Birth prevalence - [34, 36) wks, [2000, 2500) g	Mortality	Females		12.895 (10.992 to 15.284)	9.44 (7.865 to 11.209)							
Neonatal encephalopathy due to birth asphyxia and trauma	Birth prevalence - [37, 38) wks, [2000, 2500) g	Mortality	Males		14.003 (12.407 to 15.831)	9.977 (8.758 to 11.444)							
Neonatal encephalopathy due to birth asphyxia and trauma	Birth prevalence - [37, 38) wks, [2000, 2500) g	Mortality	Females		10.456 (8.899 to 12.149)	9.83 (8.317 to 11.54)							
Neonatal encephalopathy due to birth asphyxia and trauma	Birth prevalence - [36, 37) wks, [2000, 2500) g	Mortality	Males		14.873 (13.083 to 16.88)	9.756 (8.38 to 11.214)							
Neonatal encephalopathy due to birth asphyxia and trauma	Birth prevalence - [36, 37) wks, [2000, 2500) g	Mortality	Females		10.517 (8.897 to 12.379)	9.233 (7.691 to 10.92)							
Neonatal encephalopathy due to birth asphyxia and trauma	Birth prevalence - [34, 36) wks, [2500, 3000) g	Mortality	Males		8.216 (7.133 to 9.378)	5.419 (4.631 to 6.282)							
Neonatal encephalopathy due to birth asphyxia and trauma	Birth prevalence - [34, 36) wks, [2500, 3000) g	Mortality	Females		6.037 (5.044 to 7.094)	5.198 (4.31 to 6.293)							
Neonatal encephalopathy due to birth asphyxia and trauma	Birth prevalence - [34, 36) wks, [4000, 4500) g	Mortality	Males		2.68 (2.245 to 3.183)	1.532 (1.254 to 1.867)							
Neonatal encephalopathy due to birth asphyxia and trauma	Birth prevalence - [34, 36) wks, [4000, 4500) g	Mortality	Females		2.458 (1.967 to 3.074)	1.673 (1.344 to 2.093)							
Neonatal encephalopathy due to birth asphyxia and trauma	Birth prevalence - [34, 36) wks, [3000, 3500) g	Mortality	Males		3.961 (3.433 to 4.521)	3.194 (2.71 to 3.696)							
Neonatal encephalopathy due to birth asphyxia and trauma	Birth prevalence - [34, 36) wks, [3000, 3500) g	Mortality	Females		3.267 (2.738 to 3.876)	2.783 (2.273 to 3.408)							
Neonatal encephalopathy due to birth asphyxia and trauma	Birth prevalence - [36, 37) wks, [2500, 3000) g	Mortality	Males		5.363 (4.69 to 6.082)	4.559 (3.945 to 5.248)							

Ages													
30-34 years	35-39 years	40-44 years	45-49 years	50-54 years	55-59 years	60-64 years	65-69 years	70-74 years	75-79 years	80-84 years	85-89 years	90-94 years	95+ years

Appendix Table 1a. Relative risks used by age and sex for each outcome for all risk factors except for ambient air pollution and alcohol.

Risk - Outcome	Category / Units	Morbidity / Mortality	Sex	All-age	0-6 days	7-27 days	28-364 days	1-4 years	5-9 years	10-14 years	15-19 years	20-24 years	25-29 years
Neonatal encephalopathy due to birth asphyxia and trauma	Birth prevalence - [36, 37) wks, [2500, 3000) g	Mortality	Females		3.969 (3.371 to 4.675)	4.138 (3.469 to 4.868)							
Neonatal encephalopathy due to birth asphyxia and trauma	Birth prevalence - [34, 36) wks, [3500, 4000) g	Mortality	Males		2.678 (2.289 to 3.086)	2.014 (1.69 to 2.365)							
Neonatal encephalopathy due to birth asphyxia and trauma	Birth prevalence - [34, 36) wks, [3500, 4000) g	Mortality	Females		2.342 (1.908 to 2.845)	1.756 (1.426 to 2.126)							
Neonatal encephalopathy due to birth asphyxia and trauma	Birth prevalence - [37, 38) wks, [2500, 3000) g	Mortality	Males		4.093 (3.581 to 4.626)	4.139 (3.631 to 4.74)							
Neonatal encephalopathy due to birth asphyxia and trauma	Birth prevalence - [37, 38) wks, [2500, 3000) g	Mortality	Females		3.07 (2.612 to 3.603)	3.618 (3.045 to 4.25)							
Neonatal encephalopathy due to birth asphyxia and trauma	Birth prevalence - [40, 42) wks, [2500, 3000) g	Mortality	Males		3.833 (3.364 to 4.388)	3.828 (3.271 to 4.426)							
Neonatal encephalopathy due to birth asphyxia and trauma	Birth prevalence - [40, 42) wks, [2500, 3000) g	Mortality	Females		2.699 (2.264 to 3.202)	3.379 (2.825 to 4.042)							
Neonatal encephalopathy due to birth asphyxia and trauma	Birth prevalence - [38, 40) wks, [2500, 3000) g	Mortality	Males		3.396 (3.002 to 3.839)	3.858 (3.346 to 4.424)							
Neonatal encephalopathy due to birth asphyxia and trauma	Birth prevalence - [38, 40) wks, [2500, 3000) g	Mortality	Females		2.382 (2.04 to 2.802)	3.27 (2.774 to 3.927)							
Neonatal encephalopathy due to birth asphyxia and trauma	Birth prevalence - [36, 37) wks, [3000, 3500) g	Mortality	Males		2.819 (2.433 to 3.229)	2.683 (2.299 to 3.079)							
Neonatal encephalopathy due to birth asphyxia and trauma	Birth prevalence - [36, 37) wks, [3000, 3500) g	Mortality	Females		2.335 (1.952 to 2.784)	2.434 (2.007 to 2.884)							
Neonatal encephalopathy due to birth asphyxia and trauma	Birth prevalence - [36, 37) wks, [4000, 4500) g	Mortality	Males		2.331 (1.993 to 2.727)	1.471 (1.221 to 1.731)							
Neonatal encephalopathy due to birth asphyxia and trauma	Birth prevalence - [36, 37) wks, [4000, 4500) g	Mortality	Females		2.141 (1.729 to 2.577)	1.601 (1.291 to 1.97)							
Neonatal encephalopathy due to birth asphyxia and trauma	Birth prevalence - [36, 37) wks, [3500, 4000) g	Mortality	Males		2.084 (1.789 to 2.418)	1.923 (1.624 to 2.252)							
Neonatal encephalopathy due to birth asphyxia and trauma	Birth prevalence - [36, 37) wks, [3500, 4000) g	Mortality	Females		1.845 (1.527 to 2.216)	1.731 (1.426 to 2.071)							
Neonatal encephalopathy due to birth asphyxia and trauma	Birth prevalence - [37, 38) wks, [3000, 3500) g	Mortality	Males		2.027 (1.788 to 2.287)	2.236 (1.926 to 2.542)							
Neonatal encephalopathy due to birth asphyxia and trauma	Birth prevalence - [37, 38) wks, [3000, 3500) g	Mortality	Females		1.667 (1.411 to 1.947)	1.989 (1.672 to 2.366)							
Neonatal encephalopathy due to birth asphyxia and trauma	Birth prevalence - [37, 38) wks, [4000, 4500) g	Mortality	Males		1.572 (1.352 to 1.813)	1.28 (1.095 to 1.481)							
Neonatal encephalopathy due to birth asphyxia and trauma	Birth prevalence - [37, 38) wks, [4000, 4500) g	Mortality	Females		1.647 (1.37 to 1.98)	1.396 (1.167 to 1.674)							
Neonatal encephalopathy due to birth asphyxia and trauma	Birth prevalence - [37, 38) wks, [3500, 4000) g	Mortality	Males		1.502 (1.309 to 1.712)	1.563 (1.353 to 1.807)							
Neonatal encephalopathy due to birth asphyxia and trauma	Birth prevalence - [37, 38) wks, [3500, 4000) g	Mortality	Females		1.275 (1.075 to 1.501)	1.444 (1.206 to 1.711)							
Neonatal encephalopathy due to birth asphyxia and trauma	Birth prevalence - [40, 42) wks, [3000, 3500) g	Mortality	Males		1.446 (1.258 to 1.639)	1.79 (1.546 to 2.08)							
Neonatal encephalopathy due to birth asphyxia and trauma	Birth prevalence - [40, 42) wks, [3000, 3500) g	Mortality	Females		1.103 (0.922 to 1.305)	1.491 (1.233 to 1.776)							
Neonatal encephalopathy due to birth asphyxia and trauma	Birth prevalence - [38, 40) wks, [3000, 3500) g	Mortality	Males		1.414 (1.242 to 1.593)	1.874 (1.636 to 2.156)							
Neonatal encephalopathy due to birth asphyxia and trauma	Birth prevalence - [38, 40) wks, [3000, 3500) g	Mortality	Females		1.096 (0.927 to 1.289)	1.617 (1.352 to 1.907)							
Neonatal encephalopathy due to birth asphyxia and trauma	Birth prevalence - [38, 40) wks, [4000, 4500) g	Mortality	Males		1.052 (0.92 to 1.203)	1.093 (0.939 to 1.261)							
Neonatal encephalopathy due to birth asphyxia and trauma	Birth prevalence - [38, 40) wks, [4000, 4500) g	Mortality	Females		1.121 (0.925 to 1.31)	1.149 (0.955 to 1.367)							
Neonatal encephalopathy due to birth asphyxia and trauma	Birth prevalence - [38, 40) wks, [3500, 4000) g	Mortality	Males		1.009 (0.883 to 1.143)	1.243 (1.078 to 1.421)							
Neonatal encephalopathy due to birth asphyxia and trauma	Birth prevalence - [38, 40) wks, [3500, 4000) g	Mortality	Females		0.828 (0.702 to 0.972)	1.155 (0.967 to 1.369)							
Neonatal encephalopathy due to birth asphyxia and trauma	Birth prevalence - [40, 42) wks, [3500, 4000) g	Mortality	Males		0.98 (0.854 to 1.123)	1.155 (0.983 to 1.334)							

Ages													
30-34 years	35-39 years	40-44 years	45-49 years	50-54 years	55-59 years	60-64 years	65-69 years	70-74 years	75-79 years	80-84 years	85-89 years	90-94 years	95+ years

Appendix Table 1a. Relative risks used by age and sex for each outcome for all risk factors except for ambient air pollution and alcohol.

Risk - Outcome	Category / Units	Morbidity / Mortality	Sex	All-age	0-6 days	7-27 days	28-364 days	1-4 years	5-9 years	10-14 years	15-19 years	20-24 years	25-29 years
Neonatal encephalopathy due to birth asphyxia and trauma	Birth prevalence - [40, 42) wks, [3500, 4000) g	Mortality	Females		0.819 (0.679 to 0.974)	1.045 (0.874 to 1.26)							
Neonatal sepsis and other neonatal infections	Birth prevalence - [22, 24) wks, [0, 500) g	Mortality	Males		3273.944 (2987.306 to 3600.961)	599.42 (512.777 to 692.914)							
Neonatal sepsis and other neonatal infections	Birth prevalence - [22, 24) wks, [0, 500) g	Mortality	Females		3353.667 (2964.862 to 3798.637)	771.075 (635.721 to 931.62)							
Neonatal sepsis and other neonatal infections	Birth prevalence - [24, 26) wks, [0, 500) g	Mortality	Males		2454.576 (2216.544 to 2713.168)	884.902 (766.03 to 1021.494)							
Neonatal sepsis and other neonatal infections	Birth prevalence - [24, 26) wks, [0, 500) g	Mortality	Females		2376.85 (2080.354 to 2699.254)	915.957 (765.805 to 1079.243)							
Neonatal sepsis and other neonatal infections	Birth prevalence - [26, 28) wks, [0, 500) g	Mortality	Males		1870.684 (1676.978 to 2075.277)	847.512 (727.279 to 983.252)							
Neonatal sepsis and other neonatal infections	Birth prevalence - [26, 28) wks, [0, 500) g	Mortality	Females		1708.132 (1477.512 to 1960.75)	822.906 (677.101 to 990.491)							
Neonatal sepsis and other neonatal infections	Birth prevalence - [28, 30) wks, [0, 500) g	Mortality	Males		1805.939 (1612.157 to 2006.088)	583.881 (485.096 to 690.434)							
Neonatal sepsis and other neonatal infections	Birth prevalence - [28, 30) wks, [0, 500) g	Mortality	Females		1585.788 (1377.653 to 1836.365)	636.164 (520.511 to 760.561)							
Neonatal sepsis and other neonatal infections	Birth prevalence - [22, 24) wks, [500, 1000) g	Mortality	Males		2037.627 (1843.599 to 2249.686)	720.117 (628.641 to 820.917)							
Neonatal sepsis and other neonatal infections	Birth prevalence - [22, 24) wks, [500, 1000) g	Mortality	Females		1833.266 (1596.246 to 2105.971)	610.368 (510.239 to 722.278)							
Neonatal sepsis and other neonatal infections	Birth prevalence - [24, 26) wks, [500, 1000) g	Mortality	Males		852.585 (753.604 to 959.026)	642.627 (558.091 to 730.626)							
Neonatal sepsis and other neonatal infections	Birth prevalence - [24, 26) wks, [500, 1000) g	Mortality	Females		715.379 (610.81 to 830.296)	546.057 (463.282 to 637.815)							
Neonatal sepsis and other neonatal infections	Birth prevalence - [26, 28) wks, [500, 1000) g	Mortality	Males		411.87 (365.037 to 467.411)	353.266 (305.647 to 405.904)							
Neonatal sepsis and other neonatal infections	Birth prevalence - [26, 28) wks, [500, 1000) g	Mortality	Females		308.092 (262.47 to 362.302)	292.936 (243.986 to 349.019)							
Neonatal sepsis and other neonatal infections	Birth prevalence - [32, 34) wks, [500, 1000) g	Mortality	Males		344.501 (301.197 to 397.343)	194.954 (163.844 to 231.771)							
Neonatal sepsis and other neonatal infections	Birth prevalence - [32, 34) wks, [500, 1000) g	Mortality	Females		299.264 (251.468 to 353.995)	161.548 (130.866 to 196.323)							
Neonatal sepsis and other neonatal infections	Birth prevalence - [22, 24) wks, [1000, 1500) g	Mortality	Males		673.302 (584.187 to 768.258)	213.181 (179.555 to 254.572)							
Neonatal sepsis and other neonatal infections	Birth prevalence - [22, 24) wks, [1000, 1500) g	Mortality	Females		620.446 (522.864 to 734.748)	186.215 (149.772 to 227.046)							
Neonatal sepsis and other neonatal infections	Birth prevalence - [30, 32) wks, [500, 1000) g	Mortality	Males		332.681 (293.52 to 377.86)	212.332 (181.213 to 245.271)							
Neonatal sepsis and other neonatal infections	Birth prevalence - [30, 32) wks, [500, 1000) g	Mortality	Females		239.082 (200.416 to 284.597)	163.95 (135.165 to 197.261)							
Neonatal sepsis and other neonatal infections	Birth prevalence - [28, 30) wks, [500, 1000) g	Mortality	Males		326.557 (286.01 to 366.811)	249.246 (216.248 to 289.232)							
Neonatal sepsis and other neonatal infections	Birth prevalence - [28, 30) wks, [500, 1000) g	Mortality	Females		231.533 (194.62 to 271.396)	187.47 (157.953 to 224.552)							
Neonatal sepsis and other neonatal infections	Birth prevalence - [24, 26) wks, [1000, 1500) g	Mortality	Males		273.536 (236.795 to 312.421)	182.743 (155.477 to 212.281)							
Neonatal sepsis and other neonatal infections	Birth prevalence - [24, 26) wks, [1000, 1500) g	Mortality	Females		268.033 (223.95 to 318.815)	157.928 (130.936 to 190.298)							
Neonatal sepsis and other neonatal infections	Birth prevalence - [26, 28) wks, [1000, 1500) g	Mortality	Males		164.685 (145.457 to 186.571)	120.878 (102.672 to 140.967)							
Neonatal sepsis and other neonatal infections	Birth prevalence - [26, 28) wks, [1000, 1500) g	Mortality	Females		142.274 (121.33 to 168.246)	97.496 (80.858 to 116.625)							
Neonatal sepsis and other neonatal infections	Birth prevalence - [26, 28) wks, [1500, 2000) g	Mortality	Males		113.21 (97.079 to 130.806)	49.449 (40.908 to 59.113)							
Neonatal sepsis and other neonatal infections	Birth prevalence - [26, 28) wks, [1500, 2000) g	Mortality	Females		108.393 (89.864 to 129.883)	49.979 (40.484 to 60.784)							
Neonatal sepsis and other neonatal infections	Birth prevalence - [34, 36) wks, [1000, 1500) g	Mortality	Males		119.602 (104.655 to 136.729)	56.519 (48.084 to 66.018)							

Ages													
30-34 years	35-39 years	40-44 years	45-49 years	50-54 years	55-59 years	60-64 years	65-69 years	70-74 years	75-79 years	80-84 years	85-89 years	90-94 years	95+ years

Appendix Table 1a. Relative risks used by age and sex for each outcome for all risk factors except for ambient air pollution and alcohol.

Risk - Outcome	Category / Units	Morbidity / Mortality	Sex	All-age	0-6 days	7-27 days	28-364 days	1-4 years	5-9 years	10-14 years	15-19 years	20-24 years	25-29 years
Neonatal sepsis and other neonatal infections	Birth prevalence - [34, 36) wks, [1000, 1500) g	Mortality	Females		109.231 (91.177 to 128.378)	52.834 (43.454 to 63.459)							
Neonatal sepsis and other neonatal infections	Birth prevalence - [28, 30) wks, [1500, 2000) g	Mortality	Males		87.285 (76.012 to 100.83)	37.15 (30.87 to 43.664)							
Neonatal sepsis and other neonatal infections	Birth prevalence - [28, 30) wks, [1500, 2000) g	Mortality	Females		86.639 (71.639 to 103.632)	38.3 (31.326 to 45.531)							
Neonatal sepsis and other neonatal infections	Birth prevalence - [28, 30) wks, [1000, 1500) g	Mortality	Males		107.077 (93.214 to 122.668)	81.407 (70.511 to 93.12)							
Neonatal sepsis and other neonatal infections	Birth prevalence - [28, 30) wks, [1000, 1500) g	Mortality	Females		87.409 (73.438 to 104.222)	64.981 (53.651 to 77.029)							
Neonatal sepsis and other neonatal infections	Birth prevalence - [32, 34) wks, [1000, 1500) g	Mortality	Males		98.982 (86.649 to 112.931)	51.159 (43.522 to 59.402)							
Neonatal sepsis and other neonatal infections	Birth prevalence - [32, 34) wks, [1000, 1500) g	Mortality	Females		84.081 (70.672 to 99.726)	44.695 (37.129 to 53.257)							
Neonatal sepsis and other neonatal infections	Birth prevalence - [30, 32) wks, [1000, 1500) g	Mortality	Males		90.239 (78.583 to 103.215)	59.239 (50.511 to 69.508)							
Neonatal sepsis and other neonatal infections	Birth prevalence - [30, 32) wks, [1000, 1500) g	Mortality	Females		70.119 (59.11 to 81.846)	47.493 (39.526 to 56.547)							
Neonatal sepsis and other neonatal infections	Birth prevalence - [37, 38) wks, [1500, 2000) g	Mortality	Males		55.659 (48.726 to 62.963)	25.45 (21.867 to 29.451)							
Neonatal sepsis and other neonatal infections	Birth prevalence - [37, 38) wks, [1500, 2000) g	Mortality	Females		54.453 (46.308 to 63.524)	29.339 (24.736 to 34.558)							
Neonatal sepsis and other neonatal infections	Birth prevalence - [36, 37) wks, [1500, 2000) g	Mortality	Males		53.885 (47.099 to 61.414)	23.622 (20.373 to 27.298)							
Neonatal sepsis and other neonatal infections	Birth prevalence - [36, 37) wks, [1500, 2000) g	Mortality	Females		47.995 (40.82 to 56.378)	23.905 (19.8 to 29.023)							
Neonatal sepsis and other neonatal infections	Birth prevalence - [30, 32) wks, [2000, 2500) g	Mortality	Males		39.701 (34.573 to 45.311)	16.391 (13.683 to 19.411)							
Neonatal sepsis and other neonatal infections	Birth prevalence - [30, 32) wks, [2000, 2500) g	Mortality	Females		34.894 (28.953 to 41.934)	16.549 (13.438 to 19.916)							
Neonatal sepsis and other neonatal infections	Birth prevalence - [30, 32) wks, [1500, 2000) g	Mortality	Males		55.142 (48.379 to 62.989)	26.391 (22.751 to 30.584)							
Neonatal sepsis and other neonatal infections	Birth prevalence - [30, 32) wks, [1500, 2000) g	Mortality	Females		48.414 (40.924 to 57.128)	26.986 (22.299 to 32.305)							
Neonatal sepsis and other neonatal infections	Birth prevalence - [34, 36) wks, [1500, 2000) g	Mortality	Males		45.579 (39.817 to 51.922)	19.836 (16.94 to 23.141)							
Neonatal sepsis and other neonatal infections	Birth prevalence - [34, 36) wks, [1500, 2000) g	Mortality	Females		38.405 (32.273 to 45.04)	18.409 (15.134 to 21.755)							
Neonatal sepsis and other neonatal infections	Birth prevalence - [32, 34) wks, [1500, 2000) g	Mortality	Males		41.734 (36.381 to 47.516)	19.083 (16.193 to 21.909)							
Neonatal sepsis and other neonatal infections	Birth prevalence - [32, 34) wks, [1500, 2000) g	Mortality	Females		36.162 (30.231 to 42.934)	19.528 (16.278 to 23.535)							
Neonatal sepsis and other neonatal infections	Birth prevalence - [32, 34) wks, [2000, 2500) g	Mortality	Males		22.379 (19.576 to 25.426)	11.555 (9.874 to 13.332)							
Neonatal sepsis and other neonatal infections	Birth prevalence - [32, 34) wks, [2000, 2500) g	Mortality	Females		20.326 (17.055 to 23.945)	12.376 (10.192 to 14.834)							
Neonatal sepsis and other neonatal infections	Birth prevalence - [40, 42) wks, [2000, 2500) g	Mortality	Males		17.218 (14.893 to 19.961)	11.519 (9.766 to 13.495)							
Neonatal sepsis and other neonatal infections	Birth prevalence - [40, 42) wks, [2000, 2500) g	Mortality	Females		13.555 (11.337 to 16.132)	11.838 (9.792 to 14.338)							
Neonatal sepsis and other neonatal infections	Birth prevalence - [38, 40) wks, [2000, 2500) g	Mortality	Males		14.227 (12.521 to 16.06)	10.475 (9.122 to 12.085)							
Neonatal sepsis and other neonatal infections	Birth prevalence - [38, 40) wks, [2000, 2500) g	Mortality	Females		10.867 (9.199 to 12.777)	10.493 (8.801 to 12.342)							
Neonatal sepsis and other neonatal infections	Birth prevalence - [32, 34) wks, [2500, 3000) g	Mortality	Males		12.775 (11.146 to 14.607)	6.296 (5.269 to 7.299)							
Neonatal sepsis and other neonatal infections	Birth prevalence - [32, 34) wks, [2500, 3000) g	Mortality	Females		9.041 (7.504 to 10.706)	6.205 (5.041 to 7.52)							
Neonatal sepsis and other neonatal infections	Birth prevalence - [34, 36) wks, [2000, 2500) g	Mortality	Males		16.472 (14.422 to 18.7)	9.777 (8.456 to 11.277)							

Ages													
30-34 years	35-39 years	40-44 years	45-49 years	50-54 years	55-59 years	60-64 years	65-69 years	70-74 years	75-79 years	80-84 years	85-89 years	90-94 years	95+ years

Appendix Table 1a. Relative risks used by age and sex for each outcome for all risk factors except for ambient air pollution and alcohol.

Risk - Outcome	Category / Units	Morbidity / Mortality	Sex	All-age	0-6 days	7-27 days	28-364 days	1-4 years	5-9 years	10-14 years	15-19 years	20-24 years	25-29 years
Neonatal sepsis and other neonatal infections	Birth prevalence - [34, 36) wks, [2000, 2500) g	Mortality	Females		12.895 (10.992 to 15.284)	9.44 (7.865 to 11.209)							
Neonatal sepsis and other neonatal infections	Birth prevalence - [37, 38) wks, [2000, 2500) g	Mortality	Males		14.003 (12.407 to 15.831)	9.977 (8.758 to 11.444)							
Neonatal sepsis and other neonatal infections	Birth prevalence - [37, 38) wks, [2000, 2500) g	Mortality	Females		10.456 (8.899 to 12.149)	9.83 (8.317 to 11.54)							
Neonatal sepsis and other neonatal infections	Birth prevalence - [36, 37) wks, [2000, 2500) g	Mortality	Males		14.873 (13.083 to 16.88)	9.756 (8.38 to 11.214)							
Neonatal sepsis and other neonatal infections	Birth prevalence - [36, 37) wks, [2000, 2500) g	Mortality	Females		10.517 (8.897 to 12.379)	9.233 (7.691 to 10.92)							
Neonatal sepsis and other neonatal infections	Birth prevalence - [34, 36) wks, [2500, 3000) g	Mortality	Males		8.216 (7.133 to 9.378)	5.419 (4.631 to 6.282)							
Neonatal sepsis and other neonatal infections	Birth prevalence - [34, 36) wks, [2500, 3000) g	Mortality	Females		6.037 (5.044 to 7.094)	5.198 (4.31 to 6.293)							
Neonatal sepsis and other neonatal infections	Birth prevalence - [34, 36) wks, [4000, 4500) g	Mortality	Males		2.68 (2.245 to 3.183)	1.532 (1.254 to 1.867)							
Neonatal sepsis and other neonatal infections	Birth prevalence - [34, 36) wks, [4000, 4500) g	Mortality	Females		2.458 (1.967 to 3.074)	1.673 (1.344 to 2.093)							
Neonatal sepsis and other neonatal infections	Birth prevalence - [34, 36) wks, [3000, 3500) g	Mortality	Males		3.961 (3.433 to 4.521)	3.194 (2.71 to 3.696)							
Neonatal sepsis and other neonatal infections	Birth prevalence - [34, 36) wks, [3000, 3500) g	Mortality	Females		3.267 (2.738 to 3.876)	2.783 (2.273 to 3.408)							
Neonatal sepsis and other neonatal infections	Birth prevalence - [36, 37) wks, [2500, 3000) g	Mortality	Males		5.363 (4.69 to 6.082)	4.559 (3.945 to 5.248)							
Neonatal sepsis and other neonatal infections	Birth prevalence - [36, 37) wks, [2500, 3000) g	Mortality	Females		3.969 (3.371 to 4.675)	4.138 (3.469 to 4.868)							
Neonatal sepsis and other neonatal infections	Birth prevalence - [34, 36) wks, [3500, 4000) g	Mortality	Males		2.678 (2.289 to 3.086)	2.014 (1.69 to 2.365)							
Neonatal sepsis and other neonatal infections	Birth prevalence - [34, 36) wks, [3500, 4000) g	Mortality	Females		2.342 (1.908 to 2.845)	1.756 (1.426 to 2.126)							
Neonatal sepsis and other neonatal infections	Birth prevalence - [37, 38) wks, [2500, 3000) g	Mortality	Males		4.093 (3.581 to 4.626)	4.139 (3.631 to 4.74)							
Neonatal sepsis and other neonatal infections	Birth prevalence - [37, 38) wks, [2500, 3000) g	Mortality	Females		3.07 (2.612 to 3.603)	3.618 (3.045 to 4.25)							
Neonatal sepsis and other neonatal infections	Birth prevalence - [40, 42) wks, [2500, 3000) g	Mortality	Males		3.833 (3.364 to 4.388)	3.828 (3.271 to 4.426)							
Neonatal sepsis and other neonatal infections	Birth prevalence - [40, 42) wks, [2500, 3000) g	Mortality	Females		2.699 (2.264 to 3.202)	3.379 (2.825 to 4.042)							
Neonatal sepsis and other neonatal infections	Birth prevalence - [38, 40) wks, [2500, 3000) g	Mortality	Males		3.396 (3.002 to 3.839)	3.858 (3.346 to 4.424)							
Neonatal sepsis and other neonatal infections	Birth prevalence - [38, 40) wks, [2500, 3000) g	Mortality	Females		2.382 (2.04 to 2.802)	3.27 (2.774 to 3.927)							
Neonatal sepsis and other neonatal infections	Birth prevalence - [36, 37) wks, [3000, 3500) g	Mortality	Males		2.819 (2.433 to 3.229)	2.683 (2.299 to 3.079)							
Neonatal sepsis and other neonatal infections	Birth prevalence - [36, 37) wks, [3000, 3500) g	Mortality	Females		2.335 (1.952 to 2.784)	2.434 (2.007 to 2.884)							
Neonatal sepsis and other neonatal infections	Birth prevalence - [36, 37) wks, [4000, 4500) g	Mortality	Males		2.331 (1.993 to 2.727)	1.471 (1.221 to 1.731)							
Neonatal sepsis and other neonatal infections	Birth prevalence - [36, 37) wks, [4000, 4500) g	Mortality	Females		2.141 (1.729 to 2.577)	1.601 (1.291 to 1.97)							
Neonatal sepsis and other neonatal infections	Birth prevalence - [36, 37) wks, [3500, 4000) g	Mortality	Males		2.084 (1.789 to 2.418)	1.923 (1.624 to 2.252)							
Neonatal sepsis and other neonatal infections	Birth prevalence - [36, 37) wks, [3500, 4000) g	Mortality	Females		1.845 (1.527 to 2.216)	1.731 (1.426 to 2.071)							
Neonatal sepsis and other neonatal infections	Birth prevalence - [37, 38) wks, [3000, 3500) g	Mortality	Males		2.027 (1.788 to 2.287)	2.236 (1.926 to 2.542)							
Neonatal sepsis and other neonatal infections	Birth prevalence - [37, 38) wks, [3000, 3500) g	Mortality	Females		1.667 (1.411 to 1.947)	1.989 (1.672 to 2.366)							
Neonatal sepsis and other neonatal infections	Birth prevalence - [37, 38) wks, [4000, 4500) g	Mortality	Males		1.572 (1.352 to 1.813)	1.28 (1.095 to 1.481)							

Ages													
30-34 years	35-39 years	40-44 years	45-49 years	50-54 years	55-59 years	60-64 years	65-69 years	70-74 years	75-79 years	80-84 years	85-89 years	90-94 years	95+ years

Appendix Table 1a. Relative risks used by age and sex for each outcome for all risk factors except for ambient air pollution and alcohol.

Risk - Outcome	Category / Units	Morbidity / Mortality	Sex	All-age	0-6 days	7-27 days	28-364 days	1-4 years	5-9 years	10-14 years	15-19 years	20-24 years	25-29 years
Neonatal sepsis and other neonatal infections	Birth prevalence - [37, 38) wks, [4000, 4500) g	Mortality	Females		1.647 (1.37 to 1.98)	1.396 (1.167 to 1.674)							
Neonatal sepsis and other neonatal infections	Birth prevalence - [37, 38) wks, [3500, 4000) g	Mortality	Males		1.502 (1.309 to 1.712)	1.563 (1.353 to 1.807)							
Neonatal sepsis and other neonatal infections	Birth prevalence - [37, 38) wks, [3500, 4000) g	Mortality	Females		1.275 (1.075 to 1.501)	1.444 (1.206 to 1.711)							
Neonatal sepsis and other neonatal infections	Birth prevalence - [40, 42) wks, [3000, 3500) g	Mortality	Males		1.446 (1.258 to 1.639)	1.79 (1.546 to 2.08)							
Neonatal sepsis and other neonatal infections	Birth prevalence - [40, 42) wks, [3000, 3500) g	Mortality	Females		1.103 (0.922 to 1.305)	1.491 (1.233 to 1.776)							
Neonatal sepsis and other neonatal infections	Birth prevalence - [38, 40) wks, [3000, 3500) g	Mortality	Males		1.414 (1.242 to 1.593)	1.874 (1.636 to 2.156)							
Neonatal sepsis and other neonatal infections	Birth prevalence - [38, 40) wks, [3000, 3500) g	Mortality	Females		1.096 (0.927 to 1.289)	1.617 (1.352 to 1.907)							
Neonatal sepsis and other neonatal infections	Birth prevalence - [38, 40) wks, [4000, 4500) g	Mortality	Males		1.052 (0.92 to 1.203)	1.093 (0.939 to 1.261)							
Neonatal sepsis and other neonatal infections	Birth prevalence - [38, 40) wks, [4000, 4500) g	Mortality	Females		1.121 (0.925 to 1.31)	1.149 (0.955 to 1.367)							
Neonatal sepsis and other neonatal infections	Birth prevalence - [38, 40) wks, [3500, 4000) g	Mortality	Males		1.009 (0.883 to 1.143)	1.243 (1.078 to 1.421)							
Neonatal sepsis and other neonatal infections	Birth prevalence - [38, 40) wks, [3500, 4000) g	Mortality	Females		0.828 (0.702 to 0.972)	1.155 (0.967 to 1.369)							
Neonatal sepsis and other neonatal infections	Birth prevalence - [40, 42) wks, [3500, 4000) g	Mortality	Males		0.98 (0.854 to 1.123)	1.155 (0.983 to 1.334)							
Neonatal sepsis and other neonatal infections	Birth prevalence - [40, 42) wks, [3500, 4000) g	Mortality	Females		0.819 (0.679 to 0.974)	1.045 (0.874 to 1.26)							
Hemolytic disease and other neonatal jaundice	Birth prevalence - [22, 24) wks, [0, 500) g	Mortality	Males		3273.944 (2987.306 to 3600.961)	599.42 (512.777 to 692.914)							
Hemolytic disease and other neonatal jaundice	Birth prevalence - [22, 24) wks, [0, 500) g	Mortality	Females		3353.667 (2964.862 to 3798.637)	771.075 (635.721 to 931.62)							
Hemolytic disease and other neonatal jaundice	Birth prevalence - [24, 26) wks, [0, 500) g	Mortality	Males		2454.576 (2216.544 to 2713.168)	884.902 (766.03 to 1021.494)							
Hemolytic disease and other neonatal jaundice	Birth prevalence - [24, 26) wks, [0, 500) g	Mortality	Females		2376.85 (2080.354 to 2699.254)	915.957 (765.805 to 1079.243)							
Hemolytic disease and other neonatal jaundice	Birth prevalence - [26, 28) wks, [0, 500) g	Mortality	Males		1870.684 (1676.978 to 2075.277)	847.512 (727.279 to 983.252)							
Hemolytic disease and other neonatal jaundice	Birth prevalence - [26, 28) wks, [0, 500) g	Mortality	Females		1708.132 (1477.512 to 1960.75)	822.906 (677.101 to 990.491)							
Hemolytic disease and other neonatal jaundice	Birth prevalence - [28, 30) wks, [0, 500) g	Mortality	Males		1805.939 (1612.157 to 2006.088)	583.881 (485.096 to 690.434)							
Hemolytic disease and other neonatal jaundice	Birth prevalence - [28, 30) wks, [0, 500) g	Mortality	Females		1585.788 (1377.653 to 1836.365)	636.164 (520.511 to 760.561)							
Hemolytic disease and other neonatal jaundice	Birth prevalence - [22, 24) wks, [500, 1000) g	Mortality	Males		2037.627 (1843.599 to 2249.686)	720.117 (628.641 to 820.917)							
Hemolytic disease and other neonatal jaundice	Birth prevalence - [22, 24) wks, [500, 1000) g	Mortality	Females		1833.266 (1596.246 to 2105.971)	610.368 (510.239 to 722.278)							
Hemolytic disease and other neonatal jaundice	Birth prevalence - [24, 26) wks, [500, 1000) g	Mortality	Males		852.585 (753.604 to 959.026)	642.627 (558.091 to 730.626)							
Hemolytic disease and other neonatal jaundice	Birth prevalence - [24, 26) wks, [500, 1000) g	Mortality	Females		715.379 (610.81 to 830.296)	546.057 (463.282 to 637.815)							
Hemolytic disease and other neonatal jaundice	Birth prevalence - [26, 28) wks, [500, 1000) g	Mortality	Males		411.87 (365.037 to 467.411)	353.266 (305.647 to 405.904)							
Hemolytic disease and other neonatal jaundice	Birth prevalence - [26, 28) wks, [500, 1000) g	Mortality	Females		308.092 (262.47 to 362.302)	292.936 (243.986 to 349.019)							
Hemolytic disease and other neonatal jaundice	Birth prevalence - [32, 34) wks, [500, 1000) g	Mortality	Males		344.501 (301.197 to 397.343)	194.954 (163.844 to 231.771)							
Hemolytic disease and other neonatal jaundice	Birth prevalence - [32, 34) wks, [500, 1000) g	Mortality	Females		299.264 (251.468 to 353.995)	161.548 (130.866 to 196.323)							
Hemolytic disease and other neonatal jaundice	Birth prevalence - [22, 24) wks, [1000, 1500) g	Mortality	Males		673.302 (584.187 to 768.258)	213.181 (179.555 to 254.572)							

Ages													
30-34 years	35-39 years	40-44 years	45-49 years	50-54 years	55-59 years	60-64 years	65-69 years	70-74 years	75-79 years	80-84 years	85-89 years	90-94 years	95+ years

Appendix Table 1a. Relative risks used by age and sex for each outcome for all risk factors except for ambient air pollution and alcohol.

Risk - Outcome	Category / Units	Morbidity / Mortality	Sex	All-age	0-6 days	7-27 days	28-364 days	1-4 years	5-9 years	10-14 years	15-19 years	20-24 years	25-29 years
Hemolytic disease and other neonatal jaundice	Birth prevalence - [22, 24) wks, [1000, 1500) g	Mortality	Females		620.446 (522.864 to 734.748)	186.215 (149.772 to 227.046)							
Hemolytic disease and other neonatal jaundice	Birth prevalence - [30, 32) wks, [500, 1000) g	Mortality	Males		332.681 (293.52 to 377.86)	212.332 (181.213 to 245.271)							
Hemolytic disease and other neonatal jaundice	Birth prevalence - [30, 32) wks, [500, 1000) g	Mortality	Females		239.082 (200.416 to 284.597)	163.95 (135.165 to 197.261)							
Hemolytic disease and other neonatal jaundice	Birth prevalence - [28, 30) wks, [500, 1000) g	Mortality	Males		326.557 (286.01 to 366.811)	249.246 (216.248 to 289.232)							
Hemolytic disease and other neonatal jaundice	Birth prevalence - [28, 30) wks, [500, 1000) g	Mortality	Females		231.533 (194.62 to 271.396)	187.47 (157.953 to 224.552)							
Hemolytic disease and other neonatal jaundice	Birth prevalence - [24, 26) wks, [1000, 1500) g	Mortality	Males		273.536 (236.795 to 312.421)	182.743 (155.477 to 212.281)							
Hemolytic disease and other neonatal jaundice	Birth prevalence - [24, 26) wks, [1000, 1500) g	Mortality	Females		268.033 (223.95 to 318.815)	157.928 (130.936 to 190.298)							
Hemolytic disease and other neonatal jaundice	Birth prevalence - [26, 28) wks, [1000, 1500) g	Mortality	Males		164.685 (145.457 to 186.571)	120.878 (102.672 to 140.967)							
Hemolytic disease and other neonatal jaundice	Birth prevalence - [26, 28) wks, [1000, 1500) g	Mortality	Females		142.274 (121.33 to 168.246)	97.496 (80.858 to 116.625)							
Hemolytic disease and other neonatal jaundice	Birth prevalence - [26, 28) wks, [1500, 2000) g	Mortality	Males		113.21 (97.079 to 130.806)	49.449 (40.908 to 59.113)							
Hemolytic disease and other neonatal jaundice	Birth prevalence - [26, 28) wks, [1500, 2000) g	Mortality	Females		108.393 (89.864 to 129.883)	49.979 (40.484 to 60.784)							
Hemolytic disease and other neonatal jaundice	Birth prevalence - [34, 36) wks, [1000, 1500) g	Mortality	Males		119.602 (104.655 to 136.729)	56.519 (48.084 to 66.018)							
Hemolytic disease and other neonatal jaundice	Birth prevalence - [34, 36) wks, [1000, 1500) g	Mortality	Females		109.231 (91.177 to 128.378)	52.834 (43.454 to 63.459)							
Hemolytic disease and other neonatal jaundice	Birth prevalence - [28, 30) wks, [1500, 2000) g	Mortality	Males		87.285 (76.012 to 100.83)	37.15 (30.87 to 43.664)							
Hemolytic disease and other neonatal jaundice	Birth prevalence - [28, 30) wks, [1500, 2000) g	Mortality	Females		86.639 (71.639 to 103.632)	38.3 (31.326 to 45.531)							
Hemolytic disease and other neonatal jaundice	Birth prevalence - [28, 30) wks, [1000, 1500) g	Mortality	Males		107.077 (93.214 to 122.668)	81.407 (70.511 to 93.12)							
Hemolytic disease and other neonatal jaundice	Birth prevalence - [28, 30) wks, [1000, 1500) g	Mortality	Females		87.409 (73.438 to 104.222)	64.981 (53.651 to 77.029)							
Hemolytic disease and other neonatal jaundice	Birth prevalence - [32, 34) wks, [1000, 1500) g	Mortality	Males		98.982 (86.649 to 112.931)	51.159 (43.522 to 59.402)							
Hemolytic disease and other neonatal jaundice	Birth prevalence - [32, 34) wks, [1000, 1500) g	Mortality	Females		84.081 (70.672 to 99.726)	44.695 (37.129 to 53.257)							
Hemolytic disease and other neonatal jaundice	Birth prevalence - [30, 32) wks, [1000, 1500) g	Mortality	Males		90.239 (78.583 to 103.215)	59.239 (50.511 to 69.508)							
Hemolytic disease and other neonatal jaundice	Birth prevalence - [30, 32) wks, [1000, 1500) g	Mortality	Females		70.119 (59.11 to 81.846)	47.493 (39.526 to 56.547)							
Hemolytic disease and other neonatal jaundice	Birth prevalence - [37, 38) wks, [1500, 2000) g	Mortality	Males		55.659 (48.726 to 62.963)	25.45 (21.867 to 29.451)							
Hemolytic disease and other neonatal jaundice	Birth prevalence - [37, 38) wks, [1500, 2000) g	Mortality	Females		54.453 (46.308 to 63.524)	29.339 (24.736 to 34.558)							
Hemolytic disease and other neonatal jaundice	Birth prevalence - [36, 37) wks, [1500, 2000) g	Mortality	Males		53.885 (47.099 to 61.414)	23.622 (20.373 to 27.298)							
Hemolytic disease and other neonatal jaundice	Birth prevalence - [36, 37) wks, [1500, 2000) g	Mortality	Females		47.995 (40.82 to 56.378)	23.905 (19.8 to 29.023)							
Hemolytic disease and other neonatal jaundice	Birth prevalence - [30, 32) wks, [2000, 2500) g	Mortality	Males		39.701 (34.573 to 45.311)	16.391 (13.683 to 19.411)							
Hemolytic disease and other neonatal jaundice	Birth prevalence - [30, 32) wks, [2000, 2500) g	Mortality	Females		34.894 (28.953 to 41.934)	16.549 (13.438 to 19.916)							
Hemolytic disease and other neonatal jaundice	Birth prevalence - [30, 32) wks, [1500, 2000) g	Mortality	Males		55.142 (48.379 to 62.989)	26.391 (22.751 to 30.584)							
Hemolytic disease and other neonatal jaundice	Birth prevalence - [30, 32) wks, [1500, 2000) g	Mortality	Females		48.414 (40.924 to 57.128)	26.986 (22.299 to 32.305)							
Hemolytic disease and other neonatal jaundice	Birth prevalence - [34, 36) wks, [1500, 2000) g	Mortality	Males		45.579 (39.817 to 51.922)	19.836 (16.94 to 23.141)							

Ages													
30-34 years	35-39 years	40-44 years	45-49 years	50-54 years	55-59 years	60-64 years	65-69 years	70-74 years	75-79 years	80-84 years	85-89 years	90-94 years	95+ years

Appendix Table 1a. Relative risks used by age and sex for each outcome for all risk factors except for ambient air pollution and alcohol.													
**Risk - Outcome**	**Category / Units**	**Morbidity / Mortality**	**Sex**	**All-age**	**0-6 days**	**7-27 days**	**28-364 days**	**1-4 years**	**5-9 years**	**10-14 years**	**15-19 years**	**20-24 years**	**25-29 years**
Hemolytic disease and other neonatal jaundice	Birth prevalence - [34, 36) wks, [1500, 2000) g	Mortality	Females		38.405<br>(32.273 to 45.04)	18.409<br>(15.134 to 21.755)							
Hemolytic disease and other neonatal jaundice	Birth prevalence - [32, 34) wks, [1500, 2000) g	Mortality	Males		41.734<br>(36.381 to 47.516)	19.083<br>(16.193 to 21.909)							
Hemolytic disease and other neonatal jaundice	Birth prevalence - [32, 34) wks, [1500, 2000) g	Mortality	Females		36.162<br>(30.231 to 42.934)	19.528<br>(16.278 to 23.535)							
Hemolytic disease and other neonatal jaundice	Birth prevalence - [32, 34) wks, [2000, 2500) g	Mortality	Males		22.379<br>(19.576 to 25.426)	11.555<br>(9.874 to 13.332)							
Hemolytic disease and other neonatal jaundice	Birth prevalence - [32, 34) wks, [2000, 2500) g	Mortality	Females		20.326<br>(17.055 to 23.945)	12.376<br>(10.192 to 14.834)							
Hemolytic disease and other neonatal jaundice	Birth prevalence - [40, 42) wks, [2000, 2500) g	Mortality	Males		17.218<br>(14.893 to 19.961)	11.519<br>(9.766 to 13.495)							
Hemolytic disease and other neonatal jaundice	Birth prevalence - [40, 42) wks, [2000, 2500) g	Mortality	Females		13.555<br>(11.337 to 16.132)	11.838<br>(9.792 to 14.338)							
Hemolytic disease and other neonatal jaundice	Birth prevalence - [38, 40) wks, [2000, 2500) g	Mortality	Males		14.227<br>(12.521 to 16.06)	10.475<br>(9.122 to 12.085)							
Hemolytic disease and other neonatal jaundice	Birth prevalence - [38, 40) wks, [2000, 2500) g	Mortality	Females		10.867<br>(9.199 to 12.777)	10.493<br>(8.801 to 12.342)							
Hemolytic disease and other neonatal jaundice	Birth prevalence - [32, 34) wks, [2500, 3000) g	Mortality	Males		12.775<br>(11.146 to 14.607)	6.296<br>(5.269 to 7.299)							
Hemolytic disease and other neonatal jaundice	Birth prevalence - [32, 34) wks, [2500, 3000) g	Mortality	Females		9.041<br>(7.504 to 10.706)	6.205<br>(5.041 to 7.52)							
Hemolytic disease and other neonatal jaundice	Birth prevalence - [34, 36) wks, [2000, 2500) g	Mortality	Males		16.472<br>(14.422 to 18.7)	9.777<br>(8.456 to 11.277)							
Hemolytic disease and other neonatal jaundice	Birth prevalence - [34, 36) wks, [2000, 2500) g	Mortality	Females		12.895<br>(10.992 to 15.284)	9.44<br>(7.865 to 11.209)							
Hemolytic disease and other neonatal jaundice	Birth prevalence - [37, 38) wks, [2000, 2500) g	Mortality	Males		14.003<br>(12.407 to 15.831)	9.977<br>(8.758 to 11.444)							
Hemolytic disease and other neonatal jaundice	Birth prevalence - [37, 38) wks, [2000, 2500) g	Mortality	Females		10.456<br>(8.899 to 12.149)	9.83<br>(8.317 to 11.54)							
Hemolytic disease and other neonatal jaundice	Birth prevalence - [36, 37) wks, [2000, 2500) g	Mortality	Males		14.873<br>(13.083 to 16.88)	9.756<br>(8.38 to 11.214)							
Hemolytic disease and other neonatal jaundice	Birth prevalence - [36, 37) wks, [2000, 2500) g	Mortality	Females		10.517<br>(8.897 to 12.379)	9.233<br>(7.691 to 10.92)							
Hemolytic disease and other neonatal jaundice	Birth prevalence - [34, 36) wks, [2500, 3000) g	Mortality	Males		8.216<br>(7.133 to 9.378)	5.419<br>(4.631 to 6.282)							
Hemolytic disease and other neonatal jaundice	Birth prevalence - [34, 36) wks, [2500, 3000) g	Mortality	Females		6.037<br>(5.044 to 7.094)	5.198<br>(4.31 to 6.293)							
Hemolytic disease and other neonatal jaundice	Birth prevalence - [34, 36) wks, [4000, 4500) g	Mortality	Males		2.68<br>(2.245 to 3.183)	1.532<br>(1.254 to 1.867)							
Hemolytic disease and other neonatal jaundice	Birth prevalence - [34, 36) wks, [4000, 4500) g	Mortality	Females		2.458<br>(1.967 to 3.074)	1.673<br>(1.344 to 2.093)							
Hemolytic disease and other neonatal jaundice	Birth prevalence - [34, 36) wks, [3000, 3500) g	Mortality	Males		3.961<br>(3.433 to 4.521)	3.194<br>(2.71 to 3.696)							
Hemolytic disease and other neonatal jaundice	Birth prevalence - [34, 36) wks, [3000, 3500) g	Mortality	Females		3.267<br>(2.738 to 3.876)	2.783<br>(2.273 to 3.408)							
Hemolytic disease and other neonatal jaundice	Birth prevalence - [36, 37) wks, [2500, 3000) g	Mortality	Males		5.363<br>(4.69 to 6.082)	4.559<br>(3.945 to 5.248)							
Hemolytic disease and other neonatal jaundice	Birth prevalence - [36, 37) wks, [2500, 3000) g	Mortality	Females		3.969<br>(3.371 to 4.675)	4.138<br>(3.469 to 4.868)							
Hemolytic disease and other neonatal jaundice	Birth prevalence - [34, 36) wks, [3500, 4000) g	Mortality	Males		2.678<br>(2.289 to 3.086)	2.014<br>(1.69 to 2.365)							
Hemolytic disease and other neonatal jaundice	Birth prevalence - [34, 36) wks, [3500, 4000) g	Mortality	Females		2.342<br>(1.908 to 2.845)	1.756<br>(1.426 to 2.126)							
Hemolytic disease and other neonatal jaundice	Birth prevalence - [37, 38) wks, [2500, 3000) g	Mortality	Males		4.093<br>(3.581 to 4.626)	4.139<br>(3.631 to 4.74)							
Hemolytic disease and other neonatal jaundice	Birth prevalence - [37, 38) wks, [2500, 3000) g	Mortality	Females		3.07<br>(2.612 to 3.603)	3.618<br>(3.045 to 4.25)							
Hemolytic disease and other neonatal jaundice	Birth prevalence - [40, 42) wks, [2500, 3000) g	Mortality	Males		3.833<br>(3.364 to 4.388)	3.828<br>(3.271 to 4.426)							

Ages													
30-34 years	35-39 years	40-44 years	45-49 years	50-54 years	55-59 years	60-64 years	65-69 years	70-74 years	75-79 years	80-84 years	85-89 years	90-94 years	95+ years

Appendix Table 1a. Relative risks used by age and sex for each outcome for all risk factors except for ambient air pollution and alcohol.

Risk - Outcome	Category / Units	Morbidity / Mortality	Sex	All-age	0-6 days	7-27 days	28-364 days	1-4 years	5-9 years	10-14 years	15-19 years	20-24 years	25-29 years
Hemolytic disease and other neonatal jaundice	Birth prevalence - [40, 42) wks, [2500, 3000) g	Mortality	Females		2.699 (2.264 to 3.202)	3.379 (2.825 to 4.042)							
Hemolytic disease and other neonatal jaundice	Birth prevalence - [38, 40) wks, [2500, 3000) g	Mortality	Males		3.396 (3.002 to 3.839)	3.858 (3.346 to 4.424)							
Hemolytic disease and other neonatal jaundice	Birth prevalence - [38, 40) wks, [2500, 3000) g	Mortality	Females		2.382 (2.04 to 2.802)	3.27 (2.774 to 3.927)							
Hemolytic disease and other neonatal jaundice	Birth prevalence - [36, 37) wks, [3000, 3500) g	Mortality	Males		2.819 (2.433 to 3.229)	2.683 (2.299 to 3.079)							
Hemolytic disease and other neonatal jaundice	Birth prevalence - [36, 37) wks, [3000, 3500) g	Mortality	Females		2.335 (1.952 to 2.784)	2.434 (2.007 to 2.884)							
Hemolytic disease and other neonatal jaundice	Birth prevalence - [36, 37) wks, [4000, 4500) g	Mortality	Males		2.331 (1.993 to 2.727)	1.471 (1.221 to 1.731)							
Hemolytic disease and other neonatal jaundice	Birth prevalence - [36, 37) wks, [4000, 4500) g	Mortality	Females		2.141 (1.729 to 2.577)	1.601 (1.291 to 1.97)							
Hemolytic disease and other neonatal jaundice	Birth prevalence - [36, 37) wks, [3500, 4000) g	Mortality	Males		2.084 (1.789 to 2.418)	1.923 (1.624 to 2.252)							
Hemolytic disease and other neonatal jaundice	Birth prevalence - [36, 37) wks, [3500, 4000) g	Mortality	Females		1.845 (1.527 to 2.216)	1.731 (1.426 to 2.071)							
Hemolytic disease and other neonatal jaundice	Birth prevalence - [37, 38) wks, [3000, 3500) g	Mortality	Males		2.027 (1.788 to 2.287)	2.236 (1.926 to 2.542)							
Hemolytic disease and other neonatal jaundice	Birth prevalence - [37, 38) wks, [3000, 3500) g	Mortality	Females		1.667 (1.411 to 1.947)	1.989 (1.672 to 2.366)							
Hemolytic disease and other neonatal jaundice	Birth prevalence - [37, 38) wks, [4000, 4500) g	Mortality	Males		1.572 (1.352 to 1.813)	1.28 (1.095 to 1.481)							
Hemolytic disease and other neonatal jaundice	Birth prevalence - [37, 38) wks, [4000, 4500) g	Mortality	Females		1.647 (1.37 to 1.98)	1.396 (1.167 to 1.674)							
Hemolytic disease and other neonatal jaundice	Birth prevalence - [37, 38) wks, [3500, 4000) g	Mortality	Males		1.502 (1.309 to 1.712)	1.563 (1.353 to 1.807)							
Hemolytic disease and other neonatal jaundice	Birth prevalence - [37, 38) wks, [3500, 4000) g	Mortality	Females		1.275 (1.075 to 1.501)	1.444 (1.206 to 1.711)							
Hemolytic disease and other neonatal jaundice	Birth prevalence - [40, 42) wks, [3000, 3500) g	Mortality	Males		1.446 (1.258 to 1.639)	1.79 (1.546 to 2.08)							
Hemolytic disease and other neonatal jaundice	Birth prevalence - [40, 42) wks, [3000, 3500) g	Mortality	Females		1.103 (0.922 to 1.305)	1.491 (1.233 to 1.776)							
Hemolytic disease and other neonatal jaundice	Birth prevalence - [38, 40) wks, [3000, 3500) g	Mortality	Males		1.414 (1.242 to 1.593)	1.874 (1.636 to 2.156)							
Hemolytic disease and other neonatal jaundice	Birth prevalence - [38, 40) wks, [3000, 3500) g	Mortality	Females		1.096 (0.927 to 1.289)	1.617 (1.352 to 1.907)							
Hemolytic disease and other neonatal jaundice	Birth prevalence - [38, 40) wks, [4000, 4500) g	Mortality	Males		1.052 (0.92 to 1.203)	1.093 (0.939 to 1.261)							
Hemolytic disease and other neonatal jaundice	Birth prevalence - [38, 40) wks, [4000, 4500) g	Mortality	Females		1.121 (0.925 to 1.31)	1.149 (0.955 to 1.367)							
Hemolytic disease and other neonatal jaundice	Birth prevalence - [38, 40) wks, [3500, 4000) g	Mortality	Males		1.009 (0.883 to 1.143)	1.243 (1.078 to 1.421)							
Hemolytic disease and other neonatal jaundice	Birth prevalence - [38, 40) wks, [3500, 4000) g	Mortality	Females		0.828 (0.702 to 0.972)	1.155 (0.967 to 1.369)							
Hemolytic disease and other neonatal jaundice	Birth prevalence - [40, 42) wks, [3500, 4000) g	Mortality	Males		0.98 (0.854 to 1.123)	1.155 (0.983 to 1.334)							
Hemolytic disease and other neonatal jaundice	Birth prevalence - [40, 42) wks, [3500, 4000) g	Mortality	Females		0.819 (0.679 to 0.974)	1.045 (0.874 to 1.26)							
Other neonatal disorders	Birth prevalence - [22, 24) wks, [0, 500) g	Mortality	Males		3273.944 (2987.306 to 3600.961)	599.42 (512.777 to 692.914)							
Other neonatal disorders	Birth prevalence - [22, 24) wks, [0, 500) g	Mortality	Females		3353.667 (2964.862 to 3798.637)	771.075 (635.721 to 931.62)							
Other neonatal disorders	Birth prevalence - [24, 26) wks, [0, 500) g	Mortality	Males		2454.576 (2216.544 to 2713.168)	884.902 (766.03 to 1021.494)							
Other neonatal disorders	Birth prevalence - [24, 26) wks, [0, 500) g	Mortality	Females		2376.85 (2080.354 to 2699.254)	915.957 (765.805 to 1079.243)							
Other neonatal disorders	Birth prevalence - [26, 28) wks, [0, 500) g	Mortality	Males		1870.684 (1676.978 to 2075.277)	847.512 (727.279 to 983.252)							

Ages													
**30-34 years**	**35-39 years**	**40-44 years**	**45-49 years**	**50-54 years**	**55-59 years**	**60-64 years**	**65-69 years**	**70-74 years**	**75-79 years**	**80-84 years**	**85-89 years**	**90-94 years**	**95+ years**

Appendix Table 1a. Relative risks used by age and sex for each outcome for all risk factors except for ambient air pollution and alcohol.

Risk - Outcome	Category / Units	Morbidity / Mortality	Sex	All-age	0-6 days	7-27 days	28-364 days	1-4 years	5-9 years	10-14 years	15-19 years	20-24 years	25-29 years
Other neonatal disorders	Birth prevalence - [26, 28) wks, [0, 500) g	Mortality	Females		1708.132 (1477.512 to 1960.75)	822.906 (677.101 to 990.491)							
Other neonatal disorders	Birth prevalence - [28, 30) wks, [0, 500) g	Mortality	Males		1805.939 (1612.157 to 2006.088)	583.881 (485.096 to 690.434)							
Other neonatal disorders	Birth prevalence - [28, 30) wks, [0, 500) g	Mortality	Females		1585.788 (1377.653 to 1836.365)	636.164 (520.511 to 760.561)							
Other neonatal disorders	Birth prevalence - [22, 24) wks, [500, 1000) g	Mortality	Males		2037.627 (1843.599 to 2249.686)	720.117 (628.641 to 820.917)							
Other neonatal disorders	Birth prevalence - [22, 24) wks, [500, 1000) g	Mortality	Females		1833.266 (1596.246 to 2105.971)	610.368 (510.239 to 722.278)							
Other neonatal disorders	Birth prevalence - [24, 26) wks, [500, 1000) g	Mortality	Males		852.585 (753.604 to 959.026)	642.627 (558.091 to 730.626)							
Other neonatal disorders	Birth prevalence - [24, 26) wks, [500, 1000) g	Mortality	Females		715.379 (610.81 to 830.296)	546.057 (463.282 to 637.815)							
Other neonatal disorders	Birth prevalence - [26, 28) wks, [500, 1000) g	Mortality	Males		411.87 (365.037 to 467.411)	353.266 (305.647 to 405.904)							
Other neonatal disorders	Birth prevalence - [26, 28) wks, [500, 1000) g	Mortality	Females		308.092 (262.47 to 362.302)	292.936 (243.986 to 349.019)							
Other neonatal disorders	Birth prevalence - [32, 34) wks, [500, 1000) g	Mortality	Males		344.501 (301.197 to 397.343)	194.954 (163.844 to 231.771)							
Other neonatal disorders	Birth prevalence - [32, 34) wks, [500, 1000) g	Mortality	Females		299.264 (251.468 to 353.995)	161.548 (130.866 to 196.323)							
Other neonatal disorders	Birth prevalence - [22, 24) wks, [1000, 1500) g	Mortality	Males		673.302 (584.187 to 768.258)	213.181 (179.555 to 254.572)							
Other neonatal disorders	Birth prevalence - [22, 24) wks, [1000, 1500) g	Mortality	Females		620.446 (522.864 to 734.748)	186.215 (149.772 to 227.046)							
Other neonatal disorders	Birth prevalence - [30, 32) wks, [500, 1000) g	Mortality	Males		332.681 (293.52 to 377.86)	212.332 (181.213 to 245.271)							
Other neonatal disorders	Birth prevalence - [30, 32) wks, [500, 1000) g	Mortality	Females		239.082 (200.416 to 284.597)	163.95 (135.165 to 197.261)							
Other neonatal disorders	Birth prevalence - [28, 30) wks, [500, 1000) g	Mortality	Males		326.557 (286.01 to 366.811)	249.246 (216.248 to 289.232)							
Other neonatal disorders	Birth prevalence - [28, 30) wks, [500, 1000) g	Mortality	Females		231.533 (194.62 to 271.396)	187.47 (157.953 to 224.552)							
Other neonatal disorders	Birth prevalence - [24, 26) wks, [1000, 1500) g	Mortality	Males		273.536 (236.795 to 312.421)	182.743 (155.477 to 212.281)							
Other neonatal disorders	Birth prevalence - [24, 26) wks, [1000, 1500) g	Mortality	Females		268.033 (223.95 to 318.815)	157.928 (130.936 to 190.298)							
Other neonatal disorders	Birth prevalence - [26, 28) wks, [1000, 1500) g	Mortality	Males		164.685 (145.457 to 186.571)	120.878 (102.672 to 140.967)							
Other neonatal disorders	Birth prevalence - [26, 28) wks, [1000, 1500) g	Mortality	Females		142.274 (121.33 to 168.246)	97.496 (80.858 to 116.625)							
Other neonatal disorders	Birth prevalence - [26, 28) wks, [1500, 2000) g	Mortality	Males		113.21 (97.079 to 130.806)	49.449 (40.908 to 59.113)							
Other neonatal disorders	Birth prevalence - [26, 28) wks, [1500, 2000) g	Mortality	Females		108.393 (89.864 to 129.883)	49.979 (40.484 to 60.784)							
Other neonatal disorders	Birth prevalence - [34, 36) wks, [1000, 1500) g	Mortality	Males		119.602 (104.655 to 136.729)	56.519 (48.084 to 66.018)							
Other neonatal disorders	Birth prevalence - [34, 36) wks, [1000, 1500) g	Mortality	Females		109.231 (91.177 to 128.378)	52.834 (43.454 to 63.459)							
Other neonatal disorders	Birth prevalence - [28, 30) wks, [1500, 2000) g	Mortality	Males		87.285 (76.012 to 100.83)	37.15 (30.87 to 43.664)							
Other neonatal disorders	Birth prevalence - [28, 30) wks, [1500, 2000) g	Mortality	Females		86.639 (71.639 to 103.632)	38.3 (31.326 to 45.531)							
Other neonatal disorders	Birth prevalence - [28, 30) wks, [1000, 1500) g	Mortality	Males		107.077 (93.214 to 122.668)	81.407 (70.511 to 93.12)							
Other neonatal disorders	Birth prevalence - [28, 30) wks, [1000, 1500) g	Mortality	Females		87.409 (73.438 to 104.222)	64.981 (53.651 to 77.029)							
Other neonatal disorders	Birth prevalence - [32, 34) wks, [1000, 1500) g	Mortality	Males		98.982 (86.649 to 112.931)	51.159 (43.522 to 59.402)							

Ages													
30-34 years	35-39 years	40-44 years	45-49 years	50-54 years	55-59 years	60-64 years	65-69 years	70-74 years	75-79 years	80-84 years	85-89 years	90-94 years	95+ years

Appendix Table 1a. Relative risks used by age and sex for each outcome for all risk factors except for ambient air pollution and alcohol.

Risk - Outcome	Category / Units	Morbidity / Mortality	Sex	All-age	0-6 days	7-27 days	28-364 days	1-4 years	5-9 years	10-14 years	15-19 years	20-24 years	25-29 years
Other neonatal disorders	Birth prevalence - [32, 34) wks, [1000, 1500) g	Mortality	Females		84.081 (70.672 to 99.726)	44.695 (37.129 to 53.257)							
Other neonatal disorders	Birth prevalence - [30, 32) wks, [1000, 1500) g	Mortality	Males		90.239 (78.583 to 103.215)	59.239 (50.511 to 69.508)							
Other neonatal disorders	Birth prevalence - [30, 32) wks, [1000, 1500) g	Mortality	Females		70.119 (59.11 to 81.846)	47.493 (39.526 to 56.547)							
Other neonatal disorders	Birth prevalence - [37, 38) wks, [1500, 2000) g	Mortality	Males		55.659 (48.726 to 62.963)	25.45 (21.867 to 29.451)							
Other neonatal disorders	Birth prevalence - [37, 38) wks, [1500, 2000) g	Mortality	Females		54.453 (46.308 to 63.524)	29.339 (24.736 to 34.558)							
Other neonatal disorders	Birth prevalence - [36, 37) wks, [1500, 2000) g	Mortality	Males		53.885 (47.099 to 61.414)	23.622 (20.373 to 27.298)							
Other neonatal disorders	Birth prevalence - [36, 37) wks, [1500, 2000) g	Mortality	Females		47.995 (40.82 to 56.378)	23.905 (19.8 to 29.023)							
Other neonatal disorders	Birth prevalence - [30, 32) wks, [2000, 2500) g	Mortality	Males		39.701 (34.573 to 45.311)	16.391 (13.683 to 19.411)							
Other neonatal disorders	Birth prevalence - [30, 32) wks, [2000, 2500) g	Mortality	Females		34.894 (28.953 to 41.934)	16.549 (13.438 to 19.916)							
Other neonatal disorders	Birth prevalence - [30, 32) wks, [1500, 2000) g	Mortality	Males		55.142 (48.379 to 62.989)	26.391 (22.751 to 30.584)							
Other neonatal disorders	Birth prevalence - [30, 32) wks, [1500, 2000) g	Mortality	Females		48.414 (40.924 to 57.128)	26.986 (22.299 to 32.305)							
Other neonatal disorders	Birth prevalence - [34, 36) wks, [1500, 2000) g	Mortality	Males		45.579 (39.817 to 51.922)	19.836 (16.94 to 23.141)							
Other neonatal disorders	Birth prevalence - [34, 36) wks, [1500, 2000) g	Mortality	Females		38.405 (32.273 to 45.04)	18.409 (15.134 to 21.755)							
Other neonatal disorders	Birth prevalence - [32, 34) wks, [1500, 2000) g	Mortality	Males		41.734 (36.381 to 47.516)	19.083 (16.193 to 21.909)							
Other neonatal disorders	Birth prevalence - [32, 34) wks, [1500, 2000) g	Mortality	Females		36.162 (30.231 to 42.934)	19.528 (16.278 to 23.535)							
Other neonatal disorders	Birth prevalence - [32, 34) wks, [2000, 2500) g	Mortality	Males		22.379 (19.576 to 25.426)	11.555 (9.874 to 13.332)							
Other neonatal disorders	Birth prevalence - [32, 34) wks, [2000, 2500) g	Mortality	Females		20.326 (17.055 to 23.945)	12.376 (10.192 to 14.834)							
Other neonatal disorders	Birth prevalence - [40, 42) wks, [2000, 2500) g	Mortality	Males		17.218 (14.893 to 19.961)	11.519 (9.766 to 13.495)							
Other neonatal disorders	Birth prevalence - [40, 42) wks, [2000, 2500) g	Mortality	Females		13.555 (11.337 to 16.132)	11.838 (9.792 to 14.338)							
Other neonatal disorders	Birth prevalence - [38, 40) wks, [2000, 2500) g	Mortality	Males		14.227 (12.521 to 16.06)	10.475 (9.122 to 12.085)							
Other neonatal disorders	Birth prevalence - [38, 40) wks, [2000, 2500) g	Mortality	Females		10.867 (9.199 to 12.777)	10.493 (8.801 to 12.342)							
Other neonatal disorders	Birth prevalence - [32, 34) wks, [2500, 3000) g	Mortality	Males		12.775 (11.146 to 14.607)	6.296 (5.269 to 7.299)							
Other neonatal disorders	Birth prevalence - [32, 34) wks, [2500, 3000) g	Mortality	Females		9.041 (7.504 to 10.706)	6.205 (5.041 to 7.52)							
Other neonatal disorders	Birth prevalence - [34, 36) wks, [2000, 2500) g	Mortality	Males		16.472 (14.422 to 18.7)	9.777 (8.456 to 11.277)							
Other neonatal disorders	Birth prevalence - [34, 36) wks, [2000, 2500) g	Mortality	Females		12.895 (10.992 to 15.284)	9.44 (7.865 to 11.209)							
Other neonatal disorders	Birth prevalence - [37, 38) wks, [2000, 2500) g	Mortality	Males		14.003 (12.407 to 15.831)	9.977 (8.758 to 11.444)							
Other neonatal disorders	Birth prevalence - [37, 38) wks, [2000, 2500) g	Mortality	Females		10.456 (8.899 to 12.149)	9.83 (8.317 to 11.54)							
Other neonatal disorders	Birth prevalence - [36, 37) wks, [2000, 2500) g	Mortality	Males		14.873 (13.083 to 16.88)	9.756 (8.38 to 11.214)							
Other neonatal disorders	Birth prevalence - [36, 37) wks, [2000, 2500) g	Mortality	Females		10.517 (8.897 to 12.379)	9.233 (7.691 to 10.92)							
Other neonatal disorders	Birth prevalence - [34, 36) wks, [2500, 3000) g	Mortality	Males		8.216 (7.133 to 9.378)	5.419 (4.631 to 6.282)							

Ages													
30-34 years	35-39 years	40-44 years	45-49 years	50-54 years	55-59 years	60-64 years	65-69 years	70-74 years	75-79 years	80-84 years	85-89 years	90-94 years	95+ years

Appendix Table 1a. Relative risks used by age and sex for each outcome for all risk factors except for ambient air pollution and alcohol.

Risk - Outcome	Category / Units	Morbidity / Mortality	Sex	All-age	0-6 days	7-27 days	28-364 days	1-4 years	5-9 years	10-14 years	15-19 years	20-24 years	25-29 years
Other neonatal disorders	Birth prevalence - [34, 36) wks, [2500, 3000) g	Mortality	Females		6.037 (5.044 to 7.094)	5.198 (4.31 to 6.293)							
Other neonatal disorders	Birth prevalence - [34, 36) wks, [4000, 4500) g	Mortality	Males		2.68 (2.245 to 3.183)	1.532 (1.254 to 1.867)							
Other neonatal disorders	Birth prevalence - [34, 36) wks, [4000, 4500) g	Mortality	Females		2.458 (1.967 to 3.074)	1.673 (1.344 to 2.093)							
Other neonatal disorders	Birth prevalence - [34, 36) wks, [3000, 3500) g	Mortality	Males		3.961 (3.433 to 4.521)	3.194 (2.71 to 3.696)							
Other neonatal disorders	Birth prevalence - [34, 36) wks, [3000, 3500) g	Mortality	Females		3.267 (2.738 to 3.876)	2.783 (2.273 to 3.408)							
Other neonatal disorders	Birth prevalence - [36, 37) wks, [2500, 3000) g	Mortality	Males		5.363 (4.69 to 6.082)	4.559 (3.945 to 5.248)							
Other neonatal disorders	Birth prevalence - [36, 37) wks, [2500, 3000) g	Mortality	Females		3.969 (3.371 to 4.675)	4.138 (3.469 to 4.868)							
Other neonatal disorders	Birth prevalence - [34, 36) wks, [3500, 4000) g	Mortality	Males		2.678 (2.289 to 3.086)	2.014 (1.69 to 2.365)							
Other neonatal disorders	Birth prevalence - [34, 36) wks, [3500, 4000) g	Mortality	Females		2.342 (1.908 to 2.845)	1.756 (1.426 to 2.126)							
Other neonatal disorders	Birth prevalence - [37, 38) wks, [2500, 3000) g	Mortality	Males		4.093 (3.581 to 4.626)	4.139 (3.631 to 4.74)							
Other neonatal disorders	Birth prevalence - [37, 38) wks, [2500, 3000) g	Mortality	Females		3.07 (2.612 to 3.603)	3.618 (3.045 to 4.25)							
Other neonatal disorders	Birth prevalence - [40, 42) wks, [2500, 3000) g	Mortality	Males		3.833 (3.364 to 4.388)	3.828 (3.271 to 4.426)							
Other neonatal disorders	Birth prevalence - [40, 42) wks, [2500, 3000) g	Mortality	Females		2.699 (2.264 to 3.202)	3.379 (2.825 to 4.042)							
Other neonatal disorders	Birth prevalence - [38, 40) wks, [2500, 3000) g	Mortality	Males		3.396 (3.002 to 3.839)	3.858 (3.346 to 4.424)							
Other neonatal disorders	Birth prevalence - [38, 40) wks, [2500, 3000) g	Mortality	Females		2.382 (2.04 to 2.802)	3.27 (2.774 to 3.927)							
Other neonatal disorders	Birth prevalence - [36, 37) wks, [3000, 3500) g	Mortality	Males		2.819 (2.433 to 3.229)	2.683 (2.299 to 3.079)							
Other neonatal disorders	Birth prevalence - [36, 37) wks, [3000, 3500) g	Mortality	Females		2.335 (1.952 to 2.784)	2.434 (2.007 to 2.884)							
Other neonatal disorders	Birth prevalence - [36, 37) wks, [4000, 4500) g	Mortality	Males		2.331 (1.993 to 2.727)	1.471 (1.221 to 1.731)							
Other neonatal disorders	Birth prevalence - [36, 37) wks, [4000, 4500) g	Mortality	Females		2.141 (1.729 to 2.577)	1.601 (1.291 to 1.97)							
Other neonatal disorders	Birth prevalence - [36, 37) wks, [3500, 4000) g	Mortality	Males		2.084 (1.789 to 2.418)	1.923 (1.624 to 2.252)							
Other neonatal disorders	Birth prevalence - [36, 37) wks, [3500, 4000) g	Mortality	Females		1.845 (1.527 to 2.216)	1.731 (1.426 to 2.071)							
Other neonatal disorders	Birth prevalence - [37, 38) wks, [3000, 3500) g	Mortality	Males		2.027 (1.788 to 2.287)	2.236 (1.926 to 2.542)							
Other neonatal disorders	Birth prevalence - [37, 38) wks, [3000, 3500) g	Mortality	Females		1.667 (1.411 to 1.947)	1.989 (1.672 to 2.366)							
Other neonatal disorders	Birth prevalence - [37, 38) wks, [4000, 4500) g	Mortality	Males		1.572 (1.352 to 1.813)	1.28 (1.095 to 1.481)							
Other neonatal disorders	Birth prevalence - [37, 38) wks, [4000, 4500) g	Mortality	Females		1.647 (1.37 to 1.98)	1.396 (1.167 to 1.674)							
Other neonatal disorders	Birth prevalence - [37, 38) wks, [3500, 4000) g	Mortality	Males		1.502 (1.309 to 1.712)	1.563 (1.353 to 1.807)							
Other neonatal disorders	Birth prevalence - [37, 38) wks, [3500, 4000) g	Mortality	Females		1.275 (1.075 to 1.501)	1.444 (1.206 to 1.711)							
Other neonatal disorders	Birth prevalence - [40, 42) wks, [3000, 3500) g	Mortality	Males		1.446 (1.258 to 1.639)	1.79 (1.546 to 2.08)							
Other neonatal disorders	Birth prevalence - [40, 42) wks, [3000, 3500) g	Mortality	Females		1.103 (0.922 to 1.305)	1.491 (1.233 to 1.776)							
Other neonatal disorders	Birth prevalence - [38, 40) wks, [3000, 3500) g	Mortality	Males		1.414 (1.242 to 1.593)	1.874 (1.636 to 2.156)							

Ages													
**30-34 years**	**35-39 years**	**40-44 years**	**45-49 years**	**50-54 years**	**55-59 years**	**60-64 years**	**65-69 years**	**70-74 years**	**75-79 years**	**80-84 years**	**85-89 years**	**90-94 years**	**95+ years**

Appendix Table 1a. Relative risks used by age and sex for each outcome for all risk factors except for ambient air pollution and alcohol.

Risk - Outcome	Category / Units	Morbidity / Mortality	Sex	All-age	0-6 days	7-27 days	28-364 days	1-4 years	5-9 years	10-14 years	15-19 years	20-24 years	25-29 years
Other neonatal disorders	Birth prevalence - [38, 40) wks, [3000, 3500) g	Mortality	Females		1.096<br>(0.927 to 1.289)	1.617<br>(1.352 to 1.907)							
Other neonatal disorders	Birth prevalence - [38, 40) wks, [4000, 4500) g	Mortality	Males		1.052<br>(0.92 to 1.203)	1.093<br>(0.939 to 1.261)							
Other neonatal disorders	Birth prevalence - [38, 40) wks, [4000, 4500) g	Mortality	Females		1.121<br>(0.925 to 1.31)	1.149<br>(0.955 to 1.367)							
Other neonatal disorders	Birth prevalence - [38, 40) wks, [3500, 4000) g	Mortality	Males		1.009<br>(0.883 to 1.143)	1.243<br>(1.078 to 1.421)							
Other neonatal disorders	Birth prevalence - [38, 40) wks, [3500, 4000) g	Mortality	Females		0.828<br>(0.702 to 0.972)	1.155<br>(0.967 to 1.369)							
Other neonatal disorders	Birth prevalence - [40, 42) wks, [3500, 4000) g	Mortality	Males		0.98<br>(0.854 to 1.123)	1.155<br>(0.983 to 1.334)							
Other neonatal disorders	Birth prevalence - [40, 42) wks, [3500, 4000) g	Mortality	Females		0.819<br>(0.679 to 0.974)	1.045<br>(0.874 to 1.26)							
Sudden infant death syndrome	Birth prevalence - [22, 24) wks, [0, 500) g	Mortality	Males		3273.944<br>(2987.306 to 3600.961)	599.42<br>(512.777 to 692.914)							
Sudden infant death syndrome	Birth prevalence - [22, 24) wks, [0, 500) g	Mortality	Females		3353.667<br>(2964.862 to 3798.637)	771.075<br>(635.721 to 931.62)							
Sudden infant death syndrome	Birth prevalence - [24, 26) wks, [0, 500) g	Mortality	Males		2454.576<br>(2216.544 to 2713.168)	884.902<br>(766.03 to 1021.494)							
Sudden infant death syndrome	Birth prevalence - [24, 26) wks, [0, 500) g	Mortality	Females		2376.85<br>(2080.354 to 2699.254)	915.957<br>(765.805 to 1079.243)							
Sudden infant death syndrome	Birth prevalence - [26, 28) wks, [0, 500) g	Mortality	Males		1870.684<br>(1676.978 to 2075.277)	847.512<br>(727.279 to 983.252)							
Sudden infant death syndrome	Birth prevalence - [26, 28) wks, [0, 500) g	Mortality	Females		1708.132<br>(1477.512 to 1960.75)	822.906<br>(677.101 to 990.491)							
Sudden infant death syndrome	Birth prevalence - [28, 30) wks, [0, 500) g	Mortality	Males		1805.939<br>(1612.157 to 2006.088)	583.881<br>(485.096 to 690.434)							
Sudden infant death syndrome	Birth prevalence - [28, 30) wks, [0, 500) g	Mortality	Females		1585.788<br>(1377.653 to 1836.365)	636.164<br>(520.511 to 760.561)							
Sudden infant death syndrome	Birth prevalence - [22, 24) wks, [500, 1000) g	Mortality	Males		2037.627<br>(1843.599 to 2249.686)	720.117<br>(628.641 to 820.917)							
Sudden infant death syndrome	Birth prevalence - [22, 24) wks, [500, 1000) g	Mortality	Females		1833.266<br>(1596.246 to 2105.971)	610.368<br>(510.239 to 722.278)							
Sudden infant death syndrome	Birth prevalence - [24, 26) wks, [500, 1000) g	Mortality	Males		852.585<br>(753.604 to 959.026)	642.627<br>(558.091 to 730.626)							
Sudden infant death syndrome	Birth prevalence - [24, 26) wks, [500, 1000) g	Mortality	Females		715.379<br>(610.81 to 830.296)	546.057<br>(463.282 to 637.815)							
Sudden infant death syndrome	Birth prevalence - [26, 28) wks, [500, 1000) g	Mortality	Males		411.87<br>(365.037 to 467.411)	353.266<br>(305.647 to 405.904)							
Sudden infant death syndrome	Birth prevalence - [26, 28) wks, [500, 1000) g	Mortality	Females		308.092<br>(262.47 to 362.302)	292.936<br>(243.986 to 349.019)							
Sudden infant death syndrome	Birth prevalence - [32, 34) wks, [500, 1000) g	Mortality	Males		344.501<br>(301.197 to 397.343)	194.954<br>(163.844 to 231.771)							
Sudden infant death syndrome	Birth prevalence - [32, 34) wks, [500, 1000) g	Mortality	Females		299.264<br>(251.468 to 353.995)	161.548<br>(130.866 to 196.323)							
Sudden infant death syndrome	Birth prevalence - [22, 24) wks, [1000, 1500) g	Mortality	Males		673.302<br>(584.187 to 768.258)	213.181<br>(179.555 to 254.572)							
Sudden infant death syndrome	Birth prevalence - [22, 24) wks, [1000, 1500) g	Mortality	Females		620.446<br>(522.864 to 734.748)	186.215<br>(149.772 to 227.046)							
Sudden infant death syndrome	Birth prevalence - [30, 32) wks, [500, 1000) g	Mortality	Males		332.681<br>(293.52 to 377.86)	212.332<br>(181.213 to 245.271)							
Sudden infant death syndrome	Birth prevalence - [30, 32) wks, [500, 1000) g	Mortality	Females		239.082<br>(200.416 to 284.597)	163.95<br>(135.165 to 197.261)							
Sudden infant death syndrome	Birth prevalence - [28, 30) wks, [500, 1000) g	Mortality	Males		326.557<br>(286.01 to 366.811)	249.246<br>(216.248 to 289.232)							
Sudden infant death syndrome	Birth prevalence - [28, 30) wks, [500, 1000) g	Mortality	Females		231.533<br>(194.62 to 271.396)	187.47<br>(157.953 to 224.552)							
Sudden infant death syndrome	Birth prevalence - [24, 26) wks, [1000, 1500) g	Mortality	Males		273.536<br>(236.795 to 312.421)	182.743<br>(155.477 to 212.281)							

Ages													
30-34 years	35-39 years	40-44 years	45-49 years	50-54 years	55-59 years	60-64 years	65-69 years	70-74 years	75-79 years	80-84 years	85-89 years	90-94 years	95+ years

Appendix Table 1a. Relative risks used by age and sex for each outcome for all risk factors except for ambient air pollution and alcohol.

Risk - Outcome	Category / Units	Morbidity / Mortality	Sex	All-age	0-6 days	7-27 days	28-364 days	1-4 years	5-9 years	10-14 years	15-19 years	20-24 years	25-29 years
Sudden infant death syndrome	Birth prevalence - [24, 26) wks, [1000, 1500) g	Mortality	Females		268.033 (223.95 to 318.815)	157.928 (130.936 to 190.298)							
Sudden infant death syndrome	Birth prevalence - [26, 28) wks, [1000, 1500) g	Mortality	Males		164.685 (145.457 to 186.571)	120.878 (102.672 to 140.967)							
Sudden infant death syndrome	Birth prevalence - [26, 28) wks, [1000, 1500) g	Mortality	Females		142.274 (121.33 to 168.246)	97.496 (80.858 to 116.625)							
Sudden infant death syndrome	Birth prevalence - [26, 28) wks, [1500, 2000) g	Mortality	Males		113.21 (97.079 to 130.806)	49.449 (40.908 to 59.113)							
Sudden infant death syndrome	Birth prevalence - [26, 28) wks, [1500, 2000) g	Mortality	Females		108.393 (89.864 to 129.883)	49.979 (40.484 to 60.784)							
Sudden infant death syndrome	Birth prevalence - [34, 36) wks, [1000, 1500) g	Mortality	Males		119.602 (104.655 to 136.729)	56.519 (48.084 to 66.018)							
Sudden infant death syndrome	Birth prevalence - [34, 36) wks, [1000, 1500) g	Mortality	Females		109.231 (91.177 to 128.378)	52.834 (43.454 to 63.459)							
Sudden infant death syndrome	Birth prevalence - [28, 30) wks, [1500, 2000) g	Mortality	Males		87.285 (76.012 to 100.83)	37.15 (30.87 to 43.664)							
Sudden infant death syndrome	Birth prevalence - [28, 30) wks, [1500, 2000) g	Mortality	Females		86.639 (71.639 to 103.632)	38.3 (31.326 to 45.531)							
Sudden infant death syndrome	Birth prevalence - [28, 30) wks, [1000, 1500) g	Mortality	Males		107.077 (93.214 to 122.668)	81.407 (70.511 to 93.12)							
Sudden infant death syndrome	Birth prevalence - [28, 30) wks, [1000, 1500) g	Mortality	Females		87.409 (73.438 to 104.222)	64.981 (53.651 to 77.029)							
Sudden infant death syndrome	Birth prevalence - [32, 34) wks, [1000, 1500) g	Mortality	Males		98.982 (86.649 to 112.931)	51.159 (43.522 to 59.402)							
Sudden infant death syndrome	Birth prevalence - [32, 34) wks, [1000, 1500) g	Mortality	Females		84.081 (70.672 to 99.726)	44.695 (37.129 to 53.257)							
Sudden infant death syndrome	Birth prevalence - [30, 32) wks, [1000, 1500) g	Mortality	Males		90.239 (78.583 to 103.215)	59.239 (50.511 to 69.508)							
Sudden infant death syndrome	Birth prevalence - [30, 32) wks, [1000, 1500) g	Mortality	Females		70.119 (59.11 to 81.846)	47.493 (39.526 to 56.547)							
Sudden infant death syndrome	Birth prevalence - [37, 38) wks, [1500, 2000) g	Mortality	Males		55.659 (48.726 to 62.963)	25.45 (21.867 to 29.451)							
Sudden infant death syndrome	Birth prevalence - [37, 38) wks, [1500, 2000) g	Mortality	Females		54.453 (46.308 to 63.524)	29.339 (24.736 to 34.558)							
Sudden infant death syndrome	Birth prevalence - [36, 37) wks, [1500, 2000) g	Mortality	Males		53.885 (47.099 to 61.414)	23.622 (20.373 to 27.298)							
Sudden infant death syndrome	Birth prevalence - [36, 37) wks, [1500, 2000) g	Mortality	Females		47.995 (40.82 to 56.378)	23.905 (19.8 to 29.023)							
Sudden infant death syndrome	Birth prevalence - [30, 32) wks, [2000, 2500) g	Mortality	Males		39.701 (34.573 to 45.311)	16.391 (13.683 to 19.411)							
Sudden infant death syndrome	Birth prevalence - [30, 32) wks, [2000, 2500) g	Mortality	Females		34.894 (28.953 to 41.934)	16.549 (13.438 to 19.916)							
Sudden infant death syndrome	Birth prevalence - [30, 32) wks, [1500, 2000) g	Mortality	Males		55.142 (48.379 to 62.989)	26.391 (22.751 to 30.584)							
Sudden infant death syndrome	Birth prevalence - [30, 32) wks, [1500, 2000) g	Mortality	Females		48.414 (40.924 to 57.128)	26.986 (22.299 to 32.305)							
Sudden infant death syndrome	Birth prevalence - [34, 36) wks, [1500, 2000) g	Mortality	Males		45.579 (39.817 to 51.922)	19.836 (16.94 to 23.141)							
Sudden infant death syndrome	Birth prevalence - [34, 36) wks, [1500, 2000) g	Mortality	Females		38.405 (32.273 to 45.04)	18.409 (15.134 to 21.755)							
Sudden infant death syndrome	Birth prevalence - [32, 34) wks, [1500, 2000) g	Mortality	Males		41.734 (36.381 to 47.516)	19.083 (16.193 to 21.909)							
Sudden infant death syndrome	Birth prevalence - [32, 34) wks, [1500, 2000) g	Mortality	Females		36.162 (30.231 to 42.934)	19.528 (16.278 to 23.535)							
Sudden infant death syndrome	Birth prevalence - [32, 34) wks, [2000, 2500) g	Mortality	Males		22.379 (19.576 to 25.426)	11.555 (9.874 to 13.332)							
Sudden infant death syndrome	Birth prevalence - [32, 34) wks, [2000, 2500) g	Mortality	Females		20.326 (17.055 to 23.945)	12.376 (10.192 to 14.834)							
Sudden infant death syndrome	Birth prevalence - [40, 42) wks, [2000, 2500) g	Mortality	Males		17.218 (14.893 to 19.961)	11.519 (9.766 to 13.495)							

Ages													
30-34 years	35-39 years	40-44 years	45-49 years	50-54 years	55-59 years	60-64 years	65-69 years	70-74 years	75-79 years	80-84 years	85-89 years	90-94 years	95+ years

Appendix Table 1a. Relative risks used by age and sex for each outcome for all risk factors except for ambient air pollution and alcohol.

Risk - Outcome	Category / Units	Morbidity / Mortality	Sex	All-age	0-6 days	7-27 days	28-364 days	1-4 years	5-9 years	10-14 years	15-19 years	20-24 years	25-29 years
Sudden infant death syndrome	Birth prevalence - [40, 42) wks, [2000, 2500) g	Mortality	Females		13.555 (11.337 to 16.132)	11.838 (9.792 to 14.338)							
Sudden infant death syndrome	Birth prevalence - [38, 40) wks, [2000, 2500) g	Mortality	Males		14.227 (12.521 to 16.06)	10.475 (9.122 to 12.085)							
Sudden infant death syndrome	Birth prevalence - [38, 40) wks, [2000, 2500) g	Mortality	Females		10.867 (9.199 to 12.777)	10.493 (8.801 to 12.342)							
Sudden infant death syndrome	Birth prevalence - [32, 34) wks, [2500, 3000) g	Mortality	Males		12.775 (11.146 to 14.607)	6.296 (5.269 to 7.299)							
Sudden infant death syndrome	Birth prevalence - [32, 34) wks, [2500, 3000) g	Mortality	Females		9.041 (7.504 to 10.706)	6.205 (5.041 to 7.52)							
Sudden infant death syndrome	Birth prevalence - [34, 36) wks, [2000, 2500) g	Mortality	Males		16.472 (14.422 to 18.7)	9.777 (8.456 to 11.277)							
Sudden infant death syndrome	Birth prevalence - [34, 36) wks, [2000, 2500) g	Mortality	Females		12.895 (10.992 to 15.284)	9.44 (7.865 to 11.209)							
Sudden infant death syndrome	Birth prevalence - [37, 38) wks, [2000, 2500) g	Mortality	Males		14.003 (12.407 to 15.831)	9.977 (8.758 to 11.444)							
Sudden infant death syndrome	Birth prevalence - [37, 38) wks, [2000, 2500) g	Mortality	Females		10.456 (8.899 to 12.149)	9.83 (8.317 to 11.54)							
Sudden infant death syndrome	Birth prevalence - [36, 37) wks, [2000, 2500) g	Mortality	Males		14.873 (13.083 to 16.88)	9.756 (8.38 to 11.214)							
Sudden infant death syndrome	Birth prevalence - [36, 37) wks, [2000, 2500) g	Mortality	Females		10.517 (8.897 to 12.379)	9.233 (7.691 to 10.92)							
Sudden infant death syndrome	Birth prevalence - [34, 36) wks, [2500, 3000) g	Mortality	Males		8.216 (7.133 to 9.378)	5.419 (4.631 to 6.282)							
Sudden infant death syndrome	Birth prevalence - [34, 36) wks, [2500, 3000) g	Mortality	Females		6.037 (5.044 to 7.094)	5.198 (4.31 to 6.293)							
Sudden infant death syndrome	Birth prevalence - [34, 36) wks, [4000, 4500) g	Mortality	Males		2.68 (2.245 to 3.183)	1.532 (1.254 to 1.867)							
Sudden infant death syndrome	Birth prevalence - [34, 36) wks, [4000, 4500) g	Mortality	Females		2.458 (1.967 to 3.074)	1.673 (1.344 to 2.093)							
Sudden infant death syndrome	Birth prevalence - [34, 36) wks, [3000, 3500) g	Mortality	Males		3.961 (3.433 to 4.521)	3.194 (2.71 to 3.696)							
Sudden infant death syndrome	Birth prevalence - [34, 36) wks, [3000, 3500) g	Mortality	Females		3.267 (2.738 to 3.876)	2.783 (2.273 to 3.408)							
Sudden infant death syndrome	Birth prevalence - [36, 37) wks, [2500, 3000) g	Mortality	Males		5.363 (4.69 to 6.082)	4.559 (3.945 to 5.248)							
Sudden infant death syndrome	Birth prevalence - [36, 37) wks, [2500, 3000) g	Mortality	Females		3.969 (3.371 to 4.675)	4.138 (3.469 to 4.868)							
Sudden infant death syndrome	Birth prevalence - [34, 36) wks, [3500, 4000) g	Mortality	Males		2.678 (2.289 to 3.086)	2.014 (1.69 to 2.365)							
Sudden infant death syndrome	Birth prevalence - [34, 36) wks, [3500, 4000) g	Mortality	Females		2.342 (1.908 to 2.845)	1.756 (1.426 to 2.126)							
Sudden infant death syndrome	Birth prevalence - [37, 38) wks, [2500, 3000) g	Mortality	Males		4.093 (3.581 to 4.626)	4.139 (3.631 to 4.74)							
Sudden infant death syndrome	Birth prevalence - [37, 38) wks, [2500, 3000) g	Mortality	Females		3.07 (2.612 to 3.603)	3.618 (3.045 to 4.25)							
Sudden infant death syndrome	Birth prevalence - [40, 42) wks, [2500, 3000) g	Mortality	Males		3.833 (3.364 to 4.388)	3.828 (3.271 to 4.426)							
Sudden infant death syndrome	Birth prevalence - [40, 42) wks, [2500, 3000) g	Mortality	Females		2.699 (2.264 to 3.202)	3.379 (2.825 to 4.042)							
Sudden infant death syndrome	Birth prevalence - [38, 40) wks, [2500, 3000) g	Mortality	Males		3.396 (3.002 to 3.839)	3.858 (3.346 to 4.424)							
Sudden infant death syndrome	Birth prevalence - [38, 40) wks, [2500, 3000) g	Mortality	Females		2.382 (2.04 to 2.802)	3.27 (2.774 to 3.927)							
Sudden infant death syndrome	Birth prevalence - [36, 37) wks, [3000, 3500) g	Mortality	Males		2.819 (2.433 to 3.229)	2.683 (2.299 to 3.079)							
Sudden infant death syndrome	Birth prevalence - [36, 37) wks, [3000, 3500) g	Mortality	Females		2.335 (1.952 to 2.784)	2.434 (2.007 to 2.884)							
Sudden infant death syndrome	Birth prevalence - [36, 37) wks, [4000, 4500) g	Mortality	Males		2.331 (1.993 to 2.727)	1.471 (1.221 to 1.731)							

Ages													
30-34 years	35-39 years	40-44 years	45-49 years	50-54 years	55-59 years	60-64 years	65-69 years	70-74 years	75-79 years	80-84 years	85-89 years	90-94 years	95+ years

Appendix Table 1a. Relative risks used by age and sex for each outcome for all risk factors except for ambient air pollution and alcohol.

Risk - Outcome	Category / Units	Morbidity / Mortality	Sex	All-age	0-6 days	7-27 days	28-364 days	1-4 years	5-9 years	10-14 years	15-19 years	20-24 years	25-29 years
Sudden infant death syndrome	Birth prevalence - [36, 37) wks, [4000, 4500) g	Mortality	Females		2.141 (1.729 to 2.577)	1.601 (1.291 to 1.97)							
Sudden infant death syndrome	Birth prevalence - [36, 37) wks, [3500, 4000) g	Mortality	Males		2.084 (1.789 to 2.418)	1.923 (1.624 to 2.252)							
Sudden infant death syndrome	Birth prevalence - [36, 37) wks, [3500, 4000) g	Mortality	Females		1.845 (1.527 to 2.216)	1.731 (1.426 to 2.071)							
Sudden infant death syndrome	Birth prevalence - [37, 38) wks, [3000, 3500) g	Mortality	Males		2.027 (1.788 to 2.287)	2.236 (1.926 to 2.542)							
Sudden infant death syndrome	Birth prevalence - [37, 38) wks, [3000, 3500) g	Mortality	Females		1.667 (1.411 to 1.947)	1.989 (1.672 to 2.366)							
Sudden infant death syndrome	Birth prevalence - [37, 38) wks, [4000, 4500) g	Mortality	Males		1.572 (1.352 to 1.813)	1.28 (1.095 to 1.481)							
Sudden infant death syndrome	Birth prevalence - [37, 38) wks, [4000, 4500) g	Mortality	Females		1.647 (1.37 to 1.98)	1.396 (1.167 to 1.674)							
Sudden infant death syndrome	Birth prevalence - [37, 38) wks, [3500, 4000) g	Mortality	Males		1.502 (1.309 to 1.712)	1.563 (1.353 to 1.807)							
Sudden infant death syndrome	Birth prevalence - [37, 38) wks, [3500, 4000) g	Mortality	Females		1.275 (1.075 to 1.501)	1.444 (1.206 to 1.711)							
Sudden infant death syndrome	Birth prevalence - [40, 42) wks, [3000, 3500) g	Mortality	Males		1.446 (1.258 to 1.639)	1.79 (1.546 to 2.08)							
Sudden infant death syndrome	Birth prevalence - [40, 42) wks, [3000, 3500) g	Mortality	Females		1.103 (0.922 to 1.305)	1.491 (1.233 to 1.776)							
Sudden infant death syndrome	Birth prevalence - [38, 40) wks, [3000, 3500) g	Mortality	Males		1.414 (1.242 to 1.593)	1.874 (1.636 to 2.156)							
Sudden infant death syndrome	Birth prevalence - [38, 40) wks, [3000, 3500) g	Mortality	Females		1.096 (0.927 to 1.289)	1.617 (1.352 to 1.907)							
Sudden infant death syndrome	Birth prevalence - [38, 40) wks, [4000, 4500) g	Mortality	Males		1.052 (0.92 to 1.203)	1.093 (0.939 to 1.261)							
Sudden infant death syndrome	Birth prevalence - [38, 40) wks, [4000, 4500) g	Mortality	Females		1.121 (0.925 to 1.31)	1.149 (0.955 to 1.367)							
Sudden infant death syndrome	Birth prevalence - [38, 40) wks, [3500, 4000) g	Mortality	Males		1.009 (0.883 to 1.143)	1.243 (1.078 to 1.421)							
Sudden infant death syndrome	Birth prevalence - [38, 40) wks, [3500, 4000) g	Mortality	Females		0.828 (0.702 to 0.972)	1.155 (0.967 to 1.369)							
Sudden infant death syndrome	Birth prevalence - [40, 42) wks, [3500, 4000) g	Mortality	Males		0.98 (0.854 to 1.123)	1.155 (0.983 to 1.334)							
Sudden infant death syndrome	Birth prevalence - [40, 42) wks, [3500, 4000) g	Mortality	Females		0.819 (0.679 to 0.974)	1.045 (0.874 to 1.26)							
**Iron deficiency**													
Maternal haemorrhage	1 g/dL	Both	Both								1.252 (1.087 to 1.425)	1.252 (1.087 to 1.425)	1.252 (1.087 to 1.425)
Maternal sepsis and other pregnancy related infections	1 g/dL	Both	Both								1.252 (1.087 to 1.425)	1.252 (1.087 to 1.425)	1.252 (1.087 to 1.425)
**Vitamin A deficiency**													
Diarrhoeal diseases	Vitamin A deficient	Both	Both				1.323 (1.109 to 1.577)	1.595 (1.214 to 2.021)					
Diarrhoeal diseases	Not deficient	Both	Both				1.0 (1.0 to 1.0)	1.0 (1.0 to 1.0)					
Measles	Vitamin A deficient	Both	Both				1.766 (1.327 to 2.317)	2.402 (1.608 to 3.479)					
Measles	Not deficient	Both	Both				1.0 (1.0 to 1.0)	1.0 (1.0 to 1.0)					
**Zinc deficiency**													
Diarrhoeal diseases	Zinc deficient	Morbidity	Both					1.903 (1.517 to 2.335)					
Diarrhoeal diseases	Zinc deficient	Mortality	Both					1.951 (0.905 to 3.909)					

Ages													
30-34 years	35-39 years	40-44 years	45-49 years	50-54 years	55-59 years	60-64 years	65-69 years	70-74 years	75-79 years	80-84 years	85-89 years	90-94 years	95+ years
1.252<br>(1.087 to 1.425)<br>1.252<br>(1.087 to 1.425)	1.252<br>(1.087 to 1.425)<br>1.252<br>(1.087 to 1.425)	1.252<br>(1.087 to 1.425)<br>1.252<br>(1.087 to 1.425)	1.252<br>(1.087 to 1.425)<br>1.252<br>(1.087 to 1.425)										

Appendix Table 1a. Relative risks used by age and sex for each outcome for all risk factors except for ambient air pollution and alcohol.

Risk - Outcome	Category / Units	Morbidity / Mortality	Sex	All-age	0-6 days	7-27 days	28-364 days	1-4 years	5-9 years	10-14 years	15-19 years	20-24 years	25-29 years
Diarrhoeal diseases	Not deficient	Both	Both					1.0<br>(1.0 to 1.0)					
Lower respiratory infections	Zinc deficient	Morbidity	Both					1.837<br>(1.275 to 2.523)					
Lower respiratory infections	Zinc deficient	Mortality	Both					1.672<br>(0.458 to 4.135)					
Lower respiratory infections	Not deficient	Both	Both					1.0<br>(1.0 to 1.0)					
**Smoking (SIR approach)**													
Lip and oral cavity cancer	SIR	Both	Males										
Lip and oral cavity cancer	SIR	Both	Females										
Lip and oral cavity cancer	1-SIR	Both	Males										
Lip and oral cavity cancer	1-SIR	Both	Females										
Nasopharynx cancer	SIR	Both	Males										
Nasopharynx cancer	SIR	Both	Females										
Nasopharynx cancer	1-SIR	Both	Males										
Nasopharynx cancer	1-SIR	Both	Females										
Oesophageal cancer	SIR	Both	Males										
Oesophageal cancer	SIR	Both	Females										
Oesophageal cancer	1-SIR	Both	Males										
Oesophageal cancer	1-SIR	Both	Females										
Stomach cancer	SIR	Both	Males										
Stomach cancer	SIR	Both	Females										
Stomach cancer	1-SIR	Both	Males										
Stomach cancer	1-SIR	Both	Females										
Colon and rectum cancer	SIR	Both	Males										
Colon and rectum cancer	SIR	Both	Females										
Colon and rectum cancer	1-SIR	Both	Males										
Colon and rectum cancer	1-SIR	Both	Females										
Liver cancer due to hepatitis B	SIR	Both	Males										
Liver cancer due to hepatitis B	SIR	Both	Females										
Liver cancer due to hepatitis B	1-SIR	Both	Males										
Liver cancer due to hepatitis B	1-SIR	Both	Females										
Liver cancer due to hepatitis C	SIR	Both	Males										

Ages													
30-34 years	35-39 years	40-44 years	45-49 years	50-54 years	55-59 years	60-64 years	65-69 years	70-74 years	75-79 years	80-84 years	85-89 years	90-94 years	95+ years
8.162 (5.628 to 11.373)	8.162 (5.628 to 11.373)	8.162 (5.628 to 11.373)	8.162 (5.628 to 11.373)	8.162 (5.628 to 11.373)	8.162 (5.628 to 11.373)	8.162 (5.628 to 11.373)	8.162 (5.628 to 11.373)	8.162 (5.628 to 11.373)	8.162 (5.628 to 11.373)	8.162 (5.628 to 11.373)	8.162 (5.628 to 11.373)	8.162 (5.628 to 11.373)	8.162 (5.628 to 11.373)
6.056 (4.243 to 8.515)	6.056 (4.243 to 8.515)	6.056 (4.243 to 8.515)	6.056 (4.243 to 8.515)	6.056 (4.243 to 8.515)	6.056 (4.243 to 8.515)	6.056 (4.243 to 8.515)	6.056 (4.243 to 8.515)	6.056 (4.243 to 8.515)	6.056 (4.243 to 8.515)	6.056 (4.243 to 8.515)	6.056 (4.243 to 8.515)	6.056 (4.243 to 8.515)	6.056 (4.243 to 8.515)
1.0 (1.0 to 1.0)	1.0 (1.0 to 1.0)	1.0 (1.0 to 1.0)	1.0 (1.0 to 1.0)	1.0 (1.0 to 1.0)	1.0 (1.0 to 1.0)	1.0 (1.0 to 1.0)	1.0 (1.0 to 1.0)	1.0 (1.0 to 1.0)	1.0 (1.0 to 1.0)	1.0 (1.0 to 1.0)	1.0 (1.0 to 1.0)	1.0 (1.0 to 1.0)	1.0 (1.0 to 1.0)
1.0 (1.0 to 1.0)	1.0 (1.0 to 1.0)	1.0 (1.0 to 1.0)	1.0 (1.0 to 1.0)	1.0 (1.0 to 1.0)	1.0 (1.0 to 1.0)	1.0 (1.0 to 1.0)	1.0 (1.0 to 1.0)	1.0 (1.0 to 1.0)	1.0 (1.0 to 1.0)	1.0 (1.0 to 1.0)	1.0 (1.0 to 1.0)	1.0 (1.0 to 1.0)	1.0 (1.0 to 1.0)
8.227 (5.677 to 11.495)	8.227 (5.677 to 11.495)	8.227 (5.677 to 11.495)	8.227 (5.677 to 11.495)	8.227 (5.677 to 11.495)	8.227 (5.677 to 11.495)	8.227 (5.677 to 11.495)	8.227 (5.677 to 11.495)	8.227 (5.677 to 11.495)	8.227 (5.677 to 11.495)	8.227 (5.677 to 11.495)	8.227 (5.677 to 11.495)	8.227 (5.677 to 11.495)	8.227 (5.677 to 11.495)
6.089 (4.29 to 8.47)	6.089 (4.29 to 8.47)	6.089 (4.29 to 8.47)	6.089 (4.29 to 8.47)	6.089 (4.29 to 8.47)	6.089 (4.29 to 8.47)	6.089 (4.29 to 8.47)	6.089 (4.29 to 8.47)	6.089 (4.29 to 8.47)	6.089 (4.29 to 8.47)	6.089 (4.29 to 8.47)	6.089 (4.29 to 8.47)	6.089 (4.29 to 8.47)	6.089 (4.29 to 8.47)
1.0 (1.0 to 1.0)	1.0 (1.0 to 1.0)	1.0 (1.0 to 1.0)	1.0 (1.0 to 1.0)	1.0 (1.0 to 1.0)	1.0 (1.0 to 1.0)	1.0 (1.0 to 1.0)	1.0 (1.0 to 1.0)	1.0 (1.0 to 1.0)	1.0 (1.0 to 1.0)	1.0 (1.0 to 1.0)	1.0 (1.0 to 1.0)	1.0 (1.0 to 1.0)	1.0 (1.0 to 1.0)
1.0 (1.0 to 1.0)	1.0 (1.0 to 1.0)	1.0 (1.0 to 1.0)	1.0 (1.0 to 1.0)	1.0 (1.0 to 1.0)	1.0 (1.0 to 1.0)	1.0 (1.0 to 1.0)	1.0 (1.0 to 1.0)	1.0 (1.0 to 1.0)	1.0 (1.0 to 1.0)	1.0 (1.0 to 1.0)	1.0 (1.0 to 1.0)	1.0 (1.0 to 1.0)	1.0 (1.0 to 1.0)
6.676 (4.147 to 10.226)	6.676 (4.147 to 10.226)	6.676 (4.147 to 10.226)	6.676 (4.147 to 10.226)	6.676 (4.147 to 10.226)	6.676 (4.147 to 10.226)	6.676 (4.147 to 10.226)	6.676 (4.147 to 10.226)	6.676 (4.147 to 10.226)	6.676 (4.147 to 10.226)	6.676 (4.147 to 10.226)	6.676 (4.147 to 10.226)	6.676 (4.147 to 10.226)	6.676 (4.147 to 10.226)
6.357 (4.444 to 8.631)	6.357 (4.444 to 8.631)	6.357 (4.444 to 8.631)	6.357 (4.444 to 8.631)	6.357 (4.444 to 8.631)	6.357 (4.444 to 8.631)	6.357 (4.444 to 8.631)	6.357 (4.444 to 8.631)	6.357 (4.444 to 8.631)	6.357 (4.444 to 8.631)	6.357 (4.444 to 8.631)	6.357 (4.444 to 8.631)	6.357 (4.444 to 8.631)	6.357 (4.444 to 8.631)
1.0 (1.0 to 1.0)	1.0 (1.0 to 1.0)	1.0 (1.0 to 1.0)	1.0 (1.0 to 1.0)	1.0 (1.0 to 1.0)	1.0 (1.0 to 1.0)	1.0 (1.0 to 1.0)	1.0 (1.0 to 1.0)	1.0 (1.0 to 1.0)	1.0 (1.0 to 1.0)	1.0 (1.0 to 1.0)	1.0 (1.0 to 1.0)	1.0 (1.0 to 1.0)	1.0 (1.0 to 1.0)
1.0 (1.0 to 1.0)	1.0 (1.0 to 1.0)	1.0 (1.0 to 1.0)	1.0 (1.0 to 1.0)	1.0 (1.0 to 1.0)	1.0 (1.0 to 1.0)	1.0 (1.0 to 1.0)	1.0 (1.0 to 1.0)	1.0 (1.0 to 1.0)	1.0 (1.0 to 1.0)	1.0 (1.0 to 1.0)	1.0 (1.0 to 1.0)	1.0 (1.0 to 1.0)	1.0 (1.0 to 1.0)
1.927 (1.445 to 2.532)	1.927 (1.445 to 2.532)	1.927 (1.445 to 2.532)	1.927 (1.445 to 2.532)	1.927 (1.445 to 2.532)	1.927 (1.445 to 2.532)	1.927 (1.445 to 2.532)	1.927 (1.445 to 2.532)	1.927 (1.445 to 2.532)	1.927 (1.445 to 2.532)	1.927 (1.445 to 2.532)	1.927 (1.445 to 2.532)	1.927 (1.445 to 2.532)	1.927 (1.445 to 2.532)
1.57 (1.246 to 1.924)	1.57 (1.246 to 1.924)	1.57 (1.246 to 1.924)	1.57 (1.246 to 1.924)	1.57 (1.246 to 1.924)	1.57 (1.246 to 1.924)	1.57 (1.246 to 1.924)	1.57 (1.246 to 1.924)	1.57 (1.246 to 1.924)	1.57 (1.246 to 1.924)	1.57 (1.246 to 1.924)	1.57 (1.246 to 1.924)	1.57 (1.246 to 1.924)	1.57 (1.246 to 1.924)
1.0 (1.0 to 1.0)	1.0 (1.0 to 1.0)	1.0 (1.0 to 1.0)	1.0 (1.0 to 1.0)	1.0 (1.0 to 1.0)	1.0 (1.0 to 1.0)	1.0 (1.0 to 1.0)	1.0 (1.0 to 1.0)	1.0 (1.0 to 1.0)	1.0 (1.0 to 1.0)	1.0 (1.0 to 1.0)	1.0 (1.0 to 1.0)	1.0 (1.0 to 1.0)	1.0 (1.0 to 1.0)
1.0 (1.0 to 1.0)	1.0 (1.0 to 1.0)	1.0 (1.0 to 1.0)	1.0 (1.0 to 1.0)	1.0 (1.0 to 1.0)	1.0 (1.0 to 1.0)	1.0 (1.0 to 1.0)	1.0 (1.0 to 1.0)	1.0 (1.0 to 1.0)	1.0 (1.0 to 1.0)	1.0 (1.0 to 1.0)	1.0 (1.0 to 1.0)	1.0 (1.0 to 1.0)	1.0 (1.0 to 1.0)
1.325 (1.195 to 1.471)	1.325 (1.195 to 1.471)	1.325 (1.195 to 1.471)	1.325 (1.195 to 1.471)	1.325 (1.195 to 1.471)	1.325 (1.195 to 1.471)	1.325 (1.195 to 1.471)	1.325 (1.195 to 1.471)	1.325 (1.195 to 1.471)	1.325 (1.195 to 1.471)	1.325 (1.195 to 1.471)	1.325 (1.195 to 1.471)	1.325 (1.195 to 1.471)	1.325 (1.195 to 1.471)
1.418 (1.279 to 1.57)	1.418 (1.279 to 1.57)	1.418 (1.279 to 1.57)	1.418 (1.279 to 1.57)	1.418 (1.279 to 1.57)	1.418 (1.279 to 1.57)	1.418 (1.279 to 1.57)	1.418 (1.279 to 1.57)	1.418 (1.279 to 1.57)	1.418 (1.279 to 1.57)	1.418 (1.279 to 1.57)	1.418 (1.279 to 1.57)	1.418 (1.279 to 1.57)	1.418 (1.279 to 1.57)
1.0 (1.0 to 1.0)	1.0 (1.0 to 1.0)	1.0 (1.0 to 1.0)	1.0 (1.0 to 1.0)	1.0 (1.0 to 1.0)	1.0 (1.0 to 1.0)	1.0 (1.0 to 1.0)	1.0 (1.0 to 1.0)	1.0 (1.0 to 1.0)	1.0 (1.0 to 1.0)	1.0 (1.0 to 1.0)	1.0 (1.0 to 1.0)	1.0 (1.0 to 1.0)	1.0 (1.0 to 1.0)
1.0 (1.0 to 1.0)	1.0 (1.0 to 1.0)	1.0 (1.0 to 1.0)	1.0 (1.0 to 1.0)	1.0 (1.0 to 1.0)	1.0 (1.0 to 1.0)	1.0 (1.0 to 1.0)	1.0 (1.0 to 1.0)	1.0 (1.0 to 1.0)	1.0 (1.0 to 1.0)	1.0 (1.0 to 1.0)	1.0 (1.0 to 1.0)	1.0 (1.0 to 1.0)	1.0 (1.0 to 1.0)
2.54 (1.658 to 3.741)	2.54 (1.658 to 3.741)	2.54 (1.658 to 3.741)	2.54 (1.658 to 3.741)	2.54 (1.658 to 3.741)	2.54 (1.658 to 3.741)	2.54 (1.658 to 3.741)	2.54 (1.658 to 3.741)	2.54 (1.658 to 3.741)	2.54 (1.658 to 3.741)	2.54 (1.658 to 3.741)	2.54 (1.658 to 3.741)	2.54 (1.658 to 3.741)	2.54 (1.658 to 3.741)
1.724 (1.252 to 2.323)	1.724 (1.252 to 2.323)	1.724 (1.252 to 2.323)	1.724 (1.252 to 2.323)	1.724 (1.252 to 2.323)	1.724 (1.252 to 2.323)	1.724 (1.252 to 2.323)	1.724 (1.252 to 2.323)	1.724 (1.252 to 2.323)	1.724 (1.252 to 2.323)	1.724 (1.252 to 2.323)	1.724 (1.252 to 2.323)	1.724 (1.252 to 2.323)	1.724 (1.252 to 2.323)
1.0 (1.0 to 1.0)	1.0 (1.0 to 1.0)	1.0 (1.0 to 1.0)	1.0 (1.0 to 1.0)	1.0 (1.0 to 1.0)	1.0 (1.0 to 1.0)	1.0 (1.0 to 1.0)	1.0 (1.0 to 1.0)	1.0 (1.0 to 1.0)	1.0 (1.0 to 1.0)	1.0 (1.0 to 1.0)	1.0 (1.0 to 1.0)	1.0 (1.0 to 1.0)	1.0 (1.0 to 1.0)
1.0 (1.0 to 1.0)	1.0 (1.0 to 1.0)	1.0 (1.0 to 1.0)	1.0 (1.0 to 1.0)	1.0 (1.0 to 1.0)	1.0 (1.0 to 1.0)	1.0 (1.0 to 1.0)	1.0 (1.0 to 1.0)	1.0 (1.0 to 1.0)	1.0 (1.0 to 1.0)	1.0 (1.0 to 1.0)	1.0 (1.0 to 1.0)	1.0 (1.0 to 1.0)	1.0 (1.0 to 1.0)
2.54 (1.658 to 3.741)	2.54 (1.658 to 3.741)	2.54 (1.658 to 3.741)	2.54 (1.658 to 3.741)	2.54 (1.658 to 3.741)	2.54 (1.658 to 3.741)	2.54 (1.658 to 3.741)	2.54 (1.658 to 3.741)	2.54 (1.658 to 3.741)	2.54 (1.658 to 3.741)	2.54 (1.658 to 3.741)	2.54 (1.658 to 3.741)	2.54 (1.658 to 3.741)	2.54 (1.658 to 3.741)

Appendix Table 1a. Relative risks used by age and sex for each outcome for all risk factors except for ambient air pollution and alcohol.

Risk - Outcome	Category / Units	Morbidity / Mortality	Sex	All-age	0-6 days	7-27 days	28-364 days	1-4 years	5-9 years	10-14 years	15-19 years	20-24 years	25-29 years
Liver cancer due to hepatitis C	SIR	Both	Females										
Liver cancer due to hepatitis C	1-SIR	Both	Males										
Liver cancer due to hepatitis C	1-SIR	Both	Females										
Liver cancer due to alcohol use	SIR	Both	Males										
Liver cancer due to alcohol use	SIR	Both	Females										
Liver cancer due to alcohol use	1-SIR	Both	Males										
Liver cancer due to alcohol use	1-SIR	Both	Females										
Liver cancer due to other causes	SIR	Both	Males										
Liver cancer due to other causes	SIR	Both	Females										
Liver cancer due to other causes	1-SIR	Both	Males										
Liver cancer due to other causes	1-SIR	Both	Females										
Pancreatic cancer	SIR	Both	Males										
Pancreatic cancer	SIR	Both	Females										
Pancreatic cancer	1-SIR	Both	Males										
Pancreatic cancer	1-SIR	Both	Females										
Larynx cancer	SIR	Both	Males										
Larynx cancer	SIR	Both	Females										
Larynx cancer	1-SIR	Both	Males										
Larynx cancer	1-SIR	Both	Females										
Tracheal, bronchus, and lung cancer	SIR	Both	Males										
Tracheal, bronchus, and lung cancer	SIR	Both	Females										
Tracheal, bronchus, and lung cancer	1-SIR	Both	Males										
Tracheal, bronchus, and lung cancer	1-SIR	Both	Females										
Breast cancer	SIR	Both	Both										
Breast cancer	1-SIR	Both	Both										
Cervical cancer	SIR	Both	Both										
Cervical cancer	1-SIR	Both	Both										
Prostate cancer	SIR	Both	Both										
Prostate cancer	1-SIR	Both	Both										
Kidney cancer	SIR	Both	Males										

Ages													
**30-34 years**	**35-39 years**	**40-44 years**	**45-49 years**	**50-54 years**	**55-59 years**	**60-64 years**	**65-69 years**	**70-74 years**	**75-79 years**	**80-84 years**	**85-89 years**	**90-94 years**	**95+ years**
1.724 (1.252 to 2.323)	1.724 (1.252 to 2.323)	1.724 (1.252 to 2.323)	1.724 (1.252 to 2.323)	1.724 (1.252 to 2.323)	1.724 (1.252 to 2.323)	1.724 (1.252 to 2.323)	1.724 (1.252 to 2.323)	1.724 (1.252 to 2.323)	1.724 (1.252 to 2.323)	1.724 (1.252 to 2.323)	1.724 (1.252 to 2.323)	1.724 (1.252 to 2.323)	1.724 (1.252 to 2.323)
1.0 (1.0 to 1.0)	1.0 (1.0 to 1.0)	1.0 (1.0 to 1.0)	1.0 (1.0 to 1.0)	1.0 (1.0 to 1.0)	1.0 (1.0 to 1.0)	1.0 (1.0 to 1.0)	1.0 (1.0 to 1.0)	1.0 (1.0 to 1.0)	1.0 (1.0 to 1.0)	1.0 (1.0 to 1.0)	1.0 (1.0 to 1.0)	1.0 (1.0 to 1.0)	1.0 (1.0 to 1.0)
1.0 (1.0 to 1.0)	1.0 (1.0 to 1.0)	1.0 (1.0 to 1.0)	1.0 (1.0 to 1.0)	1.0 (1.0 to 1.0)	1.0 (1.0 to 1.0)	1.0 (1.0 to 1.0)	1.0 (1.0 to 1.0)	1.0 (1.0 to 1.0)	1.0 (1.0 to 1.0)	1.0 (1.0 to 1.0)	1.0 (1.0 to 1.0)	1.0 (1.0 to 1.0)	1.0 (1.0 to 1.0)
2.54 (1.658 to 3.741)	2.54 (1.658 to 3.741)	2.54 (1.658 to 3.741)	2.54 (1.658 to 3.741)	2.54 (1.658 to 3.741)	2.54 (1.658 to 3.741)	2.54 (1.658 to 3.741)	2.54 (1.658 to 3.741)	2.54 (1.658 to 3.741)	2.54 (1.658 to 3.741)	2.54 (1.658 to 3.741)	2.54 (1.658 to 3.741)	2.54 (1.658 to 3.741)	2.54 (1.658 to 3.741)
1.724 (1.252 to 2.323)	1.724 (1.252 to 2.323)	1.724 (1.252 to 2.323)	1.724 (1.252 to 2.323)	1.724 (1.252 to 2.323)	1.724 (1.252 to 2.323)	1.724 (1.252 to 2.323)	1.724 (1.252 to 2.323)	1.724 (1.252 to 2.323)	1.724 (1.252 to 2.323)	1.724 (1.252 to 2.323)	1.724 (1.252 to 2.323)	1.724 (1.252 to 2.323)	1.724 (1.252 to 2.323)
1.0 (1.0 to 1.0)	1.0 (1.0 to 1.0)	1.0 (1.0 to 1.0)	1.0 (1.0 to 1.0)	1.0 (1.0 to 1.0)	1.0 (1.0 to 1.0)	1.0 (1.0 to 1.0)	1.0 (1.0 to 1.0)	1.0 (1.0 to 1.0)	1.0 (1.0 to 1.0)	1.0 (1.0 to 1.0)	1.0 (1.0 to 1.0)	1.0 (1.0 to 1.0)	1.0 (1.0 to 1.0)
1.0 (1.0 to 1.0)	1.0 (1.0 to 1.0)	1.0 (1.0 to 1.0)	1.0 (1.0 to 1.0)	1.0 (1.0 to 1.0)	1.0 (1.0 to 1.0)	1.0 (1.0 to 1.0)	1.0 (1.0 to 1.0)	1.0 (1.0 to 1.0)	1.0 (1.0 to 1.0)	1.0 (1.0 to 1.0)	1.0 (1.0 to 1.0)	1.0 (1.0 to 1.0)	1.0 (1.0 to 1.0)
2.54 (1.658 to 3.741)	2.54 (1.658 to 3.741)	2.54 (1.658 to 3.741)	2.54 (1.658 to 3.741)	2.54 (1.658 to 3.741)	2.54 (1.658 to 3.741)	2.54 (1.658 to 3.741)	2.54 (1.658 to 3.741)	2.54 (1.658 to 3.741)	2.54 (1.658 to 3.741)	2.54 (1.658 to 3.741)	2.54 (1.658 to 3.741)	2.54 (1.658 to 3.741)	2.54 (1.658 to 3.741)
1.724 (1.252 to 2.323)	1.724 (1.252 to 2.323)	1.724 (1.252 to 2.323)	1.724 (1.252 to 2.323)	1.724 (1.252 to 2.323)	1.724 (1.252 to 2.323)	1.724 (1.252 to 2.323)	1.724 (1.252 to 2.323)	1.724 (1.252 to 2.323)	1.724 (1.252 to 2.323)	1.724 (1.252 to 2.323)	1.724 (1.252 to 2.323)	1.724 (1.252 to 2.323)	1.724 (1.252 to 2.323)
1.0 (1.0 to 1.0)	1.0 (1.0 to 1.0)	1.0 (1.0 to 1.0)	1.0 (1.0 to 1.0)	1.0 (1.0 to 1.0)	1.0 (1.0 to 1.0)	1.0 (1.0 to 1.0)	1.0 (1.0 to 1.0)	1.0 (1.0 to 1.0)	1.0 (1.0 to 1.0)	1.0 (1.0 to 1.0)	1.0 (1.0 to 1.0)	1.0 (1.0 to 1.0)	1.0 (1.0 to 1.0)
1.0 (1.0 to 1.0)	1.0 (1.0 to 1.0)	1.0 (1.0 to 1.0)	1.0 (1.0 to 1.0)	1.0 (1.0 to 1.0)	1.0 (1.0 to 1.0)	1.0 (1.0 to 1.0)	1.0 (1.0 to 1.0)	1.0 (1.0 to 1.0)	1.0 (1.0 to 1.0)	1.0 (1.0 to 1.0)	1.0 (1.0 to 1.0)	1.0 (1.0 to 1.0)	1.0 (1.0 to 1.0)
2.506 (1.962 to 3.109)	2.506 (1.962 to 3.109)	2.506 (1.962 to 3.109)	2.506 (1.962 to 3.109)	2.506 (1.962 to 3.109)	2.506 (1.962 to 3.109)	2.506 (1.962 to 3.109)	2.506 (1.962 to 3.109)	2.506 (1.962 to 3.109)	2.506 (1.962 to 3.109)	2.506 (1.962 to 3.109)	2.506 (1.962 to 3.109)	2.506 (1.962 to 3.109)	2.506 (1.962 to 3.109)
2.098 (1.838 to 2.37)	2.098 (1.838 to 2.37)	2.098 (1.838 to 2.37)	2.098 (1.838 to 2.37)	2.098 (1.838 to 2.37)	2.098 (1.838 to 2.37)	2.098 (1.838 to 2.37)	2.098 (1.838 to 2.37)	2.098 (1.838 to 2.37)	2.098 (1.838 to 2.37)	2.098 (1.838 to 2.37)	2.098 (1.838 to 2.37)	2.098 (1.838 to 2.37)	2.098 (1.838 to 2.37)
1.0 (1.0 to 1.0)	1.0 (1.0 to 1.0)	1.0 (1.0 to 1.0)	1.0 (1.0 to 1.0)	1.0 (1.0 to 1.0)	1.0 (1.0 to 1.0)	1.0 (1.0 to 1.0)	1.0 (1.0 to 1.0)	1.0 (1.0 to 1.0)	1.0 (1.0 to 1.0)	1.0 (1.0 to 1.0)	1.0 (1.0 to 1.0)	1.0 (1.0 to 1.0)	1.0 (1.0 to 1.0)
1.0 (1.0 to 1.0)	1.0 (1.0 to 1.0)	1.0 (1.0 to 1.0)	1.0 (1.0 to 1.0)	1.0 (1.0 to 1.0)	1.0 (1.0 to 1.0)	1.0 (1.0 to 1.0)	1.0 (1.0 to 1.0)	1.0 (1.0 to 1.0)	1.0 (1.0 to 1.0)	1.0 (1.0 to 1.0)	1.0 (1.0 to 1.0)	1.0 (1.0 to 1.0)	1.0 (1.0 to 1.0)
14.602 (8.533 to 23.315)	14.602 (8.533 to 23.315)	14.602 (8.533 to 23.315)	14.602 (8.533 to 23.315)	14.602 (8.533 to 23.315)	14.602 (8.533 to 23.315)	14.602 (8.533 to 23.315)	14.602 (8.533 to 23.315)	14.602 (8.533 to 23.315)	14.602 (8.533 to 23.315)	14.602 (8.533 to 23.315)	14.602 (8.533 to 23.315)	14.602 (8.533 to 23.315)	14.602 (8.533 to 23.315)
135.959 (23.441 to 462.178)	135.959 (23.441 to 462.178)	135.959 (23.441 to 462.178)	135.959 (23.441 to 462.178)	135.959 (23.441 to 462.178)	135.959 (23.441 to 462.178)	135.959 (23.441 to 462.178)	135.959 (23.441 to 462.178)	135.959 (23.441 to 462.178)	135.959 (23.441 to 462.178)	135.959 (23.441 to 462.178)	135.959 (23.441 to 462.178)	135.959 (23.441 to 462.178)	135.959 (23.441 to 462.178)
1.0 (1.0 to 1.0)	1.0 (1.0 to 1.0)	1.0 (1.0 to 1.0)	1.0 (1.0 to 1.0)	1.0 (1.0 to 1.0)	1.0 (1.0 to 1.0)	1.0 (1.0 to 1.0)	1.0 (1.0 to 1.0)	1.0 (1.0 to 1.0)	1.0 (1.0 to 1.0)	1.0 (1.0 to 1.0)	1.0 (1.0 to 1.0)	1.0 (1.0 to 1.0)	1.0 (1.0 to 1.0)
1.0 (1.0 to 1.0)	1.0 (1.0 to 1.0)	1.0 (1.0 to 1.0)	1.0 (1.0 to 1.0)	1.0 (1.0 to 1.0)	1.0 (1.0 to 1.0)	1.0 (1.0 to 1.0)	1.0 (1.0 to 1.0)	1.0 (1.0 to 1.0)	1.0 (1.0 to 1.0)	1.0 (1.0 to 1.0)	1.0 (1.0 to 1.0)	1.0 (1.0 to 1.0)	1.0 (1.0 to 1.0)
22.511 (19.065 to 26.704)	22.511 (19.065 to 26.704)	22.511 (19.065 to 26.704)	22.511 (19.065 to 26.704)	22.511 (19.065 to 26.704)	22.511 (19.065 to 26.704)	22.511 (19.065 to 26.704)	22.511 (19.065 to 26.704)	22.511 (19.065 to 26.704)	22.511 (19.065 to 26.704)	22.511 (19.065 to 26.704)	22.511 (19.065 to 26.704)	22.511 (19.065 to 26.704)	22.511 (19.065 to 26.704)
14.095 (13.049 to 15.352)	14.095 (13.049 to 15.352)	14.095 (13.049 to 15.352)	14.095 (13.049 to 15.352)	14.095 (13.049 to 15.352)	14.095 (13.049 to 15.352)	14.095 (13.049 to 15.352)	14.095 (13.049 to 15.352)	14.095 (13.049 to 15.352)	14.095 (13.049 to 15.352)	14.095 (13.049 to 15.352)	14.095 (13.049 to 15.352)	14.095 (13.049 to 15.352)	14.095 (13.049 to 15.352)
1.0 (1.0 to 1.0)	1.0 (1.0 to 1.0)	1.0 (1.0 to 1.0)	1.0 (1.0 to 1.0)	1.0 (1.0 to 1.0)	1.0 (1.0 to 1.0)	1.0 (1.0 to 1.0)	1.0 (1.0 to 1.0)	1.0 (1.0 to 1.0)	1.0 (1.0 to 1.0)	1.0 (1.0 to 1.0)	1.0 (1.0 to 1.0)	1.0 (1.0 to 1.0)	1.0 (1.0 to 1.0)
1.0 (1.0 to 1.0)	1.0 (1.0 to 1.0)	1.0 (1.0 to 1.0)	1.0 (1.0 to 1.0)	1.0 (1.0 to 1.0)	1.0 (1.0 to 1.0)	1.0 (1.0 to 1.0)	1.0 (1.0 to 1.0)	1.0 (1.0 to 1.0)	1.0 (1.0 to 1.0)	1.0 (1.0 to 1.0)	1.0 (1.0 to 1.0)	1.0 (1.0 to 1.0)	1.0 (1.0 to 1.0)
1.29 (1.08 to 1.549)	1.29 (1.08 to 1.549)	1.29 (1.08 to 1.549)	1.29 (1.08 to 1.549)	1.29 (1.08 to 1.549)	1.29 (1.08 to 1.549)	1.29 (1.08 to 1.549)	1.29 (1.08 to 1.549)	1.29 (1.08 to 1.549)	1.29 (1.08 to 1.549)	1.29 (1.08 to 1.549)	1.29 (1.08 to 1.549)	1.29 (1.08 to 1.549)	1.29 (1.08 to 1.549)
1.0 (1.0 to 1.0)	1.0 (1.0 to 1.0)	1.0 (1.0 to 1.0)	1.0 (1.0 to 1.0)	1.0 (1.0 to 1.0)	1.0 (1.0 to 1.0)	1.0 (1.0 to 1.0)	1.0 (1.0 to 1.0)	1.0 (1.0 to 1.0)	1.0 (1.0 to 1.0)	1.0 (1.0 to 1.0)	1.0 (1.0 to 1.0)	1.0 (1.0 to 1.0)	1.0 (1.0 to 1.0)
1.679 (1.21 to 2.239)	1.679 (1.21 to 2.239)	1.679 (1.21 to 2.239)	1.679 (1.21 to 2.239)	1.679 (1.21 to 2.239)	1.679 (1.21 to 2.239)	1.679 (1.21 to 2.239)	1.679 (1.21 to 2.239)	1.679 (1.21 to 2.239)	1.679 (1.21 to 2.239)	1.679 (1.21 to 2.239)	1.679 (1.21 to 2.239)	1.679 (1.21 to 2.239)	1.679 (1.21 to 2.239)
1.0 (1.0 to 1.0)	1.0 (1.0 to 1.0)	1.0 (1.0 to 1.0)	1.0 (1.0 to 1.0)	1.0 (1.0 to 1.0)	1.0 (1.0 to 1.0)	1.0 (1.0 to 1.0)	1.0 (1.0 to 1.0)	1.0 (1.0 to 1.0)	1.0 (1.0 to 1.0)	1.0 (1.0 to 1.0)	1.0 (1.0 to 1.0)	1.0 (1.0 to 1.0)	1.0 (1.0 to 1.0)
1.239 (1.174 to 1.308)	1.239 (1.174 to 1.308)	1.239 (1.174 to 1.308)	1.239 (1.174 to 1.308)	1.239 (1.174 to 1.308)	1.239 (1.174 to 1.308)	1.239 (1.174 to 1.308)	1.239 (1.174 to 1.308)	1.239 (1.174 to 1.308)	1.239 (1.174 to 1.308)	1.239 (1.174 to 1.308)	1.239 (1.174 to 1.308)	1.239 (1.174 to 1.308)	1.239 (1.174 to 1.308)
1.0 (1.0 to 1.0)	1.0 (1.0 to 1.0)	1.0 (1.0 to 1.0)	1.0 (1.0 to 1.0)	1.0 (1.0 to 1.0)	1.0 (1.0 to 1.0)	1.0 (1.0 to 1.0)	1.0 (1.0 to 1.0)	1.0 (1.0 to 1.0)	1.0 (1.0 to 1.0)	1.0 (1.0 to 1.0)	1.0 (1.0 to 1.0)	1.0 (1.0 to 1.0)	1.0 (1.0 to 1.0)
2.293 (1.682 to 3.038)	2.293 (1.682 to 3.038)	2.293 (1.682 to 3.038)	2.293 (1.682 to 3.038)	2.293 (1.682 to 3.038)	2.293 (1.682 to 3.038)	2.293 (1.682 to 3.038)	2.293 (1.682 to 3.038)	2.293 (1.682 to 3.038)	2.293 (1.682 to 3.038)	2.293 (1.682 to 3.038)	2.293 (1.682 to 3.038)	2.293 (1.682 to 3.038)	2.293 (1.682 to 3.038)

Appendix Table 1a. Relative risks used by age and sex for each outcome for all risk factors except for ambient air pollution and alcohol.

Risk - Outcome	Category / Units	Morbidity / Mortality	Sex	All-age	0-6 days	7-27 days	28-364 days	1-4 years	5-9 years	10-14 years	15-19 years	20-24 years	25-29 years
Kidney cancer	SIR	Both	Females										
Kidney cancer	1-SIR	Both	Males										
Kidney cancer	1-SIR	Both	Females										
Bladder cancer	SIR	Both	Males										
Bladder cancer	SIR	Both	Females										
Bladder cancer	1-SIR	Both	Males										
Bladder cancer	1-SIR	Both	Females										
Acute lymphoid leukaemia	SIR	Both	Males										
Acute lymphoid leukaemia	SIR	Both	Females										
Acute lymphoid leukaemia	1-SIR	Both	Males										
Acute lymphoid leukaemia	1-SIR	Both	Females										
Chronic lymphoid leukaemia	SIR	Both	Males										
Chronic lymphoid leukaemia	SIR	Both	Females										
Chronic lymphoid leukaemia	1-SIR	Both	Males										
Chronic lymphoid leukaemia	1-SIR	Both	Females										
Acute myeloid leukaemia	SIR	Both	Males										
Acute myeloid leukaemia	SIR	Both	Females										
Acute myeloid leukaemia	1-SIR	Both	Males										
Acute myeloid leukaemia	1-SIR	Both	Females										
Chronic myeloid leukaemia	SIR	Both	Males										
Chronic myeloid leukaemia	SIR	Both	Females										
Chronic myeloid leukaemia	1-SIR	Both	Males										
Chronic myeloid leukaemia	1-SIR	Both	Females										
Other leukaemia	SIR	Both	Males										
Other leukaemia	SIR	Both	Females										
Other leukaemia	1-SIR	Both	Males										
Other leukaemia	1-SIR	Both	Females										
Chronic obstructive pulmonary disease	SIR	Both	Males										
Chronic obstructive pulmonary disease	SIR	Both	Females										
Chronic obstructive pulmonary disease	1-SIR	Both	Males										

Ages													
30-34 years	35-39 years	40-44 years	45-49 years	50-54 years	55-59 years	60-64 years	65-69 years	70-74 years	75-79 years	80-84 years	85-89 years	90-94 years	95+ years
1.518 (1.205 to 1.874)	1.518 (1.205 to 1.874)	1.518 (1.205 to 1.874)	1.518 (1.205 to 1.874)	1.518 (1.205 to 1.874)	1.518 (1.205 to 1.874)	1.518 (1.205 to 1.874)	1.518 (1.205 to 1.874)	1.518 (1.205 to 1.874)	1.518 (1.205 to 1.874)	1.518 (1.205 to 1.874)	1.518 (1.205 to 1.874)	1.518 (1.205 to 1.874)	1.518 (1.205 to 1.874)
1.0 (1.0 to 1.0)	1.0 (1.0 to 1.0)	1.0 (1.0 to 1.0)	1.0 (1.0 to 1.0)	1.0 (1.0 to 1.0)	1.0 (1.0 to 1.0)	1.0 (1.0 to 1.0)	1.0 (1.0 to 1.0)	1.0 (1.0 to 1.0)	1.0 (1.0 to 1.0)	1.0 (1.0 to 1.0)	1.0 (1.0 to 1.0)	1.0 (1.0 to 1.0)	1.0 (1.0 to 1.0)
1.0 (1.0 to 1.0)	1.0 (1.0 to 1.0)	1.0 (1.0 to 1.0)	1.0 (1.0 to 1.0)	1.0 (1.0 to 1.0)	1.0 (1.0 to 1.0)	1.0 (1.0 to 1.0)	1.0 (1.0 to 1.0)	1.0 (1.0 to 1.0)	1.0 (1.0 to 1.0)	1.0 (1.0 to 1.0)	1.0 (1.0 to 1.0)	1.0 (1.0 to 1.0)	1.0 (1.0 to 1.0)
3.332 (2.367 to 4.556)	3.332 (2.367 to 4.556)	3.332 (2.367 to 4.556)	3.332 (2.367 to 4.556)	3.332 (2.367 to 4.556)	3.332 (2.367 to 4.556)	3.332 (2.367 to 4.556)	3.332 (2.367 to 4.556)	3.332 (2.367 to 4.556)	3.332 (2.367 to 4.556)	3.332 (2.367 to 4.556)	3.332 (2.367 to 4.556)	3.332 (2.367 to 4.556)	3.332 (2.367 to 4.556)
2.582 (1.926 to 3.42)	2.582 (1.926 to 3.42)	2.582 (1.926 to 3.42)	2.582 (1.926 to 3.42)	2.582 (1.926 to 3.42)	2.582 (1.926 to 3.42)	2.582 (1.926 to 3.42)	2.582 (1.926 to 3.42)	2.582 (1.926 to 3.42)	2.582 (1.926 to 3.42)	2.582 (1.926 to 3.42)	2.582 (1.926 to 3.42)	2.582 (1.926 to 3.42)	2.582 (1.926 to 3.42)
1.0 (1.0 to 1.0)	1.0 (1.0 to 1.0)	1.0 (1.0 to 1.0)	1.0 (1.0 to 1.0)	1.0 (1.0 to 1.0)	1.0 (1.0 to 1.0)	1.0 (1.0 to 1.0)	1.0 (1.0 to 1.0)	1.0 (1.0 to 1.0)	1.0 (1.0 to 1.0)	1.0 (1.0 to 1.0)	1.0 (1.0 to 1.0)	1.0 (1.0 to 1.0)	1.0 (1.0 to 1.0)
1.0 (1.0 to 1.0)	1.0 (1.0 to 1.0)	1.0 (1.0 to 1.0)	1.0 (1.0 to 1.0)	1.0 (1.0 to 1.0)	1.0 (1.0 to 1.0)	1.0 (1.0 to 1.0)	1.0 (1.0 to 1.0)	1.0 (1.0 to 1.0)	1.0 (1.0 to 1.0)	1.0 (1.0 to 1.0)	1.0 (1.0 to 1.0)	1.0 (1.0 to 1.0)	1.0 (1.0 to 1.0)
2.013 (1.391 to 2.858)	2.013 (1.391 to 2.858)	2.013 (1.391 to 2.858)	2.013 (1.391 to 2.858)	2.013 (1.391 to 2.858)	2.013 (1.391 to 2.858)	2.013 (1.391 to 2.858)	2.013 (1.391 to 2.858)	2.013 (1.391 to 2.858)	2.013 (1.391 to 2.858)	2.013 (1.391 to 2.858)	2.013 (1.391 to 2.858)	2.013 (1.391 to 2.858)	2.013 (1.391 to 2.858)
1.163 (0.895 to 1.478)	1.163 (0.895 to 1.478)	1.163 (0.895 to 1.478)	1.163 (0.895 to 1.478)	1.163 (0.895 to 1.478)	1.163 (0.895 to 1.478)	1.163 (0.895 to 1.478)	1.163 (0.895 to 1.478)	1.163 (0.895 to 1.478)	1.163 (0.895 to 1.478)	1.163 (0.895 to 1.478)	1.163 (0.895 to 1.478)	1.163 (0.895 to 1.478)	1.163 (0.895 to 1.478)
1.0 (1.0 to 1.0)	1.0 (1.0 to 1.0)	1.0 (1.0 to 1.0)	1.0 (1.0 to 1.0)	1.0 (1.0 to 1.0)	1.0 (1.0 to 1.0)	1.0 (1.0 to 1.0)	1.0 (1.0 to 1.0)	1.0 (1.0 to 1.0)	1.0 (1.0 to 1.0)	1.0 (1.0 to 1.0)	1.0 (1.0 to 1.0)	1.0 (1.0 to 1.0)	1.0 (1.0 to 1.0)
1.0 (1.0 to 1.0)	1.0 (1.0 to 1.0)	1.0 (1.0 to 1.0)	1.0 (1.0 to 1.0)	1.0 (1.0 to 1.0)	1.0 (1.0 to 1.0)	1.0 (1.0 to 1.0)	1.0 (1.0 to 1.0)	1.0 (1.0 to 1.0)	1.0 (1.0 to 1.0)	1.0 (1.0 to 1.0)	1.0 (1.0 to 1.0)	1.0 (1.0 to 1.0)	1.0 (1.0 to 1.0)
2.013 (1.391 to 2.858)	2.013 (1.391 to 2.858)	2.013 (1.391 to 2.858)	2.013 (1.391 to 2.858)	2.013 (1.391 to 2.858)	2.013 (1.391 to 2.858)	2.013 (1.391 to 2.858)	2.013 (1.391 to 2.858)	2.013 (1.391 to 2.858)	2.013 (1.391 to 2.858)	2.013 (1.391 to 2.858)	2.013 (1.391 to 2.858)	2.013 (1.391 to 2.858)	2.013 (1.391 to 2.858)
1.163 (0.895 to 1.478)	1.163 (0.895 to 1.478)	1.163 (0.895 to 1.478)	1.163 (0.895 to 1.478)	1.163 (0.895 to 1.478)	1.163 (0.895 to 1.478)	1.163 (0.895 to 1.478)	1.163 (0.895 to 1.478)	1.163 (0.895 to 1.478)	1.163 (0.895 to 1.478)	1.163 (0.895 to 1.478)	1.163 (0.895 to 1.478)	1.163 (0.895 to 1.478)	1.163 (0.895 to 1.478)
1.0 (1.0 to 1.0)	1.0 (1.0 to 1.0)	1.0 (1.0 to 1.0)	1.0 (1.0 to 1.0)	1.0 (1.0 to 1.0)	1.0 (1.0 to 1.0)	1.0 (1.0 to 1.0)	1.0 (1.0 to 1.0)	1.0 (1.0 to 1.0)	1.0 (1.0 to 1.0)	1.0 (1.0 to 1.0)	1.0 (1.0 to 1.0)	1.0 (1.0 to 1.0)	1.0 (1.0 to 1.0)
1.0 (1.0 to 1.0)	1.0 (1.0 to 1.0)	1.0 (1.0 to 1.0)	1.0 (1.0 to 1.0)	1.0 (1.0 to 1.0)	1.0 (1.0 to 1.0)	1.0 (1.0 to 1.0)	1.0 (1.0 to 1.0)	1.0 (1.0 to 1.0)	1.0 (1.0 to 1.0)	1.0 (1.0 to 1.0)	1.0 (1.0 to 1.0)	1.0 (1.0 to 1.0)	1.0 (1.0 to 1.0)
2.013 (1.391 to 2.858)	2.013 (1.391 to 2.858)	2.013 (1.391 to 2.858)	2.013 (1.391 to 2.858)	2.013 (1.391 to 2.858)	2.013 (1.391 to 2.858)	2.013 (1.391 to 2.858)	2.013 (1.391 to 2.858)	2.013 (1.391 to 2.858)	2.013 (1.391 to 2.858)	2.013 (1.391 to 2.858)	2.013 (1.391 to 2.858)	2.013 (1.391 to 2.858)	2.013 (1.391 to 2.858)
1.163 (0.895 to 1.478)	1.163 (0.895 to 1.478)	1.163 (0.895 to 1.478)	1.163 (0.895 to 1.478)	1.163 (0.895 to 1.478)	1.163 (0.895 to 1.478)	1.163 (0.895 to 1.478)	1.163 (0.895 to 1.478)	1.163 (0.895 to 1.478)	1.163 (0.895 to 1.478)	1.163 (0.895 to 1.478)	1.163 (0.895 to 1.478)	1.163 (0.895 to 1.478)	1.163 (0.895 to 1.478)
1.0 (1.0 to 1.0)	1.0 (1.0 to 1.0)	1.0 (1.0 to 1.0)	1.0 (1.0 to 1.0)	1.0 (1.0 to 1.0)	1.0 (1.0 to 1.0)	1.0 (1.0 to 1.0)	1.0 (1.0 to 1.0)	1.0 (1.0 to 1.0)	1.0 (1.0 to 1.0)	1.0 (1.0 to 1.0)	1.0 (1.0 to 1.0)	1.0 (1.0 to 1.0)	1.0 (1.0 to 1.0)
1.0 (1.0 to 1.0)	1.0 (1.0 to 1.0)	1.0 (1.0 to 1.0)	1.0 (1.0 to 1.0)	1.0 (1.0 to 1.0)	1.0 (1.0 to 1.0)	1.0 (1.0 to 1.0)	1.0 (1.0 to 1.0)	1.0 (1.0 to 1.0)	1.0 (1.0 to 1.0)	1.0 (1.0 to 1.0)	1.0 (1.0 to 1.0)	1.0 (1.0 to 1.0)	1.0 (1.0 to 1.0)
2.013 (1.391 to 2.858)	2.013 (1.391 to 2.858)	2.013 (1.391 to 2.858)	2.013 (1.391 to 2.858)	2.013 (1.391 to 2.858)	2.013 (1.391 to 2.858)	2.013 (1.391 to 2.858)	2.013 (1.391 to 2.858)	2.013 (1.391 to 2.858)	2.013 (1.391 to 2.858)	2.013 (1.391 to 2.858)	2.013 (1.391 to 2.858)	2.013 (1.391 to 2.858)	2.013 (1.391 to 2.858)
1.163 (0.895 to 1.478)	1.163 (0.895 to 1.478)	1.163 (0.895 to 1.478)	1.163 (0.895 to 1.478)	1.163 (0.895 to 1.478)	1.163 (0.895 to 1.478)	1.163 (0.895 to 1.478)	1.163 (0.895 to 1.478)	1.163 (0.895 to 1.478)	1.163 (0.895 to 1.478)	1.163 (0.895 to 1.478)	1.163 (0.895 to 1.478)	1.163 (0.895 to 1.478)	1.163 (0.895 to 1.478)
1.0 (1.0 to 1.0)	1.0 (1.0 to 1.0)	1.0 (1.0 to 1.0)	1.0 (1.0 to 1.0)	1.0 (1.0 to 1.0)	1.0 (1.0 to 1.0)	1.0 (1.0 to 1.0)	1.0 (1.0 to 1.0)	1.0 (1.0 to 1.0)	1.0 (1.0 to 1.0)	1.0 (1.0 to 1.0)	1.0 (1.0 to 1.0)	1.0 (1.0 to 1.0)	1.0 (1.0 to 1.0)
1.0 (1.0 to 1.0)	1.0 (1.0 to 1.0)	1.0 (1.0 to 1.0)	1.0 (1.0 to 1.0)	1.0 (1.0 to 1.0)	1.0 (1.0 to 1.0)	1.0 (1.0 to 1.0)	1.0 (1.0 to 1.0)	1.0 (1.0 to 1.0)	1.0 (1.0 to 1.0)	1.0 (1.0 to 1.0)	1.0 (1.0 to 1.0)	1.0 (1.0 to 1.0)	1.0 (1.0 to 1.0)
2.013 (1.391 to 2.858)	2.013 (1.391 to 2.858)	2.013 (1.391 to 2.858)	2.013 (1.391 to 2.858)	2.013 (1.391 to 2.858)	2.013 (1.391 to 2.858)	2.013 (1.391 to 2.858)	2.013 (1.391 to 2.858)	2.013 (1.391 to 2.858)	2.013 (1.391 to 2.858)	2.013 (1.391 to 2.858)	2.013 (1.391 to 2.858)	2.013 (1.391 to 2.858)	2.013 (1.391 to 2.858)
1.163 (0.895 to 1.478)	1.163 (0.895 to 1.478)	1.163 (0.895 to 1.478)	1.163 (0.895 to 1.478)	1.163 (0.895 to 1.478)	1.163 (0.895 to 1.478)	1.163 (0.895 to 1.478)	1.163 (0.895 to 1.478)	1.163 (0.895 to 1.478)	1.163 (0.895 to 1.478)	1.163 (0.895 to 1.478)	1.163 (0.895 to 1.478)	1.163 (0.895 to 1.478)	1.163 (0.895 to 1.478)
1.0 (1.0 to 1.0)	1.0 (1.0 to 1.0)	1.0 (1.0 to 1.0)	1.0 (1.0 to 1.0)	1.0 (1.0 to 1.0)	1.0 (1.0 to 1.0)	1.0 (1.0 to 1.0)	1.0 (1.0 to 1.0)	1.0 (1.0 to 1.0)	1.0 (1.0 to 1.0)	1.0 (1.0 to 1.0)	1.0 (1.0 to 1.0)	1.0 (1.0 to 1.0)	1.0 (1.0 to 1.0)
1.0 (1.0 to 1.0)	1.0 (1.0 to 1.0)	1.0 (1.0 to 1.0)	1.0 (1.0 to 1.0)	1.0 (1.0 to 1.0)	1.0 (1.0 to 1.0)	1.0 (1.0 to 1.0)	1.0 (1.0 to 1.0)	1.0 (1.0 to 1.0)	1.0 (1.0 to 1.0)	1.0 (1.0 to 1.0)	1.0 (1.0 to 1.0)	1.0 (1.0 to 1.0)	1.0 (1.0 to 1.0)
11.546 (8.921 to 14.93)	11.546 (8.921 to 14.93)	11.546 (8.921 to 14.93)	11.546 (8.921 to 14.93)	11.546 (8.921 to 14.93)	11.546 (8.921 to 14.93)	11.546 (8.921 to 14.93)	11.546 (8.921 to 14.93)	11.546 (8.921 to 14.93)	11.546 (8.921 to 14.93)	11.546 (8.921 to 14.93)	11.546 (8.921 to 14.93)	11.546 (8.921 to 14.93)	11.546 (8.921 to 14.93)
15.257 (13.638 to 17.149)	15.257 (13.638 to 17.149)	15.257 (13.638 to 17.149)	15.257 (13.638 to 17.149)	15.257 (13.638 to 17.149)	15.257 (13.638 to 17.149)	15.257 (13.638 to 17.149)	15.257 (13.638 to 17.149)	15.257 (13.638 to 17.149)	15.257 (13.638 to 17.149)	15.257 (13.638 to 17.149)	15.257 (13.638 to 17.149)	15.257 (13.638 to 17.149)	15.257 (13.638 to 17.149)
1.0 (1.0 to 1.0)	1.0 (1.0 to 1.0)	1.0 (1.0 to 1.0)	1.0 (1.0 to 1.0)	1.0 (1.0 to 1.0)	1.0 (1.0 to 1.0)	1.0 (1.0 to 1.0)	1.0 (1.0 to 1.0)	1.0 (1.0 to 1.0)	1.0 (1.0 to 1.0)	1.0 (1.0 to 1.0)	1.0 (1.0 to 1.0)	1.0 (1.0 to 1.0)	1.0 (1.0 to 1.0)

Appendix Table 1a. Relative risks used by age and sex for each outcome for all risk factors except for ambient air pollution and alcohol.

Risk - Outcome	Category / Units	Morbidity / Mortality	Sex	All-age	0-6 days	7-27 days	28-364 days	1-4 years	5-9 years	10-14 years	15-19 years	20-24 years	25-29 years
Chronic obstructive pulmonary disease	1-SIR	Both	Females										
Other chronic respiratory diseases	SIR	Both	Males										
Other chronic respiratory diseases	SIR	Both	Females										
Other chronic respiratory diseases	1-SIR	Both	Males										
Other chronic respiratory diseases	1-SIR	Both	Females										
**Smoking (prevalence approach)**													
Tuberculosis	Smoker (5 year lag)	Both	Both										
Tuberculosis	Nonsmoker (5 year lag)	Both	Both										
Drug-susceptible tuberculosis	Smoker (5 year lag)	Both	Both										
Drug-susceptible tuberculosis	Nonsmoker (5 year lag)	Both	Both										
Multidrug-resistant tuberculosis without extensive drug resistance	Smoker (5 year lag)	Both	Both										
Multidrug-resistant tuberculosis without extensive drug resistance	Nonsmoker (5 year lag)	Both	Both										
Extensively drug-resistant tuberculosis	Smoker (5 year lag)	Both	Both										
Extensively drug-resistant tuberculosis	Nonsmoker (5 year lag)	Both	Both										
Lower respiratory infections	Smoker (5 year lag)	Both	Both										
Lower respiratory infections	Nonsmoker (5 year lag)	Both	Both										
Ischaemic heart disease	Smoker (5 year lag)	Both	Males										
Ischaemic heart disease	Smoker (5 year lag)	Both	Females										
Ischaemic heart disease	Nonsmoker (5 year lag)	Both	Males										
Ischaemic heart disease	Nonsmoker (5 year lag)	Both	Females										
Ischaemic stroke	Smoker (5 year lag)	Both	Males										
Ischaemic stroke	Smoker (5 year lag)	Both	Females										
Ischaemic stroke	Nonsmoker (5 year lag)	Both	Males										
Ischaemic stroke	Nonsmoker (5 year lag)	Both	Females										
Hemorrhagic stroke	Smoker (5 year lag)	Both	Males										
Hemorrhagic stroke	Smoker (5 year lag)	Both	Females										
Hemorrhagic stroke	Nonsmoker (5 year lag)	Both	Males										
Hemorrhagic stroke	Nonsmoker (5 year lag)	Both	Females										
Hypertensive heart disease	Smoker (5 year lag)	Both	Males										
Hypertensive heart disease	Smoker (5 year lag)	Both	Females										

Ages													
30-34 years	35-39 years	40-44 years	45-49 years	50-54 years	55-59 years	60-64 years	65-69 years	70-74 years	75-79 years	80-84 years	85-89 years	90-94 years	95+ years
1.0 (1.0 to 1.0)	1.0 (1.0 to 1.0)	1.0 (1.0 to 1.0)	1.0 (1.0 to 1.0)	1.0 (1.0 to 1.0)	1.0 (1.0 to 1.0)	1.0 (1.0 to 1.0)	1.0 (1.0 to 1.0)	1.0 (1.0 to 1.0)	1.0 (1.0 to 1.0)	1.0 (1.0 to 1.0)	1.0 (1.0 to 1.0)	1.0 (1.0 to 1.0)	1.0 (1.0 to 1.0)
2.1 (1.775 to 2.461)	2.1 (1.775 to 2.461)	2.1 (1.775 to 2.461)	2.1 (1.775 to 2.461)	2.1 (1.775 to 2.461)	2.1 (1.775 to 2.461)	2.1 (1.775 to 2.461)	2.1 (1.775 to 2.461)	2.1 (1.775 to 2.461)	2.1 (1.775 to 2.461)	2.1 (1.775 to 2.461)	2.1 (1.775 to 2.461)	2.1 (1.775 to 2.461)	2.1 (1.775 to 2.461)
1.982 (1.8 to 2.172)	1.982 (1.8 to 2.172)	1.982 (1.8 to 2.172)	1.982 (1.8 to 2.172)	1.982 (1.8 to 2.172)	1.982 (1.8 to 2.172)	1.982 (1.8 to 2.172)	1.982 (1.8 to 2.172)	1.982 (1.8 to 2.172)	1.982 (1.8 to 2.172)	1.982 (1.8 to 2.172)	1.982 (1.8 to 2.172)	1.982 (1.8 to 2.172)	1.982 (1.8 to 2.172)
1.0 (1.0 to 1.0)	1.0 (1.0 to 1.0)	1.0 (1.0 to 1.0)	1.0 (1.0 to 1.0)	1.0 (1.0 to 1.0)	1.0 (1.0 to 1.0)	1.0 (1.0 to 1.0)	1.0 (1.0 to 1.0)	1.0 (1.0 to 1.0)	1.0 (1.0 to 1.0)	1.0 (1.0 to 1.0)	1.0 (1.0 to 1.0)	1.0 (1.0 to 1.0)	1.0 (1.0 to 1.0)
1.0 (1.0 to 1.0)	1.0 (1.0 to 1.0)	1.0 (1.0 to 1.0)	1.0 (1.0 to 1.0)	1.0 (1.0 to 1.0)	1.0 (1.0 to 1.0)	1.0 (1.0 to 1.0)	1.0 (1.0 to 1.0)	1.0 (1.0 to 1.0)	1.0 (1.0 to 1.0)	1.0 (1.0 to 1.0)	1.0 (1.0 to 1.0)	1.0 (1.0 to 1.0)	1.0 (1.0 to 1.0)
1.588 (1.243 to 2.035)	1.588 (1.243 to 2.035)	1.588 (1.243 to 2.035)	1.588 (1.243 to 2.035)	1.588 (1.243 to 2.035)	1.588 (1.243 to 2.035)	1.588 (1.243 to 2.035)	1.588 (1.243 to 2.035)	1.588 (1.243 to 2.035)	1.588 (1.243 to 2.035)	1.588 (1.243 to 2.035)	1.588 (1.243 to 2.035)	1.588 (1.243 to 2.035)	1.588 (1.243 to 2.035)
1.0 (1.0 to 1.0)	1.0 (1.0 to 1.0)	1.0 (1.0 to 1.0)	1.0 (1.0 to 1.0)	1.0 (1.0 to 1.0)	1.0 (1.0 to 1.0)	1.0 (1.0 to 1.0)	1.0 (1.0 to 1.0)	1.0 (1.0 to 1.0)	1.0 (1.0 to 1.0)	1.0 (1.0 to 1.0)	1.0 (1.0 to 1.0)	1.0 (1.0 to 1.0)	1.0 (1.0 to 1.0)
1.599 (1.259 to 2.023)	1.599 (1.259 to 2.023)	1.599 (1.259 to 2.023)	1.599 (1.259 to 2.023)	1.599 (1.259 to 2.023)	1.599 (1.259 to 2.023)	1.599 (1.259 to 2.023)	1.599 (1.259 to 2.023)	1.599 (1.259 to 2.023)	1.599 (1.259 to 2.023)	1.599 (1.259 to 2.023)	1.599 (1.259 to 2.023)	1.599 (1.259 to 2.023)	1.599 (1.259 to 2.023)
1.0 (1.0 to 1.0)	1.0 (1.0 to 1.0)	1.0 (1.0 to 1.0)	1.0 (1.0 to 1.0)	1.0 (1.0 to 1.0)	1.0 (1.0 to 1.0)	1.0 (1.0 to 1.0)	1.0 (1.0 to 1.0)	1.0 (1.0 to 1.0)	1.0 (1.0 to 1.0)	1.0 (1.0 to 1.0)	1.0 (1.0 to 1.0)	1.0 (1.0 to 1.0)	1.0 (1.0 to 1.0)
1.599 (1.259 to 2.023)	1.599 (1.259 to 2.023)	1.599 (1.259 to 2.023)	1.599 (1.259 to 2.023)	1.599 (1.259 to 2.023)	1.599 (1.259 to 2.023)	1.599 (1.259 to 2.023)	1.599 (1.259 to 2.023)	1.599 (1.259 to 2.023)	1.599 (1.259 to 2.023)	1.599 (1.259 to 2.023)	1.599 (1.259 to 2.023)	1.599 (1.259 to 2.023)	1.599 (1.259 to 2.023)
1.0 (1.0 to 1.0)	1.0 (1.0 to 1.0)	1.0 (1.0 to 1.0)	1.0 (1.0 to 1.0)	1.0 (1.0 to 1.0)	1.0 (1.0 to 1.0)	1.0 (1.0 to 1.0)	1.0 (1.0 to 1.0)	1.0 (1.0 to 1.0)	1.0 (1.0 to 1.0)	1.0 (1.0 to 1.0)	1.0 (1.0 to 1.0)	1.0 (1.0 to 1.0)	1.0 (1.0 to 1.0)
1.599 (1.259 to 2.023)	1.599 (1.259 to 2.023)	1.599 (1.259 to 2.023)	1.599 (1.259 to 2.023)	1.599 (1.259 to 2.023)	1.599 (1.259 to 2.023)	1.599 (1.259 to 2.023)	1.599 (1.259 to 2.023)	1.599 (1.259 to 2.023)	1.599 (1.259 to 2.023)	1.599 (1.259 to 2.023)	1.599 (1.259 to 2.023)	1.599 (1.259 to 2.023)	1.599 (1.259 to 2.023)
1.0 (1.0 to 1.0)	1.0 (1.0 to 1.0)	1.0 (1.0 to 1.0)	1.0 (1.0 to 1.0)	1.0 (1.0 to 1.0)	1.0 (1.0 to 1.0)	1.0 (1.0 to 1.0)	1.0 (1.0 to 1.0)	1.0 (1.0 to 1.0)	1.0 (1.0 to 1.0)	1.0 (1.0 to 1.0)	1.0 (1.0 to 1.0)	1.0 (1.0 to 1.0)	1.0 (1.0 to 1.0)
3.484 (2.802 to 4.299)	3.484 (2.802 to 4.299)	3.484 (2.802 to 4.299)	3.484 (2.802 to 4.299)	3.484 (2.802 to 4.299)	3.484 (2.802 to 4.299)	3.484 (2.802 to 4.299)	3.484 (2.802 to 4.299)	3.484 (2.802 to 4.299)	3.484 (2.802 to 4.299)	3.484 (2.802 to 4.299)	3.484 (2.802 to 4.299)	3.484 (2.802 to 4.299)	3.484 (2.802 to 4.299)
1.0 (1.0 to 1.0)	1.0 (1.0 to 1.0)	1.0 (1.0 to 1.0)	1.0 (1.0 to 1.0)	1.0 (1.0 to 1.0)	1.0 (1.0 to 1.0)	1.0 (1.0 to 1.0)	1.0 (1.0 to 1.0)	1.0 (1.0 to 1.0)	1.0 (1.0 to 1.0)	1.0 (1.0 to 1.0)	1.0 (1.0 to 1.0)	1.0 (1.0 to 1.0)	1.0 (1.0 to 1.0)
4.316 (3.129 to 5.802)	3.924 (2.907 to 5.18)	3.569 (2.701 to 4.624)	3.246 (2.509 to 4.128)	2.952 (2.331 to 3.686)	2.685 (2.166 to 3.29)	2.443 (2.012 to 2.938)	2.223 (1.869 to 2.623)	2.023 (1.737 to 2.341)	1.841 (1.613 to 2.09)	1.598 (1.445 to 1.763)	1.598 (1.445 to 1.763)	1.598 (1.445 to 1.763)	1.598 (1.445 to 1.763)
6.145 (5.066 to 7.41)	5.464 (4.563 to 6.511)	4.859 (4.109 to 5.722)	4.321 (3.701 to 5.029)	3.843 (3.333 to 4.419)	3.417 (3.002 to 3.883)	3.039 (2.703 to 3.413)	2.703 (2.435 to 2.999)	2.404 (2.193 to 2.636)	2.139 (1.975 to 2.316)	1.794 (1.688 to 1.908)	1.794 (1.688 to 1.908)	1.794 (1.688 to 1.908)	1.794 (1.688 to 1.908)
1.0 (1.0 to 1.0)	1.0 (1.0 to 1.0)	1.0 (1.0 to 1.0)	1.0 (1.0 to 1.0)	1.0 (1.0 to 1.0)	1.0 (1.0 to 1.0)	1.0 (1.0 to 1.0)	1.0 (1.0 to 1.0)	1.0 (1.0 to 1.0)	1.0 (1.0 to 1.0)	1.0 (1.0 to 1.0)	1.0 (1.0 to 1.0)	1.0 (1.0 to 1.0)	1.0 (1.0 to 1.0)
1.0 (1.0 to 1.0)	1.0 (1.0 to 1.0)	1.0 (1.0 to 1.0)	1.0 (1.0 to 1.0)	1.0 (1.0 to 1.0)	1.0 (1.0 to 1.0)	1.0 (1.0 to 1.0)	1.0 (1.0 to 1.0)	1.0 (1.0 to 1.0)	1.0 (1.0 to 1.0)	1.0 (1.0 to 1.0)	1.0 (1.0 to 1.0)	1.0 (1.0 to 1.0)	1.0 (1.0 to 1.0)
4.175 (3.167 to 5.444)	3.805 (2.94 to 4.88)	3.468 (2.73 to 4.375)	3.161 (2.534 to 3.922)	2.882 (2.352 to 3.516)	2.627 (2.184 to 3.152)	2.395 (2.027 to 2.825)	2.184 (1.882 to 2.533)	1.992 (1.747 to 2.27)	1.816 (1.622 to 2.035)	1.582 (1.45 to 1.727)	1.582 (1.45 to 1.727)	1.582 (1.45 to 1.727)	1.582 (1.45 to 1.727)
6.02 (4.253 to 8.384)	5.357 (3.874 to 7.309)	4.767 (3.529 to 6.372)	4.243 (3.214 to 5.555)	3.777 (2.927 to 4.843)	3.363 (2.666 to 4.222)	2.994 (2.429 to 3.681)	2.666 (2.212 to 3.209)	2.375 (2.015 to 2.798)	2.115 (1.835 to 2.439)	1.778 (1.595 to 1.986)	1.778 (1.595 to 1.986)	1.778 (1.595 to 1.986)	1.778 (1.595 to 1.986)
1.0 (1.0 to 1.0)	1.0 (1.0 to 1.0)	1.0 (1.0 to 1.0)	1.0 (1.0 to 1.0)	1.0 (1.0 to 1.0)	1.0 (1.0 to 1.0)	1.0 (1.0 to 1.0)	1.0 (1.0 to 1.0)	1.0 (1.0 to 1.0)	1.0 (1.0 to 1.0)	1.0 (1.0 to 1.0)	1.0 (1.0 to 1.0)	1.0 (1.0 to 1.0)	1.0 (1.0 to 1.0)
1.0 (1.0 to 1.0)	1.0 (1.0 to 1.0)	1.0 (1.0 to 1.0)	1.0 (1.0 to 1.0)	1.0 (1.0 to 1.0)	1.0 (1.0 to 1.0)	1.0 (1.0 to 1.0)	1.0 (1.0 to 1.0)	1.0 (1.0 to 1.0)	1.0 (1.0 to 1.0)	1.0 (1.0 to 1.0)	1.0 (1.0 to 1.0)	1.0 (1.0 to 1.0)	1.0 (1.0 to 1.0)
4.175 (3.167 to 5.444)	3.805 (2.94 to 4.88)	3.468 (2.73 to 4.375)	3.161 (2.534 to 3.922)	2.882 (2.352 to 3.516)	2.627 (2.184 to 3.152)	2.395 (2.027 to 2.825)	2.184 (1.882 to 2.533)	1.992 (1.747 to 2.27)	1.816 (1.622 to 2.035)	1.582 (1.45 to 1.727)	1.582 (1.45 to 1.727)	1.582 (1.45 to 1.727)	1.582 (1.45 to 1.727)
6.02 (4.253 to 8.384)	5.357 (3.874 to 7.309)	4.767 (3.529 to 6.372)	4.243 (3.214 to 5.555)	3.777 (2.927 to 4.843)	3.363 (2.666 to 4.222)	2.994 (2.429 to 3.681)	2.666 (2.212 to 3.209)	2.375 (2.015 to 2.798)	2.115 (1.835 to 2.439)	1.778 (1.595 to 1.986)	1.778 (1.595 to 1.986)	1.778 (1.595 to 1.986)	1.778 (1.595 to 1.986)
1.0 (1.0 to 1.0)	1.0 (1.0 to 1.0)	1.0 (1.0 to 1.0)	1.0 (1.0 to 1.0)	1.0 (1.0 to 1.0)	1.0 (1.0 to 1.0)	1.0 (1.0 to 1.0)	1.0 (1.0 to 1.0)	1.0 (1.0 to 1.0)	1.0 (1.0 to 1.0)	1.0 (1.0 to 1.0)	1.0 (1.0 to 1.0)	1.0 (1.0 to 1.0)	1.0 (1.0 to 1.0)
1.0 (1.0 to 1.0)	1.0 (1.0 to 1.0)	1.0 (1.0 to 1.0)	1.0 (1.0 to 1.0)	1.0 (1.0 to 1.0)	1.0 (1.0 to 1.0)	1.0 (1.0 to 1.0)	1.0 (1.0 to 1.0)	1.0 (1.0 to 1.0)	1.0 (1.0 to 1.0)	1.0 (1.0 to 1.0)	1.0 (1.0 to 1.0)	1.0 (1.0 to 1.0)	1.0 (1.0 to 1.0)
4.153 (2.999 to 5.658)	3.785 (2.793 to 5.059)	3.451 (2.602 to 4.524)	3.146 (2.424 to 4.046)	2.868 (2.259 to 3.618)	2.616 (2.104 to 3.235)	2.386 (1.96 to 2.893)	2.176 (1.826 to 2.587)	1.985 (1.701 to 2.313)	1.811 (1.585 to 2.068)	1.578 (1.425 to 1.749)	1.578 (1.425 to 1.749)	1.578 (1.425 to 1.749)	1.578 (1.425 to 1.749)
4.11 (2.077 to 7.156)	3.74 (1.981 to 6.303)	3.405 (1.89 to 5.551)	3.102 (1.803 to 4.889)	2.826 (1.72 to 4.306)	2.576 (1.641 to 3.793)	2.35 (1.565 to 3.341)	2.144 (1.493 to 2.942)	1.957 (1.424 to 2.592)	1.787 (1.359 to 2.283)	1.56 (1.266 to 1.887)	1.56 (1.266 to 1.887)	1.56 (1.266 to 1.887)	1.56 (1.266 to 1.887)

Appendix Table 1a. Relative risks used by age and sex for each outcome for all risk factors except for ambient air pollution and alcohol.

Risk - Outcome	Category / Units	Morbidity / Mortality	Sex	All-age	0-6 days	7-27 days	28-364 days	1-4 years	5-9 years	10-14 years	15-19 years	20-24 years	25-29 years
Hypertensive heart disease	Nonsmoker (5 year lag)	Both	Males										
Hypertensive heart disease	Nonsmoker (5 year lag)	Both	Females										
Atrial fibrillation and flutter	Smoker (5 year lag)	Both	Males										
Atrial fibrillation and flutter	Smoker (5 year lag)	Both	Females										
Atrial fibrillation and flutter	Nonsmoker (5 year lag)	Both	Males										
Atrial fibrillation and flutter	Nonsmoker (5 year lag)	Both	Females										
Aortic aneurysm	Smoker (5 year lag)	Both	Males										
Aortic aneurysm	Smoker (5 year lag)	Both	Females										
Aortic aneurysm	Nonsmoker (5 year lag)	Both	Males										
Aortic aneurysm	Nonsmoker (5 year lag)	Both	Females										
Peripheral vascular disease	Smoker (5 year lag)	Both	Males										
Peripheral vascular disease	Smoker (5 year lag)	Both	Females										
Peripheral vascular disease	Nonsmoker (5 year lag)	Both	Males										
Peripheral vascular disease	Nonsmoker (5 year lag)	Both	Females										
Other cardiovascular and circulatory diseases	Smoker (5 year lag)	Both	Males										
Other cardiovascular and circulatory diseases	Smoker (5 year lag)	Both	Females										
Other cardiovascular and circulatory diseases	Nonsmoker (5 year lag)	Both	Males										
Other cardiovascular and circulatory diseases	Nonsmoker (5 year lag)	Both	Females										
Asthma	Smoker (5 year lag)	Both	Males										
Asthma	Smoker (5 year lag)	Both	Females										
Asthma	Nonsmoker (5 year lag)	Both	Males										
Asthma	Nonsmoker (5 year lag)	Both	Females										
Peptic ulcer disease	Smoker (5 year lag)	Both	Both										
Peptic ulcer disease	Nonsmoker (5 year lag)	Both	Both										
Gallbladder and biliary diseases	Smoker (5 year lag)	Both	Males										
Gallbladder and biliary diseases	Smoker (5 year lag)	Both	Females										
Gallbladder and biliary diseases	Nonsmoker (5 year lag)	Both	Males										
Gallbladder and biliary diseases	Nonsmoker (5 year lag)	Both	Females										
Alzheimer's disease and other dementias	Smoker (5 year lag)	Both	Males										
Alzheimer's disease and other dementias	Smoker (5 year lag)	Both	Females										

Ages													
30-34 years	35-39 years	40-44 years	45-49 years	50-54 years	55-59 years	60-64 years	65-69 years	70-74 years	75-79 years	80-84 years	85-89 years	90-94 years	95+ years
1.0 (1.0 to 1.0)	1.0 (1.0 to 1.0)	1.0 (1.0 to 1.0)	1.0 (1.0 to 1.0)	1.0 (1.0 to 1.0)	1.0 (1.0 to 1.0)	1.0 (1.0 to 1.0)	1.0 (1.0 to 1.0)	1.0 (1.0 to 1.0)	1.0 (1.0 to 1.0)	1.0 (1.0 to 1.0)	1.0 (1.0 to 1.0)	1.0 (1.0 to 1.0)	1.0 (1.0 to 1.0)
1.0 (1.0 to 1.0)	1.0 (1.0 to 1.0)	1.0 (1.0 to 1.0)	1.0 (1.0 to 1.0)	1.0 (1.0 to 1.0)	1.0 (1.0 to 1.0)	1.0 (1.0 to 1.0)	1.0 (1.0 to 1.0)	1.0 (1.0 to 1.0)	1.0 (1.0 to 1.0)	1.0 (1.0 to 1.0)	1.0 (1.0 to 1.0)	1.0 (1.0 to 1.0)	1.0 (1.0 to 1.0)
4.153 (2.999 to 5.658)	3.785 (2.793 to 5.059)	3.451 (2.602 to 4.524)	3.146 (2.424 to 4.046)	2.868 (2.259 to 3.618)	2.616 (2.104 to 3.235)	2.386 (1.96 to 2.893)	2.176 (1.826 to 2.587)	1.985 (1.701 to 2.313)	1.811 (1.585 to 2.068)	1.578 (1.425 to 1.749)	1.578 (1.425 to 1.749)	1.578 (1.425 to 1.749)	1.578 (1.425 to 1.749)
4.11 (2.077 to 7.156)	3.74 (1.981 to 6.303)	3.405 (1.89 to 5.551)	3.102 (1.803 to 4.889)	2.826 (1.72 to 4.306)	2.576 (1.641 to 3.793)	2.35 (1.565 to 3.341)	2.144 (1.493 to 2.942)	1.957 (1.424 to 2.592)	1.787 (1.359 to 2.283)	1.56 (1.266 to 1.887)	1.56 (1.266 to 1.887)	1.56 (1.266 to 1.887)	1.56 (1.266 to 1.887)
1.0 (1.0 to 1.0)	1.0 (1.0 to 1.0)	1.0 (1.0 to 1.0)	1.0 (1.0 to 1.0)	1.0 (1.0 to 1.0)	1.0 (1.0 to 1.0)	1.0 (1.0 to 1.0)	1.0 (1.0 to 1.0)	1.0 (1.0 to 1.0)	1.0 (1.0 to 1.0)	1.0 (1.0 to 1.0)	1.0 (1.0 to 1.0)	1.0 (1.0 to 1.0)	1.0 (1.0 to 1.0)
1.0 (1.0 to 1.0)	1.0 (1.0 to 1.0)	1.0 (1.0 to 1.0)	1.0 (1.0 to 1.0)	1.0 (1.0 to 1.0)	1.0 (1.0 to 1.0)	1.0 (1.0 to 1.0)	1.0 (1.0 to 1.0)	1.0 (1.0 to 1.0)	1.0 (1.0 to 1.0)	1.0 (1.0 to 1.0)	1.0 (1.0 to 1.0)	1.0 (1.0 to 1.0)	1.0 (1.0 to 1.0)
4.153 (2.999 to 5.658)	3.785 (2.793 to 5.059)	3.451 (2.602 to 4.524)	3.146 (2.424 to 4.046)	2.868 (2.259 to 3.618)	2.616 (2.104 to 3.235)	2.386 (1.96 to 2.893)	2.176 (1.826 to 2.587)	1.985 (1.701 to 2.313)	1.811 (1.585 to 2.068)	1.578 (1.425 to 1.749)	1.578 (1.425 to 1.749)	1.578 (1.425 to 1.749)	1.578 (1.425 to 1.749)
4.11 (2.077 to 7.156)	3.74 (1.981 to 6.303)	3.405 (1.89 to 5.551)	3.102 (1.803 to 4.889)	2.826 (1.72 to 4.306)	2.576 (1.641 to 3.793)	2.35 (1.565 to 3.341)	2.144 (1.493 to 2.942)	1.957 (1.424 to 2.592)	1.787 (1.359 to 2.283)	1.56 (1.266 to 1.887)	1.56 (1.266 to 1.887)	1.56 (1.266 to 1.887)	1.56 (1.266 to 1.887)
1.0 (1.0 to 1.0)	1.0 (1.0 to 1.0)	1.0 (1.0 to 1.0)	1.0 (1.0 to 1.0)	1.0 (1.0 to 1.0)	1.0 (1.0 to 1.0)	1.0 (1.0 to 1.0)	1.0 (1.0 to 1.0)	1.0 (1.0 to 1.0)	1.0 (1.0 to 1.0)	1.0 (1.0 to 1.0)	1.0 (1.0 to 1.0)	1.0 (1.0 to 1.0)	1.0 (1.0 to 1.0)
1.0 (1.0 to 1.0)	1.0 (1.0 to 1.0)	1.0 (1.0 to 1.0)	1.0 (1.0 to 1.0)	1.0 (1.0 to 1.0)	1.0 (1.0 to 1.0)	1.0 (1.0 to 1.0)	1.0 (1.0 to 1.0)	1.0 (1.0 to 1.0)	1.0 (1.0 to 1.0)	1.0 (1.0 to 1.0)	1.0 (1.0 to 1.0)	1.0 (1.0 to 1.0)	1.0 (1.0 to 1.0)
4.153 (2.999 to 5.658)	3.785 (2.793 to 5.059)	3.451 (2.602 to 4.524)	3.146 (2.424 to 4.046)	2.868 (2.259 to 3.618)	2.616 (2.104 to 3.235)	2.386 (1.96 to 2.893)	2.176 (1.826 to 2.587)	1.985 (1.701 to 2.313)	1.811 (1.585 to 2.068)	1.578 (1.425 to 1.749)	1.578 (1.425 to 1.749)	1.578 (1.425 to 1.749)	1.578 (1.425 to 1.749)
4.11 (2.077 to 7.156)	3.74 (1.981 to 6.303)	3.405 (1.89 to 5.551)	3.102 (1.803 to 4.889)	2.826 (1.72 to 4.306)	2.576 (1.641 to 3.793)	2.35 (1.565 to 3.341)	2.144 (1.493 to 2.942)	1.957 (1.424 to 2.592)	1.787 (1.359 to 2.283)	1.56 (1.266 to 1.887)	1.56 (1.266 to 1.887)	1.56 (1.266 to 1.887)	1.56 (1.266 to 1.887)
1.0 (1.0 to 1.0)	1.0 (1.0 to 1.0)	1.0 (1.0 to 1.0)	1.0 (1.0 to 1.0)	1.0 (1.0 to 1.0)	1.0 (1.0 to 1.0)	1.0 (1.0 to 1.0)	1.0 (1.0 to 1.0)	1.0 (1.0 to 1.0)	1.0 (1.0 to 1.0)	1.0 (1.0 to 1.0)	1.0 (1.0 to 1.0)	1.0 (1.0 to 1.0)	1.0 (1.0 to 1.0)
1.0 (1.0 to 1.0)	1.0 (1.0 to 1.0)	1.0 (1.0 to 1.0)	1.0 (1.0 to 1.0)	1.0 (1.0 to 1.0)	1.0 (1.0 to 1.0)	1.0 (1.0 to 1.0)	1.0 (1.0 to 1.0)	1.0 (1.0 to 1.0)	1.0 (1.0 to 1.0)	1.0 (1.0 to 1.0)	1.0 (1.0 to 1.0)	1.0 (1.0 to 1.0)	1.0 (1.0 to 1.0)
4.153 (2.999 to 5.658)	3.785 (2.793 to 5.059)	3.451 (2.602 to 4.524)	3.146 (2.424 to 4.046)	2.868 (2.259 to 3.618)	2.616 (2.104 to 3.235)	2.386 (1.96 to 2.893)	2.176 (1.826 to 2.587)	1.985 (1.701 to 2.313)	1.811 (1.585 to 2.068)	1.578 (1.425 to 1.749)	1.578 (1.425 to 1.749)	1.578 (1.425 to 1.749)	1.578 (1.425 to 1.749)
4.11 (2.077 to 7.156)	3.74 (1.981 to 6.303)	3.405 (1.89 to 5.551)	3.102 (1.803 to 4.889)	2.826 (1.72 to 4.306)	2.576 (1.641 to 3.793)	2.35 (1.565 to 3.341)	2.144 (1.493 to 2.942)	1.957 (1.424 to 2.592)	1.787 (1.359 to 2.283)	1.56 (1.266 to 1.887)	1.56 (1.266 to 1.887)	1.56 (1.266 to 1.887)	1.56 (1.266 to 1.887)
1.0 (1.0 to 1.0)	1.0 (1.0 to 1.0)	1.0 (1.0 to 1.0)	1.0 (1.0 to 1.0)	1.0 (1.0 to 1.0)	1.0 (1.0 to 1.0)	1.0 (1.0 to 1.0)	1.0 (1.0 to 1.0)	1.0 (1.0 to 1.0)	1.0 (1.0 to 1.0)	1.0 (1.0 to 1.0)	1.0 (1.0 to 1.0)	1.0 (1.0 to 1.0)	1.0 (1.0 to 1.0)
1.0 (1.0 to 1.0)	1.0 (1.0 to 1.0)	1.0 (1.0 to 1.0)	1.0 (1.0 to 1.0)	1.0 (1.0 to 1.0)	1.0 (1.0 to 1.0)	1.0 (1.0 to 1.0)	1.0 (1.0 to 1.0)	1.0 (1.0 to 1.0)	1.0 (1.0 to 1.0)	1.0 (1.0 to 1.0)	1.0 (1.0 to 1.0)	1.0 (1.0 to 1.0)	1.0 (1.0 to 1.0)
2.098 (1.762 to 2.46)	2.098 (1.762 to 2.46)	2.098 (1.762 to 2.46)	2.098 (1.762 to 2.46)	2.098 (1.762 to 2.46)	2.098 (1.762 to 2.46)	2.098 (1.762 to 2.46)	2.098 (1.762 to 2.46)	2.098 (1.762 to 2.46)	2.098 (1.762 to 2.46)	2.098 (1.762 to 2.46)	2.098 (1.762 to 2.46)	2.098 (1.762 to 2.46)	2.098 (1.762 to 2.46)
1.976 (1.788 to 2.18)	1.976 (1.788 to 2.18)	1.976 (1.788 to 2.18)	1.976 (1.788 to 2.18)	1.976 (1.788 to 2.18)	1.976 (1.788 to 2.18)	1.976 (1.788 to 2.18)	1.976 (1.788 to 2.18)	1.976 (1.788 to 2.18)	1.976 (1.788 to 2.18)	1.976 (1.788 to 2.18)	1.976 (1.788 to 2.18)	1.976 (1.788 to 2.18)	1.976 (1.788 to 2.18)
1.0 (1.0 to 1.0)	1.0 (1.0 to 1.0)	1.0 (1.0 to 1.0)	1.0 (1.0 to 1.0)	1.0 (1.0 to 1.0)	1.0 (1.0 to 1.0)	1.0 (1.0 to 1.0)	1.0 (1.0 to 1.0)	1.0 (1.0 to 1.0)	1.0 (1.0 to 1.0)	1.0 (1.0 to 1.0)	1.0 (1.0 to 1.0)	1.0 (1.0 to 1.0)	1.0 (1.0 to 1.0)
1.0 (1.0 to 1.0)	1.0 (1.0 to 1.0)	1.0 (1.0 to 1.0)	1.0 (1.0 to 1.0)	1.0 (1.0 to 1.0)	1.0 (1.0 to 1.0)	1.0 (1.0 to 1.0)	1.0 (1.0 to 1.0)	1.0 (1.0 to 1.0)	1.0 (1.0 to 1.0)	1.0 (1.0 to 1.0)	1.0 (1.0 to 1.0)	1.0 (1.0 to 1.0)	1.0 (1.0 to 1.0)
2.04 (1.684 to 2.479)	2.04 (1.684 to 2.479)	2.04 (1.684 to 2.479)	2.04 (1.684 to 2.479)	2.04 (1.684 to 2.479)	2.04 (1.684 to 2.479)	2.04 (1.684 to 2.479)	2.04 (1.684 to 2.479)	2.04 (1.684 to 2.479)	2.04 (1.684 to 2.479)	2.04 (1.684 to 2.479)	2.04 (1.684 to 2.479)	2.04 (1.684 to 2.479)	2.04 (1.684 to 2.479)
1.0 (1.0 to 1.0)	1.0 (1.0 to 1.0)	1.0 (1.0 to 1.0)	1.0 (1.0 to 1.0)	1.0 (1.0 to 1.0)	1.0 (1.0 to 1.0)	1.0 (1.0 to 1.0)	1.0 (1.0 to 1.0)	1.0 (1.0 to 1.0)	1.0 (1.0 to 1.0)	1.0 (1.0 to 1.0)	1.0 (1.0 to 1.0)	1.0 (1.0 to 1.0)	1.0 (1.0 to 1.0)
1.189 (1.114 to 1.266)	1.189 (1.114 to 1.266)	1.189 (1.114 to 1.266)	1.189 (1.114 to 1.266)	1.189 (1.114 to 1.266)	1.189 (1.114 to 1.266)	1.189 (1.114 to 1.266)	1.189 (1.114 to 1.266)	1.189 (1.114 to 1.266)	1.189 (1.114 to 1.266)	1.189 (1.114 to 1.266)	1.189 (1.114 to 1.266)	1.189 (1.114 to 1.266)	1.189 (1.114 to 1.266)
1.19 (1.115 to 1.264)	1.19 (1.115 to 1.264)	1.19 (1.115 to 1.264)	1.19 (1.115 to 1.264)	1.19 (1.115 to 1.264)	1.19 (1.115 to 1.264)	1.19 (1.115 to 1.264)	1.19 (1.115 to 1.264)	1.19 (1.115 to 1.264)	1.19 (1.115 to 1.264)	1.19 (1.115 to 1.264)	1.19 (1.115 to 1.264)	1.19 (1.115 to 1.264)	1.19 (1.115 to 1.264)
1.0 (1.0 to 1.0)	1.0 (1.0 to 1.0)	1.0 (1.0 to 1.0)	1.0 (1.0 to 1.0)	1.0 (1.0 to 1.0)	1.0 (1.0 to 1.0)	1.0 (1.0 to 1.0)	1.0 (1.0 to 1.0)	1.0 (1.0 to 1.0)	1.0 (1.0 to 1.0)	1.0 (1.0 to 1.0)	1.0 (1.0 to 1.0)	1.0 (1.0 to 1.0)	1.0 (1.0 to 1.0)
1.0 (1.0 to 1.0)	1.0 (1.0 to 1.0)	1.0 (1.0 to 1.0)	1.0 (1.0 to 1.0)	1.0 (1.0 to 1.0)	1.0 (1.0 to 1.0)	1.0 (1.0 to 1.0)	1.0 (1.0 to 1.0)	1.0 (1.0 to 1.0)	1.0 (1.0 to 1.0)	1.0 (1.0 to 1.0)	1.0 (1.0 to 1.0)	1.0 (1.0 to 1.0)	1.0 (1.0 to 1.0)
1.405 (1.113 to 1.741)	1.405 (1.113 to 1.741)	1.405 (1.113 to 1.741)	1.405 (1.113 to 1.741)	1.405 (1.113 to 1.741)	1.405 (1.113 to 1.741)	1.405 (1.113 to 1.741)	1.405 (1.113 to 1.741)	1.405 (1.113 to 1.741)	1.405 (1.113 to 1.741)	1.405 (1.113 to 1.741)	1.405 (1.113 to 1.741)	1.405 (1.113 to 1.741)	1.405 (1.113 to 1.741)
1.401 (1.125 to 1.718)	1.401 (1.125 to 1.718)	1.401 (1.125 to 1.718)	1.401 (1.125 to 1.718)	1.401 (1.125 to 1.718)	1.401 (1.125 to 1.718)	1.401 (1.125 to 1.718)	1.401 (1.125 to 1.718)	1.401 (1.125 to 1.718)	1.401 (1.125 to 1.718)	1.401 (1.125 to 1.718)	1.401 (1.125 to 1.718)	1.401 (1.125 to 1.718)	1.401 (1.125 to 1.718)

Appendix Table 1a. Relative risks used by age and sex for each outcome for all risk factors except for ambient air pollution and alcohol.

Risk - Outcome	Category / Units	Morbidity / Mortality	Sex	All-age	0-6 days	7-27 days	28-364 days	1-4 years	5-9 years	10-14 years	15-19 years	20-24 years	25-29 years
Alzheimer's disease and other dementias	Nonsmoker (5 year lag)	Both	Males										
Alzheimer's disease and other dementias	Nonsmoker (5 year lag)	Both	Females										
Parkinson's disease	Smoker (5 year lag)	Both	Males										
Parkinson's disease	Smoker (5 year lag)	Both	Females										
Parkinson's disease	Nonsmoker (5 year lag)	Both	Males										
Parkinson's disease	Nonsmoker (5 year lag)	Both	Females										
Multiple sclerosis	Smoker (5 year lag)	Both	Males										
Multiple sclerosis	Smoker (5 year lag)	Both	Females										
Multiple sclerosis	Nonsmoker (5 year lag)	Both	Males										
Multiple sclerosis	Nonsmoker (5 year lag)	Both	Females										
Diabetes mellitus	Smoker (5 year lag)	Both	Males										
Diabetes mellitus	Smoker (5 year lag)	Both	Females										
Diabetes mellitus	Nonsmoker (5 year lag)	Both	Males										
Diabetes mellitus	Nonsmoker (5 year lag)	Both	Females										
Rheumatoid arthritis	Smoker (5 year lag)	Both	Both										
Rheumatoid arthritis	Nonsmoker (5 year lag)	Both	Both										
Low back pain	Smoker (5 year lag)	Both	Males										
Low back pain	Smoker (5 year lag)	Both	Females										
Low back pain	Nonsmoker (5 year lag)	Both	Males										
Low back pain	Nonsmoker (5 year lag)	Both	Females										
Cataract	Smoker (5 year lag)	Both	Both										
Cataract	Nonsmoker (5 year lag)	Both	Both										
Macular degeneration	Smoker (5 year lag)	Both	Both										
Macular degeneration	Nonsmoker (5 year lag)	Both	Both										
**Smokeless tobacco**													
Lip and oral cavity cancer	Exposed	Both	Males										
Lip and oral cavity cancer	Exposed	Both	Females										
Oesophageal cancer	Exposed	Both	Both										
**Second-hand smoke**													
Otitis media	Exposed	Morbidity	Both		1.37 (1.238 to 1.493)	1.37 (1.238 to 1.493)	1.37 (1.238 to 1.493)	1.37 (1.238 to 1.493)	1.37 (1.238 to 1.493)	1.37 (1.238 to 1.493)			

Ages													
**30-34 years**	**35-39 years**	**40-44 years**	**45-49 years**	**50-54 years**	**55-59 years**	**60-64 years**	**65-69 years**	**70-74 years**	**75-79 years**	**80-84 years**	**85-89 years**	**90-94 years**	**95+ years**
1.0 (1.0 to 1.0)	1.0 (1.0 to 1.0)	1.0 (1.0 to 1.0)	1.0 (1.0 to 1.0)	1.0 (1.0 to 1.0)	1.0 (1.0 to 1.0)	1.0 (1.0 to 1.0)	1.0 (1.0 to 1.0)	1.0 (1.0 to 1.0)	1.0 (1.0 to 1.0)	1.0 (1.0 to 1.0)	1.0 (1.0 to 1.0)	1.0 (1.0 to 1.0)	1.0 (1.0 to 1.0)
1.0 (1.0 to 1.0)	1.0 (1.0 to 1.0)	1.0 (1.0 to 1.0)	1.0 (1.0 to 1.0)	1.0 (1.0 to 1.0)	1.0 (1.0 to 1.0)	1.0 (1.0 to 1.0)	1.0 (1.0 to 1.0)	1.0 (1.0 to 1.0)	1.0 (1.0 to 1.0)	1.0 (1.0 to 1.0)	1.0 (1.0 to 1.0)	1.0 (1.0 to 1.0)	1.0 (1.0 to 1.0)
0.335 (0.236 to 0.463)	0.335 (0.236 to 0.463)	0.335 (0.236 to 0.463)	0.335 (0.236 to 0.463)	0.335 (0.236 to 0.463)	0.335 (0.236 to 0.463)	0.335 (0.236 to 0.463)	0.335 (0.236 to 0.463)	0.335 (0.236 to 0.463)	0.335 (0.236 to 0.463)	0.335 (0.236 to 0.463)	0.335 (0.236 to 0.463)	0.335 (0.236 to 0.463)	0.335 (0.236 to 0.463)
0.332 (0.232 to 0.459)	0.332 (0.232 to 0.459)	0.332 (0.232 to 0.459)	0.332 (0.232 to 0.459)	0.332 (0.232 to 0.459)	0.332 (0.232 to 0.459)	0.332 (0.232 to 0.459)	0.332 (0.232 to 0.459)	0.332 (0.232 to 0.459)	0.332 (0.232 to 0.459)	0.332 (0.232 to 0.459)	0.332 (0.232 to 0.459)	0.332 (0.232 to 0.459)	0.332 (0.232 to 0.459)
1.0 (1.0 to 1.0)	1.0 (1.0 to 1.0)	1.0 (1.0 to 1.0)	1.0 (1.0 to 1.0)	1.0 (1.0 to 1.0)	1.0 (1.0 to 1.0)	1.0 (1.0 to 1.0)	1.0 (1.0 to 1.0)	1.0 (1.0 to 1.0)	1.0 (1.0 to 1.0)	1.0 (1.0 to 1.0)	1.0 (1.0 to 1.0)	1.0 (1.0 to 1.0)	1.0 (1.0 to 1.0)
1.0 (1.0 to 1.0)	1.0 (1.0 to 1.0)	1.0 (1.0 to 1.0)	1.0 (1.0 to 1.0)	1.0 (1.0 to 1.0)	1.0 (1.0 to 1.0)	1.0 (1.0 to 1.0)	1.0 (1.0 to 1.0)	1.0 (1.0 to 1.0)	1.0 (1.0 to 1.0)	1.0 (1.0 to 1.0)	1.0 (1.0 to 1.0)	1.0 (1.0 to 1.0)	1.0 (1.0 to 1.0)
1.565 (1.27 to 1.907)	1.565 (1.27 to 1.907)	1.565 (1.27 to 1.907)	1.565 (1.27 to 1.907)	1.565 (1.27 to 1.907)	1.565 (1.27 to 1.907)	1.565 (1.27 to 1.907)	1.565 (1.27 to 1.907)	1.565 (1.27 to 1.907)	1.565 (1.27 to 1.907)	1.565 (1.27 to 1.907)	1.565 (1.27 to 1.907)	1.565 (1.27 to 1.907)	1.565 (1.27 to 1.907)
1.567 (1.275 to 1.901)	1.567 (1.275 to 1.901)	1.567 (1.275 to 1.901)	1.567 (1.275 to 1.901)	1.567 (1.275 to 1.901)	1.567 (1.275 to 1.901)	1.567 (1.275 to 1.901)	1.567 (1.275 to 1.901)	1.567 (1.275 to 1.901)	1.567 (1.275 to 1.901)	1.567 (1.275 to 1.901)	1.567 (1.275 to 1.901)	1.567 (1.275 to 1.901)	1.567 (1.275 to 1.901)
1.0 (1.0 to 1.0)	1.0 (1.0 to 1.0)	1.0 (1.0 to 1.0)	1.0 (1.0 to 1.0)	1.0 (1.0 to 1.0)	1.0 (1.0 to 1.0)	1.0 (1.0 to 1.0)	1.0 (1.0 to 1.0)	1.0 (1.0 to 1.0)	1.0 (1.0 to 1.0)	1.0 (1.0 to 1.0)	1.0 (1.0 to 1.0)	1.0 (1.0 to 1.0)	1.0 (1.0 to 1.0)
1.0 (1.0 to 1.0)	1.0 (1.0 to 1.0)	1.0 (1.0 to 1.0)	1.0 (1.0 to 1.0)	1.0 (1.0 to 1.0)	1.0 (1.0 to 1.0)	1.0 (1.0 to 1.0)	1.0 (1.0 to 1.0)	1.0 (1.0 to 1.0)	1.0 (1.0 to 1.0)	1.0 (1.0 to 1.0)	1.0 (1.0 to 1.0)	1.0 (1.0 to 1.0)	1.0 (1.0 to 1.0)
1.426 (1.094 to 1.84)	1.426 (1.094 to 1.84)	1.426 (1.094 to 1.84)	1.426 (1.094 to 1.84)	1.426 (1.094 to 1.84)	1.426 (1.094 to 1.84)	1.426 (1.094 to 1.84)	1.426 (1.094 to 1.84)	1.426 (1.094 to 1.84)	1.426 (1.094 to 1.84)	1.426 (1.094 to 1.84)	1.426 (1.094 to 1.84)	1.426 (1.094 to 1.84)	1.426 (1.094 to 1.84)
1.102 (0.954 to 1.275)	1.102 (0.954 to 1.275)	1.102 (0.954 to 1.275)	1.102 (0.954 to 1.275)	1.102 (0.954 to 1.275)	1.102 (0.954 to 1.275)	1.102 (0.954 to 1.275)	1.102 (0.954 to 1.275)	1.102 (0.954 to 1.275)	1.102 (0.954 to 1.275)	1.102 (0.954 to 1.275)	1.102 (0.954 to 1.275)	1.102 (0.954 to 1.275)	1.102 (0.954 to 1.275)
1.0 (1.0 to 1.0)	1.0 (1.0 to 1.0)	1.0 (1.0 to 1.0)	1.0 (1.0 to 1.0)	1.0 (1.0 to 1.0)	1.0 (1.0 to 1.0)	1.0 (1.0 to 1.0)	1.0 (1.0 to 1.0)	1.0 (1.0 to 1.0)	1.0 (1.0 to 1.0)	1.0 (1.0 to 1.0)	1.0 (1.0 to 1.0)	1.0 (1.0 to 1.0)	1.0 (1.0 to 1.0)
1.0 (1.0 to 1.0)	1.0 (1.0 to 1.0)	1.0 (1.0 to 1.0)	1.0 (1.0 to 1.0)	1.0 (1.0 to 1.0)	1.0 (1.0 to 1.0)	1.0 (1.0 to 1.0)	1.0 (1.0 to 1.0)	1.0 (1.0 to 1.0)	1.0 (1.0 to 1.0)	1.0 (1.0 to 1.0)	1.0 (1.0 to 1.0)	1.0 (1.0 to 1.0)	1.0 (1.0 to 1.0)
1.375 (1.143 to 1.651)	1.375 (1.143 to 1.651)	1.375 (1.143 to 1.651)	1.375 (1.143 to 1.651)	1.375 (1.143 to 1.651)	1.375 (1.143 to 1.651)	1.375 (1.143 to 1.651)	1.375 (1.143 to 1.651)	1.375 (1.143 to 1.651)	1.375 (1.143 to 1.651)	1.375 (1.143 to 1.651)	1.375 (1.143 to 1.651)	1.375 (1.143 to 1.651)	1.375 (1.143 to 1.651)
1.0 (1.0 to 1.0)	1.0 (1.0 to 1.0)	1.0 (1.0 to 1.0)	1.0 (1.0 to 1.0)	1.0 (1.0 to 1.0)	1.0 (1.0 to 1.0)	1.0 (1.0 to 1.0)	1.0 (1.0 to 1.0)	1.0 (1.0 to 1.0)	1.0 (1.0 to 1.0)	1.0 (1.0 to 1.0)	1.0 (1.0 to 1.0)	1.0 (1.0 to 1.0)	1.0 (1.0 to 1.0)
1.313 (1.103 to 1.561)	1.313 (1.103 to 1.561)	1.313 (1.103 to 1.561)	1.313 (1.103 to 1.561)	1.313 (1.103 to 1.561)	1.313 (1.103 to 1.561)	1.313 (1.103 to 1.561)	1.313 (1.103 to 1.561)	1.313 (1.103 to 1.561)	1.313 (1.103 to 1.561)	1.313 (1.103 to 1.561)	1.313 (1.103 to 1.561)	1.313 (1.103 to 1.561)	1.313 (1.103 to 1.561)
1.317 (1.105 to 1.539)	1.317 (1.105 to 1.539)	1.317 (1.105 to 1.539)	1.317 (1.105 to 1.539)	1.317 (1.105 to 1.539)	1.317 (1.105 to 1.539)	1.317 (1.105 to 1.539)	1.317 (1.105 to 1.539)	1.317 (1.105 to 1.539)	1.317 (1.105 to 1.539)	1.317 (1.105 to 1.539)	1.317 (1.105 to 1.539)	1.317 (1.105 to 1.539)	1.317 (1.105 to 1.539)
1.0 (1.0 to 1.0)	1.0 (1.0 to 1.0)	1.0 (1.0 to 1.0)	1.0 (1.0 to 1.0)	1.0 (1.0 to 1.0)	1.0 (1.0 to 1.0)	1.0 (1.0 to 1.0)	1.0 (1.0 to 1.0)	1.0 (1.0 to 1.0)	1.0 (1.0 to 1.0)	1.0 (1.0 to 1.0)	1.0 (1.0 to 1.0)	1.0 (1.0 to 1.0)	1.0 (1.0 to 1.0)
1.0 (1.0 to 1.0)	1.0 (1.0 to 1.0)	1.0 (1.0 to 1.0)	1.0 (1.0 to 1.0)	1.0 (1.0 to 1.0)	1.0 (1.0 to 1.0)	1.0 (1.0 to 1.0)	1.0 (1.0 to 1.0)	1.0 (1.0 to 1.0)	1.0 (1.0 to 1.0)	1.0 (1.0 to 1.0)	1.0 (1.0 to 1.0)	1.0 (1.0 to 1.0)	1.0 (1.0 to 1.0)
1.671 (1.48 to 1.874)	1.671 (1.48 to 1.874)	1.671 (1.48 to 1.874)	1.671 (1.48 to 1.874)	1.671 (1.48 to 1.874)	1.671 (1.48 to 1.874)	1.671 (1.48 to 1.874)	1.671 (1.48 to 1.874)	1.671 (1.48 to 1.874)	1.671 (1.48 to 1.874)	1.671 (1.48 to 1.874)	1.671 (1.48 to 1.874)	1.671 (1.48 to 1.874)	1.671 (1.48 to 1.874)
1.0 (1.0 to 1.0)	1.0 (1.0 to 1.0)	1.0 (1.0 to 1.0)	1.0 (1.0 to 1.0)	1.0 (1.0 to 1.0)	1.0 (1.0 to 1.0)	1.0 (1.0 to 1.0)	1.0 (1.0 to 1.0)	1.0 (1.0 to 1.0)	1.0 (1.0 to 1.0)	1.0 (1.0 to 1.0)	1.0 (1.0 to 1.0)	1.0 (1.0 to 1.0)	1.0 (1.0 to 1.0)
1.911 (1.265 to 2.735)	1.911 (1.265 to 2.735)	1.911 (1.265 to 2.735)	1.911 (1.265 to 2.735)	1.911 (1.265 to 2.735)	1.911 (1.265 to 2.735)	1.911 (1.265 to 2.735)	1.911 (1.265 to 2.735)	1.911 (1.265 to 2.735)	1.911 (1.265 to 2.735)	1.911 (1.265 to 2.735)	1.911 (1.265 to 2.735)	1.911 (1.265 to 2.735)	1.911 (1.265 to 2.735)
1.0 (1.0 to 1.0)	1.0 (1.0 to 1.0)	1.0 (1.0 to 1.0)	1.0 (1.0 to 1.0)	1.0 (1.0 to 1.0)	1.0 (1.0 to 1.0)	1.0 (1.0 to 1.0)	1.0 (1.0 to 1.0)	1.0 (1.0 to 1.0)	1.0 (1.0 to 1.0)	1.0 (1.0 to 1.0)	1.0 (1.0 to 1.0)	1.0 (1.0 to 1.0)	1.0 (1.0 to 1.0)
3.021 (2.015 to 4.47)	3.021 (2.015 to 4.47)	3.021 (2.015 to 4.47)	3.021 (2.015 to 4.47)	3.021 (2.015 to 4.47)	3.021 (2.015 to 4.47)	3.021 (2.015 to 4.47)	3.021 (2.015 to 4.47)	3.021 (2.015 to 4.47)	3.021 (2.015 to 4.47)	3.021 (2.015 to 4.47)	3.021 (2.015 to 4.47)	3.021 (2.015 to 4.47)	3.021 (2.015 to 4.47)
8.09 (5.463 to 11.767)	8.09 (5.463 to 11.767)	8.09 (5.463 to 11.767)	8.09 (5.463 to 11.767)	8.09 (5.463 to 11.767)	8.09 (5.463 to 11.767)	8.09 (5.463 to 11.767)	8.09 (5.463 to 11.767)	8.09 (5.463 to 11.767)	8.09 (5.463 to 11.767)	8.09 (5.463 to 11.767)	8.09 (5.463 to 11.767)	8.09 (5.463 to 11.767)	8.09 (5.463 to 11.767)
2.571 (1.854 to 3.416)	2.571 (1.854 to 3.416)	2.571 (1.854 to 3.416)	2.571 (1.854 to 3.416)	2.571 (1.854 to 3.416)	2.571 (1.854 to 3.416)	2.571 (1.854 to 3.416)	2.571 (1.854 to 3.416)	2.571 (1.854 to 3.416)	2.571 (1.854 to 3.416)	2.571 (1.854 to 3.416)	2.571 (1.854 to 3.416)	2.571 (1.854 to 3.416)	2.571 (1.854 to 3.416)

Appendix Table 1a. Relative risks used by age and sex for each outcome for all risk factors except for ambient air pollution and alcohol.

Risk - Outcome	Category / Units	Morbidity / Mortality	Sex	All-age	0-6 days	7-27 days	28-364 days	1-4 years	5-9 years	10-14 years	15-19 years	20-24 years	25-29 years
Otitis media	Exposed	Mortality	Both		1.37 (1.238 to 1.493)	1.37 (1.238 to 1.493)	1.37 (1.238 to 1.493)	1.37 (1.238 to 1.493)	1.37 (1.238 to 1.493)	1.37 (1.238 to 1.493)			
Otitis media	Not exposed	Morbidity	Both		1.0 (1.0 to 1.0)	1.0 (1.0 to 1.0)	1.0 (1.0 to 1.0)	1.0 (1.0 to 1.0)	1.0 (1.0 to 1.0)	1.0 (1.0 to 1.0)			
Otitis media	Not exposed	Mortality	Both		1.0 (1.0 to 1.0)	1.0 (1.0 to 1.0)	1.0 (1.0 to 1.0)	1.0 (1.0 to 1.0)	1.0 (1.0 to 1.0)	1.0 (1.0 to 1.0)			
Breast cancer	Exposed	Morbidity	Both										1.072 (1.017 to 1.126)
Breast cancer	Exposed	Mortality	Both										1.072 (1.017 to 1.126)
Breast cancer	Not exposed	Morbidity	Both										1.0 (1.0 to 1.0)
Breast cancer	Not exposed	Mortality	Both										1.0 (1.0 to 1.0)
Diabetes mellitus	Exposed	Morbidity	Both										1.337 (1.121 to 1.542)
Diabetes mellitus	Exposed	Mortality	Both										1.337 (1.121 to 1.542)
Diabetes mellitus	Not exposed	Morbidity	Both										1.0 (1.0 to 1.0)
Diabetes mellitus	Not exposed	Mortality	Both										1.0 (1.0 to 1.0)
**Diet low in fruits**													
Lip and oral cavity cancer	100 g/day	Both	Both										1.042 (0.994 to 1.091)
Nasopharynx cancer	100 g/day	Both	Both										1.043 (0.992 to 1.092)
Other pharynx cancer	100 g/day	Both	Both										1.042 (0.996 to 1.095)
Oesophageal cancer	100 g/day	Both	Both										1.153 (1.033 to 1.288)
Larynx cancer	100 g/day	Both	Both										1.042 (0.995 to 1.095)
Tracheal, bronchus, and lung cancer	100 g/day	Both	Both										1.076 (1.031 to 1.123)
Ischaemic heart disease	100 g/day	Both	Both										1.254 (1.083 to 1.442)
Ischaemic stroke	100 g/day	Both	Both										2.024 (1.465 to 2.818)
Hemorrhagic stroke	100 g/day	Both	Both										1.688 (1.319 to 2.182)
Diabetes mellitus	100 g/day	Both	Both										1.125 (1.027 to 1.238)
**Diet low in vegetables**													
Ischaemic heart disease	100 g/day	Both	Both										1.249 (1.089 to 1.446)
Ischaemic stroke	100 g/day	Both	Both										1.249 (1.049 to 1.463)
Hemorrhagic stroke	100 g/day	Both	Both										1.177 (1.046 to 1.326)
**Diet low in legumes**													
Ischaemic heart disease	50 g/day	Both	Both										1.499 (1.18 to 1.89)
**Diet low in whole grains**													
Ischaemic heart disease	50 g/day	Both	Both										1.478 (1.274 to 1.722)

Ages													
**30-34 years**	**35-39 years**	**40-44 years**	**45-49 years**	**50-54 years**	**55-59 years**	**60-64 years**	**65-69 years**	**70-74 years**	**75-79 years**	**80-84 years**	**85-89 years**	**90-94 years**	**95+ years**
1.072<br>(1.017 to 1.126)	1.072<br>(1.017 to 1.126)	1.072<br>(1.017 to 1.126)	1.072<br>(1.017 to 1.126)	1.072<br>(1.017 to 1.126)	1.072<br>(1.017 to 1.126)	1.072<br>(1.017 to 1.126)	1.072<br>(1.017 to 1.126)	1.072<br>(1.017 to 1.126)	1.072<br>(1.017 to 1.126)	1.072<br>(1.017 to 1.126)	1.072<br>(1.017 to 1.126)	1.072<br>(1.017 to 1.126)	1.072<br>(1.017 to 1.126)
1.072<br>(1.017 to 1.126)	1.072<br>(1.017 to 1.126)	1.072<br>(1.017 to 1.126)	1.072<br>(1.017 to 1.126)	1.072<br>(1.017 to 1.126)	1.072<br>(1.017 to 1.126)	1.072<br>(1.017 to 1.126)	1.072<br>(1.017 to 1.126)	1.072<br>(1.017 to 1.126)	1.072<br>(1.017 to 1.126)	1.072<br>(1.017 to 1.126)	1.072<br>(1.017 to 1.126)	1.072<br>(1.017 to 1.126)	1.072<br>(1.017 to 1.126)
1.0<br>(1.0 to 1.0)	1.0<br>(1.0 to 1.0)	1.0<br>(1.0 to 1.0)	1.0<br>(1.0 to 1.0)	1.0<br>(1.0 to 1.0)	1.0<br>(1.0 to 1.0)	1.0<br>(1.0 to 1.0)	1.0<br>(1.0 to 1.0)	1.0<br>(1.0 to 1.0)	1.0<br>(1.0 to 1.0)	1.0<br>(1.0 to 1.0)	1.0<br>(1.0 to 1.0)	1.0<br>(1.0 to 1.0)	1.0<br>(1.0 to 1.0)
1.0<br>(1.0 to 1.0)	1.0<br>(1.0 to 1.0)	1.0<br>(1.0 to 1.0)	1.0<br>(1.0 to 1.0)	1.0<br>(1.0 to 1.0)	1.0<br>(1.0 to 1.0)	1.0<br>(1.0 to 1.0)	1.0<br>(1.0 to 1.0)	1.0<br>(1.0 to 1.0)	1.0<br>(1.0 to 1.0)	1.0<br>(1.0 to 1.0)	1.0<br>(1.0 to 1.0)	1.0<br>(1.0 to 1.0)	1.0<br>(1.0 to 1.0)
1.337<br>(1.121 to 1.542)	1.337<br>(1.121 to 1.542)	1.337<br>(1.121 to 1.542)	1.337<br>(1.121 to 1.542)	1.337<br>(1.121 to 1.542)	1.337<br>(1.121 to 1.542)	1.337<br>(1.121 to 1.542)	1.337<br>(1.121 to 1.542)	1.337<br>(1.121 to 1.542)	1.337<br>(1.121 to 1.542)	1.337<br>(1.121 to 1.542)	1.337<br>(1.121 to 1.542)	1.337<br>(1.121 to 1.542)	1.337<br>(1.121 to 1.542)
1.337<br>(1.121 to 1.542)	1.337<br>(1.121 to 1.542)	1.337<br>(1.121 to 1.542)	1.337<br>(1.121 to 1.542)	1.337<br>(1.121 to 1.542)	1.337<br>(1.121 to 1.542)	1.337<br>(1.121 to 1.542)	1.337<br>(1.121 to 1.542)	1.337<br>(1.121 to 1.542)	1.337<br>(1.121 to 1.542)	1.337<br>(1.121 to 1.542)	1.337<br>(1.121 to 1.542)	1.337<br>(1.121 to 1.542)	1.337<br>(1.121 to 1.542)
1.0<br>(1.0 to 1.0)	1.0<br>(1.0 to 1.0)	1.0<br>(1.0 to 1.0)	1.0<br>(1.0 to 1.0)	1.0<br>(1.0 to 1.0)	1.0<br>(1.0 to 1.0)	1.0<br>(1.0 to 1.0)	1.0<br>(1.0 to 1.0)	1.0<br>(1.0 to 1.0)	1.0<br>(1.0 to 1.0)	1.0<br>(1.0 to 1.0)	1.0<br>(1.0 to 1.0)	1.0<br>(1.0 to 1.0)	1.0<br>(1.0 to 1.0)
1.0<br>(1.0 to 1.0)	1.0<br>(1.0 to 1.0)	1.0<br>(1.0 to 1.0)	1.0<br>(1.0 to 1.0)	1.0<br>(1.0 to 1.0)	1.0<br>(1.0 to 1.0)	1.0<br>(1.0 to 1.0)	1.0<br>(1.0 to 1.0)	1.0<br>(1.0 to 1.0)	1.0<br>(1.0 to 1.0)	1.0<br>(1.0 to 1.0)	1.0<br>(1.0 to 1.0)	1.0<br>(1.0 to 1.0)	1.0<br>(1.0 to 1.0)
1.042<br>(0.994 to 1.091)	1.042<br>(0.994 to 1.091)	1.042<br>(0.994 to 1.091)	1.042<br>(0.994 to 1.091)	1.042<br>(0.994 to 1.091)	1.042<br>(0.994 to 1.091)	1.042<br>(0.994 to 1.091)	1.042<br>(0.994 to 1.091)	1.042<br>(0.994 to 1.091)	1.042<br>(0.994 to 1.091)	1.042<br>(0.994 to 1.091)	1.042<br>(0.994 to 1.091)	1.042<br>(0.994 to 1.091)	1.042<br>(0.994 to 1.091)
1.043<br>(0.992 to 1.092)	1.043<br>(0.992 to 1.092)	1.043<br>(0.992 to 1.092)	1.043<br>(0.992 to 1.092)	1.043<br>(0.992 to 1.092)	1.043<br>(0.992 to 1.092)	1.043<br>(0.992 to 1.092)	1.043<br>(0.992 to 1.092)	1.043<br>(0.992 to 1.092)	1.043<br>(0.992 to 1.092)	1.043<br>(0.992 to 1.092)	1.043<br>(0.992 to 1.092)	1.043<br>(0.992 to 1.092)	1.043<br>(0.992 to 1.092)
1.042<br>(0.996 to 1.095)	1.042<br>(0.996 to 1.095)	1.042<br>(0.996 to 1.095)	1.042<br>(0.996 to 1.095)	1.042<br>(0.996 to 1.095)	1.042<br>(0.996 to 1.095)	1.042<br>(0.996 to 1.095)	1.042<br>(0.996 to 1.095)	1.042<br>(0.996 to 1.095)	1.042<br>(0.996 to 1.095)	1.042<br>(0.996 to 1.095)	1.042<br>(0.996 to 1.095)	1.042<br>(0.996 to 1.095)	1.042<br>(0.996 to 1.095)
1.153<br>(1.033 to 1.288)	1.153<br>(1.033 to 1.288)	1.153<br>(1.033 to 1.288)	1.153<br>(1.033 to 1.288)	1.153<br>(1.033 to 1.288)	1.153<br>(1.033 to 1.288)	1.153<br>(1.033 to 1.288)	1.153<br>(1.033 to 1.288)	1.153<br>(1.033 to 1.288)	1.153<br>(1.033 to 1.288)	1.153<br>(1.033 to 1.288)	1.153<br>(1.033 to 1.288)	1.153<br>(1.033 to 1.288)	1.153<br>(1.033 to 1.288)
1.042<br>(0.995 to 1.095)	1.042<br>(0.995 to 1.095)	1.042<br>(0.995 to 1.095)	1.042<br>(0.995 to 1.095)	1.042<br>(0.995 to 1.095)	1.042<br>(0.995 to 1.095)	1.042<br>(0.995 to 1.095)	1.042<br>(0.995 to 1.095)	1.042<br>(0.995 to 1.095)	1.042<br>(0.995 to 1.095)	1.042<br>(0.995 to 1.095)	1.042<br>(0.995 to 1.095)	1.042<br>(0.995 to 1.095)	1.042<br>(0.995 to 1.095)
1.076<br>(1.031 to 1.123)	1.076<br>(1.031 to 1.123)	1.076<br>(1.031 to 1.123)	1.076<br>(1.031 to 1.123)	1.076<br>(1.031 to 1.123)	1.076<br>(1.031 to 1.123)	1.076<br>(1.031 to 1.123)	1.076<br>(1.031 to 1.123)	1.076<br>(1.031 to 1.123)	1.076<br>(1.031 to 1.123)	1.076<br>(1.031 to 1.123)	1.076<br>(1.031 to 1.123)	1.076<br>(1.031 to 1.123)	1.076<br>(1.031 to 1.123)
1.209<br>(1.07 to 1.361)	1.159<br>(1.054 to 1.271)	1.131<br>(1.045 to 1.221)	1.125<br>(1.043 to 1.211)	1.114<br>(1.039 to 1.193)	1.099<br>(1.034 to 1.167)	1.087<br>(1.03 to 1.146)	1.078<br>(1.027 to 1.13)	1.07<br>(1.025 to 1.117)	1.064<br>(1.022 to 1.106)	1.057<br>(1.02 to 1.095)	1.057<br>(1.02 to 1.095)	1.057<br>(1.02 to 1.095)	1.057<br>(1.02 to 1.095)
1.834<br>(1.39 to 2.444)	1.621<br>(1.301 to 2.043)	1.48<br>(1.239 to 1.787)	1.403<br>(1.204 to 1.653)	1.333<br>(1.171 to 1.533)	1.272<br>(1.142 to 1.432)	1.222<br>(1.116 to 1.348)	1.181<br>(1.096 to 1.283)	1.145<br>(1.078 to 1.225)	1.114<br>(1.061 to 1.175)	1.054<br>(1.029 to 1.082)	1.054<br>(1.029 to 1.082)	1.054<br>(1.029 to 1.082)	1.054<br>(1.029 to 1.082)
1.576<br>(1.273 to 1.972)	1.444<br>(1.215 to 1.732)	1.365<br>(1.18 to 1.595)	1.336<br>(1.167 to 1.544)	1.3<br>(1.15 to 1.483)	1.26<br>(1.131 to 1.415)	1.226<br>(1.115 to 1.358)	1.193<br>(1.099 to 1.305)	1.164<br>(1.084 to 1.256)	1.133<br>(1.069 to 1.207)	1.065<br>(1.034 to 1.1)	1.065<br>(1.034 to 1.1)	1.065<br>(1.034 to 1.1)	1.065<br>(1.034 to 1.1)
1.122<br>(1.026 to 1.232)	1.119<br>(1.026 to 1.226)	1.113<br>(1.024 to 1.214)	1.102<br>(1.022 to 1.194)	1.093<br>(1.02 to 1.176)	1.085<br>(1.019 to 1.16)	1.076<br>(1.017 to 1.143)	1.068<br>(1.015 to 1.128)	1.061<br>(1.014 to 1.114)	1.052<br>(1.012 to 1.098)	1.036<br>(1.008 to 1.066)	1.036<br>(1.008 to 1.066)	1.036<br>(1.008 to 1.066)	1.036<br>(1.008 to 1.066)
1.205<br>(1.074 to 1.362)	1.154<br>(1.057 to 1.269)	1.126<br>(1.047 to 1.219)	1.121<br>(1.045 to 1.21)	1.111<br>(1.042 to 1.193)	1.098<br>(1.037 to 1.168)	1.086<br>(1.032 to 1.148)	1.077<br>(1.029 to 1.133)	1.07<br>(1.027 to 1.12)	1.064<br>(1.024 to 1.109)	1.057<br>(1.022 to 1.097)	1.057<br>(1.022 to 1.097)	1.057<br>(1.022 to 1.097)	1.057<br>(1.022 to 1.097)
1.211<br>(1.042 to 1.388)	1.165<br>(1.033 to 1.3)	1.132<br>(1.027 to 1.238)	1.113<br>(1.023 to 1.203)	1.095<br>(1.02 to 1.17)	1.079<br>(1.017 to 1.141)	1.065<br>(1.014 to 1.116)	1.054<br>(1.012 to 1.096)	1.044<br>(1.009 to 1.077)	1.035<br>(1.007 to 1.061)	1.017<br>(1.004 to 1.029)	1.017<br>(1.004 to 1.029)	1.017<br>(1.004 to 1.029)	1.017<br>(1.004 to 1.029)
1.153<br>(1.04 to 1.278)	1.122<br>(1.032 to 1.22)	1.102<br>(1.027 to 1.184)	1.095<br>(1.025 to 1.17)	1.086<br>(1.023 to 1.153)	1.075<br>(1.02 to 1.134)	1.066<br>(1.018 to 1.117)	1.057<br>(1.015 to 1.101)	1.049<br>(1.013 to 1.086)	1.04<br>(1.011 to 1.071)	1.02<br>(1.005 to 1.035)	1.02<br>(1.005 to 1.035)	1.02<br>(1.005 to 1.035)	1.02<br>(1.005 to 1.035)
1.453<br>(1.166 to 1.801)	1.388<br>(1.144 to 1.677)	1.332<br>(1.125 to 1.573)	1.287<br>(1.11 to 1.49)	1.237<br>(1.092 to 1.401)	1.181<br>(1.071 to 1.303)	1.139<br>(1.055 to 1.23)	1.111<br>(1.045 to 1.183)	1.089<br>(1.036 to 1.146)	1.074<br>(1.03 to 1.12)	1.101<br>(1.041 to 1.165)	1.101<br>(1.041 to 1.165)	1.101<br>(1.041 to 1.165)	1.101<br>(1.041 to 1.165)
1.387<br>(1.225 to 1.578)	1.285<br>(1.168 to 1.418)	1.228<br>(1.136 to 1.333)	1.216<br>(1.129 to 1.313)	1.194<br>(1.117 to 1.281)	1.165<br>(1.1 to 1.238)	1.141<br>(1.086 to 1.203)	1.125<br>(1.076 to 1.179)	1.112<br>(1.068 to 1.16)	1.102<br>(1.062 to 1.145)	1.097<br>(1.059 to 1.138)	1.097<br>(1.059 to 1.138)	1.097<br>(1.059 to 1.138)	1.097<br>(1.059 to 1.138)

Appendix Table 1a. Relative risks used by age and sex for each outcome for all risk factors except for ambient air pollution and alcohol.

Risk - Outcome	Category / Units	Morbidity / Mortality	Sex	All-age	0-6 days	7-27 days	28-364 days	1-4 years	5-9 years	10-14 years	15-19 years	20-24 years	25-29 years
Ischaemic stroke	50 g/day	Both	Both										2.075 (1.669 to 2.517)
Hemorrhagic stroke	50 g/day	Both	Both										1.596 (1.406 to 1.825)
Diabetes mellitus	50 g/day	Both	Both										1.231 (1.125 to 1.349)
**Diet low in nuts and seeds**													
Ischaemic heart disease	4.05 g/day	Morbidity	Both										1.176 (1.055 to 1.322)
Ischaemic heart disease	4.05 g/day	Mortality	Both										1.209 (1.128 to 1.296)
Diabetes mellitus	4.05 g/day	Both	Both										1.05 (1.025 to 1.075)
**Diet low in milk**													
Colon and rectum cancer	226.8 g/day	Both	Both										1.113 (1.039 to 1.202)
**Diet high in red meat**													
Colon and rectum cancer	100 g/day	Both	Both										1.167 (1.033 to 1.309)
Diabetes mellitus	100 g/day	Both	Both										1.322 (1.037 to 1.603)
**Diet high in processed meat**													
Colon and rectum cancer	50 g/day	Both	Both										1.179 (1.093 to 1.267)
Ischaemic heart disease	50 g/day	Both	Both										2.568 (1.047 to 4.657)
Diabetes mellitus	50 g/day	Both	Both										1.94 (1.395 to 2.545)
**Diet high in sugar-sweetened beverages ***													
	BMI > 25	Both	Both										0.236 (0.146 to 0.36)
	BMI < 25	Both	Both										0.094 (0.055 to 0.149)
**Diet low in fibre**													
Colon and rectum cancer	20 g/day	Both	Both										1.236 (1.133 to 1.35)
Ischaemic heart disease	20 g/day	Both	Both										1.688 (1.415 to 2.028)
**Diet low in calcium**													
Colon and rectum cancer	1 g/day	Both	Both										1.372 (1.268 to 1.485)
**Diet low in seafood omega-3 fatty acids**													
Ischaemic heart disease	100 mg/day	Morbidity	Both										1.0 (1.0 to 1.0)
Ischaemic heart disease	100 mg/day	Mortality	Both										1.291 (1.109 to 1.505)
**Diet low in polyunsaturated fatty acids**													
Ischaemic heart disease	5% energy/day	Both	Both										1.267 (1.098 to 1.452)
**Diet high in trans fatty acids**													

Ages													
**30-34 years**	**35-39 years**	**40-44 years**	**45-49 years**	**50-54 years**	**55-59 years**	**60-64 years**	**65-69 years**	**70-74 years**	**75-79 years**	**80-84 years**	**85-89 years**	**90-94 years**	**95+ years**
1.863 (1.548 to 2.199)	1.624 (1.406 to 1.849)	1.466 (1.309 to 1.625)	1.38 (1.255 to 1.505)	1.304 (1.206 to 1.401)	1.241 (1.165 to 1.316)	1.189 (1.13 to 1.247)	1.15 (1.104 to 1.195)	1.117 (1.081 to 1.151)	1.09 (1.063 to 1.116)	1.041 (1.029 to 1.053)	1.041 (1.029 to 1.053)	1.041 (1.029 to 1.053)	1.041 (1.029 to 1.053)
1.484 (1.333 to 1.662)	1.349 (1.244 to 1.471)	1.276 (1.194 to 1.369)	1.258 (1.182 to 1.344)	1.232 (1.165 to 1.309)	1.201 (1.143 to 1.267)	1.176 (1.126 to 1.233)	1.15 (1.108 to 1.198)	1.128 (1.092 to 1.169)	1.106 (1.076 to 1.139)	1.05 (1.036 to 1.065)	1.05 (1.036 to 1.065)	1.05 (1.036 to 1.065)	1.05 (1.036 to 1.065)
1.226 (1.122 to 1.341)	1.22 (1.119 to 1.331)	1.208 (1.113 to 1.313)	1.189 (1.103 to 1.283)	1.172 (1.094 to 1.256)	1.156 (1.085 to 1.232)	1.139 (1.077 to 1.207)	1.125 (1.069 to 1.184)	1.111 (1.061 to 1.163)	1.095 (1.053 to 1.14)	1.064 (1.036 to 1.094)	1.064 (1.036 to 1.094)	1.064 (1.036 to 1.094)	1.064 (1.036 to 1.094)
1.143 (1.045 to 1.259)	1.105 (1.033 to 1.188)	1.084 (1.027 to 1.15)	1.081 (1.026 to 1.144)	1.074 (1.024 to 1.132)	1.064 (1.021 to 1.114)	1.056 (1.018 to 1.099)	1.05 (1.016 to 1.089)	1.046 (1.015 to 1.081)	1.042 (1.014 to 1.075)	1.039 (1.013 to 1.069)	1.039 (1.013 to 1.069)	1.039 (1.013 to 1.069)	1.039 (1.013 to 1.069)
1.169 (1.105 to 1.239)	1.124 (1.077 to 1.174)	1.099 (1.062 to 1.138)	1.095 (1.06 to 1.133)	1.088 (1.055 to 1.122)	1.076 (1.048 to 1.105)	1.066 (1.042 to 1.092)	1.059 (1.037 to 1.082)	1.054 (1.034 to 1.075)	1.05 (1.032 to 1.069)	1.046 (1.029 to 1.064)	1.046 (1.029 to 1.064)	1.046 (1.029 to 1.064)	1.046 (1.029 to 1.064)
1.049 (1.025 to 1.073)	1.048 (1.024 to 1.071)	1.045 (1.023 to 1.068)	1.041 (1.021 to 1.062)	1.038 (1.019 to 1.056)	1.035 (1.018 to 1.052)	1.031 (1.016 to 1.046)	1.028 (1.014 to 1.042)	1.025 (1.013 to 1.037)	1.022 (1.011 to 1.032)	1.015 (1.007 to 1.022)	1.015 (1.007 to 1.022)	1.015 (1.007 to 1.022)	1.015 (1.007 to 1.022)
1.113 (1.039 to 1.202)	1.113 (1.039 to 1.202)	1.113 (1.039 to 1.202)	1.113 (1.039 to 1.202)	1.113 (1.039 to 1.202)	1.113 (1.039 to 1.202)	1.113 (1.039 to 1.202)	1.113 (1.039 to 1.202)	1.113 (1.039 to 1.202)	1.113 (1.039 to 1.202)	1.113 (1.039 to 1.202)	1.113 (1.039 to 1.202)	1.113 (1.039 to 1.202)	1.113 (1.039 to 1.202)
1.167 (1.033 to 1.309)	1.167 (1.033 to 1.309)	1.167 (1.033 to 1.309)	1.167 (1.033 to 1.309)	1.167 (1.033 to 1.309)	1.167 (1.033 to 1.309)	1.167 (1.033 to 1.309)	1.167 (1.033 to 1.309)	1.167 (1.033 to 1.309)	1.167 (1.033 to 1.309)	1.167 (1.033 to 1.309)	1.167 (1.033 to 1.309)	1.167 (1.033 to 1.309)	1.167 (1.033 to 1.309)
1.314 (1.036 to 1.588)	1.305 (1.035 to 1.569)	1.288 (1.034 to 1.536)	1.26 (1.031 to 1.48)	1.236 (1.028 to 1.433)	1.213 (1.026 to 1.389)	1.19 (1.023 to 1.345)	1.169 (1.021 to 1.306)	1.15 (1.019 to 1.269)	1.128 (1.016 to 1.229)	1.086 (1.011 to 1.152)	1.086 (1.011 to 1.152)	1.086 (1.011 to 1.152)	1.086 (1.011 to 1.152)
1.179 (1.093 to 1.267)	1.179 (1.093 to 1.267)	1.179 (1.093 to 1.267)	1.179 (1.093 to 1.267)	1.179 (1.093 to 1.267)	1.179 (1.093 to 1.267)	1.179 (1.093 to 1.267)	1.179 (1.093 to 1.267)	1.179 (1.093 to 1.267)	1.179 (1.093 to 1.267)	1.179 (1.093 to 1.267)	1.179 (1.093 to 1.267)	1.179 (1.093 to 1.267)	1.179 (1.093 to 1.267)
2.124 (1.038 to 3.478)	1.72 (1.028 to 2.489)	1.545 (1.022 to 2.093)	1.547 (1.022 to 2.097)	1.52 (1.022 to 2.037)	1.467 (1.02 to 1.922)	1.422 (1.018 to 1.826)	1.386 (1.017 to 1.75)	1.354 (1.016 to 1.683)	1.325 (1.015 to 1.622)	1.252 (1.012 to 1.475)	1.252 (1.012 to 1.475)	1.252 (1.012 to 1.475)	1.252 (1.012 to 1.475)
1.913 (1.386 to 2.496)	1.881 (1.375 to 2.439)	1.824 (1.354 to 2.337)	1.731 (1.319 to 2.173)	1.653 (1.289 to 2.038)	1.583 (1.261 to 1.918)	1.512 (1.233 to 1.798)	1.45 (1.207 to 1.696)	1.393 (1.183 to 1.603)	1.332 (1.157 to 1.505)	1.216 (1.105 to 1.323)	1.216 (1.105 to 1.323)	1.216 (1.105 to 1.323)	1.216 (1.105 to 1.323)
0.236 (0.146 to 0.36)	0.236 (0.146 to 0.36)	0.236 (0.146 to 0.36)	0.236 (0.146 to 0.36)	0.236 (0.146 to 0.36)	0.236 (0.146 to 0.36)	0.236 (0.146 to 0.36)	0.236 (0.146 to 0.36)	0.236 (0.146 to 0.36)	0.236 (0.146 to 0.36)	0.236 (0.146 to 0.36)	0.236 (0.146 to 0.36)	0.236 (0.146 to 0.36)	0.236 (0.146 to 0.36)
0.094 (0.055 to 0.149)	0.094 (0.055 to 0.149)	0.094 (0.055 to 0.149)	0.094 (0.055 to 0.149)	0.094 (0.055 to 0.149)	0.094 (0.055 to 0.149)	0.094 (0.055 to 0.149)	0.094 (0.055 to 0.149)	0.094 (0.055 to 0.149)	0.094 (0.055 to 0.149)	0.094 (0.055 to 0.149)	0.094 (0.055 to 0.149)	0.094 (0.055 to 0.149)	0.094 (0.055 to 0.149)
1.236 (1.133 to 1.35)	1.236 (1.133 to 1.35)	1.236 (1.133 to 1.35)	1.236 (1.133 to 1.35)	1.236 (1.133 to 1.35)	1.236 (1.133 to 1.35)	1.236 (1.133 to 1.35)	1.236 (1.133 to 1.35)	1.236 (1.133 to 1.35)	1.236 (1.133 to 1.35)	1.236 (1.133 to 1.35)	1.236 (1.133 to 1.35)	1.236 (1.133 to 1.35)	1.236 (1.133 to 1.35)
1.622 (1.379 to 1.922)	1.529 (1.326 to 1.776)	1.45 (1.28 to 1.654)	1.387 (1.243 to 1.558)	1.318 (1.202 to 1.455)	1.242 (1.156 to 1.342)	1.184 (1.119 to 1.258)	1.147 (1.096 to 1.205)	1.118 (1.077 to 1.163)	1.097 (1.064 to 1.135)	1.133 (1.087 to 1.185)	1.133 (1.087 to 1.185)	1.133 (1.087 to 1.185)	1.133 (1.087 to 1.185)
1.372 (1.268 to 1.485)	1.372 (1.268 to 1.485)	1.372 (1.268 to 1.485)	1.372 (1.268 to 1.485)	1.372 (1.268 to 1.485)	1.372 (1.268 to 1.485)	1.372 (1.268 to 1.485)	1.372 (1.268 to 1.485)	1.372 (1.268 to 1.485)	1.372 (1.268 to 1.485)	1.372 (1.268 to 1.485)	1.372 (1.268 to 1.485)	1.372 (1.268 to 1.485)	1.372 (1.268 to 1.485)
1.0 (1.0 to 1.0)	1.0 (1.0 to 1.0)	1.0 (1.0 to 1.0)	1.0 (1.0 to 1.0)	1.0 (1.0 to 1.0)	1.0 (1.0 to 1.0)	1.0 (1.0 to 1.0)	1.0 (1.0 to 1.0)	1.0 (1.0 to 1.0)	1.0 (1.0 to 1.0)	1.0 (1.0 to 1.0)	1.0 (1.0 to 1.0)	1.0 (1.0 to 1.0)	1.0 (1.0 to 1.0)
1.249 (1.094 to 1.428)	1.199 (1.077 to 1.338)	1.173 (1.067 to 1.293)	1.165 (1.064 to 1.279)	1.154 (1.06 to 1.26)	1.14 (1.055 to 1.235)	1.126 (1.05 to 1.211)	1.113 (1.045 to 1.189)	1.101 (1.04 to 1.167)	1.088 (1.035 to 1.145)	1.062 (1.025 to 1.102)	1.062 (1.025 to 1.102)	1.062 (1.025 to 1.102)	1.062 (1.025 to 1.102)
1.211 (1.079 to 1.352)	1.148 (1.056 to 1.244)	1.114 (1.044 to 1.186)	1.111 (1.043 to 1.181)	1.101 (1.039 to 1.165)	1.086 (1.033 to 1.14)	1.075 (1.029 to 1.121)	1.068 (1.026 to 1.11)	1.063 (1.025 to 1.102)	1.06 (1.024 to 1.097)	1.063 (1.025 to 1.102)	1.063 (1.025 to 1.102)	1.063 (1.025 to 1.102)	1.063 (1.025 to 1.102)

Appendix Table 1a. Relative risks used by age and sex for each outcome for all risk factors except for ambient air pollution and alcohol.

Risk - Outcome	Category / Units	Morbidity / Mortality	Sex	All-age	0-6 days	7-27 days	28-364 days	1-4 years	5-9 years	10-14 years	15-19 years	20-24 years	25-29 years
Ischaemic heart disease	2% energy/day	Both	Both										1.901 (1.591 to 2.275)
**Diet high in sodium ****													
	Non-Black, Non-Hypertensive	Both	Both										-1.366 (-1.937 to -0.795)
	Non-Black, Hypertensive	Both	Both										-3.300 (-4.147 to -2.454)
	Black, Non-Hypertensive	Both	Both										-3.910 (-5.065 to -2.755)
	Black, Hypertensive	Both	Both										-5.844 (-7.222 to -4.467)
**Childhood sexual abuse**													
Alcohol use disorders	Exposed	Both	Both		1.55 (1.186 to 1.952)	1.549 (1.194 to 2.024)	1.551 (1.208 to 1.983)	1.55 (1.184 to 1.941)	1.551 (1.172 to 2.013)	1.55 (1.202 to 1.965)	1.553 (1.182 to 2.008)	1.545 (1.194 to 1.939)	1.557 (1.202 to 1.994)
Alcohol use disorders	Not exposed	Both	Both		1.0 (1.0 to 1.0)	1.0 (1.0 to 1.0)	1.0 (1.0 to 1.0)	1.0 (1.0 to 1.0)	1.0 (1.0 to 1.0)	1.0 (1.0 to 1.0)	1.0 (1.0 to 1.0)	1.0 (1.0 to 1.0)	1.0 (1.0 to 1.0)
Major depressive disorder	Exposed	Morbidity	Both		1.631 (1.405 to 1.877)	1.631 (1.422 to 1.881)	1.633 (1.409 to 1.893)	1.63 (1.404 to 1.896)	1.627 (1.396 to 1.863)	1.63 (1.407 to 1.881)	1.635 (1.401 to 1.894)	1.637 (1.41 to 1.879)	1.638 (1.42 to 1.888)
Major depressive disorder	Not exposed	Morbidity	Both		1.0 (1.0 to 1.0)	1.0 (1.0 to 1.0)	1.0 (1.0 to 1.0)	1.0 (1.0 to 1.0)	1.0 (1.0 to 1.0)	1.0 (1.0 to 1.0)	1.0 (1.0 to 1.0)	1.0 (1.0 to 1.0)	1.0 (1.0 to 1.0)
**Intimate partner violence (exposure approach)**													
Maternal abortion, miscarriage, and ectopic pregnancy	Exposed	Both	Both		1.971 (1.154 to 3.098)	1.96 (1.145 to 3.167)	1.949 (1.169 to 3.094)	1.981 (1.151 to 3.204)	1.981 (1.137 to 3.211)	1.967 (1.144 to 3.144)	1.945 (1.168 to 3.019)	1.997 (1.124 to 3.269)	1.977 (1.161 to 3.187)
Maternal abortion, miscarriage, and ectopic pregnancy	Not exposed	Both	Both		1.0 (1.0 to 1.0)	1.0 (1.0 to 1.0)	1.0 (1.0 to 1.0)	1.0 (1.0 to 1.0)	1.0 (1.0 to 1.0)	1.0 (1.0 to 1.0)	1.0 (1.0 to 1.0)	1.0 (1.0 to 1.0)	1.0 (1.0 to 1.0)
Major depressive disorder	Exposed	Morbidity	Both		1.474 (1.099 to 1.949)	1.456 (1.051 to 1.898)	1.464 (1.099 to 1.914)	1.454 (1.103 to 1.922)	1.457 (1.088 to 1.917)	1.46 (1.106 to 1.92)	1.454 (1.094 to 1.872)	1.455 (1.098 to 1.92)	1.459 (1.091 to 1.917)
Major depressive disorder	Not exposed	Morbidity	Both		1.0 (1.0 to 1.0)	1.0 (1.0 to 1.0)	1.0 (1.0 to 1.0)	1.0 (1.0 to 1.0)	1.0 (1.0 to 1.0)	1.0 (1.0 to 1.0)	1.0 (1.0 to 1.0)	1.0 (1.0 to 1.0)	1.0 (1.0 to 1.0)
**Low physical activity**													
Colon and rectum cancer	<600 METs	Both	Both										1.293 (1.211 to 1.381)
Colon and rectum cancer	600-3,999 METs	Both	Both										1.172 (1.095 to 1.26)
Colon and rectum cancer	4,000-7,999 METs	Both	Both										1.067 (0.966 to 1.18)
Colon and rectum cancer	>=8,000 METs	Both	Both										1.0 (1.0 to 1.0)
Breast cancer	<600 METs	Both	Both										1.159 (1.111 to 1.207)
Breast cancer	600-3,999 METs	Both	Both										1.12 (1.081 to 1.162)
Breast cancer	4,000-7,999 METs	Both	Both										1.09 (1.047 to 1.135)
Breast cancer	>=8,000 METs	Both	Both										1.0 (1.0 to 1.0)
Ischaemic heart disease	<600 METs	Both	Both										1.565 (1.398 to 1.74)
Ischaemic heart disease	600-3,999 METs	Both	Both										1.181 (1.063 to 1.309)
Ischaemic heart disease	4,000-7,999 METs	Both	Both										1.034 (0.878 to 1.206)
Ischaemic heart disease	>=8,000 METs	Both	Both										1.0 (1.0 to 1.0)
Ischaemic stroke	<600 METs	Both	Both										1.666 (1.412 to 1.988)

Ages													
**30-34 years**	**35-39 years**	**40-44 years**	**45-49 years**	**50-54 years**	**55-59 years**	**60-64 years**	**65-69 years**	**70-74 years**	**75-79 years**	**80-84 years**	**85-89 years**	**90-94 years**	**95+ years**
1.775 (1.514 to 2.085)	1.615 (1.415 to 1.848)	1.517 (1.352 to 1.707)	1.461 (1.316 to 1.627)	1.396 (1.274 to 1.535)	1.323 (1.225 to 1.433)	1.264 (1.186 to 1.352)	1.222 (1.157 to 1.294)	1.186 (1.132 to 1.246)	1.158 (1.112 to 1.207)	1.15 (1.107 to 1.197)	1.15 (1.107 to 1.197)	1.15 (1.107 to 1.197)	1.15 (1.107 to 1.197)
-1.882 (-2.434 to -1.330)	-2.397 (-2.967 to -1.828)	-2.913 (-3.533 to -2.292)	-3.428 (-4.126 to -2.730)	-3.944 (-4.738 to -3.150)	-4.459 (-5.362 to -3.556)	-4.975 (-5.995 to -3.954)	-5.490 (-6.634 to -4.347)	-5.490 (-6.634 to -4.347)	-5.490 (-6.634 to -4.347)	-5.490 (-6.634 to -4.347)	-5.490 (-6.634 to -4.347)	-5.490 (-6.634 to -4.347)	-5.490 (-6.634 to -4.347)
-3.816 (-4.547 to -3.085)	-4.331 (-4.959 to -3.704)	-4.847 (-5.389 to -4.305)	-5.363 (-5.848 to -4.877)	-5.878 (-6.346 to -5.411)	-6.394 (-6.886 to -5.901)	-6.909 (-7.464 to -6.354)	-7.425 (-8.069 to -6.781)	-7.425 (-8.069 to -6.781)	-7.425 (-8.069 to -6.781)	-7.425 (-8.069 to -6.781)	-7.425 (-8.069 to -6.781)	-7.425 (-8.069 to -6.781)	-7.425 (-8.069 to -6.781)
-4.426 (-5.564 to -3.287)	-4.941 (-6.081 to -3.802)	-5.457 (-6.616 to -4.298)	-5.972 (-7.168 to -4.777)	-6.488 (-7.735 to -5.241)	-7.004 (-8.316 to -5.691)	-7.519 (-8.909 to -6.129)	-8.035 (-9.512 to -6.557)	-8.035 (-9.512 to -6.557)	-8.035 (-9.512 to -6.557)	-8.035 (-9.512 to -6.557)	-8.035 (-9.512 to -6.557)	-8.035 (-9.512 to -6.557)	-8.035 (-9.512 to -6.557)
-6.360 (-7.663 to -5.057)	-6.876 (-8.117 to -5.635)	-7.391 (-8.584 to -6.198)	-7.907 (-9.068 to -6.745)	-8.422 (-9.569 to -7.275)	-8.938 (-10.088 to -7.788)	-9.453 (-10.624 to -8.282)	-9.969 (-11.178 to -8.760)	-9.969 (-11.178 to -8.760)	-9.969 (-11.178 to -8.760)	-9.969 (-11.178 to -8.760)	-9.969 (-11.178 to -8.760)	-9.969 (-11.178 to -8.760)	-9.969 (-11.178 to -8.760)
1.54 (1.167 to 2.047)	1.541 (1.184 to 1.953)	1.554 (1.187 to 1.97)	1.552 (1.196 to 1.997)	1.549 (1.188 to 2.0)	1.55 (1.183 to 1.971)	1.562 (1.206 to 1.992)	1.546 (1.206 to 1.973)	1.556 (1.19 to 1.971)	1.562 (1.179 to 1.982)	1.545 (1.187 to 1.961)	1.549 (1.2 to 1.967)	1.545 (1.211 to 1.967)	1.549 (1.19 to 1.944)
1.0 (1.0 to 1.0)	1.0 (1.0 to 1.0)	1.0 (1.0 to 1.0)	1.0 (1.0 to 1.0)	1.0 (1.0 to 1.0)	1.0 (1.0 to 1.0)	1.0 (1.0 to 1.0)	1.0 (1.0 to 1.0)	1.0 (1.0 to 1.0)	1.0 (1.0 to 1.0)	1.0 (1.0 to 1.0)	1.0 (1.0 to 1.0)	1.0 (1.0 to 1.0)	1.0 (1.0 to 1.0)
1.633 (1.416 to 1.876)	1.633 (1.416 to 1.874)	1.634 (1.417 to 1.882)	1.632 (1.411 to 1.91)	1.631 (1.416 to 1.889)	1.634 (1.402 to 1.883)	1.634 (1.408 to 1.866)	1.634 (1.422 to 1.886)	1.634 (1.412 to 1.878)	1.633 (1.406 to 1.886)	1.634 (1.419 to 1.899)	1.637 (1.412 to 1.856)	1.636 (1.401 to 1.877)	1.635 (1.402 to 1.903)
1.0 (1.0 to 1.0)	1.0 (1.0 to 1.0)	1.0 (1.0 to 1.0)	1.0 (1.0 to 1.0)	1.0 (1.0 to 1.0)	1.0 (1.0 to 1.0)	1.0 (1.0 to 1.0)	1.0 (1.0 to 1.0)	1.0 (1.0 to 1.0)	1.0 (1.0 to 1.0)	1.0 (1.0 to 1.0)	1.0 (1.0 to 1.0)	1.0 (1.0 to 1.0)	1.0 (1.0 to 1.0)
1.994 (1.154 to 3.119)	1.944 (1.128 to 3.04)	2.0 (1.175 to 3.159)	1.965 (1.175 to 3.202)	1.971 (1.156 to 3.107)	1.957 (1.132 to 3.217)	1.975 (1.164 to 3.09)	1.995 (1.146 to 3.147)	1.975 (1.154 to 3.189)	1.969 (1.188 to 3.11)	1.978 (1.176 to 3.148)	1.969 (1.192 to 3.065)	1.975 (1.149 to 3.087)	1.976 (1.136 to 3.152)
1.0 (1.0 to 1.0)	1.0 (1.0 to 1.0)	1.0 (1.0 to 1.0)	1.0 (1.0 to 1.0)	1.0 (1.0 to 1.0)	1.0 (1.0 to 1.0)	1.0 (1.0 to 1.0)	1.0 (1.0 to 1.0)	1.0 (1.0 to 1.0)	1.0 (1.0 to 1.0)	1.0 (1.0 to 1.0)	1.0 (1.0 to 1.0)	1.0 (1.0 to 1.0)	1.0 (1.0 to 1.0)
1.447 (1.079 to 1.885)	1.458 (1.099 to 1.921)	1.442 (1.068 to 1.924)	1.449 (1.061 to 1.942)	1.448 (1.104 to 1.875)	1.458 (1.111 to 1.905)	1.461 (1.083 to 1.92)	1.458 (1.094 to 1.928)	1.456 (1.09 to 1.912)	1.462 (1.097 to 1.914)	1.47 (1.116 to 1.892)	1.447 (1.061 to 1.898)	1.448 (1.064 to 1.879)	1.45 (1.081 to 1.915)
1.0 (1.0 to 1.0)	1.0 (1.0 to 1.0)	1.0 (1.0 to 1.0)	1.0 (1.0 to 1.0)	1.0 (1.0 to 1.0)	1.0 (1.0 to 1.0)	1.0 (1.0 to 1.0)	1.0 (1.0 to 1.0)	1.0 (1.0 to 1.0)	1.0 (1.0 to 1.0)	1.0 (1.0 to 1.0)	1.0 (1.0 to 1.0)	1.0 (1.0 to 1.0)	1.0 (1.0 to 1.0)
1.293 (1.211 to 1.381)	1.293 (1.211 to 1.381)	1.293 (1.211 to 1.381)	1.293 (1.211 to 1.381)	1.293 (1.211 to 1.381)	1.293 (1.211 to 1.381)	1.293 (1.211 to 1.381)	1.293 (1.211 to 1.381)	1.293 (1.211 to 1.381)	1.293 (1.211 to 1.381)	1.293 (1.211 to 1.381)	1.293 (1.211 to 1.381)	1.293 (1.211 to 1.381)	1.293 (1.211 to 1.381)
1.172 (1.095 to 1.26)	1.172 (1.095 to 1.26)	1.172 (1.095 to 1.26)	1.172 (1.095 to 1.26)	1.172 (1.095 to 1.26)	1.172 (1.095 to 1.26)	1.172 (1.095 to 1.26)	1.172 (1.095 to 1.26)	1.172 (1.095 to 1.26)	1.172 (1.095 to 1.26)	1.172 (1.095 to 1.26)	1.172 (1.095 to 1.26)	1.172 (1.095 to 1.26)	1.172 (1.095 to 1.26)
1.067 (0.966 to 1.18)	1.067 (0.966 to 1.18)	1.067 (0.966 to 1.18)	1.067 (0.966 to 1.18)	1.067 (0.966 to 1.18)	1.067 (0.966 to 1.18)	1.067 (0.966 to 1.18)	1.067 (0.966 to 1.18)	1.067 (0.966 to 1.18)	1.067 (0.966 to 1.18)	1.067 (0.966 to 1.18)	1.067 (0.966 to 1.18)	1.067 (0.966 to 1.18)	1.067 (0.966 to 1.18)
1.0 (1.0 to 1.0)	1.0 (1.0 to 1.0)	1.0 (1.0 to 1.0)	1.0 (1.0 to 1.0)	1.0 (1.0 to 1.0)	1.0 (1.0 to 1.0)	1.0 (1.0 to 1.0)	1.0 (1.0 to 1.0)	1.0 (1.0 to 1.0)	1.0 (1.0 to 1.0)	1.0 (1.0 to 1.0)	1.0 (1.0 to 1.0)	1.0 (1.0 to 1.0)	1.0 (1.0 to 1.0)
1.159 (1.111 to 1.207)	1.159 (1.111 to 1.207)	1.159 (1.111 to 1.207)	1.159 (1.111 to 1.207)	1.159 (1.111 to 1.207)	1.159 (1.111 to 1.207)	1.159 (1.111 to 1.207)	1.159 (1.111 to 1.207)	1.159 (1.111 to 1.207)	1.159 (1.111 to 1.207)	1.159 (1.111 to 1.207)	1.159 (1.111 to 1.207)	1.159 (1.111 to 1.207)	1.159 (1.111 to 1.207)
1.12 (1.081 to 1.162)	1.12 (1.081 to 1.162)	1.12 (1.081 to 1.162)	1.12 (1.081 to 1.162)	1.12 (1.081 to 1.162)	1.12 (1.081 to 1.162)	1.12 (1.081 to 1.162)	1.12 (1.081 to 1.162)	1.12 (1.081 to 1.162)	1.12 (1.081 to 1.162)	1.12 (1.081 to 1.162)	1.12 (1.081 to 1.162)	1.12 (1.081 to 1.162)	1.12 (1.081 to 1.162)
1.09 (1.047 to 1.135)	1.09 (1.047 to 1.135)	1.09 (1.047 to 1.135)	1.09 (1.047 to 1.135)	1.09 (1.047 to 1.135)	1.09 (1.047 to 1.135)	1.09 (1.047 to 1.135)	1.09 (1.047 to 1.135)	1.09 (1.047 to 1.135)	1.09 (1.047 to 1.135)	1.09 (1.047 to 1.135)	1.09 (1.047 to 1.135)	1.09 (1.047 to 1.135)	1.09 (1.047 to 1.135)
1.0 (1.0 to 1.0)	1.0 (1.0 to 1.0)	1.0 (1.0 to 1.0)	1.0 (1.0 to 1.0)	1.0 (1.0 to 1.0)	1.0 (1.0 to 1.0)	1.0 (1.0 to 1.0)	1.0 (1.0 to 1.0)	1.0 (1.0 to 1.0)	1.0 (1.0 to 1.0)	1.0 (1.0 to 1.0)	1.0 (1.0 to 1.0)	1.0 (1.0 to 1.0)	1.0 (1.0 to 1.0)
1.524 (1.37 to 1.684)	1.484 (1.344 to 1.63)	1.445 (1.317 to 1.578)	1.408 (1.292 to 1.528)	1.371 (1.267 to 1.479)	1.336 (1.242 to 1.431)	1.301 (1.218 to 1.385)	1.267 (1.194 to 1.341)	1.234 (1.171 to 1.298)	1.202 (1.148 to 1.256)	1.171 (1.125 to 1.216)	1.171 (1.125 to 1.216)	1.171 (1.125 to 1.216)	1.171 (1.125 to 1.216)
1.17 (1.059 to 1.288)	1.158 (1.056 to 1.268)	1.147 (1.052 to 1.248)	1.136 (1.048 to 1.229)	1.125 (1.044 to 1.209)	1.114 (1.04 to 1.19)	1.103 (1.037 to 1.172)	1.092 (1.033 to 1.153)	1.081 (1.029 to 1.135)	1.071 (1.026 to 1.117)	1.06 (1.022 to 1.1)	1.06 (1.022 to 1.1)	1.06 (1.022 to 1.1)	1.06 (1.022 to 1.1)
1.032 (0.885 to 1.192)	1.03 (0.891 to 1.179)	1.028 (0.898 to 1.166)	1.025 (0.905 to 1.154)	1.023 (0.912 to 1.141)	1.021 (0.919 to 1.129)	1.019 (0.926 to 1.116)	1.017 (0.933 to 1.104)	1.015 (0.941 to 1.092)	1.013 (0.948 to 1.08)	1.011 (0.955 to 1.068)	1.011 (0.955 to 1.068)	1.011 (0.955 to 1.068)	1.011 (0.955 to 1.068)
1.0 (1.0 to 1.0)	1.0 (1.0 to 1.0)	1.0 (1.0 to 1.0)	1.0 (1.0 to 1.0)	1.0 (1.0 to 1.0)	1.0 (1.0 to 1.0)	1.0 (1.0 to 1.0)	1.0 (1.0 to 1.0)	1.0 (1.0 to 1.0)	1.0 (1.0 to 1.0)	1.0 (1.0 to 1.0)	1.0 (1.0 to 1.0)	1.0 (1.0 to 1.0)	1.0 (1.0 to 1.0)
1.617 (1.383 to 1.91)	1.569 (1.356 to 1.834)	1.522 (1.328 to 1.761)	1.477 (1.302 to 1.691)	1.433 (1.276 to 1.624)	1.39 (1.25 to 1.56)	1.349 (1.225 to 1.498)	1.309 (1.2 to 1.439)	1.27 (1.176 to 1.382)	1.233 (1.153 to 1.327)	1.196 (1.129 to 1.275)	1.196 (1.129 to 1.275)	1.196 (1.129 to 1.275)	1.196 (1.129 to 1.275)

Appendix Table 1a. Relative risks used by age and sex for each outcome for all risk factors except for ambient air pollution and alcohol.

Risk - Outcome	Category / Units	Morbidity / Mortality	Sex	All-age	0-6 days	7-27 days	28-364 days	1-4 years	5-9 years	10-14 years	15-19 years	20-24 years	25-29 years
Ischaemic stroke	600-3,999 METs	Both	Both										1.255 (1.056 to 1.51)
Ischaemic stroke	4,000-7,999 METs	Both	Both										1.177 (0.879 to 1.53)
Ischaemic stroke	>=8,000 METs	Both	Both										1.0 (1.0 to 1.0)
Diabetes mellitus	<600 METs	Both	Both										1.387 (1.301 to 1.475)
Diabetes mellitus	600-3,999 METs	Both	Both										1.189 (1.12 to 1.263)
Diabetes mellitus	4,000-7,999 METs	Both	Both										1.037 (0.96 to 1.119)
Diabetes mellitus	>=8,000 METs	Both	Both										1.0 (1.0 to 1.0)
**High fasting plasma glucose (continuous)**													
Ischaemic heart disease	mmol/L	Both	Both										1.471 (1.147 to 2.099)
Ischaemic stroke	mmol/L	Both	Both										1.526 (1.11 to 2.227)
Hemorrhagic stroke	mmol/L	Both	Both										1.506 (1.112 to 2.221)
Chronic kidney disease due to diabetes mellitus	mmol/L	Both	Both										1.388 (1.272 to 1.512)
Chronic kidney disease due to hypertension	mmol/L	Both	Both										1.388 (1.272 to 1.512)
Chronic kidney disease due to glomerulonephritis	mmol/L	Both	Both										1.388 (1.272 to 1.512)
Chronic kidney disease due to other causes	mmol/L	Both	Both										1.388 (1.272 to 1.512)
**High fasting plasma glucose (categorical)**													
Drug-susceptible tuberculosis	Diabetic	Both	Both										2.73 (1.973 to 3.602)
Drug-susceptible tuberculosis	Not diabetic	Both	Both										1.0 (1.0 to 1.0)
Multidrug-resistant tuberculosis without extensive drug resistance	Diabetic	Both	Both										2.73 (1.973 to 3.602)
Multidrug-resistant tuberculosis without extensive drug resistance	Not diabetic	Both	Both										1.0 (1.0 to 1.0)
Extensively drug-resistant tuberculosis	Diabetic	Both	Both										2.73 (1.973 to 3.602)
Extensively drug-resistant tuberculosis	Not diabetic	Both	Both										1.0 (1.0 to 1.0)
Latent tuberculosis infection	Diabetic	Both	Both										2.73 (1.973 to 3.602)
Latent tuberculosis infection	Not diabetic	Both	Both										1.0 (1.0 to 1.0)
Colon and rectum cancer	Diabetic	Both	Males										1.527 (1.081 to 2.304)
Colon and rectum cancer	Diabetic	Both	Females										1.527 (1.086 to 2.315)
Colon and rectum cancer	Not diabetic	Both	Males										1.0 (1.0 to 1.0)
Colon and rectum cancer	Not diabetic	Both	Females										1.0 (1.0 to 1.0)
Liver cancer due to other causes	Diabetic	Both	Males										1.523 (1.093 to 2.3)
Liver cancer due to other causes	Diabetic	Both	Females										1.512 (1.083 to 2.293)

Ages													
**30-34 years**	**35-39 years**	**40-44 years**	**45-49 years**	**50-54 years**	**55-59 years**	**60-64 years**	**65-69 years**	**70-74 years**	**75-79 years**	**80-84 years**	**85-89 years**	**90-94 years**	**95+ years**
1.238 (1.053 to 1.474)	1.221 (1.049 to 1.439)	1.205 (1.046 to 1.404)	1.189 (1.043 to 1.371)	1.173 (1.039 to 1.338)	1.157 (1.036 to 1.306)	1.142 (1.033 to 1.274)	1.127 (1.029 to 1.244)	1.112 (1.026 to 1.214)	1.097 (1.023 to 1.185)	1.082 (1.02 to 1.157)	1.082 (1.02 to 1.157)	1.082 (1.02 to 1.157)	1.082 (1.02 to 1.157)
1.166 (0.886 to 1.492)	1.154 (0.892 to 1.455)	1.142 (0.899 to 1.419)	1.131 (0.906 to 1.384)	1.12 (0.913 to 1.35)	1.109 (0.92 to 1.317)	1.098 (0.927 to 1.284)	1.088 (0.934 to 1.252)	1.077 (0.941 to 1.222)	1.067 (0.948 to 1.191)	1.057 (0.955 to 1.162)	1.057 (0.955 to 1.162)	1.057 (0.955 to 1.162)	1.057 (0.955 to 1.162)
1.0 (1.0 to 1.0)	1.0 (1.0 to 1.0)	1.0 (1.0 to 1.0)	1.0 (1.0 to 1.0)	1.0 (1.0 to 1.0)	1.0 (1.0 to 1.0)	1.0 (1.0 to 1.0)	1.0 (1.0 to 1.0)	1.0 (1.0 to 1.0)	1.0 (1.0 to 1.0)	1.0 (1.0 to 1.0)	1.0 (1.0 to 1.0)	1.0 (1.0 to 1.0)	1.0 (1.0 to 1.0)
1.387 (1.301 to 1.475)	1.387 (1.301 to 1.475)	1.387 (1.301 to 1.475)	1.387 (1.301 to 1.475)	1.387 (1.301 to 1.475)	1.387 (1.301 to 1.475)	1.387 (1.301 to 1.475)	1.387 (1.301 to 1.475)	1.387 (1.301 to 1.475)	1.387 (1.301 to 1.475)	1.387 (1.301 to 1.475)	1.387 (1.301 to 1.475)	1.387 (1.301 to 1.475)	1.387 (1.301 to 1.475)
1.189 (1.12 to 1.263)	1.189 (1.12 to 1.263)	1.189 (1.12 to 1.263)	1.189 (1.12 to 1.263)	1.189 (1.12 to 1.263)	1.189 (1.12 to 1.263)	1.189 (1.12 to 1.263)	1.189 (1.12 to 1.263)	1.189 (1.12 to 1.263)	1.189 (1.12 to 1.263)	1.189 (1.12 to 1.263)	1.189 (1.12 to 1.263)	1.189 (1.12 to 1.263)	1.189 (1.12 to 1.263)
1.037 (0.96 to 1.119)	1.037 (0.96 to 1.119)	1.037 (0.96 to 1.119)	1.037 (0.96 to 1.119)	1.037 (0.96 to 1.119)	1.037 (0.96 to 1.119)	1.037 (0.96 to 1.119)	1.037 (0.96 to 1.119)	1.037 (0.96 to 1.119)	1.037 (0.96 to 1.119)	1.037 (0.96 to 1.119)	1.037 (0.96 to 1.119)	1.037 (0.96 to 1.119)	1.037 (0.96 to 1.119)
1.0 (1.0 to 1.0)	1.0 (1.0 to 1.0)	1.0 (1.0 to 1.0)	1.0 (1.0 to 1.0)	1.0 (1.0 to 1.0)	1.0 (1.0 to 1.0)	1.0 (1.0 to 1.0)	1.0 (1.0 to 1.0)	1.0 (1.0 to 1.0)	1.0 (1.0 to 1.0)	1.0 (1.0 to 1.0)	1.0 (1.0 to 1.0)	1.0 (1.0 to 1.0)	1.0 (1.0 to 1.0)
1.373 (1.153 to 1.741)	1.274 (1.13 to 1.451)	1.22 (1.085 to 1.365)	1.211 (1.111 to 1.327)	1.201 (1.121 to 1.297)	1.192 (1.123 to 1.269)	1.182 (1.111 to 1.267)	1.173 (1.085 to 1.271)	1.168 (1.08 to 1.273)	1.168 (1.097 to 1.264)	1.169 (1.071 to 1.316)	1.169 (1.071 to 1.316)	1.169 (1.071 to 1.316)	1.169 (1.071 to 1.316)
1.4 (1.101 to 1.85)	1.275 (1.081 to 1.561)	1.21 (1.044 to 1.441)	1.204 (1.075 to 1.383)	1.199 (1.095 to 1.334)	1.194 (1.108 to 1.302)	1.188 (1.101 to 1.295)	1.183 (1.069 to 1.308)	1.174 (1.056 to 1.309)	1.162 (1.075 to 1.295)	1.133 (1.055 to 1.332)	1.133 (1.055 to 1.332)	1.133 (1.055 to 1.332)	1.133 (1.055 to 1.332)
1.382 (1.111 to 1.843)	1.258 (1.085 to 1.487)	1.196 (1.053 to 1.371)	1.193 (1.082 to 1.333)	1.191 (1.106 to 1.298)	1.189 (1.115 to 1.262)	1.187 (1.115 to 1.252)	1.184 (1.097 to 1.264)	1.175 (1.087 to 1.263)	1.158 (1.09 to 1.233)	1.116 (1.056 to 1.25)	1.116 (1.056 to 1.25)	1.116 (1.056 to 1.25)	1.116 (1.056 to 1.25)
1.388 (1.272 to 1.512)	1.388 (1.272 to 1.512)	1.388 (1.272 to 1.512)	1.388 (1.272 to 1.512)	1.388 (1.272 to 1.512)	1.388 (1.272 to 1.512)	1.388 (1.272 to 1.512)	1.388 (1.272 to 1.512)	1.388 (1.272 to 1.512)	1.388 (1.272 to 1.512)	1.388 (1.272 to 1.512)	1.388 (1.272 to 1.512)	1.388 (1.272 to 1.512)	1.388 (1.272 to 1.512)
1.388 (1.272 to 1.512)	1.388 (1.272 to 1.512)	1.388 (1.272 to 1.512)	1.388 (1.272 to 1.512)	1.388 (1.272 to 1.512)	1.388 (1.272 to 1.512)	1.388 (1.272 to 1.512)	1.388 (1.272 to 1.512)	1.388 (1.272 to 1.512)	1.388 (1.272 to 1.512)	1.388 (1.272 to 1.512)	1.388 (1.272 to 1.512)	1.388 (1.272 to 1.512)	1.388 (1.272 to 1.512)
1.388 (1.272 to 1.512)	1.388 (1.272 to 1.512)	1.388 (1.272 to 1.512)	1.388 (1.272 to 1.512)	1.388 (1.272 to 1.512)	1.388 (1.272 to 1.512)	1.388 (1.272 to 1.512)	1.388 (1.272 to 1.512)	1.388 (1.272 to 1.512)	1.388 (1.272 to 1.512)	1.388 (1.272 to 1.512)	1.388 (1.272 to 1.512)	1.388 (1.272 to 1.512)	1.388 (1.272 to 1.512)
1.388 (1.272 to 1.512)	1.388 (1.272 to 1.512)	1.388 (1.272 to 1.512)	1.388 (1.272 to 1.512)	1.388 (1.272 to 1.512)	1.388 (1.272 to 1.512)	1.388 (1.272 to 1.512)	1.388 (1.272 to 1.512)	1.388 (1.272 to 1.512)	1.388 (1.272 to 1.512)	1.388 (1.272 to 1.512)	1.388 (1.272 to 1.512)	1.388 (1.272 to 1.512)	1.388 (1.272 to 1.512)
2.801 (2.056 to 3.658)	2.871 (2.047 to 3.698)	2.798 (1.97 to 3.63)	2.581 (1.91 to 3.268)	2.364 (1.817 to 2.943)	2.147 (1.692 to 2.673)	1.93 (1.485 to 2.44)	1.713 (1.231 to 2.317)	1.598 (1.125 to 2.239)	1.587 (1.184 to 2.115)	1.559 (1.182 to 2.174)	1.559 (1.182 to 2.174)	1.559 (1.182 to 2.174)	1.559 (1.182 to 2.174)
1.0 (1.0 to 1.0)	1.0 (1.0 to 1.0)	1.0 (1.0 to 1.0)	1.0 (1.0 to 1.0)	1.0 (1.0 to 1.0)	1.0 (1.0 to 1.0)	1.0 (1.0 to 1.0)	1.0 (1.0 to 1.0)	1.0 (1.0 to 1.0)	1.0 (1.0 to 1.0)	1.0 (1.0 to 1.0)	1.0 (1.0 to 1.0)	1.0 (1.0 to 1.0)	1.0 (1.0 to 1.0)
2.801 (2.056 to 3.658)	2.871 (2.047 to 3.698)	2.798 (1.97 to 3.63)	2.581 (1.91 to 3.268)	2.364 (1.817 to 2.943)	2.147 (1.692 to 2.673)	1.93 (1.485 to 2.44)	1.713 (1.231 to 2.317)	1.598 (1.125 to 2.239)	1.587 (1.184 to 2.115)	1.559 (1.182 to 2.174)	1.559 (1.182 to 2.174)	1.559 (1.182 to 2.174)	1.559 (1.182 to 2.174)
1.0 (1.0 to 1.0)	1.0 (1.0 to 1.0)	1.0 (1.0 to 1.0)	1.0 (1.0 to 1.0)	1.0 (1.0 to 1.0)	1.0 (1.0 to 1.0)	1.0 (1.0 to 1.0)	1.0 (1.0 to 1.0)	1.0 (1.0 to 1.0)	1.0 (1.0 to 1.0)	1.0 (1.0 to 1.0)	1.0 (1.0 to 1.0)	1.0 (1.0 to 1.0)	1.0 (1.0 to 1.0)
2.801 (2.056 to 3.658)	2.871 (2.047 to 3.698)	2.798 (1.97 to 3.63)	2.581 (1.91 to 3.268)	2.364 (1.817 to 2.943)	2.147 (1.692 to 2.673)	1.93 (1.485 to 2.44)	1.713 (1.231 to 2.317)	1.598 (1.125 to 2.239)	1.587 (1.184 to 2.115)	1.559 (1.182 to 2.174)	1.559 (1.182 to 2.174)	1.559 (1.182 to 2.174)	1.559 (1.182 to 2.174)
1.0 (1.0 to 1.0)	1.0 (1.0 to 1.0)	1.0 (1.0 to 1.0)	1.0 (1.0 to 1.0)	1.0 (1.0 to 1.0)	1.0 (1.0 to 1.0)	1.0 (1.0 to 1.0)	1.0 (1.0 to 1.0)	1.0 (1.0 to 1.0)	1.0 (1.0 to 1.0)	1.0 (1.0 to 1.0)	1.0 (1.0 to 1.0)	1.0 (1.0 to 1.0)	1.0 (1.0 to 1.0)
2.801 (2.056 to 3.658)	2.871 (2.047 to 3.698)	2.798 (1.97 to 3.63)	2.581 (1.91 to 3.268)	2.364 (1.817 to 2.943)	2.147 (1.692 to 2.673)	1.93 (1.485 to 2.44)	1.713 (1.231 to 2.317)	1.598 (1.125 to 2.239)	1.587 (1.184 to 2.115)	1.559 (1.182 to 2.174)	1.559 (1.182 to 2.174)	1.559 (1.182 to 2.174)	1.559 (1.182 to 2.174)
1.0 (1.0 to 1.0)	1.0 (1.0 to 1.0)	1.0 (1.0 to 1.0)	1.0 (1.0 to 1.0)	1.0 (1.0 to 1.0)	1.0 (1.0 to 1.0)	1.0 (1.0 to 1.0)	1.0 (1.0 to 1.0)	1.0 (1.0 to 1.0)	1.0 (1.0 to 1.0)	1.0 (1.0 to 1.0)	1.0 (1.0 to 1.0)	1.0 (1.0 to 1.0)	1.0 (1.0 to 1.0)
1.527 (1.081 to 2.304)	1.527 (1.081 to 2.304)	1.527 (1.081 to 2.304)	1.527 (1.081 to 2.304)	1.527 (1.081 to 2.304)	1.527 (1.081 to 2.304)	1.527 (1.081 to 2.304)	1.527 (1.081 to 2.304)	1.527 (1.081 to 2.304)	1.527 (1.081 to 2.304)	1.527 (1.081 to 2.304)	1.527 (1.081 to 2.304)	1.527 (1.081 to 2.304)	1.527 (1.081 to 2.304)
1.527 (1.086 to 2.315)	1.527 (1.086 to 2.315)	1.527 (1.086 to 2.315)	1.527 (1.086 to 2.315)	1.527 (1.086 to 2.315)	1.527 (1.086 to 2.315)	1.527 (1.086 to 2.315)	1.527 (1.086 to 2.315)	1.527 (1.086 to 2.315)	1.527 (1.086 to 2.315)	1.527 (1.086 to 2.315)	1.527 (1.086 to 2.315)	1.527 (1.086 to 2.315)	1.527 (1.086 to 2.315)
1.0 (1.0 to 1.0)	1.0 (1.0 to 1.0)	1.0 (1.0 to 1.0)	1.0 (1.0 to 1.0)	1.0 (1.0 to 1.0)	1.0 (1.0 to 1.0)	1.0 (1.0 to 1.0)	1.0 (1.0 to 1.0)	1.0 (1.0 to 1.0)	1.0 (1.0 to 1.0)	1.0 (1.0 to 1.0)	1.0 (1.0 to 1.0)	1.0 (1.0 to 1.0)	1.0 (1.0 to 1.0)
1.0 (1.0 to 1.0)	1.0 (1.0 to 1.0)	1.0 (1.0 to 1.0)	1.0 (1.0 to 1.0)	1.0 (1.0 to 1.0)	1.0 (1.0 to 1.0)	1.0 (1.0 to 1.0)	1.0 (1.0 to 1.0)	1.0 (1.0 to 1.0)	1.0 (1.0 to 1.0)	1.0 (1.0 to 1.0)	1.0 (1.0 to 1.0)	1.0 (1.0 to 1.0)	1.0 (1.0 to 1.0)
1.523 (1.093 to 2.3)	1.523 (1.093 to 2.3)	1.523 (1.093 to 2.3)	1.523 (1.093 to 2.3)	1.523 (1.093 to 2.3)	1.523 (1.093 to 2.3)	1.523 (1.093 to 2.3)	1.523 (1.093 to 2.3)	1.523 (1.093 to 2.3)	1.523 (1.093 to 2.3)	1.523 (1.093 to 2.3)	1.523 (1.093 to 2.3)	1.523 (1.093 to 2.3)	1.523 (1.093 to 2.3)
1.512 (1.083 to 2.293)	1.512 (1.083 to 2.293)	1.512 (1.083 to 2.293)	1.512 (1.083 to 2.293)	1.512 (1.083 to 2.293)	1.512 (1.083 to 2.293)	1.512 (1.083 to 2.293)	1.512 (1.083 to 2.293)	1.512 (1.083 to 2.293)	1.512 (1.083 to 2.293)	1.512 (1.083 to 2.293)	1.512 (1.083 to 2.293)	1.512 (1.083 to 2.293)	1.512 (1.083 to 2.293)

Appendix Table 1a. Relative risks used by age and sex for each outcome for all risk factors except for ambient air pollution and alcohol.

Risk - Outcome	Category / Units	Morbidity / Mortality	Sex	All-age	0-6 days	7-27 days	28-364 days	1-4 years	5-9 years	10-14 years	15-19 years	20-24 years	25-29 years
Liver cancer due to other causes	Not diabetic	Both	Males										1.0 (1.0 to 1.0)
Liver cancer due to other causes	Not diabetic	Both	Females										1.0 (1.0 to 1.0)
Pancreatic cancer	Diabetic	Both	Males										1.517 (1.085 to 2.314)
Pancreatic cancer	Diabetic	Both	Females										1.518 (1.075 to 2.311)
Pancreatic cancer	Not diabetic	Both	Males										1.0 (1.0 to 1.0)
Pancreatic cancer	Not diabetic	Both	Females										1.0 (1.0 to 1.0)
Tracheal, bronchus, and lung cancer	Diabetic	Both	Males										1.515 (1.076 to 2.309)
Tracheal, bronchus, and lung cancer	Diabetic	Both	Females										1.512 (1.087 to 2.299)
Tracheal, bronchus, and lung cancer	Not diabetic	Both	Males										1.0 (1.0 to 1.0)
Tracheal, bronchus, and lung cancer	Not diabetic	Both	Females										1.0 (1.0 to 1.0)
Breast cancer	Diabetic	Both	Both										1.513 (1.087 to 2.206)
Breast cancer	Not diabetic	Both	Both										1.0 (1.0 to 1.0)
Ovarian cancer	Diabetic	Both	Both										1.522 (1.092 to 2.32)
Ovarian cancer	Not diabetic	Both	Both										1.0 (1.0 to 1.0)
Bladder cancer	Diabetic	Both	Males										1.514 (1.076 to 2.256)
Bladder cancer	Diabetic	Both	Females										1.511 (1.082 to 2.253)
Bladder cancer	Not diabetic	Both	Males										1.0 (1.0 to 1.0)
Bladder cancer	Not diabetic	Both	Females										1.0 (1.0 to 1.0)
Peripheral vascular disease	Diabetic	Both	Both										8.264 (6.044 to 9.303)
Peripheral vascular disease	Not diabetic	Both	Both										1.0 (1.0 to 1.0)
Alzheimer's disease and other dementias	Diabetic	Both	Males										1.516 (1.084 to 2.295)
Alzheimer's disease and other dementias	Diabetic	Both	Females										1.52 (1.08 to 2.301)
Alzheimer's disease and other dementias	Not diabetic	Both	Males										1.0 (1.0 to 1.0)
Alzheimer's disease and other dementias	Not diabetic	Both	Females										1.0 (1.0 to 1.0)
Glaucoma	Diabetic	Both	Males										1.52 (1.098 to 2.325)
Glaucoma	Diabetic	Both	Females										1.516 (1.08 to 2.328)
Glaucoma	Not diabetic	Both	Males										1.0 (1.0 to 1.0)
Glaucoma	Not diabetic	Both	Females										1.0 (1.0 to 1.0)
Cataract	Diabetic	Both	Males										1.52 (1.09 to 2.259)
Cataract	Diabetic	Both	Females										1.522 (1.094 to 2.289)

Ages													
**30-34 years**	**35-39 years**	**40-44 years**	**45-49 years**	**50-54 years**	**55-59 years**	**60-64 years**	**65-69 years**	**70-74 years**	**75-79 years**	**80-84 years**	**85-89 years**	**90-94 years**	**95+ years**
1.0 (1.0 to 1.0)	1.0 (1.0 to 1.0)	1.0 (1.0 to 1.0)	1.0 (1.0 to 1.0)	1.0 (1.0 to 1.0)	1.0 (1.0 to 1.0)	1.0 (1.0 to 1.0)	1.0 (1.0 to 1.0)	1.0 (1.0 to 1.0)	1.0 (1.0 to 1.0)	1.0 (1.0 to 1.0)	1.0 (1.0 to 1.0)	1.0 (1.0 to 1.0)	1.0 (1.0 to 1.0)
1.0 (1.0 to 1.0)	1.0 (1.0 to 1.0)	1.0 (1.0 to 1.0)	1.0 (1.0 to 1.0)	1.0 (1.0 to 1.0)	1.0 (1.0 to 1.0)	1.0 (1.0 to 1.0)	1.0 (1.0 to 1.0)	1.0 (1.0 to 1.0)	1.0 (1.0 to 1.0)	1.0 (1.0 to 1.0)	1.0 (1.0 to 1.0)	1.0 (1.0 to 1.0)	1.0 (1.0 to 1.0)
1.517 (1.085 to 2.314)	1.517 (1.085 to 2.314)	1.517 (1.085 to 2.314)	1.517 (1.085 to 2.314)	1.517 (1.085 to 2.314)	1.517 (1.085 to 2.314)	1.517 (1.085 to 2.314)	1.517 (1.085 to 2.314)	1.517 (1.085 to 2.314)	1.517 (1.085 to 2.314)	1.517 (1.085 to 2.314)	1.517 (1.085 to 2.314)	1.517 (1.085 to 2.314)	1.517 (1.085 to 2.314)
1.518 (1.075 to 2.311)	1.518 (1.075 to 2.311)	1.518 (1.075 to 2.311)	1.518 (1.075 to 2.311)	1.518 (1.075 to 2.311)	1.518 (1.075 to 2.311)	1.518 (1.075 to 2.311)	1.518 (1.075 to 2.311)	1.518 (1.075 to 2.311)	1.518 (1.075 to 2.311)	1.518 (1.075 to 2.311)	1.518 (1.075 to 2.311)	1.518 (1.075 to 2.311)	1.518 (1.075 to 2.311)
1.0 (1.0 to 1.0)	1.0 (1.0 to 1.0)	1.0 (1.0 to 1.0)	1.0 (1.0 to 1.0)	1.0 (1.0 to 1.0)	1.0 (1.0 to 1.0)	1.0 (1.0 to 1.0)	1.0 (1.0 to 1.0)	1.0 (1.0 to 1.0)	1.0 (1.0 to 1.0)	1.0 (1.0 to 1.0)	1.0 (1.0 to 1.0)	1.0 (1.0 to 1.0)	1.0 (1.0 to 1.0)
1.0 (1.0 to 1.0)	1.0 (1.0 to 1.0)	1.0 (1.0 to 1.0)	1.0 (1.0 to 1.0)	1.0 (1.0 to 1.0)	1.0 (1.0 to 1.0)	1.0 (1.0 to 1.0)	1.0 (1.0 to 1.0)	1.0 (1.0 to 1.0)	1.0 (1.0 to 1.0)	1.0 (1.0 to 1.0)	1.0 (1.0 to 1.0)	1.0 (1.0 to 1.0)	1.0 (1.0 to 1.0)
1.515 (1.076 to 2.309)	1.515 (1.076 to 2.309)	1.515 (1.076 to 2.309)	1.515 (1.076 to 2.309)	1.515 (1.076 to 2.309)	1.515 (1.076 to 2.309)	1.515 (1.076 to 2.309)	1.515 (1.076 to 2.309)	1.515 (1.076 to 2.309)	1.515 (1.076 to 2.309)	1.515 (1.076 to 2.309)	1.515 (1.076 to 2.309)	1.515 (1.076 to 2.309)	1.515 (1.076 to 2.309)
1.512 (1.087 to 2.299)	1.512 (1.087 to 2.299)	1.512 (1.087 to 2.299)	1.512 (1.087 to 2.299)	1.512 (1.087 to 2.299)	1.512 (1.087 to 2.299)	1.512 (1.087 to 2.299)	1.512 (1.087 to 2.299)	1.512 (1.087 to 2.299)	1.512 (1.087 to 2.299)	1.512 (1.087 to 2.299)	1.512 (1.087 to 2.299)	1.512 (1.087 to 2.299)	1.512 (1.087 to 2.299)
1.0 (1.0 to 1.0)	1.0 (1.0 to 1.0)	1.0 (1.0 to 1.0)	1.0 (1.0 to 1.0)	1.0 (1.0 to 1.0)	1.0 (1.0 to 1.0)	1.0 (1.0 to 1.0)	1.0 (1.0 to 1.0)	1.0 (1.0 to 1.0)	1.0 (1.0 to 1.0)	1.0 (1.0 to 1.0)	1.0 (1.0 to 1.0)	1.0 (1.0 to 1.0)	1.0 (1.0 to 1.0)
1.0 (1.0 to 1.0)	1.0 (1.0 to 1.0)	1.0 (1.0 to 1.0)	1.0 (1.0 to 1.0)	1.0 (1.0 to 1.0)	1.0 (1.0 to 1.0)	1.0 (1.0 to 1.0)	1.0 (1.0 to 1.0)	1.0 (1.0 to 1.0)	1.0 (1.0 to 1.0)	1.0 (1.0 to 1.0)	1.0 (1.0 to 1.0)	1.0 (1.0 to 1.0)	1.0 (1.0 to 1.0)
1.513 (1.087 to 2.206)	1.513 (1.087 to 2.206)	1.513 (1.087 to 2.206)	1.513 (1.087 to 2.206)	1.513 (1.087 to 2.206)	1.513 (1.087 to 2.206)	1.513 (1.087 to 2.206)	1.513 (1.087 to 2.206)	1.513 (1.087 to 2.206)	1.513 (1.087 to 2.206)	1.513 (1.087 to 2.206)	1.513 (1.087 to 2.206)	1.513 (1.087 to 2.206)	1.513 (1.087 to 2.206)
1.0 (1.0 to 1.0)	1.0 (1.0 to 1.0)	1.0 (1.0 to 1.0)	1.0 (1.0 to 1.0)	1.0 (1.0 to 1.0)	1.0 (1.0 to 1.0)	1.0 (1.0 to 1.0)	1.0 (1.0 to 1.0)	1.0 (1.0 to 1.0)	1.0 (1.0 to 1.0)	1.0 (1.0 to 1.0)	1.0 (1.0 to 1.0)	1.0 (1.0 to 1.0)	1.0 (1.0 to 1.0)
1.522 (1.092 to 2.32)	1.522 (1.092 to 2.32)	1.522 (1.092 to 2.32)	1.522 (1.092 to 2.32)	1.522 (1.092 to 2.32)	1.522 (1.092 to 2.32)	1.522 (1.092 to 2.32)	1.522 (1.092 to 2.32)	1.522 (1.092 to 2.32)	1.522 (1.092 to 2.32)	1.522 (1.092 to 2.32)	1.522 (1.092 to 2.32)	1.522 (1.092 to 2.32)	1.522 (1.092 to 2.32)
1.0 (1.0 to 1.0)	1.0 (1.0 to 1.0)	1.0 (1.0 to 1.0)	1.0 (1.0 to 1.0)	1.0 (1.0 to 1.0)	1.0 (1.0 to 1.0)	1.0 (1.0 to 1.0)	1.0 (1.0 to 1.0)	1.0 (1.0 to 1.0)	1.0 (1.0 to 1.0)	1.0 (1.0 to 1.0)	1.0 (1.0 to 1.0)	1.0 (1.0 to 1.0)	1.0 (1.0 to 1.0)
1.514 (1.076 to 2.256)	1.514 (1.076 to 2.256)	1.514 (1.076 to 2.256)	1.514 (1.076 to 2.256)	1.514 (1.076 to 2.256)	1.514 (1.076 to 2.256)	1.514 (1.076 to 2.256)	1.514 (1.076 to 2.256)	1.514 (1.076 to 2.256)	1.514 (1.076 to 2.256)	1.514 (1.076 to 2.256)	1.514 (1.076 to 2.256)	1.514 (1.076 to 2.256)	1.514 (1.076 to 2.256)
1.511 (1.082 to 2.253)	1.511 (1.082 to 2.253)	1.511 (1.082 to 2.253)	1.511 (1.082 to 2.253)	1.511 (1.082 to 2.253)	1.511 (1.082 to 2.253)	1.511 (1.082 to 2.253)	1.511 (1.082 to 2.253)	1.511 (1.082 to 2.253)	1.511 (1.082 to 2.253)	1.511 (1.082 to 2.253)	1.511 (1.082 to 2.253)	1.511 (1.082 to 2.253)	1.511 (1.082 to 2.253)
1.0 (1.0 to 1.0)	1.0 (1.0 to 1.0)	1.0 (1.0 to 1.0)	1.0 (1.0 to 1.0)	1.0 (1.0 to 1.0)	1.0 (1.0 to 1.0)	1.0 (1.0 to 1.0)	1.0 (1.0 to 1.0)	1.0 (1.0 to 1.0)	1.0 (1.0 to 1.0)	1.0 (1.0 to 1.0)	1.0 (1.0 to 1.0)	1.0 (1.0 to 1.0)	1.0 (1.0 to 1.0)
1.0 (1.0 to 1.0)	1.0 (1.0 to 1.0)	1.0 (1.0 to 1.0)	1.0 (1.0 to 1.0)	1.0 (1.0 to 1.0)	1.0 (1.0 to 1.0)	1.0 (1.0 to 1.0)	1.0 (1.0 to 1.0)	1.0 (1.0 to 1.0)	1.0 (1.0 to 1.0)	1.0 (1.0 to 1.0)	1.0 (1.0 to 1.0)	1.0 (1.0 to 1.0)	1.0 (1.0 to 1.0)
6.651 (5.375 to 7.452)	5.039 (4.436 to 5.673)	4.138 (3.561 to 4.76)	3.947 (3.447 to 4.504)	3.756 (3.347 to 4.232)	3.565 (3.214 to 3.972)	3.374 (3.079 to 3.714)	3.183 (2.92 to 3.466)	2.992 (2.76 to 3.25)	2.801 (2.558 to 3.05)	2.324 (1.977 to 2.671)	2.324 (1.977 to 2.671)	2.324 (1.977 to 2.671)	2.324 (1.977 to 2.671)
1.0 (1.0 to 1.0)	1.0 (1.0 to 1.0)	1.0 (1.0 to 1.0)	1.0 (1.0 to 1.0)	1.0 (1.0 to 1.0)	1.0 (1.0 to 1.0)	1.0 (1.0 to 1.0)	1.0 (1.0 to 1.0)	1.0 (1.0 to 1.0)	1.0 (1.0 to 1.0)	1.0 (1.0 to 1.0)	1.0 (1.0 to 1.0)	1.0 (1.0 to 1.0)	1.0 (1.0 to 1.0)
1.516 (1.084 to 2.295)	1.516 (1.084 to 2.295)	1.516 (1.084 to 2.295)	1.516 (1.084 to 2.295)	1.516 (1.084 to 2.295)	1.516 (1.084 to 2.295)	1.516 (1.084 to 2.295)	1.516 (1.084 to 2.295)	1.516 (1.084 to 2.295)	1.516 (1.084 to 2.295)	1.516 (1.084 to 2.295)	1.516 (1.084 to 2.295)	1.516 (1.084 to 2.295)	1.516 (1.084 to 2.295)
1.52 (1.08 to 2.301)	1.52 (1.08 to 2.301)	1.52 (1.08 to 2.301)	1.52 (1.08 to 2.301)	1.52 (1.08 to 2.301)	1.52 (1.08 to 2.301)	1.52 (1.08 to 2.301)	1.52 (1.08 to 2.301)	1.52 (1.08 to 2.301)	1.52 (1.08 to 2.301)	1.52 (1.08 to 2.301)	1.52 (1.08 to 2.301)	1.52 (1.08 to 2.301)	1.52 (1.08 to 2.301)
1.0 (1.0 to 1.0)	1.0 (1.0 to 1.0)	1.0 (1.0 to 1.0)	1.0 (1.0 to 1.0)	1.0 (1.0 to 1.0)	1.0 (1.0 to 1.0)	1.0 (1.0 to 1.0)	1.0 (1.0 to 1.0)	1.0 (1.0 to 1.0)	1.0 (1.0 to 1.0)	1.0 (1.0 to 1.0)	1.0 (1.0 to 1.0)	1.0 (1.0 to 1.0)	1.0 (1.0 to 1.0)
1.0 (1.0 to 1.0)	1.0 (1.0 to 1.0)	1.0 (1.0 to 1.0)	1.0 (1.0 to 1.0)	1.0 (1.0 to 1.0)	1.0 (1.0 to 1.0)	1.0 (1.0 to 1.0)	1.0 (1.0 to 1.0)	1.0 (1.0 to 1.0)	1.0 (1.0 to 1.0)	1.0 (1.0 to 1.0)	1.0 (1.0 to 1.0)	1.0 (1.0 to 1.0)	1.0 (1.0 to 1.0)
1.52 (1.098 to 2.325)	1.52 (1.098 to 2.325)	1.52 (1.098 to 2.325)	1.52 (1.098 to 2.325)	1.52 (1.098 to 2.325)	1.52 (1.098 to 2.325)	1.52 (1.098 to 2.325)	1.52 (1.098 to 2.325)	1.52 (1.098 to 2.325)	1.52 (1.098 to 2.325)	1.52 (1.098 to 2.325)	1.52 (1.098 to 2.325)	1.52 (1.098 to 2.325)	1.52 (1.098 to 2.325)
1.516 (1.08 to 2.328)	1.516 (1.08 to 2.328)	1.516 (1.08 to 2.328)	1.516 (1.08 to 2.328)	1.516 (1.08 to 2.328)	1.516 (1.08 to 2.328)	1.516 (1.08 to 2.328)	1.516 (1.08 to 2.328)	1.516 (1.08 to 2.328)	1.516 (1.08 to 2.328)	1.516 (1.08 to 2.328)	1.516 (1.08 to 2.328)	1.516 (1.08 to 2.328)	1.516 (1.08 to 2.328)
1.0 (1.0 to 1.0)	1.0 (1.0 to 1.0)	1.0 (1.0 to 1.0)	1.0 (1.0 to 1.0)	1.0 (1.0 to 1.0)	1.0 (1.0 to 1.0)	1.0 (1.0 to 1.0)	1.0 (1.0 to 1.0)	1.0 (1.0 to 1.0)	1.0 (1.0 to 1.0)	1.0 (1.0 to 1.0)	1.0 (1.0 to 1.0)	1.0 (1.0 to 1.0)	1.0 (1.0 to 1.0)
1.0 (1.0 to 1.0)	1.0 (1.0 to 1.0)	1.0 (1.0 to 1.0)	1.0 (1.0 to 1.0)	1.0 (1.0 to 1.0)	1.0 (1.0 to 1.0)	1.0 (1.0 to 1.0)	1.0 (1.0 to 1.0)	1.0 (1.0 to 1.0)	1.0 (1.0 to 1.0)	1.0 (1.0 to 1.0)	1.0 (1.0 to 1.0)	1.0 (1.0 to 1.0)	1.0 (1.0 to 1.0)
1.52 (1.09 to 2.259)	1.52 (1.09 to 2.259)	1.52 (1.09 to 2.259)	1.52 (1.09 to 2.259)	1.52 (1.09 to 2.259)	1.52 (1.09 to 2.259)	1.52 (1.09 to 2.259)	1.52 (1.09 to 2.259)	1.52 (1.09 to 2.259)	1.52 (1.09 to 2.259)	1.52 (1.09 to 2.259)	1.52 (1.09 to 2.259)	1.52 (1.09 to 2.259)	1.52 (1.09 to 2.259)
1.522 (1.094 to 2.289)	1.522 (1.094 to 2.289)	1.522 (1.094 to 2.289)	1.522 (1.094 to 2.289)	1.522 (1.094 to 2.289)	1.522 (1.094 to 2.289)	1.522 (1.094 to 2.289)	1.522 (1.094 to 2.289)	1.522 (1.094 to 2.289)	1.522 (1.094 to 2.289)	1.522 (1.094 to 2.289)	1.522 (1.094 to 2.289)	1.522 (1.094 to 2.289)	1.522 (1.094 to 2.289)

Appendix Table 1a. Relative risks used by age and sex for each outcome for all risk factors except for ambient air pollution and alcohol.

Risk - Outcome	Category / Units	Morbidity / Mortality	Sex	All-age	0-6 days	7-27 days	28-364 days	1-4 years	5-9 years	10-14 years	15-19 years	20-24 years	25-29 years
Cataract	Not diabetic	Both	Males										1.0 (1.0 to 1.0)
Cataract	Not diabetic	Both	Females										1.0 (1.0 to 1.0)
**High total cholesterol**													
Ischaemic heart disease	mmol/L	Both	Both										2.016 (1.684 to 2.544)
Ischaemic stroke	mmol/L	Both	Both										1.67 (1.334 to 2.339)
**High systolic blood pressure**													
Rheumatic heart disease	10 mmHg	Both	Both										1.631 (1.174 to 2.306)
Ischaemic heart disease	10 mmHg	Both	Both										1.972 (1.44 to 2.596)
Ischaemic stroke	10 mmHg	Both	Both										1.854 (1.395 to 2.588)
Hemorrhagic stroke	10 mmHg	Both	Both										2.134 (1.555 to 2.919)
Hypertensive heart disease	10 mmHg	Both	Both										2.862 (1.829 to 4.108)
Other cardiomyopathy	10 mmHg	Both	Both										1.755 (1.266 to 2.423)
Atrial fibrillation and flutter	10 mmHg	Both	Both										1.76 (1.336 to 2.43)
Aortic aneurysm	10 mmHg	Both	Both										1.544 (1.259 to 2.164)
Peripheral vascular disease	10 mmHg	Both	Both										1.728 (1.203 to 2.428)
Endocarditis	10 mmHg	Both	Both										1.755 (1.266 to 2.423)
Other cardiovascular and circulatory diseases	10 mmHg	Both	Both										1.744 (1.339 to 2.396)
Chronic kidney disease due to diabetes mellitus	10 mmHg	Both	Both										1.283 (1.186 to 1.397)
Chronic kidney disease due to hypertension	10 mmHg	Both	Both										1.281 (1.18 to 1.385)
Chronic kidney disease due to glomerulonephritis	10 mmHg	Both	Both										1.281 (1.182 to 1.383)
Chronic kidney disease due to other causes	10 mmHg	Both	Both										1.282 (1.181 to 1.395)
**High body-mass index**													
Oesophageal cancer	5 kg/m2	Both	Males									1.391 (1.077 to 1.754)	1.391 (1.077 to 1.754)
Oesophageal cancer	5 kg/m2	Both	Females									1.351 (1.012 to 1.745)	1.351 (1.012 to 1.745)
Colon and rectum cancer	5 kg/m2	Both	Males									1.177 (1.145 to 1.208)	1.177 (1.145 to 1.208)
Colon and rectum cancer	5 kg/m2	Both	Females									1.059 (1.031 to 1.083)	1.059 (1.031 to 1.083)
Liver cancer due to hepatitis B	5 kg/m2	Both	Males									1.289 (1.109 to 1.491)	1.289 (1.109 to 1.491)
Liver cancer due to hepatitis B	5 kg/m2	Both	Females									1.176 (1.03 to 1.334)	1.176 (1.03 to 1.334)
Liver cancer due to hepatitis C	5 kg/m2	Both	Males									1.289 (1.109 to 1.491)	1.289 (1.109 to 1.491)
Liver cancer due to hepatitis C	5 kg/m2	Both	Females									1.176 (1.03 to 1.334)	1.176 (1.03 to 1.334)

Ages													
**30-34 years**	**35-39 years**	**40-44 years**	**45-49 years**	**50-54 years**	**55-59 years**	**60-64 years**	**65-69 years**	**70-74 years**	**75-79 years**	**80-84 years**	**85-89 years**	**90-94 years**	**95+ years**
1.0 (1.0 to 1.0)	1.0 (1.0 to 1.0)	1.0 (1.0 to 1.0)	1.0 (1.0 to 1.0)	1.0 (1.0 to 1.0)	1.0 (1.0 to 1.0)	1.0 (1.0 to 1.0)	1.0 (1.0 to 1.0)	1.0 (1.0 to 1.0)	1.0 (1.0 to 1.0)	1.0 (1.0 to 1.0)	1.0 (1.0 to 1.0)	1.0 (1.0 to 1.0)	1.0 (1.0 to 1.0)
1.0 (1.0 to 1.0)	1.0 (1.0 to 1.0)	1.0 (1.0 to 1.0)	1.0 (1.0 to 1.0)	1.0 (1.0 to 1.0)	1.0 (1.0 to 1.0)	1.0 (1.0 to 1.0)	1.0 (1.0 to 1.0)	1.0 (1.0 to 1.0)	1.0 (1.0 to 1.0)	1.0 (1.0 to 1.0)	1.0 (1.0 to 1.0)	1.0 (1.0 to 1.0)	1.0 (1.0 to 1.0)
2.027 (1.768 to 2.354)	2.038 (1.831 to 2.273)	1.971 (1.775 to 2.191)	1.828 (1.676 to 2.004)	1.685 (1.561 to 1.815)	1.541 (1.446 to 1.648)	1.398 (1.306 to 1.494)	1.254 (1.141 to 1.372)	1.193 (1.088 to 1.312)	1.213 (1.124 to 1.321)	1.262 (1.11 to 1.465)	1.262 (1.11 to 1.465)	1.262 (1.11 to 1.465)	1.262 (1.11 to 1.465)
1.626 (1.352 to 2.041)	1.583 (1.342 to 1.849)	1.518 (1.287 to 1.76)	1.434 (1.242 to 1.636)	1.35 (1.212 to 1.514)	1.265 (1.164 to 1.391)	1.181 (1.109 to 1.299)	1.096 (1.043 to 1.223)	1.062 (1.008 to 1.193)	1.077 (1.012 to 1.216)	1.116 (1.014 to 1.344)	1.116 (1.014 to 1.344)	1.116 (1.014 to 1.344)	1.116 (1.014 to 1.344)
1.474 (1.17 to 1.898)	1.317 (1.144 to 1.575)	1.229 (1.089 to 1.422)	1.211 (1.101 to 1.367)	1.193 (1.107 to 1.328)	1.175 (1.101 to 1.287)	1.157 (1.086 to 1.265)	1.139 (1.055 to 1.248)	1.127 (1.048 to 1.241)	1.12 (1.06 to 1.238)	1.104 (1.04 to 1.28)	1.104 (1.04 to 1.28)	1.104 (1.04 to 1.28)	1.104 (1.04 to 1.28)
1.818 (1.458 to 2.207)	1.665 (1.461 to 1.911)	1.568 (1.398 to 1.799)	1.527 (1.393 to 1.705)	1.487 (1.385 to 1.619)	1.446 (1.368 to 1.535)	1.405 (1.332 to 1.488)	1.364 (1.257 to 1.456)	1.33 (1.224 to 1.424)	1.303 (1.225 to 1.404)	1.266 (1.134 to 1.437)	1.266 (1.134 to 1.437)	1.266 (1.134 to 1.437)	1.266 (1.134 to 1.437)
1.774 (1.427 to 2.252)	1.694 (1.404 to 2.035)	1.628 (1.354 to 1.95)	1.574 (1.36 to 1.823)	1.521 (1.361 to 1.698)	1.468 (1.344 to 1.596)	1.414 (1.302 to 1.524)	1.361 (1.214 to 1.49)	1.318 (1.168 to 1.451)	1.284 (1.179 to 1.389)	1.201 (1.109 to 1.37)	1.201 (1.109 to 1.37)	1.201 (1.109 to 1.37)	1.201 (1.109 to 1.37)
2.05 (1.593 to 2.648)	1.966 (1.589 to 2.465)	1.874 (1.492 to 2.302)	1.775 (1.484 to 2.114)	1.676 (1.446 to 1.932)	1.577 (1.402 to 1.754)	1.478 (1.331 to 1.619)	1.379 (1.207 to 1.54)	1.323 (1.162 to 1.495)	1.311 (1.193 to 1.45)	1.279 (1.126 to 1.515)	1.279 (1.126 to 1.515)	1.279 (1.126 to 1.515)	1.279 (1.126 to 1.515)
2.838 (1.857 to 4.187)	2.814 (1.802 to 4.337)	2.703 (1.762 to 4.186)	2.504 (1.804 to 3.758)	2.304 (1.772 to 3.464)	2.105 (1.645 to 3.336)	1.905 (1.447 to 3.171)	1.706 (1.188 to 3.215)	1.619 (1.053 to 3.136)	1.644 (1.078 to 3.091)	1.708 (1.103 to 3.258)	1.708 (1.103 to 3.258)	1.708 (1.103 to 3.258)	1.708 (1.103 to 3.258)
1.605 (1.293 to 2.011)	1.455 (1.278 to 1.642)	1.365 (1.232 to 1.51)	1.335 (1.222 to 1.449)	1.306 (1.219 to 1.394)	1.276 (1.212 to 1.342)	1.247 (1.183 to 1.303)	1.217 (1.131 to 1.284)	1.193 (1.116 to 1.263)	1.175 (1.12 to 1.237)	1.128 (1.071 to 1.235)	1.128 (1.071 to 1.235)	1.128 (1.071 to 1.235)	1.128 (1.071 to 1.235)
1.631 (1.379 to 2.026)	1.503 (1.396 to 1.644)	1.423 (1.34 to 1.505)	1.392 (1.328 to 1.457)	1.361 (1.313 to 1.411)	1.33 (1.293 to 1.369)	1.299 (1.265 to 1.333)	1.268 (1.233 to 1.308)	1.237 (1.202 to 1.277)	1.208 (1.177 to 1.238)	1.134 (1.092 to 1.185)	1.134 (1.092 to 1.185)	1.134 (1.092 to 1.185)	1.134 (1.092 to 1.185)
1.469 (1.29 to 1.816)	1.394 (1.3 to 1.535)	1.345 (1.227 to 1.451)	1.321 (1.233 to 1.405)	1.296 (1.229 to 1.362)	1.272 (1.218 to 1.327)	1.248 (1.191 to 1.299)	1.223 (1.16 to 1.286)	1.2 (1.137 to 1.261)	1.177 (1.126 to 1.229)	1.119 (1.071 to 1.184)	1.119 (1.071 to 1.184)	1.119 (1.071 to 1.184)	1.119 (1.071 to 1.184)
1.491 (1.206 to 1.87)	1.254 (1.182 to 1.329)	1.138 (1.019 to 1.263)	1.142 (1.047 to 1.243)	1.146 (1.071 to 1.224)	1.15 (1.094 to 1.208)	1.154 (1.11 to 1.199)	1.159 (1.113 to 1.207)	1.152 (1.104 to 1.201)	1.136 (1.098 to 1.176)	1.095 (1.054 to 1.154)	1.095 (1.054 to 1.154)	1.095 (1.054 to 1.154)	1.095 (1.054 to 1.154)
1.605 (1.293 to 2.011)	1.455 (1.278 to 1.642)	1.365 (1.232 to 1.51)	1.335 (1.222 to 1.449)	1.306 (1.219 to 1.394)	1.276 (1.212 to 1.342)	1.247 (1.183 to 1.303)	1.217 (1.131 to 1.284)	1.193 (1.116 to 1.263)	1.175 (1.12 to 1.237)	1.128 (1.071 to 1.235)	1.128 (1.071 to 1.235)	1.128 (1.071 to 1.235)	1.128 (1.071 to 1.235)
1.624 (1.384 to 2.006)	1.504 (1.405 to 1.626)	1.427 (1.354 to 1.498)	1.395 (1.336 to 1.452)	1.363 (1.318 to 1.406)	1.33 (1.296 to 1.365)	1.298 (1.266 to 1.332)	1.265 (1.231 to 1.303)	1.235 (1.201 to 1.27)	1.207 (1.177 to 1.235)	1.137 (1.095 to 1.187)	1.137 (1.095 to 1.187)	1.137 (1.095 to 1.187)	1.137 (1.095 to 1.187)
1.283 (1.186 to 1.397)	1.283 (1.186 to 1.397)	1.283 (1.186 to 1.397)	1.283 (1.186 to 1.397)	1.283 (1.186 to 1.397)	1.283 (1.186 to 1.397)	1.283 (1.186 to 1.397)	1.283 (1.186 to 1.397)	1.283 (1.186 to 1.397)	1.283 (1.186 to 1.397)	1.283 (1.186 to 1.397)	1.283 (1.186 to 1.397)	1.283 (1.186 to 1.397)	1.283 (1.186 to 1.397)
1.281 (1.18 to 1.385)	1.281 (1.18 to 1.385)	1.281 (1.18 to 1.385)	1.281 (1.18 to 1.385)	1.281 (1.18 to 1.385)	1.281 (1.18 to 1.385)	1.281 (1.18 to 1.385)	1.281 (1.18 to 1.385)	1.281 (1.18 to 1.385)	1.281 (1.18 to 1.385)	1.281 (1.18 to 1.385)	1.281 (1.18 to 1.385)	1.281 (1.18 to 1.385)	1.281 (1.18 to 1.385)
1.281 (1.182 to 1.383)	1.281 (1.182 to 1.383)	1.281 (1.182 to 1.383)	1.281 (1.182 to 1.383)	1.281 (1.182 to 1.383)	1.281 (1.182 to 1.383)	1.281 (1.182 to 1.383)	1.281 (1.182 to 1.383)	1.281 (1.182 to 1.383)	1.281 (1.182 to 1.383)	1.281 (1.182 to 1.383)	1.281 (1.182 to 1.383)	1.281 (1.182 to 1.383)	1.281 (1.182 to 1.383)
1.282 (1.181 to 1.395)	1.282 (1.181 to 1.395)	1.282 (1.181 to 1.395)	1.282 (1.181 to 1.395)	1.282 (1.181 to 1.395)	1.282 (1.181 to 1.395)	1.282 (1.181 to 1.395)	1.282 (1.181 to 1.395)	1.282 (1.181 to 1.395)	1.282 (1.181 to 1.395)	1.282 (1.181 to 1.395)	1.282 (1.181 to 1.395)	1.282 (1.181 to 1.395)	1.282 (1.181 to 1.395)
1.391 (1.077 to 1.754)	1.391 (1.077 to 1.754)	1.391 (1.077 to 1.754)	1.391 (1.077 to 1.754)	1.391 (1.077 to 1.754)	1.391 (1.077 to 1.754)	1.391 (1.077 to 1.754)	1.391 (1.077 to 1.754)	1.391 (1.077 to 1.754)	1.391 (1.077 to 1.754)	1.391 (1.077 to 1.754)	1.391 (1.077 to 1.754)	1.391 (1.077 to 1.754)	1.391 (1.077 to 1.754)
1.351 (1.012 to 1.745)	1.351 (1.012 to 1.745)	1.351 (1.012 to 1.745)	1.351 (1.012 to 1.745)	1.351 (1.012 to 1.745)	1.351 (1.012 to 1.745)	1.351 (1.012 to 1.745)	1.351 (1.012 to 1.745)	1.351 (1.012 to 1.745)	1.351 (1.012 to 1.745)	1.351 (1.012 to 1.745)	1.351 (1.012 to 1.745)	1.351 (1.012 to 1.745)	1.351 (1.012 to 1.745)
1.177 (1.145 to 1.208)	1.177 (1.145 to 1.208)	1.177 (1.145 to 1.208)	1.177 (1.145 to 1.208)	1.177 (1.145 to 1.208)	1.177 (1.145 to 1.208)	1.177 (1.145 to 1.208)	1.177 (1.145 to 1.208)	1.177 (1.145 to 1.208)	1.177 (1.145 to 1.208)	1.177 (1.145 to 1.208)	1.177 (1.145 to 1.208)	1.177 (1.145 to 1.208)	1.177 (1.145 to 1.208)
1.059 (1.031 to 1.083)	1.059 (1.031 to 1.083)	1.059 (1.031 to 1.083)	1.059 (1.031 to 1.083)	1.059 (1.031 to 1.083)	1.059 (1.031 to 1.083)	1.059 (1.031 to 1.083)	1.059 (1.031 to 1.083)	1.059 (1.031 to 1.083)	1.059 (1.031 to 1.083)	1.059 (1.031 to 1.083)	1.059 (1.031 to 1.083)	1.059 (1.031 to 1.083)	1.059 (1.031 to 1.083)
1.289 (1.109 to 1.491)	1.289 (1.109 to 1.491)	1.289 (1.109 to 1.491)	1.289 (1.109 to 1.491)	1.289 (1.109 to 1.491)	1.289 (1.109 to 1.491)	1.289 (1.109 to 1.491)	1.289 (1.109 to 1.491)	1.289 (1.109 to 1.491)	1.289 (1.109 to 1.491)	1.289 (1.109 to 1.491)	1.289 (1.109 to 1.491)	1.289 (1.109 to 1.491)	1.289 (1.109 to 1.491)
1.176 (1.03 to 1.334)	1.176 (1.03 to 1.334)	1.176 (1.03 to 1.334)	1.176 (1.03 to 1.334)	1.176 (1.03 to 1.334)	1.176 (1.03 to 1.334)	1.176 (1.03 to 1.334)	1.176 (1.03 to 1.334)	1.176 (1.03 to 1.334)	1.176 (1.03 to 1.334)	1.176 (1.03 to 1.334)	1.176 (1.03 to 1.334)	1.176 (1.03 to 1.334)	1.176 (1.03 to 1.334)
1.289 (1.109 to 1.491)	1.289 (1.109 to 1.491)	1.289 (1.109 to 1.491)	1.289 (1.109 to 1.491)	1.289 (1.109 to 1.491)	1.289 (1.109 to 1.491)	1.289 (1.109 to 1.491)	1.289 (1.109 to 1.491)	1.289 (1.109 to 1.491)	1.289 (1.109 to 1.491)	1.289 (1.109 to 1.491)	1.289 (1.109 to 1.491)	1.289 (1.109 to 1.491)	1.289 (1.109 to 1.491)
1.176 (1.03 to 1.334)	1.176 (1.03 to 1.334)	1.176 (1.03 to 1.334)	1.176 (1.03 to 1.334)	1.176 (1.03 to 1.334)	1.176 (1.03 to 1.334)	1.176 (1.03 to 1.334)	1.176 (1.03 to 1.334)	1.176 (1.03 to 1.334)	1.176 (1.03 to 1.334)	1.176 (1.03 to 1.334)	1.176 (1.03 to 1.334)	1.176 (1.03 to 1.334)	1.176 (1.03 to 1.334)

Appendix Table 1a. Relative risks used by age and sex for each outcome for all risk factors except for ambient air pollution and alcohol.

Risk - Outcome	Category / Units	Morbidity / Mortality	Sex	All-age	0-6 days	7-27 days	28-364 days	1-4 years	5-9 years	10-14 years	15-19 years	20-24 years	25-29 years
Liver cancer due to alcohol use	5 kg/m2	Both	Males									1.289 (1.109 to 1.491)	1.289 (1.109 to 1.491)
Liver cancer due to alcohol use	5 kg/m2	Both	Females									1.176 (1.03 to 1.334)	1.176 (1.03 to 1.334)
Liver cancer due to other causes	5 kg/m2	Both	Males									1.289 (1.109 to 1.491)	1.289 (1.109 to 1.491)
Liver cancer due to other causes	5 kg/m2	Both	Females									1.176 (1.03 to 1.334)	1.176 (1.03 to 1.334)
Gallbladder and biliary tract cancer	5 kg/m2	Both	Males									1.155 (1.033 to 1.281)	1.155 (1.033 to 1.281)
Gallbladder and biliary tract cancer	5 kg/m2	Both	Females									1.344 (1.223 to 1.477)	1.344 (1.223 to 1.477)
Pancreatic cancer	5 kg/m2	Both	Males									1.071 (0.999 to 1.153)	1.071 (0.999 to 1.153)
Pancreatic cancer	5 kg/m2	Both	Females									1.092 (1.037 to 1.144)	1.092 (1.037 to 1.144)
Breast cancer	Premenopausal	Both	Both									0.89 (0.869 to 0.914)	0.89 (0.869 to 0.914)
Breast cancer	Postmenopausal	Both	Both										
Uterine cancer	5 kg/m2	Both	Both									1.613 (1.543 to 1.681)	1.613 (1.543 to 1.681)
Ovarian cancer	5 kg/m2	Both	Both									1.038 (0.999 to 1.077)	1.038 (0.999 to 1.077)
Kidney cancer	5 kg/m2	Both	Males									1.24 (1.171 to 1.313)	1.24 (1.171 to 1.313)
Kidney cancer	5 kg/m2	Both	Females									1.32 (1.254 to 1.394)	1.32 (1.254 to 1.394)
Thyroid cancer	5 kg/m2	Both	Males									1.221 (1.068 to 1.381)	1.221 (1.068 to 1.381)
Thyroid cancer	5 kg/m2	Both	Females									1.136 (1.094 to 1.178)	1.136 (1.094 to 1.178)
Non-Hodgkin's lymphoma	5 kg/m2	Both	Males									1.089 (1.038 to 1.143)	1.089 (1.038 to 1.143)
Non-Hodgkin's lymphoma	5 kg/m2	Both	Females									1.068 (1.01 to 1.125)	1.068 (1.01 to 1.125)
Multiple myeloma	5 kg/m2	Both	Both									1.089 (1.027 to 1.153)	1.089 (1.027 to 1.153)
Acute lymphoid leukaemia	5 kg/m2	Both	Males									1.086 (1.053 to 1.119)	1.086 (1.053 to 1.119)
Acute lymphoid leukaemia	5 kg/m2	Both	Females									1.131 (1.061 to 1.208)	1.131 (1.061 to 1.208)
Chronic lymphoid leukaemia	5 kg/m2	Both	Males									1.086 (1.053 to 1.119)	1.086 (1.053 to 1.119)
Chronic lymphoid leukaemia	5 kg/m2	Both	Females									1.131 (1.061 to 1.208)	1.131 (1.061 to 1.208)
Acute myeloid leukaemia	5 kg/m2	Both	Males									1.086 (1.053 to 1.119)	1.086 (1.053 to 1.119)
Acute myeloid leukaemia	5 kg/m2	Both	Females									1.131 (1.061 to 1.208)	1.131 (1.061 to 1.208)
Chronic myeloid leukaemia	5 kg/m2	Both	Males									1.086 (1.053 to 1.119)	1.086 (1.053 to 1.119)
Chronic myeloid leukaemia	5 kg/m2	Both	Females									1.131 (1.061 to 1.208)	1.131 (1.061 to 1.208)
Other leukaemia	5 kg/m2	Both	Males									1.086 (1.053 to 1.119)	1.086 (1.053 to 1.119)
Other leukaemia	5 kg/m2	Both	Females									1.131 (1.061 to 1.208)	1.131 (1.061 to 1.208)
Ischaemic heart disease	5 kg/m2	Both	Both									2.274 (1.259 to 3.683)	2.274 (1.259 to 3.683)

Ages													
**30-34 years**	**35-39 years**	**40-44 years**	**45-49 years**	**50-54 years**	**55-59 years**	**60-64 years**	**65-69 years**	**70-74 years**	**75-79 years**	**80-84 years**	**85-89 years**	**90-94 years**	**95+ years**
1.289 (1.109 to 1.491)	1.289 (1.109 to 1.491)	1.289 (1.109 to 1.491)	1.289 (1.109 to 1.491)	1.289 (1.109 to 1.491)	1.289 (1.109 to 1.491)	1.289 (1.109 to 1.491)	1.289 (1.109 to 1.491)	1.289 (1.109 to 1.491)	1.289 (1.109 to 1.491)	1.289 (1.109 to 1.491)	1.289 (1.109 to 1.491)	1.289 (1.109 to 1.491)	1.289 (1.109 to 1.491)
1.176 (1.03 to 1.334)	1.176 (1.03 to 1.334)	1.176 (1.03 to 1.334)	1.176 (1.03 to 1.334)	1.176 (1.03 to 1.334)	1.176 (1.03 to 1.334)	1.176 (1.03 to 1.334)	1.176 (1.03 to 1.334)	1.176 (1.03 to 1.334)	1.176 (1.03 to 1.334)	1.176 (1.03 to 1.334)	1.176 (1.03 to 1.334)	1.176 (1.03 to 1.334)	1.176 (1.03 to 1.334)
1.289 (1.109 to 1.491)	1.289 (1.109 to 1.491)	1.289 (1.109 to 1.491)	1.289 (1.109 to 1.491)	1.289 (1.109 to 1.491)	1.289 (1.109 to 1.491)	1.289 (1.109 to 1.491)	1.289 (1.109 to 1.491)	1.289 (1.109 to 1.491)	1.289 (1.109 to 1.491)	1.289 (1.109 to 1.491)	1.289 (1.109 to 1.491)	1.289 (1.109 to 1.491)	1.289 (1.109 to 1.491)
1.176 (1.03 to 1.334)	1.176 (1.03 to 1.334)	1.176 (1.03 to 1.334)	1.176 (1.03 to 1.334)	1.176 (1.03 to 1.334)	1.176 (1.03 to 1.334)	1.176 (1.03 to 1.334)	1.176 (1.03 to 1.334)	1.176 (1.03 to 1.334)	1.176 (1.03 to 1.334)	1.176 (1.03 to 1.334)	1.176 (1.03 to 1.334)	1.176 (1.03 to 1.334)	1.176 (1.03 to 1.334)
1.155 (1.033 to 1.281)	1.155 (1.033 to 1.281)	1.155 (1.033 to 1.281)	1.155 (1.033 to 1.281)	1.155 (1.033 to 1.281)	1.155 (1.033 to 1.281)	1.155 (1.033 to 1.281)	1.155 (1.033 to 1.281)	1.155 (1.033 to 1.281)	1.155 (1.033 to 1.281)	1.155 (1.033 to 1.281)	1.155 (1.033 to 1.281)	1.155 (1.033 to 1.281)	1.155 (1.033 to 1.281)
1.344 (1.223 to 1.477)	1.344 (1.223 to 1.477)	1.344 (1.223 to 1.477)	1.344 (1.223 to 1.477)	1.344 (1.223 to 1.477)	1.344 (1.223 to 1.477)	1.344 (1.223 to 1.477)	1.344 (1.223 to 1.477)	1.344 (1.223 to 1.477)	1.344 (1.223 to 1.477)	1.344 (1.223 to 1.477)	1.344 (1.223 to 1.477)	1.344 (1.223 to 1.477)	1.344 (1.223 to 1.477)
1.071 (0.999 to 1.153)	1.071 (0.999 to 1.153)	1.071 (0.999 to 1.153)	1.071 (0.999 to 1.153)	1.071 (0.999 to 1.153)	1.071 (0.999 to 1.153)	1.071 (0.999 to 1.153)	1.071 (0.999 to 1.153)	1.071 (0.999 to 1.153)	1.071 (0.999 to 1.153)	1.071 (0.999 to 1.153)	1.071 (0.999 to 1.153)	1.071 (0.999 to 1.153)	1.071 (0.999 to 1.153)
1.092 (1.037 to 1.144)	1.092 (1.037 to 1.144)	1.092 (1.037 to 1.144)	1.092 (1.037 to 1.144)	1.092 (1.037 to 1.144)	1.092 (1.037 to 1.144)	1.092 (1.037 to 1.144)	1.092 (1.037 to 1.144)	1.092 (1.037 to 1.144)	1.092 (1.037 to 1.144)	1.092 (1.037 to 1.144)	1.092 (1.037 to 1.144)	1.092 (1.037 to 1.144)	1.092 (1.037 to 1.144)
0.89 (0.869 to 0.914)	0.89 (0.869 to 0.914)	0.89 (0.869 to 0.914)	0.89 (0.869 to 0.914)										
				1.089 (1.037 to 1.14)	1.089 (1.037 to 1.14)	1.089 (1.037 to 1.14)	1.089 (1.037 to 1.14)	1.089 (1.037 to 1.14)	1.089 (1.037 to 1.14)	1.089 (1.037 to 1.14)	1.089 (1.037 to 1.14)	1.089 (1.037 to 1.14)	1.089 (1.037 to 1.14)
1.613 (1.543 to 1.681)	1.613 (1.543 to 1.681)	1.613 (1.543 to 1.681)	1.613 (1.543 to 1.681)	1.613 (1.543 to 1.681)	1.613 (1.543 to 1.681)	1.613 (1.543 to 1.681)	1.613 (1.543 to 1.681)	1.613 (1.543 to 1.681)	1.613 (1.543 to 1.681)	1.613 (1.543 to 1.681)	1.613 (1.543 to 1.681)	1.613 (1.543 to 1.681)	1.613 (1.543 to 1.681)
1.038 (0.999 to 1.077)	1.038 (0.999 to 1.077)	1.038 (0.999 to 1.077)	1.038 (0.999 to 1.077)	1.038 (0.999 to 1.077)	1.038 (0.999 to 1.077)	1.038 (0.999 to 1.077)	1.038 (0.999 to 1.077)	1.038 (0.999 to 1.077)	1.038 (0.999 to 1.077)	1.038 (0.999 to 1.077)	1.038 (0.999 to 1.077)	1.038 (0.999 to 1.077)	1.038 (0.999 to 1.077)
1.24 (1.171 to 1.313)	1.24 (1.171 to 1.313)	1.24 (1.171 to 1.313)	1.24 (1.171 to 1.313)	1.24 (1.171 to 1.313)	1.24 (1.171 to 1.313)	1.24 (1.171 to 1.313)	1.24 (1.171 to 1.313)	1.24 (1.171 to 1.313)	1.24 (1.171 to 1.313)	1.24 (1.171 to 1.313)	1.24 (1.171 to 1.313)	1.24 (1.171 to 1.313)	1.24 (1.171 to 1.313)
1.32 (1.254 to 1.394)	1.32 (1.254 to 1.394)	1.32 (1.254 to 1.394)	1.32 (1.254 to 1.394)	1.32 (1.254 to 1.394)	1.32 (1.254 to 1.394)	1.32 (1.254 to 1.394)	1.32 (1.254 to 1.394)	1.32 (1.254 to 1.394)	1.32 (1.254 to 1.394)	1.32 (1.254 to 1.394)	1.32 (1.254 to 1.394)	1.32 (1.254 to 1.394)	1.32 (1.254 to 1.394)
1.221 (1.068 to 1.381)	1.221 (1.068 to 1.381)	1.221 (1.068 to 1.381)	1.221 (1.068 to 1.381)	1.221 (1.068 to 1.381)	1.221 (1.068 to 1.381)	1.221 (1.068 to 1.381)	1.221 (1.068 to 1.381)	1.221 (1.068 to 1.381)	1.221 (1.068 to 1.381)	1.221 (1.068 to 1.381)	1.221 (1.068 to 1.381)	1.221 (1.068 to 1.381)	1.221 (1.068 to 1.381)
1.136 (1.094 to 1.178)	1.136 (1.094 to 1.178)	1.136 (1.094 to 1.178)	1.136 (1.094 to 1.178)	1.136 (1.094 to 1.178)	1.136 (1.094 to 1.178)	1.136 (1.094 to 1.178)	1.136 (1.094 to 1.178)	1.136 (1.094 to 1.178)	1.136 (1.094 to 1.178)	1.136 (1.094 to 1.178)	1.136 (1.094 to 1.178)	1.136 (1.094 to 1.178)	1.136 (1.094 to 1.178)
1.089 (1.038 to 1.143)	1.089 (1.038 to 1.143)	1.089 (1.038 to 1.143)	1.089 (1.038 to 1.143)	1.089 (1.038 to 1.143)	1.089 (1.038 to 1.143)	1.089 (1.038 to 1.143)	1.089 (1.038 to 1.143)	1.089 (1.038 to 1.143)	1.089 (1.038 to 1.143)	1.089 (1.038 to 1.143)	1.089 (1.038 to 1.143)	1.089 (1.038 to 1.143)	1.089 (1.038 to 1.143)
1.068 (1.01 to 1.125)	1.068 (1.01 to 1.125)	1.068 (1.01 to 1.125)	1.068 (1.01 to 1.125)	1.068 (1.01 to 1.125)	1.068 (1.01 to 1.125)	1.068 (1.01 to 1.125)	1.068 (1.01 to 1.125)	1.068 (1.01 to 1.125)	1.068 (1.01 to 1.125)	1.068 (1.01 to 1.125)	1.068 (1.01 to 1.125)	1.068 (1.01 to 1.125)	1.068 (1.01 to 1.125)
1.089 (1.027 to 1.153)	1.089 (1.027 to 1.153)	1.089 (1.027 to 1.153)	1.089 (1.027 to 1.153)	1.089 (1.027 to 1.153)	1.089 (1.027 to 1.153)	1.089 (1.027 to 1.153)	1.089 (1.027 to 1.153)	1.089 (1.027 to 1.153)	1.089 (1.027 to 1.153)	1.089 (1.027 to 1.153)	1.089 (1.027 to 1.153)	1.089 (1.027 to 1.153)	1.089 (1.027 to 1.153)
1.086 (1.053 to 1.119)	1.086 (1.053 to 1.119)	1.086 (1.053 to 1.119)	1.086 (1.053 to 1.119)	1.086 (1.053 to 1.119)	1.086 (1.053 to 1.119)	1.086 (1.053 to 1.119)	1.086 (1.053 to 1.119)	1.086 (1.053 to 1.119)	1.086 (1.053 to 1.119)	1.086 (1.053 to 1.119)	1.086 (1.053 to 1.119)	1.086 (1.053 to 1.119)	1.086 (1.053 to 1.119)
1.131 (1.061 to 1.208)	1.131 (1.061 to 1.208)	1.131 (1.061 to 1.208)	1.131 (1.061 to 1.208)	1.131 (1.061 to 1.208)	1.131 (1.061 to 1.208)	1.131 (1.061 to 1.208)	1.131 (1.061 to 1.208)	1.131 (1.061 to 1.208)	1.131 (1.061 to 1.208)	1.131 (1.061 to 1.208)	1.131 (1.061 to 1.208)	1.131 (1.061 to 1.208)	1.131 (1.061 to 1.208)
1.086 (1.053 to 1.119)	1.086 (1.053 to 1.119)	1.086 (1.053 to 1.119)	1.086 (1.053 to 1.119)	1.086 (1.053 to 1.119)	1.086 (1.053 to 1.119)	1.086 (1.053 to 1.119)	1.086 (1.053 to 1.119)	1.086 (1.053 to 1.119)	1.086 (1.053 to 1.119)	1.086 (1.053 to 1.119)	1.086 (1.053 to 1.119)	1.086 (1.053 to 1.119)	1.086 (1.053 to 1.119)
1.131 (1.061 to 1.208)	1.131 (1.061 to 1.208)	1.131 (1.061 to 1.208)	1.131 (1.061 to 1.208)	1.131 (1.061 to 1.208)	1.131 (1.061 to 1.208)	1.131 (1.061 to 1.208)	1.131 (1.061 to 1.208)	1.131 (1.061 to 1.208)	1.131 (1.061 to 1.208)	1.131 (1.061 to 1.208)	1.131 (1.061 to 1.208)	1.131 (1.061 to 1.208)	1.131 (1.061 to 1.208)
1.086 (1.053 to 1.119)	1.086 (1.053 to 1.119)	1.086 (1.053 to 1.119)	1.086 (1.053 to 1.119)	1.086 (1.053 to 1.119)	1.086 (1.053 to 1.119)	1.086 (1.053 to 1.119)	1.086 (1.053 to 1.119)	1.086 (1.053 to 1.119)	1.086 (1.053 to 1.119)	1.086 (1.053 to 1.119)	1.086 (1.053 to 1.119)	1.086 (1.053 to 1.119)	1.086 (1.053 to 1.119)
1.131 (1.061 to 1.208)	1.131 (1.061 to 1.208)	1.131 (1.061 to 1.208)	1.131 (1.061 to 1.208)	1.131 (1.061 to 1.208)	1.131 (1.061 to 1.208)	1.131 (1.061 to 1.208)	1.131 (1.061 to 1.208)	1.131 (1.061 to 1.208)	1.131 (1.061 to 1.208)	1.131 (1.061 to 1.208)	1.131 (1.061 to 1.208)	1.131 (1.061 to 1.208)	1.131 (1.061 to 1.208)
1.086 (1.053 to 1.119)	1.086 (1.053 to 1.119)	1.086 (1.053 to 1.119)	1.086 (1.053 to 1.119)	1.086 (1.053 to 1.119)	1.086 (1.053 to 1.119)	1.086 (1.053 to 1.119)	1.086 (1.053 to 1.119)	1.086 (1.053 to 1.119)	1.086 (1.053 to 1.119)	1.086 (1.053 to 1.119)	1.086 (1.053 to 1.119)	1.086 (1.053 to 1.119)	1.086 (1.053 to 1.119)
1.131 (1.061 to 1.208)	1.131 (1.061 to 1.208)	1.131 (1.061 to 1.208)	1.131 (1.061 to 1.208)	1.131 (1.061 to 1.208)	1.131 (1.061 to 1.208)	1.131 (1.061 to 1.208)	1.131 (1.061 to 1.208)	1.131 (1.061 to 1.208)	1.131 (1.061 to 1.208)	1.131 (1.061 to 1.208)	1.131 (1.061 to 1.208)	1.131 (1.061 to 1.208)	1.131 (1.061 to 1.208)
1.086 (1.053 to 1.119)	1.086 (1.053 to 1.119)	1.086 (1.053 to 1.119)	1.086 (1.053 to 1.119)	1.086 (1.053 to 1.119)	1.086 (1.053 to 1.119)	1.086 (1.053 to 1.119)	1.086 (1.053 to 1.119)	1.086 (1.053 to 1.119)	1.086 (1.053 to 1.119)	1.086 (1.053 to 1.119)	1.086 (1.053 to 1.119)	1.086 (1.053 to 1.119)	1.086 (1.053 to 1.119)
1.131 (1.061 to 1.208)	1.131 (1.061 to 1.208)	1.131 (1.061 to 1.208)	1.131 (1.061 to 1.208)	1.131 (1.061 to 1.208)	1.131 (1.061 to 1.208)	1.131 (1.061 to 1.208)	1.131 (1.061 to 1.208)	1.131 (1.061 to 1.208)	1.131 (1.061 to 1.208)	1.131 (1.061 to 1.208)	1.131 (1.061 to 1.208)	1.131 (1.061 to 1.208)	1.131 (1.061 to 1.208)
1.086 (1.053 to 1.119)	1.086 (1.053 to 1.119)	1.086 (1.053 to 1.119)	1.086 (1.053 to 1.119)	1.086 (1.053 to 1.119)	1.086 (1.053 to 1.119)	1.086 (1.053 to 1.119)	1.086 (1.053 to 1.119)	1.086 (1.053 to 1.119)	1.086 (1.053 to 1.119)	1.086 (1.053 to 1.119)	1.086 (1.053 to 1.119)	1.086 (1.053 to 1.119)	1.086 (1.053 to 1.119)
1.131 (1.061 to 1.208)	1.131 (1.061 to 1.208)	1.131 (1.061 to 1.208)	1.131 (1.061 to 1.208)	1.131 (1.061 to 1.208)	1.131 (1.061 to 1.208)	1.131 (1.061 to 1.208)	1.131 (1.061 to 1.208)	1.131 (1.061 to 1.208)	1.131 (1.061 to 1.208)	1.131 (1.061 to 1.208)	1.131 (1.061 to 1.208)	1.131 (1.061 to 1.208)	1.131 (1.061 to 1.208)
2.018 (1.3 to 3.099)	1.724 (1.533 to 1.93)	1.599 (1.418 to 1.784)	1.567 (1.458 to 1.68)	1.52 (1.417 to 1.631)	1.466 (1.372 to 1.557)	1.414 (1.325 to 1.504)	1.364 (1.287 to 1.448)	1.319 (1.242 to 1.4)	1.274 (1.187 to 1.365)	1.17 (1.091 to 1.252)	1.17 (1.091 to 1.252)	1.17 (1.091 to 1.252)	1.17 (1.091 to 1.252)

Appendix Table 1a. Relative risks used by age and sex for each outcome for all risk factors except for ambient air pollution and alcohol.

Risk - Outcome	Category / Units	Morbidity / Mortality	Sex	All-age	0-6 days	7-27 days	28-364 days	1-4 years	5-9 years	10-14 years	15-19 years	20-24 years	25-29 years
Ischaemic stroke	5 kg/m2	Both	Both									2.472 (1.4 to 3.975)	2.472 (1.4 to 3.975)
Hemorrhagic stroke	5 kg/m2	Both	Both									3.066 (1.751 to 5.334)	3.066 (1.751 to 5.334)
Hypertensive heart disease	5 kg/m2	Both	Both									3.122 (1.588 to 5.498)	3.122 (1.588 to 5.498)
Atrial fibrillation and flutter	5 kg/m2	Both	Both									1.344 (1.231 to 1.473)	1.344 (1.231 to 1.473)
Asthma	Obese	Both	Both					2.2 (1.51 to 3.19)	2.2 (1.51 to 3.19)	2.2 (1.51 to 3.19)	2.2 (1.51 to 3.19)		
Asthma	Overweight	Both	Both					1.33 (1.11 to 1.6)	1.33 (1.11 to 1.6)	1.33 (1.11 to 1.6)	1.33 (1.11 to 1.6)		
Asthma	5 kg/m2	Both	Both									1.402 (1.275 to 1.532)	1.402 (1.275 to 1.532)
Gallbladder and biliary diseases	5 kg/m2	Both	Males									1.464 (1.291 to 1.64)	1.464 (1.291 to 1.64)
Gallbladder and biliary diseases	5 kg/m2	Both	Females									1.729 (1.571 to 1.893)	1.729 (1.571 to 1.893)
Alzheimer's disease and other dementias	5 kg/m2	Both	Both									1.214 (1.047 to 1.404)	1.214 (1.047 to 1.404)
Diabetes mellitus	5 kg/m2	Both	Both									3.547 (2.314 to 5.219)	3.547 (2.314 to 5.219)
Chronic kidney disease due to diabetes mellitus	5 kg/m2	Both	Both										
Chronic kidney disease due to hypertension	5 kg/m2	Both	Both										
Chronic kidney disease due to glomerulonephritis	5 kg/m2	Both	Both										
Chronic kidney disease due to other causes	5 kg/m2	Both	Both										
Low back pain	5 kg/m2	Morbidity	Both									1.1 (1.073 to 1.126)	1.1 (1.073 to 1.126)
Gout	5 kg/m2	Both	Males									1.628 (1.34 to 1.964)	1.628 (1.34 to 1.964)
Gout	5 kg/m2	Both	Females									1.493 (1.322 to 1.677)	1.493 (1.322 to 1.677)
Cataract	5 kg/m2	Both	Both									1.104 (1.051 to 1.156)	1.104 (1.051 to 1.156)
Osteoarthritis of the hip	5 kg/m2	Morbidity	Both									1.11 (1.06 to 1.157)	1.11 (1.06 to 1.157)
Osteoarthritis of the knee	5 kg/m2	Morbidity	Both									1.37 (1.201 to 1.538)	1.37 (1.201 to 1.538)
**Low bone mineral density**													
Hip	0.1 g/cm2	Both	Males										
Hip	0.1 g/cm2	Both	Females										
Non-hip	0.1 g/cm2	Both	Males										
Non-hip	0.1 g/cm2	Both	Females										
**Impaired kidney function**													
Ischaemic heart disease	Stage 5 CKD	Both	Both										6.403 (1.632 to 17.373)
Ischaemic heart disease	Stage 4 CKD	Both	Both										4.187 (1.636 to 8.618)
Ischaemic heart disease	Stage 3 CKD	Both	Both										1.508 (1.187 to 1.905)

Ages													
**30-34 years**	**35-39 years**	**40-44 years**	**45-49 years**	**50-54 years**	**55-59 years**	**60-64 years**	**65-69 years**	**70-74 years**	**75-79 years**	**80-84 years**	**85-89 years**	**90-94 years**	**95+ years**
2.235 (1.457 to 3.329)	1.979 (1.699 to 2.313)	1.826 (1.6 to 2.075)	1.733 (1.581 to 1.898)	1.635 (1.479 to 1.795)	1.543 (1.441 to 1.653)	1.455 (1.345 to 1.566)	1.38 (1.31 to 1.458)	1.304 (1.234 to 1.376)	1.228 (1.16 to 1.304)	1.068 (0.992 to 1.143)	1.068 (0.992 to 1.143)	1.068 (0.992 to 1.143)	1.068 (0.992 to 1.143)
2.913 (1.862 to 4.395)	2.597 (1.974 to 3.375)	2.389 (1.87 to 2.998)	2.199 (1.822 to 2.672)	1.996 (1.626 to 2.416)	1.805 (1.574 to 2.06)	1.665 (1.438 to 1.932)	1.523 (1.377 to 1.683)	1.41 (1.266 to 1.57)	1.295 (1.162 to 1.438)	1.07 (0.928 to 1.219)	1.07 (0.928 to 1.219)	1.07 (0.928 to 1.219)	1.07 (0.928 to 1.219)
3.0 (1.783 to 4.902)	2.769 (1.816 to 4.211)	2.573 (1.743 to 3.636)	2.407 (1.717 to 3.293)	2.281 (1.602 to 3.188)	2.159 (1.505 to 3.036)	2.035 (1.452 to 2.822)	1.955 (1.343 to 2.698)	1.86 (1.296 to 2.617)	1.792 (1.17 to 2.553)	1.697 (1.069 to 2.618)	1.697 (1.069 to 2.618)	1.697 (1.069 to 2.618)	1.697 (1.069 to 2.618)
1.344 (1.231 to 1.473)	1.344 (1.231 to 1.473)	1.344 (1.231 to 1.473)	1.344 (1.231 to 1.473)	1.344 (1.231 to 1.473)	1.344 (1.231 to 1.473)	1.344 (1.231 to 1.473)	1.344 (1.231 to 1.473)	1.344 (1.231 to 1.473)	1.344 (1.231 to 1.473)	1.344 (1.231 to 1.473)	1.344 (1.231 to 1.473)	1.344 (1.231 to 1.473)	1.344 (1.231 to 1.473)
1.402 (1.275 to 1.532)	1.402 (1.275 to 1.532)	1.402 (1.275 to 1.532)	1.402 (1.275 to 1.532)	1.402 (1.275 to 1.532)	1.402 (1.275 to 1.532)	1.402 (1.275 to 1.532)	1.402 (1.275 to 1.532)	1.402 (1.275 to 1.532)	1.402 (1.275 to 1.532)	1.402 (1.275 to 1.532)	1.402 (1.275 to 1.532)	1.402 (1.275 to 1.532)	1.402 (1.275 to 1.532)
1.464 (1.291 to 1.64)	1.464 (1.291 to 1.64)	1.464 (1.291 to 1.64)	1.464 (1.291 to 1.64)	1.464 (1.291 to 1.64)	1.464 (1.291 to 1.64)	1.464 (1.291 to 1.64)	1.464 (1.291 to 1.64)	1.464 (1.291 to 1.64)	1.464 (1.291 to 1.64)	1.464 (1.291 to 1.64)	1.464 (1.291 to 1.64)	1.464 (1.291 to 1.64)	1.464 (1.291 to 1.64)
1.729 (1.571 to 1.893)	1.729 (1.571 to 1.893)	1.729 (1.571 to 1.893)	1.729 (1.571 to 1.893)	1.729 (1.571 to 1.893)	1.729 (1.571 to 1.893)	1.729 (1.571 to 1.893)	1.729 (1.571 to 1.893)	1.729 (1.571 to 1.893)	1.729 (1.571 to 1.893)	1.729 (1.571 to 1.893)	1.729 (1.571 to 1.893)	1.729 (1.571 to 1.893)	1.729 (1.571 to 1.893)
1.214 (1.047 to 1.404)	1.214 (1.047 to 1.404)	1.214 (1.047 to 1.404)	1.214 (1.047 to 1.404)	1.214 (1.047 to 1.404)	1.214 (1.047 to 1.404)	1.214 (1.047 to 1.404)	1.214 (1.047 to 1.404)	1.214 (1.047 to 1.404)	1.214 (1.047 to 1.404)	1.214 (1.047 to 1.404)	1.214 (1.047 to 1.404)	1.214 (1.047 to 1.404)	1.214 (1.047 to 1.404)
3.455 (2.516 to 4.691)	3.349 (2.803 to 3.918)	3.16 (2.697 to 3.699)	2.864 (2.453 to 3.312)	2.624 (2.224 to 3.038)	2.417 (2.089 to 2.779)	2.215 (1.868 to 2.606)	2.046 (1.724 to 2.379)	1.896 (1.597 to 2.228)	1.74 (1.446 to 2.074)	1.461 (1.207 to 1.758)	1.461 (1.207 to 1.758)	1.461 (1.207 to 1.758)	1.461 (1.207 to 1.758)
	1.746 (1.054 to 2.746)	1.746 (1.054 to 2.746)	1.746 (1.054 to 2.746)	1.746 (1.054 to 2.746)	1.746 (1.054 to 2.746)	2.036 (1.298 to 3.044)	2.036 (1.298 to 3.044)	1.621 (1.063 to 2.378)	1.621 (1.063 to 2.378)	1.431 (0.802 to 2.396)	1.431 (0.802 to 2.396)	1.431 (0.802 to 2.396)	1.431 (0.802 to 2.396)
	1.763 (1.09 to 2.755)	1.763 (1.09 to 2.755)	1.763 (1.09 to 2.755)	1.763 (1.09 to 2.755)	1.763 (1.09 to 2.755)	2.044 (1.305 to 3.082)	2.044 (1.305 to 3.082)	1.605 (1.067 to 2.31)	1.605 (1.067 to 2.31)	1.437 (0.829 to 2.415)	1.437 (0.829 to 2.415)	1.437 (0.829 to 2.415)	1.437 (0.829 to 2.415)
	1.742 (1.021 to 2.775)	1.742 (1.021 to 2.775)	1.742 (1.021 to 2.775)	1.742 (1.021 to 2.775)	1.742 (1.021 to 2.775)	2.044 (1.254 to 3.154)	2.044 (1.254 to 3.154)	1.604 (1.109 to 2.254)	1.604 (1.109 to 2.254)	1.452 (0.851 to 2.345)	1.452 (0.851 to 2.345)	1.452 (0.851 to 2.345)	1.452 (0.851 to 2.345)
	1.732 (1.052 to 2.681)	1.732 (1.052 to 2.681)	1.732 (1.052 to 2.681)	1.732 (1.052 to 2.681)	1.732 (1.052 to 2.681)	2.032 (1.216 to 3.101)	2.032 (1.216 to 3.101)	1.625 (1.068 to 2.365)	1.625 (1.068 to 2.365)	1.433 (0.778 to 2.344)	1.433 (0.778 to 2.344)	1.433 (0.778 to 2.344)	1.433 (0.778 to 2.344)
1.1 (1.073 to 1.127)	1.101 (1.076 to 1.128)	1.1 (1.074 to 1.126)	1.099 (1.075 to 1.123)	1.1 (1.075 to 1.128)	1.1 (1.075 to 1.126)	1.101 (1.077 to 1.126)	1.1 (1.075 to 1.126)	1.1 (1.076 to 1.124)	1.1 (1.075 to 1.124)	1.1 (1.074 to 1.125)	1.1 (1.074 to 1.125)	1.1 (1.074 to 1.125)	1.1 (1.074 to 1.125)
1.628 (1.34 to 1.964)	1.628 (1.34 to 1.964)	1.628 (1.34 to 1.964)	1.628 (1.34 to 1.964)	1.628 (1.34 to 1.964)	1.628 (1.34 to 1.964)	1.628 (1.34 to 1.964)	1.628 (1.34 to 1.964)	1.628 (1.34 to 1.964)	1.628 (1.34 to 1.964)	1.628 (1.34 to 1.964)	1.628 (1.34 to 1.964)	1.628 (1.34 to 1.964)	1.628 (1.34 to 1.964)
1.493 (1.322 to 1.677)	1.493 (1.322 to 1.677)	1.493 (1.322 to 1.677)	1.493 (1.322 to 1.677)	1.493 (1.322 to 1.677)	1.493 (1.322 to 1.677)	1.493 (1.322 to 1.677)	1.493 (1.322 to 1.677)	1.493 (1.322 to 1.677)	1.493 (1.322 to 1.677)	1.493 (1.322 to 1.677)	1.493 (1.322 to 1.677)	1.493 (1.322 to 1.677)	1.493 (1.322 to 1.677)
1.104 (1.051 to 1.156)	1.104 (1.051 to 1.156)	1.104 (1.051 to 1.156)	1.104 (1.051 to 1.156)	1.104 (1.051 to 1.156)	1.104 (1.051 to 1.156)	1.104 (1.051 to 1.156)	1.104 (1.051 to 1.156)	1.104 (1.051 to 1.156)	1.104 (1.051 to 1.156)	1.104 (1.051 to 1.156)	1.104 (1.051 to 1.156)	1.104 (1.051 to 1.156)	1.104 (1.051 to 1.156)
1.11 (1.06 to 1.157)	1.11 (1.06 to 1.157)	1.11 (1.06 to 1.157)	1.11 (1.06 to 1.157)	1.11 (1.06 to 1.157)	1.11 (1.06 to 1.157)	1.11 (1.06 to 1.157)	1.11 (1.06 to 1.157)	1.11 (1.06 to 1.157)	1.11 (1.06 to 1.157)	1.11 (1.06 to 1.157)	1.11 (1.06 to 1.157)	1.11 (1.06 to 1.157)	1.11 (1.06 to 1.157)
1.37 (1.201 to 1.538)	1.37 (1.201 to 1.538)	1.37 (1.201 to 1.538)	1.37 (1.201 to 1.538)	1.37 (1.201 to 1.538)	1.37 (1.201 to 1.538)	1.37 (1.201 to 1.538)	1.37 (1.201 to 1.538)	1.37 (1.201 to 1.538)	1.37 (1.201 to 1.538)	1.37 (1.201 to 1.538)	1.37 (1.201 to 1.538)	1.37 (1.201 to 1.538)	1.37 (1.201 to 1.538)
		2.945 (2.121 to 3.906)	2.85 (2.133 to 3.819)	2.614 (2.019 to 3.326)	2.438 (2.002 to 2.966)	2.286 (1.964 to 2.662)	2.184 (1.912 to 2.473)	2.102 (1.888 to 2.322)	1.921 (1.786 to 2.084)	1.732 (1.631 to 1.84)	1.732 (1.631 to 1.84)	1.732 (1.631 to 1.84)	1.732 (1.631 to 1.84)
		3.255 (2.261 to 4.486)	2.94 (2.146 to 3.899)	2.713 (2.074 to 3.427)	2.642 (2.094 to 3.271)	2.474 (2.061 to 2.95)	2.412 (2.058 to 2.768)	2.32 (2.077 to 2.571)	2.118 (1.938 to 2.299)	1.876 (1.748 to 2.001)	1.876 (1.748 to 2.001)	1.876 (1.748 to 2.001)	1.876 (1.748 to 2.001)
		1.077 (1.074 to 1.08)	1.114 (1.112 to 1.115)	1.151 (1.057 to 1.258)	1.182 (1.1 to 1.264)	1.214 (1.148 to 1.284)	1.247 (1.186 to 1.309)	1.297 (1.241 to 1.353)	1.339 (1.278 to 1.399)	1.37 (1.297 to 1.448)	1.37 (1.297 to 1.448)	1.37 (1.297 to 1.448)	1.37 (1.297 to 1.448)
		1.083 (1.08 to 1.087)	1.118 (1.116 to 1.12)	1.163 (1.065 to 1.273)	1.203 (1.119 to 1.294)	1.239 (1.161 to 1.317)	1.287 (1.216 to 1.361)	1.343 (1.273 to 1.418)	1.401 (1.33 to 1.479)	1.437 (1.352 to 1.524)	1.437 (1.352 to 1.524)	1.437 (1.352 to 1.524)	1.437 (1.352 to 1.524)
6.579 (1.642 to 17.922)	6.222 (1.812 to 16.832)	6.454 (1.486 to 18.425)	6.576 (1.712 to 19.892)	6.638 (1.619 to 18.398)	6.447 (1.575 to 17.801)	3.682 (2.162 to 6.067)	3.674 (2.083 to 6.132)	3.058 (1.883 to 4.917)	3.066 (1.895 to 4.796)	2.611 (1.17 to 5.088)	2.546 (1.085 to 4.94)	2.593 (1.177 to 5.083)	2.545 (1.026 to 4.924)
4.138 (1.626 to 9.02)	4.158 (1.576 to 9.128)	4.096 (1.662 to 8.682)	4.106 (1.554 to 8.24)	4.15 (1.564 to 9.113)	4.15 (1.579 to 9.015)	2.596 (1.79 to 3.639)	2.54 (1.751 to 3.555)	2.263 (1.621 to 3.112)	2.272 (1.624 to 3.038)	2.01 (1.167 to 3.381)	2.01 (1.18 to 3.267)	2.041 (1.192 to 3.214)	2.029 (1.172 to 3.309)
1.51 (1.183 to 1.902)	1.509 (1.195 to 1.905)	1.509 (1.195 to 1.893)	1.519 (1.201 to 1.94)	1.506 (1.169 to 1.856)	1.507 (1.182 to 1.887)	1.406 (1.233 to 1.597)	1.403 (1.227 to 1.615)	1.375 (1.205 to 1.564)	1.381 (1.23 to 1.544)	1.316 (1.12 to 1.534)	1.321 (1.146 to 1.532)	1.312 (1.129 to 1.525)	1.312 (1.125 to 1.522)

Appendix Table 1a. Relative risks used by age and sex for each outcome for all risk factors except for ambient air pollution and alcohol.

Risk - Outcome	Category / Units	Morbidity / Mortality	Sex	All-age	0-6 days	7-27 days	28-364 days	1-4 years	5-9 years	10-14 years	15-19 years	20-24 years	25-29 years
Ischaemic heart disease	Albuminira	Both	Both										1.627 (1.249 to 2.107)
Ischaemic heart disease	None	Both	Both										1.0 (1.0 to 1.0)
Ischaemic stroke	Stage 5 CKD	Both	Both										6.665 (0.633 to 28.506)
Ischaemic stroke	Stage 4 CKD	Both	Both										3.642 (0.769 to 10.195)
Ischaemic stroke	Stage 3 CKD	Both	Both										1.063 (0.699 to 1.513)
Ischaemic stroke	Albuminira	Both	Both										2.09 (1.406 to 3.11)
Ischaemic stroke	None	Both	Both										1.0 (1.0 to 1.0)
Hemorrhagic stroke	Stage 5 CKD	Both	Both										6.665 (0.633 to 28.506)
Hemorrhagic stroke	Stage 4 CKD	Both	Both										3.642 (0.769 to 10.195)
Hemorrhagic stroke	Stage 3 CKD	Both	Both										1.063 (0.699 to 1.513)
Hemorrhagic stroke	Albuminira	Both	Both										2.09 (1.406 to 3.11)
Hemorrhagic stroke	None	Both	Both										1.0 (1.0 to 1.0)
Peripheral vascular disease	Stage 5 CKD	Both	Both										207.753 (30.856 to 704.06)
Peripheral vascular disease	Stage 4 CKD	Both	Both										58.362 (15.568 to 149.001)
Peripheral vascular disease	Stage 3 CKD	Both	Both										3.083 (1.826 to 4.774)
Peripheral vascular disease	Albuminira	Both	Both										3.542 (1.986 to 5.721)
Peripheral vascular disease	None	Both	Both										1.0 (1.0 to 1.0)
Gout	Stage 5 CKD	Both	Both										2.749 (2.491 to 3.017)
Gout	Stage 4 CKD	Both	Both										2.745 (2.473 to 3.032)
Gout	Stage 3 CKD	Both	Both										2.748 (2.457 to 3.035)
Gout	Albuminira	Both	Both										1.0 (1.0 to 1.0)
Gout	None	Both	Both										1.0 (1.0 to 1.0)

* Shifts are reported for diet high in sugar-sweetened beverages as the estimation is based on mediation through body-mass index.

** Shifts are reported for diet high in sodium as the estimation is based on mediation through high systolic blood pressure.

Risk-outcome pairs with 100% attribution

Alcohol use
Liver cancer due to alcohol use
Cirrhosis due to alcohol use
Alcohol use disorders

Childhood underweight
Protein-energy malnutrition

Childhood wasting
Protein-energy malnutrition

High fasting plasma glucose
Diabetes mellitus
Chronic kidney disease due to diabetes mellitus

High systolic blood pressure
Hypertensive heart disease
Chronic kidney disease due to hypertension

Iron deficiency
Iron-deficiency anaemia

Occupational particulate matter, gases, and fumes
Coal workers pneumoconiosis

Unsafe sex
Syphilis
Chlamydial infection
Gonococcal infection
Trichomoniasis
Genital herpes

Ages													
**30-34 years**	**35-39 years**	**40-44 years**	**45-49 years**	**50-54 years**	**55-59 years**	**60-64 years**	**65-69 years**	**70-74 years**	**75-79 years**	**80-84 years**	**85-89 years**	**90-94 years**	**95+ years**
1.64 (1.252 to 2.097)	1.63 (1.216 to 2.103)	1.638 (1.26 to 2.081)	1.636 (1.255 to 2.08)	1.632 (1.246 to 2.074)	1.628 (1.253 to 2.071)	1.172 (1.072 to 1.272)	1.173 (1.067 to 1.287)	1.041 (0.918 to 1.178)	1.041 (0.914 to 1.173)	0.923 (0.749 to 1.107)	0.927 (0.762 to 1.115)	0.923 (0.754 to 1.125)	0.921 (0.75 to 1.122)
1.0 (1.0 to 1.0)	1.0 (1.0 to 1.0)	1.0 (1.0 to 1.0)	1.0 (1.0 to 1.0)	1.0 (1.0 to 1.0)	1.0 (1.0 to 1.0)	1.0 (1.0 to 1.0)	1.0 (1.0 to 1.0)	1.0 (1.0 to 1.0)	1.0 (1.0 to 1.0)	1.0 (1.0 to 1.0)	1.0 (1.0 to 1.0)	1.0 (1.0 to 1.0)	1.0 (1.0 to 1.0)
6.631 (0.586 to 26.898)	6.571 (0.671 to 25.958)	6.66 (0.529 to 28.335)	6.568 (0.674 to 27.459)	7.071 (0.699 to 27.677)	6.859 (0.656 to 28.559)	3.305 (1.133 to 7.402)	3.34 (1.183 to 7.005)	2.175 (1.085 to 4.053)	2.154 (1.054 to 4.058)	1.608 (0.478 to 3.837)	1.567 (0.488 to 3.819)	1.539 (0.499 to 3.799)	1.544 (0.454 to 3.893)
3.665 (0.716 to 11.442)	3.493 (0.721 to 10.629)	3.634 (0.744 to 10.727)	3.518 (0.743 to 9.943)	3.579 (0.832 to 10.234)	3.576 (0.774 to 10.582)	2.291 (1.209 to 3.935)	2.287 (1.13 to 4.048)	1.708 (1.023 to 2.625)	1.67 (1.026 to 2.52)	1.298 (0.628 to 2.387)	1.331 (0.65 to 2.537)	1.323 (0.61 to 2.458)	1.301 (0.624 to 2.507)
1.073 (0.733 to 1.534)	1.071 (0.73 to 1.524)	1.072 (0.717 to 1.565)	1.081 (0.721 to 1.571)	1.077 (0.725 to 1.55)	1.077 (0.724 to 1.542)	1.324 (1.072 to 1.619)	1.323 (1.069 to 1.629)	1.255 (1.064 to 1.462)	1.254 (1.08 to 1.46)	1.155 (0.925 to 1.405)	1.148 (0.936 to 1.393)	1.147 (0.931 to 1.385)	1.156 (0.932 to 1.41)
2.077 (1.425 to 2.946)	2.074 (1.405 to 2.996)	2.093 (1.413 to 3.032)	2.092 (1.365 to 3.032)	2.085 (1.391 to 2.983)	2.079 (1.4 to 2.988)	1.478 (1.289 to 1.683)	1.487 (1.319 to 1.692)	1.272 (1.093 to 1.49)	1.27 (1.076 to 1.497)	1.11 (0.854 to 1.433)	1.102 (0.834 to 1.419)	1.107 (0.835 to 1.446)	1.109 (0.822 to 1.429)
1.0 (1.0 to 1.0)	1.0 (1.0 to 1.0)	1.0 (1.0 to 1.0)	1.0 (1.0 to 1.0)	1.0 (1.0 to 1.0)	1.0 (1.0 to 1.0)	1.0 (1.0 to 1.0)	1.0 (1.0 to 1.0)	1.0 (1.0 to 1.0)	1.0 (1.0 to 1.0)	1.0 (1.0 to 1.0)	1.0 (1.0 to 1.0)	1.0 (1.0 to 1.0)	1.0 (1.0 to 1.0)
6.631 (0.586 to 26.898)	6.571 (0.671 to 25.958)	6.66 (0.529 to 28.335)	6.568 (0.674 to 27.459)	7.071 (0.699 to 27.677)	6.859 (0.656 to 28.559)	3.305 (1.133 to 7.402)	3.34 (1.183 to 7.005)	2.175 (1.085 to 4.053)	2.154 (1.054 to 4.058)	1.608 (0.478 to 3.837)	1.567 (0.488 to 3.819)	1.539 (0.499 to 3.799)	1.544 (0.454 to 3.893)
3.665 (0.716 to 11.442)	3.493 (0.721 to 10.629)	3.634 (0.744 to 10.727)	3.518 (0.743 to 9.943)	3.579 (0.832 to 10.234)	3.576 (0.774 to 10.582)	2.291 (1.209 to 3.935)	2.287 (1.13 to 4.048)	1.708 (1.023 to 2.625)	1.67 (1.026 to 2.52)	1.298 (0.628 to 2.387)	1.331 (0.65 to 2.537)	1.323 (0.61 to 2.458)	1.301 (0.624 to 2.507)
1.073 (0.733 to 1.534)	1.071 (0.73 to 1.524)	1.072 (0.717 to 1.565)	1.081 (0.721 to 1.571)	1.077 (0.725 to 1.55)	1.077 (0.724 to 1.542)	1.324 (1.072 to 1.619)	1.323 (1.069 to 1.629)	1.255 (1.064 to 1.462)	1.254 (1.08 to 1.46)	1.155 (0.925 to 1.405)	1.148 (0.936 to 1.393)	1.147 (0.931 to 1.385)	1.156 (0.932 to 1.41)
2.077 (1.425 to 2.946)	2.074 (1.405 to 2.996)	2.093 (1.413 to 3.032)	2.092 (1.365 to 3.032)	2.085 (1.391 to 2.983)	2.079 (1.4 to 2.988)	1.478 (1.289 to 1.683)	1.487 (1.319 to 1.692)	1.272 (1.093 to 1.49)	1.27 (1.076 to 1.497)	1.11 (0.854 to 1.433)	1.102 (0.834 to 1.419)	1.107 (0.835 to 1.446)	1.109 (0.822 to 1.429)
1.0 (1.0 to 1.0)	1.0 (1.0 to 1.0)	1.0 (1.0 to 1.0)	1.0 (1.0 to 1.0)	1.0 (1.0 to 1.0)	1.0 (1.0 to 1.0)	1.0 (1.0 to 1.0)	1.0 (1.0 to 1.0)	1.0 (1.0 to 1.0)	1.0 (1.0 to 1.0)	1.0 (1.0 to 1.0)	1.0 (1.0 to 1.0)	1.0 (1.0 to 1.0)	1.0 (1.0 to 1.0)
200.617 (34.114 to 695.696)	211.239 (32.055 to 800.634)	205.926 (31.084 to 751.759)	200.856 (33.786 to 732.241)	207.342 (30.718 to 650.978)	202.164 (32.222 to 701.663)	17.647 (7.939 to 32.318)	17.353 (8.097 to 31.55)	5.347 (2.334 to 10.628)	5.427 (2.272 to 10.749)	1.895 (0.429 to 5.39)	1.852 (0.444 to 5.291)	1.856 (0.437 to 5.035)	1.925 (0.427 to 5.377)
54.062 (15.177 to 136.039)	54.638 (14.697 to 139.549)	55.201 (16.279 to 139.398)	54.066 (14.955 to 142.728)	55.805 (15.506 to 137.121)	55.87 (15.838 to 139.768)	7.713 (4.647 to 11.89)	7.845 (4.686 to 12.511)	3.141 (1.836 to 5.218)	3.213 (1.87 to 5.196)	1.464 (0.558 to 3.037)	1.47 (0.601 to 3.006)	1.462 (0.599 to 3.073)	1.488 (0.593 to 3.038)
3.102 (1.839 to 4.864)	3.045 (1.792 to 4.748)	3.114 (1.906 to 4.86)	3.039 (1.817 to 4.807)	3.117 (1.883 to 4.881)	3.113 (1.839 to 4.781)	2.055 (1.57 to 2.665)	2.05 (1.584 to 2.635)	1.525 (1.216 to 1.891)	1.531 (1.221 to 1.92)	1.142 (0.868 to 1.461)	1.158 (0.875 to 1.513)	1.147 (0.871 to 1.476)	1.158 (0.879 to 1.484)
3.572 (1.98 to 5.768)	3.57 (1.977 to 5.944)	3.569 (1.991 to 5.91)	3.543 (1.934 to 5.923)	3.607 (1.98 to 6.203)	3.552 (1.902 to 5.879)	1.758 (1.442 to 2.135)	1.759 (1.451 to 2.101)	1.336 (1.033 to 1.695)	1.327 (1.018 to 1.727)	1.037 (0.664 to 1.542)	1.029 (0.687 to 1.478)	1.033 (0.646 to 1.496)	1.038 (0.68 to 1.548)
1.0 (1.0 to 1.0)	1.0 (1.0 to 1.0)	1.0 (1.0 to 1.0)	1.0 (1.0 to 1.0)	1.0 (1.0 to 1.0)	1.0 (1.0 to 1.0)	1.0 (1.0 to 1.0)	1.0 (1.0 to 1.0)	1.0 (1.0 to 1.0)	1.0 (1.0 to 1.0)	1.0 (1.0 to 1.0)	1.0 (1.0 to 1.0)	1.0 (1.0 to 1.0)	1.0 (1.0 to 1.0)
2.747 (2.47 to 3.035)	2.747 (2.482 to 3.035)	2.736 (2.466 to 3.014)	2.741 (2.48 to 3.053)	2.729 (2.47 to 3.007)	2.74 (2.479 to 3.038)	2.747 (2.485 to 3.049)	2.742 (2.479 to 3.021)	2.742 (2.496 to 3.039)	2.74 (2.48 to 3.042)	2.736 (2.468 to 3.013)	2.736 (2.468 to 3.013)	2.736 (2.468 to 3.013)	2.736 (2.468 to 3.013)
2.743 (2.484 to 3.014)	2.742 (2.473 to 3.034)	2.742 (2.442 to 3.029)	2.746 (2.494 to 3.045)	2.748 (2.483 to 3.045)	2.744 (2.462 to 3.029)	2.749 (2.477 to 3.018)	2.755 (2.495 to 3.038)	2.747 (2.473 to 3.034)	2.738 (2.463 to 3.041)	2.744 (2.478 to 3.021)	2.744 (2.478 to 3.021)	2.744 (2.478 to 3.021)	2.744 (2.478 to 3.021)
2.753 (2.48 to 3.038)	2.743 (2.465 to 3.024)	2.748 (2.491 to 3.029)	2.743 (2.467 to 3.051)	2.749 (2.473 to 3.042)	2.744 (2.475 to 3.017)	2.741 (2.464 to 3.034)	2.745 (2.478 to 3.037)	2.739 (2.465 to 3.006)	2.745 (2.473 to 3.029)	2.747 (2.475 to 3.028)	2.747 (2.475 to 3.028)	2.747 (2.475 to 3.028)	2.747 (2.475 to 3.028)
1.0 (1.0 to 1.0)	1.0 (1.0 to 1.0)	1.0 (1.0 to 1.0)	1.0 (1.0 to 1.0)	1.0 (1.0 to 1.0)	1.0 (1.0 to 1.0)	1.0 (1.0 to 1.0)	1.0 (1.0 to 1.0)	1.0 (1.0 to 1.0)	1.0 (1.0 to 1.0)	1.0 (1.0 to 1.0)	1.0 (1.0 to 1.0)	1.0 (1.0 to 1.0)	1.0 (1.0 to 1.0)
1.0 (1.0 to 1.0)	1.0 (1.0 to 1.0)	1.0 (1.0 to 1.0)	1.0 (1.0 to 1.0)	1.0 (1.0 to 1.0)	1.0 (1.0 to 1.0)	1.0 (1.0 to 1.0)	1.0 (1.0 to 1.0)	1.0 (1.0 to 1.0)	1.0 (1.0 to 1.0)	1.0 (1.0 to 1.0)	1.0 (1.0 to 1.0)	1.0 (1.0 to 1.0)	1.0 (1.0 to 1.0)

Appendix Table 1a. Relative risks used by age and sex for each outcome for all risk factors except for ambient air pollution and alcohol.

Risk - Outcome	Category / Units	Morbidity / Mortality	Sex	All-age	0-6 days	7-27 days	28-364 days	1-4 years	5-9 years	10-14 years	15-19 years	20-24 years	25-29 years

Drug use

Opiod use disorders

Cocaine use disorders

Amphetamine use disorders

Cannabis use disorders

Other drug use disorders

Low glomerular filtration rate

Chronic kidney disease due to diabetes mellitus

Chronic kidney disease due to hypertension

Chronic kidney disease due to glomerulonephritis

Chronic kidney disease due to other causes

Other sexually transmitted diseases

Cervical cancer

Sexually transmitted diseases excluding HIV

Vitamin A deficiency

Vitamin A deficiency

Ages													
30-34 years	35-39 years	40-44 years	45-49 years	50-54 years	55-59 years	60-64 years	65-69 years	70-74 years	75-79 years	80-84 years	85-89 years	90-94 years	95+ years

Appendix Table 1b. Relative risks used by age and sex for each outcome for the particulate matter integrated exposure response curve.

Risk - Outcome	Category / Units	Morbidity / Mortality	Sex	25-29 years	30-34 years	35-39 years	40-44 years	45-49 years	50-54 years	55-59 years	60-64 years	65-69 years	70-74 years	75-79 years	80-84 years	85-89 years	90-94 years	95+ years
**Ambient particulate matter pollution (PM2.5)**																		
Lower respiratory infections	600 $\mu g/m^3$	Both	Both	2.568 (2.002 to 3.192)	2.568 (2.002 to 3.192)	2.568 (2.002 to 3.192)	2.568 (2.002 to 3.192)	2.568 (2.002 to 3.192)	2.568 (2.002 to 3.192)	2.568 (2.002 to 3.192)	2.568 (2.002 to 3.192)	2.568 (2.002 to 3.192)	2.568 (2.002 to 3.192)	2.568 (2.002 to 3.192)	2.568 (2.002 to 3.192)	2.568 (2.002 to 3.192)	2.568 (2.002 to 3.192)	2.568 (2.002 to 3.192)
Lower respiratory infections	500 $\mu g/m^3$	Both	Both	2.517 (1.961 to 3.152)	2.517 (1.961 to 3.152)	2.517 (1.961 to 3.152)	2.517 (1.961 to 3.152)	2.517 (1.961 to 3.152)	2.517 (1.961 to 3.152)	2.517 (1.961 to 3.152)	2.517 (1.961 to 3.152)	2.517 (1.961 to 3.152)	2.517 (1.961 to 3.152)	2.517 (1.961 to 3.152)	2.517 (1.961 to 3.152)	2.517 (1.961 to 3.152)	2.517 (1.961 to 3.152)	2.517 (1.961 to 3.152)
Lower respiratory infections	400 $\mu g/m^3$	Both	Both	2.445 (1.91 to 3.046)	2.445 (1.91 to 3.046)	2.445 (1.91 to 3.046)	2.445 (1.91 to 3.046)	2.445 (1.91 to 3.046)	2.445 (1.91 to 3.046)	2.445 (1.91 to 3.046)	2.445 (1.91 to 3.046)	2.445 (1.91 to 3.046)	2.445 (1.91 to 3.046)	2.445 (1.91 to 3.046)	2.445 (1.91 to 3.046)	2.445 (1.91 to 3.046)	2.445 (1.91 to 3.046)	2.445 (1.91 to 3.046)
Lower respiratory infections	300 $\mu g/m^3$	Both	Both	2.332 (1.836 to 2.894)	2.332 (1.836 to 2.894)	2.332 (1.836 to 2.894)	2.332 (1.836 to 2.894)	2.332 (1.836 to 2.894)	2.332 (1.836 to 2.894)	2.332 (1.836 to 2.894)	2.332 (1.836 to 2.894)	2.332 (1.836 to 2.894)	2.332 (1.836 to 2.894)	2.332 (1.836 to 2.894)	2.332 (1.836 to 2.894)	2.332 (1.836 to 2.894)	2.332 (1.836 to 2.894)	2.332 (1.836 to 2.894)
Lower respiratory infections	200 $\mu g/m^3$	Both	Both	2.139 (1.711 to 2.642)	2.139 (1.711 to 2.642)	2.139 (1.711 to 2.642)	2.139 (1.711 to 2.642)	2.139 (1.711 to 2.642)	2.139 (1.711 to 2.642)	2.139 (1.711 to 2.642)	2.139 (1.711 to 2.642)	2.139 (1.711 to 2.642)	2.139 (1.711 to 2.642)	2.139 (1.711 to 2.642)	2.139 (1.711 to 2.642)	2.139 (1.711 to 2.642)	2.139 (1.711 to 2.642)	2.139 (1.711 to 2.642)
Lower respiratory infections	150 $\mu g/m^3$	Both	Both	1.987 (1.63 to 2.417)	1.987 (1.63 to 2.417)	1.987 (1.63 to 2.417)	1.987 (1.63 to 2.417)	1.987 (1.63 to 2.417)	1.987 (1.63 to 2.417)	1.987 (1.63 to 2.417)	1.987 (1.63 to 2.417)	1.987 (1.63 to 2.417)	1.987 (1.63 to 2.417)	1.987 (1.63 to 2.417)	1.987 (1.63 to 2.417)	1.987 (1.63 to 2.417)	1.987 (1.63 to 2.417)	1.987 (1.63 to 2.417)
Lower respiratory infections	135 $\mu g/m^3$	Both	Both	1.93 (1.6 to 2.333)	1.93 (1.6 to 2.333)	1.93 (1.6 to 2.333)	1.93 (1.6 to 2.333)	1.93 (1.6 to 2.333)	1.93 (1.6 to 2.333)	1.93 (1.6 to 2.333)	1.93 (1.6 to 2.333)	1.93 (1.6 to 2.333)	1.93 (1.6 to 2.333)	1.93 (1.6 to 2.333)	1.93 (1.6 to 2.333)	1.93 (1.6 to 2.333)	1.93 (1.6 to 2.333)	1.93 (1.6 to 2.333)
Lower respiratory infections	120 $\mu g/m^3$	Both	Both	1.867 (1.565 to 2.23)	1.867 (1.565 to 2.23)	1.867 (1.565 to 2.23)	1.867 (1.565 to 2.23)	1.867 (1.565 to 2.23)	1.867 (1.565 to 2.23)	1.867 (1.565 to 2.23)	1.867 (1.565 to 2.23)	1.867 (1.565 to 2.23)	1.867 (1.565 to 2.23)	1.867 (1.565 to 2.23)	1.867 (1.565 to 2.23)	1.867 (1.565 to 2.23)	1.867 (1.565 to 2.23)	1.867 (1.565 to 2.23)
Lower respiratory infections	105 $\mu g/m^3$	Both	Both	1.797 (1.528 to 2.131)	1.797 (1.528 to 2.131)	1.797 (1.528 to 2.131)	1.797 (1.528 to 2.131)	1.797 (1.528 to 2.131)	1.797 (1.528 to 2.131)	1.797 (1.528 to 2.131)	1.797 (1.528 to 2.131)	1.797 (1.528 to 2.131)	1.797 (1.528 to 2.131)	1.797 (1.528 to 2.131)	1.797 (1.528 to 2.131)	1.797 (1.528 to 2.131)	1.797 (1.528 to 2.131)	1.797 (1.528 to 2.131)
Lower respiratory infections	90 $\mu g/m^3$	Both	Both	1.719 (1.483 to 2.022)	1.719 (1.483 to 2.022)	1.719 (1.483 to 2.022)	1.719 (1.483 to 2.022)	1.719 (1.483 to 2.022)	1.719 (1.483 to 2.022)	1.719 (1.483 to 2.022)	1.719 (1.483 to 2.022)	1.719 (1.483 to 2.022)	1.719 (1.483 to 2.022)	1.719 (1.483 to 2.022)	1.719 (1.483 to 2.022)	1.719 (1.483 to 2.022)	1.719 (1.483 to 2.022)	1.719 (1.483 to 2.022)
Lower respiratory infections	75 $\mu g/m^3$	Both	Both	1.631 (1.433 to 1.885)	1.631 (1.433 to 1.885)	1.631 (1.433 to 1.885)	1.631 (1.433 to 1.885)	1.631 (1.433 to 1.885)	1.631 (1.433 to 1.885)	1.631 (1.433 to 1.885)	1.631 (1.433 to 1.885)	1.631 (1.433 to 1.885)	1.631 (1.433 to 1.885)	1.631 (1.433 to 1.885)	1.631 (1.433 to 1.885)	1.631 (1.433 to 1.885)	1.631 (1.433 to 1.885)	1.631 (1.433 to 1.885)
Lower respiratory infections	60 $\mu g/m^3$	Both	Both	1.533 (1.365 to 1.769)	1.533 (1.365 to 1.769)	1.533 (1.365 to 1.769)	1.533 (1.365 to 1.769)	1.533 (1.365 to 1.769)	1.533 (1.365 to 1.769)	1.533 (1.365 to 1.769)	1.533 (1.365 to 1.769)	1.533 (1.365 to 1.769)	1.533 (1.365 to 1.769)	1.533 (1.365 to 1.769)	1.533 (1.365 to 1.769)	1.533 (1.365 to 1.769)	1.533 (1.365 to 1.769)	1.533 (1.365 to 1.769)
Lower respiratory infections	45 $\mu g/m^3$	Both	Both	1.421 (1.271 to 1.614)	1.421 (1.271 to 1.614)	1.421 (1.271 to 1.614)	1.421 (1.271 to 1.614)	1.421 (1.271 to 1.614)	1.421 (1.271 to 1.614)	1.421 (1.271 to 1.614)	1.421 (1.271 to 1.614)	1.421 (1.271 to 1.614)	1.421 (1.271 to 1.614)	1.421 (1.271 to 1.614)	1.421 (1.271 to 1.614)	1.421 (1.271 to 1.614)	1.421 (1.271 to 1.614)	1.421 (1.271 to 1.614)
Lower respiratory infections	30 $\mu g/m^3$	Both	Both	1.292 (1.159 to 1.451)	1.292 (1.159 to 1.451)	1.292 (1.159 to 1.451)	1.292 (1.159 to 1.451)	1.292 (1.159 to 1.451)	1.292 (1.159 to 1.451)	1.292 (1.159 to 1.451)	1.292 (1.159 to 1.451)	1.292 (1.159 to 1.451)	1.292 (1.159 to 1.451)	1.292 (1.159 to 1.451)	1.292 (1.159 to 1.451)	1.292 (1.159 to 1.451)	1.292 (1.159 to 1.451)	1.292 (1.159 to 1.451)
Lower respiratory infections	25 $\mu g/m^3$	Both	Both	1.244 (1.118 to 1.403)	1.244 (1.118 to 1.403)	1.244 (1.118 to 1.403)	1.244 (1.118 to 1.403)	1.244 (1.118 to 1.403)	1.244 (1.118 to 1.403)	1.244 (1.118 to 1.403)	1.244 (1.118 to 1.403)	1.244 (1.118 to 1.403)	1.244 (1.118 to 1.403)	1.244 (1.118 to 1.403)	1.244 (1.118 to 1.403)	1.244 (1.118 to 1.403)	1.244 (1.118 to 1.403)	1.244 (1.118 to 1.403)
Lower respiratory infections	20 $\mu g/m^3$	Both	Both	1.191 (1.078 to 1.344)	1.191 (1.078 to 1.344)	1.191 (1.078 to 1.344)	1.191 (1.078 to 1.344)	1.191 (1.078 to 1.344)	1.191 (1.078 to 1.344)	1.191 (1.078 to 1.344)	1.191 (1.078 to 1.344)	1.191 (1.078 to 1.344)	1.191 (1.078 to 1.344)	1.191 (1.078 to 1.344)	1.191 (1.078 to 1.344)	1.191 (1.078 to 1.344)	1.191 (1.078 to 1.344)	1.191 (1.078 to 1.344)
Lower respiratory infections	15 $\mu g/m^3$	Both	Both	1.133 (1.041 to 1.278)	1.133 (1.041 to 1.278)	1.133 (1.041 to 1.278)	1.133 (1.041 to 1.278)	1.133 (1.041 to 1.278)	1.133 (1.041 to 1.278)	1.133 (1.041 to 1.278)	1.133 (1.041 to 1.278)	1.133 (1.041 to 1.278)	1.133 (1.041 to 1.278)	1.133 (1.041 to 1.278)	1.133 (1.041 to 1.278)	1.133 (1.041 to 1.278)	1.133 (1.041 to 1.278)	1.133 (1.041 to 1.278)
Lower respiratory infections	10 $\mu g/m^3$	Both	Both	1.063 (1.007 to 1.189)	1.063 (1.007 to 1.189)	1.063 (1.007 to 1.189)	1.063 (1.007 to 1.189)	1.063 (1.007 to 1.189)	1.063 (1.007 to 1.189)	1.063 (1.007 to 1.189)	1.063 (1.007 to 1.189)	1.063 (1.007 to 1.189)	1.063 (1.007 to 1.189)	1.063 (1.007 to 1.189)	1.063 (1.007 to 1.189)	1.063 (1.007 to 1.189)	1.063 (1.007 to 1.189)	1.063 (1.007 to 1.189)
Lower respiratory infections	5 $\mu g/m^3$	Both	Both	1.0 (1.0 to 1.0)	1.0 (1.0 to 1.0)	1.0 (1.0 to 1.0)	1.0 (1.0 to 1.0)	1.0 (1.0 to 1.0)	1.0 (1.0 to 1.0)	1.0 (1.0 to 1.0)	1.0 (1.0 to 1.0)	1.0 (1.0 to 1.0)	1.0 (1.0 to 1.0)	1.0 (1.0 to 1.0)	1.0 (1.0 to 1.0)	1.0 (1.0 to 1.0)	1.0 (1.0 to 1.0)	1.0 (1.0 to 1.0)
Lower respiratory infections	0 $\mu g/m^3$	Both	Both	1.0 (1.0 to 1.0)	1.0 (1.0 to 1.0)	1.0 (1.0 to 1.0)	1.0 (1.0 to 1.0)	1.0 (1.0 to 1.0)	1.0 (1.0 to 1.0)	1.0 (1.0 to 1.0)	1.0 (1.0 to 1.0)	1.0 (1.0 to 1.0)	1.0 (1.0 to 1.0)	1.0 (1.0 to 1.0)	1.0 (1.0 to 1.0)	1.0 (1.0 to 1.0)	1.0 (1.0 to 1.0)	1.0 (1.0 to 1.0)
Tracheal, bronchus, and lung cancer	600 $\mu g/m^3$	Both	Both	3.146 (2.611 to 3.719)	3.146 (2.611 to 3.719)	3.146 (2.611 to 3.719)	3.146 (2.611 to 3.719)	3.146 (2.611 to 3.719)	3.146 (2.611 to 3.719)	3.146 (2.611 to 3.719)	3.146 (2.611 to 3.719)	3.146 (2.611 to 3.719)	3.146 (2.611 to 3.719)	3.146 (2.611 to 3.719)	3.146 (2.611 to 3.719)	3.146 (2.611 to 3.719)	3.146 (2.611 to 3.719)	3.146 (2.611 to 3.719)
Tracheal, bronchus, and lung cancer	500 $\mu g/m^3$	Both	Both	2.864 (2.367 to 3.38)	2.864 (2.367 to 3.38)	2.864 (2.367 to 3.38)	2.864 (2.367 to 3.38)	2.864 (2.367 to 3.38)	2.864 (2.367 to 3.38)	2.864 (2.367 to 3.38)	2.864 (2.367 to 3.38)	2.864 (2.367 to 3.38)	2.864 (2.367 to 3.38)	2.864 (2.367 to 3.38)	2.864 (2.367 to 3.38)	2.864 (2.367 to 3.38)	2.864 (2.367 to 3.38)	2.864 (2.367 to 3.38)
Tracheal, bronchus, and lung cancer	400 $\mu g/m^3$	Both	Both	2.567 (2.126 to 3.012)	2.567 (2.126 to 3.012)	2.567 (2.126 to 3.012)	2.567 (2.126 to 3.012)	2.567 (2.126 to 3.012)	2.567 (2.126 to 3.012)	2.567 (2.126 to 3.012)	2.567 (2.126 to 3.012)	2.567 (2.126 to 3.012)	2.567 (2.126 to 3.012)	2.567 (2.126 to 3.012)	2.567 (2.126 to 3.012)	2.567 (2.126 to 3.012)	2.567 (2.126 to 3.012)	2.567 (2.126 to 3.012)
Tracheal, bronchus, and lung cancer	300 $\mu g/m^3$	Both	Both	2.251 (1.882 to 2.637)	2.251 (1.882 to 2.637)	2.251 (1.882 to 2.637)	2.251 (1.882 to 2.637)	2.251 (1.882 to 2.637)	2.251 (1.882 to 2.637)	2.251 (1.882 to 2.637)	2.251 (1.882 to 2.637)	2.251 (1.882 to 2.637)	2.251 (1.882 to 2.637)	2.251 (1.882 to 2.637)	2.251 (1.882 to 2.637)	2.251 (1.882 to 2.637)	2.251 (1.882 to 2.637)	2.251 (1.882 to 2.637)
Tracheal, bronchus, and lung cancer	200 $\mu g/m^3$	Both	Both	1.908 (1.622 to 2.228)	1.908 (1.622 to 2.228)	1.908 (1.622 to 2.228)	1.908 (1.622 to 2.228)	1.908 (1.622 to 2.228)	1.908 (1.622 to 2.228)	1.908 (1.622 to 2.228)	1.908 (1.622 to 2.228)	1.908 (1.622 to 2.228)	1.908 (1.622 to 2.228)	1.908 (1.622 to 2.228)	1.908 (1.622 to 2.228)	1.908 (1.622 to 2.228)	1.908 (1.622 to 2.228)	1.908 (1.622 to 2.228)
Tracheal, bronchus, and lung cancer	150 $\mu g/m^3$	Both	Both	1.721 (1.481 to 1.998)	1.721 (1.481 to 1.998)	1.721 (1.481 to 1.998)	1.721 (1.481 to 1.998)	1.721 (1.481 to 1.998)	1.721 (1.481 to 1.998)	1.721 (1.481 to 1.998)	1.721 (1.481 to 1.998)	1.721 (1.481 to 1.998)	1.721 (1.481 to 1.998)	1.721 (1.481 to 1.998)	1.721 (1.481 to 1.998)	1.721 (1.481 to 1.998)	1.721 (1.481 to 1.998)	1.721 (1.481 to 1.998)
Tracheal, bronchus, and lung cancer	135 $\mu g/m^3$	Both	Both	1.662 (1.437 to 1.926)	1.662 (1.437 to 1.926)	1.662 (1.437 to 1.926)	1.662 (1.437 to 1.926)	1.662 (1.437 to 1.926)	1.662 (1.437 to 1.926)	1.662 (1.437 to 1.926)	1.662 (1.437 to 1.926)	1.662 (1.437 to 1.926)	1.662 (1.437 to 1.926)	1.662 (1.437 to 1.926)	1.662 (1.437 to 1.926)	1.662 (1.437 to 1.926)	1.662 (1.437 to 1.926)	1.662 (1.437 to 1.926)
Tracheal, bronchus, and lung cancer	120 $\mu g/m^3$	Both	Both	1.601 (1.391 to 1.851)	1.601 (1.391 to 1.851)	1.601 (1.391 to 1.851)	1.601 (1.391 to 1.851)	1.601 (1.391 to 1.851)	1.601 (1.391 to 1.851)	1.601 (1.391 to 1.851)	1.601 (1.391 to 1.851)	1.601 (1.391 to 1.851)	1.601 (1.391 to 1.851)	1.601 (1.391 to 1.851)	1.601 (1.391 to 1.851)	1.601 (1.391 to 1.851)	1.601 (1.391 to 1.851)	1.601 (1.391 to 1.851)
Tracheal, bronchus, and lung cancer	105 $\mu g/m^3$	Both	Both	1.539 (1.345 to 1.772)	1.539 (1.345 to 1.772)	1.539 (1.345 to 1.772)	1.539 (1.345 to 1.772)	1.539 (1.345 to 1.772)	1.539 (1.345 to 1.772)	1.539 (1.345 to 1.772)	1.539 (1.345 to 1.772)	1.539 (1.345 to 1.772)	1.539 (1.345 to 1.772)	1.539 (1.345 to 1.772)	1.539 (1.345 to 1.772)	1.539 (1.345 to 1.772)	1.539 (1.345 to 1.772)	1.539 (1.345 to 1.772)
Tracheal, bronchus, and lung cancer	90 $\mu g/m^3$	Both	Both	1.474 (1.297 to 1.686)	1.474 (1.297 to 1.686)	1.474 (1.297 to 1.686)	1.474 (1.297 to 1.686)	1.474 (1.297 to 1.686)	1.474 (1.297 to 1.686)	1.474 (1.297 to 1.686)	1.474 (1.297 to 1.686)	1.474 (1.297 to 1.686)	1.474 (1.297 to 1.686)	1.474 (1.297 to 1.686)	1.474 (1.297 to 1.686)	1.474 (1.297 to 1.686)	1.474 (1.297 to 1.686)	1.474 (1.297 to 1.686)

Appendix Table 1b. Relative risks used by age and sex for each outcome for the particulate matter integrated exposure response curve.

Risk - Outcome	Category / Units	Morbidity / Mortality	Sex	25-29 years	30-34 years	35-39 years	40-44 years	45-49 years	50-54 years	55-59 years	60-64 years	65-69 years	70-74 years	75-79 years	80-84 years	85-89 years	90-94 years	95+ years
**Ambient particulate matter pollution (PM2.5)**																		
Tracheal, bronchus, and lung cancer	75 µg/m³	Both	Both	1.406 (1.248 to 1.598)	1.406 (1.248 to 1.598)	1.406 (1.248 to 1.598)	1.406 (1.248 to 1.598)	1.406 (1.248 to 1.598)	1.406 (1.248 to 1.598)	1.406 (1.248 to 1.598)	1.406 (1.248 to 1.598)	1.406 (1.248 to 1.598)	1.406 (1.248 to 1.598)	1.406 (1.248 to 1.598)	1.406 (1.248 to 1.598)	1.406 (1.248 to 1.598)	1.406 (1.248 to 1.598)	1.406 (1.248 to 1.598)
Tracheal, bronchus, and lung cancer	60 µg/m³	Both	Both	1.335 (1.199 to 1.505)	1.335 (1.199 to 1.505)	1.335 (1.199 to 1.505)	1.335 (1.199 to 1.505)	1.335 (1.199 to 1.505)	1.335 (1.199 to 1.505)	1.335 (1.199 to 1.505)	1.335 (1.199 to 1.505)	1.335 (1.199 to 1.505)	1.335 (1.199 to 1.505)	1.335 (1.199 to 1.505)	1.335 (1.199 to 1.505)	1.335 (1.199 to 1.505)	1.335 (1.199 to 1.505)	1.335 (1.199 to 1.505)
Tracheal, bronchus, and lung cancer	45 µg/m³	Both	Both	1.259 (1.148 to 1.403)	1.259 (1.148 to 1.403)	1.259 (1.148 to 1.403)	1.259 (1.148 to 1.403)	1.259 (1.148 to 1.403)	1.259 (1.148 to 1.403)	1.259 (1.148 to 1.403)	1.259 (1.148 to 1.403)	1.259 (1.148 to 1.403)	1.259 (1.148 to 1.403)	1.259 (1.148 to 1.403)	1.259 (1.148 to 1.403)	1.259 (1.148 to 1.403)	1.259 (1.148 to 1.403)	1.259 (1.148 to 1.403)
Tracheal, bronchus, and lung cancer	30 µg/m³	Both	Both	1.176 (1.094 to 1.284)	1.176 (1.094 to 1.284)	1.176 (1.094 to 1.284)	1.176 (1.094 to 1.284)	1.176 (1.094 to 1.284)	1.176 (1.094 to 1.284)	1.176 (1.094 to 1.284)	1.176 (1.094 to 1.284)	1.176 (1.094 to 1.284)	1.176 (1.094 to 1.284)	1.176 (1.094 to 1.284)	1.176 (1.094 to 1.284)	1.176 (1.094 to 1.284)	1.176 (1.094 to 1.284)	1.176 (1.094 to 1.284)
Tracheal, bronchus, and lung cancer	25 µg/m³	Both	Both	1.145 (1.075 to 1.241)	1.145 (1.075 to 1.241)	1.145 (1.075 to 1.241)	1.145 (1.075 to 1.241)	1.145 (1.075 to 1.241)	1.145 (1.075 to 1.241)	1.145 (1.075 to 1.241)	1.145 (1.075 to 1.241)	1.145 (1.075 to 1.241)	1.145 (1.075 to 1.241)	1.145 (1.075 to 1.241)	1.145 (1.075 to 1.241)	1.145 (1.075 to 1.241)	1.145 (1.075 to 1.241)	1.145 (1.075 to 1.241)
Tracheal, bronchus, and lung cancer	20 µg/m³	Both	Both	1.112 (1.056 to 1.195)	1.112 (1.056 to 1.195)	1.112 (1.056 to 1.195)	1.112 (1.056 to 1.195)	1.112 (1.056 to 1.195)	1.112 (1.056 to 1.195)	1.112 (1.056 to 1.195)	1.112 (1.056 to 1.195)	1.112 (1.056 to 1.195)	1.112 (1.056 to 1.195)	1.112 (1.056 to 1.195)	1.112 (1.056 to 1.195)	1.112 (1.056 to 1.195)	1.112 (1.056 to 1.195)	1.112 (1.056 to 1.195)
Tracheal, bronchus, and lung cancer	15 µg/m³	Both	Both	1.076 (1.036 to 1.14)	1.076 (1.036 to 1.14)	1.076 (1.036 to 1.14)	1.076 (1.036 to 1.14)	1.076 (1.036 to 1.14)	1.076 (1.036 to 1.14)	1.076 (1.036 to 1.14)	1.076 (1.036 to 1.14)	1.076 (1.036 to 1.14)	1.076 (1.036 to 1.14)	1.076 (1.036 to 1.14)	1.076 (1.036 to 1.14)	1.076 (1.036 to 1.14)	1.076 (1.036 to 1.14)	1.076 (1.036 to 1.14)
Tracheal, bronchus, and lung cancer	10 µg/m³	Both	Both	1.034 (1.012 to 1.075)	1.034 (1.012 to 1.075)	1.034 (1.012 to 1.075)	1.034 (1.012 to 1.075)	1.034 (1.012 to 1.075)	1.034 (1.012 to 1.075)	1.034 (1.012 to 1.075)	1.034 (1.012 to 1.075)	1.034 (1.012 to 1.075)	1.034 (1.012 to 1.075)	1.034 (1.012 to 1.075)	1.034 (1.012 to 1.075)	1.034 (1.012 to 1.075)	1.034 (1.012 to 1.075)	1.034 (1.012 to 1.075)
Tracheal, bronchus, and lung cancer	5 µg/m³	Both	Both	1.0 (1.0 to 1.0)	1.0 (1.0 to 1.0)	1.0 (1.0 to 1.0)	1.0 (1.0 to 1.0)	1.0 (1.0 to 1.0)	1.0 (1.0 to 1.0)	1.0 (1.0 to 1.0)	1.0 (1.0 to 1.0)	1.0 (1.0 to 1.0)	1.0 (1.0 to 1.0)	1.0 (1.0 to 1.0)	1.0 (1.0 to 1.0)	1.0 (1.0 to 1.0)	1.0 (1.0 to 1.0)	1.0 (1.0 to 1.0)
Tracheal, bronchus, and lung cancer	0 µg/m³	Both	Both	1.0 (1.0 to 1.0)	1.0 (1.0 to 1.0)	1.0 (1.0 to 1.0)	1.0 (1.0 to 1.0)	1.0 (1.0 to 1.0)	1.0 (1.0 to 1.0)	1.0 (1.0 to 1.0)	1.0 (1.0 to 1.0)	1.0 (1.0 to 1.0)	1.0 (1.0 to 1.0)	1.0 (1.0 to 1.0)	1.0 (1.0 to 1.0)	1.0 (1.0 to 1.0)	1.0 (1.0 to 1.0)	1.0 (1.0 to 1.0)
Ischaemic heart disease	600 µg/m³	Both	Both	2.534 (1.953 to 3.574)	2.461 (1.902 to 3.954)	2.31 (1.857 to 3.495)	2.156 (1.748 to 3.079)	2.025 (1.68 to 2.823)	1.904 (1.596 to 2.559)	1.82 (1.519 to 2.516)	1.71 (1.438 to 2.322)	1.62 (1.408 to 2.105)	1.512 (1.344 to 1.781)	1.445 (1.295 to 1.814)	1.369 (1.262 to 1.612)	1.29 (1.205 to 1.421)	1.231 (1.164 to 1.394)	1.164 (1.122 to 1.236)
Ischaemic heart disease	500 µg/m³	Both	Both	2.441 (1.875 to 3.447)	2.378 (1.824 to 3.931)	2.237 (1.79 to 3.416)	2.091 (1.689 to 3.034)	1.969 (1.623 to 2.772)	1.855 (1.548 to 2.49)	1.778 (1.479 to 2.493)	1.674 (1.4 to 2.291)	1.589 (1.38 to 2.093)	1.486 (1.321 to 1.747)	1.424 (1.276 to 1.804)	1.352 (1.245 to 1.606)	1.277 (1.192 to 1.404)	1.221 (1.153 to 1.382)	1.157 (1.113 to 1.229)
Ischaemic heart disease	400 µg/m³	Both	Both	2.333 (1.784 to 3.287)	2.28 (1.744 to 3.869)	2.151 (1.71 to 3.292)	2.017 (1.615 to 2.957)	1.903 (1.563 to 2.7)	1.798 (1.498 to 2.404)	1.728 (1.435 to 2.48)	1.632 (1.359 to 2.25)	1.554 (1.346 to 2.073)	1.456 (1.293 to 1.707)	1.399 (1.254 to 1.79)	1.332 (1.225 to 1.595)	1.261 (1.177 to 1.386)	1.209 (1.142 to 1.366)	1.149 (1.105 to 1.22)
Ischaemic heart disease	300 µg/m³	Both	Both	2.204 (1.678 to 3.074)	2.162 (1.647 to 3.714)	2.048 (1.618 to 3.189)	1.926 (1.535 to 2.866)	1.824 (1.489 to 2.599)	1.729 (1.435 to 2.289)	1.667 (1.383 to 2.42)	1.58 (1.315 to 2.184)	1.509 (1.308 to 2.018)	1.42 (1.263 to 1.655)	1.369 (1.227 to 1.765)	1.308 (1.203 to 1.579)	1.241 (1.158 to 1.362)	1.194 (1.127 to 1.344)	1.138 (1.095 to 1.208)
Ischaemic heart disease	200 µg/m³	Both	Both	2.038 (1.553 to 2.792)	2.008 (1.521 to 3.417)	1.913 (1.505 to 2.953)	1.807 (1.438 to 2.67)	1.721 (1.403 to 2.399)	1.639 (1.361 to 2.13)	1.587 (1.317 to 2.298)	1.512 (1.257 to 2.047)	1.451 (1.257 to 1.919)	1.372 (1.222 to 1.594)	1.329 (1.194 to 1.693)	1.275 (1.172 to 1.538)	1.215 (1.136 to 1.33)	1.174 (1.109 to 1.308)	1.124 (1.082 to 1.188)
Ischaemic heart disease	150 µg/m³	Both	Both	1.931 (1.475 to 2.614)	1.907 (1.445 to 3.155)	1.824 (1.437 to 2.745)	1.73 (1.371 to 2.494)	1.653 (1.351 to 2.287)	1.58 (1.314 to 2.026)	1.534 (1.277 to 2.173)	1.467 (1.221 to 1.942)	1.412 (1.227 to 1.842)	1.341 (1.195 to 1.555)	1.302 (1.173 to 1.629)	1.253 (1.151 to 1.497)	1.198 (1.122 to 1.308)	1.161 (1.098 to 1.282)	1.115 (1.074 to 1.176)
Ischaemic heart disease	135 µg/m³	Both	Both	1.894 (1.449 to 2.547)	1.871 (1.419 to 3.056)	1.793 (1.413 to 2.669)	1.703 (1.348 to 2.427)	1.629 (1.334 to 2.239)	1.559 (1.299 to 1.988)	1.516 (1.263 to 2.119)	1.451 (1.21 to 1.904)	1.399 (1.216 to 1.808)	1.33 (1.186 to 1.541)	1.292 (1.166 to 1.604)	1.245 (1.144 to 1.485)	1.192 (1.117 to 1.302)	1.156 (1.094 to 1.272)	1.112 (1.071 to 1.172)
Ischaemic heart disease	120 µg/m³	Both	Both	1.853 (1.423 to 2.494)	1.832 (1.392 to 2.944)	1.76 (1.388 to 2.585)	1.674 (1.325 to 2.352)	1.604 (1.315 to 2.174)	1.537 (1.283 to 1.948)	1.495 (1.249 to 2.058)	1.434 (1.197 to 1.863)	1.384 (1.206 to 1.77)	1.318 (1.176 to 1.525)	1.281 (1.159 to 1.576)	1.237 (1.137 to 1.465)	1.185 (1.11 to 1.297)	1.151 (1.089 to 1.261)	1.108 (1.068 to 1.168)
Ischaemic heart disease	105 µg/m³	Both	Both	1.809 (1.395 to 2.446)	1.79 (1.363 to 2.819)	1.723 (1.362 to 2.49)	1.641 (1.301 to 2.268)	1.575 (1.295 to 2.1)	1.512 (1.266 to 1.904)	1.472 (1.232 to 1.99)	1.415 (1.183 to 1.817)	1.367 (1.194 to 1.726)	1.305 (1.166 to 1.508)	1.27 (1.15 to 1.549)	1.228 (1.129 to 1.44)	1.178 (1.104 to 1.286)	1.145 (1.085 to 1.248)	1.104 (1.065 to 1.164)
Ischaemic heart disease	90 µg/m³	Both	Both	1.761 (1.361 to 2.393)	1.743 (1.332 to 2.675)	1.681 (1.334 to 2.382)	1.605 (1.276 to 2.165)	1.543 (1.272 to 2.018)	1.484 (1.247 to 1.863)	1.447 (1.214 to 1.916)	1.393 (1.168 to 1.76)	1.348 (1.18 to 1.676)	1.29 (1.155 to 1.488)	1.257 (1.14 to 1.517)	1.217 (1.121 to 1.41)	1.17 (1.097 to 1.277)	1.139 (1.079 to 1.234)	1.1 (1.061 to 1.16)
Ischaemic heart disease	75 µg/m³	Both	Both	1.706 (1.325 to 2.331)	1.689 (1.299 to 2.502)	1.634 (1.304 to 2.245)	1.564 (1.249 to 2.054)	1.507 (1.245 to 1.931)	1.452 (1.224 to 1.779)	1.418 (1.194 to 1.842)	1.368 (1.153 to 1.688)	1.327 (1.165 to 1.619)	1.273 (1.142 to 1.458)	1.241 (1.128 to 1.477)	1.205 (1.111 to 1.376)	1.16 (1.09 to 1.258)	1.131 (1.074 to 1.219)	1.094 (1.056 to 1.155)
Ischaemic heart disease	60 µg/m³	Both	Both	1.642 (1.28 to 2.213)	1.626 (1.262 to 2.298)	1.579 (1.271 to 2.086)	1.516 (1.219 to 1.933)	1.465 (1.216 to 1.848)	1.416 (1.196 to 1.706)	1.384 (1.173 to 1.753)	1.34 (1.136 to 1.604)	1.302 (1.149 to 1.557)	1.253 (1.127 to 1.43)	1.223 (1.116 to 1.425)	1.19 (1.101 to 1.336)	1.149 (1.082 to 1.243)	1.122 (1.067 to 1.204)	1.088 (1.051 to 1.149)
Ischaemic heart disease	45 µg/m³	Both	Both	1.566 (1.231 to 2.004)	1.551 (1.224 to 2.054)	1.513 (1.231 to 1.912)	1.458 (1.186 to 1.795)	1.414 (1.181 to 1.718)	1.371 (1.166 to 1.649)	1.342 (1.148 to 1.64)	1.304 (1.115 to 1.529)	1.271 (1.128 to 1.484)	1.228 (1.11 to 1.382)	1.201 (1.101 to 1.362)	1.173 (1.088 to 1.291)	1.135 (1.072 to 1.221)	1.111 (1.058 to 1.191)	1.081 (1.045 to 1.133)
Ischaemic heart disease	30 µg/m³	Both	Both	1.466 (1.176 to 1.838)	1.454 (1.178 to 1.798)	1.426 (1.181 to 1.763)	1.382 (1.145 to 1.648)	1.346 (1.14 to 1.59)	1.311 (1.131 to 1.531)	1.288 (1.115 to 1.509)	1.257 (1.089 to 1.446)	1.23 (1.101 to 1.382)	1.195 (1.086 to 1.33)	1.171 (1.082 to 1.283)	1.149 (1.071 to 1.242)	1.117 (1.058 to 1.188)	1.096 (1.047 to 1.163)	1.07 (1.037 to 1.114)
Ischaemic heart disease	25 µg/m³	Both	Both	1.424 (1.154 to 1.733)	1.412 (1.159 to 1.708)	1.389 (1.16 to 1.691)	1.349 (1.128 to 1.592)	1.317 (1.122 to 1.542)	1.286 (1.117 to 1.485)	1.264 (1.103 to 1.479)	1.237 (1.079 to 1.4)	1.212 (1.089 to 1.351)	1.181 (1.076 to 1.305)	1.158 (1.073 to 1.261)	1.138 (1.064 to 1.22)	1.109 (1.052 to 1.178)	1.09 (1.042 to 1.155)	1.066 (1.034 to 1.106)
Ischaemic heart disease	20 µg/m³	Both	Both	1.374 (1.128 to 1.66)	1.363 (1.137 to 1.613)	1.345 (1.133 to 1.623)	1.31 (1.107 to 1.539)	1.282 (1.102 to 1.494)	1.255 (1.099 to 1.446)	1.235 (1.089 to 1.427)	1.213 (1.067 to 1.36)	1.191 (1.076 to 1.325)	1.163 (1.065 to 1.283)	1.142 (1.062 to 1.233)	1.125 (1.056 to 1.197)	1.099 (1.045 to 1.164)	1.082 (1.037 to 1.143)	1.06 (1.03 to 1.097)
Ischaemic heart disease	15 µg/m³	Both	Both	1.309 (1.097 to 1.575)	1.3 (1.109 to 1.532)	1.287 (1.103 to 1.541)	1.259 (1.082 to 1.468)	1.237 (1.079 to 1.427)	1.215 (1.078 to 1.393)	1.198 (1.069 to 1.378)	1.181 (1.052 to 1.318)	1.162 (1.059 to 1.287)	1.14 (1.05 to 1.256)	1.121 (1.049 to 1.204)	1.108 (1.045 to 1.176)	1.085 (1.037 to 1.146)	1.071 (1.03 to 1.126)	1.052 (1.024 to 1.089)
Ischaemic heart disease	10 µg/m³	Both	Both	1.207 (1.05 to 1.433)	1.201 (1.062 to 1.408)	1.195 (1.058 to 1.404)	1.178 (1.046 to 1.353)	1.164 (1.046 to 1.324)	1.15 (1.046 to 1.304)	1.138 (1.04 to 1.291)	1.128 (1.03 to 1.258)	1.115 (1.034 to 1.222)	1.101 (1.03 to 1.202)	1.087 (1.03 to 1.166)	1.079 (1.029 to 1.142)	1.063 (1.021 to 1.12)	1.052 (1.019 to 1.102)	1.039 (1.015 to 1.072)
Ischaemic heart disease	5 µg/m³	Both	Both	1.0 (1.0 to 1.0)	1.0 (1.0 to 1.0)	1.0 (1.0 to 1.0)	1.0 (1.0 to 1.0)	1.0 (1.0 to 1.0)	1.0 (1.0 to 1.0)	1.0 (1.0 to 1.0)	1.0 (1.0 to 1.0)	1.0 (1.0 to 1.0)	1.0 (1.0 to 1.0)	1.0 (1.0 to 1.0)	1.0 (1.0 to 1.0)	1.0 (1.0 to 1.0)	1.0 (1.0 to 1.0)	1.0 (1.0 to 1.0)
Ischaemic heart disease	0 µg/m³	Both	Both	1.0 (1.0 to 1.0)	1.0 (1.0 to 1.0)	1.0 (1.0 to 1.0)	1.0 (1.0 to 1.0)	1.0 (1.0 to 1.0)	1.0 (1.0 to 1.0)	1.0 (1.0 to 1.0)	1.0 (1.0 to 1.0)	1.0 (1.0 to 1.0)	1.0 (1.0 to 1.0)	1.0 (1.0 to 1.0)	1.0 (1.0 to 1.0)	1.0 (1.0 to 1.0)	1.0 (1.0 to 1.0)	1.0 (1.0 to 1.0)

Appendix Table 1b. Relative risks used by age and sex for each outcome for the particulate matter integrated exposure response curve.

Risk - Outcome	Category / Units	Morbidity / Mortality	Sex	25-29 years	30-34 years	35-39 years	40-44 years	45-49 years	50-54 years	55-59 years	60-64 years	65-69 years	70-74 years	75-79 years	80-84 years	85-89 years	90-94 years	95+ years
**Ambient particulate matter pollution (PM2.5)**																		
Cerebrovascular disease	600 μg/m^3	Both	Both	2.509 (1.832 to 4.018)	2.35 (1.817 to 3.51)	1.988 (1.268 to 3.236)	2.092 (1.665 to 3.085)	1.999 (1.592 to 2.931)	1.899 (1.53 to 2.749)	1.785 (1.472 to 2.422)	1.678 (1.405 to 2.191)	1.598 (1.383 to 2.02)	1.531 (1.305 to 1.935)	1.44 (1.255 to 1.754)	1.365 (1.235 to 1.611)	1.287 (1.176 to 1.522)	1.231 (1.138 to 1.396)	1.168 (1.105 to 1.286)
Cerebrovascular disease	500 μg/m^3	Both	Both	2.405 (1.761 to 3.831)	2.257 (1.759 to 3.347)	1.946 (1.268 to 3.102)	2.019 (1.602 to 2.986)	1.934 (1.543 to 2.843)	1.843 (1.489 to 2.665)	1.737 (1.435 to 2.347)	1.636 (1.375 to 2.133)	1.562 (1.352 to 1.962)	1.501 (1.282 to 1.9)	1.415 (1.234 to 1.718)	1.345 (1.219 to 1.6)	1.272 (1.165 to 1.506)	1.219 (1.129 to 1.375)	1.159 (1.098 to 1.276)
Cerebrovascular disease	400 μg/m^3	Both	Both	2.284 (1.683 to 3.501)	2.149 (1.683 to 3.173)	1.897 (1.268 to 3.102)	1.935 (1.537 to 2.818)	1.858 (1.486 to 2.716)	1.776 (1.44 to 2.525)	1.68 (1.392 to 2.269)	1.587 (1.341 to 2.039)	1.519 (1.316 to 1.897)	1.464 (1.256 to 1.844)	1.385 (1.213 to 1.665)	1.321 (1.199 to 1.575)	1.253 (1.15 to 1.476)	1.205 (1.118 to 1.35)	1.149 (1.091 to 1.258)
Cerebrovascular disease	300 μg/m^3	Both	Both	2.136 (1.596 to 3.227)	2.018 (1.588 to 2.946)	1.837 (1.268 to 3.085)	1.832 (1.465 to 2.583)	1.765 (1.418 to 2.511)	1.695 (1.388 to 2.347)	1.609 (1.342 to 2.123)	1.528 (1.3 to 1.907)	1.467 (1.28 to 1.799)	1.419 (1.226 to 1.761)	1.348 (1.185 to 1.606)	1.291 (1.175 to 1.526)	1.23 (1.133 to 1.426)	1.187 (1.107 to 1.326)	1.136 (1.08 to 1.239)
Cerebrovascular disease	200 μg/m^3	Both	Both	1.946 (1.481 to 2.857)	1.852 (1.478 to 2.558)	1.761 (1.268 to 2.96)	1.7 (1.381 to 2.254)	1.644 (1.336 to 2.206)	1.589 (1.317 to 2.101)	1.517 (1.278 to 1.938)	1.45 (1.241 to 1.74)	1.398 (1.233 to 1.671)	1.358 (1.189 to 1.632)	1.299 (1.154 to 1.516)	1.251 (1.145 to 1.443)	1.199 (1.111 to 1.348)	1.162 (1.092 to 1.278)	1.118 (1.067 to 1.207)
Cerebrovascular disease	150 μg/m^3	Both	Both	1.826 (1.409 to 2.604)	1.746 (1.401 to 2.304)	1.712 (1.265 to 2.905)	1.615 (1.326 to 2.07)	1.566 (1.286 to 2.013)	1.52 (1.274 to 1.926)	1.457 (1.237 to 1.802)	1.4 (1.205 to 1.658)	1.354 (1.2 to 1.586)	1.318 (1.166 to 1.542)	1.267 (1.133 to 1.45)	1.225 (1.128 to 1.384)	1.178 (1.098 to 1.3)	1.145 (1.08 to 1.241)	1.107 (1.058 to 1.185)
Cerebrovascular disease	135 μg/m^3	Both	Both	1.784 (1.383 to 2.475)	1.711 (1.376 to 2.219)	1.695 (1.262 to 2.859)	1.586 (1.308 to 2.027)	1.54 (1.266 to 1.975)	1.496 (1.258 to 1.886)	1.437 (1.224 to 1.739)	1.382 (1.194 to 1.63)	1.339 (1.186 to 1.56)	1.305 (1.157 to 1.518)	1.255 (1.126 to 1.426)	1.215 (1.119 to 1.36)	1.171 (1.093 to 1.286)	1.14 (1.076 to 1.227)	1.103 (1.054 to 1.176)
Cerebrovascular disease	120 μg/m^3	Both	Both	1.74 (1.355 to 2.357)	1.672 (1.35 to 2.152)	1.677 (1.261 to 2.81)	1.555 (1.287 to 1.994)	1.511 (1.25 to 1.924)	1.471 (1.243 to 1.816)	1.415 (1.209 to 1.696)	1.363 (1.183 to 1.599)	1.322 (1.175 to 1.529)	1.29 (1.148 to 1.49)	1.243 (1.12 to 1.401)	1.205 (1.112 to 1.339)	1.163 (1.088 to 1.269)	1.133 (1.072 to 1.214)	1.098 (1.05 to 1.166)
Cerebrovascular disease	105 μg/m^3	Both	Both	1.692 (1.323 to 2.295)	1.63 (1.324 to 2.087)	1.658 (1.257 to 2.81)	1.521 (1.266 to 1.908)	1.48 (1.233 to 1.857)	1.443 (1.227 to 1.781)	1.39 (1.193 to 1.656)	1.343 (1.167 to 1.562)	1.304 (1.164 to 1.502)	1.274 (1.139 to 1.453)	1.23 (1.112 to 1.38)	1.194 (1.105 to 1.319)	1.155 (1.083 to 1.251)	1.126 (1.068 to 1.201)	1.093 (1.046 to 1.154)
Cerebrovascular disease	90 μg/m^3	Both	Both	1.64 (1.291 to 2.165)	1.585 (1.292 to 2.019)	1.636 (1.241 to 2.81)	1.484 (1.241 to 1.834)	1.446 (1.215 to 1.776)	1.413 (1.208 to 1.723)	1.364 (1.176 to 1.616)	1.32 (1.148 to 1.52)	1.285 (1.15 to 1.466)	1.256 (1.13 to 1.423)	1.215 (1.1 to 1.355)	1.182 (1.096 to 1.285)	1.145 (1.077 to 1.23)	1.119 (1.063 to 1.184)	1.088 (1.042 to 1.14)
Cerebrovascular disease	75 μg/m^3	Both	Both	1.582 (1.256 to 2.024)	1.535 (1.262 to 1.943)	1.613 (1.231 to 2.81)	1.443 (1.213 to 1.75)	1.409 (1.194 to 1.695)	1.379 (1.187 to 1.638)	1.334 (1.157 to 1.555)	1.295 (1.133 to 1.476)	1.263 (1.134 to 1.434)	1.236 (1.117 to 1.386)	1.199 (1.09 to 1.331)	1.169 (1.087 to 1.26)	1.134 (1.07 to 1.209)	1.11 (1.056 to 1.175)	1.081 (1.038 to 1.129)
Cerebrovascular disease	60 μg/m^3	Both	Both	1.517 (1.219 to 1.944)	1.478 (1.226 to 1.848)	1.587 (1.199 to 2.81)	1.396 (1.183 to 1.674)	1.366 (1.165 to 1.625)	1.341 (1.163 to 1.583)	1.301 (1.137 to 1.502)	1.266 (1.115 to 1.435)	1.237 (1.111 to 1.401)	1.213 (1.102 to 1.347)	1.18 (1.079 to 1.301)	1.153 (1.076 to 1.242)	1.122 (1.061 to 1.192)	1.1 (1.049 to 1.162)	1.074 (1.034 to 1.119)
Cerebrovascular disease	45 μg/m^3	Both	Both	1.443 (1.17 to 1.849)	1.411 (1.183 to 1.752)	1.556 (1.175 to 2.81)	1.342 (1.146 to 1.608)	1.316 (1.133 to 1.549)	1.295 (1.133 to 1.522)	1.261 (1.115 to 1.454)	1.232 (1.094 to 1.387)	1.207 (1.089 to 1.363)	1.186 (1.085 to 1.318)	1.158 (1.067 to 1.263)	1.134 (1.064 to 1.222)	1.107 (1.05 to 1.176)	1.088 (1.042 to 1.146)	1.066 (1.028 to 1.111)
Cerebrovascular disease	30 μg/m^3	Both	Both	1.351 (1.117 to 1.721)	1.328 (1.131 to 1.618)	1.518 (1.121 to 2.81)	1.274 (1.108 to 1.511)	1.253 (1.093 to 1.457)	1.239 (1.095 to 1.45)	1.212 (1.08 to 1.386)	1.189 (1.067 to 1.342)	1.169 (1.062 to 1.312)	1.152 (1.062 to 1.28)	1.129 (1.048 to 1.235)	1.11 (1.048 to 1.195)	1.089 (1.036 to 1.156)	1.073 (1.031 to 1.13)	1.055 (1.021 to 1.096)
Cerebrovascular disease	25 μg/m^3	Both	Both	1.313 (1.097 to 1.67)	1.294 (1.108 to 1.568)	1.503 (1.103 to 2.81)	1.246 (1.095 to 1.473)	1.227 (1.075 to 1.423)	1.215 (1.082 to 1.417)	1.191 (1.065 to 1.362)	1.171 (1.057 to 1.323)	1.153 (1.054 to 1.292)	1.137 (1.05 to 1.261)	1.118 (1.039 to 1.22)	1.1 (1.039 to 1.181)	1.081 (1.029 to 1.144)	1.067 (1.026 to 1.124)	1.05 (1.018 to 1.091)
Cerebrovascular disease	20 μg/m^3	Both	Both	1.27 (1.072 to 1.6)	1.254 (1.083 to 1.507)	1.485 (1.083 to 2.81)	1.213 (1.079 to 1.425)	1.197 (1.059 to 1.383)	1.188 (1.061 to 1.379)	1.167 (1.049 to 1.322)	1.15 (1.044 to 1.296)	1.134 (1.043 to 1.262)	1.121 (1.04 to 1.242)	1.104 (1.031 to 1.206)	1.088 (1.03 to 1.167)	1.071 (1.023 to 1.134)	1.059 (1.021 to 1.116)	1.044 (1.014 to 1.085)
Cerebrovascular disease	15 μg/m^3	Both	Both	1.216 (1.049 to 1.505)	1.204 (1.053 to 1.441)	1.462 (1.059 to 2.81)	1.172 (1.051 to 1.359)	1.159 (1.04 to 1.331)	1.153 (1.042 to 1.326)	1.136 (1.035 to 1.283)	1.123 (1.03 to 1.266)	1.11 (1.029 to 1.231)	1.099 (1.026 to 1.217)	1.086 (1.02 to 1.176)	1.073 (1.021 to 1.149)	1.059 (1.016 to 1.119)	1.049 (1.014 to 1.105)	1.037 (1.01 to 1.077)
Cerebrovascular disease	10 μg/m^3	Both	Both	1.136 (1.02 to 1.351)	1.129 (1.021 to 1.334)	1.429 (1.024 to 2.81)	1.109 (1.02 to 1.259)	1.102 (1.016 to 1.252)	1.1 (1.016 to 1.242)	1.089 (1.012 to 1.213)	1.081 (1.01 to 1.207)	1.073 (1.012 to 1.179)	1.067 (1.01 to 1.169)	1.058 (1.008 to 1.143)	1.049 (1.008 to 1.119)	1.041 (1.006 to 1.093)	1.034 (1.005 to 1.085)	1.025 (1.005 to 1.062)
Cerebrovascular disease	5 μg/m^3	Both	Both	1.0 (1.0 to 1.0)	1.0 (1.0 to 1.0)	1.0 (1.0 to 1.0)	1.0 (1.0 to 1.0)	1.0 (1.0 to 1.0)	1.0 (1.0 to 1.0)	1.0 (1.0 to 1.0)	1.0 (1.0 to 1.0)	1.0 (1.0 to 1.0)	1.0 (1.0 to 1.0)	1.0 (1.0 to 1.0)	1.0 (1.0 to 1.0)	1.0 (1.0 to 1.0)	1.0 (1.0 to 1.0)	1.0 (1.0 to 1.0)
Cerebrovascular disease	0 μg/m^3	Both	Both	1.0 (1.0 to 1.0)	1.0 (1.0 to 1.0)	1.0 (1.0 to 1.0)	1.0 (1.0 to 1.0)	1.0 (1.0 to 1.0)	1.0 (1.0 to 1.0)	1.0 (1.0 to 1.0)	1.0 (1.0 to 1.0)	1.0 (1.0 to 1.0)	1.0 (1.0 to 1.0)	1.0 (1.0 to 1.0)	1.0 (1.0 to 1.0)	1.0 (1.0 to 1.0)	1.0 (1.0 to 1.0)	1.0 (1.0 to 1.0)
Chronic obstructive pulmonary disease	600 μg/m^3	Both	Both	2.922 (2.191 to 5.737)	2.922 (2.191 to 5.737)	2.922 (2.191 to 5.737)	2.922 (2.191 to 5.737)	2.922 (2.191 to 5.737)	2.922 (2.191 to 5.737)	2.922 (2.191 to 5.737)	2.922 (2.191 to 5.737)	2.922 (2.191 to 5.737)	2.922 (2.191 to 5.737)	2.922 (2.191 to 5.737)	2.922 (2.191 to 5.737)	2.922 (2.191 to 5.737)	2.922 (2.191 to 5.737)	2.922 (2.191 to 5.737)
Chronic obstructive pulmonary disease	500 μg/m^3	Both	Both	2.738 (2.07 to 5.149)	2.738 (2.07 to 5.149)	2.738 (2.07 to 5.149)	2.738 (2.07 to 5.149)	2.738 (2.07 to 5.149)	2.738 (2.07 to 5.149)	2.738 (2.07 to 5.149)	2.738 (2.07 to 5.149)	2.738 (2.07 to 5.149)	2.738 (2.07 to 5.149)	2.738 (2.07 to 5.149)	2.738 (2.07 to 5.149)	2.738 (2.07 to 5.149)	2.738 (2.07 to 5.149)	2.738 (2.07 to 5.149)
Chronic obstructive pulmonary disease	400 μg/m^3	Both	Both	2.53 (1.931 to 4.534)	2.53 (1.931 to 4.534)	2.53 (1.931 to 4.534)	2.53 (1.931 to 4.534)	2.53 (1.931 to 4.534)	2.53 (1.931 to 4.534)	2.53 (1.931 to 4.534)	2.53 (1.931 to 4.534)	2.53 (1.931 to 4.534)	2.53 (1.931 to 4.534)	2.53 (1.931 to 4.534)	2.53 (1.931 to 4.534)	2.53 (1.931 to 4.534)	2.53 (1.931 to 4.534)	2.53 (1.931 to 4.534)
Chronic obstructive pulmonary disease	300 μg/m^3	Both	Both	2.291 (1.785 to 3.841)	2.291 (1.785 to 3.841)	2.291 (1.785 to 3.841)	2.291 (1.785 to 3.841)	2.291 (1.785 to 3.841)	2.291 (1.785 to 3.841)	2.291 (1.785 to 3.841)	2.291 (1.785 to 3.841)	2.291 (1.785 to 3.841)	2.291 (1.785 to 3.841)	2.291 (1.785 to 3.841)	2.291 (1.785 to 3.841)	2.291 (1.785 to 3.841)	2.291 (1.785 to 3.841)	2.291 (1.785 to 3.841)
Chronic obstructive pulmonary disease	200 μg/m^3	Both	Both	2.007 (1.606 to 3.131)	2.007 (1.606 to 3.131)	2.007 (1.606 to 3.131)	2.007 (1.606 to 3.131)	2.007 (1.606 to 3.131)	2.007 (1.606 to 3.131)	2.007 (1.606 to 3.131)	2.007 (1.606 to 3.131)	2.007 (1.606 to 3.131)	2.007 (1.606 to 3.131)	2.007 (1.606 to 3.131)	2.007 (1.606 to 3.131)	2.007 (1.606 to 3.131)	2.007 (1.606 to 3.131)	2.007 (1.606 to 3.131)
Chronic obstructive pulmonary disease	150 μg/m^3	Both	Both	1.84 (1.498 to 2.709)	1.84 (1.498 to 2.709)	1.84 (1.498 to 2.709)	1.84 (1.498 to 2.709)	1.84 (1.498 to 2.709)	1.84 (1.498 to 2.709)	1.84 (1.498 to 2.709)	1.84 (1.498 to 2.709)	1.84 (1.498 to 2.709)	1.84 (1.498 to 2.709)	1.84 (1.498 to 2.709)	1.84 (1.498 to 2.709)	1.84 (1.498 to 2.709)	1.84 (1.498 to 2.709)	1.84 (1.498 to 2.709)
Chronic obstructive pulmonary disease	135 μg/m^3	Both	Both	1.786 (1.462 to 2.536)	1.786 (1.462 to 2.536)	1.786 (1.462 to 2.536)	1.786 (1.462 to 2.536)	1.786 (1.462 to 2.536)	1.786 (1.462 to 2.536)	1.786 (1.462 to 2.536)	1.786 (1.462 to 2.536)	1.786 (1.462 to 2.536)	1.786 (1.462 to 2.536)	1.786 (1.462 to 2.536)	1.786 (1.462 to 2.536)	1.786 (1.462 to 2.536)	1.786 (1.462 to 2.536)	1.786 (1.462 to 2.536)
Chronic obstructive pulmonary disease	120 μg/m^3	Both	Both	1.728 (1.427 to 2.351)	1.728 (1.427 to 2.351)	1.728 (1.427 to 2.351)	1.728 (1.427 to 2.351)	1.728 (1.427 to 2.351)	1.728 (1.427 to 2.351)	1.728 (1.427 to 2.351)	1.728 (1.427 to 2.351)	1.728 (1.427 to 2.351)	1.728 (1.427 to 2.351)	1.728 (1.427 to 2.351)	1.728 (1.427 to 2.351)	1.728 (1.427 to 2.351)	1.728 (1.427 to 2.351)	1.728 (1.427 to 2.351)
Chronic obstructive pulmonary disease	105 μg/m^3	Both	Both	1.668 (1.39 to 2.211)	1.668 (1.39 to 2.211)	1.668 (1.39 to 2.211)	1.668 (1.39 to 2.211)	1.668 (1.39 to 2.211)	1.668 (1.39 to 2.211)	1.668 (1.39 to 2.211)	1.668 (1.39 to 2.211)	1.668 (1.39 to 2.211)	1.668 (1.39 to 2.211)	1.668 (1.39 to 2.211)	1.668 (1.39 to 2.211)	1.668 (1.39 to 2.211)	1.668 (1.39 to 2.211)	1.668 (1.39 to 2.211)
Chronic obstructive pulmonary disease	90 μg/m^3	Both	Both	1.604 (1.35 to 2.065)	1.604 (1.35 to 2.065)	1.604 (1.35 to 2.065)	1.604 (1.35 to 2.065)	1.604 (1.35 to 2.065)	1.604 (1.35 to 2.065)	1.604 (1.35 to 2.065)	1.604 (1.35 to 2.065)	1.604 (1.35 to 2.065)	1.604 (1.35 to 2.065)	1.604 (1.35 to 2.065)	1.604 (1.35 to 2.065)	1.604 (1.35 to 2.065)	1.604 (1.35 to 2.065)	1.604 (1.35 to 2.065)

Appendix Table 1b. Relative risks used by age and sex for each outcome for the particulate matter integrated exposure response curve.

Risk - Outcome	Category / Units	Morbidity / Mortality	Sex	25-29 years	30-34 years	35-39 years	40-44 years	45-49 years	50-54 years	55-59 years	60-64 years	65-69 years	70-74 years	75-79 years	80-84 years	85-89 years	90-94 years	95+ years
**Ambient particulate matter pollution (PM2.5)**																		
Chronic obstructive pulmonary disease	75 µg/m³	Both	Both	1.536 (1.308 to 1.915)	1.536 (1.308 to 1.915)	1.536 (1.308 to 1.915)	1.536 (1.308 to 1.915)	1.536 (1.308 to 1.915)	1.536 (1.308 to 1.915)	1.536 (1.308 to 1.915)	1.536 (1.308 to 1.915)	1.536 (1.308 to 1.915)	1.536 (1.308 to 1.915)	1.536 (1.308 to 1.915)	1.536 (1.308 to 1.915)	1.536 (1.308 to 1.915)	1.536 (1.308 to 1.915)	1.536 (1.308 to 1.915)
Chronic obstructive pulmonary disease	60 µg/m³	Both	Both	1.461 (1.261 to 1.787)	1.461 (1.261 to 1.787)	1.461 (1.261 to 1.787)	1.461 (1.261 to 1.787)	1.461 (1.261 to 1.787)	1.461 (1.261 to 1.787)	1.461 (1.261 to 1.787)	1.461 (1.261 to 1.787)	1.461 (1.261 to 1.787)	1.461 (1.261 to 1.787)	1.461 (1.261 to 1.787)	1.461 (1.261 to 1.787)	1.461 (1.261 to 1.787)	1.461 (1.261 to 1.787)	1.461 (1.261 to 1.787)
Chronic obstructive pulmonary disease	45 µg/m³	Both	Both	1.378 (1.205 to 1.65)	1.378 (1.205 to 1.65)	1.378 (1.205 to 1.65)	1.378 (1.205 to 1.65)	1.378 (1.205 to 1.65)	1.378 (1.205 to 1.65)	1.378 (1.205 to 1.65)	1.378 (1.205 to 1.65)	1.378 (1.205 to 1.65)	1.378 (1.205 to 1.65)	1.378 (1.205 to 1.65)	1.378 (1.205 to 1.65)	1.378 (1.205 to 1.65)	1.378 (1.205 to 1.65)	1.378 (1.205 to 1.65)
Chronic obstructive pulmonary disease	30 µg/m³	Both	Both	1.28 (1.138 to 1.498)	1.28 (1.138 to 1.498)	1.28 (1.138 to 1.498)	1.28 (1.138 to 1.498)	1.28 (1.138 to 1.498)	1.28 (1.138 to 1.498)	1.28 (1.138 to 1.498)	1.28 (1.138 to 1.498)	1.28 (1.138 to 1.498)	1.28 (1.138 to 1.498)	1.28 (1.138 to 1.498)	1.28 (1.138 to 1.498)	1.28 (1.138 to 1.498)	1.28 (1.138 to 1.498)	1.28 (1.138 to 1.498)
Chronic obstructive pulmonary disease	25 µg/m³	Both	Both	1.242 (1.114 to 1.439)	1.242 (1.114 to 1.439)	1.242 (1.114 to 1.439)	1.242 (1.114 to 1.439)	1.242 (1.114 to 1.439)	1.242 (1.114 to 1.439)	1.242 (1.114 to 1.439)	1.242 (1.114 to 1.439)	1.242 (1.114 to 1.439)	1.242 (1.114 to 1.439)	1.242 (1.114 to 1.439)	1.242 (1.114 to 1.439)	1.242 (1.114 to 1.439)	1.242 (1.114 to 1.439)	1.242 (1.114 to 1.439)
Chronic obstructive pulmonary disease	20 µg/m³	Both	Both	1.199 (1.083 to 1.378)	1.199 (1.083 to 1.378)	1.199 (1.083 to 1.378)	1.199 (1.083 to 1.378)	1.199 (1.083 to 1.378)	1.199 (1.083 to 1.378)	1.199 (1.083 to 1.378)	1.199 (1.083 to 1.378)	1.199 (1.083 to 1.378)	1.199 (1.083 to 1.378)	1.199 (1.083 to 1.378)	1.199 (1.083 to 1.378)	1.199 (1.083 to 1.378)	1.199 (1.083 to 1.378)	1.199 (1.083 to 1.378)
Chronic obstructive pulmonary disease	15 µg/m³	Both	Both	1.149 (1.055 to 1.304)	1.149 (1.055 to 1.304)	1.149 (1.055 to 1.304)	1.149 (1.055 to 1.304)	1.149 (1.055 to 1.304)	1.149 (1.055 to 1.304)	1.149 (1.055 to 1.304)	1.149 (1.055 to 1.304)	1.149 (1.055 to 1.304)	1.149 (1.055 to 1.304)	1.149 (1.055 to 1.304)	1.149 (1.055 to 1.304)	1.149 (1.055 to 1.304)	1.149 (1.055 to 1.304)	1.149 (1.055 to 1.304)
Chronic obstructive pulmonary disease	10 µg/m³	Both	Both	1.081 (1.016 to 1.189)	1.081 (1.016 to 1.189)	1.081 (1.016 to 1.189)	1.081 (1.016 to 1.189)	1.081 (1.016 to 1.189)	1.081 (1.016 to 1.189)	1.081 (1.016 to 1.189)	1.081 (1.016 to 1.189)	1.081 (1.016 to 1.189)	1.081 (1.016 to 1.189)	1.081 (1.016 to 1.189)	1.081 (1.016 to 1.189)	1.081 (1.016 to 1.189)	1.081 (1.016 to 1.189)	1.081 (1.016 to 1.189)
Chronic obstructive pulmonary disease	5 µg/m³	Both	Both	1.0 (1.0 to 1.0)	1.0 (1.0 to 1.0)	1.0 (1.0 to 1.0)	1.0 (1.0 to 1.0)	1.0 (1.0 to 1.0)	1.0 (1.0 to 1.0)	1.0 (1.0 to 1.0)	1.0 (1.0 to 1.0)	1.0 (1.0 to 1.0)	1.0 (1.0 to 1.0)	1.0 (1.0 to 1.0)	1.0 (1.0 to 1.0)	1.0 (1.0 to 1.0)	1.0 (1.0 to 1.0)	1.0 (1.0 to 1.0)
Chronic obstructive pulmonary disease	0 µg/m³	Both	Both	1.0 (1.0 to 1.0)	1.0 (1.0 to 1.0)	1.0 (1.0 to 1.0)	1.0 (1.0 to 1.0)	1.0 (1.0 to 1.0)	1.0 (1.0 to 1.0)	1.0 (1.0 to 1.0)	1.0 (1.0 to 1.0)	1.0 (1.0 to 1.0)	1.0 (1.0 to 1.0)	1.0 (1.0 to 1.0)	1.0 (1.0 to 1.0)	1.0 (1.0 to 1.0)	1.0 (1.0 to 1.0)	1.0 (1.0 to 1.0)

Appendix Table 1c. Relative risks used by age and sex for each outcome for alcohol use globally.

Risk - Outcome	Category / Units	Morbidity / Mortality	Sex	15-19 years	20-24 years	25-29 years	30-34 years	35-39 years	40-44 years	45-49 years	50-54 years	55-59 years	60-64 years	65-69 years	70-74 years	75-79 years	80-84 years	85-89 years	90-94 years	95+ years
**Alcohol use**																				
Tuberculosis	72 g/day	Both	Both	3.507 (2.596 to 4.474)	3.507 (2.596 to 4.474)	3.507 (2.596 to 4.474)	3.507 (2.596 to 4.474)	3.507 (2.596 to 4.474)	3.507 (2.596 to 4.474)	3.507 (2.596 to 4.474)	3.507 (2.596 to 4.474)	3.507 (2.596 to 4.474)	3.507 (2.596 to 4.474)	3.507 (2.596 to 4.474)	3.507 (2.596 to 4.474)	3.507 (2.596 to 4.474)	3.507 (2.596 to 4.474)	3.507 (2.596 to 4.474)	3.507 (2.596 to 4.474)	3.507 (2.596 to 4.474)
Tuberculosis	60 g/day	Both	Both	2.994 (1.972 to 4.204)	2.994 (1.972 to 4.204)	2.994 (1.972 to 4.204)	2.994 (1.972 to 4.204)	2.994 (1.972 to 4.204)	2.994 (1.972 to 4.204)	2.994 (1.972 to 4.204)	2.994 (1.972 to 4.204)	2.994 (1.972 to 4.204)	2.994 (1.972 to 4.204)	2.994 (1.972 to 4.204)	2.994 (1.972 to 4.204)	2.994 (1.972 to 4.204)	2.994 (1.972 to 4.204)	2.994 (1.972 to 4.204)	2.994 (1.972 to 4.204)	2.994 (1.972 to 4.204)
Tuberculosis	48 g/day	Both	Both	2.535 (1.701 to 3.51)	2.535 (1.701 to 3.51)	2.535 (1.701 to 3.51)	2.535 (1.701 to 3.51)	2.535 (1.701 to 3.51)	2.535 (1.701 to 3.51)	2.535 (1.701 to 3.51)	2.535 (1.701 to 3.51)	2.535 (1.701 to 3.51)	2.535 (1.701 to 3.51)	2.535 (1.701 to 3.51)	2.535 (1.701 to 3.51)	2.535 (1.701 to 3.51)	2.535 (1.701 to 3.51)	2.535 (1.701 to 3.51)	2.535 (1.701 to 3.51)	2.535 (1.701 to 3.51)
Tuberculosis	36 g/day	Both	Both	2.058 (1.485 to 2.795)	2.058 (1.485 to 2.795)	2.058 (1.485 to 2.795)	2.058 (1.485 to 2.795)	2.058 (1.485 to 2.795)	2.058 (1.485 to 2.795)	2.058 (1.485 to 2.795)	2.058 (1.485 to 2.795)	2.058 (1.485 to 2.795)	2.058 (1.485 to 2.795)	2.058 (1.485 to 2.795)	2.058 (1.485 to 2.795)	2.058 (1.485 to 2.795)	2.058 (1.485 to 2.795)	2.058 (1.485 to 2.795)	2.058 (1.485 to 2.795)	2.058 (1.485 to 2.795)
Tuberculosis	24 g/day	Both	Both	1.531 (1.165 to 1.98)	1.531 (1.165 to 1.98)	1.531 (1.165 to 1.98)	1.531 (1.165 to 1.98)	1.531 (1.165 to 1.98)	1.531 (1.165 to 1.98)	1.531 (1.165 to 1.98)	1.531 (1.165 to 1.98)	1.531 (1.165 to 1.98)	1.531 (1.165 to 1.98)	1.531 (1.165 to 1.98)	1.531 (1.165 to 1.98)	1.531 (1.165 to 1.98)	1.531 (1.165 to 1.98)	1.531 (1.165 to 1.98)	1.531 (1.165 to 1.98)	1.531 (1.165 to 1.98)
Tuberculosis	12 g/day	Both	Both	1.101 (0.815 to 1.425)	1.101 (0.815 to 1.425)	1.101 (0.815 to 1.425)	1.101 (0.815 to 1.425)	1.101 (0.815 to 1.425)	1.101 (0.815 to 1.425)	1.101 (0.815 to 1.425)	1.101 (0.815 to 1.425)	1.101 (0.815 to 1.425)	1.101 (0.815 to 1.425)	1.101 (0.815 to 1.425)	1.101 (0.815 to 1.425)	1.101 (0.815 to 1.425)	1.101 (0.815 to 1.425)	1.101 (0.815 to 1.425)	1.101 (0.815 to 1.425)	1.101 (0.815 to 1.425)
Tuberculosis	0 g/day	Both	Both	1.0 (1.0 to 1.0)	1.0 (1.0 to 1.0)	1.0 (1.0 to 1.0)	1.0 (1.0 to 1.0)	1.0 (1.0 to 1.0)	1.0 (1.0 to 1.0)	1.0 (1.0 to 1.0)	1.0 (1.0 to 1.0)	1.0 (1.0 to 1.0)	1.0 (1.0 to 1.0)	1.0 (1.0 to 1.0)	1.0 (1.0 to 1.0)	1.0 (1.0 to 1.0)	1.0 (1.0 to 1.0)	1.0 (1.0 to 1.0)	1.0 (1.0 to 1.0)	1.0 (1.0 to 1.0)
Lower respiratory infections	72 g/day	Both	Both	1.357 (1.113 to 1.648)	1.357 (1.113 to 1.648)	1.357 (1.113 to 1.648)	1.357 (1.113 to 1.648)	1.357 (1.113 to 1.648)	1.357 (1.113 to 1.648)	1.357 (1.113 to 1.648)	1.357 (1.113 to 1.648)	1.357 (1.113 to 1.648)	1.357 (1.113 to 1.648)	1.357 (1.113 to 1.648)	1.357 (1.113 to 1.648)	1.357 (1.113 to 1.648)	1.357 (1.113 to 1.648)	1.357 (1.113 to 1.648)	1.357 (1.113 to 1.648)	1.357 (1.113 to 1.648)
Lower respiratory infections	60 g/day	Both	Both	1.226 (1.036 to 1.423)	1.226 (1.036 to 1.423)	1.226 (1.036 to 1.423)	1.226 (1.036 to 1.423)	1.226 (1.036 to 1.423)	1.226 (1.036 to 1.423)	1.226 (1.036 to 1.423)	1.226 (1.036 to 1.423)	1.226 (1.036 to 1.423)	1.226 (1.036 to 1.423)	1.226 (1.036 to 1.423)	1.226 (1.036 to 1.423)	1.226 (1.036 to 1.423)	1.226 (1.036 to 1.423)	1.226 (1.036 to 1.423)	1.226 (1.036 to 1.423)	1.226 (1.036 to 1.423)
Lower respiratory infections	48 g/day	Both	Both	1.127 (0.936 to 1.327)	1.127 (0.936 to 1.327)	1.127 (0.936 to 1.327)	1.127 (0.936 to 1.327)	1.127 (0.936 to 1.327)	1.127 (0.936 to 1.327)	1.127 (0.936 to 1.327)	1.127 (0.936 to 1.327)	1.127 (0.936 to 1.327)	1.127 (0.936 to 1.327)	1.127 (0.936 to 1.327)	1.127 (0.936 to 1.327)	1.127 (0.936 to 1.327)	1.127 (0.936 to 1.327)	1.127 (0.936 to 1.327)	1.127 (0.936 to 1.327)	1.127 (0.936 to 1.327)
Lower respiratory infections	36 g/day	Both	Both	1.064 (0.928 to 1.219)	1.064 (0.928 to 1.219)	1.064 (0.928 to 1.219)	1.064 (0.928 to 1.219)	1.064 (0.928 to 1.219)	1.064 (0.928 to 1.219)	1.064 (0.928 to 1.219)	1.064 (0.928 to 1.219)	1.064 (0.928 to 1.219)	1.064 (0.928 to 1.219)	1.064 (0.928 to 1.219)	1.064 (0.928 to 1.219)	1.064 (0.928 to 1.219)	1.064 (0.928 to 1.219)	1.064 (0.928 to 1.219)	1.064 (0.928 to 1.219)	1.064 (0.928 to 1.219)
Lower respiratory infections	24 g/day	Both	Both	1.026 (0.901 to 1.167)	1.026 (0.901 to 1.167)	1.026 (0.901 to 1.167)	1.026 (0.901 to 1.167)	1.026 (0.901 to 1.167)	1.026 (0.901 to 1.167)	1.026 (0.901 to 1.167)	1.026 (0.901 to 1.167)	1.026 (0.901 to 1.167)	1.026 (0.901 to 1.167)	1.026 (0.901 to 1.167)	1.026 (0.901 to 1.167)	1.026 (0.901 to 1.167)	1.026 (0.901 to 1.167)	1.026 (0.901 to 1.167)	1.026 (0.901 to 1.167)	1.026 (0.901 to 1.167)
Lower respiratory infections	12 g/day	Both	Both	1.013 (0.951 to 1.084)	1.013 (0.951 to 1.084)	1.013 (0.951 to 1.084)	1.013 (0.951 to 1.084)	1.013 (0.951 to 1.084)	1.013 (0.951 to 1.084)	1.013 (0.951 to 1.084)	1.013 (0.951 to 1.084)	1.013 (0.951 to 1.084)	1.013 (0.951 to 1.084)	1.013 (0.951 to 1.084)	1.013 (0.951 to 1.084)	1.013 (0.951 to 1.084)	1.013 (0.951 to 1.084)	1.013 (0.951 to 1.084)	1.013 (0.951 to 1.084)	1.013 (0.951 to 1.084)
Lower respiratory infections	0 g/day	Both	Both	1.0 (1.0 to 1.0)	1.0 (1.0 to 1.0)	1.0 (1.0 to 1.0)	1.0 (1.0 to 1.0)	1.0 (1.0 to 1.0)	1.0 (1.0 to 1.0)	1.0 (1.0 to 1.0)	1.0 (1.0 to 1.0)	1.0 (1.0 to 1.0)	1.0 (1.0 to 1.0)	1.0 (1.0 to 1.0)	1.0 (1.0 to 1.0)	1.0 (1.0 to 1.0)	1.0 (1.0 to 1.0)	1.0 (1.0 to 1.0)	1.0 (1.0 to 1.0)	1.0 (1.0 to 1.0)
Oesophageal cancer	72 g/day	Both	Both	2.669 (2.074 to 3.348)	2.669 (2.074 to 3.348)	2.669 (2.074 to 3.348)	2.669 (2.074 to 3.348)	2.669 (2.074 to 3.348)	2.669 (2.074 to 3.348)	2.669 (2.074 to 3.348)	2.669 (2.074 to 3.348)	2.669 (2.074 to 3.348)	2.669 (2.074 to 3.348)	2.669 (2.074 to 3.348)	2.669 (2.074 to 3.348)	2.669 (2.074 to 3.348)	2.669 (2.074 to 3.348)	2.669 (2.074 to 3.348)	2.669 (2.074 to 3.348)	2.669 (2.074 to 3.348)
Oesophageal cancer	60 g/day	Both	Both	2.452 (1.905 to 3.094)	2.452 (1.905 to 3.094)	2.452 (1.905 to 3.094)	2.452 (1.905 to 3.094)	2.452 (1.905 to 3.094)	2.452 (1.905 to 3.094)	2.452 (1.905 to 3.094)	2.452 (1.905 to 3.094)	2.452 (1.905 to 3.094)	2.452 (1.905 to 3.094)	2.452 (1.905 to 3.094)	2.452 (1.905 to 3.094)	2.452 (1.905 to 3.094)	2.452 (1.905 to 3.094)	2.452 (1.905 to 3.094)	2.452 (1.905 to 3.094)	2.452 (1.905 to 3.094)
Oesophageal cancer	48 g/day	Both	Both	2.202 (1.73 to 2.703)	2.202 (1.73 to 2.703)	2.202 (1.73 to 2.703)	2.202 (1.73 to 2.703)	2.202 (1.73 to 2.703)	2.202 (1.73 to 2.703)	2.202 (1.73 to 2.703)	2.202 (1.73 to 2.703)	2.202 (1.73 to 2.703)	2.202 (1.73 to 2.703)	2.202 (1.73 to 2.703)	2.202 (1.73 to 2.703)	2.202 (1.73 to 2.703)	2.202 (1.73 to 2.703)	2.202 (1.73 to 2.703)	2.202 (1.73 to 2.703)	2.202 (1.73 to 2.703)
Oesophageal cancer	36 g/day	Both	Both	1.815 (1.468 to 2.222)	1.815 (1.468 to 2.222)	1.815 (1.468 to 2.222)	1.815 (1.468 to 2.222)	1.815 (1.468 to 2.222)	1.815 (1.468 to 2.222)	1.815 (1.468 to 2.222)	1.815 (1.468 to 2.222)	1.815 (1.468 to 2.222)	1.815 (1.468 to 2.222)	1.815 (1.468 to 2.222)	1.815 (1.468 to 2.222)	1.815 (1.468 to 2.222)	1.815 (1.468 to 2.222)	1.815 (1.468 to 2.222)	1.815 (1.468 to 2.222)	1.815 (1.468 to 2.222)
Oesophageal cancer	24 g/day	Both	Both	1.466 (1.209 to 1.764)	1.466 (1.209 to 1.764)	1.466 (1.209 to 1.764)	1.466 (1.209 to 1.764)	1.466 (1.209 to 1.764)	1.466 (1.209 to 1.764)	1.466 (1.209 to 1.764)	1.466 (1.209 to 1.764)	1.466 (1.209 to 1.764)	1.466 (1.209 to 1.764)	1.466 (1.209 to 1.764)	1.466 (1.209 to 1.764)	1.466 (1.209 to 1.764)	1.466 (1.209 to 1.764)	1.466 (1.209 to 1.764)	1.466 (1.209 to 1.764)	1.466 (1.209 to 1.764)
Oesophageal cancer	12 g/day	Both	Both	1.212 (1.031 to 1.439)	1.212 (1.031 to 1.439)	1.212 (1.031 to 1.439)	1.212 (1.031 to 1.439)	1.212 (1.031 to 1.439)	1.212 (1.031 to 1.439)	1.212 (1.031 to 1.439)	1.212 (1.031 to 1.439)	1.212 (1.031 to 1.439)	1.212 (1.031 to 1.439)	1.212 (1.031 to 1.439)	1.212 (1.031 to 1.439)	1.212 (1.031 to 1.439)	1.212 (1.031 to 1.439)	1.212 (1.031 to 1.439)	1.212 (1.031 to 1.439)	1.212 (1.031 to 1.439)
Oesophageal cancer	0 g/day	Both	Both	1.0 (1.0 to 1.0)	1.0 (1.0 to 1.0)	1.0 (1.0 to 1.0)	1.0 (1.0 to 1.0)	1.0 (1.0 to 1.0)	1.0 (1.0 to 1.0)	1.0 (1.0 to 1.0)	1.0 (1.0 to 1.0)	1.0 (1.0 to 1.0)	1.0 (1.0 to 1.0)	1.0 (1.0 to 1.0)	1.0 (1.0 to 1.0)	1.0 (1.0 to 1.0)	1.0 (1.0 to 1.0)	1.0 (1.0 to 1.0)	1.0 (1.0 to 1.0)	1.0 (1.0 to 1.0)
Liver cancer due to alcohol use	72 g/day	Both	Both	1.424 (1.088 to 1.855)	1.424 (1.088 to 1.855)	1.424 (1.088 to 1.855)	1.424 (1.088 to 1.855)	1.424 (1.088 to 1.855)	1.424 (1.088 to 1.855)	1.424 (1.088 to 1.855)	1.424 (1.088 to 1.855)	1.424 (1.088 to 1.855)	1.424 (1.088 to 1.855)	1.424 (1.088 to 1.855)	1.424 (1.088 to 1.855)	1.424 (1.088 to 1.855)	1.424 (1.088 to 1.855)	1.424 (1.088 to 1.855)	1.424 (1.088 to 1.855)	1.424 (1.088 to 1.855)
Liver cancer due to alcohol use	60 g/day	Both	Both	1.372 (1.093 to 1.692)	1.372 (1.093 to 1.692)	1.372 (1.093 to 1.692)	1.372 (1.093 to 1.692)	1.372 (1.093 to 1.692)	1.372 (1.093 to 1.692)	1.372 (1.093 to 1.692)	1.372 (1.093 to 1.692)	1.372 (1.093 to 1.692)	1.372 (1.093 to 1.692)	1.372 (1.093 to 1.692)	1.372 (1.093 to 1.692)	1.372 (1.093 to 1.692)	1.372 (1.093 to 1.692)	1.372 (1.093 to 1.692)	1.372 (1.093 to 1.692)	1.372 (1.093 to 1.692)
Liver cancer due to alcohol use	48 g/day	Both	Both	1.31 (1.036 to 1.639)	1.31 (1.036 to 1.639)	1.31 (1.036 to 1.639)	1.31 (1.036 to 1.639)	1.31 (1.036 to 1.639)	1.31 (1.036 to 1.639)	1.31 (1.036 to 1.639)	1.31 (1.036 to 1.639)	1.31 (1.036 to 1.639)	1.31 (1.036 to 1.639)	1.31 (1.036 to 1.639)	1.31 (1.036 to 1.639)	1.31 (1.036 to 1.639)	1.31 (1.036 to 1.639)	1.31 (1.036 to 1.639)	1.31 (1.036 to 1.639)	1.31 (1.036 to 1.639)
Liver cancer due to alcohol use	36 g/day	Both	Both	1.225 (1.009 to 1.455)	1.225 (1.009 to 1.455)	1.225 (1.009 to 1.455)	1.225 (1.009 to 1.455)	1.225 (1.009 to 1.455)	1.225 (1.009 to 1.455)	1.225 (1.009 to 1.455)	1.225 (1.009 to 1.455)	1.225 (1.009 to 1.455)	1.225 (1.009 to 1.455)	1.225 (1.009 to 1.455)	1.225 (1.009 to 1.455)	1.225 (1.009 to 1.455)	1.225 (1.009 to 1.455)	1.225 (1.009 to 1.455)	1.225 (1.009 to 1.455)	1.225 (1.009 to 1.455)
Liver cancer due to alcohol use	24 g/day	Both	Both	1.14 (0.934 to 1.359)	1.14 (0.934 to 1.359)	1.14 (0.934 to 1.359)	1.14 (0.934 to 1.359)	1.14 (0.934 to 1.359)	1.14 (0.934 to 1.359)	1.14 (0.934 to 1.359)	1.14 (0.934 to 1.359)	1.14 (0.934 to 1.359)	1.14 (0.934 to 1.359)	1.14 (0.934 to 1.359)	1.14 (0.934 to 1.359)	1.14 (0.934 to 1.359)	1.14 (0.934 to 1.359)	1.14 (0.934 to 1.359)	1.14 (0.934 to 1.359)	1.14 (0.934 to 1.359)
Liver cancer due to alcohol use	12 g/day	Both	Both	1.067 (0.936 to 1.207)	1.067 (0.936 to 1.207)	1.067 (0.936 to 1.207)	1.067 (0.936 to 1.207)	1.067 (0.936 to 1.207)	1.067 (0.936 to 1.207)	1.067 (0.936 to 1.207)	1.067 (0.936 to 1.207)	1.067 (0.936 to 1.207)	1.067 (0.936 to 1.207)	1.067 (0.936 to 1.207)	1.067 (0.936 to 1.207)	1.067 (0.936 to 1.207)	1.067 (0.936 to 1.207)	1.067 (0.936 to 1.207)	1.067 (0.936 to 1.207)	1.067 (0.936 to 1.207)
Liver cancer due to alcohol use	0 g/day	Both	Both	1.0 (1.0 to 1.0)	1.0 (1.0 to 1.0)	1.0 (1.0 to 1.0)	1.0 (1.0 to 1.0)	1.0 (1.0 to 1.0)	1.0 (1.0 to 1.0)	1.0 (1.0 to 1.0)	1.0 (1.0 to 1.0)	1.0 (1.0 to 1.0)	1.0 (1.0 to 1.0)	1.0 (1.0 to 1.0)	1.0 (1.0 to 1.0)	1.0 (1.0 to 1.0)	1.0 (1.0 to 1.0)	1.0 (1.0 to 1.0)	1.0 (1.0 to 1.0)	1.0 (1.0 to 1.0)
Larynx cancer	72 g/day	Both	Both	2.461 (1.758 to 3.228)	2.461 (1.758 to 3.228)	2.461 (1.758 to 3.228)	2.461 (1.758 to 3.228)	2.461 (1.758 to 3.228)	2.461 (1.758 to 3.228)	2.461 (1.758 to 3.228)	2.461 (1.758 to 3.228)	2.461 (1.758 to 3.228)	2.461 (1.758 to 3.228)	2.461 (1.758 to 3.228)	2.461 (1.758 to 3.228)	2.461 (1.758 to 3.228)	2.461 (1.758 to 3.228)	2.461 (1.758 to 3.228)	2.461 (1.758 to 3.228)	2.461 (1.758 to 3.228)
Larynx cancer	60 g/day	Both	Both	2.144 (1.46 to 2.935)	2.144 (1.46 to 2.935)	2.144 (1.46 to 2.935)	2.144 (1.46 to 2.935)	2.144 (1.46 to 2.935)	2.144 (1.46 to 2.935)	2.144 (1.46 to 2.935)	2.144 (1.46 to 2.935)	2.144 (1.46 to 2.935)	2.144 (1.46 to 2.935)	2.144 (1.46 to 2.935)	2.144 (1.46 to 2.935)	2.144 (1.46 to 2.935)	2.144 (1.46 to 2.935)	2.144 (1.46 to 2.935)	2.144 (1.46 to 2.935)	2.144 (1.46 to 2.935)
Larynx cancer	48 g/day	Both	Both	1.813 (1.3 to 2.421)	1.813 (1.3 to 2.421)	1.813 (1.3 to 2.421)	1.813 (1.3 to 2.421)	1.813 (1.3 to 2.421)	1.813 (1.3 to 2.421)	1.813 (1.3 to 2.421)	1.813 (1.3 to 2.421)	1.813 (1.3 to 2.421)	1.813 (1.3 to 2.421)	1.813 (1.3 to 2.421)	1.813 (1.3 to 2.421)	1.813 (1.3 to 2.421)	1.813 (1.3 to 2.421)	1.813 (1.3 to 2.421)	1.813 (1.3 to 2.421)	1.813 (1.3 to 2.421)
Larynx cancer	36 g/day	Both	Both	1.531 (1.126 to 2.061)	1.531 (1.126 to 2.061)	1.531 (1.126 to 2.061)	1.531 (1.126 to 2.061)	1.531 (1.126 to 2.061)	1.531 (1.126 to 2.061)	1.531 (1.126 to 2.061)	1.531 (1.126 to 2.061)	1.531 (1.126 to 2.061)	1.531 (1.126 to 2.061)	1.531 (1.126 to 2.061)	1.531 (1.126 to 2.061)	1.531 (1.126 to 2.061)	1.531 (1.126 to 2.061)	1.531 (1.126 to 2.061)	1.531 (1.126 to 2.061)	1.531 (1.126 to 2.061)
Larynx cancer	24 g/day	Both	Both	1.304 (1.006 to 1.659)	1.304 (1.006 to 1.659)	1.304 (1.006 to 1.659)	1.304 (1.006 to 1.659)	1.304 (1.006 to 1.659)	1.304 (1.006 to 1.659)	1.304 (1.006 to 1.659)	1.304 (1.006 to 1.659)	1.304 (1.006 to 1.659)	1.304 (1.006 to 1.659)	1.304 (1.006 to 1.659)	1.304 (1.006 to 1.659)	1.304 (1.006 to 1.659)	1.304 (1.006 to 1.659)	1.304 (1.006 to 1.659)	1.304 (1.006 to 1.659)	1.304 (1.006 to 1.659)
Larynx cancer	12 g/day	Both	Both	1.12 (0.903 to 1.386)	1.12 (0.903 to 1.386)	1.12 (0.903 to 1.386)	1.12 (0.903 to 1.386)	1.12 (0.903 to 1.386)	1.12 (0.903 to 1.386)	1.12 (0.903 to 1.386)	1.12 (0.903 to 1.386)	1.12 (0.903 to 1.386)	1.12 (0.903 to 1.386)	1.12 (0.903 to 1.386)	1.12 (0.903 to 1.386)	1.12 (0.903 to 1.386)	1.12 (0.903 to 1.386)	1.12 (0.903 to 1.386)	1.12 (0.903 to 1.386)	1.12 (0.903 to 1.386)
Larynx cancer	0 g/day	Both	Both	1.0 (1.0 to 1.0)	1.0 (1.0 to 1.0)	1.0 (1.0 to 1.0)	1.0 (1.0 to 1.0)	1.0 (1.0 to 1.0)	1.0 (1.0 to 1.0)	1.0 (1.0 to 1.0)	1.0 (1.0 to 1.0)	1.0 (1.0 to 1.0)	1.0 (1.0 to 1.0)	1.0 (1.0 to 1.0)	1.0 (1.0 to 1.0)	1.0 (1.0 to 1.0)	1.0 (1.0 to 1.0)	1.0 (1.0 to 1.0)	1.0 (1.0 to 1.0)	1.0 (1.0 to 1.0)

Appendix Table 1c. Relative risks used by age and sex for each outcome for alcohol use globally.

Risk - Outcome	Category / Units	Morbidity / Mortality	Sex	15-19 years	20-24 years	25-29 years	30-34 years	35-39 years	40-44 years	45-49 years	50-54 years	55-59 years	60-64 years	65-69 years	70-74 years	75-79 years	80-84 years	85-89 years	90-94 years	95+ years
**Alcohol use**																				
Breast cancer	72 g/day	Both	Both	1.476 (1.282 to 1.691)	1.476 (1.282 to 1.691)	1.476 (1.282 to 1.691)	1.476 (1.282 to 1.691)	1.476 (1.282 to 1.691)	1.476 (1.282 to 1.691)	1.476 (1.282 to 1.691)	1.476 (1.282 to 1.691)	1.476 (1.282 to 1.691)	1.476 (1.282 to 1.691)	1.476 (1.282 to 1.691)	1.476 (1.282 to 1.691)	1.476 (1.282 to 1.691)	1.476 (1.282 to 1.691)	1.476 (1.282 to 1.691)	1.476 (1.282 to 1.691)	1.476 (1.282 to 1.691)
Breast cancer	60 g/day	Both	Both	1.452 (1.312 to 1.599)	1.452 (1.312 to 1.599)	1.452 (1.312 to 1.599)	1.452 (1.312 to 1.599)	1.452 (1.312 to 1.599)	1.452 (1.312 to 1.599)	1.452 (1.312 to 1.599)	1.452 (1.312 to 1.599)	1.452 (1.312 to 1.599)	1.452 (1.312 to 1.599)	1.452 (1.312 to 1.599)	1.452 (1.312 to 1.599)	1.452 (1.312 to 1.599)	1.452 (1.312 to 1.599)	1.452 (1.312 to 1.599)	1.452 (1.312 to 1.599)	1.452 (1.312 to 1.599)
Breast cancer	48 g/day	Both	Both	1.443 (1.348 to 1.542)	1.443 (1.348 to 1.542)	1.443 (1.348 to 1.542)	1.443 (1.348 to 1.542)	1.443 (1.348 to 1.542)	1.443 (1.348 to 1.542)	1.443 (1.348 to 1.542)	1.443 (1.348 to 1.542)	1.443 (1.348 to 1.542)	1.443 (1.348 to 1.542)	1.443 (1.348 to 1.542)	1.443 (1.348 to 1.542)	1.443 (1.348 to 1.542)	1.443 (1.348 to 1.542)	1.443 (1.348 to 1.542)	1.443 (1.348 to 1.542)	1.443 (1.348 to 1.542)
Breast cancer	36 g/day	Both	Both	1.433 (1.311 to 1.551)	1.433 (1.311 to 1.551)	1.433 (1.311 to 1.551)	1.433 (1.311 to 1.551)	1.433 (1.311 to 1.551)	1.433 (1.311 to 1.551)	1.433 (1.311 to 1.551)	1.433 (1.311 to 1.551)	1.433 (1.311 to 1.551)	1.433 (1.311 to 1.551)	1.433 (1.311 to 1.551)	1.433 (1.311 to 1.551)	1.433 (1.311 to 1.551)	1.433 (1.311 to 1.551)	1.433 (1.311 to 1.551)	1.433 (1.311 to 1.551)	1.433 (1.311 to 1.551)
Breast cancer	24 g/day	Both	Both	1.329 (1.237 to 1.419)	1.329 (1.237 to 1.419)	1.329 (1.237 to 1.419)	1.329 (1.237 to 1.419)	1.329 (1.237 to 1.419)	1.329 (1.237 to 1.419)	1.329 (1.237 to 1.419)	1.329 (1.237 to 1.419)	1.329 (1.237 to 1.419)	1.329 (1.237 to 1.419)	1.329 (1.237 to 1.419)	1.329 (1.237 to 1.419)	1.329 (1.237 to 1.419)	1.329 (1.237 to 1.419)	1.329 (1.237 to 1.419)	1.329 (1.237 to 1.419)	1.329 (1.237 to 1.419)
Breast cancer	12 g/day	Both	Both	1.17 (1.081 to 1.265)	1.17 (1.081 to 1.265)	1.17 (1.081 to 1.265)	1.17 (1.081 to 1.265)	1.17 (1.081 to 1.265)	1.17 (1.081 to 1.265)	1.17 (1.081 to 1.265)	1.17 (1.081 to 1.265)	1.17 (1.081 to 1.265)	1.17 (1.081 to 1.265)	1.17 (1.081 to 1.265)	1.17 (1.081 to 1.265)	1.17 (1.081 to 1.265)	1.17 (1.081 to 1.265)	1.17 (1.081 to 1.265)	1.17 (1.081 to 1.265)	1.17 (1.081 to 1.265)
Breast cancer	0 g/day	Both	Both	1.0 (1.0 to 1.0)	1.0 (1.0 to 1.0)	1.0 (1.0 to 1.0)	1.0 (1.0 to 1.0)	1.0 (1.0 to 1.0)	1.0 (1.0 to 1.0)	1.0 (1.0 to 1.0)	1.0 (1.0 to 1.0)	1.0 (1.0 to 1.0)	1.0 (1.0 to 1.0)	1.0 (1.0 to 1.0)	1.0 (1.0 to 1.0)	1.0 (1.0 to 1.0)	1.0 (1.0 to 1.0)	1.0 (1.0 to 1.0)	1.0 (1.0 to 1.0)	1.0 (1.0 to 1.0)
Colon and rectum cancer	72 g/day	Both	Both	1.616 (1.38 to 1.861)	1.616 (1.38 to 1.861)	1.616 (1.38 to 1.861)	1.616 (1.38 to 1.861)	1.616 (1.38 to 1.861)	1.616 (1.38 to 1.861)	1.616 (1.38 to 1.861)	1.616 (1.38 to 1.861)	1.616 (1.38 to 1.861)	1.616 (1.38 to 1.861)	1.616 (1.38 to 1.861)	1.616 (1.38 to 1.861)	1.616 (1.38 to 1.861)	1.616 (1.38 to 1.861)	1.616 (1.38 to 1.861)	1.616 (1.38 to 1.861)	1.616 (1.38 to 1.861)
Colon and rectum cancer	60 g/day	Both	Both	1.468 (1.329 to 1.615)	1.468 (1.329 to 1.615)	1.468 (1.329 to 1.615)	1.468 (1.329 to 1.615)	1.468 (1.329 to 1.615)	1.468 (1.329 to 1.615)	1.468 (1.329 to 1.615)	1.468 (1.329 to 1.615)	1.468 (1.329 to 1.615)	1.468 (1.329 to 1.615)	1.468 (1.329 to 1.615)	1.468 (1.329 to 1.615)	1.468 (1.329 to 1.615)	1.468 (1.329 to 1.615)	1.468 (1.329 to 1.615)	1.468 (1.329 to 1.615)	1.468 (1.329 to 1.615)
Colon and rectum cancer	48 g/day	Both	Both	1.323 (1.156 to 1.501)	1.323 (1.156 to 1.501)	1.323 (1.156 to 1.501)	1.323 (1.156 to 1.501)	1.323 (1.156 to 1.501)	1.323 (1.156 to 1.501)	1.323 (1.156 to 1.501)	1.323 (1.156 to 1.501)	1.323 (1.156 to 1.501)	1.323 (1.156 to 1.501)	1.323 (1.156 to 1.501)	1.323 (1.156 to 1.501)	1.323 (1.156 to 1.501)	1.323 (1.156 to 1.501)	1.323 (1.156 to 1.501)	1.323 (1.156 to 1.501)	1.323 (1.156 to 1.501)
Colon and rectum cancer	36 g/day	Both	Both	1.237 (1.148 to 1.336)	1.237 (1.148 to 1.336)	1.237 (1.148 to 1.336)	1.237 (1.148 to 1.336)	1.237 (1.148 to 1.336)	1.237 (1.148 to 1.336)	1.237 (1.148 to 1.336)	1.237 (1.148 to 1.336)	1.237 (1.148 to 1.336)	1.237 (1.148 to 1.336)	1.237 (1.148 to 1.336)	1.237 (1.148 to 1.336)	1.237 (1.148 to 1.336)	1.237 (1.148 to 1.336)	1.237 (1.148 to 1.336)	1.237 (1.148 to 1.336)	1.237 (1.148 to 1.336)
Colon and rectum cancer	24 g/day	Both	Both	1.156 (1.067 to 1.248)	1.156 (1.067 to 1.248)	1.156 (1.067 to 1.248)	1.156 (1.067 to 1.248)	1.156 (1.067 to 1.248)	1.156 (1.067 to 1.248)	1.156 (1.067 to 1.248)	1.156 (1.067 to 1.248)	1.156 (1.067 to 1.248)	1.156 (1.067 to 1.248)	1.156 (1.067 to 1.248)	1.156 (1.067 to 1.248)	1.156 (1.067 to 1.248)	1.156 (1.067 to 1.248)	1.156 (1.067 to 1.248)	1.156 (1.067 to 1.248)	1.156 (1.067 to 1.248)
Colon and rectum cancer	12 g/day	Both	Both	1.078 (1.034 to 1.124)	1.078 (1.034 to 1.124)	1.078 (1.034 to 1.124)	1.078 (1.034 to 1.124)	1.078 (1.034 to 1.124)	1.078 (1.034 to 1.124)	1.078 (1.034 to 1.124)	1.078 (1.034 to 1.124)	1.078 (1.034 to 1.124)	1.078 (1.034 to 1.124)	1.078 (1.034 to 1.124)	1.078 (1.034 to 1.124)	1.078 (1.034 to 1.124)	1.078 (1.034 to 1.124)	1.078 (1.034 to 1.124)	1.078 (1.034 to 1.124)	1.078 (1.034 to 1.124)
Colon and rectum cancer	0 g/day	Both	Both	1.0 (1.0 to 1.0)	1.0 (1.0 to 1.0)	1.0 (1.0 to 1.0)	1.0 (1.0 to 1.0)	1.0 (1.0 to 1.0)	1.0 (1.0 to 1.0)	1.0 (1.0 to 1.0)	1.0 (1.0 to 1.0)	1.0 (1.0 to 1.0)	1.0 (1.0 to 1.0)	1.0 (1.0 to 1.0)	1.0 (1.0 to 1.0)	1.0 (1.0 to 1.0)	1.0 (1.0 to 1.0)	1.0 (1.0 to 1.0)	1.0 (1.0 to 1.0)	1.0 (1.0 to 1.0)
Lip and oral cavity cancer	72 g/day	Both	Both	4.858 (3.74 to 6.076)	4.858 (3.74 to 6.076)	4.858 (3.74 to 6.076)	4.858 (3.74 to 6.076)	4.858 (3.74 to 6.076)	4.858 (3.74 to 6.076)	4.858 (3.74 to 6.076)	4.858 (3.74 to 6.076)	4.858 (3.74 to 6.076)	4.858 (3.74 to 6.076)	4.858 (3.74 to 6.076)	4.858 (3.74 to 6.076)	4.858 (3.74 to 6.076)	4.858 (3.74 to 6.076)	4.858 (3.74 to 6.076)	4.858 (3.74 to 6.076)	4.858 (3.74 to 6.076)
Lip and oral cavity cancer	60 g/day	Both	Both	3.766 (2.839 to 4.9)	3.766 (2.839 to 4.9)	3.766 (2.839 to 4.9)	3.766 (2.839 to 4.9)	3.766 (2.839 to 4.9)	3.766 (2.839 to 4.9)	3.766 (2.839 to 4.9)	3.766 (2.839 to 4.9)	3.766 (2.839 to 4.9)	3.766 (2.839 to 4.9)	3.766 (2.839 to 4.9)	3.766 (2.839 to 4.9)	3.766 (2.839 to 4.9)	3.766 (2.839 to 4.9)	3.766 (2.839 to 4.9)	3.766 (2.839 to 4.9)	3.766 (2.839 to 4.9)
Lip and oral cavity cancer	48 g/day	Both	Both	2.991 (2.283 to 3.896)	2.991 (2.283 to 3.896)	2.991 (2.283 to 3.896)	2.991 (2.283 to 3.896)	2.991 (2.283 to 3.896)	2.991 (2.283 to 3.896)	2.991 (2.283 to 3.896)	2.991 (2.283 to 3.896)	2.991 (2.283 to 3.896)	2.991 (2.283 to 3.896)	2.991 (2.283 to 3.896)	2.991 (2.283 to 3.896)	2.991 (2.283 to 3.896)	2.991 (2.283 to 3.896)	2.991 (2.283 to 3.896)	2.991 (2.283 to 3.896)	2.991 (2.283 to 3.896)
Lip and oral cavity cancer	36 g/day	Both	Both	2.311 (1.757 to 2.929)	2.311 (1.757 to 2.929)	2.311 (1.757 to 2.929)	2.311 (1.757 to 2.929)	2.311 (1.757 to 2.929)	2.311 (1.757 to 2.929)	2.311 (1.757 to 2.929)	2.311 (1.757 to 2.929)	2.311 (1.757 to 2.929)	2.311 (1.757 to 2.929)	2.311 (1.757 to 2.929)	2.311 (1.757 to 2.929)	2.311 (1.757 to 2.929)	2.311 (1.757 to 2.929)	2.311 (1.757 to 2.929)	2.311 (1.757 to 2.929)	2.311 (1.757 to 2.929)
Lip and oral cavity cancer	24 g/day	Both	Both	1.738 (1.383 to 2.161)	1.738 (1.383 to 2.161)	1.738 (1.383 to 2.161)	1.738 (1.383 to 2.161)	1.738 (1.383 to 2.161)	1.738 (1.383 to 2.161)	1.738 (1.383 to 2.161)	1.738 (1.383 to 2.161)	1.738 (1.383 to 2.161)	1.738 (1.383 to 2.161)	1.738 (1.383 to 2.161)	1.738 (1.383 to 2.161)	1.738 (1.383 to 2.161)	1.738 (1.383 to 2.161)	1.738 (1.383 to 2.161)	1.738 (1.383 to 2.161)	1.738 (1.383 to 2.161)
Lip and oral cavity cancer	12 g/day	Both	Both	1.293 (1.076 to 1.551)	1.293 (1.076 to 1.551)	1.293 (1.076 to 1.551)	1.293 (1.076 to 1.551)	1.293 (1.076 to 1.551)	1.293 (1.076 to 1.551)	1.293 (1.076 to 1.551)	1.293 (1.076 to 1.551)	1.293 (1.076 to 1.551)	1.293 (1.076 to 1.551)	1.293 (1.076 to 1.551)	1.293 (1.076 to 1.551)	1.293 (1.076 to 1.551)	1.293 (1.076 to 1.551)	1.293 (1.076 to 1.551)	1.293 (1.076 to 1.551)	1.293 (1.076 to 1.551)
Lip and oral cavity cancer	0 g/day	Both	Both	1.0 (1.0 to 1.0)	1.0 (1.0 to 1.0)	1.0 (1.0 to 1.0)	1.0 (1.0 to 1.0)	1.0 (1.0 to 1.0)	1.0 (1.0 to 1.0)	1.0 (1.0 to 1.0)	1.0 (1.0 to 1.0)	1.0 (1.0 to 1.0)	1.0 (1.0 to 1.0)	1.0 (1.0 to 1.0)	1.0 (1.0 to 1.0)	1.0 (1.0 to 1.0)	1.0 (1.0 to 1.0)	1.0 (1.0 to 1.0)	1.0 (1.0 to 1.0)	1.0 (1.0 to 1.0)
Nasopharynx cancer	72 g/day	Both	Both	4.545 (4.1 to 4.982)	4.545 (4.1 to 4.982)	4.545 (4.1 to 4.982)	4.545 (4.1 to 4.982)	4.545 (4.1 to 4.982)	4.545 (4.1 to 4.982)	4.545 (4.1 to 4.982)	4.545 (4.1 to 4.982)	4.545 (4.1 to 4.982)	4.545 (4.1 to 4.982)	4.545 (4.1 to 4.982)	4.545 (4.1 to 4.982)	4.545 (4.1 to 4.982)	4.545 (4.1 to 4.982)	4.545 (4.1 to 4.982)	4.545 (4.1 to 4.982)	4.545 (4.1 to 4.982)
Nasopharynx cancer	60 g/day	Both	Both	3.803 (3.509 to 4.102)	3.803 (3.509 to 4.102)	3.803 (3.509 to 4.102)	3.803 (3.509 to 4.102)	3.803 (3.509 to 4.102)	3.803 (3.509 to 4.102)	3.803 (3.509 to 4.102)	3.803 (3.509 to 4.102)	3.803 (3.509 to 4.102)	3.803 (3.509 to 4.102)	3.803 (3.509 to 4.102)	3.803 (3.509 to 4.102)	3.803 (3.509 to 4.102)	3.803 (3.509 to 4.102)	3.803 (3.509 to 4.102)	3.803 (3.509 to 4.102)	3.803 (3.509 to 4.102)
Nasopharynx cancer	48 g/day	Both	Both	3.062 (2.873 to 3.258)	3.062 (2.873 to 3.258)	3.062 (2.873 to 3.258)	3.062 (2.873 to 3.258)	3.062 (2.873 to 3.258)	3.062 (2.873 to 3.258)	3.062 (2.873 to 3.258)	3.062 (2.873 to 3.258)	3.062 (2.873 to 3.258)	3.062 (2.873 to 3.258)	3.062 (2.873 to 3.258)	3.062 (2.873 to 3.258)	3.062 (2.873 to 3.258)	3.062 (2.873 to 3.258)	3.062 (2.873 to 3.258)	3.062 (2.873 to 3.258)	3.062 (2.873 to 3.258)
Nasopharynx cancer	36 g/day	Both	Both	2.385 (2.25 to 2.552)	2.385 (2.25 to 2.552)	2.385 (2.25 to 2.552)	2.385 (2.25 to 2.552)	2.385 (2.25 to 2.552)	2.385 (2.25 to 2.552)	2.385 (2.25 to 2.552)	2.385 (2.25 to 2.552)	2.385 (2.25 to 2.552)	2.385 (2.25 to 2.552)	2.385 (2.25 to 2.552)	2.385 (2.25 to 2.552)	2.385 (2.25 to 2.552)	2.385 (2.25 to 2.552)	2.385 (2.25 to 2.552)	2.385 (2.25 to 2.552)	2.385 (2.25 to 2.552)
Nasopharynx cancer	24 g/day	Both	Both	1.839 (1.77 to 1.907)	1.839 (1.77 to 1.907)	1.839 (1.77 to 1.907)	1.839 (1.77 to 1.907)	1.839 (1.77 to 1.907)	1.839 (1.77 to 1.907)	1.839 (1.77 to 1.907)	1.839 (1.77 to 1.907)	1.839 (1.77 to 1.907)	1.839 (1.77 to 1.907)	1.839 (1.77 to 1.907)	1.839 (1.77 to 1.907)	1.839 (1.77 to 1.907)	1.839 (1.77 to 1.907)	1.839 (1.77 to 1.907)	1.839 (1.77 to 1.907)	1.839 (1.77 to 1.907)
Nasopharynx cancer	12 g/day	Both	Both	1.371 (1.341 to 1.398)	1.371 (1.341 to 1.398)	1.371 (1.341 to 1.398)	1.371 (1.341 to 1.398)	1.371 (1.341 to 1.398)	1.371 (1.341 to 1.398)	1.371 (1.341 to 1.398)	1.371 (1.341 to 1.398)	1.371 (1.341 to 1.398)	1.371 (1.341 to 1.398)	1.371 (1.341 to 1.398)	1.371 (1.341 to 1.398)	1.371 (1.341 to 1.398)	1.371 (1.341 to 1.398)	1.371 (1.341 to 1.398)	1.371 (1.341 to 1.398)	1.371 (1.341 to 1.398)
Nasopharynx cancer	0 g/day	Both	Both	1.0 (1.0 to 1.0)	1.0 (1.0 to 1.0)	1.0 (1.0 to 1.0)	1.0 (1.0 to 1.0)	1.0 (1.0 to 1.0)	1.0 (1.0 to 1.0)	1.0 (1.0 to 1.0)	1.0 (1.0 to 1.0)	1.0 (1.0 to 1.0)	1.0 (1.0 to 1.0)	1.0 (1.0 to 1.0)	1.0 (1.0 to 1.0)	1.0 (1.0 to 1.0)	1.0 (1.0 to 1.0)	1.0 (1.0 to 1.0)	1.0 (1.0 to 1.0)	1.0 (1.0 to 1.0)
Other pharynx cancer	72 g/day	Both	Both	4.764 (3.315 to 6.576)	4.764 (3.315 to 6.576)	4.764 (3.315 to 6.576)	4.764 (3.315 to 6.576)	4.764 (3.315 to 6.576)	4.764 (3.315 to 6.576)	4.764 (3.315 to 6.576)	4.764 (3.315 to 6.576)	4.764 (3.315 to 6.576)	4.764 (3.315 to 6.576)	4.764 (3.315 to 6.576)	4.764 (3.315 to 6.576)	4.764 (3.315 to 6.576)	4.764 (3.315 to 6.576)	4.764 (3.315 to 6.576)	4.764 (3.315 to 6.576)	4.764 (3.315 to 6.576)
Other pharynx cancer	60 g/day	Both	Both	3.972 (2.813 to 5.354)	3.972 (2.813 to 5.354)	3.972 (2.813 to 5.354)	3.972 (2.813 to 5.354)	3.972 (2.813 to 5.354)	3.972 (2.813 to 5.354)	3.972 (2.813 to 5.354)	3.972 (2.813 to 5.354)	3.972 (2.813 to 5.354)	3.972 (2.813 to 5.354)	3.972 (2.813 to 5.354)	3.972 (2.813 to 5.354)	3.972 (2.813 to 5.354)	3.972 (2.813 to 5.354)	3.972 (2.813 to 5.354)	3.972 (2.813 to 5.354)	3.972 (2.813 to 5.354)
Other pharynx cancer	48 g/day	Both	Both	3.199 (2.202 to 4.407)	3.199 (2.202 to 4.407)	3.199 (2.202 to 4.407)	3.199 (2.202 to 4.407)	3.199 (2.202 to 4.407)	3.199 (2.202 to 4.407)	3.199 (2.202 to 4.407)	3.199 (2.202 to 4.407)	3.199 (2.202 to 4.407)	3.199 (2.202 to 4.407)	3.199 (2.202 to 4.407)	3.199 (2.202 to 4.407)	3.199 (2.202 to 4.407)	3.199 (2.202 to 4.407)	3.199 (2.202 to 4.407)	3.199 (2.202 to 4.407)	3.199 (2.202 to 4.407)
Other pharynx cancer	36 g/day	Both	Both	2.519 (1.843 to 3.299)	2.519 (1.843 to 3.299)	2.519 (1.843 to 3.299)	2.519 (1.843 to 3.299)	2.519 (1.843 to 3.299)	2.519 (1.843 to 3.299)	2.519 (1.843 to 3.299)	2.519 (1.843 to 3.299)	2.519 (1.843 to 3.299)	2.519 (1.843 to 3.299)	2.519 (1.843 to 3.299)	2.519 (1.843 to 3.299)	2.519 (1.843 to 3.299)	2.519 (1.843 to 3.299)	2.519 (1.843 to 3.299)	2.519 (1.843 to 3.299)	2.519 (1.843 to 3.299)
Other pharynx cancer	24 g/day	Both	Both	1.943 (1.467 to 2.484)	1.943 (1.467 to 2.484)	1.943 (1.467 to 2.484)	1.943 (1.467 to 2.484)	1.943 (1.467 to 2.484)	1.943 (1.467 to 2.484)	1.943 (1.467 to 2.484)	1.943 (1.467 to 2.484)	1.943 (1.467 to 2.484)	1.943 (1.467 to 2.484)	1.943 (1.467 to 2.484)	1.943 (1.467 to 2.484)	1.943 (1.467 to 2.484)	1.943 (1.467 to 2.484)	1.943 (1.467 to 2.484)	1.943 (1.467 to 2.484)	1.943 (1.467 to 2.484)
Other pharynx cancer	12 g/day	Both	Both	1.472 (1.234 to 1.742)	1.472 (1.234 to 1.742)	1.472 (1.234 to 1.742)	1.472 (1.234 to 1.742)	1.472 (1.234 to 1.742)	1.472 (1.234 to 1.742)	1.472 (1.234 to 1.742)	1.472 (1.234 to 1.742)	1.472 (1.234 to 1.742)	1.472 (1.234 to 1.742)	1.472 (1.234 to 1.742)	1.472 (1.234 to 1.742)	1.472 (1.234 to 1.742)	1.472 (1.234 to 1.742)	1.472 (1.234 to 1.742)	1.472 (1.234 to 1.742)	1.472 (1.234 to 1.742)
Other pharynx cancer	0 g/day	Both	Both	1.0 (1.0 to 1.0)	1.0 (1.0 to 1.0)	1.0 (1.0 to 1.0)	1.0 (1.0 to 1.0)	1.0 (1.0 to 1.0)	1.0 (1.0 to 1.0)	1.0 (1.0 to 1.0)	1.0 (1.0 to 1.0)	1.0 (1.0 to 1.0)	1.0 (1.0 to 1.0)	1.0 (1.0 to 1.0)	1.0 (1.0 to 1.0)	1.0 (1.0 to 1.0)	1.0 (1.0 to 1.0)	1.0 (1.0 to 1.0)	1.0 (1.0 to 1.0)	1.0 (1.0 to 1.0)

Appendix Table 1c. Relative risks used by age and sex for each outcome for alcohol use globally.

Risk - Outcome	Category / Units	Morbidity / Mortality	Sex	15-19 years	20-24 years	25-29 years	30-34 years	35-39 years	40-44 years	45-49 years	50-54 years	55-59 years	60-64 years	65-69 years	70-74 years	75-79 years	80-84 years	85-89 years	90-94 years	95+ years
**Alcohol use**																				
Ischaemic heart disease	72 g/day	Both	Male	1.091 (0.933 to 1.271)	1.091 (0.933 to 1.271)	1.091 (0.933 to 1.271)	1.091 (0.933 to 1.271)	1.091 (0.933 to 1.271)	1.091 (0.933 to 1.271)	1.091 (0.933 to 1.271)	1.091 (0.933 to 1.271)	1.091 (0.933 to 1.271)	1.091 (0.933 to 1.271)	1.091 (0.933 to 1.271)	1.091 (0.933 to 1.271)	1.091 (0.933 to 1.271)	1.091 (0.933 to 1.271)	1.091 (0.933 to 1.271)	1.091 (0.933 to 1.271)	1.091 (0.933 to 1.271)
Ischaemic heart disease	60 g/day	Both	Male	0.993 (0.883 to 1.105)	0.993 (0.883 to 1.105)	0.993 (0.883 to 1.105)	0.993 (0.883 to 1.105)	0.993 (0.883 to 1.105)	0.993 (0.883 to 1.105)	0.993 (0.883 to 1.105)	0.993 (0.883 to 1.105)	0.993 (0.883 to 1.105)	0.993 (0.883 to 1.105)	0.993 (0.883 to 1.105)	0.993 (0.883 to 1.105)	0.993 (0.883 to 1.105)	0.993 (0.883 to 1.105)	0.993 (0.883 to 1.105)	0.993 (0.883 to 1.105)	0.993 (0.883 to 1.105)
Ischaemic heart disease	48 g/day	Both	Male	0.906 (0.797 to 1.035)	0.906 (0.797 to 1.035)	0.906 (0.797 to 1.035)	0.906 (0.797 to 1.035)	0.906 (0.797 to 1.035)	0.906 (0.797 to 1.035)	0.906 (0.797 to 1.035)	0.906 (0.797 to 1.035)	0.906 (0.797 to 1.035)	0.906 (0.797 to 1.035)	0.906 (0.797 to 1.035)	0.906 (0.797 to 1.035)	0.906 (0.797 to 1.035)	0.906 (0.797 to 1.035)	0.906 (0.797 to 1.035)	0.906 (0.797 to 1.035)	0.906 (0.797 to 1.035)
Ischaemic heart disease	36 g/day	Both	Male	0.871 (0.788 to 0.964)	0.871 (0.788 to 0.964)	0.871 (0.788 to 0.964)	0.871 (0.788 to 0.964)	0.871 (0.788 to 0.964)	0.871 (0.788 to 0.964)	0.871 (0.788 to 0.964)	0.871 (0.788 to 0.964)	0.871 (0.788 to 0.964)	0.871 (0.788 to 0.964)	0.871 (0.788 to 0.964)	0.871 (0.788 to 0.964)	0.871 (0.788 to 0.964)	0.871 (0.788 to 0.964)	0.871 (0.788 to 0.964)	0.871 (0.788 to 0.964)	0.871 (0.788 to 0.964)
Ischaemic heart disease	24 g/day	Both	Male	0.857 (0.779 to 0.943)	0.857 (0.779 to 0.943)	0.857 (0.779 to 0.943)	0.857 (0.779 to 0.943)	0.857 (0.779 to 0.943)	0.857 (0.779 to 0.943)	0.857 (0.779 to 0.943)	0.857 (0.779 to 0.943)	0.857 (0.779 to 0.943)	0.857 (0.779 to 0.943)	0.857 (0.779 to 0.943)	0.857 (0.779 to 0.943)	0.857 (0.779 to 0.943)	0.857 (0.779 to 0.943)	0.857 (0.779 to 0.943)	0.857 (0.779 to 0.943)	0.857 (0.779 to 0.943)
Ischaemic heart disease	12 g/day	Both	Male	0.865 (0.79 to 0.948)	0.865 (0.79 to 0.948)	0.865 (0.79 to 0.948)	0.865 (0.79 to 0.948)	0.865 (0.79 to 0.948)	0.865 (0.79 to 0.948)	0.865 (0.79 to 0.948)	0.865 (0.79 to 0.948)	0.865 (0.79 to 0.948)	0.865 (0.79 to 0.948)	0.865 (0.79 to 0.948)	0.865 (0.79 to 0.948)	0.865 (0.79 to 0.948)	0.865 (0.79 to 0.948)	0.865 (0.79 to 0.948)	0.865 (0.79 to 0.948)	0.865 (0.79 to 0.948)
Ischaemic heart disease	0 g/day	Both	Male	1.0 (1.0 to 1.0)	1.0 (1.0 to 1.0)	1.0 (1.0 to 1.0)	1.0 (1.0 to 1.0)	1.0 (1.0 to 1.0)	1.0 (1.0 to 1.0)	1.0 (1.0 to 1.0)	1.0 (1.0 to 1.0)	1.0 (1.0 to 1.0)	1.0 (1.0 to 1.0)	1.0 (1.0 to 1.0)	1.0 (1.0 to 1.0)	1.0 (1.0 to 1.0)	1.0 (1.0 to 1.0)	1.0 (1.0 to 1.0)	1.0 (1.0 to 1.0)	1.0 (1.0 to 1.0)
Ischaemic heart disease	72 g/day	Both	Female	1.107 (0.894 to 1.341)	1.107 (0.894 to 1.341)	1.107 (0.894 to 1.341)	1.107 (0.894 to 1.341)	1.107 (0.894 to 1.341)	1.107 (0.894 to 1.341)	1.107 (0.894 to 1.341)	1.107 (0.894 to 1.341)	1.107 (0.894 to 1.341)	1.107 (0.894 to 1.341)	1.107 (0.894 to 1.341)	1.107 (0.894 to 1.341)	1.107 (0.894 to 1.341)	1.107 (0.894 to 1.341)	1.107 (0.894 to 1.341)	1.107 (0.894 to 1.341)	1.107 (0.894 to 1.341)
Ischaemic heart disease	60 g/day	Both	Female	1.012 (0.869 to 1.174)	1.012 (0.869 to 1.174)	1.012 (0.869 to 1.174)	1.012 (0.869 to 1.174)	1.012 (0.869 to 1.174)	1.012 (0.869 to 1.174)	1.012 (0.869 to 1.174)	1.012 (0.869 to 1.174)	1.012 (0.869 to 1.174)	1.012 (0.869 to 1.174)	1.012 (0.869 to 1.174)	1.012 (0.869 to 1.174)	1.012 (0.869 to 1.174)	1.012 (0.869 to 1.174)	1.012 (0.869 to 1.174)	1.012 (0.869 to 1.174)	1.012 (0.869 to 1.174)
Ischaemic heart disease	48 g/day	Both	Female	0.932 (0.786 to 1.113)	0.932 (0.786 to 1.113)	0.932 (0.786 to 1.113)	0.932 (0.786 to 1.113)	0.932 (0.786 to 1.113)	0.932 (0.786 to 1.113)	0.932 (0.786 to 1.113)	0.932 (0.786 to 1.113)	0.932 (0.786 to 1.113)	0.932 (0.786 to 1.113)	0.932 (0.786 to 1.113)	0.932 (0.786 to 1.113)	0.932 (0.786 to 1.113)	0.932 (0.786 to 1.113)	0.932 (0.786 to 1.113)	0.932 (0.786 to 1.113)	0.932 (0.786 to 1.113)
Ischaemic heart disease	36 g/day	Both	Female	0.882 (0.781 to 0.997)	0.882 (0.781 to 0.997)	0.882 (0.781 to 0.997)	0.882 (0.781 to 0.997)	0.882 (0.781 to 0.997)	0.882 (0.781 to 0.997)	0.882 (0.781 to 0.997)	0.882 (0.781 to 0.997)	0.882 (0.781 to 0.997)	0.882 (0.781 to 0.997)	0.882 (0.781 to 0.997)	0.882 (0.781 to 0.997)	0.882 (0.781 to 0.997)	0.882 (0.781 to 0.997)	0.882 (0.781 to 0.997)	0.882 (0.781 to 0.997)	0.882 (0.781 to 0.997)
Ischaemic heart disease	24 g/day	Both	Female	0.846 (0.749 to 0.948)	0.846 (0.749 to 0.948)	0.846 (0.749 to 0.948)	0.846 (0.749 to 0.948)	0.846 (0.749 to 0.948)	0.846 (0.749 to 0.948)	0.846 (0.749 to 0.948)	0.846 (0.749 to 0.948)	0.846 (0.749 to 0.948)	0.846 (0.749 to 0.948)	0.846 (0.749 to 0.948)	0.846 (0.749 to 0.948)	0.846 (0.749 to 0.948)	0.846 (0.749 to 0.948)	0.846 (0.749 to 0.948)	0.846 (0.749 to 0.948)	0.846 (0.749 to 0.948)
Ischaemic heart disease	12 g/day	Both	Female	0.823 (0.733 to 0.926)	0.823 (0.733 to 0.926)	0.823 (0.733 to 0.926)	0.823 (0.733 to 0.926)	0.823 (0.733 to 0.926)	0.823 (0.733 to 0.926)	0.823 (0.733 to 0.926)	0.823 (0.733 to 0.926)	0.823 (0.733 to 0.926)	0.823 (0.733 to 0.926)	0.823 (0.733 to 0.926)	0.823 (0.733 to 0.926)	0.823 (0.733 to 0.926)	0.823 (0.733 to 0.926)	0.823 (0.733 to 0.926)	0.823 (0.733 to 0.926)	0.823 (0.733 to 0.926)
Ischaemic heart disease	0 g/day	Both	Female	1.0 (1.0 to 1.0)	1.0 (1.0 to 1.0)	1.0 (1.0 to 1.0)	1.0 (1.0 to 1.0)	1.0 (1.0 to 1.0)	1.0 (1.0 to 1.0)	1.0 (1.0 to 1.0)	1.0 (1.0 to 1.0)	1.0 (1.0 to 1.0)	1.0 (1.0 to 1.0)	1.0 (1.0 to 1.0)	1.0 (1.0 to 1.0)	1.0 (1.0 to 1.0)	1.0 (1.0 to 1.0)	1.0 (1.0 to 1.0)	1.0 (1.0 to 1.0)	1.0 (1.0 to 1.0)
Ischaemic stroke	72 g/day	Both	Male	1.451 (1.228 to 1.69)	1.451 (1.228 to 1.69)	1.451 (1.228 to 1.69)	1.451 (1.228 to 1.69)	1.451 (1.228 to 1.69)	1.451 (1.228 to 1.69)	1.451 (1.228 to 1.69)	1.451 (1.228 to 1.69)	1.451 (1.228 to 1.69)	1.451 (1.228 to 1.69)	1.451 (1.228 to 1.69)	1.451 (1.228 to 1.69)	1.451 (1.228 to 1.69)	1.451 (1.228 to 1.69)	1.451 (1.228 to 1.69)	1.451 (1.228 to 1.69)	1.451 (1.228 to 1.69)
Ischaemic stroke	60 g/day	Both	Male	1.312 (1.167 to 1.471)	1.312 (1.167 to 1.471)	1.312 (1.167 to 1.471)	1.312 (1.167 to 1.471)	1.312 (1.167 to 1.471)	1.312 (1.167 to 1.471)	1.312 (1.167 to 1.471)	1.312 (1.167 to 1.471)	1.312 (1.167 to 1.471)	1.312 (1.167 to 1.471)	1.312 (1.167 to 1.471)	1.312 (1.167 to 1.471)	1.312 (1.167 to 1.471)	1.312 (1.167 to 1.471)	1.312 (1.167 to 1.471)	1.312 (1.167 to 1.471)	1.312 (1.167 to 1.471)
Ischaemic stroke	48 g/day	Both	Male	1.159 (0.98 to 1.353)	1.159 (0.98 to 1.353)	1.159 (0.98 to 1.353)	1.159 (0.98 to 1.353)	1.159 (0.98 to 1.353)	1.159 (0.98 to 1.353)	1.159 (0.98 to 1.353)	1.159 (0.98 to 1.353)	1.159 (0.98 to 1.353)	1.159 (0.98 to 1.353)	1.159 (0.98 to 1.353)	1.159 (0.98 to 1.353)	1.159 (0.98 to 1.353)	1.159 (0.98 to 1.353)	1.159 (0.98 to 1.353)	1.159 (0.98 to 1.353)	1.159 (0.98 to 1.353)
Ischaemic stroke	36 g/day	Both	Male	1.057 (0.931 to 1.192)	1.057 (0.931 to 1.192)	1.057 (0.931 to 1.192)	1.057 (0.931 to 1.192)	1.057 (0.931 to 1.192)	1.057 (0.931 to 1.192)	1.057 (0.931 to 1.192)	1.057 (0.931 to 1.192)	1.057 (0.931 to 1.192)	1.057 (0.931 to 1.192)	1.057 (0.931 to 1.192)	1.057 (0.931 to 1.192)	1.057 (0.931 to 1.192)	1.057 (0.931 to 1.192)	1.057 (0.931 to 1.192)	1.057 (0.931 to 1.192)	1.057 (0.931 to 1.192)
Ischaemic stroke	24 g/day	Both	Male	0.97 (0.862 to 1.088)	0.97 (0.862 to 1.088)	0.97 (0.862 to 1.088)	0.97 (0.862 to 1.088)	0.97 (0.862 to 1.088)	0.97 (0.862 to 1.088)	0.97 (0.862 to 1.088)	0.97 (0.862 to 1.088)	0.97 (0.862 to 1.088)	0.97 (0.862 to 1.088)	0.97 (0.862 to 1.088)	0.97 (0.862 to 1.088)	0.97 (0.862 to 1.088)	0.97 (0.862 to 1.088)	0.97 (0.862 to 1.088)	0.97 (0.862 to 1.088)	0.97 (0.862 to 1.088)
Ischaemic stroke	12 g/day	Both	Male	0.938 (0.83 to 1.054)	0.938 (0.83 to 1.054)	0.938 (0.83 to 1.054)	0.938 (0.83 to 1.054)	0.938 (0.83 to 1.054)	0.938 (0.83 to 1.054)	0.938 (0.83 to 1.054)	0.938 (0.83 to 1.054)	0.938 (0.83 to 1.054)	0.938 (0.83 to 1.054)	0.938 (0.83 to 1.054)	0.938 (0.83 to 1.054)	0.938 (0.83 to 1.054)	0.938 (0.83 to 1.054)	0.938 (0.83 to 1.054)	0.938 (0.83 to 1.054)	0.938 (0.83 to 1.054)
Ischaemic stroke	0 g/day	Both	Male	1.0 (1.0 to 1.0)	1.0 (1.0 to 1.0)	1.0 (1.0 to 1.0)	1.0 (1.0 to 1.0)	1.0 (1.0 to 1.0)	1.0 (1.0 to 1.0)	1.0 (1.0 to 1.0)	1.0 (1.0 to 1.0)	1.0 (1.0 to 1.0)	1.0 (1.0 to 1.0)	1.0 (1.0 to 1.0)	1.0 (1.0 to 1.0)	1.0 (1.0 to 1.0)	1.0 (1.0 to 1.0)	1.0 (1.0 to 1.0)	1.0 (1.0 to 1.0)	1.0 (1.0 to 1.0)
Ischaemic stroke	72 g/day	Both	Female	1.43 (1.147 to 1.771)	1.43 (1.147 to 1.771)	1.43 (1.147 to 1.771)	1.43 (1.147 to 1.771)	1.43 (1.147 to 1.771)	1.43 (1.147 to 1.771)	1.43 (1.147 to 1.771)	1.43 (1.147 to 1.771)	1.43 (1.147 to 1.771)	1.43 (1.147 to 1.771)	1.43 (1.147 to 1.771)	1.43 (1.147 to 1.771)	1.43 (1.147 to 1.771)	1.43 (1.147 to 1.771)	1.43 (1.147 to 1.771)	1.43 (1.147 to 1.771)	1.43 (1.147 to 1.771)
Ischaemic stroke	60 g/day	Both	Female	1.3 (1.121 to 1.496)	1.3 (1.121 to 1.496)	1.3 (1.121 to 1.496)	1.3 (1.121 to 1.496)	1.3 (1.121 to 1.496)	1.3 (1.121 to 1.496)	1.3 (1.121 to 1.496)	1.3 (1.121 to 1.496)	1.3 (1.121 to 1.496)	1.3 (1.121 to 1.496)	1.3 (1.121 to 1.496)	1.3 (1.121 to 1.496)	1.3 (1.121 to 1.496)	1.3 (1.121 to 1.496)	1.3 (1.121 to 1.496)	1.3 (1.121 to 1.496)	1.3 (1.121 to 1.496)
Ischaemic stroke	48 g/day	Both	Female	1.145 (0.946 to 1.359)	1.145 (0.946 to 1.359)	1.145 (0.946 to 1.359)	1.145 (0.946 to 1.359)	1.145 (0.946 to 1.359)	1.145 (0.946 to 1.359)	1.145 (0.946 to 1.359)	1.145 (0.946 to 1.359)	1.145 (0.946 to 1.359)	1.145 (0.946 to 1.359)	1.145 (0.946 to 1.359)	1.145 (0.946 to 1.359)	1.145 (0.946 to 1.359)	1.145 (0.946 to 1.359)	1.145 (0.946 to 1.359)	1.145 (0.946 to 1.359)	1.145 (0.946 to 1.359)
Ischaemic stroke	36 g/day	Both	Female	0.985 (0.834 to 1.149)	0.985 (0.834 to 1.149)	0.985 (0.834 to 1.149)	0.985 (0.834 to 1.149)	0.985 (0.834 to 1.149)	0.985 (0.834 to 1.149)	0.985 (0.834 to 1.149)	0.985 (0.834 to 1.149)	0.985 (0.834 to 1.149)	0.985 (0.834 to 1.149)	0.985 (0.834 to 1.149)	0.985 (0.834 to 1.149)	0.985 (0.834 to 1.149)	0.985 (0.834 to 1.149)	0.985 (0.834 to 1.149)	0.985 (0.834 to 1.149)	0.985 (0.834 to 1.149)
Ischaemic stroke	24 g/day	Both	Female	0.85 (0.726 to 0.985)	0.85 (0.726 to 0.985)	0.85 (0.726 to 0.985)	0.85 (0.726 to 0.985)	0.85 (0.726 to 0.985)	0.85 (0.726 to 0.985)	0.85 (0.726 to 0.985)	0.85 (0.726 to 0.985)	0.85 (0.726 to 0.985)	0.85 (0.726 to 0.985)	0.85 (0.726 to 0.985)	0.85 (0.726 to 0.985)	0.85 (0.726 to 0.985)	0.85 (0.726 to 0.985)	0.85 (0.726 to 0.985)	0.85 (0.726 to 0.985)	0.85 (0.726 to 0.985)
Ischaemic stroke	12 g/day	Both	Female	0.824 (0.718 to 0.939)	0.824 (0.718 to 0.939)	0.824 (0.718 to 0.939)	0.824 (0.718 to 0.939)	0.824 (0.718 to 0.939)	0.824 (0.718 to 0.939)	0.824 (0.718 to 0.939)	0.824 (0.718 to 0.939)	0.824 (0.718 to 0.939)	0.824 (0.718 to 0.939)	0.824 (0.718 to 0.939)	0.824 (0.718 to 0.939)	0.824 (0.718 to 0.939)	0.824 (0.718 to 0.939)	0.824 (0.718 to 0.939)	0.824 (0.718 to 0.939)	0.824 (0.718 to 0.939)
Ischaemic stroke	0 g/day	Both	Female	1.0 (1.0 to 1.0)	1.0 (1.0 to 1.0)	1.0 (1.0 to 1.0)	1.0 (1.0 to 1.0)	1.0 (1.0 to 1.0)	1.0 (1.0 to 1.0)	1.0 (1.0 to 1.0)	1.0 (1.0 to 1.0)	1.0 (1.0 to 1.0)	1.0 (1.0 to 1.0)	1.0 (1.0 to 1.0)	1.0 (1.0 to 1.0)	1.0 (1.0 to 1.0)	1.0 (1.0 to 1.0)	1.0 (1.0 to 1.0)	1.0 (1.0 to 1.0)	1.0 (1.0 to 1.0)
Hemorrhagic stroke	72 g/day	Both	Male	1.971 (1.663 to 2.316)	1.971 (1.663 to 2.316)	1.971 (1.663 to 2.316)	1.971 (1.663 to 2.316)	1.971 (1.663 to 2.316)	1.971 (1.663 to 2.316)	1.971 (1.663 to 2.316)	1.971 (1.663 to 2.316)	1.971 (1.663 to 2.316)	1.971 (1.663 to 2.316)	1.971 (1.663 to 2.316)	1.971 (1.663 to 2.316)	1.971 (1.663 to 2.316)	1.971 (1.663 to 2.316)	1.971 (1.663 to 2.316)	1.971 (1.663 to 2.316)	1.971 (1.663 to 2.316)
Hemorrhagic stroke	60 g/day	Both	Male	1.705 (1.45 to 1.991)	1.705 (1.45 to 1.991)	1.705 (1.45 to 1.991)	1.705 (1.45 to 1.991)	1.705 (1.45 to 1.991)	1.705 (1.45 to 1.991)	1.705 (1.45 to 1.991)	1.705 (1.45 to 1.991)	1.705 (1.45 to 1.991)	1.705 (1.45 to 1.991)	1.705 (1.45 to 1.991)	1.705 (1.45 to 1.991)	1.705 (1.45 to 1.991)	1.705 (1.45 to 1.991)	1.705 (1.45 to 1.991)	1.705 (1.45 to 1.991)	1.705 (1.45 to 1.991)
Hemorrhagic stroke	48 g/day	Both	Male	1.458 (1.182 to 1.768)	1.458 (1.182 to 1.768)	1.458 (1.182 to 1.768)	1.458 (1.182 to 1.768)	1.458 (1.182 to 1.768)	1.458 (1.182 to 1.768)	1.458 (1.182 to 1.768)	1.458 (1.182 to 1.768)	1.458 (1.182 to 1.768)	1.458 (1.182 to 1.768)	1.458 (1.182 to 1.768)	1.458 (1.182 to 1.768)	1.458 (1.182 to 1.768)	1.458 (1.182 to 1.768)	1.458 (1.182 to 1.768)	1.458 (1.182 to 1.768)	1.458 (1.182 to 1.768)
Hemorrhagic stroke	36 g/day	Both	Male	1.31 (1.105 to 1.539)	1.31 (1.105 to 1.539)	1.31 (1.105 to 1.539)	1.31 (1.105 to 1.539)	1.31 (1.105 to 1.539)	1.31 (1.105 to 1.539)	1.31 (1.105 to 1.539)	1.31 (1.105 to 1.539)	1.31 (1.105 to 1.539)	1.31 (1.105 to 1.539)	1.31 (1.105 to 1.539)	1.31 (1.105 to 1.539)	1.31 (1.105 to 1.539)	1.31 (1.105 to 1.539)	1.31 (1.105 to 1.539)	1.31 (1.105 to 1.539)	1.31 (1.105 to 1.539)
Hemorrhagic stroke	24 g/day	Both	Male	1.162 (0.973 to 1.385)	1.162 (0.973 to 1.385)	1.162 (0.973 to 1.385)	1.162 (0.973 to 1.385)	1.162 (0.973 to 1.385)	1.162 (0.973 to 1.385)	1.162 (0.973 to 1.385)	1.162 (0.973 to 1.385)	1.162 (0.973 to 1.385)	1.162 (0.973 to 1.385)	1.162 (0.973 to 1.385)	1.162 (0.973 to 1.385)	1.162 (0.973 to 1.385)	1.162 (0.973 to 1.385)	1.162 (0.973 to 1.385)	1.162 (0.973 to 1.385)	1.162 (0.973 to 1.385)
Hemorrhagic stroke	12 g/day	Both	Male	1.068 (0.945 to 1.214)	1.068 (0.945 to 1.214)	1.068 (0.945 to 1.214)	1.068 (0.945 to 1.214)	1.068 (0.945 to 1.214)	1.068 (0.945 to 1.214)	1.068 (0.945 to 1.214)	1.068 (0.945 to 1.214)	1.068 (0.945 to 1.214)	1.068 (0.945 to 1.214)	1.068 (0.945 to 1.214)	1.068 (0.945 to 1.214)	1.068 (0.945 to 1.214)	1.068 (0.945 to 1.214)	1.068 (0.945 to 1.214)	1.068 (0.945 to 1.214)	1.068 (0.945 to 1.214)
Hemorrhagic stroke	0 g/day	Both	Male	1.0 (1.0 to 1.0)	1.0 (1.0 to 1.0)	1.0 (1.0 to 1.0)	1.0 (1.0 to 1.0)	1.0 (1.0 to 1.0)	1.0 (1.0 to 1.0)	1.0 (1.0 to 1.0)	1.0 (1.0 to 1.0)	1.0 (1.0 to 1.0)	1.0 (1.0 to 1.0)	1.0 (1.0 to 1.0)	1.0 (1.0 to 1.0)	1.0 (1.0 to 1.0)	1.0 (1.0 to 1.0)	1.0 (1.0 to 1.0)	1.0 (1.0 to 1.0)	1.0 (1.0 to 1.0)

Appendix Table 1c. Relative risks used by age and sex for each outcome for alcohol use globally.

Risk - Outcome	Category / Units	Morbidity / Mortality	Sex	15-19 years	20-24 years	25-29 years	30-34 years	35-39 years	40-44 years	45-49 years	50-54 years	55-59 years	60-64 years	65-69 years	70-74 years	75-79 years	80-84 years	85-89 years	90-94 years	95+ years
**Alcohol use**																				
Hemorrhagic stroke	72 g/day	Both	Female	2.276 (1.701 to 2.934)	2.276 (1.701 to 2.934)	2.276 (1.701 to 2.934)	2.276 (1.701 to 2.934)	2.276 (1.701 to 2.934)	2.276 (1.701 to 2.934)	2.276 (1.701 to 2.934)	2.276 (1.701 to 2.934)	2.276 (1.701 to 2.934)	2.276 (1.701 to 2.934)	2.276 (1.701 to 2.934)	2.276 (1.701 to 2.934)	2.276 (1.701 to 2.934)	2.276 (1.701 to 2.934)	2.276 (1.701 to 2.934)	2.276 (1.701 to 2.934)	2.276 (1.701 to 2.934)
Hemorrhagic stroke	60 g/day	Both	Female	1.964 (1.536 to 2.464)	1.964 (1.536 to 2.464)	1.964 (1.536 to 2.464)	1.964 (1.536 to 2.464)	1.964 (1.536 to 2.464)	1.964 (1.536 to 2.464)	1.964 (1.536 to 2.464)	1.964 (1.536 to 2.464)	1.964 (1.536 to 2.464)	1.964 (1.536 to 2.464)	1.964 (1.536 to 2.464)	1.964 (1.536 to 2.464)	1.964 (1.536 to 2.464)	1.964 (1.536 to 2.464)	1.964 (1.536 to 2.464)	1.964 (1.536 to 2.464)	1.964 (1.536 to 2.464)
Hemorrhagic stroke	48 g/day	Both	Female	1.614 (1.245 to 2.048)	1.614 (1.245 to 2.048)	1.614 (1.245 to 2.048)	1.614 (1.245 to 2.048)	1.614 (1.245 to 2.048)	1.614 (1.245 to 2.048)	1.614 (1.245 to 2.048)	1.614 (1.245 to 2.048)	1.614 (1.245 to 2.048)	1.614 (1.245 to 2.048)	1.614 (1.245 to 2.048)	1.614 (1.245 to 2.048)	1.614 (1.245 to 2.048)	1.614 (1.245 to 2.048)	1.614 (1.245 to 2.048)	1.614 (1.245 to 2.048)	1.614 (1.245 to 2.048)
Hemorrhagic stroke	36 g/day	Both	Female	1.337 (1.065 to 1.664)	1.337 (1.065 to 1.664)	1.337 (1.065 to 1.664)	1.337 (1.065 to 1.664)	1.337 (1.065 to 1.664)	1.337 (1.065 to 1.664)	1.337 (1.065 to 1.664)	1.337 (1.065 to 1.664)	1.337 (1.065 to 1.664)	1.337 (1.065 to 1.664)	1.337 (1.065 to 1.664)	1.337 (1.065 to 1.664)	1.337 (1.065 to 1.664)	1.337 (1.065 to 1.664)	1.337 (1.065 to 1.664)	1.337 (1.065 to 1.664)	1.337 (1.065 to 1.664)
Hemorrhagic stroke	24 g/day	Both	Female	1.11 (0.884 to 1.367)	1.11 (0.884 to 1.367)	1.11 (0.884 to 1.367)	1.11 (0.884 to 1.367)	1.11 (0.884 to 1.367)	1.11 (0.884 to 1.367)	1.11 (0.884 to 1.367)	1.11 (0.884 to 1.367)	1.11 (0.884 to 1.367)	1.11 (0.884 to 1.367)	1.11 (0.884 to 1.367)	1.11 (0.884 to 1.367)	1.11 (0.884 to 1.367)	1.11 (0.884 to 1.367)	1.11 (0.884 to 1.367)	1.11 (0.884 to 1.367)	1.11 (0.884 to 1.367)
Hemorrhagic stroke	12 g/day	Both	Female	1.031 (0.897 to 1.18)	1.031 (0.897 to 1.18)	1.031 (0.897 to 1.18)	1.031 (0.897 to 1.18)	1.031 (0.897 to 1.18)	1.031 (0.897 to 1.18)	1.031 (0.897 to 1.18)	1.031 (0.897 to 1.18)	1.031 (0.897 to 1.18)	1.031 (0.897 to 1.18)	1.031 (0.897 to 1.18)	1.031 (0.897 to 1.18)	1.031 (0.897 to 1.18)	1.031 (0.897 to 1.18)	1.031 (0.897 to 1.18)	1.031 (0.897 to 1.18)	1.031 (0.897 to 1.18)
Hemorrhagic stroke	0 g/day	Both	Female	1.0 (1.0 to 1.0)	1.0 (1.0 to 1.0)	1.0 (1.0 to 1.0)	1.0 (1.0 to 1.0)	1.0 (1.0 to 1.0)	1.0 (1.0 to 1.0)	1.0 (1.0 to 1.0)	1.0 (1.0 to 1.0)	1.0 (1.0 to 1.0)	1.0 (1.0 to 1.0)	1.0 (1.0 to 1.0)	1.0 (1.0 to 1.0)	1.0 (1.0 to 1.0)	1.0 (1.0 to 1.0)	1.0 (1.0 to 1.0)	1.0 (1.0 to 1.0)	1.0 (1.0 to 1.0)
Hypertensive heart disease	72 g/day	Both	Both	1.86 (1.445 to 2.358)	1.86 (1.445 to 2.358)	1.86 (1.445 to 2.358)	1.86 (1.445 to 2.358)	1.86 (1.445 to 2.358)	1.86 (1.445 to 2.358)	1.86 (1.445 to 2.358)	1.86 (1.445 to 2.358)	1.86 (1.445 to 2.358)	1.86 (1.445 to 2.358)	1.86 (1.445 to 2.358)	1.86 (1.445 to 2.358)	1.86 (1.445 to 2.358)	1.86 (1.445 to 2.358)	1.86 (1.445 to 2.358)	1.86 (1.445 to 2.358)	1.86 (1.445 to 2.358)
Hypertensive heart disease	60 g/day	Both	Both	1.705 (1.297 to 2.175)	1.705 (1.297 to 2.175)	1.705 (1.297 to 2.175)	1.705 (1.297 to 2.175)	1.705 (1.297 to 2.175)	1.705 (1.297 to 2.175)	1.705 (1.297 to 2.175)	1.705 (1.297 to 2.175)	1.705 (1.297 to 2.175)	1.705 (1.297 to 2.175)	1.705 (1.297 to 2.175)	1.705 (1.297 to 2.175)	1.705 (1.297 to 2.175)	1.705 (1.297 to 2.175)	1.705 (1.297 to 2.175)	1.705 (1.297 to 2.175)	1.705 (1.297 to 2.175)
Hypertensive heart disease	48 g/day	Both	Both	1.614 (1.25 to 2.049)	1.614 (1.25 to 2.049)	1.614 (1.25 to 2.049)	1.614 (1.25 to 2.049)	1.614 (1.25 to 2.049)	1.614 (1.25 to 2.049)	1.614 (1.25 to 2.049)	1.614 (1.25 to 2.049)	1.614 (1.25 to 2.049)	1.614 (1.25 to 2.049)	1.614 (1.25 to 2.049)	1.614 (1.25 to 2.049)	1.614 (1.25 to 2.049)	1.614 (1.25 to 2.049)	1.614 (1.25 to 2.049)	1.614 (1.25 to 2.049)	1.614 (1.25 to 2.049)
Hypertensive heart disease	36 g/day	Both	Both	1.479 (1.232 to 1.759)	1.479 (1.232 to 1.759)	1.479 (1.232 to 1.759)	1.479 (1.232 to 1.759)	1.479 (1.232 to 1.759)	1.479 (1.232 to 1.759)	1.479 (1.232 to 1.759)	1.479 (1.232 to 1.759)	1.479 (1.232 to 1.759)	1.479 (1.232 to 1.759)	1.479 (1.232 to 1.759)	1.479 (1.232 to 1.759)	1.479 (1.232 to 1.759)	1.479 (1.232 to 1.759)	1.479 (1.232 to 1.759)	1.479 (1.232 to 1.759)	1.479 (1.232 to 1.759)
Hypertensive heart disease	24 g/day	Both	Both	1.315 (1.136 to 1.526)	1.315 (1.136 to 1.526)	1.315 (1.136 to 1.526)	1.315 (1.136 to 1.526)	1.315 (1.136 to 1.526)	1.315 (1.136 to 1.526)	1.315 (1.136 to 1.526)	1.315 (1.136 to 1.526)	1.315 (1.136 to 1.526)	1.315 (1.136 to 1.526)	1.315 (1.136 to 1.526)	1.315 (1.136 to 1.526)	1.315 (1.136 to 1.526)	1.315 (1.136 to 1.526)	1.315 (1.136 to 1.526)	1.315 (1.136 to 1.526)	1.315 (1.136 to 1.526)
Hypertensive heart disease	12 g/day	Both	Both	1.046 (0.913 to 1.198)	1.046 (0.913 to 1.198)	1.046 (0.913 to 1.198)	1.046 (0.913 to 1.198)	1.046 (0.913 to 1.198)	1.046 (0.913 to 1.198)	1.046 (0.913 to 1.198)	1.046 (0.913 to 1.198)	1.046 (0.913 to 1.198)	1.046 (0.913 to 1.198)	1.046 (0.913 to 1.198)	1.046 (0.913 to 1.198)	1.046 (0.913 to 1.198)	1.046 (0.913 to 1.198)	1.046 (0.913 to 1.198)	1.046 (0.913 to 1.198)	1.046 (0.913 to 1.198)
Hypertensive heart disease	0 g/day	Both	Both	1.0 (1.0 to 1.0)	1.0 (1.0 to 1.0)	1.0 (1.0 to 1.0)	1.0 (1.0 to 1.0)	1.0 (1.0 to 1.0)	1.0 (1.0 to 1.0)	1.0 (1.0 to 1.0)	1.0 (1.0 to 1.0)	1.0 (1.0 to 1.0)	1.0 (1.0 to 1.0)	1.0 (1.0 to 1.0)	1.0 (1.0 to 1.0)	1.0 (1.0 to 1.0)	1.0 (1.0 to 1.0)	1.0 (1.0 to 1.0)	1.0 (1.0 to 1.0)	1.0 (1.0 to 1.0)
Atrial fibrillation and flutter	72 g/day	Both	Both	1.535 (1.348 to 1.728)	1.535 (1.348 to 1.728)	1.535 (1.348 to 1.728)	1.535 (1.348 to 1.728)	1.535 (1.348 to 1.728)	1.535 (1.348 to 1.728)	1.535 (1.348 to 1.728)	1.535 (1.348 to 1.728)	1.535 (1.348 to 1.728)	1.535 (1.348 to 1.728)	1.535 (1.348 to 1.728)	1.535 (1.348 to 1.728)	1.535 (1.348 to 1.728)	1.535 (1.348 to 1.728)	1.535 (1.348 to 1.728)	1.535 (1.348 to 1.728)	1.535 (1.348 to 1.728)
Atrial fibrillation and flutter	60 g/day	Both	Both	1.411 (1.26 to 1.569)	1.411 (1.26 to 1.569)	1.411 (1.26 to 1.569)	1.411 (1.26 to 1.569)	1.411 (1.26 to 1.569)	1.411 (1.26 to 1.569)	1.411 (1.26 to 1.569)	1.411 (1.26 to 1.569)	1.411 (1.26 to 1.569)	1.411 (1.26 to 1.569)	1.411 (1.26 to 1.569)	1.411 (1.26 to 1.569)	1.411 (1.26 to 1.569)	1.411 (1.26 to 1.569)	1.411 (1.26 to 1.569)	1.411 (1.26 to 1.569)	1.411 (1.26 to 1.569)
Atrial fibrillation and flutter	48 g/day	Both	Both	1.312 (1.218 to 1.407)	1.312 (1.218 to 1.407)	1.312 (1.218 to 1.407)	1.312 (1.218 to 1.407)	1.312 (1.218 to 1.407)	1.312 (1.218 to 1.407)	1.312 (1.218 to 1.407)	1.312 (1.218 to 1.407)	1.312 (1.218 to 1.407)	1.312 (1.218 to 1.407)	1.312 (1.218 to 1.407)	1.312 (1.218 to 1.407)	1.312 (1.218 to 1.407)	1.312 (1.218 to 1.407)	1.312 (1.218 to 1.407)	1.312 (1.218 to 1.407)	1.312 (1.218 to 1.407)
Atrial fibrillation and flutter	36 g/day	Both	Both	1.214 (1.145 to 1.29)	1.214 (1.145 to 1.29)	1.214 (1.145 to 1.29)	1.214 (1.145 to 1.29)	1.214 (1.145 to 1.29)	1.214 (1.145 to 1.29)	1.214 (1.145 to 1.29)	1.214 (1.145 to 1.29)	1.214 (1.145 to 1.29)	1.214 (1.145 to 1.29)	1.214 (1.145 to 1.29)	1.214 (1.145 to 1.29)	1.214 (1.145 to 1.29)	1.214 (1.145 to 1.29)	1.214 (1.145 to 1.29)	1.214 (1.145 to 1.29)	1.214 (1.145 to 1.29)
Atrial fibrillation and flutter	24 g/day	Both	Both	1.131 (1.067 to 1.204)	1.131 (1.067 to 1.204)	1.131 (1.067 to 1.204)	1.131 (1.067 to 1.204)	1.131 (1.067 to 1.204)	1.131 (1.067 to 1.204)	1.131 (1.067 to 1.204)	1.131 (1.067 to 1.204)	1.131 (1.067 to 1.204)	1.131 (1.067 to 1.204)	1.131 (1.067 to 1.204)	1.131 (1.067 to 1.204)	1.131 (1.067 to 1.204)	1.131 (1.067 to 1.204)	1.131 (1.067 to 1.204)	1.131 (1.067 to 1.204)	1.131 (1.067 to 1.204)
Atrial fibrillation and flutter	12 g/day	Both	Both	1.066 (1.034 to 1.102)	1.066 (1.034 to 1.102)	1.066 (1.034 to 1.102)	1.066 (1.034 to 1.102)	1.066 (1.034 to 1.102)	1.066 (1.034 to 1.102)	1.066 (1.034 to 1.102)	1.066 (1.034 to 1.102)	1.066 (1.034 to 1.102)	1.066 (1.034 to 1.102)	1.066 (1.034 to 1.102)	1.066 (1.034 to 1.102)	1.066 (1.034 to 1.102)	1.066 (1.034 to 1.102)	1.066 (1.034 to 1.102)	1.066 (1.034 to 1.102)	1.066 (1.034 to 1.102)
Atrial fibrillation and flutter	0 g/day	Both	Both	1.0 (1.0 to 1.0)	1.0 (1.0 to 1.0)	1.0 (1.0 to 1.0)	1.0 (1.0 to 1.0)	1.0 (1.0 to 1.0)	1.0 (1.0 to 1.0)	1.0 (1.0 to 1.0)	1.0 (1.0 to 1.0)	1.0 (1.0 to 1.0)	1.0 (1.0 to 1.0)	1.0 (1.0 to 1.0)	1.0 (1.0 to 1.0)	1.0 (1.0 to 1.0)	1.0 (1.0 to 1.0)	1.0 (1.0 to 1.0)	1.0 (1.0 to 1.0)	1.0 (1.0 to 1.0)
Cirrhosis and other chronic liver diseases due	72 g/day	Both	Both	9.427 (6.131 to 13.804)	9.427 (6.131 to 13.804)	9.427 (6.131 to 13.804)	9.427 (6.131 to 13.804)	9.427 (6.131 to 13.804)	9.427 (6.131 to 13.804)	9.427 (6.131 to 13.804)	9.427 (6.131 to 13.804)	9.427 (6.131 to 13.804)	9.427 (6.131 to 13.804)	9.427 (6.131 to 13.804)	9.427 (6.131 to 13.804)	9.427 (6.131 to 13.804)	9.427 (6.131 to 13.804)	9.427 (6.131 to 13.804)	9.427 (6.131 to 13.804)	9.427 (6.131 to 13.804)
Cirrhosis and other chronic liver diseases due	60 g/day	Both	Both	6.274 (3.958 to 9.319)	6.274 (3.958 to 9.319)	6.274 (3.958 to 9.319)	6.274 (3.958 to 9.319)	6.274 (3.958 to 9.319)	6.274 (3.958 to 9.319)	6.274 (3.958 to 9.319)	6.274 (3.958 to 9.319)	6.274 (3.958 to 9.319)	6.274 (3.958 to 9.319)	6.274 (3.958 to 9.319)	6.274 (3.958 to 9.319)	6.274 (3.958 to 9.319)	6.274 (3.958 to 9.319)	6.274 (3.958 to 9.319)	6.274 (3.958 to 9.319)	6.274 (3.958 to 9.319)
Cirrhosis and other chronic liver diseases due	48 g/day	Both	Both	4.673 (3.25 to 6.717)	4.673 (3.25 to 6.717)	4.673 (3.25 to 6.717)	4.673 (3.25 to 6.717)	4.673 (3.25 to 6.717)	4.673 (3.25 to 6.717)	4.673 (3.25 to 6.717)	4.673 (3.25 to 6.717)	4.673 (3.25 to 6.717)	4.673 (3.25 to 6.717)	4.673 (3.25 to 6.717)	4.673 (3.25 to 6.717)	4.673 (3.25 to 6.717)	4.673 (3.25 to 6.717)	4.673 (3.25 to 6.717)	4.673 (3.25 to 6.717)	4.673 (3.25 to 6.717)
Cirrhosis and other chronic liver diseases due	36 g/day	Both	Both	3.274 (2.309 to 4.485)	3.274 (2.309 to 4.485)	3.274 (2.309 to 4.485)	3.274 (2.309 to 4.485)	3.274 (2.309 to 4.485)	3.274 (2.309 to 4.485)	3.274 (2.309 to 4.485)	3.274 (2.309 to 4.485)	3.274 (2.309 to 4.485)	3.274 (2.309 to 4.485)	3.274 (2.309 to 4.485)	3.274 (2.309 to 4.485)	3.274 (2.309 to 4.485)	3.274 (2.309 to 4.485)	3.274 (2.309 to 4.485)	3.274 (2.309 to 4.485)	3.274 (2.309 to 4.485)
Cirrhosis and other chronic liver diseases due	24 g/day	Both	Both	2.055 (1.521 to 2.688)	2.055 (1.521 to 2.688)	2.055 (1.521 to 2.688)	2.055 (1.521 to 2.688)	2.055 (1.521 to 2.688)	2.055 (1.521 to 2.688)	2.055 (1.521 to 2.688)	2.055 (1.521 to 2.688)	2.055 (1.521 to 2.688)	2.055 (1.521 to 2.688)	2.055 (1.521 to 2.688)	2.055 (1.521 to 2.688)	2.055 (1.521 to 2.688)	2.055 (1.521 to 2.688)	2.055 (1.521 to 2.688)	2.055 (1.521 to 2.688)	2.055 (1.521 to 2.688)
Cirrhosis and other chronic liver diseases due	12 g/day	Both	Both	1.243 (0.943 to 1.611)	1.243 (0.943 to 1.611)	1.243 (0.943 to 1.611)	1.243 (0.943 to 1.611)	1.243 (0.943 to 1.611)	1.243 (0.943 to 1.611)	1.243 (0.943 to 1.611)	1.243 (0.943 to 1.611)	1.243 (0.943 to 1.611)	1.243 (0.943 to 1.611)	1.243 (0.943 to 1.611)	1.243 (0.943 to 1.611)	1.243 (0.943 to 1.611)	1.243 (0.943 to 1.611)	1.243 (0.943 to 1.611)	1.243 (0.943 to 1.611)	1.243 (0.943 to 1.611)
Cirrhosis and other chronic liver diseases due	0 g/day	Both	Both	1.0 (1.0 to 1.0)	1.0 (1.0 to 1.0)	1.0 (1.0 to 1.0)	1.0 (1.0 to 1.0)	1.0 (1.0 to 1.0)	1.0 (1.0 to 1.0)	1.0 (1.0 to 1.0)	1.0 (1.0 to 1.0)	1.0 (1.0 to 1.0)	1.0 (1.0 to 1.0)	1.0 (1.0 to 1.0)	1.0 (1.0 to 1.0)	1.0 (1.0 to 1.0)	1.0 (1.0 to 1.0)	1.0 (1.0 to 1.0)	1.0 (1.0 to 1.0)	1.0 (1.0 to 1.0)
Pancreatitis	72 g/day	Both	Both	3.298 (2.473 to 4.458)	3.298 (2.473 to 4.458)	3.298 (2.473 to 4.458)	3.298 (2.473 to 4.458)	3.298 (2.473 to 4.458)	3.298 (2.473 to 4.458)	3.298 (2.473 to 4.458)	3.298 (2.473 to 4.458)	3.298 (2.473 to 4.458)	3.298 (2.473 to 4.458)	3.298 (2.473 to 4.458)	3.298 (2.473 to 4.458)	3.298 (2.473 to 4.458)	3.298 (2.473 to 4.458)	3.298 (2.473 to 4.458)	3.298 (2.473 to 4.458)	3.298 (2.473 to 4.458)
Pancreatitis	60 g/day	Both	Both	2.217 (1.415 to 3.389)	2.217 (1.415 to 3.389)	2.217 (1.415 to 3.389)	2.217 (1.415 to 3.389)	2.217 (1.415 to 3.389)	2.217 (1.415 to 3.389)	2.217 (1.415 to 3.389)	2.217 (1.415 to 3.389)	2.217 (1.415 to 3.389)	2.217 (1.415 to 3.389)	2.217 (1.415 to 3.389)	2.217 (1.415 to 3.389)	2.217 (1.415 to 3.389)	2.217 (1.415 to 3.389)	2.217 (1.415 to 3.389)	2.217 (1.415 to 3.389)	2.217 (1.415 to 3.389)
Pancreatitis	48 g/day	Both	Both	1.717 (1.199 to 2.477)	1.717 (1.199 to 2.477)	1.717 (1.199 to 2.477)	1.717 (1.199 to 2.477)	1.717 (1.199 to 2.477)	1.717 (1.199 to 2.477)	1.717 (1.199 to 2.477)	1.717 (1.199 to 2.477)	1.717 (1.199 to 2.477)	1.717 (1.199 to 2.477)	1.717 (1.199 to 2.477)	1.717 (1.199 to 2.477)	1.717 (1.199 to 2.477)	1.717 (1.199 to 2.477)	1.717 (1.199 to 2.477)	1.717 (1.199 to 2.477)	1.717 (1.199 to 2.477)
Pancreatitis	36 g/day	Both	Both	1.471 (1.062 to 2.021)	1.471 (1.062 to 2.021)	1.471 (1.062 to 2.021)	1.471 (1.062 to 2.021)	1.471 (1.062 to 2.021)	1.471 (1.062 to 2.021)	1.471 (1.062 to 2.021)	1.471 (1.062 to 2.021)	1.471 (1.062 to 2.021)	1.471 (1.062 to 2.021)	1.471 (1.062 to 2.021)	1.471 (1.062 to 2.021)	1.471 (1.062 to 2.021)	1.471 (1.062 to 2.021)	1.471 (1.062 to 2.021)	1.471 (1.062 to 2.021)	1.471 (1.062 to 2.021)
Pancreatitis	24 g/day	Both	Both	1.228 (0.874 to 1.67)	1.228 (0.874 to 1.67)	1.228 (0.874 to 1.67)	1.228 (0.874 to 1.67)	1.228 (0.874 to 1.67)	1.228 (0.874 to 1.67)	1.228 (0.874 to 1.67)	1.228 (0.874 to 1.67)	1.228 (0.874 to 1.67)	1.228 (0.874 to 1.67)	1.228 (0.874 to 1.67)	1.228 (0.874 to 1.67)	1.228 (0.874 to 1.67)	1.228 (0.874 to 1.67)	1.228 (0.874 to 1.67)	1.228 (0.874 to 1.67)	1.228 (0.874 to 1.67)
Pancreatitis	12 g/day	Both	Both	1.073 (0.791 to 1.481)	1.073 (0.791 to 1.481)	1.073 (0.791 to 1.481)	1.073 (0.791 to 1.481)	1.073 (0.791 to 1.481)	1.073 (0.791 to 1.481)	1.073 (0.791 to 1.481)	1.073 (0.791 to 1.481)	1.073 (0.791 to 1.481)	1.073 (0.791 to 1.481)	1.073 (0.791 to 1.481)	1.073 (0.791 to 1.481)	1.073 (0.791 to 1.481)	1.073 (0.791 to 1.481)	1.073 (0.791 to 1.481)	1.073 (0.791 to 1.481)	1.073 (0.791 to 1.481)
Pancreatitis	0 g/day	Both	Both	1.0 (1.0 to 1.0)	1.0 (1.0 to 1.0)	1.0 (1.0 to 1.0)	1.0 (1.0 to 1.0)	1.0 (1.0 to 1.0)	1.0 (1.0 to 1.0)	1.0 (1.0 to 1.0)	1.0 (1.0 to 1.0)	1.0 (1.0 to 1.0)	1.0 (1.0 to 1.0)	1.0 (1.0 to 1.0)	1.0 (1.0 to 1.0)	1.0 (1.0 to 1.0)	1.0 (1.0 to 1.0)	1.0 (1.0 to 1.0)	1.0 (1.0 to 1.0)	1.0 (1.0 to 1.0)

Appendix Table 1c. Relative risks used by age and sex for each outcome for alcohol use globally.

Risk - Outcome	Category / Units	Morbidity / Mortality	Sex	15-19 years	20-24 years	25-29 years	30-34 years	35-39 years	40-44 years	45-49 years	50-54 years	55-59 years	60-64 years	65-69 years	70-74 years	75-79 years	80-84 years	85-89 years	90-94 years	95+ years
**Alcohol use**																				
Epilepsy	72 g/day	Both	Both	2.48 (1.929 to 3.144)	2.48 (1.929 to 3.144)	2.48 (1.929 to 3.144)	2.48 (1.929 to 3.144)	2.48 (1.929 to 3.144)	2.48 (1.929 to 3.144)	2.48 (1.929 to 3.144)	2.48 (1.929 to 3.144)	2.48 (1.929 to 3.144)	2.48 (1.929 to 3.144)	2.48 (1.929 to 3.144)	2.48 (1.929 to 3.144)	2.48 (1.929 to 3.144)	2.48 (1.929 to 3.144)	2.48 (1.929 to 3.144)	2.48 (1.929 to 3.144)	2.48 (1.929 to 3.144)
Epilepsy	60 g/day	Both	Both	2.186 (1.781 to 2.622)	2.186 (1.781 to 2.622)	2.186 (1.781 to 2.622)	2.186 (1.781 to 2.622)	2.186 (1.781 to 2.622)	2.186 (1.781 to 2.622)	2.186 (1.781 to 2.622)	2.186 (1.781 to 2.622)	2.186 (1.781 to 2.622)	2.186 (1.781 to 2.622)	2.186 (1.781 to 2.622)	2.186 (1.781 to 2.622)	2.186 (1.781 to 2.622)	2.186 (1.781 to 2.622)	2.186 (1.781 to 2.622)	2.186 (1.781 to 2.622)	2.186 (1.781 to 2.622)
Epilepsy	48 g/day	Both	Both	1.872 (1.438 to 2.369)	1.872 (1.438 to 2.369)	1.872 (1.438 to 2.369)	1.872 (1.438 to 2.369)	1.872 (1.438 to 2.369)	1.872 (1.438 to 2.369)	1.872 (1.438 to 2.369)	1.872 (1.438 to 2.369)	1.872 (1.438 to 2.369)	1.872 (1.438 to 2.369)	1.872 (1.438 to 2.369)	1.872 (1.438 to 2.369)	1.872 (1.438 to 2.369)	1.872 (1.438 to 2.369)	1.872 (1.438 to 2.369)	1.872 (1.438 to 2.369)	1.872 (1.438 to 2.369)
Epilepsy	36 g/day	Both	Both	1.585 (1.303 to 1.898)	1.585 (1.303 to 1.898)	1.585 (1.303 to 1.898)	1.585 (1.303 to 1.898)	1.585 (1.303 to 1.898)	1.585 (1.303 to 1.898)	1.585 (1.303 to 1.898)	1.585 (1.303 to 1.898)	1.585 (1.303 to 1.898)	1.585 (1.303 to 1.898)	1.585 (1.303 to 1.898)	1.585 (1.303 to 1.898)	1.585 (1.303 to 1.898)	1.585 (1.303 to 1.898)	1.585 (1.303 to 1.898)	1.585 (1.303 to 1.898)	1.585 (1.303 to 1.898)
Epilepsy	24 g/day	Both	Both	1.353 (1.118 to 1.633)	1.353 (1.118 to 1.633)	1.353 (1.118 to 1.633)	1.353 (1.118 to 1.633)	1.353 (1.118 to 1.633)	1.353 (1.118 to 1.633)	1.353 (1.118 to 1.633)	1.353 (1.118 to 1.633)	1.353 (1.118 to 1.633)	1.353 (1.118 to 1.633)	1.353 (1.118 to 1.633)	1.353 (1.118 to 1.633)	1.353 (1.118 to 1.633)	1.353 (1.118 to 1.633)	1.353 (1.118 to 1.633)	1.353 (1.118 to 1.633)	1.353 (1.118 to 1.633)
Epilepsy	12 g/day	Both	Both	1.177 (1.059 to 1.316)	1.177 (1.059 to 1.316)	1.177 (1.059 to 1.316)	1.177 (1.059 to 1.316)	1.177 (1.059 to 1.316)	1.177 (1.059 to 1.316)	1.177 (1.059 to 1.316)	1.177 (1.059 to 1.316)	1.177 (1.059 to 1.316)	1.177 (1.059 to 1.316)	1.177 (1.059 to 1.316)	1.177 (1.059 to 1.316)	1.177 (1.059 to 1.316)	1.177 (1.059 to 1.316)	1.177 (1.059 to 1.316)	1.177 (1.059 to 1.316)	1.177 (1.059 to 1.316)
Epilepsy	0 g/day	Both	Both	1.0 (1.0 to 1.0)	1.0 (1.0 to 1.0)	1.0 (1.0 to 1.0)	1.0 (1.0 to 1.0)	1.0 (1.0 to 1.0)	1.0 (1.0 to 1.0)	1.0 (1.0 to 1.0)	1.0 (1.0 to 1.0)	1.0 (1.0 to 1.0)	1.0 (1.0 to 1.0)	1.0 (1.0 to 1.0)	1.0 (1.0 to 1.0)	1.0 (1.0 to 1.0)	1.0 (1.0 to 1.0)	1.0 (1.0 to 1.0)	1.0 (1.0 to 1.0)	1.0 (1.0 to 1.0)
Diabetes mellitus	72 g/day	Both	Male	1.198 (1.065 to 1.337)	1.198 (1.065 to 1.337)	1.198 (1.065 to 1.337)	1.198 (1.065 to 1.337)	1.198 (1.065 to 1.337)	1.198 (1.065 to 1.337)	1.198 (1.065 to 1.337)	1.198 (1.065 to 1.337)	1.198 (1.065 to 1.337)	1.198 (1.065 to 1.337)	1.198 (1.065 to 1.337)	1.198 (1.065 to 1.337)	1.198 (1.065 to 1.337)	1.198 (1.065 to 1.337)	1.198 (1.065 to 1.337)	1.198 (1.065 to 1.337)	1.198 (1.065 to 1.337)
Diabetes mellitus	60 g/day	Both	Male	1.165 (0.998 to 1.342)	1.165 (0.998 to 1.342)	1.165 (0.998 to 1.342)	1.165 (0.998 to 1.342)	1.165 (0.998 to 1.342)	1.165 (0.998 to 1.342)	1.165 (0.998 to 1.342)	1.165 (0.998 to 1.342)	1.165 (0.998 to 1.342)	1.165 (0.998 to 1.342)	1.165 (0.998 to 1.342)	1.165 (0.998 to 1.342)	1.165 (0.998 to 1.342)	1.165 (0.998 to 1.342)	1.165 (0.998 to 1.342)	1.165 (0.998 to 1.342)	1.165 (0.998 to 1.342)
Diabetes mellitus	48 g/day	Both	Male	1.084 (0.933 to 1.239)	1.084 (0.933 to 1.239)	1.084 (0.933 to 1.239)	1.084 (0.933 to 1.239)	1.084 (0.933 to 1.239)	1.084 (0.933 to 1.239)	1.084 (0.933 to 1.239)	1.084 (0.933 to 1.239)	1.084 (0.933 to 1.239)	1.084 (0.933 to 1.239)	1.084 (0.933 to 1.239)	1.084 (0.933 to 1.239)	1.084 (0.933 to 1.239)	1.084 (0.933 to 1.239)	1.084 (0.933 to 1.239)	1.084 (0.933 to 1.239)	1.084 (0.933 to 1.239)
Diabetes mellitus	36 g/day	Both	Male	1.0 (0.891 to 1.119)	1.0 (0.891 to 1.119)	1.0 (0.891 to 1.119)	1.0 (0.891 to 1.119)	1.0 (0.891 to 1.119)	1.0 (0.891 to 1.119)	1.0 (0.891 to 1.119)	1.0 (0.891 to 1.119)	1.0 (0.891 to 1.119)	1.0 (0.891 to 1.119)	1.0 (0.891 to 1.119)	1.0 (0.891 to 1.119)	1.0 (0.891 to 1.119)	1.0 (0.891 to 1.119)	1.0 (0.891 to 1.119)	1.0 (0.891 to 1.119)	1.0 (0.891 to 1.119)
Diabetes mellitus	24 g/day	Both	Male	0.932 (0.841 to 1.03)	0.932 (0.841 to 1.03)	0.932 (0.841 to 1.03)	0.932 (0.841 to 1.03)	0.932 (0.841 to 1.03)	0.932 (0.841 to 1.03)	0.932 (0.841 to 1.03)	0.932 (0.841 to 1.03)	0.932 (0.841 to 1.03)	0.932 (0.841 to 1.03)	0.932 (0.841 to 1.03)	0.932 (0.841 to 1.03)	0.932 (0.841 to 1.03)	0.932 (0.841 to 1.03)	0.932 (0.841 to 1.03)	0.932 (0.841 to 1.03)	0.932 (0.841 to 1.03)
Diabetes mellitus	12 g/day	Both	Male	0.921 (0.833 to 1.015)	0.921 (0.833 to 1.015)	0.921 (0.833 to 1.015)	0.921 (0.833 to 1.015)	0.921 (0.833 to 1.015)	0.921 (0.833 to 1.015)	0.921 (0.833 to 1.015)	0.921 (0.833 to 1.015)	0.921 (0.833 to 1.015)	0.921 (0.833 to 1.015)	0.921 (0.833 to 1.015)	0.921 (0.833 to 1.015)	0.921 (0.833 to 1.015)	0.921 (0.833 to 1.015)	0.921 (0.833 to 1.015)	0.921 (0.833 to 1.015)	0.921 (0.833 to 1.015)
Diabetes mellitus	0 g/day	Both	Male	1.0 (1.0 to 1.0)	1.0 (1.0 to 1.0)	1.0 (1.0 to 1.0)	1.0 (1.0 to 1.0)	1.0 (1.0 to 1.0)	1.0 (1.0 to 1.0)	1.0 (1.0 to 1.0)	1.0 (1.0 to 1.0)	1.0 (1.0 to 1.0)	1.0 (1.0 to 1.0)	1.0 (1.0 to 1.0)	1.0 (1.0 to 1.0)	1.0 (1.0 to 1.0)	1.0 (1.0 to 1.0)	1.0 (1.0 to 1.0)	1.0 (1.0 to 1.0)	1.0 (1.0 to 1.0)
Diabetes mellitus	72 g/day	Both	Female	1.172 (0.81 to 1.652)	1.172 (0.81 to 1.652)	1.172 (0.81 to 1.652)	1.172 (0.81 to 1.652)	1.172 (0.81 to 1.652)	1.172 (0.81 to 1.652)	1.172 (0.81 to 1.652)	1.172 (0.81 to 1.652)	1.172 (0.81 to 1.652)	1.172 (0.81 to 1.652)	1.172 (0.81 to 1.652)	1.172 (0.81 to 1.652)	1.172 (0.81 to 1.652)	1.172 (0.81 to 1.652)	1.172 (0.81 to 1.652)	1.172 (0.81 to 1.652)	1.172 (0.81 to 1.652)
Diabetes mellitus	60 g/day	Both	Female	1.074 (0.765 to 1.443)	1.074 (0.765 to 1.443)	1.074 (0.765 to 1.443)	1.074 (0.765 to 1.443)	1.074 (0.765 to 1.443)	1.074 (0.765 to 1.443)	1.074 (0.765 to 1.443)	1.074 (0.765 to 1.443)	1.074 (0.765 to 1.443)	1.074 (0.765 to 1.443)	1.074 (0.765 to 1.443)	1.074 (0.765 to 1.443)	1.074 (0.765 to 1.443)	1.074 (0.765 to 1.443)	1.074 (0.765 to 1.443)	1.074 (0.765 to 1.443)	1.074 (0.765 to 1.443)
Diabetes mellitus	48 g/day	Both	Female	0.945 (0.737 to 1.173)	0.945 (0.737 to 1.173)	0.945 (0.737 to 1.173)	0.945 (0.737 to 1.173)	0.945 (0.737 to 1.173)	0.945 (0.737 to 1.173)	0.945 (0.737 to 1.173)	0.945 (0.737 to 1.173)	0.945 (0.737 to 1.173)	0.945 (0.737 to 1.173)	0.945 (0.737 to 1.173)	0.945 (0.737 to 1.173)	0.945 (0.737 to 1.173)	0.945 (0.737 to 1.173)	0.945 (0.737 to 1.173)	0.945 (0.737 to 1.173)	0.945 (0.737 to 1.173)
Diabetes mellitus	36 g/day	Both	Female	0.836 (0.702 to 0.981)	0.836 (0.702 to 0.981)	0.836 (0.702 to 0.981)	0.836 (0.702 to 0.981)	0.836 (0.702 to 0.981)	0.836 (0.702 to 0.981)	0.836 (0.702 to 0.981)	0.836 (0.702 to 0.981)	0.836 (0.702 to 0.981)	0.836 (0.702 to 0.981)	0.836 (0.702 to 0.981)	0.836 (0.702 to 0.981)	0.836 (0.702 to 0.981)	0.836 (0.702 to 0.981)	0.836 (0.702 to 0.981)	0.836 (0.702 to 0.981)	0.836 (0.702 to 0.981)
Diabetes mellitus	24 g/day	Both	Female	0.76 (0.66 to 0.872)	0.76 (0.66 to 0.872)	0.76 (0.66 to 0.872)	0.76 (0.66 to 0.872)	0.76 (0.66 to 0.872)	0.76 (0.66 to 0.872)	0.76 (0.66 to 0.872)	0.76 (0.66 to 0.872)	0.76 (0.66 to 0.872)	0.76 (0.66 to 0.872)	0.76 (0.66 to 0.872)	0.76 (0.66 to 0.872)	0.76 (0.66 to 0.872)	0.76 (0.66 to 0.872)	0.76 (0.66 to 0.872)	0.76 (0.66 to 0.872)	0.76 (0.66 to 0.872)
Diabetes mellitus	12 g/day	Both	Female	0.733 (0.658 to 0.826)	0.733 (0.658 to 0.826)	0.733 (0.658 to 0.826)	0.733 (0.658 to 0.826)	0.733 (0.658 to 0.826)	0.733 (0.658 to 0.826)	0.733 (0.658 to 0.826)	0.733 (0.658 to 0.826)	0.733 (0.658 to 0.826)	0.733 (0.658 to 0.826)	0.733 (0.658 to 0.826)	0.733 (0.658 to 0.826)	0.733 (0.658 to 0.826)	0.733 (0.658 to 0.826)	0.733 (0.658 to 0.826)	0.733 (0.658 to 0.826)	0.733 (0.658 to 0.826)
Diabetes mellitus	0 g/day	Both	Female	1.0 (1.0 to 1.0)	1.0 (1.0 to 1.0)	1.0 (1.0 to 1.0)	1.0 (1.0 to 1.0)	1.0 (1.0 to 1.0)	1.0 (1.0 to 1.0)	1.0 (1.0 to 1.0)	1.0 (1.0 to 1.0)	1.0 (1.0 to 1.0)	1.0 (1.0 to 1.0)	1.0 (1.0 to 1.0)	1.0 (1.0 to 1.0)	1.0 (1.0 to 1.0)	1.0 (1.0 to 1.0)	1.0 (1.0 to 1.0)	1.0 (1.0 to 1.0)	1.0 (1.0 to 1.0)
Transport injuries	72 g/day	Both	Both	1.552 (1.201 to 2.032)	1.552 (1.201 to 2.032)	1.552 (1.201 to 2.032)	1.552 (1.201 to 2.032)	1.552 (1.201 to 2.032)	1.552 (1.201 to 2.032)	1.552 (1.201 to 2.032)	1.552 (1.201 to 2.032)	1.552 (1.201 to 2.032)	1.552 (1.201 to 2.032)	1.552 (1.201 to 2.032)	1.552 (1.201 to 2.032)	1.552 (1.201 to 2.032)	1.552 (1.201 to 2.032)	1.552 (1.201 to 2.032)	1.552 (1.201 to 2.032)	1.552 (1.201 to 2.032)
Transport injuries	60 g/day	Both	Both	1.456 (1.186 to 1.818)	1.456 (1.186 to 1.818)	1.456 (1.186 to 1.818)	1.456 (1.186 to 1.818)	1.456 (1.186 to 1.818)	1.456 (1.186 to 1.818)	1.456 (1.186 to 1.818)	1.456 (1.186 to 1.818)	1.456 (1.186 to 1.818)	1.456 (1.186 to 1.818)	1.456 (1.186 to 1.818)	1.456 (1.186 to 1.818)	1.456 (1.186 to 1.818)	1.456 (1.186 to 1.818)	1.456 (1.186 to 1.818)	1.456 (1.186 to 1.818)	1.456 (1.186 to 1.818)
Transport injuries	48 g/day	Both	Both	1.366 (1.101 to 1.692)	1.366 (1.101 to 1.692)	1.366 (1.101 to 1.692)	1.366 (1.101 to 1.692)	1.366 (1.101 to 1.692)	1.366 (1.101 to 1.692)	1.366 (1.101 to 1.692)	1.366 (1.101 to 1.692)	1.366 (1.101 to 1.692)	1.366 (1.101 to 1.692)	1.366 (1.101 to 1.692)	1.366 (1.101 to 1.692)	1.366 (1.101 to 1.692)	1.366 (1.101 to 1.692)	1.366 (1.101 to 1.692)	1.366 (1.101 to 1.692)	1.366 (1.101 to 1.692)
Transport injuries	36 g/day	Both	Both	1.288 (1.089 to 1.534)	1.288 (1.089 to 1.534)	1.288 (1.089 to 1.534)	1.288 (1.089 to 1.534)	1.288 (1.089 to 1.534)	1.288 (1.089 to 1.534)	1.288 (1.089 to 1.534)	1.288 (1.089 to 1.534)	1.288 (1.089 to 1.534)	1.288 (1.089 to 1.534)	1.288 (1.089 to 1.534)	1.288 (1.089 to 1.534)	1.288 (1.089 to 1.534)	1.288 (1.089 to 1.534)	1.288 (1.089 to 1.534)	1.288 (1.089 to 1.534)	1.288 (1.089 to 1.534)
Transport injuries	24 g/day	Both	Both	1.22 (1.062 to 1.4)	1.22 (1.062 to 1.4)	1.22 (1.062 to 1.4)	1.22 (1.062 to 1.4)	1.22 (1.062 to 1.4)	1.22 (1.062 to 1.4)	1.22 (1.062 to 1.4)	1.22 (1.062 to 1.4)	1.22 (1.062 to 1.4)	1.22 (1.062 to 1.4)	1.22 (1.062 to 1.4)	1.22 (1.062 to 1.4)	1.22 (1.062 to 1.4)	1.22 (1.062 to 1.4)	1.22 (1.062 to 1.4)	1.22 (1.062 to 1.4)	1.22 (1.062 to 1.4)
Transport injuries	12 g/day	Both	Both	1.163 (1.021 to 1.346)	1.163 (1.021 to 1.346)	1.163 (1.021 to 1.346)	1.163 (1.021 to 1.346)	1.163 (1.021 to 1.346)	1.163 (1.021 to 1.346)	1.163 (1.021 to 1.346)	1.163 (1.021 to 1.346)	1.163 (1.021 to 1.346)	1.163 (1.021 to 1.346)	1.163 (1.021 to 1.346)	1.163 (1.021 to 1.346)	1.163 (1.021 to 1.346)	1.163 (1.021 to 1.346)	1.163 (1.021 to 1.346)	1.163 (1.021 to 1.346)	1.163 (1.021 to 1.346)
Transport injuries	0 g/day	Both	Both	1.0 (1.0 to 1.0)	1.0 (1.0 to 1.0)	1.0 (1.0 to 1.0)	1.0 (1.0 to 1.0)	1.0 (1.0 to 1.0)	1.0 (1.0 to 1.0)	1.0 (1.0 to 1.0)	1.0 (1.0 to 1.0)	1.0 (1.0 to 1.0)	1.0 (1.0 to 1.0)	1.0 (1.0 to 1.0)	1.0 (1.0 to 1.0)	1.0 (1.0 to 1.0)	1.0 (1.0 to 1.0)	1.0 (1.0 to 1.0)	1.0 (1.0 to 1.0)	1.0 (1.0 to 1.0)
Unintentional injuries	72 g/day	Both	Both	1.266 (1.063 to 1.555)	1.266 (1.063 to 1.555)	1.266 (1.063 to 1.555)	1.266 (1.063 to 1.555)	1.266 (1.063 to 1.555)	1.266 (1.063 to 1.555)	1.266 (1.063 to 1.555)	1.266 (1.063 to 1.555)	1.266 (1.063 to 1.555)	1.266 (1.063 to 1.555)	1.266 (1.063 to 1.555)	1.266 (1.063 to 1.555)	1.266 (1.063 to 1.555)	1.266 (1.063 to 1.555)	1.266 (1.063 to 1.555)	1.266 (1.063 to 1.555)	1.266 (1.063 to 1.555)
Unintentional injuries	60 g/day	Both	Both	1.221 (1.059 to 1.46)	1.221 (1.059 to 1.46)	1.221 (1.059 to 1.46)	1.221 (1.059 to 1.46)	1.221 (1.059 to 1.46)	1.221 (1.059 to 1.46)	1.221 (1.059 to 1.46)	1.221 (1.059 to 1.46)	1.221 (1.059 to 1.46)	1.221 (1.059 to 1.46)	1.221 (1.059 to 1.46)	1.221 (1.059 to 1.46)	1.221 (1.059 to 1.46)	1.221 (1.059 to 1.46)	1.221 (1.059 to 1.46)	1.221 (1.059 to 1.46)	1.221 (1.059 to 1.46)
Unintentional injuries	48 g/day	Both	Both	1.182 (1.024 to 1.428)	1.182 (1.024 to 1.428)	1.182 (1.024 to 1.428)	1.182 (1.024 to 1.428)	1.182 (1.024 to 1.428)	1.182 (1.024 to 1.428)	1.182 (1.024 to 1.428)	1.182 (1.024 to 1.428)	1.182 (1.024 to 1.428)	1.182 (1.024 to 1.428)	1.182 (1.024 to 1.428)	1.182 (1.024 to 1.428)	1.182 (1.024 to 1.428)	1.182 (1.024 to 1.428)	1.182 (1.024 to 1.428)	1.182 (1.024 to 1.428)	1.182 (1.024 to 1.428)
Unintentional injuries	36 g/day	Both	Both	1.168 (1.054 to 1.347)	1.168 (1.054 to 1.347)	1.168 (1.054 to 1.347)	1.168 (1.054 to 1.347)	1.168 (1.054 to 1.347)	1.168 (1.054 to 1.347)	1.168 (1.054 to 1.347)	1.168 (1.054 to 1.347)	1.168 (1.054 to 1.347)	1.168 (1.054 to 1.347)	1.168 (1.054 to 1.347)	1.168 (1.054 to 1.347)	1.168 (1.054 to 1.347)	1.168 (1.054 to 1.347)	1.168 (1.054 to 1.347)	1.168 (1.054 to 1.347)	1.168 (1.054 to 1.347)
Unintentional injuries	24 g/day	Both	Both	1.154 (1.046 to 1.319)	1.154 (1.046 to 1.319)	1.154 (1.046 to 1.319)	1.154 (1.046 to 1.319)	1.154 (1.046 to 1.319)	1.154 (1.046 to 1.319)	1.154 (1.046 to 1.319)	1.154 (1.046 to 1.319)	1.154 (1.046 to 1.319)	1.154 (1.046 to 1.319)	1.154 (1.046 to 1.319)	1.154 (1.046 to 1.319)	1.154 (1.046 to 1.319)	1.154 (1.046 to 1.319)	1.154 (1.046 to 1.319)	1.154 (1.046 to 1.319)	1.154 (1.046 to 1.319)
Unintentional injuries	12 g/day	Both	Both	1.09 (1.016 to 1.187)	1.09 (1.016 to 1.187)	1.09 (1.016 to 1.187)	1.09 (1.016 to 1.187)	1.09 (1.016 to 1.187)	1.09 (1.016 to 1.187)	1.09 (1.016 to 1.187)	1.09 (1.016 to 1.187)	1.09 (1.016 to 1.187)	1.09 (1.016 to 1.187)	1.09 (1.016 to 1.187)	1.09 (1.016 to 1.187)	1.09 (1.016 to 1.187)	1.09 (1.016 to 1.187)	1.09 (1.016 to 1.187)	1.09 (1.016 to 1.187)	1.09 (1.016 to 1.187)
Unintentional injuries	0 g/day	Both	Both	1.0 (1.0 to 1.0)	1.0 (1.0 to 1.0)	1.0 (1.0 to 1.0)	1.0 (1.0 to 1.0)	1.0 (1.0 to 1.0)	1.0 (1.0 to 1.0)	1.0 (1.0 to 1.0)	1.0 (1.0 to 1.0)	1.0 (1.0 to 1.0)	1.0 (1.0 to 1.0)	1.0 (1.0 to 1.0)	1.0 (1.0 to 1.0)	1.0 (1.0 to 1.0)	1.0 (1.0 to 1.0)	1.0 (1.0 to 1.0)	1.0 (1.0 to 1.0)	1.0 (1.0 to 1.0)

Appendix Table 1c. Relative risks used by age and sex for each outcome for alcohol use globally.

Risk - Outcome	Category / Units	Morbidity / Mortality	Sex	15-19 years	20-24 years	25-29 years	30-34 years	35-39 years	40-44 years	45-49 years	50-54 years	55-59 years	60-64 years	65-69 years	70-74 years	75-79 years	80-84 years	85-89 years	90-94 years	95+ years
**Alcohol use**																				
Self-harm	72 g/day	Both	Both	1.927 (1.398 to 2.665)	1.927 (1.398 to 2.665)	1.927 (1.398 to 2.665)	1.927 (1.398 to 2.665)	1.927 (1.398 to 2.665)	1.927 (1.398 to 2.665)	1.927 (1.398 to 2.665)	1.927 (1.398 to 2.665)	1.927 (1.398 to 2.665)	1.927 (1.398 to 2.665)	1.927 (1.398 to 2.665)	1.927 (1.398 to 2.665)	1.927 (1.398 to 2.665)	1.927 (1.398 to 2.665)	1.927 (1.398 to 2.665)	1.927 (1.398 to 2.665)	1.927 (1.398 to 2.665)
Self-harm	60 g/day	Both	Both	1.734 (1.29 to 2.308)	1.734 (1.29 to 2.308)	1.734 (1.29 to 2.308)	1.734 (1.29 to 2.308)	1.734 (1.29 to 2.308)	1.734 (1.29 to 2.308)	1.734 (1.29 to 2.308)	1.734 (1.29 to 2.308)	1.734 (1.29 to 2.308)	1.734 (1.29 to 2.308)	1.734 (1.29 to 2.308)	1.734 (1.29 to 2.308)	1.734 (1.29 to 2.308)	1.734 (1.29 to 2.308)	1.734 (1.29 to 2.308)	1.734 (1.29 to 2.308)	1.734 (1.29 to 2.308)
Self-harm	48 g/day	Both	Both	1.545 (1.132 to 2.048)	1.545 (1.132 to 2.048)	1.545 (1.132 to 2.048)	1.545 (1.132 to 2.048)	1.545 (1.132 to 2.048)	1.545 (1.132 to 2.048)	1.545 (1.132 to 2.048)	1.545 (1.132 to 2.048)	1.545 (1.132 to 2.048)	1.545 (1.132 to 2.048)	1.545 (1.132 to 2.048)	1.545 (1.132 to 2.048)	1.545 (1.132 to 2.048)	1.545 (1.132 to 2.048)	1.545 (1.132 to 2.048)	1.545 (1.132 to 2.048)	1.545 (1.132 to 2.048)
Self-harm	36 g/day	Both	Both	1.376 (1.05 to 1.751)	1.376 (1.05 to 1.751)	1.376 (1.05 to 1.751)	1.376 (1.05 to 1.751)	1.376 (1.05 to 1.751)	1.376 (1.05 to 1.751)	1.376 (1.05 to 1.751)	1.376 (1.05 to 1.751)	1.376 (1.05 to 1.751)	1.376 (1.05 to 1.751)	1.376 (1.05 to 1.751)	1.376 (1.05 to 1.751)	1.376 (1.05 to 1.751)	1.376 (1.05 to 1.751)	1.376 (1.05 to 1.751)	1.376 (1.05 to 1.751)	1.376 (1.05 to 1.751)
Self-harm	24 g/day	Both	Both	1.23 (0.972 to 1.533)	1.23 (0.972 to 1.533)	1.23 (0.972 to 1.533)	1.23 (0.972 to 1.533)	1.23 (0.972 to 1.533)	1.23 (0.972 to 1.533)	1.23 (0.972 to 1.533)	1.23 (0.972 to 1.533)	1.23 (0.972 to 1.533)	1.23 (0.972 to 1.533)	1.23 (0.972 to 1.533)	1.23 (0.972 to 1.533)	1.23 (0.972 to 1.533)	1.23 (0.972 to 1.533)	1.23 (0.972 to 1.533)	1.23 (0.972 to 1.533)	1.23 (0.972 to 1.533)
Self-harm	12 g/day	Both	Both	1.107 (0.908 to 1.343)	1.107 (0.908 to 1.343)	1.107 (0.908 to 1.343)	1.107 (0.908 to 1.343)	1.107 (0.908 to 1.343)	1.107 (0.908 to 1.343)	1.107 (0.908 to 1.343)	1.107 (0.908 to 1.343)	1.107 (0.908 to 1.343)	1.107 (0.908 to 1.343)	1.107 (0.908 to 1.343)	1.107 (0.908 to 1.343)	1.107 (0.908 to 1.343)	1.107 (0.908 to 1.343)	1.107 (0.908 to 1.343)	1.107 (0.908 to 1.343)	1.107 (0.908 to 1.343)
Self-harm	0 g/day	Both	Both	1.0 (1.0 to 1.0)	1.0 (1.0 to 1.0)	1.0 (1.0 to 1.0)	1.0 (1.0 to 1.0)	1.0 (1.0 to 1.0)	1.0 (1.0 to 1.0)	1.0 (1.0 to 1.0)	1.0 (1.0 to 1.0)	1.0 (1.0 to 1.0)	1.0 (1.0 to 1.0)	1.0 (1.0 to 1.0)	1.0 (1.0 to 1.0)	1.0 (1.0 to 1.0)	1.0 (1.0 to 1.0)	1.0 (1.0 to 1.0)	1.0 (1.0 to 1.0)	1.0 (1.0 to 1.0)
Interpersonal violence	72 g/day	Both	Both	1.516 (1.255 to 1.867)	1.516 (1.255 to 1.867)	1.516 (1.255 to 1.867)	1.516 (1.255 to 1.867)	1.516 (1.255 to 1.867)	1.516 (1.255 to 1.867)	1.516 (1.255 to 1.867)	1.516 (1.255 to 1.867)	1.516 (1.255 to 1.867)	1.516 (1.255 to 1.867)	1.516 (1.255 to 1.867)	1.516 (1.255 to 1.867)	1.516 (1.255 to 1.867)	1.516 (1.255 to 1.867)	1.516 (1.255 to 1.867)	1.516 (1.255 to 1.867)	1.516 (1.255 to 1.867)
Interpersonal violence	60 g/day	Both	Both	1.452 (1.215 to 1.719)	1.452 (1.215 to 1.719)	1.452 (1.215 to 1.719)	1.452 (1.215 to 1.719)	1.452 (1.215 to 1.719)	1.452 (1.215 to 1.719)	1.452 (1.215 to 1.719)	1.452 (1.215 to 1.719)	1.452 (1.215 to 1.719)	1.452 (1.215 to 1.719)	1.452 (1.215 to 1.719)	1.452 (1.215 to 1.719)	1.452 (1.215 to 1.719)	1.452 (1.215 to 1.719)	1.452 (1.215 to 1.719)	1.452 (1.215 to 1.719)	1.452 (1.215 to 1.719)
Interpersonal violence	48 g/day	Both	Both	1.396 (1.118 to 1.739)	1.396 (1.118 to 1.739)	1.396 (1.118 to 1.739)	1.396 (1.118 to 1.739)	1.396 (1.118 to 1.739)	1.396 (1.118 to 1.739)	1.396 (1.118 to 1.739)	1.396 (1.118 to 1.739)	1.396 (1.118 to 1.739)	1.396 (1.118 to 1.739)	1.396 (1.118 to 1.739)	1.396 (1.118 to 1.739)	1.396 (1.118 to 1.739)	1.396 (1.118 to 1.739)	1.396 (1.118 to 1.739)	1.396 (1.118 to 1.739)	1.396 (1.118 to 1.739)
Interpersonal violence	36 g/day	Both	Both	1.345 (1.14 to 1.585)	1.345 (1.14 to 1.585)	1.345 (1.14 to 1.585)	1.345 (1.14 to 1.585)	1.345 (1.14 to 1.585)	1.345 (1.14 to 1.585)	1.345 (1.14 to 1.585)	1.345 (1.14 to 1.585)	1.345 (1.14 to 1.585)	1.345 (1.14 to 1.585)	1.345 (1.14 to 1.585)	1.345 (1.14 to 1.585)	1.345 (1.14 to 1.585)	1.345 (1.14 to 1.585)	1.345 (1.14 to 1.585)	1.345 (1.14 to 1.585)	1.345 (1.14 to 1.585)
Interpersonal violence	24 g/day	Both	Both	1.256 (1.055 to 1.46)	1.256 (1.055 to 1.46)	1.256 (1.055 to 1.46)	1.256 (1.055 to 1.46)	1.256 (1.055 to 1.46)	1.256 (1.055 to 1.46)	1.256 (1.055 to 1.46)	1.256 (1.055 to 1.46)	1.256 (1.055 to 1.46)	1.256 (1.055 to 1.46)	1.256 (1.055 to 1.46)	1.256 (1.055 to 1.46)	1.256 (1.055 to 1.46)	1.256 (1.055 to 1.46)	1.256 (1.055 to 1.46)	1.256 (1.055 to 1.46)	1.256 (1.055 to 1.46)
Interpersonal violence	12 g/day	Both	Both	1.129 (0.963 to 1.317)	1.129 (0.963 to 1.317)	1.129 (0.963 to 1.317)	1.129 (0.963 to 1.317)	1.129 (0.963 to 1.317)	1.129 (0.963 to 1.317)	1.129 (0.963 to 1.317)	1.129 (0.963 to 1.317)	1.129 (0.963 to 1.317)	1.129 (0.963 to 1.317)	1.129 (0.963 to 1.317)	1.129 (0.963 to 1.317)	1.129 (0.963 to 1.317)	1.129 (0.963 to 1.317)	1.129 (0.963 to 1.317)	1.129 (0.963 to 1.317)	1.129 (0.963 to 1.317)
Interpersonal violence	0 g/day	Both	Both	1.0 (1.0 to 1.0)	1.0 (1.0 to 1.0)	1.0 (1.0 to 1.0)	1.0 (1.0 to 1.0)	1.0 (1.0 to 1.0)	1.0 (1.0 to 1.0)	1.0 (1.0 to 1.0)	1.0 (1.0 to 1.0)	1.0 (1.0 to 1.0)	1.0 (1.0 to 1.0)	1.0 (1.0 to 1.0)	1.0 (1.0 to 1.0)	1.0 (1.0 to 1.0)	1.0 (1.0 to 1.0)	1.0 (1.0 to 1.0)	1.0 (1.0 to 1.0)	1.0 (1.0 to 1.0)

Appendix Table 1d. Relative risks used for IQ shifts by age and sex for lead exposure blood.

Risk - Outcome	Category / Units	Morbidity / Mortality	Sex	15-19 years	20-24 years	25-29 years	30-34 years	35-39 years	40-44 years	45-49 years	50-54 years	55-59 years	60-64 years	65-69 years	70-74 years	75-79 years	80-84 years	85-89 years	90-94 years	95+ years
**Lead exposure blood**																				
IQ shift	40 µg/dL	Morbidity	Both	7.26 (2.662 to 11.857)	7.26 (2.662 to 11.857)	7.26 (2.662 to 11.857)	7.26 (2.662 to 11.857)	7.26 (2.662 to 11.857)	7.26 (2.662 to 11.857)	7.26 (2.662 to 11.857)	7.26 (2.662 to 11.857)	7.26 (2.662 to 11.857)	7.26 (2.662 to 11.857)	7.26 (2.662 to 11.857)	7.26 (2.662 to 11.857)	7.26 (2.662 to 11.857)	7.26 (2.662 to 11.857)	7.26 (2.662 to 11.857)	7.26 (2.662 to 11.857)	7.26 (2.662 to 11.857)
IQ shift	35 µg/dL	Morbidity	Both	7.006 (2.569 to 11.442)	7.006 (2.569 to 11.442)	7.006 (2.569 to 11.442)	7.006 (2.569 to 11.442)	7.006 (2.569 to 11.442)	7.006 (2.569 to 11.442)	7.006 (2.569 to 11.442)	7.006 (2.569 to 11.442)	7.006 (2.569 to 11.442)	7.006 (2.569 to 11.442)	7.006 (2.569 to 11.442)	7.006 (2.569 to 11.442)	7.006 (2.569 to 11.442)	7.006 (2.569 to 11.442)	7.006 (2.569 to 11.442)	7.006 (2.569 to 11.442)	7.006 (2.569 to 11.442)
IQ shift	30 µg/dL	Morbidity	Both	6.713 (2.462 to 10.965)	6.713 (2.462 to 10.965)	6.713 (2.462 to 10.965)	6.713 (2.462 to 10.965)	6.713 (2.462 to 10.965)	6.713 (2.462 to 10.965)	6.713 (2.462 to 10.965)	6.713 (2.462 to 10.965)	6.713 (2.462 to 10.965)	6.713 (2.462 to 10.965)	6.713 (2.462 to 10.965)	6.713 (2.462 to 10.965)	6.713 (2.462 to 10.965)	6.713 (2.462 to 10.965)	6.713 (2.462 to 10.965)	6.713 (2.462 to 10.965)	6.713 (2.462 to 10.965)
IQ shift	25 µg/dL	Morbidity	Both	6.37 (2.336 to 10.403)	6.37 (2.336 to 10.403)	6.37 (2.336 to 10.403)	6.37 (2.336 to 10.403)	6.37 (2.336 to 10.403)	6.37 (2.336 to 10.403)	6.37 (2.336 to 10.403)	6.37 (2.336 to 10.403)	6.37 (2.336 to 10.403)	6.37 (2.336 to 10.403)	6.37 (2.336 to 10.403)	6.37 (2.336 to 10.403)	6.37 (2.336 to 10.403)	6.37 (2.336 to 10.403)	6.37 (2.336 to 10.403)	6.37 (2.336 to 10.403)	6.37 (2.336 to 10.403)
IQ shift	20 µg/dL	Morbidity	Both	5.952 (2.183 to 9.721)	5.952 (2.183 to 9.721)	5.952 (2.183 to 9.721)	5.952 (2.183 to 9.721)	5.952 (2.183 to 9.721)	5.952 (2.183 to 9.721)	5.952 (2.183 to 9.721)	5.952 (2.183 to 9.721)	5.952 (2.183 to 9.721)	5.952 (2.183 to 9.721)	5.952 (2.183 to 9.721)	5.952 (2.183 to 9.721)	5.952 (2.183 to 9.721)	5.952 (2.183 to 9.721)	5.952 (2.183 to 9.721)	5.952 (2.183 to 9.721)	5.952 (2.183 to 9.721)
IQ shift	15 µg/dL	Morbidity	Both	5.42 (1.988 to 8.853)	5.42 (1.988 to 8.853)	5.42 (1.988 to 8.853)	5.42 (1.988 to 8.853)	5.42 (1.988 to 8.853)	5.42 (1.988 to 8.853)	5.42 (1.988 to 8.853)	5.42 (1.988 to 8.853)	5.42 (1.988 to 8.853)	5.42 (1.988 to 8.853)	5.42 (1.988 to 8.853)	5.42 (1.988 to 8.853)	5.42 (1.988 to 8.853)	5.42 (1.988 to 8.853)	5.42 (1.988 to 8.853)	5.42 (1.988 to 8.853)	5.42 (1.988 to 8.853)
IQ shift	12 µg/dL	Morbidity	Both	5.014 (1.839 to 8.19)	5.014 (1.839 to 8.19)	5.014 (1.839 to 8.19)	5.014 (1.839 to 8.19)	5.014 (1.839 to 8.19)	5.014 (1.839 to 8.19)	5.014 (1.839 to 8.19)	5.014 (1.839 to 8.19)	5.014 (1.839 to 8.19)	5.014 (1.839 to 8.19)	5.014 (1.839 to 8.19)	5.014 (1.839 to 8.19)	5.014 (1.839 to 8.19)	5.014 (1.839 to 8.19)	5.014 (1.839 to 8.19)	5.014 (1.839 to 8.19)	5.014 (1.839 to 8.19)
IQ shift	10 µg/dL	Morbidity	Both	4.688 (1.719 to 7.656)	4.688 (1.719 to 7.656)	4.688 (1.719 to 7.656)	4.688 (1.719 to 7.656)	4.688 (1.719 to 7.656)	4.688 (1.719 to 7.656)	4.688 (1.719 to 7.656)	4.688 (1.719 to 7.656)	4.688 (1.719 to 7.656)	4.688 (1.719 to 7.656)	4.688 (1.719 to 7.656)	4.688 (1.719 to 7.656)	4.688 (1.719 to 7.656)	4.688 (1.719 to 7.656)	4.688 (1.719 to 7.656)	4.688 (1.719 to 7.656)	4.688 (1.719 to 7.656)
IQ shift	8 µg/dL	Morbidity	Both	4.296 (1.575 to 7.016)	4.296 (1.575 to 7.016)	4.296 (1.575 to 7.016)	4.296 (1.575 to 7.016)	4.296 (1.575 to 7.016)	4.296 (1.575 to 7.016)	4.296 (1.575 to 7.016)	4.296 (1.575 to 7.016)	4.296 (1.575 to 7.016)	4.296 (1.575 to 7.016)	4.296 (1.575 to 7.016)	4.296 (1.575 to 7.016)	4.296 (1.575 to 7.016)	4.296 (1.575 to 7.016)	4.296 (1.575 to 7.016)	4.296 (1.575 to 7.016)	4.296 (1.575 to 7.016)
IQ shift	6 µg/dL	Morbidity	Both	3.804 (1.395 to 6.213)	3.804 (1.395 to 6.213)	3.804 (1.395 to 6.213)	3.804 (1.395 to 6.213)	3.804 (1.395 to 6.213)	3.804 (1.395 to 6.213)	3.804 (1.395 to 6.213)	3.804 (1.395 to 6.213)	3.804 (1.395 to 6.213)	3.804 (1.395 to 6.213)	3.804 (1.395 to 6.213)	3.804 (1.395 to 6.213)	3.804 (1.395 to 6.213)	3.804 (1.395 to 6.213)	3.804 (1.395 to 6.213)	3.804 (1.395 to 6.213)	3.804 (1.395 to 6.213)
IQ shift	4 µg/dL	Morbidity	Both	3.146 (1.154 to 5.139)	3.146 (1.154 to 5.139)	3.146 (1.154 to 5.139)	3.146 (1.154 to 5.139)	3.146 (1.154 to 5.139)	3.146 (1.154 to 5.139)	3.146 (1.154 to 5.139)	3.146 (1.154 to 5.139)	3.146 (1.154 to 5.139)	3.146 (1.154 to 5.139)	3.146 (1.154 to 5.139)	3.146 (1.154 to 5.139)	3.146 (1.154 to 5.139)	3.146 (1.154 to 5.139)	3.146 (1.154 to 5.139)	3.146 (1.154 to 5.139)	3.146 (1.154 to 5.139)
IQ shift	2 µg/dL	Morbidity	Both	0.0 (0.0 to 0.0)	0.0 (0.0 to 0.0)	0.0 (0.0 to 0.0)	0.0 (0.0 to 0.0)	0.0 (0.0 to 0.0)	0.0 (0.0 to 0.0)	0.0 (0.0 to 0.0)	0.0 (0.0 to 0.0)	0.0 (0.0 to 0.0)	0.0 (0.0 to 0.0)	0.0 (0.0 to 0.0)	0.0 (0.0 to 0.0)	0.0 (0.0 to 0.0)	0.0 (0.0 to 0.0)	0.0 (0.0 to 0.0)	0.0 (0.0 to 0.0)	0.0 (0.0 to 0.0)

Appendix Table 2: Supplemental information for Figure 1: Epidemiological evidence supporting causality between risk-outcome pairs included in the Global Burden of Disease 2016 study including 2A. Citations and 2B. Additional information 1a. Citations		
**Risk**	**Outcome**	**Citation/Note**
Unsafe water	Diarrhoeal diseases	Cairncross S, Valdmanis V. Water Supply, Sanitation, and Hygiene Promotion. In: Jamison DT, Breman JG, Measham AR, et al., eds. Disease Control Priorities in Developing Countries, 2nd edn. Washington (DC): World Bank, 2006.
Unsafe water	Diarrhoeal diseases	Wolf J, Prüss-Ustün A, Cumming O, et al. Assessing the impact of drinking water and sanitation on diarrhoeal disease in low- and middle-income settings: systematic review and meta-regression. Trop Med Int Health 2014; 19: 928–42.
Unsafe water	Diarrhoeal diseases	Fewtrell, L., Kaufmann, R. B., Kay, D., Enanoria, W., Haller, L., & Colford, J. M. (2005). Water, sanitation, and hygiene interventions to reduce diarrhoea in less developed countries: a systematic review and meta-analysis. The Lancet Infectious Diseases, 5(1), 42-52. doi:10.1016/s1473-3099(04)01253-8
Unsafe sanitation - improved sanitation	Diarrhoeal diseases	Wolf J, Prüss-Ustün A, Cumming O, et al. Assessing the impact of drinking water and sanitation on diarrhoeal disease in low- and middle-income settings: systematic review and meta-regression. Trop Med Int Health 2014; 19: 928–42.
Unsafe sanitation - improved sanitation	Diarrhoeal diseases	Fewtrell, L., Kaufmann, R. B., Kay, D., Enanoria, W., Haller, L., & Colford, J. M. (2005). Water, sanitation, and hygiene interventions to reduce diarrhoea in less developed countries: a systematic review and meta-analysis. The Lancet Infectious Diseases, 5(1), 42-52. doi:10.1016/s1473-3099(04)01253-8
Unsafe sanitation - improved sanitation	Diarrhoeal diseases	Baker, K. K., O'Reilly, C. E., Levine, M. M., Kotloff, K. L., Nataro, J. P., Ayers, T. L., . . . Mintz, E. D. (2016). Sanitation and Hygiene-Specific Risk Factors for Moderate-to-Severe Diarrhea in Young Children in the Global Enteric Multicenter Study, 2007–2011: Case-Control Study. PLOS Medicine,13(5). doi:10.1371/journal.pmed.1002010
Unsafe sanitation - piped	Diarrhoeal diseases	Genser, B., Strina, A., Santos, L. A., Teles, C. A., Prado, M. S., Cairncross, S., & Barreto, M. L. (2008). Impact of a city-wide sanitation intervention in a large urban centre on social, environmental and behavioural determinants of childhood diarrhoea: analysis of two cohort studies. International Journal of Epidemiology, 37(4), 831-840. doi:10.1093/ije/dyn101
Unsafe sanitation - piped	Diarrhoeal diseases	Norman, G., Pedley, S., & Takkouche, B. (2010). Effects of sewerage on diarrhoea and enteric infections: a systematic review and meta-analysis. The Lancet Infectious Diseases, 10(8), 536-544. doi:10.1016/s1473-3099(10)70123-7
Unsafe sanitation - piped	Diarrhoeal diseases	Wolf J, Prüss-Ustün A, Cumming O, et al. Assessing the impact of drinking water and sanitation on diarrhoeal disease in low- and middle-income settings: systematic review and meta-regression. Trop Med Int Health 2014; 19: 928–42.
No access to handwashing facility	Diarrhoeal diseases	Ejemot-Nwadiaro RI, Ehiri JE, Arikpo D, Meremikwu MM, Critchley JA. Hand washing promotion for preventing diarrhoea. Cochrane Database Syst Rev 2015; : CD004265.
No access to handwashing facility	Lower respiratory infections	Rabie T, Curtis V. Handwashing and risk of respiratory infections: a quantitative systematic review. Trop Med Int Health Tropical Medicine and International Health. 2006;11(3):258–67.
No access to handwashing facility	Lower respiratory infections	Aiello, A. E., Coulborn, R. M., Perez, V., & Larson, E. L. (2008). Effect of Hand Hygiene on Infectious Disease Risk in the Community Setting: A Meta-Analysis. American Journal of Public Health, 98(8), 1372-1381. doi:10.2105/ajph.2007.124610
Ambient particulate matter pollution	Lower respiratory infections	Hoek G, Krishnan RM, Beelen R, et al. Long-term air pollution exposure and cardio- respiratory mortality: a review. Environ Health 2013; 12: 43.
Ambient particulate matter pollution	Lower respiratory infections	Mehta S, Shin H, Burnett R, North T, Cohen AJ. Ambient particulate air pollution and acute lower respiratory infections: a systematic review and implications for estimating the global burden of disease. Air Qual Atmos Health 2013; 6: 69–83.
Ambient particulate matter pollution	Lower respiratory infections	Fuertes E, Macintyre E, Agius R, Beelen R, Brunekreef B, Bucci S, et al. Associations between particulate matter elements and early-life pneumonia in seven birth cohorts: Results from the ESCAPE and TRANSPHORM projects. International Journal of Hygiene and Environmental Health. 2014Nov;217(8):819–29.
Ambient particulate matter pollution	Tracheal, bronchus and lung cancer	Burnett RT, Pope CA, Ezzati M, et al. An integrated risk function for estimating the global burden of disease attributable to ambient fine particulate matter exposure. Environ Health Perspect 2014; 122: 397–403.
Ambient particulate matter pollution	Ischaemic heart disease	Hoek G, Krishnan RM, Beelen R, et al. Long-term air pollution exposure and cardio- respiratory mortality: a review. Environ Health 2013; 12: 43.
Ambient particulate matter pollution	Ischaemic heart disease	Newby DE, Mannucci PM, Tell GS, et al. Expert position paper on air pollution and cardiovascular disease. Eur Heart J 2015; 36: 83–93b.
Ambient particulate matter pollution	Cerebrovascular disease	Hoek G, Krishnan RM, Beelen R, et al. Long-term air pollution exposure and cardio- respiratory mortality: a review. Environ Health 2013; 12: 43.
Ambient particulate matter pollution	Cerebrovascular disease	Hong Y-C, Lee J-T, Kim H, Kwon H-J. Air pollution: a new risk factor in ischemic stroke mortality. Stroke 2002; 33: 2165–9.
Ambient particulate matter pollution	Cerebrovascular disease	Newby DE, Mannucci PM, Tell GS, et al. Expert position paper on air pollution and cardiovascular disease. Eur Heart J 2015; 36: 83–93b.
Ambient particulate matter pollution	Chronic obstructive pulmonary disease	Hu G, Zhou Y, Tian J, Yao W, Li J, Li B, et al. Risk of COPD From Exposure to Biomass Smoke. Chest. 2010;138(1):20–31.
Ambient particulate matter pollution	Chronic obstructive pulmonary disease	Laumbach RJ, Kipen HM. Respiratory health effects of air pollution: update on biomass smoke and traffic pollution. J Allergy Clin Immunol 2012; 129: 3-11-13.
Ambient particulate matter pollution	Chronic obstructive pulmonary disease	Newby DE, Mannucci PM, Tell GS, et al. Expert position paper on air pollution and cardiovascular disease. Eur Heart J 2015; 36: 83–93b.
Household air pollution from solid fuels	Lower respiratory infections	Dherani M, Pope D, Mascarenhas M, Smith KR, Weber M, Bruce N. Indoor air pollution from unprocessed solid fuel use and pneumonia risk in children aged under five years: a systematic review and meta-analysis. Bull World Health Organ 2008; 86: 390–398C.
Household air pollution from solid fuels	Lower respiratory infections	Marbury MC, Maldonado G, Waller L. The indoor air and children's health study: methods and incidence rates. Epidemiology 1996; 7: 166–74.

Appendix Table 2. Supplemental information for Figure 1: Epidemiological evidence supporting causality between risk-outcome pairs included in the Global Burden of Disease 2016 study including 2A. Citations and 2B. Additional information
6A. Citations

Risk	Outcome	Citation/Note
Household air pollution from solid fuels	Lower respiratory infections	Jary, H., Simpson, H., Havens, D., Manda, G., Pope, D., Bruce, N., & Mortimer, K. (2016). Household Air Pollution and Acute Lower Respiratory Infections in Adults: A Systematic Review. Plos One, 11(12). doi:10.1371/journal.pone.0167656
Household air pollution from solid fuels	Lower respiratory infections	Smith, K. R. (2000). Indoor air pollution in developing countries and acute lower respiratory infections in children. Thorax, 55(6), 518-532. doi:10.1136/thorax.55.6.518
Household air pollution from solid fuels	Tracheal, bronchus and lung cancer	Josyula S, Lin J, Xue X, et al. Household air pollution and cancers other than lung: a meta-analysis. Environ Health 2015; 14: 24.
Household air pollution from solid fuels	Tracheal, bronchus and lung cancer	Smith KR, Bruce N, Balakrishnan K, et al. Millions dead: how do we know and what does it mean? Methods used in the comparative risk assessment of household air pollution. Annu Rev Public Health 2014; 35: 185–206.
Household air pollution from solid fuels	Tracheal, bronchus and lung cancer	Hosgood, H. D., Wei, H., Sapkota, A., Choudhury, I., Bruce, N., Smith, K. R., . . . Lan, Q. (2011). Household coal use and lung cancer: systematic review and meta-analysis of case-control studies, with an emphasis on geographic variation. International Journal of Epidemiology, 40(3), 719-728. doi:10.1093/ije/dyq259
Household air pollution from solid fuels	Chronic obstructive pulmonary disease	Rivera RM, Cosio MG, Ghezzo H, Salazar M, Pérez-Padilla R. Comparison of lung morphology in COPD secondary to cigarette and biomass smoke. Int J Tuberc Lung Dis 2008; 12: 972–7.
Household air pollution from solid fuels	Chronic obstructive pulmonary disease	Po, J. Y., Fitzgerald, J. M., & Carlsten, C. (2011). Respiratory disease associated with solid biomass fuel exposure in rural women and children: systematic review and meta-analysis. Thorax, 66(3), 232-239. doi:10.1136/thx.2010.147884
Household air pollution from solid fuels	Chronic obstructive pulmonary disease	Smith KR, Bruce N, Balakrishnan K, et al. Millions dead: how do we know and what does it mean? Methods used in the comparative risk assessment of household air pollution. Annu Rev Public Health 2014; 35: 185–206.
Household air pollution from solid fuels	Cataract	Smith KR, Bruce N, Balakrishnan K, et al. Millions dead: how do we know and what does it mean? Methods used in the comparative risk assessment of household air pollution. Annu Rev Public Health 2014; 35: 185–206.
Household air pollution from solid fuels	Cataract	West, S., Bates, M., Lee, J., Schaumberg, D., Lee, D., Adair-Rohani, H., . . . Araj, H. (2013). Is Household Air Pollution a Risk Factor for Eye Disease? International Journal of Environmental Research and Public Health, 10(11), 5378-5398. doi:10.3390/ijerph10115378
Ambient ozone pollution	Chronic obstructive pulmonary disease	Jerrett M, Burnett RT, Pope CA, et al. Long-term ozone exposure and mortality. N Engl J Med 2009; 360: 1085–95.
Ambient ozone pollution	Chronic obstructive pulmonary disease	Turner MC, Jerrett M, Pope CA, et al. Long-Term Ozone Exposure and Mortality in a Large Prospective Study. Am J Respir Crit Care Med 2016; 193: 1134–42.
Residential radon	Tracheal, bronchus and lung cancer	Torres-Durán MCAD, Barros-Dios JM, Fernández-Villar A, Ruano-Ravina A. Residential radon and lung cancer in never smokers. A systematic review. Cancer Letters. 2014;345(1):21–6.
Residential radon	Tracheal, bronchus and lung cancer	Turner MC, Krewski D, Chen Y, Pope CA, Gapstur S, Thun MJ. Radon and Lung Cancer in the American Cancer Society Cohort. Cancer Epidemiology Biomarkers & Prevention. 2011Jun;20(3):438–48.
Residential radon	Tracheal, bronchus and lung cancer	Krewski D, Lubin JH, Zielinski JM, Alavanja M, Catalan VS, Field RW, et al. Residential Radon and Risk of Lung Cancer. Epidemiology. 2005;16(2):137–45.
Residential radon	Tracheal, bronchus and lung cancer	Darby S. Radon in homes and risk of lung cancer: collaborative analysis of individual data from 13 European case-control studies. Bmj. 2005;330(7485):223.
Residential radon	Tracheal, bronchus and lung cancer	Lubin JH. Studies of radon and lung cancer in North America and China. Radiation Protection Dosimetry. 2003Jan;104(4):315–9.
Residential radon	Tracheal, bronchus and lung cancer	Kreuzer M, Gerken M, Kreienbrock L, Wellmann J, Wichmann HE. Lung cancer in lifetime nonsmoking men – results of a case-control study in Germany. British Journal of Cancer. 2001May;84(1):134–40.
Residential radon	Tracheal, bronchus and lung cancer	Kreuzer M, Heinrich J, Kreienbrock L, Rosario AS, Gerken M, Wichmann HE. Risk factors for lung cancer among nonsmoking women. International Journal of Cancer. 2002;100(6):706–13.
Residential radon	Tracheal, bronchus and lung cancer	Field R, Steck DJ, Smith BJ, Brus CP, Fisher EL, Neuberger JS, et al. The Iowa radon lung cancer study — phase I: residential radon gas exposure and lung cancer. Science of The Total Environment. 2001;272(1-3):67–72.
Residential radon	Tracheal, bronchus and lung cancer	Wang Z. Residential Radon and Lung Cancer Risk in a High-exposure Area of Gansu Province, China. American Journal of Epidemiology. 2002;155(6):554–64.
Residential radon	Tracheal, bronchus and lung cancer	Lagarde F, Axelsson G, Damber L, Mellander H, Nyberg F, Pershagen G. Residential Radon and Lung Cancer among Never-Smokers in Sweden. Epidemiology. 2001;12(4):396–404.
Residential radon	Tracheal, bronchus and lung cancer	Pershagen G, Akerblom G, Axelson O, Clavensjo B, Damber L, Desai G, et al. Residential Radon Exposure and Lung Cancer in Sweden. New England Journal of Medicine. 1994;330(3):159–64.
Residential radon	Tracheal, bronchus and lung cancer	Alavanja MCR, Brownson RC, Lubin JH, Berger E, Chang J, Boice JD. Residential Radon Exposure and Lung Cancer Among Nonsmoking Women. JNCI Journal of the National Cancer Institute. 1994;86(24):1829–37.
Residential radon	Tracheal, bronchus and lung cancer	Schoenberg JB, Klotz JB, Wilcox HB, Nicholls GP, Gil-del-Real MT, Stemhagen A. Case-control study of residential radon and lung cancer among New Jersey women. Cancer Research. 1990Oct15;50(20):6520–4.
Lead exposure	Systolic blood pressure	Navas-Acien A, Schwartz BS, Rothenberg SJ, Hu H, Silbergeld EK, Guallar E. Bone lead levels and blood pressure endpoints: a meta-analysis. Epidemiology 2008; 19: 496–504.
Lead exposure	Systolic blood pressure	Glenn BS, Stewart WF, Links JM, Todd AC, Schwartz BS. The Longitudinal Association of Lead with Blood Pressure. Epidemiology. 2003;14(1):30–6.
Lead exposure	Systolic blood pressure	Glenn BS, Bandeen-Roche K, Lee B-K, Weaver VM, Todd AC, Schwartz BS. Changes in Systolic Blood Pressure Associated With Lead in Blood and Bone. Epidemiology. 2006;17(5):538–44.

Appendix Table 2. Supplemental information for Figure 1: Epidemiological evidence supporting causality between risk-outcome pairs included in the Global Burden of Disease 2016 study including 2A. Citations and 2B. Additional information
6A. Citations

Risk	Outcome	Citation/Note
Lead exposure	Systolic blood pressure	Korrick SA, Hunter DJ, Rotnitzky A, Hu H, Speizer FE. Lead and hypertension in a sample of middle-aged women. American Journal of Public Health. 1999;89(3):330–5.
Lead exposure	Systolic blood pressure	Cheng Y, Schwartz J, Sparrow D, Aro A, Weiss ST, Hu H. Bone Lead and Blood Lead Levels in Relation to Baseline Blood Pressure and the Prospective Development of Hypertension The Normative Aging Study. American Journal of Epidemiology. 2001;153(2):164–71.
Lead exposure	Idiopathic intellectual disability	Lanphear BP, Hornung R, Khoury J, et al. Low-level environmental lead exposure and children's intellectual function: an international pooled analysis. Environ Health Perspect 2005; 113: 894–9.
Lead exposure	Idiopathic intellectual disability	Liu J, Li L, Wang Y, Yan C, Liu X. Impact of low blood lead concentrations on IQ and school performance in Chinese children. PLoS ONE 2013; 8: e65230.
Lead exposure	Idiopathic intellectual disability	Lanphear BP, Hornung R, Khoury J, et al. Low-level environmental lead exposure and children's intellectual function: an international pooled analysis. Environ Health Perspect 2005; 113: 894–9.
Lead exposure	Idiopathic intellectual disability	Needleman HL, Schell A, Bellinger D, Leviton A, Allred EN. The Long-Term Effects of Exposure to Low Doses of Lead in Childhood. New England Journal of Medicine. 1990Nov;322(2):83–8.
Occupational exposure to asbestos	Larynx cancer	Goodman M, Morgan RW, Ray R, Malloy CD, Zhao K. Cancer in asbestos-exposed occupational cohorts: a meta-analysis. Cancer Causes Control 1999; 10: 453–65.
Occupational exposure to asbestos	Tracheal, bronchus and lung cancer	Lenters V, Vermeulen R, Dogger S, et al. A meta-analysis of asbestos and lung cancer: is better quality exposure assessment associated with steeper slopes of the exposure-response relationships? Environ Health Perspect 2011; 119: 1547–55.
Occupational exposure to asbestos	Ovarian cancer	Camargo MC, Stayner LT, Straif K, et al. Occupational exposure to asbestos and ovarian cancer: a meta-analysis. Environ Health Perspect 2011; 119: 1211–7.
Occupational exposure to asbestos	Mesothelioma	Bourdès V, Boffetta P, Pisani P. Environmental exposure to asbestos and risk of pleural mesothelioma: review and meta-analysis. Eur J Epidemiol 2000; 16: 411–7.
Occupational exposure to arsenic	Tracheal, bronchus and lung cancer	Lenters V, Vermeulen R, Dogger S, et al. A meta-analysis of asbestos and lung cancer: is better quality exposure assessment associated with steeper slopes of the exposure-response relationships? Environ Health Perspect 2011; 119: 1547–55.
Occupational exposure to benzene	Leukaemia	Khalade A, Jaakkola MS, Pukkala E, Jaakkola JJK. Exposure to benzene at work and the risk of leukemia: a systematic review and meta-analysis. Environ Health 2010; 9: 31.
Occupational exposure to beryllium	Tracheal, bronchus and lung cancer	Boffetta P, Fryzek JP, Mandel JS. Occupational exposure to beryllium and cancer risk: a review of the epidemiologic evidence. Crit Rev Toxicol 2012; 42: 107–18.
Occupational exposure to cadmium	Tracheal, bronchus and lung cancer	Verougstraete V, Lison D, Hotz P. Cadmium, lung and prostate cancer: a systematic review of recent epidemiological data. J Toxicol Environ Health B Crit Rev 2003; 6: 227–55.
Occupational exposure to chromium	Tracheal, bronchus and lung cancer	Denis Ambroise, Pascal Wild and Jean-Jacques Moulin, Scandinavian Journal of Work, Environment & Health, Vol. 32, No. 1 (February 2006), pp. 22-31
Occupational exposure to diesel engine exhaust	Tracheal, bronchus and lung cancer	Lipsett M, Campleman S. Occupational exposure to diesel exhaust and lung cancer: a meta-analysis. Am J Public Health 1999; 89: 1009–17.
Occupational exposure to second-hand smoke	Tracheal, bronchus and lung cancer	Stayner L, Bena J, Sasco AJ, et al. Lung cancer risk and workplace exposure to environmental tobacco smoke. Am J Public Health 2007; 97: 545–51.
Occupational exposure to formaldehyde	Nasopharynx cancer	Hauptmann M, Lubin JH, Stewart PA, Hayes RB, Blair A. Mortality from solid cancers among workers in formaldehyde industries. Am J Epidemiol 2004; 159: 1117–30.
Occupational exposure to formaldehyde	Leukaemia	Collins JJ, Lineker GA. A review and meta-analysis of formaldehyde exposure and leukemia. Regul Toxicol Pharmacol 2004; 40: 81–91.
Occupational exposure to nickel	Tracheal, bronchus and lung cancer	Grimsrud TK, Berge SR, Haldorsen T, Andersen A. Can lung cancer risk among nickel refinery workers be explained by occupational exposures other than nickel? Epidemiology 2005; 16: 146–54.
Occupational exposure to polycyclic aromatic hydrocarbons	Tracheal, bronchus and lung cancer	Armstrong B, Hutchinson E, Unwin J, Fletcher T. Lung cancer risk after exposure to polycyclic aromatic hydrocarbons: a review and meta-analysis. Environ Health Perspect 2004; 112: 970–8.
Occupational exposure to silica	Tracheal, bronchus and lung cancer	Stayner L, Bena J, Sasco AJ, et al. Lung cancer risk and workplace exposure to environmental tobacco smoke. Am J Public Health 2007; 97: 545–51.
Occupational exposure to sulfuric acid	Larynx cancer	Soskolne CL, Jhangri GS, Siemiatycki J, et al. Occupational exposure to sulfuric acid in southern Ontario, Canada, in association with laryngeal cancer. Scand J Work Environ Health 1992; 18: 225–32.
Occupational exposure to trichloroethylene	Kidney cancer	Kelsh MA, Alexander DD, Mink PJ, Mandel JH. Occupational trichloroethylene exposure and kidney cancer: a meta-analysis. Epidemiology 2010; 21: 95–102.
Occupational asthmagens	Asthma	Karjalainen A, Kurppa K, Martikainen R, Klaukka T, Karjalainen J. Work is related to a substantial portion of adult-onset asthma incidence in the Finnish population. Am J Respir Crit Care Med 2001; 164: 565–8.
Occupational particulate matter, gases, and fumes	Chronic obstructive pulmonary disease	Blanc PD, Iribarren C, Trupin L, et al. Occupational exposures and the risk of COPD: dusty trades revisited. Thorax 2009; 64: 6–12.
Occupational noise	Age-related and other hearing loss	Agrawal Y, Platz EA, Niparko JK. Prevalence of hearing loss and differences by demographic characteristics among US adults: data from the National Health and Nutrition Examination Survey, 1999-2004. Arch Intern Med 2008; 168: 1522–30.
Occupational noise	Age-related and other hearing loss	Davis A. The prevalence of hearing impairment and reported hearing disability among adults in Great Britain. International Journal of Epidemiology 1989,18: 911-917.
Occupational noise	Age-related and other hearing loss	Wilson D, Walsh P, Sanchez L, Davis A, Taylor A, Tucker G, Meagher I. The epidemiology of hearing impairment in an Australian adult population. International Journal of Epidemiology 1999;28:247-252.
Occupational injuries	Injuries	International Labour Organization. Resolution concerning statistics of occupational injuries (resulting from occupational accidents). 1998; published online Oct. http://www.ilo.org/global/statistics-and-databases/standards-and-guidelines/resolutions-adopted-by-international-conferences-of-labour-statisticians/WCMS_087528/lang--en/index.htm.
Occupational injuries	Injuries	Eurostat. Accidents at work statistics. http://ec.europa.eu/eurostat/statistics-explained/index.php/Accidents_at_work_statistics.

Appendix Table 2. Supplemental information for Figure 1: Epidemiological evidence supporting causality between risk-outcome pairs included in the Global Burden of Disease 2016 study including 2A. Citations and 2B. Additional information
6A. Citations

Risk	Outcome	Citation/Note
Occupational ergonomic factors	Low back pain	Driscoll T, Jacklyn G, Orchard J, et al. The global burden of occupationally related low back pain: estimates from the Global Burden of Disease 2010 study. Ann Rheum Dis 2014; 73: 975–81.
Non-exclusive breastfeeding	Lower respiratory infections	Horta BL, Victora CG. Short-term effects of breastfeeding: a systematic review on the benefits of breastfeeding on diarrhoea and pneumonia mortality. World Health Organization, 2013 http://allattamento.sip.it/wp-content/uploads/2014/03/WHO_breve-termine.pdf.
Discontinued breastfeeding	Diarrhoeal diseases	Olofin I, McDonald CM, Ezzati M, et al. Associations of suboptimal growth with all-cause and cause-specific mortality in children under five years: a pooled analysis of ten prospective studies. PLoS ONE 2013; 8: e64636.
Discontinued breastfeeding	Diarrhoeal diseases	Genser, B., Strina, A., Santos, L. A., Teles, C. A., Prado, M. S., Cairncross, S., & Barreto, M. L. (2008). Impact of a city-wide sanitation intervention in a large urban centre on social, environmental and behavioural determinants of childhood diarrhoea: analysis of two cohort studies. International Journal of Epidemiology, 37(4), 831-840. doi:10.1093/ije/dyn101
Childhood underweight	Diarrhoeal diseases	Olofin I, McDonald CM, Ezzati M, et al. Associations of suboptimal growth with all-cause and cause-specific mortality in children under five years: a pooled analysis of ten prospective studies. PLoS ONE 2013; 8: e64636.
Childhood underweight	Lower respiratory infections	Olofin I, McDonald CM, Ezzati M, et al. Associations of suboptimal growth with all-cause and cause-specific mortality in children under five years: a pooled analysis of ten prospective studies. PLoS ONE 2013; 8: e64636.
Childhood underweight	Measles	Olofin I, McDonald CM, Ezzati M, et al. Associations of suboptimal growth with all-cause and cause-specific mortality in children under five years: a pooled analysis of ten prospective studies. PLoS ONE 2013; 8: e64636.
Childhood wasting	Diarrhoeal diseases	Olofin I, McDonald CM, Ezzati M, et al. Associations of suboptimal growth with all-cause and cause-specific mortality in children under five years: a pooled analysis of ten prospective studies. PLoS ONE 2013; 8: e64636.
Childhood wasting	Lower respiratory infections	Olofin I, McDonald CM, Ezzati M, et al. Associations of suboptimal growth with all-cause and cause-specific mortality in children under five years: a pooled analysis of ten prospective studies. PLoS ONE 2013; 8: e64636.
Childhood wasting	Measles	Olofin I, McDonald CM, Ezzati M, et al. Associations of suboptimal growth with all-cause and cause-specific mortality in children under five years: a pooled analysis of ten prospective studies. PLoS ONE 2013; 8: e64636.
Childhood stunting	Diarrhoeal diseases	Olofin I, McDonald CM, Ezzati M, et al. Associations of suboptimal growth with all-cause and cause-specific mortality in children under five years: a pooled analysis of ten prospective studies. PLoS ONE 2013; 8: e64636.
Childhood stunting	Lower respiratory infections	Olofin I, McDonald CM, Ezzati M, et al. Associations of suboptimal growth with all-cause and cause-specific mortality in children under five years: a pooled analysis of ten prospective studies. PLoS ONE 2013; 8: e64636.
Childhood stunting	Measles	Olofin I, McDonald CM, Ezzati M, et al. Associations of suboptimal growth with all-cause and cause-specific mortality in children under five years: a pooled analysis of ten prospective studies. PLoS ONE 2013; 8: e64636.
Iron deficiency	Maternal hemorrhage	Murray-Kolb LE, Chen L, Chen P, Shapiro M, Caulfield L. CHERG Iron Report: Maternal Mortality, Child Mortality, Perinatal Mortality, Child Cognition, and Estimates of Prevalence of Anemia due to Iron Deficiency. Baltimore, USA: CHERG, 2012.
Vitamin A deficiency	Diarrhoeal diseases	Awasthi S, Peto R, Read S, et al. Vitamin A supplementation every 6 months with retinol in 1 million pre-school children in north India: DEVTA, a cluster-randomised trial. Lancet 2013; 381: 1469–77.
Vitamin A deficiency	Diarrhoeal diseases	Diness BR, Christoffersen D, Pedersen UB, Rodrigues A, Fischer TK, Andersen A, Whittle H, Yazdanbakhsh M, Aaby P, Benn CS. The effect of high-dose vitamin A supplementation given with bacille Calmette-Guérin vaccine at birth on infant rotavirus infection and diarrhea: a randomized prospective study from Guinea-Bissau. J Infect Dis. 2010; S243-251.
Vitamin A deficiency	Diarrhoeal diseases	Imdad A, Herzer K, Mayo-Wilson E, Yakoob MY, Bhutta ZA. Vitamin A supplementation for preventing morbidity and mortality in children from 6 months to 5 years of age. Cochrane Database Syst Rev. 2010; CD008524.
Vitamin A deficiency	Diarrhoeal diseases	Imdad A, Yakoob MY, Sudfeld C, Haider BA, Black RE, Bhutta ZA. Impact of vitamin A supplementation on infant and childhood mortality. BMC Public Health. 2011; S20.
Vitamin A deficiency	Measles	Awasthi S, Peto R, Read S, et al. Vitamin A supplementation every 6 months with retinol in 1 million pre-school children in north India: DEVTA, a cluster-randomised trial. Lancet 2013; 381: 1469–77.
Vitamin A deficiency	Measles	Imdad A, Herzer K, Mayo-Wilson E, Yakoob MY, Bhutta ZA. Vitamin A supplementation for preventing morbidity and mortality in children from 6 months to 5 years of age. Cochrane Database Syst Rev. 2010; CD008524.
Vitamin A deficiency	Measles	Imdad A, Yakoob MY, Sudfeld C, Haider BA, Black RE, Bhutta ZA. Impact of vitamin A supplementation on infant and childhood mortality. BMC Public Health. 2011; S20.
Zinc deficiency	Diarrhoeal diseases	Shankar AH, Prasad AS. Zinc and immune function: the biological basis of altered resistance to infection. Am J Clin Nutr 1998; 68: 447S–463S.
Zinc deficiency	Diarrhoeal diseases	Yakoob MY, Theodoratou E, Jabeen A, et al. Preventive zinc supplementation in developing countries: impact on mortality and morbidity due to diarrhea, pneumonia and malaria. BMC Public Health 2011; 11 Suppl 3: S23.
Zinc deficiency	Lower respiratory infections	Shankar AH, Prasad AS. Zinc and immune function: the biological basis of altered resistance to infection. Am J Clin Nutr 1998; 68: 447S–463S.

Appendix Table 2. Supplemental information for Figure 1: Epidemiological evidence supporting causality between risk-outcome pairs included in the Global Burden of Disease 2016 study including 2A. Citations and 2B. Additional information<br>6A. Citations		
**Risk**	**Outcome**	**Citation/Note**
Zinc deficiency	Lower respiratory infections	Yakoob MY, Theodoratou E, Jabeen A, et al. Preventive zinc supplementation in developing countries: impact on mortality and morbidity due to diarrhea, pneumonia and malaria. BMC Public Health 2011; 11 Suppl 3: S23.
Smoking	Tuberculosis	Bates MN, Khalakdina A, Pai M, Chang L, Lessa F, Smith KR. Risk of tuberculosis from exposure to tobacco smoke: a systematic review and meta-analysis. Arch Intern Med 2007; 167: 335–42.
Smoking	Tuberculosis	Lin H-H, Ezzati M, Murray M. Tobacco smoke, indoor air pollution and tuberculosis: a systematic review and meta-analysis. PLoS Med 2007; 4: e20.
Smoking	Tuberculosis	Slama K, Chiang C-Y, Enarson DA, et al. Tobacco and tuberculosis: a qualitative systematic review and meta-analysis. Int J Tuberc Lung Dis 2007; 11: 1049–61.
Smoking	Lower respiratory infections	Surgeon General’s Report - The Health Consequences of Smoking. U.S. Department of Health & Human Services, 2004 http://www.cdc.gov/tobacco/data_statistics/sgr/2004/.
Smoking	Larynx cancer	Carter BD, Abnet CC, Feskanich D, et al. Smoking and mortality--beyond established causes. N Engl J Med 2015; 372: 631–40.
Smoking	Lip and oral cavity cancer	Carter BD, Abnet CC, Feskanich D, et al. Smoking and mortality--beyond established causes. N Engl J Med 2015; 372: 631–40.
Smoking	Tracheal, bronchus and lung cancer	International Agency for Research on Cancer Working Group on the Evaluation of Carcinogenic Risks to Humans. IARC monographs on the evaluation of carcinogenic risks to humans: Tobacco Smoke and Involuntary Smoking. Lyon: IARC, 2004.
Smoking	Breast cancer	Ordóñez-Mena JM, Schöttker B, Mons U, et al. Quantification of the smoking-associated cancer risk with rate advancement periods: meta-analysis of individual participant data from cohorts of the CHANCES consortium. BMC Medicine 2016; 14: 62.
Smoking	Cervical cancer	International Agency for Research on Cancer Working Group on the Evaluation of Carcinogenic Risks to Humans. IARC monographs on the evaluation of carcinogenic risks to humans: Tobacco Smoke and Involuntary Smoking. Lyon: IARC, 2004.
Smoking	Prostate cancer	Islami F, Moreira DM, Boffetta P, Freedland SJ. A Systematic Review and Meta-analysis of Tobacco Use and Prostate Cancer Mortality and Incidence in Prospective Cohort Studies. European Urology 2014; 66: 1054–64.
Smoking	Kidney cancer	International Agency for Research on Cancer Working Group on the Evaluation of Carcinogenic Risks to Humans. IARC monographs on the evaluation of carcinogenic risks to humans: Tobacco Smoke and Involuntary Smoking. Lyon: IARC, 2004.
Smoking	Nasopharynx cancer	Xue W-Q, Qin H-D, Ruan H-L, Shugart YY, Jia W-H. Quantitative Association of Tobacco Smoking With the Risk of Nasopharyngeal Carcinoma: A Comprehensive Meta-Analysis of Studies Conducted Between 1979 and 2011. Am J Epidemiol 2013; 178: 325–38.
Smoking	Bladder cancer	Cumberbatch MG, Rota M, Catto JWF, La Vecchia C. The Role of Tobacco Smoke in Bladder and Kidney Carcinogenesis: A Comparison of Exposures and Meta-analysis of Incidence and Mortality Risks. Eur Urol 2016; 70: 458–66.
Smoking	Leukaemia	Colamesta V, D’Aguanno S, Breccia M, Bruffa S, Cartoni C, Torre GL. Do the smoking intensity and duration, the years since quitting, the methodological quality and the year of publication of the studies affect the results of the meta-analysis on cigarette smoking and Acute Myeloid Leukemia (AML) in adults? Critical Reviews in Oncology / Hematology 2016; 99: 376–88.
Smoking	Oesophageal cancer	Surgeon General’s Report - The Health Consequences of Smoking. U.S. Department of Health & Human Services, 2004 http://www.cdc.gov/tobacco/data_statistics/sgr/2004/.
Smoking	Stomach cancer	Ordóñez-Mena JM, Schöttker B, Mons U, et al. Quantification of the smoking-associated cancer risk with rate advancement periods: meta-analysis of individual participant data from cohorts of the CHANCES consortium. BMC Medicine 2016; 14: 62.
Smoking	Colon and rectum cancer	Surgeon General’s Report - The Health Consequences of Smoking. U.S. Department of Health & Human Services, 2004 http://www.cdc.gov/tobacco/data_statistics/sgr/2004/.
Smoking	Liver cancer	Surgeon General’s Report - The Health Consequences of Smoking. U.S. Department of Health & Human Services, 2004 http://www.cdc.gov/tobacco/data_statistics/sgr/2004/.
Smoking	Pancreatic cancer	Ordóñez-Mena JM, Schöttker B, Mons U, et al. Quantification of the smoking-associated cancer risk with rate advancement periods: meta-analysis of individual participant data from cohorts of the CHANCES consortium. BMC Medicine 2016; 14: 62.
Smoking	Ischaemic heart disease	Huxley RR, Woodward M. Cigarette smoking as a risk factor for coronary heart disease in women compared with men: a systematic review and meta-analysis of prospective cohort studies. Lancet 2011; 378: 1297–305.
Smoking	Cerebrovascular disease	Peters SAE, Huxley RR, Woodward M. Smoking as a risk factor for stroke in women compared with men: a systematic review and meta-analysis of 81 cohorts, including 3,980,359 individuals and 42,401 strokes. Stroke 2013; 44: 2821–8.
Smoking	Hypertensive heart disease	Carter BD, Abnet CC, Feskanich D, et al. Smoking and mortality--beyond established causes. N Engl J Med 2015; 372: 631–40.
Smoking	Atrial fibrillation and flutter	Zhu W, Yuan P, Shen Y, Wan R, Hong K. Association of smoking with the risk of incident atrial fibrillation: A meta-analysis of prospective studies. Int J Cardiol 2016; 218: 259–66.
Smoking	Aortic aneurysm	Lederle FA, Nelson DB, Joseph AM. Smokers’ relative risk for aortic aneurysm compared with other smoking-related diseases: a systematic review. J Vasc Surg 2003; 38: 329–34.
Smoking	Peripheral vascular disease	Lu L, Mackay DF, Pell JP. Meta-analysis of the association between cigarette smoking and peripheral arterial disease. Heart 2014; 100: 414–23.
Smoking	Other cardiovascular and circulatory diseases	Carter BD, Abnet CC, Feskanich D, et al. Smoking and mortality--beyond established causes. N Engl J Med 2015; 372: 631–40.
Smoking	Chronic obstructive pulmonary disease	Forey BA, Thornton AJ, Lee PN. Systematic review with meta-analysis of the epidemiological evidence relating smoking to COPD, chronic bronchitis and emphysema. BMC Pulm Med 2011; 11: 36.

Appendix Table 2. Supplemental information for Figure 1: Epidemiological evidence supporting causality between risk-outcome pairs included in the Global Burden of Disease 2016 study including 2A. Citations and 2B. Additional information<br>6A. Citations		
**Risk**	**Outcome**	**Citation/Note**
Smoking	Asthma	Jayes L, Haslam PL, Gratziou CG, et al. SmokeHaz: Systematic Reviews and Meta-analyses of the Effects of Smoking on Respiratory Health. Chest 2016; 150: 164–79.
Smoking	Other chronic respiratory diseases	Carter BD, Abnet CC, Feskanich D, et al. Smoking and mortality--beyond established causes. N Engl J Med 2015; 372: 631–40.
Smoking	Peptic ulcer disease	Kurata JH, Nogawa AN. Meta-analysis of risk factors for peptic ulcer. Nonsteroidal antiinflammatory drugs, Helicobacter pylori, and smoking. J Clin Gastroenterol 1997; 24: 2–17.
Smoking	Gallbladder and biliary tract disease	Aune D, Vatten LJ, Boffetta P. Tobacco smoking and the risk of gallbladder disease. Eur J Epidemiol 2016; 31: 643–53.
Smoking	Alzheimer's disease and other dimentias	Zhong G, Wang Y, Zhang Y, Guo JJ, Zhao Y. Smoking Is Associated with an Increased Risk of Dementia: A Meta-Analysis of Prospective Cohort Studies with Investigation of Potential Effect Modifiers. PLoS One 2015; 10. DOI:10.1371/journal.pone.0118333.
Smoking	Parkinson's disease	Li X, Li W, Liu G, Shen X, Tang Y. Association between cigarette smoking and Parkinson's disease: A meta-analysis. Arch Gerontol Geriatr 2015; 61: 510–6.
Smoking	Multiple sclerosis	O'Gorman C, Broadley SA. Smoking and multiple sclerosis: evidence for latitudinal and temporal variation. J Neurol 2014; 261: 1677–83.
Smoking	Diabetes mellitus	Pan A, Wang Y, Talaei M, Hu FB, Wu T. Relation of active, passive, and quitting smoking with incident type 2 diabetes: a systematic review and meta-analysis. The Lancet Diabetes & Endocrinology 2015; 3: 958–67.
Smoking	Rheumatoid arthritis	Sugiyama D, Nishimura K, Tamaki K, et al. Impact of smoking as a risk factor for developing rheumatoid arthritis: a meta-analysis of observational studies. Ann Rheum Dis 2010; 69: 70–81.
Smoking	Low back pain	Shiri R, Karppinen J, Leino-Arjas P, Solovieva S, Viikari-Juntura E. The Association between Smoking and Low Back Pain: A Meta-analysis. The American Journal of Medicine 2010; 123: 87.e7-87.e35.
Smoking	Cataract	Ye J, He J, Wang C, et al. Smoking and risk of age-related cataract: a meta-analysis. Invest Ophthalmol Vis Sci 2012; 53: 3885–95.
Smoking	Macular degeneration	Chakravarthy U, Wong TY, Fletcher A, et al. Clinical risk factors for age-related macular degeneration: a systematic review and meta-analysis. BMC Ophthalmol 2010; 10: 31.
Smoking	Injuries	Vestergaard P, Mosekilde L. Fracture risk associated with smoking: a meta-analysis. J Intern Med 2003; 254: 572–83.
Smokeless tobacco	Lip and oral cavity cancer	Siddiqi K, Shah S, Abbas SM, et al. Global burden of disease due to smokeless tobacco consumption in adults: analysis of data from 113 countries. BMC Med 2015; 13: 194.
Smokeless tobacco	Oesophageal cancer	Siddiqi K, Shah S, Abbas SM, et al. Global burden of disease due to smokeless tobacco consumption in adults: analysis of data from 113 countries. BMC Med 2015; 13: 194.
Second-hand smoke	Diabetes mellitus	Zhu B, Wu X, Wang X, Zheng Q, Sun G. The association between passive smoking and type 2 diabetes: a meta-analysis. Asia Pac J Public Health 2014; 26: 226–37.
Second-hand smoke	Tuberculosis	Dogar OF, Pillai N, Safdar N, Shah SK, Zahid R, Siddiqi K. Second-hand smoke and the risk of tuberculosis: a systematic review and a meta-analysis. Epidemiol Infect 2015; 143: 3158–72.
Second-hand smoke	Lower respiratory infections	Baker RJ, Hertz-Picciotto I, Dostal M, Keller JA, Nozicka J, Kotesovec F, Dejmek J, Loomis D, Sram RJ. Coal home heating and environmental tobacco smoke in relation to lower respiratory illness in Czech children, from birth to 3 years of age. Environ Health Perspect. 2006; 1126-32.
Second-hand smoke	Lower respiratory infections	Blizzard L, Ponsonby A-L, Dwyer T, Venn A, Cochrane JA. Parental smoking and infant respiratory infection: how important is not smoking in the same room with the baby?. Am J Public Health. 2003; 482-8.
Second-hand smoke	Lower respiratory infections	Bonu S, Rani M, Jha P, Peters DH, Nguyen SN. Household tobacco and alcohol use, and child health: an exploratory study from India. Health Policy. 2004; 67-83.
Second-hand smoke	Lower respiratory infections	Broor S, Pandey RM, Ghosh M, Maitreyi RS, Lodha R, Singhal T, Kabra SK. Risk factors for severe acute lower respiratory tract infection in under-five children. Indian Pediatr. 2001; 1361-9.
Second-hand smoke	Lower respiratory infections	Chen Y, Li WX, Yu SZ, Qian WH. Chang-Ning epidemiological study of children's health: I: Passive smoking and children's respiratory diseases. Int J Epidemiol. 1988; 348-55.
Second-hand smoke	Lower respiratory infections	Duijts L, Jaddoe VWV, Hofman A, Steegers EAP, Mackenbach JP, de Jongste JC, Moll HA. Maternal smoking in pre-natal and early post-natal life and the risk of respiratory tract infections in infancy. The Generation R study. Eur J Epidemiol. 2008; 547-55.
Second-hand smoke	Lower respiratory infections	Ekwo EE, Weinberger MM, Lachenbruch PA, Huntley WH. Relationship of parental smoking and gas cooking to respiratory disease in children. Chest. 1983; 662-8.
Second-hand smoke	Lower respiratory infections	Etiler N, Velipasaoglu S, Aktekin M. Incidence of acute respiratory infections and the relationship with some factors in infancy in Antalya, Turkey. Pediatr Int 2002; 44: 64–9.
Second-hand smoke	Lower respiratory infections	Ferris BG, Ware JH, Berkey CS, Dockery DW, Spiro A, Speizer FE. Effects of passive smoking on health of children. Environ Health Perspect. 1985; 289-95.
Second-hand smoke	Lower respiratory infections	Forastiere F, Corbo GM, Michelozzi P, Pistelli R, Agabiti N, Brancato G, Ciappi G, Perucci CA. Effects of environment and passive smoking on the respiratory health of children. Int J Epidemiol. 1992; 66-73.
Second-hand smoke	Lower respiratory infections	Gardner G, Frank AL, Taber LH. Effects of social and family factors on viral respiratory infection and illness in the first year of life. J Epidemiol Community Health. 1984; 42-8.
Second-hand smoke	Lower respiratory infections	Hassan MK, Al-Sadoon I. Risk factors for severe pneumonia in children in Basrah. Trop Doct. 2001; 139-41.
Second-hand smoke	Lower respiratory infections	Koch A, Molbak K, Homoe P, Sorensen P, Hjuler T, Olesen ME, Pejl J, Pedersen FK, Olsen OR, Melbye M. Risk factors for acute respiratory tract infections in young Greenlandic children. Am J Epidemiol. 2003; 374-84.
Second-hand smoke	Lower respiratory infections	Kristensen IA, Olsen J. Determinants of acute respiratory infections in Soweto--a population-based birth cohort. S Afr Med J. 2006; 633-40.
Second-hand smoke	Lower respiratory infections	Margolis PA, Keyes LL, Greenberg RA, Bauman KE, LaVange LM. Urinary cotinine and parent history (questionnaire) as indicators of passive smoking and predictors of lower respiratory illness in infants. Pediatr Pulmonol. 1997; 417-23.

Appendix Table 2. Supplemental information for Figure 1: Epidemiological evidence supporting causality between risk-outcome pairs included in the Global Burden of Disease 2016 study including 2A. Citations and 2B. Additional information
6A. Citations

Risk	Outcome	Citation/Note
Second-hand smoke	Lower respiratory infections	Nuesslein TG, Beckers D, Rieger CH. Cotinine in meconium indicates risk for early respiratory tract infections. Hum Exp Toxicol. 1999; 283-90.
Second-hand smoke	Lower respiratory infections	Ogston SA, Florey CD, Walker CH. The Tayside infant morbidity and mortality study: effect on health of using gas for cooking. BMJ. 1985; 957-60.
Second-hand smoke	Lower respiratory infections	Ogston SA, Florey CD, Walker CH. Association of infant alimentary and respiratory illness with parental smoking and other environmental factors. J Epidemiol Community Health. 1987; 21-5.
Second-hand smoke	Lower respiratory infections	Pedreira FA, Guandolo VL, Feroli EJ, Mella GW, Weiss IP. Involuntary smoking and incidence of respiratory illness during the first year of life. Pediatrics. 1985; 594-7.
Second-hand smoke	Lower respiratory infections	Rylander E, Pershagen G, Eriksson M, Bermann G. Parental smoking, urinary cotinine, and wheezing bronchitis in children. Epidemiology. 1995; 289-93.
Second-hand smoke	Lower respiratory infections	Suzuki M, Thiem VD, Yanai H, Matsubayashi T, Yoshida LM, Tho LH, Minh TT, Anh DD, Kilgore PE, Ariyoshi K. Association of environmental tobacco smoking exposure with an increased risk of hospital admissions for pneumonia in children under 5 years of age in Vietnam. Thorax. 2009; 484-9.
Second-hand smoke	Lower respiratory infections	Taylor B, Wadsworth J. Maternal smoking during pregnancy and lower respiratory tract illness in early life. Arch Dis Child. 1987; 786-91.
Second-hand smoke	Lower respiratory infections	Victora CG, Fuchs SC, Flores JA, Fonseca W, Kirkwood B. Risk factors for pneumonia among children in a Brazilian metropolitan area. Pediatrics. 1994; 977-85.
Second-hand smoke	Otitis media	Jones LL, Hassanien A, Cook DG, Britton J, Leonardi-Bee J. Parental smoking and the risk of middle ear disease in children: a systematic review and meta-analysis. Arch Pediatr Adolesc Med 2012; 166: 18–27.
Second-hand smoke	Tracheal, bronchus, and lung cancer	Jayes L, Haslam PL, Gratziou CG, et al. SmokeHaz: Systematic Reviews and Meta-analyses of the Effects of Smoking on Respiratory Health. Chest 2016; 150: 164–79.
Second-hand smoke	Ischaemic heart disease	Fischer F, Kraemer A. Meta-analysis of the association between second-hand smoke exposure and ischaemic heart diseases, COPD and stroke. BMC Public Health 2015; 15: 1202.
Second-hand smoke	Ischaemic stroke	Oono IP, Mackay DF, Pell JP. Meta-analysis of the association between secondhand smoke exposure and stroke. J Public Health (Oxf) 2011; 33: 496–502.
Alcohol use	Tuberculosis	Lönnroth K, Williams BG, Stadlin S, Jaramillo E, Dye C. Alcohol use as a risk factor for tuberculosis - a systematic review. BMC Public Health 2008; 8: 289.
Alcohol use	Tuberculosis	Rehm J, Samokhvalov AV, Neuman MG, et al. The association between alcohol use, alcohol use disorders and tuberculosis (TB). A systematic review. BMC Public Health 2009; 9: 450.
Alcohol use	Tuberculosis	Hemilä H, Kaprio J, Pietinen P, Albanes D, Heinonen OP. Vitamin C and other compounds in vitamin C rich food in relation to risk of tuberculosis in male smokers. Am J Epidemiol 1999; 150: 632–41.
Alcohol use	Tuberculosis	Brown KE, Campbell AH. Tobacco, alcohol and tuberculosis. British Journal of Diseases of the Chest 1961; 55: 150–8.
Alcohol use	Tuberculosis	Buskin SE, Gale JL, Weiss NS, Nolan CM. Tuberculosis risk factors in adults in King County, Washington, 1988 through 1990. Am J Public Health 1994; 84: 1750–6.
Alcohol use	Tuberculosis	Crampin AC, Glynn JR, Floyd S, et al. Tuberculosis and gender: exploring the patterns in a case control study in Malawi. Int J Tuberc Lung Dis 2004; 8: 194–203.
Alcohol use	Tuberculosis	Lewis JG, Chamberlain DA. Alcohol consumption and smoking habits in male patients with pulmonary tuberculosis. Br J Prev Soc Med 1963; 17: 149–52.
Alcohol use	Tuberculosis	Rosenman KD, Hall N. Occupational risk factors for developing tuberculosis. Am J Ind Med 1996; 30: 148–54.
Alcohol use	Tuberculosis	Tekkel M, Rahu M, Loit HM, Baburin A. Risk factors for pulmonary tuberculosis in Estonia. Int J Tuberc Lung Dis 2002; 6: 887–94.
Alcohol use	Tuberculosis	Tocque K, Bellis MA, Beeching NJ, Syed Q, Remmington T, Davies PD. A case-control study of lifestyle risk factors associated with tuberculosis in Liverpool, North-West England. Eur Respir J 2001; 18: 959–64.
Alcohol use	Tuberculosis	Zaridze D, Brennan P, Boreham J, et al. Alcohol and cause-specific mortality in Russia: a retrospective case-control study of 48,557 adult deaths. Lancet 2009; 373: 2201–14.
Alcohol use	Lower respiratory infections	Samokhvalov AV, Irving HM, Rehm J. Alcohol consumption as a risk factor for pneumonia: a systematic review and meta-analysis. Epidemiol Infect 2010; 138: 1789–95.
Alcohol use	Lower respiratory infections	Baik I, Curhan GC, Rimm EB, Bendich A, Willett WC, Fawzi WW. A prospective study of age and lifestyle factors in relation to community-acquired pneumonia in US men and women. Arch Intern Med 2000; 160: 3082–8.
Alcohol use	Lower respiratory infections	Kornum JB, Due KM, Nørgaard M, et al. Alcohol drinking and risk of subsequent hospitalisation with pneumonia. Eur Respir J 2012; 39: 149–55.
Alcohol use	Lower respiratory infections	Shen C, Ni MY, Schooling CM, Chan WM, Lee SY, Lam TH. Alcohol use and death from respiratory disease in a prospective Chinese elderly cohort study in Hong Kong. Prev Med 2013; 57: 819–23.
Alcohol use	Lower respiratory infections	Almirall J, Bolíbar I, Serra-Prat M, et al. New evidence of risk factors for community-acquired pneumonia: a population-based study. Eur Respir J 2008; 31: 1274–84.
Alcohol use	Lower respiratory infections	Almirall J, Bolíbar I, Balanzó X, González CA. Risk factors for community-acquired pneumonia in adults: a population-based case-control study. Eur Respir J 1999; 13: 349–55.
Alcohol use	Lip and oral cavity cancer	Bagnardi V, Blangiardo M, La Vecchia C, Corrao G. A meta-analysis of alcohol drinking and cancer risk. Br J Cancer 2001; 85: 1700–5.
Alcohol use	Lip and oral cavity cancer	Jayasekara H, MacInnis RJ, Room R, English DR. Long-Term Alcohol Consumption and Breast, Upper Aero-Digestive Tract and Colorectal Cancer Risk: A Systematic Review and Meta-Analysis. Alcohol Alcohol 2016; 51: 315–30.
Alcohol use	Lip and oral cavity cancer	Turati F, Garavello W, Tramacere I, et al. A meta-analysis of alcohol drinking and oral and pharyngeal cancers. Part 2: results by subsites. Oral Oncol 2010; 46: 720–6.
Alcohol use	Lip and oral cavity cancer	Shanmugham JR, Zavras AI, Rosner BA, Giovannucci EL. Alcohol-folate interactions in the risk of oral cancer in women: a prospective cohort study. Cancer Epidemiol Biomarkers Prev 2010; 19: 2516–24.

Appendix Table 2. Supplemental information for Figure 1: Epidemiological evidence supporting causality between risk-outcome pairs included in the Global Burden of Disease 2016 study including 2A. Citations and 2B. Additional information
6A. Citations

Risk	Outcome	Citation/Note
Alcohol use	Lip and oral cavity cancer	Weikert C, Dietrich T, Boeing H, et al. Lifetime and baseline alcohol intake and risk of cancer of the upper aero-digestive tract in the European Prospective Investigation into Cancer and Nutrition (EPIC) study. Int J Cancer 2009; 125: 406–12.
Alcohol use	Lip and oral cavity cancer	Martinez I. Factors associated with cancer of the esophagus, mouth, and pharynx in Puerto Rico. J Natl Cancer Inst 1969; 42: 1069–94.
Alcohol use	Nasopharynx cancer	Bagnardi V, Blangiardo M, La Vecchia C, Corrao G. A meta-analysis of alcohol drinking and cancer risk. Br J Cancer 2001; 85: 1700–5.
Alcohol use	Other pharynx cancer	Bagnardi V, Blangiardo M, La Vecchia C, Corrao G. A meta-analysis of alcohol drinking and cancer risk. Br J Cancer 2001; 85: 1700–5.
Alcohol use	Oesophageal cancer	Bagnardi V, Blangiardo M, La Vecchia C, Corrao G. A meta-analysis of alcohol drinking and cancer risk. Br J Cancer 2001; 85: 1700–5.
Alcohol use	Oesophageal cancer	Allen NE, Beral V, Casabonne D, et al. Moderate alcohol intake and cancer incidence in women. J Natl Cancer Inst 2009; 101: 296–305.
Alcohol use	Oesophageal cancer	Boffetta P, Garfinkel L. Alcohol drinking and mortality among men enrolled in an American Cancer Society prospective study. Epidemiology 1990; 1: 342–8.
Alcohol use	Oesophageal cancer	Fan Y, Yuan J-M, Wang R, Gao Y-T, Yu MC. Alcohol, tobacco, and diet in relation to esophageal cancer: the Shanghai Cohort Study. Nutr Cancer 2008; 60: 354–63.
Alcohol use	Oesophageal cancer	Freedman ND, Abnet CC, Leitzmann MF, et al. A prospective study of tobacco, alcohol, and the risk of esophageal and gastric cancer subtypes. Am J Epidemiol 2007; 165: 1424–33.
Alcohol use	Oesophageal cancer	Ishiguro S, Sasazuki S, Inoue M, Kurahashi N, Iwasaki M, Tsugane S. Effect of alcohol consumption, cigarette smoking and flushing response on esophageal cancer risk: a population-based cohort study (JPHC study). Cancer Lett 2009; 275: 240–6.
Alcohol use	Oesophageal cancer	Kim MK, Ko MJ, Han JT. Alcohol consumption and mortality from all-cause and cancers among 1.34 million Koreans: the results from the Korea national health insurance corporation's health examinee cohort in 2000. Cancer Causes Control 2010; 21: 2295–302.
Alcohol use	Oesophageal cancer	Kimm H, Kim S, Jee SH. The independent effects of cigarette smoking, alcohol consumption, and serum aspartate aminotransferase on the alanine aminotransferase ratio in korean men for the risk for esophageal cancer. Yonsei Med J 2010; 51: 310–7.
Alcohol use	Oesophageal cancer	Kono S, Ikeda M, Tokudome S, Nishizumi M, Kuratsune M. Cigarette smoking, alcohol and cancer mortality: a cohort study of male Japanese physicians. Jpn J Cancer Res 1987; 78: 1323–8.
Alcohol use	Oesophageal cancer	Nakaya N, Tsubono Y, Kuriyama S, et al. Alcohol consumption and the risk of cancer in Japanese men: the Miyagi cohort study. Eur J Cancer Prev 2005; 14: 169–74.
Alcohol use	Oesophageal cancer	Ozasa K. Alcohol use and mortality in the Japan Collaborative Cohort Study for Evaluation of Cancer (JACC). Asian Pac J Cancer Prev 2007; 8 Suppl: 81–8.
Alcohol use	Oesophageal cancer	Smith M, Zhou M, Whitlock G, et al. Esophageal cancer and body mass index: results from a prospective study of 220,000 men in China and a meta-analysis of published studies. Int J Cancer 2008; 122: 1604–10.
Alcohol use	Oesophageal cancer	Wu M, Zhang Z-F, Kampman E, et al. Does family history of cancer modify the effects of lifestyle risk factors on esophageal cancer? A population-based case-control study in China. Int J Cancer 2011; 128: 2147–57.
Alcohol use	Colon and rectum cancer	Bagnardi V, Blangiardo M, La Vecchia C, Corrao G. A meta-analysis of alcohol drinking and cancer risk. Br J Cancer 2001; 85: 1700–5.
Alcohol use	Colon and rectum cancer	Akhter M, Kuriyama S, Nakaya N, et al. Alcohol consumption is associated with an increased risk of distal colon and rectal cancer in Japanese men: the Miyagi Cohort Study. Eur J Cancer 2007; 43: 383–90.
Alcohol use	Colon and rectum cancer	Allen NE, Beral V, Casabonne D, et al. Moderate alcohol intake and cancer incidence in women. J Natl Cancer Inst 2009; 101: 296–305.
Alcohol use	Colon and rectum cancer	Breslow RA, Chen CM, Graubard BI, Mukamal KJ. Prospective study of alcohol consumption quantity and frequency and cancer-specific mortality in the US population. Am J Epidemiol 2011; 174: 1044–53.
Alcohol use	Colon and rectum cancer	Chen K, Jiang Q, Ma X, et al. Alcohol drinking and colorectal cancer: a population-based prospective cohort study in China. Eur J Epidemiol 2005; 20: 149–54.
Alcohol use	Colon and rectum cancer	Chyou PH, Nomura AM, Stemmermann GN. A prospective study of colon and rectal cancer among Hawaii Japanese men. Ann Epidemiol 1996; 6: 276–82.
Alcohol use	Colon and rectum cancer	Crockett SD, Long MD, Dellon ES, Martin CF, Galanko JA, Sandler RS. Inverse relationship between moderate alcohol intake and rectal cancer: analysis of the North Carolina Colon Cancer Study. Dis Colon Rectum 2011; 54: 887–94.
Alcohol use	Colon and rectum cancer	Ferrari P, Jenab M, Norat T, et al. Lifetime and baseline alcohol intake and risk of colon and rectal cancers in the European prospective investigation into cancer and nutrition (EPIC). Int J Cancer 2007; 121: 2065–72.
Alcohol use	Colon and rectum cancer	Flood A, Caprario L, Chaterjee N, Lacey JVJ, Schairer C, Schatzkin A. Folate, methionine, alcohol, and colorectal cancer in a prospective study of women in the USA. Cancer Causes Control 2002; 13: 551–61.
Alcohol use	Colon and rectum cancer	Gaziano JM, Gaziano TA, Glynn RJ, et al. Light-to-moderate alcohol consumption and mortality in the Physicians' Health Study enrollment cohort. J Am Coll Cardiol 2000; 35: 96–105.
Alcohol use	Colon and rectum cancer	Kabat GC, Miller AB, Jain M, Rohan TE. Dietary intake of selected B vitamins in relation to risk of major cancers in women. Br J Cancer 2008; 99: 816–21.
Alcohol use	Colon and rectum cancer	Kim MK, Ko MJ, Han JT. Alcohol consumption and mortality from all-cause and cancers among 1.34 million Koreans: the results from the Korea national health insurance corporation's health examinee cohort in 2000. Cancer Causes Control 2010; 21: 2295–302.
Alcohol use	Colon and rectum cancer	Klatsky AL, Armstrong MA, Friedman GD, Hiatt RA. The relations of alcoholic beverage use to colon and rectal cancer. Am J Epidemiol 1988; 128: 1007–15.

Appendix Table 2. Supplemental information for Figure 1: Epidemiological evidence supporting causality between risk-outcome pairs included in the Global Burden of Disease 2016 study including 2A. Citations and 2B. Additional information
6A. Citations

Risk	Outcome	Citation/Note
Alcohol use	Colon and rectum cancer	Kono S, Ikeda M, Tokudome S, Nishizumi M, Kuratsune M. Cigarette smoking, alcohol and cancer mortality: a cohort study of male Japanese physicians. Jpn J Cancer Res 1987; 78: 1323–8.
Alcohol use	Colon and rectum cancer	Lim HJ, Park BJ. [Cohort study on the association between alcohol consumption and the risk of colorectal cancer in the Korean elderly]. J Prev Med Public Health 2008; 41: 23–9.
Alcohol use	Colon and rectum cancer	Nakaya N, Tsubono Y, Kuriyama S, et al. Alcohol consumption and the risk of cancer in Japanese men: the Miyagi cohort study. Eur J Cancer Prev 2005; 14: 169–74.
Alcohol use	Colon and rectum cancer	Otani T, Iwasaki M, Yamamoto S, et al. Alcohol consumption, smoking, and subsequent risk of colorectal cancer in middle-aged and elderly Japanese men and women: Japan Public Health Center-based prospective study. Cancer Epidemiol Biomarkers Prev 2003; 12: 1492–500.
Alcohol use	Colon and rectum cancer	Ozasa K. Alcohol use and mortality in the Japan Collaborative Cohort Study for Evaluation of Cancer (JACC). Asian Pac J Cancer Prev 2007; 8 Suppl: 81–8.
Alcohol use	Colon and rectum cancer	Pedersen A, Johansen C, Gronbaek M. Relations between amount and type of alcohol and colon and rectal cancer in a Danish population based cohort study. Gut 2003; 52: 861–7.
Alcohol use	Colon and rectum cancer	Sanjoaquin MA, Appleby PN, Thorogood M, Mann JI, Key TJ. Nutrition, lifestyle and colorectal cancer incidence: a prospective investigation of 10998 vegetarians and non-vegetarians in the United Kingdom. Br J Cancer 2004; 90: 118–21.
Alcohol use	Colon and rectum cancer	Thun MJ, Peto R, Lopez AD, et al. Alcohol consumption and mortality among middle-aged and elderly U.S. adults. N Engl J Med 1997; 337: 1705–14.
Alcohol use	Colon and rectum cancer	Thygesen LC, Wu K, Gronbaek M, Fuchs CS, Willett WC, Giovannucci E. Alcohol intake and colorectal cancer: a comparison of approaches for including repeated measures of alcohol consumption. Epidemiology 2008; 19: 258–64.
Alcohol use	Colon and rectum cancer	Toriola AT, Kurl S, Laukanen JA, Mazengo C, Kauhanen J. Alcohol consumption and risk of colorectal cancer: the Findrink study. Eur J Epidemiol 2008; 23: 395–401.
Alcohol use	Colon and rectum cancer	Wakai K, Kojima M, Tamakoshi K, et al. Alcohol consumption and colorectal cancer risk: findings from the JACC Study. J Epidemiol 2005; 15 Suppl 2: S173-179.
Alcohol use	Colon and rectum cancer	Wu AH, Paganini-Hill A, Ross RK, Henderson BE. Alcohol, physical activity and other risk factors for colorectal cancer: a prospective study. Br J Cancer 1987; 55: 687–94.
Alcohol use	Colon and rectum cancer	Yamamoto S, Nakagawa T, Matsushita Y, et al. Visceral fat area and markers of insulin resistance in relation to colorectal neoplasia. Diabetes Care 2010; 33: 184–9.
Alcohol use	Colon and rectum cancer	Yi S-W, Sull JW, Linton JA, Nam CM, Ohrr H. Alcohol consumption and digestive cancer mortality in Koreans: the Kangwha Cohort Study. J Epidemiol 2010; 20: 204–11.
Alcohol use	Colon and rectum cancer	Yuan JM, Ross RK, Gao YT, Henderson BE, Yu MC. Follow up study of moderate alcohol intake and mortality among middle aged men in Shanghai, China. BMJ 1997; 314: 18–23.
Alcohol use	Larynx cancer	Bagnardi V, Blangiardo M, La Vecchia C, Corrao G. A meta-analysis of alcohol drinking and cancer risk. Br J Cancer 2001; 85: 1700–5.
Alcohol use	Larynx cancer	Allen NE, Beral V, Casabonne D, et al. Moderate alcohol intake and cancer incidence in women. J Natl Cancer Inst 2009; 101: 296–305.
Alcohol use	Larynx cancer	Freedman ND, Schatzkin A, Leitzmann MF, Hollenbeck AR, Abnet CC. Alcohol and head and neck cancer risk in a prospective study. Br J Cancer 2007; 96: 1469–74.
Alcohol use	Larynx cancer	Garavello W, Bosetti C, Gallus S, et al. Type of alcoholic beverage and the risk of laryngeal cancer. Eur J Cancer Prev 2006; 15: 69–73.
Alcohol use	Larynx cancer	Kim MK, Ko MJ, Han JT. Alcohol consumption and mortality from all-cause and cancers among 1.34 million Koreans: the results from the Korea national health insurance corporation's health examinee cohort in 2000. Cancer Causes Control 2010; 21: 2295–302.
Alcohol use	Breast cancer	Bagnardi V, Blangiardo M, La Vecchia C, Corrao G. A meta-analysis of alcohol drinking and cancer risk. Br J Cancer 2001; 85: 1700–5.
Alcohol use	Breast cancer	Allen NE, Beral V, Casabonne D, et al. Moderate alcohol intake and cancer incidence in women. J Natl Cancer Inst 2009; 101: 296–305.
Alcohol use	Breast cancer	Baglietto L, English DR, Gertig DM, Hopper JL, Giles GG. Does dietary folate intake modify effect of alcohol consumption on breast cancer risk? Prospective cohort study. BMJ 2005; 331: 807.
Alcohol use	Breast cancer	Breslow RA, Chen CM, Graubard BI, Mukamal KJ. Prospective study of alcohol consumption quantity and frequency and cancer-specific mortality in the US population. Am J Epidemiol 2011; 174: 1044–53.
Alcohol use	Breast cancer	Chen WY, Colditz GA, Rosner B, et al. Use of postmenopausal hormones, alcohol, and risk for invasive breast cancer. Ann Intern Med 2002; 137: 798–804.
Alcohol use	Ischaemic heart disease	Roerecke M, Rehm J. The cardioprotective association of average alcohol consumption and ischaemic heart disease: a systematic review and meta-analysis. Addiction 2012; 107: 1246–60.
Alcohol use	Ischaemic heart disease	Ronksley PE, Brien SE, Turner BJ, Mukamal KJ, Ghali WA. Association of alcohol consumption with selected cardiovascular disease outcomes: a systematic review and meta-analysis. BMJ 2011; 342: d671.
Alcohol use	Ischaemic heart disease	Corrao G, Rubbiati L, Bagnardi V, Zambon A, Poikolainen K. Alcohol and coronary heart disease: a meta-analysis. Addiction 2000; 95: 1505–23.
Alcohol use	Ischaemic heart disease	Zhao J, Stockwell T, Roemer A, Naimi T, Chikritzhs T. Alcohol Consumption and Mortality From Coronary Heart Disease: An Updated Meta-Analysis of Cohort Studies. J Stud Alcohol Drugs 2017; 78: 375–86.
Alcohol use	Ischaemic heart disease	Zhang X-Y, Shu L, Si C-J, et al. Dietary Patterns, Alcohol Consumption and Risk of Coronary Heart Disease in Adults: A Meta-Analysis. Nutrients 2015; 7: 6582–605.
Alcohol use	Ischaemic heart disease	Zheng Y-L, Lian F, Shi Q, et al. Alcohol intake and associated risk of major cardiovascular outcomes in women compared with men: a systematic review and meta-analysis of prospective observational studies. BMC Public Health 2015; 15: 773.
Alcohol use	Cerebrovascular disease	Patra J, Taylor B, Irving H, et al. Alcohol consumption and the risk of morbidity and mortality for different stroke types--a systematic review and meta-analysis. BMC Public Health 2010; 10: 258.

Appendix Table 2. Supplemental information for Figure 1: Epidemiological evidence supporting causality between risk-outcome pairs included in the Global Burden of Disease 2016 study including 2A. Citations and 2B. Additional information
6A. Citations

Risk	Outcome	Citation/Note
Alcohol use	Hypertensive heart disease	Briasoulis A, Agarwal V, Messerli FH. Alcohol consumption and the risk of hypertension in men and women: a systematic review and meta-analysis. J Clin Hypertens (Greenwich) 2012; 14: 792–8.
Alcohol use	Hypertensive heart disease	Taylor B, Irving HM, Baliunas D, et al. Alcohol and hypertension: gender differences in dose-response relationships determined through systematic review and meta-analysis. Addiction 2009; 104: 1981–90.
Alcohol use	Atrial fibrillation and flutter	Kodama S, Saito K, Tanaka S, et al. Alcohol consumption and risk of atrial fibrillation: a meta-analysis. J Am Coll Cardiol 2011; 57: 427–36.
Alcohol use	Atrial fibrillation and flutter	Samokhvalov AV, Irving HM, Rehm J. Alcohol consumption as a risk factor for atrial fibrillation: a systematic review and meta-analysis. Eur J Cardiovasc Prev Rehabil 2010; 17: 706–12.
Alcohol use	Cirrhosis and other chronic liver diseases	Rehm J, Taylor B, Mohapatra S, et al. Alcohol as a risk factor for liver cirrhosis: a systematic review and meta-analysis. Drug Alcohol Rev 2010; 29: 437–45.
Alcohol use	Cirrhosis and other chronic liver diseases	Becker U, Grønbaek M, Johansen D, Sørensen TIA. Lower risk for alcohol-induced cirrhosis in wine drinkers. Hepatology 2002; 35: 868–75.
Alcohol use	Cirrhosis and other chronic liver diseases	Becker U, Deis A, Sørensen TI, et al. Prediction of risk of liver disease by alcohol intake, sex, and age: a prospective population study. Hepatology 1996; 23: 1025–9.
Alcohol use	Cirrhosis and other chronic liver diseases	Bellentani S, Saccoccio G, Costa G, et al. Drinking habits as cofactors of risk for alcohol induced liver damage. The Dionysos Study Group. Gut 1997; 41: 845–50.
Alcohol use	Cirrhosis and other chronic liver diseases	Blackwelder WC, Yano K, Rhoads GG, Kagan A, Gordon T, Palesch Y. Alcohol and mortality: the Honolulu Heart Study. Am J Med 1980; 68: 164–9.
Alcohol use	Cirrhosis and other chronic liver diseases	Boffetta P, Garfinkel L. Alcohol drinking and mortality among men enrolled in an American Cancer Society prospective study. Epidemiology 1990; 1: 342–8.
Alcohol use	Cirrhosis and other chronic liver diseases	Fuchs CS, Stampfer MJ, Colditz GA, et al. Alcohol consumption and mortality among women. N Engl J Med 1995; 332: 1245–50.
Alcohol use	Cirrhosis and other chronic liver diseases	Garfinkel L, Boffetta P, Stellman SD. Alcohol and breast cancer: a cohort study. Prev Med 1988; 17: 686–93.
Alcohol use	Cirrhosis and other chronic liver diseases	Gordon T, Doyle JT. Drinking and mortality. The Albany Study. Am J Epidemiol 1987; 125: 263–70.
Alcohol use	Cirrhosis and other chronic liver diseases	Gordon T, Kannel WB. Drinking and mortality. The Framingham Study. Am J Epidemiol 1984; 120: 97–107.
Alcohol use	Cirrhosis and other chronic liver diseases	Kono S, Ikeda M, Tokudome S, Nishizumi M, Kuratsune M. Alcohol and mortality: a cohort study of male Japanese physicians. Int J Epidemiol 1986; 15: 527–32.
Alcohol use	Cirrhosis and other chronic liver diseases	Thun MJ, Peto R, Lopez AD, et al. Alcohol consumption and mortality among middle-aged and elderly U.S. adults. N Engl J Med 1997; 337: 1705–14.
Alcohol use	Cirrhosis and other chronic liver diseases	Yuan JM, Ross RK, Gao YT, Henderson BE, Yu MC. Follow up study of moderate alcohol intake and mortality among middle aged men in Shanghai, China. BMJ 1997; 314: 18–23.
Alcohol use	Liver cancer	Bagnardi V, Rota M, Botteri E, et al. Alcohol consumption and site-specific cancer risk: a comprehensive dose-response meta-analysis. Br J Cancer 2015; 112: 580–93.
Alcohol use	Liver cancer	Allen NE, Beral V, Casabonne D, et al. Moderate alcohol intake and cancer incidence in women. J Natl Cancer Inst 2009; 101: 296–305.
Alcohol use	Liver cancer	Jee SH, Ohrr H, Sull JW, Samet JM. Cigarette smoking, alcohol drinking, hepatitis B, and risk for hepatocellular carcinoma in Korea. J Natl Cancer Inst 2004; 96: 1851–6.
Alcohol use	Liver cancer	Joshi S, Song Y-M, Kim T-H, Cho S-I. Socio-economic status and the risk of liver cancer mortality: a prospective study in Korean men. Public Health 2008; 122: 1144–51.
Alcohol use	Liver cancer	Kim MK, Ko MJ, Han JT. Alcohol consumption and mortality from all-cause and cancers among 1.34 million Koreans: the results from the Korea national health insurance corporation's health examinee cohort in 2000. Cancer Causes Control 2010; 21: 2295–302.
Alcohol use	Liver cancer	Koh W-P, Robien K, Wang R, Govindarajan S, Yuan J-M, Yu MC. Smoking as an independent risk factor for hepatocellular carcinoma: the Singapore Chinese Health Study. Br J Cancer 2011; 105: 1430–5.
Alcohol use	Liver cancer	Kono S, Ikeda M, Tokudome S, Nishizumi M, Kuratsune M. Cigarette smoking, alcohol and cancer mortality: a cohort study of male Japanese physicians. Jpn J Cancer Res 1987; 78: 1323–8.
Alcohol use	Liver cancer	Nakaya N, Tsubono Y, Kuriyama S, et al. Alcohol consumption and the risk of cancer in Japanese men: the Miyagi cohort study. Eur J Cancer Prev 2005; 14: 169–74.
Alcohol use	Liver cancer	Ozasa K. Alcohol use and mortality in the Japan Collaborative Cohort Study for Evaluation of Cancer (JACC). Asian Pac J Cancer Prev 2007; 8 Suppl: 81–8.
Alcohol use	Liver cancer	Yi S-W, Sull JW, Linton JA, Nam CM, Ohrr H. Alcohol consumption and digestive cancer mortality in Koreans: the Kangwha Cohort Study. J Epidemiol 2010; 20: 204–11.
Alcohol use	Pancreatitis	Alsamarrai A, Das SLM, Windsor JA, Petrov MS. Factors that affect risk for pancreatic disease in the general population: a systematic review and meta-analysis of prospective cohort studies. Clin Gastroenterol Hepatol 2014; 12: 1635–1644.e5; quiz e103.
Alcohol use	Pancreatitis	Irving HM, Samokhvalov AV, Rehm J. Alcohol as a risk factor for pancreatitis. A systematic review and meta-analysis. JOP 2009; 10: 387–92.
Alcohol use	Pancreatitis	Samokhvalov AV, Rehm J, Roerecke M. Alcohol Consumption as a Risk Factor for Acute and Chronic Pancreatitis: A Systematic Review and a Series of Meta-analyses. EBioMedicine 2015; 2: 1996–2002.
Alcohol use	Epilepsy	Samokhvalov AV, Irving H, Mohapatra S, Rehm J. Alcohol consumption, unprovoked seizures, and epilepsy: a systematic review and meta-analysis. Epilepsia 2010; 51: 1177–84.
Alcohol use	Epilepsy	Dworetzky BA, Bromfield EB, Townsend MK, Kang JH. A prospective study of smoking, caffeine, and alcohol as risk factors for seizures or epilepsy in young adult women: data from the Nurses' Health Study II. Epilepsia 2010; 51: 198–205.
Alcohol use	Diabetes mellitus	Carlsson S, Hammar N, Grill V. Alcohol consumption and type 2 diabetes Meta-analysis of epidemiological studies indicates a U-shaped relationship. Diabetologia 2005; 48: 1051–4.

Appendix Table 2. Supplemental information for Figure 1: Epidemiological evidence supporting causality between risk-outcome pairs included in the Global Burden of Disease 2016 study including 2A. Citations and 2B. Additional information<br>6A. Citations		
**Risk**	**Outcome**	**Citation/Note**
Alcohol use	Diabetes mellitus	Li X-H, Yu F-F, Zhou Y-H, He J. Association between alcohol consumption and the risk of incident type 2 diabetes: a systematic review and dose-response meta-analysis. Am J Clin Nutr 2016; 103: 818–29.
Alcohol use	Injuries	Beghi M, Rosenbaum JF, Cerri C, Cornaggia CM. Risk factors for fatal and nonfatal repetition of suicide attempts: a literature review. Neuropsychiatr Dis Treat2013;9: 1725–36.
Alcohol use	Injuries	Borges G, Bagge CL, Cherpitel CJ, Conner KR, Orozco R, Rossow I. A meta-analysis of acute use of alcohol and the risk of suicide attempt. Psychol Med 2017; 47: 949–57.
Alcohol use	Injuries	Cherpitel CJ, Borges GLG, Wilcox HC. Acute alcohol use and suicidal behavior: a review of the literature. Alcohol Clin Exp Res 2004; 28: 18S–28S.
Alcohol use	Injuries	Cherpitel CJ, Ye Y, Bond J, et al. Alcohol Attributable Fraction for Injury Morbidity from the Dose-Response Relationship of Acute Alcohol Consumption: Emergency Department Data from 18 Countries. Addiction 2015; 110: 1724–32.
Alcohol use	Injuries	Devries KM, Mak JY, Bacchus LJ, et al. Intimate partner violence and incident depressive symptoms and suicide attempts: a systematic review of longitudinal studies. PLoS Med 2013; 10: e1001439.
Alcohol use	Injuries	Taylor B, Irving HM, Kanteres F, et al. The more you drink, the harder you fall: a systematic review and meta analysis of how acute alcohol consumption and injury or collision risk increase together. Drug Alcohol Depend 2010; 110: 108–16.
Alcohol use	Self-harm	Haw C, Hawton K, Casey D, Bale E, Shepherd A. Alcohol dependence, excessive drinking and deliberate self-harm: trends and patterns in Oxford, 1989-2002. Soc Psychiatry Psychiatr Epidemiol 2005; 40: 964–71.
Alcohol use	Interpersonal violence	Corrao G, Bagnardi V, Zambon A, La Vecchia C. A meta-analysis of alcohol consumption and the risk of 15 diseases. Prev Med 2004; 38: 613–9.
Alcohol use	Ischaemic and haemorrhagic stroke	Patra J, Taylor B, Irving H, et al. Alcohol consumption and the risk of morbidity and mortality for different stroke types--a systematic review and meta-analysis. BMC Public Health 2010; 10: 258.
Alcohol use	Ischaemic and haemorrhagic stroke	Ronksley PE, Brien SE, Turner BJ, Mukamal KJ, Ghali WA. Association of alcohol consumption with selected cardiovascular disease outcomes: a systematic review and meta-analysis. BMJ 2011; 342: d671.
Alcohol use	Ischaemic and haemorrhagic stroke	Bazzano LA, Gu D, Reynolds K, et al. Alcohol consumption and risk for stroke among Chinese men. Ann Neurol 2007; 62: 569–78.
Alcohol use	Ischaemic and haemorrhagic stroke	Berger K, Ajani UA, Kase CS, et al. Light-to-moderate alcohol consumption and the risk of stroke among U.S. male physicians. N Engl J Med 1999; 341: 1557–64.
Alcohol use	Ischaemic and haemorrhagic stroke	Chiuve SE, Rexrode KM, Spiegelman D, Logroscino G, Manson JE, Rimm EB. Primary prevention of stroke by healthy lifestyle. Circulation 2008; 118: 947–54.
Alcohol use	Ischaemic and haemorrhagic stroke	Djoussé L, Ellison RC, Beiser A, Scaramucci A, D'Agostino RB, Wolf PA. Alcohol consumption and risk of ischemic stroke: The Framingham Study. Stroke 2002; 33: 907–12.
Alcohol use	Ischaemic and haemorrhagic stroke	Donahue RP, Abbott RD, Reed DM, Yano K. Alcohol and hemorrhagic stroke. The Honolulu Heart Program. JAMA 1986; 255: 2311–4.
Alcohol use	Ischaemic and haemorrhagic stroke	Elkind MSV, Sciacca R, Boden-Albala B, Rundek T, Paik MC, Sacco RL. Moderate alcohol consumption reduces risk of ischemic stroke: the Northern Manhattan Study. Stroke 2006; 37: 13–9.
Alcohol use	Ischaemic and haemorrhagic stroke	Hansagi H, Romelsjö A, Gerhardsson de Verdier M, Andréasson S, Leifman A. Alcohol consumption and stroke mortality. 20-year follow-up of 15,077 men and women. Stroke 1995; 26: 1768–73.
Alcohol use	Ischaemic and haemorrhagic stroke	Higashiyama A, Wakabayashi I, Ono Y, et al. Association with serum gamma-glutamyltransferase levels and alcohol consumption on stroke and coronary artery disease: the Suita study. Stroke 2011; 42: 1764–7.
Alcohol use	Ischaemic and haemorrhagic stroke	Ikehara S, Iso H, Toyoshima H, et al. Alcohol consumption and mortality from stroke and coronary heart disease among Japanese men and women: the Japan collaborative cohort study. Stroke 2008; 39: 2936–42.
Alcohol use	Ischaemic and haemorrhagic stroke	Ikehara S, Iso H, Yamagishi K, et al. Alcohol consumption and risk of stroke and coronary heart disease among Japanese women: the Japan Public Health Center-based prospective study. Prev Med 2013; 57: 505–10.
Alcohol use	Ischaemic and haemorrhagic stroke	Ikehara S, Iso H, Yamagishi K, et al. Alcohol consumption, social support, and risk of stroke and coronary heart disease among Japanese men: the JPHC Study. Alcohol Clin Exp Res 2009; 33: 1025–32.
Alcohol use	Ischaemic and haemorrhagic stroke	Iso H, Kitamura A, Shimamoto T, et al. Alcohol intake and the risk of cardiovascular disease in middle-aged Japanese men. Stroke 1995; 26: 767–73.
Alcohol use	Ischaemic and haemorrhagic stroke	Jones SB, Loehr L, Avery CL, et al. Midlife Alcohol Consumption and the Risk of Stroke in the Atherosclerosis Risk in Communities Study. Stroke 2015; 46: 3124–30.
Alcohol use	Ischaemic and haemorrhagic stroke	Klatsky AL, Armstrong MA, Friedman GD, Sidney S. Alcohol drinking and risk of hemorrhagic stroke. Neuroepidemiology 2002; 21: 115–22.
Alcohol use	Ischaemic and haemorrhagic stroke	Klatsky AL, Armstrong MA, Friedman GD, Sidney S. Alcohol drinking and risk of hospitalization for ischemic stroke. Am J Cardiol 2001; 88: 703–6.
Alcohol use	Ischaemic and haemorrhagic stroke	Klatsky AL, Armstrong MA, Friedman GD. Alcohol use and subsequent cerebrovascular disease hospitalizations. Stroke 1989; 20: 741–6.
Alcohol use	Ischaemic and haemorrhagic stroke	Kono S, Ikeda M, Tokudome S, Nishizumi M, Kuratsune M. Alcohol and mortality: a cohort study of male Japanese physicians. Int J Epidemiol 1986; 15: 527–32.
Alcohol use	Ischaemic and haemorrhagic stroke	Leppälä JM, Paunio M, Virtamo J, et al. Alcohol consumption and stroke incidence in male smokers. Circulation 1999; 100: 1209–14.
Alcohol use	Ischaemic and haemorrhagic stroke	Mukamal KJ, Ascherio A, Mittleman MA, et al. Alcohol and risk for ischemic stroke in men: the role of drinking patterns and usual beverage. Ann Intern Med 2005; 142: 11–9.
Alcohol use	Ischaemic and haemorrhagic stroke	Mukamal KJ, Chung H, Jenny NS, et al. Alcohol use and risk of ischemic stroke among older adults: the cardiovascular health study. Stroke 2005; 36: 1830–4.
Alcohol use	Ischaemic and haemorrhagic stroke	Nielsen NR, Truelsen T, Barefoot JC, et al. Is the effect of alcohol on risk of stroke confined to highly stressed persons? Neuroepidemiology 2005; 25: 105–13.

Appendix Table 2. Supplemental information for Figure 1: Epidemiological evidence supporting causality between risk-outcome pairs included in the Global Burden of Disease 2016 study including 2A. Citations and 2B. Additional information
6A. Citations

Risk	Outcome	Citation/Note
Alcohol use	Ischaemic and haemorrhagic stroke	Sankai T, Iso H, Shimamoto T, et al. Prospective study on alcohol intake and risk of subarachnoid hemorrhage among Japanese men and women. Alcohol Clin Exp Res 2000; 24: 386–9.
Alcohol use	Ischaemic and haemorrhagic stroke	Stampfer MJ, Colditz GA, Willett WC, Speizer FE, Hennekens CH. A prospective study of moderate alcohol consumption and the risk of coronary disease and stroke in women. N Engl J Med 1988; 319: 267–73.
Alcohol use	Ischaemic and haemorrhagic stroke	Suh I, Jee SH, Kim HC, Nam CM, Kim IS, Appel LJ. Low serum cholesterol and haemorrhagic stroke in men: Korea Medical Insurance Corporation Study. Lancet 2001; 357: 922–5.
Alcohol use	Ischaemic and haemorrhagic stroke	Yamada S, Koizumi A, Iso H, et al. Risk factors for fatal subarachnoid hemorrhage: the Japan Collaborative Cohort Study. Stroke 2003; 34: 2781–7.
Drug use	Hepatitis B	Blomé MA, Björkman P, Flamholc L, Jacobsson H, Molnegren V, Widell A. Minimal transmission of HIV despite persistently high transmission of hepatitis C virus in a Swedish needle exchange program. J Viral Hepat 2011; 18: 831–9.
Drug use	Hepatitis B	Crofts N, Aitken CK. Incidence of bloodborne virus infection and risk behaviours in a cohort of injecting drug users in Victoria, 1990-1995. Med J Aust 1997; 167: 17–20.
Drug use	Hepatitis B	Hagan H, McGough JP, Thiede H, Weiss NS, Hopkins S, Alexander ER. Syringe Exchange and Risk of Infection with Hepatitis B and C Viruses, Am. J. Epi. 1999; 149(3): 203–213.
Drug use	Hepatitis B	Jackson JB, Wei L, Liping F, et al. Prevalence and seroincidence of hepatitis B and hepatitis C infection in high risk people who inject drugs in china and Thailand. Hepat Res Treat 2014; 2014: 296958.
Drug use	Hepatitis B	Månsson AS, Moestrup T, Nordenfelt E, Widell A. Continued transmission of hepatitis B and C viruses, but no transmission of human immunodeficiency virus among intravenous drug users participating in a syringe/needle exchange program. Scand J Infect Dis 2000; 32: 253–8.
Drug use	Hepatitis C	Abou-Saleh M, Davis P, Rice P, et al. The effectiveness of behavioural interventions in the primary prevention of hepatitis C amongst injecting drug users: a randomised controlled trial and lessons learned. Harm Reduct J 2008; 5: 25.
Drug use	Hepatitis C	Blomé MA, Björkman P, Flamholc L, Jacobsson H, Molnegren V, Widell A. Minimal transmission of HIV despite persistently high transmission of hepatitis C virus in a Swedish needle exchange program. J Viral Hepat 2011; 18: 831–9.
Drug use	Hepatitis C	Craine N, Hickman M, Parry JV, et al. Incidence of hepatitis C in drug injectors: the role of homelessness, opiate substitution treatment, equipment sharing, and community size. Epidemiol Infect 2009; 137: 1255–65.
Drug use	Hepatitis C	Crofts N, Aitken CK. Incidence of bloodborne virus infection and risk behaviours in a cohort of injecting drug users in Victoria, 1990-1995. Med J Aust 1997; 167: 17–20.
Drug use	Hepatitis C	Foley SB, Abou-Saleh MT. Risk Behaviors and Transmission of Hepatitis C in Injecting Drug Users. Addictive Disorders & Their Treatment 2009; 8: 13–21.
Drug use	Hepatitis C	Grebely J, Lima VD, Marshall BDL, et al. Declining incidence of hepatitis C virus infection among people who inject drugs in a Canadian setting, 1996-2012. PLoS ONE 2014; 9: e97726.
Drug use	Hepatitis C	Hagan H, McGough JP, Thiede H, Weiss NS, Hopkins S, Alexander ER. Syringe exchange and risk of infection with hepatitis B and C viruses. Am J Epidemiol 1999; 149: 203–13.
Drug use	Hepatitis C	Jackson JB, Wei L, Liping F, et al. Prevalence and seroincidence of hepatitis B and hepatitis C infection in high risk people who inject drugs in china and Thailand. Hepat Res Treat 2014; 2014: 296958.
Drug use	Hepatitis C	Lucidarme D, Bruandet A, Ilef D, et al. Incidence and risk factors of HCV and HIV infections in a cohort of intravenous drug users in the North and East of France. Epidemiol Infect 2004; 132: 699–708.
Drug use	Hepatitis C	Maher L, Jalaludin B, Chant KG, et al. Incidence and risk factors for hepatitis C seroconversion in injecting drug users in Australia. Addiction 2006; 101: 1499–508.
Drug use	Hepatitis C	Månsson AS, Moestrup T, Nordenfelt E, Widell A. Continued transmission of hepatitis B and C viruses, but no transmission of human immunodeficiency virus among intravenous drug users participating in a syringe/needle exchange program. Scand J Infect Dis 2000; 32: 253–8.
Drug use	Hepatitis C	Partanen A, Malin K, Perälä R, Harju O, Holopainen A, Holmström P, et al. Riski-tutkimus 2000-2003. Pistämällä huumeita käyttävien seurantatutkimus. A-Klinikkasäätiön Raporttisarja nro 52. Helsinki: A-Klinikkasäätiön, 2006.
Drug use	Hepatitis C	Roy KM, Goldberg D, Taylor A, et al. A method to detect the incidence of hepatitis C infection among injecting drug users in Glasgow 1993-98. J Infect 2001; 43: 200–5.
Drug use	Hepatitis C	Turner KME, Hutchinson S, Vickerman P, et al. The impact of needle and syringe provision and opiate substitution therapy on the incidence of hepatitis C virus in injecting drug users: pooling of UK evidence. Addiction 2011; 106: 1978–88.
Drug use	Hepatitis C	Van Den Berg C, Smit C, Van Brussel G, Coutinho R, Prins M, Amsterdam Cohort. Full participation in harm reduction programmes is associated with decreased risk for human immunodeficiency virus and hepatitis C virus: evidence from the Amsterdam Cohort Studies among drug users. Addiction 2007; 102: 1454–62.
Drug use	Hepatitis C	Villano SA, Vlahov D, Nelson KE, Lyles CM, Cohn S, Thomas DL. Incidence and risk factors for hepatitis C among injection drug users in Baltimore, Maryland. J Clin Microbiol 1997; 35: 3274–7.
Diet low in fruits	Lip and oral cavity cancer	Key TJ. Fruit and vegetables and cancer risk. British Journal of Cancer 2011; 104: 6–11.
Diet low in fruits	Lip and oral cavity cancer	Jin, Jian, Zhiguo Ouyang, and Zhaoyan Wang. 2014. "Association of Fruit and Vegetables with the Risk of Nasopharyngeal Cancer: Evidence from a Meta-Analysis." Scientific Reports4 (July): srep05229. doi:10.1038/srep05229.
Diet low in fruits	Lip and oral cavity cancer	Pavia, Maria, Claudia Pileggi, Carmelo GA Nobile, and Italo F. Angelillo. 2006. "Association between Fruit and Vegetable Consumption and Oral Cancer: A Meta-Analysis of Observational Studies." The American Journal of Clinical Nutrition83 (5): 1126–34.
Diet low in fruits	Nasopharynx cancer	Key TJ. Fruit and vegetables and cancer risk. British Journal of Cancer 2011; 104: 6–11.

Appendix Table 2. Supplemental information for Figure 1: Epidemiological evidence supporting causality between risk-outcome pairs included in the Global Burden of Disease 2016 study including 2A. Citations and 2B. Additional information<br>6A. Citations		
**Risk**	**Outcome**	**Citation/Note**
Diet low in fruits	Nasopharynx cancer	American Institute for Cancer Research, and World Cancer Research Fund, eds. 2007. Food, Nutrition, Physical Activity and the Prevention of Cancer: A Global Perspective: A Project of World Cancer Research Fund International. Washington, D.C: American Institute for Cancer Research.
Diet low in fruits	Nasopharynx cancer	Pavia, Maria, Claudia Pileggi, Carmelo GA Nobile, and Italo F. Angelillo. 2006. "Association between Fruit and Vegetable Consumption and Oral Cancer: A Meta-Analysis of Observational Studies." The American Journal of Clinical Nutrition83 (5): 1126–34.
Diet low in fruits	Other pharynx cancer	Key TJ. Fruit and vegetables and cancer risk. British Journal of Cancer 2011; 104: 6–11.
Diet low in fruits	Other pharynx cancer	American Institute for Cancer Research, and World Cancer Research Fund, eds. 2007. Food, Nutrition, Physical Activity and the Prevention of Cancer: A Global Perspective: A Project of World Cancer Research Fund International. Washington, D.C: American Institute for Cancer Research.
Diet low in fruits	Other pharynx cancer	Pavia, Maria, Claudia Pileggi, Carmelo GA Nobile, and Italo F. Angelillo. 2006. "Association between Fruit and Vegetable Consumption and Oral Cancer: A Meta-Analysis of Observational Studies." The American Journal of Clinical Nutrition83 (5): 1126–34.
Diet low in fruits	Larynx cancer	Key TJ. Fruit and vegetables and cancer risk. British Journal of Cancer 2011; 104: 6–11.
Diet low in fruits	Larynx cancer	American Institute for Cancer Research, and World Cancer Research Fund, eds. 2007. Food, Nutrition, Physical Activity and the Prevention of Cancer: A Global Perspective: A Project of World Cancer Research Fund International. Washington, D.C: American Institute for Cancer Research.
Diet low in fruits	Larynx cancer	Pavia, Maria, Claudia Pileggi, Carmelo GA Nobile, and Italo F. Angelillo. 2006. "Association between Fruit and Vegetable Consumption and Oral Cancer: A Meta-Analysis of Observational Studies." The American Journal of Clinical Nutrition83 (5): 1126–34.
Diet low in fruits	Oesophageal cancer	Liu J, Wang J, Leng Y, Lv C. Intake of fruit and vegetables and risk of esophageal squamous cell carcinoma: a meta-analysis of observational studies. Int J Cancer 2013; 133: 473–85.
Diet low in fruits	Tracheal, bronchus and lung cancer	Vieira AR, Abar L, Vingeliene S, et al. Fruits, vegetables and lung cancer risk: a systematic review and meta-analysis. Ann Oncol 2016; 27: 81–96.
Diet low in fruits	Ischaemic heart disease	Wang X, Ouyang Y, Liu J, et al. Fruit and vegetable consumption and mortality from all causes, cardiovascular disease, and cancer: systematic review and dose-response meta-analysis of prospective cohort studies. BMJ 2014; 349: g4490.
Diet low in fruits	Ischaemic stroke	Hu D, Huang J, Wang Y, Zhang D, Qu Y. Fruits and vegetables consumption and risk of stroke: a meta-analysis of prospective cohort studies. Stroke 2014; 45: 1613–9.
Diet low in fruits	Hemorrhagic stroke	Hu D, Huang J, Wang Y, Zhang D, Qu Y. Fruits and vegetables consumption and risk of stroke: a meta-analysis of prospective cohort studies. Stroke 2014; 45: 1613–9.
Diet low in fruits	Diabetes	Li M, Fan Y, Zhang X, Hou W, Tang Z. Fruit and vegetable intake and risk of type 2 diabetes mellitus: meta-analysis of prospective cohort studies. BMJ open 2014; 4(11): e005497.
Diet low in vegetables	Oesophageal cancer	Liu J, Wang J, Leng Y, Lv C. Intake of fruit and vegetables and risk of esophageal squamous cell carcinoma: a meta-analysis of observational studies. Int J Cancer 2013; 133: 473–85.
Diet low in vegetables	Ischaemic heart disease	Wang X, Ouyang Y, Liu J, et al. Fruit and vegetable consumption and mortality from all causes, cardiovascular disease, and cancer: systematic review and dose-response meta-analysis of prospective cohort studies. BMJ 2014; 349: g4490.
Diet low in vegetables	Ischaemic stroke	Hu D, Huang J, Wang Y, Zhang D, Qu Y. Fruits and vegetables consumption and risk of stroke: a meta-analysis of prospective cohort studies. Stroke 2014; 45: 1613–9.
Diet low in vegetables	Hemorrhagic stroke	Hu D, Huang J, Wang Y, Zhang D, Qu Y. Fruits and vegetables consumption and risk of stroke: a meta-analysis of prospective cohort studies. Stroke 2014; 45: 1613–9.
Diet low in whole grains	Diabetes	Aune D, Norat T, Romundstad P, Vatten LJ. Whole grain and refined grain consumption and the risk of type 2 diabetes: a systematic review and dose-response meta-analysis of cohort studies. Eur J Epidemiol 2013; 28: 845–58.
Diet low in whole grains	Ischaemic heart disease	Aune D, Keum N, Giovannucci E, et al. Whole grain consumption and risk of cardiovascular disease, cancer, and all cause and cause specific mortality: systematic review and dose-response meta-analysis of prospective studies. BMJ 2016; 353: i2716.
Diet low in nuts and seeds	Ischaemic heart disease	Afshin A, Micha R, Khatibzadeh S, Mozaffarian D. Consumption of nuts and legumes and risk of incident ischemic heart disease, stroke, and diabetes: a systematic review and meta-analysis. Am J Clin Nutr 2014; 100: 278–88.
Diet low in nuts and seeds	Diabetes	Afshin A, Micha R, Khatibzadeh S, Mozaffarian D. Consumption of nuts and legumes and risk of incident ischemic heart disease, stroke, and diabetes: a systematic review and meta-analysis. Am J Clin Nutr 2014; 100: 278–88.
Diet low in milk	Colon and rectum cancer	World Cancer Research Fund, American Institute for Cancer Research, Imperial College London. WCRF/AICR Systematic Literature Review Continuous Update Project Report: The Associations between Food, Nutrition and Physical Activity and the Risk of Colorectal Cancer. Oct 2010.
Diet high in red meat	Colon and rectum cancer	World Cancer Research Fund, American Institute for Cancer Research, Imperial College London. WCRF/AICR Systematic Literature Review Continuous Update Project Report: The Associations between Food, Nutrition and Physical Activity and the Risk of Colorectal Cancer. Oct 2010.
Diet high in red meat	Diabetes	Pan A, Sun Q, Bernstein AM, et al. Red meat consumption and risk of type 2 diabetes: 3 cohorts of US adults and an updated meta-analysis. Am J Clin Nutr 2011; 94: 1088–96.
Diet high in processed meat	Colon and rectum cancer	World Cancer Research Fund, American Institute for Cancer Research, Imperial College London. WCRF/AICR Systematic Literature Review Continuous Update Project Report: The Associations between Food, Nutrition and Physical Activity and the Risk of Colorectal Cancer. Oct 2010.
Diet high in processed meat	Ischaemic heart disease	Micha R, Wallace SK, Mozaffarian D. Red and processed meat consumption and risk of incident coronary heart disease, stroke, and diabetes mellitus: a systematic review and meta-analysis. Circulation 2010; 121: 2271–83.
Diet high in processed meat	Diabetes	Pan A, Sun Q, Bernstein AM, et al. Red meat consumption and risk of type 2 diabetes: 3 cohorts of US adults and an updated meta-analysis. Am J Clin Nutr 2011; 94: 1088–96.

Appendix Table 2. Supplemental information for Figure 1: Epidemiological evidence supporting causality between risk-outcome pairs included in the Global Burden of Disease 2016 study including 2A. Citations and 2B. Additional information
6A. Citations

Risk	Outcome	Citation/Note
Diet high in sugar-sweetened beverages and high body-mass index	n/a	Malik VS, Pan A, Willett WC, Hu FB. Sugar-sweetened beverages and weight gain in children and adults: a systematic review and meta-analysis. Am J Clin Nutr 2013; 98: 1084–102.
Diet low fibre	Colon and rectum cancer	World Cancer Research Fund, American Institute for Cancer Research, Imperial College London. WCRF/AICR Systematic Literature Review Continuous Update Project Report: The Associations between Food, Nutrition and Physical Activity and the Risk of Colorectal Cancer. Oct 2010.
Diet low fibre	Ischaemic heart disease	Threapleton DE, Greenwood DC, Evans CE, et al. Dietary fibre intake and risk of cardiovascular disease: systematic review and meta-analysis. BMJ (Clinical research ed) 2013; 347: f6879.
Diet low in calcium	Colon and rectum cancer	World Cancer Research Fund, American Institute for Cancer Research, Imperial College London. WCRF/AICR Systematic Literature Review Continuous Update Project Report: The Associations between Food, Nutrition and Physical Activity and the Risk of Colorectal Cancer. Oct 2010.
Diet low in seafood omega-3 fats	Ischaemic heart disease	Chowdhury R, Stevens S, Gorman D, et al. Association between fish consumption, long chain omega 3 fatty acids, and risk of cerebrovascular disease: systematic review and meta-analysis. BMJ (Clinical research ed) 2012; 345: e6698.
Diet low in polyunsaturated fats	Ischaemic heart disease	Farvid MS, Ding M, Pan A, et al. Dietary linoleic acid and risk of coronary heart disease: a systematic review and meta-analysis of prospective cohort studies. Circulation 2014; 130: 1568–78.
Diet low in polyunsaturated fats	Ischaemic heart disease	Mozaffarian D, Micha R, Wallace S. Effects on coronary heart disease of increasing polyunsaturated fat in place of saturated fat: a systematic review and meta-analysis of randomized controlled trials. PLoS Med 2010; 7: e1000252.
Diet high in trans fats	Ischaemic heart disease	Mozaffarian D, Clarke R. Quantitative effects on cardiovascular risk factors and coronary heart disease risk of replacing partially hydrogenated vegetable oils with other fats and oils. Eur J Clin Nutr. 2009; 63(Suppl 2): S22-33.
Diet high in trans fats	Ischaemic heart disease	http://www.bmj.com/content/bmj/suppl/2015/08/11/bmj.h3978.DC1/sour025275.ww2_default.pdf; pg. 44
Diet high in sodium and high systolic blood pressure	n/a	Aburto NJ, Ziolkovska A, Hooper L, Elliott P, Cappuccio FP, Meerpohl JJ. Effect of lower sodium intake on health: systematic review and meta-analyses. BMJ 2013; 346: f1326.
Diet high in sodium	Stomach cancer	World Cancer Research Fund, American Institute for Cancer Research. Food, Nutrition, Physical Activity, and the Prevention of Cancer: a Global Perspective. Washington DC: AICR, 2007.
Diet high in sodium	Stomach cancer	D'Elia, Lanfranco, Giovanni Rossi, Renato Ippolito, Francesco P. Cappuccio, and Pasquale Strazzullo. 2012. "Habitual Salt Intake and Risk of Gastric Cancer: A Meta-Analysis of Prospective Studies." Clinical Nutrition31 (4): 489–98. doi:10.1016/j.clnu.2012.01.003.
Childhood sexual abuse	Major depressive disorder	Brown J, Cohen P, Johnson JG, Smailes EM. Childhood abuse and neglect: specificity of effects on adolescent and young adult depression and suicidality. J Am Acad Child Adolesc Psychiatry 1999; 38: 1490–6.
Childhood sexual abuse	Major depressive disorder	Chapman, D.P., Whitfield, C.L., Felitti, V.J., Dube, S.R., Edwards, V.J. and Anda, R.F., 2004. Adverse childhood experiences and the risk of depressive disorders in adulthood. Journal of affective disorders, 82(2), pp.217-225.
Childhood sexual abuse	Major depressive disorder	Cheasty, M., Clare, A.W. and Collins, C., 1998. Relation between sexual abuse in childhood and adult depression: case-control study. Bmj, 316(7126), pp.198-201.
Childhood sexual abuse	Major depressive disorder	Dinwiddie S, Heath AC, Dunne MP, Bucholz KK, Madden PA, Slutske WS, Bierut LJ, Statham DB, Martin NG. Early sexual abuse and lifetime psychopathology: a co-twin-control study. Psychol Med. 2000; 30(1): 41–52.
Childhood sexual abuse	Major depressive disorder	Dube, S.R., Anda, R.F., Whitfield, C.L., Brown, D.W., Felitti, V.J., Dong, M. and Giles, W.H., 2005. Long-term consequences of childhood sexual abuse by gender of victim. American journal of preventive medicine, 28(5), pp.430-438.
Childhood sexual abuse	Major depressive disorder	Ernst C, Angst J, Földényi M. The Zurich Study. XVII. Sexual abuse in childhood. Frequency and relevance for adult morbidity data of a longitudinal epidemiological study. Eur Arch Psychiatry Clin Neurosci. 1993; 242(5): 293–300.
Childhood sexual abuse	Major depressive disorder	Jaffee SR, Moffitt TE, Caspi A, Fombonne E, Poulton R, Martin J. Differences in early childhood risk factors for juvenile-onset and adult-onset depression. Arch Gen Psychiatry. 2002; 59(3): 215-22.
Childhood sexual abuse	Major depressive disorder	Kendler KS, Bulik CM, Silberg J, Hettema JM, Myers J, Prescott CA. Childhood sexual abuse and adult psychiatric and substance use disorders in women: an epidemiological and cotwin control analysis. Arch Gen Psychiatry. 2000; 57(10): 953–9.
Childhood sexual abuse	Major depressive disorder	Molnar, B.E., Buka, S.L. and Kessler, R.C., 2001. Child sexual abuse and subsequent psychopathology: results from the National Comorbidity Survey. American journal of public health, 91(5), p.753.
Childhood sexual abuse	Major depressive disorder	Nelson EC, Heath AC, Madden PA, Cooper ML, Dinwiddie SH, Bucholz KK, Glowinski A, McLaughlin T, Dunne MP, Statham DJ, Martin NG. Association between self-reported sexual abuse and adverse psychosocial outcomes: results from a twin study. Arch Gen Psychiatry. 2002; 59(2): 139-45.
Childhood sexual abuse	Major depressive disorder	Peleikis, D.E., Mykletun, A. and Dahl, A.A., 2004. The relative influence of childhood sexual abuse and other family background risk factors on adult adversities in female outpatients treated for anxiety disorders and depression. Child Abuse & Neglect, 28(1), pp.61-76.
Childhood sexual abuse	Major depressive disorder	Silverman, A.B., Reinherz, H.Z. and Giaconia, R.M., 1996. The long-term sequelae of child and adolescent abuse: A longitudinal community study. Child abuse & neglect, 20(8), pp.709-723.
Childhood sexual abuse	Major depressive disorder	Widom, C.S., DuMont, K. and Czaja, S.J., 2007. A prospective investigation of major depressive disorder and comorbidity in abused and neglected children grown up. Archives of general psychiatry, 64(1), pp.49-56.
Childhood sexual abuse	Alcohol use disorders	Dinwiddie S, Heath AC, Dunne MP, Bucholz KK, Madden PA, Slutske WS, Bierut LJ, Statham DB, Martin NG. Early sexual abuse and lifetime psychopathology: a co-twin-control study. Psychol Med. 2000; 30(1): 41–52.

Appendix Table 2. Supplemental information for Figure 1: Epidemiological evidence supporting causality between risk-outcome pairs included in the Global Burden of Disease 2016 study including 2A. Citations and 2B. Additional information
6A. Citations

Risk	Outcome	Citation/Note
Childhood sexual abuse	Alcohol use disorders	Dube, S.R., Anda, R.F., Whitfield, C.L., Brown, D.W., Felitti, V.J., Dong, M. and Giles, W.H., 2005. Long-term consequences of childhood sexual abuse by gender of victim. American journal of preventive medicine, 28(5), pp.430-438.
Childhood sexual abuse	Alcohol use disorders	Fleming, J., Mullen, P.E., Sibthorpe, B., Attewell, R. and Bammer, G., 1998. The relationship between childhood sexual abuse and alcohol abuse in women-a case-control study. Addiction, 93(12), pp.1787-1798.
Childhood sexual abuse	Alcohol use disorders	Kendler KS, Bulik CM, Silberg J, Hettema JM, Myers J, Prescott CA. Childhood sexual abuse and adult psychiatric and substance use disorders in women: an epidemiological and cotwin control analysis. Arch Gen Psychiatry. 2000; 57(10): 953–9.
Childhood sexual abuse	Alcohol use disorders	Molnar, B.E., Buka, S.L. and Kessler, R.C., 2001. Child sexual abuse and subsequent psychopathology: results from the National Comorbidity Survey. American journal of public health, 91(5), p.753.
Childhood sexual abuse	Alcohol use disorders	Nelson EC, Heath AC, Madden PA, Cooper ML, Dinwiddie SH, Bucholz KK, Glowinski A, McLaughlin T, Dunne MP, Statham DJ, Martin NG. Association between self-reported sexual abuse and adverse psychosocial outcomes: results from a twin study. Arch Gen Psychiatry. 2002; 59(2): 139-45.
Childhood sexual abuse	Alcohol use disorders	Sartor CE, Lynskey MT, Bucholz KK, McCutcheon VV, Nelson EC, Waldron M, Heath AC. Childhood sexual abuse and the course of alcohol dependence development: findings from a female twin sample. Drug Alcohol Depend. 2007; 89(2-3): 139–44.
Childhood sexual abuse	Alcohol use disorders	Silverman, A.B., Reinherz, H.Z. and Giaconia, R.M., 1996. The long-term sequelae of child and adolescent abuse: A longitudinal community study. Child abuse & neglect, 20(8), pp.709-723.
Intimate partner violence	HIV/AIDS	Jewkes RK, Dunkle K, Nduna M, Shai N. Intimate partner violence, relationship power inequity, and incidence of HIV infection in young women in South Africa: a cohort study. Lancet 2010; 376: 41–8.
Intimate partner violence	HIV/AIDS	Kouyoumdjian FG, Calzavara LM, Bondy SJ, et al. Intimate partner violence is associated with incident HIV infection in women in Uganda. AIDS 2013; 27: 1331–8.
Intimate partner violence	Major depressive disorder	Chowdhary N, Patel V. The effect of spousal violence on women’s health: findings from the Stree Arogya Shodh in Goa, India. J Postgrad Med. 2008; 54(4): 306–12.
Intimate partner violence	Major depressive disorder	Lipsky S, Caetano R, Roy-Byrne P. Racial and ethnic disparities in police-reported intimate partner violence and risk of hospitalization among women. Womens Health Issues. 2009; 19(2):109–118.
Intimate partner violence	Major depressive disorder	Ouellet-Morin I, Fisher HL, York-Smith M, Fincham-Campbell S, Moffitt TE, Arseneault L. Intimate partner violence and new-onset depression: a longitudinal study of women's childhood and adult histories of abuse. Depression and anxiety. 2015;32(5):316-324.
Intimate partner violence	Major depressive disorder	Suglia SF, Duarte CS, Sandel MT. Housing quality, housing instability, and maternal mental health. J Urban Health. 2011; 88(6): 1105–16.
Intimate partner violence	Induced abortion	Bourassa D, Bérubé J. The prevalence of intimate partner violence among women and teenagers seeking abortion compared with those continuing pregnancy. J Obstet Gynaecol Can 2007; 29: 415–23.
Intimate partner violence	Induced abortion	Leung TW, Leung WC, Chan PL, Ho PC. A comparison of the prevalence of domestic violence between patients seeking termination of pregnancy and other general gynecology patients. Int J Gynaecol Obstet 2002; 77: 47–54.
Intimate partner violence	Induced abortion	Romito P, Escribà-Agüir V, Pomicino L, Lucchetta C, Scrimin F, Molzan Turan J. Violence in the lives of women in Italy who have an elective abortion. Womens Health Issues 2009; 19: 335–43.
Intimate partner violence	Induced abortion	Taft AJ, Watson LF. Termination of pregnancy: associations with partner violence and other factors in a national cohort of young Australian women. Aust N Z J Public Health 2007; 31: 135–42.
Low physical activity	Colon and rectum cancer	Bostick RM, Potter JD, Kushi LH, et al. Sugar, meat, and fat intake, and non-dietary risk factors for colon cancer incidence in Iowa women (United States). Cancer Causes Control 1994; 5: 38–52.
Low physical activity	Colon and rectum cancer	Calton BA, Lacey JV, Schatzkin A, et al. Physical activity and the risk of colon cancer among women: a prospective cohort study (United States). Int J Cancer 2006; 119: 385–91.
Low physical activity	Colon and rectum cancer	Chao A, Connell CJ, Jacobs EJ, et al. Amount, type, and timing of recreational physical activity in relation to colon and rectal cancer in older adults: the Cancer Prevention Study II Nutrition Cohort. Cancer Epidemiol Biomarkers Prev 2004; 13: 2187–95.
Low physical activity	Colon and rectum cancer	Colbert LH, Hartman TJ, Malila N, et al. Physical activity in relation to cancer of the colon and rectum in a cohort of male smokers. Cancer Epidemiol Biomarkers Prev 2001; 10: 265–8.
Low physical activity	Colon and rectum cancer	Fraser G, Pearce N. Occupational physical activity and risk of cancer of the colon and rectum in New Zealand males. Cancer Causes Control 1993; 4: 45–50.
Low physical activity	Colon and rectum cancer	Friedenreich C, Norat T, Steindorf K, et al. Physical activity and risk of colon and rectal cancers: the European prospective investigation into cancer and nutrition. Cancer Epidemiol Biomarkers Prev 2006; 15: 2398–407.
Low physical activity	Colon and rectum cancer	Garabrant DH, Peters JM, Mack TM, Bernstein L. Job activity and colon cancer risk. Am J Epidemiol 1984; 119: 1005–14.
Low physical activity	Colon and rectum cancer	Gerhardsson M, Norell SE, Kiviranta H, Pedersen NL, Ahlbom A. Sedentary jobs and colon cancer. Am J Epidemiol 1986; 123: 775–80.
Low physical activity	Colon and rectum cancer	Giovannucci E, Ascherio A, Rimm EB, Colditz GA, Stampfer MJ, Willett WC. Physical activity, obesity, and risk for colon cancer and adenoma in men. Ann Intern Med 1995; 122: 327–34.
Low physical activity	Colon and rectum cancer	Howard RA, Freedman DM, Park Y, Hollenbeck A, Schatzkin A, Leitzmann MF. Physical activity, sedentary behavior, and the risk of colon and rectal cancer in the NIH-AARP Diet and Health Study. Cancer Causes Control 2008; 19: 939–53.
Low physical activity	Colon and rectum cancer	Larsson SC, Rutegård J, Bergkvist L, Wolk A. Physical activity, obesity, and risk of colon and rectal cancer in a cohort of Swedish men. Eur J Cancer 2006; 42: 2590–7.
Low physical activity	Colon and rectum cancer	Lee IM, Manson JE, Ajani U, Paffenbarger RS, Hennekens CH, Buring JE. Physical activity and risk of colon cancer: the Physicians’ Health Study (United States). Cancer Causes Control 1997; 8: 568–74.
Low physical activity	Colon and rectum cancer	Lee IM, Paffenbarger RS. Physical activity and its relation to cancer risk: a prospective study of college alumni. Med Sci Sports Exerc 1994; 26: 831–7.

Appendix Table 2. Supplemental information for Figure 1: Epidemiological evidence supporting causality between risk-outcome pairs included in the Global Burden of Disease 2016 study including 2A. Citations and 2B. Additional information
6A. Citations

Risk	Outcome	Citation/Note
Low physical activity	Colon and rectum cancer	Lee K-J, Inoue M, Otani T, et al. Physical activity and risk of colorectal cancer in Japanese men and women: the Japan Public Health Center-based prospective study. Cancer Causes Control 2007; 18: 199–209.
Low physical activity	Colon and rectum cancer	Mai PL, Sullivan-Halley J, Ursin G, et al. Physical activity and colon cancer risk among women in the California Teachers Study. Cancer Epidemiol Biomarkers Prev 2007; 16: 517–25.
Low physical activity	Colon and rectum cancer	Moradi T, Gridley G, Björk J, et al. Occupational physical activity and risk for cancer of the colon and rectum in Sweden among men and women by anatomic subsite. Eur J Cancer Prev 2008; 17: 201–8.
Low physical activity	Colon and rectum cancer	Nilsen TIL, Romundstad PR, Petersen H, Gunnell D, Vatten LJ. Recreational physical activity and cancer risk in subsites of the colon (the Nord-Trøndelag Health Study). Cancer Epidemiol Biomarkers Prev 2008; 17: 183–8.
Low physical activity	Colon and rectum cancer	Severson RK, Nomura AM, Grove JS, Stemmermann GN. A prospective analysis of physical activity and cancer. Am J Epidemiol 1989; 130: 522–9.
Low physical activity	Colon and rectum cancer	Thune I, Lund E. Physical activity and risk of colorectal cancer in men and women. Br J Cancer 1996; 73: 1134–40.
Low physical activity	Colon and rectum cancer	Wolin KY, Lee I-M, Colditz GA, Glynn RJ, Fuchs C, Giovannucci E. Leisure-time physical activity patterns and risk of colon cancer in women. Int J Cancer 2007; 121: 2776–81.
Low physical activity	Breast cancer	Bardia A, Hartmann LC, Vachon CM, et al. Recreational physical activity and risk of postmenopausal breast cancer based on hormone receptor status. Arch Intern Med 2006; 166: 2478–83.
Low physical activity	Breast cancer	Borch KB, Lund E, Braaten T, Weiderpass E. Physical activity and the risk of postmenopausal breast cancer - the Norwegian Women and Cancer Study. J Negat Results Biomed 2014; 13: 3.
Low physical activity	Breast cancer	Breslow RA, Ballard-Barbash R, Munoz K, Graubard BI. Long-term recreational physical activity and breast cancer in the National Health and Nutrition Examination Survey I epidemiologic follow-up study. Cancer Epidemiol Biomarkers Prev 2001; 10: 805–8.
Low physical activity	Breast cancer	Cerhan JR, Chiu BC, Wallace RB, et al. Physical activity, physical function, and the risk of breast cancer in a prospective study among elderly women. J Gerontol A Biol Sci Med Sci 1998; 53: M251-256.
Low physical activity	Breast cancer	Chang S-C, Ziegler RG, Dunn B, et al. Association of energy intake and energy balance with postmenopausal breast cancer in the prostate, lung, colorectal, and ovarian cancer screening trial. Cancer Epidemiol Biomarkers Prev 2006; 15: 334–41.
Low physical activity	Breast cancer	Colditz GA, Feskanich D, Chen WY, Hunter DJ, Willett WC. Physical activity and risk of breast cancer in premenopausal women. Br J Cancer 2003; 89: 847–51.
Low physical activity	Breast cancer	Dallal CM, Sullivan-Halley J, Ross RK, et al. Long-term recreational physical activity and risk of invasive and in situ breast cancer: the California teachers study. Arch Intern Med 2007; 167: 408–15.
Low physical activity	Breast cancer	Dorgan JF, Brown C, Barrett M, et al. Physical activity and risk of breast cancer in the Framingham Heart Study. Am J Epidemiol 1994; 139: 662–9.
Low physical activity	Breast cancer	Eliassen AH, Hankinson SE, Rosner B, Holmes MD, Willett WC. Physical activity and risk of breast cancer among postmenopausal women. Arch Intern Med 2010; 170: 1758–64.
Low physical activity	Breast cancer	Frisch RE, Wyshak G, Witschi J, Albright NL, Albright TE, Schiff I. Lower lifetime occurrence of breast cancer and cancers of the reproductive system among former college athletes. Int J Fertil 1987; 32: 217–25.
Low physical activity	Breast cancer	Hastert TA, Beresford SAA, Patterson RE, Kristal AR, White E. Adherence to WCRF/AICR cancer prevention recommendations and risk of postmenopausal breast cancer. Cancer Epidemiol Biomarkers Prev 2013; 22: 1498–508.
Low physical activity	Breast cancer	Hildebrand JS, Gapstur SM, Campbell PT, Gaudet MM, Patel AV. Recreational physical activity and leisure-time sitting in relation to postmenopausal breast cancer risk. Cancer Epidemiol Biomarkers Prev 2013; 22: 1906–12.
Low physical activity	Breast cancer	Howard RA, Leitzmann MF, Linet MS, Freedman DM. Physical activity and breast cancer risk among pre- and postmenopausal women in the U.S. Radiologic Technologists cohort. Cancer Causes Control 2009; 20: 323–33.
Low physical activity	Breast cancer	Leitzmann MF, Moore SC, Peters TM, et al. Prospective study of physical activity and risk of postmenopausal breast cancer. Breast Cancer Res 2008; 10: R92.
Low physical activity	Breast cancer	Luoto R, Latikka P, Pukkala E, Hakulinen T, Vihko V. The effect of physical activity on breast cancer risk: a cohort study of 30,548 women. Eur J Epidemiol 2000; 16: 973–80.
Low physical activity	Breast cancer	Margolis KL, Mucci L, Braaten T, et al. Physical activity in different periods of life and the risk of breast cancer: the Norwegian-Swedish Women's Lifestyle and Health cohort study. Cancer Epidemiol Biomarkers Prev 2005; 14: 27–32.
Low physical activity	Breast cancer	Mertens AJ, Sweeney C, Shahar E, Rosamond WD, Folsom AR. Physical activity and breast cancer incidence in middle-aged women: a prospective cohort study. Breast Cancer Res Treat 2006; 97: 209–14.
Low physical activity	Breast cancer	Ministry of Health (Benin), National Institute of Statistics and Economic Analysis (INSAE) (Benin). Benin Health Statistical Yearbook 2005. Porto-Novo, Benin: Ministry of Health (Benin), 2006.
Low physical activity	Breast cancer	Ministry of Health (Burkina Faso). Burkina Faso Health Statistical Yearbook 2007. Ouagadougou, Burkina Faso: Ministry of Health (Burkina Faso), 2008.
Low physical activity	Breast cancer	Ministry of Health (Burkina Faso). Burkina Faso Health Statistical Yearbook 2008. Ouagadougou, Burkina Faso: Ministry of Health (Burkina Faso), 2009.
Low physical activity	Breast cancer	Moradi T, Adami HO, Bergström R, et al. Occupational physical activity and risk for breast cancer in a nationwide cohort study in Sweden. Cancer Causes Control 1999; 10: 423–30.
Low physical activity	Breast cancer	Moradi T, Adami H-O, Ekbom A, et al. Physical activity and risk for breast cancer a prospective cohort study among Swedish twins. Int J Cancer 2002; 100: 76–81.
Low physical activity	Breast cancer	Peters TM, Schatzkin A, Gierach GL, et al. Physical activity and postmenopausal breast cancer risk in the NIH-AARP diet and health study. Cancer Epidemiol Biomarkers Prev 2009; 18: 289–96.
Low physical activity	Breast cancer	Pronk A, Ji B-T, Shu X-O, et al. Physical activity and breast cancer risk in Chinese women. Br J Cancer 2011; 105: 1443–50.

Appendix Table 2. Supplemental information for Figure 1: Epidemiological evidence supporting causality between risk-outcome pairs included in the Global Burden of Disease 2016 study including 2A. Citations and 2B. Additional information 6A. Citations		
**Risk**	**Outcome**	**Citation/Note**
Low physical activity	Breast cancer	Rintala by PE, Pukkala E, Paakkulainen HT, Vihko VJ. Self-experienced physical workload and risk of breast cancer. Scand J Work Environ Health 2002; 28: 158–62.
Low physical activity	Breast cancer	Rockhill B, Willett WC, Hunter DJ, Manson JE, Hankinson SE, Colditz GA. A prospective study of recreational physical activity and breast cancer risk. Arch Intern Med 1999; 159: 2290–6.
Low physical activity	Breast cancer	Rosenberg L, Palmer JR, Bethea TN, Ban Y, Kipping-Ruane K, Adams-Campbell LL. A prospective study of physical activity and breast cancer incidence in African-American women. Cancer Epidemiol Biomarkers Prev 2014; 23: 2522–31.
Low physical activity	Breast cancer	Sesso HD, Paffenbarger RS, Lee IM. Physical activity and breast cancer risk in the College Alumni Health Study (United States). Cancer Causes Control 1998; 9: 433–9.
Low physical activity	Breast cancer	Silvera SAN, Jain M, Howe GR, Miller AB, Rohan TE. Energy balance and breast cancer risk: a prospective cohort study. Breast Cancer Res Treat 2006; 97: 97–106.
Low physical activity	Breast cancer	Suzuki R, Iwasaki M, Yamamoto S, et al. Leisure-time physical activity and breast cancer risk defined by estrogen and progesterone receptor status--the Japan Public Health Center-based Prospective Study. Prev Med 2011; 52: 227–33.
Low physical activity	Breast cancer	Suzuki S, Kojima M, Tokudome S, et al. Effect of physical activity on breast cancer risk: findings of the Japan collaborative cohort study. Cancer Epidemiol Biomarkers Prev 2008; 17: 3396–401.
Low physical activity	Breast cancer	Thune I, Brenn T, Lund E, Gaard M. Physical activity and the risk of breast cancer. N Engl J Med 1997; 336: 1269–75.
Low physical activity	Breast cancer	Wyrwich KW, Wolinsky FD. Physical activity, disability, and the risk of hospitalization for breast cancer among older women. J Gerontol A Biol Sci Med Sci 2000; 55: M418-421.
Low physical activity	Breast cancer	Wyshak G, Frisch RE. Breast cancer among former college athletes compared to non-athletes: a 15-year follow-up. Br J Cancer 2000; 82: 726–30.
Low physical activity	Ischaemic stroke	Abbott RD, Rodriguez BL, Burchfiel CM, Curb JD. Physical activity in older middle-aged men and reduced risk of stroke: the Honolulu Heart Program. Am J Epidemiol 1994; 139: 881–93.
Low physical activity	Ischaemic stroke	Agnarsson U, Thorgeirsson G, Sigvaldason H, Sigfusson N. Effects of leisure-time physical activity and ventilatory function on risk for stroke in men: the Reykjavík Study. Ann Intern Med 1999; 130: 987–90.
Low physical activity	Ischaemic stroke	Autenrieth CS, Evenson KR, Yatsuya H, Shahar E, Baggett C, Rosamond WD. Association between physical activity and risk of stroke subtypes: the atherosclerosis risk in communities study. Neuroepidemiology 2013; 40: 109–16.
Low physical activity	Ischaemic stroke	Bijnen FC, Caspersen CJ, Feskens EJ, Saris WH, Mosterd WL, Kromhout D. Physical activity and 10-year mortality from cardiovascular diseases and all causes: The Zutphen Elderly Study. Arch Intern Med 1998; 158: 1499–505.
Low physical activity	Ischaemic stroke	Calling S, Hedblad B, Engström G, Berglund G, Janzon L. Effects of body fatness and physical activity on cardiovascular risk: risk prediction using the bioelectrical impedance method. Scand J Public Health 2006; 34: 568–75.
Low physical activity	Ischaemic stroke	Chiuve SE, Rexrode KM, Spiegelman D, Logroscino G, Manson JE, Rimm EB. Primary prevention of stroke by healthy lifestyle. Circulation 2008; 118: 947–54.
Low physical activity	Ischaemic stroke	Ellekjaer H, Holmen J, Ellekjaer E, Vatten L. Physical activity and stroke mortality in women. Ten-year follow-up of the Nord-Trondelag health survey, 1984-1986. Stroke 2000; 31: 14–8.
Low physical activity	Ischaemic stroke	Gulsvik AK, Thelle DS, Samuelsen SO, Myrstad M, Mowé M, Wyller TB. Ageing, physical activity and mortality--a 42-year follow-up study. Int J Epidemiol 2012; 41: 521–30.
Low physical activity	Ischaemic stroke	Håheim LL, Holme I, Hjermann I, Leren P. Risk factors of stroke incidence and mortality. A 12-year follow-up of the Oslo Study. Stroke 1993; 24: 1484–9.
Low physical activity	Ischaemic stroke	Hu FB, Stampfer MJ, Colditz GA, et al. Physical activity and risk of stroke in women. JAMA 2000; 283: 2961–7.
Low physical activity	Ischaemic stroke	Hu G, Sarti C, Jousilahti P, Silventoinen K, Barengo NC, Tuomilehto J. Leisure time, occupational, and commuting physical activity and the risk of stroke. Stroke 2005; 36: 1994–9.
Low physical activity	Ischaemic stroke	Lapidus L, Bengtsson C. Socioeconomic factors and physical activity in relation to cardiovascular disease and death. A 12 year follow up of participants in a population study of women in Gothenburg, Sweden. Br Heart J 1986; 55: 295–301.
Low physical activity	Ischaemic stroke	Lee IM, Hennekens CH, Berger K, Buring JE, Manson JE. Exercise and risk of stroke in male physicians. Stroke 1999; 30: 1–6.
Low physical activity	Ischaemic stroke	Lee IM, Paffenbarger RS. Physical activity and stroke incidence: the Harvard Alumni Health Study. Stroke 1998; 29: 2049–54.
Low physical activity	Ischaemic stroke	Lindenstrøm E, Boysen G, Nyboe J. Lifestyle factors and risk of cerebrovascular disease in women. The Copenhagen City Heart Study. Stroke 1993; 24: 1468–72.
Low physical activity	Ischaemic stroke	Myint PK, Luben RN, Wareham NJ, et al. Combined work and leisure physical activity and risk of stroke in men and women in the European prospective investigation into Cancer-Norfolk Prospective Population Study. Neuroepidemiology 2006; 27: 122–9.
Low physical activity	Ischaemic stroke	Okada H, Horibe H, Yoshiyuki O, Hayakawa N, Aoki N. A prospective study of cerebrovascular disease in Japanese rural communities, Akabane and Asahi. Part 1: evaluation of risk factors in the occurrence of cerebral hemorrhage and thrombosis. Stroke 1976; 7: 599–607.
Low physical activity	Ischaemic stroke	Paffenbarger RS, Brand RJ, Sholtz RI, Jung DL. Energy expenditure, cigarette smoking, and blood pressure level as related to death from specific diseases. Am J Epidemiol 1978; 108: 12–8.
Low physical activity	Ischaemic stroke	Paganini-Hill A, Perez Barreto M. Stroke risk in older men and women: aspirin, estrogen, exercise, vitamins, and other factors. J Gend Specif Med 2001; 4: 18–28.
Low physical activity	Ischaemic stroke	Salonen JT, Puska P, Tuomilehto J. Physical activity and risk of myocardial infarction, cerebral stroke and death: a longitudinal study in Eastern Finland. Am J Epidemiol 1982; 115: 526–37.
Low physical activity	Ischaemic stroke	Sattelmair JR, Kurth T, Buring JE, Lee I-M. Physical activity and risk of stroke in women. Stroke 2010; 41: 1243–50.

Appendix Table 2. Supplemental information for Figure 1: Epidemiological evidence supporting causality between risk-outcome pairs included in the Global Burden of Disease 2016 study including 2A. Citations and 2B. Additional information
6A. Citations

Risk	Outcome	Citation/Note
Low physical activity	Ischaemic stroke	Simonsick EM, Lafferty ME, Phillips CL, et al. Risk due to inactivity in physically capable older adults. Am J Public Health 1993; 83: 1443–50.
Low physical activity	Ischaemic stroke	Wannamethee G, Shaper AG. Physical activity and stroke in British middle aged men. BMJ 1992; 304: 597–601.
Low physical activity	Ischaemic stroke	Willey JZ, Moon YP, Paik MC, Boden-Albala B, Sacco RL, Elkind MSV. Physical activity and risk of ischemic stroke in the Northern Manhattan Study. Neurology 2009; 73: 1774–9.
Low physical activity	Ischaemic stroke	Zhang Q, Zhou Y, Gao X, et al. Ideal cardiovascular health metrics and the risks of ischemic and intracerebral hemorrhagic stroke. Stroke 2013; 44: 2451–6.
Low physical activity	Diabetes mellitus	Baan CA, Stolk RP, Grobbee DE, Witteman JC, Feskens EJ. Physical activity in elderly subjects with impaired glucose tolerance and newly diagnosed diabetes mellitus. Am J Epidemiol 1999; 149: 219–27.
Low physical activity	Diabetes mellitus	Bonora E, Kiechl S, Willeit J, et al. Population-based incidence rates and risk factors for type 2 diabetes in white individuals: the Bruneck study. Diabetes 2004; 53: 1782–9.
Low physical activity	Diabetes mellitus	Burchfiel CM, Sharp DS, Curb JD, et al. Physical activity and incidence of diabetes: the Honolulu Heart Program. Am J Epidemiol 1995; 141: 360–8.
Low physical activity	Diabetes mellitus	Carlsson S, Ahlbom A, Lichtenstein P, Andersson T. Shared genetic influence of BMI, physical activity and type 2 diabetes: a twin study. Diabetologia 2013; 56: 1031–5.
Low physical activity	Diabetes mellitus	Carlsson S, Midthjell K, Tesfamarian MY, Grill V. Age, overweight and physical inactivity increase the risk of latent autoimmune diabetes in adults: results from the Nord-Trøndelag health study. Diabetologia 2007; 50: 55–8.
Low physical activity	Diabetes mellitus	Chien K-L, Chen M-F, Hsu H-C, Su T-C, Lee Y-T. Sports activity and risk of type 2 diabetes in Chinese. Diabetes Res Clin Pract 2009; 84: 311–8.
Low physical activity	Diabetes mellitus	Demakakos P, Hamer M, Stamatakis E, Steptoe A. Low-intensity physical activity is associated with reduced risk of incident type 2 diabetes in older adults: evidence from the English Longitudinal Study of Ageing. Diabetologia 2010; 53: 1877–85.
Low physical activity	Diabetes mellitus	Doi Y, Ninomiya T, Hata J, et al. Two risk score models for predicting incident Type 2 diabetes in Japan. Diabet Med 2012; 29: 107–14.
Low physical activity	Diabetes mellitus	Dotevall A, Johansson S, Wilhelmsen L, Rosengren A. Increased levels of triglycerides, BMI and blood pressure and low physical activity increase the risk of diabetes in Swedish women. A prospective 18-year follow-up of the BEDA study. Diabet Med 2004; 21: 615–22.
Low physical activity	Diabetes mellitus	Elwood P, Galante J, Pickering J, et al. Healthy lifestyles reduce the incidence of chronic diseases and dementia: evidence from the Caerphilly cohort study. PLoS ONE 2013; 8: e81877.
Low physical activity	Diabetes mellitus	Fan S, Chen J, Huang J, et al. Physical activity level and incident type 2 diabetes among Chinese adults. Med Sci Sports Exerc 2015; 47: 751–6.
Low physical activity	Diabetes mellitus	Folsom AR, Kushi LH, Hong CP. Physical activity and incident diabetes mellitus in postmenopausal women. Am J Public Health 2000; 90: 134–8.
Low physical activity	Diabetes mellitus	Fretts AM, Howard BV, Kriska AM, et al. Physical activity and incident diabetes in American Indians: the Strong Heart Study. Am J Epidemiol 2009; 170: 632–9.
Low physical activity	Diabetes mellitus	Grøntved A, Pan A, Mekary RA, et al. Muscle-strengthening and conditioning activities and risk of type 2 diabetes: a prospective study in two cohorts of US women. PLoS Med 2014; 11: e1001587.
Low physical activity	Diabetes mellitus	Gurwitz JH, Field TS, Glynn RJ, et al. Risk factors for non-insulin-dependent diabetes mellitus requiring treatment in the elderly. J Am Geriatr Soc 1994; 42: 1235–40.
Low physical activity	Diabetes mellitus	Haapanen N, Miilunpalo S, Vuori I, Oja P, Pasanen M. Association of leisure time physical activity with the risk of coronary heart disease, hypertension and diabetes in middle-aged men and women. Int J Epidemiol 1997; 26: 739–47.
Low physical activity	Diabetes mellitus	Helmrich SP, Ragland DR, Paffenbarger RS. Prevention of non-insulin-dependent diabetes mellitus with physical activity. Med Sci Sports Exerc 1994; 26: 824–30.
Low physical activity	Diabetes mellitus	Holme I, Tonstad S, Sogaard AJ, Larsen PGL, Haheim LL. Leisure time physical activity in middle age predicts the metabolic syndrome in old age: results of a 28-year follow-up of men in the Oslo study. BMC Public Health 2007; 7: 154.
Low physical activity	Diabetes mellitus	Hsia J, Wu L, Allen C, et al. Physical activity and diabetes risk in postmenopausal women. Am J Prev Med 2005; 28: 19–25.
Low physical activity	Diabetes mellitus	Hu FB, Leitzmann MF, Stampfer MJ, Colditz GA, Willett WC, Rimm EB. Physical activity and television watching in relation to risk for type 2 diabetes mellitus in men. Arch Intern Med 2001; 161: 1542–8.
Low physical activity	Diabetes mellitus	Hu FB, Sigal RJ, Rich-Edwards JW, et al. Walking compared with vigorous physical activity and risk of type 2 diabetes in women: a prospective study. JAMA 1999; 282: 1433–9.
Low physical activity	Diabetes mellitus	Hu G, Qiao Q, Silventoinen K, et al. Occupational, commuting, and leisure-time physical activity in relation to risk for Type 2 diabetes in middle-aged Finnish men and women. Diabetologia 2003; 46: 322–9.
Low physical activity	Diabetes mellitus	James SA, Jamjoum L, Raghunathan TE, Strogatz DS, Furth ED, Khazanie PG. Physical activity and NIDDM in African-Americans. The Pitt County Study. Diabetes Care 1998; 21: 555–62.
Low physical activity	Diabetes mellitus	Jefferis BJ, Whincup PH, Lennon L, Wannamethee SG. Longitudinal associations between changes in physical activity and onset of type 2 diabetes in older British men: the influence of adiposity. Diabetes Care 2012; 35: 1876–83.
Low physical activity	Diabetes mellitus	Joseph J, Svartberg J, Njølstad I, Schirmer H. Incidence of and risk factors for type-2 diabetes in a general population: the Tromsø Study. Scand J Public Health 2010; 38: 768–75.
Low physical activity	Diabetes mellitus	Koloverou E, Panagiotakos DB, Pitsavos C, et al. 10-year incidence of diabetes and associated risk factors in Greece: the ATTICA study (2002-2012). Rev Diabet Stud 2014; 11: 181–9.
Low physical activity	Diabetes mellitus	Krishnan S, Rosenberg L, Palmer JR. Physical activity and television watching in relation to risk of type 2 diabetes: the Black Women's Health Study. Am J Epidemiol 2009; 169: 428–34.

Appendix Table 2. Supplemental information for Figure 1: Epidemiological evidence supporting causality between risk-outcome pairs included in the Global Burden of Disease 2016 study including 2A. Citations and 2B. Additional information
6A. Citations

Risk	Outcome	Citation/Note
Low physical activity	Diabetes mellitus	Laaksonen MA, Knekt P, Rissanen H, et al. The relative importance of modifiable potential risk factors of type 2 diabetes: a meta-analysis of two cohorts. Eur J Epidemiol 2010; 25: 115–24.
Low physical activity	Diabetes mellitus	Lee D, Park I, Jun T-W, et al. Physical activity and body mass index and their associations with the development of type 2 diabetes in korean men. Am J Epidemiol 2012; 176: 43–51.
Low physical activity	Diabetes mellitus	Longo-Mbenza B, On'kin JBKL, Okwe AN, Kabangu NK, Fuele SM. Metabolic syndrome, aging, physical inactivity, and incidence of type 2 diabetes in general African population. Diab Vasc Dis Res 2010; 7: 28–39.
Low physical activity	Diabetes mellitus	Lucke J, Waters B, Hockey R, et al. Trends in women's risk factors and chronic conditions: findings from the Australian Longitudinal Study on Women's Health. Womens Health (Lond) 2007; 3: 423–32.
Low physical activity	Diabetes mellitus	Magliano DJ, Barr ELM, Zimmet PZ, et al. Glucose indices, health behaviors, and incidence of diabetes in Australia: the Australian Diabetes, Obesity and Lifestyle Study. Diabetes Care 2008; 31: 267–72.
Low physical activity	Diabetes mellitus	Manson JE, Nathan DM, Krolewski AS, Stampfer MJ, Willett WC, Hennekens CH. A prospective study of exercise and incidence of diabetes among US male physicians. JAMA 1992; 268: 63–7.
Low physical activity	Diabetes mellitus	Manson JE, Rimm EB, Stampfer MJ, et al. Physical activity and incidence of non-insulin-dependent diabetes mellitus in women. Lancet 1991; 338: 774–8.
Low physical activity	Diabetes mellitus	Meisinger C, Löwel H, Thorand B, Döring A. Leisure time physical activity and the risk of type 2 diabetes in men and women from the general population. The MONICA/KORA Augsburg Cohort Study. Diabetologia 2005; 48: 27–34.
Low physical activity	Diabetes mellitus	Mozaffarian D, Kamineni A, Carnethon M, Djoussé L, Mukamal KJ, Siscovick D. Lifestyle risk factors and new-onset diabetes mellitus in older adults: the cardiovascular health study. Arch Intern Med 2009; 169: 798–807.
Low physical activity	Diabetes mellitus	Okada K, Hayashi T, Tsumura K, Suematsu C, Endo G, Fujii S. Leisure-time physical activity at weekends and the risk of Type 2 diabetes mellitus in Japanese men: the Osaka Health Survey. Diabet Med 2000; 17: 53–8.
Low physical activity	Diabetes mellitus	Panagiotakos DB, Pitsavos C, Skoumas Y, Lentzas Y, Stefanadis C. Five-year incidence of type 2 diabetes mellitus among cardiovascular disease-free Greek adults: findings from the ATTICA study. Vasc Health Risk Manag 2008; 4: 691–8.
Low physical activity	Diabetes mellitus	Rathmann W, Strassburger K, Heier M, et al. Incidence of Type 2 diabetes in the elderly German population and the effect of clinical and lifestyle risk factors: KORA S4/F4 cohort study. Diabet Med 2009; 26: 1212–9.
Low physical activity	Diabetes mellitus	Reis JP, Loria CM, Sorlie PD, Park Y, Hollenbeck A, Schatzkin A. Lifestyle factors and risk for new-onset diabetes: a population-based cohort study. Ann Intern Med 2011; 155: 292–9.
Low physical activity	Diabetes mellitus	Shi L, Shu X-O, Li H, et al. Physical activity, smoking, and alcohol consumption in association with incidence of type 2 diabetes among middle-aged and elderly Chinese men. PLoS ONE 2013; 8: e77919.
Low physical activity	Diabetes mellitus	Siegel LC, Sesso HD, Bowman TS, Lee I-M, Manson JE, Gaziano JM. Physical activity, body mass index, and diabetes risk in men: a prospective study. Am J Med 2009; 122: 1115–21.
Low physical activity	Diabetes mellitus	Simonsick EM, Lafferty ME, Phillips CL, et al. Risk due to inactivity in physically capable older adults. Am J Public Health 1993; 83: 1443–50.
Low physical activity	Diabetes mellitus	Steinbrecher A, Erber E, Grandinetti A, Nigg C, Kolonel LN, Maskarinec G. Physical activity and risk of type 2 diabetes among Native Hawaiians, Japanese Americans, and Caucasians: the Multiethnic Cohort. J Phys Act Health 2012; 9: 634–41.
Low physical activity	Diabetes mellitus	Stringhini S, Tabak AG, Akbaraly TN, et al. Contribution of modifiable risk factors to social inequalities in type 2 diabetes: prospective Whitehall II cohort study. BMJ 2012; 345: e5452.
Low physical activity	Diabetes mellitus	Sun F, Tao Q, Zhan S. An accurate risk score for estimation 5-year risk of type 2 diabetes based on a health screening population in Taiwan (Province of China). Diabetes Res Clin Pract 2009; 85: 228–34.
Low physical activity	Diabetes mellitus	Tsai AC, Lee S-H. Determinants of new-onset diabetes in older adults—Results of a national cohort study. Clin Nutr 2015; 34: 937–42.
Low physical activity	Diabetes mellitus	Villegas R, Shu X-O, Li H, et al. Physical activity and the incidence of type 2 diabetes in the Shanghai women's health study. Int J Epidemiol 2006; 35: 1553–62.
Low physical activity	Diabetes mellitus	Waki K, Noda M, Sasaki S, et al. Alcohol consumption and other risk factors for self-reported diabetes among middle-aged Japanese: a population-based prospective study in the JPHC study cohort I. Diabet Med 2005; 22: 323–31.
Low physical activity	Diabetes mellitus	Waller K, Kaprio J, Lehtovirta M, Silventoinen K, Koskenvuo M, Kujala UM. Leisure-time physical activity and type 2 diabetes during a 28 year follow-up in twins. Diabetologia 2010; 53: 2531–7.
Low physical activity	Diabetes mellitus	Wannamethee SG, Shaper AG, Alberti KG. Physical activity, metabolic factors, and the incidence of coronary heart disease and type 2 diabetes. Arch Intern Med 2000; 160: 2108–16.
Low physical activity	Diabetes mellitus	Weinstein AR, Sesso HD, Lee IM, et al. Relationship of physical activity vs body mass index with type 2 diabetes in women. JAMA 2004; 292: 1188–94.
Low physical activity	Diabetes mellitus	Williams PT, Thompson PD. Walking versus running for hypertension, cholesterol, and diabetes mellitus risk reduction. Arterioscler Thromb Vasc Biol 2013; 33: 1085–91.
Low physical activity	Diabetes mellitus	Xu F, Ware RS, Tse LA, et al. Joint associations of physical activity and hypertension with the development of type 2 diabetes among urban men and women in Mainland China. PLoS ONE 2014; 9: e88719.
High fasting plasma glucose	Ischaemic heart disease	Boussageon R, Bejan-Angoulvant T, Saadatian-Elahi M, et al. Effect of intensive glucose lowering treatment on all cause mortality, cardiovascular death, and microvascular events in type 2 diabetes: meta-analysis of randomised controlled trials. BMJ 2011; 343: d4169.
High fasting plasma glucose	Ischaemic heart disease	Singh GM, Danaei G, Farzadfar F, et al. The age-specific quantitative effects of metabolic risk factors on cardiovascular diseases and diabetes: a pooled analysis. PLoS ONE 2013; 8: e65174.
High fasting plasma glucose	Ischaemic stroke	Singh GM, Danaei G, Farzadfar F, et al. The age-specific quantitative effects of metabolic risk factors on cardiovascular diseases and diabetes: a pooled analysis. PLoS ONE 2013; 8: e65174.

Appendix Table 2. Supplemental information for Figure 1: Epidemiological evidence supporting causality between risk-outcome pairs included in the Global Burden of Disease 2016 study including 2A. Citations and 2B. Additional information
6A. Citations

Risk	Outcome	Citation/Note
High fasting plasma glucose	Ischaemic stroke	Zhang C, Zhou Y-H, Xu C-L, Chi F-L, Ju H-N. Efficacy of intensive control of glucose in stroke prevention: a meta-analysis of data from 59,197 participants in 9 randomized controlled trials. PloS One 2013; 8: e54465.
High fasting plasma glucose	Hemorrhagic stroke	Singh GM, Danaei G, Farzadfar F, et al. The age-specific quantitative effects of metabolic risk factors on cardiovascular diseases and diabetes: a pooled analysis. PLoS ONE 2013; 8: e65174.
High fasting plasma glucose	Hemorrhagic stroke	Zhang C, Zhou Y-H, Xu C-L, Chi F-L, Ju H-N. Efficacy of intensive control of glucose in stroke prevention: a meta-analysis of data from 59,197 participants in 9 randomized controlled trials. PloS One 2013; 8: e54465.
High fasting plasma glucose	Chronic kidney disease due to diabetes mellitus	Coca SG, Ismail-Beigi F, Haq N, Krumholz HM, Parikh CR. Role of intensive glucose control in development of renal end points in type 2 diabetes mellitus: systematic review and meta-analysis intensive glucose control in type 2 diabetes. Arch Intern Med 2012; 172: 761–9.
High fasting plasma glucose	Chronic kidney disease due to diabetes mellitus	O'Seaghdha CM, Perkovic V, Lam TH, et al. Blood Pressure Is a Major Risk Factor for Renal Death An Analysis of 560 352 Participants From the Asia-Pacific Region. Hypertension 2009; 54: 509–15.
High fasting plasma glucose	Chronic kidney disease due to hypertension	Coca SG, Ismail-Beigi F, Haq N, Krumholz HM, Parikh CR. Role of intensive glucose control in development of renal end points in type 2 diabetes mellitus: systematic review and meta-analysis intensive glucose control in type 2 diabetes. Arch Intern Med 2012; 172: 761–9.
High fasting plasma glucose	Chronic kidney disease due to hypertension	O'Seaghdha CM, Perkovic V, Lam TH, et al. Blood Pressure Is a Major Risk Factor for Renal Death An Analysis of 560 352 Participants From the Asia-Pacific Region. Hypertension 2009; 54: 509–15.
High fasting plasma glucose	Chronic kidney disease due to glomerulonephritis	Coca SG, Ismail-Beigi F, Haq N, Krumholz HM, Parikh CR. Role of intensive glucose control in development of renal end points in type 2 diabetes mellitus: systematic review and meta-analysis intensive glucose control in type 2 diabetes. Arch Intern Med 2012; 172: 761–9.
High fasting plasma glucose	Chronic kidney disease due to glomerulonephritis	O'Seaghdha CM, Perkovic V, Lam TH, et al. Blood Pressure Is a Major Risk Factor for Renal Death An Analysis of 560 352 Participants From the Asia-Pacific Region. Hypertension 2009; 54: 509–15.
High fasting plasma glucose	Chronic kidney disease due to other causes	Coca SG, Ismail-Beigi F, Haq N, Krumholz HM, Parikh CR. Role of intensive glucose control in development of renal end points in type 2 diabetes mellitus: systematic review and meta-analysis intensive glucose control in type 2 diabetes. Arch Intern Med 2012; 172: 761–9.
High fasting plasma glucose	Chronic kidney disease due to other causes	O'Seaghdha CM, Perkovic V, Lam TH, et al. Blood Pressure Is a Major Risk Factor for Renal Death An Analysis of 560 352 Participants From the Asia-Pacific Region. Hypertension 2009; 54: 509–15.
High fasting plasma glucose	Tuberculosis	Young F, Wotton CJ, Critchley JA, Unwin NC, Goldacre MJ. Increased risk of tuberculosis disease in people with diabetes mellitus: record-linkage study in a UK population. J Epidemiol Community Health. 2012; 66(6): 519-23.
High fasting plasma glucose	Tuberculosis	Kuo MC, Lin SH, Lin CH, Mao IC, Chang SJ, Hsieh MC. Type 2 diabetes: an independent risk factor for tuberculosis: a nationwide population-based study. PLoS One. 2013; 8((11):e78924).
High fasting plasma glucose	Tuberculosis	Dobler CC, Flack JR, Marks GB. Risk of tuberculosis among people with diabetes mellitus: an Australian nationwide cohort study. BMJ Open. 2((1):e000666).
High fasting plasma glucose	Tuberculosis	Baker MA, Lin HH, Chang HY, Murray MB. The risk of tuberculosis disease among persons with diabetes mellitus: a prospective cohort study. Clin Infect Dis. 2012; 54(6): 818-25.
High fasting plasma glucose	Tuberculosis	Leung CC, Lam TH, Chan WM, Yew WW, Ho KS, Leung GM, Law WS, Tam CM, Chan CK, Chang KC. Diabetic control and risk of tuberculosis: a cohort study. Am J Epidemiol. 2008; 167(12): 1486-94.
High fasting plasma glucose	Tuberculosis	Kim SJ, Hong YP, Lew WJ, Yang SC, Lee EG. Incidence of pulmonary tuberculosis among diabetics. Tuber Lung Dis. 1995; 76(6): 529-33.
High fasting plasma glucose	Tuberculosis	Pealing L, Wing K, Mathur R, Prieto-Merino D, Smeeth L, Moore DA. Risk of tuberculosis in patients with diabetes: population based cohort study using the UK Clinical Practice Research Datalink. BMC Med. 2015; 13(135).
High fasting plasma glucose	Dementia/Alzheimer's	Zhang J, Chen C, Hua S, Liao H, Wang M, Xiong Y, Cao F. An updated meta-analysis of cohort studies: diabetes and risk of Alzheimer's disease. Diabetes Res Clin Pract. 2017; Feb (124): 41-47.
High fasting plasma glucose	Cataracts	Li L, Wan XH, Zhao GH. Meta-analysis of the risk of cataract in type 2 diabetes. BMC Ophthalmol. 2014; 14(94).
High fasting plasma glucose	Glaucoma	Zhao D, Cho J, Kim MH, Friedman DS, Guallar E. Diabetes, fasting glucose, and the risk of glaucoma: a meta-analysis. Ophthalmology. 2015; 122(1): 72-8.
High fasting plasma glucose	Lung cancer	Zhu L, Cao H, Zhang T, Shen H, Dong W, Wang L, Du J. The effect of diabetes mellitus on lung cancer prognosis: a PRISMA-compliant meta-analysis of cohort studies. Medicine (Baltimore). 2016; 95(17): :e3528.
High fasting plasma glucose	Bladder cancer	Fang H, Yao B, Yan Y, Xu H, Liu Y, Tang H, Zhou J, Cao L, Wang W, Zhang J, Zhao L, Chen X, Zhang F, Zhao Y. Diabetes mellitus increases the risk of bladder cancer: an updated meta-analysis. Diabetes Technol Ther. 2013; 15(11): 914-22.
High fasting plasma glucose	Colon cancer	Shi J, Xiong L, Li J, Cao H, Jiang W, Liu B, Chen X, Liu C, Liu K, Wang G, Cai K . A linear dose-response relationship between fasting plasma glucose and colorectal cancer risk: systematic review and meta-analysis. Sci Rep. 2015; 5(17591).
High fasting plasma glucose	Ovarian cancer	Lee JY1, Jeon I, Kim JW, Song YS, Yoon JM, Park SM.. Diabetes mellitus and ovarian cancer risk: a systematic review and meta-analysis of observational studies. Int J Gynaecol Obstet. 2013; 23(3): 402-12.
High fasting plasma glucose	Pancreatic cancer	Ben Q, Xu M, Ning X, Liu J, Hong S, Huang W, Zhang H, Li Z. Diabetes mellitus and risk of pancreatic cancer: A meta-analysis of cohort studies. Eur J Cancer. 2011; 47(13): 1928-1937.
High fasting plasma glucose	Liver cancer	Yang WS, Va P, Bray F, Gao S, Gao J, Li HL, Xiang YB. The role of pre-existing diabetes mellitus on hepatocellular carcinoma occurrence and prognosis: a meta-analysis of prospective cohort studies. PLoS One. 2011; 6(12): :e27326.
High fasting plasma glucose	Peripheral vascular disease	Rapsomaniki E, Timmis A, George J, Pujades-Rodriguez M, Shah AD, Denaxas S, White IR, Caulfield MJ, Deanfield JE, Smeeth L, Williams B, Hingorani A, Hemingway H. Blood pressure and incidence of twelve cardiovascular diseases: lifetime risks, healthy life-years lost, and age-specific associations in 1·25 million people. Lancet. 2014; 31(383): 1899-911.

Appendix Table 2. Supplemental information for Figure 1: Epidemiological evidence supporting causality between risk-outcome pairs included in the Global Burden of Disease 2016 study including 2A. Citations and 2B. Additional information
6A. Citations

Risk	Outcome	Citation/Note
High fasting plasma glucose	Ischaemic stroke	Rapsomaniki E, Timmis A, George J, Pujades-Rodriguez M, Shah AD, Denaxas S, White IR, Caulfield MJ, Deanfield JE, Smeeth L, Williams B, Hingorani A, Hemingway H. Blood pressure and incidence of twelve cardiovascular diseases: lifetime risks, healthy life-years lost, and age-specific associations in 1·25 million people. Lancet. 2014; 31(383): 1899-911.
High fasting plasma glucose	Hemorrhagic stroke	Rapsomaniki E, Timmis A, George J, Pujades-Rodriguez M, Shah AD, Denaxas S, White IR, Caulfield MJ, Deanfield JE, Smeeth L, Williams B, Hingorani A, Hemingway H. Blood pressure and incidence of twelve cardiovascular diseases: lifetime risks, healthy life-years lost, and age-specific associations in 1·25 million people. Lancet. 2014; 31(383): 1899-911.
High total cholesterol	Ischaemic heart disease	Cholesterol Treatment Trialists' (CTT) Collaboration, Baigent C, Blackwell L, et al. Efficacy and safety of more intensive lowering of LDL cholesterol: a meta-analysis of data from 170,000 participants in 26 randomised trials. Lancet Lond Engl 2010; 376: 1670–81.
High total cholesterol	Ischaemic heart disease	Singh GM, Danaei G, Farzadfar F, et al. The age-specific quantitative effects of metabolic risk factors on cardiovascular diseases and diabetes: a pooled analysis. PLoS ONE 2013; 8: e65174.
High total cholesterol	Ischaemic stroke	Cholesterol Treatment Trialists' (CTT) Collaboration, Baigent C, Blackwell L, et al. Efficacy and safety of more intensive lowering of LDL cholesterol: a meta-analysis of data from 170,000 participants in 26 randomised trials. Lancet Lond Engl 2010; 376: 1670–81.
High total cholesterol	Ischaemic stroke	Singh GM, Danaei G, Farzadfar F, et al. The age-specific quantitative effects of metabolic risk factors on cardiovascular diseases and diabetes: a pooled analysis. PLoS ONE 2013; 8: e65174.
High systolic blood pressure	Rheumatic heart disease	Singh GM, Danaei G, Farzadfar F, et al. The age-specific quantitative effects of metabolic risk factors on cardiovascular diseases and diabetes: a pooled analysis. PLoS ONE 2013; 8: e65174.
High systolic blood pressure	Ischaemic heart disease	Singh GM, Danaei G, Farzadfar F, et al. The age-specific quantitative effects of metabolic risk factors on cardiovascular diseases and diabetes: a pooled analysis. PLoS ONE 2013; 8: e65174.
High systolic blood pressure	Ischaemic heart disease	Thomopoulos C, Parati G, Zanchetti A. Effects of blood pressure lowering on outcome incidence in hypertension. 1. Overview, meta-analyses, and meta-regression analyses of randomized trials. J Hypertens 2014; 32: 2285–95.
High systolic blood pressure	Ischaemic stroke	Singh GM, Danaei G, Farzadfar F, et al. The age-specific quantitative effects of metabolic risk factors on cardiovascular diseases and diabetes: a pooled analysis. PLoS ONE 2013; 8: e65174.
High systolic blood pressure	Ischaemic stroke	Thomopoulos C, Parati G, Zanchetti A. Effects of blood pressure lowering on outcome incidence in hypertension. 1. Overview, meta-analyses, and meta-regression analyses of randomized trials. J Hypertens 2014; 32: 2285–95.
High systolic blood pressure	Hemorrhagic stroke	Singh GM, Danaei G, Farzadfar F, et al. The age-specific quantitative effects of metabolic risk factors on cardiovascular diseases and diabetes: a pooled analysis. PLoS ONE 2013; 8: e65174.
High systolic blood pressure	Hemorrhagic stroke	Thomopoulos C, Parati G, Zanchetti A. Effects of blood pressure lowering on outcome incidence in hypertension. 1. Overview, meta-analyses, and meta-regression analyses of randomized trials. J Hypertens 2014; 32: 2285–95.
High systolic blood pressure	Hypertensive heart disease	Singh GM, Danaei G, Farzadfar F, et al. The age-specific quantitative effects of metabolic risk factors on cardiovascular diseases and diabetes: a pooled analysis. PLoS ONE 2013; 8: e65174.
High systolic blood pressure	Hypertensive heart disease	Thomopoulos C, Parati G, Zanchetti A. Effects of blood pressure lowering on outcome incidence in hypertension. 1. Overview, meta-analyses, and meta-regression analyses of randomized trials. J Hypertens 2014; 32: 2285–95.
High systolic blood pressure	Cardiomyopathy and myocarditis	Singh GM, Danaei G, Farzadfar F, et al. The age-specific quantitative effects of metabolic risk factors on cardiovascular diseases and diabetes: a pooled analysis. PLoS ONE 2013; 8: e65174.
High systolic blood pressure	Atrial fibrillation and flutter	Emdin CA, Callender T, Cao J, Rahimi K. Effect of antihypertensive agents on risk of atrial fibrillation: a meta-analysis of large-scale randomized trials. Eur Eur Pacing Arrhythm Card Electrophysiol J Work Groups Card Pacing Arrhythm Card Cell Electrophysiol Eur Soc Cardiol 2015; 17: 701–10.
High systolic blood pressure	Atrial fibrillation and flutter	Singh GM, Danaei G, Farzadfar F, et al. The age-specific quantitative effects of metabolic risk factors on cardiovascular diseases and diabetes: a pooled analysis. PLoS ONE 2013; 8: e65174.
High systolic blood pressure	Aortic aneurysm	Singh GM, Danaei G, Farzadfar F, et al. The age-specific quantitative effects of metabolic risk factors on cardiovascular diseases and diabetes: a pooled analysis. PLoS ONE 2013; 8: e65174.
High systolic blood pressure	Peripheral vascular disease	Singh GM, Danaei G, Farzadfar F, et al. The age-specific quantitative effects of metabolic risk factors on cardiovascular diseases and diabetes: a pooled analysis. PLoS ONE 2013; 8: e65174.
High systolic blood pressure	Endocarditis	Singh GM, Danaei G, Farzadfar F, et al. The age-specific quantitative effects of metabolic risk factors on cardiovascular diseases and diabetes: a pooled analysis. PLoS ONE 2013; 8: e65174.
High systolic blood pressure	Other cardiovascular and circulatory diseases	Singh GM, Danaei G, Farzadfar F, et al. The age-specific quantitative effects of metabolic risk factors on cardiovascular diseases and diabetes: a pooled analysis. PLoS ONE 2013; 8: e65174.
High systolic blood pressure	Chronic kidney disease due to diabetes mellitus	The Renal Risk Collaboration, Foote C, Lin J, et al. The effect of Blood Pressure on Kidney Failure: a systematic review and meta-analysis in 2.7 million participants (unpublished).
High systolic blood pressure	Chronic kidney disease due to diabetes mellitus	Xie X, Atkins E, Lv J, et al. Effects of intensive blood pressure lowering on cardiovascular and renal outcomes: updated systematic review and meta-analysis. Lancet Lond Engl 2016; 387: 435–43.
High systolic blood pressure	Chronic kidney disease due to hypertension	The Renal Risk Collaboration, Foote C, Lin J, et al. The effect of Blood Pressure on Kidney Failure: a systematic review and meta-analysis in 2.7 million participants (unpublished).
High systolic blood pressure	Chronic kidney disease due to hypertension	Xie X, Atkins E, Lv J, et al. Effects of intensive blood pressure lowering on cardiovascular and renal outcomes: updated systematic review and meta-analysis. Lancet Lond Engl 2016; 387: 435–43.
High systolic blood pressure	Chronic kidney disease due to glomerulonephritis	The Renal Risk Collaboration, Foote C, Lin J, et al. The effect of Blood Pressure on Kidney Failure: a systematic review and meta-analysis in 2.7 million participants (unpublished).
High systolic blood pressure	Chronic kidney disease due to glomerulonephritis	Xie X, Atkins E, Lv J, et al. Effects of intensive blood pressure lowering on cardiovascular and renal outcomes: updated systematic review and meta-analysis. Lancet Lond Engl 2016; 387: 435–43.
High systolic blood pressure	Chronic kidney disease due to other causes	The Renal Risk Collaboration, Foote C, Lin J, et al. The effect of Blood Pressure on Kidney Failure: a systematic review and meta-analysis in 2.7 million participants (unpublished).
High systolic blood pressure	Chronic kidney disease due to other causes	Xie X, Atkins E, Lv J, et al. Effects of intensive blood pressure lowering on cardiovascular and renal outcomes: updated systematic review and meta-analysis. Lancet Lond Engl 2016; 387: 435–43.

Appendix Table 2. Supplemental information for Figure 1: Epidemiological evidence supporting causality between risk-outcome pairs included in the Global Burden of Disease 2016 study including 2A. Citations and 2B. Additional information
6A. Citations

Risk	Outcome	Citation/Note
High body-mass index	Oesophageal cancer	Renehan AG, Tyson M, Egger M, Heller RF, Zwahlen M. Body-mass index and incidence of cancer: a systematic review and meta-analysis of prospective observational studies. Lancet 2008; 371: 569–78.
High body-mass index	Colon and rectum cancer	Karahalios A, English DR, Simpson JA. Weight change and risk of colorectal cancer: a systematic review and meta-analysis. Am J Epidemiol 2015; 181: 832–45.
High body-mass index	Colon and rectum cancer	Renehan AG, Tyson M, Egger M, Heller RF, Zwahlen M. Body-mass index and incidence of cancer: a systematic review and meta-analysis of prospective observational studies. Lancet 2008; 371: 569–78.
High body-mass index	Colon and rectum cancer	Schlesinger S, Lieb W, Koch M, et al. Body weight gain and risk of colorectal cancer: a systematic review and meta-analysis of observational studies. Obes Rev 2015; 16: 607–19.
High body-mass index	Liver cancer	Chen Y, Wang X, Wang J, Yan Z, Luo J. Excess body weight and the risk of primary liver cancer: an updated meta-analysis of prospective studies. Eur J Cancer 2012; 48: 2137–45.
High body-mass index	Liver cancer	Renehan AG, Tyson M, Egger M, Heller RF, Zwahlen M. Body-mass index and incidence of cancer: a systematic review and meta-analysis of prospective observational studies. Lancet 2008; 371: 569–78.
High body-mass index	Liver cancer	Rui R, Lou J, Zou L, et al. Excess body mass index and risk of liver cancer: a nonlinear dose-response meta-analysis of prospective studies. PLoS ONE 2012; 7: e44522.
High body-mass index	Liver cancer	Tanaka K, Tsuji I, Tamakoshi A, et al. Obesity and liver cancer risk: an evaluation based on a systematic review of epidemiologic evidence among the Japanese population. Jpn J Clin Oncol 2012; 42: 212–21.
High body-mass index	Liver cancer	Wang Y, Wang B, Shen F, Fan J, Cao H. Body mass index and risk of primary liver cancer: a meta-analysis of prospective studies. Oncologist 2012; 17: 1461–8.
High body-mass index	Gallbladder and biliary tract cancer	Park M, Song DY, Je Y, Lee JE. Body mass index and biliary tract disease: a systematic review and meta-analysis of prospective studies. Prev Med 2014; 65: 13–22.
High body-mass index	Gallbladder and biliary tract cancer	Renehan AG, Tyson M, Egger M, Heller RF, Zwahlen M. Body-mass index and incidence of cancer: a systematic review and meta-analysis of prospective observational studies. Lancet 2008; 371: 569–78.
High body-mass index	Pancreatic cancer	Alsamarrai A, Das SLM, Windsor JA, Petrov MS. Factors that affect risk for pancreatic disease in the general population: a systematic review and meta-analysis of prospective cohort studies. Clin Gastroenterol Hepatol 2014; 12: 1635–1644.e5; quiz e103.
High body-mass index	Pancreatic cancer	Renehan AG, Tyson M, Egger M, Heller RF, Zwahlen M. Body-mass index and incidence of cancer: a systematic review and meta-analysis of prospective observational studies. Lancet 2008; 371: 569–78.
High body-mass index	Breast cancer (Pre-menopause)	Renehan AG, Tyson M, Egger M, Heller RF, Zwahlen M. Body-mass index and incidence of cancer: a systematic review and meta-analysis of prospective observational studies. Lancet 2008; 371: 569–78.
High body-mass index	Breast cancer (Pre-menopause)	Xia X, Chen W, Li J, et al. Body mass index and risk of breast cancer: a nonlinear dose-response meta-analysis of prospective studies. Sci Rep 2014; 4: 7480.
High body-mass index	Breast cancer (Post-menopause)	Renehan AG, Tyson M, Egger M, Heller RF, Zwahlen M. Body-mass index and incidence of cancer: a systematic review and meta-analysis of prospective observational studies. Lancet 2008; 371: 569–78.
High body-mass index	Breast cancer (Post-menopause)	Xia X, Chen W, Li J, et al. Body mass index and risk of breast cancer: a nonlinear dose-response meta-analysis of prospective studies. Sci Rep 2014; 4: 7480.
High body-mass index	Uterine cancer	Aune D, Greenwood DC, Chan DSM, et al. Body mass index, abdominal fatness and pancreatic cancer risk: a systematic review and non-linear dose-response meta-analysis of prospective studies. Ann Oncol 2012; 23: 843–52.
High body-mass index	Uterine cancer	Jenabi E, Poorolajal J. The effect of body mass index on endometrial cancer: a meta-analysis. Public Health 2015; 129: 872–80.
High body-mass index	Uterine cancer	Renehan AG, Tyson M, Egger M, Heller RF, Zwahlen M. Body-mass index and incidence of cancer: a systematic review and meta-analysis of prospective observational studies. Lancet 2008; 371: 569–78.
High body-mass index	Ovarian cancer	Aune D, Navarro Rosenblatt DA, Chan DSM, et al. Anthropometric factors and ovarian cancer risk: a systematic review and nonlinear dose-response meta-analysis of prospective studies. Int J Cancer 2015; 136: 1888–98.
High body-mass index	Ovarian cancer	Collaborative Group on Epidemiological Studies of Ovarian Cancer. Ovarian cancer and body size: individual participant meta-analysis including 25,157 women with ovarian cancer from 47 epidemiological studies. PLoS Med 2012; 9: e1001200.
High body-mass index	Ovarian cancer	Liu Z, Zhang T-T, Zhao J-J, et al. The association between overweight, obesity and ovarian cancer: a meta-analysis. Jpn J Clin Oncol 2015; 45: 1107–15.
High body-mass index	Ovarian cancer	Renehan AG, Tyson M, Egger M, Heller RF, Zwahlen M. Body-mass index and incidence of cancer: a systematic review and meta-analysis of prospective observational studies. Lancet 2008; 371: 569–78.
High body-mass index	Kidney cancer	Renehan AG, Tyson M, Egger M, Heller RF, Zwahlen M. Body-mass index and incidence of cancer: a systematic review and meta-analysis of prospective observational studies. Lancet 2008; 371: 569–78.
High body-mass index	Kidney cancer	Wang F, Xu Y. Body mass index and risk of renal cell cancer: a dose-response meta-analysis of published cohort studies. Int J Cancer 2014; 135: 1673–86.
High body-mass index	Thyroid cancer	Ma J, Huang M, Wang L, Ye W, Tong Y, Wang H. Obesity and risk of thyroid cancer: evidence from a meta-analysis of 21 observational studies. Med Sci Monit 2015; 21: 283–91.
High body-mass index	Thyroid cancer	Renehan AG, Tyson M, Egger M, Heller RF, Zwahlen M. Body-mass index and incidence of cancer: a systematic review and meta-analysis of prospective observational studies. Lancet. 2008; 371(9612): 569-78.
High body-mass index	Non-hodgkin lymphoma	Larsson SC, Wolk A. Body mass index and risk of non-Hodgkin's and Hodgkin's lymphoma: A meta-analysis of prospective studies. European Journal of Cancer 2011; 47: 2422–30.
High body-mass index	Leukaemia	Castillo JJ, Reagan JL, Ingham RR, et al. Obesity but not overweight increases the incidence and mortality of leukemia in adults: a meta-analysis of prospective cohort studies. Leuk Res 2012; 36: 868–75.
High body-mass index	Leukaemia	Renehan AG, Tyson M, Egger M, Heller RF, Zwahlen M. Body-mass index and incidence of cancer: a systematic review and meta-analysis of prospective observational studies. Lancet. 2008; 371(9612): 569-78.
High body-mass index	Multiple myeloma	Teras LR, Kitahara CM, Birmann BM, et al. Body Size and Multiple Myeloma Mortality: a pooled analysis of 20 prospective studies. Br J Haematol 2014; 166: 667–76.

Appendix Table 2. Supplemental information for Figure 1: Epidemiological evidence supporting causality between risk-outcome pairs included in the Global Burden of Disease 2016 study including 2A. Citations and 2B. Additional information
6A. Citations

Risk	Outcome	Citation/Note
High body-mass index	Ischaemic heart disease	Singh GM, Danaei G, Farzadfar F, et al. The age-specific quantitative effects of metabolic risk factors on cardiovascular diseases and diabetes: a pooled analysis. PLoS ONE 2013; 8: e65174.
High body-mass index	Cerebrovascular disease	Singh GM, Danaei G, Farzadfar F, et al. The age-specific quantitative effects of metabolic risk factors on cardiovascular diseases and diabetes: a pooled analysis. PLoS ONE 2013; 8: e65174.
High body-mass index	Hypertensive heart disease	Singh GM, Danaei G, Farzadfar F, et al. The age-specific quantitative effects of metabolic risk factors on cardiovascular diseases and diabetes: a pooled analysis. PLoS ONE 2013; 8: e65174.
High body-mass index	Atrial fibrillation and flutter	Wanahita N, Messerli FH, Bangalore S, Gami AS, Somers VK, Steinberg JS. Atrial fibrillation and obesity—results of a meta-analysis. American Heart Journal 2008; 155: 310–5.
High body-mass index	Asthma	Beuther DA, Sutherland ER. Overweight, Obesity, and Incident Asthma. Am J Respir Crit Care Med 2007; 175: 661–6.
High body-mass index	Gallbladder and biliary tract diseases	Aune D, Norat T, Vatten LJ. Body mass index, abdominal fatness and the risk of gallbladder disease. Eur J Epidemiol 2015; 30: 1009–19.
High body-mass index	Alzheimer's disease and other dementias	Profenno LA, Porsteinsson AP, Faraone SV. Meta-Analysis of Alzheimer's Disease Risk with Obesity, Diabetes, and Related Disorders. Biological Psychiatry 2010; 67: 505–12.
High body-mass index	Diabetes mellitus	Singh GM, Danaei G, Farzadfar F, et al. The age-specific quantitative effects of metabolic risk factors on cardiovascular diseases and diabetes: a pooled analysis. PLoS ONE 2013; 8: e65174.
High body-mass index	Chronic kidney disease	Emerging Risk Factors Collaboration, Wormser D, Kaptoge S, et al. Separate and combined associations of body-mass index and abdominal adiposity with cardiovascular disease: collaborative analysis of 58 prospective studies. Lancet 2011; 377: 1085–95.
High body-mass index	Chronic kidney disease	Ni Mhurchu C, Rodgers A, Pan WH, Gu DF, Woodward M, Asia Pacific Cohort Studies Collaboration. Body mass index and cardiovascular disease in the Asia-Pacific Region: an overview of 33 cohorts involving 310 000 participants. Int J Epidemiol 2004; 33: 751–8.
High body-mass index	Chronic kidney disease	Prospective Studies Collaboration, Whitlock G, Lewington S, et al. Body-mass index and cause-specific mortality in 900 000 adults: collaborative analyses of 57 prospective studies. Lancet 2009; 373: 1083–96.
High body-mass index	Osteoarthritis	Jiang L, Rong J, Wang Y, et al. The relationship between body mass index and hip osteoarthritis: a systematic review and meta-analysis. Joint Bone Spine 2011; 78: 150–5.
High body-mass index	Osteoarthritis	Jiang L, Tian W, Wang Y, et al. Body mass index and susceptibility to knee osteoarthritis: a systematic review and meta-analysis. Joint Bone Spine 2012; 79: 291–7.
High body-mass index	Osteoarthritis	Silverwood V, Blagojevic-Bucknall M, Jinks C, Jordan JL, Protheroe J, Jordan KP. Current evidence on risk factors for knee osteoarthritis in older adults: a systematic review and meta-analysis. Osteoarthr Cartil 2015; 23: 507–15.
High body-mass index	Low back pain	Shiri R, Karppinen J, Leino-Arjas P, Solovieva S, Viikari-Juntura E. The association between obesity and low back pain: a meta-analysis. Am J Epidemiol 2010; 171: 135–54.
High body-mass index	Gout	Aune D, Norat T, Vatten LJ. Body mass index and the risk of gout: a systematic review and dose-response meta-analysis of prospective studies. Eur J Nutr 2014; 53: 1591–601.
High body-mass index	Cataract	Ye J, Lou L-X, He J-J, Xu Y-F. Body Mass Index and Risk of Age-Related Cataract: A Meta-Analysis of Prospective Cohort Studies. PLOS ONE 2014; 9: e89923.
Childhood overweight and obesity	Asthma	Mebrahtu TF, Feltbower RG, Greenwood DC, Parslow RC. Childhood body mass index and wheezing disorders: a systematic review and meta-analysis. Pediatr Allergy Immunol 2015; 26: 62–72.
Low bone mineral density	Injuries	Johnell O, Kanis JA, Oden A, et al. Predictive value of BMD for hip and other fractures. J Bone Miner Res 2005; 20: 1185–94.
Impaired kidney function	Ischaemic heart disease	Chronic Kidney Disease Prognosis Consortium (CKD-PC). Chronic Kidney Disease Prognosis Consortium GBD 2016 Impaired Kidney Function Relative Risk Meta-Analysis.
Impaired kidney function	Ischaemic heart disease	National Heart, Lung, and Blood Institute, National Institutes of Health (NIH). United States Atherosclerosis Risk in Communities Study. Bethesda, United States: National Heart, Lung, and Blood Institute, National Institutes of Health (NIH).
Impaired kidney function	Ischaemic heart disease	International Diabetes Institute (IDI). Australia Diabetes, Obesity and Lifestyle Study 1999-2000. Melbourne, Australia: International Diabetes Institute (IDI).
Impaired kidney function	Ischaemic heart disease	Boston University, School of Medicine, Framingham Heart Study, National Heart, Lung, and Blood Institute, National Institutes of Health (NIH). United States Framingham Heart Study .
Impaired kidney function	Ischaemic heart disease	Association for Cardiac Research, Rome (Italy). The Gubbio Population Study.
Impaired kidney function	Ischaemic heart disease	National Heart, Lung, and Blood Institute, National Institutes of Health, University of California, Los Angeles (UCLA), University of Minnesota. United States Multi-Ethnic Study of Atherosclerosis First Examination 2000-2002. Bethesda, United States: National Heart, Lung, and Blood Institute, National Institutes of Health.
Impaired kidney function	Ischaemic heart disease	Uppsala University. Sweden Uppsala Longitudinal Study of Adult Men.
Impaired kidney function	Cerebrovascular disease	Chronic Kidney Disease Prognosis Consortium (CKD-PC). Chronic Kidney Disease Prognosis Consortium GBD 2016 Impaired Kidney Function Relative Risk Meta-Analysis.
Impaired kidney function	Cerebrovascular disease	National Heart, Lung, and Blood Institute, National Institutes of Health (NIH). United States Atherosclerosis Risk in Communities Study. Bethesda, United States: National Heart, Lung, and Blood Institute, National Institutes of Health (NIH).
Impaired kidney function	Cerebrovascular disease	International Diabetes Institute (IDI). Australia Diabetes, Obesity and Lifestyle Study 1999-2000. Melbourne, Australia: International Diabetes Institute (IDI).
Impaired kidney function	Cerebrovascular disease	National Heart, Lung, and Blood Institute, National Institutes of Health, University of California, Los Angeles (UCLA), University of Minnesota. United States Multi-Ethnic Study of Atherosclerosis First Examination 2000-2002. Bethesda, United States: National Heart, Lung, and Blood Institute, National Institutes of Health.
Impaired kidney function	Cerebrovascular disease	Uppsala University. Sweden Uppsala Longitudinal Study of Adult Men.

Appendix Table 2. Supplemental information for Figure 1: Epidemiological evidence supporting causality between risk-outcome pairs included in the Global Burden of Disease 2016 study including 2A. Citations and 2B. Additional information<br>6A. Citations		
**Risk**	**Outcome**	**Citation/Note**
Impaired kidney function	Peripheral vascular disease	Chronic Kidney Disease Prognosis Consortium (CKD-PC). Chronic Kidney Disease Prognosis Consortium GBD 2016 Impaired Kidney Function Relative Risk Meta-Analysis.
Impaired kidney function	Peripheral vascular disease	National Heart, Lung, and Blood Institute, National Institutes of Health (NIH). United States Atherosclerosis Risk in Communities Study. Bethesda, United States: National Heart, Lung, and Blood Institute, National Institutes of Health (NIH).
Impaired kidney function	Peripheral vascular disease	Association for Cardiac Research, Rome (Italy). The Gubbio Population Study.
Impaired kidney function	Peripheral vascular disease	National Heart, Lung, and Blood Institute, National Institutes of Health, University of California, Los Angeles (UCLA), University of Minnesota. United States Multi-Ethnic Study of Atherosclerosis First Examination 2000-2002. Bethesda, United States: National Heart, Lung, and Blood Institute, National Institutes of Health.
Impaired kidney function	Gout	Cea Soriano L, Rothenbacher D, Choi HK, García Rodríguez LA. Contemporary epidemiology of gout in the UK general population. Arthritis Res Ther 2011; 13: R39.
Impaired kidney function	Gout	Krishnan E. Chronic kidney disease and the risk of incident gout among middle-aged men: a seven-year prospective observational study. Arthritis Rheum 2013; 65: 3271–8.
Impaired kidney function	Gout	McAdams-DeMarco MA, Maynard JW, Baer AN, Coresh J. Hypertension and the risk of incident gout in a population-based study: the atherosclerosis risk in communities cohort. J Clin Hypertens (Greenwich) 2012; 14: 675–9.
Impaired kidney function	Gout	Trifirò G, Morabito P, Cavagna L, et al. Epidemiology of gout and hyperuricaemia in Italy during the years 2005-2009: a nationwide population-based study. Ann Rheum Dis 2013; 72: 694–700.
Short gestation for birth weight	Diarrheal diseases	Katz J, Lee AC, Kozuki N, Lawn JE, Cousens S, Blencowe H, Ezzati M, Bhutta ZA, Marchant T, Willey BA, Adair L, Barros F, Baqui AH, Christian P, Fawzi W, Gonzalez R, Humphrey J, Huybregts L, Kolsteren P, Mongkolchati A, Mullany LC, Ndyomugyenyi R, Nien JK, Osrin D, Roberfroid D, Sania A, Schmiegelow C, Silveira MF, Tielsch J, Vaidya A, Velaphi SC, Victora CG, Watson-Jones D, Black RE; CHERG Small-for-Gestational-Age-Preterm Birth Working Group. Mortality risk in preterm and small-for-gestational-age infants in low-income and middle-income countries: a pooled country analysis. Lancet. 2013; 382(9890): 417-25.
Short gestation for birth weight	Lower respiratory infections	Katz J, Lee AC, Kozuki N, Lawn JE, Cousens S, Blencowe H, Ezzati M, Bhutta ZA, Marchant T, Willey BA, Adair L, Barros F, Baqui AH, Christian P, Fawzi W, Gonzalez R, Humphrey J, Huybregts L, Kolsteren P, Mongkolchati A, Mullany LC, Ndyomugyenyi R, Nien JK, Osrin D, Roberfroid D, Sania A, Schmiegelow C, Silveira MF, Tielsch J, Vaidya A, Velaphi SC, Victora CG, Watson-Jones D, Black RE; CHERG Small-for-Gestational-Age-Preterm Birth Working Group. Mortality risk in preterm and small-for-gestational-age infants in low-income and middle-income countries: a pooled country analysis. Lancet. 2013; 382(9890): 417-25.
Short gestation for birth weight	Upper respiratory infections	Katz J, Lee AC, Kozuki N, Lawn JE, Cousens S, Blencowe H, Ezzati M, Bhutta ZA, Marchant T, Willey BA, Adair L, Barros F, Baqui AH, Christian P, Fawzi W, Gonzalez R, Humphrey J, Huybregts L, Kolsteren P, Mongkolchati A, Mullany LC, Ndyomugyenyi R, Nien JK, Osrin D, Roberfroid D, Sania A, Schmiegelow C, Silveira MF, Tielsch J, Vaidya A, Velaphi SC, Victora CG, Watson-Jones D, Black RE; CHERG Small-for-Gestational-Age-Preterm Birth Working Group. Mortality risk in preterm and small-for-gestational-age infants in low-income and middle-income countries: a pooled country analysis. Lancet. 2013; 382(9890): 417-25.
Short gestation for birth weight	Otitis media	Katz J, Lee AC, Kozuki N, Lawn JE, Cousens S, Blencowe H, Ezzati M, Bhutta ZA, Marchant T, Willey BA, Adair L, Barros F, Baqui AH, Christian P, Fawzi W, Gonzalez R, Humphrey J, Huybregts L, Kolsteren P, Mongkolchati A, Mullany LC, Ndyomugyenyi R, Nien JK, Osrin D, Roberfroid D, Sania A, Schmiegelow C, Silveira MF, Tielsch J, Vaidya A, Velaphi SC, Victora CG, Watson-Jones D, Black RE; CHERG Small-for-Gestational-Age-Preterm Birth Working Group. Mortality risk in preterm and small-for-gestational-age infants in low-income and middle-income countries: a pooled country analysis. Lancet. 2013; 382(9890): 417-25.
Short gestation for birth weight	Pneumococcal meningitis	Katz J, Lee AC, Kozuki N, Lawn JE, Cousens S, Blencowe H, Ezzati M, Bhutta ZA, Marchant T, Willey BA, Adair L, Barros F, Baqui AH, Christian P, Fawzi W, Gonzalez R, Humphrey J, Huybregts L, Kolsteren P, Mongkolchati A, Mullany LC, Ndyomugyenyi R, Nien JK, Osrin D, Roberfroid D, Sania A, Schmiegelow C, Silveira MF, Tielsch J, Vaidya A, Velaphi SC, Victora CG, Watson-Jones D, Black RE; CHERG Small-for-Gestational-Age-Preterm Birth Working Group. Mortality risk in preterm and small-for-gestational-age infants in low-income and middle-income countries: a pooled country analysis. Lancet. 2013; 382(9890): 417-25.
Short gestation for birth weight	H influenzae type B meningitis	Katz J, Lee AC, Kozuki N, Lawn JE, Cousens S, Blencowe H, Ezzati M, Bhutta ZA, Marchant T, Willey BA, Adair L, Barros F, Baqui AH, Christian P, Fawzi W, Gonzalez R, Humphrey J, Huybregts L, Kolsteren P, Mongkolchati A, Mullany LC, Ndyomugyenyi R, Nien JK, Osrin D, Roberfroid D, Sania A, Schmiegelow C, Silveira MF, Tielsch J, Vaidya A, Velaphi SC, Victora CG, Watson-Jones D, Black RE; CHERG Small-for-Gestational-Age-Preterm Birth Working Group. Mortality risk in preterm and small-for-gestational-age infants in low-income and middle-income countries: a pooled country analysis. Lancet. 2013; 382(9890): 417-25.
Short gestation for birth weight	Meningococcal meningitis	Katz J, Lee AC, Kozuki N, Lawn JE, Cousens S, Blencowe H, Ezzati M, Bhutta ZA, Marchant T, Willey BA, Adair L, Barros F, Baqui AH, Christian P, Fawzi W, Gonzalez R, Humphrey J, Huybregts L, Kolsteren P, Mongkolchati A, Mullany LC, Ndyomugyenyi R, Nien JK, Osrin D, Roberfroid D, Sania A, Schmiegelow C, Silveira MF, Tielsch J, Vaidya A, Velaphi SC, Victora CG, Watson-Jones D, Black RE; CHERG Small-for-Gestational-Age-Preterm Birth Working Group. Mortality risk in preterm and small-for-gestational-age infants in low-income and middle-income countries: a pooled country analysis. Lancet. 2013; 382(9890): 417-25.

Appendix Table 2. Supplemental information for Figure 1: Epidemiological evidence supporting causality between risk-outcome pairs included in the Global Burden of Disease 2016 study including 2A. Citations and 2B. Additional information
6A. Citations

Risk	Outcome	Citation/Note
Short gestation for birth weight	Other meningitis	Katz J, Lee AC, Kozuki N, Lawn JE, Cousens S, Blencowe H, Ezzati M, Bhutta ZA, Marchant T, Willey BA, Adair L, Barros F, Baqui AH, Christian P, Fawzi W, Gonzalez R, Humphrey J, Huybregts L, Kolsteren P, Mongkolchati A, Mullany LC, Ndyomugyenyi R, Nien JK, Osrin D, Roberfroid D, Sania A, Schmiegelow C, Silveira MF, Tielsch J, Vaidya A, Velaphi SC, Victora CG, Watson-Jones D, Black RE; CHERG Small-for-Gestational-Age-Preterm Birth Working Group. Mortality risk in preterm and small-for-gestational-age infants in low-income and middle-income countries: a pooled country analysis. Lancet. 2013; 382(9890): 417-25.
Short gestation for birth weight	Encephalitis	Katz J, Lee AC, Kozuki N, Lawn JE, Cousens S, Blencowe H, Ezzati M, Bhutta ZA, Marchant T, Willey BA, Adair L, Barros F, Baqui AH, Christian P, Fawzi W, Gonzalez R, Humphrey J, Huybregts L, Kolsteren P, Mongkolchati A, Mullany LC, Ndyomugyenyi R, Nien JK, Osrin D, Roberfroid D, Sania A, Schmiegelow C, Silveira MF, Tielsch J, Vaidya A, Velaphi SC, Victora CG, Watson-Jones D, Black RE; CHERG Small-for-Gestational-Age-Preterm Birth Working Group. Mortality risk in preterm and small-for-gestational-age infants in low-income and middle-income countries: a pooled country analysis. Lancet. 2013; 382(9890): 417-25.
Short gestation for birth weight	Neonatal preterm birth complications	Katz J, Lee AC, Kozuki N, Lawn JE, Cousens S, Blencowe H, Ezzati M, Bhutta ZA, Marchant T, Willey BA, Adair L, Barros F, Baqui AH, Christian P, Fawzi W, Gonzalez R, Humphrey J, Huybregts L, Kolsteren P, Mongkolchati A, Mullany LC, Ndyomugyenyi R, Nien JK, Osrin D, Roberfroid D, Sania A, Schmiegelow C, Silveira MF, Tielsch J, Vaidya A, Velaphi SC, Victora CG, Watson-Jones D, Black RE; CHERG Small-for-Gestational-Age-Preterm Birth Working Group. Mortality risk in preterm and small-for-gestational-age infants in low-income and middle-income countries: a pooled country analysis. Lancet. 2013; 382(9890): 417-25.
Short gestation for birth weight	Neonatal encephalopathy due to birth asphyxia and trauma	Katz J, Lee AC, Kozuki N, Lawn JE, Cousens S, Blencowe H, Ezzati M, Bhutta ZA, Marchant T, Willey BA, Adair L, Barros F, Baqui AH, Christian P, Fawzi W, Gonzalez R, Humphrey J, Huybregts L, Kolsteren P, Mongkolchati A, Mullany LC, Ndyomugyenyi R, Nien JK, Osrin D, Roberfroid D, Sania A, Schmiegelow C, Silveira MF, Tielsch J, Vaidya A, Velaphi SC, Victora CG, Watson-Jones D, Black RE; CHERG Small-for-Gestational-Age-Preterm Birth Working Group. Mortality risk in preterm and small-for-gestational-age infants in low-income and middle-income countries: a pooled country analysis. Lancet. 2013; 382(9890): 417-25.
Short gestation for birth weight	Neonatal sepsis and other neonatal infections	Katz J, Lee AC, Kozuki N, Lawn JE, Cousens S, Blencowe H, Ezzati M, Bhutta ZA, Marchant T, Willey BA, Adair L, Barros F, Baqui AH, Christian P, Fawzi W, Gonzalez R, Humphrey J, Huybregts L, Kolsteren P, Mongkolchati A, Mullany LC, Ndyomugyenyi R, Nien JK, Osrin D, Roberfroid D, Sania A, Schmiegelow C, Silveira MF, Tielsch J, Vaidya A, Velaphi SC, Victora CG, Watson-Jones D, Black RE; CHERG Small-for-Gestational-Age-Preterm Birth Working Group. Mortality risk in preterm and small-for-gestational-age infants in low-income and middle-income countries: a pooled country analysis. Lancet. 2013; 382(9890): 417-25.
Short gestation for birth weight	Hemolytic disease and other neonatal jaundice	Katz J, Lee AC, Kozuki N, Lawn JE, Cousens S, Blencowe H, Ezzati M, Bhutta ZA, Marchant T, Willey BA, Adair L, Barros F, Baqui AH, Christian P, Fawzi W, Gonzalez R, Humphrey J, Huybregts L, Kolsteren P, Mongkolchati A, Mullany LC, Ndyomugyenyi R, Nien JK, Osrin D, Roberfroid D, Sania A, Schmiegelow C, Silveira MF, Tielsch J, Vaidya A, Velaphi SC, Victora CG, Watson-Jones D, Black RE; CHERG Small-for-Gestational-Age-Preterm Birth Working Group. Mortality risk in preterm and small-for-gestational-age infants in low-income and middle-income countries: a pooled country analysis. Lancet. 2013; 382(9890): 417-25.
Short gestation for birth weight	Other neonatal disorders	Katz J, Lee AC, Kozuki N, Lawn JE, Cousens S, Blencowe H, Ezzati M, Bhutta ZA, Marchant T, Willey BA, Adair L, Barros F, Baqui AH, Christian P, Fawzi W, Gonzalez R, Humphrey J, Huybregts L, Kolsteren P, Mongkolchati A, Mullany LC, Ndyomugyenyi R, Nien JK, Osrin D, Roberfroid D, Sania A, Schmiegelow C, Silveira MF, Tielsch J, Vaidya A, Velaphi SC, Victora CG, Watson-Jones D, Black RE; CHERG Small-for-Gestational-Age-Preterm Birth Working Group. Mortality risk in preterm and small-for-gestational-age infants in low-income and middle-income countries: a pooled country analysis. Lancet. 2013; 382(9890): 417-25.
Short gestation for birth weight	Sudden infant death syndrome	Katz J, Lee AC, Kozuki N, Lawn JE, Cousens S, Blencowe H, Ezzati M, Bhutta ZA, Marchant T, Willey BA, Adair L, Barros F, Baqui AH, Christian P, Fawzi W, Gonzalez R, Humphrey J, Huybregts L, Kolsteren P, Mongkolchati A, Mullany LC, Ndyomugyenyi R, Nien JK, Osrin D, Roberfroid D, Sania A, Schmiegelow C, Silveira MF, Tielsch J, Vaidya A, Velaphi SC, Victora CG, Watson-Jones D, Black RE; CHERG Small-for-Gestational-Age-Preterm Birth Working Group. Mortality risk in preterm and small-for-gestational-age infants in low-income and middle-income countries: a pooled country analysis. Lancet. 2013; 382(9890): 417-25.
Low birth weight for gestation	Diarrheal diseases	Katz J, Lee AC, Kozuki N, Lawn JE, Cousens S, Blencowe H, Ezzati M, Bhutta ZA, Marchant T, Willey BA, Adair L, Barros F, Baqui AH, Christian P, Fawzi W, Gonzalez R, Humphrey J, Huybregts L, Kolsteren P, Mongkolchati A, Mullany LC, Ndyomugyenyi R, Nien JK, Osrin D, Roberfroid D, Sania A, Schmiegelow C, Silveira MF, Tielsch J, Vaidya A, Velaphi SC, Victora CG, Watson-Jones D, Black RE; CHERG Small-for-Gestational-Age-Preterm Birth Working Group. Mortality risk in preterm and small-for-gestational-age infants in low-income and middle-income countries: a pooled country analysis. Lancet. 2013; 382(9890): 417-25.
Low birth weight for gestation	Lower respiratory infections	Katz J, Lee AC, Kozuki N, Lawn JE, Cousens S, Blencowe H, Ezzati M, Bhutta ZA, Marchant T, Willey BA, Adair L, Barros F, Baqui AH, Christian P, Fawzi W, Gonzalez R, Humphrey J, Huybregts L, Kolsteren P, Mongkolchati A, Mullany LC, Ndyomugyenyi R, Nien JK, Osrin D, Roberfroid D, Sania A, Schmiegelow C, Silveira MF, Tielsch J, Vaidya A, Velaphi SC, Victora CG, Watson-Jones D, Black RE; CHERG Small-for-Gestational-Age-Preterm Birth Working Group. Mortality risk in preterm and small-for-gestational-age infants in low-income and middle-income countries: a pooled country analysis. Lancet. 2013; 382(9890): 417-25.

Appendix Table 2. Supplemental information for Figure 1: Epidemiological evidence supporting causality between risk-outcome pairs included in the Global Burden of Disease 2016 study including 2A. Citations and 2B. Additional information
6A. Citations

Risk	Outcome	Citation/Note
Low birth weight for gestation	Upper respiratory infections	Katz J, Lee AC, Kozuki N, Lawn JE, Cousens S, Blencowe H, Ezzati M, Bhutta ZA, Marchant T, Willey BA, Adair L, Barros F, Baqui AH, Christian P, Fawzi W, Gonzalez R, Humphrey J, Huybregts L, Kolsteren P, Mongkolchati A, Mullany LC, Ndyomugyenyi R, Nien JK, Osrin D, Roberfroid D, Sania A, Schmiegelow C, Silveira MF, Tielsch J, Vaidya A, Velaphi SC, Victora CG, Watson-Jones D, Black RE; CHERG Small-for-Gestational-Age-Preterm Birth Working Group. Mortality risk in preterm and small-for-gestational-age infants in low-income and middle-income countries: a pooled country analysis. Lancet. 2013; 382(9890): 417-25.
Low birth weight for gestation	Otitis media	Katz J, Lee AC, Kozuki N, Lawn JE, Cousens S, Blencowe H, Ezzati M, Bhutta ZA, Marchant T, Willey BA, Adair L, Barros F, Baqui AH, Christian P, Fawzi W, Gonzalez R, Humphrey J, Huybregts L, Kolsteren P, Mongkolchati A, Mullany LC, Ndyomugyenyi R, Nien JK, Osrin D, Roberfroid D, Sania A, Schmiegelow C, Silveira MF, Tielsch J, Vaidya A, Velaphi SC, Victora CG, Watson-Jones D, Black RE; CHERG Small-for-Gestational-Age-Preterm Birth Working Group. Mortality risk in preterm and small-for-gestational-age infants in low-income and middle-income countries: a pooled country analysis. Lancet. 2013; 382(9890): 417-25.
Low birth weight for gestation	Pneumococcal meningitis	Katz J, Lee AC, Kozuki N, Lawn JE, Cousens S, Blencowe H, Ezzati M, Bhutta ZA, Marchant T, Willey BA, Adair L, Barros F, Baqui AH, Christian P, Fawzi W, Gonzalez R, Humphrey J, Huybregts L, Kolsteren P, Mongkolchati A, Mullany LC, Ndyomugyenyi R, Nien JK, Osrin D, Roberfroid D, Sania A, Schmiegelow C, Silveira MF, Tielsch J, Vaidya A, Velaphi SC, Victora CG, Watson-Jones D, Black RE; CHERG Small-for-Gestational-Age-Preterm Birth Working Group. Mortality risk in preterm and small-for-gestational-age infants in low-income and middle-income countries: a pooled country analysis. Lancet. 2013; 382(9890): 417-25.
Low birth weight for gestation	H influenzae type B meningitis	Katz J, Lee AC, Kozuki N, Lawn JE, Cousens S, Blencowe H, Ezzati M, Bhutta ZA, Marchant T, Willey BA, Adair L, Barros F, Baqui AH, Christian P, Fawzi W, Gonzalez R, Humphrey J, Huybregts L, Kolsteren P, Mongkolchati A, Mullany LC, Ndyomugyenyi R, Nien JK, Osrin D, Roberfroid D, Sania A, Schmiegelow C, Silveira MF, Tielsch J, Vaidya A, Velaphi SC, Victora CG, Watson-Jones D, Black RE; CHERG Small-for-Gestational-Age-Preterm Birth Working Group. Mortality risk in preterm and small-for-gestational-age infants in low-income and middle-income countries: a pooled country analysis. Lancet. 2013; 382(9890): 417-25.
Low birth weight for gestation	Meningococcal meningitis	Katz J, Lee AC, Kozuki N, Lawn JE, Cousens S, Blencowe H, Ezzati M, Bhutta ZA, Marchant T, Willey BA, Adair L, Barros F, Baqui AH, Christian P, Fawzi W, Gonzalez R, Humphrey J, Huybregts L, Kolsteren P, Mongkolchati A, Mullany LC, Ndyomugyenyi R, Nien JK, Osrin D, Roberfroid D, Sania A, Schmiegelow C, Silveira MF, Tielsch J, Vaidya A, Velaphi SC, Victora CG, Watson-Jones D, Black RE; CHERG Small-for-Gestational-Age-Preterm Birth Working Group. Mortality risk in preterm and small-for-gestational-age infants in low-income and middle-income countries: a pooled country analysis. Lancet. 2013; 382(9890): 417-25.
Low birth weight for gestation	Other meningitis	Katz J, Lee AC, Kozuki N, Lawn JE, Cousens S, Blencowe H, Ezzati M, Bhutta ZA, Marchant T, Willey BA, Adair L, Barros F, Baqui AH, Christian P, Fawzi W, Gonzalez R, Humphrey J, Huybregts L, Kolsteren P, Mongkolchati A, Mullany LC, Ndyomugyenyi R, Nien JK, Osrin D, Roberfroid D, Sania A, Schmiegelow C, Silveira MF, Tielsch J, Vaidya A, Velaphi SC, Victora CG, Watson-Jones D, Black RE; CHERG Small-for-Gestational-Age-Preterm Birth Working Group. Mortality risk in preterm and small-for-gestational-age infants in low-income and middle-income countries: a pooled country analysis. Lancet. 2013; 382(9890): 417-25.
Low birth weight for gestation	Encephalitis	Katz J, Lee AC, Kozuki N, Lawn JE, Cousens S, Blencowe H, Ezzati M, Bhutta ZA, Marchant T, Willey BA, Adair L, Barros F, Baqui AH, Christian P, Fawzi W, Gonzalez R, Humphrey J, Huybregts L, Kolsteren P, Mongkolchati A, Mullany LC, Ndyomugyenyi R, Nien JK, Osrin D, Roberfroid D, Sania A, Schmiegelow C, Silveira MF, Tielsch J, Vaidya A, Velaphi SC, Victora CG, Watson-Jones D, Black RE; CHERG Small-for-Gestational-Age-Preterm Birth Working Group. Mortality risk in preterm and small-for-gestational-age infants in low-income and middle-income countries: a pooled country analysis. Lancet. 2013; 382(9890): 417-25.
Low birth weight for gestation	Neonatal preterm birth complications	Katz J, Lee AC, Kozuki N, Lawn JE, Cousens S, Blencowe H, Ezzati M, Bhutta ZA, Marchant T, Willey BA, Adair L, Barros F, Baqui AH, Christian P, Fawzi W, Gonzalez R, Humphrey J, Huybregts L, Kolsteren P, Mongkolchati A, Mullany LC, Ndyomugyenyi R, Nien JK, Osrin D, Roberfroid D, Sania A, Schmiegelow C, Silveira MF, Tielsch J, Vaidya A, Velaphi SC, Victora CG, Watson-Jones D, Black RE; CHERG Small-for-Gestational-Age-Preterm Birth Working Group. Mortality risk in preterm and small-for-gestational-age infants in low-income and middle-income countries: a pooled country analysis. Lancet. 2013; 382(9890): 417-25.
Low birth weight for gestation	Neonatal encephalopathy due to birth asphyxia and trauma	Katz J, Lee AC, Kozuki N, Lawn JE, Cousens S, Blencowe H, Ezzati M, Bhutta ZA, Marchant T, Willey BA, Adair L, Barros F, Baqui AH, Christian P, Fawzi W, Gonzalez R, Humphrey J, Huybregts L, Kolsteren P, Mongkolchati A, Mullany LC, Ndyomugyenyi R, Nien JK, Osrin D, Roberfroid D, Sania A, Schmiegelow C, Silveira MF, Tielsch J, Vaidya A, Velaphi SC, Victora CG, Watson-Jones D, Black RE; CHERG Small-for-Gestational-Age-Preterm Birth Working Group. Mortality risk in preterm and small-for-gestational-age infants in low-income and middle-income countries: a pooled country analysis. Lancet. 2013; 382(9890): 417-25.
Low birth weight for gestation	Neonatal sepsis and other neonatal infections	Katz J, Lee AC, Kozuki N, Lawn JE, Cousens S, Blencowe H, Ezzati M, Bhutta ZA, Marchant T, Willey BA, Adair L, Barros F, Baqui AH, Christian P, Fawzi W, Gonzalez R, Humphrey J, Huybregts L, Kolsteren P, Mongkolchati A, Mullany LC, Ndyomugyenyi R, Nien JK, Osrin D, Roberfroid D, Sania A, Schmiegelow C, Silveira MF, Tielsch J, Vaidya A, Velaphi SC, Victora CG, Watson-Jones D, Black RE; CHERG Small-for-Gestational-Age-Preterm Birth Working Group. Mortality risk in preterm and small-for-gestational-age infants in low-income and middle-income countries: a pooled country analysis. Lancet. 2013; 382(9890): 417-25.

Appendix Table 2. Supplemental information for Figure 1: Epidemiological evidence supporting causality between risk-outcome pairs included in the Global Burden of Disease 2016 study including 2A. Citations and 2B. Additional information 6A. Citations		
**Risk**	**Outcome**	**Citation/Note**
Low birth weight for gestation	Hemolytic disease and other neonatal jaundice	Katz J, Lee AC, Kozuki N, Lawn JE, Cousens S, Blencowe H, Ezzati M, Bhutta ZA, Marchant T, Willey BA, Adair L, Barros F, Baqui AH, Christian P, Fawzi W, Gonzalez R, Humphrey J, Huybregts L, Kolsteren P, Mongkolchati A, Mullany LC, Ndyomugyenyi R, Nien JK, Osrin D, Roberfroid D, Sania A, Schmiegelow C, Silveira MF, Tielsch J, Vaidya A, Velaphi SC, Victora CG, Watson-Jones D, Black RE; CHERG Small-for-Gestational-Age-Preterm Birth Working Group. Mortality risk in preterm and small-for-gestational-age infants in low-income and middle-income countries: a pooled country analysis. Lancet. 2013; 382(9890): 417-25.
Low birth weight for gestation	Other neonatal disorders	Katz J, Lee AC, Kozuki N, Lawn JE, Cousens S, Blencowe H, Ezzati M, Bhutta ZA, Marchant T, Willey BA, Adair L, Barros F, Baqui AH, Christian P, Fawzi W, Gonzalez R, Humphrey J, Huybregts L, Kolsteren P, Mongkolchati A, Mullany LC, Ndyomugyenyi R, Nien JK, Osrin D, Roberfroid D, Sania A, Schmiegelow C, Silveira MF, Tielsch J, Vaidya A, Velaphi SC, Victora CG, Watson-Jones D, Black RE; CHERG Small-for-Gestational-Age-Preterm Birth Working Group. Mortality risk in preterm and small-for-gestational-age infants in low-income and middle-income countries: a pooled country analysis. Lancet. 2013; 382(9890): 417-25.
Low birth weight for gestation	Sudden infant death syndrome	Katz J, Lee AC, Kozuki N, Lawn JE, Cousens S, Blencowe H, Ezzati M, Bhutta ZA, Marchant T, Willey BA, Adair L, Barros F, Baqui AH, Christian P, Fawzi W, Gonzalez R, Humphrey J, Huybregts L, Kolsteren P, Mongkolchati A, Mullany LC, Ndyomugyenyi R, Nien JK, Osrin D, Roberfroid D, Sania A, Schmiegelow C, Silveira MF, Tielsch J, Vaidya A, Velaphi SC, Victora CG, Watson-Jones D, Black RE; CHERG Small-for-Gestational-Age-Preterm Birth Working Group. Mortality risk in preterm and small-for-gestational-age infants in low-income and middle-income countries: a pooled country analysis. Lancet. 2013; 382(9890): 417-25.
Low birth weight and short gestation	Diarrheal diseases	Katz J, Lee AC, Kozuki N, Lawn JE, Cousens S, Blencowe H, Ezzati M, Bhutta ZA, Marchant T, Willey BA, Adair L, Barros F, Baqui AH, Christian P, Fawzi W, Gonzalez R, Humphrey J, Huybregts L, Kolsteren P, Mongkolchati A, Mullany LC, Ndyomugyenyi R, Nien JK, Osrin D, Roberfroid D, Sania A, Schmiegelow C, Silveira MF, Tielsch J, Vaidya A, Velaphi SC, Victora CG, Watson-Jones D, Black RE; CHERG Small-for-Gestational-Age-Preterm Birth Working Group. Mortality risk in preterm and small-for-gestational-age infants in low-income and middle-income countries: a pooled country analysis. Lancet. 2013; 382(9890): 417-25.
Low birth weight and short gestation	Lower respiratory infections	Katz J, Lee AC, Kozuki N, Lawn JE, Cousens S, Blencowe H, Ezzati M, Bhutta ZA, Marchant T, Willey BA, Adair L, Barros F, Baqui AH, Christian P, Fawzi W, Gonzalez R, Humphrey J, Huybregts L, Kolsteren P, Mongkolchati A, Mullany LC, Ndyomugyenyi R, Nien JK, Osrin D, Roberfroid D, Sania A, Schmiegelow C, Silveira MF, Tielsch J, Vaidya A, Velaphi SC, Victora CG, Watson-Jones D, Black RE; CHERG Small-for-Gestational-Age-Preterm Birth Working Group. Mortality risk in preterm and small-for-gestational-age infants in low-income and middle-income countries: a pooled country analysis. Lancet. 2013; 382(9890): 417-25.
Low birth weight and short gestation	Upper respiratory infections	Katz J, Lee AC, Kozuki N, Lawn JE, Cousens S, Blencowe H, Ezzati M, Bhutta ZA, Marchant T, Willey BA, Adair L, Barros F, Baqui AH, Christian P, Fawzi W, Gonzalez R, Humphrey J, Huybregts L, Kolsteren P, Mongkolchati A, Mullany LC, Ndyomugyenyi R, Nien JK, Osrin D, Roberfroid D, Sania A, Schmiegelow C, Silveira MF, Tielsch J, Vaidya A, Velaphi SC, Victora CG, Watson-Jones D, Black RE; CHERG Small-for-Gestational-Age-Preterm Birth Working Group. Mortality risk in preterm and small-for-gestational-age infants in low-income and middle-income countries: a pooled country analysis. Lancet. 2013; 382(9890): 417-25.
Low birth weight and short gestation	Otitis media	Katz J, Lee AC, Kozuki N, Lawn JE, Cousens S, Blencowe H, Ezzati M, Bhutta ZA, Marchant T, Willey BA, Adair L, Barros F, Baqui AH, Christian P, Fawzi W, Gonzalez R, Humphrey J, Huybregts L, Kolsteren P, Mongkolchati A, Mullany LC, Ndyomugyenyi R, Nien JK, Osrin D, Roberfroid D, Sania A, Schmiegelow C, Silveira MF, Tielsch J, Vaidya A, Velaphi SC, Victora CG, Watson-Jones D, Black RE; CHERG Small-for-Gestational-Age-Preterm Birth Working Group. Mortality risk in preterm and small-for-gestational-age infants in low-income and middle-income countries: a pooled country analysis. Lancet. 2013; 382(9890): 417-25.
Low birth weight and short gestation	Pneumococcal meningitis	Katz J, Lee AC, Kozuki N, Lawn JE, Cousens S, Blencowe H, Ezzati M, Bhutta ZA, Marchant T, Willey BA, Adair L, Barros F, Baqui AH, Christian P, Fawzi W, Gonzalez R, Humphrey J, Huybregts L, Kolsteren P, Mongkolchati A, Mullany LC, Ndyomugyenyi R, Nien JK, Osrin D, Roberfroid D, Sania A, Schmiegelow C, Silveira MF, Tielsch J, Vaidya A, Velaphi SC, Victora CG, Watson-Jones D, Black RE; CHERG Small-for-Gestational-Age-Preterm Birth Working Group. Mortality risk in preterm and small-for-gestational-age infants in low-income and middle-income countries: a pooled country analysis. Lancet. 2013; 382(9890): 417-25.
Low birth weight and short gestation	H influenzae type B meningitis	Katz J, Lee AC, Kozuki N, Lawn JE, Cousens S, Blencowe H, Ezzati M, Bhutta ZA, Marchant T, Willey BA, Adair L, Barros F, Baqui AH, Christian P, Fawzi W, Gonzalez R, Humphrey J, Huybregts L, Kolsteren P, Mongkolchati A, Mullany LC, Ndyomugyenyi R, Nien JK, Osrin D, Roberfroid D, Sania A, Schmiegelow C, Silveira MF, Tielsch J, Vaidya A, Velaphi SC, Victora CG, Watson-Jones D, Black RE; CHERG Small-for-Gestational-Age-Preterm Birth Working Group. Mortality risk in preterm and small-for-gestational-age infants in low-income and middle-income countries: a pooled country analysis. Lancet. 2013; 382(9890): 417-25.
Low birth weight and short gestation	Meningococcal meningitis	Katz J, Lee AC, Kozuki N, Lawn JE, Cousens S, Blencowe H, Ezzati M, Bhutta ZA, Marchant T, Willey BA, Adair L, Barros F, Baqui AH, Christian P, Fawzi W, Gonzalez R, Humphrey J, Huybregts L, Kolsteren P, Mongkolchati A, Mullany LC, Ndyomugyenyi R, Nien JK, Osrin D, Roberfroid D, Sania A, Schmiegelow C, Silveira MF, Tielsch J, Vaidya A, Velaphi SC, Victora CG, Watson-Jones D, Black RE; CHERG Small-for-Gestational-Age-Preterm Birth Working Group. Mortality risk in preterm and small-for-gestational-age infants in low-income and middle-income countries: a pooled country analysis. Lancet. 2013; 382(9890): 417-25.

Appendix Table 2. Supplemental information for Figure 1: Epidemiological evidence supporting causality between risk-outcome pairs included in the Global Burden of Disease 2016 study including 2A. Citations and 2B. Additional information
6A. Citations

Risk	Outcome	Citation/Note
Low birth weight and short gestation	Other meningitis	Katz J, Lee AC, Kozuki N, Lawn JE, Cousens S, Blencowe H, Ezzati M, Bhutta ZA, Marchant T, Willey BA, Adair L, Barros F, Baqui AH, Christian P, Fawzi W, Gonzalez R, Humphrey J, Huybregts L, Kolsteren P, Mongkolchati A, Mullany LC, Ndyomugyenyi R, Nien JK, Osrin D, Roberfroid D, Sania A, Schmiegelow C, Silveira MF, Tielsch J, Vaidya A, Velaphi SC, Victora CG, Watson-Jones D, Black RE; CHERG Small-for-Gestational-Age-Preterm Birth Working Group. Mortality risk in preterm and small-for-gestational-age infants in low-income and middle-income countries: a pooled country analysis. Lancet. 2013; 382(9890): 417-25.
Low birth weight and short gestation	Encephalitis	Katz J, Lee AC, Kozuki N, Lawn JE, Cousens S, Blencowe H, Ezzati M, Bhutta ZA, Marchant T, Willey BA, Adair L, Barros F, Baqui AH, Christian P, Fawzi W, Gonzalez R, Humphrey J, Huybregts L, Kolsteren P, Mongkolchati A, Mullany LC, Ndyomugyenyi R, Nien JK, Osrin D, Roberfroid D, Sania A, Schmiegelow C, Silveira MF, Tielsch J, Vaidya A, Velaphi SC, Victora CG, Watson-Jones D, Black RE; CHERG Small-for-Gestational-Age-Preterm Birth Working Group. Mortality risk in preterm and small-for-gestational-age infants in low-income and middle-income countries: a pooled country analysis. Lancet. 2013; 382(9890): 417-25.
Low birth weight and short gestation	Neonatal preterm birth complications	Katz J, Lee AC, Kozuki N, Lawn JE, Cousens S, Blencowe H, Ezzati M, Bhutta ZA, Marchant T, Willey BA, Adair L, Barros F, Baqui AH, Christian P, Fawzi W, Gonzalez R, Humphrey J, Huybregts L, Kolsteren P, Mongkolchati A, Mullany LC, Ndyomugyenyi R, Nien JK, Osrin D, Roberfroid D, Sania A, Schmiegelow C, Silveira MF, Tielsch J, Vaidya A, Velaphi SC, Victora CG, Watson-Jones D, Black RE; CHERG Small-for-Gestational-Age-Preterm Birth Working Group. Mortality risk in preterm and small-for-gestational-age infants in low-income and middle-income countries: a pooled country analysis. Lancet. 2013; 382(9890): 417-25.
Low birth weight and short gestation	Neonatal encephalopathy due to birth asphyxia and trauma	Katz J, Lee AC, Kozuki N, Lawn JE, Cousens S, Blencowe H, Ezzati M, Bhutta ZA, Marchant T, Willey BA, Adair L, Barros F, Baqui AH, Christian P, Fawzi W, Gonzalez R, Humphrey J, Huybregts L, Kolsteren P, Mongkolchati A, Mullany LC, Ndyomugyenyi R, Nien JK, Osrin D, Roberfroid D, Sania A, Schmiegelow C, Silveira MF, Tielsch J, Vaidya A, Velaphi SC, Victora CG, Watson-Jones D, Black RE; CHERG Small-for-Gestational-Age-Preterm Birth Working Group. Mortality risk in preterm and small-for-gestational-age infants in low-income and middle-income countries: a pooled country analysis. Lancet. 2013; 382(9890): 417-25.
Low birth weight and short gestation	Neonatal sepsis and other neonatal infections	Katz J, Lee AC, Kozuki N, Lawn JE, Cousens S, Blencowe H, Ezzati M, Bhutta ZA, Marchant T, Willey BA, Adair L, Barros F, Baqui AH, Christian P, Fawzi W, Gonzalez R, Humphrey J, Huybregts L, Kolsteren P, Mongkolchati A, Mullany LC, Ndyomugyenyi R, Nien JK, Osrin D, Roberfroid D, Sania A, Schmiegelow C, Silveira MF, Tielsch J, Vaidya A, Velaphi SC, Victora CG, Watson-Jones D, Black RE; CHERG Small-for-Gestational-Age-Preterm Birth Working Group. Mortality risk in preterm and small-for-gestational-age infants in low-income and middle-income countries: a pooled country analysis. Lancet. 2013; 382(9890): 417-25.
Low birth weight and short gestation	Hemolytic disease and other neonatal jaundice	Katz J, Lee AC, Kozuki N, Lawn JE, Cousens S, Blencowe H, Ezzati M, Bhutta ZA, Marchant T, Willey BA, Adair L, Barros F, Baqui AH, Christian P, Fawzi W, Gonzalez R, Humphrey J, Huybregts L, Kolsteren P, Mongkolchati A, Mullany LC, Ndyomugyenyi R, Nien JK, Osrin D, Roberfroid D, Sania A, Schmiegelow C, Silveira MF, Tielsch J, Vaidya A, Velaphi SC, Victora CG, Watson-Jones D, Black RE; CHERG Small-for-Gestational-Age-Preterm Birth Working Group. Mortality risk in preterm and small-for-gestational-age infants in low-income and middle-income countries: a pooled country analysis. Lancet. 2013; 382(9890): 417-25.
Low birth weight and short gestation	Other neonatal disorders	Katz J, Lee AC, Kozuki N, Lawn JE, Cousens S, Blencowe H, Ezzati M, Bhutta ZA, Marchant T, Willey BA, Adair L, Barros F, Baqui AH, Christian P, Fawzi W, Gonzalez R, Humphrey J, Huybregts L, Kolsteren P, Mongkolchati A, Mullany LC, Ndyomugyenyi R, Nien JK, Osrin D, Roberfroid D, Sania A, Schmiegelow C, Silveira MF, Tielsch J, Vaidya A, Velaphi SC, Victora CG, Watson-Jones D, Black RE; CHERG Small-for-Gestational-Age-Preterm Birth Working Group. Mortality risk in preterm and small-for-gestational-age infants in low-income and middle-income countries: a pooled country analysis. Lancet. 2013; 382(9890): 417-25.
Low birth weight and short gestation	Sudden infant death syndrome	Katz J, Lee AC, Kozuki N, Lawn JE, Cousens S, Blencowe H, Ezzati M, Bhutta ZA, Marchant T, Willey BA, Adair L, Barros F, Baqui AH, Christian P, Fawzi W, Gonzalez R, Humphrey J, Huybregts L, Kolsteren P, Mongkolchati A, Mullany LC, Ndyomugyenyi R, Nien JK, Osrin D, Roberfroid D, Sania A, Schmiegelow C, Silveira MF, Tielsch J, Vaidya A, Velaphi SC, Victora CG, Watson-Jones D, Black RE; CHERG Small-for-Gestational-Age-Preterm Birth Working Group. Mortality risk in preterm and small-for-gestational-age infants in low-income and middle-income countries: a pooled country analysis. Lancet. 2013; 382(9890): 417-25.

Appendix Table 2. Supplemental information for Figure 1: Epidemiological evidence supporting causality between risk-outcome pairs included in the Global Burden of Disease 2016 study including 2A. Citations and 2B. Additional information

6A. Citations

Risk	Outcome	Citation/Note

6B. Supplemental information

“RCTs (Number)” represents the total number of independent randomized controlled trials evaluating the relationship of each risk-outcome pair. “RCTs with significant effect in the opposite direction (%)” represents the percentage of randomized controlled trials showing a significant effect in the opposite direction. “Prospective observational studies (Number)” shows the total number of independent prospective cohort studies or non-randomized interventions evaluating the relationship of the risk-outcome pair. “Prospective observational studies with significant association in the opposite direction (%)” represents the percentage of prospective cohort studies or non-randomized interventions reporting a significant association in the opposite direction. “Lower limit of RR > 1.5” shows whether the lower limit of the 95% confidence interval for the relative risk of the risk-outcome pair is greater than 1.5. “Dose-response relationship” shows whether there is any evidence of linear or non-linear dose-response relationship between the risk and the outcome. “Biologic plausibility” shows whether there is any biologic or mechanistic pathway that could potentially explain the relationship of the risk-outcome pair. “Analogy” shows whether the risk is associated with another outcome from the same category and there is evidence that it can cause the current outcome through the same pathway. The numbers in the table represent the independent RCTs and prospective observational studies evaluated the relationship between each risk-outcome pairs. If there were multiple reports from one study, they were counted as one study. Dose-response relationship was only assessed for continuous risks. To evaluate the magnitude of the effect size for continuous risks, we evaluated the RR comparing the 75th percentile to the 25th percentile of the exposure distribution at the global level .

Unsafe water, sanitation, and handwashing	Typhoid and paratyphoid fever	Typhoid and paratyphoid were included as outcome for unsafe water and sanitation by analogy to diarrhoeal diseases
Household air pollution from solid fuels	Cataract	Evidence on the relationship between household air pollution and cataract was from 6 case-control and 1 cross-sectional studies
Air pollution	--	The relationships of cerebrovascular disease, chronic obstructive pulmonary disease, ischaemic heart disease, and lung cancer with ambient air pollution, second-hand smoke, and active smoking were used to interpolate their relationship with household air pollution. We considered the biological pathway for health impact of all four sources to be PM2.5 exposure, with the effect size being a function of the level of PM2.5. As such, we presented data from cohorts reporting on ambient PM2.5 and the outcome was used to inform the strength of evidence for household air pollution.
Other environmental risks and dietary risks	Cardiovascular diseases and chronic kidney disease	The health effects of lead and sodium on cardiovascular outcomes and chronic kidney disease were assessed through systolic blood pressure and the health effects of sugar sweetened beverages were assessed through body mass index.
Residential Radon	Tracheal, bronchus, and lung cancer	In evaluation of evidence on the relationship of residential radon and lung cancer, we excluded evidence from cohorts o miners as they were not from a representative population. Evidence on this risk-outcome pair mostly comes from case-control studies
Occupational injuries	Injuries	Evidence from International Labour Organization Safety and Health and Eurostat Safety and Health was used to establish causality between occupational injuries and injurie
Child and maternal malnutrition	--	Evidence on the causal relationship of childhood stunting, underweight, and wasting was from a pooled analysis of 7 prospective cohorts
Child and maternal malnutrition	--	For the following risk-outcome pairs, the risk factor was considered as the necessary cause: childhood underweight and protein-energy malnutrition; childhood wasting and protein-energy malnutrition; vitamin A deficiency and vitamin A deficiency; alcohol use and cirrhosis due to alcohol use; alcohol use and alcohol use disorders; alcohol use and liver cancer due to alcohol use; drug use and amphetamine use disorders; drug use and cannabis use disorders; drug use and cocaine use disorders; drug use and opioid use disorders; drug use and other drug use disorders; iron deficiency and iron deficiency anemia; unsafe sex and cervical cancer; unsafe sex and syphilis; unsafe sex and chlamydial infection; unsafe sex and gonococcal infection; unsafe sex and trichomoniasis; unsafe sex and genital herpes; unsafe sex and other sexually transmitted diseases; high systolic blood pressure and hypertensive heart disease; high systolic blood pressure and chronic kidney disease due to hypertension; high fasting plasma glucose and chronic kidney disease due to diabetes mellitus; high fasting plasma glucose and diabetes mellitus; low glomerular filtration rate and chronic kidney disease
Iron deficiency	Maternal haemorrhage	Evidence on the relationship of iron deficiency with maternal haemorrhage and maternal sepsis mainly came 10 observational studies evaluating the association between low hemoglobin and maternal mortality using hospital records
Smoking, alcohol use, and high body mass index	--	For smoking, alcohol use, and high body mass index evidence from risk reduction trials has not been included
Smoking, alcohol use, and high body mass index	Liver cancer	Liver cancer included liver cancer due to alcohol use, hepatitis B, hepatitis C, and other causes
Smoking	Lower respiratory infections	Evidence on the relationship between smoking and lower respiratory infections comes 10 case-control or cross-sectional studies
Smoking, alcohol use	Nasopharynx cancer	The evidence on causal relationship of alcohol and smoking with nasopharynx cancer was from the studies evaluating oral cavity and pharyngeal cancers as outcome
Smoking	Bladder cancer	The evidence on causal relationship of smoking and bladder cancer was based on the studies evaluating the lower urinary tract as outcome
Smoking	Asbestosis	Asbestosis, coal workers pneumoconiosis, other pneumoconiosis, silicosis were included as outcomes for smoking as they were included in the other chronic respiratory diseases categor
Alcohol use	Ischaemic heart disease, cerebrovascular disease, hypertensive heart disease, and diabetes mellitus	Alcohol was included as both a protective and harmful risk factor for ischaemic heart disease, cerebrovascular disease, hypertensive heart disease, and diabetes mellitus
Alcohol use	Cirrhosis	Cirrhosis included cirrhosis due to alcohol use, hepatitis B, hepatitis C, and other causes
Alcohol use	Self-harm	Self-harm was included as an outcome for alcohol use by analogy to injury
Alcohol use	Injuries	Injuries included pedestrian road injuries, cyclist road injuries, motorcyclist road injuries, motor vehicle road injuries, drowning, falls, fire, heat, hot substances, poisonings, unintentional firearm injuries, unintentional suffocation, other exposure to mechanical forces
Alcohol use	Interpersonal violence	Interpersonal violence included assault by firearm, sharp object, other means
Diet low in nuts and seeds	Ischaemic heart disease and diabetes mellitus	Experimental evidence on the relationship of nuts with ischaemic heart disease and diabetes mellitus come from the PREDIMED trial; a randomized trial consisting of three arms: a Mediterranean diet with extra-virgin olive oil, a Mediterranean diet with nuts, and a control diet. Given that the intake of dietary factors other than nuts changed in the intervention arms of this trial, the observed effect might be fully attributable to nuts.
Diet high in sugar sweetened beverages and body mass index	--	Evidence on the relationship between sugar-sweetened beverages and body mass index comes from the interventional and prospective observational studies evaluating the relationship of sugar-sweetened beverages with weight change

Appendix Table 2. Supplemental information for Figure 1: Epidemiological evidence supporting causality between risk-outcome pairs included in the Global Burden of Disease 2016 study including 2A. Citations and 2B. Additional information
6A. Citations

Risk	Outcome	Citation/Note
Diet high in sodium	Cardiovascular diseases	Evidence on the direct effect of sodium on cardiovascular disease mainly comes from prospective cohort studies. Considering that, in GBD, we have only evaluated the effect of sodium mediated through systolic blood pressure, we di not present epidemiologic evidence on the direct effect of sodium on cardiovascular disease in this table. Evidence on the effect of sodium on systolic blood pressure mostly comes from randomized controlled trials. While some cohort studies evaluated the relationship between sodium and systolic blood pressure, we did not identify a systematic evaluation of these studies.
Drug use	Hepatitis B and C	We included liver cancer due to Hepatitis B and Hepatitis C and cirrhosis due to Hepatitis B and Hepatitis C as outcomes for drug use because these were considered secondary outcomes of Hepatitis B and Hepatitis C
Drug use, unsafe sex	HIV/AIDS	For the following risk-outcome pairs, the risk factor was considered as the sufficient cause: drug use and HIV/AIDS and unsafe sex and HIV/AIDS
Metabolic risks	Chronic kidney disease	Chronic kidney disease included chronic kidney disease due to diabetes mellitus, hypertension, glomerulonephritis, or
High fasting plasma glucose	Cerebrovascular disease, chronic kidney disease, ischaemic heart disease	Evidence on the relationship of high fasting plasma glucose with stroke (DECODE, APCSC, ERFC); chronic kidney disease (APCSC), and ischaemic heart disease (DECODE, APCSC, ERFC) was from pooled analysis of cohorts
High systolic blood pressure	Atrial fibrillation and flutter, peripheral vascular disease	Evidence on the relationship of high systolic blood pressure with atrial fibrillation and peripheral vascular disease was from two pooled cohort analysis (APCSC and PSC)
High systolic blood pressure	Rheumatic heart disease, cardiomyopathy and myocarditis, aortic aneurysm, endocarditis, and other cardiovascular diseases	Evidence on the relationship of high systolic blood pressure with rheumatic heart disease, cardiomyopathy and myocarditis, aortic aneurysm, endocarditis, and other cardiovascular diseases came from a pooled cohort analysis (PSC)
High body-mass index	Ischaemic heart disease	Evidence on the relationship of high body-mass index with ischaemic heart disease (APCSC, ERFC, PSC) and stroke (ischaemic: APCSC, ERFC, PSC; hemorrhagic: PSC and ERFC) came from three pooled cohort analysis
High body-mass index	Diabetes mellitus, hypertensive heart disease	Evidence on the relationship of high body-mass index with diabetes mellitus and hypertensive heart disease came from two pooled cohort analysis (APCSC and PSC)
High body-mass index	Chronic kidney disease	Evidence on the relationship of high body-mass index with chronic kidney disease was from a pooled cohort analysis (PSC)
High total cholesterol	Ischaemic heart disease, ischaemic stroke	Evidence on the relationship of high total cholesterol with ischaemic heart disease and ischaemic stroke came from two pooled cohort analysis (APCSC and PSC)
Impaired kidney function	Tuberculosis	Glycemic Control and the Risk of Tuberculosis: A Cohort Study.

**Appendix Table 3. GBD 2016 risk factor hierarchy with levels, modeling strategies, and the main type of data sources used to estimate exposure levels**<br>GBD=Global Burden of Disease.			
**Risk factor**	**Level**	**Model type**	**Main data source for exposure**
**All risk factors**	**0**		
**Environmental/occupational risks**	**1**		
**Unsafe water, sanitation, and handwashing**	**2**		
Unsafe water source	3	Spatiotemporal Gauissian process regression (ST-GPR)	Population surveys and censuses
Unsafe sanitation	3	ST-GPR	Population surveys and censuses
No handwashing with soap	3	ST-GPR	Population surveys, censuses, and epidemiological studies
**Air pollution**	**2**		
Ambient particulate matter pollution	3	Regression crosswalk between grid-level fusion of satellite/chemical transport models and ground level monitoring data	Atmospheric chemical transport models, satellite measurements of aerosols in the atmosphere, data from ground-level monitoring sites
Household air pollution from solid fuels	3	ST-GPR	Population surveys and censuses, WHO Energy Database, literature review
Ambient ozone pollution	3	Chemical transport model	Atmospheric chemical transport models
**Other environmental risks**	**2**		
Residential radon	3	ST-GPR	Literature review, government agencies, and monitoring stations
Lead exposure	3	ST-GPR	Literature review, blood lead surveys
**Occupational risks**	**2**		
Occupational carcinogens	3		
Occupational exposure to asbestos	4	Asbestos Impact Ratio approach	GBD cause-specific mortality data for mesothelioma, epidemiological studies
Occupational exposure to arsenic	4	ST-GPR	Labor force surveys, censuses, and international information system on occupational exposure to carcinogens
Occupational exposure to benzene	4	ST-GPR	Labor force surveys, censuses, and international information system on occupational exposure to carcinogens
Occupational exposure to beryllium	4	ST-GPR	Labor force surveys, censuses, and international information system on occupational exposure to carcinogens
Occupational exposure to cadmium	4	ST-GPR	Labor force surveys, censuses, and international information system on occupational exposure to carcinogens
Occupational exposure to chromium	4	ST-GPR	Labor force surveys, censuses, and international information system on occupational exposure to carcinogens
Occupational exposure to diesel engine exhaust	4	ST-GPR	Labor force surveys, censuses, and international information system on occupational exposure to carcinogens
Occupational exposure to second-hand smoke	4	ST-GPR	Labor force surveys, censuses, and international information system on occupational exposure to carcinogens
Occupational exposure to formaldehyde	4	ST-GPR	Labor force surveys, censuses, and international information system on occupational exposure to carcinogens
Occupational exposure to nickel	4	ST-GPR	Labor force surveys, censuses, and international information system on occupational exposure to carcinogens
Occupational exposure to polycyclic aromatic hydrocarbons	4	ST-GPR	Labor force surveys, censuses, and international information system on occupational exposure to carcinogens
Occupational exposure to silica	4	ST-GPR	Labor force surveys, censuses, and international information system on occupational exposure to carcinogens
Occupational exposure to sulfphuric acid	4	ST-GPR	Labor force surveys, censuses, and international information system on occupational exposure to carcinogens
Occupational exposure to trichloroethylene	4	ST-GPR	Labor force surveys, censuses, and international information system on occupational exposure to carcinogens
Occupational asthmagens	3	ST-GPR	Labor force surveys and censuses
Occupational particulate matter, gases, and fumes	3	ST-GPR	Labor force surveys and censuses
Occupational noise	3	ST-GPR	Labor force surveys and censuses, industry-based surveys of noise exposure
Occupational injuries	3	ST-GPR	International Labor Organization injury database
Occupational ergonomic factors	3	ST-GPR	Labor force surveys and censuses
**Behavioural risks**	**1**		
**Child and maternal malnutrition**	**2**		
Suboptimal breastfeeding	3		
Non-exclusive breastfeeding	4	ST-GPR	Population surveys
Discontinued breastfeeding	4	ST-GPR	Population surveys
Child growth failure	3		
Childhood underweight	4	DisMod-MR 2.1	Population surveys, literature review, and WHO Global Database on Child Growth and Malnutrition
Childhood wasting	4	DisMod-MR 2.1	Population surveys, literature review, and WHO Global Database on Child Growth and Malnutrition
Childhood stunting	4	DisMod-MR 2.1	Population surveys, literature review, and WHO Global Database on Child Growth and Malnutrition
Low birth weight and short gestation	3		
Low birth weight for gestation	4	Joint distribution using Copula optimization	Population surveys and censuses, vital registration
Short gestation for birth weight	4	Joint distribution using Copula optimization	Population surveys and censuses, vital registration
Iron deficiency	3	Mixed effect regression	Population surveys, examination surveys, and epidemiological studies
Vitamin A deficiency	3	DisMod-MR 2.1	Population surveys, examination surveys, and epidemiological studies
Zinc deficiency	3	Mixed effect regression based on stunting prevalence and dietary composition	FAO food balance sheets
**Tobacco smoke**	**2**		
Smoking	3	• Smoking Impact Ratio (SIR) calculated from lung cancer mortality rates<br>• Smoking prevalence estimated using ST-GPR	SIR input data: mortality and cause of death data including vital registration and verbal autopsy<br>Smoking prevalence input data: nationally representative survey and report data and WHO InfoBase and International Smoking Statistics Database
Second-hand smoke	3	ST-GPR	Household surveys and national health surveys
Smokeless tobacco	3	ST-GPR	Nationall representative survey report and report data, literature review

Risk factor	Level	Model type	Main data source for exposure
**Alcohol and drug use**	**2**		
Alcohol use	3	• Alcohol consumption per capita obtained from the FAO and the WHO Global Information System on Alcohol and Health (GISAH)<br>• ST-GPR used to integrate the data and to derive coherent time series for each country<br>• Prevalence of current alcohol drinkers, lifetime abstainers, former drinkers, and binge drinkers estimated using DisMod-MR 2.1<br>• DisMod-MR 2.1 used to estimate the relative sex- and age-specific pattern of alcohol consumption in current drinkers	Population surveys, alcohol sales, production, and other economic statistics
Drug use	3	DisMod-MR 2.1	Systematic review of published literature, reports from governments and international organizations, which include data from: school surveys, population surveys, registration data, and indirect estimates of prevalence
**Dietary risks**	**2**		
Diet low in fruits	3	DisMod-MR 2.1	Nutrition and health surveys, FAO food balance sheets
Diet low in vegetables	3	DisMod-MR 2.1	Nutrition and health surveys, FAO food balance sheets
Diet low in legumes	3	DisMod-MR 2.1	Nutrition and health surveys
Diet low in whole grains	3	DisMod-MR 2.1	Nutrition and health surveys
Diet low in nuts and seeds	3	DisMod-MR 2.1	Nutrition and health surveys, FAO food balance sheets
Diet low in milk	3	DisMod-MR 2.1	Nutrition and health surveys, FAO food balance sheets
Diet high in red meat	3	DisMod-MR 2.1	Nutrition and health surveys, FAO food balance sheets
Diet high in processed meat	3	DisMod-MR 2.1	Nutrition and health surveys
Diet high in sugar-sweetened beverages	3	DisMod-MR 2.1	Nutrition and health surveys
Diet low in fibre	3	DisMod-MR 2.1	Nutrition and health surveys, FAO SUA/USDA
Diet suboptimal in calcium	3	DisMod-MR 2.1	Nutrition and health surveys, FAO SUA/USDA
Diet low in seafood omega-3 fatty acids	3	DisMod-MR 2.1	Nutrition and health surveys, FAO SUA/USDA
Diet low in polyunsaturated fatty acids	3	DisMod-MR 2.1	Nutrition and health surveys, FAO SUA/USDA
Diet high in trans fatty acids	3	DisMod-MR 2.1	Nutrition and health surveys, availability of partially hydrogenated vegetable oil in packaged foods
Diet high in sodium	3	DisMod-MR 2.1	Nutrition and health surveys, 24-hour urinary sodium
**Sexual abuse and violence**	**2**		
Childhood sexual abuse	3	DisMod-MR 2.1	Systematic review of published literature, national health surveys, violence-specific surveys
Intimate partner violence	3	DisMod-MR 2.1	Systematic review of published literature, national health surveys, violence-specific surveys
**Unsafe sex**	2	DisMod-MR 2.1	UNAIDS country progress reports, disease surveillance reports
**Low physical activity**	2	DisMod-MR 2.1	Surveys of the adult population that capture reported frequency, duration and intensity of physical activity undertaken in the past seven days across all domains of life (work, transport, recreation or house/yard work)
**Metabolic risks**	**1**		
High fasting plasma glucose	2	ST-GPR	Examination surveys and epidemiological studies
High total cholesterol	2	ST-GPR	Examination surveys and epidemiological studies
High systolic blood pressure	2	ST-GPR	Examination surveys and epidemiological studies
High body-mass index	2	ST-GPR	Examination surveys and epidemiological studies
Low bone mineral density	2	DisMod-MR 2.1	Examination surveys and epidemiological studies
Impaired kidney function	2	DisMod-MR 2.1	Examination surveys and epidemiological studies

Appendix Table 4. Types of Comparative Risk Assessments (CRA) based on the time perspective and the nature of the counterfactual level or distribution of exposure. The shaded box represents the type of CRA currently undertaken in GBD 2016. GBD=Global Burden of Disease.

	**Counterfactual distributions of exposure**			
Construct	Theoretical minimum risk: level of risk with the lowest level of burden	Plausible minimum risk: level of risk with the lowest level of burden that could be imagined with current technology and knowledge	Feasible minimum risk: level of risk with the lowest level of burden that has been achieved in any population	Cost-effective minimum risk: lowest level of risk that can be achieved cost-effectively in a given population
**Attributable burden:** burden of disease today that would be avoided if each individual in the past had been exposed to the counterfactual level of exposure	**Currently in GBD**			
**Avoidable burden:** burden of disease in the future that would be avoided if each individual today was shifted to the counterfactual level of exposure				

Appendix Table 5. Guidelines for Accurate and Transparent Health Estimates Reporting (GATHER) 18-items checklist with description of compliance and location of information for GBD 2016 risk factors capstone

#	GATHER checklist item	Description of compliance	Reference
**Objectives and funding**			
1	Define the indicators, populations, and time periods for which estimates were made.	Narrative provided in paper and appendix describing indicators, definitions, and populations.	Main text; Tables & Figures; and Appendix, Section 1. GBD Overview
2	List the funding sources for the work.	Funding sources listed in paper.	Main text, Summary, Funding.
**Data Inputs**			
For all data inputs from multiple sources that are synthesized as part of the study:			
3	Describe how the data were identified and how the data were accessed.	Narrative description of data seeking methodology provided.	Main text, Methods, Estimation process, Effect size estimation and Exposure estimation; and Appendix, Section 2. Risk factor estimation, Step 1. Effect size estimation, 1a. Collate relative risk data and Step 2. Exposure estimation, 2a collate exposure data; and Section 3. Risk-specific estimation
4	Specify the inclusion and exclusion criteria. Identify all ad-hoc exclusions.	Narrative about inclusion and exclusion criteria by data type provided.	Main text, Methods, Estimation process, Effect size estimation and Exposure estimation; and Appendix, Section 2. Risk factor estimation, Step 1. Effect size estimation, 1a. Collate relative risk data and Step 2. Exposure estimation, 2a collate exposure data; and Section 3. Risk-specific estimation
5	Provide information on all included data sources and their main characteristics. For each data source used, report reference information or contact name/institution, population represented, data collection method, year(s) of data collection, sex and age range, diagnostic criteria or measurement method, and sample size, as relevant.	Interactive, online data source tool that provides metadata for data sources by component, geography, cause, risk, or impairment has been developed.	http://ghdx.healthdata.org/node/311220
6	Identify and describe any categories of input data that have potentially important biases (e.g., based on characteristics listed in item 5).	Summary of known biases by risk included in methodological appendix.	Appendix, Section 3. Risk-specific estimation

For data inputs that contribute to the analysis but were not synthesized as part of the study:			
7	Describe and give sources for any other data inputs.	Interactive, online data source tool that provides metadata for data sources by component, geography, cause, risk, or impairment has been developed.	http://ghdx.healthdata.org/node/311220
For all data inputs:			
8	Provide all data inputs in a file format from which data can be efficiently extracted (e.g., a spreadsheet as opposed to a PDF), including all relevant meta-data listed in item 5. For any data inputs that cannot be shared due to ethical or legal reasons, such as third-party ownership, provide a contact name or the name of the institution that retains the right to the data.	Downloads of input data will be available through online tools, including data visualization tools and data query tools. Input data not available in tools will be made available upon request.	http://ghdx.healthdata.org/node/311220
**Data analysis**			
9	Provide a conceptual overview of the data analysis method. A diagram may be helpful.	Flow diagrams of the overall methodological processes, as well as risk-specific modelling processes have been provided.	Main text, Methods; Appendix, Section 2, DisMod-MR 2.1 Estimation and spatiotemporal Gaussian process regression estimation; Appendix, Section 3. Risk-specific estimation; and Appendix Figure 2. Analytical flowchart of the comparative risk assessment for the estimation of population attributable fractions by geography, age, sex, and year for GBD 2016
10	Provide a detailed description of all steps of the analysis, including mathematical formulae. This description should cover, as relevant, data cleaning, data pre-processing, data adjustments and weighting of data sources, and mathematical or statistical model(s).	Flow diagrams and corresponding methodological write-ups for each risk and modelling processes have been provided.	Appendix, Section 2, DisMod-MR 2.1 Estimation and spatiotemporal Gaussian process regression estimation; and Appendix, Section 3. Risk-specific estimation
11	Describe how candidate models were evaluated and how the final model(s) were selected.	Provided in the methodological write-ups.	Appendix, Section 3. Risk-specific estimation
12	Provide the results of an evaluation of model performance, if done, as well as the results of any relevant sensitivity analysis.	Provided in the methodological write-ups.	Appendix, Section 3. Risk-specific estimation
13	Describe methods for calculating uncertainty of the estimates. State which sources of uncertainty were, and were not, accounted for in the uncertainty analysis.	Provided in the methodological write-ups.	Appendix, Section 3. Risk-specific estimation
14	State how analytic or statistical source code used to generate estimates can be accessed.	Access statement provided.	Code will be provided in an online repository
**Results and Discussion**			

15	Provide published estimates in a file format from which data can be efficiently extracted.	GBD 2016 results will be made available through online data visualization tools, the Global Health Data Exchange, and the online data query tool (these tools are already available for GBD 2015 results).	Main text; Supplemental results; and online data tools: http://www.healthdata.org/results/data-visualizations; http://ghdx.healthdata.org/
16	Report a quantitative measure of the uncertainty of the estimates (e.g. uncertainty intervals).	Uncertainty intervals are provided with all results.	Main text; Appendix, Section 3. Risk-specific estimation; and online tools: http://www.healthdata.org/results/data-visualizations; http://ghdx.healthdata.org/
17	Interpret results in light of existing evidence. If updating a previous set of estimates, describe the reasons for changes in estimates.	Discussion of methodological changes between GBD rounds provided in the narrative of the paper and appendix.	Main text, Methods; Appendix, Section 2, Step 1. Effect size estimation, Step 2 Exposure estimation; and Appendix, Section 3. Risk-specific estimation
18	Discuss limitations of the estimates. Include a discussion of any modelling assumptions or data limitations that affect interpretation of the estimates.	Discussion of limitations provided in the narrative of the main paper as well as in the methodological write-ups in the appendix.	Main text, Limitations; and Appendix, Section 3. Risk-specific estimation

Appendix Table 6: GBD 2016 location hierarchy with levels	
**Location**	**Level**
Global	0
Southeast Asia, East Asia, and Oceania	1
East Asia	2
China	3
Anhui	4
Beijing	4
Chongqing	4
Fujian	4
Gansu	4
Guangdong	4
Guangxi	4
Guizhou	4
Hainan	4
Hebei	4
Heilongjiang	4
Henan	4
Hong Kong Special Administrative Region of China	4
Hubei	4
Hunan	4
Inner Mongolia	4
Jiangsu	4
Jiangxi	4
Jilin	4
Liaoning	4
Macao Special Administrative Region of China	4
Ningxia	4
Qinghai	4
Shaanxi	4
Shandong	4
Shanghai	4
Shanxi	4
Sichuan	4
Tianjin	4
Tibet	4
Xinjiang	4
Yunnan	4
Zhejiang	4
North Korea	3
Taiwan (Province of China)	3
Southeast Asia	2
Cambodia	3
Indonesia	3
Aceh	4
Bali	4
Bangka Belitung	4
Banten	4

Appendix Table 6 GBD 2016 location hierarchy with levels

Location	Level
Bengkulu	4
Gorontalo	4
Jakarta	4
Jambi	4
Cental Java	4
West Java	4
East Java	4
West Kalimantan	4
South Kalimantan	4
Central Kalimantan	4
East Kalimantan	4
North Kalimantan	4
Riau Islands	4
Lampung	4
Maluku	4
North Maluku	4
Nusa Tenggara Barat	4
East Nusa Tenggara	4
Papua	4
West Papua	4
Riau	4
West Sulawesi	4
South Sumatera	4
Central Sulawesi	4
Southeast Sulawesi	4
North Sulawesi	4
West Sumatera	4
South Sumatera	4
North Sumatera	4
Yogyakarta	4
Laos	3
Malaysia	3
Maldives	3
Mauritius	3
Myanmar	3
Philippines	3
Sri Lanka	3
Seychelles	3
Thailand	3
Timor-Leste	3
Vietnam	3
Oceania	2
American Samoa	3
Federated States of Micronesia	3
Fiji	3
Guam	3

Appendix Table 6 GBD 2016 location hierarchy with levels

Location	Level
Kiribati	3
Marshall Islands	3
Northern Mariana Islands	3
Papua New Guinea	3
Samoa	3
Solomon Islands	3
Tonga	3
Vanuatu	3
Central Europe, Eastern Europe, and Central Asia	1
Central Asia	2
Armenia	3
Azerbaijan	3
Georgia	4
Georgia	3
Kazakhstan	3
Kyrgyzstan	3
Mongolia	3
Tajikistan	3
Turkmenistan	3
Uzbekistan	3
Central Europe	2
Albania	3
Bosnia and Herzegovina	3
Bulgaria	3
Croatia	3
Czech Republic	3
Hungary	3
Macedonia	3
Montenegro	3
Poland	3
Romania	3
Serbia	3
Slovakia	3
Slovenia	3
Eastern Europe	2
Belarus	3
Estonia	3
Latvia	3
Lithuania	3
Moldova	3
Russia	3
Ukraine	3
High-income	1
High-income Asia Pacific	2
Brunei	3
Japan	3

Appendix Table 6 GBD 2016 location hierarchy with levels	
**Location**	**Level**
Aichi	4
Akita	4
Aomori	4
Chiba	4
Ehime	4
Fukui	4
Fukuoka	4
Fukushima	4
Gifu	4
Gunma	4
Hiroshima	4
Hokkaidō	4
Hyōgo	4
Ibaraki	4
Ishikawa	4
Iwate	4
Kagawa	4
Kagoshima	4
Kanagawa	4
Kōchi	4
Kumamoto	4
Kyōto	4
Mie	4
Miyagi	4
Miyazaki	4
Nagano	4
Nagasaki	4
Nara	4
Niigata	4
Ôita	4
Okayama	4
Okinawa	4
Ōsaka	4
Saga	4
Saitama	4
Shiga	4
Shimane	4
Shizuoka	4
Tochigi	4
Tokushima	4
Tōkyō	4
Tottori	4
Toyama	4
Wakayama	4
Yamagata	4
Yamaguchi	4

Appendix Table 6 GBD 2016 location hierarchy with levels

Location	Level
Yamanashi	4
South Korea	3
Singapore	3
Australasia	2
Australia	3
New Zealand	3
Western Europe	2
Andorra	3
Austria	3
Belgium	3
Cyprus	3
Denmark	3
Finland	3
France	3
Germany	3
Greece	3
Iceland	3
Ireland	3
Israel	3
Italy	3
Luxembourg	3
Malta	3
Netherlands	3
Norway	3
Portugal	3
Spain	3
Sweden	3
Stockholm	4
Sweden except Stockholm	4
Switzerland	3
United Kingdom	3
England	4
East Midlands	5
Derby	6
Derbyshire	6
Leicester	6
Leicestershire	6
Lincolnshire	6
Northamptonshire	6
Nottingham	6
Nottinghamshire	6
Rutland	6
East of England	5
Bedford	6
Cambridgeshire	6
Central Bedfordshire	6

Appendix Table 6 GBD 2016 location hierarchy with levels	
**Location**	**Level**
Essex	6
Hertfordshire	6
Luton	6
Norfolk	6
Peterborough	6
Southend-on-Sea	6
Suffolk	6
Thurrock	6
Greater London	5
Barking and Dagenham	6
Barnet	6
Bexley	6
Brent	6
Bromley	6
Camden	6
Croydon	6
Ealing	6
Enfield	6
Greenwich	6
Hackney	6
Hammersmith and Fulham	6
Haringey	6
Harrow	6
Havering	6
Hillingdon	6
Hounslow	6
Islington	6
Kensington and Chelsea	6
Kingston upon Thames	6
Lambeth	6
Lewisham	6
Merton	6
Newham	6
Redbridge	6
Richmond upon Thames	6
Southwark	6
Sutton	6
Tower Hamlets	6
Waltham Forest	6
Wandsworth	6
Westminster	6
North East England	5
County Durham	6
Darlington	6
Gateshead	6
Hartlepool	6

Appendix Table 6 GBD 2016 location hierarchy with levels

Location	Level
Middlesbrough	6
Newcastle upon Tyne	6
North Tyneside	6
Northumberland	6
Redcar and Cleveland	6
South Tyneside	6
Stockton-on-Tees	6
Sunderland	6
North West England	5
Blackburn with Darwen	6
Blackpool	6
Bolton	6
Bury	6
Cheshire East	6
Cheshire West and Chester	6
Cumbria	6
Halton	6
Knowsley	6
Lancashire	6
Liverpool	6
Manchester	6
Oldham	6
Rochdale	6
Salford	6
Sefton	6
St Helens	6
Stockport	6
Tameside	6
Trafford	6
Warrington	6
Wigan	6
Wirral	6
South East England	5
Bracknell Forest	6
Brighton and Hove	6
Buckinghamshire	6
East Sussex	6
Hampshire	6
Isle of Wight	6
Kent	6
Medway	6
Milton Keynes	6
Oxfordshire	6
Portsmouth	6
Reading	6
Slough	6

Appendix Table 6 GBD 2016 location hierarchy with levels	
**Location**	**Level**
Southampton	6
Surrey	6
West Berkshire	6
West Sussex	6
Windsor and Maidenhead	6
Wokingham	6
South West England	5
Bath and North East Somerset	6
Bournemouth	6
Bristol, City of	6
Cornwall	6
Devon	6
Dorset	6
Gloucestershire	6
North Somerset	6
Plymouth	6
Poole	6
Somerset	6
South Gloucestershire	6
Swindon	6
Torbay	6
Wiltshire	6
West Midlands	5
Birmingham	6
Coventry	6
Dudley	6
Herefordshire, County of	6
Sandwell	6
Shropshire	6
Solihull	6
Staffordshire	6
Stoke-on-Trent	6
Telford and Wrekin	6
Walsall	6
Warwickshire	6
Wolverhampton	6
Worcestershire	6
Yorkshire and the Humber	5
Barnsley	6
Bradford	6
Calderdale	6
Doncaster	6
East Riding of Yorkshire	6
Kingston upon Hull, City of	6
Kirklees	6
Leeds	6

Appendix Table 6 GBD 2016 location hierarchy with levels	
**Location**	**Level**
North East Lincolnshire	6
North Lincolnshire	6
North Yorkshire	6
Rotherham	6
Sheffield	6
Wakefield	6
York	6
Northern Ireland	4
Scotland	4
Wales	4
Southern Latin America	2
Argentina	3
Chile	3
Uruguay	3
High-income North America	2
Canada	3
Greenland	3
USA	3
Alabama	4
Alaska	4
Arizona	4
Arkansas	4
California	4
Colorado	4
Connecticut	4
Delaware	4
District of Columbia	4
Florida	4
Georgia	3
Georgia	4
Hawaii	4
Idaho	4
Illinois	4
Indiana	4
Iowa	4
Kansas	4
Kentucky	4
Louisiana	4
Maine	4
Maryland	4
Massachusetts	4
Michigan	4
Minnesota	4
Mississippi	4
Missouri	4
Montana	4

Appendix Table 6 GBD 2016 location hierarchy with levels	
**Location**	**Level**
Nebraska	4
Nevada	4
New Hampshire	4
New Jersey	4
New Mexico	4
New York	4
North Carolina	4
North Dakota	4
Ohio	4
Oklahoma	4
Oregon	4
Pennsylvania	4
Rhode Island	4
South Carolina	4
South Dakota	4
Tennessee	4
Texas	4
Utah	4
Vermont	4
Virginia	4
Washington	4
West Virginia	4
Wisconsin	4
Wyoming	4
Latin America and Caribbean	1
Caribbean	2
Antigua and Barbuda	3
The Bahamas	3
Barbados	3
Belize	3
Bermuda	3
Cuba	3
Dominica	3
Dominican Republic	3
Grenada	3
Guyana	3
Haiti	3
Jamaica	3
Puerto Rico	3
Saint Lucia	3
Saint Vincent and the Grenadines	3
Suriname	3
Trinidad and Tobago	3
Virgin Islands, U.S.	3
Andean Latin America	2
Bolivia	3

Appendix Table 6 GBD 2016 location hierarchy with levels	
**Location**	**Level**
Ecuador	3
Peru	3
Central Latin America	2
Colombia	3
Costa Rica	3
El Salvador	3
Guatemala	3
Honduras	3
Mexico	3
Aguascalientes	4
Baja California	4
Baja California Sur	4
Campeche	4
Chiapas	4
Chihuahua	4
Coahuila	4
Colima	4
Distrito Federal	4
Distrito Federal	4
Durango	4
Guanajuato	4
Guerrero	4
Hidalgo	4
Jalisco	4
México	4
Michoacán de Ocampo	4
Morelos	4
Nayarit	4
Nuevo León	4
Oaxaca	4
Puebla	4
Querétaro	4
Quintana Roo	4
San Luis Potosí	4
Sinaloa	4
Sonora	4
Tabasco	4
Tamaulipas	4
Tlaxcala	4
Veracruz de Ignacio de la Llave	4
Yucatán	4
Zacatecas	4
Nicaragua	3
Panama	3
Venezuela	3
Tropical Latin America	2

Appendix Table 6 GBD 2016 location hierarchy with levels	
**Location**	**Level**
Brazil	3
Acre	4
Alagoas	4
Amapá	4
Amazonas	4
Bahia	4
Ceará	4
Distrito Federal	4
Distrito Federal	4
Espírito Santo	4
Goiás	4
Maranhão	4
Mato Grosso	4
Mato Grosso do Sul	4
Minas Gerais	4
Pará	4
Paraíba	4
Paraná	4
Pernambuco	4
Piaui	4
Rio de Janeiro	4
Rio Grande do Norte	4
Rio Grande do Sul	4
Rondônia	4
Roraima	4
Santa Catarina	4
São Paulo	4
Sergipe	4
Tocantins	4
Paraguay	3
North Africa and Middle East	1
North Africa and Middle East	2
Afghanistan	3
Algeria	3
Bahrain	3
Egypt	3
Iran	3
Iraq	3
Jordan	3
Kuwait	3
Lebanon	3
Libya	3
Morocco	3
Palestine	3
Oman	3
Qatar	3

Appendix Table 6 GBD 2016 location hierarchy with levels	
**Location**	**Level**
Saudi Arabia	3
'Asir	4
Bahah	4
Eastern Province	4
Ha'il	4
Jawf	4
Jizan	4
Madinah	4
Makkah	4
Najran	4
Northern Borders	4
Qassim	4
Riyadh	4
Tabuk	4
Sudan	3
Syria	3
Tunisia	3
Turkey	3
United Arab Emirates	3
Yemen	3
South Asia	1
South Asia	2
Bangladesh	3
Bhutan	3
India	3
Andhra Pradesh	4
Andhra Pradesh, Rural	5
Andhra Pradesh, Urban	5
Arunāchal Pradesh	4
Arunāchal Pradesh, Rural	5
Arunāchal Pradesh, Urban	5
Assam	4
Assam, Rural	5
Assam, Urban	5
Bihār	4
Bihār, Rural	5
Bihār, Urban	5
Chhattīsgarh	4
Chhattīsgarh, Rural	5
Chhattīsgarh, Urban	5
Delhi	4
Delhi, Rural	5
Delhi, Urban	5
Goa	4
Goa, Rural	5
Goa, Urban	5

Appendix Table 6 GBD 2016 location hierarchy with levels	
**Location**	**Level**
Gujarāt	4
Gujarāt, Rural	5
Gujarāt, Urban	5
Haryāna	4
Haryāna, Rural	5
Haryāna, Urban	5
Himachal Pradesh	4
Himachal Pradesh, Rural	5
Himachal Pradesh, Urban	5
Jammu and Kashmīr	4
Jammu and Kashmīr, Rural	5
Jammu and Kashmīr, Urban	5
Jharkhand	4
Jharkhand, Rural	5
Jharkhand, Urban	5
Karnātaka	4
Karnātaka, Rural	5
Karnātaka, Urban	5
Kerala	4
Kerala, Rural	5
Kerala, Urban	5
Madhya Pradesh	4
Madhya Pradesh, Rural	5
Madhya Pradesh, Urban	5
Mahārāshtra	4
Mahārāshtra, Rural	5
Mahārāshtra, Urban	5
Manipur	4
Manipur, Rural	5
Manipur, Urban	5
Meghālaya	4
Meghālaya, Rural	5
Meghālaya, Urban	5
Mizoram	4
Mizoram, Rural	5
Mizoram, Urban	5
Nāgāland	4
Nāgāland, Rural	5
Nāgāland, Urban	5
Orissa	4
Orissa, Rural	5
Orissa, Urban	5
Punjab	4
Punjab, Rural	5
Punjab, Urban	5
Rājasthān	4

Appendix Table 6 GBD 2016 location hierarchy with levels	
**Location**	**Level**
Rājasthān, Rural	5
Rājasthān, Urban	5
Sikkim	4
Sikkim, Rural	5
Sikkim, Urban	5
Tamil Nādu	4
Tamil Nādu, Rural	5
Tamil Nādu, Urban	5
Telangana	4
Telangana, Rural	5
Telangana, Urban	5
Tripura	4
Tripura, Rural	5
Tripura, Urban	5
Uttar Pradesh	4
Uttar Pradesh, Rural	5
Uttar Pradesh, Urban	5
Uttarakhand	4
Uttarakhand, Rural	5
Uttarakhand, Urban	5
West Bengal	4
West Bengal, Rural	5
West Bengal, Urban	5
The Six Minor Territories	4
The Six Minor Territories, Rural	5
The Six Minor Territories, Urban	5
Nepal	3
Pakistan	3
Sub-Saharan Africa	1
Central Sub-Saharan Africa	2
Angola	3
the Central African Republic	3
Congo (Brazzaville)	3
Democratic Republic of the Congo	3
Equatorial Guinea	3
Gabon	3
Eastern Sub-Saharan Africa	2
Burundi	3
Comoros	3
Djibouti	3
Eritrea	3
Ethiopia	3
Kenya	3
Baringo	4
Bomet	4
Bungoma	4

Appendix Table 6 GBD 2016 location hierarchy with levels

Location	Level
Busia	4
Elgeyo-Marakwet	4
Embu	4
Garissa	4
HomaBay	4
Isiolo	4
Kajiado	4
Kakamega	4
Kericho	4
Kiambu	4
Kilifi	4
Kirinyaga	4
Kisii	4
Kisumu	4
Kitui	4
Kwale	4
Laikipia	4
Lamu	4
Machakos	4
Makueni	4
Mandera	4
Marsabit	4
Meru	4
Migori	4
Mombasa	4
Murang’a	4
Nairobi	4
Nakuru	4
Nandi	4
Narok	4
Nyamira	4
Nyandarua	4
Nyeri	4
Samburu	4
Siaya	4
TaitaTaveta	4
TanaRiver	4
TharakaNithi	4
TransNzoia	4
Turkana	4
UasinGishu	4
Vihiga	4
Wajir	4
WestPokot	4
Madagascar	3
Malawi	3

Appendix Table 6 GBD 2016 location hierarchy with levels	
**Location**	**Level**
Mozambique	3
Rwanda	3
Somalia	3
South Sudan	3
Tanzania	3
Uganda	3
Zambia	3
Southern Sub-Saharan Africa	2
Botswana	3
Lesotho	3
Namibia	3
South Africa	3
Eastern Cape	4
Free State	4
Gauteng	4
KwaZulu-Natal	4
Limpopo	4
Mpumalanga	4
North-West	4
Northern Cape	4
Western Cape	4
Swaziland	3
Zimbabwe	3
Western Sub-Saharan Africa	2
Benin	3
Burkina Faso	3
Cameroon	3
Cape Verde	3
Chad	3
Cote d'Ivoire	3
The Gambia	3
Ghana	3
Guinea	3
Guinea-Bissau	3
Liberia	3
Mali	3
Mauritania	3
Niger	3
Nigeria	3
Sao Tome and Principe	3
Senegal	3
Sierra Leone	3
Togo	3

Appendix Table 7. Mediation factors

For IHD, stroke, and diabetes we pooled all available cohorts and estimated relative risks with and without adjustment across all combinations of metabolic risk factors. We then computed the excess attenuated risk for each mediation-risk-cause set.

Cause	Risk Factor	Mediator	Cohort	Mediation Factor
Ischemic heart disease	Diet low in fiber	Diet low in fruits	0	1<br>(1 to 1)
Ischemic heart disease	Diet low in fiber	Diet low in whole grains	0	1<br>(1 to 1)
Ischemic heart disease	Diet low in fiber	Diet low in vegetables	0	1<br>(1 to 1)
Ischemic heart disease	Diet low in nuts and seeds	High body-mass index	0	0.01<br>(0 to 0.02)
Ischemic heart disease	Diet low in seafood omega-3 fatty acids	High body-mass index	0	0.03<br>(0.02 to 0.05)
Ischemic heart disease	Diet low in whole grains	High body-mass index	0	0.04<br>(0.03 to 0.05)
Hemorrhagic stroke	Diet low in whole grains	High body-mass index	0	0.03<br>(0.02 to 0.04)
Ischemic stroke	Diet low in whole grains	High body-mass index	0	0.04<br>(0.03 to 0.05)
Ischemic heart disease	Diet high in processed meat	High body-mass index	0	0.03<br>(0.02 to 0.04)
Ischemic heart disease	Diet high in sugar-sweetened beverages	High body-mass index	0	1<br>(1 to 1)
Hemorrhagic stroke	Diet high in sugar-sweetened beverages	High body-mass index	0	1<br>(1 to 1)
Ischemic stroke	Diet high in sugar-sweetened beverages	High body-mass index	0	1<br>(1 to 1)
Chronic kidney disease due to diabetes mellitus	Diet high in sugar-sweetened beverages	High body-mass index	0	1<br>(1 to 1)
Chronic kidney disease due to glomerulonephritis	Diet high in sugar-sweetened beverages	High body-mass index	0	1<br>(1 to 1)
Chronic kidney disease due to hypertension	Diet high in sugar-sweetened beverages	High body-mass index	0	1<br>(1 to 1)
Chronic kidney disease due to other causes	Diet high in sugar-sweetened beverages	High body-mass index	0	1<br>(1 to 1)
Atrial fibrillation and flutter	Diet high in sugar-sweetened beverages	High body-mass index	0	1<br>(1 to 1)
Cardiomyopathy and myocarditis	Diet high in sugar-sweetened beverages	High body-mass index	0	1<br>(1 to 1)
Hypertensive heart disease	Diet high in sugar-sweetened beverages	High body-mass index	0	1<br>(1 to 1)
Other cardiovascular and circulatory diseases	Diet high in sugar-sweetened beverages	High body-mass index	0	1<br>(1 to 1)
Peripheral artery disease	Diet high in sugar-sweetened beverages	High body-mass index	0	1<br>(1 to 1)
Diabetes mellitus	Diet high in sugar-sweetened beverages	High body-mass index	0	1<br>(1 to 1)
Osteoarthritis	Diet high in sugar-sweetened beverages	High body-mass index	0	1<br>(1 to 1)
Low back pain	Diet high in sugar-sweetened beverages	High body-mass index	0	1<br>(1 to 1)
Breast cancer	Diet high in sugar-sweetened beverages	High body-mass index	0	1<br>(1 to 1)
Colon and rectum cancer	Diet high in sugar-sweetened beverages	High body-mass index	0	1<br>(1 to 1)
Esophageal cancer	Diet high in sugar-sweetened beverages	High body-mass index	0	1<br>(1 to 1)
Gallbladder and biliary tract cancer	Diet high in sugar-sweetened beverages	High body-mass index	0	1<br>(1 to 1)

Appendix Table 7. Mediation factors

For IHD, stroke, and diabetes we pooled all available cohorts and estimated relative risks with and without adjustment across all combinations of metabolic risk factors. We then computed the excess attenuated risk for each mediation-risk-cause set.

Cause	Risk Factor	Mediator	Cohort	Mediation Factor
Kidney cancer	Diet high in sugar-sweetened beverages	High body-mass index	0	1<br>(1 to 1)
Pancreatic cancer	Diet high in sugar-sweetened beverages	High body-mass index	0	1<br>(1 to 1)
Uterine cancer	Diet high in sugar-sweetened beverages	High body-mass index	0	1<br>(1 to 1)
Ischemic heart disease	Diet low in nuts and seeds	High total cholesterol	1	0.13<br>(0 to 0.24)
Ischemic heart disease	Diet low in vegetables	High total cholesterol	0	0.04<br>(0.03 to 0.05)
Ischemic stroke	Diet low in vegetables	High total cholesterol	1	0.08<br>(0.03 to 0.13)
Ischemic heart disease	High fasting plasma glucose	High total cholesterol	1	0.03<br>(0.02 to 0.05)
Ischemic stroke	High fasting plasma glucose	High total cholesterol	1	0.04<br>(0.03 to 0.06)
Ischemic heart disease	Diet low in whole grains	High total cholesterol	0	0.39<br>(0.17 to 0.54)
Ischemic stroke	Diet low in whole grains	High total cholesterol	1	0.15<br>(0.04 to 0.24)
Ischemic heart disease	Diet high in sugar-sweetened beverages	High total cholesterol	0	0.1<br>(0.05 to 0.15)
Ischemic stroke	Diet high in sugar-sweetened beverages	High total cholesterol	0	0.03<br>(0 to 0.08)
Atrial fibrillation and flutter	Diet high in sugar-sweetened beverages	High total cholesterol	0	0.1<br>(0.05 to 0.15)
Cardiomyopathy and myocarditis	Diet high in sugar-sweetened beverages	High total cholesterol	0	0.1<br>(0.05 to 0.15)
Peripheral artery disease	Diet high in sugar-sweetened beverages	High total cholesterol	0	0.1<br>(0.05 to 0.15)
Ischemic heart disease	Diet low in fruits	High total cholesterol	0	0.06<br>(0.05 to 0.08)
Ischemic stroke	Diet low in fruits	High total cholesterol	0	0.05<br>(0.04 to 0.06)
Ischemic heart disease	Diet high in trans fatty acids	High total cholesterol	0	0.15<br>(0.02 to 0.24)
Ischemic heart disease	High body-mass index	High total cholesterol	0	0.1<br>(0.05 to 0.15)
Ischemic stroke	High body-mass index	High total cholesterol	0	0.03<br>(0 to 0.08)
Atrial fibrillation and flutter	High body-mass index	High total cholesterol	0	0.1<br>(0.05 to 0.15)
Cardiomyopathy and myocarditis	High body-mass index	High total cholesterol	0	0.1<br>(0.05 to 0.15)
Peripheral artery disease	High body-mass index	High total cholesterol	0	0.1<br>(0.05 to 0.15)
Ischemic heart disease	Diet low in nuts and seeds	High fasting plasma glucose	0	0.03<br>(0.02 to 0.06)
Diabetes mellitus	Diet low in nuts and seeds	High fasting plasma glucose	0	0.99<br>(0.99 to 0.99)
Ischemic heart disease	Diet low in polyunsaturated fatty acids	High fasting plasma glucose	0	0.57<br>(0.39 to 0.77)
Ischemic heart disease	Diet low in vegetables	High fasting plasma glucose	1	0.05<br>(0.01 to 0.09)
Hemorrhagic stroke	Diet low in vegetables	High fasting plasma glucose	1	0.08<br>(0.03 to 0.12)

Appendix Table 7. Mediation factors

For IHD, stroke, and diabetes we pooled all available cohorts and estimated relative risks with and without adjustment across all combinations of metabolic risk factors. We then computed the excess attenuated risk for each mediation-risk-cause set.

Cause	Risk Factor	Mediator	Cohort	Mediation Factor
Ischemic stroke	Diet low in vegetables	High fasting plasma glucose	1	0.08<br>(0.03 to 0.12)
Diabetes mellitus	Diet low in whole grains	High fasting plasma glucose	0	1<br>(1 to 1)
Ischemic heart disease	Diet high in processed meat	High fasting plasma glucose	0	0.01<br>(0.01 to 0.02)
Diabetes mellitus	Diet high in processed meat	High fasting plasma glucose	0	1<br>(1 to 1)
Ischemic heart disease	Diet high in sugar-sweetened beverages	High fasting plasma glucose	0	0.15<br>(0.1 to 0.2)
Hemorrhagic stroke	Diet high in sugar-sweetened beverages	High fasting plasma glucose	0	0.22<br>(0.12 to 0.33)
Ischemic stroke	Diet high in sugar-sweetened beverages	High fasting plasma glucose	0	0.22<br>(0.12 to 0.31)
Atrial fibrillation and flutter	Diet high in sugar-sweetened beverages	High fasting plasma glucose	0	0.15<br>(0.09 to 0.2)
Cardiomyopathy and myocarditis	Diet high in sugar-sweetened beverages	High fasting plasma glucose	0	0.15<br>(0.1 to 0.2)
Peripheral artery disease	Diet high in sugar-sweetened beverages	High fasting plasma glucose	0	0.15<br>(0.1 to 0.2)
Diabetes mellitus	Diet high in sugar-sweetened beverages	High fasting plasma glucose	0	1<br>(1 to 1)
Ischemic stroke	Diet low in fruits	High fasting plasma glucose	0	0.05<br>(0.04 to 0.06)
Diabetes mellitus	Diet low in fruits	High fasting plasma glucose	0	0.99<br>(0.99 to 0.99)
Ischemic heart disease	High body-mass index	High fasting plasma glucose	0	0.15<br>(0.1 to 0.2)
Hemorrhagic stroke	High body-mass index	High fasting plasma glucose	0	0.22<br>(0.12 to 0.33)
Ischemic stroke	High body-mass index	High fasting plasma glucose	0	0.22<br>(0.12 to 0.31)
Atrial fibrillation and flutter	High body-mass index	High fasting plasma glucose	0	0.15<br>(0.09 to 0.2)
Cardiomyopathy and myocarditis	High body-mass index	High fasting plasma glucose	0	0.15<br>(0.1 to 0.2)
Peripheral artery disease	High body-mass index	High fasting plasma glucose	0	0.15<br>(0.1 to 0.2)
Diabetes mellitus	High body-mass index	High fasting plasma glucose	0	1<br>(1 to 1)
Ischemic heart disease	Low physical activity	High fasting plasma glucose	0	0.14<br>(0.11 to 0.18)
Ischemic stroke	Low physical activity	High fasting plasma glucose	0	0.08<br>(0.03 to 0.14)
Diabetes mellitus	Low physical activity	High fasting plasma glucose	0	1<br>(1 to 1)
Diabetes mellitus	Diet high in red meat	High fasting plasma glucose	0	1<br>(1 to 1)
Ischemic heart disease	Diet low in vegetables	High systolic blood pressure	0	0.04<br>(0.03 to 0.05)
Hemorrhagic stroke	Diet low in vegetables	High systolic blood pressure	0	0.04<br>(0.02 to 0.05)
Ischemic stroke	Diet low in vegetables	High systolic blood pressure	0	0.03<br>(0.02 to 0.04)
Ischemic heart disease	Diet low in seafood omega-3 fatty acids	High systolic blood pressure	0	0.01<br>(0 to 0.02)

Appendix Table 7. Mediation factors<br>For IHD, stroke, and diabetes we pooled all available cohorts and estimated relative risks with and without adjustment across all combinations of metabolic risk factors. We then computed the excess attenuated risk for each mediation-risk-cause set.				
**Cause**	**Risk Factor**	**Mediator**	**Cohort**	**Mediation Factor**
Ischemic heart disease	Diet high in sugar-sweetened beverages	High systolic blood pressure	0	0.31<br>(0.28 to 0.34)
Hemorrhagic stroke	Diet high in sugar-sweetened beverages	High systolic blood pressure	0	0.65<br>(0.58 to 0.73)
Ischemic stroke	Diet high in sugar-sweetened beverages	High systolic blood pressure	0	0.65<br>(0.57 to 0.72)
Atrial fibrillation and flutter	Diet high in sugar-sweetened beverages	High systolic blood pressure	0	0.31<br>(0.28 to 0.34)
Cardiomyopathy and myocarditis	Diet high in sugar-sweetened beverages	High systolic blood pressure	0	0.31<br>(0.28 to 0.34)
Hypertensive heart disease	Diet high in sugar-sweetened beverages	High systolic blood pressure	0	1<br>(1 to 1)
Other cardiovascular and circulatory diseases	Diet high in sugar-sweetened beverages	High systolic blood pressure	0	0.31<br>(0.28 to 0.34)
Peripheral artery disease	Diet high in sugar-sweetened beverages	High systolic blood pressure	0	0.31<br>(0.28 to 0.34)
Ischemic heart disease	Diet low in fruits	High systolic blood pressure	0	0.06<br>(0.05 to 0.08)
Hemorrhagic stroke	Diet low in fruits	High systolic blood pressure	0	0.02<br>(0.02 to 0.03)
Ischemic stroke	Diet low in fruits	High systolic blood pressure	0	0.05<br>(0.04 to 0.06)
Ischemic heart disease	Diet high in trans fatty acids	High systolic blood pressure	0	0.15<br>(0.02 to 0.24)
Ischemic heart disease	High body-mass index	High systolic blood pressure	0	0.31<br>(0.28 to 0.34)
Hemorrhagic stroke	High body-mass index	High systolic blood pressure	0	0.65<br>(0.58 to 0.73)
Ischemic stroke	High body-mass index	High systolic blood pressure	0	0.65<br>(0.57 to 0.72)
Atrial fibrillation and flutter	High body-mass index	High systolic blood pressure	0	0.31<br>(0.28 to 0.34)
Cardiomyopathy and myocarditis	High body-mass index	High systolic blood pressure	0	0.31<br>(0.28 to 0.34)
Hypertensive heart disease	High body-mass index	High systolic blood pressure	0	1<br>(1 to 1)
Other cardiovascular and circulatory diseases	High body-mass index	High systolic blood pressure	0	0.31<br>(0.28 to 0.34)
Peripheral artery disease	High body-mass index	High systolic blood pressure	0	0.31<br>(0.28 to 0.34)
Ischemic heart disease	Diet high in sodium	High systolic blood pressure	0	1<br>(1 to 1)
Hemorrhagic stroke	Diet high in sodium	High systolic blood pressure	0	1<br>(1 to 1)
Ischemic stroke	Diet high in sodium	High systolic blood pressure	0	1<br>(1 to 1)
Chronic kidney disease due to diabetes mellitus	Diet high in sodium	High systolic blood pressure	0	1<br>(1 to 1)
Chronic kidney disease due to glomerulonephritis	Diet high in sodium	High systolic blood pressure	0	1<br>(1 to 1)
Chronic kidney disease due to hypertension	Diet high in sodium	High systolic blood pressure	0	1<br>(1 to 1)
Chronic kidney disease due to other causes	Diet high in sodium	High systolic blood pressure	0	1<br>(1 to 1)
Atrial fibrillation and flutter	Diet high in sodium	High systolic blood pressure	0	1<br>(1 to 1)

Appendix Table 7. Mediation factors
For IHD, stroke, and diabetes we pooled all available cohorts and estimated relative risks with and without adjustment across all combinations of metabolic risk factors. We then computed the excess attenuated risk for each mediation-risk-cause set.

Cause	Risk Factor	Mediator	Cohort	Mediation Factor
Cardiomyopathy and myocarditis	Diet high in sodium	High systolic blood pressure	0	1<br>(1 to 1)
Hypertensive heart disease	Diet high in sodium	High systolic blood pressure	0	1<br>(1 to 1)
Other cardiovascular and circulatory diseases	Diet high in sodium	High systolic blood pressure	0	1<br>(1 to 1)
Peripheral artery disease	Diet high in sodium	High systolic blood pressure	0	1<br>(1 to 1)
Aortic aneurysm	Diet high in sodium	High systolic blood pressure	0	1<br>(1 to 1)
Rheumatic heart disease	Diet high in sodium	High systolic blood pressure	0	1<br>(1 to 1)
Alcohol use disorders	Childhood sexual abuse	Alcohol use	0	1<br>(1 to 1)

Appendix Table 8: Socio-demographic Index groupings by location, based on 2016 values	
**Location**	**SDI Level**
Aichi	High SDI
Akita	High SDI
Alabama	High SDI
Alaska	High SDI
Andorra	High SDI
Aomori	High SDI
Arizona	High SDI
Arkansas	High SDI
Australia	High SDI
Austria	High SDI
Barking and Dagenham	High SDI
Barnet	High SDI
Barnsley	High SDI
Bath and North East Somerset	High SDI
Bedford	High SDI
Belgium	High SDI
Bexley	High SDI
Birmingham	High SDI
Blackburn with Darwen	High SDI
Blackpool	High SDI
Bolton	High SDI
Bournemouth	High SDI
Bracknell Forest	High SDI
Bradford	High SDI
Brent	High SDI
Brighton and Hove	High SDI
Bristol, City of	High SDI
Bromley	High SDI
Brunei	High SDI
Buckinghamshire	High SDI
Bury	High SDI
Calderdale	High SDI
California	High SDI
Cambridgeshire	High SDI
Camden	High SDI
Canada	High SDI
Central Bedfordshire	High SDI
Cheshire East	High SDI
Cheshire West and Chester	High SDI
Chiba	High SDI
Colorado	High SDI
Connecticut	High SDI
Cornwall	High SDI
County Durham	High SDI
Coventry	High SDI

Appendix Table 8: Socio-demographic Index groupings by location, based on 2016 values	
**Location**	**SDI Level**
Croatia	High SDI
Croydon	High SDI
Cumbria	High SDI
Cyprus	High SDI
Czech Republic	High SDI
Darlington	High SDI
Delaware	High SDI
Denmark	High SDI
Derby	High SDI
Derbyshire	High SDI
Devon	High SDI
District of Columbia	High SDI
Doncaster	High SDI
Dorset	High SDI
Dudley	High SDI
Ealing	High SDI
East Riding of Yorkshire	High SDI
East Sussex	High SDI
Ehime	High SDI
Enfield	High SDI
Essex	High SDI
Estonia	High SDI
Finland	High SDI
Florida	High SDI
France	High SDI
Fukui	High SDI
Fukuoka	High SDI
Fukushima	High SDI
Gateshead	High SDI
Georgia	High SDI
Germany	High SDI
Gifu	High SDI
Gloucestershire	High SDI
Greece	High SDI
Greenwich	High SDI
Gunma	High SDI
Hackney	High SDI
Halton	High SDI
Hammersmith and Fulham	High SDI
Hampshire	High SDI
Haringey	High SDI
Harrow	High SDI
Hartlepool	High SDI
Havering	High SDI
Hawaii	High SDI

Appendix Table 8: Socio-demographic Index groupings by location, based on 2016 values	
**Location**	**SDI Level**
Herefordshire, County of	High SDI
Hertfordshire	High SDI
Hillingdon	High SDI
Hiroshima	High SDI
Hokkaidō	High SDI
Hounslow	High SDI
Hyōgo	High SDI
Ibaraki	High SDI
Iceland	High SDI
Idaho	High SDI
Illinois	High SDI
Indiana	High SDI
Iowa	High SDI
Ireland	High SDI
Ishikawa	High SDI
Isle of Wight	High SDI
Islington	High SDI
Italy	High SDI
Iwate	High SDI
Kagawa	High SDI
Kagoshima	High SDI
Kanagawa	High SDI
Kansas	High SDI
Kensington and Chelsea	High SDI
Kent	High SDI
Kentucky	High SDI
Kingston upon Hull, City of	High SDI
Kingston upon Thames	High SDI
Kirklees	High SDI
Knowsley	High SDI
Kumamoto	High SDI
Kyōto	High SDI
Kōchi	High SDI
Lambeth	High SDI
Lancashire	High SDI
Latvia	High SDI
Leeds	High SDI
Leicester	High SDI
Leicestershire	High SDI
Lewisham	High SDI
Lincolnshire	High SDI
Lithuania	High SDI
Liverpool	High SDI
Louisiana	High SDI
Luton	High SDI

Appendix Table 8: Socio-demographic Index groupings by location, based on 2016 values	
**Location**	**SDI Level**
Luxembourg	High SDI
Maine	High SDI
Malta	High SDI
Manchester	High SDI
Maryland	High SDI
Massachusetts	High SDI
Medway	High SDI
Merton	High SDI
Michigan	High SDI
Middlesbrough	High SDI
Mie	High SDI
Milton Keynes	High SDI
Minnesota	High SDI
Mississippi	High SDI
Missouri	High SDI
Miyagi	High SDI
Miyazaki	High SDI
Montana	High SDI
Nagano	High SDI
Nagasaki	High SDI
Nara	High SDI
Nebraska	High SDI
Netherlands	High SDI
Nevada	High SDI
New Hampshire	High SDI
New Jersey	High SDI
New Mexico	High SDI
New York	High SDI
New Zealand	High SDI
Newcastle upon Tyne	High SDI
Newham	High SDI
Niigata	High SDI
Norfolk	High SDI
North Carolina	High SDI
North Dakota	High SDI
North East Lincolnshire	High SDI
North Lincolnshire	High SDI
North Somerset	High SDI
North Tyneside	High SDI
North Yorkshire	High SDI
Northamptonshire	High SDI
Northern Ireland	High SDI
Northumberland	High SDI
Norway	High SDI
Nottingham	High SDI

Appendix Table 8: Socio-demographic Index groupings by location, based on 2016 values	
**Location**	**SDI Level**
Nottinghamshire	High SDI
Ohio	High SDI
Okayama	High SDI
Okinawa	High SDI
Oklahoma	High SDI
Oldham	High SDI
Oregon	High SDI
Oxfordshire	High SDI
Pennsylvania	High SDI
Peterborough	High SDI
Plymouth	High SDI
Poland	High SDI
Poole	High SDI
Portsmouth	High SDI
Puerto Rico	High SDI
Reading	High SDI
Redbridge	High SDI
Redcar and Cleveland	High SDI
Rhode Island	High SDI
Richmond upon Thames	High SDI
Rochdale	High SDI
Rotherham	High SDI
Rutland	High SDI
Saga	High SDI
Saitama	High SDI
Salford	High SDI
Sandwell	High SDI
Scotland	High SDI
Sefton	High SDI
Sheffield	High SDI
Shiga	High SDI
Shimane	High SDI
Shizuoka	High SDI
Shropshire	High SDI
Singapore	High SDI
Slough	High SDI
Slovakia	High SDI
Slovenia	High SDI
Solihull	High SDI
Somerset	High SDI
South Carolina	High SDI
South Dakota	High SDI
South Gloucestershire	High SDI
South Korea	High SDI
South Tyneside	High SDI

Appendix Table 8: Socio-demographic Index groupings by location, based on 2016 values	
**Location**	**SDI Level**
Southampton	High SDI
Southend-on-Sea Southwark	High SDI
St Helens	High SDI
Staffordshire Stockholm	High SDI
Stockport	High SDI
Stockton-on-Tees Stoke-on-	High SDI
Trent	High SDI
Suffolk	High SDI
Sunderland	High SDI
Surrey	High SDI
Sutton	High SDI
Sweden except Stockholm	High SDI
Swindon	High SDI
Switzerland	High SDI
Taiwan (Province of China)	High SDI
Tameside	High SDI
Telford and Wrekin	High SDI
Tennessee	High SDI
Texas	High SDI
Thurrock	High SDI
Tochigi	High SDI
Tokushima	High SDI
Torbay	High SDI
Tottori	High SDI
Tower Hamlets	High SDI
Toyama	High SDI
Trafford	High SDI
Tōkyō	High SDI
Utah	High SDI
Vermont	High SDI
Virgin Islands, U.S. Virginia	High SDI
Wakayama	High SDI
Wakefield	High SDI
Wales	High SDI
Walsall	High SDI
Waltham Forest	High SDI
Wandsworth Warrington	High SDI
Warwickshire Washington	High SDI
West Berkshire	High SDI
West Sussex	High SDI
	High SDI
	High SDI
	High SDI
	High SDI
	High SDI

Appendix Table 8: Socio-demographic Index groupings by location, based on 2016 values	
**Location**	**SDI Level**
West Virginia	High SDI
Westminster	High SDI
Wigan	High SDI
Wiltshire	High SDI
Windsor and Maidenhead	High SDI
Wirral	High SDI
Wisconsin	High SDI
Wokingham	High SDI
Wolverhampton	High SDI
Worcestershire	High SDI
Wyoming	High SDI
Yamagata	High SDI
Yamaguchi	High SDI
Yamanashi	High SDI
York	High SDI
Ôita	High SDI
Ōsaka	High SDI
'Asir	High-middle SDI
Antigua and Barbuda	High-middle SDI
Argentina	High-middle SDI
Armenia	High-middle SDI
Azerbaijan	High-middle SDI
Bahah	High-middle SDI
Barbados	High-middle SDI
Beijing	High-middle SDI
Belarus	High-middle SDI
Bermuda	High-middle SDI
Bulgaria	High-middle SDI
Chile	High-middle SDI
Cuba	High-middle SDI
Delhi, Urban	High-middle SDI
Distrito Federal	High-middle SDI
Eastern Province	High-middle SDI
Georgia	High-middle SDI
Goa, Urban	High-middle SDI
Greenland	High-middle SDI
Guam	High-middle SDI
Guangdong	High-middle SDI
Ha'il	High-middle SDI
Himachal Pradesh, Urban	High-middle SDI
Hong Kong Special Administrative Region of China	High-middle SDI
Hungary	High-middle SDI
Iran	High-middle SDI
Israel	High-middle SDI

Appendix Table 8: Socio-demographic Index groupings by location, based on 2016 values	
**Location**	**SDI Level**
Jakarta	High-middle SDI
Jawf	High-middle SDI
Jiangsu	High-middle SDI
Jizan	High-middle SDI
East Kalimantan	High-middle SDI
North Kalimantan	High-middle SDI
Kazakhstan	High-middle SDI
Riau Islands	High-middle SDI
Kuwait	High-middle SDI
Lebanon	High-middle SDI
Libya	High-middle SDI
Macao Special Administrative Region of China	High-middle SDI
Macedonia	High-middle SDI
Madinah	High-middle SDI
Makkah	High-middle SDI
Malaysia	High-middle SDI
Mauritius	High-middle SDI
Montenegro	High-middle SDI
Najran	High-middle SDI
Northern Borders	High-middle SDI
Northern Mariana Islands	High-middle SDI
Panama	High-middle SDI
Portugal	High-middle SDI
Qassim	High-middle SDI
Qatar	High-middle SDI
Rio de Janeiro	High-middle SDI
Riyadh	High-middle SDI
Romania	High-middle SDI
Russia	High-middle SDI
Serbia	High-middle SDI
Shanghai	High-middle SDI
Spain	High-middle SDI
São Paulo	High-middle SDI
Tabuk	High-middle SDI
The Bahamas	High-middle SDI
Tianjin	High-middle SDI
Trinidad and Tobago	High-middle SDI
Turkey	High-middle SDI
Turkmenistan	High-middle SDI
Ukraine	High-middle SDI
United Arab Emirates	High-middle SDI
Zhejiang	High-middle SDI
Aceh	Middle SDI
Acre	Middle SDI

Appendix Table 8: Socio-demographic Index groupings by location, based on 2016 values	
**Location**	**SDI Level**
Aguascalientes	Middle SDI
Albania	Middle SDI
Algeria	Middle SDI
Amapá	Middle SDI
Amazonas	Middle SDI
American Samoa	Middle SDI
Andhra Pradesh, Urban	Middle SDI
Anhui	Middle SDI
Assam, Urban	Middle SDI
Bahia	Middle SDI
Bahrain	Middle SDI
Baja California	Middle SDI
Baja California Sur	Middle SDI
Bali	Middle SDI
Bangka Belitung	Middle SDI
Banten	Middle SDI
Bengkulu	Middle SDI
Bosnia and Herzegovina	Middle SDI
Botswana	Middle SDI
Campeche	Middle SDI
Chhattisgarh, Urban	Middle SDI
Chiapas	Middle SDI
Chihuahua	Middle SDI
Chongqing	Middle SDI
Coahuila	Middle SDI
Colima	Middle SDI
Colombia	Middle SDI
Costa Rica	Middle SDI
Delhi, Rural	Middle SDI
Distrito Federal	Middle SDI
Dominica	Middle SDI
Dominican Republic	Middle SDI
Durango	Middle SDI
Eastern Cape	Middle SDI
Ecuador	Middle SDI
Egypt	Middle SDI
El Salvador	Middle SDI
Equatorial Guinea	Middle SDI
Espírito Santo	Middle SDI
Fiji	Middle SDI
Free State	Middle SDI
Fujian	Middle SDI
Gauteng	Middle SDI
Goa, Rural	Middle SDI
Goiás	Middle SDI

Appendix Table 8: Socio-demographic Index groupings by location, based on 2016 values	
**Location**	**SDI Level**
Grenada	Middle SDI
Guanajuato	Middle SDI
Guangxi	Middle SDI
Guerrero	Middle SDI
Gujarat, Urban	Middle SDI
Guyana	Middle SDI
Hainan	Middle SDI
Haryana, Urban	Middle SDI
Hebei	Middle SDI
Heilongjiang	Middle SDI
Henan	Middle SDI
Hidalgo	Middle SDI
Himachal Pradesh, Rural	Middle SDI
Hubei	Middle SDI
Hunan	Middle SDI
Inner Mongolia	Middle SDI
Jalisco	Middle SDI
Jamaica	Middle SDI
Jambi	Middle SDI
Jammu and Kashmir, Urban	Middle SDI
Cental Java	Middle SDI
East Java	Middle SDI
Jharkhand, Urban	Middle SDI
Jiangxi	Middle SDI
Jilin	Middle SDI
Jordan	Middle SDI
South Kalimantan	Middle SDI
Central Kalimantan	Middle SDI
Karnataka, Urban	Middle SDI
Kerala, Rural	Middle SDI
Kerala, Urban	Middle SDI
KwaZulu-Natal	Middle SDI
Lampung	Middle SDI
Liaoning	Middle SDI
Limpopo	Middle SDI
Madhya Pradesh, Urban	Middle SDI
Maharashtra, Urban	Middle SDI
Maldives	Middle SDI
Manipur, Urban	Middle SDI
Mato Grosso	Middle SDI
Mato Grosso do Sul	Middle SDI
Meghalaya, Urban	Middle SDI
Michoacán de Ocampo	Middle SDI
Minas Gerais	Middle SDI
Mizoram, Urban	Middle SDI

Appendix Table 8: Socio-demographic Index groupings by location, based on 2016 values	
**Location**	**SDI Level**
Moldova	Middle SDI
Mongolia	Middle SDI
Morelos	Middle SDI
Mpumalanga	Middle SDI
México	Middle SDI
Nagaland, Urban	Middle SDI
Nayarit	Middle SDI
Ningxia	Middle SDI
North-West	Middle SDI
Northern Cape	Middle SDI
Nuevo León	Middle SDI
Oaxaca	Middle SDI
Oman	Middle SDI
West Papua	Middle SDI
Paraguay	Middle SDI
Paraná	Middle SDI
Pernambuco	Middle SDI
Peru	Middle SDI
Philippines	Middle SDI
Puebla	Middle SDI
Punjab, Urban	Middle SDI
Qinghai	Middle SDI
Querétaro	Middle SDI
Quintana Roo	Middle SDI
Riau	Middle SDI
Rio Grande do Norte	Middle SDI
Rio Grande do Sul	Middle SDI
Rondônia	Middle SDI
Roraima	Middle SDI
Saint Lucia	Middle SDI
Saint Vincent and the Grenadines	Middle SDI
San Luis Potosí	Middle SDI
Santa Catarina	Middle SDI
Sergipe	Middle SDI
Seychelles	Middle SDI
Shaanxi	Middle SDI
Shandong	Middle SDI
Shanxi	Middle SDI
Sichuan	Middle SDI
Sikkim, Urban	Middle SDI
Sinaloa	Middle SDI
Sonora	Middle SDI
Sri Lanka	Middle SDI
South Sumatera	Middle SDI
North Sulawesi	Middle SDI

Appendix Table 8: Socio-demographic Index groupings by location, based on 2016 values

Location	SDI Level
West Sumatera	Middle SDI
South Sumatera	Middle SDI
North Sumatera	Middle SDI
Suriname	Middle SDI
Tabasco	Middle SDI
Tamaulipas	Middle SDI
Tamil Nadu, Urban	Middle SDI
Telangana, Urban	Middle SDI
Thailand	Middle SDI
The Six Minor Territories, Urban	Middle SDI
Tlaxcala	Middle SDI
Tocantins	Middle SDI
Tunisia	Middle SDI
Uruguay	Middle SDI
Uttarakhand, Urban	Middle SDI
Uzbekistan	Middle SDI
Venezuela	Middle SDI
Veracruz de Ignacio de la Llave	Middle SDI
Vietnam	Middle SDI
West Bengal, Urban	Middle SDI
Western Cape	Middle SDI
Xinjiang	Middle SDI
Yogyakarta	Middle SDI
Yucatán	Middle SDI
Yunnan	Middle SDI
Zacatecas	Middle SDI
Alagoas	Low-middle SDI
Andhra Pradesh, Rural	Low-middle SDI
Arunachal Pradesh, Rural	Low-middle SDI
Arunachal Pradesh, Urban	Low-middle SDI
Assam, Rural	Low-middle SDI
Bangladesh	Low-middle SDI
Baringo	Low-middle SDI
Belize	Low-middle SDI
Bhutan	Low-middle SDI
Bihar, Urban	Low-middle SDI
Bolivia	Low-middle SDI
Bomet	Low-middle SDI
Bungoma	Low-middle SDI
Busia	Low-middle SDI
Cambodia	Low-middle SDI
Cameroon	Low-middle SDI
Cape Verde	Low-middle SDI
Ceará	Low-middle SDI
Chhattisgarh, Rural	Low-middle SDI

Appendix Table 8: Socio-demographic Index groupings by location, based on 2016 values	
**Location**	**SDI Level**
Congo (Brazzaville)	Low-middle SDI
Elgeyo-Marakwet	Low-middle SDI
Embu	Low-middle SDI
Federated States of Micronesia	Low-middle SDI
Gabon	Low-middle SDI
Gansu	Low-middle SDI
Garissa	Low-middle SDI
Ghana	Low-middle SDI
Gorontalo	Low-middle SDI
Guatemala	Low-middle SDI
Guizhou	Low-middle SDI
Gujarat, Rural	Low-middle SDI
Haryana, Rural	Low-middle SDI
HomaBay	Low-middle SDI
Honduras	Low-middle SDI
Iraq	Low-middle SDI
Isiolo	Low-middle SDI
Jammu and Kashmir, Rural	Low-middle SDI
West Java	Low-middle SDI
Jharkhand, Rural	Low-middle SDI
Kajiado	Low-middle SDI
Kakamega	Low-middle SDI
West Kalimantan	Low-middle SDI
Karnataka, Rural	Low-middle SDI
Kericho	Low-middle SDI
Kiambu	Low-middle SDI
Kilifi	Low-middle SDI
Kirinyaga	Low-middle SDI
Kisii	Low-middle SDI
Kisumu	Low-middle SDI
Kitui	Low-middle SDI
Kwale	Low-middle SDI
Kyrgyzstan	Low-middle SDI
Laikipia	Low-middle SDI
Lamu	Low-middle SDI
Laos	Low-middle SDI
Lesotho	Low-middle SDI
Machakos	Low-middle SDI
Madhya Pradesh, Rural	Low-middle SDI
Maharashtra, Rural	Low-middle SDI
Makueni	Low-middle SDI
Maluku	Low-middle SDI
North Maluku	Low-middle SDI
Mandera	Low-middle SDI
Manipur, Rural	Low-middle SDI

Appendix Table 8: Socio-demographic Index groupings by location, based on 2016 values	
**Location**	**SDI Level**
Maranhão	Low-middle SDI
Marsabit	Low-middle SDI
Marshall Islands	Low-middle SDI
Mauritania	Low-middle SDI
Meghalaya, Rural	Low-middle SDI
Meru	Low-middle SDI
Migori	Low-middle SDI
Mizoram, Rural	Low-middle SDI
Mombasa	Low-middle SDI
Morocco	Low-middle SDI
Murang'a	Low-middle SDI
Myanmar	Low-middle SDI
Nagaland, Rural	Low-middle SDI
Nairobi	Low-middle SDI
Nakuru	Low-middle SDI
Namibia	Low-middle SDI
Nandi	Low-middle SDI
Narok	Low-middle SDI
Nepal	Low-middle SDI
Nicaragua	Low-middle SDI
Nigeria	Low-middle SDI
North Korea	Low-middle SDI
Nusa Tenggara Barat	Low-middle SDI
East Nusa Tenggara	Low-middle SDI
Nyamira Nyandarua	Low-middle SDI
Nyeri	Low-middle SDI
Odisha, Rural Odisha,	Low-middle SDI
Urban Pakistan	Low-middle SDI
Papua	Low-middle SDI
Paraíba	Low-middle SDI
Pará	Low-middle SDI
Piaui	Low-middle SDI
Punjab, Rural	Low-middle SDI
Rajasthan, Rural	Low-middle SDI
Rajasthan, Urban	Low-middle SDI
Samburu	Low-middle SDI
Samoa	Low-middle SDI
Siaya	Low-middle SDI
Sikkim, Rural Sudan	Low-middle SDI
West Sulawesi	Low-middle SDI
Central Sulawesi	Low-middle SDI
Southeast Sulawesi	Low-middle SDI
	Low-middle SDI
	Low-middle SDI
	Low-middle SDI

Appendix Table 8: Socio-demographic Index groupings by location, based on 2016 values	
**Location**	**SDI Level**
Swaziland	Low-middle SDI
Syria	Low-middle SDI
TaitaTaveta	Low-middle SDI
Tajikistan	Low-middle SDI
Tamil Nadu, Rural	Low-middle SDI
TanaRiver	Low-middle SDI
Telangana, Rural	Low-middle SDI
TharakaNithi	Low-middle SDI
The Six Minor Territories, Rural	Low-middle SDI
Tibet	Low-middle SDI
Timor-Leste	Low-middle SDI
Tonga	Low-middle SDI
TransNzoia	Low-middle SDI
Tripura, Rural	Low-middle SDI
Tripura, Urban	Low-middle SDI
Turkana	Low-middle SDI
UasinGishu	Low-middle SDI
Uttar Pradesh, Rural	Low-middle SDI
Uttar Pradesh, Urban	Low-middle SDI
Uttarakhand, Rural	Low-middle SDI
Vanuatu	Low-middle SDI
Vihiga	Low-middle SDI
Wajir	Low-middle SDI
West Bengal, Rural	Low-middle SDI
WestPokot	Low-middle SDI
Zambia	Low-middle SDI
Zimbabwe	Low-middle SDI
Afghanistan	Low SDI
Angola	Low SDI
Benin	Low SDI
Bihar, Rural	Low SDI
Burkina Faso	Low SDI
Burundi	Low SDI
the Central African Republic	Low SDI
Chad	Low SDI
Comoros	Low SDI
Cote d'Ivoire	Low SDI
Democratic Republic of the Congo	Low SDI
Djibouti	Low SDI
Eritrea	Low SDI
Ethiopia	Low SDI
Guinea	Low SDI
Guinea-Bissau	Low SDI
Haiti	Low SDI
Kiribati	Low SDI

Appendix Table 8: Socio-demographic Index groupings by location, based on 2016 values	
**Location**	**SDI Level**
Liberia	Low SDI
Madagascar	Low SDI
Malawi	Low SDI
Mali	Low SDI
Mozambique	Low SDI
Niger	Low SDI
Palestine	Low SDI
Papua New Guinea	Low SDI
Rwanda	Low SDI
Sao Tome and Principe	Low SDI
Senegal	Low SDI
Sierra Leone	Low SDI
Solomon Islands	Low SDI
Somalia	Low SDI
South Sudan	Low SDI
Tanzania	Low SDI
The Gambia	Low SDI
Togo	Low SDI
Uganda	Low SDI
Yemen	Low SDI

Appendix Table 9: Socio-demographic Index values for all estimated GBD 2016 locations, 1980–2016

Location	1980	1981	1982	1983	1984	1985	1986	1987	1988	1989	1990	1991	1992	1993	1994	1995	1996	1997	1998	1999	2000	2001	2002	2003	2004	2005	2006	2007	2008	2009	2010	2011	2012	2013	2014	2015	2016
Global	0.526	0.531	0.535	0.54	0.544	0.549	0.554	0.559	0.564	0.569	0.575	0.58	0.586	0.591	0.596	0.6	0.605	0.609	0.614	0.618	0.622	0.626	0.63	0.634	0.639	0.643	0.648	0.652	0.657	0.662	0.666	0.671	0.676	0.681	0.686	0.691	0.696
Southeast Asia, East Asia, and Oceania	0.438	0.445	0.453	0.461	0.47	0.478	0.486	0.495	0.504	0.512	0.521	0.53	0.539	0.548	0.556	0.565	0.573	0.581	0.587	0.594	0.6	0.606	0.612	0.619	0.626	0.633	0.641	0.65	0.658	0.666	0.675	0.683	0.691	0.699	0.706	0.712	0.719
East Asia	0.436	0.443	0.45	0.458	0.467	0.477	0.486	0.496	0.506	0.515	0.524	0.533	0.543	0.552	0.562	0.571	0.58	0.588	0.594	0.601	0.608	0.614	0.621	0.629	0.636	0.644	0.653	0.661	0.67	0.678	0.686	0.694	0.701	0.709	0.715	0.722	0.729
China	0.42	0.428	0.435	0.444	0.453	0.463	0.473	0.483	0.494	0.503	0.512	0.521	0.531	0.542	0.552	0.562	0.57	0.578	0.585	0.592	0.6	0.607	0.614	0.622	0.63	0.638	0.647	0.657	0.665	0.674	0.682	0.691	0.698	0.706	0.713	0.72	0.727
North Korea	0.471	0.476	0.48	0.485	0.488	0.491	0.494	0.497	0.499	0.502	0.505	0.508	0.51	0.511	0.51	0.506	0.501	0.495	0.489	0.483	0.478	0.473	0.47	0.468	0.468	0.469	0.47	0.471	0.473	0.474	0.476	0.478	0.481	0.484	0.488	0.491	0.494
Taiwan (Province of China)	0.636	0.645	0.657	0.669	0.681	0.695	0.706	0.71	0.718	0.727	0.734	0.743	0.749	0.756	0.762	0.768	0.775	0.788	0.798	0.8	0.808	0.815	0.82	0.824	0.829	0.833	0.837	0.842	0.845	0.849	0.853	0.857	0.86	0.864	0.868	0.871	0.875
Hong Kong Special Administrative Region of China	0.633	0.643	0.652	0.662	0.671	0.677	0.682	0.688	0.694	0.699	0.704	0.708	0.713	0.718	0.723	0.727	0.731	0.734	0.737	0.74	0.743	0.746	0.749	0.752	0.756	0.761	0.765	0.77	0.775	0.779	0.783	0.787	0.791	0.795	0.798	0.801	0.805
Macao Special Administrative Region of China	0.638	0.646	0.654	0.66	0.667	0.672	0.678	0.684	0.689	0.695	0.7	0.705	0.711	0.716	0.721	0.725	0.728	0.731	0.732	0.734	0.735	0.738	0.742	0.747	0.754	0.761	0.768	0.776	0.784	0.789	0.793	0.796	0.799	0.802	0.806	0.809	0.811
China (without Hong Kong and Macao)	0.413	0.42	0.428	0.436	0.446	0.456	0.467	0.477	0.487	0.497	0.506	0.515	0.525	0.536	0.546	0.556	0.565	0.574	0.581	0.588	0.596	0.603	0.611	0.619	0.627	0.635	0.645	0.654	0.663	0.672	0.68	0.689	0.697	0.704	0.712	0.718	0.725
Anhui	0.353	0.36	0.367	0.375	0.385	0.396	0.406	0.417	0.427	0.437	0.446	0.455	0.464	0.474	0.483	0.493	0.502	0.51	0.518	0.525	0.532	0.538	0.545	0.552	0.559	0.567	0.576	0.585	0.594	0.603	0.611	0.619	0.626	0.633	0.639	0.645	0.651
Beijing	0.606	0.612	0.618	0.624	0.631	0.638	0.645	0.653	0.659	0.665	0.671	0.676	0.682	0.689	0.695	0.702	0.709	0.715	0.721	0.726	0.732	0.738	0.744	0.75	0.757	0.764	0.772	0.78	0.787	0.795	0.802	0.809	0.816	0.823	0.829	0.835	0.842
Chongqing	0.367	0.375	0.384	0.394	0.407	0.419	0.431	0.443	0.454	0.464	0.474	0.485	0.496	0.507	0.519	0.53	0.539	0.549	0.557	0.565	0.574	0.582	0.591	0.599	0.607	0.616	0.625	0.635	0.643	0.652	0.66	0.668	0.675	0.682	0.689	0.694	0.7
Fujian	0.372	0.378	0.386	0.396	0.408	0.42	0.433	0.445	0.458	0.469	0.48	0.492	0.504	0.518	0.531	0.544	0.555	0.564	0.573	0.581	0.59	0.598	0.606	0.615	0.623	0.632	0.642	0.652	0.662	0.671	0.68	0.689	0.698	0.706	0.713	0.72	0.727
Gansu	0.353	0.358	0.364	0.372	0.382	0.391	0.401	0.411	0.421	0.431	0.438	0.447	0.456	0.465	0.473	0.482	0.49	0.498	0.506	0.513	0.52	0.526	0.533	0.539	0.546	0.553	0.56	0.568	0.575	0.583	0.59	0.597	0.604	0.61	0.616	0.622	0.628
Guangdong	0.444	0.451	0.458	0.467	0.476	0.487	0.497	0.507	0.518	0.527	0.536	0.546	0.557	0.567	0.578	0.588	0.598	0.607	0.615	0.623	0.632	0.64	0.648	0.658	0.667	0.678	0.688	0.7	0.71	0.72	0.729	0.737	0.745	0.753	0.761	0.768	0.775
Guangxi	0.361	0.369	0.376	0.385	0.395	0.406	0.417	0.428	0.439	0.449	0.458	0.468	0.479	0.49	0.501	0.511	0.521	0.531	0.539	0.547	0.555	0.562	0.569	0.577	0.585	0.594	0.603	0.613	0.622	0.632	0.641	0.651	0.661	0.67	0.679	0.686	0.693
Guizhou	0.279	0.287	0.295	0.303	0.314	0.325	0.336	0.347	0.358	0.368	0.376	0.385	0.394	0.403	0.413	0.422	0.431	0.439	0.446	0.454	0.462	0.469	0.477	0.485	0.494	0.503	0.512	0.522	0.531	0.54	0.55	0.559	0.567	0.575	0.581	0.587	0.594
Hainan	0.438	0.443	0.448	0.454	0.462	0.47	0.478	0.486	0.495	0.502	0.511	0.52	0.529	0.539	0.549	0.558	0.566	0.575	0.582	0.59	0.597	0.604	0.611	0.618	0.625	0.632	0.64	0.648	0.656	0.665	0.672	0.68	0.686	0.693	0.699	0.705	0.711
Hebei	0.42	0.427	0.435	0.443	0.452	0.462	0.472	0.482	0.492	0.501	0.509	0.517	0.527	0.537	0.547	0.556	0.565	0.573	0.581	0.588	0.596	0.603	0.611	0.619	0.627	0.635	0.644	0.654	0.662	0.671	0.679	0.687	0.695	0.702	0.709	0.716	0.722
Heilongjiang	0.477	0.482	0.488	0.495	0.504	0.513	0.522	0.531	0.54	0.548	0.557	0.566	0.575	0.582	0.588	0.594	0.6	0.606	0.611	0.617	0.623	0.628	0.634	0.639	0.644	0.649	0.655	0.662	0.668	0.674	0.679	0.685	0.69	0.695	0.7	0.704	0.709
Henan	0.368	0.375	0.384	0.394	0.405	0.417	0.429	0.441	0.453	0.463	0.471	0.481	0.491	0.502	0.513	0.525	0.535	0.545	0.554	0.562	0.57	0.578	0.587	0.595	0.604	0.613	0.623	0.633	0.643	0.652	0.661	0.67	0.678	0.685	0.693	0.699	0.706
Hubei	0.407	0.415	0.423	0.432	0.442	0.452	0.463	0.474	0.484	0.494	0.504	0.514	0.524	0.535	0.545	0.553	0.561	0.569	0.576	0.583	0.59	0.597	0.604	0.611	0.618	0.626	0.635	0.643	0.651	0.659	0.667	0.674	0.681	0.688	0.694	0.7	0.706
Hunan	0.383	0.391	0.399	0.409	0.42	0.431	0.442	0.454	0.465	0.476	0.485	0.495	0.505	0.516	0.526	0.536	0.546	0.555	0.562	0.569	0.576	0.582	0.59	0.597	0.605	0.613	0.621	0.63	0.639	0.647	0.655	0.663	0.67	0.678	0.684	0.691	0.697
Inner Mongolia	0.407	0.413	0.422	0.431	0.444	0.456	0.468	0.48	0.492	0.502	0.512	0.522	0.534	0.545	0.556	0.566	0.575	0.584	0.593	0.602	0.61	0.618	0.627	0.634	0.641	0.648	0.656	0.664	0.672	0.68	0.688	0.696	0.703	0.71	0.716	0.722	0.728
Jiangsu	0.443	0.451	0.459	0.468	0.479	0.489	0.5	0.51	0.521	0.53	0.539	0.548	0.557	0.565	0.573	0.582	0.591	0.599	0.607	0.614	0.621	0.629	0.637	0.646	0.655	0.664	0.674	0.684	0.693	0.702	0.711	0.72	0.728	0.736	0.743	0.75	0.757
Jiangxi	0.347	0.354	0.362	0.371	0.382	0.394	0.405	0.416	0.428	0.439	0.449	0.458	0.469	0.48	0.49	0.5	0.509	0.518	0.526	0.534	0.542	0.549	0.557	0.565	0.573	0.581	0.59	0.599	0.607	0.615	0.622	0.629	0.636	0.643	0.649	0.655	0.662
Jilin	0.451	0.457	0.464	0.471	0.48	0.49	0.5	0.509	0.519	0.528	0.537	0.547	0.557	0.564	0.571	0.579	0.586	0.594	0.6	0.607	0.613	0.62	0.626	0.633	0.639	0.646	0.653	0.661	0.668	0.676	0.683	0.689	0.696	0.702	0.708	0.713	0.719
Liaoning	0.521	0.527	0.533	0.539	0.547	0.555	0.563	0.571	0.579	0.587	0.592	0.597	0.602	0.608	0.614	0.621	0.627	0.634	0.639	0.645	0.651	0.656	0.662	0.668	0.674	0.68	0.686	0.693	0.7	0.707	0.713	0.719	0.725	0.73	0.736	0.741	0.746
Ningxia	0.37	0.375	0.382	0.39	0.398	0.408	0.418	0.429	0.44	0.45	0.46	0.469	0.479	0.489	0.498	0.507	0.515	0.523	0.531	0.538	0.545	0.552	0.559	0.566	0.574	0.583	0.592	0.601	0.61	0.617	0.624	0.632	0.639	0.645	0.652	0.658	0.664
Qinghai	0.354	0.36	0.366	0.375	0.386	0.397	0.408	0.42	0.431	0.442	0.452	0.462	0.473	0.482	0.492	0.501	0.509	0.517	0.524	0.531	0.537	0.544	0.55	0.557	0.564	0.571	0.578	0.586	0.593	0.6	0.607	0.614	0.62	0.627	0.633	0.639	0.645
Shaanxi	0.385	0.392	0.4	0.409	0.419	0.43	0.441	0.451	0.462	0.471	0.48	0.49	0.501	0.512	0.522	0.532	0.54	0.549	0.556	0.564	0.571	0.579	0.587	0.594	0.602	0.61	0.619	0.628	0.637	0.645	0.654	0.662	0.67	0.677	0.684	0.69	0.696
Shandong	0.413	0.421	0.431	0.44	0.451	0.463	0.474	0.485	0.497	0.507	0.516	0.525	0.535	0.544	0.553	0.563	0.572	0.581	0.589	0.596	0.604	0.612	0.62	0.629	0.638	0.647	0.657	0.668	0.678	0.687	0.696	0.705	0.713	0.721	0.729	0.736	0.742
Shanghai	0.638	0.643	0.648	0.652	0.656	0.661	0.666	0.67	0.675	0.679	0.684	0.689	0.694	0.699	0.704	0.709	0.714	0.719	0.723	0.727	0.732	0.736	0.741	0.747	0.753	0.759	0.765	0.772	0.778	0.785	0.791	0.797	0.803	0.809	0.815	0.82	0.826
Shanxi	0.42	0.426	0.434	0.442	0.452	0.462	0.472	0.482	0.493	0.502	0.511	0.52	0.53	0.54	0.55	0.559	0.568	0.577	0.585	0.593	0.6	0.607	0.614	0.621	0.628	0.635	0.643	0.651	0.659	0.666	0.674	0.681	0.688	0.694	0.7	0.706	0.712
Sichuan	0.349	0.359	0.369	0.38	0.392	0.405	0.418	0.43	0.443	0.454	0.463	0.472	0.482	0.492	0.502	0.511	0.52	0.528	0.535	0.542	0.549	0.556	0.563	0.571	0.58	0.589	0.598	0.608	0.617	0.625	0.634	0.642	0.65	0.657	0.664	0.67	0.677
Tianjin	0.569	0.575	0.582	0.589	0.596	0.604	0.611	0.618	0.625	0.63	0.636	0.641	0.647	0.653	0.66	0.666	0.673	0.679	0.685	0.69	0.696	0.702	0.709	0.715	0.722	0.729	0.737	0.745	0.753	0.76	0.768	0.775	0.782	0.789	0.795	0.801	0.807
Tibet	0.344	0.348	0.352	0.357	0.363	0.369	0.376	0.383	0.39	0.398	0.406	0.414	0.422	0.429	0.437	0.444	0.45	0.456	0.462	0.468	0.474	0.48	0.487	0.495	0.503	0.512	0.519	0.526	0.532	0.538	0.544	0.55	0.556	0.561	0.566	0.571	0.577
Xinjiang	0.377	0.383	0.39	0.399	0.41	0.423	0.435	0.448	0.462	0.474	0.486	0.499	0.512	0.524	0.536	0.548	0.558	0.569	0.578	0.588	0.597	0.606	0.614	0.622	0.629	0.636	0.644	0.652	0.66	0.668	0.676	0.683	0.69	0.696	0.702	0.707	0.713
Yunnan	0.311	0.319	0.327	0.336	0.346	0.358	0.369	0.38	0.391	0.402	0.412	0.422	0.432	0.443	0.453	0.464	0.473	0.482	0.491	0.499	0.507	0.515	0.523	0.532	0.541	0.55	0.56	0.57	0.579	0.587	0.596	0.604	0.611	0.618	0.625	0.632	0.639
Zhejiang	0.418	0.426	0.435	0.445	0.457	0.469	0.481	0.493	0.505	0.515	0.524	0.534	0.544	0.552	0.561	0.57	0.579	0.588	0.596	0.604	0.612	0.62	0.629	0.637	0.647	0.656	0.666	0.677	0.686	0.695	0.705	0.714	0.722	0.73	0.738	0.745	0.753
Southeast Asia	0.411	0.421	0.431	0.441	0.45	0.459	0.467	0.475	0.483	0.491	0.499	0.507	0.515	0.523	0.531	0.539	0.547	0.554	0.56	0.566	0.571	0.577	0.582	0.587	0.593	0.598	0.604	0.61	0.616	0.623	0.629	0.636	0.644	0.651	0.659	0.666	0.672
Cambodia	0.258	0.261	0.264	0.267	0.271	0.275	0.279	0.284	0.29	0.296	0.301	0.306	0.312	0.316	0.321	0.326	0.331	0.336	0.342	0.349	0.357	0.365	0.373	0.382	0.391	0.401	0.412	0.423	0.433	0.443	0.452	0.461	0.47	0.479	0.487	0.496	0.503
Indonesia	0.403	0.414	0.425	0.436	0.446	0.457	0.467	0.476	0.485	0.493	0.501	0.51	0.518	0.526	0.534	0.541	0.549	0.557	0.562	0.567	0.572	0.577	0.582	0.586	0.591	0.596	0.601	0.607	0.613	0.62	0.627	0.635	0.643	0.652	0.66	0.669	0.676
Laos	0.239	0.245	0.252	0.259	0.265	0.272	0.279	0.286	0.291	0.297	0.303	0.309	0.315	0.321	0.327	0.335	0.344	0.354	0.363	0.372	0.382	0.391	0.4	0.409	0.418	0.426	0.434	0.441	0.448	0.456	0.463	0.471	0.48	0.488	0.496	0.505	0.512
Malaysia	0.495	0.502	0.51	0.515	0.518	0.523	0.531	0.54	0.549	0.557	0.564	0.569	0.575	0.583	0.593	0.603	0.613	0.625	0.632	0.637	0.648	0.661	0.672	0.679	0.687	0.694	0.701	0.707	0.712	0.718	0.726	0.734	0.742	0.75	0.758	0.766	0.773
Maldives	0.344	0.353	0.362	0.371	0.379	0.389	0.397	0.406	0.415	0.424	0.433	0.442	0.451	0.46	0.469	0.479	0.489	0.5	0.511	0.522	0.534	0.545	0.555	0.565	0.576	0.583	0.592	0.602	0.611	0.621	0.63	0.641	0.651	0.661	0.67	0.68	0.687
Myanmar	0.295	0.301	0.308	0.315	0.321	0.328	0.334	0.339	0.342	0.346	0.349	0.352	0.356	0.36	0.366	0.372	0.379	0.386	0.393	0.401	0.411	0.42	0.43	0.441	0.453	0.465	0.478	0.49	0.502	0.513	0.524	0.536	0.547	0.558	0.569	0.58	0.59
Philippines	0.457	0.465	0.474	0.482	0.489	0.495	0.5	0.505	0.51	0.515	0.52	0.525	0.529	0.534	0.539	0.544	0.549	0.554	0.558	0.562	0.566	0.57	0.573	0.577	0.581	0.585	0.589	0.594	0.598	0.603	0.608	0.613	0.619	0.625	0.631	0.637	0.642
Sri Lanka	0.43	0.437	0.445	0.453	0.463	0.473	0.484	0.492	0.5	0.506	0.512	0.519	0.526	0.533	0.541	0.55	0.559	0.568	0.576	0.581	0.584	0.588	0.592	0.598	0.604	0.609	0.615	0.621	0.628	0.636	0.646	0.656	0.666	0.676	0.685	0.695	0.703
Thailand	0.435	0.449	0.462	0.475	0.486	0.497	0.507	0.517	0.526	0.535	0.544	0.554	0.563	0.572	0.581	0.591	0.6	0.608	0.615	0.621	0.628	0.635	0.642	0.649	0.657	0.664	0.672	0.68	0.686	0.691	0.697	0.703	0.709	0.714	0.719	0.724	0.729
Timor-Leste	0.236	0.245	0.253	0.261	0.269	0.278	0.286	0.295	0.304	0.314	0.323	0.332	0.342	0.351	0.36	0.368	0.376	0.383	0.389	0.39	0.392	0.394	0.396	0.395	0.404	0.416	0.429	0.437	0.444	0.452	0.462	0.475	0.488	0.502	0.514	0.525	0.532
Vietnam	0.354	0.362	0.37	0.378	0.387	0.395	0.403	0.411	0.421	0.43	0.439	0.448	0.458	0.467	0.476	0.486	0.495	0.505	0.514	0.522	0.53	0.538	0.546	0.552	0.559	0.566	0.573	0.579	0.586	0.592	0.599	0.606	0.613	0.619	0.626	0.633	0.639
Mauritius	0.532	0.546	0.559	0.569	0.578	0.587	0.59	0.591	0.595	0.602	0.61	0.616	0.621	0.63	0.639	0.647	0.654	0.662	0.667	0.671	0.678	0.684	0.688	0.693	0.698	0.703	0.71	0.715	0.722	0.726	0.73	0.735	0.739	0.743	0.747	0.752	0.756
Seychelles	0.492	0.51	0.524	0.531	0.538	0.548	0.56	0.574	0.586	0.595	0.603	0.61	0.618	0.625	0.634	0.644	0.654	0.663	0.671	0.677	0.682	0.687	0.691	0.693	0.694	0.696	0.697	0.698	0.7	0.703	0.706	0.708	0.711	0.716	0.723	0.73	0.736
Aceh	0.418	0.43	0.443	0.456	0.469	0.482	0.494	0.505	0.516	0.527	0.537	0.547	0.557	0.566	0.575	0.583	0.591	0.599	0.604	0.609	0.612	0.614	0.616	0.618	0.62	0.622	0.625	0.629	0.633	0.637	0.642	0.647	0.653	0.66	0.667	0.674	0.681
North Sumatera	0.407	0.418	0.429	0.439	0.45	0.46	0.47	0.478	0.485	0.492	0.499	0.506	0.513	0.52	0.527	0.533	0.54	0.547	0.552	0.558	0.562	0.567	0.572	0.578	0.583	0.588	0.593	0.6	0.607	0.616	0.625	0.635	0.645	0.654	0.663	0.672	0.68

Appendix Table 9: Socio-demographic Index values for all estimated GBD 2016 locations, 1980–2016

Location	1980	1981	1982	1983	1984	1985	1986	1987	1988	1989	1990	1991	1992	1993	1994	1995	1996	1997	1998	1999	2000	2001	2002	2003	2004	2005	2006	2007	2008	2009	2010	2011	2012	2013	2014	2015	2016
West Sumatera	0.387	0.398	0.408	0.418	0.429	0.439	0.448	0.458	0.466	0.475	0.483	0.491	0.498	0.506	0.513	0.519	0.526	0.532	0.536	0.541	0.546	0.551	0.557	0.563	0.568	0.574	0.58	0.587	0.594	0.602	0.611	0.62	0.629	0.638	0.647	0.656	0.663
Riau	0.438	0.451	0.465	0.479	0.494	0.507	0.521	0.533	0.545	0.556	0.567	0.577	0.586	0.595	0.602	0.609	0.616	0.622	0.628	0.632	0.639	0.645	0.652	0.656	0.661	0.665	0.67	0.675	0.682	0.688	0.695	0.702	0.711	0.72	0.728	0.737	0.744
Jambi	0.355	0.366	0.376	0.387	0.398	0.408	0.418	0.428	0.438	0.447	0.456	0.465	0.474	0.483	0.492	0.501	0.51	0.519	0.526	0.532	0.537	0.542	0.549	0.555	0.562	0.568	0.575	0.583	0.592	0.6	0.61	0.62	0.63	0.64	0.649	0.659	0.667
South Sumatera	0.416	0.426	0.436	0.446	0.456	0.466	0.476	0.485	0.495	0.503	0.512	0.521	0.53	0.537	0.544	0.55	0.557	0.563	0.569	0.572	0.575	0.578	0.581	0.584	0.587	0.592	0.596	0.602	0.608	0.615	0.622	0.629	0.638	0.646	0.655	0.663	0.671
Bengkulu	0.348	0.359	0.369	0.38	0.391	0.402	0.413	0.423	0.433	0.443	0.452	0.462	0.471	0.48	0.489	0.498	0.507	0.515	0.521	0.526	0.53	0.534	0.538	0.543	0.548	0.554	0.561	0.567	0.575	0.582	0.589	0.598	0.607	0.616	0.625	0.634	0.641
Lampung	0.344	0.355	0.365	0.376	0.386	0.396	0.405	0.414	0.421	0.429	0.437	0.444	0.453	0.46	0.469	0.478	0.489	0.498	0.507	0.516	0.522	0.527	0.532	0.536	0.54	0.544	0.549	0.555	0.562	0.571	0.581	0.592	0.603	0.613	0.623	0.633	0.641
Bangka Belitung	0.398	0.408	0.417	0.427	0.437	0.447	0.456	0.466	0.475	0.483	0.492	0.499	0.507	0.514	0.521	0.528	0.535	0.542	0.548	0.553	0.557	0.562	0.567	0.572	0.578	0.584	0.589	0.595	0.602	0.608	0.615	0.623	0.631	0.639	0.647	0.656	0.663
Riau Islands	0.439	0.453	0.467	0.482	0.498	0.513	0.528	0.542	0.555	0.568	0.581	0.593	0.606	0.617	0.626	0.635	0.644	0.653	0.659	0.665	0.669	0.672	0.675	0.678	0.681	0.684	0.688	0.692	0.698	0.704	0.711	0.718	0.726	0.734	0.742	0.751	0.758
North Kalimantan	0.455	0.469	0.483	0.498	0.513	0.527	0.541	0.554	0.566	0.577	0.588	0.599	0.61	0.62	0.63	0.64	0.649	0.658	0.666	0.673	0.679	0.684	0.689	0.693	0.698	0.704	0.709	0.713	0.72	0.726	0.731	0.739	0.746	0.754	0.761	0.769	0.775
Jakarta	0.554	0.566	0.576	0.587	0.599	0.609	0.619	0.629	0.638	0.646	0.654	0.663	0.67	0.68	0.689	0.697	0.706	0.714	0.72	0.725	0.73	0.735	0.74	0.745	0.75	0.754	0.759	0.764	0.769	0.775	0.782	0.789	0.795	0.803	0.81	0.818	0.824
Cental Java	0.406	0.416	0.426	0.436	0.446	0.456	0.465	0.474	0.481	0.488	0.496	0.504	0.512	0.52	0.529	0.538	0.546	0.554	0.56	0.563	0.566	0.569	0.571	0.575	0.578	0.582	0.587	0.593	0.6	0.608	0.615	0.623	0.631	0.639	0.648	0.656	0.663
West Java	0.363	0.374	0.384	0.395	0.405	0.415	0.425	0.433	0.442	0.449	0.457	0.466	0.476	0.483	0.492	0.5	0.508	0.516	0.521	0.526	0.529	0.533	0.536	0.54	0.545	0.549	0.555	0.561	0.568	0.575	0.582	0.591	0.599	0.608	0.616	0.625	0.632
Yogyakarta	0.393	0.405	0.417	0.429	0.44	0.451	0.462	0.472	0.481	0.49	0.499	0.508	0.517	0.53	0.543	0.554	0.563	0.572	0.576	0.58	0.584	0.587	0.591	0.595	0.599	0.603	0.607	0.612	0.618	0.624	0.631	0.639	0.647	0.655	0.663	0.672	0.679
East Java	0.384	0.395	0.406	0.416	0.427	0.437	0.447	0.456	0.465	0.473	0.482	0.49	0.498	0.506	0.514	0.522	0.53	0.538	0.545	0.55	0.556	0.562	0.567	0.573	0.579	0.584	0.59	0.596	0.603	0.61	0.617	0.625	0.633	0.642	0.651	0.659	0.667
Banten	0.398	0.408	0.418	0.428	0.439	0.449	0.459	0.469	0.478	0.486	0.495	0.504	0.513	0.521	0.529	0.536	0.544	0.553	0.561	0.568	0.573	0.577	0.581	0.586	0.59	0.593	0.598	0.602	0.607	0.612	0.618	0.626	0.635	0.644	0.652	0.661	0.669
Bali	0.394	0.406	0.418	0.43	0.442	0.454	0.466	0.477	0.486	0.496	0.506	0.515	0.524	0.534	0.544	0.552	0.561	0.57	0.575	0.579	0.582	0.585	0.589	0.592	0.596	0.6	0.604	0.609	0.614	0.621	0.628	0.636	0.644	0.653	0.662	0.67	0.678
Nusa Tenggara Barat	0.285	0.297	0.307	0.318	0.328	0.338	0.346	0.354	0.361	0.367	0.374	0.383	0.392	0.403	0.413	0.424	0.434	0.444	0.452	0.458	0.466	0.475	0.483	0.49	0.498	0.505	0.511	0.518	0.524	0.532	0.539	0.547	0.554	0.562	0.569	0.577	0.584
East Nusa Tenggara	0.301	0.311	0.319	0.327	0.335	0.343	0.351	0.359	0.366	0.373	0.381	0.388	0.395	0.402	0.409	0.415	0.42	0.426	0.427	0.427	0.429	0.432	0.436	0.439	0.443	0.447	0.451	0.457	0.463	0.47	0.479	0.488	0.497	0.507	0.516	0.525	0.534
West Kalimantan Central Kalimantan	0.319	0.33	0.341	0.353	0.365	0.377	0.388	0.398	0.408	0.419	0.429	0.439	0.45	0.461	0.472	0.483	0.494	0.505	0.513	0.52	0.525	0.53	0.534	0.538	0.542	0.546	0.55	0.555	0.559	0.565	0.571	0.578	0.586	0.594	0.602	0.61	0.618
South Kalimantan	0.41	0.42	0.429	0.439	0.449	0.459	0.469	0.479	0.488	0.497	0.506	0.515	0.523	0.534	0.545	0.554	0.564	0.573	0.579	0.583	0.585	0.586	0.587	0.588	0.59	0.591	0.594	0.598	0.603	0.61	0.617	0.625	0.633	0.642	0.65	0.659	0.667
East Kalimantan	0.399	0.41	0.421	0.432	0.442	0.453	0.463	0.472	0.48	0.487	0.494	0.502	0.51	0.519	0.527	0.534	0.542	0.549	0.554	0.558	0.561	0.564	0.567	0.57	0.574	0.577	0.58	0.584	0.589	0.594	0.601	0.609	0.617	0.625	0.633	0.642	0.649
North Sulawesi	0.463	0.478	0.492	0.507	0.522	0.536	0.55	0.563	0.575	0.587	0.598	0.607	0.617	0.625	0.632	0.64	0.647	0.654	0.661	0.667	0.673	0.68	0.686	0.691	0.696	0.702	0.707	0.711	0.717	0.722	0.726	0.733	0.739	0.747	0.755	0.763	0.77
Central Sulawesi	0.44	0.45	0.46	0.469	0.478	0.487	0.495	0.503	0.51	0.517	0.524	0.532	0.539	0.548	0.556	0.564	0.573	0.581	0.588	0.591	0.593	0.595	0.596	0.597	0.599	0.601	0.603	0.607	0.612	0.619	0.626	0.634	0.642	0.651	0.659	0.668	0.675
South Sumatera	0.363	0.373	0.383	0.392	0.402	0.411	0.42	0.429	0.437	0.444	0.452	0.46	0.468	0.476	0.485	0.493	0.502	0.509	0.516	0.523	0.527	0.533	0.538	0.542	0.547	0.551	0.555	0.559	0.565	0.572	0.579	0.588	0.597	0.606	0.616	0.625	0.633
Southeast Sulawesi	0.354	0.366	0.377	0.388	0.399	0.409	0.42	0.429	0.438	0.447	0.456	0.465	0.474	0.482	0.489	0.496	0.504	0.51	0.516	0.52	0.525	0.53	0.535	0.54	0.546	0.55	0.554	0.56	0.566	0.574	0.583	0.593	0.603	0.613	0.623	0.632	0.64
Gorontalo	0.327	0.339	0.35	0.361	0.373	0.384	0.395	0.405	0.414	0.423	0.432	0.442	0.45	0.458	0.465	0.473	0.48	0.487	0.494	0.499	0.503	0.508	0.513	0.519	0.525	0.531	0.537	0.544	0.552	0.561	0.57	0.58	0.589	0.599	0.608	0.617	0.625
West Sulawesi	0.328	0.338	0.346	0.354	0.362	0.37	0.378	0.385	0.392	0.399	0.407	0.415	0.424	0.43	0.437	0.444	0.45	0.457	0.462	0.466	0.467	0.47	0.473	0.478	0.482	0.488	0.493	0.5	0.507	0.517	0.526	0.536	0.546	0.555	0.564	0.573	0.581
Maluku	0.318	0.329	0.338	0.348	0.358	0.368	0.378	0.387	0.396	0.405	0.414	0.422	0.43	0.436	0.442	0.447	0.453	0.458	0.462	0.466	0.469	0.473	0.477	0.481	0.485	0.489	0.493	0.498	0.507	0.516	0.525	0.536	0.547	0.558	0.568	0.579	0.587
North Maluku	0.446	0.453	0.46	0.468	0.476	0.483	0.491	0.499	0.506	0.512	0.519	0.526	0.533	0.539	0.545	0.551	0.557	0.562	0.566	0.567	0.564	0.559	0.554	0.548	0.542	0.536	0.531	0.527	0.527	0.529	0.534	0.541	0.551	0.561	0.571	0.581	0.59
West Papua	0.364	0.374	0.383	0.393	0.404	0.414	0.423	0.432	0.441	0.449	0.458	0.467	0.476	0.483	0.491	0.498	0.506	0.514	0.518	0.523	0.524	0.524	0.523	0.521	0.518	0.515	0.511	0.508	0.507	0.51	0.515	0.522	0.531	0.541	0.552	0.562	0.571
Papua	0.413	0.425	0.436	0.448	0.461	0.472	0.484	0.495	0.505	0.515	0.525	0.534	0.543	0.55	0.558	0.565	0.572	0.578	0.582	0.585	0.587	0.589	0.59	0.592	0.594	0.596	0.598	0.601	0.607	0.615	0.626	0.643	0.657	0.671	0.683	0.695	0.706
Oceania	0.442	0.45	0.457	0.465	0.474	0.481	0.488	0.494	0.5	0.505	0.51	0.516	0.522	0.529	0.534	0.54	0.546	0.552	0.558	0.562	0.565	0.569	0.571	0.573	0.574	0.577	0.58	0.583	0.584	0.585	0.588	0.589	0.591	0.595	0.6	0.605	0.61
Fiji	0.332	0.338	0.343	0.348	0.353	0.358	0.363	0.368	0.373	0.378	0.383	0.388	0.393	0.398	0.403	0.407	0.412	0.416	0.42	0.424	0.428	0.433	0.436	0.44	0.443	0.447	0.45	0.454	0.457	0.461	0.464	0.468	0.472	0.476	0.481	0.485	0.489
Kiribati	0.525	0.534	0.542	0.55	0.558	0.565	0.572	0.579	0.585	0.592	0.6	0.607	0.614	0.62	0.627	0.634	0.64	0.646	0.652	0.658	0.663	0.669	0.674	0.679	0.684	0.689	0.694	0.699	0.704	0.708	0.712	0.716	0.721	0.725	0.729	0.733	0.736
Marshall Islands	0.261	0.267	0.273	0.279	0.284	0.288	0.292	0.295	0.299	0.303	0.307	0.312	0.316	0.32	0.324	0.328	0.333	0.338	0.345	0.35	0.357	0.363	0.37	0.376	0.383	0.389	0.395	0.401	0.406	0.412	0.416	0.421	0.426	0.431	0.437	0.442	0.446
Federated States of Micronesia	0.399	0.406	0.413	0.42	0.427	0.432	0.439	0.446	0.453	0.46	0.466	0.47	0.473	0.474	0.475	0.476	0.477	0.479	0.482	0.487	0.492	0.499	0.504	0.51	0.514	0.518	0.522	0.527	0.531	0.536	0.541	0.547	0.553	0.559	0.565	0.571	0.575
Papua New Guinea	0.274	0.292	0.31	0.328	0.346	0.363	0.379	0.394	0.409	0.422	0.434	0.446	0.456	0.466	0.475	0.485	0.495	0.503	0.512	0.521	0.53	0.539	0.547	0.555	0.561	0.568	0.573	0.578	0.582	0.586	0.59	0.595	0.599	0.603	0.607	0.61	0.612
Samoa	0.256	0.26	0.263	0.267	0.271	0.274	0.278	0.283	0.287	0.292	0.296	0.3	0.304	0.31	0.315	0.32	0.325	0.33	0.334	0.338	0.342	0.345	0.348	0.352	0.355	0.359	0.362	0.367	0.371	0.376	0.382	0.387	0.394	0.4	0.406	0.413	0.418
Solomon Islands	0.372	0.381	0.389	0.397	0.405	0.412	0.42	0.428	0.435	0.443	0.451	0.458	0.465	0.472	0.477	0.482	0.487	0.492	0.498	0.501	0.504	0.507	0.511	0.514	0.518	0.521	0.525	0.529	0.533	0.537	0.54	0.542	0.545	0.548	0.553	0.558	0.562
Tonga	0.269	0.275	0.28	0.284	0.289	0.294	0.298	0.303	0.308	0.313	0.318	0.323	0.329	0.335	0.34	0.346	0.352	0.357	0.363	0.368	0.371	0.373	0.374	0.375	0.377	0.379	0.383	0.387	0.393	0.398	0.404	0.411	0.418	0.425	0.432	0.439	0.444
Vanuatu	0.382	0.389	0.397	0.403	0.412	0.419	0.427	0.435	0.442	0.448	0.454	0.461	0.466	0.471	0.476	0.481	0.486	0.491	0.496	0.501	0.506	0.511	0.516	0.522	0.527	0.532	0.537	0.541	0.546	0.551	0.555	0.561	0.566	0.571	0.576	0.582	0.586
American Samoa	0.277	0.283	0.288	0.294	0.3	0.306	0.312	0.318	0.324	0.329	0.335	0.341	0.347	0.353	0.359	0.364	0.37	0.376	0.382	0.387	0.393	0.399	0.405	0.41	0.415	0.42	0.426	0.431	0.437	0.443	0.449	0.454	0.46	0.465	0.471	0.476	0.479
Guam	0.551	0.561	0.569	0.576	0.583	0.589	0.595	0.601	0.606	0.612	0.617	0.622	0.626	0.631	0.635	0.639	0.643	0.648	0.652	0.656	0.661	0.666	0.671	0.676	0.682	0.688	0.695	0.7	0.705	0.711	0.716	0.721	0.726	0.73	0.735	0.739	0.742
Northern Mariana Islands	0.718	0.726	0.733	0.738	0.742	0.743	0.743	0.743	0.746	0.75	0.755	0.761	0.765	0.77	0.774	0.778	0.782	0.785	0.788	0.791	0.794	0.797	0.799	0.801	0.802	0.804	0.806	0.807	0.809	0.81	0.812	0.813	0.815	0.817	0.819	0.821	0.822
	0.61	0.622	0.633	0.643	0.653	0.662	0.671	0.68	0.689	0.697	0.705	0.713	0.72	0.727	0.733	0.739	0.744	0.75	0.755	0.761	0.766	0.772	0.777	0.782	0.787	0.79	0.792	0.794	0.795	0.795	0.795	0.794	0.793	0.793	0.794	0.796	0.797
Central Europe, Eastern Europe, and Central Asia	0.706	0.708	0.71	0.711	0.714	0.716	0.717	0.721	0.727	0.733	0.739	0.746	0.751	0.754	0.755	0.757	0.758	0.758	0.758	0.758	0.759	0.762	0.765	0.769	0.773	0.778	0.782	0.786	0.79	0.793	0.797	0.801	0.804	0.806	0.809	0.812	0.816
Central Asia	0.576	0.582	0.587	0.591	0.594	0.598	0.602	0.607	0.612	0.618	0.624	0.63	0.635	0.638	0.641	0.642	0.643	0.643	0.643	0.643	0.645	0.647	0.65	0.654	0.66	0.666	0.672	0.679	0.685	0.691	0.697	0.703	0.708	0.714	0.719	0.724	0.729
Armenia	0.602	0.606	0.61	0.615	0.619	0.622	0.626	0.631	0.635	0.64	0.644	0.648	0.648	0.647	0.645	0.643	0.642	0.641	0.642	0.643	0.646	0.651	0.659	0.668	0.676	0.686	0.695	0.705	0.714	0.719	0.724	0.73	0.735	0.74	0.745	0.75	0.755
Azerbaijan	0.606	0.614	0.622	0.63	0.637	0.643	0.648	0.654	0.659	0.664	0.668	0.673	0.676	0.678	0.678	0.677	0.674	0.67	0.667	0.664	0.662	0.662	0.664	0.667	0.671	0.678	0.688	0.7	0.712	0.722	0.731	0.739	0.747	0.753	0.759	0.763	0.767
Georgia	0.679	0.684	0.688	0.691	0.695	0.698	0.702	0.706	0.71	0.715	0.72	0.723	0.722	0.718	0.713	0.705	0.698	0.689	0.682	0.677	0.674	0.676	0.679	0.684	0.69	0.695	0.701	0.708	0.714	0.719	0.723	0.727	0.731	0.736	0.741	0.746	0.751
Kazakhstan	0.629	0.636	0.641	0.645	0.649	0.653	0.658	0.663	0.669	0.675	0.681	0.687	0.692	0.696	0.699	0.701	0.703	0.704	0.704	0.704	0.705	0.706	0.708	0.71	0.712	0.715	0.718	0.721	0.725	0.728	0.731	0.736	0.74	0.744	0.748	0.753	0.757
Kyrgyzstan	0.556	0.562	0.567	0.571	0.575	0.578	0.582	0.587	0.592	0.598	0.604	0.61	0.614	0.618	0.619	0.618	0.617	0.615	0.612	0.609	0.607	0.606	0.605	0.605	0.606	0.606	0.606	0.607	0.608	0.609	0.61	0.612	0.614	0.618	0.622	0.627	0.632
Mongolia	0.403	0.417	0.431	0.445	0.458	0.473	0.489	0.506	0.523	0.539	0.557	0.573	0.586	0.597	0.604	0.611	0.615	0.619	0.623	0.627	0.631	0.635	0.639	0.643	0.647	0.65	0.653	0.656	0.659	0.66	0.662	0.664	0.667	0.67	0.674	0.678	0.683
Tajikistan	0.462	0.469	0.474	0.478	0.481	0.484	0.488	0.494	0.501	0.51	0.519	0.53	0.537	0.542	0.543	0.542	0.537	0.532	0.525	0.519	0.515	0.513	0.515	0.52	0.527	0.535	0.542	0.548	0.552	0.556	0.559	0.561	0.564	0.567	0.571	0.574	0.577
Turkmenistan	0.53	0.536	0.541	0.545	0.548	0.552	0.555	0.56	0.565	0.571	0.577	0.584	0.59	0.598	0.605	0.612	0.618	0.623	0.627	0.63	0.636	0.641	0.647	0.653	0.66	0.668	0.676	0.684	0.692	0.701	0.709	0.717	0.726	0.735	0.743	0.752	0.759
Uzbekistan	0.493	0.498	0.503	0.506	0.51	0.513	0.518	0.523	0.53	0.537	0.544	0.551	0.558	0.565	0.571	0.577	0.582	0.587	0.591	0.594	0.597	0.6	0.604	0.609	0.616	0.623	0.629	0.636	0.642	0.649	0.656	0.664	0.671	0.678	0.685	0.692	0.698
Central Europe	0.701	0.705	0.709	0.711	0.713	0.717	0.72	0.724	0.729	0.736	0.741	0.746	0.75	0.753	0.758	0.763	0.766	0.77	0.773	0.777	0.781	0.785	0.79	0.794	0.799	0.803	0.808	0.814	0.819	0.823	0.828	0.832	0.836	0.839	0.842	0.846	0.849

Appendix Table 9: Socio-demographic Index values for all estimated GBD 2016 locations, 1980–2016

Location	1980	1981	1982	1983	1984	1985	1986	1987	1988	1989	1990	1991	1992	1993	1994	1995	1996	1997	1998	1999	2000	2001	2002	2003	2004	2005	2006	2007	2008	2009	2010	2011	2012	2013	2014	2015	2016
Albania	0.54	0.545	0.55	0.555	0.56	0.564	0.569	0.573	0.577	0.581	0.584	0.585	0.584	0.585	0.588	0.593	0.601	0.607	0.614	0.623	0.633	0.642	0.651	0.659	0.667	0.674	0.68	0.686	0.692	0.697	0.702	0.706	0.711	0.714	0.718	0.721	0.725
Bosnia and Herzegovina	0.502	0.508	0.514	0.518	0.522	0.527	0.532	0.535	0.539	0.542	0.542	0.542	0.543	0.543	0.546	0.552	0.573	0.597	0.619	0.635	0.648	0.658	0.667	0.675	0.682	0.688	0.694	0.699	0.705	0.71	0.714	0.717	0.721	0.724	0.727	0.73	0.733
Bulgaria	0.705	0.71	0.715	0.72	0.724	0.728	0.733	0.738	0.744	0.752	0.76	0.767	0.771	0.771	0.771	0.772	0.771	0.77	0.771	0.771	0.773	0.776	0.779	0.784	0.789	0.794	0.8	0.807	0.813	0.818	0.824	0.828	0.832	0.836	0.839	0.843	0.846
Croatia	0.746	0.751	0.755	0.759	0.763	0.767	0.772	0.775	0.779	0.782	0.786	0.789	0.788	0.785	0.782	0.779	0.779	0.785	0.79	0.793	0.797	0.801	0.806	0.811	0.815	0.819	0.824	0.828	0.833	0.836	0.838	0.841	0.843	0.845	0.846	0.848	0.85
Czech Republic	0.786	0.79	0.793	0.796	0.798	0.8	0.803	0.805	0.807	0.81	0.812	0.816	0.82	0.826	0.829	0.83	0.832	0.834	0.835	0.837	0.839	0.842	0.844	0.847	0.85	0.853	0.856	0.86	0.863	0.866	0.869	0.871	0.873	0.875	0.875	0.878	0.881
Hungary	0.711	0.719	0.727	0.732	0.733	0.735	0.739	0.744	0.748	0.751	0.753	0.756	0.761	0.766	0.77	0.777	0.78	0.783	0.786	0.79	0.794	0.798	0.803	0.807	0.812	0.816	0.821	0.825	0.828	0.831	0.834	0.836	0.838	0.841	0.843	0.846	0.849
Macedonia	0.664	0.669	0.673	0.677	0.681	0.686	0.69	0.695	0.7	0.702	0.7	0.699	0.701	0.701	0.701	0.703	0.708	0.712	0.716	0.718	0.723	0.73	0.737	0.742	0.745	0.749	0.753	0.757	0.762	0.765	0.77	0.775	0.779	0.782	0.786	0.789	0.793
Montenegro	0.725	0.728	0.732	0.734	0.737	0.739	0.742	0.744	0.746	0.748	0.75	0.751	0.75	0.747	0.743	0.74	0.74	0.741	0.743	0.745	0.748	0.753	0.759	0.764	0.769	0.773	0.777	0.781	0.786	0.79	0.795	0.8	0.803	0.806	0.809	0.812	0.815
Poland	0.702	0.702	0.699	0.699	0.703	0.708	0.714	0.719	0.724	0.73	0.735	0.739	0.745	0.751	0.759	0.767	0.773	0.778	0.783	0.789	0.795	0.8	0.805	0.81	0.815	0.82	0.824	0.83	0.835	0.84	0.845	0.85	0.855	0.86	0.864	0.868	0.872
Romania	0.661	0.672	0.684	0.688	0.687	0.688	0.689	0.695	0.703	0.715	0.731	0.738	0.738	0.739	0.74	0.742	0.744	0.747	0.749	0.751	0.755	0.759	0.764	0.769	0.774	0.78	0.786	0.794	0.802	0.808	0.813	0.818	0.822	0.827	0.83	0.833	0.838
Serbia	0.678	0.68	0.682	0.683	0.684	0.686	0.689	0.691	0.694	0.697	0.7	0.703	0.704	0.701	0.699	0.697	0.698	0.702	0.707	0.709	0.712	0.714	0.717	0.722	0.728	0.733	0.737	0.742	0.747	0.752	0.755	0.759	0.761	0.764	0.767	0.769	0.771
Slovakia	0.749	0.753	0.756	0.759	0.762	0.766	0.77	0.774	0.778	0.781	0.785	0.788	0.792	0.797	0.805	0.81	0.812	0.814	0.817	0.819	0.822	0.825	0.828	0.831	0.835	0.839	0.843	0.848	0.854	0.858	0.86	0.865	0.868	0.871	0.874	0.877	0.88
Slovenia	0.766	0.774	0.782	0.789	0.793	0.797	0.801	0.803	0.808	0.813	0.813	0.813	0.812	0.812	0.813	0.815	0.817	0.82	0.823	0.827	0.831	0.835	0.839	0.843	0.847	0.851	0.856	0.86	0.863	0.865	0.867	0.869	0.871	0.873	0.874	0.877	0.881
Eastern Europe	0.737	0.738	0.738	0.739	0.742	0.743	0.744	0.747	0.753	0.76	0.767	0.775	0.782	0.782	0.78	0.777	0.774	0.771	0.768	0.766	0.766	0.768	0.77	0.774	0.78	0.786	0.792	0.8	0.806	0.81	0.813	0.817	0.819	0.821	0.823	0.826	0.829
Belarus	0.703	0.706	0.708	0.711	0.713	0.716	0.719	0.723	0.727	0.733	0.738	0.743	0.748	0.751	0.752	0.748	0.744	0.742	0.741	0.74	0.741	0.744	0.747	0.752	0.758	0.765	0.772	0.78	0.788	0.795	0.8	0.805	0.81	0.814	0.818	0.822	0.826
Estonia	0.775	0.776	0.776	0.776	0.779	0.78	0.779	0.778	0.781	0.788	0.799	0.807	0.813	0.814	0.813	0.812	0.812	0.813	0.816	0.818	0.822	0.827	0.832	0.837	0.843	0.847	0.85	0.854	0.859	0.861	0.866	0.872	0.877	0.88	0.883	0.885	0.887
Latvia	0.759	0.759	0.758	0.758	0.759	0.76	0.762	0.765	0.77	0.776	0.783	0.789	0.793	0.794	0.79	0.786	0.783	0.781	0.781	0.783	0.785	0.79	0.795	0.801	0.808	0.815	0.822	0.831	0.837	0.841	0.843	0.847	0.85	0.851	0.851	0.851	0.853
Lithuania	0.768	0.771	0.772	0.772	0.774	0.776	0.777	0.781	0.787	0.79	0.792	0.795	0.8	0.805	0.805	0.804	0.801	0.801	0.801	0.803	0.805	0.809	0.814	0.819	0.825	0.832	0.838	0.845	0.851	0.853	0.856	0.858	0.862	0.865	0.869	0.872	0.876
Moldova	0.614	0.617	0.619	0.621	0.623	0.625	0.628	0.632	0.636	0.641	0.647	0.651	0.652	0.654	0.652	0.65	0.647	0.643	0.637	0.631	0.626	0.624	0.625	0.629	0.634	0.64	0.646	0.653	0.66	0.665	0.671	0.678	0.683	0.689	0.695	0.699	0.703
Russia	0.745	0.746	0.745	0.745	0.749	0.749	0.748	0.751	0.758	0.766	0.774	0.782	0.788	0.787	0.786	0.784	0.781	0.778	0.775	0.774	0.774	0.775	0.778	0.782	0.787	0.793	0.799	0.806	0.811	0.814	0.818	0.821	0.823	0.824	0.826	0.829	0.832
Ukraine	0.712	0.715	0.717	0.719	0.721	0.724	0.727	0.731	0.737	0.742	0.748	0.753	0.757	0.76	0.758	0.753	0.748	0.742	0.737	0.732	0.729	0.728	0.729	0.733	0.74	0.747	0.755	0.763	0.77	0.774	0.778	0.782	0.785	0.789	0.791	0.792	0.793
High-income	0.773	0.778	0.782	0.787	0.791	0.795	0.799	0.803	0.806	0.809	0.813	0.817	0.822	0.826	0.83	0.834	0.837	0.84	0.843	0.845	0.848	0.852	0.854	0.856	0.859	0.86	0.861	0.863	0.866	0.868	0.871	0.873	0.875	0.877	0.879	0.881	0.882
High-income Asia Pacific	0.745	0.75	0.756	0.763	0.769	0.775	0.781	0.787	0.793	0.799	0.805	0.809	0.816	0.821	0.826	0.831	0.835	0.839	0.841	0.844	0.847	0.849	0.851	0.854	0.856	0.859	0.861	0.864	0.867	0.868	0.871	0.873	0.875	0.877	0.879	0.882	0.884
Brunei	0.674	0.681	0.687	0.694	0.7	0.706	0.713	0.721	0.729	0.737	0.744	0.751	0.756	0.761	0.766	0.772	0.777	0.783	0.789	0.796	0.803	0.809	0.815	0.821	0.827	0.833	0.839	0.844	0.849	0.853	0.856	0.858	0.861	0.863	0.866	0.869	0.871
Japan	0.776	0.779	0.781	0.784	0.788	0.793	0.798	0.803	0.81	0.817	0.822	0.828	0.833	0.837	0.84	0.844	0.847	0.85	0.852	0.854	0.856	0.858	0.859	0.861	0.863	0.865	0.867	0.869	0.871	0.872	0.873	0.875	0.877	0.878	0.88	0.882	0.884
South Korea	0.618	0.631	0.649	0.669	0.684	0.693	0.704	0.714	0.723	0.732	0.738	0.744	0.753	0.764	0.772	0.781	0.791	0.8	0.805	0.81	0.816	0.821	0.826	0.831	0.835	0.84	0.845	0.849	0.853	0.857	0.861	0.864	0.868	0.871	0.874	0.878	0.881
Singapore	0.678	0.686	0.695	0.704	0.711	0.717	0.718	0.711	0.719	0.734	0.741	0.748	0.754	0.76	0.768	0.776	0.784	0.795	0.802	0.805	0.812	0.818	0.823	0.828	0.834	0.84	0.846	0.852	0.858	0.863	0.867	0.869	0.873	0.876	0.879	0.883	0.886
Hokkaidō	0.771	0.775	0.778	0.78	0.784	0.79	0.794	0.799	0.804	0.809	0.813	0.818	0.822	0.826	0.83	0.833	0.836	0.839	0.841	0.843	0.845	0.847	0.848	0.85	0.852	0.853	0.855	0.857	0.859	0.86	0.861	0.862	0.864	0.866	0.867	0.869	0.87
Aomori	0.76	0.764	0.766	0.769	0.773	0.778	0.784	0.789	0.795	0.801	0.805	0.81	0.814	0.818	0.822	0.827	0.831	0.835	0.838	0.84	0.842	0.843	0.845	0.847	0.848	0.85	0.852	0.854	0.855	0.856	0.858	0.859	0.861	0.862	0.864	0.865	0.867
Iwate	0.752	0.756	0.758	0.761	0.766	0.771	0.776	0.78	0.786	0.792	0.797	0.802	0.807	0.811	0.816	0.821	0.826	0.83	0.833	0.835	0.838	0.84	0.842	0.844	0.846	0.848	0.849	0.851	0.853	0.854	0.856	0.857	0.859	0.86	0.862	0.864	0.865
Miyagi	0.763	0.767	0.77	0.773	0.778	0.783	0.789	0.794	0.8	0.807	0.813	0.818	0.824	0.828	0.832	0.836	0.839	0.842	0.844	0.846	0.848	0.85	0.851	0.853	0.855	0.857	0.859	0.861	0.862	0.863	0.865	0.866	0.868	0.869	0.871	0.873	0.874
Akita	0.759	0.763	0.766	0.769	0.773	0.778	0.782	0.787	0.792	0.798	0.802	0.806	0.811	0.815	0.819	0.823	0.828	0.832	0.834	0.836	0.838	0.84	0.842	0.843	0.845	0.847	0.849	0.851	0.852	0.853	0.855	0.856	0.858	0.86	0.861	0.863	0.865
Yamagata	0.75	0.755	0.757	0.76	0.764	0.768	0.772	0.777	0.782	0.788	0.793	0.798	0.803	0.807	0.812	0.817	0.821	0.825	0.829	0.831	0.834	0.837	0.84	0.843	0.845	0.847	0.849	0.851	0.853	0.854	0.855	0.857	0.858	0.86	0.862	0.863	0.865
Fukushima	0.755	0.759	0.761	0.763	0.768	0.773	0.778	0.783	0.79	0.796	0.801	0.806	0.811	0.815	0.82	0.825	0.829	0.833	0.836	0.838	0.841	0.844	0.847	0.85	0.853	0.855	0.857	0.858	0.86	0.861	0.863	0.864	0.865	0.866	0.868	0.87	0.873
Ibaraki	0.768	0.772	0.775	0.777	0.781	0.787	0.792	0.797	0.804	0.811	0.817	0.822	0.828	0.832	0.837	0.842	0.846	0.849	0.851	0.853	0.855	0.857	0.858	0.86	0.862	0.863	0.865	0.867	0.869	0.87	0.872	0.873	0.875	0.876	0.878	0.879	0.881
Tochigi	0.771	0.774	0.776	0.778	0.782	0.787	0.792	0.798	0.805	0.812	0.818	0.823	0.829	0.834	0.839	0.844	0.848	0.851	0.853	0.855	0.857	0.859	0.861	0.862	0.864	0.866	0.868	0.87	0.872	0.873	0.874	0.876	0.877	0.879	0.881	0.882	0.884
Gunma	0.767	0.771	0.773	0.775	0.779	0.784	0.789	0.794	0.8	0.807	0.812	0.818	0.823	0.827	0.832	0.837	0.841	0.845	0.847	0.849	0.851	0.853	0.855	0.856	0.858	0.86	0.862	0.864	0.866	0.867	0.868	0.87	0.871	0.873	0.875	0.876	0.878
Saitama	0.763	0.767	0.769	0.772	0.776	0.781	0.786	0.791	0.798	0.804	0.81	0.815	0.82	0.824	0.827	0.831	0.834	0.837	0.839	0.84	0.842	0.844	0.845	0.847	0.848	0.85	0.852	0.853	0.855	0.856	0.857	0.859	0.86	0.862	0.863	0.865	0.866
Chiba	0.766	0.77	0.771	0.774	0.778	0.784	0.79	0.796	0.803	0.81	0.815	0.82	0.824	0.828	0.831	0.835	0.838	0.841	0.843	0.845	0.847	0.848	0.85	0.851	0.853	0.854	0.856	0.858	0.86	0.861	0.862	0.863	0.865	0.866	0.868	0.869	0.871
Tōkyō	0.824	0.828	0.832	0.835	0.839	0.842	0.846	0.849	0.853	0.858	0.862	0.867	0.871	0.875	0.878	0.881	0.885	0.887	0.89	0.892	0.893	0.895	0.897	0.899	0.901	0.902	0.905	0.906	0.908	0.909	0.911	0.912	0.914	0.915	0.917	0.918	0.92
Kanagawa	0.775	0.779	0.781	0.784	0.788	0.793	0.798	0.803	0.81	0.817	0.821	0.826	0.83	0.834	0.838	0.841	0.844	0.847	0.849	0.851	0.853	0.854	0.856	0.858	0.859	0.861	0.863	0.865	0.866	0.867	0.869	0.87	0.871	0.873	0.875	0.876	0.878
Niigata	0.761	0.765	0.768	0.77	0.774	0.778	0.783	0.788	0.794	0.8	0.805	0.811	0.816	0.821	0.825	0.83	0.835	0.839	0.843	0.845	0.847	0.849	0.851	0.853	0.854	0.856	0.858	0.86	0.862	0.863	0.865	0.866	0.868	0.87	0.871	0.873	0.875
Toyama	0.771	0.774	0.776	0.778	0.782	0.787	0.792	0.798	0.804	0.811	0.817	0.823	0.828	0.832	0.836	0.84	0.844	0.847	0.849	0.851	0.853	0.855	0.857	0.858	0.86	0.862	0.864	0.866	0.868	0.869	0.871	0.872	0.874	0.876	0.877	0.879	0.881
Ishikawa	0.766	0.77	0.773	0.776	0.78	0.786	0.791	0.796	0.802	0.809	0.815	0.82	0.826	0.831	0.836	0.84	0.843	0.846	0.848	0.85	0.852	0.854	0.856	0.857	0.859	0.861	0.863	0.865	0.867	0.868	0.869	0.871	0.873	0.874	0.876	0.878	0.88
Fukui	0.765	0.768	0.77	0.772	0.776	0.781	0.786	0.791	0.798	0.804	0.81	0.815	0.821	0.825	0.829	0.834	0.838	0.842	0.846	0.849	0.851	0.854	0.857	0.859	0.861	0.863	0.864	0.865	0.866	0.866	0.867	0.868	0.869	0.871	0.873	0.876	0.878
Yamanashi	0.763	0.767	0.77	0.772	0.775	0.78	0.785	0.79	0.796	0.803	0.808	0.813	0.818	0.822	0.827	0.832	0.837	0.841	0.844	0.846	0.848	0.85	0.852	0.853	0.855	0.857	0.859	0.861	0.863	0.864	0.865	0.867	0.868	0.87	0.872	0.874	0.875
Nagano	0.761	0.765	0.768	0.77	0.774	0.779	0.783	0.788	0.794	0.8	0.805	0.81	0.816	0.82	0.825	0.83	0.834	0.838	0.841	0.844	0.847	0.849	0.852	0.854	0.856	0.857	0.859	0.861	0.863	0.864	0.866	0.867	0.868	0.869	0.871	0.874	0.876
Gifu	0.764	0.768	0.77	0.772	0.776	0.781	0.787	0.792	0.799	0.806	0.811	0.817	0.822	0.826	0.831	0.835	0.838	0.841	0.843	0.845	0.847	0.849	0.85	0.852	0.854	0.856	0.857	0.859	0.861	0.862	0.864	0.865	0.867	0.868	0.87	0.872	0.873
Shizuoka	0.777	0.78	0.781	0.783	0.787	0.793	0.798	0.803	0.81	0.817	0.823	0.829	0.834	0.839	0.843	0.847	0.851	0.854	0.856	0.858	0.86	0.861	0.863	0.865	0.866	0.868	0.87	0.872	0.874	0.874	0.875	0.876	0.877	0.879	0.881	0.883	0.886
Aichi	0.786	0.79	0.792	0.794	0.797	0.803	0.808	0.813	0.82	0.828	0.833	0.839	0.845	0.849	0.852	0.856	0.859	0.862	0.864	0.866	0.868	0.87	0.871	0.873	0.875	0.877	0.878	0.88	0.882	0.883	0.885	0.886	0.887	0.888	0.89	0.893	0.895
Mie	0.77	0.774	0.776	0.778	0.782	0.788	0.793	0.798	0.805	0.812	0.818	0.824	0.83	0.834	0.839	0.843	0.846	0.849	0.851	0.853	0.855	0.857	0.859	0.86	0.862	0.864	0.866	0.868	0.87	0.871	0.873	0.874	0.875	0.877	0.879	0.881	0.883
Shiga	0.77	0.773	0.775	0.777	0.781	0.787	0.792	0.798	0.805	0.812	0.819	0.825	0.831	0.835	0.84	0.845	0.849	0.853	0.857	0.859	0.862	0.864	0.865	0.867	0.869	0.871	0.873	0.875	0.876	0.877	0.878	0.879	0.88	0.881	0.883	0.886	0.889
Kyōto	0.776	0.779	0.781	0.783	0.787	0.792	0.797	0.803	0.81	0.816	0.821	0.825	0.83	0.834	0.838	0.841	0.845	0.848	0.85	0.852	0.854	0.855	0.857	0.859	0.861	0.862	0.864	0.866	0.868	0.869	0.871	0.872	0.874	0.876	0.877	0.879	0.881
Ōsaka	0.788	0.791	0.793	0.795	0.799	0.804	0.809	0.814	0.821	0.827	0.832	0.836	0.841	0.845	0.848	0.852	0.855	0.858	0.86	0.861	0.863	0.865	0.866	0.868	0.869	0.871	0.873	0.875	0.877	0.878	0.879	0.88	0.882	0.884	0.885	0.887	0.888
Hyōgo	0.765	0.768	0.77	0.773	0.777	0.783	0.788	0.794	0.801	0.808	0.814	0.818	0.823	0.827	0.831	0.834	0.838	0.841	0.843	0.845	0.847	0.848	0.85	0.851	0.853	0.855	0.857	0.859	0.861	0.862	0.863	0.865	0.866	0.868	0.869	0.871	0.873
Nara	0.749	0.753	0.754	0.757	0.762	0.768	0.773	0.779	0.786	0.792	0.797	0.802	0.807	0.811	0.814	0.818	0.821	0.824	0.826	0.828	0.83	0.832	0.833	0.835	0.836	0.838	0.84	0.842	0.843	0.844	0.846	0.847	0.849	0.85	0.852	0.853	0.855
Wakayama	0.757	0.761	0.763	0.766	0.77	0.775	0.781	0.787	0.793	0.8	0.806	0.812	0.817	0.821	0.826	0.829	0.833	0.836	0.838	0.84	0.842	0.844	0.845	0.847	0.849	0.85	0.852	0.854	0.856	0.857	0.859	0.86	0.862	0.864	0.865	0.867	0.869

Appendix Table 9: Socio-demographic Index values for all estimated GBD 2016 locations, 1980–2016

Location	1980	1981	1982	1983	1984	1985	1986	1987	1988	1989	1990	1991	1992	1993	1994	1995	1996	1997	1998	1999	2000	2001	2002	2003	2004	2005	2006	2007	2008	2009	2010	2011	2012	2013	2014	2015	2016
Tottori	0.747	0.751	0.753	0.755	0.759	0.764	0.769	0.774	0.78	0.786	0.791	0.797	0.803	0.807	0.812	0.817	0.822	0.826	0.83	0.832	0.835	0.837	0.84	0.843	0.845	0.847	0.849	0.851	0.853	0.854	0.856	0.857	0.858	0.859	0.861	0.864	0.866
Shimane	0.744	0.748	0.75	0.752	0.756	0.761	0.766	0.771	0.777	0.783	0.788	0.794	0.799	0.803	0.808	0.813	0.818	0.823	0.827	0.83	0.833	0.836	0.839	0.842	0.845	0.847	0.847	0.849	0.85	0.85	0.851	0.852	0.853	0.854	0.856	0.859	0.862
Okayama	0.762	0.765	0.767	0.769	0.773	0.779	0.784	0.79	0.797	0.804	0.809	0.815	0.821	0.825	0.83	0.835	0.839	0.843	0.845	0.847	0.849	0.851	0.853	0.855	0.857	0.859	0.861	0.863	0.865	0.866	0.867	0.869	0.871	0.872	0.874	0.876	0.878
Hiroshima	0.77	0.773	0.775	0.777	0.781	0.787	0.792	0.797	0.804	0.811	0.816	0.822	0.828	0.833	0.837	0.842	0.845	0.848	0.85	0.852	0.854	0.856	0.857	0.859	0.861	0.863	0.865	0.867	0.869	0.869	0.869	0.87	0.871	0.872	0.874	0.877	0.881
Yamaguchi	0.767	0.771	0.773	0.775	0.779	0.784	0.789	0.795	0.801	0.808	0.813	0.818	0.824	0.828	0.832	0.837	0.84	0.843	0.846	0.848	0.85	0.851	0.853	0.855	0.857	0.859	0.861	0.863	0.865	0.866	0.867	0.867	0.868	0.87	0.872	0.875	0.878
Tokushima	0.762	0.765	0.768	0.77	0.774	0.78	0.785	0.79	0.796	0.803	0.808	0.814	0.82	0.824	0.828	0.832	0.835	0.838	0.841	0.843	0.845	0.847	0.849	0.85	0.852	0.854	0.856	0.858	0.86	0.861	0.862	0.864	0.866	0.867	0.869	0.871	0.873
Kagawa	0.764	0.768	0.77	0.772	0.776	0.781	0.786	0.791	0.798	0.805	0.811	0.817	0.822	0.827	0.831	0.835	0.839	0.842	0.844	0.846	0.848	0.85	0.852	0.854	0.855	0.857	0.859	0.861	0.863	0.863	0.864	0.865	0.866	0.867	0.869	0.872	0.875
Ehime	0.757	0.761	0.764	0.767	0.771	0.776	0.781	0.786	0.792	0.799	0.804	0.809	0.815	0.819	0.823	0.828	0.832	0.835	0.837	0.839	0.841	0.843	0.845	0.847	0.849	0.851	0.852	0.855	0.856	0.857	0.859	0.861	0.862	0.864	0.866	0.867	0.869
Kōchi	0.746	0.749	0.751	0.753	0.757	0.763	0.768	0.774	0.78	0.787	0.792	0.796	0.801	0.805	0.81	0.815	0.819	0.823	0.825	0.827	0.829	0.831	0.833	0.835	0.837	0.839	0.84	0.843	0.844	0.845	0.847	0.848	0.85	0.852	0.853	0.855	0.857
Fukuoka	0.769	0.773	0.775	0.777	0.782	0.787	0.792	0.798	0.804	0.811	0.816	0.822	0.826	0.83	0.834	0.837	0.841	0.844	0.846	0.848	0.85	0.852	0.853	0.855	0.857	0.859	0.861	0.863	0.865	0.866	0.867	0.869	0.87	0.872	0.874	0.876	0.877
Saga	0.751	0.755	0.757	0.76	0.764	0.769	0.775	0.78	0.786	0.793	0.798	0.803	0.808	0.812	0.816	0.821	0.825	0.828	0.831	0.834	0.836	0.839	0.843	0.846	0.849	0.85	0.852	0.853	0.854	0.855	0.856	0.857	0.857	0.859	0.861	0.863	0.866
Nagasaki	0.749	0.753	0.755	0.757	0.761	0.767	0.772	0.777	0.783	0.79	0.794	0.799	0.804	0.808	0.813	0.818	0.822	0.826	0.829	0.832	0.834	0.837	0.839	0.841	0.843	0.845	0.847	0.848	0.849	0.849	0.849	0.85	0.851	0.852	0.854	0.857	0.859
Kumamoto	0.75	0.754	0.757	0.759	0.763	0.768	0.773	0.778	0.784	0.791	0.796	0.801	0.806	0.81	0.814	0.819	0.823	0.827	0.83	0.832	0.835	0.837	0.839	0.841	0.843	0.845	0.846	0.846	0.847	0.848	0.849	0.85	0.851	0.852	0.854	0.857	0.86
Ôita	0.761	0.765	0.768	0.771	0.775	0.78	0.785	0.789	0.795	0.802	0.807	0.812	0.817	0.821	0.825	0.83	0.834	0.838	0.841	0.843	0.845	0.847	0.849	0.85	0.852	0.854	0.856	0.858	0.86	0.86	0.861	0.862	0.863	0.865	0.867	0.87	0.872
Miyazaki	0.749	0.752	0.755	0.757	0.762	0.767	0.773	0.778	0.784	0.79	0.795	0.799	0.804	0.808	0.812	0.816	0.821	0.825	0.828	0.831	0.833	0.836	0.839	0.841	0.844	0.845	0.845	0.846	0.847	0.847	0.847	0.848	0.849	0.85	0.853	0.855	0.858
Kagoshima	0.744	0.748	0.751	0.754	0.759	0.765	0.77	0.775	0.782	0.788	0.793	0.799	0.804	0.808	0.813	0.818	0.822	0.826	0.83	0.832	0.834	0.837	0.84	0.842	0.844	0.846	0.846	0.847	0.848	0.849	0.85	0.851	0.852	0.853	0.855	0.858	0.861
Okinawa	0.73	0.734	0.736	0.738	0.743	0.75	0.756	0.762	0.769	0.777	0.782	0.787	0.793	0.797	0.802	0.808	0.812	0.816	0.819	0.821	0.823	0.825	0.828	0.832	0.835	0.836	0.836	0.837	0.837	0.837	0.837	0.838	0.838	0.84	0.842	0.845	0.848
Australasia	0.787	0.79	0.793	0.796	0.799	0.802	0.805	0.808	0.811	0.813	0.815	0.818	0.821	0.824	0.828	0.832	0.836	0.839	0.843	0.846	0.849	0.853	0.856	0.858	0.86	0.862	0.861	0.861	0.864	0.867	0.87	0.872	0.875	0.88	0.884	0.887	0.889
Australia	0.787	0.789	0.792	0.796	0.799	0.802	0.806	0.81	0.813	0.815	0.818	0.82	0.823	0.826	0.83	0.834	0.838	0.842	0.845	0.849	0.853	0.856	0.859	0.862	0.864	0.865	0.865	0.865	0.868	0.872	0.875	0.876	0.879	0.883	0.887	0.89	0.892
New Zealand	0.786	0.79	0.794	0.797	0.799	0.8	0.8	0.8	0.8	0.8	0.803	0.807	0.81	0.813	0.816	0.819	0.822	0.826	0.828	0.828	0.831	0.836	0.839	0.84	0.841	0.843	0.84	0.838	0.841	0.844	0.847	0.851	0.856	0.86	0.863	0.865	0.867
Western Europe	0.754	0.759	0.765	0.771	0.775	0.779	0.784	0.788	0.792	0.797	0.801	0.807	0.812	0.817	0.821	0.825	0.828	0.832	0.835	0.838	0.842	0.845	0.848	0.85	0.852	0.854	0.856	0.858	0.859	0.861	0.864	0.866	0.869	0.871	0.873	0.874	0.876
Andorra	0.845	0.848	0.85	0.852	0.855	0.857	0.859	0.862	0.864	0.866	0.868	0.87	0.872	0.873	0.875	0.876	0.878	0.881	0.884	0.887	0.889	0.891	0.892	0.894	0.896	0.897	0.899	0.901	0.902	0.904	0.906	0.907	0.909	0.91	0.912	0.913	0.915
Austria	0.801	0.803	0.807	0.812	0.816	0.818	0.82	0.822	0.824	0.827	0.83	0.832	0.836	0.838	0.841	0.844	0.846	0.849	0.851	0.854	0.857	0.86	0.862	0.864	0.867	0.869	0.871	0.874	0.876	0.878	0.88	0.882	0.885	0.887	0.889	0.89	0.892
Belgium	0.813	0.818	0.823	0.827	0.831	0.833	0.835	0.837	0.84	0.841	0.843	0.846	0.85	0.855	0.859	0.861	0.862	0.865	0.867	0.869	0.871	0.874	0.877	0.877	0.877	0.878	0.879	0.88	0.882	0.883	0.886	0.89	0.893	0.896	0.897	0.898	0.899
Cyprus	0.692	0.696	0.699	0.704	0.712	0.72	0.726	0.73	0.734	0.739	0.745	0.748	0.754	0.765	0.774	0.783	0.79	0.798	0.806	0.812	0.819	0.826	0.831	0.835	0.839	0.843	0.847	0.852	0.856	0.86	0.863	0.866	0.868	0.869	0.87	0.871	0.872
Denmark	0.866	0.867	0.869	0.871	0.873	0.876	0.879	0.88	0.879	0.879	0.88	0.88	0.88	0.881	0.882	0.885	0.889	0.893	0.895	0.897	0.9	0.903	0.905	0.905	0.906	0.906	0.907	0.908	0.909	0.911	0.914	0.919	0.922	0.925	0.925	0.925	0.925
Finland	0.815	0.816	0.818	0.821	0.826	0.832	0.836	0.837	0.838	0.84	0.841	0.841	0.842	0.843	0.845	0.849	0.852	0.856	0.86	0.863	0.867	0.871	0.874	0.876	0.878	0.88	0.883	0.886	0.888	0.89	0.892	0.896	0.9	0.904	0.906	0.907	0.907
France	0.743	0.749	0.758	0.765	0.769	0.773	0.777	0.783	0.788	0.793	0.799	0.805	0.812	0.817	0.821	0.823	0.826	0.829	0.832	0.833	0.836	0.84	0.843	0.846	0.848	0.849	0.85	0.853	0.855	0.856	0.858	0.861	0.863	0.865	0.867	0.868	0.869
Germany	0.801	0.804	0.807	0.809	0.812	0.814	0.817	0.82	0.822	0.826	0.829	0.832	0.835	0.838	0.841	0.843	0.845	0.847	0.85	0.852	0.854	0.857	0.859	0.86	0.862	0.864	0.866	0.869	0.871	0.873	0.875	0.877	0.88	0.882	0.884	0.886	0.889
Greece	0.699	0.708	0.714	0.722	0.731	0.739	0.745	0.751	0.756	0.761	0.765	0.77	0.775	0.779	0.783	0.787	0.792	0.796	0.801	0.806	0.81	0.815	0.82	0.825	0.829	0.834	0.838	0.843	0.847	0.85	0.852	0.853	0.853	0.852	0.852	0.852	0.853
Iceland	0.808	0.817	0.823	0.83	0.841	0.848	0.847	0.842	0.842	0.844	0.847	0.852	0.852	0.855	0.861	0.862	0.865	0.869	0.873	0.875	0.879	0.886	0.888	0.888	0.889	0.892	0.894	0.895	0.894	0.894	0.901	0.906	0.909	0.914	0.918	0.92	0.921
Ireland	0.684	0.694	0.705	0.717	0.727	0.733	0.74	0.75	0.759	0.764	0.768	0.775	0.784	0.792	0.798	0.802	0.806	0.811	0.818	0.826	0.832	0.836	0.842	0.848	0.856	0.861	0.861	0.861	0.863	0.864	0.867	0.871	0.874	0.877	0.879	0.883	0.885
Israel	0.691	0.698	0.7	0.7	0.702	0.708	0.716	0.723	0.727	0.731	0.736	0.743	0.748	0.753	0.757	0.761	0.764	0.766	0.767	0.769	0.772	0.775	0.777	0.777	0.779	0.781	0.784	0.786	0.786	0.788	0.791	0.794	0.796	0.797	0.798	0.799	0.801
Italy	0.748	0.755	0.761	0.767	0.772	0.776	0.781	0.785	0.789	0.794	0.798	0.803	0.807	0.81	0.814	0.818	0.821	0.824	0.828	0.831	0.834	0.837	0.84	0.843	0.846	0.848	0.85	0.853	0.855	0.857	0.858	0.86	0.862	0.863	0.864	0.865	0.867
Luxembourg	0.848	0.851	0.853	0.855	0.858	0.861	0.864	0.868	0.871	0.873	0.876	0.88	0.882	0.885	0.888	0.891	0.893	0.898	0.9	0.9	0.904	0.909	0.912	0.912	0.913	0.915	0.917	0.919	0.921	0.922	0.924	0.928	0.929	0.932	0.934	0.935	0.936
Malta	0.717	0.723	0.728	0.733	0.738	0.741	0.743	0.743	0.743	0.747	0.752	0.754	0.759	0.768	0.775	0.777	0.778	0.786	0.795	0.803	0.814	0.819	0.822	0.825	0.827	0.829	0.831	0.834	0.836	0.838	0.84	0.842	0.845	0.848	0.85	0.853	0.856
Netherlands	0.825	0.83	0.833	0.835	0.838	0.839	0.84	0.843	0.846	0.848	0.85	0.854	0.859	0.862	0.866	0.871	0.873	0.874	0.876	0.878	0.88	0.884	0.886	0.889	0.893	0.896	0.898	0.9	0.901	0.902	0.905	0.909	0.913	0.915	0.917	0.917	0.918
Norway	0.824	0.828	0.833	0.837	0.84	0.843	0.845	0.845	0.845	0.845	0.847	0.851	0.856	0.859	0.862	0.864	0.868	0.874	0.877	0.88	0.885	0.891	0.894	0.894	0.896	0.897	0.899	0.9	0.901	0.903	0.907	0.912	0.916	0.92	0.922	0.922	0.922
Portugal	0.61	0.618	0.626	0.634	0.643	0.651	0.658	0.665	0.672	0.68	0.687	0.694	0.701	0.708	0.714	0.719	0.725	0.73	0.735	0.74	0.746	0.751	0.755	0.759	0.763	0.766	0.77	0.773	0.777	0.779	0.782	0.785	0.787	0.789	0.791	0.794	0.796
Spain	0.67	0.68	0.689	0.698	0.705	0.713	0.721	0.727	0.733	0.739	0.746	0.752	0.759	0.764	0.77	0.775	0.78	0.786	0.791	0.796	0.801	0.806	0.811	0.816	0.82	0.824	0.828	0.833	0.836	0.838	0.84	0.842	0.843	0.844	0.845	0.847	0.848
Sweden	0.799	0.803	0.806	0.808	0.808	0.808	0.809	0.808	0.807	0.806	0.807	0.81	0.816	0.822	0.829	0.838	0.845	0.849	0.853	0.855	0.857	0.857	0.857	0.857	0.858	0.858	0.858	0.859	0.86	0.859	0.86	0.864	0.867	0.869	0.87	0.871	0.872
Switzerland	0.867	0.867	0.868	0.868	0.869	0.869	0.869	0.869	0.869	0.869	0.869	0.87	0.872	0.874	0.874	0.874	0.874	0.874	0.875	0.875	0.876	0.877	0.878	0.879	0.88	0.881	0.883	0.885	0.887	0.888	0.889	0.891	0.893	0.895	0.896	0.899	0.901
United Kingdom	0.711	0.717	0.722	0.727	0.732	0.737	0.742	0.746	0.753	0.758	0.763	0.769	0.775	0.781	0.787	0.793	0.797	0.802	0.808	0.815	0.822	0.827	0.83	0.831	0.834	0.837	0.838	0.84	0.842	0.843	0.846	0.849	0.853	0.857	0.86	0.862	0.863
Sweden except Stockholm	0.774	0.778	0.782	0.784	0.784	0.784	0.785	0.785	0.784	0.783	0.784	0.788	0.794	0.801	0.809	0.819	0.826	0.831	0.834	0.837	0.839	0.84	0.839	0.84	0.841	0.841	0.841	0.843	0.845	0.844	0.846	0.85	0.854	0.856	0.858	0.86	0.861
Stockholm	0.866	0.868	0.87	0.871	0.873	0.875	0.877	0.879	0.88	0.879	0.879	0.882	0.885	0.889	0.891	0.892	0.893	0.895	0.897	0.899	0.901	0.903	0.905	0.906	0.908	0.909	0.908	0.907	0.906	0.904	0.904	0.905	0.906	0.906	0.905	0.905	0.905
Northern Ireland	0.718	0.726	0.732	0.737	0.742	0.747	0.752	0.759	0.768	0.775	0.782	0.788	0.795	0.802	0.807	0.811	0.815	0.82	0.826	0.832	0.838	0.842	0.846	0.848	0.85	0.852	0.852	0.851	0.853	0.855	0.856	0.857	0.859	0.863	0.866	0.87	0.872
Scotland	0.706	0.712	0.718	0.724	0.728	0.732	0.737	0.742	0.748	0.752	0.755	0.759	0.764	0.77	0.775	0.779	0.782	0.786	0.792	0.797	0.801	0.805	0.809	0.811	0.813	0.815	0.816	0.816	0.818	0.821	0.823	0.826	0.83	0.832	0.834	0.835	0.837
Wales	0.681	0.686	0.692	0.697	0.702	0.708	0.713	0.718	0.726	0.733	0.737	0.742	0.749	0.755	0.762	0.767	0.772	0.778	0.785	0.792	0.8	0.807	0.811	0.813	0.816	0.819	0.821	0.822	0.825	0.827	0.829	0.833	0.837	0.842	0.845	0.847	0.848
England	0.712	0.718	0.723	0.729	0.733	0.738	0.743	0.747	0.753	0.759	0.765	0.771	0.777	0.783	0.789	0.794	0.799	0.804	0.81	0.817	0.824	0.829	0.832	0.834	0.837	0.839	0.841	0.842	0.844	0.846	0.848	0.851	0.855	0.86	0.863	0.865	0.866
Darlington	0.695	0.701	0.708	0.714	0.719	0.725	0.731	0.735	0.741	0.744	0.746	0.75	0.756	0.764	0.772	0.779	0.785	0.791	0.796	0.802	0.808	0.812	0.813	0.814	0.817	0.82	0.823	0.824	0.824	0.825	0.831	0.836	0.841	0.846	0.848	0.848	0.849
Northumberland	0.683	0.689	0.695	0.701	0.707	0.713	0.72	0.726	0.733	0.738	0.742	0.746	0.75	0.756	0.762	0.769	0.775	0.781	0.787	0.792	0.797	0.8	0.802	0.804	0.806	0.808	0.81	0.812	0.815	0.817	0.819	0.821	0.825	0.828	0.83	0.831	0.832
Stockton-on-Tees	0.692	0.698	0.703	0.708	0.714	0.721	0.727	0.733	0.74	0.745	0.748	0.753	0.759	0.766	0.774	0.78	0.786	0.792	0.798	0.806	0.812	0.815	0.817	0.818	0.82	0.822	0.824	0.825	0.827	0.829	0.831	0.833	0.835	0.84	0.843	0.846	0.848
Newcastle upon Tyne	0.705	0.711	0.717	0.722	0.726	0.731	0.737	0.743	0.75	0.756	0.763	0.771	0.78	0.787	0.792	0.798	0.802	0.806	0.811	0.819	0.827	0.833	0.84	0.846	0.852	0.858	0.861	0.865	0.868	0.871	0.873	0.876	0.879	0.882	0.885	0.887	0.889
North Tyneside	0.682	0.687	0.692	0.697	0.701	0.706	0.711	0.718	0.725	0.731	0.737	0.742	0.749	0.756	0.762	0.768	0.772	0.778	0.784	0.79	0.797	0.802	0.805	0.807	0.811	0.816	0.82	0.824	0.827	0.828	0.831	0.834	0.837	0.84	0.842	0.844	0.846
Redcar and Cleveland	0.659	0.665	0.67	0.676	0.68	0.686	0.691	0.697	0.704	0.71	0.714	0.718	0.723	0.727	0.733	0.739	0.744	0.75	0.757	0.766	0.774	0.779	0.782	0.783	0.786	0.789	0.791	0.793	0.795	0.795	0.796	0.797	0.802	0.808	0.813	0.816	0.819
County Durham	0.673	0.679	0.685	0.69	0.696	0.702	0.708	0.713	0.719	0.724	0.728	0.733	0.74	0.746	0.753	0.759	0.764	0.771	0.777	0.784	0.791	0.796	0.799	0.8	0.803	0.805	0.807	0.809	0.812	0.813	0.816	0.818	0.822	0.827	0.83	0.832	0.833
Gateshead	0.687	0.692	0.698	0.703	0.708	0.713	0.719	0.725	0.732	0.738	0.744	0.749	0.755	0.761	0.768	0.774	0.779	0.785	0.791	0.797	0.803	0.808	0.811	0.814	0.818	0.821	0.824	0.826	0.828	0.83	0.833	0.837	0.84	0.844	0.847	0.849	0.851

Appendix Table 9: Socio-demographic Index values for all estimated GBD 2016 locations, 1980–2016

Location	1980	1981	1982	1983	1984	1985	1986	1987	1988	1989	1990	1991	1992	1993	1994	1995	1996	1997	1998	1999	2000	2001	2002	2003	2004	2005	2006	2007	2008	2009	2010	2011	2012	2013	2014	2015	2016
Middlesbrough	0.668	0.674	0.68	0.686	0.69	0.695	0.699	0.705	0.713	0.72	0.727	0.735	0.741	0.748	0.755	0.761	0.767	0.772	0.778	0.785	0.791	0.794	0.795	0.795	0.8	0.804	0.808	0.813	0.816	0.817	0.818	0.819	0.821	0.824	0.826	0.827	0.829
South Tyneside	0.657	0.662	0.666	0.669	0.672	0.675	0.68	0.686	0.694	0.701	0.707	0.713	0.719	0.725	0.731	0.736	0.741	0.746	0.753	0.761	0.768	0.775	0.78	0.784	0.789	0.793	0.796	0.799	0.802	0.803	0.805	0.807	0.81	0.814	0.817	0.818	0.82
Sunderland	0.676	0.683	0.689	0.694	0.7	0.706	0.712	0.718	0.725	0.731	0.736	0.742	0.748	0.754	0.761	0.767	0.772	0.778	0.785	0.791	0.798	0.804	0.809	0.812	0.816	0.82	0.823	0.826	0.829	0.83	0.831	0.832	0.835	0.838	0.841	0.844	0.845
Hartlepool	0.672	0.678	0.683	0.687	0.691	0.695	0.7	0.704	0.711	0.718	0.723	0.728	0.734	0.74	0.746	0.753	0.759	0.765	0.77	0.777	0.782	0.785	0.787	0.788	0.79	0.793	0.795	0.799	0.802	0.805	0.807	0.81	0.814	0.819	0.82	0.821	0.821
Cheshire East	0.726	0.732	0.737	0.742	0.747	0.753	0.758	0.764	0.769	0.774	0.778	0.783	0.788	0.795	0.802	0.809	0.815	0.821	0.826	0.832	0.837	0.842	0.845	0.845	0.847	0.848	0.848	0.847	0.848	0.851	0.854	0.858	0.863	0.869	0.872	0.873	0.874
Stockport	0.702	0.708	0.714	0.719	0.724	0.73	0.734	0.74	0.747	0.753	0.757	0.763	0.77	0.778	0.784	0.789	0.794	0.8	0.806	0.813	0.82	0.825	0.828	0.829	0.831	0.833	0.835	0.836	0.837	0.838	0.839	0.841	0.844	0.849	0.853	0.855	0.857
Trafford	0.726	0.732	0.737	0.741	0.745	0.75	0.755	0.76	0.767	0.772	0.777	0.784	0.791	0.799	0.806	0.812	0.816	0.821	0.828	0.835	0.841	0.846	0.849	0.851	0.854	0.855	0.855	0.856	0.858	0.86	0.864	0.868	0.872	0.877	0.88	0.882	0.883
Cheshire West and Chester	0.714	0.72	0.726	0.731	0.736	0.741	0.747	0.751	0.756	0.761	0.766	0.773	0.78	0.787	0.792	0.795	0.796	0.799	0.803	0.812	0.822	0.831	0.837	0.839	0.841	0.843	0.845	0.846	0.847	0.849	0.852	0.857	0.862	0.866	0.868	0.869	0.869
Sefton	0.682	0.688	0.693	0.698	0.704	0.709	0.715	0.72	0.725	0.73	0.734	0.74	0.747	0.754	0.761	0.767	0.771	0.777	0.783	0.789	0.795	0.8	0.803	0.805	0.809	0.811	0.812	0.813	0.813	0.814	0.816	0.818	0.82	0.823	0.824	0.825	0.826
Lancashire	0.694	0.7	0.705	0.711	0.716	0.721	0.727	0.732	0.738	0.744	0.749	0.755	0.761	0.768	0.775	0.781	0.786	0.792	0.798	0.804	0.81	0.815	0.817	0.819	0.822	0.824	0.826	0.827	0.829	0.83	0.832	0.834	0.838	0.842	0.845	0.847	0.848
Cumbria	0.702	0.707	0.713	0.718	0.723	0.73	0.736	0.741	0.747	0.751	0.754	0.757	0.763	0.77	0.777	0.783	0.789	0.794	0.8	0.804	0.809	0.812	0.814	0.815	0.818	0.82	0.821	0.821	0.823	0.825	0.828	0.832	0.837	0.842	0.845	0.846	0.848
Bolton	0.678	0.684	0.69	0.695	0.701	0.707	0.713	0.719	0.725	0.73	0.735	0.741	0.747	0.754	0.761	0.766	0.77	0.774	0.78	0.786	0.791	0.795	0.797	0.798	0.8	0.8	0.8	0.8	0.802	0.804	0.807	0.81	0.814	0.819	0.821	0.823	0.824
Wirral	0.677	0.682	0.687	0.692	0.696	0.7	0.705	0.71	0.716	0.722	0.727	0.733	0.739	0.746	0.753	0.76	0.765	0.771	0.777	0.783	0.787	0.79	0.791	0.792	0.795	0.797	0.798	0.798	0.799	0.8	0.803	0.808	0.812	0.817	0.819	0.82	0.82
Bury	0.683	0.688	0.693	0.697	0.7	0.704	0.709	0.714	0.72	0.727	0.733	0.739	0.747	0.754	0.761	0.766	0.77	0.774	0.78	0.787	0.793	0.797	0.799	0.8	0.803	0.806	0.807	0.806	0.806	0.805	0.808	0.812	0.818	0.824	0.828	0.83	0.831
St Helens	0.67	0.675	0.681	0.686	0.691	0.697	0.703	0.709	0.715	0.72	0.722	0.725	0.731	0.738	0.745	0.75	0.755	0.76	0.766	0.772	0.78	0.786	0.79	0.792	0.796	0.799	0.801	0.804	0.806	0.808	0.81	0.812	0.816	0.822	0.826	0.828	0.83
Warrington	0.719	0.724	0.73	0.735	0.741	0.747	0.753	0.759	0.766	0.771	0.774	0.777	0.783	0.789	0.796	0.802	0.807	0.812	0.817	0.824	0.83	0.836	0.841	0.844	0.847	0.85	0.852	0.852	0.852	0.854	0.858	0.862	0.867	0.873	0.875	0.877	0.878
Oldham	0.669	0.674	0.679	0.684	0.689	0.694	0.699	0.704	0.71	0.715	0.72	0.725	0.731	0.738	0.743	0.747	0.751	0.754	0.758	0.763	0.769	0.773	0.775	0.776	0.778	0.78	0.782	0.784	0.787	0.789	0.792	0.796	0.8	0.804	0.806	0.807	0.808
Rochdale	0.668	0.673	0.679	0.684	0.688	0.694	0.7	0.705	0.712	0.716	0.72	0.725	0.731	0.737	0.744	0.75	0.755	0.76	0.767	0.773	0.779	0.783	0.784	0.785	0.787	0.788	0.788	0.788	0.789	0.791	0.795	0.8	0.805	0.81	0.813	0.814	0.815
Wigan	0.671	0.676	0.682	0.687	0.692	0.698	0.704	0.71	0.716	0.722	0.727	0.733	0.739	0.746	0.752	0.758	0.763	0.769	0.774	0.779	0.785	0.789	0.791	0.793	0.795	0.797	0.798	0.799	0.8	0.801	0.804	0.807	0.811	0.816	0.82	0.822	0.824
Halton	0.69	0.695	0.7	0.704	0.707	0.711	0.715	0.72	0.727	0.734	0.739	0.745	0.752	0.759	0.767	0.773	0.778	0.782	0.786	0.792	0.8	0.806	0.81	0.811	0.812	0.814	0.816	0.818	0.82	0.822	0.827	0.832	0.836	0.842	0.847	0.85	0.853
Liverpool	0.688	0.693	0.698	0.702	0.705	0.709	0.712	0.716	0.722	0.729	0.738	0.75	0.76	0.767	0.773	0.777	0.781	0.787	0.794	0.803	0.812	0.818	0.824	0.83	0.836	0.839	0.842	0.844	0.848	0.852	0.855	0.859	0.862	0.865	0.867	0.868	0.87
Tameside	0.672	0.678	0.683	0.688	0.692	0.697	0.702	0.707	0.713	0.719	0.724	0.73	0.737	0.744	0.751	0.756	0.761	0.766	0.771	0.778	0.786	0.792	0.795	0.797	0.8	0.802	0.801	0.801	0.8	0.8	0.802	0.805	0.809	0.813	0.816	0.818	0.82
Salford	0.691	0.696	0.701	0.706	0.71	0.714	0.719	0.723	0.728	0.732	0.737	0.744	0.752	0.761	0.769	0.775	0.78	0.784	0.791	0.799	0.807	0.814	0.818	0.821	0.825	0.828	0.831	0.832	0.834	0.835	0.839	0.843	0.848	0.853	0.856	0.859	0.861
Blackburn with Darwen	0.669	0.675	0.68	0.685	0.689	0.694	0.698	0.702	0.709	0.715	0.72	0.726	0.733	0.739	0.745	0.75	0.755	0.76	0.765	0.77	0.775	0.779	0.781	0.782	0.784	0.786	0.786	0.787	0.791	0.794	0.798	0.802	0.807	0.812	0.816	0.818	0.82
Knowsley	0.665	0.672	0.678	0.683	0.689	0.694	0.7	0.704	0.71	0.715	0.72	0.727	0.733	0.739	0.744	0.75	0.755	0.762	0.768	0.774	0.781	0.787	0.791	0.792	0.795	0.799	0.803	0.807	0.811	0.813	0.816	0.819	0.822	0.828	0.832	0.834	0.837
Blackpool	0.667	0.673	0.679	0.685	0.688	0.69	0.691	0.693	0.699	0.704	0.711	0.722	0.732	0.74	0.746	0.751	0.754	0.759	0.766	0.773	0.778	0.782	0.782	0.78	0.782	0.786	0.788	0.788	0.787	0.788	0.792	0.796	0.799	0.801	0.803	0.806	0.809
Manchester	0.715	0.721	0.726	0.731	0.734	0.739	0.744	0.749	0.755	0.761	0.768	0.775	0.781	0.789	0.796	0.801	0.806	0.812	0.819	0.829	0.839	0.847	0.853	0.857	0.862	0.866	0.869	0.872	0.875	0.878	0.882	0.884	0.888	0.892	0.895	0.897	0.899
North Yorkshire	0.707	0.712	0.718	0.724	0.73	0.736	0.742	0.747	0.754	0.759	0.762	0.765	0.769	0.774	0.78	0.785	0.79	0.796	0.802	0.808	0.815	0.819	0.821	0.822	0.826	0.829	0.831	0.833	0.834	0.835	0.838	0.841	0.844	0.848	0.849	0.849	0.85
East Riding of Yorkshire	0.696	0.702	0.708	0.714	0.72	0.724	0.727	0.726	0.73	0.738	0.746	0.752	0.758	0.765	0.771	0.777	0.782	0.788	0.793	0.799	0.804	0.808	0.81	0.811	0.815	0.818	0.819	0.821	0.823	0.825	0.827	0.828	0.831	0.834	0.836	0.837	0.839
York	0.729	0.735	0.74	0.745	0.749	0.753	0.756	0.76	0.766	0.773	0.781	0.79	0.799	0.804	0.81	0.815	0.819	0.824	0.831	0.836	0.842	0.848	0.854	0.86	0.865	0.87	0.873	0.877	0.88	0.881	0.883	0.885	0.886	0.888	0.89	0.892	0.894
North East Lincolnshire	0.693	0.698	0.703	0.706	0.709	0.714	0.721	0.728	0.736	0.74	0.743	0.748	0.755	0.762	0.769	0.773	0.776	0.779	0.784	0.789	0.795	0.801	0.802	0.802	0.803	0.805	0.808	0.813	0.817	0.822	0.825	0.828	0.831	0.833	0.834	0.834	0.834
Calderdale	0.688	0.694	0.699	0.704	0.709	0.716	0.722	0.729	0.736	0.741	0.746	0.75	0.755	0.761	0.767	0.773	0.777	0.783	0.788	0.795	0.801	0.805	0.806	0.807	0.81	0.814	0.816	0.818	0.82	0.821	0.824	0.828	0.833	0.839	0.842	0.845	0.847
North Lincolnshire	0.691	0.696	0.701	0.706	0.71	0.715	0.72	0.724	0.731	0.736	0.741	0.746	0.751	0.757	0.763	0.769	0.775	0.781	0.787	0.792	0.796	0.8	0.802	0.802	0.803	0.804	0.805	0.806	0.809	0.812	0.816	0.821	0.826	0.831	0.833	0.834	0.835
Bradford	0.669	0.675	0.681	0.686	0.69	0.696	0.702	0.707	0.714	0.72	0.726	0.732	0.738	0.744	0.75	0.755	0.759	0.763	0.767	0.773	0.779	0.784	0.786	0.787	0.79	0.792	0.795	0.798	0.802	0.805	0.808	0.81	0.812	0.815	0.817	0.818	0.819
Kirklees	0.676	0.682	0.688	0.694	0.699	0.705	0.711	0.716	0.723	0.729	0.735	0.741	0.747	0.753	0.759	0.763	0.768	0.772	0.778	0.785	0.79	0.795	0.796	0.797	0.8	0.803	0.805	0.807	0.81	0.812	0.814	0.817	0.821	0.825	0.828	0.83	0.832
Leeds	0.707	0.713	0.719	0.724	0.729	0.734	0.739	0.743	0.75	0.756	0.763	0.771	0.778	0.785	0.791	0.796	0.802	0.807	0.814	0.822	0.83	0.836	0.842	0.847	0.852	0.857	0.861	0.865	0.869	0.869	0.869	0.868	0.869	0.872	0.874	0.876	0.878
Sheffield	0.702	0.707	0.713	0.718	0.723	0.727	0.732	0.737	0.743	0.748	0.753	0.759	0.764	0.77	0.776	0.781	0.786	0.792	0.798	0.805	0.812	0.818	0.823	0.826	0.831	0.835	0.838	0.843	0.848	0.851	0.853	0.855	0.857	0.861	0.863	0.865	0.867
Wakefield	0.68	0.685	0.691	0.696	0.701	0.706	0.711	0.716	0.723	0.728	0.733	0.738	0.744	0.75	0.756	0.761	0.766	0.771	0.777	0.784	0.792	0.797	0.8	0.801	0.804	0.806	0.807	0.808	0.809	0.81	0.812	0.815	0.82	0.825	0.828	0.831	0.833
Rotherham	0.667	0.673	0.678	0.684	0.689	0.694	0.699	0.704	0.71	0.715	0.72	0.725	0.731	0.737	0.743	0.749	0.753	0.758	0.763	0.769	0.776	0.781	0.785	0.788	0.791	0.794	0.795	0.797	0.799	0.801	0.804	0.807	0.809	0.813	0.816	0.818	0.82
Doncaster	0.663	0.668	0.674	0.679	0.683	0.689	0.694	0.7	0.707	0.712	0.716	0.721	0.725	0.73	0.736	0.741	0.745	0.75	0.756	0.763	0.769	0.774	0.777	0.779	0.782	0.785	0.787	0.79	0.793	0.796	0.801	0.805	0.809	0.813	0.816	0.818	0.819
Kingston upon Hull, City of	0.675	0.68	0.685	0.688	0.69	0.694	0.7	0.709	0.718	0.721	0.725	0.731	0.74	0.747	0.754	0.759	0.764	0.77	0.776	0.783	0.791	0.797	0.8	0.803	0.808	0.812	0.817	0.821	0.825	0.826	0.825	0.825	0.827	0.831	0.835	0.838	0.84
Barnsley	0.66	0.665	0.671	0.676	0.681	0.687	0.692	0.697	0.703	0.708	0.712	0.718	0.724	0.73	0.737	0.742	0.747	0.753	0.76	0.766	0.772	0.776	0.778	0.78	0.782	0.784	0.786	0.789	0.791	0.792	0.794	0.797	0.801	0.806	0.81	0.812	0.814
Northamptonshire	0.698	0.704	0.71	0.716	0.72	0.725	0.73	0.734	0.74	0.746	0.751	0.757	0.764	0.771	0.777	0.783	0.788	0.793	0.799	0.805	0.811	0.816	0.818	0.819	0.821	0.824	0.825	0.827	0.828	0.83	0.833	0.835	0.839	0.843	0.845	0.845	0.846
Leicestershire	0.705	0.711	0.717	0.723	0.728	0.733	0.739	0.744	0.75	0.756	0.76	0.765	0.77	0.776	0.782	0.787	0.792	0.798	0.804	0.81	0.816	0.821	0.823	0.826	0.829	0.833	0.836	0.839	0.842	0.843	0.845	0.847	0.85	0.854	0.857	0.859	0.861
Lincolnshire	0.687	0.693	0.699	0.705	0.71	0.716	0.722	0.727	0.733	0.738	0.741	0.744	0.749	0.755	0.761	0.768	0.773	0.779	0.784	0.79	0.796	0.802	0.805	0.808	0.81	0.811	0.811	0.811	0.812	0.813	0.816	0.819	0.823	0.828	0.831	0.832	0.833
Rutland	0.703	0.709	0.715	0.721	0.728	0.736	0.744	0.751	0.76	0.765	0.771	0.772	0.776	0.784	0.79	0.796	0.801	0.806	0.811	0.815	0.82	0.814	0.802	0.782	0.776	0.788	0.797	0.804	0.809	0.817	0.831	0.839	0.839	0.839	0.838	0.839	0.84
Derby	0.689	0.696	0.703	0.71	0.716	0.723	0.728	0.733	0.738	0.743	0.748	0.756	0.764	0.773	0.782	0.789	0.794	0.798	0.803	0.81	0.816	0.822	0.826	0.829	0.834	0.837	0.838	0.841	0.844	0.845	0.846	0.846	0.849	0.855	0.858	0.861	0.864
Derbyshire	0.683	0.689	0.695	0.7	0.705	0.711	0.716	0.722	0.728	0.733	0.737	0.742	0.747	0.754	0.76	0.766	0.772	0.777	0.784	0.79	0.796	0.801	0.803	0.805	0.807	0.81	0.812	0.814	0.815	0.816	0.818	0.822	0.826	0.832	0.836	0.839	0.841
Nottinghamshire	0.68	0.686	0.692	0.698	0.703	0.709	0.714	0.719	0.725	0.731	0.735	0.74	0.745	0.751	0.757	0.762	0.766	0.771	0.778	0.786	0.794	0.8	0.805	0.807	0.811	0.814	0.816	0.817	0.819	0.819	0.82	0.821	0.824	0.828	0.831	0.834	0.835
Nottingham	0.705	0.711	0.718	0.724	0.729	0.733	0.737	0.741	0.746	0.753	0.76	0.769	0.777	0.785	0.793	0.801	0.807	0.815	0.822	0.828	0.834	0.84	0.846	0.85	0.855	0.86	0.863	0.867	0.87	0.871	0.871	0.87	0.871	0.873	0.875	0.877	0.878
Leicester	0.674	0.68	0.686	0.692	0.696	0.702	0.707	0.711	0.717	0.724	0.733	0.744	0.753	0.761	0.768	0.774	0.778	0.781	0.786	0.793	0.8	0.807	0.811	0.814	0.818	0.822	0.825	0.828	0.831	0.834	0.836	0.837	0.84	0.843	0.845	0.847	0.849
Warwickshire	0.714	0.72	0.725	0.731	0.735	0.741	0.746	0.75	0.756	0.761	0.766	0.772	0.778	0.784	0.791	0.796	0.801	0.806	0.812	0.819	0.825	0.83	0.833	0.836	0.838	0.84	0.841	0.842	0.844	0.846	0.849	0.852	0.856	0.861	0.863	0.864	0.865
Herefordshire, County of	0.692	0.698	0.704	0.709	0.715	0.72	0.725	0.729	0.733	0.738	0.741	0.746	0.752	0.759	0.765	0.77	0.775	0.78	0.786	0.793	0.801	0.807	0.809	0.811	0.813	0.815	0.815	0.814	0.815	0.816	0.821	0.828	0.836	0.842	0.846	0.848	0.85
Solihull	0.723	0.728	0.733	0.738	0.742	0.746	0.751	0.755	0.761	0.765	0.768	0.773	0.779	0.787	0.794	0.801	0.807	0.814	0.82	0.826	0.83	0.834	0.836	0.839	0.842	0.846	0.849	0.851	0.852	0.851	0.852	0.853	0.857	0.861	0.863	0.864	0.864
Shropshire	0.694	0.7	0.705	0.71	0.716	0.722	0.727	0.733	0.738	0.743	0.746	0.75	0.755	0.761	0.768	0.773	0.778	0.783	0.79	0.796	0.802	0.805	0.808	0.81	0.814	0.817	0.818	0.818	0.82	0.824	0.829	0.834	0.839	0.843	0.845	0.846	0.847
Worcestershire	0.688	0.694	0.699	0.705	0.71	0.716	0.721	0.727	0.733	0.738	0.742	0.747	0.753	0.759	0.765	0.77	0.775	0.78	0.787	0.796	0.804	0.81	0.813	0.815	0.819	0.821	0.822	0.823	0.824	0.825	0.828	0.832	0.836	0.841	0.844	0.846	0.847
Staffordshire	0.69	0.695	0.701	0.706	0.711	0.716	0.721	0.727	0.733	0.739	0.744	0.749	0.756	0.762	0.768	0.773	0.778	0.783	0.79	0.796	0.802	0.806	0.809	0.81	0.813	0.816	0.818	0.821	0.824	0.826	0.829	0.832	0.835	0.839	0.841	0.842	0.843

Appendix Table 9: Socio-demographic Index values for all estimated GBD 2016 locations, 1980–2016

Location	1980	1981	1982	1983	1984	1985	1986	1987	1988	1989	1990	1991	1992	1993	1994	1995	1996	1997	1998	1999	2000	2001	2002	2003	2004	2005	2006	2007	2008	2009	2010	2011	2012	2013	2014	2015	2016
Dudley	0.677	0.682	0.688	0.693	0.698	0.703	0.708	0.714	0.721	0.726	0.731	0.736	0.741	0.747	0.753	0.757	0.762	0.767	0.772	0.778	0.784	0.789	0.792	0.794	0.797	0.8	0.802	0.804	0.806	0.806	0.807	0.808	0.81	0.814	0.816	0.817	0.818
Coventry	0.697	0.702	0.707	0.711	0.715	0.719	0.724	0.729	0.735	0.742	0.75	0.757	0.764	0.771	0.777	0.782	0.787	0.792	0.797	0.805	0.812	0.819	0.823	0.826	0.83	0.833	0.835	0.836	0.837	0.838	0.84	0.843	0.848	0.855	0.86	0.863	0.866
Telford and Wrekin	0.698	0.704	0.71	0.716	0.721	0.726	0.731	0.736	0.742	0.747	0.752	0.757	0.763	0.769	0.775	0.78	0.785	0.792	0.798	0.804	0.81	0.815	0.817	0.817	0.818	0.818	0.818	0.818	0.821	0.822	0.824	0.828	0.833	0.838	0.842	0.845	0.846
Stoke-on-Trent	0.67	0.676	0.681	0.686	0.691	0.697	0.701	0.705	0.71	0.716	0.724	0.733	0.742	0.749	0.756	0.761	0.766	0.772	0.777	0.783	0.788	0.79	0.789	0.788	0.789	0.791	0.791	0.79	0.793	0.796	0.8	0.804	0.809	0.815	0.819	0.823	0.827
Walsall	0.667	0.672	0.677	0.682	0.686	0.691	0.697	0.702	0.709	0.714	0.718	0.722	0.728	0.735	0.741	0.744	0.746	0.747	0.752	0.759	0.766	0.771	0.773	0.773	0.776	0.779	0.781	0.784	0.786	0.788	0.792	0.796	0.8	0.804	0.807	0.808	0.809
Wolverhampton	0.678	0.684	0.689	0.693	0.698	0.703	0.707	0.712	0.717	0.723	0.728	0.734	0.741	0.747	0.753	0.757	0.761	0.766	0.772	0.78	0.788	0.795	0.799	0.8	0.803	0.806	0.807	0.808	0.809	0.81	0.813	0.816	0.821	0.825	0.827	0.829	0.83
Birmingham	0.68	0.686	0.691	0.696	0.7	0.705	0.71	0.714	0.72	0.727	0.733	0.742	0.75	0.757	0.763	0.767	0.771	0.775	0.781	0.788	0.795	0.801	0.805	0.808	0.812	0.817	0.819	0.822	0.826	0.829	0.832	0.833	0.836	0.84	0.843	0.845	0.847
Sandwell	0.666	0.671	0.677	0.682	0.686	0.691	0.696	0.701	0.708	0.714	0.719	0.725	0.732	0.739	0.745	0.749	0.753	0.758	0.763	0.768	0.773	0.778	0.781	0.782	0.785	0.787	0.788	0.789	0.79	0.791	0.794	0.797	0.803	0.808	0.812	0.813	0.815
Bedford	0.711	0.717	0.723	0.728	0.733	0.739	0.745	0.75	0.757	0.764	0.771	0.776	0.781	0.784	0.788	0.791	0.795	0.801	0.807	0.814	0.822	0.828	0.831	0.833	0.836	0.839	0.841	0.842	0.843	0.844	0.845	0.846	0.848	0.85	0.85	0.851	0.851
Central Bedfordshire	0.709	0.715	0.72	0.725	0.73	0.735	0.741	0.747	0.754	0.759	0.765	0.77	0.775	0.781	0.788	0.794	0.8	0.806	0.811	0.815	0.818	0.82	0.822	0.823	0.827	0.829	0.83	0.831	0.831	0.831	0.832	0.834	0.838	0.844	0.848	0.85	0.853
Suffolk	0.703	0.709	0.715	0.72	0.725	0.731	0.736	0.74	0.746	0.752	0.757	0.763	0.768	0.773	0.78	0.786	0.791	0.797	0.802	0.807	0.811	0.814	0.814	0.814	0.817	0.82	0.822	0.823	0.824	0.825	0.827	0.83	0.833	0.837	0.838	0.839	0.84
Hertfordshire	0.731	0.737	0.743	0.748	0.753	0.757	0.76	0.764	0.769	0.775	0.781	0.788	0.795	0.801	0.806	0.812	0.816	0.822	0.828	0.836	0.843	0.848	0.851	0.853	0.855	0.858	0.858	0.859	0.861	0.862	0.864	0.866	0.869	0.874	0.876	0.878	0.879
Essex	0.701	0.706	0.712	0.717	0.722	0.726	0.73	0.734	0.739	0.745	0.75	0.757	0.763	0.768	0.774	0.779	0.784	0.79	0.796	0.802	0.808	0.812	0.814	0.816	0.819	0.823	0.825	0.828	0.83	0.83	0.831	0.833	0.836	0.84	0.842	0.844	0.845
Cambridgeshire	0.728	0.734	0.74	0.745	0.749	0.755	0.76	0.765	0.771	0.777	0.783	0.789	0.796	0.801	0.807	0.814	0.82	0.827	0.833	0.838	0.844	0.849	0.854	0.856	0.86	0.862	0.862	0.862	0.862	0.863	0.867	0.87	0.874	0.878	0.88	0.881	0.882
Thurrock	0.702	0.708	0.711	0.715	0.717	0.72	0.723	0.726	0.733	0.741	0.75	0.759	0.765	0.77	0.775	0.78	0.783	0.787	0.793	0.799	0.806	0.811	0.812	0.812	0.813	0.813	0.813	0.814	0.816	0.819	0.821	0.822	0.823	0.825	0.826	0.825	0.824
Norfolk	0.702	0.708	0.714	0.719	0.723	0.728	0.733	0.737	0.743	0.749	0.754	0.759	0.764	0.77	0.775	0.781	0.786	0.792	0.798	0.804	0.81	0.814	0.817	0.818	0.821	0.824	0.826	0.828	0.83	0.831	0.832	0.833	0.836	0.841	0.844	0.846	0.848
Southend-on-Sea	0.683	0.688	0.693	0.696	0.699	0.703	0.709	0.716	0.724	0.731	0.736	0.742	0.749	0.757	0.765	0.772	0.778	0.785	0.79	0.794	0.796	0.797	0.798	0.799	0.802	0.804	0.804	0.806	0.809	0.813	0.817	0.819	0.822	0.825	0.827	0.827	0.828
Peterborough	0.713	0.719	0.724	0.728	0.732	0.737	0.742	0.747	0.753	0.759	0.763	0.767	0.773	0.778	0.785	0.79	0.794	0.798	0.803	0.809	0.815	0.819	0.818	0.816	0.816	0.816	0.816	0.816	0.816	0.818	0.823	0.828	0.831	0.835	0.837	0.838	0.839
Luton	0.692	0.698	0.703	0.707	0.71	0.713	0.715	0.717	0.725	0.733	0.743	0.752	0.759	0.765	0.771	0.775	0.779	0.783	0.787	0.793	0.798	0.803	0.804	0.804	0.806	0.808	0.811	0.816	0.823	0.829	0.834	0.837	0.84	0.843	0.844	0.844	0.845
Richmond upon Thames	0.76	0.766	0.771	0.775	0.779	0.783	0.787	0.791	0.798	0.805	0.813	0.82	0.827	0.833	0.839	0.845	0.85	0.855	0.861	0.868	0.873	0.878	0.882	0.885	0.887	0.888	0.887	0.887	0.888	0.888	0.889	0.891	0.895	0.9	0.905	0.908	0.91
Kensington and Chelsea	0.798	0.804	0.809	0.815	0.82	0.826	0.832	0.837	0.84	0.848	0.855	0.861	0.866	0.871	0.875	0.88	0.884	0.889	0.894	0.9	0.906	0.91	0.914	0.917	0.921	0.924	0.927	0.929	0.932	0.934	0.936	0.938	0.94	0.941	0.942	0.943	0.945
Barnet	0.729	0.735	0.742	0.748	0.753	0.759	0.764	0.769	0.775	0.782	0.787	0.792	0.797	0.803	0.81	0.816	0.823	0.829	0.835	0.841	0.846	0.851	0.853	0.854	0.855	0.855	0.854	0.853	0.855	0.857	0.86	0.865	0.87	0.875	0.878	0.879	0.88
Westminster	0.808	0.812	0.817	0.821	0.825	0.829	0.831	0.832	0.837	0.844	0.853	0.86	0.865	0.869	0.873	0.877	0.882	0.886	0.891	0.896	0.901	0.906	0.91	0.914	0.918	0.921	0.924	0.927	0.929	0.931	0.933	0.935	0.936	0.938	0.939	0.941	0.943
Bromley	0.73	0.736	0.741	0.746	0.749	0.753	0.757	0.761	0.767	0.774	0.779	0.784	0.789	0.793	0.799	0.804	0.809	0.815	0.821	0.829	0.835	0.839	0.842	0.844	0.846	0.847	0.848	0.85	0.851	0.851	0.851	0.851	0.853	0.857	0.86	0.862	0.863
Bexley	0.701	0.707	0.713	0.718	0.722	0.726	0.73	0.733	0.739	0.744	0.749	0.756	0.762	0.768	0.774	0.778	0.781	0.784	0.789	0.795	0.801	0.806	0.809	0.811	0.814	0.815	0.815	0.817	0.819	0.821	0.823	0.824	0.828	0.833	0.836	0.838	0.84
Redbridge	0.702	0.708	0.713	0.718	0.723	0.729	0.734	0.74	0.746	0.752	0.757	0.761	0.767	0.772	0.777	0.782	0.787	0.792	0.797	0.804	0.81	0.815	0.818	0.82	0.822	0.823	0.824	0.827	0.83	0.83	0.831	0.831	0.834	0.837	0.84	0.841	0.843
Merton	0.726	0.732	0.737	0.74	0.743	0.747	0.752	0.757	0.765	0.773	0.782	0.792	0.8	0.807	0.813	0.818	0.823	0.828	0.836	0.845	0.852	0.857	0.859	0.861	0.863	0.865	0.866	0.867	0.869	0.87	0.87	0.871	0.874	0.879	0.881	0.882	0.884
Brent	0.724	0.73	0.736	0.741	0.746	0.752	0.759	0.765	0.773	0.779	0.784	0.788	0.792	0.797	0.802	0.807	0.811	0.815	0.82	0.826	0.834	0.841	0.845	0.846	0.847	0.846	0.843	0.836	0.832	0.832	0.84	0.85	0.858	0.864	0.866	0.865	0.865
Hillingdon	0.756	0.762	0.767	0.772	0.776	0.78	0.784	0.788	0.795	0.801	0.807	0.814	0.821	0.827	0.834	0.84	0.844	0.848	0.853	0.859	0.864	0.866	0.868	0.869	0.871	0.871	0.87	0.87	0.872	0.874	0.876	0.878	0.882	0.888	0.892	0.895	0.897
Havering	0.703	0.709	0.715	0.72	0.724	0.728	0.732	0.735	0.74	0.745	0.75	0.756	0.762	0.768	0.774	0.778	0.783	0.787	0.792	0.798	0.803	0.808	0.81	0.812	0.815	0.818	0.82	0.822	0.825	0.827	0.828	0.829	0.83	0.833	0.835	0.836	0.837
Kingston upon Thames	0.746	0.752	0.758	0.762	0.766	0.77	0.774	0.779	0.788	0.796	0.804	0.811	0.818	0.823	0.828	0.832	0.837	0.842	0.85	0.858	0.864	0.869	0.874	0.878	0.881	0.884	0.886	0.888	0.891	0.892	0.892	0.892	0.895	0.898	0.9	0.901	0.902
Sutton	0.714	0.72	0.726	0.73	0.734	0.737	0.739	0.743	0.749	0.756	0.762	0.769	0.776	0.783	0.79	0.796	0.802	0.807	0.814	0.821	0.828	0.832	0.834	0.834	0.836	0.838	0.84	0.842	0.845	0.846	0.847	0.847	0.85	0.854	0.856	0.857	0.858
Harrow	0.719	0.726	0.732	0.737	0.741	0.745	0.749	0.751	0.757	0.764	0.772	0.781	0.789	0.796	0.802	0.806	0.81	0.814	0.821	0.829	0.836	0.84	0.841	0.842	0.844	0.847	0.847	0.848	0.849	0.849	0.851	0.852	0.854	0.856	0.856	0.856	0.856
Enfield	0.716	0.722	0.728	0.732	0.736	0.741	0.745	0.749	0.755	0.761	0.767	0.773	0.778	0.783	0.787	0.79	0.793	0.798	0.804	0.812	0.82	0.826	0.828	0.828	0.828	0.827	0.826	0.826	0.828	0.831	0.837	0.842	0.847	0.851	0.853	0.853	0.854
Croydon	0.721	0.727	0.732	0.737	0.741	0.745	0.749	0.753	0.759	0.765	0.772	0.78	0.787	0.794	0.801	0.806	0.81	0.815	0.82	0.826	0.832	0.836	0.839	0.841	0.843	0.843	0.841	0.84	0.841	0.841	0.843	0.845	0.848	0.85	0.85	0.849	0.849
Hammersmith and Fulham	0.784	0.789	0.795	0.801	0.807	0.814	0.82	0.826	0.833	0.841	0.849	0.856	0.862	0.867	0.872	0.877	0.883	0.887	0.891	0.897	0.901	0.906	0.909	0.912	0.916	0.919	0.92	0.922	0.924	0.927	0.932	0.936	0.937	0.938	0.94	0.941	0.942
Ealing	0.729	0.736	0.743	0.749	0.757	0.765	0.773	0.779	0.786	0.791	0.795	0.8	0.805	0.81	0.816	0.822	0.827	0.833	0.839	0.847	0.853	0.858	0.86	0.86	0.861	0.86	0.859	0.858	0.857	0.857	0.859	0.863	0.868	0.873	0.876	0.877	0.879
Greenwich	0.701	0.707	0.713	0.719	0.723	0.727	0.732	0.735	0.74	0.746	0.753	0.76	0.768	0.775	0.78	0.784	0.788	0.793	0.798	0.806	0.814	0.821	0.825	0.826	0.826	0.824	0.821	0.82	0.822	0.825	0.83	0.835	0.841	0.846	0.848	0.848	0.848
Wandsworth	0.746	0.752	0.758	0.764	0.77	0.777	0.784	0.791	0.799	0.808	0.816	0.825	0.833	0.839	0.844	0.849	0.854	0.86	0.865	0.872	0.879	0.885	0.89	0.895	0.899	0.902	0.906	0.907	0.909	0.911	0.912	0.915	0.919	0.922	0.924	0.926	0.928
Waltham Forest	0.681	0.687	0.694	0.699	0.705	0.71	0.715	0.72	0.726	0.733	0.739	0.746	0.753	0.76	0.766	0.771	0.774	0.777	0.783	0.79	0.797	0.803	0.806	0.806	0.807	0.805	0.802	0.798	0.798	0.8	0.807	0.814	0.822	0.828	0.832	0.834	0.835
Camden	0.81	0.815	0.819	0.823	0.828	0.833	0.838	0.843	0.848	0.852	0.857	0.862	0.867	0.872	0.876	0.881	0.883	0.884	0.888	0.897	0.904	0.909	0.912	0.915	0.919	0.922	0.924	0.927	0.929	0.931	0.933	0.935	0.937	0.939	0.94	0.942	0.943
Lambeth	0.738	0.745	0.751	0.756	0.761	0.766	0.771	0.773	0.779	0.787	0.798	0.811	0.821	0.828	0.834	0.838	0.842	0.847	0.853	0.86	0.867	0.871	0.872	0.873	0.876	0.881	0.885	0.891	0.898	0.903	0.908	0.914	0.919	0.921	0.922	0.924	0.926
Lewisham	0.702	0.708	0.714	0.719	0.724	0.728	0.732	0.736	0.741	0.747	0.755	0.766	0.775	0.782	0.787	0.79	0.793	0.796	0.802	0.81	0.819	0.826	0.828	0.828	0.829	0.83	0.832	0.833	0.835	0.836	0.84	0.844	0.85	0.856	0.86	0.863	0.865
Hounslow	0.745	0.75	0.753	0.755	0.755	0.757	0.761	0.766	0.773	0.784	0.795	0.805	0.81	0.815	0.818	0.822	0.825	0.829	0.836	0.847	0.857	0.864	0.866	0.864	0.864	0.865	0.867	0.869	0.872	0.874	0.876	0.878	0.883	0.889	0.891	0.891	0.892
Southwark	0.753	0.76	0.766	0.772	0.778	0.782	0.787	0.791	0.798	0.806	0.816	0.825	0.833	0.838	0.841	0.844	0.845	0.848	0.854	0.864	0.875	0.883	0.885	0.885	0.888	0.892	0.896	0.9	0.904	0.907	0.911	0.916	0.923	0.929	0.933	0.935	0.937
Newham	0.69	0.696	0.701	0.705	0.708	0.712	0.716	0.722	0.728	0.734	0.74	0.747	0.754	0.759	0.76	0.759	0.758	0.76	0.765	0.775	0.79	0.803	0.808	0.808	0.81	0.811	0.809	0.803	0.795	0.795	0.813	0.833	0.844	0.85	0.852	0.853	0.853
Barking and Dagenham	0.692	0.698	0.702	0.705	0.707	0.711	0.715	0.719	0.724	0.728	0.733	0.739	0.745	0.752	0.758	0.763	0.765	0.768	0.775	0.784	0.793	0.799	0.801	0.798	0.794	0.79	0.787	0.787	0.791	0.794	0.798	0.8	0.804	0.81	0.814	0.815	0.815
Haringey	0.73	0.736	0.741	0.745	0.747	0.748	0.75	0.752	0.758	0.765	0.777	0.79	0.799	0.805	0.809	0.813	0.817	0.821	0.825	0.831	0.837	0.843	0.846	0.847	0.849	0.851	0.85	0.849	0.848	0.848	0.855	0.865	0.872	0.878	0.88	0.88	0.88
Hackney	0.755	0.761	0.766	0.771	0.777	0.782	0.784	0.787	0.794	0.8	0.806	0.814	0.822	0.828	0.832	0.832	0.829	0.829	0.834	0.844	0.855	0.863	0.866	0.866	0.868	0.871	0.875	0.881	0.887	0.894	0.902	0.91	0.915	0.92	0.923	0.923	0.924
Islington	0.786	0.792	0.797	0.802	0.807	0.813	0.817	0.821	0.827	0.831	0.837	0.844	0.851	0.858	0.864	0.869	0.873	0.877	0.882	0.888	0.894	0.898	0.902	0.905	0.909	0.912	0.915	0.919	0.922	0.925	0.927	0.93	0.932	0.934	0.936	0.938	0.94
Tower Hamlets	0.727	0.732	0.736	0.74	0.747	0.757	0.768	0.777	0.785	0.79	0.794	0.797	0.801	0.805	0.809	0.813	0.819	0.826	0.834	0.847	0.859	0.869	0.876	0.88	0.885	0.891	0.896	0.902	0.907	0.912	0.916	0.919	0.924	0.927	0.929	0.931	0.933
Wokingham	0.758	0.763	0.768	0.773	0.778	0.784	0.79	0.796	0.803	0.806	0.809	0.81	0.812	0.815	0.817	0.82	0.824	0.829	0.836	0.846	0.857	0.865	0.87	0.872	0.875	0.879	0.881	0.883	0.884	0.883	0.882	0.88	0.882	0.887	0.891	0.893	0.895
Buckinghamshire	0.74	0.746	0.752	0.758	0.763	0.768	0.773	0.776	0.781	0.786	0.79	0.795	0.801	0.806	0.812	0.818	0.823	0.828	0.834	0.839	0.845	0.849	0.85	0.851	0.854	0.856	0.856	0.856	0.856	0.856	0.858	0.861	0.865	0.869	0.872	0.873	0.874
Surrey	0.749	0.755	0.76	0.764	0.767	0.77	0.773	0.775	0.781	0.787	0.793	0.8	0.806	0.811	0.816	0.821	0.825	0.831	0.837	0.846	0.854	0.859	0.861	0.863	0.865	0.866	0.867	0.868	0.869	0.87	0.871	0.872	0.875	0.88	0.882	0.884	0.886
Windsor and Maidenhead	0.76	0.766	0.77	0.774	0.776	0.779	0.782	0.783	0.786	0.79	0.799	0.809	0.817	0.824	0.83	0.837	0.841	0.846	0.85	0.856	0.861	0.865	0.868	0.872	0.876	0.879	0.88	0.881	0.883	0.884	0.885	0.888	0.891	0.896	0.9	0.903	0.905
West Berkshire	0.755	0.76	0.765	0.77	0.774	0.78	0.786	0.791	0.798	0.803	0.809	0.814	0.82	0.827	0.833	0.838	0.842	0.846	0.851	0.858	0.865	0.868	0.869	0.869	0.869	0.869	0.866	0.863	0.862	0.863	0.868	0.873	0.878	0.883	0.885	0.886	0.887
Hampshire	0.725	0.731	0.737	0.742	0.747	0.752	0.756	0.76	0.766	0.771	0.776	0.781	0.786	0.791	0.797	0.803	0.808	0.813	0.819	0.824	0.83	0.834	0.836	0.837	0.839	0.841	0.842	0.842	0.844	0.845	0.847	0.85	0.855	0.859	0.862	0.864	0.866

Appendix Table 9: Socio-demographic Index values for all estimated GBD 2016 locations, 1980–2016

Location	1980	1981	1982	1983	1984	1985	1986	1987	1988	1989	1990	1991	1992	1993	1994	1995	1996	1997	1998	1999	2000	2001	2002	2003	2004	2005	2006	2007	2008	2009	2010	2011	2012	2013	2014	2015	2016
Bracknell Forest	0.733	0.74	0.746	0.753	0.76	0.766	0.771	0.777	0.784	0.791	0.796	0.799	0.804	0.808	0.814	0.82	0.824	0.828	0.833	0.842	0.849	0.855	0.858	0.86	0.863	0.866	0.868	0.87	0.871	0.872	0.873	0.875	0.878	0.882	0.885	0.887	0.888
West Sussex	0.72	0.726	0.731	0.737	0.741	0.746	0.75	0.753	0.759	0.764	0.769	0.774	0.78	0.786	0.792	0.797	0.803	0.808	0.814	0.819	0.824	0.827	0.828	0.829	0.831	0.832	0.834	0.835	0.836	0.838	0.841	0.844	0.847	0.852	0.854	0.855	0.856
Oxfordshire	0.745	0.751	0.757	0.762	0.767	0.773	0.778	0.782	0.786	0.79	0.794	0.799	0.805	0.81	0.816	0.822	0.827	0.832	0.838	0.845	0.851	0.856	0.858	0.86	0.863	0.865	0.866	0.866	0.867	0.868	0.87	0.874	0.879	0.884	0.887	0.889	0.89
Reading	0.753	0.759	0.764	0.769	0.772	0.775	0.779	0.783	0.79	0.798	0.806	0.815	0.822	0.827	0.832	0.837	0.842	0.847	0.853	0.861	0.869	0.876	0.88	0.882	0.885	0.886	0.884	0.883	0.884	0.886	0.89	0.893	0.897	0.901	0.904	0.905	0.906
Kent	0.71	0.716	0.721	0.726	0.729	0.733	0.736	0.74	0.745	0.75	0.755	0.76	0.765	0.771	0.776	0.781	0.786	0.791	0.797	0.803	0.808	0.812	0.815	0.816	0.819	0.822	0.823	0.824	0.826	0.827	0.829	0.832	0.835	0.839	0.842	0.844	0.845
Brighton and Hove	0.73	0.735	0.74	0.743	0.745	0.748	0.751	0.755	0.763	0.771	0.781	0.79	0.797	0.803	0.808	0.813	0.819	0.824	0.829	0.834	0.839	0.845	0.851	0.857	0.862	0.867	0.872	0.874	0.877	0.88	0.884	0.887	0.889	0.891	0.894	0.896	0.899
Medway	0.686	0.691	0.697	0.701	0.706	0.71	0.715	0.72	0.727	0.733	0.738	0.744	0.749	0.753	0.758	0.763	0.769	0.774	0.78	0.785	0.791	0.796	0.799	0.801	0.804	0.807	0.81	0.811	0.813	0.813	0.815	0.816	0.82	0.824	0.827	0.829	0.831
East Sussex	0.696	0.702	0.708	0.713	0.718	0.723	0.728	0.732	0.737	0.742	0.746	0.75	0.755	0.759	0.765	0.77	0.776	0.781	0.786	0.791	0.796	0.799	0.8	0.801	0.803	0.806	0.807	0.808	0.809	0.81	0.813	0.817	0.821	0.826	0.83	0.832	0.834
Portsmouth	0.728	0.734	0.74	0.744	0.746	0.748	0.751	0.754	0.76	0.768	0.776	0.784	0.791	0.797	0.802	0.806	0.81	0.815	0.821	0.828	0.836	0.843	0.849	0.853	0.858	0.861	0.864	0.867	0.869	0.87	0.87	0.868	0.869	0.871	0.873	0.876	0.878
Isle of Wight	0.695	0.7	0.706	0.71	0.714	0.719	0.724	0.73	0.737	0.741	0.744	0.746	0.748	0.751	0.756	0.762	0.768	0.774	0.78	0.787	0.795	0.804	0.809	0.811	0.812	0.813	0.814	0.816	0.818	0.819	0.821	0.823	0.826	0.829	0.831	0.833	0.835
Milton Keynes	0.757	0.761	0.762	0.762	0.763	0.768	0.775	0.783	0.79	0.795	0.796	0.799	0.804	0.811	0.818	0.825	0.83	0.835	0.839	0.843	0.847	0.85	0.85	0.85	0.853	0.855	0.856	0.855	0.855	0.856	0.862	0.868	0.873	0.878	0.88	0.88	0.88
Southampton	0.728	0.734	0.739	0.743	0.746	0.748	0.749	0.75	0.754	0.761	0.771	0.782	0.79	0.798	0.804	0.81	0.814	0.819	0.825	0.832	0.84	0.845	0.849	0.854	0.858	0.863	0.866	0.868	0.869	0.868	0.867	0.866	0.867	0.871	0.874	0.876	0.878
Slough	0.749	0.753	0.756	0.757	0.759	0.762	0.766	0.771	0.779	0.787	0.795	0.805	0.812	0.818	0.824	0.83	0.836	0.84	0.844	0.847	0.852	0.855	0.854	0.851	0.848	0.846	0.843	0.845	0.85	0.855	0.861	0.866	0.871	0.874	0.875	0.874	0.873
South Gloucestershire	0.731	0.737	0.742	0.747	0.751	0.756	0.762	0.767	0.774	0.779	0.783	0.787	0.791	0.795	0.799	0.803	0.809	0.816	0.824	0.832	0.839	0.844	0.846	0.848	0.851	0.855	0.858	0.861	0.865	0.866	0.867	0.868	0.871	0.875	0.878	0.88	0.882
Dorset	0.704	0.71	0.715	0.721	0.726	0.731	0.737	0.742	0.747	0.752	0.754	0.757	0.762	0.768	0.775	0.781	0.788	0.794	0.801	0.807	0.811	0.813	0.814	0.813	0.815	0.816	0.815	0.814	0.814	0.816	0.821	0.827	0.833	0.837	0.839	0.841	0.842
Wiltshire	0.713	0.719	0.725	0.731	0.736	0.741	0.746	0.75	0.756	0.761	0.766	0.77	0.775	0.781	0.787	0.793	0.798	0.804	0.81	0.815	0.821	0.825	0.826	0.827	0.829	0.829	0.828	0.826	0.825	0.826	0.83	0.835	0.84	0.844	0.846	0.846	0.847
North Somerset	0.695	0.701	0.708	0.714	0.721	0.727	0.734	0.74	0.746	0.751	0.753	0.756	0.761	0.766	0.771	0.776	0.781	0.787	0.793	0.799	0.805	0.809	0.811	0.813	0.817	0.821	0.823	0.825	0.827	0.826	0.827	0.829	0.833	0.838	0.841	0.843	0.844
Devon	0.701	0.707	0.712	0.717	0.721	0.726	0.731	0.736	0.742	0.748	0.753	0.757	0.762	0.767	0.772	0.777	0.782	0.788	0.794	0.802	0.809	0.815	0.818	0.821	0.825	0.829	0.831	0.833	0.834	0.835	0.836	0.837	0.84	0.845	0.849	0.851	0.853
Poole	0.708	0.714	0.719	0.724	0.729	0.735	0.741	0.748	0.755	0.761	0.766	0.771	0.776	0.782	0.789	0.796	0.802	0.808	0.814	0.82	0.826	0.83	0.83	0.83	0.832	0.834	0.835	0.835	0.836	0.836	0.84	0.845	0.851	0.856	0.859	0.861	0.862
Bath and North East Somerset	0.722	0.727	0.732	0.737	0.74	0.745	0.75	0.756	0.762	0.768	0.772	0.777	0.782	0.787	0.793	0.798	0.803	0.808	0.816	0.824	0.83	0.837	0.843	0.849	0.855	0.86	0.863	0.867	0.87	0.871	0.871	0.872	0.874	0.878	0.882	0.884	0.886
Gloucestershire	0.716	0.722	0.728	0.733	0.738	0.744	0.749	0.754	0.76	0.765	0.77	0.775	0.779	0.785	0.79	0.796	0.801	0.806	0.812	0.819	0.826	0.832	0.835	0.838	0.842	0.844	0.844	0.844	0.845	0.846	0.849	0.851	0.855	0.859	0.861	0.862	0.863
Somerset	0.701	0.708	0.714	0.719	0.725	0.73	0.734	0.738	0.743	0.748	0.752	0.756	0.762	0.767	0.774	0.78	0.786	0.791	0.795	0.801	0.807	0.811	0.812	0.813	0.816	0.818	0.819	0.82	0.82	0.821	0.823	0.827	0.83	0.834	0.835	0.835	0.836
Swindon	0.736	0.742	0.748	0.753	0.757	0.761	0.766	0.77	0.777	0.784	0.79	0.796	0.801	0.806	0.81	0.815	0.819	0.824	0.831	0.838	0.844	0.849	0.851	0.851	0.851	0.852	0.851	0.853	0.855	0.856	0.858	0.859	0.861	0.865	0.866	0.866	0.867
Torbay	0.684	0.69	0.696	0.701	0.706	0.711	0.715	0.721	0.728	0.734	0.738	0.743	0.749	0.755	0.761	0.767	0.772	0.777	0.782	0.787	0.793	0.795	0.794	0.792	0.794	0.797	0.798	0.8	0.802	0.802	0.802	0.801	0.803	0.806	0.809	0.811	0.813
Bristol, City of	0.738	0.744	0.749	0.753	0.756	0.759	0.763	0.766	0.772	0.779	0.787	0.796	0.804	0.811	0.817	0.822	0.827	0.832	0.839	0.847	0.854	0.859	0.863	0.865	0.869	0.872	0.875	0.878	0.881	0.882	0.881	0.88	0.882	0.887	0.891	0.895	0.898
Bournemouth	0.705	0.71	0.715	0.72	0.726	0.732	0.737	0.742	0.747	0.752	0.759	0.767	0.775	0.78	0.786	0.791	0.797	0.802	0.808	0.814	0.82	0.826	0.831	0.837	0.841	0.847	0.846	0.846	0.847	0.849	0.853	0.858	0.862	0.866	0.869	0.871	0.872
Cornwall	0.685	0.691	0.697	0.702	0.708	0.713	0.718	0.723	0.729	0.735	0.739	0.743	0.748	0.754	0.76	0.766	0.771	0.776	0.782	0.789	0.796	0.801	0.805	0.808	0.812	0.815	0.818	0.819	0.821	0.821	0.822	0.823	0.826	0.83	0.832	0.833	0.835
Plymouth	0.699	0.705	0.711	0.718	0.723	0.729	0.736	0.741	0.747	0.752	0.757	0.762	0.769	0.776	0.783	0.789	0.793	0.798	0.804	0.81	0.815	0.819	0.822	0.823	0.827	0.83	0.833	0.836	0.839	0.84	0.84	0.841	0.843	0.847	0.85	0.851	0.853
North East England	0.681	0.687	0.692	0.698	0.702	0.708	0.714	0.72	0.727	0.732	0.737	0.743	0.749	0.755	0.762	0.768	0.773	0.779	0.785	0.792	0.798	0.804	0.807	0.809	0.813	0.816	0.819	0.822	0.824	0.826	0.828	0.83	0.834	0.838	0.841	0.842	0.844
North West England	0.693	0.699	0.704	0.709	0.714	0.719	0.724	0.729	0.735	0.741	0.746	0.752	0.759	0.766	0.773	0.778	0.783	0.788	0.794	0.801	0.807	0.813	0.816	0.818	0.821	0.823	0.825	0.826	0.828	0.83	0.833	0.836	0.84	0.845	0.848	0.85	0.851
Yorkshire and the Humber	0.69	0.695	0.701	0.706	0.711	0.716	0.721	0.726	0.733	0.738	0.743	0.749	0.755	0.761	0.767	0.773	0.777	0.783	0.789	0.795	0.802	0.808	0.811	0.813	0.816	0.82	0.822	0.825	0.828	0.83	0.832	0.834	0.837	0.841	0.843	0.845	0.846
East Midlands	0.691	0.697	0.703	0.709	0.714	0.719	0.725	0.73	0.736	0.741	0.746	0.751	0.758	0.764	0.771	0.777	0.782	0.787	0.793	0.8	0.806	0.811	0.815	0.817	0.82	0.823	0.824	0.826	0.828	0.83	0.832	0.834	0.837	0.841	0.844	0.846	0.848
West Midlands	0.688	0.694	0.699	0.704	0.709	0.714	0.719	0.724	0.73	0.736	0.741	0.747	0.754	0.76	0.766	0.771	0.776	0.781	0.787	0.793	0.8	0.805	0.808	0.81	0.813	0.816	0.817	0.819	0.821	0.823	0.826	0.829	0.833	0.837	0.84	0.842	0.844
East of England	0.711	0.716	0.722	0.727	0.732	0.736	0.741	0.745	0.751	0.757	0.762	0.769	0.775	0.78	0.786	0.791	0.797	0.802	0.808	0.815	0.82	0.824	0.826	0.828	0.831	0.833	0.835	0.836	0.838	0.839	0.841	0.843	0.846	0.85	0.853	0.854	0.855
Greater London	0.751	0.757	0.763	0.768	0.772	0.777	0.782	0.786	0.792	0.799	0.807	0.815	0.821	0.827	0.833	0.837	0.841	0.846	0.852	0.86	0.868	0.875	0.878	0.88	0.883	0.885	0.886	0.886	0.888	0.89	0.894	0.899	0.904	0.909	0.912	0.914	0.915
South East England	0.729	0.735	0.74	0.745	0.749	0.753	0.757	0.761	0.767	0.772	0.777	0.783	0.789	0.794	0.8	0.805	0.81	0.816	0.821	0.828	0.834	0.839	0.841	0.842	0.845	0.847	0.848	0.849	0.85	0.851	0.854	0.856	0.86	0.864	0.867	0.869	0.87
South West England	0.711	0.716	0.722	0.727	0.732	0.737	0.742	0.747	0.753	0.758	0.763	0.768	0.773	0.779	0.785	0.79	0.795	0.801	0.807	0.814	0.82	0.825	0.828	0.829	0.832	0.835	0.836	0.837	0.839	0.84	0.841	0.844	0.847	0.851	0.854	0.856	0.857
Southern Latin America	0.599	0.604	0.607	0.613	0.618	0.621	0.627	0.631	0.634	0.637	0.641	0.647	0.654	0.662	0.671	0.678	0.684	0.688	0.693	0.698	0.702	0.706	0.709	0.712	0.716	0.72	0.725	0.729	0.734	0.738	0.743	0.749	0.754	0.76	0.764	0.768	0.771
Argentina	0.595	0.6	0.602	0.608	0.613	0.617	0.624	0.629	0.634	0.636	0.639	0.645	0.652	0.66	0.67	0.677	0.681	0.684	0.687	0.691	0.695	0.699	0.701	0.703	0.706	0.71	0.714	0.719	0.723	0.727	0.732	0.739	0.745	0.75	0.754	0.758	0.761
Chile	0.603	0.606	0.613	0.619	0.623	0.627	0.63	0.631	0.633	0.638	0.645	0.652	0.66	0.668	0.677	0.685	0.694	0.702	0.71	0.716	0.722	0.727	0.732	0.737	0.744	0.749	0.754	0.759	0.763	0.767	0.772	0.777	0.782	0.787	0.792	0.796	0.8
Uruguay	0.61	0.615	0.619	0.622	0.624	0.626	0.629	0.632	0.635	0.638	0.641	0.642	0.645	0.648	0.652	0.656	0.661	0.667	0.673	0.678	0.682	0.685	0.688	0.69	0.693	0.696	0.7	0.704	0.709	0.714	0.719	0.724	0.729	0.734	0.739	0.743	0.747
High-income North America	0.836	0.839	0.842	0.845	0.847	0.849	0.851	0.852	0.852	0.851	0.852	0.855	0.858	0.86	0.863	0.865	0.867	0.868	0.87	0.871	0.873	0.876	0.878	0.878	0.88	0.88	0.88	0.882	0.885	0.89	0.893	0.895	0.896	0.897	0.898	0.899	0.9
Canada	0.815	0.82	0.823	0.826	0.83	0.834	0.839	0.843	0.845	0.845	0.847	0.849	0.853	0.855	0.858	0.862	0.867	0.87	0.873	0.878	0.881	0.884	0.887	0.889	0.891	0.892	0.892	0.892	0.894	0.897	0.899	0.901	0.903	0.904	0.905	0.907	0.908
USA	0.838	0.841	0.844	0.847	0.849	0.85	0.853	0.853	0.852	0.851	0.852	0.855	0.858	0.86	0.863	0.865	0.867	0.868	0.869	0.87	0.872	0.875	0.876	0.877	0.878	0.879	0.878	0.88	0.884	0.889	0.892	0.894	0.895	0.896	0.897	0.898	0.899
Greenland	0.733	0.739	0.745	0.75	0.752	0.754	0.755	0.755	0.754	0.752	0.75	0.75	0.749	0.749	0.75	0.75	0.75	0.753	0.758	0.762	0.763	0.764	0.766	0.771	0.775	0.779	0.783	0.786	0.788	0.792	0.798	0.804	0.808	0.811	0.813	0.814	0.816
Alabama	0.808	0.812	0.816	0.82	0.823	0.826	0.828	0.829	0.829	0.83	0.832	0.835	0.839	0.842	0.845	0.847	0.849	0.85	0.851	0.853	0.856	0.86	0.862	0.864	0.865	0.865	0.864	0.865	0.869	0.874	0.878	0.88	0.882	0.882	0.883	0.884	0.884
Alaska	0.849	0.844	0.843	0.844	0.843	0.846	0.85	0.851	0.85	0.847	0.845	0.846	0.848	0.852	0.856	0.859	0.861	0.862	0.863	0.863	0.863	0.863	0.863	0.864	0.868	0.871	0.872	0.874	0.878	0.881	0.885	0.887	0.89	0.891	0.894	0.896	0.897
Arizona	0.829	0.832	0.834	0.836	0.836	0.837	0.839	0.84	0.839	0.838	0.837	0.84	0.842	0.844	0.846	0.847	0.848	0.848	0.848	0.848	0.849	0.852	0.853	0.853	0.855	0.855	0.856	0.861	0.868	0.875	0.879	0.88	0.881	0.881	0.881	0.881	0.881
Arkansas	0.799	0.803	0.807	0.811	0.814	0.816	0.819	0.82	0.82	0.82	0.822	0.826	0.83	0.833	0.836	0.837	0.839	0.84	0.842	0.844	0.846	0.849	0.85	0.851	0.853	0.853	0.852	0.854	0.859	0.863	0.867	0.869	0.871	0.872	0.874	0.875	0.876
California	0.851	0.853	0.854	0.856	0.856	0.856	0.856	0.854	0.849	0.844	0.841	0.843	0.846	0.848	0.851	0.854	0.857	0.861	0.863	0.866	0.869	0.872	0.873	0.872	0.873	0.873	0.872	0.875	0.88	0.885	0.889	0.891	0.893	0.894	0.896	0.897	0.898
Colorado	0.859	0.861	0.863	0.865	0.865	0.866	0.869	0.87	0.87	0.869	0.87	0.873	0.875	0.878	0.881	0.883	0.884	0.883	0.883	0.882	0.883	0.885	0.887	0.888	0.891	0.892	0.893	0.895	0.899	0.903	0.906	0.908	0.91	0.912	0.913	0.914	0.915
Connecticut	0.87	0.873	0.875	0.878	0.879	0.881	0.883	0.885	0.886	0.887	0.889	0.892	0.895	0.897	0.899	0.9	0.902	0.903	0.904	0.905	0.907	0.91	0.911	0.911	0.912	0.912	0.912	0.913	0.917	0.921	0.924	0.926	0.928	0.929	0.93	0.93	0.93
Delaware	0.854	0.857	0.859	0.862	0.863	0.865	0.867	0.867	0.866	0.866	0.867	0.871	0.875	0.878	0.88	0.882	0.883	0.882	0.882	0.882	0.884	0.887	0.888	0.889	0.89	0.889	0.887	0.888	0.892	0.897	0.901	0.903	0.905	0.907	0.908	0.909	0.909
District of Columbia	0.881	0.883	0.884	0.886	0.887	0.888	0.889	0.887	0.882	0.876	0.874	0.877	0.883	0.89	0.898	0.905	0.911	0.917	0.921	0.924	0.927	0.929	0.93	0.927	0.926	0.925	0.925	0.925	0.928	0.932	0.935	0.938	0.941	0.944	0.946	0.947	0.947
Florida	0.843	0.846	0.847	0.849	0.85	0.851	0.853	0.853	0.852	0.851	0.854	0.857	0.86	0.863	0.865	0.867	0.869	0.87	0.87	0.871	0.873	0.875	0.876	0.877	0.878	0.878	0.878	0.881	0.886	0.892	0.895	0.897	0.898	0.898	0.898	0.898	0.898
Georgia	0.82	0.825	0.829	0.832	0.835	0.837	0.84	0.841	0.841	0.841	0.843	0.848	0.851	0.855	0.857	0.859	0.86	0.86	0.86	0.86	0.862	0.865	0.867	0.868	0.869	0.868	0.867	0.869	0.874	0.88	0.884	0.886	0.887	0.887	0.888	0.888	0.888
Hawaii	0.843	0.844	0.845	0.848	0.85	0.852	0.855	0.855	0.854	0.853	0.854	0.857	0.86	0.863	0.866	0.869	0.872	0.874	0.875	0.875	0.875	0.876	0.874	0.873	0.874	0.875	0.875	0.877	0.881	0.885	0.889	0.891	0.894	0.896	0.898	0.9	0.901

Appendix Table 9: Socio-demographic Index values for all estimated GBD 2016 locations, 1980–2016

Location	1980	1981	1982	1983	1984	1985	1986	1987	1988	1989	1990	1991	1992	1993	1994	1995	1996	1997	1998	1999	2000	2001	2002	2003	2004	2005	2006	2007	2008	2009	2010	2011	2012	2013	2014	2015	2016
Idaho	0.809	0.813	0.816	0.822	0.825	0.829	0.833	0.834	0.834	0.832	0.833	0.835	0.837	0.84	0.842	0.845	0.846	0.847	0.848	0.849	0.851	0.854	0.854	0.854	0.855	0.854	0.853	0.854	0.859	0.864	0.867	0.869	0.87	0.871	0.872	0.872	0.872
Illinois	0.843	0.846	0.849	0.852	0.854	0.856	0.857	0.858	0.856	0.855	0.856	0.858	0.86	0.863	0.866	0.868	0.871	0.872	0.874	0.875	0.878	0.881	0.883	0.884	0.886	0.888	0.888	0.891	0.895	0.899	0.902	0.903	0.905	0.906	0.907	0.908	0.908
Indiana	0.833	0.837	0.84	0.843	0.844	0.846	0.848	0.849	0.848	0.847	0.848	0.85	0.853	0.855	0.858	0.859	0.86	0.86	0.86	0.861	0.863	0.866	0.867	0.867	0.869	0.869	0.868	0.869	0.872	0.875	0.878	0.879	0.88	0.881	0.882	0.883	0.883
Iowa	0.834	0.838	0.842	0.845	0.848	0.85	0.853	0.854	0.854	0.853	0.854	0.856	0.858	0.86	0.862	0.864	0.865	0.866	0.867	0.868	0.87	0.873	0.874	0.875	0.876	0.876	0.874	0.876	0.88	0.884	0.887	0.888	0.89	0.891	0.891	0.892	0.893
Kansas	0.835	0.839	0.842	0.846	0.848	0.851	0.854	0.855	0.856	0.855	0.857	0.859	0.861	0.862	0.864	0.865	0.866	0.866	0.866	0.866	0.868	0.871	0.873	0.874	0.875	0.875	0.874	0.875	0.879	0.883	0.886	0.888	0.89	0.892	0.893	0.895	0.895
Kentucky	0.805	0.81	0.814	0.818	0.821	0.824	0.827	0.828	0.828	0.829	0.83	0.833	0.836	0.839	0.841	0.843	0.845	0.846	0.847	0.849	0.851	0.855	0.856	0.857	0.858	0.858	0.857	0.859	0.862	0.865	0.868	0.869	0.87	0.87	0.871	0.873	0.874
Louisiana	0.796	0.801	0.806	0.811	0.814	0.817	0.82	0.822	0.822	0.822	0.824	0.827	0.83	0.833	0.837	0.84	0.842	0.844	0.846	0.847	0.85	0.854	0.856	0.857	0.86	0.86	0.861	0.864	0.869	0.873	0.876	0.878	0.88	0.882	0.883	0.885	0.885
Maine	0.832	0.834	0.836	0.839	0.841	0.844	0.848	0.85	0.853	0.855	0.858	0.862	0.866	0.869	0.872	0.874	0.876	0.877	0.878	0.88	0.882	0.885	0.886	0.887	0.889	0.89	0.891	0.893	0.896	0.899	0.901	0.902	0.902	0.903	0.904	0.905	0.906
Maryland	0.865	0.867	0.868	0.87	0.871	0.872	0.875	0.875	0.875	0.875	0.877	0.88	0.883	0.886	0.889	0.891	0.892	0.892	0.892	0.893	0.895	0.897	0.899	0.899	0.901	0.902	0.901	0.903	0.907	0.912	0.915	0.917	0.919	0.92	0.92	0.921	0.92
Massachusetts	0.865	0.868	0.871	0.874	0.877	0.88	0.882	0.884	0.886	0.886	0.889	0.892	0.895	0.897	0.9	0.902	0.904	0.905	0.906	0.907	0.91	0.913	0.915	0.916	0.918	0.919	0.919	0.921	0.923	0.926	0.928	0.929	0.931	0.932	0.933	0.935	0.935
Michigan	0.845	0.847	0.849	0.852	0.853	0.855	0.857	0.857	0.856	0.854	0.855	0.858	0.861	0.864	0.867	0.87	0.871	0.872	0.873	0.875	0.877	0.88	0.882	0.883	0.886	0.887	0.887	0.888	0.89	0.891	0.892	0.893	0.893	0.894	0.895	0.896	0.897
Minnesota	0.839	0.844	0.848	0.852	0.856	0.859	0.863	0.866	0.867	0.868	0.87	0.873	0.875	0.877	0.879	0.88	0.882	0.882	0.883	0.884	0.886	0.889	0.89	0.89	0.891	0.891	0.89	0.892	0.896	0.9	0.903	0.904	0.905	0.906	0.907	0.909	0.909
Mississippi	0.784	0.789	0.794	0.799	0.803	0.806	0.81	0.811	0.811	0.811	0.813	0.816	0.82	0.824	0.828	0.831	0.833	0.835	0.836	0.838	0.841	0.845	0.847	0.848	0.848	0.847	0.846	0.848	0.853	0.859	0.864	0.867	0.869	0.871	0.872	0.873	0.874
Missouri	0.826	0.83	0.833	0.837	0.839	0.842	0.845	0.846	0.846	0.846	0.847	0.85	0.853	0.856	0.859	0.86	0.862	0.863	0.864	0.865	0.868	0.871	0.872	0.873	0.875	0.875	0.874	0.876	0.879	0.883	0.886	0.887	0.888	0.89	0.891	0.892	0.893
Montana	0.829	0.831	0.834	0.837	0.838	0.84	0.843	0.845	0.844	0.844	0.845	0.847	0.849	0.852	0.855	0.858	0.86	0.862	0.863	0.865	0.866	0.868	0.869	0.869	0.872	0.872	0.873	0.875	0.879	0.882	0.885	0.886	0.888	0.889	0.891	0.892	0.893
Nebraska	0.832	0.835	0.838	0.842	0.845	0.848	0.852	0.853	0.854	0.853	0.855	0.858	0.86	0.862	0.863	0.865	0.866	0.867	0.867	0.867	0.868	0.87	0.87	0.871	0.873	0.873	0.871	0.873	0.877	0.881	0.884	0.886	0.888	0.888	0.889	0.89	0.89
Nevada	0.855	0.857	0.858	0.86	0.86	0.86	0.86	0.857	0.854	0.85	0.85	0.853	0.855	0.857	0.858	0.859	0.861	0.862	0.862	0.863	0.865	0.868	0.868	0.868	0.869	0.867	0.865	0.868	0.875	0.882	0.887	0.889	0.889	0.889	0.888	0.887	0.886
New Hampshire	0.852	0.856	0.859	0.862	0.864	0.866	0.869	0.871	0.873	0.876	0.879	0.882	0.885	0.887	0.889	0.891	0.893	0.894	0.896	0.897	0.9	0.903	0.904	0.905	0.908	0.91	0.911	0.914	0.916	0.919	0.921	0.922	0.924	0.926	0.928	0.93	0.931
New Jersey	0.858	0.861	0.864	0.867	0.868	0.87	0.872	0.873	0.873	0.873	0.875	0.878	0.881	0.883	0.885	0.887	0.889	0.891	0.891	0.892	0.894	0.896	0.896	0.897	0.899	0.9	0.899	0.9	0.904	0.908	0.912	0.914	0.915	0.916	0.917	0.918	0.918
New Mexico	0.809	0.812	0.814	0.818	0.819	0.822	0.825	0.826	0.826	0.824	0.825	0.826	0.829	0.832	0.835	0.838	0.841	0.843	0.844	0.845	0.847	0.85	0.852	0.852	0.854	0.855	0.855	0.858	0.862	0.867	0.871	0.873	0.875	0.877	0.879	0.88	0.881
New York	0.854	0.856	0.858	0.861	0.862	0.864	0.866	0.866	0.866	0.866	0.868	0.871	0.874	0.877	0.879	0.882	0.885	0.887	0.888	0.89	0.892	0.895	0.896	0.897	0.899	0.899	0.899	0.901	0.904	0.907	0.91	0.912	0.915	0.916	0.918	0.919	0.92
North Carolina	0.825	0.829	0.832	0.835	0.837	0.84	0.842	0.842	0.842	0.841	0.844	0.848	0.851	0.855	0.858	0.86	0.86	0.86	0.86	0.86	0.861	0.865	0.867	0.868	0.869	0.868	0.866	0.868	0.873	0.878	0.883	0.885	0.886	0.886	0.887	0.887	0.887
North Dakota	0.816	0.818	0.822	0.827	0.83	0.835	0.839	0.842	0.842	0.843	0.846	0.848	0.85	0.852	0.854	0.857	0.86	0.863	0.866	0.869	0.871	0.874	0.874	0.875	0.875	0.874	0.872	0.874	0.879	0.883	0.887	0.888	0.891	0.892	0.893	0.895	0.896
Ohio	0.838	0.841	0.844	0.847	0.848	0.85	0.853	0.853	0.853	0.852	0.854	0.856	0.858	0.86	0.863	0.865	0.866	0.866	0.867	0.868	0.87	0.874	0.875	0.876	0.878	0.879	0.879	0.88	0.883	0.885	0.887	0.888	0.889	0.89	0.891	0.892	0.893
Oklahoma	0.82	0.824	0.828	0.833	0.836	0.84	0.843	0.844	0.843	0.842	0.843	0.844	0.846	0.847	0.848	0.849	0.849	0.849	0.85	0.85	0.853	0.856	0.857	0.858	0.86	0.861	0.861	0.862	0.866	0.869	0.872	0.874	0.877	0.879	0.88	0.882	0.883
Oregon	0.85	0.852	0.854	0.857	0.857	0.858	0.859	0.858	0.857	0.856	0.857	0.86	0.863	0.866	0.868	0.869	0.87	0.871	0.873	0.875	0.878	0.882	0.884	0.885	0.887	0.887	0.886	0.887	0.89	0.894	0.897	0.898	0.899	0.9	0.901	0.903	0.903
Pennsylvania	0.842	0.845	0.847	0.85	0.851	0.853	0.855	0.856	0.856	0.856	0.858	0.861	0.864	0.867	0.87	0.873	0.875	0.877	0.878	0.879	0.881	0.884	0.885	0.885	0.887	0.887	0.887	0.888	0.892	0.896	0.899	0.9	0.902	0.904	0.905	0.907	0.908
Rhode Island	0.847	0.851	0.853	0.857	0.859	0.861	0.863	0.864	0.865	0.865	0.867	0.87	0.873	0.877	0.88	0.883	0.885	0.886	0.887	0.889	0.89	0.893	0.894	0.895	0.898	0.9	0.9	0.902	0.906	0.909	0.912	0.913	0.915	0.916	0.918	0.92	0.921
South Carolina	0.814	0.818	0.821	0.825	0.828	0.83	0.833	0.833	0.832	0.831	0.834	0.839	0.844	0.849	0.853	0.855	0.856	0.857	0.857	0.858	0.86	0.864	0.866	0.866	0.866	0.864	0.862	0.863	0.868	0.874	0.879	0.881	0.883	0.884	0.884	0.885	0.886
South Dakota	0.806	0.81	0.814	0.818	0.822	0.825	0.83	0.832	0.832	0.832	0.834	0.836	0.839	0.842	0.846	0.849	0.852	0.853	0.855	0.857	0.859	0.861	0.861	0.862	0.863	0.862	0.861	0.863	0.868	0.872	0.876	0.877	0.878	0.879	0.88	0.881	0.882
Tennessee	0.818	0.822	0.826	0.829	0.831	0.833	0.836	0.836	0.836	0.835	0.837	0.841	0.844	0.848	0.851	0.853	0.855	0.856	0.857	0.858	0.861	0.864	0.866	0.866	0.867	0.867	0.865	0.867	0.871	0.875	0.879	0.88	0.882	0.882	0.883	0.884	0.885
Texas	0.815	0.819	0.823	0.827	0.829	0.831	0.834	0.834	0.834	0.832	0.832	0.834	0.835	0.837	0.839	0.84	0.842	0.843	0.843	0.844	0.846	0.849	0.85	0.851	0.853	0.853	0.853	0.855	0.86	0.866	0.87	0.873	0.875	0.876	0.877	0.878	0.878
Utah	0.779	0.786	0.794	0.803	0.809	0.814	0.819	0.82	0.82	0.819	0.822	0.827	0.831	0.833	0.834	0.835	0.834	0.834	0.834	0.836	0.839	0.843	0.846	0.847	0.849	0.849	0.848	0.85	0.855	0.859	0.863	0.864	0.866	0.867	0.869	0.87	0.87
Vermont	0.843	0.846	0.848	0.851	0.853	0.855	0.859	0.861	0.863	0.866	0.869	0.872	0.876	0.879	0.882	0.885	0.887	0.889	0.89	0.893	0.895	0.898	0.899	0.9	0.902	0.904	0.905	0.907	0.909	0.912	0.913	0.915	0.916	0.917	0.918	0.919	0.92
Virginia	0.849	0.852	0.855	0.858	0.86	0.862	0.864	0.866	0.866	0.867	0.868	0.871	0.874	0.877	0.88	0.882	0.883	0.883	0.884	0.884	0.885	0.888	0.888	0.888	0.89	0.891	0.891	0.894	0.899	0.903	0.907	0.909	0.91	0.911	0.912	0.913	0.913
Washington	0.857	0.859	0.862	0.865	0.866	0.867	0.869	0.869	0.868	0.867	0.868	0.87	0.873	0.876	0.878	0.88	0.882	0.883	0.885	0.887	0.891	0.894	0.896	0.896	0.898	0.897	0.896	0.897	0.899	0.902	0.904	0.906	0.907	0.908	0.91	0.911	0.912
West Virginia	0.809	0.813	0.818	0.822	0.824	0.827	0.83	0.831	0.831	0.83	0.832	0.834	0.837	0.84	0.843	0.845	0.847	0.849	0.85	0.851	0.852	0.854	0.856	0.857	0.859	0.859	0.859	0.859	0.862	0.865	0.867	0.869	0.87	0.872	0.873	0.875	0.876
Wisconsin	0.836	0.839	0.842	0.845	0.847	0.85	0.853	0.855	0.856	0.857	0.859	0.861	0.864	0.867	0.869	0.871	0.873	0.874	0.875	0.876	0.878	0.88	0.881	0.882	0.884	0.884	0.884	0.885	0.888	0.892	0.894	0.895	0.897	0.898	0.899	0.901	0.902
Wyoming	0.824	0.827	0.83	0.835	0.837	0.842	0.847	0.85	0.851	0.851	0.852	0.854	0.857	0.86	0.863	0.865	0.867	0.868	0.871	0.874	0.876	0.878	0.878	0.878	0.878	0.877	0.877	0.879	0.886	0.893	0.898	0.901	0.902	0.903	0.904	0.905	0.905
Latin America and Caribbean	0.467	0.477	0.486	0.494	0.503	0.511	0.519	0.526	0.533	0.54	0.546	0.554	0.561	0.568	0.575	0.581	0.588	0.595	0.602	0.608	0.615	0.621	0.627	0.632	0.638	0.643	0.649	0.655	0.661	0.666	0.672	0.678	0.684	0.689	0.695	0.7	0.705
Caribbean	0.532	0.538	0.543	0.551	0.557	0.563	0.569	0.573	0.578	0.583	0.588	0.594	0.599	0.602	0.605	0.608	0.61	0.613	0.617	0.621	0.626	0.631	0.635	0.64	0.645	0.651	0.656	0.66	0.663	0.667	0.671	0.675	0.679	0.683	0.688	0.692	0.696
Antigua and Barbuda	0.611	0.619	0.627	0.634	0.64	0.647	0.654	0.662	0.67	0.678	0.684	0.691	0.696	0.7	0.704	0.707	0.71	0.713	0.716	0.719	0.722	0.723	0.725	0.728	0.731	0.735	0.74	0.746	0.752	0.756	0.759	0.761	0.765	0.767	0.771	0.774	0.778
The Bahamas	0.659	0.666	0.674	0.681	0.689	0.697	0.704	0.709	0.713	0.716	0.719	0.72	0.721	0.722	0.724	0.728	0.733	0.739	0.745	0.751	0.757	0.761	0.765	0.767	0.769	0.77	0.771	0.773	0.774	0.775	0.775	0.776	0.777	0.779	0.78	0.781	0.783
Barbados	0.662	0.668	0.673	0.679	0.685	0.691	0.699	0.706	0.713	0.72	0.726	0.732	0.737	0.741	0.744	0.748	0.751	0.755	0.759	0.762	0.766	0.769	0.771	0.772	0.773	0.776	0.779	0.782	0.784	0.786	0.788	0.789	0.79	0.791	0.793	0.795	0.797
Belize	0.344	0.348	0.353	0.36	0.369	0.379	0.391	0.404	0.419	0.435	0.452	0.469	0.487	0.502	0.515	0.525	0.532	0.537	0.542	0.546	0.552	0.557	0.563	0.569	0.574	0.58	0.585	0.589	0.593	0.596	0.6	0.604	0.608	0.612	0.617	0.621	0.625
Cuba	0.631	0.634	0.637	0.645	0.65	0.658	0.666	0.668	0.673	0.679	0.685	0.693	0.698	0.697	0.696	0.695	0.695	0.692	0.693	0.694	0.697	0.701	0.705	0.712	0.719	0.725	0.732	0.739	0.739	0.743	0.747	0.751	0.757	0.761	0.766	0.772	0.778
Dominica	0.529	0.534	0.541	0.548	0.556	0.563	0.57	0.577	0.584	0.59	0.596	0.6	0.605	0.612	0.62	0.629	0.637	0.645	0.653	0.659	0.665	0.67	0.674	0.678	0.682	0.686	0.69	0.695	0.701	0.705	0.71	0.714	0.718	0.721	0.725	0.729	0.734
Dominican Republic	0.435	0.449	0.463	0.477	0.49	0.499	0.506	0.513	0.519	0.524	0.527	0.53	0.535	0.54	0.545	0.551	0.558	0.566	0.574	0.582	0.589	0.596	0.603	0.609	0.615	0.622	0.629	0.637	0.643	0.649	0.656	0.662	0.668	0.675	0.682	0.69	0.696
Grenada	0.442	0.448	0.453	0.458	0.463	0.468	0.473	0.479	0.485	0.492	0.5	0.51	0.519	0.527	0.536	0.544	0.552	0.561	0.571	0.581	0.591	0.6	0.609	0.618	0.627	0.637	0.646	0.654	0.662	0.668	0.674	0.679	0.684	0.688	0.694	0.699	0.704
Guyana	0.496	0.503	0.509	0.514	0.517	0.518	0.519	0.519	0.519	0.518	0.517	0.518	0.52	0.525	0.53	0.537	0.545	0.552	0.559	0.565	0.569	0.573	0.576	0.579	0.582	0.584	0.587	0.592	0.596	0.601	0.607	0.614	0.621	0.628	0.636	0.643	0.649
Haiti	0.257	0.261	0.264	0.267	0.271	0.275	0.279	0.284	0.29	0.297	0.303	0.31	0.315	0.319	0.321	0.325	0.329	0.334	0.339	0.346	0.352	0.358	0.363	0.368	0.372	0.376	0.381	0.386	0.391	0.397	0.402	0.407	0.413	0.418	0.424	0.428	0.432
Jamaica	0.557	0.563	0.568	0.573	0.578	0.58	0.583	0.587	0.591	0.595	0.599	0.604	0.609	0.615	0.62	0.625	0.629	0.633	0.636	0.64	0.643	0.647	0.651	0.655	0.659	0.664	0.669	0.674	0.679	0.682	0.685	0.688	0.691	0.694	0.696	0.699	0.701
Saint Lucia	0.453	0.464	0.473	0.482	0.492	0.502	0.513	0.523	0.534	0.544	0.555	0.565	0.574	0.583	0.591	0.598	0.606	0.614	0.621	0.629	0.635	0.641	0.647	0.654	0.662	0.668	0.674	0.679	0.684	0.689	0.693	0.697	0.7	0.703	0.707	0.71	0.713
Saint Vincent and the Grenadines	0.418	0.428	0.438	0.449	0.46	0.471	0.48	0.489	0.499	0.508	0.516	0.524	0.533	0.541	0.547	0.555	0.562	0.569	0.576	0.583	0.589	0.595	0.602	0.608	0.615	0.621	0.627	0.633	0.638	0.642	0.645	0.648	0.651	0.655	0.659	0.663	0.667
Suriname	0.51	0.515	0.519	0.524	0.528	0.535	0.541	0.546	0.553	0.56	0.566	0.569	0.572	0.574	0.575	0.577	0.58	0.583	0.587	0.589	0.592	0.597	0.601	0.607	0.614	0.621	0.628	0.636	0.643	0.651	0.659	0.666	0.673	0.68	0.686	0.691	0.696
Trinidad and Tobago	0.592	0.595	0.599	0.603	0.608	0.615	0.624	0.633	0.642	0.649	0.655	0.659	0.663	0.666	0.668	0.669	0.671	0.675	0.68	0.687	0.694	0.7	0.706	0.713	0.719	0.725	0.731	0.738	0.745	0.75	0.755	0.758	0.76	0.762	0.764	0.765	0.766
Bermuda	0.68	0.687	0.692	0.698	0.703	0.708	0.713	0.719	0.724	0.729	0.733	0.737	0.741	0.745	0.749	0.752	0.756	0.76	0.763	0.767	0.77	0.774	0.777	0.781	0.785	0.789	0.793	0.796	0.799	0.801	0.803	0.806	0.808	0.811	0.813	0.816	0.819

Appendix Table 9: Socio-demographic Index values for all estimated GBD 2016 locations, 1980–2016

Location	1980	1981	1982	1983	1984	1985	1986	1987	1988	1989	1990	1991	1992	1993	1994	1995	1996	1997	1998	1999	2000	2001	2002	2003	2004	2005	2006	2007	2008	2009	2010	2011	2012	2013	2014	2015	2016
Puerto Rico	0.699	0.71	0.719	0.727	0.733	0.739	0.743	0.748	0.753	0.76	0.767	0.774	0.781	0.788	0.793	0.799	0.805	0.81	0.817	0.822	0.828	0.834	0.84	0.845	0.85	0.854	0.858	0.86	0.863	0.864	0.866	0.867	0.868	0.87	0.871	0.873	0.875
Virgin Islands, U.S.	0.723	0.732	0.738	0.743	0.749	0.755	0.76	0.764	0.767	0.771	0.777	0.782	0.789	0.796	0.802	0.808	0.814	0.82	0.825	0.829	0.833	0.836	0.839	0.842	0.845	0.85	0.855	0.861	0.864	0.868	0.871	0.874	0.875	0.876	0.876	0.877	0.877
Andean Latin America	0.45	0.457	0.465	0.473	0.481	0.488	0.496	0.504	0.511	0.518	0.524	0.53	0.536	0.541	0.548	0.555	0.561	0.568	0.575	0.582	0.588	0.594	0.6	0.605	0.61	0.615	0.62	0.625	0.63	0.635	0.64	0.644	0.649	0.653	0.657	0.662	0.666
Bolivia	0.354	0.361	0.368	0.376	0.383	0.39	0.397	0.405	0.412	0.418	0.425	0.432	0.439	0.446	0.453	0.461	0.469	0.477	0.485	0.494	0.502	0.51	0.518	0.525	0.531	0.539	0.546	0.553	0.56	0.567	0.573	0.58	0.586	0.593	0.599	0.605	0.61
Ecuador	0.45	0.458	0.467	0.475	0.484	0.493	0.501	0.509	0.516	0.523	0.53	0.537	0.543	0.55	0.556	0.563	0.569	0.576	0.583	0.588	0.593	0.598	0.603	0.608	0.613	0.619	0.624	0.629	0.635	0.64	0.645	0.65	0.655	0.66	0.665	0.67	0.674
Peru	0.478	0.485	0.493	0.5	0.507	0.514	0.521	0.529	0.537	0.543	0.548	0.553	0.558	0.563	0.569	0.576	0.582	0.59	0.597	0.603	0.61	0.615	0.621	0.626	0.63	0.634	0.639	0.644	0.648	0.652	0.656	0.66	0.663	0.666	0.67	0.674	0.678
Central Latin America	0.466	0.476	0.485	0.493	0.502	0.51	0.518	0.525	0.531	0.537	0.544	0.551	0.56	0.568	0.575	0.581	0.588	0.595	0.602	0.609	0.615	0.622	0.627	0.633	0.639	0.645	0.652	0.659	0.665	0.671	0.676	0.682	0.689	0.695	0.701	0.707	0.711
Colombia	0.485	0.493	0.5	0.506	0.513	0.519	0.526	0.532	0.538	0.544	0.55	0.556	0.562	0.568	0.574	0.581	0.588	0.595	0.601	0.606	0.611	0.616	0.62	0.624	0.629	0.634	0.639	0.645	0.652	0.658	0.664	0.672	0.679	0.686	0.694	0.701	0.707
Costa Rica	0.527	0.532	0.535	0.537	0.539	0.541	0.546	0.552	0.557	0.563	0.57	0.577	0.584	0.591	0.598	0.603	0.608	0.614	0.62	0.627	0.634	0.64	0.646	0.651	0.657	0.662	0.668	0.674	0.679	0.685	0.691	0.697	0.704	0.71	0.716	0.722	0.726
El Salvador	0.383	0.392	0.401	0.408	0.414	0.419	0.424	0.429	0.434	0.439	0.444	0.451	0.458	0.465	0.472	0.48	0.488	0.496	0.504	0.513	0.521	0.529	0.537	0.545	0.552	0.56	0.568	0.576	0.584	0.592	0.6	0.609	0.617	0.625	0.632	0.639	0.645
Guatemala	0.325	0.33	0.335	0.338	0.342	0.345	0.348	0.351	0.355	0.36	0.365	0.37	0.376	0.383	0.389	0.396	0.404	0.412	0.42	0.429	0.438	0.446	0.455	0.463	0.472	0.48	0.489	0.498	0.506	0.514	0.522	0.53	0.537	0.543	0.55	0.556	0.561
Honduras	0.295	0.304	0.313	0.321	0.329	0.337	0.345	0.353	0.362	0.372	0.381	0.39	0.4	0.409	0.418	0.427	0.435	0.443	0.451	0.459	0.466	0.474	0.481	0.488	0.495	0.502	0.508	0.515	0.522	0.527	0.533	0.539	0.545	0.551	0.556	0.562	0.567
Mexico	0.458	0.47	0.481	0.491	0.501	0.511	0.521	0.53	0.538	0.547	0.555	0.564	0.573	0.582	0.591	0.598	0.606	0.614	0.622	0.629	0.637	0.644	0.651	0.657	0.664	0.671	0.678	0.684	0.691	0.695	0.701	0.706	0.712	0.718	0.723	0.729	0.734
Nicaragua	0.409	0.412	0.415	0.418	0.421	0.424	0.427	0.43	0.431	0.433	0.434	0.436	0.438	0.44	0.443	0.447	0.453	0.459	0.466	0.473	0.481	0.488	0.496	0.502	0.509	0.516	0.523	0.53	0.537	0.543	0.549	0.555	0.562	0.569	0.576	0.583	0.588
Panama	0.542	0.552	0.56	0.568	0.574	0.581	0.588	0.594	0.597	0.6	0.603	0.607	0.611	0.615	0.62	0.624	0.628	0.633	0.64	0.647	0.653	0.66	0.666	0.67	0.674	0.679	0.683	0.688	0.693	0.698	0.704	0.71	0.718	0.726	0.735	0.744	0.752
Venezuela	0.533	0.54	0.547	0.557	0.568	0.577	0.583	0.588	0.594	0.592	0.59	0.602	0.619	0.628	0.629	0.632	0.637	0.642	0.647	0.65	0.654	0.657	0.66	0.662	0.667	0.673	0.681	0.688	0.696	0.702	0.708	0.714	0.721	0.728	0.734	0.738	0.742
Aguascalientes	0.49	0.502	0.512	0.521	0.53	0.538	0.548	0.558	0.567	0.576	0.586	0.597	0.608	0.617	0.624	0.631	0.639	0.648	0.656	0.663	0.67	0.675	0.681	0.687	0.693	0.699	0.705	0.711	0.715	0.719	0.723	0.728	0.733	0.739	0.744	0.749	0.754
Baja California	0.523	0.536	0.548	0.56	0.572	0.585	0.597	0.608	0.617	0.624	0.629	0.635	0.642	0.65	0.658	0.666	0.672	0.676	0.68	0.687	0.695	0.703	0.709	0.713	0.715	0.718	0.722	0.728	0.735	0.74	0.745	0.751	0.758	0.764	0.769	0.773	0.776
Baja California Sur	0.535	0.547	0.559	0.571	0.584	0.596	0.605	0.615	0.627	0.635	0.644	0.654	0.663	0.669	0.674	0.679	0.686	0.691	0.692	0.689	0.689	0.694	0.698	0.701	0.707	0.712	0.711	0.707	0.706	0.71	0.718	0.729	0.738	0.744	0.748	0.753	0.757
Campeche	0.437	0.449	0.459	0.468	0.477	0.485	0.494	0.503	0.511	0.52	0.53	0.541	0.551	0.56	0.567	0.574	0.582	0.593	0.603	0.614	0.625	0.635	0.643	0.651	0.658	0.665	0.671	0.677	0.682	0.685	0.688	0.693	0.7	0.707	0.713	0.72	0.726
Coahuila	0.499	0.512	0.523	0.533	0.543	0.552	0.562	0.572	0.582	0.593	0.605	0.617	0.63	0.64	0.649	0.656	0.663	0.671	0.679	0.685	0.691	0.697	0.702	0.708	0.714	0.72	0.726	0.733	0.739	0.743	0.747	0.751	0.754	0.758	0.761	0.766	0.77
Colima	0.472	0.485	0.496	0.506	0.517	0.528	0.54	0.553	0.565	0.579	0.591	0.604	0.616	0.625	0.632	0.638	0.645	0.652	0.659	0.666	0.672	0.678	0.683	0.689	0.695	0.7	0.706	0.712	0.716	0.719	0.723	0.727	0.732	0.737	0.742	0.748	0.753
Chiapas	0.3	0.313	0.323	0.332	0.338	0.342	0.345	0.345	0.342	0.339	0.342	0.355	0.375	0.393	0.41	0.427	0.444	0.459	0.472	0.483	0.491	0.499	0.508	0.517	0.527	0.536	0.546	0.555	0.563	0.57	0.577	0.585	0.593	0.6	0.608	0.616	0.622
Chihuahua	0.49	0.503	0.513	0.523	0.531	0.54	0.549	0.559	0.568	0.578	0.587	0.597	0.607	0.614	0.62	0.623	0.628	0.634	0.639	0.645	0.651	0.658	0.666	0.673	0.682	0.689	0.697	0.705	0.713	0.719	0.726	0.734	0.743	0.75	0.758	0.764	0.769
Distrito Federal	0.592	0.605	0.615	0.624	0.631	0.635	0.638	0.642	0.649	0.66	0.671	0.68	0.686	0.692	0.702	0.713	0.723	0.729	0.733	0.738	0.743	0.749	0.755	0.761	0.766	0.771	0.776	0.779	0.782	0.783	0.785	0.79	0.796	0.802	0.808	0.814	0.819
Durango	0.459	0.47	0.48	0.488	0.495	0.502	0.509	0.516	0.522	0.529	0.536	0.544	0.553	0.559	0.563	0.568	0.576	0.586	0.596	0.605	0.615	0.624	0.632	0.641	0.65	0.657	0.665	0.672	0.678	0.681	0.684	0.688	0.691	0.696	0.7	0.705	0.709
Guanajuato	0.384	0.396	0.407	0.417	0.427	0.437	0.448	0.461	0.472	0.485	0.497	0.511	0.524	0.534	0.543	0.551	0.562	0.573	0.583	0.593	0.603	0.611	0.619	0.627	0.634	0.642	0.65	0.658	0.666	0.671	0.677	0.683	0.688	0.694	0.7	0.706	0.711
Guerrero	0.355	0.367	0.378	0.387	0.395	0.404	0.413	0.423	0.431	0.437	0.441	0.444	0.445	0.442	0.438	0.439	0.445	0.454	0.464	0.473	0.483	0.494	0.505	0.517	0.529	0.54	0.551	0.562	0.57	0.577	0.585	0.595	0.606	0.617	0.626	0.634	0.639
Hidalgo	0.383	0.395	0.406	0.416	0.426	0.437	0.449	0.46	0.47	0.48	0.49	0.501	0.513	0.521	0.527	0.533	0.541	0.55	0.56	0.569	0.577	0.585	0.593	0.6	0.608	0.616	0.624	0.633	0.64	0.646	0.651	0.656	0.662	0.668	0.674	0.68	0.685
Jalisco	0.463	0.475	0.486	0.496	0.505	0.514	0.524	0.535	0.544	0.554	0.565	0.577	0.589	0.599	0.607	0.615	0.624	0.633	0.642	0.65	0.658	0.664	0.671	0.677	0.684	0.69	0.697	0.703	0.709	0.712	0.716	0.719	0.723	0.727	0.732	0.736	0.741
México	0.447	0.463	0.481	0.502	0.528	0.554	0.571	0.582	0.592	0.595	0.597	0.598	0.6	0.616	0.637	0.646	0.651	0.655	0.66	0.666	0.673	0.679	0.684	0.687	0.692	0.697	0.701	0.703	0.706	0.711	0.717	0.723	0.728	0.733	0.739	0.745	0.75
Michoacán de Ocampo	0.368	0.38	0.391	0.401	0.41	0.42	0.431	0.443	0.453	0.464	0.476	0.488	0.5	0.51	0.517	0.525	0.535	0.547	0.558	0.568	0.579	0.588	0.597	0.607	0.616	0.624	0.633	0.641	0.648	0.653	0.657	0.661	0.665	0.67	0.675	0.681	0.685
Morelos	0.479	0.492	0.503	0.513	0.523	0.533	0.543	0.554	0.564	0.576	0.587	0.599	0.611	0.62	0.627	0.634	0.64	0.647	0.653	0.659	0.664	0.67	0.675	0.68	0.686	0.692	0.698	0.705	0.71	0.714	0.718	0.722	0.726	0.73	0.734	0.737	0.741
Nayarit	0.428	0.442	0.454	0.464	0.473	0.482	0.491	0.501	0.511	0.522	0.533	0.545	0.557	0.567	0.575	0.582	0.592	0.602	0.612	0.621	0.63	0.638	0.645	0.652	0.658	0.664	0.67	0.675	0.679	0.683	0.687	0.692	0.698	0.705	0.711	0.718	0.723
Nuevo León	0.582	0.594	0.603	0.612	0.619	0.626	0.632	0.638	0.644	0.652	0.66	0.668	0.678	0.686	0.692	0.697	0.703	0.709	0.715	0.72	0.725	0.73	0.734	0.739	0.744	0.749	0.755	0.76	0.765	0.767	0.77	0.772	0.775	0.777	0.78	0.784	0.787
Oaxaca	0.351	0.362	0.371	0.38	0.388	0.396	0.406	0.417	0.427	0.437	0.446	0.456	0.466	0.471	0.474	0.476	0.481	0.487	0.494	0.501	0.508	0.515	0.523	0.533	0.544	0.555	0.568	0.581	0.592	0.601	0.609	0.617	0.623	0.629	0.634	0.639	0.643
Puebla	0.404	0.416	0.426	0.436	0.445	0.454	0.465	0.475	0.485	0.495	0.505	0.515	0.526	0.532	0.537	0.54	0.545	0.552	0.558	0.565	0.572	0.578	0.586	0.595	0.604	0.614	0.625	0.637	0.647	0.655	0.662	0.669	0.674	0.679	0.683	0.688	0.692
Querétaro	0.445	0.456	0.463	0.465	0.461	0.453	0.452	0.47	0.495	0.515	0.53	0.54	0.549	0.558	0.572	0.589	0.606	0.619	0.627	0.636	0.649	0.661	0.669	0.673	0.677	0.682	0.687	0.693	0.699	0.705	0.71	0.715	0.722	0.728	0.734	0.74	0.744
Quintana Roo	0.49	0.503	0.514	0.524	0.532	0.54	0.547	0.553	0.559	0.567	0.576	0.586	0.598	0.609	0.617	0.625	0.634	0.642	0.65	0.658	0.665	0.671	0.677	0.683	0.688	0.693	0.698	0.704	0.709	0.713	0.718	0.724	0.731	0.738	0.744	0.75	0.755
San Luis Potosí	0.375	0.39	0.403	0.414	0.425	0.436	0.449	0.462	0.474	0.487	0.5	0.513	0.526	0.536	0.544	0.552	0.561	0.572	0.583	0.593	0.604	0.613	0.622	0.631	0.64	0.648	0.656	0.665	0.672	0.678	0.685	0.694	0.703	0.711	0.72	0.728	0.734
Sinaloa	0.491	0.503	0.514	0.523	0.531	0.539	0.547	0.556	0.564	0.573	0.581	0.589	0.597	0.603	0.607	0.613	0.62	0.629	0.639	0.649	0.658	0.666	0.674	0.682	0.689	0.697	0.704	0.712	0.718	0.723	0.727	0.732	0.736	0.74	0.744	0.748	0.751
Sonora	0.528	0.541	0.552	0.561	0.569	0.577	0.584	0.592	0.598	0.606	0.614	0.623	0.632	0.64	0.645	0.65	0.656	0.663	0.67	0.677	0.683	0.69	0.696	0.702	0.709	0.716	0.723	0.731	0.737	0.742	0.746	0.751	0.756	0.761	0.767	0.772	0.777
Tabasco	0.42	0.432	0.443	0.452	0.461	0.469	0.478	0.487	0.496	0.506	0.515	0.525	0.536	0.545	0.552	0.561	0.572	0.584	0.597	0.607	0.616	0.624	0.63	0.636	0.642	0.648	0.654	0.66	0.665	0.669	0.674	0.68	0.685	0.691	0.696	0.701	0.706
Tamaulipas	0.504	0.516	0.526	0.535	0.544	0.553	0.562	0.572	0.582	0.593	0.605	0.617	0.629	0.638	0.646	0.653	0.66	0.668	0.674	0.68	0.685	0.689	0.693	0.697	0.701	0.706	0.712	0.719	0.726	0.732	0.738	0.744	0.751	0.756	0.761	0.766	0.77
Tlaxcala	0.359	0.371	0.384	0.397	0.413	0.432	0.455	0.479	0.499	0.514	0.524	0.533	0.538	0.545	0.559	0.573	0.585	0.593	0.599	0.605	0.612	0.62	0.628	0.635	0.644	0.651	0.657	0.664	0.672	0.677	0.681	0.685	0.693	0.701	0.708	0.715	0.72
Veracruz de Ignacio de la Llave	0.408	0.42	0.431	0.44	0.449	0.458	0.467	0.477	0.484	0.493	0.501	0.51	0.519	0.526	0.531	0.537	0.545	0.554	0.564	0.573	0.582	0.59	0.597	0.605	0.613	0.621	0.63	0.638	0.646	0.653	0.659	0.665	0.67	0.675	0.68	0.686	0.69
Yucatán	0.466	0.477	0.486	0.493	0.501	0.508	0.515	0.522	0.528	0.536	0.545	0.555	0.566	0.575	0.582	0.59	0.599	0.608	0.617	0.625	0.633	0.641	0.647	0.654	0.661	0.667	0.674	0.68	0.685	0.689	0.693	0.697	0.701	0.706	0.71	0.715	0.719
Zacatecas	0.381	0.393	0.405	0.415	0.425	0.435	0.446	0.459	0.471	0.484	0.498	0.512	0.526	0.537	0.546	0.554	0.564	0.574	0.583	0.592	0.601	0.608	0.615	0.622	0.629	0.635	0.641	0.647	0.652	0.656	0.66	0.666	0.672	0.679	0.686	0.693	0.699
Tropical Latin America	0.449	0.461	0.471	0.48	0.489	0.498	0.507	0.515	0.524	0.532	0.539	0.546	0.553	0.56	0.568	0.576	0.583	0.591	0.598	0.605	0.613	0.62	0.626	0.631	0.637	0.642	0.648	0.654	0.66	0.666	0.672	0.679	0.684	0.69	0.696	0.702	0.707
Brazil	0.449	0.461	0.471	0.48	0.49	0.499	0.508	0.516	0.525	0.533	0.54	0.548	0.554	0.562	0.569	0.577	0.585	0.592	0.6	0.607	0.614	0.621	0.627	0.633	0.638	0.644	0.65	0.656	0.662	0.667	0.673	0.68	0.686	0.691	0.697	0.703	0.708
Paraguay	0.437	0.444	0.448	0.451	0.454	0.456	0.459	0.461	0.465	0.469	0.473	0.478	0.484	0.49	0.496	0.504	0.511	0.519	0.527	0.535	0.543	0.55	0.558	0.565	0.573	0.58	0.588	0.596	0.604	0.611	0.62	0.628	0.636	0.645	0.653	0.661	0.668
Acre	0.332	0.346	0.358	0.369	0.38	0.392	0.404	0.416	0.428	0.439	0.449	0.459	0.467	0.474	0.483	0.492	0.501	0.509	0.517	0.522	0.528	0.534	0.539	0.543	0.548	0.555	0.563	0.57	0.578	0.586	0.595	0.604	0.612	0.621	0.629	0.636	0.642
Alagoas	0.314	0.325	0.334	0.342	0.35	0.358	0.368	0.378	0.388	0.397	0.406	0.414	0.422	0.431	0.441	0.452	0.461	0.469	0.477	0.485	0.493	0.501	0.508	0.515	0.522	0.529	0.536	0.544	0.553	0.56	0.569	0.577	0.584	0.592	0.599	0.605	0.61
Amazonas	0.367	0.382	0.394	0.405	0.416	0.427	0.439	0.452	0.464	0.475	0.486	0.497	0.506	0.513	0.523	0.534	0.544	0.552	0.56	0.569	0.578	0.586	0.592	0.597	0.603	0.609	0.615	0.622	0.63	0.637	0.645	0.652	0.659	0.666	0.674	0.68	0.686
Amapá	0.352	0.366	0.38	0.393	0.406	0.419	0.432	0.445	0.457	0.468	0.477	0.486	0.494	0.501	0.509	0.517	0.528	0.54	0.554	0.567	0.577	0.586	0.594	0.603	0.611	0.617	0.623	0.63	0.638	0.645	0.652	0.66	0.666	0.67	0.675	0.679	0.684
Bahia	0.356	0.365	0.373	0.381	0.389	0.397	0.406	0.417	0.428	0.438	0.447	0.456	0.464	0.472	0.481	0.49	0.498	0.507	0.517	0.524	0.532	0.539	0.545	0.552	0.558	0.564	0.571	0.578	0.584	0.591	0.598	0.606	0.613	0.62	0.627	0.633	0.638
Ceará	0.346	0.355	0.362	0.369	0.376	0.382	0.392	0.406	0.417	0.429	0.439	0.448	0.455	0.463	0.472	0.481	0.492	0.501	0.509	0.516	0.523	0.53	0.538	0.545	0.552	0.558	0.565	0.573	0.581	0.588	0.596	0.604	0.612	0.619	0.626	0.632	0.637

Appendix Table 9: Socio-demographic Index values for all estimated GBD 2016 locations, 1980–2016

Location	1980	1981	1982	1983	1984	1985	1986	1987	1988	1989	1990	1991	1992	1993	1994	1995	1996	1997	1998	1999	2000	2001	2002	2003	2004	2005	2006	2007	2008	2009	2010	2011	2012	2013	2014	2015	2016
Distrito Federal	0.597	0.607	0.617	0.625	0.634	0.643	0.652	0.659	0.666	0.673	0.68	0.686	0.692	0.698	0.704	0.71	0.717	0.723	0.729	0.735	0.741	0.747	0.753	0.758	0.764	0.769	0.775	0.781	0.787	0.793	0.8	0.806	0.812	0.818	0.824	0.829	0.832
Espírito Santo	0.459	0.47	0.481	0.49	0.499	0.509	0.518	0.527	0.536	0.545	0.553	0.561	0.568	0.577	0.584	0.592	0.6	0.607	0.614	0.621	0.628	0.635	0.642	0.648	0.654	0.66	0.665	0.671	0.677	0.682	0.688	0.694	0.699	0.705	0.711	0.717	0.721
Goiás	0.396	0.411	0.425	0.437	0.45	0.462	0.473	0.483	0.493	0.502	0.511	0.52	0.528	0.537	0.546	0.555	0.565	0.574	0.582	0.59	0.597	0.604	0.611	0.617	0.623	0.628	0.635	0.641	0.647	0.653	0.659	0.665	0.67	0.676	0.682	0.689	0.693
Maranhão	0.309	0.314	0.319	0.324	0.329	0.333	0.341	0.352	0.361	0.371	0.38	0.387	0.393	0.403	0.413	0.424	0.435	0.446	0.457	0.468	0.479	0.488	0.496	0.503	0.511	0.517	0.525	0.533	0.541	0.549	0.557	0.566	0.574	0.581	0.588	0.595	0.6
Minas Gerais	0.45	0.462	0.473	0.483	0.493	0.503	0.512	0.518	0.525	0.532	0.539	0.546	0.552	0.559	0.566	0.574	0.582	0.589	0.596	0.603	0.609	0.615	0.621	0.627	0.632	0.638	0.643	0.65	0.656	0.661	0.668	0.674	0.68	0.686	0.692	0.697	0.702
Mato Grosso do Sul	0.401	0.418	0.432	0.445	0.457	0.471	0.481	0.489	0.498	0.506	0.514	0.522	0.53	0.539	0.548	0.558	0.567	0.576	0.584	0.591	0.598	0.604	0.61	0.615	0.62	0.626	0.632	0.638	0.644	0.649	0.654	0.659	0.664	0.669	0.675	0.681	0.686
Mato Grosso	0.419	0.433	0.445	0.456	0.467	0.48	0.49	0.498	0.507	0.516	0.524	0.532	0.54	0.549	0.559	0.569	0.578	0.587	0.595	0.602	0.609	0.615	0.621	0.626	0.631	0.636	0.642	0.647	0.653	0.658	0.664	0.67	0.675	0.68	0.687	0.693	0.699
Pará	0.364	0.375	0.385	0.393	0.402	0.411	0.42	0.428	0.436	0.444	0.451	0.458	0.465	0.473	0.483	0.493	0.503	0.511	0.519	0.526	0.532	0.537	0.541	0.545	0.549	0.554	0.56	0.566	0.574	0.581	0.589	0.597	0.605	0.612	0.619	0.625	0.63
Paraíba	0.325	0.337	0.347	0.356	0.365	0.372	0.384	0.399	0.412	0.424	0.436	0.444	0.451	0.459	0.467	0.476	0.486	0.495	0.504	0.512	0.519	0.527	0.533	0.54	0.546	0.553	0.559	0.567	0.574	0.581	0.589	0.596	0.603	0.61	0.616	0.622	0.627
Paraná	0.443	0.458	0.471	0.483	0.494	0.507	0.517	0.524	0.533	0.541	0.548	0.556	0.564	0.572	0.58	0.589	0.597	0.606	0.614	0.622	0.629	0.636	0.643	0.649	0.654	0.66	0.665	0.671	0.677	0.682	0.688	0.694	0.7	0.706	0.712	0.718	0.722
Pernambuco	0.364	0.377	0.387	0.396	0.405	0.414	0.425	0.437	0.448	0.458	0.468	0.477	0.485	0.492	0.5	0.508	0.516	0.523	0.53	0.536	0.543	0.549	0.555	0.561	0.567	0.573	0.579	0.585	0.592	0.598	0.605	0.611	0.617	0.623	0.63	0.636	0.64
Piaui	0.321	0.329	0.337	0.344	0.351	0.358	0.367	0.378	0.388	0.398	0.408	0.415	0.421	0.428	0.436	0.445	0.455	0.464	0.473	0.482	0.49	0.498	0.505	0.512	0.519	0.526	0.533	0.54	0.548	0.555	0.563	0.571	0.578	0.585	0.591	0.598	0.602
Rio de Janeiro	0.531	0.545	0.557	0.567	0.577	0.587	0.595	0.602	0.608	0.615	0.62	0.626	0.631	0.637	0.642	0.648	0.653	0.659	0.665	0.67	0.676	0.682	0.688	0.692	0.696	0.701	0.705	0.71	0.714	0.718	0.723	0.727	0.732	0.736	0.74	0.745	0.749
Rio Grande do Norte	0.357	0.369	0.38	0.39	0.4	0.409	0.42	0.431	0.442	0.452	0.461	0.469	0.477	0.485	0.494	0.504	0.515	0.524	0.531	0.539	0.546	0.554	0.562	0.568	0.575	0.582	0.589	0.596	0.603	0.61	0.618	0.625	0.632	0.639	0.645	0.651	0.656
Rondônia	0.358	0.373	0.386	0.398	0.41	0.422	0.434	0.446	0.457	0.467	0.477	0.486	0.494	0.502	0.511	0.519	0.528	0.536	0.545	0.552	0.56	0.568	0.575	0.582	0.589	0.597	0.604	0.612	0.619	0.626	0.633	0.64	0.646	0.653	0.66	0.666	0.671
Roraima	0.401	0.411	0.421	0.43	0.44	0.45	0.461	0.472	0.483	0.493	0.503	0.512	0.52	0.527	0.535	0.542	0.548	0.552	0.554	0.558	0.569	0.581	0.589	0.595	0.604	0.612	0.62	0.628	0.636	0.644	0.651	0.657	0.661	0.666	0.672	0.678	0.683
Rio Grande do Sul	0.506	0.516	0.525	0.533	0.541	0.549	0.557	0.565	0.572	0.579	0.585	0.592	0.598	0.604	0.61	0.617	0.622	0.628	0.635	0.641	0.647	0.654	0.66	0.665	0.671	0.676	0.682	0.688	0.693	0.698	0.703	0.708	0.712	0.717	0.722	0.727	0.732
Santa Catarina	0.477	0.489	0.499	0.508	0.517	0.526	0.536	0.544	0.553	0.561	0.569	0.577	0.584	0.592	0.6	0.609	0.617	0.624	0.632	0.639	0.647	0.654	0.66	0.665	0.671	0.676	0.681	0.687	0.693	0.698	0.704	0.711	0.716	0.722	0.729	0.735	0.74
Sergipe	0.336	0.35	0.361	0.372	0.382	0.391	0.403	0.417	0.43	0.442	0.453	0.463	0.472	0.481	0.49	0.501	0.511	0.52	0.529	0.537	0.546	0.554	0.562	0.569	0.576	0.582	0.589	0.596	0.604	0.611	0.619	0.627	0.634	0.641	0.648	0.654	0.659
São Paulo	0.53	0.542	0.552	0.561	0.571	0.581	0.589	0.596	0.602	0.609	0.615	0.621	0.628	0.634	0.64	0.647	0.653	0.659	0.666	0.672	0.679	0.686	0.692	0.697	0.702	0.706	0.711	0.716	0.721	0.726	0.73	0.736	0.74	0.745	0.75	0.756	0.76
Tocantins	0.38	0.389	0.398	0.406	0.415	0.424	0.434	0.443	0.452	0.461	0.469	0.477	0.486	0.494	0.502	0.511	0.521	0.53	0.539	0.547	0.556	0.563	0.571	0.577	0.584	0.591	0.597	0.604	0.612	0.619	0.626	0.634	0.642	0.649	0.656	0.663	0.668
North Africa and Middle East	0.382	0.392	0.401	0.41	0.418	0.427	0.436	0.446	0.455	0.465	0.475	0.485	0.495	0.505	0.513	0.522	0.531	0.539	0.548	0.556	0.564	0.571	0.578	0.586	0.593	0.601	0.608	0.615	0.622	0.628	0.634	0.64	0.647	0.654	0.661	0.669	0.674
North Africa and Middle East	0.382	0.392	0.401	0.41	0.418	0.427	0.436	0.446	0.455	0.465	0.475	0.485	0.495	0.505	0.513	0.522	0.531	0.539	0.548	0.556	0.564	0.571	0.578	0.586	0.593	0.601	0.608	0.615	0.622	0.628	0.634	0.64	0.647	0.654	0.661	0.669	0.674
Algeria	0.356	0.367	0.379	0.39	0.401	0.412	0.423	0.433	0.443	0.453	0.463	0.472	0.479	0.485	0.49	0.495	0.5	0.505	0.51	0.516	0.523	0.53	0.538	0.549	0.559	0.57	0.581	0.591	0.602	0.612	0.622	0.632	0.642	0.651	0.66	0.669	0.676
Bahrain	0.498	0.513	0.526	0.536	0.54	0.542	0.55	0.559	0.565	0.571	0.586	0.599	0.605	0.613	0.623	0.633	0.641	0.644	0.646	0.65	0.663	0.681	0.694	0.699	0.701	0.703	0.707	0.711	0.716	0.719	0.72	0.719	0.718	0.721	0.726	0.732	0.738
Egypt	0.347	0.358	0.369	0.38	0.391	0.402	0.413	0.425	0.435	0.446	0.458	0.469	0.48	0.49	0.499	0.509	0.518	0.527	0.535	0.543	0.551	0.558	0.566	0.573	0.579	0.585	0.591	0.596	0.601	0.607	0.612	0.617	0.623	0.629	0.637	0.645	0.653
Iran	0.379	0.387	0.395	0.404	0.412	0.422	0.434	0.449	0.466	0.487	0.51	0.536	0.562	0.585	0.607	0.625	0.642	0.656	0.667	0.676	0.683	0.689	0.695	0.701	0.707	0.713	0.719	0.725	0.731	0.736	0.742	0.748	0.752	0.757	0.762	0.766	0.769
Iraq	0.289	0.299	0.31	0.32	0.331	0.341	0.351	0.361	0.372	0.381	0.391	0.398	0.405	0.412	0.418	0.423	0.429	0.437	0.447	0.459	0.472	0.485	0.495	0.498	0.498	0.492	0.485	0.475	0.461	0.447	0.434	0.429	0.432	0.44	0.45	0.463	0.474
Jordan	0.264	0.294	0.322	0.346	0.369	0.389	0.409	0.428	0.448	0.466	0.483	0.497	0.511	0.524	0.536	0.548	0.558	0.568	0.577	0.586	0.595	0.603	0.61	0.615	0.621	0.626	0.632	0.637	0.643	0.649	0.656	0.663	0.669	0.676	0.683	0.689	0.694
Kuwait	0.46	0.476	0.494	0.515	0.54	0.566	0.59	0.611	0.628	0.642	0.652	0.657	0.66	0.659	0.653	0.649	0.655	0.671	0.689	0.702	0.711	0.717	0.721	0.726	0.732	0.738	0.747	0.758	0.77	0.781	0.791	0.8	0.808	0.816	0.823	0.829	0.832
Lebanon	0.51	0.525	0.536	0.547	0.561	0.575	0.588	0.602	0.613	0.621	0.627	0.636	0.645	0.653	0.661	0.669	0.678	0.687	0.697	0.706	0.715	0.723	0.73	0.738	0.744	0.751	0.757	0.763	0.77	0.777	0.785	0.792	0.797	0.801	0.804	0.807	0.81
Libya	0.565	0.57	0.575	0.579	0.583	0.588	0.591	0.595	0.598	0.601	0.605	0.611	0.617	0.623	0.63	0.636	0.643	0.651	0.659	0.667	0.675	0.684	0.692	0.702	0.712	0.723	0.734	0.746	0.757	0.768	0.781	0.785	0.793	0.8	0.804	0.807	0.806
Morocco	0.287	0.295	0.303	0.312	0.321	0.329	0.339	0.349	0.359	0.37	0.381	0.393	0.404	0.415	0.427	0.438	0.449	0.46	0.471	0.481	0.49	0.499	0.507	0.516	0.524	0.531	0.54	0.548	0.557	0.565	0.573	0.581	0.589	0.597	0.604	0.611	0.617
Palestine	0.328	0.341	0.351	0.36	0.369	0.376	0.384	0.391	0.397	0.404	0.411	0.417	0.424	0.43	0.437	0.446	0.457	0.467	0.478	0.488	0.497	0.504	0.51	0.513	0.51	0.502	0.49	0.472	0.452	0.432	0.418	0.413	0.412	0.416	0.425	0.438	0.45
Oman	0.107	0.109	0.111	0.113	0.115	0.135	0.156	0.176	0.194	0.214	0.234	0.256	0.277	0.298	0.318	0.337	0.356	0.376	0.396	0.417	0.438	0.458	0.479	0.499	0.52	0.54	0.561	0.581	0.602	0.621	0.639	0.658	0.676	0.691	0.704	0.714	0.72
Qatar	0.52	0.54	0.558	0.575	0.587	0.596	0.601	0.605	0.609	0.615	0.622	0.631	0.64	0.65	0.659	0.669	0.676	0.684	0.69	0.696	0.702	0.708	0.715	0.722	0.728	0.735	0.74	0.745	0.75	0.756	0.761	0.765	0.769	0.774	0.779	0.784	0.788
Saudi Arabia	0.21	0.225	0.242	0.26	0.277	0.293	0.308	0.323	0.338	0.353	0.368	0.383	0.398	0.414	0.429	0.445	0.462	0.479	0.497	0.515	0.533	0.551	0.569	0.587	0.605	0.623	0.642	0.66	0.679	0.696	0.713	0.73	0.746	0.761	0.775	0.788	0.797
Syria	0.28	0.29	0.301	0.311	0.32	0.33	0.339	0.348	0.357	0.366	0.376	0.386	0.397	0.408	0.419	0.43	0.441	0.451	0.462	0.472	0.483	0.493	0.503	0.514	0.525	0.537	0.549	0.56	0.571	0.582	0.593	0.603	0.611	0.618	0.625	0.632	0.638
Tunisia	0.353	0.364	0.376	0.388	0.4	0.412	0.425	0.437	0.45	0.462	0.476	0.489	0.502	0.515	0.527	0.539	0.551	0.563	0.573	0.583	0.591	0.599	0.606	0.612	0.618	0.623	0.629	0.635	0.64	0.646	0.653	0.659	0.667	0.674	0.682	0.69	0.696
Turkey	0.479	0.489	0.498	0.508	0.517	0.527	0.537	0.547	0.557	0.566	0.576	0.584	0.592	0.599	0.605	0.612	0.619	0.627	0.634	0.641	0.649	0.656	0.663	0.67	0.677	0.684	0.691	0.698	0.705	0.711	0.717	0.724	0.731	0.738	0.746	0.754	0.762
United Arab Emirates	0.34	0.365	0.389	0.413	0.436	0.458	0.48	0.501	0.521	0.539	0.557	0.574	0.59	0.606	0.62	0.633	0.645	0.656	0.667	0.677	0.686	0.695	0.703	0.71	0.716	0.721	0.725	0.729	0.732	0.736	0.74	0.745	0.751	0.756	0.762	0.768	0.772
Yemen	0.155	0.159	0.163	0.166	0.168	0.168	0.168	0.167	0.168	0.17	0.173	0.177	0.185	0.195	0.206	0.217	0.229	0.24	0.252	0.264	0.276	0.288	0.302	0.317	0.335	0.351	0.362	0.367	0.371	0.374	0.379	0.386	0.398	0.417	0.434	0.445	0.45
Afghanistan	0.134	0.136	0.139	0.142	0.145	0.147	0.15	0.153	0.154	0.156	0.158	0.16	0.163	0.165	0.165	0.166	0.166	0.167	0.168	0.169	0.17	0.171	0.175	0.181	0.188	0.195	0.203	0.211	0.22	0.229	0.238	0.247	0.255	0.262	0.269	0.276	0.281
Sudan	0.24	0.245	0.252	0.258	0.263	0.268	0.274	0.279	0.285	0.29	0.295	0.3	0.304	0.309	0.313	0.317	0.32	0.326	0.332	0.34	0.351	0.361	0.372	0.383	0.393	0.404	0.415	0.426	0.437	0.448	0.459	0.47	0.48	0.49	0.501	0.51	0.518
Ha'il	0.205	0.22	0.236	0.252	0.267	0.283	0.297	0.312	0.326	0.341	0.355	0.369	0.384	0.399	0.414	0.429	0.446	0.463	0.479	0.496	0.514	0.532	0.549	0.567	0.585	0.603	0.621	0.639	0.657	0.674	0.691	0.708	0.724	0.739	0.752	0.765	0.774
Qassim	0.15	0.169	0.19	0.211	0.231	0.251	0.269	0.288	0.306	0.324	0.341	0.357	0.373	0.389	0.405	0.422	0.439	0.458	0.476	0.495	0.515	0.534	0.554	0.573	0.592	0.61	0.629	0.647	0.666	0.684	0.701	0.717	0.733	0.748	0.762	0.776	0.785
Riyadh	0.185	0.203	0.223	0.244	0.264	0.283	0.301	0.319	0.337	0.353	0.369	0.385	0.401	0.417	0.434	0.451	0.468	0.486	0.505	0.523	0.543	0.562	0.58	0.599	0.618	0.637	0.656	0.675	0.693	0.711	0.727	0.742	0.756	0.769	0.783	0.795	0.804
Tabuk	0.194	0.21	0.229	0.248	0.267	0.285	0.301	0.316	0.332	0.347	0.361	0.376	0.391	0.406	0.422	0.438	0.454	0.471	0.489	0.507	0.525	0.543	0.562	0.58	0.598	0.62	0.64	0.659	0.678	0.695	0.712	0.729	0.745	0.76	0.775	0.79	0.8
Madinah	0.199	0.215	0.232	0.251	0.269	0.285	0.3	0.316	0.331	0.347	0.362	0.377	0.392	0.408	0.424	0.44	0.457	0.474	0.492	0.51	0.528	0.547	0.565	0.583	0.601	0.62	0.638	0.656	0.675	0.693	0.71	0.727	0.744	0.76	0.773	0.786	0.795
Makkah	0.231	0.246	0.261	0.277	0.293	0.308	0.323	0.337	0.352	0.367	0.382	0.396	0.411	0.427	0.442	0.458	0.474	0.491	0.508	0.525	0.543	0.56	0.577	0.594	0.611	0.628	0.645	0.662	0.679	0.695	0.71	0.726	0.741	0.755	0.768	0.779	0.788
Bahah	0.219	0.232	0.246	0.26	0.273	0.287	0.299	0.312	0.325	0.338	0.351	0.364	0.378	0.392	0.406	0.42	0.436	0.452	0.468	0.484	0.5	0.516	0.532	0.549	0.565	0.582	0.599	0.616	0.634	0.65	0.666	0.683	0.699	0.714	0.726	0.737	0.746
Northern Borders	0.264	0.278	0.293	0.308	0.323	0.337	0.351	0.364	0.378	0.392	0.406	0.42	0.434	0.45	0.465	0.48	0.497	0.513	0.53	0.546	0.563	0.58	0.596	0.612	0.629	0.647	0.665	0.681	0.698	0.713	0.728	0.742	0.755	0.766	0.779	0.79	0.797
Jawf	0.236	0.253	0.272	0.291	0.311	0.33	0.347	0.364	0.381	0.398	0.414	0.431	0.448	0.465	0.482	0.499	0.517	0.536	0.555	0.573	0.592	0.61	0.628	0.645	0.663	0.681	0.699	0.717	0.735	0.752	0.768	0.784	0.799	0.814	0.826	0.838	0.846
Jizan	0.202	0.212	0.223	0.234	0.245	0.255	0.265	0.275	0.285	0.296	0.306	0.317	0.329	0.341	0.353	0.365	0.379	0.393	0.407	0.421	0.436	0.45	0.465	0.48	0.496	0.513	0.53	0.546	0.564	0.581	0.597	0.614	0.63	0.645	0.658	0.67	0.679
'Asir	0.167	0.181	0.197	0.213	0.229	0.243	0.258	0.272	0.286	0.301	0.314	0.327	0.341	0.355	0.369	0.384	0.401	0.417	0.434	0.451	0.469	0.487	0.504	0.522	0.541	0.559	0.578	0.598	0.617	0.636	0.654	0.672	0.69	0.706	0.721	0.735	0.744
Najran	0.187	0.199	0.212	0.224	0.237	0.249	0.261	0.273	0.285	0.297	0.309	0.32	0.332	0.345	0.357	0.371	0.385	0.4	0.415	0.431	0.447	0.463	0.479	0.496	0.513	0.53	0.548	0.566	0.585	0.602	0.62	0.637	0.654	0.671	0.685	0.699	0.709
Eastern Province	0.209	0.225	0.243	0.261	0.28	0.297	0.313	0.329	0.346	0.362	0.377	0.393	0.409	0.425	0.441	0.458	0.475	0.493	0.511	0.529	0.548	0.566	0.584	0.603	0.621	0.639	0.658	0.676	0.694	0.711	0.728	0.745	0.761	0.776	0.79	0.803	0.813

Appendix Table 9: Socio-demographic Index values for all estimated GBD 2016 locations, 1980–2016

Location	1980	1981	1982	1983	1984	1985	1986	1987	1988	1989	1990	1991	1992	1993	1994	1995	1996	1997	1998	1999	2000	2001	2002	2003	2004	2005	2006	2007	2008	2009	2010	2011	2012	2013	2014	2015	2016
South Asia	0.288	0.293	0.298	0.303	0.309	0.314	0.32	0.327	0.334	0.341	0.349	0.356	0.364	0.371	0.378	0.386	0.394	0.402	0.409	0.417	0.425	0.432	0.44	0.447	0.455	0.464	0.473	0.482	0.491	0.5	0.51	0.519	0.529	0.539	0.549	0.56	0.569
South Asia	0.288	0.293	0.298	0.303	0.309	0.314	0.32	0.327	0.334	0.341	0.349	0.356	0.364	0.371	0.378	0.386	0.394	0.402	0.409	0.417	0.425	0.432	0.44	0.447	0.455	0.464	0.473	0.482	0.491	0.5	0.51	0.519	0.529	0.539	0.549	0.56	0.569
Bangladesh	0.254	0.258	0.263	0.269	0.275	0.281	0.287	0.294	0.3	0.307	0.315	0.323	0.331	0.339	0.347	0.355	0.363	0.37	0.377	0.384	0.39	0.396	0.402	0.407	0.414	0.42	0.427	0.434	0.442	0.45	0.459	0.467	0.476	0.485	0.494	0.503	0.511
Bhutan	0.237	0.242	0.247	0.253	0.259	0.265	0.272	0.279	0.286	0.293	0.301	0.31	0.318	0.327	0.337	0.348	0.359	0.37	0.382	0.394	0.406	0.418	0.431	0.444	0.457	0.47	0.482	0.494	0.506	0.518	0.53	0.541	0.552	0.563	0.572	0.581	0.588
India	0.298	0.303	0.308	0.313	0.318	0.324	0.329	0.336	0.342	0.35	0.357	0.364	0.371	0.378	0.385	0.393	0.401	0.408	0.416	0.424	0.432	0.44	0.447	0.455	0.464	0.473	0.482	0.492	0.502	0.512	0.522	0.533	0.543	0.553	0.564	0.575	0.584
Nepal	0.194	0.198	0.202	0.206	0.21	0.214	0.219	0.224	0.23	0.236	0.242	0.25	0.258	0.266	0.276	0.285	0.293	0.302	0.31	0.319	0.328	0.337	0.345	0.354	0.362	0.37	0.377	0.384	0.391	0.398	0.404	0.411	0.417	0.424	0.433	0.443	0.452
Pakistan	0.216	0.218	0.22	0.223	0.227	0.234	0.243	0.255	0.268	0.282	0.297	0.309	0.32	0.33	0.339	0.349	0.36	0.371	0.383	0.392	0.4	0.408	0.416	0.424	0.431	0.438	0.443	0.448	0.453	0.458	0.464	0.471	0.478	0.488	0.499	0.51	0.519
Andhra Pradesh	0.276	0.281	0.285	0.29	0.294	0.299	0.303	0.309	0.315	0.322	0.329	0.337	0.345	0.354	0.363	0.373	0.383	0.393	0.403	0.414	0.424	0.434	0.444	0.453	0.463	0.472	0.481	0.493	0.503	0.514	0.525	0.536	0.546	0.556	0.566	0.577	0.585
Arunachal Pradesh	0.263	0.269	0.274	0.28	0.286	0.292	0.3	0.307	0.315	0.322	0.33	0.338	0.346	0.354	0.362	0.371	0.38	0.389	0.397	0.405	0.413	0.422	0.43	0.438	0.447	0.457	0.466	0.477	0.487	0.5	0.513	0.526	0.539	0.552	0.565	0.578	0.589
Assam	0.292	0.298	0.304	0.311	0.318	0.325	0.332	0.339	0.346	0.353	0.361	0.367	0.373	0.378	0.383	0.389	0.394	0.4	0.405	0.413	0.421	0.429	0.437	0.446	0.454	0.462	0.471	0.479	0.487	0.496	0.504	0.514	0.523	0.532	0.542	0.552	0.562
Bihar	0.25	0.255	0.26	0.264	0.27	0.275	0.281	0.287	0.294	0.3	0.306	0.312	0.317	0.318	0.319	0.318	0.318	0.319	0.317	0.317	0.318	0.321	0.325	0.329	0.334	0.338	0.346	0.354	0.363	0.371	0.38	0.39	0.4	0.411	0.421	0.431	0.44
Chhattisgarh	0.256	0.26	0.264	0.269	0.273	0.277	0.282	0.287	0.292	0.298	0.304	0.31	0.316	0.323	0.329	0.336	0.344	0.352	0.36	0.369	0.376	0.386	0.395	0.405	0.414	0.425	0.436	0.449	0.461	0.473	0.485	0.497	0.509	0.521	0.534	0.547	0.558
Delhi	0.471	0.478	0.484	0.49	0.495	0.5	0.505	0.51	0.515	0.52	0.524	0.529	0.533	0.538	0.544	0.55	0.557	0.565	0.573	0.582	0.592	0.602	0.612	0.623	0.633	0.643	0.653	0.664	0.674	0.684	0.694	0.705	0.715	0.724	0.734	0.744	0.752
Goa	0.394	0.401	0.409	0.416	0.424	0.432	0.44	0.448	0.456	0.465	0.473	0.481	0.49	0.5	0.511	0.523	0.535	0.547	0.561	0.573	0.586	0.597	0.608	0.616	0.623	0.631	0.638	0.645	0.654	0.664	0.673	0.686	0.696	0.707	0.718	0.729	0.738
Gujarat	0.319	0.326	0.331	0.338	0.345	0.352	0.359	0.365	0.373	0.38	0.389	0.395	0.403	0.411	0.42	0.429	0.439	0.448	0.457	0.465	0.473	0.48	0.487	0.495	0.503	0.512	0.522	0.532	0.542	0.551	0.561	0.571	0.58	0.589	0.598	0.607	0.615
Haryana	0.317	0.324	0.331	0.338	0.345	0.353	0.361	0.368	0.377	0.386	0.395	0.405	0.413	0.422	0.431	0.439	0.449	0.458	0.467	0.475	0.484	0.494	0.503	0.513	0.524	0.534	0.545	0.557	0.569	0.58	0.591	0.602	0.613	0.624	0.635	0.646	0.655
Himachal Pradesh	0.289	0.296	0.303	0.311	0.318	0.326	0.334	0.343	0.353	0.363	0.374	0.385	0.396	0.407	0.419	0.43	0.443	0.456	0.469	0.483	0.496	0.508	0.52	0.531	0.541	0.553	0.564	0.575	0.587	0.598	0.61	0.622	0.633	0.644	0.654	0.662	0.669
Jammu and Kashmir	0.276	0.282	0.288	0.295	0.301	0.308	0.316	0.322	0.329	0.336	0.343	0.349	0.353	0.361	0.369	0.377	0.384	0.395	0.405	0.417	0.429	0.441	0.452	0.464	0.475	0.486	0.497	0.507	0.518	0.528	0.539	0.549	0.559	0.569	0.579	0.589	0.598
Jharkhand	0.259	0.262	0.266	0.269	0.273	0.277	0.281	0.285	0.29	0.295	0.3	0.306	0.311	0.316	0.322	0.329	0.334	0.342	0.35	0.358	0.365	0.373	0.381	0.389	0.397	0.406	0.415	0.424	0.433	0.442	0.453	0.464	0.475	0.487	0.499	0.512	0.523
Karnataka	0.296	0.301	0.306	0.312	0.318	0.324	0.33	0.337	0.344	0.352	0.36	0.368	0.376	0.385	0.395	0.405	0.415	0.425	0.436	0.447	0.457	0.465	0.474	0.481	0.49	0.499	0.509	0.521	0.532	0.543	0.554	0.565	0.576	0.586	0.596	0.606	0.614
Kerala	0.369	0.374	0.379	0.385	0.391	0.396	0.403	0.409	0.415	0.422	0.429	0.436	0.444	0.454	0.464	0.476	0.488	0.5	0.512	0.524	0.535	0.545	0.553	0.562	0.57	0.58	0.589	0.599	0.608	0.619	0.628	0.638	0.648	0.657	0.667	0.676	0.683
Madhya Pradesh	0.271	0.275	0.28	0.286	0.291	0.297	0.303	0.309	0.317	0.324	0.332	0.339	0.345	0.35	0.355	0.36	0.365	0.37	0.375	0.38	0.385	0.391	0.397	0.404	0.411	0.418	0.425	0.433	0.441	0.45	0.459	0.47	0.481	0.494	0.507	0.52	0.532
Maharashtra	0.339	0.346	0.353	0.36	0.368	0.375	0.383	0.391	0.399	0.408	0.418	0.426	0.435	0.444	0.454	0.464	0.475	0.485	0.494	0.503	0.512	0.519	0.526	0.534	0.543	0.553	0.564	0.576	0.588	0.599	0.61	0.62	0.63	0.639	0.648	0.658	0.666
Manipur	0.28	0.288	0.295	0.302	0.31	0.318	0.326	0.336	0.346	0.355	0.364	0.372	0.38	0.386	0.391	0.395	0.4	0.406	0.413	0.423	0.433	0.444	0.455	0.467	0.48	0.494	0.507	0.518	0.529	0.54	0.549	0.56	0.571	0.581	0.591	0.601	0.61
Meghalaya	0.287	0.291	0.296	0.3	0.305	0.31	0.316	0.323	0.328	0.335	0.342	0.348	0.353	0.358	0.36	0.363	0.367	0.372	0.379	0.389	0.402	0.417	0.431	0.443	0.455	0.467	0.477	0.488	0.498	0.508	0.519	0.53	0.541	0.552	0.563	0.575	0.585
Mizoram	0.334	0.34	0.346	0.352	0.359	0.369	0.378	0.39	0.399	0.407	0.414	0.422	0.429	0.436	0.44	0.446	0.453	0.459	0.465	0.473	0.482	0.492	0.502	0.511	0.52	0.528	0.537	0.545	0.553	0.562	0.572	0.581	0.591	0.601	0.611	0.621	0.63
Nagaland	0.343	0.348	0.354	0.36	0.366	0.371	0.377	0.383	0.389	0.395	0.401	0.406	0.409	0.413	0.416	0.419	0.422	0.428	0.434	0.442	0.456	0.473	0.49	0.505	0.519	0.533	0.545	0.559	0.572	0.585	0.597	0.61	0.622	0.634	0.645	0.654	0.661
Odisha	0.251	0.255	0.259	0.264	0.269	0.274	0.28	0.285	0.292	0.299	0.305	0.312	0.318	0.324	0.33	0.338	0.344	0.351	0.359	0.368	0.376	0.385	0.393	0.402	0.413	0.422	0.433	0.445	0.457	0.468	0.48	0.491	0.503	0.514	0.526	0.537	0.547
Punjab	0.329	0.337	0.344	0.352	0.359	0.367	0.375	0.383	0.392	0.401	0.409	0.418	0.427	0.436	0.445	0.455	0.465	0.474	0.484	0.493	0.503	0.511	0.519	0.527	0.535	0.545	0.555	0.566	0.576	0.587	0.598	0.608	0.618	0.627	0.636	0.643	0.65
Rajasthan	0.254	0.259	0.263	0.27	0.275	0.28	0.286	0.292	0.299	0.306	0.315	0.322	0.329	0.335	0.341	0.348	0.355	0.362	0.368	0.375	0.382	0.39	0.397	0.405	0.414	0.422	0.43	0.44	0.449	0.457	0.468	0.479	0.49	0.5	0.511	0.521	0.53
Sikkim	0.268	0.273	0.279	0.284	0.29	0.297	0.304	0.313	0.321	0.329	0.337	0.346	0.352	0.361	0.369	0.378	0.388	0.398	0.408	0.418	0.43	0.441	0.453	0.466	0.478	0.49	0.501	0.511	0.52	0.536	0.551	0.565	0.579	0.593	0.607	0.62	0.632
Tamil Nadu	0.309	0.315	0.319	0.325	0.33	0.337	0.343	0.351	0.359	0.367	0.376	0.385	0.395	0.405	0.417	0.428	0.44	0.452	0.464	0.476	0.487	0.496	0.505	0.513	0.521	0.531	0.541	0.552	0.563	0.575	0.587	0.599	0.61	0.621	0.632	0.64	0.648
Telangana	0.265	0.269	0.273	0.278	0.282	0.286	0.291	0.296	0.302	0.309	0.317	0.325	0.333	0.342	0.352	0.362	0.373	0.383	0.395	0.406	0.417	0.428	0.438	0.449	0.459	0.47	0.482	0.495	0.509	0.521	0.535	0.549	0.562	0.574	0.587	0.599	0.609
Tripura	0.315	0.319	0.324	0.328	0.331	0.335	0.339	0.343	0.348	0.353	0.358	0.362	0.366	0.372	0.378	0.386	0.395	0.404	0.412	0.425	0.438	0.451	0.462	0.47	0.478	0.485	0.491	0.497	0.504	0.511	0.518	0.527	0.536	0.545	0.554	0.564	0.573
Uttar Pradesh	0.265	0.269	0.273	0.278	0.282	0.287	0.293	0.299	0.306	0.312	0.32	0.326	0.332	0.336	0.34	0.344	0.348	0.351	0.355	0.358	0.362	0.368	0.374	0.382	0.39	0.398	0.407	0.415	0.423	0.432	0.441	0.451	0.462	0.473	0.485	0.498	0.508
Uttarakhand	0.257	0.262	0.267	0.272	0.277	0.283	0.289	0.295	0.302	0.309	0.317	0.324	0.332	0.34	0.349	0.358	0.368	0.379	0.389	0.4	0.412	0.424	0.436	0.449	0.463	0.477	0.491	0.505	0.519	0.535	0.551	0.568	0.583	0.598	0.613	0.628	0.639
West Bengal	0.324	0.328	0.331	0.335	0.339	0.343	0.347	0.352	0.356	0.361	0.366	0.371	0.376	0.382	0.389	0.396	0.404	0.412	0.42	0.429	0.438	0.446	0.454	0.462	0.47	0.478	0.486	0.496	0.506	0.516	0.526	0.536	0.546	0.555	0.565	0.573	0.58
The Six Minor Territories	0.38	0.388	0.397	0.405	0.413	0.42	0.429	0.437	0.445	0.452	0.461	0.468	0.475	0.482	0.49	0.498	0.506	0.516	0.525	0.534	0.543	0.552	0.562	0.572	0.581	0.592	0.602	0.613	0.623	0.634	0.644	0.654	0.663	0.672	0.682	0.691	0.698
Andhra Pradesh, Urban	0.377	0.382	0.387	0.392	0.397	0.403	0.408	0.413	0.42	0.427	0.434	0.441	0.447	0.454	0.462	0.47	0.479	0.488	0.498	0.51	0.521	0.532	0.541	0.55	0.559	0.567	0.576	0.586	0.595	0.604	0.613	0.623	0.632	0.641	0.65	0.66	0.668
Andhra Pradesh, Rural	0.239	0.242	0.246	0.249	0.253	0.257	0.261	0.265	0.271	0.277	0.284	0.291	0.298	0.307	0.317	0.327	0.337	0.347	0.358	0.368	0.379	0.389	0.398	0.407	0.417	0.425	0.435	0.445	0.455	0.465	0.475	0.485	0.495	0.506	0.516	0.528	0.537
Arunachal Pradesh, Urban	0.405	0.411	0.416	0.421	0.427	0.433	0.439	0.445	0.452	0.457	0.463	0.469	0.474	0.481	0.486	0.493	0.499	0.506	0.511	0.516	0.52	0.525	0.528	0.532	0.536	0.541	0.546	0.552	0.557	0.565	0.573	0.582	0.59	0.599	0.61	0.621	0.631
Arunachal Pradesh, Rural	0.251	0.256	0.261	0.265	0.271	0.276	0.283	0.289	0.296	0.302	0.309	0.316	0.323	0.331	0.338	0.346	0.353	0.361	0.369	0.376	0.384	0.392	0.401	0.409	0.419	0.428	0.438	0.45	0.46	0.474	0.488	0.501	0.515	0.528	0.541	0.554	0.565
Assam, Urban	0.443	0.449	0.454	0.46	0.467	0.473	0.478	0.484	0.489	0.494	0.499	0.504	0.508	0.513	0.519	0.525	0.531	0.536	0.541	0.549	0.556	0.564	0.571	0.579	0.586	0.592	0.599	0.606	0.612	0.618	0.624	0.63	0.636	0.643	0.65	0.657	0.663
Assam, Rural	0.274	0.28	0.286	0.292	0.299	0.306	0.313	0.32	0.327	0.334	0.342	0.348	0.353	0.358	0.363	0.368	0.374	0.379	0.384	0.391	0.399	0.407	0.415	0.423	0.432	0.44	0.449	0.457	0.466	0.474	0.483	0.492	0.501	0.511	0.521	0.532	0.542
Bihar, Urban	0.37	0.377	0.384	0.391	0.399	0.406	0.413	0.42	0.428	0.434	0.441	0.447	0.453	0.456	0.459	0.46	0.463	0.466	0.467	0.468	0.471	0.474	0.479	0.482	0.487	0.491	0.498	0.505	0.513	0.52	0.529	0.537	0.546	0.554	0.561	0.568	0.575
Bihar, Rural	0.231	0.235	0.239	0.243	0.248	0.253	0.258	0.264	0.27	0.275	0.281	0.287	0.292	0.294	0.294	0.294	0.294	0.296	0.295	0.295	0.297	0.299	0.304	0.308	0.313	0.317	0.325	0.333	0.342	0.35	0.359	0.368	0.379	0.389	0.4	0.41	0.419
Chhattisgarh, Urban	0.456	0.462	0.468	0.472	0.477	0.481	0.485	0.489	0.492	0.496	0.5	0.503	0.507	0.51	0.513	0.517	0.521	0.527	0.533	0.539	0.543	0.55	0.557	0.565	0.572	0.581	0.59	0.601	0.611	0.62	0.628	0.637	0.645	0.653	0.661	0.669	0.677
Chhattisgarh, Rural	0.203	0.206	0.21	0.214	0.218	0.223	0.228	0.233	0.238	0.244	0.251	0.257	0.264	0.271	0.278	0.286	0.294	0.302	0.31	0.319	0.328	0.337	0.346	0.356	0.365	0.376	0.387	0.399	0.412	0.424	0.435	0.448	0.461	0.474	0.488	0.502	0.514
Delhi, Urban	0.478	0.485	0.491	0.497	0.502	0.506	0.512	0.517	0.521	0.526	0.53	0.535	0.539	0.543	0.549	0.555	0.561	0.569	0.577	0.586	0.596	0.606	0.616	0.626	0.636	0.646	0.656	0.666	0.676	0.686	0.696	0.706	0.716	0.726	0.736	0.746	0.753
Delhi, Rural	0.388	0.395	0.402	0.409	0.415	0.422	0.429	0.436	0.443	0.45	0.457	0.464	0.471	0.477	0.483	0.49	0.497	0.505	0.515	0.525	0.536	0.547	0.558	0.569	0.58	0.591	0.603	0.614	0.624	0.636	0.647	0.658	0.669	0.681	0.692	0.703	0.713
Goa, Urban	0.459	0.464	0.471	0.476	0.483	0.489	0.495	0.501	0.507	0.514	0.52	0.526	0.533	0.541	0.551	0.561	0.572	0.582	0.595	0.606	0.616	0.626	0.635	0.642	0.648	0.654	0.66	0.665	0.672	0.681	0.69	0.701	0.711	0.721	0.732	0.744	0.753
Goa, Rural	0.365	0.372	0.38	0.386	0.394	0.401	0.409	0.417	0.424	0.433	0.441	0.449	0.458	0.468	0.479	0.491	0.503	0.515	0.529	0.542	0.554	0.566	0.577	0.585	0.592	0.6	0.608	0.616	0.625	0.636	0.646	0.658	0.669	0.679	0.69	0.701	0.71
Gujarat, Urban	0.408	0.415	0.422	0.431	0.439	0.446	0.454	0.459	0.467	0.474	0.482	0.487	0.494	0.501	0.51	0.519	0.529	0.538	0.547	0.555	0.562	0.569	0.575	0.581	0.587	0.594	0.602	0.611	0.618	0.627	0.635	0.643	0.65	0.657	0.664	0.671	0.677
Gujarat, Rural	0.273	0.278	0.283	0.289	0.294	0.3	0.306	0.312	0.319	0.326	0.334	0.341	0.349	0.356	0.364	0.373	0.382	0.39	0.399	0.407	0.414	0.421	0.428	0.435	0.443	0.452	0.461	0.471	0.48	0.49	0.5	0.51	0.52	0.53	0.541	0.552	0.561
Haryana, Urban	0.428	0.436	0.444	0.452	0.46	0.468	0.476	0.483	0.491	0.498	0.506	0.514	0.521	0.528	0.536	0.543	0.552	0.56	0.567	0.575	0.582	0.59	0.596	0.603	0.61	0.617	0.626	0.635	0.645	0.655	0.665	0.674	0.683	0.691	0.7	0.709	0.717
Haryana, Rural	0.284	0.29	0.296	0.302	0.308	0.315	0.322	0.329	0.337	0.346	0.354	0.364	0.372	0.38	0.389	0.397	0.407	0.416	0.424	0.433	0.441	0.45	0.459	0.469	0.478	0.488	0.499	0.511	0.523	0.535	0.547	0.559	0.57	0.581	0.592	0.604	0.614
Himachal Pradesh, Urban	0.433	0.441	0.449	0.457	0.464	0.472	0.48	0.488	0.497	0.505	0.514	0.522	0.531	0.54	0.549	0.558	0.569	0.579	0.59	0.602	0.614	0.624	0.635	0.645	0.655	0.665	0.675	0.685	0.692	0.699	0.708	0.715	0.723	0.729	0.736	0.743	0.75

Appendix Table 9: Socio-demographic Index values for all estimated GBD 2016 locations, 1980–2016

Location	1980	1981	1982	1983	1984	1985	1986	1987	1988	1989	1990	1991	1992	1993	1994	1995	1996	1997	1998	1999	2000	2001	2002	2003	2004	2005	2006	2007	2008	2009	2010	2011	2012	2013	2014	2015	2016
Himachal Pradesh, Rural	0.275	0.282	0.289	0.297	0.304	0.312	0.32	0.329	0.339	0.349	0.36	0.37	0.381	0.393	0.404	0.416	0.429	0.442	0.455	0.469	0.482	0.495	0.506	0.517	0.528	0.539	0.551	0.563	0.574	0.585	0.598	0.61	0.621	0.632	0.643	0.652	0.659
Jammu and Kashmir, Urban	0.366	0.374	0.383	0.392	0.401	0.411	0.42	0.428	0.437	0.444	0.451	0.457	0.462	0.47	0.478	0.486	0.494	0.506	0.516	0.528	0.54	0.551	0.562	0.572	0.581	0.59	0.599	0.607	0.615	0.623	0.629	0.636	0.643	0.65	0.657	0.664	0.671
Jammu and Kashmir, Rural	0.247	0.252	0.258	0.263	0.269	0.275	0.282	0.287	0.294	0.301	0.307	0.312	0.317	0.324	0.332	0.339	0.346	0.357	0.366	0.378	0.389	0.4	0.412	0.423	0.435	0.446	0.458	0.468	0.479	0.49	0.5	0.511	0.522	0.532	0.543	0.553	0.562
Jharkhand, Urban	0.453	0.457	0.462	0.466	0.469	0.472	0.476	0.479	0.483	0.487	0.492	0.497	0.502	0.508	0.514	0.521	0.527	0.536	0.545	0.554	0.561	0.569	0.577	0.584	0.591	0.599	0.607	0.615	0.622	0.63	0.638	0.646	0.653	0.659	0.666	0.673	0.679
Jharkhand, Rural	0.233	0.236	0.239	0.242	0.245	0.248	0.251	0.255	0.259	0.264	0.269	0.274	0.279	0.285	0.292	0.299	0.305	0.313	0.321	0.33	0.338	0.346	0.354	0.362	0.37	0.379	0.387	0.397	0.405	0.415	0.425	0.436	0.448	0.459	0.472	0.485	0.496
Karnataka, Urban	0.391	0.398	0.404	0.412	0.42	0.427	0.434	0.441	0.449	0.456	0.463	0.471	0.478	0.486	0.495	0.505	0.515	0.525	0.536	0.546	0.557	0.565	0.574	0.581	0.589	0.598	0.607	0.616	0.624	0.631	0.639	0.647	0.655	0.664	0.672	0.681	0.688
Karnataka, Rural	0.251	0.256	0.26	0.264	0.269	0.274	0.28	0.286	0.292	0.299	0.306	0.314	0.322	0.33	0.339	0.348	0.357	0.367	0.377	0.387	0.397	0.406	0.415	0.423	0.432	0.442	0.453	0.464	0.475	0.485	0.496	0.507	0.518	0.529	0.54	0.552	0.561
Kerala, Urban	0.408	0.412	0.417	0.422	0.428	0.432	0.438	0.443	0.448	0.454	0.46	0.466	0.474	0.483	0.494	0.506	0.518	0.53	0.542	0.554	0.565	0.574	0.582	0.589	0.597	0.605	0.613	0.621	0.629	0.638	0.646	0.655	0.664	0.673	0.683	0.692	0.699
Kerala, Rural	0.358	0.363	0.368	0.374	0.38	0.385	0.391	0.397	0.404	0.411	0.418	0.425	0.433	0.442	0.452	0.464	0.476	0.488	0.5	0.513	0.524	0.534	0.543	0.551	0.559	0.568	0.577	0.586	0.595	0.605	0.613	0.623	0.632	0.642	0.653	0.662	0.669
Madhya Pradesh, Urban	0.403	0.409	0.415	0.422	0.428	0.435	0.441	0.448	0.455	0.461	0.469	0.476	0.483	0.49	0.497	0.504	0.511	0.517	0.522	0.527	0.531	0.535	0.538	0.541	0.545	0.55	0.557	0.564	0.572	0.581	0.591	0.6	0.609	0.618	0.626	0.635	0.643
Madhya Pradesh, Rural	0.229	0.232	0.236	0.24	0.245	0.249	0.254	0.26	0.266	0.272	0.28	0.286	0.292	0.296	0.3	0.305	0.309	0.314	0.318	0.322	0.325	0.331	0.336	0.342	0.349	0.355	0.362	0.37	0.378	0.388	0.399	0.411	0.423	0.436	0.451	0.465	0.478
Maharashtra, Urban	0.421	0.428	0.436	0.444	0.451	0.458	0.465	0.472	0.478	0.486	0.493	0.499	0.506	0.514	0.522	0.531	0.54	0.55	0.558	0.568	0.576	0.584	0.591	0.598	0.606	0.616	0.627	0.638	0.648	0.658	0.669	0.678	0.687	0.695	0.703	0.712	0.719
Maharashtra, Rural	0.288	0.295	0.301	0.308	0.314	0.322	0.329	0.337	0.345	0.354	0.364	0.373	0.382	0.391	0.4	0.41	0.421	0.43	0.439	0.449	0.457	0.464	0.472	0.48	0.488	0.498	0.51	0.522	0.533	0.544	0.556	0.567	0.577	0.586	0.596	0.607	0.615
Manipur, Urban	0.346	0.353	0.361	0.369	0.378	0.385	0.393	0.402	0.411	0.419	0.427	0.434	0.441	0.447	0.453	0.458	0.465	0.472	0.479	0.489	0.498	0.509	0.519	0.53	0.541	0.554	0.564	0.574	0.582	0.592	0.6	0.609	0.618	0.627	0.636	0.643	0.65
Manipur, Rural	0.265	0.272	0.278	0.285	0.292	0.299	0.306	0.315	0.324	0.332	0.341	0.349	0.356	0.362	0.367	0.371	0.375	0.381	0.387	0.397	0.408	0.419	0.43	0.442	0.455	0.469	0.482	0.494	0.504	0.515	0.525	0.535	0.545	0.555	0.565	0.575	0.585
Meghalaya, Urban	0.456	0.459	0.46	0.462	0.463	0.465	0.466	0.468	0.469	0.471	0.473	0.474	0.474	0.476	0.479	0.484	0.49	0.498	0.506	0.516	0.529	0.545	0.559	0.572	0.584	0.595	0.605	0.613	0.621	0.63	0.638	0.648	0.657	0.667	0.677	0.687	0.696
Meghalaya, Rural	0.25	0.254	0.258	0.263	0.268	0.273	0.279	0.286	0.292	0.299	0.307	0.313	0.319	0.323	0.325	0.328	0.331	0.336	0.342	0.352	0.365	0.379	0.393	0.406	0.417	0.429	0.44	0.451	0.461	0.472	0.483	0.495	0.505	0.516	0.528	0.54	0.55
Mizoram, Urban	0.383	0.389	0.395	0.401	0.408	0.417	0.426	0.437	0.446	0.453	0.459	0.466	0.472	0.479	0.485	0.492	0.5	0.508	0.514	0.522	0.531	0.54	0.548	0.555	0.562	0.568	0.575	0.581	0.587	0.593	0.601	0.608	0.616	0.625	0.633	0.643	0.651
Mizoram, Rural	0.292	0.298	0.303	0.309	0.315	0.325	0.334	0.346	0.355	0.363	0.37	0.378	0.385	0.392	0.395	0.398	0.404	0.408	0.413	0.42	0.43	0.44	0.451	0.461	0.472	0.482	0.491	0.502	0.512	0.523	0.535	0.546	0.557	0.568	0.58	0.591	0.6
Nagaland, Urban	0.446	0.451	0.458	0.466	0.473	0.48	0.486	0.494	0.501	0.507	0.513	0.517	0.521	0.525	0.527	0.53	0.532	0.536	0.54	0.544	0.553	0.564	0.576	0.586	0.595	0.604	0.612	0.622	0.632	0.641	0.65	0.66	0.67	0.68	0.691	0.702	0.711
Nagaland, Rural	0.328	0.333	0.338	0.343	0.348	0.353	0.357	0.363	0.368	0.374	0.379	0.383	0.387	0.39	0.393	0.395	0.399	0.405	0.412	0.42	0.435	0.452	0.469	0.484	0.498	0.512	0.524	0.537	0.55	0.563	0.575	0.588	0.6	0.611	0.622	0.629	0.637
Odisha, Urban	0.37	0.376	0.382	0.389	0.396	0.403	0.411	0.417	0.424	0.432	0.438	0.444	0.45	0.455	0.461	0.469	0.474	0.481	0.488	0.496	0.504	0.512	0.519	0.527	0.536	0.544	0.552	0.561	0.569	0.575	0.583	0.591	0.599	0.608	0.617	0.627	0.635
Odisha, Rural	0.233	0.237	0.24	0.245	0.248	0.253	0.259	0.263	0.269	0.276	0.281	0.287	0.293	0.299	0.305	0.312	0.318	0.325	0.333	0.341	0.35	0.358	0.366	0.376	0.387	0.397	0.408	0.42	0.432	0.443	0.455	0.467	0.479	0.49	0.502	0.514	0.524
Punjab, Urban	0.441	0.449	0.456	0.463	0.47	0.477	0.484	0.491	0.498	0.506	0.513	0.519	0.526	0.533	0.541	0.548	0.556	0.564	0.571	0.578	0.585	0.592	0.598	0.605	0.611	0.619	0.627	0.636	0.645	0.654	0.662	0.671	0.679	0.688	0.696	0.703	0.709
Punjab, Rural	0.286	0.293	0.3	0.307	0.314	0.322	0.33	0.338	0.346	0.355	0.364	0.373	0.381	0.39	0.399	0.408	0.418	0.427	0.437	0.447	0.457	0.466	0.474	0.483	0.492	0.502	0.513	0.524	0.535	0.546	0.556	0.566	0.577	0.587	0.596	0.604	0.611
Rajasthan, Urban	0.374	0.379	0.384	0.39	0.396	0.401	0.407	0.412	0.419	0.425	0.433	0.44	0.446	0.452	0.459	0.465	0.473	0.481	0.489	0.499	0.507	0.516	0.523	0.531	0.539	0.546	0.554	0.561	0.569	0.576	0.585	0.594	0.602	0.61	0.618	0.626	0.632
Rajasthan, Rural	0.218	0.222	0.226	0.232	0.236	0.241	0.246	0.251	0.258	0.265	0.273	0.28	0.286	0.292	0.299	0.305	0.312	0.318	0.324	0.331	0.337	0.345	0.352	0.36	0.368	0.376	0.385	0.395	0.404	0.412	0.423	0.434	0.445	0.456	0.467	0.478	0.487
Sikkim, Urban	0.371	0.377	0.386	0.394	0.404	0.414	0.426	0.437	0.448	0.459	0.469	0.477	0.483	0.491	0.498	0.504	0.511	0.518	0.525	0.533	0.542	0.551	0.56	0.57	0.579	0.588	0.595	0.601	0.605	0.618	0.628	0.639	0.649	0.66	0.672	0.685	0.695
Sikkim, Rural	0.257	0.261	0.266	0.271	0.276	0.283	0.29	0.297	0.305	0.313	0.321	0.329	0.335	0.343	0.352	0.36	0.37	0.38	0.39	0.4	0.411	0.422	0.434	0.445	0.457	0.469	0.479	0.489	0.496	0.513	0.527	0.542	0.555	0.569	0.584	0.598	0.61
Tamil Nadu, Urban	0.391	0.398	0.403	0.408	0.413	0.419	0.425	0.432	0.439	0.446	0.453	0.459	0.467	0.476	0.486	0.495	0.505	0.515	0.526	0.537	0.547	0.557	0.565	0.573	0.581	0.59	0.599	0.609	0.617	0.627	0.636	0.646	0.656	0.667	0.678	0.686	0.693
Tamil Nadu, Rural	0.264	0.27	0.274	0.279	0.285	0.292	0.299	0.307	0.315	0.324	0.333	0.341	0.35	0.359	0.369	0.379	0.389	0.399	0.411	0.422	0.433	0.443	0.453	0.462	0.472	0.483	0.494	0.506	0.516	0.528	0.539	0.551	0.562	0.574	0.586	0.595	0.603
Telangana, Urban	0.37	0.376	0.382	0.389	0.396	0.402	0.409	0.416	0.424	0.432	0.44	0.449	0.457	0.465	0.474	0.483	0.494	0.503	0.514	0.526	0.539	0.55	0.56	0.569	0.578	0.587	0.596	0.607	0.618	0.626	0.637	0.647	0.658	0.668	0.679	0.689	0.698
Telangana, Rural	0.225	0.228	0.231	0.234	0.237	0.24	0.244	0.248	0.253	0.259	0.265	0.272	0.28	0.289	0.298	0.308	0.32	0.33	0.342	0.353	0.364	0.375	0.385	0.396	0.406	0.417	0.429	0.441	0.454	0.466	0.48	0.493	0.506	0.519	0.533	0.547	0.558
Tripura, Urban	0.447	0.45	0.453	0.455	0.456	0.458	0.459	0.46	0.463	0.465	0.467	0.469	0.469	0.474	0.479	0.485	0.493	0.501	0.507	0.519	0.53	0.542	0.549	0.555	0.559	0.563	0.567	0.57	0.574	0.579	0.584	0.592	0.599	0.608	0.617	0.627	0.635
Tripura, Rural	0.294	0.298	0.302	0.306	0.31	0.313	0.317	0.321	0.326	0.331	0.336	0.34	0.344	0.351	0.357	0.365	0.373	0.382	0.39	0.403	0.416	0.429	0.439	0.448	0.455	0.461	0.467	0.472	0.479	0.486	0.493	0.501	0.509	0.518	0.528	0.538	0.546
Uttar Pradesh, Urban	0.367	0.371	0.377	0.382	0.387	0.393	0.399	0.404	0.41	0.417	0.423	0.43	0.435	0.44	0.446	0.451	0.457	0.463	0.468	0.473	0.478	0.484	0.49	0.496	0.502	0.509	0.516	0.524	0.531	0.538	0.546	0.554	0.562	0.57	0.578	0.587	0.594
Uttar Pradesh, Rural	0.24	0.243	0.247	0.251	0.255	0.259	0.264	0.27	0.276	0.282	0.289	0.295	0.3	0.304	0.308	0.311	0.314	0.317	0.32	0.323	0.327	0.332	0.339	0.347	0.355	0.364	0.372	0.381	0.389	0.398	0.407	0.417	0.429	0.441	0.454	0.468	0.479
Uttarakhand, Urban	0.401	0.406	0.412	0.417	0.422	0.427	0.432	0.437	0.442	0.448	0.454	0.459	0.464	0.469	0.476	0.482	0.489	0.496	0.503	0.511	0.519	0.528	0.537	0.547	0.556	0.567	0.578	0.589	0.6	0.615	0.628	0.642	0.656	0.669	0.682	0.695	0.706
Uttarakhand, Rural	0.223	0.227	0.231	0.235	0.24	0.245	0.25	0.256	0.263	0.269	0.277	0.284	0.292	0.3	0.309	0.318	0.328	0.338	0.349	0.36	0.372	0.385	0.397	0.411	0.424	0.439	0.453	0.468	0.482	0.499	0.515	0.531	0.547	0.562	0.577	0.592	0.604
West Bengal, Urban	0.44	0.444	0.45	0.455	0.461	0.466	0.471	0.476	0.481	0.486	0.491	0.496	0.501	0.507	0.513	0.52	0.527	0.535	0.543	0.552	0.56	0.568	0.574	0.58	0.586	0.591	0.597	0.604	0.612	0.619	0.625	0.632	0.638	0.644	0.651	0.658	0.665
West Bengal, Rural	0.275	0.279	0.281	0.284	0.288	0.291	0.295	0.299	0.303	0.307	0.312	0.317	0.323	0.329	0.335	0.343	0.351	0.358	0.367	0.376	0.384	0.392	0.4	0.407	0.414	0.422	0.43	0.441	0.451	0.462	0.473	0.483	0.493	0.504	0.514	0.525	0.533
The Six Minor Territories, Rural	0.293	0.299	0.306	0.312	0.319	0.326	0.333	0.34	0.348	0.355	0.363	0.371	0.379	0.387	0.397	0.406	0.416	0.428	0.439	0.451	0.462	0.474	0.487	0.499	0.512	0.525	0.538	0.552	0.565	0.579	0.591	0.603	0.61	0.617	0.623	0.63	0.637
The Six Minor Territories, Urban	0.477	0.483	0.488	0.493	0.497	0.501	0.506	0.51	0.514	0.519	0.523	0.528	0.533	0.538	0.544	0.55	0.557	0.565	0.572	0.579	0.586	0.593	0.6	0.607	0.614	0.622	0.63	0.638	0.645	0.654	0.661	0.669	0.677	0.685	0.693	0.702	0.71
Sub-Saharan Africa	0.263	0.268	0.272	0.277	0.282	0.287	0.291	0.295	0.298	0.302	0.307	0.311	0.316	0.321	0.325	0.329	0.334	0.339	0.343	0.346	0.35	0.354	0.358	0.362	0.366	0.371	0.377	0.383	0.389	0.395	0.401	0.408	0.416	0.423	0.43	0.437	0.442
Central Sub-Saharan Africa	0.259	0.262	0.266	0.271	0.275	0.28	0.283	0.287	0.291	0.295	0.298	0.302	0.305	0.306	0.307	0.308	0.31	0.312	0.314	0.315	0.316	0.317	0.32	0.322	0.327	0.332	0.338	0.345	0.352	0.359	0.366	0.374	0.382	0.388	0.394	0.401	0.406
Angola	0.218	0.22	0.223	0.225	0.229	0.232	0.235	0.239	0.243	0.248	0.252	0.256	0.26	0.262	0.265	0.268	0.272	0.276	0.281	0.286	0.289	0.294	0.299	0.305	0.312	0.32	0.329	0.339	0.349	0.357	0.366	0.377	0.388	0.4	0.411	0.422	0.431
the Central African Republic	0.212	0.216	0.22	0.222	0.226	0.23	0.233	0.236	0.24	0.243	0.247	0.25	0.252	0.254	0.258	0.261	0.263	0.266	0.269	0.273	0.276	0.279	0.283	0.285	0.288	0.291	0.295	0.299	0.303	0.307	0.311	0.316	0.322	0.32	0.319	0.318	0.316
Congo (Brazzaville)	0.3	0.308	0.317	0.326	0.335	0.344	0.351	0.358	0.365	0.372	0.379	0.386	0.393	0.399	0.404	0.41	0.415	0.421	0.426	0.43	0.434	0.439	0.444	0.45	0.456	0.462	0.467	0.471	0.475	0.48	0.487	0.495	0.503	0.511	0.519	0.526	0.531
Democratic Republic of the Congo	0.246	0.249	0.253	0.257	0.262	0.267	0.271	0.274	0.277	0.281	0.283	0.284	0.285	0.284	0.282	0.28	0.277	0.273	0.268	0.264	0.257	0.251	0.246	0.243	0.242	0.243	0.242	0.241	0.242	0.244	0.251	0.26	0.27	0.282	0.292	0.304	0.312
Equatorial Guinea	0.242	0.252	0.26	0.268	0.275	0.279	0.282	0.284	0.286	0.289	0.29	0.292	0.297	0.303	0.311	0.321	0.343	0.382	0.409	0.433	0.459	0.486	0.509	0.528	0.548	0.565	0.579	0.592	0.605	0.614	0.624	0.633	0.643	0.651	0.658	0.665	0.669
Gabon	0.38	0.389	0.397	0.406	0.414	0.422	0.429	0.435	0.442	0.45	0.458	0.466	0.474	0.482	0.49	0.498	0.506	0.514	0.521	0.527	0.531	0.534	0.539	0.545	0.551	0.558	0.564	0.57	0.576	0.581	0.586	0.592	0.599	0.605	0.611	0.617	0.622
Eastern Sub-Saharan Africa	0.176	0.181	0.185	0.19	0.196	0.201	0.207	0.212	0.217	0.222	0.228	0.234	0.239	0.244	0.249	0.254	0.259	0.264	0.268	0.273	0.277	0.282	0.286	0.291	0.296	0.302	0.308	0.314	0.321	0.329	0.337	0.345	0.354	0.363	0.371	0.38	0.387
Burundi	0.131	0.137	0.142	0.148	0.154	0.16	0.166	0.172	0.178	0.183	0.188	0.193	0.197	0.2	0.202	0.202	0.202	0.201	0.202	0.202	0.204	0.205	0.208	0.21	0.214	0.217	0.221	0.224	0.229	0.234	0.24	0.246	0.252	0.259	0.266	0.272	0.277
Comoros	0.208	0.213	0.219	0.225	0.232	0.239	0.246	0.253	0.26	0.268	0.275	0.282	0.289	0.297	0.303	0.309	0.315	0.321	0.325	0.33	0.335	0.34	0.346	0.352	0.359	0.366	0.373	0.381	0.388	0.395	0.403	0.41	0.419	0.427	0.436	0.443	0.449
Djibouti	0.089	0.1	0.11	0.121	0.135	0.148	0.16	0.171	0.181	0.19	0.199	0.207	0.215	0.223	0.23	0.237	0.244	0.252	0.258	0.265	0.272	0.28	0.288	0.296	0.304	0.312	0.321	0.331	0.34	0.35	0.359	0.369	0.379	0.389	0.399	0.409	0.416
Eritrea	0.143	0.146	0.15	0.154	0.158	0.163	0.167	0.172	0.177	0.182	0.187	0.193	0.2	0.208	0.218	0.229	0.241	0.254	0.267	0.279	0.29	0.3	0.309	0.315	0.321	0.326	0.33	0.335	0.338	0.342	0.346	0.352	0.359	0.367	0.375	0.383	0.389
Ethiopia	0.122	0.125	0.128	0.132	0.136	0.139	0.142	0.145	0.148	0.15	0.153	0.156	0.158	0.161	0.163	0.166	0.171	0.175	0.179	0.182	0.187	0.192	0.196	0.2	0.205	0.212	0.22	0.229	0.239	0.25	0.26	0.272	0.284	0.295	0.306	0.317	0.326

Appendix Table 9: Socio-demographic Index values for all estimated GBD 2016 locations, 1980–2016

Location	1980	1981	1982	1983	1984	1985	1986	1987	1988	1989	1990	1991	1992	1993	1994	1995	1996	1997	1998	1999	2000	2001	2002	2003	2004	2005	2006	2007	2008	2009	2010	2011	2012	2013	2014	2015	2016
Kenya	0.22	0.232	0.245	0.257	0.269	0.28	0.291	0.302	0.313	0.323	0.335	0.346	0.357	0.367	0.376	0.384	0.393	0.4	0.405	0.41	0.415	0.42	0.425	0.429	0.434	0.44	0.446	0.452	0.459	0.465	0.473	0.481	0.489	0.498	0.506	0.514	0.52
Madagascar	0.195	0.201	0.207	0.213	0.219	0.226	0.232	0.238	0.244	0.249	0.255	0.26	0.264	0.267	0.269	0.272	0.275	0.278	0.282	0.286	0.289	0.294	0.297	0.302	0.306	0.311	0.315	0.319	0.323	0.327	0.331	0.338	0.344	0.35	0.355	0.361	0.365
Malawi	0.143	0.149	0.155	0.161	0.167	0.175	0.182	0.188	0.194	0.199	0.205	0.21	0.215	0.219	0.223	0.227	0.232	0.238	0.243	0.249	0.254	0.258	0.263	0.268	0.273	0.279	0.284	0.29	0.296	0.302	0.308	0.314	0.32	0.326	0.334	0.342	0.348
Mozambique	0.131	0.133	0.135	0.136	0.137	0.138	0.138	0.139	0.14	0.142	0.145	0.149	0.151	0.153	0.157	0.158	0.165	0.172	0.18	0.186	0.193	0.2	0.207	0.214	0.22	0.226	0.231	0.236	0.242	0.247	0.253	0.26	0.268	0.277	0.287	0.297	0.306
Rwanda	0.065	0.067	0.068	0.069	0.07	0.071	0.089	0.12	0.144	0.164	0.181	0.197	0.208	0.217	0.22	0.223	0.226	0.23	0.234	0.237	0.24	0.244	0.25	0.256	0.263	0.272	0.282	0.293	0.305	0.318	0.33	0.342	0.354	0.365	0.374	0.383	0.39
Somalia	0.161	0.162	0.164	0.166	0.168	0.171	0.173	0.176	0.18	0.183	0.187	0.19	0.192	0.194	0.195	0.196	0.197	0.198	0.199	0.2	0.201	0.203	0.205	0.208	0.212	0.216	0.22	0.225	0.23	0.235	0.24	0.245	0.251	0.256	0.26	0.265	0.268
Tanzania	0.176	0.183	0.19	0.197	0.205	0.213	0.22	0.226	0.233	0.239	0.245	0.251	0.256	0.259	0.263	0.271	0.279	0.288	0.295	0.302	0.308	0.314	0.32	0.326	0.332	0.339	0.345	0.352	0.359	0.367	0.375	0.384	0.392	0.402	0.411	0.421	0.428
Uganda	0.101	0.11	0.117	0.125	0.134	0.142	0.149	0.156	0.163	0.17	0.177	0.184	0.19	0.196	0.203	0.209	0.215	0.22	0.226	0.232	0.238	0.244	0.251	0.257	0.265	0.272	0.28	0.289	0.298	0.308	0.317	0.328	0.338	0.348	0.359	0.37	0.377
Zambia	0.193	0.205	0.216	0.228	0.239	0.251	0.261	0.271	0.281	0.291	0.299	0.307	0.315	0.321	0.326	0.331	0.336	0.34	0.343	0.346	0.35	0.354	0.357	0.362	0.366	0.371	0.377	0.385	0.393	0.401	0.41	0.419	0.428	0.437	0.446	0.454	0.46
South Sudan	0.053	0.054	0.054	0.055	0.056	0.056	0.057	0.062	0.07	0.077	0.082	0.088	0.092	0.095	0.098	0.102	0.105	0.108	0.111	0.114	0.117	0.12	0.123	0.127	0.13	0.134	0.138	0.143	0.148	0.152	0.158	0.164	0.17	0.176	0.182	0.188	0.192
Central	0.292	0.305	0.318	0.33	0.343	0.356	0.368	0.379	0.39	0.401	0.413	0.425	0.435	0.444	0.453	0.462	0.47	0.476	0.482	0.487	0.492	0.497	0.501	0.505	0.51	0.515	0.521	0.527	0.533	0.54	0.546	0.553	0.56	0.566	0.573	0.58	0.585
Coast	0.146	0.16	0.172	0.185	0.196	0.205	0.214	0.222	0.231	0.241	0.252	0.264	0.275	0.286	0.296	0.305	0.313	0.321	0.327	0.332	0.337	0.343	0.348	0.354	0.361	0.368	0.376	0.385	0.394	0.403	0.413	0.423	0.434	0.444	0.455	0.465	0.473
Eastern	0.196	0.209	0.221	0.233	0.245	0.256	0.266	0.277	0.287	0.298	0.31	0.322	0.333	0.344	0.353	0.362	0.371	0.379	0.385	0.391	0.396	0.401	0.407	0.412	0.418	0.424	0.431	0.438	0.445	0.452	0.46	0.467	0.475	0.483	0.49	0.498	0.503
Nairobi	0.42	0.431	0.442	0.453	0.464	0.475	0.486	0.496	0.506	0.516	0.526	0.536	0.545	0.553	0.56	0.567	0.574	0.58	0.585	0.589	0.593	0.598	0.602	0.607	0.611	0.617	0.623	0.63	0.637	0.644	0.652	0.66	0.669	0.677	0.685	0.694	0.701
North Eastern	0.049	0.05	0.051	0.052	0.053	0.053	0.054	0.055	0.056	0.057	0.058	0.059	0.061	0.062	0.067	0.075	0.08	0.081	0.076	0.068	0.069	0.07	0.071	0.072	0.073	0.074	0.075	0.077	0.078	0.079	0.08	0.081	0.083	0.084	0.092	0.112	0.124
Nyanza	0.204	0.216	0.229	0.241	0.253	0.264	0.274	0.285	0.295	0.306	0.318	0.331	0.343	0.353	0.364	0.373	0.383	0.391	0.398	0.404	0.411	0.417	0.423	0.429	0.436	0.443	0.45	0.458	0.466	0.475	0.483	0.491	0.5	0.508	0.516	0.524	0.53
Rift Valley	0.173	0.188	0.202	0.216	0.228	0.239	0.249	0.259	0.269	0.28	0.292	0.305	0.316	0.327	0.337	0.347	0.356	0.363	0.37	0.375	0.381	0.386	0.392	0.397	0.403	0.41	0.417	0.424	0.432	0.44	0.448	0.457	0.465	0.474	0.482	0.491	0.497
Western	0.228	0.24	0.251	0.263	0.274	0.284	0.294	0.303	0.312	0.322	0.332	0.343	0.353	0.362	0.37	0.378	0.385	0.391	0.396	0.4	0.404	0.408	0.412	0.416	0.421	0.426	0.431	0.437	0.443	0.45	0.456	0.463	0.47	0.477	0.484	0.491	0.496
Kiambu	0.313	0.327	0.341	0.355	0.368	0.382	0.394	0.406	0.418	0.429	0.441	0.452	0.462	0.47	0.478	0.486	0.493	0.498	0.503	0.507	0.511	0.515	0.519	0.523	0.527	0.531	0.536	0.541	0.547	0.553	0.559	0.566	0.574	0.581	0.588	0.596	0.601
Kirinyaga	0.28	0.292	0.303	0.314	0.326	0.337	0.348	0.358	0.369	0.379	0.389	0.399	0.408	0.417	0.427	0.437	0.446	0.454	0.461	0.467	0.473	0.479	0.484	0.49	0.495	0.501	0.508	0.514	0.521	0.527	0.533	0.54	0.546	0.552	0.558	0.564	0.569
Murang'a	0.278	0.29	0.303	0.315	0.328	0.34	0.352	0.363	0.374	0.384	0.395	0.406	0.415	0.424	0.432	0.44	0.447	0.454	0.461	0.466	0.472	0.477	0.482	0.487	0.492	0.497	0.503	0.509	0.515	0.52	0.526	0.531	0.537	0.542	0.547	0.552	0.556
Nyandarua	0.263	0.275	0.287	0.3	0.312	0.325	0.337	0.348	0.36	0.371	0.383	0.395	0.405	0.415	0.423	0.431	0.439	0.446	0.452	0.457	0.462	0.466	0.47	0.474	0.479	0.484	0.489	0.496	0.503	0.51	0.518	0.526	0.534	0.542	0.55	0.558	0.564
Nyeri	0.292	0.305	0.318	0.332	0.345	0.358	0.37	0.382	0.394	0.407	0.419	0.432	0.443	0.453	0.462	0.47	0.477	0.483	0.488	0.492	0.496	0.5	0.503	0.507	0.512	0.517	0.524	0.531	0.538	0.546	0.553	0.56	0.566	0.572	0.577	0.583	0.587
Kilifi	0.085	0.086	0.087	0.088	0.089	0.09	0.098	0.111	0.122	0.134	0.15	0.166	0.182	0.197	0.212	0.226	0.238	0.249	0.258	0.266	0.273	0.28	0.286	0.291	0.295	0.301	0.306	0.313	0.321	0.33	0.341	0.352	0.364	0.375	0.387	0.398	0.406
Kwale	0.13	0.141	0.151	0.161	0.17	0.177	0.185	0.192	0.2	0.209	0.221	0.234	0.246	0.255	0.261	0.266	0.271	0.275	0.278	0.28	0.281	0.283	0.285	0.289	0.294	0.3	0.306	0.314	0.324	0.334	0.346	0.357	0.369	0.381	0.393	0.404	0.412
Lamu	0.168	0.181	0.193	0.205	0.215	0.224	0.233	0.241	0.249	0.255	0.262	0.269	0.276	0.283	0.292	0.3	0.309	0.317	0.325	0.332	0.338	0.344	0.349	0.355	0.36	0.365	0.371	0.376	0.381	0.388	0.397	0.406	0.415	0.425	0.434	0.444	0.45
Mombasa	0.246	0.259	0.271	0.283	0.294	0.305	0.316	0.326	0.337	0.347	0.36	0.372	0.384	0.394	0.404	0.413	0.421	0.429	0.435	0.44	0.444	0.448	0.452	0.457	0.463	0.47	0.477	0.484	0.491	0.499	0.508	0.518	0.529	0.539	0.549	0.56	0.568
TaitaTaveta	0.156	0.172	0.186	0.2	0.212	0.223	0.233	0.242	0.252	0.263	0.276	0.29	0.303	0.315	0.327	0.339	0.349	0.359	0.367	0.374	0.38	0.386	0.392	0.399	0.408	0.417	0.426	0.435	0.444	0.453	0.464	0.474	0.484	0.494	0.504	0.513	0.52
TanaRiver	0.064	0.065	0.066	0.077	0.084	0.088	0.09	0.092	0.093	0.097	0.107	0.12	0.132	0.145	0.157	0.167	0.176	0.183	0.188	0.189	0.187	0.185	0.182	0.179	0.175	0.172	0.169	0.168	0.171	0.178	0.191	0.204	0.219	0.234	0.248	0.261	0.271
Embu	0.237	0.25	0.262	0.274	0.287	0.298	0.31	0.32	0.331	0.342	0.353	0.365	0.375	0.386	0.396	0.407	0.417	0.426	0.434	0.441	0.449	0.456	0.463	0.469	0.474	0.48	0.486	0.493	0.499	0.506	0.514	0.521	0.529	0.536	0.543	0.55	0.555
Isiolo	0.205	0.212	0.219	0.226	0.233	0.24	0.246	0.252	0.258	0.264	0.27	0.277	0.284	0.289	0.295	0.3	0.306	0.311	0.315	0.32	0.325	0.33	0.334	0.336	0.336	0.335	0.334	0.334	0.336	0.339	0.343	0.348	0.354	0.36	0.366	0.372	0.377
Kitui	0.145	0.158	0.171	0.183	0.195	0.207	0.217	0.227	0.237	0.246	0.255	0.265	0.274	0.283	0.293	0.302	0.311	0.319	0.327	0.333	0.339	0.345	0.351	0.357	0.364	0.371	0.378	0.386	0.394	0.402	0.411	0.42	0.429	0.438	0.447	0.455	0.461
Machakos	0.194	0.21	0.225	0.24	0.253	0.266	0.278	0.29	0.302	0.315	0.33	0.345	0.358	0.371	0.382	0.393	0.403	0.412	0.42	0.426	0.432	0.437	0.442	0.447	0.453	0.459	0.466	0.474	0.482	0.49	0.497	0.505	0.512	0.519	0.526	0.533	0.538
Makueni	0.154	0.175	0.193	0.21	0.225	0.239	0.252	0.264	0.276	0.288	0.301	0.314	0.325	0.336	0.344	0.351	0.358	0.363	0.367	0.369	0.369	0.369	0.37	0.373	0.378	0.385	0.393	0.402	0.411	0.422	0.432	0.442	0.451	0.46	0.468	0.475	0.48
Marsabit	0.184	0.189	0.193	0.198	0.203	0.207	0.212	0.216	0.22	0.224	0.229	0.234	0.238	0.242	0.246	0.25	0.254	0.257	0.261	0.264	0.268	0.272	0.277	0.282	0.288	0.293	0.299	0.304	0.308	0.312	0.315	0.318	0.322	0.326	0.33	0.335	0.339
Meru	0.209	0.221	0.232	0.243	0.254	0.264	0.274	0.284	0.294	0.305	0.318	0.332	0.345	0.356	0.367	0.377	0.386	0.395	0.402	0.408	0.413	0.419	0.425	0.43	0.435	0.44	0.445	0.451	0.455	0.461	0.467	0.475	0.482	0.49	0.498	0.505	0.512
TharakaNithi	0.204	0.216	0.227	0.238	0.25	0.26	0.271	0.282	0.293	0.303	0.315	0.327	0.338	0.349	0.359	0.368	0.377	0.386	0.393	0.4	0.407	0.413	0.42	0.428	0.435	0.444	0.452	0.462	0.471	0.48	0.489	0.497	0.506	0.514	0.522	0.53	0.536
Nairobi	0.42	0.431	0.442	0.453	0.464	0.475	0.486	0.496	0.506	0.516	0.526	0.536	0.545	0.553	0.56	0.567	0.574	0.58	0.585	0.589	0.593	0.598	0.602	0.607	0.611	0.617	0.623	0.63	0.637	0.644	0.652	0.66	0.669	0.677	0.685	0.694	0.701
Garissa	0.052	0.053	0.054	0.055	0.056	0.057	0.058	0.059	0.06	0.061	0.062	0.064	0.065	0.069	0.075	0.077	0.077	0.074	0.073	0.074	0.075	0.077	0.078	0.079	0.08	0.081	0.083	0.084	0.106	0.127	0.141	0.154	0.166	0.178	0.19	0.202	0.21
Mandera	0.045	0.046	0.047	0.048	0.049	0.05	0.051	0.052	0.053	0.054	0.055	0.057	0.065	0.079	0.088	0.095	0.1	0.103	0.102	0.095	0.086	0.074	0.07	0.072	0.073	0.074	0.075	0.076	0.077	0.077	0.079	0.08	0.081	0.083	0.085	0.087	0.089
Wajir	0.048	0.049	0.05	0.05	0.051	0.052	0.052	0.053	0.054	0.055	0.056	0.057	0.058	0.059	0.06	0.061	0.062	0.062	0.063	0.064	0.065	0.065	0.066	0.067	0.067	0.068	0.069	0.07	0.071	0.072	0.073	0.074	0.075	0.083	0.101	0.115	0.124
HomaBay	0.173	0.188	0.202	0.216	0.229	0.24	0.251	0.261	0.271	0.281	0.291	0.301	0.311	0.32	0.329	0.338	0.347	0.354	0.361	0.368	0.374	0.38	0.385	0.39	0.395	0.401	0.408	0.416	0.425	0.435	0.445	0.455	0.464	0.473	0.481	0.488	0.493
Kisii	0.206	0.218	0.23	0.242	0.254	0.264	0.275	0.285	0.296	0.308	0.322	0.336	0.349	0.362	0.375	0.387	0.399	0.409	0.417	0.425	0.432	0.439	0.447	0.454	0.462	0.47	0.478	0.487	0.495	0.502	0.51	0.518	0.526	0.534	0.542	0.55	0.556
Kisumu	0.214	0.228	0.241	0.254	0.266	0.278	0.29	0.301	0.313	0.326	0.341	0.355	0.369	0.38	0.388	0.396	0.403	0.409	0.413	0.417	0.421	0.425	0.43	0.435	0.441	0.448	0.456	0.465	0.475	0.485	0.495	0.504	0.512	0.521	0.529	0.536	0.542
Migori	0.18	0.194	0.208	0.22	0.233	0.244	0.255	0.264	0.274	0.283	0.294	0.304	0.314	0.324	0.334	0.343	0.353	0.362	0.37	0.378	0.386	0.395	0.404	0.411	0.417	0.422	0.428	0.435	0.44	0.445	0.451	0.458	0.465	0.473	0.48	0.488	0.494
Nyamira	0.268	0.28	0.292	0.304	0.316	0.328	0.34	0.351	0.363	0.374	0.385	0.397	0.409	0.419	0.43	0.44	0.449	0.458	0.464	0.47	0.476	0.483	0.49	0.496	0.501	0.507	0.514	0.521	0.528	0.537	0.545	0.555	0.563	0.572	0.58	0.588	0.594
Siaya	0.166	0.176	0.186	0.196	0.206	0.215	0.225	0.234	0.244	0.253	0.264	0.275	0.285	0.295	0.305	0.314	0.324	0.333	0.34	0.347	0.355	0.363	0.371	0.38	0.39	0.401	0.411	0.422	0.432	0.442	0.451	0.46	0.47	0.479	0.489	0.498	0.505
Baringo	0.151	0.169	0.185	0.2	0.213	0.225	0.236	0.247	0.258	0.268	0.28	0.292	0.304	0.313	0.322	0.329	0.337	0.344	0.351	0.358	0.366	0.373	0.379	0.383	0.385	0.387	0.39	0.393	0.398	0.406	0.414	0.422	0.432	0.441	0.45	0.458	0.465
Bomet	0.099	0.108	0.143	0.169	0.189	0.204	0.218	0.231	0.243	0.254	0.268	0.281	0.293	0.305	0.316	0.327	0.338	0.348	0.357	0.366	0.377	0.388	0.398	0.409	0.418	0.427	0.436	0.446	0.454	0.462	0.47	0.479	0.488	0.497	0.506	0.514	0.521
Elgeyo-Marakwet	0.149	0.167	0.183	0.198	0.211	0.223	0.234	0.245	0.255	0.266	0.278	0.291	0.303	0.313	0.322	0.331	0.34	0.348	0.355	0.362	0.371	0.379	0.388	0.395	0.401	0.408	0.415	0.424	0.433	0.444	0.453	0.463	0.473	0.483	0.492	0.501	0.508
Kajiado	0.197	0.213	0.227	0.241	0.254	0.266	0.278	0.29	0.301	0.313	0.325	0.338	0.35	0.36	0.368	0.376	0.383	0.389	0.393	0.395	0.399	0.403	0.409	0.416	0.426	0.437	0.448	0.46	0.471	0.48	0.489	0.498	0.506	0.515	0.524	0.533	0.539
Kericho	0.094	0.096	0.108	0.135	0.154	0.169	0.183	0.196	0.208	0.224	0.242	0.261	0.278	0.295	0.313	0.328	0.343	0.356	0.368	0.378	0.388	0.397	0.406	0.413	0.42	0.428	0.436	0.445	0.455	0.466	0.476	0.487	0.498	0.509	0.519	0.529	0.536
Laikipia	0.229	0.241	0.253	0.265	0.276	0.287	0.297	0.307	0.317	0.327	0.338	0.349	0.36	0.372	0.386	0.398	0.41	0.42	0.428	0.435	0.442	0.449	0.456	0.463	0.47	0.478	0.487	0.496	0.507	0.518	0.527	0.537	0.547	0.556	0.566	0.575	0.582
Nakuru	0.208	0.221	0.232	0.244	0.255	0.266	0.277	0.287	0.298	0.309	0.321	0.333	0.345	0.355	0.365	0.373	0.382	0.389	0.394	0.398	0.403	0.408	0.413	0.42	0.43	0.441	0.453	0.466	0.479	0.492	0.503	0.515	0.526	0.537	0.546	0.556	0.563
Nandi	0.099	0.124	0.152	0.173	0.191	0.206	0.219	0.232	0.244	0.256	0.27	0.285	0.299	0.313	0.327	0.339	0.35	0.36	0.368	0.374	0.382	0.39	0.399	0.408	0.417	0.427	0.436	0.446	0.455	0.465	0.474	0.483	0.491	0.5	0.508	0.516	0.522
Narok	0.106	0.125	0.141	0.155	0.168	0.178	0.188	0.198	0.208	0.219	0.231	0.244	0.257	0.269	0.282	0.293	0.305	0.315	0.323	0.328	0.332	0.336	0.339	0.345	0.353	0.361	0.368	0.375	0.381	0.387	0.394	0.402	0.41	0.418	0.427	0.435	0.441
Samburu	0.169	0.175	0.181	0.187	0.192	0.196	0.201	0.205	0.209	0.212	0.216	0.22	0.224	0.227	0.229	0.231	0.233	0.235	0.235	0.235	0.236	0.238	0.241	0.245	0.251	0.257	0.264	0.27	0.275	0.281	0.286	0.292	0.297	0.302	0.308	0.313	0.316

Appendix Table 9: Socio-demographic Index values for all estimated GBD 2016 locations, 1980–2016

Location	1980	1981	1982	1983	1984	1985	1986	1987	1988	1989	1990	1991	1992	1993	1994	1995	1996	1997	1998	1999	2000	2001	2002	2003	2004	2005	2006	2007	2008	2009	2010	2011	2012	2013	2014	2015	2016
TransNzoia	0.103	0.137	0.161	0.18	0.197	0.21	0.222	0.234	0.246	0.26	0.278	0.296	0.313	0.328	0.341	0.353	0.364	0.374	0.382	0.39	0.398	0.405	0.412	0.418	0.422	0.427	0.432	0.438	0.445	0.453	0.462	0.471	0.48	0.489	0.498	0.506	0.513
Turkana	0.185	0.19	0.195	0.2	0.205	0.209	0.213	0.217	0.22	0.223	0.227	0.231	0.235	0.238	0.241	0.244	0.247	0.249	0.251	0.25	0.249	0.248	0.248	0.249	0.251	0.253	0.256	0.26	0.265	0.268	0.271	0.273	0.276	0.279	0.283	0.287	0.289
UasinGishu	0.183	0.202	0.22	0.237	0.252	0.266	0.279	0.291	0.303	0.316	0.329	0.342	0.355	0.366	0.376	0.386	0.395	0.404	0.413	0.422	0.432	0.443	0.453	0.462	0.47	0.478	0.486	0.495	0.504	0.513	0.522	0.53	0.539	0.548	0.556	0.564	0.57
WestPokot	0.153	0.16	0.168	0.176	0.183	0.19	0.196	0.203	0.211	0.219	0.23	0.24	0.25	0.258	0.264	0.27	0.275	0.279	0.28	0.281	0.283	0.284	0.286	0.289	0.292	0.296	0.301	0.308	0.314	0.321	0.328	0.335	0.343	0.351	0.359	0.367	0.373
Bungoma	0.199	0.214	0.228	0.242	0.254	0.266	0.277	0.287	0.296	0.305	0.316	0.327	0.336	0.345	0.354	0.362	0.37	0.378	0.385	0.392	0.399	0.406	0.413	0.42	0.426	0.432	0.438	0.444	0.45	0.456	0.462	0.469	0.477	0.484	0.492	0.499	0.505
Busia	0.235	0.246	0.256	0.266	0.276	0.285	0.294	0.303	0.312	0.321	0.331	0.341	0.35	0.359	0.365	0.371	0.377	0.382	0.385	0.387	0.39	0.393	0.396	0.399	0.402	0.406	0.41	0.415	0.421	0.427	0.433	0.44	0.447	0.454	0.461	0.468	0.473
Kakamega	0.23	0.241	0.252	0.263	0.273	0.283	0.292	0.302	0.311	0.321	0.332	0.344	0.355	0.365	0.374	0.383	0.39	0.397	0.402	0.405	0.408	0.411	0.414	0.417	0.421	0.426	0.431	0.438	0.444	0.45	0.457	0.463	0.47	0.477	0.484	0.491	0.496
Vihiga	0.258	0.27	0.281	0.293	0.304	0.315	0.325	0.334	0.344	0.352	0.361	0.37	0.378	0.385	0.391	0.397	0.401	0.405	0.408	0.409	0.41	0.412	0.415	0.418	0.422	0.428	0.436	0.446	0.456	0.467	0.476	0.485	0.493	0.5	0.507	0.513	0.517
Southern Sub-Saharan Africa	0.505	0.511	0.516	0.521	0.527	0.531	0.536	0.541	0.546	0.552	0.558	0.564	0.57	0.577	0.583	0.589	0.595	0.601	0.606	0.611	0.615	0.62	0.625	0.63	0.635	0.64	0.646	0.651	0.656	0.661	0.666	0.672	0.677	0.681	0.685	0.689	0.691
Botswana	0.404	0.414	0.423	0.432	0.441	0.451	0.461	0.471	0.484	0.497	0.508	0.518	0.527	0.536	0.544	0.552	0.56	0.569	0.578	0.587	0.595	0.602	0.609	0.616	0.623	0.631	0.638	0.645	0.65	0.656	0.662	0.669	0.675	0.682	0.689	0.695	0.701
Lesotho	0.323	0.328	0.333	0.337	0.341	0.345	0.349	0.353	0.358	0.364	0.37	0.376	0.383	0.39	0.397	0.403	0.41	0.417	0.423	0.429	0.435	0.442	0.448	0.455	0.462	0.469	0.475	0.482	0.489	0.496	0.504	0.513	0.521	0.529	0.538	0.545	0.552
Namibia	0.374	0.38	0.385	0.39	0.395	0.399	0.404	0.41	0.416	0.422	0.427	0.433	0.441	0.45	0.459	0.469	0.478	0.488	0.497	0.505	0.514	0.522	0.531	0.539	0.547	0.555	0.563	0.569	0.575	0.58	0.586	0.591	0.597	0.604	0.611	0.618	0.624
South Africa	0.542	0.548	0.554	0.559	0.564	0.569	0.574	0.579	0.584	0.59	0.596	0.601	0.606	0.613	0.619	0.624	0.63	0.636	0.64	0.645	0.649	0.653	0.658	0.663	0.669	0.675	0.682	0.688	0.695	0.701	0.707	0.714	0.719	0.724	0.727	0.73	0.734
Swaziland	0.384	0.391	0.398	0.404	0.41	0.415	0.421	0.429	0.437	0.446	0.454	0.462	0.47	0.477	0.484	0.491	0.498	0.504	0.509	0.514	0.518	0.522	0.525	0.528	0.531	0.534	0.537	0.538	0.538	0.539	0.544	0.553	0.562	0.571	0.578	0.584	0.589
Zimbabwe	0.357	0.364	0.37	0.377	0.384	0.39	0.397	0.403	0.412	0.422	0.432	0.443	0.452	0.461	0.47	0.477	0.486	0.493	0.498	0.502	0.505	0.507	0.508	0.506	0.503	0.498	0.492	0.485	0.477	0.472	0.469	0.47	0.474	0.479	0.485	0.491	0.497
Eastern Cape	0.495	0.501	0.506	0.511	0.516	0.521	0.524	0.528	0.533	0.538	0.543	0.547	0.551	0.557	0.562	0.567	0.573	0.578	0.583	0.587	0.591	0.596	0.601	0.606	0.612	0.618	0.626	0.633	0.64	0.647	0.653	0.66	0.665	0.67	0.673	0.676	0.679
Free State	0.546	0.551	0.556	0.56	0.564	0.568	0.572	0.576	0.581	0.586	0.591	0.597	0.602	0.609	0.617	0.624	0.631	0.637	0.641	0.645	0.649	0.652	0.656	0.661	0.666	0.672	0.678	0.684	0.69	0.696	0.702	0.708	0.714	0.718	0.721	0.724	0.727
Gauteng	0.656	0.66	0.664	0.667	0.671	0.674	0.677	0.681	0.684	0.688	0.692	0.695	0.698	0.702	0.705	0.707	0.709	0.712	0.715	0.718	0.721	0.724	0.728	0.731	0.735	0.74	0.744	0.749	0.754	0.759	0.763	0.768	0.773	0.775	0.778	0.78	0.782
KwaZulu-Natal	0.521	0.527	0.532	0.537	0.542	0.546	0.551	0.555	0.56	0.566	0.571	0.576	0.581	0.588	0.594	0.599	0.605	0.61	0.615	0.619	0.624	0.629	0.634	0.639	0.645	0.651	0.658	0.665	0.672	0.679	0.685	0.692	0.698	0.702	0.706	0.71	0.713
Limpopo	0.447	0.454	0.461	0.467	0.473	0.479	0.484	0.49	0.497	0.504	0.511	0.518	0.524	0.532	0.54	0.547	0.554	0.562	0.568	0.572	0.577	0.582	0.588	0.594	0.602	0.61	0.618	0.627	0.636	0.643	0.651	0.659	0.665	0.671	0.675	0.679	0.682
Mpumalanga	0.495	0.502	0.508	0.514	0.52	0.525	0.531	0.537	0.543	0.55	0.557	0.563	0.57	0.577	0.584	0.589	0.595	0.601	0.606	0.61	0.615	0.62	0.625	0.631	0.637	0.644	0.652	0.659	0.667	0.674	0.681	0.688	0.695	0.7	0.703	0.707	0.71
North-West	0.523	0.529	0.534	0.539	0.545	0.55	0.555	0.56	0.565	0.571	0.576	0.58	0.585	0.59	0.594	0.597	0.6	0.604	0.608	0.613	0.618	0.623	0.628	0.633	0.639	0.645	0.651	0.658	0.664	0.671	0.677	0.683	0.689	0.693	0.696	0.699	0.702
Northern Cape	0.545	0.55	0.555	0.559	0.564	0.568	0.572	0.576	0.58	0.585	0.59	0.594	0.598	0.603	0.607	0.61	0.614	0.619	0.623	0.626	0.63	0.633	0.637	0.641	0.646	0.652	0.658	0.664	0.67	0.675	0.681	0.688	0.693	0.697	0.7	0.704	0.707
Western Cape	0.653	0.657	0.66	0.662	0.665	0.668	0.67	0.672	0.675	0.678	0.681	0.684	0.687	0.691	0.695	0.698	0.703	0.708	0.711	0.713	0.715	0.717	0.72	0.722	0.725	0.728	0.732	0.736	0.74	0.744	0.748	0.752	0.756	0.758	0.76	0.762	0.764
Western Sub-Saharan Africa	0.254	0.259	0.263	0.268	0.272	0.276	0.279	0.281	0.282	0.285	0.287	0.29	0.293	0.297	0.301	0.305	0.31	0.314	0.318	0.322	0.326	0.33	0.335	0.339	0.345	0.351	0.357	0.364	0.37	0.376	0.383	0.391	0.399	0.407	0.415	0.423	0.428
Benin	0.171	0.175	0.178	0.182	0.187	0.192	0.197	0.202	0.207	0.212	0.217	0.222	0.227	0.233	0.237	0.242	0.247	0.252	0.257	0.262	0.267	0.273	0.278	0.283	0.288	0.293	0.298	0.304	0.31	0.315	0.32	0.326	0.33	0.335	0.34	0.346	0.351
Burkina Faso	0.103	0.106	0.108	0.109	0.111	0.113	0.115	0.117	0.12	0.123	0.125	0.13	0.136	0.141	0.146	0.15	0.155	0.16	0.165	0.169	0.173	0.178	0.182	0.186	0.191	0.195	0.2	0.206	0.212	0.217	0.225	0.234	0.244	0.253	0.262	0.269	0.274
Cameroon	0.283	0.289	0.296	0.302	0.309	0.316	0.322	0.327	0.332	0.337	0.341	0.345	0.349	0.353	0.356	0.36	0.364	0.369	0.374	0.379	0.383	0.388	0.392	0.396	0.4	0.403	0.406	0.409	0.412	0.415	0.419	0.423	0.428	0.433	0.439	0.445	0.451
Cape Verde	0.241	0.248	0.256	0.263	0.271	0.279	0.287	0.294	0.302	0.31	0.317	0.325	0.332	0.34	0.349	0.358	0.367	0.377	0.387	0.399	0.41	0.421	0.431	0.442	0.452	0.462	0.473	0.484	0.495	0.505	0.514	0.524	0.534	0.542	0.551	0.559	0.566
Chad	0.158	0.159	0.159	0.161	0.163	0.166	0.169	0.172	0.175	0.179	0.183	0.187	0.191	0.194	0.197	0.2	0.203	0.206	0.209	0.212	0.214	0.217	0.219	0.222	0.228	0.235	0.241	0.247	0.252	0.257	0.262	0.268	0.274	0.28	0.285	0.29	0.295
Cote d'Ivoire	0.225	0.226	0.227	0.228	0.23	0.234	0.241	0.249	0.258	0.267	0.277	0.287	0.297	0.306	0.313	0.32	0.327	0.332	0.337	0.341	0.345	0.348	0.351	0.353	0.355	0.358	0.36	0.363	0.366	0.369	0.373	0.377	0.382	0.388	0.394	0.401	0.407
The Gambia	0.205	0.21	0.215	0.221	0.226	0.232	0.237	0.242	0.247	0.251	0.254	0.255	0.255	0.255	0.254	0.254	0.255	0.259	0.264	0.269	0.274	0.28	0.285	0.29	0.295	0.3	0.305	0.31	0.315	0.32	0.325	0.329	0.334	0.339	0.344	0.349	0.352
Ghana	0.309	0.313	0.315	0.317	0.32	0.323	0.326	0.33	0.338	0.347	0.356	0.366	0.375	0.384	0.392	0.401	0.409	0.416	0.421	0.426	0.429	0.431	0.433	0.436	0.438	0.44	0.443	0.446	0.448	0.449	0.451	0.457	0.466	0.477	0.486	0.494	0.499
Guinea	0.181	0.184	0.188	0.191	0.194	0.197	0.2	0.203	0.207	0.21	0.213	0.217	0.22	0.223	0.226	0.23	0.233	0.237	0.24	0.243	0.246	0.249	0.251	0.254	0.257	0.26	0.264	0.268	0.273	0.277	0.282	0.288	0.293	0.299	0.305	0.311	0.316
Guinea-Bissau	0.176	0.179	0.182	0.184	0.187	0.19	0.193	0.197	0.2	0.204	0.208	0.213	0.217	0.222	0.226	0.231	0.236	0.242	0.246	0.25	0.254	0.258	0.262	0.266	0.27	0.274	0.279	0.283	0.288	0.293	0.298	0.305	0.31	0.316	0.322	0.328	0.333
Liberia	0.198	0.201	0.203	0.205	0.207	0.209	0.211	0.213	0.216	0.219	0.218	0.218	0.215	0.211	0.204	0.196	0.186	0.183	0.184	0.19	0.204	0.218	0.234	0.241	0.247	0.253	0.258	0.264	0.27	0.276	0.282	0.289	0.298	0.307	0.314	0.321	0.327
Mali	0.114	0.117	0.118	0.12	0.122	0.124	0.127	0.129	0.131	0.135	0.138	0.142	0.146	0.15	0.153	0.157	0.161	0.164	0.168	0.172	0.176	0.18	0.185	0.19	0.195	0.2	0.205	0.211	0.217	0.223	0.23	0.237	0.243	0.249	0.256	0.262	0.267
Mauritania	0.273	0.279	0.283	0.288	0.293	0.297	0.302	0.307	0.312	0.317	0.322	0.327	0.332	0.338	0.344	0.349	0.356	0.361	0.366	0.372	0.376	0.381	0.386	0.391	0.396	0.402	0.41	0.417	0.424	0.431	0.438	0.445	0.452	0.46	0.468	0.475	0.482
Niger	0.119	0.121	0.123	0.126	0.127	0.129	0.131	0.133	0.135	0.136	0.138	0.139	0.14	0.141	0.141	0.141	0.142	0.143	0.145	0.146	0.148	0.149	0.152	0.154	0.156	0.158	0.161	0.163	0.166	0.169	0.172	0.175	0.179	0.183	0.188	0.192	0.195
Nigeria	0.289	0.296	0.303	0.309	0.314	0.32	0.323	0.323	0.323	0.322	0.322	0.322	0.324	0.327	0.33	0.333	0.337	0.341	0.345	0.349	0.353	0.358	0.364	0.369	0.377	0.385	0.392	0.4	0.407	0.414	0.423	0.433	0.443	0.454	0.464	0.473	0.479
Sao Tome and Principe	0.237	0.243	0.248	0.254	0.259	0.265	0.27	0.275	0.279	0.284	0.289	0.294	0.299	0.304	0.31	0.315	0.32	0.326	0.331	0.336	0.34	0.346	0.351	0.357	0.363	0.369	0.376	0.382	0.388	0.393	0.398	0.403	0.408	0.413	0.42	0.427	0.434
Senegal	0.197	0.2	0.204	0.208	0.211	0.216	0.22	0.225	0.23	0.235	0.24	0.245	0.251	0.257	0.263	0.27	0.276	0.283	0.29	0.295	0.299	0.302	0.304	0.307	0.309	0.312	0.314	0.317	0.32	0.324	0.327	0.331	0.336	0.34	0.345	0.35	0.354
Sierra Leone	0.186	0.189	0.192	0.195	0.198	0.201	0.204	0.207	0.21	0.214	0.217	0.222	0.225	0.229	0.233	0.237	0.241	0.243	0.246	0.247	0.249	0.252	0.257	0.262	0.268	0.273	0.279	0.286	0.292	0.299	0.306	0.313	0.322	0.332	0.341	0.348	0.352
Togo	0.208	0.212	0.215	0.218	0.222	0.225	0.228	0.232	0.236	0.242	0.248	0.255	0.261	0.267	0.273	0.28	0.288	0.296	0.301	0.307	0.312	0.317	0.321	0.326	0.331	0.336	0.341	0.346	0.351	0.356	0.361	0.367	0.373	0.379	0.385	0.392	0.397

Appendix Figure 1. A more general causal web of the causes of health outcomes with the categories of causes included in this analysis shown in blue.

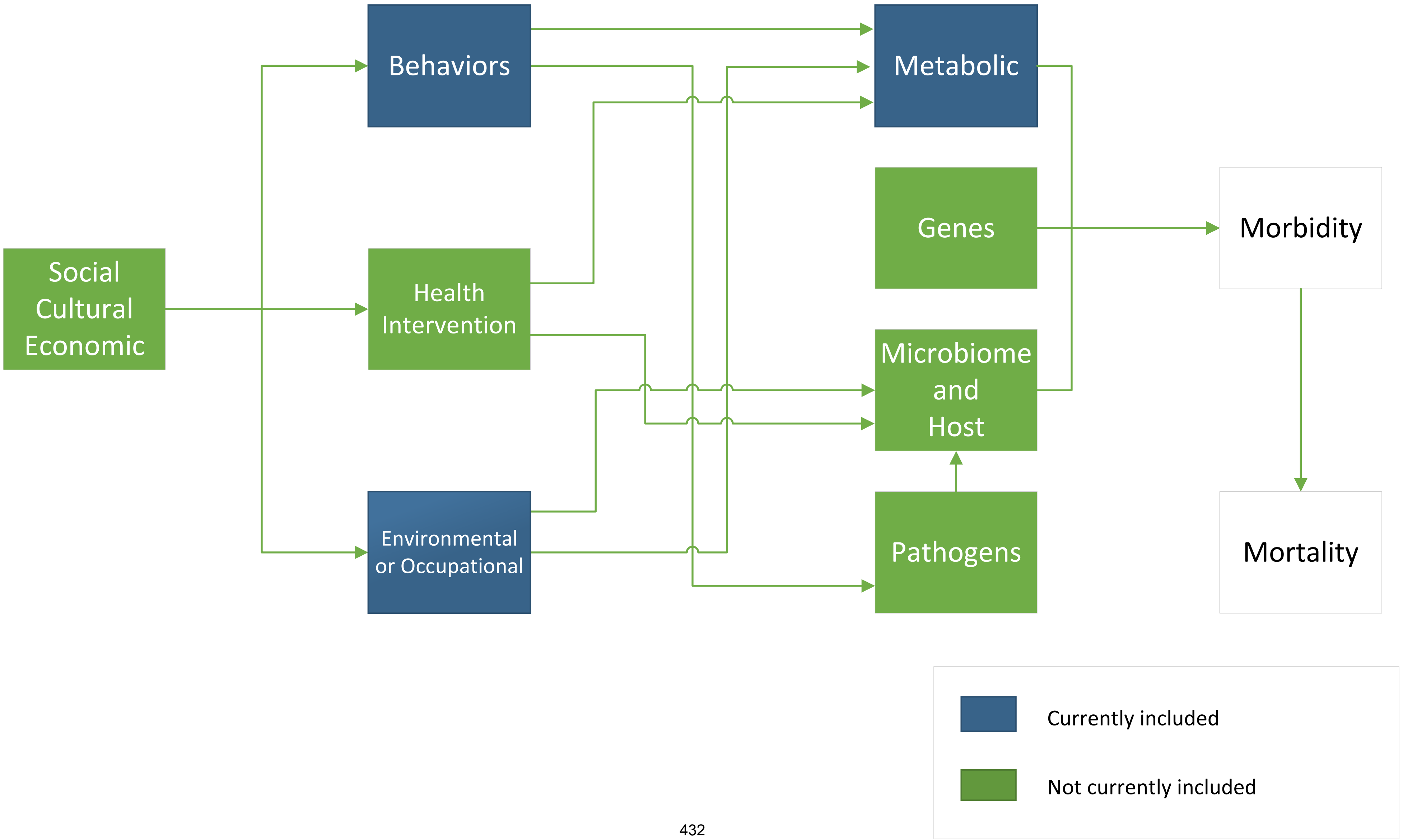

Appendix Figure 2. Analytical flowchart of the comparative risk assessment for the estimation of population attributable fractions by geography, age, sex, and year for GBD 2016. Ovals represent data inputs, rectangular boxes represent analytical steps, cylinders represent databases, and parallelograms represent intermediate and final results. GBD=Global Burden of Disease. SEVs=Summary exposure values. TMREL=Theoretical minimum-risk exposure level. PAFs=Population attributable fractions. YLLs=years of life lost. YLDs=years lived with disability. DALYs=disability-adjusted life-years.

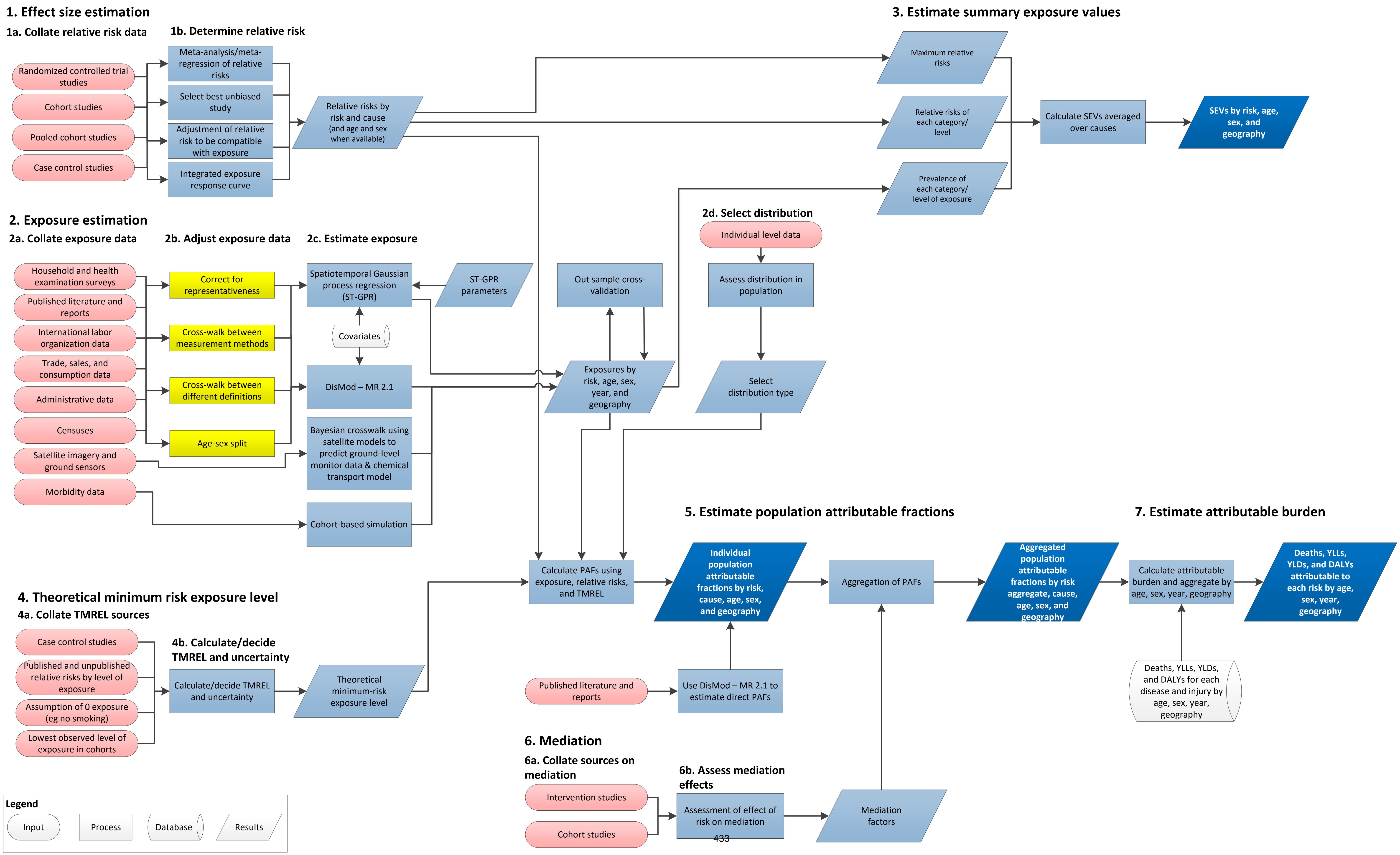

Appendix Figure 3 - GBD 2016 DisMod-MR 2.1 analytical cascade

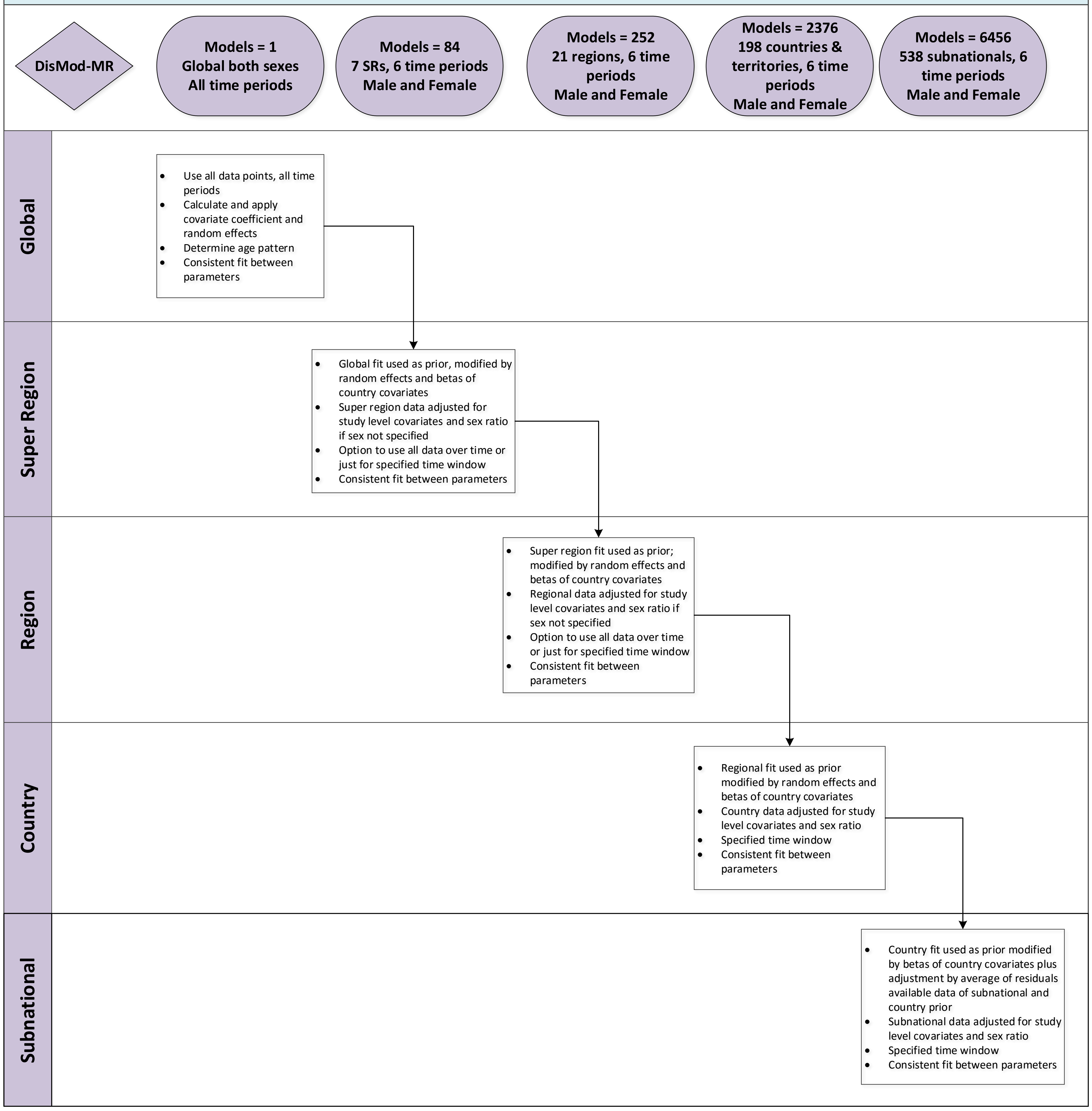

Appendix Figure 4: ST-GPR flowchart

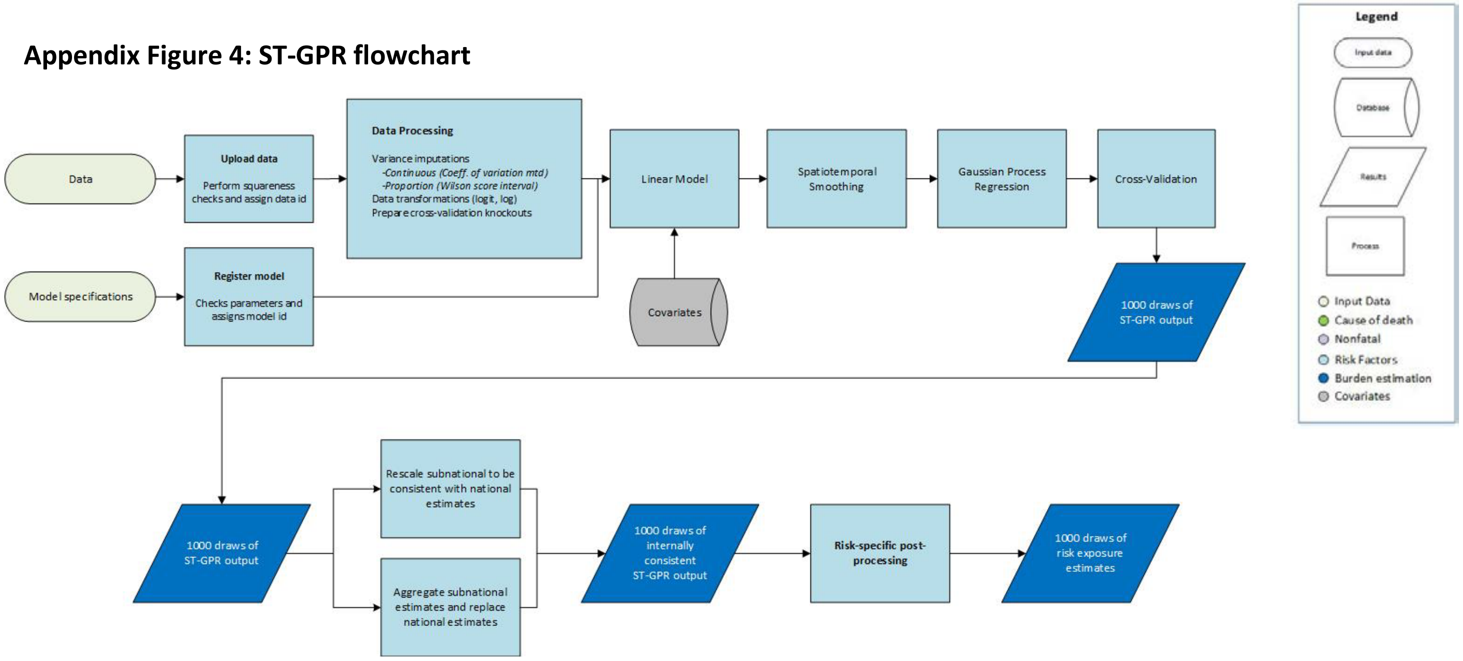

Supplement: Supplementary appendix 1 [file mmc1.pdf]
